# Neuronal megalin mediates synaptic plasticity—a novel mechanism underlying intellectual disabilities in megalin gene pathologies

**DOI:** 10.1093/braincomms/fcaa135

**Published:** 2020-08-25

**Authors:** João R Gomes, Andrea Lobo, Renata Nogueira, Ana F Terceiro, Susete Costelha, Igor M Lopes, Ana Magalhães, Teresa Summavielle, Maria J Saraiva

**Affiliations:** f1 Molecular Neurobiology Unit, IBMC- Instituto de Biologia Molecular e Celular, 4200-135 Porto, Portugal; f2 I3S—Instituto de Investigação e Inovação em Saúde, Universidade do Porto, 4200-135 Porto, Portugal; f3 Addiction Biology Group, IBMC- Instituto de Biologia Molecular e Celular, 4200-135 Porto, Portugal

**Keywords:** megalin, hippocampus, synaptic plasticity, learning and memory, Donnai-Barrow syndrome

## Abstract

Donnai-Barrow syndrome, a genetic disorder associated to LRP2 (low-density lipoprotein receptor 2/megalin) mutations, is characterized by unexplained neurological symptoms and intellectual deficits. Megalin is a multifunctional endocytic clearance cell-surface receptor, mostly described in epithelial cells. This receptor is also expressed in the CNS, mainly in neurons, being involved in neurite outgrowth and neuroprotective mechanisms. Yet, the mechanisms involved in the regulation of megalin in the CNS are poorly understood. Using transthyretin knockout mice, a megalin ligand, we found that transthyretin positively regulates neuronal megalin levels in different CNS areas, particularly in the hippocampus. Transthyretin is even able to rescue megalin downregulation in transthyretin knockout hippocampal neuronal cultures, in a positive feedback mechanism via megalin. Importantly, transthyretin activates a regulated intracellular proteolysis mechanism of neuronal megalin, producing an intracellular domain, which is translocated to the nucleus, unveiling megalin C-terminal as a potential transcription factor, able to regulate gene expression. We unveil that neuronal megalin reduction affects physiological neuronal activity, leading to decreased neurite number, length and branching, and increasing neuronal susceptibility to a toxic insult. Finally, we unravel a new unexpected role of megalin in synaptic plasticity, by promoting the formation and maturation of dendritic spines, and contributing for the establishment of active synapses, both in *in vitro* and *in vivo* hippocampal neurons. Moreover, these structural and synaptic roles of megalin impact on learning and memory mechanisms, since megalin heterozygous mice show hippocampal-related memory and learning deficits in several behaviour tests. Altogether, we unveil a complete novel role of megalin in the physiological neuronal activity, mainly in synaptic plasticity with impact in learning and memory. Importantly, we contribute to disclose the molecular mechanisms underlying the cognitive and intellectual disabilities related to megalin gene pathologies.

## Teaser

Donnai-Barrow syndrome, genetic disorder linked to megalin mutations, shows unexplained intellectual deficits. We describe that megalin, a multifunctional receptor, via its ligand TTR, regulates neuronal activity and survival. Additionally, we unravel a novel role of megalin in synaptic plasticity, with impact on learning and memory, explaining the described intellectual disabilities.

## Graphical Abstract

**Graphical Abstract:**
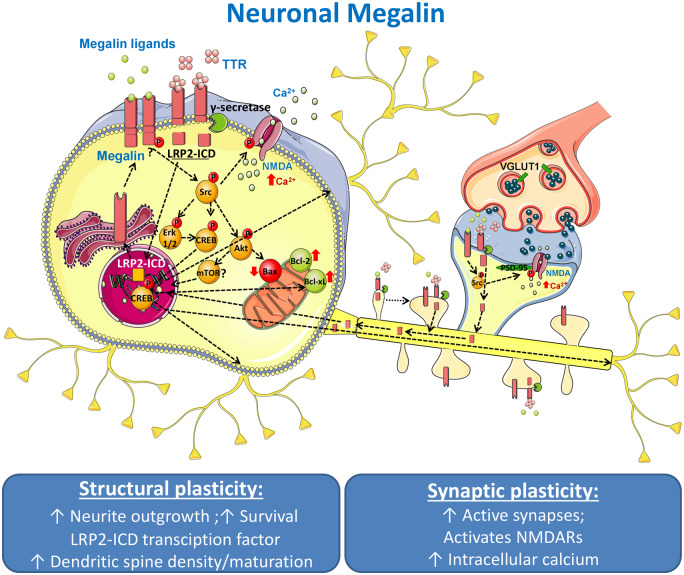

## Introduction

Low-density lipoprotein receptor-related protein 2 (LRP2), aka megalin, mutations lead to the protein loss of function, underlying an autosomal recessive disorder, the Donnai-Barrow syndrome (Kantarci *et al.*, 2007), characterized by several CNS functional defects, particularly myopia and ocular complications, sensorineural hearing loss, mild to moderate intellectual disability, development delay and, in some cases, agenesis of the corpus callosum (Pober *et al.*, 2009), and other symptoms such as specific craniofacial features, intestinal and heart abnormalities. Moreover, LRP2 polymorphisms were recently linked to non-syndromic cognitive impairment and intellectual disabilities (Beydoun *et al.*, 2012; Dietrich *et al.*, 2014; Vasli *et al.*, 2016; Beydoun *et al.*, 2017). Animal models of megalin deficiency recapitulate several Donnai-Barrow syndrome symptoms, such as forebrain abnormalities, agenesis of corpus callosum, holoprosencephaly, eye defects, defects in cardiovascular development (Willnow *et al.*, 1996; Kantarci *et al.*, 2007).

Megalin is a membrane glycoprotein with a molecular weight of 600 kDa (517 kDa non-glycosylated), standing as one of the largest glycoproteins in vertebrates. It belongs to the low-density lipoprotein receptor family (Saito *et al.*, 1994) and is constituted by a large extracellular domain (490 kDa) with four cysteine-rich complement-type ligand-binding repeats, involved in ligand binding (Russell *et al.*, 1989; Fass *et al.*, 1997), one transmembrane domain and an intracellular C-terminal tail of about 23 kDa (Saito *et al.*, 1994; Hjalm *et al.*, 1996). The cytoplasmatic domain contains several motifs that regulate receptor trafficking and endocytosis, ranging from NPXY sequences, which mediate internalization through clathrin-dependent mechanisms, to SH2/SH3 domains involved in tyrosine kinases signalling (Marzolo and Farfan, 2011).

Megalin was firstly identified as an auto-antigen in a kidney pathology denominated Heyman nephritis (Kerjaschki and Farquhar, 1982), and is currently recognized as a multiligand receptor, which binds and internalizes a wide variety of molecules, such as hormones, carrier proteins, lipoproteins, drugs and enzymes, mostly in the epithelial cells of the renal tubules (Christensen *et al.*, 2012). Moreover, megalin is also expressed in the epithelial cells of the choroid plexus (Carro *et al.*, 2005) and lateral ventricles (Gajera *et al.*, 2010), and in different CNS cells, ranging from oligodendrocytes (Wicher *et al.*, 2006), retinal ganglion cells (Fitzgerald *et al.*, 2007), cerebellar granule neurons (Ambjorn *et al.*, 2008), astrocytes (Bento-Abreu *et al.*, 2008), to hippocampal neurons (Gomes *et al.*, 2016). Megalin complete knockout led to abnormal development of the forebrain (Willnow *et al.*, 1996), and conditional knockouts of this protein unveiled its role in adult brain neurogenesis and presented cognitive impairment (Spoelgen *et al.*, 2005; Gajera *et al.*, 2010; Dietrich *et al.*, 2014*b*). Megalin’s major role in the CNS, through the binding of several ligands, has been as a mediator of neuronal survival and regeneration (Marzolo and Farfan, 2011). However, among its ligands, only metallothioneins (Pedersen *et al.*, 2009) and transthyretin (Fleming *et al.*, 2009; Gomes *et al.*, 2016) have shown a neuroprotective and neuroregenerative role in the nervous system. Many other ligands have shown regenerative properties involving megalin, but not in the nervous system (Min *et al.*, 2003; Zang *et al.*, 2014).

Megalin signal transduction in CNS is still poorly understood. After extracellular ligand binding, phosphorylation of the receptor in the cytoplasmic domain seems to occur (Yuseff *et al.*, 2007), followed by either receptor-mediated endocytosis, direct activation of signalling pathways and/or regulation of gene expression via regulated intramembrane proteolysis (RIP) of the intracellular domain (LRP2-ICD). Metallothioneins have shown to promote neurite outgrowth and neuronal survival involving different signalling pathways, such as ERK, Akt and CREB, via megalin receptor (Fitzgerald *et al.*, 2007; Ambjorn *et al.*, 2008; Chung *et al.*, 2008). Transthyretin has proven its neurotrophic properties independently of its ligands, through megalin binding (Gomes *et al.*, 2019) and activation of signalling pathways Src/Erk/Akt/CREB, leading to neurite outgrowth and neuroprotection, both *in vitro* and *in vivo*, in the CNS and PNS (Sousa *et al.*, 2000; Sousa and Saraiva, 2001; Gomes *et al.*, 2016). APP (amyloid precursor protein) was also shown to interact with megalin in neurons, leading to changes in neurite outgrowth and Aβ-neurotoxicity (LaFerla *et al.*, 1997; Alvira-Botero *et al.*, 2010). However, megalin RIP processing and gene expression controlled by megalin remain understudied. It has been demonstrated in kidney cells and rat yolk sac cells that megalin, like Notch (Schroeter *et al.*, 1998), is subjected to RIP, with ectodomain shedding mediated by a metalloprotease, producing a membrane-bound megalin C-terminal fragment (MCTF or LRP-2_ECD). Subsequently, a γ-secretase cleaves these MCTF to a soluble megalin intracellular domain (LRP-2_ICD) (Biemesderfer, 2006; Li *et al.*, 2008; Shah *et al.*, 2013). However, the RIP processing remains to be addressed in neurons.

In the present study, we identify TTR, a megalin ligand, as a fine regulator of megalin expression, mostly in CNS hippocampal neurons. We show that TTR regulates megalin mRNA/protein levels in the hippocampus/hippocampal neuronal cultures, in a megalin-dependent positive feedback mechanism. Additionally, we unveil that TTR does not only activate signalling pathways via megalin, but also through RIP, producing an LRP2-ICD, which is translocated to the nucleus. Importantly, we describe that megalin, in a mechanism independent of TTR, promotes neurite outgrowth, increases dendritic spine density and improves neuronal survival, in neuronal cultures. In addition, we unclose a new role of megalin in synaptic plasticity, by increasing dendritic spine number and maturation, and the number of active synapses, in megalin heterozygous mice. Accordingly, these mice present learning and memory deficits in several behavioural tests associated to hippocampal function. These findings suggest novel mechanisms that underlie the cognitive and intellectual disabilities related to megalin gene pathologies, particularly the Donnai-Barrow syndrome.

## Material and methods

### Mice

The number of mice handled for this research was approved by the Institutional and National General Veterinary Board Ethical Committees (approval reference number 003424), according to National and European Union rules. Three- to six-month-old TTR wild type (TTR^+/+^), TTR KO (TTR^−/−^) (Episkopou *et al.*, 1993), Megalin heterozygous (Meg^+/−^) and Meg^+/−^ TTR KO mice, in a 129/svJ background were used to access megalin levels in different areas and for hippocampal neuronal cultures. Megalin heterozygous mice were kindly provided by Dr Thomas Willnow, Max-Delbrueck Center for Molecular Medicine, Berlin, Germany (Willnow *et al.*, 1996). The animals were reproduced, maintained (regular rodents chow and tap water *ad libitum*) and experimentally manipulated under a 12 h light/dark cycle in type II cages in specific pathogen-free conditions in the animal facility (microbiological health status available). The method of euthanasia used was cervical displacement. Charles River Laboratories is the external animal facility used to acquire animals. Genotypes were determined from tail extracted genomic DNA, using primers for the detection of exon 2 of TTR (which is disrupted in TTR^−/−^ by insertion of a neomycin resistance gene), Megalin and neomycin as previously described (Episkopou *et al.*, 1993; Willnow *et al.*, 1996). Physical randomization, for group selection, was performed by doing a lottery with the earplug numbers, using small papers. The order by which the animals in the different experimental groups were assessed was random, testing all the groups in each experimental session. ARRIVE guidelines were taken into consideration in experimental reporting. This study was not pre-registered.

### Recombinant TTR production and purification

Recombinant mouse and human TTR were produced in a bacterial expression system using *Escherichia coli* BL21 (Furuya *et al.*, 1991) and purified as previously described (Almeida *et al.*, 1997). Briefly, after growing the bacteria, the protein was isolated and purified by preparative gel electrophoresis after ion-exchange chromatography. Protein concentration was determined using the Lowry method (Lowry *et al.*, 1951). To remove endotoxins from recombinant TTR, a polymixin B column (Thermo Scientific, Detoxi-Gel™ Endotoxin Removing Gel, #20339) was used. Briefly, the column was regenerated with 1% sodium deoxycholate (Sigma) and washed with pyrogen-free buffer to remove detergent. Recombinant TTR was applied to the column and incubated during 1 h at room temperature. Aliquots of pyrogen-free buffer were added and the flow-through was collected. Protein concentration was determined using the Bradford method (Hammond and Kruger, 1988). The SDS-PAGE of recombinant TTR proteins used (purity of protein preparations) is presented in Supplementary material.

### Primary hippocampal neuronal cultures

Primary cultures of mouse hippocampal neurons were prepared from the hippocampus of E17-E18 WT, TTR KO and Megalin^+/−^ or Megalin^+/+^ mice embryos (129/svJ background), as previously described (Almeida *et al.*, 2005; Gomes *et al.*, 2011). Neuronal cultures were maintained in serum-free Neurobasal medium (Gibco, Life Technologies), supplemented with B27 (Gibco, Life Technologies), glutamate (25 μM), glutamine (0.5 mM) and gentamicin (0.12 mg/ml). Cells were kept at 37°C in a humidified incubator with 5% CO2/95% air, for 1 DIV/13 DIV, for the neurite outgrowth experiments or 7 days for the western blot experiments, the time required for maturation of hippocampal neurons. Cells were cultured at a density of 9 × 10^4^ cells/cm^2^ or 8 × 10^4^ cells/cm^2^ on poly-d-lysine-coated six-well microplates (MW6) (for western blot and real-time PCR experiments) or glass coverslips (for immunocytochemistry studies), respectively. Megalin mice embryos were genotyped (5 h express protocol) to separate the Meg^+/−^ from the Meg^+/+^ animals. Meanwhile, hippocampi were hibernated using Hibernate E medium (Gibco, Life Technologies) supplemented with diluted 1:10 B27 (Gibco, Life Technologies) and kept at 4°C. These hippocampal neuronal cultures were performed using a serum-free medium (B27), which does not contain T4 or RBP, TTR ligands. The following pre-established exclusion criteria were used: cell cultures with high % of cell death (>30%) and/or neurite network undeveloped or damaged.

### Western blot analysis

For cultured hippocampal neurons, cells were homogenized in lysis buffer containing 20 mM MOPS, 2 mM EGTA, 5 mM EDTA, 30 mM sodium fluoride, 60 mM β-glycerophosphate, 20 mM sodium pyrophosphate, 1 mM sodium orthovanadate, 1 mM phenylmethylsulphonyl fluoride, 1% Triton X-100 and 1× protease inhibitors mixture (GE Healthcare). For whole tissue hippocampal extracts, tissue was dissociated in the same lysis buffer used for cultures, using an Eppendorf shape tissue homogenizer at 3000 rpm, with samples frozen. Total protein concentration was determined using the Bradford method. Fifty to 150 µg of protein was applied and separated by 10% SDS-PAGE and transferred to a nitrocellulose Hybond-C membrane (GE Healthcare), using a wet system, with Tris/Glycine/methanol buffer (Bio-Rad). Membranes were blocked at least 1 h at room temperature in blocking buffer, 5% bovine serum albumin (BSA) in phosphate-buffered saline Tween-20 (PBST), and then incubated overnight at 4°C with primary antibodies diluted in blocking buffer, namely sheep polyclonal anti-Megalin (1:1000; custom made), rabbit monoclonal C-terminal anti-Megalin (1:750, Abcam, ab24640), rabbit polyclonal C-terminal anti-Megalin (1:500, Abcam, ab76969), rabbit anti-vinculin (1:1500, Abcam, ab129002), mouse anti-α-tubulin (1:10 000, Sigma, T8203), rabbit anti-MAP2 (1:800, Abcam, ab24640), mouse anti-GFP (1:2000, 11814460001, Roche, Sigma), mouse anti-Gapdh (1:20 000, Abcam, ab9484), goat anti-Histone H2A.X (1:500, Sta. Cruz, sc–4606), rabbit VGLUT1 (1:5000, Sysy, 135 303), mouse PSD95 (1:2000, ThermoFisher Scientific, MA1-045), GAPDH (1:20 000, Abcam, ab9484). Membranes were then incubated with anti-rabbit IgG-HRP (1:10 000; Binding Site), anti-mouse IgG-HRP (1:5000; Binding Site) and anti-goat IgG-HRP (1:5000; Binding Site), for 1 h at room temperature. Blots were developed using Immun-Star WesternC Chemiluminescent kit (Bio-Rad) and exposed to Bio-Rad ChemiDoc XRS system or ECL Hyperfilm (GE Healthcare), if the signal was too low. Quantitative analyses were performed using the Quantity One software or ImageLab from Biorad^®^ Laboratories. The experimental unit in western blot assays was each individual culture (always performed with different breeding females in independent neuronal culture isolation procedures or different animals).

### Subcellular fractionation protocol

In order to separate nuclear and cytoplasmatic fractions from TTR KO hippocampal neurons cellular extracts (13 DIV), cultured neurons were homogenized in buffer containing 10 mM HEPES, 1.5 mM MgCl_2_, 10 mM KCl, 0.5 mM DTT and 0.05% NP40 at pH 7.9, supplemented with 1× protease inhibitors mixture (GE Healthcare). Neurons were extracted in 80 µl of chilled supplemented buffer, using a cell scraper, and kept on ice for 10 min. Then, neurons were centrifuged at 720 × g, 4°C for 5 min. The pellet containing the nuclear fraction was resuspended in TBS (Tris-buffered saline) with 0.1% SDS, while the supernatant was centrifuged again for 10 000 × g for 5 min. The obtained supernatant contained mostly the cytoplasmatic fraction (with some membranes). Both fractions were then sonicated on ice, which is particularly important for nuclear fractions, in order to shear genomic DNA and homogenize the lysate. Western blot confirmed nuclear fraction enrichment by Histone H2Ax in opposition to GAPDH. This protocol was based on Abcam ‘Nuclear Extraction and fractionation protocol’ and ‘Subcellular fractionation protocol’.

### mRNA semi-quantification through real-time PCR

Total RNA was extracted from either 7 DIV cultured hippocampal neurons or dissected brain/organ areas from WT or TTR KO mice using TRIzol Reagent (Invitrogen), as previously described (Santos and Duarte, 2008). RNA quality and integrity were assessed using the Experion automated gel electrophoresis system (Bio-Rad, Portugal), as previously described (Santos and Duarte, 2008). Samples showing RNA degradation or contamination by DNA were discarded. RNA concentration was determined using NanoDrop 1000 (Thermo Scientific). The samples were aliquoted and stored at −80°C until further use. cDNA synthesis was performed using 1 µg of total RNA and the SuperScript^®^ cDNA synthesis (random primers) (Invitrogen, Portugal), as previously described (Santos and Duarte, 2008). Samples were stored at −80°C until further use (1 week—1 month). Primers used for real-time PCR were designed using ‘Beacon Designer’ software (Premier Biosoft International) as described previously (Santos and Duarte, 2008). Oligonucleotides used for Megalin real-time PCR were: forward, 5ʹGGCTCACTCAAGTCCGCATCTTCC3ʹ and reverse 5ʹACTCAACGGTGCTGCCAGTTACG3ʹ. For LRP1: forward, 5ʹCGAGGAGCAGGTTGTTAG3ʹ and reverse 5ʹCAGAAGCAGCAGGAGAAG3ʹ. 18S RNA was used as reference gene with the following primers: forward, 5ʹAAATCAGTTATGGTTCCTTTGGTC3ʹ and reverse 5ʹGCTCTAGAATTACCACAGTTATCCAA3ʹ; and for GAPDH, forward, 5ʹGCCTTCCGTGTTCCTACC3ʹ, and reverse, 5ʹAGAGTGGGAGTTGCTGTTG3ʹ. The annealing temperature was 60°C. For gene expression analysis, 1 μl of stock to 1:1000 diluted cDNA was added to 10 μl of 2× SYBR Green Master Mix (Bio-Rad) and the final concentration of each primer was 250 nM in 20 μl total volume. The thermocycling reaction was initiated through activation of Taq DNA polymerase by heating at 95°C during 3 min, followed by 45 cycles of a 15s denaturation step at 95°C and a 20s annealing/elongation step at 60°C. The fluorescence was measured after the extension step, using the iQ5 Multicolor Real-Time PCR Detection System (Bio-Rad). After the thermocycling reaction, the melting step was performed with slow heating, starting at 55°C and with a rate of 0.5°C per 10 s, up to 95°C, with continuous measurement of fluorescence. Data analysis was performed using Pfaffl method for efficiency correction (Pfaffl, 2001). Results were normalized with either 18S or GAPDH mRNA, as internal reference gene, since these genes show a stable expression in the conditions tested (we tested the different reference genes). We took into consideration MIQE guidelines, for minimum information for Publication of Quantitative Real-Time PCR Experiments (Bustin *et al.*, 2009).

### Immunocytochemistry

Neurons were fixed in 4% sucrose/4% paraformaldehyde (PFA) and permeabilized with 0.3% Triton X-100 in PBS for 10 min. Neurons were then incubated with 5% BSA (Sigma) in PBS with 0.1% Tween 20 (PBST), for 1 h at 37°C, to block non-specific binding, and incubated with primary antibodies, overnight at 4°C. Cells were then washed five times with 0.5% BSA in PBST, and incubated with the appropriate secondary antibodies, for 1 h at 37°C. The coverslips were mounted in a fluorescent mounting medium (DAKO, Denmark) and imaging was performed on a laser scanning Confocal Microscope Leica SP5 AOBS SE, using the 40× water or 63× oil objective. Primary antibodies used were anti-MAP2 (1:800; Abcam, ab24640), mouse anti-GFP (1:500, 11814460001, Roche, Sigma) and rabbit polyclonal C-terminal anti-Megalin (1:500, Abcam, ab76969); the secondary antibodies were Alexa Fluor 488 or 568 (1:750, Life technologies). The fluorescent dye Hoechst 33342 (0.5 µg/ml, 10 min room temperature) was used to stain nuclei. In each set of experiments, the same batch of antibodies (primary and secondary) was used, and imagens were taken using the same settings. The quantification of nuclear megalin C-terminus fused with green fluorescent protein (GFP) was performed by quantifying GFP nuclear fluorescence, and co-localizing with Hoechst stain, using ImageJ software. At least 12–13 transfected neurons were counted for each experimental condition in a blind way for the condition, from three independent preparations. The experimental unit in these assays was each independent culture.

### Transfection

Transfection of cultured hippocampal neurons was performed through the calcium phosphate co-precipitation method as previously described, with minor modifications (Dudek *et al.*, 2001; Gomes *et al.*, 2011). Briefly, 2 µg of single plasmid DNA (or mixture of equal amounts of two plasmids) were diluted in Tris-EDTA (TE) pH 7.3 and mixed with HEPES calcium chloride pH 7.2 (2.5 M CaCl2, 10 mM HEPES). This DNA/TE/Calcium mix was added to 2× HEPES Buffered Saline solution (270 mM NaCl, 10 mM KCl, 1.4 mM Na2HPO4, 11 mM Dextrose, 42 mM HEPES), pH 7.2. The precipitates were allowed to form for 30 min, with vortex mixing every 5 min, to ensure that the precipitates had similar small sizes. Meanwhile, coverslips with cultured neurons were incubated with cultured conditioned medium with 2 mM of Kynurenic acid. The precipitate was added dropwise to each coverslip and incubated at 37°C, 5% CO_2_, for 3 h. Cells were then washed with acidic (10% CO_2_) equilibrated culture medium containing 2 mM Kynurenic acid and returned to the 37°C/5% CO_2_ incubator for 15 min. Finally, the medium was replaced with the initial culture-conditioned medium, and the cells were further incubated in a 37°C/5% CO_2_ incubator for 48 h to allow protein expression.

### cDNA constructs

Three plasmids were used: pEGFP-N1, the expression vector containing the GFP gene only (BD Biosciences, Clontech); pLNCX-M4 that generates functional megalin mini-receptor; and pEGFP_N1-C-terminal megalin that briefly contains the entire cytoplasmatic region of the megalin gene fused with GFP. The megalin mini-receptor pLNCX-M4 was a kind gift from Akihiko Saito (Takeda *et al.*, 2003) and was generated by a PCR fragment containing the fourth ligand-binding motif till the end of the C-terminal of the megalin gene (rat origin—95% identity with mouse), and cloned into the pLNCX2 retroviral vector. In this work, no retroviruses were produced as a transient transfection was the objective, and a more physiological overexpression of the protein (CMV promoter). The pEGFP_N1-C-terminal Megalin plasmid was a gift from Ignacio Torres-Aleman (Bolos *et al.*, 2010). The cDNA of this plasmid contains a small extracellular region with two perimembrane extracellular cysteine-rich domains, the transmembrane part and the complete cytoplasmatic region of megalin gene (from human origin—C terminal with 75% identity with mouse), fused C terminally with the GFP cDNA of the pEGFP vector. All the plasmids were sequenced by DNA-sequencing reactions to confirm their identity and integrity.

### *In vitro* neuronal morphological analysis

Cultured hippocampal neurons from WT, TTR KO and Meg^+/−^ mice embryos were isolated under the above-described conditions, plated at a density of 5 × 10^4^ cells/cm^2^ (24h) or 8 × 10^4^ cells/cm^2^ (15DIV). Cells were maintained in culture 24 h, approximately, in order to allow the precise tracing of all the neurites per neuron. For the analysis of mature neurons (10–15 DIV), transfection with a GFP plasmid was performed, to allow the analysis of individual neurons. In these experiments, TTR KO cultured hippocampal neurons (11 DIV) were transfected with either GFP plasmid (pEGFP) or co-transfected with GFP (pEGFP) and mini-megalin plasmid (pLNCX-M4), protein expression was allowed for 48 h, and then neurons were stimulated, or not, with recombinant mouse TTR (55 µg/ml) for 24 h, in cultured conditioned medium. Cells were fixed with 4% paraformaldehyde and immunofluorescence was performed using rabbit anti-MAP_2_ (1:800, ab24640, Abcam) or mouse anti-GFP (1:500, 11814460001, Roche, Sigma). The coverslips were mounted in a fluorescent mounting medium (DAKO, Denmark) and imaging was performed on a Zeiss AxioImager Z1microscope, using a 20× oil objective, for the 1 DIV cultures, or Confocal Microscope Leica SP5 AOBS SE, for mature neurons. For 1 DIV neurons, 15–20 images were randomly acquired throughout the coverslip, in a blind way for the condition; in the case of mature transfected neurons, pictures were taken from GFP-transfected neurons that were alive, in a blind way for the condition. Regarding the 1 DIV neurons, at least 60–80 cells were analysed per experimental condition in a blind way, and the experiments were repeated in at least three independent preparations (indicated in figure legends); for the transfected neurons (14 DIV), a total of 15–20 neurons were analysed in each preparation, from at least three independent preparations. The experimental unit in these assays was the independent culture.

Morphological measurements of neurite outgrowth (number of neurites and total neurite length per cell) were performed using the plugin NeuronJ for ImageJ software (Meijering *et al.*, 2004). The analysis of neurite branching (Sholl analysis) was performed as previously described (Braz *et al.*, 2017), using Bonfire scripts for MATLAB (Mathworks). After neurites were defined in NeuronJ, data were converted to SWC using Bonfire, and the dendritic arbour of each neuron was determined by defining the tracings connectivity using NeuronStudio software. Then, Sholl analysis was performed using Bonfire, by drawing concentric circles around the cell body with incremental radii, separated from each other by 6.0 µm, and counting the number of times each circle crosses a neurite. The number of intersections was represented in function of the distance from the cell body. In this case, graphics display individual neuron behaviour for better data visualization. For the analysis of total dendritic spine density, dendritic segments were defined using ImageJ software, and the total number of dendritic spines was defined by the user, blind to the treatment. The number of dendritic spines was represented in function of 10 µm of dendritic length.

### Immunofluorescence of brain choroid plexus

Tissue samples for immunohistochemistry were collected after mice were perfused with PBS and 4% paraformaldehyde. Five millimetre thick tissue sections were deparaffinated in Histoclear and hydrated in a descending alcohol concentration series. Then, slides were incubated with TBS, and permeabilized with 0.2% Triton X-100 in TBS solution for 10 min and rinsed in TBS 0.025% Triton X-100. Blocking was performed with 10% foetal bovine serum, plus 1% BSA and 0.3 M glycine, in TBS, for 2 h at room temperature. Primary antibodies were always incubated overnight at 4°C, in 1% BSA in TBS. Secondary antibodies were incubated 1 h at room temperature. The slides were mounted in a fluorescent mounting medium (DAKO, Denmark) and imaging was performed on a laser scanning Confocal Microscope Leica SP5 AOBS SE, using the 40×/63× oil objective. In each set of experiments, the same batch of antibodies (primary and secondary) was used, and images were taken using the same settings, such as camera exposure times. Primary antibodies used were rabbit monoclonal C-terminal anti-Megalin (1:750, Abcam, ab24640) and anti-mouse recombinant TTR (1:250, custom made, Quantum Appligene, Illkirch, France). As secondary antibodies, Alexa Fluor 488 and 568 (1:750, Life Technologies) were employed.

### Cell death assay

Hippocampal neurons from TTR KO and Meg^+/−^ TTR KO were cultured for 7 days on poly-d-lysine-coated glass coverslips as previously described. After excitotoxic stimulation with glutamate (125 µM glutamate, 20 min), and further incubation in cultured conditioned medium (14 h), cells were fixed in 4% sucrose/4% paraformaldehyde (in PBS) and the nucleus was stained with Hoechst as previously described. Analysis of the nuclear morphology was performed on Zeiss AxioImager Z1 fluorescence microscope, under a 40× oil objective. Live and dead cells were counted blind, using ImageJ. The experimental unit in these assays was each individual culture (always performed with different breeding females in independent neuronal cultures).

### FRET assay—intracellular calcium concentration

TTR KO cultured neurons (8 × 10^4^ cells/cm^2^) were plated in poly-d-lysine-coated glass-bottom dishes (ibidi GmbH), transfected at 7 DIV and imaged 48 h later, with yellow cameleon-Nano 15 (YC-Nano15), an ultrasensitive Ca^2+^ FRET probe (Horikawa *et al.*, 2010), as previously well described (Gomes *et al.*, 2016). Fluorescence imaging of cells was performed using an epifluorescence inverted microscope (DMI 6000B, Leica Microsystems) with a PlanApo 63× (N.A. 1.4) glycerol immersion objective. Data acquisition and processing were based on (Ferraz-Nogueira *et al.*, 2014). The FRET/donor change was calculated using ImageJ software. The experiments performed without calcium used a Krebs-Ringer solution: 119 mM NaCl, 2.5 mM KCl, 1.0 mM NaH_2_PO_4_, 2 mM EGTA, 1.3 mM MgCl_2_.6H_2_0, 20 mM HEPES and 11 mM D-glucose, pH 7.4. The experimental unit in FRET assays was each individual culture (12–16 neurons were analysed in each individual culture), although the figure displays individual neuron behaviour for better data visualization.

### AAV *in vivo* delivery

To achieve a sparse labelling of neurons in the CNS, we performed injections in the tail vein of mice-Meg^+/+^ and Meg^+/−^—with AAV_(PHP.eB)-CAG-GFP (Addgene viral prep # 37825-PHPeB) in a final titre of 0.5 × 10^11^, diluted in sterile PBS to a final volume of 150 µl. Twenty-one days post-injection, mice were deeply anaesthetized with ketamine/medetomidine 170 mg/kg (ketamine) + 2 mg/kg (medetomidine), intraperitoneal and perfused transcardially with PBS followed by 4% PFA with 4% sucrose in PBS, pH 7.4. Whole brains were removed and fixed with 4% PFA with 4% sucrose for 48 h, followed by transfer to 20% sucrose in PBS, until they moved to the bottom of the tube (around 24 h). Sixty to 80 µm serial coronal sections of the hippocampus were obtained using a Cryostat (Leica CM 3050S, Leica Microsystems, USA), and kept in PBS with 0.2% sodium azide at 4°C until further use.

### Immunocytochemistry of hippocampal slices

For the analysis of neuronal morphology and synapse quantification (co-localization of PSD95 and VGLUT1) in brain slices, we proceed as follows. Brain slices from different parts of the hippocampus (anterior/posterior) were selected and processed two slices per well in a 48-well plate. Unspecific staining was blocked by incubating with 10% horse serum in PBS containing 0.2% Triton X-100, and primary antibodies (GFP 1:500 for neuronal morphological analysis; PSD95 1:500 and VGLUT1 1:1000 for synapse analysis) were incubated in blocking solution for 72 h at 4°C with agitation. After washing with PBS (3 times, 15 min each), slices were incubated with secondary antibodies (Alexa 488 for GFP, Alexa 594 for PSD95 and Alexa 647 for VGLUT1) 24 h at 4°C with agitation. Slices were then washed in PBS, and mounted in gelatinized slides in DAKO mounting medium, and sealed with nail polish. Slices were kept at 4°C protected from light.

### Neuronal morphological analysis *in vivo*

Images of neurons in the CA1 and DG regions of the hippocampus were acquired in Leica TCS SP5II confocal microscope (Leica microsystems, Germany), with a 40×/1.10 NA Water objective, using an argon 488 nm laser, keeping the settings equal for all images and animals. Neurons expressing GFP were randomly chosen for quantification from at least four different sections from the region of interest, and three animals were analysed per genotype, with at least 10 neurons per region (CA1 or DG) analysed per animal. Neuron tracing was performed using NeuronJ plugin for ImageJ software, and Sholl analysis as described for the *in vitro* analysis of neuronal morphology in hippocampal neuronal cultures. For the analysis of dendritic spine density, images from secondary dendrites of hippocampal neurons were acquired in Leica TCS SP5II confocal microscope (Leica microsystems, Germany), with a 63×/1.3 NA Glycerol objective, using Ar 488 nm laser. Spine density and morphology were defined as immature spines, which included filopodia (without a defined head), or mature, which included thin (with a long neck and a small head), mushroom (with a small neck and a large head) and stubby (without a defined neck) using ImageJ software. The number of dendritic spines was represented in function of 10 µm of dendritic length. Experiments were performed by a user blind to the genotype, both in image acquisition and analysis.

### Synapse quantification *in vivo*

The quantification of protein co-localization at dendrites was performed as previously described (Catarino *et al.*, 2013). Briefly, images from the CA1 region of the hippocampus were acquired in a confocal microscope Leica TCS SP5II confocal microscope (Leica microsystems, Germany), with a 63×/1.3 NA Glycerol objective, maintaining the settings between slices and animals. Seven animals were analysed per genotype, and 2–10 images were acquired per animal. The acquired images had 20–40 µm of depth and were analysed using ImageJ software. In each image, two to four regions of interest were selected as rectangles (19.26 width × 15.89 high µm) in regions containing staining of both PSD95 and VGLUT1, and multiplied by the stack depth. The signal was subjected to a user-defined intensity threshold to select recognizable puncta in the region of interest. The thresholded signals were then used to determine the co-localization. For this, PSD95 signal was set as binary, whereas VGLUT1 signal was used to define the total synaptic puncta, and the puncta positive for PSD95 were defined as co-localized. Finally, all the values acquired were normalized to the mean values of the control group (if near 0, values were excluded, meaning signal was undetectable). Experiments were performed by a user blind to the genotype, both in image acquisition and analysis.

### Behavioural tests

Prior to the beginning of the behavioural tests, mice were allowed a 2-week adaptation period to the behavioural tests facilities. Tests were conducted in the dark (active) phase. All materials were cleaned with a neutral detergent without smell between animals. The same groups of animals performed the elevated plus maze (EPM) and Morris Water Maze (MWM) test, and different groups performed the Open field followed by the NOR test (a 3R strategy). Two-way ANOVA was performed to assess both sex and genotype effects, and no differences between sexes were observed. Therefore, the statistical analysis is presented with both sexes together.

### Elevated plus maze

EPM apparatus was placed at 50 cm from the ground, and is composed of a cross from of two open arms (30 × 5 cm) and two closed arms (30 × 5 cm, surrounded by 15 cm-high wall, opaque), with the two pairs of identical arms in opposite positions to each other, with the arms emerging from a central platform (5 × 5 cm). The test was initiated by placing the mouse in centre of the apparatus facing an open arm and allowed to move freely during 5 min. The lights were on and placed above the apparatus. Mouse behaviour was continuously videotaped by a camera placed above the apparatus. We evaluated the number of entries, time spent in open or closed arms, time spent in the central platform, and total distance travelled, using a tracking system (Smart Video Tracking Software v 2.5, Panlab).

### Open field

Each mouse was placed in the centre of an opaque arena (43 × 43 cm) and allowed to move freely for 10 min. The total distance travelled, peripheral activity (locomotion along the walls) and centre activity (locomotion in the central zone) were automatically obtained through video tracking (Smart Video Tracking Software v 2.5, Panlab).

### Morris water maze

The test was performed as previously described, with minor modifications (Ribeiro *et al.*, 2014). A circular pool (110 cm in diameter) filled with water (27°C ± 2°C) to a depth of 18.5 cm was placed in a quiet room decorated with contrast visual cues. Water was made opaque by the addition of white non-toxic ink. Abstractly, the pool was divided into four quadrants, and eight start locations were defined—north (N), south (S), east (E), west (W), northeast (NE), southeast (SE), northwest (NW) and southwest (SW)—at equal distances to the centre. An escape platform (10 × 10 cm) was immersed 0.5 cm below the waterline. On the first 2 days, mice were subjected to cue learning in order to test them for their ability to learn to swim to a cued goal. For this procedure, curtains were closed around the maze to reduce the availability of distal cues, and a flag was attached to the hidden platform. Animals were given four 60 s trials per day, each trial with different start and goal positions. Between each trial, mice were allowed to stay on the platform for 15 s. After the cued learning, mice were tested for their visual acuity. For this, a large plastic cue was placed on the platform and each mouse was scored for its latency to reach the platform. Twenty-four hours before this test, an identical plastic cue was placed in each of the mouse housing cages to minimize the possible effects of exposure to a novel object. After the cued learning, a 7-day hidden-platform learning phase was initiated. The platform was placed in the SW quadrant and the animals were scored for their latency to find the hidden platform. Mice were given four 60 s trials per day, each trial with different start locations, and inter-trial intervals on the platform of 30 and 15 s on Days 1 and 2–7, respectively. Twenty-four hours following Day 7 of the hidden-platform learning phase, the platform was removed and each mouse was subjected to a 30 s probe trial starting 180° (NE) from the original platform position (SW). The number of platform-site crossovers, the latency to first target-site crossover, the per cent time spent in the target quadrant (and also in the opposite quadrant) compared with the other quadrants were evaluated using SMART software.

### Novel object recognition

The NOR test was performed in an arena (43 × 43 cm) and consisted in three phases: the habituation phase, in which mice were allowed explored the apparatus for 10 min; the acquisition/sample phase 24 h after the habituation, where mice were placed in the apparatus with two identical objects (familiar object) for 10 min; and the retention/choice session, performed 6 h after (inter-trial interval), in which a novel object and a familiar object were used, and mice were allowed to explore the objects for 3 min. The objects chosen for this experiment were approximately the same height and weight, with different shapes, colours or materials (legos, glass cup or toy). Object exploration was defined as mice nose touching or directed towards the object at a distance shorter than 2 cm, and the duration of time mice spent exploring each object was recorded by the observer, blind to the genotype, using Observer 7 XT software (Noldus Information Technology, Wageningen, The Netherlands). The discrimination index (DI) was calculated as the ratio TN / (TN + TF) [TN = time exploring the novel (N) object; TF = time exploring the familiar (F) object]. As exclusion criteria, mice exploring the objects for periods lower than 7 s at 3 min of choice session were removed.

### Nuclear-localizing signals, nuclear-exporting signals, protein DNA and metal-binding residues—bioinformatic tools

The following bioinformatics tools were used to access nuclear-localizing signals (NLS) and nuclear-exporting signals (NES), in the mouse megalin C terminal. For NLS:: NucPred, website: https://nucpred.bioinfo.se/cgi-bin/single.cgi (Brameier *et al.*, 2007), cNLS Mapper, website http://nls-mapper.iab.keio.ac.jp/cgi-bin/NLS_Mapper_form.cgi (Kosugi *et al.*, 2009), PSORT II server, website  https://psort.hgc.jp/form2.html (Reinhardt and Hubbard, 1998) and NLStradamus, website http://www.moseslab.csb.utoronto.ca/NLStradamus/ (Nguyen Ba *et al.*, 2009). NLStradamus; cNLS Mapper identify classical NLS’s, sequences enriched in basic residues K (lysine) and R (arginine); NucPred, PSORTII identify sequences that to be nuclear/non-nuclear and have increased potential to unveil novel NLS’s.

Regarding NES, we used the computational tools Nespredictor NetNES, website http://www.cbs.dtu.dk/services/NetNES/ (la Cour *et al.*, 2004) and LocNES, website http://prodata.swmed.edu/LocNES/LocNES.php (Xu *et al.*, 2015). These tools not only identify potential leucine-rich NES, but also take into account the accessibility and flexibility of the protein sequence, to allow exporting proteins to interact with the predicted NES region.

Concerning protein DNA-binding residues we used, DRNApred (Yan and Kurgan, 2017), website http://biomine.cs.vcu.edu/servers/DRNApred/ and DP-Bind (Hwang *et al.*, 2007), website http://lcg.rit.albany.edu/dp-bind/. For the metal-binding residues Metaldetector (Passerini *et al.*, 2011), website http://metaldetector.dsi.unifi.it/v2.0/ and ZincBinder (Srivastava and Kumar, 2018), website  http://proteininformatics.org/mkumar/znbinder/prediction.html, were used. All URL's were up-to-date at 26th August 2020. The results are in the Supplementary information.

### Statistical analysis

Data presentation and experimental unit are described in figure legends. A previous power analysis was performed in order to obtain a 25% difference (10% SD) among two groups, with 90–95% power and sample sizes between three to six animals or individual cultures were determined. Statistical analysis of the results was performed using one-way ANOVA followed by Bonferroni multiple comparison test, when three groups were present. Unpaired Student's *t*-test was used when the comparisons were only between two groups. When indicated, a linear mixed model analysis was performed using SAS^®^ Visual Statistics with REML function, followed by Tukey–Kramer multiple comparison test. In that case, the culture or the animal is considered a random variable, and both individual cells (circles) and culture/mice averages (triangles) are presented in the graphs. For all statistical analysis: ***P < 0.001, **P < 0.01, *P < 0.05, ns (not significant). Identification and removal of outliers were performed using automatic Graphpad prism software 7.0/8.0, using the ROUT (robust non-linear regression) method, with a *Q* = 5%, for removing likely outliers (Motulsky and Brown, 2006). Supplementary Table 1 at the end of the Supplementary information file compiles a summary of all the statistical analysis used in this study. Moreover, upon request, all the analysis done for the linear mixed model approaches is available.

### Data availability analysis

Raw data were generated at I3S. The authors confirm that the data supporting the findings of this study are available within the article and Supplementary data. Derived data supporting the findings of this study are available from the corresponding author João Gomes upon request.

## Results

### TTR regulates megalin levels: TTR KO versus WT mice

**Figure 1:**
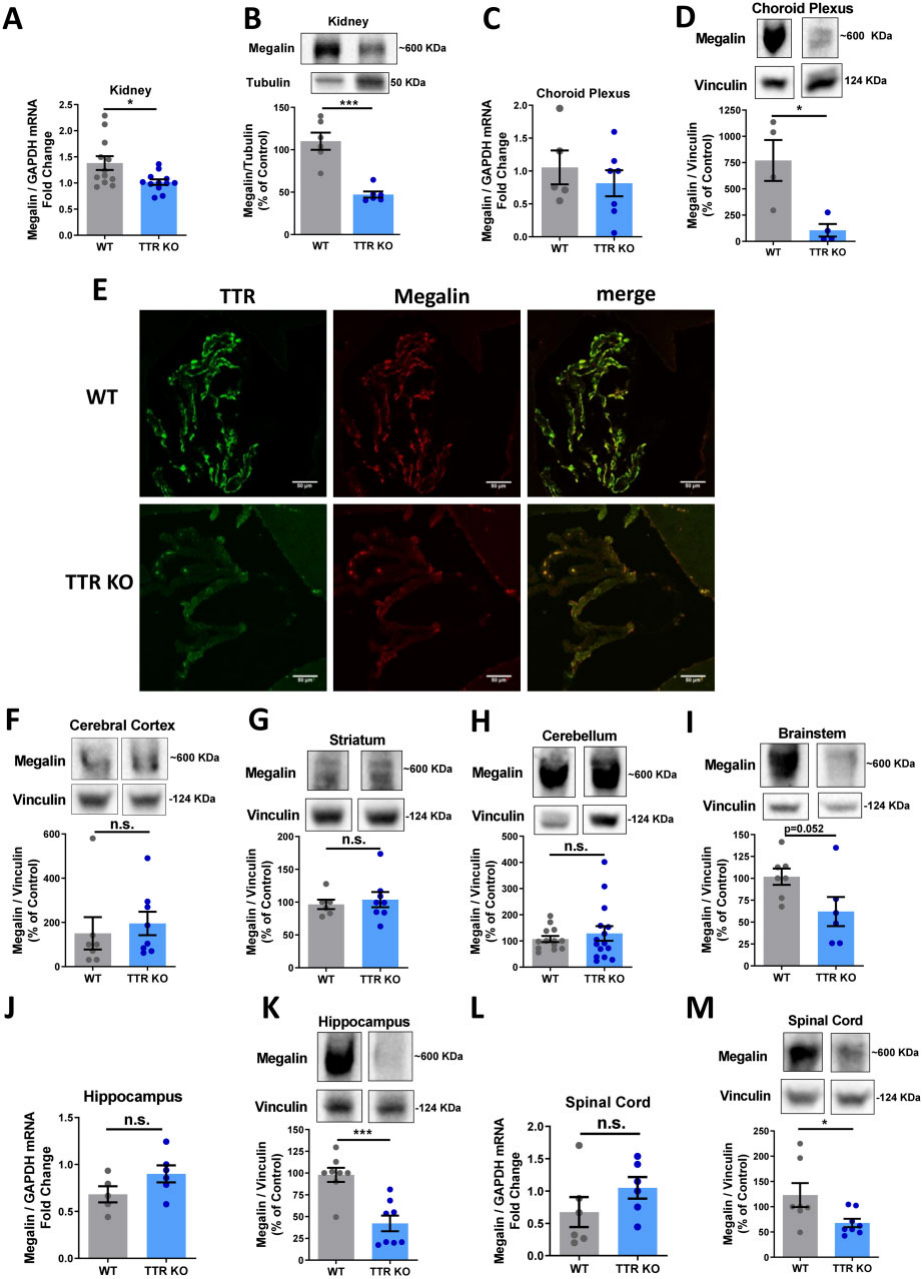
**TTR regulates megalin levels: TTR KO versus WT mice.** Total RNA was extracted from kidney (**A**, WT *n* = 12 mice: 9 males, 3 females; TTRKO *n* = 12 mice: 8 males, 4 females), choroid plexus (**C**, WT *n* = 5 male mice; TTRKO *n* = 7 mice: 5 males, 2 females), hippocampus (**J**, WT *n* = 7 mice: 6 males, 1 female; TTRKO *n* = 8: 5 males, 3 females) and spinal cord (**L**, WT *n* = 7 mice: 6 males, 1 female; TTRKO *n* = 8 mice: 5 males, 3 females) of WT and TTR KO mice, and megalin and GAPDH mRNA levels were semi-quantified by real-time PCR. Megalin mRNA levels are reduced in the kidney of TTR KO mice. Megalin protein levels were determined by western blot in the Kidney (**B,** WT *n* = 6 mice: 4 males, 2 females; TTRKO *n* = 6 mice: 3 males, 3 females), Choroid Plexus (**D,** WT *n* = 4 mice: 3 males, 1 female; TTRKO *n* = 6 mice: 1 male, 3 females), and in different brain regions of WT and TTR KO mice: Cerebral cortex (**F,** WT *n* = 7 mice: 6 males, 1 female; TTRKO *n* = 8 mice: 5 males, 3 females) Striatum (**G,** WT *n* = 6 mice: 5 males, 1 female; TTRKO *n* = 8 mice: 5 males, 3 females), Cerebellum (**H,** WT *n* = 13 mice: 12 males, 1 female; TTRKO *n* = 15 mice: 10 males, 5 females), Brainstem (**I,** WT *n* = 7 mice: 6 males, 1 female; TTRKO *n* = 8 mice: 5 males, 3 females), Hippocampus (**K,** WT *n* = 7 mice: 6 males, 1 female; TTRKO *n* = 8 mice: 5 males, 3 females) and spinal cord (**M**, WT *n* = 7 mice: 6 males, 1 female; TTRKO *n* = 8 mice: 5 males, 3 females). Megalin protein levels are reduced in the kidney, choroid plexus, hippocampus and spinal cord of TTR KO mice. Statistical analysis was performed using Student’s unpaired *t*-test. ***P < 0.001, *P < 0.05. **(E)** Representative images of immunofluorescence of choroid plexus from WT and TTR KO mice stained for TTR and megalin (three mice/genotype), showing a reduction of both TTR and megalin expression in TTR KO mice. Scale bar corresponds to 50 μm.

Megalin is described as a TTR receptor, and the interaction between TTR and megalin underlies TTR neuroprotective role in *in vitro* and *in vivo* cerebral ischaemia (Santos *et al.*, 2010; Gomes *et al.*, 2016). Moreover, megalin is the receptor regulating TTR-induced effect in neurite outgrowth, in physiological conditions (Gomes *et al.*, 2016). Thus, in order to explore the interplay between megalin and TTR, we assessed megalin expression levels in the CNS of WT mice versus TTR KO mice, in organs where megalin is highly expressed: kidney and epithelial cells of choroid plexus. We found that both megalin mRNA (Fig. 1A) and protein (Fig. 1B) levels were downregulated in the kidney of TTR KO mice, compared to WT mice. Regarding the choroid plexus, no differences were observed in mRNA levels (Fig. 1C), but, importantly, megalin protein levels were downregulated in TTR KO mice (Fig. 1D and E). When comparing megalin protein expression in the CNS of TTR KO versus WT mice, levels were reduced in the brainstem, hippocampus and spinal cord of TTR KO mice (Fig.1I, K and M), whereas the cerebral cortex, striatum and cerebellum had no significant differences (Fig.1F–H). In the hippocampus and spinal cord, despite the downregulation of megalin protein levels, no changes in mRNA were observed (Fig. 1J and L). Hence, the hippocampus stood out as the brain region where megalin expression is mostly affected by TTR absence.

### TTR rescues megalin downregulation in hippocampal neuronal cultures, in a megalin-dependent way

**Figure 2:**
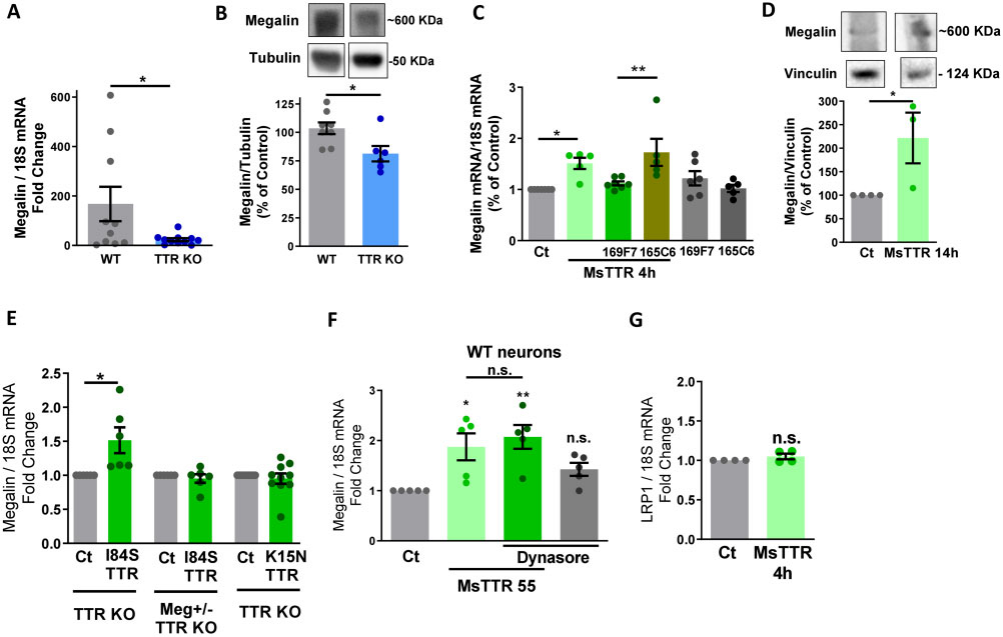
**TTR rescues megalin downregulation in TTR KO hippocampal neuronal cultures, in a megalin-dependent way.** (**A**) Total RNA was extracted from WT (*n* = 11) and TTR KO (*n* = 10) hippocampal neuronal cultures (7 DIV), and megalin and 18S mRNA levels were semi-quantified through real-time PCR, showing a reduction in megalin mRNA levels in hippocampal neurons of TTR KO mice. (**B**) Megalin and tubulin protein levels were determined by western blot in TTR KO (*n* = 6) and WT (*n* = 8) hippocampal neuronal cultures, with a decrease in megalin protein levels observed in TTR KO neurons. TTR KO cultured hippocampal neurons were stimulated with recombinant mouse TTR [55 µg/ml (1 µM)] for 4 h (mRNA) (**C**) or 14 h (protein) (**D**). When indicated, neurons were treated with TTR, in presence or absence of anti-TTR Nanobodies 169F7 and 165C6 (2 µM), or treated only with the nanobodies (**C**). TTR treatment rescued megalin mRNA (**C**, *n* = 5–7 independent cultures) and protein levels (**D**, *n* = 3 independent cultures) in TTR KO neurons, and the effect was abolished in the presence of the 169F7 nanobody, specific for TTR-megalin interaction epitope (**C**). Megalin (+/−) TTR KO and TTR KO cultured hippocampal neurons (7 DIV) were stimulated with recombinant mutated forms of mouse TTR, I84S (a TTR with low affinity for its ligands) and K15N (55 µg/ml, for 4 h; a TTR mutated in the 169F7 nanobody epitope, affecting TTR-megalin interaction), and megalin and 18S mRNA levels were assessed (**E**, *n* = 6–10 neuronal cultures). TTR-induced effect in increasing megalin levels is independent of TTR ligands but depends on TTR binding to megalin and on megalin levels. **(F)** WT hippocampal neuronal cultures were stimulated with recombinant mouse TTR (55 µg/ml, for 4 h) in the presence or absence of the inhibitor clathrin-mediated endocytosis Dynasore (80 μM) and megalin mRNA levels were assessed (*n* = 5 neuronal cultures). No effect was observed in the presence of the inhibitor, indicating that TTR-induced increase in megalin levels does not involve receptor internalization. (**G**) LRP1 and 18S mRNA levels were determined in TTR KO hippocampal neurons (*n* = 4 neuronal cultures), in the presence or absence of recombinant mouse TTR (55 µg/ml, for 4 h). LRP1 levels are not affected by TTR stimulation, indicating a specific effect for LRP2 (megalin). Statistical analysis was performed using Student’s unpaired *t*-test or one-way ANOVA followed by Bonferroni’s multiple comparison test. *P < 0.05, **P < 0.01, ***P < 0.001.

In order to explore how TTR regulates megalin levels in the hippocampus, we compared megalin expression in TTR KO versus WT hippocampal neuronal cultures at 7 DIV. We found that in TTR KO cultures, both mRNA (Fig. 2A) and protein (Fig. 2B) megalin levels were downregulated, compared to WT cultures, suggesting that hippocampal megalin levels in these mice models (Fig. 1K) derive from neuronal cells. For this reason, cultured hippocampal neurons are a suitable model to study the signalling pathways involved in the regulation of megalin levels. Since in TTR KO neurons the absence of TTR resulted in a downregulation of megalin levels, we hypothesized that treating neurons with recombinant TTR could revert this effect. For that, TTR KO hippocampal neuronal cultures were incubated with recombinant mouse TTR [55 µg/ml—physiological concentration of TTR in CSF (Ribeiro *et al.*, 2014)], which significantly upregulated megalin mRNA and protein levels (Fig. 2C and D). In addition, we observed that a specific-TTR nanobody (Nb) (Gomes *et al.*, 2018)—169F7 [described to bind/block the epitope responsible for TTR-megalin interaction (Gomes *et al.*, 2019)], was able to block TTR-induced megalin upregulation (Fig. 2C). Accordingly, a mouse TTR mutated in the epitope tagged by the 169F7Nb (K15N TTR) could not trigger megalin upregulation (Fig. 2E). However, another TTR Nb-165C6, described to bind TTR without blocking its interaction with megalin (Gomes *et al.*, 2019), did not affect megalin upregulation promoted by TTR (Fig. 2C). When TTR KO neurons were incubated with a TTR variant (I84S) with low affinity for TTR ligands (RBP/T4) (Refetoff *et al.*, 1986; Berni *et al.*, 1994), megalin mRNA upregulation was still observed, indicating that this effect is independent of TTR ligands (Fig. 2E). Moreover, we observed that TTR-induced megalin upregulation was dependent on megalin signalling, since in TTR KO megalin heterozygous neurons, TTR stimulation was not able to upregulate megalin mRNA levels (Fig. 2E). This transcription control of megalin levels by TTR was also observed in WT cultured hippocampal neurons (Fig. 2F), and is specific for megalin (LRP2) receptor, since LRP1 mRNA levels were not affected, in TTR KO neurons (Fig. 2G). Megalin levels regulation is not dependent on its internalization, since when neurons were treated with Dynasore, to block clathrin-mediated endocytosis (Macia *et al.*, 2006; Kirchhausen *et al.*, 2008; Vieira *et al.*, 2015), megalin upregulation triggered by TTR still occurred (Fig. 2F). The effects observed are probably due to megalin triggered intracellular signalling pathways and/or C-terminus nuclear translocation, as demonstrated for other LRP members (Derocq *et al.*, 2012).

### TTR leads to LRP2-ICD formation, probably via γ-secretase activity

**Figure 3:**
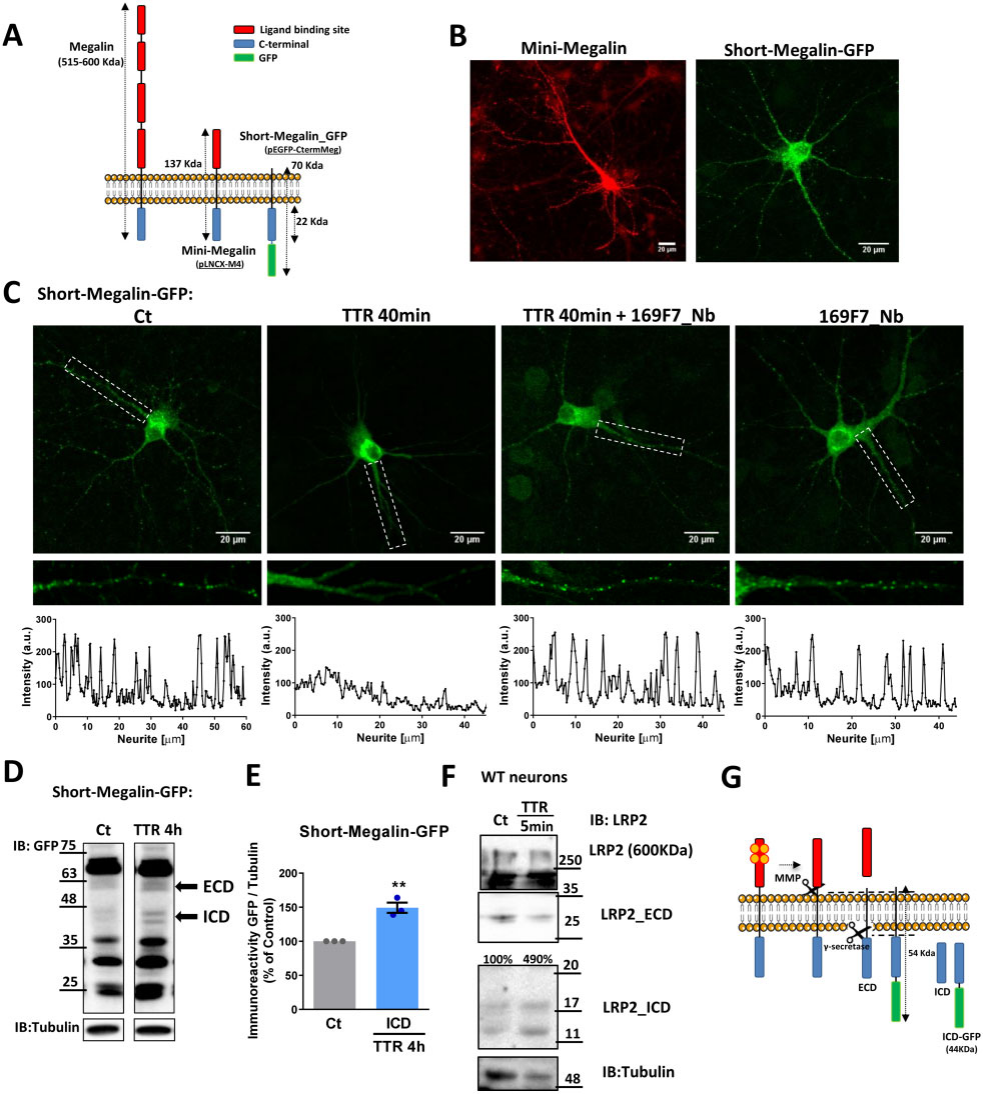
**TTR leads to LRP2-ICD formation, probably via γ-secretase activity.** (**A**) Cartoons describing mouse megalin and megalin plasmids, representing its structural differences and predicted molecular weights. (**B**) Representative images of TTR KO cultured hippocampal neurons 11 DIV transfected with either mini-megalin plasmid (pLNCX-M4) or with short-megalin fused with GFP (Short-megalin-GFP) for 48 h, stained with megalin and GFP antibodies, respectively, showing both plasmids with overlapping intracellular distribution. (**C**) Representative images of TTR KO cultured hippocampal neurons transfected with short-megalin-GFP for 48 h, and stimulated with recombinant mouse TTR in the presence or absence of specific-TTR nanobody 169F7. Quantification of GFP fluorescence intensity in neurites in the different experimental conditions is shown. TTR stimulation reduces the expression of megalin in neurites, and this effect is abolished in the presence of the nanobody. The results represent three independent neuronal cultures. (**D**) TTR KO neurons transfected with short-megalin-GFP were stimulated, or not, with recombinant mouse TTR (55 µg/ml) for 4 h. Western blot was performed, using an antibody for GFP. The LRP2-ICD immunoreactivity was quantified in Control and TTR exposed neurons, and show the increase in LRP2-ICD upon TTR stimulation. The results represent three independent neuronal cultures (**E**). (**F**) The same result was observed in WT cultured hippocampal neurons (15 DIV) stimulated, or not, with recombinant mouse TTR (55 µg/ml) for 5 min. Western blot was performed to assess megalin protein levels using an antibody for megalin. (**G)** Cartoon describing mouse megalin and megalin constructs being RIP processed, upon ligand (TTR) binding. Statistical analysis was performed using Student’s unpaired *t*-test. **P < 0.01.

Previous studies identified several mechanisms for megalin signal transduction, including the regulation of gene expression involving RIP of megalin intracellular domain (LRP2-ICD) (Biemesderfer, 2006; Li *et al.*, 2008), but never in the CNS. We hypothesized that megalin RIP was also occurring in neurons, with megalin C-terminal translocation to the nucleus (Zou *et al.*, 2004; Li *et al.*, 2008). To assess this hypothesis, we used a plasmid encoding a short form of megalin (short-megalin-GFP), constituted by megalin C-terminal region fused with GFP, the transmembrane region, and only a short part of the N-terminal domain (Bolos *et al.*, 2010). This short-megalin mimics native megalin (and mini-megalin), e.g. enhances IGF-I internalization (Bolos *et al.*, 2010), presenting subcellular localization in neurons, with punctate distribution along neurites (Fig. 3A and B). We observed that in TTR KO hippocampal neurons expressing short-megalin-GFP, and stimulated with recombinant mouse TTR (55 µg/ml, 40 min), TTR stimulation of endogenous megalin (since short-megalin does not contain the ligand-binding domain, working as a decoy) promoted a translocation of short-megalin-GFP from neurites towards cell body (Fig. 3C). Furthermore, we detected increased levels of megalin ICD in TTR KO neurons, upon TTR stimulation (50% increase compared to control neurons, not exposed to TTR; Fig. 3D and E). In non-transfected WT neurons (15 DIV), we also observed the upregulation of LRP2-ICD, after 5 min incubation with recombinant mouse TTR (∼5-fold, compared to neurons not treated with TTR) (Fig. 3F). This effect was previously demonstrated in the kidney, where upon ligand binding, MMP and γ-secretase were activated, creating ECD, and then ICD megalin domains (Li *et al.*, 2008) (Fig. 3G, Supplementary Fig. 1). Taken together, these results indicate that TTR regulates megalin RIP processing, a novel signal transduction mechanism in neurons.

### TTR induces LRP2-ICD nuclear translocation, as a putative transcription regulation mechanism

**Figure 4:**
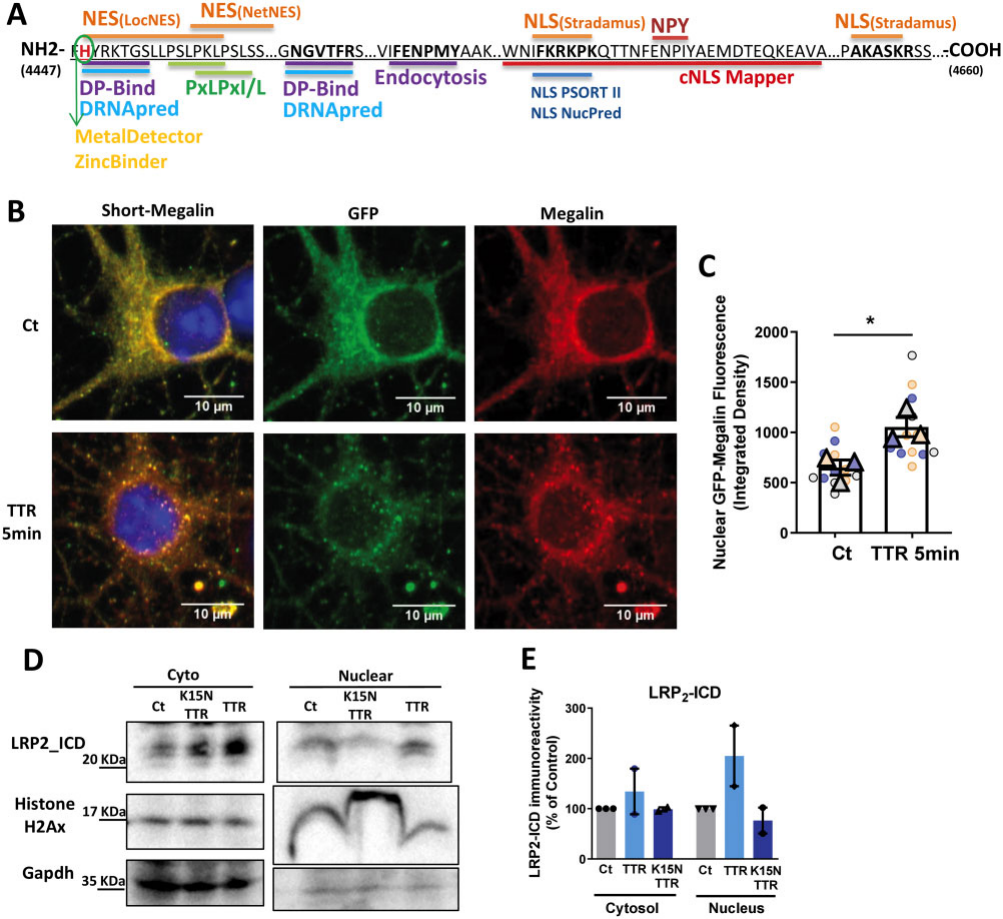
**TTR leads to LRP2-ICD nuclear translocation, as a putative transcription regulation mechanism.** (**A**) Mouse megalin C-terminal with highlighted consensus sequences for NLS (nuclear-localizing signals), NES (nuclear-exporting signals), protein DNA-binding residues and metal-binding sites, predicted by different bioinformatics tools. (**B**) Representative images of TTR KO cultured hippocampal neurons (11 DIV) transfected with short-megalin-GFP, and stimulated, or not, with recombinant mouse TTR (55 µg/ml) for 5 min. (**C**) Quantification of GFP fluorescence intensity in the nucleus (DAPI co-localization) indicates the nuclear translocation of megalin. The results are the average ±SEM of three independent cultures (Control: 12 neurons, MsTTR = 13 neurons). Statistical analysis was performed using unpaired Student’s *t*-test. *P < 0.05. Symbol triangle represents the average of each culture, and circles represent each hippocampal neuron of each individual culture, each colour represents each group culture/neuron. (**D, E**) Nuclear and cytosolic fractions isolated from TTR KO cultured hippocampal neurons (11 DIV) transfected with mini-megalin plasmid (pLNCX-M4) for 48 h and stimulated with recombinant mouse TTR or K15N TTR (55 µg/ml) for 20 min, and analysed by western blot showing the nuclear translocation of megalin. The effect was blocked when neurons were treated with the TTR mutated form that targets the site for TTR–megalin interaction. Histone H2Ax (nuclear fraction) and GAPDH (cytosolic fraction) were used to confirm cytosolic and nuclear fraction separation. The results are representative of two independent neuronal cultures.

Given that TTR leads to megalin RIP in hippocampal neurons, with LRP2-ICD formation, we assessed if LRP2-ICD participates in a similar regulatory pathway as other LRP-family members, such as LRP1, which has RIP activity with ICD translocation to the nucleus, affecting transcriptional activity (May *et al.*, 2002; Liu *et al.*, 2007; Polavarapu *et al.*, 2008; Zurhove *et al.*, 2008). Using several bioinformatics tools to predict nuclear localization of proteins, we looked for short binding sites on the C-terminus of mouse megalin, described to mediate transport to the nucleus (NLS-nuclear-localizing signals), and for motifs associated with transport from the nucleus to the cytoplasm (NES), as it would be expected in a nucleo-cytoplasmatic shuttling (Kosugi *et al.*, 2009). All the platforms (described in Materials and methods section) identified a putative NLS and NES sequence, with high probability, in the C-terminal region of megalin (Fig. 4A, methods section and Supplementary information). In addition, to understand if megalin C-terminal region could interact with DNA and regulate gene transcription, we looked for protein DNA-binding residues and identified two sequences in the C-terminal region of mouse megalin that display a high probability of binding to DNA (Fig. 4A, methods section and Supplementary information). In agreement with these data, a metal ion binding site, in the first protein DNA-binding region, was identified (Fig. 4A, methods section and Supplementary information). The identification of histidine residue that possibly binds zinc ions indicates that megalin C-terminal might be a metalloprotein with a Zinc finger, potentially functioning as a transcription factor in the nucleus. ICD as a transcription factor, regulating gene expression paradigm has been described for other receptors (Neuhaus-Follini and Bashaw, 2015; Scholer *et al.*, 2015; Gil-Yarom *et al.*, 2017). To investigate if this bioinformatic predictions corresponded to a real translocation of megalin to the nucleus, TTR KO hippocampal neurons (11 DIV) were transfected with short-megalin-GFP (Fig. 3A and B), and treated with recombinant mouse TTR for 5 min. In fact, we observed that TTR induced a translocation and accumulation in the nucleus of megalin C-terminal fused with GFP (Fig. 4B and C). In a complementary approach, TTR KO neurons were transfected with the plasmid encoding the mini-megalin, and treated with recombinant mouse TTR, 48 h later, during 20 min (Figs 4B and 6A). Total neuronal extracts were subjected to a nuclear fractionation protocol, allowing separation of cytoplasm from nuclear fractions. TTR stimulation leads to a translocation of megalin C-terminal (LRP2-ICD) to the nucleus, an effect that was abolished when mutated TTR form (K15N), in the epitope responsible for TTR interaction with megalin (Gomes *et al.*, 2019), was used (Fig. 4D and E, Supplementary Fig. 2). Thus, the experimental data confirm that megalin C-terminal is translocated to the nucleus upon TTR stimuli, in hippocampal neurons.

### Reduction of megalin levels impairs neurite outgrowth and survival of hippocampal neurons

**Figure 5:**
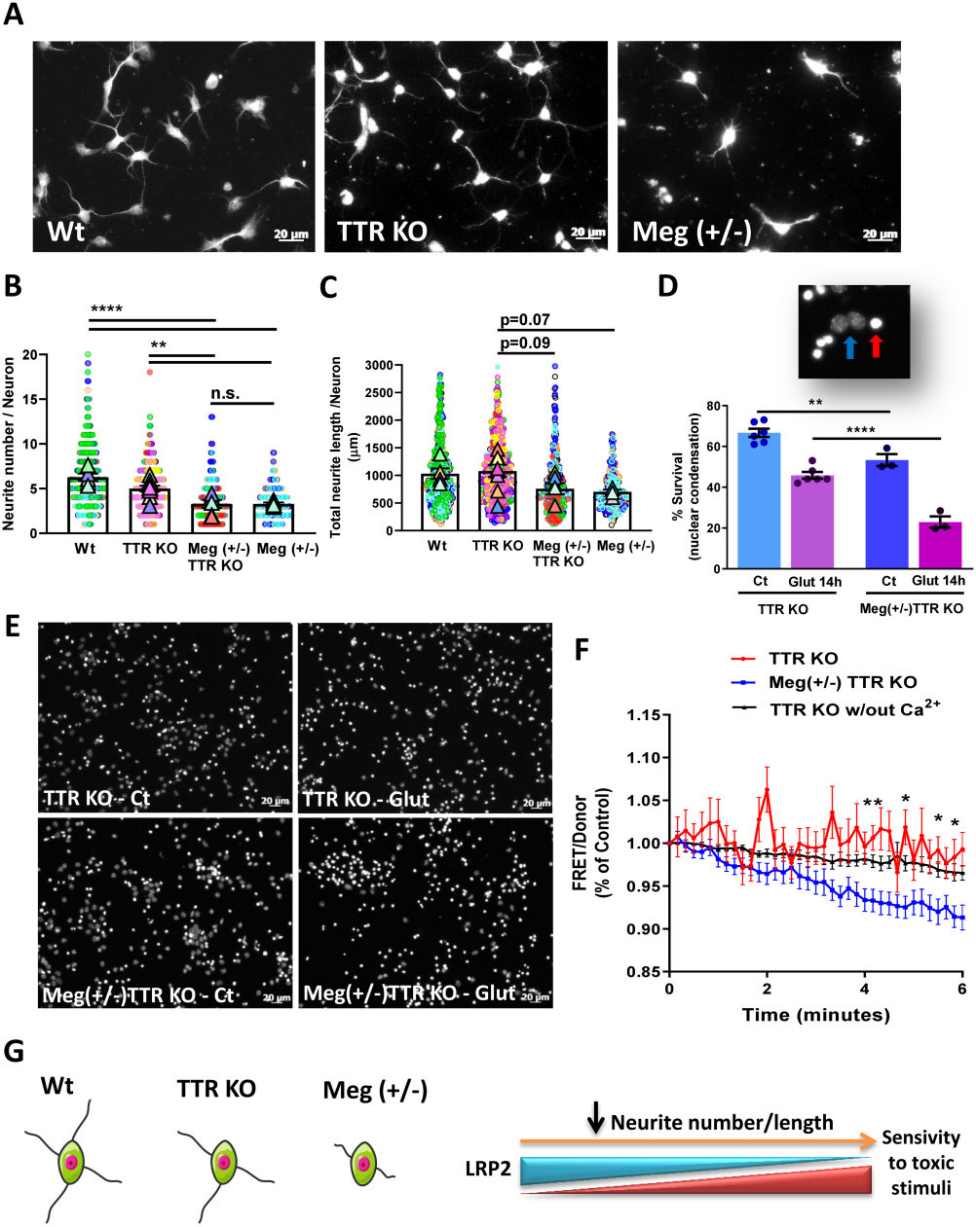
**Reduction of megalin levels impairs neurite outgrowth and survival of hippocampal neurons.** (**A**) Representative MAP2 staining of WT, TTR KO and Meg^+/−^ cultured hippocampal neurons (1 DIV). (**B**) Neurite number and (**C**) total neurite length of hippocampal neurons were determined (WT, *n* = 5 cultures (507 neurons)); TTR KO, *n* = 9 cultures (613 neurons); Meg^+/−^ TTR KO, *n* = 6 cultures (359 neurons); Meg^+/−^, *n* = 4 cultures (220 neurons), showing that decreased megalin expression reduces neurite number and length. (**D, E**) TTR KO and Meg^+/−^ TTR KO cultured hippocampal neurons (7 DIV) were subjected to excitotoxic stimulation with glutamate. Neuronal survival was assessed 14 h after the excitotoxic insult through nuclear condensation (*n* = 3–6 independent cultures), demonstrating that megalin deficiency reduces neuronal survival of hippocampal neurons in physiological and toxic conditions. (**F**) Time course of normalized FRET/Donor values in the cell body of YC-Nano15 transfected TTR KO or Meg^+/−^ TTR KO cultured hippocampal neurons (7 DIV) under physiologic conditions, or in absence of extracellular calcium (extended results from (Gomes *et al.*, 2016)) (TTR KO, *n* = 3 cultures (16 neurons), Meg^+/−^ TTR KO, *n* = 3 cultures (15 neurons)); TTR KO w/out Ca^2+^, *n* = 2 cultures (12 neurons) indicating that megalin seems to be required for physiological neuronal activity. (**G**) Pictorial figure summarizing the association between megalin expression levels and hippocampal neuronal viability. Statistical analysis was performed using one-way ANOVA followed by Bonferroni’s multiple comparison test (**D**), and a linear mixed model followed by Tukey–Kramer multiple comparison test (**B**, **C**, **F**). *P < 0.05, **P < 0.01, ****P < 0.0001. Symbol triangle represents the average of each culture, and circles represent each hippocampal neuron of each individual culture, each colour represents each group culture/neuron.

Megalin was shown to be involved in neurite outgrowth, not only through TTR signalling, but also by metallothioneins (Fitzgerald *et al.*, 2007). Therefore, due to its prominent role in the CNS, we analysed megalin’s effect in neurite outgrowth using cultured hippocampal neurons from WT mice and mice with reduced expression of TTR and/or megalin. To assess neurite effects, we quantified neurite number and length per neuron in neuronal cultures at 1 DIV. Representative images are shown in Fig. 5A, and neurite tracing in Supplementary Fig. 3. We observed that megalin reduction significantly decreased neurite number (Fig. 5B—WT versus Meg^+/−^). This effect was also observed when comparing cultures from megalin heterozygous mice with TTRKO mice (Fig. 5B—TTR KO versus Meg^+/−^ TTR KO), although to a lesser extent, indicating that TTR might also contribute to this effect. Neurite length was affected by megalin reduction (TTR KO versus Meg^+/−^ TTR KO, *P* = 0.09; TTRKO versus Meg^+/−^, *P* = 0.07), but not by TTR (WT versus TTR KO) (Fig. 5C, Supplementary Fig. 4). Additionally, to address the role of megalin in pathological toxic conditions, we subjected TTR KO and Meg^+/−^ TTR KO neurons to excitotoxic conditions, consisting of a transient incubation with a high glutamate concentration (125 µM, 20 min), an *in vitro* model of neuronal death in cerebral ischaemia/stroke (Dwyer *et al.*, 2008), and further incubation in culture-conditioned medium for 14 h (40–50% apoptotic neuronal death) (Almeida *et al.*, 2005). Neuronal death/survival was assessed using Hoechst 33342 staining to analyse nuclear morphology. Alive and dead (apoptotic) neurons show distinct nuclear staining, with apoptotic nuclei appearing smaller and brighter (red arrow in the magnified image Fig. 5D), whereas live nuclei are larger and with diffuse staining (blue arrow, Fig. 5D). Meg^+/−^ TTR KO hippocampal neuronal cultures have increased neuronal death compared to TTR KO neuronal cultures, in physiological control conditions [53% versus 67% of neuronal survival, respectively (Fig. 5D and E)]. Importantly, Meg^+/−^ TTR KO hippocampal neuronal cultures are clearly more sensitive to an excitotoxic glutamate insult than TTR KO cultures [23% neuronal survival, compared to 46% in TTR KO neurons (Fig. 5D and E)]. Thus, other megalin ligands, besides TTR, present in culture media, seem to be crucial to induce neurite outgrowth and survival via megalin (Fig. 5G). In addition, since megalin affects the neurite outgrowth, we wondered whether intracellular signalling dynamics and neuronal activity were also being affected. For that, we used an ultrasensitive Ca^2+^ sensitive indicator—yellow Cameleon-nano FRET probe (Horikawa *et al.*, 2010)—used in Gomes *et al.* to address TTR signalling via megalin [Fig. 2 in Gomes *et al.*, (2016)], that can detect action potentials, and allows the visualization of spontaneous neuronal activity (Yamada *et al.*, 2011; Kanemaru *et al.*, 2014). We found that in physiologic conditions, Meg^+/−^ TTR KO neuronal cultures have decreased neuronal activity (blue line), meaning less Ca^2+^ transients, than full megalin neuronal cultures Meg^+/+^ TTR KO (red line), observed by the number of intracellular calcium fluctuations. Moreover, as expected and previously shown (Gomes *et al.*, 2016), the absence of calcium in the cell culture medium abolishes Ca^2+^ fluctuations, an indicator of neuronal activity (black line) (Fig. 5F). Together, these data indicate that megalin seems to be required for physiological neuronal activity, and for neuronal survival.

### Megalin overexpression rescues neurite outgrowth and increases dendritic spine density

**Figure 6:**
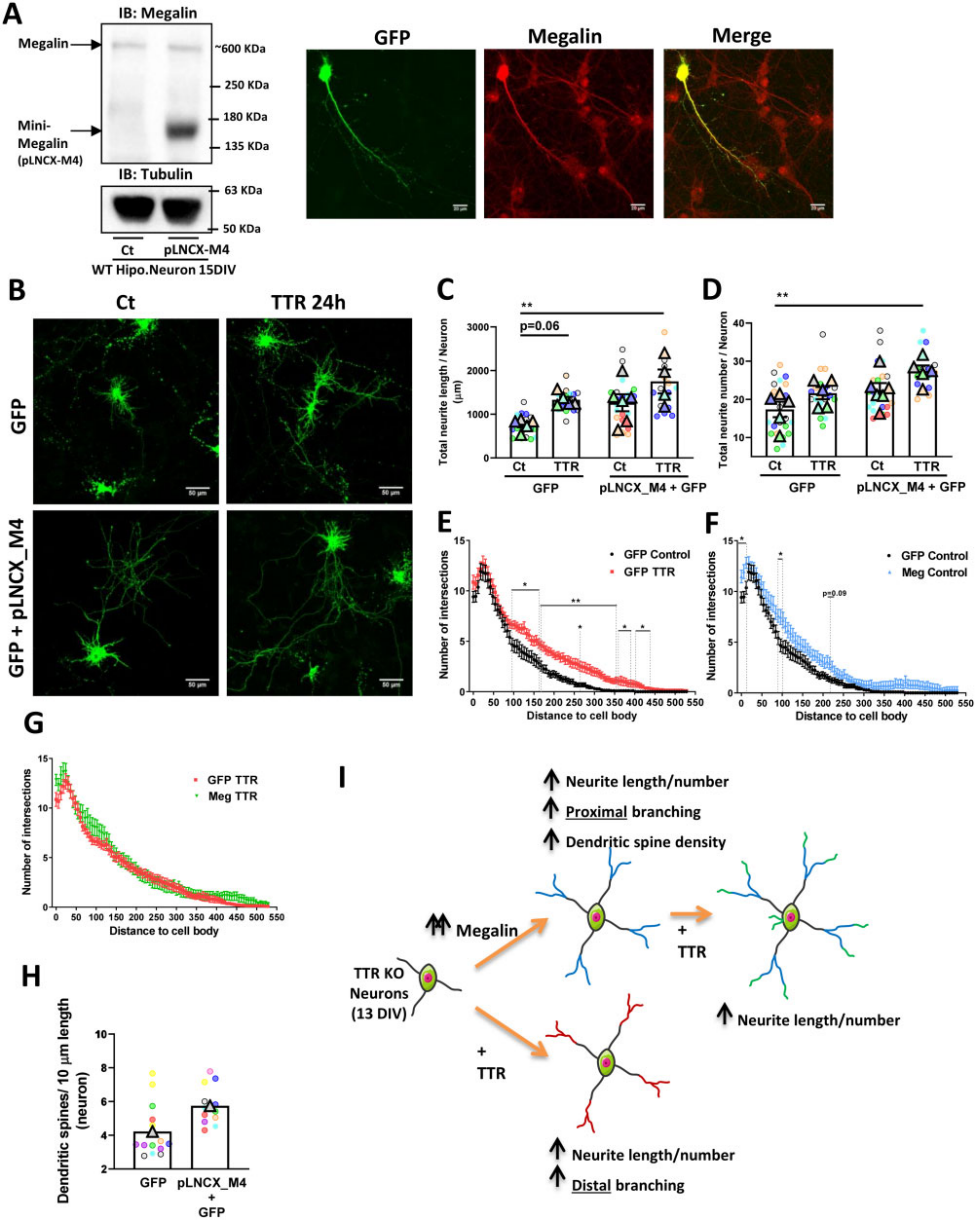
**Megalin overexpression rescues neurite outgrowth and increases dendritic spine density.** (**A**) WT cultured hippocampal neurons (11 DIV) were transfected with a functional mini-megalin plasmid (pLNCX-M4) for 48/72 h. Megalin protein levels in transfected and untransfected neurons were assessed by western blot (**A**) and immunocytochemistry. (**B–H**) TTR KO cultured hippocampal neurons (11 DIV) were transfected with either GFP plasmid (pEGFP) or co-transfected with GFP (pEGFP) and mini-megalin plasmid (pLNCX-M4) for 48 h, and stimulated, or not, as indicated, with recombinant mouse TTR (55 µg/ml) for 24 h. An immunocytochemistry was performed using GFP antibody. (**B**) Representative images of neurons expressing GFP. (**C**) Total neurite length, (**D**) neurite number [GFP Ct, *n* = 5 (21–23 neurons); GFP TTR, *n* = 5 (19–20 neurons); pLNCX_M4+GFP Ct, *n* = 6 (23 neurons); pLNCX_M4+GFP TTR, *n* = 4/5 (17 neurons)] and (**E–G**) branching were increased when megalin is overexpressed and or neurons are treated with TTR. (GFP Ct, *n* = 7 (22 neurons); GFP TTR, *n* = 5 (22 neurons); Meg Ct, *n* = 6 (23 neurons); Meg TTR, *n* = 5 (19 neurons). (**H**) Dendritic spine density is increased in neurons overexpressing megalin (*n* = 1 culture, 13–14 dendrites from 8 to 10 neurons. (**I**) Pictorial figure summarizes the effects in neurite outgrowth triggered by overexpression of megalin versus TTR stimulation. Scale bar in A corresponds to 20 μm and in B to 50 μm. Statistical analysis was performed using linear mixed model followed by Tukey–Kramer multiple comparison test (**C–G**). *P < 0.05, **P < 0.01. Symbol triangle represents the average of each culture, and circles represent each hippocampal neuron of each individual culture, each colour represents each group culture/neuron.

To further explore the role of megalin in neurite outgrowth in the absence of TTR, we overexpressed megalin in TTR KO (to avoid potential TTR synthesis/presence in culture) hippocampal neurons, by transfecting neurons with fully functional megalin receptor (mini-megalin—pLNCX-M4 plasmid) (Fig. 3B) (Takeda *et al.*, 2003). Mini-megalin has the expected size of ∼150 kDa (Fig. 6A), and since it is under the CMV promoter, overexpression is observed, when compared to full-length megalin endogenous expression (Figs 3A and 6A for schematic representation of plasmids). In WT neurons co-transfected with a plasmid encoding GFP (pEGFP) and mini-megalin, megalin overexpression was also observed when compared to non-transfected neighbour neurons, both at the cell body and neurites (Fig. 6A), as previously described (Gomes *et al.*, 2016). In cultured hippocampal neurons from TTR KO mice expressing only GFP compared to neurons stimulated with recombinant mouse TTR, total neurite length (Fig. 6C, GFP Control versus GFP TTR, *P* = 0.06) and branching (distal branching: 100–450 µm from cell body) were increased, in a TTR-dependent effect (Fig. 6B, C and E, Supplementary Fig. 5). This is the first time that TTR neuritogenic activity is described in mature neurons, but it was previously observed in immature TTR KO (1 DIV) hippocampal neurons (Gomes *et al.*, 2016). Of relevance is the fact that, when we compare the control conditions of GFP and GFP plus mini-megalin expressing neurons, there is an increase of neuronal branching (proximal branching: 0–200 µm from cell body, Fig. 6F) (total neurite length also increases ∼50%, although without statistical significance, Fig. 6C GFP Control versus pLNCX_M4 Control), indicating that megalin has a role in neurite outgrowth independent of TTR, probably depending on other endogenous megalin ligands, present in neuronal cultures under physiologic conditions. When comparing GFP expressing neurons with GFP plus mini-megalin expressing neurons exposed to TTR, there is a clear increase of both neurite number and length/branching, indicating that this pathway megalin-TTR is relevant to promote neurite outgrowth (Fig. 6C and D, GFP Control versus pLNCX_M4 TTR). Finally, when comparing neurons expressing GFP versus GFP plus mini-megalin, in the presence of TTR, there is no cumulative effect of megalin and TTR (Fig. 6G). Similarly, in GFP plus mini-megalin expressing neurons, TTR was unable to further increase neuronal branching (Supplementary Fig. 6), probably due to megalin endogenous activation. Importantly, we also demonstrate that overexpressing megalin increases the density of dendritic spines (Fig. 6H). In WT GFP-transfected neurons (Supplementary Fig. 7), the density of dendritic spines is ∼8 spines/10 µm dendritic length, showing that in TTR KO mice spine density is reduced, even though megalin is still able to increase dendritic spines density (Fig. 6H), in a complementary multi neuron single culture approach. A pictorial figure summarizes this effects triggered by overexpression of megalin and TTR stimulation (Fig. 6I).

### Megalin heterozygous mice show structural alterations in hippocampal neurons

**Figure 7:**
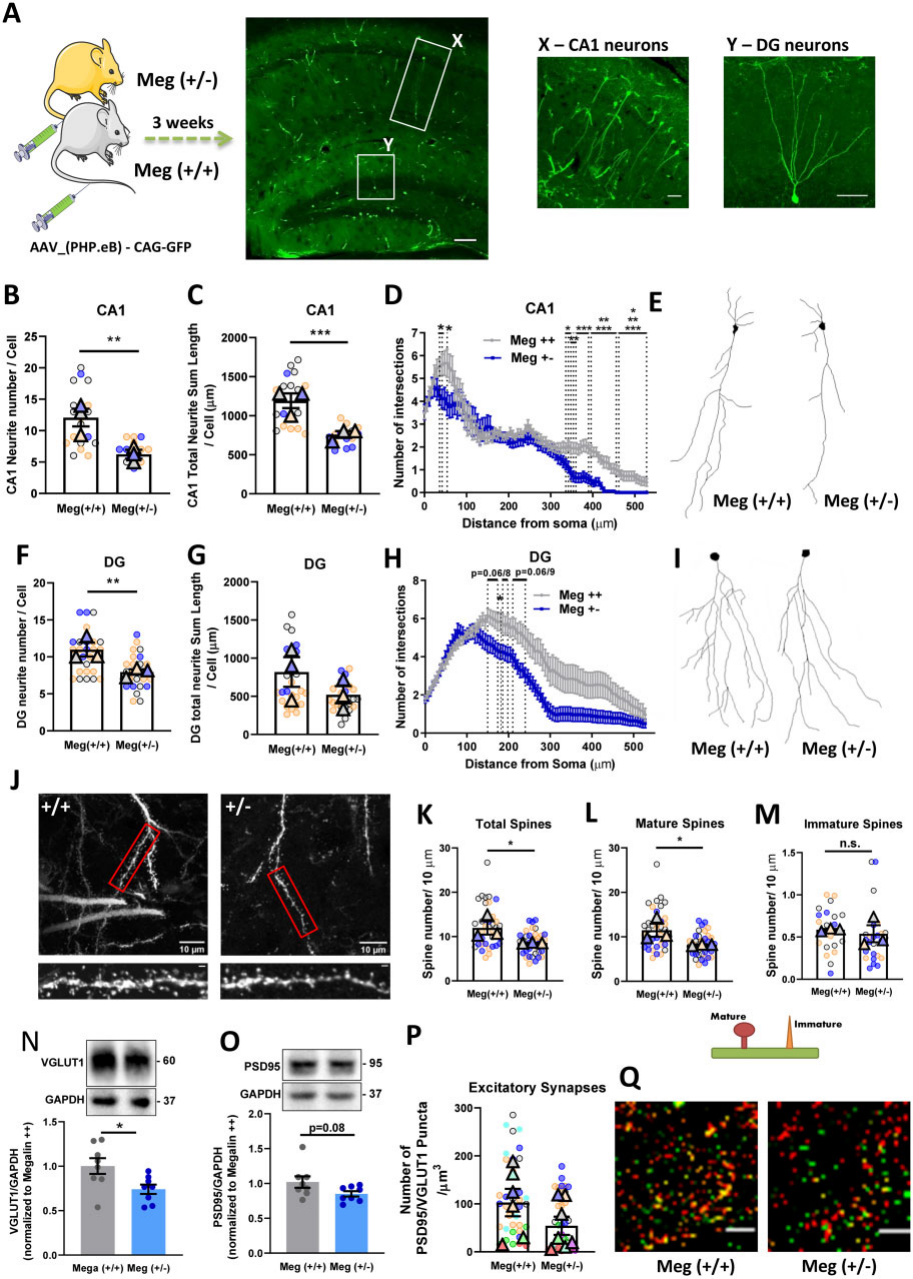
**Megalin heterozygous mice show structural alterations in hippocampal neurons.** (**A**) Schematic representation of mice intravenously injected with AAV.PHP.eB_GFP with representative images of the hippocampus (Scale bar: 100 µm), and high-magnification images of CA1 and DG neurons (Scale bar: 50 µm). Neurite number (**B, F**), total neurite length (**C, G**) and branching (Sholl) (**D, H**) analysis in neurons from CA1 and DG regions of the hippocampus, respectively, show a decrease in neuronal complexity in Meg^+/−^ mice, compared to WT littermates [CA1 neurons: Meg^+/+^  *n* = 3 mice (2 females, 1 male), 18 neurons; Meg^+/−^  *n* = 3 mice (2 females, 1 male), 15 neurons; DG neurons: Meg^+/+^  *n* = 3 mice (2 females, 1 male) 26 neurons; Meg^+/−^  *n* = 3 mice (2 females, 1 male), 26 neurons]. Schematic representations of CA1 (**E**) and DG (**I**) neurons are shown. (**L**) Representative images from secondary dendrites of CA1 pyramidal neurons from Meg^+/−^ and WT littermates [Meg^+/+^  *n* = 3 mice (2 females, 1 male) 13 neurons, 37 dendrites; Meg^+/−^  *n* = 3 mice (2 females, 1 male), 15 neurons, 37 dendrites] (**J**). The values are represented by 10 µm of dendritic length. Scale bar: 1 µm. (**J–M**) The density of dendritic spines in secondary branches of hippocampal CA1 neurons of Meg^+/−^ was reduced, when compared to WT littermates (**K**). This effect was observed only in mature spines, whereas immatures spines were not affected (**L, M**, and schematic representation bellow graphics). Levels of VGLUT1 are decreased in whole tissue hippocampal extracts in Meg^+/−^ mice (**N**), and PSD95 levels show a statistical tendency to decrease (*P* = 0.08) (**O**), as determined by western blot analysis. Meg^+/+^  *n* = 8 mice (4 females, 4 males), Meg^+/−^  *n* = 8 mice (4 females, 4 males). (**P, Q**) The number of excitatory synapses, defined by the co-localization of VGLUT1 and PSD95 puncta, is decreased in Meg^+/−^ mice. Meg^+/+^  *n* = 7 mice (4 females, 3 males), 42 hippocampal segments; Meg^+/−^  *n* = 8 mice (4 females, 4 males), 45 hippocampal segments. Values are normalized to Meg^+/+^ mice. Representative images are shown in **R**. Scale bar: 5 µm. Statistical analysis was performed using Student’s unpaired *t*-test (**N**, **O**) or linear mixed model followed by Tukey–Kramer multiple comparison test. *P < 0.05, **P < 0.01, ***P < 0.001. Symbol triangle represents the average of each mice, and circles represent each hippocampal neuron/region of each mice, each colour represents each group mice/neuron or hippocampal region.

To address whether neurite outgrowth triggered by megalin in cultured hippocampal neurons were also occurring *in vivo*, we evaluated hippocampal neuronal morphology from Meg^+/−^ mice versus Meg^+/+^ corresponding littermates, for neurite length and branching both in CA1 and DG regions of the hippocampus, and dendritic spine density in CA1 region. For that, mice were injected intravenously with AAV produced with PHP.eB serotype for efficient transduction of CNS neurons (Chan *et al.*, 2017), with GFP under the CAG promoter, for high levels of gene expression (Fig. 7A). We observed that Meg^+/−^ mice show reduced neurite number, neurite length, and decreased dendritic arborization, when compared to Meg^+/+^ mice littermates, in neurons from both the CA1 (Fig. 7B–E) and DG (Fig. 7F–I, (for DG neurite length there is ∼50% decrease although with no statistical significance, considering animal as experimental unit)) regions. In CA1 neurons, the effect in dendritic ramification was more pronounced in proximal (between 36–60 µm from cell body) and distal neurites (340–530 µm from cell body) (Fig. 7D), with no effect on intermediate neurites; whereas for DG neurons the effect was only observed on more distal dendrites (150–240 µm from cell body) (Fig. 7H). Moreover, to understand if megalin is required for dendritic spine formation and maturation *in vivo*, we analysed spine density in GFP-labelled CA1 pyramidal neurons. We found that Meg^+/−^ mice have decreased dendritic spine density, and this effect was due to mature spines, whereas immature spines were not altered (Fig. 7J–M). This indicates that megalin is probably mainly required for dendritic spine maturation, and not for the formation of novel dendritic spines. Up on that, we evaluated if the effects at dendritic spines were associated to changes in synaptic activity, by analysing the expression levels of synaptic proteins in the hippocampus (pre-synaptic-VGLUT1 and post-synaptic-PSD95), as well as the number of synapses, defined as the co-localization of pre- and post-synaptic proteins that appear at a punctate distribution (McLeod *et al.*, 2017). In total hippocampal extracts, VGLUT1 levels are decreased in Meg^+/−^ mice compared to Meg^+/+^ littermates, and PSD95 shows a tendency to decrease (*P* = 0.08) (Fig. 7N and O, respectively), although no significant changes (*P* = 0.13, with a ∼46% reduction in the average of the animals when comparing Meg^+/+^ to Meg^+/−^) were observed for PSD95/VGLUT1 co-localized puncta number, as a marker of excitatory synapses. Taken together, these data regarding dendritic spine maturation and expression of synaptic proteins in Meg^+/+^ versus Meg^+/−^ mice indicates that megalin plays a relevant role in hippocampal synaptic function, with potential impact on learning and memory behaviours (Fig. 7P and Q, Supplementary Fig. 8).

### Megalin heterozygous mice show cognitive deficits, but no effects in anxiety-like behaviour or locomotor activity

**Figure 8:**
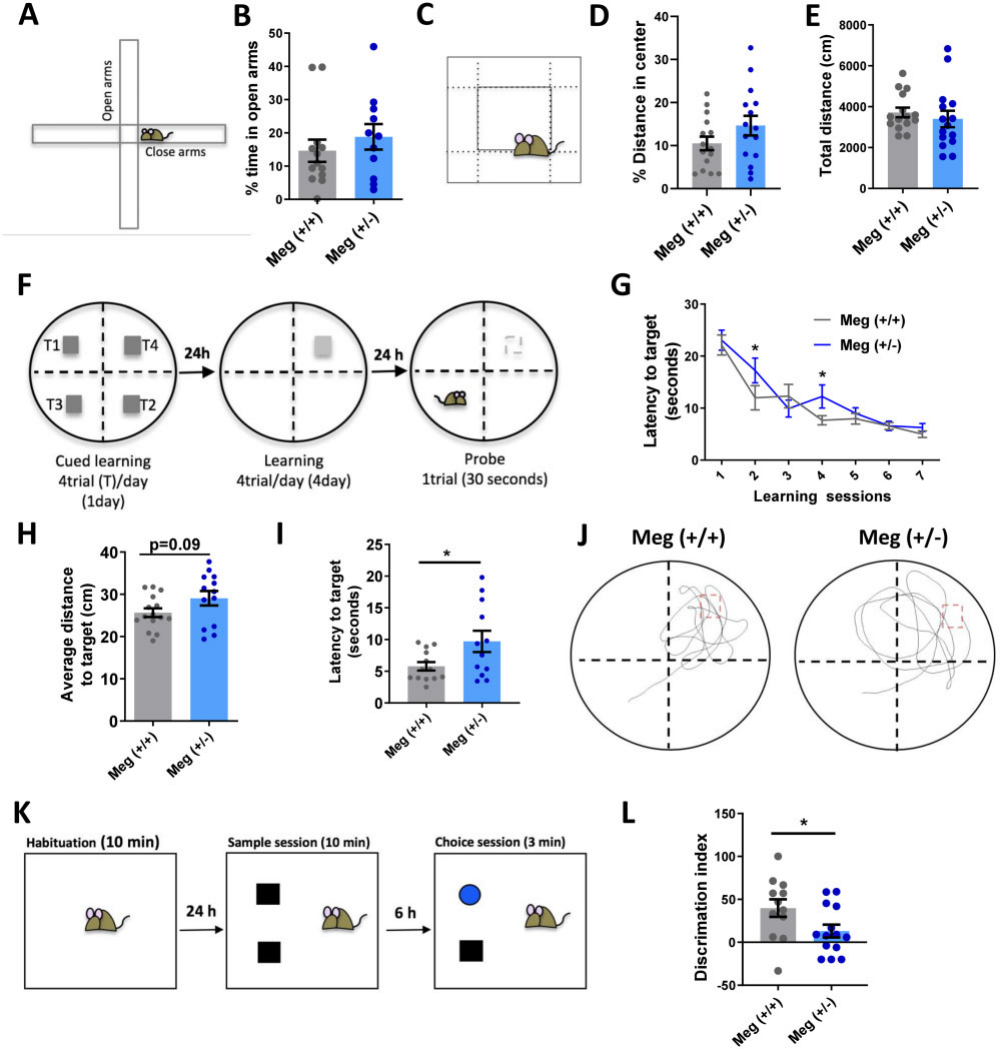
**Megalin heterozygous mice show cognitive deficits, but no effects in anxiety-like behaviour or locomotor activity.** (**A**) Schematic representation of the EPM test. (**B**) Anxiety-like behaviour was not altered in Meg^+/−^ mice, compared to WT littermates, as shown by the % of time spent in open arms in EPM test. Meg^+/+^= 13 mice (4 females, 9 males), Meg^+/−^ = 11 mice (7 females, 4 males). (**C**) Schematic design of the open field test. (**D, E**) Open field reinforces the absence of effects in anxiety in Meg^+/−^ mice compared to Meg^+/+^ mice (% of distance in centre-**D**), and demonstrates no impairment in locomotor activity in Meg^+/−^ (total distance travelled-**E**). Meg^+/+^  *n* = 15 mice (7 females, 8 males), Meg^+/−^  *n* = 15 mice (8 females, 7 males). (**F**) Schematic diagram of MWM. (**G**) Meg^+/−^ mice display increased latency to reach the platform during training sessions 2 and 4. (**H, I**) During trial session, Meg^+/−^ mice show a tendency for an increased average distance travelled (**H**) and a higher latency to reach the platform (**I**). (**J**) Representative tracing of probe trials of Meg^+/−^ and WT littermates. Meg^+/+^  *n* = 15 mice (6 females, 9 males); Meg^+/−^  *n* = 13 mice (8 females, 5 males). (**K**) Schematic representation of Novel Object Recognition test. (**L**) Meg^+/−^ mice display a reduction of the DI of the novel object versus the familiar object, compared to WT littermates. (**M**) No differences were observed for the objects total exploration time between genotypes. (**N**) Representative tracing of choice session of Meg^+/−^ and WT littermates. Meg^+/+^  *n* = 12 mice (7 females, 5 males), Meg^+/−^  *n* = 14 mice (7 females, 7 males). Statistical analysis was performed using Student’s *t*-test, except in G, where two-way ANOVA was used. *P < 0.05.

Considering the *in vivo* effects observed in hippocampal neuronal morphology, dendritic spines and synaptic number for Meg^+/−^ mice, we addressed if these might impact on mice behaviours particularly learning and memory, anxiety-like and locomotor behaviour. We found that reduced levels of megalin did not affect anxiety-like behaviour, as determined by the percentage of time spent in the open arms using the EPM (Fig. 8A) test, and distance travelled in the centre of the arena in the open field (OF, Fig. 8C) test (Fig. 8B and D, respectively). Moreover, megalin deficits did not affect locomotor activity, as determined by the total distance travelled in the arena of the OF test (Fig. 8E). Importantly, when performing the MWM (Fig. 8F) test, the megalin heterozygous animals showed impairment in the learning process, with increased latency to find the platform at learning sessions 2 and 4 (Fig. 8G), when compared to Meg^+/+^ littermates. Moreover, Meg^+/−^ mice show an impairment in reference memory, presenting a tendency to an increase in the average distance to the target (Fig. 8H, *P* = 0.09) and a higher latency to the target (Fig.8I) in the probe session. Representative images of the track performed by the mice in the MWM clearly demonstrate the differences between the two genotypes (Fig. 8J). In the Novel Object Recognition (NOR, Fig. 8K) test, to assess memory and cognitive capabilities, Meg^+/−^ mice showed a reduction in time exploring the novel object, as assessed by the discrimination index (DI), that evaluates the preference to explore the novel object versus the familiar object (Fig. 8L). Moreover, total exploration time of the objects by both genotypes was not different, indicating that the differences in exploring novel objects are not due to distinct locomotion or motivation effects to explore the objects (Fig. 8M). Together, our data suggest that Meg^+/−^ mice display learning and memory deficits that correlate with structural and functional hippocampal synaptic dysfunction, unveiling a relevant role of megalin in these mechanisms (see also, Supplementary Figs 9–11).

## Discussion

In this work, we report that transthyretin is a fine regulator of megalin expression, affecting both its mRNA and protein levels in areas characterized by high megalin expression, such as the kidney and the choroid plexus, and, importantly, in the CNS, the hippocampus. We describe that megalin levels in hippocampal neuronal cultures are fundamental for neurite outgrowth and neuronal survival, as decreasing megalin levels results in neurons with impaired neurite length and branching, with reduced number of neurites, and, consequently, more susceptible to insults, such as excitotoxic conditions. Moreover, we found that TTR controls megalin levels through a regulated intramembrane proteolysis (RIP) processing, and the ICD domain formed is translocated to the nucleus. In addition, we disclose a novel role for megalin in learning and memory mechanisms, as megalin heterozygous mice show hippocampal-related memory deficits, in several behaviour tests, that were in agreement with the neurite, dendritic spine density/maturation and synaptic impairments observed in megalin deficient hippocampal neurons, both *in vitro* and *in vivo*.

The regulation of megalin expression by TTR described in this work (Figs 1 and 2) is in agreement with the effect of other megalin ligands that also regulate the receptor expression. These megalin ligands control mRNA and/or protein levels of megalin, via the receptor itself (Marzolo and Farfan, 2011), in a positive feedback mechanism. For instance, exposure to vitamin A and D, both megalin ligands, leads to increased megalin protein and mRNA levels (Liu *et al.*, 1998). Clusterin overexpression also upregulates megalin levels (mRNA and protein), which in turn confers clusterin an antiapoptotic role in prostate cancer cells (Ammar and Closset, 2008).

Regarding the expression levels of megalin in CNS, our results are also in agreement with several reports: choroid plexus (Chun *et al.*, 1999; Carro *et al.*, 2005); spinal cord in either embryonic (Wicher *et al.*, 2005) or adult stages (Wicher *et al.*, 2006); different brain areas such as the cerebral cortex, hippocampus, striatum and the cerebellum (Alvira-Botero *et al.*, 2010). Moreover, megalin is mainly expressed in neurons in the CNS (Fass *et al.*, 1997; LaFerla *et al.*, 1997; Gomes *et al.*, 2016), although it was also shown to be present in astrocytes (Bento-Abreu *et al.*, 2008) and olygodendrocytes in the spinal cord (Wicher *et al.*, 2006). Nonetheless, in this study, we demonstrate that TTR controls megalin levels, besides kidney and CP, in the CNS, including hippocampus, brainstem and spinal cord.

The fact that TTR is affecting/regulating megalin levels mainly in the hippocampus and spinal cord, and not in other CNS areas, such as the cerebral cortex or the cerebellum (Fig. 1F–I), is probably related to the fact that TTR has been described to be either synthesized by hippocampal/motor neurons or uptaken from the CSF TTR pool, making these regions more prone to the effects of TTR over megalin (Stein and Johnson, 2002; Sousa *et al.*, 2007*a*; Buxbaum *et al.*, 2008; Li *et al.*, 2011; Gomes *et al.*, 2018). TTR does not seem to affect megalin mRNA expression levels *in vivo*, although it affects megalin protein levels (Fig. 1). This could be due to megalin’s long half-life period (Perez Bay *et al.*, 2016), and also to the fact that proteins in the brain usually have almost triple average lifetimes compared to proteins in other tissues (Alvarez-Castelao and Schuman, 2015). Proteins may also display different turnover rates depending on their location in the neuron (synapses, dendritic branch or axons). Supporting this idea, data in cultured hippocampal neurons (Fig. 2A and C) showed that TTR was able to regulate megalin mRNA in cell body and dendrites, and to a lower extent in axons (early mature neurons) (Gomes *et al.*, 2016). In a study by Alvira-Botero, the presence of megalin in neurons throughout the brain was observed, with synaptic localization in axon terminals near synaptic vesicles (Alvira-Botero *et al.*, 2010). Moreover, it described a possible role of megalin in neurite development, independent of supplemented ligands, in agreement with our results. Furthermore, the authors reported that knocking down megalin in hippocampal neuronal cultures significantly increased neurite branching. This goes in the opposite direction of our results, as we describe that decreasing levels of megalin led to decrease in number and length of neurites per cell, either in developing (1 DIV, Fig. 5A–C) or in mature hippocampal neurons (13 DIV) (Fig. 6B–G). Megalin was also shown to be the receptor mediating the neuritogenic activity induced by different molecules, such as metallothioneins (Fitzgerald *et al.*, 2007), α2-macroglobulin (Qiu *et al.*, 2004) and TTR (Gomes *et al.*, 2016). Altogether, these data highlight the important role of megalin in neurite outgrowth, which we have now explored in more detail.

After establishing this critical role of megalin in neurite development, we assessed whether neuronal cultures could be susceptible to stressful conditions, such as excitotoxicity. We observed that megalin heterozygous TTR KO neurons are more sensitive to neuronal death induced by an excitotoxic insult compared to TTR KO neurons. A previous work (Gomes *et al.*, 2016), also found that TTR KO neurons were more sensitive to this toxic stimuli than WT neurons; we can now indicate that decreased megalin receptor levels are likely related to this increased neuronal death (Gomes *et al.*, 2016). In addition, we found that decreasing megalin levels in neuronal cultures also decreases their activity, using an ultrasensitive Ca^2+^ indicator. Taken together, we clearly indicate that reduced levels of megalin impair neuronal viability and reduce neurite outgrowth, making neurons more susceptible to toxic insults. In agreement with our results, Cases *et al.* (2015) established a link between megalin and congenital high myopia, in a megalin conditional KO in the mouse forebrain, including neural retina, which resulted in increased cell death in the retinal ganglion cell layer, and decreased axon number.

The signalling functions of megalin were addressed for several ligands, including TTR in physiologic and pathologic conditions (Gomes *et al.*, 2016). TTR (or other megalin ligands) binding to megalin initiates/activates adaptor proteins in the cytoplasm that recognize specific motifs within megalin C-terminal, activating different signalling pathways (Spuch *et al.*, 2012). However, as we describe here, megalin is also able to initiate signalling events through a different mechanism, RIP, already widely described for APP and Notch (Ebinu and Yankner, 2002). In this mechanism, upon ligand binding, the receptor is proteolytically cleaved, by a metalloprotease, producing a membrane-bound megalin C-terminal fragment (LRP2-ECD), followed by an internal cleavage of the C-terminus by a γ-secretase enzyme, forming a soluble megalin intracellular domain (LRP2-ICD). This intracellular domain will target the nucleus where it regulates gene expression. Li *et al.* have shown that megalin is subjected to RIP processing in opossum kidney cells, demonstrating that megalin C-terminal regulates gene expression (Biemesderfer, 2006; Li *et al.*, 2008). However, details of this signalling are completely unknown, and in neurons are not even described (Carlos and Carmen, 2010). Here, we demonstrated that, upon TTR binding, megalin C-terminal domain translocates from neurites to the cell body, specifically to the nucleus of hippocampal neurons (Fig. 3C). Moreover, using TTR ligand we demonstrate megalin RIP processing and its ICD formation [using several approaches (Fig. 3D–F)].

Using several bioinformatics tools, we identified putative-specific motifs involved in nucleo-cytoplasmatic shuttling in megalin C-terminus, and also protein DNA-binding residues, as well as a metal-binding site, indicating that LRP2-ICD (megalin C terminal) could regulate directly gene expression, as a transcription factor (Fig. 4A). We validate this bioinformatic data, by a biochemical approach, and found that LRP2-ICD translocates to the nucleus (Fig. 4B–E). These results shed more light into recent works where megalin was shown to be an extremely important receptor for cell proliferation and survival, e.g. in melanoma cells, in which sustained megalin expression was crucial for cell maintenance and proliferation (mainly overexpressed), being a target for therapy (Andersen *et al.*, 2015); in non-Hodgkin-lymphoma, in which LRP2 has increased expression mainly in neurons (Pedersen *et al.*, 2010). This role of megalin C-terminal as a transcription factor might be the source of this sustained support of survival. Likewise, Notch, upon RIP, also forms an ICD domain which translocates to the nucleus, where it acts as a transcriptional co-factor to modulate gene expression (De Strooper *et al.*, 1999; Bray, 2006).

We found that megalin heterozygous mice show clear hippocampal neuronal deficits, particularly reduced neurite complexity, dendritic spine density/maturation and synaptic density (Fig. 7), linked to learning and memory deficits, as assessed by the Morris Water Maze (MWM) and novel object recognition tests (Fig. 8F–N). Additionally, in an *in vitro* approach, overexpressing megalin in hippocampal neuronal cultures resulted in increased dendritic spines density (Fig. 6H). Consistent with our results, some reports establish a link between megalin and learning/memory processes. In an exome sequencing screening for non-syndromic intellectual disability (ID) mutations, megalin was detected in a consanguineous family of two boys with mild ID (Vasli *et al.*, 2016). Also, there is both impaired learning ability and recognition memory (Dietrich *et al.*, 2014), in endothelial-specific megalin null mice, in which megalin expression is blocked only in endothelial cells, also affecting the choroid plexus through the Tie-Cre promoter (Theis *et al.*, 2003). Moreover, degenerating neurons in the cerebral cortex were observed in these mice that also display cortical and hippocampal exacerbated inflammation processes (Bartolome *et al.*, 2017). LRP8 (low-density lipoprotein receptor-related protein 8), another member of the LDL receptor family, is also RIP processed, triggered by Reelin, with intracellular domain cross-talking with NMDA receptor, in a similar way that we have described for LRP2 (Gomes *et al.*, 2016); and after going from the synapse to the nucleus, LRP8 regulates epigenetic events that culminate in the regulation of memory formation *in vivo* (Telese *et al.*, 2015). TTR KO mice also show some spatial learning and memory deficits (Sousa *et al.*, 2007*b*; Brouillette and Quirion, 2008; Buxbaum *et al.*, 2014). Given the fact that these TTR KO mice have downregulated megalin levels in the hippocampus (Fig. 1), these data should now be re-analysed taking into account megalin’s regulation of these processes. However, in megalin gene pathologies, in which megalin mutations lead to reduced expression of the protein, and contribute to cognitive deficits, as we demonstrate here, TTR supplementation/overexpression could potentially be used as a therapeutic strategy, based in our results showing that TTR is able to revert megalin downregulation. An increase in megalin levels and function induced by TTR treatment, or other strategies, could potentially mitigate some of the pathology symptoms.

In conclusion (graphical abstract), our data demonstrate that: (i) TTR is a fine and positive regulator of megalin levels, mainly in hippocampal neurons; (ii) TTR signalling through neuronal megalin does not only rely on the signalling pathways Src/Erk/Akt, but also in a RIP process, with ICD formation and translocation to the nucleus; (iii) megalin C-terminal has full potential for being considered a transcription factor, with all the motifs associated; (iv) megalin regulates neuronal activity and neuronal survival; (v) megalin has a role in synaptic plasticity, regulating neurite outgrowth, dendritic spine maturation and synaptic density; (vi) we disclose a new megalin role in learning and memory mechanisms; (vii) finally, we have contributed to unveil the molecular route underlying the cognitive and intellectual disabilities occurring in megalin gene pathologies, particularly the Donnai-Barrow syndrome; (viii) neuronal megalin should now be considered as a relevant player in the mechanisms regulating synaptic neurotransmission and learning and memory . In summary, upon TTR (or other ligand) binding to megalin, a signalling pathway involving Src, NMDARs activation, Erk1/2, CREB and Akt and/or a pathway involving RIP and formation of LRP2-ICD can contribute to increase megalin levels, in a mechanism important for physiological structural and synaptic plasticity. First and foremost these mechanisms shed a light on the knowledge of cognitive and intellectual disabilities that occur in megalin gene pathologies, and on potential therapeutic targets.

## Supplementary material

Supplementary material is available at *Brain Communications* online.

## Supplementary Material

fcaa135_Supplementary_Data

## Abbreviations

Akt =: serine/threonine kinase
CMV =: Citomegalovírus
CREB =: cAMP-response element-binding protein
ECD =: extracellular domain
EPM: = elevated plus maze
Erk =: extracellular-signal-regulated kinase; FRET = fluorescence resonance energy transfer; GAPDH = glyceraldehyde 3-phosphate dehydrogenase
GFP =: green fluorescent protein
ICD =: intracellular domain
kDa =: KiloDalton
KO =: knockout
LRP =: low-density lipoprotein receptor; MAP2 = microtubule associated protein 2
MCTF =: membrane-bound megalin C-terminal fragment
min =: minutes
MWM =: Morris Water Maze test
NES =: nuclear-exporting sequence
NLS =: nuclear-localizing sequence
NOR =: Novel Object Recognition test
OF =: Open Field test
PSD =: post-synaptic density
RBP =: retinol-binding protein
RIP =: regulated intramembrane proteolysis
SH =: Src-homology domain
Src =: tyrosine-protein kinase
TTR =: transthyretin
T4 =: thyroxine
VGLUT1 =: vesicular glutamate transporter 1
WT =: wild type
