# Supplementary material for: Neuronal megalin mediates synaptic plasticity—a novel mechanism underlying intellectual disabilities in megalin gene pathologies
Source: Brain Commun. 2020 Aug 25;2(2):fcaa135. doi: 10.1093/braincomms/fcaa135 (PMC7667529; doi:10.1093/braincomms/fcaa135)
Supplement: fcaa135_Supplementary_Data [file fcaa135_supplementary_data.zip › Linear Mixed Model All statistical analysis_Fig.5_7_Supp.6_8.pdf]

**Data Set Name**	WORK.FIG5B	**Observations**	1698
**Member Type**	DATA	**Variables**	3
**Engine**	V9	**Indexes**	0
**Created**	07/03/2020 01:00:55	**Observation Length**	32
**Last Modified**	07/03/2020 01:00:55	**Deleted Observations**	0
**Protection**		**Compressed**	NO
**Data Set Type**		**Sorted**	NO
**Label**			
**Data Representation**	SOLARIS_X86_64, LINUX_X86_64, ALPHA_TRU64, LINUX_IA64		
**Encoding**	utf-8 Unicode (UTF-8)		

**Engine/Host Dependent Information**	
**Data Set Page Size**	65536
**Number of Data Set Pages**	1
**First Data Page**	1
**Max Obs per Page**	2038
**Obs in First Data Page**	1698
**Number of Data Set Repairs**	0
**Filename**	/tmp/SAS_work110900000989_localhost.localdomain/SAS_work03CC00000989_localhost.localdomain/fig5b.sas7bdat
**Release Created**	9.0401M6
**Host Created**	Linux
**Inode Number**	672501
**Access Permission**	rw-rw-r--
**Owner Name**	sasdemo
**File Size**	128KB
**File Size (bytes)**	131072

Alphabetic List of Variables and Attributes

#	Variable	Type	Len	Format	Informat	Label
**2**	Culture	Num	8	BEST.		Culture
**1**	Genotype	Char	10	$10.	$10.	Genotype
**3**	Neurite_number	Num	8	BEST.		Neurite number

Model Information	
**Data Set**	WORK.FIG5B
**Dependent Variable**	Neurite_number
**Covariance Structure**	Variance Components
**Estimation Method**	REML
**Residual Variance Method**	Profile
**Fixed Effects SE Method**	Model-Based
**Degrees of Freedom Method**	Containment

Class Level Information		
**Class**	**Levels**	**Values**
**Genotype**	4	Meg+- Meg+-TTRKO TTR KO Wt
**Culture**	24	1 2 3 4 5 6 7 8 9 10 11 12 13 14 15 16 17 18 19 20 21 22 23 24

Dimensions	
**Covariance Parameters**	2
**Columns in X**	5
**Columns in Z**	24
**Subjects**	1
**Max Obs per Subject**	1698

Number of Observations	
**Number of Observations Read**	1698
**Number of Observations Used**	1698
**Number of Observations Not Used**	0

Iteration History			
**Iteration**	**Evaluations**	**-2 Res Log Like**	**Criterion**
**0**	1	8080.94042173	
**1**	2	7956.10662307	0.00000003
**2**	1	7956.10655530	0.00000000

Convergence criteria met.

Covariance Parameter Estimates							
**Cov Parm**	**Estimate**	**Standard Error**	**Z Value**	**Pr > Z**	**Alpha**	**Lower**	**Upper**
**Culture**	0.7968	0.2816	2.83	0.0023	0.05	0.4421	1.8449
**Residual**	6.1573	0.2128	28.93	<.0001	0.05	5.7605	6.5967

Fit Statistics	
**-2 Res Log Likelihood**	7956.1
**AIC (Smaller is Better)**	7960.1
**AICC (Smaller is Better)**	7960.1
**BIC (Smaller is Better)**	7962.5

Solution for Fixed Effects

Effect	Genotype	Estimate	Standard Error	DF	t Value	Pr > |t|	Alpha	Lower	Upper
**Intercept**		6.2666	0.4143	20	15.12	<.0001	0.05	5.4023	7.1309
**Genotype**	Meg+-	-2.9715	0.6318	1674	-4.70	<.0001	0.05	-4.2107	-1.7322
**Genotype**	Meg+-TTRKO	-2.9389	0.5678	1674	-5.18	<.0001	0.05	-4.0526	-1.8253
**Genotype**	TTR KO	-1.1682	0.5200	1674	-2.25	0.0248	0.05	-2.1881	-0.1483
**Genotype**	Wt	0	.	.	.	.	.	.	.

Solution for Random Effects

Effect	Culture	Estimate	Std Err Pred	DF	t Value	Pr > |t|	Alpha	Lower	Upper
**Culture**	1	-0.5667	0.4464	1674	-1.27	0.2044	0.05	-1.4423	0.3089
**Culture**	2	-0.1684	0.4512	1674	-0.37	0.7089	0.05	-1.0534	0.7165
**Culture**	3	0.3435	0.4551	1674	0.75	0.4505	0.05	-0.5491	1.2361
**Culture**	4	-0.8656	0.4572	1674	-1.89	0.0585	0.05	-1.7622	0.03111
**Culture**	5	1.2572	0.4543	1674	2.77	0.0057	0.05	0.3661	2.1484
**Culture**	6	0.5715	0.3927	1674	1.46	0.1457	0.05	-0.1987	1.3418
**Culture**	7	-0.9021	0.4091	1674	-2.21	0.0276	0.05	-1.7045	-0.09972
**Culture**	8	-1.8219	0.4001	1674	-4.55	<.0001	0.05	-2.6067	-1.0371
**Culture**	9	1.2940	0.3910	1674	3.31	0.0010	0.05	0.5271	2.0610
**Culture**	10	0.2202	0.3991	1674	0.55	0.5812	0.05	-0.5626	1.0030
**Culture**	11	0.6790	0.4172	1674	1.63	0.1038	0.05	-0.1393	1.4973
**Culture**	12	-0.2995	0.3919	1674	-0.76	0.4448	0.05	-1.0681	0.4691
**Culture**	13	0.2533	0.4144	1674	0.61	0.5411	0.05	-0.5594	1.0660
**Culture**	14	0.005476	0.4022	1674	0.01	0.9891	0.05	-0.7834	0.7943
**Culture**	15	0.6939	0.4447	1674	1.56	0.1188	0.05	-0.1782	1.5661
**Culture**	16	0.5477	0.4594	1674	1.19	0.2334	0.05	-0.3534	1.4488
**Culture**	17	0.9881	0.4460	1674	2.22	0.0269	0.05	0.1134	1.8629
**Culture**	18	0.08157	0.4555	1674	0.18	0.8579	0.05	-0.8118	0.9750
**Culture**	19	-1.0939	0.4701	1674	-2.33	0.0201	0.05	-2.0160	-0.1718
**Culture**	20	-1.2174	0.4846	1674	-2.51	0.0121	0.05	-2.1680	-0.2669
**Culture**	21	-0.3056	0.5185	1674	-0.59	0.5557	0.05	-1.3225	0.7114
**Culture**	22	0.3517	0.5241	1674	0.67	0.5022	0.05	-0.6762	1.3797

Solution for Random Effects									
Effect	Culture	Estimate	Std Err Pred	DF	t Value	Pr > |t|	Alpha	Lower	Upper
Culture	23	0.08237	0.5305	1674	0.16	0.8766	0.05	-0.9580	1.1228
Culture	24	-0.1285	0.5191	1674	-0.25	0.8045	0.05	-1.1467	0.8896

Type 3 Tests of Fixed Effects				
Effect	Num DF	Den DF	F Value	Pr > F
Genotype	3	1674	12.30	<.0001

Least Squares Means									
Effect	Genotype	Estimate	Standard Error	DF	t Value	Pr > |t|	Alpha	Lower	Upper
Genotype	Meg+-	3.2951	0.4770	1674	6.91	<.0001	0.05	2.3595	4.2306
Genotype	Meg+-TTRKO	3.3276	0.3882	1674	8.57	<.0001	0.05	2.5661	4.0891
Genotype	TTR KO	5.0984	0.3142	1674	16.23	<.0001	0.05	4.4821	5.7147
Genotype	Wt	6.2666	0.4143	1674	15.12	<.0001	0.05	5.4539	7.0792

Differences of Least Squares Means												
Effect	Genotype	Genotype	Estimate	Standard Error	DF	t Value	Pr > |t|	Adjustment	Adj P	Alpha	Lower	Upper
Genotype	Meg+-	Meg+-TTRKO	-0.03252	0.6150	1674	-0.05	0.9578	Tukey-Kramer	0.9999	0.05	-1.2388	1.1738
Genotype	Meg+-	TTR KO	-1.8033	0.5712	1674	-3.16	0.0016	Tukey-Kramer	0.0088	0.05	-2.9236	-0.6830
Genotype	Meg+-	Wt	-2.9715	0.6318	1674	-4.70	<.0001	Tukey-Kramer	<.0001	0.05	-4.2107	-1.7322
Genotype	Meg+-TTRKO	TTR KO	-1.7708	0.4995	1674	-3.55	0.0004	Tukey-Kramer	0.0023	0.05	-2.7504	-0.7911
Genotype	Meg+-TTRKO	Wt	-2.9389	0.5678	1674	-5.18	<.0001	Tukey-Kramer	<.0001	0.05	-4.0526	-1.8253
Genotype	TTR KO	Wt	-1.1682	0.5200	1674	-2.25	0.0248	Tukey-Kramer	0.1113	0.05	-2.1881	-0.1483

Differences of Least Squares Means				
Effect	Genotype	Genotype	Adj Lower	Adj Upper
Genotype	Meg+-	Meg+-TTRKO	-1.6141	1.5490
Genotype	Meg+-	TTR KO	-3.2721	-0.3344
Genotype	Meg+-	Wt	-4.5962	-1.3467
Genotype	Meg+-TTRKO	TTR KO	-3.0551	-0.4864
Genotype	Meg+-TTRKO	Wt	-4.3991	-1.4788
Genotype	TTR KO	Wt	-2.5054	0.1691

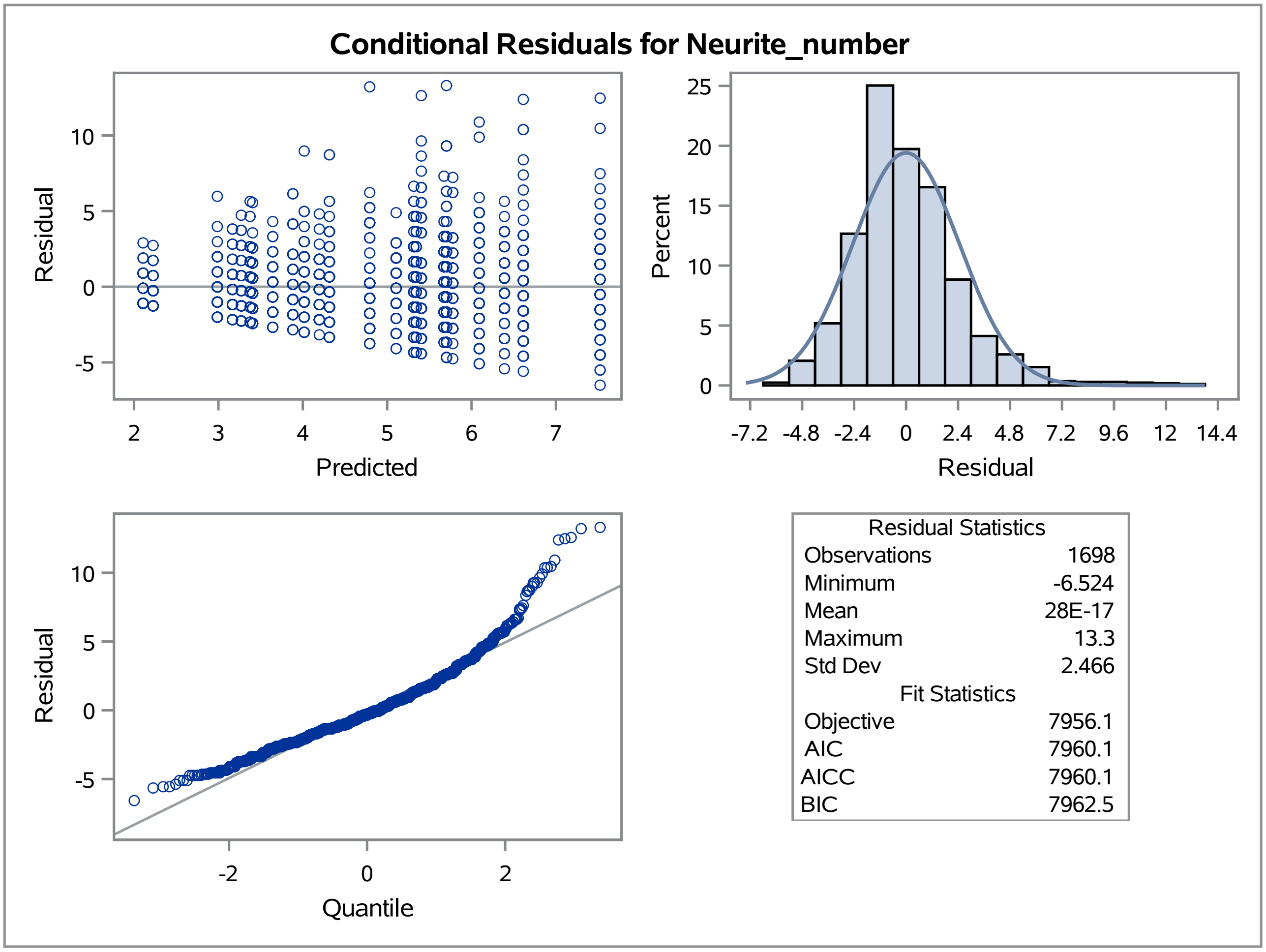
Conditional Residuals for Neurite_number
Residual
Predicted
Percent
Residual
Residual
Quantile
Residual Statistics
Observations 1698
Minimum -6.524
Mean 28E-17
Maximum 13.3
Std Dev 2.466
Fit Statistics
Objective 7956.1
AIC 7960.1
AICC 7960.1
BIC 7962.5

Data Set Name	WORK.FIG5C	Observations	1698
Member Type	DATA	Variables	3
Engine	V9	Indexes	0
Created	07/03/2020 01:00:57	Observation Length	32
Last Modified	07/03/2020 01:00:57	Deleted Observations	0
Protection		Compressed	NO
Data Set Type		Sorted	NO
Label			
Data Representation	SOLARIS_X86_64, LINUX_X86_64, ALPHA_TRU64, LINUX_IA64		
Encoding	utf-8 Unicode (UTF-8)		

Engine/Host Dependent Information	
Data Set Page Size	65536
Number of Data Set Pages	1
First Data Page	1
Max Obs per Page	2038
Obs in First Data Page	1698
Number of Data Set Repairs	0
Filename	/tmp/SAS_work110900000989_localhost.localdomain/SAS_work03CC00000989_localhost.localdomain/fig5c.sas7bdat
Release Created	9.0401M6
Host Created	Linux
Inode Number	672503
Access Permission	rw-rw-r--
Owner Name	sasdemo
File Size	128KB
File Size (bytes)	131072

Alphabetic List of Variables and Attributes

#	Variable	Type	Len	Format	Informat	Label
2	Culture	Num	8	BEST.		Culture
1	Genotype	Char	10	$10.	$10.	Genotype
3	Neurite_length	Num	8	BEST.		Neurite length

Model Information	
**Data Set**	WORK.FIG5C
**Dependent Variable**	Neurite_length
**Covariance Structure**	Variance Components
**Estimation Method**	REML
**Residual Variance Method**	Profile
**Fixed Effects SE Method**	Model-Based
**Degrees of Freedom Method**	Containment

Class Level Information		
**Class**	**Levels**	**Values**
**Genotype**	4	Meg+- Meg+-TTRKO TTR KO Wt
**Culture**	24	1 2 3 4 5 6 7 8 9 10 11 12 13 14 15 16 17 18 19 20 21 22 23 24

Dimensions	
**Covariance Parameters**	2
**Columns in X**	5
**Columns in Z**	24
**Subjects**	1
**Max Obs per Subject**	1698

Number of Observations	
**Number of Observations Read**	1698
**Number of Observations Used**	1698
**Number of Observations Not Used**	0

Iteration History			
**Iteration**	**Evaluations**	**-2 Res Log Like**	**Criterion**
**0**	1	26182.53709814	
**1**	2	25925.15078862	0.00000060
**2**	1	25925.14370994	0.00000000

Convergence criteria met.

Covariance Parameter Estimates							
**Cov Parm**	**Estimate**	**Standard Error**	**Z Value**	**Pr > Z**	**Alpha**	**Lower**	**Upper**
**Culture**	63912	21397	2.99	0.0014	0.05	36414	140346
**Residual**	247100	8541.20	28.93	<.0001	0.05	231178	264734

Fit Statistics	
**-2 Res Log Likelihood**	25925.1
**AIC (Smaller is Better)**	25929.1
**AICC (Smaller is Better)**	25929.2
**BIC (Smaller is Better)**	25931.5

Solution for Fixed Effects

Effect	Genotype	Estimate	Standard Error	DF	t Value	Pr > |t|	Alpha	Lower	Upper
**Intercept**		1032.20	115.22	20	8.96	<.0001	0.05	791.84	1272.55
**Genotype**	Meg+-	-328.14	174.33	1674	-1.88	0.0600	0.05	-670.07	13.7841
**Genotype**	Meg+-TTRKO	-270.87	157.00	1674	-1.73	0.0847	0.05	-578.81	37.0680
**Genotype**	TTR KO	47.1523	144.18	1674	0.33	0.7437	0.05	-235.63	329.94
**Genotype**	Wt	0	.	.	.	.	.	.	.

Solution for Random Effects

Effect	Culture	Estimate	Std Err Pred	DF	t Value	Pr > |t|	Alpha	Lower	Upper
**Culture**	1	-90.3500	120.15	1674	-0.75	0.4522	0.05	-326.01	145.31
**Culture**	2	28.3697	120.91	1674	0.23	0.8145	0.05	-208.79	265.53
**Culture**	3	-102.08	121.54	1674	-0.84	0.4011	0.05	-340.48	136.31
**Culture**	4	-176.32	121.88	1674	-1.45	0.1482	0.05	-415.38	62.7305
**Culture**	5	340.38	121.42	1674	2.80	0.0051	0.05	102.23	578.53
**Culture**	6	-2.9532	99.3239	1674	-0.03	0.9763	0.05	-197.77	191.86
**Culture**	7	-342.47	102.23	1674	-3.35	0.0008	0.05	-542.98	-141.95
**Culture**	8	-603.84	100.63	1674	-6.00	<.0001	0.05	-801.21	-406.48
**Culture**	9	247.08	99.0354	1674	2.49	0.0127	0.05	52.8300	441.32
**Culture**	10	148.16	100.45	1674	1.47	0.1404	0.05	-48.8653	345.18
**Culture**	11	366.98	103.70	1674	3.54	0.0004	0.05	163.58	570.38
**Culture**	12	-76.7493	99.1780	1674	-0.77	0.4391	0.05	-271.28	117.78
**Culture**	13	222.35	103.18	1674	2.15	0.0313	0.05	19.9725	424.73
**Culture**	14	41.4490	101.00	1674	0.41	0.6816	0.05	-156.64	239.54
**Culture**	15	274.95	115.73	1674	2.38	0.0176	0.05	47.9627	501.93
**Culture**	16	73.3552	118.28	1674	0.62	0.5352	0.05	-158.64	305.35
**Culture**	17	189.34	115.95	1674	1.63	0.1027	0.05	-38.0921	416.77
**Culture**	18	14.4923	117.60	1674	0.12	0.9019	0.05	-216.16	245.14
**Culture**	19	-268.94	120.19	1674	-2.24	0.0254	0.05	-504.67	-33.2063
**Culture**	20	-283.20	122.84	1674	-2.31	0.0213	0.05	-524.13	-42.2581
**Culture**	21	-99.9218	137.64	1674	-0.73	0.4680	0.05	-369.89	170.04
**Culture**	22	47.1349	138.61	1674	0.34	0.7339	0.05	-224.73	319.00

Solution for Random Effects

Effect	Culture	Estimate	Std Err Pred	DF	t Value	Pr > |t|	Alpha	Lower	Upper
Culture	23	66.9717	139.71	1674	0.48	0.6317	0.05	-207.05	341.00
Culture	24	-14.1847	137.75	1674	-0.10	0.9180	0.05	-284.36	255.99

Type 3 Tests of Fixed Effects

Effect	Num DF	Den DF	F Value	Pr > F
Genotype	3	1674	3.08	0.0265

Least Squares Means

Effect	Genotype	Estimate	Standard Error	DF	t Value	Pr > |t|	Alpha	Lower	Upper
Genotype	Meg+-	704.05	130.82	1674	5.38	<.0001	0.05	447.46	960.64
Genotype	Meg+-TTRKO	761.32	106.65	1674	7.14	<.0001	0.05	552.15	970.49
Genotype	TTR KO	1079.35	86.6636	1674	12.45	<.0001	0.05	909.37	1249.33
Genotype	Wt	1032.20	115.22	1674	8.96	<.0001	0.05	806.20	1258.19

Differences of Least Squares Means

Effect	Genotype	Genotype	Estimate	Standard Error	DF	t Value	Pr > |t|	Adjustment	Adj P	Alpha	Lower	Upper
Genotype	Meg+-	Meg+-TTRKO	-57.2708	168.78	1674	-0.34	0.7344	Tukey-Kramer	0.9866	0.05	-388.32	273.78
Genotype	Meg+-	TTR KO	-375.30	156.92	1674	-2.39	0.0169	Tukey-Kramer	0.0791	0.05	-683.08	-67.5082
Genotype	Meg+-	Wt	-328.14	174.33	1674	-1.88	0.0600	Tukey-Kramer	0.2360	0.05	-670.07	13.7841
Genotype	Meg+-TTRKO	TTR KO	-318.03	137.42	1674	-2.31	0.0208	Tukey-Kramer	0.0952	0.05	-587.56	-48.4954
Genotype	Meg+-TTRKO	Wt	-270.87	157.00	1674	-1.73	0.0847	Tukey-Kramer	0.3108	0.05	-578.81	37.0680
Genotype	TTR KO	Wt	47.1523	144.18	1674	0.33	0.7437	Tukey-Kramer	0.9879	0.05	-235.63	329.94

Differences of Least Squares Means

Effect	Genotype	Genotype	Adj Lower	Adj Upper
Genotype	Meg+-	Meg+-TTRKO	-491.31	376.77
Genotype	Meg+-	TTR KO	-778.84	28.2473
Genotype	Meg+-	Wt	-776.45	120.16
Genotype	Meg+-TTRKO	TTR KO	-671.41	35.3577
Genotype	Meg+-TTRKO	Wt	-674.62	132.87
Genotype	TTR KO	Wt	-323.61	417.92

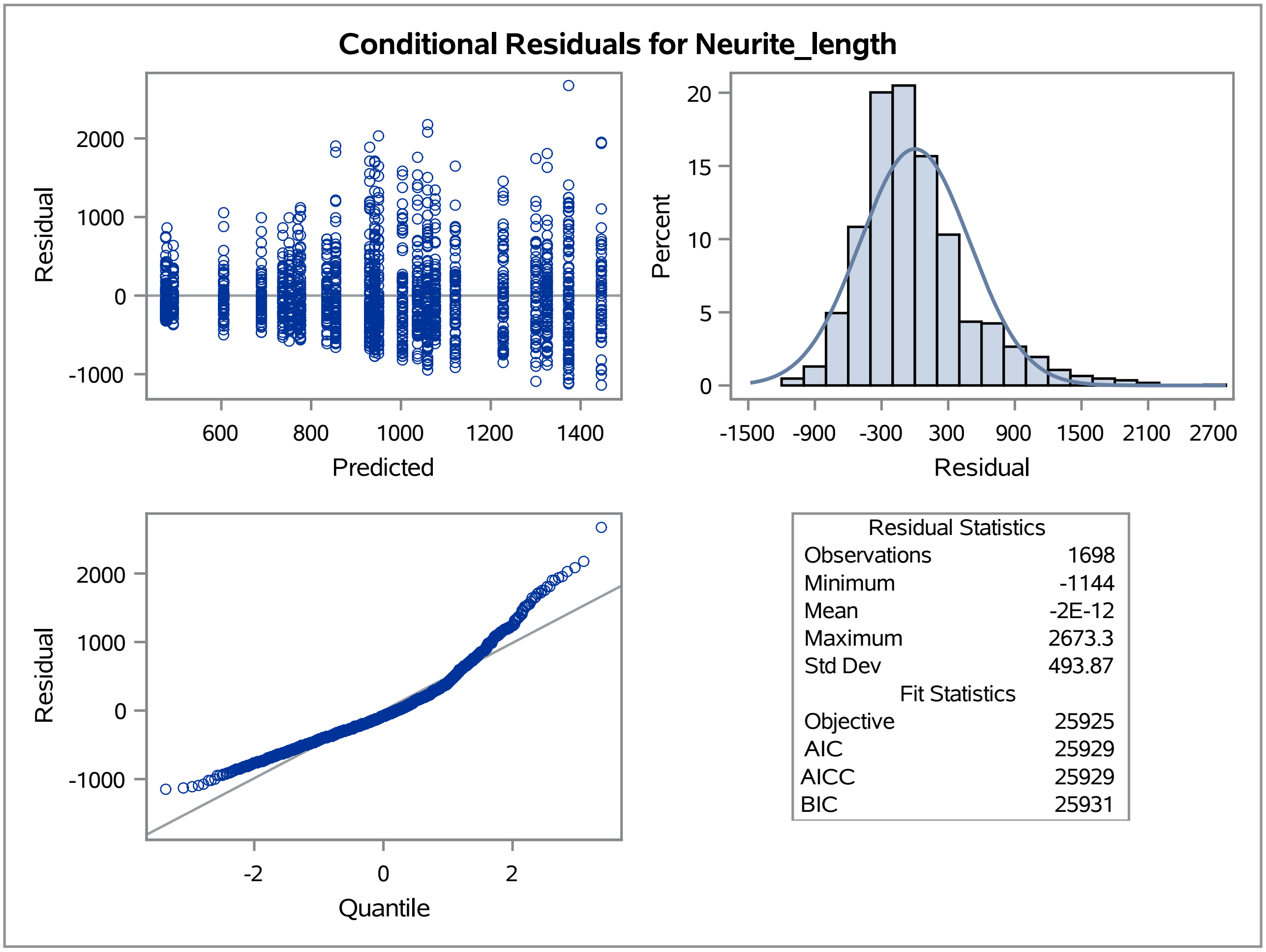
Conditional Residuals for Neurite_length
Residual
2000
1000
0
-1000
600
800
1000
1200
1400
Predicted
Percent
20
15
10
5
0
-1500
-900
-300
300
900
1500
2100
2700
Residual
Residual
2000
1000
0
-1000
-2
0
2
Quantile
Residual Statistics
Observations 1698
Minimum -1144
Mean -2E-12
Maximum 2673.3
Std Dev 493.87
Fit Statistics
Objective 25925
AIC 25929
AICC 25929
BIC 25931

The CONTENTS Procedure

**Data Set Name**	WORK.FIG5F	**Observations**	1147
**Member Type**	DATA	**Variables**	4
**Engine**	V9	**Indexes**	0
**Created**	07/05/2020 02:05:52	**Observation Length**	40
**Last Modified**	07/05/2020 02:05:52	**Deleted Observations**	0
**Protection**		**Compressed**	NO
**Data Set Type**		**Sorted**	NO
**Label**			
**Data Representation**	SOLARIS_X86_64, LINUX_X86_64, ALPHA_TRU64, LINUX_IA64		
**Encoding**	utf-8 Unicode (UTF-8)		

Engine/Host Dependent Information	
**Data Set Page Size**	65536
**Number of Data Set Pages**	1
**First Data Page**	1
**Max Obs per Page**	1632
**Obs in First Data Page**	1147
**Number of Data Set Repairs**	0
**Filename**	/tmp/SAS_workDA2300000A26_localhost.localdomain/SAS_workDFC600000A26_localhost.localdomain/fig5f.sas7bdat
**Release Created**	9.0401M6
**Host Created**	Linux
**Inode Number**	672548
**Access Permission**	rw-rw-r--
**Owner Name**	sasdemo
**File Size**	128KB
**File Size (bytes)**	131072

Alphabetic List of Variables and Attributes

#	Variable	Type	Len	Format	Informat	Label
3	Culture	Num	8	BEST.		Culture
4	FretDonor	Num	8	BEST.		FretDonor
2	Genotype	Char	13	$13.	$13.	Genotype
1	Time	Num	8	BEST.		Time

Time=0

Model Information	
**Data Set**	WORK.TEMPDATASORTED
**Dependent Variable**	FretDonor
**Covariance Structure**	Variance Components
**Estimation Method**	REML
**Residual Variance Method**	Profile
**Fixed Effects SE Method**	Model-Based
**Degrees of Freedom Method**	Containment

Class Level Information		
**Class**	**Levels**	**Values**
**Genotype**	2	Meg+/- TTR KO TTR KO
**Culture**	6	1 2 3 4 5 6

Dimensions	
**Covariance Parameters**	2
**Columns in X**	3
**Columns in Z**	6
**Subjects**	1
**Max Obs per Subject**	31

Number of Observations	
**Number of Observations Read**	31
**Number of Observations Used**	31
**Number of Observations Not Used**	0

Iteration History			
**Iteration**	**Evaluations**	**-2 Res Log Like**	**Criterion**
**0**	0	1.797693135E308	

WARNING: Stopped because of infinite likelihood.

Covariance Parameter Values At Last Iteration	
**Cov Parm**	**Estimate**
**Culture**	0
**Residual**	0

Time=0.166667

Model Information	
**Data Set**	WORK.TEMPDATASORTED
**Dependent Variable**	FretDonor
**Covariance Structure**	Variance Components
**Estimation Method**	REML
**Residual Variance Method**	Profile
**Fixed Effects SE Method**	Model-Based
**Degrees of Freedom Method**	Containment

Class Level Information		
**Class**	**Levels**	**Values**
**Genotype**	2	Meg+/- TTR KO TTR KO
**Culture**	6	1 2 3 4 5 6

Dimensions	
**Covariance Parameters**	2
**Columns in X**	3
**Columns in Z**	6
**Subjects**	1
**Max Obs per Subject**	31

Number of Observations	
**Number of Observations Read**	31
**Number of Observations Used**	31
**Number of Observations Not Used**	0

Iteration History			
**Iteration**	**Evaluations**	**-2 Res Log Like**	**Criterion**
**0**	1	-66.98254963	
**1**	2	-76.18476746	0.00000366
**2**	1	-76.18500757	0.00000000

Convergence criteria met.

Covariance Parameter Estimates				
**Cov Parm**	**Estimate**	**Alpha**	**Lower**	**Upper**
**Culture**	0.003068	0.05	0.000970	0.04623
**Residual**	0.002685	0.05	0.001651	0.005119

Time=0.166667

Fit Statistics	
**-2 Res Log Likelihood**	-76.2
**AIC (Smaller is Better)**	-72.2
**AICC (Smaller is Better)**	-71.7
**BIC (Smaller is Better)**	-72.6

Solution for Fixed Effects

Effect	Genotype	Estimate	Standard Error	DF	t Value	Pr > |t|	Alpha	Lower	Upper
**Intercept**		1.0068	0.03452	4	29.17	<.0001	0.05	0.9110	1.1027
**Genotype**	Meg+/- TTR KO	0.000664	0.04892	25	0.01	0.9893	0.05	-0.1001	0.1014
**Genotype**	TTR KO	0	.	.	.	.	.	.	.

Solution for Random Effects

Effect	Culture	Estimate	Std Err Pred	DF	t Value	Pr > |t|	Alpha	Lower	Upper
**Culture**	1	0.06625	0.03633	25	1.82	0.0802	0.05	-0.00858	0.1411
**Culture**	2	0.007244	0.03603	25	0.20	0.8423	0.05	-0.06696	0.08145
**Culture**	3	-0.07349	0.03633	25	-2.02	0.0539	0.05	-0.1483	0.001337
**Culture**	4	-0.01941	0.03643	25	-0.53	0.5990	0.05	-0.09444	0.05563
**Culture**	5	0.003234	0.03643	25	0.09	0.9300	0.05	-0.07180	0.07827
**Culture**	6	0.01617	0.03643	25	0.44	0.6609	0.05	-0.05886	0.09121

Type 3 Tests of Fixed Effects

Effect	Num DF	Den DF	F Value	Pr > F
**Genotype**	1	25	0.00	0.9893

Least Squares Means

Effect	Genotype	Estimate	Standard Error	DF	t Value	Pr > |t|	Alpha	Lower	Upper
**Genotype**	Meg+/- TTR KO	1.0075	0.03467	25	29.06	<.0001	0.05	0.9361	1.0789
**Genotype**	TTR KO	1.0068	0.03452	25	29.17	<.0001	0.05	0.9357	1.0779

Time=0.166667

Differences of Least Squares Means												
**Effect**	**Genotype**	**Genotype**	**Estimate**	**Standard Error**	**DF**	**t Value**	**Pr > |t|**	**Adjustment**	**Adj P**	**Alpha**	**Lower**	**Upper**
**Genotype**	Meg+/- TTR KO	TTR KO	0.000664	0.04892	25	0.01	0.9893	Tukey-Kramer	0.9893	0.05	-0.1001	0.1014

Differences of Least Squares Means				
**Effect**	**Genotype**	**Genotype**	**Adj Lower**	**Adj Upper**
**Genotype**	Meg+/- TTR KO	TTR KO	-0.1001	0.1014

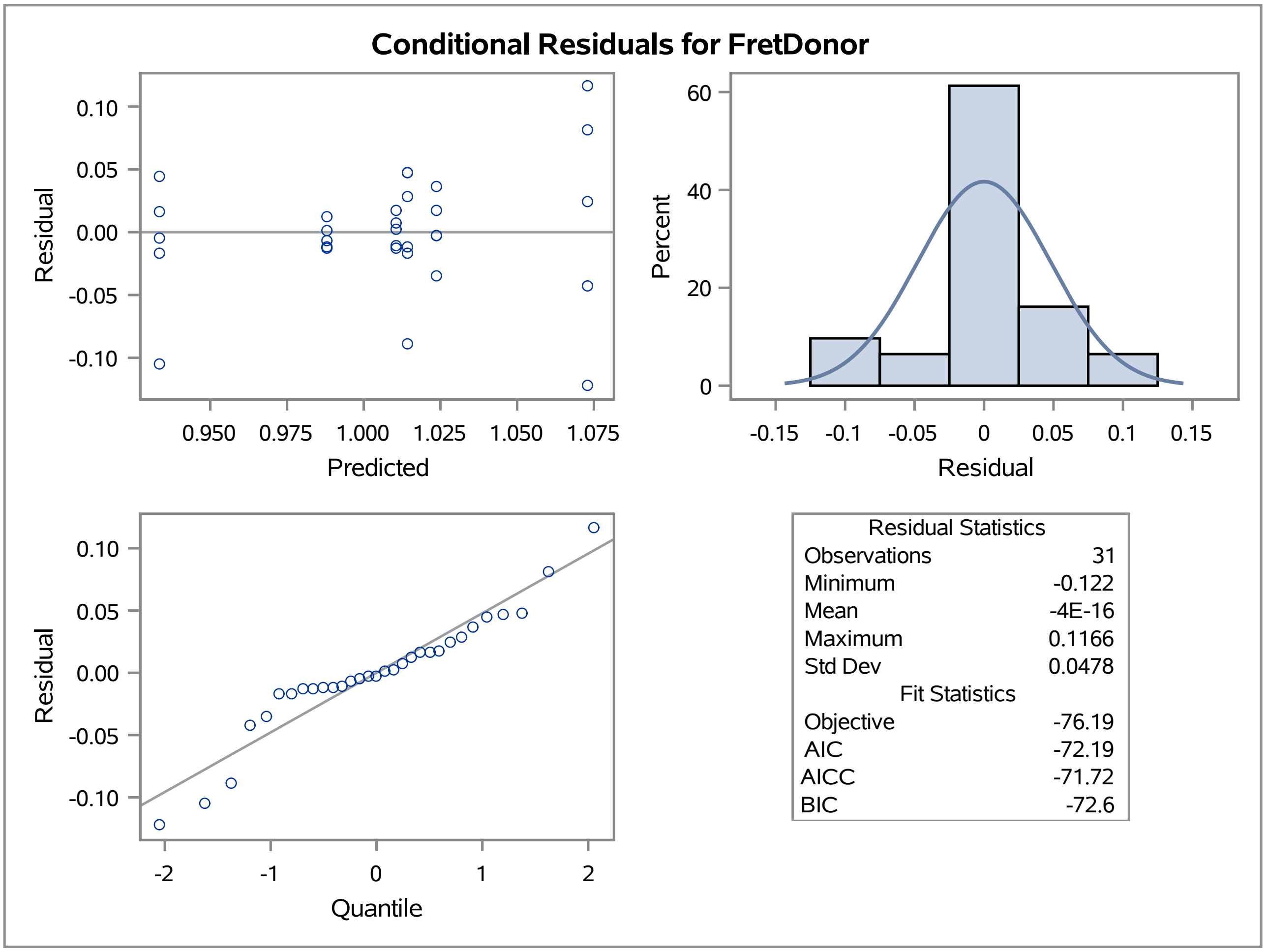

Time=0.333333

Model Information	
**Data Set**	WORK.TEMPDATASORTED
**Dependent Variable**	FretDonor
**Covariance Structure**	Variance Components
**Estimation Method**	REML
**Residual Variance Method**	Profile
**Fixed Effects SE Method**	Model-Based
**Degrees of Freedom Method**	Containment

Class Level Information		
**Class**	**Levels**	**Values**
**Genotype**	2	Meg+/- TTR KO TTR KO
**Culture**	6	1 2 3 4 5 6

Dimensions	
**Covariance Parameters**	2
**Columns in X**	3
**Columns in Z**	6
**Subjects**	1
**Max Obs per Subject**	31

Number of Observations	
**Number of Observations Read**	31
**Number of Observations Used**	31
**Number of Observations Not Used**	0

Iteration History			
**Iteration**	**Evaluations**	**-2 Res Log Like**	**Criterion**
**0**	1	-64.69492465	
**1**	2	-67.04129868	0.00000061
**2**	1	-67.04133550	0.00000000

Convergence criteria met.

Covariance Parameter Estimates				
**Cov Parm**	**Estimate**	**Alpha**	**Lower**	**Upper**
**Culture**	0.001516	0.05	0.000377	0.1214
**Residual**	0.004150	0.05	0.002551	0.007918

Time=0.333333

Fit Statistics	
-2 Res Log Likelihood	-67.0
AIC (Smaller is Better)	-63.0
AICC (Smaller is Better)	-62.6
BIC (Smaller is Better)	-63.5

Solution for Fixed Effects

Effect	Genotype	Estimate	Standard Error	DF	t Value	Pr > |t|	Alpha	Lower	Upper
Intercept		1.0145	0.02768	4	36.66	<.0001	0.05	0.9376	1.0913
Genotype	Meg+/- TTR KO	-0.01777	0.03934	25	-0.45	0.6554	0.05	-0.09880	0.06326
Genotype	TTR KO	0	.	.	.	.	.	.	.

Solution for Random Effects

Effect	Culture	Estimate	Std Err Pred	DF	t Value	Pr > |t|	Alpha	Lower	Upper
Culture	1	0.03977	0.02926	25	1.36	0.1862	0.05	-0.02049	0.1000
Culture	2	0.005305	0.02892	25	0.18	0.8559	0.05	-0.05425	0.06486
Culture	3	-0.04508	0.02926	25	-1.54	0.1360	0.05	-0.1053	0.01518
Culture	4	-0.01410	0.02937	25	-0.48	0.6355	0.05	-0.07459	0.04640
Culture	5	0.004062	0.02937	25	0.14	0.8911	0.05	-0.05643	0.06456
Culture	6	0.01004	0.02937	25	0.34	0.7355	0.05	-0.05046	0.07053

Type 3 Tests of Fixed Effects

Effect	Num DF	Den DF	F Value	Pr > F
Genotype	1	25	0.20	0.6554

Least Squares Means

Effect	Genotype	Estimate	Standard Error	DF	t Value	Pr > |t|	Alpha	Lower	Upper
Genotype	Meg+/- TTR KO	0.9967	0.02796	25	35.64	<.0001	0.05	0.9391	1.0543
Genotype	TTR KO	1.0145	0.02768	25	36.66	<.0001	0.05	0.9575	1.0715

Time=0.333333

Differences of Least Squares Means												
**Effect**	**Genotype**	**Genotype**	**Estimate**	**Standard Error**	**DF**	**t Value**	**Pr > |t|**	**Adjustment**	**Adj P**	**Alpha**	**Lower**	**Upper**
**Genotype**	Meg+/- TTR KO	TTR KO	-0.01777	0.03934	25	-0.45	0.6554	Tukey-Kramer	0.6554	0.05	-0.09880	0.06326

Differences of Least Squares Means				
**Effect**	**Genotype**	**Genotype**	**Adj Lower**	**Adj Upper**
**Genotype**	Meg+/- TTR KO	TTR KO	-0.09880	0.06326

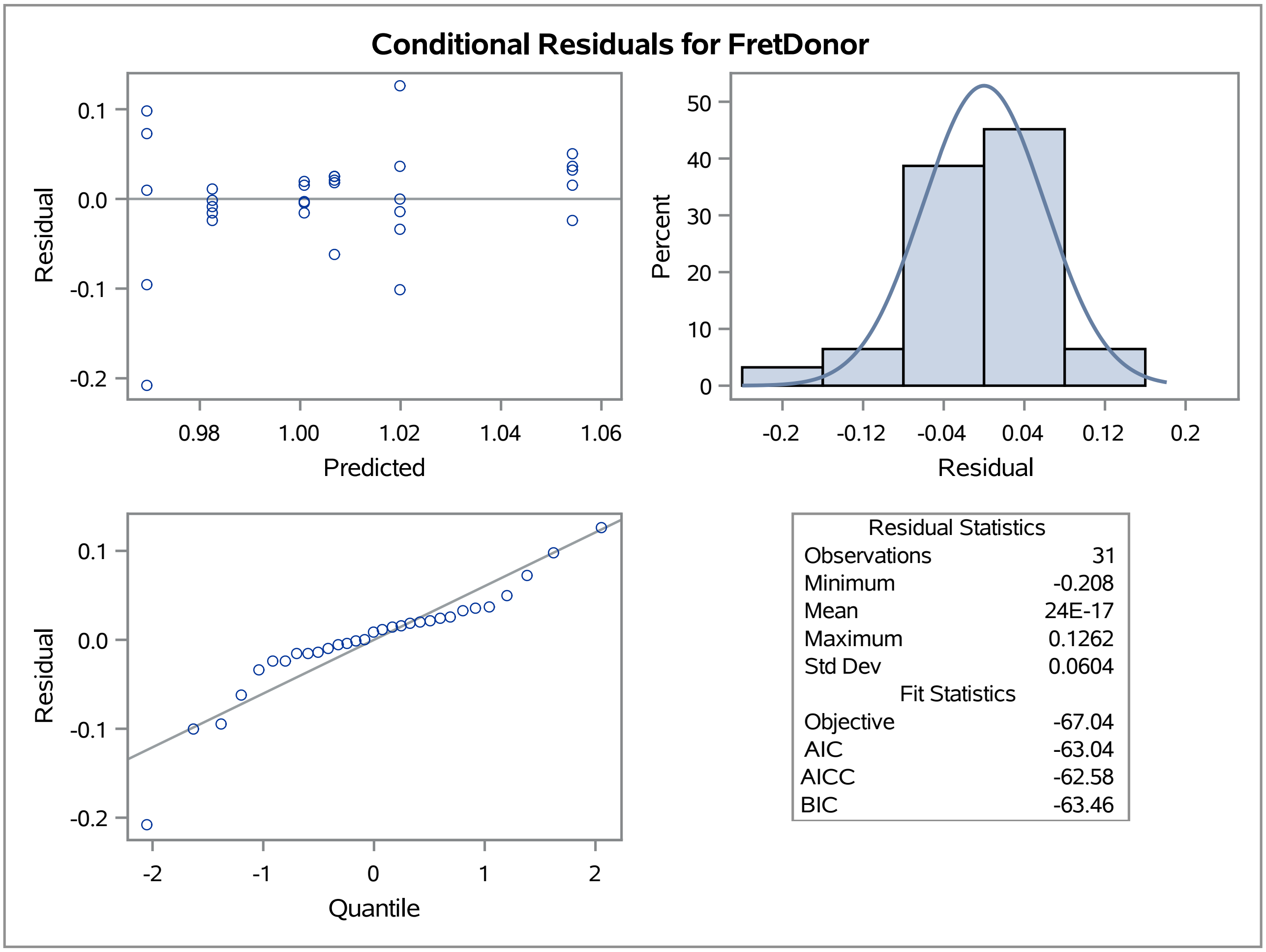

Time=0.5

Model Information	
**Data Set**	WORK.TEMPDATASORTED
**Dependent Variable**	FretDonor
**Covariance Structure**	Variance Components
**Estimation Method**	REML
**Residual Variance Method**	Profile
**Fixed Effects SE Method**	Model-Based
**Degrees of Freedom Method**	Containment

Class Level Information		
**Class**	**Levels**	**Values**
**Genotype**	2	Meg+/- TTR KO TTR KO
**Culture**	6	1 2 3 4 5 6

Dimensions	
**Covariance Parameters**	2
**Columns in X**	3
**Columns in Z**	6
**Subjects**	1
**Max Obs per Subject**	31

Number of Observations	
**Number of Observations Read**	31
**Number of Observations Used**	31
**Number of Observations Not Used**	0

Iteration History			
**Iteration**	**Evaluations**	**-2 Res Log Like**	**Criterion**
**0**	1	-83.15172664	
**1**	2	-90.93569839	0.00000239
**2**	1	-90.93587295	0.00000000

Convergence criteria met.

Covariance Parameter Estimates				
**Cov Parm**	**Estimate**	**Alpha**	**Lower**	**Upper**
**Culture**	0.001600	0.05	0.000495	0.02709
**Residual**	0.001645	0.05	0.001012	0.003137

Time=0.5

Fit Statistics	
**-2 Res Log Likelihood**	-90.9
**AIC (Smaller is Better)**	-86.9
**AICC (Smaller is Better)**	-86.5
**BIC (Smaller is Better)**	-87.4

Solution for Fixed Effects

Effect	Genotype	Estimate	Standard Error	DF	t Value	Pr > |t|	Alpha	Lower	Upper
**Intercept**		1.0055	0.02524	4	39.84	<.0001	0.05	0.9354	1.0755
**Genotype**	Meg+/- TTR KO	-0.01523	0.03578	25	-0.43	0.6740	0.05	-0.08891	0.05845
**Genotype**	TTR KO	0	.	.	.	.	.	.	.

Solution for Random Effects

Effect	Culture	Estimate	Std Err Pred	DF	t Value	Pr > |t|	Alpha	Lower	Upper
**Culture**	1	0.04591	0.02666	25	1.72	0.0975	0.05	-0.00901	0.1008
**Culture**	2	0.006466	0.02642	25	0.24	0.8087	0.05	-0.04795	0.06089
**Culture**	3	-0.05238	0.02666	25	-1.96	0.0607	0.05	-0.1073	0.002540
**Culture**	4	-0.01682	0.02674	25	-0.63	0.5351	0.05	-0.07190	0.03826
**Culture**	5	0.005441	0.02674	25	0.20	0.8404	0.05	-0.04964	0.06052
**Culture**	6	0.01138	0.02674	25	0.43	0.6741	0.05	-0.04370	0.06646

Type 3 Tests of Fixed Effects

Effect	Num DF	Den DF	F Value	Pr > F
**Genotype**	1	25	0.18	0.6740

Least Squares Means

Effect	Genotype	Estimate	Standard Error	DF	t Value	Pr > |t|	Alpha	Lower	Upper
**Genotype**	Meg+/- TTR KO	0.9902	0.02536	25	39.05	<.0001	0.05	0.9380	1.0425
**Genotype**	TTR KO	1.0055	0.02524	25	39.84	<.0001	0.05	0.9535	1.0574

Time=0.5

Differences of Least Squares Means												
**Effect**	**Genotype**	**Genotype**	**Estimate**	**Standard Error**	**DF**	**t Value**	**Pr > |t|**	**Adjustment**	**Adj P**	**Alpha**	**Lower**	**Upper**
**Genotype**	Meg+/- TTR KO	TTR KO	-0.01523	0.03578	25	-0.43	0.6740	Tukey-Kramer	0.6740	0.05	-0.08891	0.05845

Differences of Least Squares Means				
**Effect**	**Genotype**	**Genotype**	**Adj Lower**	**Adj Upper**
**Genotype**	Meg+/- TTR KO	TTR KO	-0.08891	0.05845

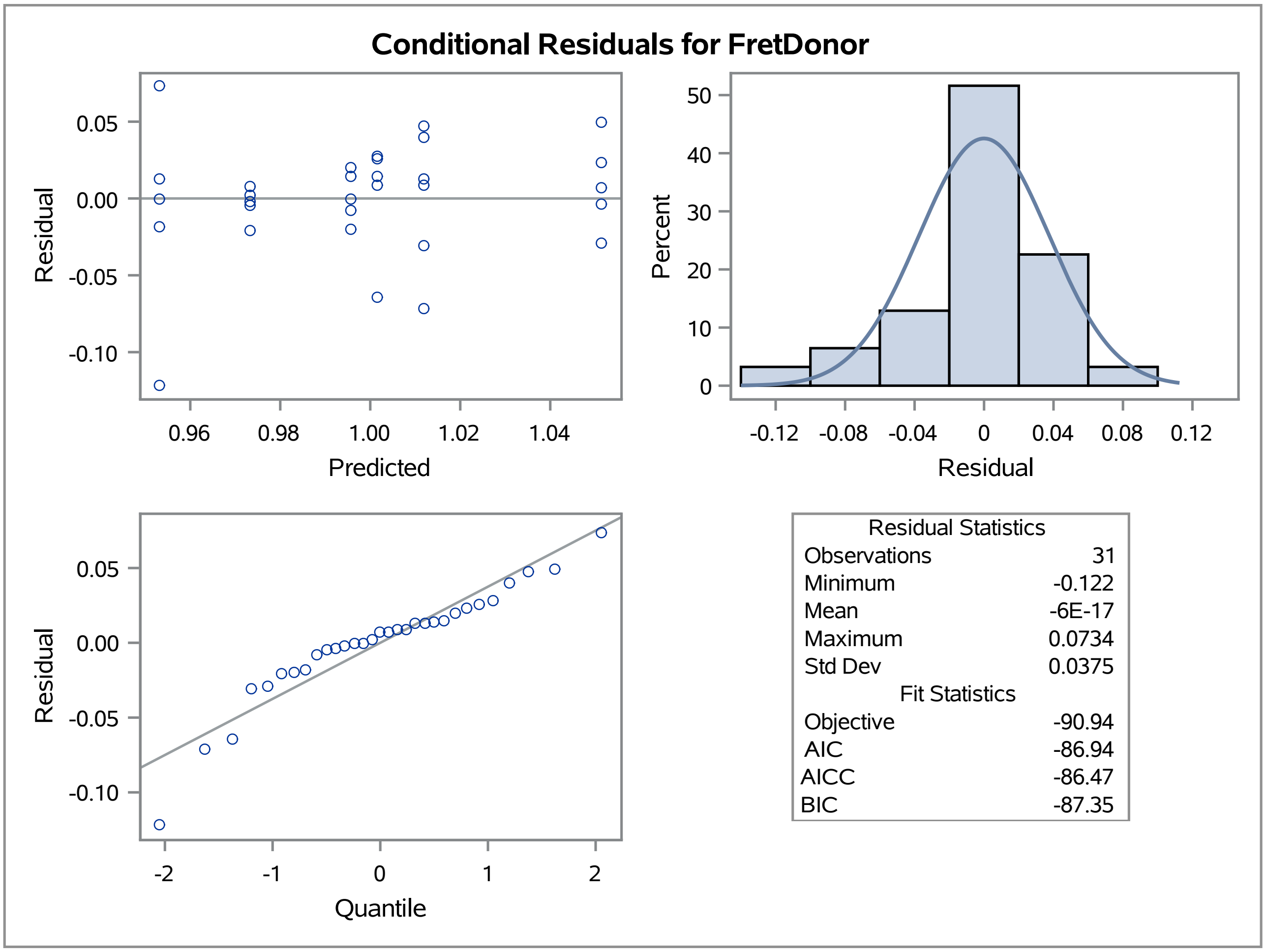

Time=0.666667

Model Information	
Data Set	WORK.TEMPDATASORTED
Dependent Variable	FretDonor
Covariance Structure	Variance Components
Estimation Method	REML
Residual Variance Method	Profile
Fixed Effects SE Method	Model-Based
Degrees of Freedom Method	Containment

Class Level Information		
Class	Levels	Values
Genotype	2	Meg+/- TTR KO TTR KO
Culture	6	1 2 3 4 5 6

Dimensions	
Covariance Parameters	2
Columns in X	3
Columns in Z	6
Subjects	1
Max Obs per Subject	31

Number of Observations	
Number of Observations Read	31
Number of Observations Used	31
Number of Observations Not Used	0

Iteration History			
Iteration	Evaluations	-2 Res Log Like	Criterion
0	1	-73.18086467	
1	2	-76.70771379	0.00000012
2	1	-76.70772189	0.00000000

Convergence criteria met.

Covariance Parameter Estimates				
Cov Parm	Estimate	Alpha	Lower	Upper
Culture	0.001364	0.05	0.000369	0.05429
Residual	0.002904	0.05	0.001787	0.005531

Time=0.666667

Fit Statistics	
**-2 Res Log Likelihood**	-76.7
**AIC (Smaller is Better)**	-72.7
**AICC (Smaller is Better)**	-72.2
**BIC (Smaller is Better)**	-73.1

Solution for Fixed Effects

Effect	Genotype	Estimate	Standard Error	DF	t Value	Pr > |t|	Alpha	Lower	Upper
**Intercept**		1.0138	0.02524	4	40.16	<.0001	0.05	0.9437	1.0839
**Genotype**	Meg+/- TTR KO	-0.02385	0.03585	25	-0.67	0.5120	0.05	-0.09769	0.04999
**Genotype**	TTR KO	0	.	.	.	.	.	.	.

Solution for Random Effects

Effect	Culture	Estimate	Std Err Pred	DF	t Value	Pr > |t|	Alpha	Lower	Upper
**Culture**	1	0.01328	0.02685	25	0.49	0.6251	0.05	-0.04201	0.06858
**Culture**	2	0.03192	0.02654	25	1.20	0.2404	0.05	-0.02274	0.08658
**Culture**	3	-0.04520	0.02685	25	-1.68	0.1047	0.05	-0.1005	0.01009
**Culture**	4	-0.02034	0.02695	25	-0.75	0.4574	0.05	-0.07585	0.03516
**Culture**	5	0.01044	0.02695	25	0.39	0.7018	0.05	-0.04506	0.06594
**Culture**	6	0.009905	0.02695	25	0.37	0.7163	0.05	-0.04560	0.06541

Type 3 Tests of Fixed Effects

Effect	Num DF	Den DF	F Value	Pr > F
**Genotype**	1	25	0.44	0.5120

Least Squares Means

Effect	Genotype	Estimate	Standard Error	DF	t Value	Pr > |t|	Alpha	Lower	Upper
**Genotype**	Meg+/- TTR KO	0.9899	0.02546	25	38.88	<.0001	0.05	0.9375	1.0424
**Genotype**	TTR KO	1.0138	0.02524	25	40.16	<.0001	0.05	0.9618	1.0658

Time=0.666667

Differences of Least Squares Means												
**Effect**	**Genotype**	**Genotype**	**Estimate**	**Standard Error**	**DF**	**t Value**	**Pr > |t|**	**Adjustment**	**Adj P**	**Alpha**	**Lower**	**Upper**
**Genotype**	Meg+/- TTR KO	TTR KO	-0.02385	0.03585	25	-0.67	0.5120	Tukey-Kramer	0.5120	0.05	-0.09769	0.04999

Differences of Least Squares Means				
**Effect**	**Genotype**	**Genotype**	**Adj Lower**	**Adj Upper**
**Genotype**	Meg+/- TTR KO	TTR KO	-0.09769	0.04999

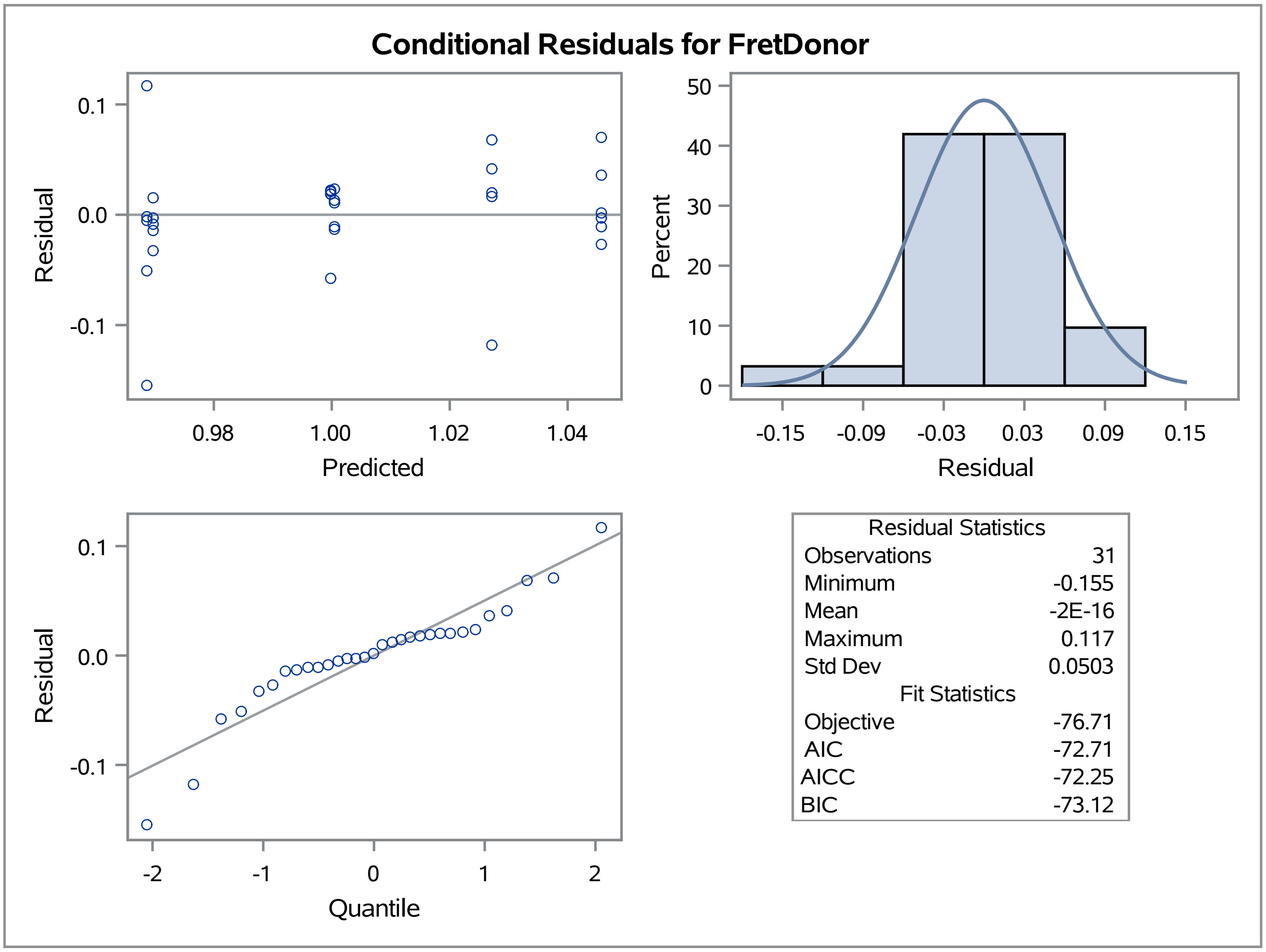

Time=0.833333

Model Information	
**Data Set**	WORK.TEMPDATASORTED
**Dependent Variable**	FretDonor
**Covariance Structure**	Variance Components
**Estimation Method**	REML
**Residual Variance Method**	Profile
**Fixed Effects SE Method**	Model-Based
**Degrees of Freedom Method**	Containment

Class Level Information		
**Class**	**Levels**	**Values**
**Genotype**	2	Meg+/- TTR KO TTR KO
**Culture**	6	1 2 3 4 5 6

Dimensions	
**Covariance Parameters**	2
**Columns in X**	3
**Columns in Z**	6
**Subjects**	1
**Max Obs per Subject**	31

Number of Observations	
**Number of Observations Read**	31
**Number of Observations Used**	31
**Number of Observations Not Used**	0

Iteration History			
**Iteration**	**Evaluations**	**-2 Res Log Like**	**Criterion**
**0**	1	-52.34864574	
**1**	2	-53.13789298	0.00000012
**2**	1	-53.13789930	0.00000000

Convergence criteria met.

Covariance Parameter Estimates				
**Cov Parm**	**Estimate**	**Alpha**	**Lower**	**Upper**
**Culture**	0.001244	0.05	0.000228	4.7875
**Residual**	0.007098	0.05	0.004361	0.01355

Time=0.833333

Fit Statistics	
**-2 Res Log Likelihood**	-53.1
**AIC (Smaller is Better)**	-49.1
**AICC (Smaller is Better)**	-48.7
**BIC (Smaller is Better)**	-49.6

Solution for Fixed Effects

Effect	Genotype	Estimate	Standard Error	DF	t Value	Pr > |t|	Alpha	Lower	Upper
**Intercept**		1.0236	0.02932	4	34.91	<.0001	0.05	0.9422	1.1050
**Genotype**	Meg+/- TTR KO	-0.02870	0.04180	25	-0.69	0.4988	0.05	-0.1148	0.05740
**Genotype**	TTR KO	0	.	.	.	.	.	.	.

Solution for Random Effects

Effect	Culture	Estimate	Std Err Pred	DF	t Value	Pr > |t|	Alpha	Lower	Upper
**Culture**	1	0.03469	0.02916	25	1.19	0.2453	0.05	-0.02537	0.09475
**Culture**	2	-0.00680	0.02885	25	-0.24	0.8155	0.05	-0.06621	0.05261
**Culture**	3	-0.02789	0.02916	25	-0.96	0.3480	0.05	-0.08795	0.03217
**Culture**	4	-0.01442	0.02926	25	-0.49	0.6264	0.05	-0.07470	0.04585
**Culture**	5	0.004095	0.02926	25	0.14	0.8898	0.05	-0.05618	0.06437
**Culture**	6	0.01033	0.02926	25	0.35	0.7271	0.05	-0.04994	0.07060

Type 3 Tests of Fixed Effects

Effect	Num DF	Den DF	F Value	Pr > F
**Genotype**	1	25	0.47	0.4988

Least Squares Means

Effect	Genotype	Estimate	Standard Error	DF	t Value	Pr > |t|	Alpha	Lower	Upper
**Genotype**	Meg+/- TTR KO	0.9949	0.02979	25	33.39	<.0001	0.05	0.9336	1.0563
**Genotype**	TTR KO	1.0236	0.02932	25	34.91	<.0001	0.05	0.9632	1.0840

Time=0.833333

Differences of Least Squares Means												
Effect	Genotype	Genotype	Estimate	Standard Error	DF	t Value	Pr > |t|	Adjustment	Adj P	Alpha	Lower	Upper
**Genotype**	Meg+/- TTR KO	TTR KO	-0.02870	0.04180	25	-0.69	0.4988	Tukey-Kramer	0.4988	0.05	-0.1148	0.05740

Differences of Least Squares Means				
Effect	Genotype	Genotype	Adj Lower	Adj Upper
**Genotype**	Meg+/- TTR KO	TTR KO	-0.1148	0.05740

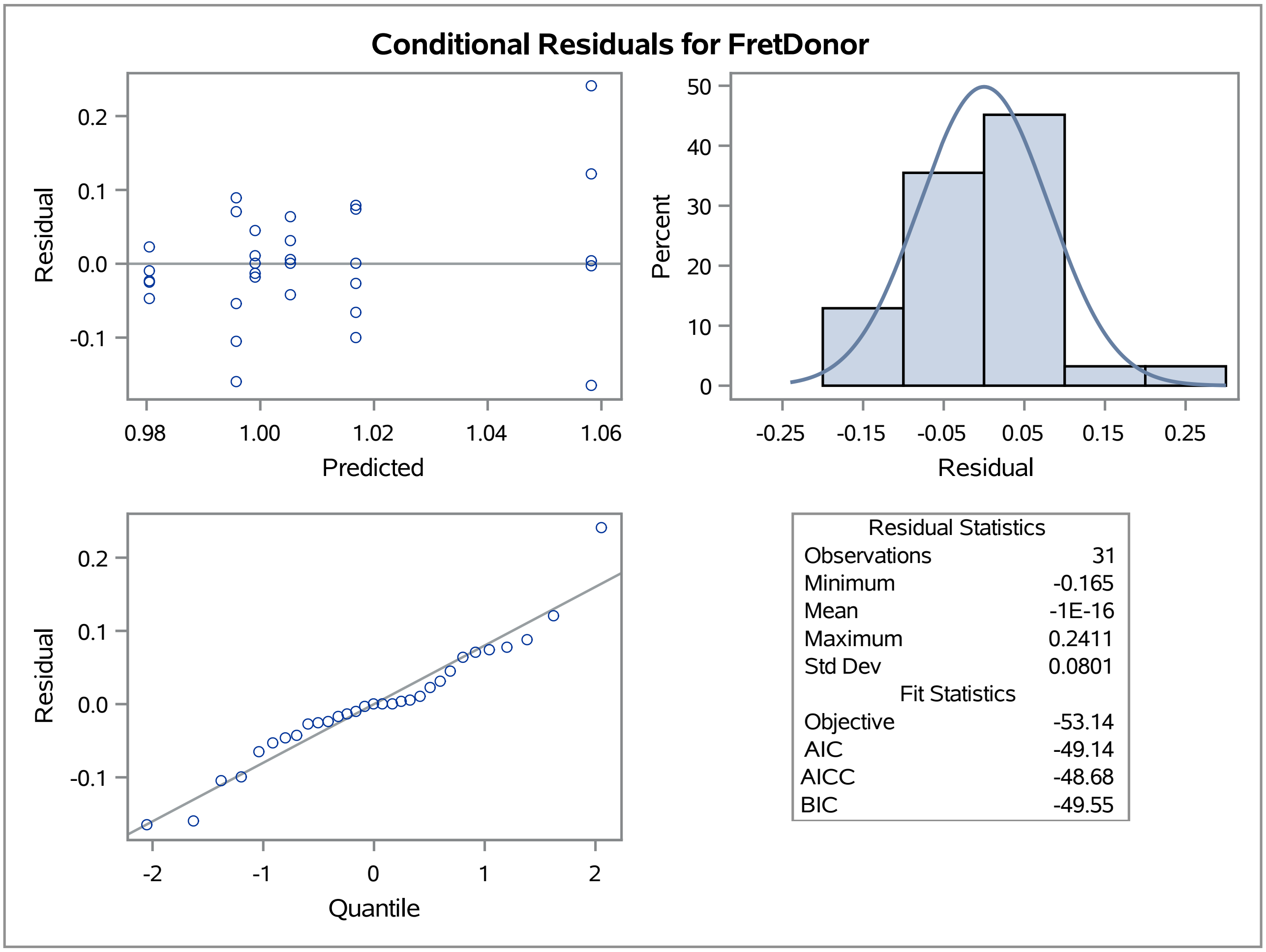

Time=1

Model Information	
**Data Set**	WORK.TEMPDATASORTED
**Dependent Variable**	FretDonor
**Covariance Structure**	Variance Components
**Estimation Method**	REML
**Residual Variance Method**	Profile
**Fixed Effects SE Method**	Model-Based
**Degrees of Freedom Method**	Containment

Class Level Information		
**Class**	**Levels**	**Values**
**Genotype**	2	Meg+/- TTR KO TTR KO
**Culture**	6	1 2 3 4 5 6

Dimensions	
**Covariance Parameters**	2
**Columns in X**	3
**Columns in Z**	6
**Subjects**	1
**Max Obs per Subject**	31

Number of Observations	
**Number of Observations Read**	31
**Number of Observations Used**	31
**Number of Observations Not Used**	0

Iteration History			
**Iteration**	**Evaluations**	**-2 Res Log Like**	**Criterion**
**0**	1	-58.81490224	
**1**	2	-71.87415696	0.00000729
**2**	1	-71.87462220	0.00000001

Convergence criteria met.

Covariance Parameter Estimates				
**Cov Parm**	**Estimate**	**Alpha**	**Lower**	**Upper**
**Culture**	0.004906	0.05	0.001611	0.06076
**Residual**	0.002981	0.05	0.001833	0.005683

Time=1

Fit Statistics	
**-2 Res Log Likelihood**	-71.9
**AIC (Smaller is Better)**	-67.9
**AICC (Smaller is Better)**	-67.4
**BIC (Smaller is Better)**	-68.3

Solution for Fixed Effects

Effect	Genotype	Estimate	Standard Error	DF	t Value	Pr > |t|	Alpha	Lower	Upper
**Intercept**		1.0253	0.04270	4	24.01	<.0001	0.05	0.9068	1.1439
**Genotype**	Meg+/- TTR KO	-0.04395	0.06047	25	-0.73	0.4741	0.05	-0.1685	0.08060
**Genotype**	TTR KO	0	.	.	.	.	.	.	.

Solution for Random Effects

Effect	Culture	Estimate	Std Err Pred	DF	t Value	Pr > |t|	Alpha	Lower	Upper
**Culture**	1	0.09412	0.04451	25	2.11	0.0446	0.05	0.002454	0.1858
**Culture**	2	-0.00613	0.04421	25	-0.14	0.8909	0.05	-0.09717	0.08492
**Culture**	3	-0.08799	0.04451	25	-1.98	0.0592	0.05	-0.1797	0.003673
**Culture**	4	-0.02392	0.04461	25	-0.54	0.5965	0.05	-0.1158	0.06795
**Culture**	5	0.006376	0.04461	25	0.14	0.8875	0.05	-0.08549	0.09825
**Culture**	6	0.01754	0.04461	25	0.39	0.6974	0.05	-0.07433	0.1094

Type 3 Tests of Fixed Effects

Effect	Num DF	Den DF	F Value	Pr > F
**Genotype**	1	25	0.53	0.4741

Least Squares Means

Effect	Genotype	Estimate	Standard Error	DF	t Value	Pr > |t|	Alpha	Lower	Upper
**Genotype**	Meg+/- TTR KO	0.9814	0.04283	25	22.91	<.0001	0.05	0.8932	1.0696
**Genotype**	TTR KO	1.0253	0.04270	25	24.01	<.0001	0.05	0.9374	1.1132

Time=1

Differences of Least Squares Means												
**Effect**	**Genotype**	**Genotype**	**Estimate**	**Standard Error**	**DF**	**t Value**	**Pr > |t|**	**Adjustment**	**Adj P**	**Alpha**	**Lower**	**Upper**
**Genotype**	Meg+/- TTR KO	TTR KO	-0.04395	0.06047	25	-0.73	0.4741	Tukey-Kramer	0.4741	0.05	-0.1685	0.08060

Differences of Least Squares Means				
**Effect**	**Genotype**	**Genotype**	**Adj Lower**	**Adj Upper**
**Genotype**	Meg+/- TTR KO	TTR KO	-0.1685	0.08060

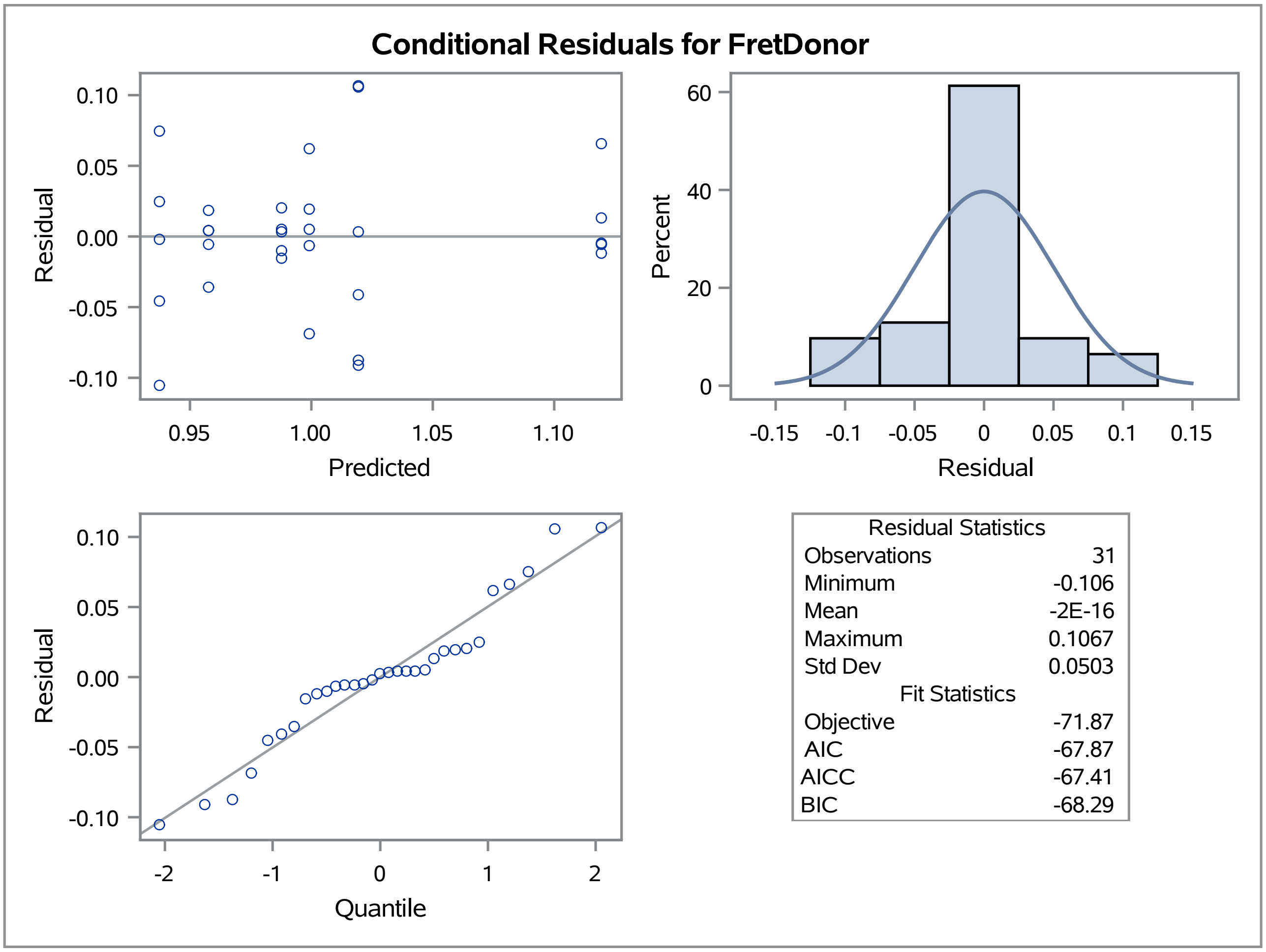

Time=1.166667

Model Information	
**Data Set**	WORK.TEMPDATASORTED
**Dependent Variable**	FretDonor
**Covariance Structure**	Variance Components
**Estimation Method**	REML
**Residual Variance Method**	Profile
**Fixed Effects SE Method**	Model-Based
**Degrees of Freedom Method**	Containment

Class Level Information		
**Class**	**Levels**	**Values**
**Genotype**	2	Meg+/- TTR KO TTR KO
**Culture**	6	1 2 3 4 5 6

Dimensions	
**Covariance Parameters**	2
**Columns in X**	3
**Columns in Z**	6
**Subjects**	1
**Max Obs per Subject**	31

Number of Observations	
**Number of Observations Read**	31
**Number of Observations Used**	31
**Number of Observations Not Used**	0

Iteration History			
**Iteration**	**Evaluations**	**-2 Res Log Like**	**Criterion**
**0**	1	-71.20434935	
**1**	2	-75.46846937	0.00000044
**2**	1	-75.46849795	0.00000000

Convergence criteria met.

Covariance Parameter Estimates				
**Cov Parm**	**Estimate**	**Alpha**	**Lower**	**Upper**
**Culture**	0.001700	0.05	0.000478	0.05175
**Residual**	0.002972	0.05	0.001827	0.005667

Time=1.166667

Fit Statistics	
**-2 Res Log Likelihood**	-75.5
**AIC (Smaller is Better)**	-71.5
**AICC (Smaller is Better)**	-71.0
**BIC (Smaller is Better)**	-71.9

Solution for Fixed Effects

Effect	Genotype	Estimate	Standard Error	DF	t Value	Pr > |t|	Alpha	Lower	Upper
**Intercept**		0.9987	0.02745	4	36.38	<.0001	0.05	0.9225	1.0749
**Genotype**	Meg+/- TTR KO	-0.02317	0.03896	25	-0.59	0.5574	0.05	-0.1034	0.05708
**Genotype**	TTR KO	0	.	.	.	.	.	.	.

Solution for Random Effects

Effect	Culture	Estimate	Std Err Pred	DF	t Value	Pr > |t|	Alpha	Lower	Upper
**Culture**	1	0.03776	0.02922	25	1.29	0.2082	0.05	-0.02243	0.09794
**Culture**	2	0.01505	0.02890	25	0.52	0.6071	0.05	-0.04447	0.07458
**Culture**	3	-0.05281	0.02922	25	-1.81	0.0828	0.05	-0.1130	0.007379
**Culture**	4	-0.01996	0.02933	25	-0.68	0.5023	0.05	-0.08037	0.04044
**Culture**	5	0.005678	0.02933	25	0.19	0.8481	0.05	-0.05473	0.06608
**Culture**	6	0.01429	0.02933	25	0.49	0.6304	0.05	-0.04612	0.07469

Type 3 Tests of Fixed Effects

Effect	Num DF	Den DF	F Value	Pr > F
**Genotype**	1	25	0.35	0.5574

Least Squares Means

Effect	Genotype	Estimate	Standard Error	DF	t Value	Pr > |t|	Alpha	Lower	Upper
**Genotype**	Meg+/- TTR KO	0.9755	0.02765	25	35.28	<.0001	0.05	0.9186	1.0325
**Genotype**	TTR KO	0.9987	0.02745	25	36.38	<.0001	0.05	0.9422	1.0552

Time=1.166667

Differences of Least Squares Means												
**Effect**	**Genotype**	**Genotype**	**Estimate**	**Standard Error**	**DF**	**t Value**	**Pr > |t|**	**Adjustment**	**Adj P**	**Alpha**	**Lower**	**Upper**
**Genotype**	Meg+/- TTR KO	TTR KO	-0.02317	0.03896	25	-0.59	0.5574	Tukey-Kramer	0.5574	0.05	-0.1034	0.05708

Differences of Least Squares Means				
**Effect**	**Genotype**	**Genotype**	**Adj Lower**	**Adj Upper**
**Genotype**	Meg+/- TTR KO	TTR KO	-0.1034	0.05708

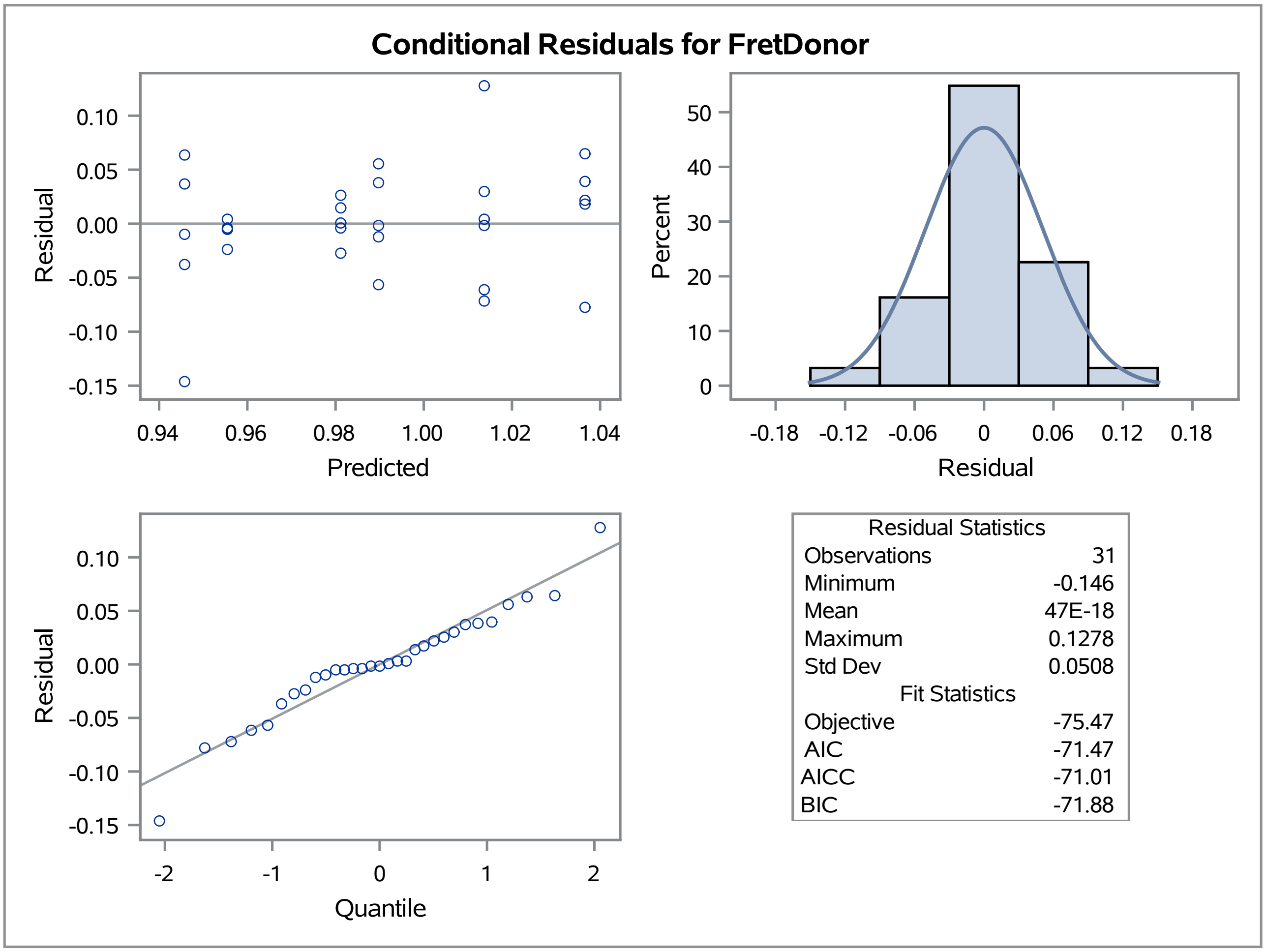

Time=1.333333

Model Information	
**Data Set**	WORK.TEMPDATASORTED
**Dependent Variable**	FretDonor
**Covariance Structure**	Variance Components
**Estimation Method**	REML
**Residual Variance Method**	Profile
**Fixed Effects SE Method**	Model-Based
**Degrees of Freedom Method**	Containment

Class Level Information		
**Class**	**Levels**	**Values**
**Genotype**	2	Meg+/- TTR KO TTR KO
**Culture**	6	1 2 3 4 5 6

Dimensions	
**Covariance Parameters**	2
**Columns in X**	3
**Columns in Z**	6
**Subjects**	1
**Max Obs per Subject**	31

Number of Observations	
**Number of Observations Read**	31
**Number of Observations Used**	31
**Number of Observations Not Used**	0

Iteration History			
**Iteration**	**Evaluations**	**-2 Res Log Like**	**Criterion**
**0**	1	-87.83966736	
**1**	2	-89.85764388	0.00000022
**2**	1	-89.85765944	0.00000000

Convergence criteria met.

Covariance Parameter Estimates				
**Cov Parm**	**Estimate**	**Alpha**	**Lower**	**Upper**
**Culture**	0.000622	0.05	0.000149	0.06993
**Residual**	0.001909	0.05	0.001173	0.003641

Time=1.333333

Fit Statistics	
**-2 Res Log Likelihood**	-89.9
**AIC (Smaller is Better)**	-85.9
**AICC (Smaller is Better)**	-85.4
**BIC (Smaller is Better)**	-86.3

Solution for Fixed Effects

Effect	Genotype	Estimate	Standard Error	DF	t Value	Pr > |t|	Alpha	Lower	Upper
**Intercept**		0.9997	0.01809	4	55.26	<.0001	0.05	0.9494	1.0499
**Genotype**	Meg+/- TTR KO	-0.02674	0.02573	25	-1.04	0.3086	0.05	-0.07972	0.02625
**Genotype**	TTR KO	0	.	.	.	.	.	.	.

Solution for Random Effects

Effect	Culture	Estimate	Std Err Pred	DF	t Value	Pr > |t|	Alpha	Lower	Upper
**Culture**	1	0.01589	0.01903	25	0.83	0.4116	0.05	-0.02331	0.05509
**Culture**	2	0.007184	0.01881	25	0.38	0.7057	0.05	-0.03155	0.04592
**Culture**	3	-0.02308	0.01903	25	-1.21	0.2367	0.05	-0.06228	0.01612
**Culture**	4	-0.02192	0.01911	25	-1.15	0.2622	0.05	-0.06127	0.01744
**Culture**	5	0.01202	0.01911	25	0.63	0.5349	0.05	-0.02733	0.05138
**Culture**	6	0.009894	0.01911	25	0.52	0.6092	0.05	-0.02946	0.04925

Type 3 Tests of Fixed Effects

Effect	Num DF	Den DF	F Value	Pr > F
**Genotype**	1	25	1.08	0.3086

Least Squares Means

Effect	Genotype	Estimate	Standard Error	DF	t Value	Pr > |t|	Alpha	Lower	Upper
**Genotype**	Meg+/- TTR KO	0.9729	0.01829	25	53.18	<.0001	0.05	0.9353	1.0106
**Genotype**	TTR KO	0.9997	0.01809	25	55.26	<.0001	0.05	0.9624	1.0369

Time=1.333333

Differences of Least Squares Means												
**Effect**	**Genotype**	**Genotype**	**Estimate**	**Standard Error**	**DF**	**t Value**	**Pr > |t|**	**Adjustment**	**Adj P**	**Alpha**	**Lower**	**Upper**
**Genotype**	Meg+/- TTR KO	TTR KO	-0.02674	0.02573	25	-1.04	0.3086	Tukey-Kramer	0.3086	0.05	-0.07972	0.02625

Differences of Least Squares Means				
**Effect**	**Genotype**	**Genotype**	**Adj Lower**	**Adj Upper**
**Genotype**	Meg+/- TTR KO	TTR KO	-0.07972	0.02625

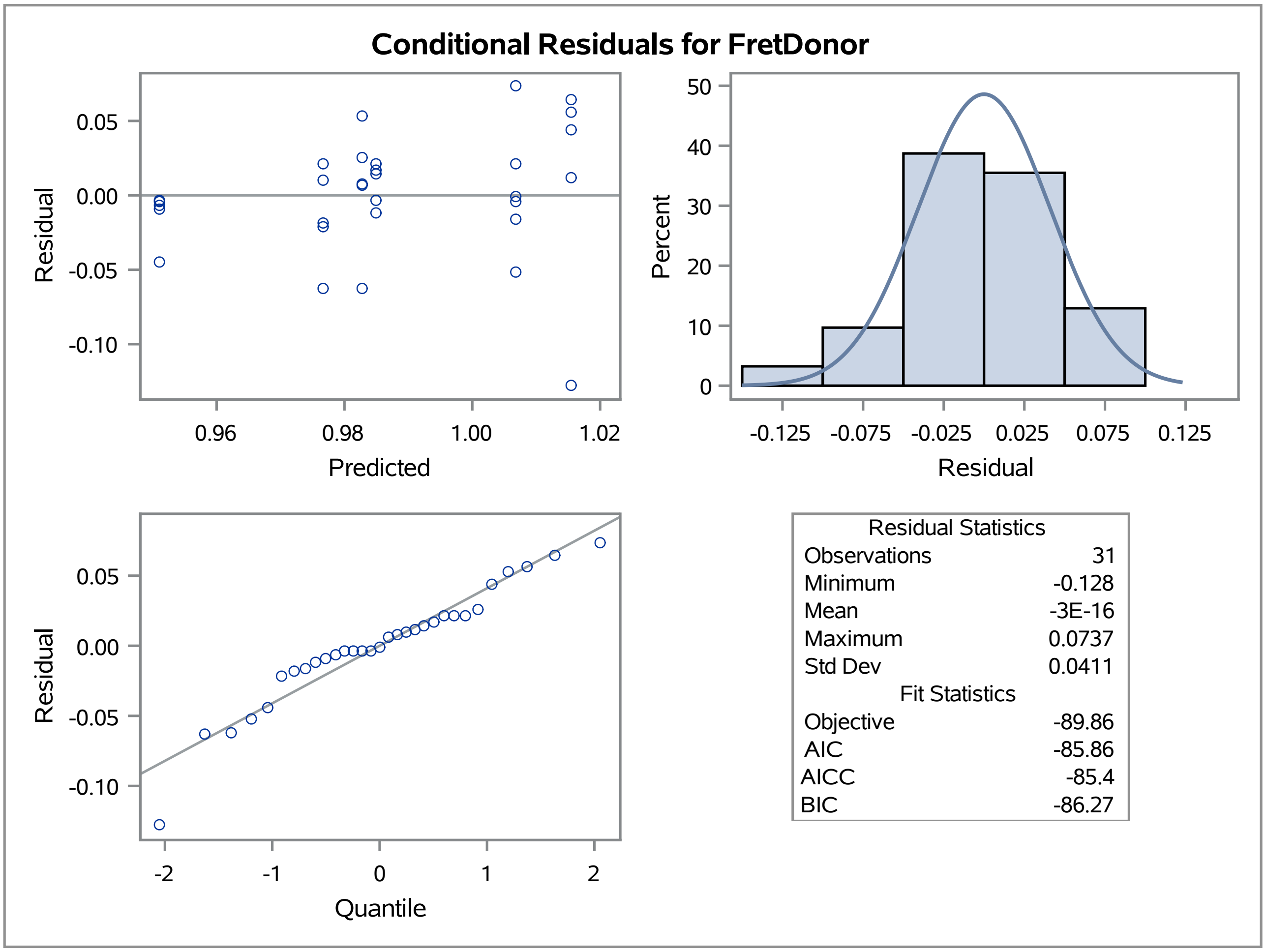

Time=1.5

Model Information	
**Data Set**	WORK.TEMPDATASORTED
**Dependent Variable**	FretDonor
**Covariance Structure**	Variance Components
**Estimation Method**	REML
**Residual Variance Method**	Profile
**Fixed Effects SE Method**	Model-Based
**Degrees of Freedom Method**	Containment

Class Level Information		
**Class**	**Levels**	**Values**
**Genotype**	2	Meg+/- TTR KO TTR KO
**Culture**	6	1 2 3 4 5 6

Dimensions	
**Covariance Parameters**	2
**Columns in X**	3
**Columns in Z**	6
**Subjects**	1
**Max Obs per Subject**	31

Number of Observations	
**Number of Observations Read**	31
**Number of Observations Used**	31
**Number of Observations Not Used**	0

Iteration History			
**Iteration**	**Evaluations**	**-2 Res Log Like**	**Criterion**
**0**	1	-73.71289519	
**1**	2	-77.77426752	0.00000118
**2**	1	-77.77434558	0.00000000

Convergence criteria met.

Covariance Parameter Estimates				
**Cov Parm**	**Estimate**	**Alpha**	**Lower**	**Upper**
**Culture**	0.001527	0.05	0.000426	0.04934
**Residual**	0.002753	0.05	0.001693	0.005251

Time=1.5

Fit Statistics	
**-2 Res Log Likelihood**	-77.8
**AIC (Smaller is Better)**	-73.8
**AICC (Smaller is Better)**	-73.3
**BIC (Smaller is Better)**	-74.2

Solution for Fixed Effects

Effect	Genotype	Estimate	Standard Error	DF	t Value	Pr > |t|	Alpha	Lower	Upper
**Intercept**		0.9709	0.02612	4	37.17	<.0001	0.05	0.8984	1.0434
**Genotype**	Meg+/- TTR KO	0.002685	0.03708	25	0.07	0.9429	0.05	-0.07368	0.07905
**Genotype**	TTR KO	0	.	.	.	.	.	.	.

Solution for Random Effects

Effect	Culture	Estimate	Std Err Pred	DF	t Value	Pr > |t|	Alpha	Lower	Upper
**Culture**	1	0.04312	0.02781	25	1.55	0.1336	0.05	-0.01415	0.1004
**Culture**	2	0.004648	0.02750	25	0.17	0.8671	0.05	-0.05199	0.06129
**Culture**	3	-0.04777	0.02781	25	-1.72	0.0982	0.05	-0.1050	0.009504
**Culture**	4	-0.01549	0.02791	25	-0.55	0.5839	0.05	-0.07297	0.04199
**Culture**	5	0.006600	0.02791	25	0.24	0.8150	0.05	-0.05088	0.06408
**Culture**	6	0.008888	0.02791	25	0.32	0.7528	0.05	-0.04859	0.06637

Type 3 Tests of Fixed Effects

Effect	Num DF	Den DF	F Value	Pr > F
**Genotype**	1	25	0.01	0.9429

Least Squares Means

Effect	Genotype	Estimate	Standard Error	DF	t Value	Pr > |t|	Alpha	Lower	Upper
**Genotype**	Meg+/- TTR KO	0.9736	0.02632	25	36.99	<.0001	0.05	0.9194	1.0278
**Genotype**	TTR KO	0.9709	0.02612	25	37.17	<.0001	0.05	0.9171	1.0247

Time=1.5

Differences of Least Squares Means												
Effect	Genotype	Genotype	Estimate	Standard Error	DF	t Value	Pr > |t|	Adjustment	Adj P	Alpha	Lower	Upper
Genotype	Meg+/- TTR KO	TTR KO	0.002685	0.03708	25	0.07	0.9429	Tukey-Kramer	0.9429	0.05	-0.07368	0.07905

Differences of Least Squares Means				
Effect	Genotype	Genotype	Adj Lower	Adj Upper
Genotype	Meg+/- TTR KO	TTR KO	-0.07368	0.07905

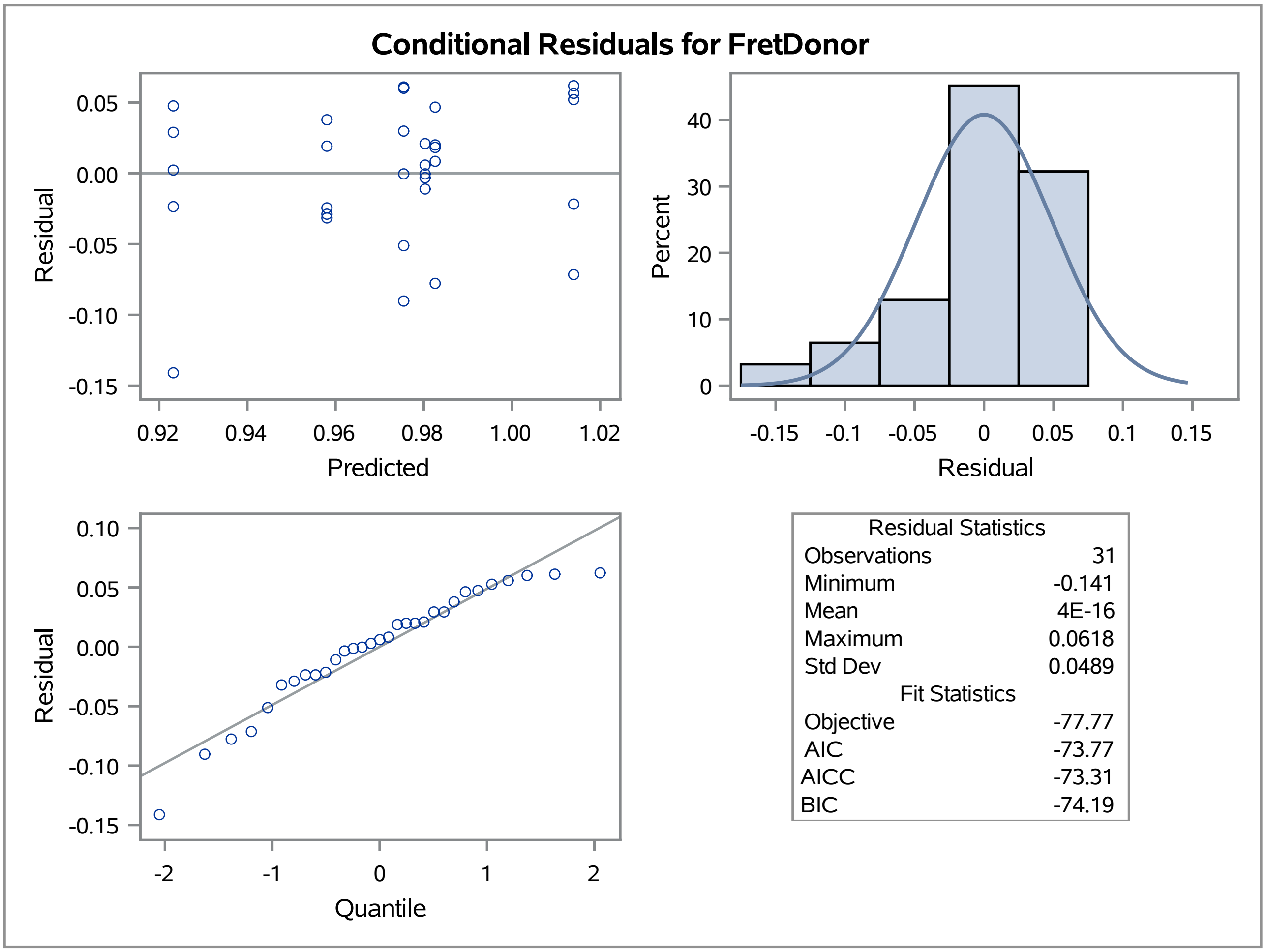

Time=1.666667

Model Information	
**Data Set**	WORK.TEMPDATASORTED
**Dependent Variable**	FretDonor
**Covariance Structure**	Variance Components
**Estimation Method**	REML
**Residual Variance Method**	Profile
**Fixed Effects SE Method**	Model-Based
**Degrees of Freedom Method**	Containment

Class Level Information		
**Class**	**Levels**	**Values**
**Genotype**	2	Meg+/- TTR KO TTR KO
**Culture**	6	1 2 3 4 5 6

Dimensions	
**Covariance Parameters**	2
**Columns in X**	3
**Columns in Z**	6
**Subjects**	1
**Max Obs per Subject**	31

Number of Observations	
**Number of Observations Read**	31
**Number of Observations Used**	31
**Number of Observations Not Used**	0

Iteration History			
**Iteration**	**Evaluations**	**-2 Res Log Like**	**Criterion**
**0**	1	-78.20972889	
**1**	2	-79.75780804	0.00000007
**2**	1	-79.75781299	0.00000000

Convergence criteria met.

Covariance Parameter Estimates				
**Cov Parm**	**Estimate**	**Alpha**	**Lower**	**Upper**
**Culture**	0.000741	0.05	0.000166	0.1706
**Residual**	0.002747	0.05	0.001689	0.005239

Time=1.666667

Fit Statistics	
-2 Res Log Likelihood	-79.8
AIC (Smaller is Better)	-75.8
AICC (Smaller is Better)	-75.3
BIC (Smaller is Better)	-76.2

Solution for Fixed Effects

Effect	Genotype	Estimate	Standard Error	DF	t Value	Pr > |t|	Alpha	Lower	Upper
Intercept		0.9744	0.02048	4	47.58	<.0001	0.05	0.9176	1.0313
Genotype	Meg+/- TTR KO	-0.00323	0.02915	25	-0.11	0.9127	0.05	-0.06325	0.05680
Genotype	TTR KO	0	.	.	.	.	.	.	.

Solution for Random Effects

Effect	Culture	Estimate	Std Err Pred	DF	t Value	Pr > |t|	Alpha	Lower	Upper
Culture	1	0.02760	0.02130	25	1.30	0.2069	0.05	-0.01627	0.07147
Culture	2	-0.01007	0.02105	25	-0.48	0.6366	0.05	-0.05343	0.03329
Culture	3	-0.01753	0.02130	25	-0.82	0.4182	0.05	-0.06140	0.02634
Culture	4	-0.01892	0.02138	25	-0.88	0.3846	0.05	-0.06296	0.02512
Culture	5	0.01219	0.02138	25	0.57	0.5738	0.05	-0.03185	0.05623
Culture	6	0.006736	0.02138	25	0.32	0.7554	0.05	-0.03730	0.05078

Type 3 Tests of Fixed Effects

Effect	Num DF	Den DF	F Value	Pr > F
Genotype	1	25	0.01	0.9127

Least Squares Means

Effect	Genotype	Estimate	Standard Error	DF	t Value	Pr > |t|	Alpha	Lower	Upper
Genotype	Meg+/- TTR KO	0.9712	0.02074	25	46.83	<.0001	0.05	0.9285	1.0139
Genotype	TTR KO	0.9744	0.02048	25	47.58	<.0001	0.05	0.9323	1.0166

Time=1.666667

Differences of Least Squares Means												
**Effect**	**Genotype**	**Genotype**	**Estimate**	**Standard Error**	**DF**	**t Value**	**Pr > |t|**	**Adjustment**	**Adj P**	**Alpha**	**Lower**	**Upper**
**Genotype**	Meg+/- TTR KO	TTR KO	-0.00323	0.02915	25	-0.11	0.9127	Tukey-Kramer	0.9127	0.05	-0.06325	0.05680

Differences of Least Squares Means				
**Effect**	**Genotype**	**Genotype**	**Adj Lower**	**Adj Upper**
**Genotype**	Meg+/- TTR KO	TTR KO	-0.06325	0.05680

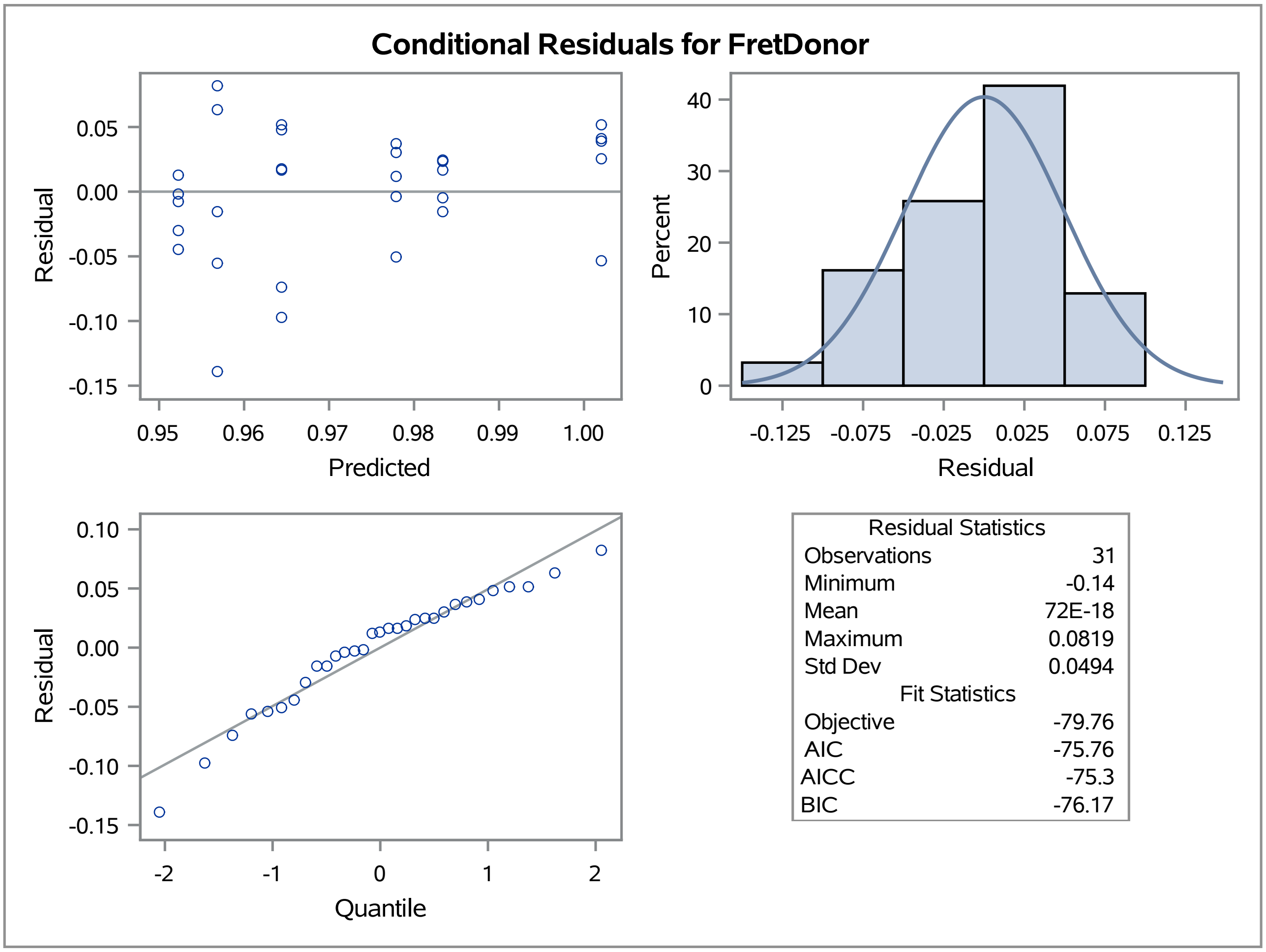

Time=1.833333

Model Information	
Data Set	WORK.TEMPDATASORTED
Dependent Variable	FretDonor
Covariance Structure	Variance Components
Estimation Method	REML
Residual Variance Method	Profile
Fixed Effects SE Method	Model-Based
Degrees of Freedom Method	Containment

Class Level Information		
Class	Levels	Values
Genotype	2	Meg+/- TTR KO TTR KO
Culture	6	1 2 3 4 5 6

Dimensions	
Covariance Parameters	2
Columns in X	3
Columns in Z	6
Subjects	1
Max Obs per Subject	31

Number of Observations	
Number of Observations Read	31
Number of Observations Used	31
Number of Observations Not Used	0

Iteration History			
Iteration	Evaluations	-2 Res Log Like	Criterion
0	1	-58.90776931	
1	2	-61.19126748	0.00000038
2	1	-61.19128926	0.00000000

Convergence criteria met.

Covariance Parameter Estimates				
Cov Parm	Estimate	Alpha	Lower	Upper
Culture	0.001815	0.05	0.000448	0.1542
Residual	0.005089	0.05	0.003128	0.009707

Time=1.833333

Fit Statistics	
**-2 Res Log Likelihood**	-61.2
**AIC (Smaller is Better)**	-57.2
**AICC (Smaller is Better)**	-56.7
**BIC (Smaller is Better)**	-57.6

Solution for Fixed Effects

Effect	Genotype	Estimate	Standard Error	DF	t Value	Pr > |t|	Alpha	Lower	Upper
**Intercept**		1.0284	0.03041	4	33.82	<.0001	0.05	0.9440	1.1128
**Genotype**	Meg+/- TTR KO	-0.06227	0.04323	25	-1.44	0.1621	0.05	-0.1513	0.02676
**Genotype**	TTR KO	0	.	.	.	.	.	.	.

Solution for Random Effects

Effect	Culture	Estimate	Std Err Pred	DF	t Value	Pr > |t|	Alpha	Lower	Upper
**Culture**	1	0.04867	0.03212	25	1.52	0.1422	0.05	-0.01748	0.1148
**Culture**	2	-0.01118	0.03174	25	-0.35	0.7276	0.05	-0.07655	0.05419
**Culture**	3	-0.03749	0.03212	25	-1.17	0.2541	0.05	-0.1036	0.02866
**Culture**	4	-0.02283	0.03224	25	-0.71	0.4855	0.05	-0.08923	0.04358
**Culture**	5	0.01442	0.03224	25	0.45	0.6586	0.05	-0.05199	0.08082
**Culture**	6	0.008409	0.03224	25	0.26	0.7964	0.05	-0.05800	0.07481

Type 3 Tests of Fixed Effects

Effect	Num DF	Den DF	F Value	Pr > F
**Genotype**	1	25	2.08	0.1621

Least Squares Means

Effect	Genotype	Estimate	Standard Error	DF	t Value	Pr > |t|	Alpha	Lower	Upper
**Genotype**	Meg+/- TTR KO	0.9661	0.03073	25	31.44	<.0001	0.05	0.9029	1.0294
**Genotype**	TTR KO	1.0284	0.03041	25	33.82	<.0001	0.05	0.9658	1.0910

Time=1.833333

Differences of Least Squares Means												
Effect	Genotype	Genotype	Estimate	Standard Error	DF	t Value	Pr > |t|	Adjustment	Adj P	Alpha	Lower	Upper
**Genotype**	Meg+/- TTR KO	TTR KO	-0.06227	0.04323	25	-1.44	0.1621	Tukey-Kramer	0.1621	0.05	-0.1513	0.02676

Differences of Least Squares Means				
Effect	Genotype	Genotype	Adj Lower	Adj Upper
**Genotype**	Meg+/- TTR KO	TTR KO	-0.1513	0.02676

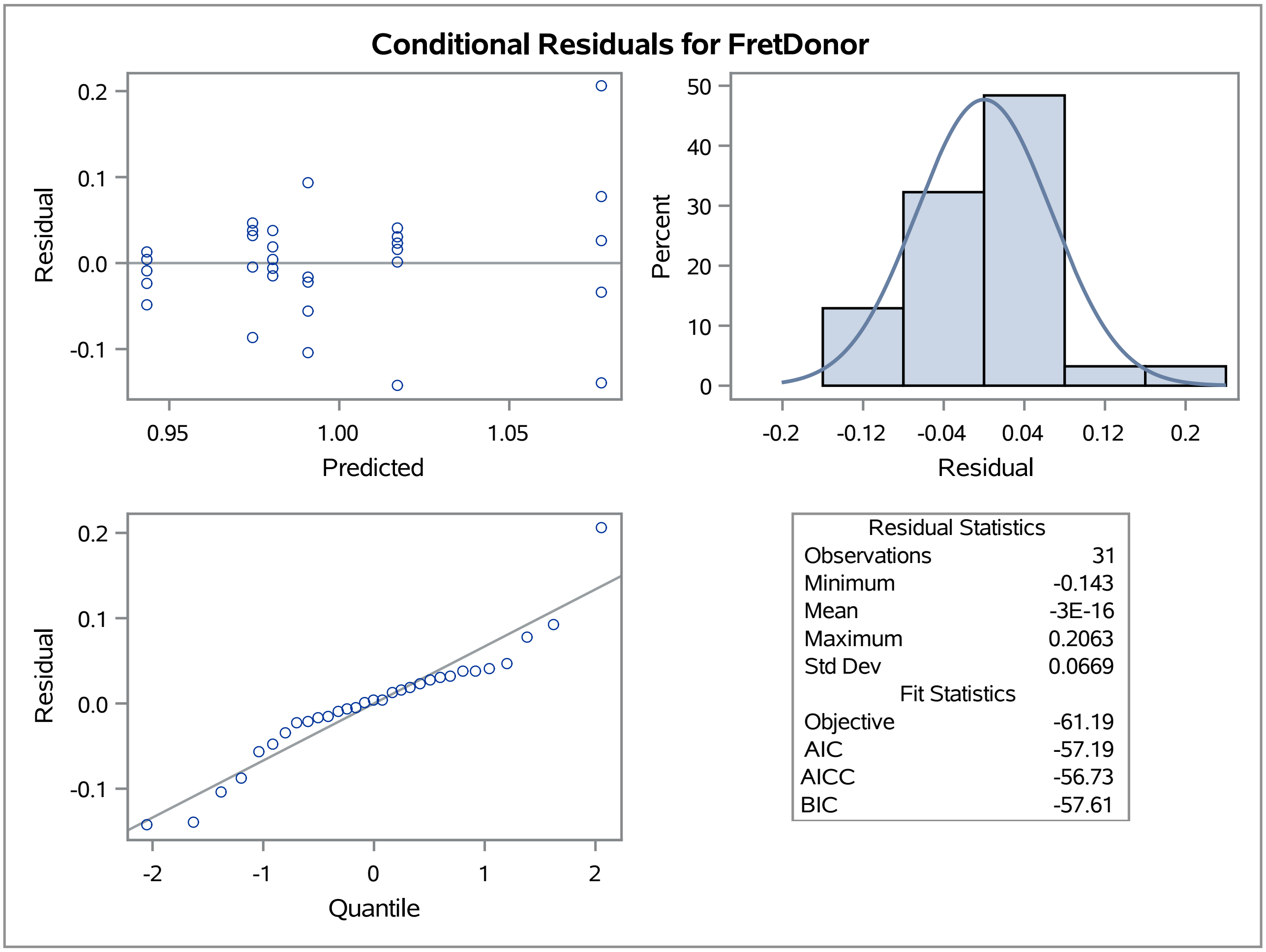

Time=2

Model Information	
**Data Set**	WORK.TEMPDATASORTED
**Dependent Variable**	FretDonor
**Covariance Structure**	Variance Components
**Estimation Method**	REML
**Residual Variance Method**	Profile
**Fixed Effects SE Method**	Model-Based
**Degrees of Freedom Method**	Containment

Class Level Information		
**Class**	**Levels**	**Values**
**Genotype**	2	Meg+/- TTR KO TTR KO
**Culture**	6	1 2 3 4 5 6

Dimensions	
**Covariance Parameters**	2
**Columns in X**	3
**Columns in Z**	6
**Subjects**	1
**Max Obs per Subject**	31

Number of Observations	
**Number of Observations Read**	31
**Number of Observations Used**	31
**Number of Observations Not Used**	0

Iteration History			
**Iteration**	**Evaluations**	**-2 Res Log Like**	**Criterion**
**0**	1	-56.60584342	
**1**	2	-71.18223733	0.00000787
**2**	1	-71.18273697	0.00000001

Convergence criteria met.

Covariance Parameter Estimates				
**Cov Parm**	**Estimate**	**Alpha**	**Lower**	**Upper**
**Culture**	0.005594	0.05	0.001855	0.06590
**Residual**	0.003007	0.05	0.001849	0.005732

Time=2

Fit Statistics	
**-2 Res Log Likelihood**	-71.2
**AIC (Smaller is Better)**	-67.2
**AICC (Smaller is Better)**	-66.7
**BIC (Smaller is Better)**	-67.6

Solution for Fixed Effects

Effect	Genotype	Estimate	Standard Error	DF	t Value	Pr > |t|	Alpha	Lower	Upper
**Intercept**		1.0633	0.04532	4	23.46	<.0001	0.05	0.9375	1.1891
**Genotype**	Meg+/- TTR KO	-0.09913	0.06418	25	-1.54	0.1350	0.05	-0.2313	0.03305
**Genotype**	TTR KO	0	.	.	.	.	.	.	.

Solution for Random Effects

Effect	Culture	Estimate	Std Err Pred	DF	t Value	Pr > |t|	Alpha	Lower	Upper
**Culture**	1	0.1004	0.04709	25	2.13	0.0430	0.05	0.003436	0.1974
**Culture**	2	-0.01299	0.04680	25	-0.28	0.7836	0.05	-0.1094	0.08339
**Culture**	3	-0.08743	0.04709	25	-1.86	0.0752	0.05	-0.1844	0.009554
**Culture**	4	-0.03887	0.04719	25	-0.82	0.4179	0.05	-0.1361	0.05831
**Culture**	5	0.02666	0.04719	25	0.56	0.5771	0.05	-0.07052	0.1238
**Culture**	6	0.01221	0.04719	25	0.26	0.7979	0.05	-0.08497	0.1094

Type 3 Tests of Fixed Effects

Effect	Num DF	Den DF	F Value	Pr > F
**Genotype**	1	25	2.39	0.1350

Least Squares Means

Effect	Genotype	Estimate	Standard Error	DF	t Value	Pr > |t|	Alpha	Lower	Upper
**Genotype**	Meg+/- TTR KO	0.9642	0.04544	25	21.22	<.0001	0.05	0.8706	1.0578
**Genotype**	TTR KO	1.0633	0.04532	25	23.46	<.0001	0.05	0.9700	1.1566

Time=2

Differences of Least Squares Means												
**Effect**	**Genotype**	**Genotype**	**Estimate**	**Standard Error**	**DF**	**t Value**	**Pr > |t|**	**Adjustment**	**Adj P**	**Alpha**	**Lower**	**Upper**
**Genotype**	Meg+/- TTR KO	TTR KO	-0.09913	0.06418	25	-1.54	0.1350	Tukey-Kramer	0.1350	0.05	-0.2313	0.03305

Differences of Least Squares Means				
**Effect**	**Genotype**	**Genotype**	**Adj Lower**	**Adj Upper**
**Genotype**	Meg+/- TTR KO	TTR KO	-0.2313	0.03305

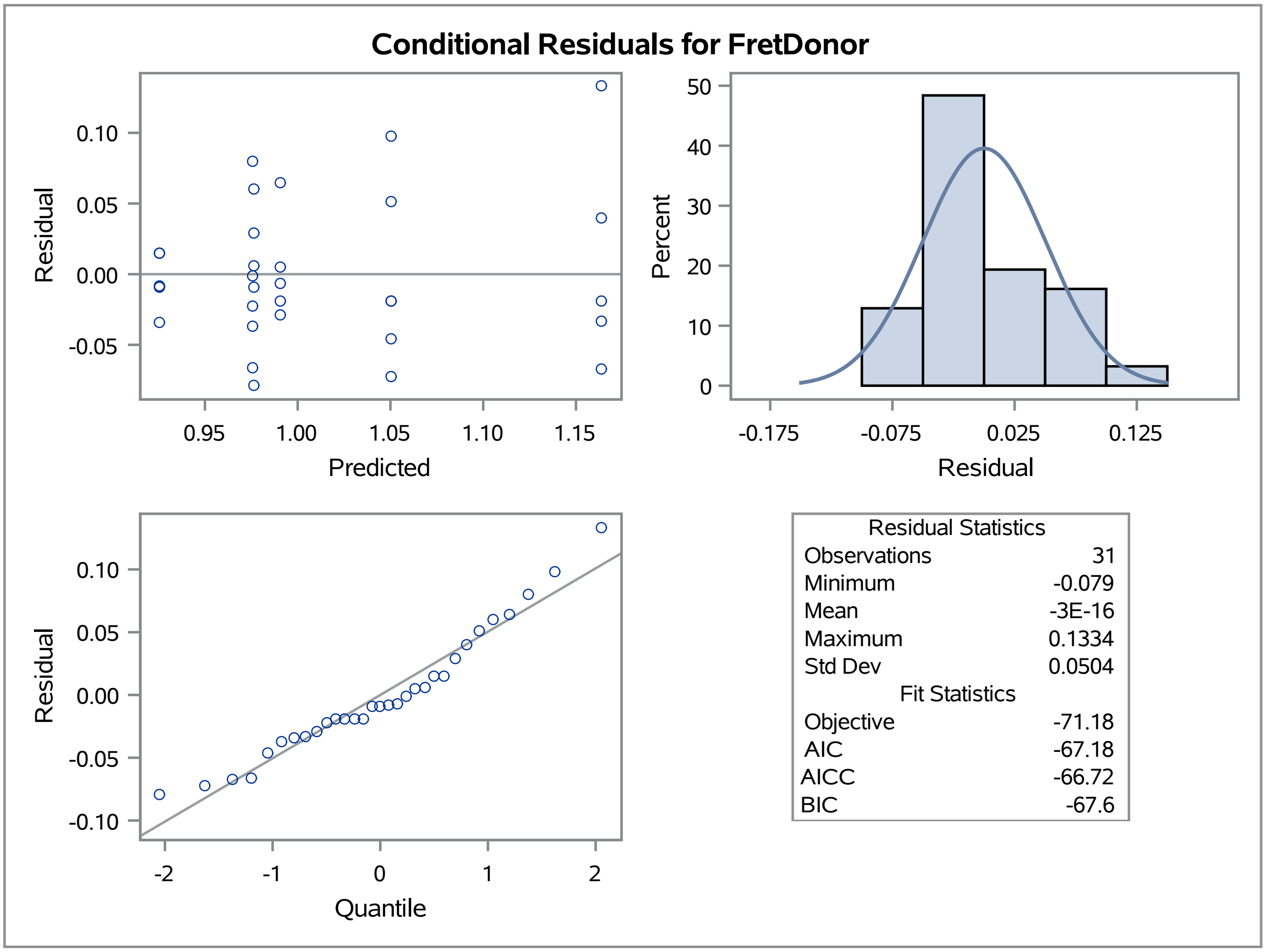

Time=2.166667

Model Information	
Data Set	WORK.TEMPDATASORTED
Dependent Variable	FretDonor
Covariance Structure	Variance Components
Estimation Method	REML
Residual Variance Method	Profile
Fixed Effects SE Method	Model-Based
Degrees of Freedom Method	Containment

Class Level Information		
Class	Levels	Values
Genotype	2	Meg+/- TTR KO TTR KO
Culture	6	1 2 3 4 5 6

Dimensions	
Covariance Parameters	2
Columns in X	3
Columns in Z	6
Subjects	1
Max Obs per Subject	31

Number of Observations	
Number of Observations Read	31
Number of Observations Used	31
Number of Observations Not Used	0

Iteration History			
Iteration	Evaluations	-2 Res Log Like	Criterion
0	1	-63.70091349	
1	2	-65.94468379	0.00000008
2	1	-65.94468873	0.00000000

Convergence criteria met.

Covariance Parameter Estimates				
Cov Parm	Estimate	Alpha	Lower	Upper
Culture	0.001509	0.05	0.000371	0.1338
Residual	0.004328	0.05	0.002661	0.008253

Time=2.166667

Fit Statistics	
-2 Res Log Likelihood	-65.9
AIC (Smaller is Better)	-61.9
AICC (Smaller is Better)	-61.5
BIC (Smaller is Better)	-62.4

Solution for Fixed Effects

Effect	Genotype	Estimate	Standard Error	DF	t Value	Pr > |t|	Alpha	Lower	Upper
Intercept		0.9939	0.02784	4	35.71	<.0001	0.05	0.9166	1.0712
Genotype	Meg+/- TTR KO	-0.02446	0.03958	25	-0.62	0.5421	0.05	-0.1060	0.05705
Genotype	TTR KO	0	.	.	.	.	.	.	.

Solution for Random Effects

Effect	Culture	Estimate	Std Err Pred	DF	t Value	Pr > |t|	Alpha	Lower	Upper
Culture	1	0.04281	0.02938	25	1.46	0.1575	0.05	-0.01769	0.1033
Culture	2	-0.01669	0.02903	25	-0.57	0.5705	0.05	-0.07648	0.04310
Culture	3	-0.02612	0.02938	25	-0.89	0.3824	0.05	-0.08663	0.03438
Culture	4	-0.02687	0.02949	25	-0.91	0.3710	0.05	-0.08761	0.03387
Culture	5	0.01340	0.02949	25	0.45	0.6536	0.05	-0.04734	0.07413
Culture	6	0.01347	0.02949	25	0.46	0.6517	0.05	-0.04727	0.07421

Type 3 Tests of Fixed Effects

Effect	Num DF	Den DF	F Value	Pr > F
Genotype	1	25	0.38	0.5421

Least Squares Means

Effect	Genotype	Estimate	Standard Error	DF	t Value	Pr > |t|	Alpha	Lower	Upper
Genotype	Meg+/- TTR KO	0.9694	0.02813	25	34.46	<.0001	0.05	0.9115	1.0274
Genotype	TTR KO	0.9939	0.02784	25	35.71	<.0001	0.05	0.9366	1.0512

Time=2.166667

Differences of Least Squares Means												
**Effect**	**Genotype**	**Genotype**	**Estimate**	**Standard Error**	**DF**	**t Value**	**Pr > |t|**	**Adjustment**	**Adj P**	**Alpha**	**Lower**	**Upper**
**Genotype**	Meg+/- TTR KO	TTR KO	-0.02446	0.03958	25	-0.62	0.5421	Tukey-Kramer	0.5421	0.05	-0.1060	0.05705

Differences of Least Squares Means				
**Effect**	**Genotype**	**Genotype**	**Adj Lower**	**Adj Upper**
**Genotype**	Meg+/- TTR KO	TTR KO	-0.1060	0.05705

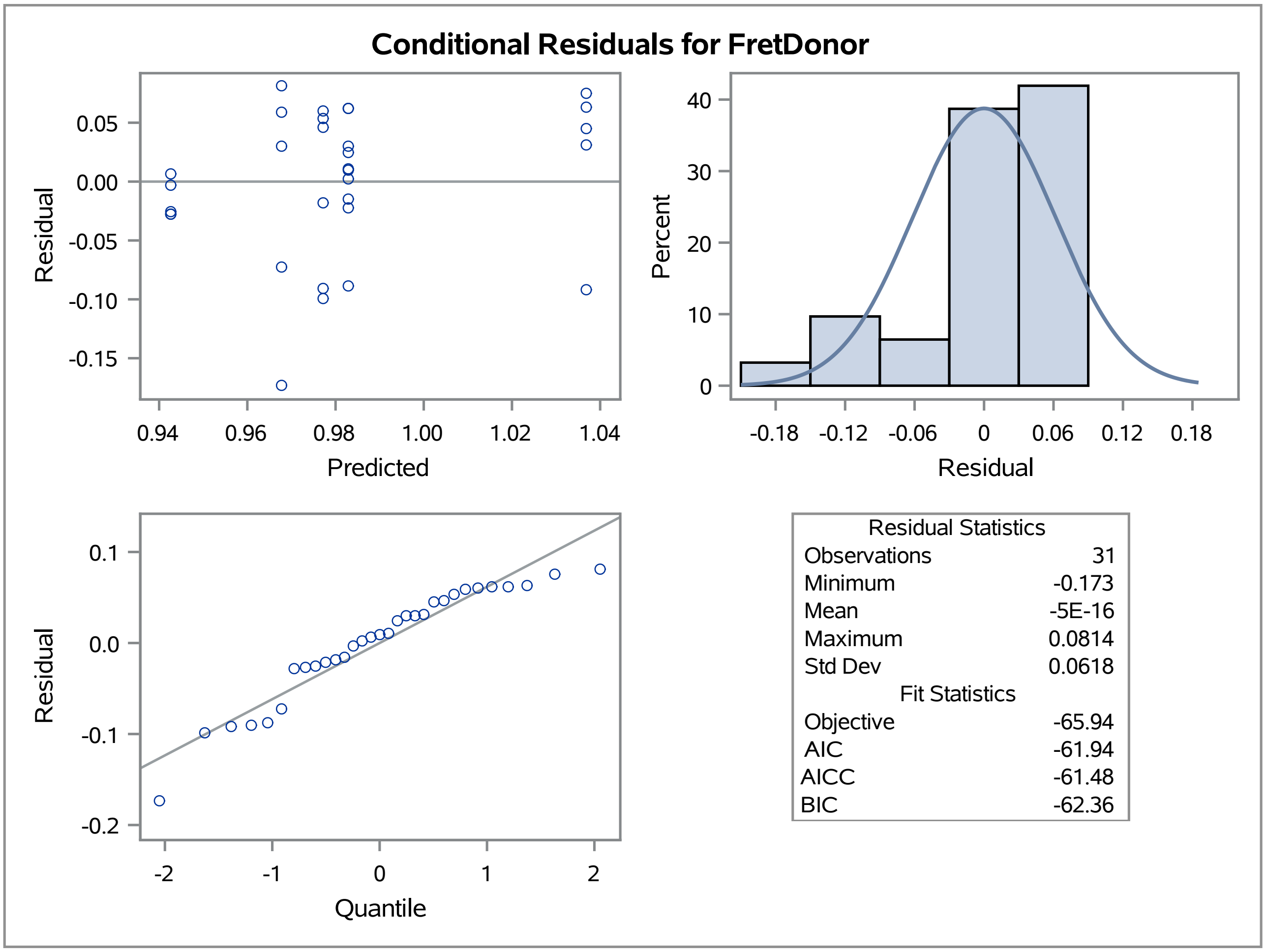

Time=2.333333

Model Information	
**Data Set**	WORK.TEMPDATASORTED
**Dependent Variable**	FretDonor
**Covariance Structure**	Variance Components
**Estimation Method**	REML
**Residual Variance Method**	Profile
**Fixed Effects SE Method**	Model-Based
**Degrees of Freedom Method**	Containment

Class Level Information		
**Class**	**Levels**	**Values**
**Genotype**	2	Meg+/- TTR KO TTR KO
**Culture**	6	1 2 3 4 5 6

Dimensions	
**Covariance Parameters**	2
**Columns in X**	3
**Columns in Z**	6
**Subjects**	1
**Max Obs per Subject**	31

Number of Observations	
**Number of Observations Read**	31
**Number of Observations Used**	31
**Number of Observations Not Used**	0

Iteration History			
**Iteration**	**Evaluations**	**-2 Res Log Like**	**Criterion**
**0**	1	-68.96496683	
**1**	2	-69.63712912	0.00000009
**2**	1	-69.63713483	0.00000000

Convergence criteria met.

Covariance Parameter Estimates				
**Cov Parm**	**Estimate**	**Alpha**	**Lower**	**Upper**
**Culture**	0.000642	0.05	0.000112	6.5453
**Residual**	0.004044	0.05	0.002484	0.007720

Time=2.333333

Fit Statistics	
-2 Res Log Likelihood	-69.6
AIC (Smaller is Better)	-65.6
AICC (Smaller is Better)	-65.2
BIC (Smaller is Better)	-66.1

Solution for Fixed Effects

Effect	Genotype	Estimate	Standard Error	DF	t Value	Pr > |t|	Alpha	Lower	Upper
Intercept		0.9982	0.02162	4	46.17	<.0001	0.05	0.9382	1.0583
Genotype	Meg+/- TTR KO	-0.03230	0.03084	25	-1.05	0.3049	0.05	-0.09581	0.03121
Genotype	TTR KO	0	.	.	.	.	.	.	.

Solution for Random Effects

Effect	Culture	Estimate	Std Err Pred	DF	t Value	Pr > |t|	Alpha	Lower	Upper
Culture	1	0.01465	0.02120	25	0.69	0.4958	0.05	-0.02901	0.05831
Culture	2	0.004084	0.02097	25	0.19	0.8472	0.05	-0.03911	0.04728
Culture	3	-0.01873	0.02120	25	-0.88	0.3852	0.05	-0.06239	0.02492
Culture	4	-0.01949	0.02127	25	-0.92	0.3683	0.05	-0.06330	0.02432
Culture	5	0.01085	0.02127	25	0.51	0.6146	0.05	-0.03296	0.05465
Culture	6	0.008642	0.02127	25	0.41	0.6880	0.05	-0.03517	0.05245

Type 3 Tests of Fixed Effects

Effect	Num DF	Den DF	F Value	Pr > F
Genotype	1	25	1.10	0.3049

Least Squares Means

Effect	Genotype	Estimate	Standard Error	DF	t Value	Pr > |t|	Alpha	Lower	Upper
Genotype	Meg+/- TTR KO	0.9659	0.02199	25	43.93	<.0001	0.05	0.9206	1.0112
Genotype	TTR KO	0.9982	0.02162	25	46.17	<.0001	0.05	0.9537	1.0428

Time=2.333333

Differences of Least Squares Means												
**Effect**	**Genotype**	**Genotype**	**Estimate**	**Standard Error**	**DF**	**t Value**	**Pr > |t|**	**Adjustment**	**Adj P**	**Alpha**	**Lower**	**Upper**
**Genotype**	Meg+/- TTR KO	TTR KO	-0.03230	0.03084	25	-1.05	0.3049	Tukey-Kramer	0.3049	0.05	-0.09581	0.03121

Differences of Least Squares Means				
**Effect**	**Genotype**	**Genotype**	**Adj Lower**	**Adj Upper**
**Genotype**	Meg+/- TTR KO	TTR KO	-0.09581	0.03121

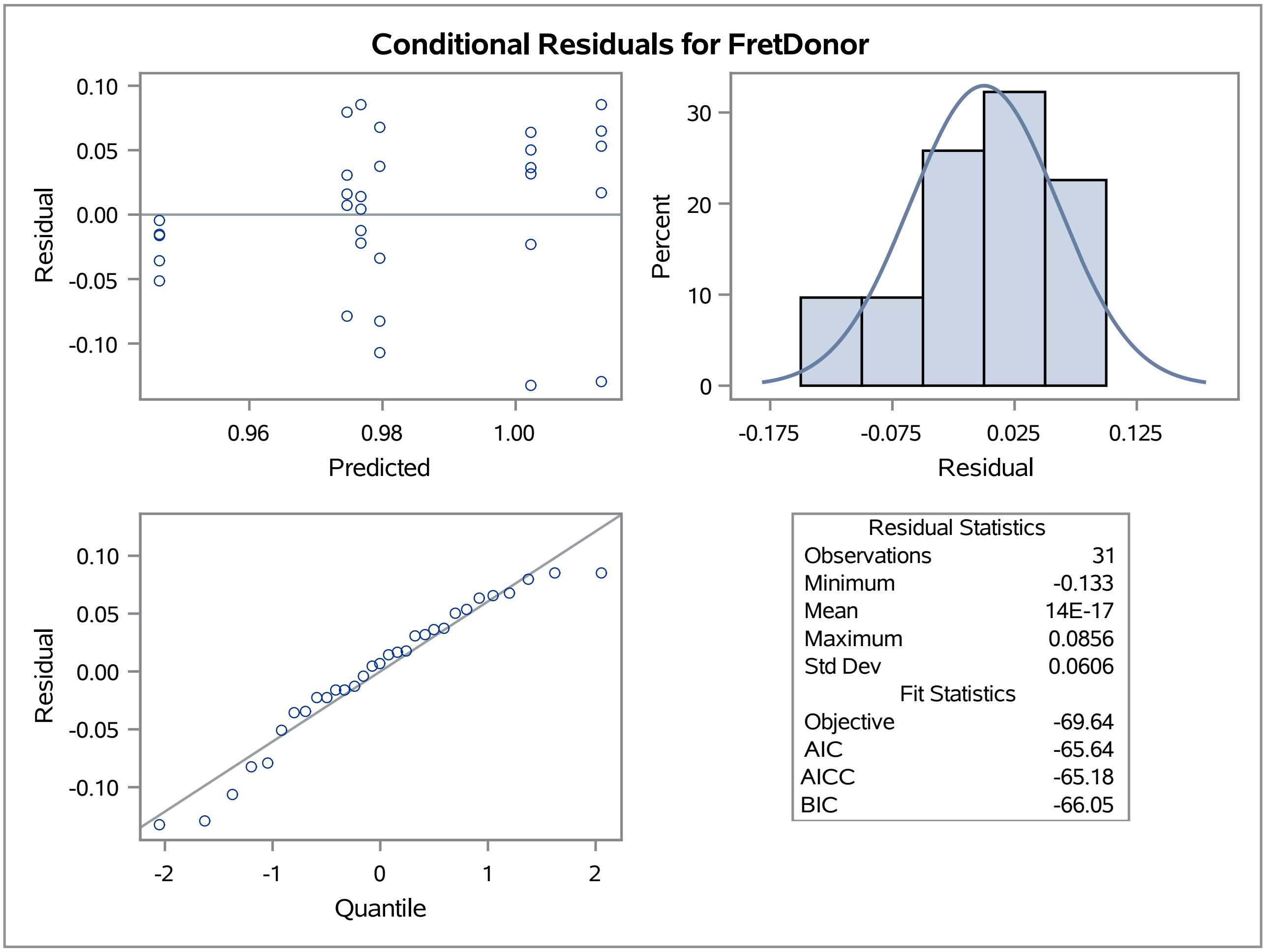

Time=2.5

Model Information	
**Data Set**	WORK.TEMPDATASORTED
**Dependent Variable**	FretDonor
**Covariance Structure**	Variance Components
**Estimation Method**	REML
**Residual Variance Method**	Profile
**Fixed Effects SE Method**	Model-Based
**Degrees of Freedom Method**	Containment

Class Level Information		
**Class**	**Levels**	**Values**
**Genotype**	2	Meg+/- TTR KO TTR KO
**Culture**	6	1 2 3 4 5 6

Dimensions	
**Covariance Parameters**	2
**Columns in X**	3
**Columns in Z**	6
**Subjects**	1
**Max Obs per Subject**	31

Number of Observations	
**Number of Observations Read**	31
**Number of Observations Used**	31
**Number of Observations Not Used**	0

Iteration History			
**Iteration**	**Evaluations**	**-2 Res Log Like**	**Criterion**
**0**	1	-63.98715444	
**1**	2	-70.66846784	0.00000268
**2**	1	-70.66863584	0.00000000

Convergence criteria met.

Covariance Parameter Estimates				
**Cov Parm**	**Estimate**	**Alpha**	**Lower**	**Upper**
**Culture**	0.002847	0.05	0.000862	0.05440
**Residual**	0.003362	0.05	0.002067	0.006410

Time=2.5

Fit Statistics	
-2 Res Log Likelihood	-70.7
AIC (Smaller is Better)	-66.7
AICC (Smaller is Better)	-66.2
BIC (Smaller is Better)	-67.1

Solution for Fixed Effects									
Effect	Genotype	Estimate	Standard Error	DF	t Value	Pr > |t|	Alpha	Lower	Upper
Intercept		0.9771	0.03406	4	28.69	<.0001	0.05	0.8826	1.0717
Genotype	Meg+/- TTR KO	-0.00573	0.04830	25	-0.12	0.9066	0.05	-0.1052	0.09376
Genotype	TTR KO	0	.	.	.	.	.	.	.

Solution for Random Effects									
Effect	Culture	Estimate	Std Err Pred	DF	t Value	Pr > |t|	Alpha	Lower	Upper
Culture	1	0.05325	0.03610	25	1.48	0.1527	0.05	-0.02110	0.1276
Culture	2	0.000889	0.03575	25	0.02	0.9803	0.05	-0.07274	0.07452
Culture	3	-0.05414	0.03610	25	-1.50	0.1462	0.05	-0.1285	0.02021
Culture	4	-0.04416	0.03621	25	-1.22	0.2341	0.05	-0.1187	0.03042
Culture	5	0.03897	0.03621	25	1.08	0.2921	0.05	-0.03561	0.1136
Culture	6	0.005186	0.03621	25	0.14	0.8873	0.05	-0.06940	0.07977

Type 3 Tests of Fixed Effects				
Effect	Num DF	Den DF	F Value	Pr > F
Genotype	1	25	0.01	0.9066

Least Squares Means									
Effect	Genotype	Estimate	Standard Error	DF	t Value	Pr > |t|	Alpha	Lower	Upper
Genotype	Meg+/- TTR KO	0.9714	0.03425	25	28.36	<.0001	0.05	0.9009	1.0419
Genotype	TTR KO	0.9771	0.03406	25	28.69	<.0001	0.05	0.9070	1.0473

Time=2.5

Differences of Least Squares Means												
**Effect**	**Genotype**	**Genotype**	**Estimate**	**Standard Error**	**DF**	**t Value**	**Pr > |t|**	**Adjustment**	**Adj P**	**Alpha**	**Lower**	**Upper**
**Genotype**	Meg+/- TTR KO	TTR KO	-0.00573	0.04830	25	-0.12	0.9066	Tukey-Kramer	0.9066	0.05	-0.1052	0.09376

Differences of Least Squares Means				
**Effect**	**Genotype**	**Genotype**	**Adj Lower**	**Adj Upper**
**Genotype**	Meg+/- TTR KO	TTR KO	-0.1052	0.09376

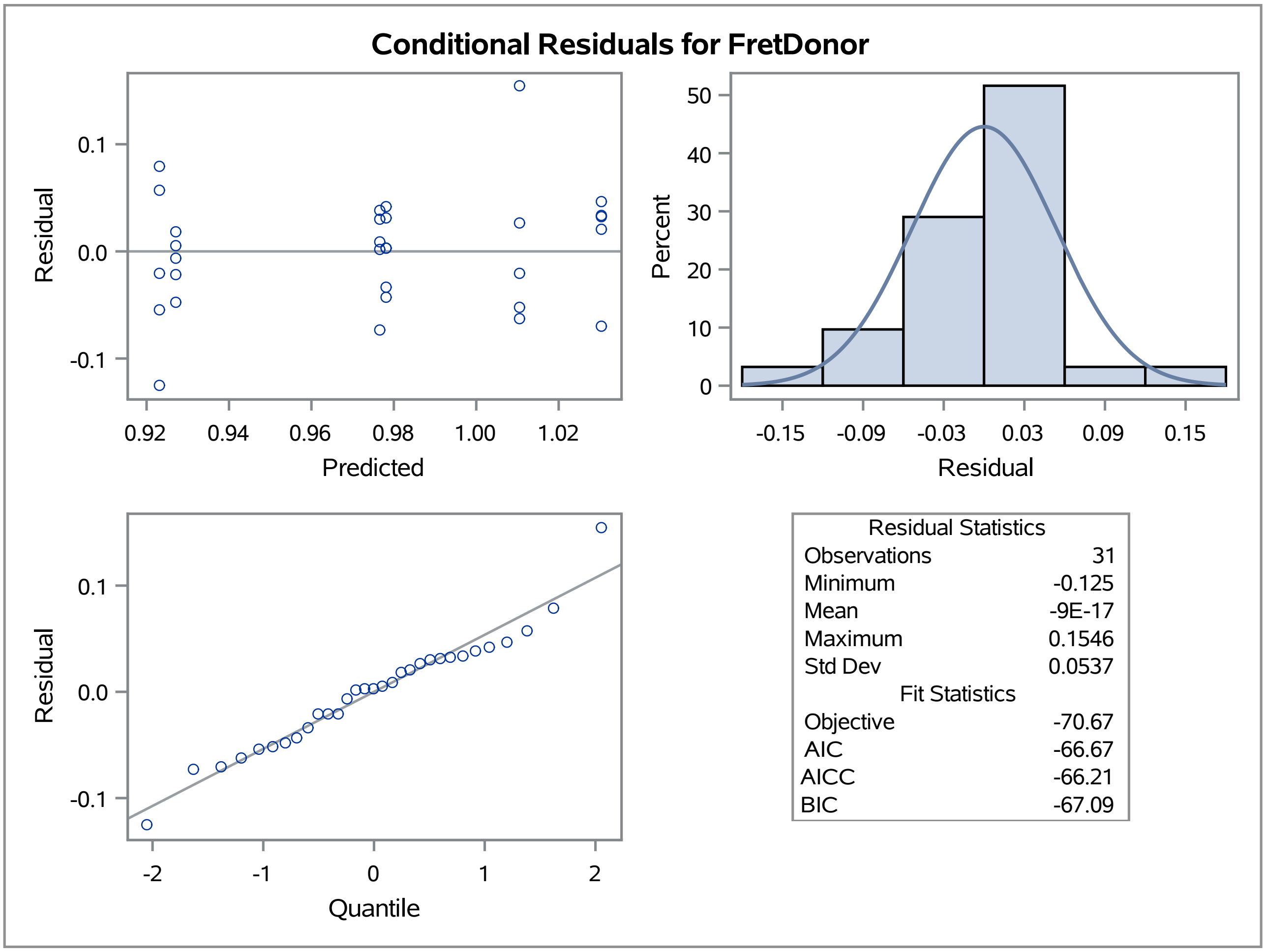

Time=2.666667

Model Information	
**Data Set**	WORK.TEMPDATASORTED
**Dependent Variable**	FretDonor
**Covariance Structure**	Variance Components
**Estimation Method**	REML
**Residual Variance Method**	Profile
**Fixed Effects SE Method**	Model-Based
**Degrees of Freedom Method**	Containment

Class Level Information		
**Class**	**Levels**	**Values**
**Genotype**	2	Meg+/- TTR KO TTR KO
**Culture**	6	1 2 3 4 5 6

Dimensions	
**Covariance Parameters**	2
**Columns in X**	3
**Columns in Z**	6
**Subjects**	1
**Max Obs per Subject**	31

Number of Observations	
**Number of Observations Read**	31
**Number of Observations Used**	31
**Number of Observations Not Used**	0

Iteration History			
**Iteration**	**Evaluations**	**-2 Res Log Like**	**Criterion**
**0**	1	-71.12370977	
**1**	2	-75.47593846	0.00000000

Convergence criteria met.

Covariance Parameter Estimates				
**Cov Parm**	**Estimate**	**Alpha**	**Lower**	**Upper**
**Culture**	0.001685	0.05	0.000475	0.05034
**Residual**	0.002974	0.05	0.001829	0.005667

Time=2.666667

Fit Statistics	
-2 Res Log Likelihood	-75.5
AIC (Smaller is Better)	-71.5
AICC (Smaller is Better)	-71.0
BIC (Smaller is Better)	-71.9

Solution for Fixed Effects

Effect	Genotype	Estimate	Standard Error	DF	t Value	Pr > |t|	Alpha	Lower	Upper
Intercept		1.0013	0.02736	4	36.59	<.0001	0.05	0.9253	1.0773
Genotype	Meg+/- TTR KO	-0.03975	0.03884	25	-1.02	0.3160	0.05	-0.1197	0.04025
Genotype	TTR KO	0	.	.	.	.	.	.	.

Solution for Random Effects

Effect	Culture	Estimate	Std Err Pred	DF	t Value	Pr > |t|	Alpha	Lower	Upper
Culture	1	0.04500	0.02913	25	1.54	0.1350	0.05	-0.01500	0.1050
Culture	2	-0.02890	0.02881	25	-1.00	0.3255	0.05	-0.08823	0.03044
Culture	3	-0.01610	0.02913	25	-0.55	0.5854	0.05	-0.07610	0.04390
Culture	4	-0.03452	0.02924	25	-1.18	0.2488	0.05	-0.09474	0.02569
Culture	5	0.02477	0.02924	25	0.85	0.4049	0.05	-0.03544	0.08499
Culture	6	0.009750	0.02924	25	0.33	0.7415	0.05	-0.05046	0.06997

Type 3 Tests of Fixed Effects

Effect	Num DF	Den DF	F Value	Pr > F
Genotype	1	25	1.05	0.3160

Least Squares Means

Effect	Genotype	Estimate	Standard Error	DF	t Value	Pr > |t|	Alpha	Lower	Upper
Genotype	Meg+/- TTR KO	0.9615	0.02757	25	34.88	<.0001	0.05	0.9048	1.0183
Genotype	TTR KO	1.0013	0.02736	25	36.59	<.0001	0.05	0.9449	1.0576

Time=2.666667

Differences of Least Squares Means												
**Effect**	**Genotype**	**Genotype**	**Estimate**	**Standard Error**	**DF**	**t Value**	**Pr > |t|**	**Adjustment**	**Adj P**	**Alpha**	**Lower**	**Upper**
**Genotype**	Meg+/- TTR KO	TTR KO	-0.03975	0.03884	25	-1.02	0.3160	Tukey-Kramer	0.3160	0.05	-0.1197	0.04025

Differences of Least Squares Means				
**Effect**	**Genotype**	**Genotype**	**Adj Lower**	**Adj Upper**
**Genotype**	Meg+/- TTR KO	TTR KO	-0.1197	0.04025

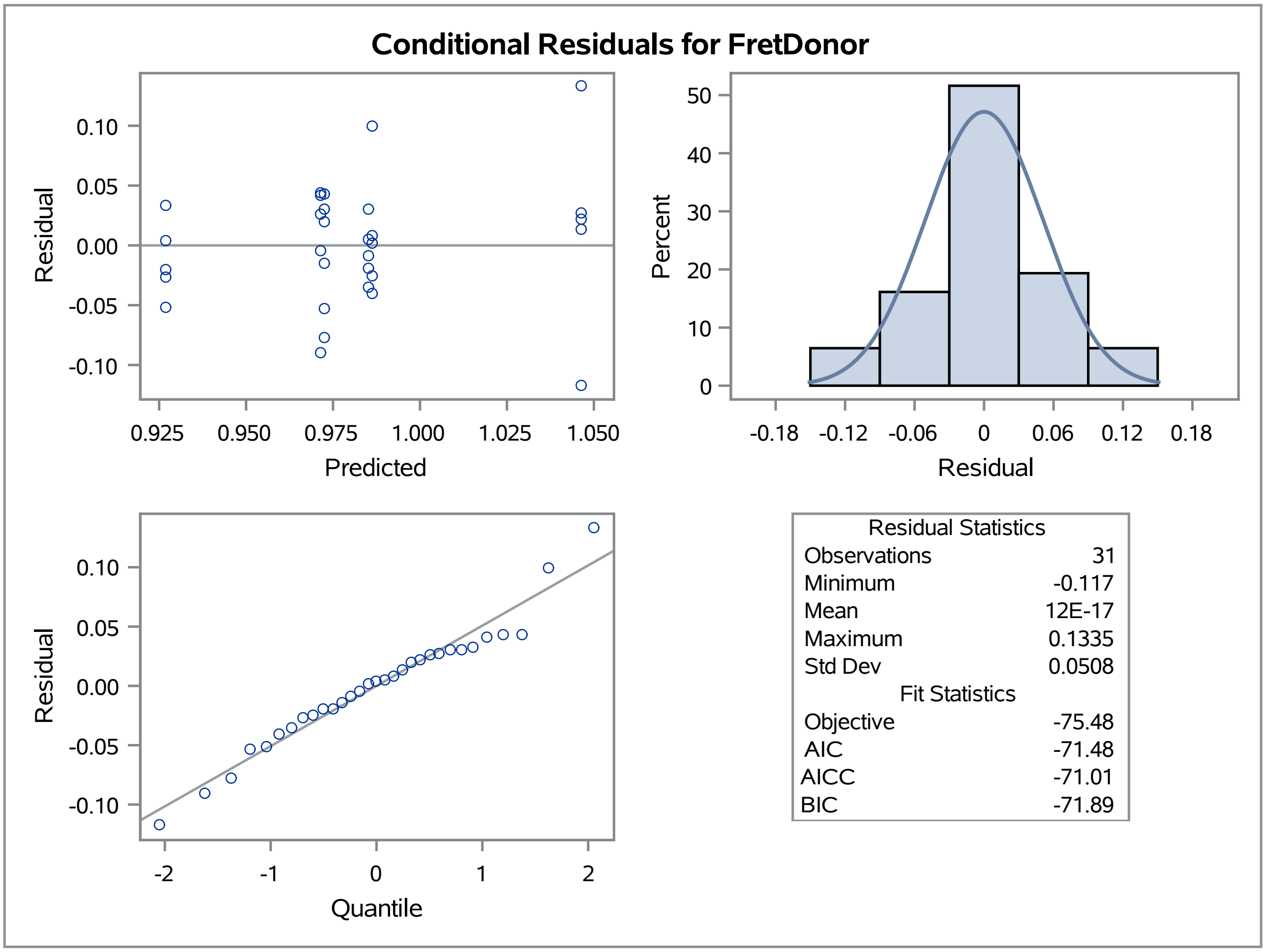

Time=2.833333

Model Information	
**Data Set**	WORK.TEMPDATASORTED
**Dependent Variable**	FretDonor
**Covariance Structure**	Variance Components
**Estimation Method**	REML
**Residual Variance Method**	Profile
**Fixed Effects SE Method**	Model-Based
**Degrees of Freedom Method**	Containment

Class Level Information		
**Class**	**Levels**	**Values**
**Genotype**	2	Meg+/- TTR KO TTR KO
**Culture**	6	1 2 3 4 5 6

Dimensions	
**Covariance Parameters**	2
**Columns in X**	3
**Columns in Z**	6
**Subjects**	1
**Max Obs per Subject**	31

Number of Observations	
**Number of Observations Read**	31
**Number of Observations Used**	31
**Number of Observations Not Used**	0

Iteration History			
**Iteration**	**Evaluations**	**-2 Res Log Like**	**Criterion**
**0**	1	-63.03005402	
**1**	2	-83.79545865	0.00001866
**2**	1	-83.79677818	0.00000005
**3**	1	-83.79678131	0.00000000

Convergence criteria met.

Time=2.833333

Covariance Parameter Estimates				
**Cov Parm**	**Estimate**	**Alpha**	**Lower**	**Upper**
**Culture**	0.005317	0.05	0.001813	0.05502
**Residual**	0.001840	0.05	0.001132	0.003508

Fit Statistics	
**-2 Res Log Likelihood**	-83.8
**AIC (Smaller is Better)**	-79.8
**AICC (Smaller is Better)**	-79.3
**BIC (Smaller is Better)**	-80.2

Solution for Fixed Effects									
**Effect**	**Genotype**	**Estimate**	**Standard Error**	**DF**	**t Value**	**Pr > |t|**	**Alpha**	**Lower**	**Upper**
**Intercept**		0.9950	0.04345	4	22.90	<.0001	0.05	0.8743	1.1156
**Genotype**	Meg+/- TTR KO	-0.03636	0.06151	25	-0.59	0.5597	0.05	-0.1630	0.09032
**Genotype**	TTR KO	0	.	.	.	.	.	.	.

Solution for Random Effects									
**Effect**	**Culture**	**Estimate**	**Std Err Pred**	**DF**	**t Value**	**Pr > |t|**	**Alpha**	**Lower**	**Upper**
**Culture**	1	0.09050	0.04468	25	2.03	0.0536	0.05	-0.00151	0.1825
**Culture**	2	0.004743	0.04447	25	0.11	0.9159	0.05	-0.08685	0.09634
**Culture**	3	-0.09525	0.04468	25	-2.13	0.0430	0.05	-0.1873	-0.00323
**Culture**	4	-0.04192	0.04474	25	-0.94	0.3578	0.05	-0.1341	0.05023
**Culture**	5	0.02293	0.04474	25	0.51	0.6128	0.05	-0.06922	0.1151
**Culture**	6	0.01898	0.04474	25	0.42	0.6750	0.05	-0.07317	0.1111

Type 3 Tests of Fixed Effects				
**Effect**	**Num DF**	**Den DF**	**F Value**	**Pr > F**
**Genotype**	1	25	0.35	0.5597

Least Squares Means									
**Effect**	**Genotype**	**Estimate**	**Standard Error**	**DF**	**t Value**	**Pr > |t|**	**Alpha**	**Lower**	**Upper**
**Genotype**	Meg+/- TTR KO	0.9586	0.04353	25	22.02	<.0001	0.05	0.8690	1.0483
**Genotype**	TTR KO	0.9950	0.04345	25	22.90	<.0001	0.05	0.9055	1.0845

Time=2.833333

Differences of Least Squares Means												
Effect	Genotype	Genotype	Estimate	Standard Error	DF	t Value	Pr > |t|	Adjustment	Adj P	Alpha	Lower	Upper
**Genotype**	Meg+/- TTR KO	TTR KO	-0.03636	0.06151	25	-0.59	0.5597	Tukey-Kramer	0.5597	0.05	-0.1630	0.09032

Differences of Least Squares Means				
Effect	Genotype	Genotype	Adj Lower	Adj Upper
**Genotype**	Meg+/- TTR KO	TTR KO	-0.1630	0.09032

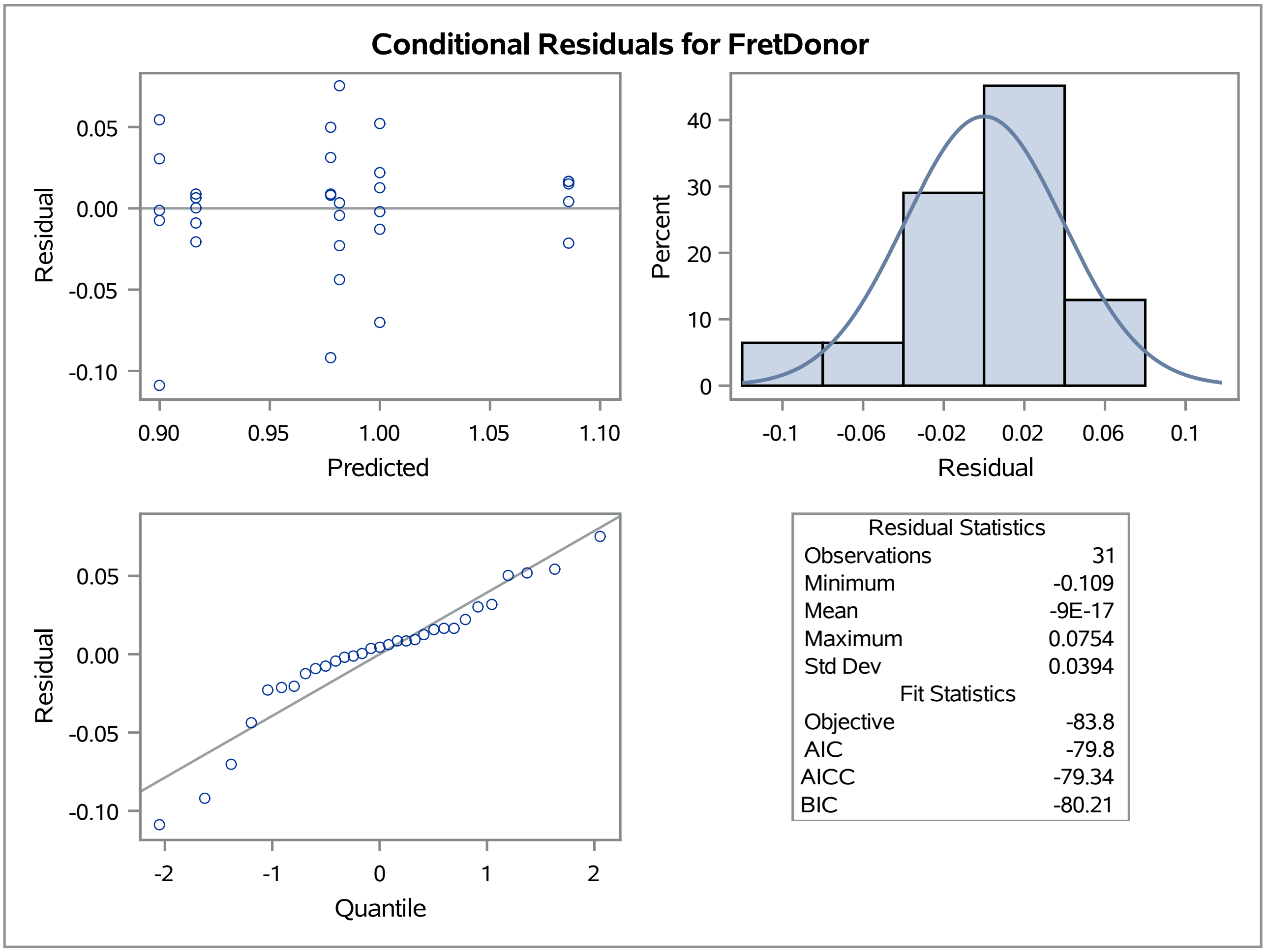

Time=3

Model Information	
**Data Set**	WORK.TEMPDATASORTED
**Dependent Variable**	FretDonor
**Covariance Structure**	Variance Components
**Estimation Method**	REML
**Residual Variance Method**	Profile
**Fixed Effects SE Method**	Model-Based
**Degrees of Freedom Method**	Containment

Class Level Information		
**Class**	**Levels**	**Values**
**Genotype**	2	Meg+/- TTR KO TTR KO
**Culture**	6	1 2 3 4 5 6

Dimensions	
**Covariance Parameters**	2
**Columns in X**	3
**Columns in Z**	6
**Subjects**	1
**Max Obs per Subject**	31

Number of Observations	
**Number of Observations Read**	31
**Number of Observations Used**	31
**Number of Observations Not Used**	0

Iteration History			
**Iteration**	**Evaluations**	**-2 Res Log Like**	**Criterion**
**0**	1	-69.99801384	
**1**	2	-78.84269716	0.00000023
**2**	1	-78.84271268	0.00000000

Convergence criteria met.

Covariance Parameter Estimates				
**Cov Parm**	**Estimate**	**Alpha**	**Lower**	**Upper**
**Culture**	0.002664	0.05	0.000837	0.04132
**Residual**	0.002466	0.05	0.001517	0.004701

Time=3

Fit Statistics	
-2 Res Log Likelihood	-78.8
AIC (Smaller is Better)	-74.8
AICC (Smaller is Better)	-74.4
BIC (Smaller is Better)	-75.3

Solution for Fixed Effects

Effect	Genotype	Estimate	Standard Error	DF	t Value	Pr > |t|	Alpha	Lower	Upper
Intercept		0.9927	0.03230	4	30.74	<.0001	0.05	0.9031	1.0824
Genotype	Meg+/- TTR KO	-0.03866	0.04578	25	-0.84	0.4064	0.05	-0.1329	0.05562
Genotype	TTR KO	0	.	.	.	.	.	.	.

Solution for Random Effects

Effect	Culture	Estimate	Std Err Pred	DF	t Value	Pr > |t|	Alpha	Lower	Upper
Culture	1	0.04005	0.03404	25	1.18	0.2504	0.05	-0.03006	0.1102
Culture	2	0.02805	0.03375	25	0.83	0.4138	0.05	-0.04146	0.09756
Culture	3	-0.06810	0.03404	25	-2.00	0.0564	0.05	-0.1382	0.002007
Culture	4	-0.03652	0.03414	25	-1.07	0.2950	0.05	-0.1068	0.03379
Culture	5	0.01913	0.03414	25	0.56	0.5803	0.05	-0.05118	0.08944
Culture	6	0.01739	0.03414	25	0.51	0.6149	0.05	-0.05292	0.08770

Type 3 Tests of Fixed Effects

Effect	Num DF	Den DF	F Value	Pr > F
Genotype	1	25	0.71	0.4064

Least Squares Means

Effect	Genotype	Estimate	Standard Error	DF	t Value	Pr > |t|	Alpha	Lower	Upper
Genotype	Meg+/- TTR KO	0.9541	0.03244	25	29.41	<.0001	0.05	0.8873	1.0209
Genotype	TTR KO	0.9927	0.03230	25	30.74	<.0001	0.05	0.9262	1.0592

Time=3

Differences of Least Squares Means												
**Effect**	**Genotype**	**Genotype**	**Estimate**	**Standard Error**	**DF**	**t Value**	**Pr > |t|**	**Adjustment**	**Adj P**	**Alpha**	**Lower**	**Upper**
**Genotype**	Meg+/- TTR KO	TTR KO	-0.03866	0.04578	25	-0.84	0.4064	Tukey-Kramer	0.4064	0.05	-0.1329	0.05562

Differences of Least Squares Means				
**Effect**	**Genotype**	**Genotype**	**Adj Lower**	**Adj Upper**
**Genotype**	Meg+/- TTR KO	TTR KO	-0.1329	0.05562

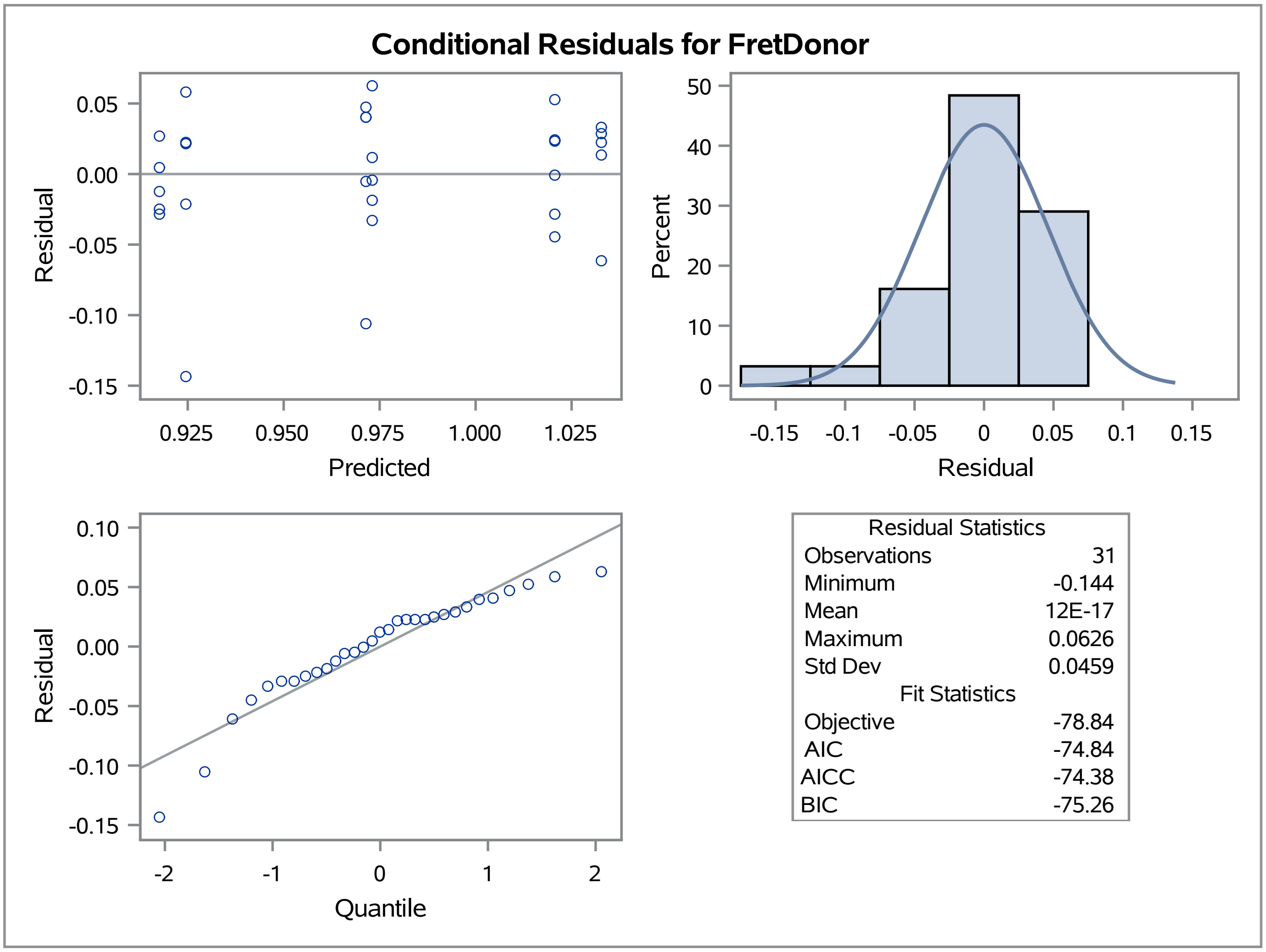

Time=3.166667

Model Information	
**Data Set**	WORK.TEMPDATASORTED
**Dependent Variable**	FretDonor
**Covariance Structure**	Variance Components
**Estimation Method**	REML
**Residual Variance Method**	Profile
**Fixed Effects SE Method**	Model-Based
**Degrees of Freedom Method**	Containment

Class Level Information		
**Class**	**Levels**	**Values**
**Genotype**	2	Meg+/- TTR KO TTR KO
**Culture**	6	1 2 3 4 5 6

Dimensions	
**Covariance Parameters**	2
**Columns in X**	3
**Columns in Z**	6
**Subjects**	1
**Max Obs per Subject**	31

Number of Observations	
**Number of Observations Read**	31
**Number of Observations Used**	31
**Number of Observations Not Used**	0

Iteration History			
**Iteration**	**Evaluations**	**-2 Res Log Like**	**Criterion**
**0**	1	-65.17447918	
**1**	2	-73.69151281	0.00000002
**2**	1	-73.69151437	0.00000000

Convergence criteria met.

Covariance Parameter Estimates				
**Cov Parm**	**Estimate**	**Alpha**	**Lower**	**Upper**
**Culture**	0.003060	0.05	0.000957	0.04876
**Residual**	0.002961	0.05	0.001821	0.005642

Time=3.166667

Fit Statistics	
-2 Res Log Likelihood	-73.7
AIC (Smaller is Better)	-69.7
AICC (Smaller is Better)	-69.2
BIC (Smaller is Better)	-70.1

Solution for Fixed Effects

Effect	Genotype	Estimate	Standard Error	DF	t Value	Pr > |t|	Alpha	Lower	Upper
Intercept		0.9986	0.03473	4	28.75	<.0001	0.05	0.9022	1.0950
Genotype	Meg+/- TTR KO	-0.04397	0.04923	25	-0.89	0.3803	0.05	-0.1454	0.05742
Genotype	TTR KO	0	.	.	.	.	.	.	.

Solution for Random Effects

Effect	Culture	Estimate	Std Err Pred	DF	t Value	Pr > |t|	Alpha	Lower	Upper
Culture	1	0.06742	0.03665	25	1.84	0.0777	0.05	-0.00805	0.1429
Culture	2	-0.03556	0.03632	25	-0.98	0.3370	0.05	-0.1104	0.03926
Culture	3	-0.03187	0.03665	25	-0.87	0.3928	0.05	-0.1073	0.04361
Culture	4	-0.04559	0.03675	25	-1.24	0.2263	0.05	-0.1213	0.03010
Culture	5	0.009393	0.03675	25	0.26	0.8004	0.05	-0.06630	0.08509
Culture	6	0.03620	0.03675	25	0.98	0.3341	0.05	-0.03949	0.1119

Type 3 Tests of Fixed Effects

Effect	Num DF	Den DF	F Value	Pr > F
Genotype	1	25	0.80	0.3803

Least Squares Means

Effect	Genotype	Estimate	Standard Error	DF	t Value	Pr > |t|	Alpha	Lower	Upper
Genotype	Meg+/- TTR KO	0.9546	0.03489	25	27.36	<.0001	0.05	0.8828	1.0265
Genotype	TTR KO	0.9986	0.03473	25	28.75	<.0001	0.05	0.9271	1.0701

Time=3.166667

Differences of Least Squares Means												
**Effect**	**Genotype**	**Genotype**	**Estimate**	**Standard Error**	**DF**	**t Value**	**Pr > |t|**	**Adjustment**	**Adj P**	**Alpha**	**Lower**	**Upper**
**Genotype**	Meg+/- TTR KO	TTR KO	-0.04397	0.04923	25	-0.89	0.3803	Tukey-Kramer	0.3803	0.05	-0.1454	0.05742

Differences of Least Squares Means				
**Effect**	**Genotype**	**Genotype**	**Adj Lower**	**Adj Upper**
**Genotype**	Meg+/- TTR KO	TTR KO	-0.1454	0.05742

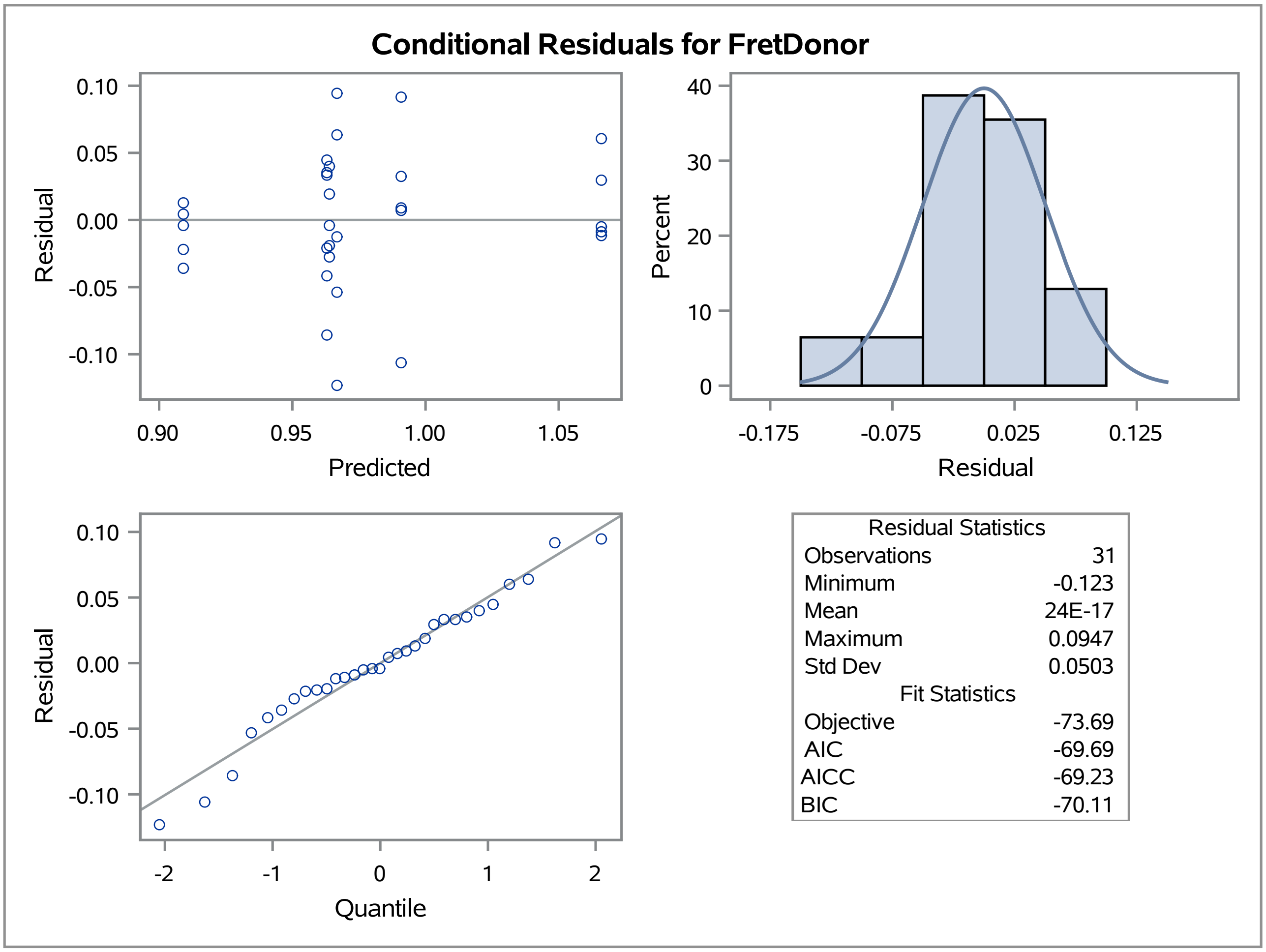

Time=3.333333

Model Information	
**Data Set**	WORK.TEMPDATASORTED
**Dependent Variable**	FretDonor
**Covariance Structure**	Variance Components
**Estimation Method**	REML
**Residual Variance Method**	Profile
**Fixed Effects SE Method**	Model-Based
**Degrees of Freedom Method**	Containment

Class Level Information		
**Class**	**Levels**	**Values**
**Genotype**	2	Meg+/- TTR KO TTR KO
**Culture**	6	1 2 3 4 5 6

Dimensions	
**Covariance Parameters**	2
**Columns in X**	3
**Columns in Z**	6
**Subjects**	1
**Max Obs per Subject**	31

Number of Observations	
**Number of Observations Read**	31
**Number of Observations Used**	31
**Number of Observations Not Used**	0

Iteration History			
**Iteration**	**Evaluations**	**-2 Res Log Like**	**Criterion**
**0**	1	-50.04226641	
**1**	2	-64.87464934	0.00000885
**2**	1	-64.87518336	0.00000001

Convergence criteria met.

Covariance Parameter Estimates				
**Cov Parm**	**Estimate**	**Alpha**	**Lower**	**Upper**
**Culture**	0.007078	0.05	0.002351	0.08274
**Residual**	0.003728	0.05	0.002293	0.007106

Time=3.333333

Fit Statistics	
-2 Res Log Likelihood	-64.9
AIC (Smaller is Better)	-60.9
AICC (Smaller is Better)	-60.4
BIC (Smaller is Better)	-61.3

Solution for Fixed Effects

Effect	Genotype	Estimate	Standard Error	DF	t Value	Pr > |t|	Alpha	Lower	Upper
Intercept		1.0368	0.05093	4	20.36	<.0001	0.05	0.8954	1.1782
Genotype	Meg+/- TTR KO	-0.09169	0.07212	25	-1.27	0.2153	0.05	-0.2402	0.05685
Genotype	TTR KO	0	.	.	.	.	.	.	.

Solution for Random Effects

Effect	Culture	Estimate	Std Err Pred	DF	t Value	Pr > |t|	Alpha	Lower	Upper
Culture	1	0.1146	0.05289	25	2.17	0.0400	0.05	0.005670	0.2235
Culture	2	-0.01339	0.05257	25	-0.25	0.8010	0.05	-0.1217	0.09487
Culture	3	-0.1012	0.05289	25	-1.91	0.0672	0.05	-0.2101	0.007722
Culture	4	-0.03659	0.05300	25	-0.69	0.4963	0.05	-0.1457	0.07256
Culture	5	0.01029	0.05300	25	0.19	0.8477	0.05	-0.09887	0.1194
Culture	6	0.02631	0.05300	25	0.50	0.6240	0.05	-0.08285	0.1355

Type 3 Tests of Fixed Effects

Effect	Num DF	Den DF	F Value	Pr > F
Genotype	1	25	1.62	0.2153

Least Squares Means

Effect	Genotype	Estimate	Standard Error	DF	t Value	Pr > |t|	Alpha	Lower	Upper
Genotype	Meg+/- TTR KO	0.9451	0.05107	25	18.51	<.0001	0.05	0.8400	1.0503
Genotype	TTR KO	1.0368	0.05093	25	20.36	<.0001	0.05	0.9319	1.1417

Time=3.333333

Differences of Least Squares Means												
Effect	Genotype	Genotype	Estimate	Standard Error	DF	t Value	Pr > |t|	Adjustment	Adj P	Alpha	Lower	Upper
Genotype	Meg+/- TTR KO	TTR KO	-0.09169	0.07212	25	-1.27	0.2153	Tukey-Kramer	0.2153	0.05	-0.2402	0.05685

Differences of Least Squares Means				
Effect	Genotype	Genotype	Adj Lower	Adj Upper
Genotype	Meg+/- TTR KO	TTR KO	-0.2402	0.05685

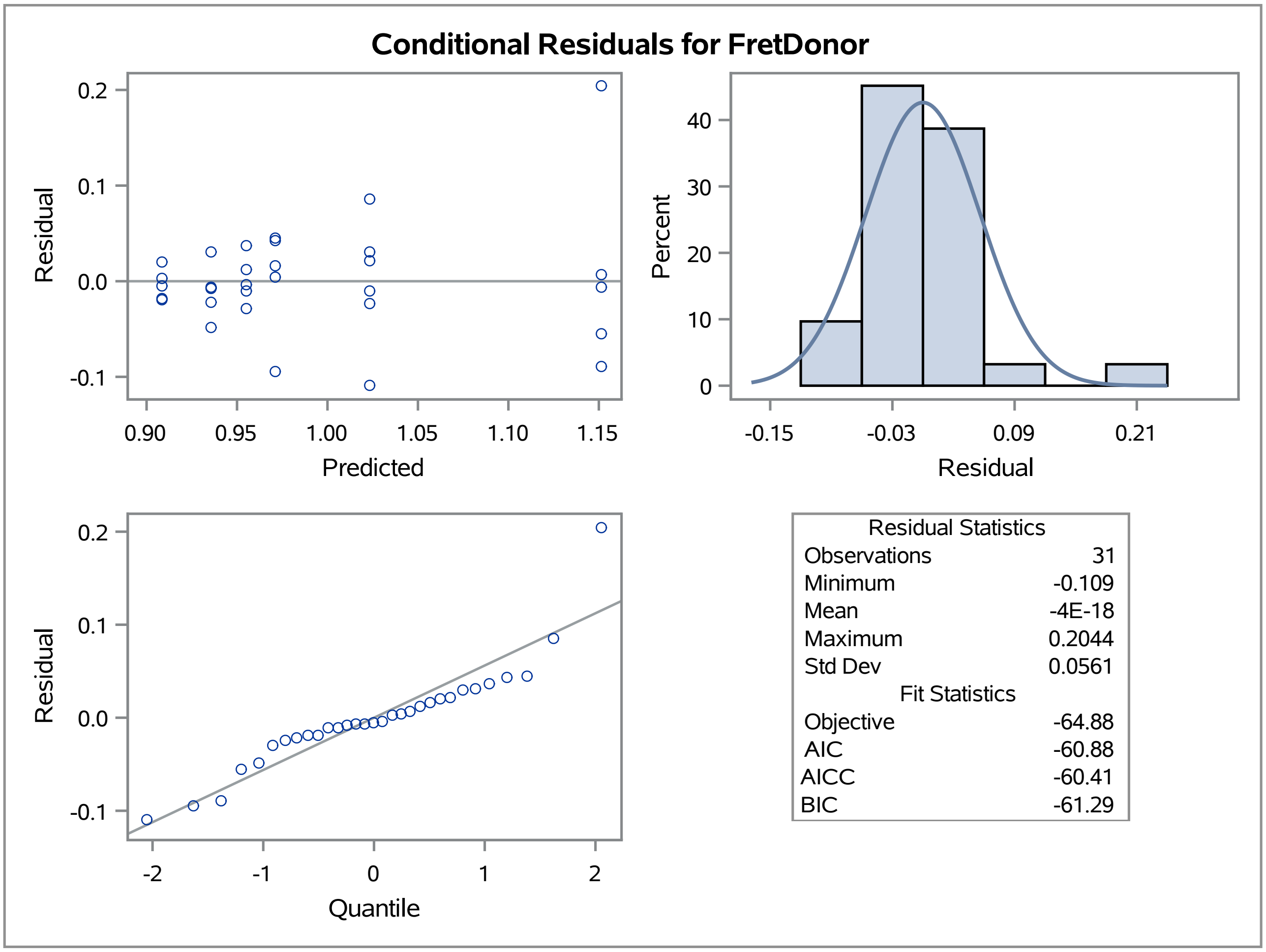

Time=3.5

Model Information	
**Data Set**	WORK.TEMPDATASORTED
**Dependent Variable**	FretDonor
**Covariance Structure**	Variance Components
**Estimation Method**	REML
**Residual Variance Method**	Profile
**Fixed Effects SE Method**	Model-Based
**Degrees of Freedom Method**	Containment

Class Level Information		
**Class**	**Levels**	**Values**
**Genotype**	2	Meg+/- TTR KO TTR KO
**Culture**	6	1 2 3 4 5 6

Dimensions	
**Covariance Parameters**	2
**Columns in X**	3
**Columns in Z**	6
**Subjects**	1
**Max Obs per Subject**	31

Number of Observations	
**Number of Observations Read**	31
**Number of Observations Used**	31
**Number of Observations Not Used**	0

Iteration History			
**Iteration**	**Evaluations**	**-2 Res Log Like**	**Criterion**
**0**	1	-71.29885742	
**1**	2	-89.88596925	0.00001393
**2**	1	-89.88699459	0.00000003
**3**	1	-89.88699651	0.00000000

Convergence criteria met.

Time=3.5

Covariance Parameter Estimates				
**Cov Parm**	**Estimate**	**Alpha**	**Lower**	**Upper**
**Culture**	0.003801	0.05	0.001286	0.04079
**Residual**	0.001520	0.05	0.000935	0.002897

Fit Statistics	
**-2 Res Log Likelihood**	-89.9
**AIC (Smaller is Better)**	-85.9
**AICC (Smaller is Better)**	-85.4
**BIC (Smaller is Better)**	-86.3

Solution for Fixed Effects									
**Effect**	**Genotype**	**Estimate**	**Standard Error**	**DF**	**t Value**	**Pr > |t|**	**Alpha**	**Lower**	**Upper**
**Intercept**		0.9983	0.03691	4	27.04	<.0001	0.05	0.8958	1.1007
**Genotype**	Meg+/- TTR KO	-0.06043	0.05226	25	-1.16	0.2585	0.05	-0.1681	0.04720
**Genotype**	TTR KO	0	.	.	.	.	.	.	.

Solution for Random Effects									
**Effect**	**Culture**	**Estimate**	**Std Err Pred**	**DF**	**t Value**	**Pr > |t|**	**Alpha**	**Lower**	**Upper**
**Culture**	1	0.07798	0.03808	25	2.05	0.0512	0.05	-0.00044	0.1564
**Culture**	2	-0.00090	0.03788	25	-0.02	0.9813	0.05	-0.07892	0.07713
**Culture**	3	-0.07708	0.03808	25	-2.02	0.0537	0.05	-0.1555	0.001342
**Culture**	4	-0.03513	0.03814	25	-0.92	0.3658	0.05	-0.1137	0.04342
**Culture**	5	0.006740	0.03814	25	0.18	0.8612	0.05	-0.07181	0.08529
**Culture**	6	0.02839	0.03814	25	0.74	0.4635	0.05	-0.05016	0.1069

Type 3 Tests of Fixed Effects				
**Effect**	**Num DF**	**Den DF**	**F Value**	**Pr > F**
**Genotype**	1	25	1.34	0.2585

Least Squares Means									
**Effect**	**Genotype**	**Estimate**	**Standard Error**	**DF**	**t Value**	**Pr > |t|**	**Alpha**	**Lower**	**Upper**
**Genotype**	Meg+/- TTR KO	0.9378	0.03699	25	25.35	<.0001	0.05	0.8616	1.0140
**Genotype**	TTR KO	0.9983	0.03691	25	27.04	<.0001	0.05	0.9222	1.0743

Time=3.5

Differences of Least Squares Means												
**Effect**	**Genotype**	**Genotype**	**Estimate**	**Standard Error**	**DF**	**t Value**	**Pr > |t|**	**Adjustment**	**Adj P**	**Alpha**	**Lower**	**Upper**
**Genotype**	Meg+/- TTR KO	TTR KO	-0.06043	0.05226	25	-1.16	0.2585	Tukey-Kramer	0.2585	0.05	-0.1681	0.04720

Differences of Least Squares Means				
**Effect**	**Genotype**	**Genotype**	**Adj Lower**	**Adj Upper**
**Genotype**	Meg+/- TTR KO	TTR KO	-0.1681	0.04720

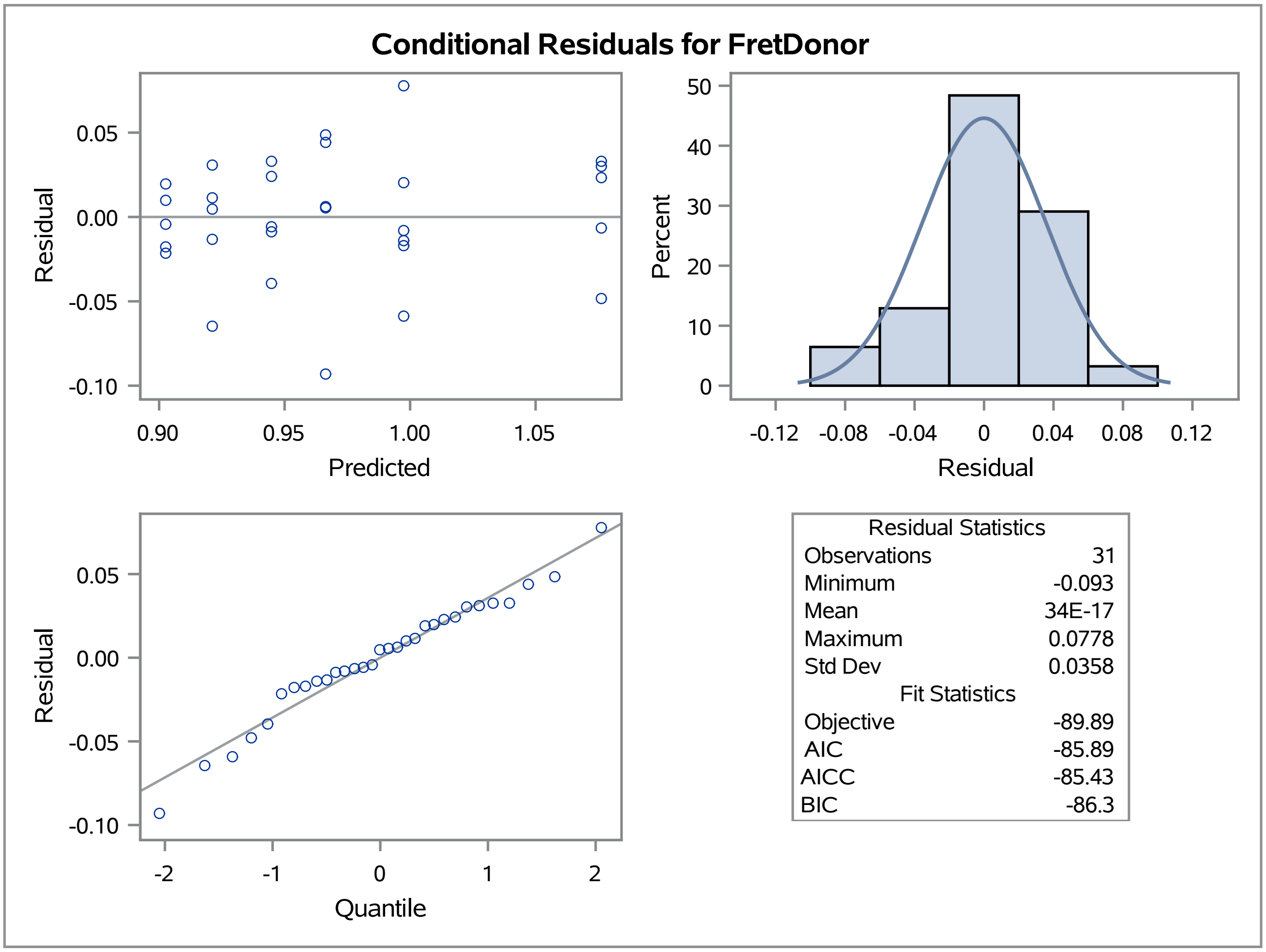

Time=3.666667

Model Information	
Data Set	WORK.TEMPDATASORTED
Dependent Variable	FretDonor
Covariance Structure	Variance Components
Estimation Method	REML
Residual Variance Method	Profile
Fixed Effects SE Method	Model-Based
Degrees of Freedom Method	Containment

Class Level Information		
Class	Levels	Values
Genotype	2	Meg+/- TTR KO TTR KO
Culture	6	1 2 3 4 5 6

Dimensions	
Covariance Parameters	2
Columns in X	3
Columns in Z	6
Subjects	1
Max Obs per Subject	31

Number of Observations	
Number of Observations Read	31
Number of Observations Used	31
Number of Observations Not Used	0

Iteration History			
Iteration	Evaluations	-2 Res Log Like	Criterion
0	1	-57.32913434	
1	2	-71.34707809	0.00000744
2	1	-71.34755069	0.00000001

Convergence criteria met.

Covariance Parameter Estimates				
Cov Parm	Estimate	Alpha	Lower	Upper
Culture	0.005350	0.05	0.001768	0.06412
Residual	0.003007	0.05	0.001849	0.005731

Time=3.666667

Fit Statistics	
-2 Res Log Likelihood	-71.3
AIC (Smaller is Better)	-67.3
AICC (Smaller is Better)	-66.9
BIC (Smaller is Better)	-67.8

Solution for Fixed Effects

Effect	Genotype	Estimate	Standard Error	DF	t Value	Pr > |t|	Alpha	Lower	Upper
Intercept		1.0033	0.04441	4	22.59	<.0001	0.05	0.8800	1.1266
Genotype	Meg+/- TTR KO	-0.05596	0.06290	25	-0.89	0.3821	0.05	-0.1855	0.07358
Genotype	TTR KO	0	.	.	.	.	.	.	.

Solution for Random Effects

Effect	Culture	Estimate	Std Err Pred	DF	t Value	Pr > |t|	Alpha	Lower	Upper
Culture	1	0.09886	0.04620	25	2.14	0.0423	0.05	0.003708	0.1940
Culture	2	-0.01194	0.04591	25	-0.26	0.7970	0.05	-0.1065	0.08261
Culture	3	-0.08693	0.04620	25	-1.88	0.0716	0.05	-0.1821	0.008227
Culture	4	-0.03487	0.04630	25	-0.75	0.4584	0.05	-0.1302	0.06049
Culture	5	0.01592	0.04630	25	0.34	0.7338	0.05	-0.07943	0.1113
Culture	6	0.01894	0.04630	25	0.41	0.6859	0.05	-0.07641	0.1143

Type 3 Tests of Fixed Effects

Effect	Num DF	Den DF	F Value	Pr > F
Genotype	1	25	0.79	0.3821

Least Squares Means

Effect	Genotype	Estimate	Standard Error	DF	t Value	Pr > |t|	Alpha	Lower	Upper
Genotype	Meg+/- TTR KO	0.9473	0.04454	25	21.27	<.0001	0.05	0.8556	1.0391
Genotype	TTR KO	1.0033	0.04441	25	22.59	<.0001	0.05	0.9118	1.0948

Time=3.666667

Differences of Least Squares Means												
**Effect**	**Genotype**	**Genotype**	**Estimate**	**Standard Error**	**DF**	**t Value**	**Pr > |t|**	**Adjustment**	**Adj P**	**Alpha**	**Lower**	**Upper**
**Genotype**	Meg+/- TTR KO	TTR KO	-0.05596	0.06290	25	-0.89	0.3821	Tukey-Kramer	0.3821	0.05	-0.1855	0.07358

Differences of Least Squares Means				
**Effect**	**Genotype**	**Genotype**	**Adj Lower**	**Adj Upper**
**Genotype**	Meg+/- TTR KO	TTR KO	-0.1855	0.07358

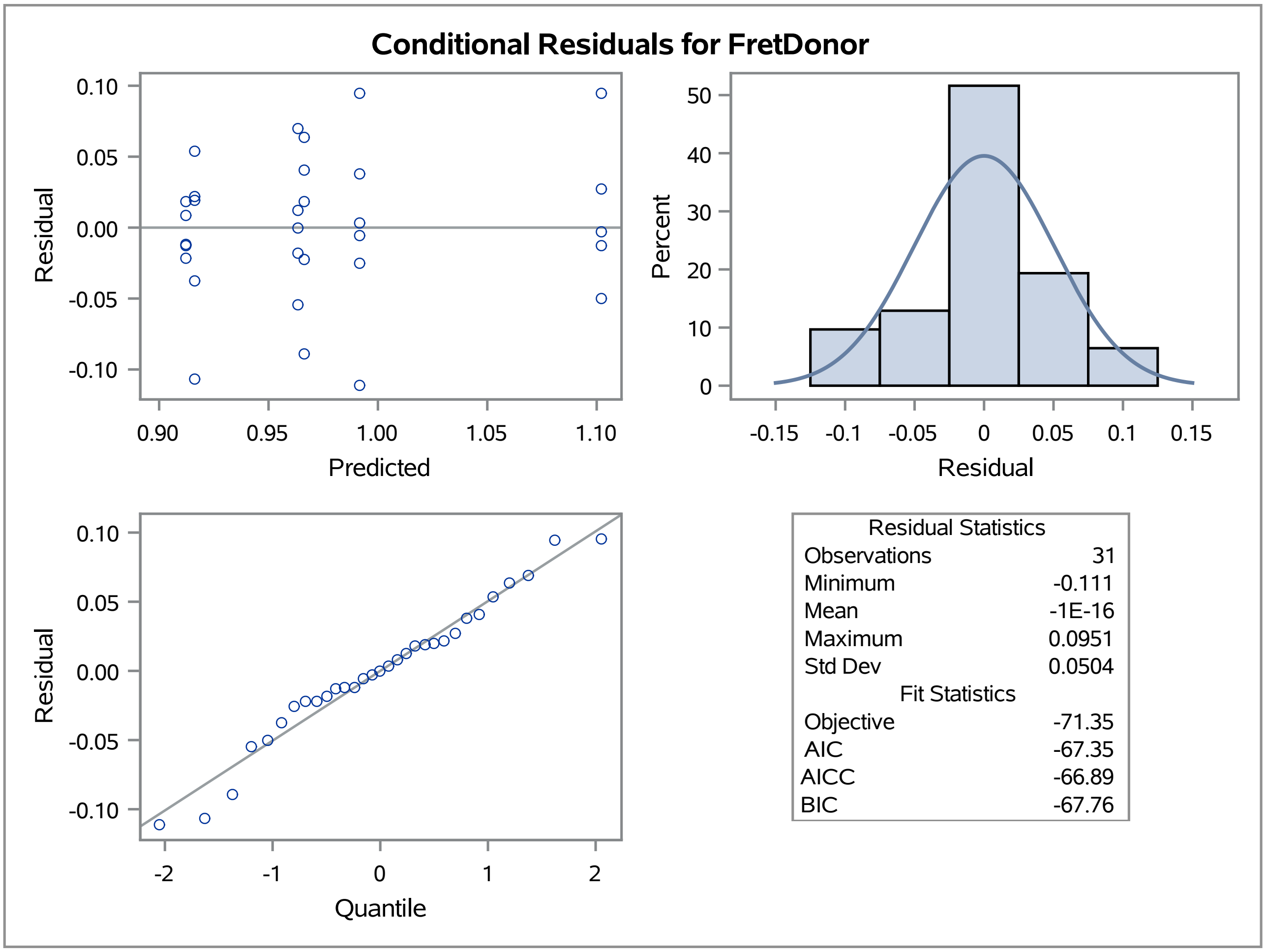

Time=3.833333

Model Information	
**Data Set**	WORK.TEMPDATASORTED
**Dependent Variable**	FretDonor
**Covariance Structure**	Variance Components
**Estimation Method**	REML
**Residual Variance Method**	Profile
**Fixed Effects SE Method**	Model-Based
**Degrees of Freedom Method**	Containment

Class Level Information		
**Class**	**Levels**	**Values**
**Genotype**	2	Meg+/- TTR KO TTR KO
**Culture**	6	1 2 3 4 5 6

Dimensions	
**Covariance Parameters**	2
**Columns in X**	3
**Columns in Z**	6
**Subjects**	1
**Max Obs per Subject**	31

Number of Observations	
**Number of Observations Read**	31
**Number of Observations Used**	31
**Number of Observations Not Used**	0

Iteration History			
**Iteration**	**Evaluations**	**-2 Res Log Like**	**Criterion**
**0**	1	-52.42235530	
**1**	2	-70.02634435	0.00001332
**2**	1	-70.02718688	0.00000002
**3**	1	-70.02718818	0.00000000

Convergence criteria met.

Time=3.833333

Covariance Parameter Estimates				
**Cov Parm**	**Estimate**	**Alpha**	**Lower**	**Upper**
**Culture**	0.007099	0.05	0.002391	0.07768
**Residual**	0.003041	0.05	0.001870	0.005796

Fit Statistics	
**-2 Res Log Likelihood**	-70.0
**AIC (Smaller is Better)**	-66.0
**AICC (Smaller is Better)**	-65.6
**BIC (Smaller is Better)**	-66.4

Solution for Fixed Effects									
**Effect**	**Genotype**	**Estimate**	**Standard Error**	**DF**	**t Value**	**Pr > |t|**	**Alpha**	**Lower**	**Upper**
**Intercept**		1.0189	0.05057	4	20.15	<.0001	0.05	0.8785	1.1594
**Genotype**	Meg+/- TTR KO	-0.07853	0.07160	25	-1.10	0.2832	0.05	-0.2260	0.06894
**Genotype**	TTR KO	0	.	.	.	.	.	.	.

Solution for Random Effects									
**Effect**	**Culture**	**Estimate**	**Std Err Pred**	**DF**	**t Value**	**Pr > |t|**	**Alpha**	**Lower**	**Upper**
**Culture**	1	0.1136	0.05225	25	2.17	0.0394	0.05	0.005940	0.2212
**Culture**	2	-0.00916	0.05197	25	-0.18	0.8616	0.05	-0.1162	0.09789
**Culture**	3	-0.1044	0.05225	25	-2.00	0.0567	0.05	-0.2120	0.003217
**Culture**	4	-0.03947	0.05234	25	-0.75	0.4579	0.05	-0.1473	0.06834
**Culture**	5	0.02028	0.05234	25	0.39	0.7017	0.05	-0.08752	0.1281
**Culture**	6	0.01919	0.05234	25	0.37	0.7170	0.05	-0.08862	0.1270

Type 3 Tests of Fixed Effects				
**Effect**	**Num DF**	**Den DF**	**F Value**	**Pr > F**
**Genotype**	1	25	1.20	0.2832

Least Squares Means									
**Effect**	**Genotype**	**Estimate**	**Standard Error**	**DF**	**t Value**	**Pr > |t|**	**Alpha**	**Lower**	**Upper**
**Genotype**	Meg+/- TTR KO	0.9404	0.05069	25	18.55	<.0001	0.05	0.8360	1.0448
**Genotype**	TTR KO	1.0189	0.05057	25	20.15	<.0001	0.05	0.9148	1.1231

Time=3.833333

Differences of Least Squares Means												
Effect	Genotype	Genotype	Estimate	Standard Error	DF	t Value	Pr > |t|	Adjustment	Adj P	Alpha	Lower	Upper
Genotype	Meg+/- TTR KO	TTR KO	-0.07853	0.07160	25	-1.10	0.2832	Tukey-Kramer	0.2832	0.05	-0.2260	0.06894

Differences of Least Squares Means				
Effect	Genotype	Genotype	Adj Lower	Adj Upper
Genotype	Meg+/- TTR KO	TTR KO	-0.2260	0.06894

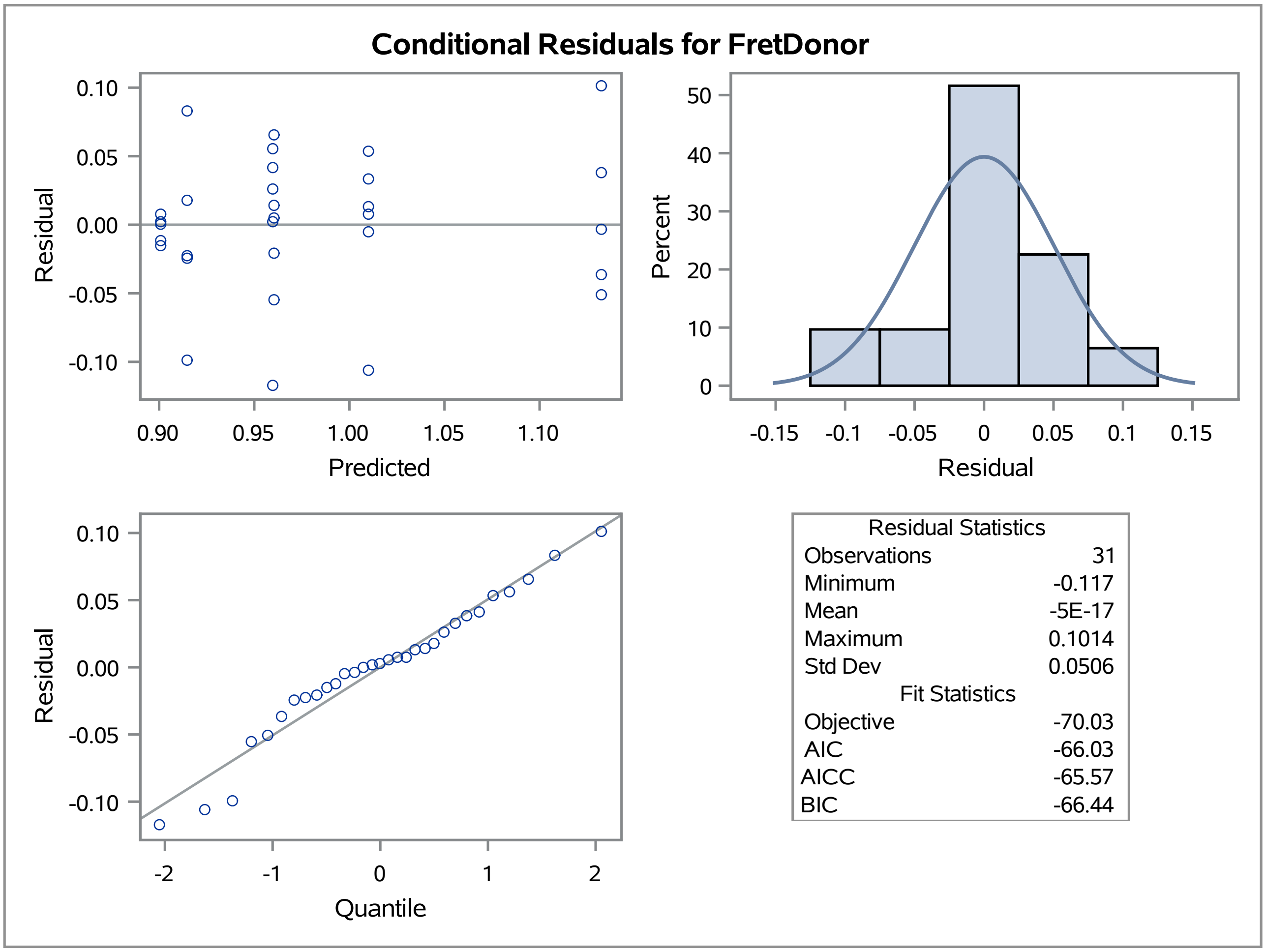

Time=4

Model Information	
**Data Set**	WORK.TEMPDATASORTED
**Dependent Variable**	FretDonor
**Covariance Structure**	Variance Components
**Estimation Method**	REML
**Residual Variance Method**	Profile
**Fixed Effects SE Method**	Model-Based
**Degrees of Freedom Method**	Containment

Class Level Information		
**Class**	**Levels**	**Values**
**Genotype**	2	Meg+/- TTR KO TTR KO
**Culture**	6	1 2 3 4 5 6

Dimensions	
**Covariance Parameters**	2
**Columns in X**	3
**Columns in Z**	6
**Subjects**	1
**Max Obs per Subject**	31

Number of Observations	
**Number of Observations Read**	31
**Number of Observations Used**	31
**Number of Observations Not Used**	0

Iteration History			
**Iteration**	**Evaluations**	**-2 Res Log Like**	**Criterion**
**0**	1	-75.01119038	
**1**	2	-84.04431010	0.00000341
**2**	1	-84.04454742	0.00000000

Convergence criteria met.

Covariance Parameter Estimates				
**Cov Parm**	**Estimate**	**Alpha**	**Lower**	**Upper**
**Culture**	0.002303	0.05	0.000726	0.03512
**Residual**	0.002052	0.05	0.001262	0.003912

Time=4

Fit Statistics	
-2 Res Log Likelihood	-84.0
AIC (Smaller is Better)	-80.0
AICC (Smaller is Better)	-79.6
BIC (Smaller is Better)	-80.5

Solution for Fixed Effects

Effect	Genotype	Estimate	Standard Error	DF	t Value	Pr > |t|	Alpha	Lower	Upper
Intercept		0.9956	0.02995	4	33.25	<.0001	0.05	0.9125	1.0788
Genotype	Meg+/- TTR KO	-0.06194	0.04244	25	-1.46	0.1569	0.05	-0.1493	0.02547
Genotype	TTR KO	0	.	.	.	.	.	.	.

Solution for Random Effects

Effect	Culture	Estimate	Std Err Pred	DF	t Value	Pr > |t|	Alpha	Lower	Upper
Culture	1	0.05205	0.03153	25	1.65	0.1113	0.05	-0.01289	0.1170
Culture	2	0.005786	0.03127	25	0.19	0.8547	0.05	-0.05861	0.07018
Culture	3	-0.05784	0.03153	25	-1.83	0.0786	0.05	-0.1228	0.007107
Culture	4	-0.03371	0.03162	25	-1.07	0.2965	0.05	-0.09884	0.03141
Culture	5	0.01132	0.03162	25	0.36	0.7234	0.05	-0.05381	0.07644
Culture	6	0.02239	0.03162	25	0.71	0.4853	0.05	-0.04273	0.08752

Type 3 Tests of Fixed Effects

Effect	Num DF	Den DF	F Value	Pr > F
Genotype	1	25	2.13	0.1569

Least Squares Means

Effect	Genotype	Estimate	Standard Error	DF	t Value	Pr > |t|	Alpha	Lower	Upper
Genotype	Meg+/- TTR KO	0.9337	0.03007	25	31.05	<.0001	0.05	0.8718	0.9956
Genotype	TTR KO	0.9956	0.02995	25	33.25	<.0001	0.05	0.9340	1.0573

Time=4

Differences of Least Squares Means												
Effect	Genotype	Genotype	Estimate	Standard Error	DF	t Value	Pr > |t|	Adjustment	Adj P	Alpha	Lower	Upper
Genotype	Meg+/- TTR KO	TTR KO	-0.06194	0.04244	25	-1.46	0.1569	Tukey-Kramer	0.1569	0.05	-0.1493	0.02547

Differences of Least Squares Means				
Effect	Genotype	Genotype	Adj Lower	Adj Upper
Genotype	Meg+/- TTR KO	TTR KO	-0.1493	0.02547

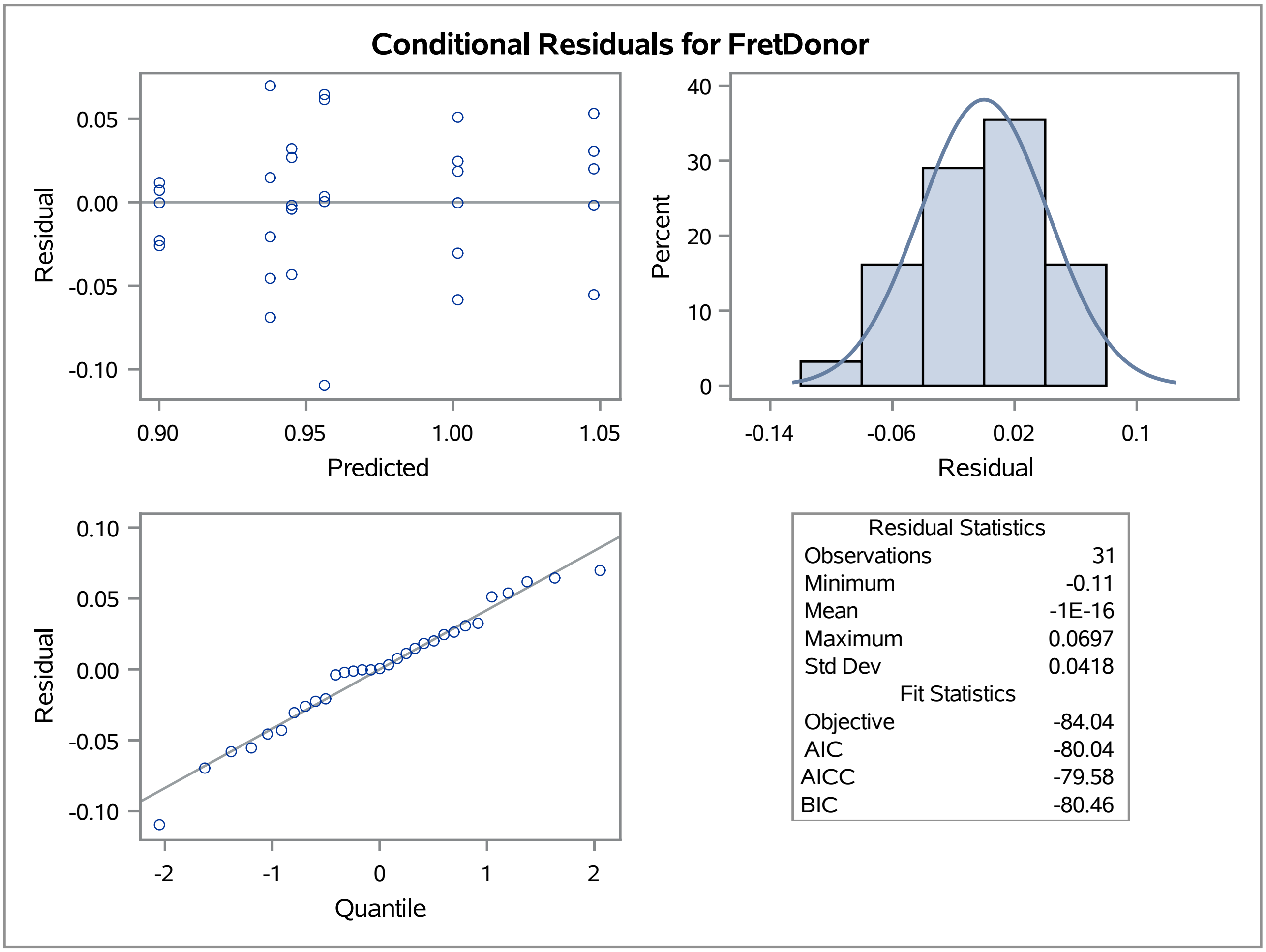

Time=4.166667

Model Information	
**Data Set**	WORK.TEMPDATASORTED
**Dependent Variable**	FretDonor
**Covariance Structure**	Variance Components
**Estimation Method**	REML
**Residual Variance Method**	Profile
**Fixed Effects SE Method**	Model-Based
**Degrees of Freedom Method**	Containment

Class Level Information		
**Class**	**Levels**	**Values**
**Genotype**	2	Meg+/- TTR KO TTR KO
**Culture**	6	1 2 3 4 5 6

Dimensions	
**Covariance Parameters**	2
**Columns in X**	3
**Columns in Z**	6
**Subjects**	1
**Max Obs per Subject**	31

Number of Observations	
**Number of Observations Read**	31
**Number of Observations Used**	31
**Number of Observations Not Used**	0

Iteration History			
**Iteration**	**Evaluations**	**-2 Res Log Like**	**Criterion**
**0**	1	-81.05807203	
**1**	2	-81.72855197	0.00000008
**2**	1	-81.72855769	0.00000000

Convergence criteria met.

Covariance Parameter Estimates				
**Cov Parm**	**Estimate**	**Alpha**	**Lower**	**Upper**
**Culture**	0.000422	0.05	0.000073	4.3787
**Residual**	0.002665	0.05	0.001638	0.005089

Time=4.166667

Fit Statistics	
**-2 Res Log Likelihood**	-81.7
**AIC (Smaller is Better)**	-77.7
**AICC (Smaller is Better)**	-77.3
**BIC (Smaller is Better)**	-78.1

Solution for Fixed Effects

Effect	Genotype	Estimate	Standard Error	DF	t Value	Pr > |t|	Alpha	Lower	Upper
**Intercept**		1.0064	0.01755	4	57.35	<.0001	0.05	0.9576	1.0551
**Genotype**	Meg+/- TTR KO	-0.07326	0.02503	25	-2.93	0.0072	0.05	-0.1248	-0.02171
**Genotype**	TTR KO	0	.	.	.	.	.	.	.

Solution for Random Effects

Effect	Culture	Estimate	Std Err Pred	DF	t Value	Pr > |t|	Alpha	Lower	Upper
**Culture**	1	0.01365	0.01720	25	0.79	0.4348	0.05	-0.02177	0.04907
**Culture**	2	-0.00328	0.01702	25	-0.19	0.8487	0.05	-0.03833	0.03177
**Culture**	3	-0.01037	0.01720	25	-0.60	0.5520	0.05	-0.04579	0.02505
**Culture**	4	-0.01725	0.01726	25	-1.00	0.3270	0.05	-0.05280	0.01829
**Culture**	5	0.006642	0.01726	25	0.38	0.7036	0.05	-0.02890	0.04219
**Culture**	6	0.01061	0.01726	25	0.61	0.5442	0.05	-0.02493	0.04616

Type 3 Tests of Fixed Effects

Effect	Num DF	Den DF	F Value	Pr > F
**Genotype**	1	25	8.57	0.0072

Least Squares Means

Effect	Genotype	Estimate	Standard Error	DF	t Value	Pr > |t|	Alpha	Lower	Upper
**Genotype**	Meg+/- TTR KO	0.9331	0.01785	25	52.29	<.0001	0.05	0.8964	0.9699
**Genotype**	TTR KO	1.0064	0.01755	25	57.35	<.0001	0.05	0.9702	1.0425

Time=4.166667

Differences of Least Squares Means												
Effect	Genotype	Genotype	Estimate	Standard Error	DF	t Value	Pr > |t|	Adjustment	Adj P	Alpha	Lower	Upper
Genotype	Meg+/- TTR KO	TTR KO	-0.07326	0.02503	25	-2.93	0.0072	Tukey-Kramer	0.0072	0.05	-0.1248	-0.02171

Differences of Least Squares Means				
Effect	Genotype	Genotype	Adj Lower	Adj Upper
Genotype	Meg+/- TTR KO	TTR KO	-0.1248	-0.02171

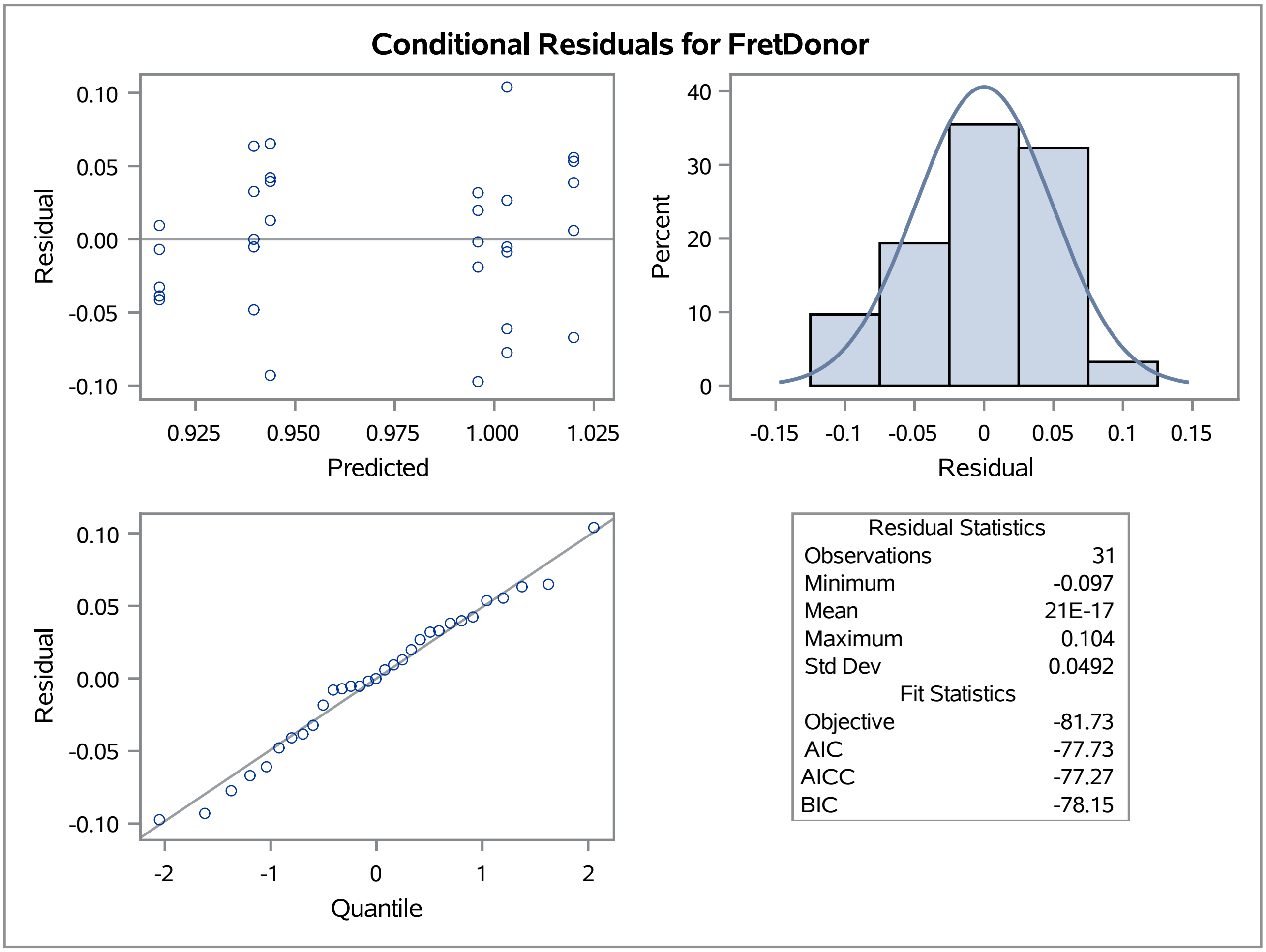

Time=4.333333

Model Information	
**Data Set**	WORK.TEMPDATASORTED
**Dependent Variable**	FretDonor
**Covariance Structure**	Variance Components
**Estimation Method**	REML
**Residual Variance Method**	Profile
**Fixed Effects SE Method**	Model-Based
**Degrees of Freedom Method**	Containment

Class Level Information		
**Class**	**Levels**	**Values**
**Genotype**	2	Meg+/- TTR KO TTR KO
**Culture**	6	1 2 3 4 5 6

Dimensions	
**Covariance Parameters**	2
**Columns in X**	3
**Columns in Z**	6
**Subjects**	1
**Max Obs per Subject**	31

Number of Observations	
**Number of Observations Read**	31
**Number of Observations Used**	31
**Number of Observations Not Used**	0

Iteration History			
**Iteration**	**Evaluations**	**-2 Res Log Like**	**Criterion**
**0**	1	-58.60184714	
**1**	2	-68.65067806	0.00000486
**2**	1	-68.65097936	0.00000000

Convergence criteria met.

Covariance Parameter Estimates				
**Cov Parm**	**Estimate**	**Alpha**	**Lower**	**Upper**
**Culture**	0.004301	0.05	0.001374	0.06134
**Residual**	0.003445	0.05	0.002118	0.006568

Time=4.333333

Fit Statistics	
**-2 Res Log Likelihood**	-68.7
**AIC (Smaller is Better)**	-64.7
**AICC (Smaller is Better)**	-64.2
**BIC (Smaller is Better)**	-65.1

Solution for Fixed Effects

Effect	Genotype	Estimate	Standard Error	DF	t Value	Pr > |t|	Alpha	Lower	Upper
**Intercept**		1.0165	0.04063	4	25.02	<.0001	0.05	0.9037	1.1293
**Genotype**	Meg+/- TTR KO	-0.08644	0.05757	25	-1.50	0.1457	0.05	-0.2050	0.03212
**Genotype**	TTR KO	0	.	.	.	.	.	.	.

Solution for Random Effects

Effect	Culture	Estimate	Std Err Pred	DF	t Value	Pr > |t|	Alpha	Lower	Upper
**Culture**	1	0.07749	0.04266	25	1.82	0.0813	0.05	-0.01038	0.1654
**Culture**	2	0.004226	0.04232	25	0.10	0.9213	0.05	-0.08294	0.09139
**Culture**	3	-0.08171	0.04266	25	-1.92	0.0670	0.05	-0.1696	0.006153
**Culture**	4	-0.03794	0.04278	25	-0.89	0.3835	0.05	-0.1260	0.05015
**Culture**	5	0.01490	0.04278	25	0.35	0.7305	0.05	-0.07320	0.1030
**Culture**	6	0.02304	0.04278	25	0.54	0.5949	0.05	-0.06505	0.1111

Type 3 Tests of Fixed Effects

Effect	Num DF	Den DF	F Value	Pr > F
**Genotype**	1	25	2.25	0.1457

Least Squares Means

Effect	Genotype	Estimate	Standard Error	DF	t Value	Pr > |t|	Alpha	Lower	Upper
**Genotype**	Meg+/- TTR KO	0.9301	0.04079	25	22.80	<.0001	0.05	0.8461	1.0141
**Genotype**	TTR KO	1.0165	0.04063	25	25.02	<.0001	0.05	0.9328	1.1002

Time=4.333333

Differences of Least Squares Means												
**Effect**	**Genotype**	**Genotype**	**Estimate**	**Standard Error**	**DF**	**t Value**	**Pr > |t|**	**Adjustment**	**Adj P**	**Alpha**	**Lower**	**Upper**
**Genotype**	Meg+/- TTR KO	TTR KO	-0.08644	0.05757	25	-1.50	0.1457	Tukey-Kramer	0.1457	0.05	-0.2050	0.03212

Differences of Least Squares Means				
**Effect**	**Genotype**	**Genotype**	**Adj Lower**	**Adj Upper**
**Genotype**	Meg+/- TTR KO	TTR KO	-0.2050	0.03212

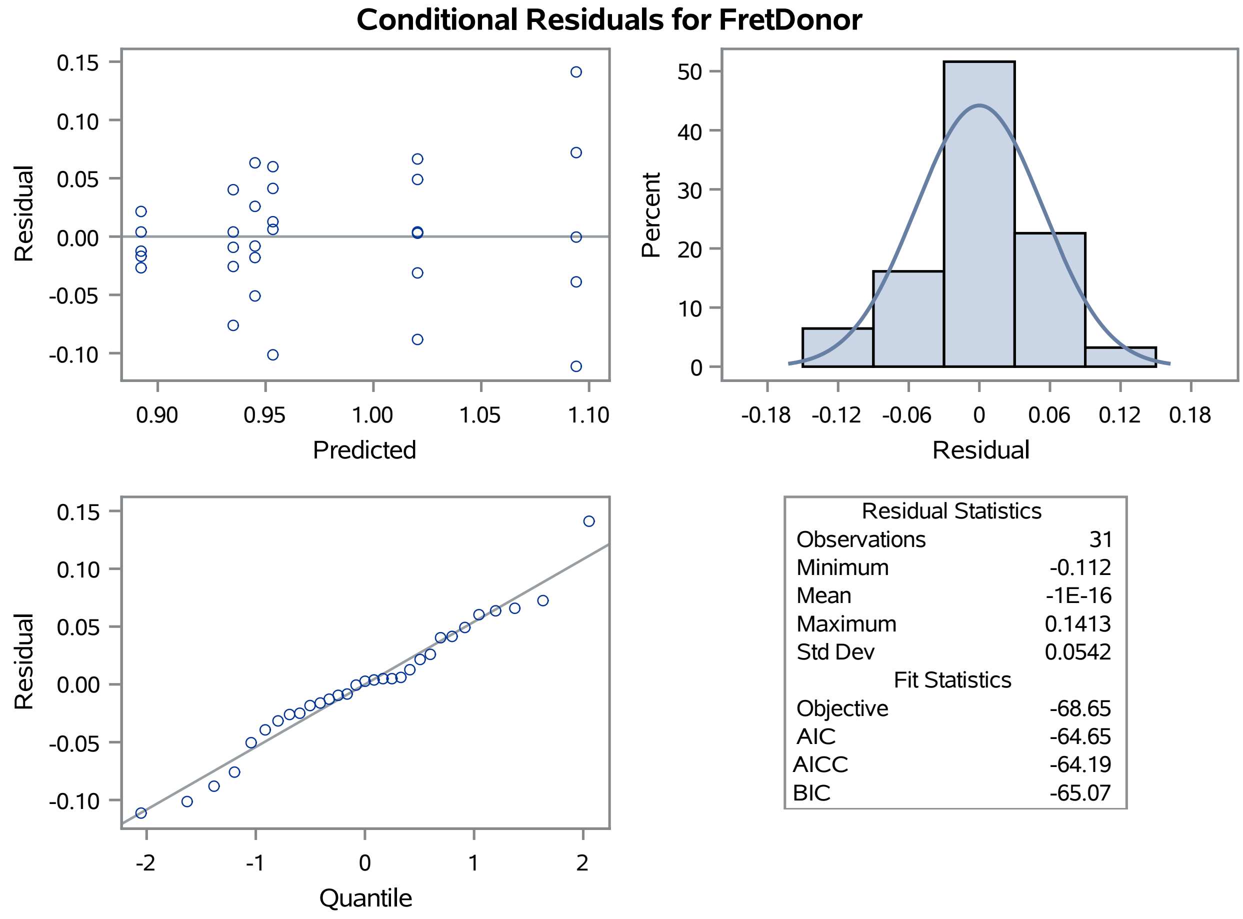

Residual Statistics	
Observations	31
Minimum	-0.112
Mean	-1E-16
Maximum	0.1413
Std Dev	0.0542
**Fit Statistics**	
Objective	-68.65
AIC	-64.65
AICC	-64.19
BIC	-65.07

Time=4.5

Model Information	
**Data Set**	WORK.TEMPDATASORTED
**Dependent Variable**	FretDonor
**Covariance Structure**	Variance Components
**Estimation Method**	REML
**Residual Variance Method**	Profile
**Fixed Effects SE Method**	Model-Based
**Degrees of Freedom Method**	Containment

Class Level Information		
**Class**	**Levels**	**Values**
**Genotype**	2	Meg+/- TTR KO TTR KO
**Culture**	6	1 2 3 4 5 6

Dimensions	
**Covariance Parameters**	2
**Columns in X**	3
**Columns in Z**	6
**Subjects**	1
**Max Obs per Subject**	31

Number of Observations	
**Number of Observations Read**	31
**Number of Observations Used**	31
**Number of Observations Not Used**	0

Iteration History			
**Iteration**	**Evaluations**	**-2 Res Log Like**	**Criterion**
**0**	1	-61.73785023	
**1**	2	-82.33570646	0.00000505
**2**	1	-82.33605505	0.00000000

Convergence criteria met.

Covariance Parameter Estimates				
**Cov Parm**	**Estimate**	**Alpha**	**Lower**	**Upper**
**Culture**	0.005485	0.05	0.001869	0.05688
**Residual**	0.001941	0.05	0.001194	0.003699

Time=4.5

Fit Statistics	
**-2 Res Log Likelihood**	-82.3
**AIC (Smaller is Better)**	-78.3
**AICC (Smaller is Better)**	-77.9
**BIC (Smaller is Better)**	-78.8

Solution for Fixed Effects

Effect	Genotype	Estimate	Standard Error	DF	t Value	Pr > |t|	Alpha	Lower	Upper
**Intercept**		1.0160	0.04417	4	23.00	<.0001	0.05	0.8934	1.1386
**Genotype**	Meg+/- TTR KO	-0.08643	0.06252	25	-1.38	0.1791	0.05	-0.2152	0.04233
**Genotype**	TTR KO	0	.	.	.	.	.	.	.

Solution for Random Effects

Effect	Culture	Estimate	Std Err Pred	DF	t Value	Pr > |t|	Alpha	Lower	Upper
**Culture**	1	0.1070	0.04543	25	2.35	0.0267	0.05	0.01339	0.2005
**Culture**	2	-0.03238	0.04522	25	-0.72	0.4806	0.05	-0.1255	0.06075
**Culture**	3	-0.07458	0.04543	25	-1.64	0.1132	0.05	-0.1681	0.01899
**Culture**	4	-0.03956	0.04550	25	-0.87	0.3928	0.05	-0.1333	0.05414
**Culture**	5	0.01146	0.04550	25	0.25	0.8032	0.05	-0.08225	0.1052
**Culture**	6	0.02810	0.04550	25	0.62	0.5424	0.05	-0.06561	0.1218

Type 3 Tests of Fixed Effects

Effect	Num DF	Den DF	F Value	Pr > F
**Genotype**	1	25	1.91	0.1791

Least Squares Means

Effect	Genotype	Estimate	Standard Error	DF	t Value	Pr > |t|	Alpha	Lower	Upper
**Genotype**	Meg+/- TTR KO	0.9296	0.04425	25	21.01	<.0001	0.05	0.8385	1.0207
**Genotype**	TTR KO	1.0160	0.04417	25	23.00	<.0001	0.05	0.9251	1.1070

Time=4.5

Differences of Least Squares Means

Effect	Genotype	Genotype	Estimate	Standard Error	DF	t Value	Pr > |t|	Adjustment	Adj P	Alpha	Lower	Upper
**Genotype**	Meg+/- TTR KO	TTR KO	-0.08643	0.06252	25	-1.38	0.1791	Tukey-Kramer	0.1791	0.05	-0.2152	0.04233

Differences of Least Squares Means

Effect	Genotype	Genotype	Adj Lower	Adj Upper
**Genotype**	Meg+/- TTR KO	TTR KO	-0.2152	0.04233

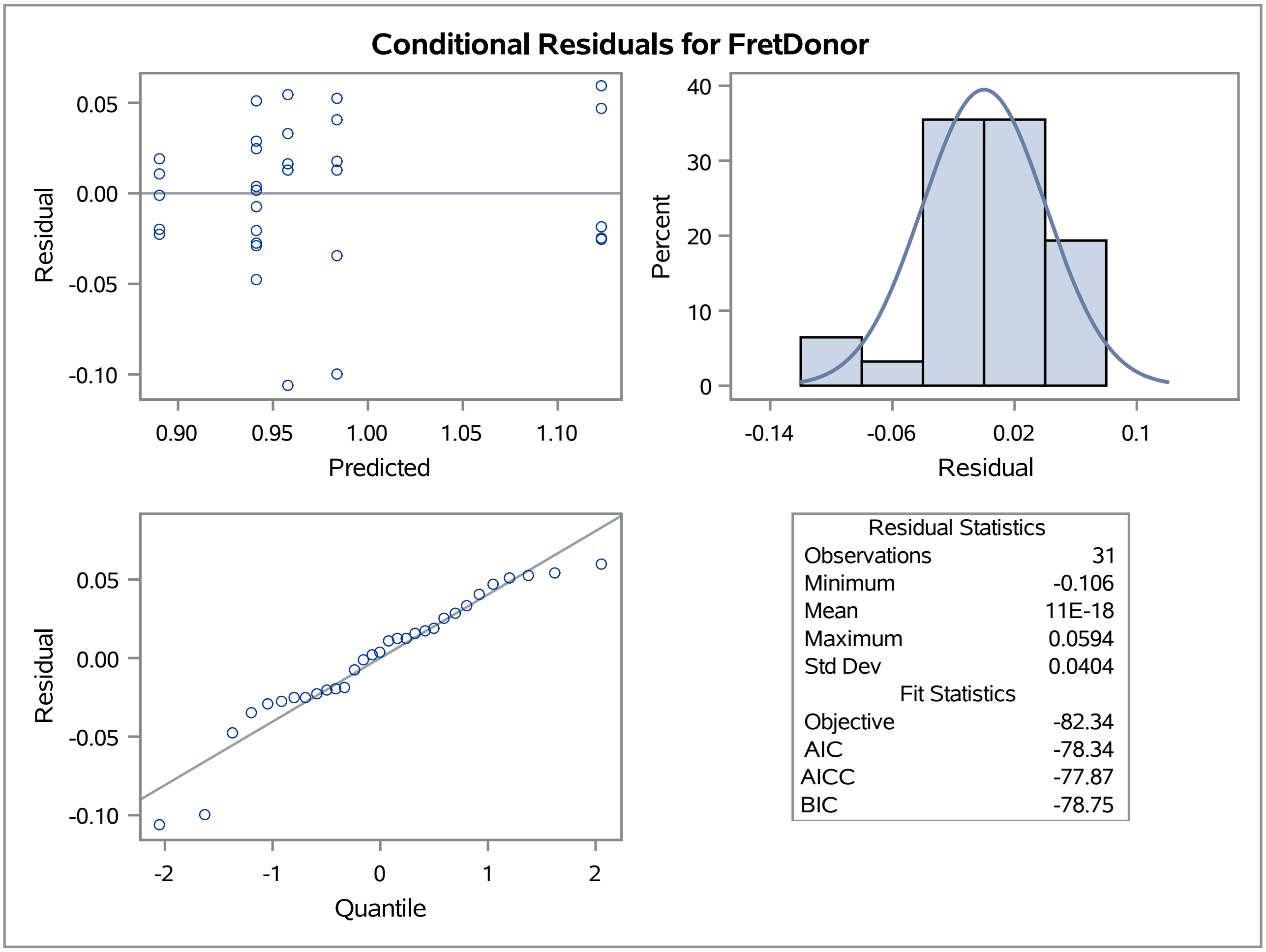

Time=4.666667

Model Information	
**Data Set**	WORK.TEMPDATASORTED
**Dependent Variable**	FretDonor
**Covariance Structure**	Variance Components
**Estimation Method**	REML
**Residual Variance Method**	Profile
**Fixed Effects SE Method**	Model-Based
**Degrees of Freedom Method**	Containment

Class Level Information		
**Class**	**Levels**	**Values**
**Genotype**	2	Meg+/- TTR KO TTR KO
**Culture**	6	1 2 3 4 5 6

Dimensions	
**Covariance Parameters**	2
**Columns in X**	3
**Columns in Z**	6
**Subjects**	1
**Max Obs per Subject**	31

Number of Observations	
**Number of Observations Read**	31
**Number of Observations Used**	31
**Number of Observations Not Used**	0

Iteration History			
**Iteration**	**Evaluations**	**-2 Res Log Like**	**Criterion**
**0**	1	-61.51414977	
**1**	2	-70.81460375	0.00000422
**2**	1	-70.81486923	0.00000000

Convergence criteria met.

Covariance Parameter Estimates				
**Cov Parm**	**Estimate**	**Alpha**	**Lower**	**Upper**
**Culture**	0.003729	0.05	0.001180	0.05580
**Residual**	0.003227	0.05	0.001984	0.006152

Time=4.666667

Fit Statistics	
-2 Res Log Likelihood	-70.8
AIC (Smaller is Better)	-66.8
AICC (Smaller is Better)	-66.4
BIC (Smaller is Better)	-67.2

Solution for Fixed Effects

Effect	Genotype	Estimate	Standard Error	DF	t Value	Pr > |t|	Alpha	Lower	Upper
Intercept		0.9657	0.03803	4	25.39	<.0001	0.05	0.8601	1.0713
Genotype	Meg+/- TTR KO	-0.03911	0.05389	25	-0.73	0.4748	0.05	-0.1501	0.07189
Genotype	TTR KO	0	.	.	.	.	.	.	.

Solution for Random Effects

Effect	Culture	Estimate	Std Err Pred	DF	t Value	Pr > |t|	Alpha	Lower	Upper
Culture	1	0.07257	0.04001	25	1.81	0.0817	0.05	-0.00984	0.1550
Culture	2	0.004146	0.03968	25	0.10	0.9176	0.05	-0.07758	0.08587
Culture	3	-0.07672	0.04001	25	-1.92	0.0667	0.05	-0.1591	0.005692
Culture	4	-0.03133	0.04012	25	-0.78	0.4422	0.05	-0.1140	0.05130
Culture	5	0.007579	0.04012	25	0.19	0.8517	0.05	-0.07506	0.09021
Culture	6	0.02375	0.04012	25	0.59	0.5592	0.05	-0.05888	0.1064

Type 3 Tests of Fixed Effects

Effect	Num DF	Den DF	F Value	Pr > F
Genotype	1	25	0.53	0.4748

Least Squares Means

Effect	Genotype	Estimate	Standard Error	DF	t Value	Pr > |t|	Alpha	Lower	Upper
Genotype	Meg+/- TTR KO	0.9266	0.03819	25	24.26	<.0001	0.05	0.8479	1.0052
Genotype	TTR KO	0.9657	0.03803	25	25.39	<.0001	0.05	0.8874	1.0440

Time=4.666667

Differences of Least Squares Means												
**Effect**	**Genotype**	**Genotype**	**Estimate**	**Standard Error**	**DF**	**t Value**	**Pr > |t|**	**Adjustment**	**Adj P**	**Alpha**	**Lower**	**Upper**
**Genotype**	Meg+/- TTR KO	TTR KO	-0.03911	0.05389	25	-0.73	0.4748	Tukey-Kramer	0.4748	0.05	-0.1501	0.07189

Differences of Least Squares Means				
**Effect**	**Genotype**	**Genotype**	**Adj Lower**	**Adj Upper**
**Genotype**	Meg+/- TTR KO	TTR KO	-0.1501	0.07189

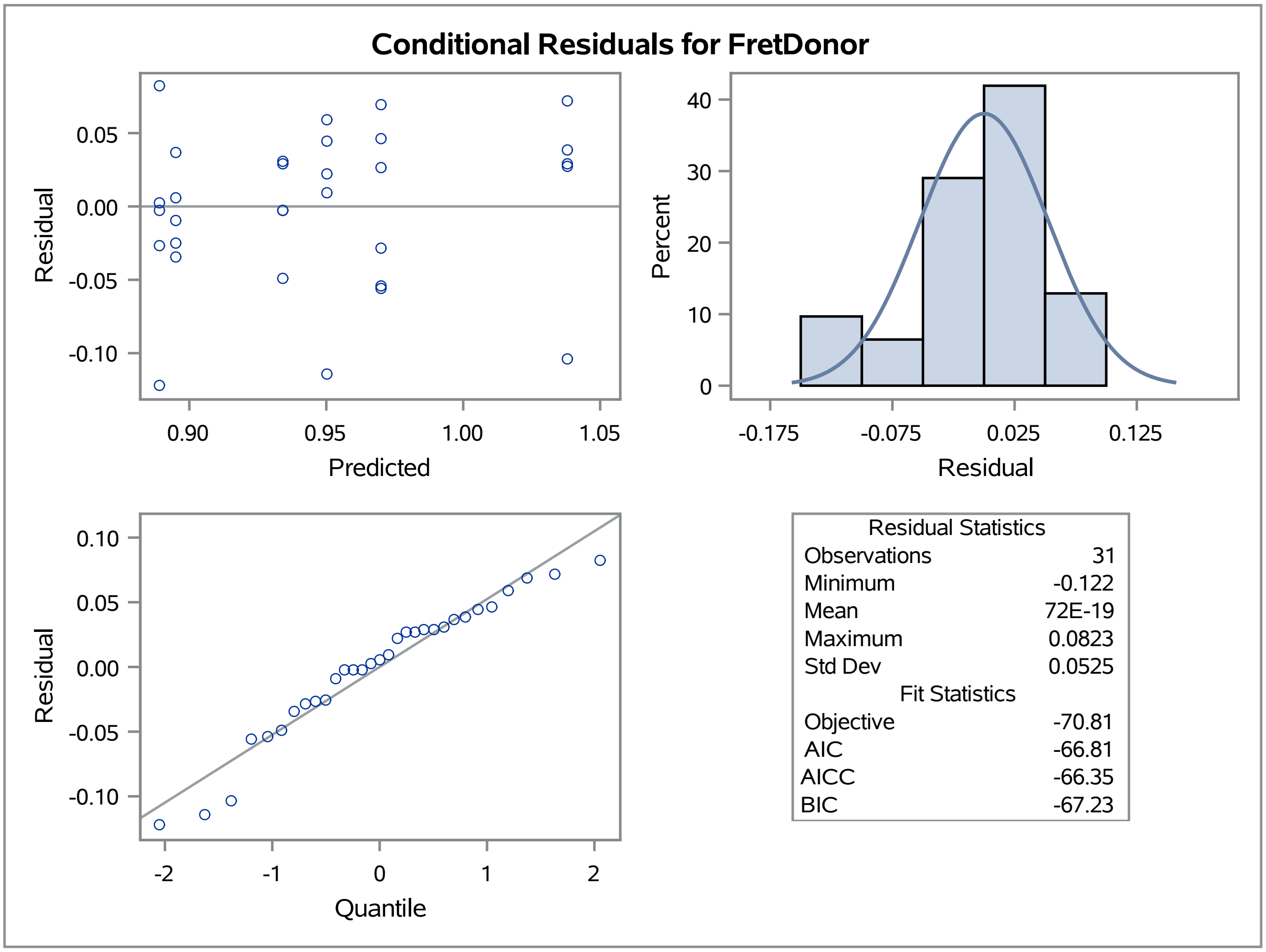

Time=4.833333

Model Information	
**Data Set**	WORK.TEMPDATASORTED
**Dependent Variable**	FretDonor
**Covariance Structure**	Variance Components
**Estimation Method**	REML
**Residual Variance Method**	Profile
**Fixed Effects SE Method**	Model-Based
**Degrees of Freedom Method**	Containment

Class Level Information		
**Class**	**Levels**	**Values**
**Genotype**	2	Meg+/- TTR KO TTR KO
**Culture**	6	1 2 3 4 5 6

Dimensions	
**Covariance Parameters**	2
**Columns in X**	3
**Columns in Z**	6
**Subjects**	1
**Max Obs per Subject**	31

Number of Observations	
**Number of Observations Read**	31
**Number of Observations Used**	31
**Number of Observations Not Used**	0

Iteration History			
**Iteration**	**Evaluations**	**-2 Res Log Like**	**Criterion**
**0**	1	-68.33900815	
**1**	2	-71.56461830	0.00000005
**2**	1	-71.56462138	0.00000000

Convergence criteria met.

Covariance Parameter Estimates				
**Cov Parm**	**Estimate**	**Alpha**	**Lower**	**Upper**
**Culture**	0.001581	0.05	0.000421	0.07145
**Residual**	0.003479	0.05	0.002139	0.006633

Time=4.833333

Fit Statistics	
**-2 Res Log Likelihood**	-71.6
**AIC (Smaller is Better)**	-67.6
**AICC (Smaller is Better)**	-67.1
**BIC (Smaller is Better)**	-68.0

Solution for Fixed Effects

Effect	Genotype	Estimate	Standard Error	DF	t Value	Pr > |t|	Alpha	Lower	Upper
**Intercept**		1.0175	0.02731	4	37.26	<.0001	0.05	0.9416	1.0933
**Genotype**	Meg+/- TTR KO	-0.09233	0.03879	25	-2.38	0.0252	0.05	-0.1722	-0.01245
**Genotype**	TTR KO	0	.	.	.	.	.	.	.

Solution for Random Effects

Effect	Culture	Estimate	Std Err Pred	DF	t Value	Pr > |t|	Alpha	Lower	Upper
**Culture**	1	0.02499	0.02903	25	0.86	0.3976	0.05	-0.03480	0.08477
**Culture**	2	0.02027	0.02869	25	0.71	0.4866	0.05	-0.03883	0.07936
**Culture**	3	-0.04525	0.02903	25	-1.56	0.1316	0.05	-0.1050	0.01453
**Culture**	4	-0.02746	0.02914	25	-0.94	0.3551	0.05	-0.08747	0.03256
**Culture**	5	0.003329	0.02914	25	0.11	0.9100	0.05	-0.05668	0.06334
**Culture**	6	0.02413	0.02914	25	0.83	0.4155	0.05	-0.03589	0.08414

Type 3 Tests of Fixed Effects

Effect	Num DF	Den DF	F Value	Pr > F
**Genotype**	1	25	5.67	0.0252

Least Squares Means

Effect	Genotype	Estimate	Standard Error	DF	t Value	Pr > |t|	Alpha	Lower	Upper
**Genotype**	Meg+/- TTR KO	0.9251	0.02755	25	33.58	<.0001	0.05	0.8684	0.9819
**Genotype**	TTR KO	1.0175	0.02731	25	37.26	<.0001	0.05	0.9612	1.0737

Time=4.833333

Differences of Least Squares Means												
Effect	Genotype	Genotype	Estimate	Standard Error	DF	t Value	Pr > |t|	Adjustment	Adj P	Alpha	Lower	Upper
Genotype	Meg+/- TTR KO	TTR KO	-0.09233	0.03879	25	-2.38	0.0252	Tukey-Kramer	0.0252	0.05	-0.1722	-0.01245

Differences of Least Squares Means				
Effect	Genotype	Genotype	Adj Lower	Adj Upper
Genotype	Meg+/- TTR KO	TTR KO	-0.1722	-0.01245

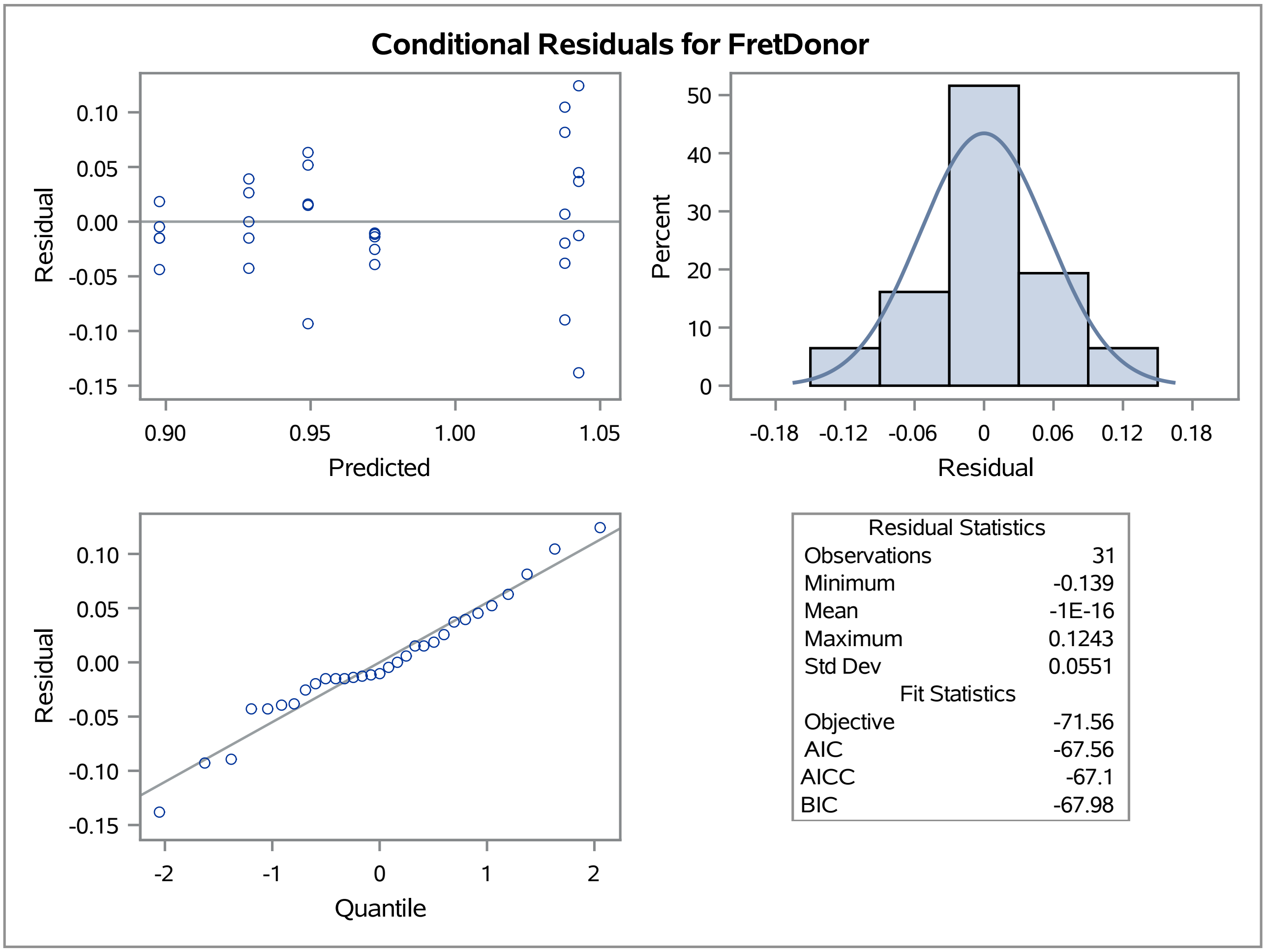

Time=5

Model Information	
**Data Set**	WORK.TEMPDATASORTED
**Dependent Variable**	FretDonor
**Covariance Structure**	Variance Components
**Estimation Method**	REML
**Residual Variance Method**	Profile
**Fixed Effects SE Method**	Model-Based
**Degrees of Freedom Method**	Containment

Class Level Information		
**Class**	**Levels**	**Values**
**Genotype**	2	Meg+/- TTR KO TTR KO
**Culture**	6	1 2 3 4 5 6

Dimensions	
**Covariance Parameters**	2
**Columns in X**	3
**Columns in Z**	6
**Subjects**	1
**Max Obs per Subject**	31

Number of Observations	
**Number of Observations Read**	31
**Number of Observations Used**	31
**Number of Observations Not Used**	0

Iteration History			
**Iteration**	**Evaluations**	**-2 Res Log Like**	**Criterion**
**0**	1	-60.47560056	
**1**	2	-75.82254967	0.00000169
**2**	1	-75.82265964	0.00000000

Convergence criteria met.

Covariance Parameter Estimates				
**Cov Parm**	**Estimate**	**Alpha**	**Lower**	**Upper**
**Culture**	0.004972	0.05	0.001656	0.05742
**Residual**	0.002547	0.05	0.001566	0.004854

Time=5

Fit Statistics	
**-2 Res Log Likelihood**	-75.8
**AIC (Smaller is Better)**	-71.8
**AICC (Smaller is Better)**	-71.4
**BIC (Smaller is Better)**	-72.2

Solution for Fixed Effects

Effect	Genotype	Estimate	Standard Error	DF	t Value	Pr > |t|	Alpha	Lower	Upper
**Intercept**		0.9780	0.04263	4	22.94	<.0001	0.05	0.8597	1.0964
**Genotype**	Meg+/- TTR KO	-0.04740	0.06037	25	-0.79	0.4397	0.05	-0.1717	0.07693
**Genotype**	TTR KO	0	.	.	.	.	.	.	.

Solution for Random Effects

Effect	Culture	Estimate	Std Err Pred	DF	t Value	Pr > |t|	Alpha	Lower	Upper
**Culture**	1	0.06446	0.04424	25	1.46	0.1576	0.05	-0.02666	0.1556
**Culture**	2	0.03406	0.04398	25	0.77	0.4459	0.05	-0.05651	0.1246
**Culture**	3	-0.09852	0.04424	25	-2.23	0.0352	0.05	-0.1896	-0.00740
**Culture**	4	-0.03906	0.04433	25	-0.88	0.3867	0.05	-0.1304	0.05224
**Culture**	5	-0.00020	0.04433	25	-0.00	0.9965	0.05	-0.09150	0.09111
**Culture**	6	0.03926	0.04433	25	0.89	0.3843	0.05	-0.05205	0.1306

Type 3 Tests of Fixed Effects

Effect	Num DF	Den DF	F Value	Pr > F
**Genotype**	1	25	0.62	0.4397

Least Squares Means

Effect	Genotype	Estimate	Standard Error	DF	t Value	Pr > |t|	Alpha	Lower	Upper
**Genotype**	Meg+/- TTR KO	0.9306	0.04274	25	21.77	<.0001	0.05	0.8426	1.0187
**Genotype**	TTR KO	0.9780	0.04263	25	22.94	<.0001	0.05	0.8902	1.0659

Time=5

Differences of Least Squares Means												
Effect	Genotype	Genotype	Estimate	Standard Error	DF	t Value	Pr > |t|	Adjustment	Adj P	Alpha	Lower	Upper
**Genotype**	Meg+/- TTR KO	TTR KO	-0.04740	0.06037	25	-0.79	0.4397	Tukey-Kramer	0.4397	0.05	-0.1717	0.07693

Differences of Least Squares Means				
Effect	Genotype	Genotype	Adj Lower	Adj Upper
**Genotype**	Meg+/- TTR KO	TTR KO	-0.1717	0.07693

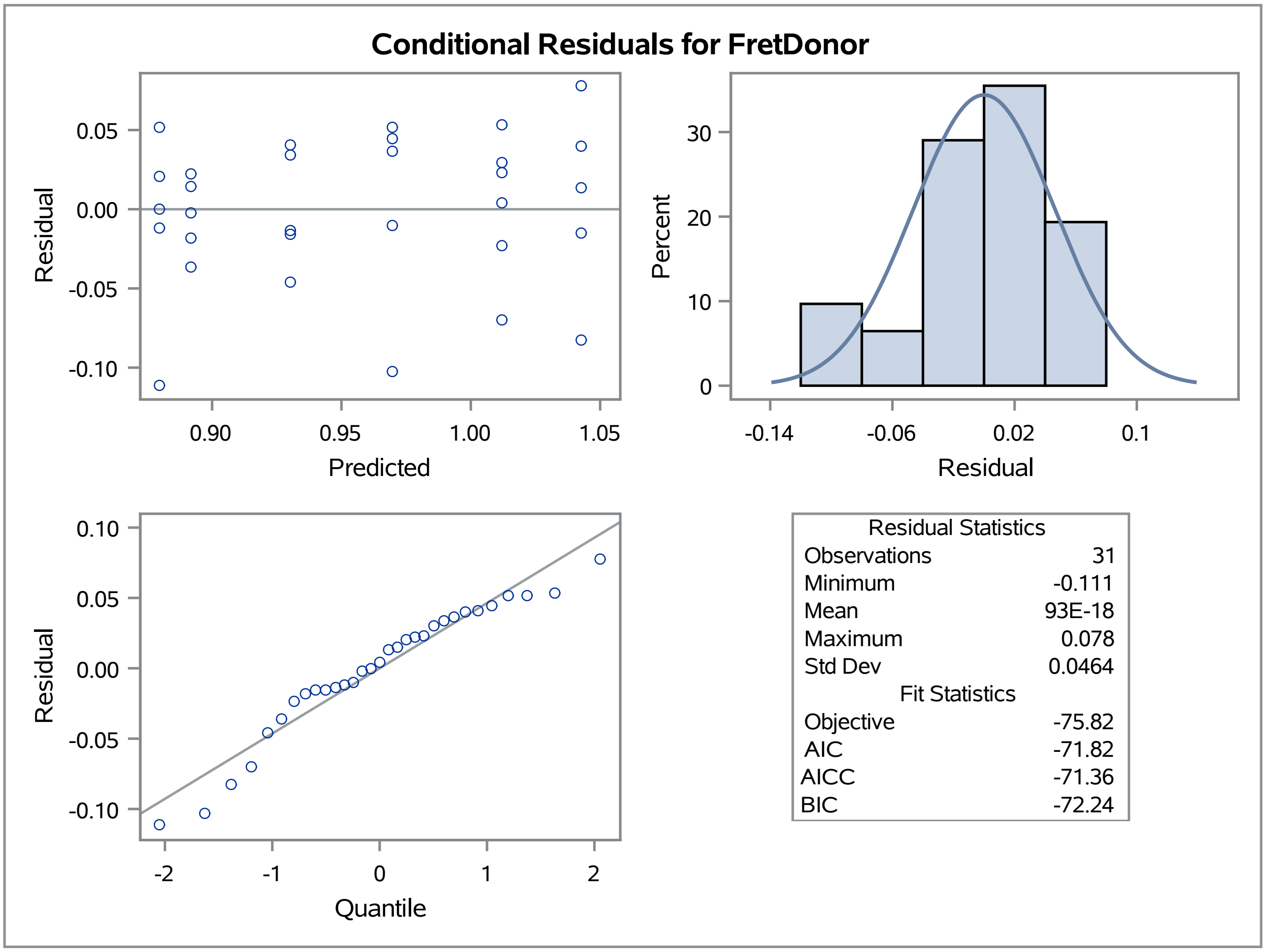

Time=5.166667

Model Information	
**Data Set**	WORK.TEMPDATASORTED
**Dependent Variable**	FretDonor
**Covariance Structure**	Variance Components
**Estimation Method**	REML
**Residual Variance Method**	Profile
**Fixed Effects SE Method**	Model-Based
**Degrees of Freedom Method**	Containment

Class Level Information		
**Class**	**Levels**	**Values**
**Genotype**	2	Meg+/- TTR KO TTR KO
**Culture**	6	1 2 3 4 5 6

Dimensions	
**Covariance Parameters**	2
**Columns in X**	3
**Columns in Z**	6
**Subjects**	1
**Max Obs per Subject**	31

Number of Observations	
**Number of Observations Read**	31
**Number of Observations Used**	31
**Number of Observations Not Used**	0

Iteration History			
**Iteration**	**Evaluations**	**-2 Res Log Like**	**Criterion**
**0**	1	-44.70616363	
**1**	2	-60.92783849	0.00000825
**2**	1	-60.92831922	0.00000001

Convergence criteria met.

Covariance Parameter Estimates				
**Cov Parm**	**Estimate**	**Alpha**	**Lower**	**Upper**
**Culture**	0.008873	0.05	0.002970	0.1000
**Residual**	0.004217	0.05	0.002593	0.008037

Time=5.166667

Fit Statistics	
-2 Res Log Likelihood	-60.9
AIC (Smaller is Better)	-56.9
AICC (Smaller is Better)	-56.5
BIC (Smaller is Better)	-57.3

Solution for Fixed Effects

Effect	Genotype	Estimate	Standard Error	DF	t Value	Pr > |t|	Alpha	Lower	Upper
Intercept		1.0102	0.05677	4	17.79	<.0001	0.05	0.8525	1.1678
Genotype	Meg+/- TTR KO	-0.07947	0.08039	25	-0.99	0.3323	0.05	-0.2450	0.08609
Genotype	TTR KO	0	.	.	.	.	.	.	.

Solution for Random Effects

Effect	Culture	Estimate	Std Err Pred	DF	t Value	Pr > |t|	Alpha	Lower	Upper
Culture	1	0.1316	0.05881	25	2.24	0.0344	0.05	0.01049	0.2527
Culture	2	-0.02498	0.05847	25	-0.43	0.6729	0.05	-0.1454	0.09544
Culture	3	-0.1066	0.05881	25	-1.81	0.0818	0.05	-0.2277	0.01449
Culture	4	-0.04186	0.05892	25	-0.71	0.4839	0.05	-0.1632	0.07948
Culture	5	0.004122	0.05892	25	0.07	0.9448	0.05	-0.1172	0.1255
Culture	6	0.03774	0.05892	25	0.64	0.5276	0.05	-0.08360	0.1591

Type 3 Tests of Fixed Effects

Effect	Num DF	Den DF	F Value	Pr > F
Genotype	1	25	0.98	0.3323

Least Squares Means

Effect	Genotype	Estimate	Standard Error	DF	t Value	Pr > |t|	Alpha	Lower	Upper
Genotype	Meg+/- TTR KO	0.9307	0.05691	25	16.35	<.0001	0.05	0.8135	1.0479
Genotype	TTR KO	1.0102	0.05677	25	17.79	<.0001	0.05	0.8932	1.1271

Time=5.166667

Differences of Least Squares Means												
**Effect**	**Genotype**	**Genotype**	**Estimate**	**Standard Error**	**DF**	**t Value**	**Pr > |t|**	**Adjustment**	**Adj P**	**Alpha**	**Lower**	**Upper**
**Genotype**	Meg+/- TTR KO	TTR KO	-0.07947	0.08039	25	-0.99	0.3323	Tukey-Kramer	0.3323	0.05	-0.2450	0.08609

Differences of Least Squares Means				
**Effect**	**Genotype**	**Genotype**	**Adj Lower**	**Adj Upper**
**Genotype**	Meg+/- TTR KO	TTR KO	-0.2450	0.08609

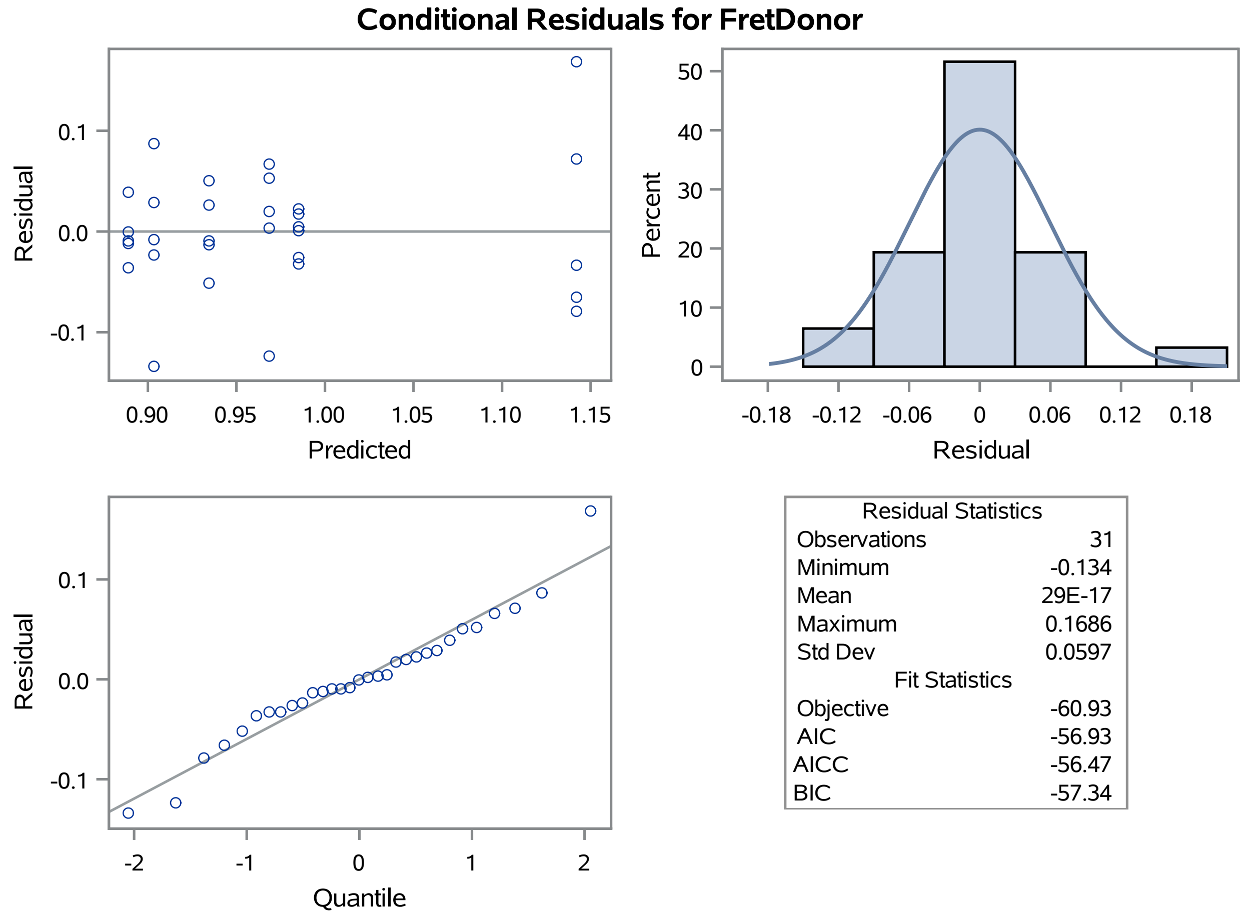

Residual Statistics	
Observations	31
Minimum	-0.134
Mean	29E-17
Maximum	0.1686
Std Dev	0.0597
**Fit Statistics**	
Objective	-60.93
AIC	-56.93
AICC	-56.47
BIC	-57.34

Time=5.333333

Model Information	
**Data Set**	WORK.TEMPDATASORTED
**Dependent Variable**	FretDonor
**Covariance Structure**	Variance Components
**Estimation Method**	REML
**Residual Variance Method**	Profile
**Fixed Effects SE Method**	Model-Based
**Degrees of Freedom Method**	Containment

Class Level Information		
**Class**	**Levels**	**Values**
**Genotype**	2	Meg+/- TTR KO TTR KO
**Culture**	6	1 2 3 4 5 6

Dimensions	
**Covariance Parameters**	2
**Columns in X**	3
**Columns in Z**	6
**Subjects**	1
**Max Obs per Subject**	31

Number of Observations	
**Number of Observations Read**	31
**Number of Observations Used**	31
**Number of Observations Not Used**	0

Iteration History			
**Iteration**	**Evaluations**	**-2 Res Log Like**	**Criterion**
**0**	1	-66.52648615	
**1**	2	-69.64650686	0.00000078
**2**	1	-69.64655529	0.00000000

Convergence criteria met.

Covariance Parameter Estimates				
**Cov Parm**	**Estimate**	**Alpha**	**Lower**	**Upper**
**Culture**	0.001677	0.05	0.000444	0.07947
**Residual**	0.003720	0.05	0.002287	0.007095

Time=5.333333

Fit Statistics	
-2 Res Log Likelihood	-69.6
AIC (Smaller is Better)	-65.6
AICC (Smaller is Better)	-65.2
BIC (Smaller is Better)	-66.1

Solution for Fixed Effects

Effect	Genotype	Estimate	Standard Error	DF	t Value	Pr > |t|	Alpha	Lower	Upper
Intercept		0.9844	0.02816	4	34.96	<.0001	0.05	0.9062	1.0626
Genotype	Meg+/- TTR KO	-0.06001	0.04000	25	-1.50	0.1461	0.05	-0.1424	0.02237
Genotype	TTR KO	0	.	.	.	.	.	.	.

Solution for Random Effects

Effect	Culture	Estimate	Std Err Pred	DF	t Value	Pr > |t|	Alpha	Lower	Upper
Culture	1	0.04289	0.02993	25	1.43	0.1643	0.05	-0.01876	0.1045
Culture	2	-0.00710	0.02959	25	-0.24	0.8122	0.05	-0.06804	0.05383
Culture	3	-0.03578	0.02993	25	-1.20	0.2431	0.05	-0.09743	0.02586
Culture	4	-0.02975	0.03005	25	-0.99	0.3315	0.05	-0.09163	0.03212
Culture	5	0.005106	0.03005	25	0.17	0.8664	0.05	-0.05677	0.06698
Culture	6	0.02465	0.03005	25	0.82	0.4198	0.05	-0.03723	0.08653

Type 3 Tests of Fixed Effects

Effect	Num DF	Den DF	F Value	Pr > F
Genotype	1	25	2.25	0.1461

Least Squares Means

Effect	Genotype	Estimate	Standard Error	DF	t Value	Pr > |t|	Alpha	Lower	Upper
Genotype	Meg+/- TTR KO	0.9244	0.02841	25	32.54	<.0001	0.05	0.8659	0.9829
Genotype	TTR KO	0.9844	0.02816	25	34.96	<.0001	0.05	0.9264	1.0424

Time=5.333333

Differences of Least Squares Means												
**Effect**	**Genotype**	**Genotype**	**Estimate**	**Standard Error**	**DF**	**t Value**	**Pr > |t|**	**Adjustment**	**Adj P**	**Alpha**	**Lower**	**Upper**
**Genotype**	Meg+/- TTR KO	TTR KO	-0.06001	0.04000	25	-1.50	0.1461	Tukey-Kramer	0.1461	0.05	-0.1424	0.02237

Differences of Least Squares Means				
**Effect**	**Genotype**	**Genotype**	**Adj Lower**	**Adj Upper**
**Genotype**	Meg+/- TTR KO	TTR KO	-0.1424	0.02237

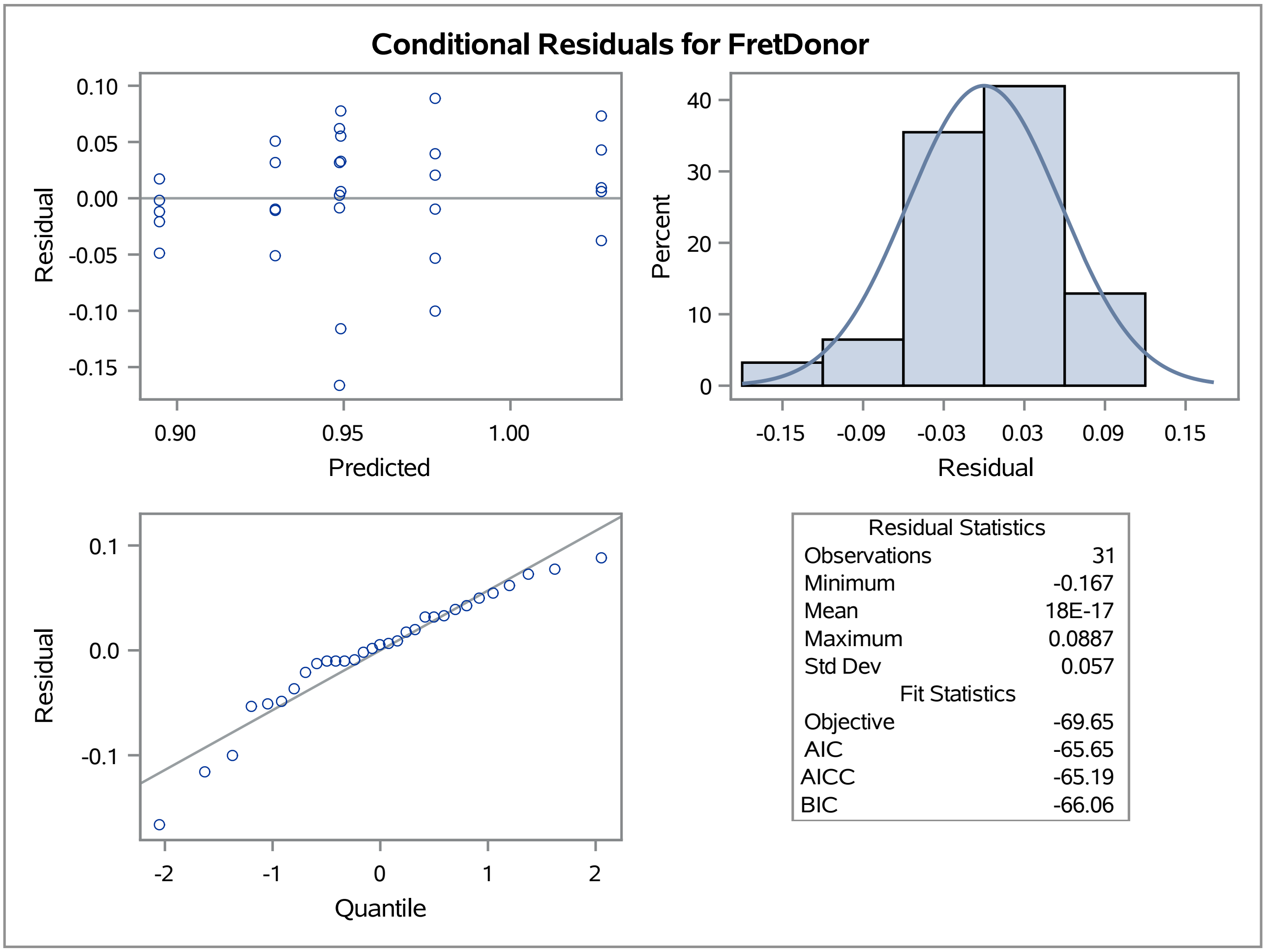

Residual Statistics	
Observations	31
Minimum	-0.167
Mean	18E-17
Maximum	0.0887
Std Dev	0.057
Fit Statistics	
Objective	-69.65
AIC	-65.65
AICC	-65.19
BIC	-66.06

Time=5.5

Model Information	
**Data Set**	WORK.TEMPDATASORTED
**Dependent Variable**	FretDonor
**Covariance Structure**	Variance Components
**Estimation Method**	REML
**Residual Variance Method**	Profile
**Fixed Effects SE Method**	Model-Based
**Degrees of Freedom Method**	Containment

Class Level Information		
**Class**	**Levels**	**Values**
**Genotype**	2	Meg+/- TTR KO TTR KO
**Culture**	6	1 2 3 4 5 6

Dimensions	
**Covariance Parameters**	2
**Columns in X**	3
**Columns in Z**	6
**Subjects**	1
**Max Obs per Subject**	31

Number of Observations	
**Number of Observations Read**	31
**Number of Observations Used**	31
**Number of Observations Not Used**	0

Iteration History			
**Iteration**	**Evaluations**	**-2 Res Log Like**	**Criterion**
**0**	1	-74.99553854	
**1**	2	-77.42120483	0.00000058
**2**	1	-77.42124304	0.00000000

Convergence criteria met.

Covariance Parameter Estimates				
**Cov Parm**	**Estimate**	**Alpha**	**Lower**	**Upper**
**Culture**	0.001083	0.05	0.000271	0.08102
**Residual**	0.002895	0.05	0.001779	0.005523

Time=5.5

Fit Statistics	
-2 Res Log Likelihood	-77.4
AIC (Smaller is Better)	-73.4
AICC (Smaller is Better)	-73.0
BIC (Smaller is Better)	-73.8

Solution for Fixed Effects

Effect	Genotype	Estimate	Standard Error	DF	t Value	Pr > |t|	Alpha	Lower	Upper
Intercept		0.9920	0.02330	4	42.57	<.0001	0.05	0.9273	1.0567
Genotype	Meg+/- TTR KO	-0.07210	0.03312	25	-2.18	0.0391	0.05	-0.1403	-0.00388
Genotype	TTR KO	0	.	.	.	.	.	.	.

Solution for Random Effects

Effect	Culture	Estimate	Std Err Pred	DF	t Value	Pr > |t|	Alpha	Lower	Upper
Culture	1	0.02630	0.02466	25	1.07	0.2963	0.05	-0.02448	0.07708
Culture	2	-0.00457	0.02437	25	-0.19	0.8527	0.05	-0.05475	0.04561
Culture	3	-0.02173	0.02466	25	-0.88	0.3865	0.05	-0.07251	0.02905
Culture	4	-0.02990	0.02475	25	-1.21	0.2384	0.05	-0.08087	0.02108
Culture	5	0.002170	0.02475	25	0.09	0.9308	0.05	-0.04881	0.05315
Culture	6	0.02773	0.02475	25	1.12	0.2733	0.05	-0.02325	0.07870

Type 3 Tests of Fixed Effects

Effect	Num DF	Den DF	F Value	Pr > F
Genotype	1	25	4.74	0.0391

Least Squares Means

Effect	Genotype	Estimate	Standard Error	DF	t Value	Pr > |t|	Alpha	Lower	Upper
Genotype	Meg+/- TTR KO	0.9199	0.02354	25	39.08	<.0001	0.05	0.8714	0.9684
Genotype	TTR KO	0.9920	0.02330	25	42.57	<.0001	0.05	0.9440	1.0400

Time=5.5

Differences of Least Squares Means												
**Effect**	**Genotype**	**Genotype**	**Estimate**	**Standard Error**	**DF**	**t Value**	**Pr > |t|**	**Adjustment**	**Adj P**	**Alpha**	**Lower**	**Upper**
**Genotype**	Meg+/- TTR KO	TTR KO	-0.07210	0.03312	25	-2.18	0.0391	Tukey-Kramer	0.0391	0.05	-0.1403	-0.00388

Differences of Least Squares Means				
**Effect**	**Genotype**	**Genotype**	**Adj Lower**	**Adj Upper**
**Genotype**	Meg+/- TTR KO	TTR KO	-0.1403	-0.00388

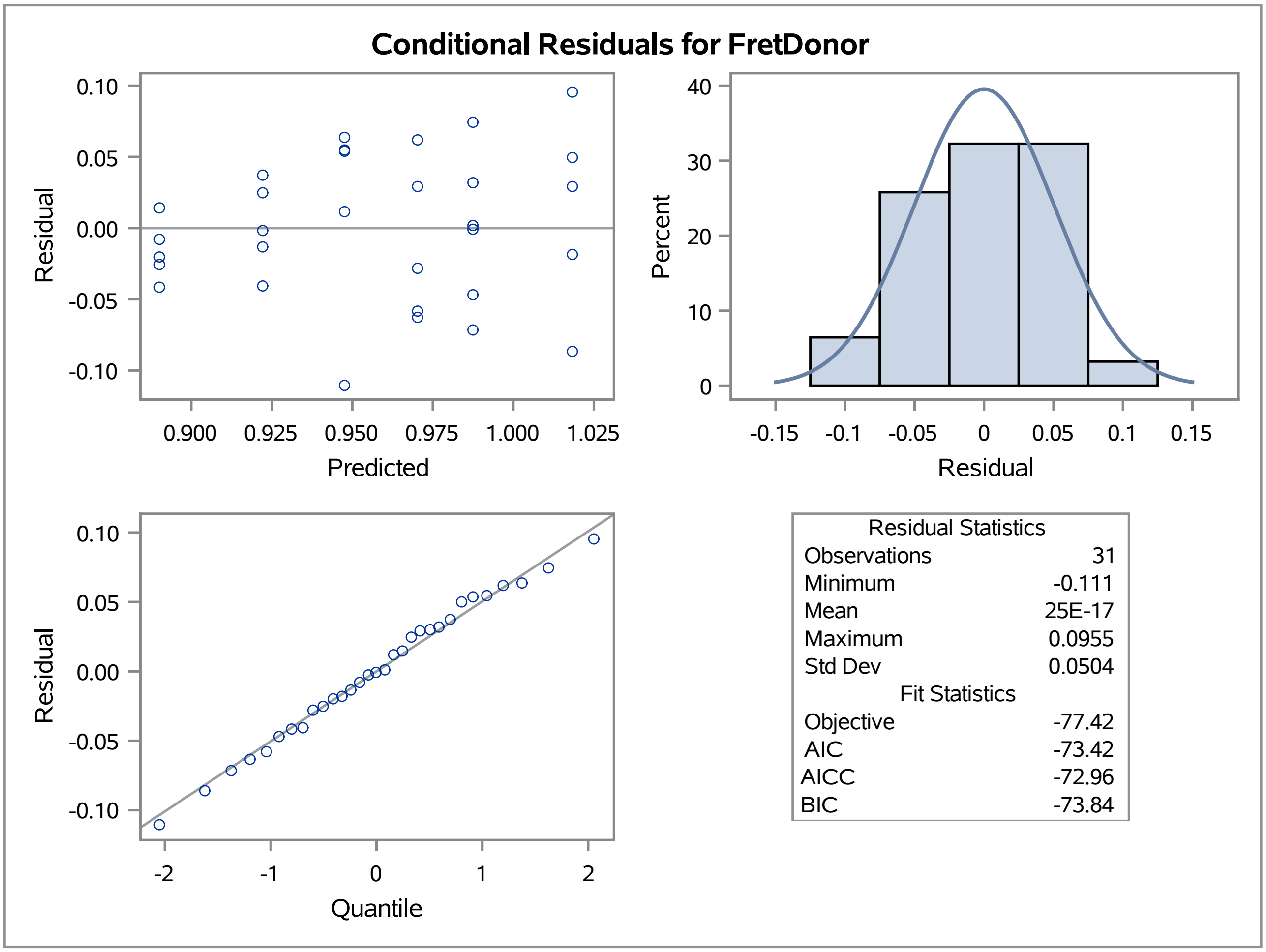

Time=5.666667

Model Information	
**Data Set**	WORK.TEMPDATASORTED
**Dependent Variable**	FretDonor
**Covariance Structure**	Variance Components
**Estimation Method**	REML
**Residual Variance Method**	Profile
**Fixed Effects SE Method**	Model-Based
**Degrees of Freedom Method**	Containment

Class Level Information		
**Class**	**Levels**	**Values**
**Genotype**	2	Meg+/- TTR KO TTR KO
**Culture**	6	1 2 3 4 5 6

Dimensions	
**Covariance Parameters**	2
**Columns in X**	3
**Columns in Z**	6
**Subjects**	1
**Max Obs per Subject**	31

Number of Observations	
**Number of Observations Read**	31
**Number of Observations Used**	31
**Number of Observations Not Used**	0

Iteration History			
**Iteration**	**Evaluations**	**-2 Res Log Like**	**Criterion**
**0**	1	-71.49781957	
**1**	2	-73.13058042	0.00000041
**2**	1	-73.13060638	0.00000000

Convergence criteria met.

Covariance Parameter Estimates				
**Cov Parm**	**Estimate**	**Alpha**	**Lower**	**Upper**
**Culture**	0.000974	0.05	0.000221	0.1901
**Residual**	0.003439	0.05	0.002113	0.006562

Time=5.666667

Fit Statistics	
-2 Res Log Likelihood	-73.1
AIC (Smaller is Better)	-69.1
AICC (Smaller is Better)	-68.7
BIC (Smaller is Better)	-69.5

Solution for Fixed Effects

Effect	Genotype	Estimate	Standard Error	DF	t Value	Pr > |t|	Alpha	Lower	Upper
Intercept		0.9769	0.02325	4	42.02	<.0001	0.05	0.9123	1.0414
Genotype	Meg+/- TTR KO	-0.05149	0.03308	25	-1.56	0.1321	0.05	-0.1196	0.01664
Genotype	TTR KO	0	.	.	.	.	.	.	.

Solution for Random Effects

Effect	Culture	Estimate	Std Err Pred	DF	t Value	Pr > |t|	Alpha	Lower	Upper
Culture	1	0.02726	0.02426	25	1.12	0.2720	0.05	-0.02272	0.07723
Culture	2	-0.00215	0.02398	25	-0.09	0.9294	0.05	-0.05153	0.04724
Culture	3	-0.02511	0.02426	25	-1.03	0.3107	0.05	-0.07508	0.02486
Culture	4	-0.02195	0.02436	25	-0.90	0.3761	0.05	-0.07212	0.02821
Culture	5	0.000737	0.02436	25	0.03	0.9761	0.05	-0.04943	0.05090
Culture	6	0.02122	0.02436	25	0.87	0.3920	0.05	-0.02895	0.07138

Type 3 Tests of Fixed Effects

Effect	Num DF	Den DF	F Value	Pr > F
Genotype	1	25	2.42	0.1321

Least Squares Means

Effect	Genotype	Estimate	Standard Error	DF	t Value	Pr > |t|	Alpha	Lower	Upper
Genotype	Meg+/- TTR KO	0.9254	0.02353	25	39.32	<.0001	0.05	0.8769	0.9739
Genotype	TTR KO	0.9769	0.02325	25	42.02	<.0001	0.05	0.9290	1.0248

Time=5.666667

Differences of Least Squares Means												
**Effect**	**Genotype**	**Genotype**	**Estimate**	**Standard Error**	**DF**	**t Value**	**Pr > |t|**	**Adjustment**	**Adj P**	**Alpha**	**Lower**	**Upper**
**Genotype**	Meg+/- TTR KO	TTR KO	-0.05149	0.03308	25	-1.56	0.1321	Tukey-Kramer	0.1321	0.05	-0.1196	0.01664

Differences of Least Squares Means				
**Effect**	**Genotype**	**Genotype**	**Adj Lower**	**Adj Upper**
**Genotype**	Meg+/- TTR KO	TTR KO	-0.1196	0.01664

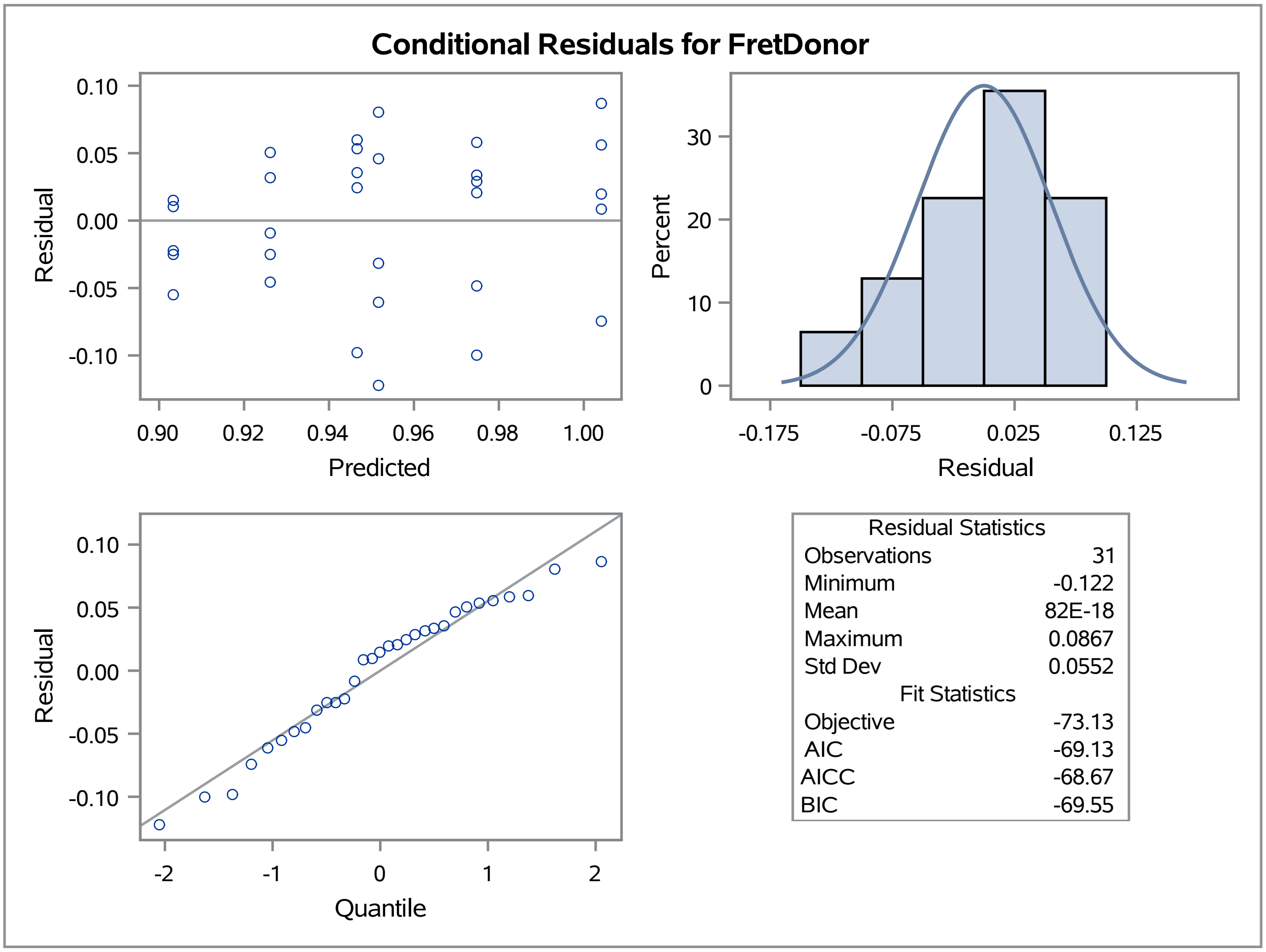

Time=5.833333

Model Information	
**Data Set**	WORK.TEMPDATASORTED
**Dependent Variable**	FretDonor
**Covariance Structure**	Variance Components
**Estimation Method**	REML
**Residual Variance Method**	Profile
**Fixed Effects SE Method**	Model-Based
**Degrees of Freedom Method**	Containment

Class Level Information		
**Class**	**Levels**	**Values**
**Genotype**	2	Meg+/- TTR KO TTR KO
**Culture**	6	1 2 3 4 5 6

Dimensions	
**Covariance Parameters**	2
**Columns in X**	3
**Columns in Z**	6
**Subjects**	1
**Max Obs per Subject**	31

Number of Observations	
**Number of Observations Read**	31
**Number of Observations Used**	31
**Number of Observations Not Used**	0

Iteration History			
**Iteration**	**Evaluations**	**-2 Res Log Like**	**Criterion**
**0**	1	-66.82902223	
**1**	2	-67.94786563	0.00000015
**2**	1	-67.94787470	0.00000000

Convergence criteria met.

Covariance Parameter Estimates				
**Cov Parm**	**Estimate**	**Alpha**	**Lower**	**Upper**
**Culture**	0.000918	0.05	0.000187	0.6604
**Residual**	0.004194	0.05	0.002578	0.008004

Time=5.833333

Fit Statistics	
-2 Res Log Likelihood	-67.9
AIC (Smaller is Better)	-63.9
AICC (Smaller is Better)	-63.5
BIC (Smaller is Better)	-64.4

Solution for Fixed Effects

Effect	Genotype	Estimate	Standard Error	DF	t Value	Pr > |t|	Alpha	Lower	Upper
Intercept		0.9831	0.02386	4	41.21	<.0001	0.05	0.9168	1.0493
Genotype	Meg+/- TTR KO	-0.06884	0.03398	25	-2.03	0.0536	0.05	-0.1388	0.001147
Genotype	TTR KO	0	.	.	.	.	.	.	.

Solution for Random Effects

Effect	Culture	Estimate	Std Err Pred	DF	t Value	Pr > |t|	Alpha	Lower	Upper
Culture	1	0.02104	0.02437	25	0.86	0.3961	0.05	-0.02914	0.07122
Culture	2	0.006881	0.02409	25	0.29	0.7775	0.05	-0.04273	0.05649
Culture	3	-0.02792	0.02437	25	-1.15	0.2627	0.05	-0.07810	0.02226
Culture	4	-0.02052	0.02446	25	-0.84	0.4094	0.05	-0.07089	0.02985
Culture	5	0.005574	0.02446	25	0.23	0.8216	0.05	-0.04480	0.05594
Culture	6	0.01495	0.02446	25	0.61	0.5466	0.05	-0.03542	0.06532

Type 3 Tests of Fixed Effects

Effect	Num DF	Den DF	F Value	Pr > F
Genotype	1	25	4.10	0.0536

Least Squares Means

Effect	Genotype	Estimate	Standard Error	DF	t Value	Pr > |t|	Alpha	Lower	Upper
Genotype	Meg+/- TTR KO	0.9142	0.02420	25	37.78	<.0001	0.05	0.8644	0.9641
Genotype	TTR KO	0.9831	0.02386	25	41.21	<.0001	0.05	0.9339	1.0322

Time=5.833333

Differences of Least Squares Means												
**Effect**	**Genotype**	**Genotype**	**Estimate**	**Standard Error**	**DF**	**t Value**	**Pr > |t|**	**Adjustment**	**Adj P**	**Alpha**	**Lower**	**Upper**
**Genotype**	Meg+/- TTR KO	TTR KO	-0.06884	0.03398	25	-2.03	0.0536	Tukey-Kramer	0.0536	0.05	-0.1388	0.001147

Differences of Least Squares Means				
**Effect**	**Genotype**	**Genotype**	**Adj Lower**	**Adj Upper**
**Genotype**	Meg+/- TTR KO	TTR KO	-0.1388	0.001147

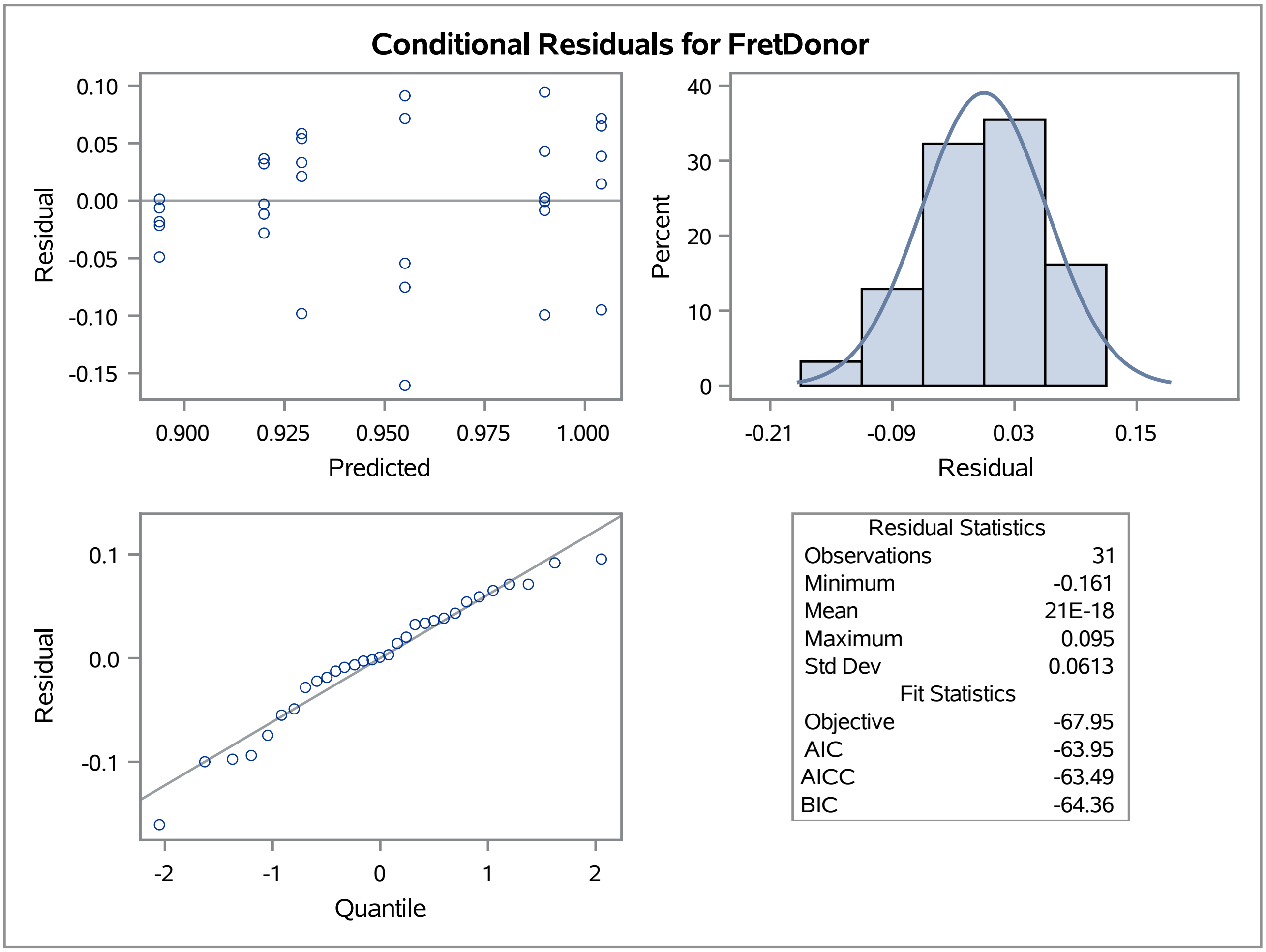

Time=6

Model Information	
**Data Set**	WORK.TEMPDATASORTED
**Dependent Variable**	FretDonor
**Covariance Structure**	Variance Components
**Estimation Method**	REML
**Residual Variance Method**	Profile
**Fixed Effects SE Method**	Model-Based
**Degrees of Freedom Method**	Containment

Class Level Information		
**Class**	**Levels**	**Values**
**Genotype**	2	Meg+/- TTR KO TTR KO
**Culture**	6	1 2 3 4 5 6

Dimensions	
**Covariance Parameters**	2
**Columns in X**	3
**Columns in Z**	6
**Subjects**	1
**Max Obs per Subject**	31

Number of Observations	
**Number of Observations Read**	31
**Number of Observations Used**	31
**Number of Observations Not Used**	0

Iteration History			
**Iteration**	**Evaluations**	**-2 Res Log Like**	**Criterion**
**0**	1	-67.80856483	
**1**	2	-83.86663620	0.00000341
**2**	1	-83.86687346	0.00000000

Convergence criteria met.

Covariance Parameter Estimates				
**Cov Parm**	**Estimate**	**Alpha**	**Lower**	**Upper**
**Culture**	0.003963	0.05	0.001325	0.04491
**Residual**	0.001916	0.05	0.001178	0.003652

Time=6

Fit Statistics	
**-2 Res Log Likelihood**	-83.9
**AIC (Smaller is Better)**	-79.9
**AICC (Smaller is Better)**	-79.4
**BIC (Smaller is Better)**	-80.3

Solution for Fixed Effects

Effect	Genotype	Estimate	Standard Error	DF	t Value	Pr > |t|	Alpha	Lower	Upper
**Intercept**		0.9913	0.03797	4	26.11	<.0001	0.05	0.8859	1.0967
**Genotype**	Meg+/- TTR KO	-0.07812	0.05376	25	-1.45	0.1587	0.05	-0.1888	0.03261
**Genotype**	TTR KO	0	.	.	.	.	.	.	.

Solution for Random Effects

Effect	Culture	Estimate	Std Err Pred	DF	t Value	Pr > |t|	Alpha	Lower	Upper
**Culture**	1	0.06102	0.03934	25	1.55	0.1335	0.05	-0.02001	0.1420
**Culture**	2	0.02526	0.03912	25	0.65	0.5244	0.05	-0.05530	0.1058
**Culture**	3	-0.08627	0.03934	25	-2.19	0.0378	0.05	-0.1673	-0.00524
**Culture**	4	-0.03885	0.03942	25	-0.99	0.3338	0.05	-0.1200	0.04234
**Culture**	5	0.004855	0.03942	25	0.12	0.9030	0.05	-0.07633	0.08604
**Culture**	6	0.03400	0.03942	25	0.86	0.3967	0.05	-0.04719	0.1152

Type 3 Tests of Fixed Effects

Effect	Num DF	Den DF	F Value	Pr > F
**Genotype**	1	25	2.11	0.1587

Least Squares Means

Effect	Genotype	Estimate	Standard Error	DF	t Value	Pr > |t|	Alpha	Lower	Upper
**Genotype**	Meg+/- TTR KO	0.9132	0.03806	25	23.99	<.0001	0.05	0.8348	0.9915
**Genotype**	TTR KO	0.9913	0.03797	25	26.11	<.0001	0.05	0.9131	1.0695

Time=6

Differences of Least Squares Means												
**Effect**	**Genotype**	**Genotype**	**Estimate**	**Standard Error**	**DF**	**t Value**	**Pr > |t|**	**Adjustment**	**Adj P**	**Alpha**	**Lower**	**Upper**
**Genotype**	Meg+/- TTR KO	TTR KO	-0.07812	0.05376	25	-1.45	0.1587	Tukey-Kramer	0.1587	0.05	-0.1888	0.03261

Differences of Least Squares Means				
**Effect**	**Genotype**	**Genotype**	**Adj Lower**	**Adj Upper**
**Genotype**	Meg+/- TTR KO	TTR KO	-0.1888	0.03261

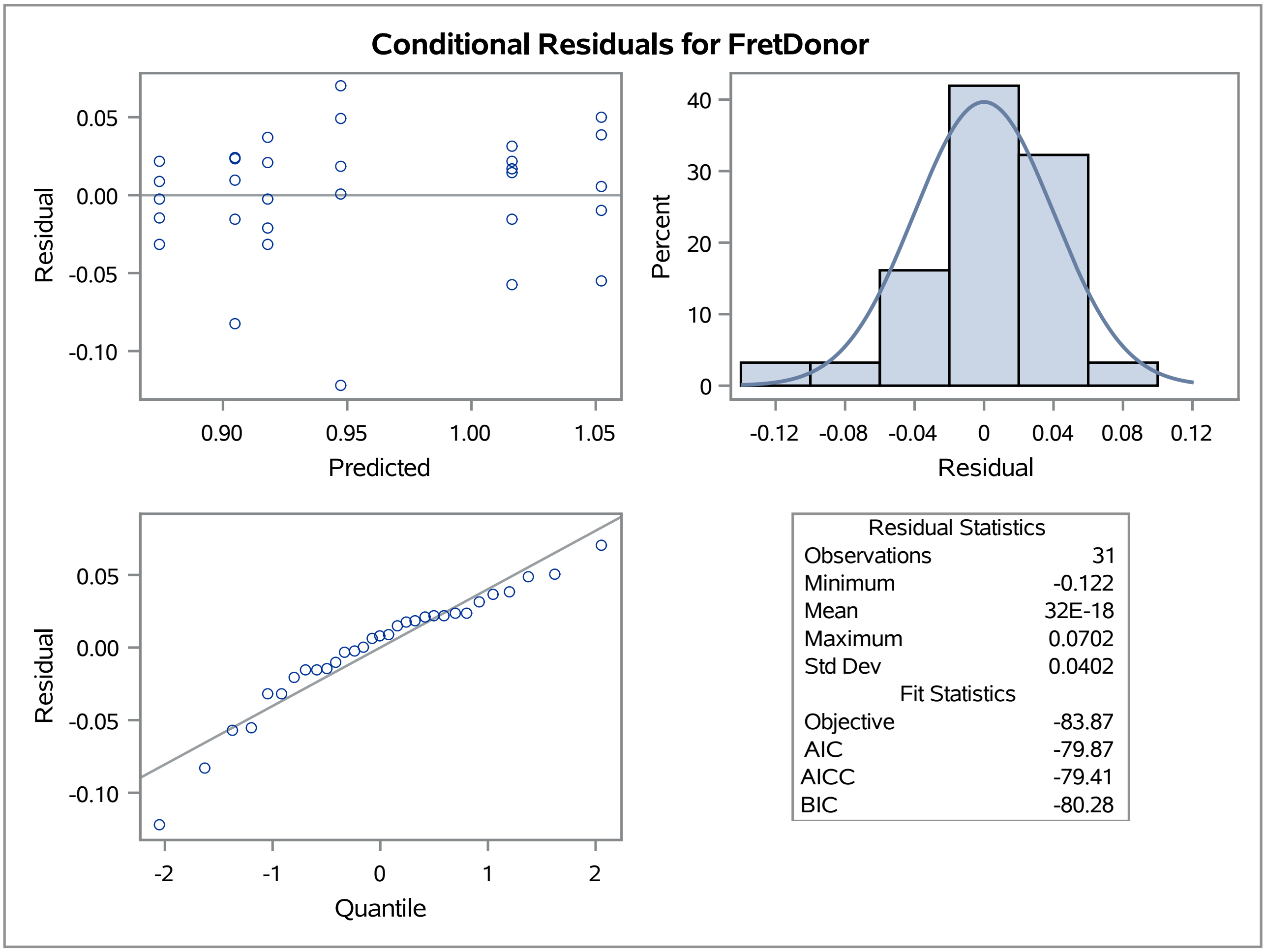

**Data Set Name**	WORK.FIG6CNOVA	**Observations**	80
**Member Type**	DATA	**Variables**	3
**Engine**	V9	**Indexes**	0
**Created**	07/10/2020 13:56:30	**Observation Length**	32
**Last Modified**	07/10/2020 13:56:30	**Deleted Observations**	0
**Protection**		**Compressed**	NO
**Data Set Type**		**Sorted**	NO
**Label**			
**Data Representation**	SOLARIS_X86_64, LINUX_X86_64, ALPHA_TRU64, LINUX_IA64		
**Encoding**	utf-8 Unicode (UTF-8)		

Engine/Host Dependent Information	
**Data Set Page Size**	65536
**Number of Data Set Pages**	1
**First Data Page**	1
**Max Obs per Page**	2038
**Obs in First Data Page**	80
**Number of Data Set Repairs**	0
**Filename**	/tmp/SAS_work3BDE00000987_localhost.localdomain/SAS_workCC9600000987_localhost.localdomain/fig6cnova.sas7bdat
**Release Created**	9.0401M6
**Host Created**	Linux
**Inode Number**	672686
**Access Permission**	rw-rw-r--
**Owner Name**	sasdemo
**File Size**	128KB
**File Size (bytes)**	131072

Alphabetic List of Variables and Attributes

#	Variable	Type	Len	Format	Informat	Label
**1**	Condition	Char	10	$10.	$10.	Condition
**2**	Culture	Num	8	BEST.		Culture
**3**	Neurite_length	Num	8	BEST.		Neurite length

Model Information	
**Data Set**	WORK.FIG6CNOVA
**Dependent Variable**	Neurite_length
**Covariance Structure**	Variance Components
**Estimation Method**	REML
**Residual Variance Method**	Profile
**Fixed Effects SE Method**	Model-Based
**Degrees of Freedom Method**	Containment

Class Level Information		
**Class**	**Levels**	**Values**
**Condition**	4	GFPMeg_Ct GFPMeg_TTR GFP_Ct GFP_TTR
**Culture**	20	1 2 3 4 5 6 7 8 9 10 11 12 13 14 15 16 17 18 19 20

Dimensions	
**Covariance Parameters**	2
**Columns in X**	5
**Columns in Z**	20
**Subjects**	1
**Max Obs per Subject**	80

Number of Observations	
**Number of Observations Read**	80
**Number of Observations Used**	80
**Number of Observations Not Used**	0

Iteration History			
**Iteration**	**Evaluations**	**-2 Res Log Like**	**Criterion**
**0**	1	1148.14454635	
**1**	3	1124.66176883	0.00003626
**2**	1	1124.64290351	0.00000030
**3**	1	1124.64275513	0.00000000

Convergence criteria met.

Covariance Parameter Estimates							
Cov Parm	Estimate	Standard Error	Z Value	Pr > Z	Alpha	Lower	Upper
**Culture**	101079	44167	2.29	0.0111	0.05	50018	301258
**Residual**	94583	17222	5.49	<.0001	0.05	68185	140022

Fit Statistics	
**-2 Res Log Likelihood**	1124.6
**AIC (Smaller is Better)**	1128.6
**AICC (Smaller is Better)**	1128.8
**BIC (Smaller is Better)**	1130.6

Solution for Fixed Effects									
Effect	Condition	Estimate	Standard Error	DF	t Value	Pr > |t|	Alpha	Lower	Upper
**Intercept**		1320.30	158.89	16	8.31	<.0001	0.05	983.47	1657.13
**Condition**	GFPMeg_Ct	-74.6653	215.20	60	-0.35	0.7298	0.05	-505.13	355.80
**Condition**	GFPMeg_TTR	378.57	237.41	60	1.59	0.1161	0.05	-96.3180	853.47
**Condition**	GFP_Ct	-571.22	224.17	60	-2.55	0.0134	0.05	-1019.61	-122.82
**Condition**	GFP_TTR	0	.	.	.	.	.	.	.

Solution for Random Effects									
Effect	Culture	Estimate	Std Err Pred	DF	t Value	Pr > |t|	Alpha	Lower	Upper
**Culture**	1	55.5630	179.86	60	0.31	0.7585	0.05	-304.21	415.34
**Culture**	2	70.8356	196.37	60	0.36	0.7196	0.05	-321.96	463.63
**Culture**	3	71.2106	188.64	60	0.38	0.7071	0.05	-306.13	448.55
**Culture**	4	-14.4496	196.37	60	-0.07	0.9416	0.05	-407.24	378.34
**Culture**	5	-183.16	183.51	60	-1.00	0.3223	0.05	-550.24	183.92
**Culture**	6	-89.9181	189.06	60	-0.48	0.6361	0.05	-468.09	288.26
**Culture**	7	195.47	196.72	60	0.99	0.3244	0.05	-198.03	588.97
**Culture**	8	-62.1776	189.06	60	-0.33	0.7434	0.05	-440.35	316.00
**Culture**	9	46.1589	189.06	60	0.24	0.8079	0.05	-332.02	424.33
**Culture**	10	-89.5332	189.06	60	-0.47	0.6375	0.05	-467.71	288.64
**Culture**	11	509.27	181.66	60	2.80	0.0068	0.05	145.90	872.63
**Culture**	12	-488.52	181.66	60	-2.69	0.0093	0.05	-851.89	-125.15
**Culture**	13	105.86	190.45	60	0.56	0.5804	0.05	-275.10	486.82
**Culture**	14	53.1533	175.73	60	0.30	0.7633	0.05	-298.37	404.67
**Culture**	15	126.30	181.66	60	0.70	0.4896	0.05	-237.07	489.67
**Culture**	16	-306.06	190.45	60	-1.61	0.1133	0.05	-687.02	74.9022

Solution for Random Effects									
**Effect**	**Culture**	**Estimate**	**Std Err Pred**	**DF**	**t Value**	**Pr > |t|**	**Alpha**	**Lower**	**Upper**
**Culture**	17	64.7623	199.00	60	0.33	0.7460	0.05	-333.30	462.82
**Culture**	18	574.11	199.00	60	2.88	0.0054	0.05	176.05	972.17
**Culture**	19	-449.40	192.16	60	-2.34	0.0227	0.05	-833.78	-65.0224
**Culture**	20	-189.47	205.21	60	-0.92	0.3596	0.05	-599.96	221.02

Type 3 Tests of Fixed Effects				
**Effect**	**Num DF**	**Den DF**	**F Value**	**Pr > F**
**Condition**	3	60	5.55	0.0020

Least Squares Means									
**Effect**	**Condition**	**Estimate**	**Standard Error**	**DF**	**t Value**	**Pr > |t|**	**Alpha**	**Lower**	**Upper**
**Condition**	GFPMeg_Ct	1245.64	145.14	60	8.58	<.0001	0.05	955.31	1535.96
**Condition**	GFPMeg_TTR	1698.88	176.41	60	9.63	<.0001	0.05	1346.01	2051.74
**Condition**	GFP_Ct	749.08	158.13	60	4.74	<.0001	0.05	432.78	1065.39
**Condition**	GFP_TTR	1320.30	158.89	60	8.31	<.0001	0.05	1002.48	1638.12

Differences of Least Squares Means												
**Effect**	**Condition**	**Condition**	**Estimate**	**Standard Error**	**DF**	**t Value**	**Pr > |t|**	**Adjustment**	**Adj P**	**Alpha**	**Lower**	**Upper**
**Condition**	GFPMeg_Ct	GFPMeg_TTR	-453.24	228.44	60	-1.98	0.0518	Tukey-Kramer	0.2054	0.05	-910.19	3.7105
**Condition**	GFPMeg_Ct	GFP_Ct	496.55	214.64	60	2.31	0.0241	Tukey-Kramer	0.1064	0.05	67.2022	925.90
**Condition**	GFPMeg_Ct	GFP_TTR	-74.6653	215.20	60	-0.35	0.7298	Tukey-Kramer	0.9855	0.05	-505.13	355.80
**Condition**	GFPMeg_TTR	GFP_Ct	949.79	236.90	60	4.01	0.0002	Tukey-Kramer	0.0010	0.05	475.91	1423.67
**Condition**	GFPMeg_TTR	GFP_TTR	378.57	237.41	60	1.59	0.1161	Tukey-Kramer	0.3894	0.05	-96.3180	853.47
**Condition**	GFP_Ct	GFP_TTR	-571.22	224.17	60	-2.55	0.0134	Tukey-Kramer	0.0627	0.05	-1019.61	-122.82

Differences of Least Squares Means				
**Effect**	**Condition**	**Condition**	**Adj Lower**	**Adj Upper**
**Condition**	GFPMeg_Ct	GFPMeg_TTR	-1056.90	150.42
**Condition**	GFPMeg_Ct	GFP_Ct	-70.6465	1063.75
**Condition**	GFPMeg_Ct	GFP_TTR	-643.34	494.01
**Condition**	GFPMeg_TTR	GFP_Ct	323.77	1575.82
**Condition**	GFPMeg_TTR	GFP_TTR	-248.79	1005.94
**Condition**	GFP_Ct	GFP_TTR	-1163.58	21.1449

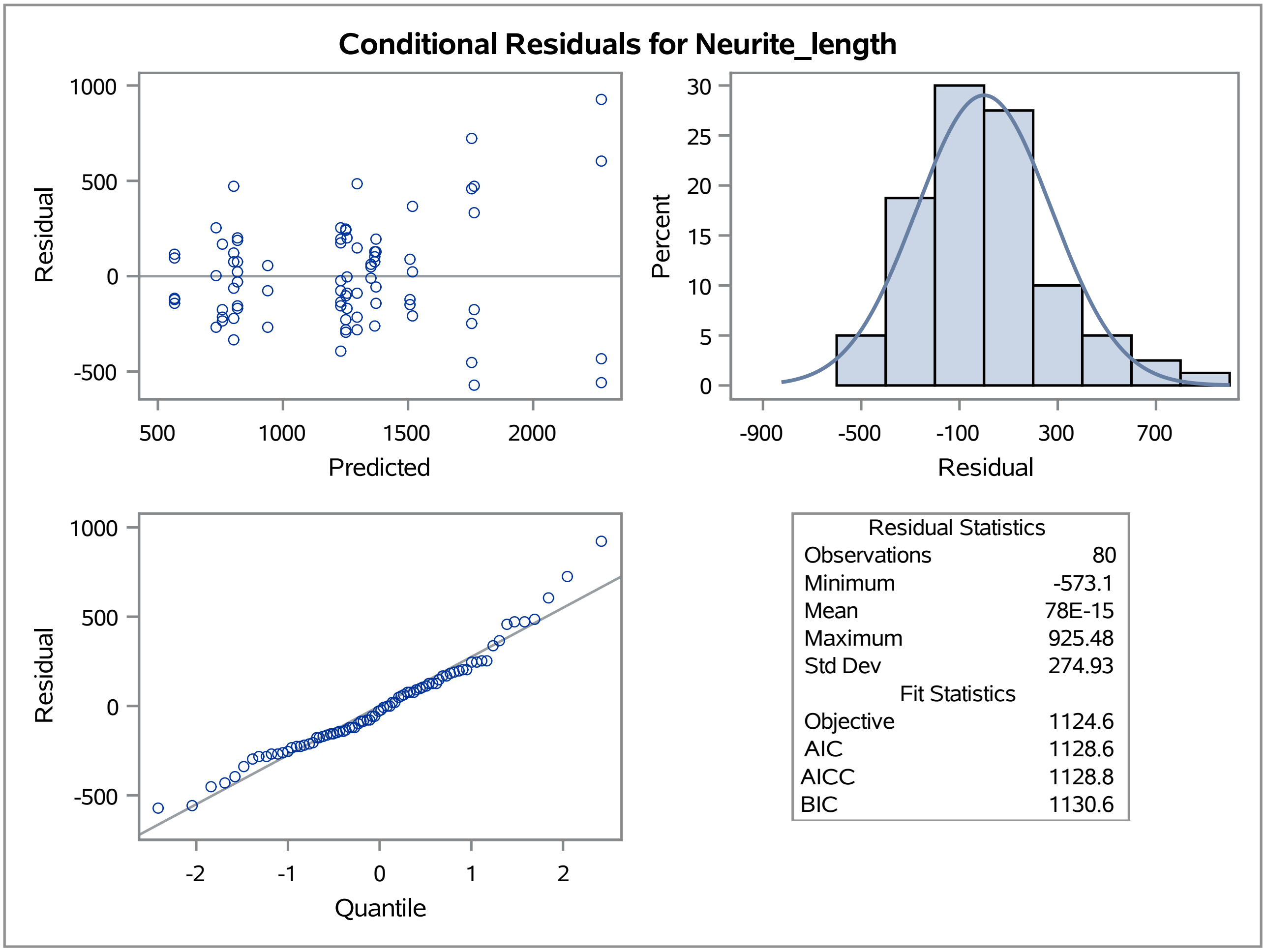

Residual Statistics	
Observations	80
Minimum	-573.1
Mean	78E-15
Maximum	925.48
Std Dev	274.93

Fit Statistics	
Objective	1124.6
AIC	1128.6
AICC	1128.8
BIC	1130.6

**Data Set Name**	WORK.FIG6DNOVA	**Observations**	83
**Member Type**	DATA	**Variables**	3
**Engine**	V9	**Indexes**	0
**Created**	07/10/2020 13:58:06	**Observation Length**	32
**Last Modified**	07/10/2020 13:58:06	**Deleted Observations**	0
**Protection**		**Compressed**	NO
**Data Set Type**		**Sorted**	NO
**Label**			
**Data Representation**	SOLARIS_X86_64, LINUX_X86_64, ALPHA_TRU64, LINUX_IA64		
**Encoding**	utf-8 Unicode (UTF-8)		

Engine/Host Dependent Information	
**Data Set Page Size**	65536
**Number of Data Set Pages**	1
**First Data Page**	1
**Max Obs per Page**	2038
**Obs in First Data Page**	83
**Number of Data Set Repairs**	0
**Filename**	/tmp/SAS_work3BDE00000987_localhost.localdomain/SAS_workCC9600000987_localhost.localdomain/fig6dnova.sas7bdat
**Release Created**	9.0401M6
**Host Created**	Linux
**Inode Number**	672693
**Access Permission**	rw-rw-r--
**Owner Name**	sasdemo
**File Size**	128KB
**File Size (bytes)**	131072

Alphabetic List of Variables and Attributes

#	Variable	Type	Len	Format	Informat	Label
**1**	Condition	Char	10	$10.	$10.	Condition
**2**	Culture	Num	8	BEST.		Culture
**3**	Neurite_number	Num	8	BEST.		Neurite number

Model Information	
**Data Set**	WORK.FIG6DNOVA
**Dependent Variable**	Neurite_number
**Covariance Structure**	Variance Components
**Estimation Method**	REML
**Residual Variance Method**	Profile
**Fixed Effects SE Method**	Model-Based
**Degrees of Freedom Method**	Containment

Class Level Information		
**Class**	**Levels**	**Values**
**Condition**	4	GFPMeg_Ct GFPMeg_TTR GFP_Ct GFP_TTR
**Culture**	21	1 2 3 4 5 6 7 8 9 10 11 12 13 14 15 16 17 18 19 20 21

Dimensions	
**Covariance Parameters**	2
**Columns in X**	5
**Columns in Z**	21
**Subjects**	1
**Max Obs per Subject**	83

Number of Observations	
**Number of Observations Read**	83
**Number of Observations Used**	83
**Number of Observations Not Used**	0

Iteration History			
**Iteration**	**Evaluations**	**-2 Res Log Like**	**Criterion**
**0**	1	515.17764629	
**1**	2	508.16728455	0.00000034
**2**	1	508.16722207	0.00000000

Convergence criteria met.

Covariance Parameter Estimates							
**Cov Parm**	**Estimate**	**Standard Error**	**Z Value**	**Pr > Z**	**Alpha**	**Lower**	**Upper**
**Culture**	9.9166	5.7270	1.73	0.0417	0.05	4.1170	48.1159
**Residual**	25.6390	4.5886	5.59	<.0001	0.05	18.5782	37.6781

Fit Statistics	
**-2 Res Log Likelihood**	508.2
**AIC (Smaller is Better)**	512.2
**AICC (Smaller is Better)**	512.3
**BIC (Smaller is Better)**	514.3

Solution for Fixed Effects

Effect	Condition	Estimate	Standard Error	DF	t Value	Pr > |t|	Alpha	Lower	Upper
**Intercept**		21.6500	1.8070	17	11.98	<.0001	0.05	17.8376	25.4624
**Condition**	GFPMeg_Ct	0.9137	2.4605	62	0.37	0.7116	0.05	-4.0048	5.8323
**Condition**	GFPMeg_TTR	5.8378	2.6101	62	2.24	0.0289	0.05	0.6203	11.0554
**Condition**	GFP_Ct	-4.3226	2.5266	62	-1.71	0.0921	0.05	-9.3731	0.7279
**Condition**	GFP_TTR	0	.	.	.	.	.	.	.

Solution for Random Effects

Effect	Culture	Estimate	Std Err Pred	DF	t Value	Pr > |t|	Alpha	Lower	Upper
**Culture**	1	1.5184	2.1235	62	0.72	0.4773	0.05	-2.7265	5.7632
**Culture**	2	2.5344	2.2458	62	1.13	0.2634	0.05	-1.9549	7.0238
**Culture**	3	1.7752	2.2458	62	0.79	0.4323	0.05	-2.7141	6.2645
**Culture**	4	-1.2618	2.2458	62	-0.56	0.5762	0.05	-5.7511	3.2275
**Culture**	5	-4.5662	2.1760	62	-2.10	0.0399	0.05	-8.9159	-0.2165
**Culture**	6	2.0348	2.2579	62	0.90	0.3710	0.05	-2.4786	6.5482
**Culture**	7	2.0348	2.2579	62	0.90	0.3710	0.05	-2.4786	6.5482
**Culture**	8	0.5163	2.2579	62	0.23	0.8199	0.05	-3.9971	5.0297
**Culture**	9	-2.3689	2.2579	62	-1.05	0.2982	0.05	-6.8823	2.1445
**Culture**	10	-2.2170	2.2579	62	-0.98	0.3300	0.05	-6.7304	2.2964
**Culture**	11	4.5168	2.2186	62	2.04	0.0460	0.05	0.08184	8.9517
**Culture**	12	0.2650	2.2186	62	0.12	0.9053	0.05	-4.1700	4.6999
**Culture**	13	0.4134	2.3227	62	0.18	0.8593	0.05	-4.2296	5.0564
**Culture**	14	-0.8989	2.1429	62	-0.42	0.6763	0.05	-5.1824	3.3846
**Culture**	15	-0.9498	2.2186	62	-0.43	0.6701	0.05	-5.3847	3.4851
**Culture**	16	-3.3464	2.3227	62	-1.44	0.1547	0.05	-7.9894	1.2966
**Culture**	17	0.4415	2.5032	62	0.18	0.8606	0.05	-4.5624	5.4454
**Culture**	18	-2.5895	2.3693	62	-1.09	0.2787	0.05	-7.3257	2.1467
**Culture**	19	0.007381	2.2808	62	0.00	0.9974	0.05	-4.5519	4.5666
**Culture**	20	2.5888	2.2808	62	1.14	0.2607	0.05	-1.9704	7.1481
**Culture**	21	-0.4482	2.2808	62	-0.20	0.8449	0.05	-5.0074	4.1111

Type 3 Tests of Fixed Effects				
Effect	Num DF	Den DF	F Value	Pr > F
Condition	3	62	5.21	0.0028

Least Squares Means									
Effect	Condition	Estimate	Standard Error	DF	t Value	Pr > |t|	Alpha	Lower	Upper
Condition	GFPMeg_Ct	22.5637	1.6700	62	13.51	<.0001	0.05	19.2254	25.9021
Condition	GFPMeg_TTR	27.4878	1.8835	62	14.59	<.0001	0.05	23.7229	31.2528
Condition	GFP_Ct	17.3274	1.7658	62	9.81	<.0001	0.05	13.7975	20.8573
Condition	GFP_TTR	21.6500	1.8070	62	11.98	<.0001	0.05	18.0378	25.2622

Differences of Least Squares Means												
Effect	Condition	Condition	Estimate	Standard Error	DF	t Value	Pr > |t|	Adjustment	Adj P	Alpha	Lower	Upper
Condition	GFPMeg_Ct	GFPMeg_TTR	-4.9241	2.5172	62	-1.96	0.0550	Tukey-Kramer	0.2158	0.05	-9.9560	0.1077
Condition	GFPMeg_Ct	GFP_Ct	5.2364	2.4305	62	2.15	0.0351	Tukey-Kramer	0.1476	0.05	0.3779	10.0948
Condition	GFPMeg_Ct	GFP_TTR	0.9137	2.4605	62	0.37	0.7116	Tukey-Kramer	0.9824	0.05	-4.0048	5.8323
Condition	GFPMeg_TTR	GFP_Ct	10.1605	2.5818	62	3.94	0.0002	Tukey-Kramer	0.0012	0.05	4.9996	15.3214
Condition	GFPMeg_TTR	GFP_TTR	5.8378	2.6101	62	2.24	0.0289	Tukey-Kramer	0.1248	0.05	0.6203	11.0554
Condition	GFP_Ct	GFP_TTR	-4.3226	2.5266	62	-1.71	0.0921	Tukey-Kramer	0.3268	0.05	-9.3731	0.7279

Differences of Least Squares Means				
Effect	Condition	Condition	Adj Lower	Adj Upper
Condition	GFPMeg_Ct	GFPMeg_TTR	-11.5698	1.7216
Condition	GFPMeg_Ct	GFP_Ct	-1.1804	11.6531
Condition	GFPMeg_Ct	GFP_TTR	-5.5824	7.4098
Condition	GFPMeg_TTR	GFP_Ct	3.3443	16.9767
Condition	GFPMeg_TTR	GFP_TTR	-1.0531	12.7288
Condition	GFP_Ct	GFP_TTR	-10.9930	2.3477

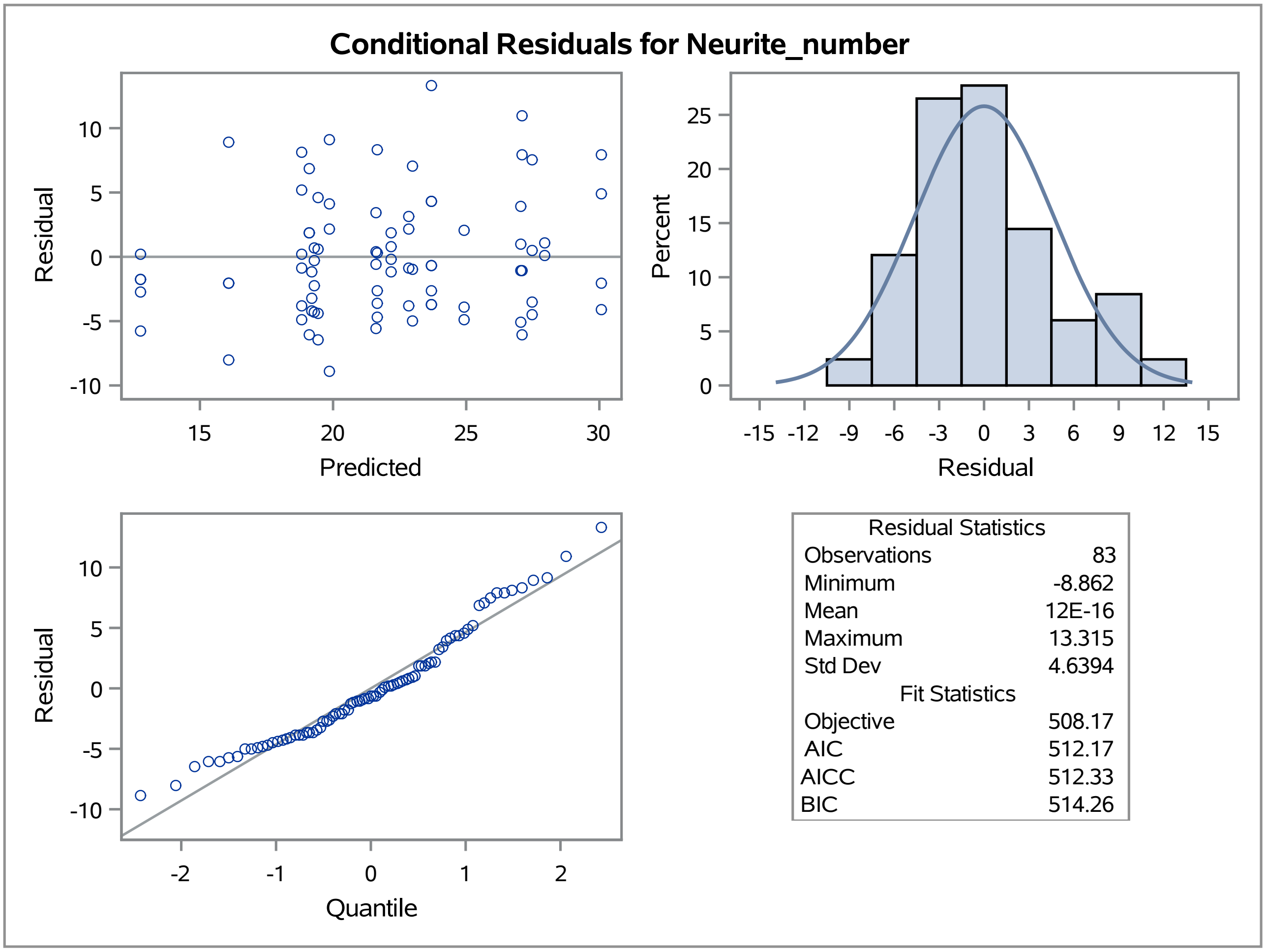
Conditional Residuals for Neurite_number
Residual
Predicted
Percent
Residual
Residual
Quantile
Residual Statistics
Observations 83
Minimum -8.862
Mean 12E-16
Maximum 13.315
Std Dev 4.6394
Fit Statistics
Objective 508.17
AIC 512.17
AICC 512.33
BIC 514.26

**Data Set Name**	WORK.FIG6E	**Observations**	3916
**Member Type**	DATA	**Variables**	50
**Engine**	V9	**Indexes**	0
**Created**	07/08/2020 00:04:20	**Observation Length**	88
**Last Modified**	07/08/2020 00:04:20	**Deleted Observations**	0
**Protection**		**Compressed**	NO
**Data Set Type**		**Sorted**	NO
**Label**			
**Data Representation**	SOLARIS_X86_64, LINUX_X86_64, ALPHA_TRU64, LINUX_IA64		
**Encoding**	utf-8 Unicode (UTF-8)		

Engine/Host Dependent Information	
**Data Set Page Size**	65536
**Number of Data Set Pages**	6
**First Data Page**	1
**Max Obs per Page**	743
**Obs in First Data Page**	646
**Number of Data Set Repairs**	0
**Filename**	/tmp/SAS_workF39800000A61_localhost.localdomain/SAS_work146A00000A61_localhost.localdomain/fig6e.sas7bdat
**Release Created**	9.0401M6
**Host Created**	Linux
**Inode Number**	672609
**Access Permission**	rw-rw-r--
**Owner Name**	sasdemo
**File Size**	448KB
**File Size (bytes)**	458752

Alphabetic List of Variables and Attributes

#	Variable	Type	Len	Format	Informat	Label
**27**	AA	Char	1	$1.	$1.	AA
**28**	AB	Char	1	$1.	$1.	AB
**30**	AD	Char	1	$1.	$1.	AD
**31**	AE	Char	1	$1.	$1.	AE
**32**	AF	Char	1	$1.	$1.	AF
**33**	AG	Char	1	$1.	$1.	AG
**35**	AI	Char	1	$1.	$1.	AI
**36**	AJ	Char	1	$1.	$1.	AJ
**37**	AK	Char	1	$1.	$1.	AK
**39**	AM	Char	1	$1.	$1.	AM

Alphabetic List of Variables and Attributes						
**#**	**Variable**	**Type**	**Len**	**Format**	**Informat**	**Label**
**40**	AN	Char	1	$1.	$1.	AN
**41**	AO	Char	1	$1.	$1.	AO
**43**	AQ	Char	1	$1.	$1.	AQ
**44**	AR	Char	1	$1.	$1.	AR
**45**	AS	Char	1	$1.	$1.	AS
**47**	AU	Char	1	$1.	$1.	AU
**48**	AV	Char	1	$1.	$1.	AV
**49**	AW	Char	1	$1.	$1.	AW
**50**	AX	Char	1	$1.	$1.	AX
**3**	Culture	Num	8	BEST.		Culture
**7**	Culture_1	Char	1	$1.	$1.	Culture 1
**10**	Culture_2	Char	1	$1.	$1.	Culture 2
**13**	Culture_3	Char	1	$1.	$1.	Culture 3
**17**	Culture_4	Char	1	$1.	$1.	Culture 4
**21**	Culture_5	Char	1	$1.	$1.	Culture 5
**25**	Culture_6	Char	1	$1.	$1.	Culture 6
**26**	Culture_7	Char	1	$1.	$1.	Culture 7
**29**	Culture_8	Char	1	$1.	$1.	Culture 8
**34**	Culture_9	Char	1	$1.	$1.	Culture 9
**38**	Culture_10	Char	1	$1.	$1.	Culture 10
**42**	Culture_11	Char	1	$1.	$1.	Culture 11
**46**	Culture_12	Char	1	$1.	$1.	Culture 12
**1**	DistSoma	Num	8	BEST.		DistSoma
**5**	E	Char	1	$1.	$1.	E
**6**	F	Char	1	$1.	$1.	F
**8**	H	Char	1	$1.	$1.	H
**9**	I	Char	1	$1.	$1.	I
**4**	Interceptions	Num	8	BEST.		Interceptions
**11**	K	Char	1	$1.	$1.	K
**12**	L	Char	1	$1.	$1.	L
**14**	N	Char	1	$1.	$1.	N
**15**	O	Char	1	$1.	$1.	O
**16**	P	Char	1	$1.	$1.	P
**18**	R	Char	1	$1.	$1.	R
**19**	S	Char	1	$1.	$1.	S
**20**	T	Char	1	$1.	$1.	T
**2**	Treatment	Char	11	$11.	$11.	Treatment

Alphabetic List of Variables and Attributes						
**#**	**Variable**	**Type**	**Len**	**Format**	**Informat**	**Label**
**22**	V	Char	1	$1.	$1.	V
**23**	W	Char	1	$1.	$1.	W
**24**	X	Char	1	$1.	$1.	X

DistSoma=0

Model Information	
**Data Set**	WORK.TEMPDATASORTED
**Dependent Variable**	Interceptions
**Covariance Structure**	Variance Components
**Estimation Method**	REML
**Residual Variance Method**	Profile
**Fixed Effects SE Method**	Model-Based
**Degrees of Freedom Method**	Containment

Class Level Information		
**Class**	**Levels**	**Values**
**Treatment**	2	Control_GFP GFP MsTTR
**Culture**	12	1 2 3 4 5 6 7 8 9 10 11 12

Dimensions	
**Covariance Parameters**	2
**Columns in X**	3
**Columns in Z**	12
**Subjects**	1
**Max Obs per Subject**	44

Number of Observations	
**Number of Observations Read**	44
**Number of Observations Used**	44
**Number of Observations Not Used**	0

Iteration History			
**Iteration**	**Evaluations**	**-2 Res Log Like**	**Criterion**
**0**	1	215.82930584	
**1**	2	215.81235487	0.00000000

Convergence criteria met.

Covariance Parameter Estimates				
**Cov Parm**	**Estimate**	**Alpha**	**Lower**	**Upper**
**Culture**	0.1627	0.05	0.01974	2.844E98
**Residual**	8.4775	0.05	5.4757	14.8618

DistSoma=0

Fit Statistics	
**-2 Res Log Likelihood**	215.8
**AIC (Smaller is Better)**	219.8
**AICC (Smaller is Better)**	220.1
**BIC (Smaller is Better)**	220.8

Solution for Fixed Effects

Effect	Treatment	Estimate	Standard Error	DF	t Value	Pr > |t|	Alpha	Lower	Upper
**Intercept**		10.8698	0.6467	10	16.81	<.0001	0.05	9.4288	12.3108
**Treatment**	Control_GFP	-1.4676	0.9105	32	-1.61	0.1168	0.05	-3.3223	0.3871
**Treatment**	GFP MsTTR	0	.	.	.	.	.	.	.

Solution for Random Effects

Effect	Culture	Estimate	Std Err Pred	DF	t Value	Pr > |t|	Alpha	Lower	Upper
**Culture**	1	0.1052	0.3938	32	0.27	0.7912	0.05	-0.6970	0.9073
**Culture**	2	0.05071	0.3938	32	0.13	0.8984	0.05	-0.7515	0.8529
**Culture**	3	0.006979	0.3914	32	0.02	0.9859	0.05	-0.7904	0.8043
**Culture**	4	0.04263	0.3914	32	0.11	0.9139	0.05	-0.7547	0.8400
**Culture**	5	-0.09999	0.3914	32	-0.26	0.8000	0.05	-0.8973	0.6973
**Culture**	6	-0.1017	0.3998	32	-0.25	0.8007	0.05	-0.9161	0.7126
**Culture**	7	-0.00375	0.3938	32	-0.01	0.9925	0.05	-0.8059	0.7984
**Culture**	8	-0.00611	0.3895	32	-0.02	0.9876	0.05	-0.7995	0.7872
**Culture**	9	-0.1155	0.3915	32	-0.30	0.7699	0.05	-0.9129	0.6819
**Culture**	10	0.1876	0.3915	32	0.48	0.6351	0.05	-0.6099	0.9850
**Culture**	11	0.06277	0.3915	32	0.16	0.8736	0.05	-0.7347	0.8602
**Culture**	12	-0.1287	0.3895	32	-0.33	0.7432	0.05	-0.9221	0.6646

Type 3 Tests of Fixed Effects

Effect	Num DF	Den DF	F Value	Pr > F
**Treatment**	1	32	2.60	0.1168

Least Squares Means

Effect	Treatment	Estimate	Standard Error	DF	t Value	Pr > |t|	Alpha	Lower	Upper
**Treatment**	Control_GFP	9.4021	0.6410	32	14.67	<.0001	0.05	8.0965	10.7077
**Treatment**	GFP MsTTR	10.8698	0.6467	32	16.81	<.0001	0.05	9.5524	12.1871

DistSoma=0

Differences of Least Squares Means												
**Effect**	**Treatment**	**Treatment**	**Estimate**	**Standard Error**	**DF**	**t Value**	**Pr > |t|**	**Adjustment**	**Adj P**	**Alpha**	**Lower**	**Upper**
**Treatment**	Control_GFP	GFP MsTTR	-1.4676	0.9105	32	-1.61	0.1168	Tukey-Kramer	0.1168	0.05	-3.3223	0.3871

Differences of Least Squares Means				
**Effect**	**Treatment**	**Treatment**	**Adj Lower**	**Adj Upper**
**Treatment**	Control_GFP	GFP MsTTR	-3.3223	0.3870

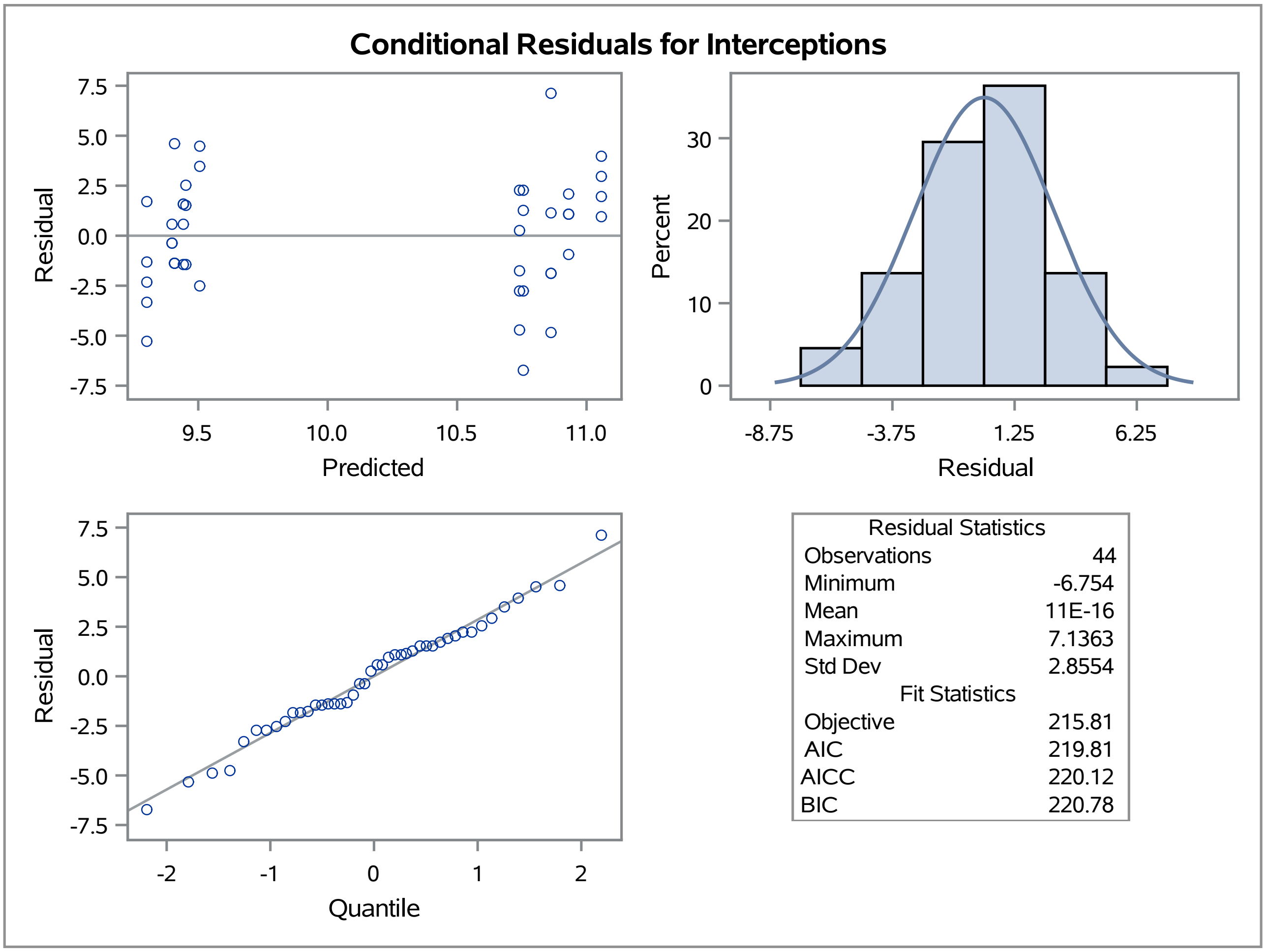

DistSoma=6

Model Information	
**Data Set**	WORK.TEMPDATASORTED
**Dependent Variable**	Interceptions
**Covariance Structure**	Variance Components
**Estimation Method**	REML
**Residual Variance Method**	Profile
**Fixed Effects SE Method**	Model-Based
**Degrees of Freedom Method**	Containment

Class Level Information		
**Class**	**Levels**	**Values**
**Treatment**	2	Control_GFP GFP MsTTR
**Culture**	12	1 2 3 4 5 6 7 8 9 10 11 12

Dimensions	
**Covariance Parameters**	2
**Columns in X**	3
**Columns in Z**	12
**Subjects**	1
**Max Obs per Subject**	44

Number of Observations	
**Number of Observations Read**	44
**Number of Observations Used**	44
**Number of Observations Not Used**	0

Iteration History			
**Iteration**	**Evaluations**	**-2 Res Log Like**	**Criterion**
**0**	1	217.00999160	
**1**	2	216.97605169	0.00000000

Convergence criteria met.

Covariance Parameter Estimates				
**Cov Parm**	**Estimate**	**Alpha**	**Lower**	**Upper**
**Culture**	0.2338	0.05	0.02088	5.339E49
**Residual**	8.6622	0.05	5.6034	15.1486

DistSoma=6

Fit Statistics	
-2 Res Log Likelihood	217.0
AIC (Smaller is Better)	221.0
AICC (Smaller is Better)	221.3
BIC (Smaller is Better)	221.9

Solution for Fixed Effects

Effect	Treatment	Estimate	Standard Error	DF	t Value	Pr > |t|	Alpha	Lower	Upper
Intercept		10.6904	0.6641	10	16.10	<.0001	0.05	9.2107	12.1701
Treatment	Control_GFP	-1.2451	0.9335	32	-1.33	0.1917	0.05	-3.1465	0.6563
Treatment	GFP MsTTR	0	.	.	.	.	.	.	.

Solution for Random Effects

Effect	Culture	Estimate	Std Err Pred	DF	t Value	Pr > |t|	Alpha	Lower	Upper
Culture	1	0.1415	0.4677	32	0.30	0.7643	0.05	-0.8112	1.0941
Culture	2	0.06654	0.4677	32	0.14	0.8878	0.05	-0.8861	1.0192
Culture	3	0.005337	0.4638	32	0.01	0.9909	0.05	-0.9395	0.9501
Culture	4	0.05407	0.4638	32	0.12	0.9079	0.05	-0.8907	0.9989
Culture	5	-0.1409	0.4638	32	-0.30	0.7633	0.05	-1.0856	0.8039
Culture	6	-0.1431	0.4775	32	-0.30	0.7663	0.05	-1.1157	0.8295
Culture	7	0.01659	0.4677	32	0.04	0.9719	0.05	-0.9361	0.9692
Culture	8	0.01304	0.4607	32	0.03	0.9776	0.05	-0.9254	0.9515
Culture	9	-0.1404	0.4639	32	-0.30	0.7642	0.05	-1.0854	0.8046
Culture	10	0.2738	0.4639	32	0.59	0.5592	0.05	-0.6712	1.2188
Culture	11	0.05454	0.4639	32	0.12	0.9071	0.05	-0.8905	0.9996
Culture	12	-0.2010	0.4607	32	-0.44	0.6655	0.05	-1.1395	0.7374

Type 3 Tests of Fixed Effects

Effect	Num DF	Den DF	F Value	Pr > F
Treatment	1	32	1.78	0.1917

Least Squares Means

Effect	Treatment	Estimate	Standard Error	DF	t Value	Pr > |t|	Alpha	Lower	Upper
Treatment	Control_GFP	9.4452	0.6560	32	14.40	<.0001	0.05	8.1090	10.7814
Treatment	GFP MsTTR	10.6904	0.6641	32	16.10	<.0001	0.05	9.3376	12.0431

DistSoma=6

Differences of Least Squares Means												
Effect	Treatment	Treatment	Estimate	Standard Error	DF	t Value	Pr > |t|	Adjustment	Adj P	Alpha	Lower	Upper
**Treatment**	Control_GFP	GFP MsTTR	-1.2451	0.9335	32	-1.33	0.1917	Tukey-Kramer	0.1917	0.05	-3.1465	0.6563

Differences of Least Squares Means				
Effect	Treatment	Treatment	Adj Lower	Adj Upper
**Treatment**	Control_GFP	GFP MsTTR	-3.1465	0.6562

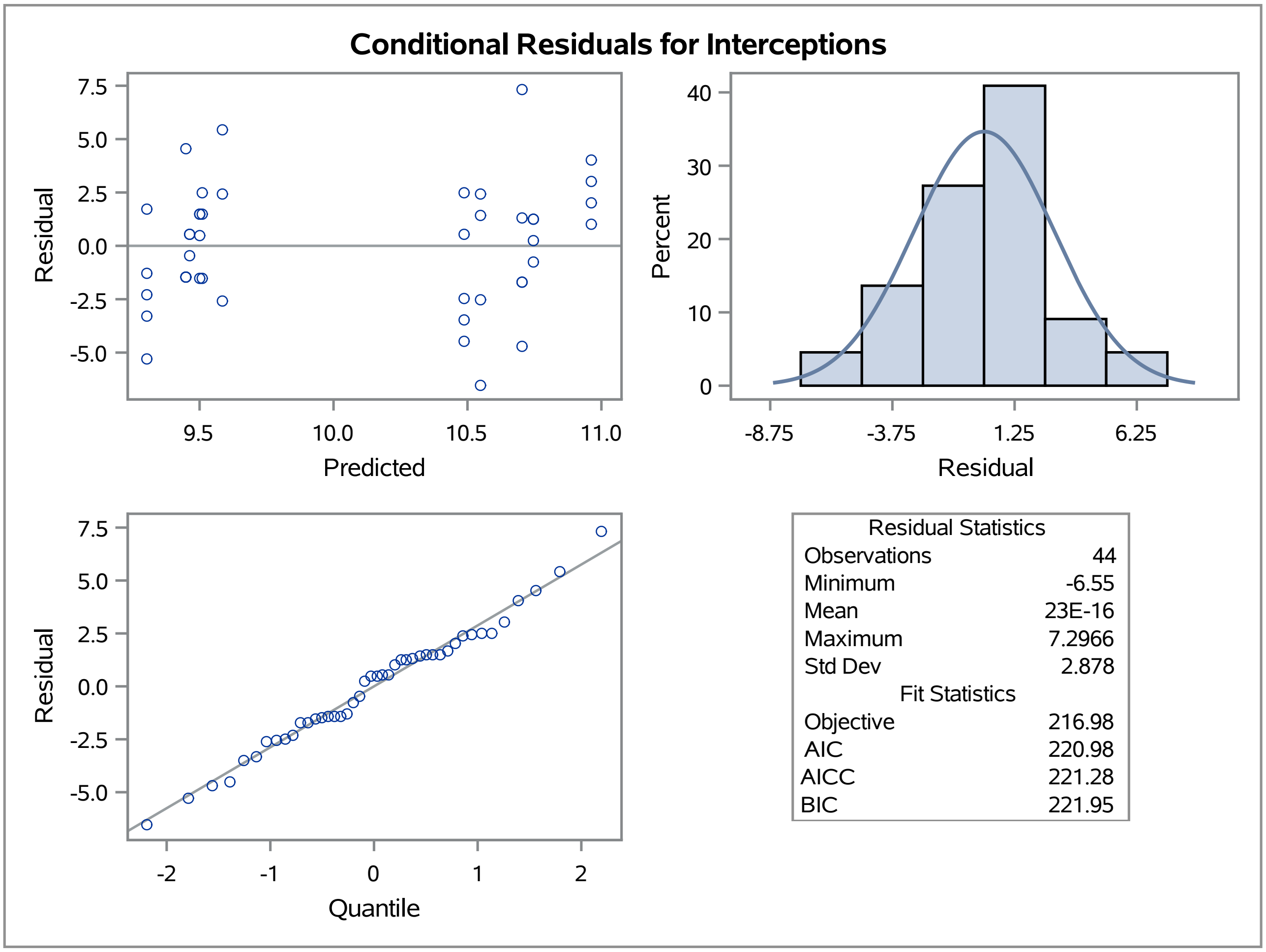

DistSoma=12

Model Information	
**Data Set**	WORK.TEMPDATASORTED
**Dependent Variable**	Interceptions
**Covariance Structure**	Variance Components
**Estimation Method**	REML
**Residual Variance Method**	Profile
**Fixed Effects SE Method**	Model-Based
**Degrees of Freedom Method**	Containment

Class Level Information		
**Class**	**Levels**	**Values**
**Treatment**	2	Control_GFP GFP MsTTR
**Culture**	12	1 2 3 4 5 6 7 8 9 10 11 12

Dimensions	
**Covariance Parameters**	2
**Columns in X**	3
**Columns in Z**	12
**Subjects**	1
**Max Obs per Subject**	44

Number of Observations	
**Number of Observations Read**	44
**Number of Observations Used**	44
**Number of Observations Not Used**	0

Iteration History			
**Iteration**	**Evaluations**	**-2 Res Log Like**	**Criterion**
**0**	1	222.13600567	
**1**	2	222.05075407	0.00000002
**2**	1	222.05075270	0.00000000

Convergence criteria met.

Covariance Parameter Estimates				
**Cov Parm**	**Estimate**	**Alpha**	**Lower**	**Upper**
**Culture**	0.4359	0.05	0.04102	2.746E20
**Residual**	9.6413	0.05	6.2179	16.9454

DistSoma=12

Fit Statistics	
-2 Res Log Likelihood	222.1
AIC (Smaller is Better)	226.1
AICC (Smaller is Better)	226.4
BIC (Smaller is Better)	227.0

Solution for Fixed Effects

Effect	Treatment	Estimate	Standard Error	DF	t Value	Pr > |t|	Alpha	Lower	Upper
Intercept		11.5565	0.7255	10	15.93	<.0001	0.05	9.9400	13.1729
Treatment	Control_GFP	-1.2212	1.0162	32	-1.20	0.2383	0.05	-3.2910	0.8487
Treatment	GFP MsTTR	0	.	.	.	.	.	.	.

Solution for Random Effects

Effect	Culture	Estimate	Std Err Pred	DF	t Value	Pr > |t|	Alpha	Lower	Upper
Culture	1	0.2386	0.6254	32	0.38	0.7053	0.05	-1.0352	1.5124
Culture	2	0.03958	0.6254	32	0.06	0.9499	0.05	-1.2342	1.3134
Culture	3	0.1018	0.6173	32	0.16	0.8700	0.05	-1.1555	1.3591
Culture	4	0.1401	0.6173	32	0.23	0.8219	0.05	-1.1172	1.3974
Culture	5	-0.1662	0.6173	32	-0.27	0.7894	0.05	-1.4236	1.0911
Culture	6	-0.2740	0.6465	32	-0.42	0.6745	0.05	-1.5910	1.0429
Culture	7	-0.07986	0.6254	32	-0.13	0.8992	0.05	-1.3537	1.1939
Culture	8	0.08178	0.6111	32	0.13	0.8944	0.05	-1.1630	1.3265
Culture	9	-0.2384	0.6177	32	-0.39	0.7021	0.05	-1.4965	1.0197
Culture	10	0.4891	0.6177	32	0.79	0.4343	0.05	-0.7690	1.7472
Culture	11	-0.00865	0.6177	32	-0.01	0.9889	0.05	-1.2668	1.2495
Culture	12	-0.3239	0.6111	32	-0.53	0.5998	0.05	-1.5686	0.9209

Type 3 Tests of Fixed Effects

Effect	Num DF	Den DF	F Value	Pr > F
Treatment	1	32	1.44	0.2383

Least Squares Means

Effect	Treatment	Estimate	Standard Error	DF	t Value	Pr > |t|	Alpha	Lower	Upper
Treatment	Control_GFP	10.3353	0.7115	32	14.53	<.0001	0.05	8.8860	11.7846
Treatment	GFP MsTTR	11.5565	0.7255	32	15.93	<.0001	0.05	10.0787	13.0342

DistSoma=12

Differences of Least Squares Means												
**Effect**	**Treatment**	**Treatment**	**Estimate**	**Standard Error**	**DF**	**t Value**	**Pr > |t|**	**Adjustment**	**Adj P**	**Alpha**	**Lower**	**Upper**
**Treatment**	Control_GFP	GFP MsTTR	-1.2212	1.0162	32	-1.20	0.2383	Tukey-Kramer	0.2383	0.05	-3.2910	0.8487

Differences of Least Squares Means				
**Effect**	**Treatment**	**Treatment**	**Adj Lower**	**Adj Upper**
**Treatment**	Control_GFP	GFP MsTTR	-3.2910	0.8487

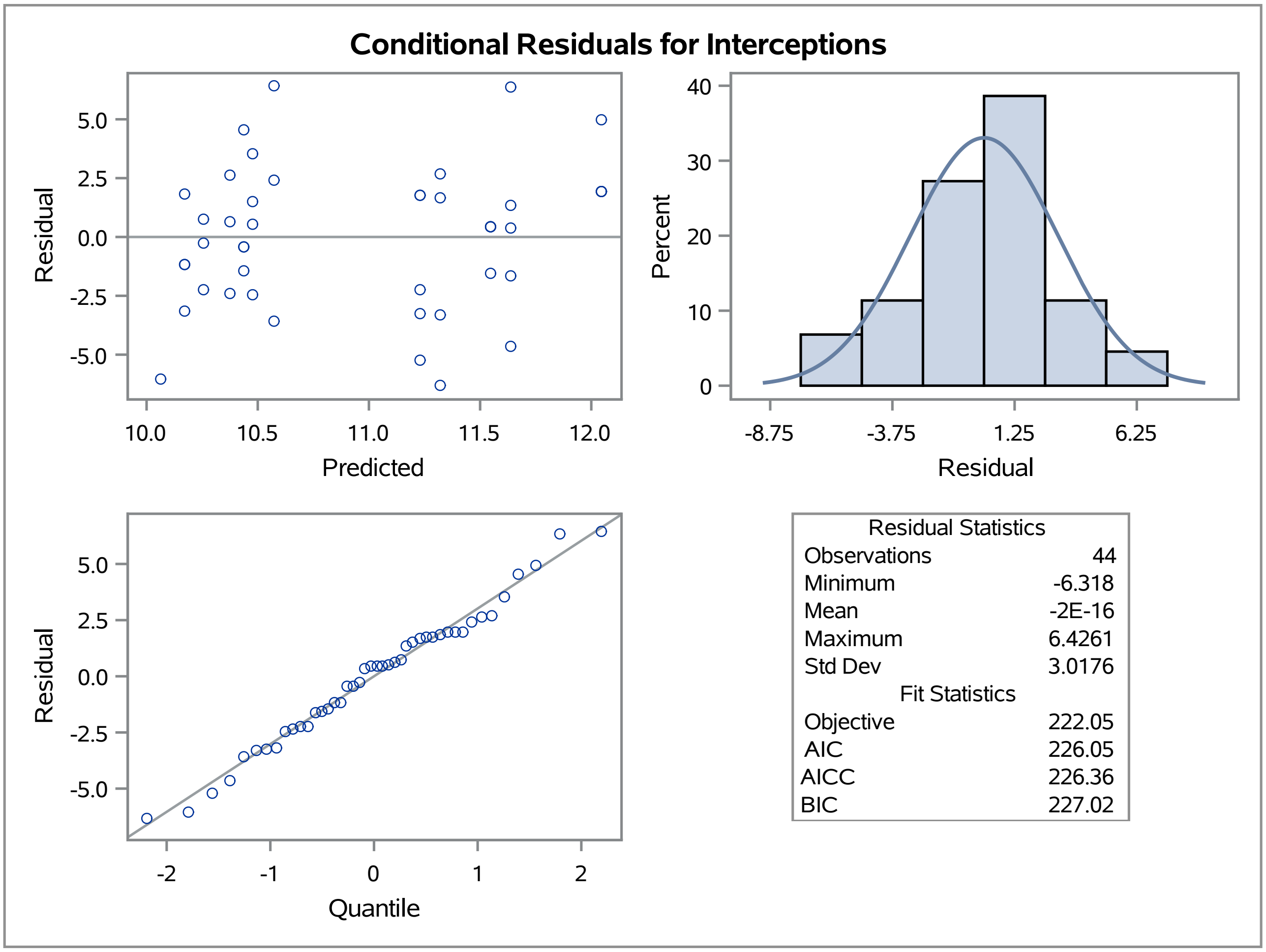

DistSoma=18

Model Information	
**Data Set**	WORK.TEMPDATASORTED
**Dependent Variable**	Interceptions
**Covariance Structure**	Variance Components
**Estimation Method**	REML
**Residual Variance Method**	Profile
**Fixed Effects SE Method**	Model-Based
**Degrees of Freedom Method**	Containment

Class Level Information		
**Class**	**Levels**	**Values**
**Treatment**	2	Control_GFP GFP MsTTR
**Culture**	12	1 2 3 4 5 6 7 8 9 10 11 12

Dimensions	
**Covariance Parameters**	2
**Columns in X**	3
**Columns in Z**	12
**Subjects**	1
**Max Obs per Subject**	44

Number of Observations	
**Number of Observations Read**	44
**Number of Observations Used**	44
**Number of Observations Not Used**	0

Iteration History			
**Iteration**	**Evaluations**	**-2 Res Log Like**	**Criterion**
**0**	1	231.13962741	
**1**	2	230.26280349	0.00001530
**2**	1	230.26160420	0.00000002
**3**	1	230.26160256	0.00000000

Convergence criteria met.

DistSoma=18

Covariance Parameter Estimates				
**Cov Parm**	**Estimate**	**Alpha**	**Lower**	**Upper**
**Culture**	2.0062	0.05	0.4164	1141.01
**Residual**	10.7686	0.05	6.8794	19.2269

Fit Statistics	
**-2 Res Log Likelihood**	230.3
**AIC (Smaller is Better)**	234.3
**AICC (Smaller is Better)**	234.6
**BIC (Smaller is Better)**	235.2

Solution for Fixed Effects									
**Effect**	**Treatment**	**Estimate**	**Standard Error**	**DF**	**t Value**	**Pr > |t|**	**Alpha**	**Lower**	**Upper**
**Intercept**		12.2268	0.9452	10	12.94	<.0001	0.05	10.1208	14.3329
**Treatment**	Control_GFP	-0.4668	1.3006	32	-0.36	0.7220	0.05	-3.1160	2.1825
**Treatment**	GFP MsTTR	0	.	.	.	.	.	.	.

Solution for Random Effects									
**Effect**	**Culture**	**Estimate**	**Std Err Pred**	**DF**	**t Value**	**Pr > |t|**	**Alpha**	**Lower**	**Upper**
**Culture**	1	0.6836	1.1788	32	0.58	0.5660	0.05	-1.7175	3.0847
**Culture**	2	0.3250	1.1788	32	0.28	0.7845	0.05	-2.0761	2.7262
**Culture**	3	1.1700	1.1380	32	1.03	0.3116	0.05	-1.1481	3.4880
**Culture**	4	0.6362	1.1380	32	0.56	0.5800	0.05	-1.6819	2.9543
**Culture**	5	-0.9651	1.1380	32	-0.85	0.4027	0.05	-3.2831	1.3530
**Culture**	6	-1.2187	1.3080	32	-0.93	0.3585	0.05	-3.8830	1.4456
**Culture**	7	-0.6310	1.1788	32	-0.54	0.5961	0.05	-3.0322	1.7701
**Culture**	8	-0.01294	1.1165	32	-0.01	0.9908	0.05	-2.2871	2.2612
**Culture**	9	-0.3104	1.1456	32	-0.27	0.7882	0.05	-2.6439	2.0232
**Culture**	10	1.2909	1.1456	32	1.13	0.2682	0.05	-1.0427	3.6245
**Culture**	11	0.009890	1.1456	32	0.01	0.9932	0.05	-2.3237	2.3435
**Culture**	12	-0.9775	1.1165	32	-0.88	0.3878	0.05	-3.2517	1.2967

Type 3 Tests of Fixed Effects				
**Effect**	**Num DF**	**Den DF**	**F Value**	**Pr > F**
**Treatment**	1	32	0.13	0.7220

DistSoma=18

Least Squares Means									
Effect	Treatment	Estimate	Standard Error	DF	t Value	Pr > |t|	Alpha	Lower	Upper
Treatment	Control_GFP	11.7601	0.8934	32	13.16	<.0001	0.05	9.9403	13.5798
Treatment	GFP MsTTR	12.2268	0.9452	32	12.94	<.0001	0.05	10.3015	14.1522

Differences of Least Squares Means												
Effect	Treatment	Treatment	Estimate	Standard Error	DF	t Value	Pr > |t|	Adjustment	Adj P	Alpha	Lower	Upper
Treatment	Control_GFP	GFP MsTTR	-0.4668	1.3006	32	-0.36	0.7220	Tukey-Kramer	0.7220	0.05	-3.1160	2.1825

Differences of Least Squares Means				
Effect	Treatment	Treatment	Adj Lower	Adj Upper
Treatment	Control_GFP	GFP MsTTR	-3.1160	2.1824

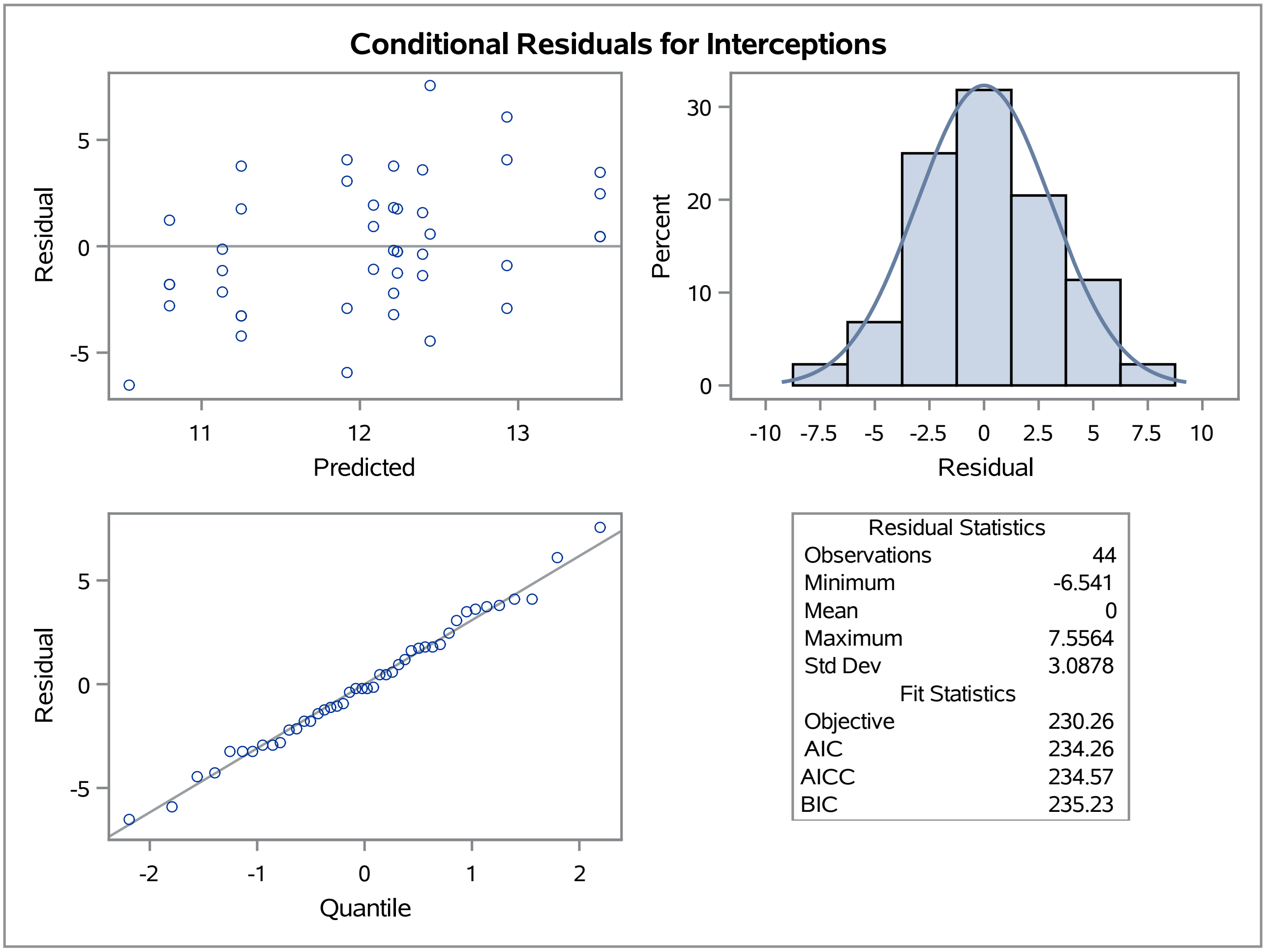

DistSoma=24

Model Information	
Data Set	WORK.TEMPDATASORTED
Dependent Variable	Interceptions
Covariance Structure	Variance Components
Estimation Method	REML
Residual Variance Method	Profile
Fixed Effects SE Method	Model-Based
Degrees of Freedom Method	Containment

Class Level Information		
Class	Levels	Values
Treatment	2	Control_GFP GFP MsTTR
Culture	12	1 2 3 4 5 6 7 8 9 10 11 12

Dimensions	
Covariance Parameters	2
Columns in X	3
Columns in Z	12
Subjects	1
Max Obs per Subject	44

Number of Observations	
Number of Observations Read	44
Number of Observations Used	44
Number of Observations Not Used	0

Iteration History			
Iteration	Evaluations	-2 Res Log Like	Criterion
0	1	233.71516044	
1	2	233.53390212	0.00000050
2	1	233.53386252	0.00000000

Convergence criteria met.

Covariance Parameter Estimates				
Cov Parm	Estimate	Alpha	Lower	Upper
Culture	0.8897	0.05	0.1045	1.249E10
Residual	12.4412	0.05	7.9916	22.0116

DistSoma=24

Fit Statistics	
-2 Res Log Likelihood	233.5
AIC (Smaller is Better)	237.5
AICC (Smaller is Better)	237.8
BIC (Smaller is Better)	238.5

Solution for Fixed Effects

Effect	Treatment	Estimate	Standard Error	DF	t Value	Pr > |t|	Alpha	Lower	Upper
Intercept		12.7035	0.8632	10	14.72	<.0001	0.05	10.7801	14.6268
Treatment	Control_GFP	-0.9411	1.2038	32	-0.78	0.4401	0.05	-3.3931	1.5109
Treatment	GFP MsTTR	0	.	.	.	.	.	.	.

Solution for Random Effects

Effect	Culture	Estimate	Std Err Pred	DF	t Value	Pr > |t|	Alpha	Lower	Upper
Culture	1	0.3364	0.8686	32	0.39	0.7011	0.05	-1.4330	2.1057
Culture	2	0.1597	0.8686	32	0.18	0.8553	0.05	-1.6096	1.9291
Culture	3	0.4977	0.8524	32	0.58	0.5634	0.05	-1.2386	2.2341
Culture	4	0.2753	0.8524	32	0.32	0.7488	0.05	-1.4611	2.0117
Culture	5	-0.4476	0.8524	32	-0.53	0.6031	0.05	-2.1840	1.2888
Culture	6	-0.4513	0.9130	32	-0.49	0.6244	0.05	-2.3110	1.4083
Culture	7	-0.3702	0.8686	32	-0.43	0.6728	0.05	-2.1396	1.3992
Culture	8	-0.1853	0.8409	32	-0.22	0.8270	0.05	-1.8981	1.5275
Culture	9	-0.2121	0.8536	32	-0.25	0.8054	0.05	-1.9509	1.5267
Culture	10	0.7889	0.8536	32	0.92	0.3623	0.05	-0.9499	2.5277
Culture	11	-0.1009	0.8536	32	-0.12	0.9067	0.05	-1.8397	1.6379
Culture	12	-0.2906	0.8409	32	-0.35	0.7319	0.05	-2.0035	1.4222

Type 3 Tests of Fixed Effects

Effect	Num DF	Den DF	F Value	Pr > F
Treatment	1	32	0.61	0.4401

Least Squares Means

Effect	Treatment	Estimate	Standard Error	DF	t Value	Pr > |t|	Alpha	Lower	Upper
Treatment	Control_GFP	11.7624	0.8390	32	14.02	<.0001	0.05	10.0534	13.4713
Treatment	GFP MsTTR	12.7035	0.8632	32	14.72	<.0001	0.05	10.9452	14.4617

DistSoma=24

Differences of Least Squares Means												
**Effect**	**Treatment**	**Treatment**	**Estimate**	**Standard Error**	**DF**	**t Value**	**Pr > |t|**	**Adjustment**	**Adj P**	**Alpha**	**Lower**	**Upper**
**Treatment**	Control_GFP	GFP MsTTR	-0.9411	1.2038	32	-0.78	0.4401	Tukey-Kramer	0.4401	0.05	-3.3931	1.5109

Differences of Least Squares Means				
**Effect**	**Treatment**	**Treatment**	**Adj Lower**	**Adj Upper**
**Treatment**	Control_GFP	GFP MsTTR	-3.3930	1.5109

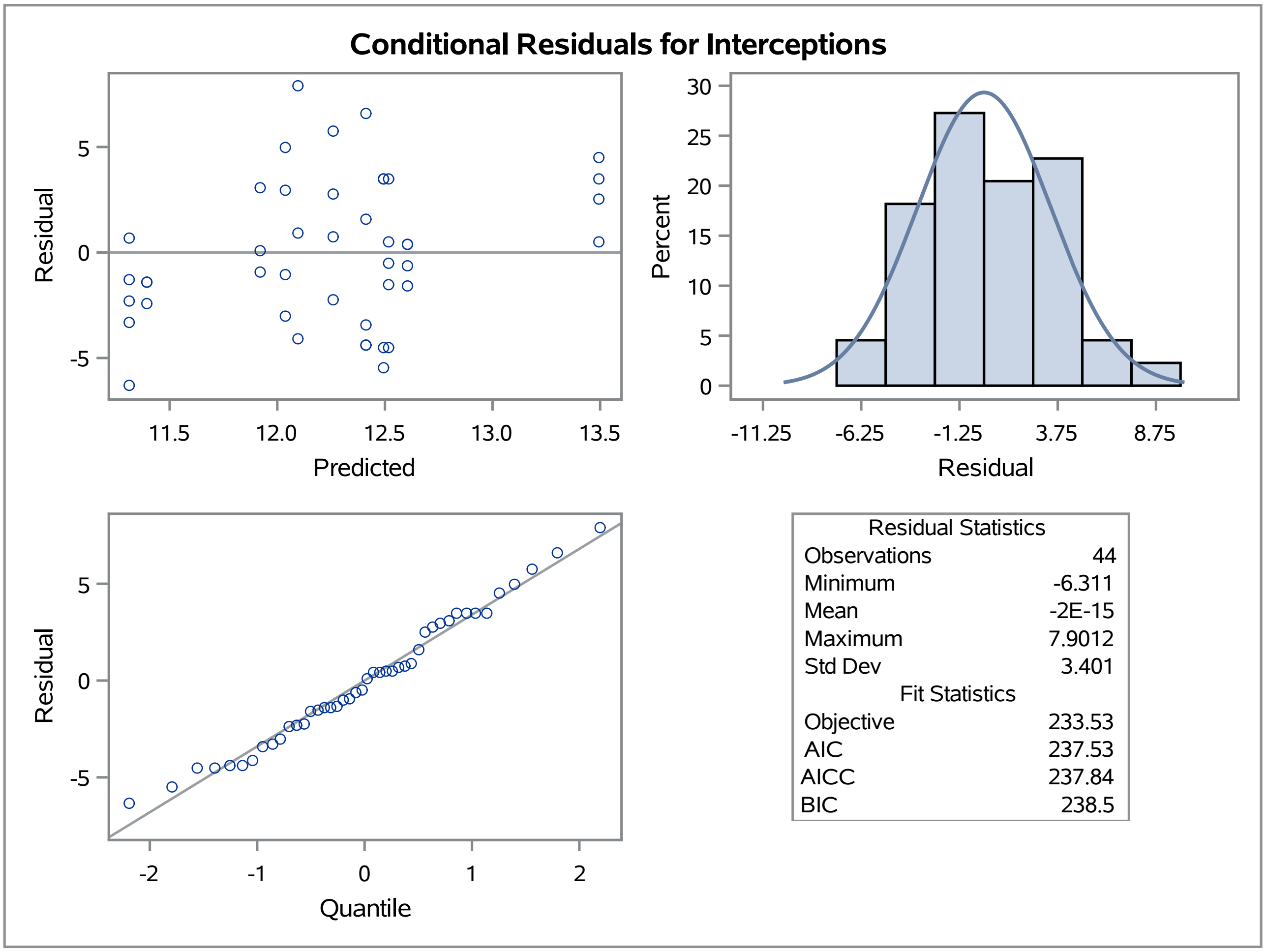

DistSoma=30

Model Information	
**Data Set**	WORK.TEMPDATASORTED
**Dependent Variable**	Interceptions
**Covariance Structure**	Variance Components
**Estimation Method**	REML
**Residual Variance Method**	Profile
**Fixed Effects SE Method**	Model-Based
**Degrees of Freedom Method**	Containment

Class Level Information		
**Class**	**Levels**	**Values**
**Treatment**	2	Control_GFP GFP MsTTR
**Culture**	12	1 2 3 4 5 6 7 8 9 10 11 12

Dimensions	
**Covariance Parameters**	2
**Columns in X**	3
**Columns in Z**	12
**Subjects**	1
**Max Obs per Subject**	44

Number of Observations	
**Number of Observations Read**	44
**Number of Observations Used**	44
**Number of Observations Not Used**	0

Iteration History			
**Iteration**	**Evaluations**	**-2 Res Log Like**	**Criterion**
**0**	1	235.17794432	
**1**	2	234.27390302	0.00000271
**2**	1	234.27368806	0.00000000

Convergence criteria met.

Covariance Parameter Estimates				
**Cov Parm**	**Estimate**	**Alpha**	**Lower**	**Upper**
**Culture**	2.0867	0.05	0.4491	747.21
**Residual**	11.9155	0.05	7.6781	20.9715

DistSoma=30

Fit Statistics	
-2 Res Log Likelihood	234.3
AIC (Smaller is Better)	238.3
AICC (Smaller is Better)	238.6
BIC (Smaller is Better)	239.2

Solution for Fixed Effects

Effect	Treatment	Estimate	Standard Error	DF	t Value	Pr > |t|	Alpha	Lower	Upper
Intercept		12.4405	0.9807	10	12.68	<.0001	0.05	10.2553	14.6257
Treatment	Control_GFP	-0.8347	1.3508	32	-0.62	0.5410	0.05	-3.5863	1.9168
Treatment	GFP MsTTR	0	.	.	.	.	.	.	.

Solution for Random Effects

Effect	Culture	Estimate	Std Err Pred	DF	t Value	Pr > |t|	Alpha	Lower	Upper
Culture	1	0.7098	1.2126	32	0.59	0.5624	0.05	-1.7601	3.1798
Culture	2	0.02097	1.2126	32	0.02	0.9863	0.05	-2.4490	2.4909
Culture	3	1.5012	1.1720	32	1.28	0.2094	0.05	-0.8861	3.8884
Culture	4	0.3684	1.1720	32	0.31	0.7553	0.05	-2.0189	2.7556
Culture	5	-0.8674	1.1720	32	-0.74	0.4646	0.05	-3.2547	1.5198
Culture	6	-0.8354	1.3397	32	-0.62	0.5373	0.05	-3.5643	1.8935
Culture	7	-0.8975	1.2126	32	-0.74	0.4646	0.05	-3.3674	1.5725
Culture	8	-0.2057	1.1498	32	-0.18	0.8592	0.05	-2.5478	2.1365
Culture	9	-0.4904	1.1791	32	-0.42	0.6802	0.05	-2.8922	1.9114
Culture	10	1.4663	1.1791	32	1.24	0.2227	0.05	-0.9355	3.8680
Culture	11	-0.2844	1.1791	32	-0.24	0.8109	0.05	-2.6862	2.1173
Culture	12	-0.4858	1.1498	32	-0.42	0.6755	0.05	-2.8279	1.8564

Type 3 Tests of Fixed Effects

Effect	Num DF	Den DF	F Value	Pr > F
Treatment	1	32	0.38	0.5410

Least Squares Means

Effect	Treatment	Estimate	Standard Error	DF	t Value	Pr > |t|	Alpha	Lower	Upper
Treatment	Control_GFP	11.6058	0.9289	32	12.49	<.0001	0.05	9.7136	13.4980
Treatment	GFP MsTTR	12.4405	0.9807	32	12.68	<.0001	0.05	10.4428	14.4382

DistSoma=30

Differences of Least Squares Means

Effect	Treatment	Treatment	Estimate	Standard Error	DF	t Value	Pr > |t|	Adjustment	Adj P	Alpha	Lower	Upper
**Treatment**	Control_GFP	GFP MsTTR	-0.8347	1.3508	32	-0.62	0.5410	Tukey-Kramer	0.5410	0.05	-3.5863	1.9168

Differences of Least Squares Means

Effect	Treatment	Treatment	Adj Lower	Adj Upper
**Treatment**	Control_GFP	GFP MsTTR	-3.5863	1.9168

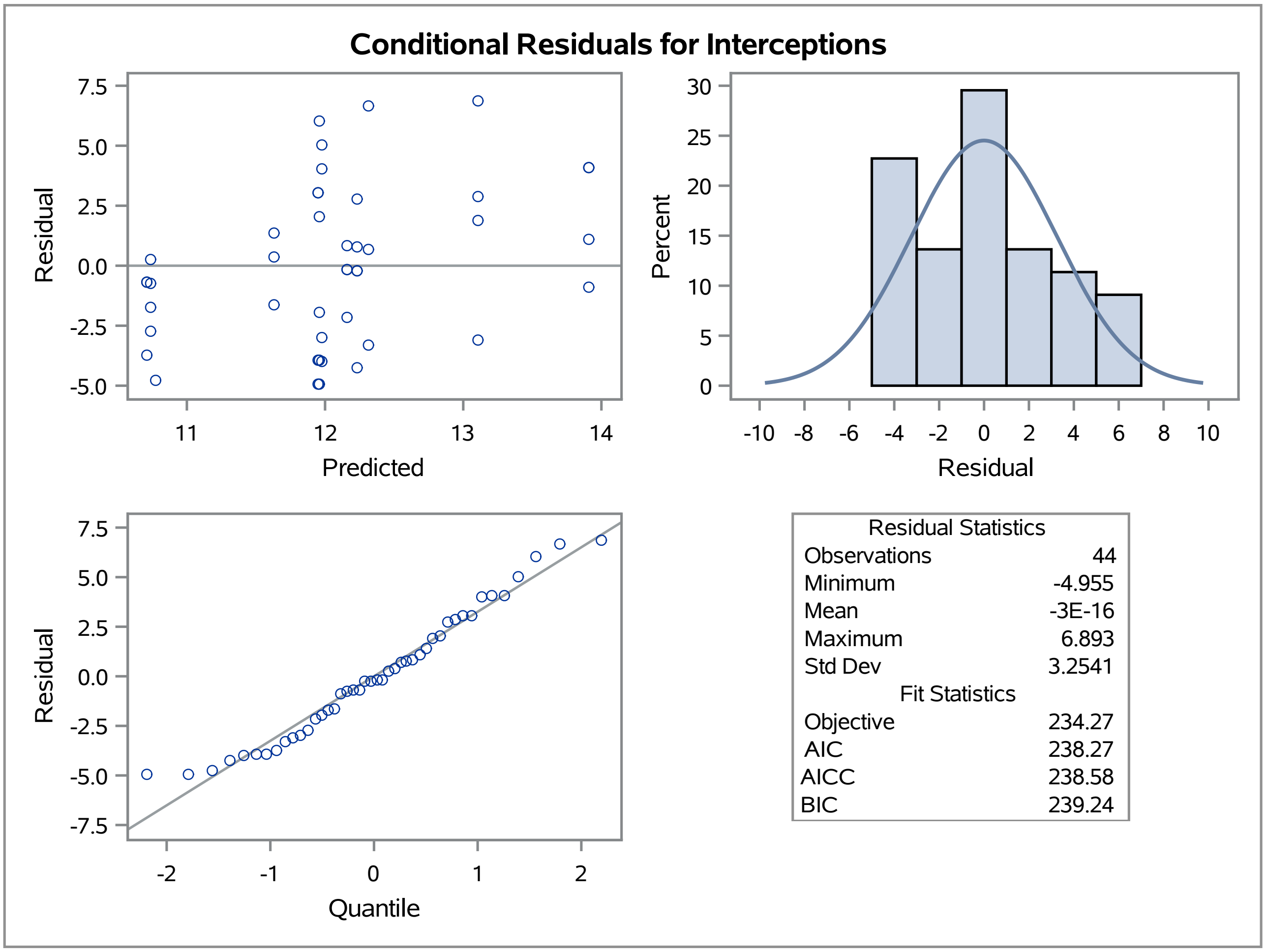

DistSoma=36

Model Information	
**Data Set**	WORK.TEMPDATASORTED
**Dependent Variable**	Interceptions
**Covariance Structure**	Variance Components
**Estimation Method**	REML
**Residual Variance Method**	Profile
**Fixed Effects SE Method**	Model-Based
**Degrees of Freedom Method**	Containment

Class Level Information		
**Class**	**Levels**	**Values**
**Treatment**	2	Control_GFP GFP MsTTR
**Culture**	12	1 2 3 4 5 6 7 8 9 10 11 12

Dimensions	
**Covariance Parameters**	2
**Columns in X**	3
**Columns in Z**	12
**Subjects**	1
**Max Obs per Subject**	44

Number of Observations	
**Number of Observations Read**	44
**Number of Observations Used**	44
**Number of Observations Not Used**	0

Iteration History			
**Iteration**	**Evaluations**	**-2 Res Log Like**	**Criterion**
**0**	1	230.54188385	
**1**	2	229.37558151	0.00000074
**2**	1	229.37552504	0.00000000

Convergence criteria met.

Covariance Parameter Estimates				
**Cov Parm**	**Estimate**	**Alpha**	**Lower**	**Upper**
**Culture**	2.0645	0.05	0.4892	256.99
**Residual**	10.4889	0.05	6.7811	18.3605

DistSoma=36

Fit Statistics	
-2 Res Log Likelihood	229.4
AIC (Smaller is Better)	233.4
AICC (Smaller is Better)	233.7
BIC (Smaller is Better)	234.3

Solution for Fixed Effects

Effect	Treatment	Estimate	Standard Error	DF	t Value	Pr > |t|	Alpha	Lower	Upper
Intercept		11.7235	0.9447	10	12.41	<.0001	0.05	9.6186	13.8283
Treatment	Control_GFP	-0.7857	1.2987	32	-0.60	0.5495	0.05	-3.4309	1.8596
Treatment	GFP MsTTR	0	.	.	.	.	.	.	.

Solution for Random Effects

Effect	Culture	Estimate	Std Err Pred	DF	t Value	Pr > |t|	Alpha	Lower	Upper
Culture	1	0.8894	1.1864	32	0.75	0.4589	0.05	-1.5272	3.3060
Culture	2	-0.4719	1.1864	32	-0.40	0.6934	0.05	-2.8885	1.9447
Culture	3	1.3489	1.1442	32	1.18	0.2471	0.05	-0.9817	3.6796
Culture	4	0.4679	1.1442	32	0.41	0.6853	0.05	-1.8627	2.7986
Culture	5	-0.7435	1.1442	32	-0.65	0.5205	0.05	-3.0741	1.5872
Culture	6	-0.6476	1.3215	32	-0.49	0.6274	0.05	-3.3395	2.0443
Culture	7	-0.8432	1.1864	32	-0.71	0.4824	0.05	-3.2598	1.5734
Culture	8	-0.5572	1.1225	32	-0.50	0.6230	0.05	-2.8437	1.7293
Culture	9	-0.6491	1.1525	32	-0.56	0.5772	0.05	-2.9966	1.6985
Culture	10	1.6636	1.1525	32	1.44	0.1586	0.05	-0.6840	4.0111
Culture	11	-0.09843	1.1525	32	-0.09	0.9325	0.05	-2.4460	2.2491
Culture	12	-0.3588	1.1225	32	-0.32	0.7513	0.05	-2.6453	1.9277

Type 3 Tests of Fixed Effects

Effect	Num DF	Den DF	F Value	Pr > F
Treatment	1	32	0.37	0.5495

Least Squares Means

Effect	Treatment	Estimate	Standard Error	DF	t Value	Pr > |t|	Alpha	Lower	Upper
Treatment	Control_GFP	10.9378	0.8911	32	12.27	<.0001	0.05	9.1226	12.7530
Treatment	GFP MsTTR	11.7235	0.9447	32	12.41	<.0001	0.05	9.7993	13.6477

DistSoma=36

Differences of Least Squares Means												
**Effect**	**Treatment**	**Treatment**	**Estimate**	**Standard Error**	**DF**	**t Value**	**Pr > |t|**	**Adjustment**	**Adj P**	**Alpha**	**Lower**	**Upper**
**Treatment**	Control_GFP	GFP MsTTR	-0.7857	1.2987	32	-0.60	0.5495	Tukey-Kramer	0.5495	0.05	-3.4309	1.8596

Differences of Least Squares Means				
**Effect**	**Treatment**	**Treatment**	**Adj Lower**	**Adj Upper**
**Treatment**	Control_GFP	GFP MsTTR	-3.4309	1.8596

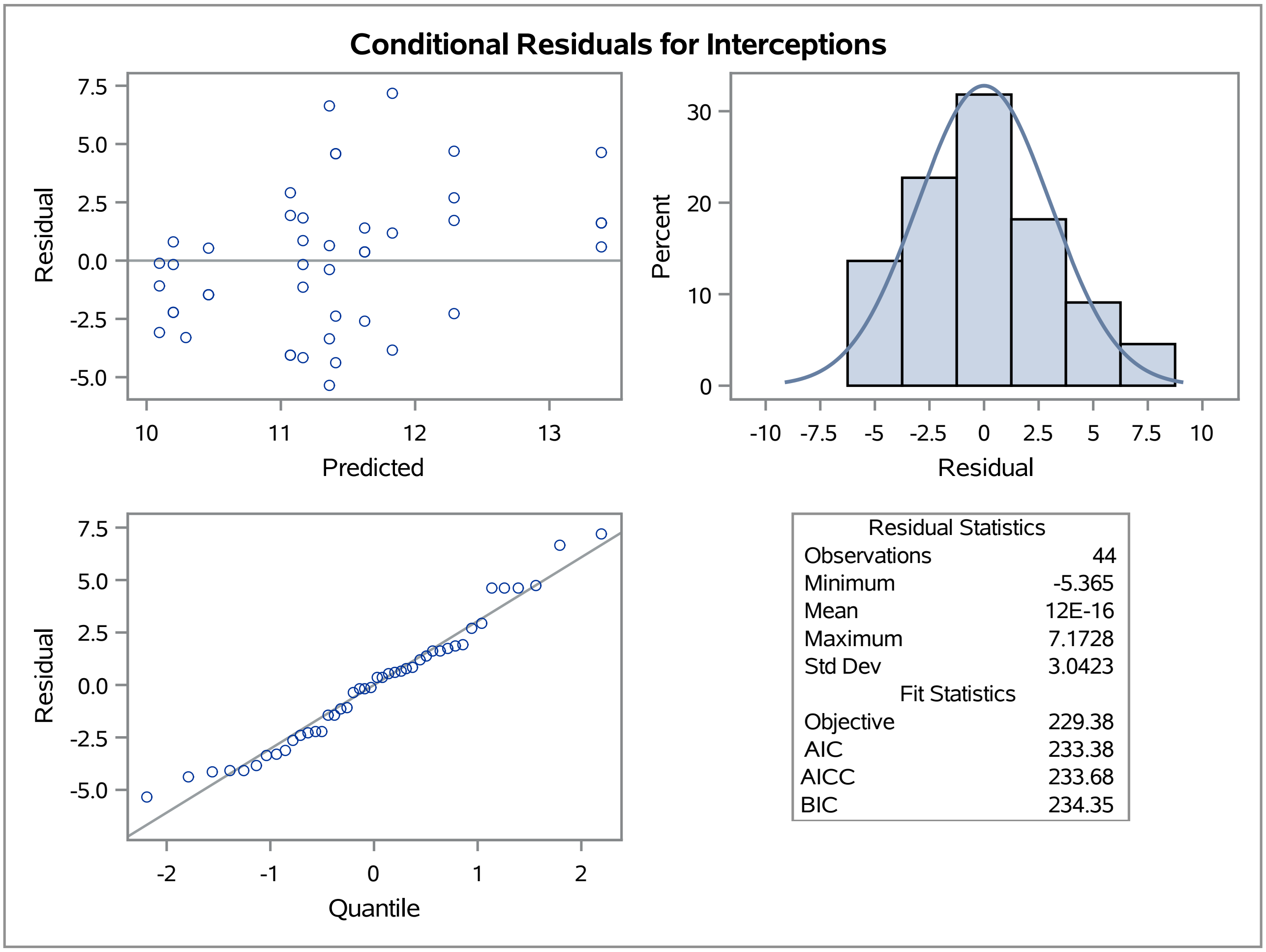

DistSoma=42

Model Information	
**Data Set**	WORK.TEMPDATASORTED
**Dependent Variable**	Interceptions
**Covariance Structure**	Variance Components
**Estimation Method**	REML
**Residual Variance Method**	Profile
**Fixed Effects SE Method**	Model-Based
**Degrees of Freedom Method**	Containment

Class Level Information		
**Class**	**Levels**	**Values**
**Treatment**	2	Control_GFP GFP MsTTR
**Culture**	12	1 2 3 4 5 6 7 8 9 10 11 12

Dimensions	
**Covariance Parameters**	2
**Columns in X**	3
**Columns in Z**	12
**Subjects**	1
**Max Obs per Subject**	44

Number of Observations	
**Number of Observations Read**	44
**Number of Observations Used**	44
**Number of Observations Not Used**	0

Iteration History			
**Iteration**	**Evaluations**	**-2 Res Log Like**	**Criterion**
**0**	1	232.50214284	
**1**	2	231.93814831	0.00000000

Convergence criteria met.

Covariance Parameter Estimates				
**Cov Parm**	**Estimate**	**Alpha**	**Lower**	**Upper**
**Culture**	1.3861	0.05	0.2523	6033.85
**Residual**	11.6228	0.05	7.5461	20.2043

DistSoma=42

Fit Statistics	
-2 Res Log Likelihood	231.9
AIC (Smaller is Better)	235.9
AICC (Smaller is Better)	236.2
BIC (Smaller is Better)	236.9

Solution for Fixed Effects

Effect	Treatment	Estimate	Standard Error	DF	t Value	Pr > |t|	Alpha	Lower	Upper
Intercept		10.9916	0.8988	10	12.23	<.0001	0.05	8.9891	12.9942
Treatment	Control_GFP	-0.6589	1.2452	32	-0.53	0.6004	0.05	-3.1953	1.8776
Treatment	GFP MsTTR	0	.	.	.	.	.	.	.

Solution for Random Effects

Effect	Culture	Estimate	Std Err Pred	DF	t Value	Pr > |t|	Alpha	Lower	Upper
Culture	1	0.1758	1.0356	32	0.17	0.8663	0.05	-1.9336	2.2853
Culture	2	-0.2633	1.0356	32	-0.25	0.8009	0.05	-2.3728	1.8461
Culture	3	1.0229	1.0079	32	1.01	0.3178	0.05	-1.0302	3.0760
Culture	4	0.5385	1.0079	32	0.53	0.5969	0.05	-1.5146	2.5916
Culture	5	-0.5919	1.0079	32	-0.59	0.5612	0.05	-2.6450	1.4612
Culture	6	-0.3551	1.1166	32	-0.32	0.7525	0.05	-2.6296	1.9194
Culture	7	-0.5268	1.0356	32	-0.51	0.6144	0.05	-2.6363	1.5826
Culture	8	-0.2957	0.9905	32	-0.30	0.7672	0.05	-2.3132	1.7218
Culture	9	-0.4817	1.0113	32	-0.48	0.6371	0.05	-2.5417	1.5782
Culture	10	1.1331	1.0113	32	1.12	0.2709	0.05	-0.9268	3.1930
Culture	11	0.1642	1.0113	32	0.16	0.8720	0.05	-1.8957	2.2241
Culture	12	-0.5198	0.9905	32	-0.52	0.6033	0.05	-2.5374	1.4977

Type 3 Tests of Fixed Effects

Effect	Num DF	Den DF	F Value	Pr > F
Treatment	1	32	0.28	0.6004

Least Squares Means

Effect	Treatment	Estimate	Standard Error	DF	t Value	Pr > |t|	Alpha	Lower	Upper
Treatment	Control_GFP	10.3327	0.8619	32	11.99	<.0001	0.05	8.5771	12.0883
Treatment	GFP MsTTR	10.9916	0.8988	32	12.23	<.0001	0.05	9.1609	12.8223

DistSoma=42

Differences of Least Squares Means												
**Effect**	**Treatment**	**Treatment**	**Estimate**	**Standard Error**	**DF**	**t Value**	**Pr > |t|**	**Adjustment**	**Adj P**	**Alpha**	**Lower**	**Upper**
**Treatment**	Control_GFP	GFP MsTTR	-0.6589	1.2452	32	-0.53	0.6004	Tukey-Kramer	0.6004	0.05	-3.1953	1.8776

Differences of Least Squares Means				
**Effect**	**Treatment**	**Treatment**	**Adj Lower**	**Adj Upper**
**Treatment**	Control_GFP	GFP MsTTR	-3.1953	1.8776

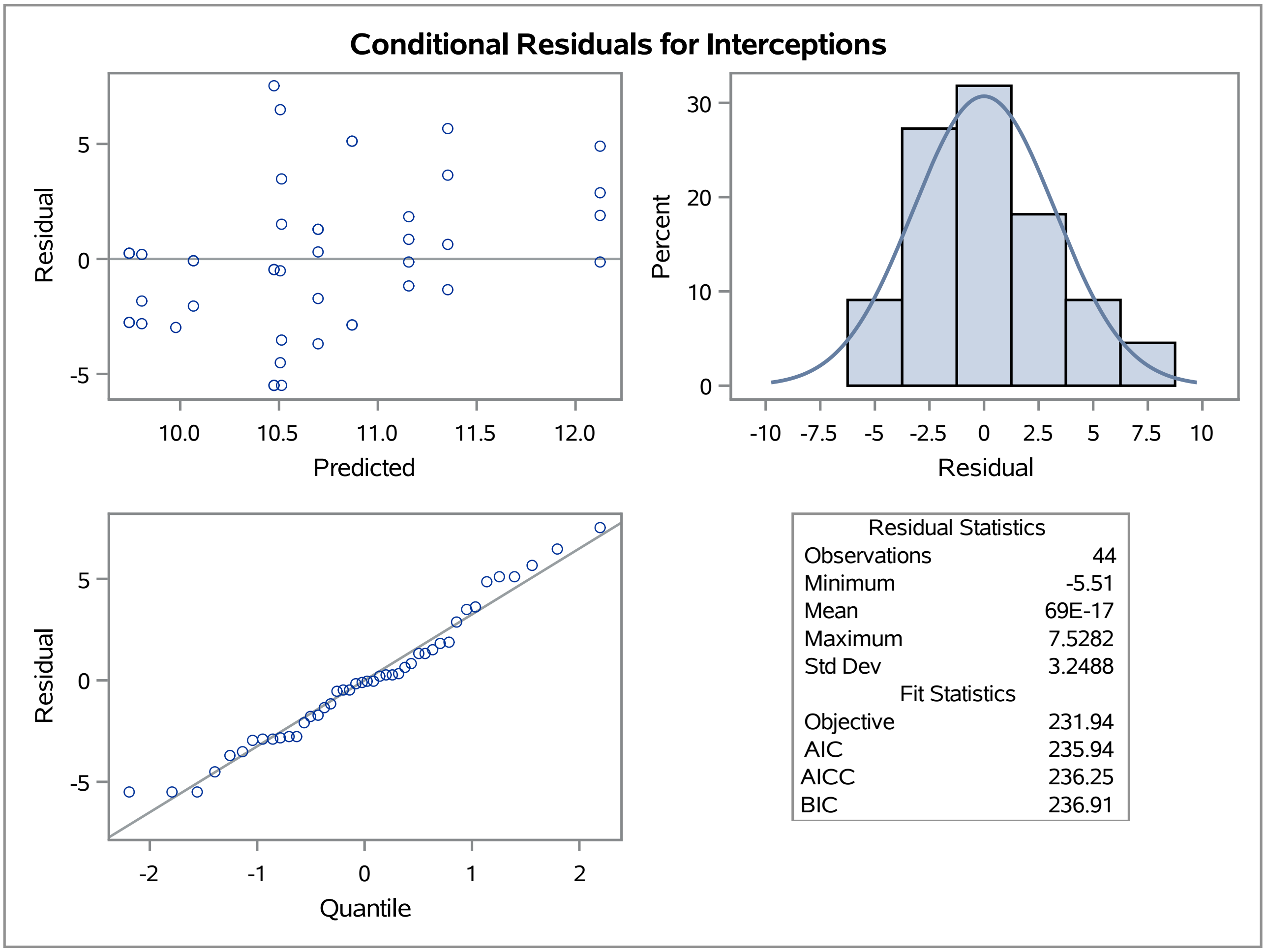

DistSoma=48

Model Information	
**Data Set**	WORK.TEMPDATASORTED
**Dependent Variable**	Interceptions
**Covariance Structure**	Variance Components
**Estimation Method**	REML
**Residual Variance Method**	Profile
**Fixed Effects SE Method**	Model-Based
**Degrees of Freedom Method**	Containment

Class Level Information		
**Class**	**Levels**	**Values**
**Treatment**	2	Control_GFP GFP MsTTR
**Culture**	12	1 2 3 4 5 6 7 8 9 10 11 12

Dimensions	
**Covariance Parameters**	2
**Columns in X**	3
**Columns in Z**	12
**Subjects**	1
**Max Obs per Subject**	44

Number of Observations	
**Number of Observations Read**	44
**Number of Observations Used**	44
**Number of Observations Not Used**	0

Iteration History			
**Iteration**	**Evaluations**	**-2 Res Log Like**	**Criterion**
**0**	1	227.59823705	
**1**	2	226.00986565	0.00000000

Convergence criteria met.

Covariance Parameter Estimates				
**Cov Parm**	**Estimate**	**Alpha**	**Lower**	**Upper**
**Culture**	2.1298	0.05	0.5615	103.89
**Residual**	9.5622	0.05	6.2090	16.6188

DistSoma=48

Fit Statistics	
-2 Res Log Likelihood	226.0
AIC (Smaller is Better)	230.0
AICC (Smaller is Better)	230.3
BIC (Smaller is Better)	231.0

Solution for Fixed Effects

Effect	Treatment	Estimate	Standard Error	DF	t Value	Pr > |t|	Alpha	Lower	Upper
Intercept		10.5604	0.9291	10	11.37	<.0001	0.05	8.4902	12.6305
Treatment	Control_GFP	-1.2303	1.2746	32	-0.97	0.3417	0.05	-3.8266	1.3660
Treatment	GFP MsTTR	0	.	.	.	.	.	.	.

Solution for Random Effects

Effect	Culture	Estimate	Std Err Pred	DF	t Value	Pr > |t|	Alpha	Lower	Upper
Culture	1	0.1348	1.1827	32	0.11	0.9100	0.05	-2.2743	2.5440
Culture	2	-0.6663	1.1827	32	-0.56	0.5771	0.05	-3.0754	1.7429
Culture	3	1.9647	1.1381	32	1.73	0.0939	0.05	-0.3536	4.2830
Culture	4	0.5512	1.1381	32	0.48	0.6315	0.05	-1.7671	2.8695
Culture	5	-0.6267	1.1381	32	-0.55	0.5857	0.05	-2.9450	1.6916
Culture	6	-0.4244	1.3293	32	-0.32	0.7516	0.05	-3.1322	2.2833
Culture	7	-0.9333	1.1827	32	-0.79	0.4359	0.05	-3.3425	1.4758
Culture	8	-0.4006	1.1168	32	-0.36	0.7222	0.05	-2.6755	1.8742
Culture	9	-0.3818	1.1480	32	-0.33	0.7416	0.05	-2.7202	1.9566
Culture	10	1.3850	1.1480	32	1.21	0.2365	0.05	-0.9534	3.7235
Culture	11	0.3249	1.1480	32	0.28	0.7790	0.05	-2.0135	2.6634
Culture	12	-0.9275	1.1168	32	-0.83	0.4124	0.05	-3.2024	1.3474

Type 3 Tests of Fixed Effects

Effect	Num DF	Den DF	F Value	Pr > F
Treatment	1	32	0.93	0.3417

Least Squares Means

Effect	Treatment	Estimate	Standard Error	DF	t Value	Pr > |t|	Alpha	Lower	Upper
Treatment	Control_GFP	9.3301	0.8726	32	10.69	<.0001	0.05	7.5527	11.1075
Treatment	GFP MsTTR	10.5604	0.9291	32	11.37	<.0001	0.05	8.6679	12.4529

DistSoma=48

Differences of Least Squares Means												
Effect	Treatment	Treatment	Estimate	Standard Error	DF	t Value	Pr > |t|	Adjustment	Adj P	Alpha	Lower	Upper
**Treatment**	Control_GFP	GFP MsTTR	-1.2303	1.2746	32	-0.97	0.3417	Tukey-Kramer	0.3417	0.05	-3.8266	1.3660

Differences of Least Squares Means				
Effect	Treatment	Treatment	Adj Lower	Adj Upper
**Treatment**	Control_GFP	GFP MsTTR	-3.8265	1.3660

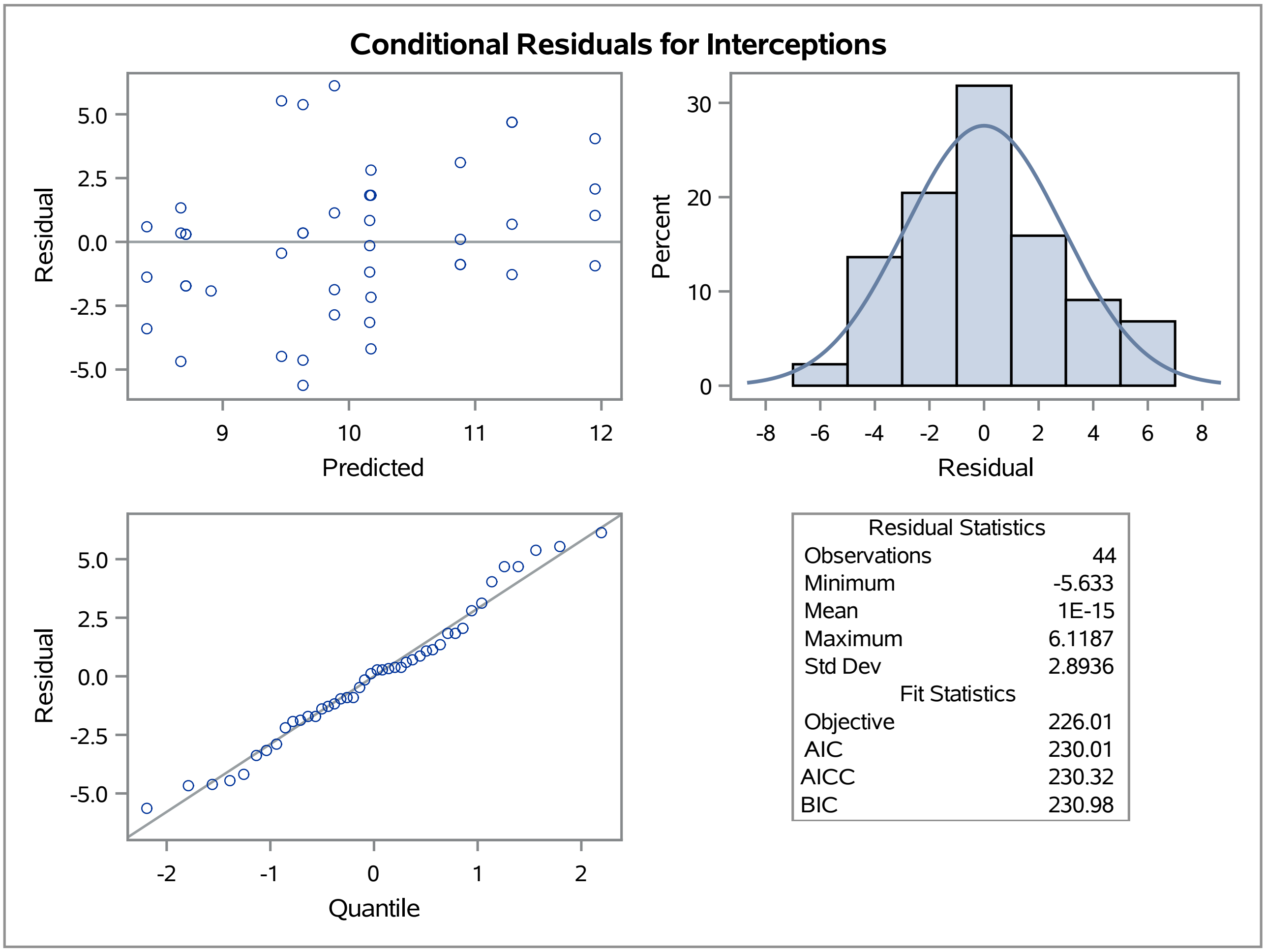

DistSoma=54

Model Information	
**Data Set**	WORK.TEMPDATASORTED
**Dependent Variable**	Interceptions
**Covariance Structure**	Variance Components
**Estimation Method**	REML
**Residual Variance Method**	Profile
**Fixed Effects SE Method**	Model-Based
**Degrees of Freedom Method**	Containment

Class Level Information		
**Class**	**Levels**	**Values**
**Treatment**	2	Control_GFP GFP MsTTR
**Culture**	12	1 2 3 4 5 6 7 8 9 10 11 12

Dimensions	
**Covariance Parameters**	2
**Columns in X**	3
**Columns in Z**	12
**Subjects**	1
**Max Obs per Subject**	44

Number of Observations	
**Number of Observations Read**	44
**Number of Observations Used**	44
**Number of Observations Not Used**	0

Iteration History			
**Iteration**	**Evaluations**	**-2 Res Log Like**	**Criterion**
**0**	1	216.91243058	
**1**	2	216.32247266	0.00000022
**2**	1	216.32245746	0.00000000

Convergence criteria met.

Covariance Parameter Estimates				
**Cov Parm**	**Estimate**	**Alpha**	**Lower**	**Upper**
**Culture**	0.9392	0.05	0.1739	3015.24
**Residual**	8.0243	0.05	5.2242	13.8861

DistSoma=54

Fit Statistics	
-2 Res Log Likelihood	216.3
AIC (Smaller is Better)	220.3
AICC (Smaller is Better)	220.6
BIC (Smaller is Better)	221.3

Solution for Fixed Effects

Effect	Treatment	Estimate	Standard Error	DF	t Value	Pr > |t|	Alpha	Lower	Upper
Intercept		9.9110	0.7444	10	13.31	<.0001	0.05	8.2524	11.5696
Treatment	Control_GFP	-1.2751	1.0316	32	-1.24	0.2255	0.05	-3.3764	0.8263
Treatment	GFP MsTTR	0	.	.	.	.	.	.	.

Solution for Random Effects

Effect	Culture	Estimate	Std Err Pred	DF	t Value	Pr > |t|	Alpha	Lower	Upper
Culture	1	0.09462	0.8542	32	0.11	0.9125	0.05	-1.6452	1.8345
Culture	2	0.007994	0.8542	32	0.01	0.9926	0.05	-1.7319	1.7479
Culture	3	0.8336	0.8316	32	1.00	0.3237	0.05	-0.8604	2.5275
Culture	4	0.1161	0.8316	32	0.14	0.8898	0.05	-1.5778	1.8101
Culture	5	-0.2825	0.8316	32	-0.34	0.7363	0.05	-1.9765	1.4114
Culture	6	-0.1714	0.9200	32	-0.19	0.8534	0.05	-2.0454	1.7026
Culture	7	-0.5984	0.8542	32	-0.70	0.4886	0.05	-2.3383	1.1415
Culture	8	-0.4102	0.8173	32	-0.50	0.6192	0.05	-2.0750	1.2547
Culture	9	-0.2108	0.8343	32	-0.25	0.8022	0.05	-1.9102	1.4886
Culture	10	0.9053	0.8343	32	1.09	0.2860	0.05	-0.7941	2.6047
Culture	11	0.3473	0.8343	32	0.42	0.6800	0.05	-1.3521	2.0467
Culture	12	-0.6317	0.8173	32	-0.77	0.4453	0.05	-2.2965	1.0332

Type 3 Tests of Fixed Effects

Effect	Num DF	Den DF	F Value	Pr > F
Treatment	1	32	1.53	0.2255

Least Squares Means

Effect	Treatment	Estimate	Standard Error	DF	t Value	Pr > |t|	Alpha	Lower	Upper
Treatment	Control_GFP	8.6359	0.7142	32	12.09	<.0001	0.05	7.1810	10.0908
Treatment	GFP MsTTR	9.9110	0.7444	32	13.31	<.0001	0.05	8.3947	11.4272

DistSoma=54

Differences of Least Squares Means												
**Effect**	**Treatment**	**Treatment**	**Estimate**	**Standard Error**	**DF**	**t Value**	**Pr > |t|**	**Adjustment**	**Adj P**	**Alpha**	**Lower**	**Upper**
**Treatment**	Control_GFP	GFP MsTTR	-1.2751	1.0316	32	-1.24	0.2255	Tukey-Kramer	0.2255	0.05	-3.3764	0.8263

Differences of Least Squares Means				
**Effect**	**Treatment**	**Treatment**	**Adj Lower**	**Adj Upper**
**Treatment**	Control_GFP	GFP MsTTR	-3.3764	0.8262

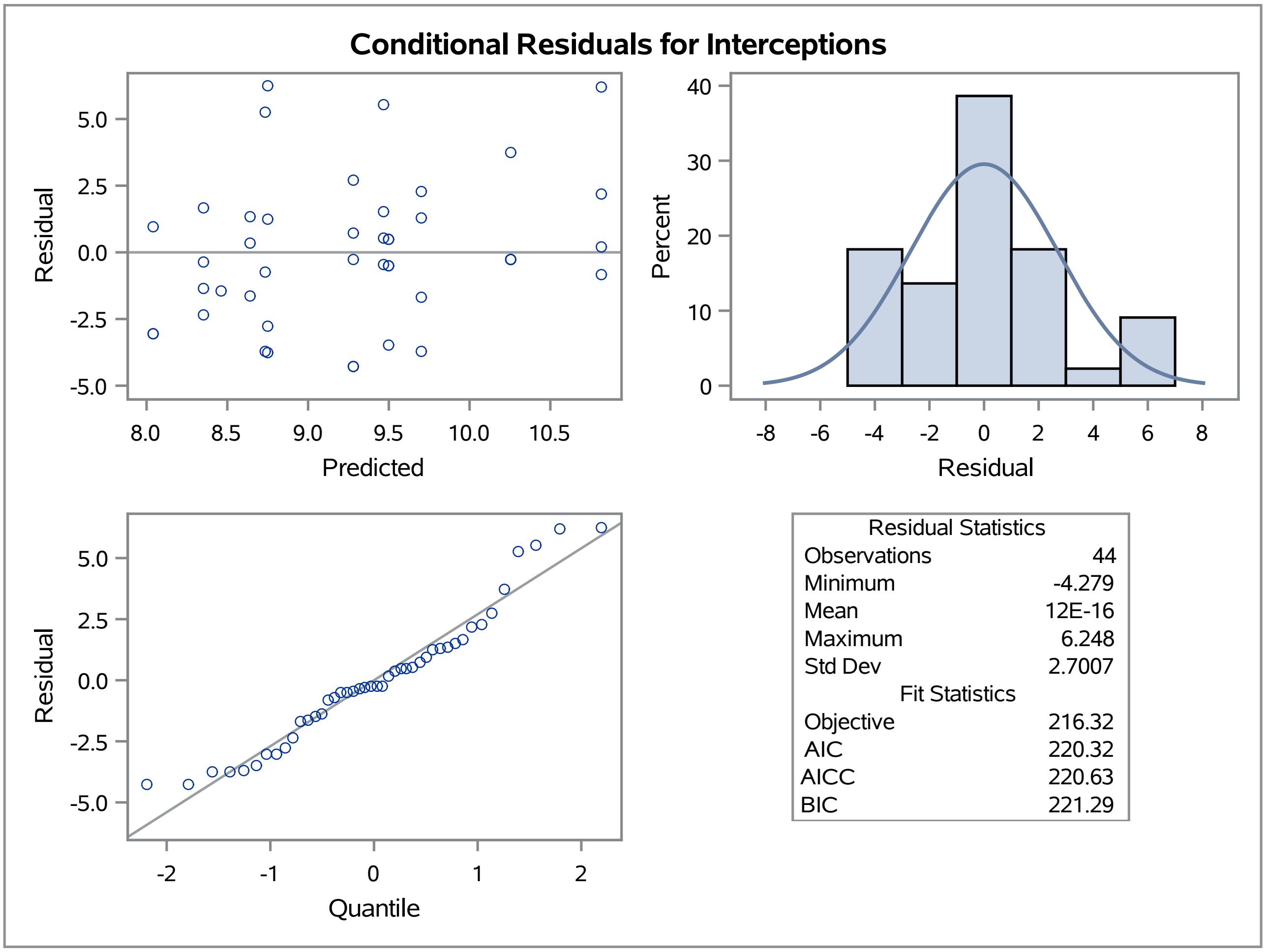

DistSoma=60

Model Information	
**Data Set**	WORK.TEMPDATASORTED
**Dependent Variable**	Interceptions
**Covariance Structure**	Variance Components
**Estimation Method**	REML
**Residual Variance Method**	Profile
**Fixed Effects SE Method**	Model-Based
**Degrees of Freedom Method**	Containment

Class Level Information		
**Class**	**Levels**	**Values**
**Treatment**	2	Control_GFP GFP MsTTR
**Culture**	12	1 2 3 4 5 6 7 8 9 10 11 12

Dimensions	
**Covariance Parameters**	2
**Columns in X**	3
**Columns in Z**	12
**Subjects**	1
**Max Obs per Subject**	44

Number of Observations	
**Number of Observations Read**	44
**Number of Observations Used**	44
**Number of Observations Not Used**	0

Iteration History			
**Iteration**	**Evaluations**	**-2 Res Log Like**	**Criterion**
**0**	1	206.45991413	
**1**	3	203.44157156	0.00002833
**2**	1	203.43973884	0.00000004
**3**	1	203.43973619	0.00000000

Convergence criteria met.

DistSoma=60

Covariance Parameter Estimates				
**Cov Parm**	**Estimate**	**Alpha**	**Lower**	**Upper**
**Culture**	1.7236	0.05	0.5450	25.9082
**Residual**	5.3575	0.05	3.4848	9.2850

Fit Statistics	
**-2 Res Log Likelihood**	203.4
**AIC (Smaller is Better)**	207.4
**AICC (Smaller is Better)**	207.7
**BIC (Smaller is Better)**	208.4

Solution for Fixed Effects									
**Effect**	**Treatment**	**Estimate**	**Standard Error**	**DF**	**t Value**	**Pr > |t|**	**Alpha**	**Lower**	**Upper**
**Intercept**		9.1491	0.7681	10	11.91	<.0001	0.05	7.4377	10.8606
**Treatment**	Control_GFP	-1.0392	1.0470	32	-0.99	0.3284	0.05	-3.1719	1.0934
**Treatment**	GFP MsTTR	0	.	.	.	.	.	.	.

Solution for Random Effects									
**Effect**	**Culture**	**Estimate**	**Std Err Pred**	**DF**	**t Value**	**Pr > |t|**	**Alpha**	**Lower**	**Upper**
**Culture**	1	-0.5451	0.9996	32	-0.55	0.5893	0.05	-2.5812	1.4910
**Culture**	2	-0.5451	0.9996	32	-0.55	0.5893	0.05	-2.5812	1.4910
**Culture**	3	1.0636	0.9560	32	1.11	0.2742	0.05	-0.8838	3.0110
**Culture**	4	0.9229	0.9560	32	0.97	0.3416	0.05	-1.0244	2.8703
**Culture**	5	-0.4839	0.9560	32	-0.51	0.6162	0.05	-2.4313	1.4635
**Culture**	6	0.4601	1.1550	32	0.40	0.6930	0.05	-1.8926	2.8128
**Culture**	7	-0.8725	0.9996	32	-0.87	0.3892	0.05	-2.9086	1.1636
**Culture**	8	-1.3253	0.9408	32	-1.41	0.1686	0.05	-3.2416	0.5911
**Culture**	9	0.1975	0.9698	32	0.20	0.8400	0.05	-1.7780	2.1729
**Culture**	10	1.4636	0.9698	32	1.51	0.1411	0.05	-0.5118	3.4390
**Culture**	11	0.6195	0.9698	32	0.64	0.5275	0.05	-1.3559	2.5949
**Culture**	12	-0.9553	0.9408	32	-1.02	0.3175	0.05	-2.8716	0.9611

Type 3 Tests of Fixed Effects				
**Effect**	**Num DF**	**Den DF**	**F Value**	**Pr > F**
**Treatment**	1	32	0.99	0.3284

DistSoma=60

Least Squares Means									
**Effect**	**Treatment**	**Estimate**	**Standard Error**	**DF**	**t Value**	**Pr > |t|**	**Alpha**	**Lower**	**Upper**
**Treatment**	Control_GFP	8.1099	0.7115	32	11.40	<.0001	0.05	6.6606	9.5591
**Treatment**	GFP MsTTR	9.1491	0.7681	32	11.91	<.0001	0.05	7.5845	10.7137

Differences of Least Squares Means												
**Effect**	**Treatment**	**Treatment**	**Estimate**	**Standard Error**	**DF**	**t Value**	**Pr > |t|**	**Adjustment**	**Adj P**	**Alpha**	**Lower**	**Upper**
**Treatment**	Control_GFP	GFP MsTTR	-1.0392	1.0470	32	-0.99	0.3284	Tukey-Kramer	0.3284	0.05	-3.1719	1.0934

Differences of Least Squares Means				
**Effect**	**Treatment**	**Treatment**	**Adj Lower**	**Adj Upper**
**Treatment**	Control_GFP	GFP MsTTR	-3.1719	1.0934

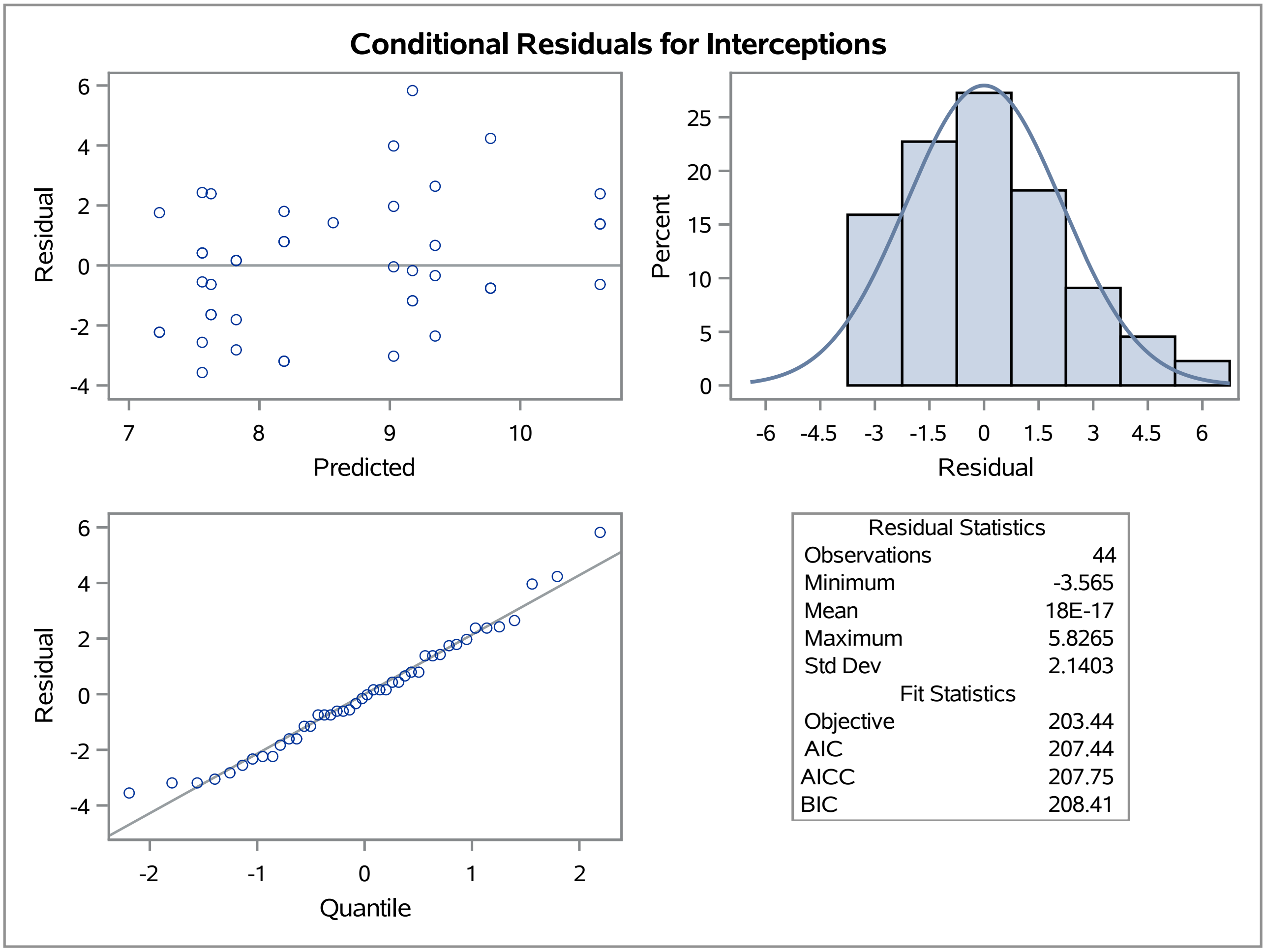

DistSoma=66

Model Information	
Data Set	WORK.TEMPDATASORTED
Dependent Variable	Interceptions
Covariance Structure	Variance Components
Estimation Method	REML
Residual Variance Method	Profile
Fixed Effects SE Method	Model-Based
Degrees of Freedom Method	Containment

Class Level Information		
Class	Levels	Values
Treatment	2	Control_GFP GFP MsTTR
Culture	12	1 2 3 4 5 6 7 8 9 10 11 12

Dimensions	
Covariance Parameters	2
Columns in X	3
Columns in Z	12
Subjects	1
Max Obs per Subject	44

Number of Observations	
Number of Observations Read	44
Number of Observations Used	44
Number of Observations Not Used	0

Iteration History			
Iteration	Evaluations	-2 Res Log Like	Criterion
0	1	204.01657584	
1	3	203.58977407	0.00000019
2	1	203.58976225	0.00000000

Convergence criteria met.

Covariance Parameter Estimates				
Cov Parm	Estimate	Alpha	Lower	Upper
Culture	0.5806	0.05	0.09484	23691
Residual	5.9994	0.05	3.9071	10.3767

DistSoma=66

Fit Statistics	
**-2 Res Log Likelihood**	203.6
**AIC (Smaller is Better)**	207.6
**AICC (Smaller is Better)**	207.9
**BIC (Smaller is Better)**	208.6

Solution for Fixed Effects

Effect	Treatment	Estimate	Standard Error	DF	t Value	Pr > |t|	Alpha	Lower	Upper
**Intercept**		8.1772	0.6244	10	13.10	<.0001	0.05	6.7860	9.5684
**Treatment**	Control_GFP	-0.3422	0.8675	32	-0.39	0.6959	0.05	-2.1093	1.4249
**Treatment**	GFP MsTTR	0	.	.	.	.	.	.	.

Solution for Random Effects

Effect	Culture	Estimate	Std Err Pred	DF	t Value	Pr > |t|	Alpha	Lower	Upper
**Culture**	1	-0.4129	0.6844	32	-0.60	0.5505	0.05	-1.8069	0.9811
**Culture**	2	-0.1879	0.6844	32	-0.27	0.7854	0.05	-1.5819	1.2061
**Culture**	3	0.3949	0.6685	32	0.59	0.5588	0.05	-0.9667	1.7565
**Culture**	4	0.4647	0.6685	32	0.70	0.4920	0.05	-0.8969	1.8263
**Culture**	5	-0.02372	0.6685	32	-0.04	0.9719	0.05	-1.3853	1.3379
**Culture**	6	0.1028	0.7295	32	0.14	0.8888	0.05	-1.3832	1.5888
**Culture**	7	-0.3379	0.6844	32	-0.49	0.6249	0.05	-1.7319	1.0561
**Culture**	8	-0.5796	0.6578	32	-0.88	0.3849	0.05	-1.9195	0.7604
**Culture**	9	0.1599	0.6700	32	0.24	0.8129	0.05	-1.2050	1.5247
**Culture**	10	0.5087	0.6700	32	0.76	0.4533	0.05	-0.8561	1.8736
**Culture**	11	0.2296	0.6700	32	0.34	0.7340	0.05	-1.1352	1.5945
**Culture**	12	-0.3187	0.6578	32	-0.48	0.6314	0.05	-1.6586	1.0213

Type 3 Tests of Fixed Effects

Effect	Num DF	Den DF	F Value	Pr > F
**Treatment**	1	32	0.16	0.6959

Least Squares Means

Effect	Treatment	Estimate	Standard Error	DF	t Value	Pr > |t|	Alpha	Lower	Upper
**Treatment**	Control_GFP	7.8350	0.6023	32	13.01	<.0001	0.05	6.6081	9.0618
**Treatment**	GFP MsTTR	8.1772	0.6244	32	13.10	<.0001	0.05	6.9054	9.4490

DistSoma=66

Differences of Least Squares Means												
Effect	Treatment	Treatment	Estimate	Standard Error	DF	t Value	Pr > |t|	Adjustment	Adj P	Alpha	Lower	Upper
**Treatment**	Control_GFP	GFP MsTTR	-0.3422	0.8675	32	-0.39	0.6959	Tukey-Kramer	0.6959	0.05	-2.1093	1.4249

Differences of Least Squares Means				
Effect	Treatment	Treatment	Adj Lower	Adj Upper
**Treatment**	Control_GFP	GFP MsTTR	-2.1093	1.4249

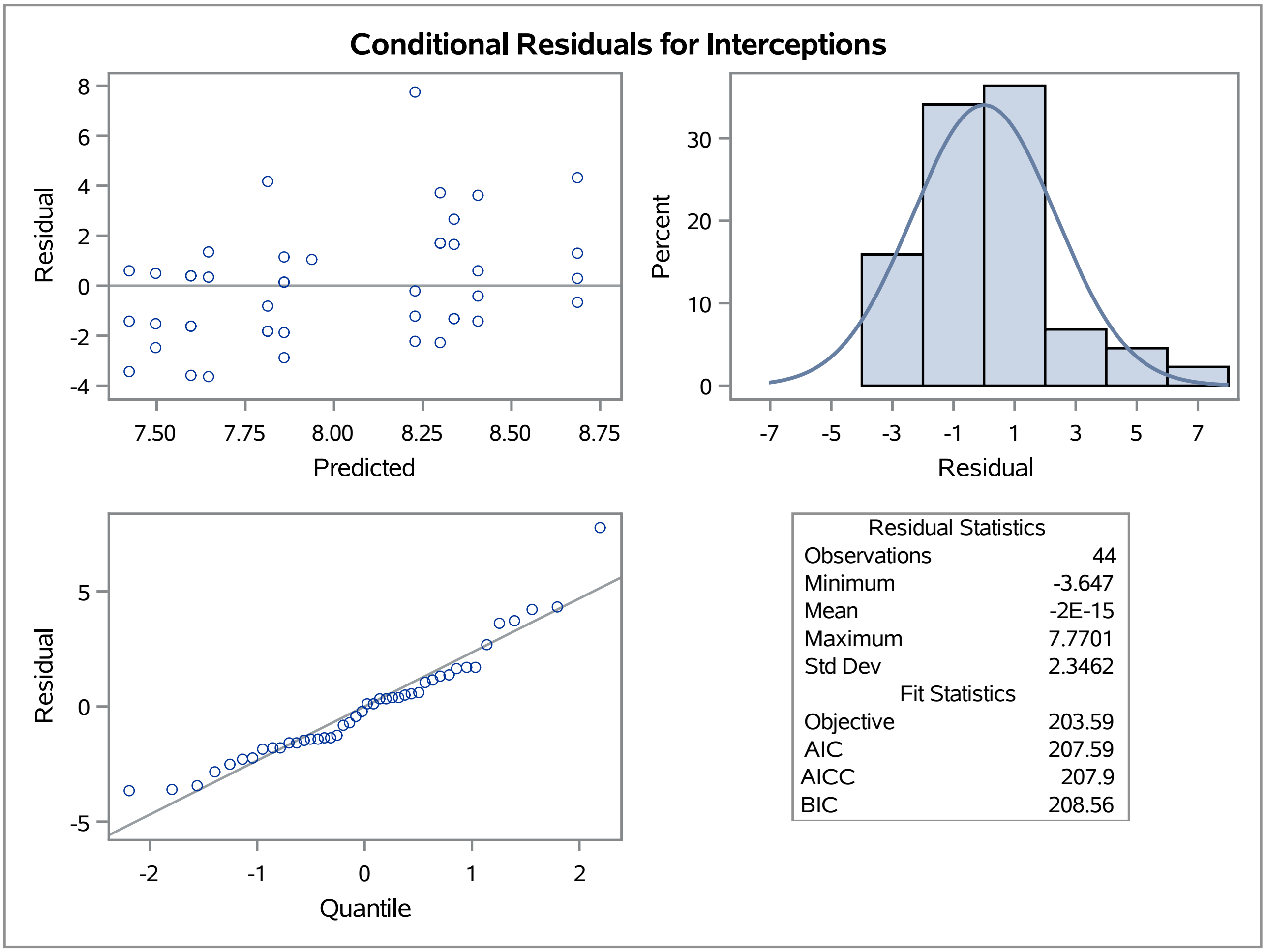

DistSoma=72

Model Information	
**Data Set**	WORK.TEMPDATASORTED
**Dependent Variable**	Interceptions
**Covariance Structure**	Variance Components
**Estimation Method**	REML
**Residual Variance Method**	Profile
**Fixed Effects SE Method**	Model-Based
**Degrees of Freedom Method**	Containment

Class Level Information		
**Class**	**Levels**	**Values**
**Treatment**	2	Control_GFP GFP MsTTR
**Culture**	12	1 2 3 4 5 6 7 8 9 10 11 12

Dimensions	
**Covariance Parameters**	2
**Columns in X**	3
**Columns in Z**	12
**Subjects**	1
**Max Obs per Subject**	44

Number of Observations	
**Number of Observations Read**	44
**Number of Observations Used**	44
**Number of Observations Not Used**	0

Iteration History			
**Iteration**	**Evaluations**	**-2 Res Log Like**	**Criterion**
**0**	1	207.46965644	
**1**	3	207.15455682	0.00000033
**2**	1	207.15453545	0.00000000

Convergence criteria met.

Covariance Parameter Estimates				
**Cov Parm**	**Estimate**	**Alpha**	**Lower**	**Upper**
**Culture**	0.5305	0.05	0.07688	590022
**Residual**	6.5998	0.05	4.3018	11.3993

DistSoma=72

Fit Statistics	
-2 Res Log Likelihood	207.2
AIC (Smaller is Better)	211.2
AICC (Smaller is Better)	211.5
BIC (Smaller is Better)	212.1

Solution for Fixed Effects

Effect	Treatment	Estimate	Standard Error	DF	t Value	Pr > |t|	Alpha	Lower	Upper
Intercept		7.9055	0.6380	10	12.39	<.0001	0.05	6.4839	9.3271
Treatment	Control_GFP	-0.9200	0.8885	32	-1.04	0.3082	0.05	-2.7299	0.8899
Treatment	GFP MsTTR	0	.	.	.	.	.	.	.

Solution for Random Effects

Effect	Culture	Estimate	Std Err Pred	DF	t Value	Pr > |t|	Alpha	Lower	Upper
Culture	1	-0.3858	0.6647	32	-0.58	0.5657	0.05	-1.7398	0.9683
Culture	2	-0.1267	0.6647	32	-0.19	0.8500	0.05	-1.4807	1.2273
Culture	3	0.3685	0.6512	32	0.57	0.5754	0.05	-0.9580	1.6950
Culture	4	0.2468	0.6512	32	0.38	0.7072	0.05	-1.0796	1.5733
Culture	5	0.003529	0.6512	32	0.01	0.9957	0.05	-1.3230	1.3300
Culture	6	0.1499	0.7023	32	0.21	0.8323	0.05	-1.2806	1.5803
Culture	7	-0.2562	0.6647	32	-0.39	0.7024	0.05	-1.6103	1.0978
Culture	8	-0.4890	0.6418	32	-0.76	0.4517	0.05	-1.7962	0.8183
Culture	9	0.2055	0.6523	32	0.31	0.7548	0.05	-1.1233	1.5342
Culture	10	0.3271	0.6523	32	0.50	0.6195	0.05	-1.0016	1.6559
Culture	11	0.3880	0.6523	32	0.59	0.5562	0.05	-0.9408	1.7167
Culture	12	-0.4316	0.6418	32	-0.67	0.5061	0.05	-1.7389	0.8757

Type 3 Tests of Fixed Effects

Effect	Num DF	Den DF	F Value	Pr > F
Treatment	1	32	1.07	0.3082

Least Squares Means

Effect	Treatment	Estimate	Standard Error	DF	t Value	Pr > |t|	Alpha	Lower	Upper
Treatment	Control_GFP	6.9855	0.6184	32	11.30	<.0001	0.05	5.7258	8.2452
Treatment	GFP MsTTR	7.9055	0.6380	32	12.39	<.0001	0.05	6.6059	9.2051

DistSoma=72

Differences of Least Squares Means												
**Effect**	**Treatment**	**Treatment**	**Estimate**	**Standard Error**	**DF**	**t Value**	**Pr > |t|**	**Adjustment**	**Adj P**	**Alpha**	**Lower**	**Upper**
**Treatment**	Control_GFP	GFP MsTTR	-0.9200	0.8885	32	-1.04	0.3082	Tukey-Kramer	0.3082	0.05	-2.7299	0.8899

Differences of Least Squares Means				
**Effect**	**Treatment**	**Treatment**	**Adj Lower**	**Adj Upper**
**Treatment**	Control_GFP	GFP MsTTR	-2.7298	0.8899

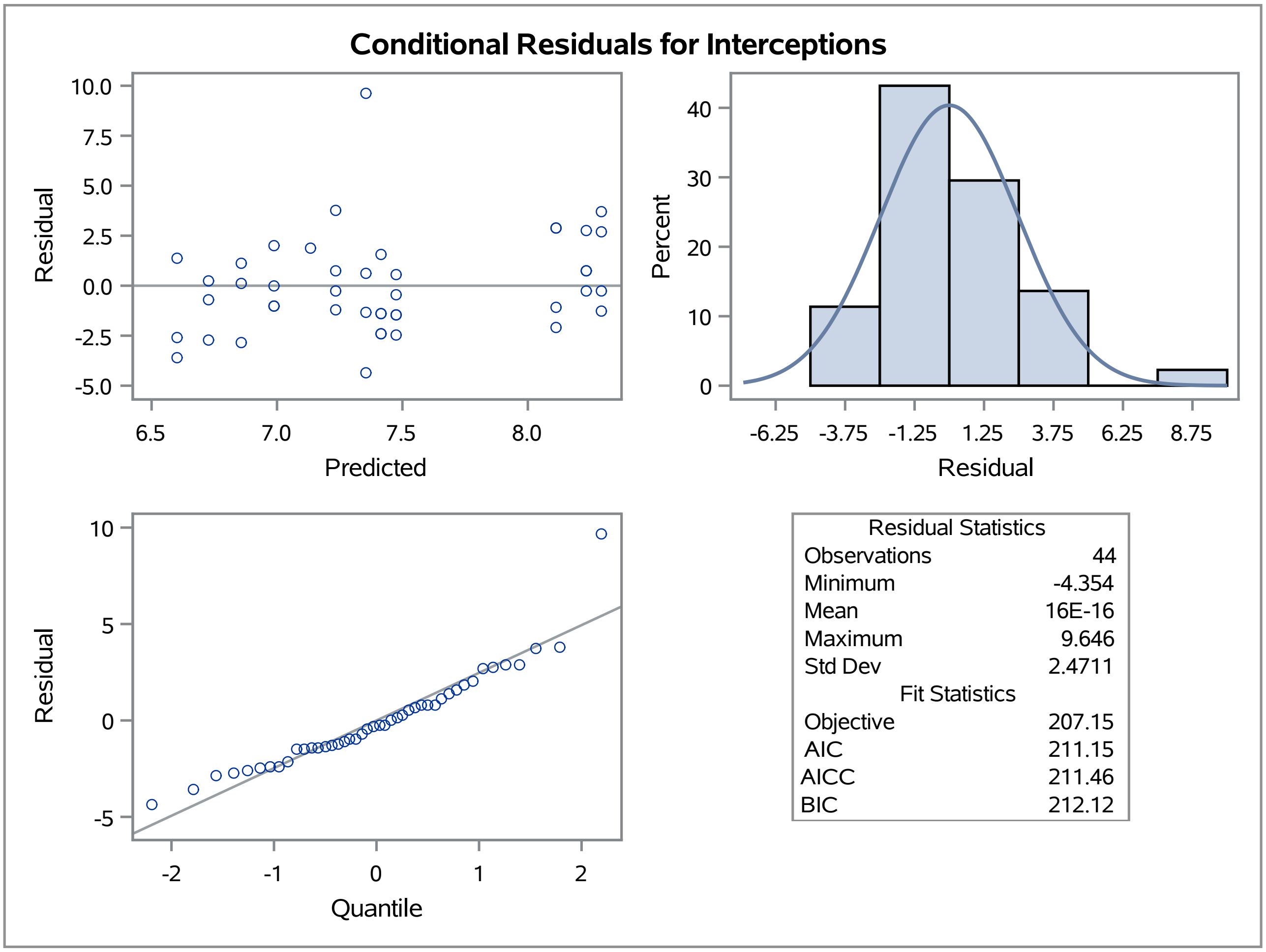

DistSoma=78

Model Information	
**Data Set**	WORK.TEMPDATASORTED
**Dependent Variable**	Interceptions
**Covariance Structure**	Variance Components
**Estimation Method**	REML
**Residual Variance Method**	Profile
**Fixed Effects SE Method**	Model-Based
**Degrees of Freedom Method**	Containment

Class Level Information		
**Class**	**Levels**	**Values**
**Treatment**	2	Control_GFP GFP MsTTR
**Culture**	12	1 2 3 4 5 6 7 8 9 10 11 12

Dimensions	
**Covariance Parameters**	2
**Columns in X**	3
**Columns in Z**	12
**Subjects**	1
**Max Obs per Subject**	44

Number of Observations	
**Number of Observations Read**	44
**Number of Observations Used**	44
**Number of Observations Not Used**	0

Iteration History			
**Iteration**	**Evaluations**	**-2 Res Log Like**	**Criterion**
**0**	1	208.58125251	
**1**	2	207.69411900	0.00001630
**2**	1	207.69303284	0.00000002
**3**	1	207.69303169	0.00000000

Convergence criteria met.

DistSoma=78

Covariance Parameter Estimates				
**Cov Parm**	**Estimate**	**Alpha**	**Lower**	**Upper**
**Culture**	1.1627	0.05	0.2471	487.99
**Residual**	6.2973	0.05	4.0374	11.1769

Fit Statistics	
**-2 Res Log Likelihood**	207.7
**AIC (Smaller is Better)**	211.7
**AICC (Smaller is Better)**	212.0
**BIC (Smaller is Better)**	212.7

Solution for Fixed Effects									
**Effect**	**Treatment**	**Estimate**	**Standard Error**	**DF**	**t Value**	**Pr > |t|**	**Alpha**	**Lower**	**Upper**
**Intercept**		7.5118	0.7214	10	10.41	<.0001	0.05	5.9045	9.1191
**Treatment**	Control_GFP	-0.8631	0.9927	32	-0.87	0.3911	0.05	-2.8852	1.1590
**Treatment**	GFP MsTTR	0	.	.	.	.	.	.	.

Solution for Random Effects									
**Effect**	**Culture**	**Estimate**	**Std Err Pred**	**DF**	**t Value**	**Pr > |t|**	**Alpha**	**Lower**	**Upper**
**Culture**	1	-1.3006	0.8985	32	-1.45	0.1575	0.05	-3.1309	0.5296
**Culture**	2	0.1252	0.8985	32	0.14	0.8900	0.05	-1.7050	1.9555
**Culture**	3	0.6802	0.8676	32	0.78	0.4388	0.05	-1.0870	2.4475
**Culture**	4	0.1492	0.8676	32	0.17	0.8645	0.05	-1.6180	1.9165
**Culture**	5	0.2554	0.8676	32	0.29	0.7703	0.05	-1.5118	2.0227
**Culture**	6	0.6782	0.9964	32	0.68	0.5010	0.05	-1.3514	2.7078
**Culture**	7	-0.5877	0.8985	32	-0.65	0.5177	0.05	-2.4179	1.2425
**Culture**	8	-0.6297	0.8512	32	-0.74	0.4648	0.05	-2.3635	1.1041
**Culture**	9	0.2074	0.8733	32	0.24	0.8138	0.05	-1.5715	1.9863
**Culture**	10	0.7384	0.8733	32	0.85	0.4041	0.05	-1.0405	2.5173
**Culture**	11	0.3136	0.8733	32	0.36	0.7219	0.05	-1.4653	2.0925
**Culture**	12	-0.6297	0.8512	32	-0.74	0.4648	0.05	-2.3635	1.1041

Type 3 Tests of Fixed Effects				
**Effect**	**Num DF**	**Den DF**	**F Value**	**Pr > F**
**Treatment**	1	32	0.76	0.3911

DistSoma=78

Least Squares Means									
**Effect**	**Treatment**	**Estimate**	**Standard Error**	**DF**	**t Value**	**Pr > |t|**	**Alpha**	**Lower**	**Upper**
**Treatment**	Control_GFP	6.6487	0.6820	32	9.75	<.0001	0.05	5.2595	8.0379
**Treatment**	GFP MsTTR	7.5118	0.7214	32	10.41	<.0001	0.05	6.0424	8.9811

Differences of Least Squares Means												
**Effect**	**Treatment**	**Treatment**	**Estimate**	**Standard Error**	**DF**	**t Value**	**Pr > |t|**	**Adjustment**	**Adj P**	**Alpha**	**Lower**	**Upper**
**Treatment**	Control_GFP	GFP MsTTR	-0.8631	0.9927	32	-0.87	0.3911	Tukey-Kramer	0.3911	0.05	-2.8852	1.1590

Differences of Least Squares Means				
**Effect**	**Treatment**	**Treatment**	**Adj Lower**	**Adj Upper**
**Treatment**	Control_GFP	GFP MsTTR	-2.8852	1.1590

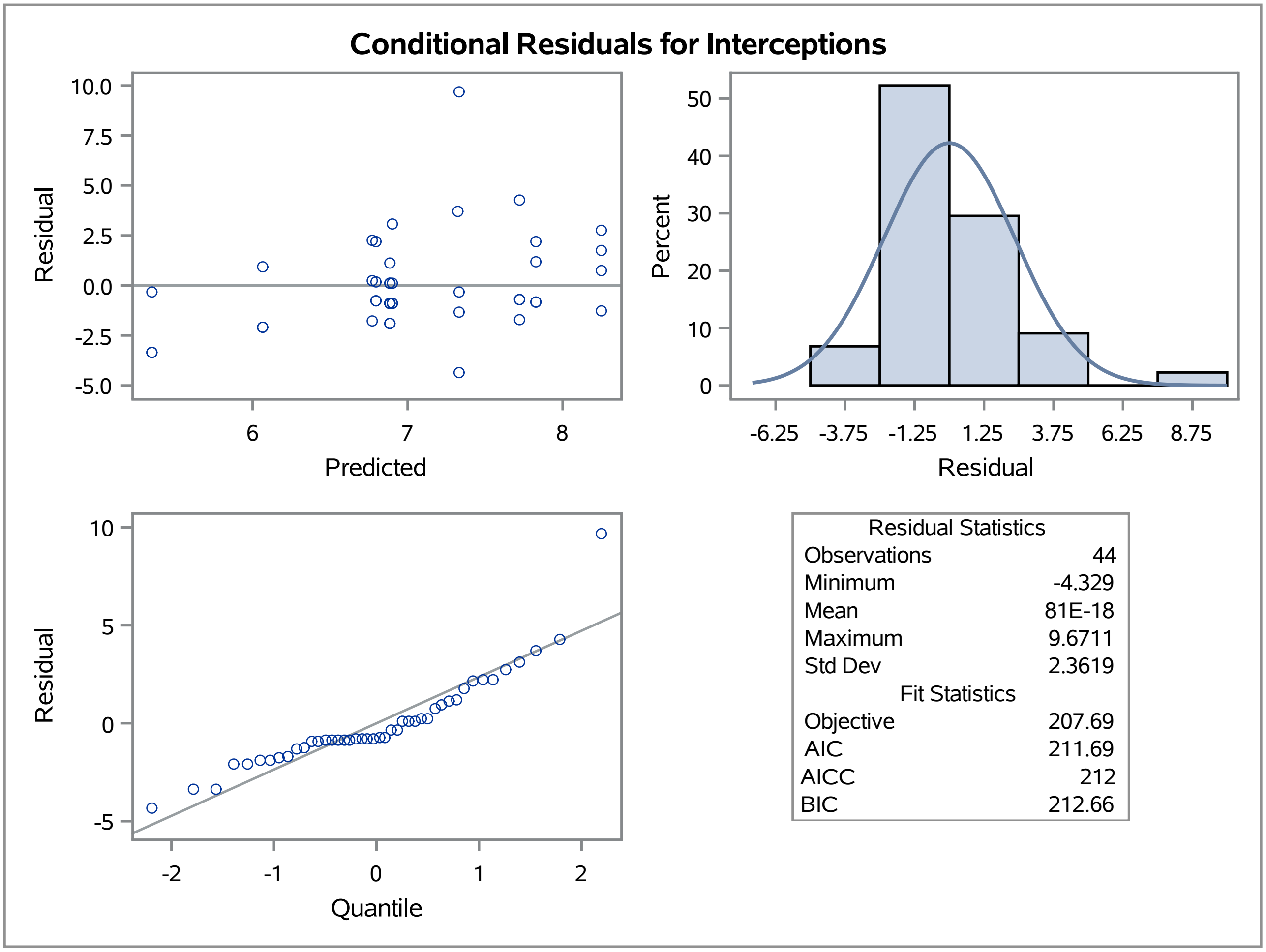

DistSoma=84

Model Information	
**Data Set**	WORK.TEMPDATASORTED
**Dependent Variable**	Interceptions
**Covariance Structure**	Variance Components
**Estimation Method**	REML
**Residual Variance Method**	Profile
**Fixed Effects SE Method**	Model-Based
**Degrees of Freedom Method**	Containment

Class Level Information		
**Class**	**Levels**	**Values**
**Treatment**	2	Control_GFP GFP MsTTR
**Culture**	12	1 2 3 4 5 6 7 8 9 10 11 12

Dimensions	
**Covariance Parameters**	2
**Columns in X**	3
**Columns in Z**	12
**Subjects**	1
**Max Obs per Subject**	44

Number of Observations	
**Number of Observations Read**	44
**Number of Observations Used**	44
**Number of Observations Not Used**	0

Iteration History			
**Iteration**	**Evaluations**	**-2 Res Log Like**	**Criterion**
**0**	1	204.86746552	
**1**	2	203.77874586	0.00000680
**2**	1	203.77830952	0.00000000

Convergence criteria met.

Covariance Parameter Estimates				
**Cov Parm**	**Estimate**	**Alpha**	**Lower**	**Upper**
**Culture**	1.1309	0.05	0.2610	183.90
**Residual**	5.6976	0.05	3.6709	10.0305

DistSoma=84

Fit Statistics	
-2 Res Log Likelihood	203.8
AIC (Smaller is Better)	207.8
AICC (Smaller is Better)	208.1
BIC (Smaller is Better)	208.7

Solution for Fixed Effects

Effect	Treatment	Estimate	Standard Error	DF	t Value	Pr > |t|	Alpha	Lower	Upper
Intercept		7.1474	0.6976	10	10.25	<.0001	0.05	5.5931	8.7017
Treatment	Control_GFP	-1.1210	0.9589	32	-1.17	0.2510	0.05	-3.0741	0.8322
Treatment	GFP MsTTR	0	.	.	.	.	.	.	.

Solution for Random Effects

Effect	Culture	Estimate	Std Err Pred	DF	t Value	Pr > |t|	Alpha	Lower	Upper
Culture	1	-1.3783	0.8770	32	-1.57	0.1259	0.05	-3.1647	0.4080
Culture	2	-0.00987	0.8770	32	-0.01	0.9911	0.05	-1.7962	1.7765
Culture	3	0.4309	0.8457	32	0.51	0.6139	0.05	-1.2917	2.1534
Culture	4	0.5415	0.8457	32	0.64	0.5265	0.05	-1.1811	2.2641
Culture	5	0.4309	0.8457	32	0.51	0.6139	0.05	-1.2917	2.1534
Culture	6	0.4924	0.9775	32	0.50	0.6179	0.05	-1.4986	2.4835
Culture	7	-0.5075	0.8770	32	-0.58	0.5669	0.05	-2.2938	1.2789
Culture	8	-0.5715	0.8296	32	-0.69	0.4959	0.05	-2.2614	1.1184
Culture	9	0.1561	0.8519	32	0.18	0.8558	0.05	-1.5792	1.8913
Culture	10	0.8199	0.8519	32	0.96	0.3430	0.05	-0.9153	2.5551
Culture	11	0.2667	0.8519	32	0.31	0.7563	0.05	-1.4685	2.0019
Culture	12	-0.6711	0.8296	32	-0.81	0.4245	0.05	-2.3611	1.0188

Type 3 Tests of Fixed Effects

Effect	Num DF	Den DF	F Value	Pr > F
Treatment	1	32	1.37	0.2510

Least Squares Means

Effect	Treatment	Estimate	Standard Error	DF	t Value	Pr > |t|	Alpha	Lower	Upper
Treatment	Control_GFP	6.0264	0.6579	32	9.16	<.0001	0.05	4.6864	7.3665
Treatment	GFP MsTTR	7.1474	0.6976	32	10.25	<.0001	0.05	5.7265	8.5683

DistSoma=84

Differences of Least Squares Means												
**Effect**	**Treatment**	**Treatment**	**Estimate**	**Standard Error**	**DF**	**t Value**	**Pr > |t|**	**Adjustment**	**Adj P**	**Alpha**	**Lower**	**Upper**
**Treatment**	Control_GFP	GFP MsTTR	-1.1210	0.9589	32	-1.17	0.2510	Tukey-Kramer	0.2510	0.05	-3.0741	0.8322

Differences of Least Squares Means				
**Effect**	**Treatment**	**Treatment**	**Adj Lower**	**Adj Upper**
**Treatment**	Control_GFP	GFP MsTTR	-3.0741	0.8322

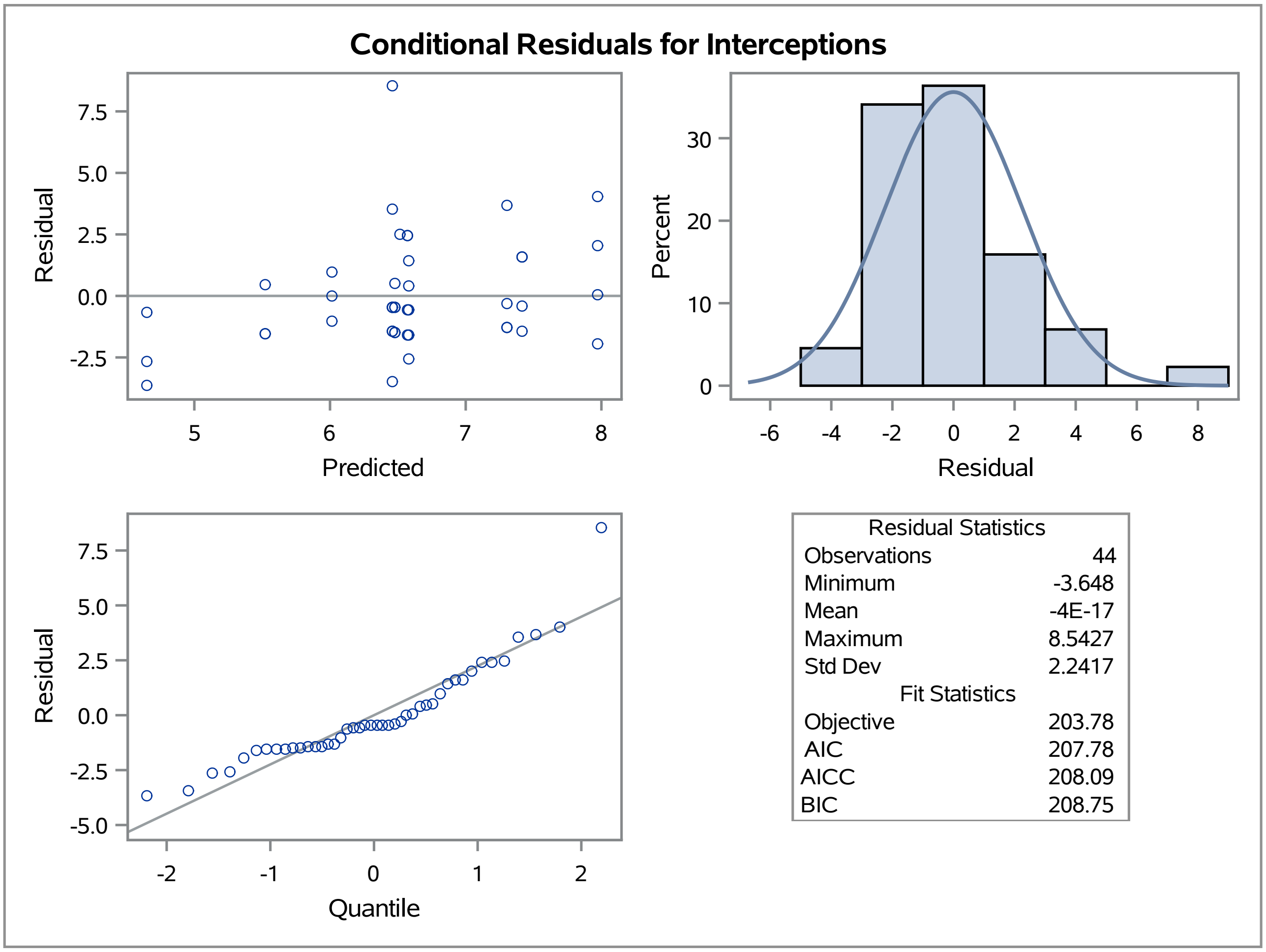

DistSoma=90

Model Information	
**Data Set**	WORK.TEMPDATASORTED
**Dependent Variable**	Interceptions
**Covariance Structure**	Variance Components
**Estimation Method**	REML
**Residual Variance Method**	Profile
**Fixed Effects SE Method**	Model-Based
**Degrees of Freedom Method**	Containment

Class Level Information		
**Class**	**Levels**	**Values**
**Treatment**	2	Control_GFP GFP MsTTR
**Culture**	12	1 2 3 4 5 6 7 8 9 10 11 12

Dimensions	
**Covariance Parameters**	2
**Columns in X**	3
**Columns in Z**	12
**Subjects**	1
**Max Obs per Subject**	44

Number of Observations	
**Number of Observations Read**	44
**Number of Observations Used**	44
**Number of Observations Not Used**	0

Iteration History			
**Iteration**	**Evaluations**	**-2 Res Log Like**	**Criterion**
**0**	1	204.70278634	
**1**	2	204.60560109	0.00000006
**2**	1	204.60559710	0.00000000

Convergence criteria met.

Covariance Parameter Estimates				
**Cov Parm**	**Estimate**	**Alpha**	**Lower**	**Upper**
**Culture**	0.3126	0.05	0.03044	3.997E17
**Residual**	6.3455	0.05	4.0894	11.1661

DistSoma=90

Fit Statistics	
-2 Res Log Likelihood	204.6
AIC (Smaller is Better)	208.6
AICC (Smaller is Better)	208.9
BIC (Smaller is Better)	209.6

Solution for Fixed Effects

Effect	Treatment	Estimate	Standard Error	DF	t Value	Pr > |t|	Alpha	Lower	Upper
Intercept		6.7479	0.5930	10	11.38	<.0001	0.05	5.4267	8.0691
Treatment	Control_GFP	-1.3443	0.8299	32	-1.62	0.1151	0.05	-3.0348	0.3462
Treatment	GFP MsTTR	0	.	.	.	.	.	.	.

Solution for Random Effects

Effect	Culture	Estimate	Std Err Pred	DF	t Value	Pr > |t|	Alpha	Lower	Upper
Culture	1	-0.3954	0.5272	32	-0.75	0.4588	0.05	-1.4693	0.6786
Culture	2	-0.09489	0.5272	32	-0.18	0.8583	0.05	-1.1688	0.9790
Culture	3	0.1393	0.5199	32	0.27	0.7904	0.05	-0.9197	1.1984
Culture	4	0.1393	0.5199	32	0.27	0.7904	0.05	-0.9197	1.1984
Culture	5	0.1805	0.5199	32	0.35	0.7307	0.05	-0.8785	1.2395
Culture	6	0.1689	0.5465	32	0.31	0.7593	0.05	-0.9444	1.2821
Culture	7	-0.1378	0.5272	32	-0.26	0.7955	0.05	-1.2117	0.9361
Culture	8	-0.2664	0.5144	32	-0.52	0.6081	0.05	-1.3142	0.7813
Culture	9	0.08266	0.5203	32	0.16	0.8748	0.05	-0.9771	1.1424
Culture	10	0.2061	0.5203	32	0.40	0.6946	0.05	-0.8537	1.2659
Culture	11	0.1650	0.5203	32	0.32	0.7532	0.05	-0.8948	1.2248
Culture	12	-0.1874	0.5144	32	-0.36	0.7181	0.05	-1.2351	0.8604

Type 3 Tests of Fixed Effects

Effect	Num DF	Den DF	F Value	Pr > F
Treatment	1	32	2.62	0.1151

Least Squares Means

Effect	Treatment	Estimate	Standard Error	DF	t Value	Pr > |t|	Alpha	Lower	Upper
Treatment	Control_GFP	5.4036	0.5807	32	9.31	<.0001	0.05	4.2208	6.5864
Treatment	GFP MsTTR	6.7479	0.5930	32	11.38	<.0001	0.05	5.5401	7.9557

DistSoma=90

Differences of Least Squares Means												
**Effect**	**Treatment**	**Treatment**	**Estimate**	**Standard Error**	**DF**	**t Value**	**Pr > |t|**	**Adjustment**	**Adj P**	**Alpha**	**Lower**	**Upper**
**Treatment**	Control_GFP	GFP MsTTR	-1.3443	0.8299	32	-1.62	0.1151	Tukey-Kramer	0.1151	0.05	-3.0348	0.3462

Differences of Least Squares Means				
**Effect**	**Treatment**	**Treatment**	**Adj Lower**	**Adj Upper**
**Treatment**	Control_GFP	GFP MsTTR	-3.0348	0.3462

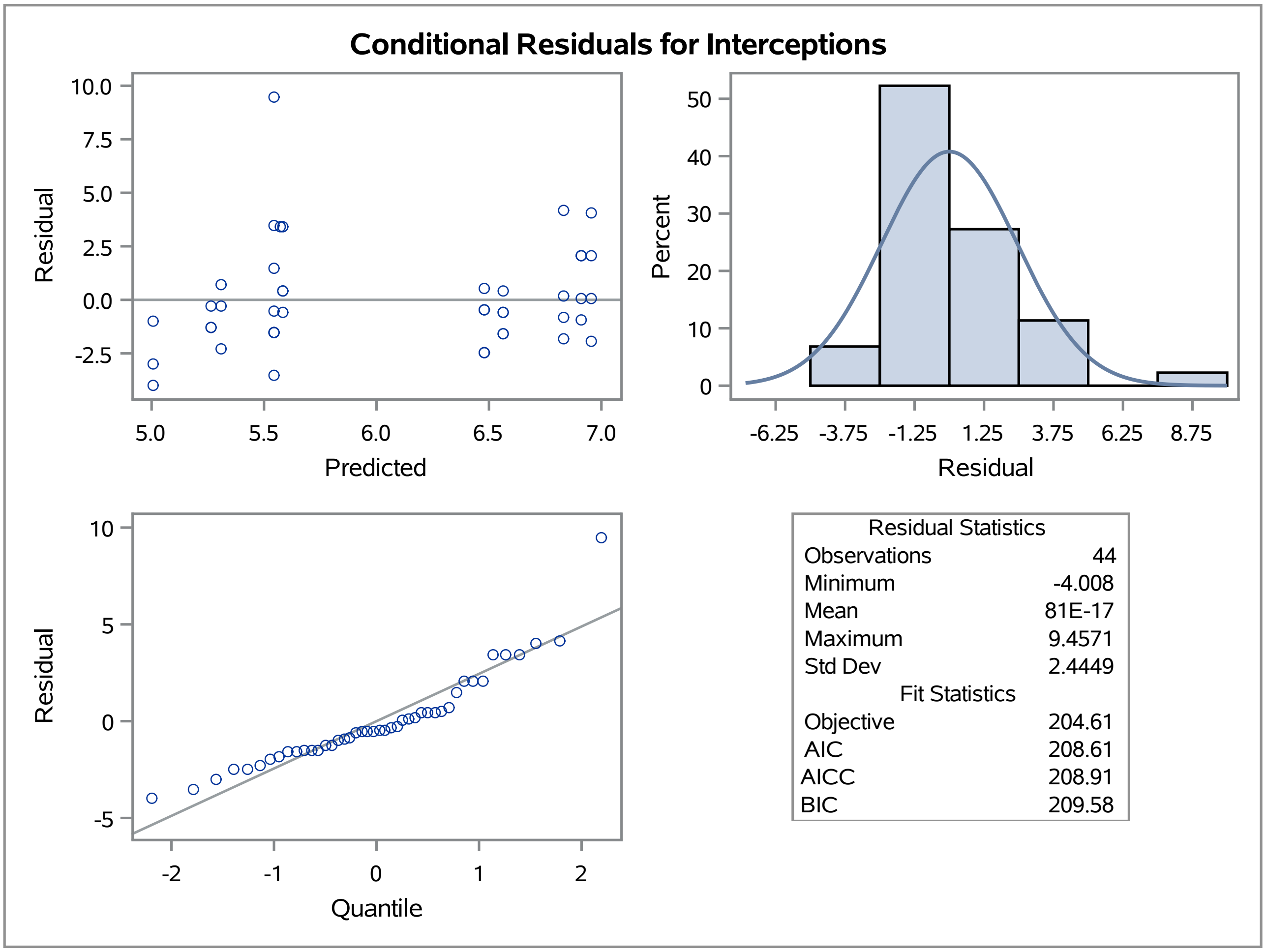

DistSoma=96

Model Information	
**Data Set**	WORK.TEMPDATASORTED
**Dependent Variable**	Interceptions
**Covariance Structure**	Variance Components
**Estimation Method**	REML
**Residual Variance Method**	Profile
**Fixed Effects SE Method**	Model-Based
**Degrees of Freedom Method**	Containment

Class Level Information		
**Class**	**Levels**	**Values**
**Treatment**	2	Control_GFP GFP MsTTR
**Culture**	12	1 2 3 4 5 6 7 8 9 10 11 12

Dimensions	
**Covariance Parameters**	2
**Columns in X**	3
**Columns in Z**	12
**Subjects**	1
**Max Obs per Subject**	44

Number of Observations	
**Number of Observations Read**	44
**Number of Observations Used**	44
**Number of Observations Not Used**	0

Iteration History			
**Iteration**	**Evaluations**	**-2 Res Log Like**	**Criterion**
**0**	1	198.90250914	
**1**	1	198.90250914	0.00000000

Convergence criteria met.

Estimated G matrix is not positive definite.

Covariance Parameter Estimates				
**Cov Parm**	**Estimate**	**Alpha**	**Lower**	**Upper**
**Culture**	0	.	.	.
**Residual**	5.7587	0.05	3.9151	9.3029

DistSoma=96

Fit Statistics	
-2 Res Log Likelihood	198.9
AIC (Smaller is Better)	200.9
AICC (Smaller is Better)	201.0
BIC (Smaller is Better)	201.4

Solution for Fixed Effects

Effect	Treatment	Estimate	Standard Error	DF	t Value	Pr > |t|	Alpha	Lower	Upper
Intercept		6.6364	0.5116	10	12.97	<.0001	0.05	5.4964	7.7763
Treatment	Control_GFP	-1.9545	0.7235	32	-2.70	0.0110	0.05	-3.4284	-0.4807
Treatment	GFP MsTTR	0	.	.	.	.	.	.	.

Solution for Random Effects

Effect	Culture	Estimate	Std Err Pred	DF	t Value	Pr > |t|	Alpha	Lower	Upper
Culture	1	0	.	.	.	.	.	.	.
Culture	2	0	.	.	.	.	.	.	.
Culture	3	0	.	.	.	.	.	.	.
Culture	4	0	.	.	.	.	.	.	.
Culture	5	0	.	.	.	.	.	.	.
Culture	6	0	.	.	.	.	.	.	.
Culture	7	0	.	.	.	.	.	.	.
Culture	8	0	.	.	.	.	.	.	.
Culture	9	0	.	.	.	.	.	.	.
Culture	10	0	.	.	.	.	.	.	.
Culture	11	0	.	.	.	.	.	.	.
Culture	12	0	.	.	.	.	.	.	.

Type 3 Tests of Fixed Effects

Effect	Num DF	Den DF	F Value	Pr > F
Treatment	1	32	7.30	0.0110

Least Squares Means

Effect	Treatment	Estimate	Standard Error	DF	t Value	Pr > |t|	Alpha	Lower	Upper
Treatment	Control_GFP	4.6818	0.5116	32	9.15	<.0001	0.05	3.6397	5.7240
Treatment	GFP MsTTR	6.6364	0.5116	32	12.97	<.0001	0.05	5.5942	7.6785

DistSoma=96

Differences of Least Squares Means												
**Effect**	**Treatment**	**Treatment**	**Estimate**	**Standard Error**	**DF**	**t Value**	**Pr > |t|**	**Adjustment**	**Adj P**	**Alpha**	**Lower**	**Upper**
**Treatment**	Control_GFP	GFP MsTTR	-1.9545	0.7235	32	-2.70	0.0110	Tukey	0.0110	0.05	-3.4284	-0.4807

Differences of Least Squares Means				
**Effect**	**Treatment**	**Treatment**	**Adj Lower**	**Adj Upper**
**Treatment**	Control_GFP	GFP MsTTR	-3.4283	-0.4808

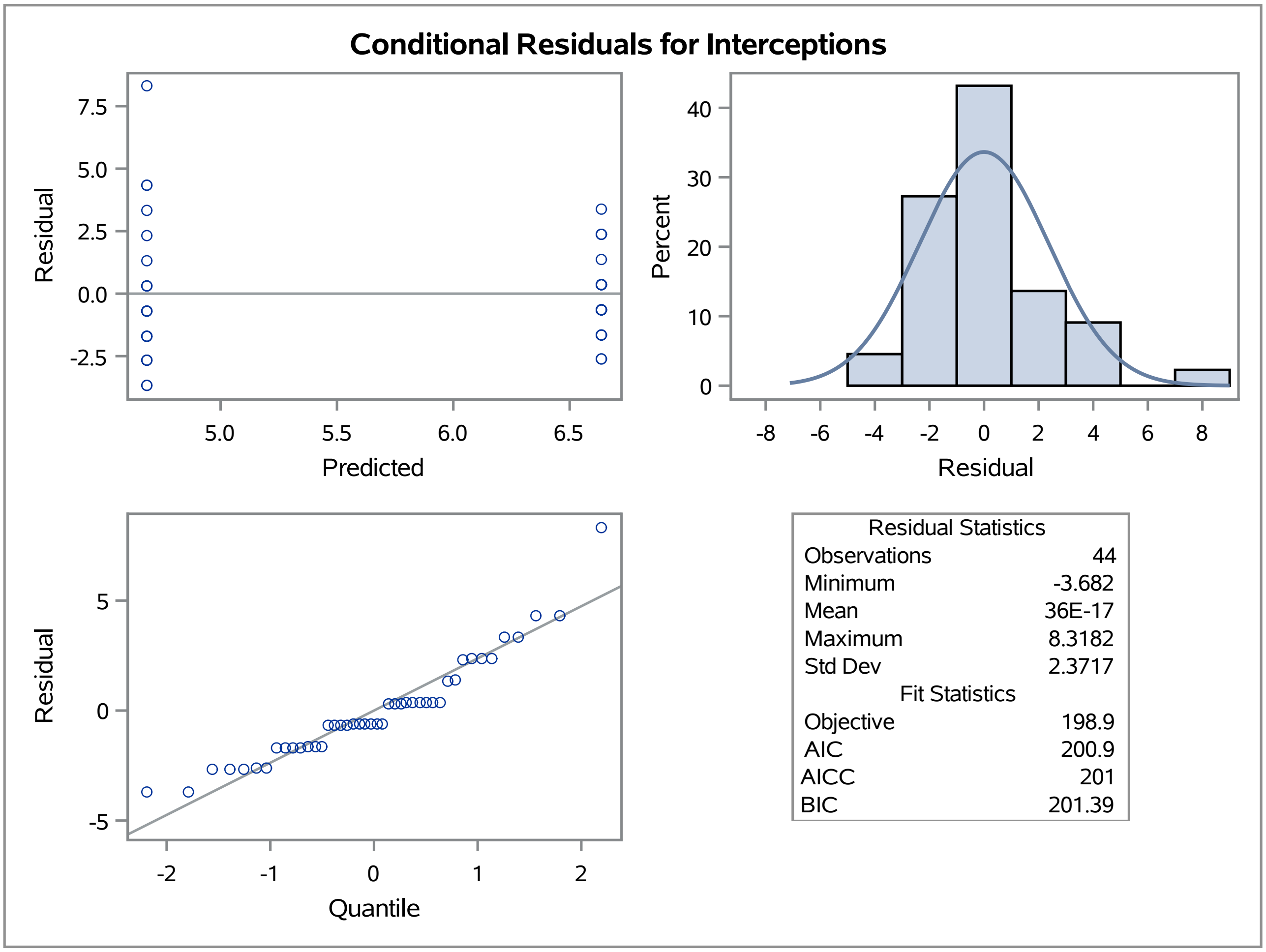

DistSoma=102

Model Information	
Data Set	WORK.TEMPDATASORTED
Dependent Variable	Interceptions
Covariance Structure	Variance Components
Estimation Method	REML
Residual Variance Method	Profile
Fixed Effects SE Method	Model-Based
Degrees of Freedom Method	Containment

Class Level Information		
Class	Levels	Values
Treatment	2	Control_GFP GFP MsTTR
Culture	12	1 2 3 4 5 6 7 8 9 10 11 12

Dimensions	
Covariance Parameters	2
Columns in X	3
Columns in Z	12
Subjects	1
Max Obs per Subject	44

Number of Observations	
Number of Observations Read	44
Number of Observations Used	44
Number of Observations Not Used	0

Iteration History			
Iteration	Evaluations	-2 Res Log Like	Criterion
0	1	200.72518896	
1	1	200.72518896	0.00000000

Convergence criteria met.

Estimated G matrix is not positive definite.

Covariance Parameter Estimates				
Cov Parm	Estimate	Alpha	Lower	Upper
Culture	0	.	.	.
Residual	6.0141	0.05	4.0888	9.7155

DistSoma=102

Fit Statistics	
-2 Res Log Likelihood	200.7
AIC (Smaller is Better)	202.7
AICC (Smaller is Better)	202.8
BIC (Smaller is Better)	203.2

Solution for Fixed Effects

Effect	Treatment	Estimate	Standard Error	DF	t Value	Pr > |t|	Alpha	Lower	Upper
Intercept		6.6364	0.5228	10	12.69	<.0001	0.05	5.4714	7.8013
Treatment	Control_GFP	-2.1364	0.7394	32	-2.89	0.0069	0.05	-3.6425	-0.6302
Treatment	GFP MsTTR	0	.	.	.	.	.	.	.

Solution for Random Effects

Effect	Culture	Estimate	Std Err Pred	DF	t Value	Pr > |t|	Alpha	Lower	Upper
Culture	1	0	.	.	.	.	.	.	.
Culture	2	0	.	.	.	.	.	.	.
Culture	3	0	.	.	.	.	.	.	.
Culture	4	0	.	.	.	.	.	.	.
Culture	5	0	.	.	.	.	.	.	.
Culture	6	0	.	.	.	.	.	.	.
Culture	7	0	.	.	.	.	.	.	.
Culture	8	0	.	.	.	.	.	.	.
Culture	9	0	.	.	.	.	.	.	.
Culture	10	0	.	.	.	.	.	.	.
Culture	11	0	.	.	.	.	.	.	.
Culture	12	0	.	.	.	.	.	.	.

Type 3 Tests of Fixed Effects

Effect	Num DF	Den DF	F Value	Pr > F
Treatment	1	32	8.35	0.0069

Least Squares Means

Effect	Treatment	Estimate	Standard Error	DF	t Value	Pr > |t|	Alpha	Lower	Upper
Treatment	Control_GFP	4.5000	0.5228	32	8.61	<.0001	0.05	3.4350	5.5650
Treatment	GFP MsTTR	6.6364	0.5228	32	12.69	<.0001	0.05	5.5714	7.7014

DistSoma=102

Differences of Least Squares Means												
**Effect**	**Treatment**	**Treatment**	**Estimate**	**Standard Error**	**DF**	**t Value**	**Pr > |t|**	**Adjustment**	**Adj P**	**Alpha**	**Lower**	**Upper**
**Treatment**	Control_GFP	GFP MsTTR	-2.1364	0.7394	32	-2.89	0.0069	Tukey	0.0069	0.05	-3.6425	-0.6302

Differences of Least Squares Means				
**Effect**	**Treatment**	**Treatment**	**Adj Lower**	**Adj Upper**
**Treatment**	Control_GFP	GFP MsTTR	-3.6425	-0.6302

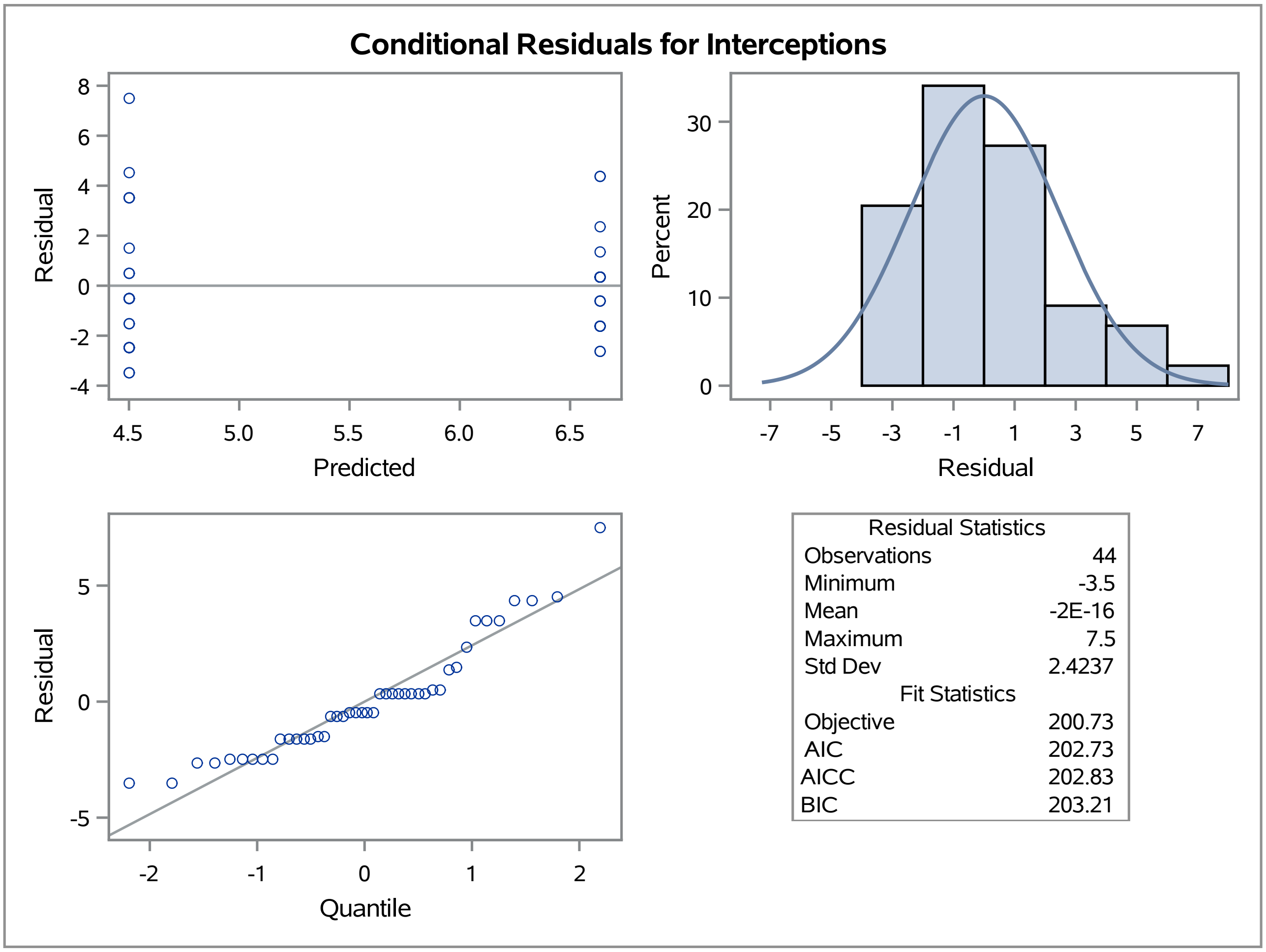

DistSoma=108

Model Information	
**Data Set**	WORK.TEMPDATASORTED
**Dependent Variable**	Interceptions
**Covariance Structure**	Variance Components
**Estimation Method**	REML
**Residual Variance Method**	Profile
**Fixed Effects SE Method**	Model-Based
**Degrees of Freedom Method**	Containment

Class Level Information		
**Class**	**Levels**	**Values**
**Treatment**	2	Control_GFP GFP MsTTR
**Culture**	12	1 2 3 4 5 6 7 8 9 10 11 12

Dimensions	
**Covariance Parameters**	2
**Columns in X**	3
**Columns in Z**	12
**Subjects**	1
**Max Obs per Subject**	44

Number of Observations	
**Number of Observations Read**	44
**Number of Observations Used**	44
**Number of Observations Not Used**	0

Iteration History			
**Iteration**	**Evaluations**	**-2 Res Log Like**	**Criterion**
**0**	1	207.23729070	
**1**	1	207.23729070	0.00000000

Convergence criteria met.

Estimated G matrix is not positive definite.

Covariance Parameter Estimates				
**Cov Parm**	**Estimate**	**Alpha**	**Lower**	**Upper**
**Culture**	0	.	.	.
**Residual**	7.0227	0.05	4.7745	11.3450

DistSoma=108

Fit Statistics	
-2 Res Log Likelihood	207.2
AIC (Smaller is Better)	209.2
AICC (Smaller is Better)	209.3
BIC (Smaller is Better)	209.7

Solution for Fixed Effects

Effect	Treatment	Estimate	Standard Error	DF	t Value	Pr > |t|	Alpha	Lower	Upper
Intercept		6.4545	0.5650	10	11.42	<.0001	0.05	5.1957	7.7134
Treatment	Control_GFP	-1.9545	0.7990	32	-2.45	0.0201	0.05	-3.5821	-0.3270
Treatment	GFP MsTTR	0	.	.	.	.	.	.	.

Solution for Random Effects

Effect	Culture	Estimate	Std Err Pred	DF	t Value	Pr > |t|	Alpha	Lower	Upper
Culture	1	0	.	.	.	.	.	.	.
Culture	2	0	.	.	.	.	.	.	.
Culture	3	0	.	.	.	.	.	.	.
Culture	4	0	.	.	.	.	.	.	.
Culture	5	0	.	.	.	.	.	.	.
Culture	6	0	.	.	.	.	.	.	.
Culture	7	0	.	.	.	.	.	.	.
Culture	8	0	.	.	.	.	.	.	.
Culture	9	0	.	.	.	.	.	.	.
Culture	10	0	.	.	.	.	.	.	.
Culture	11	0	.	.	.	.	.	.	.
Culture	12	0	.	.	.	.	.	.	.

Type 3 Tests of Fixed Effects

Effect	Num DF	Den DF	F Value	Pr > F
Treatment	1	32	5.98	0.0201

Least Squares Means

Effect	Treatment	Estimate	Standard Error	DF	t Value	Pr > |t|	Alpha	Lower	Upper
Treatment	Control_GFP	4.5000	0.5650	32	7.96	<.0001	0.05	3.3492	5.6508
Treatment	GFP MsTTR	6.4545	0.5650	32	11.42	<.0001	0.05	5.3037	7.6054

DistSoma=108

Differences of Least Squares Means												
**Effect**	**Treatment**	**Treatment**	**Estimate**	**Standard Error**	**DF**	**t Value**	**Pr > |t|**	**Adjustment**	**Adj P**	**Alpha**	**Lower**	**Upper**
**Treatment**	Control_GFP	GFP MsTTR	-1.9545	0.7990	32	-2.45	0.0201	Tukey	0.0201	0.05	-3.5821	-0.3270

Differences of Least Squares Means				
**Effect**	**Treatment**	**Treatment**	**Adj Lower**	**Adj Upper**
**Treatment**	Control_GFP	GFP MsTTR	-3.5821	-0.3270

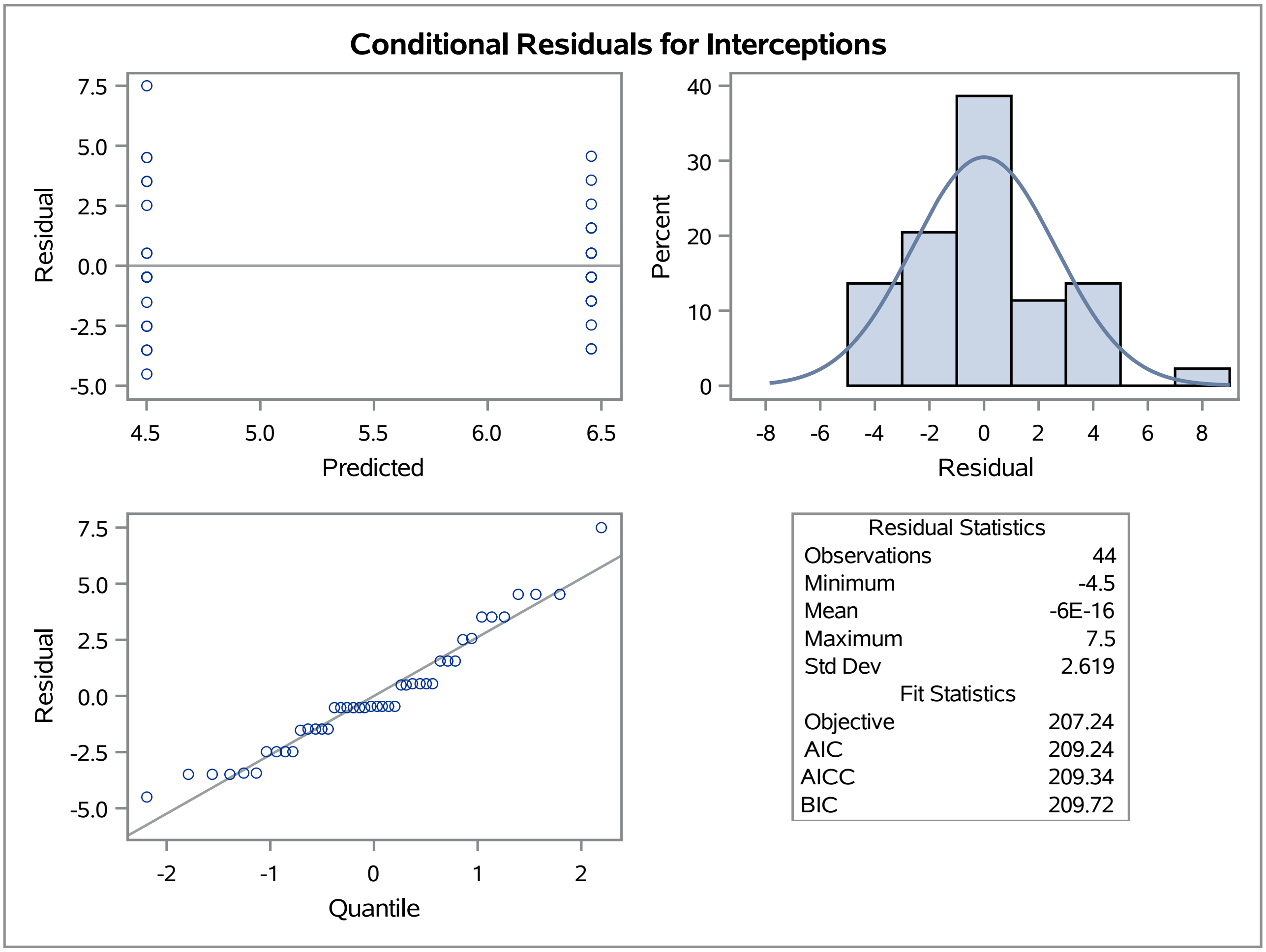

DistSoma=114

Model Information	
**Data Set**	WORK.TEMPDATASORTED
**Dependent Variable**	Interceptions
**Covariance Structure**	Variance Components
**Estimation Method**	REML
**Residual Variance Method**	Profile
**Fixed Effects SE Method**	Model-Based
**Degrees of Freedom Method**	Containment

Class Level Information		
**Class**	**Levels**	**Values**
**Treatment**	2	Control_GFP GFP MsTTR
**Culture**	12	1 2 3 4 5 6 7 8 9 10 11 12

Dimensions	
**Covariance Parameters**	2
**Columns in X**	3
**Columns in Z**	12
**Subjects**	1
**Max Obs per Subject**	44

Number of Observations	
**Number of Observations Read**	44
**Number of Observations Used**	44
**Number of Observations Not Used**	0

Iteration History			
**Iteration**	**Evaluations**	**-2 Res Log Like**	**Criterion**
**0**	1	211.98160488	
**1**	1	211.98160488	0.00000000

Convergence criteria met.

Estimated G matrix is not positive definite.

Covariance Parameter Estimates				
**Cov Parm**	**Estimate**	**Alpha**	**Lower**	**Upper**
**Culture**	0	.	.	.
**Residual**	7.8626	0.05	5.3455	12.7017

DistSoma=114

Fit Statistics	
-2 Res Log Likelihood	212.0
AIC (Smaller is Better)	214.0
AICC (Smaller is Better)	214.1
BIC (Smaller is Better)	214.5

Solution for Fixed Effects

Effect	Treatment	Estimate	Standard Error	DF	t Value	Pr > |t|	Alpha	Lower	Upper
Intercept		6.2727	0.5978	10	10.49	<.0001	0.05	4.9407	7.6048
Treatment	Control_GFP	-2.0455	0.8454	32	-2.42	0.0214	0.05	-3.7676	-0.3233
Treatment	GFP MsTTR	0	.	.	.	.	.	.	.

Solution for Random Effects

Effect	Culture	Estimate	Std Err Pred	DF	t Value	Pr > |t|	Alpha	Lower	Upper
Culture	1	0	.	.	.	.	.	.	.
Culture	2	0	.	.	.	.	.	.	.
Culture	3	0	.	.	.	.	.	.	.
Culture	4	0	.	.	.	.	.	.	.
Culture	5	0	.	.	.	.	.	.	.
Culture	6	0	.	.	.	.	.	.	.
Culture	7	0	.	.	.	.	.	.	.
Culture	8	0	.	.	.	.	.	.	.
Culture	9	0	.	.	.	.	.	.	.
Culture	10	0	.	.	.	.	.	.	.
Culture	11	0	.	.	.	.	.	.	.
Culture	12	0	.	.	.	.	.	.	.

Type 3 Tests of Fixed Effects

Effect	Num DF	Den DF	F Value	Pr > F
Treatment	1	32	5.85	0.0214

Least Squares Means

Effect	Treatment	Estimate	Standard Error	DF	t Value	Pr > |t|	Alpha	Lower	Upper
Treatment	Control_GFP	4.2273	0.5978	32	7.07	<.0001	0.05	3.0096	5.4450
Treatment	GFP MsTTR	6.2727	0.5978	32	10.49	<.0001	0.05	5.0550	7.4904

DistSoma=114

Differences of Least Squares Means												
**Effect**	**Treatment**	**Treatment**	**Estimate**	**Standard Error**	**DF**	**t Value**	**Pr > |t|**	**Adjustment**	**Adj P**	**Alpha**	**Lower**	**Upper**
**Treatment**	Control_GFP	GFP MsTTR	-2.0455	0.8454	32	-2.42	0.0214	Tukey	0.0214	0.05	-3.7676	-0.3233

Differences of Least Squares Means				
**Effect**	**Treatment**	**Treatment**	**Adj Lower**	**Adj Upper**
**Treatment**	Control_GFP	GFP MsTTR	-3.7675	-0.3234

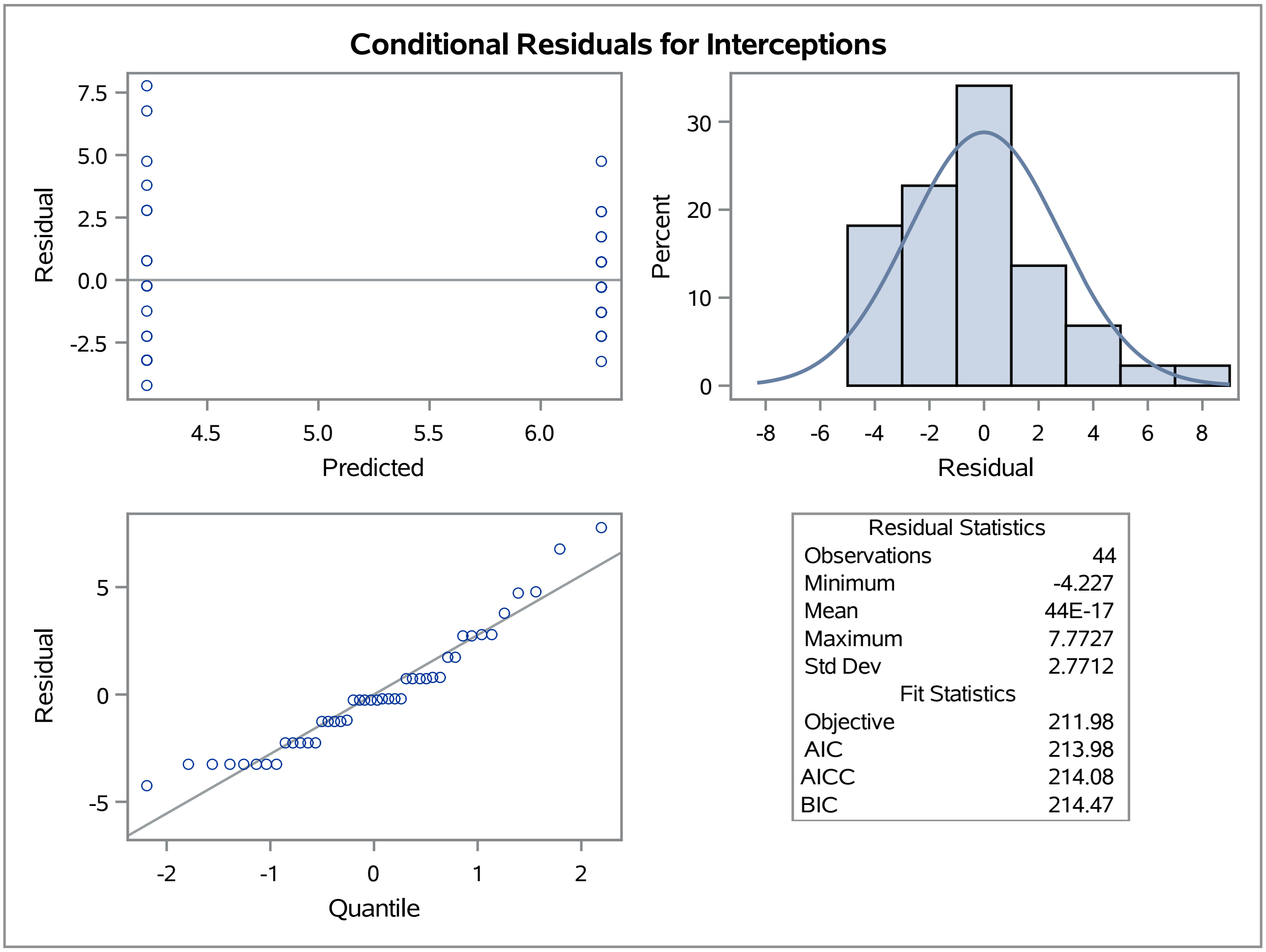

DistSoma=120

Model Information	
**Data Set**	WORK.TEMPDATASORTED
**Dependent Variable**	Interceptions
**Covariance Structure**	Variance Components
**Estimation Method**	REML
**Residual Variance Method**	Profile
**Fixed Effects SE Method**	Model-Based
**Degrees of Freedom Method**	Containment

Class Level Information		
**Class**	**Levels**	**Values**
**Treatment**	2	Control_GFP GFP MsTTR
**Culture**	12	1 2 3 4 5 6 7 8 9 10 11 12

Dimensions	
**Covariance Parameters**	2
**Columns in X**	3
**Columns in Z**	12
**Subjects**	1
**Max Obs per Subject**	44

Number of Observations	
**Number of Observations Read**	44
**Number of Observations Used**	44
**Number of Observations Not Used**	0

Iteration History			
**Iteration**	**Evaluations**	**-2 Res Log Like**	**Criterion**
**0**	1	211.80200601	
**1**	1	211.80200601	0.00000000

Convergence criteria met.

Estimated G matrix is not positive definite.

Covariance Parameter Estimates				
**Cov Parm**	**Estimate**	**Alpha**	**Lower**	**Upper**
**Culture**	0	.	.	.
**Residual**	7.8290	0.05	5.3227	12.6475

DistSoma=120

Fit Statistics	
-2 Res Log Likelihood	211.8
AIC (Smaller is Better)	213.8
AICC (Smaller is Better)	213.9
BIC (Smaller is Better)	214.3

Solution for Fixed Effects

Effect	Treatment	Estimate	Standard Error	DF	t Value	Pr > |t|	Alpha	Lower	Upper
Intercept		6.2273	0.5965	10	10.44	<.0001	0.05	4.8981	7.5565
Treatment	Control_GFP	-2.2727	0.8436	32	-2.69	0.0111	0.05	-3.9912	-0.5543
Treatment	GFP MsTTR	0	.	.	.	.	.	.	.

Solution for Random Effects

Effect	Culture	Estimate	Std Err Pred	DF	t Value	Pr > |t|	Alpha	Lower	Upper
Culture	1	0	.	.	.	.	.	.	.
Culture	2	0	.	.	.	.	.	.	.
Culture	3	0	.	.	.	.	.	.	.
Culture	4	0	.	.	.	.	.	.	.
Culture	5	0	.	.	.	.	.	.	.
Culture	6	0	.	.	.	.	.	.	.
Culture	7	0	.	.	.	.	.	.	.
Culture	8	0	.	.	.	.	.	.	.
Culture	9	0	.	.	.	.	.	.	.
Culture	10	0	.	.	.	.	.	.	.
Culture	11	0	.	.	.	.	.	.	.
Culture	12	0	.	.	.	.	.	.	.

Type 3 Tests of Fixed Effects

Effect	Num DF	Den DF	F Value	Pr > F
Treatment	1	32	7.26	0.0111

Least Squares Means

Effect	Treatment	Estimate	Standard Error	DF	t Value	Pr > |t|	Alpha	Lower	Upper
Treatment	Control_GFP	3.9545	0.5965	32	6.63	<.0001	0.05	2.7394	5.1697
Treatment	GFP MsTTR	6.2273	0.5965	32	10.44	<.0001	0.05	5.0122	7.4424

DistSoma=120

Differences of Least Squares Means												
**Effect**	**Treatment**	**Treatment**	**Estimate**	**Standard Error**	**DF**	**t Value**	**Pr > |t|**	**Adjustment**	**Adj P**	**Alpha**	**Lower**	**Upper**
**Treatment**	Control_GFP	GFP MsTTR	-2.2727	0.8436	32	-2.69	0.0111	Tukey	0.0111	0.05	-3.9912	-0.5543

Differences of Least Squares Means				
**Effect**	**Treatment**	**Treatment**	**Adj Lower**	**Adj Upper**
**Treatment**	Control_GFP	GFP MsTTR	-3.9911	-0.5543

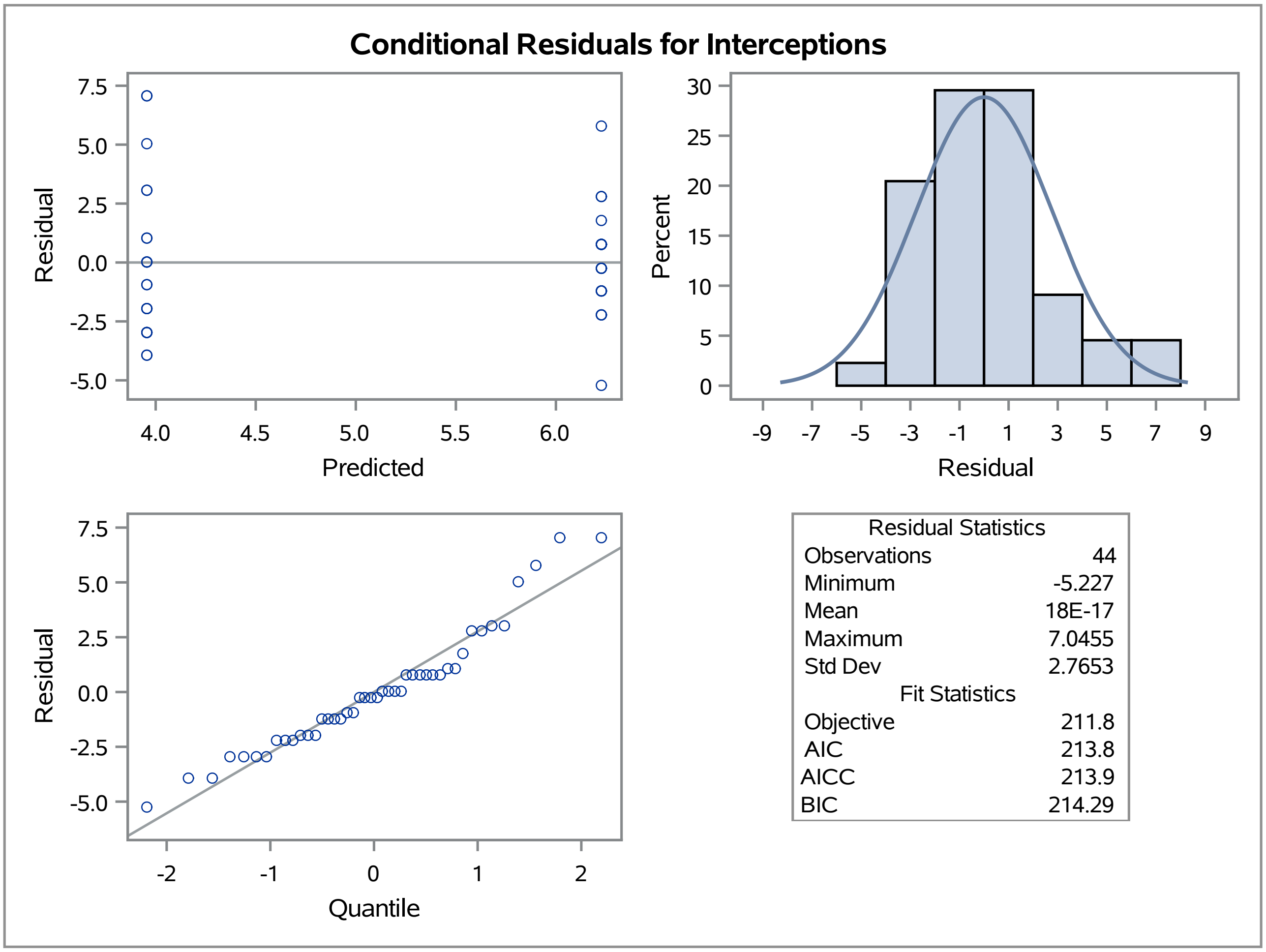

DistSoma=126

Model Information	
**Data Set**	WORK.TEMPDATASORTED
**Dependent Variable**	Interceptions
**Covariance Structure**	Variance Components
**Estimation Method**	REML
**Residual Variance Method**	Profile
**Fixed Effects SE Method**	Model-Based
**Degrees of Freedom Method**	Containment

Class Level Information		
**Class**	**Levels**	**Values**
**Treatment**	2	Control_GFP GFP MsTTR
**Culture**	12	1 2 3 4 5 6 7 8 9 10 11 12

Dimensions	
**Covariance Parameters**	2
**Columns in X**	3
**Columns in Z**	12
**Subjects**	1
**Max Obs per Subject**	44

Number of Observations	
**Number of Observations Read**	44
**Number of Observations Used**	44
**Number of Observations Not Used**	0

Iteration History			
**Iteration**	**Evaluations**	**-2 Res Log Like**	**Criterion**
**0**	1	216.07650460	
**1**	1	216.07650460	0.00000000

Convergence criteria met.

Estimated G matrix is not positive definite.

Covariance Parameter Estimates				
**Cov Parm**	**Estimate**	**Alpha**	**Lower**	**Upper**
**Culture**	0	.	.	.
**Residual**	8.6677	0.05	5.8929	14.0025

DistSoma=126

Fit Statistics	
**-2 Res Log Likelihood**	216.1
**AIC (Smaller is Better)**	218.1
**AICC (Smaller is Better)**	218.2
**BIC (Smaller is Better)**	218.6

Solution for Fixed Effects

Effect	Treatment	Estimate	Standard Error	DF	t Value	Pr > |t|	Alpha	Lower	Upper
**Intercept**		6.3182	0.6277	10	10.07	<.0001	0.05	4.9196	7.7168
**Treatment**	Control_GFP	-2.5000	0.8877	32	-2.82	0.0083	0.05	-4.3081	-0.6919
**Treatment**	GFP MsTTR	0	.	.	.	.	.	.	.

Solution for Random Effects

Effect	Culture	Estimate	Std Err Pred	DF	t Value	Pr > |t|	Alpha	Lower	Upper
**Culture**	1	0	.	.	.	.	.	.	.
**Culture**	2	0	.	.	.	.	.	.	.
**Culture**	3	0	.	.	.	.	.	.	.
**Culture**	4	0	.	.	.	.	.	.	.
**Culture**	5	0	.	.	.	.	.	.	.
**Culture**	6	0	.	.	.	.	.	.	.
**Culture**	7	0	.	.	.	.	.	.	.
**Culture**	8	0	.	.	.	.	.	.	.
**Culture**	9	0	.	.	.	.	.	.	.
**Culture**	10	0	.	.	.	.	.	.	.
**Culture**	11	0	.	.	.	.	.	.	.
**Culture**	12	0	.	.	.	.	.	.	.

Type 3 Tests of Fixed Effects

Effect	Num DF	Den DF	F Value	Pr > F
**Treatment**	1	32	7.93	0.0083

Least Squares Means

Effect	Treatment	Estimate	Standard Error	DF	t Value	Pr > |t|	Alpha	Lower	Upper
**Treatment**	Control_GFP	3.8182	0.6277	32	6.08	<.0001	0.05	2.5396	5.0967
**Treatment**	GFP MsTTR	6.3182	0.6277	32	10.07	<.0001	0.05	5.0396	7.5967

DistSoma=126

Differences of Least Squares Means												
**Effect**	**Treatment**	**Treatment**	**Estimate**	**Standard Error**	**DF**	**t Value**	**Pr > |t|**	**Adjustment**	**Adj P**	**Alpha**	**Lower**	**Upper**
**Treatment**	Control_GFP	GFP MsTTR	-2.5000	0.8877	32	-2.82	0.0083	Tukey	0.0083	0.05	-4.3081	-0.6919

Differences of Least Squares Means				
**Effect**	**Treatment**	**Treatment**	**Adj Lower**	**Adj Upper**
**Treatment**	Control_GFP	GFP MsTTR	-4.3081	-0.6919

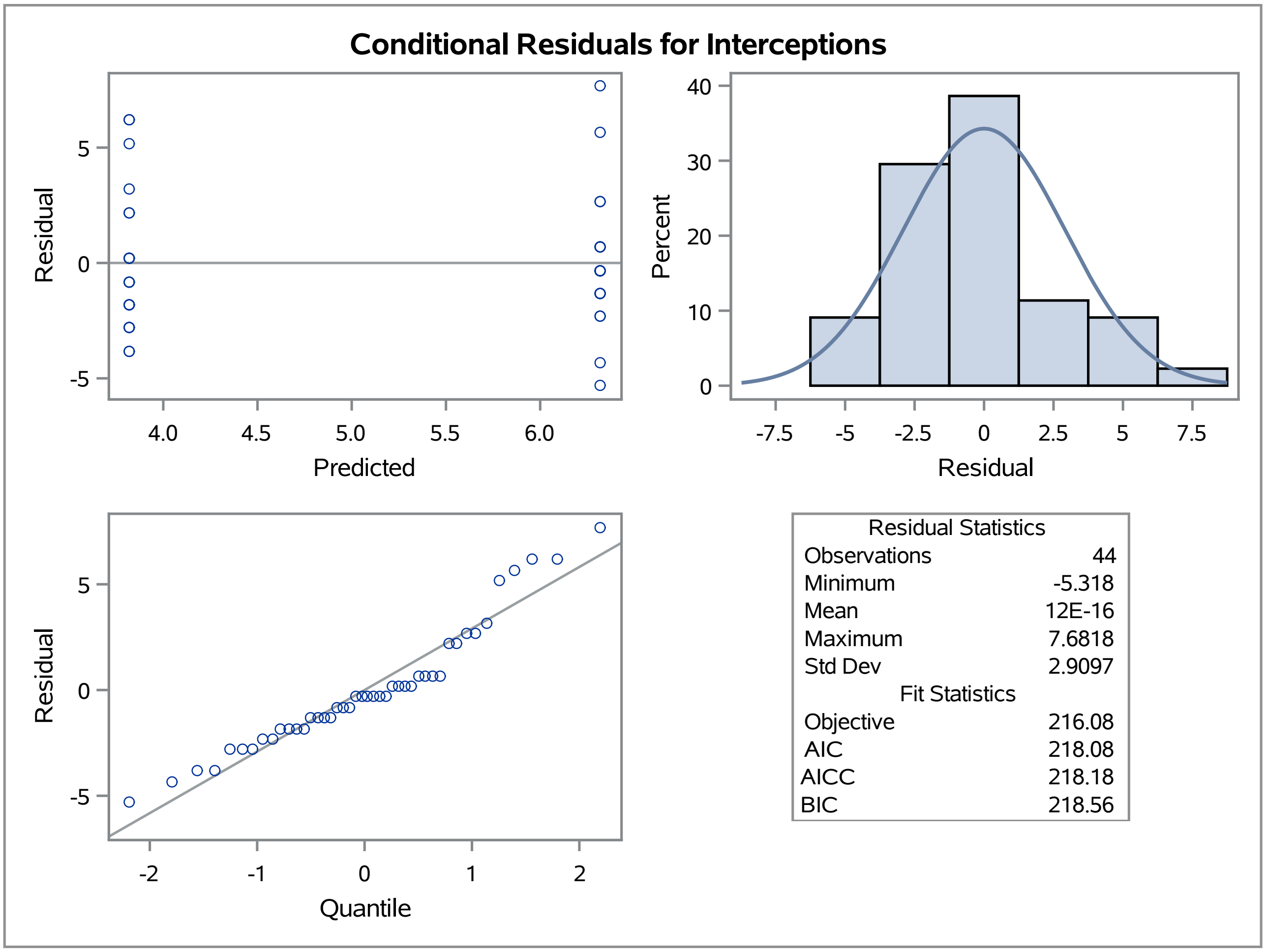

DistSoma=132

Model Information	
**Data Set**	WORK.TEMPDATASORTED
**Dependent Variable**	Interceptions
**Covariance Structure**	Variance Components
**Estimation Method**	REML
**Residual Variance Method**	Profile
**Fixed Effects SE Method**	Model-Based
**Degrees of Freedom Method**	Containment

Class Level Information		
**Class**	**Levels**	**Values**
**Treatment**	2	Control_GFP GFP MsTTR
**Culture**	12	1 2 3 4 5 6 7 8 9 10 11 12

Dimensions	
**Covariance Parameters**	2
**Columns in X**	3
**Columns in Z**	12
**Subjects**	1
**Max Obs per Subject**	44

Number of Observations	
**Number of Observations Read**	44
**Number of Observations Used**	44
**Number of Observations Not Used**	0

Iteration History			
**Iteration**	**Evaluations**	**-2 Res Log Like**	**Criterion**
**0**	1	206.90588959	
**1**	1	206.90588959	0.00000000

Convergence criteria met.

Estimated G matrix is not positive definite.

Covariance Parameter Estimates				
**Cov Parm**	**Estimate**	**Alpha**	**Lower**	**Upper**
**Culture**	0	.	.	.
**Residual**	6.9675	0.05	4.7370	11.2558

DistSoma=132

Fit Statistics	
-2 Res Log Likelihood	206.9
AIC (Smaller is Better)	208.9
AICC (Smaller is Better)	209.0
BIC (Smaller is Better)	209.4

Solution for Fixed Effects

Effect	Treatment	Estimate	Standard Error	DF	t Value	Pr > |t|	Alpha	Lower	Upper
Intercept		5.7727	0.5628	10	10.26	<.0001	0.05	4.5188	7.0266
Treatment	Control_GFP	-2.0909	0.7959	32	-2.63	0.0131	0.05	-3.7120	-0.4698
Treatment	GFP MsTTR	0	.	.	.	.	.	.	.

Solution for Random Effects

Effect	Culture	Estimate	Std Err Pred	DF	t Value	Pr > |t|	Alpha	Lower	Upper
Culture	1	0	.	.	.	.	.	.	.
Culture	2	0	.	.	.	.	.	.	.
Culture	3	0	.	.	.	.	.	.	.
Culture	4	0	.	.	.	.	.	.	.
Culture	5	0	.	.	.	.	.	.	.
Culture	6	0	.	.	.	.	.	.	.
Culture	7	0	.	.	.	.	.	.	.
Culture	8	0	.	.	.	.	.	.	.
Culture	9	0	.	.	.	.	.	.	.
Culture	10	0	.	.	.	.	.	.	.
Culture	11	0	.	.	.	.	.	.	.
Culture	12	0	.	.	.	.	.	.	.

Type 3 Tests of Fixed Effects

Effect	Num DF	Den DF	F Value	Pr > F
Treatment	1	32	6.90	0.0131

Least Squares Means

Effect	Treatment	Estimate	Standard Error	DF	t Value	Pr > |t|	Alpha	Lower	Upper
Treatment	Control_GFP	3.6818	0.5628	32	6.54	<.0001	0.05	2.5355	4.8281
Treatment	GFP MsTTR	5.7727	0.5628	32	10.26	<.0001	0.05	4.6264	6.9190

DistSoma=132

Differences of Least Squares Means												
**Effect**	**Treatment**	**Treatment**	**Estimate**	**Standard Error**	**DF**	**t Value**	**Pr > |t|**	**Adjustment**	**Adj P**	**Alpha**	**Lower**	**Upper**
**Treatment**	Control_GFP	GFP MsTTR	-2.0909	0.7959	32	-2.63	0.0131	Tukey	0.0131	0.05	-3.7120	-0.4698

Differences of Least Squares Means				
**Effect**	**Treatment**	**Treatment**	**Adj Lower**	**Adj Upper**
**Treatment**	Control_GFP	GFP MsTTR	-3.7120	-0.4698

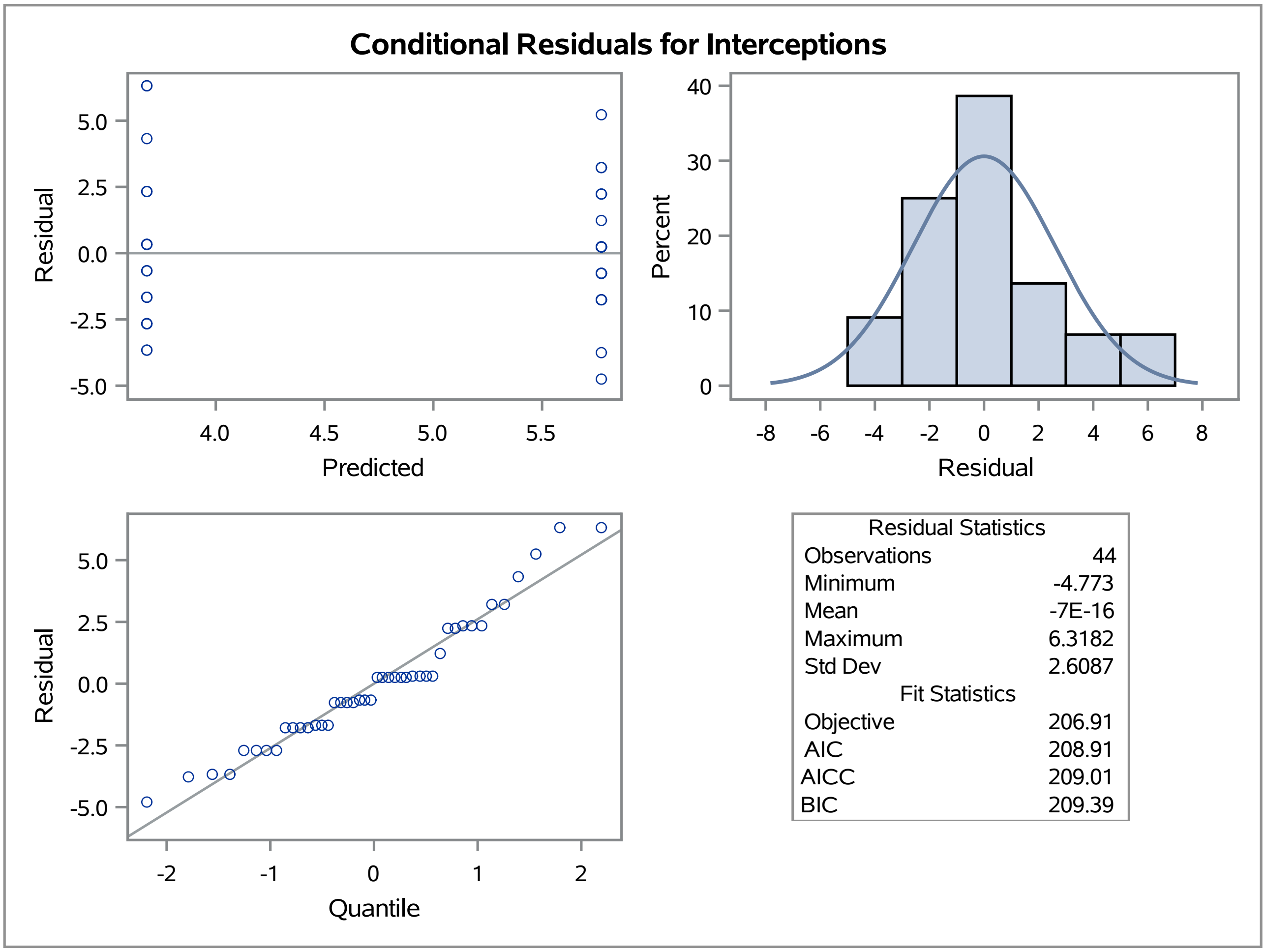

DistSoma=138

Model Information	
**Data Set**	WORK.TEMPDATASORTED
**Dependent Variable**	Interceptions
**Covariance Structure**	Variance Components
**Estimation Method**	REML
**Residual Variance Method**	Profile
**Fixed Effects SE Method**	Model-Based
**Degrees of Freedom Method**	Containment

Class Level Information		
**Class**	**Levels**	**Values**
**Treatment**	2	Control_GFP GFP MsTTR
**Culture**	12	1 2 3 4 5 6 7 8 9 10 11 12

Dimensions	
**Covariance Parameters**	2
**Columns in X**	3
**Columns in Z**	12
**Subjects**	1
**Max Obs per Subject**	44

Number of Observations	
**Number of Observations Read**	44
**Number of Observations Used**	44
**Number of Observations Not Used**	0

Iteration History			
**Iteration**	**Evaluations**	**-2 Res Log Like**	**Criterion**
**0**	1	204.57195584	
**1**	1	204.57195584	0.00000000

Convergence criteria met.

Estimated G matrix is not positive definite.

Covariance Parameter Estimates				
**Cov Parm**	**Estimate**	**Alpha**	**Lower**	**Upper**
**Culture**	0	.	.	.
**Residual**	6.5909	0.05	4.4809	10.6474

DistSoma=138

Fit Statistics	
-2 Res Log Likelihood	204.6
AIC (Smaller is Better)	206.6
AICC (Smaller is Better)	206.7
BIC (Smaller is Better)	207.1

Solution for Fixed Effects

Effect	Treatment	Estimate	Standard Error	DF	t Value	Pr > |t|	Alpha	Lower	Upper
Intercept		5.5909	0.5473	10	10.21	<.0001	0.05	4.3713	6.8105
Treatment	Control_GFP	-2.0909	0.7741	32	-2.70	0.0110	0.05	-3.6676	-0.5142
Treatment	GFP MsTTR	0	.	.	.	.	.	.	.

Solution for Random Effects

Effect	Culture	Estimate	Std Err Pred	DF	t Value	Pr > |t|	Alpha	Lower	Upper
Culture	1	0	.	.	.	.	.	.	.
Culture	2	0	.	.	.	.	.	.	.
Culture	3	0	.	.	.	.	.	.	.
Culture	4	0	.	.	.	.	.	.	.
Culture	5	0	.	.	.	.	.	.	.
Culture	6	0	.	.	.	.	.	.	.
Culture	7	0	.	.	.	.	.	.	.
Culture	8	0	.	.	.	.	.	.	.
Culture	9	0	.	.	.	.	.	.	.
Culture	10	0	.	.	.	.	.	.	.
Culture	11	0	.	.	.	.	.	.	.
Culture	12	0	.	.	.	.	.	.	.

Type 3 Tests of Fixed Effects

Effect	Num DF	Den DF	F Value	Pr > F
Treatment	1	32	7.30	0.0110

Least Squares Means

Effect	Treatment	Estimate	Standard Error	DF	t Value	Pr > |t|	Alpha	Lower	Upper
Treatment	Control_GFP	3.5000	0.5473	32	6.39	<.0001	0.05	2.3851	4.6149
Treatment	GFP MsTTR	5.5909	0.5473	32	10.21	<.0001	0.05	4.4760	6.7058

DistSoma=138

Differences of Least Squares Means												
**Effect**	**Treatment**	**Treatment**	**Estimate**	**Standard Error**	**DF**	**t Value**	**Pr > |t|**	**Adjustment**	**Adj P**	**Alpha**	**Lower**	**Upper**
**Treatment**	Control_GFP	GFP MsTTR	-2.0909	0.7741	32	-2.70	0.0110	Tukey	0.0110	0.05	-3.6676	-0.5142

Differences of Least Squares Means				
**Effect**	**Treatment**	**Treatment**	**Adj Lower**	**Adj Upper**
**Treatment**	Control_GFP	GFP MsTTR	-3.6676	-0.5142

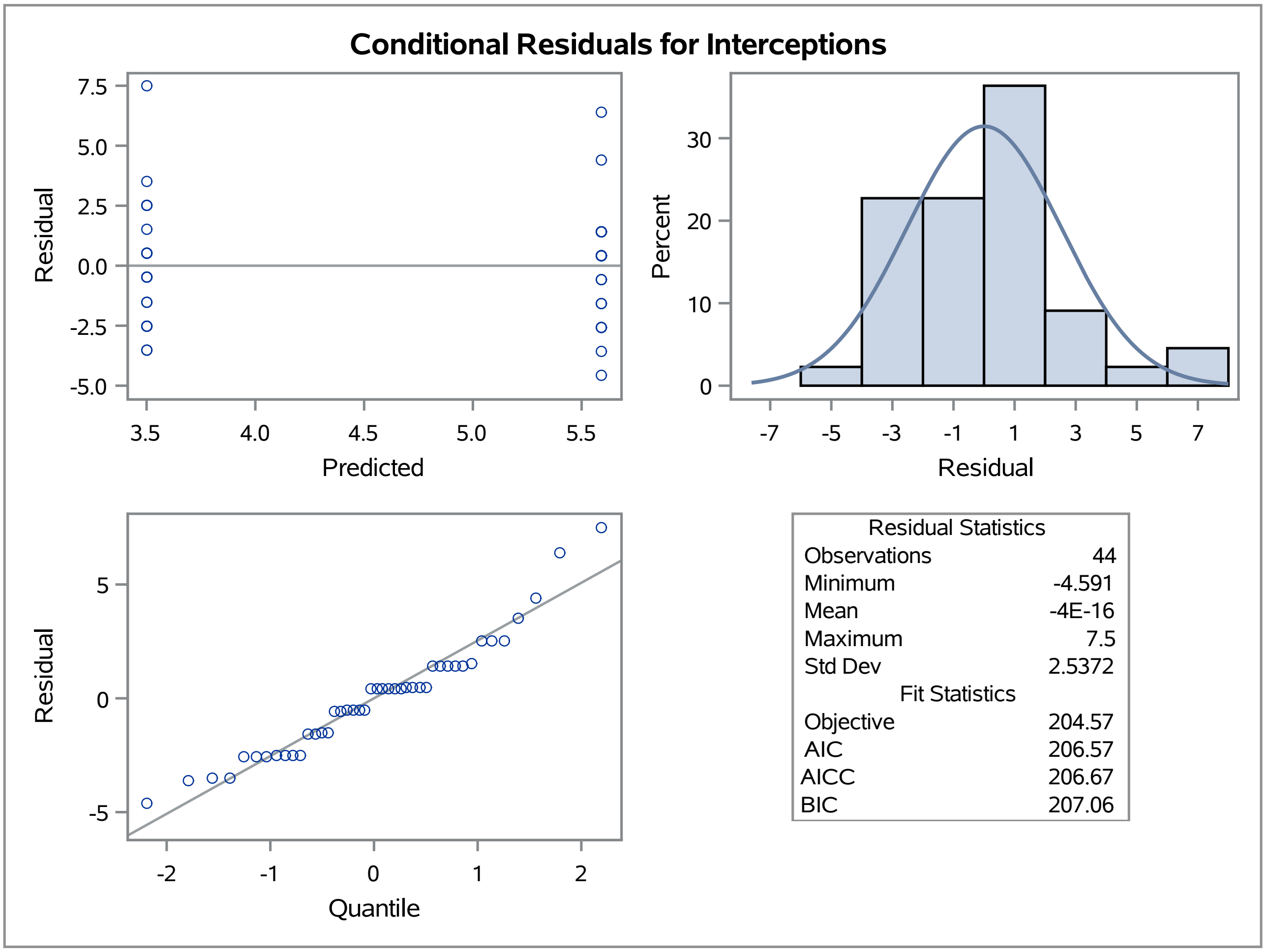

DistSoma=144

Model Information	
**Data Set**	WORK.TEMPDATASORTED
**Dependent Variable**	Interceptions
**Covariance Structure**	Variance Components
**Estimation Method**	REML
**Residual Variance Method**	Profile
**Fixed Effects SE Method**	Model-Based
**Degrees of Freedom Method**	Containment

Class Level Information		
**Class**	**Levels**	**Values**
**Treatment**	2	Control_GFP GFP MsTTR
**Culture**	12	1 2 3 4 5 6 7 8 9 10 11 12

Dimensions	
**Covariance Parameters**	2
**Columns in X**	3
**Columns in Z**	12
**Subjects**	1
**Max Obs per Subject**	44

Number of Observations	
**Number of Observations Read**	44
**Number of Observations Used**	44
**Number of Observations Not Used**	0

Iteration History			
**Iteration**	**Evaluations**	**-2 Res Log Like**	**Criterion**
**0**	1	204.03055022	
**1**	1	204.03055022	0.00000000

Convergence criteria met.

Estimated G matrix is not positive definite.

Covariance Parameter Estimates				
**Cov Parm**	**Estimate**	**Alpha**	**Lower**	**Upper**
**Culture**	0	.	.	.
**Residual**	6.5065	0.05	4.4236	10.5110

DistSoma=144

Fit Statistics	
**-2 Res Log Likelihood**	204.0
**AIC (Smaller is Better)**	206.0
**AICC (Smaller is Better)**	206.1
**BIC (Smaller is Better)**	206.5

Solution for Fixed Effects

Effect	Treatment	Estimate	Standard Error	DF	t Value	Pr > |t|	Alpha	Lower	Upper
**Intercept**		5.0909	0.5438	10	9.36	<.0001	0.05	3.8792	6.3026
**Treatment**	Control_GFP	-1.6364	0.7691	32	-2.13	0.0412	0.05	-3.2029	-0.06978
**Treatment**	GFP MsTTR	0	.	.	.	.	.	.	.

Solution for Random Effects

Effect	Culture	Estimate	Std Err Pred	DF	t Value	Pr > |t|	Alpha	Lower	Upper
**Culture**	1	0	.	.	.	.	.	.	.
**Culture**	2	0	.	.	.	.	.	.	.
**Culture**	3	0	.	.	.	.	.	.	.
**Culture**	4	0	.	.	.	.	.	.	.
**Culture**	5	0	.	.	.	.	.	.	.
**Culture**	6	0	.	.	.	.	.	.	.
**Culture**	7	0	.	.	.	.	.	.	.
**Culture**	8	0	.	.	.	.	.	.	.
**Culture**	9	0	.	.	.	.	.	.	.
**Culture**	10	0	.	.	.	.	.	.	.
**Culture**	11	0	.	.	.	.	.	.	.
**Culture**	12	0	.	.	.	.	.	.	.

Type 3 Tests of Fixed Effects

Effect	Num DF	Den DF	F Value	Pr > F
**Treatment**	1	32	4.53	0.0412

Least Squares Means

Effect	Treatment	Estimate	Standard Error	DF	t Value	Pr > |t|	Alpha	Lower	Upper
**Treatment**	Control_GFP	3.4545	0.5438	32	6.35	<.0001	0.05	2.3468	4.5623
**Treatment**	GFP MsTTR	5.0909	0.5438	32	9.36	<.0001	0.05	3.9832	6.1987

DistSoma=144

Differences of Least Squares Means												
**Effect**	**Treatment**	**Treatment**	**Estimate**	**Standard Error**	**DF**	**t Value**	**Pr > |t|**	**Adjustment**	**Adj P**	**Alpha**	**Lower**	**Upper**
**Treatment**	Control_GFP	GFP MsTTR	-1.6364	0.7691	32	-2.13	0.0412	Tukey	0.0412	0.05	-3.2029	-0.06978

Differences of Least Squares Means				
**Effect**	**Treatment**	**Treatment**	**Adj Lower**	**Adj Upper**
**Treatment**	Control_GFP	GFP MsTTR	-3.2029	-0.06980

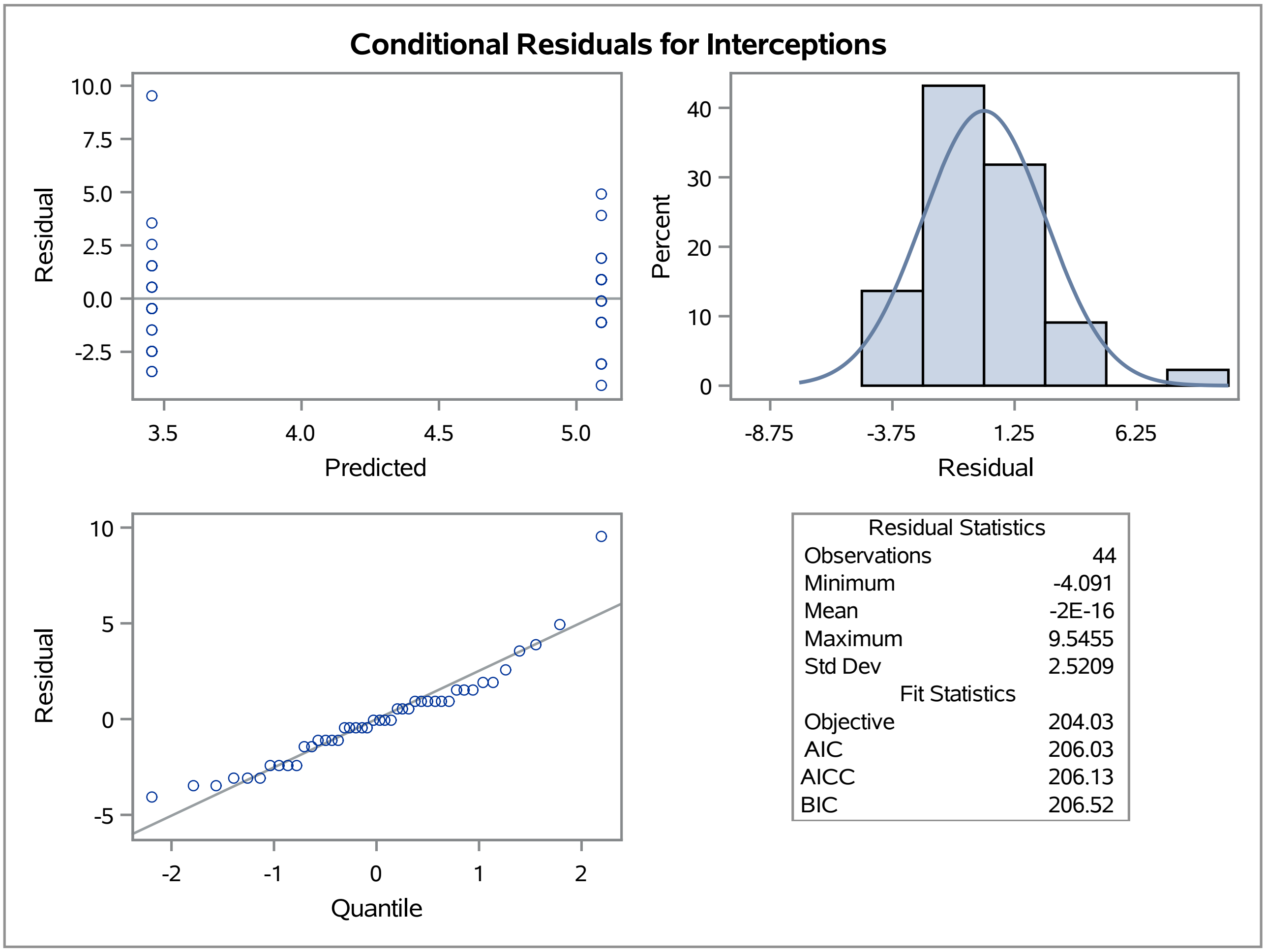

DistSoma=150

Model Information	
**Data Set**	WORK.TEMPDATASORTED
**Dependent Variable**	Interceptions
**Covariance Structure**	Variance Components
**Estimation Method**	REML
**Residual Variance Method**	Profile
**Fixed Effects SE Method**	Model-Based
**Degrees of Freedom Method**	Containment

Class Level Information		
**Class**	**Levels**	**Values**
**Treatment**	2	Control_GFP GFP MsTTR
**Culture**	12	1 2 3 4 5 6 7 8 9 10 11 12

Dimensions	
**Covariance Parameters**	2
**Columns in X**	3
**Columns in Z**	12
**Subjects**	1
**Max Obs per Subject**	44

Number of Observations	
**Number of Observations Read**	44
**Number of Observations Used**	44
**Number of Observations Not Used**	0

Iteration History			
**Iteration**	**Evaluations**	**-2 Res Log Like**	**Criterion**
**0**	1	202.46479792	
**1**	1	202.46479792	0.00000000

Convergence criteria met.

Estimated G matrix is not positive definite.

Covariance Parameter Estimates				
**Cov Parm**	**Estimate**	**Alpha**	**Lower**	**Upper**
**Culture**	0	.	.	.
**Residual**	6.2684	0.05	4.2617	10.1264

DistSoma=150

Fit Statistics	
-2 Res Log Likelihood	202.5
AIC (Smaller is Better)	204.5
AICC (Smaller is Better)	204.6
BIC (Smaller is Better)	204.9

Solution for Fixed Effects

Effect	Treatment	Estimate	Standard Error	DF	t Value	Pr > |t|	Alpha	Lower	Upper
Intercept		5.0000	0.5338	10	9.37	<.0001	0.05	3.8107	6.1893
Treatment	Control_GFP	-1.8182	0.7549	32	-2.41	0.0219	0.05	-3.3558	-0.2805
Treatment	GFP MsTTR	0	.	.	.	.	.	.	.

Solution for Random Effects

Effect	Culture	Estimate	Std Err Pred	DF	t Value	Pr > |t|	Alpha	Lower	Upper
Culture	1	0	.	.	.	.	.	.	.
Culture	2	0	.	.	.	.	.	.	.
Culture	3	0	.	.	.	.	.	.	.
Culture	4	0	.	.	.	.	.	.	.
Culture	5	0	.	.	.	.	.	.	.
Culture	6	0	.	.	.	.	.	.	.
Culture	7	0	.	.	.	.	.	.	.
Culture	8	0	.	.	.	.	.	.	.
Culture	9	0	.	.	.	.	.	.	.
Culture	10	0	.	.	.	.	.	.	.
Culture	11	0	.	.	.	.	.	.	.
Culture	12	0	.	.	.	.	.	.	.

Type 3 Tests of Fixed Effects

Effect	Num DF	Den DF	F Value	Pr > F
Treatment	1	32	5.80	0.0219

Least Squares Means

Effect	Treatment	Estimate	Standard Error	DF	t Value	Pr > |t|	Alpha	Lower	Upper
Treatment	Control_GFP	3.1818	0.5338	32	5.96	<.0001	0.05	2.0945	4.2691
Treatment	GFP MsTTR	5.0000	0.5338	32	9.37	<.0001	0.05	3.9127	6.0873

DistSoma=150

Differences of Least Squares Means												
**Effect**	**Treatment**	**Treatment**	**Estimate**	**Standard Error**	**DF**	**t Value**	**Pr > |t|**	**Adjustment**	**Adj P**	**Alpha**	**Lower**	**Upper**
**Treatment**	Control_GFP	GFP MsTTR	-1.8182	0.7549	32	-2.41	0.0219	Tukey	0.0219	0.05	-3.3558	-0.2805

Differences of Least Squares Means				
**Effect**	**Treatment**	**Treatment**	**Adj Lower**	**Adj Upper**
**Treatment**	Control_GFP	GFP MsTTR	-3.3558	-0.2805

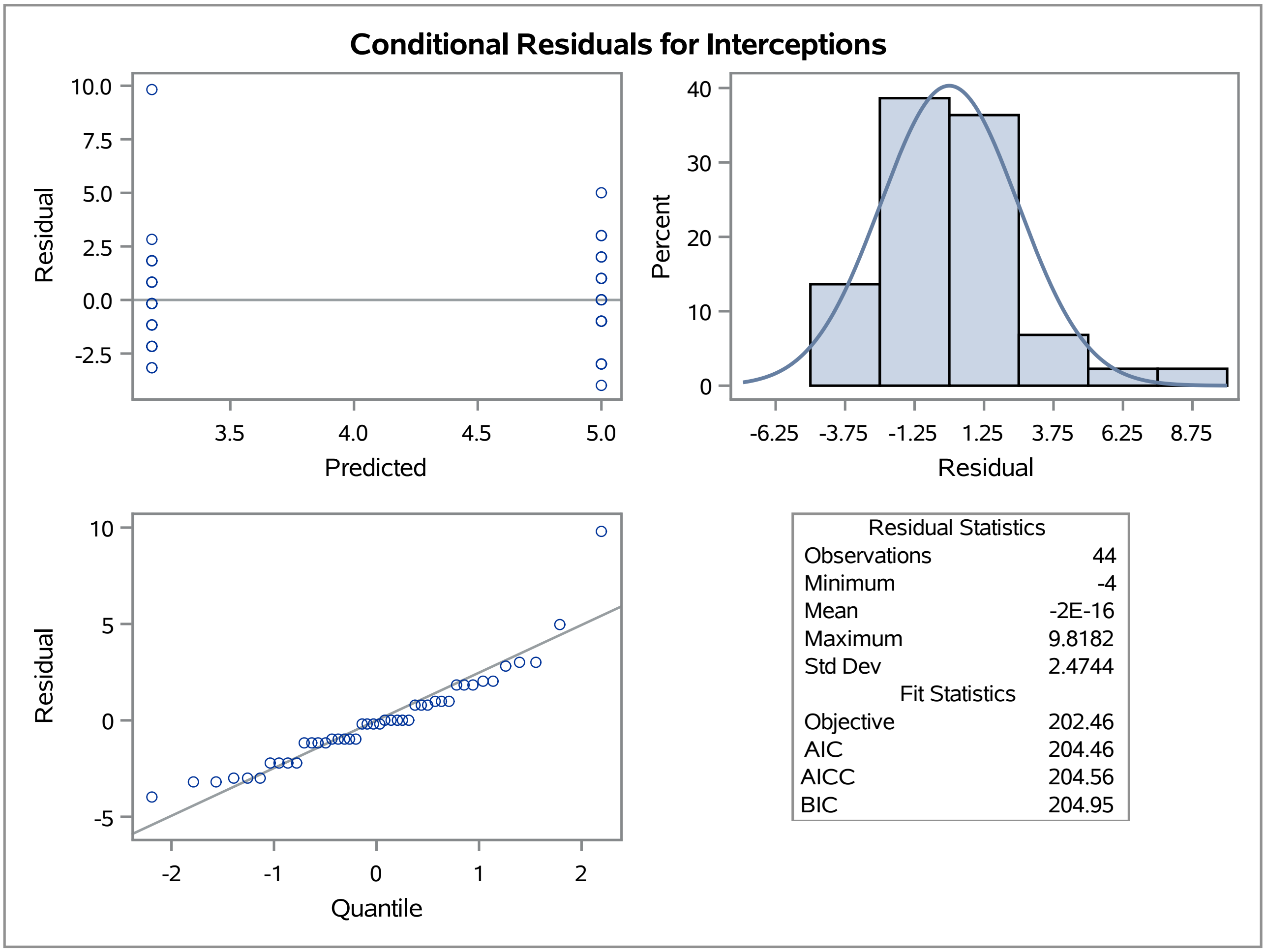

DistSoma=156

Model Information	
**Data Set**	WORK.TEMPDATASORTED
**Dependent Variable**	Interceptions
**Covariance Structure**	Variance Components
**Estimation Method**	REML
**Residual Variance Method**	Profile
**Fixed Effects SE Method**	Model-Based
**Degrees of Freedom Method**	Containment

Class Level Information		
**Class**	**Levels**	**Values**
**Treatment**	2	Control_GFP GFP MsTTR
**Culture**	12	1 2 3 4 5 6 7 8 9 10 11 12

Dimensions	
**Covariance Parameters**	2
**Columns in X**	3
**Columns in Z**	12
**Subjects**	1
**Max Obs per Subject**	44

Number of Observations	
**Number of Observations Read**	44
**Number of Observations Used**	44
**Number of Observations Not Used**	0

Iteration History			
**Iteration**	**Evaluations**	**-2 Res Log Like**	**Criterion**
**0**	1	205.16771051	
**1**	1	205.16771051	0.00000000

Convergence criteria met.

Estimated G matrix is not positive definite.

Covariance Parameter Estimates				
**Cov Parm**	**Estimate**	**Alpha**	**Lower**	**Upper**
**Culture**	0	.	.	.
**Residual**	6.6851	0.05	4.5450	10.7995

DistSoma=156

Fit Statistics	
-2 Res Log Likelihood	205.2
AIC (Smaller is Better)	207.2
AICC (Smaller is Better)	207.3
BIC (Smaller is Better)	207.7

Solution for Fixed Effects

Effect	Treatment	Estimate	Standard Error	DF	t Value	Pr > |t|	Alpha	Lower	Upper
Intercept		5.0909	0.5512	10	9.24	<.0001	0.05	3.8627	6.3192
Treatment	Control_GFP	-2.0455	0.7796	32	-2.62	0.0132	0.05	-3.6334	-0.4575
Treatment	GFP MsTTR	0	.	.	.	.	.	.	.

Solution for Random Effects

Effect	Culture	Estimate	Std Err Pred	DF	t Value	Pr > |t|	Alpha	Lower	Upper
Culture	1	0	.	.	.	.	.	.	.
Culture	2	0	.	.	.	.	.	.	.
Culture	3	0	.	.	.	.	.	.	.
Culture	4	0	.	.	.	.	.	.	.
Culture	5	0	.	.	.	.	.	.	.
Culture	6	0	.	.	.	.	.	.	.
Culture	7	0	.	.	.	.	.	.	.
Culture	8	0	.	.	.	.	.	.	.
Culture	9	0	.	.	.	.	.	.	.
Culture	10	0	.	.	.	.	.	.	.
Culture	11	0	.	.	.	.	.	.	.
Culture	12	0	.	.	.	.	.	.	.

Type 3 Tests of Fixed Effects

Effect	Num DF	Den DF	F Value	Pr > F
Treatment	1	32	6.88	0.0132

Least Squares Means

Effect	Treatment	Estimate	Standard Error	DF	t Value	Pr > |t|	Alpha	Lower	Upper
Treatment	Control_GFP	3.0455	0.5512	32	5.52	<.0001	0.05	1.9226	4.1683
Treatment	GFP MsTTR	5.0909	0.5512	32	9.24	<.0001	0.05	3.9681	6.2138

DistSoma=156

Differences of Least Squares Means												
Effect	Treatment	Treatment	Estimate	Standard Error	DF	t Value	Pr > |t|	Adjustment	Adj P	Alpha	Lower	Upper
Treatment	Control_GFP	GFP MsTTR	-2.0455	0.7796	32	-2.62	0.0132	Tukey	0.0132	0.05	-3.6334	-0.4575

Differences of Least Squares Means				
Effect	Treatment	Treatment	Adj Lower	Adj Upper
Treatment	Control_GFP	GFP MsTTR	-3.6334	-0.4575

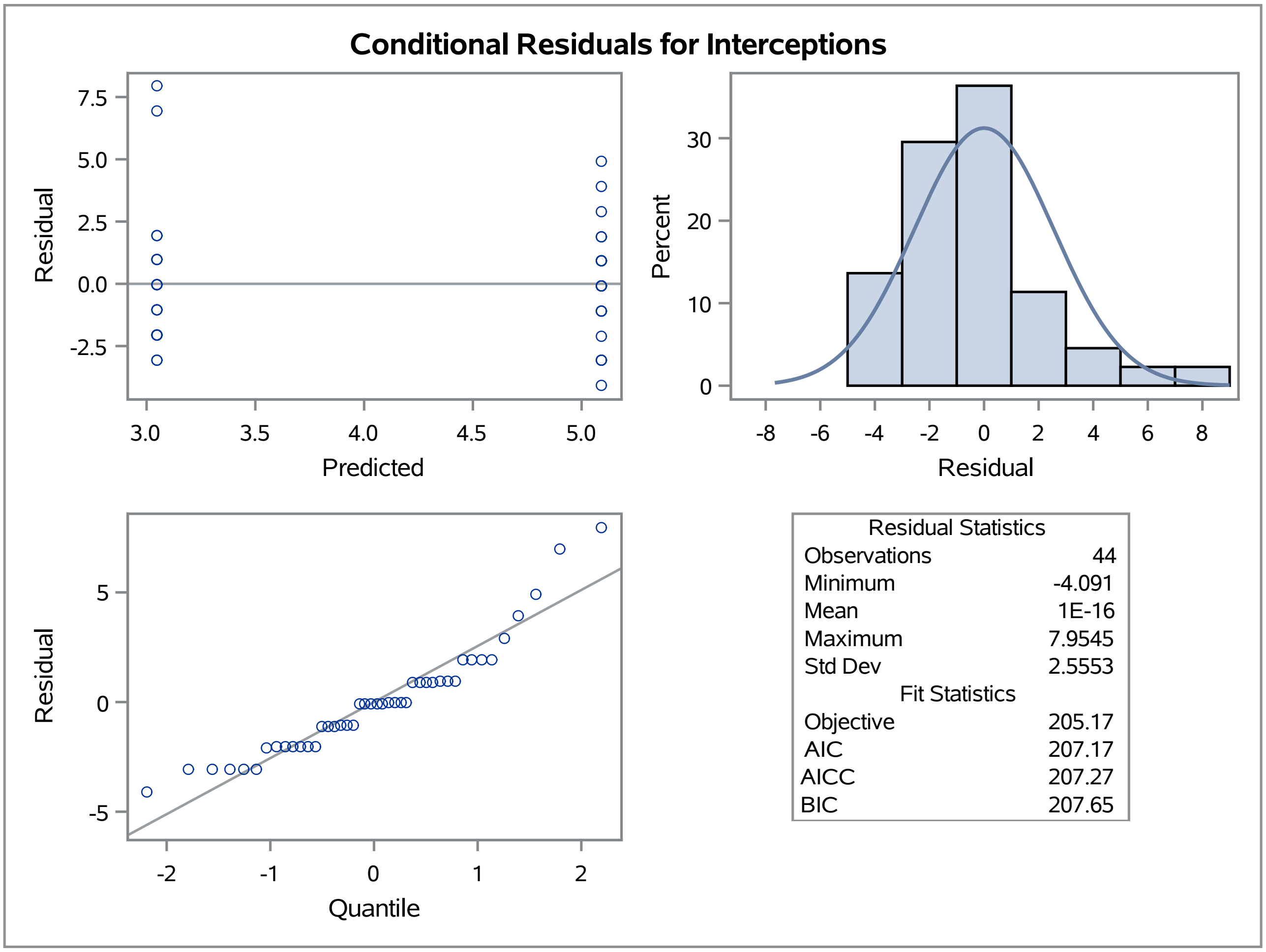

DistSoma=162

Model Information	
**Data Set**	WORK.TEMPDATASORTED
**Dependent Variable**	Interceptions
**Covariance Structure**	Variance Components
**Estimation Method**	REML
**Residual Variance Method**	Profile
**Fixed Effects SE Method**	Model-Based
**Degrees of Freedom Method**	Containment

Class Level Information		
**Class**	**Levels**	**Values**
**Treatment**	2	Control_GFP GFP MsTTR
**Culture**	12	1 2 3 4 5 6 7 8 9 10 11 12

Dimensions	
**Covariance Parameters**	2
**Columns in X**	3
**Columns in Z**	12
**Subjects**	1
**Max Obs per Subject**	44

Number of Observations	
**Number of Observations Read**	44
**Number of Observations Used**	44
**Number of Observations Not Used**	0

Iteration History			
**Iteration**	**Evaluations**	**-2 Res Log Like**	**Criterion**
**0**	1	198.07348241	
**1**	1	198.07348241	0.00000000

Convergence criteria met.

Estimated G matrix is not positive definite.

Covariance Parameter Estimates				
**Cov Parm**	**Estimate**	**Alpha**	**Lower**	**Upper**
**Culture**	0	.	.	.
**Residual**	5.6461	0.05	3.8386	9.1211

DistSoma=162

Fit Statistics	
-2 Res Log Likelihood	198.1
AIC (Smaller is Better)	200.1
AICC (Smaller is Better)	200.2
BIC (Smaller is Better)	200.6

Solution for Fixed Effects

Effect	Treatment	Estimate	Standard Error	DF	t Value	Pr > |t|	Alpha	Lower	Upper
Intercept		4.7727	0.5066	10	9.42	<.0001	0.05	3.6440	5.9015
Treatment	Control_GFP	-1.9545	0.7164	32	-2.73	0.0103	0.05	-3.4139	-0.4952
Treatment	GFP MsTTR	0	.	.	.	.	.	.	.

Solution for Random Effects

Effect	Culture	Estimate	Std Err Pred	DF	t Value	Pr > |t|	Alpha	Lower	Upper
Culture	1	0	.	.	.	.	.	.	.
Culture	2	0	.	.	.	.	.	.	.
Culture	3	0	.	.	.	.	.	.	.
Culture	4	0	.	.	.	.	.	.	.
Culture	5	0	.	.	.	.	.	.	.
Culture	6	0	.	.	.	.	.	.	.
Culture	7	0	.	.	.	.	.	.	.
Culture	8	0	.	.	.	.	.	.	.
Culture	9	0	.	.	.	.	.	.	.
Culture	10	0	.	.	.	.	.	.	.
Culture	11	0	.	.	.	.	.	.	.
Culture	12	0	.	.	.	.	.	.	.

Type 3 Tests of Fixed Effects

Effect	Num DF	Den DF	F Value	Pr > F
Treatment	1	32	7.44	0.0103

Least Squares Means

Effect	Treatment	Estimate	Standard Error	DF	t Value	Pr > |t|	Alpha	Lower	Upper
Treatment	Control_GFP	2.8182	0.5066	32	5.56	<.0001	0.05	1.7863	3.8501
Treatment	GFP MsTTR	4.7727	0.5066	32	9.42	<.0001	0.05	3.7408	5.8046

DistSoma=162

Differences of Least Squares Means												
**Effect**	**Treatment**	**Treatment**	**Estimate**	**Standard Error**	**DF**	**t Value**	**Pr > |t|**	**Adjustment**	**Adj P**	**Alpha**	**Lower**	**Upper**
**Treatment**	Control_GFP	GFP MsTTR	-1.9545	0.7164	32	-2.73	0.0103	Tukey	0.0103	0.05	-3.4139	-0.4952

Differences of Least Squares Means				
**Effect**	**Treatment**	**Treatment**	**Adj Lower**	**Adj Upper**
**Treatment**	Control_GFP	GFP MsTTR	-3.4139	-0.4952

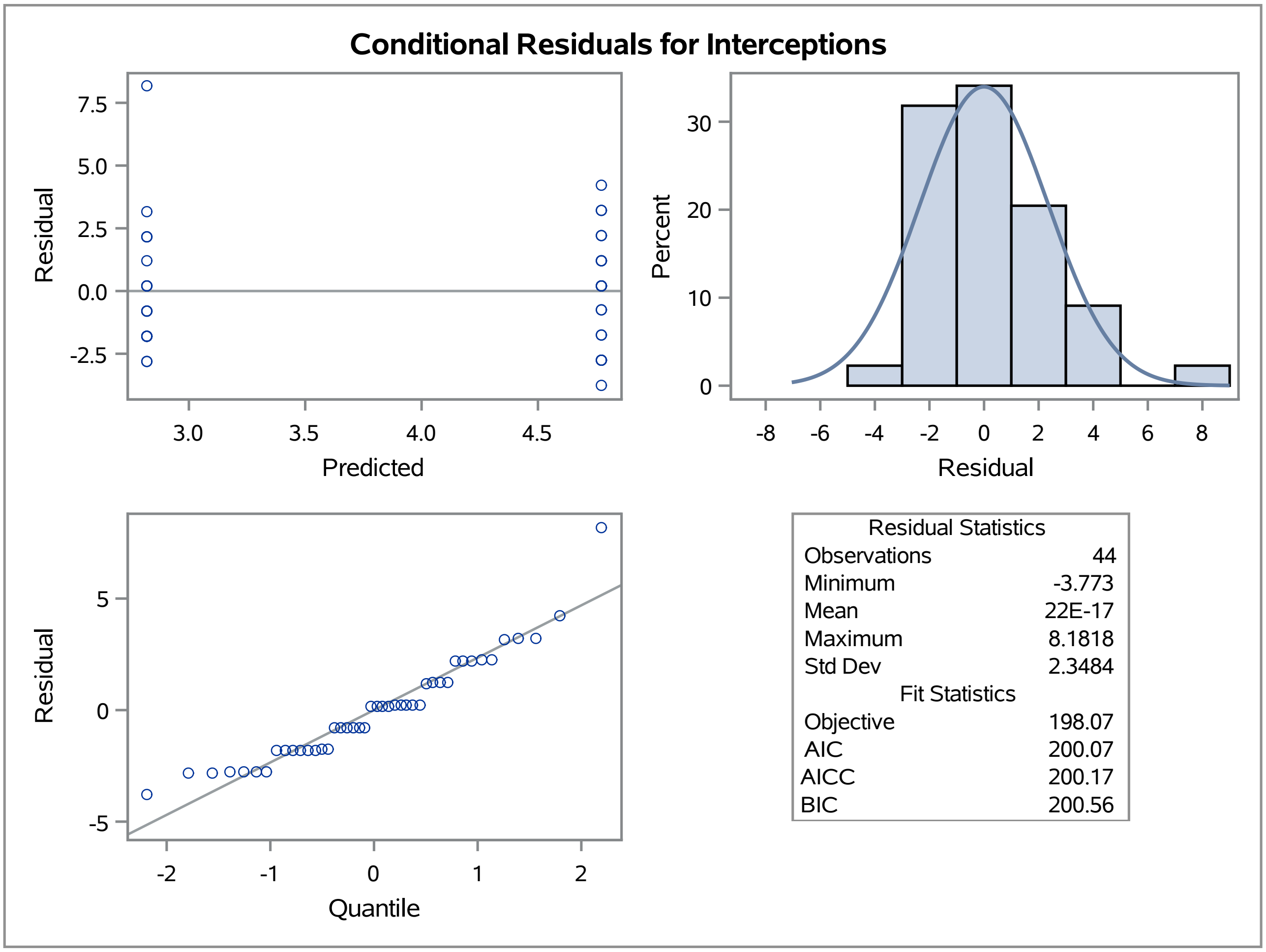

DistSoma=168

Model Information	
**Data Set**	WORK.TEMPDATASORTED
**Dependent Variable**	Interceptions
**Covariance Structure**	Variance Components
**Estimation Method**	REML
**Residual Variance Method**	Profile
**Fixed Effects SE Method**	Model-Based
**Degrees of Freedom Method**	Containment

Class Level Information		
**Class**	**Levels**	**Values**
**Treatment**	2	Control_GFP GFP MsTTR
**Culture**	12	1 2 3 4 5 6 7 8 9 10 11 12

Dimensions	
**Covariance Parameters**	2
**Columns in X**	3
**Columns in Z**	12
**Subjects**	1
**Max Obs per Subject**	44

Number of Observations	
**Number of Observations Read**	44
**Number of Observations Used**	44
**Number of Observations Not Used**	0

Iteration History			
**Iteration**	**Evaluations**	**-2 Res Log Like**	**Criterion**
**0**	1	193.12357583	
**1**	1	193.12357583	0.00000000

Convergence criteria met.

Estimated G matrix is not positive definite.

Covariance Parameter Estimates				
**Cov Parm**	**Estimate**	**Alpha**	**Lower**	**Upper**
**Culture**	0	.	.	.
**Residual**	5.0184	0.05	3.4118	8.1071

DistSoma=168

Fit Statistics	
-2 Res Log Likelihood	193.1
AIC (Smaller is Better)	195.1
AICC (Smaller is Better)	195.2
BIC (Smaller is Better)	195.6

Solution for Fixed Effects

Effect	Treatment	Estimate	Standard Error	DF	t Value	Pr > |t|	Alpha	Lower	Upper
Intercept		4.5909	0.4776	10	9.61	<.0001	0.05	3.5267	5.6551
Treatment	Control_GFP	-2.0455	0.6754	32	-3.03	0.0048	0.05	-3.4213	-0.6696
Treatment	GFP MsTTR	0	.	.	.	.	.	.	.

Solution for Random Effects

Effect	Culture	Estimate	Std Err Pred	DF	t Value	Pr > |t|	Alpha	Lower	Upper
Culture	1	0	.	.	.	.	.	.	.
Culture	2	0	.	.	.	.	.	.	.
Culture	3	0	.	.	.	.	.	.	.
Culture	4	0	.	.	.	.	.	.	.
Culture	5	0	.	.	.	.	.	.	.
Culture	6	0	.	.	.	.	.	.	.
Culture	7	0	.	.	.	.	.	.	.
Culture	8	0	.	.	.	.	.	.	.
Culture	9	0	.	.	.	.	.	.	.
Culture	10	0	.	.	.	.	.	.	.
Culture	11	0	.	.	.	.	.	.	.
Culture	12	0	.	.	.	.	.	.	.

Type 3 Tests of Fixed Effects

Effect	Num DF	Den DF	F Value	Pr > F
Treatment	1	32	9.17	0.0048

Least Squares Means

Effect	Treatment	Estimate	Standard Error	DF	t Value	Pr > |t|	Alpha	Lower	Upper
Treatment	Control_GFP	2.5455	0.4776	32	5.33	<.0001	0.05	1.5726	3.5183
Treatment	GFP MsTTR	4.5909	0.4776	32	9.61	<.0001	0.05	3.6181	5.5638

DistSoma=168

Differences of Least Squares Means												
**Effect**	**Treatment**	**Treatment**	**Estimate**	**Standard Error**	**DF**	**t Value**	**Pr > |t|**	**Adjustment**	**Adj P**	**Alpha**	**Lower**	**Upper**
**Treatment**	Control_GFP	GFP MsTTR	-2.0455	0.6754	32	-3.03	0.0048	Tukey	0.0048	0.05	-3.4213	-0.6696

Differences of Least Squares Means				
**Effect**	**Treatment**	**Treatment**	**Adj Lower**	**Adj Upper**
**Treatment**	Control_GFP	GFP MsTTR	-3.4213	-0.6696

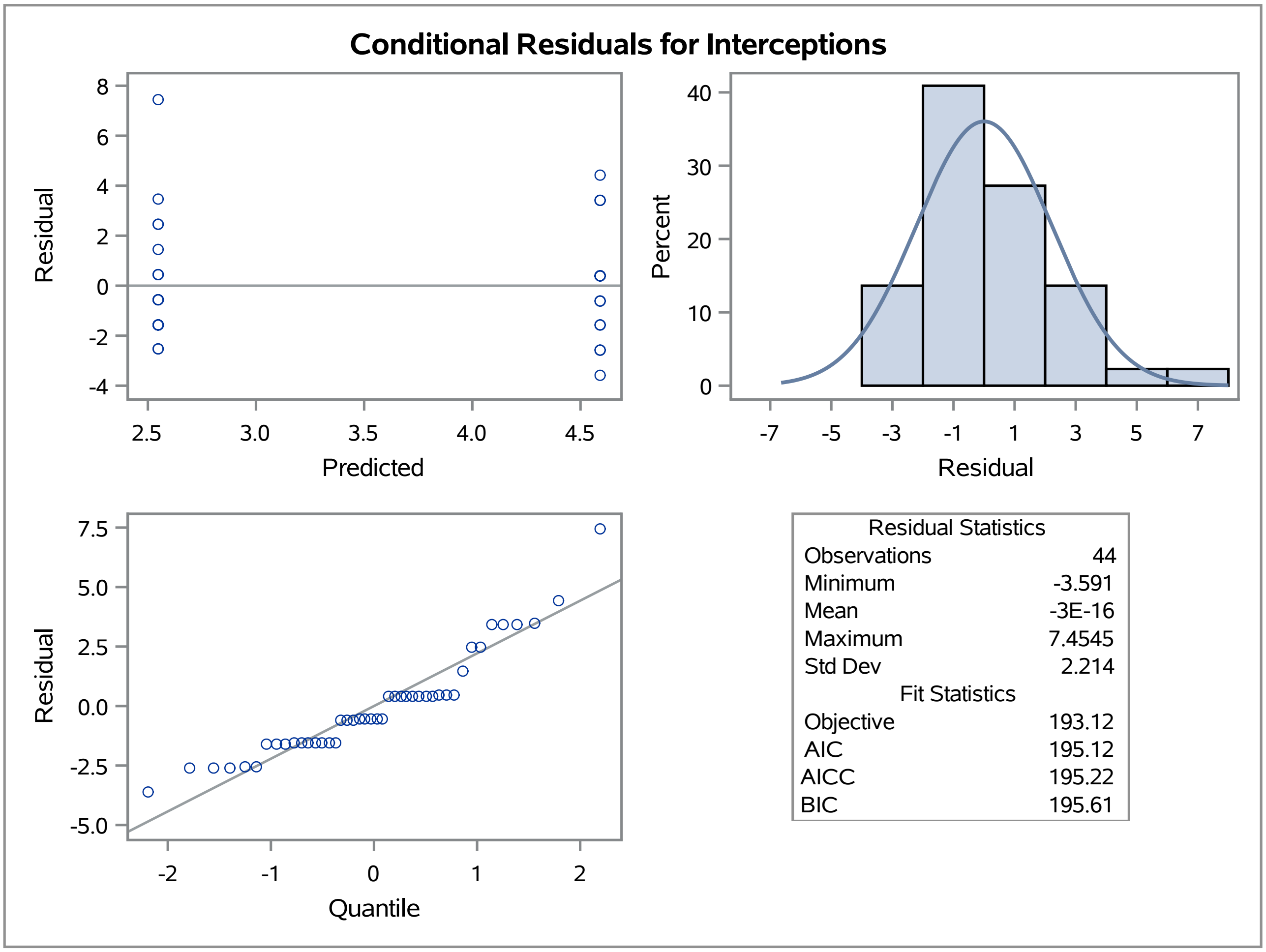

DistSoma=174

Model Information	
**Data Set**	WORK.TEMPDATASORTED
**Dependent Variable**	Interceptions
**Covariance Structure**	Variance Components
**Estimation Method**	REML
**Residual Variance Method**	Profile
**Fixed Effects SE Method**	Model-Based
**Degrees of Freedom Method**	Containment

Class Level Information		
**Class**	**Levels**	**Values**
**Treatment**	2	Control_GFP GFP MsTTR
**Culture**	12	1 2 3 4 5 6 7 8 9 10 11 12

Dimensions	
**Covariance Parameters**	2
**Columns in X**	3
**Columns in Z**	12
**Subjects**	1
**Max Obs per Subject**	44

Number of Observations	
**Number of Observations Read**	44
**Number of Observations Used**	44
**Number of Observations Not Used**	0

Iteration History			
**Iteration**	**Evaluations**	**-2 Res Log Like**	**Criterion**
**0**	1	181.50034356	
**1**	1	181.50034356	0.00000000

Convergence criteria met.

Estimated G matrix is not positive definite.

Covariance Parameter Estimates				
**Cov Parm**	**Estimate**	**Alpha**	**Lower**	**Upper**
**Culture**	0	.	.	.
**Residual**	3.8052	0.05	2.5870	6.1472

DistSoma=174

Fit Statistics	
-2 Res Log Likelihood	181.5
AIC (Smaller is Better)	183.5
AICC (Smaller is Better)	183.6
BIC (Smaller is Better)	184.0

Solution for Fixed Effects

Effect	Treatment	Estimate	Standard Error	DF	t Value	Pr > |t|	Alpha	Lower	Upper
Intercept		4.4545	0.4159	10	10.71	<.0001	0.05	3.5279	5.3812
Treatment	Control_GFP	-2.1818	0.5882	32	-3.71	0.0008	0.05	-3.3799	-0.9838
Treatment	GFP MsTTR	0	.	.	.	.	.	.	.

Solution for Random Effects

Effect	Culture	Estimate	Std Err Pred	DF	t Value	Pr > |t|	Alpha	Lower	Upper
Culture	1	0	.	.	.	.	.	.	.
Culture	2	0	.	.	.	.	.	.	.
Culture	3	0	.	.	.	.	.	.	.
Culture	4	0	.	.	.	.	.	.	.
Culture	5	0	.	.	.	.	.	.	.
Culture	6	0	.	.	.	.	.	.	.
Culture	7	0	.	.	.	.	.	.	.
Culture	8	0	.	.	.	.	.	.	.
Culture	9	0	.	.	.	.	.	.	.
Culture	10	0	.	.	.	.	.	.	.
Culture	11	0	.	.	.	.	.	.	.
Culture	12	0	.	.	.	.	.	.	.

Type 3 Tests of Fixed Effects

Effect	Num DF	Den DF	F Value	Pr > F
Treatment	1	32	13.76	0.0008

Least Squares Means

Effect	Treatment	Estimate	Standard Error	DF	t Value	Pr > |t|	Alpha	Lower	Upper
Treatment	Control_GFP	2.2727	0.4159	32	5.46	<.0001	0.05	1.4256	3.1199
Treatment	GFP MsTTR	4.4545	0.4159	32	10.71	<.0001	0.05	3.6074	5.3017

DistSoma=174

Differences of Least Squares Means												
**Effect**	**Treatment**	**Treatment**	**Estimate**	**Standard Error**	**DF**	**t Value**	**Pr > |t|**	**Adjustment**	**Adj P**	**Alpha**	**Lower**	**Upper**
**Treatment**	Control_GFP	GFP MsTTR	-2.1818	0.5882	32	-3.71	0.0008	Tukey	0.0008	0.05	-3.3799	-0.9838

Differences of Least Squares Means				
**Effect**	**Treatment**	**Treatment**	**Adj Lower**	**Adj Upper**
**Treatment**	Control_GFP	GFP MsTTR	-3.3798	-0.9838

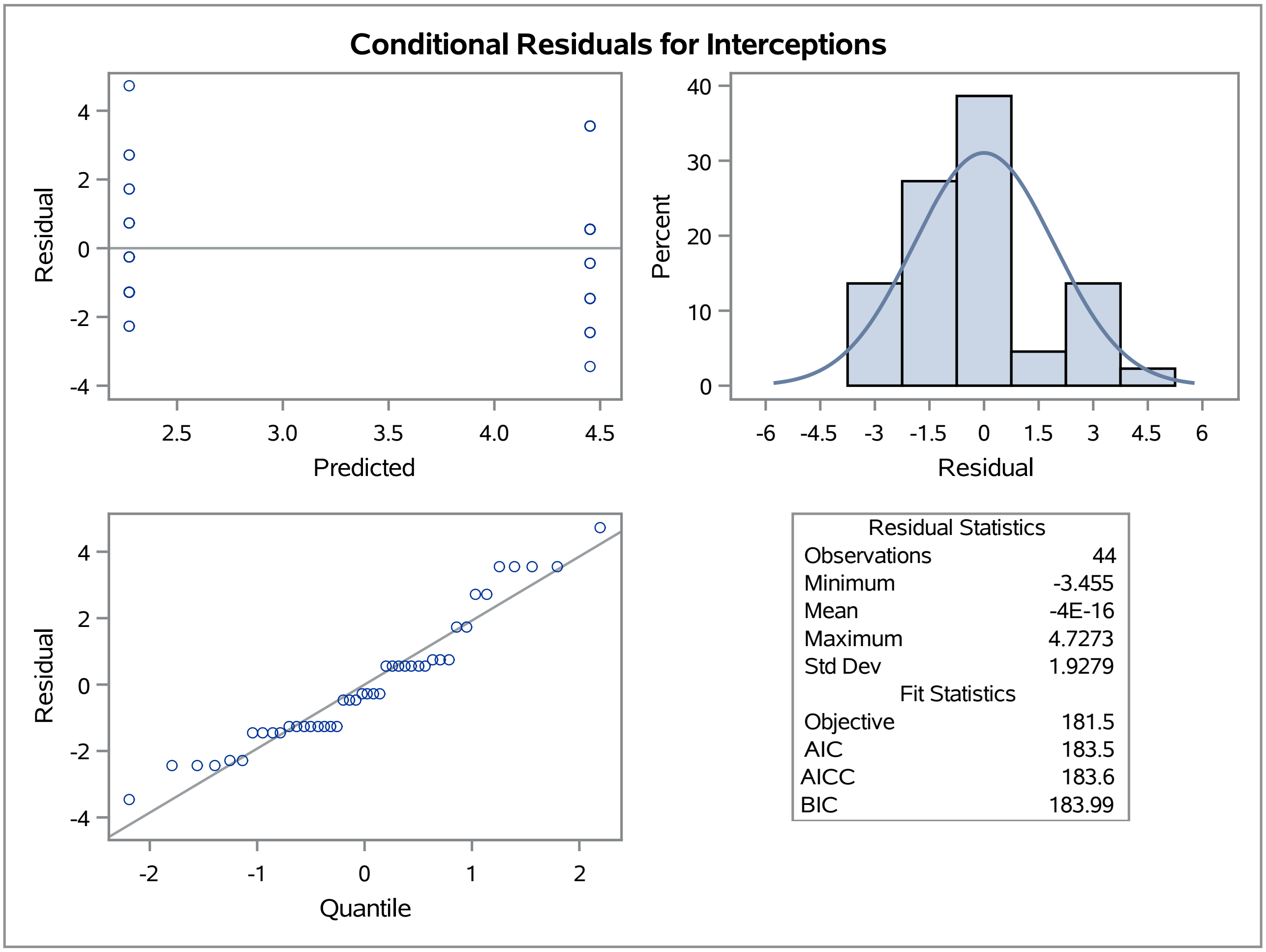

DistSoma=180

Model Information	
**Data Set**	WORK.TEMPDATASORTED
**Dependent Variable**	Interceptions
**Covariance Structure**	Variance Components
**Estimation Method**	REML
**Residual Variance Method**	Profile
**Fixed Effects SE Method**	Model-Based
**Degrees of Freedom Method**	Containment

Class Level Information		
**Class**	**Levels**	**Values**
**Treatment**	2	Control_GFP GFP MsTTR
**Culture**	12	1 2 3 4 5 6 7 8 9 10 11 12

Dimensions	
**Covariance Parameters**	2
**Columns in X**	3
**Columns in Z**	12
**Subjects**	1
**Max Obs per Subject**	44

Number of Observations	
**Number of Observations Read**	44
**Number of Observations Used**	44
**Number of Observations Not Used**	0

Iteration History			
**Iteration**	**Evaluations**	**-2 Res Log Like**	**Criterion**
**0**	1	180.60694971	
**1**	2	180.51665975	0.00000002
**2**	1	180.51665849	0.00000000

Convergence criteria met.

Covariance Parameter Estimates				
**Cov Parm**	**Estimate**	**Alpha**	**Lower**	**Upper**
**Culture**	0.1661	0.05	0.01590	3.547E18
**Residual**	3.5834	0.05	2.3141	6.2843

DistSoma=180

Fit Statistics	
-2 Res Log Likelihood	180.5
AIC (Smaller is Better)	184.5
AICC (Smaller is Better)	184.8
BIC (Smaller is Better)	185.5

Solution for Fixed Effects

Effect	Treatment	Estimate	Standard Error	DF	t Value	Pr > |t|	Alpha	Lower	Upper
Intercept		4.1422	0.4432	10	9.35	<.0001	0.05	3.1547	5.1298
Treatment	Control_GFP	-1.9005	0.6207	32	-3.06	0.0044	0.05	-3.1647	-0.6362
Treatment	GFP MsTTR	0	.	.	.	.	.	.	.

Solution for Random Effects

Effect	Culture	Estimate	Std Err Pred	DF	t Value	Pr > |t|	Alpha	Lower	Upper
Culture	1	-0.07021	0.3855	32	-0.18	0.8566	0.05	-0.8555	0.7151
Culture	2	0.1333	0.3855	32	0.35	0.7319	0.05	-0.6521	0.9186
Culture	3	-0.07692	0.3805	32	-0.20	0.8411	0.05	-0.8519	0.6980
Culture	4	-0.1551	0.3805	32	-0.41	0.6862	0.05	-0.9301	0.6198
Culture	5	0.1577	0.3805	32	0.41	0.6813	0.05	-0.6173	0.9327
Culture	6	0.1222	0.3989	32	0.31	0.7613	0.05	-0.6903	0.9347
Culture	7	-0.1109	0.3855	32	-0.29	0.7755	0.05	-0.8962	0.6744
Culture	8	-0.1020	0.3766	32	-0.27	0.7882	0.05	-0.8691	0.6650
Culture	9	0.09507	0.3807	32	0.25	0.8044	0.05	-0.6804	0.8705
Culture	10	-0.2178	0.3807	32	-0.57	0.5713	0.05	-0.9932	0.5577
Culture	11	0.2515	0.3807	32	0.66	0.5136	0.05	-0.5240	1.0270
Culture	12	-0.02676	0.3766	32	-0.07	0.9438	0.05	-0.7938	0.7403

Type 3 Tests of Fixed Effects

Effect	Num DF	Den DF	F Value	Pr > F
Treatment	1	32	9.38	0.0044

Least Squares Means

Effect	Treatment	Estimate	Standard Error	DF	t Value	Pr > |t|	Alpha	Lower	Upper
Treatment	Control_GFP	2.2418	0.4345	32	5.16	<.0001	0.05	1.3567	3.1268
Treatment	GFP MsTTR	4.1422	0.4432	32	9.35	<.0001	0.05	3.2394	5.0450

DistSoma=180

Differences of Least Squares Means												
Effect	Treatment	Treatment	Estimate	Standard Error	DF	t Value	Pr > |t|	Adjustment	Adj P	Alpha	Lower	Upper
Treatment	Control_GFP	GFP MsTTR	-1.9005	0.6207	32	-3.06	0.0044	Tukey-Kramer	0.0044	0.05	-3.1647	-0.6362

Differences of Least Squares Means				
Effect	Treatment	Treatment	Adj Lower	Adj Upper
Treatment	Control_GFP	GFP MsTTR	-3.1647	-0.6362

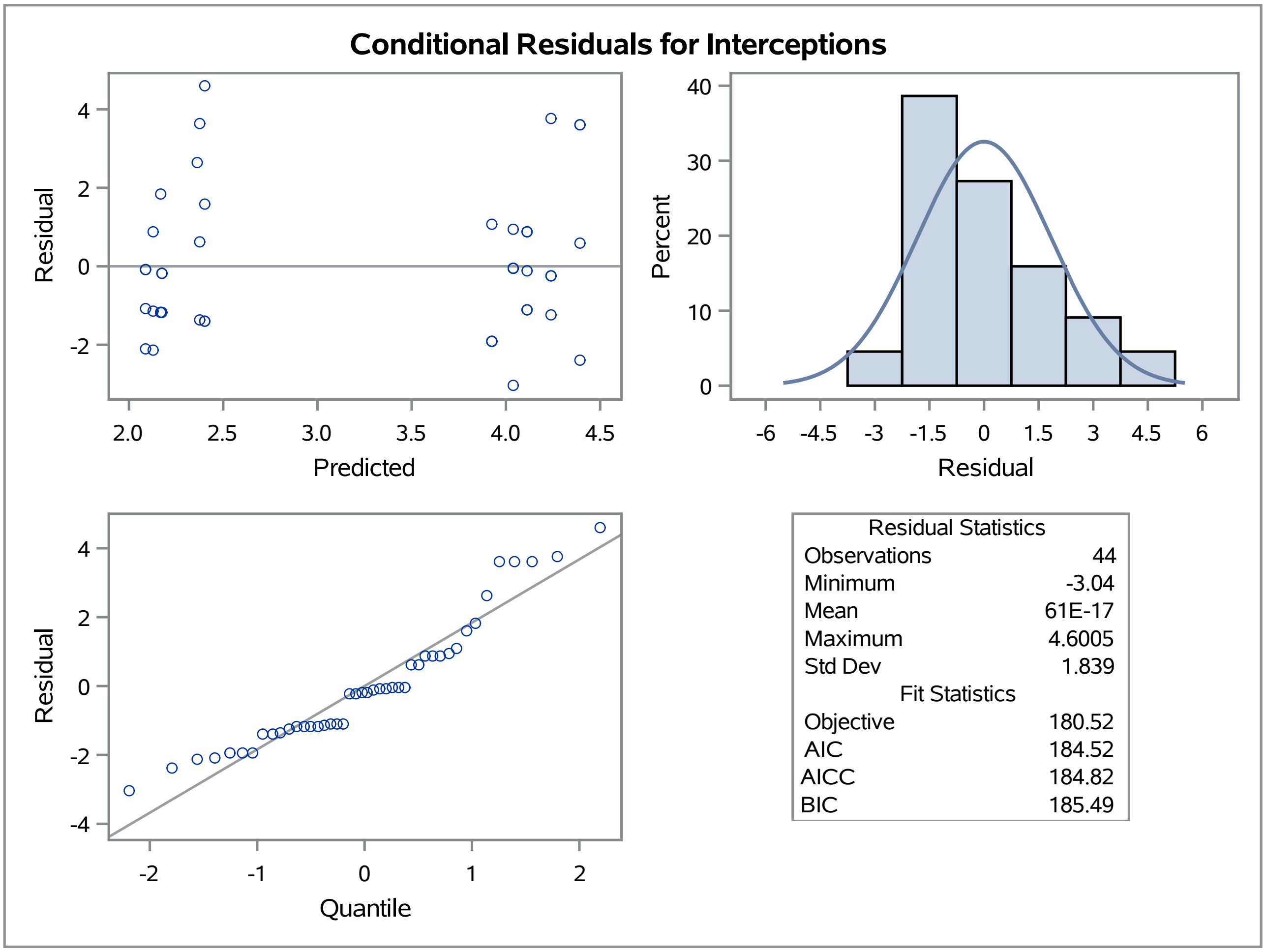

DistSoma=186

Model Information	
**Data Set**	WORK.TEMPDATASORTED
**Dependent Variable**	Interceptions
**Covariance Structure**	Variance Components
**Estimation Method**	REML
**Residual Variance Method**	Profile
**Fixed Effects SE Method**	Model-Based
**Degrees of Freedom Method**	Containment

Class Level Information		
**Class**	**Levels**	**Values**
**Treatment**	2	Control_GFP GFP MsTTR
**Culture**	12	1 2 3 4 5 6 7 8 9 10 11 12

Dimensions	
**Covariance Parameters**	2
**Columns in X**	3
**Columns in Z**	12
**Subjects**	1
**Max Obs per Subject**	44

Number of Observations	
**Number of Observations Read**	44
**Number of Observations Used**	44
**Number of Observations Not Used**	0

Iteration History			
**Iteration**	**Evaluations**	**-2 Res Log Like**	**Criterion**
**0**	1	170.59556395	
**1**	1	170.59556395	0.00000000

Convergence criteria met.

Estimated G matrix is not positive definite.

Covariance Parameter Estimates				
**Cov Parm**	**Estimate**	**Alpha**	**Lower**	**Upper**
**Culture**	0	.	.	.
**Residual**	2.9351	0.05	1.9955	4.7415

DistSoma=186

Fit Statistics	
-2 Res Log Likelihood	170.6
AIC (Smaller is Better)	172.6
AICC (Smaller is Better)	172.7
BIC (Smaller is Better)	173.1

Solution for Fixed Effects									
Effect	Treatment	Estimate	Standard Error	DF	t Value	Pr > |t|	Alpha	Lower	Upper
Intercept		4.0000	0.3653	10	10.95	<.0001	0.05	3.1862	4.8138
Treatment	Control_GFP	-2.1818	0.5166	32	-4.22	0.0002	0.05	-3.2340	-1.1296
Treatment	GFP MsTTR	0	.	.	.	.	.	.	.

Solution for Random Effects									
Effect	Culture	Estimate	Std Err Pred	DF	t Value	Pr > |t|	Alpha	Lower	Upper
Culture	1	0	.	.	.	.	.	.	.
Culture	2	0	.	.	.	.	.	.	.
Culture	3	0	.	.	.	.	.	.	.
Culture	4	0	.	.	.	.	.	.	.
Culture	5	0	.	.	.	.	.	.	.
Culture	6	0	.	.	.	.	.	.	.
Culture	7	0	.	.	.	.	.	.	.
Culture	8	0	.	.	.	.	.	.	.
Culture	9	0	.	.	.	.	.	.	.
Culture	10	0	.	.	.	.	.	.	.
Culture	11	0	.	.	.	.	.	.	.
Culture	12	0	.	.	.	.	.	.	.

Type 3 Tests of Fixed Effects				
Effect	Num DF	Den DF	F Value	Pr > F
Treatment	1	32	17.84	0.0002

Least Squares Means									
Effect	Treatment	Estimate	Standard Error	DF	t Value	Pr > |t|	Alpha	Lower	Upper
Treatment	Control_GFP	1.8182	0.3653	32	4.98	<.0001	0.05	1.0742	2.5622
Treatment	GFP MsTTR	4.0000	0.3653	32	10.95	<.0001	0.05	3.2560	4.7440

DistSoma=186

Differences of Least Squares Means												
**Effect**	**Treatment**	**Treatment**	**Estimate**	**Standard Error**	**DF**	**t Value**	**Pr > |t|**	**Adjustment**	**Adj P**	**Alpha**	**Lower**	**Upper**
**Treatment**	Control_GFP	GFP MsTTR	-2.1818	0.5166	32	-4.22	0.0002	Tukey	0.0002	0.05	-3.2340	-1.1296

Differences of Least Squares Means				
**Effect**	**Treatment**	**Treatment**	**Adj Lower**	**Adj Upper**
**Treatment**	Control_GFP	GFP MsTTR	-3.2340	-1.1297

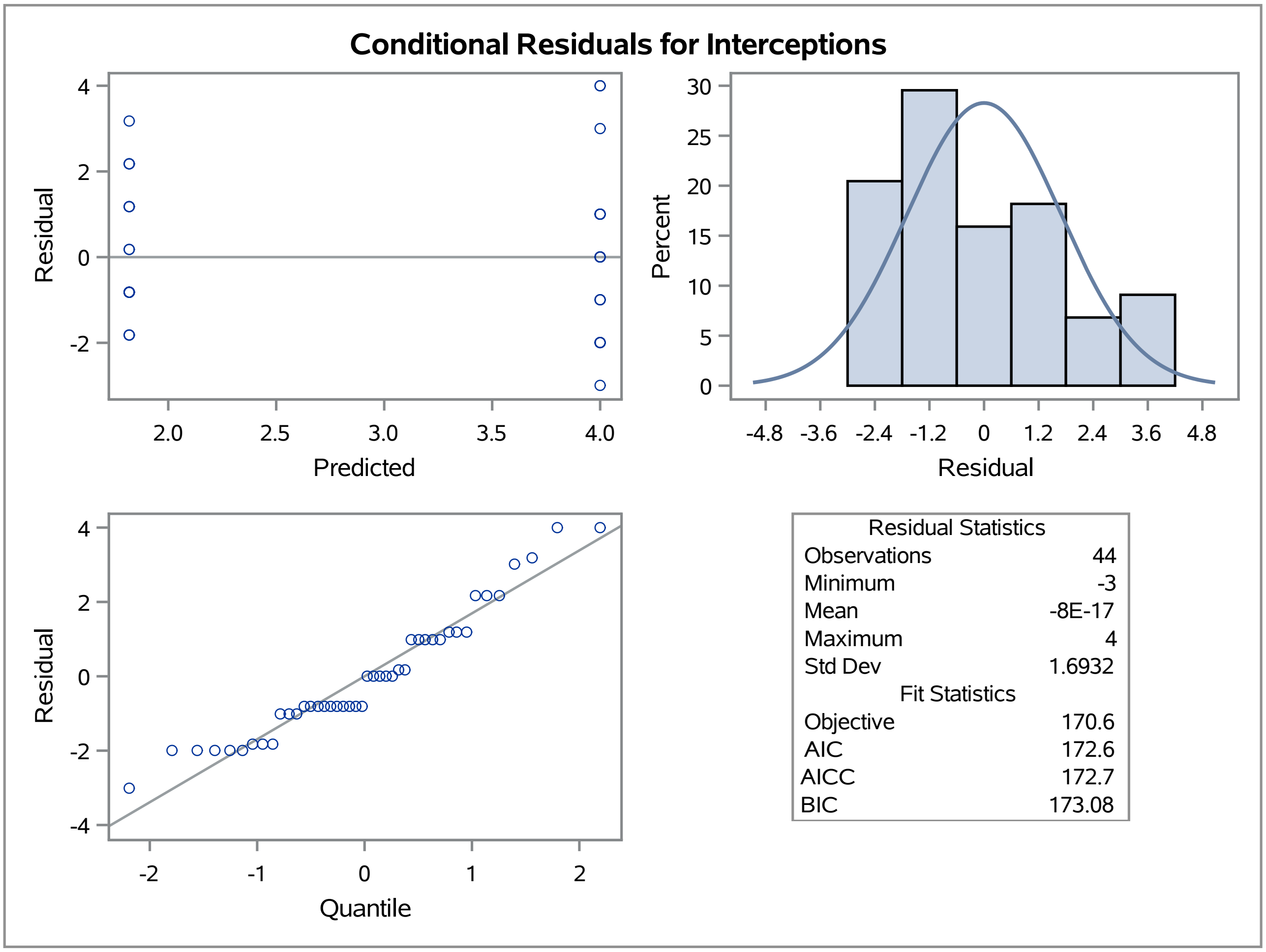

DistSoma=192

Model Information	
**Data Set**	WORK.TEMPDATASORTED
**Dependent Variable**	Interceptions
**Covariance Structure**	Variance Components
**Estimation Method**	REML
**Residual Variance Method**	Profile
**Fixed Effects SE Method**	Model-Based
**Degrees of Freedom Method**	Containment

Class Level Information		
**Class**	**Levels**	**Values**
**Treatment**	2	Control_GFP GFP MsTTR
**Culture**	12	1 2 3 4 5 6 7 8 9 10 11 12

Dimensions	
**Covariance Parameters**	2
**Columns in X**	3
**Columns in Z**	12
**Subjects**	1
**Max Obs per Subject**	44

Number of Observations	
**Number of Observations Read**	44
**Number of Observations Used**	44
**Number of Observations Not Used**	0

Iteration History			
**Iteration**	**Evaluations**	**-2 Res Log Like**	**Criterion**
**0**	1	165.70671156	
**1**	2	164.83351675	0.00000249
**2**	1	164.83340689	0.00000000

Convergence criteria met.

Covariance Parameter Estimates				
**Cov Parm**	**Estimate**	**Alpha**	**Lower**	**Upper**
**Culture**	0.3862	0.05	0.08222	158.26
**Residual**	2.2883	0.05	1.4763	4.0194

DistSoma=192

Fit Statistics	
**-2 Res Log Likelihood**	164.8
**AIC (Smaller is Better)**	168.8
**AICC (Smaller is Better)**	169.1
**BIC (Smaller is Better)**	169.8

Solution for Fixed Effects

Effect	Treatment	Estimate	Standard Error	DF	t Value	Pr > |t|	Alpha	Lower	Upper
**Intercept**		3.9656	0.4264	10	9.30	<.0001	0.05	3.0156	4.9156
**Treatment**	Control_GFP	-2.2368	0.5876	32	-3.81	0.0006	0.05	-3.4338	-1.0398
**Treatment**	GFP MsTTR	0	.	.	.	.	.	.	.

Solution for Random Effects

Effect	Culture	Estimate	Std Err Pred	DF	t Value	Pr > |t|	Alpha	Lower	Upper
**Culture**	1	-0.2450	0.5243	32	-0.47	0.6435	0.05	-1.3129	0.8229
**Culture**	2	0.4273	0.5243	32	0.82	0.4211	0.05	-0.6406	1.4952
**Culture**	3	-0.09221	0.5071	32	-0.18	0.8568	0.05	-1.1251	0.9407
**Culture**	4	-0.4952	0.5071	32	-0.98	0.3361	0.05	-1.5281	0.5376
**Culture**	5	0.2101	0.5071	32	0.41	0.6814	0.05	-0.8228	1.2429
**Culture**	6	0.3280	0.5778	32	0.57	0.5742	0.05	-0.8490	1.5049
**Culture**	7	-0.1329	0.5243	32	-0.25	0.8015	0.05	-1.2009	0.9350
**Culture**	8	-0.2589	0.4975	32	-0.52	0.6064	0.05	-1.2723	0.7546
**Culture**	9	0.4169	0.5100	32	0.82	0.4197	0.05	-0.6219	1.4557
**Culture**	10	-0.5907	0.5100	32	-1.16	0.2553	0.05	-1.6295	0.4481
**Culture**	11	0.4169	0.5100	32	0.82	0.4197	0.05	-0.6219	1.4557
**Culture**	12	0.01575	0.4975	32	0.03	0.9749	0.05	-0.9977	1.0292

Type 3 Tests of Fixed Effects

Effect	Num DF	Den DF	F Value	Pr > F
**Treatment**	1	32	14.49	0.0006

Least Squares Means

Effect	Treatment	Estimate	Standard Error	DF	t Value	Pr > |t|	Alpha	Lower	Upper
**Treatment**	Control_GFP	1.7288	0.4044	32	4.28	0.0002	0.05	0.9051	2.5525
**Treatment**	GFP MsTTR	3.9656	0.4264	32	9.30	<.0001	0.05	3.0971	4.8341

DistSoma=192

Differences of Least Squares Means												
Effect	Treatment	Treatment	Estimate	Standard Error	DF	t Value	Pr > |t|	Adjustment	Adj P	Alpha	Lower	Upper
**Treatment**	Control_GFP	GFP MsTTR	-2.2368	0.5876	32	-3.81	0.0006	Tukey-Kramer	0.0006	0.05	-3.4338	-1.0398

Differences of Least Squares Means				
Effect	Treatment	Treatment	Adj Lower	Adj Upper
**Treatment**	Control_GFP	GFP MsTTR	-3.4338	-1.0398

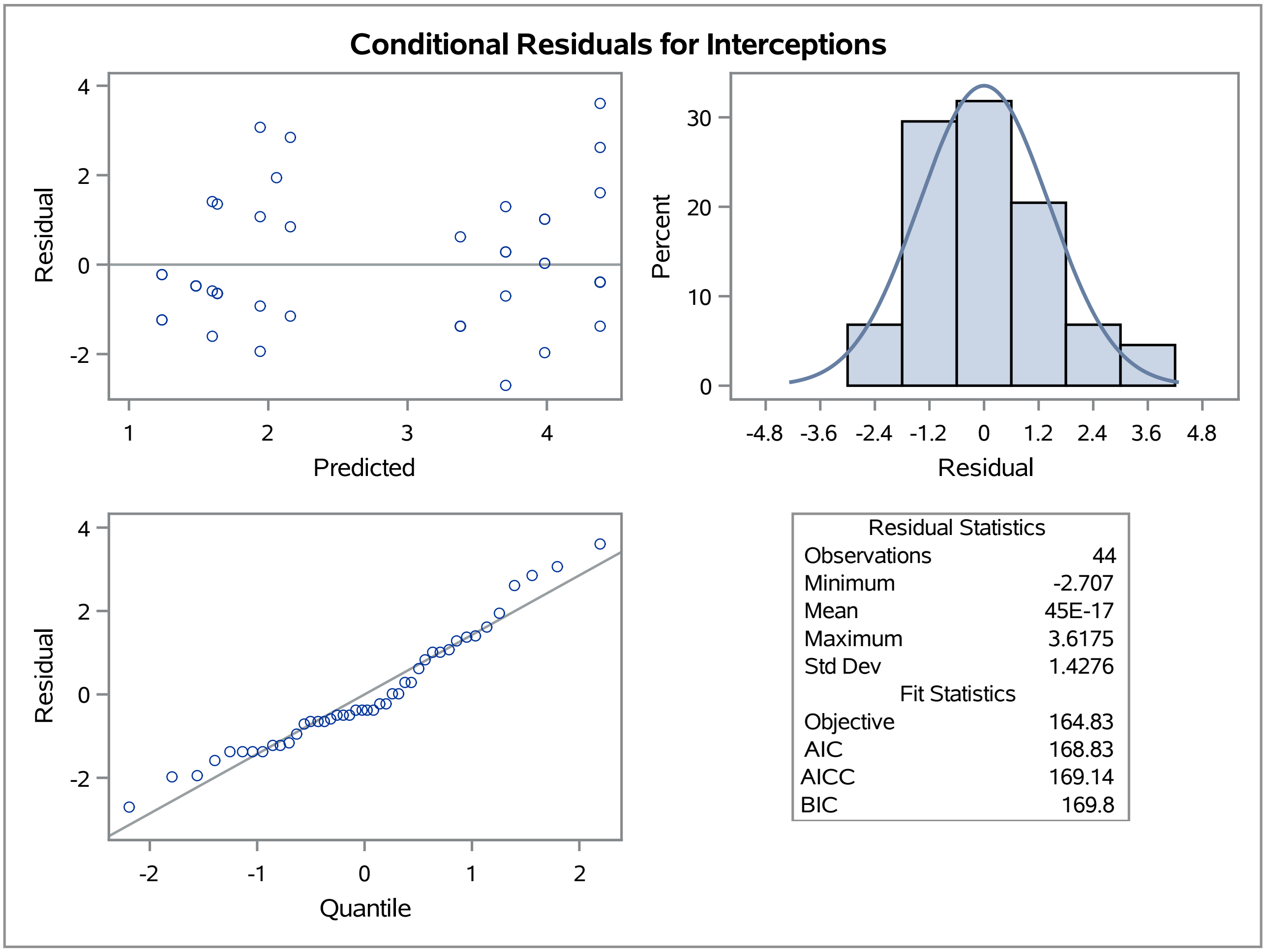

DistSoma=198

Model Information	
**Data Set**	WORK.TEMPDATASORTED
**Dependent Variable**	Interceptions
**Covariance Structure**	Variance Components
**Estimation Method**	REML
**Residual Variance Method**	Profile
**Fixed Effects SE Method**	Model-Based
**Degrees of Freedom Method**	Containment

Class Level Information		
**Class**	**Levels**	**Values**
**Treatment**	2	Control_GFP GFP MsTTR
**Culture**	12	1 2 3 4 5 6 7 8 9 10 11 12

Dimensions	
**Covariance Parameters**	2
**Columns in X**	3
**Columns in Z**	12
**Subjects**	1
**Max Obs per Subject**	44

Number of Observations	
**Number of Observations Read**	44
**Number of Observations Used**	44
**Number of Observations Not Used**	0

Iteration History			
**Iteration**	**Evaluations**	**-2 Res Log Like**	**Criterion**
**0**	1	164.34519474	
**1**	2	164.33562571	0.00000000

Convergence criteria met.

Covariance Parameter Estimates				
**Cov Parm**	**Estimate**	**Alpha**	**Lower**	**Upper**
**Culture**	0.03678	0.05	0.009143	1.02E172
**Residual**	2.4977	0.05	1.6095	4.3957

DistSoma=198

Fit Statistics	
-2 Res Log Likelihood	164.3
AIC (Smaller is Better)	168.3
AICC (Smaller is Better)	168.6
BIC (Smaller is Better)	169.3

Solution for Fixed Effects

Effect	Treatment	Estimate	Standard Error	DF	t Value	Pr > |t|	Alpha	Lower	Upper
Intercept		3.7268	0.3478	10	10.71	<.0001	0.05	2.9518	4.5018
Treatment	Control_GFP	-1.9491	0.4902	32	-3.98	0.0004	0.05	-2.9475	-0.9506
Treatment	GFP MsTTR	0	.	.	.	.	.	.	.

Solution for Random Effects

Effect	Culture	Estimate	Std Err Pred	DF	t Value	Pr > |t|	Alpha	Lower	Upper
Culture	1	-0.01880	0.1882	32	-0.10	0.9211	0.05	-0.4022	0.3646
Culture	2	0.05171	0.1882	32	0.27	0.7853	0.05	-0.3317	0.4352
Culture	3	-0.01545	0.1874	32	-0.08	0.9348	0.05	-0.3971	0.3662
Culture	4	-0.05717	0.1874	32	-0.31	0.7622	0.05	-0.4388	0.3245
Culture	5	0.02627	0.1874	32	0.14	0.8894	0.05	-0.3554	0.4079
Culture	6	0.03225	0.1904	32	0.17	0.8666	0.05	-0.3557	0.4202
Culture	7	-0.01880	0.1882	32	-0.10	0.9211	0.05	-0.4022	0.3646
Culture	8	-0.00870	0.1866	32	-0.05	0.9631	0.05	-0.3888	0.3714
Culture	9	0.02910	0.1874	32	0.16	0.8775	0.05	-0.3526	0.4108
Culture	10	-0.08215	0.1874	32	-0.44	0.6640	0.05	-0.4638	0.2995
Culture	11	0.04301	0.1874	32	0.23	0.8199	0.05	-0.3387	0.4247
Culture	12	0.01873	0.1866	32	0.10	0.9207	0.05	-0.3614	0.3989

Type 3 Tests of Fixed Effects

Effect	Num DF	Den DF	F Value	Pr > F
Treatment	1	32	15.81	0.0004

Least Squares Means

Effect	Treatment	Estimate	Standard Error	DF	t Value	Pr > |t|	Alpha	Lower	Upper
Treatment	Control_GFP	1.7778	0.3454	32	5.15	<.0001	0.05	1.0742	2.4813
Treatment	GFP MsTTR	3.7268	0.3478	32	10.71	<.0001	0.05	3.0183	4.4353

DistSoma=198

Differences of Least Squares Means												
Effect	Treatment	Treatment	Estimate	Standard Error	DF	t Value	Pr > |t|	Adjustment	Adj P	Alpha	Lower	Upper
Treatment	Control_GFP	GFP MsTTR	-1.9491	0.4902	32	-3.98	0.0004	Tukey-Kramer	0.0004	0.05	-2.9475	-0.9506

Differences of Least Squares Means				
Effect	Treatment	Treatment	Adj Lower	Adj Upper
Treatment	Control_GFP	GFP MsTTR	-2.9475	-0.9506

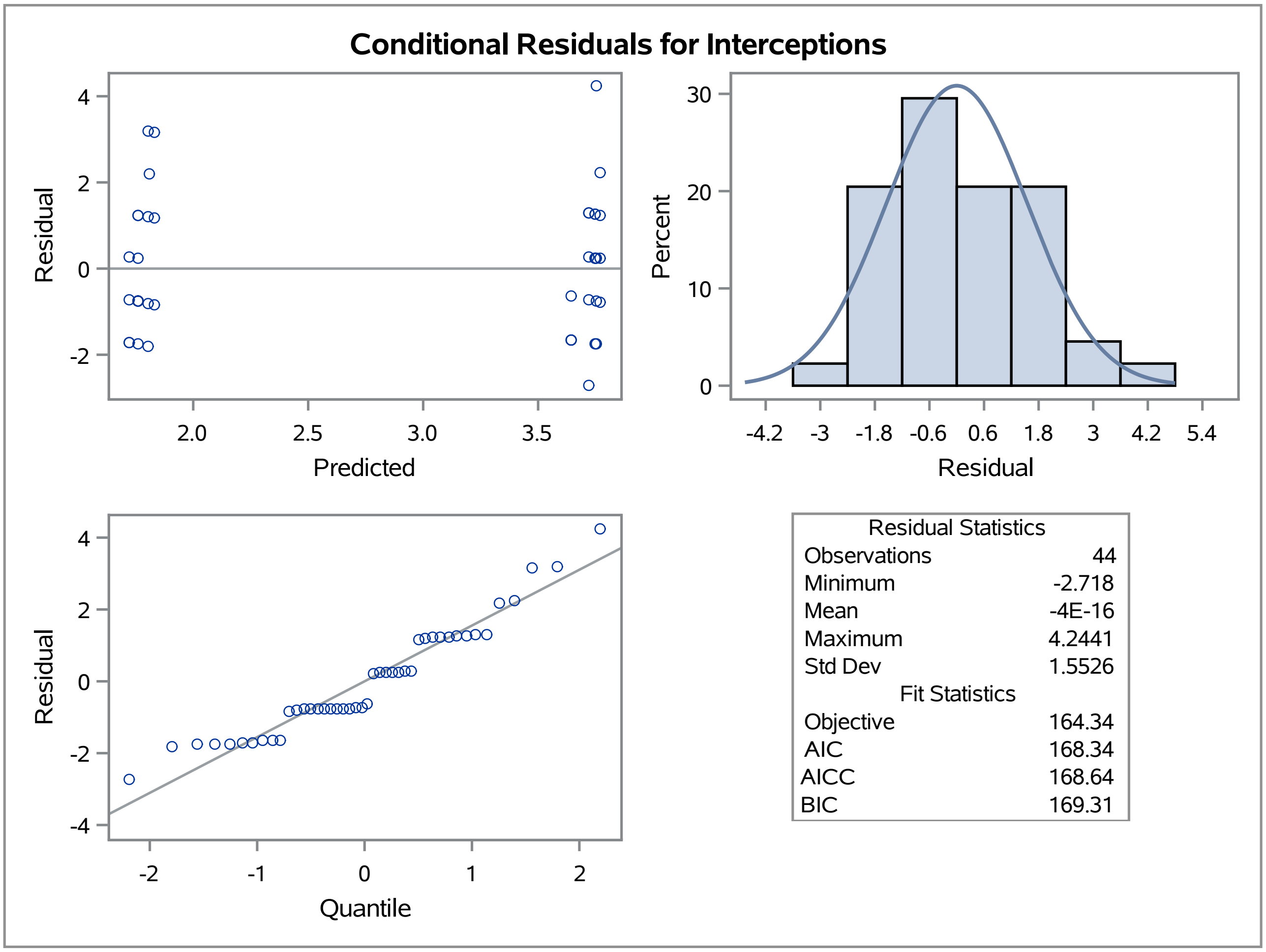

DistSoma=204

Model Information	
**Data Set**	WORK.TEMPDATASORTED
**Dependent Variable**	Interceptions
**Covariance Structure**	Variance Components
**Estimation Method**	REML
**Residual Variance Method**	Profile
**Fixed Effects SE Method**	Model-Based
**Degrees of Freedom Method**	Containment

Class Level Information		
**Class**	**Levels**	**Values**
**Treatment**	2	Control_GFP GFP MsTTR
**Culture**	12	1 2 3 4 5 6 7 8 9 10 11 12

Dimensions	
**Covariance Parameters**	2
**Columns in X**	3
**Columns in Z**	12
**Subjects**	1
**Max Obs per Subject**	44

Number of Observations	
**Number of Observations Read**	44
**Number of Observations Used**	44
**Number of Observations Not Used**	0

Iteration History			
**Iteration**	**Evaluations**	**-2 Res Log Like**	**Criterion**
**0**	1	162.73313467	
**1**	1	162.73313467	0.00000000

Convergence criteria met.

Estimated G matrix is not positive definite.

Covariance Parameter Estimates				
**Cov Parm**	**Estimate**	**Alpha**	**Lower**	**Upper**
**Culture**	0	.	.	.
**Residual**	2.4340	0.05	1.6548	3.9320

DistSoma=204

Fit Statistics	
-2 Res Log Likelihood	162.7
AIC (Smaller is Better)	164.7
AICC (Smaller is Better)	164.8
BIC (Smaller is Better)	165.2

Solution for Fixed Effects

Effect	Treatment	Estimate	Standard Error	DF	t Value	Pr > |t|	Alpha	Lower	Upper
Intercept		3.5455	0.3326	10	10.66	<.0001	0.05	2.8043	4.2866
Treatment	Control_GFP	-1.8636	0.4704	32	-3.96	0.0004	0.05	-2.8218	-0.9055
Treatment	GFP MsTTR	0	.	.	.	.	.	.	.

Solution for Random Effects

Effect	Culture	Estimate	Std Err Pred	DF	t Value	Pr > |t|	Alpha	Lower	Upper
Culture	1	0	.	.	.	.	.	.	.
Culture	2	0	.	.	.	.	.	.	.
Culture	3	0	.	.	.	.	.	.	.
Culture	4	0	.	.	.	.	.	.	.
Culture	5	0	.	.	.	.	.	.	.
Culture	6	0	.	.	.	.	.	.	.
Culture	7	0	.	.	.	.	.	.	.
Culture	8	0	.	.	.	.	.	.	.
Culture	9	0	.	.	.	.	.	.	.
Culture	10	0	.	.	.	.	.	.	.
Culture	11	0	.	.	.	.	.	.	.
Culture	12	0	.	.	.	.	.	.	.

Type 3 Tests of Fixed Effects

Effect	Num DF	Den DF	F Value	Pr > F
Treatment	1	32	15.70	0.0004

Least Squares Means

Effect	Treatment	Estimate	Standard Error	DF	t Value	Pr > |t|	Alpha	Lower	Upper
Treatment	Control_GFP	1.6818	0.3326	32	5.06	<.0001	0.05	1.0043	2.3593
Treatment	GFP MsTTR	3.5455	0.3326	32	10.66	<.0001	0.05	2.8679	4.2230

DistSoma=204

Differences of Least Squares Means												
**Effect**	**Treatment**	**Treatment**	**Estimate**	**Standard Error**	**DF**	**t Value**	**Pr > |t|**	**Adjustment**	**Adj P**	**Alpha**	**Lower**	**Upper**
**Treatment**	Control_GFP	GFP MsTTR	-1.8636	0.4704	32	-3.96	0.0004	Tukey	0.0004	0.05	-2.8218	-0.9055

Differences of Least Squares Means				
**Effect**	**Treatment**	**Treatment**	**Adj Lower**	**Adj Upper**
**Treatment**	Control_GFP	GFP MsTTR	-2.8218	-0.9055

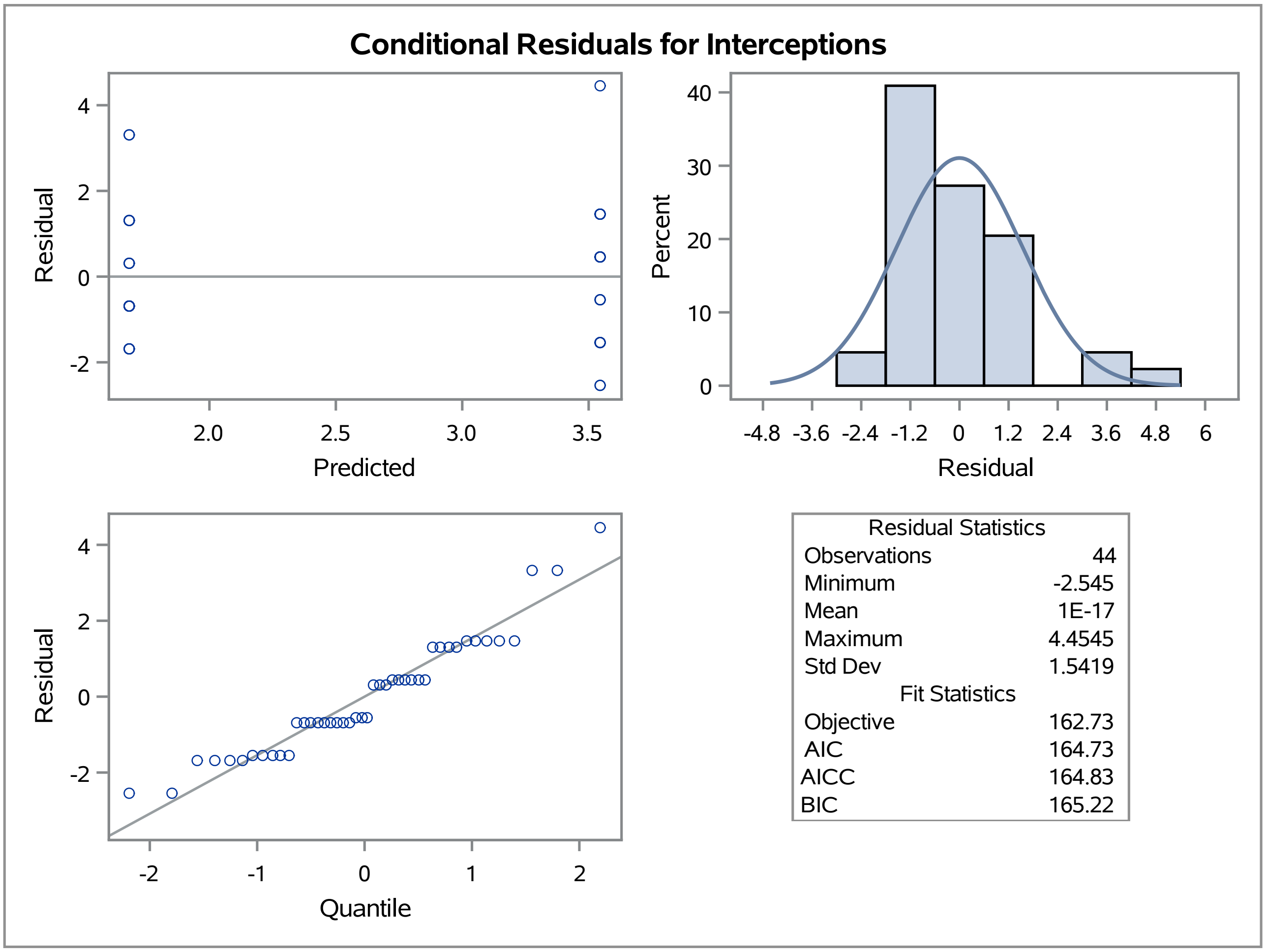

DistSoma=210

Model Information	
**Data Set**	WORK.TEMPDATASORTED
**Dependent Variable**	Interceptions
**Covariance Structure**	Variance Components
**Estimation Method**	REML
**Residual Variance Method**	Profile
**Fixed Effects SE Method**	Model-Based
**Degrees of Freedom Method**	Containment

Class Level Information		
**Class**	**Levels**	**Values**
**Treatment**	2	Control_GFP GFP MsTTR
**Culture**	12	1 2 3 4 5 6 7 8 9 10 11 12

Dimensions	
**Covariance Parameters**	2
**Columns in X**	3
**Columns in Z**	12
**Subjects**	1
**Max Obs per Subject**	44

Number of Observations	
**Number of Observations Read**	44
**Number of Observations Used**	44
**Number of Observations Not Used**	0

Iteration History			
**Iteration**	**Evaluations**	**-2 Res Log Like**	**Criterion**
**0**	1	167.61149141	
**1**	2	167.61048020	0.00000000

Convergence criteria met.

Covariance Parameter Estimates				
**Cov Parm**	**Estimate**	**Alpha**	**Lower**	**Upper**
**Culture**	0.01276	0.05	.	.
**Residual**	2.7228	0.05	1.7560	4.7856

DistSoma=210

Fit Statistics	
-2 Res Log Likelihood	167.6
AIC (Smaller is Better)	171.6
AICC (Smaller is Better)	171.9
BIC (Smaller is Better)	172.6

Solution for Fixed Effects									
Effect	Treatment	Estimate	Standard Error	DF	t Value	Pr > |t|	Alpha	Lower	Upper
Intercept		3.5911	0.3555	10	10.10	<.0001	0.05	2.7991	4.3831
Treatment	Control_GFP	-2.0907	0.5021	32	-4.16	0.0002	0.05	-3.1134	-1.0679
Treatment	GFP MsTTR	0	.	.	.	.	.	.	.

Solution for Random Effects									
Effect	Culture	Estimate	Std Err Pred	DF	t Value	Pr > |t|	Alpha	Lower	Upper
Culture	1	-0.00232	0.1123	32	-0.02	0.9837	0.05	-0.2310	0.2264
Culture	2	0.02078	0.1123	32	0.19	0.8543	0.05	-0.2079	0.2495
Culture	3	-7.76E-6	0.1121	32	-0.00	0.9999	0.05	-0.2283	0.2283
Culture	4	-0.01380	0.1121	32	-0.12	0.9028	0.05	-0.2421	0.2145
Culture	5	0.009189	0.1121	32	0.08	0.9352	0.05	-0.2191	0.2375
Culture	6	0.002329	0.1127	32	0.02	0.9836	0.05	-0.2272	0.2319
Culture	7	-0.01617	0.1123	32	-0.14	0.8863	0.05	-0.2448	0.2125
Culture	8	0.000204	0.1119	32	0.00	0.9986	0.05	-0.2278	0.2282
Culture	9	0.02132	0.1121	32	0.19	0.8504	0.05	-0.2070	0.2496
Culture	10	-0.02467	0.1121	32	-0.22	0.8272	0.05	-0.2530	0.2036
Culture	11	0.007521	0.1121	32	0.07	0.9469	0.05	-0.2208	0.2358
Culture	12	-0.00437	0.1119	32	-0.04	0.9691	0.05	-0.2324	0.2236

Type 3 Tests of Fixed Effects				
Effect	Num DF	Den DF	F Value	Pr > F
Treatment	1	32	17.34	0.0002

Least Squares Means									
Effect	Treatment	Estimate	Standard Error	DF	t Value	Pr > |t|	Alpha	Lower	Upper
Treatment	Control_GFP	1.5004	0.3546	32	4.23	0.0002	0.05	0.7781	2.2228
Treatment	GFP MsTTR	3.5911	0.3555	32	10.10	<.0001	0.05	2.8671	4.3151

DistSoma=210

Differences of Least Squares Means												
**Effect**	**Treatment**	**Treatment**	**Estimate**	**Standard Error**	**DF**	**t Value**	**Pr > |t|**	**Adjustment**	**Adj P**	**Alpha**	**Lower**	**Upper**
**Treatment**	Control_GFP	GFP MsTTR	-2.0907	0.5021	32	-4.16	0.0002	Tukey-Kramer	0.0002	0.05	-3.1134	-1.0679

Differences of Least Squares Means				
**Effect**	**Treatment**	**Treatment**	**Adj Lower**	**Adj Upper**
**Treatment**	Control_GFP	GFP MsTTR	-3.1134	-1.0679

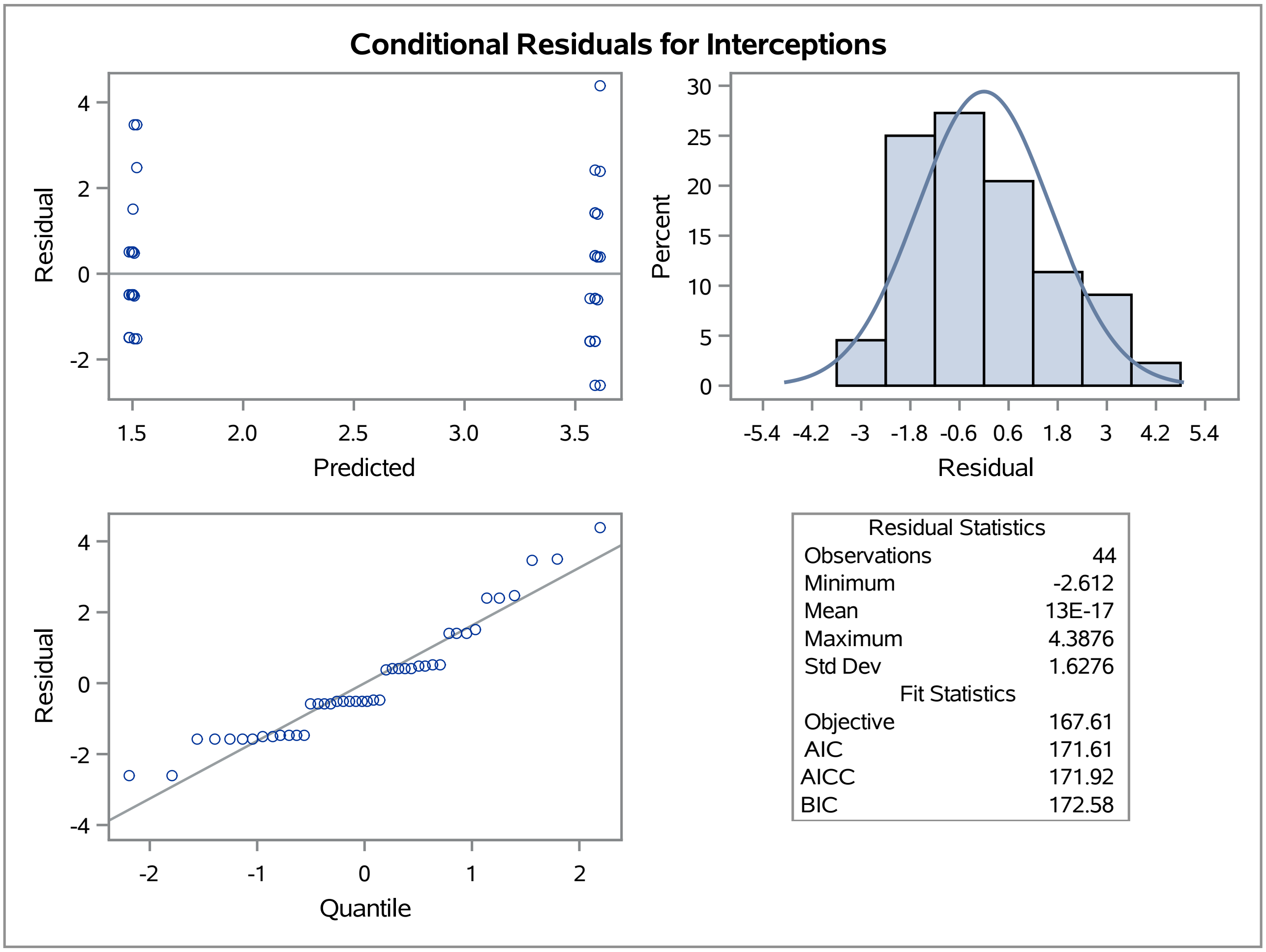

DistSoma=216

Model Information	
**Data Set**	WORK.TEMPDATASORTED
**Dependent Variable**	Interceptions
**Covariance Structure**	Variance Components
**Estimation Method**	REML
**Residual Variance Method**	Profile
**Fixed Effects SE Method**	Model-Based
**Degrees of Freedom Method**	Containment

Class Level Information		
**Class**	**Levels**	**Values**
**Treatment**	2	Control_GFP GFP MsTTR
**Culture**	12	1 2 3 4 5 6 7 8 9 10 11 12

Dimensions	
**Covariance Parameters**	2
**Columns in X**	3
**Columns in Z**	12
**Subjects**	1
**Max Obs per Subject**	44

Number of Observations	
**Number of Observations Read**	44
**Number of Observations Used**	44
**Number of Observations Not Used**	0

Iteration History			
**Iteration**	**Evaluations**	**-2 Res Log Like**	**Criterion**
**0**	1	170.22222816	
**1**	2	169.88931252	0.00000000

Convergence criteria met.

Covariance Parameter Estimates				
**Cov Parm**	**Estimate**	**Alpha**	**Lower**	**Upper**
**Culture**	0.2366	0.05	0.03525	110995
**Residual**	2.7052	0.05	1.7582	4.6946

DistSoma=216

Fit Statistics	
-2 Res Log Likelihood	169.9
AIC (Smaller is Better)	173.9
AICC (Smaller is Better)	174.2
BIC (Smaller is Better)	174.9

Solution for Fixed Effects

Effect	Treatment	Estimate	Standard Error	DF	t Value	Pr > |t|	Alpha	Lower	Upper
Intercept		3.3711	0.4132	10	8.16	<.0001	0.05	2.4506	4.2917
Treatment	Control_GFP	-2.0046	0.5748	32	-3.49	0.0014	0.05	-3.1755	-0.8338
Treatment	GFP MsTTR	0	.	.	.	.	.	.	.

Solution for Random Effects

Effect	Culture	Estimate	Std Err Pred	DF	t Value	Pr > |t|	Alpha	Lower	Upper
Culture	1	-0.00689	0.4408	32	-0.02	0.9876	0.05	-0.9048	0.8910
Culture	2	0.1317	0.4408	32	0.30	0.7671	0.05	-0.7662	1.0295
Culture	3	0.03460	0.4313	32	0.08	0.9366	0.05	-0.8438	0.9130
Culture	4	-0.1598	0.4313	32	-0.37	0.7135	0.05	-1.0382	0.7187
Culture	5	0.1642	0.4313	32	0.38	0.7060	0.05	-0.7143	1.0426
Culture	6	0.05094	0.4675	32	0.11	0.9139	0.05	-0.9014	1.0032
Culture	7	-0.2147	0.4408	32	-0.49	0.6295	0.05	-1.1126	0.6832
Culture	8	-0.1129	0.4247	32	-0.27	0.7921	0.05	-0.9780	0.7522
Culture	9	0.4869	0.4321	32	1.13	0.2682	0.05	-0.3933	1.3671
Culture	10	-0.4201	0.4321	32	-0.97	0.3382	0.05	-1.3003	0.4601
Culture	11	0.09818	0.4321	32	0.23	0.8217	0.05	-0.7820	0.9784
Culture	12	-0.05206	0.4247	32	-0.12	0.9032	0.05	-0.9172	0.8131

Type 3 Tests of Fixed Effects

Effect	Num DF	Den DF	F Value	Pr > F
Treatment	1	32	12.16	0.0014

Least Squares Means

Effect	Treatment	Estimate	Standard Error	DF	t Value	Pr > |t|	Alpha	Lower	Upper
Treatment	Control_GFP	1.3665	0.3996	32	3.42	0.0017	0.05	0.5525	2.1805
Treatment	GFP MsTTR	3.3711	0.4132	32	8.16	<.0001	0.05	2.5296	4.2127

DistSoma=216

Differences of Least Squares Means												
**Effect**	**Treatment**	**Treatment**	**Estimate**	**Standard Error**	**DF**	**t Value**	**Pr > |t|**	**Adjustment**	**Adj P**	**Alpha**	**Lower**	**Upper**
**Treatment**	Control_GFP	GFP MsTTR	-2.0046	0.5748	32	-3.49	0.0014	Tukey-Kramer	0.0014	0.05	-3.1755	-0.8338

Differences of Least Squares Means				
**Effect**	**Treatment**	**Treatment**	**Adj Lower**	**Adj Upper**
**Treatment**	Control_GFP	GFP MsTTR	-3.1755	-0.8338

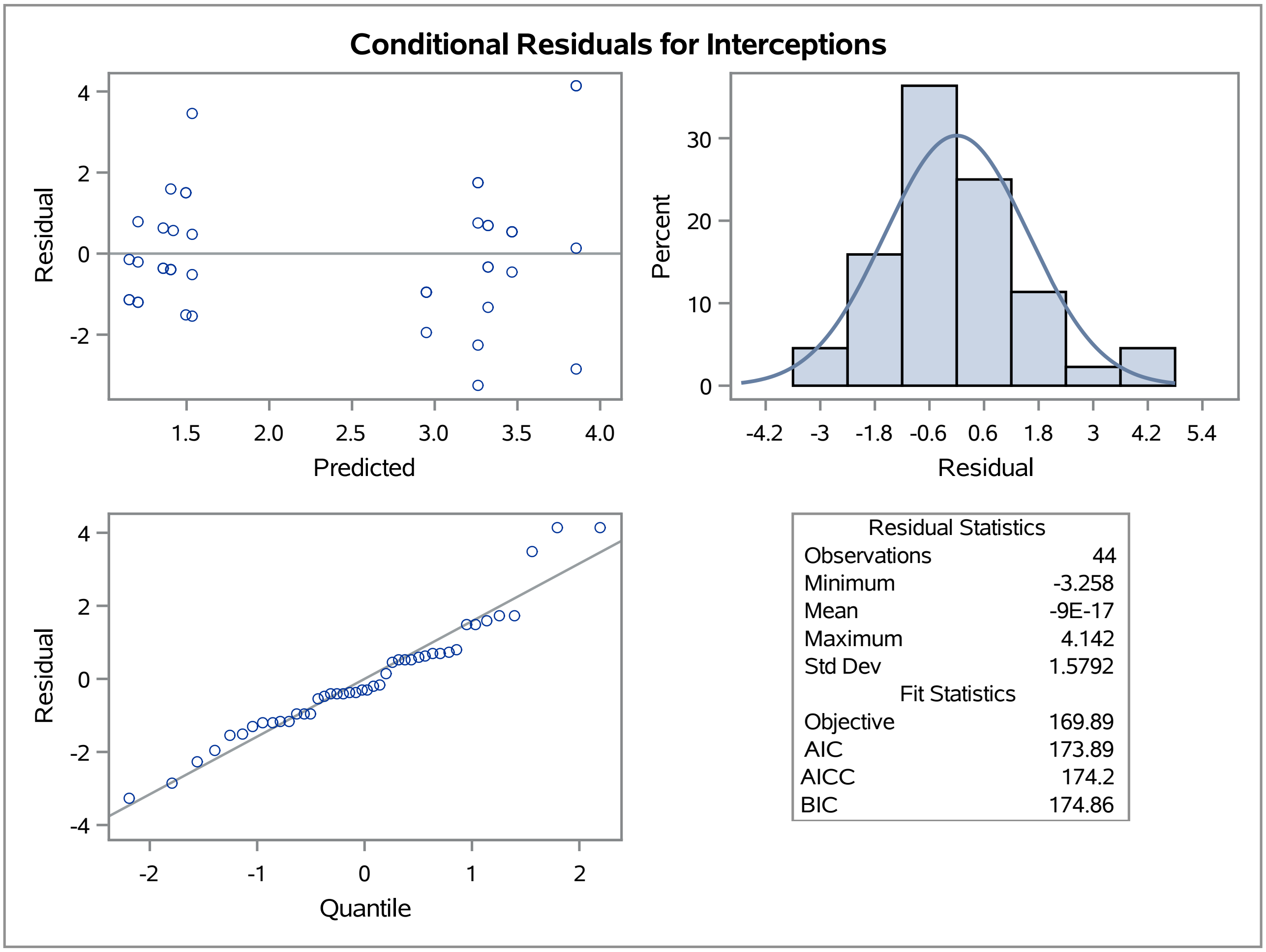

DistSoma=222

Model Information	
**Data Set**	WORK.TEMPDATASORTED
**Dependent Variable**	Interceptions
**Covariance Structure**	Variance Components
**Estimation Method**	REML
**Residual Variance Method**	Profile
**Fixed Effects SE Method**	Model-Based
**Degrees of Freedom Method**	Containment

Class Level Information		
**Class**	**Levels**	**Values**
**Treatment**	2	Control_GFP GFP MsTTR
**Culture**	12	1 2 3 4 5 6 7 8 9 10 11 12

Dimensions	
**Covariance Parameters**	2
**Columns in X**	3
**Columns in Z**	12
**Subjects**	1
**Max Obs per Subject**	44

Number of Observations	
**Number of Observations Read**	44
**Number of Observations Used**	44
**Number of Observations Not Used**	0

Iteration History			
**Iteration**	**Evaluations**	**-2 Res Log Like**	**Criterion**
**0**	1	170.56457907	
**1**	2	169.93047150	0.00000000

Convergence criteria met.

Covariance Parameter Estimates				
**Cov Parm**	**Estimate**	**Alpha**	**Lower**	**Upper**
**Culture**	0.3385	0.05	0.06470	643.63
**Residual**	2.6417	0.05	1.7154	4.5911

DistSoma=222

Fit Statistics	
-2 Res Log Likelihood	169.9
AIC (Smaller is Better)	173.9
AICC (Smaller is Better)	174.2
BIC (Smaller is Better)	174.9

Solution for Fixed Effects

Effect	Treatment	Estimate	Standard Error	DF	t Value	Pr > |t|	Alpha	Lower	Upper
Intercept		3.4205	0.4340	10	7.88	<.0001	0.05	2.4536	4.3874
Treatment	Control_GFP	-2.1860	0.6006	32	-3.64	0.0010	0.05	-3.4094	-0.9626
Treatment	GFP MsTTR	0	.	.	.	.	.	.	.

Solution for Random Effects

Effect	Culture	Estimate	Std Err Pred	DF	t Value	Pr > |t|	Alpha	Lower	Upper
Culture	1	0.02746	0.5078	32	0.05	0.9572	0.05	-1.0068	1.0617
Culture	2	0.2126	0.5078	32	0.42	0.6782	0.05	-0.8217	1.2469
Culture	3	-0.07946	0.4936	32	-0.16	0.8731	0.05	-1.0848	0.9259
Culture	4	-0.1642	0.4936	32	-0.33	0.7416	0.05	-1.1696	0.8412
Culture	5	0.2594	0.4936	32	0.53	0.6028	0.05	-0.7459	1.2648
Culture	6	0.08696	0.5498	32	0.16	0.8753	0.05	-1.0330	1.2069
Culture	7	-0.3428	0.5078	32	-0.68	0.5044	0.05	-1.3771	0.6915
Culture	8	-0.1642	0.4848	32	-0.34	0.7371	0.05	-1.1518	0.8233
Culture	9	0.6200	0.4954	32	1.25	0.2198	0.05	-0.3891	1.6292
Culture	10	-0.5661	0.4954	32	-1.14	0.2616	0.05	-1.5753	0.4430
Culture	11	0.1964	0.4954	32	0.40	0.6944	0.05	-0.8127	1.2055
Culture	12	-0.08610	0.4848	32	-0.18	0.8602	0.05	-1.0737	0.9015

Type 3 Tests of Fixed Effects

Effect	Num DF	Den DF	F Value	Pr > F
Treatment	1	32	13.25	0.0010

Least Squares Means

Effect	Treatment	Estimate	Standard Error	DF	t Value	Pr > |t|	Alpha	Lower	Upper
Treatment	Control_GFP	1.2345	0.4152	32	2.97	0.0056	0.05	0.3886	2.0803
Treatment	GFP MsTTR	3.4205	0.4340	32	7.88	<.0001	0.05	2.5365	4.3044

DistSoma=222

Differences of Least Squares Means												
**Effect**	**Treatment**	**Treatment**	**Estimate**	**Standard Error**	**DF**	**t Value**	**Pr > |t|**	**Adjustment**	**Adj P**	**Alpha**	**Lower**	**Upper**
**Treatment**	Control_GFP	GFP MsTTR	-2.1860	0.6006	32	-3.64	0.0010	Tukey-Kramer	0.0010	0.05	-3.4094	-0.9626

Differences of Least Squares Means				
**Effect**	**Treatment**	**Treatment**	**Adj Lower**	**Adj Upper**
**Treatment**	Control_GFP	GFP MsTTR	-3.4094	-0.9626

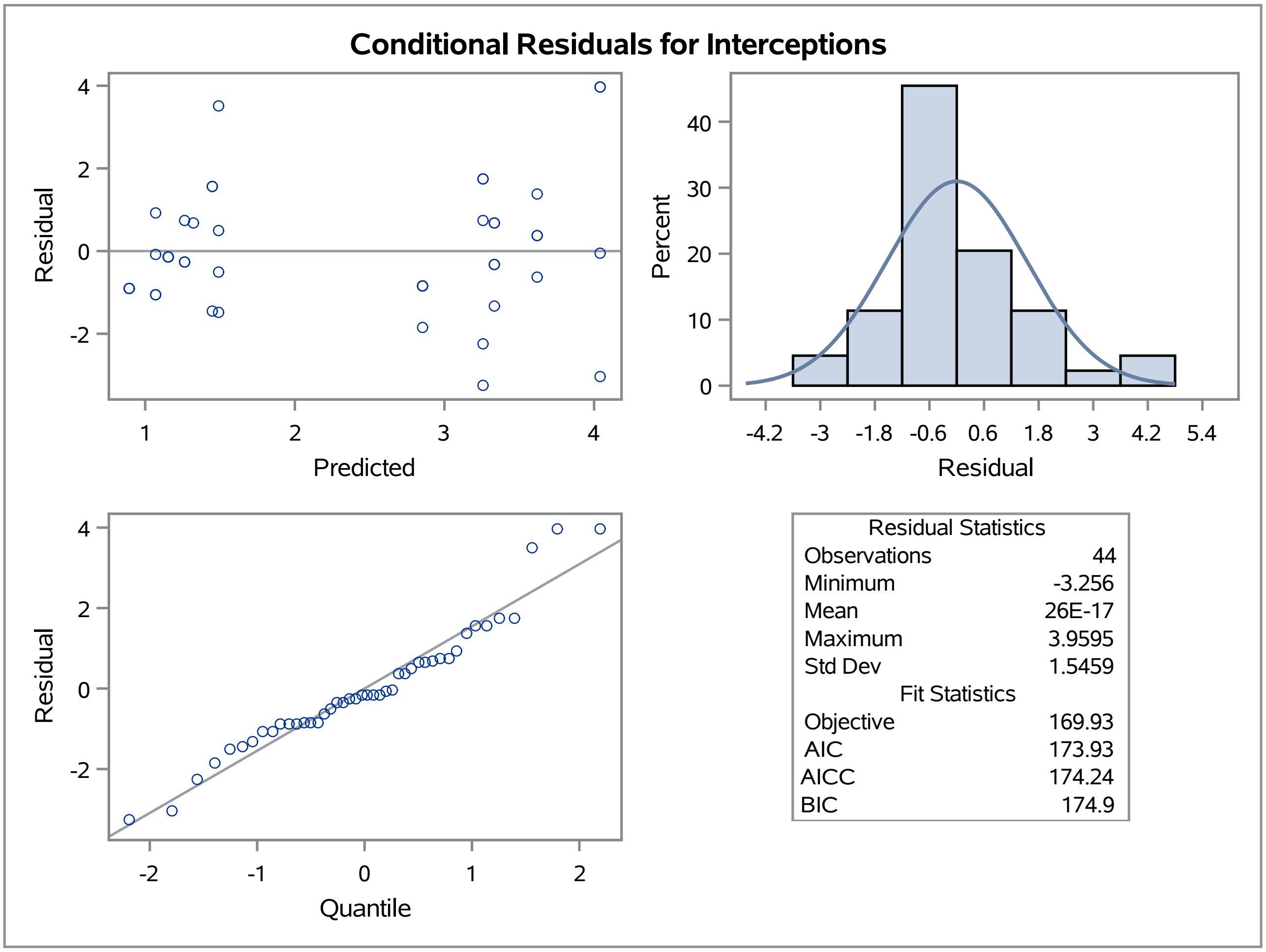

DistSoma=228

Model Information	
Data Set	WORK.TEMPDATASORTED
Dependent Variable	Interceptions
Covariance Structure	Variance Components
Estimation Method	REML
Residual Variance Method	Profile
Fixed Effects SE Method	Model-Based
Degrees of Freedom Method	Containment

Class Level Information		
Class	Levels	Values
Treatment	2	Control_GFP GFP MsTTR
Culture	12	1 2 3 4 5 6 7 8 9 10 11 12

Dimensions	
Covariance Parameters	2
Columns in X	3
Columns in Z	12
Subjects	1
Max Obs per Subject	44

Number of Observations	
Number of Observations Read	44
Number of Observations Used	44
Number of Observations Not Used	0

Iteration History			
Iteration	Evaluations	-2 Res Log Like	Criterion
0	1	169.62424128	
1	2	169.32817537	0.00000000

Convergence criteria met.

Covariance Parameter Estimates				
Cov Parm	Estimate	Alpha	Lower	Upper
Culture	0.2238	0.05	0.03187	445971
Residual	2.6759	0.05	1.7366	4.6547

DistSoma=228

Fit Statistics	
-2 Res Log Likelihood	169.3
AIC (Smaller is Better)	173.3
AICC (Smaller is Better)	173.6
BIC (Smaller is Better)	174.3

Solution for Fixed Effects									
Effect	Treatment	Estimate	Standard Error	DF	t Value	Pr > |t|	Alpha	Lower	Upper
Intercept		3.1429	0.4084	10	7.70	<.0001	0.05	2.2329	4.0529
Treatment	Control_GFP	-1.9108	0.5685	32	-3.36	0.0020	0.05	-3.0688	-0.7528
Treatment	GFP MsTTR	0	.	.	.	.	.	.	.

Solution for Random Effects									
Effect	Culture	Estimate	Std Err Pred	DF	t Value	Pr > |t|	Alpha	Lower	Upper
Culture	1	0.02032	0.4304	32	0.05	0.9626	0.05	-0.8563	0.8970
Culture	2	0.1541	0.4304	32	0.36	0.7227	0.05	-0.7226	1.0307
Culture	3	-0.05818	0.4214	32	-0.14	0.8911	0.05	-0.9165	0.8001
Culture	4	-0.1209	0.4214	32	-0.29	0.7761	0.05	-0.9792	0.7374
Culture	5	0.1925	0.4214	32	0.46	0.6508	0.05	-0.6658	1.0508
Culture	6	0.05928	0.4555	32	0.13	0.8973	0.05	-0.8686	0.9871
Culture	7	-0.2472	0.4304	32	-0.57	0.5698	0.05	-1.1238	0.6295
Culture	8	-0.1011	0.4151	32	-0.24	0.8091	0.05	-0.9467	0.7445
Culture	9	0.4029	0.4221	32	0.95	0.3470	0.05	-0.4569	1.2628
Culture	10	-0.4119	0.4221	32	-0.98	0.3365	0.05	-1.2718	0.4480
Culture	11	0.1522	0.4221	32	0.36	0.7208	0.05	-0.7077	1.0121
Culture	12	-0.04213	0.4151	32	-0.10	0.9198	0.05	-0.8877	0.8035

Type 3 Tests of Fixed Effects				
Effect	Num DF	Den DF	F Value	Pr > F
Treatment	1	32	11.30	0.0020

Least Squares Means									
Effect	Treatment	Estimate	Standard Error	DF	t Value	Pr > |t|	Alpha	Lower	Upper
Treatment	Control_GFP	1.2320	0.3955	32	3.12	0.0039	0.05	0.4265	2.0376
Treatment	GFP MsTTR	3.1429	0.4084	32	7.70	<.0001	0.05	2.3110	3.9748

DistSoma=228

Differences of Least Squares Means

Effect	Treatment	Treatment	Estimate	Standard Error	DF	t Value	Pr > |t|	Adjustment	Adj P	Alpha	Lower	Upper
**Treatment**	Control_GFP	GFP MsTTR	-1.9108	0.5685	32	-3.36	0.0020	Tukey-Kramer	0.0020	0.05	-3.0688	-0.7528

Differences of Least Squares Means

Effect	Treatment	Treatment	Adj Lower	Adj Upper
**Treatment**	Control_GFP	GFP MsTTR	-3.0688	-0.7528

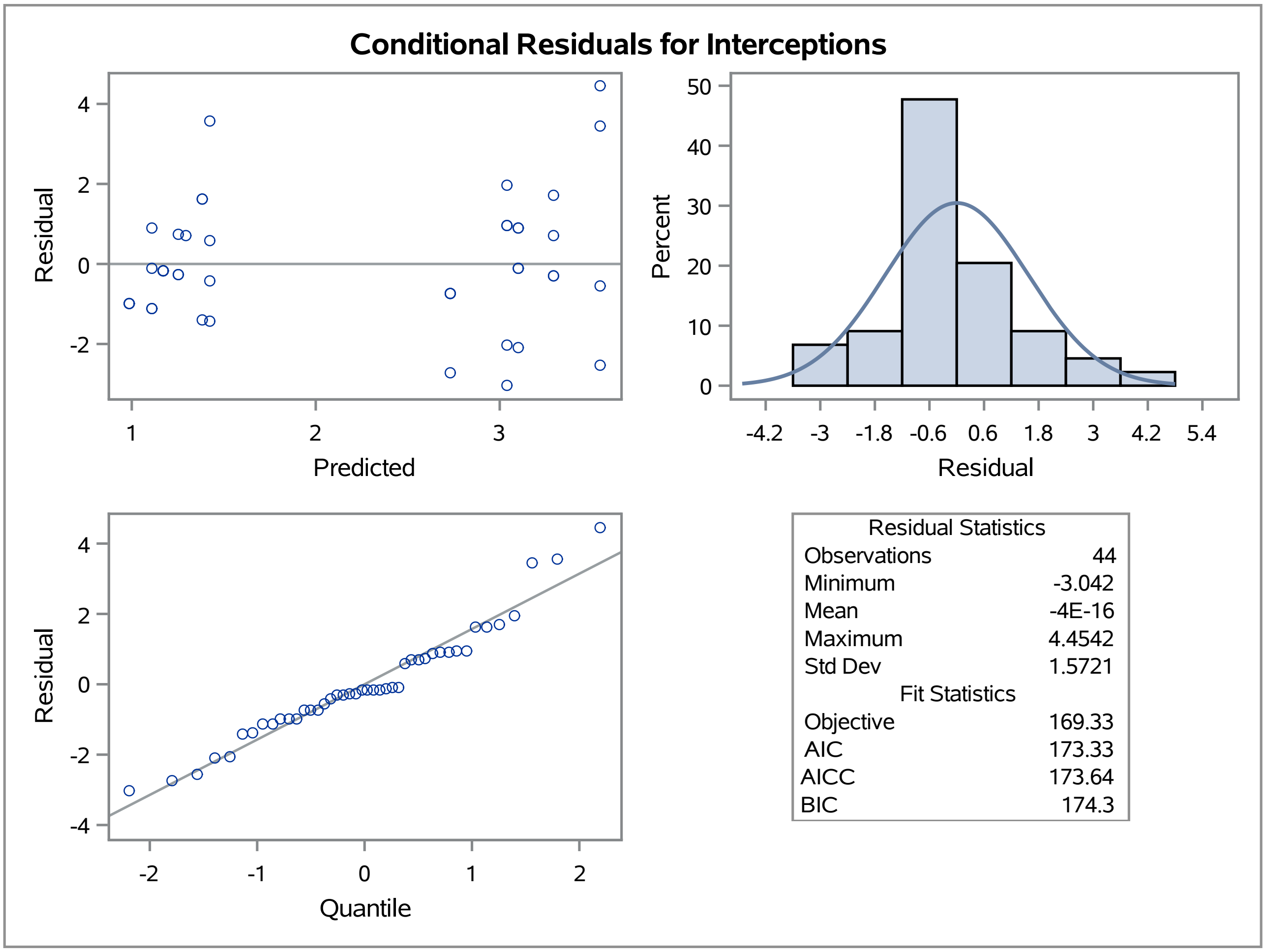

DistSoma=234

Model Information	
**Data Set**	WORK.TEMPDATASORTED
**Dependent Variable**	Interceptions
**Covariance Structure**	Variance Components
**Estimation Method**	REML
**Residual Variance Method**	Profile
**Fixed Effects SE Method**	Model-Based
**Degrees of Freedom Method**	Containment

Class Level Information		
**Class**	**Levels**	**Values**
**Treatment**	2	Control_GFP GFP MsTTR
**Culture**	12	1 2 3 4 5 6 7 8 9 10 11 12

Dimensions	
**Covariance Parameters**	2
**Columns in X**	3
**Columns in Z**	12
**Subjects**	1
**Max Obs per Subject**	44

Number of Observations	
**Number of Observations Read**	44
**Number of Observations Used**	44
**Number of Observations Not Used**	0

Iteration History			
**Iteration**	**Evaluations**	**-2 Res Log Like**	**Criterion**
**0**	1	168.18944431	
**1**	2	167.93710796	0.00000000

Convergence criteria met.

Covariance Parameter Estimates				
**Cov Parm**	**Estimate**	**Alpha**	**Lower**	**Upper**
**Culture**	0.1998	0.05	0.02675	3866589
**Residual**	2.6003	0.05	1.6870	4.5256

DistSoma=234

Fit Statistics	
-2 Res Log Likelihood	167.9
AIC (Smaller is Better)	171.9
AICC (Smaller is Better)	172.2
BIC (Smaller is Better)	172.9

Solution for Fixed Effects

Effect	Treatment	Estimate	Standard Error	DF	t Value	Pr > |t|	Alpha	Lower	Upper
Intercept		3.1399	0.3981	10	7.89	<.0001	0.05	2.2528	4.0270
Treatment	Control_GFP	-2.0427	0.5548	32	-3.68	0.0008	0.05	-3.1727	-0.9127
Treatment	GFP MsTTR	0	.	.	.	.	.	.	.

Solution for Random Effects

Effect	Culture	Estimate	Std Err Pred	DF	t Value	Pr > |t|	Alpha	Lower	Upper
Culture	1	-0.01821	0.4094	32	-0.04	0.9648	0.05	-0.8521	0.8157
Culture	2	0.1691	0.4094	32	0.41	0.6823	0.05	-0.6648	1.0030
Culture	3	-0.02285	0.4013	32	-0.06	0.9549	0.05	-0.8403	0.7946
Culture	4	-0.1404	0.4013	32	-0.35	0.7288	0.05	-0.9578	0.6771
Culture	5	0.1534	0.4013	32	0.38	0.7047	0.05	-0.6640	0.9709
Culture	6	0.06441	0.4316	32	0.15	0.8823	0.05	-0.8147	0.9435
Culture	7	-0.2055	0.4094	32	-0.50	0.6191	0.05	-1.0394	0.6284
Culture	8	0.01668	0.3956	32	0.04	0.9666	0.05	-0.7892	0.8226
Culture	9	0.3785	0.4020	32	0.94	0.3535	0.05	-0.4403	1.1972
Culture	10	-0.3855	0.4020	32	-0.96	0.3447	0.05	-1.2042	0.4333
Culture	11	0.08465	0.4020	32	0.21	0.8345	0.05	-0.7341	0.9034
Culture	12	-0.09433	0.3956	32	-0.24	0.8131	0.05	-0.9002	0.7116

Type 3 Tests of Fixed Effects

Effect	Num DF	Den DF	F Value	Pr > F
Treatment	1	32	13.56	0.0008

Least Squares Means

Effect	Treatment	Estimate	Standard Error	DF	t Value	Pr > |t|	Alpha	Lower	Upper
Treatment	Control_GFP	1.0972	0.3863	32	2.84	0.0078	0.05	0.3103	1.8841
Treatment	GFP MsTTR	3.1399	0.3981	32	7.89	<.0001	0.05	2.3289	3.9509

DistSoma=234

Differences of Least Squares Means												
**Effect**	**Treatment**	**Treatment**	**Estimate**	**Standard Error**	**DF**	**t Value**	**Pr > |t|**	**Adjustment**	**Adj P**	**Alpha**	**Lower**	**Upper**
**Treatment**	Control_GFP	GFP MsTTR	-2.0427	0.5548	32	-3.68	0.0008	Tukey-Kramer	0.0008	0.05	-3.1727	-0.9127

Differences of Least Squares Means				
**Effect**	**Treatment**	**Treatment**	**Adj Lower**	**Adj Upper**
**Treatment**	Control_GFP	GFP MsTTR	-3.1727	-0.9127

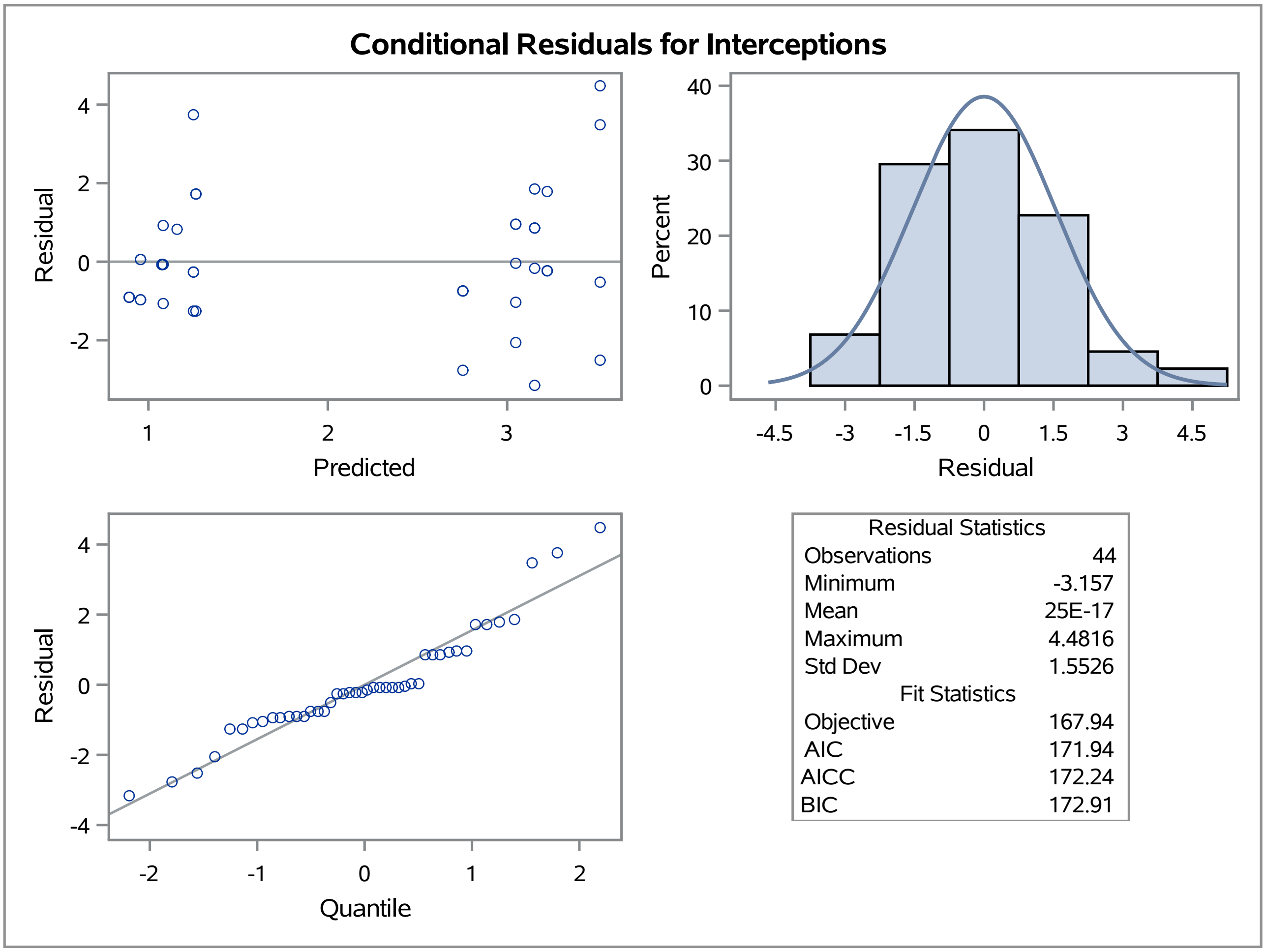

DistSoma=240

Model Information	
**Data Set**	WORK.TEMPDATASORTED
**Dependent Variable**	Interceptions
**Covariance Structure**	Variance Components
**Estimation Method**	REML
**Residual Variance Method**	Profile
**Fixed Effects SE Method**	Model-Based
**Degrees of Freedom Method**	Containment

Class Level Information		
**Class**	**Levels**	**Values**
**Treatment**	2	Control_GFP GFP MsTTR
**Culture**	12	1 2 3 4 5 6 7 8 9 10 11 12

Dimensions	
**Covariance Parameters**	2
**Columns in X**	3
**Columns in Z**	12
**Subjects**	1
**Max Obs per Subject**	44

Number of Observations	
**Number of Observations Read**	44
**Number of Observations Used**	44
**Number of Observations Not Used**	0

Iteration History			
**Iteration**	**Evaluations**	**-2 Res Log Like**	**Criterion**
**0**	1	175.01605933	
**1**	2	174.50859972	0.00000000

Convergence criteria met.

Covariance Parameter Estimates				
**Cov Parm**	**Estimate**	**Alpha**	**Lower**	**Upper**
**Culture**	0.3302	0.05	0.05793	2883.85
**Residual**	2.9759	0.05	1.9342	5.1641

DistSoma=240

Fit Statistics	
-2 Res Log Likelihood	174.5
AIC (Smaller is Better)	178.5
AICC (Smaller is Better)	178.8
BIC (Smaller is Better)	179.5

Solution for Fixed Effects

Effect	Treatment	Estimate	Standard Error	DF	t Value	Pr > |t|	Alpha	Lower	Upper
Intercept		2.9597	0.4493	10	6.59	<.0001	0.05	1.9587	3.9608
Treatment	Control_GFP	-1.9540	0.6231	32	-3.14	0.0037	0.05	-3.2232	-0.6847
Treatment	GFP MsTTR	0	.	.	.	.	.	.	.

Solution for Random Effects

Effect	Culture	Estimate	Std Err Pred	DF	t Value	Pr > |t|	Alpha	Lower	Upper
Culture	1	-0.00144	0.5093	32	-0.00	0.9978	0.05	-1.0388	1.0359
Culture	2	0.08180	0.5093	32	0.16	0.8734	0.05	-0.9555	1.1191
Culture	3	-0.00178	0.4963	32	-0.00	0.9972	0.05	-1.0127	1.0091
Culture	4	-0.1555	0.4963	32	-0.31	0.7561	0.05	-1.1664	0.8554
Culture	5	0.2288	0.4963	32	0.46	0.6480	0.05	-0.7821	1.2397
Culture	6	0.09929	0.5469	32	0.18	0.8571	0.05	-1.0146	1.2132
Culture	7	-0.2512	0.5093	32	-0.49	0.6252	0.05	-1.2885	0.7862
Culture	8	-0.05699	0.4879	32	-0.12	0.9077	0.05	-1.0509	0.9369
Culture	9	0.6272	0.4978	32	1.26	0.2168	0.05	-0.3867	1.6411
Culture	10	-0.6024	0.4978	32	-1.21	0.2351	0.05	-1.6163	0.4115
Culture	11	0.08923	0.4978	32	0.18	0.8589	0.05	-0.9247	1.1031
Culture	12	-0.05699	0.4879	32	-0.12	0.9077	0.05	-1.0509	0.9369

Type 3 Tests of Fixed Effects

Effect	Num DF	Den DF	F Value	Pr > F
Treatment	1	32	9.83	0.0037

Least Squares Means

Effect	Treatment	Estimate	Standard Error	DF	t Value	Pr > |t|	Alpha	Lower	Upper
Treatment	Control_GFP	1.0058	0.4318	32	2.33	0.0263	0.05	0.1263	1.8852
Treatment	GFP MsTTR	2.9597	0.4493	32	6.59	<.0001	0.05	2.0446	3.8749

DistSoma=240

Differences of Least Squares Means												
**Effect**	**Treatment**	**Treatment**	**Estimate**	**Standard Error**	**DF**	**t Value**	**Pr > |t|**	**Adjustment**	**Adj P**	**Alpha**	**Lower**	**Upper**
**Treatment**	Control_GFP	GFP MsTTR	-1.9540	0.6231	32	-3.14	0.0037	Tukey-Kramer	0.0037	0.05	-3.2232	-0.6847

Differences of Least Squares Means				
**Effect**	**Treatment**	**Treatment**	**Adj Lower**	**Adj Upper**
**Treatment**	Control_GFP	GFP MsTTR	-3.2232	-0.6847

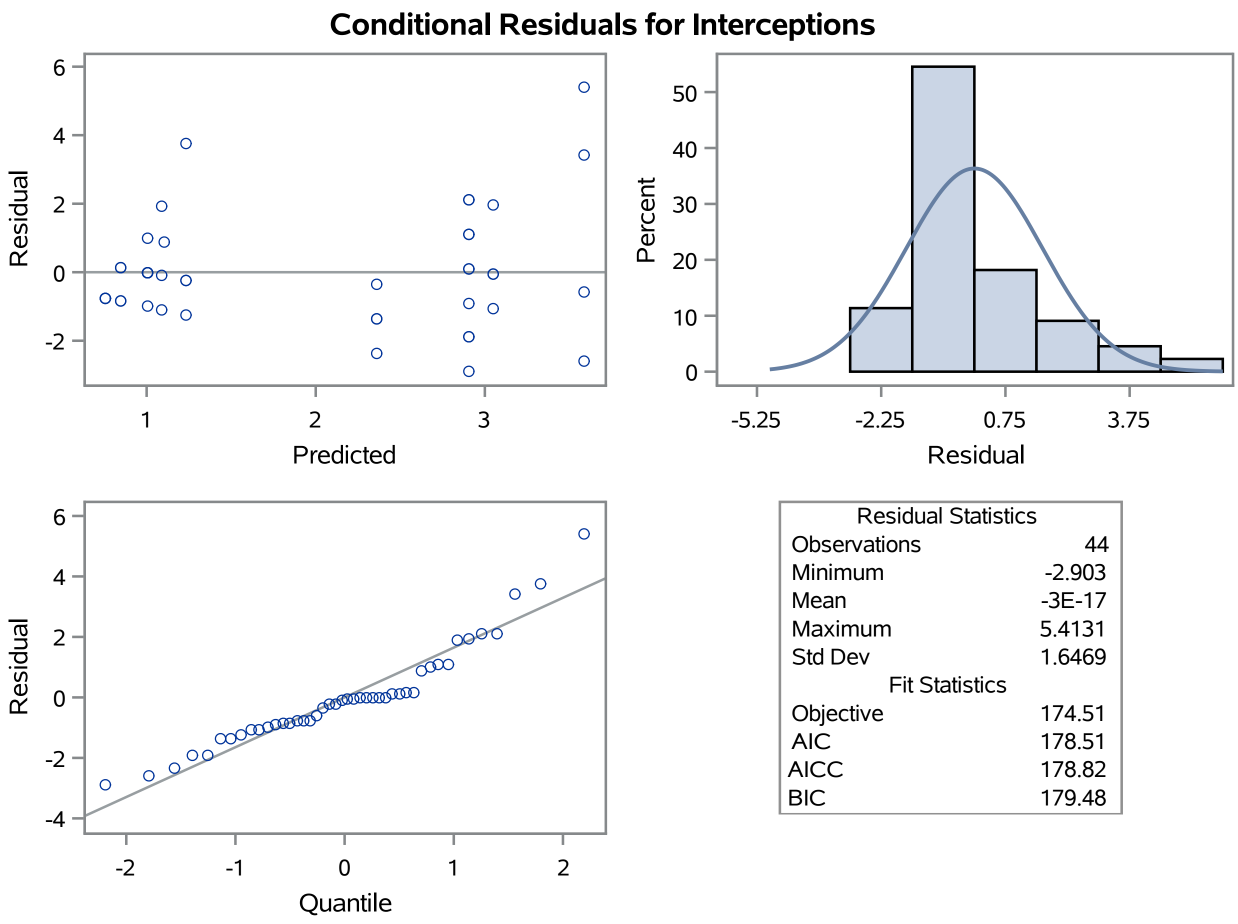

Residual Statistics	
Observations	44
Minimum	-2.903
Mean	-3E-17
Maximum	5.4131
Std Dev	1.6469
**Fit Statistics**	
Objective	174.51
AIC	178.51
AICC	178.82
BIC	179.48

DistSoma=246

Model Information	
**Data Set**	WORK.TEMPDATASORTED
**Dependent Variable**	Interceptions
**Covariance Structure**	Variance Components
**Estimation Method**	REML
**Residual Variance Method**	Profile
**Fixed Effects SE Method**	Model-Based
**Degrees of Freedom Method**	Containment

Class Level Information		
**Class**	**Levels**	**Values**
**Treatment**	2	Control_GFP GFP MsTTR
**Culture**	12	1 2 3 4 5 6 7 8 9 10 11 12

Dimensions	
**Covariance Parameters**	2
**Columns in X**	3
**Columns in Z**	12
**Subjects**	1
**Max Obs per Subject**	44

Number of Observations	
**Number of Observations Read**	44
**Number of Observations Used**	44
**Number of Observations Not Used**	0

Iteration History			
**Iteration**	**Evaluations**	**-2 Res Log Like**	**Criterion**
**0**	1	174.49710132	
**1**	2	174.30171970	0.00000000

Convergence criteria met.

Covariance Parameter Estimates				
**Cov Parm**	**Estimate**	**Alpha**	**Lower**	**Upper**
**Culture**	0.1988	0.05	0.02423	3.2532E8
**Residual**	3.0496	0.05	1.9819	5.2930

DistSoma=246

Fit Statistics	
**-2 Res Log Likelihood**	174.3
**AIC (Smaller is Better)**	178.3
**AICC (Smaller is Better)**	178.6
**BIC (Smaller is Better)**	179.3

Solution for Fixed Effects

Effect	Treatment	Estimate	Standard Error	DF	t Value	Pr > |t|	Alpha	Lower	Upper
**Intercept**		2.8209	0.4228	10	6.67	<.0001	0.05	1.8788	3.7630
**Treatment**	Control_GFP	-1.8176	0.5902	32	-3.08	0.0042	0.05	-3.0198	-0.6154
**Treatment**	GFP MsTTR	0	.	.	.	.	.	.	.

Solution for Random Effects

Effect	Culture	Estimate	Std Err Pred	DF	t Value	Pr > |t|	Alpha	Lower	Upper
**Culture**	1	-0.00054	0.4133	32	-0.00	0.9990	0.05	-0.8424	0.8414
**Culture**	2	0.05399	0.4133	32	0.13	0.8969	0.05	-0.7879	0.8959
**Culture**	3	-0.00068	0.4061	32	-0.00	0.9987	0.05	-0.8280	0.8266
**Culture**	4	-0.1041	0.4061	32	-0.26	0.7994	0.05	-0.9314	0.7232
**Culture**	5	0.1544	0.4061	32	0.38	0.7062	0.05	-0.6728	0.9817
**Culture**	6	0.06100	0.4328	32	0.14	0.8888	0.05	-0.8205	0.9425
**Culture**	7	-0.1641	0.4133	32	-0.40	0.6939	0.05	-1.0060	0.6778
**Culture**	8	-0.00513	0.4009	32	-0.01	0.9899	0.05	-0.8218	0.8115
**Culture**	9	0.3990	0.4066	32	0.98	0.3338	0.05	-0.4293	1.2273
**Culture**	10	-0.3766	0.4066	32	-0.93	0.3613	0.05	-1.2049	0.4516
**Culture**	11	0.03705	0.4066	32	0.09	0.9280	0.05	-0.7912	0.8653
**Culture**	12	-0.05430	0.4009	32	-0.14	0.8931	0.05	-0.8710	0.7624

Type 3 Tests of Fixed Effects

Effect	Num DF	Den DF	F Value	Pr > F
**Treatment**	1	32	9.48	0.0042

Least Squares Means

Effect	Treatment	Estimate	Standard Error	DF	t Value	Pr > |t|	Alpha	Lower	Upper
**Treatment**	Control_GFP	1.0033	0.4118	32	2.44	0.0206	0.05	0.1645	1.8421
**Treatment**	GFP MsTTR	2.8209	0.4228	32	6.67	<.0001	0.05	1.9597	3.6821

DistSoma=246

Differences of Least Squares Means												
**Effect**	**Treatment**	**Treatment**	**Estimate**	**Standard Error**	**DF**	**t Value**	**Pr > |t|**	**Adjustment**	**Adj P**	**Alpha**	**Lower**	**Upper**
**Treatment**	Control_GFP	GFP MsTTR	-1.8176	0.5902	32	-3.08	0.0042	Tukey-Kramer	0.0042	0.05	-3.0198	-0.6154

Differences of Least Squares Means				
**Effect**	**Treatment**	**Treatment**	**Adj Lower**	**Adj Upper**
**Treatment**	Control_GFP	GFP MsTTR	-3.0198	-0.6154

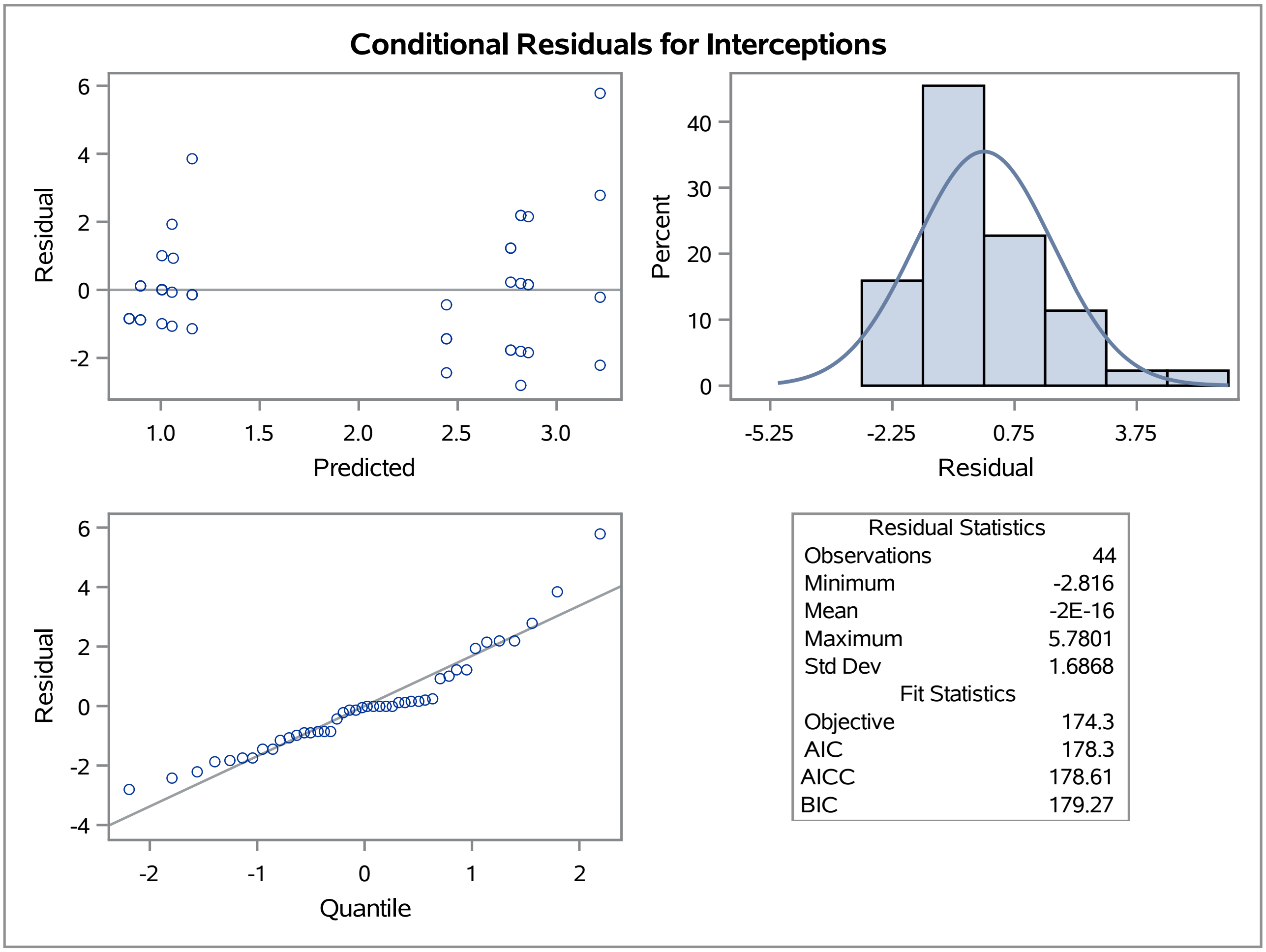

DistSoma=252

Model Information	
Data Set	WORK.TEMPDATASORTED
Dependent Variable	Interceptions
Covariance Structure	Variance Components
Estimation Method	REML
Residual Variance Method	Profile
Fixed Effects SE Method	Model-Based
Degrees of Freedom Method	Containment

Class Level Information		
Class	Levels	Values
Treatment	2	Control_GFP GFP MsTTR
Culture	12	1 2 3 4 5 6 7 8 9 10 11 12

Dimensions	
Covariance Parameters	2
Columns in X	3
Columns in Z	12
Subjects	1
Max Obs per Subject	44

Number of Observations	
Number of Observations Read	44
Number of Observations Used	44
Number of Observations Not Used	0

Iteration History			
Iteration	Evaluations	-2 Res Log Like	Criterion
0	1	173.35261633	
1	2	173.04370421	0.00000000

Convergence criteria met.

Covariance Parameter Estimates				
Cov Parm	Estimate	Alpha	Lower	Upper
Culture	0.2433	0.05	0.03514	303606
Residual	2.9242	0.05	1.9011	5.0723

DistSoma=252

Fit Statistics	
**-2 Res Log Likelihood**	173.0
**AIC (Smaller is Better)**	177.0
**AICC (Smaller is Better)**	177.4
**BIC (Smaller is Better)**	178.0

Solution for Fixed Effects

Effect	Treatment	Estimate	Standard Error	DF	t Value	Pr > |t|	Alpha	Lower	Upper
**Intercept**		2.8132	0.4266	10	6.59	<.0001	0.05	1.8626	3.7638
**Treatment**	Control_GFP	-2.0892	0.5939	32	-3.52	0.0013	0.05	-3.2990	-0.8795
**Treatment**	GFP MsTTR	0	.	.	.	.	.	.	.

Solution for Random Effects

Effect	Culture	Estimate	Std Err Pred	DF	t Value	Pr > |t|	Alpha	Lower	Upper
**Culture**	1	-0.1446	0.4489	32	-0.32	0.7494	0.05	-1.0591	0.7698
**Culture**	2	-0.07804	0.4489	32	-0.17	0.8631	0.05	-0.9925	0.8364
**Culture**	3	0.06894	0.4396	32	0.16	0.8764	0.05	-0.8264	0.9643
**Culture**	4	-0.05593	0.4396	32	-0.13	0.8996	0.05	-0.9513	0.8394
**Culture**	5	0.2562	0.4396	32	0.58	0.5640	0.05	-0.6391	1.1516
**Culture**	6	0.09803	0.4750	32	0.21	0.8378	0.05	-0.8696	1.0656
**Culture**	7	-0.1446	0.4489	32	-0.32	0.7494	0.05	-1.0591	0.7698
**Culture**	8	0.1724	0.4331	32	0.40	0.6932	0.05	-0.7097	1.0546
**Culture**	9	0.4213	0.4404	32	0.96	0.3459	0.05	-0.4757	1.3183
**Culture**	10	-0.4528	0.4404	32	-1.03	0.3115	0.05	-1.3498	0.4442
**Culture**	11	-0.07822	0.4404	32	-0.18	0.8601	0.05	-0.9752	0.8188
**Culture**	12	-0.06264	0.4331	32	-0.14	0.8859	0.05	-0.9448	0.8195

Type 3 Tests of Fixed Effects

Effect	Num DF	Den DF	F Value	Pr > F
**Treatment**	1	32	12.37	0.0013

Least Squares Means

Effect	Treatment	Estimate	Standard Error	DF	t Value	Pr > |t|	Alpha	Lower	Upper
**Treatment**	Control_GFP	0.7239	0.4132	32	1.75	0.0893	0.05	-0.1177	1.5656
**Treatment**	GFP MsTTR	2.8132	0.4266	32	6.59	<.0001	0.05	1.9441	3.6822

DistSoma=252

Differences of Least Squares Means												
**Effect**	**Treatment**	**Treatment**	**Estimate**	**Standard Error**	**DF**	**t Value**	**Pr > |t|**	**Adjustment**	**Adj P**	**Alpha**	**Lower**	**Upper**
**Treatment**	Control_GFP	GFP MsTTR	-2.0892	0.5939	32	-3.52	0.0013	Tukey-Kramer	0.0013	0.05	-3.2990	-0.8795

Differences of Least Squares Means				
**Effect**	**Treatment**	**Treatment**	**Adj Lower**	**Adj Upper**
**Treatment**	Control_GFP	GFP MsTTR	-3.2990	-0.8795

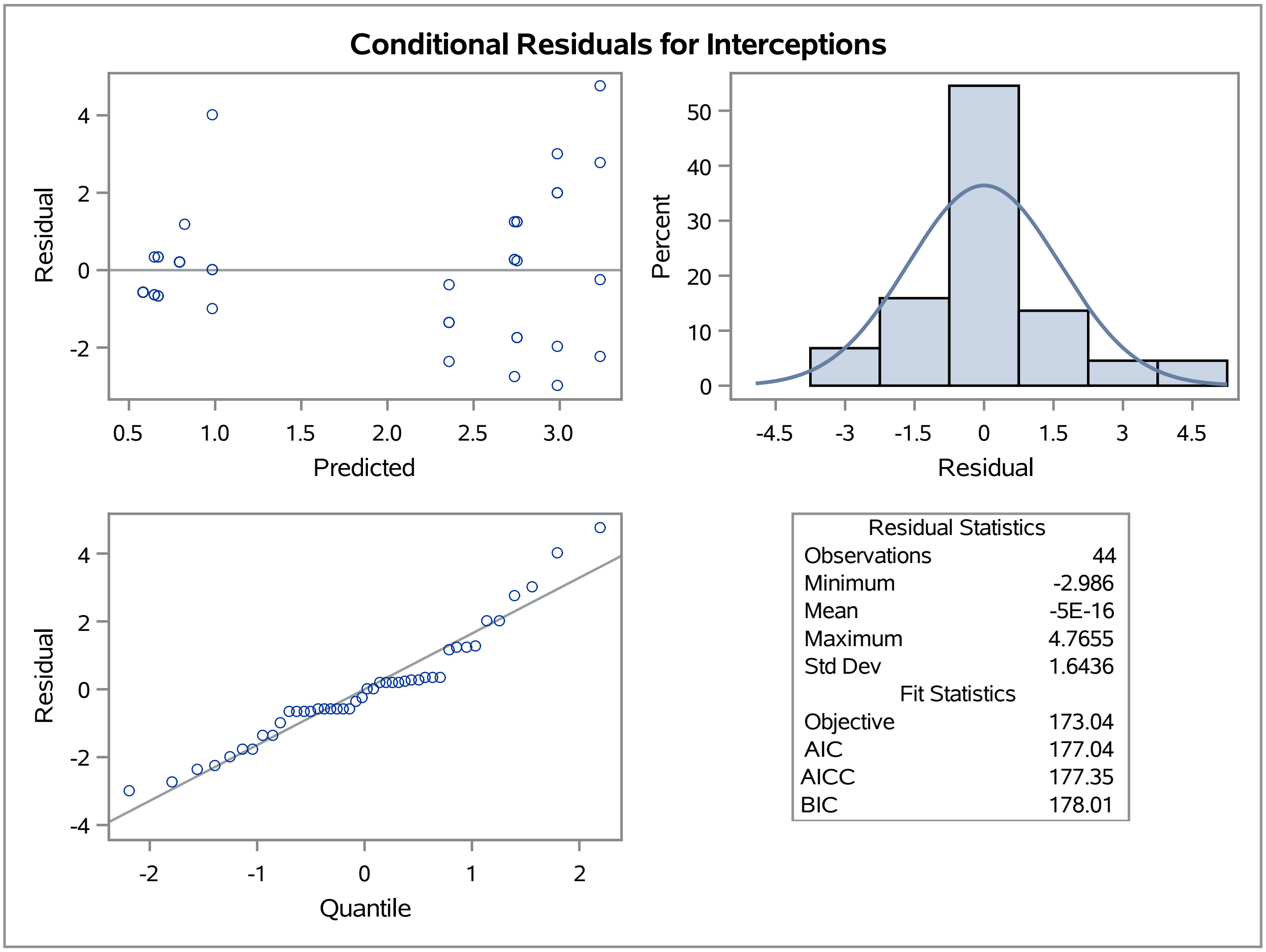

DistSoma=258

Model Information	
**Data Set**	WORK.TEMPDATASORTED
**Dependent Variable**	Interceptions
**Covariance Structure**	Variance Components
**Estimation Method**	REML
**Residual Variance Method**	Profile
**Fixed Effects SE Method**	Model-Based
**Degrees of Freedom Method**	Containment

Class Level Information		
**Class**	**Levels**	**Values**
**Treatment**	2	Control_GFP GFP MsTTR
**Culture**	12	1 2 3 4 5 6 7 8 9 10 11 12

Dimensions	
**Covariance Parameters**	2
**Columns in X**	3
**Columns in Z**	12
**Subjects**	1
**Max Obs per Subject**	44

Number of Observations	
**Number of Observations Read**	44
**Number of Observations Used**	44
**Number of Observations Not Used**	0

Iteration History			
**Iteration**	**Evaluations**	**-2 Res Log Like**	**Criterion**
**0**	1	173.82850125	
**1**	2	172.64553365	0.00000000

Convergence criteria met.

Covariance Parameter Estimates				
**Cov Parm**	**Estimate**	**Alpha**	**Lower**	**Upper**
**Culture**	0.5052	0.05	0.1209	57.1062
**Residual**	2.7334	0.05	1.7754	4.7486

DistSoma=258

Fit Statistics	
-2 Res Log Likelihood	172.6
AIC (Smaller is Better)	176.6
AICC (Smaller is Better)	177.0
BIC (Smaller is Better)	177.6

Solution for Fixed Effects

Effect	Treatment	Estimate	Standard Error	DF	t Value	Pr > |t|	Alpha	Lower	Upper
Intercept		2.7285	0.4754	10	5.74	0.0002	0.05	1.6693	3.7877
Treatment	Control_GFP	-2.0464	0.6542	32	-3.13	0.0037	0.05	-3.3790	-0.7139
Treatment	GFP MsTTR	0	.	.	.	.	.	.	.

Solution for Random Effects

Effect	Culture	Estimate	Std Err Pred	DF	t Value	Pr > |t|	Alpha	Lower	Upper
Culture	1	-0.2433	0.5922	32	-0.41	0.6839	0.05	-1.4496	0.9630
Culture	2	-0.1244	0.5922	32	-0.21	0.8350	0.05	-1.3307	1.0819
Culture	3	0.1351	0.5718	32	0.24	0.8147	0.05	-1.0296	1.2999
Culture	4	-0.07740	0.5718	32	-0.14	0.8932	0.05	-1.2422	1.0874
Culture	5	0.3477	0.5718	32	0.61	0.5475	0.05	-0.8171	1.5124
Culture	6	0.2056	0.6568	32	0.31	0.7563	0.05	-1.1322	1.5434
Culture	7	-0.2433	0.5922	32	-0.41	0.6839	0.05	-1.4496	0.9630
Culture	8	0.03433	0.5610	32	0.06	0.9516	0.05	-1.1084	1.1770
Culture	9	0.9655	0.5756	32	1.68	0.1032	0.05	-0.2069	2.1380
Culture	10	-0.8410	0.5756	32	-1.46	0.1537	0.05	-2.0135	0.3314
Culture	11	-0.09714	0.5756	32	-0.17	0.8670	0.05	-1.2696	1.0753
Culture	12	-0.06173	0.5610	32	-0.11	0.9131	0.05	-1.2044	1.0810

Type 3 Tests of Fixed Effects

Effect	Num DF	Den DF	F Value	Pr > F
Treatment	1	32	9.79	0.0037

Least Squares Means

Effect	Treatment	Estimate	Standard Error	DF	t Value	Pr > |t|	Alpha	Lower	Upper
Treatment	Control_GFP	0.6821	0.4494	32	1.52	0.1389	0.05	-0.2334	1.5975
Treatment	GFP MsTTR	2.7285	0.4754	32	5.74	<.0001	0.05	1.7602	3.6968

DistSoma=258

Differences of Least Squares Means												
Effect	Treatment	Treatment	Estimate	Standard Error	DF	t Value	Pr > |t|	Adjustment	Adj P	Alpha	Lower	Upper
**Treatment**	Control_GFP	GFP MsTTR	-2.0464	0.6542	32	-3.13	0.0037	Tukey-Kramer	0.0037	0.05	-3.3790	-0.7139

Differences of Least Squares Means				
Effect	Treatment	Treatment	Adj Lower	Adj Upper
**Treatment**	Control_GFP	GFP MsTTR	-3.3790	-0.7139

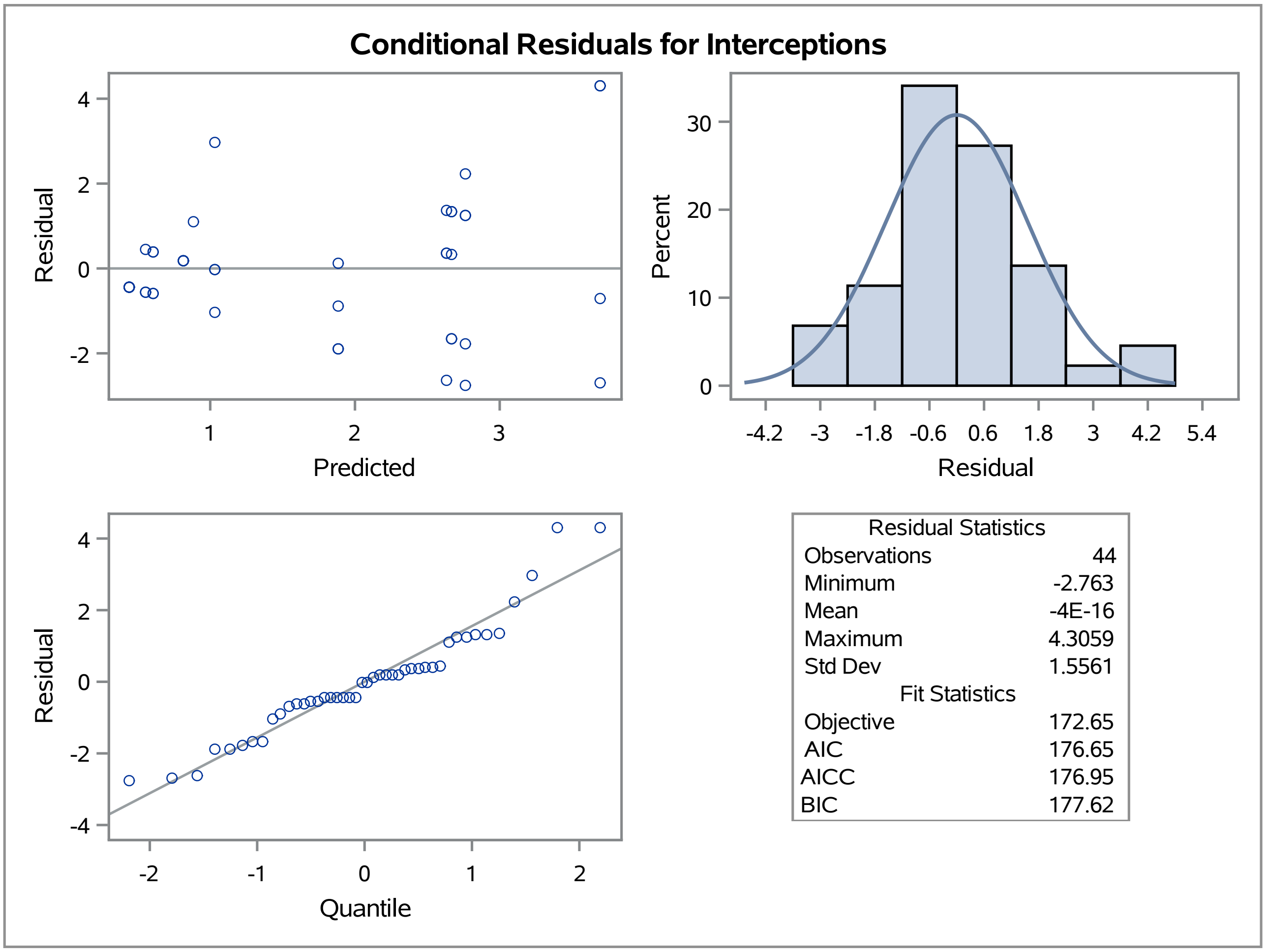

DistSoma=264

Model Information	
**Data Set**	WORK.TEMPDATASORTED
**Dependent Variable**	Interceptions
**Covariance Structure**	Variance Components
**Estimation Method**	REML
**Residual Variance Method**	Profile
**Fixed Effects SE Method**	Model-Based
**Degrees of Freedom Method**	Containment

Class Level Information		
**Class**	**Levels**	**Values**
**Treatment**	2	Control_GFP GFP MsTTR
**Culture**	12	1 2 3 4 5 6 7 8 9 10 11 12

Dimensions	
**Covariance Parameters**	2
**Columns in X**	3
**Columns in Z**	12
**Subjects**	1
**Max Obs per Subject**	44

Number of Observations	
**Number of Observations Read**	44
**Number of Observations Used**	44
**Number of Observations Not Used**	0

Iteration History			
**Iteration**	**Evaluations**	**-2 Res Log Like**	**Criterion**
**0**	1	173.54073034	
**1**	2	171.61593012	0.00000000

Convergence criteria met.

Covariance Parameter Estimates				
**Cov Parm**	**Estimate**	**Alpha**	**Lower**	**Upper**
**Culture**	0.6504	0.05	0.1822	20.3862
**Residual**	2.5848	0.05	1.6786	4.4917

DistSoma=264

Fit Statistics	
-2 Res Log Likelihood	171.6
AIC (Smaller is Better)	175.6
AICC (Smaller is Better)	175.9
BIC (Smaller is Better)	176.6

Solution for Fixed Effects

Effect	Treatment	Estimate	Standard Error	DF	t Value	Pr > |t|	Alpha	Lower	Upper
Intercept		2.5425	0.4983	10	5.10	0.0005	0.05	1.4322	3.6529
Treatment	Control_GFP	-1.8583	0.6822	32	-2.72	0.0104	0.05	-3.2479	-0.4687
Treatment	GFP MsTTR	0	.	.	.	.	.	.	.

Solution for Random Effects

Effect	Culture	Estimate	Std Err Pred	DF	t Value	Pr > |t|	Alpha	Lower	Upper
Culture	1	-0.2944	0.6409	32	-0.46	0.6492	0.05	-1.5999	1.0112
Culture	2	-0.1510	0.6409	32	-0.24	0.8153	0.05	-1.4565	1.1546
Culture	3	0.1584	0.6155	32	0.26	0.7986	0.05	-1.0953	1.4120
Culture	4	-0.09243	0.6155	32	-0.15	0.8816	0.05	-1.3461	1.1612
Culture	5	0.4092	0.6155	32	0.66	0.5109	0.05	-0.8444	1.6628
Culture	6	0.2645	0.7269	32	0.36	0.7183	0.05	-1.2162	1.7453
Culture	7	-0.2944	0.6409	32	-0.46	0.6492	0.05	-1.5999	1.0112
Culture	8	0.03201	0.6043	32	0.05	0.9581	0.05	-1.1988	1.2628
Culture	9	1.1073	0.6218	32	1.78	0.0844	0.05	-0.1593	2.3739
Culture	10	-1.1500	0.6218	32	-1.85	0.0736	0.05	-2.4166	0.1166
Culture	11	-0.02134	0.6218	32	-0.03	0.9728	0.05	-1.2879	1.2452
Culture	12	0.03201	0.6043	32	0.05	0.9581	0.05	-1.1988	1.2628

Type 3 Tests of Fixed Effects

Effect	Num DF	Den DF	F Value	Pr > F
Treatment	1	32	7.42	0.0104

Least Squares Means

Effect	Treatment	Estimate	Standard Error	DF	t Value	Pr > |t|	Alpha	Lower	Upper
Treatment	Control_GFP	0.6843	0.4659	32	1.47	0.1517	0.05	-0.2648	1.6333
Treatment	GFP MsTTR	2.5425	0.4983	32	5.10	<.0001	0.05	1.5275	3.5576

DistSoma=264

Differences of Least Squares Means												
**Effect**	**Treatment**	**Treatment**	**Estimate**	**Standard Error**	**DF**	**t Value**	**Pr > |t|**	**Adjustment**	**Adj P**	**Alpha**	**Lower**	**Upper**
**Treatment**	Control_GFP	GFP MsTTR	-1.8583	0.6822	32	-2.72	0.0104	Tukey-Kramer	0.0104	0.05	-3.2479	-0.4687

Differences of Least Squares Means				
**Effect**	**Treatment**	**Treatment**	**Adj Lower**	**Adj Upper**
**Treatment**	Control_GFP	GFP MsTTR	-3.2479	-0.4687

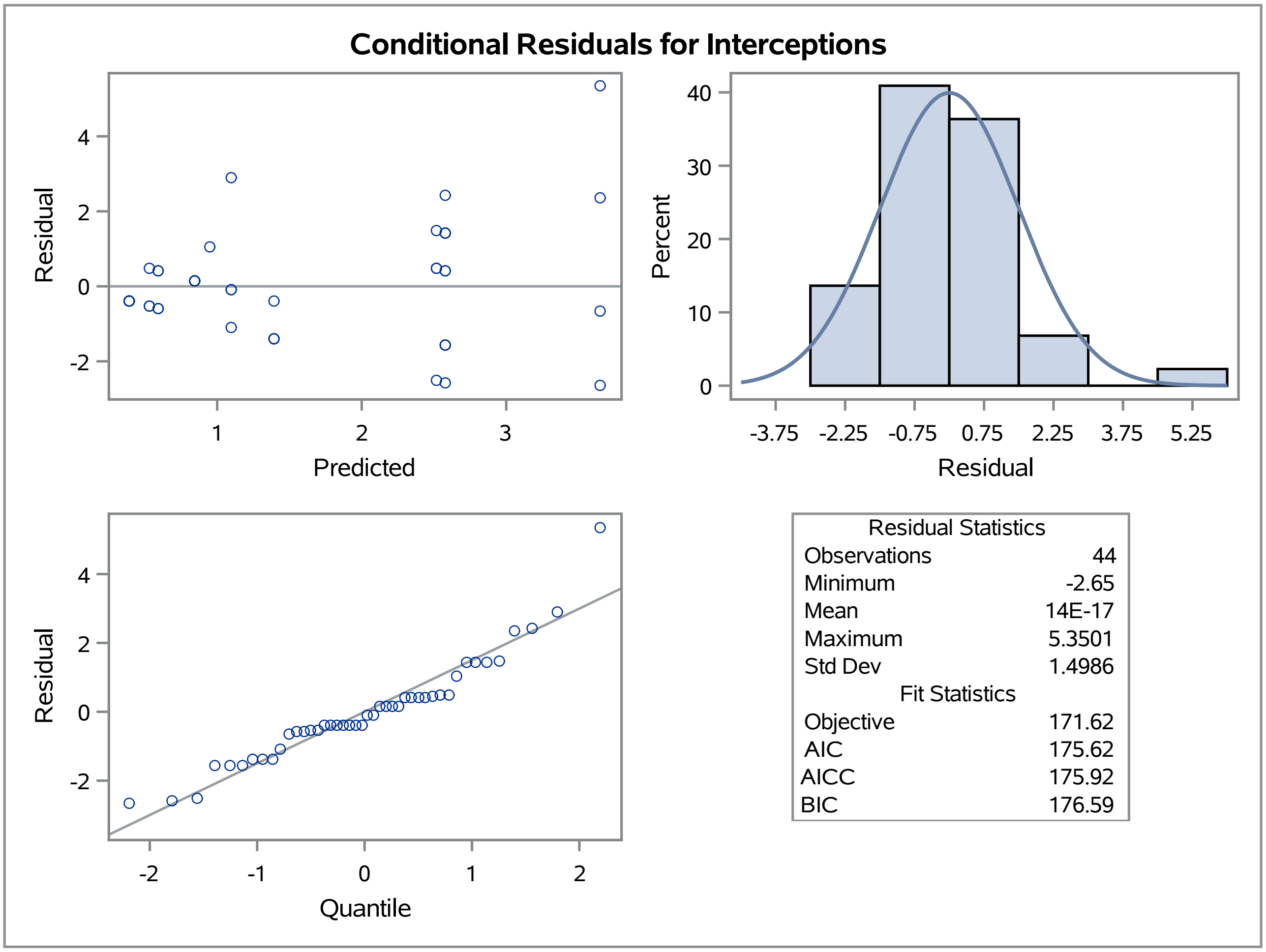

DistSoma=270

Model Information	
**Data Set**	WORK.TEMPDATASORTED
**Dependent Variable**	Interceptions
**Covariance Structure**	Variance Components
**Estimation Method**	REML
**Residual Variance Method**	Profile
**Fixed Effects SE Method**	Model-Based
**Degrees of Freedom Method**	Containment

Class Level Information		
**Class**	**Levels**	**Values**
**Treatment**	2	Control_GFP GFP MsTTR
**Culture**	12	1 2 3 4 5 6 7 8 9 10 11 12

Dimensions	
**Covariance Parameters**	2
**Columns in X**	3
**Columns in Z**	12
**Subjects**	1
**Max Obs per Subject**	44

Number of Observations	
**Number of Observations Read**	44
**Number of Observations Used**	44
**Number of Observations Not Used**	0

Iteration History			
**Iteration**	**Evaluations**	**-2 Res Log Like**	**Criterion**
**0**	1	171.59032715	
**1**	2	170.93932955	0.00000000

Convergence criteria met.

Covariance Parameter Estimates				
**Cov Parm**	**Estimate**	**Alpha**	**Lower**	**Upper**
**Culture**	0.3460	0.05	0.06660	586.01
**Residual**	2.7064	0.05	1.7588	4.6972

DistSoma=270

Fit Statistics	
-2 Res Log Likelihood	170.9
AIC (Smaller is Better)	174.9
AICC (Smaller is Better)	175.2
BIC (Smaller is Better)	175.9

Solution for Fixed Effects

Effect	Treatment	Estimate	Standard Error	DF	t Value	Pr > |t|	Alpha	Lower	Upper
Intercept		2.4455	0.4390	10	5.57	0.0002	0.05	1.4673	3.4238
Treatment	Control_GFP	-1.7648	0.6077	32	-2.90	0.0066	0.05	-3.0027	-0.5270
Treatment	GFP MsTTR	0	.	.	.	.	.	.	.

Solution for Random Effects

Effect	Culture	Estimate	Std Err Pred	DF	t Value	Pr > |t|	Alpha	Lower	Upper
Culture	1	-0.1887	0.5135	32	-0.37	0.7157	0.05	-1.2346	0.8572
Culture	2	-0.09629	0.5135	32	-0.19	0.8524	0.05	-1.1422	0.9496
Culture	3	0.1080	0.4991	32	0.22	0.8300	0.05	-0.9087	1.1248
Culture	4	-0.06113	0.4991	32	-0.12	0.9033	0.05	-1.0778	0.9556
Culture	5	0.2772	0.4991	32	0.56	0.5825	0.05	-0.7395	1.2939
Culture	6	0.1495	0.5559	32	0.27	0.7896	0.05	-0.9828	1.2819
Culture	7	-0.1887	0.5135	32	-0.37	0.7157	0.05	-1.2346	0.8572
Culture	8	0.2162	0.4903	32	0.44	0.6622	0.05	-0.7825	1.2149
Culture	9	0.5260	0.5010	32	1.05	0.3017	0.05	-0.4945	1.5465
Culture	10	-0.7429	0.5010	32	-1.48	0.1479	0.05	-1.7634	0.2776
Culture	11	0.01843	0.5010	32	0.04	0.9709	0.05	-1.0021	1.0389
Culture	12	-0.01775	0.4903	32	-0.04	0.9713	0.05	-1.0164	0.9809

Type 3 Tests of Fixed Effects

Effect	Num DF	Den DF	F Value	Pr > F
Treatment	1	32	8.43	0.0066

Least Squares Means

Effect	Treatment	Estimate	Standard Error	DF	t Value	Pr > |t|	Alpha	Lower	Upper
Treatment	Control_GFP	0.6807	0.4201	32	1.62	0.1150	0.05	-0.1751	1.5365
Treatment	GFP MsTTR	2.4455	0.4390	32	5.57	<.0001	0.05	1.5512	3.3398

DistSoma=270

Differences of Least Squares Means												
**Effect**	**Treatment**	**Treatment**	**Estimate**	**Standard Error**	**DF**	**t Value**	**Pr > |t|**	**Adjustment**	**Adj P**	**Alpha**	**Lower**	**Upper**
**Treatment**	Control_GFP	GFP MsTTR	-1.7648	0.6077	32	-2.90	0.0066	Tukey-Kramer	0.0066	0.05	-3.0027	-0.5270

Differences of Least Squares Means				
**Effect**	**Treatment**	**Treatment**	**Adj Lower**	**Adj Upper**
**Treatment**	Control_GFP	GFP MsTTR	-3.0026	-0.5270

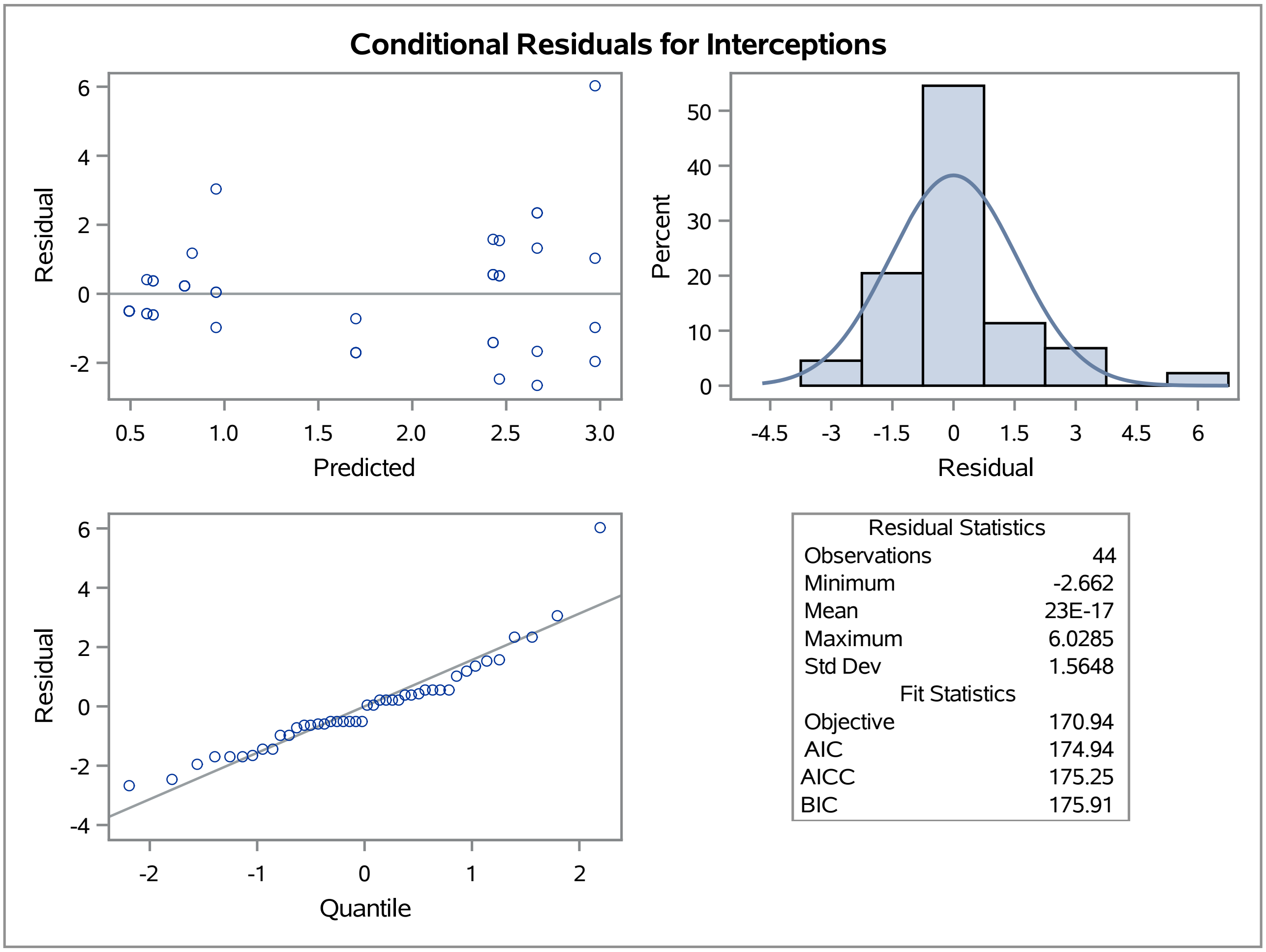

DistSoma=276

Model Information	
**Data Set**	WORK.TEMPDATASORTED
**Dependent Variable**	Interceptions
**Covariance Structure**	Variance Components
**Estimation Method**	REML
**Residual Variance Method**	Profile
**Fixed Effects SE Method**	Model-Based
**Degrees of Freedom Method**	Containment

Class Level Information		
**Class**	**Levels**	**Values**
**Treatment**	2	Control_GFP GFP MsTTR
**Culture**	12	1 2 3 4 5 6 7 8 9 10 11 12

Dimensions	
**Covariance Parameters**	2
**Columns in X**	3
**Columns in Z**	12
**Subjects**	1
**Max Obs per Subject**	44

Number of Observations	
**Number of Observations Read**	44
**Number of Observations Used**	44
**Number of Observations Not Used**	0

Iteration History			
**Iteration**	**Evaluations**	**-2 Res Log Like**	**Criterion**
**0**	1	172.85660501	
**1**	2	172.13375464	0.00000000

Convergence criteria met.

Covariance Parameter Estimates				
**Cov Parm**	**Estimate**	**Alpha**	**Lower**	**Upper**
**Culture**	0.3807	0.05	0.07634	348.68
**Residual**	2.7692	0.05	1.7987	4.8105

DistSoma=276

Fit Statistics	
**-2 Res Log Likelihood**	172.1
**AIC (Smaller is Better)**	176.1
**AICC (Smaller is Better)**	176.4
**BIC (Smaller is Better)**	177.1

Solution for Fixed Effects

Effect	Treatment	Estimate	Standard Error	DF	t Value	Pr > |t|	Alpha	Lower	Upper
**Intercept**		2.4056	0.4501	10	5.34	0.0003	0.05	1.4027	3.4085
**Treatment**	Control_GFP	-1.7247	0.6223	32	-2.77	0.0092	0.05	-2.9924	-0.4571
**Treatment**	GFP MsTTR	0	.	.	.	.	.	.	.

Solution for Random Effects

Effect	Culture	Estimate	Std Err Pred	DF	t Value	Pr > |t|	Alpha	Lower	Upper
**Culture**	1	-0.1988	0.5341	32	-0.37	0.7122	0.05	-1.2868	0.8892
**Culture**	2	-0.1015	0.5341	32	-0.19	0.8505	0.05	-1.1895	0.9865
**Culture**	3	0.1132	0.5186	32	0.22	0.8285	0.05	-0.9430	1.1695
**Culture**	4	-0.06418	0.5186	32	-0.12	0.9023	0.05	-1.1204	0.9921
**Culture**	5	0.2906	0.5186	32	0.56	0.5790	0.05	-0.7656	1.3469
**Culture**	6	0.1594	0.5809	32	0.27	0.7855	0.05	-1.0238	1.3426
**Culture**	7	-0.1988	0.5341	32	-0.37	0.7122	0.05	-1.2868	0.8892
**Culture**	8	0.07920	0.5092	32	0.16	0.8774	0.05	-0.9579	1.1163
**Culture**	9	0.6544	0.5207	32	1.26	0.2179	0.05	-0.4062	1.7151
**Culture**	10	-0.7648	0.5207	32	-1.47	0.1516	0.05	-1.8255	0.2958
**Culture**	11	0.03350	0.5207	32	0.06	0.9491	0.05	-1.0272	1.0942
**Culture**	12	-0.00228	0.5092	32	-0.00	0.9965	0.05	-1.0394	1.0349

Type 3 Tests of Fixed Effects

Effect	Num DF	Den DF	F Value	Pr > F
**Treatment**	1	32	7.68	0.0092

Least Squares Means

Effect	Treatment	Estimate	Standard Error	DF	t Value	Pr > |t|	Alpha	Lower	Upper
**Treatment**	Control_GFP	0.6809	0.4298	32	1.58	0.1230	0.05	-0.1945	1.5563
**Treatment**	GFP MsTTR	2.4056	0.4501	32	5.34	<.0001	0.05	1.4887	3.3224

DistSoma=276

Differences of Least Squares Means												
**Effect**	**Treatment**	**Treatment**	**Estimate**	**Standard Error**	**DF**	**t Value**	**Pr > |t|**	**Adjustment**	**Adj P**	**Alpha**	**Lower**	**Upper**
**Treatment**	Control_GFP	GFP MsTTR	-1.7247	0.6223	32	-2.77	0.0092	Tukey-Kramer	0.0092	0.05	-2.9924	-0.4571

Differences of Least Squares Means				
**Effect**	**Treatment**	**Treatment**	**Adj Lower**	**Adj Upper**
**Treatment**	Control_GFP	GFP MsTTR	-2.9924	-0.4571

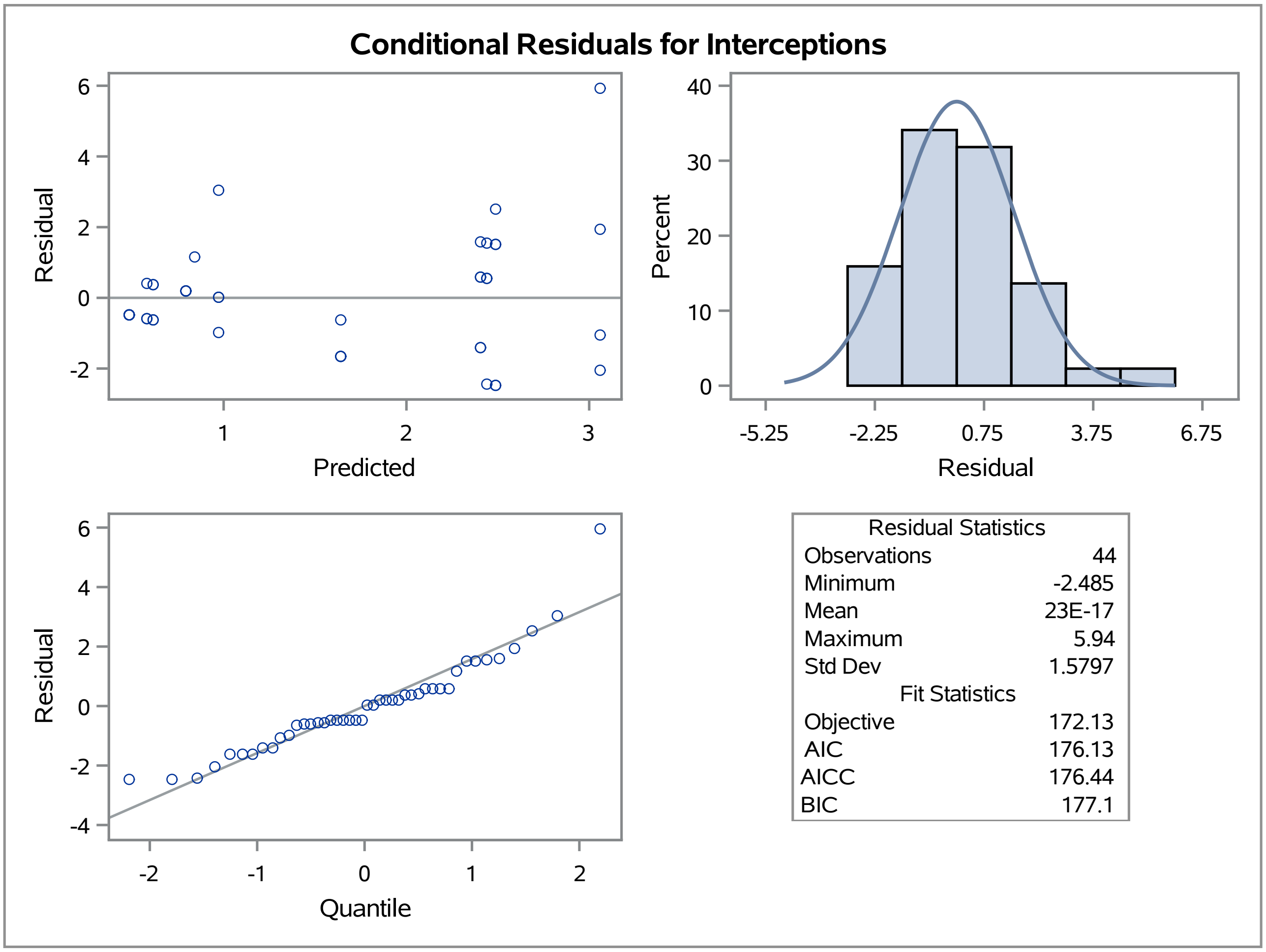

DistSoma=282

Model Information	
**Data Set**	WORK.TEMPDATASORTED
**Dependent Variable**	Interceptions
**Covariance Structure**	Variance Components
**Estimation Method**	REML
**Residual Variance Method**	Profile
**Fixed Effects SE Method**	Model-Based
**Degrees of Freedom Method**	Containment

Class Level Information		
**Class**	**Levels**	**Values**
**Treatment**	2	Control_GFP GFP MsTTR
**Culture**	12	1 2 3 4 5 6 7 8 9 10 11 12

Dimensions	
**Covariance Parameters**	2
**Columns in X**	3
**Columns in Z**	12
**Subjects**	1
**Max Obs per Subject**	44

Number of Observations	
**Number of Observations Read**	44
**Number of Observations Used**	44
**Number of Observations Not Used**	0

Iteration History			
**Iteration**	**Evaluations**	**-2 Res Log Like**	**Criterion**
**0**	1	166.51658280	
**1**	2	164.14114449	0.00000045
**2**	1	164.14112491	0.00000000

Convergence criteria met.

Covariance Parameter Estimates				
**Cov Parm**	**Estimate**	**Alpha**	**Lower**	**Upper**
**Culture**	0.6036	0.05	0.1805	12.4468
**Residual**	2.1349	0.05	1.3883	3.7016

DistSoma=282

Fit Statistics	
-2 Res Log Likelihood	164.1
AIC (Smaller is Better)	168.1
AICC (Smaller is Better)	168.4
BIC (Smaller is Better)	169.1

Solution for Fixed Effects

Effect	Treatment	Estimate	Standard Error	DF	t Value	Pr > |t|	Alpha	Lower	Upper
Intercept		2.2768	0.4673	10	4.87	0.0006	0.05	1.2355	3.3181
Treatment	Control_GFP	-1.7917	0.6385	32	-2.81	0.0085	0.05	-3.0923	-0.4911
Treatment	GFP MsTTR	0	.	.	.	.	.	.	.

Solution for Random Effects

Effect	Culture	Estimate	Std Err Pred	DF	t Value	Pr > |t|	Alpha	Lower	Upper
Culture	1	-0.2226	0.6053	32	-0.37	0.7155	0.05	-1.4557	1.0104
Culture	2	-0.2226	0.6053	32	-0.37	0.7155	0.05	-1.4557	1.0104
Culture	3	0.2733	0.5801	32	0.47	0.6408	0.05	-0.9084	1.4549
Culture	4	-0.1248	0.5801	32	-0.22	0.8311	0.05	-1.3065	1.0569
Culture	5	0.4059	0.5801	32	0.70	0.4892	0.05	-0.7758	1.5876
Culture	6	0.1135	0.6926	32	0.16	0.8709	0.05	-1.2974	1.5243
Culture	7	-0.2226	0.6053	32	-0.37	0.7155	0.05	-1.4557	1.0104
Culture	8	-0.04499	0.5701	32	-0.08	0.9376	0.05	-1.2062	1.1162
Culture	9	1.1798	0.5872	32	2.01	0.0530	0.05	-0.01618	2.3759
Culture	10	-1.0756	0.5872	32	-1.83	0.0763	0.05	-2.2717	0.1204
Culture	11	-0.01423	0.5872	32	-0.02	0.9808	0.05	-1.2103	1.1818
Culture	12	-0.04499	0.5701	32	-0.08	0.9376	0.05	-1.2062	1.1162

Type 3 Tests of Fixed Effects

Effect	Num DF	Den DF	F Value	Pr > F
Treatment	1	32	7.87	0.0085

Least Squares Means

Effect	Treatment	Estimate	Standard Error	DF	t Value	Pr > |t|	Alpha	Lower	Upper
Treatment	Control_GFP	0.4851	0.4350	32	1.12	0.2731	0.05	-0.4010	1.3712
Treatment	GFP MsTTR	2.2768	0.4673	32	4.87	<.0001	0.05	1.3249	3.2288

DistSoma=282

Differences of Least Squares Means												
**Effect**	**Treatment**	**Treatment**	**Estimate**	**Standard Error**	**DF**	**t Value**	**Pr > |t|**	**Adjustment**	**Adj P**	**Alpha**	**Lower**	**Upper**
**Treatment**	Control_GFP	GFP MsTTR	-1.7917	0.6385	32	-2.81	0.0085	Tukey-Kramer	0.0085	0.05	-3.0923	-0.4911

Differences of Least Squares Means				
**Effect**	**Treatment**	**Treatment**	**Adj Lower**	**Adj Upper**
**Treatment**	Control_GFP	GFP MsTTR	-3.0922	-0.4912

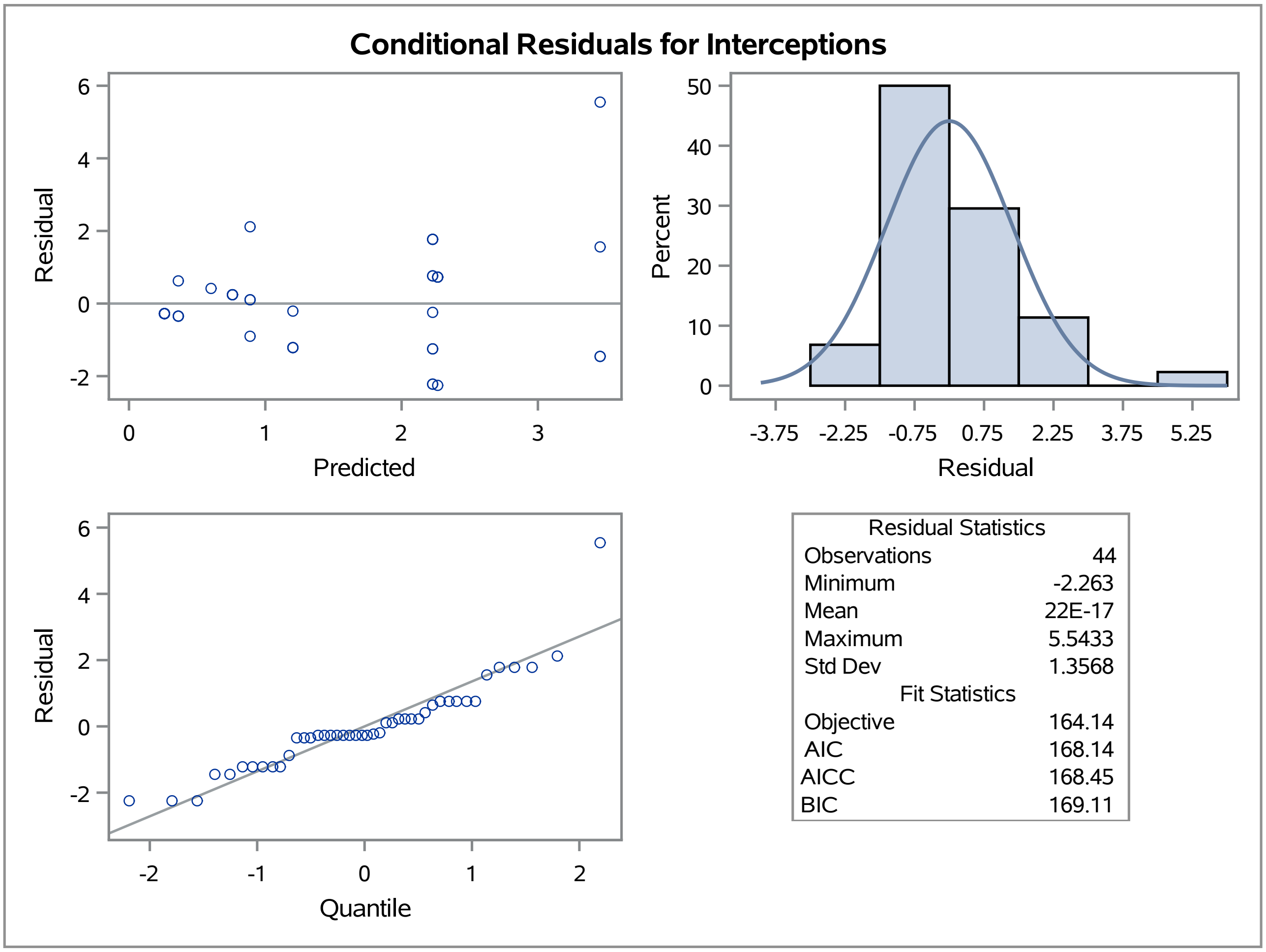

DistSoma=288

Model Information	
Data Set	WORK.TEMPDATASORTED
Dependent Variable	Interceptions
Covariance Structure	Variance Components
Estimation Method	REML
Residual Variance Method	Profile
Fixed Effects SE Method	Model-Based
Degrees of Freedom Method	Containment

Class Level Information		
Class	Levels	Values
Treatment	2	Control_GFP GFP MsTTR
Culture	12	1 2 3 4 5 6 7 8 9 10 11 12

Dimensions	
Covariance Parameters	2
Columns in X	3
Columns in Z	12
Subjects	1
Max Obs per Subject	44

Number of Observations	
Number of Observations Read	44
Number of Observations Used	44
Number of Observations Not Used	0

Iteration History			
Iteration	Evaluations	-2 Res Log Like	Criterion
0	1	166.31128708	
1	2	163.80746483	0.00000065
2	1	163.80743680	0.00000000

Convergence criteria met.

Covariance Parameter Estimates				
Cov Parm	Estimate	Alpha	Lower	Upper
Culture	0.6165	0.05	0.1872	11.6087
Residual	2.1098	0.05	1.3722	3.6572

DistSoma=288

Fit Statistics	
-2 Res Log Likelihood	163.8
AIC (Smaller is Better)	167.8
AICC (Smaller is Better)	168.1
BIC (Smaller is Better)	168.8

Solution for Fixed Effects									
Effect	Treatment	Estimate	Standard Error	DF	t Value	Pr > |t|	Alpha	Lower	Upper
Intercept		2.2288	0.4689	10	4.75	0.0008	0.05	1.1841	3.2736
Treatment	Control_GFP	-1.8135	0.6402	32	-2.83	0.0079	0.05	-3.1176	-0.5095
Treatment	GFP MsTTR	0	.	.	.	.	.	.	.

Solution for Random Effects									
Effect	Culture	Estimate	Std Err Pred	DF	t Value	Pr > |t|	Alpha	Lower	Upper
Culture	1	-0.1940	0.6082	32	-0.32	0.7519	0.05	-1.4329	1.0450
Culture	2	-0.1940	0.6082	32	-0.32	0.7519	0.05	-1.4329	1.0450
Culture	3	0.3151	0.5826	32	0.54	0.5924	0.05	-0.8716	1.5018
Culture	4	-0.08907	0.5826	32	-0.15	0.8794	0.05	-1.2758	1.0977
Culture	5	0.4498	0.5826	32	0.77	0.4457	0.05	-0.7369	1.6366
Culture	6	-0.09390	0.6977	32	-0.13	0.8938	0.05	-1.5151	1.3273
Culture	7	-0.1940	0.6082	32	-0.32	0.7519	0.05	-1.4329	1.0450
Culture	8	-0.01711	0.5727	32	-0.03	0.9763	0.05	-1.1836	1.1494
Culture	9	1.2239	0.5900	32	2.07	0.0462	0.05	0.02216	2.4257
Culture	10	-1.0664	0.5900	32	-1.81	0.0801	0.05	-2.2682	0.1354
Culture	11	-0.1233	0.5900	32	-0.21	0.8358	0.05	-1.3251	1.0785
Culture	12	-0.01711	0.5727	32	-0.03	0.9763	0.05	-1.1836	1.1494

Type 3 Tests of Fixed Effects				
Effect	Num DF	Den DF	F Value	Pr > F
Treatment	1	32	8.02	0.0079

Least Squares Means									
Effect	Treatment	Estimate	Standard Error	DF	t Value	Pr > |t|	Alpha	Lower	Upper
Treatment	Control_GFP	0.4153	0.4359	32	0.95	0.3479	0.05	-0.4726	1.3032
Treatment	GFP MsTTR	2.2288	0.4689	32	4.75	<.0001	0.05	1.2738	3.1839

DistSoma=288

Differences of Least Squares Means												
Effect	Treatment	Treatment	Estimate	Standard Error	DF	t Value	Pr > |t|	Adjustment	Adj P	Alpha	Lower	Upper
**Treatment**	Control_GFP	GFP MsTTR	-1.8135	0.6402	32	-2.83	0.0079	Tukey-Kramer	0.0079	0.05	-3.1176	-0.5095

Differences of Least Squares Means				
Effect	Treatment	Treatment	Adj Lower	Adj Upper
**Treatment**	Control_GFP	GFP MsTTR	-3.1176	-0.5095

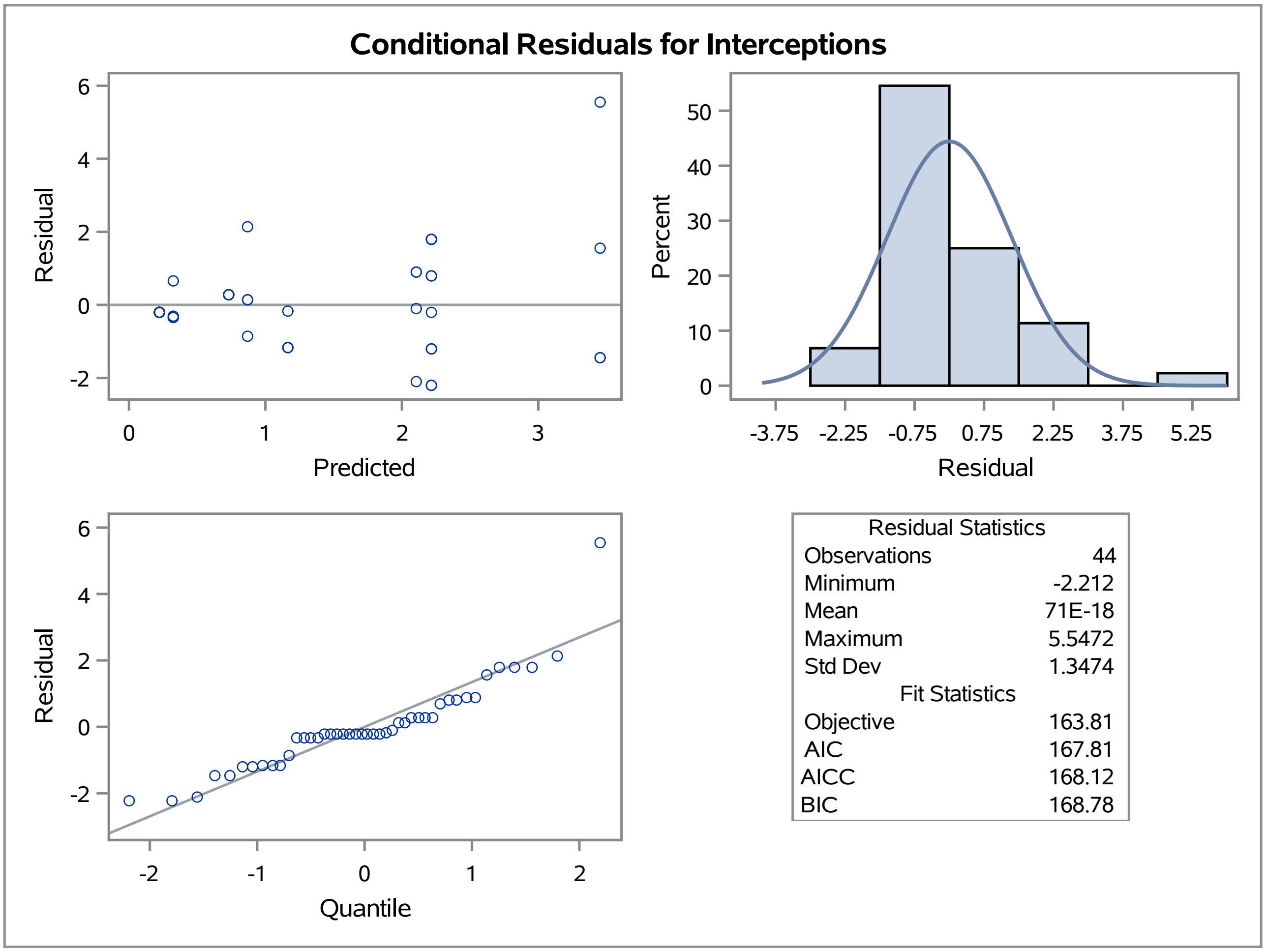

DistSoma=294

Model Information	
**Data Set**	WORK.TEMPDATASORTED
**Dependent Variable**	Interceptions
**Covariance Structure**	Variance Components
**Estimation Method**	REML
**Residual Variance Method**	Profile
**Fixed Effects SE Method**	Model-Based
**Degrees of Freedom Method**	Containment

Class Level Information		
**Class**	**Levels**	**Values**
**Treatment**	2	Control_GFP GFP MsTTR
**Culture**	12	1 2 3 4 5 6 7 8 9 10 11 12

Dimensions	
**Covariance Parameters**	2
**Columns in X**	3
**Columns in Z**	12
**Subjects**	1
**Max Obs per Subject**	44

Number of Observations	
**Number of Observations Read**	44
**Number of Observations Used**	44
**Number of Observations Not Used**	0

Iteration History			
**Iteration**	**Evaluations**	**-2 Res Log Like**	**Criterion**
**0**	1	158.43023971	
**1**	2	154.91230280	0.00000212
**2**	1	154.91221982	0.00000000

Convergence criteria met.

Covariance Parameter Estimates				
**Cov Parm**	**Estimate**	**Alpha**	**Lower**	**Upper**
**Culture**	0.6113	0.05	0.2030	7.1649
**Residual**	1.6579	0.05	1.0781	2.8746

DistSoma=294

Fit Statistics	
-2 Res Log Likelihood	154.9
AIC (Smaller is Better)	158.9
AICC (Smaller is Better)	159.2
BIC (Smaller is Better)	159.9

Solution for Fixed Effects

Effect	Treatment	Estimate	Standard Error	DF	t Value	Pr > |t|	Alpha	Lower	Upper
Intercept		2.0420	0.4452	10	4.59	0.0010	0.05	1.0501	3.0340
Treatment	Control_GFP	-1.6729	0.6054	32	-2.76	0.0094	0.05	-2.9060	-0.4399
Treatment	GFP MsTTR	0	.	.	.	.	.	.	.

Solution for Random Effects

Effect	Culture	Estimate	Std Err Pred	DF	t Value	Pr > |t|	Alpha	Lower	Upper
Culture	1	-0.1938	0.5802	32	-0.33	0.7405	0.05	-1.3757	0.9880
Culture	2	-0.1938	0.5802	32	-0.33	0.7405	0.05	-1.3757	0.9880
Culture	3	0.3760	0.5539	32	0.68	0.5021	0.05	-0.7522	1.5041
Culture	4	-0.07097	0.5539	32	-0.13	0.8988	0.05	-1.1991	1.0572
Culture	5	0.3760	0.5539	32	0.68	0.5021	0.05	-0.7522	1.5041
Culture	6	-0.09943	0.6774	32	-0.15	0.8842	0.05	-1.4792	1.2803
Culture	7	-0.1938	0.5802	32	-0.33	0.7405	0.05	-1.3757	0.9880
Culture	8	0.1024	0.5461	32	0.19	0.8524	0.05	-1.0100	1.2149
Culture	9	1.3158	0.5634	32	2.34	0.0259	0.05	0.1682	2.4633
Culture	10	-1.0679	0.5634	32	-1.90	0.0671	0.05	-2.2155	0.07962
Culture	11	-0.3230	0.5634	32	-0.57	0.5704	0.05	-1.4706	0.8245
Culture	12	-0.02725	0.5461	32	-0.05	0.9605	0.05	-1.1397	1.0852

Type 3 Tests of Fixed Effects

Effect	Num DF	Den DF	F Value	Pr > F
Treatment	1	32	7.64	0.0094

Least Squares Means

Effect	Treatment	Estimate	Standard Error	DF	t Value	Pr > |t|	Alpha	Lower	Upper
Treatment	Control_GFP	0.3691	0.4102	32	0.90	0.3750	0.05	-0.4665	1.2047
Treatment	GFP MsTTR	2.0420	0.4452	32	4.59	<.0001	0.05	1.1352	2.9488

DistSoma=294

Differences of Least Squares Means												
Effect	Treatment	Treatment	Estimate	Standard Error	DF	t Value	Pr > |t|	Adjustment	Adj P	Alpha	Lower	Upper
**Treatment**	Control_GFP	GFP MsTTR	-1.6729	0.6054	32	-2.76	0.0094	Tukey-Kramer	0.0094	0.05	-2.9060	-0.4399

Differences of Least Squares Means				
Effect	Treatment	Treatment	Adj Lower	Adj Upper
**Treatment**	Control_GFP	GFP MsTTR	-2.9060	-0.4399

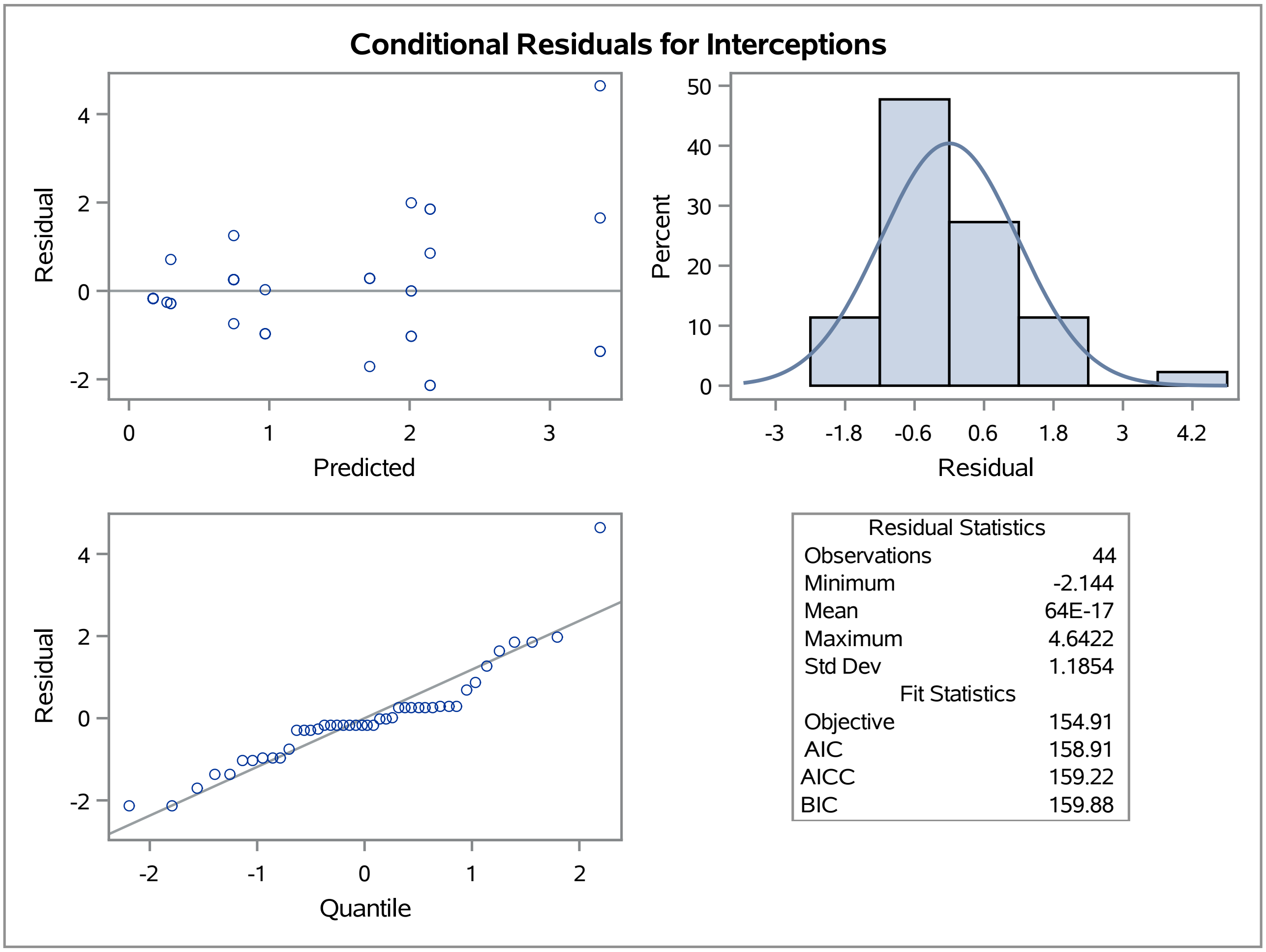

DistSoma=300

Model Information	
**Data Set**	WORK.TEMPDATASORTED
**Dependent Variable**	Interceptions
**Covariance Structure**	Variance Components
**Estimation Method**	REML
**Residual Variance Method**	Profile
**Fixed Effects SE Method**	Model-Based
**Degrees of Freedom Method**	Containment

Class Level Information		
**Class**	**Levels**	**Values**
**Treatment**	2	Control_GFP GFP MsTTR
**Culture**	12	1 2 3 4 5 6 7 8 9 10 11 12

Dimensions	
**Covariance Parameters**	2
**Columns in X**	3
**Columns in Z**	12
**Subjects**	1
**Max Obs per Subject**	44

Number of Observations	
**Number of Observations Read**	44
**Number of Observations Used**	44
**Number of Observations Not Used**	0

Iteration History			
**Iteration**	**Evaluations**	**-2 Res Log Like**	**Criterion**
**0**	1	152.29248995	
**1**	2	149.07691264	0.00000283
**2**	1	149.07681026	0.00000000

Convergence criteria met.

Covariance Parameter Estimates				
**Cov Parm**	**Estimate**	**Alpha**	**Lower**	**Upper**
**Culture**	0.5018	0.05	0.1629	6.5759
**Residual**	1.4556	0.05	0.9468	2.5228

DistSoma=300

Fit Statistics	
-2 Res Log Likelihood	149.1
AIC (Smaller is Better)	153.1
AICC (Smaller is Better)	153.4
BIC (Smaller is Better)	154.0

Solution for Fixed Effects

Effect	Treatment	Estimate	Standard Error	DF	t Value	Pr > |t|	Alpha	Lower	Upper
Intercept		1.9518	0.4087	10	4.78	0.0008	0.05	1.0412	2.8623
Treatment	Control_GFP	-1.6636	0.5564	32	-2.99	0.0053	0.05	-2.7969	-0.5304
Treatment	GFP MsTTR	0	.	.	.	.	.	.	.

Solution for Random Effects

Effect	Culture	Estimate	Std Err Pred	DF	t Value	Pr > |t|	Alpha	Lower	Upper
Culture	1	-0.1465	0.5325	32	-0.28	0.7850	0.05	-1.2311	0.9381
Culture	2	-0.1465	0.5325	32	-0.28	0.7850	0.05	-1.2311	0.9381
Culture	3	0.1228	0.5087	32	0.24	0.8108	0.05	-0.9135	1.1591
Culture	4	-0.02210	0.5087	32	-0.04	0.9656	0.05	-1.0584	1.0142
Culture	5	0.4126	0.5087	32	0.81	0.4233	0.05	-0.6236	1.4489
Culture	6	-0.07387	0.6185	32	-0.12	0.9057	0.05	-1.3337	1.1859
Culture	7	-0.1465	0.5325	32	-0.28	0.7850	0.05	-1.2311	0.9381
Culture	8	0.03052	0.5011	32	0.06	0.9518	0.05	-0.9902	1.0513
Culture	9	1.1872	0.5168	32	2.30	0.0283	0.05	0.1346	2.2399
Culture	10	-0.9864	0.5168	32	-1.91	0.0653	0.05	-2.0390	0.06618
Culture	11	-0.2619	0.5168	32	-0.51	0.6158	0.05	-1.3145	0.7907
Culture	12	0.03052	0.5011	32	0.06	0.9518	0.05	-0.9902	1.0513

Type 3 Tests of Fixed Effects

Effect	Num DF	Den DF	F Value	Pr > F
Treatment	1	32	8.94	0.0053

Least Squares Means

Effect	Treatment	Estimate	Standard Error	DF	t Value	Pr > |t|	Alpha	Lower	Upper
Treatment	Control_GFP	0.2881	0.3775	32	0.76	0.4509	0.05	-0.4809	1.0571
Treatment	GFP MsTTR	1.9518	0.4087	32	4.78	<.0001	0.05	1.1194	2.7842

DistSoma=300

Differences of Least Squares Means

Effect	Treatment	Treatment	Estimate	Standard Error	DF	t Value	Pr > |t|	Adjustment	Adj P	Alpha	Lower	Upper
**Treatment**	Control_GFP	GFP MsTTR	-1.6636	0.5564	32	-2.99	0.0053	Tukey-Kramer	0.0053	0.05	-2.7969	-0.5304

Differences of Least Squares Means

Effect	Treatment	Treatment	Adj Lower	Adj Upper
**Treatment**	Control_GFP	GFP MsTTR	-2.7969	-0.5304

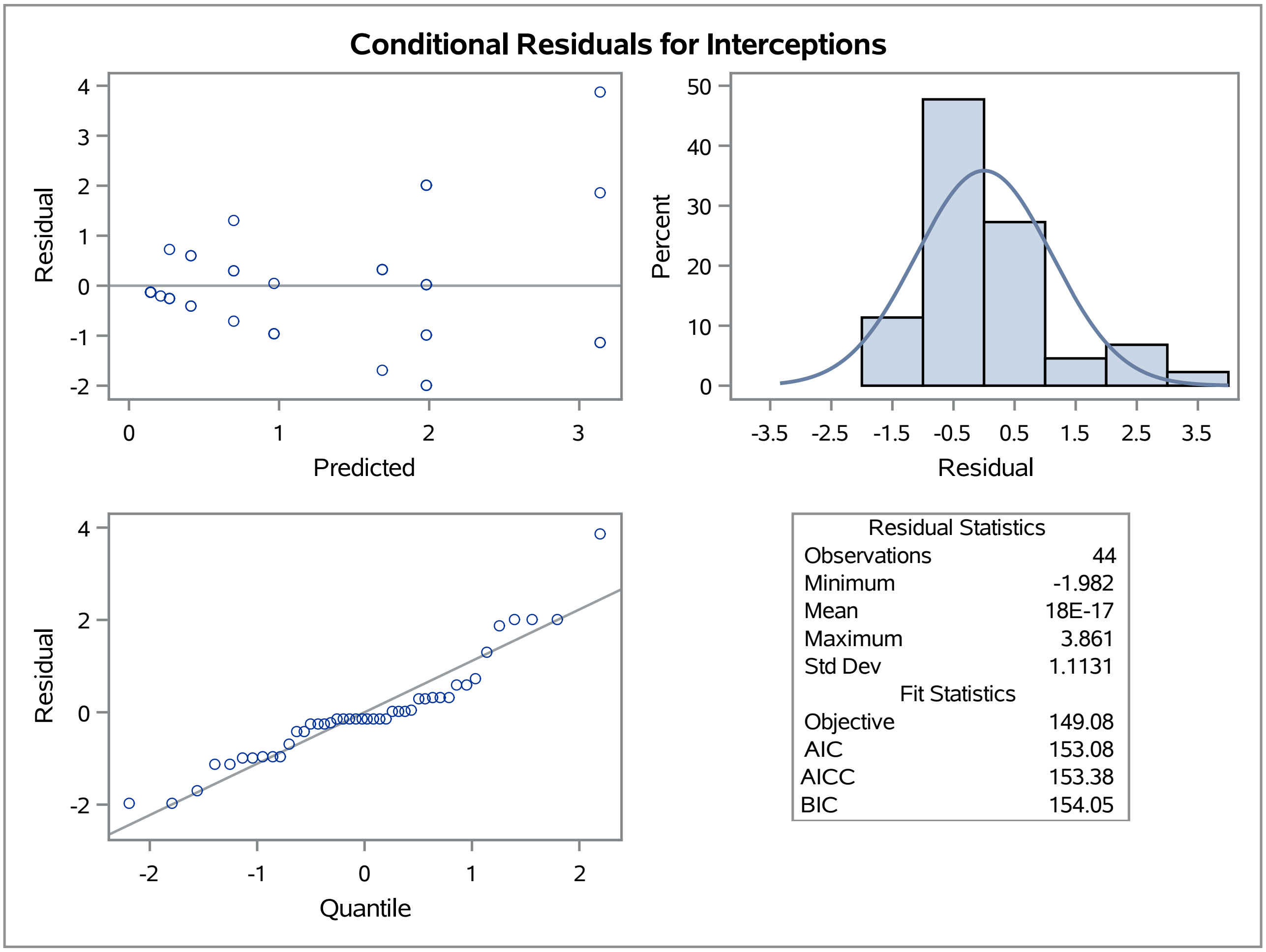

DistSoma=306

Model Information	
**Data Set**	WORK.TEMPDATASORTED
**Dependent Variable**	Interceptions
**Covariance Structure**	Variance Components
**Estimation Method**	REML
**Residual Variance Method**	Profile
**Fixed Effects SE Method**	Model-Based
**Degrees of Freedom Method**	Containment

Class Level Information		
**Class**	**Levels**	**Values**
**Treatment**	2	Control_GFP GFP MsTTR
**Culture**	12	1 2 3 4 5 6 7 8 9 10 11 12

Dimensions	
**Covariance Parameters**	2
**Columns in X**	3
**Columns in Z**	12
**Subjects**	1
**Max Obs per Subject**	44

Number of Observations	
**Number of Observations Read**	44
**Number of Observations Used**	44
**Number of Observations Not Used**	0

Iteration History			
**Iteration**	**Evaluations**	**-2 Res Log Like**	**Criterion**
**0**	1	152.53126262	
**1**	2	151.06216671	0.00000066
**2**	1	151.06214210	0.00000000

Convergence criteria met.

Covariance Parameter Estimates				
**Cov Parm**	**Estimate**	**Alpha**	**Lower**	**Upper**
**Culture**	0.3316	0.05	0.08562	19.0706
**Residual**	1.6190	0.05	1.0537	2.8034

DistSoma=306

Fit Statistics	
-2 Res Log Likelihood	151.1
AIC (Smaller is Better)	155.1
AICC (Smaller is Better)	155.4
BIC (Smaller is Better)	156.0

Solution for Fixed Effects

Effect	Treatment	Estimate	Standard Error	DF	t Value	Pr > |t|	Alpha	Lower	Upper
Intercept		1.9047	0.3746	10	5.08	0.0005	0.05	1.0700	2.7394
Treatment	Control_GFP	-1.6510	0.5146	32	-3.21	0.0030	0.05	-2.6993	-0.6027
Treatment	GFP MsTTR	0	.	.	.	.	.	.	.

Solution for Random Effects

Effect	Culture	Estimate	Std Err Pred	DF	t Value	Pr > |t|	Alpha	Lower	Upper
Culture	1	-0.09654	0.4727	32	-0.20	0.8395	0.05	-1.0593	0.8663
Culture	2	-0.09654	0.4727	32	-0.20	0.8395	0.05	-1.0593	0.8663
Culture	3	0.1109	0.4555	32	0.24	0.8092	0.05	-0.8170	1.0388
Culture	4	-0.00166	0.4555	32	-0.00	0.9971	0.05	-0.9296	0.9262
Culture	5	0.2235	0.4555	32	0.49	0.6271	0.05	-0.7044	1.1514
Culture	6	-0.04312	0.5280	32	-0.08	0.9354	0.05	-1.1187	1.0324
Culture	7	-0.09654	0.4727	32	-0.20	0.8395	0.05	-1.0593	0.8663
Culture	8	0.04821	0.4469	32	0.11	0.9148	0.05	-0.8621	0.9586
Culture	9	0.8309	0.4590	32	1.81	0.0797	0.05	-0.1041	1.7660
Culture	10	-0.7451	0.4590	32	-1.62	0.1144	0.05	-1.6801	0.1899
Culture	11	-0.1822	0.4590	32	-0.40	0.6940	0.05	-1.1173	0.7528
Culture	12	0.04821	0.4469	32	0.11	0.9148	0.05	-0.8621	0.9586

Type 3 Tests of Fixed Effects

Effect	Num DF	Den DF	F Value	Pr > F
Treatment	1	32	10.29	0.0030

Least Squares Means

Effect	Treatment	Estimate	Standard Error	DF	t Value	Pr > |t|	Alpha	Lower	Upper
Treatment	Control_GFP	0.2537	0.3529	32	0.72	0.4774	0.05	-0.4651	0.9725
Treatment	GFP MsTTR	1.9047	0.3746	32	5.08	<.0001	0.05	1.1417	2.6678

DistSoma=306

Differences of Least Squares Means												
**Effect**	**Treatment**	**Treatment**	**Estimate**	**Standard Error**	**DF**	**t Value**	**Pr > |t|**	**Adjustment**	**Adj P**	**Alpha**	**Lower**	**Upper**
**Treatment**	Control_GFP	GFP MsTTR	-1.6510	0.5146	32	-3.21	0.0030	Tukey-Kramer	0.0030	0.05	-2.6993	-0.6027

Differences of Least Squares Means				
**Effect**	**Treatment**	**Treatment**	**Adj Lower**	**Adj Upper**
**Treatment**	Control_GFP	GFP MsTTR	-2.6993	-0.6027

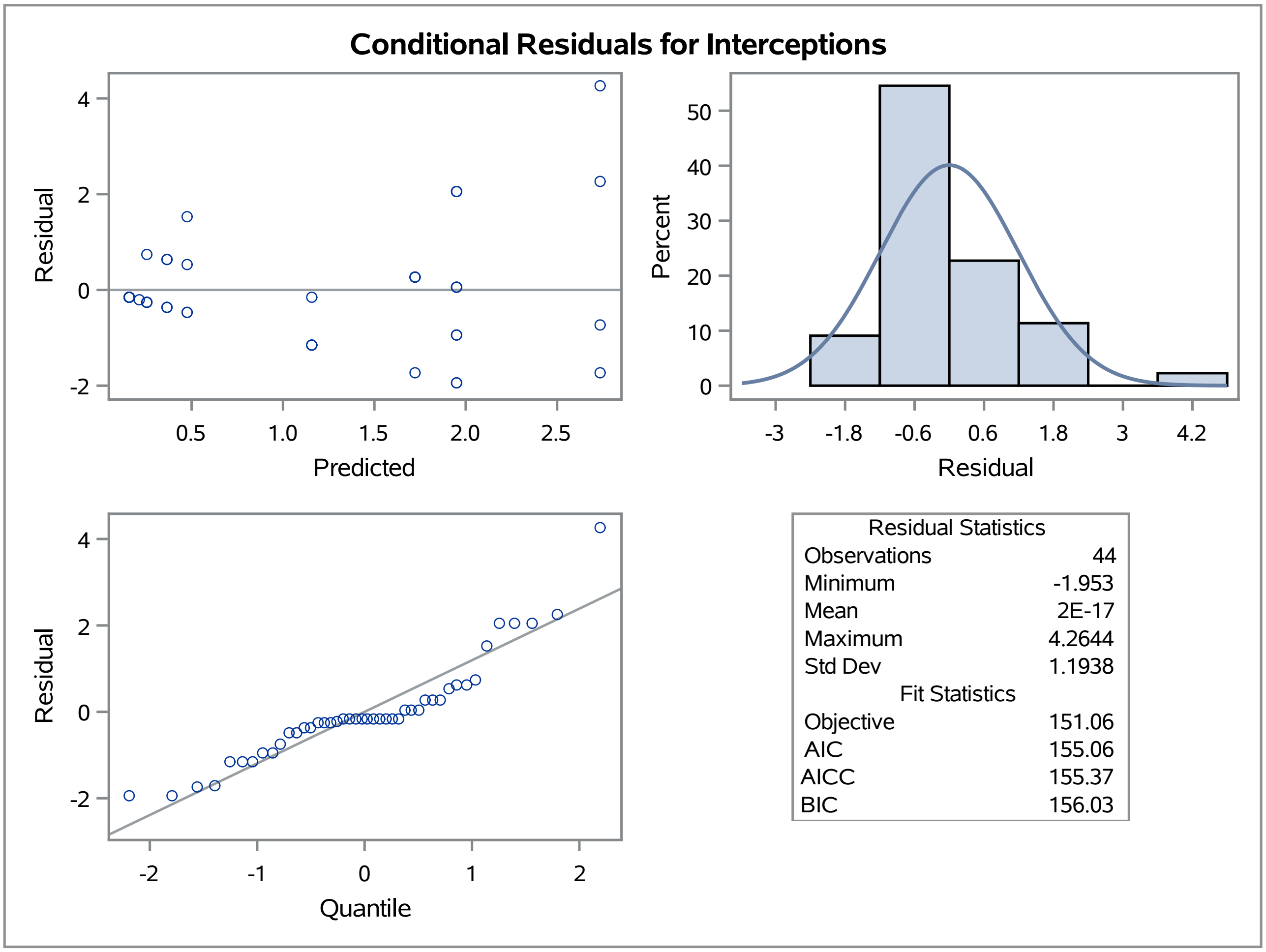

DistSoma=312

Model Information	
**Data Set**	WORK.TEMPDATASORTED
**Dependent Variable**	Interceptions
**Covariance Structure**	Variance Components
**Estimation Method**	REML
**Residual Variance Method**	Profile
**Fixed Effects SE Method**	Model-Based
**Degrees of Freedom Method**	Containment

Class Level Information		
**Class**	**Levels**	**Values**
**Treatment**	2	Control_GFP GFP MsTTR
**Culture**	12	1 2 3 4 5 6 7 8 9 10 11 12

Dimensions	
**Covariance Parameters**	2
**Columns in X**	3
**Columns in Z**	12
**Subjects**	1
**Max Obs per Subject**	44

Number of Observations	
**Number of Observations Read**	44
**Number of Observations Used**	44
**Number of Observations Not Used**	0

Iteration History			
**Iteration**	**Evaluations**	**-2 Res Log Like**	**Criterion**
**0**	1	145.75026162	
**1**	2	144.20864376	0.00000061
**2**	1	144.20862314	0.00000000

Convergence criteria met.

Covariance Parameter Estimates				
**Cov Parm**	**Estimate**	**Alpha**	**Lower**	**Upper**
**Culture**	0.2901	0.05	0.07610	14.7080
**Residual**	1.3708	0.05	0.8920	2.3741

DistSoma=312

Fit Statistics	
-2 Res Log Likelihood	144.2
AIC (Smaller is Better)	148.2
AICC (Smaller is Better)	148.5
BIC (Smaller is Better)	149.2

Solution for Fixed Effects

Effect	Treatment	Estimate	Standard Error	DF	t Value	Pr > |t|	Alpha	Lower	Upper
Intercept		1.7714	0.3474	10	5.10	0.0005	0.05	0.9973	2.5455
Treatment	Control_GFP	-1.5181	0.4770	32	-3.18	0.0032	0.05	-2.4897	-0.5465
Treatment	GFP MsTTR	0	.	.	.	.	.	.	.

Solution for Random Effects

Effect	Culture	Estimate	Std Err Pred	DF	t Value	Pr > |t|	Alpha	Lower	Upper
Culture	1	-0.09836	0.4399	32	-0.22	0.8245	0.05	-0.9945	0.7978
Culture	2	-0.09836	0.4399	32	-0.22	0.8245	0.05	-0.9945	0.7978
Culture	3	0.1131	0.4237	32	0.27	0.7912	0.05	-0.7500	0.9762
Culture	4	-0.00150	0.4237	32	-0.00	0.9972	0.05	-0.8646	0.8616
Culture	5	0.2277	0.4237	32	0.54	0.5947	0.05	-0.6354	1.0908
Culture	6	-0.04424	0.4926	32	-0.09	0.9290	0.05	-1.0477	0.9592
Culture	7	-0.09836	0.4399	32	-0.22	0.8245	0.05	-0.9945	0.7978
Culture	8	0.01471	0.4158	32	0.04	0.9720	0.05	-0.8321	0.8616
Culture	9	0.7924	0.4272	32	1.86	0.0728	0.05	-0.07766	1.6625
Culture	10	-0.6974	0.4272	32	-1.63	0.1123	0.05	-1.5675	0.1727
Culture	11	-0.1244	0.4272	32	-0.29	0.7727	0.05	-0.9945	0.7457
Culture	12	0.01471	0.4158	32	0.04	0.9720	0.05	-0.8321	0.8616

Type 3 Tests of Fixed Effects

Effect	Num DF	Den DF	F Value	Pr > F
Treatment	1	32	10.13	0.0032

Least Squares Means

Effect	Treatment	Estimate	Standard Error	DF	t Value	Pr > |t|	Alpha	Lower	Upper
Treatment	Control_GFP	0.2533	0.3269	32	0.77	0.4441	0.05	-0.4125	0.9191
Treatment	GFP MsTTR	1.7714	0.3474	32	5.10	<.0001	0.05	1.0637	2.4790

DistSoma=312

Differences of Least Squares Means												
**Effect**	**Treatment**	**Treatment**	**Estimate**	**Standard Error**	**DF**	**t Value**	**Pr > |t|**	**Adjustment**	**Adj P**	**Alpha**	**Lower**	**Upper**
**Treatment**	Control_GFP	GFP MsTTR	-1.5181	0.4770	32	-3.18	0.0032	Tukey-Kramer	0.0032	0.05	-2.4897	-0.5465

Differences of Least Squares Means				
**Effect**	**Treatment**	**Treatment**	**Adj Lower**	**Adj Upper**
**Treatment**	Control_GFP	GFP MsTTR	-2.4897	-0.5465

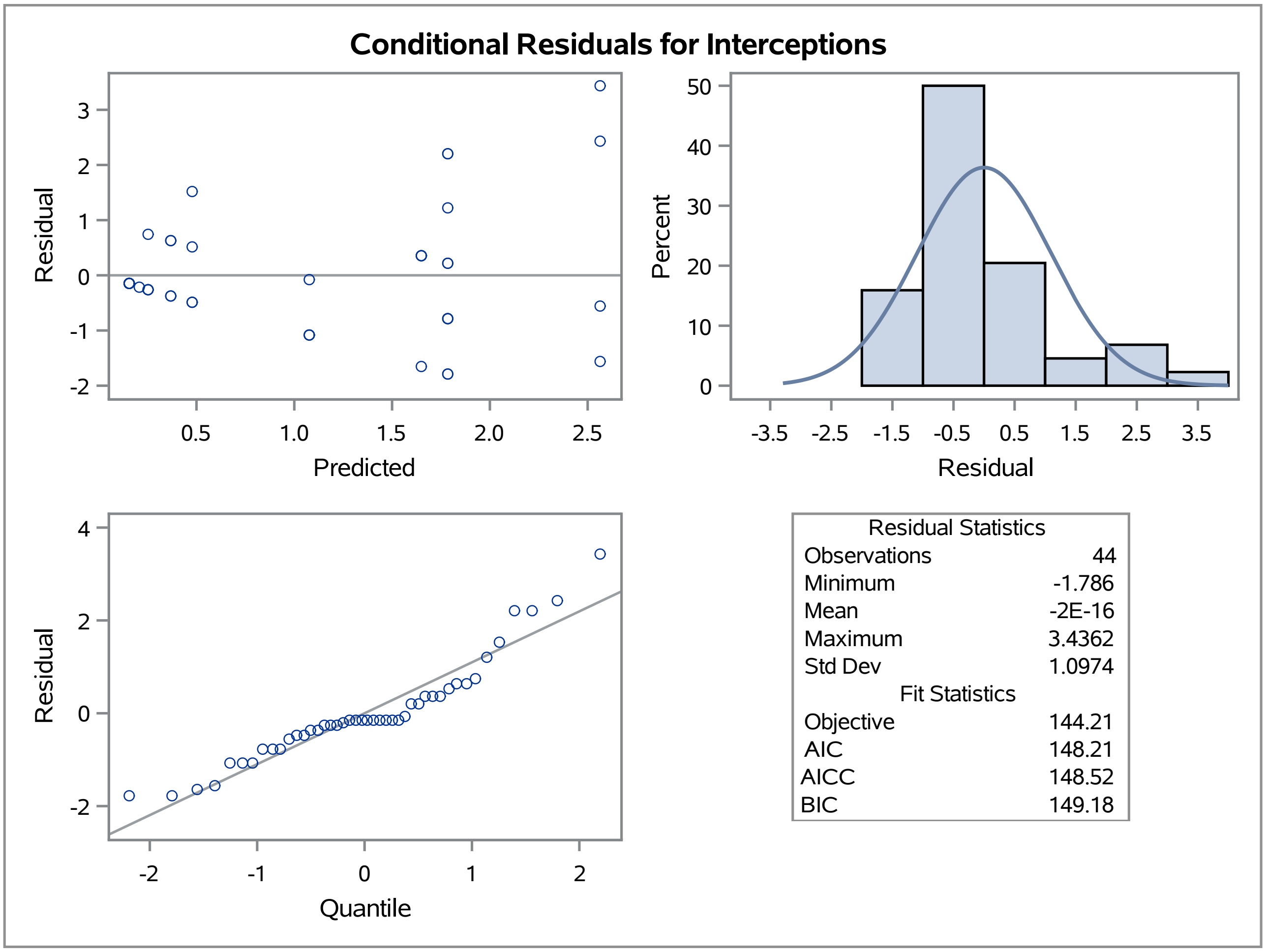

DistSoma=318

Model Information	
**Data Set**	WORK.TEMPDATASORTED
**Dependent Variable**	Interceptions
**Covariance Structure**	Variance Components
**Estimation Method**	REML
**Residual Variance Method**	Profile
**Fixed Effects SE Method**	Model-Based
**Degrees of Freedom Method**	Containment

Class Level Information		
**Class**	**Levels**	**Values**
**Treatment**	2	Control_GFP GFP MsTTR
**Culture**	12	1 2 3 4 5 6 7 8 9 10 11 12

Dimensions	
**Covariance Parameters**	2
**Columns in X**	3
**Columns in Z**	12
**Subjects**	1
**Max Obs per Subject**	44

Number of Observations	
**Number of Observations Read**	44
**Number of Observations Used**	44
**Number of Observations Not Used**	0

Iteration History			
**Iteration**	**Evaluations**	**-2 Res Log Like**	**Criterion**
**0**	1	139.19190503	
**1**	3	133.67656830	0.00003009
**2**	1	133.67570415	0.00000002
**3**	1	133.67570358	0.00000000

Convergence criteria met.

DistSoma=318

Covariance Parameter Estimates				
**Cov Parm**	**Estimate**	**Alpha**	**Lower**	**Upper**
**Culture**	0.4849	0.05	0.1782	3.6348
**Residual**	0.9559	0.05	0.6216	1.6573

Fit Statistics	
**-2 Res Log Likelihood**	133.7
**AIC (Smaller is Better)**	137.7
**AICC (Smaller is Better)**	138.0
**BIC (Smaller is Better)**	138.6

Solution for Fixed Effects									
**Effect**	**Treatment**	**Estimate**	**Standard Error**	**DF**	**t Value**	**Pr > |t|**	**Alpha**	**Lower**	**Upper**
**Intercept**		1.6459	0.3752	10	4.39	0.0014	0.05	0.8098	2.4819
**Treatment**	Control_GFP	-1.4847	0.5074	32	-2.93	0.0063	0.05	-2.5182	-0.4512
**Treatment**	GFP MsTTR	0	.	.	.	.	.	.	.

Solution for Random Effects									
**Effect**	**Culture**	**Estimate**	**Std Err Pred**	**DF**	**t Value**	**Pr > |t|**	**Alpha**	**Lower**	**Upper**
**Culture**	1	-0.09725	0.4845	32	-0.20	0.8422	0.05	-1.0842	0.8897
**Culture**	2	-0.09725	0.4845	32	-0.20	0.8422	0.05	-1.0842	0.8897
**Culture**	3	0.2270	0.4609	32	0.49	0.6257	0.05	-0.7118	1.1658
**Culture**	4	0.05951	0.4609	32	0.13	0.8981	0.05	-0.8793	0.9983
**Culture**	5	0.05951	0.4609	32	0.13	0.8981	0.05	-0.8793	0.9983
**Culture**	6	-0.05424	0.5787	32	-0.09	0.9259	0.05	-1.2331	1.1246
**Culture**	7	-0.09725	0.4845	32	-0.20	0.8422	0.05	-1.0842	0.8897
**Culture**	8	-0.1763	0.4578	32	-0.39	0.7026	0.05	-1.1088	0.7561
**Culture**	9	1.4095	0.4725	32	2.98	0.0054	0.05	0.4470	2.3719
**Culture**	10	-0.9351	0.4725	32	-1.98	0.0565	0.05	-1.8975	0.02740
**Culture**	11	-0.2652	0.4725	32	-0.56	0.5785	0.05	-1.2276	0.6973
**Culture**	12	-0.03290	0.4578	32	-0.07	0.9431	0.05	-0.9653	0.8995

Type 3 Tests of Fixed Effects				
**Effect**	**Num DF**	**Den DF**	**F Value**	**Pr > F**
**Treatment**	1	32	8.56	0.0063

DistSoma=318

Least Squares Means									
**Effect**	**Treatment**	**Estimate**	**Standard Error**	**DF**	**t Value**	**Pr > |t|**	**Alpha**	**Lower**	**Upper**
**Treatment**	Control_GFP	0.1612	0.3416	32	0.47	0.6402	0.05	-0.5346	0.8569
**Treatment**	GFP MsTTR	1.6459	0.3752	32	4.39	0.0001	0.05	0.8816	2.4102

Differences of Least Squares Means												
**Effect**	**Treatment**	**Treatment**	**Estimate**	**Standard Error**	**DF**	**t Value**	**Pr > |t|**	**Adjustment**	**Adj P**	**Alpha**	**Lower**	**Upper**
**Treatment**	Control_GFP	GFP MsTTR	-1.4847	0.5074	32	-2.93	0.0063	Tukey-Kramer	0.0063	0.05	-2.5182	-0.4512

Differences of Least Squares Means				
**Effect**	**Treatment**	**Treatment**	**Adj Lower**	**Adj Upper**
**Treatment**	Control_GFP	GFP MsTTR	-2.5182	-0.4512

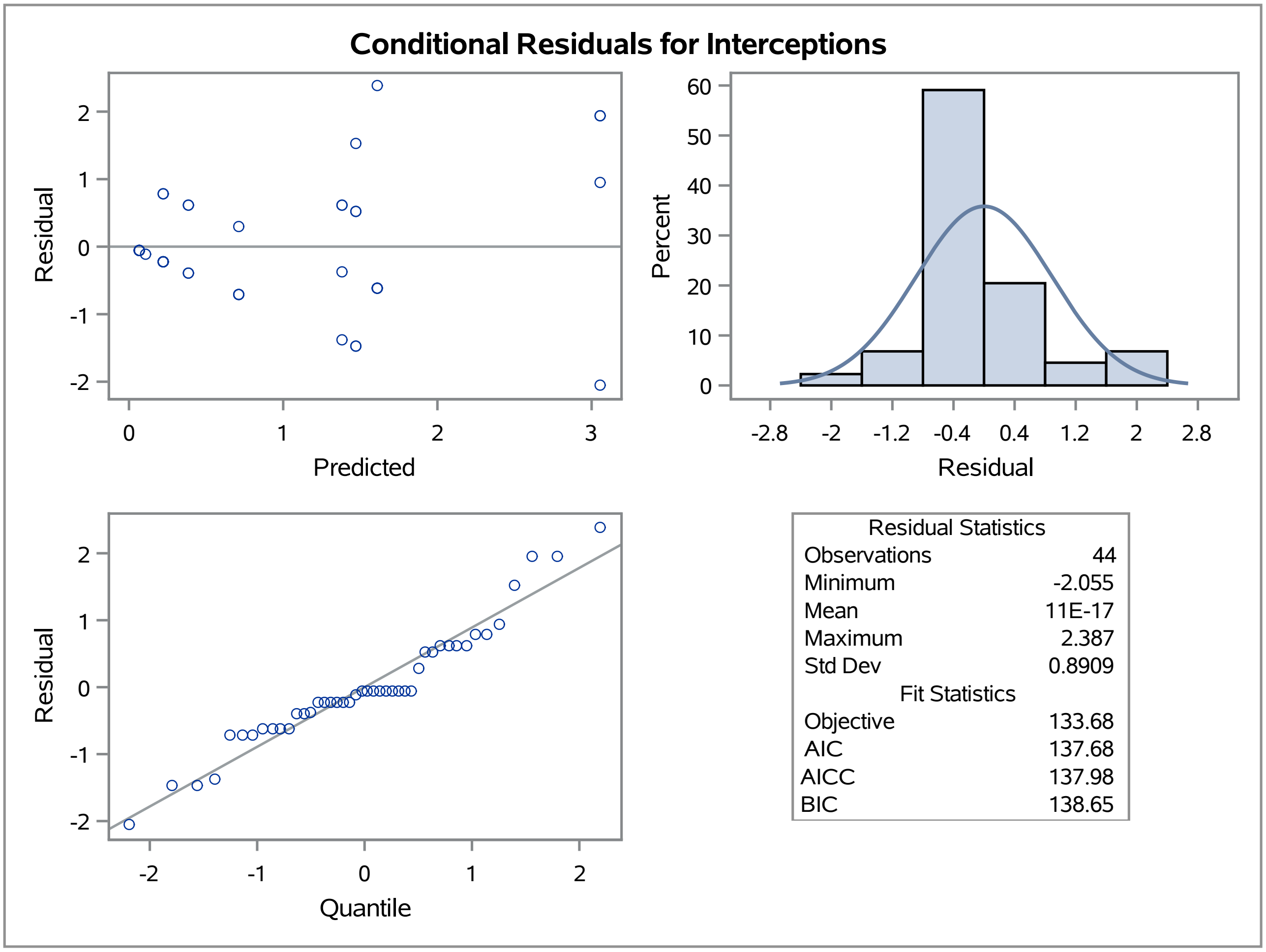

DistSoma=324

Model Information	
**Data Set**	WORK.TEMPDATASORTED
**Dependent Variable**	Interceptions
**Covariance Structure**	Variance Components
**Estimation Method**	REML
**Residual Variance Method**	Profile
**Fixed Effects SE Method**	Model-Based
**Degrees of Freedom Method**	Containment

Class Level Information		
**Class**	**Levels**	**Values**
**Treatment**	2	Control_GFP GFP MsTTR
**Culture**	12	1 2 3 4 5 6 7 8 9 10 11 12

Dimensions	
**Covariance Parameters**	2
**Columns in X**	3
**Columns in Z**	12
**Subjects**	1
**Max Obs per Subject**	44

Number of Observations	
**Number of Observations Read**	44
**Number of Observations Used**	44
**Number of Observations Not Used**	0

Iteration History			
**Iteration**	**Evaluations**	**-2 Res Log Like**	**Criterion**
**0**	1	135.85736103	
**1**	3	133.16183054	0.00001649
**2**	1	133.16136321	0.00000001

Convergence criteria met.

Covariance Parameter Estimates				
**Cov Parm**	**Estimate**	**Alpha**	**Lower**	**Upper**
**Culture**	0.3034	0.05	0.09391	5.1199
**Residual**	1.0142	0.05	0.6603	1.7552

DistSoma=324

Fit Statistics	
-2 Res Log Likelihood	133.2
AIC (Smaller is Better)	137.2
AICC (Smaller is Better)	137.5
BIC (Smaller is Better)	138.1

Solution for Fixed Effects

Effect	Treatment	Estimate	Standard Error	DF	t Value	Pr > |t|	Alpha	Lower	Upper
Intercept		1.4211	0.3273	10	4.34	0.0015	0.05	0.6920	2.1503
Treatment	Control_GFP	-1.2967	0.4467	32	-2.90	0.0066	0.05	-2.2065	-0.3869
Treatment	GFP MsTTR	0	.	.	.	.	.	.	.

Solution for Random Effects

Effect	Culture	Estimate	Std Err Pred	DF	t Value	Pr > |t|	Alpha	Lower	Upper
Culture	1	-0.05885	0.4249	32	-0.14	0.8907	0.05	-0.9244	0.8067
Culture	2	-0.05885	0.4249	32	-0.14	0.8907	0.05	-0.9244	0.8067
Culture	3	0.2046	0.4069	32	0.50	0.6185	0.05	-0.6242	1.0333
Culture	4	-0.06778	0.4069	32	-0.17	0.8687	0.05	-0.8965	0.7610
Culture	5	0.06840	0.4069	32	0.17	0.8675	0.05	-0.7604	0.8972
Culture	6	-0.02865	0.4883	32	-0.06	0.9536	0.05	-1.0233	0.9660
Culture	7	-0.05885	0.4249	32	-0.14	0.8907	0.05	-0.9244	0.8067
Culture	8	-0.2524	0.4000	32	-0.63	0.5326	0.05	-1.0672	0.5625
Culture	9	0.9962	0.4122	32	2.42	0.0215	0.05	0.1566	1.8358
Culture	10	-0.6380	0.4122	32	-1.55	0.1315	0.05	-1.4776	0.2016
Culture	11	-0.09323	0.4122	32	-0.23	0.8225	0.05	-0.9328	0.7464
Culture	12	-0.01267	0.4000	32	-0.03	0.9749	0.05	-0.8275	0.8022

Type 3 Tests of Fixed Effects

Effect	Num DF	Den DF	F Value	Pr > F
Treatment	1	32	8.43	0.0066

Least Squares Means

Effect	Treatment	Estimate	Standard Error	DF	t Value	Pr > |t|	Alpha	Lower	Upper
Treatment	Control_GFP	0.1244	0.3040	32	0.41	0.6850	0.05	-0.4947	0.7436
Treatment	GFP MsTTR	1.4211	0.3273	32	4.34	0.0001	0.05	0.7545	2.0877

DistSoma=324

Differences of Least Squares Means												
**Effect**	**Treatment**	**Treatment**	**Estimate**	**Standard Error**	**DF**	**t Value**	**Pr > |t|**	**Adjustment**	**Adj P**	**Alpha**	**Lower**	**Upper**
**Treatment**	Control_GFP	GFP MsTTR	-1.2967	0.4467	32	-2.90	0.0066	Tukey-Kramer	0.0066	0.05	-2.2065	-0.3869

Differences of Least Squares Means				
**Effect**	**Treatment**	**Treatment**	**Adj Lower**	**Adj Upper**
**Treatment**	Control_GFP	GFP MsTTR	-2.2065	-0.3869

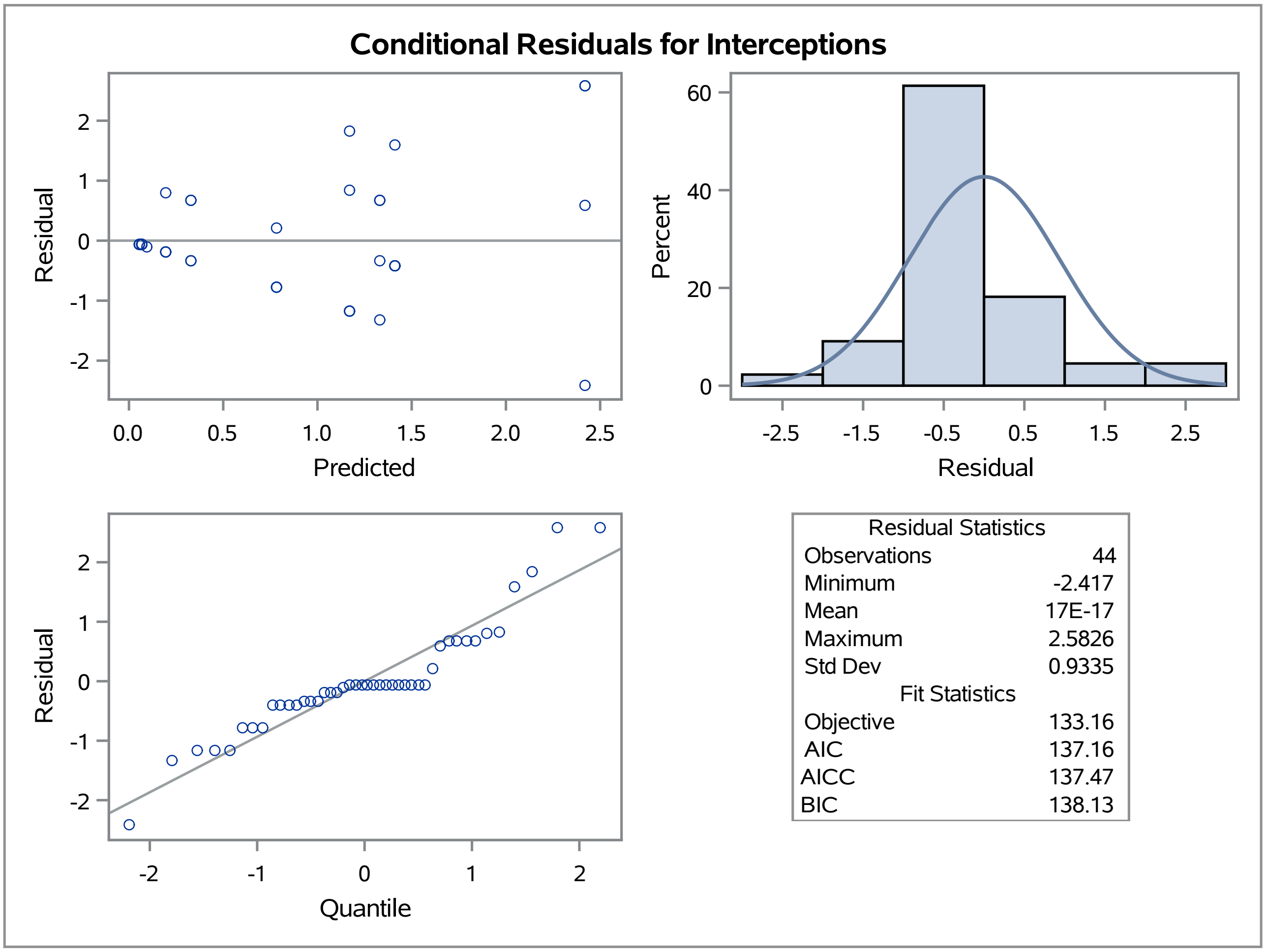

DistSoma=330

Model Information	
**Data Set**	WORK.TEMPDATASORTED
**Dependent Variable**	Interceptions
**Covariance Structure**	Variance Components
**Estimation Method**	REML
**Residual Variance Method**	Profile
**Fixed Effects SE Method**	Model-Based
**Degrees of Freedom Method**	Containment

Class Level Information		
**Class**	**Levels**	**Values**
**Treatment**	2	Control_GFP GFP MsTTR
**Culture**	12	1 2 3 4 5 6 7 8 9 10 11 12

Dimensions	
**Covariance Parameters**	2
**Columns in X**	3
**Columns in Z**	12
**Subjects**	1
**Max Obs per Subject**	44

Number of Observations	
**Number of Observations Read**	44
**Number of Observations Used**	44
**Number of Observations Not Used**	0

Iteration History			
**Iteration**	**Evaluations**	**-2 Res Log Like**	**Criterion**
**0**	1	122.21140727	
**1**	3	120.10796562	0.00000848
**2**	1	120.10778215	0.00000000

Convergence criteria met.

Covariance Parameter Estimates				
**Cov Parm**	**Estimate**	**Alpha**	**Lower**	**Upper**
**Culture**	0.1929	0.05	0.05566	4.9562
**Residual**	0.7572	0.05	0.4930	1.3105

DistSoma=330

Fit Statistics	
**-2 Res Log Likelihood**	120.1
**AIC (Smaller is Better)**	124.1
**AICC (Smaller is Better)**	124.4
**BIC (Smaller is Better)**	125.1

Solution for Fixed Effects

Effect	Treatment	Estimate	Standard Error	DF	t Value	Pr > |t|	Alpha	Lower	Upper
**Intercept**		1.2820	0.2706	10	4.74	0.0008	0.05	0.6791	1.8849
**Treatment**	Control_GFP	-1.1565	0.3704	32	-3.12	0.0038	0.05	-1.9109	-0.4021
**Treatment**	GFP MsTTR	0	.	.	.	.	.	.	.

Solution for Random Effects

Effect	Culture	Estimate	Std Err Pred	DF	t Value	Pr > |t|	Alpha	Lower	Upper
**Culture**	1	-0.05435	0.3483	32	-0.16	0.8770	0.05	-0.7639	0.6552
**Culture**	2	-0.05435	0.3483	32	-0.16	0.8770	0.05	-0.7639	0.6552
**Culture**	3	0.1890	0.3344	32	0.57	0.5759	0.05	-0.4922	0.8702
**Culture**	4	-0.06333	0.3344	32	-0.19	0.8510	0.05	-0.7445	0.6178
**Culture**	5	0.06284	0.3344	32	0.19	0.8521	0.05	-0.6183	0.7440
**Culture**	6	-0.02547	0.3954	32	-0.06	0.9490	0.05	-0.8309	0.7800
**Culture**	7	-0.05435	0.3483	32	-0.16	0.8770	0.05	-0.7639	0.6552
**Culture**	8	-0.1580	0.3283	32	-0.48	0.6337	0.05	-0.8268	0.5109
**Culture**	9	0.7409	0.3379	32	2.19	0.0357	0.05	0.05255	1.4292
**Culture**	10	-0.5208	0.3379	32	-1.54	0.1331	0.05	-1.2092	0.1675
**Culture**	11	-0.01615	0.3379	32	-0.05	0.9622	0.05	-0.7045	0.6722
**Culture**	12	-0.04593	0.3283	32	-0.14	0.8896	0.05	-0.7148	0.6229

Type 3 Tests of Fixed Effects

Effect	Num DF	Den DF	F Value	Pr > F
**Treatment**	1	32	9.75	0.0038

Least Squares Means

Effect	Treatment	Estimate	Standard Error	DF	t Value	Pr > |t|	Alpha	Lower	Upper
**Treatment**	Control_GFP	0.1255	0.2529	32	0.50	0.6231	0.05	-0.3896	0.6406
**Treatment**	GFP MsTTR	1.2820	0.2706	32	4.74	<.0001	0.05	0.7308	1.8332

DistSoma=330

Differences of Least Squares Means												
**Effect**	**Treatment**	**Treatment**	**Estimate**	**Standard Error**	**DF**	**t Value**	**Pr > |t|**	**Adjustment**	**Adj P**	**Alpha**	**Lower**	**Upper**
**Treatment**	Control_GFP	GFP MsTTR	-1.1565	0.3704	32	-3.12	0.0038	Tukey-Kramer	0.0038	0.05	-1.9109	-0.4021

Differences of Least Squares Means				
**Effect**	**Treatment**	**Treatment**	**Adj Lower**	**Adj Upper**
**Treatment**	Control_GFP	GFP MsTTR	-1.9109	-0.4021

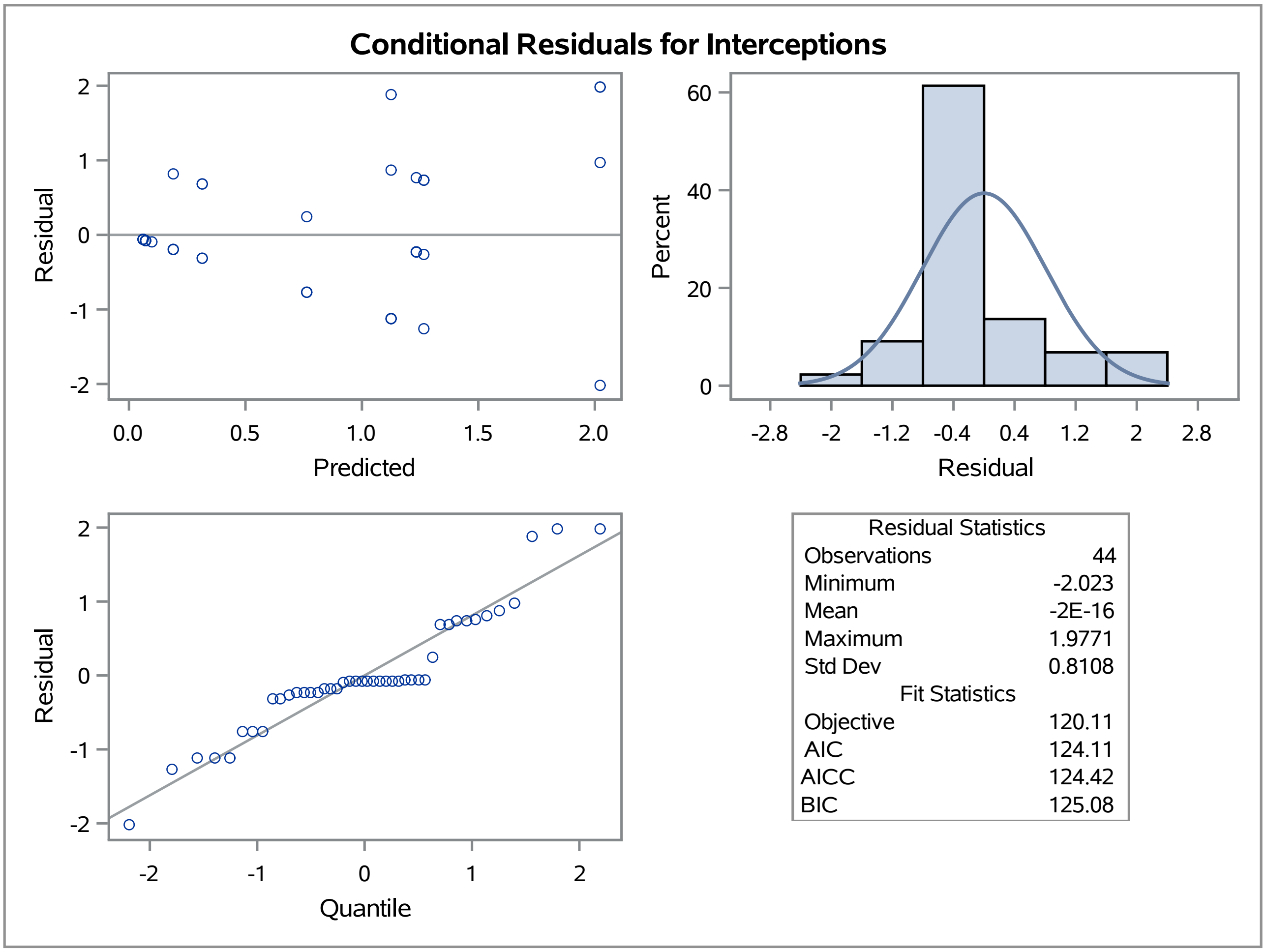

DistSoma=336

Model Information	
**Data Set**	WORK.TEMPDATASORTED
**Dependent Variable**	Interceptions
**Covariance Structure**	Variance Components
**Estimation Method**	REML
**Residual Variance Method**	Profile
**Fixed Effects SE Method**	Model-Based
**Degrees of Freedom Method**	Containment

Class Level Information		
**Class**	**Levels**	**Values**
**Treatment**	2	Control_GFP GFP MsTTR
**Culture**	12	1 2 3 4 5 6 7 8 9 10 11 12

Dimensions	
**Covariance Parameters**	2
**Columns in X**	3
**Columns in Z**	12
**Subjects**	1
**Max Obs per Subject**	44

Number of Observations	
**Number of Observations Read**	44
**Number of Observations Used**	44
**Number of Observations Not Used**	0

Iteration History			
**Iteration**	**Evaluations**	**-2 Res Log Like**	**Criterion**
**0**	1	123.79831612	
**1**	3	121.89071101	0.00000508
**2**	1	121.89059674	0.00000000

Convergence criteria met.

Covariance Parameter Estimates				
**Cov Parm**	**Estimate**	**Alpha**	**Lower**	**Upper**
**Culture**	0.1906	0.05	0.05343	5.9406
**Residual**	0.7954	0.05	0.5178	1.3765

DistSoma=336

Fit Statistics	
-2 Res Log Likelihood	121.9
AIC (Smaller is Better)	125.9
AICC (Smaller is Better)	126.2
BIC (Smaller is Better)	126.9

Solution for Fixed Effects

Effect	Treatment	Estimate	Standard Error	DF	t Value	Pr > |t|	Alpha	Lower	Upper
Intercept		1.2339	0.2730	10	4.52	0.0011	0.05	0.6257	1.8421
Treatment	Control_GFP	-1.1080	0.3740	32	-2.96	0.0057	0.05	-1.8698	-0.3463
Treatment	GFP MsTTR	0	.	.	.	.	.	.	.

Solution for Random Effects

Effect	Culture	Estimate	Std Err Pred	DF	t Value	Pr > |t|	Alpha	Lower	Upper
Culture	1	-0.05265	0.3498	32	-0.15	0.8813	0.05	-0.7651	0.6598
Culture	2	-0.05265	0.3498	32	-0.15	0.8813	0.05	-0.7651	0.6598
Culture	3	0.1831	0.3361	32	0.54	0.5897	0.05	-0.5016	0.8678
Culture	4	-0.06161	0.3361	32	-0.18	0.8557	0.05	-0.7463	0.6231
Culture	5	0.06076	0.3361	32	0.18	0.8577	0.05	-0.6239	0.7454
Culture	6	-0.02434	0.3952	32	-0.06	0.9513	0.05	-0.8294	0.7808
Culture	7	-0.05265	0.3498	32	-0.15	0.8813	0.05	-0.7651	0.6598
Culture	8	-0.1275	0.3299	32	-0.39	0.7017	0.05	-0.7996	0.5445
Culture	9	0.7421	0.3394	32	2.19	0.0362	0.05	0.05079	1.4334
Culture	10	-0.4816	0.3394	32	-1.42	0.1656	0.05	-1.1729	0.2097
Culture	11	-0.1145	0.3394	32	-0.34	0.7381	0.05	-0.8058	0.5768
Culture	12	-0.01848	0.3299	32	-0.06	0.9557	0.05	-0.6905	0.6536

Type 3 Tests of Fixed Effects

Effect	Num DF	Den DF	F Value	Pr > F
Treatment	1	32	8.78	0.0057

Least Squares Means

Effect	Treatment	Estimate	Standard Error	DF	t Value	Pr > |t|	Alpha	Lower	Upper
Treatment	Control_GFP	0.1259	0.2557	32	0.49	0.6259	0.05	-0.3949	0.6466
Treatment	GFP MsTTR	1.2339	0.2730	32	4.52	<.0001	0.05	0.6779	1.7899

DistSoma=336

Differences of Least Squares Means												
**Effect**	**Treatment**	**Treatment**	**Estimate**	**Standard Error**	**DF**	**t Value**	**Pr > |t|**	**Adjustment**	**Adj P**	**Alpha**	**Lower**	**Upper**
**Treatment**	Control_GFP	GFP MsTTR	-1.1080	0.3740	32	-2.96	0.0057	Tukey-Kramer	0.0057	0.05	-1.8698	-0.3463

Differences of Least Squares Means				
**Effect**	**Treatment**	**Treatment**	**Adj Lower**	**Adj Upper**
**Treatment**	Control_GFP	GFP MsTTR	-1.8698	-0.3463

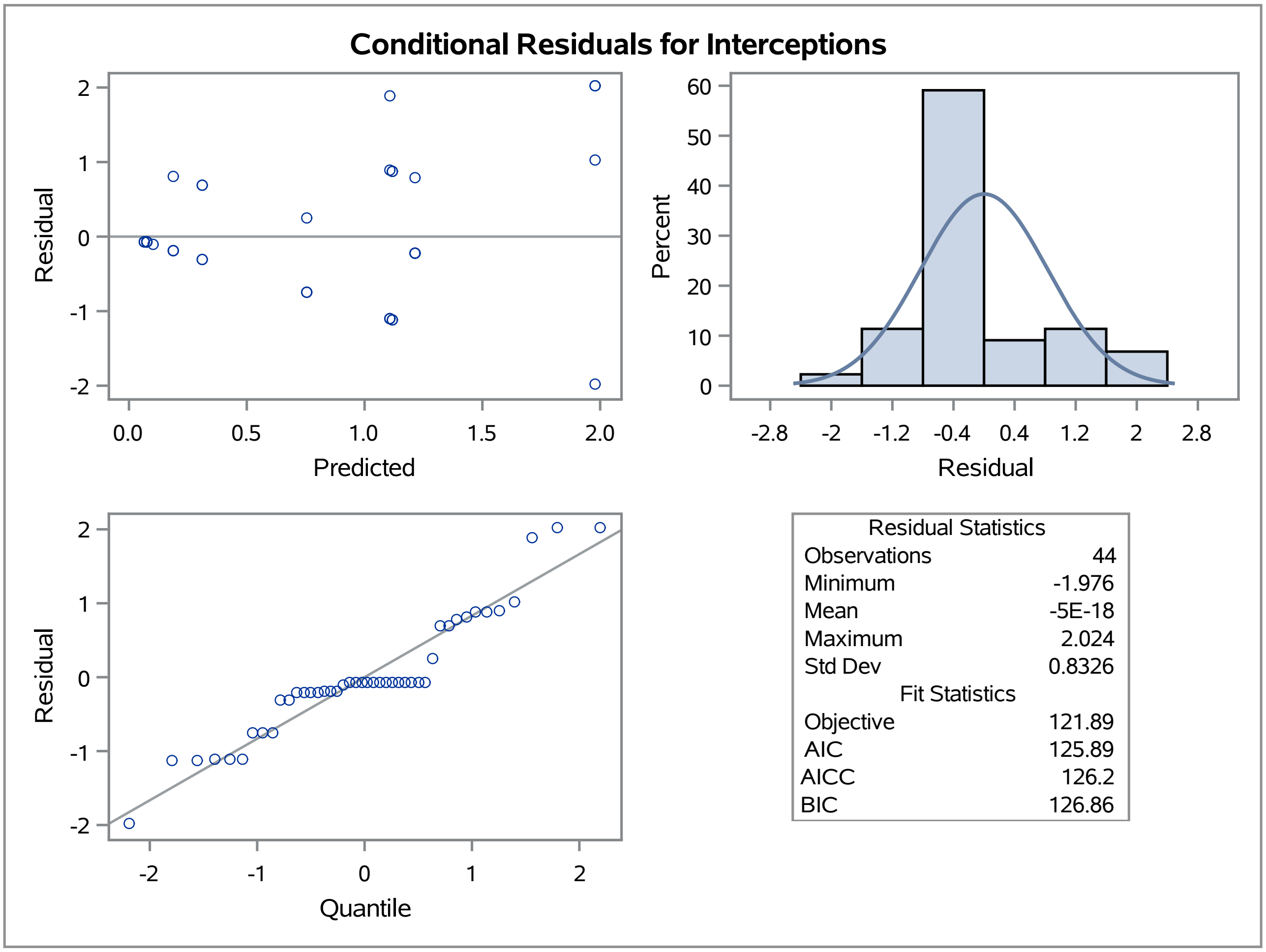

DistSoma=342

Model Information	
**Data Set**	WORK.TEMPDATASORTED
**Dependent Variable**	Interceptions
**Covariance Structure**	Variance Components
**Estimation Method**	REML
**Residual Variance Method**	Profile
**Fixed Effects SE Method**	Model-Based
**Degrees of Freedom Method**	Containment

Class Level Information		
**Class**	**Levels**	**Values**
**Treatment**	2	Control_GFP GFP MsTTR
**Culture**	12	1 2 3 4 5 6 7 8 9 10 11 12

Dimensions	
**Covariance Parameters**	2
**Columns in X**	3
**Columns in Z**	12
**Subjects**	1
**Max Obs per Subject**	44

Number of Observations	
**Number of Observations Read**	44
**Number of Observations Used**	44
**Number of Observations Not Used**	0

Iteration History			
**Iteration**	**Evaluations**	**-2 Res Log Like**	**Criterion**
**0**	1	110.72888629	
**1**	3	109.86937357	0.00000406
**2**	1	109.86930690	0.00000000

Convergence criteria met.

Covariance Parameter Estimates				
**Cov Parm**	**Estimate**	**Alpha**	**Lower**	**Upper**
**Culture**	0.09037	0.05	0.01935	34.5605
**Residual**	0.6264	0.05	0.4082	1.0824

DistSoma=342

Fit Statistics	
-2 Res Log Likelihood	109.9
AIC (Smaller is Better)	113.9
AICC (Smaller is Better)	114.2
BIC (Smaller is Better)	114.8

Solution for Fixed Effects

Effect	Treatment	Estimate	Standard Error	DF	t Value	Pr > |t|	Alpha	Lower	Upper
Intercept		1.0945	0.2161	10	5.07	0.0005	0.05	0.6131	1.5759
Treatment	Control_GFP	-1.0086	0.2985	32	-3.38	0.0019	0.05	-1.6167	-0.4006
Treatment	GFP MsTTR	0	.	.	.	.	.	.	.

Solution for Random Effects

Effect	Culture	Estimate	Std Err Pred	DF	t Value	Pr > |t|	Alpha	Lower	Upper
Culture	1	-0.02595	0.2587	32	-0.10	0.9208	0.05	-0.5530	0.5011
Culture	2	-0.02595	0.2587	32	-0.10	0.9208	0.05	-0.5530	0.5011
Culture	3	0.06005	0.2510	32	0.24	0.8124	0.05	-0.4512	0.5713
Culture	4	-0.03143	0.2510	32	-0.13	0.9011	0.05	-0.5426	0.4798
Culture	5	0.06005	0.2510	32	0.24	0.8124	0.05	-0.4512	0.5713
Culture	6	-0.01083	0.2822	32	-0.04	0.9696	0.05	-0.5857	0.5641
Culture	7	-0.02595	0.2587	32	-0.10	0.9208	0.05	-0.5530	0.5011
Culture	8	-0.1234	0.2464	32	-0.50	0.6199	0.05	-0.6253	0.3784
Culture	9	0.4228	0.2521	32	1.68	0.1033	0.05	-0.09071	0.9363
Culture	10	-0.3090	0.2521	32	-1.23	0.2292	0.05	-0.8225	0.2045
Culture	11	-0.03458	0.2521	32	-0.14	0.8918	0.05	-0.5481	0.4789
Culture	12	0.04421	0.2464	32	0.18	0.8587	0.05	-0.4576	0.5461

Type 3 Tests of Fixed Effects

Effect	Num DF	Den DF	F Value	Pr > F
Treatment	1	32	11.42	0.0019

Least Squares Means

Effect	Treatment	Estimate	Standard Error	DF	t Value	Pr > |t|	Alpha	Lower	Upper
Treatment	Control_GFP	0.08589	0.2060	32	0.42	0.6795	0.05	-0.3337	0.5055
Treatment	GFP MsTTR	1.0945	0.2161	32	5.07	<.0001	0.05	0.6544	1.5346

DistSoma=342

Differences of Least Squares Means												
**Effect**	**Treatment**	**Treatment**	**Estimate**	**Standard Error**	**DF**	**t Value**	**Pr > |t|**	**Adjustment**	**Adj P**	**Alpha**	**Lower**	**Upper**
**Treatment**	Control_GFP	GFP MsTTR	-1.0086	0.2985	32	-3.38	0.0019	Tukey-Kramer	0.0019	0.05	-1.6167	-0.4006

Differences of Least Squares Means				
**Effect**	**Treatment**	**Treatment**	**Adj Lower**	**Adj Upper**
**Treatment**	Control_GFP	GFP MsTTR	-1.6167	-0.4006

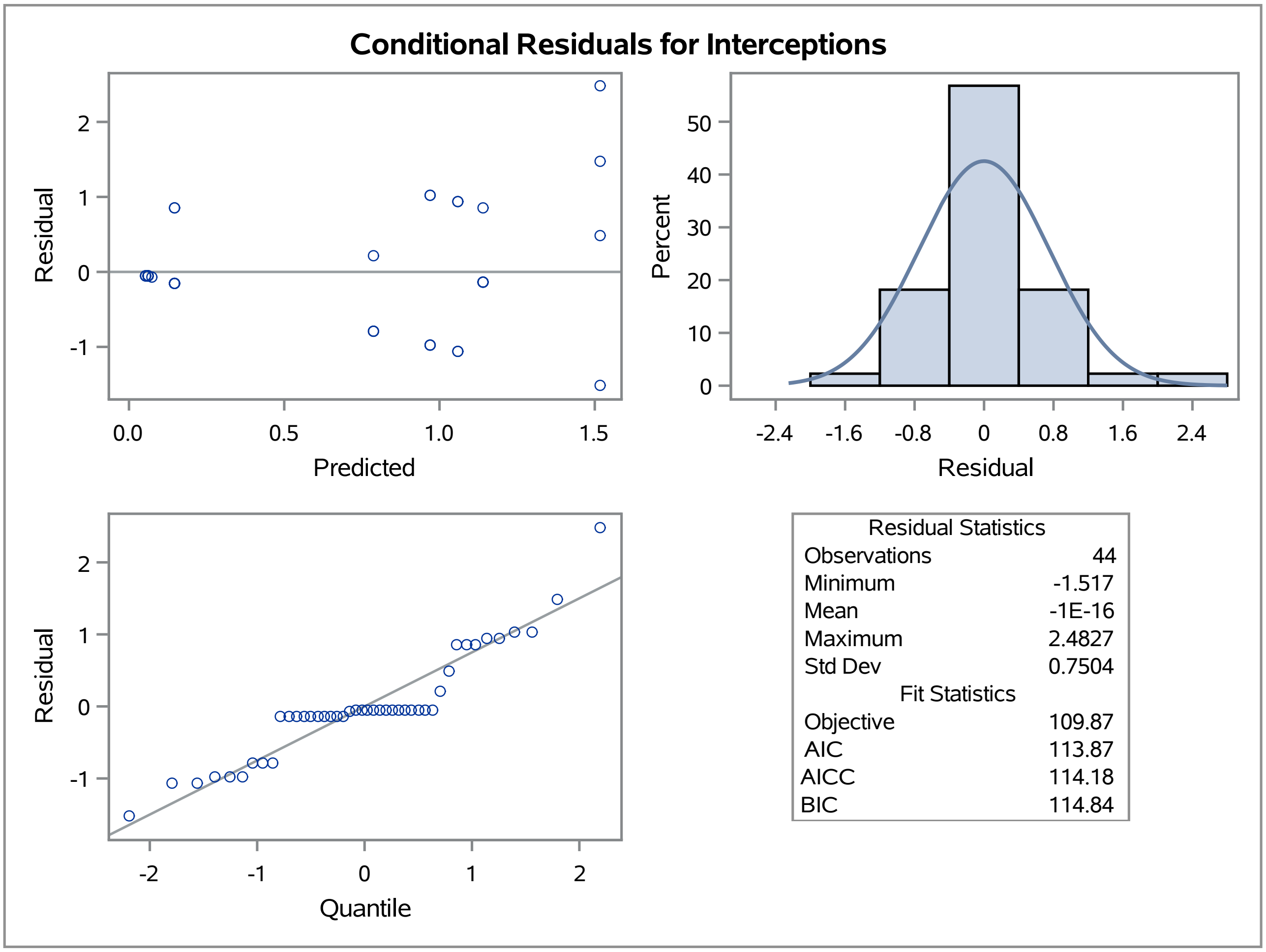

DistSoma=348

Model Information	
**Data Set**	WORK.TEMPDATASORTED
**Dependent Variable**	Interceptions
**Covariance Structure**	Variance Components
**Estimation Method**	REML
**Residual Variance Method**	Profile
**Fixed Effects SE Method**	Model-Based
**Degrees of Freedom Method**	Containment

Class Level Information		
**Class**	**Levels**	**Values**
**Treatment**	2	Control_GFP GFP MsTTR
**Culture**	12	1 2 3 4 5 6 7 8 9 10 11 12

Dimensions	
**Covariance Parameters**	2
**Columns in X**	3
**Columns in Z**	12
**Subjects**	1
**Max Obs per Subject**	44

Number of Observations	
**Number of Observations Read**	44
**Number of Observations Used**	44
**Number of Observations Not Used**	0

Iteration History			
**Iteration**	**Evaluations**	**-2 Res Log Like**	**Criterion**
**0**	1	117.44179971	
**1**	3	115.80862710	0.00001159
**2**	1	115.80840124	0.00000000

Convergence criteria met.

Covariance Parameter Estimates				
**Cov Parm**	**Estimate**	**Alpha**	**Lower**	**Upper**
**Culture**	0.1492	0.05	0.03982	6.6250
**Residual**	0.6962	0.05	0.4535	1.2037

DistSoma=348

Fit Statistics	
-2 Res Log Likelihood	115.8
AIC (Smaller is Better)	119.8
AICC (Smaller is Better)	120.1
BIC (Smaller is Better)	120.8

Solution for Fixed Effects

Effect	Treatment	Estimate	Standard Error	DF	t Value	Pr > |t|	Alpha	Lower	Upper
Intercept		1.0576	0.2483	10	4.26	0.0017	0.05	0.5042	1.6109
Treatment	Control_GFP	-0.9732	0.3409	32	-2.85	0.0075	0.05	-1.6676	-0.2787
Treatment	GFP MsTTR	0	.	.	.	.	.	.	.

Solution for Random Effects

Effect	Culture	Estimate	Std Err Pred	DF	t Value	Pr > |t|	Alpha	Lower	Upper
Culture	1	-0.03302	0.3149	32	-0.10	0.9171	0.05	-0.6745	0.6085
Culture	2	-0.03302	0.3149	32	-0.10	0.9171	0.05	-0.6745	0.6085
Culture	3	0.07645	0.3033	32	0.25	0.8026	0.05	-0.5413	0.6942
Culture	4	-0.03895	0.3033	32	-0.13	0.8986	0.05	-0.6567	0.5788
Culture	5	0.07645	0.3033	32	0.25	0.8026	0.05	-0.5413	0.6942
Culture	6	-0.01489	0.3530	32	-0.04	0.9666	0.05	-0.7339	0.7041
Culture	7	-0.03302	0.3149	32	-0.10	0.9171	0.05	-0.6745	0.6085
Culture	8	-0.1332	0.2975	32	-0.45	0.6573	0.05	-0.7393	0.4728
Culture	9	0.6658	0.3057	32	2.18	0.0369	0.05	0.04302	1.2886
Culture	10	-0.3728	0.3057	32	-1.22	0.2317	0.05	-0.9956	0.2500
Culture	11	-0.02657	0.3057	32	-0.09	0.9313	0.05	-0.6494	0.5962
Culture	12	-0.1332	0.2975	32	-0.45	0.6573	0.05	-0.7393	0.4728

Type 3 Tests of Fixed Effects

Effect	Num DF	Den DF	F Value	Pr > F
Treatment	1	32	8.15	0.0075

Least Squares Means

Effect	Treatment	Estimate	Standard Error	DF	t Value	Pr > |t|	Alpha	Lower	Upper
Treatment	Control_GFP	0.08438	0.2336	32	0.36	0.7203	0.05	-0.3914	0.5601
Treatment	GFP MsTTR	1.0576	0.2483	32	4.26	0.0002	0.05	0.5517	1.5634

DistSoma=348

Differences of Least Squares Means												
**Effect**	**Treatment**	**Treatment**	**Estimate**	**Standard Error**	**DF**	**t Value**	**Pr > |t|**	**Adjustment**	**Adj P**	**Alpha**	**Lower**	**Upper**
**Treatment**	Control_GFP	GFP MsTTR	-0.9732	0.3409	32	-2.85	0.0075	Tukey-Kramer	0.0075	0.05	-1.6676	-0.2787

Differences of Least Squares Means				
**Effect**	**Treatment**	**Treatment**	**Adj Lower**	**Adj Upper**
**Treatment**	Control_GFP	GFP MsTTR	-1.6676	-0.2788

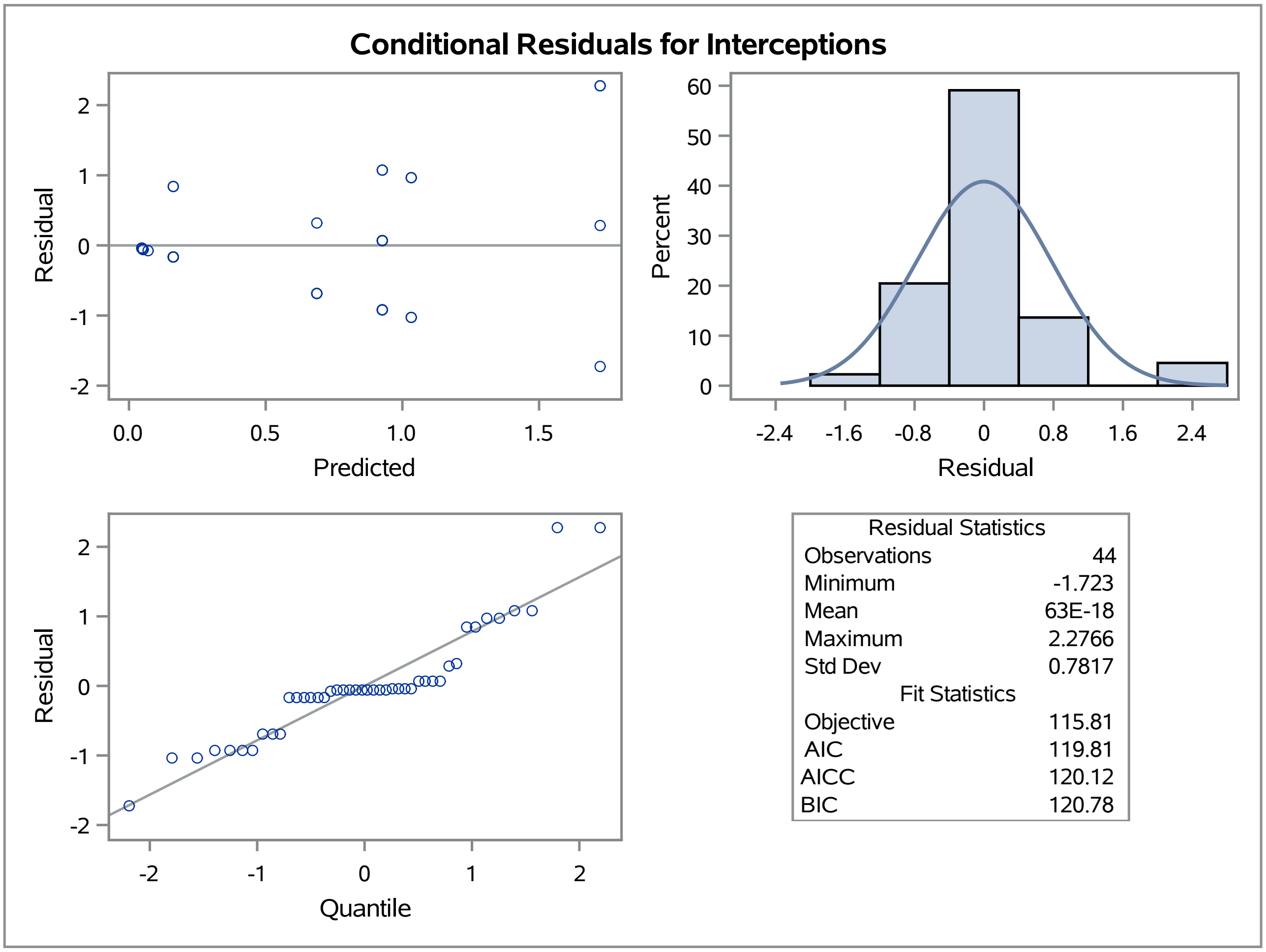

DistSoma=354

Model Information	
**Data Set**	WORK.TEMPDATASORTED
**Dependent Variable**	Interceptions
**Covariance Structure**	Variance Components
**Estimation Method**	REML
**Residual Variance Method**	Profile
**Fixed Effects SE Method**	Model-Based
**Degrees of Freedom Method**	Containment

Class Level Information		
**Class**	**Levels**	**Values**
**Treatment**	2	Control_GFP GFP MsTTR
**Culture**	12	1 2 3 4 5 6 7 8 9 10 11 12

Dimensions	
**Covariance Parameters**	2
**Columns in X**	3
**Columns in Z**	12
**Subjects**	1
**Max Obs per Subject**	44

Number of Observations	
**Number of Observations Read**	44
**Number of Observations Used**	44
**Number of Observations Not Used**	0

Iteration History			
**Iteration**	**Evaluations**	**-2 Res Log Like**	**Criterion**
**0**	1	127.71545407	
**1**	3	126.82768492	0.00001823
**2**	1	126.82722674	0.00000001

Convergence criteria met.

Covariance Parameter Estimates				
**Cov Parm**	**Estimate**	**Alpha**	**Lower**	**Upper**
**Culture**	0.1341	0.05	0.02899	45.3688
**Residual**	0.9388	0.05	0.6127	1.6184

DistSoma=354

Fit Statistics	
-2 Res Log Likelihood	126.8
AIC (Smaller is Better)	130.8
AICC (Smaller is Better)	131.1
BIC (Smaller is Better)	131.8

Solution for Fixed Effects									
Effect	Treatment	Estimate	Standard Error	DF	t Value	Pr > |t|	Alpha	Lower	Upper
Intercept		1.1535	0.2640	10	4.37	0.0014	0.05	0.5653	1.7417
Treatment	Control_GFP	-1.0676	0.3648	32	-2.93	0.0063	0.05	-1.8106	-0.3246
Treatment	GFP MsTTR	0	.	.	.	.	.	.	.

Solution for Random Effects									
Effect	Culture	Estimate	Std Err Pred	DF	t Value	Pr > |t|	Alpha	Lower	Upper
Culture	1	-0.02577	0.3155	32	-0.08	0.9354	0.05	-0.6685	0.6169
Culture	2	-0.02577	0.3155	32	-0.08	0.9354	0.05	-0.6685	0.6169
Culture	3	0.05965	0.3061	32	0.19	0.8467	0.05	-0.5639	0.6832
Culture	4	-0.03124	0.3061	32	-0.10	0.9193	0.05	-0.6548	0.5923
Culture	5	0.05965	0.3061	32	0.19	0.8467	0.05	-0.5639	0.6832
Culture	6	-0.01074	0.3440	32	-0.03	0.9753	0.05	-0.7113	0.6899
Culture	7	-0.02577	0.3155	32	-0.08	0.9354	0.05	-0.6685	0.6169
Culture	8	-0.2306	0.3005	32	-0.77	0.4485	0.05	-0.8427	0.3815
Culture	9	0.5804	0.3075	32	1.89	0.0682	0.05	-0.04590	1.2067
Culture	10	-0.1467	0.3075	32	-0.48	0.6365	0.05	-0.7730	0.4796
Culture	11	-0.05582	0.3075	32	-0.18	0.8571	0.05	-0.6821	0.5705
Culture	12	-0.1473	0.3005	32	-0.49	0.6274	0.05	-0.7594	0.4649

Type 3 Tests of Fixed Effects				
Effect	Num DF	Den DF	F Value	Pr > F
Treatment	1	32	8.57	0.0063

Least Squares Means									
Effect	Treatment	Estimate	Standard Error	DF	t Value	Pr > |t|	Alpha	Lower	Upper
Treatment	Control_GFP	0.08593	0.2517	32	0.34	0.7351	0.05	-0.4269	0.5987
Treatment	GFP MsTTR	1.1535	0.2640	32	4.37	0.0001	0.05	0.6158	1.6913

DistSoma=354

Differences of Least Squares Means												
**Effect**	**Treatment**	**Treatment**	**Estimate**	**Standard Error**	**DF**	**t Value**	**Pr > |t|**	**Adjustment**	**Adj P**	**Alpha**	**Lower**	**Upper**
**Treatment**	Control_GFP	GFP MsTTR	-1.0676	0.3648	32	-2.93	0.0063	Tukey-Kramer	0.0063	0.05	-1.8106	-0.3246

Differences of Least Squares Means				
**Effect**	**Treatment**	**Treatment**	**Adj Lower**	**Adj Upper**
**Treatment**	Control_GFP	GFP MsTTR	-1.8106	-0.3246

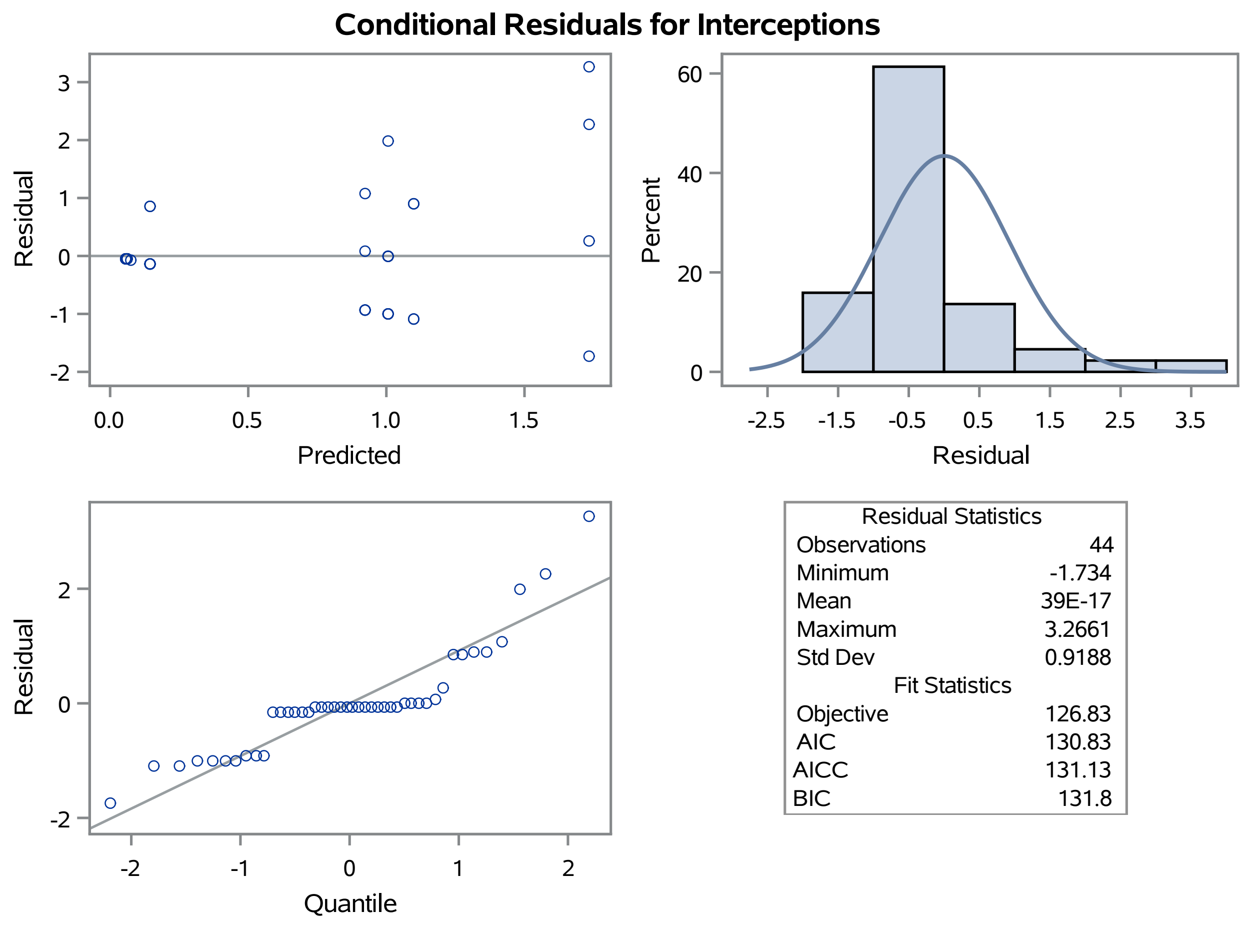

Residual Statistics	
Observations	44
Minimum	-1.734
Mean	39E-17
Maximum	3.2661
Std Dev	0.9188
**Fit Statistics**	
Objective	126.83
AIC	130.83
AICC	131.13
BIC	131.8

DistSoma=360

Model Information	
**Data Set**	WORK.TEMPDATASORTED
**Dependent Variable**	Interceptions
**Covariance Structure**	Variance Components
**Estimation Method**	REML
**Residual Variance Method**	Profile
**Fixed Effects SE Method**	Model-Based
**Degrees of Freedom Method**	Containment

Class Level Information		
**Class**	**Levels**	**Values**
**Treatment**	2	Control_GFP GFP MsTTR
**Culture**	12	1 2 3 4 5 6 7 8 9 10 11 12

Dimensions	
**Covariance Parameters**	2
**Columns in X**	3
**Columns in Z**	12
**Subjects**	1
**Max Obs per Subject**	44

Number of Observations	
**Number of Observations Read**	44
**Number of Observations Used**	44
**Number of Observations Not Used**	0

Iteration History			
**Iteration**	**Evaluations**	**-2 Res Log Like**	**Criterion**
**0**	1	138.03115211	
**1**	3	137.76637275	0.00000048
**2**	1	137.76635808	0.00000000

Convergence criteria met.

Covariance Parameter Estimates				
**Cov Parm**	**Estimate**	**Alpha**	**Lower**	**Upper**
**Culture**	0.09236	0.05	0.01259	875260
**Residual**	1.2713	0.05	0.8294	2.1928

DistSoma=360

Fit Statistics	
-2 Res Log Likelihood	137.8
AIC (Smaller is Better)	141.8
AICC (Smaller is Better)	142.1
BIC (Smaller is Better)	142.7

Solution for Fixed Effects

Effect	Treatment	Estimate	Standard Error	DF	t Value	Pr > |t|	Alpha	Lower	Upper
Intercept		1.0540	0.2765	10	3.81	0.0034	0.05	0.4380	1.6700
Treatment	Control_GFP	-0.9661	0.3855	32	-2.51	0.0175	0.05	-1.7513	-0.1809
Treatment	GFP MsTTR	0	.	.	.	.	.	.	.

Solution for Random Effects

Effect	Culture	Estimate	Std Err Pred	DF	t Value	Pr > |t|	Alpha	Lower	Upper
Culture	1	-0.01574	0.2795	32	-0.06	0.9555	0.05	-0.5852	0.5537
Culture	2	-0.01574	0.2795	32	-0.06	0.9555	0.05	-0.5852	0.5537
Culture	3	0.03649	0.2743	32	0.13	0.8950	0.05	-0.5222	0.5952
Culture	4	-0.01980	0.2743	32	-0.07	0.9429	0.05	-0.5785	0.5389
Culture	5	0.03649	0.2743	32	0.13	0.8950	0.05	-0.5222	0.5952
Culture	6	-0.00596	0.2940	32	-0.02	0.9840	0.05	-0.6048	0.5929
Culture	7	-0.01574	0.2795	32	-0.06	0.9555	0.05	-0.5852	0.5537
Culture	8	-0.1210	0.2705	32	-0.45	0.6577	0.05	-0.6720	0.4300
Culture	9	0.3819	0.2747	32	1.39	0.1740	0.05	-0.1776	0.9414
Culture	10	-0.06846	0.2747	32	-0.25	0.8048	0.05	-0.6279	0.4910
Culture	11	-0.1247	0.2747	32	-0.45	0.6528	0.05	-0.6842	0.4347
Culture	12	-0.06769	0.2705	32	-0.25	0.8040	0.05	-0.6187	0.4833

Type 3 Tests of Fixed Effects

Effect	Num DF	Den DF	F Value	Pr > F
Treatment	1	32	6.28	0.0175

Least Squares Means

Effect	Treatment	Estimate	Standard Error	DF	t Value	Pr > |t|	Alpha	Lower	Upper
Treatment	Control_GFP	0.08795	0.2686	32	0.33	0.7455	0.05	-0.4592	0.6351
Treatment	GFP MsTTR	1.0540	0.2765	32	3.81	0.0006	0.05	0.4909	1.6172

DistSoma=360

Differences of Least Squares Means												
Effect	Treatment	Treatment	Estimate	Standard Error	DF	t Value	Pr > |t|	Adjustment	Adj P	Alpha	Lower	Upper
**Treatment**	Control_GFP	GFP MsTTR	-0.9661	0.3855	32	-2.51	0.0175	Tukey-Kramer	0.0175	0.05	-1.7513	-0.1809

Differences of Least Squares Means				
Effect	Treatment	Treatment	Adj Lower	Adj Upper
**Treatment**	Control_GFP	GFP MsTTR	-1.7512	-0.1809

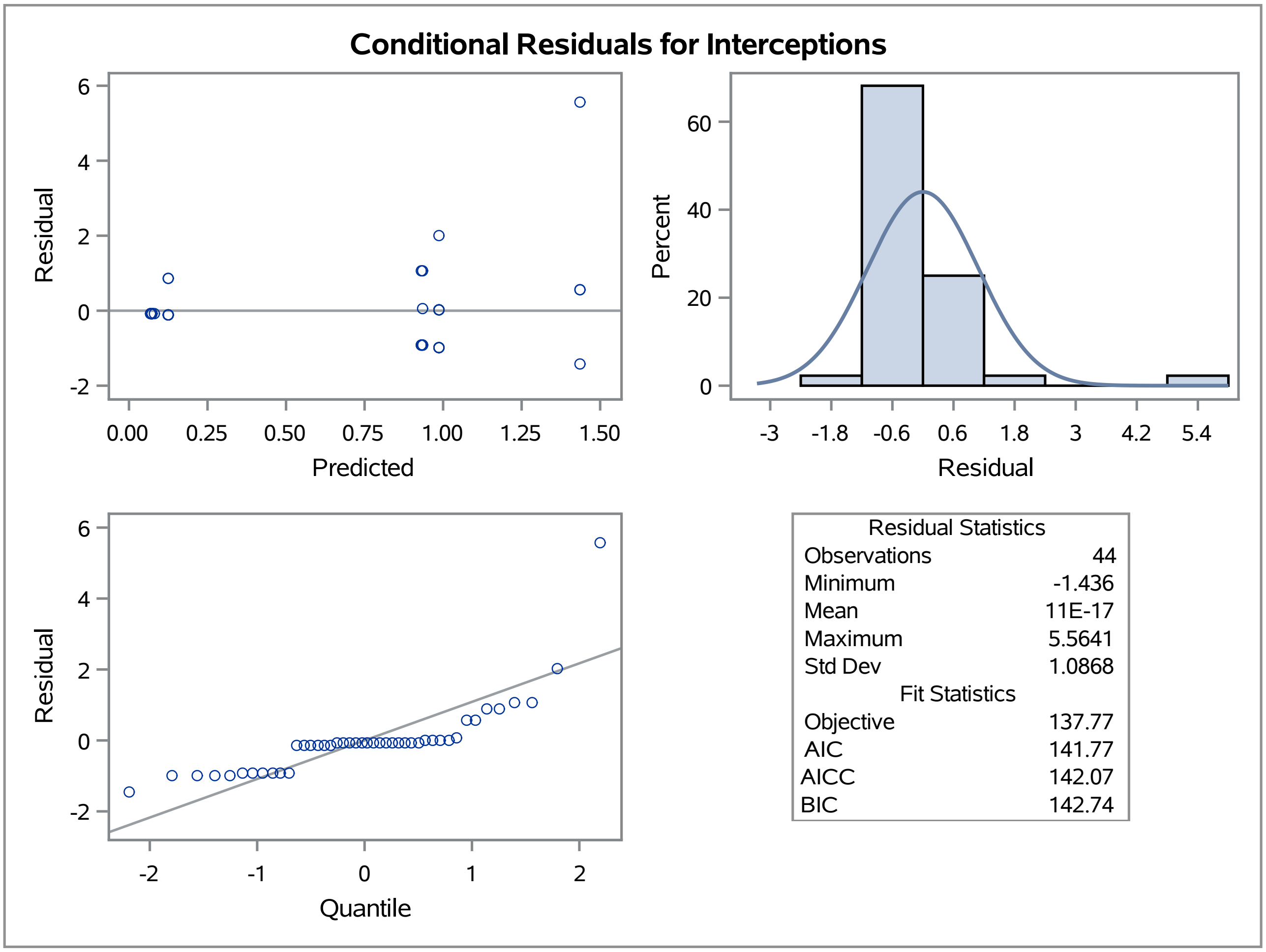

DistSoma=366

Model Information	
**Data Set**	WORK.TEMPDATASORTED
**Dependent Variable**	Interceptions
**Covariance Structure**	Variance Components
**Estimation Method**	REML
**Residual Variance Method**	Profile
**Fixed Effects SE Method**	Model-Based
**Degrees of Freedom Method**	Containment

Class Level Information		
**Class**	**Levels**	**Values**
**Treatment**	2	Control_GFP GFP MsTTR
**Culture**	12	1 2 3 4 5 6 7 8 9 10 11 12

Dimensions	
**Covariance Parameters**	2
**Columns in X**	3
**Columns in Z**	12
**Subjects**	1
**Max Obs per Subject**	44

Number of Observations	
**Number of Observations Read**	44
**Number of Observations Used**	44
**Number of Observations Not Used**	0

Iteration History			
**Iteration**	**Evaluations**	**-2 Res Log Like**	**Criterion**
**0**	1	160.80329782	
**1**	3	160.71441836	0.00000003
**2**	1	160.71441717	0.00000000

Convergence criteria met.

Covariance Parameter Estimates				
**Cov Parm**	**Estimate**	**Alpha**	**Lower**	**Upper**
**Culture**	0.09089	0.05	0.008690	2.498E18
**Residual**	2.2460	0.05	1.4651	3.8743

DistSoma=366

Fit Statistics	
-2 Res Log Likelihood	160.7
AIC (Smaller is Better)	164.7
AICC (Smaller is Better)	165.0
BIC (Smaller is Better)	165.7

Solution for Fixed Effects

Effect	Treatment	Estimate	Standard Error	DF	t Value	Pr > |t|	Alpha	Lower	Upper
Intercept		1.0970	0.3471	10	3.16	0.0101	0.05	0.3237	1.8703
Treatment	Control_GFP	-1.0079	0.4866	32	-2.07	0.0465	0.05	-1.9990	-0.01676
Treatment	GFP MsTTR	0	.	.	.	.	.	.	.

Solution for Random Effects

Effect	Culture	Estimate	Std Err Pred	DF	t Value	Pr > |t|	Alpha	Lower	Upper
Culture	1	-0.00965	0.2871	32	-0.03	0.9734	0.05	-0.5944	0.5751
Culture	2	-0.00965	0.2871	32	-0.03	0.9734	0.05	-0.5944	0.5751
Culture	3	0.02241	0.2837	32	0.08	0.9375	0.05	-0.5555	0.6003
Culture	4	-0.01242	0.2837	32	-0.04	0.9654	0.05	-0.5903	0.5655
Culture	5	0.02241	0.2837	32	0.08	0.9375	0.05	-0.5555	0.6003
Culture	6	-0.00347	0.2959	32	-0.01	0.9907	0.05	-0.6061	0.5992
Culture	7	-0.00965	0.2871	32	-0.03	0.9734	0.05	-0.5944	0.5751
Culture	8	-0.08364	0.2811	32	-0.30	0.7680	0.05	-0.6562	0.4889
Culture	9	0.3000	0.2838	32	1.06	0.2985	0.05	-0.2782	0.8781
Culture	10	-0.08317	0.2838	32	-0.29	0.7714	0.05	-0.6613	0.4950
Culture	11	-0.08317	0.2838	32	-0.29	0.7714	0.05	-0.6613	0.4950
Culture	12	-0.04998	0.2811	32	-0.18	0.8600	0.05	-0.6225	0.5226

Type 3 Tests of Fixed Effects

Effect	Num DF	Den DF	F Value	Pr > F
Treatment	1	32	4.29	0.0465

Least Squares Means

Effect	Treatment	Estimate	Standard Error	DF	t Value	Pr > |t|	Alpha	Lower	Upper
Treatment	Control_GFP	0.08912	0.3410	32	0.26	0.7955	0.05	-0.6055	0.7837
Treatment	GFP MsTTR	1.0970	0.3471	32	3.16	0.0034	0.05	0.3900	1.8040

DistSoma=366

Differences of Least Squares Means												
Effect	Treatment	Treatment	Estimate	Standard Error	DF	t Value	Pr > |t|	Adjustment	Adj P	Alpha	Lower	Upper
**Treatment**	Control_GFP	GFP MsTTR	-1.0079	0.4866	32	-2.07	0.0465	Tukey-Kramer	0.0465	0.05	-1.9990	-0.01676

Differences of Least Squares Means				
Effect	Treatment	Treatment	Adj Lower	Adj Upper
**Treatment**	Control_GFP	GFP MsTTR	-1.9990	-0.01677

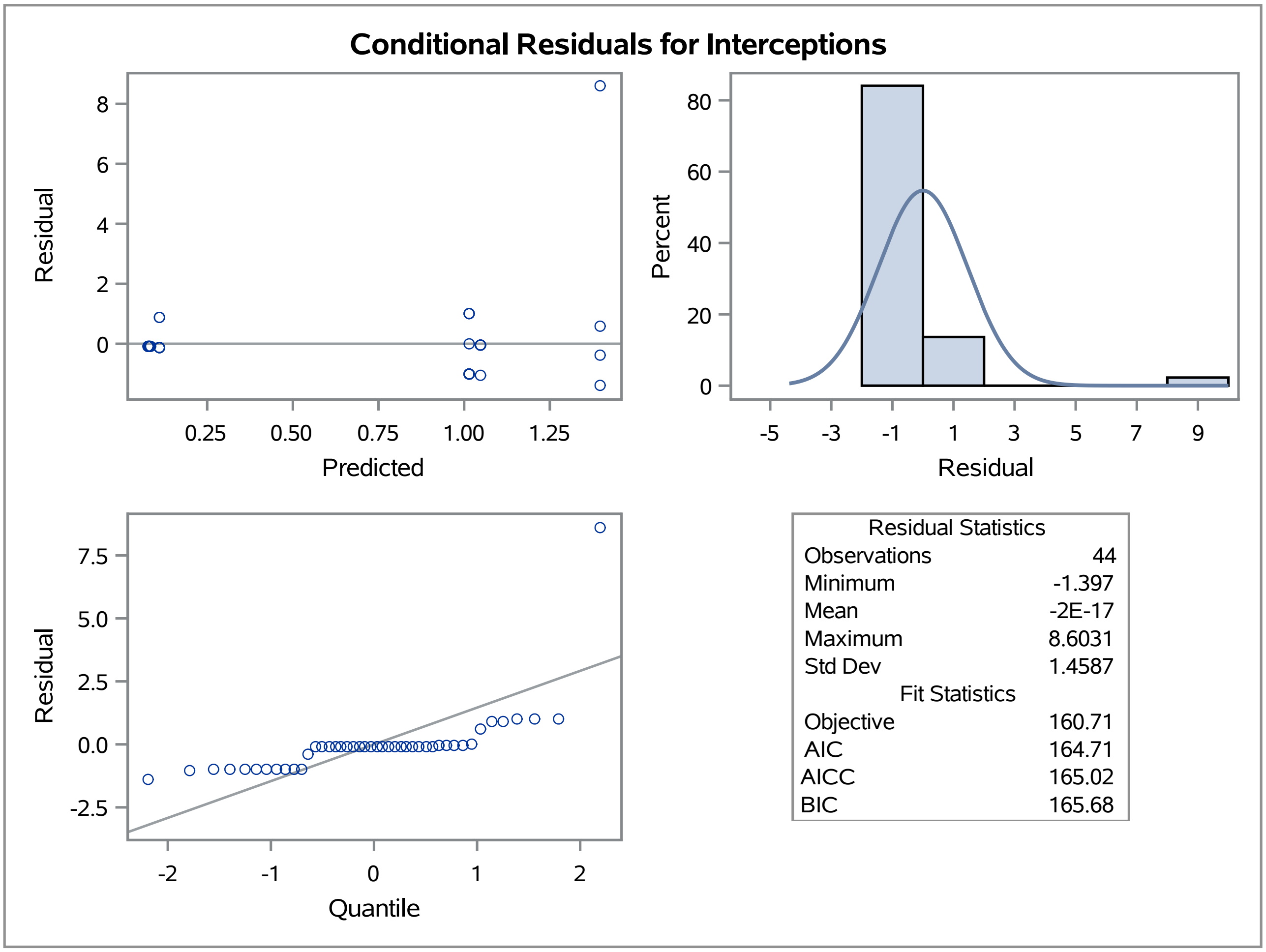

DistSoma=372

Model Information	
**Data Set**	WORK.TEMPDATASORTED
**Dependent Variable**	Interceptions
**Covariance Structure**	Variance Components
**Estimation Method**	REML
**Residual Variance Method**	Profile
**Fixed Effects SE Method**	Model-Based
**Degrees of Freedom Method**	Containment

Class Level Information		
**Class**	**Levels**	**Values**
**Treatment**	2	Control_GFP GFP MsTTR
**Culture**	12	1 2 3 4 5 6 7 8 9 10 11 12

Dimensions	
**Covariance Parameters**	2
**Columns in X**	3
**Columns in Z**	12
**Subjects**	1
**Max Obs per Subject**	44

Number of Observations	
**Number of Observations Read**	44
**Number of Observations Used**	44
**Number of Observations Not Used**	0

Iteration History			
**Iteration**	**Evaluations**	**-2 Res Log Like**	**Criterion**
**0**	1	153.37977633	
**1**	3	153.21825896	0.00000019
**2**	1	153.21825170	0.00000000

Convergence criteria met.

Covariance Parameter Estimates				
**Cov Parm**	**Estimate**	**Alpha**	**Lower**	**Upper**
**Culture**	0.1027	0.05	0.01170	1.083E10
**Residual**	1.8589	0.05	1.2132	3.2042

DistSoma=372

Fit Statistics	
-2 Res Log Likelihood	153.2
AIC (Smaller is Better)	157.2
AICC (Smaller is Better)	157.5
BIC (Smaller is Better)	158.2

Solution for Fixed Effects

Effect	Treatment	Estimate	Standard Error	DF	t Value	Pr > |t|	Alpha	Lower	Upper
Intercept		1.0080	0.3244	10	3.11	0.0111	0.05	0.2852	1.7308
Treatment	Control_GFP	-0.9195	0.4536	32	-2.03	0.0510	0.05	-1.8434	0.004423
Treatment	GFP MsTTR	0	.	.	.	.	.	.	.

Solution for Random Effects

Effect	Culture	Estimate	Std Err Pred	DF	t Value	Pr > |t|	Alpha	Lower	Upper
Culture	1	-0.01259	0.3002	32	-0.04	0.9668	0.05	-0.6240	0.5988
Culture	2	-0.01259	0.3002	32	-0.04	0.9668	0.05	-0.6240	0.5988
Culture	3	0.02921	0.2956	32	0.10	0.9219	0.05	-0.5729	0.6313
Culture	4	-0.01602	0.2956	32	-0.05	0.9571	0.05	-0.6181	0.5861
Culture	5	0.02921	0.2956	32	0.10	0.9219	0.05	-0.5729	0.6313
Culture	6	-0.00463	0.3123	32	-0.01	0.9883	0.05	-0.6408	0.6316
Culture	7	-0.01259	0.3002	32	-0.04	0.9668	0.05	-0.6240	0.5988
Culture	8	-0.1316	0.2922	32	-0.45	0.6556	0.05	-0.7267	0.4636
Culture	9	0.3604	0.2958	32	1.22	0.2321	0.05	-0.2422	0.9630
Culture	10	-0.09191	0.2958	32	-0.31	0.7581	0.05	-0.6945	0.5107
Culture	11	-0.09191	0.2958	32	-0.31	0.7581	0.05	-0.6945	0.5107
Culture	12	-0.04501	0.2922	32	-0.15	0.8785	0.05	-0.6402	0.5501

Type 3 Tests of Fixed Effects

Effect	Num DF	Den DF	F Value	Pr > F
Treatment	1	32	4.11	0.0510

Least Squares Means

Effect	Treatment	Estimate	Standard Error	DF	t Value	Pr > |t|	Alpha	Lower	Upper
Treatment	Control_GFP	0.08856	0.3170	32	0.28	0.7818	0.05	-0.5572	0.7343
Treatment	GFP MsTTR	1.0080	0.3244	32	3.11	0.0039	0.05	0.3473	1.6688

DistSoma=372

Differences of Least Squares Means												
**Effect**	**Treatment**	**Treatment**	**Estimate**	**Standard Error**	**DF**	**t Value**	**Pr > |t|**	**Adjustment**	**Adj P**	**Alpha**	**Lower**	**Upper**
**Treatment**	Control_GFP	GFP MsTTR	-0.9195	0.4536	32	-2.03	0.0510	Tukey-Kramer	0.0510	0.05	-1.8434	0.004423

Differences of Least Squares Means				
**Effect**	**Treatment**	**Treatment**	**Adj Lower**	**Adj Upper**
**Treatment**	Control_GFP	GFP MsTTR	-1.8433	0.004411

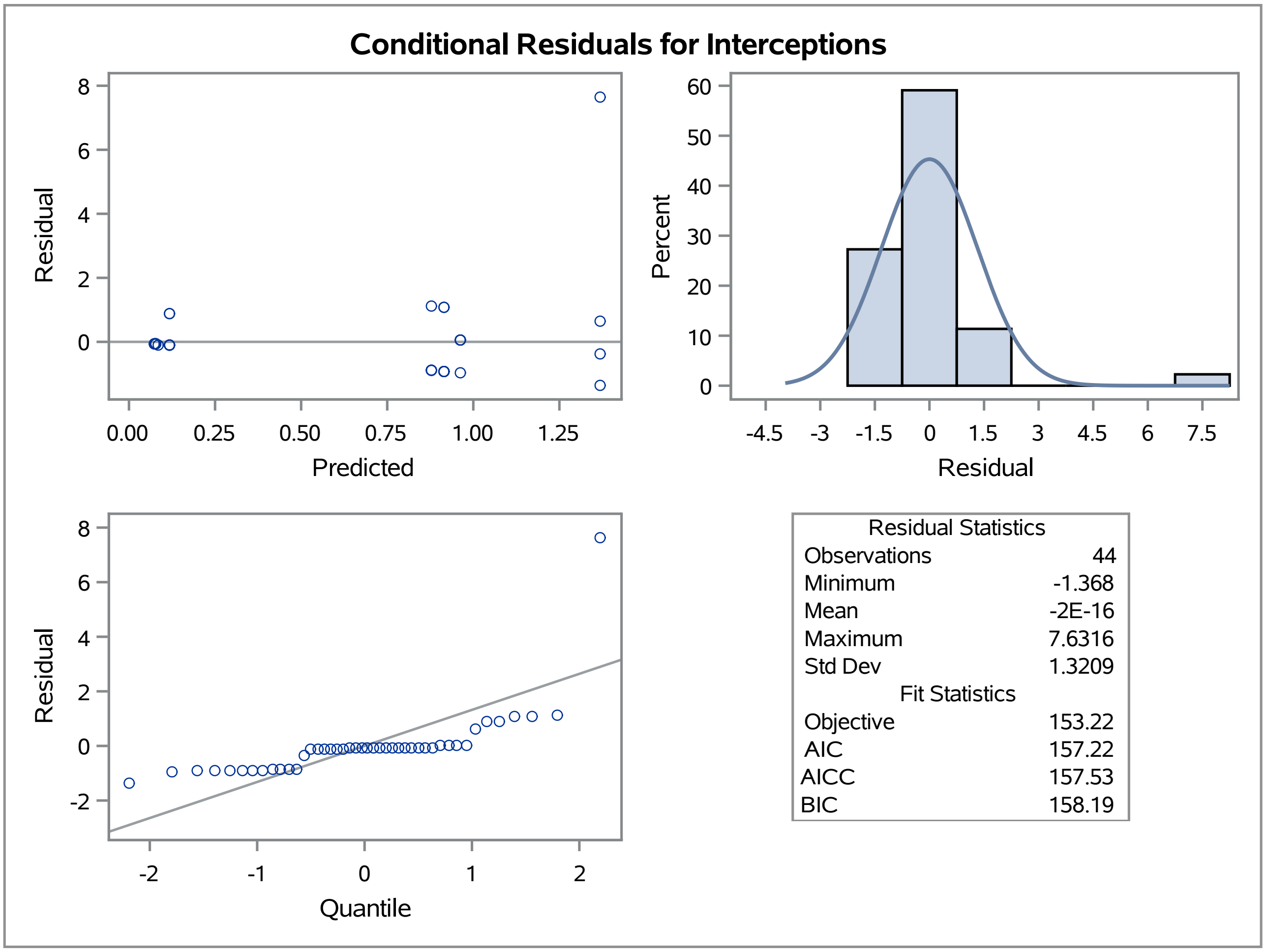

DistSoma=378

Model Information	
**Data Set**	WORK.TEMPDATASORTED
**Dependent Variable**	Interceptions
**Covariance Structure**	Variance Components
**Estimation Method**	REML
**Residual Variance Method**	Profile
**Fixed Effects SE Method**	Model-Based
**Degrees of Freedom Method**	Containment

Class Level Information		
**Class**	**Levels**	**Values**
**Treatment**	2	Control_GFP GFP MsTTR
**Culture**	12	1 2 3 4 5 6 7 8 9 10 11 12

Dimensions	
**Covariance Parameters**	2
**Columns in X**	3
**Columns in Z**	12
**Subjects**	1
**Max Obs per Subject**	44

Number of Observations	
**Number of Observations Read**	44
**Number of Observations Used**	44
**Number of Observations Not Used**	0

Iteration History			
**Iteration**	**Evaluations**	**-2 Res Log Like**	**Criterion**
**0**	1	144.84517609	
**1**	3	144.69055166	0.00000020
**2**	1	144.69054481	0.00000000

Convergence criteria met.

Covariance Parameter Estimates				
**Cov Parm**	**Estimate**	**Alpha**	**Lower**	**Upper**
**Culture**	0.08188	0.05	0.009194	2.506E10
**Residual**	1.5187	0.05	0.9912	2.6177

DistSoma=378

Fit Statistics	
-2 Res Log Likelihood	144.7
AIC (Smaller is Better)	148.7
AICC (Smaller is Better)	149.0
BIC (Smaller is Better)	149.7

Solution for Fixed Effects

Effect	Treatment	Estimate	Standard Error	DF	t Value	Pr > |t|	Alpha	Lower	Upper
Intercept		0.9615	0.2925	10	3.29	0.0082	0.05	0.3097	1.6133
Treatment	Control_GFP	-0.8729	0.4091	32	-2.13	0.0406	0.05	-1.7062	-0.03959
Treatment	GFP MsTTR	0	.	.	.	.	.	.	.

Solution for Random Effects

Effect	Culture	Estimate	Std Err Pred	DF	t Value	Pr > |t|	Alpha	Lower	Upper
Culture	1	-0.01234	0.2684	32	-0.05	0.9636	0.05	-0.5591	0.5345
Culture	2	-0.01234	0.2684	32	-0.05	0.9636	0.05	-0.5591	0.5345
Culture	3	0.02863	0.2644	32	0.11	0.9145	0.05	-0.5100	0.5673
Culture	4	-0.01572	0.2644	32	-0.06	0.9530	0.05	-0.5544	0.5229
Culture	5	0.02863	0.2644	32	0.11	0.9145	0.05	-0.5100	0.5673
Culture	6	-0.00453	0.2791	32	-0.02	0.9871	0.05	-0.5731	0.5640
Culture	7	-0.01234	0.2684	32	-0.05	0.9636	0.05	-0.5591	0.5345
Culture	8	-0.1192	0.2614	32	-0.46	0.6514	0.05	-0.6518	0.4133
Culture	9	0.3173	0.2647	32	1.20	0.2394	0.05	-0.2218	0.8564
Culture	10	-0.08187	0.2647	32	-0.31	0.7591	0.05	-0.6210	0.4572
Culture	11	-0.08187	0.2647	32	-0.31	0.7591	0.05	-0.6210	0.4572
Culture	12	-0.03430	0.2614	32	-0.13	0.8965	0.05	-0.5668	0.4982

Type 3 Tests of Fixed Effects

Effect	Num DF	Den DF	F Value	Pr > F
Treatment	1	32	4.55	0.0406

Least Squares Means

Effect	Treatment	Estimate	Standard Error	DF	t Value	Pr > |t|	Alpha	Lower	Upper
Treatment	Control_GFP	0.08861	0.2860	32	0.31	0.7587	0.05	-0.4940	0.6712
Treatment	GFP MsTTR	0.9615	0.2925	32	3.29	0.0025	0.05	0.3657	1.5574

DistSoma=378

Differences of Least Squares Means												
Effect	Treatment	Treatment	Estimate	Standard Error	DF	t Value	Pr > |t|	Adjustment	Adj P	Alpha	Lower	Upper
Treatment	Control_GFP	GFP MsTTR	-0.8729	0.4091	32	-2.13	0.0406	Tukey-Kramer	0.0406	0.05	-1.7062	-0.03959

Differences of Least Squares Means				
Effect	Treatment	Treatment	Adj Lower	Adj Upper
Treatment	Control_GFP	GFP MsTTR	-1.7062	-0.03960

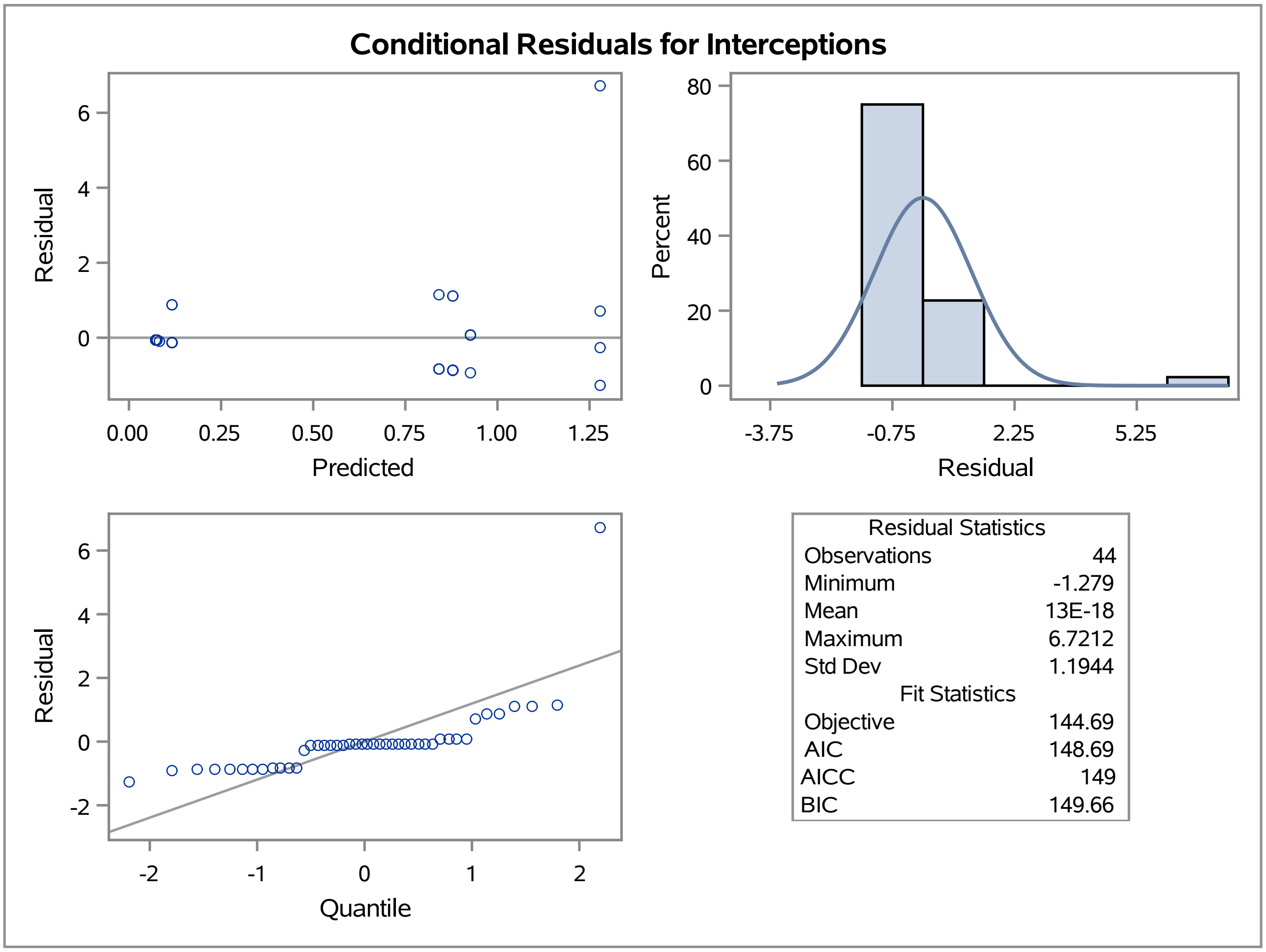

DistSoma=384

Model Information	
**Data Set**	WORK.TEMPDATASORTED
**Dependent Variable**	Interceptions
**Covariance Structure**	Variance Components
**Estimation Method**	REML
**Residual Variance Method**	Profile
**Fixed Effects SE Method**	Model-Based
**Degrees of Freedom Method**	Containment

Class Level Information		
**Class**	**Levels**	**Values**
**Treatment**	2	Control_GFP GFP MsTTR
**Culture**	12	1 2 3 4 5 6 7 8 9 10 11 12

Dimensions	
**Covariance Parameters**	2
**Columns in X**	3
**Columns in Z**	12
**Subjects**	1
**Max Obs per Subject**	44

Number of Observations	
**Number of Observations Read**	44
**Number of Observations Used**	44
**Number of Observations Not Used**	0

Iteration History			
**Iteration**	**Evaluations**	**-2 Res Log Like**	**Criterion**
**0**	1	135.21503755	
**1**	3	135.14591304	0.00000017
**2**	1	135.14590797	0.00000000

Convergence criteria met.

Covariance Parameter Estimates				
**Cov Parm**	**Estimate**	**Alpha**	**Lower**	**Upper**
**Culture**	0.04213	0.05	0.003825	3.009E23
**Residual**	1.2275	0.05	0.8024	2.1103

DistSoma=384

Fit Statistics	
**-2 Res Log Likelihood**	135.1
**AIC (Smaller is Better)**	139.1
**AICC (Smaller is Better)**	139.5
**BIC (Smaller is Better)**	140.1

Solution for Fixed Effects

Effect	Treatment	Estimate	Standard Error	DF	t Value	Pr > |t|	Alpha	Lower	Upper
**Intercept**		0.8211	0.2536	10	3.24	0.0089	0.05	0.2561	1.3862
**Treatment**	Control_GFP	-0.7318	0.3559	32	-2.06	0.0480	0.05	-1.4568	-0.00673
**Treatment**	GFP MsTTR	0	.	.	.	.	.	.	.

Solution for Random Effects

Effect	Culture	Estimate	Std Err Pred	DF	t Value	Pr > |t|	Alpha	Lower	Upper
**Culture**	1	-0.00834	0.1968	32	-0.04	0.9665	0.05	-0.4093	0.3926
**Culture**	2	-0.00834	0.1968	32	-0.04	0.9665	0.05	-0.4093	0.3926
**Culture**	3	0.01939	0.1948	32	0.10	0.9213	0.05	-0.3774	0.4162
**Culture**	4	-0.01079	0.1948	32	-0.06	0.9562	0.05	-0.4076	0.3860
**Culture**	5	0.01939	0.1948	32	0.10	0.9213	0.05	-0.3774	0.4162
**Culture**	6	-0.00297	0.2020	32	-0.01	0.9884	0.05	-0.4144	0.4085
**Culture**	7	-0.00834	0.1968	32	-0.04	0.9665	0.05	-0.4093	0.3926
**Culture**	8	-0.09098	0.1932	32	-0.47	0.6410	0.05	-0.4846	0.3026
**Culture**	9	0.1725	0.1949	32	0.89	0.3827	0.05	-0.2245	0.5695
**Culture**	10	-0.03876	0.1949	32	-0.20	0.8436	0.05	-0.4357	0.3582
**Culture**	11	-0.06894	0.1949	32	-0.35	0.7258	0.05	-0.4659	0.3280
**Culture**	12	0.02620	0.1932	32	0.14	0.8930	0.05	-0.3674	0.4198

Type 3 Tests of Fixed Effects

Effect	Num DF	Den DF	F Value	Pr > F
**Treatment**	1	32	4.23	0.0480

Least Squares Means

Effect	Treatment	Estimate	Standard Error	DF	t Value	Pr > |t|	Alpha	Lower	Upper
**Treatment**	Control_GFP	0.08937	0.2498	32	0.36	0.7228	0.05	-0.4194	0.5981
**Treatment**	GFP MsTTR	0.8211	0.2536	32	3.24	0.0028	0.05	0.3046	1.3377

DistSoma=384

Differences of Least Squares Means												
**Effect**	**Treatment**	**Treatment**	**Estimate**	**Standard Error**	**DF**	**t Value**	**Pr > |t|**	**Adjustment**	**Adj P**	**Alpha**	**Lower**	**Upper**
**Treatment**	Control_GFP	GFP MsTTR	-0.7318	0.3559	32	-2.06	0.0480	Tukey-Kramer	0.0480	0.05	-1.4568	-0.00673

Differences of Least Squares Means				
**Effect**	**Treatment**	**Treatment**	**Adj Lower**	**Adj Upper**
**Treatment**	Control_GFP	GFP MsTTR	-1.4568	-0.00674

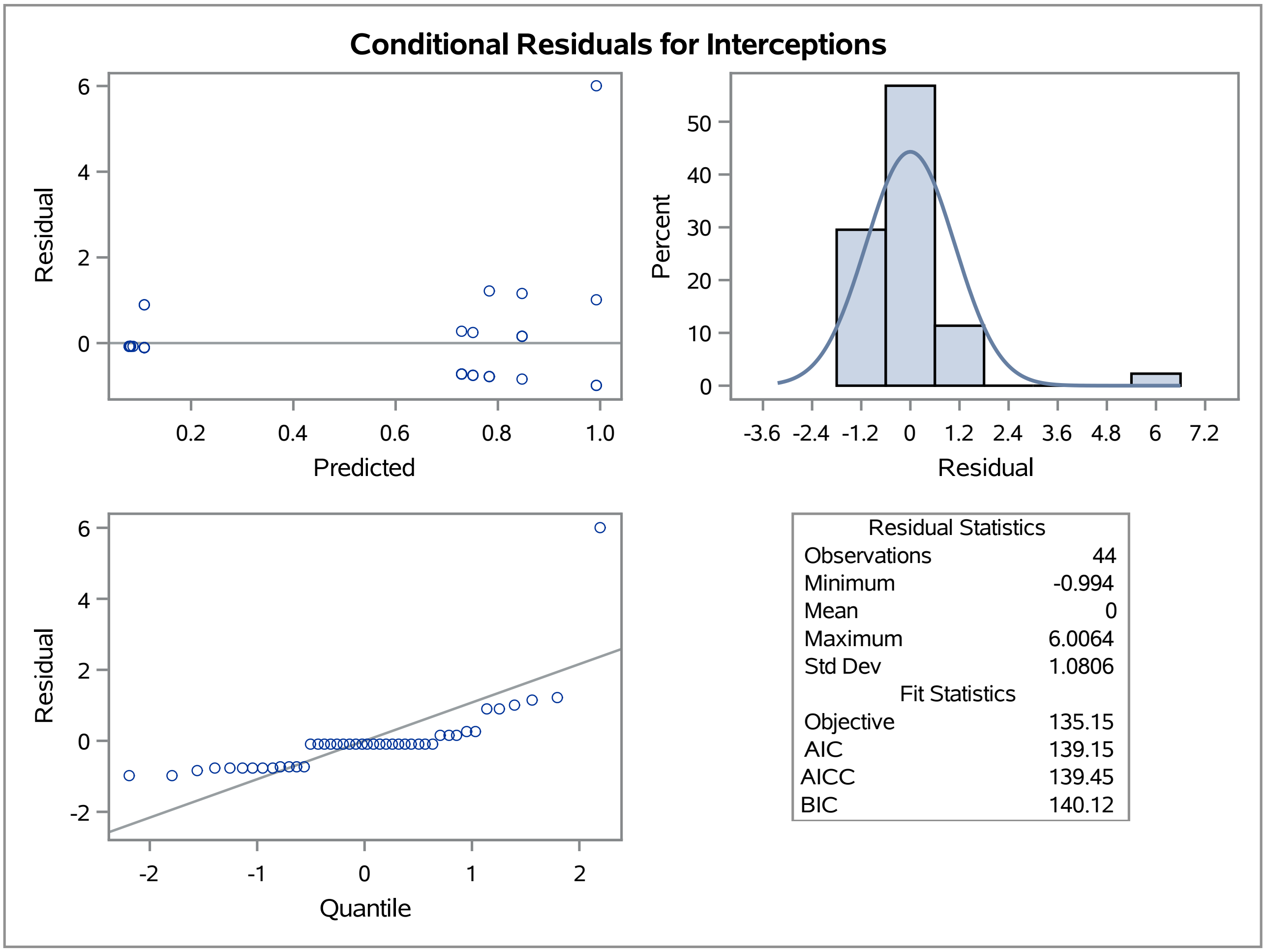

DistSoma=390

Model Information	
**Data Set**	WORK.TEMPDATASORTED
**Dependent Variable**	Interceptions
**Covariance Structure**	Variance Components
**Estimation Method**	REML
**Residual Variance Method**	Profile
**Fixed Effects SE Method**	Model-Based
**Degrees of Freedom Method**	Containment

Class Level Information		
**Class**	**Levels**	**Values**
**Treatment**	2	Control_GFP GFP MsTTR
**Culture**	12	1 2 3 4 5 6 7 8 9 10 11 12

Dimensions	
**Covariance Parameters**	2
**Columns in X**	3
**Columns in Z**	12
**Subjects**	1
**Max Obs per Subject**	44

Number of Observations	
**Number of Observations Read**	44
**Number of Observations Used**	44
**Number of Observations Not Used**	0

Iteration History			
**Iteration**	**Evaluations**	**-2 Res Log Like**	**Criterion**
**0**	1	135.21503755	
**1**	3	135.14591304	0.00000017
**2**	1	135.14590797	0.00000000

Convergence criteria met.

Covariance Parameter Estimates				
**Cov Parm**	**Estimate**	**Alpha**	**Lower**	**Upper**
**Culture**	0.04213	0.05	0.003825	3.009E23
**Residual**	1.2275	0.05	0.8024	2.1103

DistSoma=390

Fit Statistics	
-2 Res Log Likelihood	135.1
AIC (Smaller is Better)	139.1
AICC (Smaller is Better)	139.5
BIC (Smaller is Better)	140.1

Solution for Fixed Effects									
Effect	Treatment	Estimate	Standard Error	DF	t Value	Pr > |t|	Alpha	Lower	Upper
Intercept		0.8211	0.2536	10	3.24	0.0089	0.05	0.2561	1.3862
Treatment	Control_GFP	-0.7318	0.3559	32	-2.06	0.0480	0.05	-1.4568	-0.00673
Treatment	GFP MsTTR	0	.	.	.	.	.	.	.

Solution for Random Effects									
Effect	Culture	Estimate	Std Err Pred	DF	t Value	Pr > |t|	Alpha	Lower	Upper
Culture	1	-0.00834	0.1968	32	-0.04	0.9665	0.05	-0.4093	0.3926
Culture	2	-0.00834	0.1968	32	-0.04	0.9665	0.05	-0.4093	0.3926
Culture	3	0.01939	0.1948	32	0.10	0.9213	0.05	-0.3774	0.4162
Culture	4	-0.01079	0.1948	32	-0.06	0.9562	0.05	-0.4076	0.3860
Culture	5	0.01939	0.1948	32	0.10	0.9213	0.05	-0.3774	0.4162
Culture	6	-0.00297	0.2020	32	-0.01	0.9884	0.05	-0.4144	0.4085
Culture	7	-0.00834	0.1968	32	-0.04	0.9665	0.05	-0.4093	0.3926
Culture	8	-0.09098	0.1932	32	-0.47	0.6410	0.05	-0.4846	0.3026
Culture	9	0.1725	0.1949	32	0.89	0.3827	0.05	-0.2245	0.5695
Culture	10	-0.03876	0.1949	32	-0.20	0.8436	0.05	-0.4357	0.3582
Culture	11	-0.06894	0.1949	32	-0.35	0.7258	0.05	-0.4659	0.3280
Culture	12	0.02620	0.1932	32	0.14	0.8930	0.05	-0.3674	0.4198

Type 3 Tests of Fixed Effects				
Effect	Num DF	Den DF	F Value	Pr > F
Treatment	1	32	4.23	0.0480

Least Squares Means									
Effect	Treatment	Estimate	Standard Error	DF	t Value	Pr > |t|	Alpha	Lower	Upper
Treatment	Control_GFP	0.08937	0.2498	32	0.36	0.7228	0.05	-0.4194	0.5981
Treatment	GFP MsTTR	0.8211	0.2536	32	3.24	0.0028	0.05	0.3046	1.3377

DistSoma=390

Differences of Least Squares Means												
Effect	Treatment	Treatment	Estimate	Standard Error	DF	t Value	Pr > |t|	Adjustment	Adj P	Alpha	Lower	Upper
Treatment	Control_GFP	GFP MsTTR	-0.7318	0.3559	32	-2.06	0.0480	Tukey-Kramer	0.0480	0.05	-1.4568	-0.00673

Differences of Least Squares Means				
Effect	Treatment	Treatment	Adj Lower	Adj Upper
Treatment	Control_GFP	GFP MsTTR	-1.4568	-0.00674

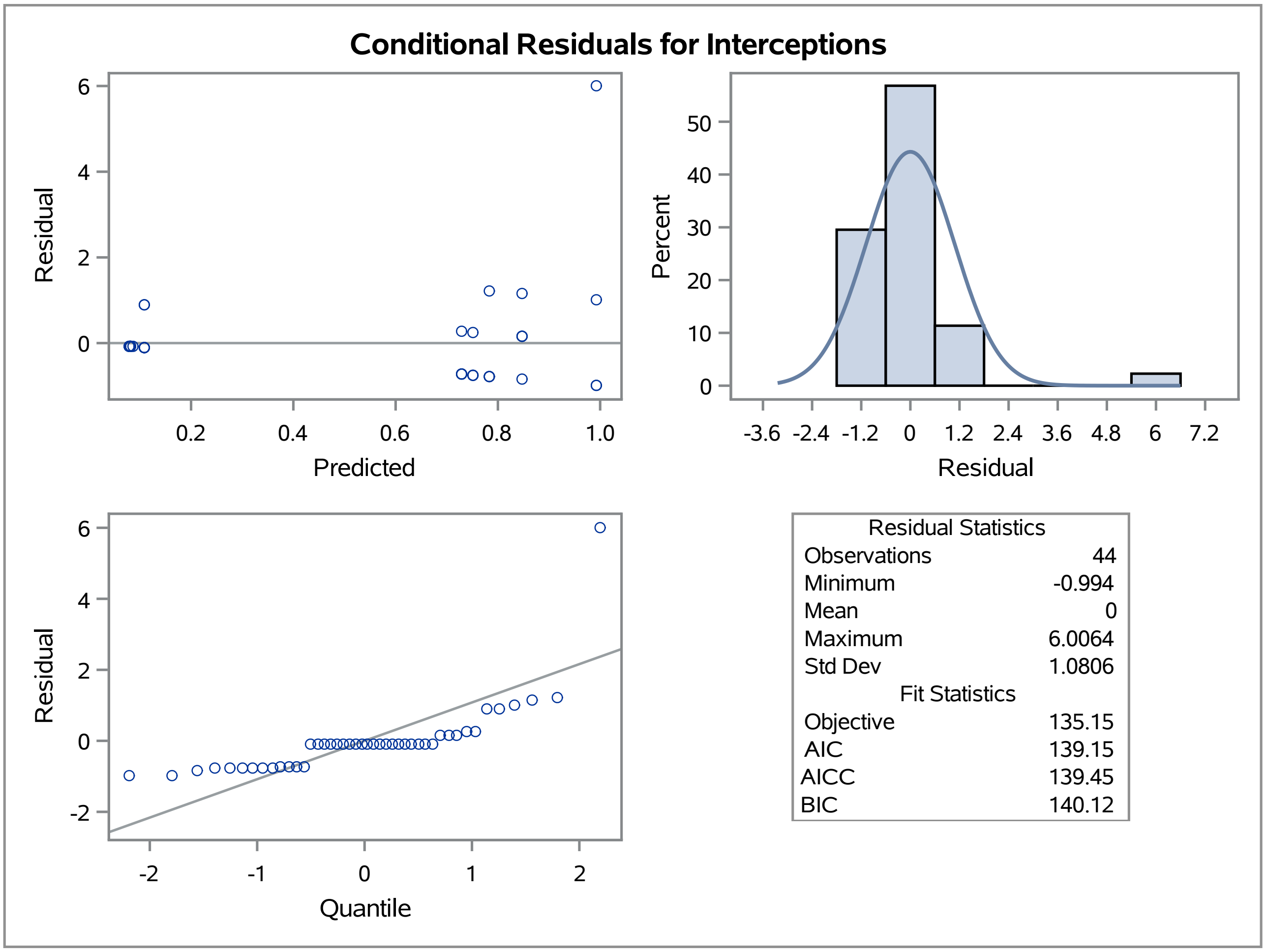

DistSoma=396

Model Information	
**Data Set**	WORK.TEMPDATASORTED
**Dependent Variable**	Interceptions
**Covariance Structure**	Variance Components
**Estimation Method**	REML
**Residual Variance Method**	Profile
**Fixed Effects SE Method**	Model-Based
**Degrees of Freedom Method**	Containment

Class Level Information		
**Class**	**Levels**	**Values**
**Treatment**	2	Control_GFP GFP MsTTR
**Culture**	12	1 2 3 4 5 6 7 8 9 10 11 12

Dimensions	
**Covariance Parameters**	2
**Columns in X**	3
**Columns in Z**	12
**Subjects**	1
**Max Obs per Subject**	44

Number of Observations	
**Number of Observations Read**	44
**Number of Observations Used**	44
**Number of Observations Not Used**	0

Iteration History			
**Iteration**	**Evaluations**	**-2 Res Log Like**	**Criterion**
**0**	1	137.21625185	
**1**	3	137.18146834	0.00000002
**2**	1	137.18146766	0.00000000

Convergence criteria met.

Covariance Parameter Estimates				
**Cov Parm**	**Estimate**	**Alpha**	**Lower**	**Upper**
**Culture**	0.03151	0.05	0.002797	8.545E46
**Residual**	1.2985	0.05	0.8483	2.2347

DistSoma=396

Fit Statistics	
-2 Res Log Likelihood	137.2
AIC (Smaller is Better)	141.2
AICC (Smaller is Better)	141.5
BIC (Smaller is Better)	142.2

Solution for Fixed Effects

Effect	Treatment	Estimate	Standard Error	DF	t Value	Pr > |t|	Alpha	Lower	Upper
Intercept		0.7754	0.2557	10	3.03	0.0126	0.05	0.2056	1.3452
Treatment	Control_GFP	-0.6856	0.3597	32	-1.91	0.0656	0.05	-1.4182	0.04694
Treatment	GFP MsTTR	0	.	.	.	.	.	.	.

Solution for Random Effects

Effect	Culture	Estimate	Std Err Pred	DF	t Value	Pr > |t|	Alpha	Lower	Upper
Culture	1	-0.00609	0.1722	32	-0.04	0.9720	0.05	-0.3569	0.3447
Culture	2	-0.00609	0.1722	32	-0.04	0.9720	0.05	-0.3569	0.3447
Culture	3	-0.00794	0.1709	32	-0.05	0.9632	0.05	-0.3561	0.3403
Culture	4	-0.00794	0.1709	32	-0.05	0.9632	0.05	-0.3561	0.3403
Culture	5	0.03629	0.1709	32	0.21	0.8332	0.05	-0.3119	0.3845
Culture	6	-0.00213	0.1755	32	-0.01	0.9904	0.05	-0.3596	0.3553
Culture	7	-0.00609	0.1722	32	-0.04	0.9720	0.05	-0.3569	0.3447
Culture	8	-0.06226	0.1699	32	-0.37	0.7164	0.05	-0.4083	0.2838
Culture	9	0.1305	0.1710	32	0.76	0.4510	0.05	-0.2178	0.4787
Culture	10	-0.02437	0.1710	32	-0.14	0.8876	0.05	-0.3726	0.3239
Culture	11	-0.04649	0.1710	32	-0.27	0.7875	0.05	-0.3948	0.3018
Culture	12	0.002658	0.1699	32	0.02	0.9876	0.05	-0.3434	0.3487

Type 3 Tests of Fixed Effects

Effect	Num DF	Den DF	F Value	Pr > F
Treatment	1	32	3.63	0.0656

Least Squares Means

Effect	Treatment	Estimate	Standard Error	DF	t Value	Pr > |t|	Alpha	Lower	Upper
Treatment	Control_GFP	0.08979	0.2529	32	0.36	0.7249	0.05	-0.4253	0.6049
Treatment	GFP MsTTR	0.7754	0.2557	32	3.03	0.0048	0.05	0.2545	1.2963

DistSoma=396

Differences of Least Squares Means												
**Effect**	**Treatment**	**Treatment**	**Estimate**	**Standard Error**	**DF**	**t Value**	**Pr > |t|**	**Adjustment**	**Adj P**	**Alpha**	**Lower**	**Upper**
**Treatment**	Control_GFP	GFP MsTTR	-0.6856	0.3597	32	-1.91	0.0656	Tukey-Kramer	0.0656	0.05	-1.4182	0.04694

Differences of Least Squares Means				
**Effect**	**Treatment**	**Treatment**	**Adj Lower**	**Adj Upper**
**Treatment**	Control_GFP	GFP MsTTR	-1.4182	0.04693

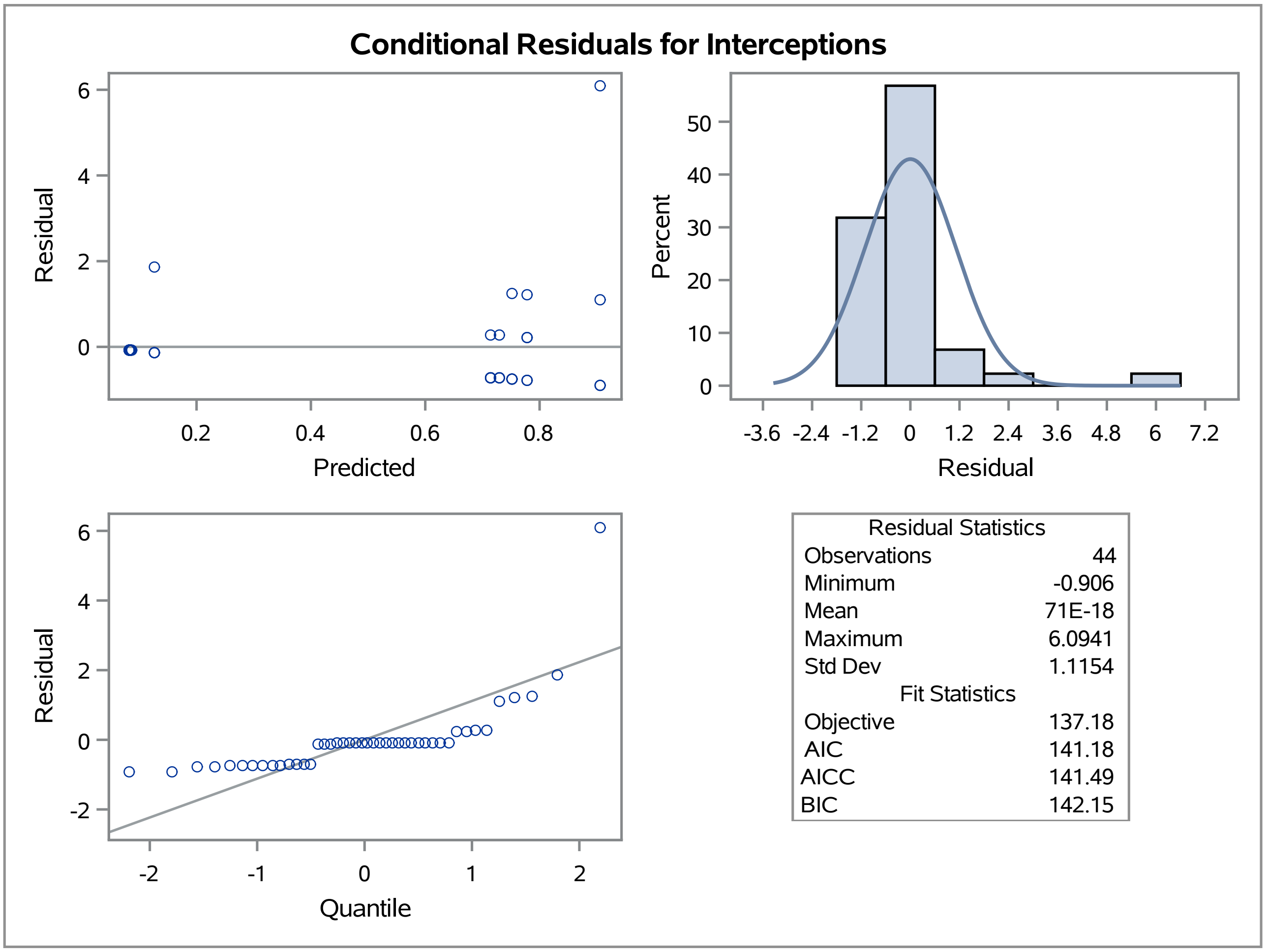

DistSoma=402

Model Information	
**Data Set**	WORK.TEMPDATASORTED
**Dependent Variable**	Interceptions
**Covariance Structure**	Variance Components
**Estimation Method**	REML
**Residual Variance Method**	Profile
**Fixed Effects SE Method**	Model-Based
**Degrees of Freedom Method**	Containment

Class Level Information		
**Class**	**Levels**	**Values**
**Treatment**	2	Control_GFP GFP MsTTR
**Culture**	12	1 2 3 4 5 6 7 8 9 10 11 12

Dimensions	
**Covariance Parameters**	2
**Columns in X**	3
**Columns in Z**	12
**Subjects**	1
**Max Obs per Subject**	44

Number of Observations	
**Number of Observations Read**	44
**Number of Observations Used**	44
**Number of Observations Not Used**	0

Iteration History			
**Iteration**	**Evaluations**	**-2 Res Log Like**	**Criterion**
**0**	1	123.70382789	
**1**	3	123.67267468	0.00000004
**2**	1	123.67267374	0.00000000

Convergence criteria met.

Covariance Parameter Estimates				
**Cov Parm**	**Estimate**	**Alpha**	**Lower**	**Upper**
**Culture**	0.02138	0.05	0.001934	2.035E52
**Residual**	0.9426	0.05	0.6162	1.6202

DistSoma=402

Fit Statistics	
-2 Res Log Likelihood	123.7
AIC (Smaller is Better)	127.7
AICC (Smaller is Better)	128.0
BIC (Smaller is Better)	128.6

Solution for Fixed Effects

Effect	Treatment	Estimate	Standard Error	DF	t Value	Pr > |t|	Alpha	Lower	Upper
Intercept		0.7294	0.2172	10	3.36	0.0073	0.05	0.2455	1.2133
Treatment	Control_GFP	-0.7294	0.3055	32	-2.39	0.0231	0.05	-1.3518	-0.1070
Treatment	GFP MsTTR	0	.	.	.	.	.	.	.

Solution for Random Effects

Effect	Culture	Estimate	Std Err Pred	DF	t Value	Pr > |t|	Alpha	Lower	Upper
Culture	1	0	0.1422	32	0.00	1.0000	0.05	-0.2896	0.2896
Culture	2	0	0.1422	32	0.00	1.0000	0.05	-0.2896	0.2896
Culture	3	0	0.1412	32	0.00	1.0000	0.05	-0.2875	0.2875
Culture	4	0	0.1412	32	0.00	1.0000	0.05	-0.2875	0.2875
Culture	5	0	0.1412	32	0.00	1.0000	0.05	-0.2875	0.2875
Culture	6	0	0.1447	32	0.00	1.0000	0.05	-0.2947	0.2947
Culture	7	0	0.1422	32	0.00	1.0000	0.05	-0.2896	0.2896
Culture	8	-0.05393	0.1403	32	-0.38	0.7033	0.05	-0.3398	0.2319
Culture	9	0.1057	0.1412	32	0.75	0.4595	0.05	-0.1819	0.3933
Culture	10	-0.01908	0.1412	32	-0.14	0.8933	0.05	-0.3067	0.2685
Culture	11	-0.03988	0.1412	32	-0.28	0.7794	0.05	-0.3274	0.2477
Culture	12	0.007193	0.1403	32	0.05	0.9594	0.05	-0.2787	0.2930

Type 3 Tests of Fixed Effects

Effect	Num DF	Den DF	F Value	Pr > F
Treatment	1	32	5.70	0.0231

Least Squares Means

Effect	Treatment	Estimate	Standard Error	DF	t Value	Pr > |t|	Alpha	Lower	Upper
Treatment	Control_GFP	0	0.2149	32	0.00	1.0000	0.05	-0.4378	0.4378
Treatment	GFP MsTTR	0.7294	0.2172	32	3.36	0.0020	0.05	0.2870	1.1718

DistSoma=402

Differences of Least Squares Means

Effect	Treatment	Treatment	Estimate	Standard Error	DF	t Value	Pr > |t|	Adjustment	Adj P	Alpha	Lower	Upper
**Treatment**	Control_GFP	GFP MsTTR	-0.7294	0.3055	32	-2.39	0.0231	Tukey-Kramer	0.0231	0.05	-1.3518	-0.1070

Differences of Least Squares Means

Effect	Treatment	Treatment	Adj Lower	Adj Upper
**Treatment**	Control_GFP	GFP MsTTR	-1.3518	-0.1070

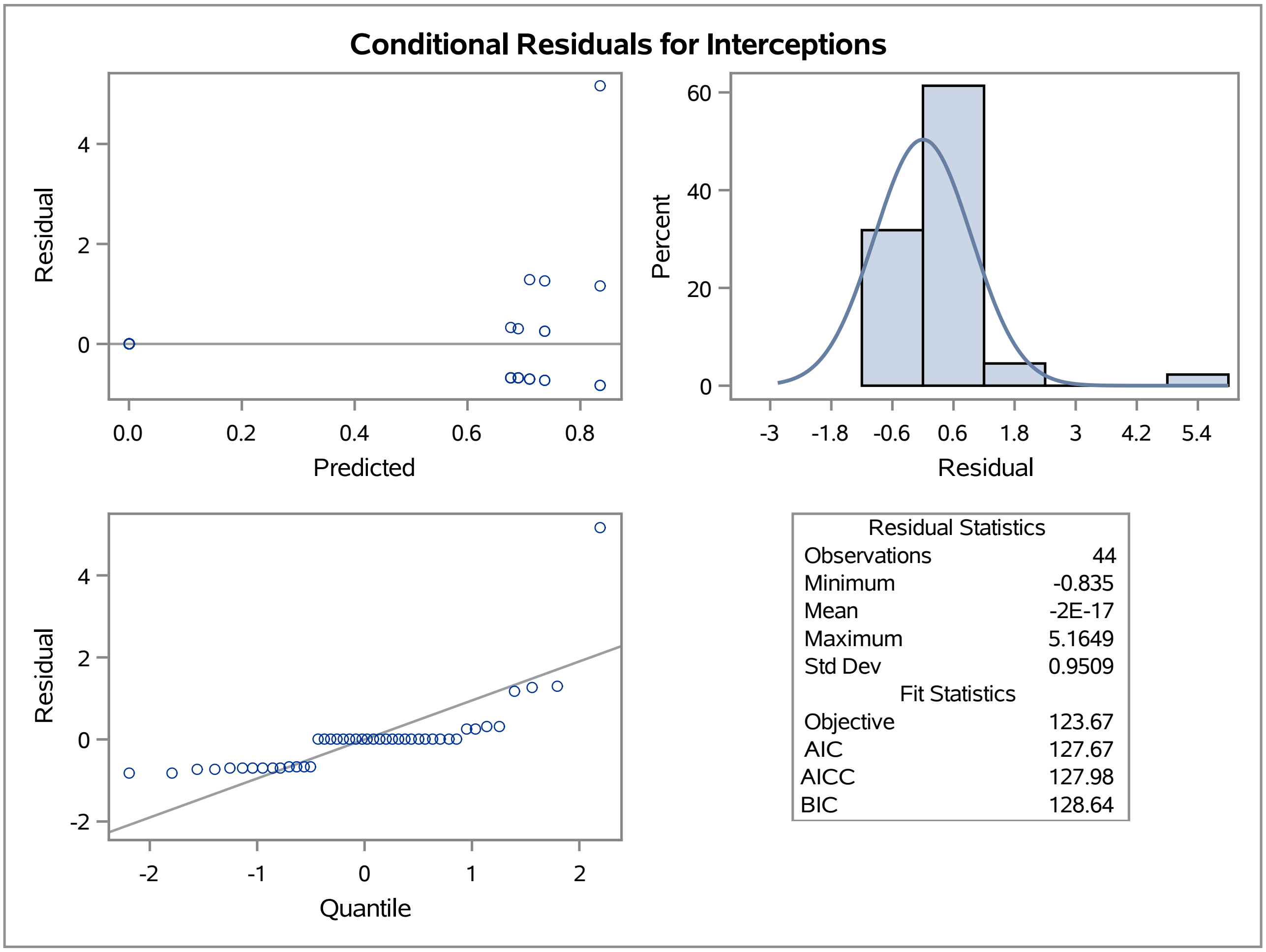

DistSoma=408

Model Information	
**Data Set**	WORK.TEMPDATASORTED
**Dependent Variable**	Interceptions
**Covariance Structure**	Variance Components
**Estimation Method**	REML
**Residual Variance Method**	Profile
**Fixed Effects SE Method**	Model-Based
**Degrees of Freedom Method**	Containment

Class Level Information		
**Class**	**Levels**	**Values**
**Treatment**	2	Control_GFP GFP MsTTR
**Culture**	12	1 2 3 4 5 6 7 8 9 10 11 12

Dimensions	
**Covariance Parameters**	2
**Columns in X**	3
**Columns in Z**	12
**Subjects**	1
**Max Obs per Subject**	44

Number of Observations	
**Number of Observations Read**	44
**Number of Observations Used**	44
**Number of Observations Not Used**	0

Iteration History			
**Iteration**	**Evaluations**	**-2 Res Log Like**	**Criterion**
**0**	1	112.30920815	
**1**	3	112.29422605	0.00000002
**2**	1	112.29422571	0.00000000

Convergence criteria met.

Covariance Parameter Estimates				
**Cov Parm**	**Estimate**	**Alpha**	**Lower**	**Upper**
**Culture**	0.01115	0.05	0.001493	7.87E108
**Residual**	0.7231	0.05	0.4730	1.2415

DistSoma=408

Fit Statistics	
-2 Res Log Likelihood	112.3
AIC (Smaller is Better)	116.3
AICC (Smaller is Better)	116.6
BIC (Smaller is Better)	117.3

Solution for Fixed Effects

Effect	Treatment	Estimate	Standard Error	DF	t Value	Pr > |t|	Alpha	Lower	Upper
Intercept		0.6830	0.1874	10	3.64	0.0045	0.05	0.2654	1.1006
Treatment	Control_GFP	-0.6830	0.2641	32	-2.59	0.0145	0.05	-1.2209	-0.1451
Treatment	GFP MsTTR	0	.	.	.	.	.	.	.

Solution for Random Effects

Effect	Culture	Estimate	Std Err Pred	DF	t Value	Pr > |t|	Alpha	Lower	Upper
Culture	1	2.49E-18	0.1036	32	0.00	1.0000	0.05	-0.2110	0.2110
Culture	2	2.49E-18	0.1036	32	0.00	1.0000	0.05	-0.2110	0.2110
Culture	3	2.47E-18	0.1031	32	0.00	1.0000	0.05	-0.2099	0.2099
Culture	4	2.47E-18	0.1031	32	0.00	1.0000	0.05	-0.2099	0.2099
Culture	5	2.47E-18	0.1031	32	0.00	1.0000	0.05	-0.2099	0.2099
Culture	6	6.46E-19	0.1048	32	0.00	1.0000	0.05	-0.2135	0.2135
Culture	7	2.49E-18	0.1036	32	0.00	1.0000	0.05	-0.2110	0.2110
Culture	8	-0.03458	0.1026	32	-0.34	0.7384	0.05	-0.2436	0.1745
Culture	9	0.06200	0.1031	32	0.60	0.5517	0.05	-0.1479	0.2719
Culture	10	-0.01063	0.1031	32	-0.10	0.9185	0.05	-0.2206	0.1993
Culture	11	-0.02516	0.1031	32	-0.24	0.8087	0.05	-0.2351	0.1848
Culture	12	0.008376	0.1026	32	0.08	0.9355	0.05	-0.2007	0.2174

Type 3 Tests of Fixed Effects

Effect	Num DF	Den DF	F Value	Pr > F
Treatment	1	32	6.69	0.0145

Least Squares Means

Effect	Treatment	Estimate	Standard Error	DF	t Value	Pr > |t|	Alpha	Lower	Upper
Treatment	Control_GFP	-111E-18	0.1860	32	-0.00	1.0000	0.05	-0.3790	0.3790
Treatment	GFP MsTTR	0.6830	0.1874	32	3.64	0.0009	0.05	0.3013	1.0648

DistSoma=408

Differences of Least Squares Means												
Effect	Treatment	Treatment	Estimate	Standard Error	DF	t Value	Pr > |t|	Adjustment	Adj P	Alpha	Lower	Upper
**Treatment**	Control_GFP	GFP MsTTR	-0.6830	0.2641	32	-2.59	0.0145	Tukey-Kramer	0.0145	0.05	-1.2209	-0.1451

Differences of Least Squares Means				
Effect	Treatment	Treatment	Adj Lower	Adj Upper
**Treatment**	Control_GFP	GFP MsTTR	-1.2209	-0.1451

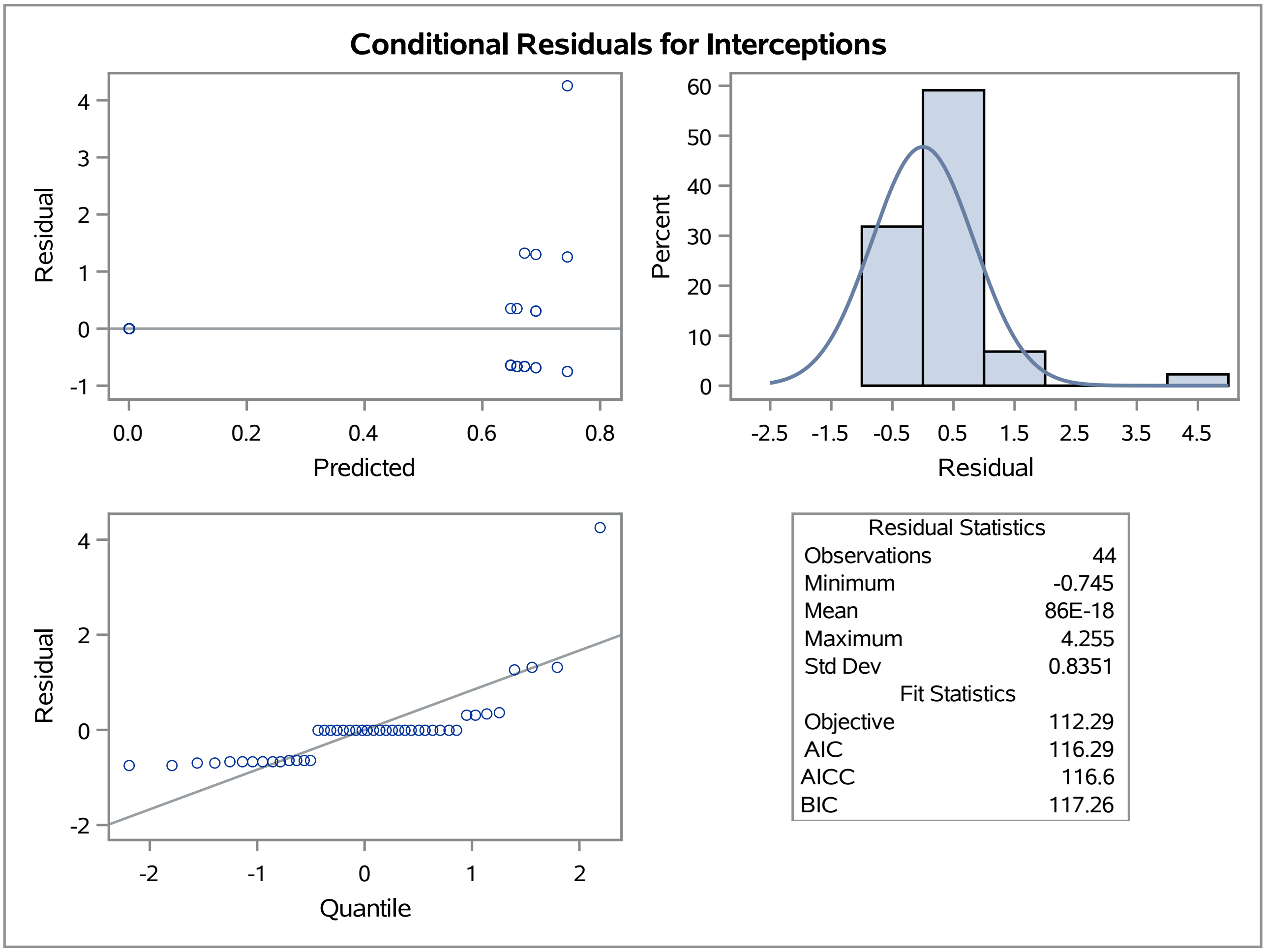

DistSoma=414

Model Information	
**Data Set**	WORK.TEMPDATASORTED
**Dependent Variable**	Interceptions
**Covariance Structure**	Variance Components
**Estimation Method**	REML
**Residual Variance Method**	Profile
**Fixed Effects SE Method**	Model-Based
**Degrees of Freedom Method**	Containment

Class Level Information		
**Class**	**Levels**	**Values**
**Treatment**	2	Control_GFP GFP MsTTR
**Culture**	12	1 2 3 4 5 6 7 8 9 10 11 12

Dimensions	
**Covariance Parameters**	2
**Columns in X**	3
**Columns in Z**	12
**Subjects**	1
**Max Obs per Subject**	44

Number of Observations	
**Number of Observations Read**	44
**Number of Observations Used**	44
**Number of Observations Not Used**	0

Iteration History			
**Iteration**	**Evaluations**	**-2 Res Log Like**	**Criterion**
**0**	1	100.65860018	
**1**	1	100.65860018	0.00000000

Convergence criteria met.

Estimated G matrix is not positive definite.

Covariance Parameter Estimates				
**Cov Parm**	**Estimate**	**Alpha**	**Lower**	**Upper**
**Culture**	0	.	.	.
**Residual**	0.5552	0.05	0.3775	0.8969

DistSoma=414

Fit Statistics	
-2 Res Log Likelihood	100.7
AIC (Smaller is Better)	102.7
AICC (Smaller is Better)	102.8
BIC (Smaller is Better)	103.1

Solution for Fixed Effects

Effect	Treatment	Estimate	Standard Error	DF	t Value	Pr > |t|	Alpha	Lower	Upper
Intercept		0.5909	0.1589	10	3.72	0.0040	0.05	0.2369	0.9449
Treatment	Control_GFP	-0.5909	0.2247	32	-2.63	0.0130	0.05	-1.0485	-0.1333
Treatment	GFP MsTTR	0	.	.	.	.	.	.	.

Solution for Random Effects

Effect	Culture	Estimate	Std Err Pred	DF	t Value	Pr > |t|	Alpha	Lower	Upper
Culture	1	0	.	.	.	.	.	.	.
Culture	2	0	.	.	.	.	.	.	.
Culture	3	0	.	.	.	.	.	.	.
Culture	4	0	.	.	.	.	.	.	.
Culture	5	0	.	.	.	.	.	.	.
Culture	6	0	.	.	.	.	.	.	.
Culture	7	0	.	.	.	.	.	.	.
Culture	8	0	.	.	.	.	.	.	.
Culture	9	0	.	.	.	.	.	.	.
Culture	10	0	.	.	.	.	.	.	.
Culture	11	0	.	.	.	.	.	.	.
Culture	12	0	.	.	.	.	.	.	.

Type 3 Tests of Fixed Effects

Effect	Num DF	Den DF	F Value	Pr > F
Treatment	1	32	6.92	0.0130

Least Squares Means

Effect	Treatment	Estimate	Standard Error	DF	t Value	Pr > |t|	Alpha	Lower	Upper
Treatment	Control_GFP	0	0.1589	32	0.00	1.0000	0.05	-0.3236	0.3236
Treatment	GFP MsTTR	0.5909	0.1589	32	3.72	0.0008	0.05	0.2673	0.9145

DistSoma=414

Differences of Least Squares Means												
**Effect**	**Treatment**	**Treatment**	**Estimate**	**Standard Error**	**DF**	**t Value**	**Pr > |t|**	**Adjustment**	**Adj P**	**Alpha**	**Lower**	**Upper**
**Treatment**	Control_GFP	GFP MsTTR	-0.5909	0.2247	32	-2.63	0.0130	Tukey	0.0130	0.05	-1.0485	-0.1333

Differences of Least Squares Means				
**Effect**	**Treatment**	**Treatment**	**Adj Lower**	**Adj Upper**
**Treatment**	Control_GFP	GFP MsTTR	-1.0485	-0.1333

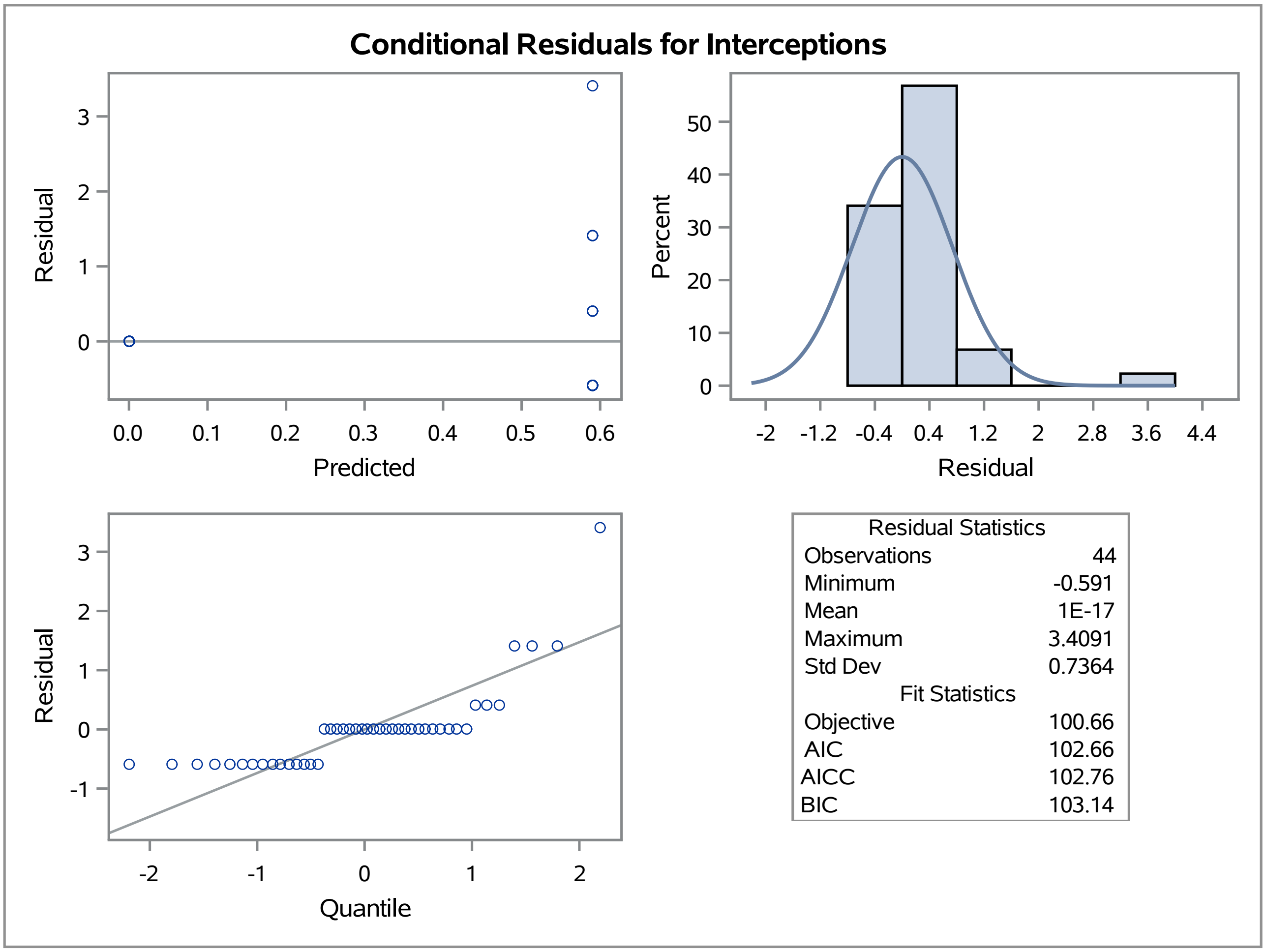

DistSoma=420

Model Information	
**Data Set**	WORK.TEMPDATASORTED
**Dependent Variable**	Interceptions
**Covariance Structure**	Variance Components
**Estimation Method**	REML
**Residual Variance Method**	Profile
**Fixed Effects SE Method**	Model-Based
**Degrees of Freedom Method**	Containment

Class Level Information		
**Class**	**Levels**	**Values**
**Treatment**	2	Control_GFP GFP MsTTR
**Culture**	12	1 2 3 4 5 6 7 8 9 10 11 12

Dimensions	
**Covariance Parameters**	2
**Columns in X**	3
**Columns in Z**	12
**Subjects**	1
**Max Obs per Subject**	44

Number of Observations	
**Number of Observations Read**	44
**Number of Observations Used**	44
**Number of Observations Not Used**	0

Iteration History			
**Iteration**	**Evaluations**	**-2 Res Log Like**	**Criterion**
**0**	1	77.70376452	
**1**	1	77.70376452	0.00000000

Convergence criteria met.

Estimated G matrix is not positive definite.

Covariance Parameter Estimates				
**Cov Parm**	**Estimate**	**Alpha**	**Lower**	**Upper**
**Culture**	0	.	.	.
**Residual**	0.3214	0.05	0.2185	0.5193

DistSoma=420

Fit Statistics	
-2 Res Log Likelihood	77.7
AIC (Smaller is Better)	79.7
AICC (Smaller is Better)	79.8
BIC (Smaller is Better)	80.2

Solution for Fixed Effects

Effect	Treatment	Estimate	Standard Error	DF	t Value	Pr > |t|	Alpha	Lower	Upper
Intercept		0.5000	0.1209	10	4.14	0.0020	0.05	0.2307	0.7693
Treatment	Control_GFP	-0.5000	0.1709	32	-2.92	0.0063	0.05	-0.8482	-0.1518
Treatment	GFP MsTTR	0	.	.	.	.	.	.	.

Solution for Random Effects

Effect	Culture	Estimate	Std Err Pred	DF	t Value	Pr > |t|	Alpha	Lower	Upper
Culture	1	0	.	.	.	.	.	.	.
Culture	2	0	.	.	.	.	.	.	.
Culture	3	0	.	.	.	.	.	.	.
Culture	4	0	.	.	.	.	.	.	.
Culture	5	0	.	.	.	.	.	.	.
Culture	6	0	.	.	.	.	.	.	.
Culture	7	0	.	.	.	.	.	.	.
Culture	8	0	.	.	.	.	.	.	.
Culture	9	0	.	.	.	.	.	.	.
Culture	10	0	.	.	.	.	.	.	.
Culture	11	0	.	.	.	.	.	.	.
Culture	12	0	.	.	.	.	.	.	.

Type 3 Tests of Fixed Effects

Effect	Num DF	Den DF	F Value	Pr > F
Treatment	1	32	8.56	0.0063

Least Squares Means

Effect	Treatment	Estimate	Standard Error	DF	t Value	Pr > |t|	Alpha	Lower	Upper
Treatment	Control_GFP	0	0.1209	32	0.00	1.0000	0.05	-0.2462	0.2462
Treatment	GFP MsTTR	0.5000	0.1209	32	4.14	0.0002	0.05	0.2538	0.7462

DistSoma=420

Differences of Least Squares Means												
**Effect**	**Treatment**	**Treatment**	**Estimate**	**Standard Error**	**DF**	**t Value**	**Pr > |t|**	**Adjustment**	**Adj P**	**Alpha**	**Lower**	**Upper**
**Treatment**	Control_GFP	GFP MsTTR	-0.5000	0.1709	32	-2.92	0.0063	Tukey	0.0063	0.05	-0.8482	-0.1518

Differences of Least Squares Means				
**Effect**	**Treatment**	**Treatment**	**Adj Lower**	**Adj Upper**
**Treatment**	Control_GFP	GFP MsTTR	-0.8482	-0.1518

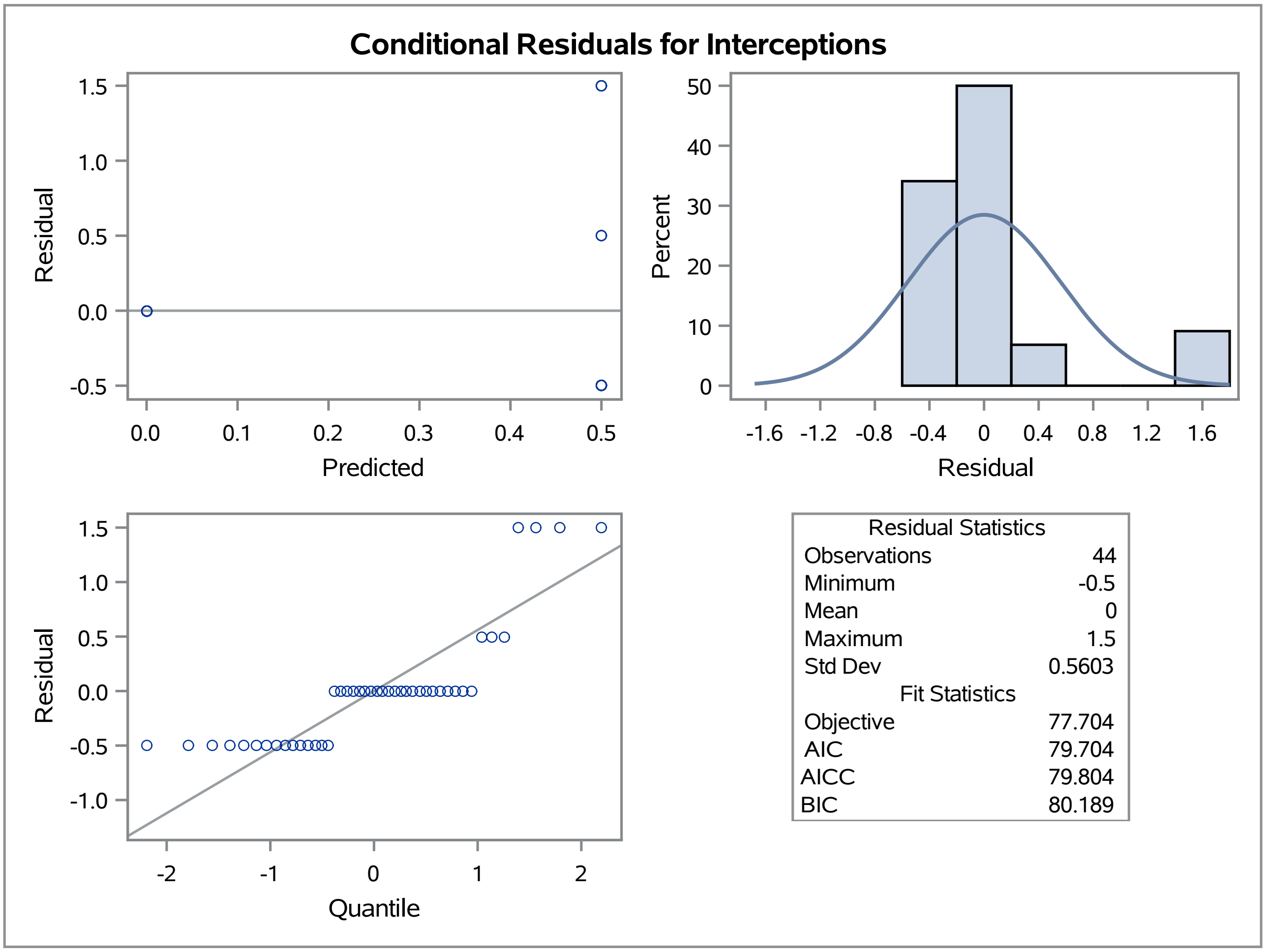

DistSoma=426

Model Information	
**Data Set**	WORK.TEMPDATASORTED
**Dependent Variable**	Interceptions
**Covariance Structure**	Variance Components
**Estimation Method**	REML
**Residual Variance Method**	Profile
**Fixed Effects SE Method**	Model-Based
**Degrees of Freedom Method**	Containment

Class Level Information		
**Class**	**Levels**	**Values**
**Treatment**	2	Control_GFP GFP MsTTR
**Culture**	12	1 2 3 4 5 6 7 8 9 10 11 12

Dimensions	
**Covariance Parameters**	2
**Columns in X**	3
**Columns in Z**	12
**Subjects**	1
**Max Obs per Subject**	44

Number of Observations	
**Number of Observations Read**	44
**Number of Observations Used**	44
**Number of Observations Not Used**	0

Iteration History			
**Iteration**	**Evaluations**	**-2 Res Log Like**	**Criterion**
**0**	1	61.09634408	
**1**	3	60.86837635	0.00000086
**2**	1	60.86836936	0.00000000

Convergence criteria met.

Covariance Parameter Estimates				
**Cov Parm**	**Estimate**	**Alpha**	**Lower**	**Upper**
**Culture**	0.01377	0.05	0.001775	1359763
**Residual**	0.2045	0.05	0.1334	0.3529

DistSoma=426

Fit Statistics	
**-2 Res Log Likelihood**	60.9
**AIC (Smaller is Better)**	64.9
**AICC (Smaller is Better)**	65.2
**BIC (Smaller is Better)**	65.8

Solution for Fixed Effects

Effect	Treatment	Estimate	Standard Error	DF	t Value	Pr > |t|	Alpha	Lower	Upper
**Intercept**		0.3651	0.1099	10	3.32	0.0077	0.05	0.1203	0.6100
**Treatment**	Control_GFP	-0.3651	0.1533	32	-2.38	0.0234	0.05	-0.6775	-0.05279
**Treatment**	GFP MsTTR	0	.	.	.	.	.	.	.

Solution for Random Effects

Effect	Culture	Estimate	Std Err Pred	DF	t Value	Pr > |t|	Alpha	Lower	Upper
**Culture**	1	0	0.1085	32	0.00	1.0000	0.05	-0.2211	0.2211
**Culture**	2	0	0.1085	32	0.00	1.0000	0.05	-0.2211	0.2211
**Culture**	3	0	0.1066	32	0.00	1.0000	0.05	-0.2171	0.2171
**Culture**	4	0	0.1066	32	0.00	1.0000	0.05	-0.2171	0.2171
**Culture**	5	0	0.1066	32	0.00	1.0000	0.05	-0.2171	0.2171
**Culture**	6	0	0.1138	32	0.00	1.0000	0.05	-0.2317	0.2317
**Culture**	7	0	0.1085	32	0.00	1.0000	0.05	-0.2211	0.2211
**Culture**	8	-0.04159	0.1052	32	-0.40	0.6952	0.05	-0.2559	0.1727
**Culture**	9	0.1347	0.1067	32	1.26	0.2160	0.05	-0.08269	0.3521
**Culture**	10	-0.07747	0.1067	32	-0.73	0.4732	0.05	-0.2949	0.1399
**Culture**	11	-0.02443	0.1067	32	-0.23	0.8204	0.05	-0.2418	0.1930
**Culture**	12	0.008783	0.1052	32	0.08	0.9340	0.05	-0.2055	0.2231

Type 3 Tests of Fixed Effects

Effect	Num DF	Den DF	F Value	Pr > F
**Treatment**	1	32	5.67	0.0234

Least Squares Means

Effect	Treatment	Estimate	Standard Error	DF	t Value	Pr > |t|	Alpha	Lower	Upper
**Treatment**	Control_GFP	0	0.1069	32	0.00	1.0000	0.05	-0.2178	0.2178
**Treatment**	GFP MsTTR	0.3651	0.1099	32	3.32	0.0022	0.05	0.1413	0.5890

DistSoma=426

Differences of Least Squares Means												
Effect	Treatment	Treatment	Estimate	Standard Error	DF	t Value	Pr > |t|	Adjustment	Adj P	Alpha	Lower	Upper
**Treatment**	Control_GFP	GFP MsTTR	-0.3651	0.1533	32	-2.38	0.0234	Tukey-Kramer	0.0234	0.05	-0.6775	-0.05279

Differences of Least Squares Means				
Effect	Treatment	Treatment	Adj Lower	Adj Upper
**Treatment**	Control_GFP	GFP MsTTR	-0.6775	-0.05279

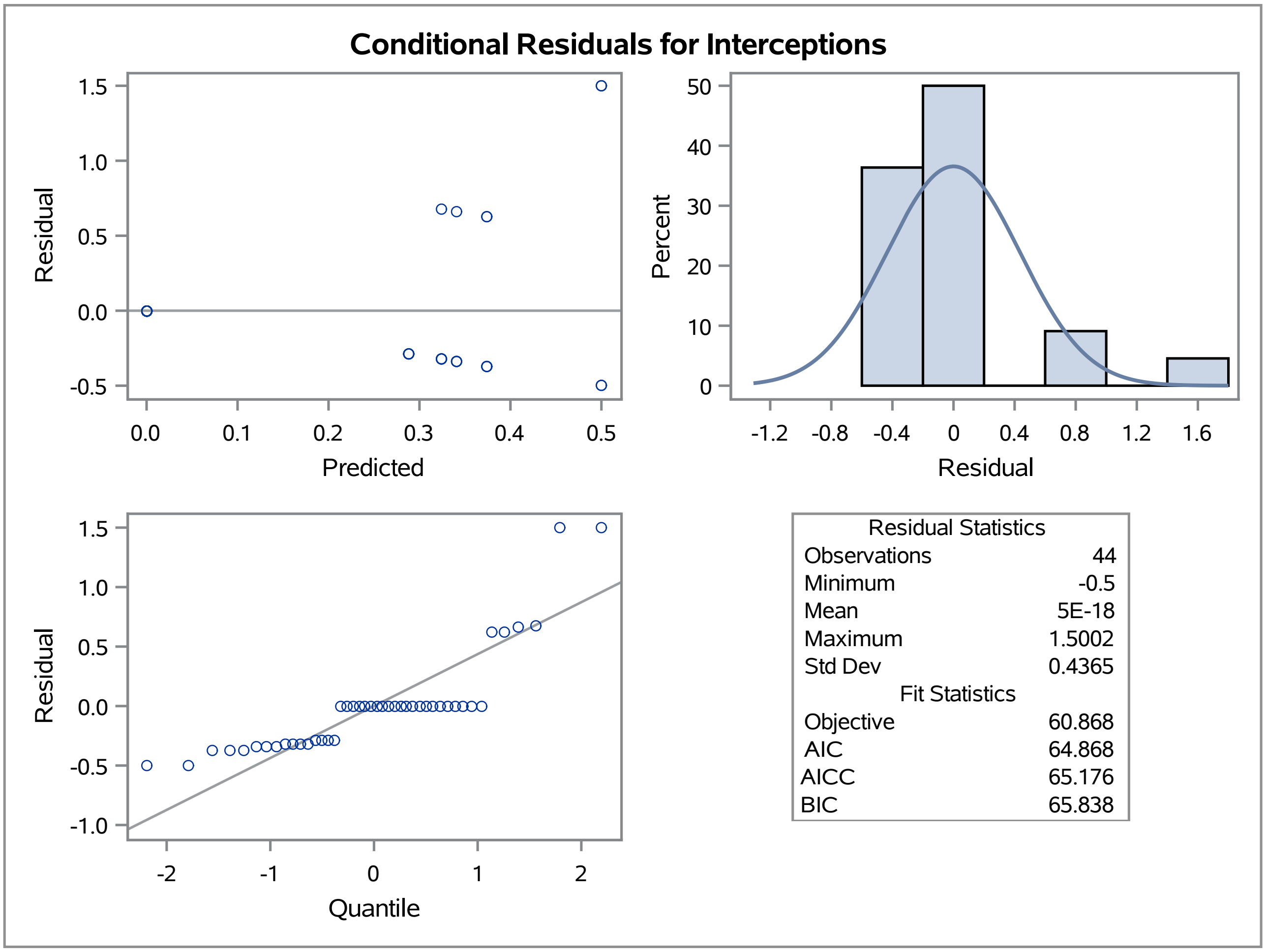

DistSoma=432

Model Information	
**Data Set**	WORK.TEMPDATASORTED
**Dependent Variable**	Interceptions
**Covariance Structure**	Variance Components
**Estimation Method**	REML
**Residual Variance Method**	Profile
**Fixed Effects SE Method**	Model-Based
**Degrees of Freedom Method**	Containment

Class Level Information		
**Class**	**Levels**	**Values**
**Treatment**	2	Control_GFP GFP MsTTR
**Culture**	12	1 2 3 4 5 6 7 8 9 10 11 12

Dimensions	
**Covariance Parameters**	2
**Columns in X**	3
**Columns in Z**	12
**Subjects**	1
**Max Obs per Subject**	44

Number of Observations	
**Number of Observations Read**	44
**Number of Observations Used**	44
**Number of Observations Not Used**	0

Iteration History			
**Iteration**	**Evaluations**	**-2 Res Log Like**	**Criterion**
**0**	1	61.09634408	
**1**	3	60.86837635	0.00000086
**2**	1	60.86836936	0.00000000

Convergence criteria met.

Covariance Parameter Estimates				
**Cov Parm**	**Estimate**	**Alpha**	**Lower**	**Upper**
**Culture**	0.01377	0.05	0.001775	1359763
**Residual**	0.2045	0.05	0.1334	0.3529

DistSoma=432

Fit Statistics	
-2 Res Log Likelihood	60.9
AIC (Smaller is Better)	64.9
AICC (Smaller is Better)	65.2
BIC (Smaller is Better)	65.8

Solution for Fixed Effects

Effect	Treatment	Estimate	Standard Error	DF	t Value	Pr > |t|	Alpha	Lower	Upper
Intercept		0.3651	0.1099	10	3.32	0.0077	0.05	0.1203	0.6100
Treatment	Control_GFP	-0.3651	0.1533	32	-2.38	0.0234	0.05	-0.6775	-0.05279
Treatment	GFP MsTTR	0	.	.	.	.	.	.	.

Solution for Random Effects

Effect	Culture	Estimate	Std Err Pred	DF	t Value	Pr > |t|	Alpha	Lower	Upper
Culture	1	0	0.1085	32	0.00	1.0000	0.05	-0.2211	0.2211
Culture	2	0	0.1085	32	0.00	1.0000	0.05	-0.2211	0.2211
Culture	3	0	0.1066	32	0.00	1.0000	0.05	-0.2171	0.2171
Culture	4	0	0.1066	32	0.00	1.0000	0.05	-0.2171	0.2171
Culture	5	0	0.1066	32	0.00	1.0000	0.05	-0.2171	0.2171
Culture	6	0	0.1138	32	0.00	1.0000	0.05	-0.2317	0.2317
Culture	7	0	0.1085	32	0.00	1.0000	0.05	-0.2211	0.2211
Culture	8	-0.04159	0.1052	32	-0.40	0.6952	0.05	-0.2559	0.1727
Culture	9	0.1347	0.1067	32	1.26	0.2160	0.05	-0.08269	0.3521
Culture	10	-0.07747	0.1067	32	-0.73	0.4732	0.05	-0.2949	0.1399
Culture	11	-0.02443	0.1067	32	-0.23	0.8204	0.05	-0.2418	0.1930
Culture	12	0.008783	0.1052	32	0.08	0.9340	0.05	-0.2055	0.2231

Type 3 Tests of Fixed Effects

Effect	Num DF	Den DF	F Value	Pr > F
Treatment	1	32	5.67	0.0234

Least Squares Means

Effect	Treatment	Estimate	Standard Error	DF	t Value	Pr > |t|	Alpha	Lower	Upper
Treatment	Control_GFP	0	0.1069	32	0.00	1.0000	0.05	-0.2178	0.2178
Treatment	GFP MsTTR	0.3651	0.1099	32	3.32	0.0022	0.05	0.1413	0.5890

DistSoma=432

Differences of Least Squares Means												
**Effect**	**Treatment**	**Treatment**	**Estimate**	**Standard Error**	**DF**	**t Value**	**Pr > |t|**	**Adjustment**	**Adj P**	**Alpha**	**Lower**	**Upper**
**Treatment**	Control_GFP	GFP MsTTR	-0.3651	0.1533	32	-2.38	0.0234	Tukey-Kramer	0.0234	0.05	-0.6775	-0.05279

Differences of Least Squares Means				
**Effect**	**Treatment**	**Treatment**	**Adj Lower**	**Adj Upper**
**Treatment**	Control_GFP	GFP MsTTR	-0.6775	-0.05279

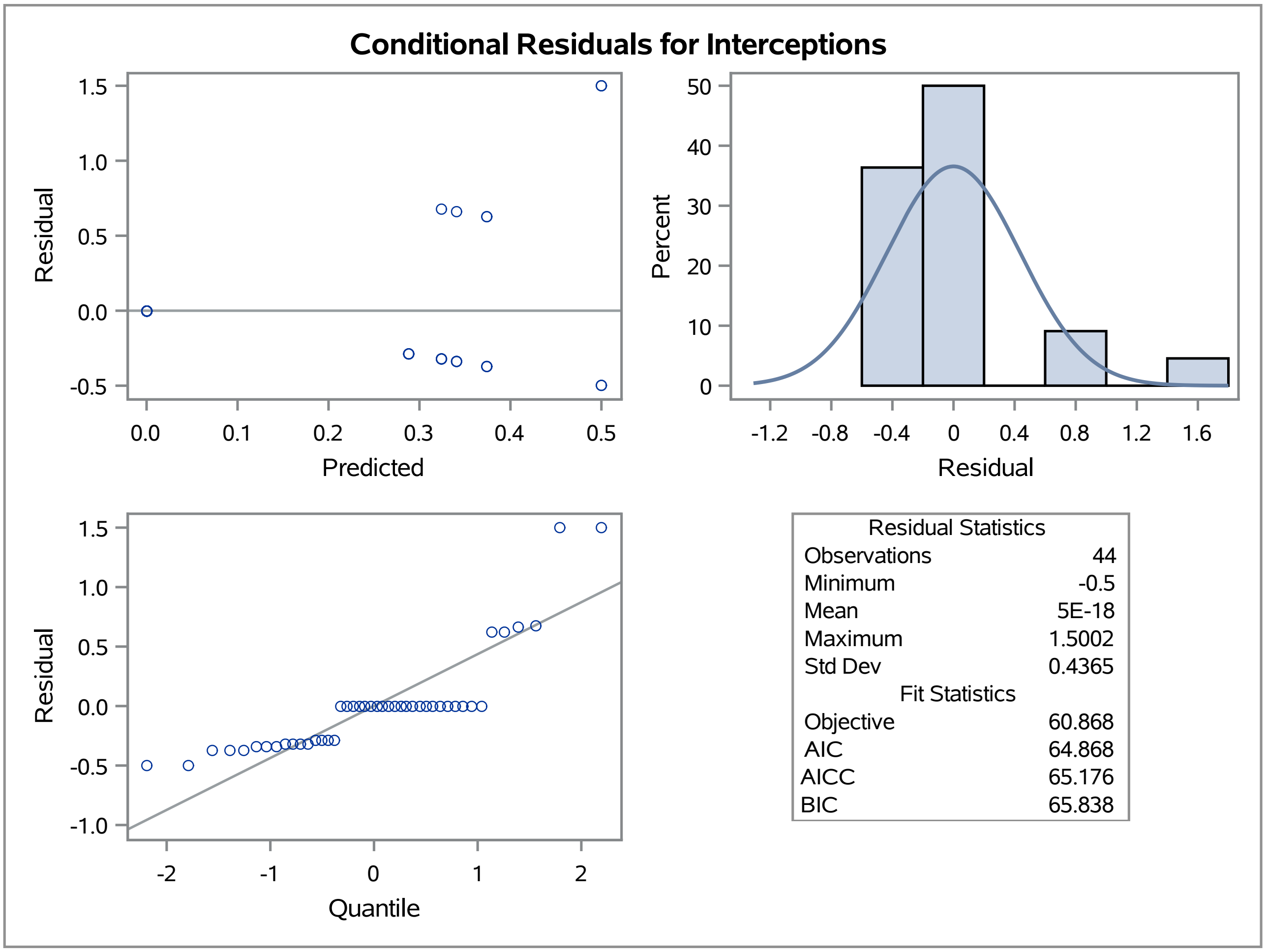

DistSoma=438

Model Information	
**Data Set**	WORK.TEMPDATASORTED
**Dependent Variable**	Interceptions
**Covariance Structure**	Variance Components
**Estimation Method**	REML
**Residual Variance Method**	Profile
**Fixed Effects SE Method**	Model-Based
**Degrees of Freedom Method**	Containment

Class Level Information		
**Class**	**Levels**	**Values**
**Treatment**	2	Control_GFP GFP MsTTR
**Culture**	12	1 2 3 4 5 6 7 8 9 10 11 12

Dimensions	
**Covariance Parameters**	2
**Columns in X**	3
**Columns in Z**	12
**Subjects**	1
**Max Obs per Subject**	44

Number of Observations	
**Number of Observations Read**	44
**Number of Observations Used**	44
**Number of Observations Not Used**	0

Iteration History			
**Iteration**	**Evaluations**	**-2 Res Log Like**	**Criterion**
**0**	1	59.60000262	
**1**	3	58.53907792	0.00000895
**2**	1	58.53899401	0.00000000

Convergence criteria met.

Covariance Parameter Estimates				
**Cov Parm**	**Estimate**	**Alpha**	**Lower**	**Upper**
**Culture**	0.02997	0.05	0.006922	4.8360
**Residual**	0.1826	0.05	0.1190	0.3156

DistSoma=438

Fit Statistics	
**-2 Res Log Likelihood**	58.5
**AIC (Smaller is Better)**	62.5
**AICC (Smaller is Better)**	62.8
**BIC (Smaller is Better)**	63.5

Solution for Fixed Effects

Effect	Treatment	Estimate	Standard Error	DF	t Value	Pr > |t|	Alpha	Lower	Upper
**Intercept**		0.3190	0.1197	10	2.66	0.0237	0.05	0.05219	0.5857
**Treatment**	Control_GFP	-0.3190	0.1651	32	-1.93	0.0622	0.05	-0.6552	0.01731
**Treatment**	GFP MsTTR	0	.	.	.	.	.	.	.

Solution for Random Effects

Effect	Culture	Estimate	Std Err Pred	DF	t Value	Pr > |t|	Alpha	Lower	Upper
**Culture**	1	-245E-20	0.1466	32	-0.00	1.0000	0.05	-0.2986	0.2986
**Culture**	2	-245E-20	0.1466	32	-0.00	1.0000	0.05	-0.2986	0.2986
**Culture**	3	-11E-18	0.1418	32	-0.00	1.0000	0.05	-0.2889	0.2889
**Culture**	4	-11E-18	0.1418	32	-0.00	1.0000	0.05	-0.2889	0.2889
**Culture**	5	-11E-18	0.1418	32	-0.00	1.0000	0.05	-0.2889	0.2889
**Culture**	6	-392E-20	0.1612	32	-0.00	1.0000	0.05	-0.3284	0.3284
**Culture**	7	-245E-20	0.1466	32	-0.00	1.0000	0.05	-0.2986	0.2986
**Culture**	8	-0.05362	0.1392	32	-0.39	0.7026	0.05	-0.3371	0.2299
**Culture**	9	0.2699	0.1426	32	1.89	0.0675	0.05	-0.02059	0.5604
**Culture**	10	-0.1264	0.1426	32	-0.89	0.3820	0.05	-0.4169	0.1641
**Culture**	11	-0.1264	0.1426	32	-0.89	0.3820	0.05	-0.4169	0.1641
**Culture**	12	0.03653	0.1392	32	0.26	0.7946	0.05	-0.2470	0.3200

Type 3 Tests of Fixed Effects

Effect	Num DF	Den DF	F Value	Pr > F
**Treatment**	1	32	3.73	0.0622

Least Squares Means

Effect	Treatment	Estimate	Standard Error	DF	t Value	Pr > |t|	Alpha	Lower	Upper
**Treatment**	Control_GFP	0	0.1137	32	0.00	1.0000	0.05	-0.2315	0.2315
**Treatment**	GFP MsTTR	0.3190	0.1197	32	2.66	0.0120	0.05	0.07508	0.5628

DistSoma=438

Differences of Least Squares Means												
**Effect**	**Treatment**	**Treatment**	**Estimate**	**Standard Error**	**DF**	**t Value**	**Pr > |t|**	**Adjustment**	**Adj P**	**Alpha**	**Lower**	**Upper**
**Treatment**	Control_GFP	GFP MsTTR	-0.3190	0.1651	32	-1.93	0.0622	Tukey-Kramer	0.0622	0.05	-0.6552	0.01731

Differences of Least Squares Means				
**Effect**	**Treatment**	**Treatment**	**Adj Lower**	**Adj Upper**
**Treatment**	Control_GFP	GFP MsTTR	-0.6552	0.01730

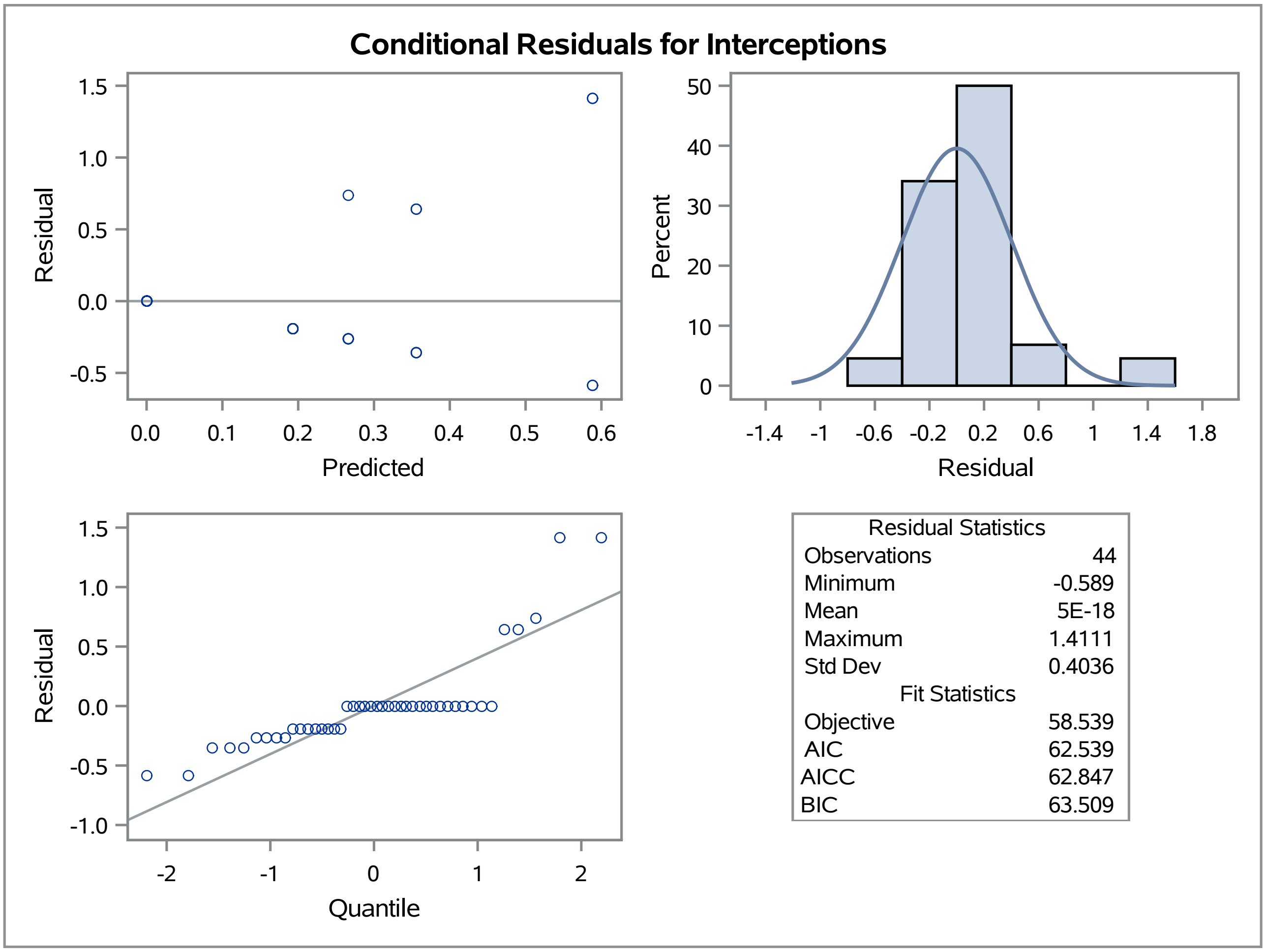

DistSoma=444

Model Information	
**Data Set**	WORK.TEMPDATASORTED
**Dependent Variable**	Interceptions
**Covariance Structure**	Variance Components
**Estimation Method**	REML
**Residual Variance Method**	Profile
**Fixed Effects SE Method**	Model-Based
**Degrees of Freedom Method**	Containment

Class Level Information		
**Class**	**Levels**	**Values**
**Treatment**	2	Control_GFP GFP MsTTR
**Culture**	12	1 2 3 4 5 6 7 8 9 10 11 12

Dimensions	
**Covariance Parameters**	2
**Columns in X**	3
**Columns in Z**	12
**Subjects**	1
**Max Obs per Subject**	44

Number of Observations	
**Number of Observations Read**	44
**Number of Observations Used**	44
**Number of Observations Not Used**	0

Iteration History			
**Iteration**	**Evaluations**	**-2 Res Log Like**	**Criterion**
**0**	1	57.59431651	
**1**	3	56.25806487	0.00000769
**2**	1	56.25798392	0.00000000

Convergence criteria met.

Covariance Parameter Estimates				
**Cov Parm**	**Estimate**	**Alpha**	**Lower**	**Upper**
**Culture**	0.03246	0.05	0.008121	2.4426
**Residual**	0.1706	0.05	0.1111	0.2951

DistSoma=444

Fit Statistics	
-2 Res Log Likelihood	56.3
AIC (Smaller is Better)	60.3
AICC (Smaller is Better)	60.6
BIC (Smaller is Better)	61.2

Solution for Fixed Effects

Effect	Treatment	Estimate	Standard Error	DF	t Value	Pr > |t|	Alpha	Lower	Upper
Intercept		0.2761	0.1195	10	2.31	0.0435	0.05	0.009730	0.5425
Treatment	Control_GFP	-0.2761	0.1644	32	-1.68	0.1029	0.05	-0.6111	0.05885
Treatment	GFP MsTTR	0	.	.	.	.	.	.	.

Solution for Random Effects

Effect	Culture	Estimate	Std Err Pred	DF	t Value	Pr > |t|	Alpha	Lower	Upper
Culture	1	-295E-20	0.1495	32	-0.00	1.0000	0.05	-0.3045	0.3045
Culture	2	-295E-20	0.1495	32	-0.00	1.0000	0.05	-0.3045	0.3045
Culture	3	-123E-19	0.1443	32	-0.00	1.0000	0.05	-0.2939	0.2939
Culture	4	-123E-19	0.1443	32	-0.00	1.0000	0.05	-0.2939	0.2939
Culture	5	-123E-19	0.1443	32	-0.00	1.0000	0.05	-0.2939	0.2939
Culture	6	-455E-20	0.1661	32	-0.00	1.0000	0.05	-0.3384	0.3384
Culture	7	-295E-20	0.1495	32	-0.00	1.0000	0.05	-0.3045	0.3045
Culture	8	-0.03710	0.1415	32	-0.26	0.7949	0.05	-0.3254	0.2512
Culture	9	0.3128	0.1453	32	2.15	0.0389	0.05	0.01694	0.6088
Culture	10	-0.1193	0.1453	32	-0.82	0.4175	0.05	-0.4152	0.1766
Culture	11	-0.1193	0.1453	32	-0.82	0.4175	0.05	-0.4152	0.1766
Culture	12	-0.03710	0.1415	32	-0.26	0.7949	0.05	-0.3254	0.2512

Type 3 Tests of Fixed Effects

Effect	Num DF	Den DF	F Value	Pr > F
Treatment	1	32	2.82	0.1029

Least Squares Means

Effect	Treatment	Estimate	Standard Error	DF	t Value	Pr > |t|	Alpha	Lower	Upper
Treatment	Control_GFP	5.55E-17	0.1129	32	0.00	1.0000	0.05	-0.2300	0.2300
Treatment	GFP MsTTR	0.2761	0.1195	32	2.31	0.0275	0.05	0.03259	0.5196

DistSoma=444

Differences of Least Squares Means												
Effect	Treatment	Treatment	Estimate	Standard Error	DF	t Value	Pr > |t|	Adjustment	Adj P	Alpha	Lower	Upper
**Treatment**	Control_GFP	GFP MsTTR	-0.2761	0.1644	32	-1.68	0.1029	Tukey-Kramer	0.1029	0.05	-0.6111	0.05885

Differences of Least Squares Means				
Effect	Treatment	Treatment	Adj Lower	Adj Upper
**Treatment**	Control_GFP	GFP MsTTR	-0.6110	0.05885

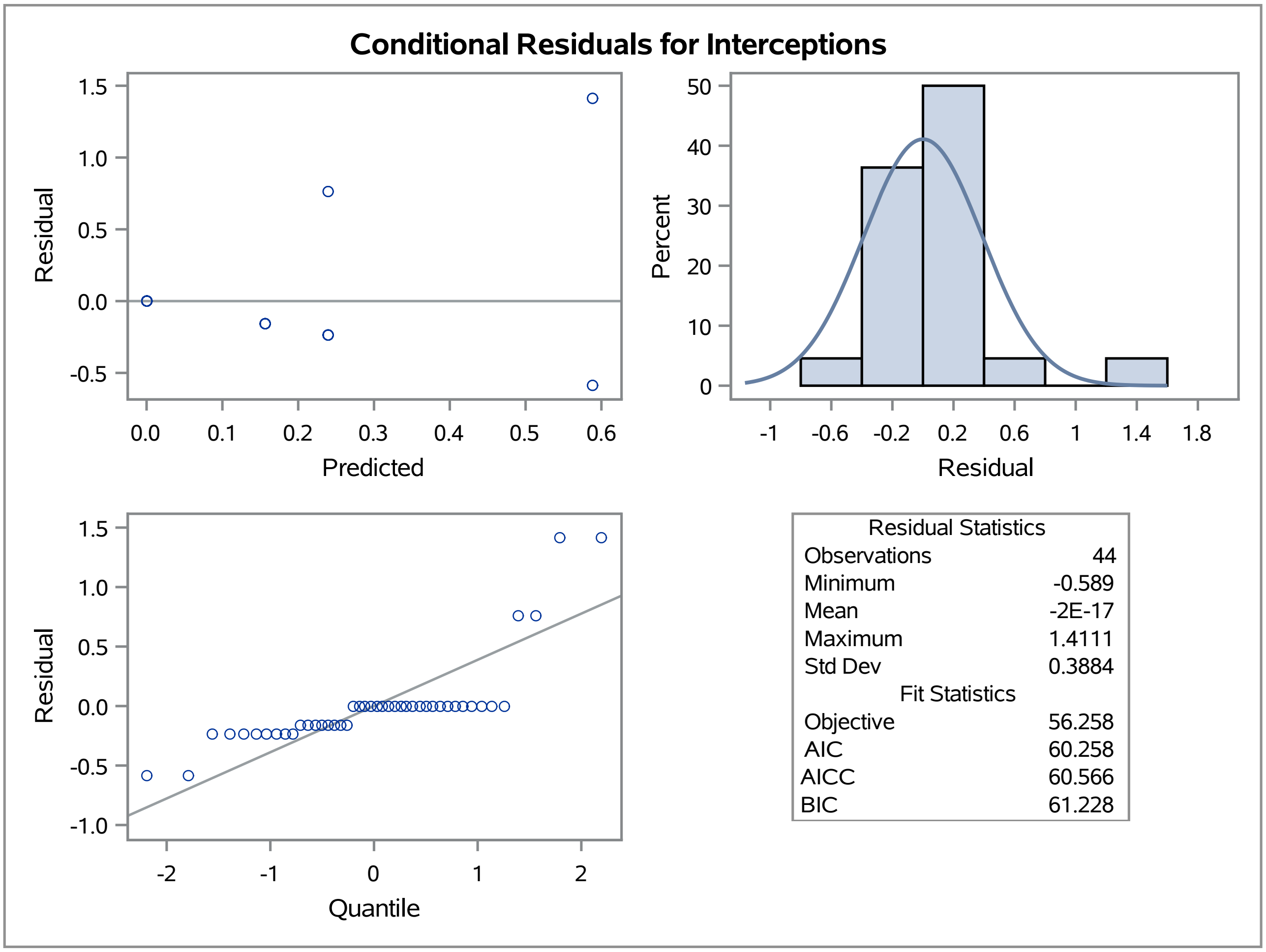

DistSoma=450

Model Information	
**Data Set**	WORK.TEMPDATASORTED
**Dependent Variable**	Interceptions
**Covariance Structure**	Variance Components
**Estimation Method**	REML
**Residual Variance Method**	Profile
**Fixed Effects SE Method**	Model-Based
**Degrees of Freedom Method**	Containment

Class Level Information		
**Class**	**Levels**	**Values**
**Treatment**	2	Control_GFP GFP MsTTR
**Culture**	12	1 2 3 4 5 6 7 8 9 10 11 12

Dimensions	
**Covariance Parameters**	2
**Columns in X**	3
**Columns in Z**	12
**Subjects**	1
**Max Obs per Subject**	44

Number of Observations	
**Number of Observations Read**	44
**Number of Observations Used**	44
**Number of Observations Not Used**	0

Iteration History			
**Iteration**	**Evaluations**	**-2 Res Log Like**	**Criterion**
**0**	1	42.67913567	
**1**	2	42.47014295	0.00000006
**2**	1	42.47014183	0.00000000

Convergence criteria met.

Covariance Parameter Estimates				
**Cov Parm**	**Estimate**	**Alpha**	**Lower**	**Upper**
**Culture**	0.008639	0.05	0.001080	3749024
**Residual**	0.1321	0.05	0.08608	0.2284

DistSoma=450

Fit Statistics	
-2 Res Log Likelihood	42.5
AIC (Smaller is Better)	46.5
AICC (Smaller is Better)	46.8
BIC (Smaller is Better)	47.4

Solution for Fixed Effects

Effect	Treatment	Estimate	Standard Error	DF	t Value	Pr > |t|	Alpha	Lower	Upper
Intercept		0.2279	0.08804	10	2.59	0.0270	0.05	0.03174	0.4241
Treatment	Control_GFP	-0.2279	0.1229	32	-1.85	0.0729	0.05	-0.4782	0.02241
Treatment	GFP MsTTR	0	.	.	.	.	.	.	.

Solution for Random Effects

Effect	Culture	Estimate	Std Err Pred	DF	t Value	Pr > |t|	Alpha	Lower	Upper
Culture	1	3.81E-18	0.08614	32	0.00	1.0000	0.05	-0.1755	0.1755
Culture	2	3.81E-18	0.08614	32	0.00	1.0000	0.05	-0.1755	0.1755
Culture	3	3.81E-18	0.08464	32	0.00	1.0000	0.05	-0.1724	0.1724
Culture	4	3.81E-18	0.08464	32	0.00	1.0000	0.05	-0.1724	0.1724
Culture	5	3.81E-18	0.08464	32	0.00	1.0000	0.05	-0.1724	0.1724
Culture	6	1.13E-18	0.09020	32	0.00	1.0000	0.05	-0.1837	0.1837
Culture	7	3.81E-18	0.08614	32	0.00	1.0000	0.05	-0.1755	0.1755
Culture	8	-0.00687	0.08355	32	-0.08	0.9350	0.05	-0.1771	0.1633
Culture	9	0.1082	0.08474	32	1.28	0.2107	0.05	-0.06438	0.2808
Culture	10	-0.04724	0.08474	32	-0.56	0.5811	0.05	-0.2199	0.1254
Culture	11	-0.04724	0.08474	32	-0.56	0.5811	0.05	-0.2199	0.1254
Culture	12	-0.00687	0.08355	32	-0.08	0.9350	0.05	-0.1771	0.1633

Type 3 Tests of Fixed Effects

Effect	Num DF	Den DF	F Value	Pr > F
Treatment	1	32	3.44	0.0729

Least Squares Means

Effect	Treatment	Estimate	Standard Error	DF	t Value	Pr > |t|	Alpha	Lower	Upper
Treatment	Control_GFP	0	0.08574	32	0.00	1.0000	0.05	-0.1746	0.1746
Treatment	GFP MsTTR	0.2279	0.08804	32	2.59	0.0144	0.05	0.04857	0.4072

DistSoma=450

Differences of Least Squares Means												
**Effect**	**Treatment**	**Treatment**	**Estimate**	**Standard Error**	**DF**	**t Value**	**Pr > |t|**	**Adjustment**	**Adj P**	**Alpha**	**Lower**	**Upper**
**Treatment**	Control_GFP	GFP MsTTR	-0.2279	0.1229	32	-1.85	0.0729	Tukey-Kramer	0.0729	0.05	-0.4782	0.02241

Differences of Least Squares Means				
**Effect**	**Treatment**	**Treatment**	**Adj Lower**	**Adj Upper**
**Treatment**	Control_GFP	GFP MsTTR	-0.4782	0.02241

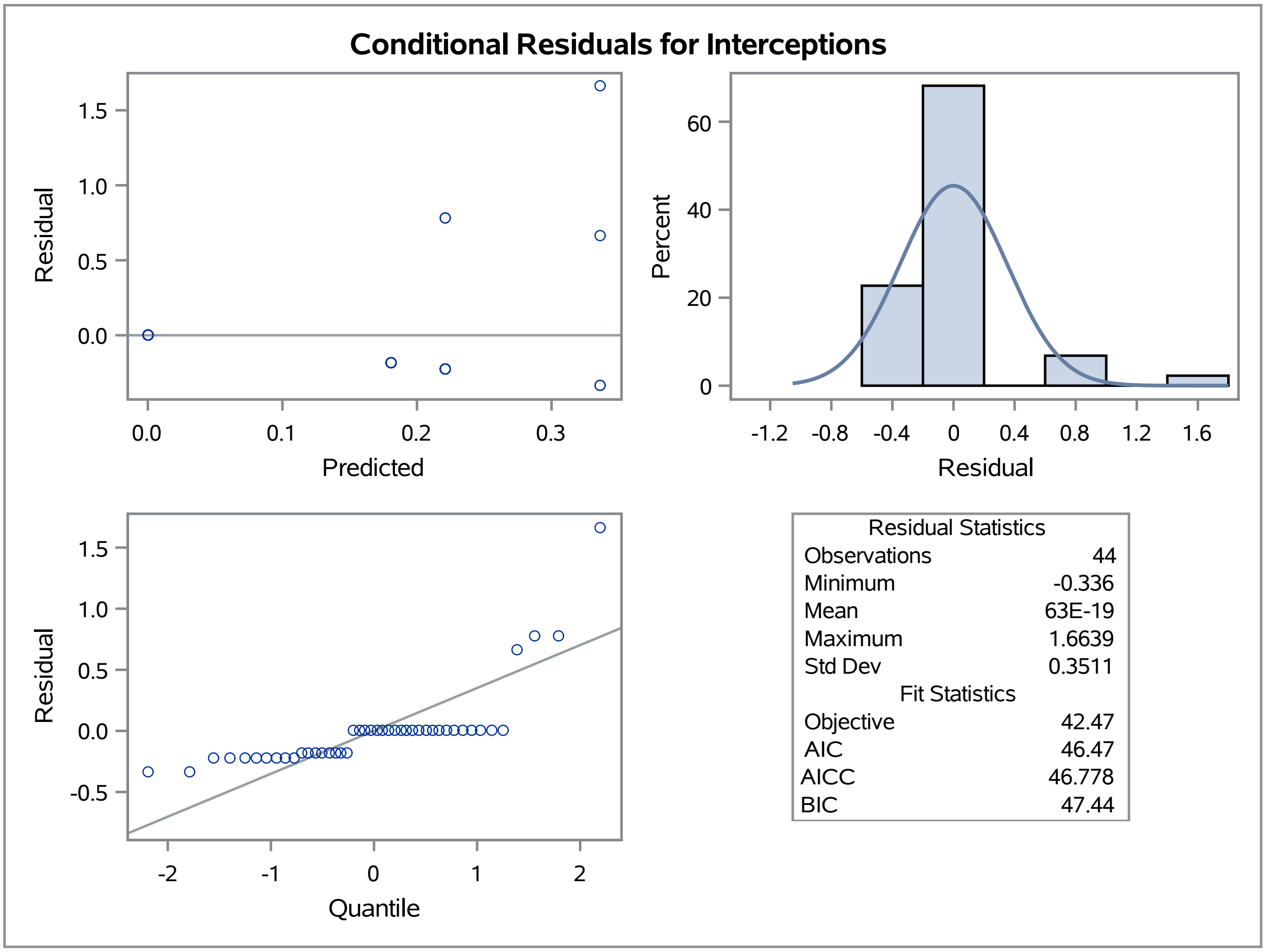

DistSoma=456

Model Information	
**Data Set**	WORK.TEMPDATASORTED
**Dependent Variable**	Interceptions
**Covariance Structure**	Variance Components
**Estimation Method**	REML
**Residual Variance Method**	Profile
**Fixed Effects SE Method**	Model-Based
**Degrees of Freedom Method**	Containment

Class Level Information		
**Class**	**Levels**	**Values**
**Treatment**	2	Control_GFP GFP MsTTR
**Culture**	12	1 2 3 4 5 6 7 8 9 10 11 12

Dimensions	
**Covariance Parameters**	2
**Columns in X**	3
**Columns in Z**	12
**Subjects**	1
**Max Obs per Subject**	44

Number of Observations	
**Number of Observations Read**	44
**Number of Observations Used**	44
**Number of Observations Not Used**	0

Iteration History			
**Iteration**	**Evaluations**	**-2 Res Log Like**	**Criterion**
**0**	1	42.67913567	
**1**	2	42.47014295	0.00000006
**2**	1	42.47014183	0.00000000

Convergence criteria met.

Covariance Parameter Estimates				
**Cov Parm**	**Estimate**	**Alpha**	**Lower**	**Upper**
**Culture**	0.008639	0.05	0.001080	3749024
**Residual**	0.1321	0.05	0.08608	0.2284

DistSoma=456

Fit Statistics	
-2 Res Log Likelihood	42.5
AIC (Smaller is Better)	46.5
AICC (Smaller is Better)	46.8
BIC (Smaller is Better)	47.4

Solution for Fixed Effects

Effect	Treatment	Estimate	Standard Error	DF	t Value	Pr > |t|	Alpha	Lower	Upper
Intercept		0.2279	0.08804	10	2.59	0.0270	0.05	0.03174	0.4241
Treatment	Control_GFP	-0.2279	0.1229	32	-1.85	0.0729	0.05	-0.4782	0.02241
Treatment	GFP MsTTR	0	.	.	.	.	.	.	.

Solution for Random Effects

Effect	Culture	Estimate	Std Err Pred	DF	t Value	Pr > |t|	Alpha	Lower	Upper
Culture	1	3.81E-18	0.08614	32	0.00	1.0000	0.05	-0.1755	0.1755
Culture	2	3.81E-18	0.08614	32	0.00	1.0000	0.05	-0.1755	0.1755
Culture	3	3.81E-18	0.08464	32	0.00	1.0000	0.05	-0.1724	0.1724
Culture	4	3.81E-18	0.08464	32	0.00	1.0000	0.05	-0.1724	0.1724
Culture	5	3.81E-18	0.08464	32	0.00	1.0000	0.05	-0.1724	0.1724
Culture	6	1.13E-18	0.09020	32	0.00	1.0000	0.05	-0.1837	0.1837
Culture	7	3.81E-18	0.08614	32	0.00	1.0000	0.05	-0.1755	0.1755
Culture	8	-0.00687	0.08355	32	-0.08	0.9350	0.05	-0.1771	0.1633
Culture	9	0.1082	0.08474	32	1.28	0.2107	0.05	-0.06438	0.2808
Culture	10	-0.04724	0.08474	32	-0.56	0.5811	0.05	-0.2199	0.1254
Culture	11	-0.04724	0.08474	32	-0.56	0.5811	0.05	-0.2199	0.1254
Culture	12	-0.00687	0.08355	32	-0.08	0.9350	0.05	-0.1771	0.1633

Type 3 Tests of Fixed Effects

Effect	Num DF	Den DF	F Value	Pr > F
Treatment	1	32	3.44	0.0729

Least Squares Means

Effect	Treatment	Estimate	Standard Error	DF	t Value	Pr > |t|	Alpha	Lower	Upper
Treatment	Control_GFP	0	0.08574	32	0.00	1.0000	0.05	-0.1746	0.1746
Treatment	GFP MsTTR	0.2279	0.08804	32	2.59	0.0144	0.05	0.04857	0.4072

DistSoma=456

Differences of Least Squares Means

Effect	Treatment	Treatment	Estimate	Standard Error	DF	t Value	Pr > |t|	Adjustment	Adj P	Alpha	Lower	Upper
**Treatment**	Control_GFP	GFP MsTTR	-0.2279	0.1229	32	-1.85	0.0729	Tukey-Kramer	0.0729	0.05	-0.4782	0.02241

Differences of Least Squares Means

Effect	Treatment	Treatment	Adj Lower	Adj Upper
**Treatment**	Control_GFP	GFP MsTTR	-0.4782	0.02241

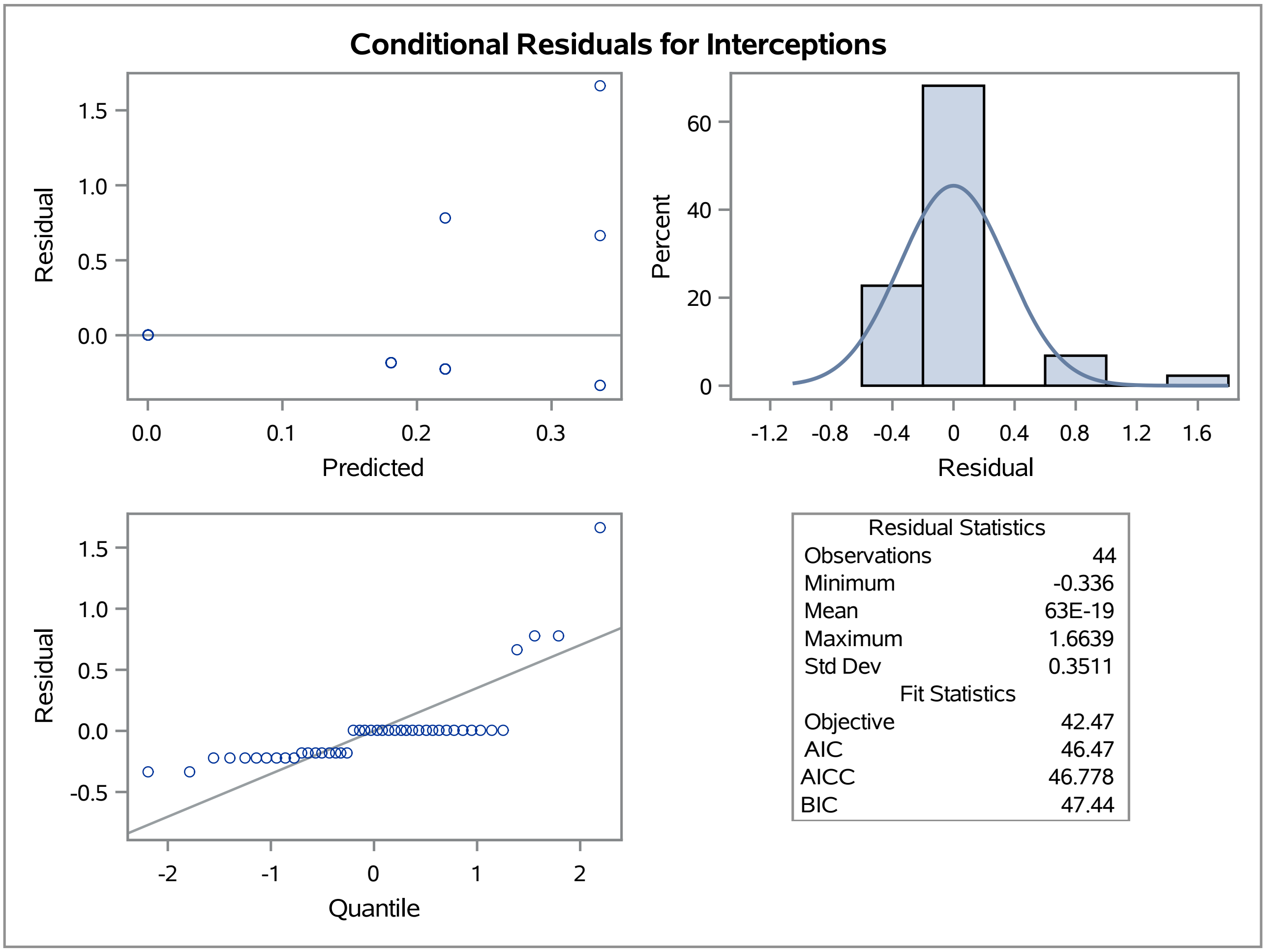

DistSoma=462

Model Information	
**Data Set**	WORK.TEMPDATASORTED
**Dependent Variable**	Interceptions
**Covariance Structure**	Variance Components
**Estimation Method**	REML
**Residual Variance Method**	Profile
**Fixed Effects SE Method**	Model-Based
**Degrees of Freedom Method**	Containment

Class Level Information		
**Class**	**Levels**	**Values**
**Treatment**	2	Control_GFP GFP MsTTR
**Culture**	12	1 2 3 4 5 6 7 8 9 10 11 12

Dimensions	
**Covariance Parameters**	2
**Columns in X**	3
**Columns in Z**	12
**Subjects**	1
**Max Obs per Subject**	44

Number of Observations	
**Number of Observations Read**	44
**Number of Observations Used**	44
**Number of Observations Not Used**	0

Iteration History			
**Iteration**	**Evaluations**	**-2 Res Log Like**	**Criterion**
**0**	1	38.21780271	
**1**	3	36.89044098	0.00002294
**2**	1	36.88997282	0.00000001

Convergence criteria met.

Covariance Parameter Estimates				
**Cov Parm**	**Estimate**	**Alpha**	**Lower**	**Upper**
**Culture**	0.01996	0.05	0.004971	1.5610
**Residual**	0.1079	0.05	0.07033	0.1863

DistSoma=462

Fit Statistics	
-2 Res Log Likelihood	36.9
AIC (Smaller is Better)	40.9
AICC (Smaller is Better)	41.2
BIC (Smaller is Better)	41.9

Solution for Fixed Effects

Effect	Treatment	Estimate	Standard Error	DF	t Value	Pr > |t|	Alpha	Lower	Upper
Intercept		0.1856	0.09446	10	1.96	0.0779	0.05	-0.02491	0.3960
Treatment	Control_GFP	-0.1856	0.1300	32	-1.43	0.1631	0.05	-0.4503	0.07922
Treatment	GFP MsTTR	0	.	.	.	.	.	.	.

Solution for Random Effects

Effect	Culture	Estimate	Std Err Pred	DF	t Value	Pr > |t|	Alpha	Lower	Upper
Culture	1	0	0.1177	32	0.00	1.0000	0.05	-0.2397	0.2397
Culture	2	0	0.1177	32	0.00	1.0000	0.05	-0.2397	0.2397
Culture	3	0	0.1136	32	0.00	1.0000	0.05	-0.2315	0.2315
Culture	4	0	0.1136	32	0.00	1.0000	0.05	-0.2315	0.2315
Culture	5	0	0.1136	32	0.00	1.0000	0.05	-0.2315	0.2315
Culture	6	0	0.1305	32	0.00	1.0000	0.05	-0.2659	0.2659
Culture	7	0	0.1177	32	0.00	1.0000	0.05	-0.2397	0.2397
Culture	8	0.006941	0.1115	32	0.06	0.9507	0.05	-0.2201	0.2340
Culture	9	0.2401	0.1144	32	2.10	0.0438	0.05	0.007080	0.4731
Culture	10	-0.07892	0.1144	32	-0.69	0.4952	0.05	-0.3119	0.1541
Culture	11	-0.07892	0.1144	32	-0.69	0.4952	0.05	-0.3119	0.1541
Culture	12	-0.08917	0.1115	32	-0.80	0.4297	0.05	-0.3163	0.1379

Type 3 Tests of Fixed Effects

Effect	Num DF	Den DF	F Value	Pr > F
Treatment	1	32	2.04	0.1631

Least Squares Means

Effect	Treatment	Estimate	Standard Error	DF	t Value	Pr > |t|	Alpha	Lower	Upper
Treatment	Control_GFP	0	0.08930	32	0.00	1.0000	0.05	-0.1819	0.1819
Treatment	GFP MsTTR	0.1856	0.09446	32	1.96	0.0582	0.05	-0.00685	0.3780

DistSoma=462

Differences of Least Squares Means												
**Effect**	**Treatment**	**Treatment**	**Estimate**	**Standard Error**	**DF**	**t Value**	**Pr > |t|**	**Adjustment**	**Adj P**	**Alpha**	**Lower**	**Upper**
**Treatment**	Control_GFP	GFP MsTTR	-0.1856	0.1300	32	-1.43	0.1631	Tukey-Kramer	0.1631	0.05	-0.4503	0.07922

Differences of Least Squares Means				
**Effect**	**Treatment**	**Treatment**	**Adj Lower**	**Adj Upper**
**Treatment**	Control_GFP	GFP MsTTR	-0.4503	0.07922

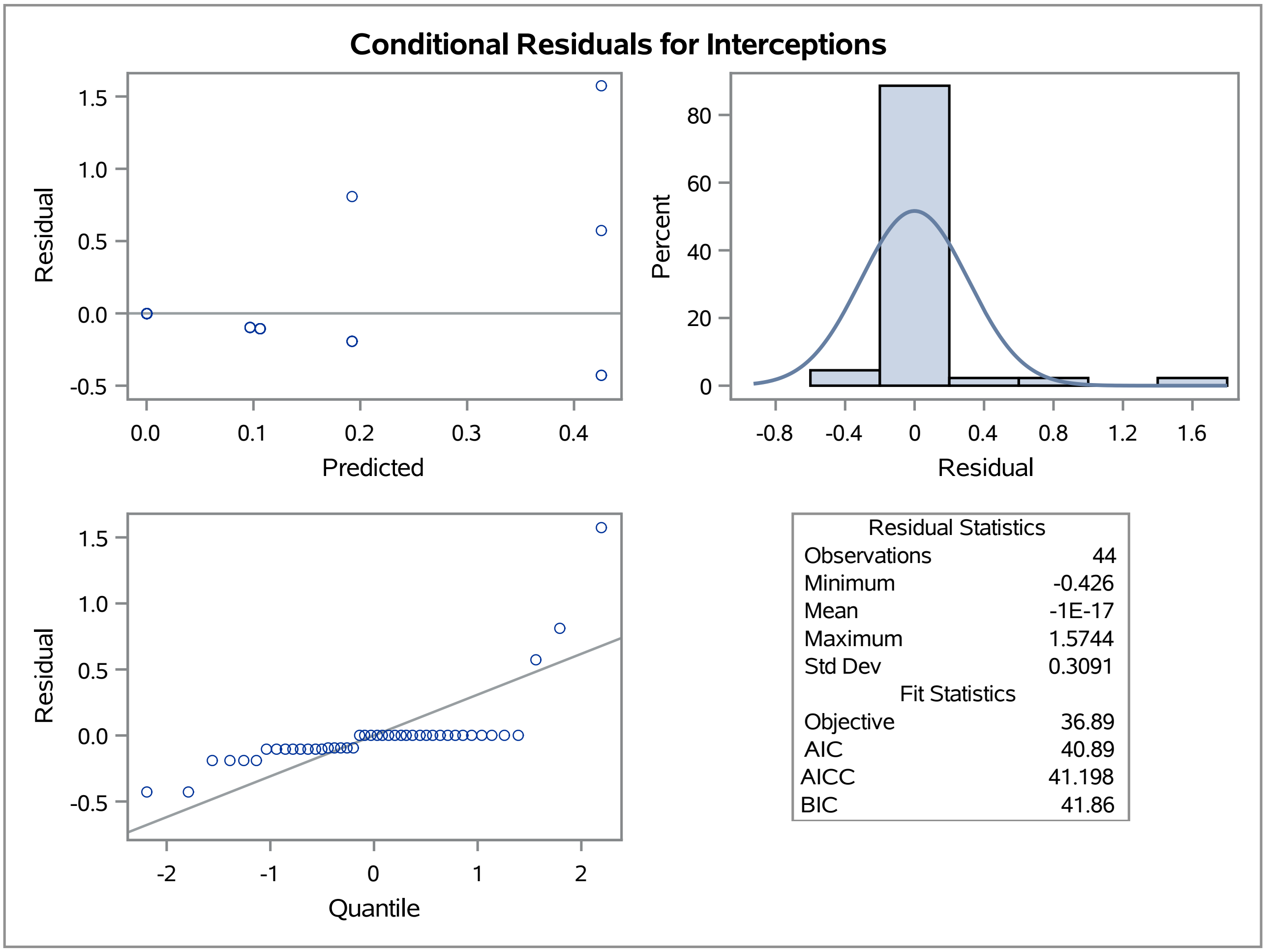

DistSoma=468

Model Information	
**Data Set**	WORK.TEMPDATASORTED
**Dependent Variable**	Interceptions
**Covariance Structure**	Variance Components
**Estimation Method**	REML
**Residual Variance Method**	Profile
**Fixed Effects SE Method**	Model-Based
**Degrees of Freedom Method**	Containment

Class Level Information		
**Class**	**Levels**	**Values**
**Treatment**	2	Control_GFP GFP MsTTR
**Culture**	12	1 2 3 4 5 6 7 8 9 10 11 12

Dimensions	
**Covariance Parameters**	2
**Columns in X**	3
**Columns in Z**	12
**Subjects**	1
**Max Obs per Subject**	44

Number of Observations	
**Number of Observations Read**	44
**Number of Observations Used**	44
**Number of Observations Not Used**	0

Iteration History			
**Iteration**	**Evaluations**	**-2 Res Log Like**	**Criterion**
**0**	1	38.21780271	
**1**	3	36.89044098	0.00002294
**2**	1	36.88997282	0.00000001

Convergence criteria met.

Covariance Parameter Estimates				
**Cov Parm**	**Estimate**	**Alpha**	**Lower**	**Upper**
**Culture**	0.01996	0.05	0.004971	1.5610
**Residual**	0.1079	0.05	0.07033	0.1863

DistSoma=468

Fit Statistics	
-2 Res Log Likelihood	36.9
AIC (Smaller is Better)	40.9
AICC (Smaller is Better)	41.2
BIC (Smaller is Better)	41.9

Solution for Fixed Effects									
Effect	Treatment	Estimate	Standard Error	DF	t Value	Pr > |t|	Alpha	Lower	Upper
Intercept		0.1856	0.09446	10	1.96	0.0779	0.05	-0.02491	0.3960
Treatment	Control_GFP	-0.1856	0.1300	32	-1.43	0.1631	0.05	-0.4503	0.07922
Treatment	GFP MsTTR	0	.	.	.	.	.	.	.

Solution for Random Effects									
Effect	Culture	Estimate	Std Err Pred	DF	t Value	Pr > |t|	Alpha	Lower	Upper
Culture	1	0	0.1177	32	0.00	1.0000	0.05	-0.2397	0.2397
Culture	2	0	0.1177	32	0.00	1.0000	0.05	-0.2397	0.2397
Culture	3	0	0.1136	32	0.00	1.0000	0.05	-0.2315	0.2315
Culture	4	0	0.1136	32	0.00	1.0000	0.05	-0.2315	0.2315
Culture	5	0	0.1136	32	0.00	1.0000	0.05	-0.2315	0.2315
Culture	6	0	0.1305	32	0.00	1.0000	0.05	-0.2659	0.2659
Culture	7	0	0.1177	32	0.00	1.0000	0.05	-0.2397	0.2397
Culture	8	0.006941	0.1115	32	0.06	0.9507	0.05	-0.2201	0.2340
Culture	9	0.2401	0.1144	32	2.10	0.0438	0.05	0.007080	0.4731
Culture	10	-0.07892	0.1144	32	-0.69	0.4952	0.05	-0.3119	0.1541
Culture	11	-0.07892	0.1144	32	-0.69	0.4952	0.05	-0.3119	0.1541
Culture	12	-0.08917	0.1115	32	-0.80	0.4297	0.05	-0.3163	0.1379

Type 3 Tests of Fixed Effects				
Effect	Num DF	Den DF	F Value	Pr > F
Treatment	1	32	2.04	0.1631

Least Squares Means									
Effect	Treatment	Estimate	Standard Error	DF	t Value	Pr > |t|	Alpha	Lower	Upper
Treatment	Control_GFP	0	0.08930	32	0.00	1.0000	0.05	-0.1819	0.1819
Treatment	GFP MsTTR	0.1856	0.09446	32	1.96	0.0582	0.05	-0.00685	0.3780

DistSoma=468

Differences of Least Squares Means												
**Effect**	**Treatment**	**Treatment**	**Estimate**	**Standard Error**	**DF**	**t Value**	**Pr > |t|**	**Adjustment**	**Adj P**	**Alpha**	**Lower**	**Upper**
**Treatment**	Control_GFP	GFP MsTTR	-0.1856	0.1300	32	-1.43	0.1631	Tukey-Kramer	0.1631	0.05	-0.4503	0.07922

Differences of Least Squares Means				
**Effect**	**Treatment**	**Treatment**	**Adj Lower**	**Adj Upper**
**Treatment**	Control_GFP	GFP MsTTR	-0.4503	0.07922

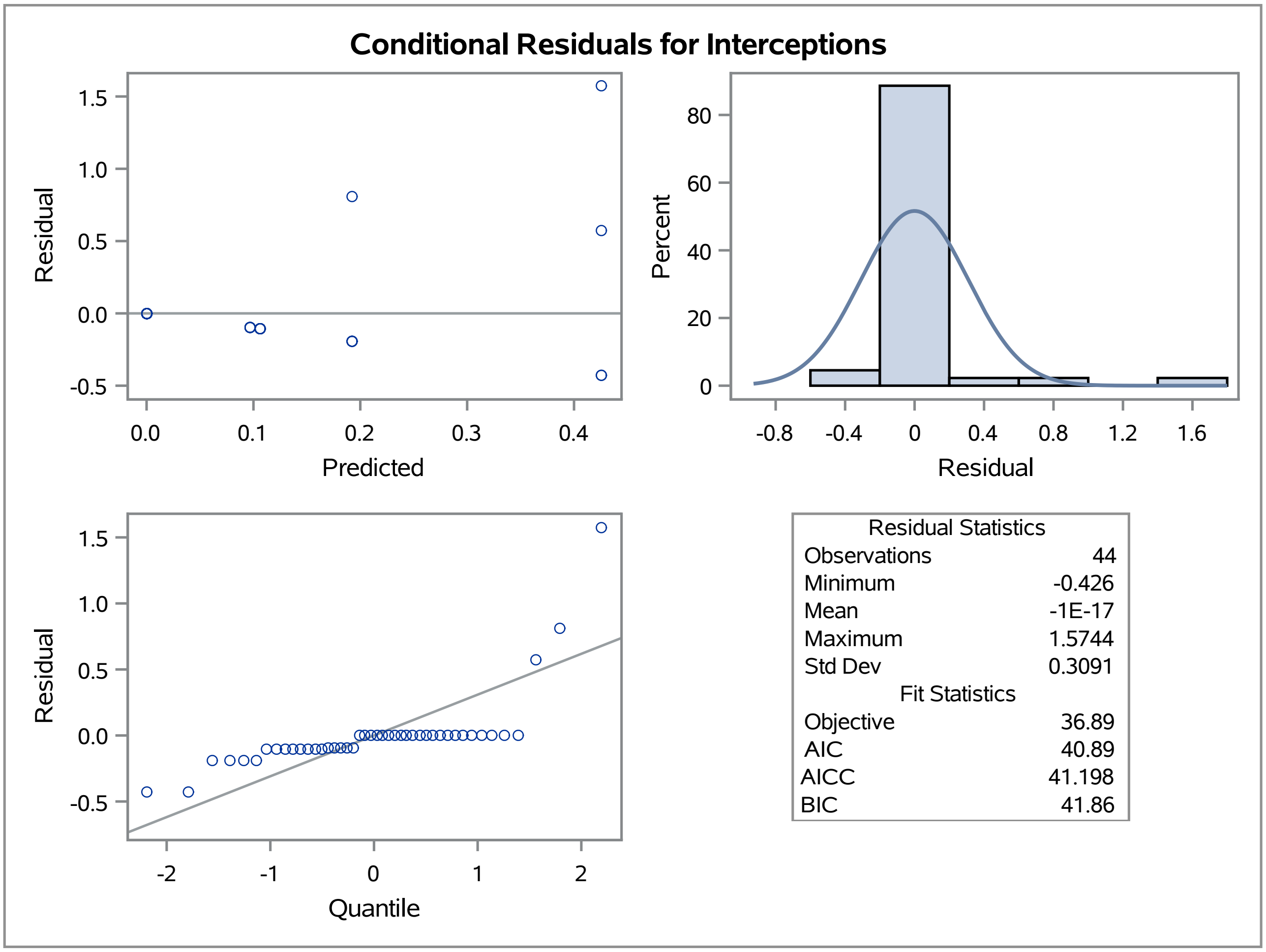

DistSoma=474

Model Information	
**Data Set**	WORK.TEMPDATASORTED
**Dependent Variable**	Interceptions
**Covariance Structure**	Variance Components
**Estimation Method**	REML
**Residual Variance Method**	Profile
**Fixed Effects SE Method**	Model-Based
**Degrees of Freedom Method**	Containment

Class Level Information		
**Class**	**Levels**	**Values**
**Treatment**	2	Control_GFP GFP MsTTR
**Culture**	12	1 2 3 4 5 6 7 8 9 10 11 12

Dimensions	
**Covariance Parameters**	2
**Columns in X**	3
**Columns in Z**	12
**Subjects**	1
**Max Obs per Subject**	44

Number of Observations	
**Number of Observations Read**	44
**Number of Observations Used**	44
**Number of Observations Not Used**	0

Iteration History			
**Iteration**	**Evaluations**	**-2 Res Log Like**	**Criterion**
**0**	1	8.37516794	
**1**	3	7.43019358	0.00001563
**2**	1	7.42964078	0.00000001

Convergence criteria met.

Covariance Parameter Estimates				
**Cov Parm**	**Estimate**	**Alpha**	**Lower**	**Upper**
**Culture**	0.008081	0.05	0.001786	2.1115
**Residual**	0.05454	0.05	0.03559	0.09402

DistSoma=474

Fit Statistics	
-2 Res Log Likelihood	7.4
AIC (Smaller is Better)	11.4
AICC (Smaller is Better)	11.7
BIC (Smaller is Better)	12.4

Solution for Fixed Effects

Effect	Treatment	Estimate	Standard Error	DF	t Value	Pr > |t|	Alpha	Lower	Upper
Intercept		0.1378	0.06409	10	2.15	0.0570	0.05	-0.00497	0.2806
Treatment	Control_GFP	-0.1378	0.08851	32	-1.56	0.1292	0.05	-0.3181	0.04245
Treatment	GFP MsTTR	0	.	.	.	.	.	.	.

Solution for Random Effects

Effect	Culture	Estimate	Std Err Pred	DF	t Value	Pr > |t|	Alpha	Lower	Upper
Culture	1	0	0.07712	32	0.00	1.0000	0.05	-0.1571	0.1571
Culture	2	0	0.07712	32	0.00	1.0000	0.05	-0.1571	0.1571
Culture	3	0	0.07476	32	0.00	1.0000	0.05	-0.1523	0.1523
Culture	4	0	0.07476	32	0.00	1.0000	0.05	-0.1523	0.1523
Culture	5	0	0.07476	32	0.00	1.0000	0.05	-0.1523	0.1523
Culture	6	0	0.08426	32	0.00	1.0000	0.05	-0.1716	0.1716
Culture	7	0	0.07712	32	0.00	1.0000	0.05	-0.1571	0.1571
Culture	8	0.02646	0.07339	32	0.36	0.7208	0.05	-0.1230	0.1759
Culture	9	0.1348	0.07512	32	1.79	0.0822	0.05	-0.01823	0.2878
Culture	10	-0.05129	0.07512	32	-0.68	0.4996	0.05	-0.2043	0.1017
Culture	11	-0.05129	0.07512	32	-0.68	0.4996	0.05	-0.2043	0.1017
Culture	12	-0.05865	0.07339	32	-0.80	0.4300	0.05	-0.2081	0.09083

Type 3 Tests of Fixed Effects

Effect	Num DF	Den DF	F Value	Pr > F
Treatment	1	32	2.43	0.1292

Least Squares Means

Effect	Treatment	Estimate	Standard Error	DF	t Value	Pr > |t|	Alpha	Lower	Upper
Treatment	Control_GFP	-278E-19	0.06104	32	-0.00	1.0000	0.05	-0.1243	0.1243
Treatment	GFP MsTTR	0.1378	0.06409	32	2.15	0.0392	0.05	0.007288	0.2684

DistSoma=474

Differences of Least Squares Means												
**Effect**	**Treatment**	**Treatment**	**Estimate**	**Standard Error**	**DF**	**t Value**	**Pr > |t|**	**Adjustment**	**Adj P**	**Alpha**	**Lower**	**Upper**
**Treatment**	Control_GFP	GFP MsTTR	-0.1378	0.08851	32	-1.56	0.1292	Tukey-Kramer	0.1292	0.05	-0.3181	0.04245

Differences of Least Squares Means				
**Effect**	**Treatment**	**Treatment**	**Adj Lower**	**Adj Upper**
**Treatment**	Control_GFP	GFP MsTTR	-0.3181	0.04245

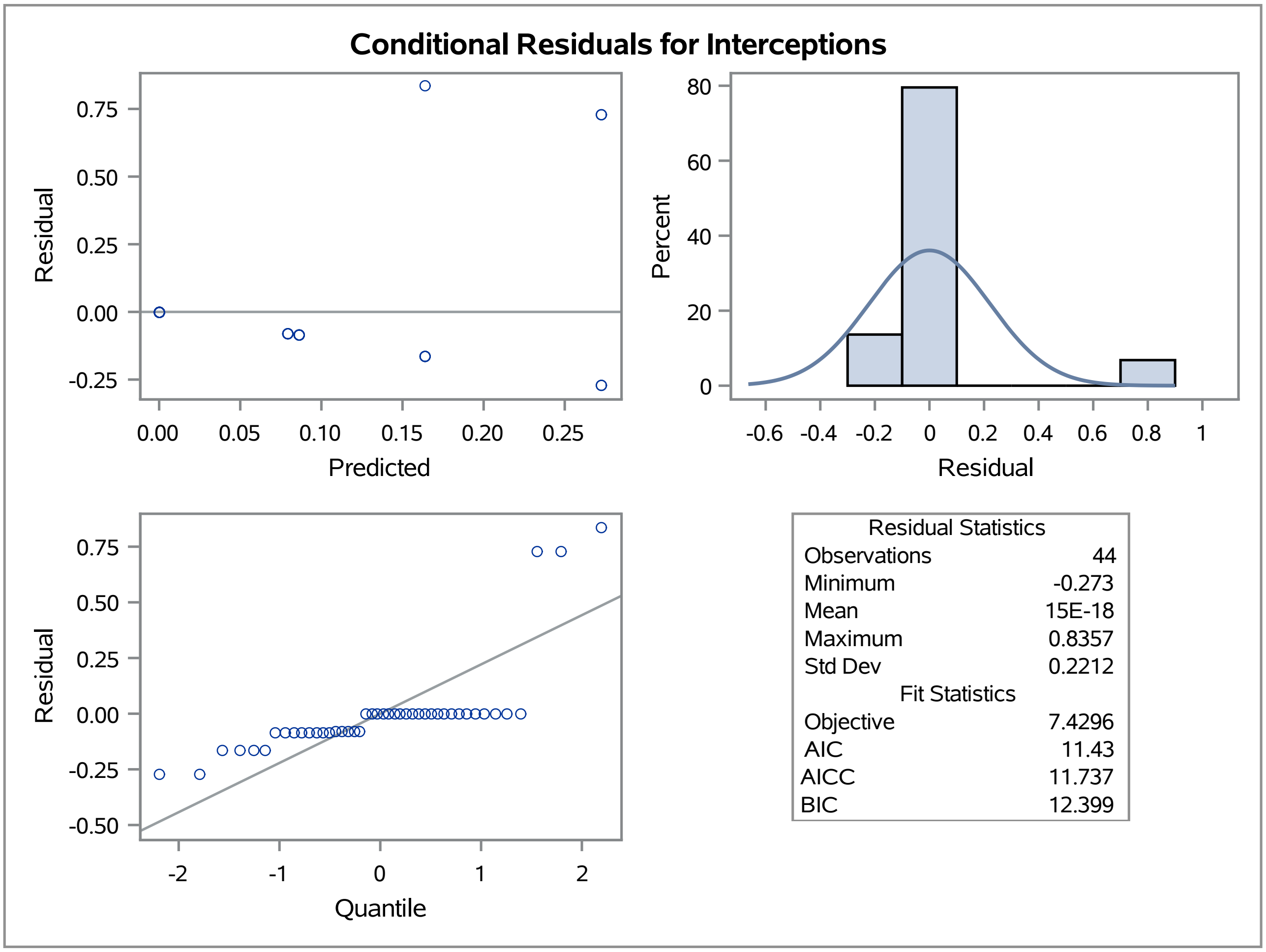

DistSoma=480

Model Information	
**Data Set**	WORK.TEMPDATASORTED
**Dependent Variable**	Interceptions
**Covariance Structure**	Variance Components
**Estimation Method**	REML
**Residual Variance Method**	Profile
**Fixed Effects SE Method**	Model-Based
**Degrees of Freedom Method**	Containment

Class Level Information		
**Class**	**Levels**	**Values**
**Treatment**	2	Control_GFP GFP MsTTR
**Culture**	12	1 2 3 4 5 6 7 8 9 10 11 12

Dimensions	
**Covariance Parameters**	2
**Columns in X**	3
**Columns in Z**	12
**Subjects**	1
**Max Obs per Subject**	44

Number of Observations	
**Number of Observations Read**	44
**Number of Observations Used**	44
**Number of Observations Not Used**	0

Iteration History			
**Iteration**	**Evaluations**	**-2 Res Log Like**	**Criterion**
**0**	1	8.37516794	
**1**	3	7.43019358	0.00001563
**2**	1	7.42964078	0.00000001

Convergence criteria met.

Covariance Parameter Estimates				
**Cov Parm**	**Estimate**	**Alpha**	**Lower**	**Upper**
**Culture**	0.008081	0.05	0.001786	2.1115
**Residual**	0.05454	0.05	0.03559	0.09402

DistSoma=480

Fit Statistics	
-2 Res Log Likelihood	7.4
AIC (Smaller is Better)	11.4
AICC (Smaller is Better)	11.7
BIC (Smaller is Better)	12.4

Solution for Fixed Effects									
Effect	Treatment	Estimate	Standard Error	DF	t Value	Pr > |t|	Alpha	Lower	Upper
Intercept		0.1378	0.06409	10	2.15	0.0570	0.05	-0.00497	0.2806
Treatment	Control_GFP	-0.1378	0.08851	32	-1.56	0.1292	0.05	-0.3181	0.04245
Treatment	GFP MsTTR	0	.	.	.	.	.	.	.

Solution for Random Effects									
Effect	Culture	Estimate	Std Err Pred	DF	t Value	Pr > |t|	Alpha	Lower	Upper
Culture	1	0	0.07712	32	0.00	1.0000	0.05	-0.1571	0.1571
Culture	2	0	0.07712	32	0.00	1.0000	0.05	-0.1571	0.1571
Culture	3	0	0.07476	32	0.00	1.0000	0.05	-0.1523	0.1523
Culture	4	0	0.07476	32	0.00	1.0000	0.05	-0.1523	0.1523
Culture	5	0	0.07476	32	0.00	1.0000	0.05	-0.1523	0.1523
Culture	6	0	0.08426	32	0.00	1.0000	0.05	-0.1716	0.1716
Culture	7	0	0.07712	32	0.00	1.0000	0.05	-0.1571	0.1571
Culture	8	0.02646	0.07339	32	0.36	0.7208	0.05	-0.1230	0.1759
Culture	9	0.1348	0.07512	32	1.79	0.0822	0.05	-0.01823	0.2878
Culture	10	-0.05129	0.07512	32	-0.68	0.4996	0.05	-0.2043	0.1017
Culture	11	-0.05129	0.07512	32	-0.68	0.4996	0.05	-0.2043	0.1017
Culture	12	-0.05865	0.07339	32	-0.80	0.4300	0.05	-0.2081	0.09083

Type 3 Tests of Fixed Effects				
Effect	Num DF	Den DF	F Value	Pr > F
Treatment	1	32	2.43	0.1292

Least Squares Means									
Effect	Treatment	Estimate	Standard Error	DF	t Value	Pr > |t|	Alpha	Lower	Upper
Treatment	Control_GFP	-278E-19	0.06104	32	-0.00	1.0000	0.05	-0.1243	0.1243
Treatment	GFP MsTTR	0.1378	0.06409	32	2.15	0.0392	0.05	0.007288	0.2684

DistSoma=480

Differences of Least Squares Means												
**Effect**	**Treatment**	**Treatment**	**Estimate**	**Standard Error**	**DF**	**t Value**	**Pr > |t|**	**Adjustment**	**Adj P**	**Alpha**	**Lower**	**Upper**
**Treatment**	Control_GFP	GFP MsTTR	-0.1378	0.08851	32	-1.56	0.1292	Tukey-Kramer	0.1292	0.05	-0.3181	0.04245

Differences of Least Squares Means				
**Effect**	**Treatment**	**Treatment**	**Adj Lower**	**Adj Upper**
**Treatment**	Control_GFP	GFP MsTTR	-0.3181	0.04245

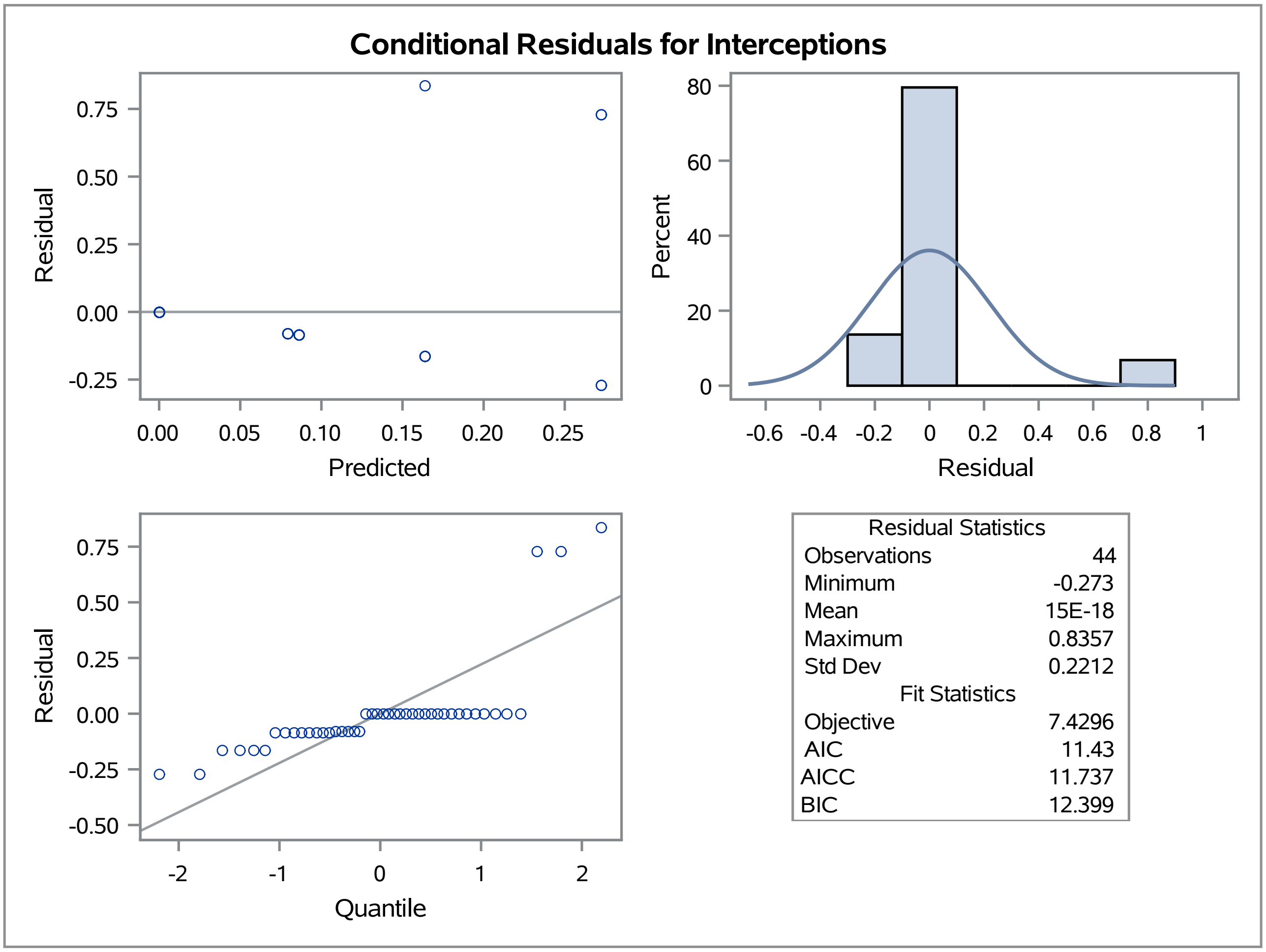

DistSoma=486

Model Information	
**Data Set**	WORK.TEMPDATASORTED
**Dependent Variable**	Interceptions
**Covariance Structure**	Variance Components
**Estimation Method**	REML
**Residual Variance Method**	Profile
**Fixed Effects SE Method**	Model-Based
**Degrees of Freedom Method**	Containment

Class Level Information		
**Class**	**Levels**	**Values**
**Treatment**	2	Control_GFP GFP MsTTR
**Culture**	12	1 2 3 4 5 6 7 8 9 10 11 12

Dimensions	
**Covariance Parameters**	2
**Columns in X**	3
**Columns in Z**	12
**Subjects**	1
**Max Obs per Subject**	44

Number of Observations	
**Number of Observations Read**	44
**Number of Observations Used**	44
**Number of Observations Not Used**	0

Iteration History			
**Iteration**	**Evaluations**	**-2 Res Log Like**	**Criterion**
**0**	1	8.37516794	
**1**	3	7.43019358	0.00001563
**2**	1	7.42964078	0.00000001

Convergence criteria met.

Covariance Parameter Estimates				
**Cov Parm**	**Estimate**	**Alpha**	**Lower**	**Upper**
**Culture**	0.008081	0.05	0.001786	2.1115
**Residual**	0.05454	0.05	0.03559	0.09402

DistSoma=486

Fit Statistics	
**-2 Res Log Likelihood**	7.4
**AIC (Smaller is Better)**	11.4
**AICC (Smaller is Better)**	11.7
**BIC (Smaller is Better)**	12.4

Solution for Fixed Effects

Effect	Treatment	Estimate	Standard Error	DF	t Value	Pr > |t|	Alpha	Lower	Upper
**Intercept**		0.1378	0.06409	10	2.15	0.0570	0.05	-0.00497	0.2806
**Treatment**	Control_GFP	-0.1378	0.08851	32	-1.56	0.1292	0.05	-0.3181	0.04245
**Treatment**	GFP MsTTR	0	.	.	.	.	.	.	.

Solution for Random Effects

Effect	Culture	Estimate	Std Err Pred	DF	t Value	Pr > |t|	Alpha	Lower	Upper
**Culture**	1	0	0.07712	32	0.00	1.0000	0.05	-0.1571	0.1571
**Culture**	2	0	0.07712	32	0.00	1.0000	0.05	-0.1571	0.1571
**Culture**	3	0	0.07476	32	0.00	1.0000	0.05	-0.1523	0.1523
**Culture**	4	0	0.07476	32	0.00	1.0000	0.05	-0.1523	0.1523
**Culture**	5	0	0.07476	32	0.00	1.0000	0.05	-0.1523	0.1523
**Culture**	6	0	0.08426	32	0.00	1.0000	0.05	-0.1716	0.1716
**Culture**	7	0	0.07712	32	0.00	1.0000	0.05	-0.1571	0.1571
**Culture**	8	0.02646	0.07339	32	0.36	0.7208	0.05	-0.1230	0.1759
**Culture**	9	0.1348	0.07512	32	1.79	0.0822	0.05	-0.01823	0.2878
**Culture**	10	-0.05129	0.07512	32	-0.68	0.4996	0.05	-0.2043	0.1017
**Culture**	11	-0.05129	0.07512	32	-0.68	0.4996	0.05	-0.2043	0.1017
**Culture**	12	-0.05865	0.07339	32	-0.80	0.4300	0.05	-0.2081	0.09083

Type 3 Tests of Fixed Effects

Effect	Num DF	Den DF	F Value	Pr > F
**Treatment**	1	32	2.43	0.1292

Least Squares Means

Effect	Treatment	Estimate	Standard Error	DF	t Value	Pr > |t|	Alpha	Lower	Upper
**Treatment**	Control_GFP	-278E-19	0.06104	32	-0.00	1.0000	0.05	-0.1243	0.1243
**Treatment**	GFP MsTTR	0.1378	0.06409	32	2.15	0.0392	0.05	0.007288	0.2684

DistSoma=486

Differences of Least Squares Means												
**Effect**	**Treatment**	**Treatment**	**Estimate**	**Standard Error**	**DF**	**t Value**	**Pr > |t|**	**Adjustment**	**Adj P**	**Alpha**	**Lower**	**Upper**
**Treatment**	Control_GFP	GFP MsTTR	-0.1378	0.08851	32	-1.56	0.1292	Tukey-Kramer	0.1292	0.05	-0.3181	0.04245

Differences of Least Squares Means				
**Effect**	**Treatment**	**Treatment**	**Adj Lower**	**Adj Upper**
**Treatment**	Control_GFP	GFP MsTTR	-0.3181	0.04245

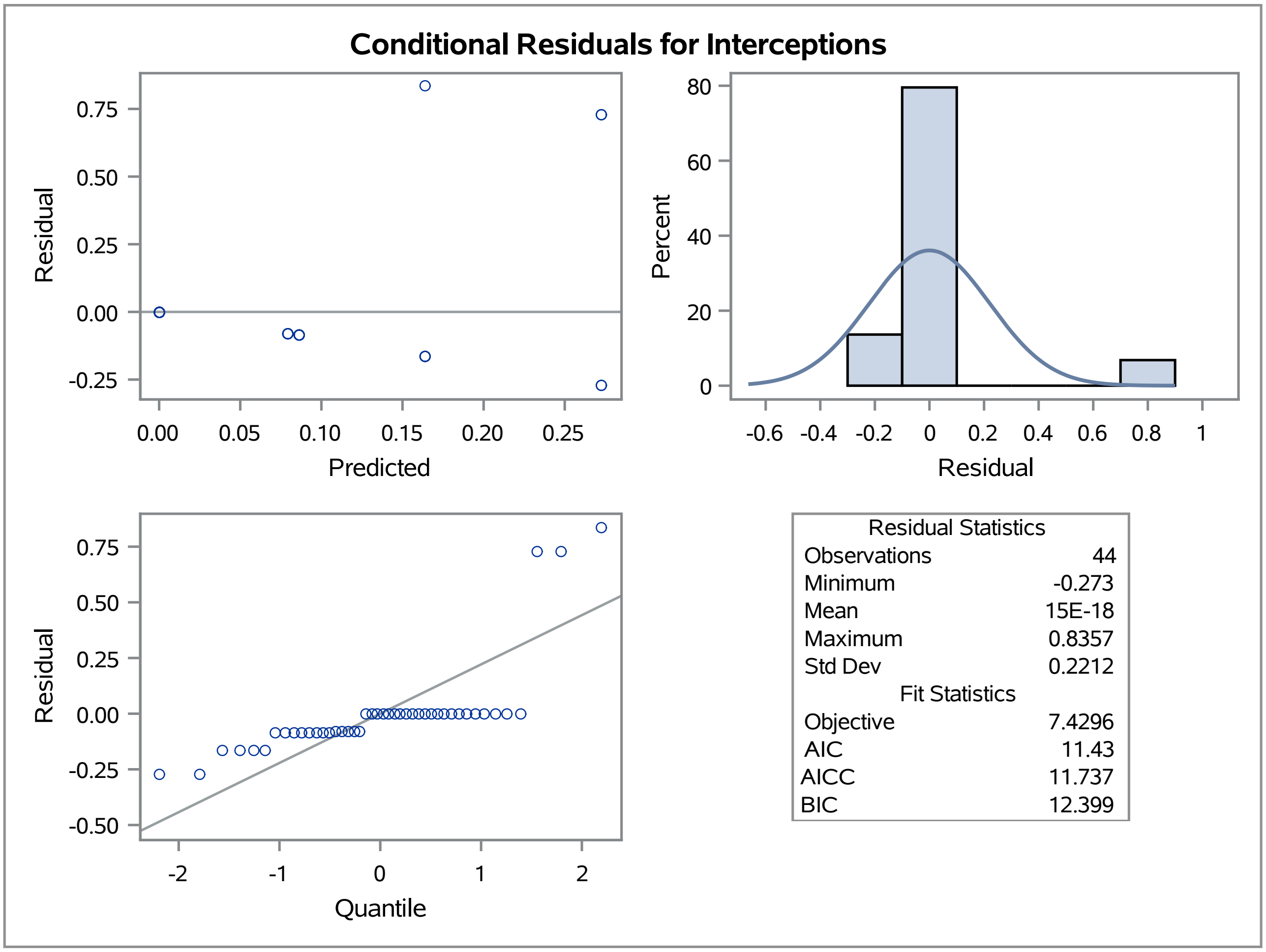

DistSoma=492

Model Information	
**Data Set**	WORK.TEMPDATASORTED
**Dependent Variable**	Interceptions
**Covariance Structure**	Variance Components
**Estimation Method**	REML
**Residual Variance Method**	Profile
**Fixed Effects SE Method**	Model-Based
**Degrees of Freedom Method**	Containment

Class Level Information		
**Class**	**Levels**	**Values**
**Treatment**	2	Control_GFP GFP MsTTR
**Culture**	12	1 2 3 4 5 6 7 8 9 10 11 12

Dimensions	
**Covariance Parameters**	2
**Columns in X**	3
**Columns in Z**	12
**Subjects**	1
**Max Obs per Subject**	44

Number of Observations	
**Number of Observations Read**	44
**Number of Observations Used**	44
**Number of Observations Not Used**	0

Iteration History			
**Iteration**	**Evaluations**	**-2 Res Log Like**	**Criterion**
**0**	1	8.37516794	
**1**	3	7.43019358	0.00001563
**2**	1	7.42964078	0.00000001

Convergence criteria met.

Covariance Parameter Estimates				
**Cov Parm**	**Estimate**	**Alpha**	**Lower**	**Upper**
**Culture**	0.008081	0.05	0.001786	2.1115
**Residual**	0.05454	0.05	0.03559	0.09402

DistSoma=492

Fit Statistics	
-2 Res Log Likelihood	7.4
AIC (Smaller is Better)	11.4
AICC (Smaller is Better)	11.7
BIC (Smaller is Better)	12.4

Solution for Fixed Effects

Effect	Treatment	Estimate	Standard Error	DF	t Value	Pr > |t|	Alpha	Lower	Upper
Intercept		0.1378	0.06409	10	2.15	0.0570	0.05	-0.00497	0.2806
Treatment	Control_GFP	-0.1378	0.08851	32	-1.56	0.1292	0.05	-0.3181	0.04245
Treatment	GFP MsTTR	0	.	.	.	.	.	.	.

Solution for Random Effects

Effect	Culture	Estimate	Std Err Pred	DF	t Value	Pr > |t|	Alpha	Lower	Upper
Culture	1	0	0.07712	32	0.00	1.0000	0.05	-0.1571	0.1571
Culture	2	0	0.07712	32	0.00	1.0000	0.05	-0.1571	0.1571
Culture	3	0	0.07476	32	0.00	1.0000	0.05	-0.1523	0.1523
Culture	4	0	0.07476	32	0.00	1.0000	0.05	-0.1523	0.1523
Culture	5	0	0.07476	32	0.00	1.0000	0.05	-0.1523	0.1523
Culture	6	0	0.08426	32	0.00	1.0000	0.05	-0.1716	0.1716
Culture	7	0	0.07712	32	0.00	1.0000	0.05	-0.1571	0.1571
Culture	8	0.02646	0.07339	32	0.36	0.7208	0.05	-0.1230	0.1759
Culture	9	0.1348	0.07512	32	1.79	0.0822	0.05	-0.01823	0.2878
Culture	10	-0.05129	0.07512	32	-0.68	0.4996	0.05	-0.2043	0.1017
Culture	11	-0.05129	0.07512	32	-0.68	0.4996	0.05	-0.2043	0.1017
Culture	12	-0.05865	0.07339	32	-0.80	0.4300	0.05	-0.2081	0.09083

Type 3 Tests of Fixed Effects

Effect	Num DF	Den DF	F Value	Pr > F
Treatment	1	32	2.43	0.1292

Least Squares Means

Effect	Treatment	Estimate	Standard Error	DF	t Value	Pr > |t|	Alpha	Lower	Upper
Treatment	Control_GFP	-278E-19	0.06104	32	-0.00	1.0000	0.05	-0.1243	0.1243
Treatment	GFP MsTTR	0.1378	0.06409	32	2.15	0.0392	0.05	0.007288	0.2684

DistSoma=492

Differences of Least Squares Means												
**Effect**	**Treatment**	**Treatment**	**Estimate**	**Standard Error**	**DF**	**t Value**	**Pr > |t|**	**Adjustment**	**Adj P**	**Alpha**	**Lower**	**Upper**
**Treatment**	Control_GFP	GFP MsTTR	-0.1378	0.08851	32	-1.56	0.1292	Tukey-Kramer	0.1292	0.05	-0.3181	0.04245

Differences of Least Squares Means				
**Effect**	**Treatment**	**Treatment**	**Adj Lower**	**Adj Upper**
**Treatment**	Control_GFP	GFP MsTTR	-0.3181	0.04245

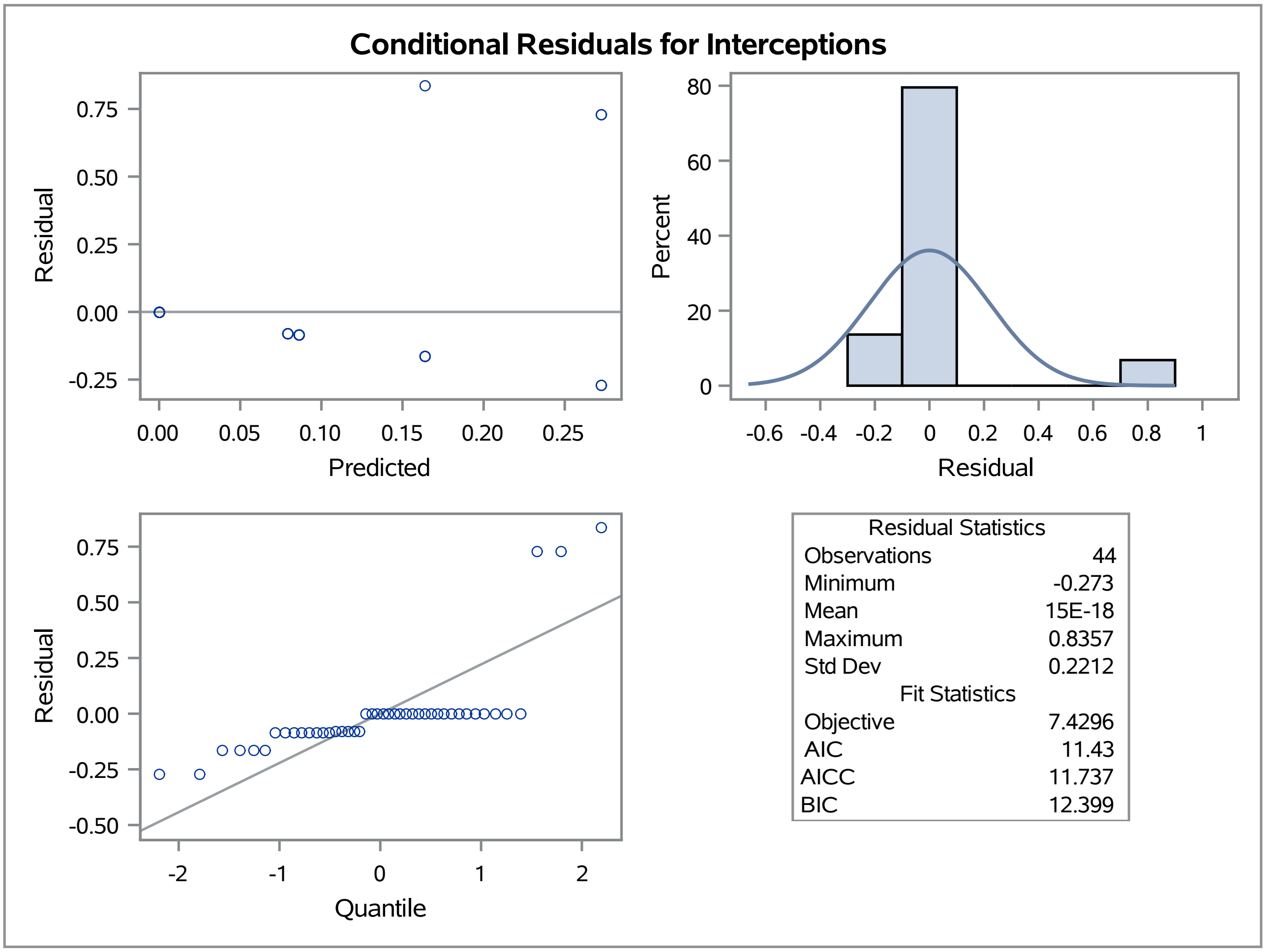

DistSoma=498

Model Information	
**Data Set**	WORK.TEMPDATASORTED
**Dependent Variable**	Interceptions
**Covariance Structure**	Variance Components
**Estimation Method**	REML
**Residual Variance Method**	Profile
**Fixed Effects SE Method**	Model-Based
**Degrees of Freedom Method**	Containment

Class Level Information		
**Class**	**Levels**	**Values**
**Treatment**	2	Control_GFP GFP MsTTR
**Culture**	12	1 2 3 4 5 6 7 8 9 10 11 12

Dimensions	
**Covariance Parameters**	2
**Columns in X**	3
**Columns in Z**	12
**Subjects**	1
**Max Obs per Subject**	44

Number of Observations	
**Number of Observations Read**	44
**Number of Observations Used**	44
**Number of Observations Not Used**	0

Iteration History			
**Iteration**	**Evaluations**	**-2 Res Log Like**	**Criterion**
**0**	1	8.37516794	
**1**	3	7.43019358	0.00001563
**2**	1	7.42964078	0.00000001

Convergence criteria met.

Covariance Parameter Estimates				
**Cov Parm**	**Estimate**	**Alpha**	**Lower**	**Upper**
**Culture**	0.008081	0.05	0.001786	2.1115
**Residual**	0.05454	0.05	0.03559	0.09402

DistSoma=498

Fit Statistics	
-2 Res Log Likelihood	7.4
AIC (Smaller is Better)	11.4
AICC (Smaller is Better)	11.7
BIC (Smaller is Better)	12.4

Solution for Fixed Effects									
Effect	Treatment	Estimate	Standard Error	DF	t Value	Pr > |t|	Alpha	Lower	Upper
Intercept		0.1378	0.06409	10	2.15	0.0570	0.05	-0.00497	0.2806
Treatment	Control_GFP	-0.1378	0.08851	32	-1.56	0.1292	0.05	-0.3181	0.04245
Treatment	GFP MsTTR	0	.	.	.	.	.	.	.

Solution for Random Effects									
Effect	Culture	Estimate	Std Err Pred	DF	t Value	Pr > |t|	Alpha	Lower	Upper
Culture	1	0	0.07712	32	0.00	1.0000	0.05	-0.1571	0.1571
Culture	2	0	0.07712	32	0.00	1.0000	0.05	-0.1571	0.1571
Culture	3	0	0.07476	32	0.00	1.0000	0.05	-0.1523	0.1523
Culture	4	0	0.07476	32	0.00	1.0000	0.05	-0.1523	0.1523
Culture	5	0	0.07476	32	0.00	1.0000	0.05	-0.1523	0.1523
Culture	6	0	0.08426	32	0.00	1.0000	0.05	-0.1716	0.1716
Culture	7	0	0.07712	32	0.00	1.0000	0.05	-0.1571	0.1571
Culture	8	0.02646	0.07339	32	0.36	0.7208	0.05	-0.1230	0.1759
Culture	9	0.1348	0.07512	32	1.79	0.0822	0.05	-0.01823	0.2878
Culture	10	-0.05129	0.07512	32	-0.68	0.4996	0.05	-0.2043	0.1017
Culture	11	-0.05129	0.07512	32	-0.68	0.4996	0.05	-0.2043	0.1017
Culture	12	-0.05865	0.07339	32	-0.80	0.4300	0.05	-0.2081	0.09083

Type 3 Tests of Fixed Effects				
Effect	Num DF	Den DF	F Value	Pr > F
Treatment	1	32	2.43	0.1292

Least Squares Means									
Effect	Treatment	Estimate	Standard Error	DF	t Value	Pr > |t|	Alpha	Lower	Upper
Treatment	Control_GFP	-278E-19	0.06104	32	-0.00	1.0000	0.05	-0.1243	0.1243
Treatment	GFP MsTTR	0.1378	0.06409	32	2.15	0.0392	0.05	0.007288	0.2684

DistSoma=498

Differences of Least Squares Means												
**Effect**	**Treatment**	**Treatment**	**Estimate**	**Standard Error**	**DF**	**t Value**	**Pr > |t|**	**Adjustment**	**Adj P**	**Alpha**	**Lower**	**Upper**
**Treatment**	Control_GFP	GFP MsTTR	-0.1378	0.08851	32	-1.56	0.1292	Tukey-Kramer	0.1292	0.05	-0.3181	0.04245

Differences of Least Squares Means				
**Effect**	**Treatment**	**Treatment**	**Adj Lower**	**Adj Upper**
**Treatment**	Control_GFP	GFP MsTTR	-0.3181	0.04245

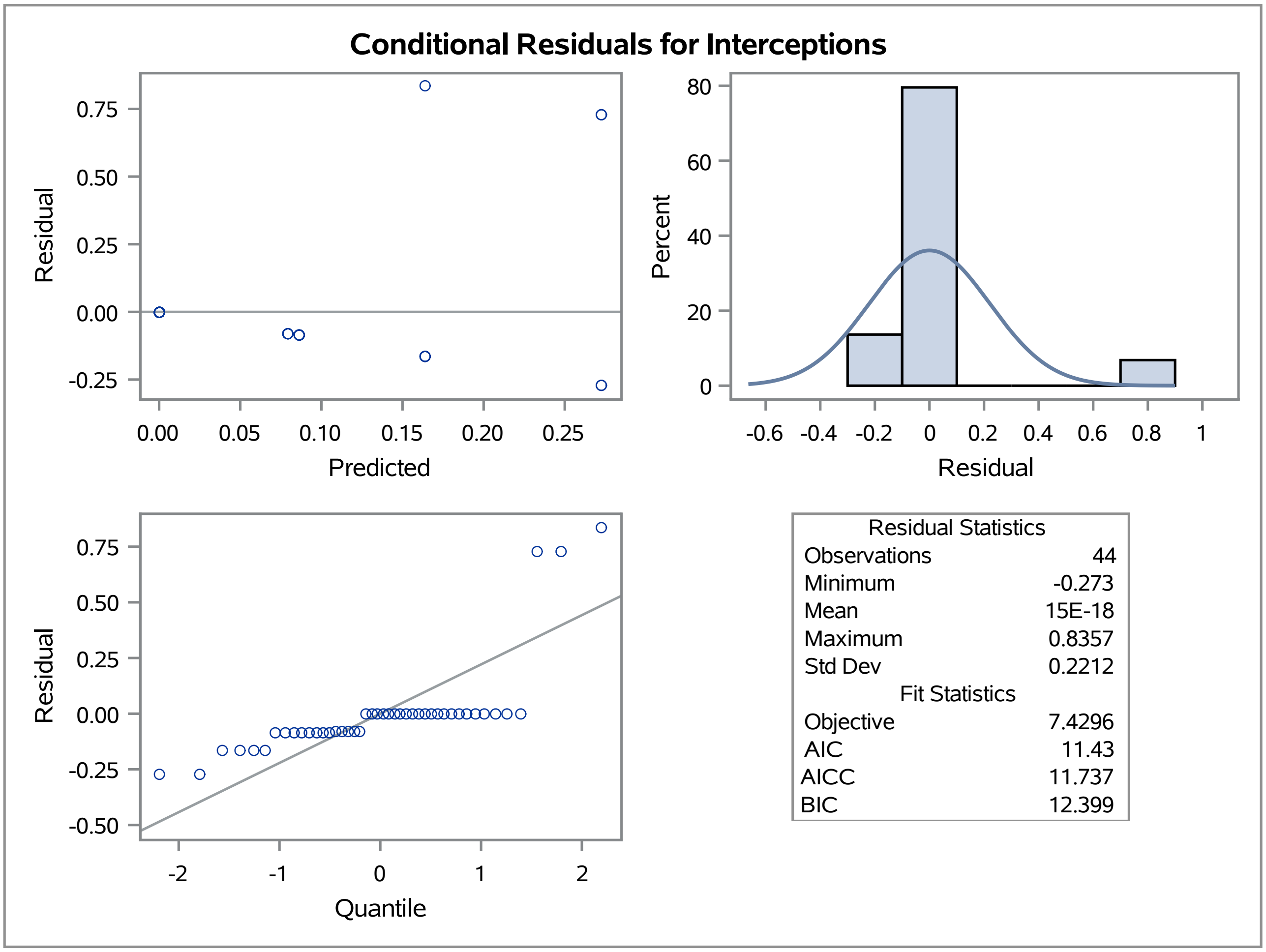

DistSoma=504

Model Information	
**Data Set**	WORK.TEMPDATASORTED
**Dependent Variable**	Interceptions
**Covariance Structure**	Variance Components
**Estimation Method**	REML
**Residual Variance Method**	Profile
**Fixed Effects SE Method**	Model-Based
**Degrees of Freedom Method**	Containment

Class Level Information		
**Class**	**Levels**	**Values**
**Treatment**	2	Control_GFP GFP MsTTR
**Culture**	12	1 2 3 4 5 6 7 8 9 10 11 12

Dimensions	
**Covariance Parameters**	2
**Columns in X**	3
**Columns in Z**	12
**Subjects**	1
**Max Obs per Subject**	44

Number of Observations	
**Number of Observations Read**	44
**Number of Observations Used**	44
**Number of Observations Not Used**	0

Iteration History			
**Iteration**	**Evaluations**	**-2 Res Log Like**	**Criterion**
**0**	1	8.37516794	
**1**	3	7.43019358	0.00001563
**2**	1	7.42964078	0.00000001

Convergence criteria met.

Covariance Parameter Estimates				
**Cov Parm**	**Estimate**	**Alpha**	**Lower**	**Upper**
**Culture**	0.008081	0.05	0.001786	2.1115
**Residual**	0.05454	0.05	0.03559	0.09402

DistSoma=504

Fit Statistics	
-2 Res Log Likelihood	7.4
AIC (Smaller is Better)	11.4
AICC (Smaller is Better)	11.7
BIC (Smaller is Better)	12.4

Solution for Fixed Effects									
Effect	Treatment	Estimate	Standard Error	DF	t Value	Pr > |t|	Alpha	Lower	Upper
Intercept		0.1378	0.06409	10	2.15	0.0570	0.05	-0.00497	0.2806
Treatment	Control_GFP	-0.1378	0.08851	32	-1.56	0.1292	0.05	-0.3181	0.04245
Treatment	GFP MsTTR	0	.	.	.	.	.	.	.

Solution for Random Effects									
Effect	Culture	Estimate	Std Err Pred	DF	t Value	Pr > |t|	Alpha	Lower	Upper
Culture	1	0	0.07712	32	0.00	1.0000	0.05	-0.1571	0.1571
Culture	2	0	0.07712	32	0.00	1.0000	0.05	-0.1571	0.1571
Culture	3	0	0.07476	32	0.00	1.0000	0.05	-0.1523	0.1523
Culture	4	0	0.07476	32	0.00	1.0000	0.05	-0.1523	0.1523
Culture	5	0	0.07476	32	0.00	1.0000	0.05	-0.1523	0.1523
Culture	6	0	0.08426	32	0.00	1.0000	0.05	-0.1716	0.1716
Culture	7	0	0.07712	32	0.00	1.0000	0.05	-0.1571	0.1571
Culture	8	0.02646	0.07339	32	0.36	0.7208	0.05	-0.1230	0.1759
Culture	9	0.1348	0.07512	32	1.79	0.0822	0.05	-0.01823	0.2878
Culture	10	-0.05129	0.07512	32	-0.68	0.4996	0.05	-0.2043	0.1017
Culture	11	-0.05129	0.07512	32	-0.68	0.4996	0.05	-0.2043	0.1017
Culture	12	-0.05865	0.07339	32	-0.80	0.4300	0.05	-0.2081	0.09083

Type 3 Tests of Fixed Effects				
Effect	Num DF	Den DF	F Value	Pr > F
Treatment	1	32	2.43	0.1292

Least Squares Means									
Effect	Treatment	Estimate	Standard Error	DF	t Value	Pr > |t|	Alpha	Lower	Upper
Treatment	Control_GFP	-278E-19	0.06104	32	-0.00	1.0000	0.05	-0.1243	0.1243
Treatment	GFP MsTTR	0.1378	0.06409	32	2.15	0.0392	0.05	0.007288	0.2684

DistSoma=504

Differences of Least Squares Means												
**Effect**	**Treatment**	**Treatment**	**Estimate**	**Standard Error**	**DF**	**t Value**	**Pr > |t|**	**Adjustment**	**Adj P**	**Alpha**	**Lower**	**Upper**
**Treatment**	Control_GFP	GFP MsTTR	-0.1378	0.08851	32	-1.56	0.1292	Tukey-Kramer	0.1292	0.05	-0.3181	0.04245

Differences of Least Squares Means				
**Effect**	**Treatment**	**Treatment**	**Adj Lower**	**Adj Upper**
**Treatment**	Control_GFP	GFP MsTTR	-0.3181	0.04245

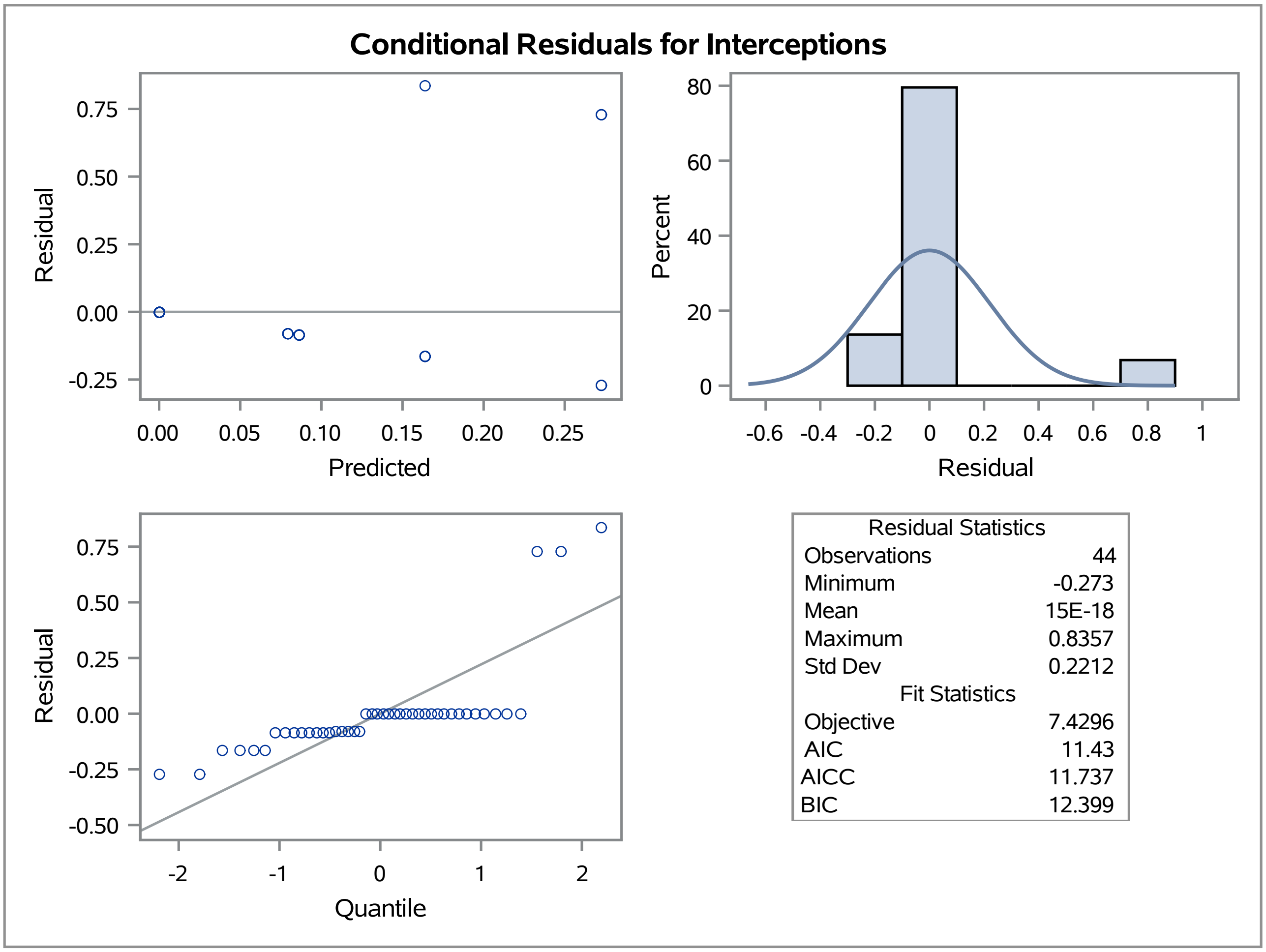

DistSoma=510

Model Information	
**Data Set**	WORK.TEMPDATASORTED
**Dependent Variable**	Interceptions
**Covariance Structure**	Variance Components
**Estimation Method**	REML
**Residual Variance Method**	Profile
**Fixed Effects SE Method**	Model-Based
**Degrees of Freedom Method**	Containment

Class Level Information		
**Class**	**Levels**	**Values**
**Treatment**	2	Control_GFP GFP MsTTR
**Culture**	12	1 2 3 4 5 6 7 8 9 10 11 12

Dimensions	
**Covariance Parameters**	2
**Columns in X**	3
**Columns in Z**	12
**Subjects**	1
**Max Obs per Subject**	44

Number of Observations	
**Number of Observations Read**	44
**Number of Observations Used**	44
**Number of Observations Not Used**	0

Iteration History			
**Iteration**	**Evaluations**	**-2 Res Log Like**	**Criterion**
**0**	1	8.37516794	
**1**	3	7.43019358	0.00001563
**2**	1	7.42964078	0.00000001

Convergence criteria met.

Covariance Parameter Estimates				
**Cov Parm**	**Estimate**	**Alpha**	**Lower**	**Upper**
**Culture**	0.008081	0.05	0.001786	2.1115
**Residual**	0.05454	0.05	0.03559	0.09402

DistSoma=510

Fit Statistics	
-2 Res Log Likelihood	7.4
AIC (Smaller is Better)	11.4
AICC (Smaller is Better)	11.7
BIC (Smaller is Better)	12.4

Solution for Fixed Effects

Effect	Treatment	Estimate	Standard Error	DF	t Value	Pr > |t|	Alpha	Lower	Upper
Intercept		0.1378	0.06409	10	2.15	0.0570	0.05	-0.00497	0.2806
Treatment	Control_GFP	-0.1378	0.08851	32	-1.56	0.1292	0.05	-0.3181	0.04245
Treatment	GFP MsTTR	0	.	.	.	.	.	.	.

Solution for Random Effects

Effect	Culture	Estimate	Std Err Pred	DF	t Value	Pr > |t|	Alpha	Lower	Upper
Culture	1	0	0.07712	32	0.00	1.0000	0.05	-0.1571	0.1571
Culture	2	0	0.07712	32	0.00	1.0000	0.05	-0.1571	0.1571
Culture	3	0	0.07476	32	0.00	1.0000	0.05	-0.1523	0.1523
Culture	4	0	0.07476	32	0.00	1.0000	0.05	-0.1523	0.1523
Culture	5	0	0.07476	32	0.00	1.0000	0.05	-0.1523	0.1523
Culture	6	0	0.08426	32	0.00	1.0000	0.05	-0.1716	0.1716
Culture	7	0	0.07712	32	0.00	1.0000	0.05	-0.1571	0.1571
Culture	8	0.02646	0.07339	32	0.36	0.7208	0.05	-0.1230	0.1759
Culture	9	0.1348	0.07512	32	1.79	0.0822	0.05	-0.01823	0.2878
Culture	10	-0.05129	0.07512	32	-0.68	0.4996	0.05	-0.2043	0.1017
Culture	11	-0.05129	0.07512	32	-0.68	0.4996	0.05	-0.2043	0.1017
Culture	12	-0.05865	0.07339	32	-0.80	0.4300	0.05	-0.2081	0.09083

Type 3 Tests of Fixed Effects

Effect	Num DF	Den DF	F Value	Pr > F
Treatment	1	32	2.43	0.1292

Least Squares Means

Effect	Treatment	Estimate	Standard Error	DF	t Value	Pr > |t|	Alpha	Lower	Upper
Treatment	Control_GFP	-278E-19	0.06104	32	-0.00	1.0000	0.05	-0.1243	0.1243
Treatment	GFP MsTTR	0.1378	0.06409	32	2.15	0.0392	0.05	0.007288	0.2684

DistSoma=510

Differences of Least Squares Means												
Effect	Treatment	Treatment	Estimate	Standard Error	DF	t Value	Pr > |t|	Adjustment	Adj P	Alpha	Lower	Upper
**Treatment**	Control_GFP	GFP MsTTR	-0.1378	0.08851	32	-1.56	0.1292	Tukey-Kramer	0.1292	0.05	-0.3181	0.04245

Differences of Least Squares Means				
Effect	Treatment	Treatment	Adj Lower	Adj Upper
**Treatment**	Control_GFP	GFP MsTTR	-0.3181	0.04245

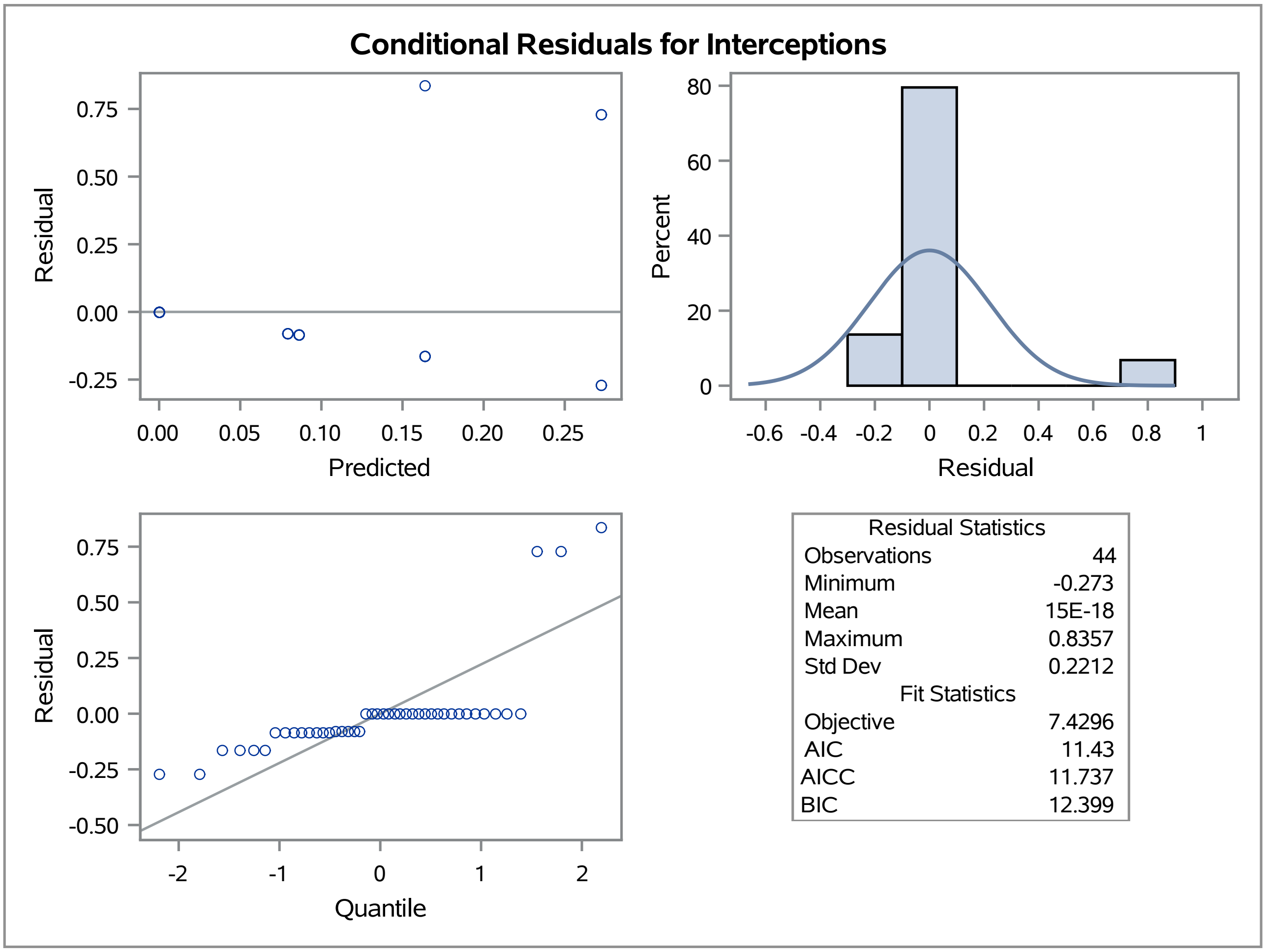

DistSoma=516

Model Information	
**Data Set**	WORK.TEMPDATASORTED
**Dependent Variable**	Interceptions
**Covariance Structure**	Variance Components
**Estimation Method**	REML
**Residual Variance Method**	Profile
**Fixed Effects SE Method**	Model-Based
**Degrees of Freedom Method**	Containment

Class Level Information		
**Class**	**Levels**	**Values**
**Treatment**	2	Control_GFP GFP MsTTR
**Culture**	12	1 2 3 4 5 6 7 8 9 10 11 12

Dimensions	
**Covariance Parameters**	2
**Columns in X**	3
**Columns in Z**	12
**Subjects**	1
**Max Obs per Subject**	44

Number of Observations	
**Number of Observations Read**	44
**Number of Observations Used**	44
**Number of Observations Not Used**	0

Iteration History			
**Iteration**	**Evaluations**	**-2 Res Log Like**	**Criterion**
**0**	1	8.37516794	
**1**	3	7.43019358	0.00001563
**2**	1	7.42964078	0.00000001

Convergence criteria met.

Covariance Parameter Estimates				
**Cov Parm**	**Estimate**	**Alpha**	**Lower**	**Upper**
**Culture**	0.008081	0.05	0.001786	2.1115
**Residual**	0.05454	0.05	0.03559	0.09402

DistSoma=516

Fit Statistics	
**-2 Res Log Likelihood**	7.4
**AIC (Smaller is Better)**	11.4
**AICC (Smaller is Better)**	11.7
**BIC (Smaller is Better)**	12.4

Solution for Fixed Effects

Effect	Treatment	Estimate	Standard Error	DF	t Value	Pr > |t|	Alpha	Lower	Upper
**Intercept**		0.1378	0.06409	10	2.15	0.0570	0.05	-0.00497	0.2806
**Treatment**	Control_GFP	-0.1378	0.08851	32	-1.56	0.1292	0.05	-0.3181	0.04245
**Treatment**	GFP MsTTR	0	.	.	.	.	.	.	.

Solution for Random Effects

Effect	Culture	Estimate	Std Err Pred	DF	t Value	Pr > |t|	Alpha	Lower	Upper
**Culture**	1	0	0.07712	32	0.00	1.0000	0.05	-0.1571	0.1571
**Culture**	2	0	0.07712	32	0.00	1.0000	0.05	-0.1571	0.1571
**Culture**	3	0	0.07476	32	0.00	1.0000	0.05	-0.1523	0.1523
**Culture**	4	0	0.07476	32	0.00	1.0000	0.05	-0.1523	0.1523
**Culture**	5	0	0.07476	32	0.00	1.0000	0.05	-0.1523	0.1523
**Culture**	6	0	0.08426	32	0.00	1.0000	0.05	-0.1716	0.1716
**Culture**	7	0	0.07712	32	0.00	1.0000	0.05	-0.1571	0.1571
**Culture**	8	0.02646	0.07339	32	0.36	0.7208	0.05	-0.1230	0.1759
**Culture**	9	0.1348	0.07512	32	1.79	0.0822	0.05	-0.01823	0.2878
**Culture**	10	-0.05129	0.07512	32	-0.68	0.4996	0.05	-0.2043	0.1017
**Culture**	11	-0.05129	0.07512	32	-0.68	0.4996	0.05	-0.2043	0.1017
**Culture**	12	-0.05865	0.07339	32	-0.80	0.4300	0.05	-0.2081	0.09083

Type 3 Tests of Fixed Effects

Effect	Num DF	Den DF	F Value	Pr > F
**Treatment**	1	32	2.43	0.1292

Least Squares Means

Effect	Treatment	Estimate	Standard Error	DF	t Value	Pr > |t|	Alpha	Lower	Upper
**Treatment**	Control_GFP	-278E-19	0.06104	32	-0.00	1.0000	0.05	-0.1243	0.1243
**Treatment**	GFP MsTTR	0.1378	0.06409	32	2.15	0.0392	0.05	0.007288	0.2684

DistSoma=516

Differences of Least Squares Means												
Effect	Treatment	Treatment	Estimate	Standard Error	DF	t Value	Pr > |t|	Adjustment	Adj P	Alpha	Lower	Upper
**Treatment**	Control_GFP	GFP MsTTR	-0.1378	0.08851	32	-1.56	0.1292	Tukey-Kramer	0.1292	0.05	-0.3181	0.04245

Differences of Least Squares Means				
Effect	Treatment	Treatment	Adj Lower	Adj Upper
**Treatment**	Control_GFP	GFP MsTTR	-0.3181	0.04245

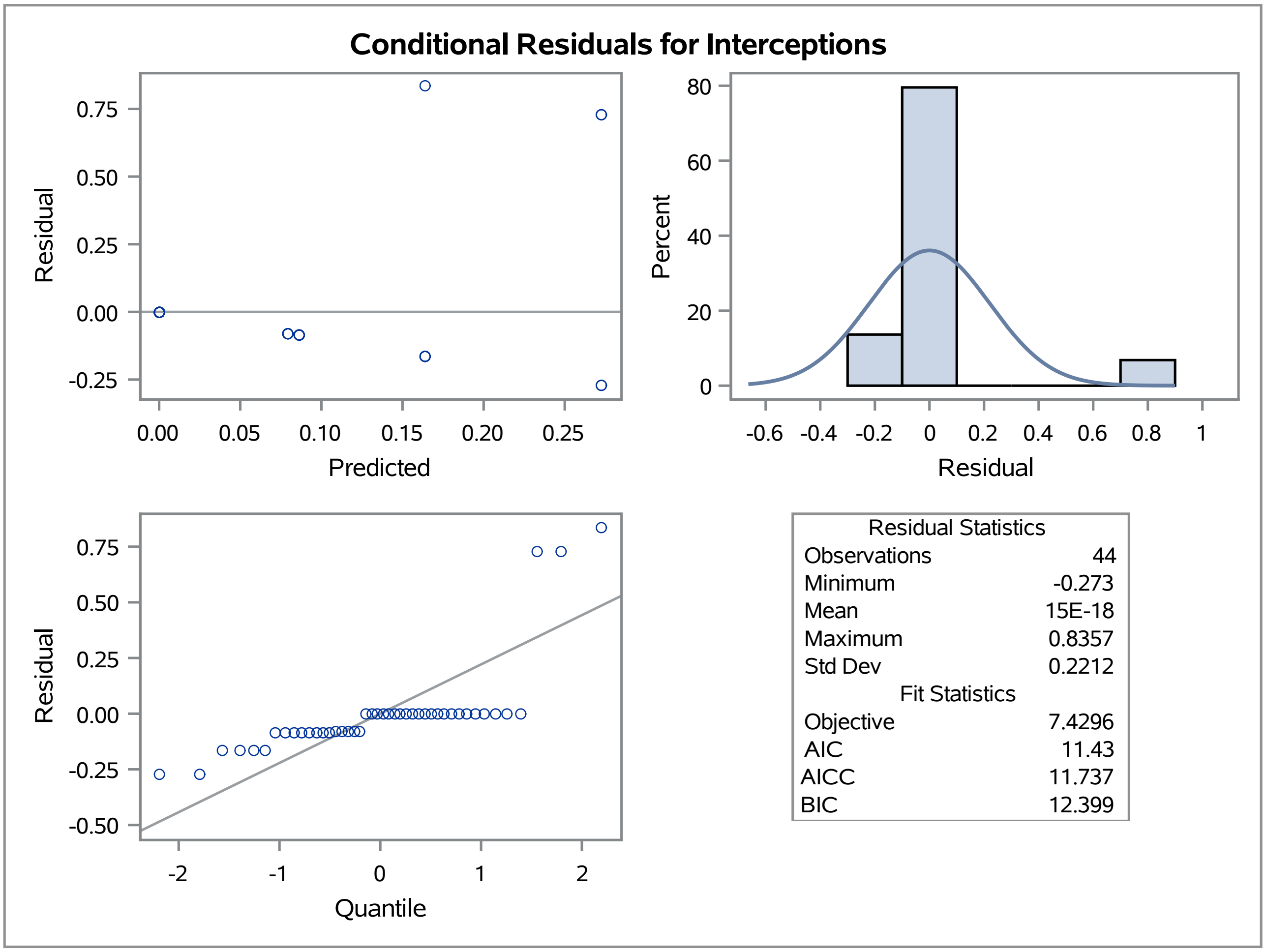

DistSoma=522

Model Information	
**Data Set**	WORK.TEMPDATASORTED
**Dependent Variable**	Interceptions
**Covariance Structure**	Variance Components
**Estimation Method**	REML
**Residual Variance Method**	Profile
**Fixed Effects SE Method**	Model-Based
**Degrees of Freedom Method**	Containment

Class Level Information		
**Class**	**Levels**	**Values**
**Treatment**	2	Control_GFP GFP MsTTR
**Culture**	12	1 2 3 4 5 6 7 8 9 10 11 12

Dimensions	
**Covariance Parameters**	2
**Columns in X**	3
**Columns in Z**	12
**Subjects**	1
**Max Obs per Subject**	44

Number of Observations	
**Number of Observations Read**	44
**Number of Observations Used**	44
**Number of Observations Not Used**	0

Iteration History			
**Iteration**	**Evaluations**	**-2 Res Log Like**	**Criterion**
**0**	1	-6.50004824	
**1**	1	-6.50004824	0.00000000

Convergence criteria met.

Estimated G matrix is not positive definite.

Covariance Parameter Estimates				
**Cov Parm**	**Estimate**	**Alpha**	**Lower**	**Upper**
**Culture**	0	.	.	.
**Residual**	0.04329	0.05	0.02943	0.06993

DistSoma=522

Fit Statistics	
-2 Res Log Likelihood	-6.5
AIC (Smaller is Better)	-4.5
AICC (Smaller is Better)	-4.4
BIC (Smaller is Better)	-4.0

Solution for Fixed Effects

Effect	Treatment	Estimate	Standard Error	DF	t Value	Pr > |t|	Alpha	Lower	Upper
Intercept		0.09091	0.04436	10	2.05	0.0676	0.05	-0.00793	0.1897
Treatment	Control_GFP	-0.09091	0.06273	32	-1.45	0.1570	0.05	-0.2187	0.03687
Treatment	GFP MsTTR	0	.	.	.	.	.	.	.

Solution for Random Effects

Effect	Culture	Estimate	Std Err Pred	DF	t Value	Pr > |t|	Alpha	Lower	Upper
Culture	1	0	.	.	.	.	.	.	.
Culture	2	0	.	.	.	.	.	.	.
Culture	3	0	.	.	.	.	.	.	.
Culture	4	0	.	.	.	.	.	.	.
Culture	5	0	.	.	.	.	.	.	.
Culture	6	0	.	.	.	.	.	.	.
Culture	7	0	.	.	.	.	.	.	.
Culture	8	0	.	.	.	.	.	.	.
Culture	9	0	.	.	.	.	.	.	.
Culture	10	0	.	.	.	.	.	.	.
Culture	11	0	.	.	.	.	.	.	.
Culture	12	0	.	.	.	.	.	.	.

Type 3 Tests of Fixed Effects

Effect	Num DF	Den DF	F Value	Pr > F
Treatment	1	32	2.10	0.1570

Least Squares Means

Effect	Treatment	Estimate	Standard Error	DF	t Value	Pr > |t|	Alpha	Lower	Upper
Treatment	Control_GFP	0	0.04436	32	0.00	1.0000	0.05	-0.09036	0.09036
Treatment	GFP MsTTR	0.09091	0.04436	32	2.05	0.0487	0.05	0.000553	0.1813

DistSoma=522

Differences of Least Squares Means												
**Effect**	**Treatment**	**Treatment**	**Estimate**	**Standard Error**	**DF**	**t Value**	**Pr > |t|**	**Adjustment**	**Adj P**	**Alpha**	**Lower**	**Upper**
**Treatment**	Control_GFP	GFP MsTTR	-0.09091	0.06273	32	-1.45	0.1570	Tukey	0.1570	0.05	-0.2187	0.03687

Differences of Least Squares Means				
**Effect**	**Treatment**	**Treatment**	**Adj Lower**	**Adj Upper**
**Treatment**	Control_GFP	GFP MsTTR	-0.2187	0.03687

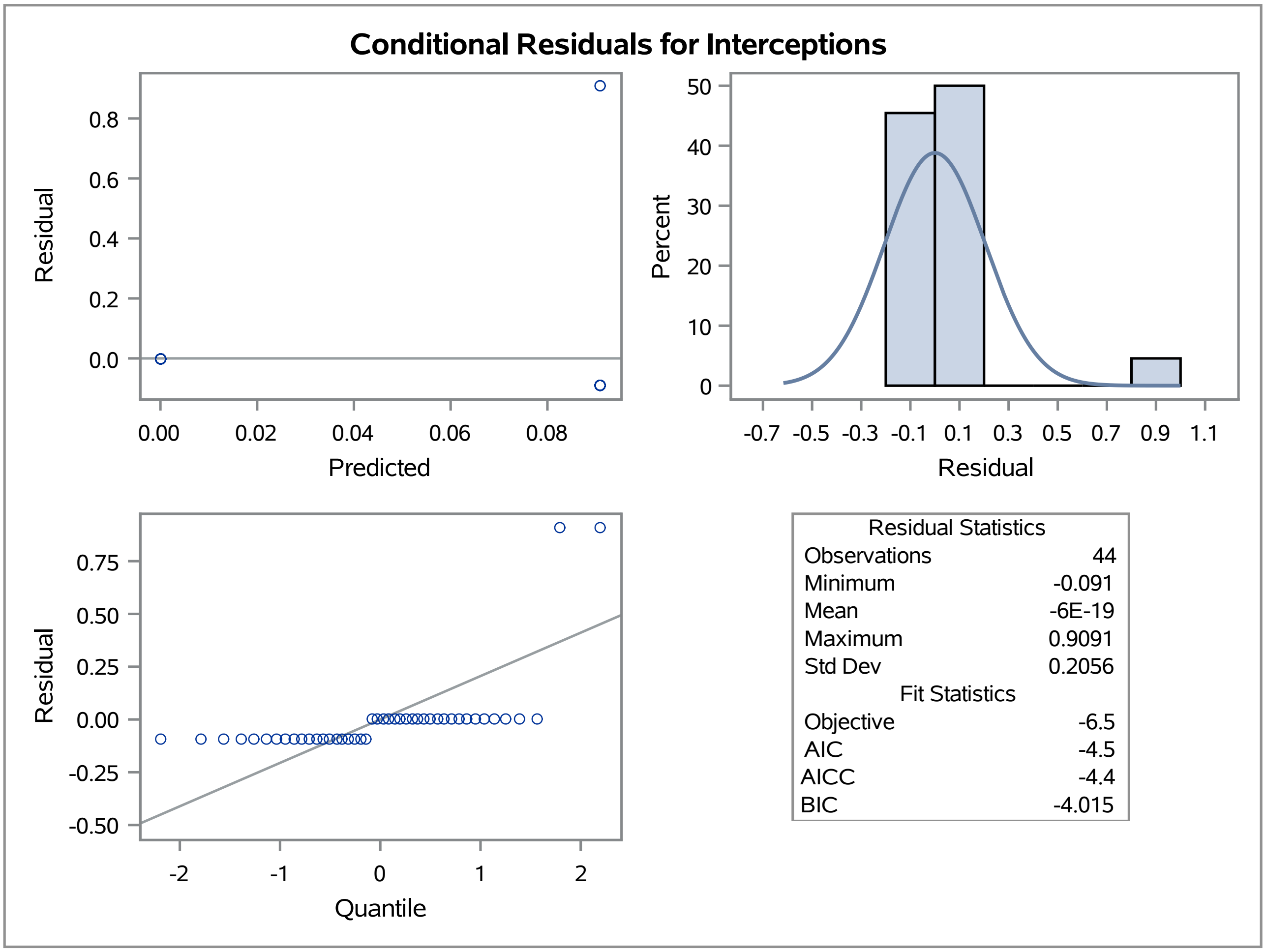

DistSoma=528

Model Information	
**Data Set**	WORK.TEMPDATASORTED
**Dependent Variable**	Interceptions
**Covariance Structure**	Variance Components
**Estimation Method**	REML
**Residual Variance Method**	Profile
**Fixed Effects SE Method**	Model-Based
**Degrees of Freedom Method**	Containment

Class Level Information		
**Class**	**Levels**	**Values**
**Treatment**	2	Control_GFP GFP MsTTR
**Culture**	12	1 2 3 4 5 6 7 8 9 10 11 12

Dimensions	
**Covariance Parameters**	2
**Columns in X**	3
**Columns in Z**	12
**Subjects**	1
**Max Obs per Subject**	44

Number of Observations	
**Number of Observations Read**	44
**Number of Observations Used**	44
**Number of Observations Not Used**	0

Iteration History			
**Iteration**	**Evaluations**	**-2 Res Log Like**	**Criterion**
**0**	1	-6.50004824	
**1**	1	-6.50004824	0.00000000

Convergence criteria met.

Estimated G matrix is not positive definite.

Covariance Parameter Estimates				
**Cov Parm**	**Estimate**	**Alpha**	**Lower**	**Upper**
**Culture**	0	.	.	.
**Residual**	0.04329	0.05	0.02943	0.06993

DistSoma=528

Fit Statistics	
-2 Res Log Likelihood	-6.5
AIC (Smaller is Better)	-4.5
AICC (Smaller is Better)	-4.4
BIC (Smaller is Better)	-4.0

Solution for Fixed Effects

Effect	Treatment	Estimate	Standard Error	DF	t Value	Pr > |t|	Alpha	Lower	Upper
Intercept		0.09091	0.04436	10	2.05	0.0676	0.05	-0.00793	0.1897
Treatment	Control_GFP	-0.09091	0.06273	32	-1.45	0.1570	0.05	-0.2187	0.03687
Treatment	GFP MsTTR	0	.	.	.	.	.	.	.

Solution for Random Effects

Effect	Culture	Estimate	Std Err Pred	DF	t Value	Pr > |t|	Alpha	Lower	Upper
Culture	1	0	.	.	.	.	.	.	.
Culture	2	0	.	.	.	.	.	.	.
Culture	3	0	.	.	.	.	.	.	.
Culture	4	0	.	.	.	.	.	.	.
Culture	5	0	.	.	.	.	.	.	.
Culture	6	0	.	.	.	.	.	.	.
Culture	7	0	.	.	.	.	.	.	.
Culture	8	0	.	.	.	.	.	.	.
Culture	9	0	.	.	.	.	.	.	.
Culture	10	0	.	.	.	.	.	.	.
Culture	11	0	.	.	.	.	.	.	.
Culture	12	0	.	.	.	.	.	.	.

Type 3 Tests of Fixed Effects

Effect	Num DF	Den DF	F Value	Pr > F
Treatment	1	32	2.10	0.1570

Least Squares Means

Effect	Treatment	Estimate	Standard Error	DF	t Value	Pr > |t|	Alpha	Lower	Upper
Treatment	Control_GFP	0	0.04436	32	0.00	1.0000	0.05	-0.09036	0.09036
Treatment	GFP MsTTR	0.09091	0.04436	32	2.05	0.0487	0.05	0.000553	0.1813

DistSoma=528

Differences of Least Squares Means												
**Effect**	**Treatment**	**Treatment**	**Estimate**	**Standard Error**	**DF**	**t Value**	**Pr > |t|**	**Adjustment**	**Adj P**	**Alpha**	**Lower**	**Upper**
**Treatment**	Control_GFP	GFP MsTTR	-0.09091	0.06273	32	-1.45	0.1570	Tukey	0.1570	0.05	-0.2187	0.03687

Differences of Least Squares Means				
**Effect**	**Treatment**	**Treatment**	**Adj Lower**	**Adj Upper**
**Treatment**	Control_GFP	GFP MsTTR	-0.2187	0.03687

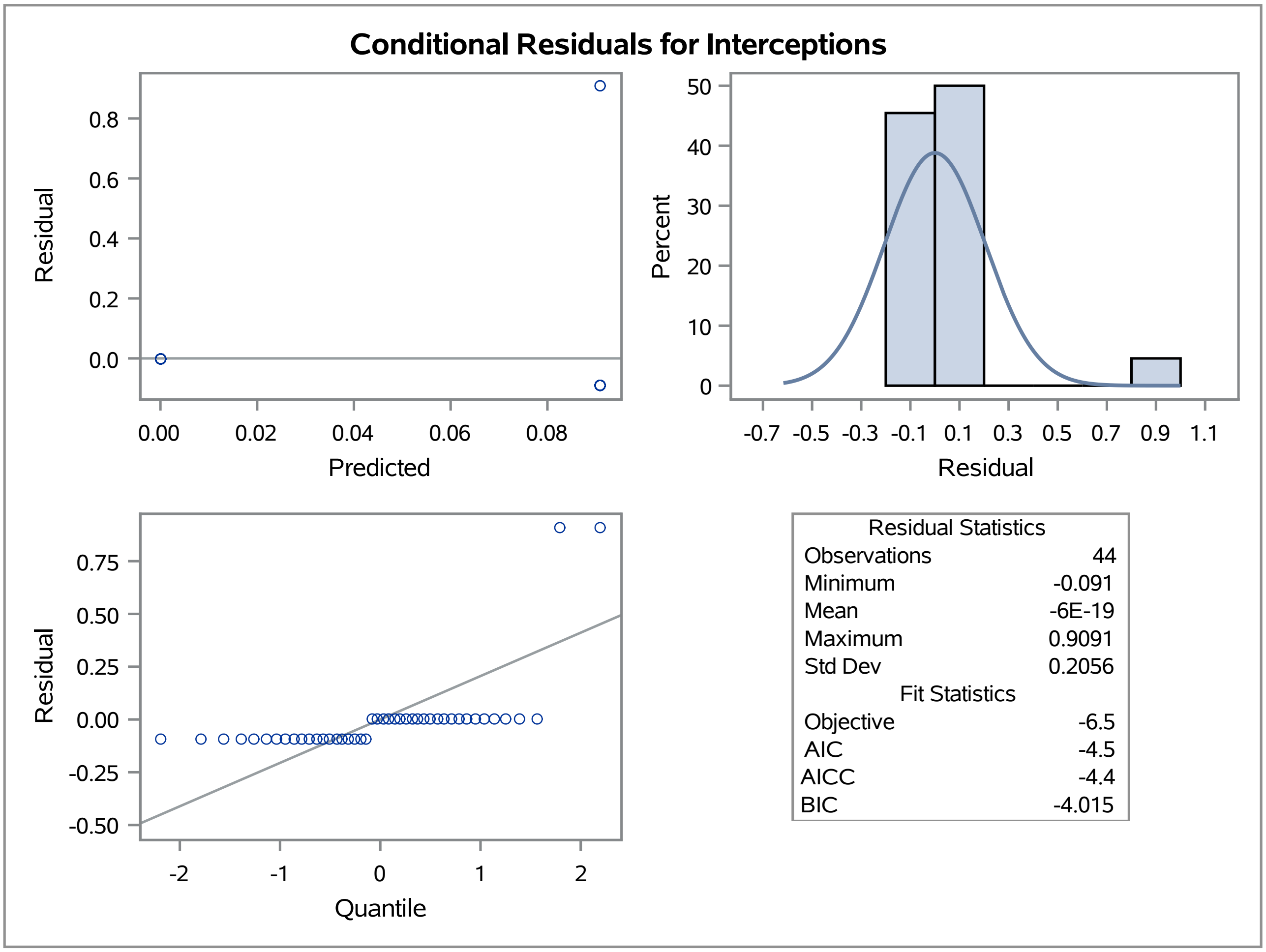

**Data Set Name**	WORK.FIG6F	**Observations**	4005
**Member Type**	DATA	**Variables**	4
**Engine**	V9	**Indexes**	0
**Created**	07/08/2020 00:54:51	**Observation Length**	40
**Last Modified**	07/08/2020 00:54:51	**Deleted Observations**	0
**Protection**		**Compressed**	NO
**Data Set Type**		**Sorted**	NO
**Label**			
**Data Representation**	SOLARIS_X86_64, LINUX_X86_64, ALPHA_TRU64, LINUX_IA64		
**Encoding**	utf-8 Unicode (UTF-8)		

Engine/Host Dependent Information	
**Data Set Page Size**	65536
**Number of Data Set Pages**	3
**First Data Page**	1
**Max Obs per Page**	1632
**Obs in First Data Page**	1573
**Number of Data Set Repairs**	0
**Filename**	/tmp/SAS_workF39800000A61_localhost.localdomain/SAS_work146A00000A61_localhost.localdomain/fig6f.sas7bdat
**Release Created**	9.0401M6
**Host Created**	Linux
**Inode Number**	672612
**Access Permission**	rw-rw-r--
**Owner Name**	sasdemo
**File Size**	256KB
**File Size (bytes)**	262144

Alphabetic List of Variables and Attributes

#	Variable	Type	Len	Format	Informat	Label
**3**	Culture	Num	8	BEST.		Culture
**1**	DistSoma	Num	8	BEST.		DistSoma
**4**	Interceptions	Num	8	BEST.		Interceptions
**2**	Treatment	Char	11	$11.	$11.	Treatment

DistSoma=0

Model Information	
**Data Set**	WORK.TEMPDATASORTED
**Dependent Variable**	Interceptions
**Covariance Structure**	Variance Components
**Estimation Method**	REML
**Residual Variance Method**	Profile
**Fixed Effects SE Method**	Model-Based
**Degrees of Freedom Method**	Containment

Class Level Information		
**Class**	**Levels**	**Values**
**Treatment**	2	Control GFP Ctr Meg
**Culture**	13	1 2 3 4 5 6 7 8 9 10 11 12 13

Dimensions	
**Covariance Parameters**	2
**Columns in X**	3
**Columns in Z**	13
**Subjects**	1
**Max Obs per Subject**	45

Number of Observations	
**Number of Observations Read**	45
**Number of Observations Used**	45
**Number of Observations Not Used**	0

Iteration History			
**Iteration**	**Evaluations**	**-2 Res Log Like**	**Criterion**
**0**	1	216.79452377	
**1**	1	216.79452377	0.00000000

Convergence criteria met.

Estimated G matrix is not positive definite.

Covariance Parameter Estimates				
**Cov Parm**	**Estimate**	**Alpha**	**Lower**	**Upper**
**Culture**	0	.	.	.
**Residual**	7.8385	0.05	5.3509	12.5836

DistSoma=0

Fit Statistics	
-2 Res Log Likelihood	216.8
AIC (Smaller is Better)	218.8
AICC (Smaller is Better)	218.9
BIC (Smaller is Better)	219.4

Solution for Fixed Effects

Effect	Treatment	Estimate	Standard Error	DF	t Value	Pr > |t|	Alpha	Lower	Upper
Intercept		11.4783	0.5838	11	19.66	<.0001	0.05	10.1934	12.7632
Treatment	Control GFP	-2.0692	0.8349	32	-2.48	0.0187	0.05	-3.7699	-0.3685
Treatment	Ctr Meg	0	.	.	.	.	.	.	.

Solution for Random Effects

Effect	Culture	Estimate	Std Err Pred	DF	t Value	Pr > |t|	Alpha	Lower	Upper
Culture	1	0	.	.	.	.	.	.	.
Culture	2	0	.	.	.	.	.	.	.
Culture	3	0	.	.	.	.	.	.	.
Culture	4	0	.	.	.	.	.	.	.
Culture	5	0	.	.	.	.	.	.	.
Culture	6	0	.	.	.	.	.	.	.
Culture	7	0	.	.	.	.	.	.	.
Culture	8	0	.	.	.	.	.	.	.
Culture	9	0	.	.	.	.	.	.	.
Culture	10	0	.	.	.	.	.	.	.
Culture	11	0	.	.	.	.	.	.	.
Culture	12	0	.	.	.	.	.	.	.
Culture	13	0	.	.	.	.	.	.	.

Type 3 Tests of Fixed Effects

Effect	Num DF	Den DF	F Value	Pr > F
Treatment	1	32	6.14	0.0187

Least Squares Means

Effect	Treatment	Estimate	Standard Error	DF	t Value	Pr > |t|	Alpha	Lower	Upper
Treatment	Control GFP	9.4091	0.5969	32	15.76	<.0001	0.05	8.1932	10.6249
Treatment	Ctr Meg	11.4783	0.5838	32	19.66	<.0001	0.05	10.2891	12.6674

DistSoma=0

Differences of Least Squares Means												
Effect	Treatment	Treatment	Estimate	Standard Error	DF	t Value	Pr > |t|	Adjustment	Adj P	Alpha	Lower	Upper
Treatment	Control GFP	Ctr Meg	-2.0692	0.8349	32	-2.48	0.0187	Tukey-Kramer	0.0187	0.05	-3.7699	-0.3685

Differences of Least Squares Means				
Effect	Treatment	Treatment	Adj Lower	Adj Upper
Treatment	Control GFP	Ctr Meg	-3.7698	-0.3685

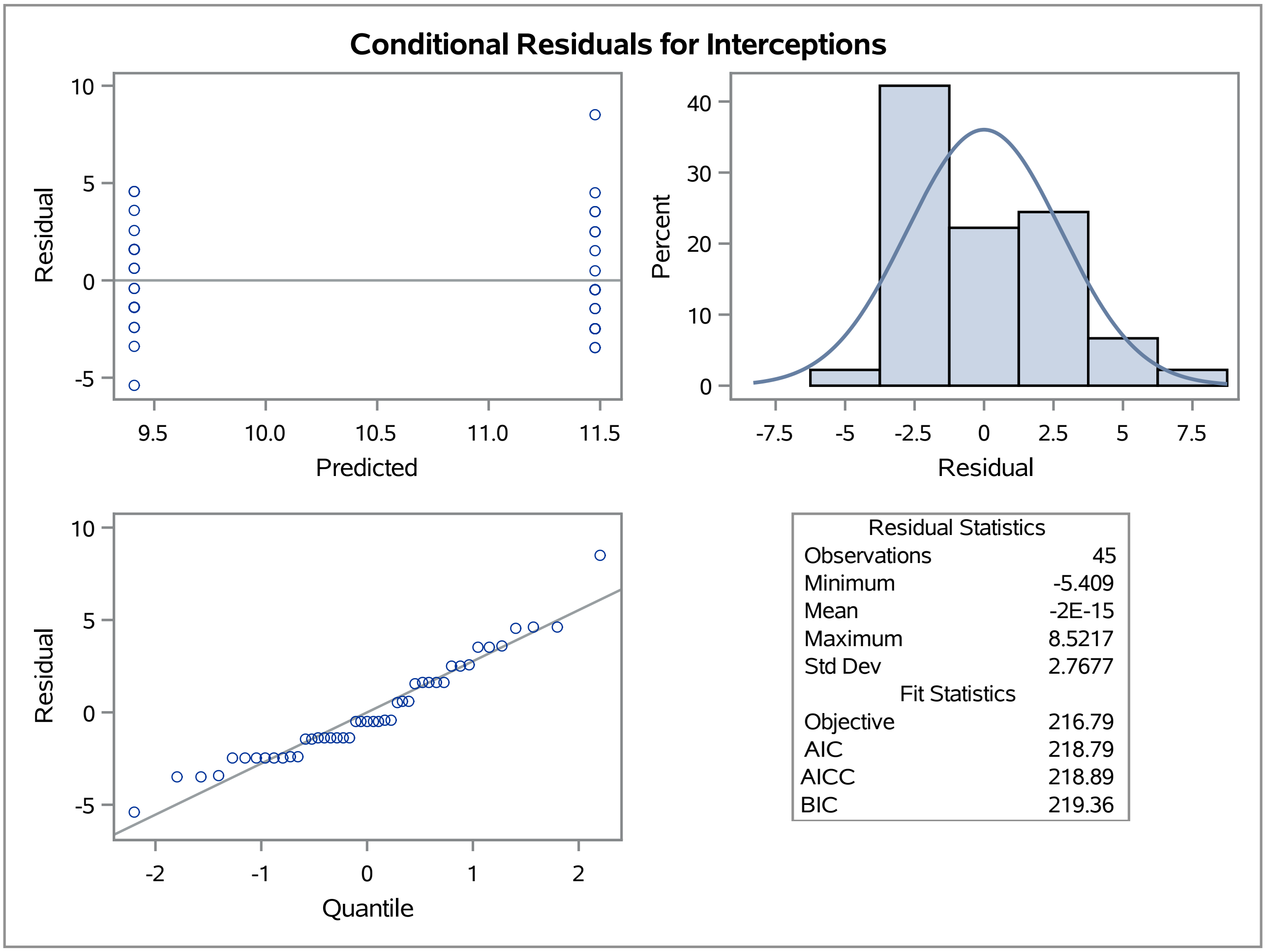

DistSoma=6

Model Information	
**Data Set**	WORK.TEMPDATASORTED
**Dependent Variable**	Interceptions
**Covariance Structure**	Variance Components
**Estimation Method**	REML
**Residual Variance Method**	Profile
**Fixed Effects SE Method**	Model-Based
**Degrees of Freedom Method**	Containment

Class Level Information		
**Class**	**Levels**	**Values**
**Treatment**	2	Control GFP Ctr Meg
**Culture**	13	1 2 3 4 5 6 7 8 9 10 11 12 13

Dimensions	
**Covariance Parameters**	2
**Columns in X**	3
**Columns in Z**	13
**Subjects**	1
**Max Obs per Subject**	45

Number of Observations	
**Number of Observations Read**	45
**Number of Observations Used**	45
**Number of Observations Not Used**	0

Iteration History			
**Iteration**	**Evaluations**	**-2 Res Log Like**	**Criterion**
**0**	1	221.54296338	
**1**	2	221.50395272	0.00000000

Convergence criteria met.

Covariance Parameter Estimates				
**Cov Parm**	**Estimate**	**Alpha**	**Lower**	**Upper**
**Culture**	0.2462	0.05	0.02161	8.221E42
**Residual**	8.5387	0.05	5.5360	14.8774

DistSoma=6

Fit Statistics	
-2 Res Log Likelihood	221.5
AIC (Smaller is Better)	225.5
AICC (Smaller is Better)	225.8
BIC (Smaller is Better)	226.6

Solution for Fixed Effects

Effect	Treatment	Estimate	Standard Error	DF	t Value	Pr > |t|	Alpha	Lower	Upper
Intercept		11.9563	0.6430	11	18.59	<.0001	0.05	10.5410	13.3716
Treatment	Control GFP	-2.5117	0.9166	32	-2.74	0.0100	0.05	-4.3787	-0.6447
Treatment	Ctr Meg	0	.	.	.	.	.	.	.

Solution for Random Effects

Effect	Culture	Estimate	Std Err Pred	DF	t Value	Pr > |t|	Alpha	Lower	Upper
Culture	1	0.1504	0.4788	32	0.31	0.7555	0.05	-0.8250	1.1257
Culture	2	0.07075	0.4788	32	0.15	0.8835	0.05	-0.9046	1.0461
Culture	3	0.005729	0.4747	32	0.01	0.9904	0.05	-0.9611	0.9726
Culture	4	0.05743	0.4747	32	0.12	0.9045	0.05	-0.9094	1.0243
Culture	5	-0.1494	0.4747	32	-0.31	0.7550	0.05	-1.1162	0.8175
Culture	6	-0.1526	0.4895	32	-0.31	0.7573	0.05	-1.1497	0.8445
Culture	7	0.01768	0.4788	32	0.04	0.9708	0.05	-0.9577	0.9931
Culture	8	0.2113	0.4745	32	0.45	0.6591	0.05	-0.7552	1.1779
Culture	9	0.2372	0.4745	32	0.50	0.6206	0.05	-0.7294	1.2037
Culture	10	-0.07613	0.4788	32	-0.16	0.8747	0.05	-1.0513	0.8991
Culture	11	-0.1205	0.4709	32	-0.26	0.7997	0.05	-1.0797	0.8387
Culture	12	-0.2023	0.4745	32	-0.43	0.6727	0.05	-1.1688	0.7642
Culture	13	-0.04959	0.4788	32	-0.10	0.9181	0.05	-1.0248	0.9256

Type 3 Tests of Fixed Effects

Effect	Num DF	Den DF	F Value	Pr > F
Treatment	1	32	7.51	0.0100

Least Squares Means

Effect	Treatment	Estimate	Standard Error	DF	t Value	Pr > |t|	Alpha	Lower	Upper
Treatment	Control GFP	9.4446	0.6532	32	14.46	<.0001	0.05	8.1141	10.7750
Treatment	Ctr Meg	11.9563	0.6430	32	18.59	<.0001	0.05	10.6465	13.2661

DistSoma=6

Differences of Least Squares Means												
Effect	Treatment	Treatment	Estimate	Standard Error	DF	t Value	Pr > |t|	Adjustment	Adj P	Alpha	Lower	Upper
Treatment	Control GFP	Ctr Meg	-2.5117	0.9166	32	-2.74	0.0100	Tukey-Kramer	0.0100	0.05	-4.3787	-0.6447

Differences of Least Squares Means				
Effect	Treatment	Treatment	Adj Lower	Adj Upper
Treatment	Control GFP	Ctr Meg	-4.3787	-0.6447

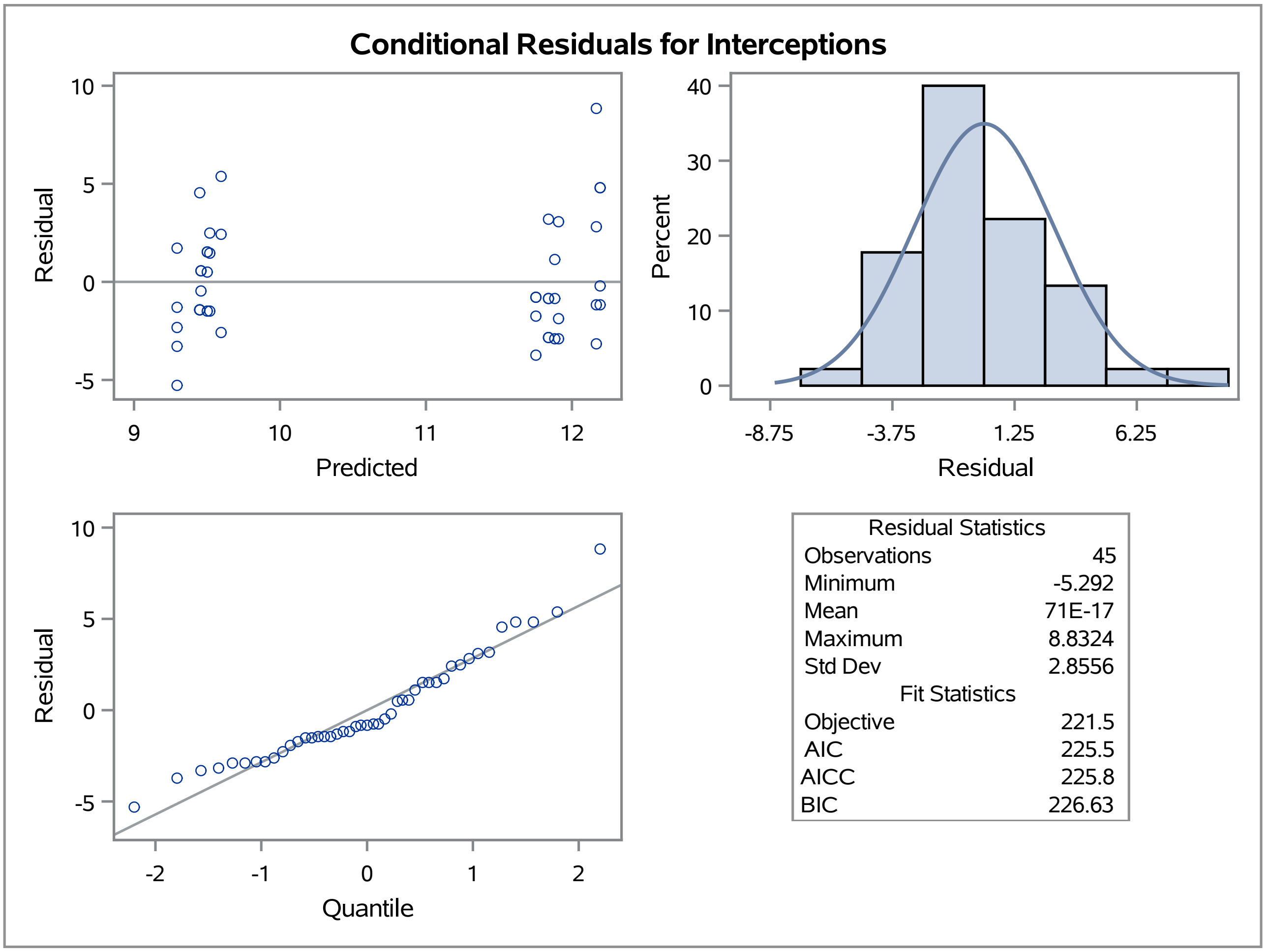

DistSoma=12

Model Information	
Data Set	WORK.TEMPDATASORTED
Dependent Variable	Interceptions
Covariance Structure	Variance Components
Estimation Method	REML
Residual Variance Method	Profile
Fixed Effects SE Method	Model-Based
Degrees of Freedom Method	Containment

Class Level Information		
Class	Levels	Values
Treatment	2	Control GFP Ctr Meg
Culture	13	1 2 3 4 5 6 7 8 9 10 11 12 13

Dimensions	
Covariance Parameters	2
Columns in X	3
Columns in Z	13
Subjects	1
Max Obs per Subject	45

Number of Observations	
Number of Observations Read	45
Number of Observations Used	45
Number of Observations Not Used	0

Iteration History			
Iteration	Evaluations	-2 Res Log Like	Criterion
0	1	216.70997917	
1	2	216.41547928	0.00000034
2	1	216.41545572	0.00000000

Convergence criteria met.

Covariance Parameter Estimates				
Cov Parm	Estimate	Alpha	Lower	Upper
Culture	0.6725	0.05	0.09411	2435574
Residual	7.2450	0.05	4.6547	12.8144

DistSoma=12

Fit Statistics	
-2 Res Log Likelihood	216.4
AIC (Smaller is Better)	220.4
AICC (Smaller is Better)	220.7
BIC (Smaller is Better)	221.5

Solution for Fixed Effects

Effect	Treatment	Estimate	Standard Error	DF	t Value	Pr > |t|	Alpha	Lower	Upper
Intercept		12.8277	0.6555	11	19.57	<.0001	0.05	11.3849	14.2706
Treatment	Control GFP	-2.5199	0.9292	32	-2.71	0.0107	0.05	-4.4126	-0.6272
Treatment	Ctr Meg	0	.	.	.	.	.	.	.

Solution for Random Effects

Effect	Culture	Estimate	Std Err Pred	DF	t Value	Pr > |t|	Alpha	Lower	Upper
Culture	1	0.4412	0.7393	32	0.60	0.5549	0.05	-1.0647	1.9471
Culture	2	0.07815	0.7393	32	0.11	0.9165	0.05	-1.4278	1.5841
Culture	3	0.1874	0.7226	32	0.26	0.7970	0.05	-1.2845	1.6593
Culture	4	0.2551	0.7226	32	0.35	0.7264	0.05	-1.2168	1.7270
Culture	5	-0.2864	0.7226	32	-0.40	0.6945	0.05	-1.7583	1.1855
Culture	6	-0.5357	0.7864	32	-0.68	0.5006	0.05	-2.1376	1.0662
Culture	7	-0.1396	0.7393	32	-0.19	0.8514	0.05	-1.6456	1.3663
Culture	8	0.3851	0.7224	32	0.53	0.5977	0.05	-1.0864	1.8566
Culture	9	0.7235	0.7224	32	1.00	0.3241	0.05	-0.7480	2.1950
Culture	10	-0.1803	0.7392	32	-0.24	0.8089	0.05	-1.6859	1.3254
Culture	11	-0.3258	0.7089	32	-0.46	0.6489	0.05	-1.7697	1.1181
Culture	12	-0.4949	0.7224	32	-0.68	0.4983	0.05	-1.9664	0.9767
Culture	13	-0.1077	0.7392	32	-0.15	0.8851	0.05	-1.6133	1.3980

Type 3 Tests of Fixed Effects

Effect	Num DF	Den DF	F Value	Pr > F
Treatment	1	32	7.35	0.0107

Least Squares Means

Effect	Treatment	Estimate	Standard Error	DF	t Value	Pr > |t|	Alpha	Lower	Upper
Treatment	Control GFP	10.3078	0.6585	32	15.65	<.0001	0.05	8.9664	11.6492
Treatment	Ctr Meg	12.8277	0.6555	32	19.57	<.0001	0.05	11.4924	14.1630

DistSoma=12

Differences of Least Squares Means

Effect	Treatment	Treatment	Estimate	Standard Error	DF	t Value	Pr > |t|	Adjustment	Adj P	Alpha	Lower	Upper
**Treatment**	Control GFP	Ctr Meg	-2.5199	0.9292	32	-2.71	0.0107	Tukey-Kramer	0.0107	0.05	-4.4126	-0.6272

Differences of Least Squares Means

Effect	Treatment	Treatment	Adj Lower	Adj Upper
**Treatment**	Control GFP	Ctr Meg	-4.4126	-0.6272

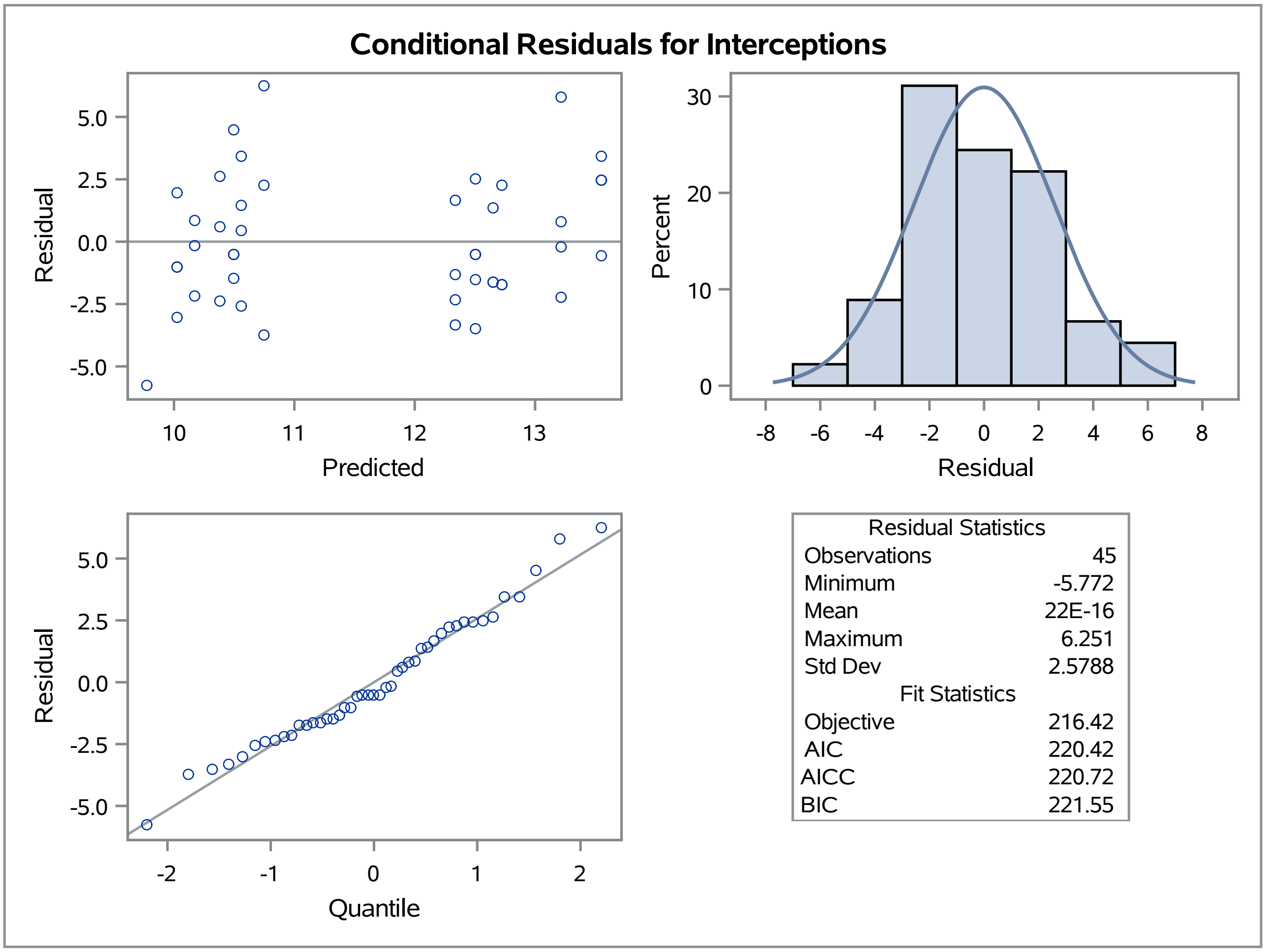

DistSoma=18

Model Information	
**Data Set**	WORK.TEMPDATASORTED
**Dependent Variable**	Interceptions
**Covariance Structure**	Variance Components
**Estimation Method**	REML
**Residual Variance Method**	Profile
**Fixed Effects SE Method**	Model-Based
**Degrees of Freedom Method**	Containment

Class Level Information		
**Class**	**Levels**	**Values**
**Treatment**	2	Control GFP Ctr Meg
**Culture**	13	1 2 3 4 5 6 7 8 9 10 11 12 13

Dimensions	
**Covariance Parameters**	2
**Columns in X**	3
**Columns in Z**	13
**Subjects**	1
**Max Obs per Subject**	45

Number of Observations	
**Number of Observations Read**	45
**Number of Observations Used**	45
**Number of Observations Not Used**	0

Iteration History			
**Iteration**	**Evaluations**	**-2 Res Log Like**	**Criterion**
**0**	1	232.09689056	
**1**	2	231.85771499	0.00000049
**2**	1	231.85767774	0.00000000

Convergence criteria met.

Covariance Parameter Estimates				
**Cov Parm**	**Estimate**	**Alpha**	**Lower**	**Upper**
**Culture**	0.8795	0.05	0.1138	74032984
**Residual**	10.4341	0.05	6.6914	18.5105

DistSoma=18

Fit Statistics	
-2 Res Log Likelihood	231.9
AIC (Smaller is Better)	235.9
AICC (Smaller is Better)	236.2
BIC (Smaller is Better)	237.0

Solution for Fixed Effects

Effect	Treatment	Estimate	Standard Error	DF	t Value	Pr > |t|	Alpha	Lower	Upper
Intercept		12.8419	0.7770	11	16.53	<.0001	0.05	11.1317	14.5522
Treatment	Control GFP	-1.0083	1.1021	32	-0.91	0.3671	0.05	-3.2532	1.2367
Treatment	Ctr Meg	0	.	.	.	.	.	.	.

Solution for Random Effects

Effect	Culture	Estimate	Std Err Pred	DF	t Value	Pr > |t|	Alpha	Lower	Upper
Culture	1	0.3700	0.8526	32	0.43	0.6672	0.05	-1.3667	2.1066
Culture	2	0.1681	0.8526	32	0.20	0.8449	0.05	-1.5685	1.9047
Culture	3	0.6723	0.8346	32	0.81	0.4265	0.05	-1.0277	2.3723
Culture	4	0.3571	0.8346	32	0.43	0.6716	0.05	-1.3429	2.0572
Culture	5	-0.5884	0.8346	32	-0.71	0.4859	0.05	-2.2885	1.1116
Culture	6	-0.6090	0.9027	32	-0.67	0.5048	0.05	-2.4476	1.2297
Culture	7	-0.3701	0.8526	32	-0.43	0.6671	0.05	-2.1067	1.3665
Culture	8	0.6072	0.8343	32	0.73	0.4721	0.05	-1.0923	2.3067
Culture	9	-0.08622	0.8343	32	-0.10	0.9183	0.05	-1.7857	1.6133
Culture	10	0.03190	0.8524	32	0.04	0.9704	0.05	-1.7044	1.7682
Culture	11	-0.3682	0.8196	32	-0.45	0.6563	0.05	-2.0378	1.3013
Culture	12	-0.1493	0.8343	32	-0.18	0.8591	0.05	-1.8487	1.5502
Culture	13	-0.03538	0.8524	32	-0.04	0.9672	0.05	-1.7716	1.7009

Type 3 Tests of Fixed Effects

Effect	Num DF	Den DF	F Value	Pr > F
Treatment	1	32	0.84	0.3671

Least Squares Means

Effect	Treatment	Estimate	Standard Error	DF	t Value	Pr > |t|	Alpha	Lower	Upper
Treatment	Control GFP	11.8337	0.7816	32	15.14	<.0001	0.05	10.2416	13.4257
Treatment	Ctr Meg	12.8419	0.7770	32	16.53	<.0001	0.05	11.2592	14.4247

DistSoma=18

Differences of Least Squares Means												
Effect	Treatment	Treatment	Estimate	Standard Error	DF	t Value	Pr > |t|	Adjustment	Adj P	Alpha	Lower	Upper
**Treatment**	Control GFP	Ctr Meg	-1.0083	1.1021	32	-0.91	0.3671	Tukey-Kramer	0.3671	0.05	-3.2532	1.2367

Differences of Least Squares Means				
Effect	Treatment	Treatment	Adj Lower	Adj Upper
**Treatment**	Control GFP	Ctr Meg	-3.2532	1.2367

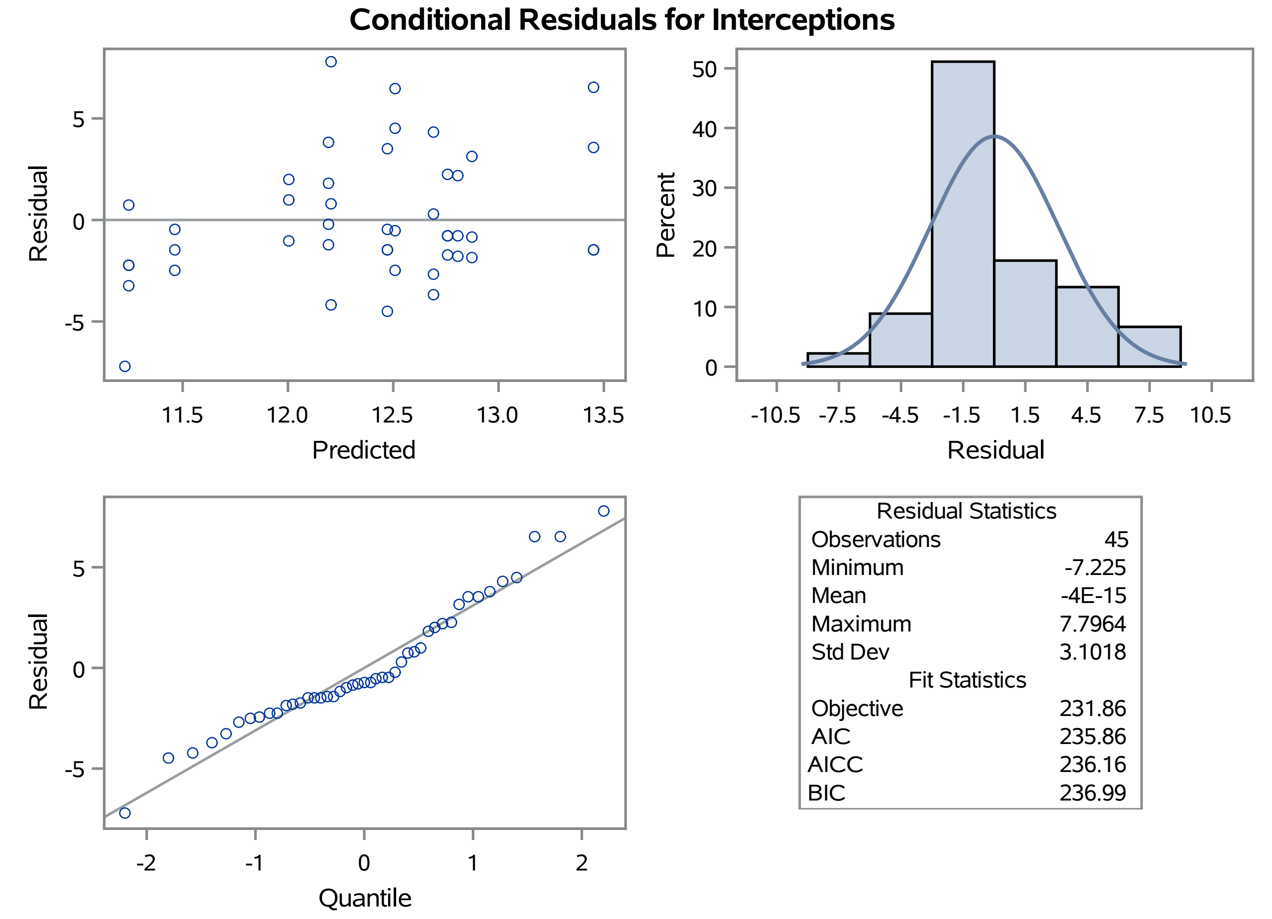

Residual Statistics	
Observations	45
Minimum	-7.225
Mean	-4E-15
Maximum	7.7964
Std Dev	3.1018
Fit Statistics	
Objective	231.86
AIC	235.86
AICC	236.16
BIC	236.99

DistSoma=24

Model Information	
**Data Set**	WORK.TEMPDATASORTED
**Dependent Variable**	Interceptions
**Covariance Structure**	Variance Components
**Estimation Method**	REML
**Residual Variance Method**	Profile
**Fixed Effects SE Method**	Model-Based
**Degrees of Freedom Method**	Containment

Class Level Information		
**Class**	**Levels**	**Values**
**Treatment**	2	Control GFP Ctr Meg
**Culture**	13	1 2 3 4 5 6 7 8 9 10 11 12 13

Dimensions	
**Covariance Parameters**	2
**Columns in X**	3
**Columns in Z**	13
**Subjects**	1
**Max Obs per Subject**	45

Number of Observations	
**Number of Observations Read**	45
**Number of Observations Used**	45
**Number of Observations Not Used**	0

Iteration History			
**Iteration**	**Evaluations**	**-2 Res Log Like**	**Criterion**
**0**	1	229.26809968	
**1**	2	228.56453316	0.00000306
**2**	1	228.56430207	0.00000000

Convergence criteria met.

Covariance Parameter Estimates				
**Cov Parm**	**Estimate**	**Alpha**	**Lower**	**Upper**
**Culture**	1.4524	0.05	0.2851	1819.46
**Residual**	9.2444	0.05	5.9302	16.3924

DistSoma=24

Fit Statistics	
-2 Res Log Likelihood	228.6
AIC (Smaller is Better)	232.6
AICC (Smaller is Better)	232.9
BIC (Smaller is Better)	233.7

Solution for Fixed Effects

Effect	Treatment	Estimate	Standard Error	DF	t Value	Pr > |t|	Alpha	Lower	Upper
Intercept		12.3808	0.8055	11	15.37	<.0001	0.05	10.6079	14.1538
Treatment	Control GFP	-0.6740	1.1371	32	-0.59	0.5575	0.05	-2.9903	1.6423
Treatment	Ctr Meg	0	.	.	.	.	.	.	.

Solution for Random Effects

Effect	Culture	Estimate	Std Err Pred	DF	t Value	Pr > |t|	Alpha	Lower	Upper
Culture	1	0.6278	1.0263	32	0.61	0.5450	0.05	-1.4626	2.7183
Culture	2	0.3075	1.0263	32	0.30	0.7664	0.05	-1.7830	2.3980
Culture	3	0.8850	0.9939	32	0.89	0.3799	0.05	-1.1395	2.9095
Culture	4	0.4991	0.9939	32	0.50	0.6190	0.05	-1.5255	2.5236
Culture	5	-0.7552	0.9939	32	-0.76	0.4529	0.05	-2.7797	1.2693
Culture	6	-0.9107	1.1257	32	-0.81	0.4245	0.05	-3.2035	1.3822
Culture	7	-0.6536	1.0263	32	-0.64	0.5288	0.05	-2.7440	1.4369
Culture	8	0.9143	0.9943	32	0.92	0.3647	0.05	-1.1109	2.9396
Culture	9	-1.0153	0.9943	32	-1.02	0.3148	0.05	-3.0405	1.0099
Culture	10	0.4119	1.0265	32	0.40	0.6909	0.05	-1.6790	2.5029
Culture	11	-0.2555	0.9690	32	-0.26	0.7937	0.05	-2.2294	1.7183
Culture	12	-0.1470	0.9943	32	-0.15	0.8834	0.05	-2.1722	1.8783
Culture	13	0.09157	1.0265	32	0.09	0.9295	0.05	-1.9994	2.1825

Type 3 Tests of Fixed Effects

Effect	Num DF	Den DF	F Value	Pr > F
Treatment	1	32	0.35	0.5575

Least Squares Means

Effect	Treatment	Estimate	Standard Error	DF	t Value	Pr > |t|	Alpha	Lower	Upper
Treatment	Control GFP	11.7068	0.8026	32	14.59	<.0001	0.05	10.0719	13.3417
Treatment	Ctr Meg	12.3808	0.8055	32	15.37	<.0001	0.05	10.7400	14.0216

DistSoma=24

Differences of Least Squares Means												
**Effect**	**Treatment**	**Treatment**	**Estimate**	**Standard Error**	**DF**	**t Value**	**Pr > |t|**	**Adjustment**	**Adj P**	**Alpha**	**Lower**	**Upper**
**Treatment**	Control GFP	Ctr Meg	-0.6740	1.1371	32	-0.59	0.5575	Tukey-Kramer	0.5575	0.05	-2.9903	1.6423

Differences of Least Squares Means				
**Effect**	**Treatment**	**Treatment**	**Adj Lower**	**Adj Upper**
**Treatment**	Control GFP	Ctr Meg	-2.9903	1.6422

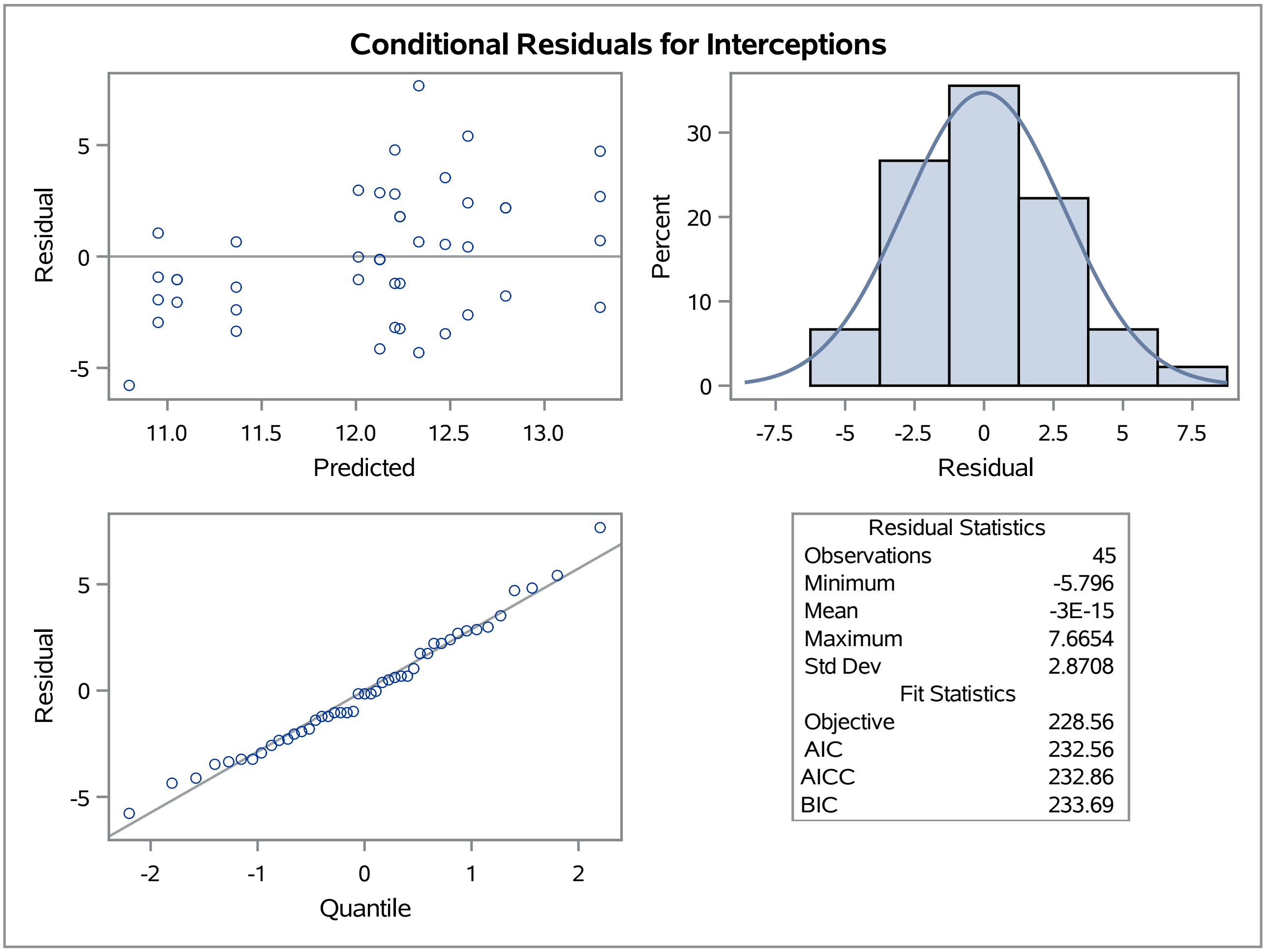

DistSoma=30

Model Information	
**Data Set**	WORK.TEMPDATASORTED
**Dependent Variable**	Interceptions
**Covariance Structure**	Variance Components
**Estimation Method**	REML
**Residual Variance Method**	Profile
**Fixed Effects SE Method**	Model-Based
**Degrees of Freedom Method**	Containment

Class Level Information		
**Class**	**Levels**	**Values**
**Treatment**	2	Control GFP Ctr Meg
**Culture**	13	1 2 3 4 5 6 7 8 9 10 11 12 13

Dimensions	
**Covariance Parameters**	2
**Columns in X**	3
**Columns in Z**	13
**Subjects**	1
**Max Obs per Subject**	45

Number of Observations	
**Number of Observations Read**	45
**Number of Observations Used**	45
**Number of Observations Not Used**	0

Iteration History			
**Iteration**	**Evaluations**	**-2 Res Log Like**	**Criterion**
**0**	1	242.87584801	
**1**	2	238.77307267	0.00000001

Convergence criteria met.

Covariance Parameter Estimates				
**Cov Parm**	**Estimate**	**Alpha**	**Lower**	**Upper**
**Culture**	4.6643	0.05	1.6127	45.2834
**Residual**	10.3340	0.05	6.6797	18.0951

DistSoma=30

Fit Statistics	
-2 Res Log Likelihood	238.8
AIC (Smaller is Better)	242.8
AICC (Smaller is Better)	243.1
BIC (Smaller is Better)	243.9

Solution for Fixed Effects

Effect	Treatment	Estimate	Standard Error	DF	t Value	Pr > |t|	Alpha	Lower	Upper
Intercept		12.1670	1.1117	11	10.94	<.0001	0.05	9.7201	14.6139
Treatment	Control GFP	-0.6762	1.5529	32	-0.44	0.6662	0.05	-3.8394	2.4870
Treatment	Ctr Meg	0	.	.	.	.	.	.	.

Solution for Random Effects

Effect	Culture	Estimate	Std Err Pred	DF	t Value	Pr > |t|	Alpha	Lower	Upper
Culture	1	1.2516	1.5396	32	0.81	0.4223	0.05	-1.8845	4.3876
Culture	2	0.1012	1.5396	32	0.07	0.9480	0.05	-3.0349	3.2372
Culture	3	2.4192	1.4661	32	1.65	0.1087	0.05	-0.5672	5.4056
Culture	4	0.6495	1.4661	32	0.44	0.6608	0.05	-2.3369	3.6359
Culture	5	-1.2812	1.4661	32	-0.87	0.3887	0.05	-4.2676	1.7052
Culture	6	-1.7076	1.8241	32	-0.94	0.3562	0.05	-5.4232	2.0081
Culture	7	-1.4327	1.5396	32	-0.93	0.3590	0.05	-4.5688	1.7034
Culture	8	1.8232	1.4746	32	1.24	0.2253	0.05	-1.1805	4.8268
Culture	9	-3.3252	1.4746	32	-2.25	0.0311	0.05	-6.3289	-0.3215
Culture	10	1.0543	1.5461	32	0.68	0.5002	0.05	-2.0949	4.2036
Culture	11	-0.1157	1.4232	32	-0.08	0.9357	0.05	-3.0148	2.7833
Culture	12	-0.1075	1.4746	32	-0.07	0.9424	0.05	-3.1111	2.8962
Culture	13	0.6709	1.5461	32	0.43	0.6673	0.05	-2.4783	3.8201

Type 3 Tests of Fixed Effects

Effect	Num DF	Den DF	F Value	Pr > F
Treatment	1	32	0.19	0.6662

Least Squares Means

Effect	Treatment	Estimate	Standard Error	DF	t Value	Pr > |t|	Alpha	Lower	Upper
Treatment	Control GFP	11.4908	1.0843	32	10.60	<.0001	0.05	9.2821	13.6994
Treatment	Ctr Meg	12.1670	1.1117	32	10.94	<.0001	0.05	9.9025	14.4315

DistSoma=30

Differences of Least Squares Means												
**Effect**	**Treatment**	**Treatment**	**Estimate**	**Standard Error**	**DF**	**t Value**	**Pr > |t|**	**Adjustment**	**Adj P**	**Alpha**	**Lower**	**Upper**
**Treatment**	Control GFP	Ctr Meg	-0.6762	1.5529	32	-0.44	0.6662	Tukey-Kramer	0.6662	0.05	-3.8394	2.4870

Differences of Least Squares Means				
**Effect**	**Treatment**	**Treatment**	**Adj Lower**	**Adj Upper**
**Treatment**	Control GFP	Ctr Meg	-3.8394	2.4870

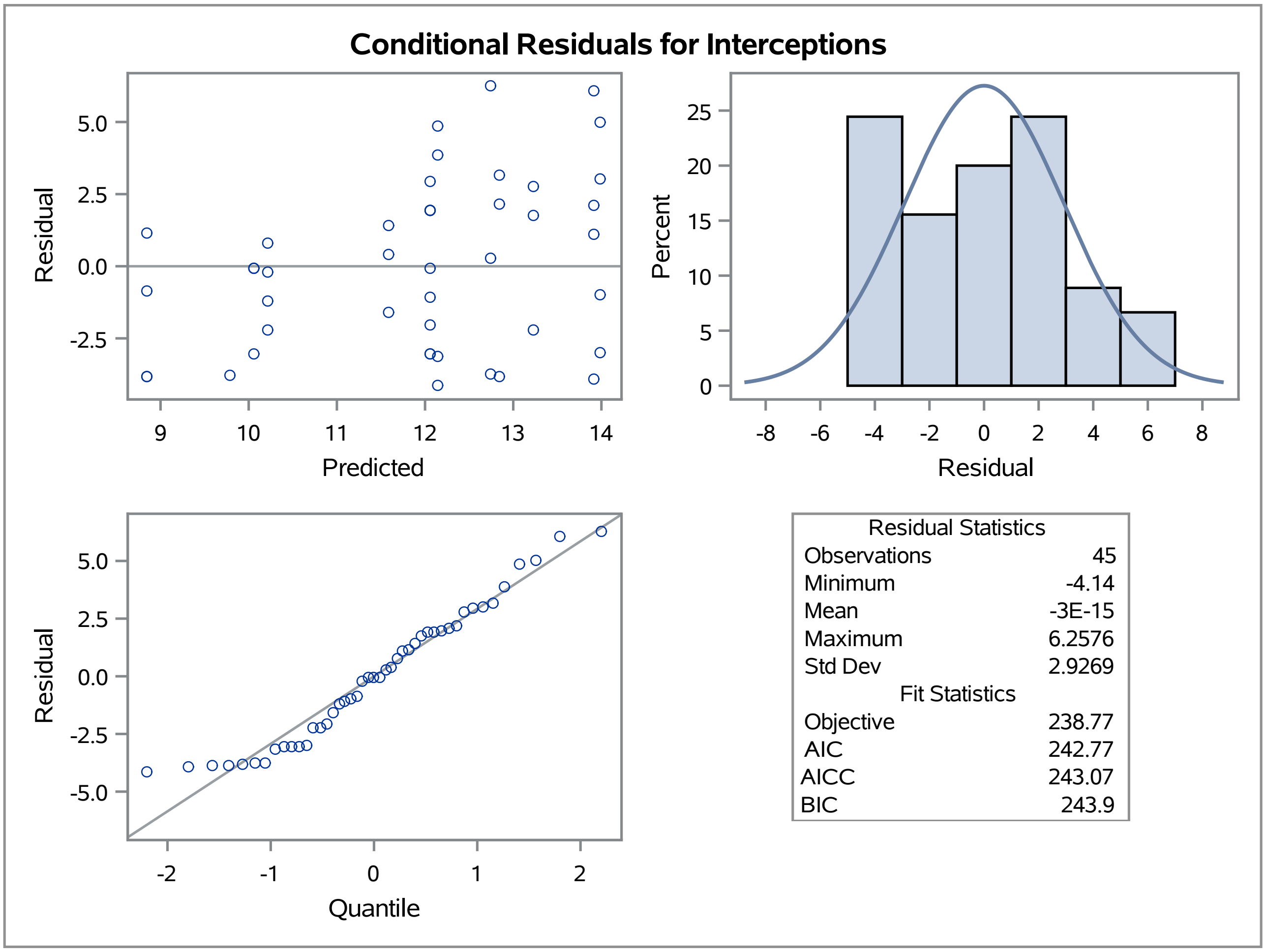

DistSoma=36

Model Information	
**Data Set**	WORK.TEMPDATASORTED
**Dependent Variable**	Interceptions
**Covariance Structure**	Variance Components
**Estimation Method**	REML
**Residual Variance Method**	Profile
**Fixed Effects SE Method**	Model-Based
**Degrees of Freedom Method**	Containment

Class Level Information		
**Class**	**Levels**	**Values**
**Treatment**	2	Control GFP Ctr Meg
**Culture**	13	1 2 3 4 5 6 7 8 9 10 11 12 13

Dimensions	
**Covariance Parameters**	2
**Columns in X**	3
**Columns in Z**	13
**Subjects**	1
**Max Obs per Subject**	45

Number of Observations	
**Number of Observations Read**	45
**Number of Observations Used**	45
**Number of Observations Not Used**	0

Iteration History			
**Iteration**	**Evaluations**	**-2 Res Log Like**	**Criterion**
**0**	1	241.64130545	
**1**	2	238.52932077	0.00000001

Convergence criteria met.

Covariance Parameter Estimates				
**Cov Parm**	**Estimate**	**Alpha**	**Lower**	**Upper**
**Culture**	3.7978	0.05	1.2356	49.2089
**Residual**	10.6287	0.05	6.8910	18.5189

DistSoma=36

Fit Statistics	
-2 Res Log Likelihood	238.5
AIC (Smaller is Better)	242.5
AICC (Smaller is Better)	242.8
BIC (Smaller is Better)	243.7

Solution for Fixed Effects

Effect	Treatment	Estimate	Standard Error	DF	t Value	Pr > |t|	Alpha	Lower	Upper
Intercept		11.5942	1.0506	11	11.04	<.0001	0.05	9.2819	13.9065
Treatment	Control GFP	-0.7113	1.4712	32	-0.48	0.6321	0.05	-3.7080	2.2855
Treatment	Ctr Meg	0	.	.	.	.	.	.	.

Solution for Random Effects

Effect	Culture	Estimate	Std Err Pred	DF	t Value	Pr > |t|	Alpha	Lower	Upper
Culture	1	1.2677	1.4549	32	0.87	0.3901	0.05	-1.6959	4.2313
Culture	2	-0.6293	1.4549	32	-0.43	0.6683	0.05	-3.5929	2.3344
Culture	3	1.8339	1.3894	32	1.32	0.1962	0.05	-0.9963	4.6641
Culture	4	0.6572	1.3894	32	0.47	0.6394	0.05	-2.1730	3.4874
Culture	5	-0.9608	1.3894	32	-0.69	0.4943	0.05	-3.7909	1.8694
Culture	6	-1.0222	1.6946	32	-0.60	0.5506	0.05	-4.4739	2.4295
Culture	7	-1.1466	1.4549	32	-0.79	0.4364	0.05	-4.1103	1.8170
Culture	8	1.2683	1.3948	32	0.91	0.3700	0.05	-1.5727	4.1094
Culture	9	-3.1443	1.3948	32	-2.25	0.0312	0.05	-5.9854	-0.3032
Culture	10	1.2446	1.4589	32	0.85	0.3999	0.05	-1.7270	4.2163
Culture	11	0.1319	1.3478	32	0.10	0.9226	0.05	-2.6135	2.8773
Culture	12	-0.05545	1.3948	32	-0.04	0.9685	0.05	-2.8965	2.7856
Culture	13	0.5548	1.4589	32	0.38	0.7062	0.05	-2.4168	3.5265

Type 3 Tests of Fixed Effects

Effect	Num DF	Den DF	F Value	Pr > F
Treatment	1	32	0.23	0.6321

Least Squares Means

Effect	Treatment	Estimate	Standard Error	DF	t Value	Pr > |t|	Alpha	Lower	Upper
Treatment	Control GFP	10.8830	1.0299	32	10.57	<.0001	0.05	8.7851	12.9808
Treatment	Ctr Meg	11.5942	1.0506	32	11.04	<.0001	0.05	9.4543	13.7342

DistSoma=36

Differences of Least Squares Means												
**Effect**	**Treatment**	**Treatment**	**Estimate**	**Standard Error**	**DF**	**t Value**	**Pr > |t|**	**Adjustment**	**Adj P**	**Alpha**	**Lower**	**Upper**
**Treatment**	Control GFP	Ctr Meg	-0.7113	1.4712	32	-0.48	0.6321	Tukey-Kramer	0.6321	0.05	-3.7080	2.2855

Differences of Least Squares Means				
**Effect**	**Treatment**	**Treatment**	**Adj Lower**	**Adj Upper**
**Treatment**	Control GFP	Ctr Meg	-3.7080	2.2854

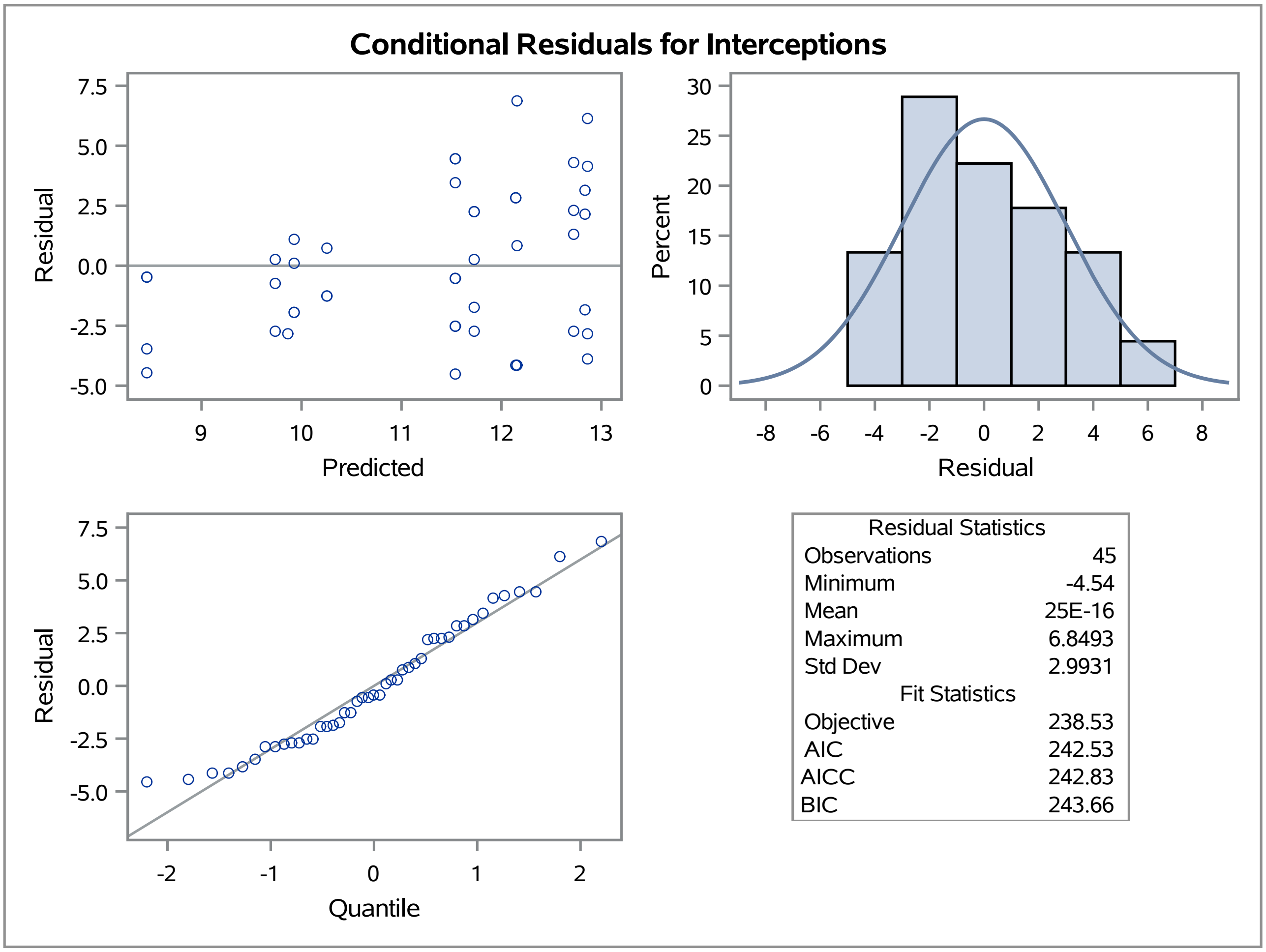

DistSoma=42

Model Information	
**Data Set**	WORK.TEMPDATASORTED
**Dependent Variable**	Interceptions
**Covariance Structure**	Variance Components
**Estimation Method**	REML
**Residual Variance Method**	Profile
**Fixed Effects SE Method**	Model-Based
**Degrees of Freedom Method**	Containment

Class Level Information		
**Class**	**Levels**	**Values**
**Treatment**	2	Control GFP Ctr Meg
**Culture**	13	1 2 3 4 5 6 7 8 9 10 11 12 13

Dimensions	
**Covariance Parameters**	2
**Columns in X**	3
**Columns in Z**	13
**Subjects**	1
**Max Obs per Subject**	45

Number of Observations	
**Number of Observations Read**	45
**Number of Observations Used**	45
**Number of Observations Not Used**	0

Iteration History			
**Iteration**	**Evaluations**	**-2 Res Log Like**	**Criterion**
**0**	1	236.92351389	
**1**	2	235.42043698	0.00000000

Convergence criteria met.

Covariance Parameter Estimates				
**Cov Parm**	**Estimate**	**Alpha**	**Lower**	**Upper**
**Culture**	2.3036	0.05	0.6054	115.15
**Residual**	10.4949	0.05	6.8108	18.2569

DistSoma=42

Fit Statistics	
-2 Res Log Likelihood	235.4
AIC (Smaller is Better)	239.4
AICC (Smaller is Better)	239.7
BIC (Smaller is Better)	240.6

Solution for Fixed Effects

Effect	Treatment	Estimate	Standard Error	DF	t Value	Pr > |t|	Alpha	Lower	Upper
Intercept		11.0151	0.9203	11	11.97	<.0001	0.05	8.9895	13.0407
Treatment	Control GFP	-0.7264	1.2952	32	-0.56	0.5788	0.05	-3.3646	1.9118
Treatment	Ctr Meg	0	.	.	.	.	.	.	.

Solution for Random Effects

Effect	Culture	Estimate	Std Err Pred	DF	t Value	Pr > |t|	Alpha	Lower	Upper
Culture	1	0.2824	1.2328	32	0.23	0.8203	0.05	-2.2288	2.7936
Culture	2	-0.3793	1.2328	32	-0.31	0.7603	0.05	-2.8905	2.1319
Culture	3	1.5013	1.1867	32	1.27	0.2149	0.05	-0.9158	3.9185
Culture	4	0.8001	1.1867	32	0.67	0.5050	0.05	-1.6171	3.2172
Culture	5	-0.8362	1.1867	32	-0.70	0.4861	0.05	-3.2534	1.5809
Culture	6	-0.5919	1.3841	32	-0.43	0.6718	0.05	-3.4113	2.2275
Culture	7	-0.7763	1.2328	32	-0.63	0.5333	0.05	-3.2876	1.7349
Culture	8	0.3436	1.1882	32	0.29	0.7743	0.05	-2.0766	2.7638
Culture	9	-1.9940	1.1882	32	-1.68	0.1030	0.05	-4.4142	0.4262
Culture	10	1.3175	1.2339	32	1.07	0.2936	0.05	-1.1959	3.8308
Culture	11	0.09675	1.1533	32	0.08	0.9337	0.05	-2.2525	2.4460
Culture	12	0.1098	1.1882	32	0.09	0.9269	0.05	-2.3104	2.5300
Culture	13	0.1264	1.2339	32	0.10	0.9191	0.05	-2.3870	2.6397

Type 3 Tests of Fixed Effects

Effect	Num DF	Den DF	F Value	Pr > F
Treatment	1	32	0.31	0.5788

Least Squares Means

Effect	Treatment	Estimate	Standard Error	DF	t Value	Pr > |t|	Alpha	Lower	Upper
Treatment	Control GFP	10.2887	0.9113	32	11.29	<.0001	0.05	8.4324	12.1450
Treatment	Ctr Meg	11.0151	0.9203	32	11.97	<.0001	0.05	9.1405	12.8897

DistSoma=42

Differences of Least Squares Means												
**Effect**	**Treatment**	**Treatment**	**Estimate**	**Standard Error**	**DF**	**t Value**	**Pr > |t|**	**Adjustment**	**Adj P**	**Alpha**	**Lower**	**Upper**
**Treatment**	Control GFP	Ctr Meg	-0.7264	1.2952	32	-0.56	0.5788	Tukey-Kramer	0.5788	0.05	-3.3646	1.9118

Differences of Least Squares Means				
**Effect**	**Treatment**	**Treatment**	**Adj Lower**	**Adj Upper**
**Treatment**	Control GFP	Ctr Meg	-3.3646	1.9117

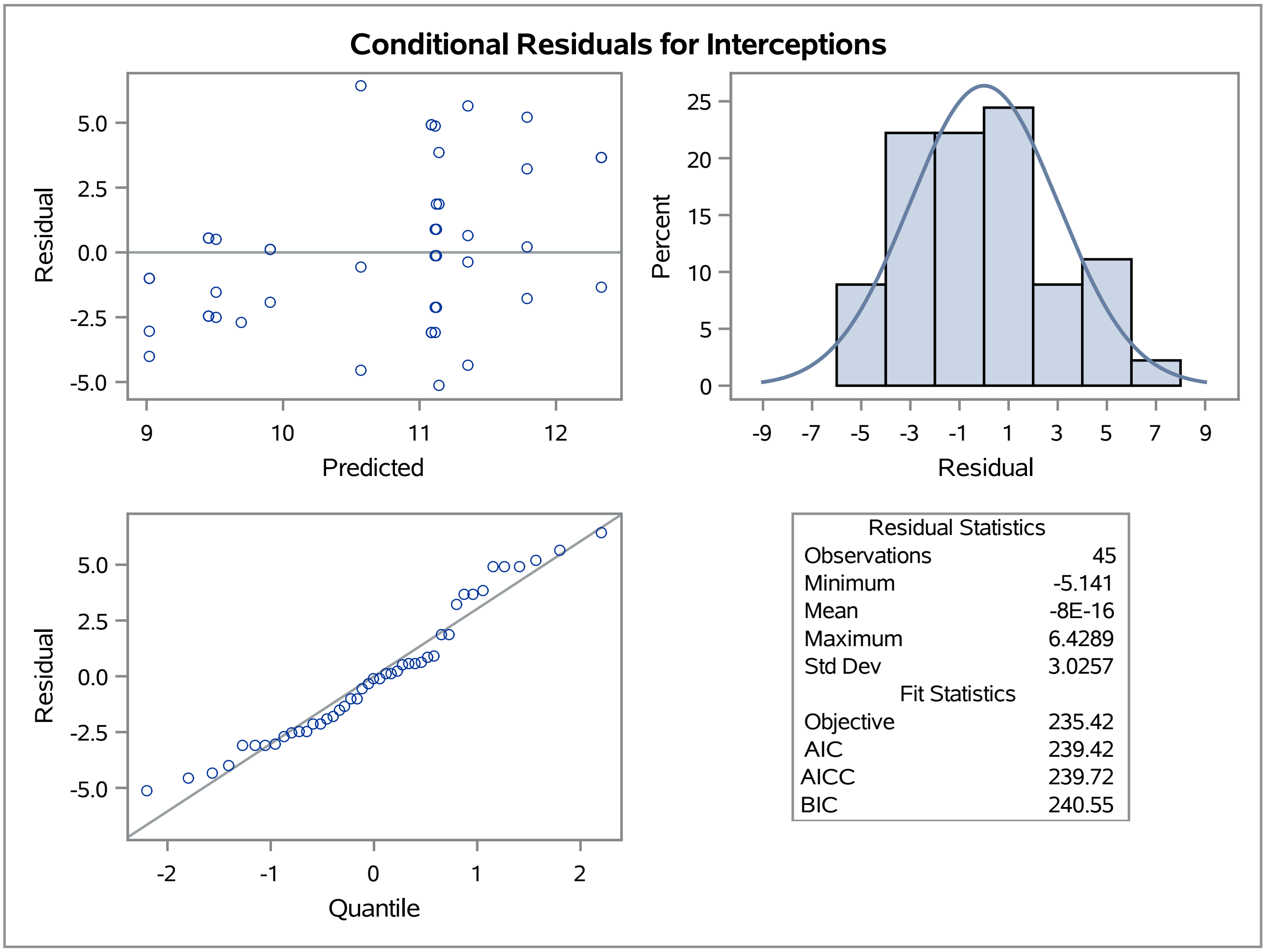

DistSoma=48

Model Information	
**Data Set**	WORK.TEMPDATASORTED
**Dependent Variable**	Interceptions
**Covariance Structure**	Variance Components
**Estimation Method**	REML
**Residual Variance Method**	Profile
**Fixed Effects SE Method**	Model-Based
**Degrees of Freedom Method**	Containment

Class Level Information		
**Class**	**Levels**	**Values**
**Treatment**	2	Control GFP Ctr Meg
**Culture**	13	1 2 3 4 5 6 7 8 9 10 11 12 13

Dimensions	
**Covariance Parameters**	2
**Columns in X**	3
**Columns in Z**	13
**Subjects**	1
**Max Obs per Subject**	45

Number of Observations	
**Number of Observations Read**	45
**Number of Observations Used**	45
**Number of Observations Not Used**	0

Iteration History			
**Iteration**	**Evaluations**	**-2 Res Log Like**	**Criterion**
**0**	1	236.52971196	
**1**	2	234.17570514	0.00000041
**2**	1	234.17567289	0.00000000

Convergence criteria met.

Covariance Parameter Estimates				
**Cov Parm**	**Estimate**	**Alpha**	**Lower**	**Upper**
**Culture**	2.8320	0.05	0.8575	54.1827
**Residual**	9.8866	0.05	6.4254	17.1577

DistSoma=48

Fit Statistics	
-2 Res Log Likelihood	234.2
AIC (Smaller is Better)	238.2
AICC (Smaller is Better)	238.5
BIC (Smaller is Better)	239.3

Solution for Fixed Effects									
Effect	Treatment	Estimate	Standard Error	DF	t Value	Pr > |t|	Alpha	Lower	Upper
Intercept		10.8467	0.9535	11	11.38	<.0001	0.05	8.7481	12.9453
Treatment	Control GFP	-1.5378	1.3383	32	-1.15	0.2591	0.05	-4.2638	1.1883
Treatment	Ctr Meg	0	.	.	.	.	.	.	.

Solution for Random Effects									
Effect	Culture	Estimate	Std Err Pred	DF	t Value	Pr > |t|	Alpha	Lower	Upper
Culture	1	0.1653	1.3082	32	0.13	0.9002	0.05	-2.4995	2.8301
Culture	2	-0.7590	1.3082	32	-0.58	0.5659	0.05	-3.4238	1.9058
Culture	3	2.2379	1.2535	32	1.79	0.0837	0.05	-0.3154	4.7912
Culture	4	0.6360	1.2535	32	0.51	0.6154	0.05	-1.9173	3.1893
Culture	5	-0.6989	1.2535	32	-0.56	0.5810	0.05	-3.2522	1.8543
Culture	6	-0.5141	1.4984	32	-0.34	0.7338	0.05	-3.5662	2.5380
Culture	7	-1.0671	1.3082	32	-0.82	0.4207	0.05	-3.7319	1.5977
Culture	8	0.3488	1.2566	32	0.28	0.7831	0.05	-2.2107	2.9084
Culture	9	-2.3210	1.2566	32	-1.85	0.0740	0.05	-4.8806	0.2386
Culture	10	1.1493	1.3105	32	0.88	0.3870	0.05	-1.5201	3.8186
Culture	11	0.2080	1.2164	32	0.17	0.8653	0.05	-2.2697	2.6857
Culture	12	0.08185	1.2566	32	0.07	0.9485	0.05	-2.4777	2.6414
Culture	13	0.5330	1.3105	32	0.41	0.6869	0.05	-2.1363	3.2023

Type 3 Tests of Fixed Effects				
Effect	Num DF	Den DF	F Value	Pr > F
Treatment	1	32	1.32	0.2591

Least Squares Means									
Effect	Treatment	Estimate	Standard Error	DF	t Value	Pr > |t|	Alpha	Lower	Upper
Treatment	Control GFP	9.3089	0.9391	32	9.91	<.0001	0.05	7.3960	11.2219
Treatment	Ctr Meg	10.8467	0.9535	32	11.38	<.0001	0.05	8.9045	12.7889

DistSoma=48

Differences of Least Squares Means												
**Effect**	**Treatment**	**Treatment**	**Estimate**	**Standard Error**	**DF**	**t Value**	**Pr > |t|**	**Adjustment**	**Adj P**	**Alpha**	**Lower**	**Upper**
**Treatment**	Control GFP	Ctr Meg	-1.5378	1.3383	32	-1.15	0.2591	Tukey-Kramer	0.2591	0.05	-4.2638	1.1883

Differences of Least Squares Means				
**Effect**	**Treatment**	**Treatment**	**Adj Lower**	**Adj Upper**
**Treatment**	Control GFP	Ctr Meg	-4.2638	1.1882

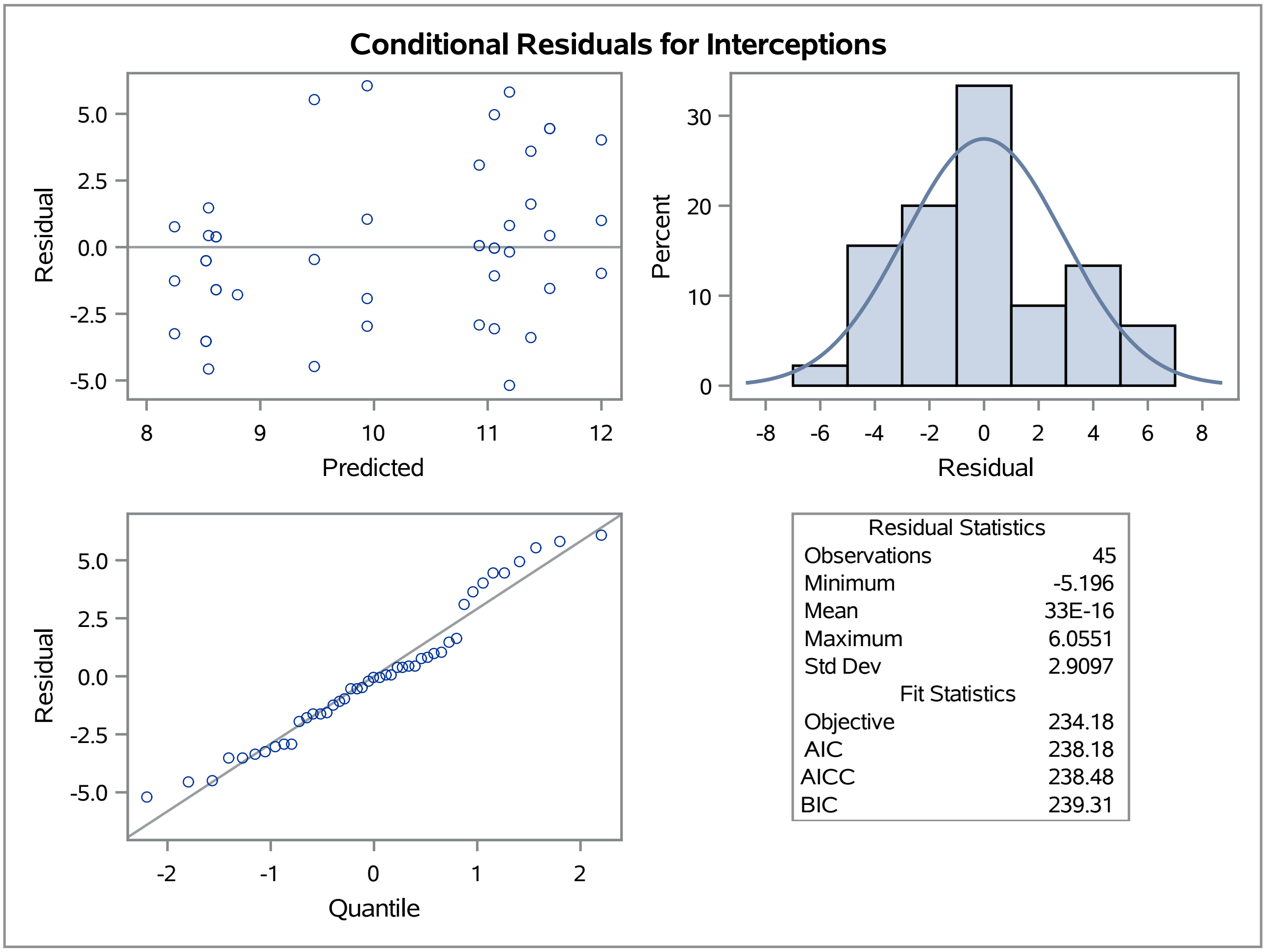

DistSoma=54

Model Information	
**Data Set**	WORK.TEMPDATASORTED
**Dependent Variable**	Interceptions
**Covariance Structure**	Variance Components
**Estimation Method**	REML
**Residual Variance Method**	Profile
**Fixed Effects SE Method**	Model-Based
**Degrees of Freedom Method**	Containment

Class Level Information		
**Class**	**Levels**	**Values**
**Treatment**	2	Control GFP Ctr Meg
**Culture**	13	1 2 3 4 5 6 7 8 9 10 11 12 13

Dimensions	
**Covariance Parameters**	2
**Columns in X**	3
**Columns in Z**	13
**Subjects**	1
**Max Obs per Subject**	45

Number of Observations	
**Number of Observations Read**	45
**Number of Observations Used**	45
**Number of Observations Not Used**	0

Iteration History			
**Iteration**	**Evaluations**	**-2 Res Log Like**	**Criterion**
**0**	1	227.58597013	
**1**	3	225.31365705	0.00000708
**2**	1	225.31313251	0.00000000

Convergence criteria met.

Covariance Parameter Estimates				
**Cov Parm**	**Estimate**	**Alpha**	**Lower**	**Upper**
**Culture**	2.1946	0.05	0.6575	44.7178
**Residual**	8.1000	0.05	5.2729	14.0198

DistSoma=54

Fit Statistics	
-2 Res Log Likelihood	225.3
AIC (Smaller is Better)	229.3
AICC (Smaller is Better)	229.6
BIC (Smaller is Better)	230.4

Solution for Fixed Effects									
Effect	Treatment	Estimate	Standard Error	DF	t Value	Pr > |t|	Alpha	Lower	Upper
Intercept		10.3002	0.8507	11	12.11	<.0001	0.05	8.4277	12.1726
Treatment	Control GFP	-1.7014	1.1948	32	-1.42	0.1641	0.05	-4.1351	0.7322
Treatment	Ctr Meg	0	.	.	.	.	.	.	.

Solution for Random Effects									
Effect	Culture	Estimate	Std Err Pred	DF	t Value	Pr > |t|	Alpha	Lower	Upper
Culture	1	0.1799	1.1628	32	0.15	0.8780	0.05	-2.1886	2.5485
Culture	2	0.03046	1.1628	32	0.03	0.9793	0.05	-2.3381	2.3990
Culture	3	1.3789	1.1152	32	1.24	0.2253	0.05	-0.8926	3.6504
Culture	4	0.2087	1.1152	32	0.19	0.8527	0.05	-2.0628	2.4802
Culture	5	-0.4414	1.1152	32	-0.40	0.6948	0.05	-2.7129	1.8301
Culture	6	-0.3408	1.3262	32	-0.26	0.7988	0.05	-3.0422	2.3605
Culture	7	-1.0157	1.1628	32	-0.87	0.3889	0.05	-3.3843	1.3528
Culture	8	0.3640	1.1176	32	0.33	0.7468	0.05	-1.9124	2.6404
Culture	9	-2.4966	1.1176	32	-2.23	0.0326	0.05	-4.7730	-0.2201
Culture	10	0.1643	1.1645	32	0.14	0.8887	0.05	-2.2077	2.5364
Culture	11	0.1725	1.0824	32	0.16	0.8744	0.05	-2.0322	2.3773
Culture	12	0.8841	1.1176	32	0.79	0.4347	0.05	-1.3923	3.1605
Culture	13	0.9116	1.1645	32	0.78	0.4395	0.05	-1.4604	3.2837

Type 3 Tests of Fixed Effects				
Effect	Num DF	Den DF	F Value	Pr > F
Treatment	1	32	2.03	0.1641

Least Squares Means									
Effect	Treatment	Estimate	Standard Error	DF	t Value	Pr > |t|	Alpha	Lower	Upper
Treatment	Control GFP	8.5987	0.8389	32	10.25	<.0001	0.05	6.8900	10.3075
Treatment	Ctr Meg	10.3002	0.8507	32	12.11	<.0001	0.05	8.5673	12.0330

DistSoma=54

Differences of Least Squares Means												
Effect	Treatment	Treatment	Estimate	Standard Error	DF	t Value	Pr > |t|	Adjustment	Adj P	Alpha	Lower	Upper
**Treatment**	Control GFP	Ctr Meg	-1.7014	1.1948	32	-1.42	0.1641	Tukey-Kramer	0.1641	0.05	-4.1351	0.7322

Differences of Least Squares Means				
Effect	Treatment	Treatment	Adj Lower	Adj Upper
**Treatment**	Control GFP	Ctr Meg	-4.1350	0.7322

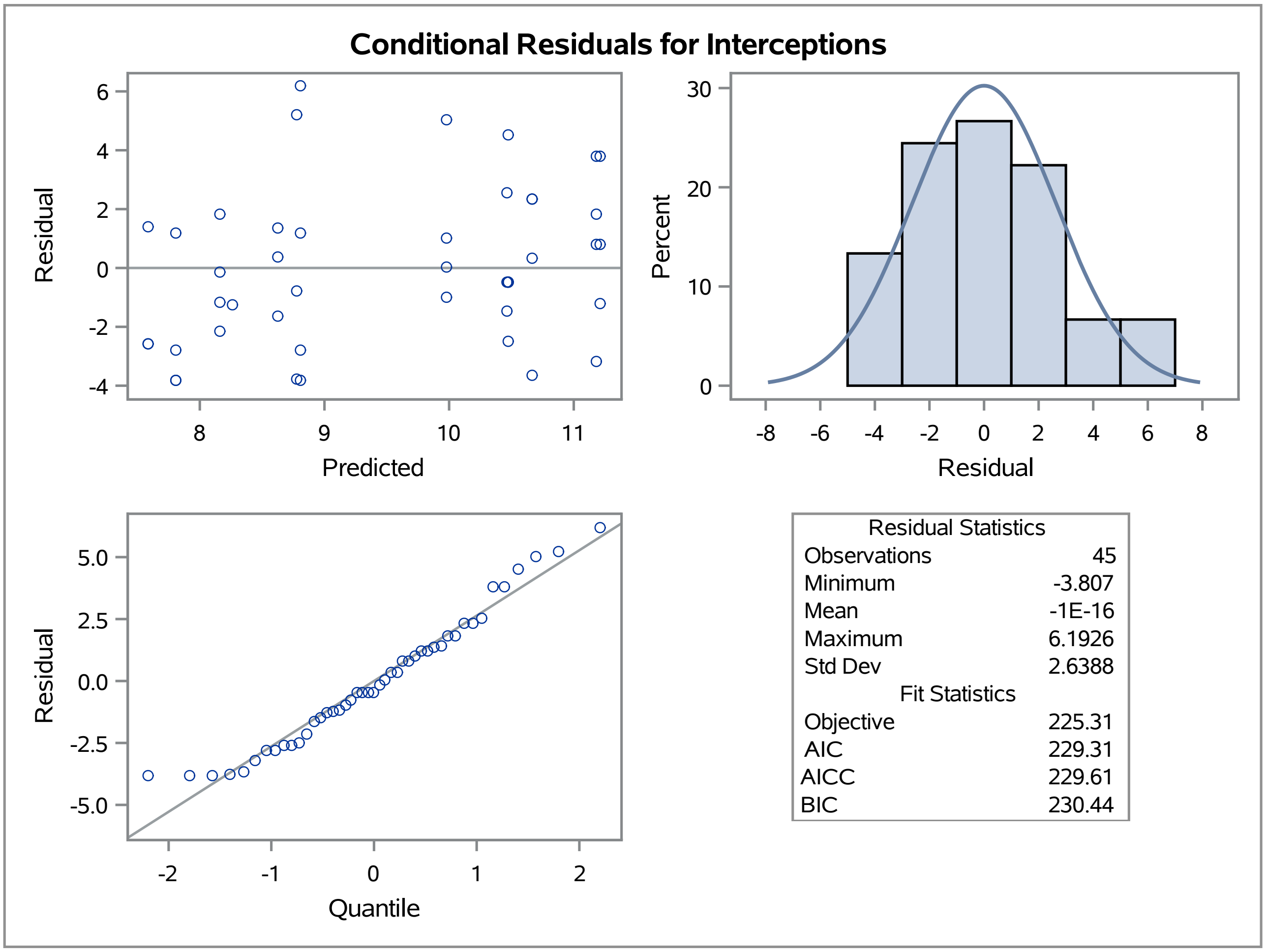

DistSoma=60

Model Information	
**Data Set**	WORK.TEMPDATASORTED
**Dependent Variable**	Interceptions
**Covariance Structure**	Variance Components
**Estimation Method**	REML
**Residual Variance Method**	Profile
**Fixed Effects SE Method**	Model-Based
**Degrees of Freedom Method**	Containment

Class Level Information		
**Class**	**Levels**	**Values**
**Treatment**	2	Control GFP Ctr Meg
**Culture**	13	1 2 3 4 5 6 7 8 9 10 11 12 13

Dimensions	
**Covariance Parameters**	2
**Columns in X**	3
**Columns in Z**	13
**Subjects**	1
**Max Obs per Subject**	45

Number of Observations	
**Number of Observations Read**	45
**Number of Observations Used**	45
**Number of Observations Not Used**	0

Iteration History			
**Iteration**	**Evaluations**	**-2 Res Log Like**	**Criterion**
**0**	1	227.49013480	
**1**	3	225.46365754	0.00001387
**2**	1	225.46262359	0.00000001
**3**	1	225.46262282	0.00000000

Convergence criteria met.

DistSoma=60

Covariance Parameter Estimates				
**Cov Parm**	**Estimate**	**Alpha**	**Lower**	**Upper**
**Culture**	2.0379	0.05	0.5890	51.8097
**Residual**	8.2127	0.05	5.3495	14.2005

Fit Statistics	
**-2 Res Log Likelihood**	225.5
**AIC (Smaller is Better)**	229.5
**AICC (Smaller is Better)**	229.8
**BIC (Smaller is Better)**	230.6

Solution for Fixed Effects									
**Effect**	**Treatment**	**Estimate**	**Standard Error**	**DF**	**t Value**	**Pr > |t|**	**Alpha**	**Lower**	**Upper**
**Intercept**		9.4495	0.8381	11	11.28	<.0001	0.05	7.6050	11.2941
**Treatment**	Control GFP	-1.3394	1.1780	32	-1.14	0.2640	0.05	-3.7390	1.0601
**Treatment**	Ctr Meg	0	.	.	.	.	.	.	.

Solution for Random Effects									
**Effect**	**Culture**	**Estimate**	**Std Err Pred**	**DF**	**t Value**	**Pr > |t|**	**Alpha**	**Lower**	**Upper**
**Culture**	1	-0.4737	1.1371	32	-0.42	0.6798	0.05	-2.7900	1.8425
**Culture**	2	-0.4737	1.1371	32	-0.42	0.6798	0.05	-2.7900	1.8425
**Culture**	3	0.9414	1.0922	32	0.86	0.3951	0.05	-1.2832	3.1661
**Culture**	4	0.8169	1.0922	32	0.75	0.4599	0.05	-1.4078	3.0416
**Culture**	5	-0.4284	1.0922	32	-0.39	0.6975	0.05	-2.6531	1.7962
**Culture**	6	0.3757	1.2883	32	0.29	0.7724	0.05	-2.2485	3.0000
**Culture**	7	-0.7582	1.1371	32	-0.67	0.5097	0.05	-3.0744	1.5580
**Culture**	8	0.1497	1.0941	32	0.14	0.8920	0.05	-2.0789	2.3783
**Culture**	9	-2.3410	1.0941	32	-2.14	0.0401	0.05	-4.5696	-0.1124
**Culture**	10	0.09267	1.1385	32	0.08	0.9356	0.05	-2.2263	2.4117
**Culture**	11	0.4156	1.0606	32	0.39	0.6978	0.05	-1.7448	2.5759
**Culture**	12	1.0214	1.0941	32	0.93	0.3575	0.05	-1.2072	3.2500
**Culture**	13	0.6617	1.1385	32	0.58	0.5652	0.05	-1.6573	2.9807

Type 3 Tests of Fixed Effects				
**Effect**	**Num DF**	**Den DF**	**F Value**	**Pr > F**
**Treatment**	1	32	1.29	0.2640

DistSoma=60

Least Squares Means									
**Effect**	**Treatment**	**Estimate**	**Standard Error**	**DF**	**t Value**	**Pr > |t|**	**Alpha**	**Lower**	**Upper**
**Treatment**	Control GFP	8.1101	0.8279	32	9.80	<.0001	0.05	6.4238	9.7964
**Treatment**	Ctr Meg	9.4495	0.8381	32	11.28	<.0001	0.05	7.7425	11.1566

Differences of Least Squares Means												
**Effect**	**Treatment**	**Treatment**	**Estimate**	**Standard Error**	**DF**	**t Value**	**Pr > |t|**	**Adjustment**	**Adj P**	**Alpha**	**Lower**	**Upper**
**Treatment**	Control GFP	Ctr Meg	-1.3394	1.1780	32	-1.14	0.2640	Tukey-Kramer	0.2640	0.05	-3.7390	1.0601

Differences of Least Squares Means				
**Effect**	**Treatment**	**Treatment**	**Adj Lower**	**Adj Upper**
**Treatment**	Control GFP	Ctr Meg	-3.7389	1.0601

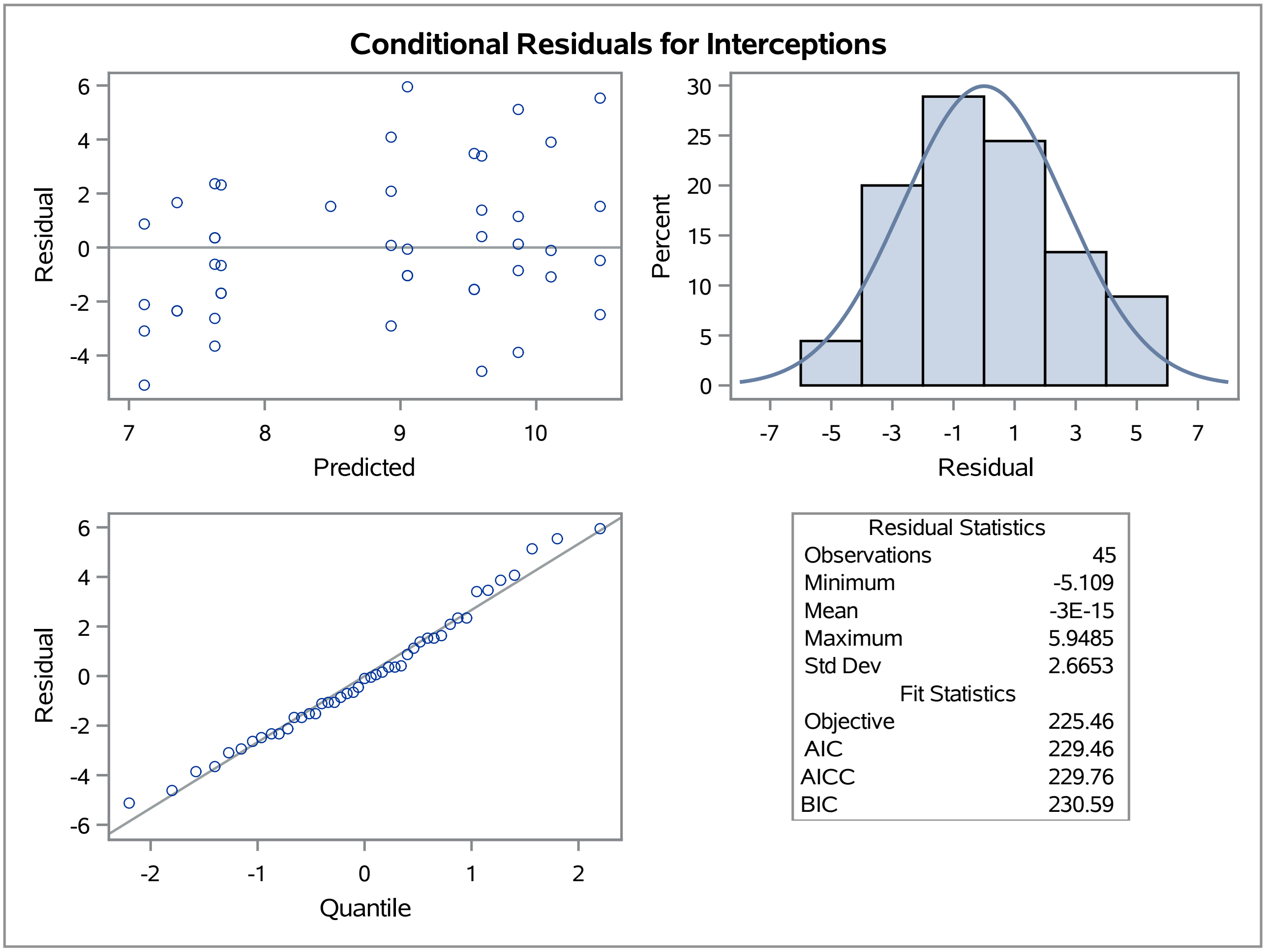

DistSoma=66

Model Information	
**Data Set**	WORK.TEMPDATASORTED
**Dependent Variable**	Interceptions
**Covariance Structure**	Variance Components
**Estimation Method**	REML
**Residual Variance Method**	Profile
**Fixed Effects SE Method**	Model-Based
**Degrees of Freedom Method**	Containment

Class Level Information		
**Class**	**Levels**	**Values**
**Treatment**	2	Control GFP Ctr Meg
**Culture**	13	1 2 3 4 5 6 7 8 9 10 11 12 13

Dimensions	
**Covariance Parameters**	2
**Columns in X**	3
**Columns in Z**	13
**Subjects**	1
**Max Obs per Subject**	45

Number of Observations	
**Number of Observations Read**	45
**Number of Observations Used**	45
**Number of Observations Not Used**	0

Iteration History			
**Iteration**	**Evaluations**	**-2 Res Log Like**	**Criterion**
**0**	1	231.62715016	
**1**	3	230.45189716	0.00000455
**2**	1	230.45154865	0.00000000

Convergence criteria met.

Covariance Parameter Estimates				
**Cov Parm**	**Estimate**	**Alpha**	**Lower**	**Upper**
**Culture**	1.6774	0.05	0.4063	168.83
**Residual**	9.5630	0.05	6.2343	16.5127

DistSoma=66

Fit Statistics	
-2 Res Log Likelihood	230.5
AIC (Smaller is Better)	234.5
AICC (Smaller is Better)	234.8
BIC (Smaller is Better)	235.6

Solution for Fixed Effects

Effect	Treatment	Estimate	Standard Error	DF	t Value	Pr > |t|	Alpha	Lower	Upper
Intercept		9.1868	0.8371	11	10.97	<.0001	0.05	7.3443	11.0292
Treatment	Control GFP	-1.3640	1.1806	32	-1.16	0.2565	0.05	-3.7687	1.0407
Treatment	Ctr Meg	0	.	.	.	.	.	.	.

Solution for Random Effects

Effect	Culture	Estimate	Std Err Pred	DF	t Value	Pr > |t|	Alpha	Lower	Upper
Culture	1	-0.6285	1.0869	32	-0.58	0.5672	0.05	-2.8425	1.5856
Culture	2	-0.2837	1.0869	32	-0.26	0.7958	0.05	-2.4977	1.9303
Culture	3	0.5885	1.0505	32	0.56	0.5793	0.05	-1.5514	2.7283
Culture	4	0.6915	1.0505	32	0.66	0.5151	0.05	-1.4483	2.8314
Culture	5	-0.03001	1.0505	32	-0.03	0.9774	0.05	-2.1698	2.1098
Culture	6	0.1757	1.2010	32	0.15	0.8846	0.05	-2.2708	2.6221
Culture	7	-0.5135	1.0869	32	-0.47	0.6398	0.05	-2.7275	1.7005
Culture	8	-0.07701	1.0511	32	-0.07	0.9421	0.05	-2.2181	2.0641
Culture	9	-1.9325	1.0511	32	-1.84	0.0753	0.05	-4.0736	0.2086
Culture	10	0.05053	1.0874	32	0.05	0.9632	0.05	-2.1643	2.2654
Culture	11	0.3800	1.0230	32	0.37	0.7128	0.05	-1.7039	2.4638
Culture	12	0.9538	1.0511	32	0.91	0.3710	0.05	-1.1873	3.0949
Culture	13	0.6252	1.0874	32	0.57	0.5694	0.05	-1.5897	2.8400

Type 3 Tests of Fixed Effects

Effect	Num DF	Den DF	F Value	Pr > F
Treatment	1	32	1.33	0.2565

Least Squares Means

Effect	Treatment	Estimate	Standard Error	DF	t Value	Pr > |t|	Alpha	Lower	Upper
Treatment	Control GFP	7.8228	0.8325	32	9.40	<.0001	0.05	6.1271	9.5184
Treatment	Ctr Meg	9.1868	0.8371	32	10.97	<.0001	0.05	7.4817	10.8919

DistSoma=66

Differences of Least Squares Means												
Effect	Treatment	Treatment	Estimate	Standard Error	DF	t Value	Pr > |t|	Adjustment	Adj P	Alpha	Lower	Upper
**Treatment**	Control GFP	Ctr Meg	-1.3640	1.1806	32	-1.16	0.2565	Tukey-Kramer	0.2565	0.05	-3.7687	1.0407

Differences of Least Squares Means				
Effect	Treatment	Treatment	Adj Lower	Adj Upper
**Treatment**	Control GFP	Ctr Meg	-3.7687	1.0407

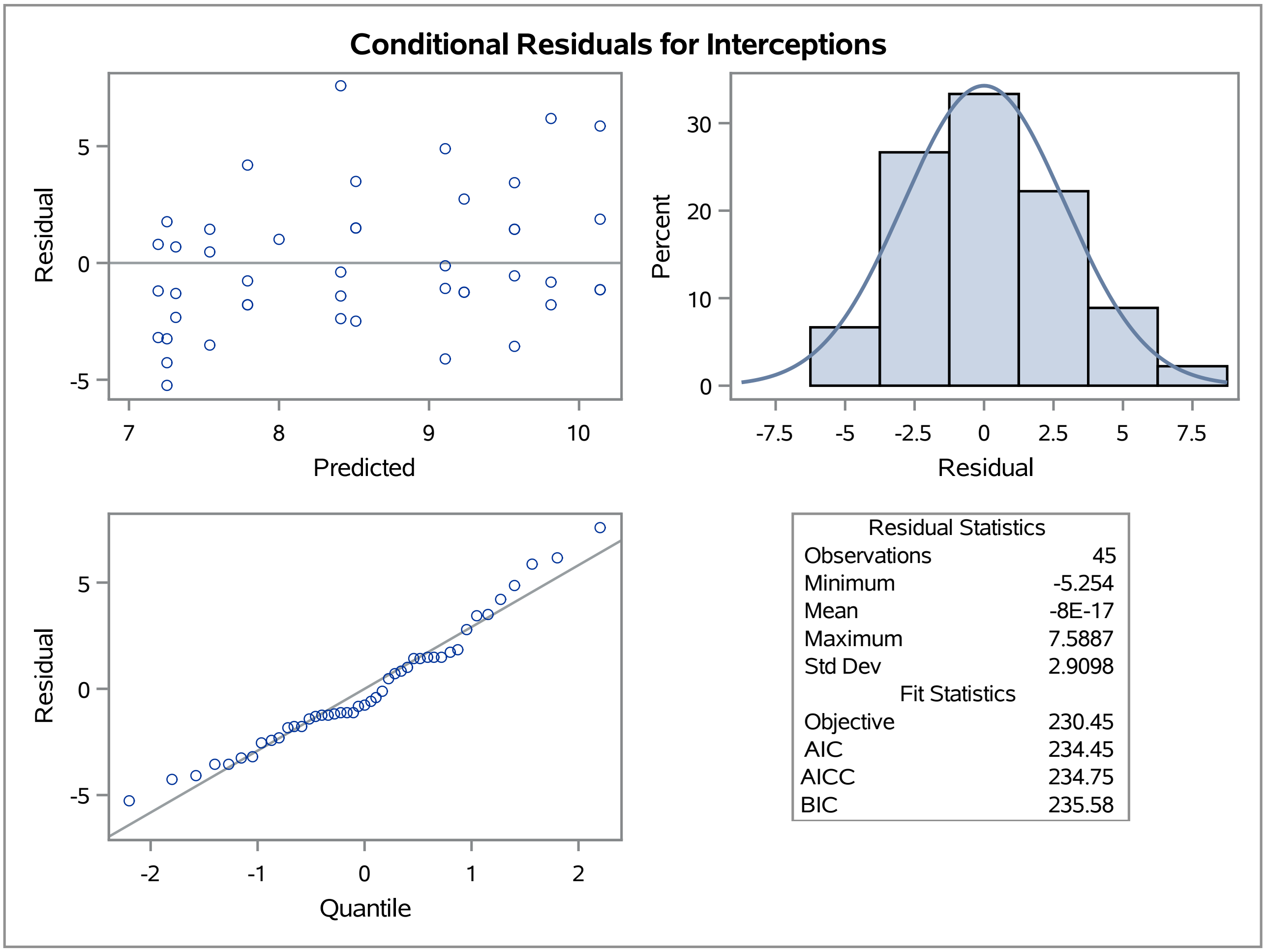

DistSoma=72

Model Information	
**Data Set**	WORK.TEMPDATASORTED
**Dependent Variable**	Interceptions
**Covariance Structure**	Variance Components
**Estimation Method**	REML
**Residual Variance Method**	Profile
**Fixed Effects SE Method**	Model-Based
**Degrees of Freedom Method**	Containment

Class Level Information		
**Class**	**Levels**	**Values**
**Treatment**	2	Control GFP Ctr Meg
**Culture**	13	1 2 3 4 5 6 7 8 9 10 11 12 13

Dimensions	
**Covariance Parameters**	2
**Columns in X**	3
**Columns in Z**	13
**Subjects**	1
**Max Obs per Subject**	45

Number of Observations	
**Number of Observations Read**	45
**Number of Observations Used**	45
**Number of Observations Not Used**	0

Iteration History			
**Iteration**	**Evaluations**	**-2 Res Log Like**	**Criterion**
**0**	1	230.70757705	
**1**	3	230.18102599	0.00000180
**2**	1	230.18088874	0.00000000

Convergence criteria met.

Covariance Parameter Estimates				
**Cov Parm**	**Estimate**	**Alpha**	**Lower**	**Upper**
**Culture**	1.0657	0.05	0.1910	6143.59
**Residual**	9.8829	0.05	6.4503	17.0331

DistSoma=72

Fit Statistics	
-2 Res Log Likelihood	230.2
AIC (Smaller is Better)	234.2
AICC (Smaller is Better)	234.5
BIC (Smaller is Better)	235.3

Solution for Fixed Effects

Effect	Treatment	Estimate	Standard Error	DF	t Value	Pr > |t|	Alpha	Lower	Upper
Intercept		8.9050	0.7819	11	11.39	<.0001	0.05	7.1841	10.6259
Treatment	Control GFP	-1.9221	1.1071	32	-1.74	0.0922	0.05	-4.1772	0.3330
Treatment	Ctr Meg	0	.	.	.	.	.	.	.

Solution for Random Effects

Effect	Culture	Estimate	Std Err Pred	DF	t Value	Pr > |t|	Alpha	Lower	Upper
Culture	1	-0.4847	0.9176	32	-0.53	0.6010	0.05	-2.3537	1.3843
Culture	2	-0.1588	0.9176	32	-0.17	0.8637	0.05	-2.0278	1.7102
Culture	3	0.4572	0.8946	32	0.51	0.6128	0.05	-1.3651	2.2794
Culture	4	0.3065	0.8946	32	0.34	0.7341	0.05	-1.5158	2.1288
Culture	5	0.005152	0.8946	32	0.01	0.9954	0.05	-1.8171	1.8274
Culture	6	0.1963	0.9838	32	0.20	0.8431	0.05	-1.8075	2.2002
Culture	7	-0.3217	0.9176	32	-0.35	0.7282	0.05	-2.1907	1.5473
Culture	8	0.1793	0.8945	32	0.20	0.8424	0.05	-1.6427	2.0013
Culture	9	-1.3274	0.8945	32	-1.48	0.1476	0.05	-3.1494	0.4945
Culture	10	0.1047	0.9175	32	0.11	0.9099	0.05	-1.7641	1.9735
Culture	11	0.3135	0.8760	32	0.36	0.7228	0.05	-1.4709	2.0979
Culture	12	0.7067	0.8945	32	0.79	0.4353	0.05	-1.1153	2.5286
Culture	13	0.02323	0.9175	32	0.03	0.9800	0.05	-1.8456	1.8920

Type 3 Tests of Fixed Effects

Effect	Num DF	Den DF	F Value	Pr > F
Treatment	1	32	3.01	0.0922

Least Squares Means

Effect	Treatment	Estimate	Standard Error	DF	t Value	Pr > |t|	Alpha	Lower	Upper
Treatment	Control GFP	6.9829	0.7838	32	8.91	<.0001	0.05	5.3863	8.5795
Treatment	Ctr Meg	8.9050	0.7819	32	11.39	<.0001	0.05	7.3123	10.4976

DistSoma=72

Differences of Least Squares Means

Effect	Treatment	Treatment	Estimate	Standard Error	DF	t Value	Pr > |t|	Adjustment	Adj P	Alpha	Lower	Upper
**Treatment**	Control GFP	Ctr Meg	-1.9221	1.1071	32	-1.74	0.0922	Tukey-Kramer	0.0922	0.05	-4.1772	0.3330

Differences of Least Squares Means

Effect	Treatment	Treatment	Adj Lower	Adj Upper
**Treatment**	Control GFP	Ctr Meg	-4.1772	0.3330

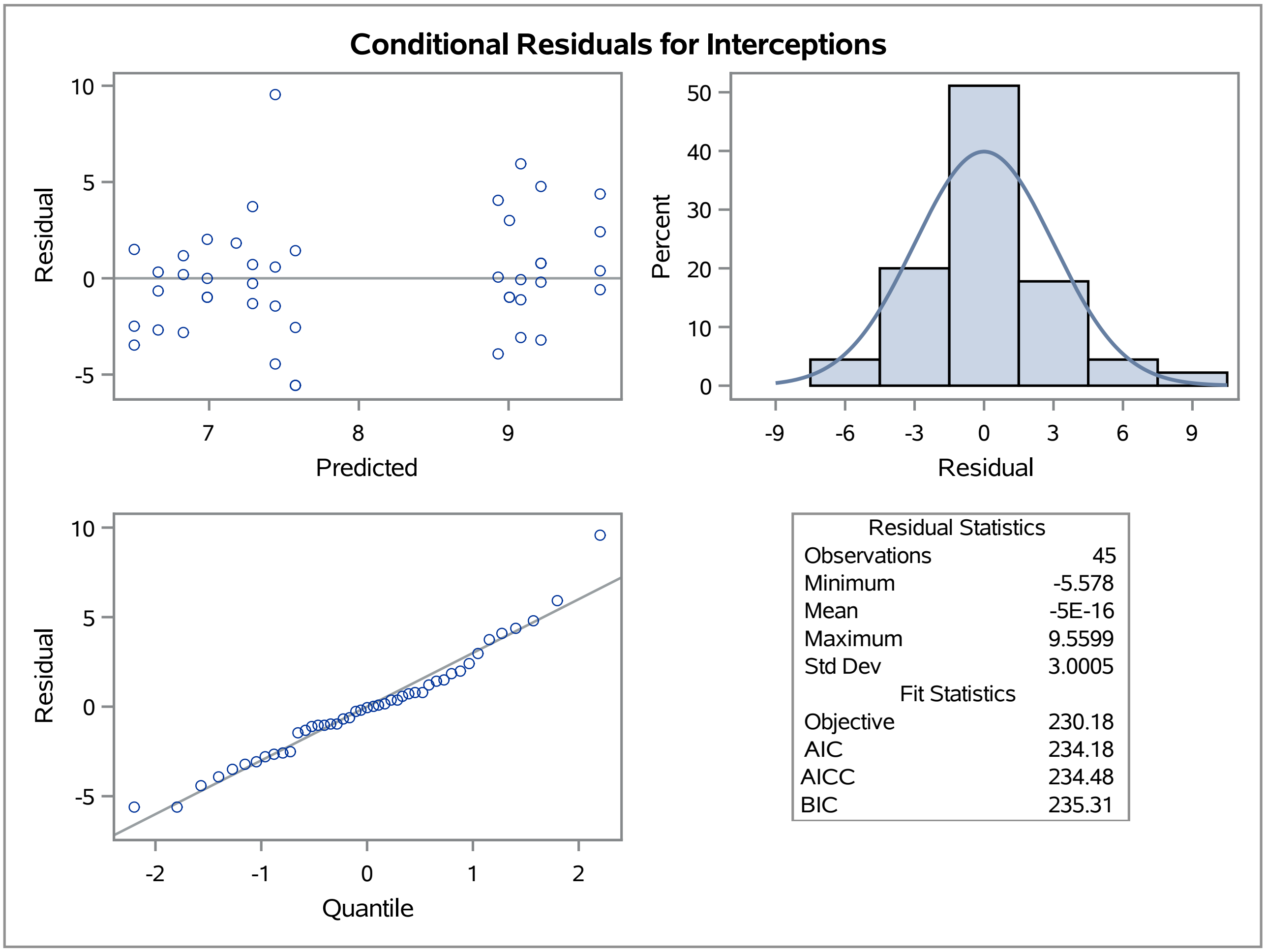

DistSoma=78

Model Information	
**Data Set**	WORK.TEMPDATASORTED
**Dependent Variable**	Interceptions
**Covariance Structure**	Variance Components
**Estimation Method**	REML
**Residual Variance Method**	Profile
**Fixed Effects SE Method**	Model-Based
**Degrees of Freedom Method**	Containment

Class Level Information		
**Class**	**Levels**	**Values**
**Treatment**	2	Control GFP Ctr Meg
**Culture**	13	1 2 3 4 5 6 7 8 9 10 11 12 13

Dimensions	
**Covariance Parameters**	2
**Columns in X**	3
**Columns in Z**	13
**Subjects**	1
**Max Obs per Subject**	45

Number of Observations	
**Number of Observations Read**	45
**Number of Observations Used**	45
**Number of Observations Not Used**	0

Iteration History			
**Iteration**	**Evaluations**	**-2 Res Log Like**	**Criterion**
**0**	1	235.56426269	
**1**	2	234.28502041	0.00000001
**2**	1	234.28501943	0.00000000

Convergence criteria met.

Covariance Parameter Estimates				
**Cov Parm**	**Estimate**	**Alpha**	**Lower**	**Upper**
**Culture**	2.0445	0.05	0.5048	172.91
**Residual**	10.3325	0.05	6.7008	17.9949

DistSoma=78

Fit Statistics	
-2 Res Log Likelihood	234.3
AIC (Smaller is Better)	238.3
AICC (Smaller is Better)	238.6
BIC (Smaller is Better)	239.4

Solution for Fixed Effects

Effect	Treatment	Estimate	Standard Error	DF	t Value	Pr > |t|	Alpha	Lower	Upper
Intercept		8.7056	0.8923	11	9.76	<.0001	0.05	6.7416	10.6695
Treatment	Control GFP	-2.0551	1.2570	32	-1.63	0.1119	0.05	-4.6156	0.5054
Treatment	Ctr Meg	0	.	.	.	.	.	.	.

Solution for Random Effects

Effect	Culture	Estimate	Std Err Pred	DF	t Value	Pr > |t|	Alpha	Lower	Upper
Culture	1	-1.3598	1.1797	32	-1.15	0.2576	0.05	-3.7628	1.0432
Culture	2	0.1302	1.1797	32	0.11	0.9128	0.05	-2.2728	2.5332
Culture	3	0.7067	1.1377	32	0.62	0.5389	0.05	-1.6107	3.0240
Culture	4	0.1544	1.1377	32	0.14	0.8929	0.05	-2.1629	2.4717
Culture	5	0.2649	1.1377	32	0.23	0.8174	0.05	-2.0525	2.5822
Culture	6	0.7185	1.3146	32	0.55	0.5885	0.05	-1.9593	3.3962
Culture	7	-0.6148	1.1797	32	-0.52	0.6059	0.05	-3.0178	1.7882
Culture	8	-0.09082	1.1387	32	-0.08	0.9369	0.05	-2.4103	2.2287
Culture	9	-1.8580	1.1387	32	-1.63	0.1125	0.05	-4.1775	0.4614
Culture	10	-0.01449	1.1804	32	-0.01	0.9903	0.05	-2.4189	2.3900
Culture	11	0.7432	1.1066	32	0.67	0.5067	0.05	-1.5109	2.9974
Culture	12	1.2346	1.1387	32	1.08	0.2864	0.05	-1.0849	3.5541
Culture	13	-0.01449	1.1804	32	-0.01	0.9903	0.05	-2.4189	2.3900

Type 3 Tests of Fixed Effects

Effect	Num DF	Den DF	F Value	Pr > F
Treatment	1	32	2.67	0.1119

Least Squares Means

Effect	Treatment	Estimate	Standard Error	DF	t Value	Pr > |t|	Alpha	Lower	Upper
Treatment	Control GFP	6.6505	0.8854	32	7.51	<.0001	0.05	4.8470	8.4540
Treatment	Ctr Meg	8.7056	0.8923	32	9.76	<.0001	0.05	6.8880	10.5232

DistSoma=78

Differences of Least Squares Means												
**Effect**	**Treatment**	**Treatment**	**Estimate**	**Standard Error**	**DF**	**t Value**	**Pr > |t|**	**Adjustment**	**Adj P**	**Alpha**	**Lower**	**Upper**
**Treatment**	Control GFP	Ctr Meg	-2.0551	1.2570	32	-1.63	0.1119	Tukey-Kramer	0.1119	0.05	-4.6156	0.5054

Differences of Least Squares Means				
**Effect**	**Treatment**	**Treatment**	**Adj Lower**	**Adj Upper**
**Treatment**	Control GFP	Ctr Meg	-4.6155	0.5054

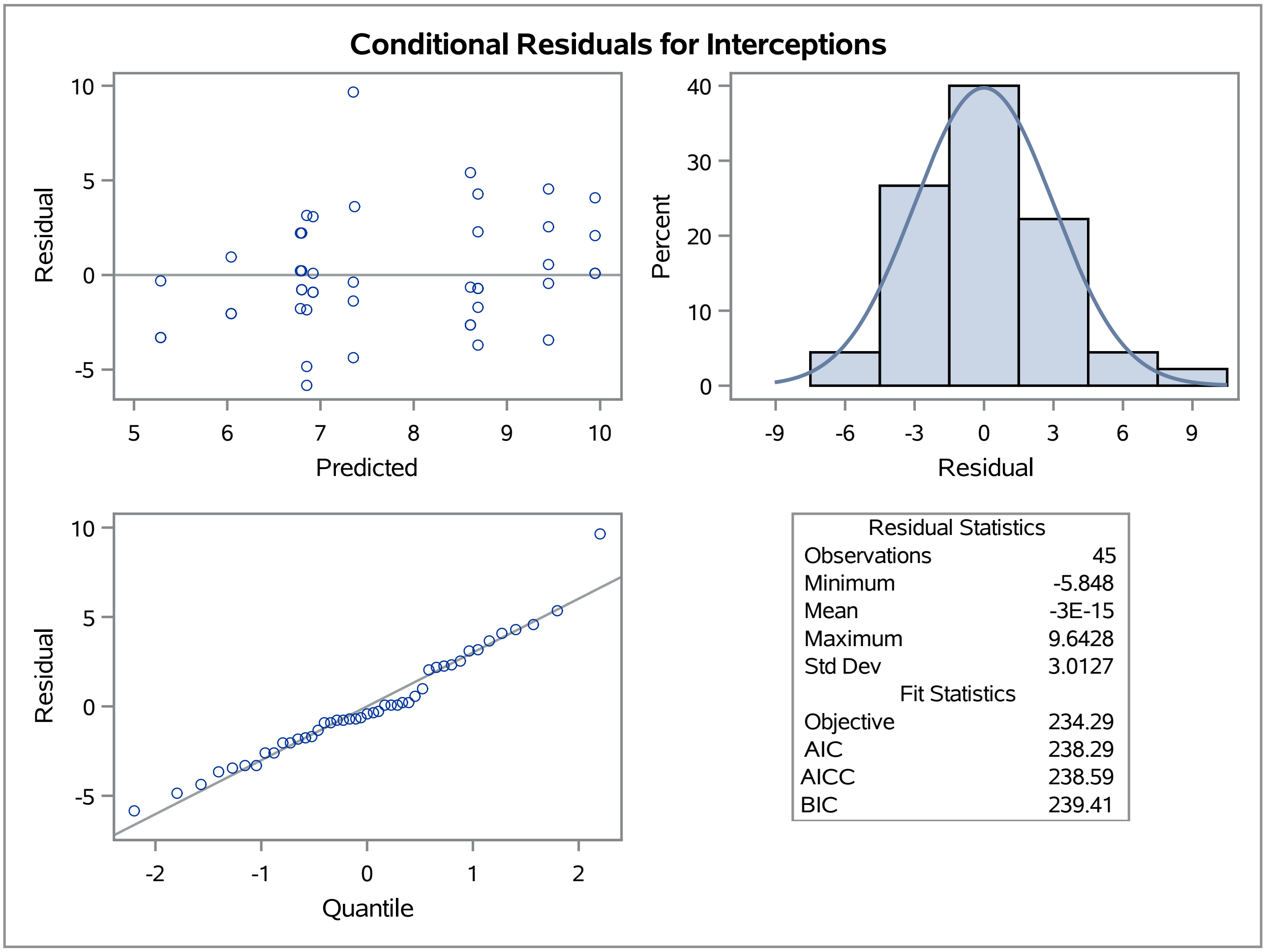

DistSoma=84

Model Information	
**Data Set**	WORK.TEMPDATASORTED
**Dependent Variable**	Interceptions
**Covariance Structure**	Variance Components
**Estimation Method**	REML
**Residual Variance Method**	Profile
**Fixed Effects SE Method**	Model-Based
**Degrees of Freedom Method**	Containment

Class Level Information		
**Class**	**Levels**	**Values**
**Treatment**	2	Control GFP Ctr Meg
**Culture**	13	1 2 3 4 5 6 7 8 9 10 11 12 13

Dimensions	
**Covariance Parameters**	2
**Columns in X**	3
**Columns in Z**	13
**Subjects**	1
**Max Obs per Subject**	45

Number of Observations	
**Number of Observations Read**	45
**Number of Observations Used**	45
**Number of Observations Not Used**	0

Iteration History			
**Iteration**	**Evaluations**	**-2 Res Log Like**	**Criterion**
**0**	1	226.94326156	
**1**	2	225.15575490	0.00000052
**2**	1	225.15571698	0.00000000

Convergence criteria met.

Covariance Parameter Estimates				
**Cov Parm**	**Estimate**	**Alpha**	**Lower**	**Upper**
**Culture**	1.9600	0.05	0.5420	67.0691
**Residual**	8.1877	0.05	5.3175	14.2259

DistSoma=84

Fit Statistics	
-2 Res Log Likelihood	225.2
AIC (Smaller is Better)	229.2
AICC (Smaller is Better)	229.5
BIC (Smaller is Better)	230.3

Solution for Fixed Effects

Effect	Treatment	Estimate	Standard Error	DF	t Value	Pr > |t|	Alpha	Lower	Upper
Intercept		7.8707	0.8295	11	9.49	<.0001	0.05	6.0449	9.6965
Treatment	Control GFP	-1.8435	1.1665	32	-1.58	0.1238	0.05	-4.2195	0.5325
Treatment	Ctr Meg	0	.	.	.	.	.	.	.

Solution for Random Effects

Effect	Culture	Estimate	Std Err Pred	DF	t Value	Pr > |t|	Alpha	Lower	Upper
Culture	1	-1.5440	1.1217	32	-1.38	0.1782	0.05	-3.8289	0.7409
Culture	2	-0.01137	1.1217	32	-0.01	0.9920	0.05	-2.2962	2.2735
Culture	3	0.4758	1.0780	32	0.44	0.6619	0.05	-1.7201	2.6718
Culture	4	0.5981	1.0780	32	0.55	0.5829	0.05	-1.5978	2.7940
Culture	5	0.4758	1.0780	32	0.44	0.6619	0.05	-1.7201	2.6718
Culture	6	0.5742	1.2675	32	0.45	0.6536	0.05	-2.0076	3.1560
Culture	7	-0.5687	1.1217	32	-0.51	0.6156	0.05	-2.8536	1.7162
Culture	8	-0.05905	1.0798	32	-0.05	0.9567	0.05	-2.2585	2.1404
Culture	9	-1.8934	1.0798	32	-1.75	0.0891	0.05	-4.0928	0.3060
Culture	10	0.1934	1.1229	32	0.17	0.8644	0.05	-2.0940	2.4807
Culture	11	0.9421	1.0471	32	0.90	0.3750	0.05	-1.1907	3.0750
Culture	12	1.0416	1.0798	32	0.96	0.3420	0.05	-1.1579	3.2410
Culture	13	-0.2246	1.1229	32	-0.20	0.8427	0.05	-2.5120	2.0627

Type 3 Tests of Fixed Effects

Effect	Num DF	Den DF	F Value	Pr > F
Treatment	1	32	2.50	0.1238

Least Squares Means

Effect	Treatment	Estimate	Standard Error	DF	t Value	Pr > |t|	Alpha	Lower	Upper
Treatment	Control GFP	6.0272	0.8201	32	7.35	<.0001	0.05	4.3568	7.6976
Treatment	Ctr Meg	7.8707	0.8295	32	9.49	<.0001	0.05	6.1810	9.5605

DistSoma=84

Differences of Least Squares Means												
**Effect**	**Treatment**	**Treatment**	**Estimate**	**Standard Error**	**DF**	**t Value**	**Pr > |t|**	**Adjustment**	**Adj P**	**Alpha**	**Lower**	**Upper**
**Treatment**	Control GFP	Ctr Meg	-1.8435	1.1665	32	-1.58	0.1238	Tukey-Kramer	0.1238	0.05	-4.2195	0.5325

Differences of Least Squares Means				
**Effect**	**Treatment**	**Treatment**	**Adj Lower**	**Adj Upper**
**Treatment**	Control GFP	Ctr Meg	-4.2195	0.5325

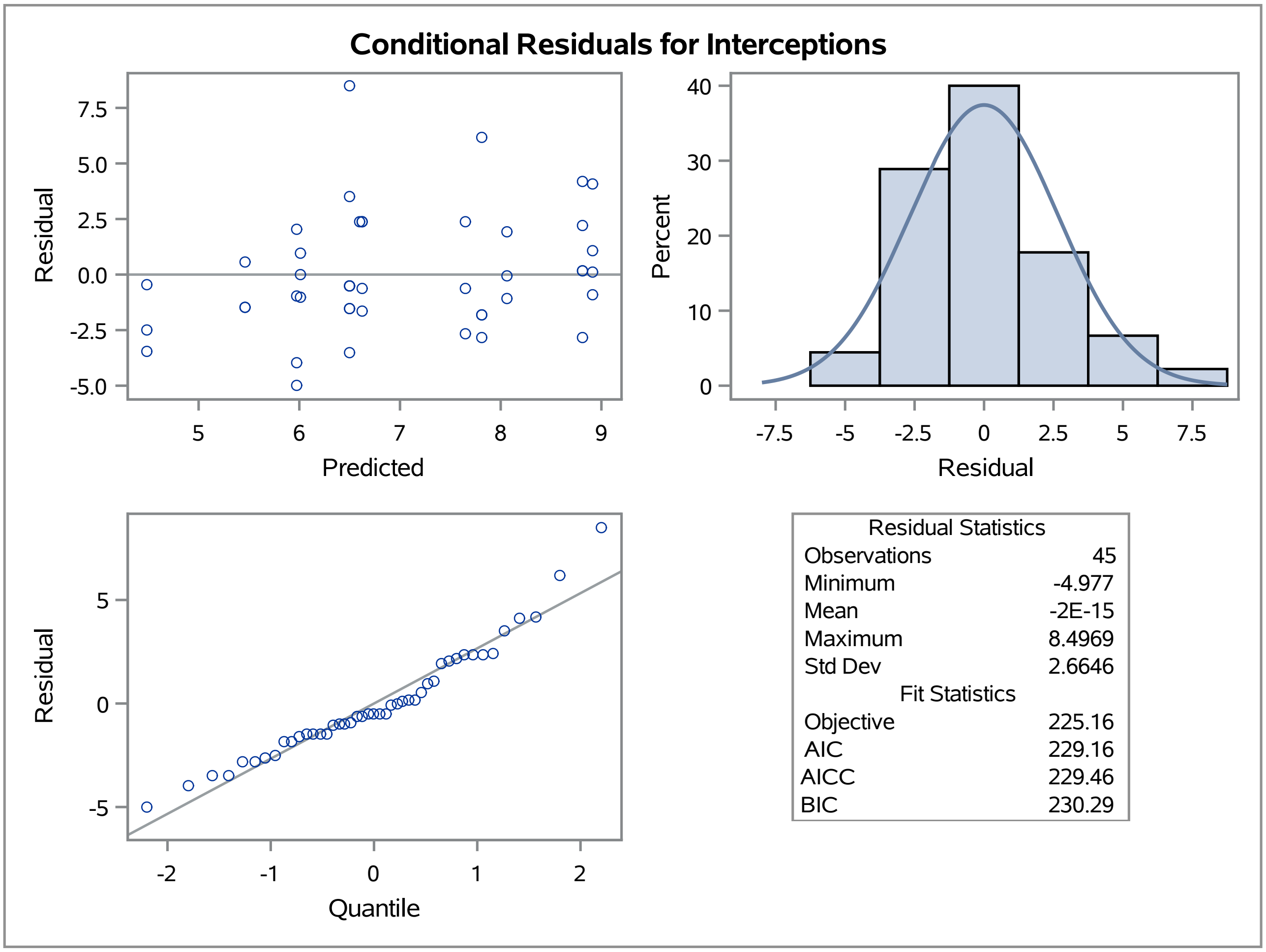

DistSoma=90

Model Information	
**Data Set**	WORK.TEMPDATASORTED
**Dependent Variable**	Interceptions
**Covariance Structure**	Variance Components
**Estimation Method**	REML
**Residual Variance Method**	Profile
**Fixed Effects SE Method**	Model-Based
**Degrees of Freedom Method**	Containment

Class Level Information		
**Class**	**Levels**	**Values**
**Treatment**	2	Control GFP Ctr Meg
**Culture**	13	1 2 3 4 5 6 7 8 9 10 11 12 13

Dimensions	
**Covariance Parameters**	2
**Columns in X**	3
**Columns in Z**	13
**Subjects**	1
**Max Obs per Subject**	45

Number of Observations	
**Number of Observations Read**	45
**Number of Observations Used**	45
**Number of Observations Not Used**	0

Iteration History			
**Iteration**	**Evaluations**	**-2 Res Log Like**	**Criterion**
**0**	1	224.66917174	
**1**	2	224.47607603	0.00000001

Convergence criteria met.

Covariance Parameter Estimates				
**Cov Parm**	**Estimate**	**Alpha**	**Lower**	**Upper**
**Culture**	0.5742	0.05	0.06954	1.3392E9
**Residual**	8.9091	0.05	5.7951	15.4398

DistSoma=90

Fit Statistics	
-2 Res Log Likelihood	224.5
AIC (Smaller is Better)	228.5
AICC (Smaller is Better)	228.8
BIC (Smaller is Better)	229.6

Solution for Fixed Effects

Effect	Treatment	Estimate	Standard Error	DF	t Value	Pr > |t|	Alpha	Lower	Upper
Intercept		7.5859	0.6968	11	10.89	<.0001	0.05	6.0523	9.1195
Treatment	Control GFP	-2.1830	0.9899	32	-2.21	0.0347	0.05	-4.1994	-0.1667
Treatment	Ctr Meg	0	.	.	.	.	.	.	.

Solution for Random Effects

Effect	Culture	Estimate	Std Err Pred	DF	t Value	Pr > |t|	Alpha	Lower	Upper
Culture	1	-0.4974	0.7030	32	-0.71	0.4844	0.05	-1.9292	0.9345
Culture	2	-0.1193	0.7030	32	-0.17	0.8663	0.05	-1.5512	1.3126
Culture	3	0.1736	0.6909	32	0.25	0.8032	0.05	-1.2336	1.5809
Culture	4	0.1736	0.6909	32	0.25	0.8032	0.05	-1.2336	1.5809
Culture	5	0.2249	0.6909	32	0.33	0.7469	0.05	-1.1824	1.6321
Culture	6	0.2178	0.7357	32	0.30	0.7691	0.05	-1.2808	1.7164
Culture	7	-0.1733	0.7030	32	-0.25	0.8068	0.05	-1.6052	1.2586
Culture	8	-0.06885	0.6906	32	-0.10	0.9212	0.05	-1.4755	1.3378
Culture	9	-0.5813	0.6906	32	-0.84	0.4062	0.05	-1.9880	0.8254
Culture	10	0.1211	0.7028	32	0.17	0.8643	0.05	-1.3105	1.5527
Culture	11	0.4421	0.6805	32	0.65	0.5205	0.05	-0.9440	1.8283
Culture	12	0.2898	0.6906	32	0.42	0.6775	0.05	-1.1169	1.6965
Culture	13	-0.2030	0.7028	32	-0.29	0.7746	0.05	-1.6345	1.2286

Type 3 Tests of Fixed Effects

Effect	Num DF	Den DF	F Value	Pr > F
Treatment	1	32	4.86	0.0347

Least Squares Means

Effect	Treatment	Estimate	Standard Error	DF	t Value	Pr > |t|	Alpha	Lower	Upper
Treatment	Control GFP	5.4029	0.7031	32	7.68	<.0001	0.05	3.9707	6.8351
Treatment	Ctr Meg	7.5859	0.6968	32	10.89	<.0001	0.05	6.1666	9.0052

DistSoma=90

Differences of Least Squares Means												
**Effect**	**Treatment**	**Treatment**	**Estimate**	**Standard Error**	**DF**	**t Value**	**Pr > |t|**	**Adjustment**	**Adj P**	**Alpha**	**Lower**	**Upper**
**Treatment**	Control GFP	Ctr Meg	-2.1830	0.9899	32	-2.21	0.0347	Tukey-Kramer	0.0347	0.05	-4.1994	-0.1667

Differences of Least Squares Means				
**Effect**	**Treatment**	**Treatment**	**Adj Lower**	**Adj Upper**
**Treatment**	Control GFP	Ctr Meg	-4.1993	-0.1667

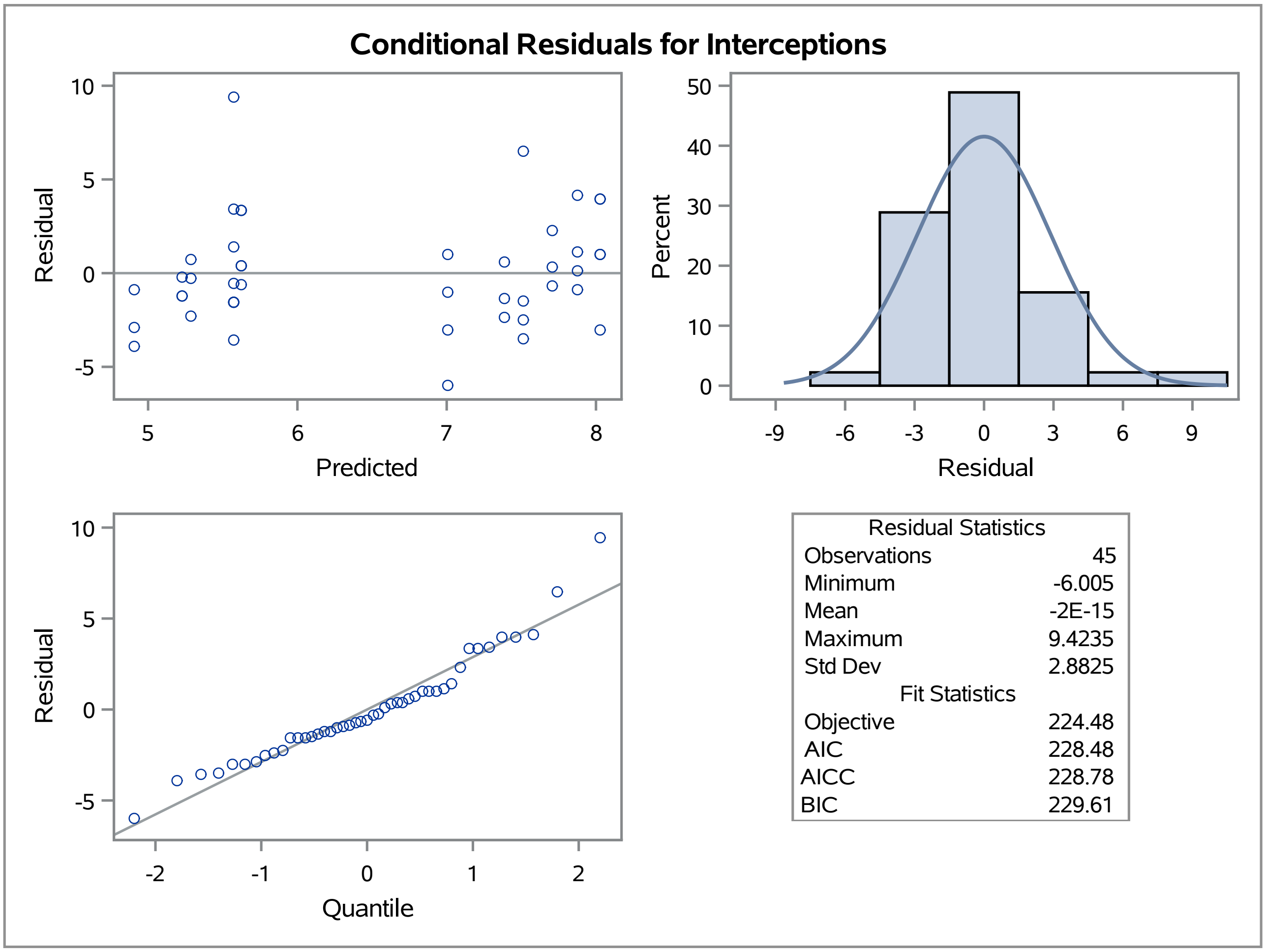

DistSoma=96

Model Information	
**Data Set**	WORK.TEMPDATASORTED
**Dependent Variable**	Interceptions
**Covariance Structure**	Variance Components
**Estimation Method**	REML
**Residual Variance Method**	Profile
**Fixed Effects SE Method**	Model-Based
**Degrees of Freedom Method**	Containment

Class Level Information		
**Class**	**Levels**	**Values**
**Treatment**	2	Control GFP Ctr Meg
**Culture**	13	1 2 3 4 5 6 7 8 9 10 11 12 13

Dimensions	
**Covariance Parameters**	2
**Columns in X**	3
**Columns in Z**	13
**Subjects**	1
**Max Obs per Subject**	45

Number of Observations	
**Number of Observations Read**	45
**Number of Observations Used**	45
**Number of Observations Not Used**	0

Iteration History			
**Iteration**	**Evaluations**	**-2 Res Log Like**	**Criterion**
**0**	1	230.77083340	
**1**	3	230.29528891	0.00000106
**2**	1	230.29520801	0.00000000

Convergence criteria met.

Covariance Parameter Estimates				
**Cov Parm**	**Estimate**	**Alpha**	**Lower**	**Upper**
**Culture**	1.0259	0.05	0.1749	16060
**Residual**	9.9381	0.05	6.4740	17.1813

DistSoma=96

Fit Statistics	
-2 Res Log Likelihood	230.3
AIC (Smaller is Better)	234.3
AICC (Smaller is Better)	234.6
BIC (Smaller is Better)	235.4

Solution for Fixed Effects

Effect	Treatment	Estimate	Standard Error	DF	t Value	Pr > |t|	Alpha	Lower	Upper
Intercept		7.4343	0.7791	11	9.54	<.0001	0.05	5.7195	9.1491
Treatment	Control GFP	-2.7448	1.1035	32	-2.49	0.0183	0.05	-4.9926	-0.4970
Treatment	Ctr Meg	0	.	.	.	.	.	.	.

Solution for Random Effects

Effect	Culture	Estimate	Std Err Pred	DF	t Value	Pr > |t|	Alpha	Lower	Upper
Culture	1	-0.6360	0.9042	32	-0.70	0.4869	0.05	-2.4777	1.2057
Culture	2	-0.2419	0.9042	32	-0.27	0.7908	0.05	-2.0836	1.5998
Culture	3	0.2369	0.8822	32	0.27	0.7900	0.05	-1.5601	2.0338
Culture	4	0.3099	0.8822	32	0.35	0.7277	0.05	-1.4870	2.1069
Culture	5	0.09074	0.8822	32	0.10	0.9187	0.05	-1.7062	1.8877
Culture	6	0.4033	0.9671	32	0.42	0.6794	0.05	-1.5666	2.3733
Culture	7	-0.1630	0.9042	32	-0.18	0.8580	0.05	-2.0047	1.6786
Culture	8	-0.1269	0.8820	32	-0.14	0.8865	0.05	-1.9235	1.6697
Culture	9	-1.0037	0.8820	32	-1.14	0.2636	0.05	-2.8003	0.7929
Culture	10	0.1338	0.9040	32	0.15	0.8833	0.05	-1.7077	1.9752
Culture	11	0.8054	0.8643	32	0.93	0.3584	0.05	-0.9552	2.5659
Culture	12	0.5306	0.8820	32	0.60	0.5517	0.05	-1.2660	2.3272
Culture	13	-0.3392	0.9040	32	-0.38	0.7100	0.05	-2.1806	1.5023

Type 3 Tests of Fixed Effects

Effect	Num DF	Den DF	F Value	Pr > F
Treatment	1	32	6.19	0.0183

Least Squares Means

Effect	Treatment	Estimate	Standard Error	DF	t Value	Pr > |t|	Alpha	Lower	Upper
Treatment	Control GFP	4.6895	0.7815	32	6.00	<.0001	0.05	3.0976	6.2814
Treatment	Ctr Meg	7.4343	0.7791	32	9.54	<.0001	0.05	5.8473	9.0213

DistSoma=96

Differences of Least Squares Means												
**Effect**	**Treatment**	**Treatment**	**Estimate**	**Standard Error**	**DF**	**t Value**	**Pr > |t|**	**Adjustment**	**Adj P**	**Alpha**	**Lower**	**Upper**
**Treatment**	Control GFP	Ctr Meg	-2.7448	1.1035	32	-2.49	0.0183	Tukey-Kramer	0.0183	0.05	-4.9926	-0.4970

Differences of Least Squares Means				
**Effect**	**Treatment**	**Treatment**	**Adj Lower**	**Adj Upper**
**Treatment**	Control GFP	Ctr Meg	-4.9926	-0.4970

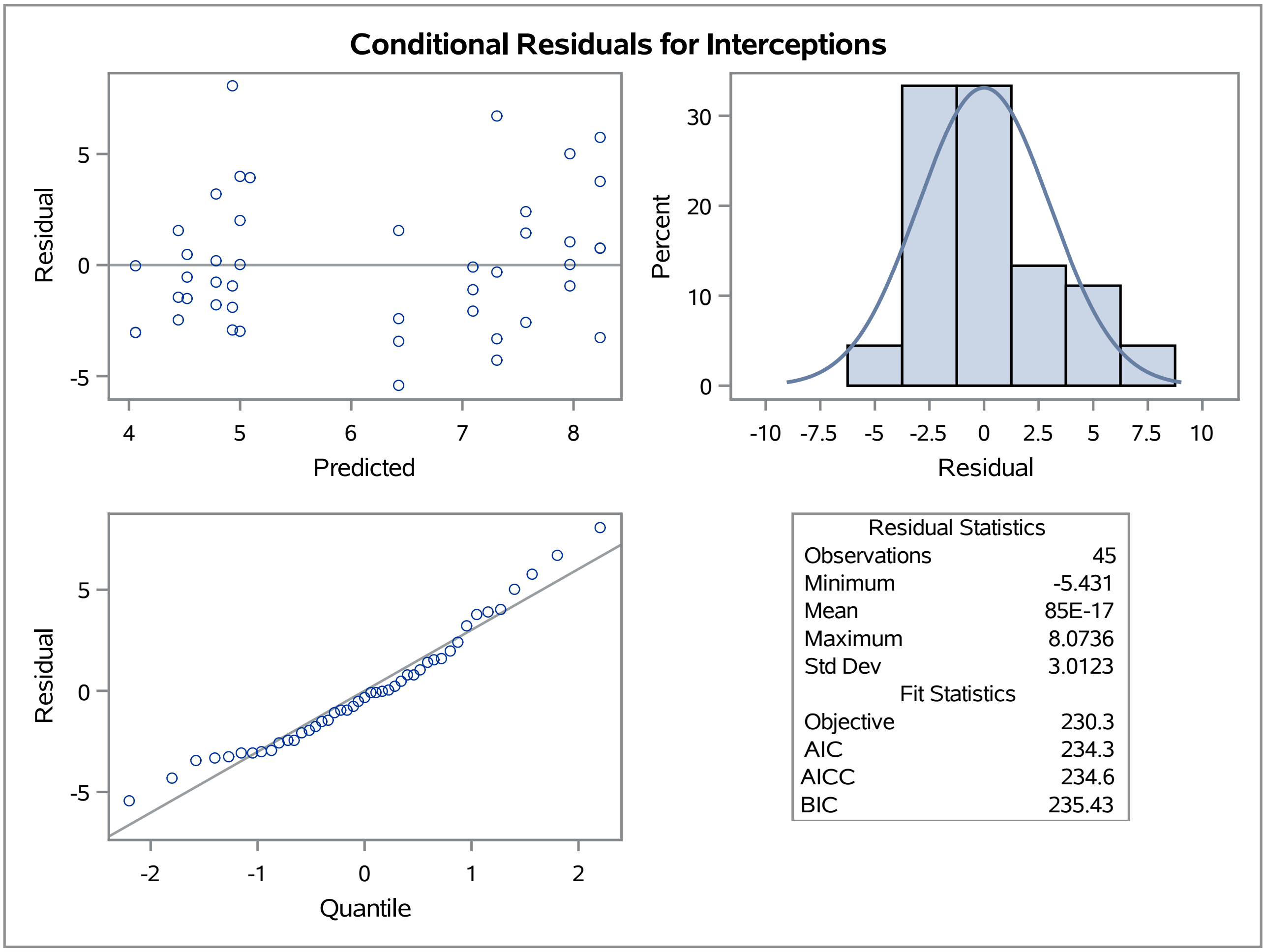

DistSoma=102

Model Information	
**Data Set**	WORK.TEMPDATASORTED
**Dependent Variable**	Interceptions
**Covariance Structure**	Variance Components
**Estimation Method**	REML
**Residual Variance Method**	Profile
**Fixed Effects SE Method**	Model-Based
**Degrees of Freedom Method**	Containment

Class Level Information		
**Class**	**Levels**	**Values**
**Treatment**	2	Control GFP Ctr Meg
**Culture**	13	1 2 3 4 5 6 7 8 9 10 11 12 13

Dimensions	
**Covariance Parameters**	2
**Columns in X**	3
**Columns in Z**	13
**Subjects**	1
**Max Obs per Subject**	45

Number of Observations	
**Number of Observations Read**	45
**Number of Observations Used**	45
**Number of Observations Not Used**	0

Iteration History			
**Iteration**	**Evaluations**	**-2 Res Log Like**	**Criterion**
**0**	1	231.88920389	
**1**	3	230.92013616	0.00003752
**2**	1	230.91719960	0.00000008
**3**	1	230.91719315	0.00000000

Convergence criteria met.

DistSoma=102

Covariance Parameter Estimates				
Cov Parm	Estimate	Alpha	Lower	Upper
Culture	1.4666	0.05	0.3282	332.24
Residual	9.8060	0.05	6.4035	16.8862

Fit Statistics	
-2 Res Log Likelihood	230.9
AIC (Smaller is Better)	234.9
AICC (Smaller is Better)	235.2
BIC (Smaller is Better)	236.0

Solution for Fixed Effects									
Effect	Treatment	Estimate	Standard Error	DF	t Value	Pr > |t|	Alpha	Lower	Upper
Intercept		7.1164	0.8221	11	8.66	<.0001	0.05	5.3070	8.9257
Treatment	Control GFP	-2.6087	1.1610	32	-2.25	0.0317	0.05	-4.9735	-0.2439
Treatment	Ctr Meg	0	.	.	.	.	.	.	.

Solution for Random Effects									
Effect	Culture	Estimate	Std Err Pred	DF	t Value	Pr > |t|	Alpha	Lower	Upper
Culture	1	-0.6734	1.0377	32	-0.65	0.5210	0.05	-2.7871	1.4403
Culture	2	-0.2605	1.0377	32	-0.25	0.8034	0.05	-2.3742	1.8532
Culture	3	0.1843	1.0059	32	0.18	0.8558	0.05	-1.8646	2.2332
Culture	4	0.4650	1.0059	32	0.46	0.6470	0.05	-1.5839	2.5139
Culture	5	0.09071	1.0059	32	0.09	0.9287	0.05	-1.9582	2.1396
Culture	6	0.4544	1.1345	32	0.40	0.6915	0.05	-1.8566	2.7653
Culture	7	-0.2605	1.0377	32	-0.25	0.8034	0.05	-2.3742	1.8532
Culture	8	-0.3243	1.0061	32	-0.32	0.7493	0.05	-2.3737	1.7251
Culture	9	-1.4472	1.0061	32	-1.44	0.1600	0.05	-3.4966	0.6022
Culture	10	0.1704	1.0379	32	0.16	0.8706	0.05	-1.9436	2.2845
Culture	11	1.1482	0.9812	32	1.17	0.2506	0.05	-0.8505	3.1469
Culture	12	0.7986	1.0061	32	0.79	0.4332	0.05	-1.2508	2.8480
Culture	13	-0.3458	1.0379	32	-0.33	0.7412	0.05	-2.4598	1.7683

Type 3 Tests of Fixed Effects				
Effect	Num DF	Den DF	F Value	Pr > F
Treatment	1	32	5.05	0.0317

DistSoma=102

Least Squares Means									
**Effect**	**Treatment**	**Estimate**	**Standard Error**	**DF**	**t Value**	**Pr > |t|**	**Alpha**	**Lower**	**Upper**
**Treatment**	Control GFP	4.5077	0.8198	32	5.50	<.0001	0.05	2.8378	6.1775
**Treatment**	Ctr Meg	7.1164	0.8221	32	8.66	<.0001	0.05	5.4419	8.7908

Differences of Least Squares Means												
**Effect**	**Treatment**	**Treatment**	**Estimate**	**Standard Error**	**DF**	**t Value**	**Pr > |t|**	**Adjustment**	**Adj P**	**Alpha**	**Lower**	**Upper**
**Treatment**	Control GFP	Ctr Meg	-2.6087	1.1610	32	-2.25	0.0317	Tukey-Kramer	0.0317	0.05	-4.9735	-0.2439

Differences of Least Squares Means				
**Effect**	**Treatment**	**Treatment**	**Adj Lower**	**Adj Upper**
**Treatment**	Control GFP	Ctr Meg	-4.9735	-0.2439

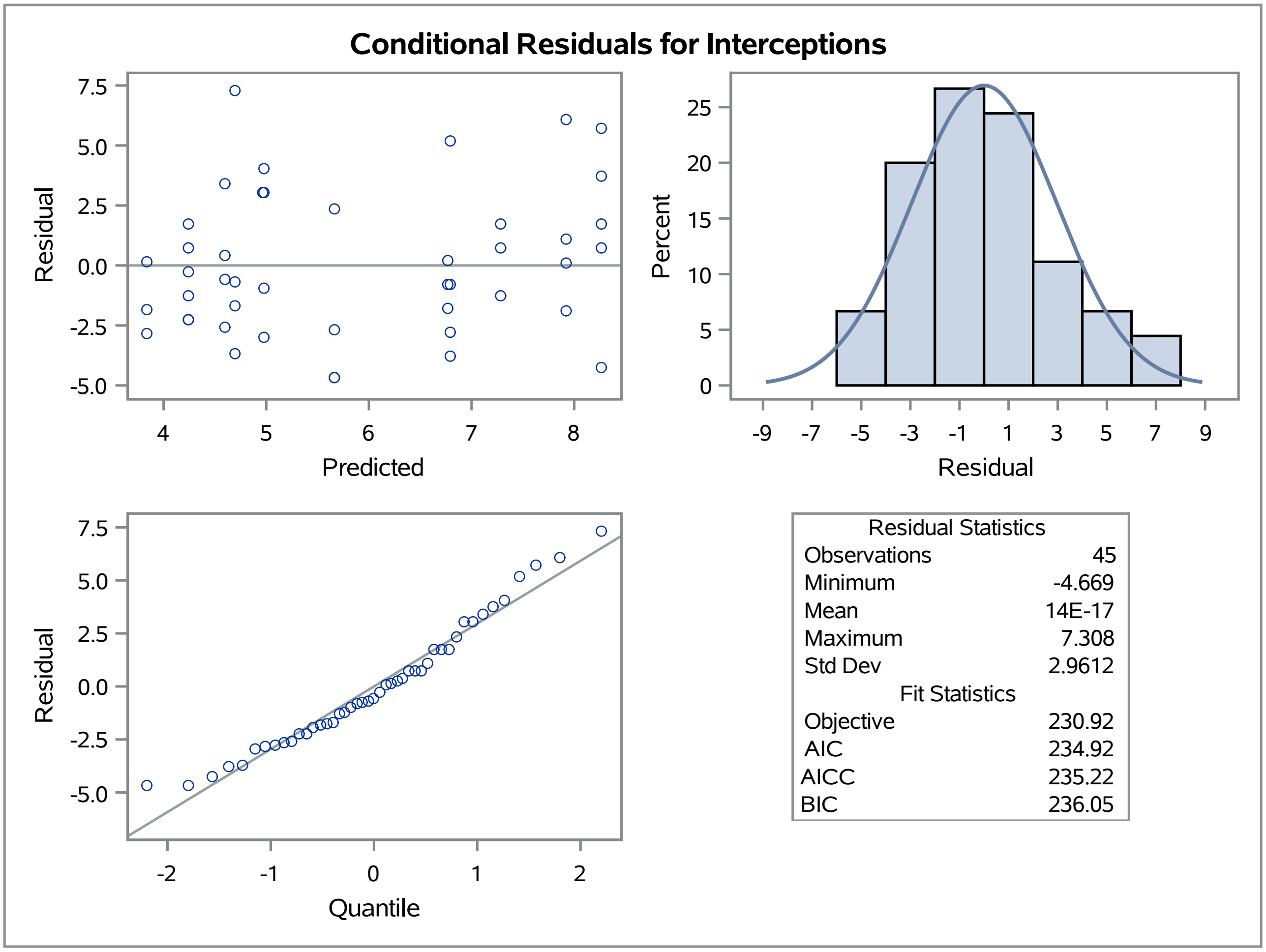

DistSoma=108

Model Information	
**Data Set**	WORK.TEMPDATASORTED
**Dependent Variable**	Interceptions
**Covariance Structure**	Variance Components
**Estimation Method**	REML
**Residual Variance Method**	Profile
**Fixed Effects SE Method**	Model-Based
**Degrees of Freedom Method**	Containment

Class Level Information		
**Class**	**Levels**	**Values**
**Treatment**	2	Control GFP Ctr Meg
**Culture**	13	1 2 3 4 5 6 7 8 9 10 11 12 13

Dimensions	
**Covariance Parameters**	2
**Columns in X**	3
**Columns in Z**	13
**Subjects**	1
**Max Obs per Subject**	45

Number of Observations	
**Number of Observations Read**	45
**Number of Observations Used**	45
**Number of Observations Not Used**	0

Iteration History			
**Iteration**	**Evaluations**	**-2 Res Log Like**	**Criterion**
**0**	1	231.88139387	
**1**	3	229.48017658	0.00010919
**2**	1	229.47155813	0.00000067
**3**	1	229.47150723	0.00000000

Convergence criteria met.

DistSoma=108

Covariance Parameter Estimates				
Cov Parm	Estimate	Alpha	Lower	Upper
Culture	2.4206	0.05	0.7289	47.8520
Residual	8.9209	0.05	5.8102	15.4276

Fit Statistics	
-2 Res Log Likelihood	229.5
AIC (Smaller is Better)	233.5
AICC (Smaller is Better)	233.8
BIC (Smaller is Better)	234.6

Solution for Fixed Effects									
Effect	Treatment	Estimate	Standard Error	DF	t Value	Pr > |t|	Alpha	Lower	Upper
Intercept		6.5770	0.8931	11	7.36	<.0001	0.05	4.6112	8.5428
Treatment	Control GFP	-2.0553	1.2543	32	-1.64	0.1111	0.05	-4.6102	0.4996
Treatment	Ctr Meg	0	.	.	.	.	.	.	.

Solution for Random Effects									
Effect	Culture	Estimate	Std Err Pred	DF	t Value	Pr > |t|	Alpha	Lower	Upper
Culture	1	-0.9820	1.2209	32	-0.80	0.4271	0.05	-3.4689	1.5048
Culture	2	-0.3837	1.2209	32	-0.31	0.7554	0.05	-2.8706	2.1032
Culture	3	0.1188	1.1708	32	0.10	0.9198	0.05	-2.2661	2.5037
Culture	4	0.3790	1.1708	32	0.32	0.7482	0.05	-2.0059	2.7640
Culture	5	0.5092	1.1708	32	0.43	0.6666	0.05	-1.8757	2.8941
Culture	6	0.7424	1.3926	32	0.53	0.5977	0.05	-2.0942	3.5790
Culture	7	-0.3837	1.2209	32	-0.31	0.7554	0.05	-2.8706	2.1032
Culture	8	-0.9509	1.1734	32	-0.81	0.4237	0.05	-3.3410	1.4392
Culture	9	-1.9918	1.1734	32	-1.70	0.0993	0.05	-4.3819	0.3983
Culture	10	0.3394	1.2227	32	0.28	0.7831	0.05	-2.1512	2.8300
Culture	11	1.5100	1.1364	32	1.33	0.1933	0.05	-0.8048	3.8248
Culture	12	1.6514	1.1734	32	1.41	0.1689	0.05	-0.7387	4.0415
Culture	13	-0.5581	1.2227	32	-0.46	0.6512	0.05	-3.0487	1.9325

Type 3 Tests of Fixed Effects				
Effect	Num DF	Den DF	F Value	Pr > F
Treatment	1	32	2.69	0.1111

DistSoma=108

Least Squares Means									
Effect	Treatment	Estimate	Standard Error	DF	t Value	Pr > |t|	Alpha	Lower	Upper
Treatment	Control GFP	4.5217	0.8807	32	5.13	<.0001	0.05	2.7279	6.3156
Treatment	Ctr Meg	6.5770	0.8931	32	7.36	<.0001	0.05	4.7578	8.3963

Differences of Least Squares Means												
Effect	Treatment	Treatment	Estimate	Standard Error	DF	t Value	Pr > |t|	Adjustment	Adj P	Alpha	Lower	Upper
Treatment	Control GFP	Ctr Meg	-2.0553	1.2543	32	-1.64	0.1111	Tukey-Kramer	0.1111	0.05	-4.6102	0.4996

Differences of Least Squares Means				
Effect	Treatment	Treatment	Adj Lower	Adj Upper
Treatment	Control GFP	Ctr Meg	-4.6102	0.4996

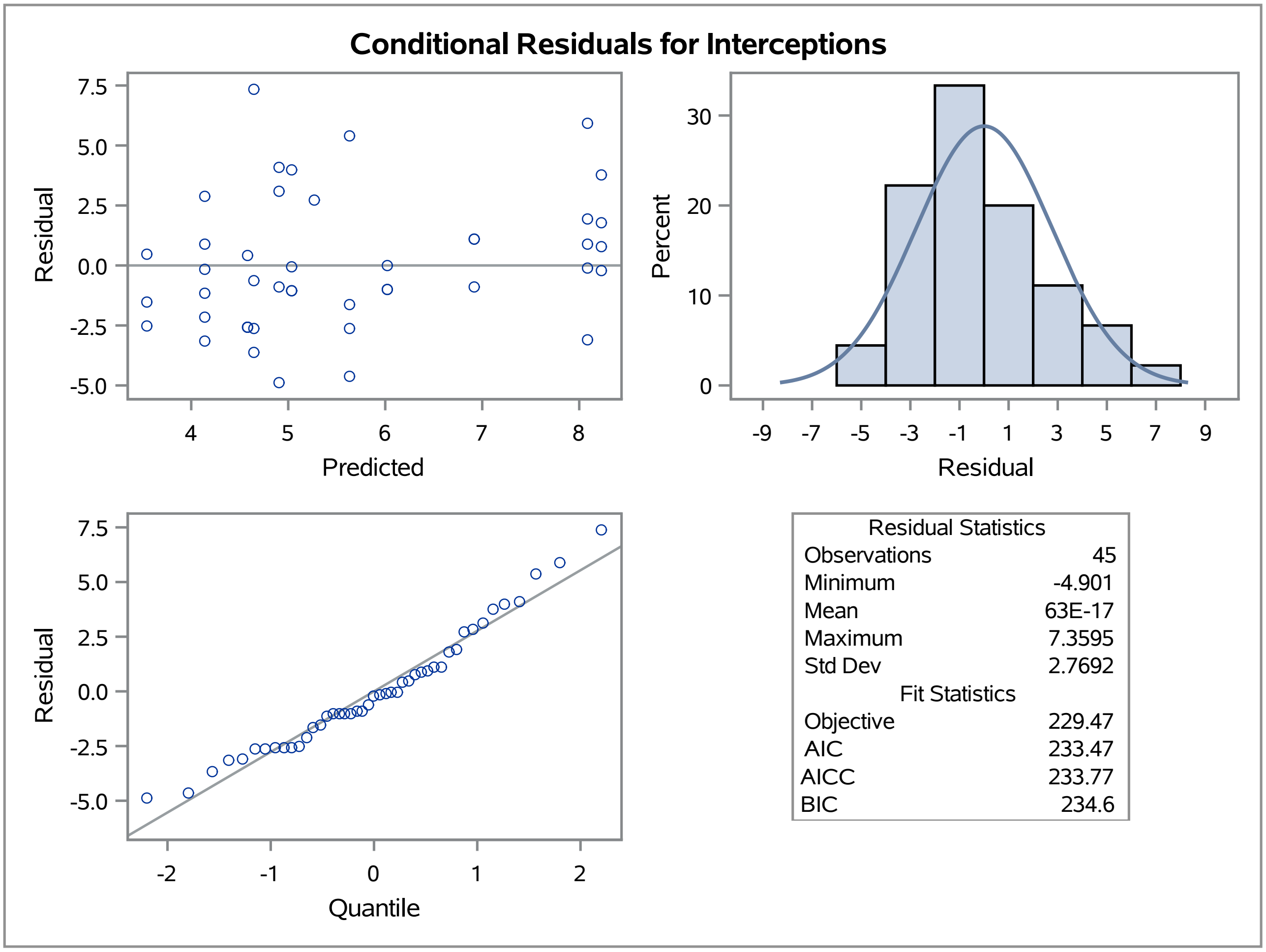

DistSoma=114

Model Information	
**Data Set**	WORK.TEMPDATASORTED
**Dependent Variable**	Interceptions
**Covariance Structure**	Variance Components
**Estimation Method**	REML
**Residual Variance Method**	Profile
**Fixed Effects SE Method**	Model-Based
**Degrees of Freedom Method**	Containment

Class Level Information		
**Class**	**Levels**	**Values**
**Treatment**	2	Control GFP Ctr Meg
**Culture**	13	1 2 3 4 5 6 7 8 9 10 11 12 13

Dimensions	
**Covariance Parameters**	2
**Columns in X**	3
**Columns in Z**	13
**Subjects**	1
**Max Obs per Subject**	45

Number of Observations	
**Number of Observations Read**	45
**Number of Observations Used**	45
**Number of Observations Not Used**	0

Iteration History			
**Iteration**	**Evaluations**	**-2 Res Log Like**	**Criterion**
**0**	1	236.67461508	
**1**	3	235.87560451	0.00002346
**2**	1	235.87372004	0.00000003
**3**	1	235.87371743	0.00000000

Convergence criteria met.

DistSoma=114

Covariance Parameter Estimates				
Cov Parm	Estimate	Alpha	Lower	Upper
Culture	1.4757	0.05	0.3091	743.25
Residual	11.1114	0.05	7.2623	19.1063

Fit Statistics	
-2 Res Log Likelihood	235.9
AIC (Smaller is Better)	239.9
AICC (Smaller is Better)	240.2
BIC (Smaller is Better)	241.0

Solution for Fixed Effects									
Effect	Treatment	Estimate	Standard Error	DF	t Value	Pr > |t|	Alpha	Lower	Upper
Intercept		6.1272	0.8569	11	7.15	<.0001	0.05	4.2412	8.0132
Treatment	Control GFP	-1.8990	1.2114	32	-1.57	0.1268	0.05	-4.3665	0.5685
Treatment	Ctr Meg	0	.	.	.	.	.	.	.

Solution for Random Effects									
Effect	Culture	Estimate	Std Err Pred	DF	t Value	Pr > |t|	Alpha	Lower	Upper
Culture	1	-0.5399	1.0558	32	-0.51	0.6126	0.05	-2.6905	1.6108
Culture	2	-0.1600	1.0558	32	-0.15	0.8805	0.05	-2.3106	1.9907
Culture	3	0.09430	1.0257	32	0.09	0.9273	0.05	-1.9949	2.1835
Culture	4	0.3545	1.0257	32	0.35	0.7319	0.05	-1.7347	2.4437
Culture	5	0.1810	1.0257	32	0.18	0.8610	0.05	-1.9082	2.2702
Culture	6	0.3250	1.1458	32	0.28	0.7785	0.05	-2.0089	2.6588
Culture	7	-0.2550	1.0558	32	-0.24	0.8107	0.05	-2.4056	1.8957
Culture	8	-0.6513	1.0257	32	-0.63	0.5300	0.05	-2.7406	1.4381
Culture	9	-1.2584	1.0257	32	-1.23	0.2288	0.05	-3.3477	0.8309
Culture	10	0.1537	1.0559	32	0.15	0.8852	0.05	-1.9970	2.3044
Culture	11	0.9070	1.0019	32	0.91	0.3721	0.05	-1.1338	2.9477
Culture	12	1.1701	1.0257	32	1.14	0.2624	0.05	-0.9192	3.2595
Culture	13	-0.3212	1.0559	32	-0.30	0.7630	0.05	-2.4719	1.8296

Type 3 Tests of Fixed Effects				
Effect	Num DF	Den DF	F Value	Pr > F
Treatment	1	32	2.46	0.1268

DistSoma=114

Least Squares Means									
**Effect**	**Treatment**	**Estimate**	**Standard Error**	**DF**	**t Value**	**Pr > |t|**	**Alpha**	**Lower**	**Upper**
**Treatment**	Control GFP	4.2282	0.8563	32	4.94	<.0001	0.05	2.4841	5.9723
**Treatment**	Ctr Meg	6.1272	0.8569	32	7.15	<.0001	0.05	4.3817	7.8727

Differences of Least Squares Means												
**Effect**	**Treatment**	**Treatment**	**Estimate**	**Standard Error**	**DF**	**t Value**	**Pr > |t|**	**Adjustment**	**Adj P**	**Alpha**	**Lower**	**Upper**
**Treatment**	Control GFP	Ctr Meg	-1.8990	1.2114	32	-1.57	0.1268	Tukey-Kramer	0.1268	0.05	-4.3665	0.5685

Differences of Least Squares Means				
**Effect**	**Treatment**	**Treatment**	**Adj Lower**	**Adj Upper**
**Treatment**	Control GFP	Ctr Meg	-4.3665	0.5685

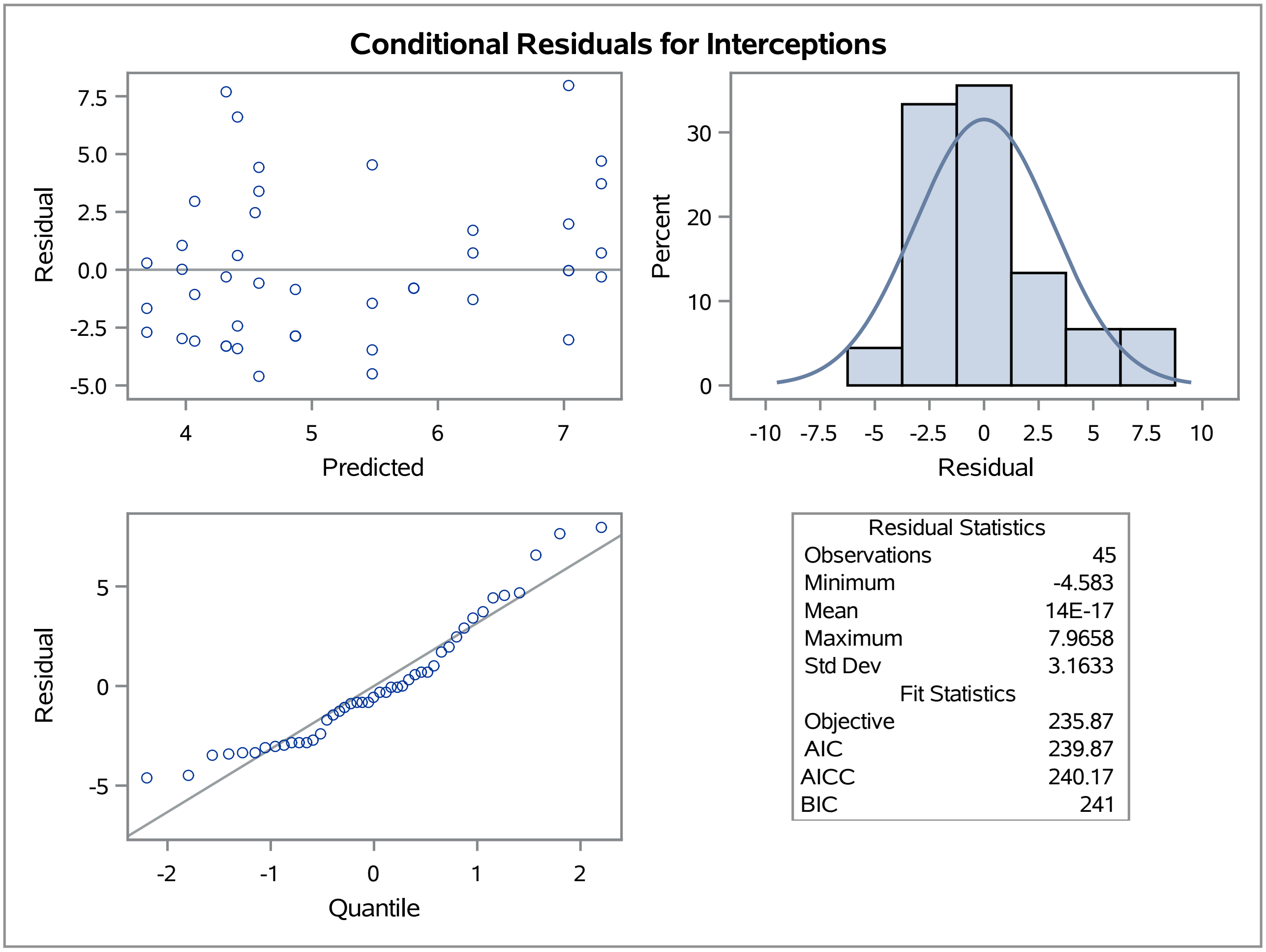

DistSoma=120

Model Information	
**Data Set**	WORK.TEMPDATASORTED
**Dependent Variable**	Interceptions
**Covariance Structure**	Variance Components
**Estimation Method**	REML
**Residual Variance Method**	Profile
**Fixed Effects SE Method**	Model-Based
**Degrees of Freedom Method**	Containment

Class Level Information		
**Class**	**Levels**	**Values**
**Treatment**	2	Control GFP Ctr Meg
**Culture**	13	1 2 3 4 5 6 7 8 9 10 11 12 13

Dimensions	
**Covariance Parameters**	2
**Columns in X**	3
**Columns in Z**	13
**Subjects**	1
**Max Obs per Subject**	45

Number of Observations	
**Number of Observations Read**	45
**Number of Observations Used**	45
**Number of Observations Not Used**	0

Iteration History			
**Iteration**	**Evaluations**	**-2 Res Log Like**	**Criterion**
**0**	1	235.00450225	
**1**	3	234.32237308	0.00006873
**2**	1	234.31682463	0.00000027
**3**	1	234.31680365	0.00000000

Convergence criteria met.

DistSoma=120

Covariance Parameter Estimates				
**Cov Parm**	**Estimate**	**Alpha**	**Lower**	**Upper**
**Culture**	1.2611	0.05	0.2495	1404.45
**Residual**	10.8220	0.05	7.0919	18.5290

Fit Statistics	
**-2 Res Log Likelihood**	234.3
**AIC (Smaller is Better)**	238.3
**AICC (Smaller is Better)**	238.6
**BIC (Smaller is Better)**	239.4

Solution for Fixed Effects									
**Effect**	**Treatment**	**Estimate**	**Standard Error**	**DF**	**t Value**	**Pr > |t|**	**Alpha**	**Lower**	**Upper**
**Intercept**		5.8542	0.8279	11	7.07	<.0001	0.05	4.0320	7.6763
**Treatment**	Control GFP	-1.9213	1.1715	32	-1.64	0.1108	0.05	-4.3077	0.4650
**Treatment**	Ctr Meg	0	.	.	.	.	.	.	.

Solution for Random Effects									
**Effect**	**Culture**	**Estimate**	**Std Err Pred**	**DF**	**t Value**	**Pr > |t|**	**Alpha**	**Lower**	**Upper**
**Culture**	1	-0.5007	0.9902	32	-0.51	0.6166	0.05	-2.5177	1.5163
**Culture**	2	0.01739	0.9902	32	0.02	0.9861	0.05	-1.9996	2.0344
**Culture**	3	0.1008	0.9642	32	0.10	0.9174	0.05	-1.8631	2.0647
**Culture**	4	0.3393	0.9642	32	0.35	0.7272	0.05	-1.6246	2.3032
**Culture**	5	0.2598	0.9642	32	0.27	0.7893	0.05	-1.7041	2.2237
**Culture**	6	0.1114	1.0663	32	0.10	0.9175	0.05	-2.0605	2.2833
**Culture**	7	-0.3280	0.9902	32	-0.33	0.7426	0.05	-2.3450	1.6890
**Culture**	8	-0.5100	0.9641	32	-0.53	0.6004	0.05	-2.4737	1.4537
**Culture**	9	-1.0664	0.9641	32	-1.11	0.2769	0.05	-3.0301	0.8974
**Culture**	10	0.1241	0.9901	32	0.13	0.9010	0.05	-1.8927	2.1410
**Culture**	11	1.0845	0.9432	32	1.15	0.2588	0.05	-0.8368	3.0058
**Culture**	12	0.7617	0.9641	32	0.79	0.4353	0.05	-1.2020	2.7254
**Culture**	13	-0.3939	0.9901	32	-0.40	0.6934	0.05	-2.4108	1.6229

Type 3 Tests of Fixed Effects				
**Effect**	**Num DF**	**Den DF**	**F Value**	**Pr > F**
**Treatment**	1	32	2.69	0.1108

DistSoma=120

Least Squares Means									
Effect	Treatment	Estimate	Standard Error	DF	t Value	Pr > |t|	Alpha	Lower	Upper
Treatment	Control GFP	3.9329	0.8289	32	4.74	<.0001	0.05	2.2444	5.6214
Treatment	Ctr Meg	5.8542	0.8279	32	7.07	<.0001	0.05	4.1678	7.5405

Differences of Least Squares Means												
Effect	Treatment	Treatment	Estimate	Standard Error	DF	t Value	Pr > |t|	Adjustment	Adj P	Alpha	Lower	Upper
Treatment	Control GFP	Ctr Meg	-1.9213	1.1715	32	-1.64	0.1108	Tukey-Kramer	0.1108	0.05	-4.3077	0.4650

Differences of Least Squares Means				
Effect	Treatment	Treatment	Adj Lower	Adj Upper
Treatment	Control GFP	Ctr Meg	-4.3076	0.4650

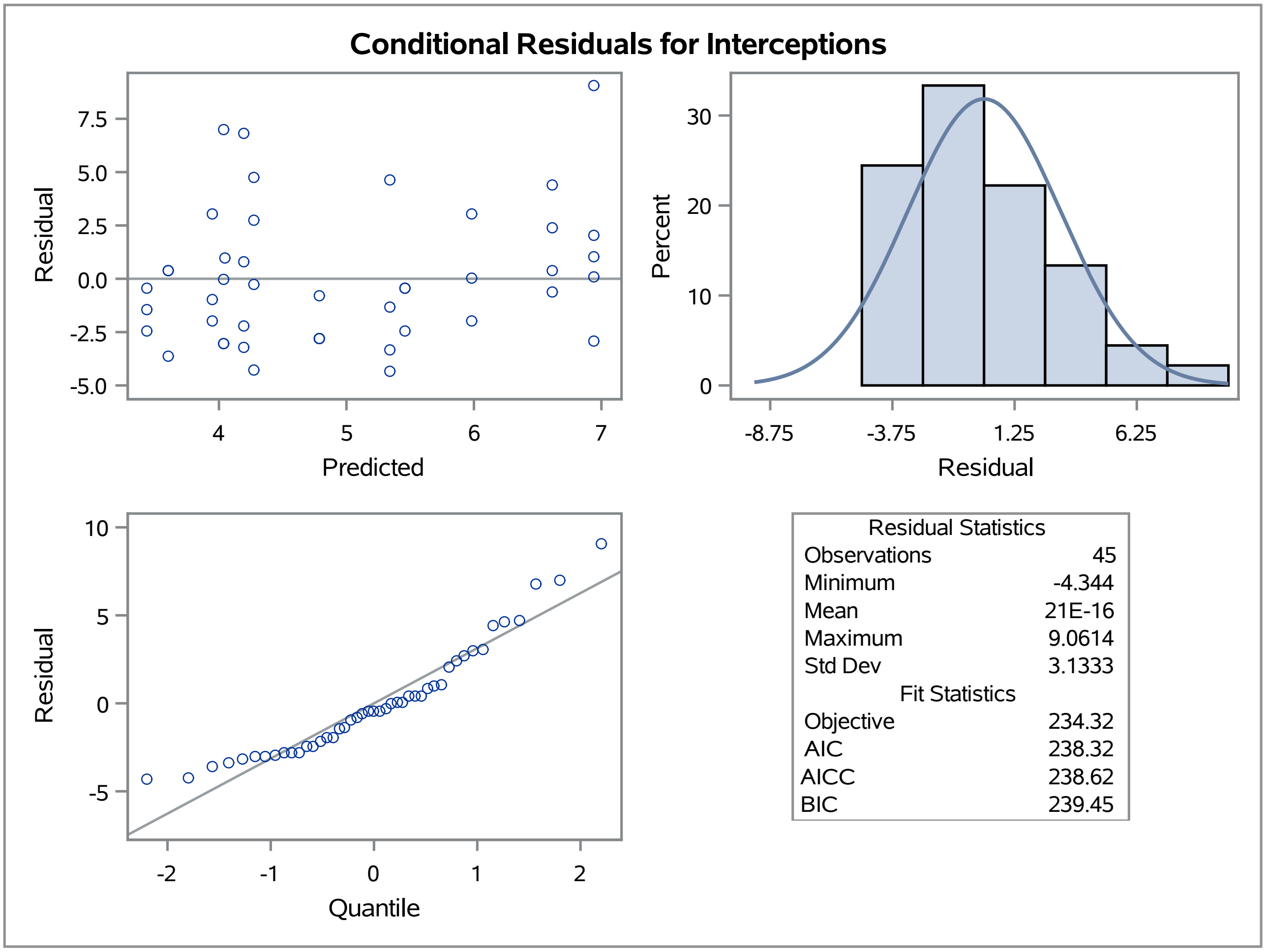

DistSoma=126

Model Information	
**Data Set**	WORK.TEMPDATASORTED
**Dependent Variable**	Interceptions
**Covariance Structure**	Variance Components
**Estimation Method**	REML
**Residual Variance Method**	Profile
**Fixed Effects SE Method**	Model-Based
**Degrees of Freedom Method**	Containment

Class Level Information		
**Class**	**Levels**	**Values**
**Treatment**	2	Control GFP Ctr Meg
**Culture**	13	1 2 3 4 5 6 7 8 9 10 11 12 13

Dimensions	
**Covariance Parameters**	2
**Columns in X**	3
**Columns in Z**	13
**Subjects**	1
**Max Obs per Subject**	45

Number of Observations	
**Number of Observations Read**	45
**Number of Observations Used**	45
**Number of Observations Not Used**	0

Iteration History			
**Iteration**	**Evaluations**	**-2 Res Log Like**	**Criterion**
**0**	1	228.69154199	
**1**	3	227.97744197	0.00001399
**2**	1	227.97638104	0.00000001
**3**	1	227.97638021	0.00000000

Convergence criteria met.

DistSoma=126

Covariance Parameter Estimates				
Cov Parm	Estimate	Alpha	Lower	Upper
Culture	1.1628	0.05	0.2339	1019.60
Residual	9.2893	0.05	6.0705	15.9773

Fit Statistics	
-2 Res Log Likelihood	228.0
AIC (Smaller is Better)	232.0
AICC (Smaller is Better)	232.3
BIC (Smaller is Better)	233.1

Solution for Fixed Effects									
Effect	Treatment	Estimate	Standard Error	DF	t Value	Pr > |t|	Alpha	Lower	Upper
Intercept		5.5978	0.7758	11	7.22	<.0001	0.05	3.8902	7.3053
Treatment	Control GFP	-1.8059	1.0973	32	-1.65	0.1096	0.05	-4.0410	0.4292
Treatment	Ctr Meg	0	.	.	.	.	.	.	.

Solution for Random Effects									
Effect	Culture	Estimate	Std Err Pred	DF	t Value	Pr > |t|	Alpha	Lower	Upper
Culture	1	-0.3982	0.9435	32	-0.42	0.6758	0.05	-2.3201	1.5237
Culture	2	0.05683	0.9435	32	0.06	0.9524	0.05	-1.8651	1.9787
Culture	3	0.06945	0.9175	32	0.08	0.9401	0.05	-1.7995	1.9384
Culture	4	0.2363	0.9175	32	0.26	0.7984	0.05	-1.6327	2.1052
Culture	5	0.3197	0.9175	32	0.35	0.7298	0.05	-1.5493	2.1887
Culture	6	0.02316	1.0203	32	0.02	0.9820	0.05	-2.0550	2.1013
Culture	7	-0.3072	0.9435	32	-0.33	0.7469	0.05	-2.2291	1.6147
Culture	8	-0.3663	0.9175	32	-0.40	0.6924	0.05	-2.2352	1.5027
Culture	9	-1.0336	0.9175	32	-1.13	0.2683	0.05	-2.9025	0.8354
Culture	10	0.3828	0.9435	32	0.41	0.6876	0.05	-1.5391	2.3047
Culture	11	0.9248	0.8969	32	1.03	0.3102	0.05	-0.9021	2.7516
Culture	12	0.8015	0.9175	32	0.87	0.3889	0.05	-1.0674	2.6705
Culture	13	-0.7092	0.9435	32	-0.75	0.4577	0.05	-2.6311	1.2127

Type 3 Tests of Fixed Effects				
Effect	Num DF	Den DF	F Value	Pr > F
Treatment	1	32	2.71	0.1096

DistSoma=126

Least Squares Means									
**Effect**	**Treatment**	**Estimate**	**Standard Error**	**DF**	**t Value**	**Pr > |t|**	**Alpha**	**Lower**	**Upper**
**Treatment**	Control GFP	3.7919	0.7760	32	4.89	<.0001	0.05	2.2113	5.3724
**Treatment**	Ctr Meg	5.5978	0.7758	32	7.22	<.0001	0.05	4.0175	7.1781

Differences of Least Squares Means												
**Effect**	**Treatment**	**Treatment**	**Estimate**	**Standard Error**	**DF**	**t Value**	**Pr > |t|**	**Adjustment**	**Adj P**	**Alpha**	**Lower**	**Upper**
**Treatment**	Control GFP	Ctr Meg	-1.8059	1.0973	32	-1.65	0.1096	Tukey-Kramer	0.1096	0.05	-4.0410	0.4292

Differences of Least Squares Means				
**Effect**	**Treatment**	**Treatment**	**Adj Lower**	**Adj Upper**
**Treatment**	Control GFP	Ctr Meg	-4.0410	0.4291

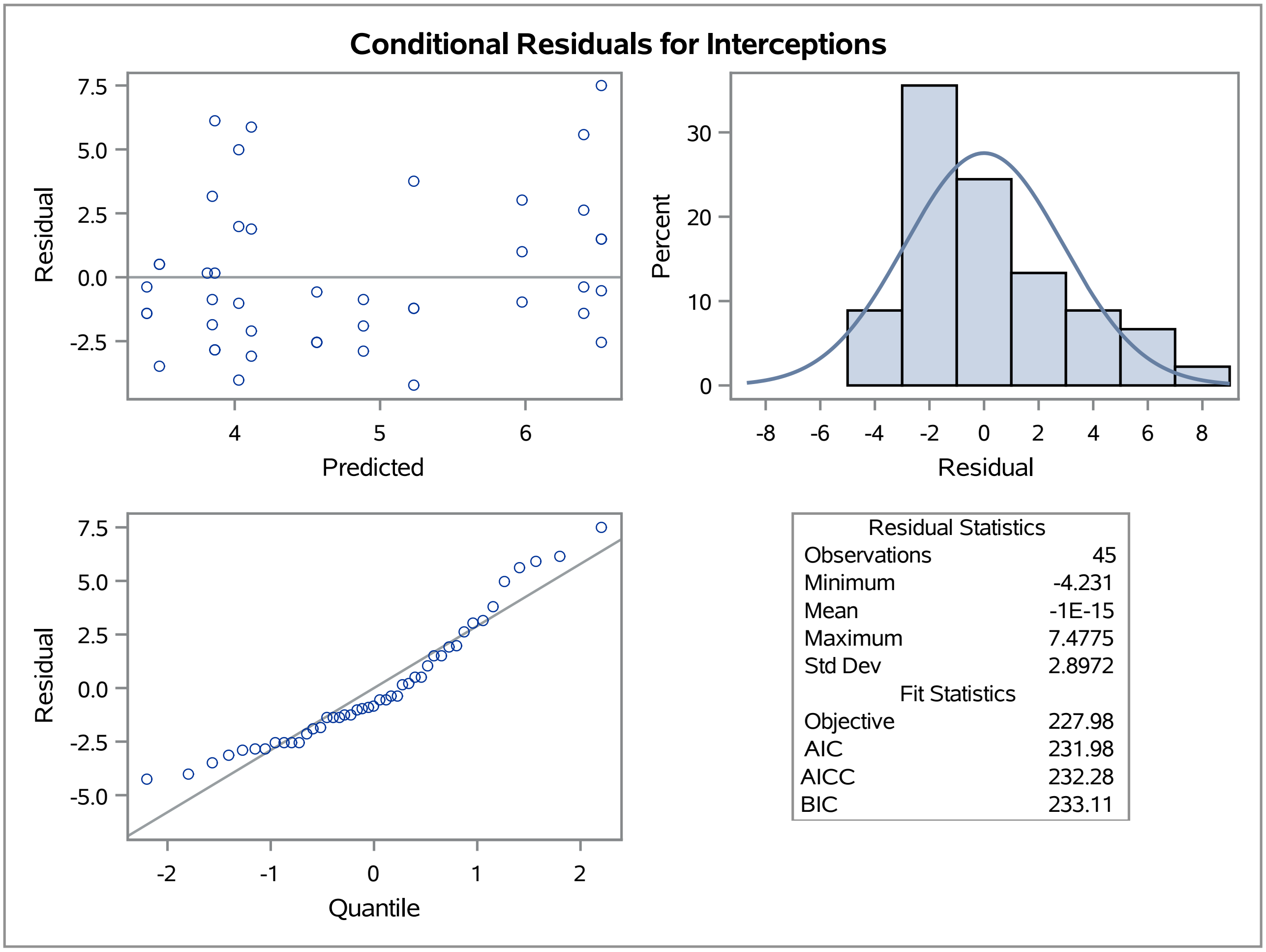

DistSoma=132

Model Information	
**Data Set**	WORK.TEMPDATASORTED
**Dependent Variable**	Interceptions
**Covariance Structure**	Variance Components
**Estimation Method**	REML
**Residual Variance Method**	Profile
**Fixed Effects SE Method**	Model-Based
**Degrees of Freedom Method**	Containment

Class Level Information		
**Class**	**Levels**	**Values**
**Treatment**	2	Control GFP Ctr Meg
**Culture**	13	1 2 3 4 5 6 7 8 9 10 11 12 13

Dimensions	
**Covariance Parameters**	2
**Columns in X**	3
**Columns in Z**	13
**Subjects**	1
**Max Obs per Subject**	45

Number of Observations	
**Number of Observations Read**	45
**Number of Observations Used**	45
**Number of Observations Not Used**	0

Iteration History			
**Iteration**	**Evaluations**	**-2 Res Log Like**	**Criterion**
**0**	1	227.92984223	
**1**	3	227.16652217	0.00000983
**2**	1	227.16578245	0.00000001

Convergence criteria met.

Covariance Parameter Estimates				
**Cov Parm**	**Estimate**	**Alpha**	**Lower**	**Upper**
**Culture**	1.1948	0.05	0.2463	743.46
**Residual**	9.0811	0.05	5.9298	15.6389

DistSoma=132

Fit Statistics	
-2 Res Log Likelihood	227.2
AIC (Smaller is Better)	231.2
AICC (Smaller is Better)	231.5
BIC (Smaller is Better)	232.3

Solution for Fixed Effects

Effect	Treatment	Estimate	Standard Error	DF	t Value	Pr > |t|	Alpha	Lower	Upper
Intercept		5.1509	0.7734	11	6.66	<.0001	0.05	3.4486	6.8533
Treatment	Control GFP	-1.4933	1.0935	32	-1.37	0.1816	0.05	-3.7207	0.7340
Treatment	Ctr Meg	0	.	.	.	.	.	.	.

Solution for Random Effects

Effect	Culture	Estimate	Std Err Pred	DF	t Value	Pr > |t|	Alpha	Lower	Upper
Culture	1	-0.3748	0.9511	32	-0.39	0.6962	0.05	-2.3120	1.5625
Culture	2	0.002571	0.9511	32	0.00	0.9979	0.05	-1.9347	1.9398
Culture	3	-0.05434	0.9240	32	-0.06	0.9535	0.05	-1.9366	1.8279
Culture	4	0.3767	0.9240	32	0.41	0.6862	0.05	-1.5055	2.2589
Culture	5	0.2905	0.9240	32	0.31	0.7553	0.05	-1.5917	2.1727
Culture	6	0.03981	1.0315	32	0.04	0.9695	0.05	-2.0613	2.1409
Culture	7	-0.2804	0.9511	32	-0.29	0.7700	0.05	-2.2177	1.6568
Culture	8	-0.2244	0.9241	32	-0.24	0.8096	0.05	-2.1068	1.6579
Culture	9	-1.0003	0.9241	32	-1.08	0.2871	0.05	-2.8826	0.8820
Culture	10	0.3346	0.9511	32	0.35	0.7273	0.05	-1.6027	2.2719
Culture	11	0.9718	0.9027	32	1.08	0.2897	0.05	-0.8669	2.8106
Culture	12	0.8100	0.9241	32	0.88	0.3873	0.05	-1.0723	2.6923
Culture	13	-0.8917	0.9511	32	-0.94	0.3555	0.05	-2.8290	1.0456

Type 3 Tests of Fixed Effects

Effect	Num DF	Den DF	F Value	Pr > F
Treatment	1	32	1.87	0.1816

Least Squares Means

Effect	Treatment	Estimate	Standard Error	DF	t Value	Pr > |t|	Alpha	Lower	Upper
Treatment	Control GFP	3.6576	0.7730	32	4.73	<.0001	0.05	2.0831	5.2321
Treatment	Ctr Meg	5.1509	0.7734	32	6.66	<.0001	0.05	3.5755	6.7264

DistSoma=132

Differences of Least Squares Means												
**Effect**	**Treatment**	**Treatment**	**Estimate**	**Standard Error**	**DF**	**t Value**	**Pr > |t|**	**Adjustment**	**Adj P**	**Alpha**	**Lower**	**Upper**
**Treatment**	Control GFP	Ctr Meg	-1.4933	1.0935	32	-1.37	0.1816	Tukey-Kramer	0.1816	0.05	-3.7207	0.7340

Differences of Least Squares Means				
**Effect**	**Treatment**	**Treatment**	**Adj Lower**	**Adj Upper**
**Treatment**	Control GFP	Ctr Meg	-3.7207	0.7340

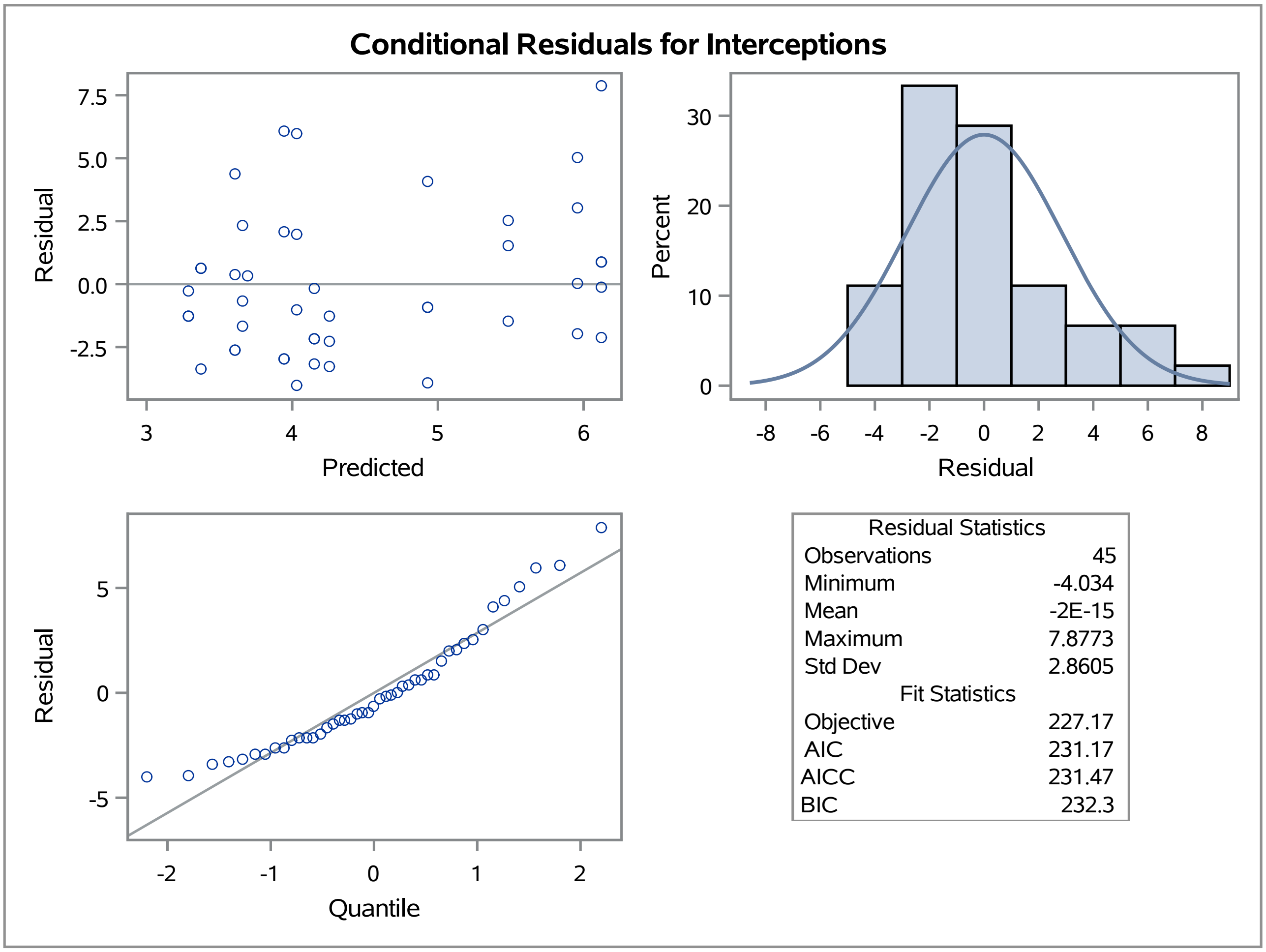

Residual Statistics	
Observations	45
Minimum	-4.034
Mean	-2E-15
Maximum	7.8773
Std Dev	2.8605
**Fit Statistics**	
Objective	227.17
AIC	231.17
AICC	231.47
BIC	232.3

DistSoma=138

Model Information	
**Data Set**	WORK.TEMPDATASORTED
**Dependent Variable**	Interceptions
**Covariance Structure**	Variance Components
**Estimation Method**	REML
**Residual Variance Method**	Profile
**Fixed Effects SE Method**	Model-Based
**Degrees of Freedom Method**	Containment

Class Level Information		
**Class**	**Levels**	**Values**
**Treatment**	2	Control GFP Ctr Meg
**Culture**	13	1 2 3 4 5 6 7 8 9 10 11 12 13

Dimensions	
**Covariance Parameters**	2
**Columns in X**	3
**Columns in Z**	13
**Subjects**	1
**Max Obs per Subject**	45

Number of Observations	
**Number of Observations Read**	45
**Number of Observations Used**	45
**Number of Observations Not Used**	0

Iteration History			
**Iteration**	**Evaluations**	**-2 Res Log Like**	**Criterion**
**0**	1	224.51269704	
**1**	3	223.47702808	0.00002778
**2**	1	223.47497139	0.00000004
**3**	1	223.47496835	0.00000000

Convergence criteria met.

DistSoma=138

Covariance Parameter Estimates				
Cov Parm	Estimate	Alpha	Lower	Upper
Culture	1.2875	0.05	0.2969	211.06
Residual	8.2143	0.05	5.3645	14.1431

Fit Statistics	
-2 Res Log Likelihood	223.5
AIC (Smaller is Better)	227.5
AICC (Smaller is Better)	227.8
BIC (Smaller is Better)	228.6

Solution for Fixed Effects									
Effect	Treatment	Estimate	Standard Error	DF	t Value	Pr > |t|	Alpha	Lower	Upper
Intercept		4.8316	0.7590	11	6.37	<.0001	0.05	3.1611	6.5021
Treatment	Control GFP	-1.3434	1.0714	32	-1.25	0.2190	0.05	-3.5259	0.8390
Treatment	Ctr Meg	0	.	.	.	.	.	.	.

Solution for Random Effects									
Effect	Culture	Estimate	Std Err Pred	DF	t Value	Pr > |t|	Alpha	Lower	Upper
Culture	1	-0.3693	0.9665	32	-0.38	0.7049	0.05	-2.3381	1.5994
Culture	2	0.1637	0.9665	32	0.17	0.8666	0.05	-1.8051	2.1325
Culture	3	-0.1881	0.9361	32	-0.20	0.8420	0.05	-2.0949	1.7186
Culture	4	0.1009	0.9361	32	0.11	0.9148	0.05	-1.8059	2.0077
Culture	5	0.4862	0.9361	32	0.52	0.6070	0.05	-1.4205	2.3930
Culture	6	0.06935	1.0600	32	0.07	0.9482	0.05	-2.0897	2.2284
Culture	7	-0.2627	0.9665	32	-0.27	0.7875	0.05	-2.2315	1.7061
Culture	8	-0.3204	0.9364	32	-0.34	0.7344	0.05	-2.2279	1.5870
Culture	9	-0.9948	0.9364	32	-1.06	0.2960	0.05	-2.9022	0.9126
Culture	10	0.2671	0.9668	32	0.28	0.7841	0.05	-1.7021	2.2363
Culture	11	1.1285	0.9127	32	1.24	0.2253	0.05	-0.7306	2.9875
Culture	12	0.9319	0.9364	32	1.00	0.3271	0.05	-0.9755	2.8394
Culture	13	-1.0122	0.9668	32	-1.05	0.3029	0.05	-2.9814	0.9570

Type 3 Tests of Fixed Effects				
Effect	Num DF	Den DF	F Value	Pr > F
Treatment	1	32	1.57	0.2190

DistSoma=138

Least Squares Means									
**Effect**	**Treatment**	**Estimate**	**Standard Error**	**DF**	**t Value**	**Pr > |t|**	**Alpha**	**Lower**	**Upper**
**Treatment**	Control GFP	3.4882	0.7563	32	4.61	<.0001	0.05	1.9477	5.0286
**Treatment**	Ctr Meg	4.8316	0.7590	32	6.37	<.0001	0.05	3.2856	6.3776

Differences of Least Squares Means												
**Effect**	**Treatment**	**Treatment**	**Estimate**	**Standard Error**	**DF**	**t Value**	**Pr > |t|**	**Adjustment**	**Adj P**	**Alpha**	**Lower**	**Upper**
**Treatment**	Control GFP	Ctr Meg	-1.3434	1.0714	32	-1.25	0.2190	Tukey-Kramer	0.2190	0.05	-3.5259	0.8390

Differences of Least Squares Means				
**Effect**	**Treatment**	**Treatment**	**Adj Lower**	**Adj Upper**
**Treatment**	Control GFP	Ctr Meg	-3.5258	0.8390

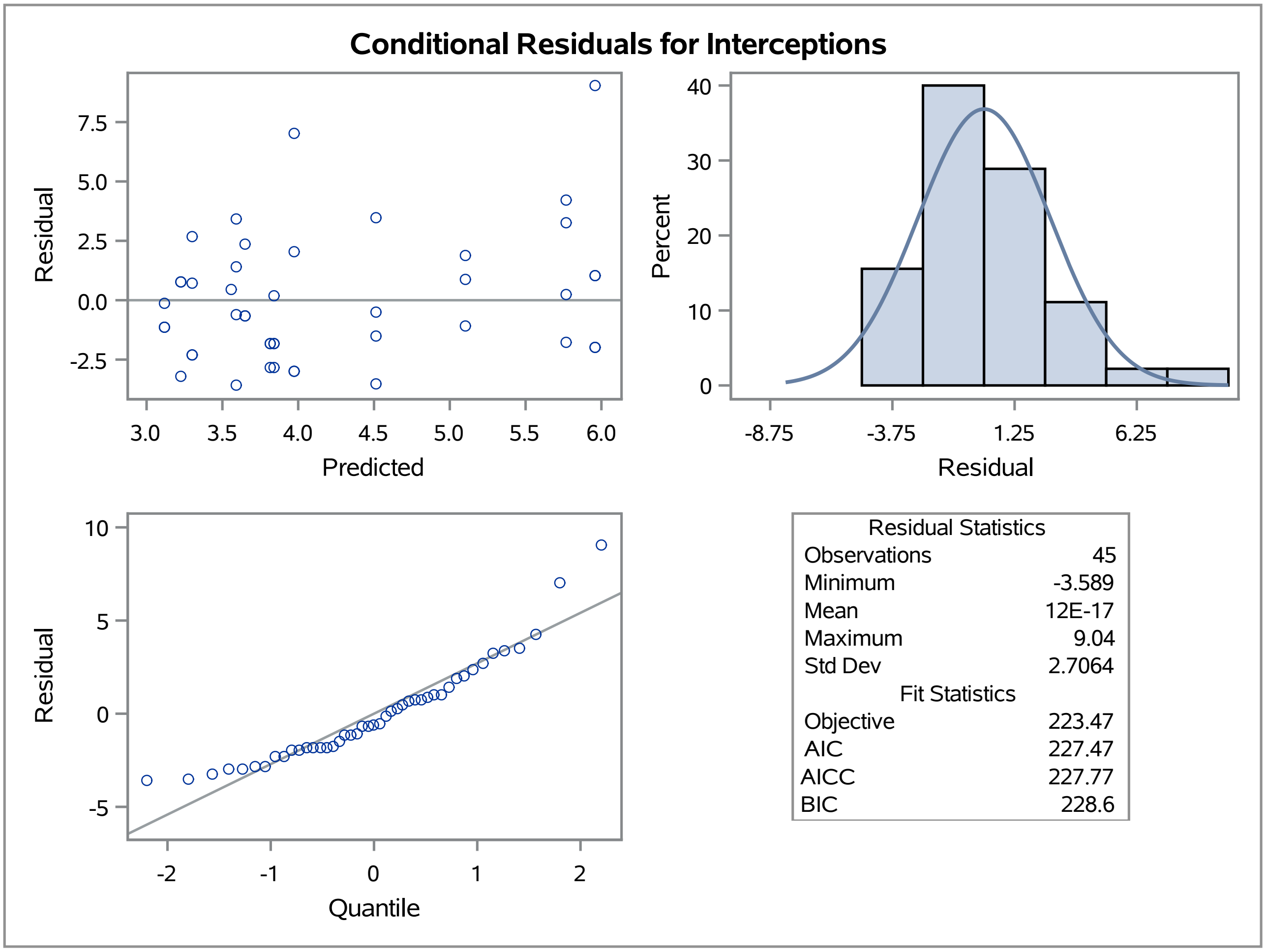

DistSoma=144

Model Information	
**Data Set**	WORK.TEMPDATASORTED
**Dependent Variable**	Interceptions
**Covariance Structure**	Variance Components
**Estimation Method**	REML
**Residual Variance Method**	Profile
**Fixed Effects SE Method**	Model-Based
**Degrees of Freedom Method**	Containment

Class Level Information		
**Class**	**Levels**	**Values**
**Treatment**	2	Control GFP Ctr Meg
**Culture**	13	1 2 3 4 5 6 7 8 9 10 11 12 13

Dimensions	
**Covariance Parameters**	2
**Columns in X**	3
**Columns in Z**	13
**Subjects**	1
**Max Obs per Subject**	45

Number of Observations	
**Number of Observations Read**	45
**Number of Observations Used**	45
**Number of Observations Not Used**	0

Iteration History			
**Iteration**	**Evaluations**	**-2 Res Log Like**	**Criterion**
**0**	1	222.85388326	
**1**	3	222.11600650	0.00000071
**2**	1	222.11595522	0.00000000

Convergence criteria met.

Covariance Parameter Estimates				
**Cov Parm**	**Estimate**	**Alpha**	**Lower**	**Upper**
**Culture**	1.0876	0.05	0.2219	780.72
**Residual**	8.0587	0.05	5.2487	13.9364

DistSoma=144

Fit Statistics	
**-2 Res Log Likelihood**	222.1
**AIC (Smaller is Better)**	226.1
**AICC (Smaller is Better)**	226.4
**BIC (Smaller is Better)**	227.2

Solution for Fixed Effects

Effect	Treatment	Estimate	Standard Error	DF	t Value	Pr > |t|	Alpha	Lower	Upper
**Intercept**		4.8178	0.7318	11	6.58	<.0001	0.05	3.2072	6.4285
**Treatment**	Control GFP	-1.3760	1.0343	32	-1.33	0.1928	0.05	-3.4829	0.7309
**Treatment**	Ctr Meg	0	.	.	.	.	.	.	.

Solution for Random Effects

Effect	Culture	Estimate	Std Err Pred	DF	t Value	Pr > |t|	Alpha	Lower	Upper
**Culture**	1	-0.3195	0.9047	32	-0.35	0.7263	0.05	-2.1624	1.5234
**Culture**	2	0.1609	0.9047	32	0.18	0.8600	0.05	-1.6820	2.0037
**Culture**	3	-0.2426	0.8786	32	-0.28	0.7843	0.05	-2.0323	1.5472
**Culture**	4	0.1080	0.8786	32	0.12	0.9029	0.05	-1.6817	1.8977
**Culture**	5	0.5463	0.8786	32	0.62	0.5385	0.05	-1.2435	2.3360
**Culture**	6	0.06637	0.9828	32	0.07	0.9466	0.05	-1.9355	2.0682
**Culture**	7	-0.3195	0.9047	32	-0.35	0.7263	0.05	-2.1624	1.5234
**Culture**	8	-0.1991	0.8787	32	-0.23	0.8222	0.05	-1.9890	1.5908
**Culture**	9	-0.9002	0.8787	32	-1.02	0.3133	0.05	-2.6901	0.8896
**Culture**	10	0.4368	0.9048	32	0.48	0.6326	0.05	-1.4062	2.2798
**Culture**	11	0.7181	0.8581	32	0.84	0.4089	0.05	-1.0298	2.4659
**Culture**	12	0.8527	0.8787	32	0.97	0.3391	0.05	-0.9372	2.6425
**Culture**	13	-0.9082	0.9048	32	-1.00	0.3230	0.05	-2.7512	0.9348

Type 3 Tests of Fixed Effects

Effect	Num DF	Den DF	F Value	Pr > F
**Treatment**	1	32	1.77	0.1928

Least Squares Means

Effect	Treatment	Estimate	Standard Error	DF	t Value	Pr > |t|	Alpha	Lower	Upper
**Treatment**	Control GFP	3.4419	0.7310	32	4.71	<.0001	0.05	1.9528	4.9309
**Treatment**	Ctr Meg	4.8178	0.7318	32	6.58	<.0001	0.05	3.3273	6.3084

DistSoma=144

Differences of Least Squares Means												
**Effect**	**Treatment**	**Treatment**	**Estimate**	**Standard Error**	**DF**	**t Value**	**Pr > |t|**	**Adjustment**	**Adj P**	**Alpha**	**Lower**	**Upper**
**Treatment**	Control GFP	Ctr Meg	-1.3760	1.0343	32	-1.33	0.1928	Tukey-Kramer	0.1928	0.05	-3.4829	0.7309

Differences of Least Squares Means				
**Effect**	**Treatment**	**Treatment**	**Adj Lower**	**Adj Upper**
**Treatment**	Control GFP	Ctr Meg	-3.4829	0.7309

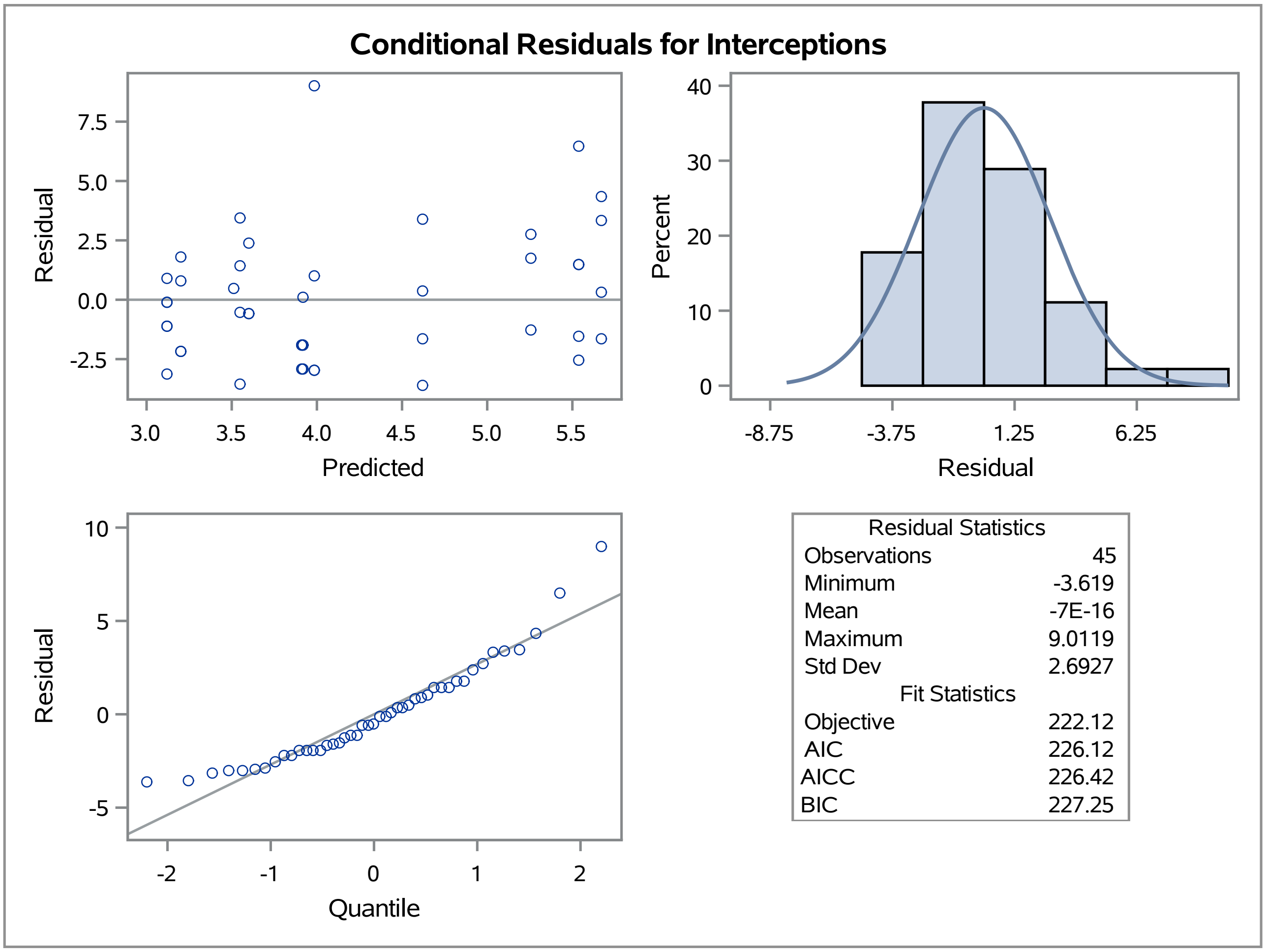

DistSoma=150

Model Information	
**Data Set**	WORK.TEMPDATASORTED
**Dependent Variable**	Interceptions
**Covariance Structure**	Variance Components
**Estimation Method**	REML
**Residual Variance Method**	Profile
**Fixed Effects SE Method**	Model-Based
**Degrees of Freedom Method**	Containment

Class Level Information		
**Class**	**Levels**	**Values**
**Treatment**	2	Control GFP Ctr Meg
**Culture**	13	1 2 3 4 5 6 7 8 9 10 11 12 13

Dimensions	
**Covariance Parameters**	2
**Columns in X**	3
**Columns in Z**	13
**Subjects**	1
**Max Obs per Subject**	45

Number of Observations	
**Number of Observations Read**	45
**Number of Observations Used**	45
**Number of Observations Not Used**	0

Iteration History			
**Iteration**	**Evaluations**	**-2 Res Log Like**	**Criterion**
**0**	1	218.47494364	
**1**	2	215.05358137	0.00000005
**2**	1	215.05357805	0.00000000

Convergence criteria met.

Covariance Parameter Estimates				
**Cov Parm**	**Estimate**	**Alpha**	**Lower**	**Upper**
**Culture**	2.3120	0.05	0.7752	25.8491
**Residual**	6.1079	0.05	3.9644	10.6225

DistSoma=150

Fit Statistics	
-2 Res Log Likelihood	215.1
AIC (Smaller is Better)	219.1
AICC (Smaller is Better)	219.4
BIC (Smaller is Better)	220.2

Solution for Fixed Effects

Effect	Treatment	Estimate	Standard Error	DF	t Value	Pr > |t|	Alpha	Lower	Upper
Intercept		4.5869	0.8099	11	5.66	0.0001	0.05	2.8042	6.3695
Treatment	Control GFP	-1.3966	1.1335	32	-1.23	0.2269	0.05	-3.7054	0.9123
Treatment	Ctr Meg	0	.	.	.	.	.	.	.

Solution for Random Effects

Effect	Culture	Estimate	Std Err Pred	DF	t Value	Pr > |t|	Alpha	Lower	Upper
Culture	1	-0.4557	1.1227	32	-0.41	0.6875	0.05	-2.7425	1.8311
Culture	2	0.7851	1.1227	32	0.70	0.4894	0.05	-1.5018	3.0719
Culture	3	-0.2652	1.0713	32	-0.25	0.8061	0.05	-2.4473	1.9170
Culture	4	-0.5663	1.0713	32	-0.53	0.6007	0.05	-2.7484	1.6159
Culture	5	1.0899	1.0713	32	1.02	0.3166	0.05	-1.0923	3.2721
Culture	6	0.2223	1.3132	32	0.17	0.8666	0.05	-2.4526	2.8973
Culture	7	-0.8102	1.1227	32	-0.72	0.4758	0.05	-3.0970	1.4767
Culture	8	-0.3534	1.0759	32	-0.33	0.7447	0.05	-2.5449	1.8381
Culture	9	-1.7085	1.0759	32	-1.59	0.1221	0.05	-3.9000	0.4830
Culture	10	1.2832	1.1261	32	1.14	0.2630	0.05	-1.0106	3.5769
Culture	11	1.0555	1.0393	32	1.02	0.3174	0.05	-1.0615	3.1724
Culture	12	1.4533	1.0759	32	1.35	0.1862	0.05	-0.7382	3.6448
Culture	13	-1.7300	1.1261	32	-1.54	0.1343	0.05	-4.0238	0.5637

Type 3 Tests of Fixed Effects

Effect	Num DF	Den DF	F Value	Pr > F
Treatment	1	32	1.52	0.2269

Least Squares Means

Effect	Treatment	Estimate	Standard Error	DF	t Value	Pr > |t|	Alpha	Lower	Upper
Treatment	Control GFP	3.1903	0.7930	32	4.02	0.0003	0.05	1.5750	4.8056
Treatment	Ctr Meg	4.5869	0.8099	32	5.66	<.0001	0.05	2.9371	6.2366

DistSoma=150

Differences of Least Squares Means												
**Effect**	**Treatment**	**Treatment**	**Estimate**	**Standard Error**	**DF**	**t Value**	**Pr > |t|**	**Adjustment**	**Adj P**	**Alpha**	**Lower**	**Upper**
**Treatment**	Control GFP	Ctr Meg	-1.3966	1.1335	32	-1.23	0.2269	Tukey-Kramer	0.2269	0.05	-3.7054	0.9123

Differences of Least Squares Means				
**Effect**	**Treatment**	**Treatment**	**Adj Lower**	**Adj Upper**
**Treatment**	Control GFP	Ctr Meg	-3.7054	0.9123

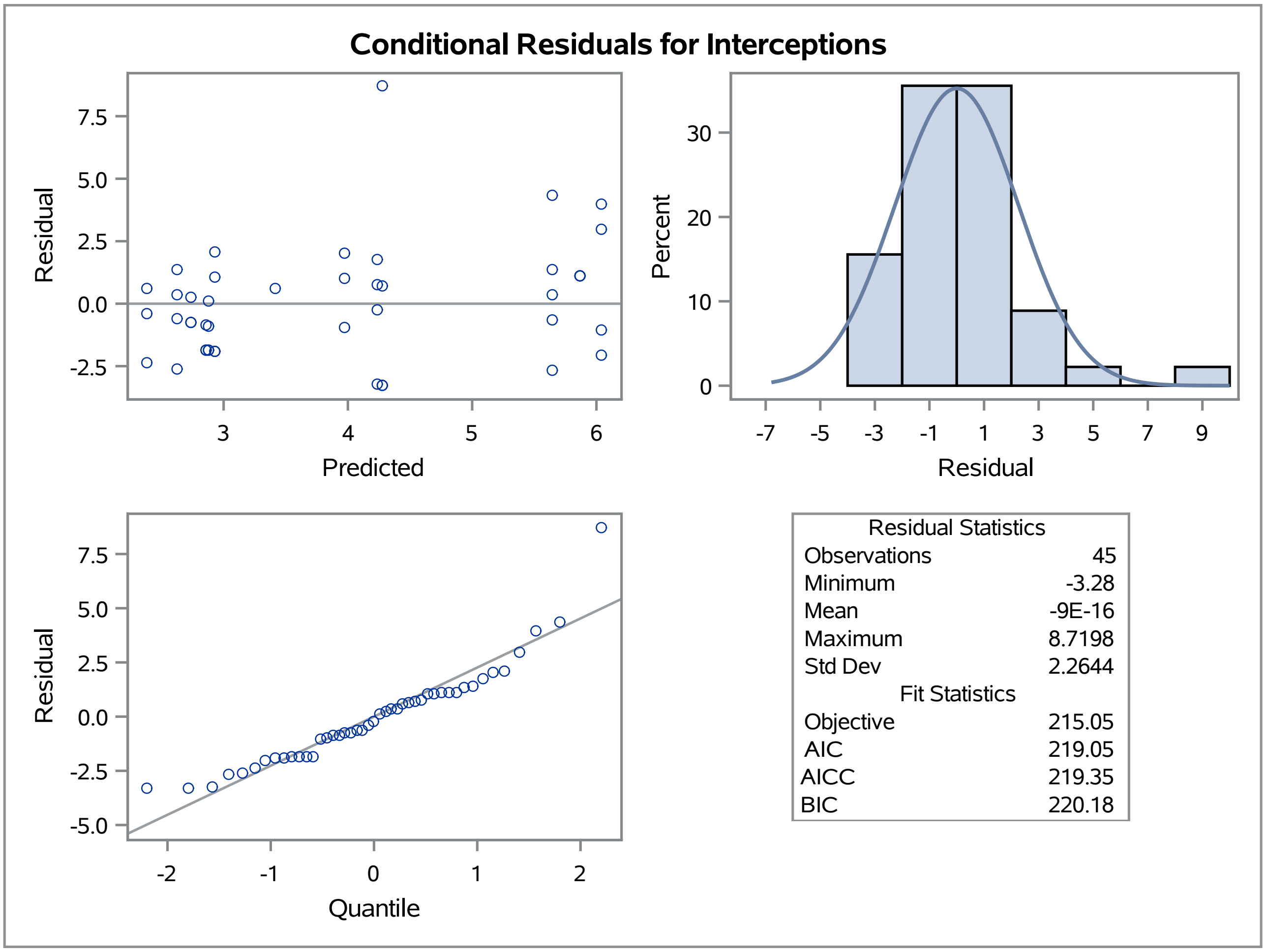

DistSoma=156

Model Information	
Data Set	WORK.TEMPDATASORTED
Dependent Variable	Interceptions
Covariance Structure	Variance Components
Estimation Method	REML
Residual Variance Method	Profile
Fixed Effects SE Method	Model-Based
Degrees of Freedom Method	Containment

Class Level Information		
Class	Levels	Values
Treatment	2	Control GFP Ctr Meg
Culture	13	1 2 3 4 5 6 7 8 9 10 11 12 13

Dimensions	
Covariance Parameters	2
Columns in X	3
Columns in Z	13
Subjects	1
Max Obs per Subject	45

Number of Observations	
Number of Observations Read	45
Number of Observations Used	45
Number of Observations Not Used	0

Iteration History			
Iteration	Evaluations	-2 Res Log Like	Criterion
0	1	220.62175465	
1	2	217.48173350	0.00000011
2	1	217.48172606	0.00000000

Convergence criteria met.

Covariance Parameter Estimates				
Cov Parm	Estimate	Alpha	Lower	Upper
Culture	2.3990	0.05	0.7886	29.5345
Residual	6.4833	0.05	4.2018	11.3029

DistSoma=156

Fit Statistics	
-2 Res Log Likelihood	217.5
AIC (Smaller is Better)	221.5
AICC (Smaller is Better)	221.8
BIC (Smaller is Better)	222.6

Solution for Fixed Effects

Effect	Treatment	Estimate	Standard Error	DF	t Value	Pr > |t|	Alpha	Lower	Upper
Intercept		4.5558	0.8289	11	5.50	0.0002	0.05	2.7314	6.3802
Treatment	Control GFP	-1.4744	1.1603	32	-1.27	0.2130	0.05	-3.8379	0.8892
Treatment	Ctr Meg	0	.	.	.	.	.	.	.

Solution for Random Effects

Effect	Culture	Estimate	Std Err Pred	DF	t Value	Pr > |t|	Alpha	Lower	Upper
Culture	1	-0.5689	1.1487	32	-0.50	0.6238	0.05	-2.9087	1.7708
Culture	2	1.3601	1.1487	32	1.18	0.2451	0.05	-0.9797	3.6998
Culture	3	-0.3470	1.0964	32	-0.32	0.7537	0.05	-2.5803	1.8863
Culture	4	-0.7946	1.0964	32	-0.72	0.4739	0.05	-3.0279	1.4387
Culture	5	0.8466	1.0964	32	0.77	0.4457	0.05	-1.3867	3.0799
Culture	6	0.2481	1.3413	32	0.18	0.8544	0.05	-2.4841	2.9803
Culture	7	-0.7443	1.1487	32	-0.65	0.5216	0.05	-3.0840	1.5954
Culture	8	-0.1825	1.1009	32	-0.17	0.8694	0.05	-2.4250	2.0600
Culture	9	-1.6745	1.1009	32	-1.52	0.1381	0.05	-3.9170	0.5680
Culture	10	1.2859	1.1520	32	1.12	0.2726	0.05	-1.0607	3.6324
Culture	11	0.8077	1.0636	32	0.76	0.4532	0.05	-1.3588	2.9742
Culture	12	1.4587	1.1009	32	1.32	0.1946	0.05	-0.7838	3.7012
Culture	13	-1.6953	1.1520	32	-1.47	0.1509	0.05	-4.0418	0.6512

Type 3 Tests of Fixed Effects

Effect	Num DF	Den DF	F Value	Pr > F
Treatment	1	32	1.61	0.2130

Least Squares Means

Effect	Treatment	Estimate	Standard Error	DF	t Value	Pr > |t|	Alpha	Lower	Upper
Treatment	Control GFP	3.0814	0.8120	32	3.79	0.0006	0.05	1.4275	4.7354
Treatment	Ctr Meg	4.5558	0.8289	32	5.50	<.0001	0.05	2.8674	6.2442

DistSoma=156

Differences of Least Squares Means												
**Effect**	**Treatment**	**Treatment**	**Estimate**	**Standard Error**	**DF**	**t Value**	**Pr > |t|**	**Adjustment**	**Adj P**	**Alpha**	**Lower**	**Upper**
**Treatment**	Control GFP	Ctr Meg	-1.4744	1.1603	32	-1.27	0.2130	Tukey-Kramer	0.2130	0.05	-3.8379	0.8892

Differences of Least Squares Means				
**Effect**	**Treatment**	**Treatment**	**Adj Lower**	**Adj Upper**
**Treatment**	Control GFP	Ctr Meg	-3.8379	0.8891

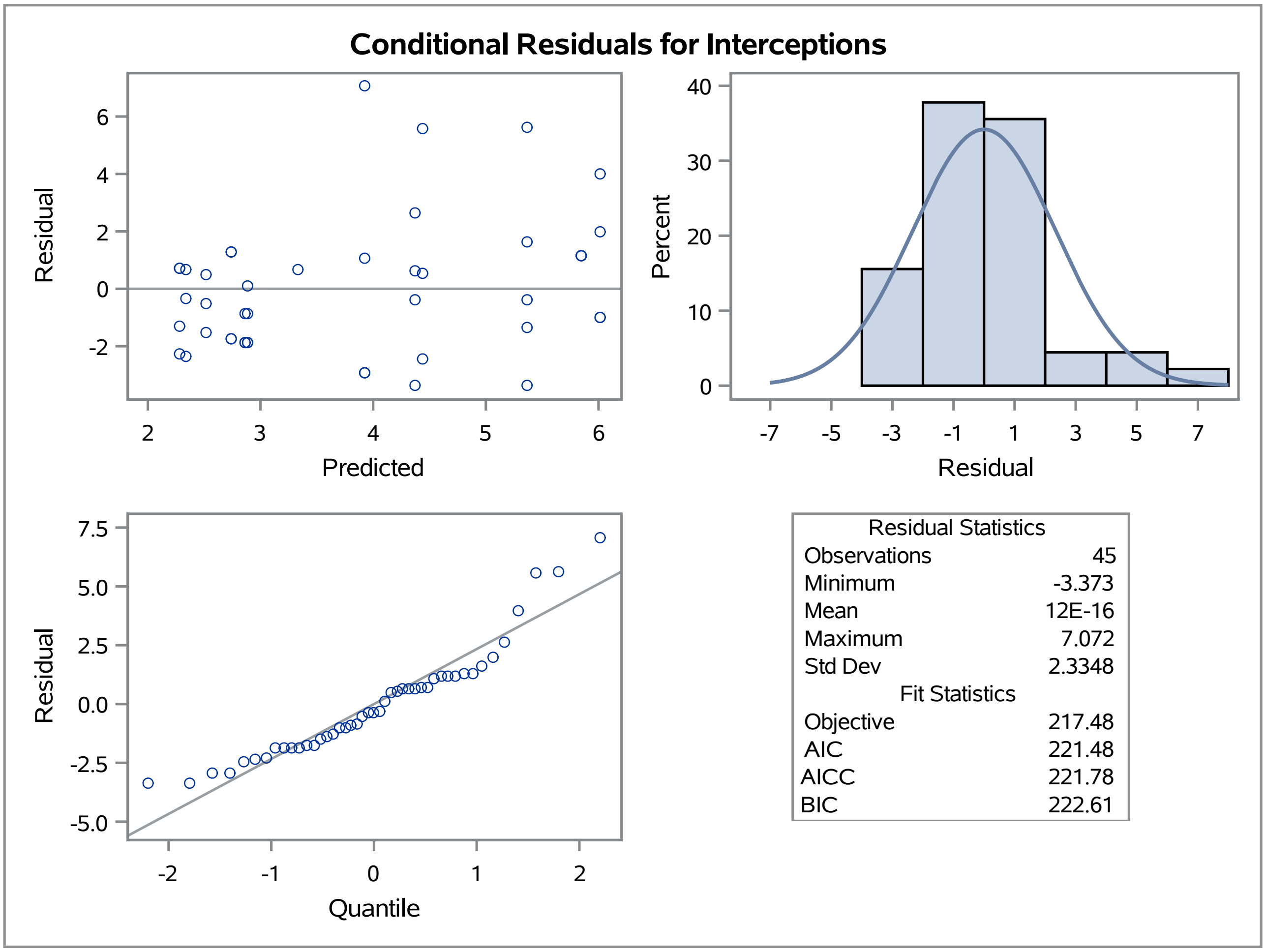

DistSoma=162

Model Information	
**Data Set**	WORK.TEMPDATASORTED
**Dependent Variable**	Interceptions
**Covariance Structure**	Variance Components
**Estimation Method**	REML
**Residual Variance Method**	Profile
**Fixed Effects SE Method**	Model-Based
**Degrees of Freedom Method**	Containment

Class Level Information		
**Class**	**Levels**	**Values**
**Treatment**	2	Control GFP Ctr Meg
**Culture**	13	1 2 3 4 5 6 7 8 9 10 11 12 13

Dimensions	
**Covariance Parameters**	2
**Columns in X**	3
**Columns in Z**	13
**Subjects**	1
**Max Obs per Subject**	45

Number of Observations	
**Number of Observations Read**	45
**Number of Observations Used**	45
**Number of Observations Not Used**	0

Iteration History			
**Iteration**	**Evaluations**	**-2 Res Log Like**	**Criterion**
**0**	1	210.34961861	
**1**	1	207.63269094	0.00000000

Convergence criteria met.

Covariance Parameter Estimates				
**Cov Parm**	**Estimate**	**Alpha**	**Lower**	**Upper**
**Culture**	1.7237	0.05	0.5415	26.8345
**Residual**	5.2392	0.05	3.3958	9.1327

DistSoma=162

Fit Statistics	
**-2 Res Log Likelihood**	207.6
**AIC (Smaller is Better)**	211.6
**AICC (Smaller is Better)**	211.9
**BIC (Smaller is Better)**	212.8

Solution for Fixed Effects

Effect	Treatment	Estimate	Standard Error	DF	t Value	Pr > |t|	Alpha	Lower	Upper
**Intercept**		4.2428	0.7205	11	5.89	0.0001	0.05	2.6569	5.8287
**Treatment**	Control GFP	-1.3670	1.0099	32	-1.35	0.1853	0.05	-3.4241	0.6901
**Treatment**	Ctr Meg	0	.	.	.	.	.	.	.

Solution for Random Effects

Effect	Culture	Estimate	Std Err Pred	DF	t Value	Pr > |t|	Alpha	Lower	Upper
**Culture**	1	-0.4350	0.9955	32	-0.44	0.6651	0.05	-2.4628	1.5928
**Culture**	2	0.7240	0.9955	32	0.73	0.4723	0.05	-1.3037	2.7518
**Culture**	3	-0.3556	0.9518	32	-0.37	0.7112	0.05	-2.2943	1.5832
**Culture**	4	-0.7817	0.9518	32	-0.82	0.4175	0.05	-2.7205	1.1570
**Culture**	5	0.9230	0.9518	32	0.97	0.3395	0.05	-1.0158	2.8617
**Culture**	6	0.5259	1.1523	32	0.46	0.6512	0.05	-1.8212	2.8729
**Culture**	7	-0.6006	0.9955	32	-0.60	0.5506	0.05	-2.6284	1.4272
**Culture**	8	0.004112	0.9549	32	0.00	0.9966	0.05	-1.9410	1.9492
**Culture**	9	-1.4165	0.9549	32	-1.48	0.1478	0.05	-3.3616	0.5287
**Culture**	10	0.8729	0.9978	32	0.87	0.3882	0.05	-1.1596	2.9053
**Culture**	11	0.8441	0.9233	32	0.91	0.3674	0.05	-1.0366	2.7248
**Culture**	12	1.1406	0.9549	32	1.19	0.2411	0.05	-0.8046	3.0857
**Culture**	13	-1.4452	0.9978	32	-1.45	0.1572	0.05	-3.4777	0.5872

Type 3 Tests of Fixed Effects

Effect	Num DF	Den DF	F Value	Pr > F
**Treatment**	1	32	1.83	0.1853

Least Squares Means

Effect	Treatment	Estimate	Standard Error	DF	t Value	Pr > |t|	Alpha	Lower	Upper
**Treatment**	Control GFP	2.8757	0.7076	32	4.06	0.0003	0.05	1.4344	4.3171
**Treatment**	Ctr Meg	4.2428	0.7205	32	5.89	<.0001	0.05	2.7751	5.7105

DistSoma=162

Differences of Least Squares Means												
**Effect**	**Treatment**	**Treatment**	**Estimate**	**Standard Error**	**DF**	**t Value**	**Pr > |t|**	**Adjustment**	**Adj P**	**Alpha**	**Lower**	**Upper**
**Treatment**	Control GFP	Ctr Meg	-1.3670	1.0099	32	-1.35	0.1853	Tukey-Kramer	0.1853	0.05	-3.4241	0.6901

Differences of Least Squares Means				
**Effect**	**Treatment**	**Treatment**	**Adj Lower**	**Adj Upper**
**Treatment**	Control GFP	Ctr Meg	-3.4241	0.6900

Conditional Residuals for Interceptions

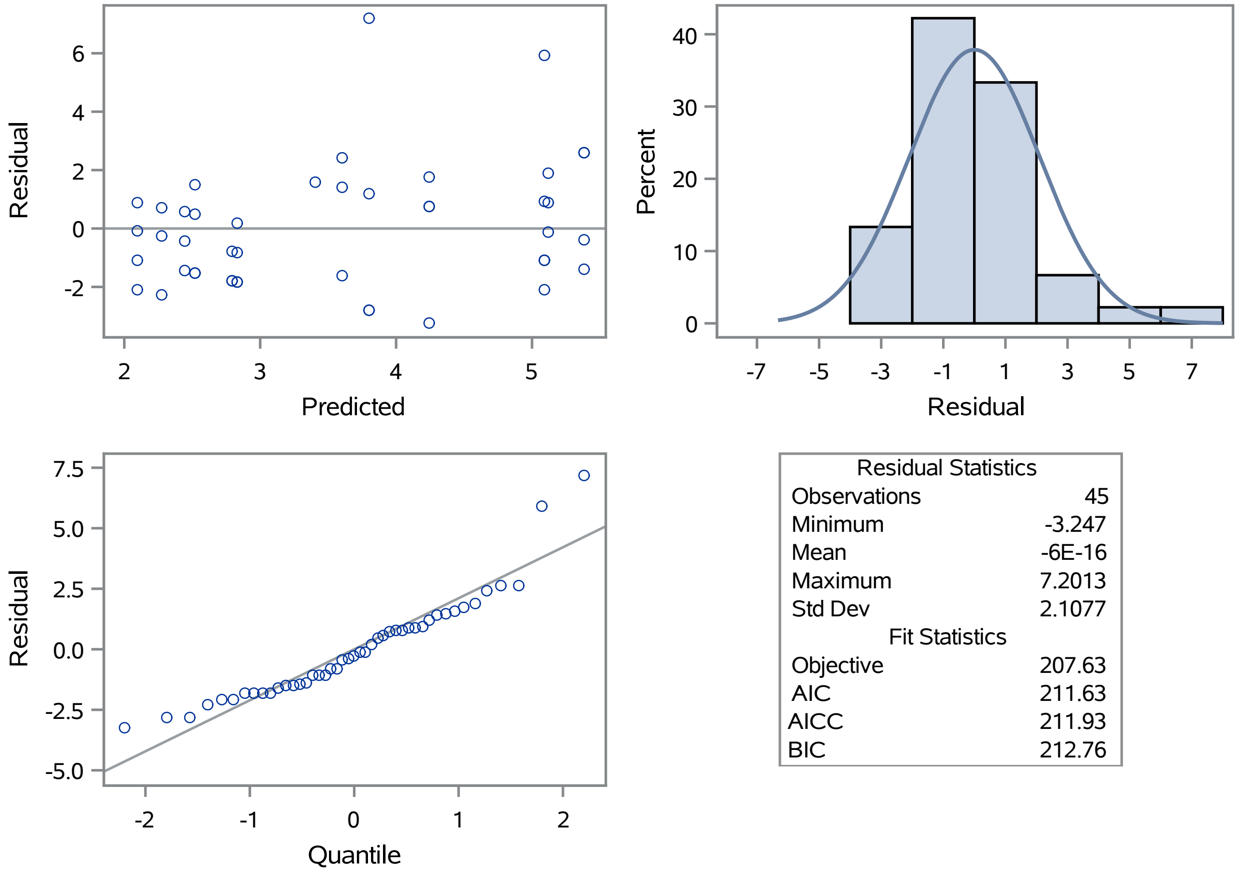

Residual Statistics	
Observations	45
Minimum	-3.247
Mean	-6E-16
Maximum	7.2013
Std Dev	2.1077
**Fit Statistics**	
Objective	207.63
AIC	211.63
AICC	211.93
BIC	212.76

DistSoma=168

Model Information	
**Data Set**	WORK.TEMPDATASORTED
**Dependent Variable**	Interceptions
**Covariance Structure**	Variance Components
**Estimation Method**	REML
**Residual Variance Method**	Profile
**Fixed Effects SE Method**	Model-Based
**Degrees of Freedom Method**	Containment

Class Level Information		
**Class**	**Levels**	**Values**
**Treatment**	2	Control GFP Ctr Meg
**Culture**	13	1 2 3 4 5 6 7 8 9 10 11 12 13

Dimensions	
**Covariance Parameters**	2
**Columns in X**	3
**Columns in Z**	13
**Subjects**	1
**Max Obs per Subject**	45

Number of Observations	
**Number of Observations Read**	45
**Number of Observations Used**	45
**Number of Observations Not Used**	0

Iteration History			
**Iteration**	**Evaluations**	**-2 Res Log Like**	**Criterion**
**0**	1	203.67229638	
**1**	2	199.26483094	0.00000073
**2**	1	199.26478677	0.00000000

Convergence criteria met.

Covariance Parameter Estimates				
**Cov Parm**	**Estimate**	**Alpha**	**Lower**	**Upper**
**Culture**	1.9671	0.05	0.6982	17.0297
**Residual**	4.0820	0.05	2.6406	7.1383

DistSoma=168

Fit Statistics	
-2 Res Log Likelihood	199.3
AIC (Smaller is Better)	203.3
AICC (Smaller is Better)	203.6
BIC (Smaller is Better)	204.4

Solution for Fixed Effects

Effect	Treatment	Estimate	Standard Error	DF	t Value	Pr > |t|	Alpha	Lower	Upper
Intercept		3.9873	0.7135	11	5.59	0.0002	0.05	2.4170	5.5577
Treatment	Control GFP	-1.3603	0.9959	32	-1.37	0.1815	0.05	-3.3890	0.6684
Treatment	Ctr Meg	0	.	.	.	.	.	.	.

Solution for Random Effects

Effect	Culture	Estimate	Std Err Pred	DF	t Value	Pr > |t|	Alpha	Lower	Upper
Culture	1	-0.5677	0.9864	32	-0.58	0.5690	0.05	-2.5769	1.4416
Culture	2	0.6146	0.9864	32	0.62	0.5377	0.05	-1.3947	2.6238
Culture	3	-0.5774	0.9387	32	-0.62	0.5428	0.05	-2.4896	1.3347
Culture	4	-0.7420	0.9387	32	-0.79	0.4351	0.05	-2.6542	1.1701
Culture	5	1.0686	0.9387	32	1.14	0.2634	0.05	-0.8436	2.9808
Culture	6	0.7717	1.1741	32	0.66	0.5157	0.05	-1.6199	3.1632
Culture	7	-0.5677	0.9864	32	-0.58	0.5690	0.05	-2.5769	1.4416
Culture	8	0.1730	0.9448	32	0.18	0.8559	0.05	-1.7515	2.0974
Culture	9	-1.6377	0.9448	32	-1.73	0.0926	0.05	-3.5621	0.2867
Culture	10	1.1897	0.9911	32	1.20	0.2388	0.05	-0.8290	3.2084
Culture	11	0.7157	0.9117	32	0.78	0.4382	0.05	-1.1414	2.5727
Culture	12	1.3252	0.9448	32	1.40	0.1703	0.05	-0.5993	3.2496
Culture	13	-1.7658	0.9911	32	-1.78	0.0843	0.05	-3.7845	0.2529

Type 3 Tests of Fixed Effects

Effect	Num DF	Den DF	F Value	Pr > F
Treatment	1	32	1.87	0.1815

Least Squares Means

Effect	Treatment	Estimate	Standard Error	DF	t Value	Pr > |t|	Alpha	Lower	Upper
Treatment	Control GFP	2.6270	0.6949	32	3.78	0.0006	0.05	1.2116	4.0424
Treatment	Ctr Meg	3.9873	0.7135	32	5.59	<.0001	0.05	2.5340	5.4406

DistSoma=168

Differences of Least Squares Means												
**Effect**	**Treatment**	**Treatment**	**Estimate**	**Standard Error**	**DF**	**t Value**	**Pr > |t|**	**Adjustment**	**Adj P**	**Alpha**	**Lower**	**Upper**
**Treatment**	Control GFP	Ctr Meg	-1.3603	0.9959	32	-1.37	0.1815	Tukey-Kramer	0.1815	0.05	-3.3890	0.6684

Differences of Least Squares Means				
**Effect**	**Treatment**	**Treatment**	**Adj Lower**	**Adj Upper**
**Treatment**	Control GFP	Ctr Meg	-3.3890	0.6684

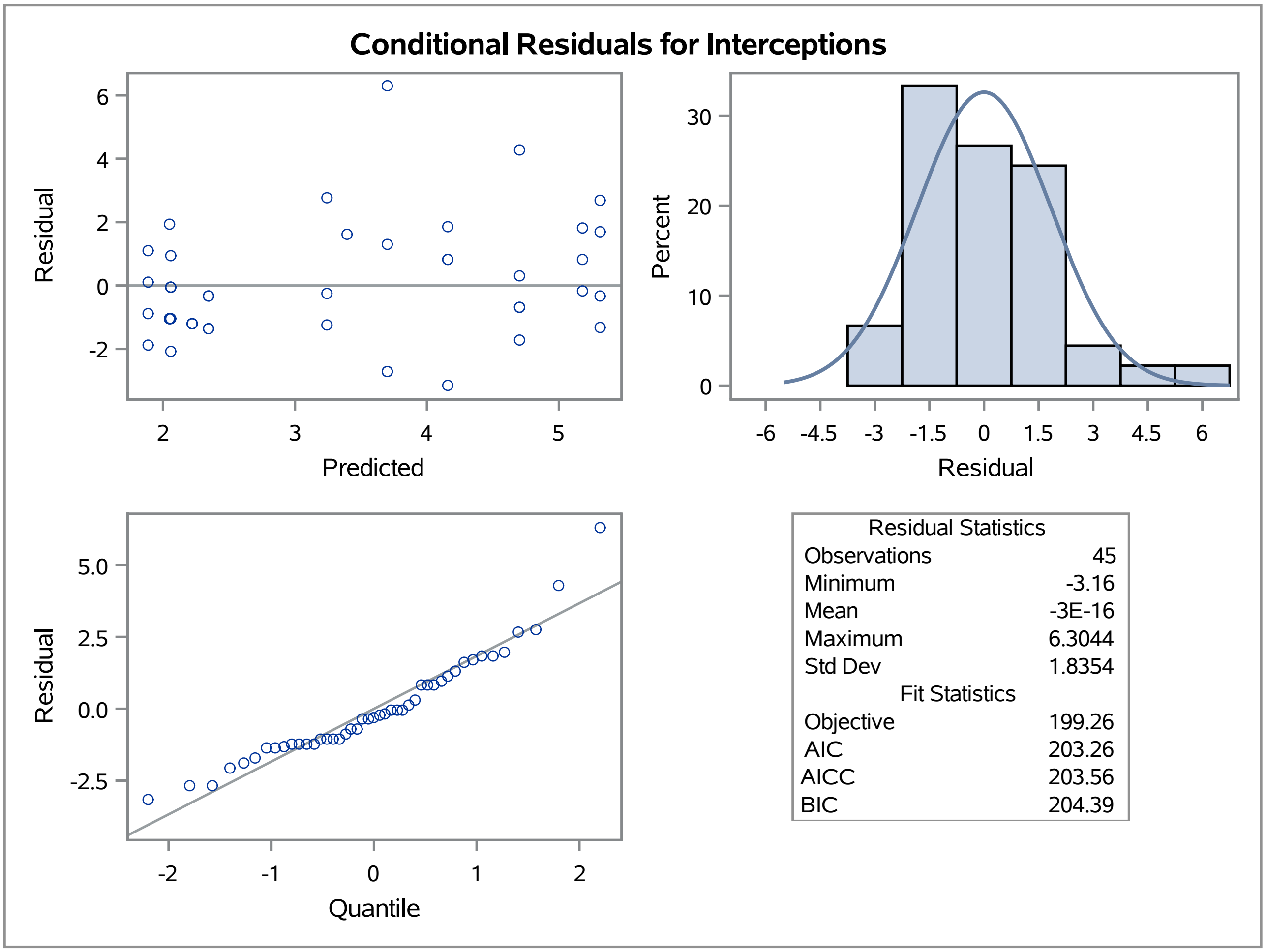

DistSoma=174

Model Information	
**Data Set**	WORK.TEMPDATASORTED
**Dependent Variable**	Interceptions
**Covariance Structure**	Variance Components
**Estimation Method**	REML
**Residual Variance Method**	Profile
**Fixed Effects SE Method**	Model-Based
**Degrees of Freedom Method**	Containment

Class Level Information		
**Class**	**Levels**	**Values**
**Treatment**	2	Control GFP Ctr Meg
**Culture**	13	1 2 3 4 5 6 7 8 9 10 11 12 13

Dimensions	
**Covariance Parameters**	2
**Columns in X**	3
**Columns in Z**	13
**Subjects**	1
**Max Obs per Subject**	45

Number of Observations	
**Number of Observations Read**	45
**Number of Observations Used**	45
**Number of Observations Not Used**	0

Iteration History			
**Iteration**	**Evaluations**	**-2 Res Log Like**	**Criterion**
**0**	1	197.81926856	
**1**	2	192.47272668	0.00001114
**2**	1	192.47208545	0.00000001

Convergence criteria met.

Covariance Parameter Estimates				
**Cov Parm**	**Estimate**	**Alpha**	**Lower**	**Upper**
**Culture**	1.9529	0.05	0.7225	14.2700
**Residual**	3.3863	0.05	2.1871	5.9374

DistSoma=174

Fit Statistics	
-2 Res Log Likelihood	192.5
AIC (Smaller is Better)	196.5
AICC (Smaller is Better)	196.8
BIC (Smaller is Better)	197.6

Solution for Fixed Effects

Effect	Treatment	Estimate	Standard Error	DF	t Value	Pr > |t|	Alpha	Lower	Upper
Intercept		3.6937	0.6899	11	5.35	0.0002	0.05	2.1752	5.2123
Treatment	Control GFP	-1.3136	0.9612	32	-1.37	0.1813	0.05	-3.2715	0.6444
Treatment	Ctr Meg	0	.	.	.	.	.	.	.

Solution for Random Effects

Effect	Culture	Estimate	Std Err Pred	DF	t Value	Pr > |t|	Alpha	Lower	Upper
Culture	1	-0.4521	0.9462	32	-0.48	0.6360	0.05	-2.3794	1.4751
Culture	2	0.3928	0.9462	32	0.42	0.6808	0.05	-1.5344	2.3201
Culture	3	-0.4396	0.8992	32	-0.49	0.6283	0.05	-2.2712	1.3920
Culture	4	-0.6140	0.8992	32	-0.68	0.4996	0.05	-2.4456	1.2176
Culture	5	0.6068	0.8992	32	0.67	0.5046	0.05	-1.2248	2.4384
Culture	6	0.9583	1.1395	32	0.84	0.4066	0.05	-1.3629	3.2794
Culture	7	-0.4521	0.9462	32	-0.48	0.6360	0.05	-2.3794	1.4751
Culture	8	0.2136	0.9068	32	0.24	0.8152	0.05	-1.6334	2.0607
Culture	9	-1.7047	0.9068	32	-1.88	0.0692	0.05	-3.5518	0.1423
Culture	10	1.2503	0.9521	32	1.31	0.1985	0.05	-0.6891	3.1896
Culture	11	0.3759	0.8748	32	0.43	0.6703	0.05	-1.4060	2.1578
Culture	12	1.7832	0.9068	32	1.97	0.0580	0.05	-0.06377	3.6302
Culture	13	-1.9183	0.9521	32	-2.01	0.0524	0.05	-3.8577	0.02104

Type 3 Tests of Fixed Effects

Effect	Num DF	Den DF	F Value	Pr > F
Treatment	1	32	1.87	0.1813

Least Squares Means

Effect	Treatment	Estimate	Standard Error	DF	t Value	Pr > |t|	Alpha	Lower	Upper
Treatment	Control GFP	2.3801	0.6693	32	3.56	0.0012	0.05	1.0169	3.7434
Treatment	Ctr Meg	3.6937	0.6899	32	5.35	<.0001	0.05	2.2884	5.0991

DistSoma=174

Differences of Least Squares Means												
**Effect**	**Treatment**	**Treatment**	**Estimate**	**Standard Error**	**DF**	**t Value**	**Pr > |t|**	**Adjustment**	**Adj P**	**Alpha**	**Lower**	**Upper**
**Treatment**	Control GFP	Ctr Meg	-1.3136	0.9612	32	-1.37	0.1813	Tukey-Kramer	0.1813	0.05	-3.2715	0.6444

Differences of Least Squares Means				
**Effect**	**Treatment**	**Treatment**	**Adj Lower**	**Adj Upper**
**Treatment**	Control GFP	Ctr Meg	-3.2715	0.6443

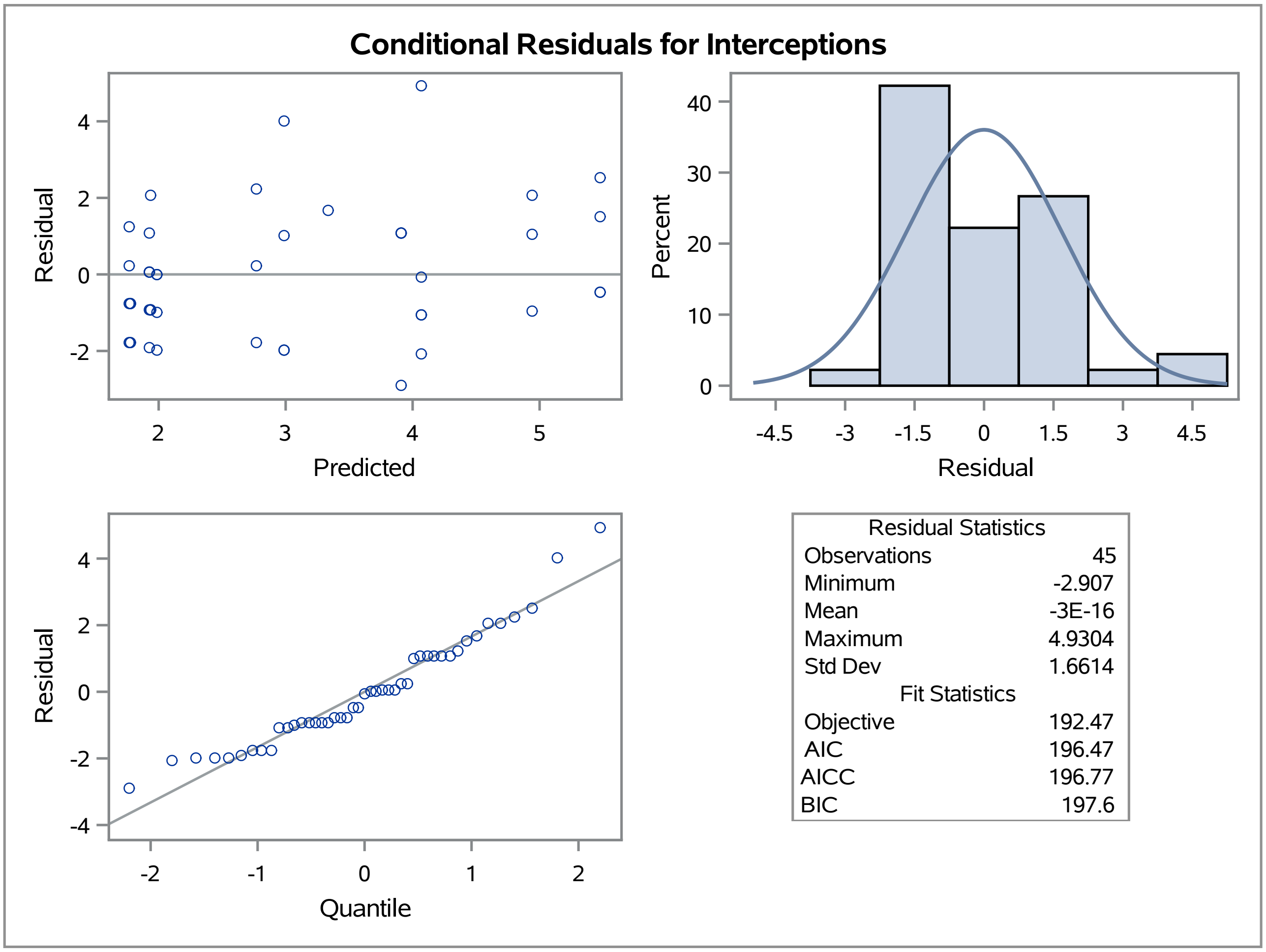

DistSoma=180

Model Information	
**Data Set**	WORK.TEMPDATASORTED
**Dependent Variable**	Interceptions
**Covariance Structure**	Variance Components
**Estimation Method**	REML
**Residual Variance Method**	Profile
**Fixed Effects SE Method**	Model-Based
**Degrees of Freedom Method**	Containment

Class Level Information		
**Class**	**Levels**	**Values**
**Treatment**	2	Control GFP Ctr Meg
**Culture**	13	1 2 3 4 5 6 7 8 9 10 11 12 13

Dimensions	
**Covariance Parameters**	2
**Columns in X**	3
**Columns in Z**	13
**Subjects**	1
**Max Obs per Subject**	45

Number of Observations	
**Number of Observations Read**	45
**Number of Observations Used**	45
**Number of Observations Not Used**	0

Iteration History			
**Iteration**	**Evaluations**	**-2 Res Log Like**	**Criterion**
**0**	1	192.75984041	
**1**	2	185.51730578	0.00002153
**2**	1	185.51613699	0.00000002
**3**	1	185.51613597	0.00000000

Convergence criteria met.

DistSoma=180

Covariance Parameter Estimates				
**Cov Parm**	**Estimate**	**Alpha**	**Lower**	**Upper**
**Culture**	2.0458	0.05	0.8029	11.9944
**Residual**	2.7579	0.05	1.7805	4.8386

Fit Statistics	
**-2 Res Log Likelihood**	185.5
**AIC (Smaller is Better)**	189.5
**AICC (Smaller is Better)**	189.8
**BIC (Smaller is Better)**	190.6

Solution for Fixed Effects									
**Effect**	**Treatment**	**Estimate**	**Standard Error**	**DF**	**t Value**	**Pr > |t|**	**Alpha**	**Lower**	**Upper**
**Intercept**		3.6284	0.6810	11	5.33	0.0002	0.05	2.1295	5.1272
**Treatment**	Control GFP	-1.2708	0.9463	32	-1.34	0.1887	0.05	-3.1983	0.6566
**Treatment**	Ctr Meg	0	.	.	.	.	.	.	.

Solution for Random Effects									
**Effect**	**Culture**	**Estimate**	**Std Err Pred**	**DF**	**t Value**	**Pr > |t|**	**Alpha**	**Lower**	**Upper**
**Culture**	1	-0.4767	0.9164	32	-0.52	0.6065	0.05	-2.3433	1.3899
**Culture**	2	0.6733	0.9164	32	0.73	0.4679	0.05	-1.1934	2.5399
**Culture**	3	-0.4544	0.8701	32	-0.52	0.6051	0.05	-2.2268	1.3180
**Culture**	4	-0.8284	0.8701	32	-0.95	0.3482	0.05	-2.6008	0.9440
**Culture**	5	0.6675	0.8701	32	0.77	0.4486	0.05	-1.1049	2.4399
**Culture**	6	1.1254	1.1193	32	1.01	0.3222	0.05	-1.1546	3.4053
**Culture**	7	-0.7067	0.9164	32	-0.77	0.4463	0.05	-2.5733	1.1599
**Culture**	8	0.6519	0.8804	32	0.74	0.4644	0.05	-1.1414	2.4452
**Culture**	9	-1.7789	0.8804	32	-2.02	0.0518	0.05	-3.5722	0.01445
**Culture**	10	0.9464	0.9247	32	1.02	0.3138	0.05	-0.9372	2.8299
**Culture**	11	0.4502	0.8498	32	0.53	0.5999	0.05	-1.2807	2.1812
**Culture**	12	1.7738	0.8804	32	2.01	0.0524	0.05	-0.01950	3.5671
**Culture**	13	-2.0435	0.9247	32	-2.21	0.0344	0.05	-3.9270	-0.1599

Type 3 Tests of Fixed Effects				
**Effect**	**Num DF**	**Den DF**	**F Value**	**Pr > F**
**Treatment**	1	32	1.80	0.1887

DistSoma=180

Least Squares Means									
**Effect**	**Treatment**	**Estimate**	**Standard Error**	**DF**	**t Value**	**Pr > |t|**	**Alpha**	**Lower**	**Upper**
**Treatment**	Control GFP	2.3575	0.6570	32	3.59	0.0011	0.05	1.0193	3.6958
**Treatment**	Ctr Meg	3.6284	0.6810	32	5.33	<.0001	0.05	2.2412	5.0155

Differences of Least Squares Means													
**Effect**	**Treatment**	**Treatment**	**Estimate**	**Standard Error**	**DF**	**t Value**	**Pr > |t|**	**Adjustment**	**Adj P**	**Alpha**	**Lower**	**Upper**
**Treatment**	Control GFP	Ctr Meg	-1.2708	0.9463	32	-1.34	0.1887	Tukey-Kramer	0.1887	0.05	-3.1983	0.6566

Differences of Least Squares Means				
**Effect**	**Treatment**	**Treatment**	**Adj Lower**	**Adj Upper**
**Treatment**	Control GFP	Ctr Meg	-3.1983	0.6566

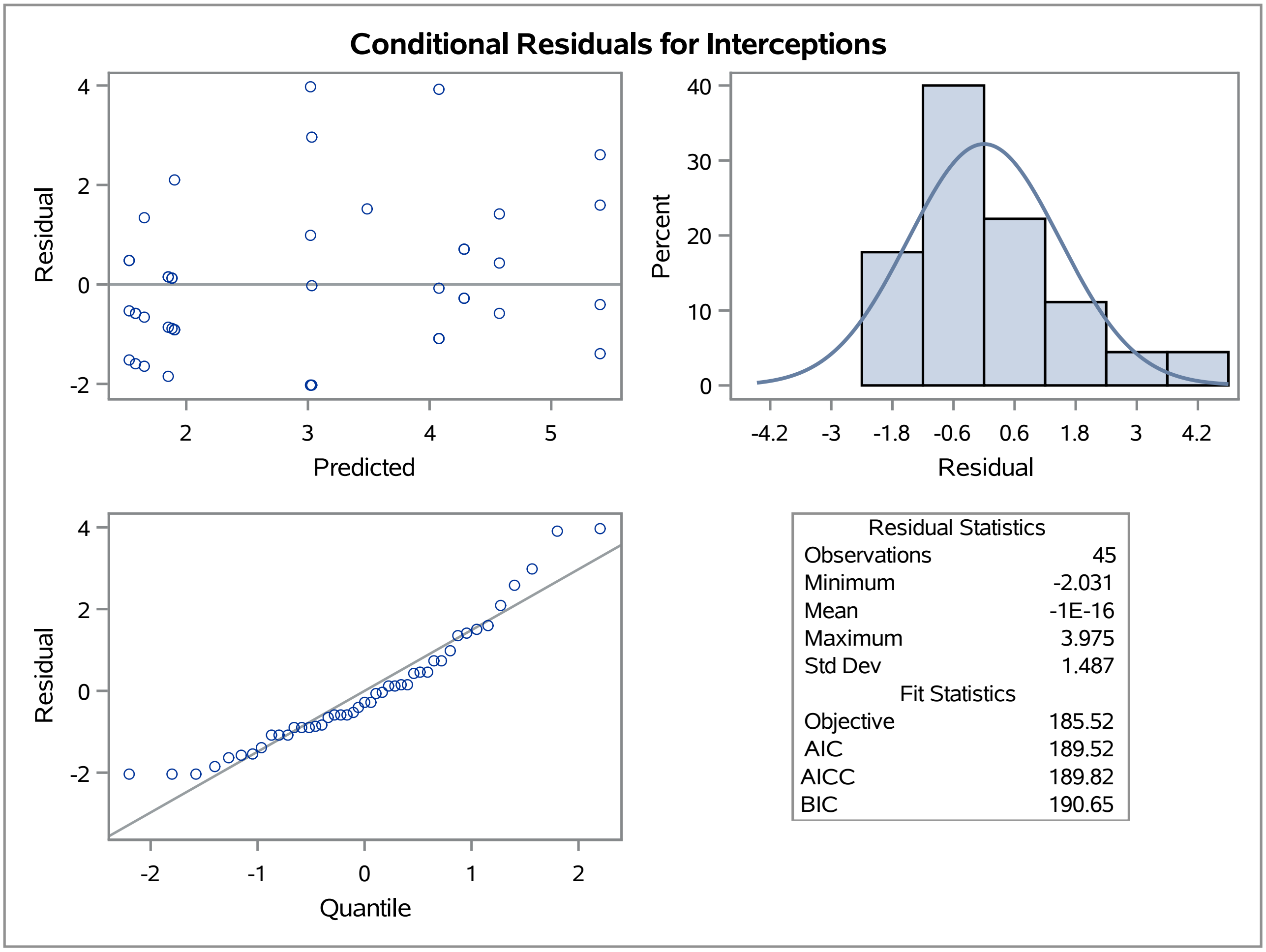

DistSoma=186

Model Information	
**Data Set**	WORK.TEMPDATASORTED
**Dependent Variable**	Interceptions
**Covariance Structure**	Variance Components
**Estimation Method**	REML
**Residual Variance Method**	Profile
**Fixed Effects SE Method**	Model-Based
**Degrees of Freedom Method**	Containment

Class Level Information		
**Class**	**Levels**	**Values**
**Treatment**	2	Control GFP Ctr Meg
**Culture**	13	1 2 3 4 5 6 7 8 9 10 11 12 13

Dimensions	
**Covariance Parameters**	2
**Columns in X**	3
**Columns in Z**	13
**Subjects**	1
**Max Obs per Subject**	45

Number of Observations	
**Number of Observations Read**	45
**Number of Observations Used**	45
**Number of Observations Not Used**	0

Iteration History			
**Iteration**	**Evaluations**	**-2 Res Log Like**	**Criterion**
**0**	1	195.81495059	
**1**	2	189.90502048	0.00000380
**2**	1	189.90480797	0.00000000

Convergence criteria met.

Covariance Parameter Estimates				
**Cov Parm**	**Estimate**	**Alpha**	**Lower**	**Upper**
**Culture**	1.9286	0.05	0.7306	12.8631
**Residual**	3.1599	0.05	2.0436	5.5281

DistSoma=186

Fit Statistics	
**-2 Res Log Likelihood**	189.9
**AIC (Smaller is Better)**	193.9
**AICC (Smaller is Better)**	194.2
**BIC (Smaller is Better)**	195.0

Solution for Fixed Effects

Effect	Treatment	Estimate	Standard Error	DF	t Value	Pr > |t|	Alpha	Lower	Upper
**Intercept**		3.4983	0.6797	11	5.15	0.0003	0.05	2.0024	4.9942
**Treatment**	Control GFP	-1.5929	0.9464	32	-1.68	0.1021	0.05	-3.5206	0.3348
**Treatment**	Ctr Meg	0	.	.	.	.	.	.	.

Solution for Random Effects

Effect	Culture	Estimate	Std Err Pred	DF	t Value	Pr > |t|	Alpha	Lower	Upper
**Culture**	1	-0.5856	0.9288	32	-0.63	0.5329	0.05	-2.4775	1.3063
**Culture**	2	0.4924	0.9288	32	0.53	0.5997	0.05	-1.3995	2.3842
**Culture**	3	-0.1102	0.8824	32	-0.12	0.9014	0.05	-1.9077	1.6872
**Culture**	4	-0.4649	0.8824	32	-0.53	0.6019	0.05	-2.2624	1.3325
**Culture**	5	0.2445	0.8824	32	0.28	0.7835	0.05	-1.5529	2.0419
**Culture**	6	0.7939	1.1225	32	0.71	0.4845	0.05	-1.4925	3.0803
**Culture**	7	-0.3700	0.9288	32	-0.40	0.6930	0.05	-2.2619	1.5219
**Culture**	8	0.1786	0.8905	32	0.20	0.8423	0.05	-1.6352	1.9924
**Culture**	9	-1.7723	0.8905	32	-1.99	0.0551	0.05	-3.5861	0.04146
**Culture**	10	1.1869	0.9351	32	1.27	0.2135	0.05	-0.7180	3.0917
**Culture**	11	0.6792	0.8591	32	0.79	0.4350	0.05	-1.0708	2.4291
**Culture**	12	1.7748	0.8905	32	1.99	0.0548	0.05	-0.03903	3.5886
**Culture**	13	-2.0470	0.9351	32	-2.19	0.0360	0.05	-3.9518	-0.1422

Type 3 Tests of Fixed Effects

Effect	Num DF	Den DF	F Value	Pr > F
**Treatment**	1	32	2.83	0.1021

Least Squares Means

Effect	Treatment	Estimate	Standard Error	DF	t Value	Pr > |t|	Alpha	Lower	Upper
**Treatment**	Control GFP	1.9054	0.6585	32	2.89	0.0068	0.05	0.5640	3.2467
**Treatment**	Ctr Meg	3.4983	0.6797	32	5.15	<.0001	0.05	2.1139	4.8827

DistSoma=186

Differences of Least Squares Means												
**Effect**	**Treatment**	**Treatment**	**Estimate**	**Standard Error**	**DF**	**t Value**	**Pr > |t|**	**Adjustment**	**Adj P**	**Alpha**	**Lower**	**Upper**
**Treatment**	Control GFP	Ctr Meg	-1.5929	0.9464	32	-1.68	0.1021	Tukey-Kramer	0.1021	0.05	-3.5206	0.3348

Differences of Least Squares Means				
**Effect**	**Treatment**	**Treatment**	**Adj Lower**	**Adj Upper**
**Treatment**	Control GFP	Ctr Meg	-3.5205	0.3347

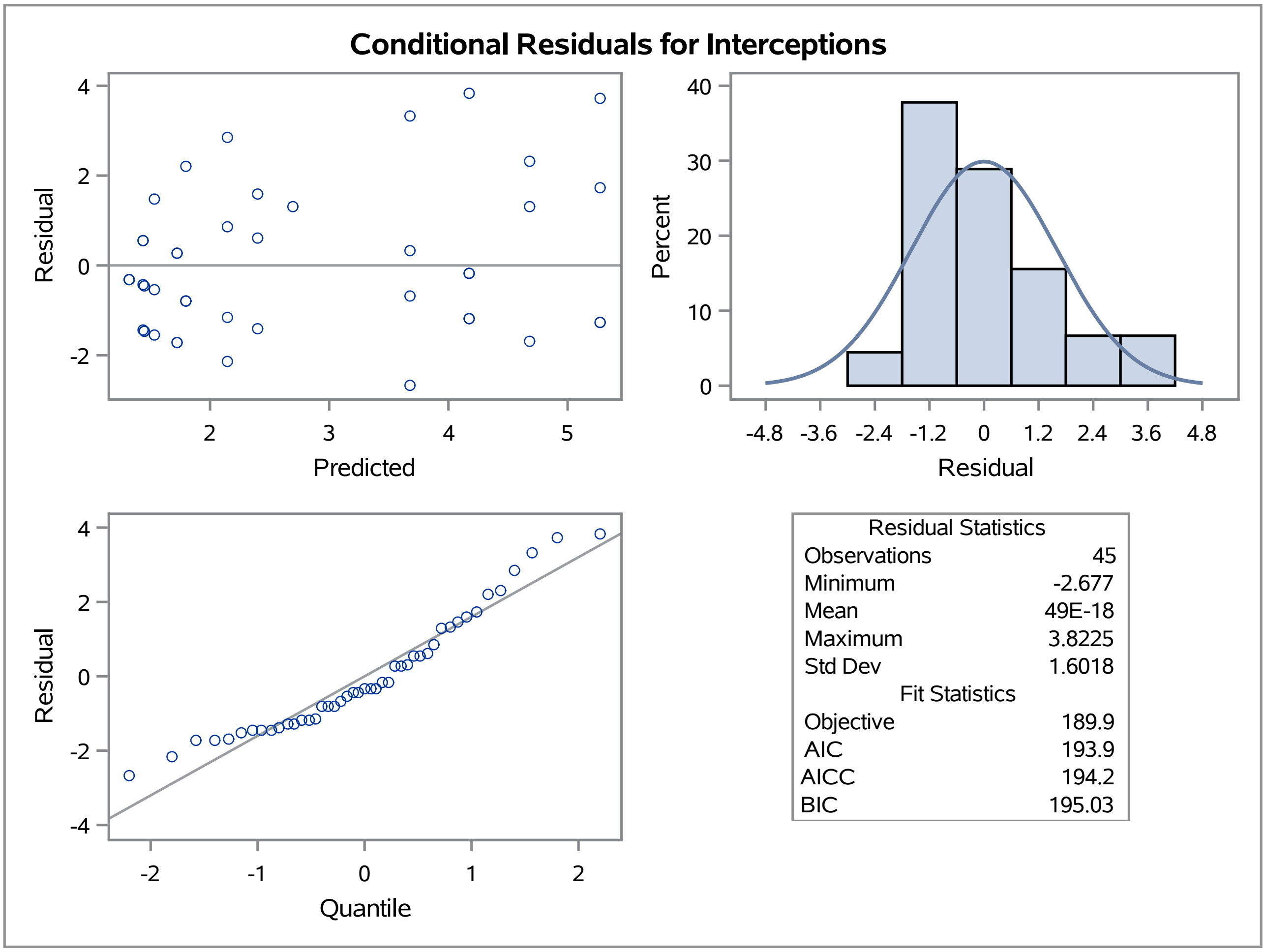

DistSoma=192

Model Information	
**Data Set**	WORK.TEMPDATASORTED
**Dependent Variable**	Interceptions
**Covariance Structure**	Variance Components
**Estimation Method**	REML
**Residual Variance Method**	Profile
**Fixed Effects SE Method**	Model-Based
**Degrees of Freedom Method**	Containment

Class Level Information		
**Class**	**Levels**	**Values**
**Treatment**	2	Control GFP Ctr Meg
**Culture**	13	1 2 3 4 5 6 7 8 9 10 11 12 13

Dimensions	
**Covariance Parameters**	2
**Columns in X**	3
**Columns in Z**	13
**Subjects**	1
**Max Obs per Subject**	45

Number of Observations	
**Number of Observations Read**	45
**Number of Observations Used**	45
**Number of Observations Not Used**	0

Iteration History			
**Iteration**	**Evaluations**	**-2 Res Log Like**	**Criterion**
**0**	1	193.84137366	
**1**	2	187.35383520	0.00002457
**2**	1	187.35247678	0.00000002
**3**	1	187.35247544	0.00000000

Convergence criteria met.

DistSoma=192

Covariance Parameter Estimates				
Cov Parm	Estimate	Alpha	Lower	Upper
Culture	1.9775	0.05	0.7636	12.2913
Residual	2.9261	0.05	1.8906	5.1270

Fit Statistics	
-2 Res Log Likelihood	187.4
AIC (Smaller is Better)	191.4
AICC (Smaller is Better)	191.7
BIC (Smaller is Better)	192.5

Solution for Fixed Effects									
Effect	Treatment	Estimate	Standard Error	DF	t Value	Pr > |t|	Alpha	Lower	Upper
Intercept		3.2359	0.6781	11	4.77	0.0006	0.05	1.7434	4.7283
Treatment	Control GFP	-1.4352	0.9431	32	-1.52	0.1379	0.05	-3.3563	0.4859
Treatment	Ctr Meg	0	.	.	.	.	.	.	.

Solution for Random Effects									
Effect	Culture	Estimate	Std Err Pred	DF	t Value	Pr > |t|	Alpha	Lower	Upper
Culture	1	-0.5362	0.9197	32	-0.58	0.5640	0.05	-2.4096	1.3372
Culture	2	0.8032	0.9197	32	0.87	0.3890	0.05	-1.0702	2.6766
Culture	3	-0.2195	0.8735	32	-0.25	0.8032	0.05	-1.9987	1.5597
Culture	4	-0.9495	0.8735	32	-1.09	0.2852	0.05	-2.7286	0.8297
Culture	5	0.3280	0.8735	32	0.38	0.7098	0.05	-1.4512	2.1072
Culture	6	0.8869	1.1180	32	0.79	0.4334	0.05	-1.3903	3.1642
Culture	7	-0.3130	0.9197	32	-0.34	0.7359	0.05	-2.1864	1.5604
Culture	8	0.3753	0.8826	32	0.43	0.6735	0.05	-1.4225	2.1731
Culture	9	-1.6321	0.8826	32	-1.85	0.0737	0.05	-3.4299	0.1657
Culture	10	1.1814	0.9270	32	1.27	0.2117	0.05	-0.7069	3.0697
Culture	11	0.5896	0.8517	32	0.69	0.4937	0.05	-1.1452	2.3245
Culture	12	1.6528	0.8826	32	1.87	0.0703	0.05	-0.1450	3.4506
Culture	13	-2.1670	0.9270	32	-2.34	0.0258	0.05	-4.0553	-0.2787

Type 3 Tests of Fixed Effects				
Effect	Num DF	Den DF	F Value	Pr > F
Treatment	1	32	2.32	0.1379

DistSoma=192

Least Squares Means									
**Effect**	**Treatment**	**Estimate**	**Standard Error**	**DF**	**t Value**	**Pr > |t|**	**Alpha**	**Lower**	**Upper**
**Treatment**	Control GFP	1.8007	0.6555	32	2.75	0.0098	0.05	0.4655	3.1359
**Treatment**	Ctr Meg	3.2359	0.6781	32	4.77	<.0001	0.05	1.8547	4.6171

Differences of Least Squares Means												
**Effect**	**Treatment**	**Treatment**	**Estimate**	**Standard Error**	**DF**	**t Value**	**Pr > |t|**	**Adjustment**	**Adj P**	**Alpha**	**Lower**	**Upper**
**Treatment**	Control GFP	Ctr Meg	-1.4352	0.9431	32	-1.52	0.1379	Tukey-Kramer	0.1379	0.05	-3.3563	0.4859

Differences of Least Squares Means				
**Effect**	**Treatment**	**Treatment**	**Adj Lower**	**Adj Upper**
**Treatment**	Control GFP	Ctr Meg	-3.3562	0.4859

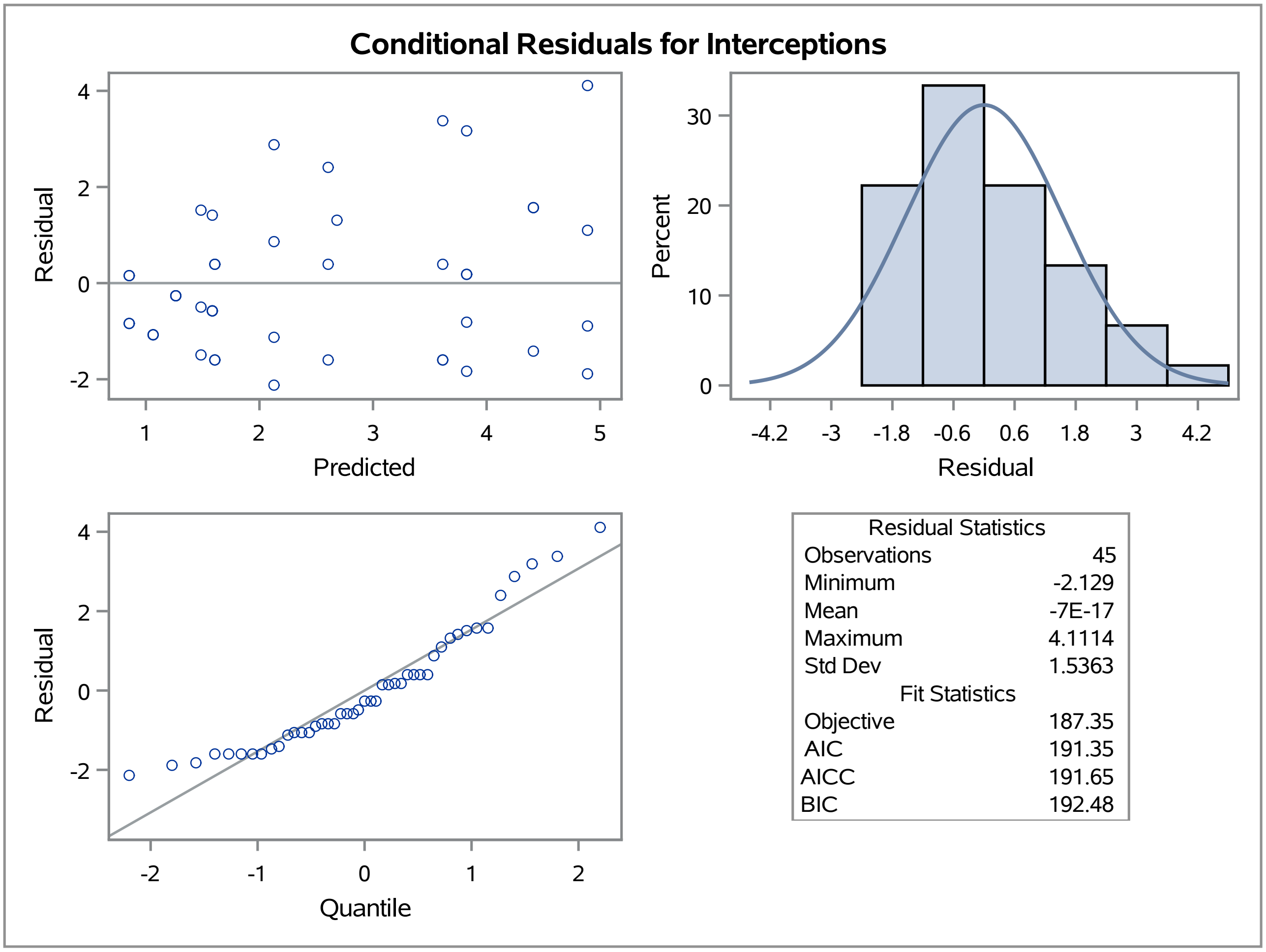

DistSoma=198

Model Information	
**Data Set**	WORK.TEMPDATASORTED
**Dependent Variable**	Interceptions
**Covariance Structure**	Variance Components
**Estimation Method**	REML
**Residual Variance Method**	Profile
**Fixed Effects SE Method**	Model-Based
**Degrees of Freedom Method**	Containment

Class Level Information		
**Class**	**Levels**	**Values**
**Treatment**	2	Control GFP Ctr Meg
**Culture**	13	1 2 3 4 5 6 7 8 9 10 11 12 13

Dimensions	
**Covariance Parameters**	2
**Columns in X**	3
**Columns in Z**	13
**Subjects**	1
**Max Obs per Subject**	45

Number of Observations	
**Number of Observations Read**	45
**Number of Observations Used**	45
**Number of Observations Not Used**	0

Iteration History			
**Iteration**	**Evaluations**	**-2 Res Log Like**	**Criterion**
**0**	1	196.68776541	
**1**	2	189.80684004	0.00001221
**2**	1	189.80615353	0.00000001

Convergence criteria met.

Covariance Parameter Estimates				
**Cov Parm**	**Estimate**	**Alpha**	**Lower**	**Upper**
**Culture**	2.1504	0.05	0.8403	12.8032
**Residual**	3.0803	0.05	1.9916	5.3913

DistSoma=198

Fit Statistics	
-2 Res Log Likelihood	189.8
AIC (Smaller is Better)	193.8
AICC (Smaller is Better)	194.1
BIC (Smaller is Better)	194.9

Solution for Fixed Effects

Effect	Treatment	Estimate	Standard Error	DF	t Value	Pr > |t|	Alpha	Lower	Upper
Intercept		3.1241	0.7039	11	4.44	0.0010	0.05	1.5748	4.6735
Treatment	Control GFP	-1.2334	0.9787	32	-1.26	0.2167	0.05	-3.2269	0.7602
Treatment	Ctr Meg	0	.	.	.	.	.	.	.

Solution for Random Effects

Effect	Culture	Estimate	Std Err Pred	DF	t Value	Pr > |t|	Alpha	Lower	Upper
Culture	1	-0.3773	0.9522	32	-0.40	0.6946	0.05	-2.3170	1.5624
Culture	2	0.7507	0.9522	32	0.79	0.4363	0.05	-1.1889	2.6904
Culture	3	-0.2878	0.9043	32	-0.32	0.7524	0.05	-2.1297	1.5542
Culture	4	-0.8400	0.9043	32	-0.93	0.3599	0.05	-2.6819	1.0020
Culture	5	0.2645	0.9043	32	0.29	0.7718	0.05	-1.5775	2.1064
Culture	6	0.8671	1.1595	32	0.75	0.4600	0.05	-1.4948	3.2290
Culture	7	-0.3773	0.9522	32	-0.40	0.6946	0.05	-2.3170	1.5624
Culture	8	0.2767	0.9141	32	0.30	0.7640	0.05	-1.5853	2.1388
Culture	9	-1.9322	0.9141	32	-2.11	0.0424	0.05	-3.7943	-0.07015
Culture	10	1.4952	0.9602	32	1.56	0.1292	0.05	-0.4606	3.4511
Culture	11	0.5253	0.8822	32	0.60	0.5557	0.05	-1.2716	2.3223
Culture	12	1.7494	0.9141	32	1.91	0.0646	0.05	-0.1127	3.6114
Culture	13	-2.1145	0.9602	32	-2.20	0.0350	0.05	-4.0703	-0.1587

Type 3 Tests of Fixed Effects

Effect	Num DF	Den DF	F Value	Pr > F
Treatment	1	32	1.59	0.2167

Least Squares Means

Effect	Treatment	Estimate	Standard Error	DF	t Value	Pr > |t|	Alpha	Lower	Upper
Treatment	Control GFP	1.8908	0.6800	32	2.78	0.0090	0.05	0.5057	3.2759
Treatment	Ctr Meg	3.1241	0.7039	32	4.44	0.0001	0.05	1.6903	4.5580

DistSoma=198

Differences of Least Squares Means												
Effect	Treatment	Treatment	Estimate	Standard Error	DF	t Value	Pr > |t|	Adjustment	Adj P	Alpha	Lower	Upper
**Treatment**	Control GFP	Ctr Meg	-1.2334	0.9787	32	-1.26	0.2167	Tukey-Kramer	0.2167	0.05	-3.2269	0.7602

Differences of Least Squares Means				
Effect	Treatment	Treatment	Adj Lower	Adj Upper
**Treatment**	Control GFP	Ctr Meg	-3.2269	0.7602

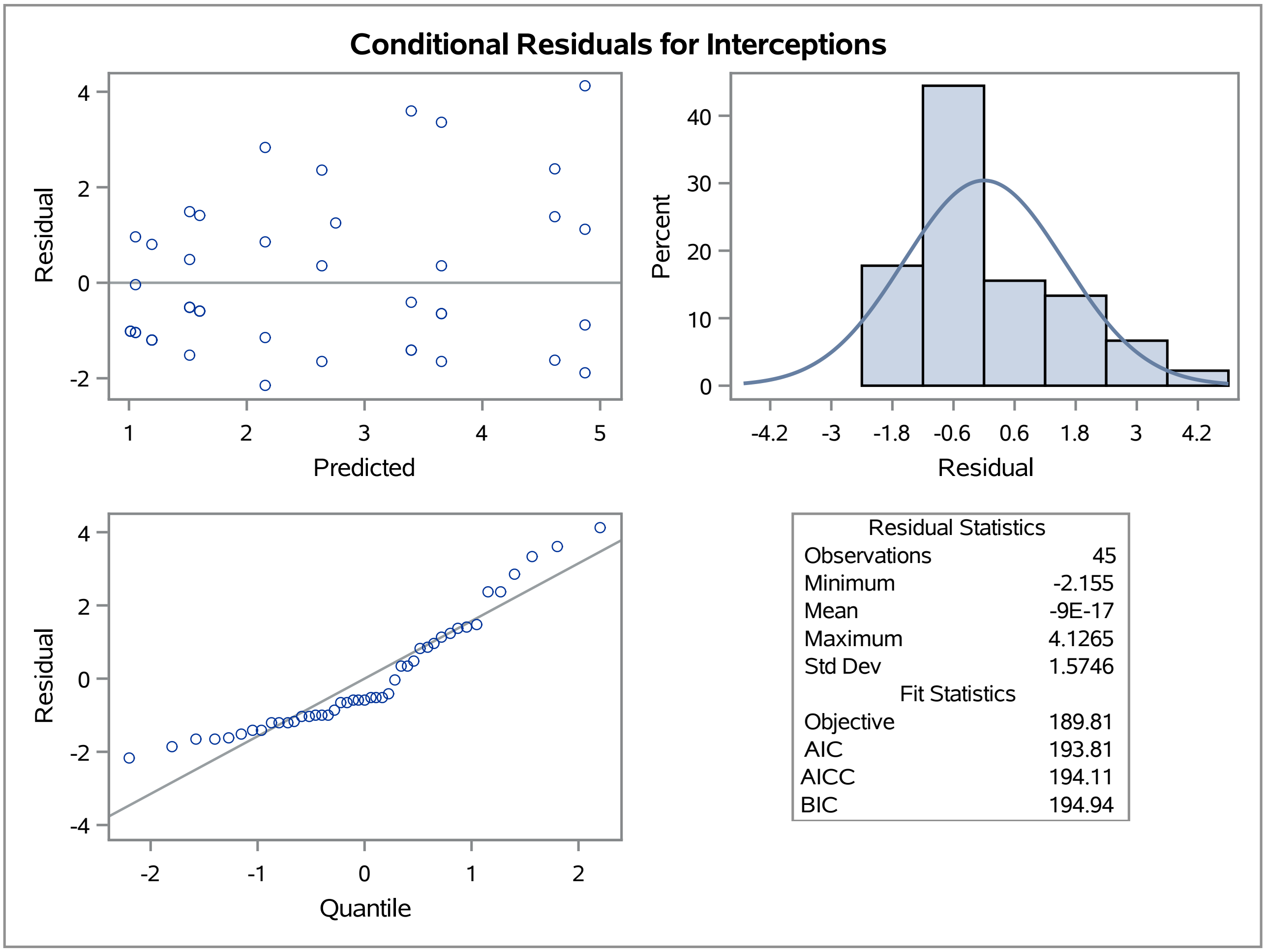

DistSoma=204

Model Information	
**Data Set**	WORK.TEMPDATASORTED
**Dependent Variable**	Interceptions
**Covariance Structure**	Variance Components
**Estimation Method**	REML
**Residual Variance Method**	Profile
**Fixed Effects SE Method**	Model-Based
**Degrees of Freedom Method**	Containment

Class Level Information		
**Class**	**Levels**	**Values**
**Treatment**	2	Control GFP Ctr Meg
**Culture**	13	1 2 3 4 5 6 7 8 9 10 11 12 13

Dimensions	
**Covariance Parameters**	2
**Columns in X**	3
**Columns in Z**	13
**Subjects**	1
**Max Obs per Subject**	45

Number of Observations	
**Number of Observations Read**	45
**Number of Observations Used**	45
**Number of Observations Not Used**	0

Iteration History			
**Iteration**	**Evaluations**	**-2 Res Log Like**	**Criterion**
**0**	1	192.53526681	
**1**	2	186.50426158	0.00000161
**2**	1	186.50417461	0.00000000

Convergence criteria met.

Covariance Parameter Estimates				
**Cov Parm**	**Estimate**	**Alpha**	**Lower**	**Upper**
**Culture**	1.7729	0.05	0.6793	11.3332
**Residual**	2.9226	0.05	1.8939	5.0966

DistSoma=204

Fit Statistics	
-2 Res Log Likelihood	186.5
AIC (Smaller is Better)	190.5
AICC (Smaller is Better)	190.8
BIC (Smaller is Better)	191.6

Solution for Fixed Effects

Effect	Treatment	Estimate	Standard Error	DF	t Value	Pr > |t|	Alpha	Lower	Upper
Intercept		2.9201	0.6523	11	4.48	0.0009	0.05	1.4845	4.3557
Treatment	Control GFP	-1.2079	0.9083	32	-1.33	0.1930	0.05	-3.0579	0.6422
Treatment	Ctr Meg	0	.	.	.	.	.	.	.

Solution for Random Effects

Effect	Culture	Estimate	Std Err Pred	DF	t Value	Pr > |t|	Alpha	Lower	Upper
Culture	1	-0.2445	0.8917	32	-0.27	0.7857	0.05	-2.0608	1.5718
Culture	2	0.8311	0.8917	32	0.93	0.3583	0.05	-0.9852	2.6474
Culture	3	-0.1503	0.8472	32	-0.18	0.8603	0.05	-1.8760	1.5754
Culture	4	-0.6814	0.8472	32	-0.80	0.4272	0.05	-2.4071	1.0443
Culture	5	0.3809	0.8472	32	0.45	0.6561	0.05	-1.3448	2.1066
Culture	6	0.1087	1.0773	32	0.10	0.9203	0.05	-2.0856	2.3030
Culture	7	-0.2445	0.8917	32	-0.27	0.7857	0.05	-2.0608	1.5718
Culture	8	0.5877	0.8549	32	0.69	0.4967	0.05	-1.1536	2.3290
Culture	9	-1.8908	0.8549	32	-2.21	0.0342	0.05	-3.6321	-0.1495
Culture	10	1.5575	0.8977	32	1.73	0.0924	0.05	-0.2712	3.3861
Culture	11	0.5113	0.8248	32	0.62	0.5397	0.05	-1.1686	2.1913
Culture	12	1.1188	0.8549	32	1.31	0.1999	0.05	-0.6224	2.8601
Culture	13	-1.8845	0.8977	32	-2.10	0.0438	0.05	-3.7132	-0.05591

Type 3 Tests of Fixed Effects

Effect	Num DF	Den DF	F Value	Pr > F
Treatment	1	32	1.77	0.1930

Least Squares Means

Effect	Treatment	Estimate	Standard Error	DF	t Value	Pr > |t|	Alpha	Lower	Upper
Treatment	Control GFP	1.7122	0.6320	32	2.71	0.0107	0.05	0.4248	2.9996
Treatment	Ctr Meg	2.9201	0.6523	32	4.48	<.0001	0.05	1.5915	4.2487

DistSoma=204

Differences of Least Squares Means												
Effect	Treatment	Treatment	Estimate	Standard Error	DF	t Value	Pr > |t|	Adjustment	Adj P	Alpha	Lower	Upper
Treatment	Control GFP	Ctr Meg	-1.2079	0.9083	32	-1.33	0.1930	Tukey-Kramer	0.1930	0.05	-3.0579	0.6422

Differences of Least Squares Means				
Effect	Treatment	Treatment	Adj Lower	Adj Upper
Treatment	Control GFP	Ctr Meg	-3.0579	0.6421

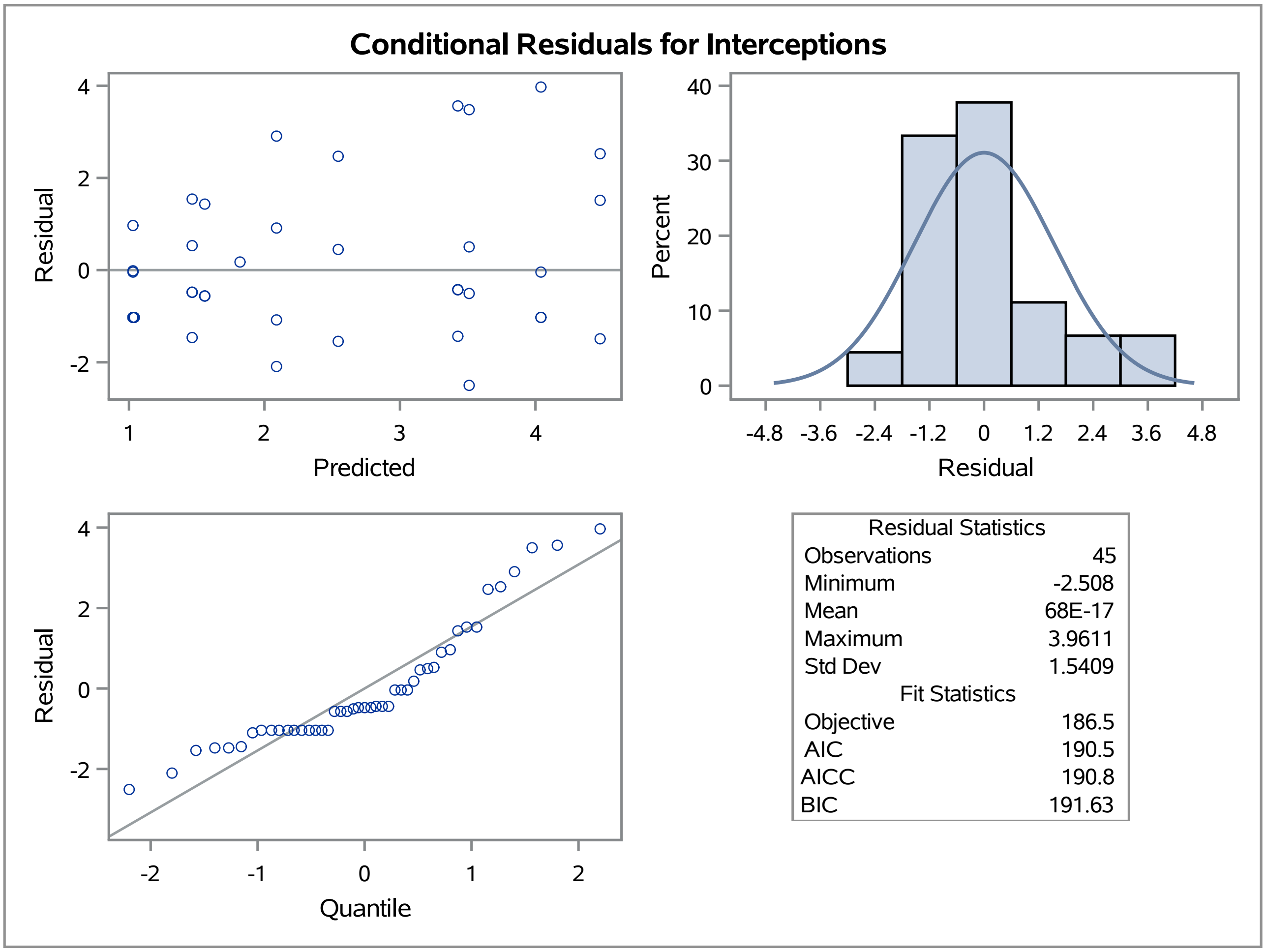

DistSoma=210

Model Information	
**Data Set**	WORK.TEMPDATASORTED
**Dependent Variable**	Interceptions
**Covariance Structure**	Variance Components
**Estimation Method**	REML
**Residual Variance Method**	Profile
**Fixed Effects SE Method**	Model-Based
**Degrees of Freedom Method**	Containment

Class Level Information		
**Class**	**Levels**	**Values**
**Treatment**	2	Control GFP Ctr Meg
**Culture**	13	1 2 3 4 5 6 7 8 9 10 11 12 13

Dimensions	
**Covariance Parameters**	2
**Columns in X**	3
**Columns in Z**	13
**Subjects**	1
**Max Obs per Subject**	45

Number of Observations	
**Number of Observations Read**	45
**Number of Observations Used**	45
**Number of Observations Not Used**	0

Iteration History			
**Iteration**	**Evaluations**	**-2 Res Log Like**	**Criterion**
**0**	1	200.60826447	
**1**	2	196.29523568	0.00000143
**2**	1	196.29515163	0.00000000

Convergence criteria met.

Covariance Parameter Estimates				
**Cov Parm**	**Estimate**	**Alpha**	**Lower**	**Upper**
**Culture**	1.8011	0.05	0.6418	15.3347
**Residual**	3.8230	0.05	2.4769	6.6683

DistSoma=210

Fit Statistics	
-2 Res Log Likelihood	196.3
AIC (Smaller is Better)	200.3
AICC (Smaller is Better)	200.6
BIC (Smaller is Better)	201.4

Solution for Fixed Effects

Effect	Treatment	Estimate	Standard Error	DF	t Value	Pr > |t|	Alpha	Lower	Upper
Intercept		3.0506	0.6855	11	4.45	0.0010	0.05	1.5419	4.5593
Treatment	Control GFP	-1.5273	0.9571	32	-1.60	0.1204	0.05	-3.4767	0.4222
Treatment	Ctr Meg	0	.	.	.	.	.	.	.

Solution for Random Effects

Effect	Culture	Estimate	Std Err Pred	DF	t Value	Pr > |t|	Alpha	Lower	Upper
Culture	1	-0.1113	0.9483	32	-0.12	0.9073	0.05	-2.0430	1.8204
Culture	2	0.8648	0.9483	32	0.91	0.3686	0.05	-1.0669	2.7964
Culture	3	-0.01528	0.9027	32	-0.02	0.9866	0.05	-1.8540	1.8234
Culture	4	-0.5053	0.9027	32	-0.56	0.5796	0.05	-2.3439	1.3334
Culture	5	0.3114	0.9027	32	0.34	0.7324	0.05	-1.5273	2.1501
Culture	6	0.1526	1.1270	32	0.14	0.8931	0.05	-2.1429	2.4482
Culture	7	-0.6969	0.9483	32	-0.73	0.4677	0.05	-2.6286	1.2347
Culture	8	1.1102	0.9083	32	1.22	0.2305	0.05	-0.7399	2.9603
Culture	9	-1.8297	0.9083	32	-2.01	0.0524	0.05	-3.6798	0.02038
Culture	10	1.3368	0.9526	32	1.40	0.1701	0.05	-0.6035	3.2772
Culture	11	0.3856	0.8765	32	0.44	0.6629	0.05	-1.3998	2.1710
Culture	12	0.7836	0.9083	32	0.86	0.3947	0.05	-1.0665	2.6336
Culture	13	-1.7866	0.9526	32	-1.88	0.0699	0.05	-3.7269	0.1538

Type 3 Tests of Fixed Effects

Effect	Num DF	Den DF	F Value	Pr > F
Treatment	1	32	2.55	0.1204

Least Squares Means

Effect	Treatment	Estimate	Standard Error	DF	t Value	Pr > |t|	Alpha	Lower	Upper
Treatment	Control GFP	1.5234	0.6679	32	2.28	0.0294	0.05	0.1629	2.8839
Treatment	Ctr Meg	3.0506	0.6855	32	4.45	<.0001	0.05	1.6544	4.4469

DistSoma=210

Differences of Least Squares Means												
**Effect**	**Treatment**	**Treatment**	**Estimate**	**Standard Error**	**DF**	**t Value**	**Pr > |t|**	**Adjustment**	**Adj P**	**Alpha**	**Lower**	**Upper**
**Treatment**	Control GFP	Ctr Meg	-1.5273	0.9571	32	-1.60	0.1204	Tukey-Kramer	0.1204	0.05	-3.4767	0.4222

Differences of Least Squares Means				
**Effect**	**Treatment**	**Treatment**	**Adj Lower**	**Adj Upper**
**Treatment**	Control GFP	Ctr Meg	-3.4767	0.4222

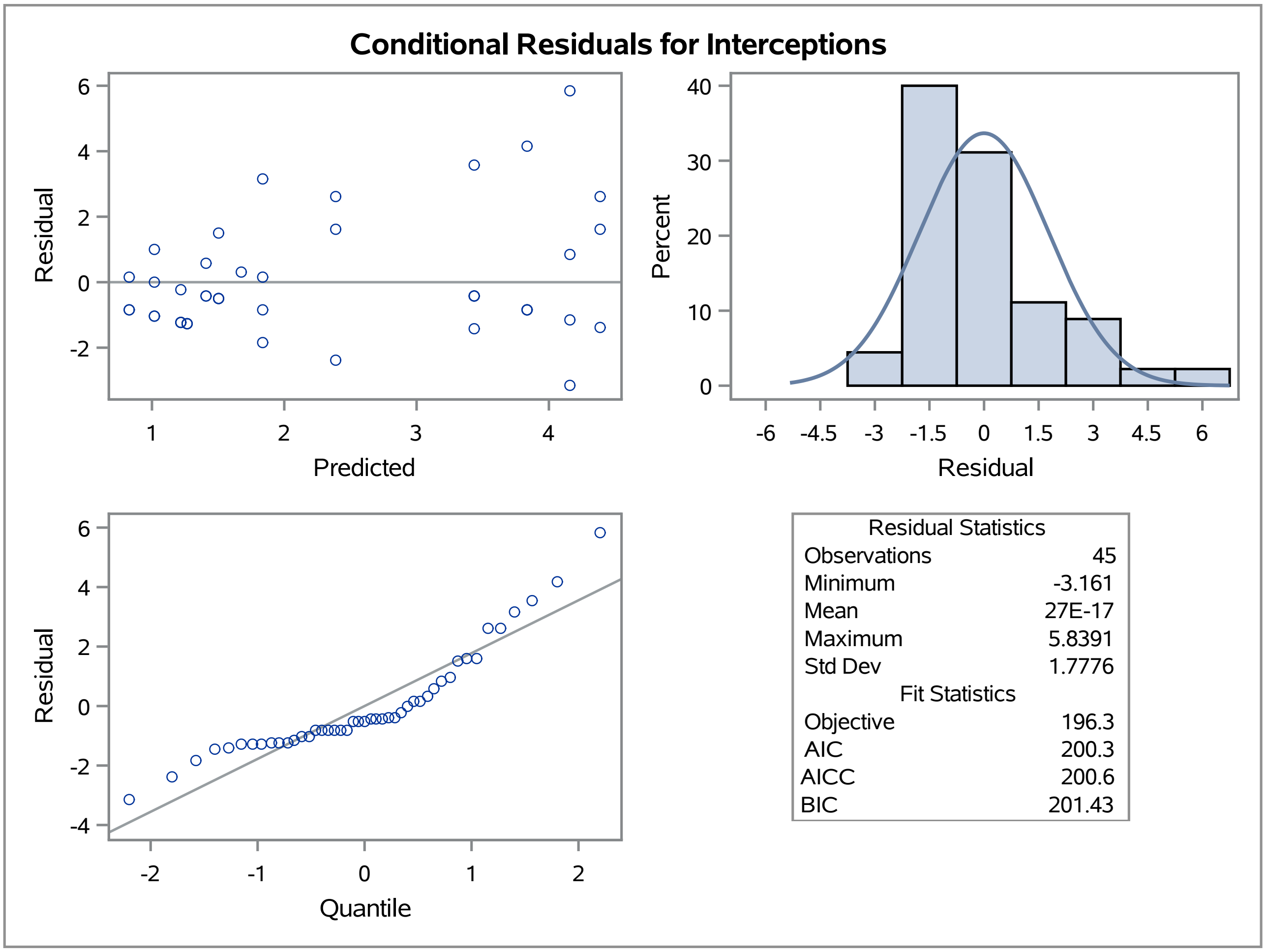

DistSoma=216

Model Information	
**Data Set**	WORK.TEMPDATASORTED
**Dependent Variable**	Interceptions
**Covariance Structure**	Variance Components
**Estimation Method**	REML
**Residual Variance Method**	Profile
**Fixed Effects SE Method**	Model-Based
**Degrees of Freedom Method**	Containment

Class Level Information		
**Class**	**Levels**	**Values**
**Treatment**	2	Control GFP Ctr Meg
**Culture**	13	1 2 3 4 5 6 7 8 9 10 11 12 13

Dimensions	
**Covariance Parameters**	2
**Columns in X**	3
**Columns in Z**	13
**Subjects**	1
**Max Obs per Subject**	45

Number of Observations	
**Number of Observations Read**	45
**Number of Observations Used**	45
**Number of Observations Not Used**	0

Iteration History			
**Iteration**	**Evaluations**	**-2 Res Log Like**	**Criterion**
**0**	1	195.81330784	
**1**	2	192.72354049	0.00000015
**2**	1	192.72353192	0.00000000

Convergence criteria met.

Covariance Parameter Estimates				
**Cov Parm**	**Estimate**	**Alpha**	**Lower**	**Upper**
**Culture**	1.3356	0.05	0.4374	16.7424
**Residual**	3.6512	0.05	2.3666	6.3646

DistSoma=216

Fit Statistics	
-2 Res Log Likelihood	192.7
AIC (Smaller is Better)	196.7
AICC (Smaller is Better)	197.0
BIC (Smaller is Better)	197.9

Solution for Fixed Effects

Effect	Treatment	Estimate	Standard Error	DF	t Value	Pr > |t|	Alpha	Lower	Upper
Intercept		2.8756	0.6200	11	4.64	0.0007	0.05	1.5111	4.2401
Treatment	Control GFP	-1.5001	0.8680	32	-1.73	0.0936	0.05	-3.2680	0.2679
Treatment	Ctr Meg	0	.	.	.	.	.	.	.

Solution for Random Effects

Effect	Culture	Estimate	Std Err Pred	DF	t Value	Pr > |t|	Alpha	Lower	Upper
Culture	1	-0.02207	0.8590	32	-0.03	0.9797	0.05	-1.7717	1.7276
Culture	2	0.3267	0.8590	32	0.38	0.7062	0.05	-1.4229	2.0764
Culture	3	0.07395	0.8200	32	0.09	0.9287	0.05	-1.5964	1.7443
Culture	4	-0.3716	0.8200	32	-0.45	0.6535	0.05	-2.0419	1.2988
Culture	5	0.3710	0.8200	32	0.45	0.6540	0.05	-1.2994	2.0413
Culture	6	0.1673	1.0022	32	0.17	0.8685	0.05	-1.8741	2.2086
Culture	7	-0.5453	0.8590	32	-0.63	0.5301	0.05	-2.2949	1.2044
Culture	8	0.8164	0.8233	32	0.99	0.3288	0.05	-0.8606	2.4935
Culture	9	-1.5597	0.8233	32	-1.89	0.0672	0.05	-3.2367	0.1174
Culture	10	1.1115	0.8614	32	1.29	0.2062	0.05	-0.6431	2.8661
Culture	11	0.4683	0.7955	32	0.59	0.5602	0.05	-1.1520	2.0886
Culture	12	0.6679	0.8233	32	0.81	0.4232	0.05	-1.0091	2.3450
Culture	13	-1.5046	0.8614	32	-1.75	0.0903	0.05	-3.2592	0.2501

Type 3 Tests of Fixed Effects

Effect	Num DF	Den DF	F Value	Pr > F
Treatment	1	32	2.99	0.0936

Least Squares Means

Effect	Treatment	Estimate	Standard Error	DF	t Value	Pr > |t|	Alpha	Lower	Upper
Treatment	Control GFP	1.3755	0.6075	32	2.26	0.0305	0.05	0.1382	2.6128
Treatment	Ctr Meg	2.8756	0.6200	32	4.64	<.0001	0.05	1.6128	4.1384

DistSoma=216

Differences of Least Squares Means												
**Effect**	**Treatment**	**Treatment**	**Estimate**	**Standard Error**	**DF**	**t Value**	**Pr > |t|**	**Adjustment**	**Adj P**	**Alpha**	**Lower**	**Upper**
**Treatment**	Control GFP	Ctr Meg	-1.5001	0.8680	32	-1.73	0.0936	Tukey-Kramer	0.0936	0.05	-3.2680	0.2679

Differences of Least Squares Means				
**Effect**	**Treatment**	**Treatment**	**Adj Lower**	**Adj Upper**
**Treatment**	Control GFP	Ctr Meg	-3.2680	0.2679

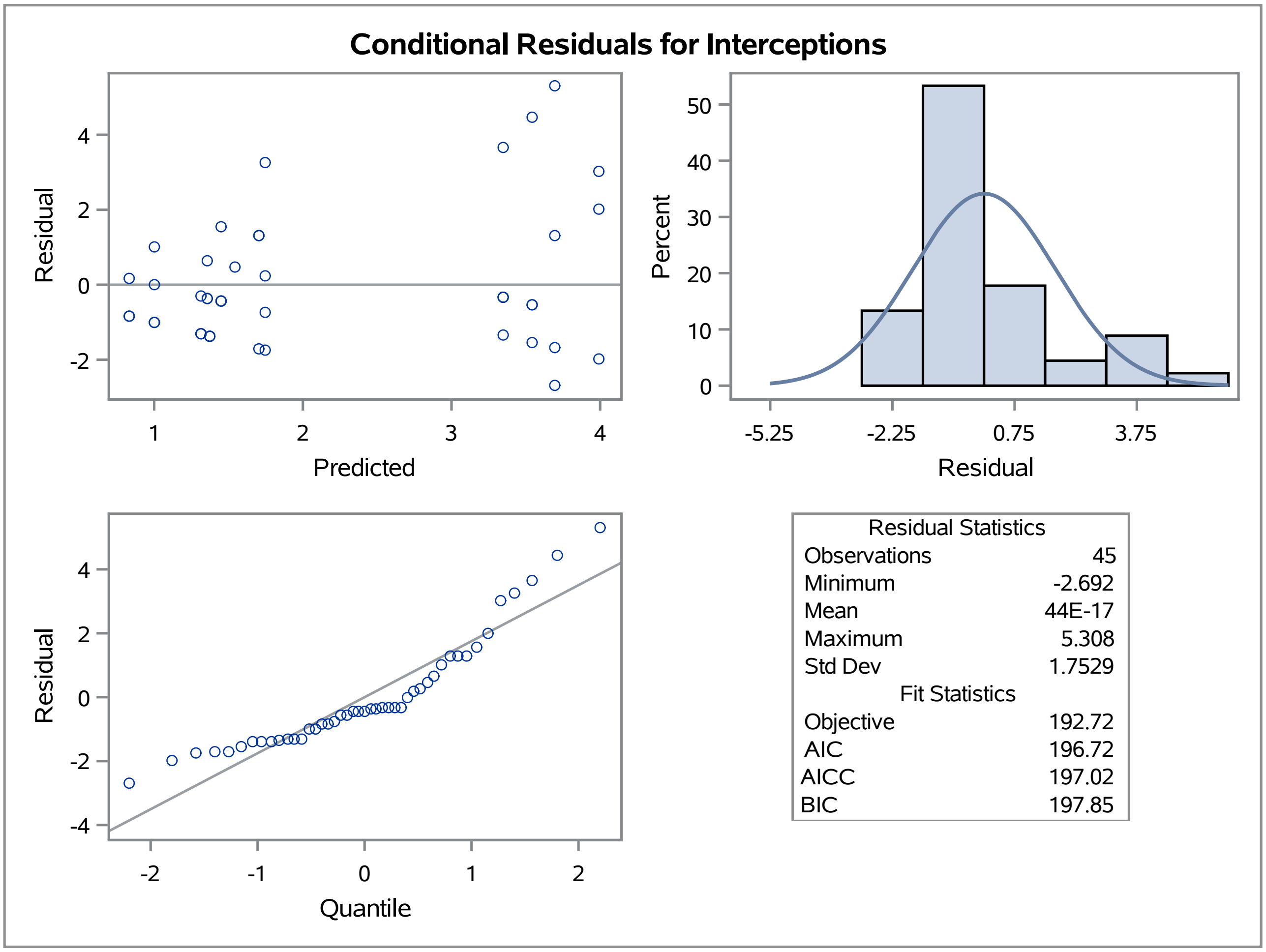

DistSoma=222

Model Information	
**Data Set**	WORK.TEMPDATASORTED
**Dependent Variable**	Interceptions
**Covariance Structure**	Variance Components
**Estimation Method**	REML
**Residual Variance Method**	Profile
**Fixed Effects SE Method**	Model-Based
**Degrees of Freedom Method**	Containment

Class Level Information		
**Class**	**Levels**	**Values**
**Treatment**	2	Control GFP Ctr Meg
**Culture**	13	1 2 3 4 5 6 7 8 9 10 11 12 13

Dimensions	
**Covariance Parameters**	2
**Columns in X**	3
**Columns in Z**	13
**Subjects**	1
**Max Obs per Subject**	45

Number of Observations	
**Number of Observations Read**	45
**Number of Observations Used**	45
**Number of Observations Not Used**	0

Iteration History			
**Iteration**	**Evaluations**	**-2 Res Log Like**	**Criterion**
**0**	1	187.70106680	
**1**	2	184.09940309	0.00000245
**2**	1	184.09927371	0.00000000

Convergence criteria met.

Covariance Parameter Estimates				
**Cov Parm**	**Estimate**	**Alpha**	**Lower**	**Upper**
**Culture**	1.2220	0.05	0.4167	12.6273
**Residual**	2.9326	0.05	1.8991	5.1194

DistSoma=222

Fit Statistics	
-2 Res Log Likelihood	184.1
AIC (Smaller is Better)	188.1
AICC (Smaller is Better)	188.4
BIC (Smaller is Better)	189.2

Solution for Fixed Effects

Effect	Treatment	Estimate	Standard Error	DF	t Value	Pr > |t|	Alpha	Lower	Upper
Intercept		2.5844	0.5777	11	4.47	0.0009	0.05	1.3129	3.8558
Treatment	Control GFP	-1.3372	0.8076	32	-1.66	0.1076	0.05	-2.9823	0.3079
Treatment	Ctr Meg	0	.	.	.	.	.	.	.

Solution for Random Effects

Effect	Culture	Estimate	Std Err Pred	DF	t Value	Pr > |t|	Alpha	Lower	Upper
Culture	1	0.04787	0.8009	32	0.06	0.9527	0.05	-1.5835	1.6792
Culture	2	0.4183	0.8009	32	0.52	0.6051	0.05	-1.2131	2.0496
Culture	3	-0.1545	0.7633	32	-0.20	0.8409	0.05	-1.7093	1.4004
Culture	4	-0.3107	0.7633	32	-0.41	0.6867	0.05	-1.8656	1.2441
Culture	5	0.4705	0.7633	32	0.62	0.5420	0.05	-1.0843	2.0254
Culture	6	0.2214	0.9435	32	0.23	0.8159	0.05	-1.7003	2.1432
Culture	7	-0.6929	0.8009	32	-0.87	0.3934	0.05	-2.3242	0.9385
Culture	8	0.7285	0.7672	32	0.95	0.3494	0.05	-0.8342	2.2913
Culture	9	-1.4590	0.7672	32	-1.90	0.0662	0.05	-3.0217	0.1037
Culture	10	1.1569	0.8038	32	1.44	0.1598	0.05	-0.4804	2.7941
Culture	11	0.2808	0.7407	32	0.38	0.7071	0.05	-1.2279	1.7896
Culture	12	0.7285	0.7672	32	0.95	0.3494	0.05	-0.8342	2.2913
Culture	13	-1.4358	0.8038	32	-1.79	0.0835	0.05	-3.0731	0.2015

Type 3 Tests of Fixed Effects

Effect	Num DF	Den DF	F Value	Pr > F
Treatment	1	32	2.74	0.1076

Least Squares Means

Effect	Treatment	Estimate	Standard Error	DF	t Value	Pr > |t|	Alpha	Lower	Upper
Treatment	Control GFP	1.2472	0.5644	32	2.21	0.0344	0.05	0.09748	2.3968
Treatment	Ctr Meg	2.5844	0.5777	32	4.47	<.0001	0.05	1.4077	3.7610

DistSoma=222

Differences of Least Squares Means												
**Effect**	**Treatment**	**Treatment**	**Estimate**	**Standard Error**	**DF**	**t Value**	**Pr > |t|**	**Adjustment**	**Adj P**	**Alpha**	**Lower**	**Upper**
**Treatment**	Control GFP	Ctr Meg	-1.3372	0.8076	32	-1.66	0.1076	Tukey-Kramer	0.1076	0.05	-2.9823	0.3079

Differences of Least Squares Means				
**Effect**	**Treatment**	**Treatment**	**Adj Lower**	**Adj Upper**
**Treatment**	Control GFP	Ctr Meg	-2.9823	0.3079

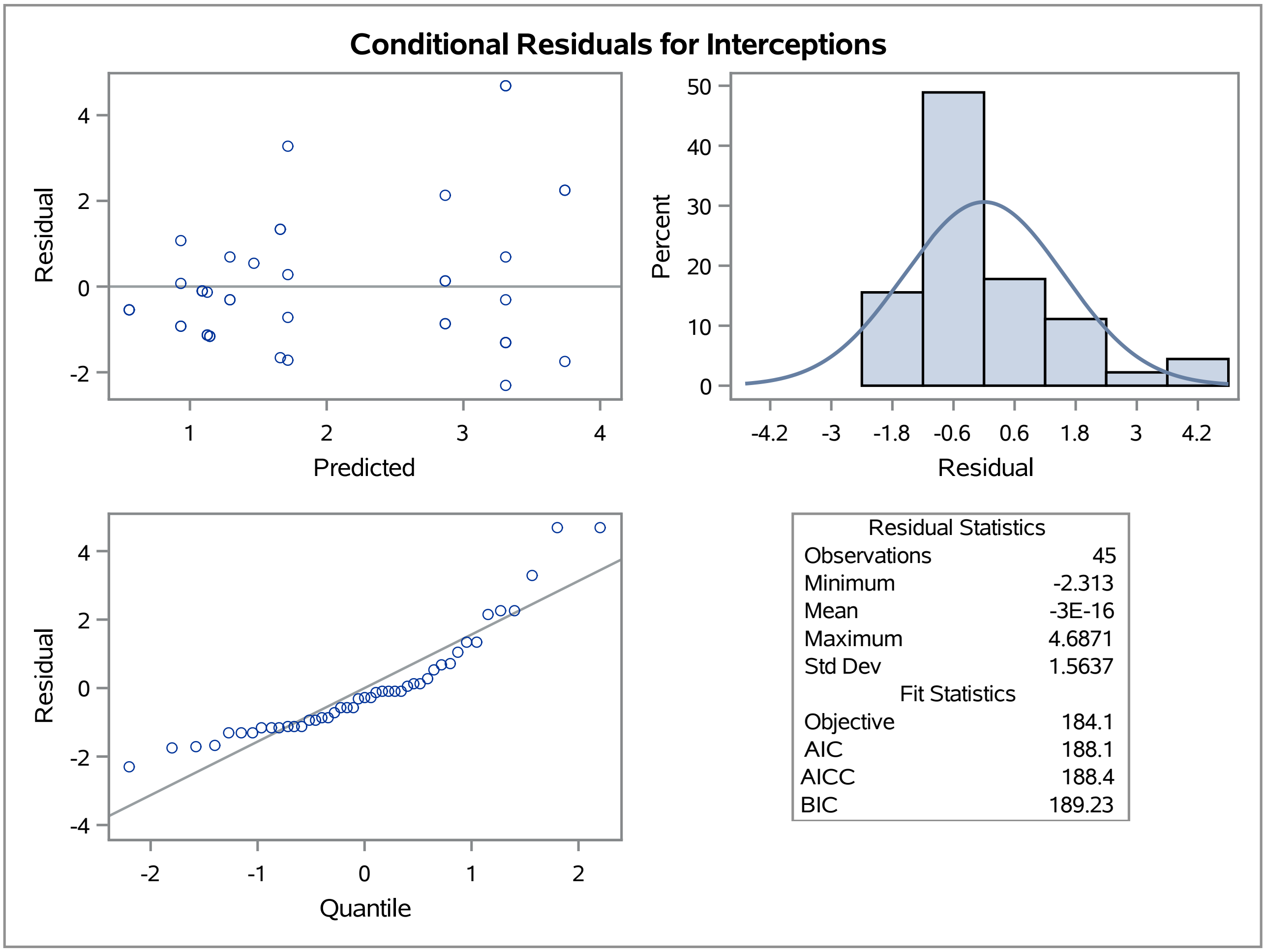

DistSoma=228

Model Information	
**Data Set**	WORK.TEMPDATASORTED
**Dependent Variable**	Interceptions
**Covariance Structure**	Variance Components
**Estimation Method**	REML
**Residual Variance Method**	Profile
**Fixed Effects SE Method**	Model-Based
**Degrees of Freedom Method**	Containment

Class Level Information		
**Class**	**Levels**	**Values**
**Treatment**	2	Control GFP Ctr Meg
**Culture**	13	1 2 3 4 5 6 7 8 9 10 11 12 13

Dimensions	
**Covariance Parameters**	2
**Columns in X**	3
**Columns in Z**	13
**Subjects**	1
**Max Obs per Subject**	45

Number of Observations	
**Number of Observations Read**	45
**Number of Observations Used**	45
**Number of Observations Not Used**	0

Iteration History			
**Iteration**	**Evaluations**	**-2 Res Log Like**	**Criterion**
**0**	1	175.75369596	
**1**	2	170.76436707	0.00000036
**2**	1	170.76435059	0.00000000

Convergence criteria met.

Covariance Parameter Estimates				
**Cov Parm**	**Estimate**	**Alpha**	**Lower**	**Upper**
**Culture**	1.0816	0.05	0.3973	8.1337
**Residual**	2.0785	0.05	1.3468	3.6252

DistSoma=228

Fit Statistics	
-2 Res Log Likelihood	170.8
AIC (Smaller is Better)	174.8
AICC (Smaller is Better)	175.1
BIC (Smaller is Better)	175.9

Solution for Fixed Effects

Effect	Treatment	Estimate	Standard Error	DF	t Value	Pr > |t|	Alpha	Lower	Upper
Intercept		2.1821	0.5221	11	4.18	0.0015	0.05	1.0329	3.3312
Treatment	Control GFP	-0.9314	0.7282	32	-1.28	0.2101	0.05	-2.4147	0.5519
Treatment	Ctr Meg	0	.	.	.	.	.	.	.

Solution for Random Effects

Effect	Culture	Estimate	Std Err Pred	DF	t Value	Pr > |t|	Alpha	Lower	Upper
Culture	1	0.05040	0.7198	32	0.07	0.9446	0.05	-1.4157	1.5165
Culture	2	0.4568	0.7198	32	0.63	0.5302	0.05	-1.0093	1.9229
Culture	3	-0.1693	0.6845	32	-0.25	0.8062	0.05	-1.5636	1.2250
Culture	4	-0.3382	0.6845	32	-0.49	0.6247	0.05	-1.7325	1.0562
Culture	5	0.5062	0.6845	32	0.74	0.4650	0.05	-0.8882	1.9005
Culture	6	0.2565	0.8612	32	0.30	0.7678	0.05	-1.4976	2.0106
Culture	7	-0.7623	0.7198	32	-1.06	0.2975	0.05	-2.2284	0.7038
Culture	8	0.7214	0.6895	32	1.05	0.3033	0.05	-0.6830	2.1258
Culture	9	-1.4739	0.6895	32	-2.14	0.0403	0.05	-2.8783	-0.06951
Culture	10	1.1081	0.7236	32	1.53	0.1355	0.05	-0.3658	2.5820
Culture	11	0.5909	0.6652	32	0.89	0.3811	0.05	-0.7642	1.9459
Culture	12	0.3836	0.6895	32	0.56	0.5818	0.05	-1.0208	1.7880
Culture	13	-1.3300	0.7236	32	-1.84	0.0753	0.05	-2.8040	0.1439

Type 3 Tests of Fixed Effects

Effect	Num DF	Den DF	F Value	Pr > F
Treatment	1	32	1.64	0.2101

Least Squares Means

Effect	Treatment	Estimate	Standard Error	DF	t Value	Pr > |t|	Alpha	Lower	Upper
Treatment	Control GFP	1.2506	0.5076	32	2.46	0.0193	0.05	0.2166	2.2847
Treatment	Ctr Meg	2.1821	0.5221	32	4.18	0.0002	0.05	1.1186	3.2455

DistSoma=228

Differences of Least Squares Means												
**Effect**	**Treatment**	**Treatment**	**Estimate**	**Standard Error**	**DF**	**t Value**	**Pr > |t|**	**Adjustment**	**Adj P**	**Alpha**	**Lower**	**Upper**
**Treatment**	Control GFP	Ctr Meg	-0.9314	0.7282	32	-1.28	0.2101	Tukey-Kramer	0.2101	0.05	-2.4147	0.5519

Differences of Least Squares Means				
**Effect**	**Treatment**	**Treatment**	**Adj Lower**	**Adj Upper**
**Treatment**	Control GFP	Ctr Meg	-2.4147	0.5519

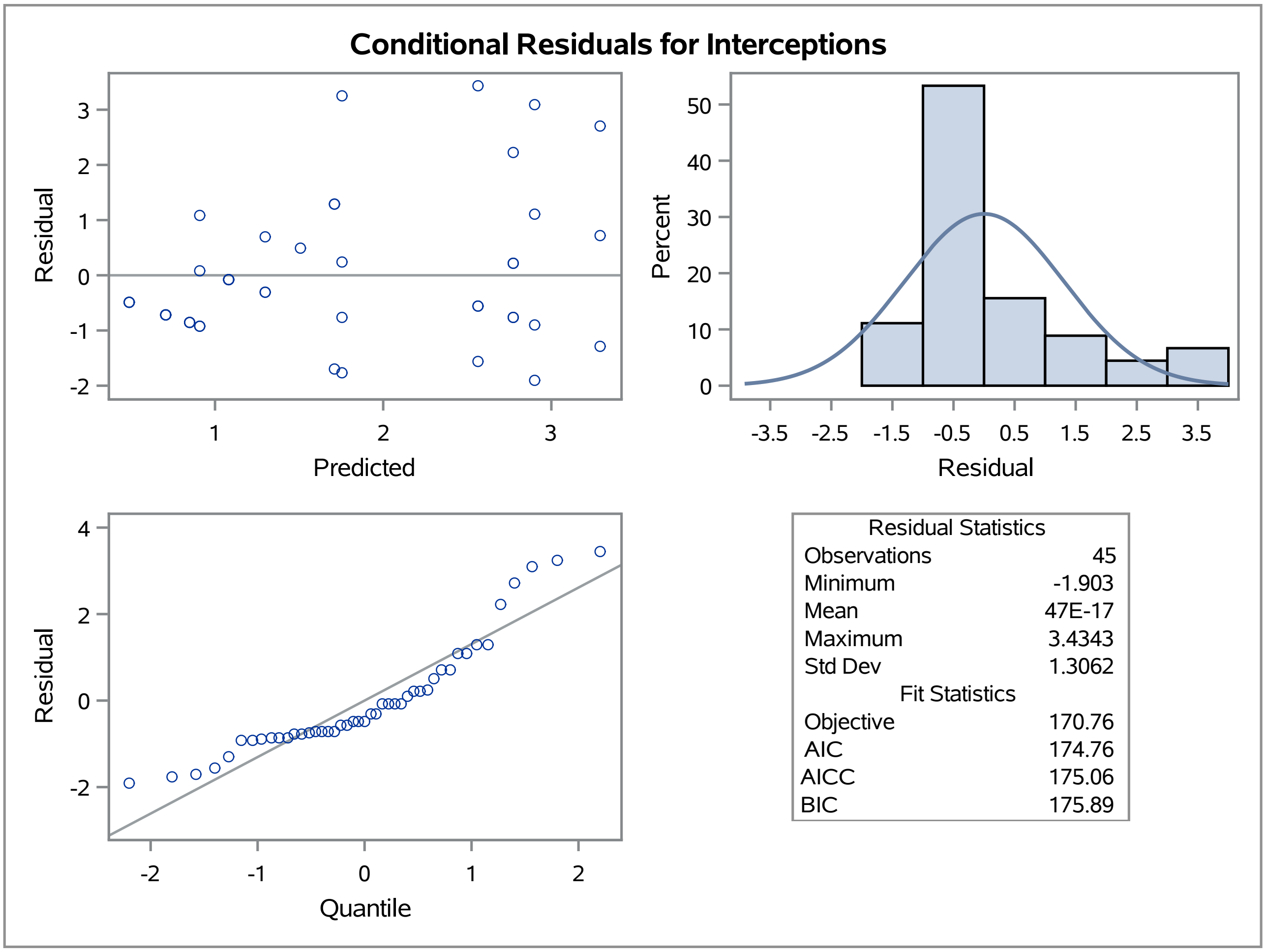

DistSoma=234

Model Information	
**Data Set**	WORK.TEMPDATASORTED
**Dependent Variable**	Interceptions
**Covariance Structure**	Variance Components
**Estimation Method**	REML
**Residual Variance Method**	Profile
**Fixed Effects SE Method**	Model-Based
**Degrees of Freedom Method**	Containment

Class Level Information		
**Class**	**Levels**	**Values**
**Treatment**	2	Control GFP Ctr Meg
**Culture**	13	1 2 3 4 5 6 7 8 9 10 11 12 13

Dimensions	
**Covariance Parameters**	2
**Columns in X**	3
**Columns in Z**	13
**Subjects**	1
**Max Obs per Subject**	45

Number of Observations	
**Number of Observations Read**	45
**Number of Observations Used**	45
**Number of Observations Not Used**	0

Iteration History			
**Iteration**	**Evaluations**	**-2 Res Log Like**	**Criterion**
**0**	1	172.32058957	
**1**	2	168.97547950	0.00000021
**2**	1	168.97547009	0.00000000

Convergence criteria met.

Covariance Parameter Estimates				
**Cov Parm**	**Estimate**	**Alpha**	**Lower**	**Upper**
**Culture**	0.8075	0.05	0.2696	9.2132
**Residual**	2.0849	0.05	1.3510	3.6358

DistSoma=234

Fit Statistics	
-2 Res Log Likelihood	169.0
AIC (Smaller is Better)	173.0
AICC (Smaller is Better)	173.3
BIC (Smaller is Better)	174.1

Solution for Fixed Effects

Effect	Treatment	Estimate	Standard Error	DF	t Value	Pr > |t|	Alpha	Lower	Upper
Intercept		1.9734	0.4764	11	4.14	0.0016	0.05	0.9248	3.0221
Treatment	Control GFP	-0.8574	0.6666	32	-1.29	0.2076	0.05	-2.2152	0.5005
Treatment	Ctr Meg	0	.	.	.	.	.	.	.

Solution for Random Effects

Effect	Culture	Estimate	Std Err Pred	DF	t Value	Pr > |t|	Alpha	Lower	Upper
Culture	1	-0.06237	0.6605	32	-0.09	0.9254	0.05	-1.4079	1.2831
Culture	2	0.4751	0.6605	32	0.72	0.4772	0.05	-0.8704	1.8206
Culture	3	-0.07053	0.6301	32	-0.11	0.9116	0.05	-1.3541	1.2130
Culture	4	-0.3744	0.6301	32	-0.59	0.5566	0.05	-1.6579	0.9091
Culture	5	0.3853	0.6301	32	0.61	0.5452	0.05	-0.8983	1.6688
Culture	6	0.2468	0.7740	32	0.32	0.7519	0.05	-1.3297	1.8233
Culture	7	-0.5998	0.6605	32	-0.91	0.3706	0.05	-1.9453	0.7456
Culture	8	0.7758	0.6329	32	1.23	0.2292	0.05	-0.5134	2.0651
Culture	9	-1.1993	0.6329	32	-1.89	0.0672	0.05	-2.4886	0.08993
Culture	10	0.7309	0.6626	32	1.10	0.2783	0.05	-0.6189	2.0806
Culture	11	0.2813	0.6113	32	0.46	0.6485	0.05	-0.9639	1.5265
Culture	12	0.4719	0.6329	32	0.75	0.4613	0.05	-0.8173	1.7612
Culture	13	-1.0606	0.6626	32	-1.60	0.1193	0.05	-2.4104	0.2891

Type 3 Tests of Fixed Effects

Effect	Num DF	Den DF	F Value	Pr > F
Treatment	1	32	1.65	0.2076

Least Squares Means

Effect	Treatment	Estimate	Standard Error	DF	t Value	Pr > |t|	Alpha	Lower	Upper
Treatment	Control GFP	1.1161	0.4662	32	2.39	0.0227	0.05	0.1663	2.0658
Treatment	Ctr Meg	1.9734	0.4764	32	4.14	0.0002	0.05	1.0030	2.9439

DistSoma=234

Differences of Least Squares Means												
**Effect**	**Treatment**	**Treatment**	**Estimate**	**Standard Error**	**DF**	**t Value**	**Pr > |t|**	**Adjustment**	**Adj P**	**Alpha**	**Lower**	**Upper**
**Treatment**	Control GFP	Ctr Meg	-0.8574	0.6666	32	-1.29	0.2076	Tukey-Kramer	0.2076	0.05	-2.2152	0.5005

Differences of Least Squares Means				
**Effect**	**Treatment**	**Treatment**	**Adj Lower**	**Adj Upper**
**Treatment**	Control GFP	Ctr Meg	-2.2152	0.5005

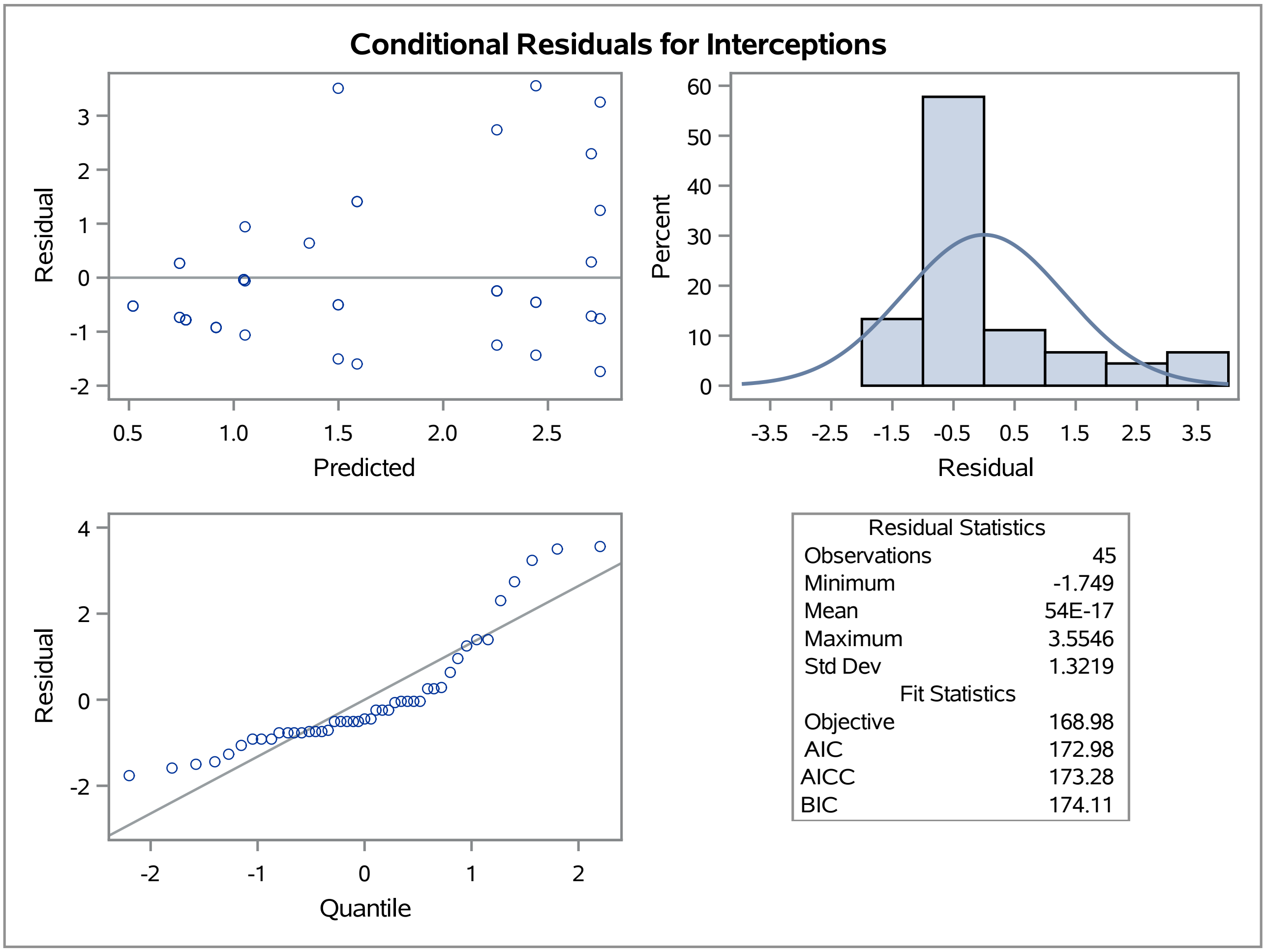

DistSoma=240

Model Information	
**Data Set**	WORK.TEMPDATASORTED
**Dependent Variable**	Interceptions
**Covariance Structure**	Variance Components
**Estimation Method**	REML
**Residual Variance Method**	Profile
**Fixed Effects SE Method**	Model-Based
**Degrees of Freedom Method**	Containment

Class Level Information		
**Class**	**Levels**	**Values**
**Treatment**	2	Control GFP Ctr Meg
**Culture**	13	1 2 3 4 5 6 7 8 9 10 11 12 13

Dimensions	
**Covariance Parameters**	2
**Columns in X**	3
**Columns in Z**	13
**Subjects**	1
**Max Obs per Subject**	45

Number of Observations	
**Number of Observations Read**	45
**Number of Observations Used**	45
**Number of Observations Not Used**	0

Iteration History			
**Iteration**	**Evaluations**	**-2 Res Log Like**	**Criterion**
**0**	1	168.88159002	
**1**	2	166.78111788	0.00000001

Convergence criteria met.

Covariance Parameter Estimates				
**Cov Parm**	**Estimate**	**Alpha**	**Lower**	**Upper**
**Culture**	0.5646	0.05	0.1651	13.3138
**Residual**	2.0754	0.05	1.3470	3.6098

DistSoma=240

Fit Statistics	
-2 Res Log Likelihood	166.8
AIC (Smaller is Better)	170.8
AICC (Smaller is Better)	171.1
BIC (Smaller is Better)	171.9

Solution for Fixed Effects

Effect	Treatment	Estimate	Standard Error	DF	t Value	Pr > |t|	Alpha	Lower	Upper
Intercept		1.8358	0.4311	11	4.26	0.0013	0.05	0.8870	2.7846
Treatment	Control GFP	-0.8215	0.6054	32	-1.36	0.1842	0.05	-2.0546	0.4116
Treatment	Ctr Meg	0	.	.	.	.	.	.	.

Solution for Random Effects

Effect	Culture	Estimate	Std Err Pred	DF	t Value	Pr > |t|	Alpha	Lower	Upper
Culture	1	-0.00641	0.5894	32	-0.01	0.9914	0.05	-1.2069	1.1941
Culture	2	0.1434	0.5894	32	0.24	0.8093	0.05	-1.0571	1.3439
Culture	3	-0.00743	0.5652	32	-0.01	0.9896	0.05	-1.1587	1.1438
Culture	4	-0.2680	0.5652	32	-0.47	0.6386	0.05	-1.4192	0.8833
Culture	5	0.3834	0.5652	32	0.68	0.5024	0.05	-0.7678	1.5346
Culture	6	0.2108	0.6724	32	0.31	0.7559	0.05	-1.1588	1.5804
Culture	7	-0.4558	0.5894	32	-0.77	0.4450	0.05	-1.6563	0.7447
Culture	8	0.6067	0.5664	32	1.07	0.2922	0.05	-0.5471	1.7604
Culture	9	-0.9566	0.5664	32	-1.69	0.1010	0.05	-2.1104	0.1971
Culture	10	0.3734	0.5903	32	0.63	0.5315	0.05	-0.8289	1.5757
Culture	11	0.3251	0.5486	32	0.59	0.5575	0.05	-0.7922	1.4425
Culture	12	0.4764	0.5664	32	0.84	0.4066	0.05	-0.6774	1.6302
Culture	13	-0.8249	0.5903	32	-1.40	0.1718	0.05	-2.0272	0.3774

Type 3 Tests of Fixed Effects

Effect	Num DF	Den DF	F Value	Pr > F
Treatment	1	32	1.84	0.1842

Least Squares Means

Effect	Treatment	Estimate	Standard Error	DF	t Value	Pr > |t|	Alpha	Lower	Upper
Treatment	Control GFP	1.0143	0.4250	32	2.39	0.0231	0.05	0.1485	1.8800
Treatment	Ctr Meg	1.8358	0.4311	32	4.26	0.0002	0.05	0.9577	2.7139

DistSoma=240

Differences of Least Squares Means

Effect	Treatment	Treatment	Estimate	Standard Error	DF	t Value	Pr > |t|	Adjustment	Adj P	Alpha	Lower	Upper
**Treatment**	Control GFP	Ctr Meg	-0.8215	0.6054	32	-1.36	0.1842	Tukey-Kramer	0.1842	0.05	-2.0546	0.4116

Differences of Least Squares Means

Effect	Treatment	Treatment	Adj Lower	Adj Upper
**Treatment**	Control GFP	Ctr Meg	-2.0546	0.4115

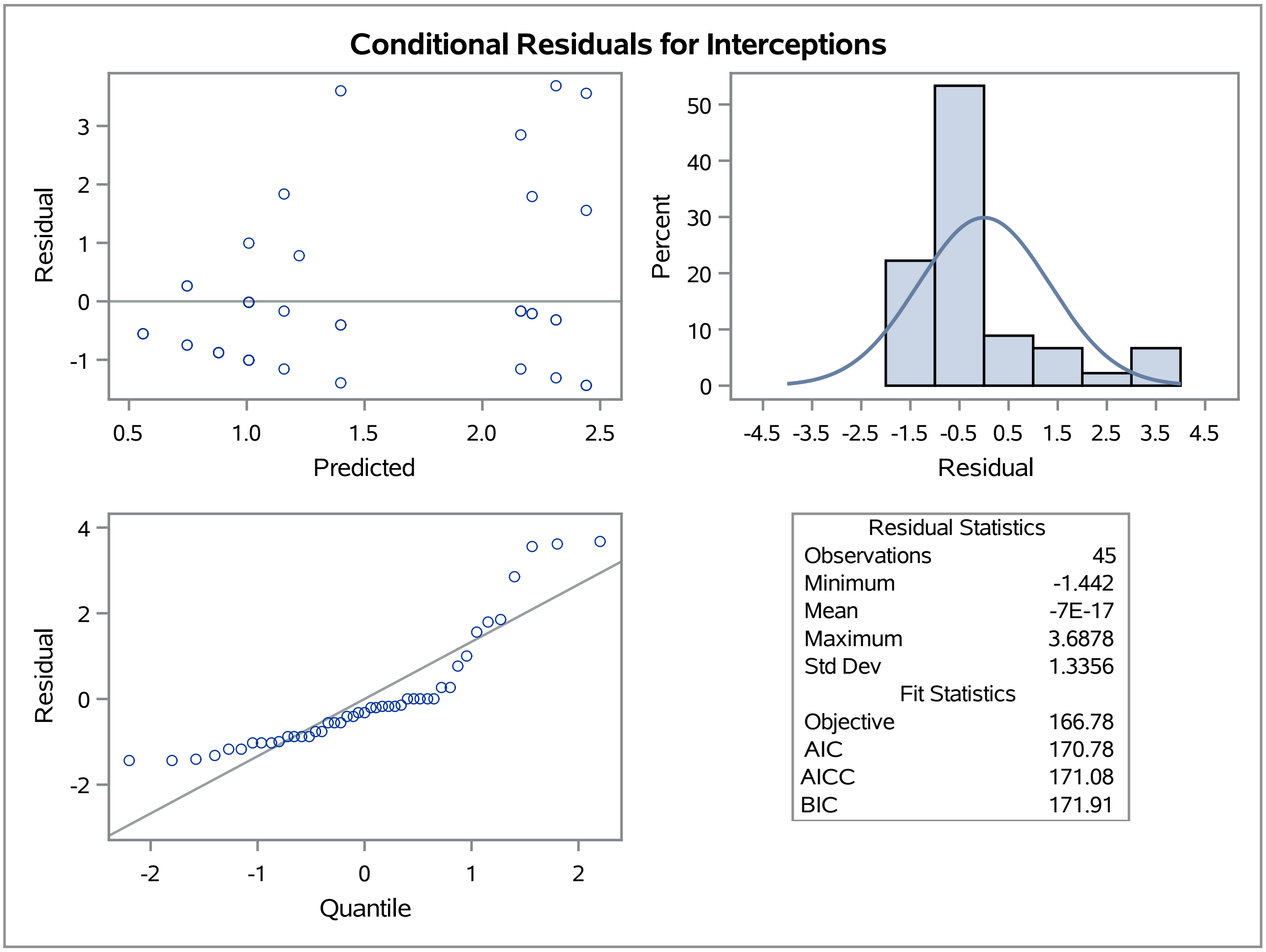

DistSoma=246

Model Information	
**Data Set**	WORK.TEMPDATASORTED
**Dependent Variable**	Interceptions
**Covariance Structure**	Variance Components
**Estimation Method**	REML
**Residual Variance Method**	Profile
**Fixed Effects SE Method**	Model-Based
**Degrees of Freedom Method**	Containment

Class Level Information		
**Class**	**Levels**	**Values**
**Treatment**	2	Control GFP Ctr Meg
**Culture**	13	1 2 3 4 5 6 7 8 9 10 11 12 13

Dimensions	
**Covariance Parameters**	2
**Columns in X**	3
**Columns in Z**	13
**Subjects**	1
**Max Obs per Subject**	45

Number of Observations	
**Number of Observations Read**	45
**Number of Observations Used**	45
**Number of Observations Not Used**	0

Iteration History			
**Iteration**	**Evaluations**	**-2 Res Log Like**	**Criterion**
**0**	1	161.16328930	
**1**	2	158.43823240	0.00000014
**2**	1	158.43822697	0.00000000

Convergence criteria met.

Covariance Parameter Estimates				
**Cov Parm**	**Estimate**	**Alpha**	**Lower**	**Upper**
**Culture**	0.5387	0.05	0.1696	8.2774
**Residual**	1.6736	0.05	1.0863	2.9106

DistSoma=246

Fit Statistics	
-2 Res Log Likelihood	158.4
AIC (Smaller is Better)	162.4
AICC (Smaller is Better)	162.7
BIC (Smaller is Better)	163.6

Solution for Fixed Effects

Effect	Treatment	Estimate	Standard Error	DF	t Value	Pr > |t|	Alpha	Lower	Upper
Intercept		1.6976	0.4048	11	4.19	0.0015	0.05	0.8067	2.5885
Treatment	Control GFP	-0.6810	0.5675	32	-1.20	0.2389	0.05	-1.8369	0.4749
Treatment	Ctr Meg	0	.	.	.	.	.	.	.

Solution for Random Effects

Effect	Culture	Estimate	Std Err Pred	DF	t Value	Pr > |t|	Alpha	Lower	Upper
Culture	1	-0.00818	0.5588	32	-0.01	0.9884	0.05	-1.1464	1.1300
Culture	2	0.1556	0.5588	32	0.28	0.7825	0.05	-0.9826	1.2938
Culture	3	-0.00937	0.5344	32	-0.02	0.9861	0.05	-1.0980	1.0792
Culture	4	-0.2908	0.5344	32	-0.54	0.5901	0.05	-1.3794	0.7978
Culture	5	0.4128	0.5344	32	0.77	0.4456	0.05	-0.6758	1.5013
Culture	6	0.2394	0.6457	32	0.37	0.7132	0.05	-1.0758	1.5547
Culture	7	-0.4994	0.5588	32	-0.89	0.3781	0.05	-1.6376	0.6388
Culture	8	0.5923	0.5361	32	1.10	0.2774	0.05	-0.4997	1.6843
Culture	9	-0.9555	0.5361	32	-1.78	0.0842	0.05	-2.0475	0.1365
Culture	10	0.3123	0.5600	32	0.56	0.5809	0.05	-0.8284	1.4530
Culture	11	0.4332	0.5184	32	0.84	0.4096	0.05	-0.6228	1.4892
Culture	12	0.4516	0.5361	32	0.84	0.4058	0.05	-0.6404	1.5436
Culture	13	-0.8340	0.5600	32	-1.49	0.1462	0.05	-1.9747	0.3067

Type 3 Tests of Fixed Effects

Effect	Num DF	Den DF	F Value	Pr > F
Treatment	1	32	1.44	0.2389

Least Squares Means

Effect	Treatment	Estimate	Standard Error	DF	t Value	Pr > |t|	Alpha	Lower	Upper
Treatment	Control GFP	1.0166	0.3977	32	2.56	0.0155	0.05	0.2065	1.8268
Treatment	Ctr Meg	1.6976	0.4048	32	4.19	0.0002	0.05	0.8731	2.5221

DistSoma=246

Differences of Least Squares Means

Effect	Treatment	Treatment	Estimate	Standard Error	DF	t Value	Pr > |t|	Adjustment	Adj P	Alpha	Lower	Upper
**Treatment**	Control GFP	Ctr Meg	-0.6810	0.5675	32	-1.20	0.2389	Tukey-Kramer	0.2389	0.05	-1.8369	0.4749

Differences of Least Squares Means

Effect	Treatment	Treatment	Adj Lower	Adj Upper
**Treatment**	Control GFP	Ctr Meg	-1.8368	0.4749

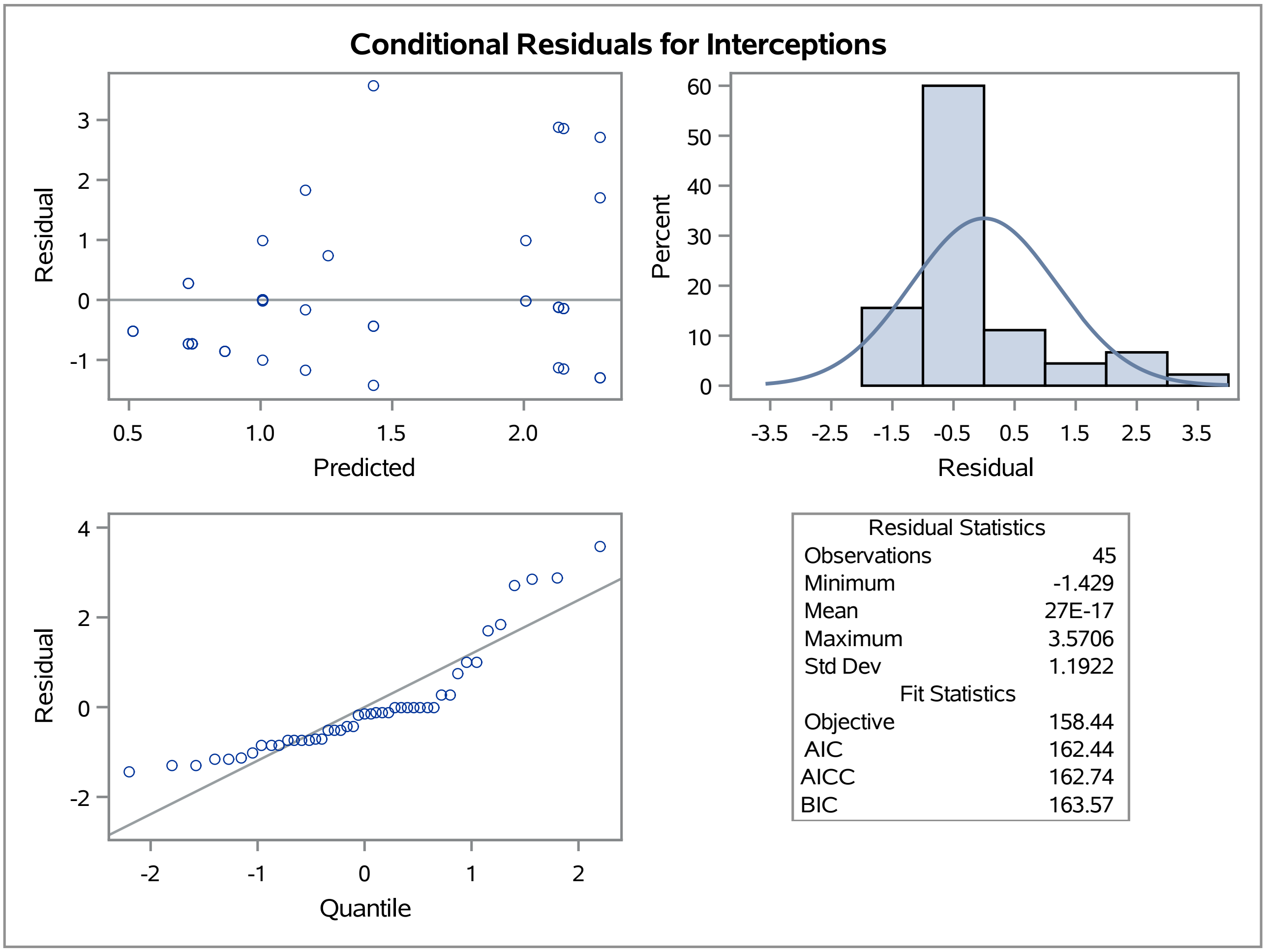

DistSoma=252

Model Information	
**Data Set**	WORK.TEMPDATASORTED
**Dependent Variable**	Interceptions
**Covariance Structure**	Variance Components
**Estimation Method**	REML
**Residual Variance Method**	Profile
**Fixed Effects SE Method**	Model-Based
**Degrees of Freedom Method**	Containment

Class Level Information		
**Class**	**Levels**	**Values**
**Treatment**	2	Control GFP Ctr Meg
**Culture**	13	1 2 3 4 5 6 7 8 9 10 11 12 13

Dimensions	
**Covariance Parameters**	2
**Columns in X**	3
**Columns in Z**	13
**Subjects**	1
**Max Obs per Subject**	45

Number of Observations	
**Number of Observations Read**	45
**Number of Observations Used**	45
**Number of Observations Not Used**	0

Iteration History			
**Iteration**	**Evaluations**	**-2 Res Log Like**	**Criterion**
**0**	1	171.28695497	
**1**	2	167.83063662	0.00000018
**2**	1	167.83062873	0.00000000

Convergence criteria met.

Covariance Parameter Estimates				
**Cov Parm**	**Estimate**	**Alpha**	**Lower**	**Upper**
**Culture**	0.7734	0.05	0.2594	8.6445
**Residual**	2.0357	0.05	1.3211	3.5414

DistSoma=252

Fit Statistics	
-2 Res Log Likelihood	167.8
AIC (Smaller is Better)	171.8
AICC (Smaller is Better)	172.1
BIC (Smaller is Better)	173.0

Solution for Fixed Effects

Effect	Treatment	Estimate	Standard Error	DF	t Value	Pr > |t|	Alpha	Lower	Upper
Intercept		1.8283	0.4681	11	3.91	0.0025	0.05	0.7981	2.8586
Treatment	Control GFP	-1.0983	0.6551	32	-1.68	0.1034	0.05	-2.4327	0.2360
Treatment	Ctr Meg	0	.	.	.	.	.	.	.

Solution for Random Effects

Effect	Culture	Estimate	Std Err Pred	DF	t Value	Pr > |t|	Alpha	Lower	Upper
Culture	1	-0.3888	0.6489	32	-0.60	0.5532	0.05	-1.7106	0.9329
Culture	2	-0.2113	0.6489	32	-0.33	0.7468	0.05	-1.5330	1.1104
Culture	3	0.1628	0.6192	32	0.26	0.7942	0.05	-1.0983	1.4240
Culture	4	-0.1387	0.6192	32	-0.22	0.8241	0.05	-1.3999	1.1224
Culture	5	0.6152	0.6192	32	0.99	0.3279	0.05	-0.6460	1.8764
Culture	6	0.3497	0.7592	32	0.46	0.6482	0.05	-1.1968	1.8961
Culture	7	-0.3888	0.6489	32	-0.60	0.5532	0.05	-1.7106	0.9329
Culture	8	1.1590	0.6218	32	1.86	0.0715	0.05	-0.1076	2.4256
Culture	9	-1.1027	0.6218	32	-1.77	0.0857	0.05	-2.3693	0.1639
Culture	10	0.2690	0.6509	32	0.41	0.6821	0.05	-1.0568	1.5948
Culture	11	0.2435	0.6006	32	0.41	0.6879	0.05	-0.9800	1.4670
Culture	12	0.4051	0.6218	32	0.65	0.5194	0.05	-0.8615	1.6717
Culture	13	-0.9739	0.6509	32	-1.50	0.1444	0.05	-2.2997	0.3519

Type 3 Tests of Fixed Effects

Effect	Num DF	Den DF	F Value	Pr > F
Treatment	1	32	2.81	0.1034

Least Squares Means

Effect	Treatment	Estimate	Standard Error	DF	t Value	Pr > |t|	Alpha	Lower	Upper
Treatment	Control GFP	0.7300	0.4583	32	1.59	0.1210	0.05	-0.2035	1.6635
Treatment	Ctr Meg	1.8283	0.4681	32	3.91	0.0005	0.05	0.8749	2.7818

DistSoma=252

Differences of Least Squares Means												
Effect	Treatment	Treatment	Estimate	Standard Error	DF	t Value	Pr > |t|	Adjustment	Adj P	Alpha	Lower	Upper
Treatment	Control GFP	Ctr Meg	-1.0983	0.6551	32	-1.68	0.1034	Tukey-Kramer	0.1034	0.05	-2.4327	0.2360

Differences of Least Squares Means				
Effect	Treatment	Treatment	Adj Lower	Adj Upper
Treatment	Control GFP	Ctr Meg	-2.4327	0.2360

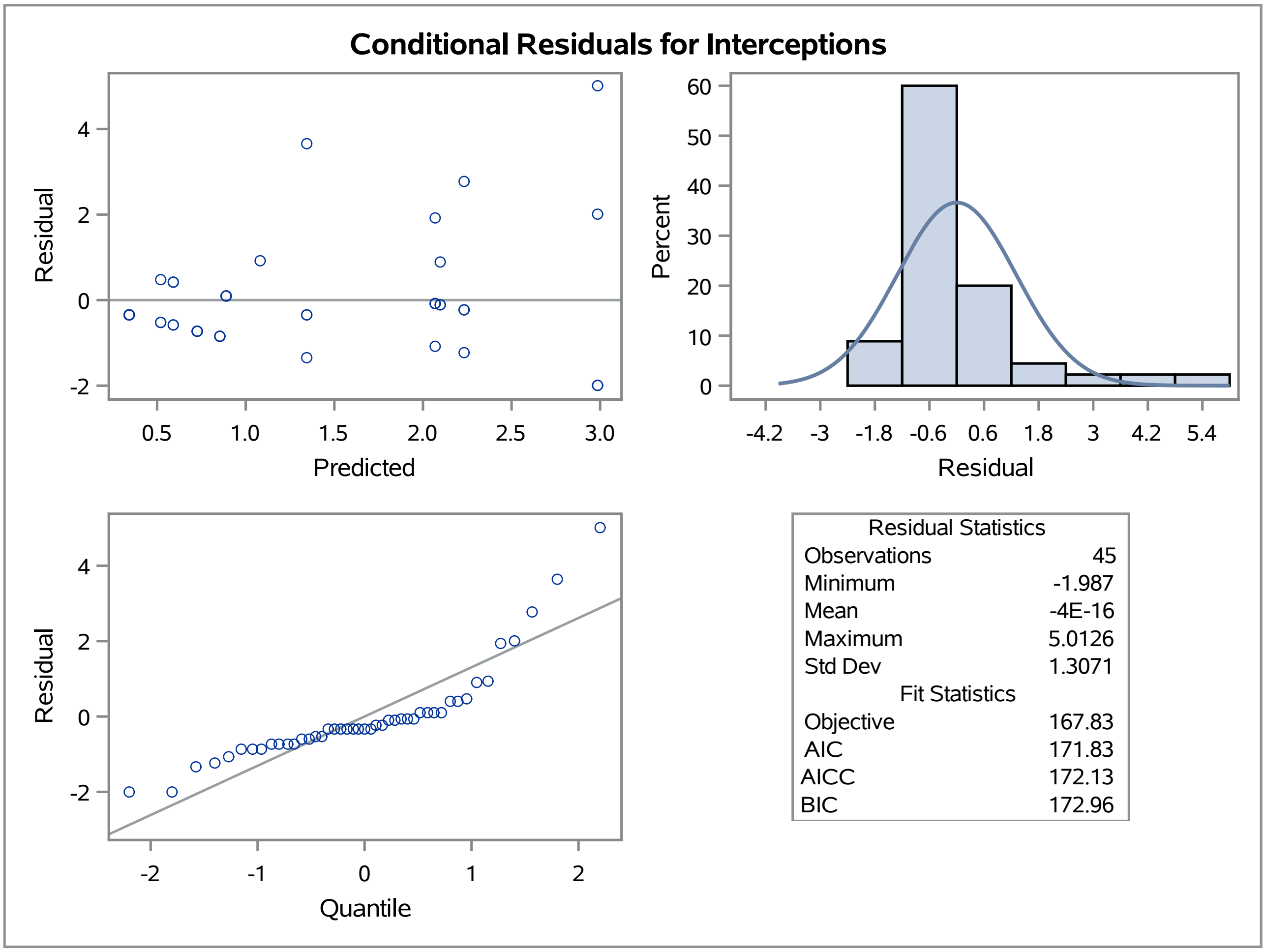

DistSoma=258

Model Information	
**Data Set**	WORK.TEMPDATASORTED
**Dependent Variable**	Interceptions
**Covariance Structure**	Variance Components
**Estimation Method**	REML
**Residual Variance Method**	Profile
**Fixed Effects SE Method**	Model-Based
**Degrees of Freedom Method**	Containment

Class Level Information		
**Class**	**Levels**	**Values**
**Treatment**	2	Control GFP Ctr Meg
**Culture**	13	1 2 3 4 5 6 7 8 9 10 11 12 13

Dimensions	
**Covariance Parameters**	2
**Columns in X**	3
**Columns in Z**	13
**Subjects**	1
**Max Obs per Subject**	45

Number of Observations	
**Number of Observations Read**	45
**Number of Observations Used**	45
**Number of Observations Not Used**	0

Iteration History			
**Iteration**	**Evaluations**	**-2 Res Log Like**	**Criterion**
**0**	1	151.89485144	
**1**	2	145.93010143	0.00000000

Convergence criteria met.

Covariance Parameter Estimates				
**Cov Parm**	**Estimate**	**Alpha**	**Lower**	**Upper**
**Culture**	0.6720	0.05	0.2545	4.4865
**Residual**	1.1437	0.05	0.7407	1.9961

DistSoma=258

Fit Statistics	
-2 Res Log Likelihood	145.9
AIC (Smaller is Better)	149.9
AICC (Smaller is Better)	150.2
BIC (Smaller is Better)	151.1

Solution for Fixed Effects

Effect	Treatment	Estimate	Standard Error	DF	t Value	Pr > |t|	Alpha	Lower	Upper
Intercept		1.4910	0.4035	11	3.69	0.0035	0.05	0.6028	2.3791
Treatment	Control GFP	-0.7943	0.5621	32	-1.41	0.1673	0.05	-1.9393	0.3507
Treatment	Ctr Meg	0	.	.	.	.	.	.	.

Solution for Random Effects

Effect	Culture	Estimate	Std Err Pred	DF	t Value	Pr > |t|	Alpha	Lower	Upper
Culture	1	-0.4445	0.5528	32	-0.80	0.4273	0.05	-1.5705	0.6815
Culture	2	-0.2318	0.5528	32	-0.42	0.6778	0.05	-1.3578	0.8942
Culture	3	0.2128	0.5253	32	0.41	0.6881	0.05	-0.8572	1.2828
Culture	4	-0.1380	0.5253	32	-0.26	0.7945	0.05	-1.2079	0.9320
Culture	5	0.5636	0.5253	32	1.07	0.2914	0.05	-0.5064	1.6336
Culture	6	0.4824	0.6665	32	0.72	0.4745	0.05	-0.8753	1.8401
Culture	7	-0.4445	0.5528	32	-0.80	0.4273	0.05	-1.5705	0.6815
Culture	8	1.2340	0.5298	32	2.33	0.0263	0.05	0.1548	2.3132
Culture	9	-1.0459	0.5298	32	-1.97	0.0571	0.05	-2.1252	0.03329
Culture	10	0.3248	0.5564	32	0.58	0.5635	0.05	-0.8085	1.4581
Culture	11	0.08135	0.5112	32	0.16	0.8746	0.05	-0.9599	1.1226
Culture	12	0.3571	0.5298	32	0.67	0.5052	0.05	-0.7221	1.4363
Culture	13	-0.9513	0.5564	32	-1.71	0.0970	0.05	-2.0846	0.1820

Type 3 Tests of Fixed Effects

Effect	Num DF	Den DF	F Value	Pr > F
Treatment	1	32	2.00	0.1673

Least Squares Means

Effect	Treatment	Estimate	Standard Error	DF	t Value	Pr > |t|	Alpha	Lower	Upper
Treatment	Control GFP	0.6967	0.3913	32	1.78	0.0845	0.05	-0.1004	1.4937
Treatment	Ctr Meg	1.4910	0.4035	32	3.69	0.0008	0.05	0.6690	2.3129

DistSoma=258

Differences of Least Squares Means												
Effect	Treatment	Treatment	Estimate	Standard Error	DF	t Value	Pr > |t|	Adjustment	Adj P	Alpha	Lower	Upper
Treatment	Control GFP	Ctr Meg	-0.7943	0.5621	32	-1.41	0.1673	Tukey-Kramer	0.1673	0.05	-1.9393	0.3507

Differences of Least Squares Means				
Effect	Treatment	Treatment	Adj Lower	Adj Upper
Treatment	Control GFP	Ctr Meg	-1.9393	0.3506

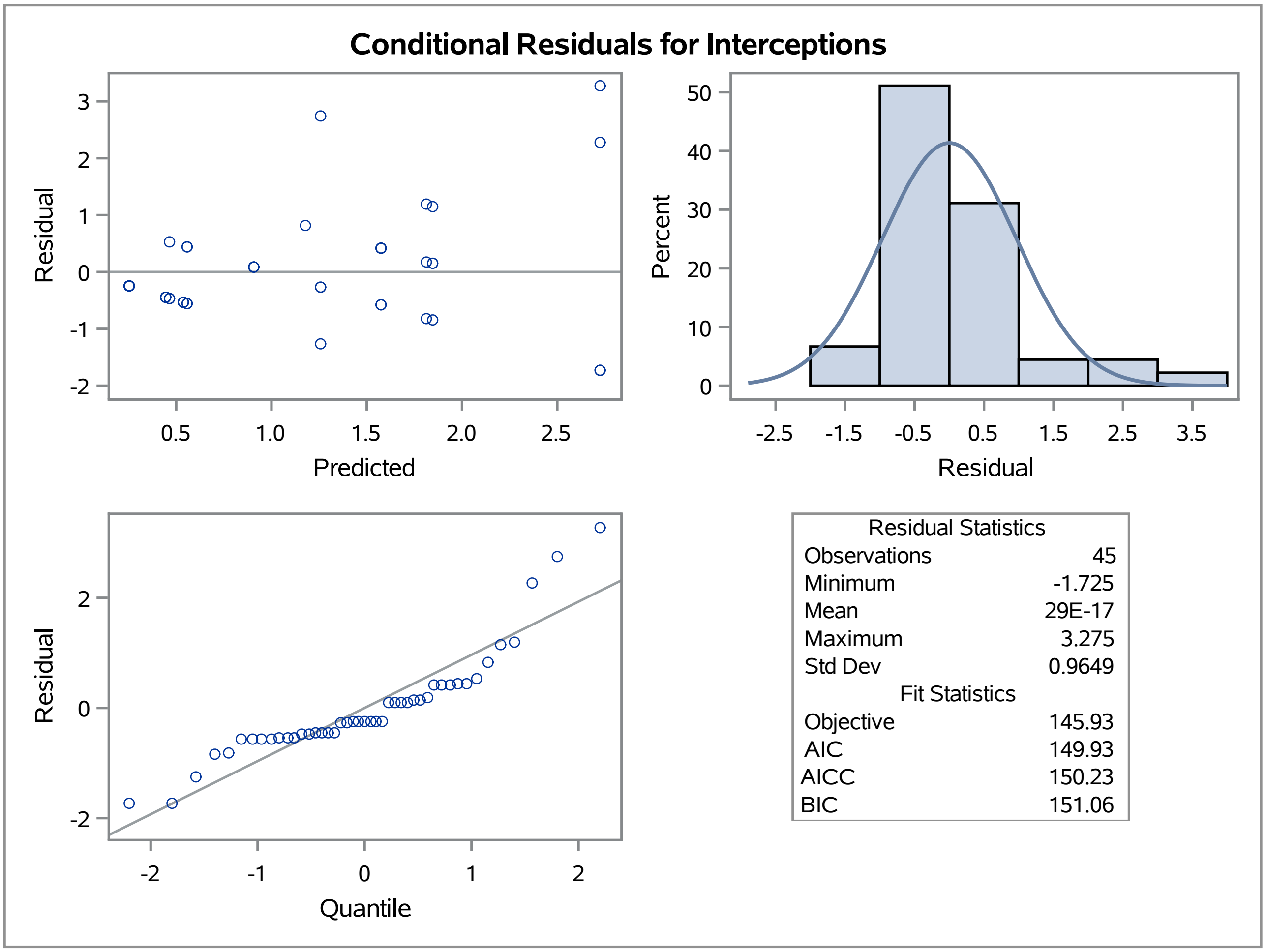

DistSoma=264

Model Information	
**Data Set**	WORK.TEMPDATASORTED
**Dependent Variable**	Interceptions
**Covariance Structure**	Variance Components
**Estimation Method**	REML
**Residual Variance Method**	Profile
**Fixed Effects SE Method**	Model-Based
**Degrees of Freedom Method**	Containment

Class Level Information		
**Class**	**Levels**	**Values**
**Treatment**	2	Control GFP Ctr Meg
**Culture**	13	1 2 3 4 5 6 7 8 9 10 11 12 13

Dimensions	
**Covariance Parameters**	2
**Columns in X**	3
**Columns in Z**	13
**Subjects**	1
**Max Obs per Subject**	45

Number of Observations	
**Number of Observations Read**	45
**Number of Observations Used**	45
**Number of Observations Not Used**	0

Iteration History			
**Iteration**	**Evaluations**	**-2 Res Log Like**	**Criterion**
**0**	1	146.84201189	
**1**	2	142.74168546	0.00000010
**2**	1	142.74168221	0.00000000

Convergence criteria met.

Covariance Parameter Estimates				
**Cov Parm**	**Estimate**	**Alpha**	**Lower**	**Upper**
**Culture**	0.4980	0.05	0.1736	4.6658
**Residual**	1.1083	0.05	0.7174	1.9363

DistSoma=264

Fit Statistics	
-2 Res Log Likelihood	142.7
AIC (Smaller is Better)	146.7
AICC (Smaller is Better)	147.0
BIC (Smaller is Better)	147.9

Solution for Fixed Effects

Effect	Treatment	Estimate	Standard Error	DF	t Value	Pr > |t|	Alpha	Lower	Upper
Intercept		1.3660	0.3636	11	3.76	0.0032	0.05	0.5658	2.1662
Treatment	Control GFP	-0.6743	0.5079	32	-1.33	0.1937	0.05	-1.7088	0.3602
Treatment	Ctr Meg	0	.	.	.	.	.	.	.

Solution for Random Effects

Effect	Culture	Estimate	Std Err Pred	DF	t Value	Pr > |t|	Alpha	Lower	Upper
Culture	1	-0.3971	0.5035	32	-0.79	0.4361	0.05	-1.4228	0.6286
Culture	2	-0.2057	0.5035	32	-0.41	0.6856	0.05	-1.2314	0.8199
Culture	3	0.1981	0.4795	32	0.41	0.6823	0.05	-0.7787	1.1749
Culture	4	-0.1232	0.4795	32	-0.26	0.7990	0.05	-1.0999	0.8536
Culture	5	0.5194	0.4795	32	1.08	0.2869	0.05	-0.4574	1.4961
Culture	6	0.4056	0.5964	32	0.68	0.5013	0.05	-0.8092	1.6204
Culture	7	-0.3971	0.5035	32	-0.79	0.4361	0.05	-1.4228	0.6286
Culture	8	0.8892	0.4823	32	1.84	0.0745	0.05	-0.09312	1.8716
Culture	9	-0.8777	0.4823	32	-1.82	0.0781	0.05	-1.8600	0.1047
Culture	10	0.3640	0.5056	32	0.72	0.4768	0.05	-0.6659	1.3939
Culture	11	0.1619	0.4655	32	0.35	0.7302	0.05	-0.7862	1.1101
Culture	12	0.2467	0.4823	32	0.51	0.6124	0.05	-0.7356	1.2291
Culture	13	-0.7842	0.5056	32	-1.55	0.1307	0.05	-1.8141	0.2457

Type 3 Tests of Fixed Effects

Effect	Num DF	Den DF	F Value	Pr > F
Treatment	1	32	1.76	0.1937

Least Squares Means

Effect	Treatment	Estimate	Standard Error	DF	t Value	Pr > |t|	Alpha	Lower	Upper
Treatment	Control GFP	0.6917	0.3546	32	1.95	0.0599	0.05	-0.03067	1.4140
Treatment	Ctr Meg	1.3660	0.3636	32	3.76	0.0007	0.05	0.6255	2.1065

DistSoma=264

Differences of Least Squares Means												
Effect	Treatment	Treatment	Estimate	Standard Error	DF	t Value	Pr > |t|	Adjustment	Adj P	Alpha	Lower	Upper
Treatment	Control GFP	Ctr Meg	-0.6743	0.5079	32	-1.33	0.1937	Tukey-Kramer	0.1937	0.05	-1.7088	0.3602

Differences of Least Squares Means				
Effect	Treatment	Treatment	Adj Lower	Adj Upper
Treatment	Control GFP	Ctr Meg	-1.7088	0.3602

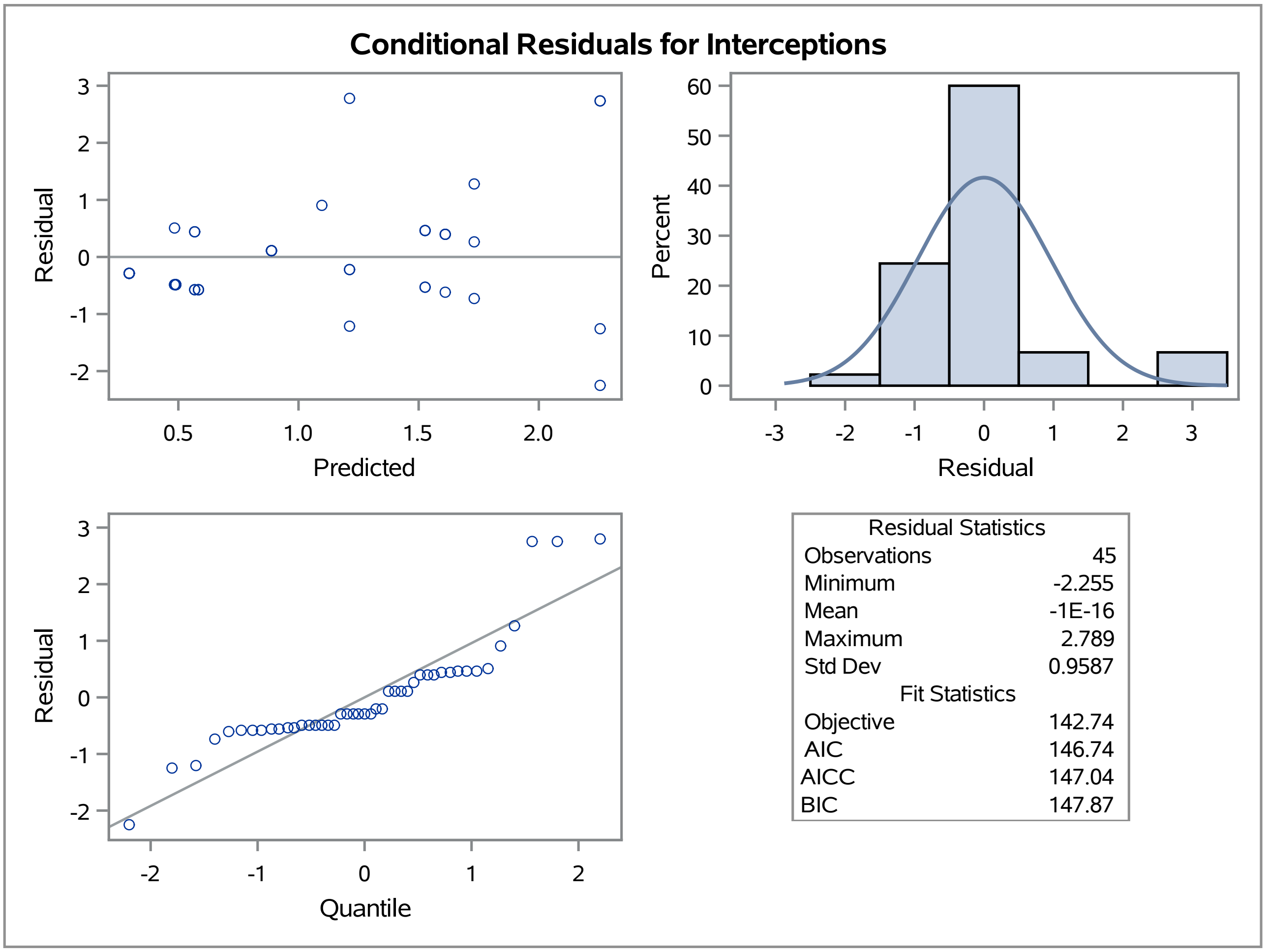

DistSoma=270

Model Information	
**Data Set**	WORK.TEMPDATASORTED
**Dependent Variable**	Interceptions
**Covariance Structure**	Variance Components
**Estimation Method**	REML
**Residual Variance Method**	Profile
**Fixed Effects SE Method**	Model-Based
**Degrees of Freedom Method**	Containment

Class Level Information		
**Class**	**Levels**	**Values**
**Treatment**	2	Control GFP Ctr Meg
**Culture**	13	1 2 3 4 5 6 7 8 9 10 11 12 13

Dimensions	
**Covariance Parameters**	2
**Columns in X**	3
**Columns in Z**	13
**Subjects**	1
**Max Obs per Subject**	45

Number of Observations	
**Number of Observations Read**	45
**Number of Observations Used**	45
**Number of Observations Not Used**	0

Iteration History			
**Iteration**	**Evaluations**	**-2 Res Log Like**	**Criterion**
**0**	1	140.85667689	
**1**	2	136.47981988	0.00000012
**2**	1	136.47981638	0.00000000

Convergence criteria met.

Covariance Parameter Estimates				
**Cov Parm**	**Estimate**	**Alpha**	**Lower**	**Upper**
**Culture**	0.4494	0.05	0.1588	3.9661
**Residual**	0.9507	0.05	0.6152	1.6619

DistSoma=270

Fit Statistics	
-2 Res Log Likelihood	136.5
AIC (Smaller is Better)	140.5
AICC (Smaller is Better)	140.8
BIC (Smaller is Better)	141.6

Solution for Fixed Effects									
Effect	Treatment	Estimate	Standard Error	DF	t Value	Pr > |t|	Alpha	Lower	Upper
Intercept		1.2720	0.3422	11	3.72	0.0034	0.05	0.5188	2.0251
Treatment	Control GFP	-0.5794	0.4778	32	-1.21	0.2341	0.05	-1.5526	0.3938
Treatment	Ctr Meg	0	.	.	.	.	.	.	.

Solution for Random Effects									
Effect	Culture	Estimate	Std Err Pred	DF	t Value	Pr > |t|	Alpha	Lower	Upper
Culture	1	-0.4061	0.4734	32	-0.86	0.3973	0.05	-1.3703	0.5581
Culture	2	-0.2107	0.4734	32	-0.45	0.6593	0.05	-1.1749	0.7536
Culture	3	0.2011	0.4506	32	0.45	0.6584	0.05	-0.7167	1.1189
Culture	4	-0.1259	0.4506	32	-0.28	0.7817	0.05	-1.0437	0.7918
Culture	5	0.5281	0.4506	32	1.17	0.2498	0.05	-0.3897	1.4459
Culture	6	0.4196	0.5627	32	0.75	0.4612	0.05	-0.7265	1.5658
Culture	7	-0.4061	0.4734	32	-0.86	0.3973	0.05	-1.3703	0.5581
Culture	8	0.8032	0.4534	32	1.77	0.0860	0.05	-0.1203	1.7267
Culture	9	-0.8319	0.4534	32	-1.83	0.0758	0.05	-1.7554	0.09156
Culture	10	0.2315	0.4755	32	0.49	0.6297	0.05	-0.7371	1.2001
Culture	11	0.2305	0.4375	32	0.53	0.6019	0.05	-0.6607	1.1217
Culture	12	0.3127	0.4534	32	0.69	0.4954	0.05	-0.6108	1.2362
Culture	13	-0.7459	0.4755	32	-1.57	0.1266	0.05	-1.7145	0.2227

Type 3 Tests of Fixed Effects				
Effect	Num DF	Den DF	F Value	Pr > F
Treatment	1	32	1.47	0.2341

Least Squares Means									
Effect	Treatment	Estimate	Standard Error	DF	t Value	Pr > |t|	Alpha	Lower	Upper
Treatment	Control GFP	0.6925	0.3334	32	2.08	0.0459	0.05	0.01341	1.3717
Treatment	Ctr Meg	1.2720	0.3422	32	3.72	0.0008	0.05	0.5749	1.9690

DistSoma=270

Differences of Least Squares Means												
**Effect**	**Treatment**	**Treatment**	**Estimate**	**Standard Error**	**DF**	**t Value**	**Pr > |t|**	**Adjustment**	**Adj P**	**Alpha**	**Lower**	**Upper**
**Treatment**	Control GFP	Ctr Meg	-0.5794	0.4778	32	-1.21	0.2341	Tukey-Kramer	0.2341	0.05	-1.5526	0.3938

Differences of Least Squares Means				
**Effect**	**Treatment**	**Treatment**	**Adj Lower**	**Adj Upper**
**Treatment**	Control GFP	Ctr Meg	-1.5526	0.3937

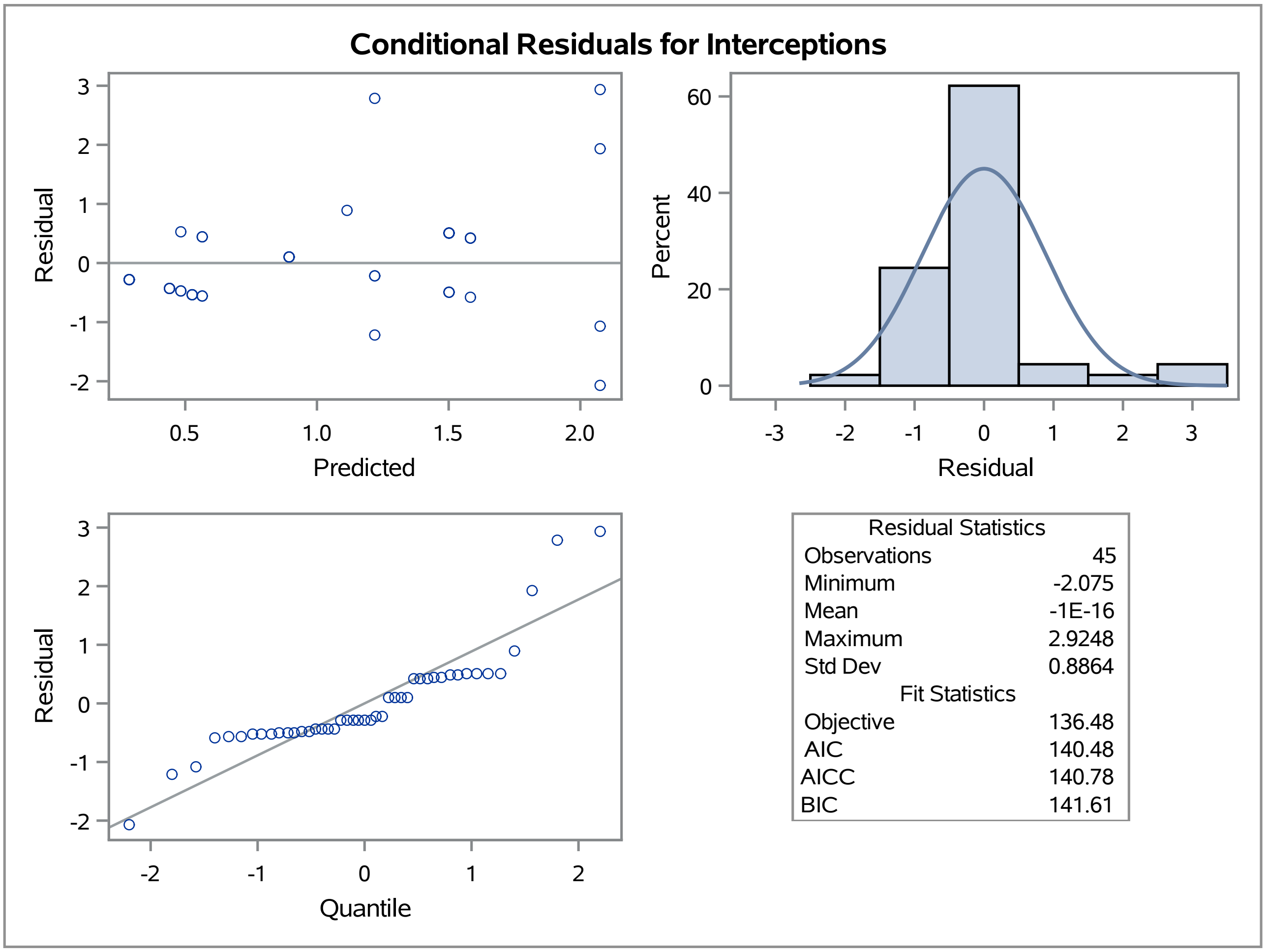

DistSoma=276

Model Information	
**Data Set**	WORK.TEMPDATASORTED
**Dependent Variable**	Interceptions
**Covariance Structure**	Variance Components
**Estimation Method**	REML
**Residual Variance Method**	Profile
**Fixed Effects SE Method**	Model-Based
**Degrees of Freedom Method**	Containment

Class Level Information		
**Class**	**Levels**	**Values**
**Treatment**	2	Control GFP Ctr Meg
**Culture**	13	1 2 3 4 5 6 7 8 9 10 11 12 13

Dimensions	
**Covariance Parameters**	2
**Columns in X**	3
**Columns in Z**	13
**Subjects**	1
**Max Obs per Subject**	45

Number of Observations	
**Number of Observations Read**	45
**Number of Observations Used**	45
**Number of Observations Not Used**	0

Iteration History			
**Iteration**	**Evaluations**	**-2 Res Log Like**	**Criterion**
**0**	1	137.55454596	
**1**	2	134.28547828	0.00000016
**2**	1	134.28547383	0.00000000

Convergence criteria met.

Covariance Parameter Estimates				
**Cov Parm**	**Estimate**	**Alpha**	**Lower**	**Upper**
**Culture**	0.3570	0.05	0.1174	4.3889
**Residual**	0.9320	0.05	0.6030	1.6293

DistSoma=276

Fit Statistics	
**-2 Res Log Likelihood**	134.3
**AIC (Smaller is Better)**	138.3
**AICC (Smaller is Better)**	138.6
**BIC (Smaller is Better)**	139.4

Solution for Fixed Effects

Effect	Treatment	Estimate	Standard Error	DF	t Value	Pr > |t|	Alpha	Lower	Upper
**Intercept**		1.1073	0.3175	11	3.49	0.0051	0.05	0.4085	1.8061
**Treatment**	Control GFP	-0.4181	0.4443	32	-0.94	0.3537	0.05	-1.3231	0.4869
**Treatment**	Ctr Meg	0	.	.	.	.	.	.	.

Solution for Random Effects

Effect	Culture	Estimate	Std Err Pred	DF	t Value	Pr > |t|	Alpha	Lower	Upper
**Culture**	1	-0.3685	0.4402	32	-0.84	0.4087	0.05	-1.2651	0.5280
**Culture**	2	-0.1903	0.4402	32	-0.43	0.6684	0.05	-1.0868	0.7063
**Culture**	3	0.1881	0.4199	32	0.45	0.6573	0.05	-0.6673	1.0435
**Culture**	4	-0.1145	0.4199	32	-0.27	0.7869	0.05	-0.9699	0.7409
**Culture**	5	0.4906	0.4199	32	1.17	0.2513	0.05	-0.3648	1.3460
**Culture**	6	0.3631	0.5153	32	0.70	0.4862	0.05	-0.6866	1.4128
**Culture**	7	-0.3685	0.4402	32	-0.84	0.4087	0.05	-1.2651	0.5280
**Culture**	8	0.6915	0.4218	32	1.64	0.1109	0.05	-0.1677	1.5506
**Culture**	9	-0.6700	0.4218	32	-1.59	0.1220	0.05	-1.5292	0.1891
**Culture**	10	0.1209	0.4415	32	0.27	0.7860	0.05	-0.7785	1.0202
**Culture**	11	0.06091	0.4074	32	0.15	0.8821	0.05	-0.7689	0.8908
**Culture**	12	0.3889	0.4218	32	0.92	0.3634	0.05	-0.4702	1.2481
**Culture**	13	-0.5921	0.4415	32	-1.34	0.1893	0.05	-1.4915	0.3072

Type 3 Tests of Fixed Effects

Effect	Num DF	Den DF	F Value	Pr > F
**Treatment**	1	32	0.89	0.3537

Least Squares Means

Effect	Treatment	Estimate	Standard Error	DF	t Value	Pr > |t|	Alpha	Lower	Upper
**Treatment**	Control GFP	0.6892	0.3108	32	2.22	0.0338	0.05	0.05613	1.3222
**Treatment**	Ctr Meg	1.1073	0.3175	32	3.49	0.0014	0.05	0.4606	1.7540

DistSoma=276

Differences of Least Squares Means												
**Effect**	**Treatment**	**Treatment**	**Estimate**	**Standard Error**	**DF**	**t Value**	**Pr > |t|**	**Adjustment**	**Adj P**	**Alpha**	**Lower**	**Upper**
**Treatment**	Control GFP	Ctr Meg	-0.4181	0.4443	32	-0.94	0.3537	Tukey-Kramer	0.3537	0.05	-1.3231	0.4869

Differences of Least Squares Means				
**Effect**	**Treatment**	**Treatment**	**Adj Lower**	**Adj Upper**
**Treatment**	Control GFP	Ctr Meg	-1.3231	0.4869

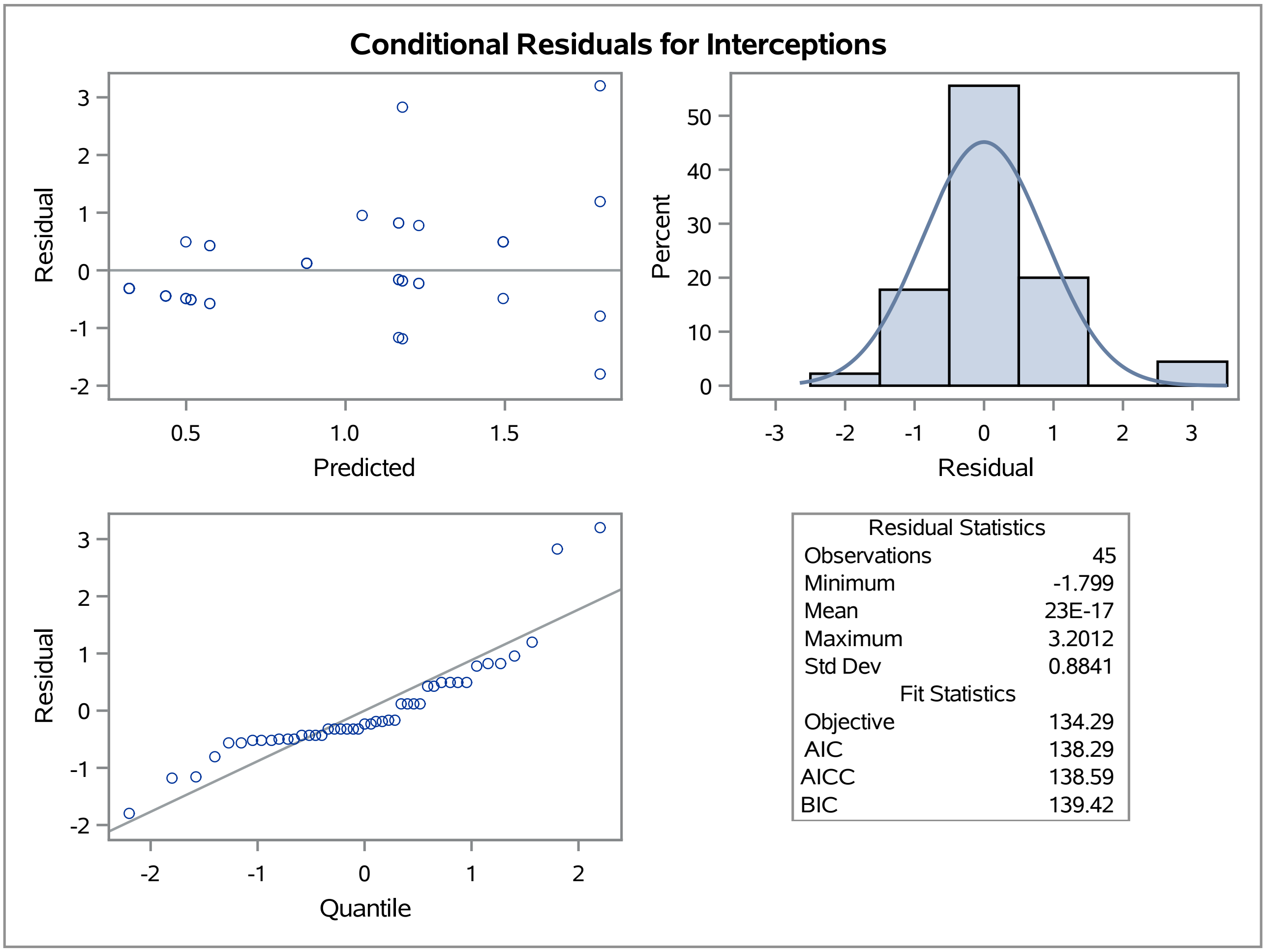

DistSoma=282

Model Information	
**Data Set**	WORK.TEMPDATASORTED
**Dependent Variable**	Interceptions
**Covariance Structure**	Variance Components
**Estimation Method**	REML
**Residual Variance Method**	Profile
**Fixed Effects SE Method**	Model-Based
**Degrees of Freedom Method**	Containment

Class Level Information		
**Class**	**Levels**	**Values**
**Treatment**	2	Control GFP Ctr Meg
**Culture**	13	1 2 3 4 5 6 7 8 9 10 11 12 13

Dimensions	
**Covariance Parameters**	2
**Columns in X**	3
**Columns in Z**	13
**Subjects**	1
**Max Obs per Subject**	45

Number of Observations	
**Number of Observations Read**	45
**Number of Observations Used**	45
**Number of Observations Not Used**	0

Iteration History			
**Iteration**	**Evaluations**	**-2 Res Log Like**	**Criterion**
**0**	1	130.52060327	
**1**	2	125.45840330	0.00000625
**2**	1	125.45825715	0.00000000

Convergence criteria met.

Covariance Parameter Estimates				
**Cov Parm**	**Estimate**	**Alpha**	**Lower**	**Upper**
**Culture**	0.3611	0.05	0.1329	2.6978
**Residual**	0.7307	0.05	0.4745	1.2695

DistSoma=282

Fit Statistics	
-2 Res Log Likelihood	125.5
AIC (Smaller is Better)	129.5
AICC (Smaller is Better)	129.8
BIC (Smaller is Better)	130.6

Solution for Fixed Effects

Effect	Treatment	Estimate	Standard Error	DF	t Value	Pr > |t|	Alpha	Lower	Upper
Intercept		1.0682	0.3043	11	3.51	0.0049	0.05	0.3983	1.7381
Treatment	Control GFP	-0.5843	0.4247	32	-1.38	0.1784	0.05	-1.4495	0.2808
Treatment	Ctr Meg	0	.	.	.	.	.	.	.

Solution for Random Effects

Effect	Culture	Estimate	Std Err Pred	DF	t Value	Pr > |t|	Alpha	Lower	Upper
Culture	1	-0.2890	0.4204	32	-0.69	0.4968	0.05	-1.1454	0.5674
Culture	2	-0.2890	0.4204	32	-0.69	0.4968	0.05	-1.1454	0.5674
Culture	3	0.3428	0.4000	32	0.86	0.3979	0.05	-0.4720	1.1576
Culture	4	-0.1553	0.4000	32	-0.39	0.7004	0.05	-0.9701	0.6595
Culture	5	0.5088	0.4000	32	1.27	0.2126	0.05	-0.3060	1.3236
Culture	6	0.1707	0.5013	32	0.34	0.7357	0.05	-0.8504	1.1918
Culture	7	-0.2890	0.4204	32	-0.69	0.4968	0.05	-1.1454	0.5674
Culture	8	0.7848	0.4027	32	1.95	0.0601	0.05	-0.03544	1.6050
Culture	9	-0.7094	0.4027	32	-1.76	0.0877	0.05	-1.5296	0.1109
Culture	10	0.1583	0.4225	32	0.37	0.7103	0.05	-0.7022	1.0189
Culture	11	-0.04856	0.3886	32	-0.12	0.9013	0.05	-0.8400	0.7429
Culture	12	0.4528	0.4027	32	1.12	0.2692	0.05	-0.3675	1.2730
Culture	13	-0.6380	0.4225	32	-1.51	0.1409	0.05	-1.4985	0.2226

Type 3 Tests of Fixed Effects

Effect	Num DF	Den DF	F Value	Pr > F
Treatment	1	32	1.89	0.1784

Least Squares Means

Effect	Treatment	Estimate	Standard Error	DF	t Value	Pr > |t|	Alpha	Lower	Upper
Treatment	Control GFP	0.4839	0.2963	32	1.63	0.1122	0.05	-0.1196	1.0873
Treatment	Ctr Meg	1.0682	0.3043	32	3.51	0.0014	0.05	0.4483	1.6882

DistSoma=282

Differences of Least Squares Means												
**Effect**	**Treatment**	**Treatment**	**Estimate**	**Standard Error**	**DF**	**t Value**	**Pr > |t|**	**Adjustment**	**Adj P**	**Alpha**	**Lower**	**Upper**
**Treatment**	Control GFP	Ctr Meg	-0.5843	0.4247	32	-1.38	0.1784	Tukey-Kramer	0.1784	0.05	-1.4495	0.2808

Differences of Least Squares Means				
**Effect**	**Treatment**	**Treatment**	**Adj Lower**	**Adj Upper**
**Treatment**	Control GFP	Ctr Meg	-1.4495	0.2808

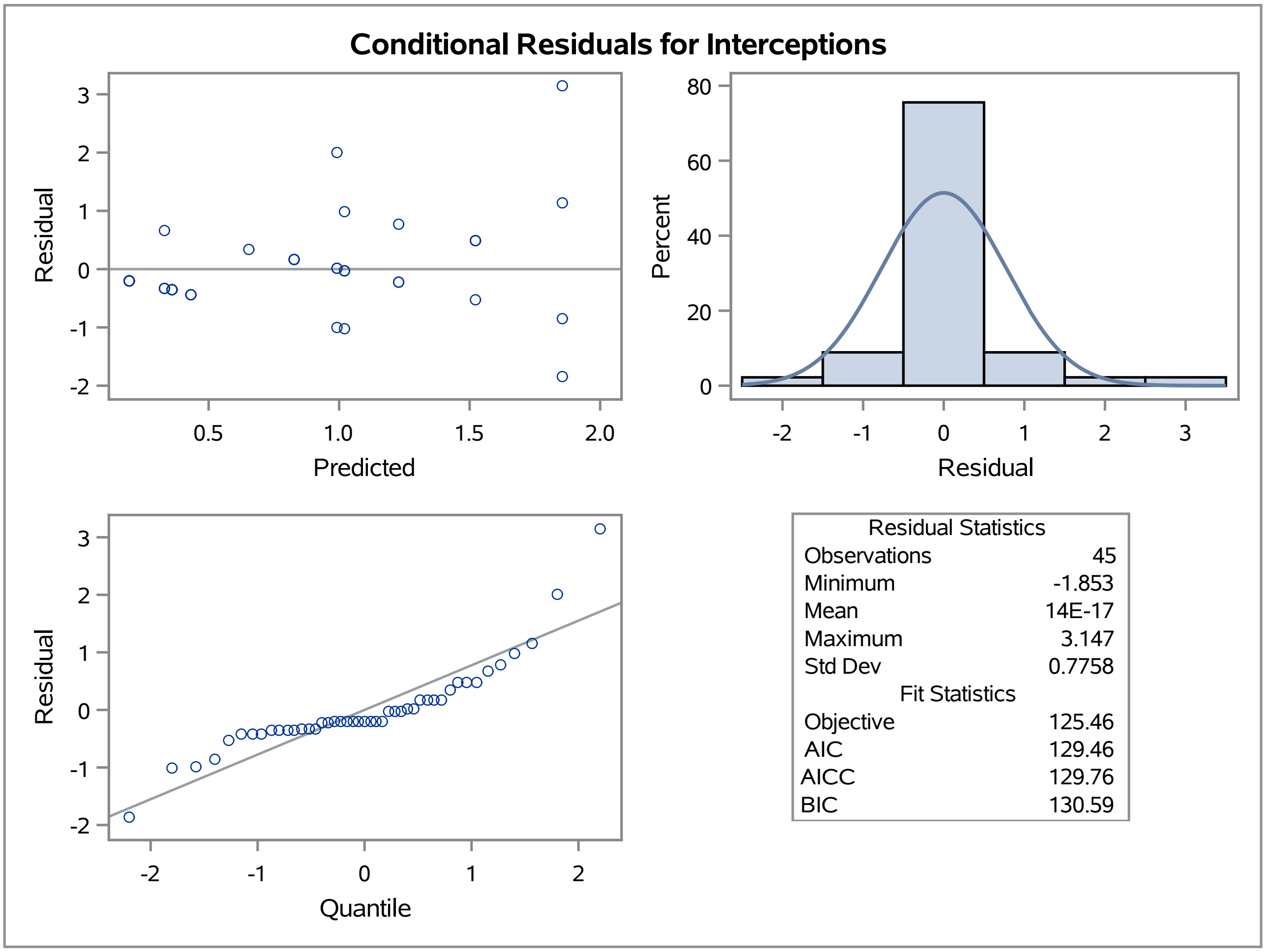

DistSoma=288

Model Information	
**Data Set**	WORK.TEMPDATASORTED
**Dependent Variable**	Interceptions
**Covariance Structure**	Variance Components
**Estimation Method**	REML
**Residual Variance Method**	Profile
**Fixed Effects SE Method**	Model-Based
**Degrees of Freedom Method**	Containment

Class Level Information		
**Class**	**Levels**	**Values**
**Treatment**	2	Control GFP Ctr Meg
**Culture**	13	1 2 3 4 5 6 7 8 9 10 11 12 13

Dimensions	
**Covariance Parameters**	2
**Columns in X**	3
**Columns in Z**	13
**Subjects**	1
**Max Obs per Subject**	45

Number of Observations	
**Number of Observations Read**	45
**Number of Observations Used**	45
**Number of Observations Not Used**	0

Iteration History			
**Iteration**	**Evaluations**	**-2 Res Log Like**	**Criterion**
**0**	1	135.09270729	
**1**	3	131.83003436	0.00000882
**2**	1	131.82979946	0.00000000

Convergence criteria met.

Covariance Parameter Estimates				
**Cov Parm**	**Estimate**	**Alpha**	**Lower**	**Upper**
**Culture**	0.3143	0.05	0.1043	3.6971
**Residual**	0.8903	0.05	0.5790	1.5435

DistSoma=288

Fit Statistics	
-2 Res Log Likelihood	131.8
AIC (Smaller is Better)	135.8
AICC (Smaller is Better)	136.1
BIC (Smaller is Better)	137.0

Solution for Fixed Effects									
Effect	Treatment	Estimate	Standard Error	DF	t Value	Pr > |t|	Alpha	Lower	Upper
Intercept		1.0208	0.3030	11	3.37	0.0063	0.05	0.3539	1.6877
Treatment	Control GFP	-0.6097	0.4244	32	-1.44	0.1605	0.05	-1.4741	0.2547
Treatment	Ctr Meg	0	.	.	.	.	.	.	.

Solution for Random Effects									
Effect	Culture	Estimate	Std Err Pred	DF	t Value	Pr > |t|	Alpha	Lower	Upper
Culture	1	-0.2114	0.4195	32	-0.50	0.6177	0.05	-1.0659	0.6431
Culture	2	-0.2114	0.4195	32	-0.50	0.6177	0.05	-1.0659	0.6431
Culture	3	0.3447	0.4007	32	0.86	0.3960	0.05	-0.4714	1.1609
Culture	4	-0.09430	0.4007	32	-0.24	0.8154	0.05	-0.9105	0.7219
Culture	5	0.4911	0.4007	32	1.23	0.2293	0.05	-0.3251	1.3072
Culture	6	-0.1072	0.4881	32	-0.22	0.8275	0.05	-1.1016	0.8871
Culture	7	-0.2114	0.4195	32	-0.50	0.6177	0.05	-1.0659	0.6431
Culture	8	0.7196	0.4022	32	1.79	0.0831	0.05	-0.09966	1.5388
Culture	9	-0.5975	0.4022	32	-1.49	0.1471	0.05	-1.4168	0.2217
Culture	10	-0.01068	0.4206	32	-0.03	0.9799	0.05	-0.8674	0.8461
Culture	11	-0.01325	0.3887	32	-0.03	0.9730	0.05	-0.8050	0.7785
Culture	12	0.4269	0.4022	32	1.06	0.2965	0.05	-0.3924	1.2461
Culture	13	-0.5250	0.4206	32	-1.25	0.2210	0.05	-1.3818	0.3318

Type 3 Tests of Fixed Effects				
Effect	Num DF	Den DF	F Value	Pr > F
Treatment	1	32	2.06	0.1605

Least Squares Means									
Effect	Treatment	Estimate	Standard Error	DF	t Value	Pr > |t|	Alpha	Lower	Upper
Treatment	Control GFP	0.4111	0.2971	32	1.38	0.1761	0.05	-0.1941	1.0163
Treatment	Ctr Meg	1.0208	0.3030	32	3.37	0.0020	0.05	0.4036	1.6379

DistSoma=288

Differences of Least Squares Means												
**Effect**	**Treatment**	**Treatment**	**Estimate**	**Standard Error**	**DF**	**t Value**	**Pr > |t|**	**Adjustment**	**Adj P**	**Alpha**	**Lower**	**Upper**
**Treatment**	Control GFP	Ctr Meg	-0.6097	0.4244	32	-1.44	0.1605	Tukey-Kramer	0.1605	0.05	-1.4741	0.2547

Differences of Least Squares Means				
**Effect**	**Treatment**	**Treatment**	**Adj Lower**	**Adj Upper**
**Treatment**	Control GFP	Ctr Meg	-1.4741	0.2547

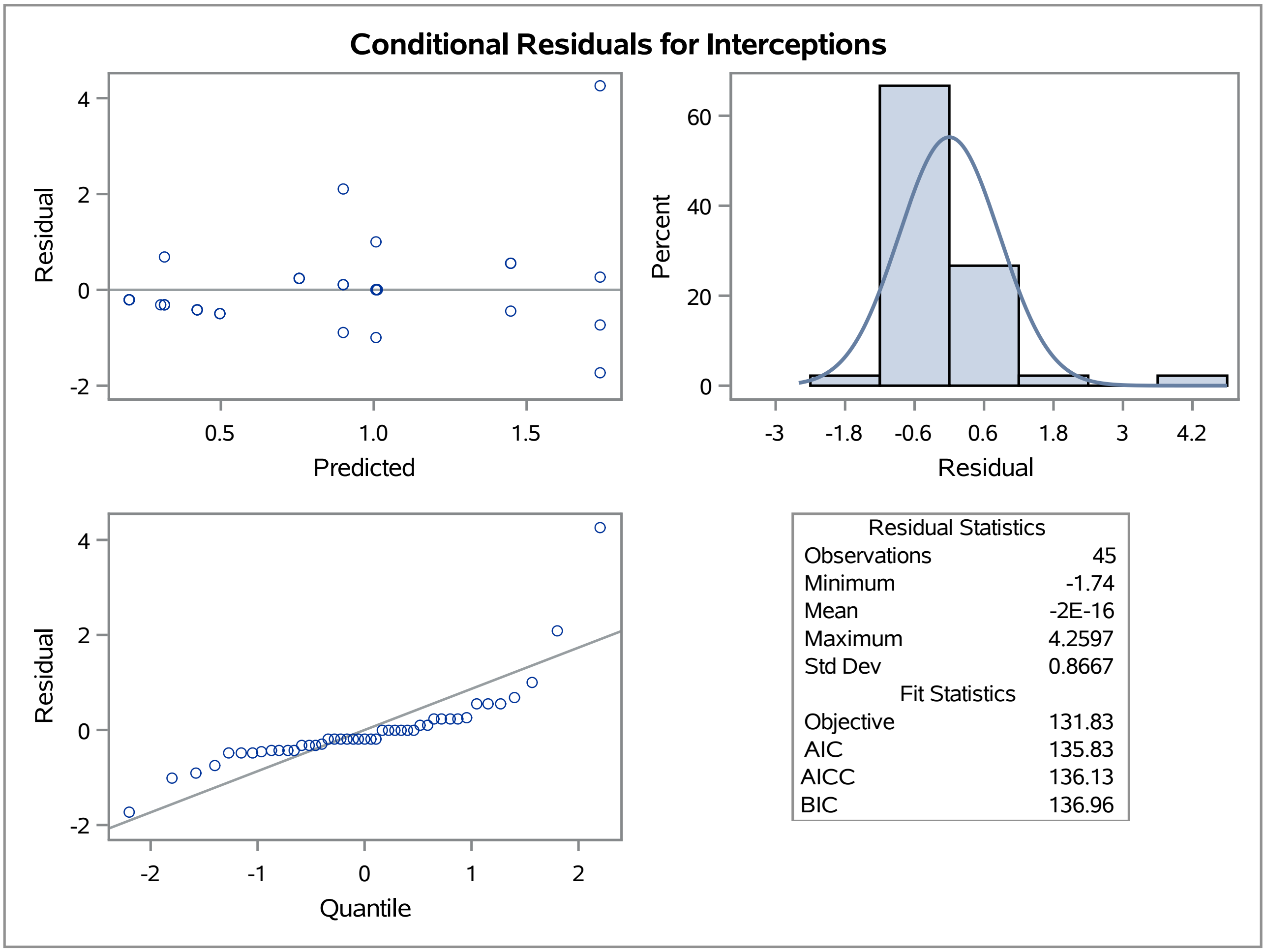

DistSoma=294

Model Information	
**Data Set**	WORK.TEMPDATASORTED
**Dependent Variable**	Interceptions
**Covariance Structure**	Variance Components
**Estimation Method**	REML
**Residual Variance Method**	Profile
**Fixed Effects SE Method**	Model-Based
**Degrees of Freedom Method**	Containment

Class Level Information		
**Class**	**Levels**	**Values**
**Treatment**	2	Control GFP Ctr Meg
**Culture**	13	1 2 3 4 5 6 7 8 9 10 11 12 13

Dimensions	
**Covariance Parameters**	2
**Columns in X**	3
**Columns in Z**	13
**Subjects**	1
**Max Obs per Subject**	45

Number of Observations	
**Number of Observations Read**	45
**Number of Observations Used**	45
**Number of Observations Not Used**	0

Iteration History			
**Iteration**	**Evaluations**	**-2 Res Log Like**	**Criterion**
**0**	1	139.82418567	
**1**	3	137.28692595	0.00004277
**2**	1	137.28565555	0.00000004
**3**	1	137.28565441	0.00000000

Convergence criteria met.

DistSoma=294

Covariance Parameter Estimates				
**Cov Parm**	**Estimate**	**Alpha**	**Lower**	**Upper**
**Culture**	0.2997	0.05	0.09272	5.0706
**Residual**	1.0376	0.05	0.6757	1.7946

Fit Statistics	
**-2 Res Log Likelihood**	137.3
**AIC (Smaller is Better)**	141.3
**AICC (Smaller is Better)**	141.6
**BIC (Smaller is Better)**	142.4

Solution for Fixed Effects									
**Effect**	**Treatment**	**Estimate**	**Standard Error**	**DF**	**t Value**	**Pr > |t|**	**Alpha**	**Lower**	**Upper**
**Intercept**		0.9474	0.3096	11	3.06	0.0108	0.05	0.2661	1.6287
**Treatment**	Control GFP	-0.5734	0.4345	32	-1.32	0.1963	0.05	-1.4584	0.3116
**Treatment**	Ctr Meg	0	.	.	.	.	.	.	.

Solution for Random Effects									
**Effect**	**Culture**	**Estimate**	**Std Err Pred**	**DF**	**t Value**	**Pr > |t|**	**Alpha**	**Lower**	**Upper**
**Culture**	1	-0.1736	0.4250	32	-0.41	0.6856	0.05	-1.0392	0.6920
**Culture**	2	-0.1736	0.4250	32	-0.41	0.6856	0.05	-1.0392	0.6920
**Culture**	3	0.3356	0.4071	32	0.82	0.4159	0.05	-0.4937	1.1648
**Culture**	4	-0.06646	0.4071	32	-0.16	0.8714	0.05	-0.8957	0.7628
**Culture**	5	0.3356	0.4071	32	0.82	0.4159	0.05	-0.4937	1.1648
**Culture**	6	-0.08381	0.4870	32	-0.17	0.8645	0.05	-1.0759	0.9082
**Culture**	7	-0.1736	0.4250	32	-0.41	0.6856	0.05	-1.0392	0.6920
**Culture**	8	0.8323	0.4081	32	2.04	0.0498	0.05	0.000900	1.6636
**Culture**	9	-0.5078	0.4081	32	-1.24	0.2224	0.05	-1.3392	0.3235
**Culture**	10	0.02443	0.4257	32	0.06	0.9546	0.05	-0.8427	0.8915
**Culture**	11	-0.2053	0.3951	32	-0.52	0.6069	0.05	-1.0100	0.5994
**Culture**	12	0.2962	0.4081	32	0.73	0.4732	0.05	-0.5351	1.1276
**Culture**	13	-0.4398	0.4257	32	-1.03	0.3093	0.05	-1.3069	0.4273

Type 3 Tests of Fixed Effects				
**Effect**	**Num DF**	**Den DF**	**F Value**	**Pr > F**
**Treatment**	1	32	1.74	0.1963

DistSoma=294

Least Squares Means									
**Effect**	**Treatment**	**Estimate**	**Standard Error**	**DF**	**t Value**	**Pr > |t|**	**Alpha**	**Lower**	**Upper**
**Treatment**	Control GFP	0.3740	0.3048	32	1.23	0.2288	0.05	-0.2470	0.9949
**Treatment**	Ctr Meg	0.9474	0.3096	32	3.06	0.0044	0.05	0.3168	1.5779

Differences of Least Squares Means												
**Effect**	**Treatment**	**Treatment**	**Estimate**	**Standard Error**	**DF**	**t Value**	**Pr > |t|**	**Adjustment**	**Adj P**	**Alpha**	**Lower**	**Upper**
**Treatment**	Control GFP	Ctr Meg	-0.5734	0.4345	32	-1.32	0.1963	Tukey-Kramer	0.1963	0.05	-1.4584	0.3116

Differences of Least Squares Means				
**Effect**	**Treatment**	**Treatment**	**Adj Lower**	**Adj Upper**
**Treatment**	Control GFP	Ctr Meg	-1.4584	0.3116

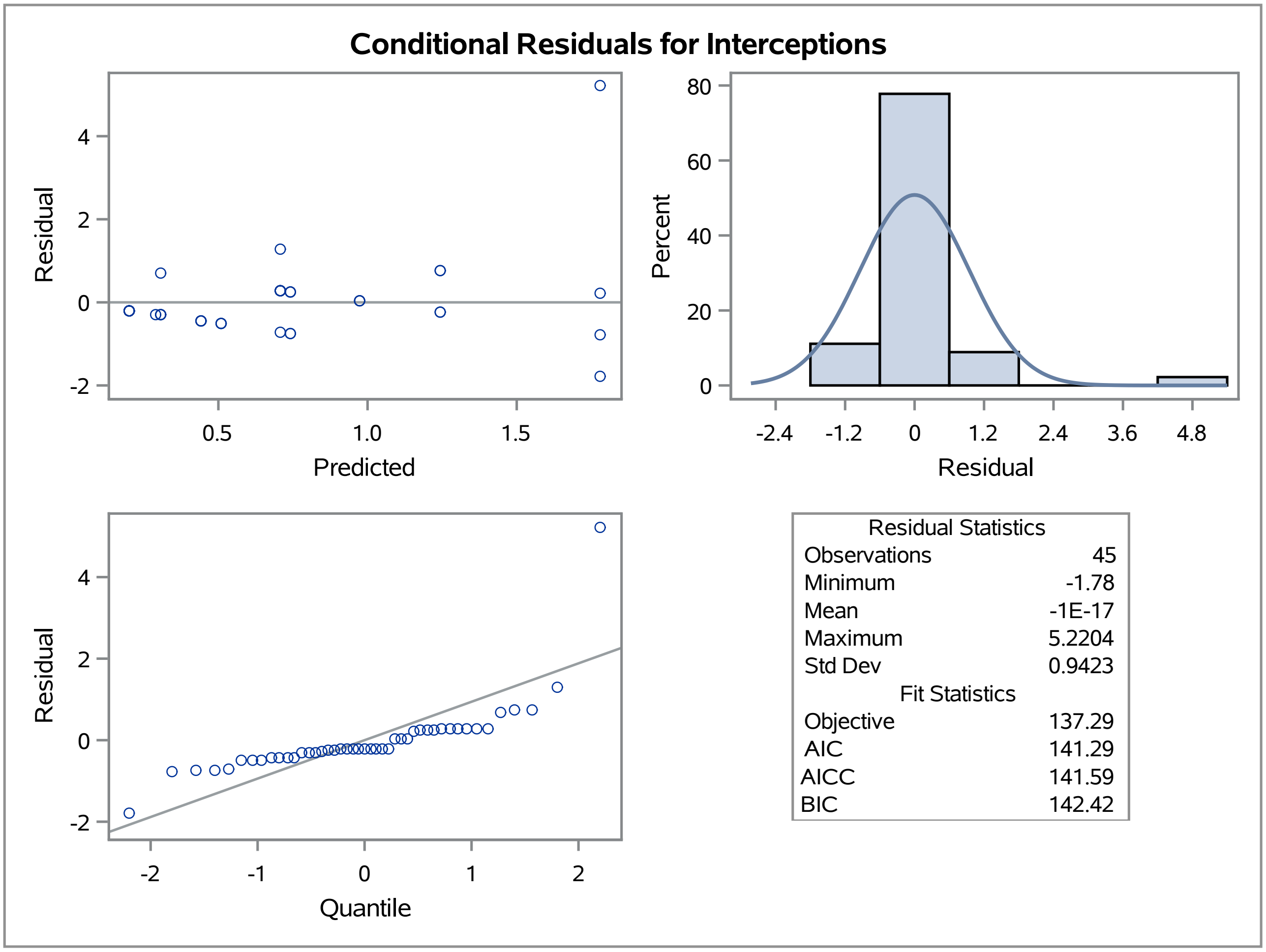

DistSoma=300

Model Information	
**Data Set**	WORK.TEMPDATASORTED
**Dependent Variable**	Interceptions
**Covariance Structure**	Variance Components
**Estimation Method**	REML
**Residual Variance Method**	Profile
**Fixed Effects SE Method**	Model-Based
**Degrees of Freedom Method**	Containment

Class Level Information		
**Class**	**Levels**	**Values**
**Treatment**	2	Control GFP Ctr Meg
**Culture**	13	1 2 3 4 5 6 7 8 9 10 11 12 13

Dimensions	
**Covariance Parameters**	2
**Columns in X**	3
**Columns in Z**	13
**Subjects**	1
**Max Obs per Subject**	45

Number of Observations	
**Number of Observations Read**	45
**Number of Observations Used**	45
**Number of Observations Not Used**	0

Iteration History			
**Iteration**	**Evaluations**	**-2 Res Log Like**	**Criterion**
**0**	1	140.66163172	
**1**	3	138.97529178	0.00006464
**2**	1	138.97330710	0.00000009
**3**	1	138.97330434	0.00000000

Convergence criteria met.

DistSoma=300

Covariance Parameter Estimates				
Cov Parm	Estimate	Alpha	Lower	Upper
Culture	0.2411	0.05	0.06589	8.9959
Residual	1.1158	0.05	0.7278	1.9250

Fit Statistics	
-2 Res Log Likelihood	139.0
AIC (Smaller is Better)	143.0
AICC (Smaller is Better)	143.3
BIC (Smaller is Better)	144.1

Solution for Fixed Effects									
Effect	Treatment	Estimate	Standard Error	DF	t Value	Pr > |t|	Alpha	Lower	Upper
Intercept		0.8676	0.2990	11	2.90	0.0144	0.05	0.2094	1.5257
Treatment	Control GFP	-0.5724	0.4209	32	-1.36	0.1834	0.05	-1.4297	0.2850
Treatment	Ctr Meg	0	.	.	.	.	.	.	.

Solution for Random Effects									
Effect	Culture	Estimate	Std Err Pred	DF	t Value	Pr > |t|	Alpha	Lower	Upper
Culture	1	-0.1161	0.3998	32	-0.29	0.7734	0.05	-0.9306	0.6983
Culture	2	-0.1161	0.3998	32	-0.29	0.7734	0.05	-0.9306	0.6983
Culture	3	0.09496	0.3850	32	0.25	0.8067	0.05	-0.6892	0.8791
Culture	4	-0.02095	0.3850	32	-0.05	0.9569	0.05	-0.8051	0.7632
Culture	5	0.3268	0.3850	32	0.85	0.4023	0.05	-0.4574	1.1109
Culture	6	-0.05246	0.4484	32	-0.12	0.9076	0.05	-0.9658	0.8609
Culture	7	-0.1161	0.3998	32	-0.29	0.7734	0.05	-0.9306	0.6983
Culture	8	0.7569	0.3854	32	1.96	0.0583	0.05	-0.02821	1.5420
Culture	9	-0.4022	0.3854	32	-1.04	0.3045	0.05	-1.1874	0.3829
Culture	10	0.05210	0.4002	32	0.13	0.8972	0.05	-0.7630	0.8672
Culture	11	-0.2428	0.3742	32	-0.65	0.5210	0.05	-1.0050	0.5194
Culture	12	0.1773	0.3854	32	0.46	0.6486	0.05	-0.6078	0.9624
Culture	13	-0.3412	0.4002	32	-0.85	0.4001	0.05	-1.1563	0.4739

Type 3 Tests of Fixed Effects				
Effect	Num DF	Den DF	F Value	Pr > F
Treatment	1	32	1.85	0.1834

DistSoma=300

Least Squares Means									
Effect	Treatment	Estimate	Standard Error	DF	t Value	Pr > |t|	Alpha	Lower	Upper
Treatment	Control GFP	0.2952	0.2962	32	1.00	0.3264	0.05	-0.3081	0.8985
Treatment	Ctr Meg	0.8676	0.2990	32	2.90	0.0067	0.05	0.2585	1.4766

Differences of Least Squares Means												
Effect	Treatment	Treatment	Estimate	Standard Error	DF	t Value	Pr > |t|	Adjustment	Adj P	Alpha	Lower	Upper
Treatment	Control GFP	Ctr Meg	-0.5724	0.4209	32	-1.36	0.1834	Tukey-Kramer	0.1834	0.05	-1.4297	0.2850

Differences of Least Squares Means				
Effect	Treatment	Treatment	Adj Lower	Adj Upper
Treatment	Control GFP	Ctr Meg	-1.4297	0.2850

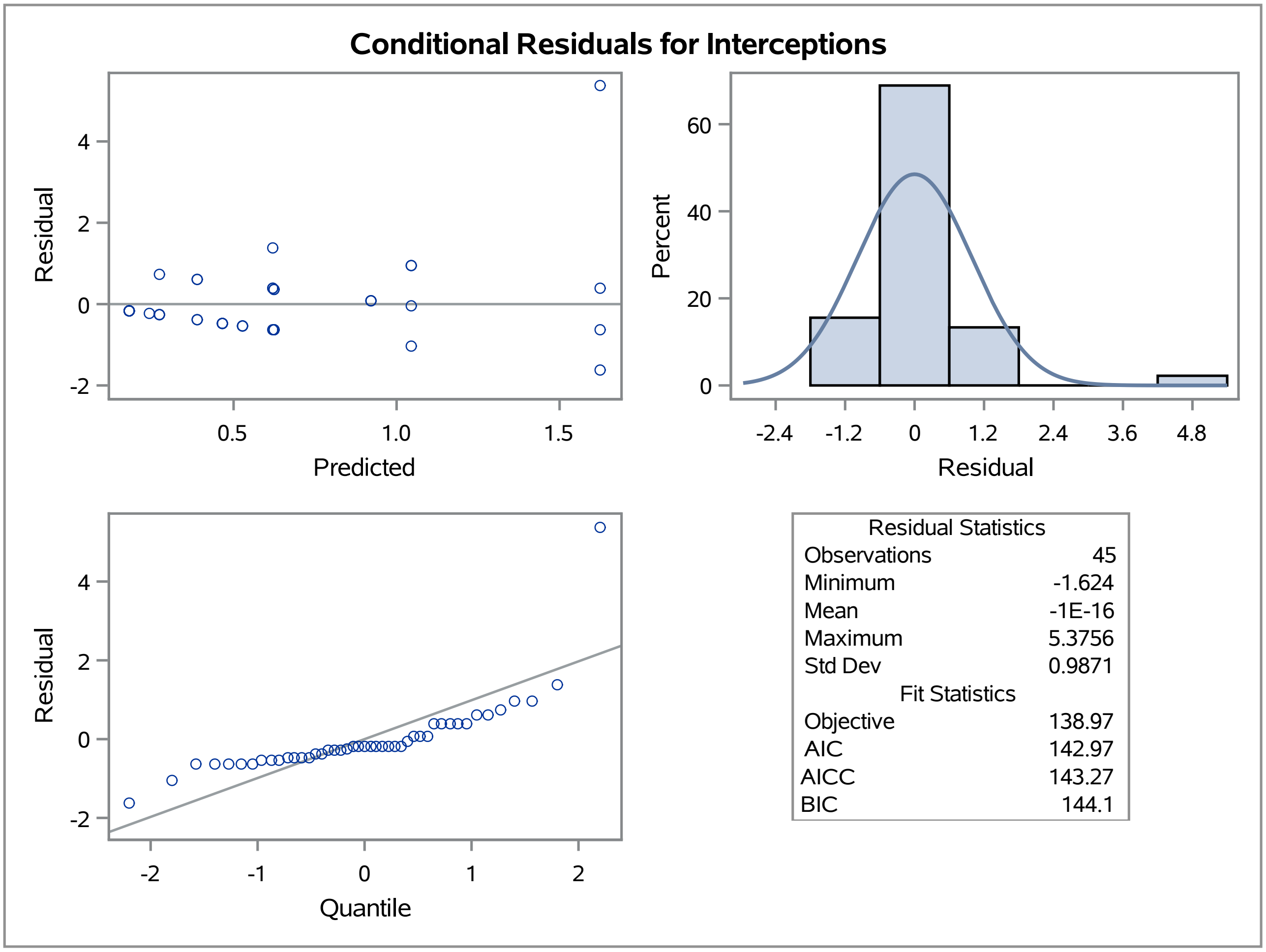

DistSoma=306

Model Information	
**Data Set**	WORK.TEMPDATASORTED
**Dependent Variable**	Interceptions
**Covariance Structure**	Variance Components
**Estimation Method**	REML
**Residual Variance Method**	Profile
**Fixed Effects SE Method**	Model-Based
**Degrees of Freedom Method**	Containment

Class Level Information		
**Class**	**Levels**	**Values**
**Treatment**	2	Control GFP Ctr Meg
**Culture**	13	1 2 3 4 5 6 7 8 9 10 11 12 13

Dimensions	
**Covariance Parameters**	2
**Columns in X**	3
**Columns in Z**	13
**Subjects**	1
**Max Obs per Subject**	45

Number of Observations	
**Number of Observations Read**	45
**Number of Observations Used**	45
**Number of Observations Not Used**	0

Iteration History			
**Iteration**	**Evaluations**	**-2 Res Log Like**	**Criterion**
**0**	1	150.22154285	
**1**	3	148.95106715	0.00009001
**2**	1	148.94782347	0.00000021
**3**	1	148.94781625	0.00000000

Convergence criteria met.

DistSoma=306

Covariance Parameter Estimates				
Cov Parm	Estimate	Alpha	Lower	Upper
Culture	0.2540	0.05	0.06313	20.2112
Residual	1.4357	0.05	0.9379	2.4708

Fit Statistics	
-2 Res Log Likelihood	148.9
AIC (Smaller is Better)	152.9
AICC (Smaller is Better)	153.2
BIC (Smaller is Better)	154.1

Solution for Fixed Effects									
Effect	Treatment	Estimate	Standard Error	DF	t Value	Pr > |t|	Alpha	Lower	Upper
Intercept		0.8240	0.3249	11	2.54	0.0277	0.05	0.1089	1.5391
Treatment	Control GFP	-0.5686	0.4582	32	-1.24	0.2236	0.05	-1.5019	0.3647
Treatment	Ctr Meg	0	.	.	.	.	.	.	.

Solution for Random Effects									
Effect	Culture	Estimate	Std Err Pred	DF	t Value	Pr > |t|	Alpha	Lower	Upper
Culture	1	-0.08855	0.4225	32	-0.21	0.8353	0.05	-0.9491	0.7720
Culture	2	-0.08855	0.4225	32	-0.21	0.8353	0.05	-0.9491	0.7720
Culture	3	0.1014	0.4082	32	0.25	0.8055	0.05	-0.7302	0.9329
Culture	4	-0.00224	0.4082	32	-0.01	0.9956	0.05	-0.8338	0.8293
Culture	5	0.2049	0.4082	32	0.50	0.6191	0.05	-0.6266	1.0365
Culture	6	-0.03839	0.4671	32	-0.08	0.9350	0.05	-0.9898	0.9130
Culture	7	-0.08855	0.4225	32	-0.21	0.8353	0.05	-0.9491	0.7720
Culture	8	0.7981	0.4085	32	1.95	0.0595	0.05	-0.03397	1.6302
Culture	9	-0.3415	0.4085	32	-0.84	0.4094	0.05	-1.1735	0.4906
Culture	10	-0.05456	0.4226	32	-0.13	0.8981	0.05	-0.9154	0.8063
Culture	11	-0.2929	0.3975	32	-0.74	0.4666	0.05	-1.1026	0.5168
Culture	12	0.1765	0.4085	32	0.43	0.6686	0.05	-0.6555	1.0086
Culture	13	-0.2857	0.4226	32	-0.68	0.5039	0.05	-1.1466	0.5752

Type 3 Tests of Fixed Effects				
Effect	Num DF	Den DF	F Value	Pr > F
Treatment	1	32	1.54	0.2236

DistSoma=306

Least Squares Means									
Effect	Treatment	Estimate	Standard Error	DF	t Value	Pr > |t|	Alpha	Lower	Upper
Treatment	Control GFP	0.2554	0.3231	32	0.79	0.4350	0.05	-0.4026	0.9135
Treatment	Ctr Meg	0.8240	0.3249	32	2.54	0.0163	0.05	0.1622	1.4858

Differences of Least Squares Means												
Effect	Treatment	Treatment	Estimate	Standard Error	DF	t Value	Pr > |t|	Adjustment	Adj P	Alpha	Lower	Upper
Treatment	Control GFP	Ctr Meg	-0.5686	0.4582	32	-1.24	0.2236	Tukey-Kramer	0.2236	0.05	-1.5019	0.3647

Differences of Least Squares Means				
Effect	Treatment	Treatment	Adj Lower	Adj Upper
Treatment	Control GFP	Ctr Meg	-1.5019	0.3647

Conditional Residuals for Interceptions

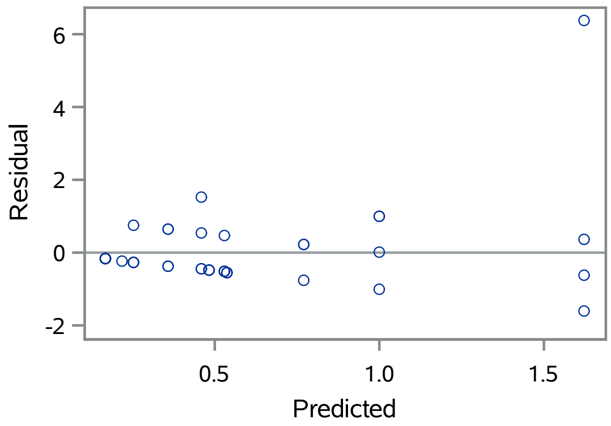

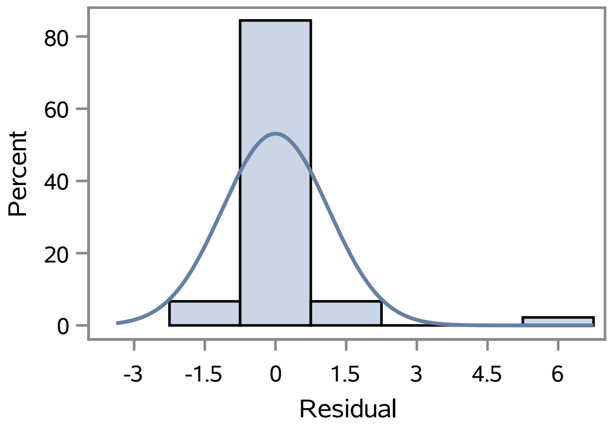

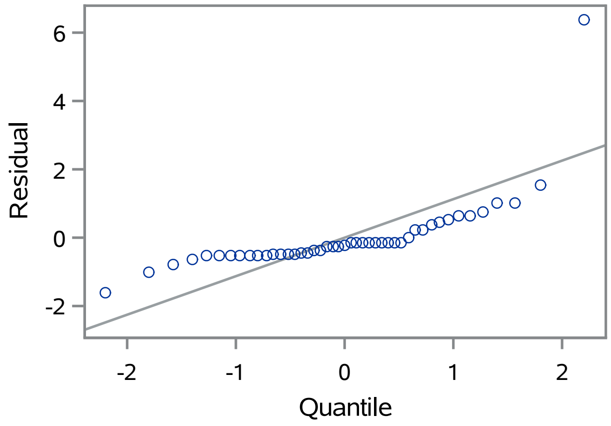

Residual Statistics	
Observations	45
Minimum	-1.622
Mean	-1E-16
Maximum	6.3779
Std Dev	1.1271
**Fit Statistics**	
Objective	148.95
AIC	152.95
AICC	153.25
BIC	154.08

DistSoma=312

Model Information	
**Data Set**	WORK.TEMPDATASORTED
**Dependent Variable**	Interceptions
**Covariance Structure**	Variance Components
**Estimation Method**	REML
**Residual Variance Method**	Profile
**Fixed Effects SE Method**	Model-Based
**Degrees of Freedom Method**	Containment

Class Level Information		
**Class**	**Levels**	**Values**
**Treatment**	2	Control GFP Ctr Meg
**Culture**	13	1 2 3 4 5 6 7 8 9 10 11 12 13

Dimensions	
**Covariance Parameters**	2
**Columns in X**	3
**Columns in Z**	13
**Subjects**	1
**Max Obs per Subject**	45

Number of Observations	
**Number of Observations Read**	45
**Number of Observations Used**	45
**Number of Observations Not Used**	0

Iteration History			
**Iteration**	**Evaluations**	**-2 Res Log Like**	**Criterion**
**0**	1	150.22154285	
**1**	3	149.78716978	0.00001182
**2**	1	149.78674682	0.00000000

Convergence criteria met.

Covariance Parameter Estimates				
**Cov Parm**	**Estimate**	**Alpha**	**Lower**	**Upper**
**Culture**	0.1438	0.05	0.02390	3967.16
**Residual**	1.5378	0.05	1.0058	2.6411

DistSoma=312

Fit Statistics	
-2 Res Log Likelihood	149.8
AIC (Smaller is Better)	153.8
AICC (Smaller is Better)	154.1
BIC (Smaller is Better)	154.9

Solution for Fixed Effects

Effect	Treatment	Estimate	Standard Error	DF	t Value	Pr > |t|	Alpha	Lower	Upper
Intercept		0.8285	0.3023	11	2.74	0.0192	0.05	0.1631	1.4939
Treatment	Control GFP	-0.5667	0.4285	32	-1.32	0.1954	0.05	-1.4395	0.3062
Treatment	Ctr Meg	0	.	.	.	.	.	.	.

Solution for Random Effects

Effect	Culture	Estimate	Std Err Pred	DF	t Value	Pr > |t|	Alpha	Lower	Upper
Culture	1	-0.05738	0.3417	32	-0.17	0.8677	0.05	-0.7534	0.6386
Culture	2	-0.05738	0.3417	32	-0.17	0.8677	0.05	-0.7534	0.6386
Culture	3	0.06485	0.3339	32	0.19	0.8473	0.05	-0.6154	0.7451
Culture	4	-0.00323	0.3339	32	-0.01	0.9924	0.05	-0.6834	0.6770
Culture	5	0.1329	0.3339	32	0.40	0.6933	0.05	-0.5473	0.8131
Culture	6	-0.02240	0.3636	32	-0.06	0.9513	0.05	-0.7631	0.7183
Culture	7	-0.05738	0.3417	32	-0.17	0.8677	0.05	-0.7534	0.6386
Culture	8	0.4551	0.3338	32	1.36	0.1823	0.05	-0.2249	1.1351
Culture	9	-0.2256	0.3338	32	-0.68	0.5041	0.05	-0.9056	0.4544
Culture	10	0.03757	0.3416	32	0.11	0.9131	0.05	-0.6583	0.7335
Culture	11	-0.2003	0.3275	32	-0.61	0.5452	0.05	-0.8675	0.4669
Culture	12	0.1148	0.3338	32	0.34	0.7333	0.05	-0.5653	0.7948
Culture	13	-0.1816	0.3416	32	-0.53	0.5988	0.05	-0.8774	0.5143

Type 3 Tests of Fixed Effects

Effect	Num DF	Den DF	F Value	Pr > F
Treatment	1	32	1.75	0.1954

Least Squares Means

Effect	Treatment	Estimate	Standard Error	DF	t Value	Pr > |t|	Alpha	Lower	Upper
Treatment	Control GFP	0.2618	0.3037	32	0.86	0.3950	0.05	-0.3567	0.8804
Treatment	Ctr Meg	0.8285	0.3023	32	2.74	0.0099	0.05	0.2127	1.4444

DistSoma=312

Differences of Least Squares Means													
**Effect**	**Treatment**	**Treatment**	**Estimate**	**Standard Error**	**DF**	**t Value**	**Pr > |t|**	**Adjustment**	**Adj P**	**Alpha**	**Lower**	**Upper**
**Treatment**	Control GFP	Ctr Meg	-0.5667	0.4285	32	-1.32	0.1954	Tukey-Kramer	0.1954	0.05	-1.4395	0.3062

Differences of Least Squares Means				
**Effect**	**Treatment**	**Treatment**	**Adj Lower**	**Adj Upper**
**Treatment**	Control GFP	Ctr Meg	-1.4395	0.3062

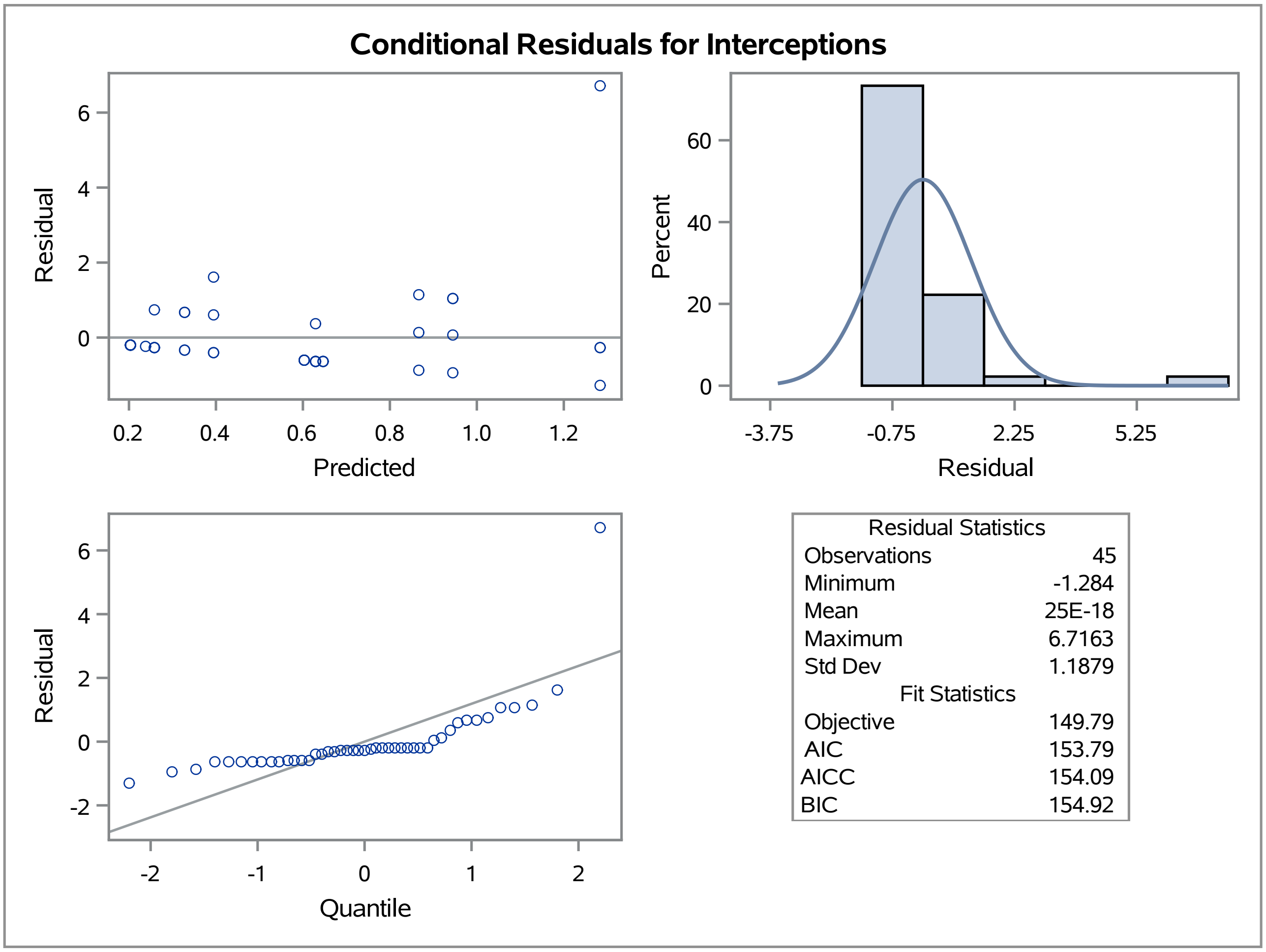

DistSoma=318

Model Information	
**Data Set**	WORK.TEMPDATASORTED
**Dependent Variable**	Interceptions
**Covariance Structure**	Variance Components
**Estimation Method**	REML
**Residual Variance Method**	Profile
**Fixed Effects SE Method**	Model-Based
**Degrees of Freedom Method**	Containment

Class Level Information		
**Class**	**Levels**	**Values**
**Treatment**	2	Control GFP Ctr Meg
**Culture**	13	1 2 3 4 5 6 7 8 9 10 11 12 13

Dimensions	
**Covariance Parameters**	2
**Columns in X**	3
**Columns in Z**	13
**Subjects**	1
**Max Obs per Subject**	45

Number of Observations	
**Number of Observations Read**	45
**Number of Observations Used**	45
**Number of Observations Not Used**	0

Iteration History			
**Iteration**	**Evaluations**	**-2 Res Log Like**	**Criterion**
**0**	1	156.98802485	
**1**	3	156.67868369	0.00000780
**2**	1	156.67837767	0.00000000

Convergence criteria met.

Covariance Parameter Estimates				
**Cov Parm**	**Estimate**	**Alpha**	**Lower**	**Upper**
**Culture**	0.1399	0.05	0.02035	138591
**Residual**	1.8256	0.05	1.1951	3.1311

DistSoma=318

Fit Statistics	
-2 Res Log Likelihood	156.7
AIC (Smaller is Better)	160.7
AICC (Smaller is Better)	161.0
BIC (Smaller is Better)	161.8

Solution for Fixed Effects

Effect	Treatment	Estimate	Standard Error	DF	t Value	Pr > |t|	Alpha	Lower	Upper
Intercept		0.8716	0.3214	11	2.71	0.0202	0.05	0.1643	1.5789
Treatment	Control GFP	-0.6960	0.4561	32	-1.53	0.1368	0.05	-1.6250	0.2330
Treatment	Ctr Meg	0	.	.	.	.	.	.	.

Solution for Random Effects

Effect	Culture	Estimate	Std Err Pred	DF	t Value	Pr > |t|	Alpha	Lower	Upper
Culture	1	-0.03283	0.3426	32	-0.10	0.9243	0.05	-0.7308	0.6651
Culture	2	-0.03283	0.3426	32	-0.10	0.9243	0.05	-0.7308	0.6651
Culture	3	0.07610	0.3359	32	0.23	0.8222	0.05	-0.6081	0.7603
Culture	4	0.01745	0.3359	32	0.05	0.9589	0.05	-0.6668	0.7017
Culture	5	0.01745	0.3359	32	0.05	0.9589	0.05	-0.6668	0.7017
Culture	6	-0.01250	0.3612	32	-0.03	0.9726	0.05	-0.7482	0.7232
Culture	7	-0.03283	0.3426	32	-0.10	0.9243	0.05	-0.7308	0.6651
Culture	8	0.4407	0.3358	32	1.31	0.1987	0.05	-0.2433	1.1247
Culture	9	-0.2045	0.3358	32	-0.61	0.5468	0.05	-0.8885	0.4795
Culture	10	0.02400	0.3426	32	0.07	0.9446	0.05	-0.6738	0.7218
Culture	11	-0.1860	0.3303	32	-0.56	0.5771	0.05	-0.8587	0.4867
Culture	12	0.08877	0.3358	32	0.26	0.7932	0.05	-0.5952	0.7728
Culture	13	-0.1629	0.3426	32	-0.48	0.6376	0.05	-0.8607	0.5349

Type 3 Tests of Fixed Effects

Effect	Num DF	Den DF	F Value	Pr > F
Treatment	1	32	2.33	0.1368

Least Squares Means

Effect	Treatment	Estimate	Standard Error	DF	t Value	Pr > |t|	Alpha	Lower	Upper
Treatment	Control GFP	0.1756	0.3236	32	0.54	0.5911	0.05	-0.4836	0.8348
Treatment	Ctr Meg	0.8716	0.3214	32	2.71	0.0107	0.05	0.2170	1.5262

DistSoma=318

Differences of Least Squares Means												
**Effect**	**Treatment**	**Treatment**	**Estimate**	**Standard Error**	**DF**	**t Value**	**Pr > |t|**	**Adjustment**	**Adj P**	**Alpha**	**Lower**	**Upper**
**Treatment**	Control GFP	Ctr Meg	-0.6960	0.4561	32	-1.53	0.1368	Tukey-Kramer	0.1368	0.05	-1.6250	0.2330

Differences of Least Squares Means				
**Effect**	**Treatment**	**Treatment**	**Adj Lower**	**Adj Upper**
**Treatment**	Control GFP	Ctr Meg	-1.6249	0.2330

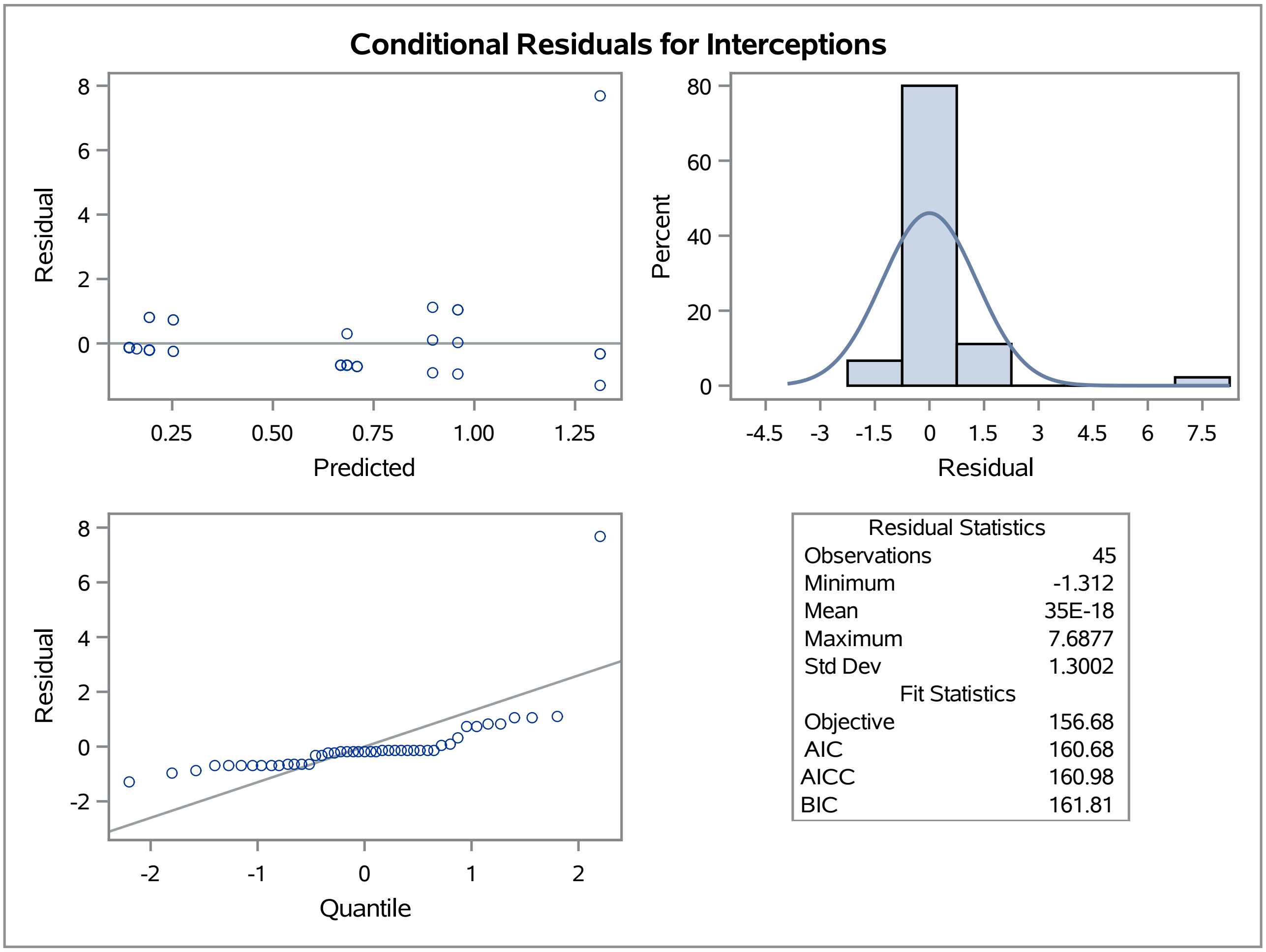

DistSoma=324

Model Information	
**Data Set**	WORK.TEMPDATASORTED
**Dependent Variable**	Interceptions
**Covariance Structure**	Variance Components
**Estimation Method**	REML
**Residual Variance Method**	Profile
**Fixed Effects SE Method**	Model-Based
**Degrees of Freedom Method**	Containment

Class Level Information		
**Class**	**Levels**	**Values**
**Treatment**	2	Control GFP Ctr Meg
**Culture**	13	1 2 3 4 5 6 7 8 9 10 11 12 13

Dimensions	
**Covariance Parameters**	2
**Columns in X**	3
**Columns in Z**	13
**Subjects**	1
**Max Obs per Subject**	45

Number of Observations	
**Number of Observations Read**	45
**Number of Observations Used**	45
**Number of Observations Not Used**	0

Iteration History			
**Iteration**	**Evaluations**	**-2 Res Log Like**	**Criterion**
**0**	1	137.61817661	
**1**	3	137.17156129	0.00001492
**2**	1	137.17112244	0.00000000

Convergence criteria met.

Covariance Parameter Estimates				
**Cov Parm**	**Estimate**	**Alpha**	**Lower**	**Upper**
**Culture**	0.1089	0.05	0.01829	2334.09
**Residual**	1.1457	0.05	0.7493	1.9676

DistSoma=324

Fit Statistics	
**-2 Res Log Likelihood**	137.2
**AIC (Smaller is Better)**	141.2
**AICC (Smaller is Better)**	141.5
**BIC (Smaller is Better)**	142.3

Solution for Fixed Effects

Effect	Treatment	Estimate	Standard Error	DF	t Value	Pr > |t|	Alpha	Lower	Upper
**Intercept**		0.6988	0.2615	11	2.67	0.0217	0.05	0.1232	1.2744
**Treatment**	Control GFP	-0.5679	0.3706	32	-1.53	0.1352	0.05	-1.3229	0.1870
**Treatment**	Ctr Meg	0	.	.	.	.	.	.	.

Solution for Random Effects

Effect	Culture	Estimate	Std Err Pred	DF	t Value	Pr > |t|	Alpha	Lower	Upper
**Culture**	1	-0.02903	0.2968	32	-0.10	0.9227	0.05	-0.6337	0.5756
**Culture**	2	-0.02903	0.2968	32	-0.10	0.9227	0.05	-0.6337	0.5756
**Culture**	3	0.1017	0.2900	32	0.35	0.7282	0.05	-0.4891	0.6925
**Culture**	4	-0.03604	0.2900	32	-0.12	0.9019	0.05	-0.6268	0.5547
**Culture**	5	0.03282	0.2900	32	0.11	0.9106	0.05	-0.5580	0.6236
**Culture**	6	-0.01136	0.3161	32	-0.04	0.9716	0.05	-0.6553	0.6326
**Culture**	7	-0.02903	0.2968	32	-0.10	0.9227	0.05	-0.6337	0.5756
**Culture**	8	0.4273	0.2900	32	1.47	0.1504	0.05	-0.1634	1.0179
**Culture**	9	-0.1925	0.2900	32	-0.66	0.5116	0.05	-0.7831	0.3982
**Culture**	10	0.06682	0.2968	32	0.23	0.8233	0.05	-0.5377	0.6714
**Culture**	11	-0.1607	0.2844	32	-0.56	0.5761	0.05	-0.7400	0.4187
**Culture**	12	0.01410	0.2900	32	0.05	0.9615	0.05	-0.5765	0.6047
**Culture**	13	-0.1550	0.2968	32	-0.52	0.6050	0.05	-0.7596	0.4495

Type 3 Tests of Fixed Effects

Effect	Num DF	Den DF	F Value	Pr > F
**Treatment**	1	32	2.35	0.1352

Least Squares Means

Effect	Treatment	Estimate	Standard Error	DF	t Value	Pr > |t|	Alpha	Lower	Upper
**Treatment**	Control GFP	0.1309	0.2626	32	0.50	0.6217	0.05	-0.4041	0.6658
**Treatment**	Ctr Meg	0.6988	0.2615	32	2.67	0.0118	0.05	0.1661	1.2315

DistSoma=324

Differences of Least Squares Means												
**Effect**	**Treatment**	**Treatment**	**Estimate**	**Standard Error**	**DF**	**t Value**	**Pr > |t|**	**Adjustment**	**Adj P**	**Alpha**	**Lower**	**Upper**
**Treatment**	Control GFP	Ctr Meg	-0.5679	0.3706	32	-1.53	0.1352	Tukey-Kramer	0.1352	0.05	-1.3229	0.1870

Differences of Least Squares Means				
**Effect**	**Treatment**	**Treatment**	**Adj Lower**	**Adj Upper**
**Treatment**	Control GFP	Ctr Meg	-1.3228	0.1870

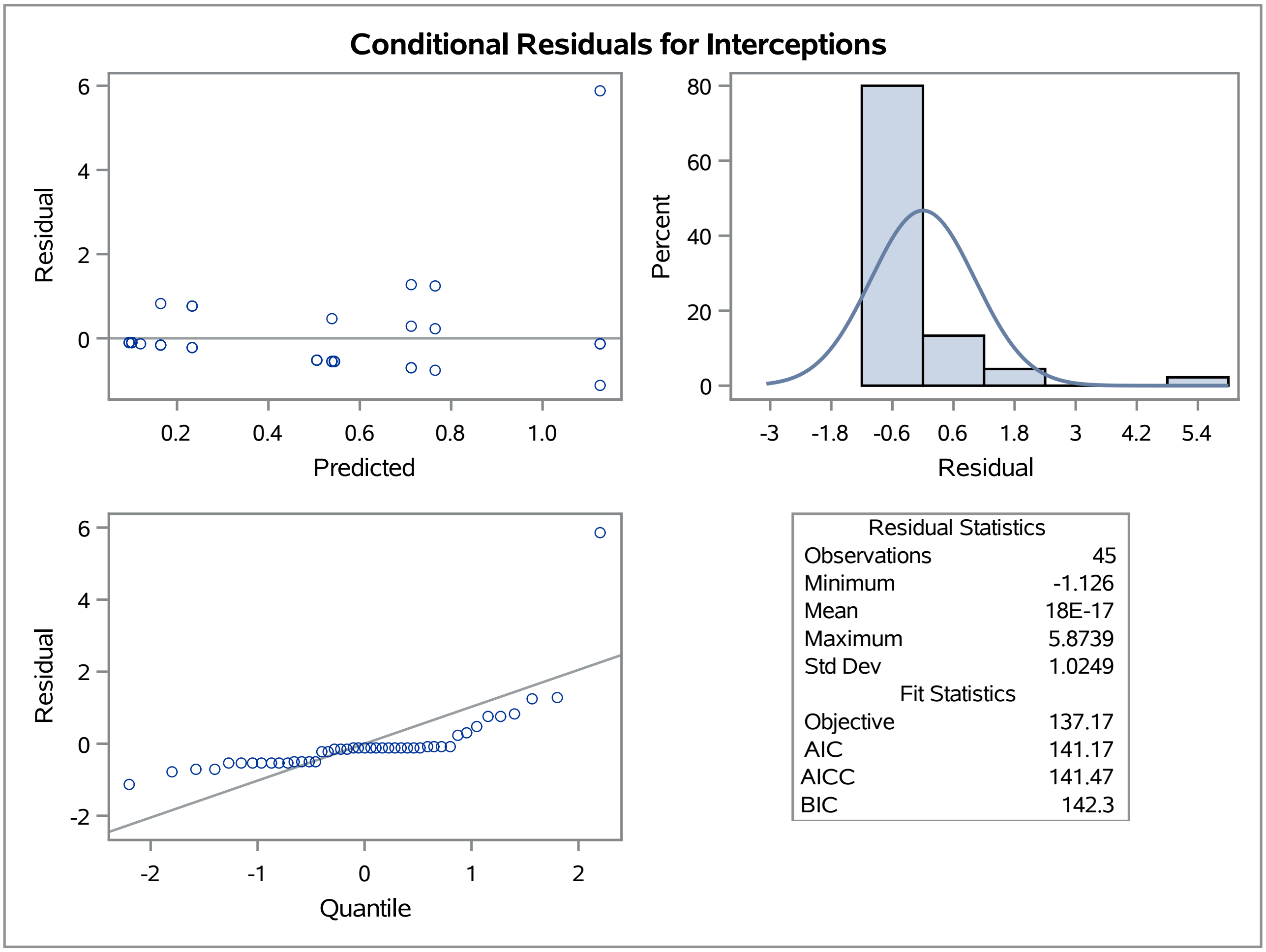

DistSoma=330

Model Information	
**Data Set**	WORK.TEMPDATASORTED
**Dependent Variable**	Interceptions
**Covariance Structure**	Variance Components
**Estimation Method**	REML
**Residual Variance Method**	Profile
**Fixed Effects SE Method**	Model-Based
**Degrees of Freedom Method**	Containment

Class Level Information		
**Class**	**Levels**	**Values**
**Treatment**	2	Control GFP Ctr Meg
**Culture**	13	1 2 3 4 5 6 7 8 9 10 11 12 13

Dimensions	
**Covariance Parameters**	2
**Columns in X**	3
**Columns in Z**	13
**Subjects**	1
**Max Obs per Subject**	45

Number of Observations	
**Number of Observations Read**	45
**Number of Observations Used**	45
**Number of Observations Not Used**	0

Iteration History			
**Iteration**	**Evaluations**	**-2 Res Log Like**	**Criterion**
**0**	1	137.61817661	
**1**	3	137.17156129	0.00001492
**2**	1	137.17112244	0.00000000

Convergence criteria met.

Covariance Parameter Estimates				
**Cov Parm**	**Estimate**	**Alpha**	**Lower**	**Upper**
**Culture**	0.1089	0.05	0.01829	2334.09
**Residual**	1.1457	0.05	0.7493	1.9676

DistSoma=330

Fit Statistics	
-2 Res Log Likelihood	137.2
AIC (Smaller is Better)	141.2
AICC (Smaller is Better)	141.5
BIC (Smaller is Better)	142.3

Solution for Fixed Effects

Effect	Treatment	Estimate	Standard Error	DF	t Value	Pr > |t|	Alpha	Lower	Upper
Intercept		0.6988	0.2615	11	2.67	0.0217	0.05	0.1232	1.2744
Treatment	Control GFP	-0.5679	0.3706	32	-1.53	0.1352	0.05	-1.3229	0.1870
Treatment	Ctr Meg	0	.	.	.	.	.	.	.

Solution for Random Effects

Effect	Culture	Estimate	Std Err Pred	DF	t Value	Pr > |t|	Alpha	Lower	Upper
Culture	1	-0.02903	0.2968	32	-0.10	0.9227	0.05	-0.6337	0.5756
Culture	2	-0.02903	0.2968	32	-0.10	0.9227	0.05	-0.6337	0.5756
Culture	3	0.1017	0.2900	32	0.35	0.7282	0.05	-0.4891	0.6925
Culture	4	-0.03604	0.2900	32	-0.12	0.9019	0.05	-0.6268	0.5547
Culture	5	0.03282	0.2900	32	0.11	0.9106	0.05	-0.5580	0.6236
Culture	6	-0.01136	0.3161	32	-0.04	0.9716	0.05	-0.6553	0.6326
Culture	7	-0.02903	0.2968	32	-0.10	0.9227	0.05	-0.6337	0.5756
Culture	8	0.4273	0.2900	32	1.47	0.1504	0.05	-0.1634	1.0179
Culture	9	-0.1925	0.2900	32	-0.66	0.5116	0.05	-0.7831	0.3982
Culture	10	0.06682	0.2968	32	0.23	0.8233	0.05	-0.5377	0.6714
Culture	11	-0.1607	0.2844	32	-0.56	0.5761	0.05	-0.7400	0.4187
Culture	12	0.01410	0.2900	32	0.05	0.9615	0.05	-0.5765	0.6047
Culture	13	-0.1550	0.2968	32	-0.52	0.6050	0.05	-0.7596	0.4495

Type 3 Tests of Fixed Effects

Effect	Num DF	Den DF	F Value	Pr > F
Treatment	1	32	2.35	0.1352

Least Squares Means

Effect	Treatment	Estimate	Standard Error	DF	t Value	Pr > |t|	Alpha	Lower	Upper
Treatment	Control GFP	0.1309	0.2626	32	0.50	0.6217	0.05	-0.4041	0.6658
Treatment	Ctr Meg	0.6988	0.2615	32	2.67	0.0118	0.05	0.1661	1.2315

DistSoma=330

Differences of Least Squares Means												
**Effect**	**Treatment**	**Treatment**	**Estimate**	**Standard Error**	**DF**	**t Value**	**Pr > |t|**	**Adjustment**	**Adj P**	**Alpha**	**Lower**	**Upper**
**Treatment**	Control GFP	Ctr Meg	-0.5679	0.3706	32	-1.53	0.1352	Tukey-Kramer	0.1352	0.05	-1.3229	0.1870

Differences of Least Squares Means				
**Effect**	**Treatment**	**Treatment**	**Adj Lower**	**Adj Upper**
**Treatment**	Control GFP	Ctr Meg	-1.3228	0.1870

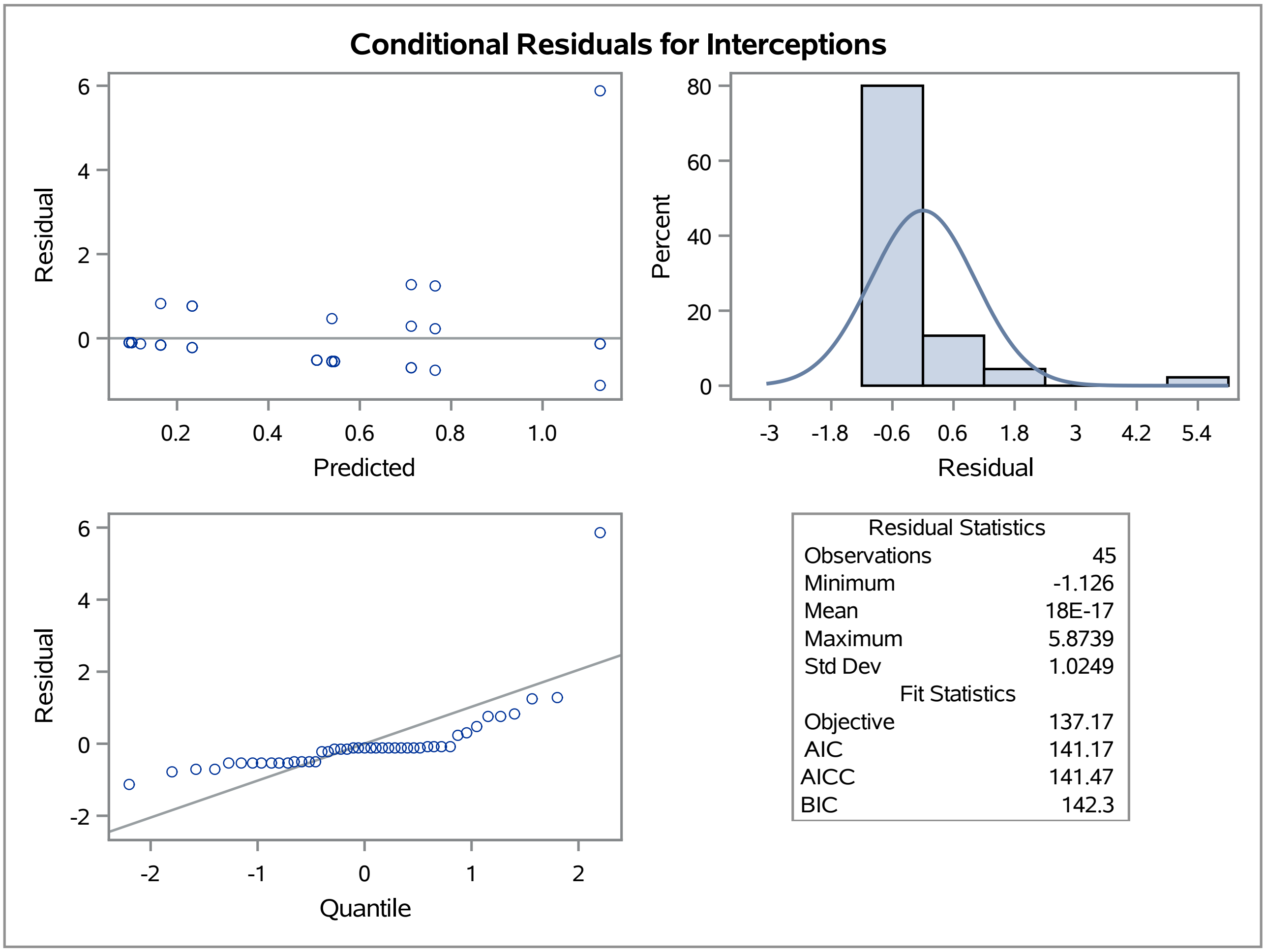

DistSoma=336

Model Information	
**Data Set**	WORK.TEMPDATASORTED
**Dependent Variable**	Interceptions
**Covariance Structure**	Variance Components
**Estimation Method**	REML
**Residual Variance Method**	Profile
**Fixed Effects SE Method**	Model-Based
**Degrees of Freedom Method**	Containment

Class Level Information		
**Class**	**Levels**	**Values**
**Treatment**	2	Control GFP Ctr Meg
**Culture**	13	1 2 3 4 5 6 7 8 9 10 11 12 13

Dimensions	
**Covariance Parameters**	2
**Columns in X**	3
**Columns in Z**	13
**Subjects**	1
**Max Obs per Subject**	45

Number of Observations	
**Number of Observations Read**	45
**Number of Observations Used**	45
**Number of Observations Not Used**	0

Iteration History			
**Iteration**	**Evaluations**	**-2 Res Log Like**	**Criterion**
**0**	1	137.61817661	
**1**	3	137.17156129	0.00001492
**2**	1	137.17112244	0.00000000

Convergence criteria met.

Covariance Parameter Estimates				
**Cov Parm**	**Estimate**	**Alpha**	**Lower**	**Upper**
**Culture**	0.1089	0.05	0.01829	2334.09
**Residual**	1.1457	0.05	0.7493	1.9676

DistSoma=336

Fit Statistics	
-2 Res Log Likelihood	137.2
AIC (Smaller is Better)	141.2
AICC (Smaller is Better)	141.5
BIC (Smaller is Better)	142.3

Solution for Fixed Effects

Effect	Treatment	Estimate	Standard Error	DF	t Value	Pr > |t|	Alpha	Lower	Upper
Intercept		0.6988	0.2615	11	2.67	0.0217	0.05	0.1232	1.2744
Treatment	Control GFP	-0.5679	0.3706	32	-1.53	0.1352	0.05	-1.3229	0.1870
Treatment	Ctr Meg	0	.	.	.	.	.	.	.

Solution for Random Effects

Effect	Culture	Estimate	Std Err Pred	DF	t Value	Pr > |t|	Alpha	Lower	Upper
Culture	1	-0.02903	0.2968	32	-0.10	0.9227	0.05	-0.6337	0.5756
Culture	2	-0.02903	0.2968	32	-0.10	0.9227	0.05	-0.6337	0.5756
Culture	3	0.1017	0.2900	32	0.35	0.7282	0.05	-0.4891	0.6925
Culture	4	-0.03604	0.2900	32	-0.12	0.9019	0.05	-0.6268	0.5547
Culture	5	0.03282	0.2900	32	0.11	0.9106	0.05	-0.5580	0.6236
Culture	6	-0.01136	0.3161	32	-0.04	0.9716	0.05	-0.6553	0.6326
Culture	7	-0.02903	0.2968	32	-0.10	0.9227	0.05	-0.6337	0.5756
Culture	8	0.4273	0.2900	32	1.47	0.1504	0.05	-0.1634	1.0179
Culture	9	-0.1925	0.2900	32	-0.66	0.5116	0.05	-0.7831	0.3982
Culture	10	0.06682	0.2968	32	0.23	0.8233	0.05	-0.5377	0.6714
Culture	11	-0.1607	0.2844	32	-0.56	0.5761	0.05	-0.7400	0.4187
Culture	12	0.01410	0.2900	32	0.05	0.9615	0.05	-0.5765	0.6047
Culture	13	-0.1550	0.2968	32	-0.52	0.6050	0.05	-0.7596	0.4495

Type 3 Tests of Fixed Effects

Effect	Num DF	Den DF	F Value	Pr > F
Treatment	1	32	2.35	0.1352

Least Squares Means

Effect	Treatment	Estimate	Standard Error	DF	t Value	Pr > |t|	Alpha	Lower	Upper
Treatment	Control GFP	0.1309	0.2626	32	0.50	0.6217	0.05	-0.4041	0.6658
Treatment	Ctr Meg	0.6988	0.2615	32	2.67	0.0118	0.05	0.1661	1.2315

DistSoma=336

Differences of Least Squares Means												
**Effect**	**Treatment**	**Treatment**	**Estimate**	**Standard Error**	**DF**	**t Value**	**Pr > |t|**	**Adjustment**	**Adj P**	**Alpha**	**Lower**	**Upper**
**Treatment**	Control GFP	Ctr Meg	-0.5679	0.3706	32	-1.53	0.1352	Tukey-Kramer	0.1352	0.05	-1.3229	0.1870

Differences of Least Squares Means				
**Effect**	**Treatment**	**Treatment**	**Adj Lower**	**Adj Upper**
**Treatment**	Control GFP	Ctr Meg	-1.3228	0.1870

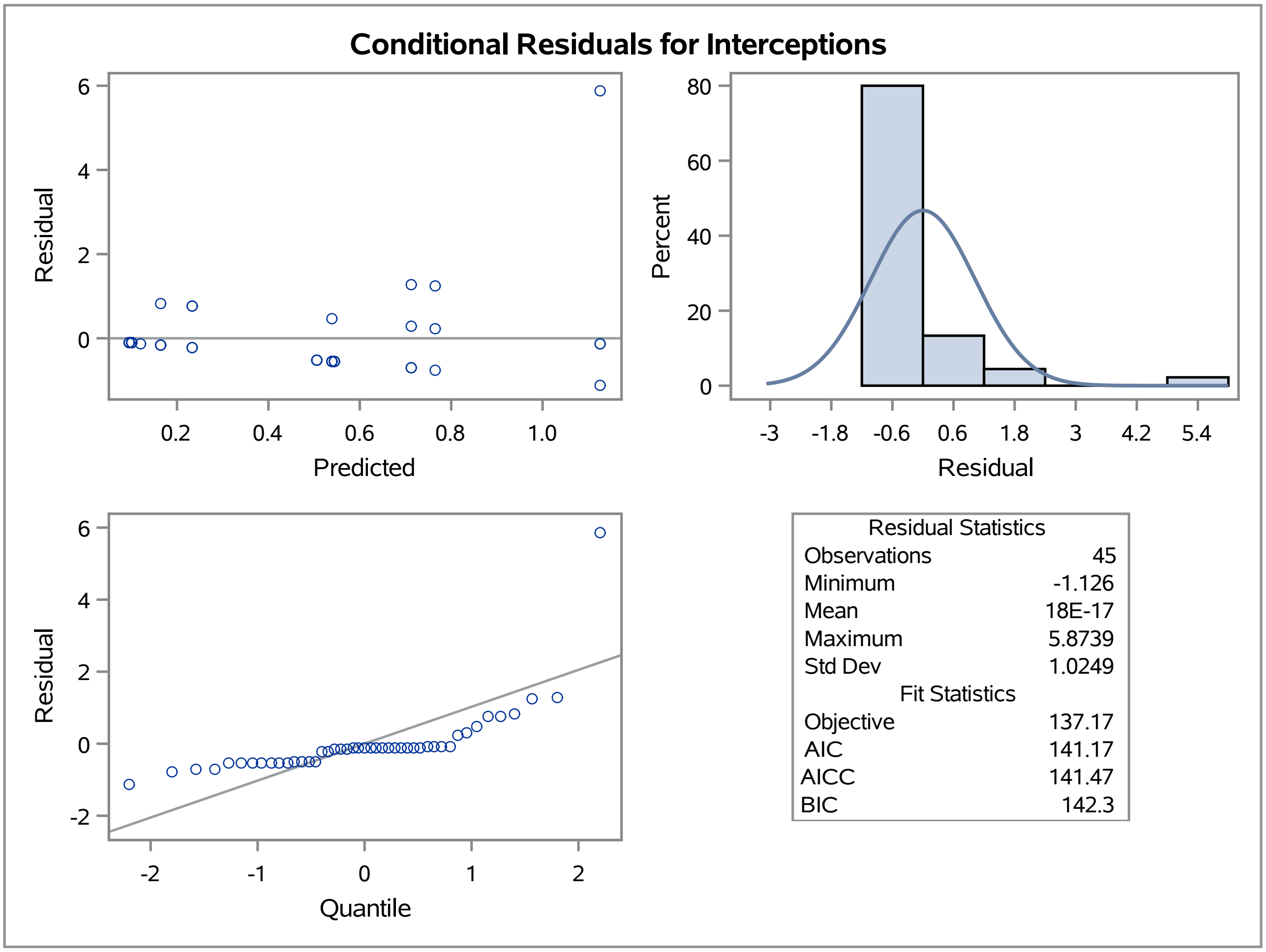

DistSoma=342

Model Information	
**Data Set**	WORK.TEMPDATASORTED
**Dependent Variable**	Interceptions
**Covariance Structure**	Variance Components
**Estimation Method**	REML
**Residual Variance Method**	Profile
**Fixed Effects SE Method**	Model-Based
**Degrees of Freedom Method**	Containment

Class Level Information		
**Class**	**Levels**	**Values**
**Treatment**	2	Control GFP Ctr Meg
**Culture**	13	1 2 3 4 5 6 7 8 9 10 11 12 13

Dimensions	
**Covariance Parameters**	2
**Columns in X**	3
**Columns in Z**	13
**Subjects**	1
**Max Obs per Subject**	45

Number of Observations	
**Number of Observations Read**	45
**Number of Observations Used**	45
**Number of Observations Not Used**	0

Iteration History			
**Iteration**	**Evaluations**	**-2 Res Log Like**	**Criterion**
**0**	1	148.12214287	
**1**	3	147.17155672	0.00007054
**2**	1	147.16908855	0.00000012
**3**	1	147.16908432	0.00000000

Convergence criteria met.

DistSoma=342

Covariance Parameter Estimates				
**Cov Parm**	**Estimate**	**Alpha**	**Lower**	**Upper**
**Culture**	0.2059	0.05	0.04620	45.2304
**Residual**	1.4005	0.05	0.9157	2.4067

Fit Statistics	
**-2 Res Log Likelihood**	147.2
**AIC (Smaller is Better)**	151.2
**AICC (Smaller is Better)**	151.5
**BIC (Smaller is Better)**	152.3

Solution for Fixed Effects									
**Effect**	**Treatment**	**Estimate**	**Standard Error**	**DF**	**t Value**	**Pr > |t|**	**Alpha**	**Lower**	**Upper**
**Intercept**		0.7383	0.3097	11	2.38	0.0363	0.05	0.05664	1.4199
**Treatment**	Control GFP	-0.6524	0.4374	32	-1.49	0.1456	0.05	-1.5435	0.2386
**Treatment**	Ctr Meg	0	.	.	.	.	.	.	.

Solution for Random Effects									
**Effect**	**Culture**	**Estimate**	**Std Err Pred**	**DF**	**t Value**	**Pr > |t|**	**Alpha**	**Lower**	**Upper**
**Culture**	1	-0.02627	0.3896	32	-0.07	0.9467	0.05	-0.8199	0.7674
**Culture**	2	-0.02627	0.3896	32	-0.07	0.9467	0.05	-0.8199	0.7674
**Culture**	3	0.06079	0.3778	32	0.16	0.8732	0.05	-0.7088	0.8303
**Culture**	4	-0.03178	0.3778	32	-0.08	0.9335	0.05	-0.8013	0.7378
**Culture**	5	0.06079	0.3778	32	0.16	0.8732	0.05	-0.7088	0.8303
**Culture**	6	-0.01100	0.4255	32	-0.03	0.9795	0.05	-0.8777	0.8557
**Culture**	7	-0.02627	0.3896	32	-0.07	0.9467	0.05	-0.8199	0.7674
**Culture**	8	0.7449	0.3779	32	1.97	0.0574	0.05	-0.02482	1.5146
**Culture**	9	-0.2734	0.3779	32	-0.72	0.4747	0.05	-1.0431	0.4963
**Culture**	10	-0.02191	0.3897	32	-0.06	0.9555	0.05	-0.8157	0.7718
**Culture**	11	-0.2280	0.3686	32	-0.62	0.5405	0.05	-0.9789	0.5228
**Culture**	12	0.004344	0.3779	32	0.01	0.9909	0.05	-0.7654	0.7741
**Culture**	13	-0.2259	0.3897	32	-0.58	0.5661	0.05	-1.0197	0.5678

Type 3 Tests of Fixed Effects				
**Effect**	**Num DF**	**Den DF**	**F Value**	**Pr > F**
**Treatment**	1	32	2.22	0.1456

DistSoma=342

Least Squares Means									
**Effect**	**Treatment**	**Estimate**	**Standard Error**	**DF**	**t Value**	**Pr > |t|**	**Alpha**	**Lower**	**Upper**
**Treatment**	Control GFP	0.08583	0.3089	32	0.28	0.7829	0.05	-0.5434	0.7151
**Treatment**	Ctr Meg	0.7383	0.3097	32	2.38	0.0232	0.05	0.1074	1.3691

Differences of Least Squares Means												
**Effect**	**Treatment**	**Treatment**	**Estimate**	**Standard Error**	**DF**	**t Value**	**Pr > |t|**	**Adjustment**	**Adj P**	**Alpha**	**Lower**	**Upper**
**Treatment**	Control GFP	Ctr Meg	-0.6524	0.4374	32	-1.49	0.1456	Tukey-Kramer	0.1456	0.05	-1.5435	0.2386

Differences of Least Squares Means				
**Effect**	**Treatment**	**Treatment**	**Adj Lower**	**Adj Upper**
**Treatment**	Control GFP	Ctr Meg	-1.5435	0.2386

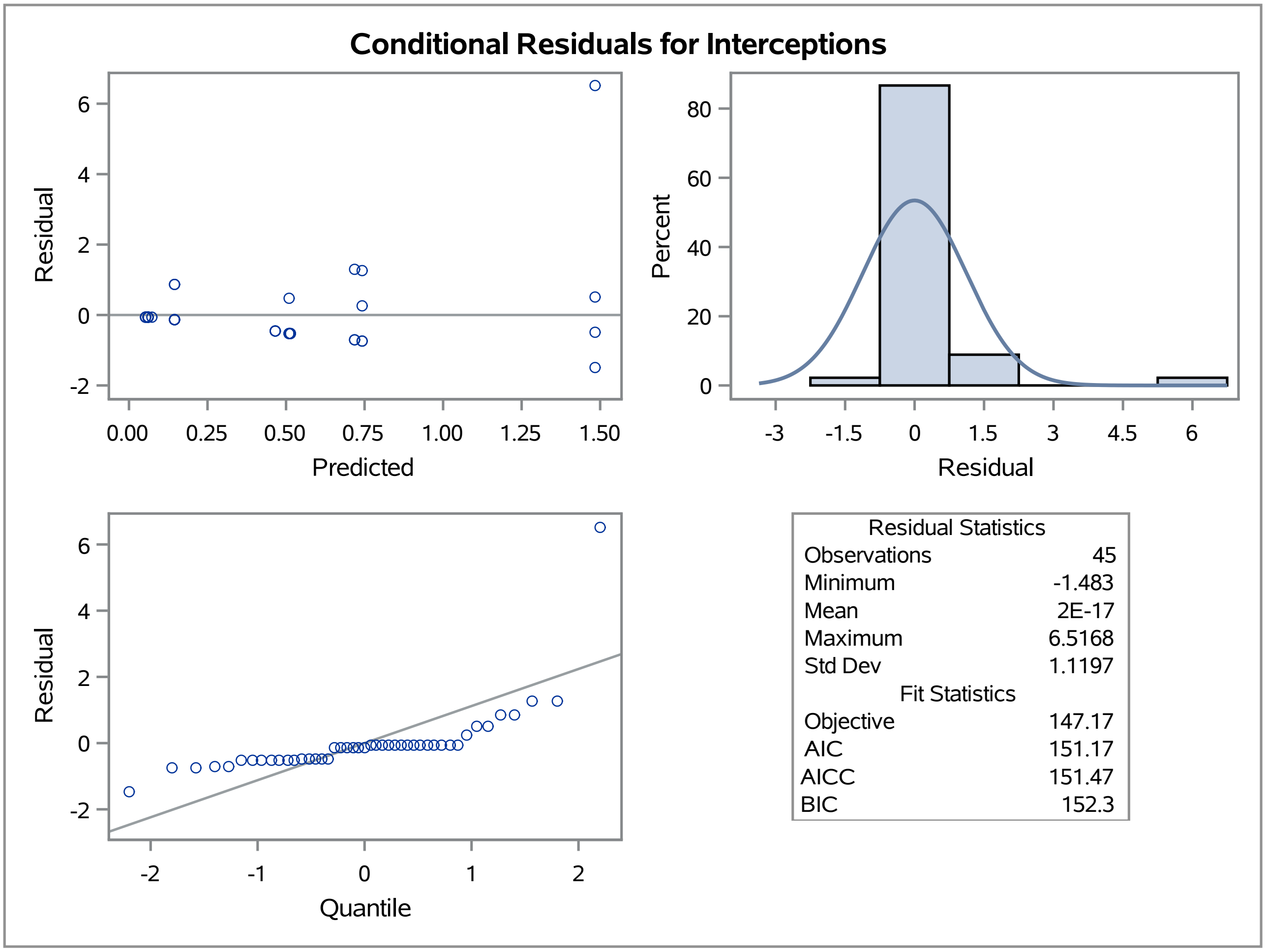

DistSoma=348

Model Information	
**Data Set**	WORK.TEMPDATASORTED
**Dependent Variable**	Interceptions
**Covariance Structure**	Variance Components
**Estimation Method**	REML
**Residual Variance Method**	Profile
**Fixed Effects SE Method**	Model-Based
**Degrees of Freedom Method**	Containment

Class Level Information		
**Class**	**Levels**	**Values**
**Treatment**	2	Control GFP Ctr Meg
**Culture**	13	1 2 3 4 5 6 7 8 9 10 11 12 13

Dimensions	
**Covariance Parameters**	2
**Columns in X**	3
**Columns in Z**	13
**Subjects**	1
**Max Obs per Subject**	45

Number of Observations	
**Number of Observations Read**	45
**Number of Observations Used**	45
**Number of Observations Not Used**	0

Iteration History			
**Iteration**	**Evaluations**	**-2 Res Log Like**	**Criterion**
**0**	1	156.14502107	
**1**	3	155.18332413	0.00006402
**2**	1	155.18082025	0.00000011
**3**	1	155.18081591	0.00000000

Convergence criteria met.

DistSoma=348

Covariance Parameter Estimates				
**Cov Parm**	**Estimate**	**Alpha**	**Lower**	**Upper**
**Culture**	0.2496	0.05	0.05628	52.1224
**Residual**	1.6864	0.05	1.1026	2.8978

Fit Statistics	
**-2 Res Log Likelihood**	155.2
**AIC (Smaller is Better)**	159.2
**AICC (Smaller is Better)**	159.5
**BIC (Smaller is Better)**	160.3

Solution for Fixed Effects									
**Effect**	**Treatment**	**Estimate**	**Standard Error**	**DF**	**t Value**	**Pr > |t|**	**Alpha**	**Lower**	**Upper**
**Intercept**		0.7383	0.3403	11	2.17	0.0528	0.05	-0.01064	1.4871
**Treatment**	Control GFP	-0.6525	0.4806	32	-1.36	0.1841	0.05	-1.6314	0.3264
**Treatment**	Ctr Meg	0	.	.	.	.	.	.	.

Solution for Random Effects									
**Effect**	**Culture**	**Estimate**	**Std Err Pred**	**DF**	**t Value**	**Pr > |t|**	**Alpha**	**Lower**	**Upper**
**Culture**	1	-0.02638	0.4286	32	-0.06	0.9513	0.05	-0.8995	0.8467
**Culture**	2	-0.02638	0.4286	32	-0.06	0.9513	0.05	-0.8995	0.8467
**Culture**	3	0.06106	0.4156	32	0.15	0.8841	0.05	-0.7854	0.9076
**Culture**	4	-0.03191	0.4156	32	-0.08	0.9393	0.05	-0.8784	0.8146
**Culture**	5	0.06106	0.4156	32	0.15	0.8841	0.05	-0.7854	0.9076
**Culture**	6	-0.01106	0.4683	32	-0.02	0.9813	0.05	-0.9650	0.9429
**Culture**	7	-0.02638	0.4286	32	-0.06	0.9513	0.05	-0.8995	0.8467
**Culture**	8	0.8411	0.4157	32	2.02	0.0515	0.05	-0.00562	1.6878
**Culture**	9	-0.2745	0.4157	32	-0.66	0.5137	0.05	-1.1212	0.5722
**Culture**	10	-0.02201	0.4287	32	-0.05	0.9594	0.05	-0.8953	0.8512
**Culture**	11	-0.2289	0.4054	32	-0.56	0.5763	0.05	-1.0548	0.5969
**Culture**	12	-0.08860	0.4157	32	-0.21	0.8326	0.05	-0.9353	0.7581
**Culture**	13	-0.2270	0.4287	32	-0.53	0.6001	0.05	-1.1003	0.6462

Type 3 Tests of Fixed Effects				
**Effect**	**Num DF**	**Den DF**	**F Value**	**Pr > F**
**Treatment**	1	32	1.84	0.1841

DistSoma=348

Least Squares Means									
**Effect**	**Treatment**	**Estimate**	**Standard Error**	**DF**	**t Value**	**Pr > |t|**	**Alpha**	**Lower**	**Upper**
**Treatment**	Control GFP	0.08580	0.3394	32	0.25	0.8020	0.05	-0.6055	0.7771
**Treatment**	Ctr Meg	0.7383	0.3403	32	2.17	0.0376	0.05	0.04518	1.4313

Differences of Least Squares Means												
**Effect**	**Treatment**	**Treatment**	**Estimate**	**Standard Error**	**DF**	**t Value**	**Pr > |t|**	**Adjustment**	**Adj P**	**Alpha**	**Lower**	**Upper**
**Treatment**	Control GFP	Ctr Meg	-0.6525	0.4806	32	-1.36	0.1841	Tukey-Kramer	0.1841	0.05	-1.6314	0.3264

Differences of Least Squares Means				
**Effect**	**Treatment**	**Treatment**	**Adj Lower**	**Adj Upper**
**Treatment**	Control GFP	Ctr Meg	-1.6313	0.3264

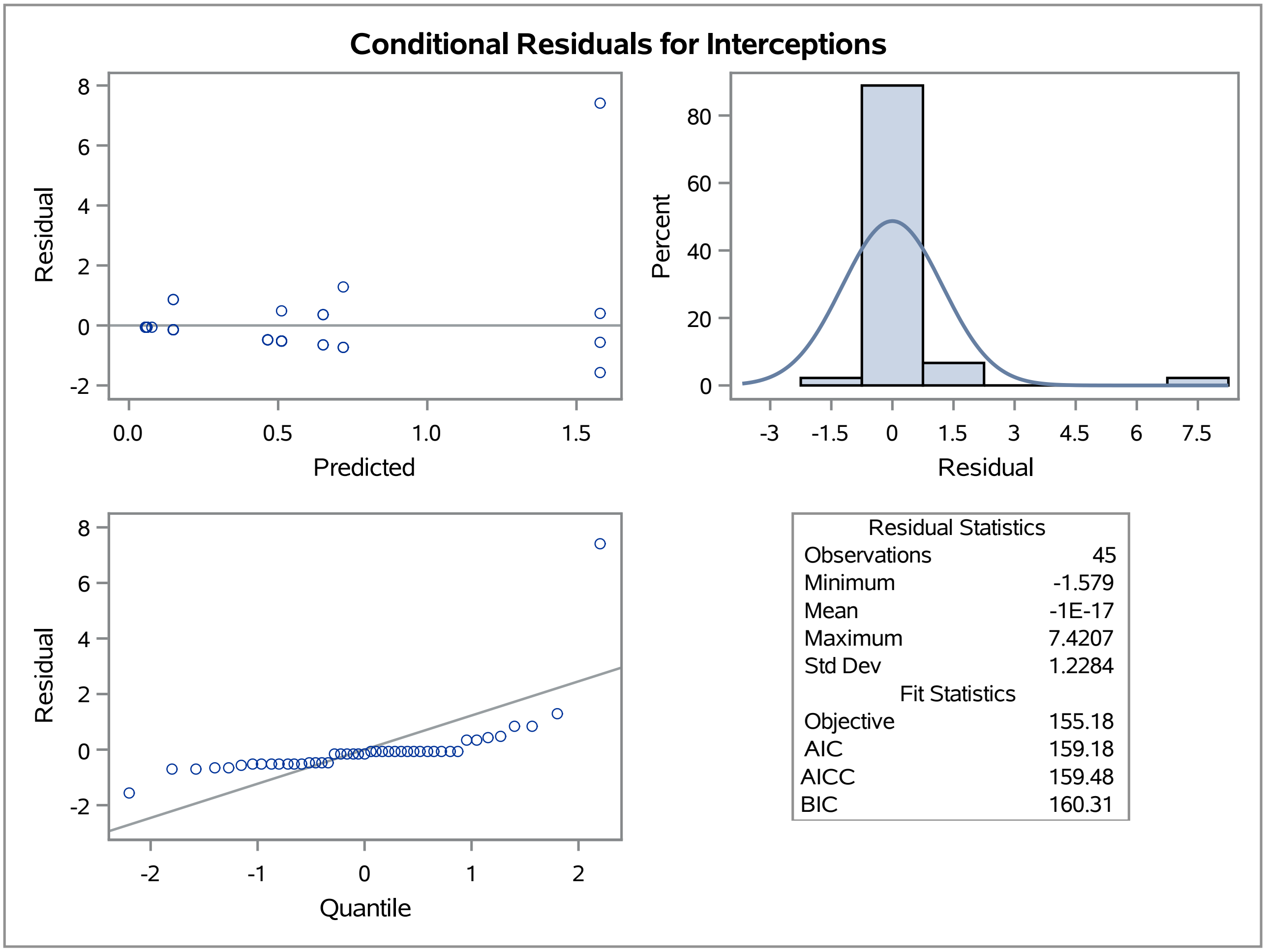

DistSoma=354

Model Information	
**Data Set**	WORK.TEMPDATASORTED
**Dependent Variable**	Interceptions
**Covariance Structure**	Variance Components
**Estimation Method**	REML
**Residual Variance Method**	Profile
**Fixed Effects SE Method**	Model-Based
**Degrees of Freedom Method**	Containment

Class Level Information		
**Class**	**Levels**	**Values**
**Treatment**	2	Control GFP Ctr Meg
**Culture**	13	1 2 3 4 5 6 7 8 9 10 11 12 13

Dimensions	
**Covariance Parameters**	2
**Columns in X**	3
**Columns in Z**	13
**Subjects**	1
**Max Obs per Subject**	45

Number of Observations	
**Number of Observations Read**	45
**Number of Observations Used**	45
**Number of Observations Not Used**	0

Iteration History			
**Iteration**	**Evaluations**	**-2 Res Log Like**	**Criterion**
**0**	1	157.17806700	
**1**	3	154.93073240	0.00033272
**2**	1	154.91737829	0.00000287
**3**	1	154.91726871	0.00000000

Convergence criteria met.

DistSoma=354

Covariance Parameter Estimates				
**Cov Parm**	**Estimate**	**Alpha**	**Lower**	**Upper**
**Culture**	0.4023	0.05	0.1202	8.3131
**Residual**	1.5884	0.05	1.0373	2.7351

Fit Statistics	
**-2 Res Log Likelihood**	154.9
**AIC (Smaller is Better)**	158.9
**AICC (Smaller is Better)**	159.2
**BIC (Smaller is Better)**	160.0

Solution for Fixed Effects									
**Effect**	**Treatment**	**Estimate**	**Standard Error**	**DF**	**t Value**	**Pr > |t|**	**Alpha**	**Lower**	**Upper**
**Intercept**		0.7370	0.3704	11	1.99	0.0721	0.05	-0.07826	1.5523
**Treatment**	Control GFP	-0.6533	0.5206	32	-1.26	0.2185	0.05	-1.7137	0.4070
**Treatment**	Ctr Meg	0	.	.	.	.	.	.	.

Solution for Random Effects									
**Effect**	**Culture**	**Estimate**	**Std Err Pred**	**DF**	**t Value**	**Pr > |t|**	**Alpha**	**Lower**	**Upper**
**Culture**	1	-0.03613	0.5035	32	-0.07	0.9432	0.05	-1.0618	0.9895
**Culture**	2	-0.03613	0.5035	32	-0.07	0.9432	0.05	-1.0618	0.9895
**Culture**	3	0.08370	0.4834	32	0.17	0.8636	0.05	-0.9011	1.0685
**Culture**	4	-0.04211	0.4834	32	-0.09	0.9311	0.05	-1.0269	0.9426
**Culture**	5	0.08370	0.4834	32	0.17	0.8636	0.05	-0.9011	1.0685
**Culture**	6	-0.01691	0.5714	32	-0.03	0.9766	0.05	-1.1807	1.1469
**Culture**	7	-0.03613	0.5035	32	-0.07	0.9432	0.05	-1.0618	0.9895
**Culture**	8	1.2647	0.4843	32	2.61	0.0136	0.05	0.2781	2.2512
**Culture**	9	-0.3709	0.4843	32	-0.77	0.4494	0.05	-1.3575	0.6157
**Culture**	10	-0.03038	0.5042	32	-0.06	0.9523	0.05	-1.0573	0.9966
**Culture**	11	-0.3001	0.4694	32	-0.64	0.5272	0.05	-1.2562	0.6561
**Culture**	12	-0.2451	0.4843	32	-0.51	0.6163	0.05	-1.2317	0.7415
**Culture**	13	-0.3182	0.5042	32	-0.63	0.5324	0.05	-1.3452	0.7087

Type 3 Tests of Fixed Effects				
**Effect**	**Num DF**	**Den DF**	**F Value**	**Pr > F**
**Treatment**	1	32	1.58	0.2185

DistSoma=354

Least Squares Means									
**Effect**	**Treatment**	**Estimate**	**Standard Error**	**DF**	**t Value**	**Pr > |t|**	**Alpha**	**Lower**	**Upper**
**Treatment**	Control GFP	0.08368	0.3658	32	0.23	0.8205	0.05	-0.6614	0.8287
**Treatment**	Ctr Meg	0.7370	0.3704	32	1.99	0.0552	0.05	-0.01749	1.4915

Differences of Least Squares Means												
**Effect**	**Treatment**	**Treatment**	**Estimate**	**Standard Error**	**DF**	**t Value**	**Pr > |t|**	**Adjustment**	**Adj P**	**Alpha**	**Lower**	**Upper**
**Treatment**	Control GFP	Ctr Meg	-0.6533	0.5206	32	-1.26	0.2185	Tukey-Kramer	0.2185	0.05	-1.7137	0.4070

Differences of Least Squares Means				
**Effect**	**Treatment**	**Treatment**	**Adj Lower**	**Adj Upper**
**Treatment**	Control GFP	Ctr Meg	-1.7137	0.4070

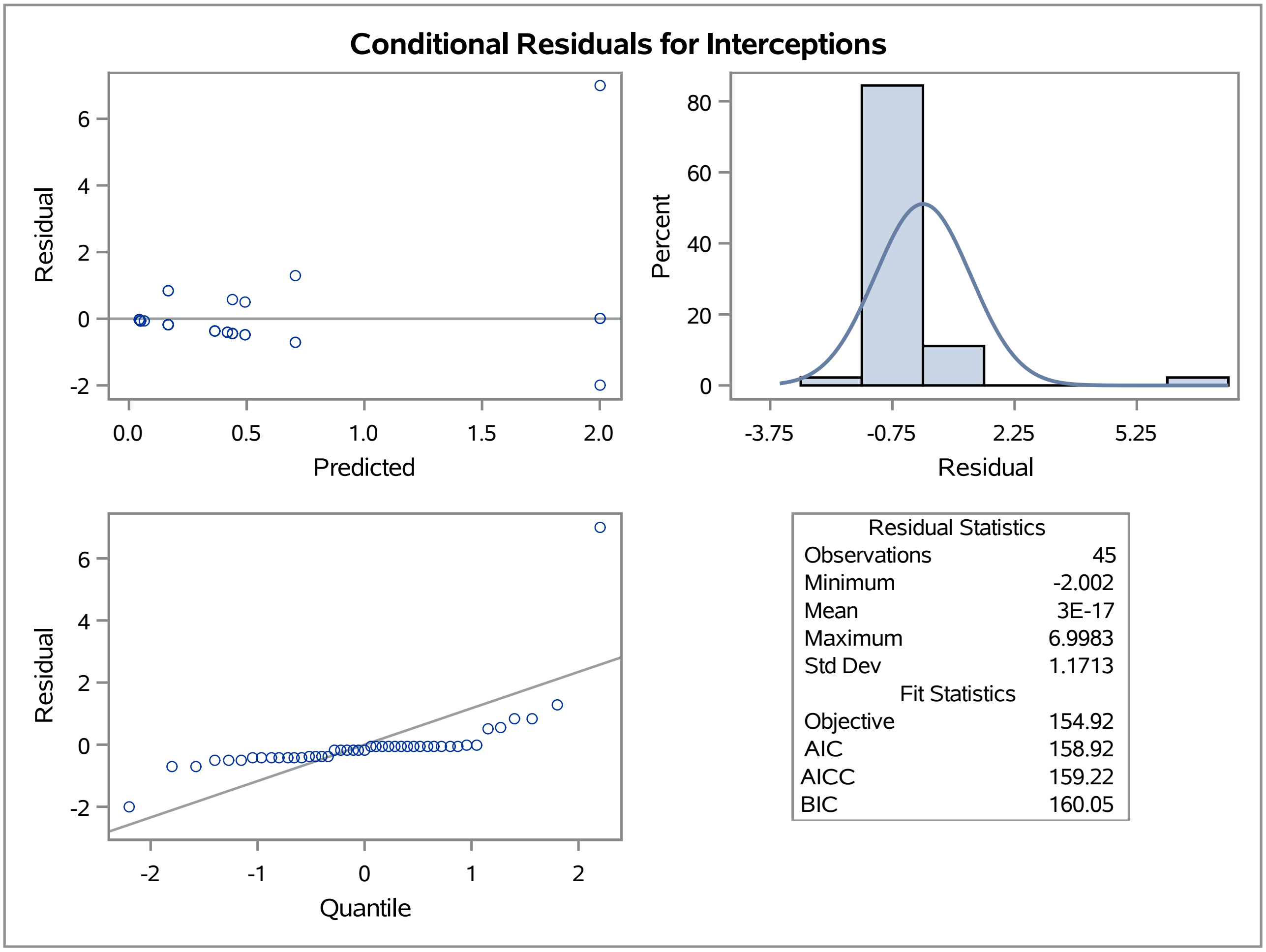

DistSoma=360

Model Information	
**Data Set**	WORK.TEMPDATASORTED
**Dependent Variable**	Interceptions
**Covariance Structure**	Variance Components
**Estimation Method**	REML
**Residual Variance Method**	Profile
**Fixed Effects SE Method**	Model-Based
**Degrees of Freedom Method**	Containment

Class Level Information		
**Class**	**Levels**	**Values**
**Treatment**	2	Control GFP Ctr Meg
**Culture**	13	1 2 3 4 5 6 7 8 9 10 11 12 13

Dimensions	
**Covariance Parameters**	2
**Columns in X**	3
**Columns in Z**	13
**Subjects**	1
**Max Obs per Subject**	45

Number of Observations	
**Number of Observations Read**	45
**Number of Observations Used**	45
**Number of Observations Not Used**	0

Iteration History			
**Iteration**	**Evaluations**	**-2 Res Log Like**	**Criterion**
**0**	1	157.17806700	
**1**	3	154.93073240	0.00033272
**2**	1	154.91737829	0.00000287
**3**	1	154.91726871	0.00000000

Convergence criteria met.

DistSoma=360

Covariance Parameter Estimates				
**Cov Parm**	**Estimate**	**Alpha**	**Lower**	**Upper**
**Culture**	0.4023	0.05	0.1202	8.3131
**Residual**	1.5884	0.05	1.0373	2.7351

Fit Statistics	
**-2 Res Log Likelihood**	154.9
**AIC (Smaller is Better)**	158.9
**AICC (Smaller is Better)**	159.2
**BIC (Smaller is Better)**	160.0

Solution for Fixed Effects									
**Effect**	**Treatment**	**Estimate**	**Standard Error**	**DF**	**t Value**	**Pr > |t|**	**Alpha**	**Lower**	**Upper**
**Intercept**		0.7370	0.3704	11	1.99	0.0721	0.05	-0.07826	1.5523
**Treatment**	Control GFP	-0.6533	0.5206	32	-1.26	0.2185	0.05	-1.7137	0.4070
**Treatment**	Ctr Meg	0	.	.	.	.	.	.	.

Solution for Random Effects									
**Effect**	**Culture**	**Estimate**	**Std Err Pred**	**DF**	**t Value**	**Pr > |t|**	**Alpha**	**Lower**	**Upper**
**Culture**	1	-0.03613	0.5035	32	-0.07	0.9432	0.05	-1.0618	0.9895
**Culture**	2	-0.03613	0.5035	32	-0.07	0.9432	0.05	-1.0618	0.9895
**Culture**	3	0.08370	0.4834	32	0.17	0.8636	0.05	-0.9011	1.0685
**Culture**	4	-0.04211	0.4834	32	-0.09	0.9311	0.05	-1.0269	0.9426
**Culture**	5	0.08370	0.4834	32	0.17	0.8636	0.05	-0.9011	1.0685
**Culture**	6	-0.01691	0.5714	32	-0.03	0.9766	0.05	-1.1807	1.1469
**Culture**	7	-0.03613	0.5035	32	-0.07	0.9432	0.05	-1.0618	0.9895
**Culture**	8	1.2647	0.4843	32	2.61	0.0136	0.05	0.2781	2.2512
**Culture**	9	-0.3709	0.4843	32	-0.77	0.4494	0.05	-1.3575	0.6157
**Culture**	10	-0.03038	0.5042	32	-0.06	0.9523	0.05	-1.0573	0.9966
**Culture**	11	-0.3001	0.4694	32	-0.64	0.5272	0.05	-1.2562	0.6561
**Culture**	12	-0.2451	0.4843	32	-0.51	0.6163	0.05	-1.2317	0.7415
**Culture**	13	-0.3182	0.5042	32	-0.63	0.5324	0.05	-1.3452	0.7087

Type 3 Tests of Fixed Effects				
**Effect**	**Num DF**	**Den DF**	**F Value**	**Pr > F**
**Treatment**	1	32	1.58	0.2185

DistSoma=360

Least Squares Means									
**Effect**	**Treatment**	**Estimate**	**Standard Error**	**DF**	**t Value**	**Pr > |t|**	**Alpha**	**Lower**	**Upper**
**Treatment**	Control GFP	0.08368	0.3658	32	0.23	0.8205	0.05	-0.6614	0.8287
**Treatment**	Ctr Meg	0.7370	0.3704	32	1.99	0.0552	0.05	-0.01749	1.4915

Differences of Least Squares Means												
**Effect**	**Treatment**	**Treatment**	**Estimate**	**Standard Error**	**DF**	**t Value**	**Pr > |t|**	**Adjustment**	**Adj P**	**Alpha**	**Lower**	**Upper**
**Treatment**	Control GFP	Ctr Meg	-0.6533	0.5206	32	-1.26	0.2185	Tukey-Kramer	0.2185	0.05	-1.7137	0.4070

Differences of Least Squares Means				
**Effect**	**Treatment**	**Treatment**	**Adj Lower**	**Adj Upper**
**Treatment**	Control GFP	Ctr Meg	-1.7137	0.4070

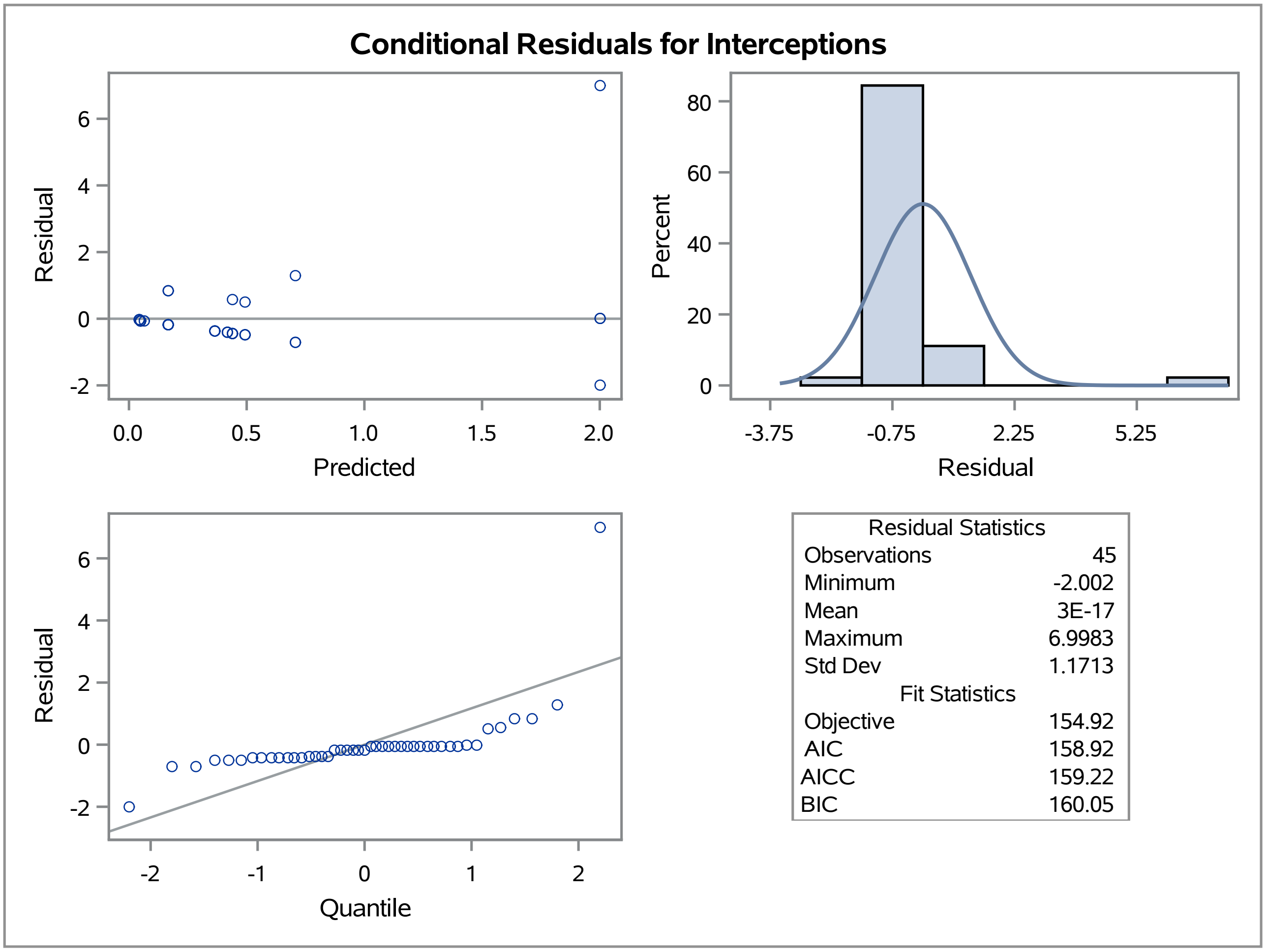

DistSoma=366

Model Information	
**Data Set**	WORK.TEMPDATASORTED
**Dependent Variable**	Interceptions
**Covariance Structure**	Variance Components
**Estimation Method**	REML
**Residual Variance Method**	Profile
**Fixed Effects SE Method**	Model-Based
**Degrees of Freedom Method**	Containment

Class Level Information		
**Class**	**Levels**	**Values**
**Treatment**	2	Control GFP Ctr Meg
**Culture**	13	1 2 3 4 5 6 7 8 9 10 11 12 13

Dimensions	
**Covariance Parameters**	2
**Columns in X**	3
**Columns in Z**	13
**Subjects**	1
**Max Obs per Subject**	45

Number of Observations	
**Number of Observations Read**	45
**Number of Observations Used**	45
**Number of Observations Not Used**	0

Iteration History			
**Iteration**	**Evaluations**	**-2 Res Log Like**	**Criterion**
**0**	1	158.91759451	
**1**	3	155.35847042	0.00086408
**2**	1	155.32263785	0.00001823
**3**	1	155.32193202	0.00000001

Convergence criteria met.

DistSoma=366

Covariance Parameter Estimates				
**Cov Parm**	**Estimate**	**Alpha**	**Lower**	**Upper**
**Culture**	0.5360	0.05	0.1818	5.6788
**Residual**	1.5406	0.05	1.0052	2.6565

Fit Statistics	
**-2 Res Log Likelihood**	155.3
**AIC (Smaller is Better)**	159.3
**AICC (Smaller is Better)**	159.6
**BIC (Smaller is Better)**	160.5

Solution for Fixed Effects									
**Effect**	**Treatment**	**Estimate**	**Standard Error**	**DF**	**t Value**	**Pr > |t|**	**Alpha**	**Lower**	**Upper**
**Intercept**		0.7788	0.3969	11	1.96	0.0755	0.05	-0.09481	1.6524
**Treatment**	Control GFP	-0.6965	0.5560	32	-1.25	0.2194	0.05	-1.8290	0.4360
**Treatment**	Ctr Meg	0	.	.	.	.	.	.	.

Solution for Random Effects									
**Effect**	**Culture**	**Estimate**	**Std Err Pred**	**DF**	**t Value**	**Pr > |t|**	**Alpha**	**Lower**	**Upper**
**Culture**	1	-0.04202	0.5494	32	-0.08	0.9395	0.05	-1.1610	1.0770
**Culture**	2	-0.04202	0.5494	32	-0.08	0.9395	0.05	-1.1610	1.0770
**Culture**	3	0.09759	0.5248	32	0.19	0.8537	0.05	-0.9714	1.1666
**Culture**	4	-0.04788	0.5248	32	-0.09	0.9279	0.05	-1.1169	1.0211
**Culture**	5	0.09759	0.5248	32	0.19	0.8537	0.05	-0.9714	1.1666
**Culture**	6	-0.02124	0.6385	32	-0.03	0.9737	0.05	-1.3219	1.2794
**Culture**	7	-0.04202	0.5494	32	-0.08	0.9395	0.05	-1.1610	1.0770
**Culture**	8	1.5834	0.5267	32	3.01	0.0051	0.05	0.5105	2.6563
**Culture**	9	-0.4532	0.5267	32	-0.86	0.3960	0.05	-1.5261	0.6197
**Culture**	10	-0.05727	0.5508	32	-0.10	0.9178	0.05	-1.1791	1.0646
**Culture**	11	-0.3675	0.5091	32	-0.72	0.4756	0.05	-1.4045	0.6694
**Culture**	12	-0.3077	0.5267	32	-0.58	0.5632	0.05	-1.3806	0.7652
**Culture**	13	-0.3977	0.5508	32	-0.72	0.4754	0.05	-1.5196	0.7241

Type 3 Tests of Fixed Effects				
**Effect**	**Num DF**	**Den DF**	**F Value**	**Pr > F**
**Treatment**	1	32	1.57	0.2194

DistSoma=366

Least Squares Means									
**Effect**	**Treatment**	**Estimate**	**Standard Error**	**DF**	**t Value**	**Pr > |t|**	**Alpha**	**Lower**	**Upper**
**Treatment**	Control GFP	0.08228	0.3893	32	0.21	0.8340	0.05	-0.7108	0.8753
**Treatment**	Ctr Meg	0.7788	0.3969	32	1.96	0.0585	0.05	-0.02969	1.5873

Differences of Least Squares Means												
**Effect**	**Treatment**	**Treatment**	**Estimate**	**Standard Error**	**DF**	**t Value**	**Pr > |t|**	**Adjustment**	**Adj P**	**Alpha**	**Lower**	**Upper**
**Treatment**	Control GFP	Ctr Meg	-0.6965	0.5560	32	-1.25	0.2194	Tukey-Kramer	0.2194	0.05	-1.8290	0.4360

Differences of Least Squares Means				
**Effect**	**Treatment**	**Treatment**	**Adj Lower**	**Adj Upper**
**Treatment**	Control GFP	Ctr Meg	-1.8290	0.4360

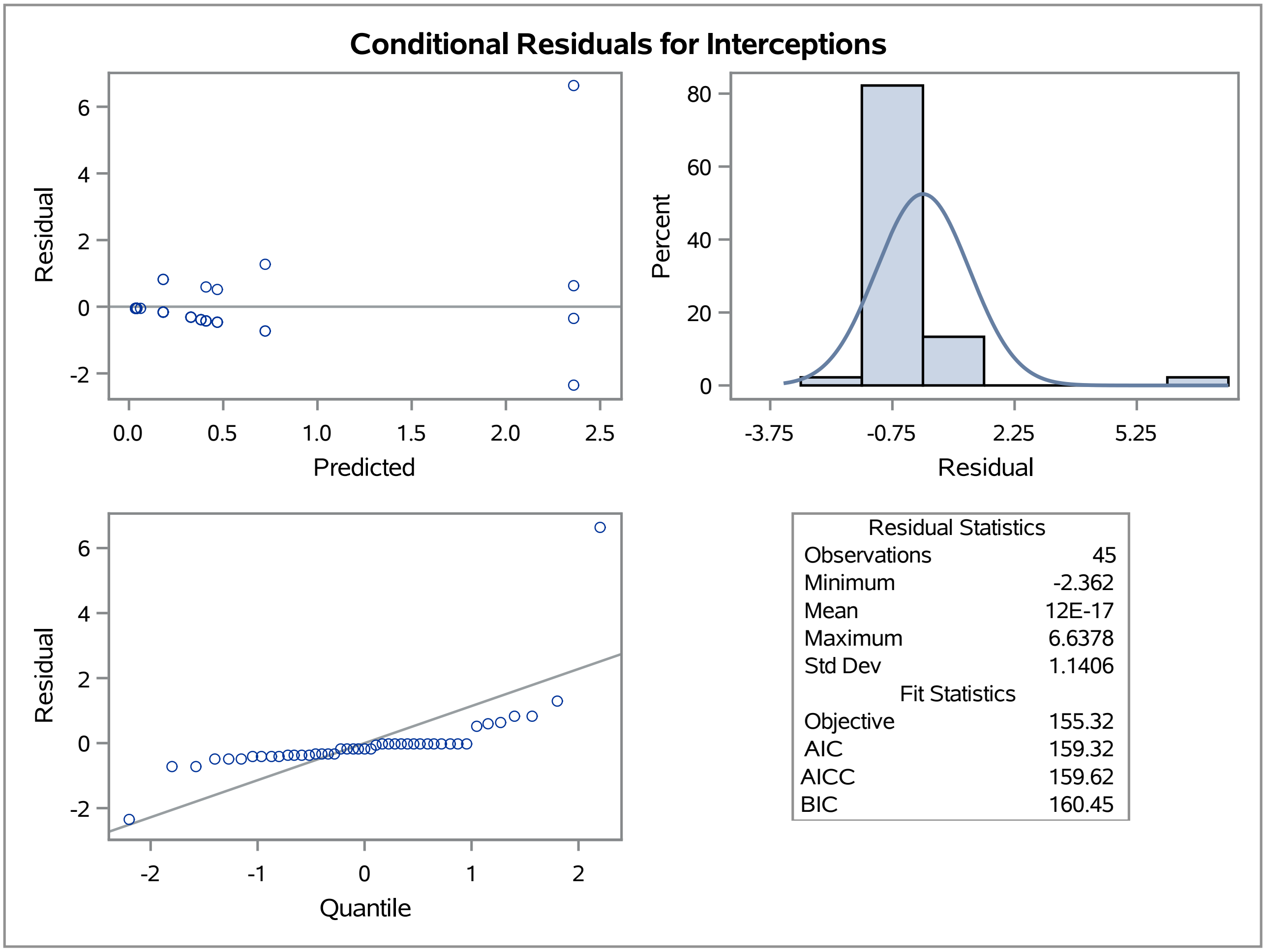

DistSoma=372

Model Information	
**Data Set**	WORK.TEMPDATASORTED
**Dependent Variable**	Interceptions
**Covariance Structure**	Variance Components
**Estimation Method**	REML
**Residual Variance Method**	Profile
**Fixed Effects SE Method**	Model-Based
**Degrees of Freedom Method**	Containment

Class Level Information		
**Class**	**Levels**	**Values**
**Treatment**	2	Control GFP Ctr Meg
**Culture**	13	1 2 3 4 5 6 7 8 9 10 11 12 13

Dimensions	
**Covariance Parameters**	2
**Columns in X**	3
**Columns in Z**	13
**Subjects**	1
**Max Obs per Subject**	45

Number of Observations	
**Number of Observations Read**	45
**Number of Observations Used**	45
**Number of Observations Not Used**	0

Iteration History			
**Iteration**	**Evaluations**	**-2 Res Log Like**	**Criterion**
**0**	1	153.71369855	
**1**	3	148.20703954	0.00250735
**2**	1	148.10939243	0.00012514
**3**	1	148.10491788	0.00000038
**4**	1	148.10490466	0.00000000

Convergence criteria met.

DistSoma=372

Covariance Parameter Estimates				
**Cov Parm**	**Estimate**	**Alpha**	**Lower**	**Upper**
**Culture**	0.6008	0.05	0.2257	4.1357
**Residual**	1.2413	0.05	0.8091	2.1438

Fit Statistics	
**-2 Res Log Likelihood**	148.1
**AIC (Smaller is Better)**	152.1
**AICC (Smaller is Better)**	152.4
**BIC (Smaller is Better)**	153.2

Solution for Fixed Effects									
**Effect**	**Treatment**	**Estimate**	**Standard Error**	**DF**	**t Value**	**Pr > |t|**	**Alpha**	**Lower**	**Upper**
**Intercept**		0.7775	0.3940	11	1.97	0.0741	0.05	-0.08969	1.6447
**Treatment**	Control GFP	-0.6967	0.5500	32	-1.27	0.2144	0.05	-1.8169	0.4235
**Treatment**	Ctr Meg	0	.	.	.	.	.	.	.

Solution for Random Effects									
**Effect**	**Culture**	**Estimate**	**Std Err Pred**	**DF**	**t Value**	**Pr > |t|**	**Alpha**	**Lower**	**Upper**
**Culture**	1	-0.04784	0.5447	32	-0.09	0.9305	0.05	-1.1573	1.0616
**Culture**	2	-0.04784	0.5447	32	-0.09	0.9305	0.05	-1.1573	1.0616
**Culture**	3	0.1116	0.5183	32	0.22	0.8309	0.05	-0.9442	1.1673
**Culture**	4	-0.05327	0.5183	32	-0.10	0.9188	0.05	-1.1090	1.0025
**Culture**	5	0.1116	0.5183	32	0.22	0.8309	0.05	-0.9442	1.1673
**Culture**	6	-0.02635	0.6485	32	-0.04	0.9678	0.05	-1.3472	1.2945
**Culture**	7	-0.04784	0.5447	32	-0.09	0.9305	0.05	-1.1573	1.0616
**Culture**	8	1.7952	0.5217	32	3.44	0.0016	0.05	0.7327	2.8578
**Culture**	9	-0.5127	0.5217	32	-0.98	0.3331	0.05	-1.5753	0.5499
**Culture**	10	-0.06564	0.5472	32	-0.12	0.9053	0.05	-1.1803	1.0490
**Culture**	11	-0.4086	0.5034	32	-0.81	0.4229	0.05	-1.4340	0.6167
**Culture**	12	-0.3478	0.5217	32	-0.67	0.5097	0.05	-1.4104	0.7147
**Culture**	13	-0.4604	0.5472	32	-0.84	0.4064	0.05	-1.5751	0.6542

Type 3 Tests of Fixed Effects				
**Effect**	**Num DF**	**Den DF**	**F Value**	**Pr > F**
**Treatment**	1	32	1.60	0.2144

DistSoma=372

Least Squares Means									
**Effect**	**Treatment**	**Estimate**	**Standard Error**	**DF**	**t Value**	**Pr > |t|**	**Alpha**	**Lower**	**Upper**
**Treatment**	Control GFP	0.08079	0.3837	32	0.21	0.8346	0.05	-0.7008	0.8623
**Treatment**	Ctr Meg	0.7775	0.3940	32	1.97	0.0571	0.05	-0.02505	1.5801

Differences of Least Squares Means												
**Effect**	**Treatment**	**Treatment**	**Estimate**	**Standard Error**	**DF**	**t Value**	**Pr > |t|**	**Adjustment**	**Adj P**	**Alpha**	**Lower**	**Upper**
**Treatment**	Control GFP	Ctr Meg	-0.6967	0.5500	32	-1.27	0.2144	Tukey-Kramer	0.2144	0.05	-1.8169	0.4235

Differences of Least Squares Means				
**Effect**	**Treatment**	**Treatment**	**Adj Lower**	**Adj Upper**
**Treatment**	Control GFP	Ctr Meg	-1.8169	0.4235

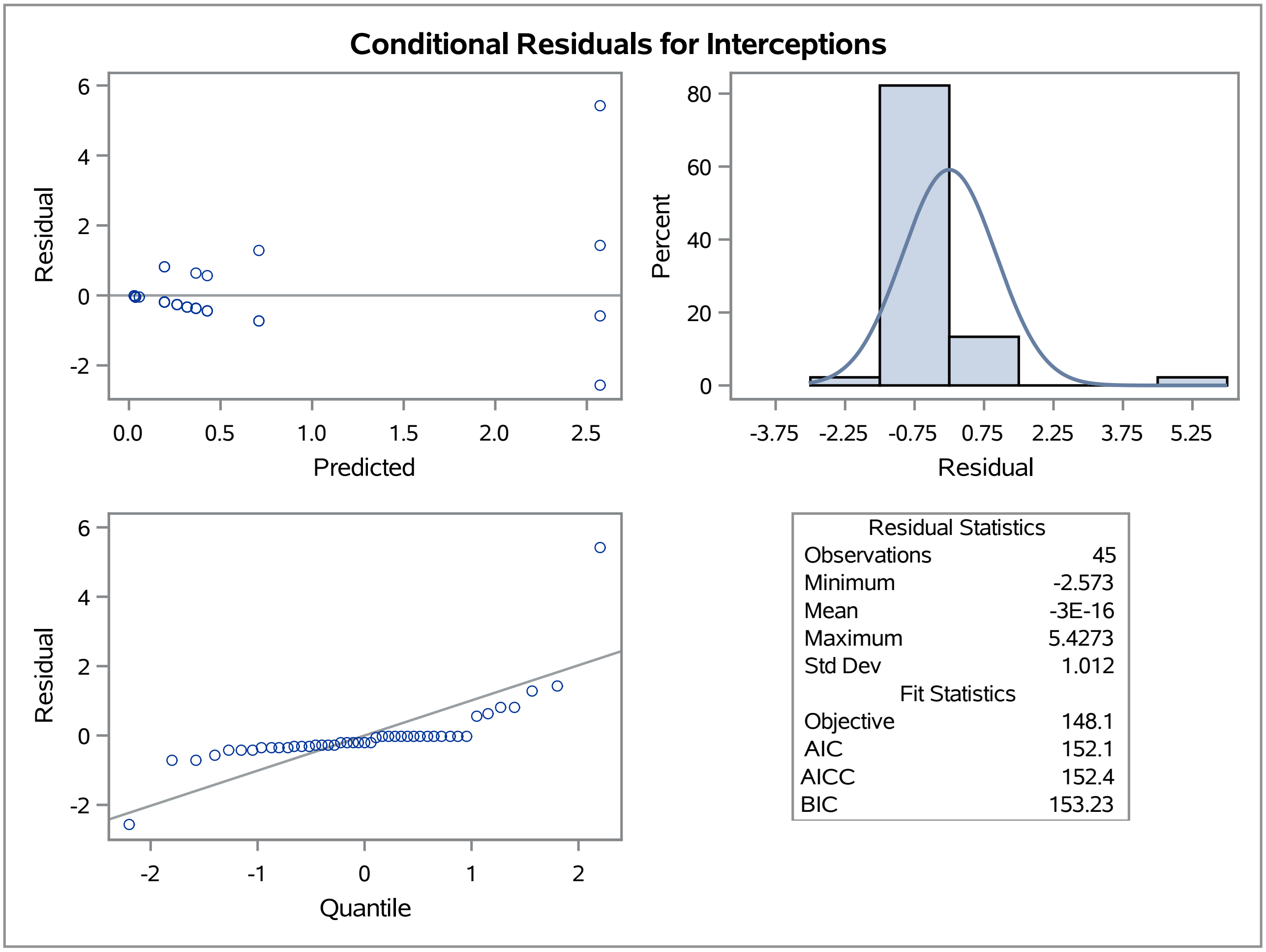

DistSoma=378

Model Information	
**Data Set**	WORK.TEMPDATASORTED
**Dependent Variable**	Interceptions
**Covariance Structure**	Variance Components
**Estimation Method**	REML
**Residual Variance Method**	Profile
**Fixed Effects SE Method**	Model-Based
**Degrees of Freedom Method**	Containment

Class Level Information		
**Class**	**Levels**	**Values**
**Treatment**	2	Control GFP Ctr Meg
**Culture**	13	1 2 3 4 5 6 7 8 9 10 11 12 13

Dimensions	
**Covariance Parameters**	2
**Columns in X**	3
**Columns in Z**	13
**Subjects**	1
**Max Obs per Subject**	45

Number of Observations	
**Number of Observations Read**	45
**Number of Observations Used**	45
**Number of Observations Not Used**	0

Iteration History			
**Iteration**	**Evaluations**	**-2 Res Log Like**	**Criterion**
**0**	1	165.72283323	
**1**	3	153.63880864	0.00448901
**2**	1	153.44492216	0.00038696
**3**	1	153.42965104	0.00000373
**4**	1	153.42951119	0.00000000

Convergence criteria met.

DistSoma=378

Covariance Parameter Estimates				
**Cov Parm**	**Estimate**	**Alpha**	**Lower**	**Upper**
**Culture**	1.1811	0.05	0.5089	5.1062
**Residual**	1.2503	0.05	0.8133	2.1667

Fit Statistics	
**-2 Res Log Likelihood**	153.4
**AIC (Smaller is Better)**	157.4
**AICC (Smaller is Better)**	157.7
**BIC (Smaller is Better)**	158.6

Solution for Fixed Effects									
**Effect**	**Treatment**	**Estimate**	**Standard Error**	**DF**	**t Value**	**Pr > |t|**	**Alpha**	**Lower**	**Upper**
**Intercept**		0.9440	0.5026	11	1.88	0.0871	0.05	-0.1622	2.0502
**Treatment**	Control GFP	-0.8661	0.6966	32	-1.24	0.2228	0.05	-2.2851	0.5529
**Treatment**	Ctr Meg	0	.	.	.	.	.	.	.

Solution for Random Effects									
**Effect**	**Culture**	**Estimate**	**Std Err Pred**	**DF**	**t Value**	**Pr > |t|**	**Alpha**	**Lower**	**Upper**
**Culture**	1	-0.05758	0.6597	32	-0.09	0.9310	0.05	-1.4014	1.2862
**Culture**	2	-0.05758	0.6597	32	-0.09	0.9310	0.05	-1.4014	1.2862
**Culture**	3	0.1361	0.6266	32	0.22	0.8295	0.05	-1.1403	1.4125
**Culture**	4	-0.06160	0.6266	32	-0.10	0.9223	0.05	-1.3380	1.2148
**Culture**	5	0.1361	0.6266	32	0.22	0.8295	0.05	-1.1403	1.4125
**Culture**	6	-0.03784	0.8138	32	-0.05	0.9632	0.05	-1.6955	1.6198
**Culture**	7	-0.05758	0.6597	32	-0.09	0.9310	0.05	-1.4014	1.2862
**Culture**	8	2.8119	0.6365	32	4.42	0.0001	0.05	1.5154	4.1083
**Culture**	9	-0.7464	0.6365	32	-1.17	0.2496	0.05	-2.0429	0.5500
**Culture**	10	-0.2050	0.6679	32	-0.31	0.7609	0.05	-1.5654	1.1555
**Culture**	11	-0.6140	0.6151	32	-1.00	0.3257	0.05	-1.8670	0.6390
**Culture**	12	-0.5487	0.6365	32	-0.86	0.3950	0.05	-1.8452	0.7477
**Culture**	13	-0.6978	0.6679	32	-1.04	0.3040	0.05	-2.0582	0.6627

Type 3 Tests of Fixed Effects				
**Effect**	**Num DF**	**Den DF**	**F Value**	**Pr > F**
**Treatment**	1	32	1.55	0.2228

DistSoma=378

Least Squares Means

Effect	Treatment	Estimate	Standard Error	DF	t Value	Pr > |t|	Alpha	Lower	Upper
**Treatment**	Control GFP	0.07790	0.4824	32	0.16	0.8727	0.05	-0.9047	1.0605
**Treatment**	Ctr Meg	0.9440	0.5026	32	1.88	0.0695	0.05	-0.07978	1.9677

Differences of Least Squares Means

Effect	Treatment	Treatment	Estimate	Standard Error	DF	t Value	Pr > |t|	Adjustment	Adj P	Alpha	Lower	Upper
**Treatment**	Control GFP	Ctr Meg	-0.8661	0.6966	32	-1.24	0.2228	Tukey-Kramer	0.2228	0.05	-2.2851	0.5529

Differences of Least Squares Means

Effect	Treatment	Treatment	Adj Lower	Adj Upper
**Treatment**	Control GFP	Ctr Meg	-2.2851	0.5529

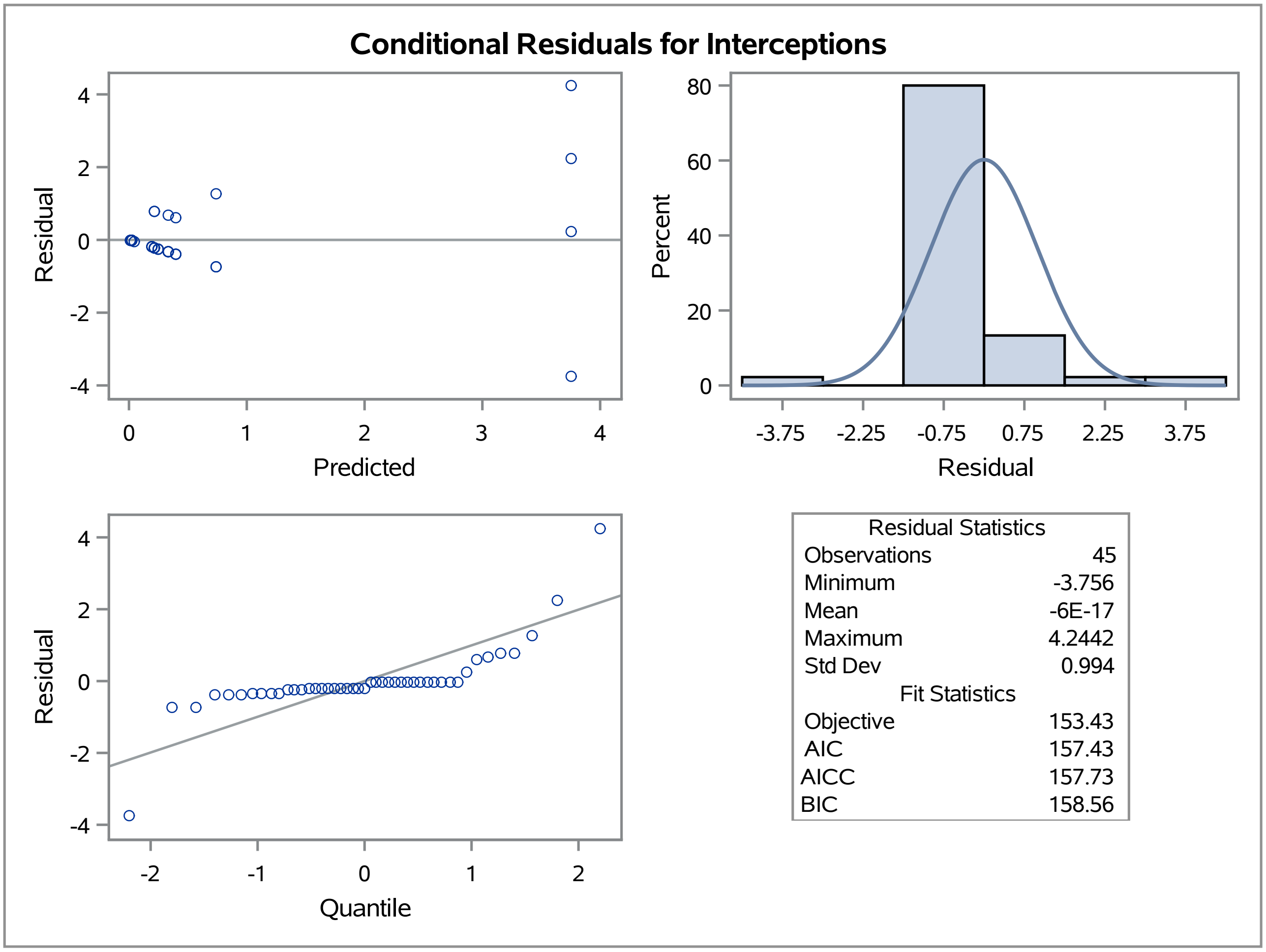

DistSoma=384

Model Information	
**Data Set**	WORK.TEMPDATASORTED
**Dependent Variable**	Interceptions
**Covariance Structure**	Variance Components
**Estimation Method**	REML
**Residual Variance Method**	Profile
**Fixed Effects SE Method**	Model-Based
**Degrees of Freedom Method**	Containment

Class Level Information		
**Class**	**Levels**	**Values**
**Treatment**	2	Control GFP Ctr Meg
**Culture**	13	1 2 3 4 5 6 7 8 9 10 11 12 13

Dimensions	
**Covariance Parameters**	2
**Columns in X**	3
**Columns in Z**	13
**Subjects**	1
**Max Obs per Subject**	45

Number of Observations	
**Number of Observations Read**	45
**Number of Observations Used**	45
**Number of Observations Not Used**	0

Iteration History			
**Iteration**	**Evaluations**	**-2 Res Log Like**	**Criterion**
**0**	1	158.27349005	
**1**	3	148.64804409	0.00521542
**2**	1	148.43708069	0.00048037
**3**	1	148.41933184	0.00000533
**4**	1	148.41914548	0.00000000

Convergence criteria met.

DistSoma=384

Covariance Parameter Estimates				
**Cov Parm**	**Estimate**	**Alpha**	**Lower**	**Upper**
**Culture**	0.8912	0.05	0.3715	4.2707
**Residual**	1.1557	0.05	0.7523	2.0008

Fit Statistics	
**-2 Res Log Likelihood**	148.4
**AIC (Smaller is Better)**	152.4
**AICC (Smaller is Better)**	152.7
**BIC (Smaller is Better)**	153.5

Solution for Fixed Effects									
**Effect**	**Treatment**	**Estimate**	**Standard Error**	**DF**	**t Value**	**Pr > |t|**	**Alpha**	**Lower**	**Upper**
**Intercept**		0.8604	0.4472	11	1.92	0.0806	0.05	-0.1240	1.8447
**Treatment**	Control GFP	-0.7816	0.6212	32	-1.26	0.2174	0.05	-2.0469	0.4837
**Treatment**	Ctr Meg	0	.	.	.	.	.	.	.

Solution for Random Effects									
**Effect**	**Culture**	**Estimate**	**Std Err Pred**	**DF**	**t Value**	**Pr > |t|**	**Alpha**	**Lower**	**Upper**
**Culture**	1	-0.05497	0.5996	32	-0.09	0.9275	0.05	-1.2764	1.1665
**Culture**	2	-0.05497	0.5996	32	-0.09	0.9275	0.05	-1.2764	1.1665
**Culture**	3	0.1293	0.5694	32	0.23	0.8218	0.05	-1.0304	1.2891
**Culture**	4	-0.05946	0.5694	32	-0.10	0.9175	0.05	-1.2192	1.1003
**Culture**	5	0.1293	0.5694	32	0.23	0.8218	0.05	-1.0304	1.2891
**Culture**	6	-0.03428	0.7338	32	-0.05	0.9630	0.05	-1.5289	1.4604
**Culture**	7	-0.05497	0.5996	32	-0.09	0.9275	0.05	-1.2764	1.1665
**Culture**	8	2.3710	0.5764	32	4.11	0.0003	0.05	1.1969	3.5451
**Culture**	9	-0.6497	0.5764	32	-1.13	0.2680	0.05	-1.8239	0.5244
**Culture**	10	-0.1352	0.6054	32	-0.22	0.8246	0.05	-1.3684	1.0979
**Culture**	11	-0.5244	0.5565	32	-0.94	0.3531	0.05	-1.6579	0.6091
**Culture**	12	-0.4609	0.5764	32	-0.80	0.4298	0.05	-1.6351	0.7132
**Culture**	13	-0.6007	0.6054	32	-0.99	0.3285	0.05	-1.8338	0.6324

Type 3 Tests of Fixed Effects				
**Effect**	**Num DF**	**Den DF**	**F Value**	**Pr > F**
**Treatment**	1	32	1.58	0.2174

DistSoma=384

Least Squares Means									
**Effect**	**Treatment**	**Estimate**	**Standard Error**	**DF**	**t Value**	**Pr > |t|**	**Alpha**	**Lower**	**Upper**
**Treatment**	Control GFP	0.07874	0.4311	32	0.18	0.8562	0.05	-0.7994	0.9569
**Treatment**	Ctr Meg	0.8604	0.4472	32	1.92	0.0633	0.05	-0.05060	1.7713

Differences of Least Squares Means												
**Effect**	**Treatment**	**Treatment**	**Estimate**	**Standard Error**	**DF**	**t Value**	**Pr > |t|**	**Adjustment**	**Adj P**	**Alpha**	**Lower**	**Upper**
**Treatment**	Control GFP	Ctr Meg	-0.7816	0.6212	32	-1.26	0.2174	Tukey-Kramer	0.2174	0.05	-2.0469	0.4837

Differences of Least Squares Means				
**Effect**	**Treatment**	**Treatment**	**Adj Lower**	**Adj Upper**
**Treatment**	Control GFP	Ctr Meg	-2.0469	0.4836

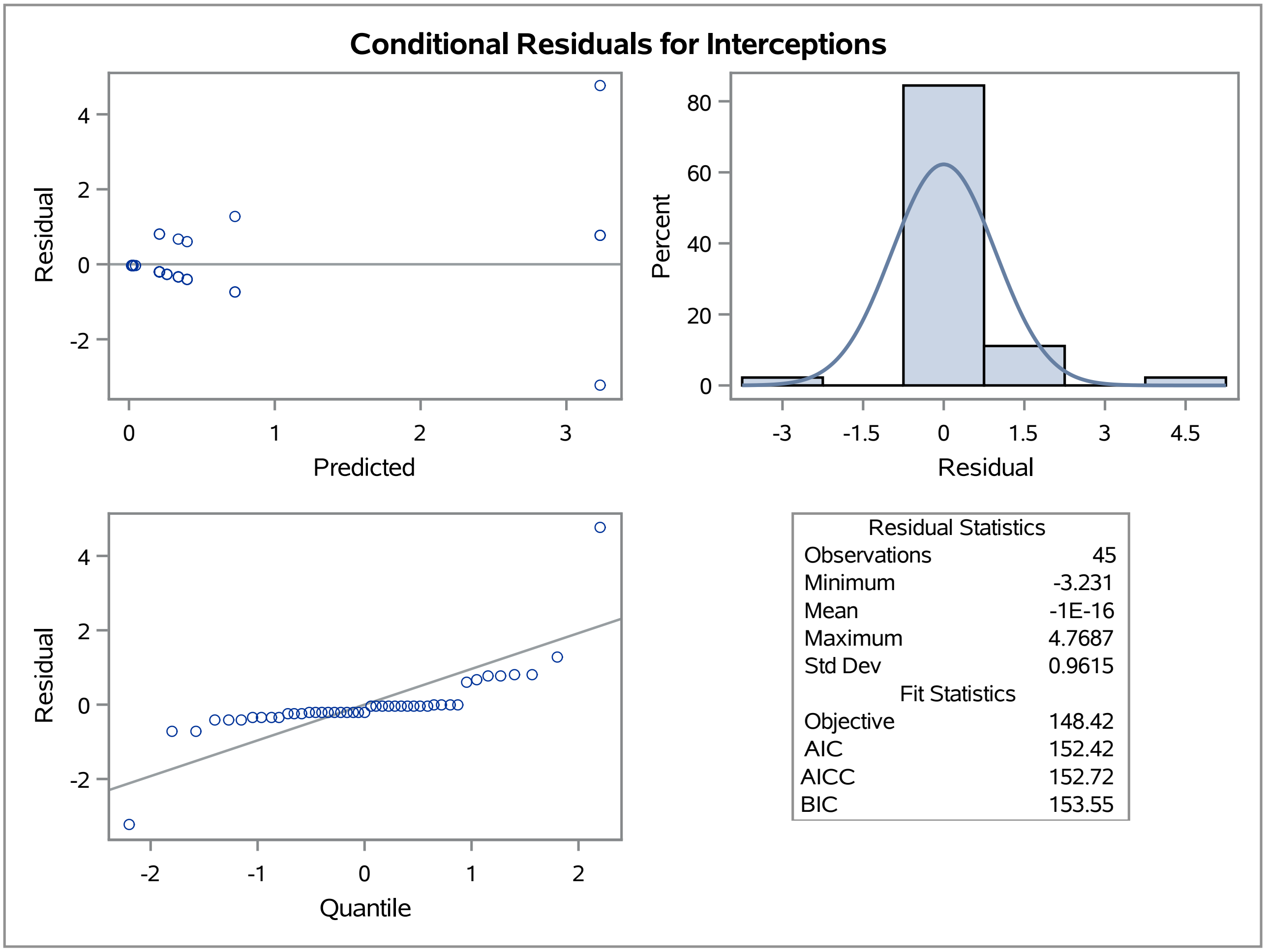

DistSoma=390

Model Information	
**Data Set**	WORK.TEMPDATASORTED
**Dependent Variable**	Interceptions
**Covariance Structure**	Variance Components
**Estimation Method**	REML
**Residual Variance Method**	Profile
**Fixed Effects SE Method**	Model-Based
**Degrees of Freedom Method**	Containment

Class Level Information		
**Class**	**Levels**	**Values**
**Treatment**	2	Control GFP Ctr Meg
**Culture**	13	1 2 3 4 5 6 7 8 9 10 11 12 13

Dimensions	
**Covariance Parameters**	2
**Columns in X**	3
**Columns in Z**	13
**Subjects**	1
**Max Obs per Subject**	45

Number of Observations	
**Number of Observations Read**	45
**Number of Observations Used**	45
**Number of Observations Not Used**	0

Iteration History			
**Iteration**	**Evaluations**	**-2 Res Log Like**	**Criterion**
**0**	1	165.24724928	
**1**	3	152.18243771	0.00477005
**2**	1	151.98006921	0.00042492
**3**	1	151.96359965	0.00000440
**4**	1	151.96343820	0.00000000

Convergence criteria met.

DistSoma=390

Covariance Parameter Estimates				
Cov Parm	Estimate	Alpha	Lower	Upper
Culture	1.2165	0.05	0.5299	5.0920
Residual	1.1901	0.05	0.7739	2.0634

Fit Statistics	
-2 Res Log Likelihood	152.0
AIC (Smaller is Better)	156.0
AICC (Smaller is Better)	156.3
BIC (Smaller is Better)	157.1

Solution for Fixed Effects									
Effect	Treatment	Estimate	Standard Error	DF	t Value	Pr > |t|	Alpha	Lower	Upper
Intercept		0.8907	0.5058	11	1.76	0.1060	0.05	-0.2226	2.0039
Treatment	Control GFP	-0.8131	0.7005	32	-1.16	0.2544	0.05	-2.2401	0.6139
Treatment	Ctr Meg	0	.	.	.	.	.	.	.

Solution for Random Effects									
Effect	Culture	Estimate	Std Err Pred	DF	t Value	Pr > |t|	Alpha	Lower	Upper
Culture	1	-0.05850	0.6578	32	-0.09	0.9297	0.05	-1.3985	1.2815
Culture	2	-0.05850	0.6578	32	-0.09	0.9297	0.05	-1.3985	1.2815
Culture	3	0.1385	0.6251	32	0.22	0.8260	0.05	-1.1347	1.4118
Culture	4	-0.06234	0.6251	32	-0.10	0.9212	0.05	-1.3356	1.2109
Culture	5	0.1385	0.6251	32	0.22	0.8260	0.05	-1.1347	1.4118
Culture	6	-0.03922	0.8134	32	-0.05	0.9618	0.05	-1.6960	1.6176
Culture	7	-0.05850	0.6578	32	-0.09	0.9297	0.05	-1.3985	1.2815
Culture	8	2.9000	0.6358	32	4.56	<.0001	0.05	1.6050	4.1951
Culture	9	-0.7156	0.6358	32	-1.13	0.2687	0.05	-2.0107	0.5794
Culture	10	-0.4203	0.6668	32	-0.63	0.5330	0.05	-1.7785	0.9380
Culture	11	-0.5777	0.6148	32	-0.94	0.3545	0.05	-1.8300	0.6747
Culture	12	-0.5148	0.6358	32	-0.81	0.4241	0.05	-1.8098	0.7803
Culture	13	-0.6716	0.6668	32	-1.01	0.3214	0.05	-2.0299	0.6866

Type 3 Tests of Fixed Effects				
Effect	Num DF	Den DF	F Value	Pr > F
Treatment	1	32	1.35	0.2544

DistSoma=390

Least Squares Means									
**Effect**	**Treatment**	**Estimate**	**Standard Error**	**DF**	**t Value**	**Pr > |t|**	**Alpha**	**Lower**	**Upper**
**Treatment**	Control GFP	0.07758	0.4847	32	0.16	0.8738	0.05	-0.9097	1.0649
**Treatment**	Ctr Meg	0.8907	0.5058	32	1.76	0.0878	0.05	-0.1396	1.9210

Differences of Least Squares Means												
**Effect**	**Treatment**	**Treatment**	**Estimate**	**Standard Error**	**DF**	**t Value**	**Pr > |t|**	**Adjustment**	**Adj P**	**Alpha**	**Lower**	**Upper**
**Treatment**	Control GFP	Ctr Meg	-0.8131	0.7005	32	-1.16	0.2544	Tukey-Kramer	0.2544	0.05	-2.2401	0.6139

Differences of Least Squares Means				
**Effect**	**Treatment**	**Treatment**	**Adj Lower**	**Adj Upper**
**Treatment**	Control GFP	Ctr Meg	-2.2400	0.6138

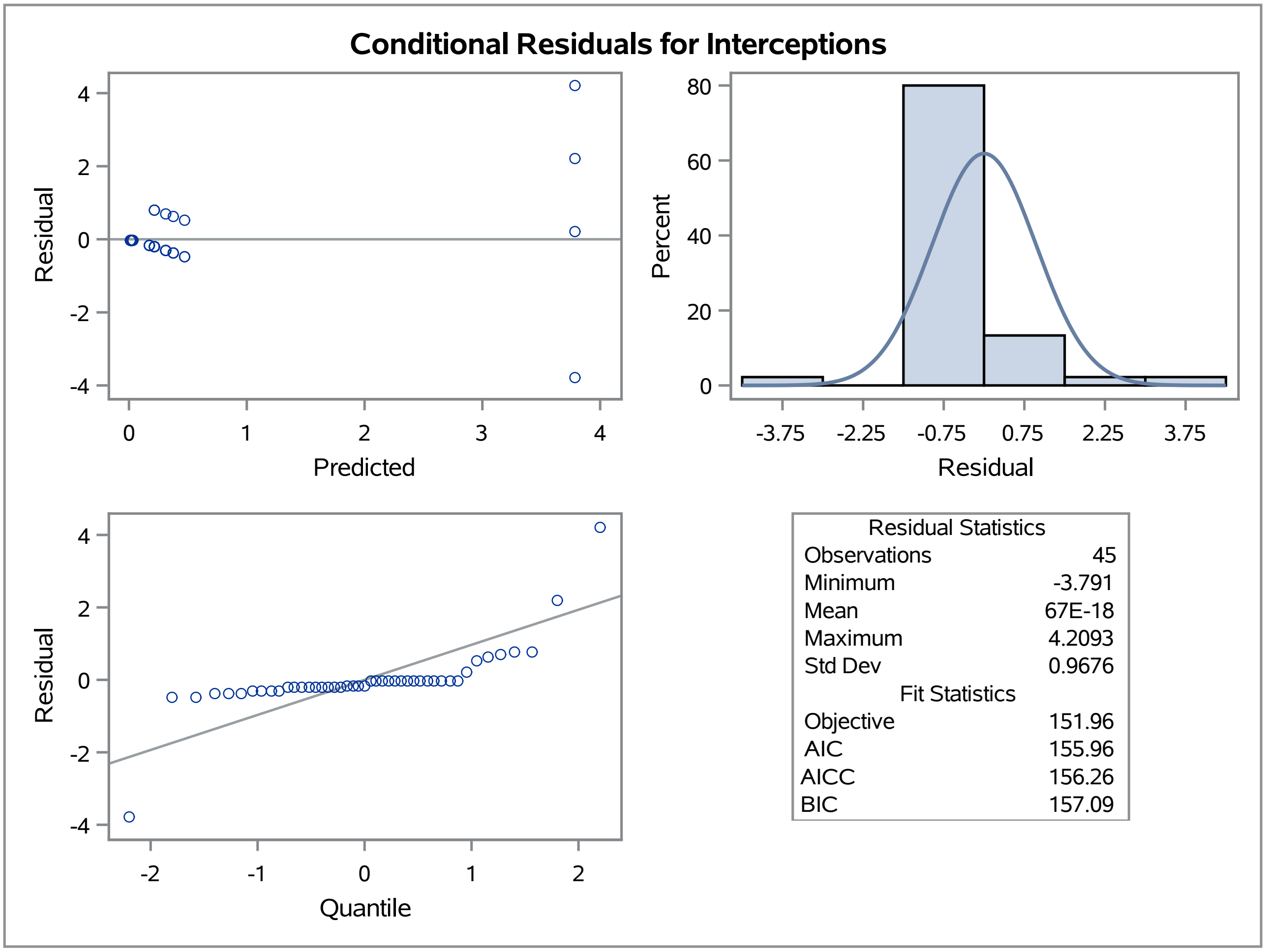

DistSoma=396

Model Information	
**Data Set**	WORK.TEMPDATASORTED
**Dependent Variable**	Interceptions
**Covariance Structure**	Variance Components
**Estimation Method**	REML
**Residual Variance Method**	Profile
**Fixed Effects SE Method**	Model-Based
**Degrees of Freedom Method**	Containment

Class Level Information		
**Class**	**Levels**	**Values**
**Treatment**	2	Control GFP Ctr Meg
**Culture**	13	1 2 3 4 5 6 7 8 9 10 11 12 13

Dimensions	
**Covariance Parameters**	2
**Columns in X**	3
**Columns in Z**	13
**Subjects**	1
**Max Obs per Subject**	45

Number of Observations	
**Number of Observations Read**	45
**Number of Observations Used**	45
**Number of Observations Not Used**	0

Iteration History			
**Iteration**	**Evaluations**	**-2 Res Log Like**	**Criterion**
**0**	1	159.59416845	
**1**	3	150.21563422	0.00546353
**2**	1	149.98896087	0.00053156
**3**	1	149.96881470	0.00000662
**4**	1	149.96857768	0.00000000

Convergence criteria met.

DistSoma=396

Covariance Parameter Estimates				
**Cov Parm**	**Estimate**	**Alpha**	**Lower**	**Upper**
**Culture**	0.9113	0.05	0.3785	4.4175
**Residual**	1.2018	0.05	0.7822	2.0806

Fit Statistics	
**-2 Res Log Likelihood**	150.0
**AIC (Smaller is Better)**	154.0
**AICC (Smaller is Better)**	154.3
**BIC (Smaller is Better)**	155.1

Solution for Fixed Effects									
**Effect**	**Treatment**	**Estimate**	**Standard Error**	**DF**	**t Value**	**Pr > |t|**	**Alpha**	**Lower**	**Upper**
**Intercept**		0.8083	0.4532	11	1.78	0.1021	0.05	-0.1893	1.8058
**Treatment**	Control GFP	-0.7294	0.6296	32	-1.16	0.2552	0.05	-2.0119	0.5530
**Treatment**	Ctr Meg	0	.	.	.	.	.	.	.

Solution for Random Effects									
**Effect**	**Culture**	**Estimate**	**Std Err Pred**	**DF**	**t Value**	**Pr > |t|**	**Alpha**	**Lower**	**Upper**
**Culture**	1	-0.05474	0.6086	32	-0.09	0.9289	0.05	-1.2945	1.1850
**Culture**	2	-0.05474	0.6086	32	-0.09	0.9289	0.05	-1.2945	1.1850
**Culture**	3	-0.05927	0.5779	32	-0.10	0.9190	0.05	-1.2364	1.1179
**Culture**	4	-0.05927	0.5779	32	-0.10	0.9190	0.05	-1.2364	1.1179
**Culture**	5	0.3168	0.5779	32	0.55	0.5874	0.05	-0.8604	1.4939
**Culture**	6	-0.03399	0.7442	32	-0.05	0.9639	0.05	-1.5499	1.4819
**Culture**	7	-0.05474	0.6086	32	-0.09	0.9289	0.05	-1.2945	1.1850
**Culture**	8	2.4004	0.5849	32	4.10	0.0003	0.05	1.2089	3.5918
**Culture**	9	-0.6079	0.5849	32	-1.04	0.3065	0.05	-1.7993	0.5836
**Culture**	10	-0.3299	0.6143	32	-0.54	0.5950	0.05	-1.5812	0.9214
**Culture**	11	-0.4813	0.5646	32	-0.85	0.4003	0.05	-1.6314	0.6688
**Culture**	12	-0.4198	0.5849	32	-0.72	0.4781	0.05	-1.6113	0.7716
**Culture**	13	-0.5615	0.6143	32	-0.91	0.3676	0.05	-1.8128	0.6899

Type 3 Tests of Fixed Effects				
**Effect**	**Num DF**	**Den DF**	**F Value**	**Pr > F**
**Treatment**	1	32	1.34	0.2552

DistSoma=396

Least Squares Means									
**Effect**	**Treatment**	**Estimate**	**Standard Error**	**DF**	**t Value**	**Pr > |t|**	**Alpha**	**Lower**	**Upper**
**Treatment**	Control GFP	0.07881	0.4370	32	0.18	0.8580	0.05	-0.8114	0.9690
**Treatment**	Ctr Meg	0.8083	0.4532	32	1.78	0.0840	0.05	-0.1149	1.7314

Differences of Least Squares Means												
**Effect**	**Treatment**	**Treatment**	**Estimate**	**Standard Error**	**DF**	**t Value**	**Pr > |t|**	**Adjustment**	**Adj P**	**Alpha**	**Lower**	**Upper**
**Treatment**	Control GFP	Ctr Meg	-0.7294	0.6296	32	-1.16	0.2552	Tukey-Kramer	0.2552	0.05	-2.0119	0.5530

Differences of Least Squares Means				
**Effect**	**Treatment**	**Treatment**	**Adj Lower**	**Adj Upper**
**Treatment**	Control GFP	Ctr Meg	-2.0119	0.5530

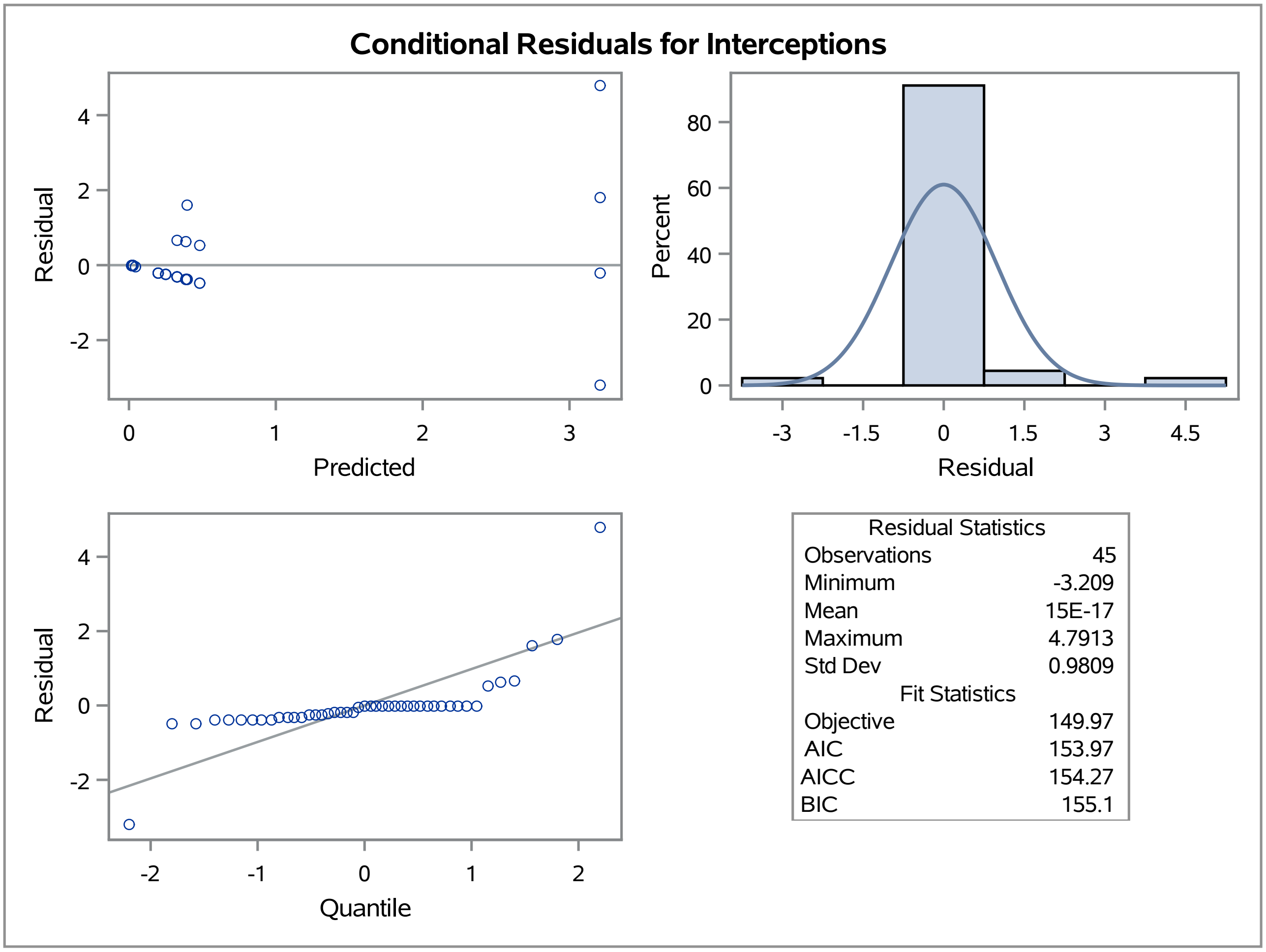

DistSoma=402

Model Information	
Data Set	WORK.TEMPDATASORTED
Dependent Variable	Interceptions
Covariance Structure	Variance Components
Estimation Method	REML
Residual Variance Method	Profile
Fixed Effects SE Method	Model-Based
Degrees of Freedom Method	Containment

Class Level Information		
Class	Levels	Values
Treatment	2	Control GFP Ctr Meg
Culture	13	1 2 3 4 5 6 7 8 9 10 11 12 13

Dimensions	
Covariance Parameters	2
Columns in X	3
Columns in Z	13
Subjects	1
Max Obs per Subject	45

Number of Observations	
Number of Observations Read	45
Number of Observations Used	45
Number of Observations Not Used	0

Iteration History			
Iteration	Evaluations	-2 Res Log Like	Criterion
0	1	187.31896606	
1	3	178.08521976	0.00385110
2	1	177.86308417	0.00036864
3	1	177.84364290	0.00000445
4	1	177.84342136	0.00000000

Convergence criteria met.

DistSoma=402

Covariance Parameter Estimates				
**Cov Parm**	**Estimate**	**Alpha**	**Lower**	**Upper**
**Culture**	1.7210	0.05	0.7129	8.4118
**Residual**	2.3044	0.05	1.5000	3.9889

Fit Statistics	
**-2 Res Log Likelihood**	177.8
**AIC (Smaller is Better)**	181.8
**AICC (Smaller is Better)**	182.1
**BIC (Smaller is Better)**	183.0

Solution for Fixed Effects									
**Effect**	**Treatment**	**Estimate**	**Standard Error**	**DF**	**t Value**	**Pr > |t|**	**Alpha**	**Lower**	**Upper**
**Intercept**		1.0627	0.6241	11	1.70	0.1166	0.05	-0.3109	2.4362
**Treatment**	Control GFP	-1.0627	0.8671	32	-1.23	0.2293	0.05	-2.8288	0.7035
**Treatment**	Ctr Meg	0	.	.	.	.	.	.	.

Solution for Random Effects									
**Effect**	**Culture**	**Estimate**	**Std Err Pred**	**DF**	**t Value**	**Pr > |t|**	**Alpha**	**Lower**	**Upper**
**Culture**	1	3.15E-16	0.8392	32	0.00	1.0000	0.05	-1.7095	1.7095
**Culture**	2	3.15E-16	0.8392	32	0.00	1.0000	0.05	-1.7095	1.7095
**Culture**	3	2.96E-16	0.7969	32	0.00	1.0000	0.05	-1.6232	1.6232
**Culture**	4	2.96E-16	0.7969	32	0.00	1.0000	0.05	-1.6232	1.6232
**Culture**	5	2.96E-16	0.7969	32	0.00	1.0000	0.05	-1.6232	1.6232
**Culture**	6	1.69E-16	1.0254	32	0.00	1.0000	0.05	-2.0887	2.0887
**Culture**	7	3.15E-16	0.8392	32	0.00	1.0000	0.05	-1.7095	1.7095
**Culture**	8	3.3245	0.8064	32	4.12	0.0002	0.05	1.6820	4.9670
**Culture**	9	-0.7962	0.8064	32	-0.99	0.3309	0.05	-2.4387	0.8463
**Culture**	10	-0.5043	0.8469	32	-0.60	0.5557	0.05	-2.2294	1.2208
**Culture**	11	-0.6805	0.7783	32	-0.87	0.3885	0.05	-2.2659	0.9050
**Culture**	12	-0.6089	0.8064	32	-0.76	0.4557	0.05	-2.2514	1.0336
**Culture**	13	-0.7347	0.8469	32	-0.87	0.3921	0.05	-2.4599	0.9904

Type 3 Tests of Fixed Effects				
**Effect**	**Num DF**	**Den DF**	**F Value**	**Pr > F**
**Treatment**	1	32	1.50	0.2293

DistSoma=402

Least Squares Means									
Effect	Treatment	Estimate	Standard Error	DF	t Value	Pr > |t|	Alpha	Lower	Upper
**Treatment**	Control GFP	-444E-18	0.6020	32	-0.00	1.0000	0.05	-1.2262	1.2262
**Treatment**	Ctr Meg	1.0627	0.6241	32	1.70	0.0983	0.05	-0.2085	2.3338

Differences of Least Squares Means												
Effect	Treatment	Treatment	Estimate	Standard Error	DF	t Value	Pr > |t|	Adjustment	Adj P	Alpha	Lower	Upper
**Treatment**	Control GFP	Ctr Meg	-1.0627	0.8671	32	-1.23	0.2293	Tukey-Kramer	0.2293	0.05	-2.8288	0.7035

Differences of Least Squares Means				
Effect	Treatment	Treatment	Adj Lower	Adj Upper
**Treatment**	Control GFP	Ctr Meg	-2.8288	0.7035

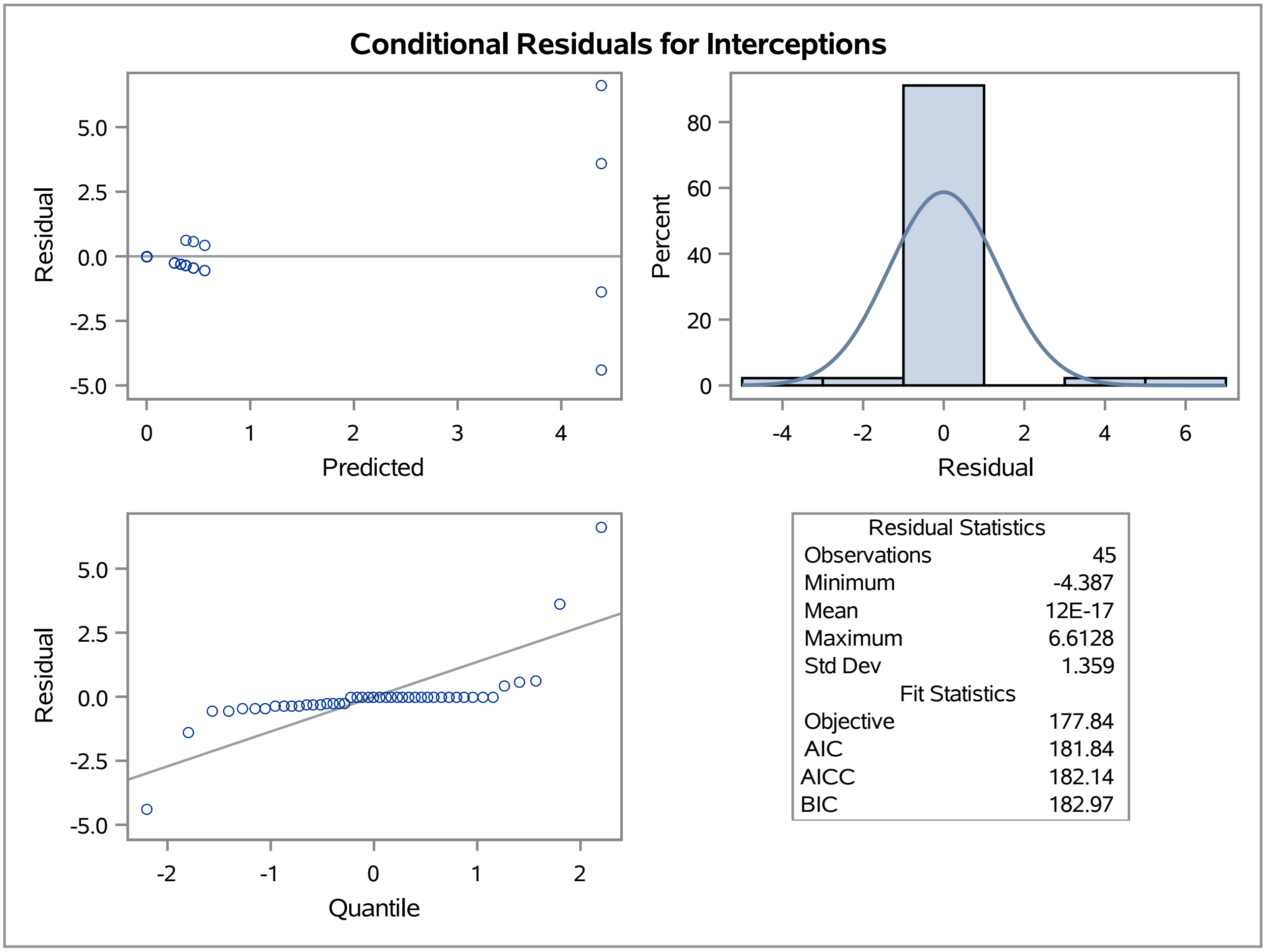

DistSoma=408

Model Information	
Data Set	WORK.TEMPDATASORTED
Dependent Variable	Interceptions
Covariance Structure	Variance Components
Estimation Method	REML
Residual Variance Method	Profile
Fixed Effects SE Method	Model-Based
Degrees of Freedom Method	Containment

Class Level Information		
Class	Levels	Values
Treatment	2	Control GFP Ctr Meg
Culture	13	1 2 3 4 5 6 7 8 9 10 11 12 13

Dimensions	
Covariance Parameters	2
Columns in X	3
Columns in Z	13
Subjects	1
Max Obs per Subject	45

Number of Observations	
Number of Observations Read	45
Number of Observations Used	45
Number of Observations Not Used	0

Iteration History			
Iteration	Evaluations	-2 Res Log Like	Criterion
0	1	161.24411639	
1	3	149.39820740	0.00516652
2	1	149.18697103	0.00047583
3	1	149.16919984	0.00000528
4	1	149.16901322	0.00000000

Convergence criteria met.

DistSoma=408

Covariance Parameter Estimates				
**Cov Parm**	**Estimate**	**Alpha**	**Lower**	**Upper**
**Culture**	1.0583	0.05	0.4549	4.6087
**Residual**	1.1352	0.05	0.7384	1.9673

Fit Statistics	
**-2 Res Log Likelihood**	149.2
**AIC (Smaller is Better)**	153.2
**AICC (Smaller is Better)**	153.5
**BIC (Smaller is Better)**	154.3

Solution for Fixed Effects									
**Effect**	**Treatment**	**Estimate**	**Standard Error**	**DF**	**t Value**	**Pr > |t|**	**Alpha**	**Lower**	**Upper**
**Intercept**		0.8491	0.4764	11	1.78	0.1023	0.05	-0.1996	1.8977
**Treatment**	Control GFP	-0.8491	0.6605	32	-1.29	0.2078	0.05	-2.1945	0.4963
**Treatment**	Ctr Meg	0	.	.	.	.	.	.	.

Solution for Random Effects									
**Effect**	**Culture**	**Estimate**	**Std Err Pred**	**DF**	**t Value**	**Pr > |t|**	**Alpha**	**Lower**	**Upper**
**Culture**	1	2.8E-16	0.6263	32	0.00	1.0000	0.05	-1.2758	1.2758
**Culture**	2	2.8E-16	0.6263	32	0.00	1.0000	0.05	-1.2758	1.2758
**Culture**	3	2.79E-16	0.5949	32	0.00	1.0000	0.05	-1.2118	1.2118
**Culture**	4	2.79E-16	0.5949	32	0.00	1.0000	0.05	-1.2118	1.2118
**Culture**	5	2.79E-16	0.5949	32	0.00	1.0000	0.05	-1.2118	1.2118
**Culture**	6	1.71E-16	0.7723	32	0.00	1.0000	0.05	-1.5731	1.5731
**Culture**	7	2.8E-16	0.6263	32	0.00	1.0000	0.05	-1.2758	1.2758
**Culture**	8	2.6818	0.6041	32	4.44	0.0001	0.05	1.4512	3.9123
**Culture**	9	-0.6695	0.6041	32	-1.11	0.2760	0.05	-1.9001	0.5610
**Culture**	10	-0.3799	0.6340	32	-0.60	0.5532	0.05	-1.6713	0.9114
**Culture**	11	-0.5344	0.5838	32	-0.92	0.3668	0.05	-1.7236	0.6547
**Culture**	12	-0.4724	0.6041	32	-0.78	0.4400	0.05	-1.7029	0.7581
**Culture**	13	-0.6255	0.6340	32	-0.99	0.3313	0.05	-1.9168	0.6659

Type 3 Tests of Fixed Effects				
**Effect**	**Num DF**	**Den DF**	**F Value**	**Pr > F**
**Treatment**	1	32	1.65	0.2078

DistSoma=408

Least Squares Means									
Effect	Treatment	Estimate	Standard Error	DF	t Value	Pr > |t|	Alpha	Lower	Upper
Treatment	Control GFP	-222E-18	0.4574	32	-0.00	1.0000	0.05	-0.9318	0.9318
Treatment	Ctr Meg	0.8491	0.4764	32	1.78	0.0842	0.05	-0.1214	1.8196

Differences of Least Squares Means												
Effect	Treatment	Treatment	Estimate	Standard Error	DF	t Value	Pr > |t|	Adjustment	Adj P	Alpha	Lower	Upper
Treatment	Control GFP	Ctr Meg	-0.8491	0.6605	32	-1.29	0.2078	Tukey-Kramer	0.2078	0.05	-2.1945	0.4963

Differences of Least Squares Means				
Effect	Treatment	Treatment	Adj Lower	Adj Upper
Treatment	Control GFP	Ctr Meg	-2.1944	0.4963

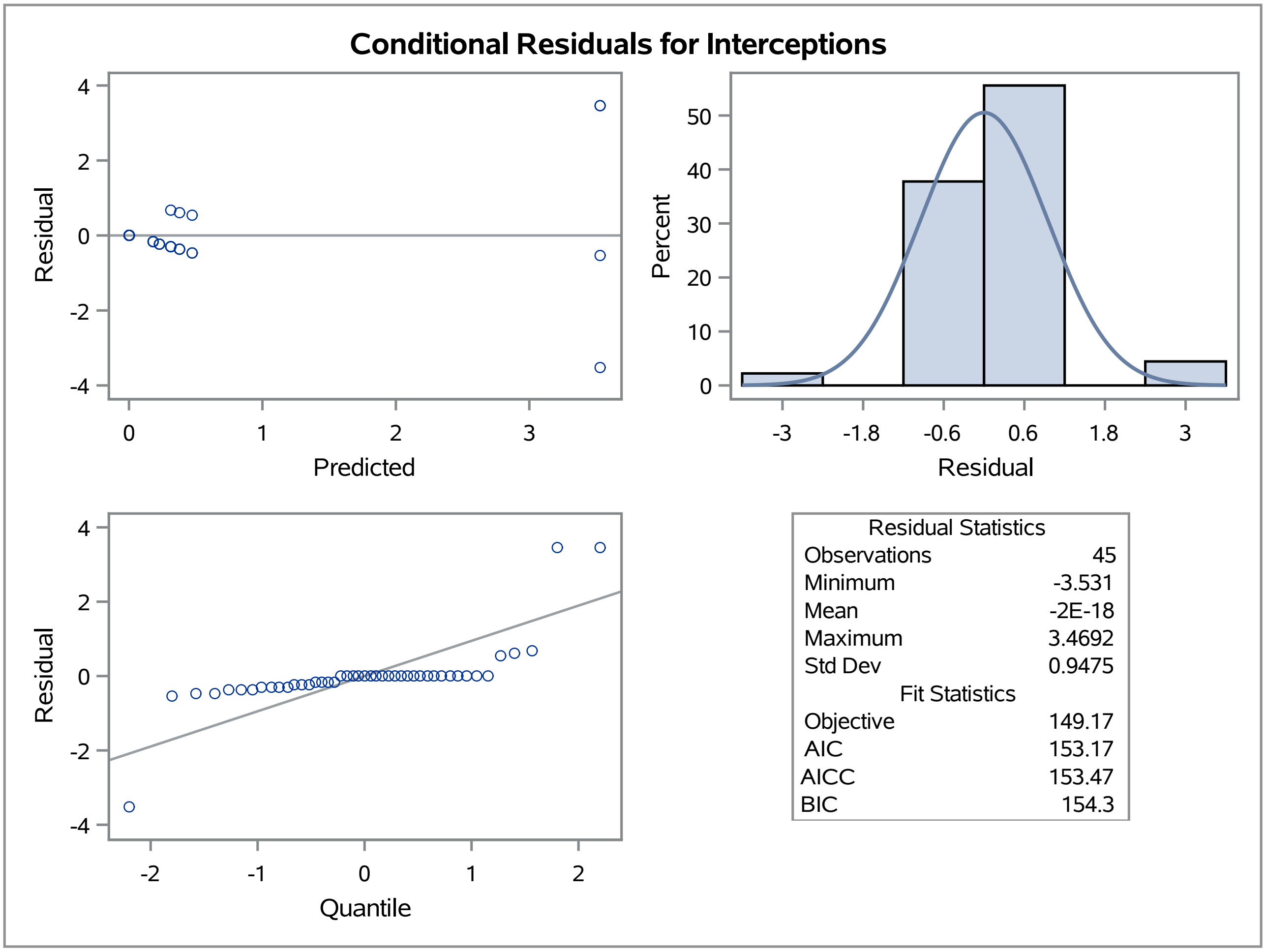

DistSoma=414

Model Information	
**Data Set**	WORK.TEMPDATASORTED
**Dependent Variable**	Interceptions
**Covariance Structure**	Variance Components
**Estimation Method**	REML
**Residual Variance Method**	Profile
**Fixed Effects SE Method**	Model-Based
**Degrees of Freedom Method**	Containment

Class Level Information		
**Class**	**Levels**	**Values**
**Treatment**	2	Control GFP Ctr Meg
**Culture**	13	1 2 3 4 5 6 7 8 9 10 11 12 13

Dimensions	
**Covariance Parameters**	2
**Columns in X**	3
**Columns in Z**	13
**Subjects**	1
**Max Obs per Subject**	45

Number of Observations	
**Number of Observations Read**	45
**Number of Observations Used**	45
**Number of Observations Not Used**	0

Iteration History			
**Iteration**	**Evaluations**	**-2 Res Log Like**	**Criterion**
**0**	1	155.64622379	
**1**	3	143.61228232	0.00561395
**2**	1	143.40165165	0.00051601
**3**	1	143.38397133	0.00000570
**4**	1	143.38378653	0.00000000

Convergence criteria met.

DistSoma=414

Covariance Parameter Estimates				
**Cov Parm**	**Estimate**	**Alpha**	**Lower**	**Upper**
**Culture**	0.9362	0.05	0.4033	4.0502
**Residual**	0.9895	0.05	0.6436	1.7150

Fit Statistics	
**-2 Res Log Likelihood**	143.4
**AIC (Smaller is Better)**	147.4
**AICC (Smaller is Better)**	147.7
**BIC (Smaller is Better)**	148.5

Solution for Fixed Effects									
**Effect**	**Treatment**	**Estimate**	**Standard Error**	**DF**	**t Value**	**Pr > |t|**	**Alpha**	**Lower**	**Upper**
**Intercept**		0.8067	0.4474	11	1.80	0.0988	0.05	-0.1780	1.7914
**Treatment**	Control GFP	-0.8067	0.6201	32	-1.30	0.2026	0.05	-2.0699	0.4564
**Treatment**	Ctr Meg	0	.	.	.	.	.	.	.

Solution for Random Effects									
**Effect**	**Culture**	**Estimate**	**Std Err Pred**	**DF**	**t Value**	**Pr > |t|**	**Alpha**	**Lower**	**Upper**
**Culture**	1	-671E-19	0.5871	32	-0.00	1.0000	0.05	-1.1960	1.1960
**Culture**	2	-671E-19	0.5871	32	-0.00	1.0000	0.05	-1.1960	1.1960
**Culture**	3	-11E-17	0.5577	32	-0.00	1.0000	0.05	-1.1360	1.1360
**Culture**	4	-11E-17	0.5577	32	-0.00	1.0000	0.05	-1.1360	1.1360
**Culture**	5	-11E-17	0.5577	32	-0.00	1.0000	0.05	-1.1360	1.1360
**Culture**	6	-678E-19	0.7243	32	-0.00	1.0000	0.05	-1.4754	1.4754
**Culture**	7	-671E-19	0.5871	32	-0.00	1.0000	0.05	-1.1960	1.1960
**Culture**	8	2.5259	0.5665	32	4.46	<.0001	0.05	1.3720	3.6798
**Culture**	9	-0.6381	0.5665	32	-1.13	0.2684	0.05	-1.7920	0.5158
**Culture**	10	-0.3500	0.5944	32	-0.59	0.5601	0.05	-1.5609	0.8608
**Culture**	11	-0.5008	0.5475	32	-0.91	0.3672	0.05	-1.6161	0.6144
**Culture**	12	-0.4404	0.5665	32	-0.78	0.4427	0.05	-1.5943	0.7136
**Culture**	13	-0.5965	0.5944	32	-1.00	0.3231	0.05	-1.8074	0.6143

Type 3 Tests of Fixed Effects				
**Effect**	**Num DF**	**Den DF**	**F Value**	**Pr > F**
**Treatment**	1	32	1.69	0.2026

DistSoma=414

Least Squares Means									
Effect	Treatment	Estimate	Standard Error	DF	t Value	Pr > |t|	Alpha	Lower	Upper
**Treatment**	Control GFP	0	0.4294	32	0.00	1.0000	0.05	-0.8747	0.8747
**Treatment**	Ctr Meg	0.8067	0.4474	32	1.80	0.0808	0.05	-0.1046	1.7180

Differences of Least Squares Means												
Effect	Treatment	Treatment	Estimate	Standard Error	DF	t Value	Pr > |t|	Adjustment	Adj P	Alpha	Lower	Upper
**Treatment**	Control GFP	Ctr Meg	-0.8067	0.6201	32	-1.30	0.2026	Tukey-Kramer	0.2026	0.05	-2.0699	0.4564

Differences of Least Squares Means				
Effect	Treatment	Treatment	Adj Lower	Adj Upper
**Treatment**	Control GFP	Ctr Meg	-2.0698	0.4564

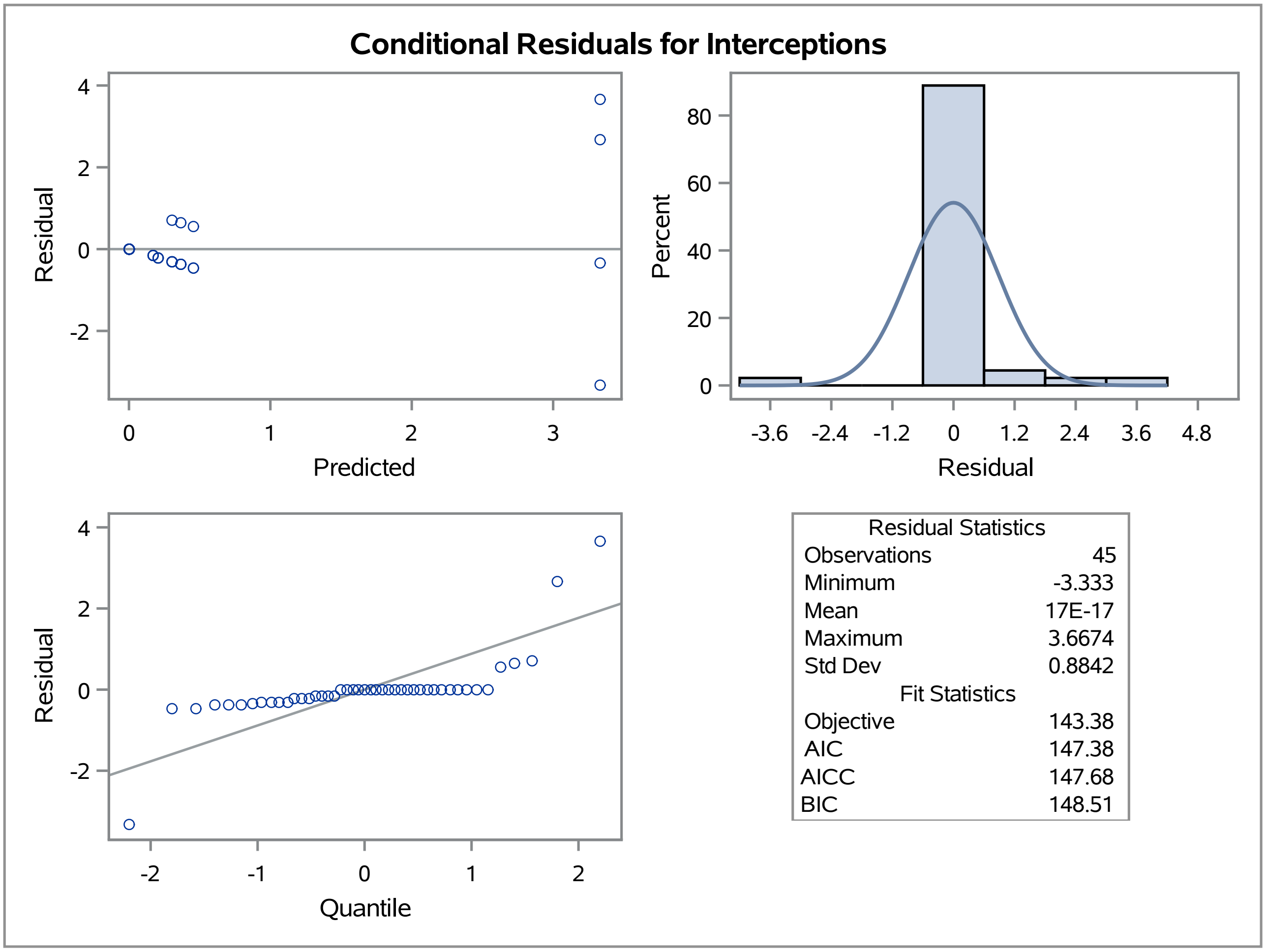

DistSoma=420

Model Information	
**Data Set**	WORK.TEMPDATASORTED
**Dependent Variable**	Interceptions
**Covariance Structure**	Variance Components
**Estimation Method**	REML
**Residual Variance Method**	Profile
**Fixed Effects SE Method**	Model-Based
**Degrees of Freedom Method**	Containment

Class Level Information		
**Class**	**Levels**	**Values**
**Treatment**	2	Control GFP Ctr Meg
**Culture**	13	1 2 3 4 5 6 7 8 9 10 11 12 13

Dimensions	
**Covariance Parameters**	2
**Columns in X**	3
**Columns in Z**	13
**Subjects**	1
**Max Obs per Subject**	45

Number of Observations	
**Number of Observations Read**	45
**Number of Observations Used**	45
**Number of Observations Not Used**	0

Iteration History			
**Iteration**	**Evaluations**	**-2 Res Log Like**	**Criterion**
**0**	1	155.64622379	
**1**	3	143.61228232	0.00561395
**2**	1	143.40165165	0.00051601
**3**	1	143.38397133	0.00000570
**4**	1	143.38378653	0.00000000

Convergence criteria met.

DistSoma=420

Covariance Parameter Estimates				
**Cov Parm**	**Estimate**	**Alpha**	**Lower**	**Upper**
**Culture**	0.9362	0.05	0.4033	4.0502
**Residual**	0.9895	0.05	0.6436	1.7150

Fit Statistics	
**-2 Res Log Likelihood**	143.4
**AIC (Smaller is Better)**	147.4
**AICC (Smaller is Better)**	147.7
**BIC (Smaller is Better)**	148.5

Solution for Fixed Effects									
**Effect**	**Treatment**	**Estimate**	**Standard Error**	**DF**	**t Value**	**Pr > |t|**	**Alpha**	**Lower**	**Upper**
**Intercept**		0.8067	0.4474	11	1.80	0.0988	0.05	-0.1780	1.7914
**Treatment**	Control GFP	-0.8067	0.6201	32	-1.30	0.2026	0.05	-2.0699	0.4564
**Treatment**	Ctr Meg	0	.	.	.	.	.	.	.

Solution for Random Effects									
**Effect**	**Culture**	**Estimate**	**Std Err Pred**	**DF**	**t Value**	**Pr > |t|**	**Alpha**	**Lower**	**Upper**
**Culture**	1	-671E-19	0.5871	32	-0.00	1.0000	0.05	-1.1960	1.1960
**Culture**	2	-671E-19	0.5871	32	-0.00	1.0000	0.05	-1.1960	1.1960
**Culture**	3	-11E-17	0.5577	32	-0.00	1.0000	0.05	-1.1360	1.1360
**Culture**	4	-11E-17	0.5577	32	-0.00	1.0000	0.05	-1.1360	1.1360
**Culture**	5	-11E-17	0.5577	32	-0.00	1.0000	0.05	-1.1360	1.1360
**Culture**	6	-678E-19	0.7243	32	-0.00	1.0000	0.05	-1.4754	1.4754
**Culture**	7	-671E-19	0.5871	32	-0.00	1.0000	0.05	-1.1960	1.1960
**Culture**	8	2.5259	0.5665	32	4.46	<.0001	0.05	1.3720	3.6798
**Culture**	9	-0.6381	0.5665	32	-1.13	0.2684	0.05	-1.7920	0.5158
**Culture**	10	-0.3500	0.5944	32	-0.59	0.5601	0.05	-1.5609	0.8608
**Culture**	11	-0.5008	0.5475	32	-0.91	0.3672	0.05	-1.6161	0.6144
**Culture**	12	-0.4404	0.5665	32	-0.78	0.4427	0.05	-1.5943	0.7136
**Culture**	13	-0.5965	0.5944	32	-1.00	0.3231	0.05	-1.8074	0.6143

Type 3 Tests of Fixed Effects				
**Effect**	**Num DF**	**Den DF**	**F Value**	**Pr > F**
**Treatment**	1	32	1.69	0.2026

DistSoma=420

Least Squares Means									
**Effect**	**Treatment**	**Estimate**	**Standard Error**	**DF**	**t Value**	**Pr > |t|**	**Alpha**	**Lower**	**Upper**
**Treatment**	Control GFP	0	0.4294	32	0.00	1.0000	0.05	-0.8747	0.8747
**Treatment**	Ctr Meg	0.8067	0.4474	32	1.80	0.0808	0.05	-0.1046	1.7180

Differences of Least Squares Means												
**Effect**	**Treatment**	**Treatment**	**Estimate**	**Standard Error**	**DF**	**t Value**	**Pr > |t|**	**Adjustment**	**Adj P**	**Alpha**	**Lower**	**Upper**
**Treatment**	Control GFP	Ctr Meg	-0.8067	0.6201	32	-1.30	0.2026	Tukey-Kramer	0.2026	0.05	-2.0699	0.4564

Differences of Least Squares Means				
**Effect**	**Treatment**	**Treatment**	**Adj Lower**	**Adj Upper**
**Treatment**	Control GFP	Ctr Meg	-2.0698	0.4564

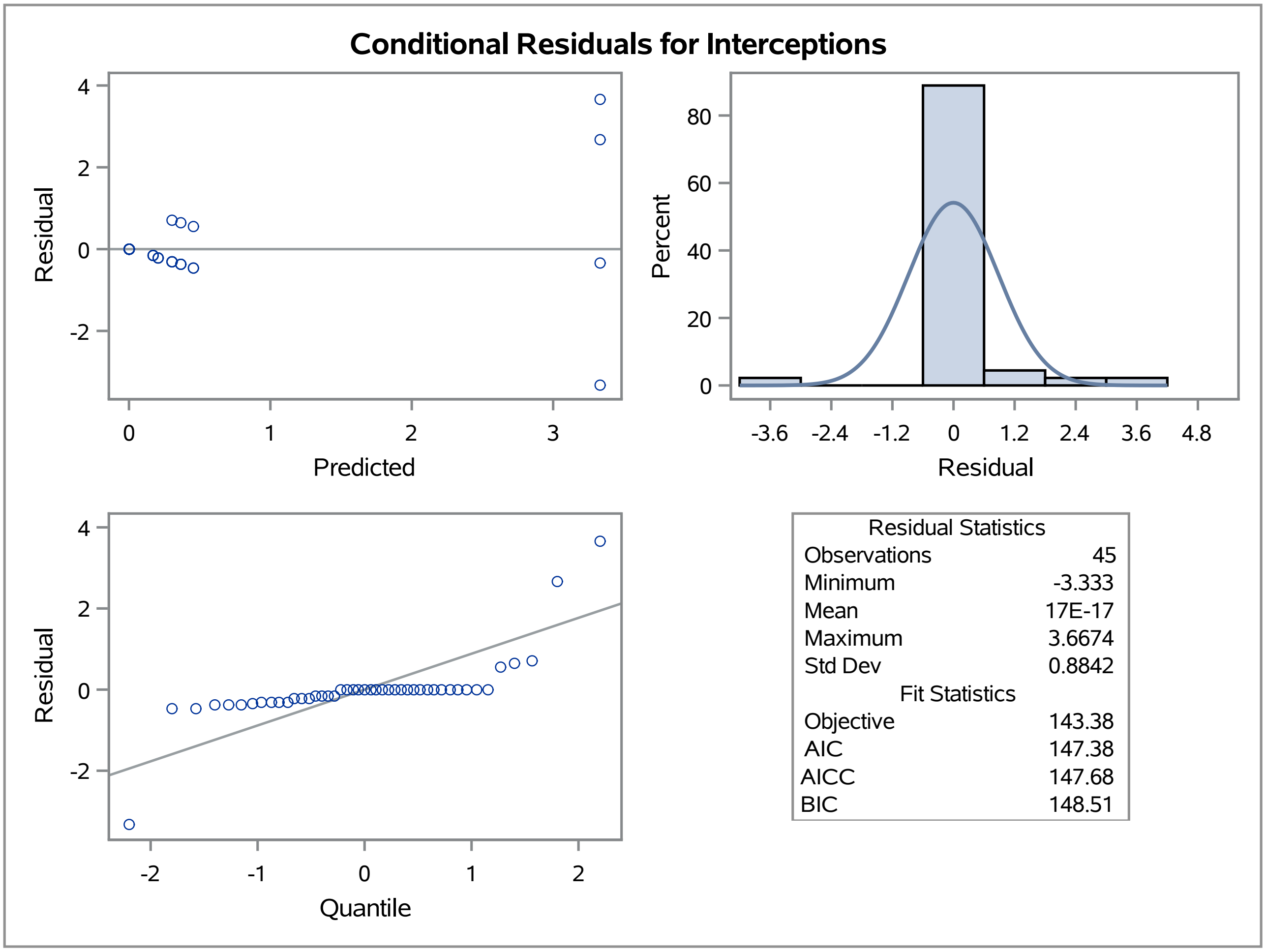

DistSoma=426

Model Information	
**Data Set**	WORK.TEMPDATASORTED
**Dependent Variable**	Interceptions
**Covariance Structure**	Variance Components
**Estimation Method**	REML
**Residual Variance Method**	Profile
**Fixed Effects SE Method**	Model-Based
**Degrees of Freedom Method**	Containment

Class Level Information		
**Class**	**Levels**	**Values**
**Treatment**	2	Control GFP Ctr Meg
**Culture**	13	1 2 3 4 5 6 7 8 9 10 11 12 13

Dimensions	
**Covariance Parameters**	2
**Columns in X**	3
**Columns in Z**	13
**Subjects**	1
**Max Obs per Subject**	45

Number of Observations	
**Number of Observations Read**	45
**Number of Observations Used**	45
**Number of Observations Not Used**	0

Iteration History			
**Iteration**	**Evaluations**	**-2 Res Log Like**	**Criterion**
**0**	1	155.64622379	
**1**	3	143.61228232	0.00561395
**2**	1	143.40165165	0.00051601
**3**	1	143.38397133	0.00000570
**4**	1	143.38378653	0.00000000

Convergence criteria met.

DistSoma=426

Covariance Parameter Estimates				
**Cov Parm**	**Estimate**	**Alpha**	**Lower**	**Upper**
**Culture**	0.9362	0.05	0.4033	4.0502
**Residual**	0.9895	0.05	0.6436	1.7150

Fit Statistics	
**-2 Res Log Likelihood**	143.4
**AIC (Smaller is Better)**	147.4
**AICC (Smaller is Better)**	147.7
**BIC (Smaller is Better)**	148.5

Solution for Fixed Effects									
**Effect**	**Treatment**	**Estimate**	**Standard Error**	**DF**	**t Value**	**Pr > |t|**	**Alpha**	**Lower**	**Upper**
**Intercept**		0.8067	0.4474	11	1.80	0.0988	0.05	-0.1780	1.7914
**Treatment**	Control GFP	-0.8067	0.6201	32	-1.30	0.2026	0.05	-2.0699	0.4564
**Treatment**	Ctr Meg	0	.	.	.	.	.	.	.

Solution for Random Effects									
**Effect**	**Culture**	**Estimate**	**Std Err Pred**	**DF**	**t Value**	**Pr > |t|**	**Alpha**	**Lower**	**Upper**
**Culture**	1	-671E-19	0.5871	32	-0.00	1.0000	0.05	-1.1960	1.1960
**Culture**	2	-671E-19	0.5871	32	-0.00	1.0000	0.05	-1.1960	1.1960
**Culture**	3	-11E-17	0.5577	32	-0.00	1.0000	0.05	-1.1360	1.1360
**Culture**	4	-11E-17	0.5577	32	-0.00	1.0000	0.05	-1.1360	1.1360
**Culture**	5	-11E-17	0.5577	32	-0.00	1.0000	0.05	-1.1360	1.1360
**Culture**	6	-678E-19	0.7243	32	-0.00	1.0000	0.05	-1.4754	1.4754
**Culture**	7	-671E-19	0.5871	32	-0.00	1.0000	0.05	-1.1960	1.1960
**Culture**	8	2.5259	0.5665	32	4.46	<.0001	0.05	1.3720	3.6798
**Culture**	9	-0.6381	0.5665	32	-1.13	0.2684	0.05	-1.7920	0.5158
**Culture**	10	-0.3500	0.5944	32	-0.59	0.5601	0.05	-1.5609	0.8608
**Culture**	11	-0.5008	0.5475	32	-0.91	0.3672	0.05	-1.6161	0.6144
**Culture**	12	-0.4404	0.5665	32	-0.78	0.4427	0.05	-1.5943	0.7136
**Culture**	13	-0.5965	0.5944	32	-1.00	0.3231	0.05	-1.8074	0.6143

Type 3 Tests of Fixed Effects				
**Effect**	**Num DF**	**Den DF**	**F Value**	**Pr > F**
**Treatment**	1	32	1.69	0.2026

DistSoma=426

Least Squares Means									
**Effect**	**Treatment**	**Estimate**	**Standard Error**	**DF**	**t Value**	**Pr > |t|**	**Alpha**	**Lower**	**Upper**
**Treatment**	Control GFP	0	0.4294	32	0.00	1.0000	0.05	-0.8747	0.8747
**Treatment**	Ctr Meg	0.8067	0.4474	32	1.80	0.0808	0.05	-0.1046	1.7180

Differences of Least Squares Means												
**Effect**	**Treatment**	**Treatment**	**Estimate**	**Standard Error**	**DF**	**t Value**	**Pr > |t|**	**Adjustment**	**Adj P**	**Alpha**	**Lower**	**Upper**
**Treatment**	Control GFP	Ctr Meg	-0.8067	0.6201	32	-1.30	0.2026	Tukey-Kramer	0.2026	0.05	-2.0699	0.4564

Differences of Least Squares Means				
**Effect**	**Treatment**	**Treatment**	**Adj Lower**	**Adj Upper**
**Treatment**	Control GFP	Ctr Meg	-2.0698	0.4564

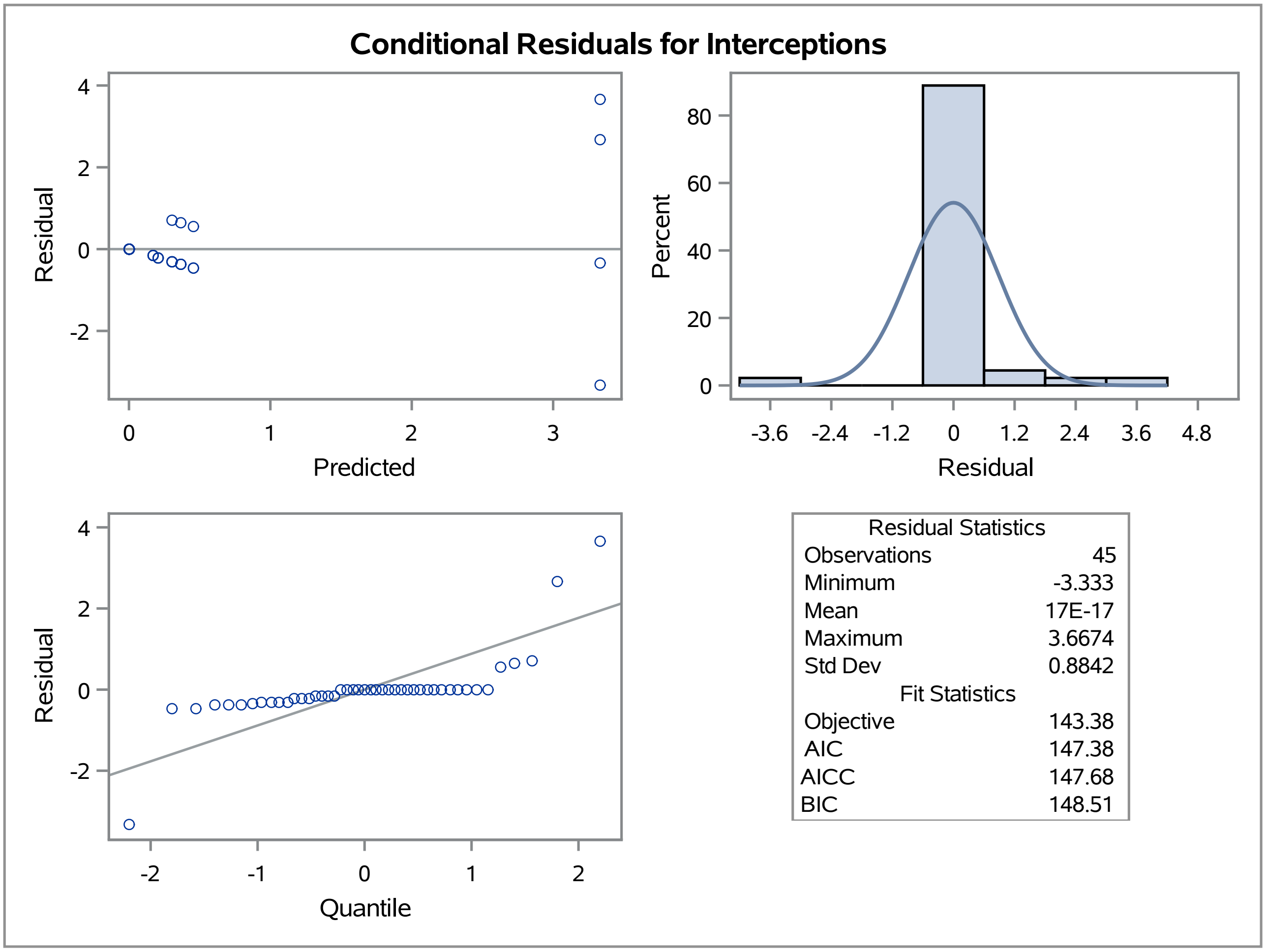

DistSoma=432

Model Information	
**Data Set**	WORK.TEMPDATASORTED
**Dependent Variable**	Interceptions
**Covariance Structure**	Variance Components
**Estimation Method**	REML
**Residual Variance Method**	Profile
**Fixed Effects SE Method**	Model-Based
**Degrees of Freedom Method**	Containment

Class Level Information		
**Class**	**Levels**	**Values**
**Treatment**	2	Control GFP Ctr Meg
**Culture**	13	1 2 3 4 5 6 7 8 9 10 11 12 13

Dimensions	
**Covariance Parameters**	2
**Columns in X**	3
**Columns in Z**	13
**Subjects**	1
**Max Obs per Subject**	45

Number of Observations	
**Number of Observations Read**	45
**Number of Observations Used**	45
**Number of Observations Not Used**	0

Iteration History			
**Iteration**	**Evaluations**	**-2 Res Log Like**	**Criterion**
**0**	1	155.64622379	
**1**	3	143.61228232	0.00561395
**2**	1	143.40165165	0.00051601
**3**	1	143.38397133	0.00000570
**4**	1	143.38378653	0.00000000

Convergence criteria met.

DistSoma=432

Covariance Parameter Estimates				
**Cov Parm**	**Estimate**	**Alpha**	**Lower**	**Upper**
**Culture**	0.9362	0.05	0.4033	4.0502
**Residual**	0.9895	0.05	0.6436	1.7150

Fit Statistics	
**-2 Res Log Likelihood**	143.4
**AIC (Smaller is Better)**	147.4
**AICC (Smaller is Better)**	147.7
**BIC (Smaller is Better)**	148.5

Solution for Fixed Effects									
**Effect**	**Treatment**	**Estimate**	**Standard Error**	**DF**	**t Value**	**Pr > |t|**	**Alpha**	**Lower**	**Upper**
**Intercept**		0.8067	0.4474	11	1.80	0.0988	0.05	-0.1780	1.7914
**Treatment**	Control GFP	-0.8067	0.6201	32	-1.30	0.2026	0.05	-2.0699	0.4564
**Treatment**	Ctr Meg	0	.	.	.	.	.	.	.

Solution for Random Effects									
**Effect**	**Culture**	**Estimate**	**Std Err Pred**	**DF**	**t Value**	**Pr > |t|**	**Alpha**	**Lower**	**Upper**
**Culture**	1	-671E-19	0.5871	32	-0.00	1.0000	0.05	-1.1960	1.1960
**Culture**	2	-671E-19	0.5871	32	-0.00	1.0000	0.05	-1.1960	1.1960
**Culture**	3	-11E-17	0.5577	32	-0.00	1.0000	0.05	-1.1360	1.1360
**Culture**	4	-11E-17	0.5577	32	-0.00	1.0000	0.05	-1.1360	1.1360
**Culture**	5	-11E-17	0.5577	32	-0.00	1.0000	0.05	-1.1360	1.1360
**Culture**	6	-678E-19	0.7243	32	-0.00	1.0000	0.05	-1.4754	1.4754
**Culture**	7	-671E-19	0.5871	32	-0.00	1.0000	0.05	-1.1960	1.1960
**Culture**	8	2.5259	0.5665	32	4.46	<.0001	0.05	1.3720	3.6798
**Culture**	9	-0.6381	0.5665	32	-1.13	0.2684	0.05	-1.7920	0.5158
**Culture**	10	-0.3500	0.5944	32	-0.59	0.5601	0.05	-1.5609	0.8608
**Culture**	11	-0.5008	0.5475	32	-0.91	0.3672	0.05	-1.6161	0.6144
**Culture**	12	-0.4404	0.5665	32	-0.78	0.4427	0.05	-1.5943	0.7136
**Culture**	13	-0.5965	0.5944	32	-1.00	0.3231	0.05	-1.8074	0.6143

Type 3 Tests of Fixed Effects				
**Effect**	**Num DF**	**Den DF**	**F Value**	**Pr > F**
**Treatment**	1	32	1.69	0.2026

DistSoma=432

Least Squares Means									
**Effect**	**Treatment**	**Estimate**	**Standard Error**	**DF**	**t Value**	**Pr > |t|**	**Alpha**	**Lower**	**Upper**
**Treatment**	Control GFP	0	0.4294	32	0.00	1.0000	0.05	-0.8747	0.8747
**Treatment**	Ctr Meg	0.8067	0.4474	32	1.80	0.0808	0.05	-0.1046	1.7180

Differences of Least Squares Means												
**Effect**	**Treatment**	**Treatment**	**Estimate**	**Standard Error**	**DF**	**t Value**	**Pr > |t|**	**Adjustment**	**Adj P**	**Alpha**	**Lower**	**Upper**
**Treatment**	Control GFP	Ctr Meg	-0.8067	0.6201	32	-1.30	0.2026	Tukey-Kramer	0.2026	0.05	-2.0699	0.4564

Differences of Least Squares Means				
**Effect**	**Treatment**	**Treatment**	**Adj Lower**	**Adj Upper**
**Treatment**	Control GFP	Ctr Meg	-2.0698	0.4564

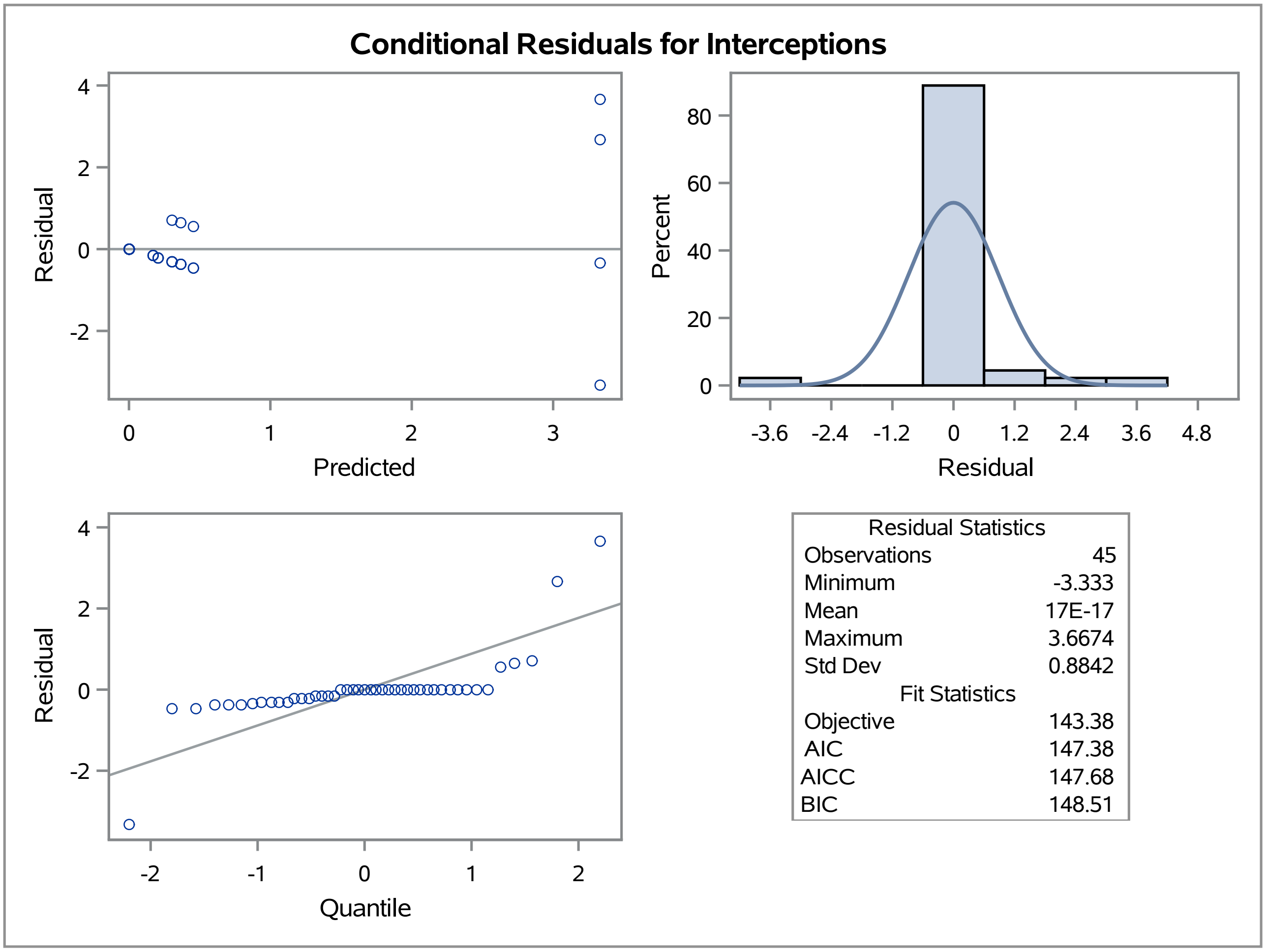

DistSoma=438

Model Information	
**Data Set**	WORK.TEMPDATASORTED
**Dependent Variable**	Interceptions
**Covariance Structure**	Variance Components
**Estimation Method**	REML
**Residual Variance Method**	Profile
**Fixed Effects SE Method**	Model-Based
**Degrees of Freedom Method**	Containment

Class Level Information		
**Class**	**Levels**	**Values**
**Treatment**	2	Control GFP Ctr Meg
**Culture**	13	1 2 3 4 5 6 7 8 9 10 11 12 13

Dimensions	
**Covariance Parameters**	2
**Columns in X**	3
**Columns in Z**	13
**Subjects**	1
**Max Obs per Subject**	45

Number of Observations	
**Number of Observations Read**	45
**Number of Observations Used**	45
**Number of Observations Not Used**	0

Iteration History			
**Iteration**	**Evaluations**	**-2 Res Log Like**	**Criterion**
**0**	1	154.10114739	
**1**	5	144.37798943	0.00022020
**2**	1	144.37118179	0.00000126
**3**	1	144.37114032	0.00000000

Convergence criteria met.

DistSoma=438

Covariance Parameter Estimates				
**Cov Parm**	**Estimate**	**Alpha**	**Lower**	**Upper**
**Culture**	0.8115	0.05	0.3376	3.9092
**Residual**	1.0518	0.05	0.6844	1.8216

Fit Statistics	
**-2 Res Log Likelihood**	144.4
**AIC (Smaller is Better)**	148.4
**AICC (Smaller is Better)**	148.7
**BIC (Smaller is Better)**	149.5

Solution for Fixed Effects									
**Effect**	**Treatment**	**Estimate**	**Standard Error**	**DF**	**t Value**	**Pr > |t|**	**Alpha**	**Lower**	**Upper**
**Intercept**		0.7135	0.4267	11	1.67	0.1227	0.05	-0.2257	1.6527
**Treatment**	Control GFP	-0.7135	0.5927	32	-1.20	0.2375	0.05	-1.9208	0.4937
**Treatment**	Ctr Meg	0	.	.	.	.	.	.	.

Solution for Random Effects									
**Effect**	**Culture**	**Estimate**	**Std Err Pred**	**DF**	**t Value**	**Pr > |t|**	**Alpha**	**Lower**	**Upper**
**Culture**	1	-19E-17	0.5721	32	-0.00	1.0000	0.05	-1.1654	1.1654
**Culture**	2	-19E-17	0.5721	32	-0.00	1.0000	0.05	-1.1654	1.1654
**Culture**	3	-138E-18	0.5432	32	-0.00	1.0000	0.05	-1.1065	1.1065
**Culture**	4	-138E-18	0.5432	32	-0.00	1.0000	0.05	-1.1065	1.1065
**Culture**	5	-138E-18	0.5432	32	-0.00	1.0000	0.05	-1.1065	1.1065
**Culture**	6	-797E-19	0.7001	32	-0.00	1.0000	0.05	-1.4261	1.4261
**Culture**	7	-19E-17	0.5721	32	-0.00	1.0000	0.05	-1.1654	1.1654
**Culture**	8	2.2933	0.5500	32	4.17	0.0002	0.05	1.1731	3.4136
**Culture**	9	-0.5389	0.5500	32	-0.98	0.3345	0.05	-1.6591	0.5813
**Culture**	10	-0.4983	0.5776	32	-0.86	0.3948	0.05	-1.6748	0.6783
**Culture**	11	-0.4078	0.5309	32	-0.77	0.4481	0.05	-1.4893	0.6737
**Culture**	12	-0.3501	0.5500	32	-0.64	0.5289	0.05	-1.4703	0.7702
**Culture**	13	-0.4983	0.5776	32	-0.86	0.3948	0.05	-1.6748	0.6783

Type 3 Tests of Fixed Effects				
**Effect**	**Num DF**	**Den DF**	**F Value**	**Pr > F**
**Treatment**	1	32	1.45	0.2375

DistSoma=438

Least Squares Means									
**Effect**	**Treatment**	**Estimate**	**Standard Error**	**DF**	**t Value**	**Pr > |t|**	**Alpha**	**Lower**	**Upper**
**Treatment**	Control GFP	1.11E-16	0.4113	32	0.00	1.0000	0.05	-0.8378	0.8378
**Treatment**	Ctr Meg	0.7135	0.4267	32	1.67	0.1042	0.05	-0.1557	1.5827

Differences of Least Squares Means													
**Effect**	**Treatment**	**Treatment**	**Estimate**	**Standard Error**	**DF**	**t Value**	**Pr > |t|**	**Adjustment**	**Adj P**	**Alpha**	**Lower**	**Upper**
**Treatment**	Control GFP	Ctr Meg	-0.7135	0.5927	32	-1.20	0.2375	Tukey-Kramer	0.2375	0.05	-1.9208	0.4937

Differences of Least Squares Means				
**Effect**	**Treatment**	**Treatment**	**Adj Lower**	**Adj Upper**
**Treatment**	Control GFP	Ctr Meg	-1.9208	0.4937

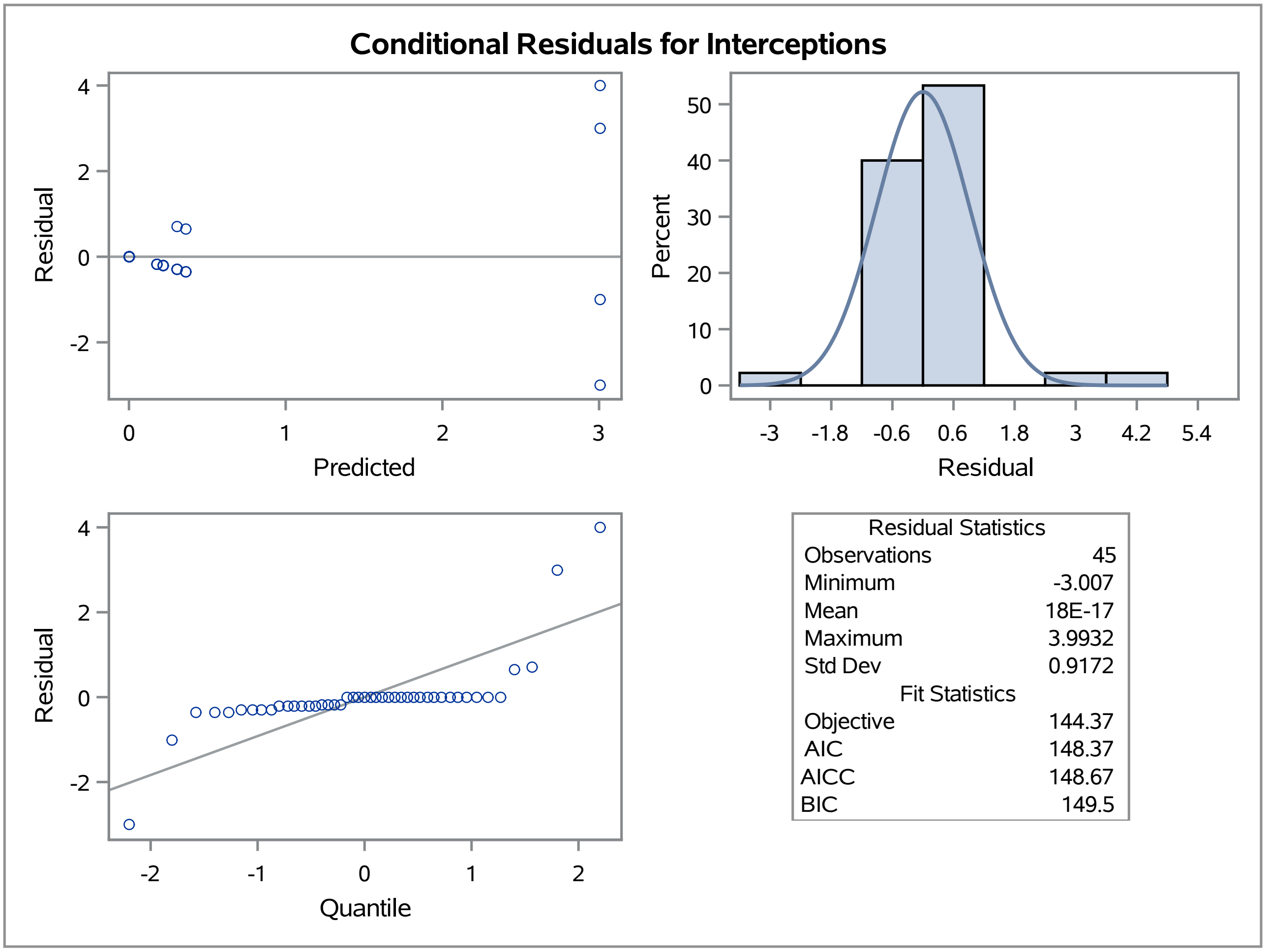

DistSoma=444

Model Information	
**Data Set**	WORK.TEMPDATASORTED
**Dependent Variable**	Interceptions
**Covariance Structure**	Variance Components
**Estimation Method**	REML
**Residual Variance Method**	Profile
**Fixed Effects SE Method**	Model-Based
**Degrees of Freedom Method**	Containment

Class Level Information		
**Class**	**Levels**	**Values**
**Treatment**	2	Control GFP Ctr Meg
**Culture**	13	1 2 3 4 5 6 7 8 9 10 11 12 13

Dimensions	
**Covariance Parameters**	2
**Columns in X**	3
**Columns in Z**	13
**Subjects**	1
**Max Obs per Subject**	45

Number of Observations	
**Number of Observations Read**	45
**Number of Observations Used**	45
**Number of Observations Not Used**	0

Iteration History			
**Iteration**	**Evaluations**	**-2 Res Log Like**	**Criterion**
**0**	1	154.10114739	
**1**	5	144.37798943	0.00022020
**2**	1	144.37118179	0.00000126
**3**	1	144.37114032	0.00000000

Convergence criteria met.

DistSoma=444

Covariance Parameter Estimates				
Cov Parm	Estimate	Alpha	Lower	Upper
Culture	0.8115	0.05	0.3376	3.9092
Residual	1.0518	0.05	0.6844	1.8216

Fit Statistics	
-2 Res Log Likelihood	144.4
AIC (Smaller is Better)	148.4
AICC (Smaller is Better)	148.7
BIC (Smaller is Better)	149.5

Solution for Fixed Effects									
Effect	Treatment	Estimate	Standard Error	DF	t Value	Pr > |t|	Alpha	Lower	Upper
Intercept		0.7135	0.4267	11	1.67	0.1227	0.05	-0.2257	1.6527
Treatment	Control GFP	-0.7135	0.5927	32	-1.20	0.2375	0.05	-1.9208	0.4937
Treatment	Ctr Meg	0	.	.	.	.	.	.	.

Solution for Random Effects									
Effect	Culture	Estimate	Std Err Pred	DF	t Value	Pr > |t|	Alpha	Lower	Upper
Culture	1	-19E-17	0.5721	32	-0.00	1.0000	0.05	-1.1654	1.1654
Culture	2	-19E-17	0.5721	32	-0.00	1.0000	0.05	-1.1654	1.1654
Culture	3	-138E-18	0.5432	32	-0.00	1.0000	0.05	-1.1065	1.1065
Culture	4	-138E-18	0.5432	32	-0.00	1.0000	0.05	-1.1065	1.1065
Culture	5	-138E-18	0.5432	32	-0.00	1.0000	0.05	-1.1065	1.1065
Culture	6	-797E-19	0.7001	32	-0.00	1.0000	0.05	-1.4261	1.4261
Culture	7	-19E-17	0.5721	32	-0.00	1.0000	0.05	-1.1654	1.1654
Culture	8	2.2933	0.5500	32	4.17	0.0002	0.05	1.1731	3.4136
Culture	9	-0.5389	0.5500	32	-0.98	0.3345	0.05	-1.6591	0.5813
Culture	10	-0.4983	0.5776	32	-0.86	0.3948	0.05	-1.6748	0.6783
Culture	11	-0.4078	0.5309	32	-0.77	0.4481	0.05	-1.4893	0.6737
Culture	12	-0.3501	0.5500	32	-0.64	0.5289	0.05	-1.4703	0.7702
Culture	13	-0.4983	0.5776	32	-0.86	0.3948	0.05	-1.6748	0.6783

Type 3 Tests of Fixed Effects				
Effect	Num DF	Den DF	F Value	Pr > F
Treatment	1	32	1.45	0.2375

DistSoma=444

Least Squares Means									
Effect	Treatment	Estimate	Standard Error	DF	t Value	Pr > |t|	Alpha	Lower	Upper
Treatment	Control GFP	1.11E-16	0.4113	32	0.00	1.0000	0.05	-0.8378	0.8378
Treatment	Ctr Meg	0.7135	0.4267	32	1.67	0.1042	0.05	-0.1557	1.5827

Differences of Least Squares Means													
Effect	Treatment	Treatment	Estimate	Standard Error	DF	t Value	Pr > |t|	Adjustment	Adj P	Alpha	Lower	Upper
Treatment	Control GFP	Ctr Meg	-0.7135	0.5927	32	-1.20	0.2375	Tukey-Kramer	0.2375	0.05	-1.9208	0.4937

Differences of Least Squares Means				
Effect	Treatment	Treatment	Adj Lower	Adj Upper
Treatment	Control GFP	Ctr Meg	-1.9208	0.4937

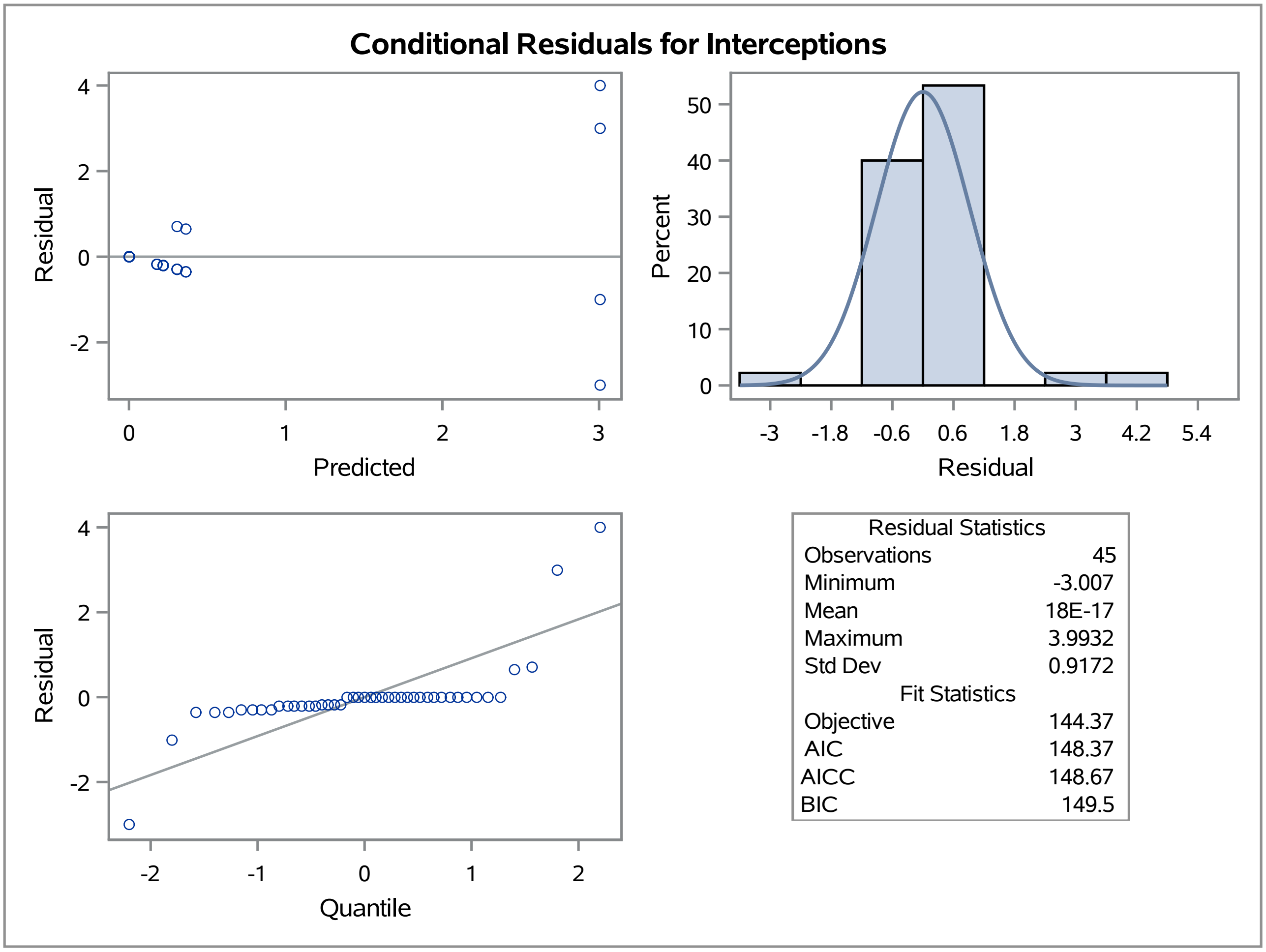

DistSoma=450

Model Information	
**Data Set**	WORK.TEMPDATASORTED
**Dependent Variable**	Interceptions
**Covariance Structure**	Variance Components
**Estimation Method**	REML
**Residual Variance Method**	Profile
**Fixed Effects SE Method**	Model-Based
**Degrees of Freedom Method**	Containment

Class Level Information		
**Class**	**Levels**	**Values**
**Treatment**	2	Control GFP Ctr Meg
**Culture**	13	1 2 3 4 5 6 7 8 9 10 11 12 13

Dimensions	
**Covariance Parameters**	2
**Columns in X**	3
**Columns in Z**	13
**Subjects**	1
**Max Obs per Subject**	45

Number of Observations	
**Number of Observations Read**	45
**Number of Observations Used**	45
**Number of Observations Not Used**	0

Iteration History			
**Iteration**	**Evaluations**	**-2 Res Log Like**	**Criterion**
**0**	1	143.44419234	
**1**	3	135.22651716	0.00467566
**2**	1	135.07600117	0.00033120
**3**	1	135.06625617	0.00000211
**4**	1	135.06619685	0.00000000

Convergence criteria met.

DistSoma=450

Covariance Parameter Estimates				
**Cov Parm**	**Estimate**	**Alpha**	**Lower**	**Upper**
**Culture**	0.5881	0.05	0.2387	3.0730
**Residual**	0.8668	0.05	0.5642	1.5003

Fit Statistics	
**-2 Res Log Likelihood**	135.1
**AIC (Smaller is Better)**	139.1
**AICC (Smaller is Better)**	139.4
**BIC (Smaller is Better)**	140.2

Solution for Fixed Effects									
**Effect**	**Treatment**	**Estimate**	**Standard Error**	**DF**	**t Value**	**Pr > |t|**	**Alpha**	**Lower**	**Upper**
**Intercept**		0.6299	0.3696	11	1.70	0.1164	0.05	-0.1836	1.4433
**Treatment**	Control GFP	-0.6299	0.5140	32	-1.23	0.2294	0.05	-1.6769	0.4171
**Treatment**	Ctr Meg	0	.	.	.	.	.	.	.

Solution for Random Effects									
**Effect**	**Culture**	**Estimate**	**Std Err Pred**	**DF**	**t Value**	**Pr > |t|**	**Alpha**	**Lower**	**Upper**
**Culture**	1	-614E-19	0.5011	32	-0.00	1.0000	0.05	-1.0208	1.0208
**Culture**	2	-614E-19	0.5011	32	-0.00	1.0000	0.05	-1.0208	1.0208
**Culture**	3	-105E-18	0.4759	32	-0.00	1.0000	0.05	-0.9694	0.9694
**Culture**	4	-105E-18	0.4759	32	-0.00	1.0000	0.05	-0.9694	0.9694
**Culture**	5	-105E-18	0.4759	32	-0.00	1.0000	0.05	-0.9694	0.9694
**Culture**	6	-578E-19	0.6093	32	-0.00	1.0000	0.05	-1.2411	1.2411
**Culture**	7	-614E-19	0.5011	32	-0.00	1.0000	0.05	-1.0208	1.0208
**Culture**	8	1.9147	0.4809	32	3.98	0.0004	0.05	0.9351	2.8943
**Culture**	9	-0.4603	0.4809	32	-0.96	0.3457	0.05	-1.4399	0.5193
**Culture**	10	-0.4224	0.5051	32	-0.84	0.4093	0.05	-1.4513	0.6065
**Culture**	11	-0.3320	0.4641	32	-0.72	0.4795	0.05	-1.2773	0.6133
**Culture**	12	-0.2776	0.4809	32	-0.58	0.5678	0.05	-1.2572	0.7020
**Culture**	13	-0.4224	0.5051	32	-0.84	0.4093	0.05	-1.4513	0.6065

Type 3 Tests of Fixed Effects				
**Effect**	**Num DF**	**Den DF**	**F Value**	**Pr > F**
**Treatment**	1	32	1.50	0.2294

DistSoma=450

Least Squares Means									
**Effect**	**Treatment**	**Estimate**	**Standard Error**	**DF**	**t Value**	**Pr > |t|**	**Alpha**	**Lower**	**Upper**
**Treatment**	Control GFP	0	0.3572	32	0.00	1.0000	0.05	-0.7277	0.7277
**Treatment**	Ctr Meg	0.6299	0.3696	32	1.70	0.0980	0.05	-0.1229	1.3827

Differences of Least Squares Means												
**Effect**	**Treatment**	**Treatment**	**Estimate**	**Standard Error**	**DF**	**t Value**	**Pr > |t|**	**Adjustment**	**Adj P**	**Alpha**	**Lower**	**Upper**
**Treatment**	Control GFP	Ctr Meg	-0.6299	0.5140	32	-1.23	0.2294	Tukey-Kramer	0.2294	0.05	-1.6769	0.4171

Differences of Least Squares Means				
**Effect**	**Treatment**	**Treatment**	**Adj Lower**	**Adj Upper**
**Treatment**	Control GFP	Ctr Meg	-1.6769	0.4171

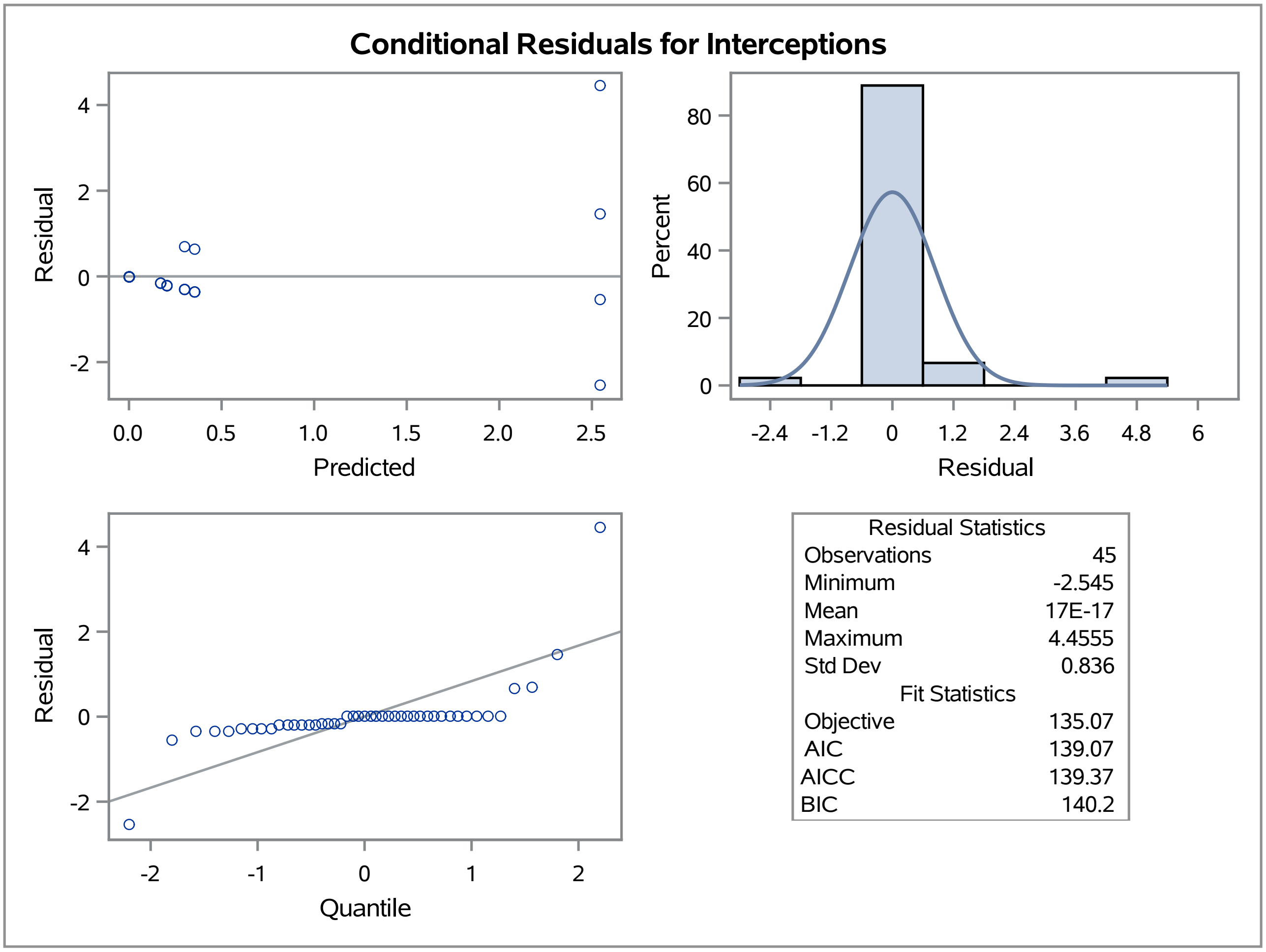

Residual Statistics	
Observations	45
Minimum	-2.545
Mean	17E-17
Maximum	4.4555
Std Dev	0.836
**Fit Statistics**	
Objective	135.07
AIC	139.07
AICC	139.37
BIC	140.2

DistSoma=456

Model Information	
**Data Set**	WORK.TEMPDATASORTED
**Dependent Variable**	Interceptions
**Covariance Structure**	Variance Components
**Estimation Method**	REML
**Residual Variance Method**	Profile
**Fixed Effects SE Method**	Model-Based
**Degrees of Freedom Method**	Containment

Class Level Information		
**Class**	**Levels**	**Values**
**Treatment**	2	Control GFP Ctr Meg
**Culture**	13	1 2 3 4 5 6 7 8 9 10 11 12 13

Dimensions	
**Covariance Parameters**	2
**Columns in X**	3
**Columns in Z**	13
**Subjects**	1
**Max Obs per Subject**	45

Number of Observations	
**Number of Observations Read**	45
**Number of Observations Used**	45
**Number of Observations Not Used**	0

Iteration History			
**Iteration**	**Evaluations**	**-2 Res Log Like**	**Criterion**
**0**	1	156.23996663	
**1**	3	148.13911355	0.00397358
**2**	1	147.98150498	0.00029184
**3**	1	147.97092253	0.00000201
**4**	1	147.97085304	0.00000000

Convergence criteria met.

DistSoma=456

Covariance Parameter Estimates				
Cov Parm	Estimate	Alpha	Lower	Upper
Culture	0.7856	0.05	0.3181	4.1371
Residual	1.1727	0.05	0.7635	2.0297

Fit Statistics	
-2 Res Log Likelihood	148.0
AIC (Smaller is Better)	152.0
AICC (Smaller is Better)	152.3
BIC (Smaller is Better)	153.1

Solution for Fixed Effects									
Effect	Treatment	Estimate	Standard Error	DF	t Value	Pr > |t|	Alpha	Lower	Upper
Intercept		0.7149	0.4279	11	1.67	0.1230	0.05	-0.2270	1.6567
Treatment	Control GFP	-0.7149	0.5952	32	-1.20	0.2386	0.05	-1.9273	0.4976
Treatment	Ctr Meg	0	.	.	.	.	.	.	.

Solution for Random Effects									
Effect	Culture	Estimate	Std Err Pred	DF	t Value	Pr > |t|	Alpha	Lower	Upper
Culture	1	3.07E-17	0.5808	32	0.00	1.0000	0.05	-1.1831	1.1831
Culture	2	3.07E-17	0.5808	32	0.00	1.0000	0.05	-1.1831	1.1831
Culture	3	5.11E-17	0.5516	32	0.00	1.0000	0.05	-1.1236	1.1236
Culture	4	5.11E-17	0.5516	32	0.00	1.0000	0.05	-1.1236	1.1236
Culture	5	5.11E-17	0.5516	32	0.00	1.0000	0.05	-1.1236	1.1236
Culture	6	2.81E-17	0.7057	32	0.00	1.0000	0.05	-1.4375	1.4375
Culture	7	3.07E-17	0.5808	32	0.00	1.0000	0.05	-1.1831	1.1831
Culture	8	2.2103	0.5573	32	3.97	0.0004	0.05	1.0750	3.3455
Culture	9	-0.5206	0.5573	32	-0.93	0.3573	0.05	-1.6558	0.6147
Culture	10	-0.4773	0.5854	32	-0.82	0.4209	0.05	-1.6697	0.7151
Culture	11	-0.3965	0.5378	32	-0.74	0.4663	0.05	-1.4919	0.6990
Culture	12	-0.3385	0.5573	32	-0.61	0.5479	0.05	-1.4738	0.7967
Culture	13	-0.4773	0.5854	32	-0.82	0.4209	0.05	-1.6697	0.7151

Type 3 Tests of Fixed Effects				
Effect	Num DF	Den DF	F Value	Pr > F
Treatment	1	32	1.44	0.2386

DistSoma=456

Least Squares Means									
**Effect**	**Treatment**	**Estimate**	**Standard Error**	**DF**	**t Value**	**Pr > |t|**	**Alpha**	**Lower**	**Upper**
**Treatment**	Control GFP	0	0.4138	32	0.00	1.0000	0.05	-0.8428	0.8428
**Treatment**	Ctr Meg	0.7149	0.4279	32	1.67	0.1046	0.05	-0.1568	1.5865

Differences of Least Squares Means												
**Effect**	**Treatment**	**Treatment**	**Estimate**	**Standard Error**	**DF**	**t Value**	**Pr > |t|**	**Adjustment**	**Adj P**	**Alpha**	**Lower**	**Upper**
**Treatment**	Control GFP	Ctr Meg	-0.7149	0.5952	32	-1.20	0.2386	Tukey-Kramer	0.2386	0.05	-1.9273	0.4976

Differences of Least Squares Means				
**Effect**	**Treatment**	**Treatment**	**Adj Lower**	**Adj Upper**
**Treatment**	Control GFP	Ctr Meg	-1.9273	0.4976

Conditional Residuals for Interceptions

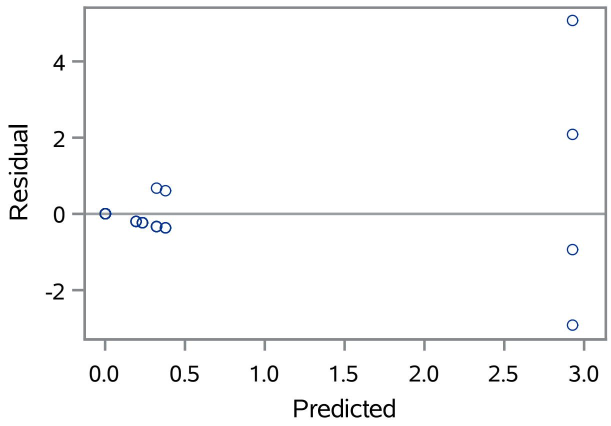

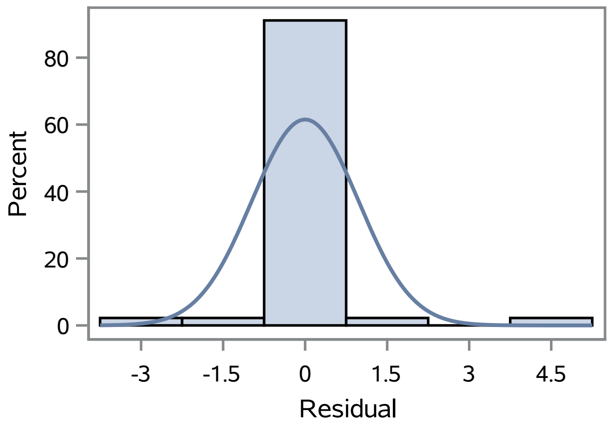

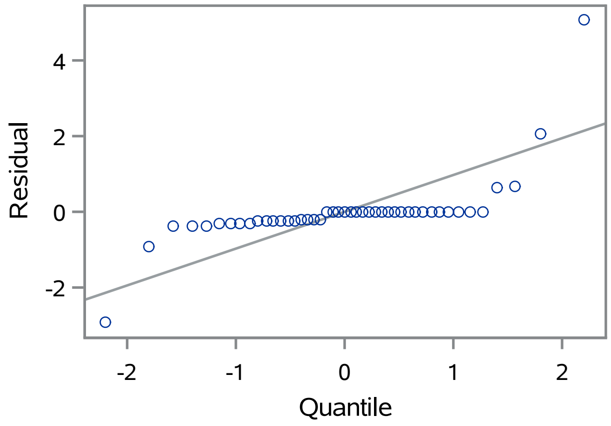

Residual Statistics	
Observations	45
Minimum	-2.925
Mean	69E-18
Maximum	5.0748
Std Dev	0.9729
**Fit Statistics**	
Objective	147.97
AIC	151.97
AICC	152.27
BIC	153.1

DistSoma=462

Model Information	
**Data Set**	WORK.TEMPDATASORTED
**Dependent Variable**	Interceptions
**Covariance Structure**	Variance Components
**Estimation Method**	REML
**Residual Variance Method**	Profile
**Fixed Effects SE Method**	Model-Based
**Degrees of Freedom Method**	Containment

Class Level Information		
**Class**	**Levels**	**Values**
**Treatment**	2	Control GFP Ctr Meg
**Culture**	13	1 2 3 4 5 6 7 8 9 10 11 12 13

Dimensions	
**Covariance Parameters**	2
**Columns in X**	3
**Columns in Z**	13
**Subjects**	1
**Max Obs per Subject**	45

Number of Observations	
**Number of Observations Read**	45
**Number of Observations Used**	45
**Number of Observations Not Used**	0

Iteration History			
**Iteration**	**Evaluations**	**-2 Res Log Like**	**Criterion**
**0**	1	148.88724370	
**1**	3	142.89561066	0.00205035
**2**	1	142.82271589	0.00007994
**3**	1	142.82009577	0.00000015
**4**	1	142.82009112	0.00000000

Convergence criteria met.

DistSoma=462

Covariance Parameter Estimates				
**Cov Parm**	**Estimate**	**Alpha**	**Lower**	**Upper**
**Culture**	0.5642	0.05	0.2156	3.6461
**Residual**	1.0854	0.05	0.7072	1.8761

Fit Statistics	
**-2 Res Log Likelihood**	142.8
**AIC (Smaller is Better)**	146.8
**AICC (Smaller is Better)**	147.1
**BIC (Smaller is Better)**	147.9

Solution for Fixed Effects									
**Effect**	**Treatment**	**Estimate**	**Standard Error**	**DF**	**t Value**	**Pr > |t|**	**Alpha**	**Lower**	**Upper**
**Intercept**		0.5897	0.3772	11	1.56	0.1462	0.05	-0.2404	1.4198
**Treatment**	Control GFP	-0.5897	0.5260	32	-1.12	0.2706	0.05	-1.6612	0.4818
**Treatment**	Ctr Meg	0	.	.	.	.	.	.	.

Solution for Random Effects									
**Effect**	**Culture**	**Estimate**	**Std Err Pred**	**DF**	**t Value**	**Pr > |t|**	**Alpha**	**Lower**	**Upper**
**Culture**	1	-919E-19	0.5200	32	-0.00	1.0000	0.05	-1.0591	1.0591
**Culture**	2	-919E-19	0.5200	32	-0.00	1.0000	0.05	-1.0591	1.0591
**Culture**	3	-113E-18	0.4945	32	-0.00	1.0000	0.05	-1.0073	1.0073
**Culture**	4	-113E-18	0.4945	32	-0.00	1.0000	0.05	-1.0073	1.0073
**Culture**	5	-113E-18	0.4945	32	-0.00	1.0000	0.05	-1.0073	1.0073
**Culture**	6	-573E-19	0.6221	32	-0.00	1.0000	0.05	-1.2671	1.2671
**Culture**	7	-919E-19	0.5200	32	-0.00	1.0000	0.05	-1.0591	1.0591
**Culture**	8	1.7964	0.4981	32	3.61	0.0010	0.05	0.7818	2.8109
**Culture**	9	-0.3982	0.4981	32	-0.80	0.4299	0.05	-1.4128	0.6164
**Culture**	10	-0.3593	0.5227	32	-0.69	0.4968	0.05	-1.4241	0.7055
**Culture**	11	-0.2814	0.4806	32	-0.59	0.5623	0.05	-1.2603	0.6975
**Culture**	12	-0.3982	0.4981	32	-0.80	0.4299	0.05	-1.4128	0.6164
**Culture**	13	-0.3593	0.5227	32	-0.69	0.4968	0.05	-1.4241	0.7055

Type 3 Tests of Fixed Effects				
**Effect**	**Num DF**	**Den DF**	**F Value**	**Pr > F**
**Treatment**	1	32	1.26	0.2706

DistSoma=462

Least Squares Means									
**Effect**	**Treatment**	**Estimate**	**Standard Error**	**DF**	**t Value**	**Pr > |t|**	**Alpha**	**Lower**	**Upper**
**Treatment**	Control GFP	0	0.3667	32	0.00	1.0000	0.05	-0.7470	0.7470
**Treatment**	Ctr Meg	0.5897	0.3772	32	1.56	0.1278	0.05	-0.1786	1.3579

Differences of Least Squares Means												
**Effect**	**Treatment**	**Treatment**	**Estimate**	**Standard Error**	**DF**	**t Value**	**Pr > |t|**	**Adjustment**	**Adj P**	**Alpha**	**Lower**	**Upper**
**Treatment**	Control GFP	Ctr Meg	-0.5897	0.5260	32	-1.12	0.2706	Tukey-Kramer	0.2706	0.05	-1.6612	0.4818

Differences of Least Squares Means				
**Effect**	**Treatment**	**Treatment**	**Adj Lower**	**Adj Upper**
**Treatment**	Control GFP	Ctr Meg	-1.6612	0.4818

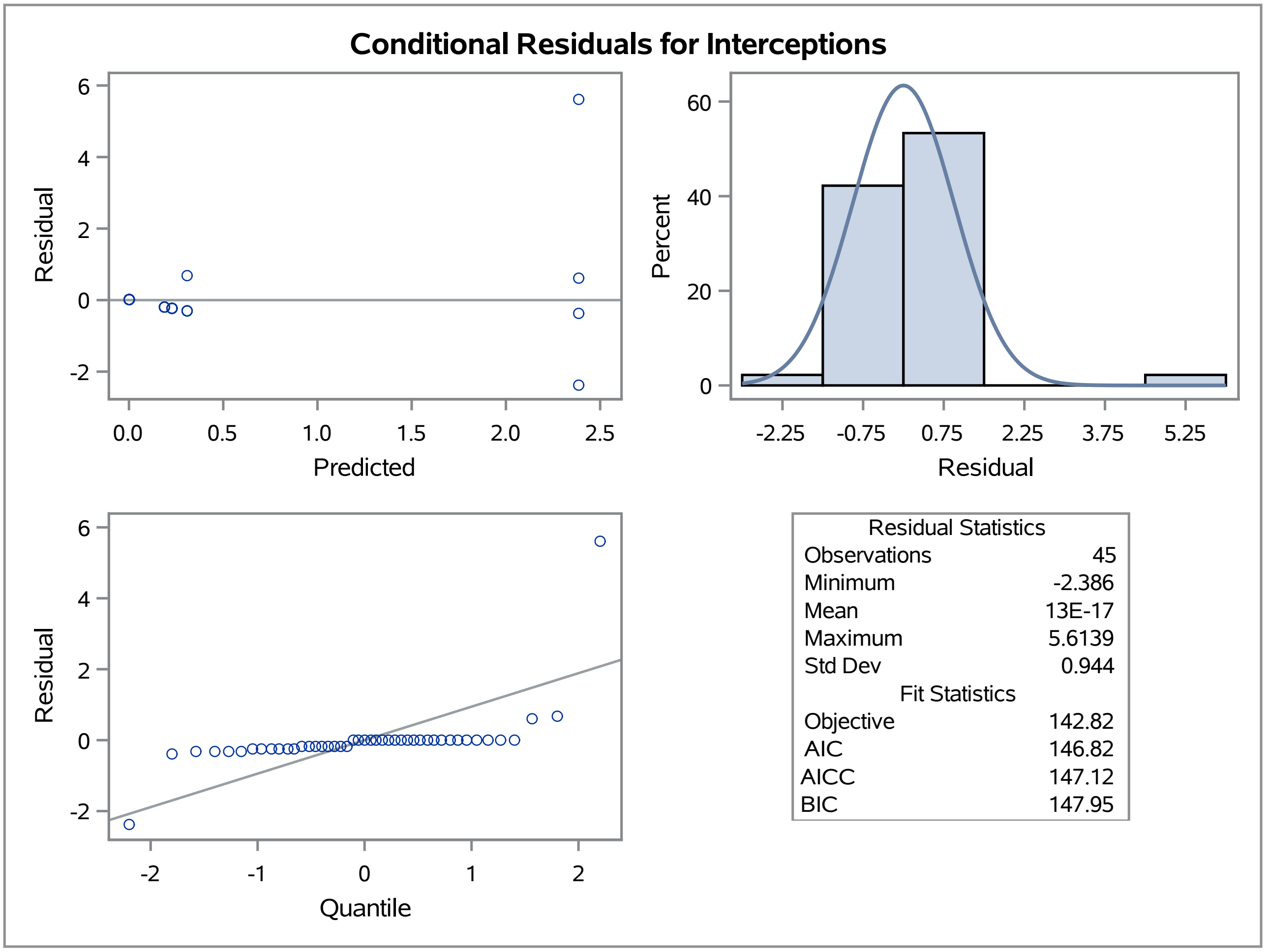

DistSoma=468

Model Information	
**Data Set**	WORK.TEMPDATASORTED
**Dependent Variable**	Interceptions
**Covariance Structure**	Variance Components
**Estimation Method**	REML
**Residual Variance Method**	Profile
**Fixed Effects SE Method**	Model-Based
**Degrees of Freedom Method**	Containment

Class Level Information		
**Class**	**Levels**	**Values**
**Treatment**	2	Control GFP Ctr Meg
**Culture**	13	1 2 3 4 5 6 7 8 9 10 11 12 13

Dimensions	
**Covariance Parameters**	2
**Columns in X**	3
**Columns in Z**	13
**Subjects**	1
**Max Obs per Subject**	45

Number of Observations	
**Number of Observations Read**	45
**Number of Observations Used**	45
**Number of Observations Not Used**	0

Iteration History			
**Iteration**	**Evaluations**	**-2 Res Log Like**	**Criterion**
**0**	1	146.45158625	
**1**	3	142.33477562	0.00087164
**2**	1	142.30496883	0.00001557
**3**	1	142.30446998	0.00000001

Convergence criteria met.

DistSoma=468

Covariance Parameter Estimates				
**Cov Parm**	**Estimate**	**Alpha**	**Lower**	**Upper**
**Culture**	0.4367	0.05	0.1535	3.9444
**Residual**	1.1203	0.05	0.7304	1.9338

Fit Statistics	
**-2 Res Log Likelihood**	142.3
**AIC (Smaller is Better)**	146.3
**AICC (Smaller is Better)**	146.6
**BIC (Smaller is Better)**	147.4

Solution for Fixed Effects									
**Effect**	**Treatment**	**Estimate**	**Standard Error**	**DF**	**t Value**	**Pr > |t|**	**Alpha**	**Lower**	**Upper**
**Intercept**		0.5495	0.3499	11	1.57	0.1446	0.05	-0.2206	1.3196
**Treatment**	Control GFP	-0.5495	0.4895	32	-1.12	0.2700	0.05	-1.5467	0.4477
**Treatment**	Ctr Meg	0	.	.	.	.	.	.	.

Solution for Random Effects									
**Effect**	**Culture**	**Estimate**	**Std Err Pred**	**DF**	**t Value**	**Pr > |t|**	**Alpha**	**Lower**	**Upper**
**Culture**	1	2.52E-17	0.4851	32	0.00	1.0000	0.05	-0.9882	0.9882
**Culture**	2	2.52E-17	0.4851	32	0.00	1.0000	0.05	-0.9882	0.9882
**Culture**	3	6.26E-18	0.4628	32	0.00	1.0000	0.05	-0.9426	0.9426
**Culture**	4	6.26E-18	0.4628	32	0.00	1.0000	0.05	-0.9426	0.9426
**Culture**	5	6.26E-18	0.4628	32	0.00	1.0000	0.05	-0.9426	0.9426
**Culture**	6	2.88E-18	0.5687	32	0.00	1.0000	0.05	-1.1584	1.1584
**Culture**	7	2.52E-17	0.4851	32	0.00	1.0000	0.05	-0.9882	0.9882
**Culture**	8	1.4930	0.4648	32	3.21	0.0030	0.05	0.5461	2.4399
**Culture**	9	-0.3348	0.4648	32	-0.72	0.4766	0.05	-1.2817	0.6121
**Culture**	10	-0.2962	0.4867	32	-0.61	0.5471	0.05	-1.2876	0.6952
**Culture**	11	-0.2310	0.4490	32	-0.51	0.6104	0.05	-1.1455	0.6835
**Culture**	12	-0.3348	0.4648	32	-0.72	0.4766	0.05	-1.2817	0.6121
**Culture**	13	-0.2962	0.4867	32	-0.61	0.5471	0.05	-1.2876	0.6952

Type 3 Tests of Fixed Effects				
**Effect**	**Num DF**	**Den DF**	**F Value**	**Pr > F**
**Treatment**	1	32	1.26	0.2700

DistSoma=468

Least Squares Means									
Effect	Treatment	Estimate	Standard Error	DF	t Value	Pr > |t|	Alpha	Lower	Upper
Treatment	Control GFP	0	0.3424	32	0.00	1.0000	0.05	-0.6974	0.6974
Treatment	Ctr Meg	0.5495	0.3499	32	1.57	0.1262	0.05	-0.1632	1.2622

Differences of Least Squares Means												
Effect	Treatment	Treatment	Estimate	Standard Error	DF	t Value	Pr > |t|	Adjustment	Adj P	Alpha	Lower	Upper
Treatment	Control GFP	Ctr Meg	-0.5495	0.4895	32	-1.12	0.2700	Tukey-Kramer	0.2700	0.05	-1.5467	0.4477

Differences of Least Squares Means				
Effect	Treatment	Treatment	Adj Lower	Adj Upper
Treatment	Control GFP	Ctr Meg	-1.5467	0.4477

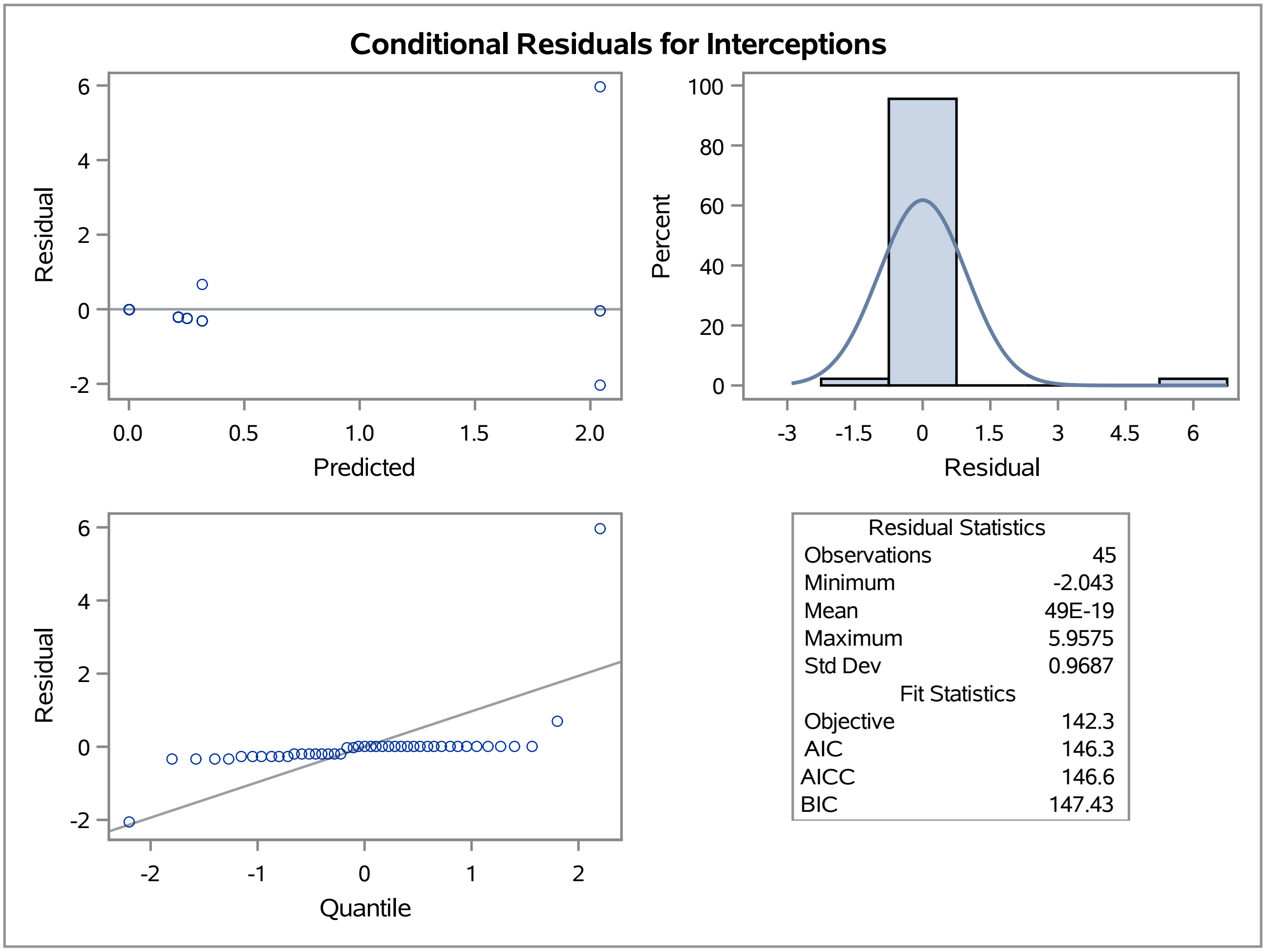

DistSoma=474

Model Information	
**Data Set**	WORK.TEMPDATASORTED
**Dependent Variable**	Interceptions
**Covariance Structure**	Variance Components
**Estimation Method**	REML
**Residual Variance Method**	Profile
**Fixed Effects SE Method**	Model-Based
**Degrees of Freedom Method**	Containment

Class Level Information		
**Class**	**Levels**	**Values**
**Treatment**	2	Control GFP Ctr Meg
**Culture**	13	1 2 3 4 5 6 7 8 9 10 11 12 13

Dimensions	
**Covariance Parameters**	2
**Columns in X**	3
**Columns in Z**	13
**Subjects**	1
**Max Obs per Subject**	45

Number of Observations	
**Number of Observations Read**	45
**Number of Observations Used**	45
**Number of Observations Not Used**	0

Iteration History			
**Iteration**	**Evaluations**	**-2 Res Log Like**	**Criterion**
**0**	1	145.17998821	
**1**	3	142.91150036	0.00024750
**2**	1	142.90322604	0.00000136
**3**	1	142.90318235	0.00000000

Convergence criteria met.

DistSoma=474

Covariance Parameter Estimates				
**Cov Parm**	**Estimate**	**Alpha**	**Lower**	**Upper**
**Culture**	0.3085	0.05	0.09248	6.2643
**Residual**	1.1990	0.05	0.7826	2.0662

Fit Statistics	
**-2 Res Log Likelihood**	142.9
**AIC (Smaller is Better)**	146.9
**AICC (Smaller is Better)**	147.2
**BIC (Smaller is Better)**	148.0

Solution for Fixed Effects									
**Effect**	**Treatment**	**Estimate**	**Standard Error**	**DF**	**t Value**	**Pr > |t|**	**Alpha**	**Lower**	**Upper**
**Intercept**		0.5100	0.3231	11	1.58	0.1428	0.05	-0.2011	1.2211
**Treatment**	Control GFP	-0.5100	0.4540	32	-1.12	0.2696	0.05	-1.4347	0.4147
**Treatment**	Ctr Meg	0	.	.	.	.	.	.	.

Solution for Random Effects									
**Effect**	**Culture**	**Estimate**	**Std Err Pred**	**DF**	**t Value**	**Pr > |t|**	**Alpha**	**Lower**	**Upper**
**Culture**	1	3.31E-17	0.4398	32	0.00	1.0000	0.05	-0.8958	0.8958
**Culture**	2	3.31E-17	0.4398	32	0.00	1.0000	0.05	-0.8958	0.8958
**Culture**	3	2.86E-17	0.4221	32	0.00	1.0000	0.05	-0.8599	0.8599
**Culture**	4	2.86E-17	0.4221	32	0.00	1.0000	0.05	-0.8599	0.8599
**Culture**	5	2.86E-17	0.4221	32	0.00	1.0000	0.05	-0.8599	0.8599
**Culture**	6	1.15E-17	0.4996	32	0.00	1.0000	0.05	-1.0177	1.0177
**Culture**	7	3.31E-17	0.4398	32	0.00	1.0000	0.05	-0.8958	0.8958
**Culture**	8	1.1362	0.4230	32	2.69	0.0114	0.05	0.2746	1.9977
**Culture**	9	-0.2587	0.4230	32	-0.61	0.5451	0.05	-1.1202	0.6029
**Culture**	10	-0.2222	0.4404	32	-0.50	0.6173	0.05	-1.1192	0.6748
**Culture**	11	-0.1744	0.4099	32	-0.43	0.6733	0.05	-1.0093	0.6604
**Culture**	12	-0.2587	0.4230	32	-0.61	0.5451	0.05	-1.1202	0.6029
**Culture**	13	-0.2222	0.4404	32	-0.50	0.6173	0.05	-1.1192	0.6748

Type 3 Tests of Fixed Effects				
**Effect**	**Num DF**	**Den DF**	**F Value**	**Pr > F**
**Treatment**	1	32	1.26	0.2696

DistSoma=474

Least Squares Means									
**Effect**	**Treatment**	**Estimate**	**Standard Error**	**DF**	**t Value**	**Pr > |t|**	**Alpha**	**Lower**	**Upper**
**Treatment**	Control GFP	0	0.3189	32	0.00	1.0000	0.05	-0.6496	0.6496
**Treatment**	Ctr Meg	0.5100	0.3231	32	1.58	0.1243	0.05	-0.1481	1.1681

Differences of Least Squares Means												
**Effect**	**Treatment**	**Treatment**	**Estimate**	**Standard Error**	**DF**	**t Value**	**Pr > |t|**	**Adjustment**	**Adj P**	**Alpha**	**Lower**	**Upper**
**Treatment**	Control GFP	Ctr Meg	-0.5100	0.4540	32	-1.12	0.2696	Tukey-Kramer	0.2696	0.05	-1.4347	0.4147

Differences of Least Squares Means				
**Effect**	**Treatment**	**Treatment**	**Adj Lower**	**Adj Upper**
**Treatment**	Control GFP	Ctr Meg	-1.4347	0.4147

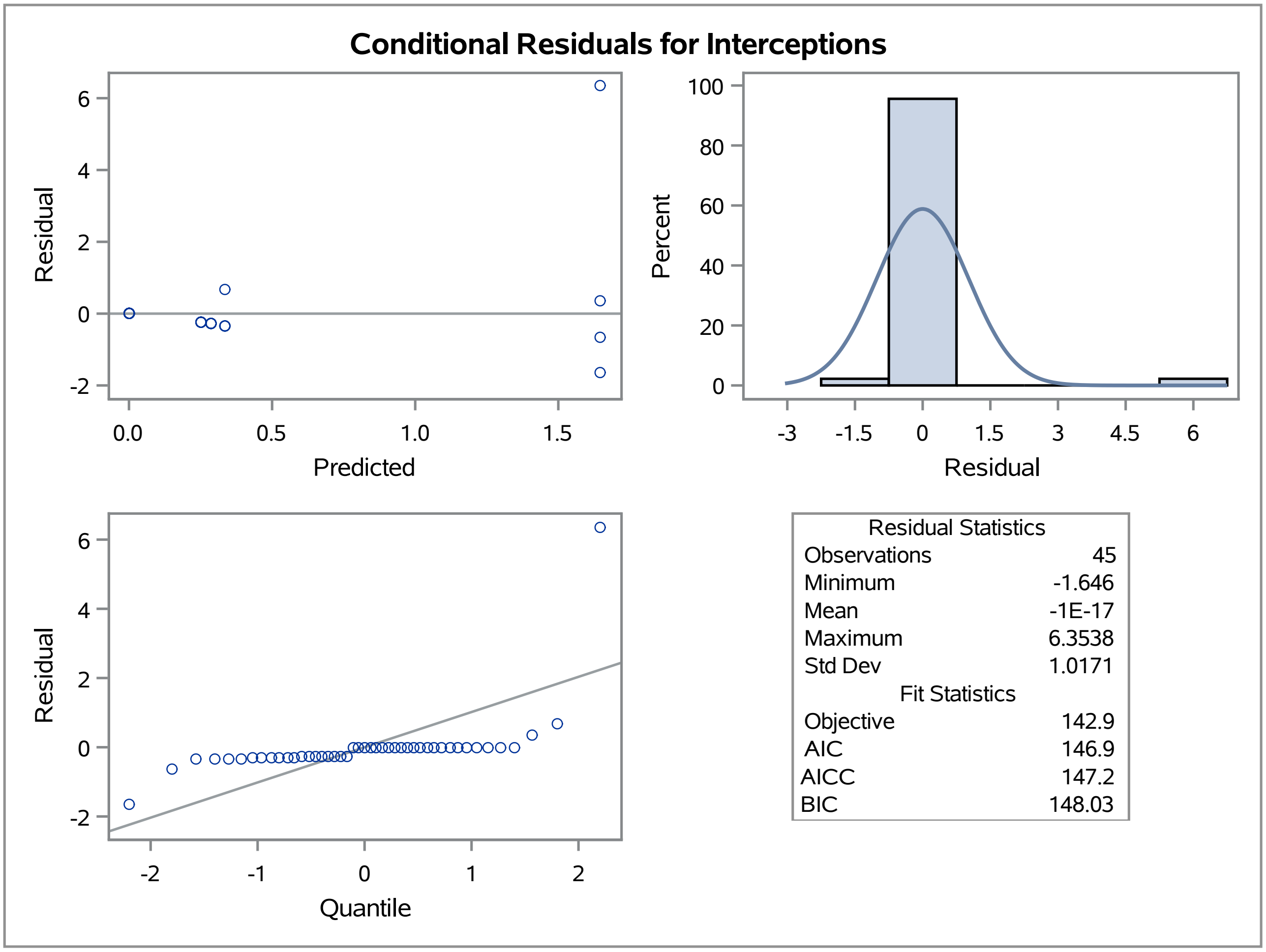

Residual Statistics	
Observations	45
Minimum	-1.646
Mean	-1E-17
Maximum	6.3538
Std Dev	1.0171
**Fit Statistics**	
Objective	142.9
AIC	146.9
AICC	147.2
BIC	148.03

DistSoma=480

Model Information	
**Data Set**	WORK.TEMPDATASORTED
**Dependent Variable**	Interceptions
**Covariance Structure**	Variance Components
**Estimation Method**	REML
**Residual Variance Method**	Profile
**Fixed Effects SE Method**	Model-Based
**Degrees of Freedom Method**	Containment

Class Level Information		
**Class**	**Levels**	**Values**
**Treatment**	2	Control GFP Ctr Meg
**Culture**	13	1 2 3 4 5 6 7 8 9 10 11 12 13

Dimensions	
**Covariance Parameters**	2
**Columns in X**	3
**Columns in Z**	13
**Subjects**	1
**Max Obs per Subject**	45

Number of Observations	
**Number of Observations Read**	45
**Number of Observations Used**	45
**Number of Observations Not Used**	0

Iteration History			
**Iteration**	**Evaluations**	**-2 Res Log Like**	**Criterion**
**0**	1	145.17998821	
**1**	3	142.91150036	0.00024750
**2**	1	142.90322604	0.00000136
**3**	1	142.90318235	0.00000000

Convergence criteria met.

DistSoma=480

Covariance Parameter Estimates				
**Cov Parm**	**Estimate**	**Alpha**	**Lower**	**Upper**
**Culture**	0.3085	0.05	0.09248	6.2643
**Residual**	1.1990	0.05	0.7826	2.0662

Fit Statistics	
**-2 Res Log Likelihood**	142.9
**AIC (Smaller is Better)**	146.9
**AICC (Smaller is Better)**	147.2
**BIC (Smaller is Better)**	148.0

Solution for Fixed Effects									
**Effect**	**Treatment**	**Estimate**	**Standard Error**	**DF**	**t Value**	**Pr > |t|**	**Alpha**	**Lower**	**Upper**
**Intercept**		0.5100	0.3231	11	1.58	0.1428	0.05	-0.2011	1.2211
**Treatment**	Control GFP	-0.5100	0.4540	32	-1.12	0.2696	0.05	-1.4347	0.4147
**Treatment**	Ctr Meg	0	.	.	.	.	.	.	.

Solution for Random Effects									
**Effect**	**Culture**	**Estimate**	**Std Err Pred**	**DF**	**t Value**	**Pr > |t|**	**Alpha**	**Lower**	**Upper**
**Culture**	1	3.31E-17	0.4398	32	0.00	1.0000	0.05	-0.8958	0.8958
**Culture**	2	3.31E-17	0.4398	32	0.00	1.0000	0.05	-0.8958	0.8958
**Culture**	3	2.86E-17	0.4221	32	0.00	1.0000	0.05	-0.8599	0.8599
**Culture**	4	2.86E-17	0.4221	32	0.00	1.0000	0.05	-0.8599	0.8599
**Culture**	5	2.86E-17	0.4221	32	0.00	1.0000	0.05	-0.8599	0.8599
**Culture**	6	1.15E-17	0.4996	32	0.00	1.0000	0.05	-1.0177	1.0177
**Culture**	7	3.31E-17	0.4398	32	0.00	1.0000	0.05	-0.8958	0.8958
**Culture**	8	1.1362	0.4230	32	2.69	0.0114	0.05	0.2746	1.9977
**Culture**	9	-0.2587	0.4230	32	-0.61	0.5451	0.05	-1.1202	0.6029
**Culture**	10	-0.2222	0.4404	32	-0.50	0.6173	0.05	-1.1192	0.6748
**Culture**	11	-0.1744	0.4099	32	-0.43	0.6733	0.05	-1.0093	0.6604
**Culture**	12	-0.2587	0.4230	32	-0.61	0.5451	0.05	-1.1202	0.6029
**Culture**	13	-0.2222	0.4404	32	-0.50	0.6173	0.05	-1.1192	0.6748

Type 3 Tests of Fixed Effects				
**Effect**	**Num DF**	**Den DF**	**F Value**	**Pr > F**
**Treatment**	1	32	1.26	0.2696

DistSoma=480

Least Squares Means									
**Effect**	**Treatment**	**Estimate**	**Standard Error**	**DF**	**t Value**	**Pr > |t|**	**Alpha**	**Lower**	**Upper**
**Treatment**	Control GFP	0	0.3189	32	0.00	1.0000	0.05	-0.6496	0.6496
**Treatment**	Ctr Meg	0.5100	0.3231	32	1.58	0.1243	0.05	-0.1481	1.1681

Differences of Least Squares Means												
**Effect**	**Treatment**	**Treatment**	**Estimate**	**Standard Error**	**DF**	**t Value**	**Pr > |t|**	**Adjustment**	**Adj P**	**Alpha**	**Lower**	**Upper**
**Treatment**	Control GFP	Ctr Meg	-0.5100	0.4540	32	-1.12	0.2696	Tukey-Kramer	0.2696	0.05	-1.4347	0.4147

Differences of Least Squares Means				
**Effect**	**Treatment**	**Treatment**	**Adj Lower**	**Adj Upper**
**Treatment**	Control GFP	Ctr Meg	-1.4347	0.4147

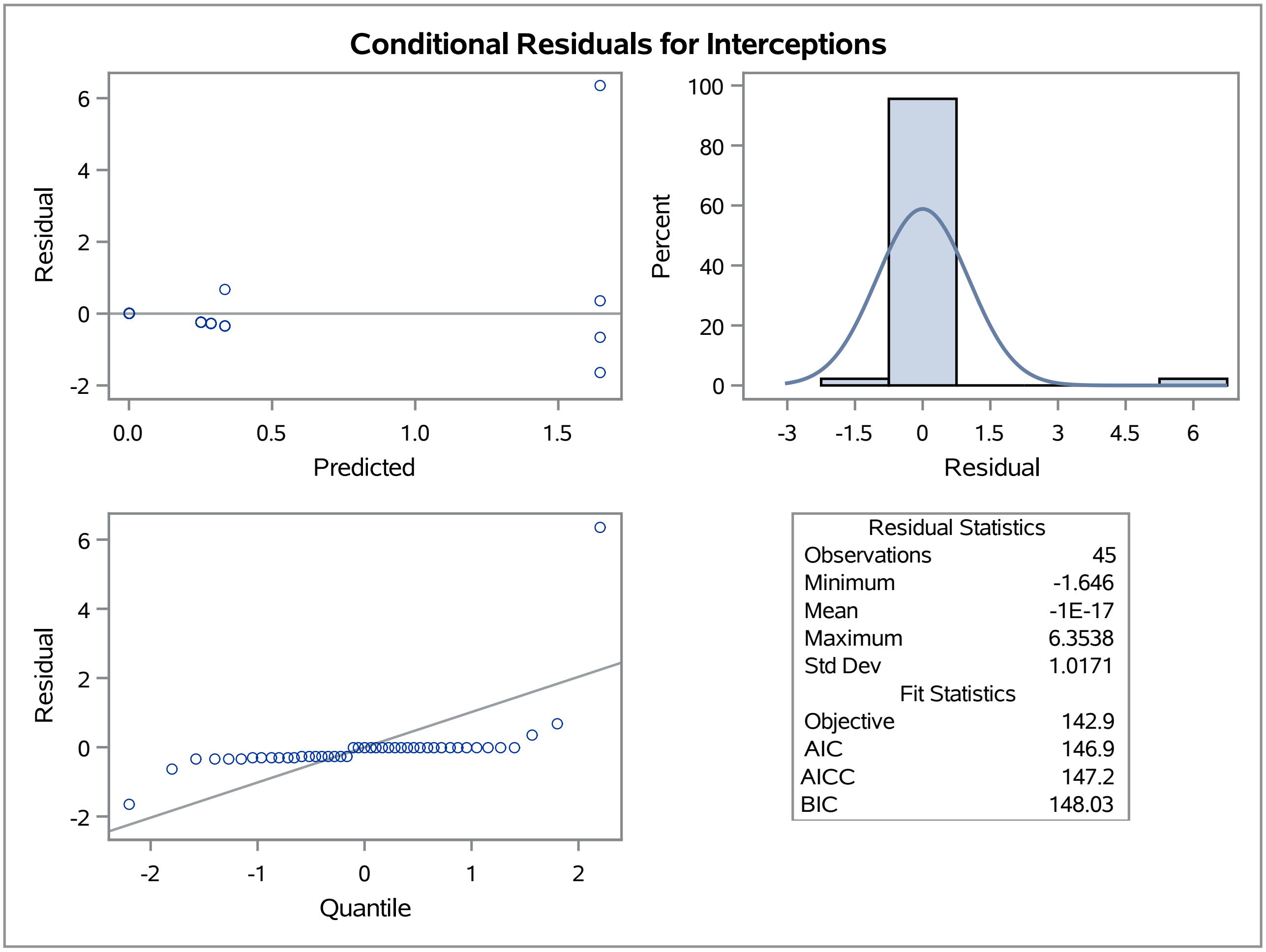

DistSoma=486

Model Information	
**Data Set**	WORK.TEMPDATASORTED
**Dependent Variable**	Interceptions
**Covariance Structure**	Variance Components
**Estimation Method**	REML
**Residual Variance Method**	Profile
**Fixed Effects SE Method**	Model-Based
**Degrees of Freedom Method**	Containment

Class Level Information		
**Class**	**Levels**	**Values**
**Treatment**	2	Control GFP Ctr Meg
**Culture**	13	1 2 3 4 5 6 7 8 9 10 11 12 13

Dimensions	
**Covariance Parameters**	2
**Columns in X**	3
**Columns in Z**	13
**Subjects**	1
**Max Obs per Subject**	45

Number of Observations	
**Number of Observations Read**	45
**Number of Observations Used**	45
**Number of Observations Not Used**	0

Iteration History			
**Iteration**	**Evaluations**	**-2 Res Log Like**	**Criterion**
**0**	1	132.67256037	
**1**	3	131.39275042	0.00008984
**2**	1	131.39033561	0.00000015
**3**	1	131.39033160	0.00000000

Convergence criteria met.

DistSoma=486

Covariance Parameter Estimates				
**Cov Parm**	**Estimate**	**Alpha**	**Lower**	**Upper**
**Culture**	0.1703	0.05	0.04251	13.0308
**Residual**	0.9536	0.05	0.6229	1.6413

Fit Statistics	
**-2 Res Log Likelihood**	131.4
**AIC (Smaller is Better)**	135.4
**AICC (Smaller is Better)**	135.7
**BIC (Smaller is Better)**	136.5

Solution for Fixed Effects									
**Effect**	**Treatment**	**Estimate**	**Standard Error**	**DF**	**t Value**	**Pr > |t|**	**Alpha**	**Lower**	**Upper**
**Intercept**		0.4265	0.2653	11	1.61	0.1362	0.05	-0.1574	1.0104
**Treatment**	Control GFP	-0.4265	0.3741	32	-1.14	0.2627	0.05	-1.1885	0.3355
**Treatment**	Ctr Meg	0	.	.	.	.	.	.	.

Solution for Random Effects									
**Effect**	**Culture**	**Estimate**	**Std Err Pred**	**DF**	**t Value**	**Pr > |t|**	**Alpha**	**Lower**	**Upper**
**Culture**	1	-634E-20	0.3454	32	-0.00	1.0000	0.05	-0.7037	0.7037
**Culture**	2	-634E-20	0.3454	32	-0.00	1.0000	0.05	-0.7037	0.7037
**Culture**	3	-15E-18	0.3338	32	-0.00	1.0000	0.05	-0.6799	0.6799
**Culture**	4	-15E-18	0.3338	32	-0.00	1.0000	0.05	-0.6799	0.6799
**Culture**	5	-15E-18	0.3338	32	-0.00	1.0000	0.05	-0.6799	0.6799
**Culture**	6	-547E-20	0.3822	32	-0.00	1.0000	0.05	-0.7785	0.7785
**Culture**	7	-634E-20	0.3454	32	-0.00	1.0000	0.05	-0.7037	0.7037
**Culture**	8	0.7597	0.3340	32	2.27	0.0298	0.05	0.07944	1.4400
**Culture**	9	-0.1777	0.3340	32	-0.53	0.5984	0.05	-0.8580	0.5026
**Culture**	10	-0.1488	0.3456	32	-0.43	0.6698	0.05	-0.8527	0.5552
**Culture**	11	-0.1068	0.3250	32	-0.33	0.7445	0.05	-0.7688	0.5551
**Culture**	12	-0.1777	0.3340	32	-0.53	0.5984	0.05	-0.8580	0.5026
**Culture**	13	-0.1488	0.3456	32	-0.43	0.6698	0.05	-0.8527	0.5552

Type 3 Tests of Fixed Effects				
**Effect**	**Num DF**	**Den DF**	**F Value**	**Pr > F**
**Treatment**	1	32	1.30	0.2627

DistSoma=486

Least Squares Means									
**Effect**	**Treatment**	**Estimate**	**Standard Error**	**DF**	**t Value**	**Pr > |t|**	**Alpha**	**Lower**	**Upper**
**Treatment**	Control GFP	0	0.2637	32	0.00	1.0000	0.05	-0.5372	0.5372
**Treatment**	Ctr Meg	0.4265	0.2653	32	1.61	0.1177	0.05	-0.1139	0.9669

Differences of Least Squares Means												
**Effect**	**Treatment**	**Treatment**	**Estimate**	**Standard Error**	**DF**	**t Value**	**Pr > |t|**	**Adjustment**	**Adj P**	**Alpha**	**Lower**	**Upper**
**Treatment**	Control GFP	Ctr Meg	-0.4265	0.3741	32	-1.14	0.2627	Tukey-Kramer	0.2627	0.05	-1.1885	0.3355

Differences of Least Squares Means				
**Effect**	**Treatment**	**Treatment**	**Adj Lower**	**Adj Upper**
**Treatment**	Control GFP	Ctr Meg	-1.1885	0.3355

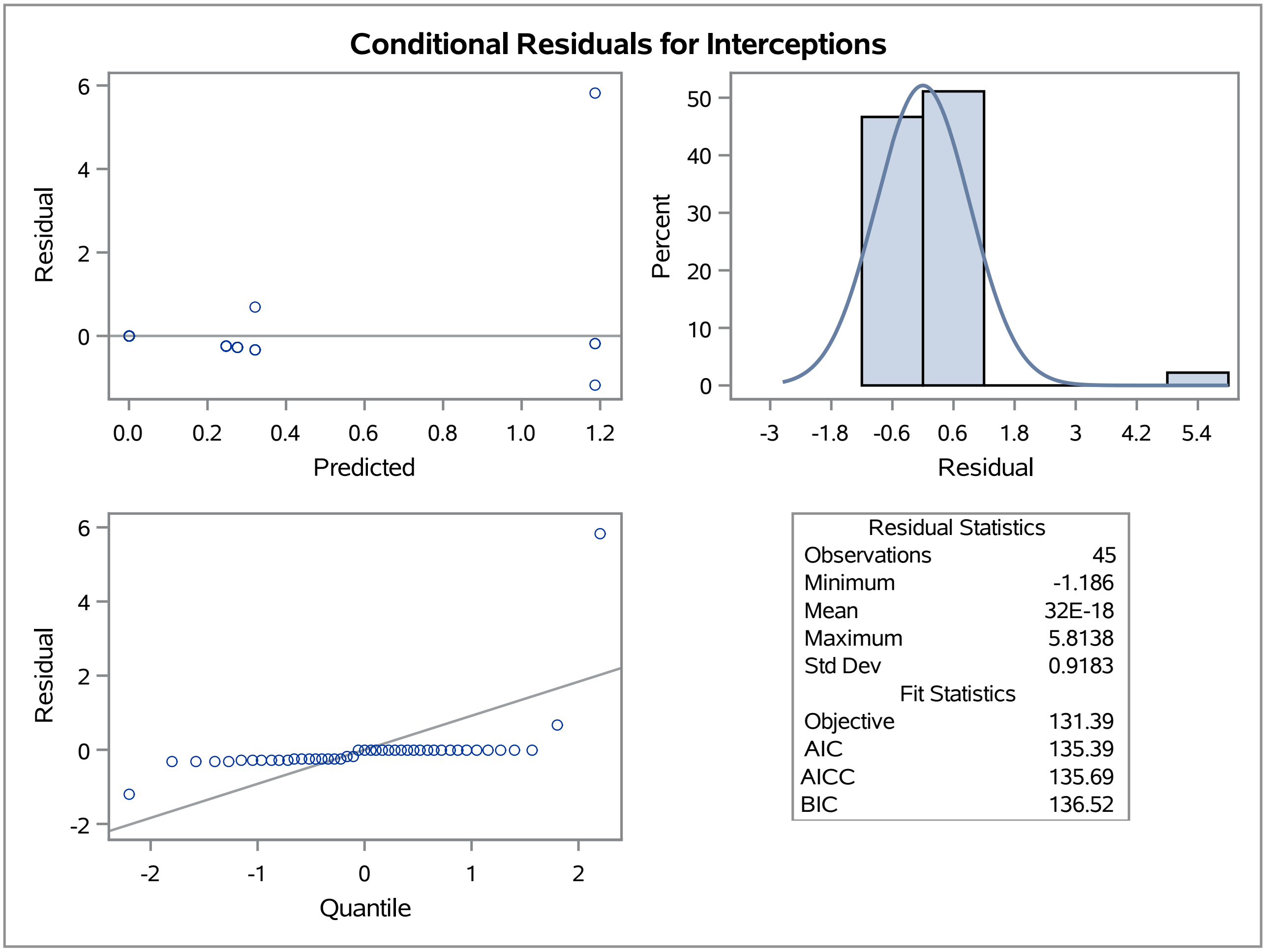

DistSoma=492

Model Information	
**Data Set**	WORK.TEMPDATASORTED
**Dependent Variable**	Interceptions
**Covariance Structure**	Variance Components
**Estimation Method**	REML
**Residual Variance Method**	Profile
**Fixed Effects SE Method**	Model-Based
**Degrees of Freedom Method**	Containment

Class Level Information		
**Class**	**Levels**	**Values**
**Treatment**	2	Control GFP Ctr Meg
**Culture**	13	1 2 3 4 5 6 7 8 9 10 11 12 13

Dimensions	
**Covariance Parameters**	2
**Columns in X**	3
**Columns in Z**	13
**Subjects**	1
**Max Obs per Subject**	45

Number of Observations	
**Number of Observations Read**	45
**Number of Observations Used**	45
**Number of Observations Not Used**	0

Iteration History			
**Iteration**	**Evaluations**	**-2 Res Log Like**	**Criterion**
**0**	1	88.02062387	
**1**	3	84.61880696	0.00446747
**2**	1	84.60560666	0.00003802
**3**	1	84.60550002	0.00000000

Convergence criteria met.

DistSoma=492

Covariance Parameter Estimates				
Cov Parm	Estimate	Alpha	Lower	Upper
Culture	0.1020	0.05	0.03419	1.1417
Residual	0.2982	0.05	0.1944	0.5146

Fit Statistics	
-2 Res Log Likelihood	84.6
AIC (Smaller is Better)	88.6
AICC (Smaller is Better)	88.9
BIC (Smaller is Better)	89.7

Solution for Fixed Effects									
Effect	Treatment	Estimate	Standard Error	DF	t Value	Pr > |t|	Alpha	Lower	Upper
Intercept		0.2940	0.1738	11	1.69	0.1188	0.05	-0.08851	0.6765
Treatment	Control GFP	-0.2940	0.2435	32	-1.21	0.2361	0.05	-0.7899	0.2019
Treatment	Ctr Meg	0	.	.	.	.	.	.	.

Solution for Random Effects									
Effect	Culture	Estimate	Std Err Pred	DF	t Value	Pr > |t|	Alpha	Lower	Upper
Culture	1	1.51E-17	0.2404	32	0.00	1.0000	0.05	-0.4897	0.4897
Culture	2	1.51E-17	0.2404	32	0.00	1.0000	0.05	-0.4897	0.4897
Culture	3	3.37E-17	0.2297	32	0.00	1.0000	0.05	-0.4679	0.4679
Culture	4	3.37E-17	0.2297	32	0.00	1.0000	0.05	-0.4679	0.4679
Culture	5	3.37E-17	0.2297	32	0.00	1.0000	0.05	-0.4679	0.4679
Culture	6	1.49E-17	0.2791	32	0.00	1.0000	0.05	-0.5685	0.5685
Culture	7	1.51E-17	0.2404	32	0.00	1.0000	0.05	-0.4897	0.4897
Culture	8	0.6968	0.2305	32	3.02	0.0049	0.05	0.2272	1.1664
Culture	9	-0.1698	0.2305	32	-0.74	0.4666	0.05	-0.6394	0.2997
Culture	10	-0.1489	0.2410	32	-0.62	0.5411	0.05	-0.6398	0.3420
Culture	11	-0.05930	0.2228	32	-0.27	0.7918	0.05	-0.5132	0.3946
Culture	12	-0.1698	0.2305	32	-0.74	0.4666	0.05	-0.6394	0.2997
Culture	13	-0.1489	0.2410	32	-0.62	0.5411	0.05	-0.6398	0.3420

Type 3 Tests of Fixed Effects				
Effect	Num DF	Den DF	F Value	Pr > F
Treatment	1	32	1.46	0.2361

DistSoma=492

Least Squares Means									
**Effect**	**Treatment**	**Estimate**	**Standard Error**	**DF**	**t Value**	**Pr > |t|**	**Alpha**	**Lower**	**Upper**
**Treatment**	Control GFP	0	0.1705	32	0.00	1.0000	0.05	-0.3473	0.3473
**Treatment**	Ctr Meg	0.2940	0.1738	32	1.69	0.1004	0.05	-0.06000	0.6480

Differences of Least Squares Means												
**Effect**	**Treatment**	**Treatment**	**Estimate**	**Standard Error**	**DF**	**t Value**	**Pr > |t|**	**Adjustment**	**Adj P**	**Alpha**	**Lower**	**Upper**
**Treatment**	Control GFP	Ctr Meg	-0.2940	0.2435	32	-1.21	0.2361	Tukey-Kramer	0.2361	0.05	-0.7899	0.2019

Differences of Least Squares Means				
**Effect**	**Treatment**	**Treatment**	**Adj Lower**	**Adj Upper**
**Treatment**	Control GFP	Ctr Meg	-0.7899	0.2019

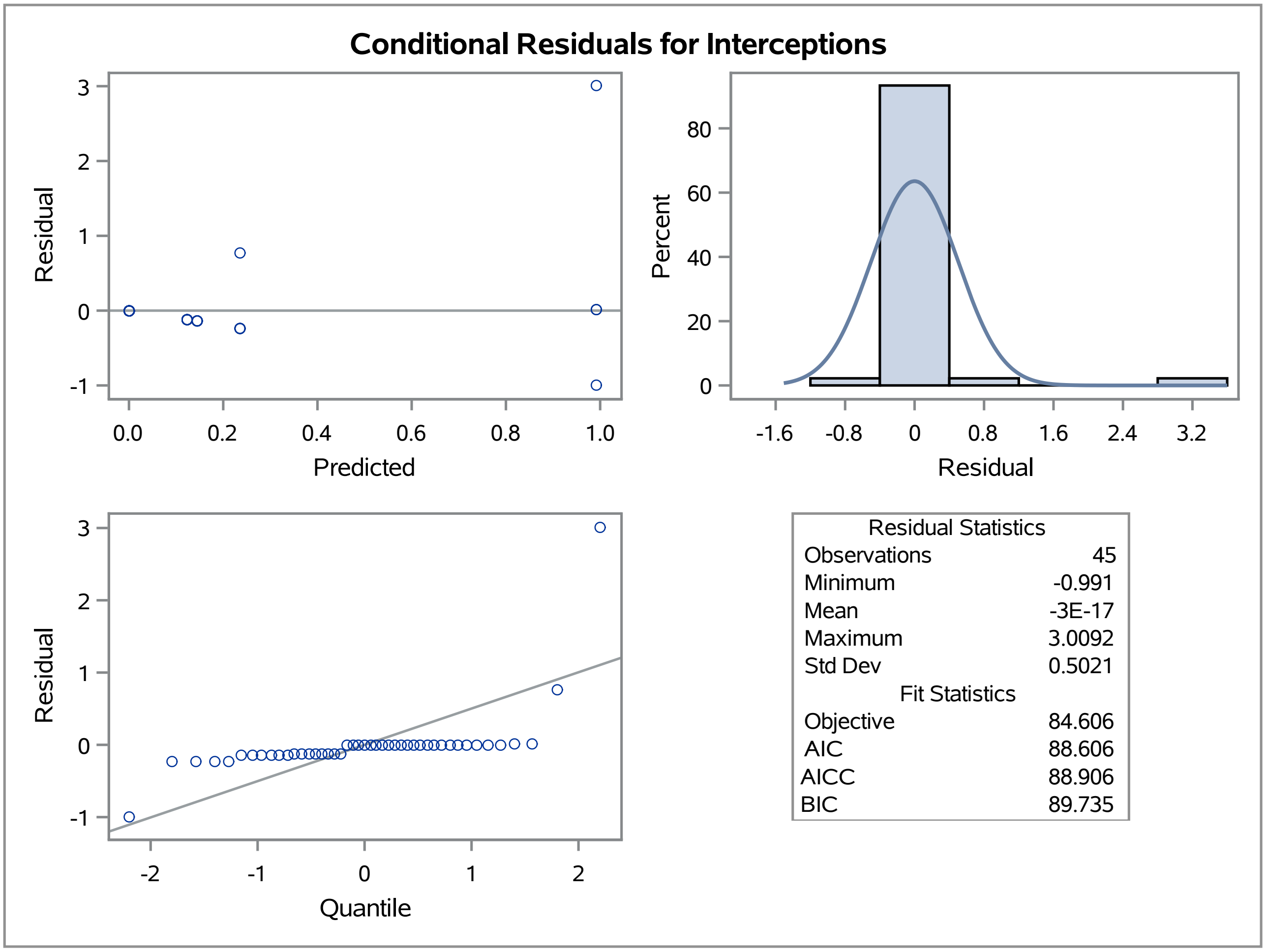

DistSoma=498

Model Information	
**Data Set**	WORK.TEMPDATASORTED
**Dependent Variable**	Interceptions
**Covariance Structure**	Variance Components
**Estimation Method**	REML
**Residual Variance Method**	Profile
**Fixed Effects SE Method**	Model-Based
**Degrees of Freedom Method**	Containment

Class Level Information		
**Class**	**Levels**	**Values**
**Treatment**	2	Control GFP Ctr Meg
**Culture**	13	1 2 3 4 5 6 7 8 9 10 11 12 13

Dimensions	
**Covariance Parameters**	2
**Columns in X**	3
**Columns in Z**	13
**Subjects**	1
**Max Obs per Subject**	45

Number of Observations	
**Number of Observations Read**	45
**Number of Observations Used**	45
**Number of Observations Not Used**	0

Iteration History			
**Iteration**	**Evaluations**	**-2 Res Log Like**	**Criterion**
**0**	1	93.62371919	
**1**	3	87.01055998	0.01462862
**2**	1	86.94584860	0.00051845
**3**	1	86.94374538	0.00000076
**4**	1	86.94374235	0.00000000

Convergence criteria met.

DistSoma=498

Covariance Parameter Estimates				
**Cov Parm**	**Estimate**	**Alpha**	**Lower**	**Upper**
**Culture**	0.1650	0.05	0.06427	0.9934
**Residual**	0.2920	0.05	0.1902	0.5050

Fit Statistics	
**-2 Res Log Likelihood**	86.9
**AIC (Smaller is Better)**	90.9
**AICC (Smaller is Better)**	91.2
**BIC (Smaller is Better)**	92.1

Solution for Fixed Effects									
**Effect**	**Treatment**	**Estimate**	**Standard Error**	**DF**	**t Value**	**Pr > |t|**	**Alpha**	**Lower**	**Upper**
**Intercept**		0.3339	0.2012	11	1.66	0.1252	0.05	-0.1089	0.7767
**Treatment**	Control GFP	-0.3339	0.2804	32	-1.19	0.2425	0.05	-0.9049	0.2372
**Treatment**	Ctr Meg	0	.	.	.	.	.	.	.

Solution for Random Effects									
**Effect**	**Culture**	**Estimate**	**Std Err Pred**	**DF**	**t Value**	**Pr > |t|**	**Alpha**	**Lower**	**Upper**
**Culture**	1	-42E-18	0.2762	32	-0.00	1.0000	0.05	-0.5627	0.5627
**Culture**	2	-42E-18	0.2762	32	-0.00	1.0000	0.05	-0.5627	0.5627
**Culture**	3	-511E-19	0.2625	32	-0.00	1.0000	0.05	-0.5348	0.5348
**Culture**	4	-511E-19	0.2625	32	-0.00	1.0000	0.05	-0.5348	0.5348
**Culture**	5	-511E-19	0.2625	32	-0.00	1.0000	0.05	-0.5348	0.5348
**Culture**	6	-266E-19	0.3323	32	-0.00	1.0000	0.05	-0.6768	0.6768
**Culture**	7	-42E-18	0.2762	32	-0.00	1.0000	0.05	-0.5627	0.5627
**Culture**	8	0.9818	0.2647	32	3.71	0.0008	0.05	0.4426	1.5209
**Culture**	9	-0.2315	0.2647	32	-0.87	0.3884	0.05	-0.7706	0.3077
**Culture**	10	-0.2100	0.2779	32	-0.76	0.4554	0.05	-0.7761	0.3561
**Culture**	11	-0.09887	0.2554	32	-0.39	0.7012	0.05	-0.6190	0.4213
**Culture**	12	-0.2315	0.2647	32	-0.87	0.3884	0.05	-0.7706	0.3077
**Culture**	13	-0.2100	0.2779	32	-0.76	0.4554	0.05	-0.7761	0.3561

Type 3 Tests of Fixed Effects				
**Effect**	**Num DF**	**Den DF**	**F Value**	**Pr > F**
**Treatment**	1	32	1.42	0.2425

DistSoma=498

Least Squares Means									
**Effect**	**Treatment**	**Estimate**	**Standard Error**	**DF**	**t Value**	**Pr > |t|**	**Alpha**	**Lower**	**Upper**
**Treatment**	Control GFP	0	0.1953	32	0.00	1.0000	0.05	-0.3977	0.3977
**Treatment**	Ctr Meg	0.3339	0.2012	32	1.66	0.1068	0.05	-0.07594	0.7437

Differences of Least Squares Means												
**Effect**	**Treatment**	**Treatment**	**Estimate**	**Standard Error**	**DF**	**t Value**	**Pr > |t|**	**Adjustment**	**Adj P**	**Alpha**	**Lower**	**Upper**
**Treatment**	Control GFP	Ctr Meg	-0.3339	0.2804	32	-1.19	0.2425	Tukey-Kramer	0.2425	0.05	-0.9049	0.2372

Differences of Least Squares Means				
**Effect**	**Treatment**	**Treatment**	**Adj Lower**	**Adj Upper**
**Treatment**	Control GFP	Ctr Meg	-0.9049	0.2372

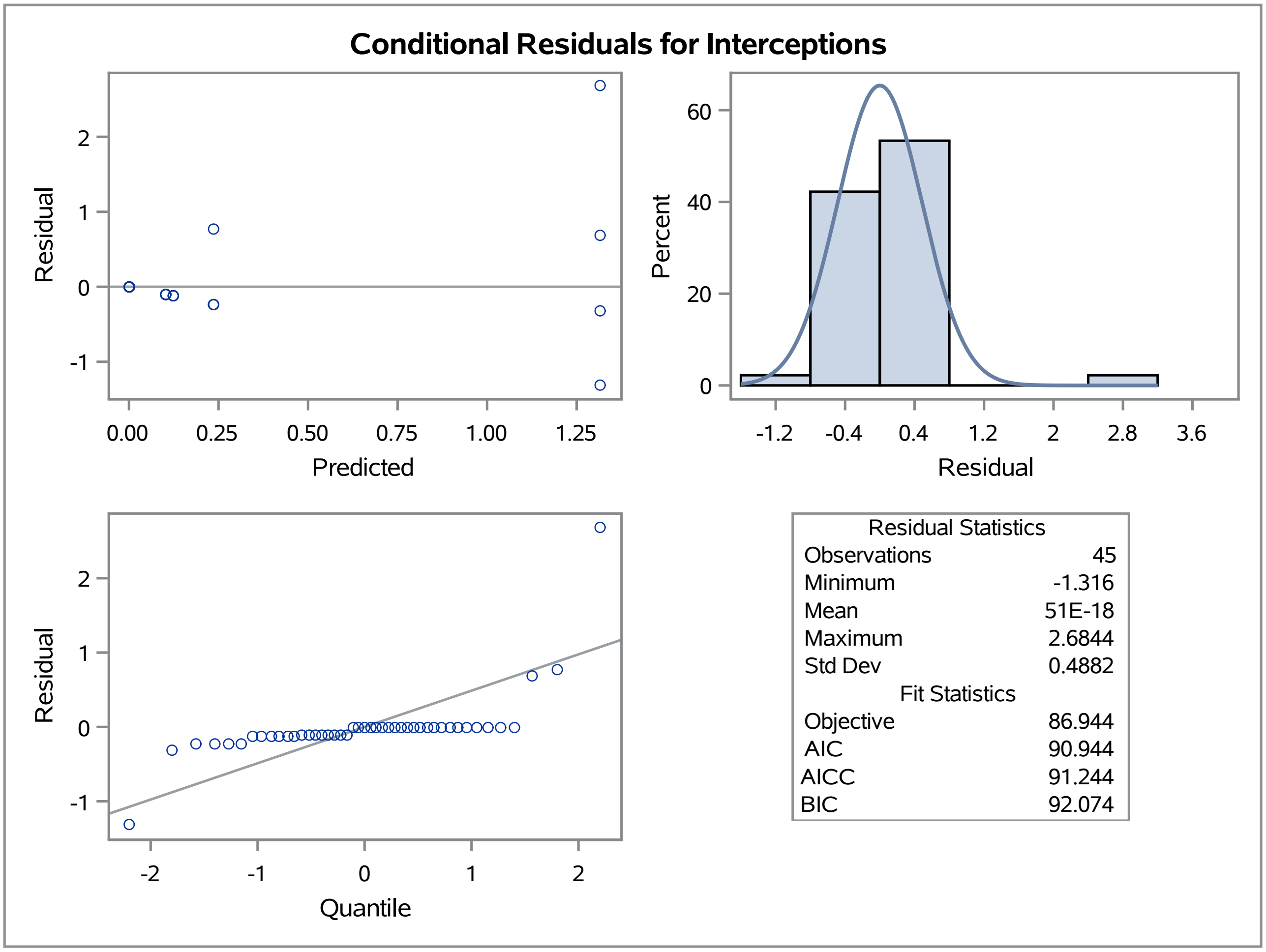

DistSoma=504

Model Information	
**Data Set**	WORK.TEMPDATASORTED
**Dependent Variable**	Interceptions
**Covariance Structure**	Variance Components
**Estimation Method**	REML
**Residual Variance Method**	Profile
**Fixed Effects SE Method**	Model-Based
**Degrees of Freedom Method**	Containment

Class Level Information		
**Class**	**Levels**	**Values**
**Treatment**	2	Control GFP Ctr Meg
**Culture**	13	1 2 3 4 5 6 7 8 9 10 11 12 13

Dimensions	
**Covariance Parameters**	2
**Columns in X**	3
**Columns in Z**	13
**Subjects**	1
**Max Obs per Subject**	45

Number of Observations	
**Number of Observations Read**	45
**Number of Observations Used**	45
**Number of Observations Not Used**	0

Iteration History			
**Iteration**	**Evaluations**	**-2 Res Log Like**	**Criterion**
**0**	1	102.23486727	
**1**	3	93.88912219	0.01528632
**2**	1	93.75981554	0.00096395
**3**	1	93.75240156	0.00000474
**4**	1	93.75236653	0.00000000

Convergence criteria met.

DistSoma=504

Covariance Parameter Estimates				
**Cov Parm**	**Estimate**	**Alpha**	**Lower**	**Upper**
**Culture**	0.2275	0.05	0.09256	1.1790
**Residual**	0.3308	0.05	0.2153	0.5727

Fit Statistics	
**-2 Res Log Likelihood**	93.8
**AIC (Smaller is Better)**	97.8
**AICC (Smaller is Better)**	98.1
**BIC (Smaller is Better)**	98.9

Solution for Fixed Effects									
**Effect**	**Treatment**	**Estimate**	**Standard Error**	**DF**	**t Value**	**Pr > |t|**	**Alpha**	**Lower**	**Upper**
**Intercept**		0.3752	0.2294	11	1.64	0.1302	0.05	-0.1297	0.8802
**Treatment**	Control GFP	-0.3752	0.3191	32	-1.18	0.2482	0.05	-1.0251	0.2747
**Treatment**	Ctr Meg	0	.	.	.	.	.	.	.

Solution for Random Effects									
**Effect**	**Culture**	**Estimate**	**Std Err Pred**	**DF**	**t Value**	**Pr > |t|**	**Alpha**	**Lower**	**Upper**
**Culture**	1	-696E-19	0.3108	32	-0.00	1.0000	0.05	-0.6330	0.6330
**Culture**	2	-696E-19	0.3108	32	-0.00	1.0000	0.05	-0.6330	0.6330
**Culture**	3	-854E-19	0.2951	32	-0.00	1.0000	0.05	-0.6011	0.6011
**Culture**	4	-854E-19	0.2951	32	-0.00	1.0000	0.05	-0.6011	0.6011
**Culture**	5	-854E-19	0.2951	32	-0.00	1.0000	0.05	-0.6011	0.6011
**Culture**	6	-474E-19	0.3781	32	-0.00	1.0000	0.05	-0.7702	0.7702
**Culture**	7	-696E-19	0.3108	32	-0.00	1.0000	0.05	-0.6330	0.6330
**Culture**	8	1.1915	0.2983	32	3.99	0.0004	0.05	0.5840	1.7991
**Culture**	9	-0.2752	0.2983	32	-0.92	0.3631	0.05	-0.8827	0.3324
**Culture**	10	-0.2527	0.3133	32	-0.81	0.4258	0.05	-0.8909	0.3854
**Culture**	11	-0.1357	0.2878	32	-0.47	0.6404	0.05	-0.7220	0.4506
**Culture**	12	-0.2752	0.2983	32	-0.92	0.3631	0.05	-0.8827	0.3324
**Culture**	13	-0.2527	0.3133	32	-0.81	0.4258	0.05	-0.8909	0.3854

Type 3 Tests of Fixed Effects				
**Effect**	**Num DF**	**Den DF**	**F Value**	**Pr > F**
**Treatment**	1	32	1.38	0.2482

DistSoma=504

Least Squares Means									
Effect	Treatment	Estimate	Standard Error	DF	t Value	Pr > |t|	Alpha	Lower	Upper
**Treatment**	Control GFP	0	0.2217	32	0.00	1.0000	0.05	-0.4516	0.4516
**Treatment**	Ctr Meg	0.3752	0.2294	32	1.64	0.1117	0.05	-0.09210	0.8426

Differences of Least Squares Means													
Effect	Treatment	Treatment	Estimate	Standard Error	DF	t Value	Pr > |t|	Adjustment	Adj P	Alpha	Lower	Upper
**Treatment**	Control GFP	Ctr Meg	-0.3752	0.3191	32	-1.18	0.2482	Tukey-Kramer	0.2482	0.05	-1.0251	0.2747

Differences of Least Squares Means				
Effect	Treatment	Treatment	Adj Lower	Adj Upper
**Treatment**	Control GFP	Ctr Meg	-1.0251	0.2746

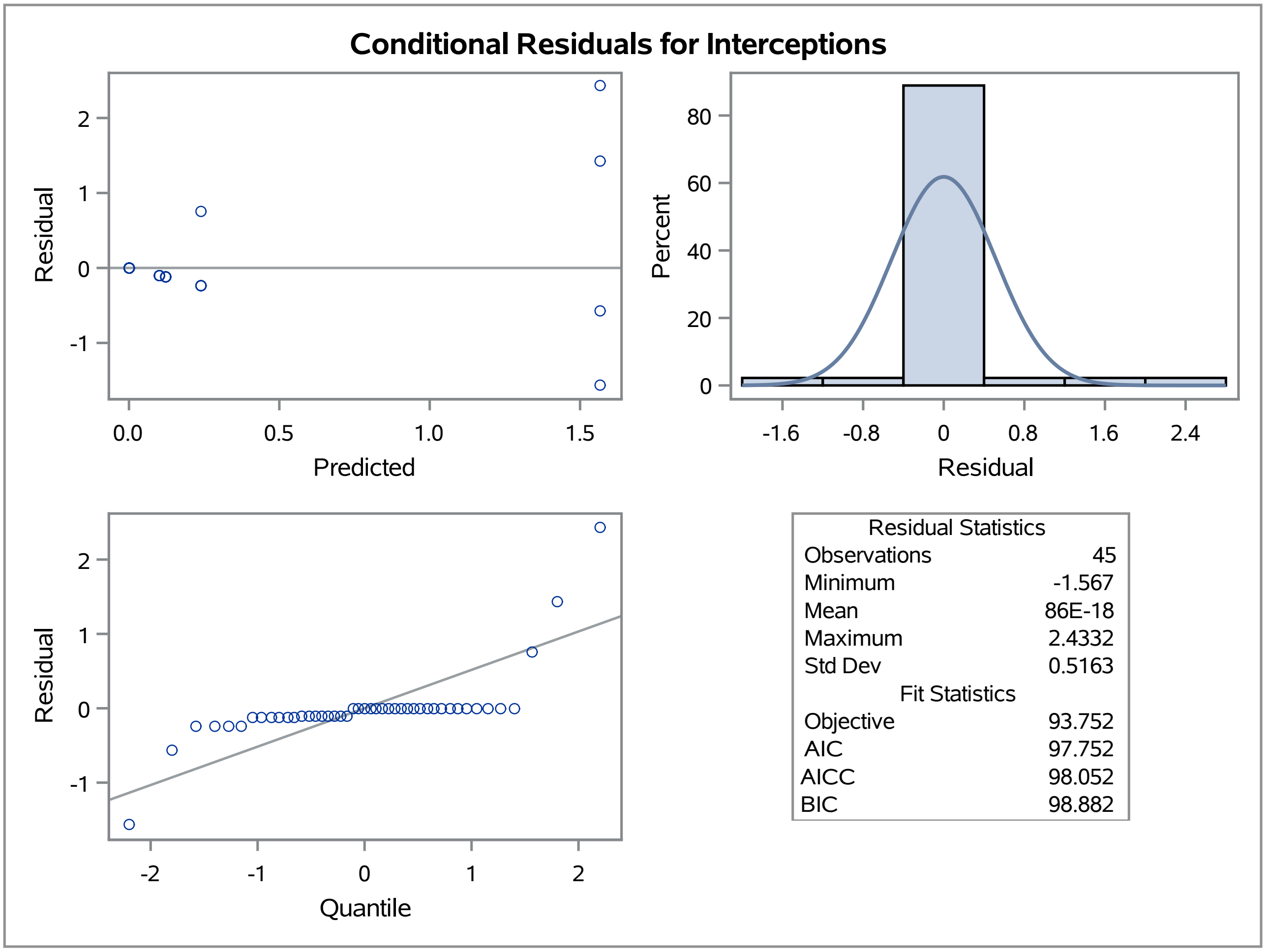

DistSoma=510

Model Information	
**Data Set**	WORK.TEMPDATASORTED
**Dependent Variable**	Interceptions
**Covariance Structure**	Variance Components
**Estimation Method**	REML
**Residual Variance Method**	Profile
**Fixed Effects SE Method**	Model-Based
**Degrees of Freedom Method**	Containment

Class Level Information		
**Class**	**Levels**	**Values**
**Treatment**	2	Control GFP Ctr Meg
**Culture**	13	1 2 3 4 5 6 7 8 9 10 11 12 13

Dimensions	
**Covariance Parameters**	2
**Columns in X**	3
**Columns in Z**	13
**Subjects**	1
**Max Obs per Subject**	45

Number of Observations	
**Number of Observations Read**	45
**Number of Observations Used**	45
**Number of Observations Not Used**	0

Iteration History			
**Iteration**	**Evaluations**	**-2 Res Log Like**	**Criterion**
**0**	1	102.23486727	
**1**	3	93.88912219	0.01528632
**2**	1	93.75981554	0.00096395
**3**	1	93.75240156	0.00000474
**4**	1	93.75236653	0.00000000

Convergence criteria met.

DistSoma=510

Covariance Parameter Estimates				
Cov Parm	Estimate	Alpha	Lower	Upper
Culture	0.2275	0.05	0.09256	1.1790
Residual	0.3308	0.05	0.2153	0.5727

Fit Statistics	
-2 Res Log Likelihood	93.8
AIC (Smaller is Better)	97.8
AICC (Smaller is Better)	98.1
BIC (Smaller is Better)	98.9

Solution for Fixed Effects									
Effect	Treatment	Estimate	Standard Error	DF	t Value	Pr > |t|	Alpha	Lower	Upper
Intercept		0.3752	0.2294	11	1.64	0.1302	0.05	-0.1297	0.8802
Treatment	Control GFP	-0.3752	0.3191	32	-1.18	0.2482	0.05	-1.0251	0.2747
Treatment	Ctr Meg	0	.	.	.	.	.	.	.

Solution for Random Effects									
Effect	Culture	Estimate	Std Err Pred	DF	t Value	Pr > |t|	Alpha	Lower	Upper
Culture	1	-696E-19	0.3108	32	-0.00	1.0000	0.05	-0.6330	0.6330
Culture	2	-696E-19	0.3108	32	-0.00	1.0000	0.05	-0.6330	0.6330
Culture	3	-854E-19	0.2951	32	-0.00	1.0000	0.05	-0.6011	0.6011
Culture	4	-854E-19	0.2951	32	-0.00	1.0000	0.05	-0.6011	0.6011
Culture	5	-854E-19	0.2951	32	-0.00	1.0000	0.05	-0.6011	0.6011
Culture	6	-474E-19	0.3781	32	-0.00	1.0000	0.05	-0.7702	0.7702
Culture	7	-696E-19	0.3108	32	-0.00	1.0000	0.05	-0.6330	0.6330
Culture	8	1.1915	0.2983	32	3.99	0.0004	0.05	0.5840	1.7991
Culture	9	-0.2752	0.2983	32	-0.92	0.3631	0.05	-0.8827	0.3324
Culture	10	-0.2527	0.3133	32	-0.81	0.4258	0.05	-0.8909	0.3854
Culture	11	-0.1357	0.2878	32	-0.47	0.6404	0.05	-0.7220	0.4506
Culture	12	-0.2752	0.2983	32	-0.92	0.3631	0.05	-0.8827	0.3324
Culture	13	-0.2527	0.3133	32	-0.81	0.4258	0.05	-0.8909	0.3854

Type 3 Tests of Fixed Effects				
Effect	Num DF	Den DF	F Value	Pr > F
Treatment	1	32	1.38	0.2482

DistSoma=510

Least Squares Means									
**Effect**	**Treatment**	**Estimate**	**Standard Error**	**DF**	**t Value**	**Pr > |t|**	**Alpha**	**Lower**	**Upper**
**Treatment**	Control GFP	0	0.2217	32	0.00	1.0000	0.05	-0.4516	0.4516
**Treatment**	Ctr Meg	0.3752	0.2294	32	1.64	0.1117	0.05	-0.09210	0.8426

Differences of Least Squares Means													
**Effect**	**Treatment**	**Treatment**	**Estimate**	**Standard Error**	**DF**	**t Value**	**Pr > |t|**	**Adjustment**	**Adj P**	**Alpha**	**Lower**	**Upper**
**Treatment**	Control GFP	Ctr Meg	-0.3752	0.3191	32	-1.18	0.2482	Tukey-Kramer	0.2482	0.05	-1.0251	0.2747

Differences of Least Squares Means				
**Effect**	**Treatment**	**Treatment**	**Adj Lower**	**Adj Upper**
**Treatment**	Control GFP	Ctr Meg	-1.0251	0.2746

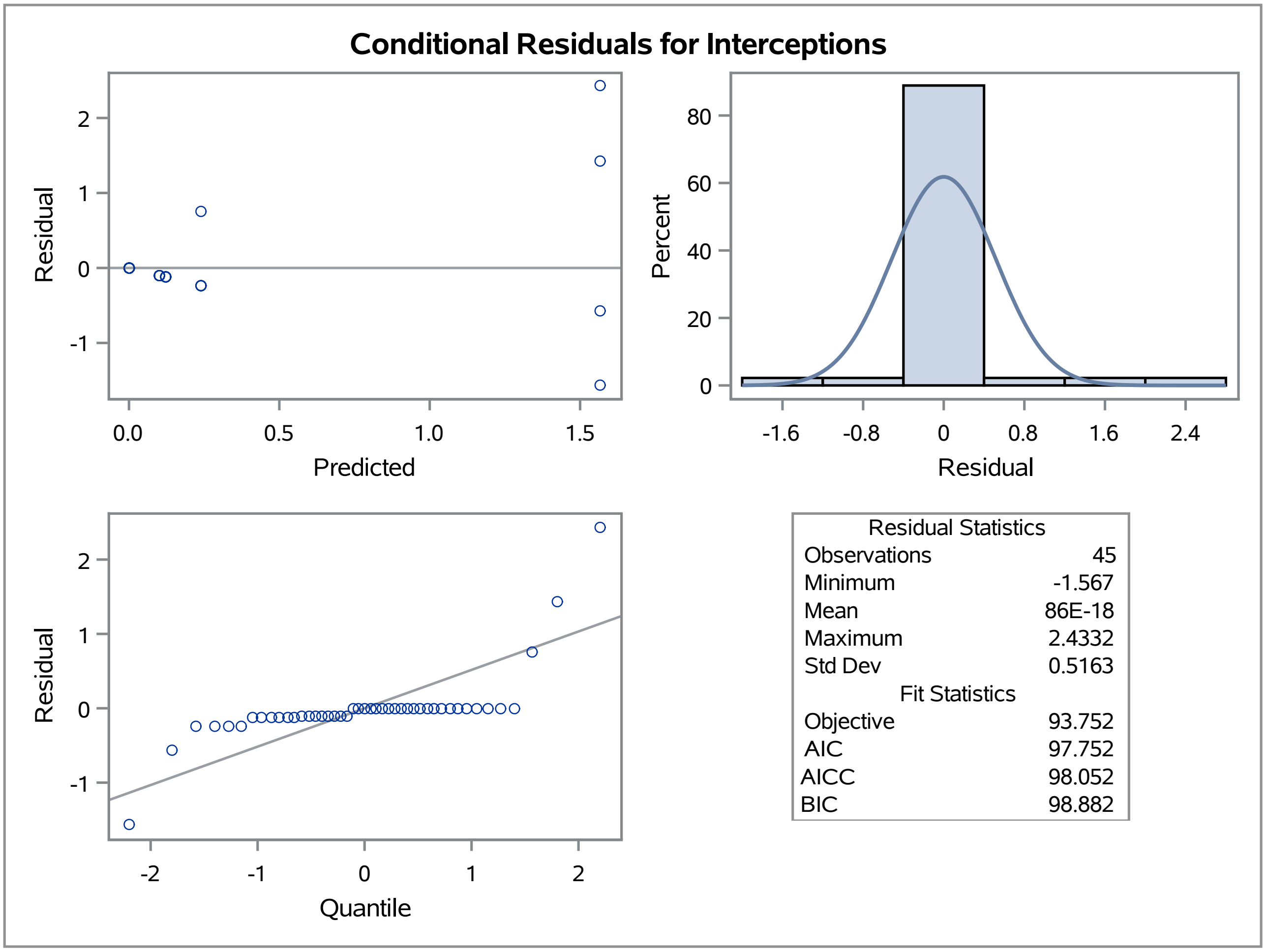

DistSoma=516

Model Information	
Data Set	WORK.TEMPDATASORTED
Dependent Variable	Interceptions
Covariance Structure	Variance Components
Estimation Method	REML
Residual Variance Method	Profile
Fixed Effects SE Method	Model-Based
Degrees of Freedom Method	Containment

Class Level Information		
Class	Levels	Values
Treatment	2	Control GFP Ctr Meg
Culture	13	1 2 3 4 5 6 7 8 9 10 11 12 13

Dimensions	
Covariance Parameters	2
Columns in X	3
Columns in Z	13
Subjects	1
Max Obs per Subject	45

Number of Observations	
Number of Observations Read	45
Number of Observations Used	45
Number of Observations Not Used	0

Iteration History			
Iteration	Evaluations	-2 Res Log Like	Criterion
0	1	108.99963821	
1	3	104.09661672	0.00259003
2	1	104.06136554	0.00005373
3	1	104.06068330	0.00000003
4	1	104.06068297	0.00000000

Convergence criteria met.

DistSoma=516

Covariance Parameter Estimates				
**Cov Parm**	**Estimate**	**Alpha**	**Lower**	**Upper**
**Culture**	0.2012	0.05	0.07364	1.5363
**Residual**	0.4513	0.05	0.2941	0.7797

Fit Statistics	
**-2 Res Log Likelihood**	104.1
**AIC (Smaller is Better)**	108.1
**AICC (Smaller is Better)**	108.4
**BIC (Smaller is Better)**	109.2

Solution for Fixed Effects									
**Effect**	**Treatment**	**Estimate**	**Standard Error**	**DF**	**t Value**	**Pr > |t|**	**Alpha**	**Lower**	**Upper**
**Intercept**		0.3778	0.2314	11	1.63	0.1308	0.05	-0.1316	0.8872
**Treatment**	Control GFP	-0.3778	0.3233	32	-1.17	0.2512	0.05	-1.0365	0.2808
**Treatment**	Ctr Meg	0	.	.	.	.	.	.	.

Solution for Random Effects									
**Effect**	**Culture**	**Estimate**	**Std Err Pred**	**DF**	**t Value**	**Pr > |t|**	**Alpha**	**Lower**	**Upper**
**Culture**	1	-153E-20	0.3206	32	-0.00	1.0000	0.05	-0.6530	0.6530
**Culture**	2	-153E-20	0.3206	32	-0.00	1.0000	0.05	-0.6530	0.6530
**Culture**	3	6.37E-18	0.3053	32	0.00	1.0000	0.05	-0.6219	0.6219
**Culture**	4	6.37E-18	0.3053	32	0.00	1.0000	0.05	-0.6219	0.6219
**Culture**	5	6.37E-18	0.3053	32	0.00	1.0000	0.05	-0.6219	0.6219
**Culture**	6	3.07E-18	0.3795	32	0.00	1.0000	0.05	-0.7730	0.7730
**Culture**	7	-153E-20	0.3206	32	-0.00	1.0000	0.05	-0.6530	0.6530
**Culture**	8	1.0394	0.3071	32	3.38	0.0019	0.05	0.4139	1.6649
**Culture**	9	-0.2421	0.3071	32	-0.79	0.4362	0.05	-0.8676	0.3834
**Culture**	10	-0.2162	0.3219	32	-0.67	0.5066	0.05	-0.8719	0.4395
**Culture**	11	-0.1228	0.2964	32	-0.41	0.6815	0.05	-0.7265	0.4809
**Culture**	12	-0.2421	0.3071	32	-0.79	0.4362	0.05	-0.8676	0.3834
**Culture**	13	-0.2162	0.3219	32	-0.67	0.5066	0.05	-0.8719	0.4395

Type 3 Tests of Fixed Effects				
**Effect**	**Num DF**	**Den DF**	**F Value**	**Pr > F**
**Treatment**	1	32	1.37	0.2512

DistSoma=516

Least Squares Means									
**Effect**	**Treatment**	**Estimate**	**Standard Error**	**DF**	**t Value**	**Pr > |t|**	**Alpha**	**Lower**	**Upper**
**Treatment**	Control GFP	0	0.2258	32	0.00	1.0000	0.05	-0.4599	0.4599
**Treatment**	Ctr Meg	0.3778	0.2314	32	1.63	0.1124	0.05	-0.09358	0.8493

Differences of Least Squares Means													
**Effect**	**Treatment**	**Treatment**	**Estimate**	**Standard Error**	**DF**	**t Value**	**Pr > |t|**	**Adjustment**	**Adj P**	**Alpha**	**Lower**	**Upper**
**Treatment**	Control GFP	Ctr Meg	-0.3778	0.3233	32	-1.17	0.2512	Tukey-Kramer	0.2512	0.05	-1.0365	0.2808

Differences of Least Squares Means				
**Effect**	**Treatment**	**Treatment**	**Adj Lower**	**Adj Upper**
**Treatment**	Control GFP	Ctr Meg	-1.0364	0.2808

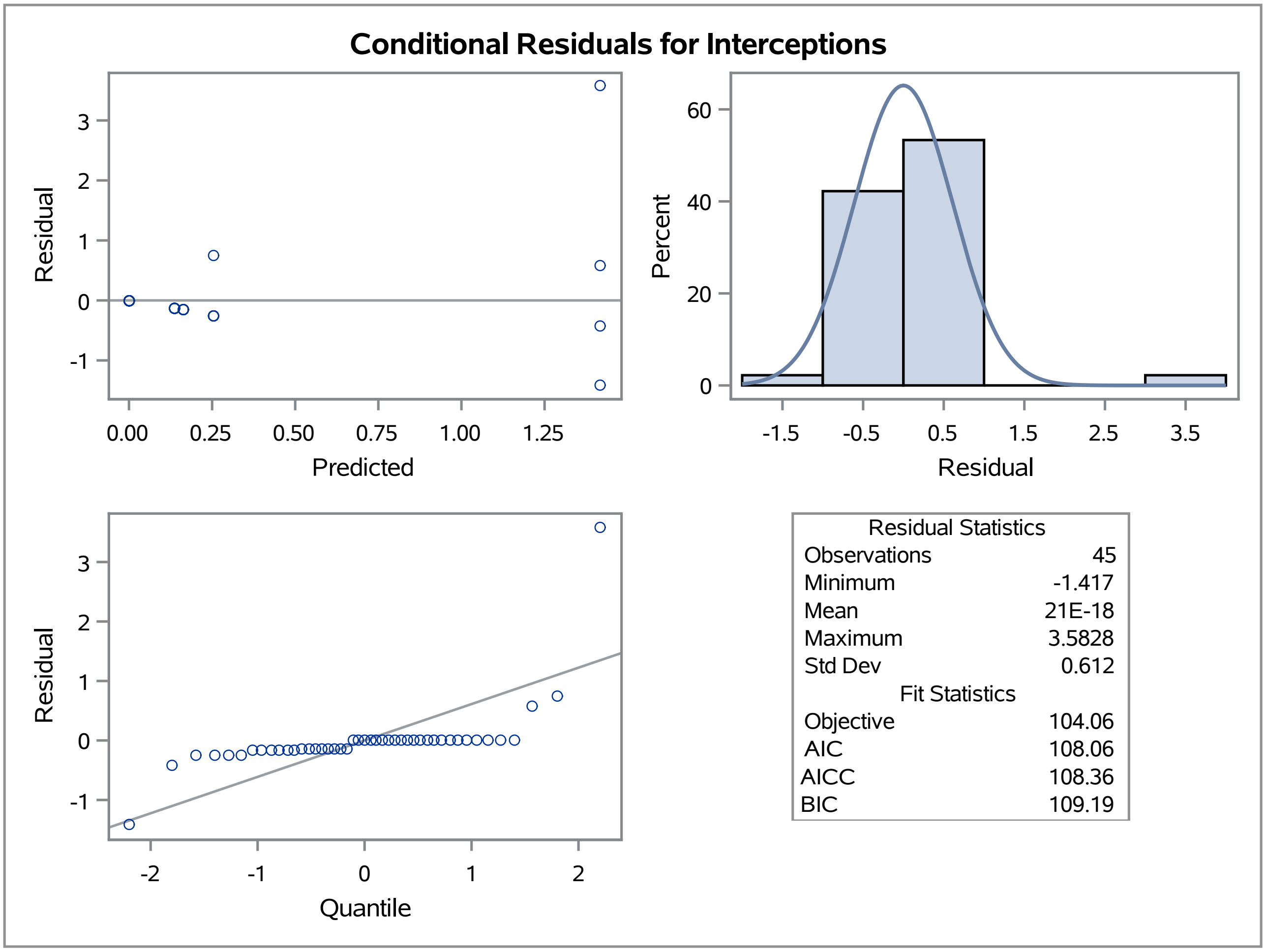

DistSoma=522

Model Information	
**Data Set**	WORK.TEMPDATASORTED
**Dependent Variable**	Interceptions
**Covariance Structure**	Variance Components
**Estimation Method**	REML
**Residual Variance Method**	Profile
**Fixed Effects SE Method**	Model-Based
**Degrees of Freedom Method**	Containment

Class Level Information		
**Class**	**Levels**	**Values**
**Treatment**	2	Control GFP Ctr Meg
**Culture**	13	1 2 3 4 5 6 7 8 9 10 11 12 13

Dimensions	
**Covariance Parameters**	2
**Columns in X**	3
**Columns in Z**	13
**Subjects**	1
**Max Obs per Subject**	45

Number of Observations	
**Number of Observations Read**	45
**Number of Observations Used**	45
**Number of Observations Not Used**	0

Iteration History			
**Iteration**	**Evaluations**	**-2 Res Log Like**	**Criterion**
**0**	1	108.99963821	
**1**	3	104.09661672	0.00259003
**2**	1	104.06136554	0.00005373
**3**	1	104.06068330	0.00000003
**4**	1	104.06068297	0.00000000

Convergence criteria met.

DistSoma=522

Covariance Parameter Estimates				
**Cov Parm**	**Estimate**	**Alpha**	**Lower**	**Upper**
**Culture**	0.2012	0.05	0.07364	1.5363
**Residual**	0.4513	0.05	0.2941	0.7797

Fit Statistics	
**-2 Res Log Likelihood**	104.1
**AIC (Smaller is Better)**	108.1
**AICC (Smaller is Better)**	108.4
**BIC (Smaller is Better)**	109.2

Solution for Fixed Effects									
**Effect**	**Treatment**	**Estimate**	**Standard Error**	**DF**	**t Value**	**Pr > |t|**	**Alpha**	**Lower**	**Upper**
**Intercept**		0.3778	0.2314	11	1.63	0.1308	0.05	-0.1316	0.8872
**Treatment**	Control GFP	-0.3778	0.3233	32	-1.17	0.2512	0.05	-1.0365	0.2808
**Treatment**	Ctr Meg	0	.	.	.	.	.	.	.

Solution for Random Effects									
**Effect**	**Culture**	**Estimate**	**Std Err Pred**	**DF**	**t Value**	**Pr > |t|**	**Alpha**	**Lower**	**Upper**
**Culture**	1	-153E-20	0.3206	32	-0.00	1.0000	0.05	-0.6530	0.6530
**Culture**	2	-153E-20	0.3206	32	-0.00	1.0000	0.05	-0.6530	0.6530
**Culture**	3	6.37E-18	0.3053	32	0.00	1.0000	0.05	-0.6219	0.6219
**Culture**	4	6.37E-18	0.3053	32	0.00	1.0000	0.05	-0.6219	0.6219
**Culture**	5	6.37E-18	0.3053	32	0.00	1.0000	0.05	-0.6219	0.6219
**Culture**	6	3.07E-18	0.3795	32	0.00	1.0000	0.05	-0.7730	0.7730
**Culture**	7	-153E-20	0.3206	32	-0.00	1.0000	0.05	-0.6530	0.6530
**Culture**	8	1.0394	0.3071	32	3.38	0.0019	0.05	0.4139	1.6649
**Culture**	9	-0.2421	0.3071	32	-0.79	0.4362	0.05	-0.8676	0.3834
**Culture**	10	-0.2162	0.3219	32	-0.67	0.5066	0.05	-0.8719	0.4395
**Culture**	11	-0.1228	0.2964	32	-0.41	0.6815	0.05	-0.7265	0.4809
**Culture**	12	-0.2421	0.3071	32	-0.79	0.4362	0.05	-0.8676	0.3834
**Culture**	13	-0.2162	0.3219	32	-0.67	0.5066	0.05	-0.8719	0.4395

Type 3 Tests of Fixed Effects				
**Effect**	**Num DF**	**Den DF**	**F Value**	**Pr > F**
**Treatment**	1	32	1.37	0.2512

DistSoma=522

Least Squares Means									
Effect	Treatment	Estimate	Standard Error	DF	t Value	Pr > |t|	Alpha	Lower	Upper
Treatment	Control GFP	0	0.2258	32	0.00	1.0000	0.05	-0.4599	0.4599
Treatment	Ctr Meg	0.3778	0.2314	32	1.63	0.1124	0.05	-0.09358	0.8493

Differences of Least Squares Means													
Effect	Treatment	Treatment	Estimate	Standard Error	DF	t Value	Pr > |t|	Adjustment	Adj P	Alpha	Lower	Upper
Treatment	Control GFP	Ctr Meg	-0.3778	0.3233	32	-1.17	0.2512	Tukey-Kramer	0.2512	0.05	-1.0365	0.2808

Differences of Least Squares Means				
Effect	Treatment	Treatment	Adj Lower	Adj Upper
Treatment	Control GFP	Ctr Meg	-1.0364	0.2808

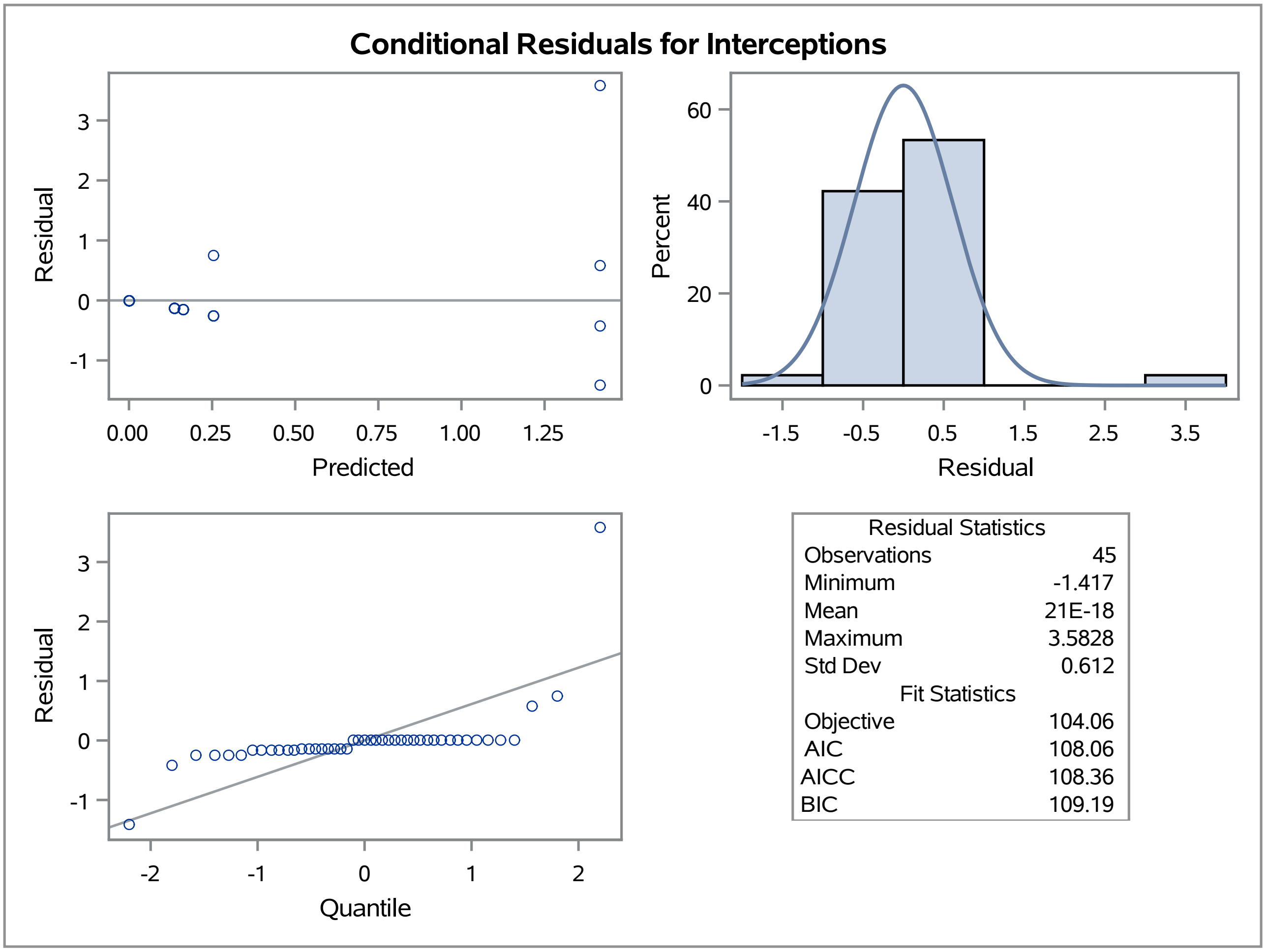

DistSoma=528

Model Information	
**Data Set**	WORK.TEMPDATASORTED
**Dependent Variable**	Interceptions
**Covariance Structure**	Variance Components
**Estimation Method**	REML
**Residual Variance Method**	Profile
**Fixed Effects SE Method**	Model-Based
**Degrees of Freedom Method**	Containment

Class Level Information		
**Class**	**Levels**	**Values**
**Treatment**	2	Control GFP Ctr Meg
**Culture**	13	1 2 3 4 5 6 7 8 9 10 11 12 13

Dimensions	
**Covariance Parameters**	2
**Columns in X**	3
**Columns in Z**	13
**Subjects**	1
**Max Obs per Subject**	45

Number of Observations	
**Number of Observations Read**	45
**Number of Observations Used**	45
**Number of Observations Not Used**	0

Iteration History			
**Iteration**	**Evaluations**	**-2 Res Log Like**	**Criterion**
**0**	1	108.99963821	
**1**	3	104.09661672	0.00259003
**2**	1	104.06136554	0.00005373
**3**	1	104.06068330	0.00000003
**4**	1	104.06068297	0.00000000

Convergence criteria met.

DistSoma=528

Covariance Parameter Estimates				
Cov Parm	Estimate	Alpha	Lower	Upper
Culture	0.2012	0.05	0.07364	1.5363
Residual	0.4513	0.05	0.2941	0.7797

Fit Statistics	
-2 Res Log Likelihood	104.1
AIC (Smaller is Better)	108.1
AICC (Smaller is Better)	108.4
BIC (Smaller is Better)	109.2

Solution for Fixed Effects									
Effect	Treatment	Estimate	Standard Error	DF	t Value	Pr > |t|	Alpha	Lower	Upper
Intercept		0.3778	0.2314	11	1.63	0.1308	0.05	-0.1316	0.8872
Treatment	Control GFP	-0.3778	0.3233	32	-1.17	0.2512	0.05	-1.0365	0.2808
Treatment	Ctr Meg	0	.	.	.	.	.	.	.

Solution for Random Effects									
Effect	Culture	Estimate	Std Err Pred	DF	t Value	Pr > |t|	Alpha	Lower	Upper
Culture	1	-153E-20	0.3206	32	-0.00	1.0000	0.05	-0.6530	0.6530
Culture	2	-153E-20	0.3206	32	-0.00	1.0000	0.05	-0.6530	0.6530
Culture	3	6.37E-18	0.3053	32	0.00	1.0000	0.05	-0.6219	0.6219
Culture	4	6.37E-18	0.3053	32	0.00	1.0000	0.05	-0.6219	0.6219
Culture	5	6.37E-18	0.3053	32	0.00	1.0000	0.05	-0.6219	0.6219
Culture	6	3.07E-18	0.3795	32	0.00	1.0000	0.05	-0.7730	0.7730
Culture	7	-153E-20	0.3206	32	-0.00	1.0000	0.05	-0.6530	0.6530
Culture	8	1.0394	0.3071	32	3.38	0.0019	0.05	0.4139	1.6649
Culture	9	-0.2421	0.3071	32	-0.79	0.4362	0.05	-0.8676	0.3834
Culture	10	-0.2162	0.3219	32	-0.67	0.5066	0.05	-0.8719	0.4395
Culture	11	-0.1228	0.2964	32	-0.41	0.6815	0.05	-0.7265	0.4809
Culture	12	-0.2421	0.3071	32	-0.79	0.4362	0.05	-0.8676	0.3834
Culture	13	-0.2162	0.3219	32	-0.67	0.5066	0.05	-0.8719	0.4395

Type 3 Tests of Fixed Effects				
Effect	Num DF	Den DF	F Value	Pr > F
Treatment	1	32	1.37	0.2512

DistSoma=528

Least Squares Means									
**Effect**	**Treatment**	**Estimate**	**Standard Error**	**DF**	**t Value**	**Pr > |t|**	**Alpha**	**Lower**	**Upper**
**Treatment**	Control GFP	0	0.2258	32	0.00	1.0000	0.05	-0.4599	0.4599
**Treatment**	Ctr Meg	0.3778	0.2314	32	1.63	0.1124	0.05	-0.09358	0.8493

Differences of Least Squares Means												
**Effect**	**Treatment**	**Treatment**	**Estimate**	**Standard Error**	**DF**	**t Value**	**Pr > |t|**	**Adjustment**	**Adj P**	**Alpha**	**Lower**	**Upper**
**Treatment**	Control GFP	Ctr Meg	-0.3778	0.3233	32	-1.17	0.2512	Tukey-Kramer	0.2512	0.05	-1.0365	0.2808

Differences of Least Squares Means				
**Effect**	**Treatment**	**Treatment**	**Adj Lower**	**Adj Upper**
**Treatment**	Control GFP	Ctr Meg	-1.0364	0.2808

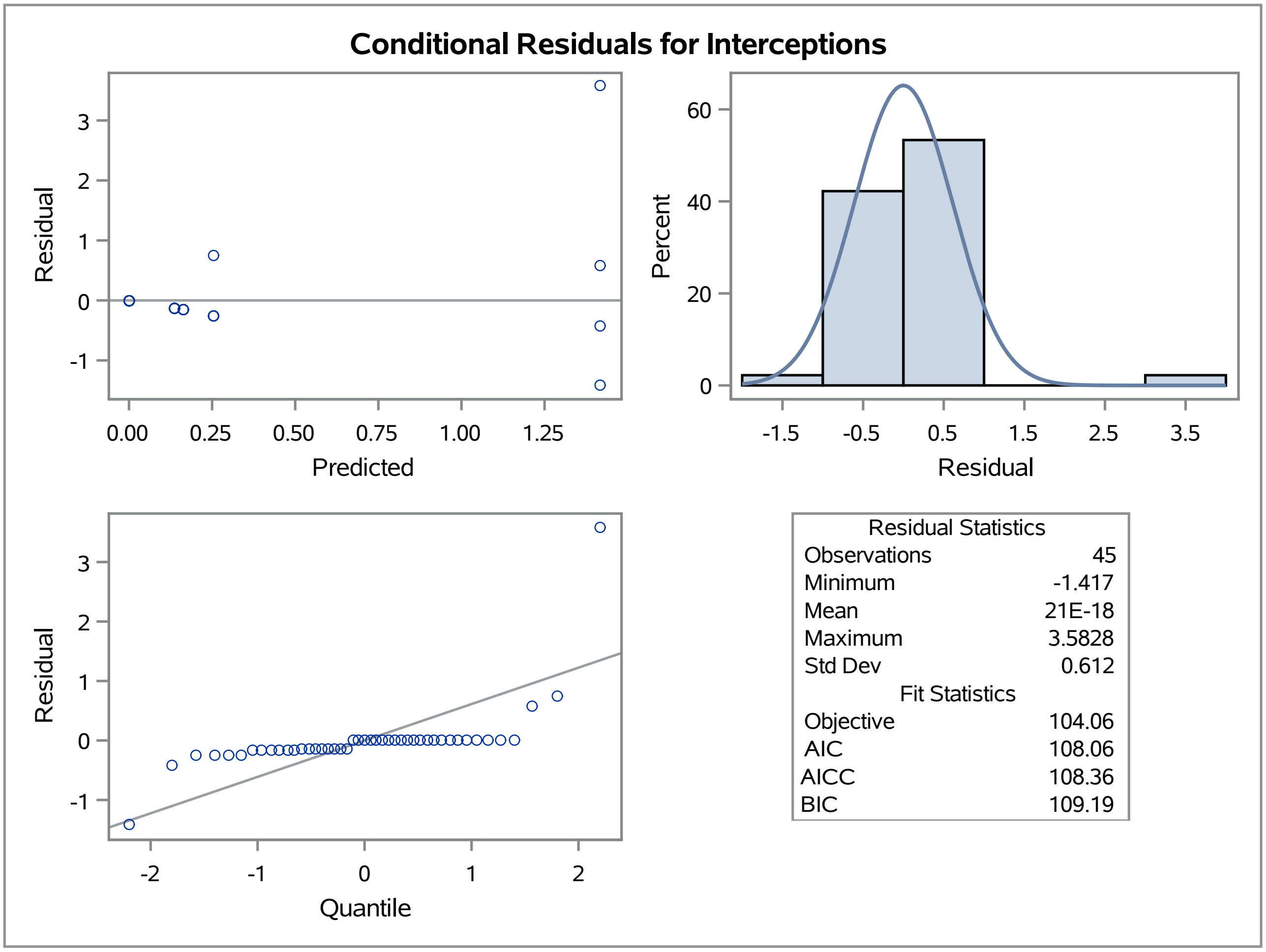

The CONTENTS Procedure

**Data Set Name**	WORK.FIG6G	**Observations**	3649
**Member Type**	DATA	**Variables**	4
**Engine**	V9	**Indexes**	0
**Created**	07/08/2020 13:59:29	**Observation Length**	40
**Last Modified**	07/08/2020 13:59:29	**Deleted Observations**	0
**Protection**		**Compressed**	NO
**Data Set Type**		**Sorted**	NO
**Label**			
**Data Representation**	SOLARIS_X86_64, LINUX_X86_64, ALPHA_TRU64, LINUX_IA64		
**Encoding**	utf-8 Unicode (UTF-8)		

Engine/Host Dependent Information	
**Data Set Page Size**	65536
**Number of Data Set Pages**	3
**First Data Page**	1
**Max Obs per Page**	1632
**Obs in First Data Page**	1573
**Number of Data Set Repairs**	0
**Filename**	/tmp/SAS_workF3DB00000EA0_localhost.localdomain/SAS_work306900000EA0_localhost.localdomain/fig6g.sas7bdat
**Release Created**	9.0401M6
**Host Created**	Linux
**Inode Number**	672645
**Access Permission**	rw-rw-r--
**Owner Name**	sasdemo
**File Size**	256KB
**File Size (bytes)**	262144

Alphabetic List of Variables and Attributes

#	Variable	Type	Len	Format	Informat	Label
**3**	Culture	Num	8	BEST.		Culture
**1**	DistSoma	Num	8	BEST.		DistSoma
**4**	Interceptions	Num	8	BEST.		Interceptions
**2**	Treatment	Char	9	$9.	$9.	Treatment

DistSoma=0

Model Information	
**Data Set**	WORK.TEMPDATASORTED
**Dependent Variable**	Interceptions
**Covariance Structure**	Variance Components
**Estimation Method**	REML
**Residual Variance Method**	Profile
**Fixed Effects SE Method**	Model-Based
**Degrees of Freedom Method**	Containment

Class Level Information		
**Class**	**Levels**	**Values**
**Treatment**	2	Meg TTR GFP MsTTR
**Culture**	10	1 2 3 4 5 6 7 8 9 10

Dimensions	
**Covariance Parameters**	2
**Columns in X**	3
**Columns in Z**	10
**Subjects**	1
**Max Obs per Subject**	41

Number of Observations	
**Number of Observations Read**	41
**Number of Observations Used**	41
**Number of Observations Not Used**	0

Iteration History			
**Iteration**	**Evaluations**	**-2 Res Log Like**	**Criterion**
**0**	1	203.81511001	
**1**	3	201.42146154	0.00005584
**2**	1	201.41770164	0.00000020
**3**	1	201.41768870	0.00000000

Convergence criteria met.

DistSoma=0

Covariance Parameter Estimates				
Cov Parm	Estimate	Alpha	Lower	Upper
Culture	2.0664	0.05	0.5897	57.0596
Residual	7.5502	0.05	4.8750	13.2445

Fit Statistics	
-2 Res Log Likelihood	201.4
AIC (Smaller is Better)	205.4
AICC (Smaller is Better)	205.8
BIC (Smaller is Better)	206.0

Solution for Fixed Effects									
Effect	Treatment	Estimate	Standard Error	DF	t Value	Pr > |t|	Alpha	Lower	Upper
Intercept		11.3115	0.9814	8	11.53	<.0001	0.05	9.0485	13.5745
Treatment	Meg TTR	0.7124	1.2809	31	0.56	0.5821	0.05	-1.9001	3.3248
Treatment	GFP MsTTR	0	.	.	.	.	.	.	.

Solution for Random Effects									
Effect	Culture	Estimate	Std Err Pred	DF	t Value	Pr > |t|	Alpha	Lower	Upper
Culture	1	-0.2955	1.0927	31	-0.27	0.7886	0.05	-2.5241	1.9330
Culture	2	-1.0774	1.1178	31	-0.96	0.3426	0.05	-3.3572	1.2024
Culture	3	1.1437	1.1178	31	1.02	0.3141	0.05	-1.1361	3.4235
Culture	4	0.2292	1.1178	31	0.21	0.8389	0.05	-2.0506	2.5090
Culture	5	-1.5160	1.0482	31	-1.45	0.1581	0.05	-3.6538	0.6218
Culture	6	1.1634	1.0824	31	1.07	0.2907	0.05	-1.0441	3.3710
Culture	7	0.2488	1.0824	31	0.23	0.8197	0.05	-1.9587	2.4564
Culture	8	1.2282	1.0217	31	1.20	0.2384	0.05	-0.8556	3.3120
Culture	9	-0.3622	1.1917	31	-0.30	0.7632	0.05	-2.7927	2.0684
Culture	10	-0.7622	1.1280	31	-0.68	0.5042	0.05	-3.0629	1.5384

Type 3 Tests of Fixed Effects				
Effect	Num DF	Den DF	F Value	Pr > F
Treatment	1	31	0.31	0.5821

DistSoma=0

Least Squares Means									
**Effect**	**Treatment**	**Estimate**	**Standard Error**	**DF**	**t Value**	**Pr > |t|**	**Alpha**	**Lower**	**Upper**
**Treatment**	Meg TTR	12.0239	0.8232	31	14.61	<.0001	0.05	10.3449	13.7029
**Treatment**	GFP MsTTR	11.3115	0.9814	31	11.53	<.0001	0.05	9.3100	13.3130

Differences of Least Squares Means												
**Effect**	**Treatment**	**Treatment**	**Estimate**	**Standard Error**	**DF**	**t Value**	**Pr > |t|**	**Adjustment**	**Adj P**	**Alpha**	**Lower**	**Upper**
**Treatment**	Meg TTR	GFP MsTTR	0.7124	1.2809	31	0.56	0.5821	Tukey-Kramer	0.5821	0.05	-1.9001	3.3248

Differences of Least Squares Means				
**Effect**	**Treatment**	**Treatment**	**Adj Lower**	**Adj Upper**
**Treatment**	Meg TTR	GFP MsTTR	-1.9001	3.3248

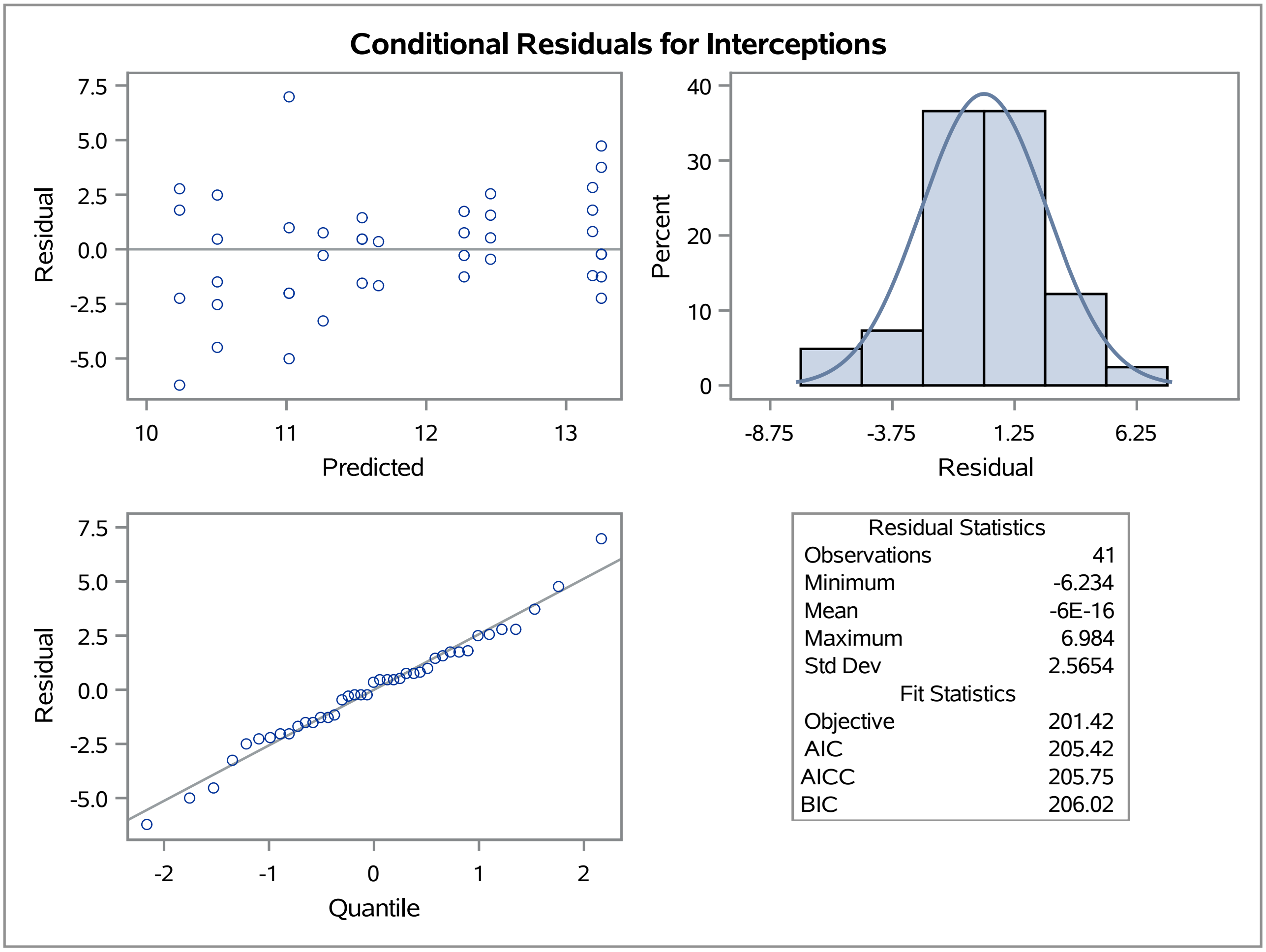

DistSoma=6

Model Information	
**Data Set**	WORK.TEMPDATASORTED
**Dependent Variable**	Interceptions
**Covariance Structure**	Variance Components
**Estimation Method**	REML
**Residual Variance Method**	Profile
**Fixed Effects SE Method**	Model-Based
**Degrees of Freedom Method**	Containment

Class Level Information		
**Class**	**Levels**	**Values**
**Treatment**	2	Meg TTR GFP MsTTR
**Culture**	10	1 2 3 4 5 6 7 8 9 10

Dimensions	
**Covariance Parameters**	2
**Columns in X**	3
**Columns in Z**	10
**Subjects**	1
**Max Obs per Subject**	41

Number of Observations	
**Number of Observations Read**	41
**Number of Observations Used**	41
**Number of Observations Not Used**	0

Iteration History			
**Iteration**	**Evaluations**	**-2 Res Log Like**	**Criterion**
**0**	1	206.26906291	
**1**	2	204.41451413	0.00000086
**2**	1	204.41445699	0.00000000

Convergence criteria met.

Covariance Parameter Estimates				
**Cov Parm**	**Estimate**	**Alpha**	**Lower**	**Upper**
**Culture**	2.0050	0.05	0.5323	92.6116
**Residual**	8.2541	0.05	5.3199	14.5221

DistSoma=6

Fit Statistics	
**-2 Res Log Likelihood**	204.4
**AIC (Smaller is Better)**	208.4
**AICC (Smaller is Better)**	208.7
**BIC (Smaller is Better)**	209.0

Solution for Fixed Effects

Effect	Treatment	Estimate	Standard Error	DF	t Value	Pr > |t|	Alpha	Lower	Upper
**Intercept**		11.1890	0.9946	8	11.25	<.0001	0.05	8.8955	13.4825
**Treatment**	Meg TTR	0.3813	1.2986	31	0.29	0.7710	0.05	-2.2673	3.0298
**Treatment**	GFP MsTTR	0	.	.	.	.	.	.	.

Solution for Random Effects

Effect	Culture	Estimate	Std Err Pred	DF	t Value	Pr > |t|	Alpha	Lower	Upper
**Culture**	1	-0.2134	1.0968	31	-0.19	0.8470	0.05	-2.4502	2.0235
**Culture**	2	-0.9556	1.1212	31	-0.85	0.4006	0.05	-3.2423	1.3312
**Culture**	3	1.1389	1.1212	31	1.02	0.3176	0.05	-1.1479	3.4256
**Culture**	4	0.03005	1.1212	31	0.03	0.9788	0.05	-2.2567	2.3168
**Culture**	5	-1.4096	1.0560	31	-1.33	0.1916	0.05	-3.5633	0.7440
**Culture**	6	1.1974	1.0891	31	1.10	0.2801	0.05	-1.0239	3.4187
**Culture**	7	0.5814	1.0891	31	0.53	0.5973	0.05	-1.6400	2.8027
**Culture**	8	0.9468	1.0301	31	0.92	0.3651	0.05	-1.1541	3.0477
**Culture**	9	-0.5134	1.1933	31	-0.43	0.6700	0.05	-2.9472	1.9203
**Culture**	10	-0.8024	1.1330	31	-0.71	0.4841	0.05	-3.1132	1.5083

Type 3 Tests of Fixed Effects

Effect	Num DF	Den DF	F Value	Pr > F
**Treatment**	1	31	0.09	0.7710

Least Squares Means

Effect	Treatment	Estimate	Standard Error	DF	t Value	Pr > |t|	Alpha	Lower	Upper
**Treatment**	Meg TTR	11.5703	0.8350	31	13.86	<.0001	0.05	9.8673	13.2733
**Treatment**	GFP MsTTR	11.1890	0.9946	31	11.25	<.0001	0.05	9.1606	13.2175

DistSoma=6

Differences of Least Squares Means												
**Effect**	**Treatment**	**Treatment**	**Estimate**	**Standard Error**	**DF**	**t Value**	**Pr > |t|**	**Adjustment**	**Adj P**	**Alpha**	**Lower**	**Upper**
**Treatment**	Meg TTR	GFP MsTTR	0.3813	1.2986	31	0.29	0.7710	Tukey-Kramer	0.7710	0.05	-2.2673	3.0298

Differences of Least Squares Means				
**Effect**	**Treatment**	**Treatment**	**Adj Lower**	**Adj Upper**
**Treatment**	Meg TTR	GFP MsTTR	-2.2673	3.0298

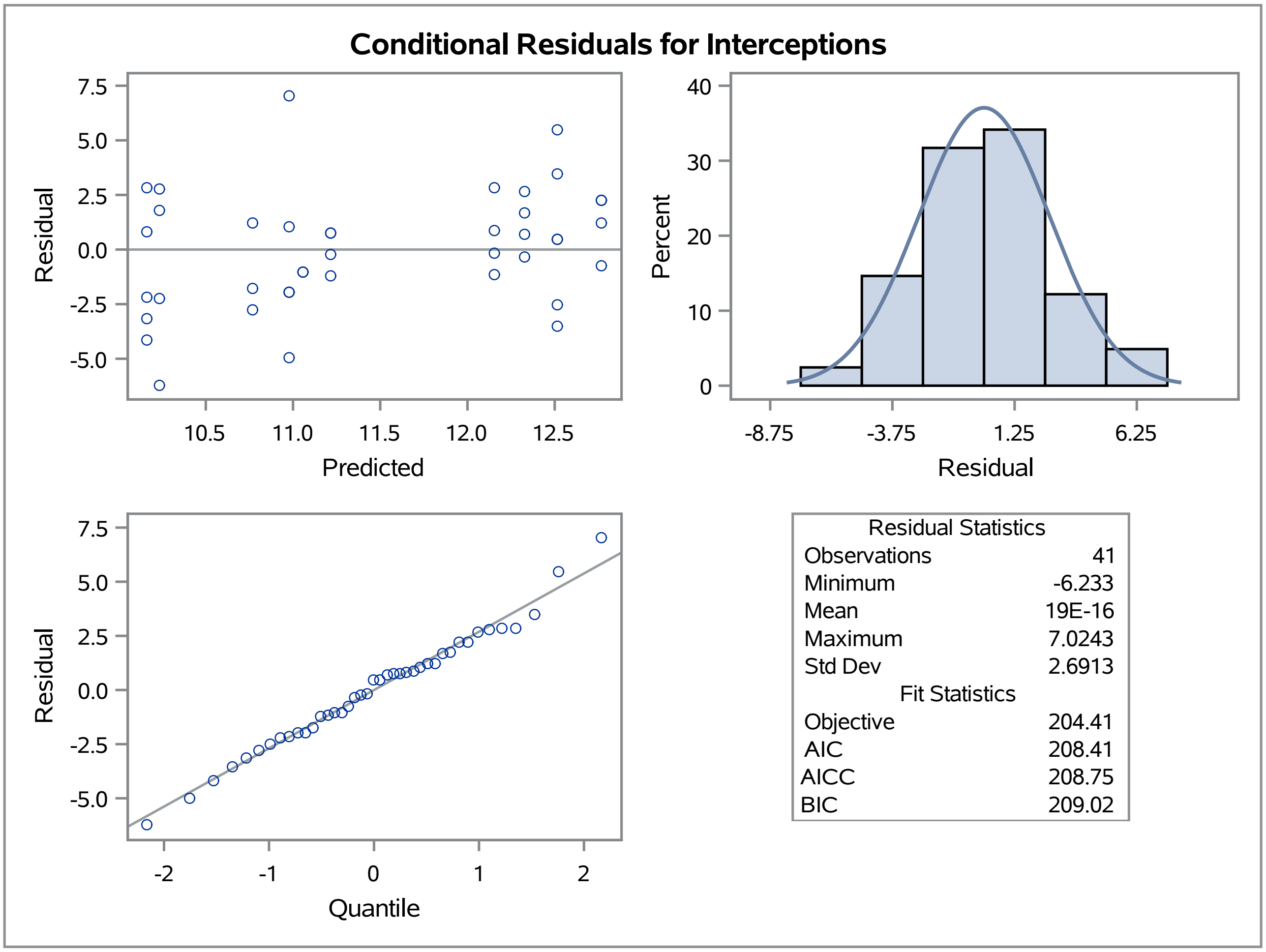

DistSoma=12

Model Information	
**Data Set**	WORK.TEMPDATASORTED
**Dependent Variable**	Interceptions
**Covariance Structure**	Variance Components
**Estimation Method**	REML
**Residual Variance Method**	Profile
**Fixed Effects SE Method**	Model-Based
**Degrees of Freedom Method**	Containment

Class Level Information		
**Class**	**Levels**	**Values**
**Treatment**	2	Meg TTR GFP MsTTR
**Culture**	10	1 2 3 4 5 6 7 8 9 10

Dimensions	
**Covariance Parameters**	2
**Columns in X**	3
**Columns in Z**	10
**Subjects**	1
**Max Obs per Subject**	41

Number of Observations	
**Number of Observations Read**	41
**Number of Observations Used**	41
**Number of Observations Not Used**	0

Iteration History			
**Iteration**	**Evaluations**	**-2 Res Log Like**	**Criterion**
**0**	1	219.38961385	
**1**	3	218.37454773	0.00000180
**2**	1	218.37441462	0.00000000

Convergence criteria met.

Covariance Parameter Estimates				
**Cov Parm**	**Estimate**	**Alpha**	**Lower**	**Upper**
**Culture**	1.9831	0.05	0.4324	606.51
**Residual**	12.2446	0.05	7.9007	21.5033

DistSoma=12

Fit Statistics	
-2 Res Log Likelihood	218.4
AIC (Smaller is Better)	222.4
AICC (Smaller is Better)	222.7
BIC (Smaller is Better)	223.0

Solution for Fixed Effects

Effect	Treatment	Estimate	Standard Error	DF	t Value	Pr > |t|	Alpha	Lower	Upper
Intercept		12.0604	1.1041	8	10.92	<.0001	0.05	9.5144	14.6064
Treatment	Meg TTR	0.3647	1.4429	31	0.25	0.8021	0.05	-2.5780	3.3075
Treatment	GFP MsTTR	0	.	.	.	.	.	.	.

Solution for Random Effects

Effect	Culture	Estimate	Std Err Pred	DF	t Value	Pr > |t|	Alpha	Lower	Upper
Culture	1	-0.02703	1.1575	31	-0.02	0.9815	0.05	-2.3878	2.3337
Culture	2	-0.8100	1.1798	31	-0.69	0.4974	0.05	-3.2162	1.5961
Culture	3	1.0574	1.1798	31	0.90	0.3770	0.05	-1.3488	3.4636
Culture	4	-0.2203	1.1798	31	-0.19	0.8531	0.05	-2.6265	2.1859
Culture	5	-1.1746	1.1263	31	-1.04	0.3051	0.05	-3.4717	1.1225
Culture	6	1.0123	1.1562	31	0.88	0.3880	0.05	-1.3458	3.3704
Culture	7	0.2260	1.1562	31	0.20	0.8463	0.05	-2.1321	2.5841
Culture	8	1.1047	1.1024	31	1.00	0.3241	0.05	-1.1437	3.3531
Culture	9	-0.5934	1.2448	31	-0.48	0.6369	0.05	-3.1322	1.9455
Culture	10	-0.5750	1.1945	31	-0.48	0.6336	0.05	-3.0113	1.8613

Type 3 Tests of Fixed Effects

Effect	Num DF	Den DF	F Value	Pr > F
Treatment	1	31	0.06	0.8021

Least Squares Means

Effect	Treatment	Estimate	Standard Error	DF	t Value	Pr > |t|	Alpha	Lower	Upper
Treatment	Meg TTR	12.4251	0.9289	31	13.38	<.0001	0.05	10.5306	14.3197
Treatment	GFP MsTTR	12.0604	1.1041	31	10.92	<.0001	0.05	9.8086	14.3122

DistSoma=12

Differences of Least Squares Means												
Effect	Treatment	Treatment	Estimate	Standard Error	DF	t Value	Pr > |t|	Adjustment	Adj P	Alpha	Lower	Upper
Treatment	Meg TTR	GFP MsTTR	0.3647	1.4429	31	0.25	0.8021	Tukey-Kramer	0.8021	0.05	-2.5780	3.3075

Differences of Least Squares Means				
Effect	Treatment	Treatment	Adj Lower	Adj Upper
Treatment	Meg TTR	GFP MsTTR	-2.5780	3.3074

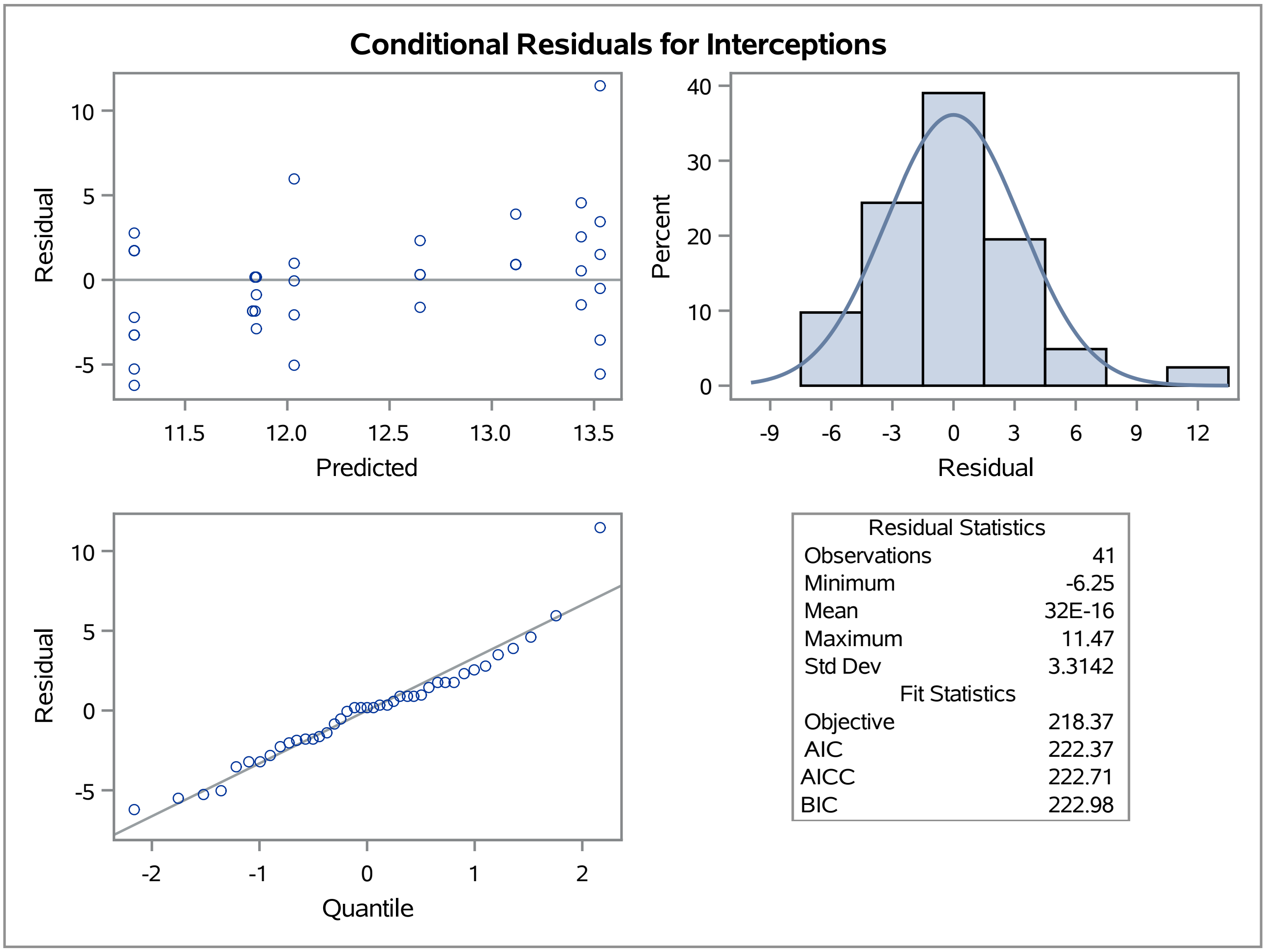

DistSoma=18

Model Information	
**Data Set**	WORK.TEMPDATASORTED
**Dependent Variable**	Interceptions
**Covariance Structure**	Variance Components
**Estimation Method**	REML
**Residual Variance Method**	Profile
**Fixed Effects SE Method**	Model-Based
**Degrees of Freedom Method**	Containment

Class Level Information		
**Class**	**Levels**	**Values**
**Treatment**	2	Meg TTR GFP MsTTR
**Culture**	10	1 2 3 4 5 6 7 8 9 10

Dimensions	
**Covariance Parameters**	2
**Columns in X**	3
**Columns in Z**	10
**Subjects**	1
**Max Obs per Subject**	41

Number of Observations	
**Number of Observations Read**	41
**Number of Observations Used**	41
**Number of Observations Not Used**	0

Iteration History			
**Iteration**	**Evaluations**	**-2 Res Log Like**	**Criterion**
**0**	1	219.24514539	
**1**	2	218.66038481	0.00000005
**2**	1	218.66038145	0.00000000

Convergence criteria met.

Covariance Parameter Estimates				
**Cov Parm**	**Estimate**	**Alpha**	**Lower**	**Upper**
**Culture**	1.5048	0.05	0.2699	8575.47
**Residual**	12.6121	0.05	8.1314	22.1775

DistSoma=18

Fit Statistics	
-2 Res Log Likelihood	218.7
AIC (Smaller is Better)	222.7
AICC (Smaller is Better)	223.0
BIC (Smaller is Better)	223.3

Solution for Fixed Effects

Effect	Treatment	Estimate	Standard Error	DF	t Value	Pr > |t|	Alpha	Lower	Upper
Intercept		12.7774	1.0586	8	12.07	<.0001	0.05	10.3363	15.2185
Treatment	Meg TTR	0.06493	1.3840	31	0.05	0.9629	0.05	-2.7577	2.8876
Treatment	GFP MsTTR	0	.	.	.	.	.	.	.

Solution for Random Effects

Effect	Culture	Estimate	Std Err Pred	DF	t Value	Pr > |t|	Alpha	Lower	Upper
Culture	1	-0.2158	1.0483	31	-0.21	0.8383	0.05	-2.3538	1.9223
Culture	2	-0.4127	1.0657	31	-0.39	0.7012	0.05	-2.5861	1.7607
Culture	3	0.7988	1.0657	31	0.75	0.4591	0.05	-1.3746	2.9723
Culture	4	-0.1704	1.0657	31	-0.16	0.8740	0.05	-2.3438	2.0030
Culture	5	-0.9873	1.0264	31	-0.96	0.3435	0.05	-3.0807	1.1060
Culture	6	1.0202	1.0496	31	0.97	0.3386	0.05	-1.1205	3.1608
Culture	7	0.2125	1.0496	31	0.20	0.8409	0.05	-1.9282	2.3531
Culture	8	0.6916	1.0076	31	0.69	0.4976	0.05	-1.3635	2.7467
Culture	9	-0.4513	1.1155	31	-0.40	0.6886	0.05	-2.7264	1.8239
Culture	10	-0.4856	1.0786	31	-0.45	0.6557	0.05	-2.6855	1.7142

Type 3 Tests of Fixed Effects

Effect	Num DF	Den DF	F Value	Pr > F
Treatment	1	31	0.00	0.9629

Least Squares Means

Effect	Treatment	Estimate	Standard Error	DF	t Value	Pr > |t|	Alpha	Lower	Upper
Treatment	Meg TTR	12.8423	0.8915	31	14.40	<.0001	0.05	11.0240	14.6606
Treatment	GFP MsTTR	12.7774	1.0586	31	12.07	<.0001	0.05	10.6184	14.9364

DistSoma=18

Differences of Least Squares Means												
Effect	Treatment	Treatment	Estimate	Standard Error	DF	t Value	Pr > |t|	Adjustment	Adj P	Alpha	Lower	Upper
**Treatment**	Meg TTR	GFP MsTTR	0.06493	1.3840	31	0.05	0.9629	Tukey-Kramer	0.9629	0.05	-2.7577	2.8876

Differences of Least Squares Means				
Effect	Treatment	Treatment	Adj Lower	Adj Upper
**Treatment**	Meg TTR	GFP MsTTR	-2.7577	2.8876

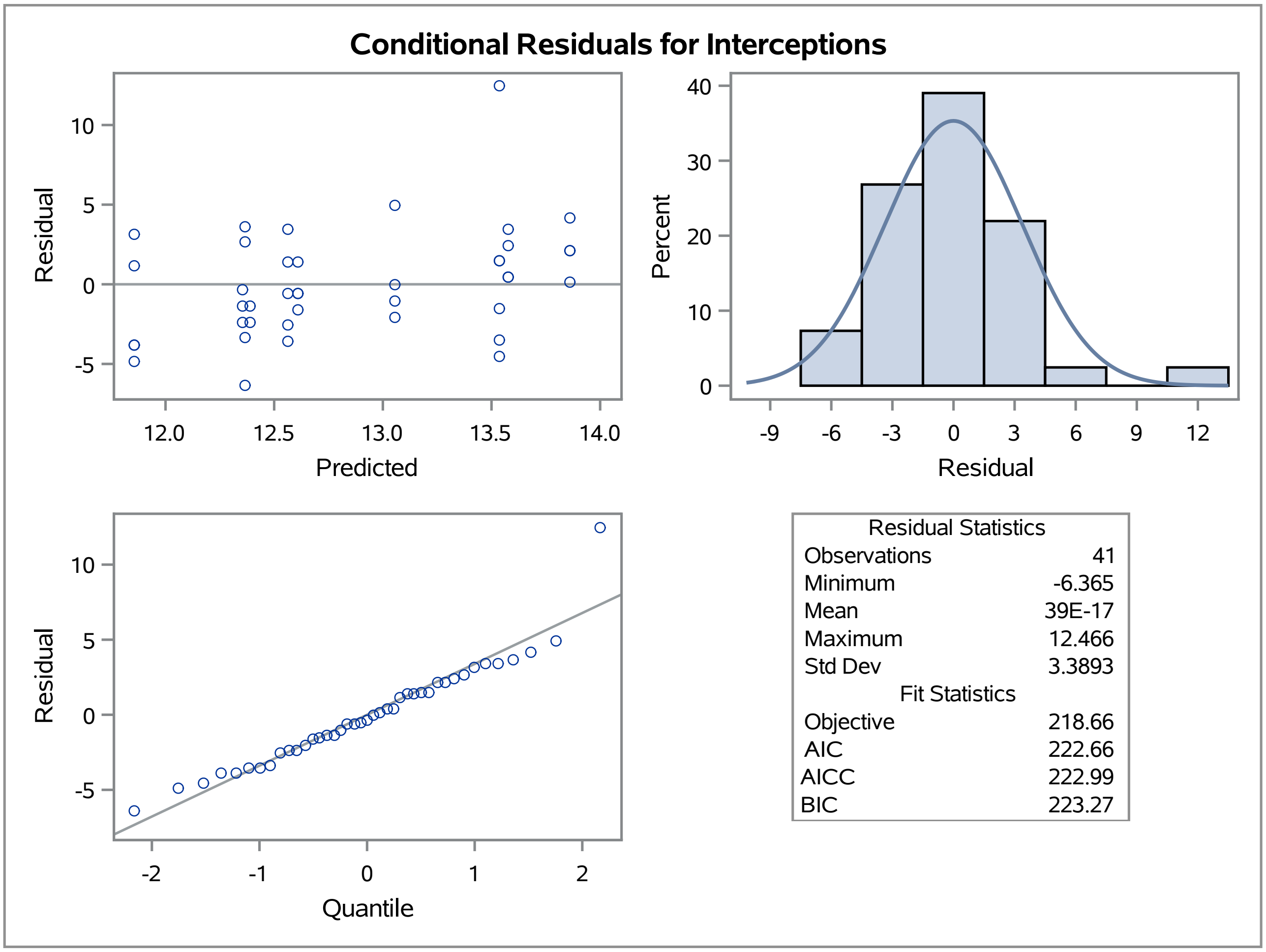

DistSoma=24

Model Information	
**Data Set**	WORK.TEMPDATASORTED
**Dependent Variable**	Interceptions
**Covariance Structure**	Variance Components
**Estimation Method**	REML
**Residual Variance Method**	Profile
**Fixed Effects SE Method**	Model-Based
**Degrees of Freedom Method**	Containment

Class Level Information		
**Class**	**Levels**	**Values**
**Treatment**	2	Meg TTR GFP MsTTR
**Culture**	10	1 2 3 4 5 6 7 8 9 10

Dimensions	
**Covariance Parameters**	2
**Columns in X**	3
**Columns in Z**	10
**Subjects**	1
**Max Obs per Subject**	41

Number of Observations	
**Number of Observations Read**	41
**Number of Observations Used**	41
**Number of Observations Not Used**	0

Iteration History			
**Iteration**	**Evaluations**	**-2 Res Log Like**	**Criterion**
**0**	1	217.44550482	
**1**	2	216.81431617	0.00000035
**2**	1	216.81429050	0.00000000

Convergence criteria met.

Covariance Parameter Estimates				
**Cov Parm**	**Estimate**	**Alpha**	**Lower**	**Upper**
**Culture**	1.6196	0.05	0.3020	4630.35
**Residual**	11.9222	0.05	7.6577	21.0955

DistSoma=24

Fit Statistics	
-2 Res Log Likelihood	216.8
AIC (Smaller is Better)	220.8
AICC (Smaller is Better)	221.1
BIC (Smaller is Better)	221.4

Solution for Fixed Effects

Effect	Treatment	Estimate	Standard Error	DF	t Value	Pr > |t|	Alpha	Lower	Upper
Intercept		13.0244	1.0530	8	12.37	<.0001	0.05	10.5962	15.4526
Treatment	Meg TTR	0.2058	1.3765	31	0.15	0.8821	0.05	-2.6015	3.0131
Treatment	GFP MsTTR	0	.	.	.	.	.	.	.

Solution for Random Effects

Effect	Culture	Estimate	Std Err Pred	DF	t Value	Pr > |t|	Alpha	Lower	Upper
Culture	1	-0.4144	1.0705	31	-0.39	0.7013	0.05	-2.5976	1.7689
Culture	2	-0.4487	1.0894	31	-0.41	0.6833	0.05	-2.6706	1.7732
Culture	3	1.1357	1.0894	31	1.04	0.3053	0.05	-1.0862	3.3575
Culture	4	-0.2726	1.0894	31	-0.25	0.8040	0.05	-2.4945	1.9492
Culture	5	-0.6594	1.0455	31	-0.63	0.5329	0.05	-2.7917	1.4729
Culture	6	1.4153	1.0709	31	1.32	0.1960	0.05	-0.7688	3.5994
Culture	7	0.09500	1.0709	31	0.09	0.9299	0.05	-2.0891	2.2791
Culture	8	-0.02852	1.0251	31	-0.03	0.9780	0.05	-2.1192	2.0621
Culture	9	-0.3697	1.1443	31	-0.32	0.7488	0.05	-2.7035	1.9642
Culture	10	-0.4527	1.1030	31	-0.41	0.6843	0.05	-2.7022	1.7968

Type 3 Tests of Fixed Effects

Effect	Num DF	Den DF	F Value	Pr > F
Treatment	1	31	0.02	0.8821

Least Squares Means

Effect	Treatment	Estimate	Standard Error	DF	t Value	Pr > |t|	Alpha	Lower	Upper
Treatment	Meg TTR	13.2302	0.8865	31	14.92	<.0001	0.05	11.4222	15.0382
Treatment	GFP MsTTR	13.0244	1.0530	31	12.37	<.0001	0.05	10.8768	15.1719

DistSoma=24

Differences of Least Squares Means												
Effect	Treatment	Treatment	Estimate	Standard Error	DF	t Value	Pr > |t|	Adjustment	Adj P	Alpha	Lower	Upper
Treatment	Meg TTR	GFP MsTTR	0.2058	1.3765	31	0.15	0.8821	Tukey-Kramer	0.8821	0.05	-2.6015	3.0131

Differences of Least Squares Means				
Effect	Treatment	Treatment	Adj Lower	Adj Upper
Treatment	Meg TTR	GFP MsTTR	-2.6015	3.0131

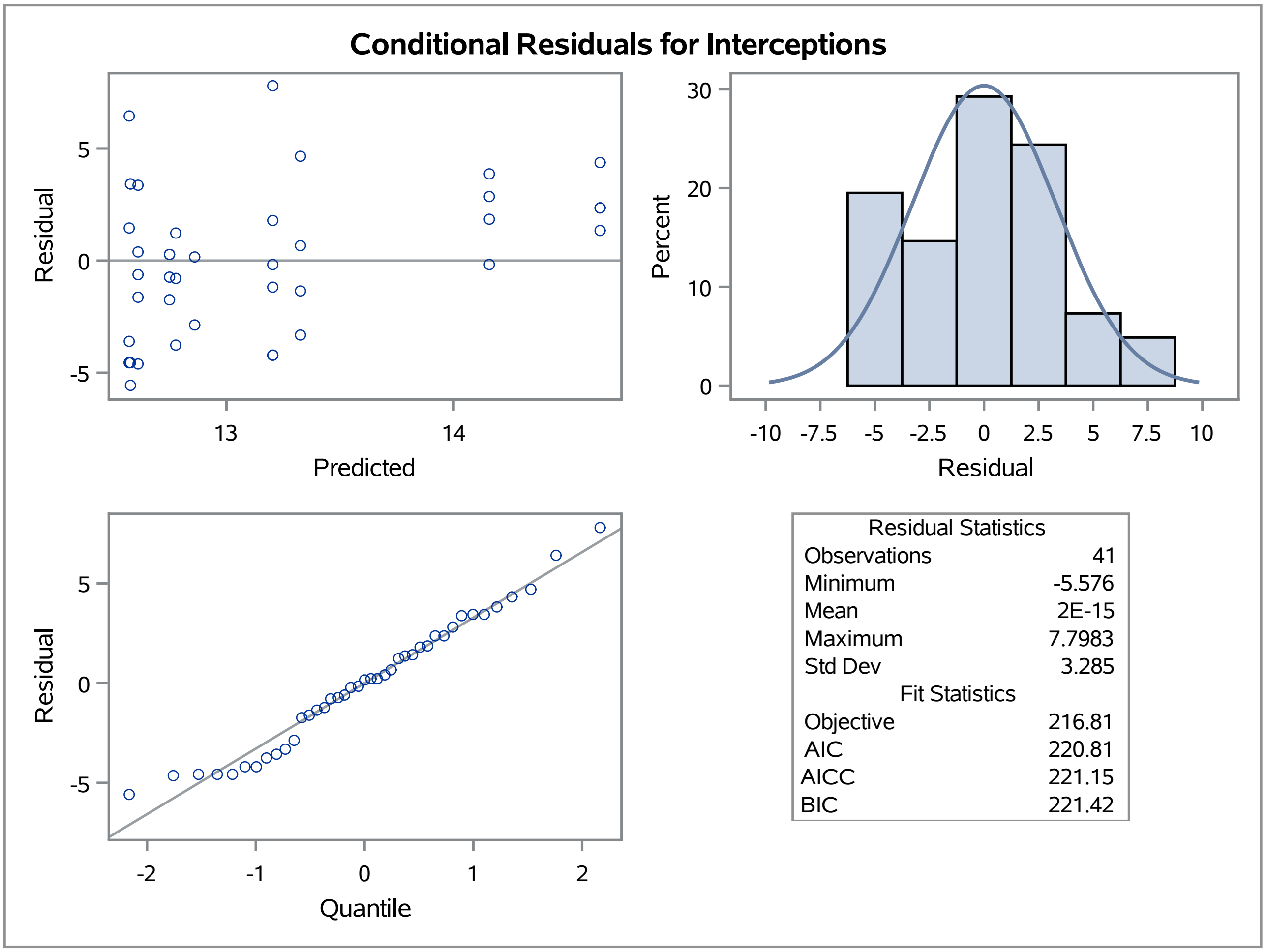

DistSoma=30

Model Information	
**Data Set**	WORK.TEMPDATASORTED
**Dependent Variable**	Interceptions
**Covariance Structure**	Variance Components
**Estimation Method**	REML
**Residual Variance Method**	Profile
**Fixed Effects SE Method**	Model-Based
**Degrees of Freedom Method**	Containment

Class Level Information		
**Class**	**Levels**	**Values**
**Treatment**	2	Meg TTR GFP MsTTR
**Culture**	10	1 2 3 4 5 6 7 8 9 10

Dimensions	
**Covariance Parameters**	2
**Columns in X**	3
**Columns in Z**	10
**Subjects**	1
**Max Obs per Subject**	41

Number of Observations	
**Number of Observations Read**	41
**Number of Observations Used**	41
**Number of Observations Not Used**	0

Iteration History			
**Iteration**	**Evaluations**	**-2 Res Log Like**	**Criterion**
**0**	1	208.89953007	
**1**	2	208.59939142	0.00000000

Convergence criteria met.

Covariance Parameter Estimates				
**Cov Parm**	**Estimate**	**Alpha**	**Lower**	**Upper**
**Culture**	0.8118	0.05	0.1140	2605905
**Residual**	9.9614	0.05	6.4262	17.4995

DistSoma=30

Fit Statistics	
-2 Res Log Likelihood	208.6
AIC (Smaller is Better)	212.6
AICC (Smaller is Better)	212.9
BIC (Smaller is Better)	213.2

Solution for Fixed Effects

Effect	Treatment	Estimate	Standard Error	DF	t Value	Pr > |t|	Alpha	Lower	Upper
Intercept		12.7181	0.8891	8	14.30	<.0001	0.05	10.6679	14.7683
Treatment	Meg TTR	-0.3231	1.1627	31	-0.28	0.7829	0.05	-2.6944	2.0482
Treatment	GFP MsTTR	0	.	.	.	.	.	.	.

Solution for Random Effects

Effect	Culture	Estimate	Std Err Pred	DF	t Value	Pr > |t|	Alpha	Lower	Upper
Culture	1	-0.2079	0.8019	31	-0.26	0.7971	0.05	-1.8434	1.4276
Culture	2	-0.3609	0.8124	31	-0.44	0.6599	0.05	-2.0179	1.2960
Culture	3	0.8069	0.8124	31	0.99	0.3283	0.05	-0.8501	2.4638
Culture	4	-0.2380	0.8124	31	-0.29	0.7715	0.05	-1.8950	1.4189
Culture	5	-0.2881	0.7898	31	-0.36	0.7178	0.05	-1.8990	1.3228
Culture	6	0.4560	0.8039	31	0.57	0.5746	0.05	-1.1834	2.0955
Culture	7	0.3331	0.8039	31	0.41	0.6814	0.05	-1.3063	1.9726
Culture	8	-0.5129	0.7783	31	-0.66	0.5148	0.05	-2.1003	1.0745
Culture	9	0.1549	0.8421	31	0.18	0.8553	0.05	-1.5626	1.8723
Culture	10	-0.1431	0.8210	31	-0.17	0.8628	0.05	-1.8175	1.5313

Type 3 Tests of Fixed Effects

Effect	Num DF	Den DF	F Value	Pr > F
Treatment	1	31	0.08	0.7829

Least Squares Means

Effect	Treatment	Estimate	Standard Error	DF	t Value	Pr > |t|	Alpha	Lower	Upper
Treatment	Meg TTR	12.3950	0.7492	31	16.54	<.0001	0.05	10.8670	13.9231
Treatment	GFP MsTTR	12.7181	0.8891	31	14.30	<.0001	0.05	10.9048	14.5314

DistSoma=30

Differences of Least Squares Means												
**Effect**	**Treatment**	**Treatment**	**Estimate**	**Standard Error**	**DF**	**t Value**	**Pr > |t|**	**Adjustment**	**Adj P**	**Alpha**	**Lower**	**Upper**
**Treatment**	Meg TTR	GFP MsTTR	-0.3231	1.1627	31	-0.28	0.7829	Tukey-Kramer	0.7829	0.05	-2.6944	2.0482

Differences of Least Squares Means				
**Effect**	**Treatment**	**Treatment**	**Adj Lower**	**Adj Upper**
**Treatment**	Meg TTR	GFP MsTTR	-2.6943	2.0482

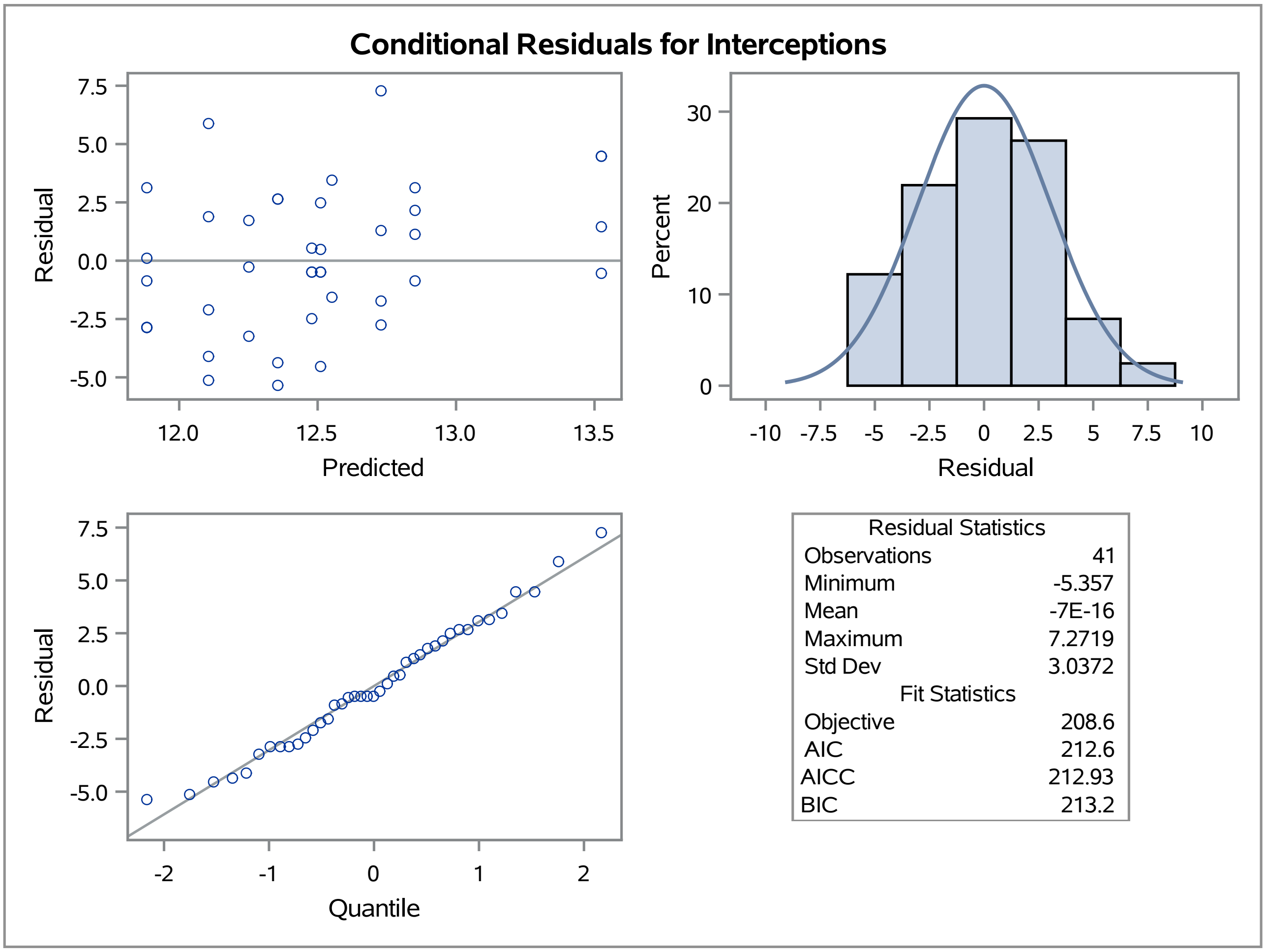

DistSoma=36

Model Information	
**Data Set**	WORK.TEMPDATASORTED
**Dependent Variable**	Interceptions
**Covariance Structure**	Variance Components
**Estimation Method**	REML
**Residual Variance Method**	Profile
**Fixed Effects SE Method**	Model-Based
**Degrees of Freedom Method**	Containment

Class Level Information		
**Class**	**Levels**	**Values**
**Treatment**	2	Meg TTR GFP MsTTR
**Culture**	10	1 2 3 4 5 6 7 8 9 10

Dimensions	
**Covariance Parameters**	2
**Columns in X**	3
**Columns in Z**	10
**Subjects**	1
**Max Obs per Subject**	41

Number of Observations	
**Number of Observations Read**	41
**Number of Observations Used**	41
**Number of Observations Not Used**	0

Iteration History			
**Iteration**	**Evaluations**	**-2 Res Log Like**	**Criterion**
**0**	1	207.44967287	
**1**	3	204.14086629	0.00009714
**2**	1	204.13411809	0.00000059
**3**	1	204.13407885	0.00000000

Convergence criteria met.

DistSoma=36

Covariance Parameter Estimates				
**Cov Parm**	**Estimate**	**Alpha**	**Lower**	**Upper**
**Culture**	2.6982	0.05	0.8408	43.8670
**Residual**	7.8969	0.05	5.0994	13.8503

Fit Statistics	
**-2 Res Log Likelihood**	204.1
**AIC (Smaller is Better)**	208.1
**AICC (Smaller is Better)**	208.5
**BIC (Smaller is Better)**	208.7

Solution for Fixed Effects									
**Effect**	**Treatment**	**Estimate**	**Standard Error**	**DF**	**t Value**	**Pr > |t|**	**Alpha**	**Lower**	**Upper**
**Intercept**		11.9318	1.0685	8	11.17	<.0001	0.05	9.4677	14.3958
**Treatment**	Meg TTR	-0.1786	1.3937	31	-0.13	0.8989	0.05	-3.0211	2.6639
**Treatment**	GFP MsTTR	0	.	.	.	.	.	.	.

Solution for Random Effects									
**Effect**	**Culture**	**Estimate**	**Std Err Pred**	**DF**	**t Value**	**Pr > |t|**	**Alpha**	**Lower**	**Upper**
**Culture**	1	-0.8400	1.2044	31	-0.70	0.4907	0.05	-3.2964	1.6163
**Culture**	2	-0.9712	1.2332	31	-0.79	0.4370	0.05	-3.4863	1.5440
**Culture**	3	2.0606	1.2332	31	1.67	0.1048	0.05	-0.4546	4.5757
**Culture**	4	-0.2493	1.2332	31	-0.20	0.8411	0.05	-2.7645	2.2658
**Culture**	5	-0.4751	1.1466	31	-0.41	0.6815	0.05	-2.8137	1.8635
**Culture**	6	1.5862	1.1862	31	1.34	0.1909	0.05	-0.8330	4.0055
**Culture**	7	0.1425	1.1862	31	0.12	0.9051	0.05	-2.2767	2.5618
**Culture**	8	-1.8505	1.1164	31	-1.66	0.1075	0.05	-4.1274	0.4264
**Culture**	9	0.3032	1.3171	31	0.23	0.8195	0.05	-2.3831	2.9895
**Culture**	10	0.2937	1.2400	31	0.24	0.8143	0.05	-2.2353	2.8226

Type 3 Tests of Fixed Effects				
**Effect**	**Num DF**	**Den DF**	**F Value**	**Pr > F**
**Treatment**	1	31	0.02	0.8989

DistSoma=36

Least Squares Means									
**Effect**	**Treatment**	**Estimate**	**Standard Error**	**DF**	**t Value**	**Pr > |t|**	**Alpha**	**Lower**	**Upper**
**Treatment**	Meg TTR	11.7532	0.8948	31	13.14	<.0001	0.05	9.9282	13.5781
**Treatment**	GFP MsTTR	11.9318	1.0685	31	11.17	<.0001	0.05	9.7525	14.1110

Differences of Least Squares Means												
**Effect**	**Treatment**	**Treatment**	**Estimate**	**Standard Error**	**DF**	**t Value**	**Pr > |t|**	**Adjustment**	**Adj P**	**Alpha**	**Lower**	**Upper**
**Treatment**	Meg TTR	GFP MsTTR	-0.1786	1.3937	31	-0.13	0.8989	Tukey-Kramer	0.8989	0.05	-3.0211	2.6639

Differences of Least Squares Means				
**Effect**	**Treatment**	**Treatment**	**Adj Lower**	**Adj Upper**
**Treatment**	Meg TTR	GFP MsTTR	-3.0210	2.6638

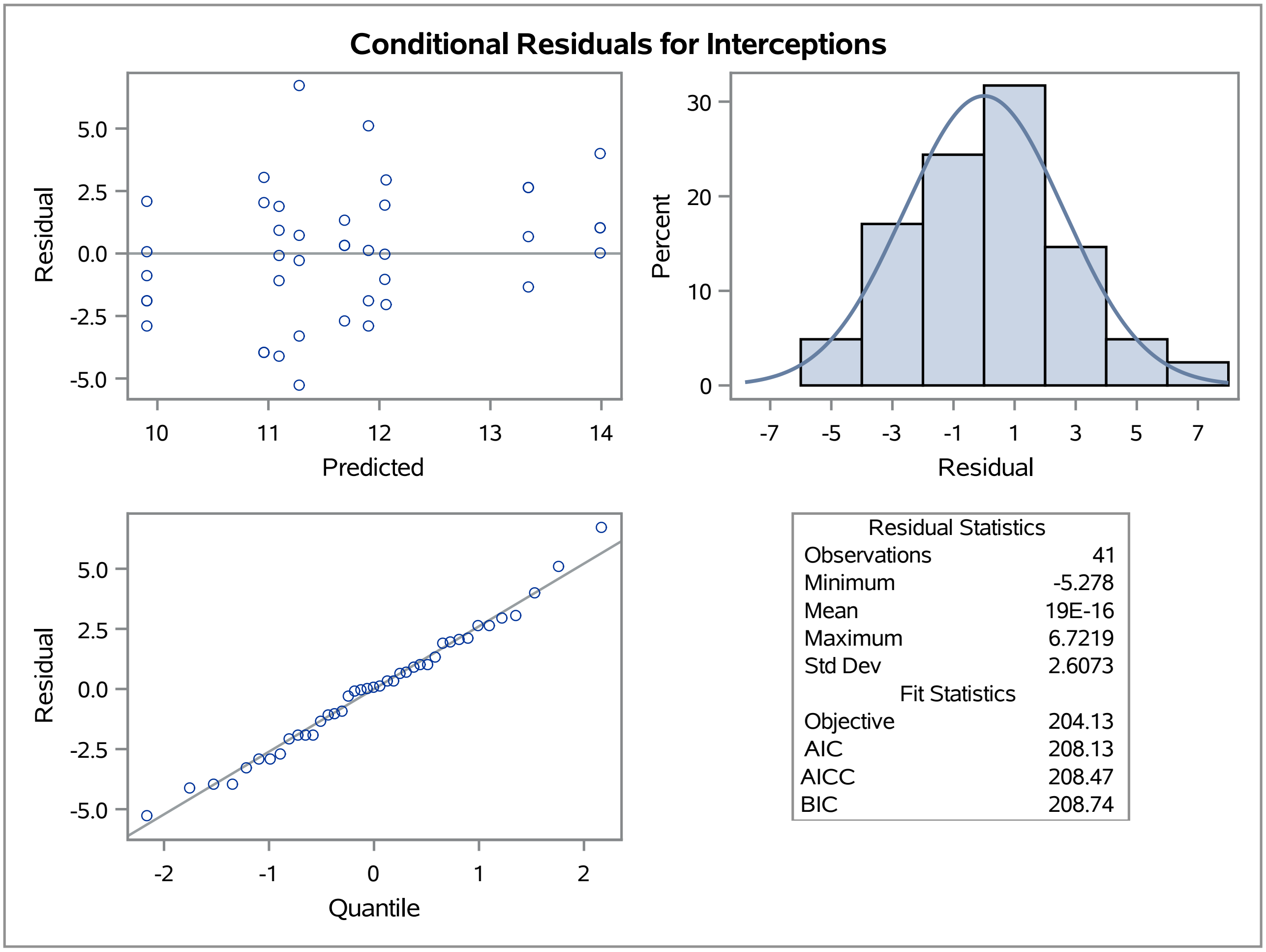

DistSoma=42

Model Information	
**Data Set**	WORK.TEMPDATASORTED
**Dependent Variable**	Interceptions
**Covariance Structure**	Variance Components
**Estimation Method**	REML
**Residual Variance Method**	Profile
**Fixed Effects SE Method**	Model-Based
**Degrees of Freedom Method**	Containment

Class Level Information		
**Class**	**Levels**	**Values**
**Treatment**	2	Meg TTR GFP MsTTR
**Culture**	10	1 2 3 4 5 6 7 8 9 10

Dimensions	
**Covariance Parameters**	2
**Columns in X**	3
**Columns in Z**	10
**Subjects**	1
**Max Obs per Subject**	41

Number of Observations	
**Number of Observations Read**	41
**Number of Observations Used**	41
**Number of Observations Not Used**	0

Iteration History			
**Iteration**	**Evaluations**	**-2 Res Log Like**	**Criterion**
**0**	1	218.41550357	
**1**	3	215.17837740	0.00076270
**2**	1	215.11712184	0.00003353
**3**	1	215.11464221	0.00000008
**4**	1	215.11463657	0.00000000

Convergence criteria met.

DistSoma=42

Covariance Parameter Estimates				
**Cov Parm**	**Estimate**	**Alpha**	**Lower**	**Upper**
**Culture**	3.4136	0.05	1.0605	56.4562
**Residual**	10.5286	0.05	6.8090	18.4201

Fit Statistics	
**-2 Res Log Likelihood**	215.1
**AIC (Smaller is Better)**	219.1
**AICC (Smaller is Better)**	219.4
**BIC (Smaller is Better)**	219.7

Solution for Fixed Effects									
**Effect**	**Treatment**	**Estimate**	**Standard Error**	**DF**	**t Value**	**Pr > |t|**	**Alpha**	**Lower**	**Upper**
**Intercept**		11.3965	1.2150	8	9.38	<.0001	0.05	8.5947	14.1983
**Treatment**	Meg TTR	-0.4802	1.5850	31	-0.30	0.7639	0.05	-3.7129	2.7525
**Treatment**	GFP MsTTR	0	.	.	.	.	.	.	.

Solution for Random Effects									
**Effect**	**Culture**	**Estimate**	**Std Err Pred**	**DF**	**t Value**	**Pr > |t|**	**Alpha**	**Lower**	**Upper**
**Culture**	1	-0.7400	1.3664	31	-0.54	0.5920	0.05	-3.5268	2.0468
**Culture**	2	-1.0708	1.3989	31	-0.77	0.4498	0.05	-3.9238	1.7822
**Culture**	3	1.7523	1.3989	31	1.25	0.2197	0.05	-1.1007	4.6054
**Culture**	4	0.05846	1.3989	31	0.04	0.9669	0.05	-2.7946	2.9115
**Culture**	5	-0.8141	1.3033	31	-0.62	0.5368	0.05	-3.4723	1.8441
**Culture**	6	1.6000	1.3478	31	1.19	0.2442	0.05	-1.1488	4.3488
**Culture**	7	0.8942	1.3478	31	0.66	0.5119	0.05	-1.8546	3.6431
**Culture**	8	-2.4765	1.2692	31	-1.95	0.0601	0.05	-5.0652	0.1121
**Culture**	9	0.4263	1.4937	31	0.29	0.7772	0.05	-2.6201	3.4727
**Culture**	10	0.3700	1.4080	31	0.26	0.7944	0.05	-2.5015	3.2416

Type 3 Tests of Fixed Effects				
**Effect**	**Num DF**	**Den DF**	**F Value**	**Pr > F**
**Treatment**	1	31	0.09	0.7639

DistSoma=42

Least Squares Means									
**Effect**	**Treatment**	**Estimate**	**Standard Error**	**DF**	**t Value**	**Pr > |t|**	**Alpha**	**Lower**	**Upper**
**Treatment**	Meg TTR	10.9162	1.0179	31	10.72	<.0001	0.05	8.8402	12.9922
**Treatment**	GFP MsTTR	11.3965	1.2150	31	9.38	<.0001	0.05	8.9184	13.8745

Differences of Least Squares Means												
**Effect**	**Treatment**	**Treatment**	**Estimate**	**Standard Error**	**DF**	**t Value**	**Pr > |t|**	**Adjustment**	**Adj P**	**Alpha**	**Lower**	**Upper**
**Treatment**	Meg TTR	GFP MsTTR	-0.4802	1.5850	31	-0.30	0.7639	Tukey-Kramer	0.7639	0.05	-3.7129	2.7525

Differences of Least Squares Means				
**Effect**	**Treatment**	**Treatment**	**Adj Lower**	**Adj Upper**
**Treatment**	Meg TTR	GFP MsTTR	-3.7129	2.7524

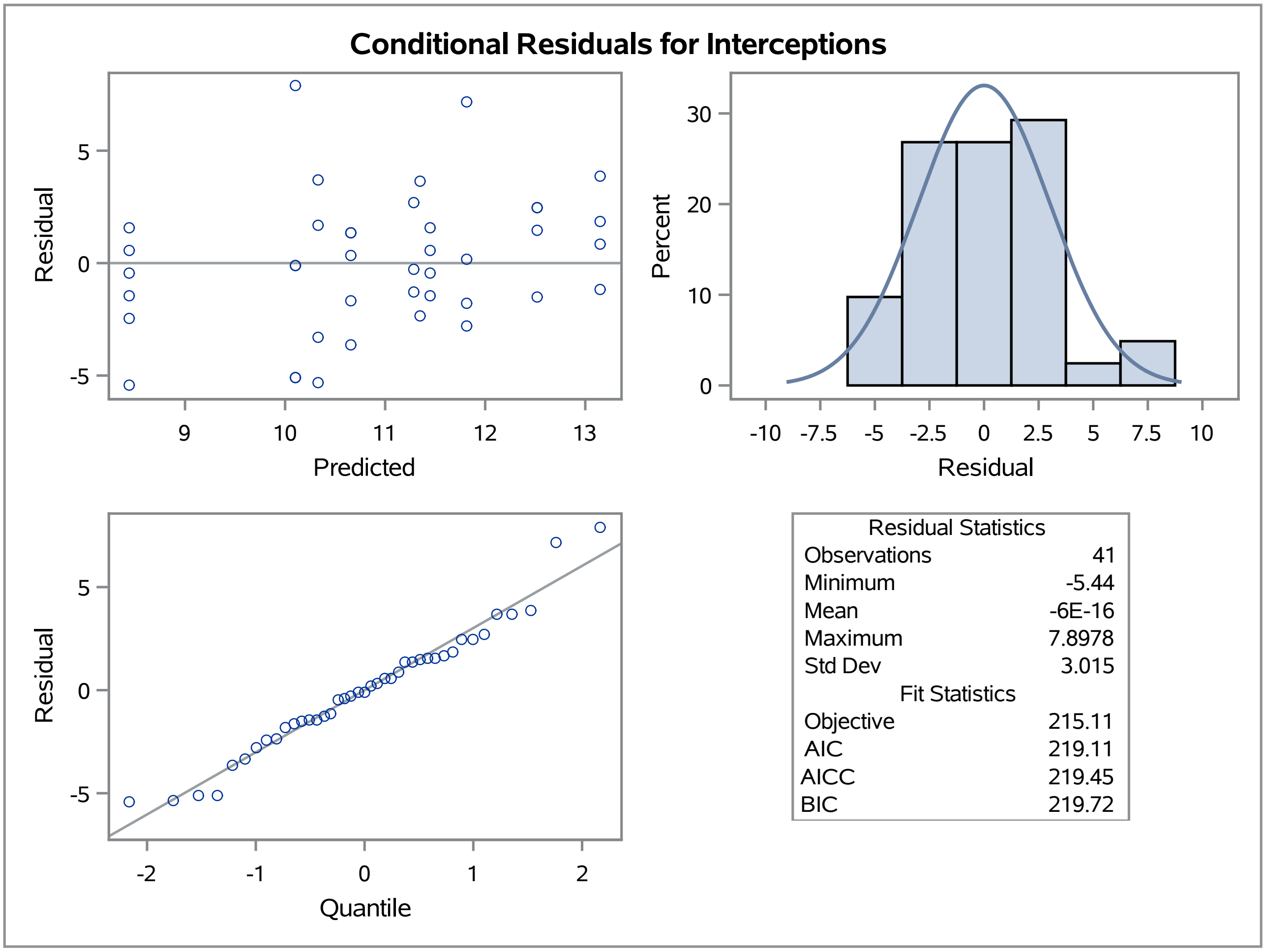

DistSoma=48

Model Information	
**Data Set**	WORK.TEMPDATASORTED
**Dependent Variable**	Interceptions
**Covariance Structure**	Variance Components
**Estimation Method**	REML
**Residual Variance Method**	Profile
**Fixed Effects SE Method**	Model-Based
**Degrees of Freedom Method**	Containment

Class Level Information		
**Class**	**Levels**	**Values**
**Treatment**	2	Meg TTR GFP MsTTR
**Culture**	10	1 2 3 4 5 6 7 8 9 10

Dimensions	
**Covariance Parameters**	2
**Columns in X**	3
**Columns in Z**	10
**Subjects**	1
**Max Obs per Subject**	41

Number of Observations	
**Number of Observations Read**	41
**Number of Observations Used**	41
**Number of Observations Not Used**	0

Iteration History			
**Iteration**	**Evaluations**	**-2 Res Log Like**	**Criterion**
**0**	1	215.39137882	
**1**	3	213.85566562	0.00005243
**2**	1	213.85179456	0.00000019
**3**	1	213.85178084	0.00000000

Convergence criteria met.

DistSoma=48

Covariance Parameter Estimates				
Cov Parm	Estimate	Alpha	Lower	Upper
Culture	2.1401	0.05	0.5368	157.22
Residual	10.7109	0.05	6.9263	18.7417

Fit Statistics	
-2 Res Log Likelihood	213.9
AIC (Smaller is Better)	217.9
AICC (Smaller is Better)	218.2
BIC (Smaller is Better)	218.5

Solution for Fixed Effects									
Effect	Treatment	Estimate	Standard Error	DF	t Value	Pr > |t|	Alpha	Lower	Upper
Intercept		11.0363	1.0807	8	10.21	<.0001	0.05	8.5442	13.5285
Treatment	Meg TTR	-0.8478	1.4118	31	-0.60	0.5525	0.05	-3.7271	2.0315
Treatment	GFP MsTTR	0	.	.	.	.	.	.	.

Solution for Random Effects									
Effect	Culture	Estimate	Std Err Pred	DF	t Value	Pr > |t|	Alpha	Lower	Upper
Culture	1	-0.6179	1.1672	31	-0.53	0.6003	0.05	-2.9983	1.7626
Culture	2	-0.5714	1.1916	31	-0.48	0.6349	0.05	-3.0017	1.8589
Culture	3	1.0944	1.1916	31	0.92	0.3655	0.05	-1.3359	3.5247
Culture	4	0.09491	1.1916	31	0.08	0.9370	0.05	-2.3354	2.5252
Culture	5	-0.6939	1.1299	31	-0.61	0.5436	0.05	-2.9984	1.6105
Culture	6	1.1378	1.1629	31	0.98	0.3354	0.05	-1.2339	3.5095
Culture	7	0.4715	1.1629	31	0.41	0.6879	0.05	-1.9002	2.8432
Culture	8	-1.6476	1.1039	31	-1.49	0.1457	0.05	-3.8990	0.6038
Culture	9	-0.1966	1.2635	31	-0.16	0.8774	0.05	-2.7734	2.3803
Culture	10	0.9287	1.2058	31	0.77	0.4470	0.05	-1.5305	3.3880

Type 3 Tests of Fixed Effects				
Effect	Num DF	Den DF	F Value	Pr > F
Treatment	1	31	0.36	0.5525

DistSoma=48

Least Squares Means									
**Effect**	**Treatment**	**Estimate**	**Standard Error**	**DF**	**t Value**	**Pr > |t|**	**Alpha**	**Lower**	**Upper**
**Treatment**	Meg TTR	10.1885	0.9084	31	11.22	<.0001	0.05	8.3359	12.0412
**Treatment**	GFP MsTTR	11.0363	1.0807	31	10.21	<.0001	0.05	8.8322	13.2405

Differences of Least Squares Means												
**Effect**	**Treatment**	**Treatment**	**Estimate**	**Standard Error**	**DF**	**t Value**	**Pr > |t|**	**Adjustment**	**Adj P**	**Alpha**	**Lower**	**Upper**
**Treatment**	Meg TTR	GFP MsTTR	-0.8478	1.4118	31	-0.60	0.5525	Tukey-Kramer	0.5525	0.05	-3.7271	2.0315

Differences of Least Squares Means				
**Effect**	**Treatment**	**Treatment**	**Adj Lower**	**Adj Upper**
**Treatment**	Meg TTR	GFP MsTTR	-3.7271	2.0315

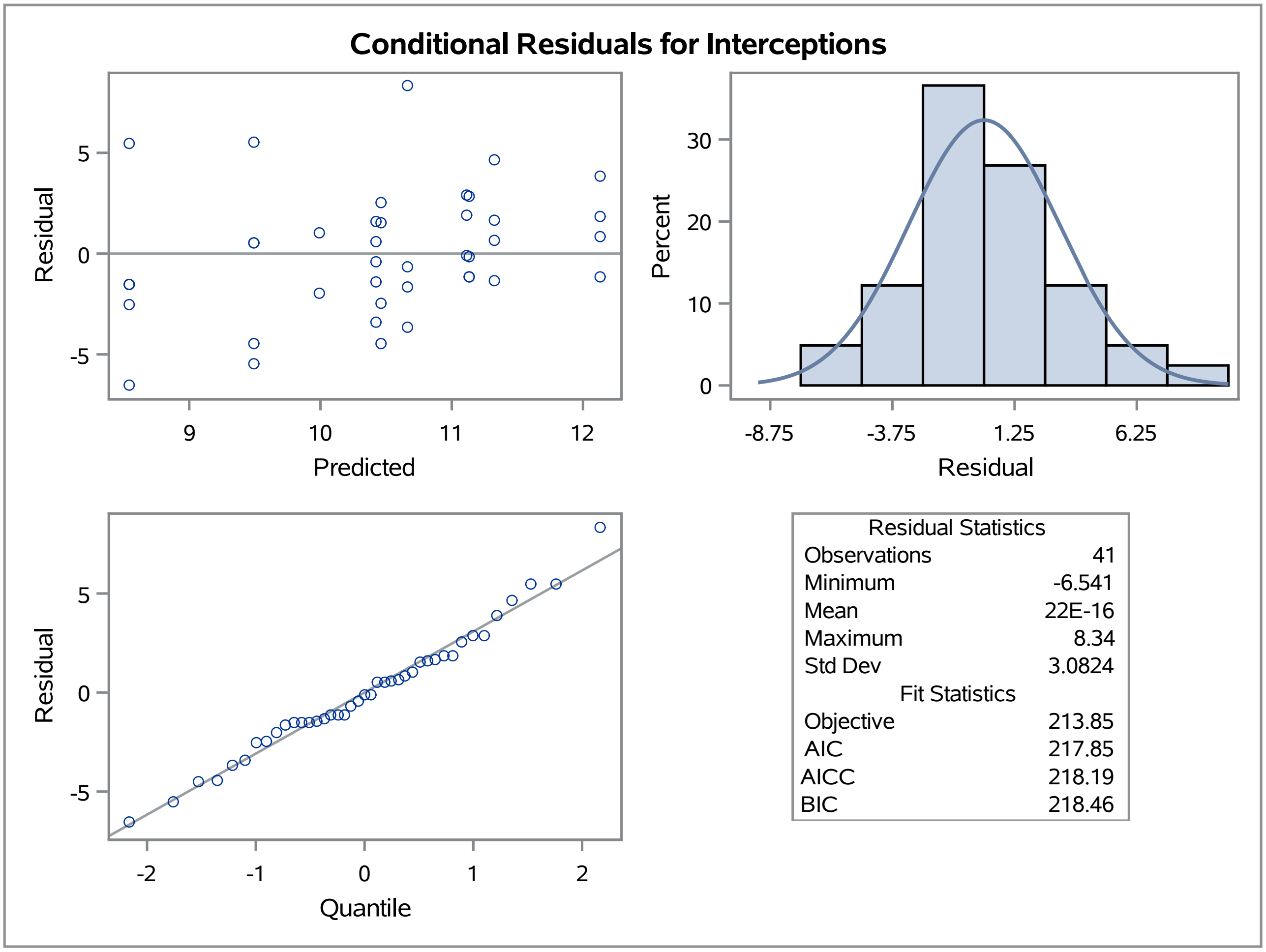

DistSoma=54

Model Information	
**Data Set**	WORK.TEMPDATASORTED
**Dependent Variable**	Interceptions
**Covariance Structure**	Variance Components
**Estimation Method**	REML
**Residual Variance Method**	Profile
**Fixed Effects SE Method**	Model-Based
**Degrees of Freedom Method**	Containment

Class Level Information		
**Class**	**Levels**	**Values**
**Treatment**	2	Meg TTR GFP MsTTR
**Culture**	10	1 2 3 4 5 6 7 8 9 10

Dimensions	
**Covariance Parameters**	2
**Columns in X**	3
**Columns in Z**	10
**Subjects**	1
**Max Obs per Subject**	41

Number of Observations	
**Number of Observations Read**	41
**Number of Observations Used**	41
**Number of Observations Not Used**	0

Iteration History			
**Iteration**	**Evaluations**	**-2 Res Log Like**	**Criterion**
**0**	1	210.10085359	
**1**	3	208.31574145	0.00008974
**2**	1	208.30931573	0.00000053
**3**	1	208.30927966	0.00000000

Convergence criteria met.

DistSoma=54

Covariance Parameter Estimates				
**Cov Parm**	**Estimate**	**Alpha**	**Lower**	**Upper**
**Culture**	2.0166	0.05	0.5308	99.5808
**Residual**	9.2140	0.05	5.9598	16.1159

Fit Statistics	
**-2 Res Log Likelihood**	208.3
**AIC (Smaller is Better)**	212.3
**AICC (Smaller is Better)**	212.6
**BIC (Smaller is Better)**	212.9

Solution for Fixed Effects									
**Effect**	**Treatment**	**Estimate**	**Standard Error**	**DF**	**t Value**	**Pr > |t|**	**Alpha**	**Lower**	**Upper**
**Intercept**		10.4022	1.0241	8	10.16	<.0001	0.05	8.0407	12.7637
**Treatment**	Meg TTR	-0.7819	1.3375	31	-0.58	0.5630	0.05	-3.5097	1.9459
**Treatment**	GFP MsTTR	0	.	.	.	.	.	.	.

Solution for Random Effects									
**Effect**	**Culture**	**Estimate**	**Std Err Pred**	**DF**	**t Value**	**Pr > |t|**	**Alpha**	**Lower**	**Upper**
**Culture**	1	-0.8372	1.1177	31	-0.75	0.4595	0.05	-3.1167	1.4424
**Culture**	2	-0.5378	1.1418	31	-0.47	0.6409	0.05	-2.8666	1.7909
**Culture**	3	1.0959	1.1418	31	0.96	0.3446	0.05	-1.2328	3.4247
**Culture**	4	0.2791	1.1418	31	0.24	0.8085	0.05	-2.0497	2.6078
**Culture**	5	-0.7421	1.0793	31	-0.69	0.4968	0.05	-2.9434	1.4592
**Culture**	6	1.3442	1.1120	31	1.21	0.2359	0.05	-0.9237	3.6122
**Culture**	7	0.5274	1.1120	31	0.47	0.6387	0.05	-1.7406	2.7953
**Culture**	8	-1.4875	1.0537	31	-1.41	0.1680	0.05	-3.6366	0.6616
**Culture**	9	-0.1888	1.2129	31	-0.16	0.8773	0.05	-2.6626	2.2850
**Culture**	10	0.5469	1.1548	31	0.47	0.6391	0.05	-1.8084	2.9021

Type 3 Tests of Fixed Effects				
**Effect**	**Num DF**	**Den DF**	**F Value**	**Pr > F**
**Treatment**	1	31	0.34	0.5630

DistSoma=54

Least Squares Means									
Effect	Treatment	Estimate	Standard Error	DF	t Value	Pr > |t|	Alpha	Lower	Upper
Treatment	Meg TTR	9.6203	0.8603	31	11.18	<.0001	0.05	7.8656	11.3749
Treatment	GFP MsTTR	10.4022	1.0241	31	10.16	<.0001	0.05	8.3136	12.4908

Differences of Least Squares Means												
Effect	Treatment	Treatment	Estimate	Standard Error	DF	t Value	Pr > |t|	Adjustment	Adj P	Alpha	Lower	Upper
Treatment	Meg TTR	GFP MsTTR	-0.7819	1.3375	31	-0.58	0.5630	Tukey-Kramer	0.5630	0.05	-3.5097	1.9459

Differences of Least Squares Means				
Effect	Treatment	Treatment	Adj Lower	Adj Upper
Treatment	Meg TTR	GFP MsTTR	-3.5097	1.9459

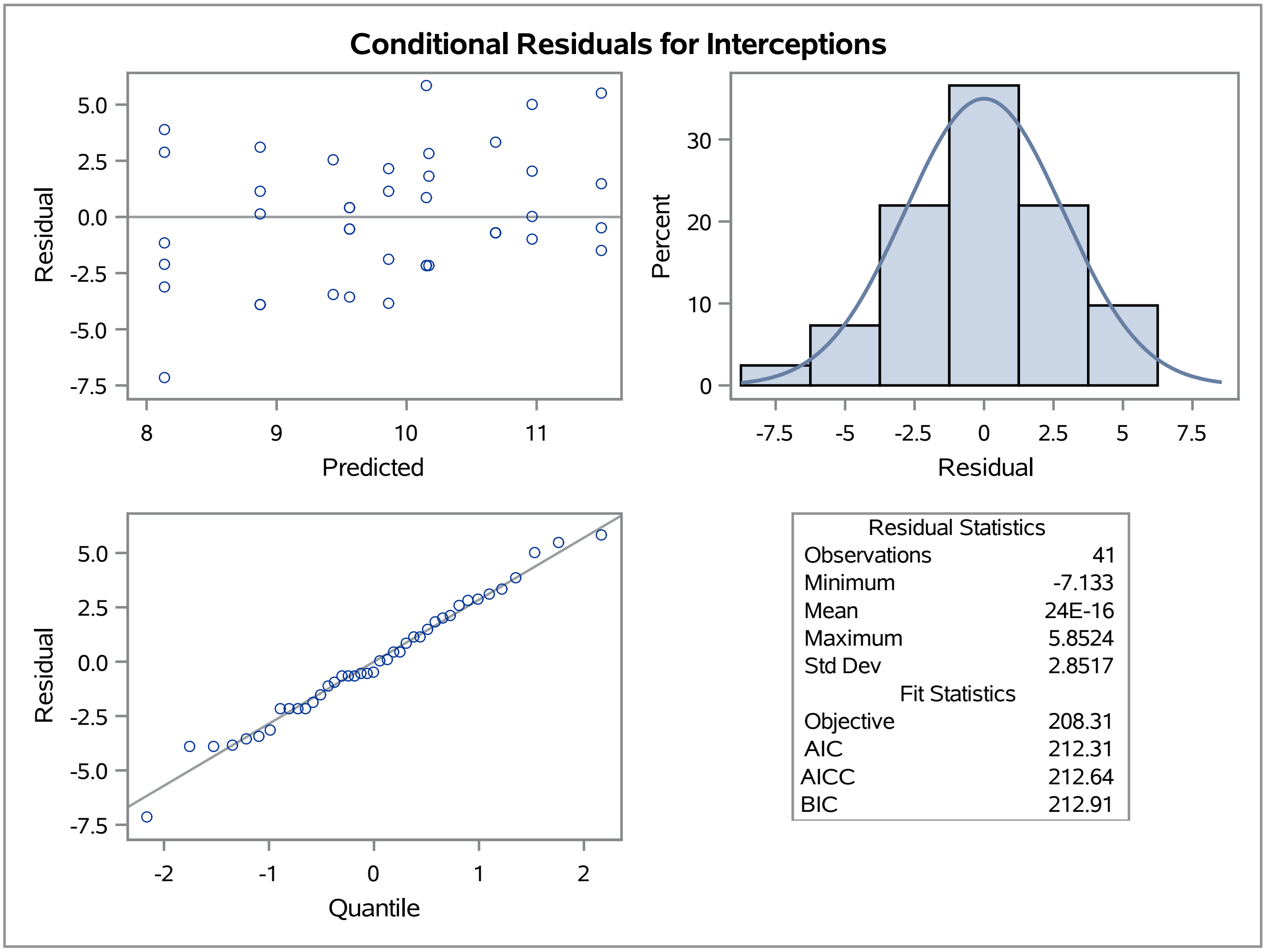

DistSoma=60

Model Information	
**Data Set**	WORK.TEMPDATASORTED
**Dependent Variable**	Interceptions
**Covariance Structure**	Variance Components
**Estimation Method**	REML
**Residual Variance Method**	Profile
**Fixed Effects SE Method**	Model-Based
**Degrees of Freedom Method**	Containment

Class Level Information		
**Class**	**Levels**	**Values**
**Treatment**	2	Meg TTR GFP MsTTR
**Culture**	10	1 2 3 4 5 6 7 8 9 10

Dimensions	
**Covariance Parameters**	2
**Columns in X**	3
**Columns in Z**	10
**Subjects**	1
**Max Obs per Subject**	41

Number of Observations	
**Number of Observations Read**	41
**Number of Observations Used**	41
**Number of Observations Not Used**	0

Iteration History			
**Iteration**	**Evaluations**	**-2 Res Log Like**	**Criterion**
**0**	1	206.97231721	
**1**	3	201.70267918	0.00103406
**2**	1	201.62678506	0.00005453
**3**	1	201.62310997	0.00000019
**4**	1	201.62309778	0.00000000

Convergence criteria met.

DistSoma=60

Covariance Parameter Estimates				
**Cov Parm**	**Estimate**	**Alpha**	**Lower**	**Upper**
**Culture**	3.3787	0.05	1.1677	32.8706
**Residual**	7.1045	0.05	4.5867	12.4646

Fit Statistics	
**-2 Res Log Likelihood**	201.6
**AIC (Smaller is Better)**	205.6
**AICC (Smaller is Better)**	206.0
**BIC (Smaller is Better)**	206.2

Solution for Fixed Effects									
**Effect**	**Treatment**	**Estimate**	**Standard Error**	**DF**	**t Value**	**Pr > |t|**	**Alpha**	**Lower**	**Upper**
**Intercept**		9.5773	1.1249	8	8.51	<.0001	0.05	6.9834	12.1712
**Treatment**	Meg TTR	-0.2482	1.4654	31	-0.17	0.8666	0.05	-3.2369	2.7404
**Treatment**	GFP MsTTR	0	.	.	.	.	.	.	.

Solution for Random Effects									
**Effect**	**Culture**	**Estimate**	**Std Err Pred**	**DF**	**t Value**	**Pr > |t|**	**Alpha**	**Lower**	**Upper**
**Culture**	1	-1.8143	1.2756	31	-1.42	0.1649	0.05	-4.4160	0.7874
**Culture**	2	-0.05067	1.3068	31	-0.04	0.9693	0.05	-2.7159	2.6146
**Culture**	3	1.4241	1.3068	31	1.09	0.2842	0.05	-1.2412	4.0893
**Culture**	4	0.4409	1.3068	31	0.34	0.7381	0.05	-2.2243	3.1062
**Culture**	5	-1.2172	1.1989	31	-1.02	0.3178	0.05	-3.6623	1.2280
**Culture**	6	1.7507	1.2422	31	1.41	0.1687	0.05	-0.7828	4.2842
**Culture**	7	1.4229	1.2422	31	1.15	0.2608	0.05	-1.1106	3.9564
**Culture**	8	-2.0949	1.1664	31	-1.80	0.0822	0.05	-4.4737	0.2839
**Culture**	9	-0.6479	1.3933	31	-0.47	0.6452	0.05	-3.4895	2.1937
**Culture**	10	0.7864	1.3028	31	0.60	0.5505	0.05	-1.8706	3.4434

Type 3 Tests of Fixed Effects				
**Effect**	**Num DF**	**Den DF**	**F Value**	**Pr > F**
**Treatment**	1	31	0.03	0.8666

DistSoma=60

Least Squares Means									
**Effect**	**Treatment**	**Estimate**	**Standard Error**	**DF**	**t Value**	**Pr > |t|**	**Alpha**	**Lower**	**Upper**
**Treatment**	Meg TTR	9.3291	0.9392	31	9.93	<.0001	0.05	7.4137	11.2445
**Treatment**	GFP MsTTR	9.5773	1.1249	31	8.51	<.0001	0.05	7.2832	11.8715

Differences of Least Squares Means												
**Effect**	**Treatment**	**Treatment**	**Estimate**	**Standard Error**	**DF**	**t Value**	**Pr > |t|**	**Adjustment**	**Adj P**	**Alpha**	**Lower**	**Upper**
**Treatment**	Meg TTR	GFP MsTTR	-0.2482	1.4654	31	-0.17	0.8666	Tukey-Kramer	0.8666	0.05	-3.2369	2.7404

Differences of Least Squares Means				
**Effect**	**Treatment**	**Treatment**	**Adj Lower**	**Adj Upper**
**Treatment**	Meg TTR	GFP MsTTR	-3.2369	2.7404

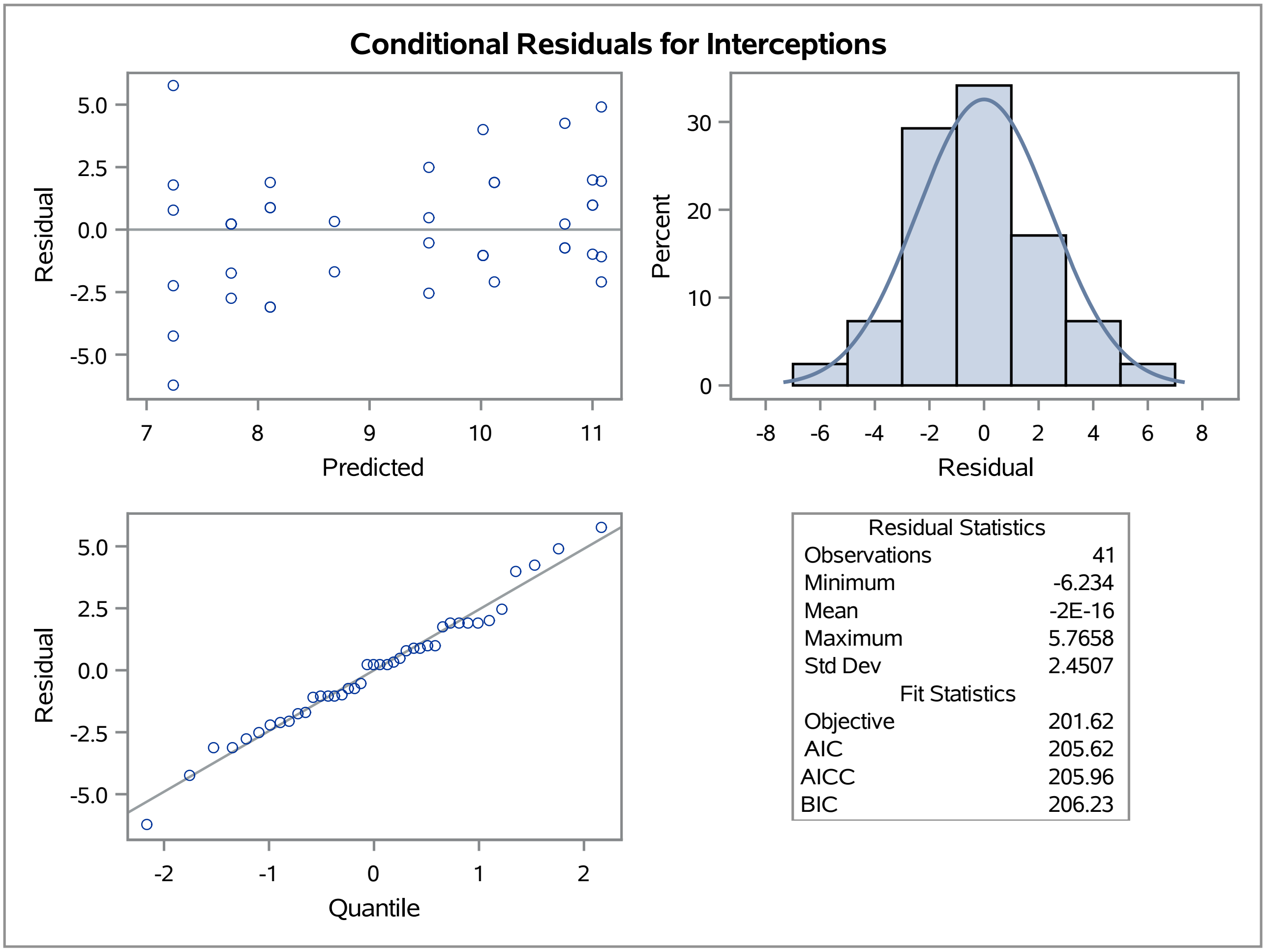

DistSoma=66

Model Information	
**Data Set**	WORK.TEMPDATASORTED
**Dependent Variable**	Interceptions
**Covariance Structure**	Variance Components
**Estimation Method**	REML
**Residual Variance Method**	Profile
**Fixed Effects SE Method**	Model-Based
**Degrees of Freedom Method**	Containment

Class Level Information		
**Class**	**Levels**	**Values**
**Treatment**	2	Meg TTR GFP MsTTR
**Culture**	10	1 2 3 4 5 6 7 8 9 10

Dimensions	
**Covariance Parameters**	2
**Columns in X**	3
**Columns in Z**	10
**Subjects**	1
**Max Obs per Subject**	41

Number of Observations	
**Number of Observations Read**	41
**Number of Observations Used**	41
**Number of Observations Not Used**	0

Iteration History			
**Iteration**	**Evaluations**	**-2 Res Log Like**	**Criterion**
**0**	1	203.38036881	
**1**	3	199.11245842	0.00063800
**2**	1	199.06746703	0.00002165
**3**	1	199.06605515	0.00000003
**4**	1	199.06605326	0.00000000

Convergence criteria met.

DistSoma=66

Covariance Parameter Estimates				
**Cov Parm**	**Estimate**	**Alpha**	**Lower**	**Upper**
**Culture**	2.7388	0.05	0.9053	32.8042
**Residual**	6.7975	0.05	4.3908	11.9161

Fit Statistics	
**-2 Res Log Likelihood**	199.1
**AIC (Smaller is Better)**	203.1
**AICC (Smaller is Better)**	203.4
**BIC (Smaller is Better)**	203.7

Solution for Fixed Effects									
**Effect**	**Treatment**	**Estimate**	**Standard Error**	**DF**	**t Value**	**Pr > |t|**	**Alpha**	**Lower**	**Upper**
**Intercept**		8.4941	1.0426	8	8.15	<.0001	0.05	6.0898	10.8983
**Treatment**	Meg TTR	0.6840	1.3591	31	0.50	0.6183	0.05	-2.0879	3.4558
**Treatment**	GFP MsTTR	0	.	.	.	.	.	.	.

Solution for Random Effects									
**Effect**	**Culture**	**Estimate**	**Std Err Pred**	**DF**	**t Value**	**Pr > |t|**	**Alpha**	**Lower**	**Upper**
**Culture**	1	-1.3994	1.1807	31	-1.19	0.2449	0.05	-3.8074	1.0086
**Culture**	2	0.1579	1.2094	31	0.13	0.8969	0.05	-2.3087	2.6245
**Culture**	3	0.9293	1.2094	31	0.77	0.4481	0.05	-1.5373	3.3959
**Culture**	4	0.3122	1.2094	31	0.26	0.7980	0.05	-2.1544	2.7788
**Culture**	5	-1.3219	1.1171	31	-1.18	0.2457	0.05	-3.6003	0.9565
**Culture**	6	1.5871	1.1568	31	1.37	0.1799	0.05	-0.7721	3.9464
**Culture**	7	1.5871	1.1568	31	1.37	0.1799	0.05	-0.7721	3.9464
**Culture**	8	-1.7765	1.0871	31	-1.63	0.1123	0.05	-3.9936	0.4406
**Culture**	9	-0.5257	1.2915	31	-0.41	0.6868	0.05	-3.1597	2.1084
**Culture**	10	0.4498	1.2115	31	0.37	0.7129	0.05	-2.0210	2.9206

Type 3 Tests of Fixed Effects				
**Effect**	**Num DF**	**Den DF**	**F Value**	**Pr > F**
**Treatment**	1	31	0.25	0.6183

DistSoma=66

Least Squares Means									
Effect	Treatment	Estimate	Standard Error	DF	t Value	Pr > |t|	Alpha	Lower	Upper
Treatment	Meg TTR	9.1781	0.8718	31	10.53	<.0001	0.05	7.4000	10.9561
Treatment	GFP MsTTR	8.4941	1.0426	31	8.15	<.0001	0.05	6.3677	10.6205

Differences of Least Squares Means												
Effect	Treatment	Treatment	Estimate	Standard Error	DF	t Value	Pr > |t|	Adjustment	Adj P	Alpha	Lower	Upper
Treatment	Meg TTR	GFP MsTTR	0.6840	1.3591	31	0.50	0.6183	Tukey-Kramer	0.6183	0.05	-2.0879	3.4558

Differences of Least Squares Means				
Effect	Treatment	Treatment	Adj Lower	Adj Upper
Treatment	Meg TTR	GFP MsTTR	-2.0878	3.4558

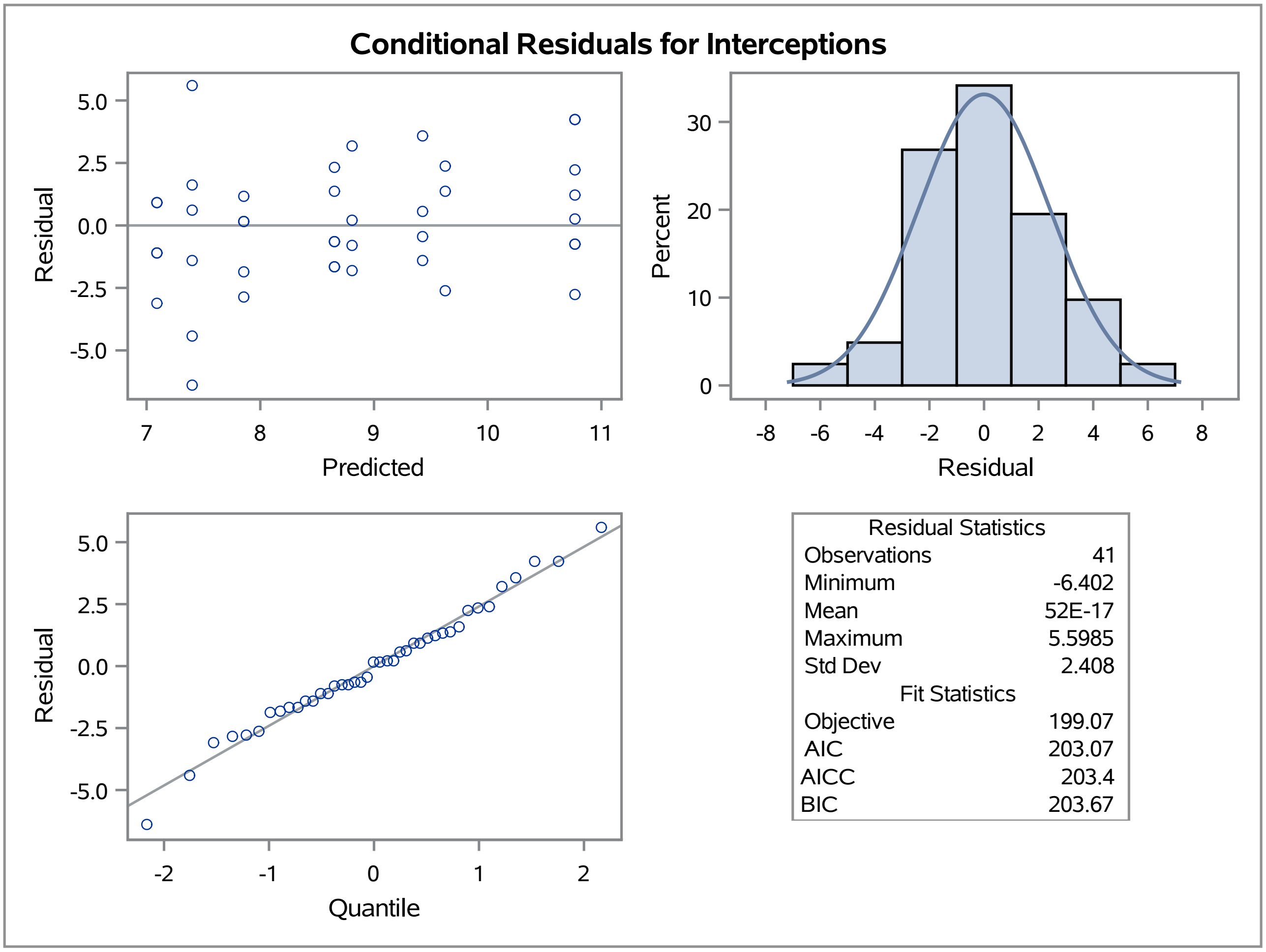

DistSoma=72

Model Information	
**Data Set**	WORK.TEMPDATASORTED
**Dependent Variable**	Interceptions
**Covariance Structure**	Variance Components
**Estimation Method**	REML
**Residual Variance Method**	Profile
**Fixed Effects SE Method**	Model-Based
**Degrees of Freedom Method**	Containment

Class Level Information		
**Class**	**Levels**	**Values**
**Treatment**	2	Meg TTR GFP MsTTR
**Culture**	10	1 2 3 4 5 6 7 8 9 10

Dimensions	
**Covariance Parameters**	2
**Columns in X**	3
**Columns in Z**	10
**Subjects**	1
**Max Obs per Subject**	41

Number of Observations	
**Number of Observations Read**	41
**Number of Observations Used**	41
**Number of Observations Not Used**	0

Iteration History			
**Iteration**	**Evaluations**	**-2 Res Log Like**	**Criterion**
**0**	1	200.60286649	
**1**	3	197.87847731	0.00011685
**2**	1	197.87071980	0.00000082
**3**	1	197.87066809	0.00000000

Convergence criteria met.

DistSoma=72

Covariance Parameter Estimates				
**Cov Parm**	**Estimate**	**Alpha**	**Lower**	**Upper**
**Culture**	2.0151	0.05	0.5974	43.7356
**Residual**	6.8392	0.05	4.4190	11.9834

Fit Statistics	
**-2 Res Log Likelihood**	197.9
**AIC (Smaller is Better)**	201.9
**AICC (Smaller is Better)**	202.2
**BIC (Smaller is Better)**	202.5

Solution for Fixed Effects									
**Effect**	**Treatment**	**Estimate**	**Standard Error**	**DF**	**t Value**	**Pr > |t|**	**Alpha**	**Lower**	**Upper**
**Intercept**		8.3702	0.9530	8	8.78	<.0001	0.05	6.1724	10.5679
**Treatment**	Meg TTR	0.1965	1.2437	31	0.16	0.8755	0.05	-2.3400	2.7330
**Treatment**	GFP MsTTR	0	.	.	.	.	.	.	.

Solution for Random Effects									
**Effect**	**Culture**	**Estimate**	**Std Err Pred**	**DF**	**t Value**	**Pr > |t|**	**Alpha**	**Lower**	**Upper**
**Culture**	1	-1.2927	1.0663	31	-1.21	0.2346	0.05	-3.4675	0.8821
**Culture**	2	0.2055	1.0912	31	0.19	0.8519	0.05	-2.0201	2.4311
**Culture**	3	0.4760	1.0912	31	0.44	0.6657	0.05	-1.7496	2.7016
**Culture**	4	0.6112	1.0912	31	0.56	0.5794	0.05	-1.6144	2.8368
**Culture**	5	-1.2906	1.0204	31	-1.26	0.2154	0.05	-3.3718	0.7906
**Culture**	6	1.3164	1.0544	31	1.25	0.2212	0.05	-0.8341	3.4669
**Culture**	7	0.9107	1.0544	31	0.86	0.3944	0.05	-1.2398	3.0612
**Culture**	8	-1.2135	0.9942	31	-1.22	0.2315	0.05	-3.2413	0.8142
**Culture**	9	-0.3955	1.1644	31	-0.34	0.7364	0.05	-2.7702	1.9792
**Culture**	10	0.6725	1.1001	31	0.61	0.5454	0.05	-1.5711	2.9162

Type 3 Tests of Fixed Effects				
**Effect**	**Num DF**	**Den DF**	**F Value**	**Pr > F**
**Treatment**	1	31	0.02	0.8755

DistSoma=72

Least Squares Means									
**Effect**	**Treatment**	**Estimate**	**Standard Error**	**DF**	**t Value**	**Pr > |t|**	**Alpha**	**Lower**	**Upper**
**Treatment**	Meg TTR	8.5666	0.7990	31	10.72	<.0001	0.05	6.9370	10.1963
**Treatment**	GFP MsTTR	8.3702	0.9530	31	8.78	<.0001	0.05	6.4264	10.3139

Differences of Least Squares Means												
**Effect**	**Treatment**	**Treatment**	**Estimate**	**Standard Error**	**DF**	**t Value**	**Pr > |t|**	**Adjustment**	**Adj P**	**Alpha**	**Lower**	**Upper**
**Treatment**	Meg TTR	GFP MsTTR	0.1965	1.2437	31	0.16	0.8755	Tukey-Kramer	0.8755	0.05	-2.3400	2.7330

Differences of Least Squares Means				
**Effect**	**Treatment**	**Treatment**	**Adj Lower**	**Adj Upper**
**Treatment**	Meg TTR	GFP MsTTR	-2.3400	2.7329

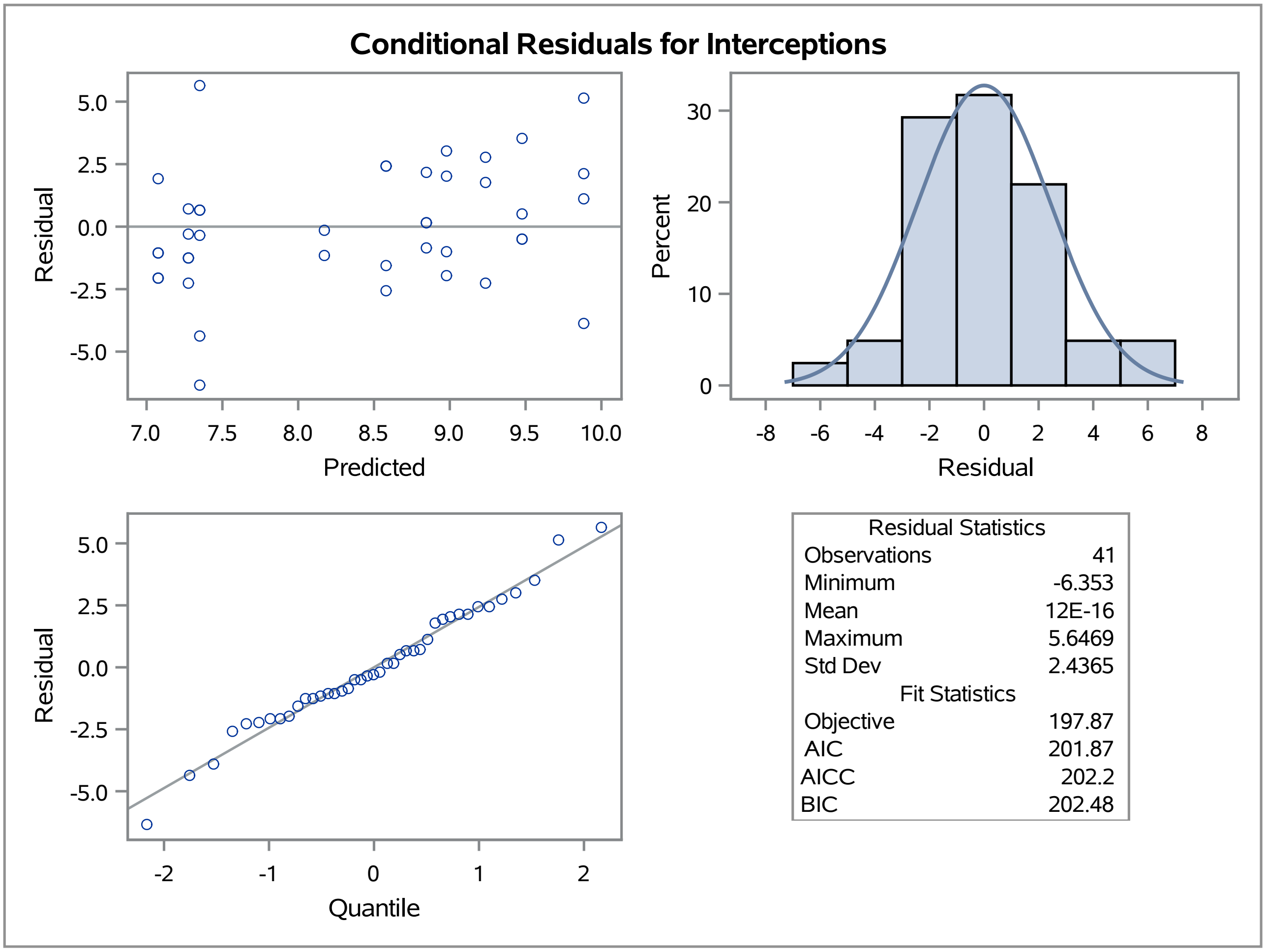

DistSoma=78

Model Information	
**Data Set**	WORK.TEMPDATASORTED
**Dependent Variable**	Interceptions
**Covariance Structure**	Variance Components
**Estimation Method**	REML
**Residual Variance Method**	Profile
**Fixed Effects SE Method**	Model-Based
**Degrees of Freedom Method**	Containment

Class Level Information		
**Class**	**Levels**	**Values**
**Treatment**	2	Meg TTR GFP MsTTR
**Culture**	10	1 2 3 4 5 6 7 8 9 10

Dimensions	
**Covariance Parameters**	2
**Columns in X**	3
**Columns in Z**	10
**Subjects**	1
**Max Obs per Subject**	41

Number of Observations	
**Number of Observations Read**	41
**Number of Observations Used**	41
**Number of Observations Not Used**	0

Iteration History			
**Iteration**	**Evaluations**	**-2 Res Log Like**	**Criterion**
**0**	1	209.03477596	
**1**	3	207.46405332	0.00001428
**2**	1	207.46306406	0.00000001
**3**	1	207.46306311	0.00000000

Convergence criteria met.

DistSoma=78

Covariance Parameter Estimates				
**Cov Parm**	**Estimate**	**Alpha**	**Lower**	**Upper**
**Culture**	1.8833	0.05	0.4764	128.73
**Residual**	9.0597	0.05	5.8529	15.8780

Fit Statistics	
**-2 Res Log Likelihood**	207.5
**AIC (Smaller is Better)**	211.5
**AICC (Smaller is Better)**	211.8
**BIC (Smaller is Better)**	212.1

Solution for Fixed Effects									
**Effect**	**Treatment**	**Estimate**	**Standard Error**	**DF**	**t Value**	**Pr > |t|**	**Alpha**	**Lower**	**Upper**
**Intercept**		7.8737	1.0031	8	7.85	<.0001	0.05	5.5605	10.1869
**Treatment**	Meg TTR	0.6595	1.3103	31	0.50	0.6183	0.05	-2.0128	3.3317
**Treatment**	GFP MsTTR	0	.	.	.	.	.	.	.

Solution for Random Effects									
**Effect**	**Culture**	**Estimate**	**Std Err Pred**	**DF**	**t Value**	**Pr > |t|**	**Alpha**	**Lower**	**Upper**
**Culture**	1	-0.8530	1.0885	31	-0.78	0.4392	0.05	-3.0730	1.3670
**Culture**	2	0.05734	1.1116	31	0.05	0.9592	0.05	-2.2098	2.3245
**Culture**	3	0.6248	1.1116	31	0.56	0.5781	0.05	-1.6423	2.8920
**Culture**	4	0.1708	1.1116	31	0.15	0.8789	0.05	-2.0963	2.4380
**Culture**	5	-1.1891	1.0526	31	-1.13	0.2673	0.05	-3.3359	0.9577
**Culture**	6	0.7794	1.0838	31	0.72	0.4774	0.05	-1.4311	2.9900
**Culture**	7	1.4604	1.0838	31	1.35	0.1876	0.05	-0.7501	3.6710
**Culture**	8	-1.0359	1.0281	31	-1.01	0.3214	0.05	-3.1327	1.0608
**Culture**	9	-0.4502	1.1796	31	-0.38	0.7053	0.05	-2.8561	1.9556
**Culture**	10	0.4354	1.1246	31	0.39	0.7013	0.05	-1.8583	2.7290

Type 3 Tests of Fixed Effects				
**Effect**	**Num DF**	**Den DF**	**F Value**	**Pr > F**
**Treatment**	1	31	0.25	0.6183

DistSoma=78

Least Squares Means									
**Effect**	**Treatment**	**Estimate**	**Standard Error**	**DF**	**t Value**	**Pr > |t|**	**Alpha**	**Lower**	**Upper**
**Treatment**	Meg TTR	8.5332	0.8429	31	10.12	<.0001	0.05	6.8140	10.2524
**Treatment**	GFP MsTTR	7.8737	1.0031	31	7.85	<.0001	0.05	5.8279	9.9195

Differences of Least Squares Means												
**Effect**	**Treatment**	**Treatment**	**Estimate**	**Standard Error**	**DF**	**t Value**	**Pr > |t|**	**Adjustment**	**Adj P**	**Alpha**	**Lower**	**Upper**
**Treatment**	Meg TTR	GFP MsTTR	0.6595	1.3103	31	0.50	0.6183	Tukey-Kramer	0.6183	0.05	-2.0128	3.3317

Differences of Least Squares Means				
**Effect**	**Treatment**	**Treatment**	**Adj Lower**	**Adj Upper**
**Treatment**	Meg TTR	GFP MsTTR	-2.0128	3.3317

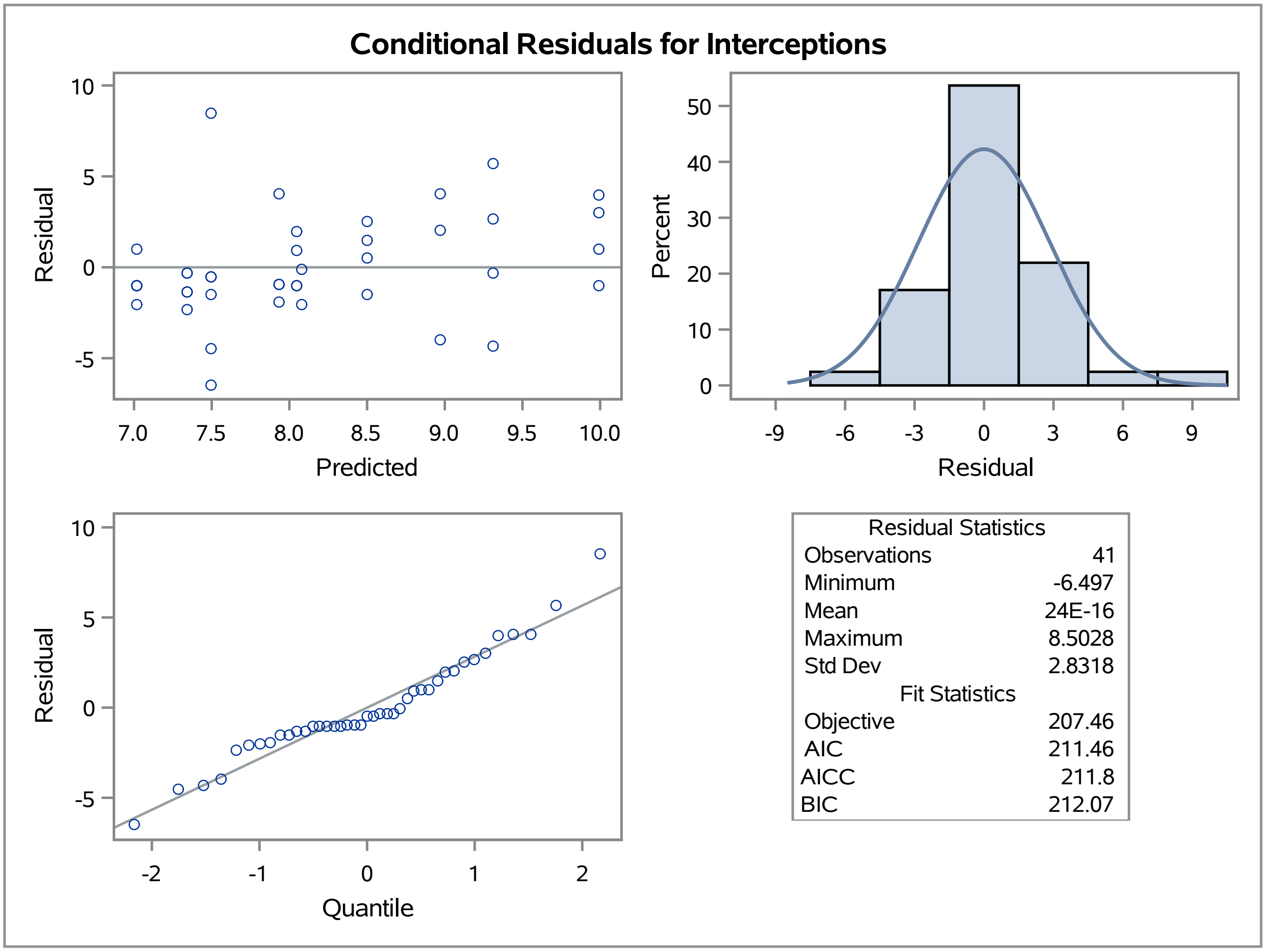

DistSoma=84

Model Information	
**Data Set**	WORK.TEMPDATASORTED
**Dependent Variable**	Interceptions
**Covariance Structure**	Variance Components
**Estimation Method**	REML
**Residual Variance Method**	Profile
**Fixed Effects SE Method**	Model-Based
**Degrees of Freedom Method**	Containment

Class Level Information		
**Class**	**Levels**	**Values**
**Treatment**	2	Meg TTR GFP MsTTR
**Culture**	10	1 2 3 4 5 6 7 8 9 10

Dimensions	
**Covariance Parameters**	2
**Columns in X**	3
**Columns in Z**	10
**Subjects**	1
**Max Obs per Subject**	41

Number of Observations	
**Number of Observations Read**	41
**Number of Observations Used**	41
**Number of Observations Not Used**	0

Iteration History			
**Iteration**	**Evaluations**	**-2 Res Log Like**	**Criterion**
**0**	1	204.94515146	
**1**	3	203.06522709	0.00002796
**2**	1	203.06333947	0.00000005
**3**	1	203.06333609	0.00000000

Convergence criteria met.

DistSoma=84

Covariance Parameter Estimates				
**Cov Parm**	**Estimate**	**Alpha**	**Lower**	**Upper**
**Culture**	1.8627	0.05	0.4973	82.4706
**Residual**	8.0072	0.05	5.1726	14.0344

Fit Statistics	
**-2 Res Log Likelihood**	203.1
**AIC (Smaller is Better)**	207.1
**AICC (Smaller is Better)**	207.4
**BIC (Smaller is Better)**	207.7

Solution for Fixed Effects									
**Effect**	**Treatment**	**Estimate**	**Standard Error**	**DF**	**t Value**	**Pr > |t|**	**Alpha**	**Lower**	**Upper**
**Intercept**		7.5186	0.9690	8	7.76	<.0001	0.05	5.2841	9.7532
**Treatment**	Meg TTR	0.5256	1.2654	31	0.42	0.6808	0.05	-2.0552	3.1063
**Treatment**	GFP MsTTR	0	.	.	.	.	.	.	.

Solution for Random Effects									
**Effect**	**Culture**	**Estimate**	**Std Err Pred**	**DF**	**t Value**	**Pr > |t|**	**Alpha**	**Lower**	**Upper**
**Culture**	1	-0.8166	1.0642	31	-0.77	0.4487	0.05	-2.9871	1.3539
**Culture**	2	-0.00898	1.0877	31	-0.01	0.9935	0.05	-2.2273	2.2093
**Culture**	3	0.7140	1.0877	31	0.66	0.5164	0.05	-1.5043	2.9323
**Culture**	4	0.1115	1.0877	31	0.10	0.9190	0.05	-2.1068	2.3298
**Culture**	5	-1.2067	1.0259	31	-1.18	0.2485	0.05	-3.2992	0.8857
**Culture**	6	1.0632	1.0577	31	1.01	0.3226	0.05	-1.0940	3.2204
**Culture**	7	0.8222	1.0577	31	0.78	0.4428	0.05	-1.3350	2.9794
**Culture**	8	-1.2880	1.0011	31	-1.29	0.2078	0.05	-3.3298	0.7538
**Culture**	9	-0.3316	1.1567	31	-0.29	0.7763	0.05	-2.6907	2.0276
**Culture**	10	0.9409	1.0995	31	0.86	0.3987	0.05	-1.3016	3.1834

Type 3 Tests of Fixed Effects				
**Effect**	**Num DF**	**Den DF**	**F Value**	**Pr > F**
**Treatment**	1	31	0.17	0.6808

DistSoma=84

Least Squares Means									
Effect	Treatment	Estimate	Standard Error	DF	t Value	Pr > |t|	Alpha	Lower	Upper
Treatment	Meg TTR	8.0442	0.8138	31	9.89	<.0001	0.05	6.3845	9.7039
Treatment	GFP MsTTR	7.5186	0.9690	31	7.76	<.0001	0.05	5.5423	9.4949

Differences of Least Squares Means												
Effect	Treatment	Treatment	Estimate	Standard Error	DF	t Value	Pr > |t|	Adjustment	Adj P	Alpha	Lower	Upper
Treatment	Meg TTR	GFP MsTTR	0.5256	1.2654	31	0.42	0.6808	Tukey-Kramer	0.6808	0.05	-2.0552	3.1063

Differences of Least Squares Means				
Effect	Treatment	Treatment	Adj Lower	Adj Upper
Treatment	Meg TTR	GFP MsTTR	-2.0552	3.1063

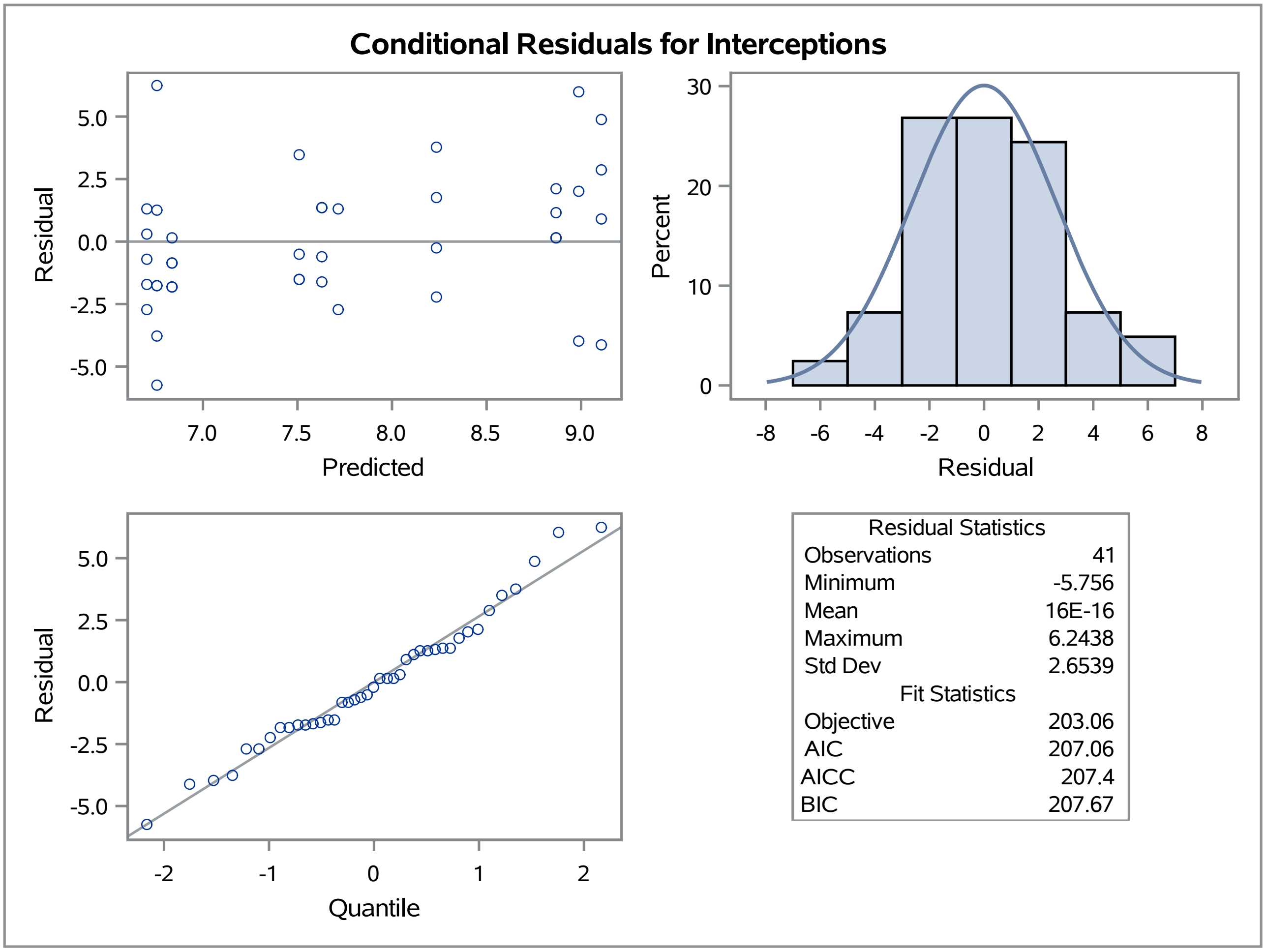

DistSoma=90

Model Information	
**Data Set**	WORK.TEMPDATASORTED
**Dependent Variable**	Interceptions
**Covariance Structure**	Variance Components
**Estimation Method**	REML
**Residual Variance Method**	Profile
**Fixed Effects SE Method**	Model-Based
**Degrees of Freedom Method**	Containment

Class Level Information		
**Class**	**Levels**	**Values**
**Treatment**	2	Meg TTR GFP MsTTR
**Culture**	10	1 2 3 4 5 6 7 8 9 10

Dimensions	
**Covariance Parameters**	2
**Columns in X**	3
**Columns in Z**	10
**Subjects**	1
**Max Obs per Subject**	41

Number of Observations	
**Number of Observations Read**	41
**Number of Observations Used**	41
**Number of Observations Not Used**	0

Iteration History			
**Iteration**	**Evaluations**	**-2 Res Log Like**	**Criterion**
**0**	1	203.29533661	
**1**	3	201.32611558	0.00011813
**2**	1	201.31805071	0.00000086
**3**	1	201.31799481	0.00000000

Convergence criteria met.

DistSoma=90

Covariance Parameter Estimates				
**Cov Parm**	**Estimate**	**Alpha**	**Lower**	**Upper**
**Culture**	1.7859	0.05	0.4838	70.8815
**Residual**	7.6545	0.05	4.9505	13.3911

Fit Statistics	
**-2 Res Log Likelihood**	201.3
**AIC (Smaller is Better)**	205.3
**AICC (Smaller is Better)**	205.7
**BIC (Smaller is Better)**	205.9

Solution for Fixed Effects									
**Effect**	**Treatment**	**Estimate**	**Standard Error**	**DF**	**t Value**	**Pr > |t|**	**Alpha**	**Lower**	**Upper**
**Intercept**		7.0523	0.9481	8	7.44	<.0001	0.05	4.8659	9.2387
**Treatment**	Meg TTR	0.7828	1.2381	31	0.63	0.5319	0.05	-1.7424	3.3079
**Treatment**	GFP MsTTR	0	.	.	.	.	.	.	.

Solution for Random Effects									
**Effect**	**Culture**	**Estimate**	**Std Err Pred**	**DF**	**t Value**	**Pr > |t|**	**Alpha**	**Lower**	**Upper**
**Culture**	1	-0.8897	1.0416	31	-0.85	0.3996	0.05	-3.0141	1.2347
**Culture**	2	0.09542	1.0646	31	0.09	0.9292	0.05	-2.0758	2.2666
**Culture**	3	0.4575	1.0646	31	0.43	0.6704	0.05	-1.7137	2.6287
**Culture**	4	0.3368	1.0646	31	0.32	0.7538	0.05	-1.8344	2.5080
**Culture**	5	-1.0958	1.0040	31	-1.09	0.2835	0.05	-3.1436	0.9520
**Culture**	6	1.1658	1.0351	31	1.13	0.2687	0.05	-0.9454	3.2770
**Culture**	7	0.8037	1.0351	31	0.78	0.4434	0.05	-1.3075	2.9149
**Culture**	8	-1.3621	0.9797	31	-1.39	0.1743	0.05	-3.3603	0.6361
**Culture**	9	-0.2657	1.1322	31	-0.23	0.8160	0.05	-2.5748	2.0434
**Culture**	10	0.7541	1.0761	31	0.70	0.4887	0.05	-1.4406	2.9489

Type 3 Tests of Fixed Effects				
**Effect**	**Num DF**	**Den DF**	**F Value**	**Pr > F**
**Treatment**	1	31	0.40	0.5319

DistSoma=90

Least Squares Means									
**Effect**	**Treatment**	**Estimate**	**Standard Error**	**DF**	**t Value**	**Pr > |t|**	**Alpha**	**Lower**	**Upper**
**Treatment**	Meg TTR	7.8351	0.7962	31	9.84	<.0001	0.05	6.2112	9.4590
**Treatment**	GFP MsTTR	7.0523	0.9481	31	7.44	<.0001	0.05	5.1186	8.9861

Differences of Least Squares Means												
**Effect**	**Treatment**	**Treatment**	**Estimate**	**Standard Error**	**DF**	**t Value**	**Pr > |t|**	**Adjustment**	**Adj P**	**Alpha**	**Lower**	**Upper**
**Treatment**	Meg TTR	GFP MsTTR	0.7828	1.2381	31	0.63	0.5319	Tukey-Kramer	0.5319	0.05	-1.7424	3.3079

Differences of Least Squares Means				
**Effect**	**Treatment**	**Treatment**	**Adj Lower**	**Adj Upper**
**Treatment**	Meg TTR	GFP MsTTR	-1.7423	3.3079

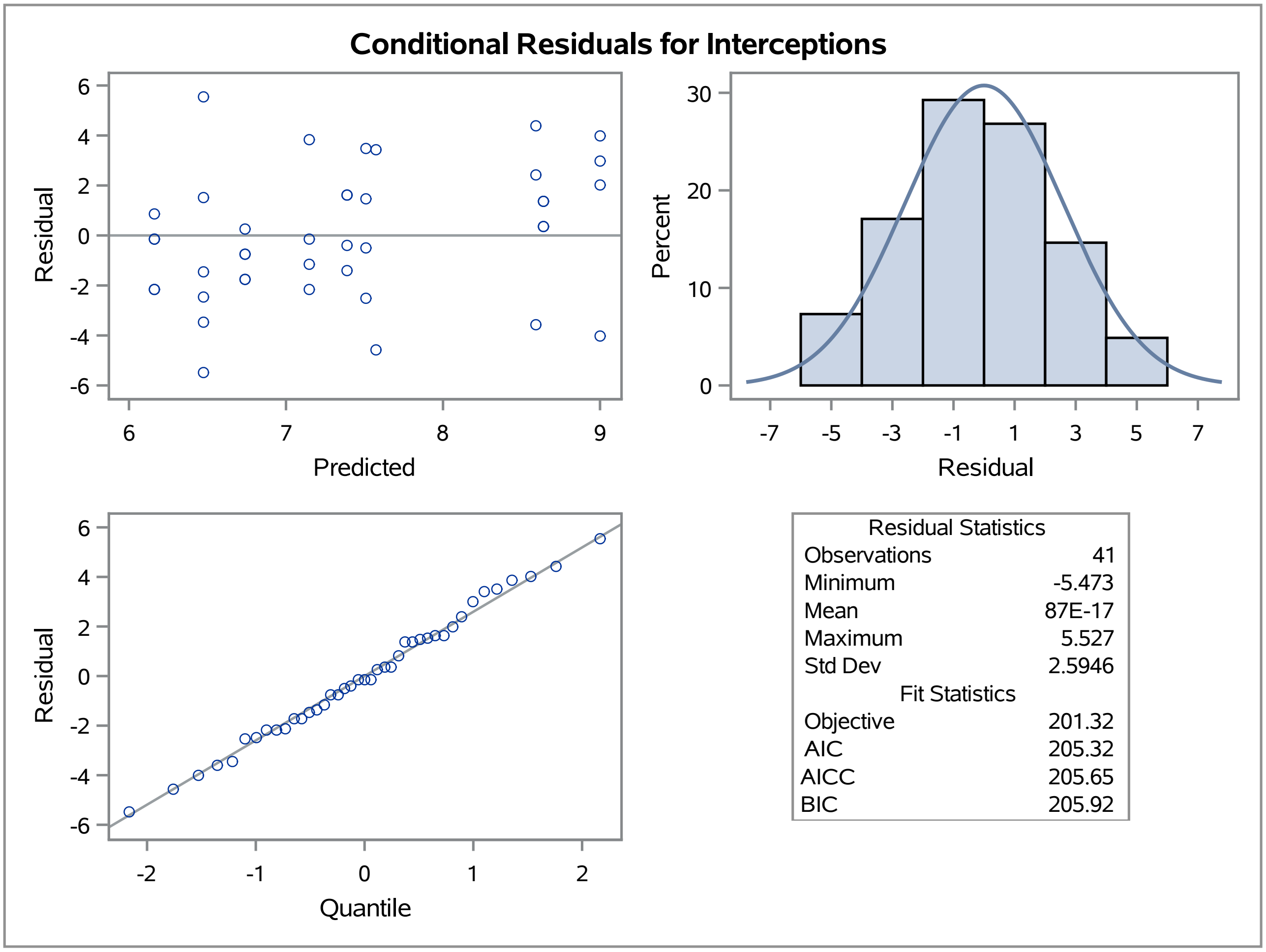

DistSoma=96

Model Information	
**Data Set**	WORK.TEMPDATASORTED
**Dependent Variable**	Interceptions
**Covariance Structure**	Variance Components
**Estimation Method**	REML
**Residual Variance Method**	Profile
**Fixed Effects SE Method**	Model-Based
**Degrees of Freedom Method**	Containment

Class Level Information		
**Class**	**Levels**	**Values**
**Treatment**	2	Meg TTR GFP MsTTR
**Culture**	10	1 2 3 4 5 6 7 8 9 10

Dimensions	
**Covariance Parameters**	2
**Columns in X**	3
**Columns in Z**	10
**Subjects**	1
**Max Obs per Subject**	41

Number of Observations	
**Number of Observations Read**	41
**Number of Observations Used**	41
**Number of Observations Not Used**	0

Iteration History			
**Iteration**	**Evaluations**	**-2 Res Log Like**	**Criterion**
**0**	1	198.58934446	
**1**	3	194.27359626	0.00126735
**2**	1	194.18532575	0.00007623
**3**	1	194.18045783	0.00000034
**4**	1	194.18043667	0.00000000

Convergence criteria met.

DistSoma=96

Covariance Parameter Estimates				
**Cov Parm**	**Estimate**	**Alpha**	**Lower**	**Upper**
**Culture**	2.4311	0.05	0.8051	28.8652
**Residual**	5.9919	0.05	3.8702	10.5046

Fit Statistics	
**-2 Res Log Likelihood**	194.2
**AIC (Smaller is Better)**	198.2
**AICC (Smaller is Better)**	198.5
**BIC (Smaller is Better)**	198.8

Solution for Fixed Effects									
**Effect**	**Treatment**	**Estimate**	**Standard Error**	**DF**	**t Value**	**Pr > |t|**	**Alpha**	**Lower**	**Upper**
**Intercept**		6.7512	0.9810	8	6.88	0.0001	0.05	4.4890	9.0135
**Treatment**	Meg TTR	1.2036	1.2788	31	0.94	0.3539	0.05	-1.4045	3.8117
**Treatment**	GFP MsTTR	0	.	.	.	.	.	.	.

Solution for Random Effects									
**Effect**	**Culture**	**Estimate**	**Std Err Pred**	**DF**	**t Value**	**Pr > |t|**	**Alpha**	**Lower**	**Upper**
**Culture**	1	-0.7711	1.1111	31	-0.69	0.4928	0.05	-3.0372	1.4949
**Culture**	2	-0.1555	1.1381	31	-0.14	0.8922	0.05	-2.4767	2.1658
**Culture**	3	0.3086	1.1381	31	0.27	0.7881	0.05	-2.0126	2.6298
**Culture**	4	0.6180	1.1381	31	0.54	0.5910	0.05	-1.7032	2.9392
**Culture**	5	-1.0415	1.0510	31	-0.99	0.3294	0.05	-3.1850	1.1020
**Culture**	6	0.9560	1.0883	31	0.88	0.3865	0.05	-1.2636	3.1757
**Culture**	7	1.4201	1.0883	31	1.30	0.2016	0.05	-0.7996	3.6398
**Culture**	8	-2.2126	1.0227	31	-2.16	0.0383	0.05	-4.2985	-0.1268
**Culture**	9	-0.4277	1.2154	31	-0.35	0.7273	0.05	-2.9065	2.0510
**Culture**	10	1.3057	1.1399	31	1.15	0.2608	0.05	-1.0190	3.6305

Type 3 Tests of Fixed Effects				
**Effect**	**Num DF**	**Den DF**	**F Value**	**Pr > F**
**Treatment**	1	31	0.89	0.3539

DistSoma=96

Least Squares Means									
**Effect**	**Treatment**	**Estimate**	**Standard Error**	**DF**	**t Value**	**Pr > |t|**	**Alpha**	**Lower**	**Upper**
**Treatment**	Meg TTR	7.9549	0.8203	31	9.70	<.0001	0.05	6.2819	9.6279
**Treatment**	GFP MsTTR	6.7512	0.9810	31	6.88	<.0001	0.05	4.7504	8.7521

Differences of Least Squares Means												
**Effect**	**Treatment**	**Treatment**	**Estimate**	**Standard Error**	**DF**	**t Value**	**Pr > |t|**	**Adjustment**	**Adj P**	**Alpha**	**Lower**	**Upper**
**Treatment**	Meg TTR	GFP MsTTR	1.2036	1.2788	31	0.94	0.3539	Tukey-Kramer	0.3539	0.05	-1.4045	3.8117

Differences of Least Squares Means				
**Effect**	**Treatment**	**Treatment**	**Adj Lower**	**Adj Upper**
**Treatment**	Meg TTR	GFP MsTTR	-1.4044	3.8117

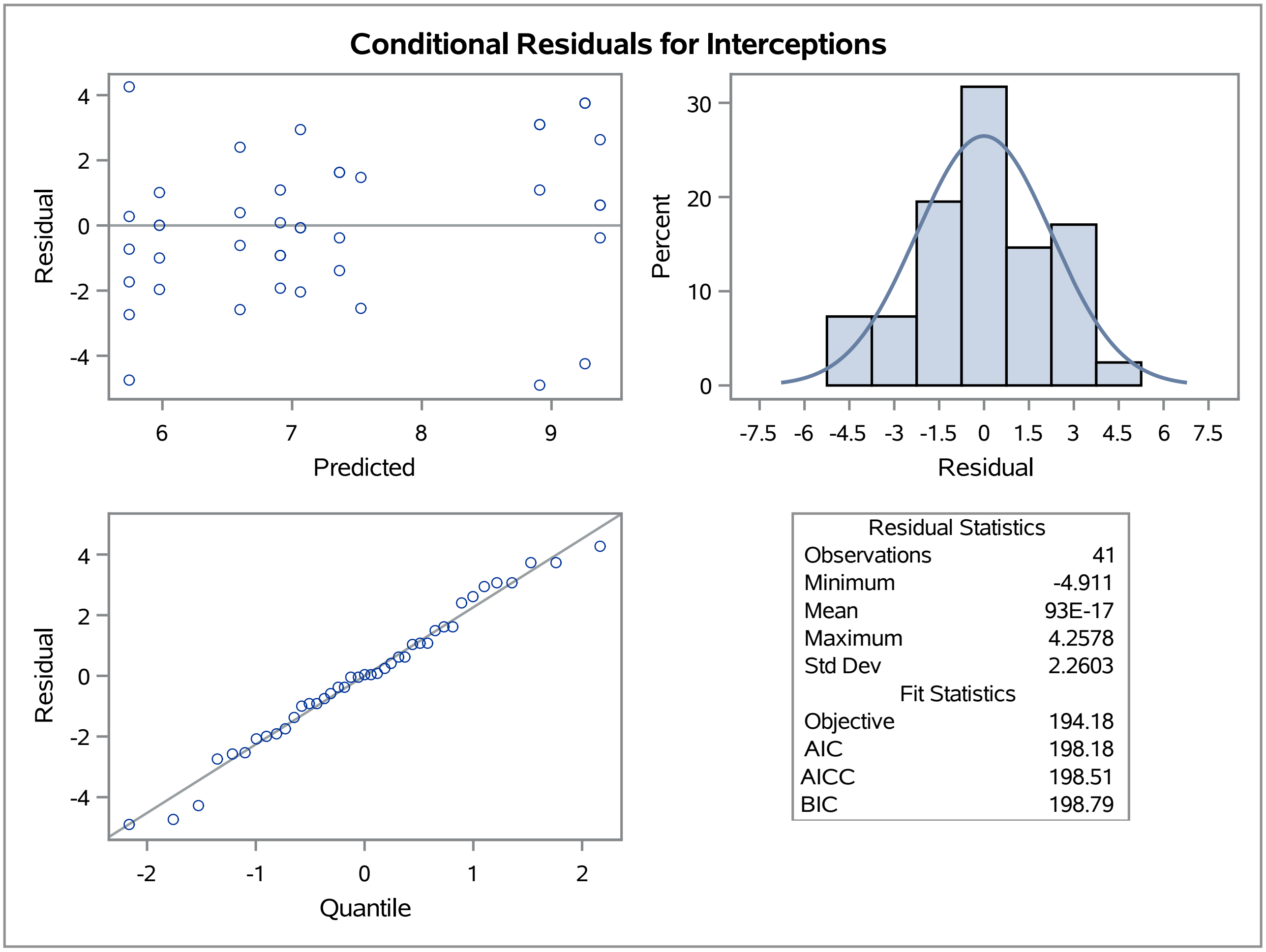

DistSoma=102

Model Information	
**Data Set**	WORK.TEMPDATASORTED
**Dependent Variable**	Interceptions
**Covariance Structure**	Variance Components
**Estimation Method**	REML
**Residual Variance Method**	Profile
**Fixed Effects SE Method**	Model-Based
**Degrees of Freedom Method**	Containment

Class Level Information		
**Class**	**Levels**	**Values**
**Treatment**	2	Meg TTR GFP MsTTR
**Culture**	10	1 2 3 4 5 6 7 8 9 10

Dimensions	
**Covariance Parameters**	2
**Columns in X**	3
**Columns in Z**	10
**Subjects**	1
**Max Obs per Subject**	41

Number of Observations	
**Number of Observations Read**	41
**Number of Observations Used**	41
**Number of Observations Not Used**	0

Iteration History			
**Iteration**	**Evaluations**	**-2 Res Log Like**	**Criterion**
**0**	1	197.36995217	
**1**	3	194.79289227	0.00086884
**2**	1	194.73308006	0.00003743
**3**	1	194.73070672	0.00000008
**4**	1	194.73070153	0.00000000

Convergence criteria met.

DistSoma=102

Covariance Parameter Estimates				
**Cov Parm**	**Estimate**	**Alpha**	**Lower**	**Upper**
**Culture**	1.7388	0.05	0.5104	40.1048
**Residual**	6.3614	0.05	4.1179	11.1123

Fit Statistics	
**-2 Res Log Likelihood**	194.7
**AIC (Smaller is Better)**	198.7
**AICC (Smaller is Better)**	199.1
**BIC (Smaller is Better)**	199.3

Solution for Fixed Effects									
**Effect**	**Treatment**	**Estimate**	**Standard Error**	**DF**	**t Value**	**Pr > |t|**	**Alpha**	**Lower**	**Upper**
**Intercept**		6.8057	0.9005	8	7.56	<.0001	0.05	4.7291	8.8822
**Treatment**	Meg TTR	1.0082	1.1754	31	0.86	0.3976	0.05	-1.3889	3.4054
**Treatment**	GFP MsTTR	0	.	.	.	.	.	.	.

Solution for Random Effects									
**Effect**	**Culture**	**Estimate**	**Std Err Pred**	**DF**	**t Value**	**Pr > |t|**	**Alpha**	**Lower**	**Upper**
**Culture**	1	-0.6962	1.0026	31	-0.69	0.4926	0.05	-2.7410	1.3485
**Culture**	2	-0.02907	1.0256	31	-0.03	0.9776	0.05	-2.1208	2.0626
**Culture**	3	0.2321	1.0256	31	0.23	0.8225	0.05	-1.8596	2.3238
**Culture**	4	0.4932	1.0256	31	0.48	0.6340	0.05	-1.5985	2.5849
**Culture**	5	-0.9320	0.9618	31	-0.97	0.3400	0.05	-2.8935	1.0296
**Culture**	6	0.7501	0.9931	31	0.76	0.4558	0.05	-1.2754	2.7756
**Culture**	7	1.4030	0.9931	31	1.41	0.1677	0.05	-0.6225	3.4285
**Culture**	8	-1.6445	0.9375	31	-1.75	0.0893	0.05	-3.5565	0.2675
**Culture**	9	-0.1109	1.0934	31	-0.10	0.9198	0.05	-2.3410	2.1191
**Culture**	10	0.5344	1.0350	31	0.52	0.6093	0.05	-1.5765	2.6453

Type 3 Tests of Fixed Effects				
**Effect**	**Num DF**	**Den DF**	**F Value**	**Pr > F**
**Treatment**	1	31	0.74	0.3976

DistSoma=102

Least Squares Means									
**Effect**	**Treatment**	**Estimate**	**Standard Error**	**DF**	**t Value**	**Pr > |t|**	**Alpha**	**Lower**	**Upper**
**Treatment**	Meg TTR	7.8139	0.7554	31	10.34	<.0001	0.05	6.2733	9.3545
**Treatment**	GFP MsTTR	6.8057	0.9005	31	7.56	<.0001	0.05	4.9691	8.6422

Differences of Least Squares Means													
**Effect**	**Treatment**	**Treatment**	**Estimate**	**Standard Error**	**DF**	**t Value**	**Pr > |t|**	**Adjustment**	**Adj P**	**Alpha**	**Lower**	**Upper**
**Treatment**	Meg TTR	GFP MsTTR	1.0082	1.1754	31	0.86	0.3976	Tukey-Kramer	0.3976	0.05	-1.3889	3.4054

Differences of Least Squares Means				
**Effect**	**Treatment**	**Treatment**	**Adj Lower**	**Adj Upper**
**Treatment**	Meg TTR	GFP MsTTR	-1.3889	3.4054

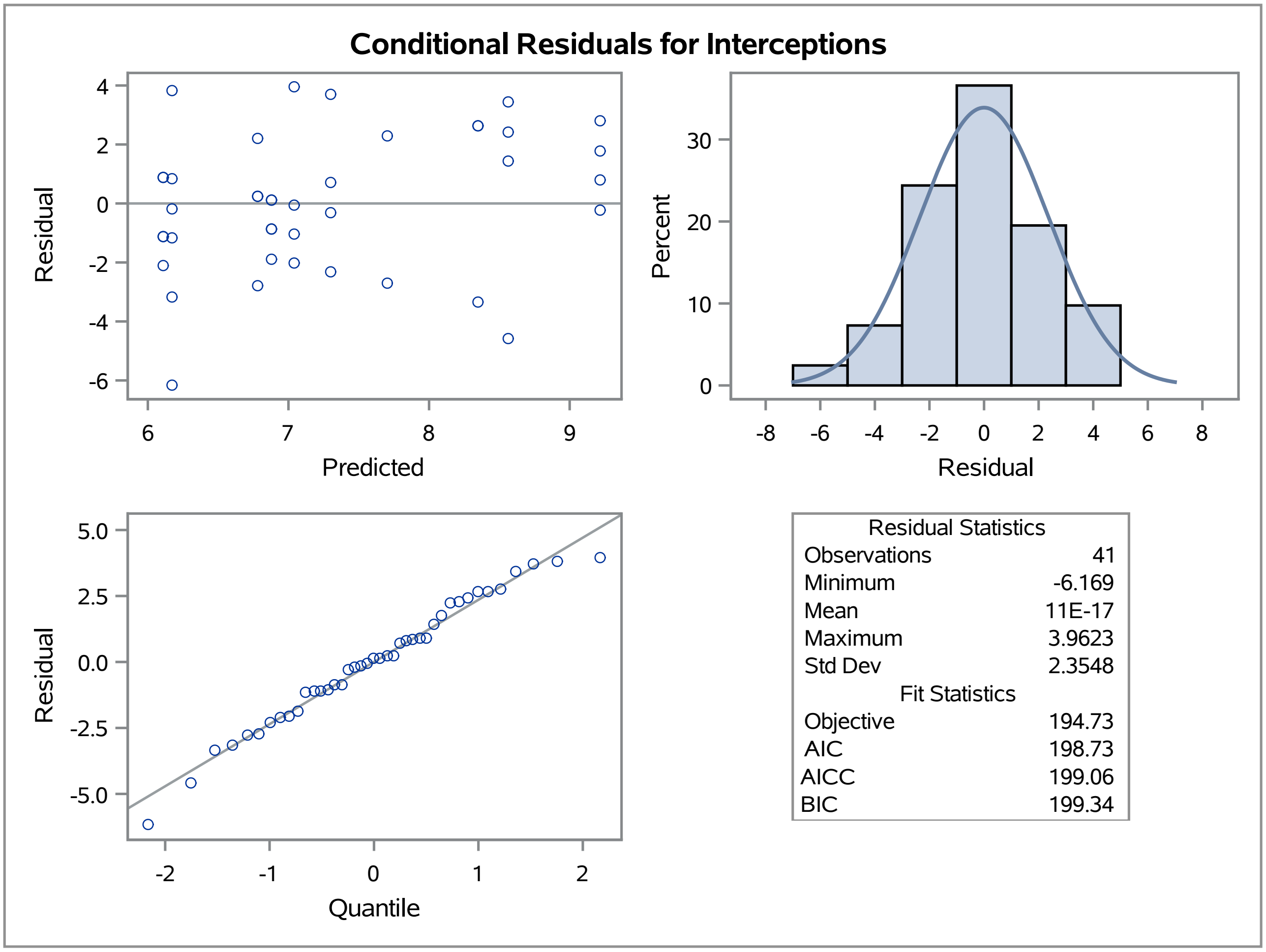

DistSoma=108

Model Information	
**Data Set**	WORK.TEMPDATASORTED
**Dependent Variable**	Interceptions
**Covariance Structure**	Variance Components
**Estimation Method**	REML
**Residual Variance Method**	Profile
**Fixed Effects SE Method**	Model-Based
**Degrees of Freedom Method**	Containment

Class Level Information		
**Class**	**Levels**	**Values**
**Treatment**	2	Meg TTR GFP MsTTR
**Culture**	10	1 2 3 4 5 6 7 8 9 10

Dimensions	
**Covariance Parameters**	2
**Columns in X**	3
**Columns in Z**	10
**Subjects**	1
**Max Obs per Subject**	41

Number of Observations	
**Number of Observations Read**	41
**Number of Observations Used**	41
**Number of Observations Not Used**	0

Iteration History			
**Iteration**	**Evaluations**	**-2 Res Log Like**	**Criterion**
**0**	1	196.39671228	
**1**	3	194.89678039	0.00020112
**2**	1	194.88357410	0.00000232
**3**	1	194.88343033	0.00000000

Convergence criteria met.

DistSoma=108

Covariance Parameter Estimates				
Cov Parm	Estimate	Alpha	Lower	Upper
Culture	1.2641	0.05	0.3154	97.2220
Residual	6.6115	0.05	4.2815	11.5419

Fit Statistics	
-2 Res Log Likelihood	194.9
AIC (Smaller is Better)	198.9
AICC (Smaller is Better)	199.2
BIC (Smaller is Better)	199.5

Solution for Fixed Effects									
Effect	Treatment	Estimate	Standard Error	DF	t Value	Pr > |t|	Alpha	Lower	Upper
Intercept		6.6234	0.8406	8	7.88	<.0001	0.05	4.6849	8.5619
Treatment	Meg TTR	0.8251	1.0982	31	0.75	0.4582	0.05	-1.4148	3.0650
Treatment	GFP MsTTR	0	.	.	.	.	.	.	.

Solution for Random Effects									
Effect	Culture	Estimate	Std Err Pred	DF	t Value	Pr > |t|	Alpha	Lower	Upper
Culture	1	-0.5979	0.9028	31	-0.66	0.5127	0.05	-2.4393	1.2434
Culture	2	0.1632	0.9214	31	0.18	0.8606	0.05	-1.7161	2.0425
Culture	3	0.05486	0.9214	31	0.06	0.9529	0.05	-1.8244	1.9341
Culture	4	0.3799	0.9214	31	0.41	0.6830	0.05	-1.4994	2.2591
Culture	5	-0.7079	0.8750	31	-0.81	0.4246	0.05	-2.4925	1.0766
Culture	6	0.5640	0.9001	31	0.63	0.5355	0.05	-1.2716	2.3997
Culture	7	0.8891	0.9001	31	0.99	0.3309	0.05	-0.9466	2.7247
Culture	8	-1.3082	0.8552	31	-1.53	0.1362	0.05	-3.0523	0.4360
Culture	9	-0.1241	0.9760	31	-0.13	0.8997	0.05	-2.1147	1.8666
Culture	10	0.6871	0.9326	31	0.74	0.4668	0.05	-1.2149	2.5891

Type 3 Tests of Fixed Effects				
Effect	Num DF	Den DF	F Value	Pr > F
Treatment	1	31	0.56	0.4582

DistSoma=108

Least Squares Means									
**Effect**	**Treatment**	**Estimate**	**Standard Error**	**DF**	**t Value**	**Pr > |t|**	**Alpha**	**Lower**	**Upper**
**Treatment**	Meg TTR	7.4485	0.7067	31	10.54	<.0001	0.05	6.0071	8.8899
**Treatment**	GFP MsTTR	6.6234	0.8406	31	7.88	<.0001	0.05	4.9089	8.3379

Differences of Least Squares Means												
**Effect**	**Treatment**	**Treatment**	**Estimate**	**Standard Error**	**DF**	**t Value**	**Pr > |t|**	**Adjustment**	**Adj P**	**Alpha**	**Lower**	**Upper**
**Treatment**	Meg TTR	GFP MsTTR	0.8251	1.0982	31	0.75	0.4582	Tukey-Kramer	0.4582	0.05	-1.4148	3.0650

Differences of Least Squares Means				
**Effect**	**Treatment**	**Treatment**	**Adj Lower**	**Adj Upper**
**Treatment**	Meg TTR	GFP MsTTR	-1.4148	3.0649

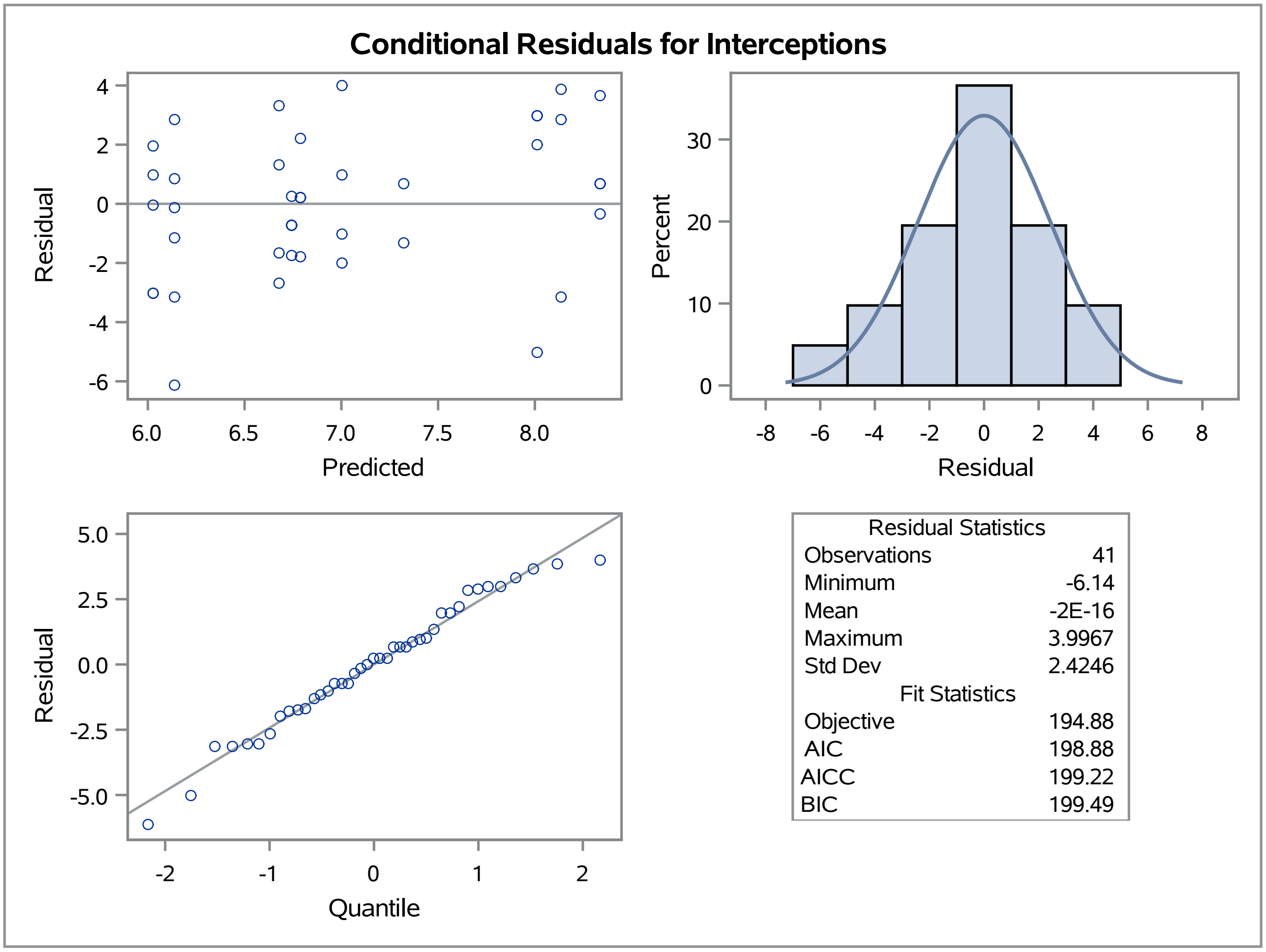

DistSoma=114

Model Information	
**Data Set**	WORK.TEMPDATASORTED
**Dependent Variable**	Interceptions
**Covariance Structure**	Variance Components
**Estimation Method**	REML
**Residual Variance Method**	Profile
**Fixed Effects SE Method**	Model-Based
**Degrees of Freedom Method**	Containment

Class Level Information		
**Class**	**Levels**	**Values**
**Treatment**	2	Meg TTR GFP MsTTR
**Culture**	10	1 2 3 4 5 6 7 8 9 10

Dimensions	
**Covariance Parameters**	2
**Columns in X**	3
**Columns in Z**	10
**Subjects**	1
**Max Obs per Subject**	41

Number of Observations	
**Number of Observations Read**	41
**Number of Observations Used**	41
**Number of Observations Not Used**	0

Iteration History			
**Iteration**	**Evaluations**	**-2 Res Log Like**	**Criterion**
**0**	1	198.91929011	
**1**	3	196.20428045	0.00081665
**2**	1	196.14752710	0.00003369
**3**	1	196.14536917	0.00000007
**4**	1	196.14536486	0.00000000

Convergence criteria met.

DistSoma=114

Covariance Parameter Estimates				
**Cov Parm**	**Estimate**	**Alpha**	**Lower**	**Upper**
**Culture**	1.8675	0.05	0.5553	39.8003
**Residual**	6.5687	0.05	4.2509	11.4799

Fit Statistics	
**-2 Res Log Likelihood**	196.1
**AIC (Smaller is Better)**	200.1
**AICC (Smaller is Better)**	200.5
**BIC (Smaller is Better)**	200.8

Solution for Fixed Effects									
**Effect**	**Treatment**	**Estimate**	**Standard Error**	**DF**	**t Value**	**Pr > |t|**	**Alpha**	**Lower**	**Upper**
**Intercept**		6.5483	0.9248	8	7.08	0.0001	0.05	4.4156	8.6810
**Treatment**	Meg TTR	0.7583	1.2070	31	0.63	0.5345	0.05	-1.7034	3.2200
**Treatment**	GFP MsTTR	0	.	.	.	.	.	.	.

Solution for Random Effects									
**Effect**	**Culture**	**Estimate**	**Std Err Pred**	**DF**	**t Value**	**Pr > |t|**	**Alpha**	**Lower**	**Upper**
**Culture**	1	-0.3219	1.0325	31	-0.31	0.7573	0.05	-2.4276	1.7838
**Culture**	2	-0.1587	1.0564	31	-0.15	0.8815	0.05	-2.3133	1.9958
**Culture**	3	-0.1587	1.0564	31	-0.15	0.8815	0.05	-2.3133	1.9958
**Culture**	4	0.6394	1.0564	31	0.61	0.5494	0.05	-1.5151	2.7939
**Culture**	5	-1.1193	0.9892	31	-1.13	0.2665	0.05	-3.1367	0.8982
**Culture**	6	0.7680	1.0218	31	0.75	0.4580	0.05	-1.3160	2.8520
**Culture**	7	1.1671	1.0218	31	1.14	0.2621	0.05	-0.9169	3.2511
**Culture**	8	-1.8744	0.9640	31	-1.94	0.0610	0.05	-3.8405	0.09161
**Culture**	9	0.4326	1.1268	31	0.38	0.7037	0.05	-1.8655	2.7306
**Culture**	10	0.6260	1.0655	31	0.59	0.5611	0.05	-1.5471	2.7992

Type 3 Tests of Fixed Effects				
**Effect**	**Num DF**	**Den DF**	**F Value**	**Pr > F**
**Treatment**	1	31	0.39	0.5345

DistSoma=114

Least Squares Means									
**Effect**	**Treatment**	**Estimate**	**Standard Error**	**DF**	**t Value**	**Pr > |t|**	**Alpha**	**Lower**	**Upper**
**Treatment**	Meg TTR	7.3066	0.7756	31	9.42	<.0001	0.05	5.7248	8.8885
**Treatment**	GFP MsTTR	6.5483	0.9248	31	7.08	<.0001	0.05	4.6621	8.4346

Differences of Least Squares Means												
**Effect**	**Treatment**	**Treatment**	**Estimate**	**Standard Error**	**DF**	**t Value**	**Pr > |t|**	**Adjustment**	**Adj P**	**Alpha**	**Lower**	**Upper**
**Treatment**	Meg TTR	GFP MsTTR	0.7583	1.2070	31	0.63	0.5345	Tukey-Kramer	0.5345	0.05	-1.7034	3.2200

Differences of Least Squares Means				
**Effect**	**Treatment**	**Treatment**	**Adj Lower**	**Adj Upper**
**Treatment**	Meg TTR	GFP MsTTR	-1.7034	3.2200

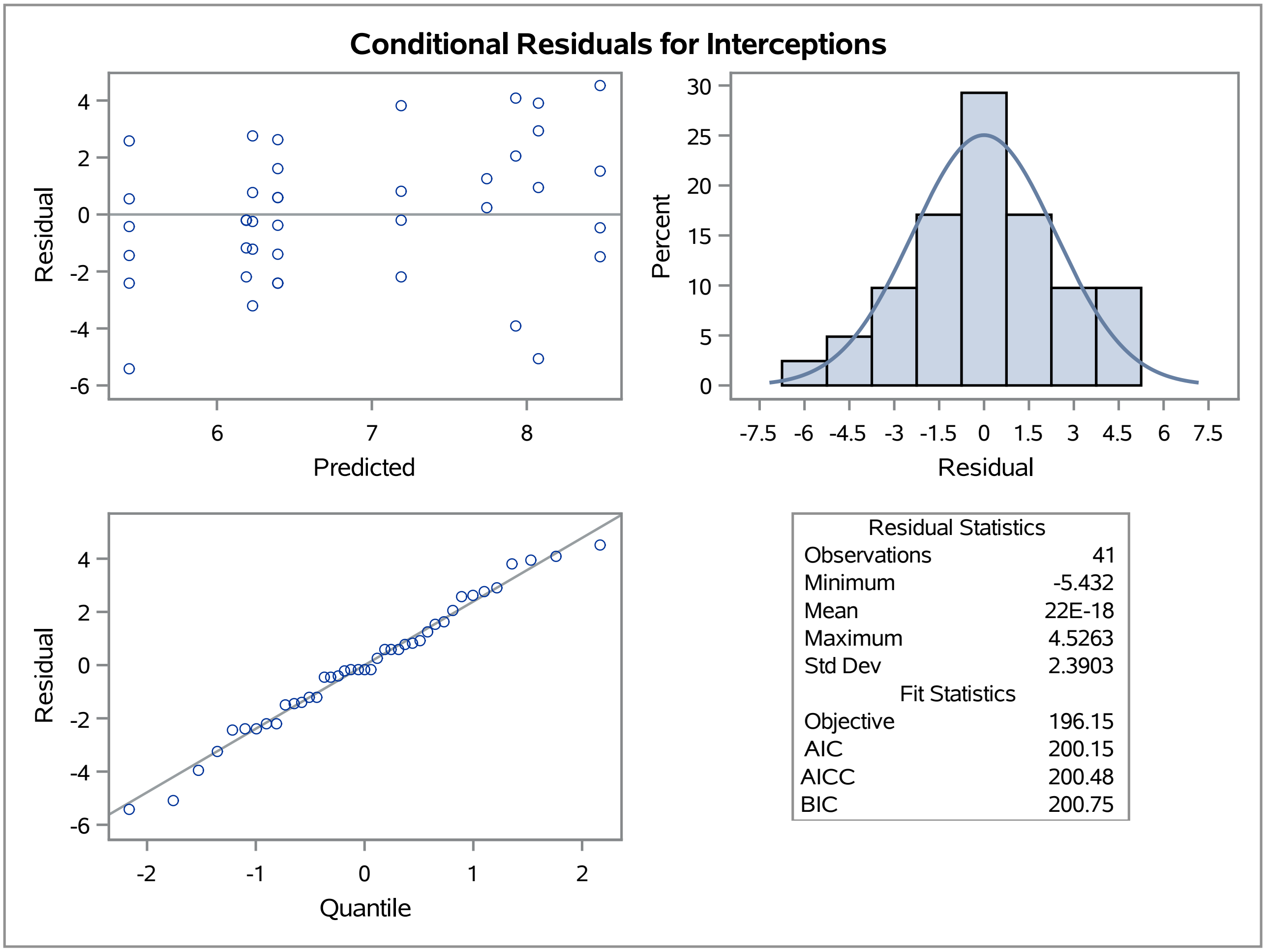

DistSoma=120

Model Information	
**Data Set**	WORK.TEMPDATASORTED
**Dependent Variable**	Interceptions
**Covariance Structure**	Variance Components
**Estimation Method**	REML
**Residual Variance Method**	Profile
**Fixed Effects SE Method**	Model-Based
**Degrees of Freedom Method**	Containment

Class Level Information		
**Class**	**Levels**	**Values**
**Treatment**	2	Meg TTR GFP MsTTR
**Culture**	10	1 2 3 4 5 6 7 8 9 10

Dimensions	
**Covariance Parameters**	2
**Columns in X**	3
**Columns in Z**	10
**Subjects**	1
**Max Obs per Subject**	41

Number of Observations	
**Number of Observations Read**	41
**Number of Observations Used**	41
**Number of Observations Not Used**	0

Iteration History			
**Iteration**	**Evaluations**	**-2 Res Log Like**	**Criterion**
**0**	1	200.10113655	
**1**	3	198.38702299	0.00055173
**2**	1	198.34855310	0.00001632
**3**	1	198.34749774	0.00000002
**4**	1	198.34749668	0.00000000

Convergence criteria met.

DistSoma=120

Covariance Parameter Estimates				
**Cov Parm**	**Estimate**	**Alpha**	**Lower**	**Upper**
**Culture**	1.4750	0.05	0.3855	76.9777
**Residual**	7.1791	0.05	4.6527	12.5166

Fit Statistics	
**-2 Res Log Likelihood**	198.3
**AIC (Smaller is Better)**	202.3
**AICC (Smaller is Better)**	202.7
**BIC (Smaller is Better)**	203.0

Solution for Fixed Effects									
**Effect**	**Treatment**	**Estimate**	**Standard Error**	**DF**	**t Value**	**Pr > |t|**	**Alpha**	**Lower**	**Upper**
**Intercept**		6.4367	0.8905	8	7.23	<.0001	0.05	4.3832	8.4902
**Treatment**	Meg TTR	0.7237	1.1632	31	0.62	0.5384	0.05	-1.6487	3.0961
**Treatment**	GFP MsTTR	0	.	.	.	.	.	.	.

Solution for Random Effects									
**Effect**	**Culture**	**Estimate**	**Std Err Pred**	**DF**	**t Value**	**Pr > |t|**	**Alpha**	**Lower**	**Upper**
**Culture**	1	-0.4240	0.9650	31	-0.44	0.6634	0.05	-2.3921	1.5441
**Culture**	2	-0.08422	0.9854	31	-0.09	0.9324	0.05	-2.0939	1.9255
**Culture**	3	-0.08422	0.9854	31	-0.09	0.9324	0.05	-2.0939	1.9255
**Culture**	4	0.5924	0.9854	31	0.60	0.5521	0.05	-1.4173	2.6022
**Culture**	5	-0.7907	0.9335	31	-0.85	0.4035	0.05	-2.6945	1.1132
**Culture**	6	0.7171	0.9610	31	0.75	0.4612	0.05	-1.2430	2.6771
**Culture**	7	0.9426	0.9610	31	0.98	0.3343	0.05	-1.0174	2.9027
**Culture**	8	-1.5609	0.9118	31	-1.71	0.0969	0.05	-3.4205	0.2987
**Culture**	9	0.2445	1.0454	31	0.23	0.8166	0.05	-1.8876	2.3767
**Culture**	10	0.4473	0.9970	31	0.45	0.6568	0.05	-1.5861	2.4807

Type 3 Tests of Fixed Effects				
**Effect**	**Num DF**	**Den DF**	**F Value**	**Pr > F**
**Treatment**	1	31	0.39	0.5384

DistSoma=120

Least Squares Means									
**Effect**	**Treatment**	**Estimate**	**Standard Error**	**DF**	**t Value**	**Pr > |t|**	**Alpha**	**Lower**	**Upper**
**Treatment**	Meg TTR	7.1604	0.7484	31	9.57	<.0001	0.05	5.6340	8.6867
**Treatment**	GFP MsTTR	6.4367	0.8905	31	7.23	<.0001	0.05	4.6205	8.2529

Differences of Least Squares Means												
**Effect**	**Treatment**	**Treatment**	**Estimate**	**Standard Error**	**DF**	**t Value**	**Pr > |t|**	**Adjustment**	**Adj P**	**Alpha**	**Lower**	**Upper**
**Treatment**	Meg TTR	GFP MsTTR	0.7237	1.1632	31	0.62	0.5384	Tukey-Kramer	0.5384	0.05	-1.6487	3.0961

Differences of Least Squares Means				
**Effect**	**Treatment**	**Treatment**	**Adj Lower**	**Adj Upper**
**Treatment**	Meg TTR	GFP MsTTR	-1.6487	3.0960

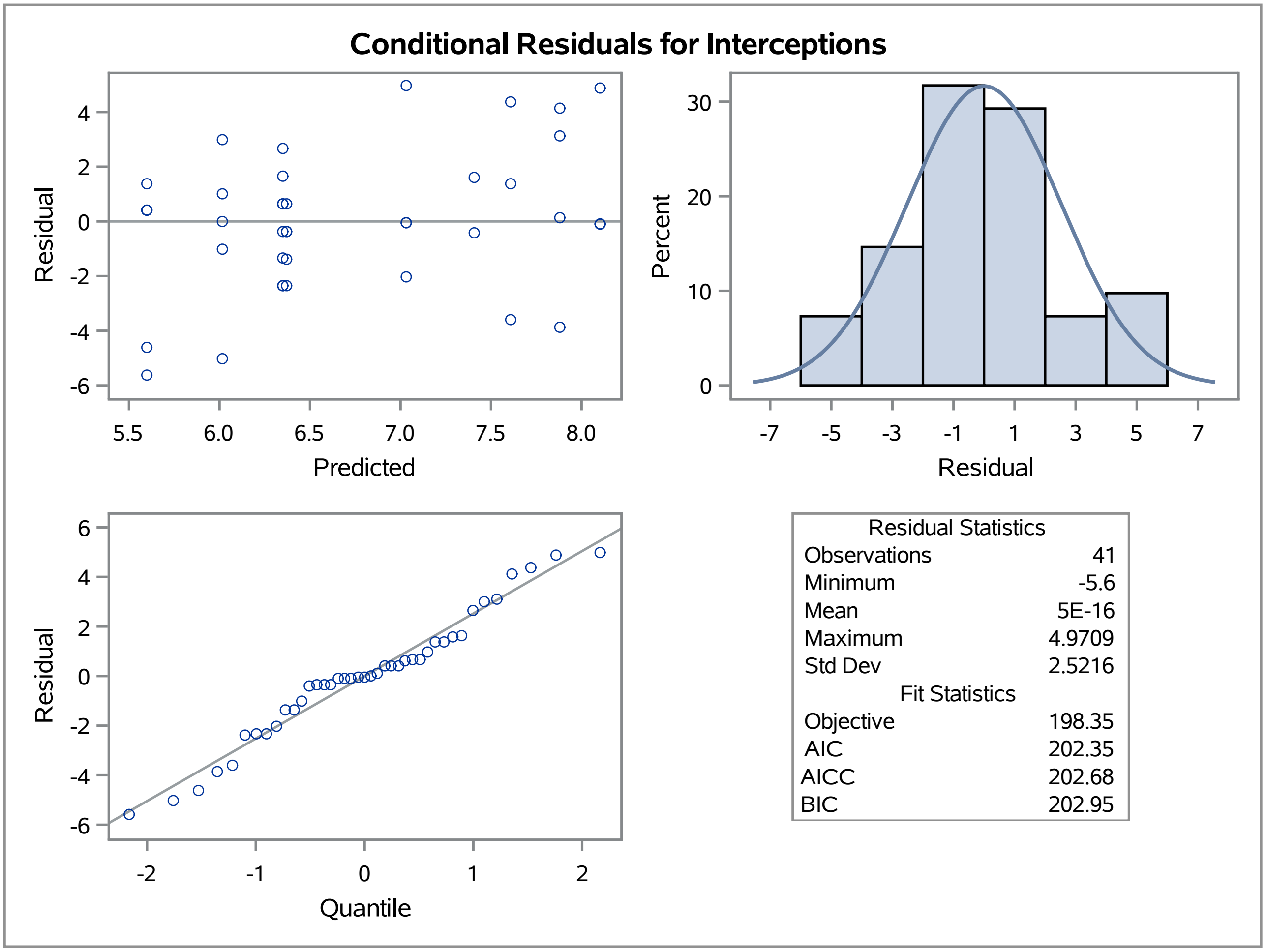

DistSoma=126

Model Information	
**Data Set**	WORK.TEMPDATASORTED
**Dependent Variable**	Interceptions
**Covariance Structure**	Variance Components
**Estimation Method**	REML
**Residual Variance Method**	Profile
**Fixed Effects SE Method**	Model-Based
**Degrees of Freedom Method**	Containment

Class Level Information		
**Class**	**Levels**	**Values**
**Treatment**	2	Meg TTR GFP MsTTR
**Culture**	10	1 2 3 4 5 6 7 8 9 10

Dimensions	
**Covariance Parameters**	2
**Columns in X**	3
**Columns in Z**	10
**Subjects**	1
**Max Obs per Subject**	41

Number of Observations	
**Number of Observations Read**	41
**Number of Observations Used**	41
**Number of Observations Not Used**	0

Iteration History			
**Iteration**	**Evaluations**	**-2 Res Log Like**	**Criterion**
**0**	1	208.06463972	
**1**	3	203.99513530	0.00144749
**2**	1	203.88540216	0.00010271
**3**	1	203.87827279	0.00000066
**4**	1	203.87822910	0.00000000

Convergence criteria met.

DistSoma=126

Covariance Parameter Estimates				
Cov Parm	Estimate	Alpha	Lower	Upper
Culture	2.9436	0.05	0.9653	36.6743
Residual	7.7461	0.05	5.0088	13.5552

Fit Statistics	
-2 Res Log Likelihood	203.9
AIC (Smaller is Better)	207.9
AICC (Smaller is Better)	208.2
BIC (Smaller is Better)	208.5

Solution for Fixed Effects									
Effect	Treatment	Estimate	Standard Error	DF	t Value	Pr > |t|	Alpha	Lower	Upper
Intercept		6.6439	1.0928	8	6.08	0.0003	0.05	4.1238	9.1640
Treatment	Meg TTR	0.1650	1.4249	31	0.12	0.9085	0.05	-2.7410	3.0711
Treatment	GFP MsTTR	0	.	.	.	.	.	.	.

Solution for Random Effects									
Effect	Culture	Estimate	Std Err Pred	DF	t Value	Pr > |t|	Alpha	Lower	Upper
Culture	1	-0.9460	1.2360	31	-0.77	0.4498	0.05	-3.4668	1.5748
Culture	2	0.9688	1.2659	31	0.77	0.4499	0.05	-1.6131	3.5507
Culture	3	-0.6900	1.2659	31	-0.55	0.5896	0.05	-3.2719	1.8919
Culture	4	0.6672	1.2659	31	0.53	0.6019	0.05	-1.9147	3.2491
Culture	5	-0.9231	1.1721	31	-0.79	0.4369	0.05	-3.3136	1.4674
Culture	6	1.0200	1.2133	31	0.84	0.4070	0.05	-1.4546	3.4947
Culture	7	0.8692	1.2133	31	0.72	0.4791	0.05	-1.6054	3.3439
Culture	8	-2.6477	1.1408	31	-2.32	0.0270	0.05	-4.9743	-0.3211
Culture	9	0.5143	1.3522	31	0.38	0.7063	0.05	-2.2434	3.2721
Culture	10	1.1672	1.2699	31	0.92	0.3651	0.05	-1.4228	3.7573

Type 3 Tests of Fixed Effects				
Effect	Num DF	Den DF	F Value	Pr > F
Treatment	1	31	0.01	0.9085

DistSoma=126

Least Squares Means									
**Effect**	**Treatment**	**Estimate**	**Standard Error**	**DF**	**t Value**	**Pr > |t|**	**Alpha**	**Lower**	**Upper**
**Treatment**	Meg TTR	6.8089	0.9143	31	7.45	<.0001	0.05	4.9442	8.6737
**Treatment**	GFP MsTTR	6.6439	1.0928	31	6.08	<.0001	0.05	4.4150	8.8727

Differences of Least Squares Means												
**Effect**	**Treatment**	**Treatment**	**Estimate**	**Standard Error**	**DF**	**t Value**	**Pr > |t|**	**Adjustment**	**Adj P**	**Alpha**	**Lower**	**Upper**
**Treatment**	Meg TTR	GFP MsTTR	0.1650	1.4249	31	0.12	0.9085	Tukey-Kramer	0.9085	0.05	-2.7410	3.0711

Differences of Least Squares Means				
**Effect**	**Treatment**	**Treatment**	**Adj Lower**	**Adj Upper**
**Treatment**	Meg TTR	GFP MsTTR	-2.7410	3.0710

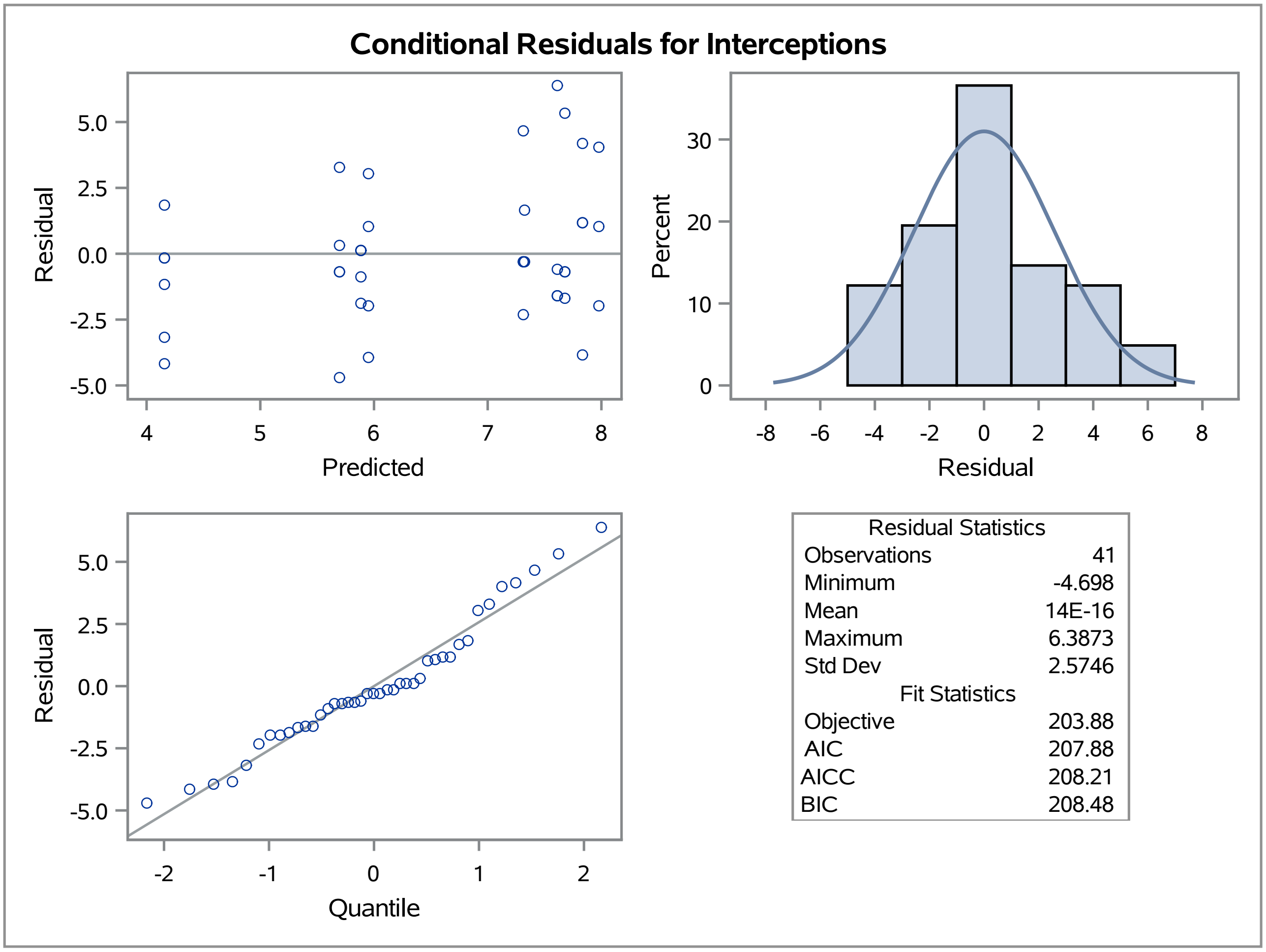

DistSoma=132

Model Information	
**Data Set**	WORK.TEMPDATASORTED
**Dependent Variable**	Interceptions
**Covariance Structure**	Variance Components
**Estimation Method**	REML
**Residual Variance Method**	Profile
**Fixed Effects SE Method**	Model-Based
**Degrees of Freedom Method**	Containment

Class Level Information		
**Class**	**Levels**	**Values**
**Treatment**	2	Meg TTR GFP MsTTR
**Culture**	10	1 2 3 4 5 6 7 8 9 10

Dimensions	
**Covariance Parameters**	2
**Columns in X**	3
**Columns in Z**	10
**Subjects**	1
**Max Obs per Subject**	41

Number of Observations	
**Number of Observations Read**	41
**Number of Observations Used**	41
**Number of Observations Not Used**	0

Iteration History			
**Iteration**	**Evaluations**	**-2 Res Log Like**	**Criterion**
**0**	1	204.93086901	
**1**	3	202.11463711	0.00099948
**2**	1	202.04113264	0.00005145
**3**	1	202.03765680	0.00000017
**4**	1	202.03764584	0.00000000

Convergence criteria met.

DistSoma=132

Covariance Parameter Estimates				
Cov Parm	Estimate	Alpha	Lower	Upper
Culture	2.1745	0.05	0.6520	44.0849
Residual	7.6390	0.05	4.9469	13.3354

Fit Statistics	
-2 Res Log Likelihood	202.0
AIC (Smaller is Better)	206.0
AICC (Smaller is Better)	206.4
BIC (Smaller is Better)	206.6

Solution for Fixed Effects									
Effect	Treatment	Estimate	Standard Error	DF	t Value	Pr > |t|	Alpha	Lower	Upper
Intercept		5.9139	0.9977	8	5.93	0.0004	0.05	3.6133	8.2146
Treatment	Meg TTR	0.6649	1.3021	31	0.51	0.6132	0.05	-1.9907	3.3205
Treatment	GFP MsTTR	0	.	.	.	.	.	.	.

Solution for Random Effects									
Effect	Culture	Estimate	Std Err Pred	DF	t Value	Pr > |t|	Alpha	Lower	Upper
Culture	1	-0.5368	1.1139	31	-0.48	0.6333	0.05	-2.8085	1.7350
Culture	2	0.1789	1.1397	31	0.16	0.8763	0.05	-2.1455	2.5034
Culture	3	-0.4866	1.1397	31	-0.43	0.6724	0.05	-2.8110	1.8378
Culture	4	0.8444	1.1397	31	0.74	0.4643	0.05	-1.4800	3.1689
Culture	5	-0.6924	1.0671	31	-0.65	0.5212	0.05	-2.8688	1.4841
Culture	6	0.7566	1.1024	31	0.69	0.4976	0.05	-1.4916	3.0049
Culture	7	1.0228	1.1024	31	0.93	0.3607	0.05	-1.2254	3.2711
Culture	8	-2.2572	1.0399	31	-2.17	0.0377	0.05	-4.3782	-0.1363
Culture	9	0.5156	1.2156	31	0.42	0.6744	0.05	-1.9637	2.9948
Culture	10	0.6546	1.1495	31	0.57	0.5732	0.05	-1.6899	2.9991

Type 3 Tests of Fixed Effects				
Effect	Num DF	Den DF	F Value	Pr > F
Treatment	1	31	0.26	0.6132

DistSoma=132

Least Squares Means									
**Effect**	**Treatment**	**Estimate**	**Standard Error**	**DF**	**t Value**	**Pr > |t|**	**Alpha**	**Lower**	**Upper**
**Treatment**	Meg TTR	6.5789	0.8367	31	7.86	<.0001	0.05	4.8724	8.2853
**Treatment**	GFP MsTTR	5.9139	0.9977	31	5.93	<.0001	0.05	3.8791	7.9487

Differences of Least Squares Means												
**Effect**	**Treatment**	**Treatment**	**Estimate**	**Standard Error**	**DF**	**t Value**	**Pr > |t|**	**Adjustment**	**Adj P**	**Alpha**	**Lower**	**Upper**
**Treatment**	Meg TTR	GFP MsTTR	0.6649	1.3021	31	0.51	0.6132	Tukey-Kramer	0.6132	0.05	-1.9907	3.3205

Differences of Least Squares Means				
**Effect**	**Treatment**	**Treatment**	**Adj Lower**	**Adj Upper**
**Treatment**	Meg TTR	GFP MsTTR	-1.9906	3.3205

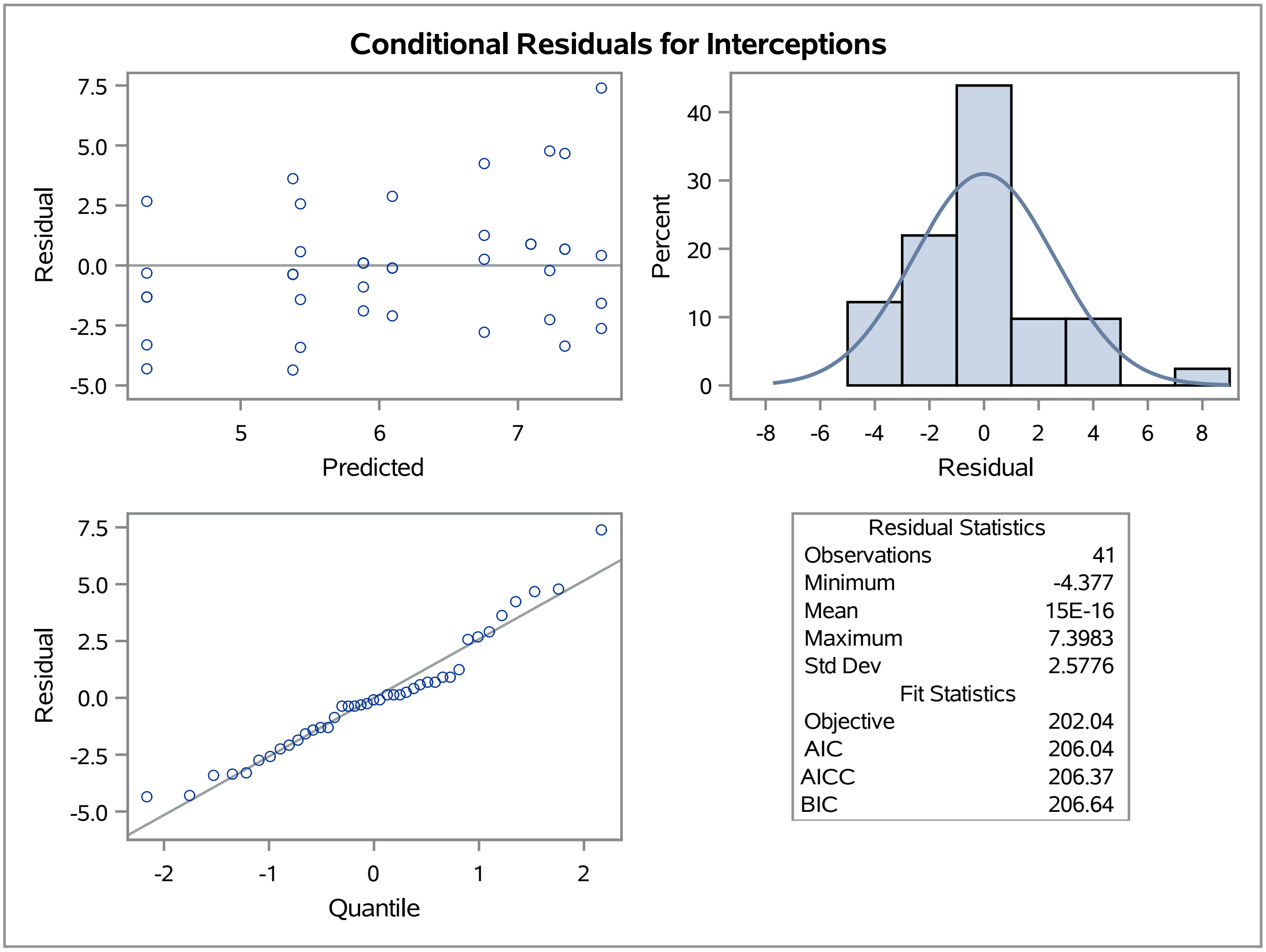

Residual Statistics	
Observations	41
Minimum	-4.377
Mean	15E-16
Maximum	7.3983
Std Dev	2.5776
**Fit Statistics**	
Objective	202.04
AIC	206.04
AICC	206.37
BIC	206.64

DistSoma=138

Model Information	
**Data Set**	WORK.TEMPDATASORTED
**Dependent Variable**	Interceptions
**Covariance Structure**	Variance Components
**Estimation Method**	REML
**Residual Variance Method**	Profile
**Fixed Effects SE Method**	Model-Based
**Degrees of Freedom Method**	Containment

Class Level Information		
**Class**	**Levels**	**Values**
**Treatment**	2	Meg TTR GFP MsTTR
**Culture**	10	1 2 3 4 5 6 7 8 9 10

Dimensions	
**Covariance Parameters**	2
**Columns in X**	3
**Columns in Z**	10
**Subjects**	1
**Max Obs per Subject**	41

Number of Observations	
**Number of Observations Read**	41
**Number of Observations Used**	41
**Number of Observations Not Used**	0

Iteration History			
**Iteration**	**Evaluations**	**-2 Res Log Like**	**Criterion**
**0**	1	198.96479683	
**1**	3	192.34578227	0.00060000
**2**	1	192.30590390	0.00001818
**3**	1	192.30478423	0.00000002
**4**	1	192.30478305	0.00000000

Convergence criteria met.

DistSoma=138

Covariance Parameter Estimates				
**Cov Parm**	**Estimate**	**Alpha**	**Lower**	**Upper**
**Culture**	2.8896	0.05	1.0405	23.5543
**Residual**	5.5229	0.05	3.5714	9.6640

Fit Statistics	
**-2 Res Log Likelihood**	192.3
**AIC (Smaller is Better)**	196.3
**AICC (Smaller is Better)**	196.6
**BIC (Smaller is Better)**	196.9

Solution for Fixed Effects									
**Effect**	**Treatment**	**Estimate**	**Standard Error**	**DF**	**t Value**	**Pr > |t|**	**Alpha**	**Lower**	**Upper**
**Intercept**		5.5885	1.0244	8	5.46	0.0006	0.05	3.2262	7.9508
**Treatment**	Meg TTR	0.5543	1.3340	31	0.42	0.6806	0.05	-2.1665	3.2750
**Treatment**	GFP MsTTR	0	.	.	.	.	.	.	.

Solution for Random Effects									
**Effect**	**Culture**	**Estimate**	**Std Err Pred**	**DF**	**t Value**	**Pr > |t|**	**Alpha**	**Lower**	**Upper**
**Culture**	1	-1.0045	1.1612	31	-0.87	0.3936	0.05	-3.3728	1.3638
**Culture**	2	0.1093	1.1895	31	0.09	0.9274	0.05	-2.3166	2.5352
**Culture**	3	-0.7366	1.1895	31	-0.62	0.5403	0.05	-3.1625	1.6894
**Culture**	4	1.6318	1.1895	31	1.37	0.1799	0.05	-0.7941	4.0577
**Culture**	5	-0.2480	1.0869	31	-0.23	0.8210	0.05	-2.4647	1.9687
**Culture**	6	1.0876	1.1263	31	0.97	0.3417	0.05	-1.2096	3.3847
**Culture**	7	1.0876	1.1263	31	0.97	0.3417	0.05	-1.2096	3.3847
**Culture**	8	-2.8891	1.0574	31	-2.73	0.0103	0.05	-5.0457	-0.7325
**Culture**	9	0.4383	1.2661	31	0.35	0.7315	0.05	-2.1438	3.0205
**Culture**	10	0.5236	1.1819	31	0.44	0.6608	0.05	-1.8869	2.9342

Type 3 Tests of Fixed Effects				
**Effect**	**Num DF**	**Den DF**	**F Value**	**Pr > F**
**Treatment**	1	31	0.17	0.6806

DistSoma=138

Least Squares Means									
**Effect**	**Treatment**	**Estimate**	**Standard Error**	**DF**	**t Value**	**Pr > |t|**	**Alpha**	**Lower**	**Upper**
**Treatment**	Meg TTR	6.1428	0.8545	31	7.19	<.0001	0.05	4.4000	7.8855
**Treatment**	GFP MsTTR	5.5885	1.0244	31	5.46	<.0001	0.05	3.4992	7.6778

Differences of Least Squares Means												
**Effect**	**Treatment**	**Treatment**	**Estimate**	**Standard Error**	**DF**	**t Value**	**Pr > |t|**	**Adjustment**	**Adj P**	**Alpha**	**Lower**	**Upper**
**Treatment**	Meg TTR	GFP MsTTR	0.5543	1.3340	31	0.42	0.6806	Tukey-Kramer	0.6806	0.05	-2.1665	3.2750

Differences of Least Squares Means				
**Effect**	**Treatment**	**Treatment**	**Adj Lower**	**Adj Upper**
**Treatment**	Meg TTR	GFP MsTTR	-2.1665	3.2750

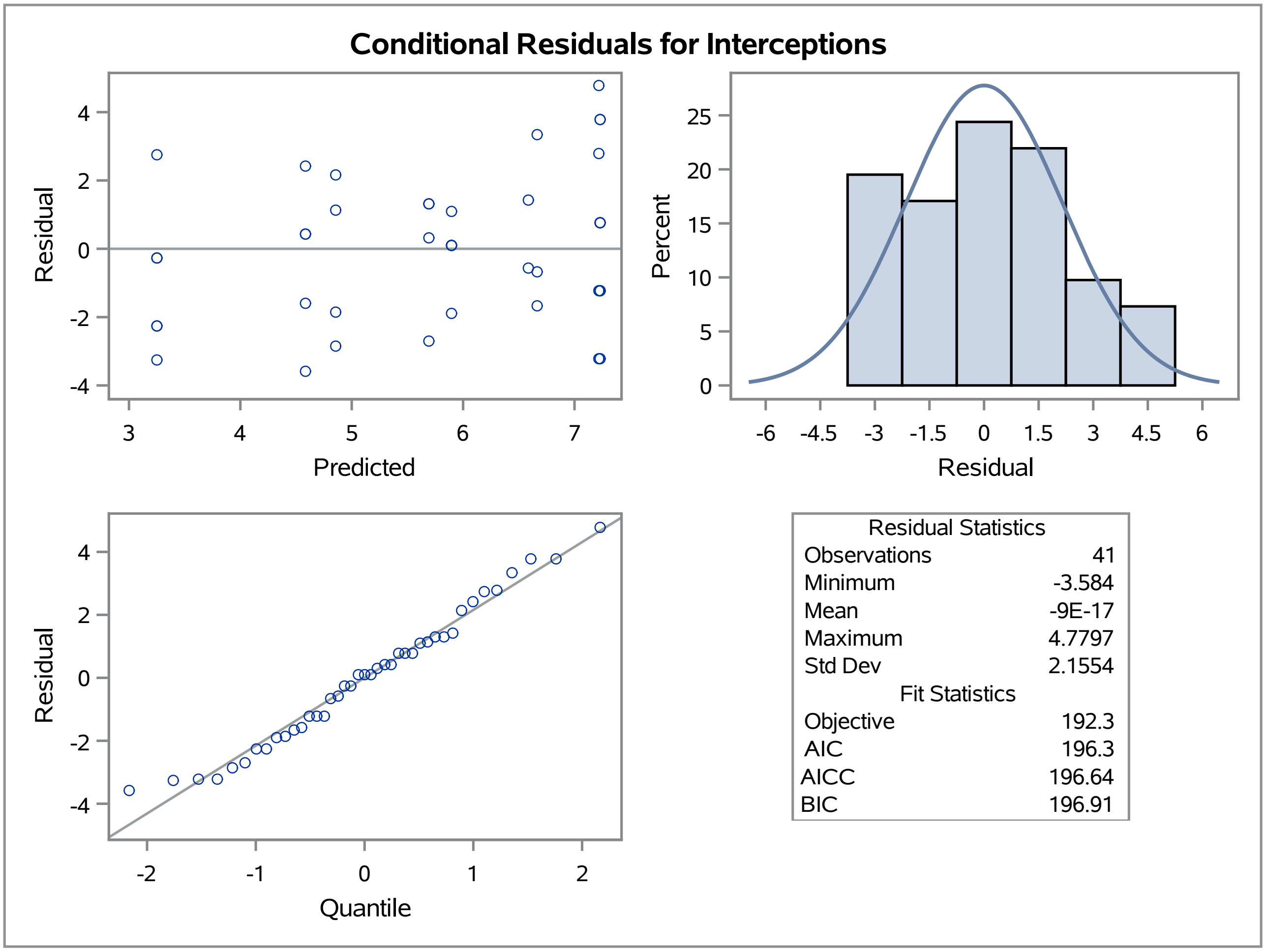

DistSoma=144

Model Information	
**Data Set**	WORK.TEMPDATASORTED
**Dependent Variable**	Interceptions
**Covariance Structure**	Variance Components
**Estimation Method**	REML
**Residual Variance Method**	Profile
**Fixed Effects SE Method**	Model-Based
**Degrees of Freedom Method**	Containment

Class Level Information		
**Class**	**Levels**	**Values**
**Treatment**	2	Meg TTR GFP MsTTR
**Culture**	10	1 2 3 4 5 6 7 8 9 10

Dimensions	
**Covariance Parameters**	2
**Columns in X**	3
**Columns in Z**	10
**Subjects**	1
**Max Obs per Subject**	41

Number of Observations	
**Number of Observations Read**	41
**Number of Observations Used**	41
**Number of Observations Not Used**	0

Iteration History			
**Iteration**	**Evaluations**	**-2 Res Log Like**	**Criterion**
**0**	1	195.57178550	
**1**	3	186.77890022	0.00031835
**2**	1	186.75916595	0.00000520
**3**	1	186.75886353	0.00000000

Convergence criteria met.

DistSoma=144

Covariance Parameter Estimates				
**Cov Parm**	**Estimate**	**Alpha**	**Lower**	**Upper**
**Culture**	3.0772	0.05	1.1600	20.9149
**Residual**	4.6303	0.05	2.9911	8.1161

Fit Statistics	
**-2 Res Log Likelihood**	186.8
**AIC (Smaller is Better)**	190.8
**AICC (Smaller is Better)**	191.1
**BIC (Smaller is Better)**	191.4

Solution for Fixed Effects									
**Effect**	**Treatment**	**Estimate**	**Standard Error**	**DF**	**t Value**	**Pr > |t|**	**Alpha**	**Lower**	**Upper**
**Intercept**		5.0900	1.0216	8	4.98	0.0011	0.05	2.7343	7.4458
**Treatment**	Meg TTR	0.7171	1.3290	31	0.54	0.5934	0.05	-1.9935	3.4276
**Treatment**	GFP MsTTR	0	.	.	.	.	.	.	.

Solution for Random Effects									
**Effect**	**Culture**	**Estimate**	**Std Err Pred**	**DF**	**t Value**	**Pr > |t|**	**Alpha**	**Lower**	**Upper**
**Culture**	1	-0.5304	1.1526	31	-0.46	0.6486	0.05	-2.8811	1.8203
**Culture**	2	0.1162	1.1799	31	0.10	0.9222	0.05	-2.2902	2.5227
**Culture**	3	-0.7921	1.1799	31	-0.67	0.5070	0.05	-3.1985	1.6144
**Culture**	4	1.2062	1.1799	31	1.02	0.3146	0.05	-1.2002	3.6127
**Culture**	5	-0.4666	1.0672	31	-0.44	0.6649	0.05	-2.6431	1.7099
**Culture**	6	1.4118	1.1058	31	1.28	0.2112	0.05	-0.8434	3.6671
**Culture**	7	1.0485	1.1058	31	0.95	0.3504	0.05	-1.2067	3.3037
**Culture**	8	-3.3102	1.0387	31	-3.19	0.0033	0.05	-5.4287	-1.1918
**Culture**	9	0.9661	1.2476	31	0.77	0.4446	0.05	-1.5784	3.5106
**Culture**	10	0.3505	1.1612	31	0.30	0.7648	0.05	-2.0178	2.7188

Type 3 Tests of Fixed Effects				
**Effect**	**Num DF**	**Den DF**	**F Value**	**Pr > F**
**Treatment**	1	31	0.29	0.5934

DistSoma=144

Least Squares Means									
Effect	Treatment	Estimate	Standard Error	DF	t Value	Pr > |t|	Alpha	Lower	Upper
Treatment	Meg TTR	5.8071	0.8501	31	6.83	<.0001	0.05	4.0733	7.5409
Treatment	GFP MsTTR	5.0900	1.0216	31	4.98	<.0001	0.05	3.0065	7.1735

Differences of Least Squares Means												
Effect	Treatment	Treatment	Estimate	Standard Error	DF	t Value	Pr > |t|	Adjustment	Adj P	Alpha	Lower	Upper
Treatment	Meg TTR	GFP MsTTR	0.7171	1.3290	31	0.54	0.5934	Tukey-Kramer	0.5934	0.05	-1.9935	3.4276

Differences of Least Squares Means				
Effect	Treatment	Treatment	Adj Lower	Adj Upper
Treatment	Meg TTR	GFP MsTTR	-1.9935	3.4276

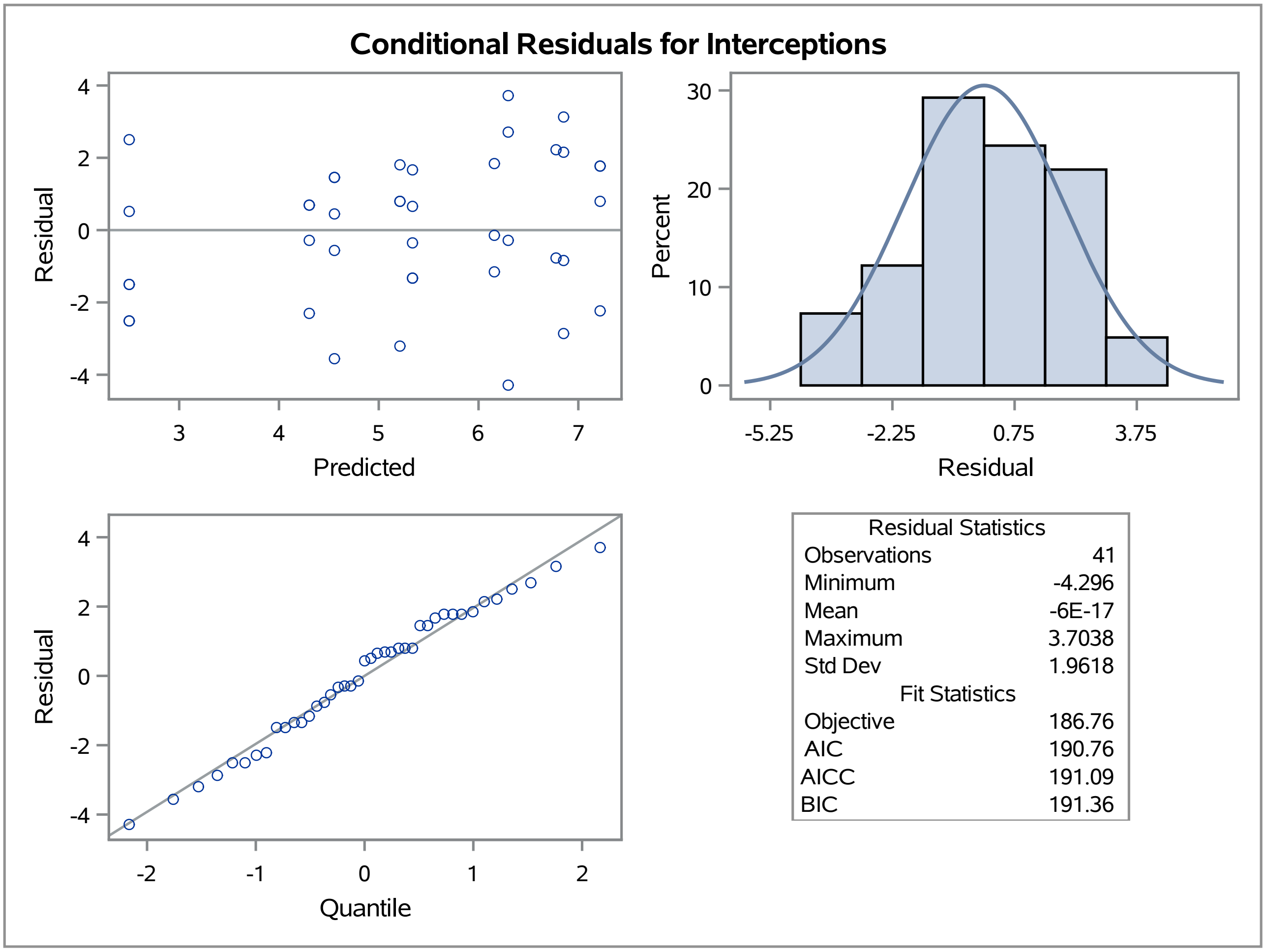

DistSoma=150

Model Information	
**Data Set**	WORK.TEMPDATASORTED
**Dependent Variable**	Interceptions
**Covariance Structure**	Variance Components
**Estimation Method**	REML
**Residual Variance Method**	Profile
**Fixed Effects SE Method**	Model-Based
**Degrees of Freedom Method**	Containment

Class Level Information		
**Class**	**Levels**	**Values**
**Treatment**	2	Meg TTR GFP MsTTR
**Culture**	10	1 2 3 4 5 6 7 8 9 10

Dimensions	
**Covariance Parameters**	2
**Columns in X**	3
**Columns in Z**	10
**Subjects**	1
**Max Obs per Subject**	41

Number of Observations	
**Number of Observations Read**	41
**Number of Observations Used**	41
**Number of Observations Not Used**	0

Iteration History			
**Iteration**	**Evaluations**	**-2 Res Log Like**	**Criterion**
**0**	1	192.53938267	
**1**	3	182.49846813	0.00042713
**2**	1	182.47276308	0.00000890
**3**	1	182.47226303	0.00000000

Convergence criteria met.

DistSoma=150

Covariance Parameter Estimates				
Cov Parm	Estimate	Alpha	Lower	Upper
Culture	3.1134	0.05	1.1946	19.8005
Residual	4.0601	0.05	2.6196	7.1308

Fit Statistics	
-2 Res Log Likelihood	182.5
AIC (Smaller is Better)	186.5
AICC (Smaller is Better)	186.8
BIC (Smaller is Better)	187.1

Solution for Fixed Effects									
Effect	Treatment	Estimate	Standard Error	DF	t Value	Pr > |t|	Alpha	Lower	Upper
Intercept		4.8962	1.0094	8	4.85	0.0013	0.05	2.5685	7.2239
Treatment	Meg TTR	0.8279	1.3124	31	0.63	0.5328	0.05	-1.8489	3.5046
Treatment	GFP MsTTR	0	.	.	.	.	.	.	.

Solution for Random Effects									
Effect	Culture	Estimate	Std Err Pred	DF	t Value	Pr > |t|	Alpha	Lower	Upper
Culture	1	-0.2349	1.1336	31	-0.21	0.8372	0.05	-2.5469	2.0771
Culture	2	-0.1102	1.1597	31	-0.10	0.9249	0.05	-2.4755	2.2550
Culture	3	-0.8644	1.1597	31	-0.75	0.4617	0.05	-3.2296	1.5009
Culture	4	1.2095	1.1597	31	1.04	0.3050	0.05	-1.1558	3.5748
Culture	5	-0.2570	1.0424	31	-0.25	0.8069	0.05	-2.3830	1.8690
Culture	6	1.3393	1.0796	31	1.24	0.2241	0.05	-0.8626	3.5412
Culture	7	0.5852	1.0796	31	0.54	0.5917	0.05	-1.6167	2.7871
Culture	8	-3.4699	1.0152	31	-3.42	0.0018	0.05	-5.5404	-1.3994
Culture	9	1.3777	1.2193	31	1.13	0.2672	0.05	-1.1090	3.8644
Culture	10	0.4247	1.1336	31	0.37	0.7105	0.05	-1.8874	2.7367

Type 3 Tests of Fixed Effects				
Effect	Num DF	Den DF	F Value	Pr > F
Treatment	1	31	0.40	0.5328

DistSoma=150

Least Squares Means									
Effect	Treatment	Estimate	Standard Error	DF	t Value	Pr > |t|	Alpha	Lower	Upper
Treatment	Meg TTR	5.7240	0.8388	31	6.82	<.0001	0.05	4.0133	7.4347
Treatment	GFP MsTTR	4.8962	1.0094	31	4.85	<.0001	0.05	2.8375	6.9549

Differences of Least Squares Means												
Effect	Treatment	Treatment	Estimate	Standard Error	DF	t Value	Pr > |t|	Adjustment	Adj P	Alpha	Lower	Upper
Treatment	Meg TTR	GFP MsTTR	0.8279	1.3124	31	0.63	0.5328	Tukey-Kramer	0.5328	0.05	-1.8489	3.5046

Differences of Least Squares Means				
Effect	Treatment	Treatment	Adj Lower	Adj Upper
Treatment	Meg TTR	GFP MsTTR	-1.8488	3.5046

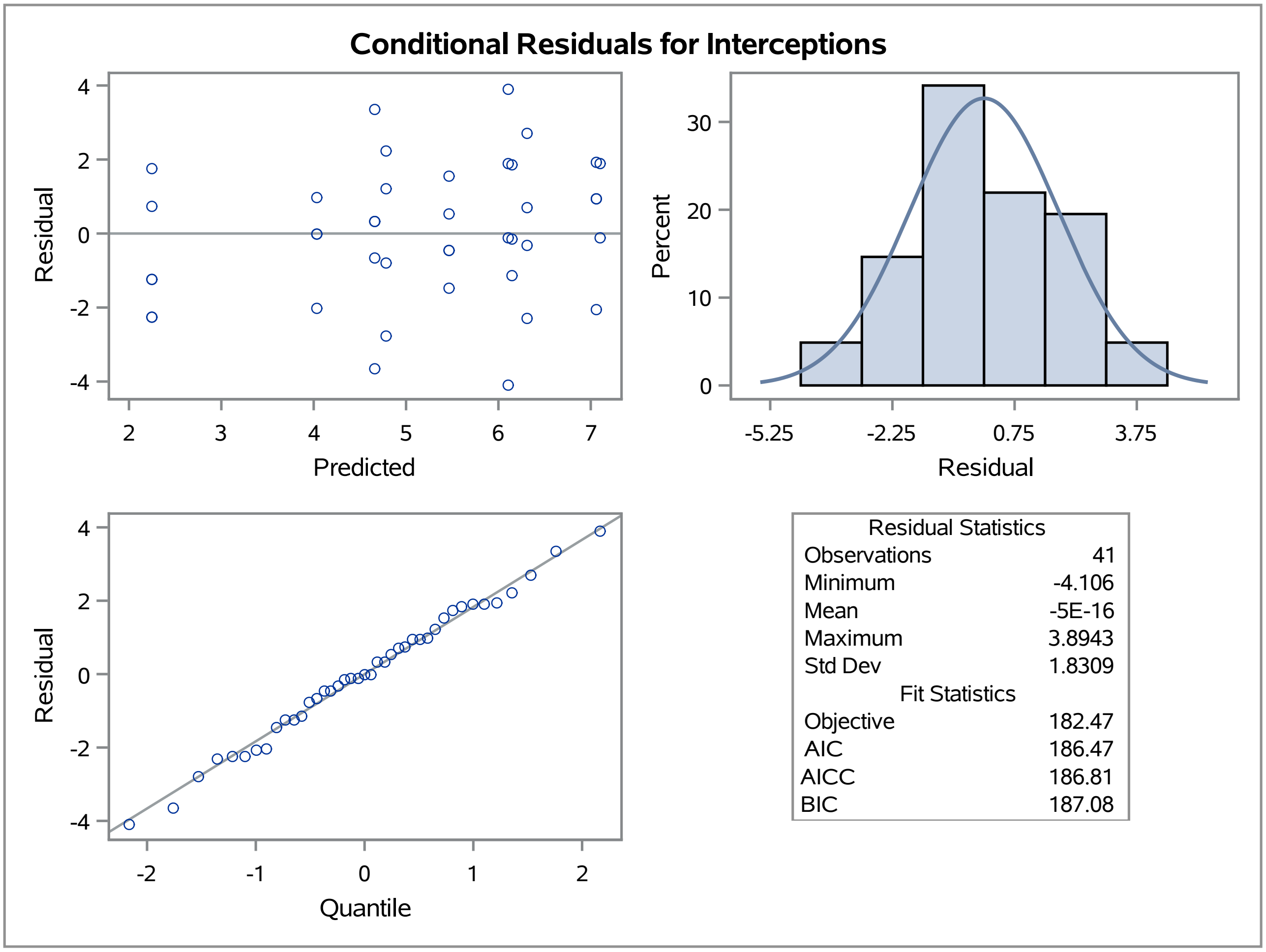

DistSoma=156

Model Information	
**Data Set**	WORK.TEMPDATASORTED
**Dependent Variable**	Interceptions
**Covariance Structure**	Variance Components
**Estimation Method**	REML
**Residual Variance Method**	Profile
**Fixed Effects SE Method**	Model-Based
**Degrees of Freedom Method**	Containment

Class Level Information		
**Class**	**Levels**	**Values**
**Treatment**	2	Meg TTR GFP MsTTR
**Culture**	10	1 2 3 4 5 6 7 8 9 10

Dimensions	
**Covariance Parameters**	2
**Columns in X**	3
**Columns in Z**	10
**Subjects**	1
**Max Obs per Subject**	41

Number of Observations	
**Number of Observations Read**	41
**Number of Observations Used**	41
**Number of Observations Not Used**	0

Iteration History			
**Iteration**	**Evaluations**	**-2 Res Log Like**	**Criterion**
**0**	1	191.70367310	
**1**	3	186.99968265	0.00048447
**2**	1	186.96916895	0.00001180
**3**	1	186.96847692	0.00000001

Convergence criteria met.

DistSoma=156

Covariance Parameter Estimates				
**Cov Parm**	**Estimate**	**Alpha**	**Lower**	**Upper**
**Culture**	1.9710	0.05	0.6609	22.0448
**Residual**	4.9980	0.05	3.2349	8.7325

Fit Statistics	
**-2 Res Log Likelihood**	187.0
**AIC (Smaller is Better)**	191.0
**AICC (Smaller is Better)**	191.3
**BIC (Smaller is Better)**	191.6

Solution for Fixed Effects									
**Effect**	**Treatment**	**Estimate**	**Standard Error**	**DF**	**t Value**	**Pr > |t|**	**Alpha**	**Lower**	**Upper**
**Intercept**		5.0612	0.8880	8	5.70	0.0005	0.05	3.0135	7.1089
**Treatment**	Meg TTR	0.2325	1.1576	31	0.20	0.8421	0.05	-2.1285	2.5935
**Treatment**	GFP MsTTR	0	.	.	.	.	.	.	.

Solution for Random Effects									
**Effect**	**Culture**	**Estimate**	**Std Err Pred**	**DF**	**t Value**	**Pr > |t|**	**Alpha**	**Lower**	**Upper**
**Culture**	1	-0.04061	1.0052	31	-0.04	0.9680	0.05	-2.0907	2.0095
**Culture**	2	-0.1905	1.0296	31	-0.18	0.8544	0.05	-2.2903	1.9094
**Culture**	3	-0.4965	1.0296	31	-0.48	0.6330	0.05	-2.5964	1.6034
**Culture**	4	0.7276	1.0296	31	0.71	0.4851	0.05	-1.3723	2.8274
**Culture**	5	-0.06217	0.9519	31	-0.07	0.9483	0.05	-2.0035	1.8792
**Culture**	6	1.1973	0.9856	31	1.21	0.2336	0.05	-0.8128	3.2073
**Culture**	7	0.2793	0.9856	31	0.28	0.7788	0.05	-1.7308	2.2893
**Culture**	8	-2.5495	0.9263	31	-2.75	0.0098	0.05	-4.4388	-0.6603
**Culture**	9	0.7524	1.0996	31	0.68	0.4989	0.05	-1.4903	2.9951
**Culture**	10	0.3828	1.0319	31	0.37	0.7132	0.05	-1.7218	2.4874

Type 3 Tests of Fixed Effects				
**Effect**	**Num DF**	**Den DF**	**F Value**	**Pr > F**
**Treatment**	1	31	0.04	0.8421

DistSoma=156

Least Squares Means									
Effect	Treatment	Estimate	Standard Error	DF	t Value	Pr > |t|	Alpha	Lower	Upper
**Treatment**	Meg TTR	5.2937	0.7427	31	7.13	<.0001	0.05	3.7790	6.8084
**Treatment**	GFP MsTTR	5.0612	0.8880	31	5.70	<.0001	0.05	3.2501	6.8723

Differences of Least Squares Means												
Effect	Treatment	Treatment	Estimate	Standard Error	DF	t Value	Pr > |t|	Adjustment	Adj P	Alpha	Lower	Upper
**Treatment**	Meg TTR	GFP MsTTR	0.2325	1.1576	31	0.20	0.8421	Tukey-Kramer	0.8421	0.05	-2.1285	2.5935

Differences of Least Squares Means				
Effect	Treatment	Treatment	Adj Lower	Adj Upper
**Treatment**	Meg TTR	GFP MsTTR	-2.1285	2.5935

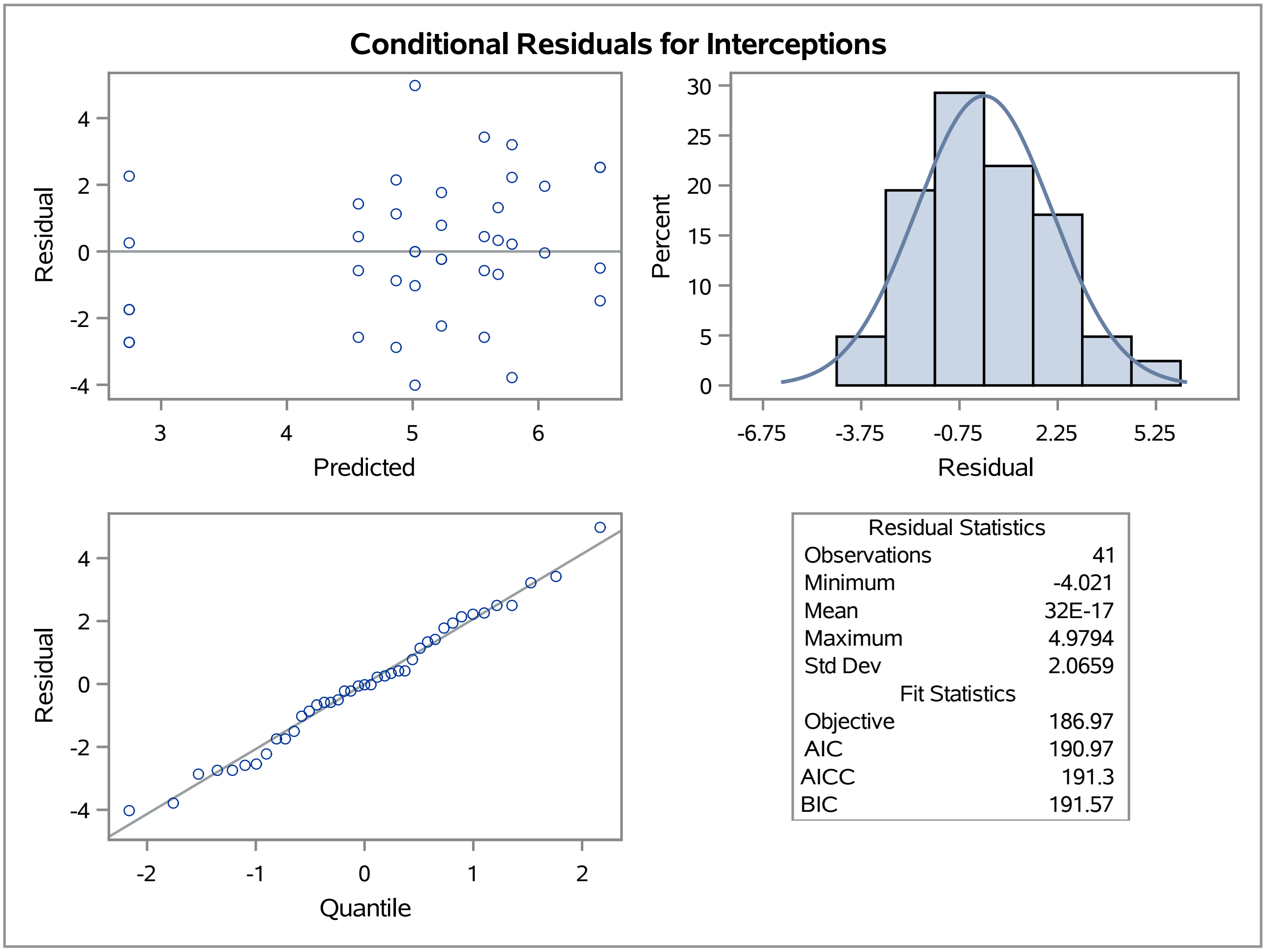

Residual Statistics	
Observations	41
Minimum	-4.021
Mean	32E-17
Maximum	4.9794
Std Dev	2.0659
**Fit Statistics**	
Objective	186.97
AIC	190.97
AICC	191.3
BIC	191.57

DistSoma=162

Model Information	
**Data Set**	WORK.TEMPDATASORTED
**Dependent Variable**	Interceptions
**Covariance Structure**	Variance Components
**Estimation Method**	REML
**Residual Variance Method**	Profile
**Fixed Effects SE Method**	Model-Based
**Degrees of Freedom Method**	Containment

Class Level Information		
**Class**	**Levels**	**Values**
**Treatment**	2	Meg TTR GFP MsTTR
**Culture**	10	1 2 3 4 5 6 7 8 9 10

Dimensions	
**Covariance Parameters**	2
**Columns in X**	3
**Columns in Z**	10
**Subjects**	1
**Max Obs per Subject**	41

Number of Observations	
**Number of Observations Read**	41
**Number of Observations Used**	41
**Number of Observations Not Used**	0

Iteration History			
**Iteration**	**Evaluations**	**-2 Res Log Like**	**Criterion**
**0**	1	193.22485117	
**1**	3	188.68710826	0.00098332
**2**	1	188.62253308	0.00004592
**3**	1	188.61975954	0.00000012
**4**	1	188.61975240	0.00000000

Convergence criteria met.

DistSoma=162

Covariance Parameter Estimates				
**Cov Parm**	**Estimate**	**Alpha**	**Lower**	**Upper**
**Culture**	2.0765	0.05	0.6917	23.9564
**Residual**	5.2069	0.05	3.3669	9.1116

Fit Statistics	
**-2 Res Log Likelihood**	188.6
**AIC (Smaller is Better)**	192.6
**AICC (Smaller is Better)**	193.0
**BIC (Smaller is Better)**	193.2

Solution for Fixed Effects									
**Effect**	**Treatment**	**Estimate**	**Standard Error**	**DF**	**t Value**	**Pr > |t|**	**Alpha**	**Lower**	**Upper**
**Intercept**		4.7551	0.9096	8	5.23	0.0008	0.05	2.6577	6.8525
**Treatment**	Meg TTR	0.2966	1.1857	31	0.25	0.8041	0.05	-2.1216	2.7148
**Treatment**	GFP MsTTR	0	.	.	.	.	.	.	.

Solution for Random Effects									
**Effect**	**Culture**	**Estimate**	**Std Err Pred**	**DF**	**t Value**	**Pr > |t|**	**Alpha**	**Lower**	**Upper**
**Culture**	1	0.1631	1.0298	31	0.16	0.8752	0.05	-1.9372	2.2634
**Culture**	2	0.1505	1.0548	31	0.14	0.8875	0.05	-2.0008	2.3019
**Culture**	3	-1.0788	1.0548	31	-1.02	0.3144	0.05	-3.2302	1.0726
**Culture**	4	0.7652	1.0548	31	0.73	0.4736	0.05	-1.3862	2.9166
**Culture**	5	-0.1676	0.9748	31	-0.17	0.8646	0.05	-2.1557	1.8204
**Culture**	6	0.8902	1.0093	31	0.88	0.3846	0.05	-1.1683	2.9487
**Culture**	7	0.5829	1.0093	31	0.58	0.5678	0.05	-1.4756	2.6414
**Culture**	8	-2.5049	0.9486	31	-2.64	0.0128	0.05	-4.4395	-0.5702
**Culture**	9	0.8645	1.1265	31	0.77	0.4487	0.05	-1.4331	3.1620
**Culture**	10	0.3350	1.0569	31	0.32	0.7534	0.05	-1.8206	2.4906

Type 3 Tests of Fixed Effects				
**Effect**	**Num DF**	**Den DF**	**F Value**	**Pr > F**
**Treatment**	1	31	0.06	0.8041

DistSoma=162

Least Squares Means									
**Effect**	**Treatment**	**Estimate**	**Standard Error**	**DF**	**t Value**	**Pr > |t|**	**Alpha**	**Lower**	**Upper**
**Treatment**	Meg TTR	5.0517	0.7606	31	6.64	<.0001	0.05	3.5004	6.6031
**Treatment**	GFP MsTTR	4.7551	0.9096	31	5.23	<.0001	0.05	2.9001	6.6102

Differences of Least Squares Means													
**Effect**	**Treatment**	**Treatment**	**Estimate**	**Standard Error**	**DF**	**t Value**	**Pr > |t|**	**Adjustment**	**Adj P**	**Alpha**	**Lower**	**Upper**
**Treatment**	Meg TTR	GFP MsTTR	0.2966	1.1857	31	0.25	0.8041	Tukey-Kramer	0.8041	0.05	-2.1216	2.7148

Differences of Least Squares Means				
**Effect**	**Treatment**	**Treatment**	**Adj Lower**	**Adj Upper**
**Treatment**	Meg TTR	GFP MsTTR	-2.1216	2.7148

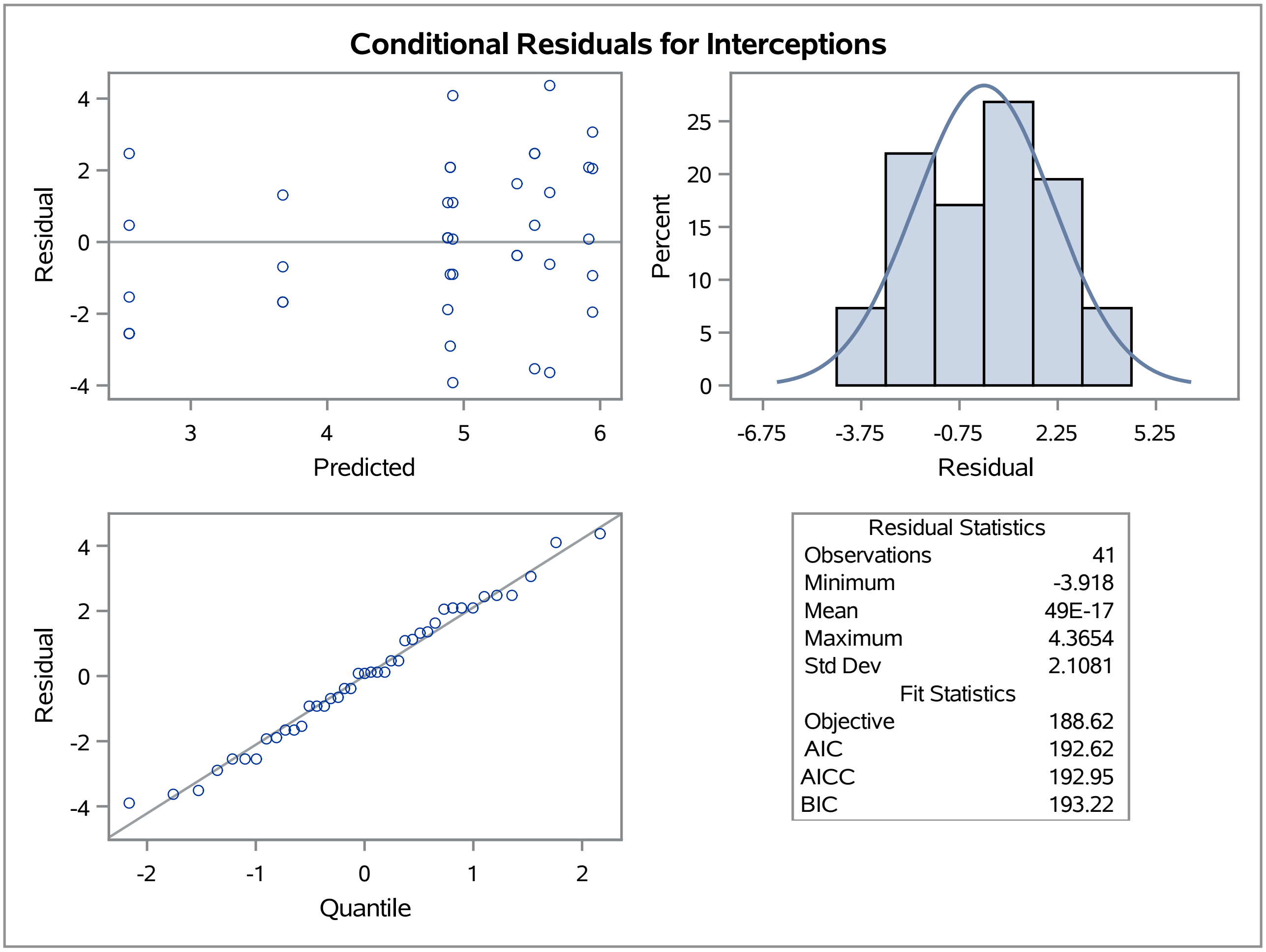

DistSoma=168

Model Information	
**Data Set**	WORK.TEMPDATASORTED
**Dependent Variable**	Interceptions
**Covariance Structure**	Variance Components
**Estimation Method**	REML
**Residual Variance Method**	Profile
**Fixed Effects SE Method**	Model-Based
**Degrees of Freedom Method**	Containment

Class Level Information		
**Class**	**Levels**	**Values**
**Treatment**	2	Meg TTR GFP MsTTR
**Culture**	10	1 2 3 4 5 6 7 8 9 10

Dimensions	
**Covariance Parameters**	2
**Columns in X**	3
**Columns in Z**	10
**Subjects**	1
**Max Obs per Subject**	41

Number of Observations	
**Number of Observations Read**	41
**Number of Observations Used**	41
**Number of Observations Not Used**	0

Iteration History			
**Iteration**	**Evaluations**	**-2 Res Log Like**	**Criterion**
**0**	1	191.36272033	
**1**	3	187.01907113	0.00086244
**2**	1	186.96348226	0.00003661
**3**	1	186.96130899	0.00000008
**4**	1	186.96130442	0.00000000

Convergence criteria met.

DistSoma=168

Covariance Parameter Estimates				
**Cov Parm**	**Estimate**	**Alpha**	**Lower**	**Upper**
**Culture**	2.0669	0.05	0.6779	25.7280
**Residual**	4.9630	0.05	3.1999	8.7267

Fit Statistics	
**-2 Res Log Likelihood**	187.0
**AIC (Smaller is Better)**	191.0
**AICC (Smaller is Better)**	191.3
**BIC (Smaller is Better)**	191.6

Solution for Fixed Effects									
**Effect**	**Treatment**	**Estimate**	**Standard Error**	**DF**	**t Value**	**Pr > |t|**	**Alpha**	**Lower**	**Upper**
**Intercept**		4.5877	0.9003	8	5.10	0.0009	0.05	2.5117	6.6638
**Treatment**	Meg TTR	0.5034	1.1734	31	0.43	0.6709	0.05	-1.8898	2.8965
**Treatment**	GFP MsTTR	0	.	.	.	.	.	.	.

Solution for Random Effects									
**Effect**	**Culture**	**Estimate**	**Std Err Pred**	**DF**	**t Value**	**Pr > |t|**	**Alpha**	**Lower**	**Upper**
**Culture**	1	0.008277	1.0200	31	0.01	0.9936	0.05	-2.0721	2.0886
**Culture**	2	0.2576	1.0449	31	0.25	0.8069	0.05	-1.8735	2.3887
**Culture**	3	-0.9922	1.0449	31	-0.95	0.3497	0.05	-3.1232	1.1389
**Culture**	4	0.7263	1.0449	31	0.70	0.4922	0.05	-1.4048	2.8573
**Culture**	5	-0.3318	0.9639	31	-0.34	0.7330	0.05	-2.2976	1.6341
**Culture**	6	0.4117	0.9982	31	0.41	0.6828	0.05	-1.6242	2.4476
**Culture**	7	0.7242	0.9982	31	0.73	0.4736	0.05	-1.3117	2.7601
**Culture**	8	-2.4457	0.9379	31	-2.61	0.0139	0.05	-4.3585	-0.5328
**Culture**	9	1.3219	1.1156	31	1.18	0.2451	0.05	-0.9535	3.5972
**Culture**	10	0.3197	1.0458	31	0.31	0.7619	0.05	-1.8131	2.4525

Type 3 Tests of Fixed Effects				
**Effect**	**Num DF**	**Den DF**	**F Value**	**Pr > F**
**Treatment**	1	31	0.18	0.6709

DistSoma=168

Least Squares Means									
**Effect**	**Treatment**	**Estimate**	**Standard Error**	**DF**	**t Value**	**Pr > |t|**	**Alpha**	**Lower**	**Upper**
**Treatment**	Meg TTR	5.0911	0.7526	31	6.76	<.0001	0.05	3.5562	6.6260
**Treatment**	GFP MsTTR	4.5877	0.9003	31	5.10	<.0001	0.05	2.7516	6.4238

Differences of Least Squares Means												
**Effect**	**Treatment**	**Treatment**	**Estimate**	**Standard Error**	**DF**	**t Value**	**Pr > |t|**	**Adjustment**	**Adj P**	**Alpha**	**Lower**	**Upper**
**Treatment**	Meg TTR	GFP MsTTR	0.5034	1.1734	31	0.43	0.6709	Tukey-Kramer	0.6709	0.05	-1.8898	2.8965

Differences of Least Squares Means				
**Effect**	**Treatment**	**Treatment**	**Adj Lower**	**Adj Upper**
**Treatment**	Meg TTR	GFP MsTTR	-1.8898	2.8965

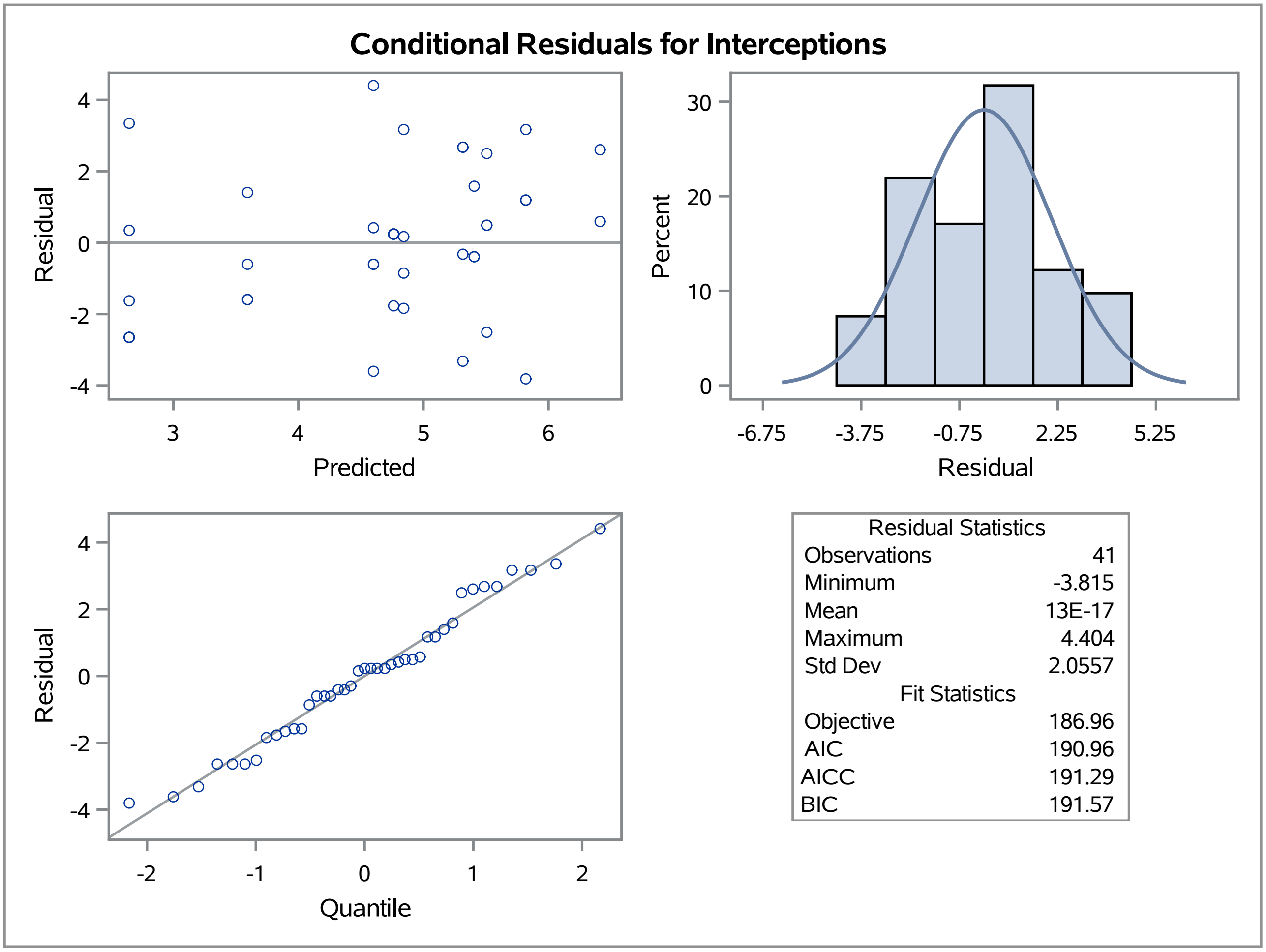

DistSoma=174

Model Information	
**Data Set**	WORK.TEMPDATASORTED
**Dependent Variable**	Interceptions
**Covariance Structure**	Variance Components
**Estimation Method**	REML
**Residual Variance Method**	Profile
**Fixed Effects SE Method**	Model-Based
**Degrees of Freedom Method**	Containment

Class Level Information		
**Class**	**Levels**	**Values**
**Treatment**	2	Meg TTR GFP MsTTR
**Culture**	10	1 2 3 4 5 6 7 8 9 10

Dimensions	
**Covariance Parameters**	2
**Columns in X**	3
**Columns in Z**	10
**Subjects**	1
**Max Obs per Subject**	41

Number of Observations	
**Number of Observations Read**	41
**Number of Observations Used**	41
**Number of Observations Not Used**	0

Iteration History			
**Iteration**	**Evaluations**	**-2 Res Log Like**	**Criterion**
**0**	1	189.17673855	
**1**	3	182.58218012	0.00203814
**2**	1	182.45163213	0.00016800
**3**	1	182.44178770	0.00000147
**4**	1	182.44170565	0.00000000

Convergence criteria met.

DistSoma=174

Covariance Parameter Estimates				
**Cov Parm**	**Estimate**	**Alpha**	**Lower**	**Upper**
**Culture**	2.3971	0.05	0.8611	19.7330
**Residual**	4.2433	0.05	2.7377	7.4530

Fit Statistics	
**-2 Res Log Likelihood**	182.4
**AIC (Smaller is Better)**	186.4
**AICC (Smaller is Better)**	186.8
**BIC (Smaller is Better)**	187.0

Solution for Fixed Effects									
**Effect**	**Treatment**	**Estimate**	**Standard Error**	**DF**	**t Value**	**Pr > |t|**	**Alpha**	**Lower**	**Upper**
**Intercept**		4.5353	0.9223	8	4.92	0.0012	0.05	2.4085	6.6620
**Treatment**	Meg TTR	0.2761	1.2006	31	0.23	0.8196	0.05	-2.1726	2.7248
**Treatment**	GFP MsTTR	0	.	.	.	.	.	.	.

Solution for Random Effects									
**Effect**	**Culture**	**Estimate**	**Std Err Pred**	**DF**	**t Value**	**Pr > |t|**	**Alpha**	**Lower**	**Upper**
**Culture**	1	-0.09992	1.0444	31	-0.10	0.9244	0.05	-2.2299	2.0301
**Culture**	2	0.3221	1.0696	31	0.30	0.7653	0.05	-1.8594	2.5037
**Culture**	3	-1.0643	1.0696	31	-0.99	0.3274	0.05	-3.2458	1.1173
**Culture**	4	0.8421	1.0696	31	0.79	0.4371	0.05	-1.3395	3.0236
**Culture**	5	-0.4515	0.9742	31	-0.46	0.6463	0.05	-2.4384	1.5354
**Culture**	6	0.1308	1.0096	31	0.13	0.8978	0.05	-1.9284	2.1899
**Culture**	7	1.5172	1.0096	31	1.50	0.1430	0.05	-0.5420	3.5763
**Culture**	8	-2.6857	0.9479	31	-2.83	0.0080	0.05	-4.6189	-0.7525
**Culture**	9	1.1610	1.1366	31	1.02	0.3149	0.05	-1.1570	3.4790
**Culture**	10	0.3283	1.0598	31	0.31	0.7588	0.05	-1.8333	2.4898

Type 3 Tests of Fixed Effects				
**Effect**	**Num DF**	**Den DF**	**F Value**	**Pr > F**
**Treatment**	1	31	0.05	0.8196

DistSoma=174

Least Squares Means									
Effect	Treatment	Estimate	Standard Error	DF	t Value	Pr > |t|	Alpha	Lower	Upper
Treatment	Meg TTR	4.8114	0.7687	31	6.26	<.0001	0.05	3.2436	6.3792
Treatment	GFP MsTTR	4.5353	0.9223	31	4.92	<.0001	0.05	2.6543	6.4163

Differences of Least Squares Means												
Effect	Treatment	Treatment	Estimate	Standard Error	DF	t Value	Pr > |t|	Adjustment	Adj P	Alpha	Lower	Upper
Treatment	Meg TTR	GFP MsTTR	0.2761	1.2006	31	0.23	0.8196	Tukey-Kramer	0.8196	0.05	-2.1726	2.7248

Differences of Least Squares Means				
Effect	Treatment	Treatment	Adj Lower	Adj Upper
Treatment	Meg TTR	GFP MsTTR	-2.1726	2.7247

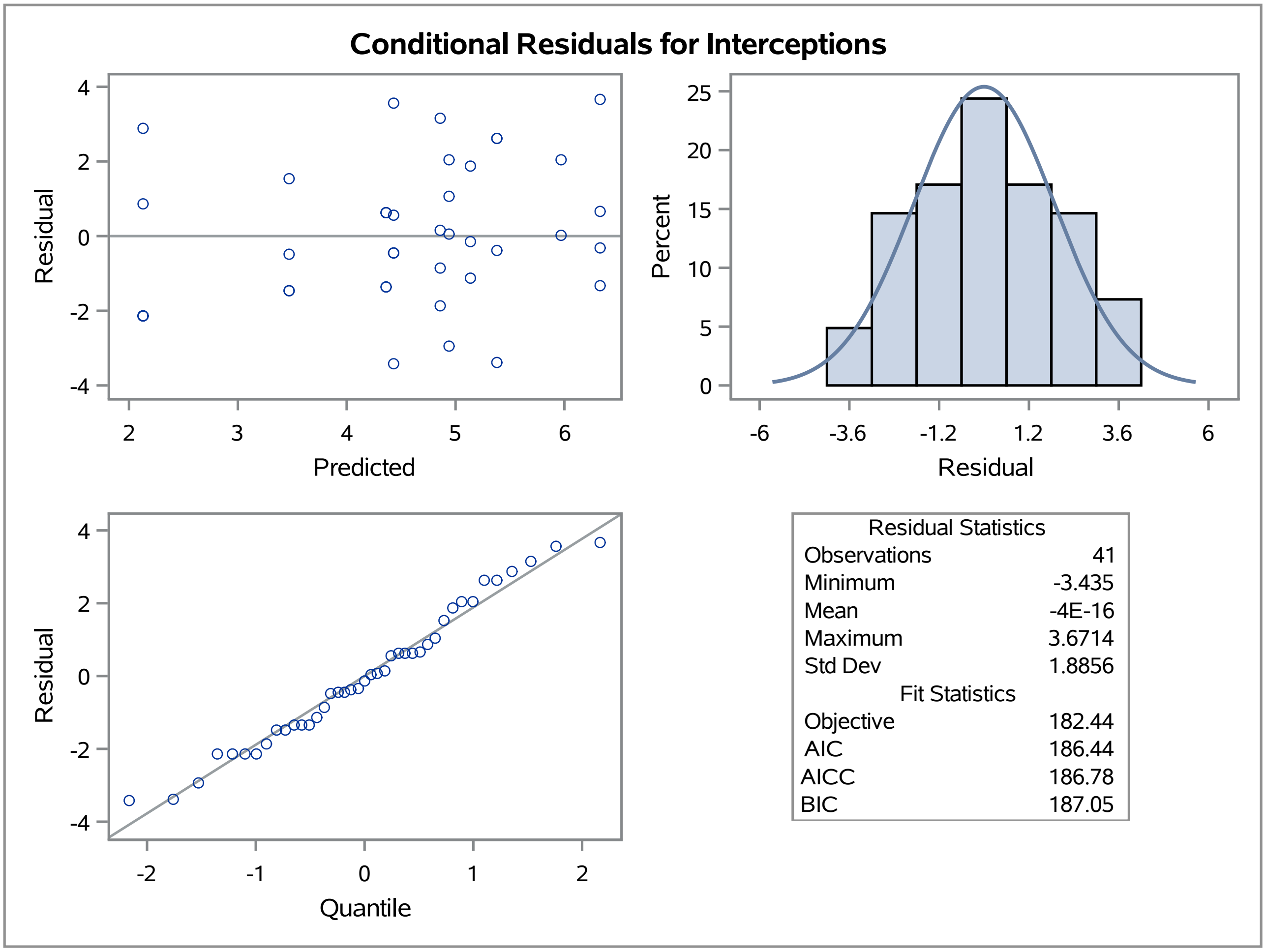

DistSoma=180

Model Information	
**Data Set**	WORK.TEMPDATASORTED
**Dependent Variable**	Interceptions
**Covariance Structure**	Variance Components
**Estimation Method**	REML
**Residual Variance Method**	Profile
**Fixed Effects SE Method**	Model-Based
**Degrees of Freedom Method**	Containment

Class Level Information		
**Class**	**Levels**	**Values**
**Treatment**	2	Meg TTR GFP MsTTR
**Culture**	10	1 2 3 4 5 6 7 8 9 10

Dimensions	
**Covariance Parameters**	2
**Columns in X**	3
**Columns in Z**	10
**Subjects**	1
**Max Obs per Subject**	41

Number of Observations	
**Number of Observations Read**	41
**Number of Observations Used**	41
**Number of Observations Not Used**	0

Iteration History			
**Iteration**	**Evaluations**	**-2 Res Log Like**	**Criterion**
**0**	1	188.84831879	
**1**	3	182.53053610	0.00062007
**2**	1	182.49271318	0.00001832
**3**	1	182.49167683	0.00000002
**4**	1	182.49167579	0.00000000

Convergence criteria met.

DistSoma=180

Covariance Parameter Estimates				
**Cov Parm**	**Estimate**	**Alpha**	**Lower**	**Upper**
**Culture**	2.4062	0.05	0.8561	20.6252
**Residual**	4.2470	0.05	2.7370	7.4735

Fit Statistics	
**-2 Res Log Likelihood**	182.5
**AIC (Smaller is Better)**	186.5
**AICC (Smaller is Better)**	186.8
**BIC (Smaller is Better)**	187.1

Solution for Fixed Effects									
**Effect**	**Treatment**	**Estimate**	**Standard Error**	**DF**	**t Value**	**Pr > |t|**	**Alpha**	**Lower**	**Upper**
**Intercept**		4.2027	0.9236	8	4.55	0.0019	0.05	2.0728	6.3326
**Treatment**	Meg TTR	0.5368	1.2024	31	0.45	0.6584	0.05	-1.9154	2.9891
**Treatment**	GFP MsTTR	0	.	.	.	.	.	.	.

Solution for Random Effects									
**Effect**	**Culture**	**Estimate**	**Std Err Pred**	**DF**	**t Value**	**Pr > |t|**	**Alpha**	**Lower**	**Upper**
**Culture**	1	-0.4454	1.0459	31	-0.43	0.6731	0.05	-2.5785	1.6876
**Culture**	2	0.3798	1.0712	31	0.35	0.7253	0.05	-1.8049	2.5644
**Culture**	3	-1.0079	1.0712	31	-0.94	0.3540	0.05	-3.1926	1.1767
**Culture**	4	1.0736	1.0712	31	1.00	0.3240	0.05	-1.1110	3.2582
**Culture**	5	-0.5466	0.9755	31	-0.56	0.5793	0.05	-2.5360	1.4429
**Culture**	6	-0.3396	1.0109	31	-0.34	0.7392	0.05	-2.4015	1.7222
**Culture**	7	1.3950	1.0109	31	1.38	0.1775	0.05	-0.6668	3.4568
**Culture**	8	-2.5032	0.9491	31	-2.64	0.0129	0.05	-4.4388	-0.5675
**Culture**	9	1.2008	1.1381	31	1.06	0.2995	0.05	-1.1204	3.5219
**Culture**	10	0.7936	1.0612	31	0.75	0.4602	0.05	-1.3708	2.9580

Type 3 Tests of Fixed Effects				
**Effect**	**Num DF**	**Den DF**	**F Value**	**Pr > F**
**Treatment**	1	31	0.20	0.6584

DistSoma=180

Least Squares Means									
**Effect**	**Treatment**	**Estimate**	**Standard Error**	**DF**	**t Value**	**Pr > |t|**	**Alpha**	**Lower**	**Upper**
**Treatment**	Meg TTR	4.7395	0.7698	31	6.16	<.0001	0.05	3.1694	6.3096
**Treatment**	GFP MsTTR	4.2027	0.9236	31	4.55	<.0001	0.05	2.3189	6.0864

Differences of Least Squares Means												
**Effect**	**Treatment**	**Treatment**	**Estimate**	**Standard Error**	**DF**	**t Value**	**Pr > |t|**	**Adjustment**	**Adj P**	**Alpha**	**Lower**	**Upper**
**Treatment**	Meg TTR	GFP MsTTR	0.5368	1.2024	31	0.45	0.6584	Tukey-Kramer	0.6584	0.05	-1.9154	2.9891

Differences of Least Squares Means				
**Effect**	**Treatment**	**Treatment**	**Adj Lower**	**Adj Upper**
**Treatment**	Meg TTR	GFP MsTTR	-1.9154	2.9891

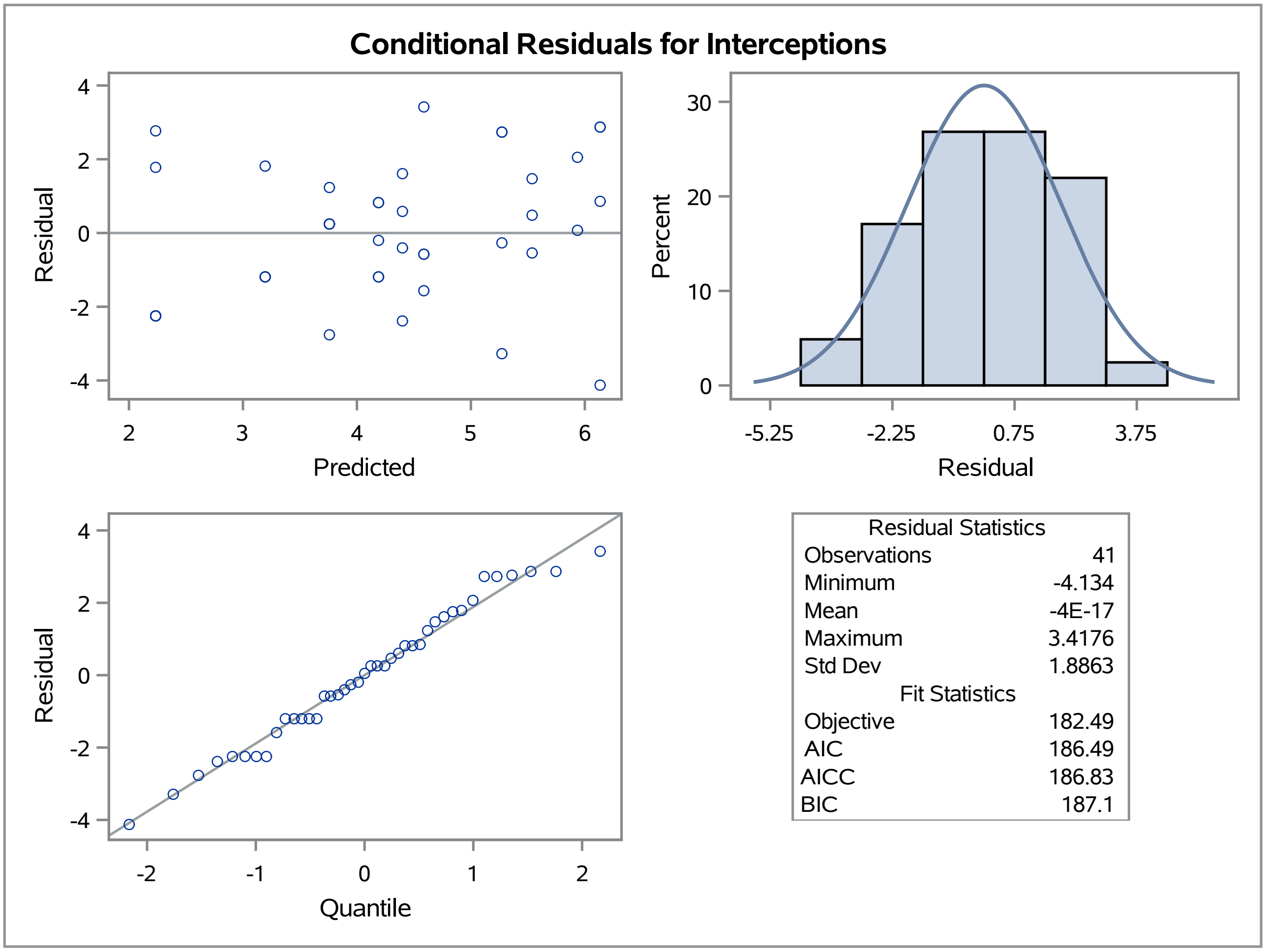

DistSoma=186

Model Information	
**Data Set**	WORK.TEMPDATASORTED
**Dependent Variable**	Interceptions
**Covariance Structure**	Variance Components
**Estimation Method**	REML
**Residual Variance Method**	Profile
**Fixed Effects SE Method**	Model-Based
**Degrees of Freedom Method**	Containment

Class Level Information		
**Class**	**Levels**	**Values**
**Treatment**	2	Meg TTR GFP MsTTR
**Culture**	10	1 2 3 4 5 6 7 8 9 10

Dimensions	
**Covariance Parameters**	2
**Columns in X**	3
**Columns in Z**	10
**Subjects**	1
**Max Obs per Subject**	41

Number of Observations	
**Number of Observations Read**	41
**Number of Observations Used**	41
**Number of Observations Not Used**	0

Iteration History			
**Iteration**	**Evaluations**	**-2 Res Log Like**	**Criterion**
**0**	1	186.49472104	
**1**	3	180.69272971	0.00039773
**2**	1	180.66923345	0.00000780
**3**	1	180.66880256	0.00000000

Convergence criteria met.

DistSoma=186

Covariance Parameter Estimates				
**Cov Parm**	**Estimate**	**Alpha**	**Lower**	**Upper**
**Culture**	2.1786	0.05	0.7606	20.2612
**Residual**	4.0878	0.05	2.6333	7.1981

Fit Statistics	
**-2 Res Log Likelihood**	180.7
**AIC (Smaller is Better)**	184.7
**AICC (Smaller is Better)**	185.0
**BIC (Smaller is Better)**	185.3

Solution for Fixed Effects									
**Effect**	**Treatment**	**Estimate**	**Standard Error**	**DF**	**t Value**	**Pr > |t|**	**Alpha**	**Lower**	**Upper**
**Intercept**		4.0793	0.8870	8	4.60	0.0018	0.05	2.0340	6.1247
**Treatment**	Meg TTR	0.3961	1.1550	31	0.34	0.7339	0.05	-1.9594	2.7517
**Treatment**	GFP MsTTR	0	.	.	.	.	.	.	.

Solution for Random Effects									
**Effect**	**Culture**	**Estimate**	**Std Err Pred**	**DF**	**t Value**	**Pr > |t|**	**Alpha**	**Lower**	**Upper**
**Culture**	1	-0.3485	1.0052	31	-0.35	0.7311	0.05	-2.3987	1.7016
**Culture**	2	0.4565	1.0296	31	0.44	0.6606	0.05	-1.6434	2.5565
**Culture**	3	-0.9049	1.0296	31	-0.88	0.3863	0.05	-3.0048	1.1951
**Culture**	4	0.7969	1.0296	31	0.77	0.4448	0.05	-1.3031	2.8969
**Culture**	5	-0.4912	0.9401	31	-0.52	0.6051	0.05	-2.4085	1.4262
**Culture**	6	-0.3237	0.9743	31	-0.33	0.7420	0.05	-2.3107	1.6634
**Culture**	7	1.3781	0.9743	31	1.41	0.1672	0.05	-0.6089	3.3651
**Culture**	8	-2.3936	0.9146	31	-2.62	0.0136	0.05	-4.2590	-0.5282
**Culture**	9	1.3025	1.0955	31	1.19	0.2435	0.05	-0.9318	3.5369
**Culture**	10	0.5278	1.0224	31	0.52	0.6094	0.05	-1.5575	2.6131

Type 3 Tests of Fixed Effects				
**Effect**	**Num DF**	**Den DF**	**F Value**	**Pr > F**
**Treatment**	1	31	0.12	0.7339

DistSoma=186

Least Squares Means									
**Effect**	**Treatment**	**Estimate**	**Standard Error**	**DF**	**t Value**	**Pr > |t|**	**Alpha**	**Lower**	**Upper**
**Treatment**	Meg TTR	4.4755	0.7397	31	6.05	<.0001	0.05	2.9668	5.9841
**Treatment**	GFP MsTTR	4.0793	0.8870	31	4.60	<.0001	0.05	2.2703	5.8883

Differences of Least Squares Means												
**Effect**	**Treatment**	**Treatment**	**Estimate**	**Standard Error**	**DF**	**t Value**	**Pr > |t|**	**Adjustment**	**Adj P**	**Alpha**	**Lower**	**Upper**
**Treatment**	Meg TTR	GFP MsTTR	0.3961	1.1550	31	0.34	0.7339	Tukey-Kramer	0.7339	0.05	-1.9594	2.7517

Differences of Least Squares Means				
**Effect**	**Treatment**	**Treatment**	**Adj Lower**	**Adj Upper**
**Treatment**	Meg TTR	GFP MsTTR	-1.9594	2.7517

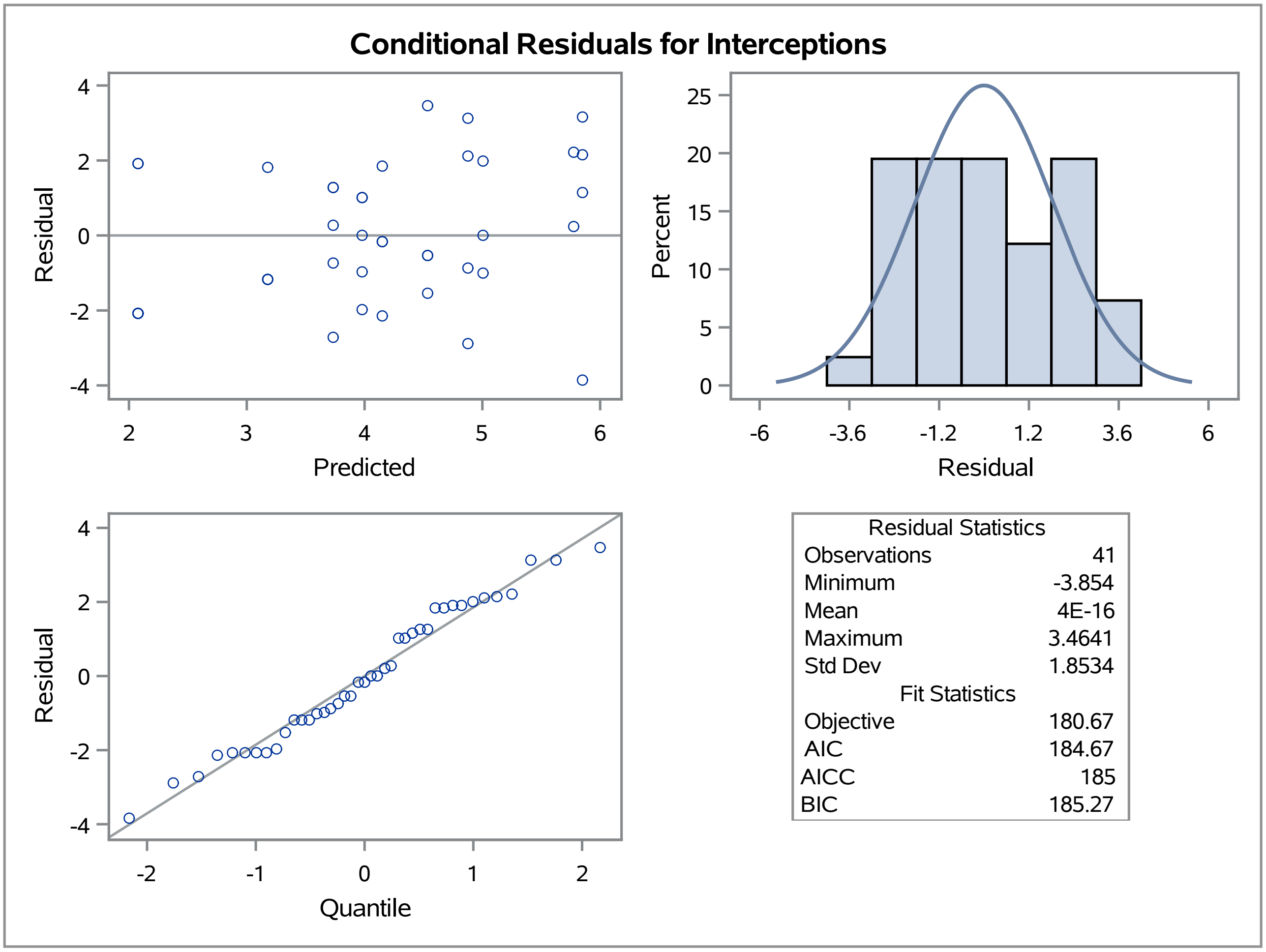

DistSoma=192

Model Information	
**Data Set**	WORK.TEMPDATASORTED
**Dependent Variable**	Interceptions
**Covariance Structure**	Variance Components
**Estimation Method**	REML
**Residual Variance Method**	Profile
**Fixed Effects SE Method**	Model-Based
**Degrees of Freedom Method**	Containment

Class Level Information		
**Class**	**Levels**	**Values**
**Treatment**	2	Meg TTR GFP MsTTR
**Culture**	10	1 2 3 4 5 6 7 8 9 10

Dimensions	
**Covariance Parameters**	2
**Columns in X**	3
**Columns in Z**	10
**Subjects**	1
**Max Obs per Subject**	41

Number of Observations	
**Number of Observations Read**	41
**Number of Observations Used**	41
**Number of Observations Not Used**	0

Iteration History			
**Iteration**	**Evaluations**	**-2 Res Log Like**	**Criterion**
**0**	1	178.19114465	
**1**	5	169.26561180	0.00012482
**2**	1	169.25988005	0.00000085
**3**	1	169.25983841	0.00000000

Convergence criteria met.

DistSoma=192

Covariance Parameter Estimates				
Cov Parm	Estimate	Alpha	Lower	Upper
Culture	2.1896	0.05	0.8246	14.9389
Residual	2.9003	0.05	1.8678	5.1093

Fit Statistics	
-2 Res Log Likelihood	169.3
AIC (Smaller is Better)	173.3
AICC (Smaller is Better)	173.6
BIC (Smaller is Better)	173.9

Solution for Fixed Effects									
Effect	Treatment	Estimate	Standard Error	DF	t Value	Pr > |t|	Alpha	Lower	Upper
Intercept		3.9676	0.8481	8	4.68	0.0016	0.05	2.0119	5.9233
Treatment	Meg TTR	0.3027	1.1027	31	0.27	0.7855	0.05	-1.9463	2.5518
Treatment	GFP MsTTR	0	.	.	.	.	.	.	.

Solution for Random Effects									
Effect	Culture	Estimate	Std Err Pred	DF	t Value	Pr > |t|	Alpha	Lower	Upper
Culture	1	-0.4487	0.9529	31	-0.47	0.6410	0.05	-2.3922	1.4948
Culture	2	0.7756	0.9750	31	0.80	0.4324	0.05	-1.2129	2.7641
Culture	3	-1.1025	0.9750	31	-1.13	0.2668	0.05	-3.0910	0.8860
Culture	4	0.7756	0.9750	31	0.80	0.4324	0.05	-1.2129	2.7641
Culture	5	-0.2137	0.8770	31	-0.24	0.8091	0.05	-2.0023	1.5749
Culture	6	-0.3909	0.9083	31	-0.43	0.6699	0.05	-2.2434	1.4617
Culture	7	1.2994	0.9083	31	1.43	0.1626	0.05	-0.5531	3.1520
Culture	8	-2.5424	0.8540	31	-2.98	0.0056	0.05	-4.2841	-0.8006
Culture	9	1.3413	1.0258	31	1.31	0.2006	0.05	-0.7507	3.4334
Culture	10	0.5062	0.9538	31	0.53	0.5994	0.05	-1.4391	2.4515

Type 3 Tests of Fixed Effects				
Effect	Num DF	Den DF	F Value	Pr > F
Treatment	1	31	0.08	0.7855

DistSoma=192

Least Squares Means									
**Effect**	**Treatment**	**Estimate**	**Standard Error**	**DF**	**t Value**	**Pr > |t|**	**Alpha**	**Lower**	**Upper**
**Treatment**	Meg TTR	4.2703	0.7048	31	6.06	<.0001	0.05	2.8328	5.7078
**Treatment**	GFP MsTTR	3.9676	0.8481	31	4.68	<.0001	0.05	2.2379	5.6973

Differences of Least Squares Means												
**Effect**	**Treatment**	**Treatment**	**Estimate**	**Standard Error**	**DF**	**t Value**	**Pr > |t|**	**Adjustment**	**Adj P**	**Alpha**	**Lower**	**Upper**
**Treatment**	Meg TTR	GFP MsTTR	0.3027	1.1027	31	0.27	0.7855	Tukey-Kramer	0.7855	0.05	-1.9463	2.5518

Differences of Least Squares Means				
**Effect**	**Treatment**	**Treatment**	**Adj Lower**	**Adj Upper**
**Treatment**	Meg TTR	GFP MsTTR	-1.9463	2.5518

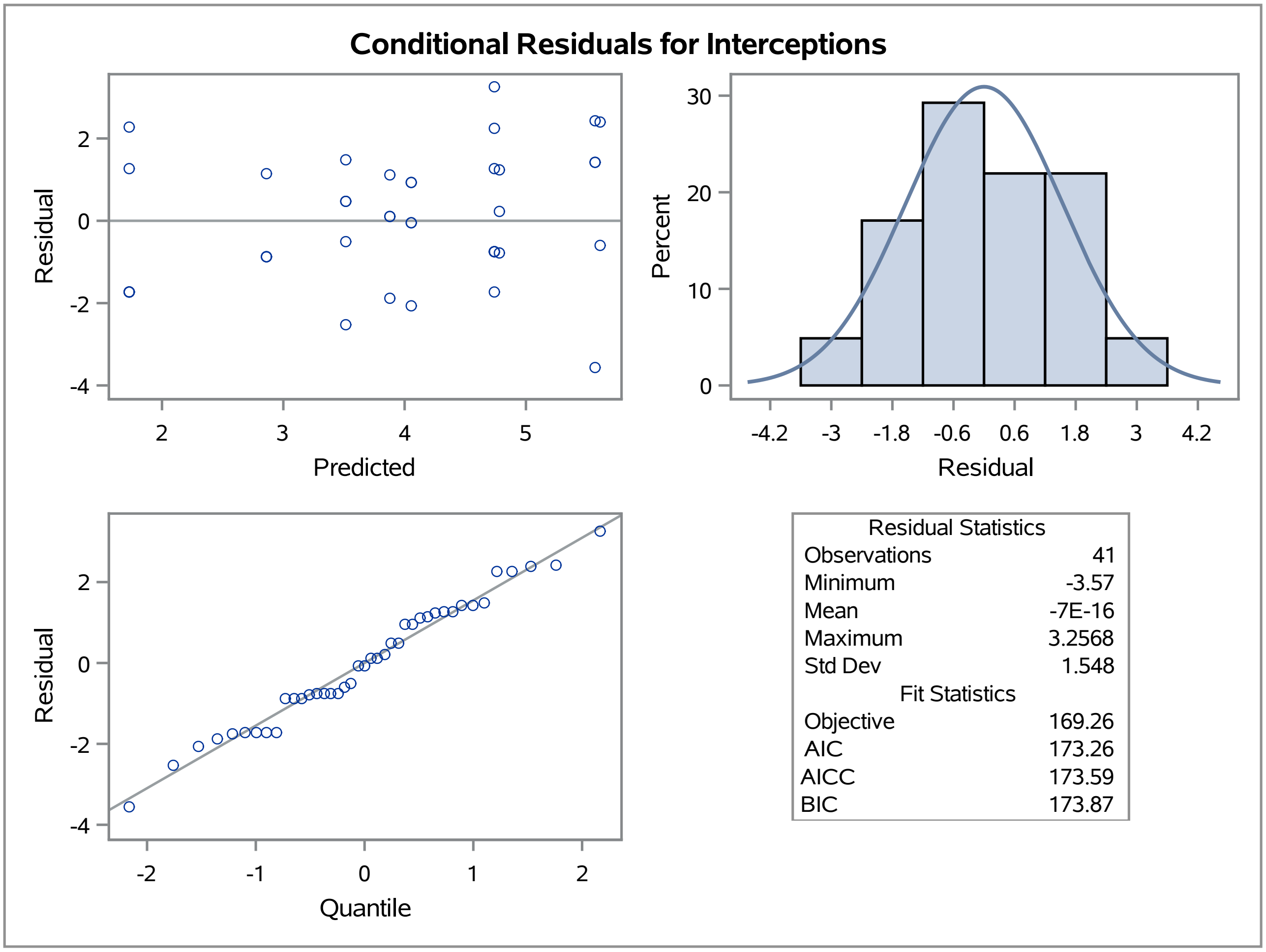

Residual Statistics	
Observations	41
Minimum	-3.57
Mean	-7E-16
Maximum	3.2568
Std Dev	1.548
**Fit Statistics**	
Objective	169.26
AIC	173.26
AICC	173.59
BIC	173.87

DistSoma=198

Model Information	
**Data Set**	WORK.TEMPDATASORTED
**Dependent Variable**	Interceptions
**Covariance Structure**	Variance Components
**Estimation Method**	REML
**Residual Variance Method**	Profile
**Fixed Effects SE Method**	Model-Based
**Degrees of Freedom Method**	Containment

Class Level Information		
**Class**	**Levels**	**Values**
**Treatment**	2	Meg TTR GFP MsTTR
**Culture**	10	1 2 3 4 5 6 7 8 9 10

Dimensions	
**Covariance Parameters**	2
**Columns in X**	3
**Columns in Z**	10
**Subjects**	1
**Max Obs per Subject**	41

Number of Observations	
**Number of Observations Read**	41
**Number of Observations Used**	41
**Number of Observations Not Used**	0

Iteration History			
**Iteration**	**Evaluations**	**-2 Res Log Like**	**Criterion**
**0**	1	179.19883899	
**1**	3	174.69732982	0.00076983
**2**	1	174.65346444	0.00002581
**3**	1	174.65210425	0.00000003
**4**	1	174.65210249	0.00000000

Convergence criteria met.

DistSoma=198

Covariance Parameter Estimates				
**Cov Parm**	**Estimate**	**Alpha**	**Lower**	**Upper**
**Culture**	1.5336	0.05	0.5109	17.6903
**Residual**	3.6107	0.05	2.3302	6.3387

Fit Statistics	
**-2 Res Log Likelihood**	174.7
**AIC (Smaller is Better)**	178.7
**AICC (Smaller is Better)**	179.0
**BIC (Smaller is Better)**	179.3

Solution for Fixed Effects									
**Effect**	**Treatment**	**Estimate**	**Standard Error**	**DF**	**t Value**	**Pr > |t|**	**Alpha**	**Lower**	**Upper**
**Intercept**		3.6490	0.7727	8	4.72	0.0015	0.05	1.8671	5.4309
**Treatment**	Meg TTR	0.5180	1.0071	31	0.51	0.6106	0.05	-1.5359	2.5720
**Treatment**	GFP MsTTR	0	.	.	.	.	.	.	.

Solution for Random Effects									
**Effect**	**Culture**	**Estimate**	**Std Err Pred**	**DF**	**t Value**	**Pr > |t|**	**Alpha**	**Lower**	**Upper**
**Culture**	1	-0.03333	0.8758	31	-0.04	0.9699	0.05	-1.8194	1.7528
**Culture**	2	0.3783	0.8971	31	0.42	0.6762	0.05	-1.4514	2.2080
**Culture**	3	-0.8807	0.8971	31	-0.98	0.3339	0.05	-2.7103	0.9490
**Culture**	4	0.5357	0.8971	31	0.60	0.5548	0.05	-1.2940	2.3654
**Culture**	5	-0.1136	0.8269	31	-0.14	0.8916	0.05	-1.8000	1.5729
**Culture**	6	-0.5773	0.8564	31	-0.67	0.5053	0.05	-2.3240	1.1695
**Culture**	7	1.1538	0.8564	31	1.35	0.1877	0.05	-0.5929	2.9005
**Culture**	8	-1.9154	0.8046	31	-2.38	0.0236	0.05	-3.5564	-0.2745
**Culture**	9	0.6122	0.9577	31	0.64	0.5273	0.05	-1.3410	2.5655
**Culture**	10	0.8402	0.8974	31	0.94	0.3564	0.05	-0.9900	2.6704

Type 3 Tests of Fixed Effects				
**Effect**	**Num DF**	**Den DF**	**F Value**	**Pr > F**
**Treatment**	1	31	0.26	0.6106

DistSoma=198

Least Squares Means									
**Effect**	**Treatment**	**Estimate**	**Standard Error**	**DF**	**t Value**	**Pr > |t|**	**Alpha**	**Lower**	**Upper**
**Treatment**	Meg TTR	4.1670	0.6458	31	6.45	<.0001	0.05	2.8498	5.4843
**Treatment**	GFP MsTTR	3.6490	0.7727	31	4.72	<.0001	0.05	2.0730	5.2250

Differences of Least Squares Means													
**Effect**	**Treatment**	**Treatment**	**Estimate**	**Standard Error**	**DF**	**t Value**	**Pr > |t|**	**Adjustment**	**Adj P**	**Alpha**	**Lower**	**Upper**
**Treatment**	Meg TTR	GFP MsTTR	0.5180	1.0071	31	0.51	0.6106	Tukey-Kramer	0.6106	0.05	-1.5359	2.5720

Differences of Least Squares Means				
**Effect**	**Treatment**	**Treatment**	**Adj Lower**	**Adj Upper**
**Treatment**	Meg TTR	GFP MsTTR	-1.5359	2.5720

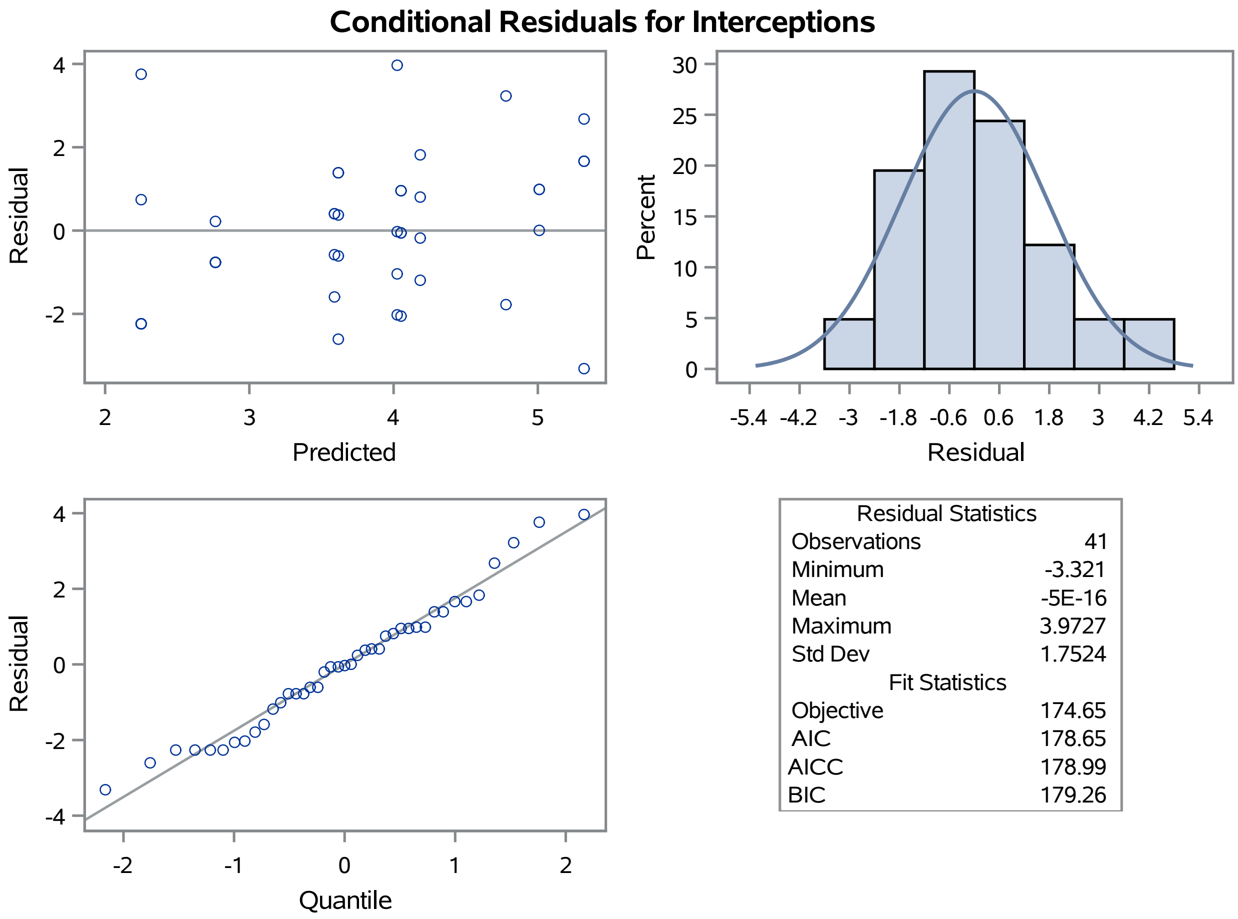

Residual Statistics	
Observations	41
Minimum	-3.321
Mean	-5E-16
Maximum	3.9727
Std Dev	1.7524
Fit Statistics	
Objective	174.65
AIC	178.65
AICC	178.99
BIC	179.26

DistSoma=204

Model Information	
**Data Set**	WORK.TEMPDATASORTED
**Dependent Variable**	Interceptions
**Covariance Structure**	Variance Components
**Estimation Method**	REML
**Residual Variance Method**	Profile
**Fixed Effects SE Method**	Model-Based
**Degrees of Freedom Method**	Containment

Class Level Information		
**Class**	**Levels**	**Values**
**Treatment**	2	Meg TTR GFP MsTTR
**Culture**	10	1 2 3 4 5 6 7 8 9 10

Dimensions	
**Covariance Parameters**	2
**Columns in X**	3
**Columns in Z**	10
**Subjects**	1
**Max Obs per Subject**	41

Number of Observations	
**Number of Observations Read**	41
**Number of Observations Used**	41
**Number of Observations Not Used**	0

Iteration History			
**Iteration**	**Evaluations**	**-2 Res Log Like**	**Criterion**
**0**	1	176.30460439	
**1**	3	172.51582649	0.00075792
**2**	1	172.47358999	0.00002489
**3**	1	172.47230669	0.00000003
**4**	1	172.47230510	0.00000000

Convergence criteria met.

DistSoma=204

Covariance Parameter Estimates				
**Cov Parm**	**Estimate**	**Alpha**	**Lower**	**Upper**
**Culture**	1.2901	0.05	0.4127	18.2318
**Residual**	3.4716	0.05	2.2411	6.0919

Fit Statistics	
**-2 Res Log Likelihood**	172.5
**AIC (Smaller is Better)**	176.5
**AICC (Smaller is Better)**	176.8
**BIC (Smaller is Better)**	177.1

Solution for Fixed Effects									
**Effect**	**Treatment**	**Estimate**	**Standard Error**	**DF**	**t Value**	**Pr > |t|**	**Alpha**	**Lower**	**Upper**
**Intercept**		3.4734	0.7266	8	4.78	0.0014	0.05	1.7978	5.1490
**Treatment**	Meg TTR	0.3742	0.9475	31	0.40	0.6955	0.05	-1.5581	2.3066
**Treatment**	GFP MsTTR	0	.	.	.	.	.	.	.

Solution for Random Effects									
**Effect**	**Culture**	**Estimate**	**Std Err Pred**	**DF**	**t Value**	**Pr > |t|**	**Alpha**	**Lower**	**Upper**
**Culture**	1	-0.04772	0.8213	31	-0.06	0.9540	0.05	-1.7228	1.6273
**Culture**	2	0.4643	0.8412	31	0.55	0.5849	0.05	-1.2513	2.1798
**Culture**	3	-0.7314	0.8412	31	-0.87	0.3913	0.05	-2.4469	0.9842
**Culture**	4	0.3148	0.8412	31	0.37	0.7108	0.05	-1.4007	2.0304
**Culture**	5	-0.03097	0.7795	31	-0.04	0.9686	0.05	-1.6208	1.5589
**Culture**	6	-0.5067	0.8068	31	-0.63	0.5346	0.05	-2.1523	1.1388
**Culture**	7	1.1373	0.8068	31	1.41	0.1686	0.05	-0.5083	2.7828
**Culture**	8	-1.7358	0.7587	31	-2.29	0.0291	0.05	-3.2832	-0.1884
**Culture**	9	0.7045	0.8985	31	0.78	0.4389	0.05	-1.1280	2.5370
**Culture**	10	0.4318	0.8443	31	0.51	0.6127	0.05	-1.2901	2.1536

Type 3 Tests of Fixed Effects				
**Effect**	**Num DF**	**Den DF**	**F Value**	**Pr > F**
**Treatment**	1	31	0.16	0.6955

DistSoma=204

Least Squares Means									
**Effect**	**Treatment**	**Estimate**	**Standard Error**	**DF**	**t Value**	**Pr > |t|**	**Alpha**	**Lower**	**Upper**
**Treatment**	Meg TTR	3.8476	0.6080	31	6.33	<.0001	0.05	2.6076	5.0877
**Treatment**	GFP MsTTR	3.4734	0.7266	31	4.78	<.0001	0.05	1.9915	4.9553

Differences of Least Squares Means												
**Effect**	**Treatment**	**Treatment**	**Estimate**	**Standard Error**	**DF**	**t Value**	**Pr > |t|**	**Adjustment**	**Adj P**	**Alpha**	**Lower**	**Upper**
**Treatment**	Meg TTR	GFP MsTTR	0.3742	0.9475	31	0.40	0.6955	Tukey-Kramer	0.6955	0.05	-1.5581	2.3066

Differences of Least Squares Means				
**Effect**	**Treatment**	**Treatment**	**Adj Lower**	**Adj Upper**
**Treatment**	Meg TTR	GFP MsTTR	-1.5581	2.3066

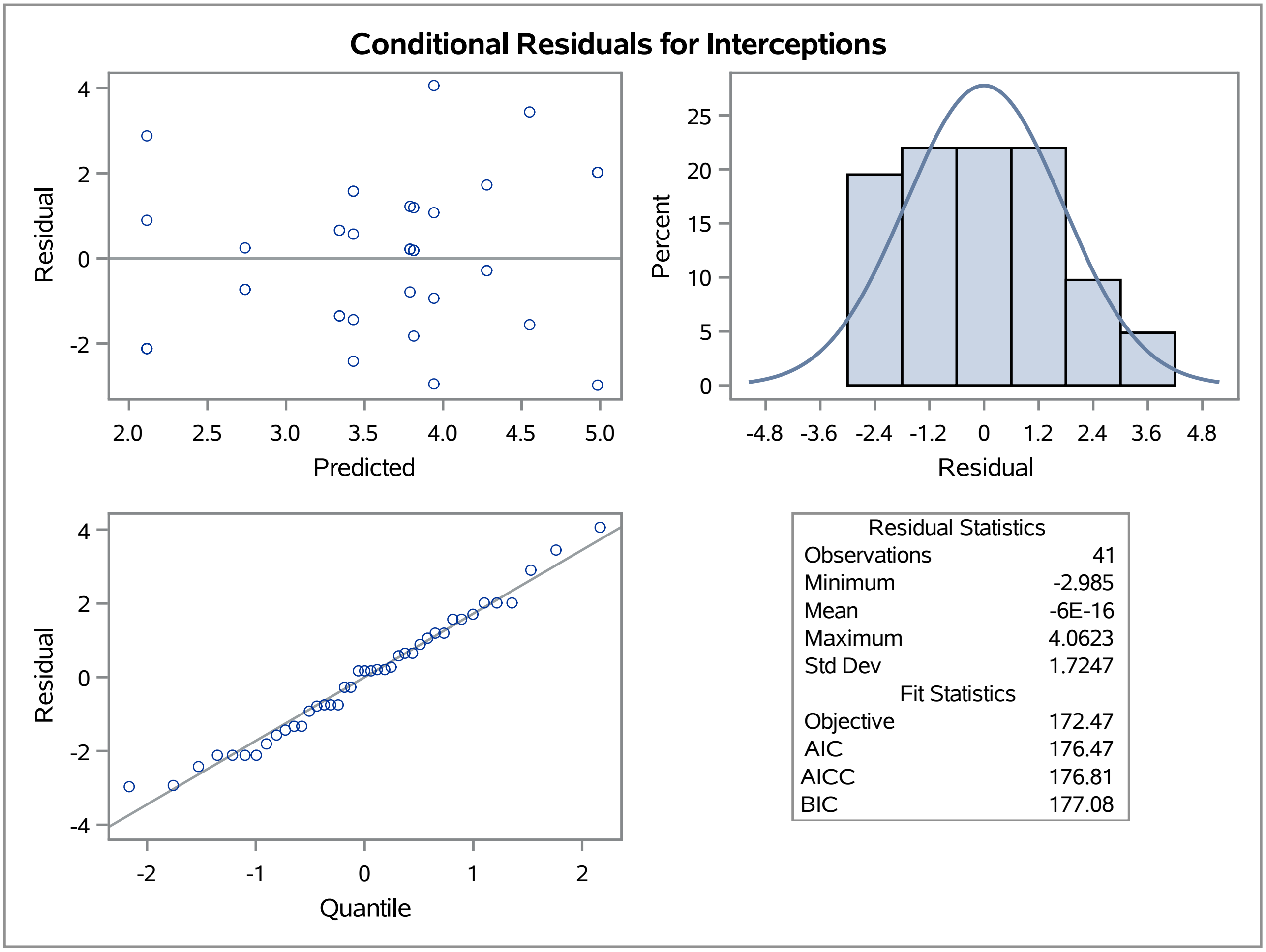

DistSoma=210

Model Information	
**Data Set**	WORK.TEMPDATASORTED
**Dependent Variable**	Interceptions
**Covariance Structure**	Variance Components
**Estimation Method**	REML
**Residual Variance Method**	Profile
**Fixed Effects SE Method**	Model-Based
**Degrees of Freedom Method**	Containment

Class Level Information		
**Class**	**Levels**	**Values**
**Treatment**	2	Meg TTR GFP MsTTR
**Culture**	10	1 2 3 4 5 6 7 8 9 10

Dimensions	
**Covariance Parameters**	2
**Columns in X**	3
**Columns in Z**	10
**Subjects**	1
**Max Obs per Subject**	41

Number of Observations	
**Number of Observations Read**	41
**Number of Observations Used**	41
**Number of Observations Not Used**	0

Iteration History			
**Iteration**	**Evaluations**	**-2 Res Log Like**	**Criterion**
**0**	1	176.51661040	
**1**	3	168.20093428	0.00165775
**2**	1	168.10996063	0.00010125
**3**	1	168.10486806	0.00000047
**4**	1	168.10484520	0.00000000

Convergence criteria met.

DistSoma=210

Covariance Parameter Estimates				
**Cov Parm**	**Estimate**	**Alpha**	**Lower**	**Upper**
**Culture**	1.9191	0.05	0.7178	13.4356
**Residual**	2.8670	0.05	1.8501	5.0338

Fit Statistics	
**-2 Res Log Likelihood**	168.1
**AIC (Smaller is Better)**	172.1
**AICC (Smaller is Better)**	172.4
**BIC (Smaller is Better)**	172.7

Solution for Fixed Effects									
**Effect**	**Treatment**	**Estimate**	**Standard Error**	**DF**	**t Value**	**Pr > |t|**	**Alpha**	**Lower**	**Upper**
**Intercept**		3.6493	0.8060	8	4.53	0.0019	0.05	1.7907	5.5079
**Treatment**	Meg TTR	-0.04789	1.0485	31	-0.05	0.9639	0.05	-2.1864	2.0906
**Treatment**	GFP MsTTR	0	.	.	.	.	.	.	.

Solution for Random Effects									
**Effect**	**Culture**	**Estimate**	**Std Err Pred**	**DF**	**t Value**	**Pr > |t|**	**Alpha**	**Lower**	**Upper**
**Culture**	1	-0.03795	0.9092	31	-0.04	0.9670	0.05	-1.8922	1.8163
**Culture**	2	0.8014	0.9307	31	0.86	0.3958	0.05	-1.0968	2.6996
**Culture**	3	-1.0188	0.9307	31	-1.09	0.2821	0.05	-2.9170	0.8794
**Culture**	4	0.2553	0.9307	31	0.27	0.7856	0.05	-1.6428	2.1535
**Culture**	5	-0.1551	0.8415	31	-0.18	0.8550	0.05	-1.8713	1.5612
**Culture**	6	-0.2558	0.8719	31	-0.29	0.7712	0.05	-2.0342	1.5225
**Culture**	7	1.3823	0.8719	31	1.59	0.1230	0.05	-0.3960	3.1606
**Culture**	8	-2.4831	0.8191	31	-3.03	0.0049	0.05	-4.1536	-0.8126
**Culture**	9	0.8006	0.9838	31	0.81	0.4220	0.05	-1.2060	2.8071
**Culture**	10	0.7111	0.9156	31	0.78	0.4433	0.05	-1.1563	2.5786

Type 3 Tests of Fixed Effects				
**Effect**	**Num DF**	**Den DF**	**F Value**	**Pr > F**
**Treatment**	1	31	0.00	0.9639

DistSoma=210

Least Squares Means									
**Effect**	**Treatment**	**Estimate**	**Standard Error**	**DF**	**t Value**	**Pr > |t|**	**Alpha**	**Lower**	**Upper**
**Treatment**	Meg TTR	3.6014	0.6707	31	5.37	<.0001	0.05	2.2336	4.9692
**Treatment**	GFP MsTTR	3.6493	0.8060	31	4.53	<.0001	0.05	2.0055	5.2931

Differences of Least Squares Means												
**Effect**	**Treatment**	**Treatment**	**Estimate**	**Standard Error**	**DF**	**t Value**	**Pr > |t|**	**Adjustment**	**Adj P**	**Alpha**	**Lower**	**Upper**
**Treatment**	Meg TTR	GFP MsTTR	-0.04789	1.0485	31	-0.05	0.9639	Tukey-Kramer	0.9639	0.05	-2.1864	2.0906

Differences of Least Squares Means				
**Effect**	**Treatment**	**Treatment**	**Adj Lower**	**Adj Upper**
**Treatment**	Meg TTR	GFP MsTTR	-2.1863	2.0905

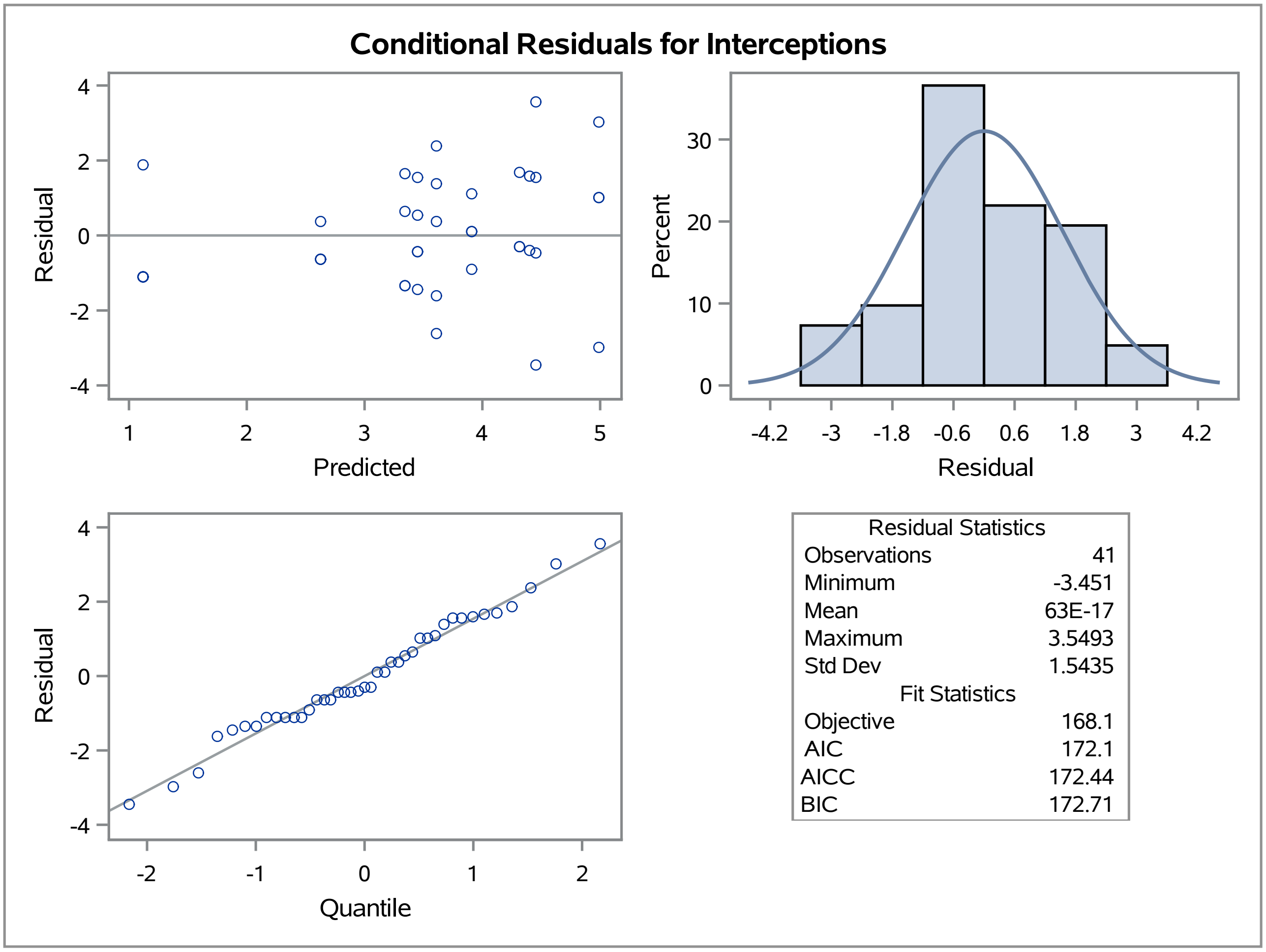

DistSoma=216

Model Information	
**Data Set**	WORK.TEMPDATASORTED
**Dependent Variable**	Interceptions
**Covariance Structure**	Variance Components
**Estimation Method**	REML
**Residual Variance Method**	Profile
**Fixed Effects SE Method**	Model-Based
**Degrees of Freedom Method**	Containment

Class Level Information		
**Class**	**Levels**	**Values**
**Treatment**	2	Meg TTR GFP MsTTR
**Culture**	10	1 2 3 4 5 6 7 8 9 10

Dimensions	
**Covariance Parameters**	2
**Columns in X**	3
**Columns in Z**	10
**Subjects**	1
**Max Obs per Subject**	41

Number of Observations	
**Number of Observations Read**	41
**Number of Observations Used**	41
**Number of Observations Not Used**	0

Iteration History			
**Iteration**	**Evaluations**	**-2 Res Log Like**	**Criterion**
**0**	1	181.49265979	
**1**	3	172.93512240	0.00106395
**2**	1	172.87484321	0.00004649
**3**	1	172.87241855	0.00000011
**4**	1	172.87241311	0.00000000

Convergence criteria met.

DistSoma=216

Covariance Parameter Estimates				
Cov Parm	Estimate	Alpha	Lower	Upper
Culture	2.3569	0.05	0.8849	16.2666
Residual	3.1926	0.05	2.0564	5.6228

Fit Statistics	
-2 Res Log Likelihood	172.9
AIC (Smaller is Better)	176.9
AICC (Smaller is Better)	177.2
BIC (Smaller is Better)	177.5

Solution for Fixed Effects									
Effect	Treatment	Estimate	Standard Error	DF	t Value	Pr > |t|	Alpha	Lower	Upper
Intercept		3.4317	0.8823	8	3.89	0.0046	0.05	1.3972	5.4663
Treatment	Meg TTR	0.07208	1.1473	31	0.06	0.9503	0.05	-2.2678	2.4120
Treatment	GFP MsTTR	0	.	.	.	.	.	.	.

Solution for Random Effects									
Effect	Culture	Estimate	Std Err Pred	DF	t Value	Pr > |t|	Alpha	Lower	Upper
Culture	1	-0.3397	0.9921	31	-0.34	0.7344	0.05	-2.3632	1.6838
Culture	2	1.3583	1.0152	31	1.34	0.1906	0.05	-0.7122	3.4288
Culture	3	-1.2563	1.0152	31	-1.24	0.2252	0.05	-3.3268	0.8142
Culture	4	0.2377	1.0152	31	0.23	0.8164	0.05	-1.8328	2.3083
Culture	5	-0.2391	0.9140	31	-0.26	0.7954	0.05	-2.1032	1.6251
Culture	6	-0.5631	0.9468	31	-0.59	0.5563	0.05	-2.4941	1.3679
Culture	7	1.4912	0.9468	31	1.57	0.1254	0.05	-0.4398	3.4222
Culture	8	-2.4506	0.8900	31	-2.75	0.0098	0.05	-4.2657	-0.6354
Culture	9	1.1901	1.0691	31	1.11	0.2742	0.05	-0.9903	3.3705
Culture	10	0.5715	0.9942	31	0.57	0.5696	0.05	-1.4562	2.5992

Type 3 Tests of Fixed Effects				
Effect	Num DF	Den DF	F Value	Pr > F
Treatment	1	31	0.00	0.9503

DistSoma=216

Least Squares Means									
**Effect**	**Treatment**	**Estimate**	**Standard Error**	**DF**	**t Value**	**Pr > |t|**	**Alpha**	**Lower**	**Upper**
**Treatment**	Meg TTR	3.5038	0.7334	31	4.78	<.0001	0.05	2.0080	4.9996
**Treatment**	GFP MsTTR	3.4317	0.8823	31	3.89	0.0005	0.05	1.6323	5.2312

Differences of Least Squares Means													
**Effect**	**Treatment**	**Treatment**	**Estimate**	**Standard Error**	**DF**	**t Value**	**Pr > |t|**	**Adjustment**	**Adj P**	**Alpha**	**Lower**	**Upper**
**Treatment**	Meg TTR	GFP MsTTR	0.07208	1.1473	31	0.06	0.9503	Tukey-Kramer	0.9503	0.05	-2.2678	2.4120

Differences of Least Squares Means				
**Effect**	**Treatment**	**Treatment**	**Adj Lower**	**Adj Upper**
**Treatment**	Meg TTR	GFP MsTTR	-2.2678	2.4120

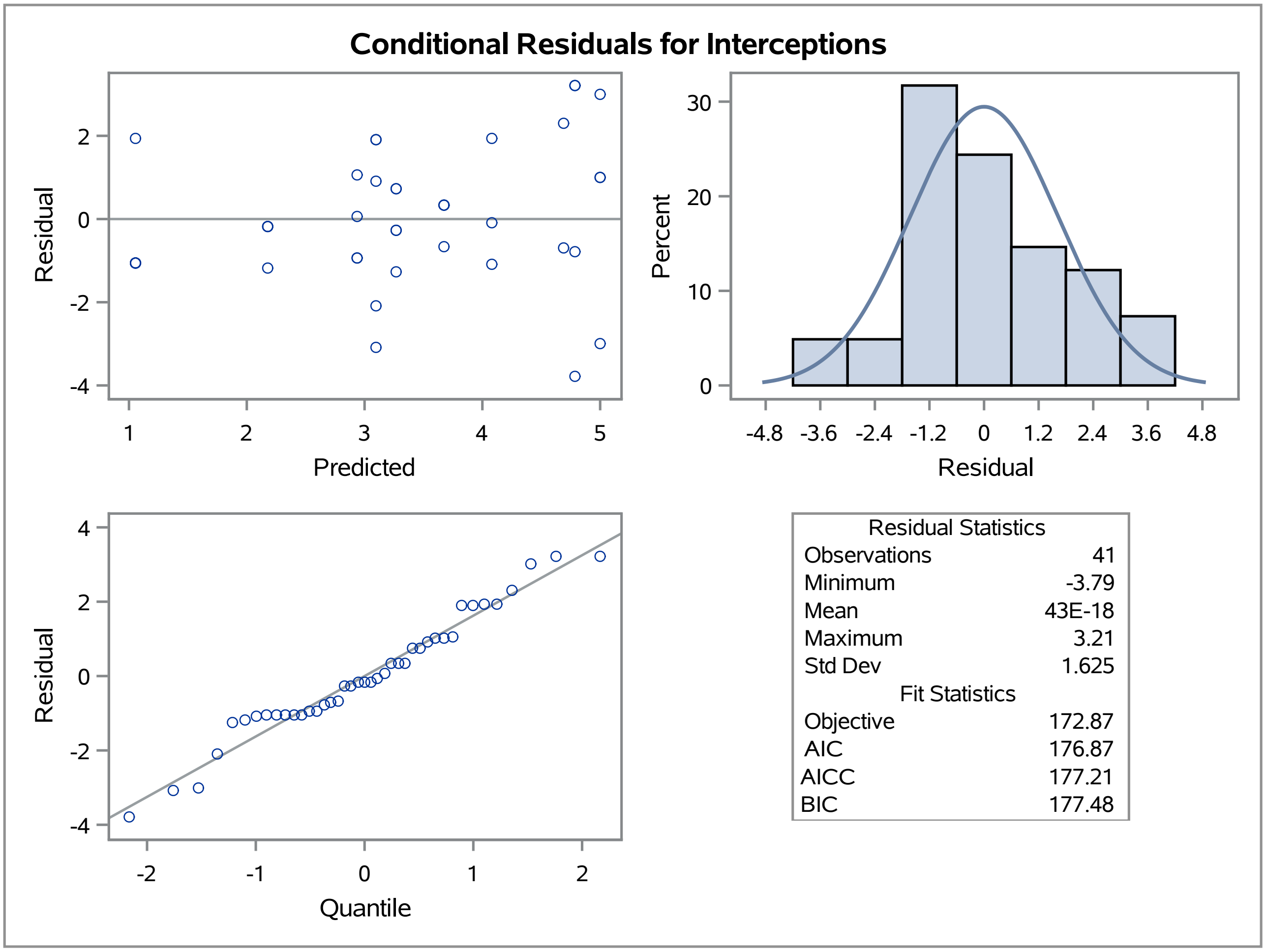

DistSoma=222

Model Information	
**Data Set**	WORK.TEMPDATASORTED
**Dependent Variable**	Interceptions
**Covariance Structure**	Variance Components
**Estimation Method**	REML
**Residual Variance Method**	Profile
**Fixed Effects SE Method**	Model-Based
**Degrees of Freedom Method**	Containment

Class Level Information		
**Class**	**Levels**	**Values**
**Treatment**	2	Meg TTR GFP MsTTR
**Culture**	10	1 2 3 4 5 6 7 8 9 10

Dimensions	
**Covariance Parameters**	2
**Columns in X**	3
**Columns in Z**	10
**Subjects**	1
**Max Obs per Subject**	41

Number of Observations	
**Number of Observations Read**	41
**Number of Observations Used**	41
**Number of Observations Not Used**	0

Iteration History			
**Iteration**	**Evaluations**	**-2 Res Log Like**	**Criterion**
**0**	1	179.89282750	
**1**	3	170.39574116	0.00272757
**2**	1	170.23918473	0.00025408
**3**	1	170.22584337	0.00000290
**4**	1	170.22569942	0.00000000

Convergence criteria met.

DistSoma=222

Covariance Parameter Estimates				
Cov Parm	Estimate	Alpha	Lower	Upper
Culture	2.3038	0.05	0.8827	14.7312
Residual	2.9590	0.05	1.9082	5.2011

Fit Statistics	
-2 Res Log Likelihood	170.2
AIC (Smaller is Better)	174.2
AICC (Smaller is Better)	174.6
BIC (Smaller is Better)	174.8

Solution for Fixed Effects									
Effect	Treatment	Estimate	Standard Error	DF	t Value	Pr > |t|	Alpha	Lower	Upper
Intercept		3.4937	0.8668	8	4.03	0.0038	0.05	1.4949	5.4925
Treatment	Meg TTR	-0.1532	1.1269	31	-0.14	0.8928	0.05	-2.4515	2.1452
Treatment	GFP MsTTR	0	.	.	.	.	.	.	.

Solution for Random Effects									
Effect	Culture	Estimate	Std Err Pred	DF	t Value	Pr > |t|	Alpha	Lower	Upper
Culture	1	-0.3928	0.9729	31	-0.40	0.6892	0.05	-2.3769	1.5913
Culture	2	1.3294	0.9952	31	1.34	0.1913	0.05	-0.7003	3.3592
Culture	3	-1.3199	0.9952	31	-1.33	0.1944	0.05	-3.3496	0.7098
Culture	4	0.3832	0.9952	31	0.39	0.7028	0.05	-1.6465	2.4130
Culture	5	-0.1118	0.8939	31	-0.13	0.9013	0.05	-1.9350	1.7114
Culture	6	-0.4470	0.9258	31	-0.48	0.6326	0.05	-2.3352	1.4412
Culture	7	1.6346	0.9258	31	1.77	0.0873	0.05	-0.2536	3.5228
Culture	8	-2.4770	0.8707	31	-2.84	0.0078	0.05	-4.2527	-0.7012
Culture	9	0.7060	1.0456	31	0.68	0.5045	0.05	-1.4264	2.8385
Culture	10	0.6952	0.9721	31	0.72	0.4799	0.05	-1.2874	2.6777

Type 3 Tests of Fixed Effects				
Effect	Num DF	Den DF	F Value	Pr > F
Treatment	1	31	0.02	0.8928

DistSoma=222

Least Squares Means									
**Effect**	**Treatment**	**Estimate**	**Standard Error**	**DF**	**t Value**	**Pr > |t|**	**Alpha**	**Lower**	**Upper**
**Treatment**	Meg TTR	3.3405	0.7201	31	4.64	<.0001	0.05	1.8718	4.8093
**Treatment**	GFP MsTTR	3.4937	0.8668	31	4.03	0.0003	0.05	1.7259	5.2615

Differences of Least Squares Means												
**Effect**	**Treatment**	**Treatment**	**Estimate**	**Standard Error**	**DF**	**t Value**	**Pr > |t|**	**Adjustment**	**Adj P**	**Alpha**	**Lower**	**Upper**
**Treatment**	Meg TTR	GFP MsTTR	-0.1532	1.1269	31	-0.14	0.8928	Tukey-Kramer	0.8928	0.05	-2.4515	2.1452

Differences of Least Squares Means				
**Effect**	**Treatment**	**Treatment**	**Adj Lower**	**Adj Upper**
**Treatment**	Meg TTR	GFP MsTTR	-2.4515	2.1452

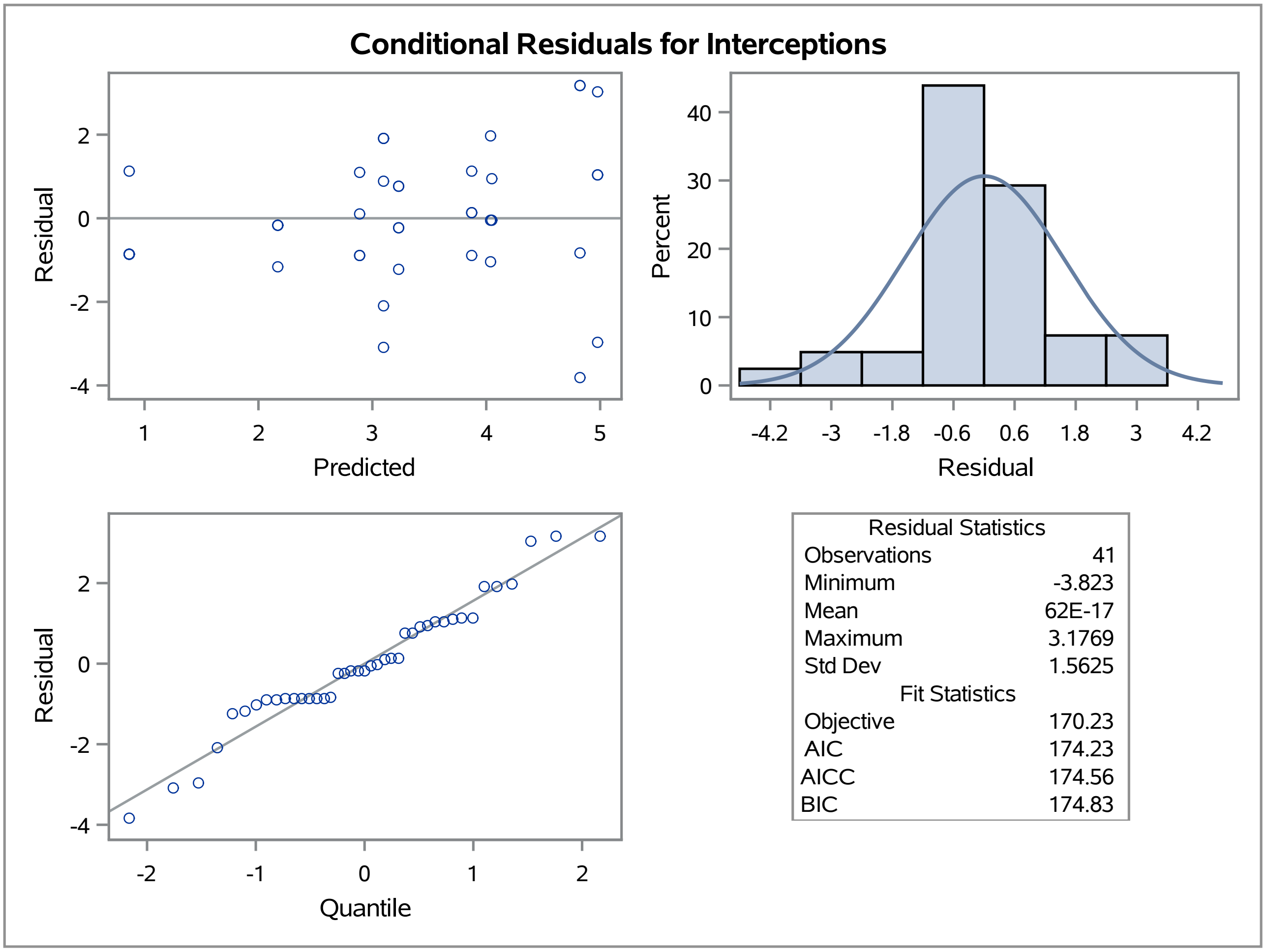

DistSoma=228

Model Information	
Data Set	WORK.TEMPDATASORTED
Dependent Variable	Interceptions
Covariance Structure	Variance Components
Estimation Method	REML
Residual Variance Method	Profile
Fixed Effects SE Method	Model-Based
Degrees of Freedom Method	Containment

Class Level Information		
Class	Levels	Values
Treatment	2	Meg TTR GFP MsTTR
Culture	10	1 2 3 4 5 6 7 8 9 10

Dimensions	
Covariance Parameters	2
Columns in X	3
Columns in Z	10
Subjects	1
Max Obs per Subject	41

Number of Observations	
Number of Observations Read	41
Number of Observations Used	41
Number of Observations Not Used	0

Iteration History			
Iteration	Evaluations	-2 Res Log Like	Criterion
0	1	184.91664364	
1	5	175.20591761	0.00011106
2	1	175.20049466	0.00000070
3	1	175.20045812	0.00000000

Convergence criteria met.

DistSoma=228

Covariance Parameter Estimates				
**Cov Parm**	**Estimate**	**Alpha**	**Lower**	**Upper**
**Culture**	2.6936	0.05	1.0327	17.1846
**Residual**	3.3440	0.05	2.1551	5.8842

Fit Statistics	
**-2 Res Log Likelihood**	175.2
**AIC (Smaller is Better)**	179.2
**AICC (Smaller is Better)**	179.5
**BIC (Smaller is Better)**	179.8

Solution for Fixed Effects									
**Effect**	**Treatment**	**Estimate**	**Standard Error**	**DF**	**t Value**	**Pr > |t|**	**Alpha**	**Lower**	**Upper**
**Intercept**		3.1951	0.9336	8	3.42	0.0091	0.05	1.0422	5.3479
**Treatment**	Meg TTR	0.2135	1.2136	31	0.18	0.8615	0.05	-2.2617	2.6886
**Treatment**	GFP MsTTR	0	.	.	.	.	.	.	.

Solution for Random Effects									
**Effect**	**Culture**	**Estimate**	**Std Err Pred**	**DF**	**t Value**	**Pr > |t|**	**Alpha**	**Lower**	**Upper**
**Culture**	1	-0.3165	1.0465	31	-0.30	0.7643	0.05	-2.4508	1.8178
**Culture**	2	1.1866	1.0703	31	1.11	0.2761	0.05	-0.9963	3.3696
**Culture**	3	-1.2936	1.0703	31	-1.21	0.2359	0.05	-3.4765	0.8893
**Culture**	4	0.4235	1.0703	31	0.40	0.6951	0.05	-1.7595	2.6064
**Culture**	5	-0.3273	0.9600	31	-0.34	0.7355	0.05	-2.2852	1.6306
**Culture**	6	-0.6934	0.9941	31	-0.70	0.4907	0.05	-2.7208	1.3340
**Culture**	7	2.1684	0.9941	31	2.18	0.0369	0.05	0.1411	4.1958
**Culture**	8	-2.5480	0.9352	31	-2.72	0.0105	0.05	-4.4553	-0.6407
**Culture**	9	0.9819	1.1227	31	0.87	0.3885	0.05	-1.3079	3.2718
**Culture**	10	0.4183	1.0436	31	0.40	0.6913	0.05	-1.7102	2.5469

Type 3 Tests of Fixed Effects				
**Effect**	**Num DF**	**Den DF**	**F Value**	**Pr > F**
**Treatment**	1	31	0.03	0.8615

DistSoma=228

Least Squares Means									
Effect	Treatment	Estimate	Standard Error	DF	t Value	Pr > |t|	Alpha	Lower	Upper
**Treatment**	Meg TTR	3.4086	0.7754	31	4.40	0.0001	0.05	1.8272	4.9900
**Treatment**	GFP MsTTR	3.1951	0.9336	31	3.42	0.0018	0.05	1.2910	5.0991

Differences of Least Squares Means												
Effect	Treatment	Treatment	Estimate	Standard Error	DF	t Value	Pr > |t|	Adjustment	Adj P	Alpha	Lower	Upper
**Treatment**	Meg TTR	GFP MsTTR	0.2135	1.2136	31	0.18	0.8615	Tukey-Kramer	0.8615	0.05	-2.2617	2.6886

Differences of Least Squares Means				
Effect	Treatment	Treatment	Adj Lower	Adj Upper
**Treatment**	Meg TTR	GFP MsTTR	-2.2616	2.6886

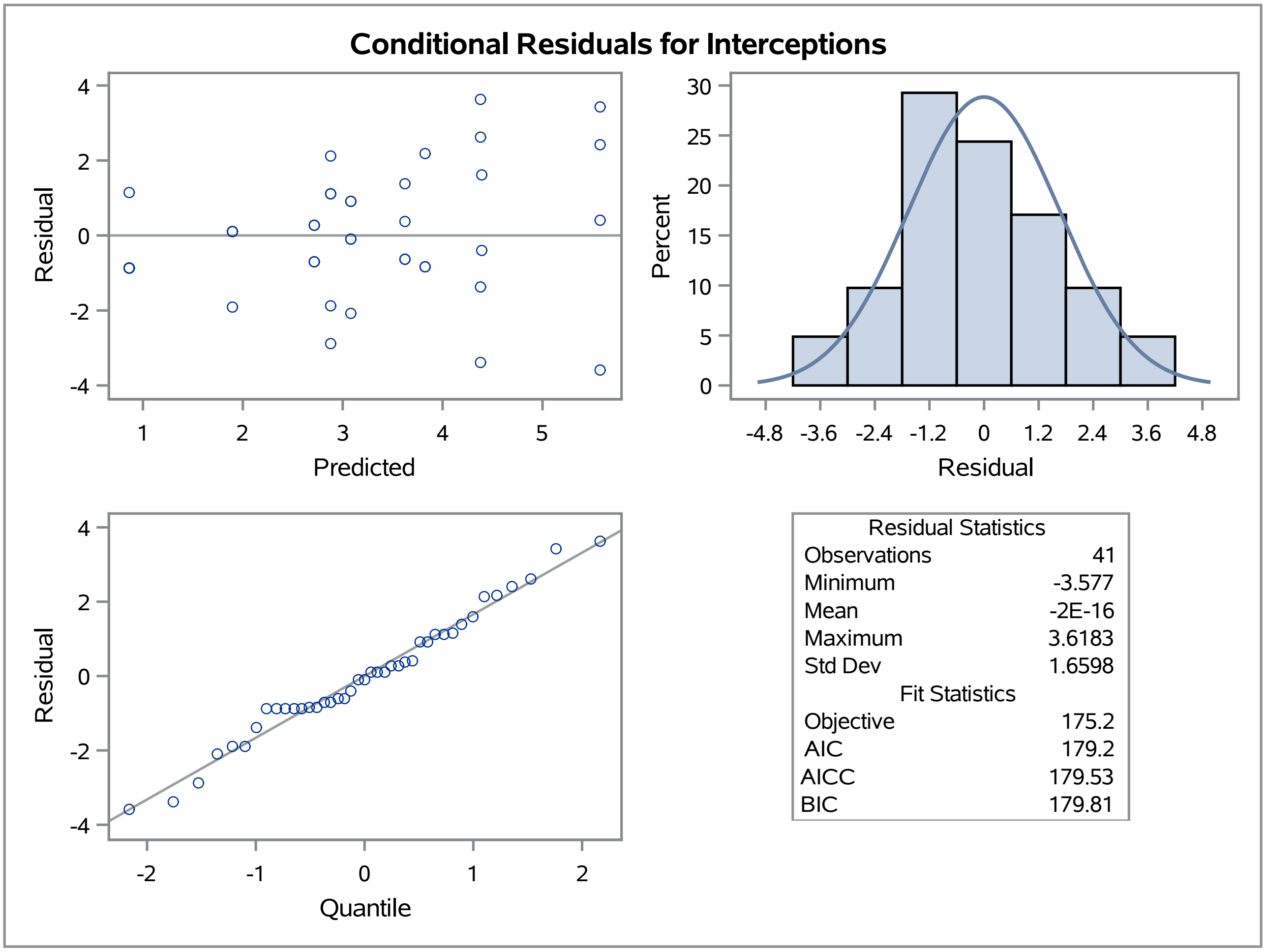

DistSoma=234

Model Information	
**Data Set**	WORK.TEMPDATASORTED
**Dependent Variable**	Interceptions
**Covariance Structure**	Variance Components
**Estimation Method**	REML
**Residual Variance Method**	Profile
**Fixed Effects SE Method**	Model-Based
**Degrees of Freedom Method**	Containment

Class Level Information		
**Class**	**Levels**	**Values**
**Treatment**	2	Meg TTR GFP MsTTR
**Culture**	10	1 2 3 4 5 6 7 8 9 10

Dimensions	
**Covariance Parameters**	2
**Columns in X**	3
**Columns in Z**	10
**Subjects**	1
**Max Obs per Subject**	41

Number of Observations	
**Number of Observations Read**	41
**Number of Observations Used**	41
**Number of Observations Not Used**	0

Iteration History			
**Iteration**	**Evaluations**	**-2 Res Log Like**	**Criterion**
**0**	1	180.25181898	
**1**	5	172.26306030	0.00011924
**2**	1	172.25741079	0.00000079
**3**	1	172.25737107	0.00000000

Convergence criteria met.

DistSoma=234

Covariance Parameter Estimates				
**Cov Parm**	**Estimate**	**Alpha**	**Lower**	**Upper**
**Culture**	2.1302	0.05	0.7929	15.1986
**Residual**	3.1902	0.05	2.0577	5.6059

Fit Statistics	
**-2 Res Log Likelihood**	172.3
**AIC (Smaller is Better)**	176.3
**AICC (Smaller is Better)**	176.6
**BIC (Smaller is Better)**	176.9

Solution for Fixed Effects									
**Effect**	**Treatment**	**Estimate**	**Standard Error**	**DF**	**t Value**	**Pr > |t|**	**Alpha**	**Lower**	**Upper**
**Intercept**		3.2370	0.8494	8	3.81	0.0052	0.05	1.2782	5.1958
**Treatment**	Meg TTR	-0.1939	1.1051	31	-0.18	0.8619	0.05	-2.4476	2.0599
**Treatment**	GFP MsTTR	0	.	.	.	.	.	.	.

Solution for Random Effects									
**Effect**	**Culture**	**Estimate**	**Std Err Pred**	**DF**	**t Value**	**Pr > |t|**	**Alpha**	**Lower**	**Upper**
**Culture**	1	-0.02845	0.9583	31	-0.03	0.9765	0.05	-1.9828	1.9259
**Culture**	2	1.1009	0.9810	31	1.12	0.2704	0.05	-0.8998	3.1015
**Culture**	3	-1.2638	0.9810	31	-1.29	0.2072	0.05	-3.2645	0.7369
**Culture**	4	0.1914	0.9810	31	0.20	0.8466	0.05	-1.8093	2.1920
**Culture**	5	-0.1871	0.8870	31	-0.21	0.8344	0.05	-1.9962	1.6220
**Culture**	6	-0.5770	0.9191	31	-0.63	0.5347	0.05	-2.4516	1.2975
**Culture**	7	1.9695	0.9191	31	2.14	0.0401	0.05	0.09495	3.8441
**Culture**	8	-2.1685	0.8634	31	-2.51	0.0174	0.05	-3.9294	-0.4076
**Culture**	9	0.5472	1.0370	31	0.53	0.6015	0.05	-1.5679	2.6622
**Culture**	10	0.4159	0.9652	31	0.43	0.6695	0.05	-1.5526	2.3845

Type 3 Tests of Fixed Effects				
**Effect**	**Num DF**	**Den DF**	**F Value**	**Pr > F**
**Treatment**	1	31	0.03	0.8619

DistSoma=234

Least Squares Means									
**Effect**	**Treatment**	**Estimate**	**Standard Error**	**DF**	**t Value**	**Pr > |t|**	**Alpha**	**Lower**	**Upper**
**Treatment**	Meg TTR	3.0431	0.7068	31	4.31	0.0002	0.05	1.6015	4.4847
**Treatment**	GFP MsTTR	3.2370	0.8494	31	3.81	0.0006	0.05	1.5045	4.9694

Differences of Least Squares Means												
**Effect**	**Treatment**	**Treatment**	**Estimate**	**Standard Error**	**DF**	**t Value**	**Pr > |t|**	**Adjustment**	**Adj P**	**Alpha**	**Lower**	**Upper**
**Treatment**	Meg TTR	GFP MsTTR	-0.1939	1.1051	31	-0.18	0.8619	Tukey-Kramer	0.8619	0.05	-2.4476	2.0599

Differences of Least Squares Means				
**Effect**	**Treatment**	**Treatment**	**Adj Lower**	**Adj Upper**
**Treatment**	Meg TTR	GFP MsTTR	-2.4476	2.0599

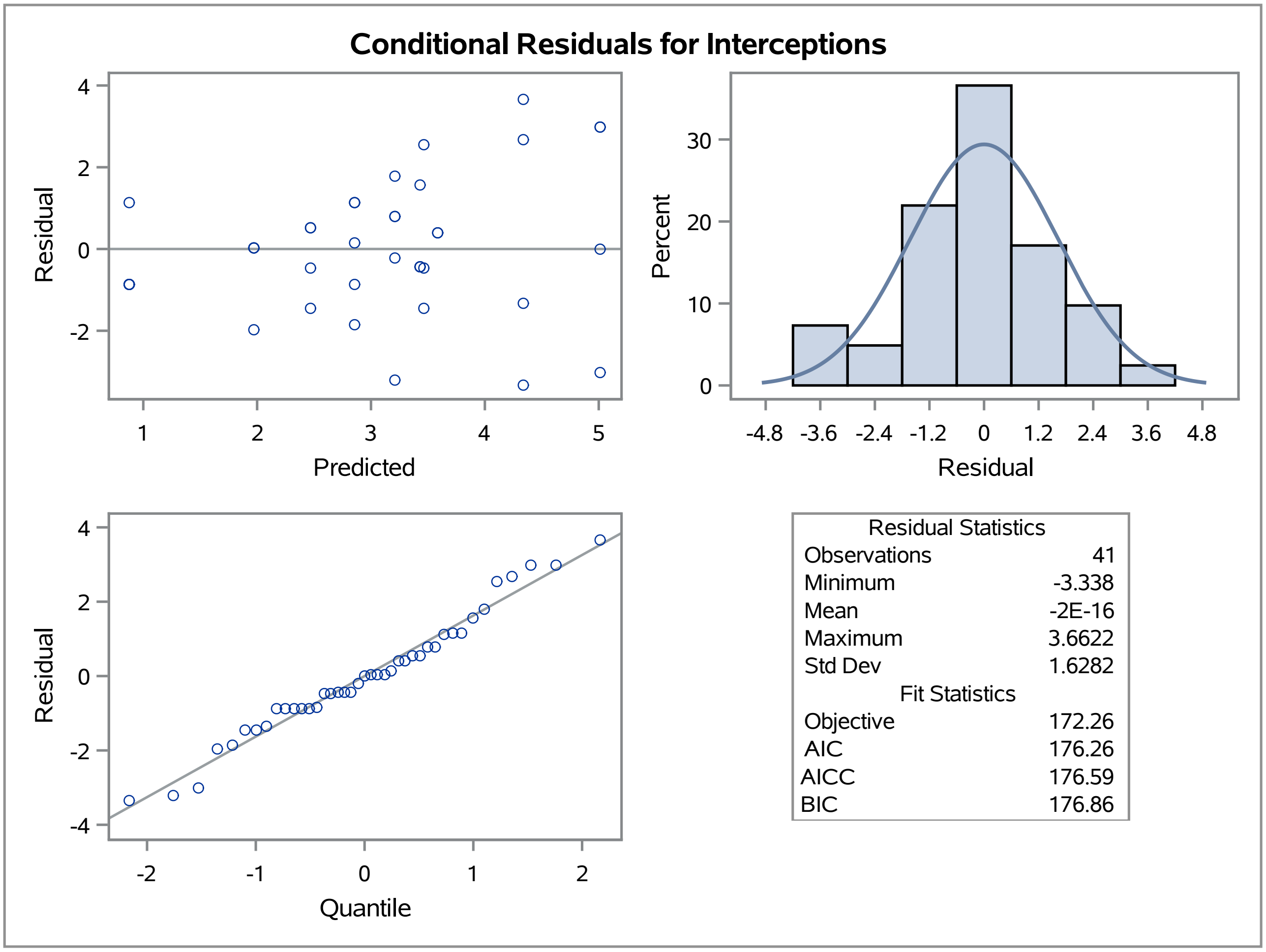

DistSoma=240

Model Information	
**Data Set**	WORK.TEMPDATASORTED
**Dependent Variable**	Interceptions
**Covariance Structure**	Variance Components
**Estimation Method**	REML
**Residual Variance Method**	Profile
**Fixed Effects SE Method**	Model-Based
**Degrees of Freedom Method**	Containment

Class Level Information		
**Class**	**Levels**	**Values**
**Treatment**	2	Meg TTR GFP MsTTR
**Culture**	10	1 2 3 4 5 6 7 8 9 10

Dimensions	
**Covariance Parameters**	2
**Columns in X**	3
**Columns in Z**	10
**Subjects**	1
**Max Obs per Subject**	41

Number of Observations	
**Number of Observations Read**	41
**Number of Observations Used**	41
**Number of Observations Not Used**	0

Iteration History			
**Iteration**	**Evaluations**	**-2 Res Log Like**	**Criterion**
**0**	1	185.18166186	
**1**	5	177.54193539	0.00011388
**2**	1	177.53625770	0.00000075
**3**	1	177.53621761	0.00000000

Convergence criteria met.

DistSoma=240

Covariance Parameter Estimates				
**Cov Parm**	**Estimate**	**Alpha**	**Lower**	**Upper**
**Culture**	2.3404	0.05	0.8649	17.1802
**Residual**	3.6786	0.05	2.3736	6.4603

Fit Statistics	
**-2 Res Log Likelihood**	177.5
**AIC (Smaller is Better)**	181.5
**AICC (Smaller is Better)**	181.9
**BIC (Smaller is Better)**	182.1

Solution for Fixed Effects									
**Effect**	**Treatment**	**Estimate**	**Standard Error**	**DF**	**t Value**	**Pr > |t|**	**Alpha**	**Lower**	**Upper**
**Intercept**		3.0094	0.8961	8	3.36	0.0100	0.05	0.9429	5.0758
**Treatment**	Meg TTR	-0.1935	1.1660	31	-0.17	0.8693	0.05	-2.5716	2.1846
**Treatment**	GFP MsTTR	0	.	.	.	.	.	.	.

Solution for Random Effects									
**Effect**	**Culture**	**Estimate**	**Std Err Pred**	**DF**	**t Value**	**Pr > |t|**	**Alpha**	**Lower**	**Upper**
**Culture**	1	-0.1593	1.0122	31	-0.16	0.8760	0.05	-2.2237	1.9052
**Culture**	2	1.4291	1.0364	31	1.38	0.1778	0.05	-0.6846	3.5428
**Culture**	3	-1.4425	1.0364	31	-1.39	0.1739	0.05	-3.5563	0.6712
**Culture**	4	0.1727	1.0364	31	0.17	0.8687	0.05	-1.9410	2.2865
**Culture**	5	-0.01206	0.9391	31	-0.01	0.9898	0.05	-1.9274	1.9033
**Culture**	6	-0.4062	0.9732	31	-0.42	0.6792	0.05	-2.3910	1.5786
**Culture**	7	1.9270	0.9732	31	1.98	0.0566	0.05	-0.05784	3.9118
**Culture**	8	-2.2313	0.9140	31	-2.44	0.0205	0.05	-4.0954	-0.3673
**Culture**	9	0.3831	1.0975	31	0.35	0.7294	0.05	-1.8552	2.6214
**Culture**	10	0.3396	1.0219	31	0.33	0.7419	0.05	-1.7446	2.4238

Type 3 Tests of Fixed Effects				
**Effect**	**Num DF**	**Den DF**	**F Value**	**Pr > F**
**Treatment**	1	31	0.03	0.8693

DistSoma=240

Least Squares Means									
**Effect**	**Treatment**	**Estimate**	**Standard Error**	**DF**	**t Value**	**Pr > |t|**	**Alpha**	**Lower**	**Upper**
**Treatment**	Meg TTR	2.8159	0.7460	31	3.77	0.0007	0.05	1.2943	4.3374
**Treatment**	GFP MsTTR	3.0094	0.8961	31	3.36	0.0021	0.05	1.1817	4.8370

Differences of Least Squares Means												
**Effect**	**Treatment**	**Treatment**	**Estimate**	**Standard Error**	**DF**	**t Value**	**Pr > |t|**	**Adjustment**	**Adj P**	**Alpha**	**Lower**	**Upper**
**Treatment**	Meg TTR	GFP MsTTR	-0.1935	1.1660	31	-0.17	0.8693	Tukey-Kramer	0.8693	0.05	-2.5716	2.1846

Differences of Least Squares Means				
**Effect**	**Treatment**	**Treatment**	**Adj Lower**	**Adj Upper**
**Treatment**	Meg TTR	GFP MsTTR	-2.5716	2.1845

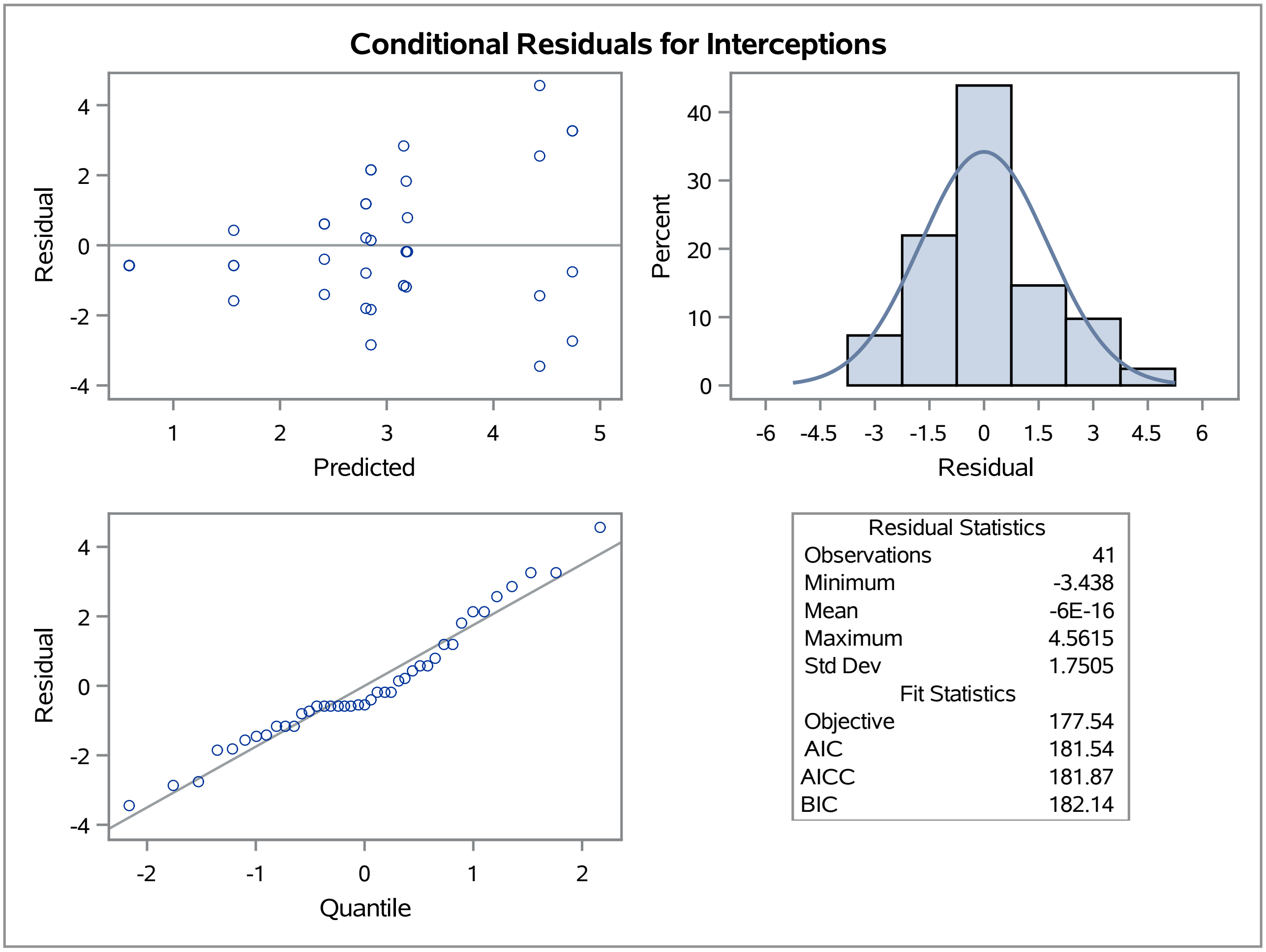

DistSoma=246

Model Information	
**Data Set**	WORK.TEMPDATASORTED
**Dependent Variable**	Interceptions
**Covariance Structure**	Variance Components
**Estimation Method**	REML
**Residual Variance Method**	Profile
**Fixed Effects SE Method**	Model-Based
**Degrees of Freedom Method**	Containment

Class Level Information		
**Class**	**Levels**	**Values**
**Treatment**	2	Meg TTR GFP MsTTR
**Culture**	10	1 2 3 4 5 6 7 8 9 10

Dimensions	
**Covariance Parameters**	2
**Columns in X**	3
**Columns in Z**	10
**Subjects**	1
**Max Obs per Subject**	41

Number of Observations	
**Number of Observations Read**	41
**Number of Observations Used**	41
**Number of Observations Not Used**	0

Iteration History			
**Iteration**	**Evaluations**	**-2 Res Log Like**	**Criterion**
**0**	1	184.47262908	
**1**	3	175.02369812	0.00213242
**2**	1	174.89672148	0.00016841
**3**	1	174.88754565	0.00000136
**4**	1	174.88747532	0.00000000

Convergence criteria met.

DistSoma=246

Covariance Parameter Estimates				
**Cov Parm**	**Estimate**	**Alpha**	**Lower**	**Upper**
**Culture**	2.6393	0.05	1.0120	16.8305
**Residual**	3.3247	0.05	2.1434	5.8472

Fit Statistics	
**-2 Res Log Likelihood**	174.9
**AIC (Smaller is Better)**	178.9
**AICC (Smaller is Better)**	179.2
**BIC (Smaller is Better)**	179.5

Solution for Fixed Effects									
**Effect**	**Treatment**	**Estimate**	**Standard Error**	**DF**	**t Value**	**Pr > |t|**	**Alpha**	**Lower**	**Upper**
**Intercept**		2.8864	0.9257	8	3.12	0.0143	0.05	0.7518	5.0210
**Treatment**	Meg TTR	-0.1566	1.2034	31	-0.13	0.8973	0.05	-2.6108	2.2977
**Treatment**	GFP MsTTR	0	.	.	.	.	.	.	.

Solution for Random Effects									
**Effect**	**Culture**	**Estimate**	**Std Err Pred**	**DF**	**t Value**	**Pr > |t|**	**Alpha**	**Lower**	**Upper**
**Culture**	1	-0.06902	1.0382	31	-0.07	0.9474	0.05	-2.1864	2.0484
**Culture**	2	1.4173	1.0619	31	1.33	0.1917	0.05	-0.7486	3.5831
**Culture**	3	-1.4346	1.0619	31	-1.35	0.1865	0.05	-3.6004	0.7312
**Culture**	4	0.08638	1.0619	31	0.08	0.9357	0.05	-2.0794	2.2522
**Culture**	5	-0.1037	0.9531	31	-0.11	0.9140	0.05	-2.0475	1.8401
**Culture**	6	-0.5551	0.9869	31	-0.56	0.5779	0.05	-2.5680	1.4578
**Culture**	7	2.4869	0.9869	31	2.52	0.0171	0.05	0.4740	4.4998
**Culture**	8	-2.2562	0.9283	31	-2.43	0.0211	0.05	-4.1496	-0.3628
**Culture**	9	0.4725	1.1147	31	0.42	0.6746	0.05	-1.8009	2.7459
**Culture**	10	-0.04451	1.0362	31	-0.04	0.9660	0.05	-2.1579	2.0689

Type 3 Tests of Fixed Effects				
**Effect**	**Num DF**	**Den DF**	**F Value**	**Pr > F**
**Treatment**	1	31	0.02	0.8973

DistSoma=246

Least Squares Means									
**Effect**	**Treatment**	**Estimate**	**Standard Error**	**DF**	**t Value**	**Pr > |t|**	**Alpha**	**Lower**	**Upper**
**Treatment**	Meg TTR	2.7299	0.7689	31	3.55	0.0013	0.05	1.1616	4.2981
**Treatment**	GFP MsTTR	2.8864	0.9257	31	3.12	0.0039	0.05	0.9985	4.7743

Differences of Least Squares Means												
**Effect**	**Treatment**	**Treatment**	**Estimate**	**Standard Error**	**DF**	**t Value**	**Pr > |t|**	**Adjustment**	**Adj P**	**Alpha**	**Lower**	**Upper**
**Treatment**	Meg TTR	GFP MsTTR	-0.1566	1.2034	31	-0.13	0.8973	Tukey-Kramer	0.8973	0.05	-2.6108	2.2977

Differences of Least Squares Means				
**Effect**	**Treatment**	**Treatment**	**Adj Lower**	**Adj Upper**
**Treatment**	Meg TTR	GFP MsTTR	-2.6108	2.2977

Conditional Residuals for Interceptions

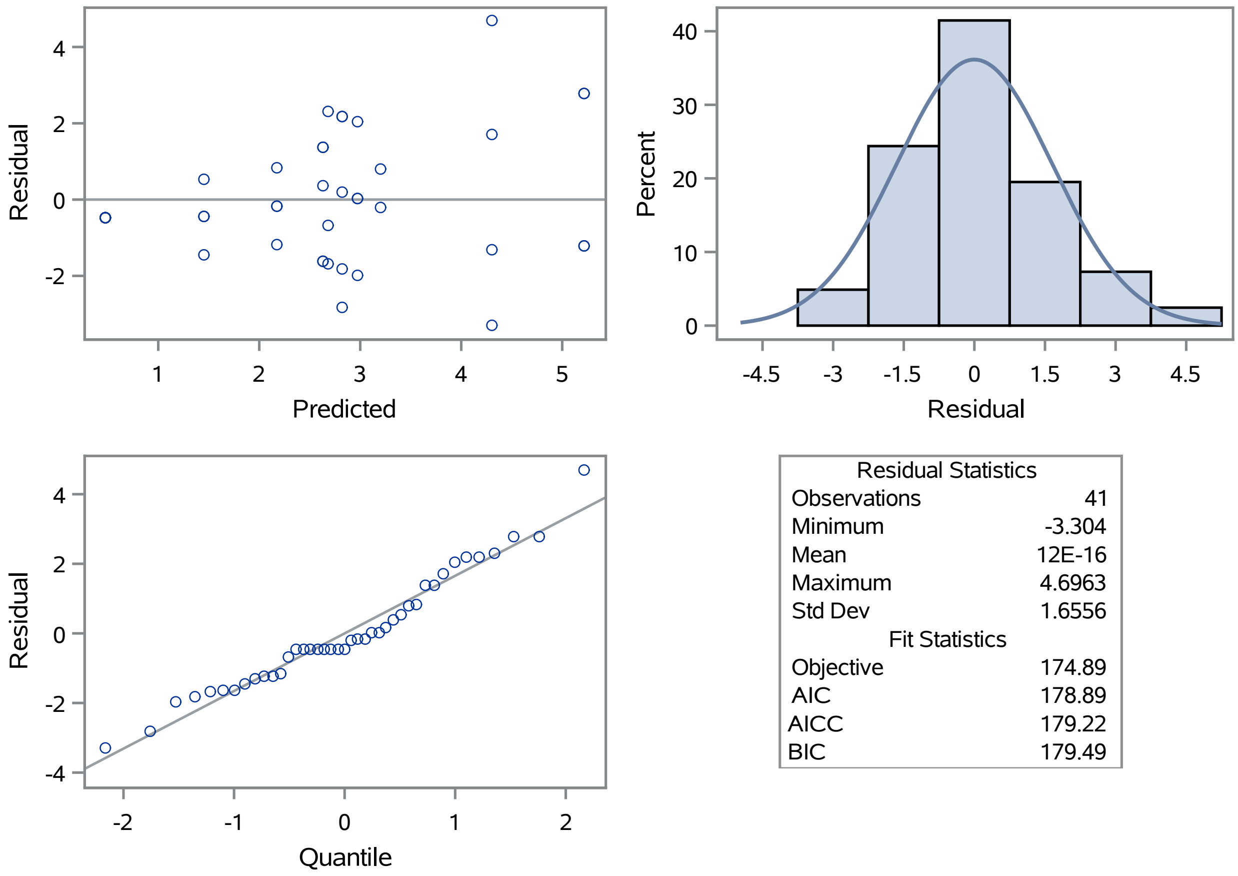

Residual Statistics	
Observations	41
Minimum	-3.304
Mean	12E-16
Maximum	4.6963
Std Dev	1.6556
**Fit Statistics**	
Objective	174.89
AIC	178.89
AICC	179.22
BIC	179.49

DistSoma=252

Model Information	
**Data Set**	WORK.TEMPDATASORTED
**Dependent Variable**	Interceptions
**Covariance Structure**	Variance Components
**Estimation Method**	REML
**Residual Variance Method**	Profile
**Fixed Effects SE Method**	Model-Based
**Degrees of Freedom Method**	Containment

Class Level Information		
**Class**	**Levels**	**Values**
**Treatment**	2	Meg TTR GFP MsTTR
**Culture**	10	1 2 3 4 5 6 7 8 9 10

Dimensions	
**Covariance Parameters**	2
**Columns in X**	3
**Columns in Z**	10
**Subjects**	1
**Max Obs per Subject**	41

Number of Observations	
**Number of Observations Read**	41
**Number of Observations Used**	41
**Number of Observations Not Used**	0

Iteration History			
**Iteration**	**Evaluations**	**-2 Res Log Like**	**Criterion**
**0**	1	184.82277667	
**1**	5	174.48254346	0.00009597
**2**	1	174.47786658	0.00000052
**3**	1	174.47784002	0.00000000

Convergence criteria met.

DistSoma=252

Covariance Parameter Estimates				
Cov Parm	Estimate	Alpha	Lower	Upper
Culture	2.7584	0.05	1.0701	16.8544
Residual	3.2570	0.05	2.0998	5.7278

Fit Statistics	
-2 Res Log Likelihood	174.5
AIC (Smaller is Better)	178.5
AICC (Smaller is Better)	178.8
BIC (Smaller is Better)	179.1

Solution for Fixed Effects									
Effect	Treatment	Estimate	Standard Error	DF	t Value	Pr > |t|	Alpha	Lower	Upper
Intercept		2.8565	0.9395	8	3.04	0.0161	0.05	0.6900	5.0229
Treatment	Meg TTR	-0.1503	1.2210	31	-0.12	0.9029	0.05	-2.6405	2.3400
Treatment	GFP MsTTR	0	.	.	.	.	.	.	.

Solution for Random Effects									
Effect	Culture	Estimate	Std Err Pred	DF	t Value	Pr > |t|	Alpha	Lower	Upper
Culture	1	0.4397	1.0510	31	0.42	0.6786	0.05	-1.7038	2.5832
Culture	2	1.2689	1.0746	31	1.18	0.2467	0.05	-0.9228	3.4607
Culture	3	-1.4334	1.0746	31	-1.33	0.1920	0.05	-3.6251	0.7584
Culture	4	-0.2752	1.0746	31	-0.26	0.7995	0.05	-2.4670	1.9165
Culture	5	-0.08593	0.9618	31	-0.09	0.9294	0.05	-2.0475	1.8756
Culture	6	-0.5453	0.9956	31	-0.55	0.5878	0.05	-2.5759	1.4853
Culture	7	2.7361	0.9956	31	2.75	0.0099	0.05	0.7055	4.7667
Culture	8	-2.2612	0.9371	31	-2.41	0.0219	0.05	-4.1725	-0.3499
Culture	9	0.1847	1.1245	31	0.16	0.8706	0.05	-2.1087	2.4781
Culture	10	-0.02839	1.0451	31	-0.03	0.9785	0.05	-2.1599	2.1031

Type 3 Tests of Fixed Effects				
Effect	Num DF	Den DF	F Value	Pr > F
Treatment	1	31	0.02	0.9029

DistSoma=252

Least Squares Means									
**Effect**	**Treatment**	**Estimate**	**Standard Error**	**DF**	**t Value**	**Pr > |t|**	**Alpha**	**Lower**	**Upper**
**Treatment**	Meg TTR	2.7062	0.7799	31	3.47	0.0016	0.05	1.1156	4.2968
**Treatment**	GFP MsTTR	2.8565	0.9395	31	3.04	0.0048	0.05	0.9404	4.7726

Differences of Least Squares Means												
**Effect**	**Treatment**	**Treatment**	**Estimate**	**Standard Error**	**DF**	**t Value**	**Pr > |t|**	**Adjustment**	**Adj P**	**Alpha**	**Lower**	**Upper**
**Treatment**	Meg TTR	GFP MsTTR	-0.1503	1.2210	31	-0.12	0.9029	Tukey-Kramer	0.9029	0.05	-2.6405	2.3400

Differences of Least Squares Means				
**Effect**	**Treatment**	**Treatment**	**Adj Lower**	**Adj Upper**
**Treatment**	Meg TTR	GFP MsTTR	-2.6405	2.3400

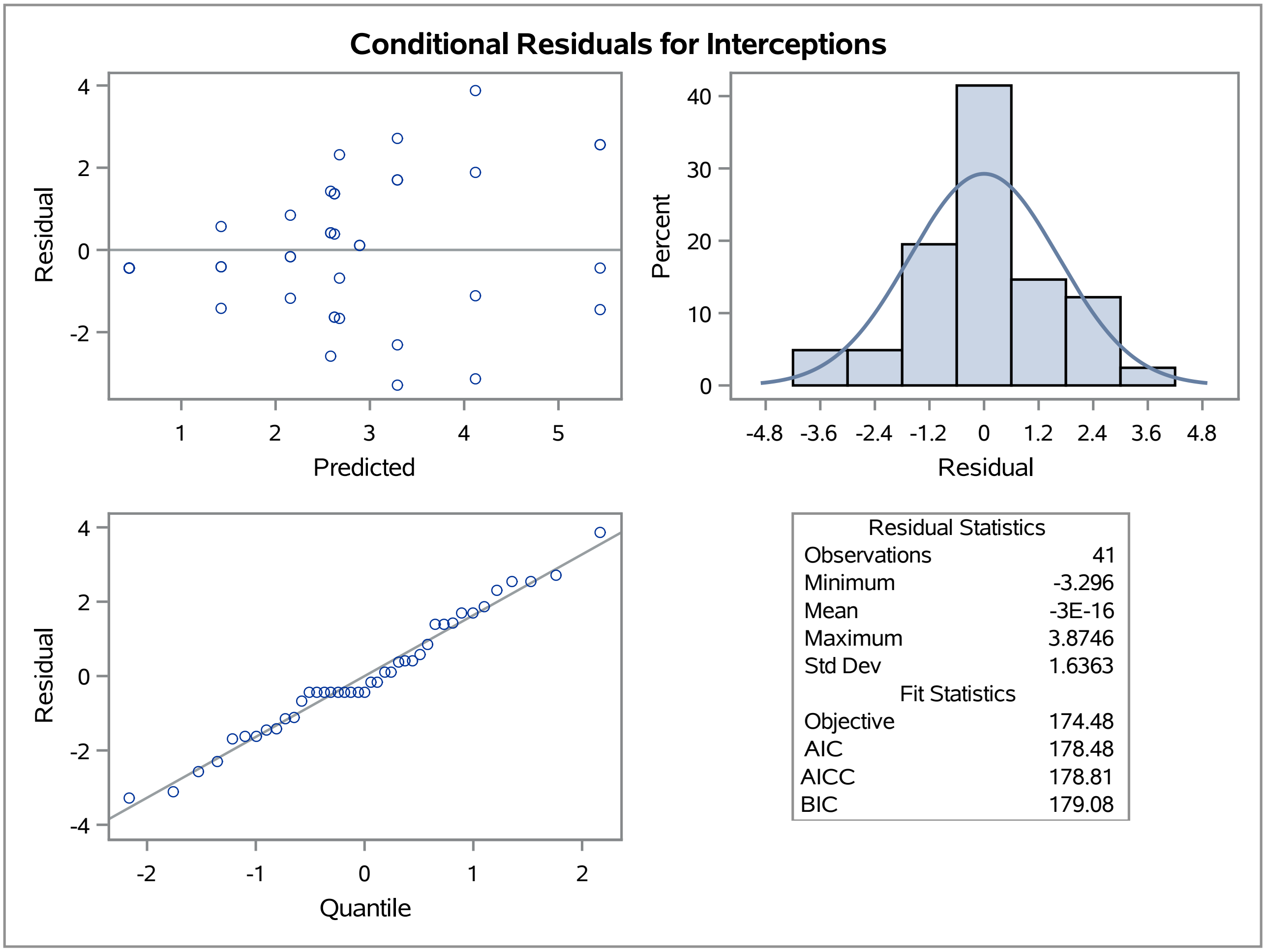

DistSoma=258

Model Information	
**Data Set**	WORK.TEMPDATASORTED
**Dependent Variable**	Interceptions
**Covariance Structure**	Variance Components
**Estimation Method**	REML
**Residual Variance Method**	Profile
**Fixed Effects SE Method**	Model-Based
**Degrees of Freedom Method**	Containment

Class Level Information		
**Class**	**Levels**	**Values**
**Treatment**	2	Meg TTR GFP MsTTR
**Culture**	10	1 2 3 4 5 6 7 8 9 10

Dimensions	
**Covariance Parameters**	2
**Columns in X**	3
**Columns in Z**	10
**Subjects**	1
**Max Obs per Subject**	41

Number of Observations	
**Number of Observations Read**	41
**Number of Observations Used**	41
**Number of Observations Not Used**	0

Iteration History			
**Iteration**	**Evaluations**	**-2 Res Log Like**	**Criterion**
**0**	1	188.04171833	
**1**	3	178.09836448	0.00111579
**2**	1	178.03171717	0.00005221
**3**	1	178.02884876	0.00000014
**4**	1	178.02884131	0.00000000

Convergence criteria met.

DistSoma=258

Covariance Parameter Estimates				
Cov Parm	Estimate	Alpha	Lower	Upper
Culture	2.9890	0.05	1.1545	18.5545
Residual	3.5747	0.05	2.3040	6.2891

Fit Statistics	
-2 Res Log Likelihood	178.0
AIC (Smaller is Better)	182.0
AICC (Smaller is Better)	182.4
BIC (Smaller is Better)	182.6

Solution for Fixed Effects									
Effect	Treatment	Estimate	Standard Error	DF	t Value	Pr > |t|	Alpha	Lower	Upper
Intercept		2.7629	0.9793	8	2.82	0.0225	0.05	0.5046	5.0213
Treatment	Meg TTR	-0.1264	1.2729	31	-0.10	0.9215	0.05	-2.7224	2.4696
Treatment	GFP MsTTR	0	.	.	.	.	.	.	.

Solution for Random Effects									
Effect	Culture	Estimate	Std Err Pred	DF	t Value	Pr > |t|	Alpha	Lower	Upper
Culture	1	0.02990	1.0961	31	0.03	0.9784	0.05	-2.2057	2.2655
Culture	2	1.7222	1.1209	31	1.54	0.1346	0.05	-0.5639	4.0082
Culture	3	-1.5496	1.1209	31	-1.38	0.1767	0.05	-3.8357	0.7364
Culture	4	-0.2024	1.1209	31	-0.18	0.8579	0.05	-2.4885	2.0836
Culture	5	-0.02948	1.0037	31	-0.03	0.9768	0.05	-2.0766	2.0176
Culture	6	-0.4900	1.0391	31	-0.47	0.6405	0.05	-2.6093	1.6293
Culture	7	2.7818	1.0391	31	2.68	0.0118	0.05	0.6625	4.9011
Culture	8	-2.1984	0.9779	31	-2.25	0.0318	0.05	-4.1929	-0.2038
Culture	9	-0.08544	1.1736	31	-0.07	0.9424	0.05	-2.4791	2.3082
Culture	10	0.02154	1.0908	31	0.02	0.9844	0.05	-2.2032	2.2463

Type 3 Tests of Fixed Effects				
Effect	Num DF	Den DF	F Value	Pr > F
Treatment	1	31	0.01	0.9215

DistSoma=258

Least Squares Means									
**Effect**	**Treatment**	**Estimate**	**Standard Error**	**DF**	**t Value**	**Pr > |t|**	**Alpha**	**Lower**	**Upper**
**Treatment**	Meg TTR	2.6365	0.8131	31	3.24	0.0028	0.05	0.9782	4.2948
**Treatment**	GFP MsTTR	2.7629	0.9793	31	2.82	0.0083	0.05	0.7656	4.7603

Differences of Least Squares Means												
**Effect**	**Treatment**	**Treatment**	**Estimate**	**Standard Error**	**DF**	**t Value**	**Pr > |t|**	**Adjustment**	**Adj P**	**Alpha**	**Lower**	**Upper**
**Treatment**	Meg TTR	GFP MsTTR	-0.1264	1.2729	31	-0.10	0.9215	Tukey-Kramer	0.9215	0.05	-2.7224	2.4696

Differences of Least Squares Means				
**Effect**	**Treatment**	**Treatment**	**Adj Lower**	**Adj Upper**
**Treatment**	Meg TTR	GFP MsTTR	-2.7224	2.4696

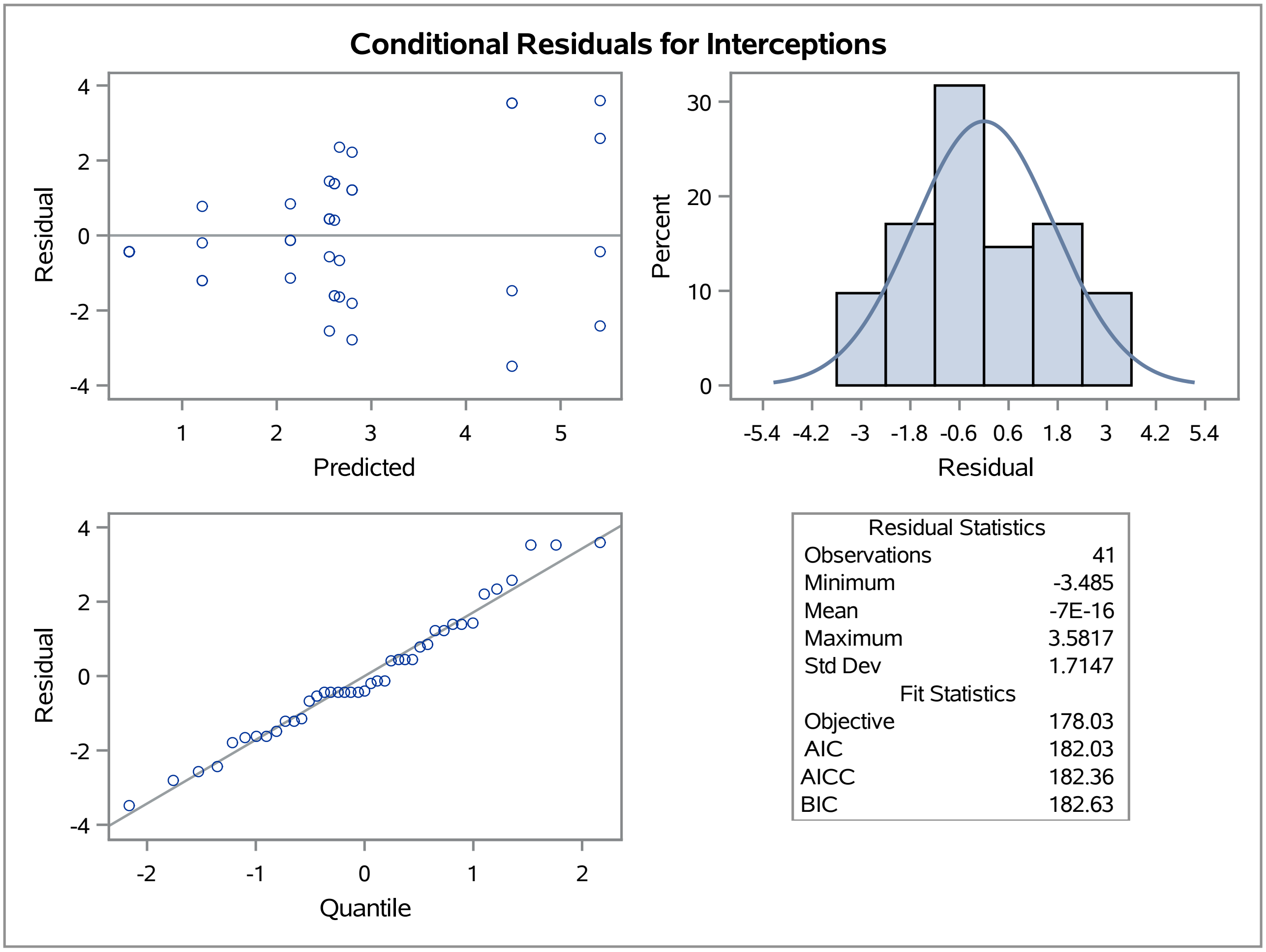

DistSoma=264

Model Information	
**Data Set**	WORK.TEMPDATASORTED
**Dependent Variable**	Interceptions
**Covariance Structure**	Variance Components
**Estimation Method**	REML
**Residual Variance Method**	Profile
**Fixed Effects SE Method**	Model-Based
**Degrees of Freedom Method**	Containment

Class Level Information		
**Class**	**Levels**	**Values**
**Treatment**	2	Meg TTR GFP MsTTR
**Culture**	10	1 2 3 4 5 6 7 8 9 10

Dimensions	
**Covariance Parameters**	2
**Columns in X**	3
**Columns in Z**	10
**Subjects**	1
**Max Obs per Subject**	41

Number of Observations	
**Number of Observations Read**	41
**Number of Observations Used**	41
**Number of Observations Not Used**	0

Iteration History			
**Iteration**	**Evaluations**	**-2 Res Log Like**	**Criterion**
**0**	1	189.46792245	
**1**	3	177.78208255	0.00054764
**2**	1	177.75032859	0.00001355
**3**	1	177.74959726	0.00000001

Convergence criteria met.

DistSoma=264

Covariance Parameter Estimates				
Cov Parm	Estimate	Alpha	Lower	Upper
Culture	3.4109	0.05	1.3480	19.5150
Residual	3.4568	0.05	2.2268	6.0870

Fit Statistics	
-2 Res Log Likelihood	177.7
AIC (Smaller is Better)	181.7
AICC (Smaller is Better)	182.1
BIC (Smaller is Better)	182.4

Solution for Fixed Effects									
Effect	Treatment	Estimate	Standard Error	DF	t Value	Pr > |t|	Alpha	Lower	Upper
Intercept		2.5258	1.0284	8	2.46	0.0396	0.05	0.1543	4.8973
Treatment	Meg TTR	0.06217	1.3358	31	0.05	0.9632	0.05	-2.6622	2.7865
Treatment	GFP MsTTR	0	.	.	.	.	.	.	.

Solution for Random Effects									
Effect	Culture	Estimate	Std Err Pred	DF	t Value	Pr > |t|	Alpha	Lower	Upper
Culture	1	0.06171	1.1428	31	0.05	0.9573	0.05	-2.2691	2.3925
Culture	2	1.7746	1.1674	31	1.52	0.1386	0.05	-0.6063	4.1555
Culture	3	-1.8157	1.1674	31	-1.56	0.1300	0.05	-4.1966	0.5651
Culture	4	-0.02057	1.1674	31	-0.02	0.9861	0.05	-2.4014	2.3603
Culture	5	0.01002	1.0379	31	0.01	0.9924	0.05	-2.1068	2.1268
Culture	6	-0.6686	1.0733	31	-0.62	0.5379	0.05	-2.8577	1.5205
Culture	7	3.1212	1.0733	31	2.91	0.0067	0.05	0.9321	5.3103
Culture	8	-2.2140	1.0123	31	-2.19	0.0364	0.05	-4.2785	-0.1494
Culture	9	-0.05837	1.2113	31	-0.05	0.9619	0.05	-2.5288	2.4120
Culture	10	-0.1903	1.1257	31	-0.17	0.8668	0.05	-2.4863	2.1057

Type 3 Tests of Fixed Effects				
Effect	Num DF	Den DF	F Value	Pr > F
Treatment	1	31	0.00	0.9632

DistSoma=264

Least Squares Means									
Effect	Treatment	Estimate	Standard Error	DF	t Value	Pr > |t|	Alpha	Lower	Upper
**Treatment**	Meg TTR	2.5880	0.8524	31	3.04	0.0048	0.05	0.8494	4.3265
**Treatment**	GFP MsTTR	2.5258	1.0284	31	2.46	0.0199	0.05	0.4283	4.6232

Differences of Least Squares Means												
Effect	Treatment	Treatment	Estimate	Standard Error	DF	t Value	Pr > |t|	Adjustment	Adj P	Alpha	Lower	Upper
**Treatment**	Meg TTR	GFP MsTTR	0.06217	1.3358	31	0.05	0.9632	Tukey-Kramer	0.9632	0.05	-2.6622	2.7865

Differences of Least Squares Means				
Effect	Treatment	Treatment	Adj Lower	Adj Upper
**Treatment**	Meg TTR	GFP MsTTR	-2.6621	2.7865

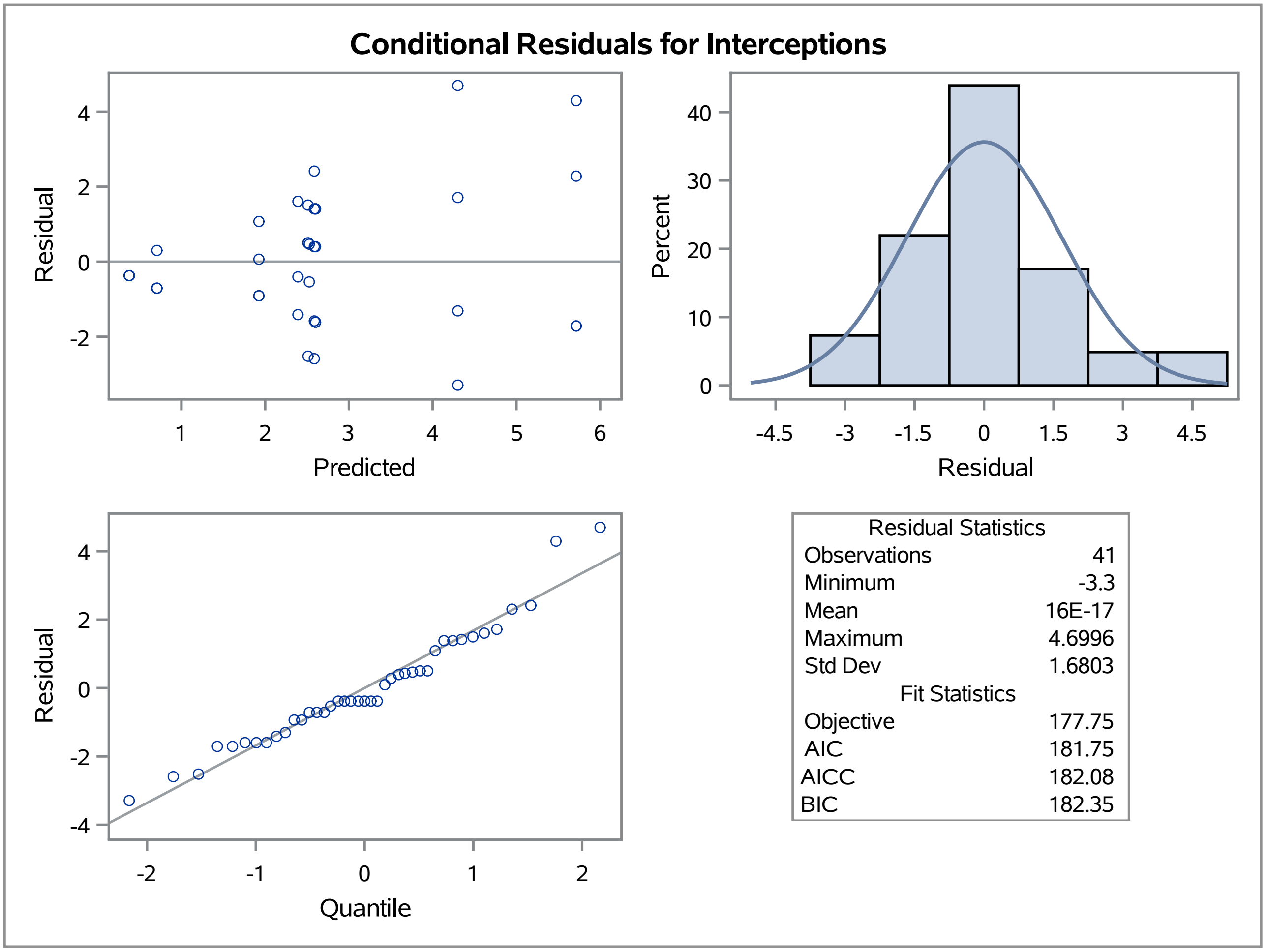

DistSoma=270

Model Information	
**Data Set**	WORK.TEMPDATASORTED
**Dependent Variable**	Interceptions
**Covariance Structure**	Variance Components
**Estimation Method**	REML
**Residual Variance Method**	Profile
**Fixed Effects SE Method**	Model-Based
**Degrees of Freedom Method**	Containment

Class Level Information		
**Class**	**Levels**	**Values**
**Treatment**	2	Meg TTR GFP MsTTR
**Culture**	10	1 2 3 4 5 6 7 8 9 10

Dimensions	
**Covariance Parameters**	2
**Columns in X**	3
**Columns in Z**	10
**Subjects**	1
**Max Obs per Subject**	41

Number of Observations	
**Number of Observations Read**	41
**Number of Observations Used**	41
**Number of Observations Not Used**	0

Iteration History			
**Iteration**	**Evaluations**	**-2 Res Log Like**	**Criterion**
**0**	1	183.36746188	
**1**	3	174.54350779	0.00044537
**2**	1	174.51866681	0.00000889
**3**	1	174.51820343	0.00000000

Convergence criteria met.

DistSoma=270

Covariance Parameter Estimates				
**Cov Parm**	**Estimate**	**Alpha**	**Lower**	**Upper**
**Culture**	2.5108	0.05	0.9519	16.6977
**Residual**	3.3177	0.05	2.1382	5.8376

Fit Statistics	
**-2 Res Log Likelihood**	174.5
**AIC (Smaller is Better)**	178.5
**AICC (Smaller is Better)**	178.9
**BIC (Smaller is Better)**	179.1

Solution for Fixed Effects									
**Effect**	**Treatment**	**Estimate**	**Standard Error**	**DF**	**t Value**	**Pr > |t|**	**Alpha**	**Lower**	**Upper**
**Intercept**		2.4448	0.9079	8	2.69	0.0274	0.05	0.3511	4.5384
**Treatment**	Meg TTR	-0.1323	1.1805	31	-0.11	0.9115	0.05	-2.5400	2.2753
**Treatment**	GFP MsTTR	0	.	.	.	.	.	.	.

Solution for Random Effects									
**Effect**	**Culture**	**Estimate**	**Std Err Pred**	**DF**	**t Value**	**Pr > |t|**	**Alpha**	**Lower**	**Upper**
**Culture**	1	0.4392	1.0201	31	0.43	0.6698	0.05	-1.6412	2.5196
**Culture**	2	1.1691	1.0436	31	1.12	0.2712	0.05	-0.9595	3.2976
**Culture**	3	-1.6498	1.0436	31	-1.58	0.1241	0.05	-3.7783	0.4788
**Culture**	4	0.04153	1.0436	31	0.04	0.9685	0.05	-2.0870	2.1701
**Culture**	5	0.06926	0.9386	31	0.07	0.9417	0.05	-1.8451	1.9836
**Culture**	6	-0.4228	0.9722	31	-0.43	0.6667	0.05	-2.4056	1.5600
**Culture**	7	2.5840	0.9722	31	2.66	0.0123	0.05	0.6012	4.5667
**Culture**	8	-1.8951	0.9141	31	-2.07	0.0465	0.05	-3.7593	-0.03085
**Culture**	9	0.1129	1.0979	31	0.10	0.9187	0.05	-2.1262	2.3521
**Culture**	10	-0.4483	1.0208	31	-0.44	0.6636	0.05	-2.5303	1.6337

Type 3 Tests of Fixed Effects				
**Effect**	**Num DF**	**Den DF**	**F Value**	**Pr > F**
**Treatment**	1	31	0.01	0.9115

DistSoma=270

Least Squares Means									
**Effect**	**Treatment**	**Estimate**	**Standard Error**	**DF**	**t Value**	**Pr > |t|**	**Alpha**	**Lower**	**Upper**
**Treatment**	Meg TTR	2.3124	0.7545	31	3.06	0.0045	0.05	0.7736	3.8513
**Treatment**	GFP MsTTR	2.4448	0.9079	31	2.69	0.0113	0.05	0.5931	4.2964

Differences of Least Squares Means												
**Effect**	**Treatment**	**Treatment**	**Estimate**	**Standard Error**	**DF**	**t Value**	**Pr > |t|**	**Adjustment**	**Adj P**	**Alpha**	**Lower**	**Upper**
**Treatment**	Meg TTR	GFP MsTTR	-0.1323	1.1805	31	-0.11	0.9115	Tukey-Kramer	0.9115	0.05	-2.5400	2.2753

Differences of Least Squares Means				
**Effect**	**Treatment**	**Treatment**	**Adj Lower**	**Adj Upper**
**Treatment**	Meg TTR	GFP MsTTR	-2.5399	2.2753

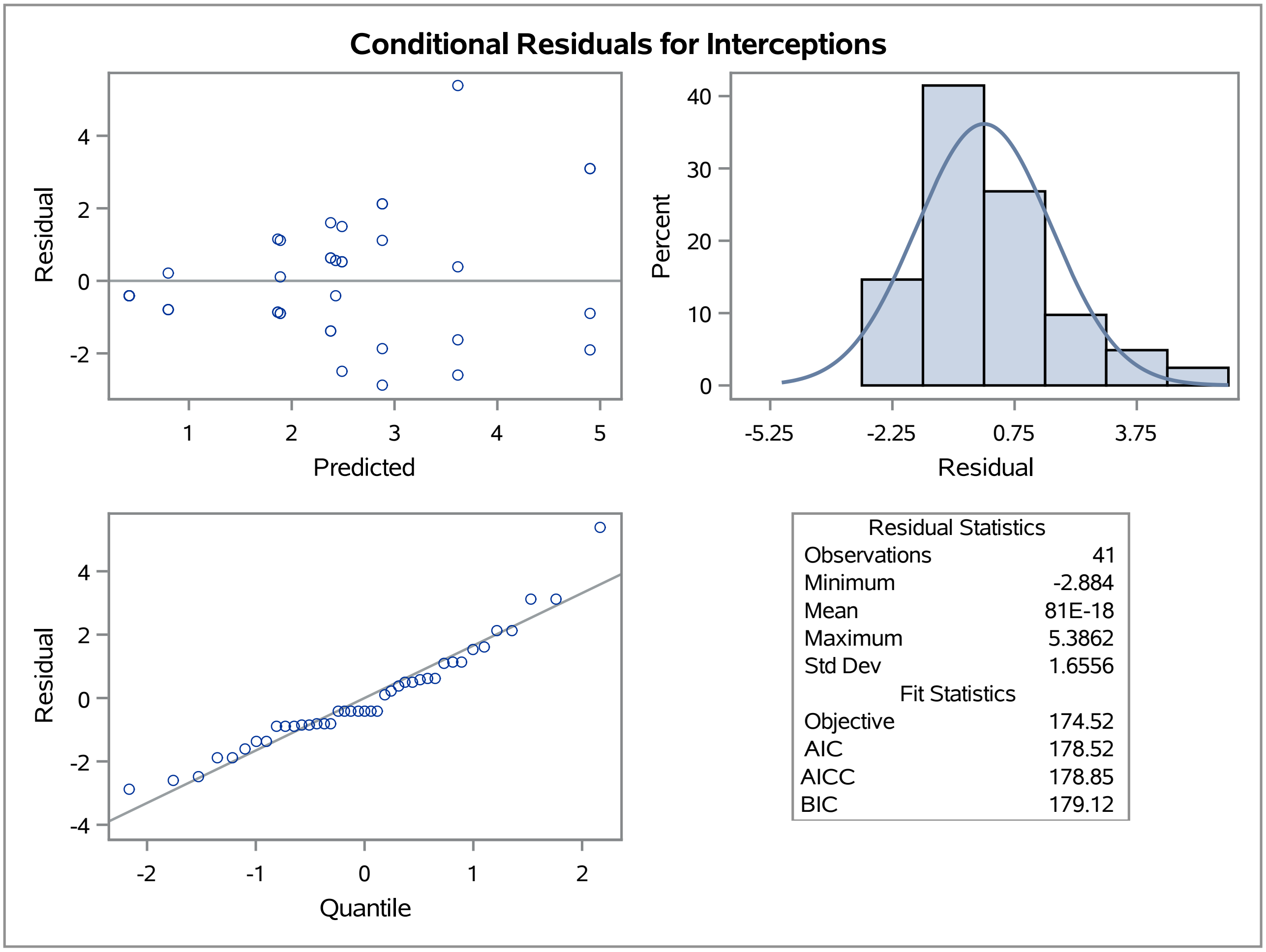

DistSoma=276

Model Information	
**Data Set**	WORK.TEMPDATASORTED
**Dependent Variable**	Interceptions
**Covariance Structure**	Variance Components
**Estimation Method**	REML
**Residual Variance Method**	Profile
**Fixed Effects SE Method**	Model-Based
**Degrees of Freedom Method**	Containment

Class Level Information		
**Class**	**Levels**	**Values**
**Treatment**	2	Meg TTR GFP MsTTR
**Culture**	10	1 2 3 4 5 6 7 8 9 10

Dimensions	
**Covariance Parameters**	2
**Columns in X**	3
**Columns in Z**	10
**Subjects**	1
**Max Obs per Subject**	41

Number of Observations	
**Number of Observations Read**	41
**Number of Observations Used**	41
**Number of Observations Not Used**	0

Iteration History			
**Iteration**	**Evaluations**	**-2 Res Log Like**	**Criterion**
**0**	1	182.33765756	
**1**	3	175.57992729	0.00034776
**2**	1	175.56047701	0.00000559
**3**	1	175.56018350	0.00000000

Convergence criteria met.

DistSoma=276

Covariance Parameter Estimates				
**Cov Parm**	**Estimate**	**Alpha**	**Lower**	**Upper**
**Culture**	2.1126	0.05	0.7653	16.7994
**Residual**	3.5276	0.05	2.2745	6.2022

Fit Statistics	
**-2 Res Log Likelihood**	175.6
**AIC (Smaller is Better)**	179.6
**AICC (Smaller is Better)**	179.9
**BIC (Smaller is Better)**	180.2

Solution for Fixed Effects									
**Effect**	**Treatment**	**Estimate**	**Standard Error**	**DF**	**t Value**	**Pr > |t|**	**Alpha**	**Lower**	**Upper**
**Intercept**		2.4031	0.8585	8	2.80	0.0232	0.05	0.4233	4.3829
**Treatment**	Meg TTR	-0.2060	1.1174	31	-0.18	0.8549	0.05	-2.4849	2.0729
**Treatment**	GFP MsTTR	0	.	.	.	.	.	.	.

Solution for Random Effects									
**Effect**	**Culture**	**Estimate**	**Std Err Pred**	**DF**	**t Value**	**Pr > |t|**	**Alpha**	**Lower**	**Upper**
**Culture**	1	0.1476	0.9711	31	0.15	0.8802	0.05	-1.8330	2.1283
**Culture**	2	1.3030	0.9945	31	1.31	0.1998	0.05	-0.7253	3.3313
**Culture**	3	-1.5190	0.9945	31	-1.53	0.1368	0.05	-3.5473	0.5093
**Culture**	4	0.06838	0.9945	31	0.07	0.9456	0.05	-1.9599	2.0967
**Culture**	5	0.1521	0.9035	31	0.17	0.8674	0.05	-1.6906	1.9948
**Culture**	6	-0.1390	0.9363	31	-0.15	0.8829	0.05	-2.0487	1.7706
**Culture**	7	2.1538	0.9363	31	2.30	0.0283	0.05	0.2441	4.0635
**Culture**	8	-1.7187	0.8792	31	-1.95	0.0597	0.05	-3.5118	0.07433
**Culture**	9	-0.1074	1.0551	31	-0.10	0.9196	0.05	-2.2592	2.0444
**Culture**	10	-0.3407	0.9831	31	-0.35	0.7312	0.05	-2.3458	1.6643

Type 3 Tests of Fixed Effects				
**Effect**	**Num DF**	**Den DF**	**F Value**	**Pr > F**
**Treatment**	1	31	0.03	0.8549

DistSoma=276

Least Squares Means									
**Effect**	**Treatment**	**Estimate**	**Standard Error**	**DF**	**t Value**	**Pr > |t|**	**Alpha**	**Lower**	**Upper**
**Treatment**	Meg TTR	2.1971	0.7152	31	3.07	0.0044	0.05	0.7385	3.6557
**Treatment**	GFP MsTTR	2.4031	0.8585	31	2.80	0.0087	0.05	0.6521	4.1541

Differences of Least Squares Means												
**Effect**	**Treatment**	**Treatment**	**Estimate**	**Standard Error**	**DF**	**t Value**	**Pr > |t|**	**Adjustment**	**Adj P**	**Alpha**	**Lower**	**Upper**
**Treatment**	Meg TTR	GFP MsTTR	-0.2060	1.1174	31	-0.18	0.8549	Tukey-Kramer	0.8549	0.05	-2.4849	2.0729

Differences of Least Squares Means				
**Effect**	**Treatment**	**Treatment**	**Adj Lower**	**Adj Upper**
**Treatment**	Meg TTR	GFP MsTTR	-2.4849	2.0729

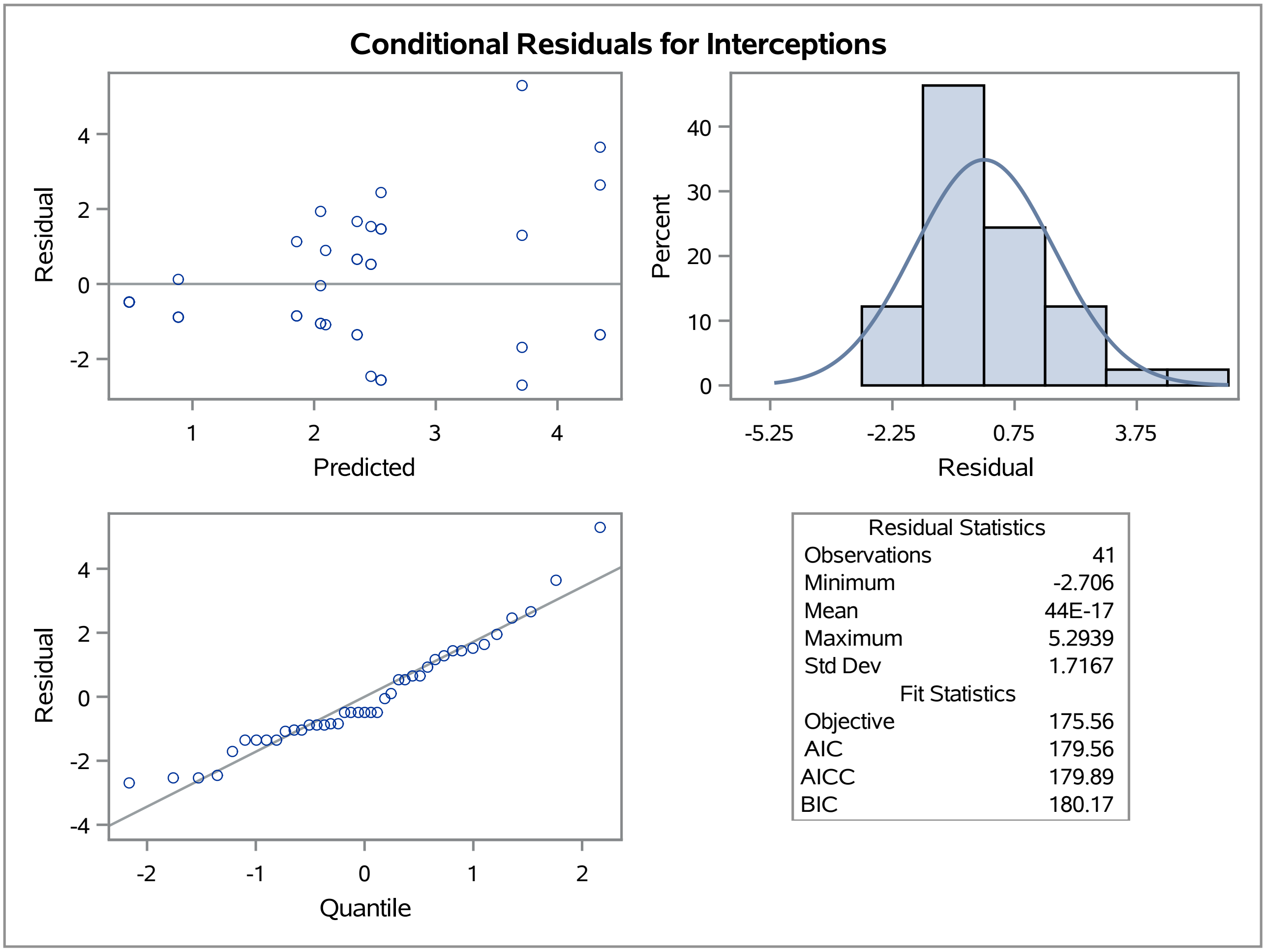

DistSoma=282

Model Information	
**Data Set**	WORK.TEMPDATASORTED
**Dependent Variable**	Interceptions
**Covariance Structure**	Variance Components
**Estimation Method**	REML
**Residual Variance Method**	Profile
**Fixed Effects SE Method**	Model-Based
**Degrees of Freedom Method**	Containment

Class Level Information		
**Class**	**Levels**	**Values**
**Treatment**	2	Meg TTR GFP MsTTR
**Culture**	10	1 2 3 4 5 6 7 8 9 10

Dimensions	
**Covariance Parameters**	2
**Columns in X**	3
**Columns in Z**	10
**Subjects**	1
**Max Obs per Subject**	41

Number of Observations	
**Number of Observations Read**	41
**Number of Observations Used**	41
**Number of Observations Not Used**	0

Iteration History			
**Iteration**	**Evaluations**	**-2 Res Log Like**	**Criterion**
**0**	1	183.83249036	
**1**	3	177.23422142	0.00034309
**2**	1	177.21472573	0.00000552
**3**	1	177.21443115	0.00000000

Convergence criteria met.

DistSoma=282

Covariance Parameter Estimates				
**Cov Parm**	**Estimate**	**Alpha**	**Lower**	**Upper**
**Culture**	2.1624	0.05	0.7803	17.4736
**Residual**	3.6921	0.05	2.3810	6.4899

Fit Statistics	
**-2 Res Log Likelihood**	177.2
**AIC (Smaller is Better)**	181.2
**AICC (Smaller is Better)**	181.5
**BIC (Smaller is Better)**	181.8

Solution for Fixed Effects									
**Effect**	**Treatment**	**Estimate**	**Standard Error**	**DF**	**t Value**	**Pr > |t|**	**Alpha**	**Lower**	**Upper**
**Intercept**		2.2984	0.8714	8	2.64	0.0298	0.05	0.2891	4.3078
**Treatment**	Meg TTR	-0.03461	1.1342	31	-0.03	0.9759	0.05	-2.3478	2.2786
**Treatment**	GFP MsTTR	0	.	.	.	.	.	.	.

Solution for Random Effects									
**Effect**	**Culture**	**Estimate**	**Std Err Pred**	**DF**	**t Value**	**Pr > |t|**	**Alpha**	**Lower**	**Upper**
**Culture**	1	-0.07338	0.9861	31	-0.07	0.9412	0.05	-2.0845	1.9378
**Culture**	2	1.5430	1.0099	31	1.53	0.1367	0.05	-0.5167	3.6026
**Culture**	3	-1.4356	1.0099	31	-1.42	0.1651	0.05	-3.4953	0.6240
**Culture**	4	-0.03394	1.0099	31	-0.03	0.9734	0.05	-2.0936	2.0257
**Culture**	5	-0.04758	0.9184	31	-0.05	0.9590	0.05	-1.9206	1.8254
**Culture**	6	-0.1849	0.9517	31	-0.19	0.8472	0.05	-2.1260	1.7562
**Culture**	7	2.0928	0.9517	31	2.20	0.0355	0.05	0.1517	4.0339
**Culture**	8	-1.7623	0.8936	31	-1.97	0.0576	0.05	-3.5848	0.06013
**Culture**	9	-0.1423	1.0720	31	-0.13	0.8952	0.05	-2.3288	2.0441
**Culture**	10	0.04430	0.9992	31	0.04	0.9649	0.05	-1.9936	2.0822

Type 3 Tests of Fixed Effects				
**Effect**	**Num DF**	**Den DF**	**F Value**	**Pr > F**
**Treatment**	1	31	0.00	0.9759

DistSoma=282

Least Squares Means

Effect	Treatment	Estimate	Standard Error	DF	t Value	Pr > |t|	Alpha	Lower	Upper
**Treatment**	Meg TTR	2.2638	0.7260	31	3.12	0.0039	0.05	0.7831	3.7446
**Treatment**	GFP MsTTR	2.2984	0.8714	31	2.64	0.0129	0.05	0.5213	4.0756

Differences of Least Squares Means

Effect	Treatment	Treatment	Estimate	Standard Error	DF	t Value	Pr > |t|	Adjustment	Adj P	Alpha	Lower	Upper
**Treatment**	Meg TTR	GFP MsTTR	-0.03461	1.1342	31	-0.03	0.9759	Tukey-Kramer	0.9759	0.05	-2.3478	2.2786

Differences of Least Squares Means

Effect	Treatment	Treatment	Adj Lower	Adj Upper
**Treatment**	Meg TTR	GFP MsTTR	-2.3478	2.2786

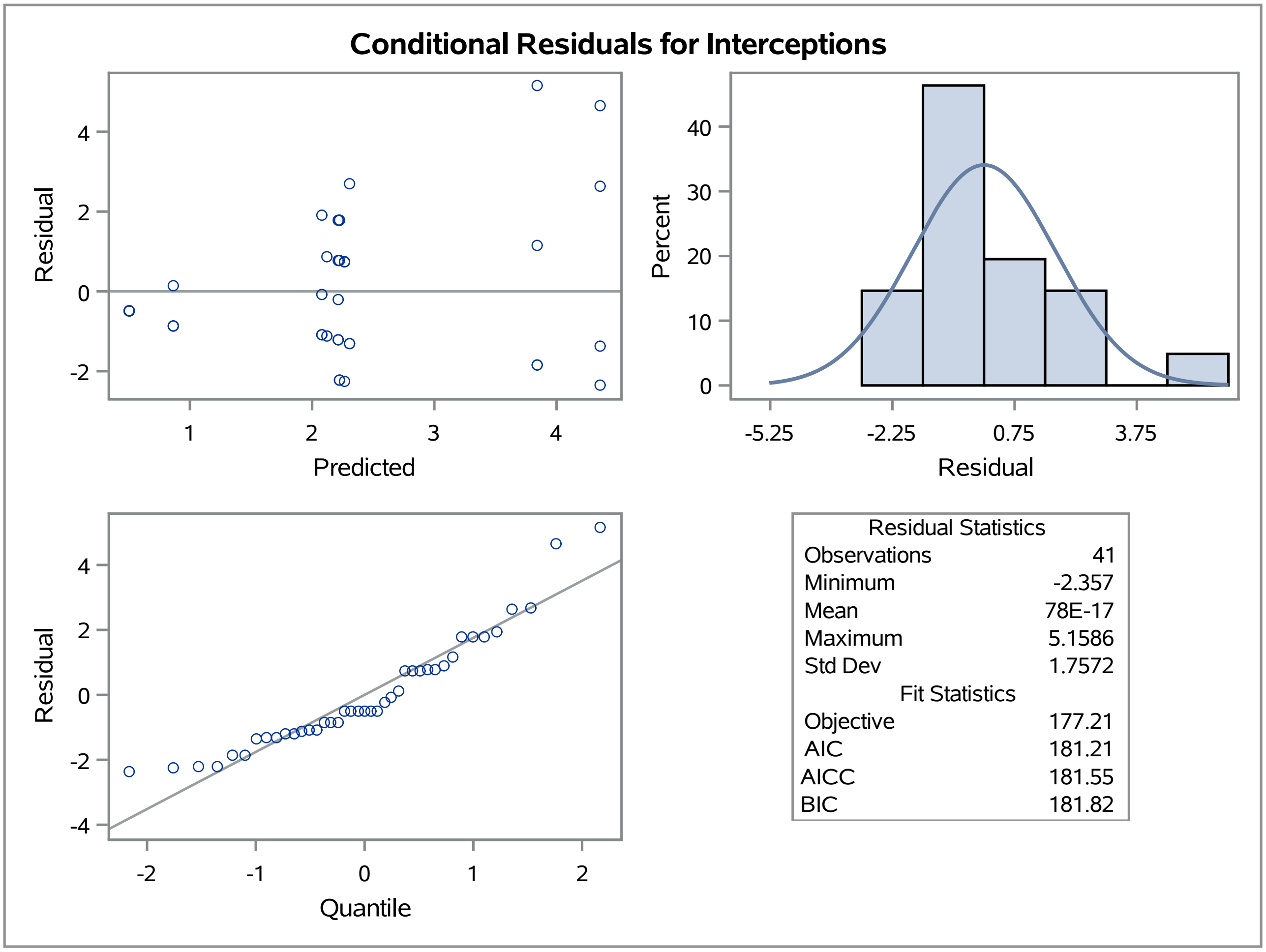

DistSoma=288

Model Information	
**Data Set**	WORK.TEMPDATASORTED
**Dependent Variable**	Interceptions
**Covariance Structure**	Variance Components
**Estimation Method**	REML
**Residual Variance Method**	Profile
**Fixed Effects SE Method**	Model-Based
**Degrees of Freedom Method**	Containment

Class Level Information		
**Class**	**Levels**	**Values**
**Treatment**	2	Meg TTR GFP MsTTR
**Culture**	10	1 2 3 4 5 6 7 8 9 10

Dimensions	
**Covariance Parameters**	2
**Columns in X**	3
**Columns in Z**	10
**Subjects**	1
**Max Obs per Subject**	41

Number of Observations	
**Number of Observations Read**	41
**Number of Observations Used**	41
**Number of Observations Not Used**	0

Iteration History			
**Iteration**	**Evaluations**	**-2 Res Log Like**	**Criterion**
**0**	1	182.34994841	
**1**	3	174.87042592	0.00021311
**2**	1	174.85875038	0.00000215
**3**	1	174.85863891	0.00000000

Convergence criteria met.

DistSoma=288

Covariance Parameter Estimates				
Cov Parm	Estimate	Alpha	Lower	Upper
Culture	2.2509	0.05	0.8304	16.6300
Residual	3.4171	0.05	2.2025	6.0114

Fit Statistics	
-2 Res Log Likelihood	174.9
AIC (Smaller is Better)	178.9
AICC (Smaller is Better)	179.2
BIC (Smaller is Better)	179.5

Solution for Fixed Effects									
Effect	Treatment	Estimate	Standard Error	DF	t Value	Pr > |t|	Alpha	Lower	Upper
Intercept		2.2370	0.8747	8	2.56	0.0338	0.05	0.2198	4.2541
Treatment	Meg TTR	-0.07408	1.1380	31	-0.07	0.9485	0.05	-2.3951	2.2469
Treatment	GFP MsTTR	0	.	.	.	.	.	.	.

Solution for Random Effects									
Effect	Culture	Estimate	Std Err Pred	DF	t Value	Pr > |t|	Alpha	Lower	Upper
Culture	1	-0.02835	0.9872	31	-0.03	0.9773	0.05	-2.0417	1.9850
Culture	2	1.6404	1.0106	31	1.62	0.1147	0.05	-0.4207	3.7016
Culture	3	-1.4403	1.0106	31	-1.43	0.1641	0.05	-3.5014	0.6208
Culture	4	-0.1718	1.0106	31	-0.17	0.8661	0.05	-2.2329	1.8894
Culture	5	0.02847	0.9144	31	0.03	0.9754	0.05	-1.8364	1.8934
Culture	6	-0.1181	0.9475	31	-0.12	0.9016	0.05	-2.0505	1.8143
Culture	7	2.2378	0.9475	31	2.36	0.0246	0.05	0.3054	4.1702
Culture	8	-1.7261	0.8900	31	-1.94	0.0616	0.05	-3.5412	0.08898
Culture	9	-0.09259	1.0689	31	-0.09	0.9315	0.05	-2.2726	2.0875
Culture	10	-0.3295	0.9950	31	-0.33	0.7428	0.05	-2.3587	1.6998

Type 3 Tests of Fixed Effects				
Effect	Num DF	Den DF	F Value	Pr > F
Treatment	1	31	0.00	0.9485

DistSoma=288

Least Squares Means									
**Effect**	**Treatment**	**Estimate**	**Standard Error**	**DF**	**t Value**	**Pr > |t|**	**Alpha**	**Lower**	**Upper**
**Treatment**	Meg TTR	2.1629	0.7280	31	2.97	0.0057	0.05	0.6782	3.6476
**Treatment**	GFP MsTTR	2.2370	0.8747	31	2.56	0.0157	0.05	0.4529	4.0210

Differences of Least Squares Means												
**Effect**	**Treatment**	**Treatment**	**Estimate**	**Standard Error**	**DF**	**t Value**	**Pr > |t|**	**Adjustment**	**Adj P**	**Alpha**	**Lower**	**Upper**
**Treatment**	Meg TTR	GFP MsTTR	-0.07408	1.1380	31	-0.07	0.9485	Tukey-Kramer	0.9485	0.05	-2.3951	2.2469

Differences of Least Squares Means				
**Effect**	**Treatment**	**Treatment**	**Adj Lower**	**Adj Upper**
**Treatment**	Meg TTR	GFP MsTTR	-2.3951	2.2469

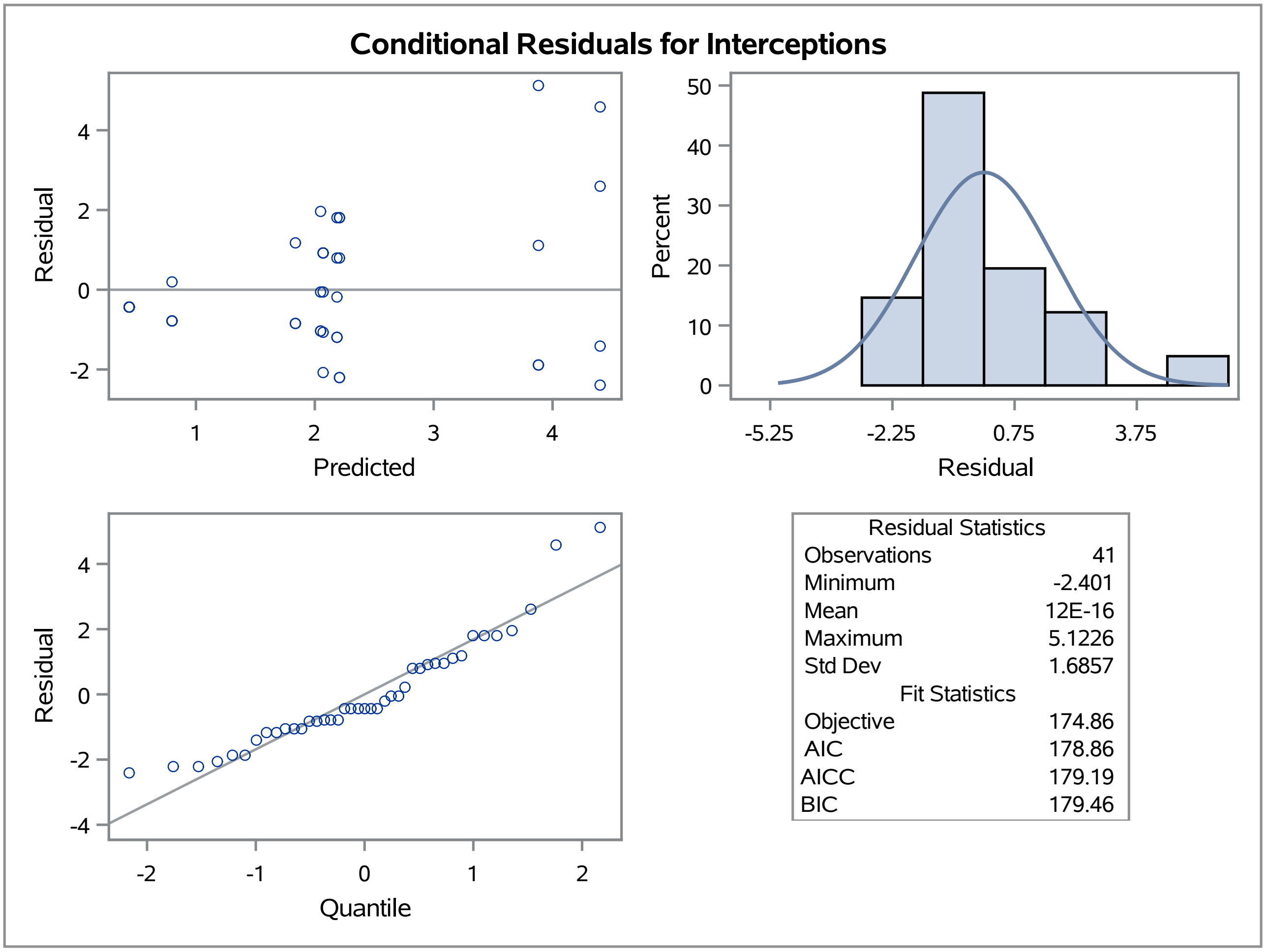

DistSoma=294

Model Information	
**Data Set**	WORK.TEMPDATASORTED
**Dependent Variable**	Interceptions
**Covariance Structure**	Variance Components
**Estimation Method**	REML
**Residual Variance Method**	Profile
**Fixed Effects SE Method**	Model-Based
**Degrees of Freedom Method**	Containment

Class Level Information		
**Class**	**Levels**	**Values**
**Treatment**	2	Meg TTR GFP MsTTR
**Culture**	10	1 2 3 4 5 6 7 8 9 10

Dimensions	
**Covariance Parameters**	2
**Columns in X**	3
**Columns in Z**	10
**Subjects**	1
**Max Obs per Subject**	41

Number of Observations	
**Number of Observations Read**	41
**Number of Observations Used**	41
**Number of Observations Not Used**	0

Iteration History			
**Iteration**	**Evaluations**	**-2 Res Log Like**	**Criterion**
**0**	1	185.34385939	
**1**	3	177.35647676	0.00012187
**2**	1	177.34972287	0.00000074
**3**	1	177.34968370	0.00000000

Convergence criteria met.

DistSoma=294

Covariance Parameter Estimates				
**Cov Parm**	**Estimate**	**Alpha**	**Lower**	**Upper**
**Culture**	2.5399	0.05	0.9471	17.9949
**Residual**	3.6065	0.05	2.3238	6.3486

Fit Statistics	
**-2 Res Log Likelihood**	177.3
**AIC (Smaller is Better)**	181.3
**AICC (Smaller is Better)**	181.7
**BIC (Smaller is Better)**	182.0

Solution for Fixed Effects									
**Effect**	**Treatment**	**Estimate**	**Standard Error**	**DF**	**t Value**	**Pr > |t|**	**Alpha**	**Lower**	**Upper**
**Intercept**		2.0520	0.9212	8	2.23	0.0565	0.05	-0.07229	4.1764
**Treatment**	Meg TTR	0.1462	1.1982	31	0.12	0.9037	0.05	-2.2975	2.5899
**Treatment**	GFP MsTTR	0	.	.	.	.	.	.	.

Solution for Random Effects									
**Effect**	**Culture**	**Estimate**	**Std Err Pred**	**DF**	**t Value**	**Pr > |t|**	**Alpha**	**Lower**	**Upper**
**Culture**	1	0.1152	1.0376	31	0.11	0.9123	0.05	-2.0009	2.2313
**Culture**	2	1.6221	1.0619	31	1.53	0.1368	0.05	-0.5437	3.7879
**Culture**	3	-1.3299	1.0619	31	-1.25	0.2198	0.05	-3.4957	0.8358
**Culture**	4	-0.4074	1.0619	31	-0.38	0.7038	0.05	-2.5732	1.7584
**Culture**	5	-0.1544	0.9580	31	-0.16	0.8730	0.05	-2.1083	1.7995
**Culture**	6	-0.1463	0.9925	31	-0.15	0.8838	0.05	-2.1706	1.8780
**Culture**	7	2.6212	0.9925	31	2.64	0.0128	0.05	0.5969	4.6455
**Culture**	8	-1.7776	0.9327	31	-1.91	0.0660	0.05	-3.6798	0.1246
**Culture**	9	-0.4083	1.1204	31	-0.36	0.7180	0.05	-2.6934	1.8767
**Culture**	10	-0.1346	1.0423	31	-0.13	0.8981	0.05	-2.2603	1.9912

Type 3 Tests of Fixed Effects				
**Effect**	**Num DF**	**Den DF**	**F Value**	**Pr > F**
**Treatment**	1	31	0.01	0.9037

DistSoma=294

Least Squares Means									
Effect	Treatment	Estimate	Standard Error	DF	t Value	Pr > |t|	Alpha	Lower	Upper
Treatment	Meg TTR	2.1983	0.7661	31	2.87	0.0073	0.05	0.6357	3.7608
Treatment	GFP MsTTR	2.0520	0.9212	31	2.23	0.0333	0.05	0.1732	3.9309

Differences of Least Squares Means												
Effect	Treatment	Treatment	Estimate	Standard Error	DF	t Value	Pr > |t|	Adjustment	Adj P	Alpha	Lower	Upper
Treatment	Meg TTR	GFP MsTTR	0.1462	1.1982	31	0.12	0.9037	Tukey-Kramer	0.9037	0.05	-2.2975	2.5899

Differences of Least Squares Means				
Effect	Treatment	Treatment	Adj Lower	Adj Upper
Treatment	Meg TTR	GFP MsTTR	-2.2975	2.5899

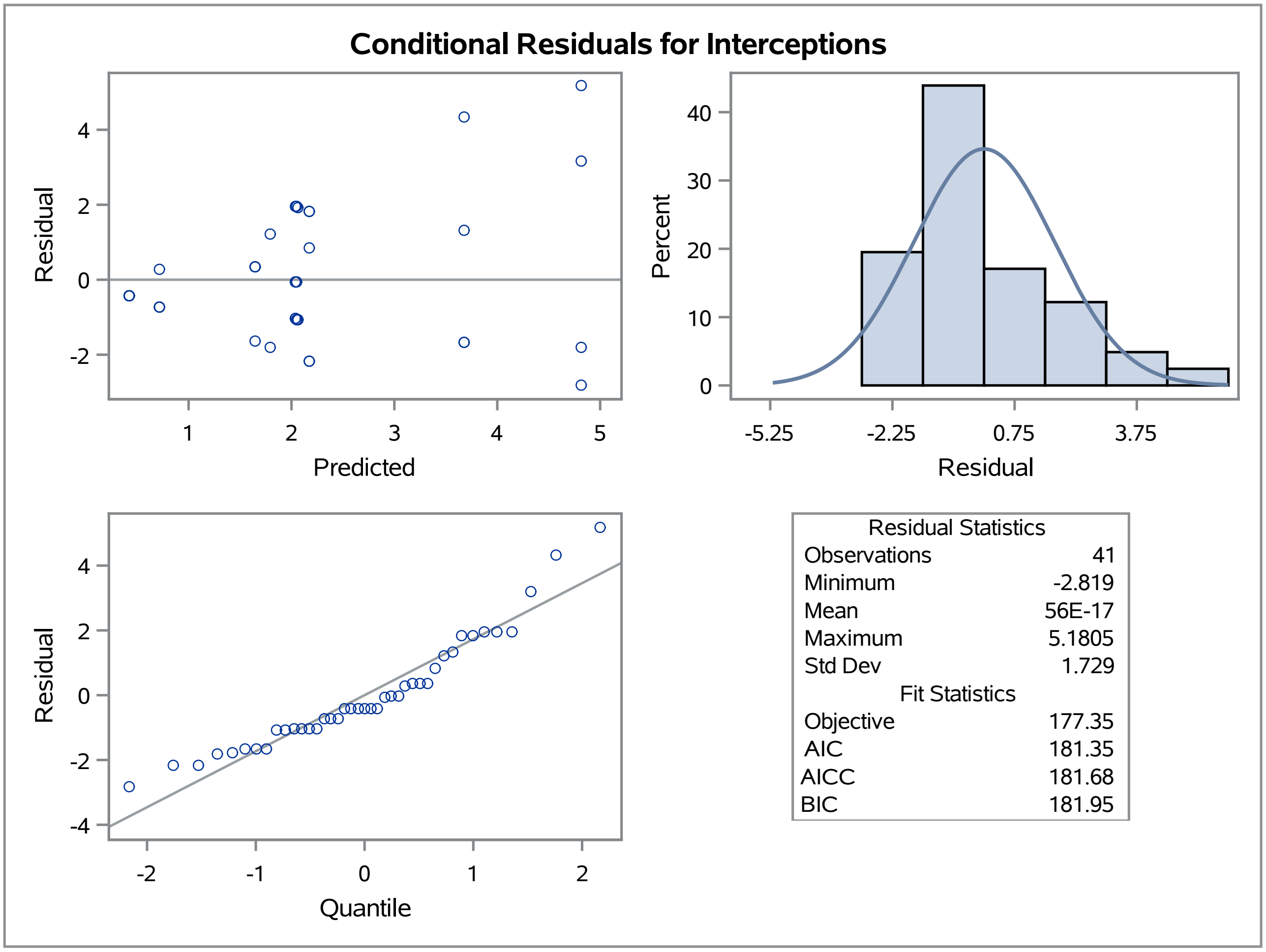

DistSoma=300

Model Information	
**Data Set**	WORK.TEMPDATASORTED
**Dependent Variable**	Interceptions
**Covariance Structure**	Variance Components
**Estimation Method**	REML
**Residual Variance Method**	Profile
**Fixed Effects SE Method**	Model-Based
**Degrees of Freedom Method**	Containment

Class Level Information		
**Class**	**Levels**	**Values**
**Treatment**	2	Meg TTR GFP MsTTR
**Culture**	10	1 2 3 4 5 6 7 8 9 10

Dimensions	
**Covariance Parameters**	2
**Columns in X**	3
**Columns in Z**	10
**Subjects**	1
**Max Obs per Subject**	41

Number of Observations	
**Number of Observations Read**	41
**Number of Observations Used**	41
**Number of Observations Not Used**	0

Iteration History			
**Iteration**	**Evaluations**	**-2 Res Log Like**	**Criterion**
**0**	1	178.49981346	
**1**	3	171.47979914	0.00005318
**2**	1	171.47705916	0.00000014
**3**	1	171.47705232	0.00000000

Convergence criteria met.

DistSoma=300

Covariance Parameter Estimates				
**Cov Parm**	**Estimate**	**Alpha**	**Lower**	**Upper**
**Culture**	2.0054	0.05	0.7314	15.5102
**Residual**	3.1488	0.05	2.0287	5.5436

Fit Statistics	
**-2 Res Log Likelihood**	171.5
**AIC (Smaller is Better)**	175.5
**AICC (Smaller is Better)**	175.8
**BIC (Smaller is Better)**	176.1

Solution for Fixed Effects									
**Effect**	**Treatment**	**Estimate**	**Standard Error**	**DF**	**t Value**	**Pr > |t|**	**Alpha**	**Lower**	**Upper**
**Intercept**		1.9384	0.8294	8	2.34	0.0476	0.05	0.02586	3.8510
**Treatment**	Meg TTR	-0.02575	1.0792	31	-0.02	0.9811	0.05	-2.2268	2.1752
**Treatment**	GFP MsTTR	0	.	.	.	.	.	.	.

Solution for Random Effects									
**Effect**	**Culture**	**Estimate**	**Std Err Pred**	**DF**	**t Value**	**Pr > |t|**	**Alpha**	**Lower**	**Upper**
**Culture**	1	0.04686	0.9368	31	0.05	0.9604	0.05	-1.8638	1.9575
**Culture**	2	1.4804	0.9592	31	1.54	0.1329	0.05	-0.4758	3.4367
**Culture**	3	-1.2125	0.9592	31	-1.26	0.2156	0.05	-3.1687	0.7438
**Culture**	4	-0.3148	0.9592	31	-0.33	0.7449	0.05	-2.2711	1.6414
**Culture**	5	0.06646	0.8691	31	0.08	0.9395	0.05	-1.7061	1.8390
**Culture**	6	0.06272	0.9006	31	0.07	0.9449	0.05	-1.7742	1.8996
**Culture**	7	2.2171	0.9006	31	2.46	0.0196	0.05	0.3802	4.0539
**Culture**	8	-1.5160	0.8458	31	-1.79	0.0829	0.05	-3.2410	0.2091
**Culture**	9	-0.2312	1.0157	31	-0.23	0.8214	0.05	-2.3027	1.8403
**Culture**	10	-0.5991	0.9457	31	-0.63	0.5311	0.05	-2.5280	1.3297

Type 3 Tests of Fixed Effects				
**Effect**	**Num DF**	**Den DF**	**F Value**	**Pr > F**
**Treatment**	1	31	0.00	0.9811

DistSoma=300

Least Squares Means

Effect	Treatment	Estimate	Standard Error	DF	t Value	Pr > |t|	Alpha	Lower	Upper
**Treatment**	Meg TTR	1.9127	0.6905	31	2.77	0.0094	0.05	0.5044	3.3209
**Treatment**	GFP MsTTR	1.9384	0.8294	31	2.34	0.0261	0.05	0.2469	3.6300

Differences of Least Squares Means

Effect	Treatment	Treatment	Estimate	Standard Error	DF	t Value	Pr > |t|	Adjustment	Adj P	Alpha	Lower	Upper
**Treatment**	Meg TTR	GFP MsTTR	-0.02575	1.0792	31	-0.02	0.9811	Tukey-Kramer	0.9811	0.05	-2.2268	2.1752

Differences of Least Squares Means

Effect	Treatment	Treatment	Adj Lower	Adj Upper
**Treatment**	Meg TTR	GFP MsTTR	-2.2267	2.1752

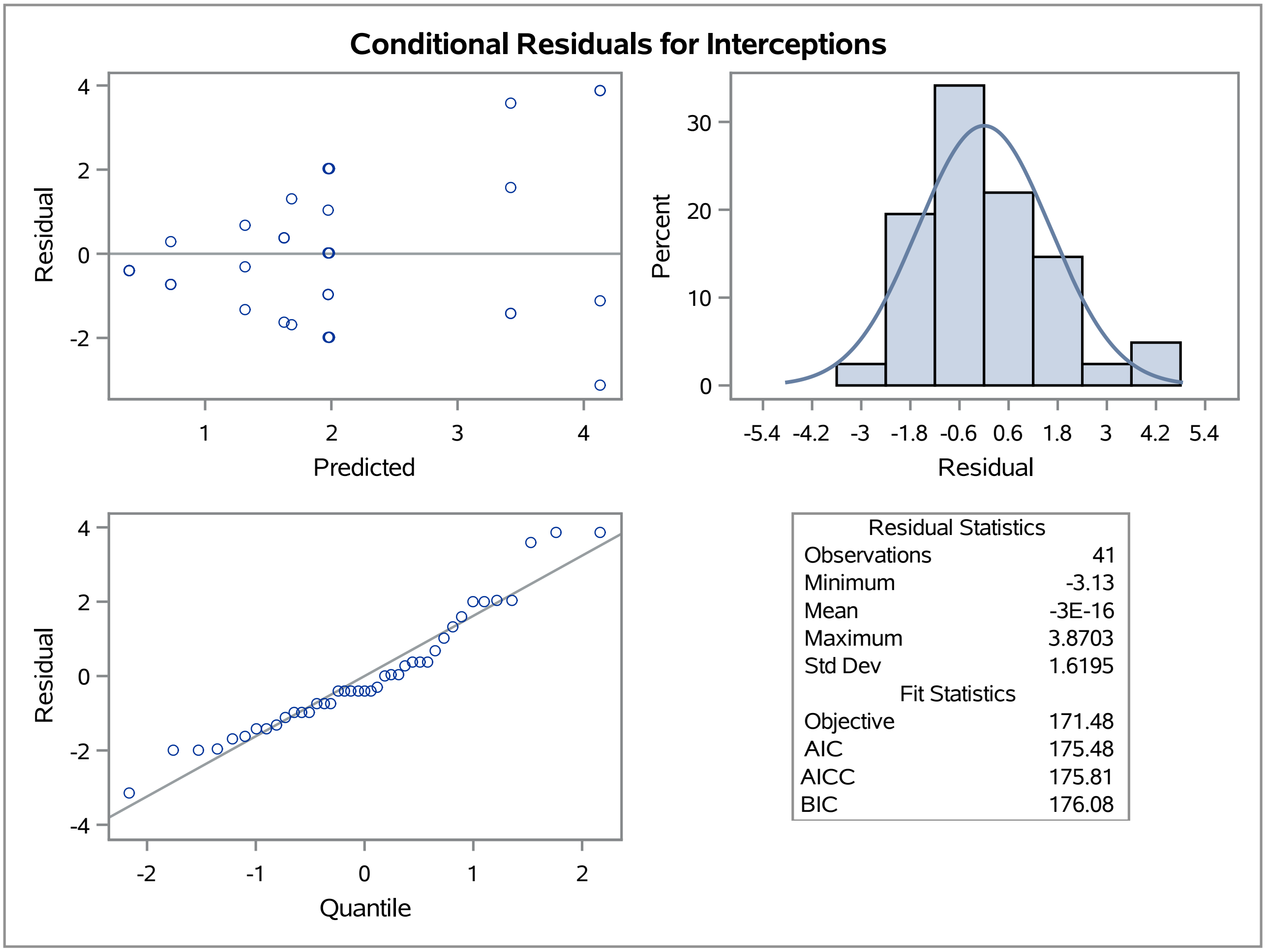

Residual Statistics	
Observations	41
Minimum	-3.13
Mean	-3E-16
Maximum	3.8703
Std Dev	1.6195
**Fit Statistics**	
Objective	171.48
AIC	175.48
AICC	175.81
BIC	176.08

DistSoma=306

Model Information	
**Data Set**	WORK.TEMPDATASORTED
**Dependent Variable**	Interceptions
**Covariance Structure**	Variance Components
**Estimation Method**	REML
**Residual Variance Method**	Profile
**Fixed Effects SE Method**	Model-Based
**Degrees of Freedom Method**	Containment

Class Level Information		
**Class**	**Levels**	**Values**
**Treatment**	2	Meg TTR GFP MsTTR
**Culture**	10	1 2 3 4 5 6 7 8 9 10

Dimensions	
**Covariance Parameters**	2
**Columns in X**	3
**Columns in Z**	10
**Subjects**	1
**Max Obs per Subject**	41

Number of Observations	
**Number of Observations Read**	41
**Number of Observations Used**	41
**Number of Observations Not Used**	0

Iteration History			
**Iteration**	**Evaluations**	**-2 Res Log Like**	**Criterion**
**0**	1	184.64080046	
**1**	3	179.17885347	0.00002964
**2**	1	179.17721965	0.00000005
**3**	1	179.17721715	0.00000000

Convergence criteria met.

DistSoma=306

Covariance Parameter Estimates				
**Cov Parm**	**Estimate**	**Alpha**	**Lower**	**Upper**
**Culture**	2.0546	0.05	0.7140	19.4930
**Residual**	3.9472	0.05	2.5432	6.9483

Fit Statistics	
**-2 Res Log Likelihood**	179.2
**AIC (Smaller is Better)**	183.2
**AICC (Smaller is Better)**	183.5
**BIC (Smaller is Better)**	183.8

Solution for Fixed Effects									
**Effect**	**Treatment**	**Estimate**	**Standard Error**	**DF**	**t Value**	**Pr > |t|**	**Alpha**	**Lower**	**Upper**
**Intercept**		1.8771	0.8645	8	2.17	0.0617	0.05	-0.1164	3.8707
**Treatment**	Meg TTR	0.1531	1.1258	31	0.14	0.8927	0.05	-2.1430	2.4492
**Treatment**	GFP MsTTR	0	.	.	.	.	.	.	.

Solution for Random Effects									
**Effect**	**Culture**	**Estimate**	**Std Err Pred**	**DF**	**t Value**	**Pr > |t|**	**Alpha**	**Lower**	**Upper**
**Culture**	1	0.08876	0.9800	31	0.09	0.9284	0.05	-1.9099	2.0874
**Culture**	2	1.2652	1.0038	31	1.26	0.2169	0.05	-0.7821	3.3125
**Culture**	3	-1.0992	1.0038	31	-1.09	0.2820	0.05	-3.1465	0.9481
**Culture**	4	-0.2548	1.0038	31	-0.25	0.8013	0.05	-2.3021	1.7926
**Culture**	5	-0.02183	0.9175	31	-0.02	0.9812	0.05	-1.8930	1.8493
**Culture**	6	0.4862	0.9508	31	0.51	0.6127	0.05	-1.4529	2.4253
**Culture**	7	2.1751	0.9508	31	2.29	0.0291	0.05	0.2360	4.1142
**Culture**	8	-1.5378	0.8926	31	-1.72	0.0949	0.05	-3.3582	0.2826
**Culture**	9	-0.2704	1.0686	31	-0.25	0.8019	0.05	-2.4499	1.9090
**Culture**	10	-0.8312	0.9977	31	-0.83	0.4111	0.05	-2.8660	1.2035

Type 3 Tests of Fixed Effects				
**Effect**	**Num DF**	**Den DF**	**F Value**	**Pr > F**
**Treatment**	1	31	0.02	0.8927

DistSoma=306

Least Squares Means									
**Effect**	**Treatment**	**Estimate**	**Standard Error**	**DF**	**t Value**	**Pr > |t|**	**Alpha**	**Lower**	**Upper**
**Treatment**	Meg TTR	2.0302	0.7212	31	2.82	0.0084	0.05	0.5594	3.5010
**Treatment**	GFP MsTTR	1.8771	0.8645	31	2.17	0.0377	0.05	0.1140	3.6403

Differences of Least Squares Means												
**Effect**	**Treatment**	**Treatment**	**Estimate**	**Standard Error**	**DF**	**t Value**	**Pr > |t|**	**Adjustment**	**Adj P**	**Alpha**	**Lower**	**Upper**
**Treatment**	Meg TTR	GFP MsTTR	0.1531	1.1258	31	0.14	0.8927	Tukey-Kramer	0.8927	0.05	-2.1430	2.4492

Differences of Least Squares Means				
**Effect**	**Treatment**	**Treatment**	**Adj Lower**	**Adj Upper**
**Treatment**	Meg TTR	GFP MsTTR	-2.1430	2.4492

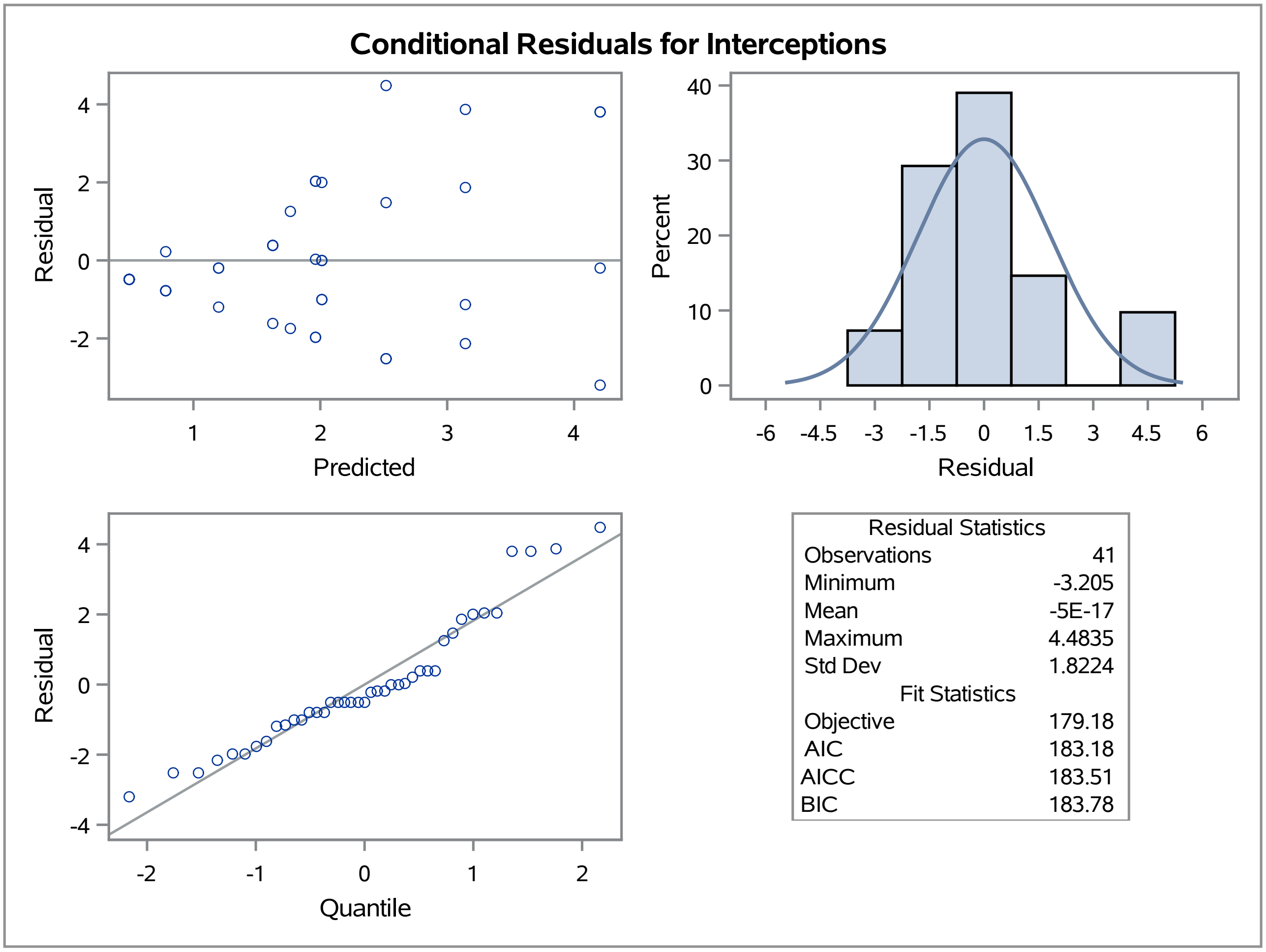

DistSoma=312

Model Information	
**Data Set**	WORK.TEMPDATASORTED
**Dependent Variable**	Interceptions
**Covariance Structure**	Variance Components
**Estimation Method**	REML
**Residual Variance Method**	Profile
**Fixed Effects SE Method**	Model-Based
**Degrees of Freedom Method**	Containment

Class Level Information		
**Class**	**Levels**	**Values**
**Treatment**	2	Meg TTR GFP MsTTR
**Culture**	10	1 2 3 4 5 6 7 8 9 10

Dimensions	
**Covariance Parameters**	2
**Columns in X**	3
**Columns in Z**	10
**Subjects**	1
**Max Obs per Subject**	41

Number of Observations	
**Number of Observations Read**	41
**Number of Observations Used**	41
**Number of Observations Not Used**	0

Iteration History			
**Iteration**	**Evaluations**	**-2 Res Log Like**	**Criterion**
**0**	1	177.69582872	
**1**	2	169.44958253	0.00000053
**2**	1	169.44955636	0.00000000

Convergence criteria met.

Covariance Parameter Estimates				
**Cov Parm**	**Estimate**	**Alpha**	**Lower**	**Upper**
**Culture**	2.2190	0.05	0.8322	15.3772
**Residual**	2.9100	0.05	1.8727	5.1325

DistSoma=312

Fit Statistics	
-2 Res Log Likelihood	169.4
AIC (Smaller is Better)	173.4
AICC (Smaller is Better)	173.8
BIC (Smaller is Better)	174.1

Solution for Fixed Effects

Effect	Treatment	Estimate	Standard Error	DF	t Value	Pr > |t|	Alpha	Lower	Upper
Intercept		1.7630	0.8527	8	2.07	0.0725	0.05	-0.2035	3.7294
Treatment	Meg TTR	-0.05811	1.1088	31	-0.05	0.9585	0.05	-2.3194	2.2032
Treatment	GFP MsTTR	0	.	.	.	.	.	.	.

Solution for Random Effects

Effect	Culture	Estimate	Std Err Pred	DF	t Value	Pr > |t|	Alpha	Lower	Upper
Culture	1	0.02933	0.9578	31	0.03	0.9758	0.05	-1.9242	1.9828
Culture	2	1.3081	0.9799	31	1.33	0.1916	0.05	-0.6905	3.3068
Culture	3	-1.1394	0.9799	31	-1.16	0.2538	0.05	-3.1380	0.8592
Culture	4	-0.1981	0.9799	31	-0.20	0.8412	0.05	-2.1967	1.8006
Culture	5	0.07536	0.8810	31	0.09	0.9324	0.05	-1.7215	1.8722
Culture	6	0.03398	0.9125	31	0.04	0.9705	0.05	-1.8271	1.8951
Culture	7	2.6698	0.9125	31	2.93	0.0064	0.05	0.8087	4.5309
Culture	8	-1.3991	0.8580	31	-1.63	0.1131	0.05	-3.1490	0.3508
Culture	9	-0.4257	1.0305	31	-0.41	0.6824	0.05	-2.5275	1.6760
Culture	10	-0.9544	0.9582	31	-1.00	0.3269	0.05	-2.9086	0.9998

Type 3 Tests of Fixed Effects

Effect	Num DF	Den DF	F Value	Pr > F
Treatment	1	31	0.00	0.9585

Least Squares Means

Effect	Treatment	Estimate	Standard Error	DF	t Value	Pr > |t|	Alpha	Lower	Upper
Treatment	Meg TTR	1.7049	0.7086	31	2.41	0.0223	0.05	0.2596	3.1501
Treatment	GFP MsTTR	1.7630	0.8527	31	2.07	0.0471	0.05	0.02379	3.5022

DistSoma=312

Differences of Least Squares Means												
Effect	Treatment	Treatment	Estimate	Standard Error	DF	t Value	Pr > |t|	Adjustment	Adj P	Alpha	Lower	Upper
**Treatment**	Meg TTR	GFP MsTTR	-0.05811	1.1088	31	-0.05	0.9585	Tukey-Kramer	0.9585	0.05	-2.3194	2.2032

Differences of Least Squares Means				
Effect	Treatment	Treatment	Adj Lower	Adj Upper
**Treatment**	Meg TTR	GFP MsTTR	-2.3194	2.2032

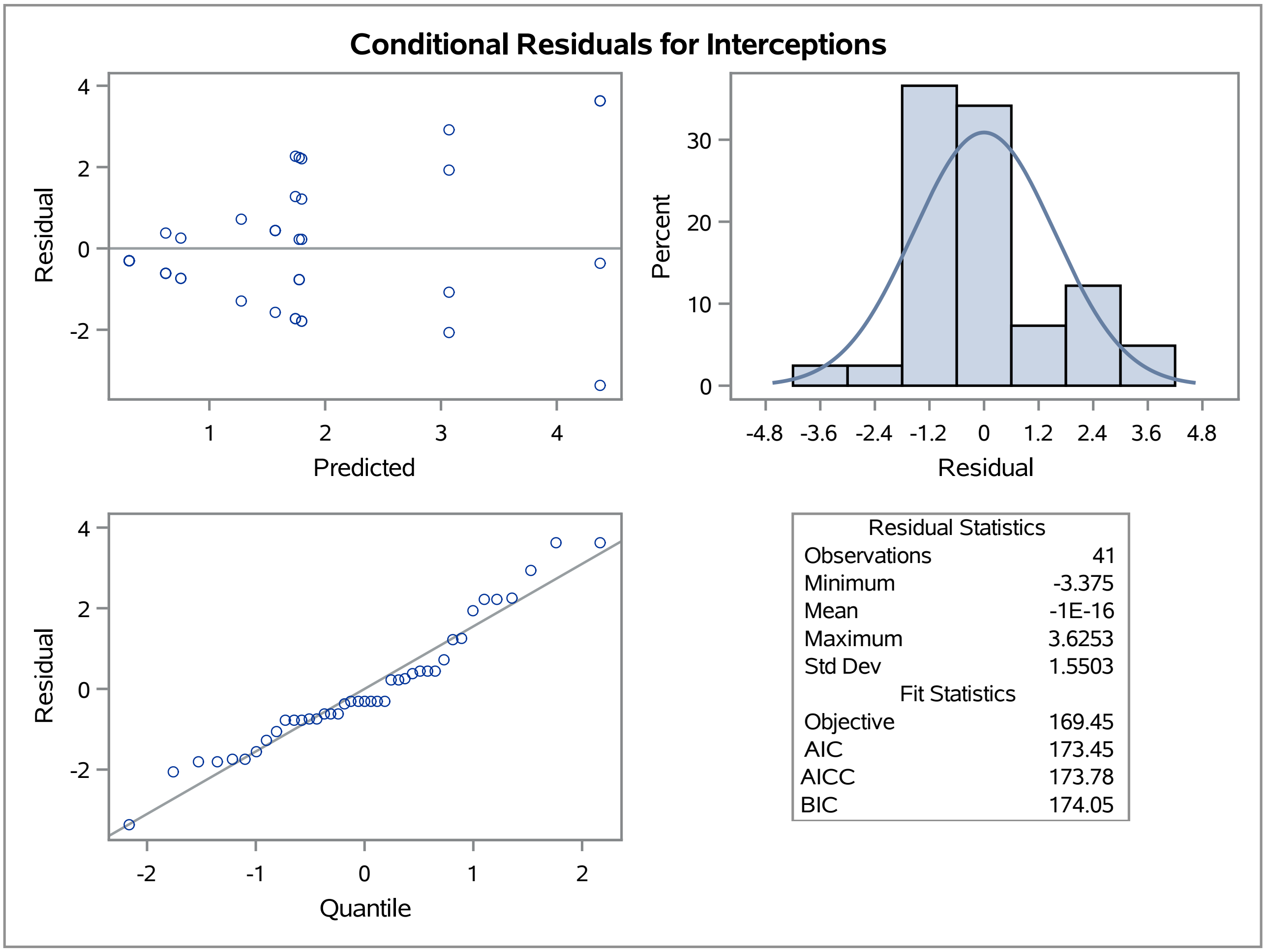

DistSoma=318

Model Information	
**Data Set**	WORK.TEMPDATASORTED
**Dependent Variable**	Interceptions
**Covariance Structure**	Variance Components
**Estimation Method**	REML
**Residual Variance Method**	Profile
**Fixed Effects SE Method**	Model-Based
**Degrees of Freedom Method**	Containment

Class Level Information		
**Class**	**Levels**	**Values**
**Treatment**	2	Meg TTR GFP MsTTR
**Culture**	10	1 2 3 4 5 6 7 8 9 10

Dimensions	
**Covariance Parameters**	2
**Columns in X**	3
**Columns in Z**	10
**Subjects**	1
**Max Obs per Subject**	41

Number of Observations	
**Number of Observations Read**	41
**Number of Observations Used**	41
**Number of Observations Not Used**	0

Iteration History			
**Iteration**	**Evaluations**	**-2 Res Log Like**	**Criterion**
**0**	1	169.70001089	
**1**	2	158.29255417	0.00000046
**2**	1	158.29253425	0.00000000

Convergence criteria met.

Covariance Parameter Estimates				
**Cov Parm**	**Estimate**	**Alpha**	**Lower**	**Upper**
**Culture**	2.1446	0.05	0.8447	12.4137
**Residual**	2.0847	0.05	1.3414	3.6781

DistSoma=318

Fit Statistics	
-2 Res Log Likelihood	158.3
AIC (Smaller is Better)	162.3
AICC (Smaller is Better)	162.6
BIC (Smaller is Better)	162.9

Solution for Fixed Effects

Effect	Treatment	Estimate	Standard Error	DF	t Value	Pr > |t|	Alpha	Lower	Upper
Intercept		1.6599	0.8122	8	2.04	0.0753	0.05	-0.2132	3.5329
Treatment	Meg TTR	-0.07729	1.0548	31	-0.07	0.9421	0.05	-2.2286	2.0740
Treatment	GFP MsTTR	0	.	.	.	.	.	.	.

Solution for Random Effects

Effect	Culture	Estimate	Std Err Pred	DF	t Value	Pr > |t|	Alpha	Lower	Upper
Culture	1	-0.2176	0.9008	31	-0.24	0.8108	0.05	-2.0548	1.6197
Culture	2	1.6815	0.9199	31	1.83	0.0772	0.05	-0.1947	3.5577
Culture	3	-1.1342	0.9199	31	-1.23	0.2269	0.05	-3.0104	0.7420
Culture	4	-0.3297	0.9199	31	-0.36	0.7225	0.05	-2.2059	1.5465
Culture	5	0.01459	0.8164	31	0.02	0.9859	0.05	-1.6505	1.6797
Culture	6	0.1347	0.8441	31	0.16	0.8742	0.05	-1.5868	1.8562
Culture	7	2.5482	0.8441	31	3.02	0.0050	0.05	0.8267	4.2696
Culture	8	-1.3619	0.7965	31	-1.71	0.0973	0.05	-2.9864	0.2626
Culture	9	-0.3920	0.9521	31	-0.41	0.6834	0.05	-2.3339	1.5498
Culture	10	-0.9435	0.8850	31	-1.07	0.2946	0.05	-2.7485	0.8614

Type 3 Tests of Fixed Effects

Effect	Num DF	Den DF	F Value	Pr > F
Treatment	1	31	0.01	0.9421

Least Squares Means

Effect	Treatment	Estimate	Standard Error	DF	t Value	Pr > |t|	Alpha	Lower	Upper
Treatment	Meg TTR	1.5826	0.6730	31	2.35	0.0252	0.05	0.2100	2.9552
Treatment	GFP MsTTR	1.6599	0.8122	31	2.04	0.0496	0.05	0.003280	3.3164

DistSoma=318

Differences of Least Squares Means												
**Effect**	**Treatment**	**Treatment**	**Estimate**	**Standard Error**	**DF**	**t Value**	**Pr > |t|**	**Adjustment**	**Adj P**	**Alpha**	**Lower**	**Upper**
**Treatment**	Meg TTR	GFP MsTTR	-0.07729	1.0548	31	-0.07	0.9421	Tukey-Kramer	0.9421	0.05	-2.2286	2.0740

Differences of Least Squares Means				
**Effect**	**Treatment**	**Treatment**	**Adj Lower**	**Adj Upper**
**Treatment**	Meg TTR	GFP MsTTR	-2.2286	2.0740

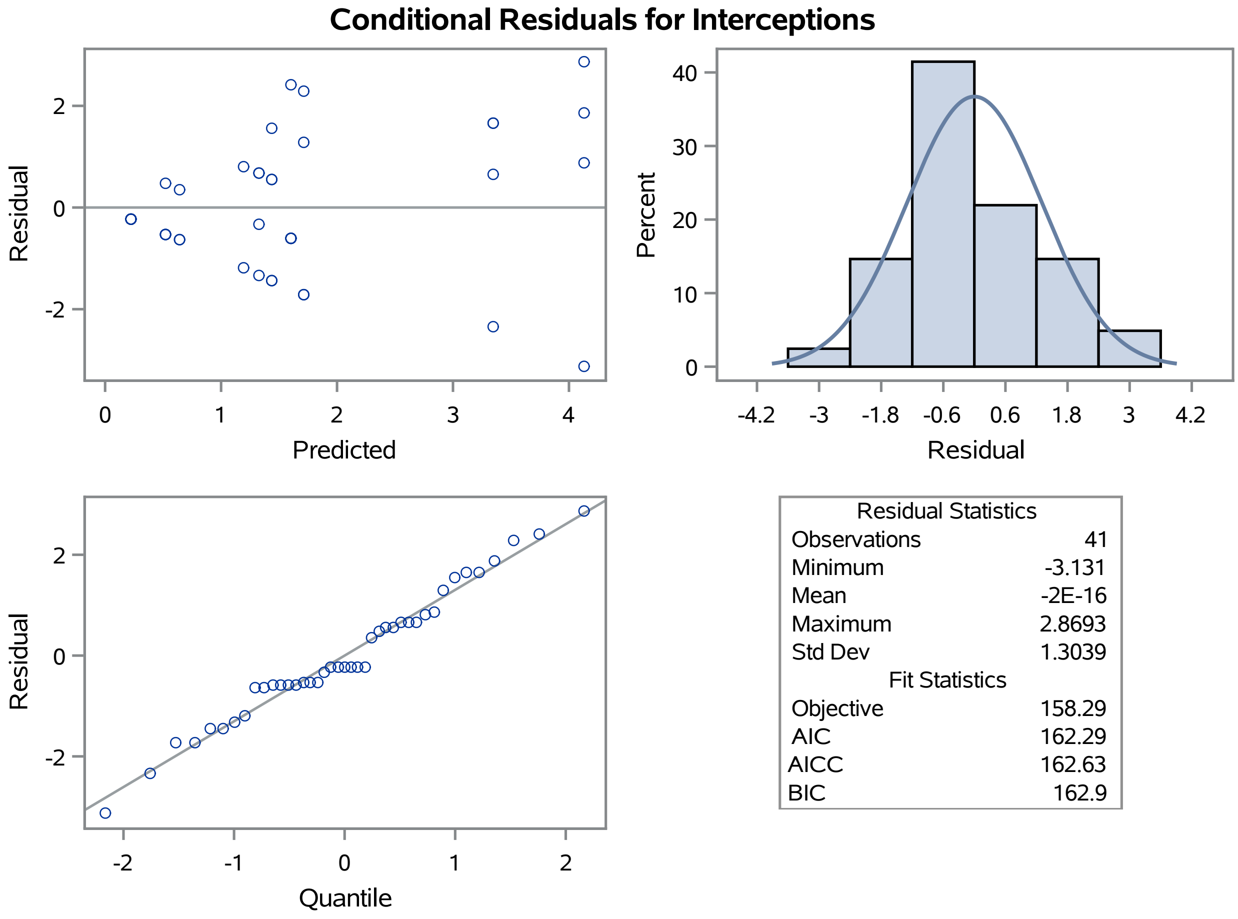

Residual Statistics	
Observations	41
Minimum	-3.131
Mean	-2E-16
Maximum	2.8693
Std Dev	1.3039
Fit Statistics	
Objective	158.29
AIC	162.29
AICC	162.63
BIC	162.9

DistSoma=324

Model Information	
**Data Set**	WORK.TEMPDATASORTED
**Dependent Variable**	Interceptions
**Covariance Structure**	Variance Components
**Estimation Method**	REML
**Residual Variance Method**	Profile
**Fixed Effects SE Method**	Model-Based
**Degrees of Freedom Method**	Containment

Class Level Information		
**Class**	**Levels**	**Values**
**Treatment**	2	Meg TTR GFP MsTTR
**Culture**	10	1 2 3 4 5 6 7 8 9 10

Dimensions	
**Covariance Parameters**	2
**Columns in X**	3
**Columns in Z**	10
**Subjects**	1
**Max Obs per Subject**	41

Number of Observations	
**Number of Observations Read**	41
**Number of Observations Used**	41
**Number of Observations Not Used**	0

Iteration History			
**Iteration**	**Evaluations**	**-2 Res Log Like**	**Criterion**
**0**	1	167.10768609	
**1**	2	158.42959913	0.00000183
**2**	1	158.42951936	0.00000000

Convergence criteria met.

Covariance Parameter Estimates				
**Cov Parm**	**Estimate**	**Alpha**	**Lower**	**Upper**
**Culture**	1.7276	0.05	0.6534	11.5932
**Residual**	2.1809	0.05	1.4037	3.8456

DistSoma=324

Fit Statistics	
-2 Res Log Likelihood	158.4
AIC (Smaller is Better)	162.4
AICC (Smaller is Better)	162.8
BIC (Smaller is Better)	163.0

Solution for Fixed Effects

Effect	Treatment	Estimate	Standard Error	DF	t Value	Pr > |t|	Alpha	Lower	Upper
Intercept		1.4321	0.7491	8	1.91	0.0923	0.05	-0.2954	3.1595
Treatment	Meg TTR	0.07323	0.9738	31	0.08	0.9405	0.05	-1.9129	2.0594
Treatment	GFP MsTTR	0	.	.	.	.	.	.	.

Solution for Random Effects

Effect	Culture	Estimate	Std Err Pred	DF	t Value	Pr > |t|	Alpha	Lower	Upper
Culture	1	-0.3450	0.8402	31	-0.41	0.6842	0.05	-2.0586	1.3687
Culture	2	1.3819	0.8594	31	1.61	0.1180	0.05	-0.3710	3.1347
Culture	3	-0.8985	0.8594	31	-1.05	0.3039	0.05	-2.6514	0.8543
Culture	4	-0.1384	0.8594	31	-0.16	0.8731	0.05	-1.8912	1.6145
Culture	5	-0.08407	0.7714	31	-0.11	0.9139	0.05	-1.6574	1.4893
Culture	6	0.1860	0.7988	31	0.23	0.8174	0.05	-1.4432	1.8153
Culture	7	2.2763	0.7988	31	2.85	0.0077	0.05	0.6471	3.9056
Culture	8	-1.2436	0.7514	31	-1.66	0.1080	0.05	-2.7761	0.2888
Culture	9	-0.3098	0.9022	31	-0.34	0.7337	0.05	-2.1499	1.5303
Culture	10	-0.8249	0.8387	31	-0.98	0.3330	0.05	-2.5355	0.8857

Type 3 Tests of Fixed Effects

Effect	Num DF	Den DF	F Value	Pr > F
Treatment	1	31	0.01	0.9405

Least Squares Means

Effect	Treatment	Estimate	Standard Error	DF	t Value	Pr > |t|	Alpha	Lower	Upper
Treatment	Meg TTR	1.5053	0.6223	31	2.42	0.0216	0.05	0.2362	2.7744
Treatment	GFP MsTTR	1.4321	0.7491	31	1.91	0.0652	0.05	-0.09573	2.9598

DistSoma=324

Differences of Least Squares Means												
Effect	Treatment	Treatment	Estimate	Standard Error	DF	t Value	Pr > |t|	Adjustment	Adj P	Alpha	Lower	Upper
Treatment	Meg TTR	GFP MsTTR	0.07323	0.9738	31	0.08	0.9405	Tukey-Kramer	0.9405	0.05	-1.9129	2.0594

Differences of Least Squares Means				
Effect	Treatment	Treatment	Adj Lower	Adj Upper
Treatment	Meg TTR	GFP MsTTR	-1.9129	2.0594

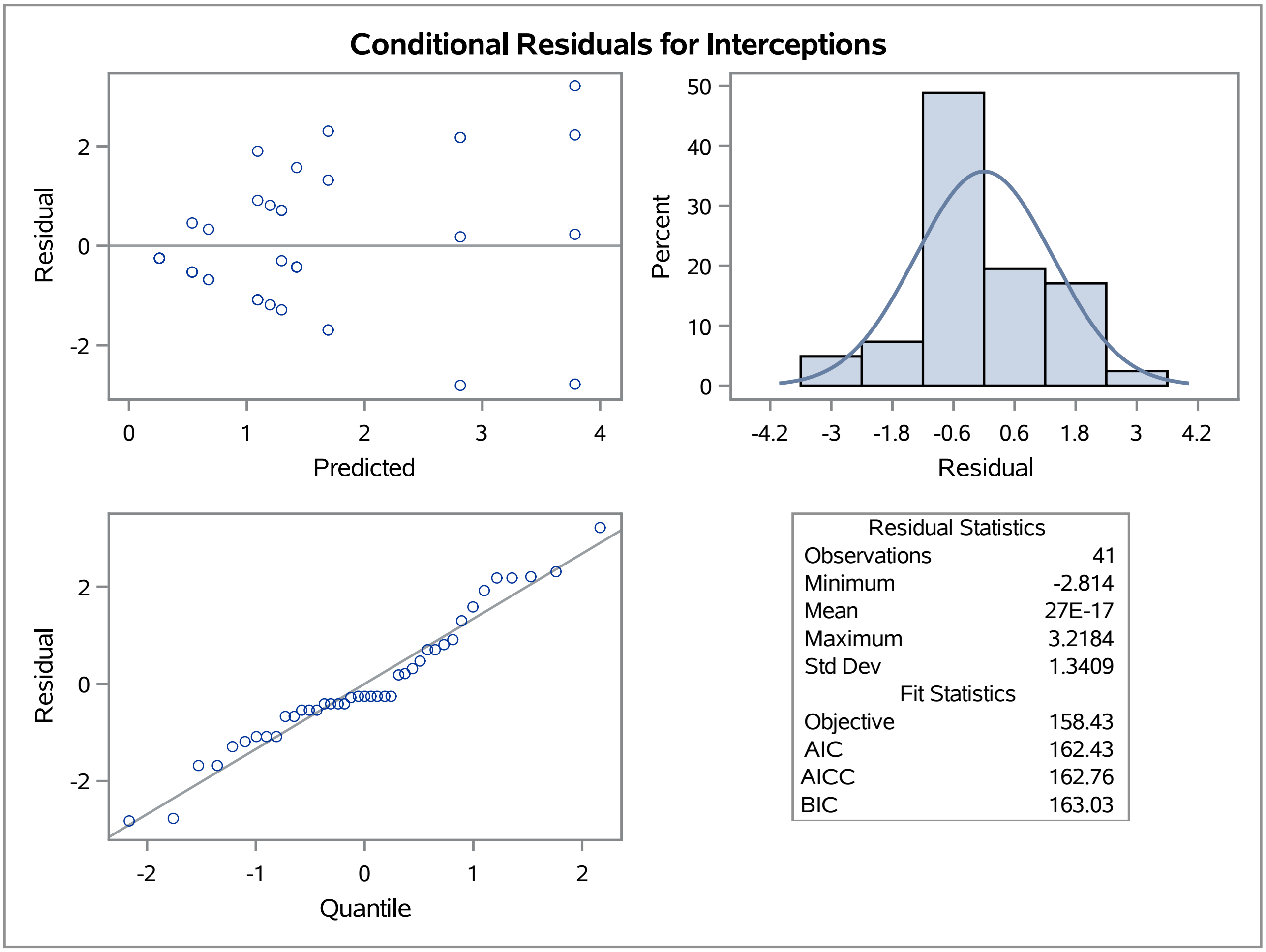

DistSoma=330

Model Information	
**Data Set**	WORK.TEMPDATASORTED
**Dependent Variable**	Interceptions
**Covariance Structure**	Variance Components
**Estimation Method**	REML
**Residual Variance Method**	Profile
**Fixed Effects SE Method**	Model-Based
**Degrees of Freedom Method**	Containment

Class Level Information		
**Class**	**Levels**	**Values**
**Treatment**	2	Meg TTR GFP MsTTR
**Culture**	10	1 2 3 4 5 6 7 8 9 10

Dimensions	
**Covariance Parameters**	2
**Columns in X**	3
**Columns in Z**	10
**Subjects**	1
**Max Obs per Subject**	41

Number of Observations	
**Number of Observations Read**	41
**Number of Observations Used**	41
**Number of Observations Not Used**	0

Iteration History			
**Iteration**	**Evaluations**	**-2 Res Log Like**	**Criterion**
**0**	1	162.84410032	
**1**	2	153.60409406	0.00000323
**2**	1	153.60396061	0.00000000

Convergence criteria met.

Covariance Parameter Estimates				
**Cov Parm**	**Estimate**	**Alpha**	**Lower**	**Upper**
**Culture**	1.5975	0.05	0.6101	10.3374
**Residual**	1.9110	0.05	1.2300	3.3698

DistSoma=330

Fit Statistics	
-2 Res Log Likelihood	153.6
AIC (Smaller is Better)	157.6
AICC (Smaller is Better)	157.9
BIC (Smaller is Better)	158.2

Solution for Fixed Effects

Effect	Treatment	Estimate	Standard Error	DF	t Value	Pr > |t|	Alpha	Lower	Upper
Intercept		1.3088	0.7160	8	1.83	0.1050	0.05	-0.3423	2.9598
Treatment	Meg TTR	0.1607	0.9306	31	0.17	0.8640	0.05	-1.7372	2.0587
Treatment	GFP MsTTR	0	.	.	.	.	.	.	.

Solution for Random Effects

Effect	Culture	Estimate	Std Err Pred	DF	t Value	Pr > |t|	Alpha	Lower	Upper
Culture	1	-0.2492	0.8014	31	-0.31	0.7579	0.05	-1.8836	1.3853
Culture	2	1.1094	0.8195	31	1.35	0.1856	0.05	-0.5619	2.7808
Culture	3	-0.8150	0.8195	31	-0.99	0.3276	0.05	-2.4864	0.8563
Culture	4	-0.04524	0.8195	31	-0.06	0.9563	0.05	-1.7166	1.6261
Culture	5	-0.2175	0.7338	31	-0.30	0.7689	0.05	-1.7141	1.2792
Culture	6	0.2159	0.7597	31	0.28	0.7781	0.05	-1.3335	1.7654
Culture	7	2.3328	0.7597	31	3.07	0.0044	0.05	0.7834	3.8823
Culture	8	-1.2252	0.7150	31	-1.71	0.0966	0.05	-2.6834	0.2330
Culture	9	-0.2938	0.8580	31	-0.34	0.7344	0.05	-2.0438	1.4562
Culture	10	-0.8123	0.7975	31	-1.02	0.3163	0.05	-2.4388	0.8142

Type 3 Tests of Fixed Effects

Effect	Num DF	Den DF	F Value	Pr > F
Treatment	1	31	0.03	0.8640

Least Squares Means

Effect	Treatment	Estimate	Standard Error	DF	t Value	Pr > |t|	Alpha	Lower	Upper
Treatment	Meg TTR	1.4695	0.5944	31	2.47	0.0191	0.05	0.2571	2.6819
Treatment	GFP MsTTR	1.3088	0.7160	31	1.83	0.0772	0.05	-0.1515	2.7690

DistSoma=330

Differences of Least Squares Means												
**Effect**	**Treatment**	**Treatment**	**Estimate**	**Standard Error**	**DF**	**t Value**	**Pr > |t|**	**Adjustment**	**Adj P**	**Alpha**	**Lower**	**Upper**
**Treatment**	Meg TTR	GFP MsTTR	0.1607	0.9306	31	0.17	0.8640	Tukey-Kramer	0.8640	0.05	-1.7372	2.0587

Differences of Least Squares Means				
**Effect**	**Treatment**	**Treatment**	**Adj Lower**	**Adj Upper**
**Treatment**	Meg TTR	GFP MsTTR	-1.7372	2.0586

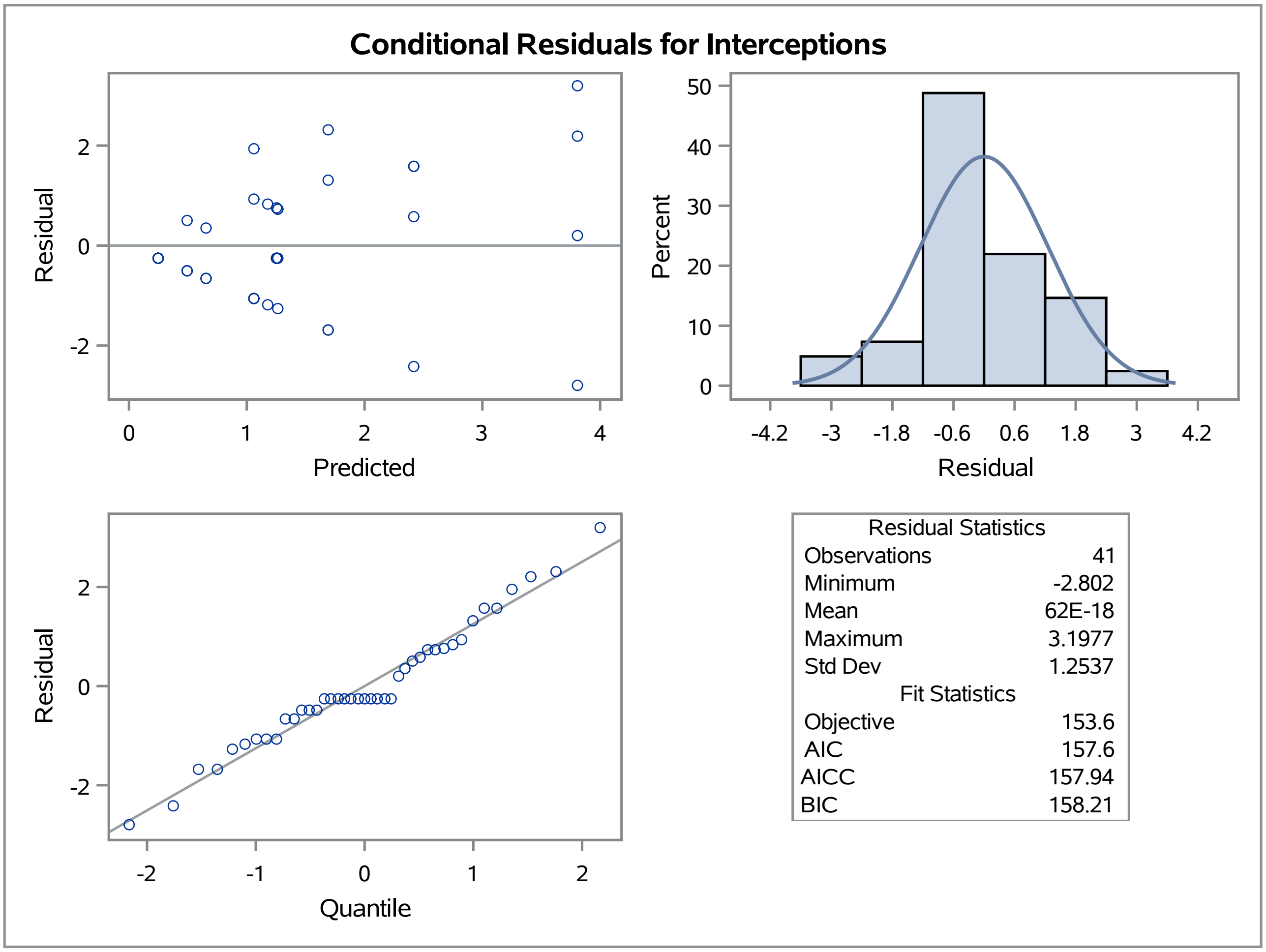

DistSoma=336

Model Information	
**Data Set**	WORK.TEMPDATASORTED
**Dependent Variable**	Interceptions
**Covariance Structure**	Variance Components
**Estimation Method**	REML
**Residual Variance Method**	Profile
**Fixed Effects SE Method**	Model-Based
**Degrees of Freedom Method**	Containment

Class Level Information		
**Class**	**Levels**	**Values**
**Treatment**	2	Meg TTR GFP MsTTR
**Culture**	10	1 2 3 4 5 6 7 8 9 10

Dimensions	
**Covariance Parameters**	2
**Columns in X**	3
**Columns in Z**	10
**Subjects**	1
**Max Obs per Subject**	41

Number of Observations	
**Number of Observations Read**	41
**Number of Observations Used**	41
**Number of Observations Not Used**	0

Iteration History			
**Iteration**	**Evaluations**	**-2 Res Log Like**	**Criterion**
**0**	1	159.99566519	
**1**	2	151.37465068	0.00000232
**2**	1	151.37455784	0.00000000

Convergence criteria met.

Covariance Parameter Estimates				
**Cov Parm**	**Estimate**	**Alpha**	**Lower**	**Upper**
**Culture**	1.4323	0.05	0.5413	9.6421
**Residual**	1.8221	0.05	1.1729	3.2127

DistSoma=336

Fit Statistics	
-2 Res Log Likelihood	151.4
AIC (Smaller is Better)	155.4
AICC (Smaller is Better)	155.7
BIC (Smaller is Better)	156.0

Solution for Fixed Effects

Effect	Treatment	Estimate	Standard Error	DF	t Value	Pr > |t|	Alpha	Lower	Upper
Intercept		1.2469	0.6827	8	1.83	0.1052	0.05	-0.3274	2.8212
Treatment	Meg TTR	0.1374	0.8875	31	0.15	0.8779	0.05	-1.6727	1.9476
Treatment	GFP MsTTR	0	.	.	.	.	.	.	.

Solution for Random Effects

Effect	Culture	Estimate	Std Err Pred	DF	t Value	Pr > |t|	Alpha	Lower	Upper
Culture	1	-0.1968	0.7660	31	-0.26	0.7989	0.05	-1.7590	1.3654
Culture	2	1.1404	0.7835	31	1.46	0.1556	0.05	-0.4576	2.7384
Culture	3	-0.7563	0.7835	31	-0.97	0.3419	0.05	-2.3543	0.8417
Culture	4	-0.1873	0.7835	31	-0.24	0.8126	0.05	-1.7853	1.4107
Culture	5	-0.1469	0.7035	31	-0.21	0.8359	0.05	-1.5817	1.2879
Culture	6	0.08778	0.7285	31	0.12	0.9049	0.05	-1.3981	1.5736
Culture	7	2.1742	0.7285	31	2.98	0.0055	0.05	0.6883	3.6601
Culture	8	-1.1421	0.6852	31	-1.67	0.1056	0.05	-2.5397	0.2554
Culture	9	-0.2349	0.8228	31	-0.29	0.7772	0.05	-1.9130	1.4433
Culture	10	-0.7380	0.7649	31	-0.96	0.3421	0.05	-2.2981	0.8221

Type 3 Tests of Fixed Effects

Effect	Num DF	Den DF	F Value	Pr > F
Treatment	1	31	0.02	0.8779

Least Squares Means

Effect	Treatment	Estimate	Standard Error	DF	t Value	Pr > |t|	Alpha	Lower	Upper
Treatment	Meg TTR	1.3843	0.5671	31	2.44	0.0206	0.05	0.2276	2.5410
Treatment	GFP MsTTR	1.2469	0.6827	31	1.83	0.0774	0.05	-0.1455	2.6392

DistSoma=336

Differences of Least Squares Means												
**Effect**	**Treatment**	**Treatment**	**Estimate**	**Standard Error**	**DF**	**t Value**	**Pr > |t|**	**Adjustment**	**Adj P**	**Alpha**	**Lower**	**Upper**
**Treatment**	Meg TTR	GFP MsTTR	0.1374	0.8875	31	0.15	0.8779	Tukey-Kramer	0.8779	0.05	-1.6727	1.9476

Differences of Least Squares Means				
**Effect**	**Treatment**	**Treatment**	**Adj Lower**	**Adj Upper**
**Treatment**	Meg TTR	GFP MsTTR	-1.6727	1.9476

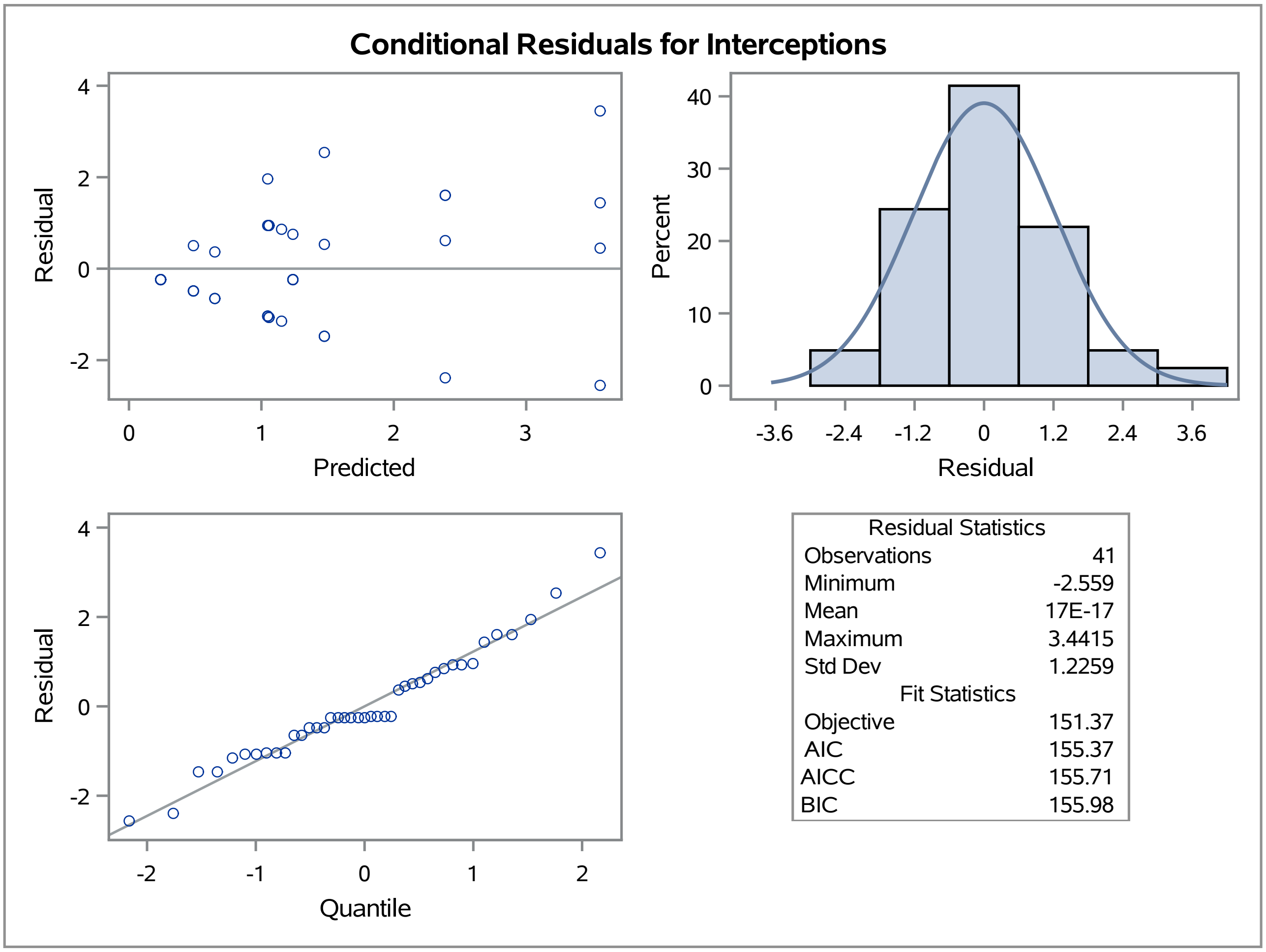

DistSoma=342

Model Information	
**Data Set**	WORK.TEMPDATASORTED
**Dependent Variable**	Interceptions
**Covariance Structure**	Variance Components
**Estimation Method**	REML
**Residual Variance Method**	Profile
**Fixed Effects SE Method**	Model-Based
**Degrees of Freedom Method**	Containment

Class Level Information		
**Class**	**Levels**	**Values**
**Treatment**	2	Meg TTR GFP MsTTR
**Culture**	10	1 2 3 4 5 6 7 8 9 10

Dimensions	
**Covariance Parameters**	2
**Columns in X**	3
**Columns in Z**	10
**Subjects**	1
**Max Obs per Subject**	41

Number of Observations	
**Number of Observations Read**	41
**Number of Observations Used**	41
**Number of Observations Not Used**	0

Iteration History			
**Iteration**	**Evaluations**	**-2 Res Log Like**	**Criterion**
**0**	1	152.58328872	
**1**	3	143.84879091	0.00001374
**2**	1	143.84828770	0.00000001

Convergence criteria met.

Covariance Parameter Estimates				
**Cov Parm**	**Estimate**	**Alpha**	**Lower**	**Upper**
**Culture**	1.1821	0.05	0.4474	7.9142
**Residual**	1.5021	0.05	0.9670	2.6478

DistSoma=342

Fit Statistics	
-2 Res Log Likelihood	143.8
AIC (Smaller is Better)	147.8
AICC (Smaller is Better)	148.2
BIC (Smaller is Better)	148.5

Solution for Fixed Effects									
Effect	Treatment	Estimate	Standard Error	DF	t Value	Pr > |t|	Alpha	Lower	Upper
Intercept		1.0716	0.6201	8	1.73	0.1223	0.05	-0.3584	2.5015
Treatment	Meg TTR	0.2702	0.8062	31	0.34	0.7398	0.05	-1.3741	1.9144
Treatment	GFP MsTTR	0	.	.	.	.	.	.	.

Solution for Random Effects									
Effect	Culture	Estimate	Std Err Pred	DF	t Value	Pr > |t|	Alpha	Lower	Upper
Culture	1	-0.2165	0.6957	31	-0.31	0.7577	0.05	-1.6355	1.2024
Culture	2	0.8943	0.7117	31	1.26	0.2183	0.05	-0.5571	2.3458
Culture	3	-0.6235	0.7117	31	-0.88	0.3877	0.05	-2.0750	0.8280
Culture	4	-0.05431	0.7117	31	-0.08	0.9397	0.05	-1.5058	1.3971
Culture	5	-0.1130	0.6390	31	-0.18	0.8608	0.05	-1.4162	1.1902
Culture	6	0.1201	0.6617	31	0.18	0.8571	0.05	-1.2294	1.4697
Culture	7	2.0174	0.6617	31	3.05	0.0047	0.05	0.6679	3.3670
Culture	8	-1.1072	0.6224	31	-1.78	0.0850	0.05	-2.3765	0.1621
Culture	9	-0.2090	0.7473	31	-0.28	0.7816	0.05	-1.7331	1.3152
Culture	10	-0.7084	0.6948	31	-1.02	0.3158	0.05	-2.1253	0.7086

Type 3 Tests of Fixed Effects				
Effect	Num DF	Den DF	F Value	Pr > F
Treatment	1	31	0.11	0.7398

Least Squares Means									
Effect	Treatment	Estimate	Standard Error	DF	t Value	Pr > |t|	Alpha	Lower	Upper
Treatment	Meg TTR	1.3417	0.5152	31	2.60	0.0140	0.05	0.2911	2.3924
Treatment	GFP MsTTR	1.0716	0.6201	31	1.73	0.0939	0.05	-0.1932	2.3363

DistSoma=342

Differences of Least Squares Means												
**Effect**	**Treatment**	**Treatment**	**Estimate**	**Standard Error**	**DF**	**t Value**	**Pr > |t|**	**Adjustment**	**Adj P**	**Alpha**	**Lower**	**Upper**
**Treatment**	Meg TTR	GFP MsTTR	0.2702	0.8062	31	0.34	0.7398	Tukey-Kramer	0.7398	0.05	-1.3741	1.9144

Differences of Least Squares Means				
**Effect**	**Treatment**	**Treatment**	**Adj Lower**	**Adj Upper**
**Treatment**	Meg TTR	GFP MsTTR	-1.3740	1.9144

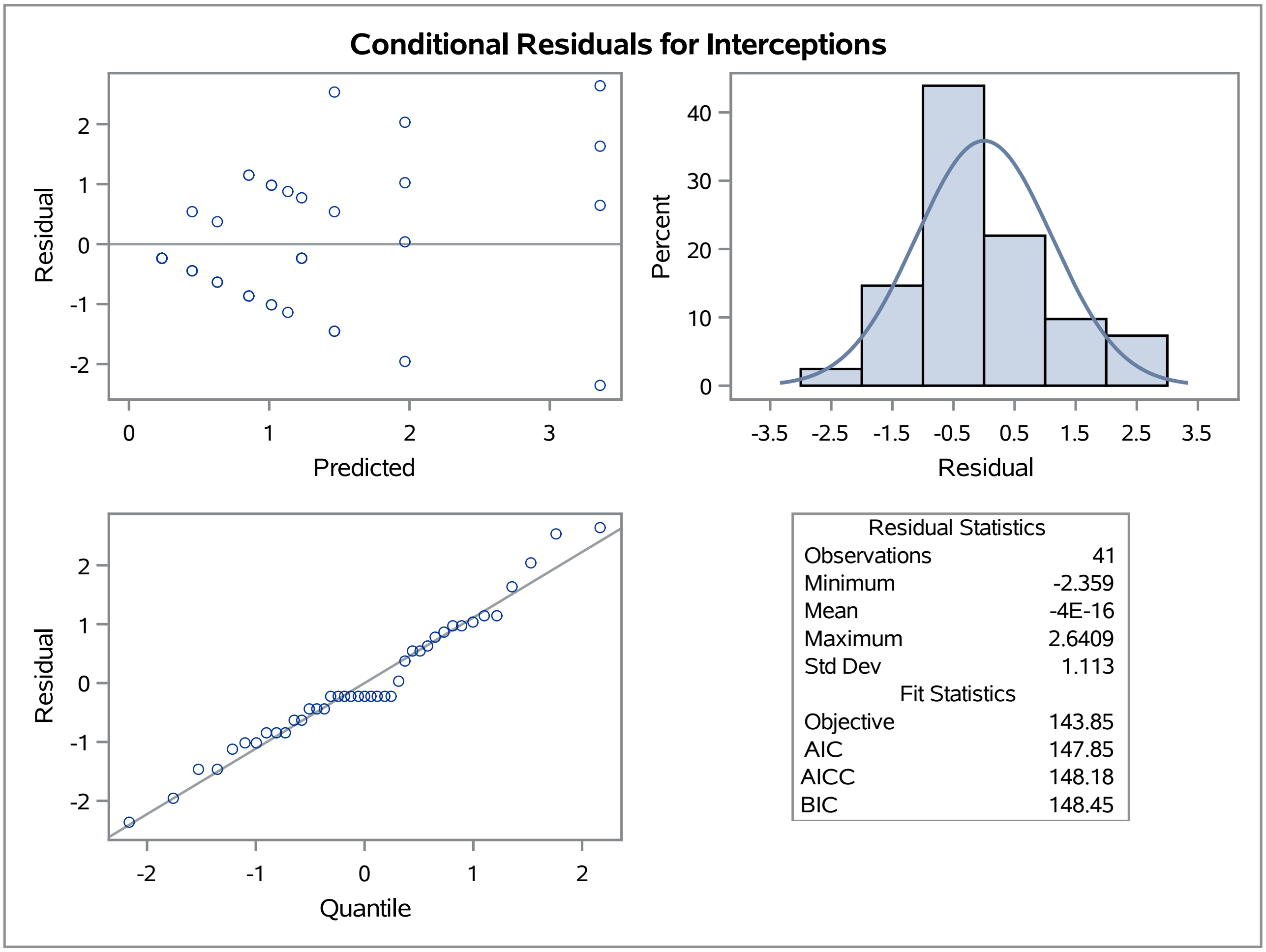

DistSoma=348

Model Information	
**Data Set**	WORK.TEMPDATASORTED
**Dependent Variable**	Interceptions
**Covariance Structure**	Variance Components
**Estimation Method**	REML
**Residual Variance Method**	Profile
**Fixed Effects SE Method**	Model-Based
**Degrees of Freedom Method**	Containment

Class Level Information		
**Class**	**Levels**	**Values**
**Treatment**	2	Meg TTR GFP MsTTR
**Culture**	10	1 2 3 4 5 6 7 8 9 10

Dimensions	
**Covariance Parameters**	2
**Columns in X**	3
**Columns in Z**	10
**Subjects**	1
**Max Obs per Subject**	41

Number of Observations	
**Number of Observations Read**	41
**Number of Observations Used**	41
**Number of Observations Not Used**	0

Iteration History			
**Iteration**	**Evaluations**	**-2 Res Log Like**	**Criterion**
**0**	1	155.35041980	
**1**	2	145.82919431	0.00000002
**2**	1	145.82919371	0.00000000

Convergence criteria met.

Covariance Parameter Estimates				
**Cov Parm**	**Estimate**	**Alpha**	**Lower**	**Upper**
**Culture**	1.3588	0.05	0.5219	8.6132
**Residual**	1.5547	0.05	1.0004	2.7426

DistSoma=348

Fit Statistics	
-2 Res Log Likelihood	145.8
AIC (Smaller is Better)	149.8
AICC (Smaller is Better)	150.2
BIC (Smaller is Better)	150.4

Solution for Fixed Effects									
Effect	Treatment	Estimate	Standard Error	DF	t Value	Pr > |t|	Alpha	Lower	Upper
Intercept		1.1336	0.6572	8	1.73	0.1228	0.05	-0.3818	2.6490
Treatment	Meg TTR	0.08417	0.8540	31	0.10	0.9221	0.05	-1.6575	1.8258
Treatment	GFP MsTTR	0	.	.	.	.	.	.	.

Solution for Random Effects									
Effect	Culture	Estimate	Std Err Pred	DF	t Value	Pr > |t|	Alpha	Lower	Upper
Culture	1	-0.2715	0.7342	31	-0.37	0.7141	0.05	-1.7689	1.2259
Culture	2	1.0625	0.7506	31	1.42	0.1669	0.05	-0.4683	2.5932
Culture	3	-0.6871	0.7506	31	-0.92	0.3670	0.05	-2.2179	0.8437
Culture	4	-0.1039	0.7506	31	-0.14	0.8908	0.05	-1.6347	1.4269
Culture	5	-0.3400	0.6708	31	-0.51	0.6159	0.05	-1.7081	1.0282
Culture	6	0.2194	0.6943	31	0.32	0.7541	0.05	-1.1966	1.6355
Culture	7	2.1634	0.6943	31	3.12	0.0039	0.05	0.7474	3.5794
Culture	8	-1.0228	0.6538	31	-1.56	0.1279	0.05	-2.3561	0.3106
Culture	9	-0.1385	0.7841	31	-0.18	0.8609	0.05	-1.7377	1.4606
Culture	10	-0.8816	0.7287	31	-1.21	0.2355	0.05	-2.3678	0.6046

Type 3 Tests of Fixed Effects				
Effect	Num DF	Den DF	F Value	Pr > F
Treatment	1	31	0.01	0.9221

Least Squares Means									
Effect	Treatment	Estimate	Standard Error	DF	t Value	Pr > |t|	Alpha	Lower	Upper
Treatment	Meg TTR	1.2178	0.5454	31	2.23	0.0329	0.05	0.1055	2.3300
Treatment	GFP MsTTR	1.1336	0.6572	31	1.73	0.0945	0.05	-0.2067	2.4739

DistSoma=348

Differences of Least Squares Means

Effect	Treatment	Treatment	Estimate	Standard Error	DF	t Value	Pr > |t|	Adjustment	Adj P	Alpha	Lower	Upper
**Treatment**	Meg TTR	GFP MsTTR	0.08417	0.8540	31	0.10	0.9221	Tukey-Kramer	0.9221	0.05	-1.6575	1.8258

Differences of Least Squares Means

Effect	Treatment	Treatment	Adj Lower	Adj Upper
**Treatment**	Meg TTR	GFP MsTTR	-1.6575	1.8258

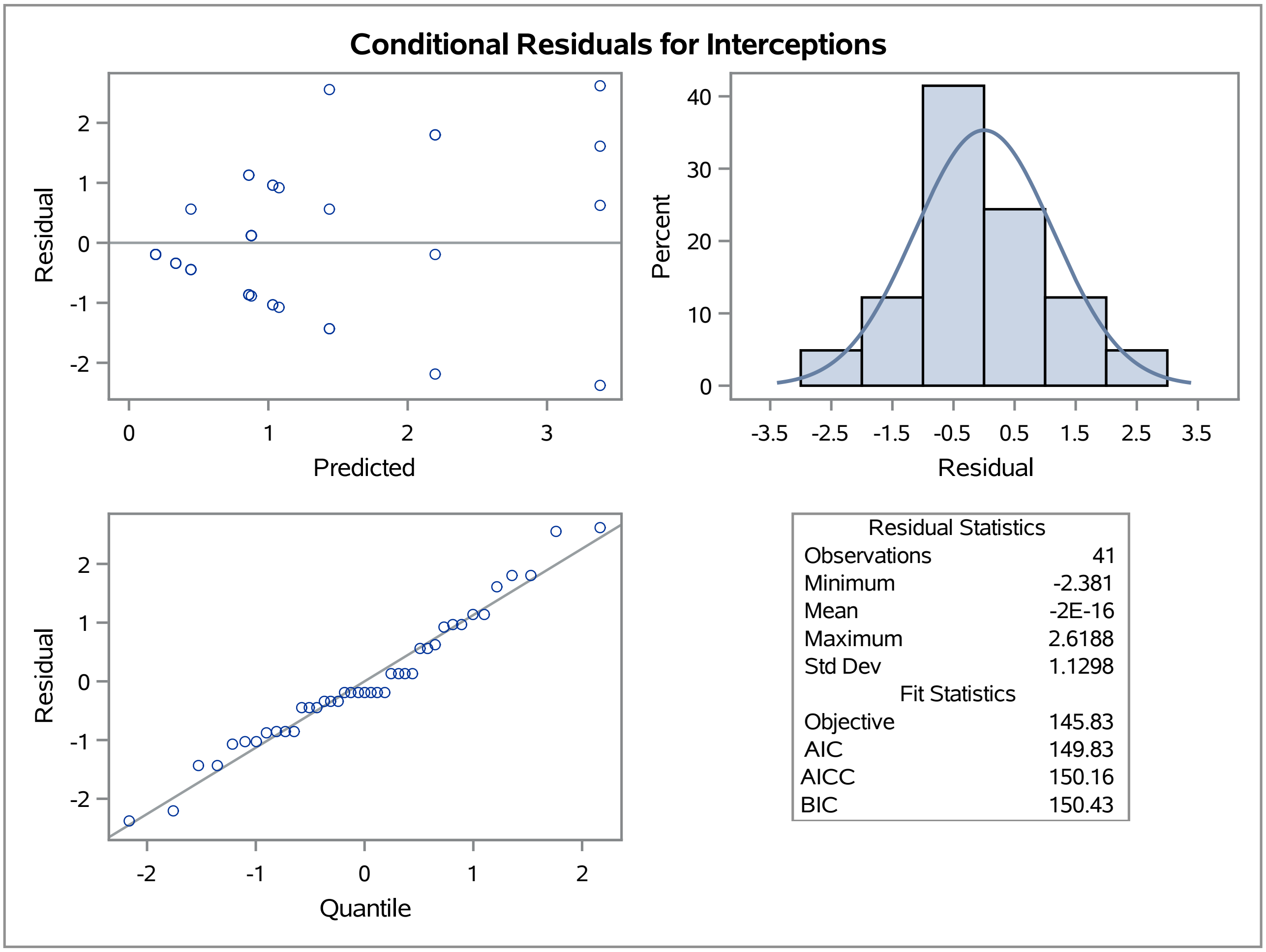

DistSoma=354

Model Information	
**Data Set**	WORK.TEMPDATASORTED
**Dependent Variable**	Interceptions
**Covariance Structure**	Variance Components
**Estimation Method**	REML
**Residual Variance Method**	Profile
**Fixed Effects SE Method**	Model-Based
**Degrees of Freedom Method**	Containment

Class Level Information		
**Class**	**Levels**	**Values**
**Treatment**	2	Meg TTR GFP MsTTR
**Culture**	10	1 2 3 4 5 6 7 8 9 10

Dimensions	
**Covariance Parameters**	2
**Columns in X**	3
**Columns in Z**	10
**Subjects**	1
**Max Obs per Subject**	41

Number of Observations	
**Number of Observations Read**	41
**Number of Observations Used**	41
**Number of Observations Not Used**	0

Iteration History			
**Iteration**	**Evaluations**	**-2 Res Log Like**	**Criterion**
**0**	1	158.65523676	
**1**	2	147.81571171	0.00000000

Convergence criteria met.

Covariance Parameter Estimates				
**Cov Parm**	**Estimate**	**Alpha**	**Lower**	**Upper**
**Culture**	1.5983	0.05	0.6252	9.4787
**Residual**	1.6015	0.05	1.0302	2.8270

DistSoma=354

Fit Statistics	
-2 Res Log Likelihood	147.8
AIC (Smaller is Better)	151.8
AICC (Smaller is Better)	152.1
BIC (Smaller is Better)	152.4

Solution for Fixed Effects

Effect	Treatment	Estimate	Standard Error	DF	t Value	Pr > |t|	Alpha	Lower	Upper
Intercept		1.2680	0.7032	8	1.80	0.1090	0.05	-0.3536	2.8896
Treatment	Meg TTR	-0.07833	0.9133	31	-0.09	0.9322	0.05	-1.9411	1.7844
Treatment	GFP MsTTR	0	.	.	.	.	.	.	.

Solution for Random Effects

Effect	Culture	Estimate	Std Err Pred	DF	t Value	Pr > |t|	Alpha	Lower	Upper
Culture	1	-0.5565	0.7810	31	-0.71	0.4815	0.05	-2.1494	1.0364
Culture	2	1.1851	0.7977	31	1.49	0.1475	0.05	-0.4419	2.8121
Culture	3	-0.4143	0.7977	31	-0.52	0.6073	0.05	-2.0413	1.2128
Culture	4	-0.2143	0.7977	31	-0.27	0.7900	0.05	-1.8414	1.4127
Culture	5	-0.3246	0.7089	31	-0.46	0.6502	0.05	-1.7705	1.1212
Culture	6	0.2481	0.7331	31	0.34	0.7373	0.05	-1.2470	1.7433
Culture	7	2.4473	0.7331	31	3.34	0.0022	0.05	0.9521	3.9424
Culture	8	-1.0194	0.6915	31	-1.47	0.1505	0.05	-2.4297	0.3908
Culture	9	-0.4595	0.8272	31	-0.56	0.5826	0.05	-2.1466	1.2276
Culture	10	-0.8918	0.7688	31	-1.16	0.2549	0.05	-2.4598	0.6762

Type 3 Tests of Fixed Effects

Effect	Num DF	Den DF	F Value	Pr > F
Treatment	1	31	0.01	0.9322

Least Squares Means

Effect	Treatment	Estimate	Standard Error	DF	t Value	Pr > |t|	Alpha	Lower	Upper
Treatment	Meg TTR	1.1897	0.5828	31	2.04	0.0498	0.05	0.001007	2.3784
Treatment	GFP MsTTR	1.2680	0.7032	31	1.80	0.0811	0.05	-0.1662	2.7022

DistSoma=354

Differences of Least Squares Means

Effect	Treatment	Treatment	Estimate	Standard Error	DF	t Value	Pr > |t|	Adjustment	Adj P	Alpha	Lower	Upper
**Treatment**	Meg TTR	GFP MsTTR	-0.07833	0.9133	31	-0.09	0.9322	Tukey-Kramer	0.9322	0.05	-1.9411	1.7844

Differences of Least Squares Means

Effect	Treatment	Treatment	Adj Lower	Adj Upper
**Treatment**	Meg TTR	GFP MsTTR	-1.9411	1.7844

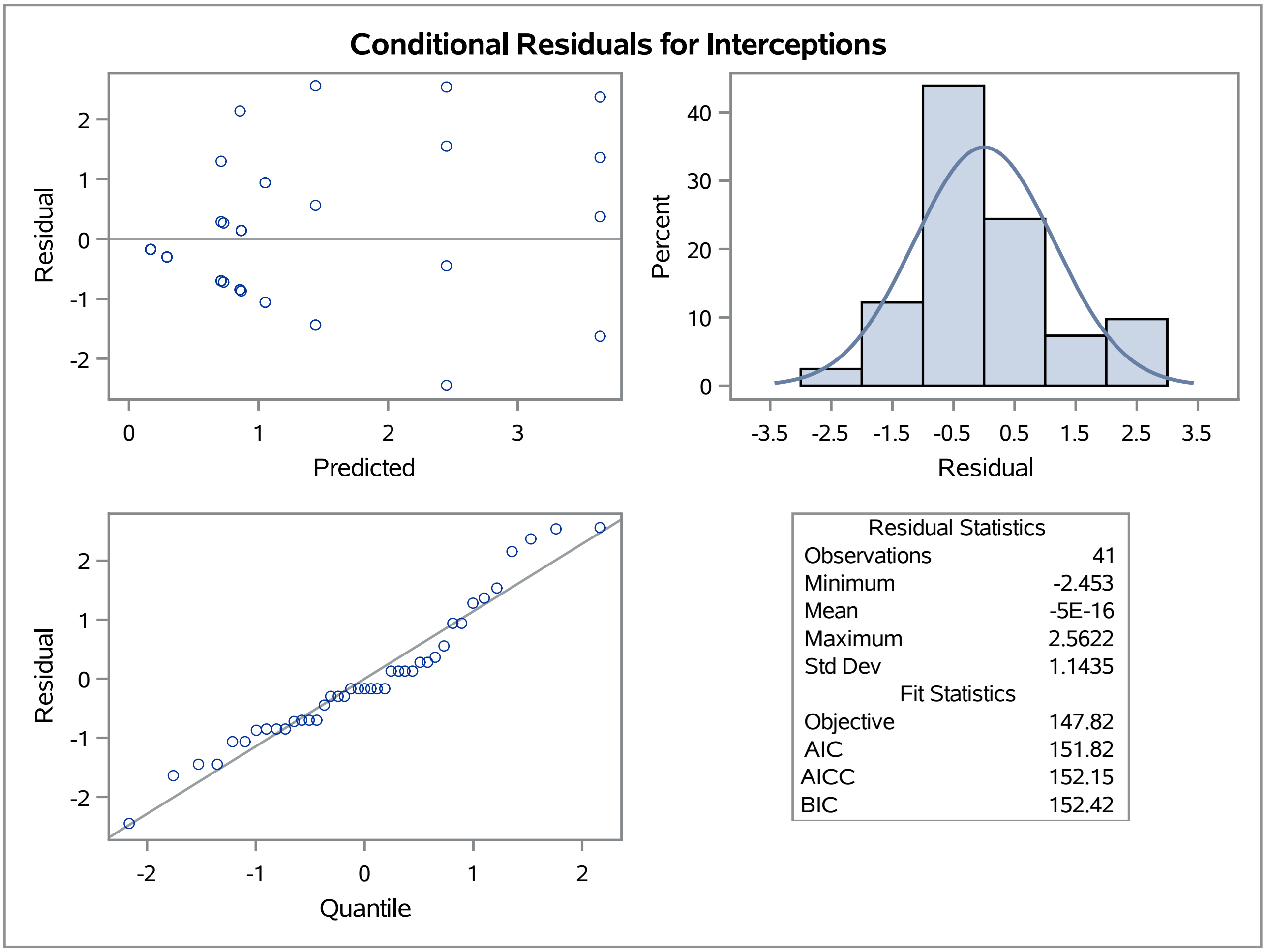

DistSoma=360

Model Information	
**Data Set**	WORK.TEMPDATASORTED
**Dependent Variable**	Interceptions
**Covariance Structure**	Variance Components
**Estimation Method**	REML
**Residual Variance Method**	Profile
**Fixed Effects SE Method**	Model-Based
**Degrees of Freedom Method**	Containment

Class Level Information		
**Class**	**Levels**	**Values**
**Treatment**	2	Meg TTR GFP MsTTR
**Culture**	10	1 2 3 4 5 6 7 8 9 10

Dimensions	
**Covariance Parameters**	2
**Columns in X**	3
**Columns in Z**	10
**Subjects**	1
**Max Obs per Subject**	41

Number of Observations	
**Number of Observations Read**	41
**Number of Observations Used**	41
**Number of Observations Not Used**	0

Iteration History			
**Iteration**	**Evaluations**	**-2 Res Log Like**	**Criterion**
**0**	1	162.76295985	
**1**	2	154.24646586	0.00000000

Convergence criteria met.

Covariance Parameter Estimates				
**Cov Parm**	**Estimate**	**Alpha**	**Lower**	**Upper**
**Culture**	1.5686	0.05	0.5920	10.6110
**Residual**	1.9552	0.05	1.2578	3.4509

DistSoma=360

Fit Statistics	
-2 Res Log Likelihood	154.2
AIC (Smaller is Better)	158.2
AICC (Smaller is Better)	158.6
BIC (Smaller is Better)	158.9

Solution for Fixed Effects									
Effect	Treatment	Estimate	Standard Error	DF	t Value	Pr > |t|	Alpha	Lower	Upper
Intercept		1.1432	0.7128	8	1.60	0.1474	0.05	-0.5004	2.7868
Treatment	Meg TTR	0.04814	0.9265	31	0.05	0.9589	0.05	-1.8416	1.9378
Treatment	GFP MsTTR	0	.	.	.	.	.	.	.

Solution for Random Effects									
Effect	Culture	Estimate	Std Err Pred	DF	t Value	Pr > |t|	Alpha	Lower	Upper
Culture	1	-0.4348	0.7991	31	-0.54	0.5902	0.05	-2.0645	1.1949
Culture	2	1.2250	0.8173	31	1.50	0.1440	0.05	-0.4419	2.8919
Culture	3	-0.2998	0.8173	31	-0.37	0.7162	0.05	-1.9667	1.3671
Culture	4	-0.4904	0.8173	31	-0.60	0.5528	0.05	-2.1573	1.1765
Culture	5	-0.3133	0.7332	31	-0.43	0.6721	0.05	-1.8086	1.1821
Culture	6	0.2353	0.7592	31	0.31	0.7587	0.05	-1.3131	1.7837
Culture	7	2.3320	0.7592	31	3.07	0.0044	0.05	0.7836	3.8804
Culture	8	-0.9864	0.7142	31	-1.38	0.1771	0.05	-2.4431	0.4702
Culture	9	-0.4259	0.8575	31	-0.50	0.6229	0.05	-2.1748	1.3229
Culture	10	-0.8417	0.7971	31	-1.06	0.2992	0.05	-2.4673	0.7840

Type 3 Tests of Fixed Effects				
Effect	Num DF	Den DF	F Value	Pr > F
Treatment	1	31	0.00	0.9589

Least Squares Means									
Effect	Treatment	Estimate	Standard Error	DF	t Value	Pr > |t|	Alpha	Lower	Upper
Treatment	Meg TTR	1.1914	0.5920	31	2.01	0.0529	0.05	-0.01603	2.3988
Treatment	GFP MsTTR	1.1432	0.7128	31	1.60	0.1189	0.05	-0.3104	2.5969

DistSoma=360

Differences of Least Squares Means													
Effect	Treatment	Treatment	Estimate	Standard Error	DF	t Value	Pr > |t|	Adjustment	Adj P	Alpha	Lower	Upper
Treatment	Meg TTR	GFP MsTTR	0.04814	0.9265	31	0.05	0.9589	Tukey-Kramer	0.9589	0.05	-1.8416	1.9378

Differences of Least Squares Means				
Effect	Treatment	Treatment	Adj Lower	Adj Upper
Treatment	Meg TTR	GFP MsTTR	-1.8415	1.9378

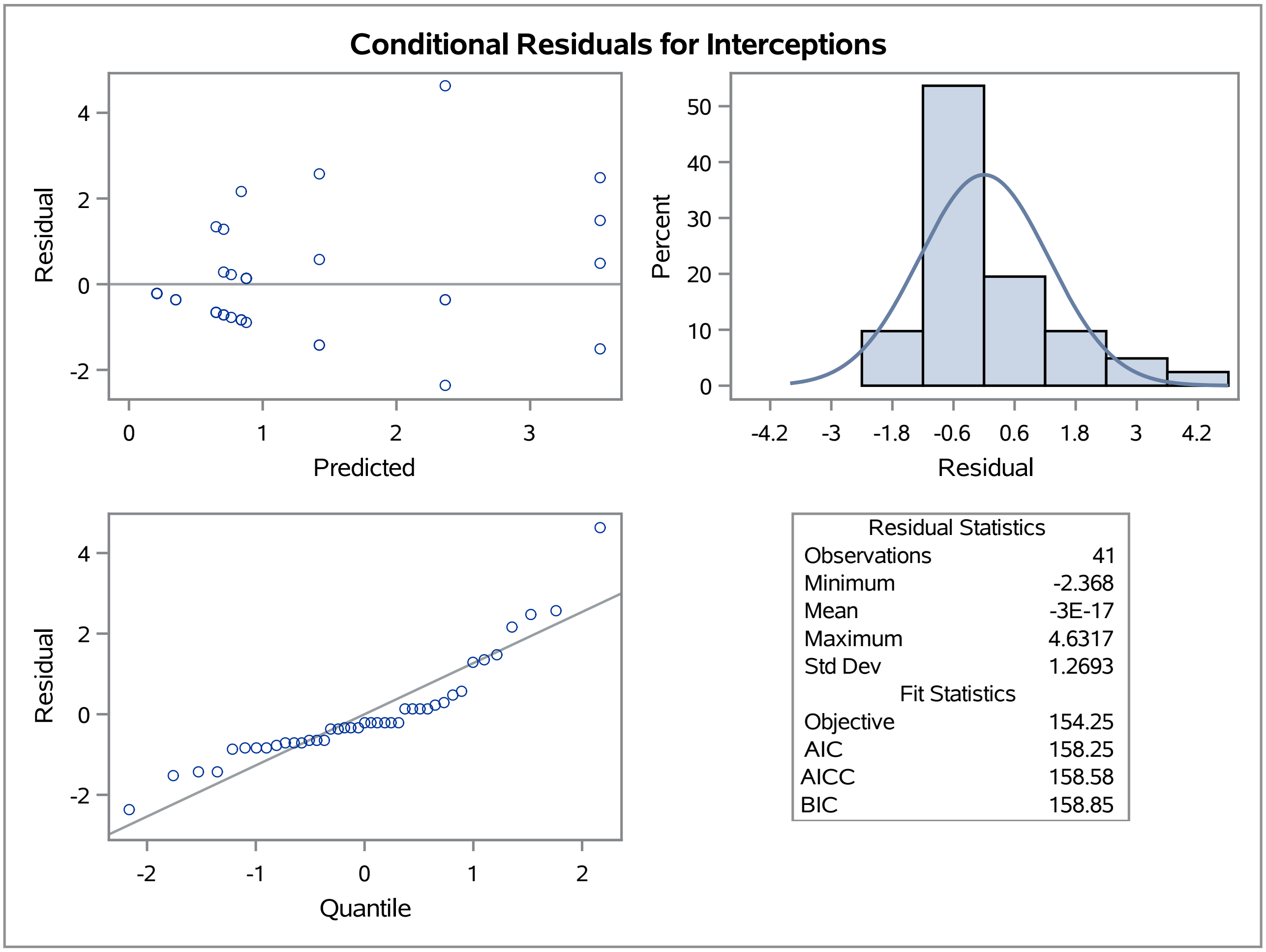

DistSoma=366

Model Information	
**Data Set**	WORK.TEMPDATASORTED
**Dependent Variable**	Interceptions
**Covariance Structure**	Variance Components
**Estimation Method**	REML
**Residual Variance Method**	Profile
**Fixed Effects SE Method**	Model-Based
**Degrees of Freedom Method**	Containment

Class Level Information		
**Class**	**Levels**	**Values**
**Treatment**	2	Meg TTR GFP MsTTR
**Culture**	10	1 2 3 4 5 6 7 8 9 10

Dimensions	
**Covariance Parameters**	2
**Columns in X**	3
**Columns in Z**	10
**Subjects**	1
**Max Obs per Subject**	41

Number of Observations	
**Number of Observations Read**	41
**Number of Observations Used**	41
**Number of Observations Not Used**	0

Iteration History			
**Iteration**	**Evaluations**	**-2 Res Log Like**	**Criterion**
**0**	1	170.82985932	
**1**	2	167.11589156	0.00000000

Convergence criteria met.

Covariance Parameter Estimates				
**Cov Parm**	**Estimate**	**Alpha**	**Lower**	**Upper**
**Culture**	1.2278	0.05	0.3941	17.0512
**Residual**	2.9888	0.05	1.9236	5.2711

DistSoma=366

Fit Statistics	
**-2 Res Log Likelihood**	167.1
**AIC (Smaller is Better)**	171.1
**AICC (Smaller is Better)**	171.4
**BIC (Smaller is Better)**	171.7

Solution for Fixed Effects

Effect	Treatment	Estimate	Standard Error	DF	t Value	Pr > |t|	Alpha	Lower	Upper
**Intercept**		1.2002	0.6956	8	1.73	0.1227	0.05	-0.4038	2.8043
**Treatment**	Meg TTR	-0.09087	0.9067	31	-0.10	0.9208	0.05	-1.9400	1.7583
**Treatment**	GFP MsTTR	0	.	.	.	.	.	.	.

Solution for Random Effects

Effect	Culture	Estimate	Std Err Pred	DF	t Value	Pr > |t|	Alpha	Lower	Upper
**Culture**	1	-0.4037	0.7880	31	-0.51	0.6121	0.05	-2.0108	1.2034
**Culture**	2	1.2743	0.8072	31	1.58	0.1246	0.05	-0.3719	2.9205
**Culture**	3	-0.4353	0.8072	31	-0.54	0.5935	0.05	-2.0815	1.2109
**Culture**	4	-0.4353	0.8072	31	-0.54	0.5935	0.05	-2.0815	1.2109
**Culture**	5	-0.2081	0.7450	31	-0.28	0.7819	0.05	-1.7275	1.3114
**Culture**	6	0.2429	0.7715	31	0.31	0.7550	0.05	-1.3306	1.8164
**Culture**	7	1.6416	0.7715	31	2.13	0.0414	0.05	0.06811	3.2151
**Culture**	8	-0.7892	0.7249	31	-1.09	0.2847	0.05	-2.2677	0.6893
**Culture**	9	-0.2748	0.8619	31	-0.32	0.7520	0.05	-2.0326	1.4830
**Culture**	10	-0.6124	0.8081	31	-0.76	0.4543	0.05	-2.2606	1.0358

Type 3 Tests of Fixed Effects

Effect	Num DF	Den DF	F Value	Pr > F
**Treatment**	1	31	0.01	0.9208

Least Squares Means

Effect	Treatment	Estimate	Standard Error	DF	t Value	Pr > |t|	Alpha	Lower	Upper
**Treatment**	Meg TTR	1.1093	0.5816	31	1.91	0.0658	0.05	-0.07675	2.2954
**Treatment**	GFP MsTTR	1.2002	0.6956	31	1.73	0.0944	0.05	-0.2185	2.6189

DistSoma=366

Differences of Least Squares Means												
**Effect**	**Treatment**	**Treatment**	**Estimate**	**Standard Error**	**DF**	**t Value**	**Pr > |t|**	**Adjustment**	**Adj P**	**Alpha**	**Lower**	**Upper**
**Treatment**	Meg TTR	GFP MsTTR	-0.09087	0.9067	31	-0.10	0.9208	Tukey-Kramer	0.9208	0.05	-1.9400	1.7583

Differences of Least Squares Means				
**Effect**	**Treatment**	**Treatment**	**Adj Lower**	**Adj Upper**
**Treatment**	Meg TTR	GFP MsTTR	-1.9400	1.7583

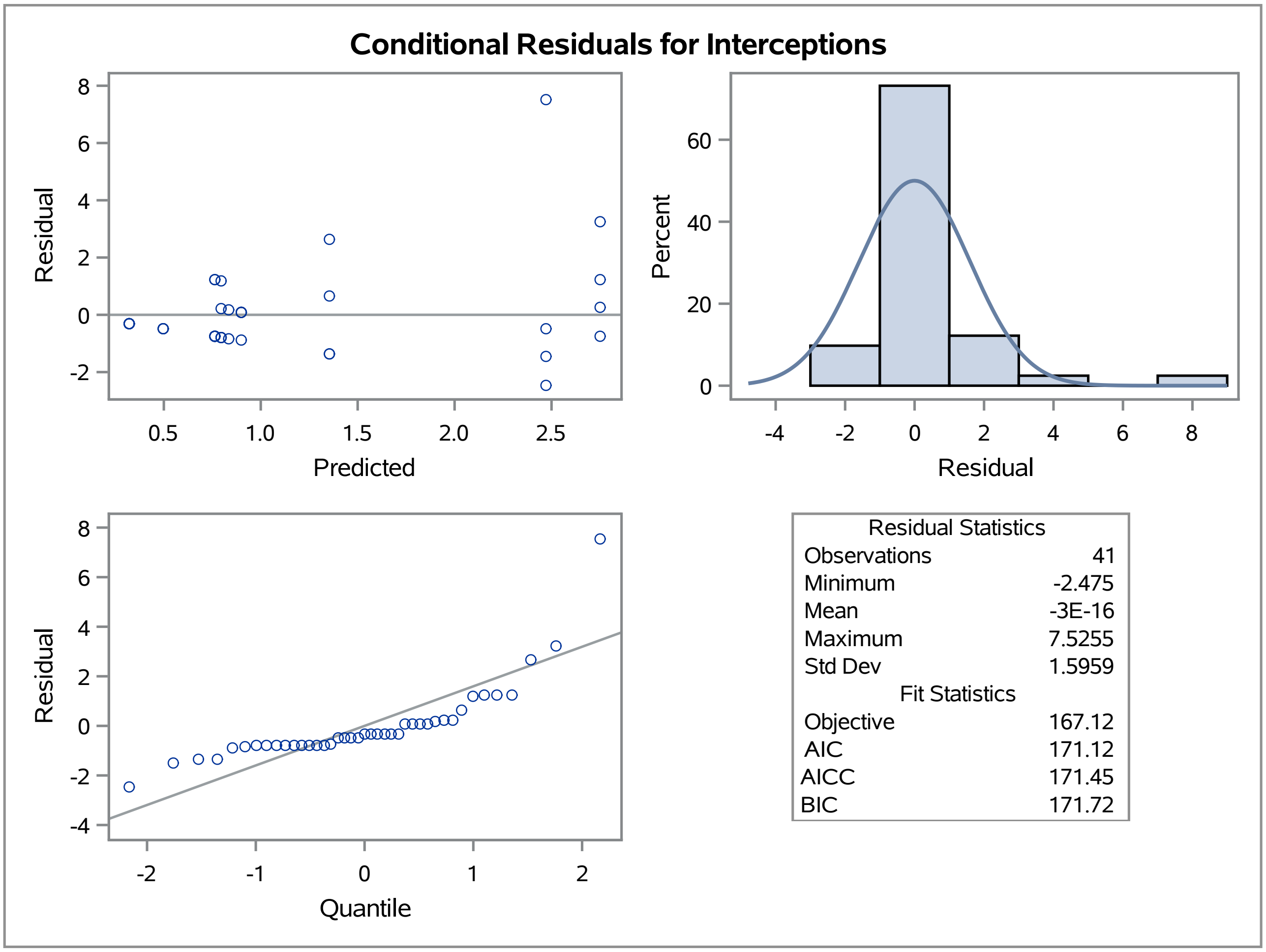

DistSoma=372

Model Information	
**Data Set**	WORK.TEMPDATASORTED
**Dependent Variable**	Interceptions
**Covariance Structure**	Variance Components
**Estimation Method**	REML
**Residual Variance Method**	Profile
**Fixed Effects SE Method**	Model-Based
**Degrees of Freedom Method**	Containment

Class Level Information		
**Class**	**Levels**	**Values**
**Treatment**	2	Meg TTR GFP MsTTR
**Culture**	10	1 2 3 4 5 6 7 8 9 10

Dimensions	
**Covariance Parameters**	2
**Columns in X**	3
**Columns in Z**	10
**Subjects**	1
**Max Obs per Subject**	41

Number of Observations	
**Number of Observations Read**	41
**Number of Observations Used**	41
**Number of Observations Not Used**	0

Iteration History			
**Iteration**	**Evaluations**	**-2 Res Log Like**	**Criterion**
**0**	1	166.74878474	
**1**	2	162.15825887	0.00000000

Convergence criteria met.

Covariance Parameter Estimates				
**Cov Parm**	**Estimate**	**Alpha**	**Lower**	**Upper**
**Culture**	1.2384	0.05	0.4166	13.6377
**Residual**	2.5794	0.05	1.6600	4.5491

DistSoma=372

Fit Statistics	
-2 Res Log Likelihood	162.2
AIC (Smaller is Better)	166.2
AICC (Smaller is Better)	166.5
BIC (Smaller is Better)	166.8

Solution for Fixed Effects

Effect	Treatment	Estimate	Standard Error	DF	t Value	Pr > |t|	Alpha	Lower	Upper
Intercept		1.0874	0.6799	8	1.60	0.1484	0.05	-0.4806	2.6553
Treatment	Meg TTR	0.02152	0.8857	31	0.02	0.9808	0.05	-1.7849	1.8280
Treatment	GFP MsTTR	0	.	.	.	.	.	.	.

Solution for Random Effects

Effect	Culture	Estimate	Std Err Pred	DF	t Value	Pr > |t|	Alpha	Lower	Upper
Culture	1	-0.4852	0.7711	31	-0.63	0.5338	0.05	-2.0579	1.0874
Culture	2	1.2577	0.7899	31	1.59	0.1215	0.05	-0.3533	2.8688
Culture	3	-0.3862	0.7899	31	-0.49	0.6283	0.05	-1.9973	1.2248
Culture	4	-0.3862	0.7899	31	-0.49	0.6283	0.05	-1.9973	1.2248
Culture	5	-0.2181	0.7244	31	-0.30	0.7654	0.05	-1.6955	1.2593
Culture	6	0.2572	0.7506	31	0.34	0.7342	0.05	-1.2736	1.7880
Culture	7	1.7368	0.7506	31	2.31	0.0275	0.05	0.2060	3.2676
Culture	8	-0.8231	0.7047	31	-1.17	0.2517	0.05	-2.2605	0.6142
Culture	9	-0.2983	0.8421	31	-0.35	0.7256	0.05	-2.0157	1.4191
Culture	10	-0.6545	0.7872	31	-0.83	0.4121	0.05	-2.2600	0.9510

Type 3 Tests of Fixed Effects

Effect	Num DF	Den DF	F Value	Pr > F
Treatment	1	31	0.00	0.9808

Least Squares Means

Effect	Treatment	Estimate	Standard Error	DF	t Value	Pr > |t|	Alpha	Lower	Upper
Treatment	Meg TTR	1.1089	0.5676	31	1.95	0.0598	0.05	-0.04880	2.2666
Treatment	GFP MsTTR	1.0874	0.6799	31	1.60	0.1199	0.05	-0.2994	2.4741

DistSoma=372

Differences of Least Squares Means												
Effect	Treatment	Treatment	Estimate	Standard Error	DF	t Value	Pr > |t|	Adjustment	Adj P	Alpha	Lower	Upper
Treatment	Meg TTR	GFP MsTTR	0.02152	0.8857	31	0.02	0.9808	Tukey-Kramer	0.9808	0.05	-1.7849	1.8280

Differences of Least Squares Means				
Effect	Treatment	Treatment	Adj Lower	Adj Upper
Treatment	Meg TTR	GFP MsTTR	-1.7849	1.8280

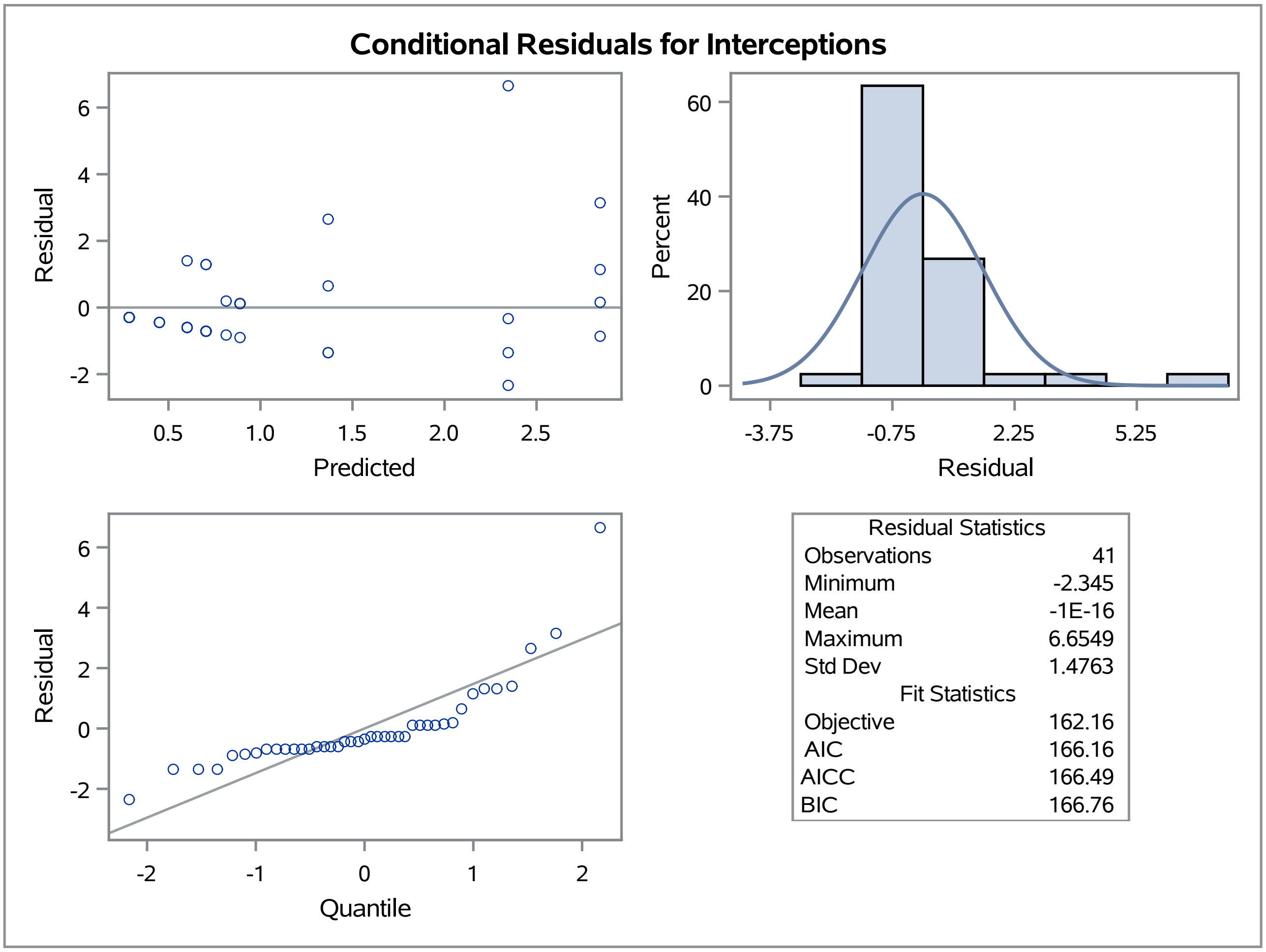

DistSoma=378

Model Information	
**Data Set**	WORK.TEMPDATASORTED
**Dependent Variable**	Interceptions
**Covariance Structure**	Variance Components
**Estimation Method**	REML
**Residual Variance Method**	Profile
**Fixed Effects SE Method**	Model-Based
**Degrees of Freedom Method**	Containment

Class Level Information		
**Class**	**Levels**	**Values**
**Treatment**	2	Meg TTR GFP MsTTR
**Culture**	10	1 2 3 4 5 6 7 8 9 10

Dimensions	
**Covariance Parameters**	2
**Columns in X**	3
**Columns in Z**	10
**Subjects**	1
**Max Obs per Subject**	41

Number of Observations	
**Number of Observations Read**	41
**Number of Observations Used**	41
**Number of Observations Not Used**	0

Iteration History			
**Iteration**	**Evaluations**	**-2 Res Log Like**	**Criterion**
**0**	1	162.37294149	
**1**	2	156.96141243	0.00000000

Convergence criteria met.

Covariance Parameter Estimates				
**Cov Parm**	**Estimate**	**Alpha**	**Lower**	**Upper**
**Culture**	1.2107	0.05	0.4212	11.4340
**Residual**	2.2185	0.05	1.4276	3.9133

DistSoma=378

Fit Statistics	
-2 Res Log Likelihood	157.0
AIC (Smaller is Better)	161.0
AICC (Smaller is Better)	161.3
BIC (Smaller is Better)	161.6

Solution for Fixed Effects

Effect	Treatment	Estimate	Standard Error	DF	t Value	Pr > |t|	Alpha	Lower	Upper
Intercept		1.0270	0.6588	8	1.56	0.1577	0.05	-0.4923	2.5463
Treatment	Meg TTR	0.08137	0.8578	31	0.09	0.9250	0.05	-1.6681	1.8309
Treatment	GFP MsTTR	0	.	.	.	.	.	.	.

Solution for Random Effects

Effect	Culture	Estimate	Std Err Pred	DF	t Value	Pr > |t|	Alpha	Lower	Upper
Culture	1	-0.4588	0.7464	31	-0.61	0.5432	0.05	-1.9812	1.0635
Culture	2	1.1817	0.7646	31	1.55	0.1324	0.05	-0.3776	2.7410
Culture	3	-0.3614	0.7646	31	-0.47	0.6397	0.05	-1.9207	1.1979
Culture	4	-0.3614	0.7646	31	-0.47	0.6397	0.05	-1.9207	1.1979
Culture	5	-0.2257	0.6974	31	-0.32	0.7484	0.05	-1.6479	1.1966
Culture	6	0.2686	0.7227	31	0.37	0.7127	0.05	-1.2054	1.7426
Culture	7	1.8117	0.7227	31	2.51	0.0176	0.05	0.3377	3.2857
Culture	8	-0.8491	0.6785	31	-1.25	0.2201	0.05	-2.2328	0.5347
Culture	9	-0.3175	0.8131	31	-0.39	0.6989	0.05	-1.9757	1.3408
Culture	10	-0.6881	0.7585	31	-0.91	0.3713	0.05	-2.2351	0.8590

Type 3 Tests of Fixed Effects

Effect	Num DF	Den DF	F Value	Pr > F
Treatment	1	31	0.01	0.9250

Least Squares Means

Effect	Treatment	Estimate	Standard Error	DF	t Value	Pr > |t|	Alpha	Lower	Upper
Treatment	Meg TTR	1.1084	0.5493	31	2.02	0.0524	0.05	-0.01199	2.2287
Treatment	GFP MsTTR	1.0270	0.6588	31	1.56	0.1292	0.05	-0.3167	2.3707

DistSoma=378

Differences of Least Squares Means												
**Effect**	**Treatment**	**Treatment**	**Estimate**	**Standard Error**	**DF**	**t Value**	**Pr > |t|**	**Adjustment**	**Adj P**	**Alpha**	**Lower**	**Upper**
**Treatment**	Meg TTR	GFP MsTTR	0.08137	0.8578	31	0.09	0.9250	Tukey-Kramer	0.9250	0.05	-1.6681	1.8309

Differences of Least Squares Means				
**Effect**	**Treatment**	**Treatment**	**Adj Lower**	**Adj Upper**
**Treatment**	Meg TTR	GFP MsTTR	-1.6681	1.8308

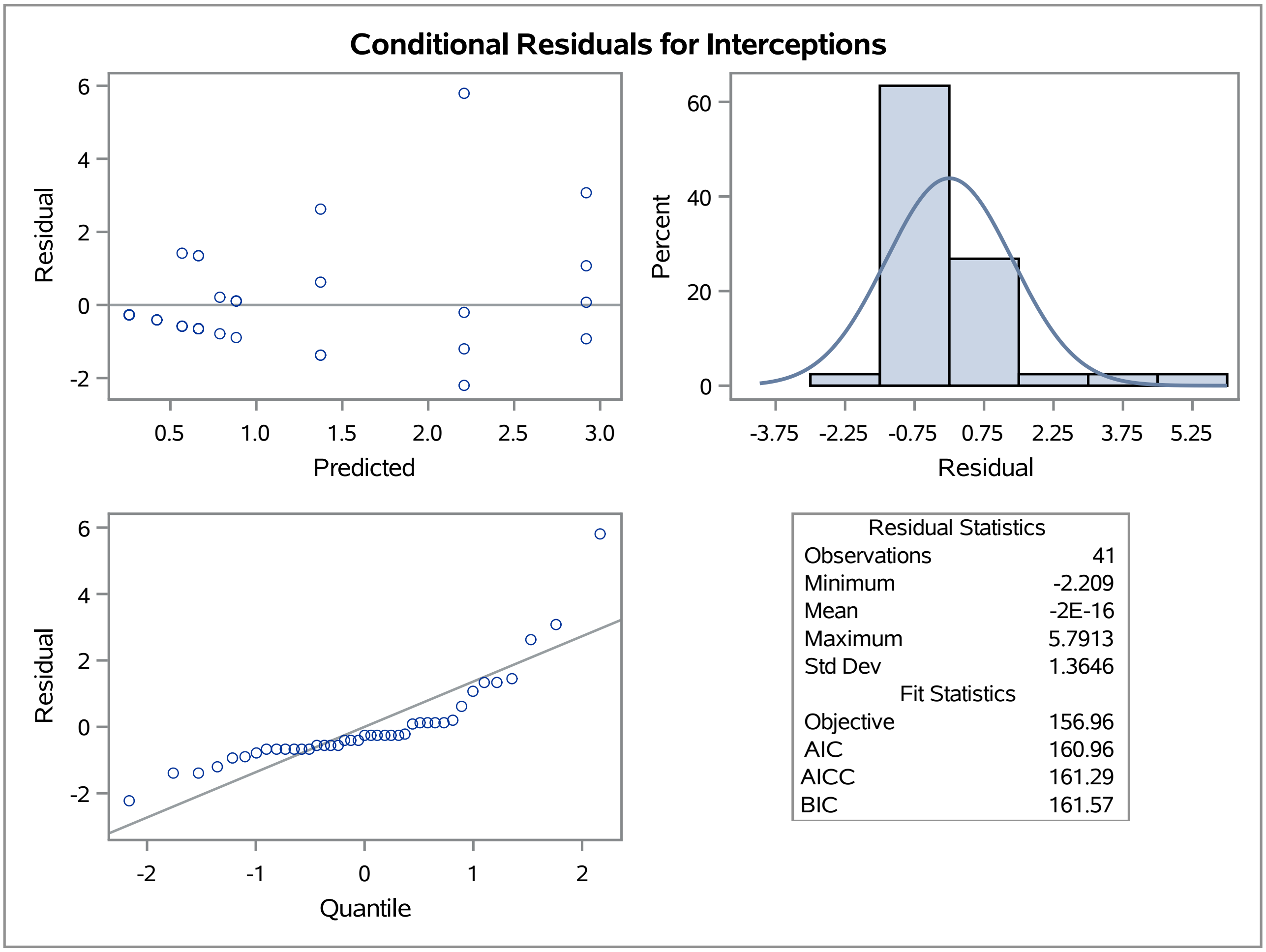

DistSoma=384

Model Information	
**Data Set**	WORK.TEMPDATASORTED
**Dependent Variable**	Interceptions
**Covariance Structure**	Variance Components
**Estimation Method**	REML
**Residual Variance Method**	Profile
**Fixed Effects SE Method**	Model-Based
**Degrees of Freedom Method**	Containment

Class Level Information		
**Class**	**Levels**	**Values**
**Treatment**	2	Meg TTR GFP MsTTR
**Culture**	10	1 2 3 4 5 6 7 8 9 10

Dimensions	
**Covariance Parameters**	2
**Columns in X**	3
**Columns in Z**	10
**Subjects**	1
**Max Obs per Subject**	41

Number of Observations	
**Number of Observations Read**	41
**Number of Observations Used**	41
**Number of Observations Not Used**	0

Iteration History			
**Iteration**	**Evaluations**	**-2 Res Log Like**	**Criterion**
**0**	1	157.72077169	
**1**	2	151.69388856	0.00000000

Convergence criteria met.

Covariance Parameter Estimates				
**Cov Parm**	**Estimate**	**Alpha**	**Lower**	**Upper**
**Culture**	1.1366	0.05	0.4034	9.8448
**Residual**	1.9155	0.05	1.2325	3.3794

DistSoma=384

Fit Statistics	
-2 Res Log Likelihood	151.7
AIC (Smaller is Better)	155.7
AICC (Smaller is Better)	156.0
BIC (Smaller is Better)	156.3

Solution for Fixed Effects

Effect	Treatment	Estimate	Standard Error	DF	t Value	Pr > |t|	Alpha	Lower	Upper
Intercept		0.7907	0.6306	8	1.25	0.2453	0.05	-0.6634	2.2448
Treatment	Meg TTR	0.3536	0.8207	31	0.43	0.6696	0.05	-1.3203	2.0274
Treatment	GFP MsTTR	0	.	.	.	.	.	.	.

Solution for Random Effects

Effect	Culture	Estimate	Std Err Pred	DF	t Value	Pr > |t|	Alpha	Lower	Upper
Culture	1	-0.4418	0.7134	31	-0.62	0.5403	0.05	-1.8968	1.0132
Culture	2	1.0267	0.7306	31	1.41	0.1699	0.05	-0.4633	2.5167
Culture	3	-0.2045	0.7306	31	-0.28	0.7814	0.05	-1.6945	1.2855
Culture	4	-0.3804	0.7306	31	-0.52	0.6063	0.05	-1.8704	1.1096
Culture	5	-0.1079	0.6640	31	-0.16	0.8720	0.05	-1.4621	1.2463
Culture	6	0.2503	0.6881	31	0.36	0.7185	0.05	-1.1532	1.6537
Culture	7	1.8333	0.6881	31	2.66	0.0121	0.05	0.4299	3.2368
Culture	8	-0.8934	0.6461	31	-1.38	0.1766	0.05	-2.2111	0.4244
Culture	9	-0.3496	0.7753	31	-0.45	0.6551	0.05	-1.9308	1.2315
Culture	10	-0.7327	0.7225	31	-1.01	0.3184	0.05	-2.2062	0.7408

Type 3 Tests of Fixed Effects

Effect	Num DF	Den DF	F Value	Pr > F
Treatment	1	31	0.19	0.6696

Least Squares Means

Effect	Treatment	Estimate	Standard Error	DF	t Value	Pr > |t|	Alpha	Lower	Upper
Treatment	Meg TTR	1.1443	0.5253	31	2.18	0.0371	0.05	0.07289	2.2157
Treatment	GFP MsTTR	0.7907	0.6306	31	1.25	0.2192	0.05	-0.4953	2.0767

DistSoma=384

Differences of Least Squares Means												
Effect	Treatment	Treatment	Estimate	Standard Error	DF	t Value	Pr > |t|	Adjustment	Adj P	Alpha	Lower	Upper
**Treatment**	Meg TTR	GFP MsTTR	0.3536	0.8207	31	0.43	0.6696	Tukey-Kramer	0.6696	0.05	-1.3203	2.0274

Differences of Least Squares Means				
Effect	Treatment	Treatment	Adj Lower	Adj Upper
**Treatment**	Meg TTR	GFP MsTTR	-1.3202	2.0274

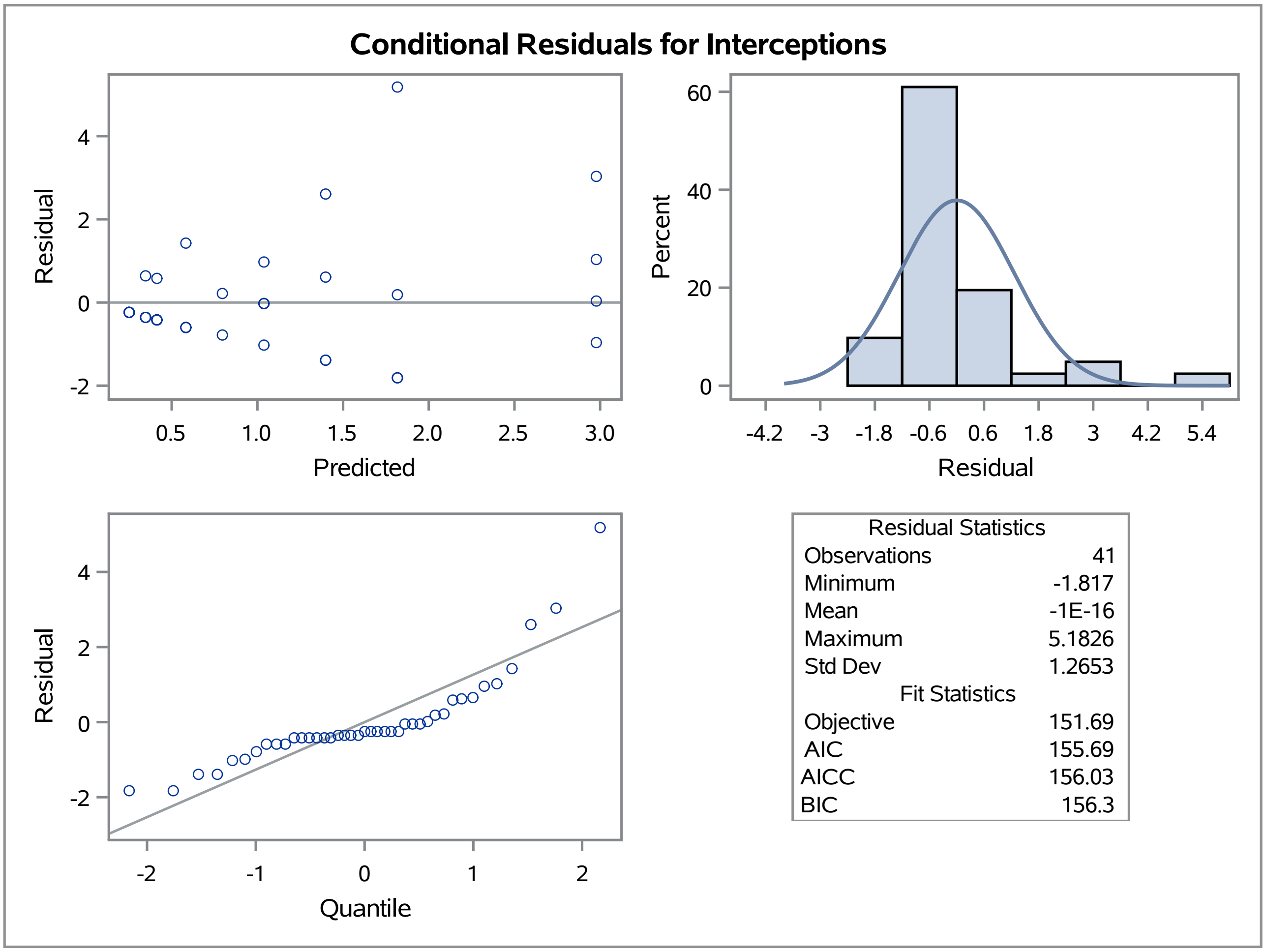

DistSoma=390

Model Information	
**Data Set**	WORK.TEMPDATASORTED
**Dependent Variable**	Interceptions
**Covariance Structure**	Variance Components
**Estimation Method**	REML
**Residual Variance Method**	Profile
**Fixed Effects SE Method**	Model-Based
**Degrees of Freedom Method**	Containment

Class Level Information		
**Class**	**Levels**	**Values**
**Treatment**	2	Meg TTR GFP MsTTR
**Culture**	10	1 2 3 4 5 6 7 8 9 10

Dimensions	
**Covariance Parameters**	2
**Columns in X**	3
**Columns in Z**	10
**Subjects**	1
**Max Obs per Subject**	41

Number of Observations	
**Number of Observations Read**	41
**Number of Observations Used**	41
**Number of Observations Not Used**	0

Iteration History			
**Iteration**	**Evaluations**	**-2 Res Log Like**	**Criterion**
**0**	1	157.72077169	
**1**	2	151.69388856	0.00000000

Convergence criteria met.

Covariance Parameter Estimates				
**Cov Parm**	**Estimate**	**Alpha**	**Lower**	**Upper**
**Culture**	1.1366	0.05	0.4034	9.8448
**Residual**	1.9155	0.05	1.2325	3.3794

DistSoma=390

Fit Statistics	
-2 Res Log Likelihood	151.7
AIC (Smaller is Better)	155.7
AICC (Smaller is Better)	156.0
BIC (Smaller is Better)	156.3

Solution for Fixed Effects

Effect	Treatment	Estimate	Standard Error	DF	t Value	Pr > |t|	Alpha	Lower	Upper
Intercept		0.7907	0.6306	8	1.25	0.2453	0.05	-0.6634	2.2448
Treatment	Meg TTR	0.3536	0.8207	31	0.43	0.6696	0.05	-1.3203	2.0274
Treatment	GFP MsTTR	0	.	.	.	.	.	.	.

Solution for Random Effects

Effect	Culture	Estimate	Std Err Pred	DF	t Value	Pr > |t|	Alpha	Lower	Upper
Culture	1	-0.4418	0.7134	31	-0.62	0.5403	0.05	-1.8968	1.0132
Culture	2	1.0267	0.7306	31	1.41	0.1699	0.05	-0.4633	2.5167
Culture	3	-0.2045	0.7306	31	-0.28	0.7814	0.05	-1.6945	1.2855
Culture	4	-0.3804	0.7306	31	-0.52	0.6063	0.05	-1.8704	1.1096
Culture	5	-0.1079	0.6640	31	-0.16	0.8720	0.05	-1.4621	1.2463
Culture	6	0.2503	0.6881	31	0.36	0.7185	0.05	-1.1532	1.6537
Culture	7	1.8333	0.6881	31	2.66	0.0121	0.05	0.4299	3.2368
Culture	8	-0.8934	0.6461	31	-1.38	0.1766	0.05	-2.2111	0.4244
Culture	9	-0.3496	0.7753	31	-0.45	0.6551	0.05	-1.9308	1.2315
Culture	10	-0.7327	0.7225	31	-1.01	0.3184	0.05	-2.2062	0.7408

Type 3 Tests of Fixed Effects

Effect	Num DF	Den DF	F Value	Pr > F
Treatment	1	31	0.19	0.6696

Least Squares Means

Effect	Treatment	Estimate	Standard Error	DF	t Value	Pr > |t|	Alpha	Lower	Upper
Treatment	Meg TTR	1.1443	0.5253	31	2.18	0.0371	0.05	0.07289	2.2157
Treatment	GFP MsTTR	0.7907	0.6306	31	1.25	0.2192	0.05	-0.4953	2.0767

DistSoma=390

Differences of Least Squares Means												
Effect	Treatment	Treatment	Estimate	Standard Error	DF	t Value	Pr > |t|	Adjustment	Adj P	Alpha	Lower	Upper
**Treatment**	Meg TTR	GFP MsTTR	0.3536	0.8207	31	0.43	0.6696	Tukey-Kramer	0.6696	0.05	-1.3203	2.0274

Differences of Least Squares Means				
Effect	Treatment	Treatment	Adj Lower	Adj Upper
**Treatment**	Meg TTR	GFP MsTTR	-1.3202	2.0274

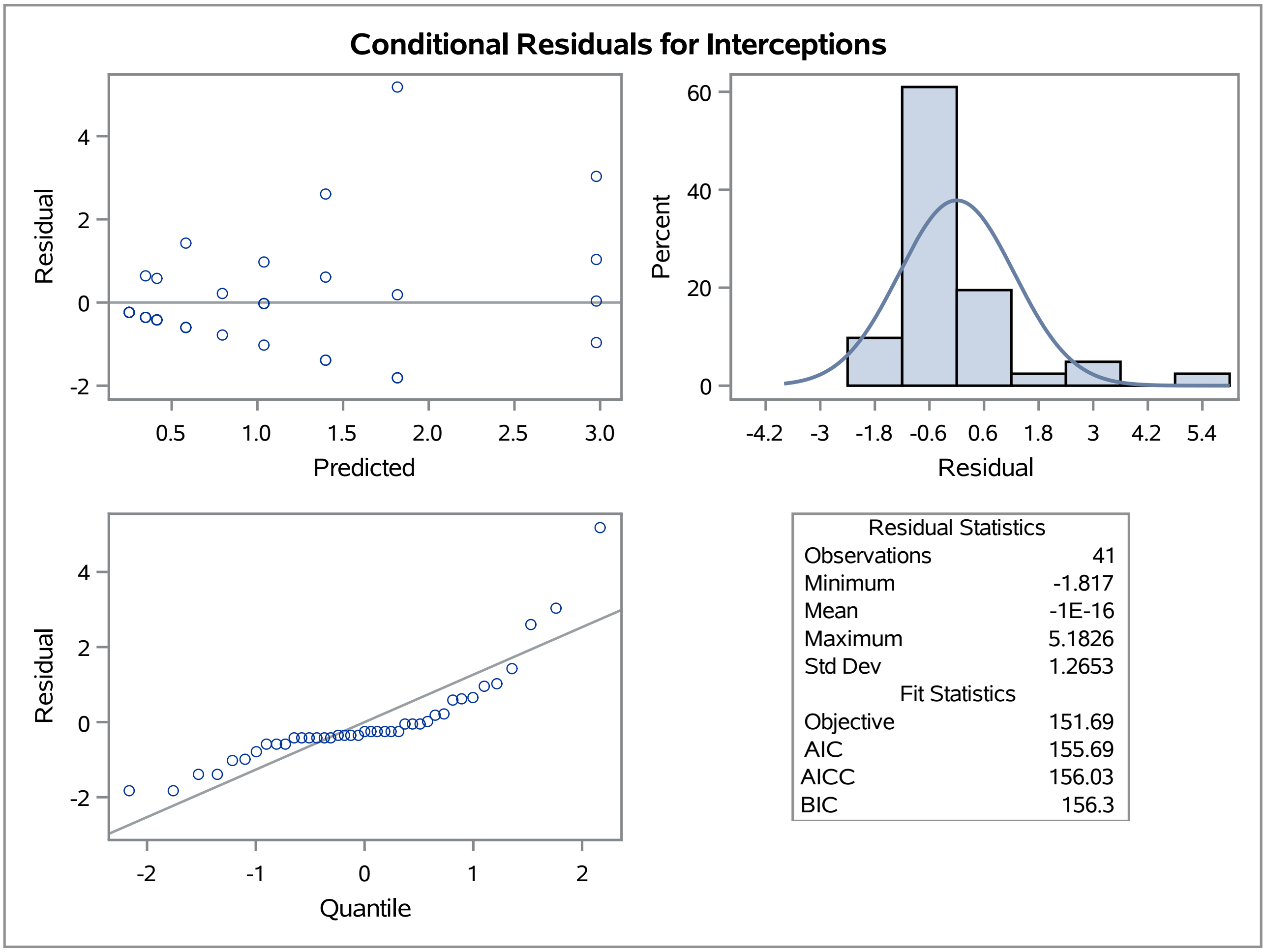

DistSoma=396

Model Information	
**Data Set**	WORK.TEMPDATASORTED
**Dependent Variable**	Interceptions
**Covariance Structure**	Variance Components
**Estimation Method**	REML
**Residual Variance Method**	Profile
**Fixed Effects SE Method**	Model-Based
**Degrees of Freedom Method**	Containment

Class Level Information		
**Class**	**Levels**	**Values**
**Treatment**	2	Meg TTR GFP MsTTR
**Culture**	10	1 2 3 4 5 6 7 8 9 10

Dimensions	
**Covariance Parameters**	2
**Columns in X**	3
**Columns in Z**	10
**Subjects**	1
**Max Obs per Subject**	41

Number of Observations	
**Number of Observations Read**	41
**Number of Observations Used**	41
**Number of Observations Not Used**	0

Iteration History			
**Iteration**	**Evaluations**	**-2 Res Log Like**	**Criterion**
**0**	1	152.24694026	
**1**	2	147.25656372	0.00000001
**2**	1	147.25656318	0.00000000

Convergence criteria met.

Covariance Parameter Estimates				
**Cov Parm**	**Estimate**	**Alpha**	**Lower**	**Upper**
**Culture**	0.8877	0.05	0.3035	9.0605
**Residual**	1.7468	0.05	1.1243	3.0805

DistSoma=396

Fit Statistics	
-2 Res Log Likelihood	147.3
AIC (Smaller is Better)	151.3
AICC (Smaller is Better)	151.6
BIC (Smaller is Better)	151.9

Solution for Fixed Effects

Effect	Treatment	Estimate	Standard Error	DF	t Value	Pr > |t|	Alpha	Lower	Upper
Intercept		0.7896	0.5704	8	1.38	0.2037	0.05	-0.5258	2.1049
Treatment	Meg TTR	0.2336	0.7429	31	0.31	0.7553	0.05	-1.2815	1.7487
Treatment	GFP MsTTR	0	.	.	.	.	.	.	.

Solution for Random Effects

Effect	Culture	Estimate	Std Err Pred	DF	t Value	Pr > |t|	Alpha	Lower	Upper
Culture	1	-0.4231	0.6467	31	-0.65	0.5178	0.05	-1.7421	0.8959
Culture	2	0.9789	0.6625	31	1.48	0.1496	0.05	-0.3723	2.3300
Culture	3	-0.1941	0.6625	31	-0.29	0.7715	0.05	-1.5452	1.1570
Culture	4	-0.3617	0.6625	31	-0.55	0.5890	0.05	-1.7128	0.9895
Culture	5	-0.1601	0.6061	31	-0.26	0.7934	0.05	-1.3962	1.0760
Culture	6	0.3196	0.6281	31	0.51	0.6144	0.05	-0.9613	1.6006
Culture	7	1.4926	0.6281	31	2.38	0.0238	0.05	0.2116	2.7735
Culture	8	-0.7705	0.5896	31	-1.31	0.2009	0.05	-1.9730	0.4321
Culture	9	-0.2637	0.7055	31	-0.37	0.7111	0.05	-1.7027	1.1753
Culture	10	-0.6179	0.6590	31	-0.94	0.3557	0.05	-1.9618	0.7261

Type 3 Tests of Fixed Effects

Effect	Num DF	Den DF	F Value	Pr > F
Treatment	1	31	0.10	0.7553

Least Squares Means

Effect	Treatment	Estimate	Standard Error	DF	t Value	Pr > |t|	Alpha	Lower	Upper
Treatment	Meg TTR	1.0232	0.4759	31	2.15	0.0395	0.05	0.05250	1.9938
Treatment	GFP MsTTR	0.7896	0.5704	31	1.38	0.1762	0.05	-0.3737	1.9529

DistSoma=396

Differences of Least Squares Means												
**Effect**	**Treatment**	**Treatment**	**Estimate**	**Standard Error**	**DF**	**t Value**	**Pr > |t|**	**Adjustment**	**Adj P**	**Alpha**	**Lower**	**Upper**
**Treatment**	Meg TTR	GFP MsTTR	0.2336	0.7429	31	0.31	0.7553	Tukey-Kramer	0.7553	0.05	-1.2815	1.7487

Differences of Least Squares Means				
**Effect**	**Treatment**	**Treatment**	**Adj Lower**	**Adj Upper**
**Treatment**	Meg TTR	GFP MsTTR	-1.2815	1.7486

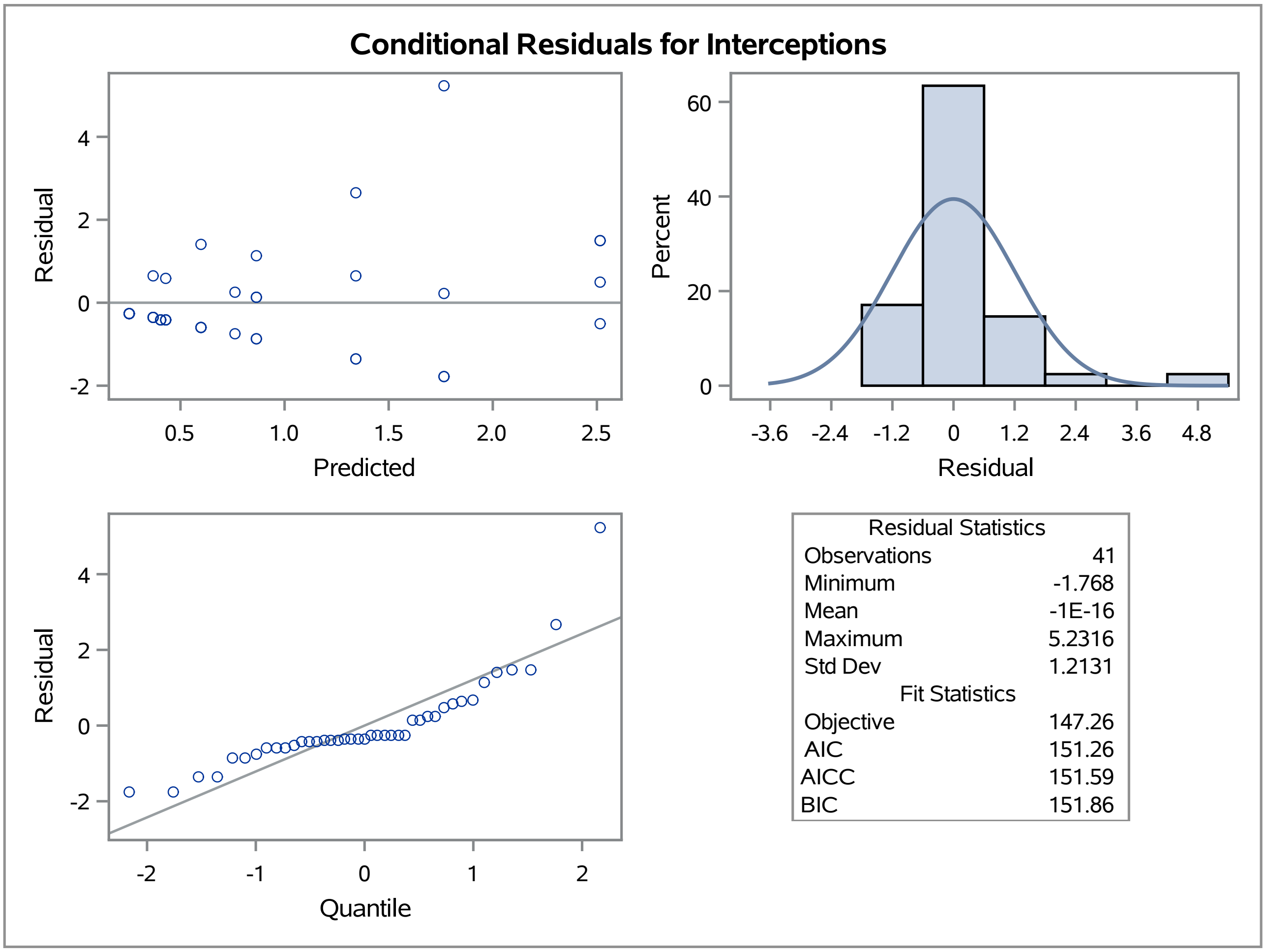

DistSoma=402

Model Information	
**Data Set**	WORK.TEMPDATASORTED
**Dependent Variable**	Interceptions
**Covariance Structure**	Variance Components
**Estimation Method**	REML
**Residual Variance Method**	Profile
**Fixed Effects SE Method**	Model-Based
**Degrees of Freedom Method**	Containment

Class Level Information		
**Class**	**Levels**	**Values**
**Treatment**	2	Meg TTR GFP MsTTR
**Culture**	10	1 2 3 4 5 6 7 8 9 10

Dimensions	
**Covariance Parameters**	2
**Columns in X**	3
**Columns in Z**	10
**Subjects**	1
**Max Obs per Subject**	41

Number of Observations	
**Number of Observations Read**	41
**Number of Observations Used**	41
**Number of Observations Not Used**	0

Iteration History			
**Iteration**	**Evaluations**	**-2 Res Log Like**	**Criterion**
**0**	1	153.93908768	
**1**	2	148.37690737	0.00000002
**2**	1	148.37690656	0.00000000

Convergence criteria met.

Covariance Parameter Estimates				
**Cov Parm**	**Estimate**	**Alpha**	**Lower**	**Upper**
**Culture**	1.0075	0.05	0.3511	9.4431
**Residual**	1.7697	0.05	1.1376	3.1269

DistSoma=402

Fit Statistics	
-2 Res Log Likelihood	148.4
AIC (Smaller is Better)	152.4
AICC (Smaller is Better)	152.7
BIC (Smaller is Better)	153.0

Solution for Fixed Effects

Effect	Treatment	Estimate	Standard Error	DF	t Value	Pr > |t|	Alpha	Lower	Upper
Intercept		0.7289	0.5972	8	1.22	0.2570	0.05	-0.6483	2.1061
Treatment	Meg TTR	0.3565	0.7775	31	0.46	0.6497	0.05	-1.2291	1.9421
Treatment	GFP MsTTR	0	.	.	.	.	.	.	.

Solution for Random Effects

Effect	Culture	Estimate	Std Err Pred	DF	t Value	Pr > |t|	Alpha	Lower	Upper
Culture	1	-0.3914	0.6762	31	-0.58	0.5669	0.05	-1.7705	0.9877
Culture	2	0.8832	0.6926	31	1.28	0.2117	0.05	-0.5292	2.2957
Culture	3	-0.1591	0.6926	31	-0.23	0.8199	0.05	-1.5715	1.2534
Culture	4	-0.3328	0.6926	31	-0.48	0.6342	0.05	-1.7453	1.0797
Culture	5	-0.2112	0.6306	31	-0.33	0.7399	0.05	-1.4972	1.0748
Culture	6	0.8092	0.6535	31	1.24	0.2249	0.05	-0.5236	2.1420
Culture	7	1.5041	0.6535	31	2.30	0.0282	0.05	0.1713	2.8369
Culture	8	-0.8396	0.6135	31	-1.37	0.1810	0.05	-2.0909	0.4117
Culture	9	-0.5779	0.7357	31	-0.79	0.4382	0.05	-2.0784	0.9227
Culture	10	-0.6846	0.6860	31	-1.00	0.3260	0.05	-2.0837	0.7145

Type 3 Tests of Fixed Effects

Effect	Num DF	Den DF	F Value	Pr > F
Treatment	1	31	0.21	0.6497

Least Squares Means

Effect	Treatment	Estimate	Standard Error	DF	t Value	Pr > |t|	Alpha	Lower	Upper
Treatment	Meg TTR	1.0854	0.4978	31	2.18	0.0369	0.05	0.07025	2.1006
Treatment	GFP MsTTR	0.7289	0.5972	31	1.22	0.2315	0.05	-0.4891	1.9470

DistSoma=402

Differences of Least Squares Means												
Effect	Treatment	Treatment	Estimate	Standard Error	DF	t Value	Pr > |t|	Adjustment	Adj P	Alpha	Lower	Upper
Treatment	Meg TTR	GFP MsTTR	0.3565	0.7775	31	0.46	0.6497	Tukey-Kramer	0.6497	0.05	-1.2291	1.9421

Differences of Least Squares Means				
Effect	Treatment	Treatment	Adj Lower	Adj Upper
Treatment	Meg TTR	GFP MsTTR	-1.2291	1.9421

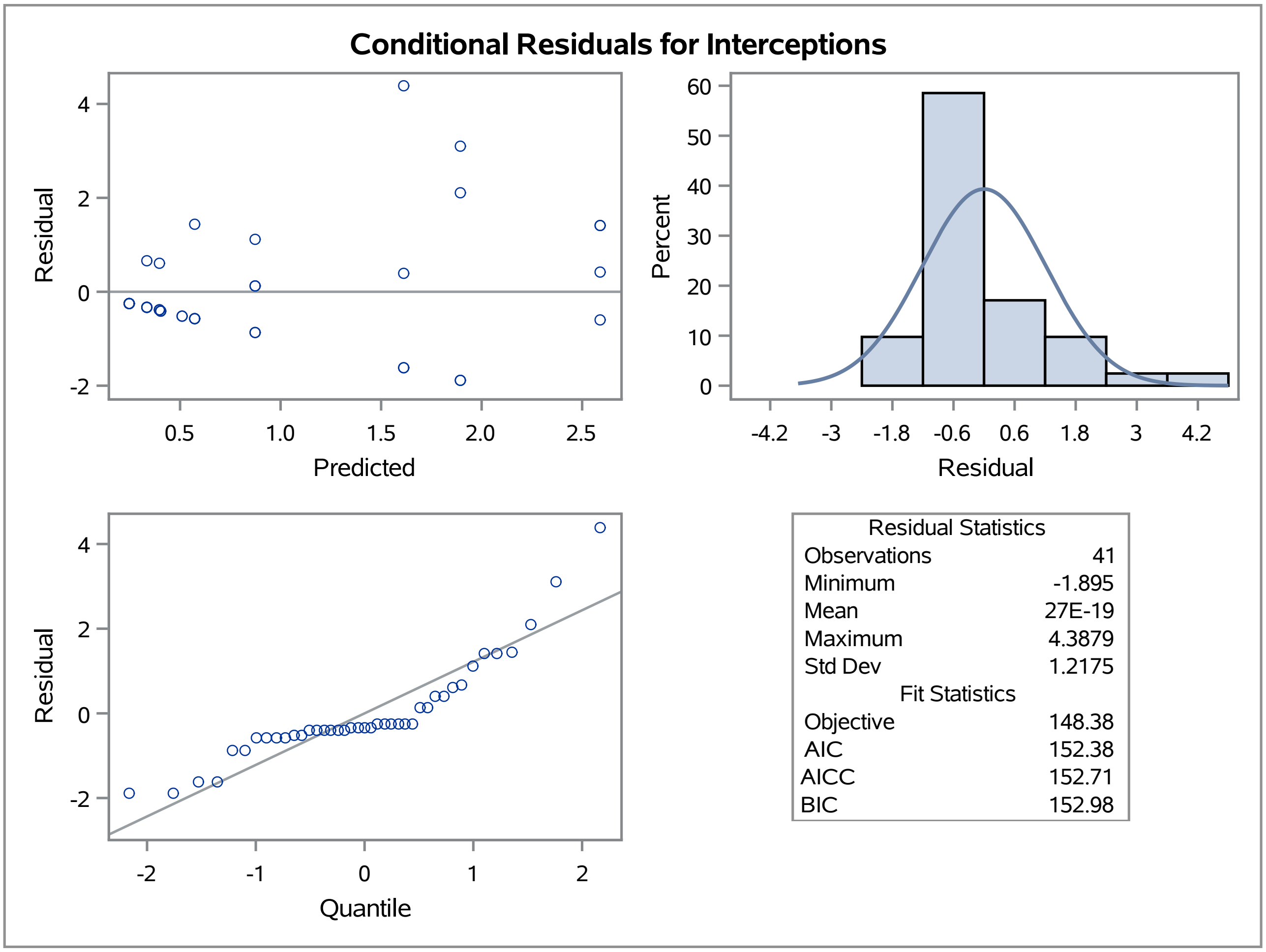

DistSoma=408

Model Information	
**Data Set**	WORK.TEMPDATASORTED
**Dependent Variable**	Interceptions
**Covariance Structure**	Variance Components
**Estimation Method**	REML
**Residual Variance Method**	Profile
**Fixed Effects SE Method**	Model-Based
**Degrees of Freedom Method**	Containment

Class Level Information		
**Class**	**Levels**	**Values**
**Treatment**	2	Meg TTR GFP MsTTR
**Culture**	10	1 2 3 4 5 6 7 8 9 10

Dimensions	
**Covariance Parameters**	2
**Columns in X**	3
**Columns in Z**	10
**Subjects**	1
**Max Obs per Subject**	41

Number of Observations	
**Number of Observations Read**	41
**Number of Observations Used**	41
**Number of Observations Not Used**	0

Iteration History			
**Iteration**	**Evaluations**	**-2 Res Log Like**	**Criterion**
**0**	1	144.65121216	
**1**	2	137.13628503	0.00000023
**2**	1	137.13627745	0.00000000

Convergence criteria met.

Covariance Parameter Estimates				
**Cov Parm**	**Estimate**	**Alpha**	**Lower**	**Upper**
**Culture**	0.9393	0.05	0.3462	6.9673
**Residual**	1.2779	0.05	0.8214	2.2585

DistSoma=408

Fit Statistics	
-2 Res Log Likelihood	137.1
AIC (Smaller is Better)	141.1
AICC (Smaller is Better)	141.5
BIC (Smaller is Better)	141.7

Solution for Fixed Effects

Effect	Treatment	Estimate	Standard Error	DF	t Value	Pr > |t|	Alpha	Lower	Upper
Intercept		0.6687	0.5573	8	1.20	0.2644	0.05	-0.6163	1.9538
Treatment	Meg TTR	0.3274	0.7247	31	0.45	0.6546	0.05	-1.1505	1.8053
Treatment	GFP MsTTR	0	.	.	.	.	.	.	.

Solution for Random Effects

Effect	Culture	Estimate	Std Err Pred	DF	t Value	Pr > |t|	Alpha	Lower	Upper
Culture	1	-0.3685	0.6267	31	-0.59	0.5608	0.05	-1.6467	0.9098
Culture	2	0.8068	0.6413	31	1.26	0.2178	0.05	-0.5011	2.1148
Culture	3	-0.1259	0.6413	31	-0.20	0.8456	0.05	-1.4339	1.1821
Culture	4	-0.3125	0.6413	31	-0.49	0.6295	0.05	-1.6204	0.9955
Culture	5	-0.1542	0.5775	31	-0.27	0.7913	0.05	-1.3320	1.0237
Culture	6	0.5625	0.5982	31	0.94	0.3543	0.05	-0.6576	1.7826
Culture	7	1.6818	0.5982	31	2.81	0.0085	0.05	0.4617	2.9019
Culture	8	-0.8120	0.5623	31	-1.44	0.1588	0.05	-1.9589	0.3349
Culture	9	-0.5928	0.6755	31	-0.88	0.3869	0.05	-1.9705	0.7848
Culture	10	-0.6853	0.6282	31	-1.09	0.2837	0.05	-1.9665	0.5959

Type 3 Tests of Fixed Effects

Effect	Num DF	Den DF	F Value	Pr > F
Treatment	1	31	0.20	0.6546

Least Squares Means

Effect	Treatment	Estimate	Standard Error	DF	t Value	Pr > |t|	Alpha	Lower	Upper
Treatment	Meg TTR	0.9961	0.4632	31	2.15	0.0394	0.05	0.05133	1.9409
Treatment	GFP MsTTR	0.6687	0.5573	31	1.20	0.2392	0.05	-0.4678	1.8053

DistSoma=408

Differences of Least Squares Means

Effect	Treatment	Treatment	Estimate	Standard Error	DF	t Value	Pr > |t|	Adjustment	Adj P	Alpha	Lower	Upper
Treatment	Meg TTR	GFP MsTTR	0.3274	0.7247	31	0.45	0.6546	Tukey-Kramer	0.6546	0.05	-1.1505	1.8053

Differences of Least Squares Means

Effect	Treatment	Treatment	Adj Lower	Adj Upper
Treatment	Meg TTR	GFP MsTTR	-1.1505	1.8053

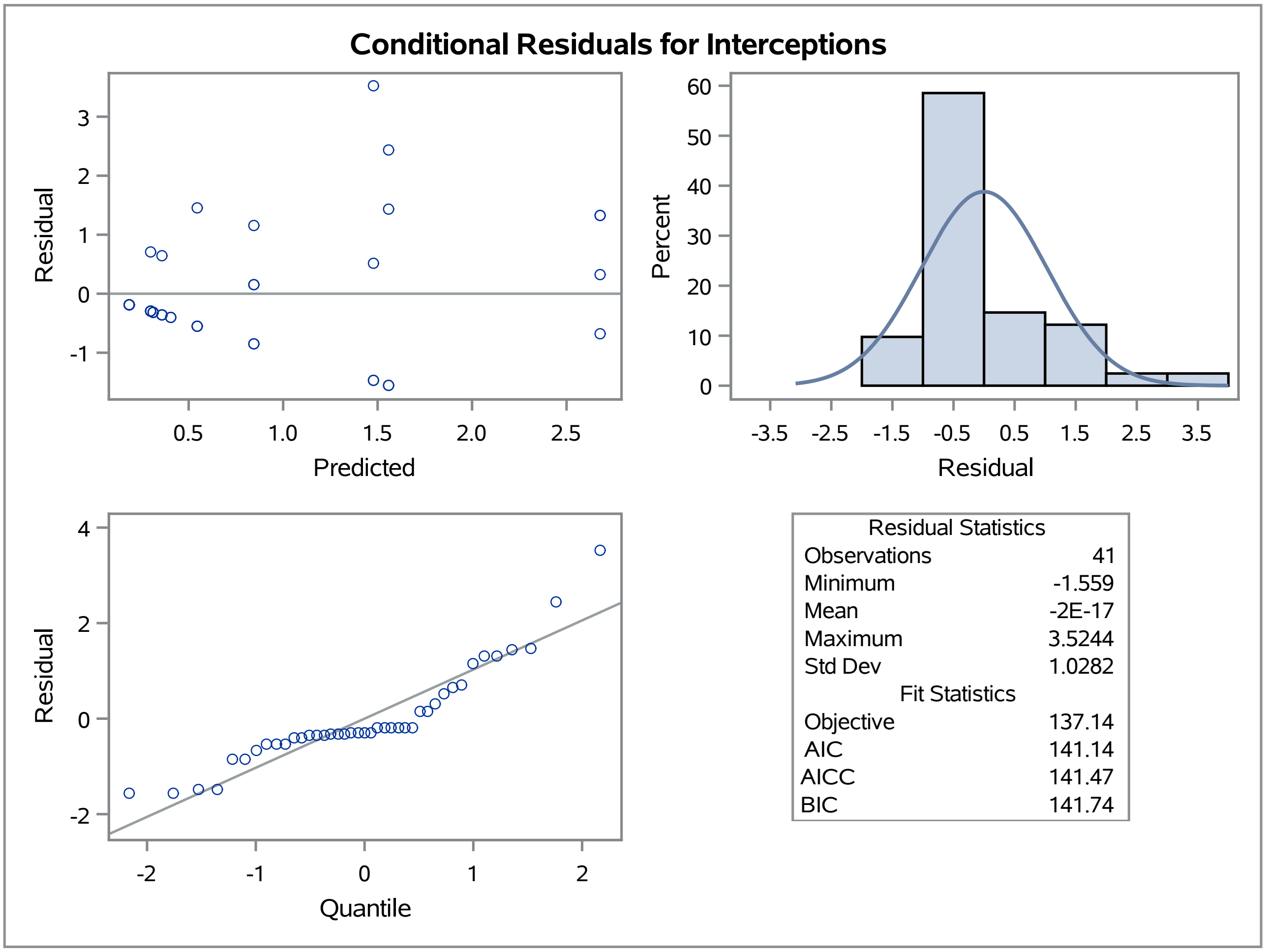

DistSoma=414

Model Information	
**Data Set**	WORK.TEMPDATASORTED
**Dependent Variable**	Interceptions
**Covariance Structure**	Variance Components
**Estimation Method**	REML
**Residual Variance Method**	Profile
**Fixed Effects SE Method**	Model-Based
**Degrees of Freedom Method**	Containment

Class Level Information		
**Class**	**Levels**	**Values**
**Treatment**	2	Meg TTR GFP MsTTR
**Culture**	10	1 2 3 4 5 6 7 8 9 10

Dimensions	
**Covariance Parameters**	2
**Columns in X**	3
**Columns in Z**	10
**Subjects**	1
**Max Obs per Subject**	41

Number of Observations	
**Number of Observations Read**	41
**Number of Observations Used**	41
**Number of Observations Not Used**	0

Iteration History			
**Iteration**	**Evaluations**	**-2 Res Log Like**	**Criterion**
**0**	1	143.77999202	
**1**	2	133.48901594	0.00000096
**2**	1	133.48898626	0.00000000

Convergence criteria met.

Covariance Parameter Estimates				
**Cov Parm**	**Estimate**	**Alpha**	**Lower**	**Upper**
**Culture**	1.0867	0.05	0.4212	6.6638
**Residual**	1.1131	0.05	0.7155	1.9673

DistSoma=414

Fit Statistics	
-2 Res Log Likelihood	133.5
AIC (Smaller is Better)	137.5
AICC (Smaller is Better)	137.8
BIC (Smaller is Better)	138.1

Solution for Fixed Effects

Effect	Treatment	Estimate	Standard Error	DF	t Value	Pr > |t|	Alpha	Lower	Upper
Intercept		0.6082	0.5811	8	1.05	0.3259	0.05	-0.7318	1.9482
Treatment	Meg TTR	0.3907	0.7548	31	0.52	0.6084	0.05	-1.1488	1.9301
Treatment	GFP MsTTR	0	.	.	.	.	.	.	.

Solution for Random Effects

Effect	Culture	Estimate	Std Err Pred	DF	t Value	Pr > |t|	Alpha	Lower	Upper
Culture	1	-0.3388	0.6460	31	-0.52	0.6038	0.05	-1.6564	0.9789
Culture	2	0.7100	0.6600	31	1.08	0.2903	0.05	-0.6360	2.0561
Culture	3	-0.08611	0.6600	31	-0.13	0.8970	0.05	-1.4321	1.2599
Culture	4	-0.2851	0.6600	31	-0.43	0.6687	0.05	-1.6312	1.0609
Culture	5	-0.3310	0.5870	31	-0.56	0.5769	0.05	-1.5283	0.8663
Culture	6	0.5980	0.6071	31	0.99	0.3322	0.05	-0.6402	1.8363
Culture	7	1.9913	0.6071	31	3.28	0.0026	0.05	0.7530	3.2296
Culture	8	-0.8532	0.5725	31	-1.49	0.1463	0.05	-2.0208	0.3145
Culture	9	-0.6605	0.6852	31	-0.96	0.3425	0.05	-2.0581	0.7370
Culture	10	-0.7446	0.6368	31	-1.17	0.2512	0.05	-2.0434	0.5542

Type 3 Tests of Fixed Effects

Effect	Num DF	Den DF	F Value	Pr > F
Treatment	1	31	0.27	0.6084

Least Squares Means

Effect	Treatment	Estimate	Standard Error	DF	t Value	Pr > |t|	Alpha	Lower	Upper
Treatment	Meg TTR	0.9988	0.4817	31	2.07	0.0465	0.05	0.01635	1.9813
Treatment	GFP MsTTR	0.6082	0.5811	31	1.05	0.3034	0.05	-0.5770	1.7933

DistSoma=414

Differences of Least Squares Means

Effect	Treatment	Treatment	Estimate	Standard Error	DF	t Value	Pr > |t|	Adjustment	Adj P	Alpha	Lower	Upper
**Treatment**	Meg TTR	GFP MsTTR	0.3907	0.7548	31	0.52	0.6084	Tukey-Kramer	0.6084	0.05	-1.1488	1.9301

Differences of Least Squares Means

Effect	Treatment	Treatment	Adj Lower	Adj Upper
**Treatment**	Meg TTR	GFP MsTTR	-1.1487	1.9301

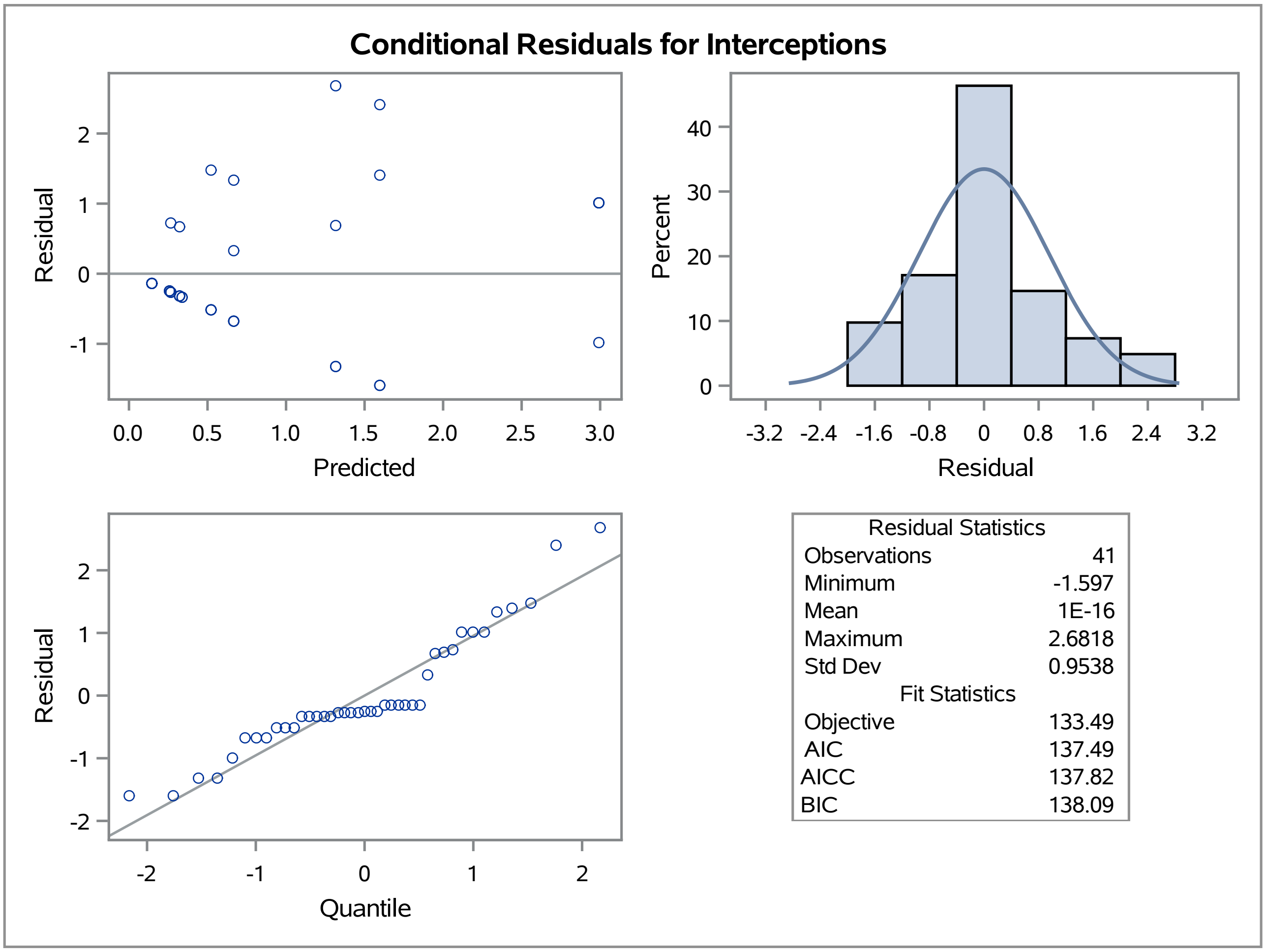

DistSoma=420

Model Information	
**Data Set**	WORK.TEMPDATASORTED
**Dependent Variable**	Interceptions
**Covariance Structure**	Variance Components
**Estimation Method**	REML
**Residual Variance Method**	Profile
**Fixed Effects SE Method**	Model-Based
**Degrees of Freedom Method**	Containment

Class Level Information		
**Class**	**Levels**	**Values**
**Treatment**	2	Meg TTR GFP MsTTR
**Culture**	10	1 2 3 4 5 6 7 8 9 10

Dimensions	
**Covariance Parameters**	2
**Columns in X**	3
**Columns in Z**	10
**Subjects**	1
**Max Obs per Subject**	41

Number of Observations	
**Number of Observations Read**	41
**Number of Observations Used**	41
**Number of Observations Not Used**	0

Iteration History			
**Iteration**	**Evaluations**	**-2 Res Log Like**	**Criterion**
**0**	1	142.29216152	
**1**	2	128.80080398	0.00000316
**2**	1	128.80071301	0.00000000

Convergence criteria met.

Covariance Parameter Estimates				
**Cov Parm**	**Estimate**	**Alpha**	**Lower**	**Upper**
**Culture**	1.2059	0.05	0.4847	6.5098
**Residual**	0.9439	0.05	0.6066	1.6686

DistSoma=420

Fit Statistics	
-2 Res Log Likelihood	128.8
AIC (Smaller is Better)	132.8
AICC (Smaller is Better)	133.1
BIC (Smaller is Better)	133.4

Solution for Fixed Effects

Effect	Treatment	Estimate	Standard Error	DF	t Value	Pr > |t|	Alpha	Lower	Upper
Intercept		0.4851	0.5979	8	0.81	0.4406	0.05	-0.8936	1.8638
Treatment	Meg TTR	0.5524	0.7758	31	0.71	0.4818	0.05	-1.0299	2.1347
Treatment	GFP MsTTR	0	.	.	.	.	.	.	.

Solution for Random Effects

Effect	Culture	Estimate	Std Err Pred	DF	t Value	Pr > |t|	Alpha	Lower	Upper
Culture	1	-0.2465	0.6561	31	-0.38	0.7097	0.05	-1.5846	1.0916
Culture	2	0.4306	0.6689	31	0.64	0.5244	0.05	-0.9335	1.7948
Culture	3	0.01247	0.6689	31	0.02	0.9852	0.05	-1.3517	1.3766
Culture	4	-0.1966	0.6689	31	-0.29	0.7708	0.05	-1.5608	1.1675
Culture	5	-0.3783	0.5882	31	-0.64	0.5249	0.05	-1.5779	0.8213
Culture	6	0.5959	0.6069	31	0.98	0.3338	0.05	-0.6419	1.8337
Culture	7	2.2686	0.6069	31	3.74	0.0008	0.05	1.0308	3.5064
Culture	8	-0.9178	0.5748	31	-1.60	0.1205	0.05	-2.0901	0.2546
Culture	9	-0.7457	0.6822	31	-1.09	0.2828	0.05	-2.1371	0.6458
Culture	10	-0.8228	0.6350	31	-1.30	0.2046	0.05	-2.1180	0.4723

Type 3 Tests of Fixed Effects

Effect	Num DF	Den DF	F Value	Pr > F
Treatment	1	31	0.51	0.4818

Least Squares Means

Effect	Treatment	Estimate	Standard Error	DF	t Value	Pr > |t|	Alpha	Lower	Upper
Treatment	Meg TTR	1.0375	0.4944	31	2.10	0.0441	0.05	0.02917	2.0458
Treatment	GFP MsTTR	0.4851	0.5979	31	0.81	0.4233	0.05	-0.7343	1.7044

DistSoma=420

Differences of Least Squares Means													
Effect	Treatment	Treatment	Estimate	Standard Error	DF	t Value	Pr > |t|	Adjustment	Adj P	Alpha	Lower	Upper
Treatment	Meg TTR	GFP MsTTR	0.5524	0.7758	31	0.71	0.4818	Tukey-Kramer	0.4818	0.05	-1.0299	2.1347

Differences of Least Squares Means				
Effect	Treatment	Treatment	Adj Lower	Adj Upper
Treatment	Meg TTR	GFP MsTTR	-1.0298	2.1346

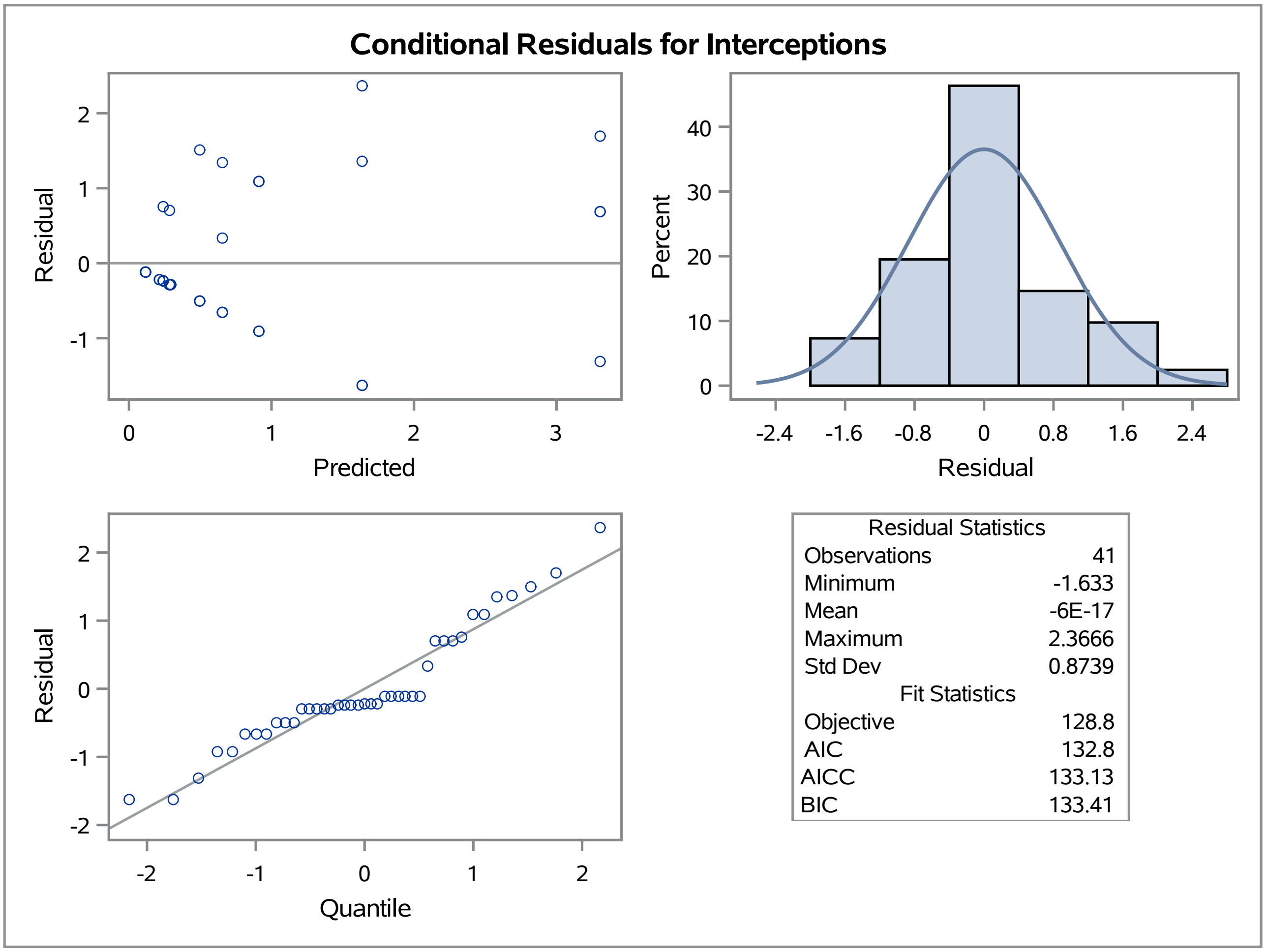

DistSoma=426

Model Information	
**Data Set**	WORK.TEMPDATASORTED
**Dependent Variable**	Interceptions
**Covariance Structure**	Variance Components
**Estimation Method**	REML
**Residual Variance Method**	Profile
**Fixed Effects SE Method**	Model-Based
**Degrees of Freedom Method**	Containment

Class Level Information		
**Class**	**Levels**	**Values**
**Treatment**	2	Meg TTR GFP MsTTR
**Culture**	10	1 2 3 4 5 6 7 8 9 10

Dimensions	
**Covariance Parameters**	2
**Columns in X**	3
**Columns in Z**	10
**Subjects**	1
**Max Obs per Subject**	41

Number of Observations	
**Number of Observations Read**	41
**Number of Observations Used**	41
**Number of Observations Not Used**	0

Iteration History			
**Iteration**	**Evaluations**	**-2 Res Log Like**	**Criterion**
**0**	1	140.53576960	
**1**	2	123.25434117	0.00000786
**2**	1	123.25413660	0.00000000

Convergence criteria met.

Covariance Parameter Estimates				
**Cov Parm**	**Estimate**	**Alpha**	**Lower**	**Upper**
**Culture**	1.2989	0.05	0.5374	6.3726
**Residual**	0.7823	0.05	0.5028	1.3828

DistSoma=426

Fit Statistics	
-2 Res Log Likelihood	123.3
AIC (Smaller is Better)	127.3
AICC (Smaller is Better)	127.6
BIC (Smaller is Better)	127.9

Solution for Fixed Effects

Effect	Treatment	Estimate	Standard Error	DF	t Value	Pr > |t|	Alpha	Lower	Upper
Intercept		0.3614	0.6092	8	0.59	0.5694	0.05	-1.0434	1.7662
Treatment	Meg TTR	0.6379	0.7898	31	0.81	0.4254	0.05	-0.9729	2.2487
Treatment	GFP MsTTR	0	.	.	.	.	.	.	.

Solution for Random Effects

Effect	Culture	Estimate	Std Err Pred	DF	t Value	Pr > |t|	Alpha	Lower	Upper
Culture	1	-0.1441	0.6597	31	-0.22	0.8286	0.05	-1.4896	1.2015
Culture	2	0.5550	0.6711	31	0.83	0.4145	0.05	-0.8136	1.9236
Culture	3	-0.3141	0.6711	31	-0.47	0.6430	0.05	-1.6828	1.0545
Culture	4	-0.09683	0.6711	31	-0.14	0.8862	0.05	-1.4655	1.2718
Culture	5	-0.5349	0.5839	31	-0.92	0.3667	0.05	-1.7256	0.6559
Culture	6	0.6525	0.6007	31	1.09	0.2858	0.05	-0.5727	1.8776
Culture	7	2.3907	0.6007	31	3.98	0.0004	0.05	1.1656	3.6158
Culture	8	-0.9081	0.5720	31	-1.59	0.1225	0.05	-2.0747	0.2584
Culture	9	-0.7680	0.6707	31	-1.15	0.2610	0.05	-2.1359	0.5999
Culture	10	-0.8322	0.6264	31	-1.33	0.1937	0.05	-2.1098	0.4454

Type 3 Tests of Fixed Effects

Effect	Num DF	Den DF	F Value	Pr > F
Treatment	1	31	0.65	0.4254

Least Squares Means

Effect	Treatment	Estimate	Standard Error	DF	t Value	Pr > |t|	Alpha	Lower	Upper
Treatment	Meg TTR	0.9993	0.5026	31	1.99	0.0557	0.05	-0.02587	2.0244
Treatment	GFP MsTTR	0.3614	0.6092	31	0.59	0.5573	0.05	-0.8810	1.6039

DistSoma=426

Differences of Least Squares Means												
Effect	Treatment	Treatment	Estimate	Standard Error	DF	t Value	Pr > |t|	Adjustment	Adj P	Alpha	Lower	Upper
**Treatment**	Meg TTR	GFP MsTTR	0.6379	0.7898	31	0.81	0.4254	Tukey-Kramer	0.4254	0.05	-0.9729	2.2487

Differences of Least Squares Means				
Effect	Treatment	Treatment	Adj Lower	Adj Upper
**Treatment**	Meg TTR	GFP MsTTR	-0.9729	2.2486

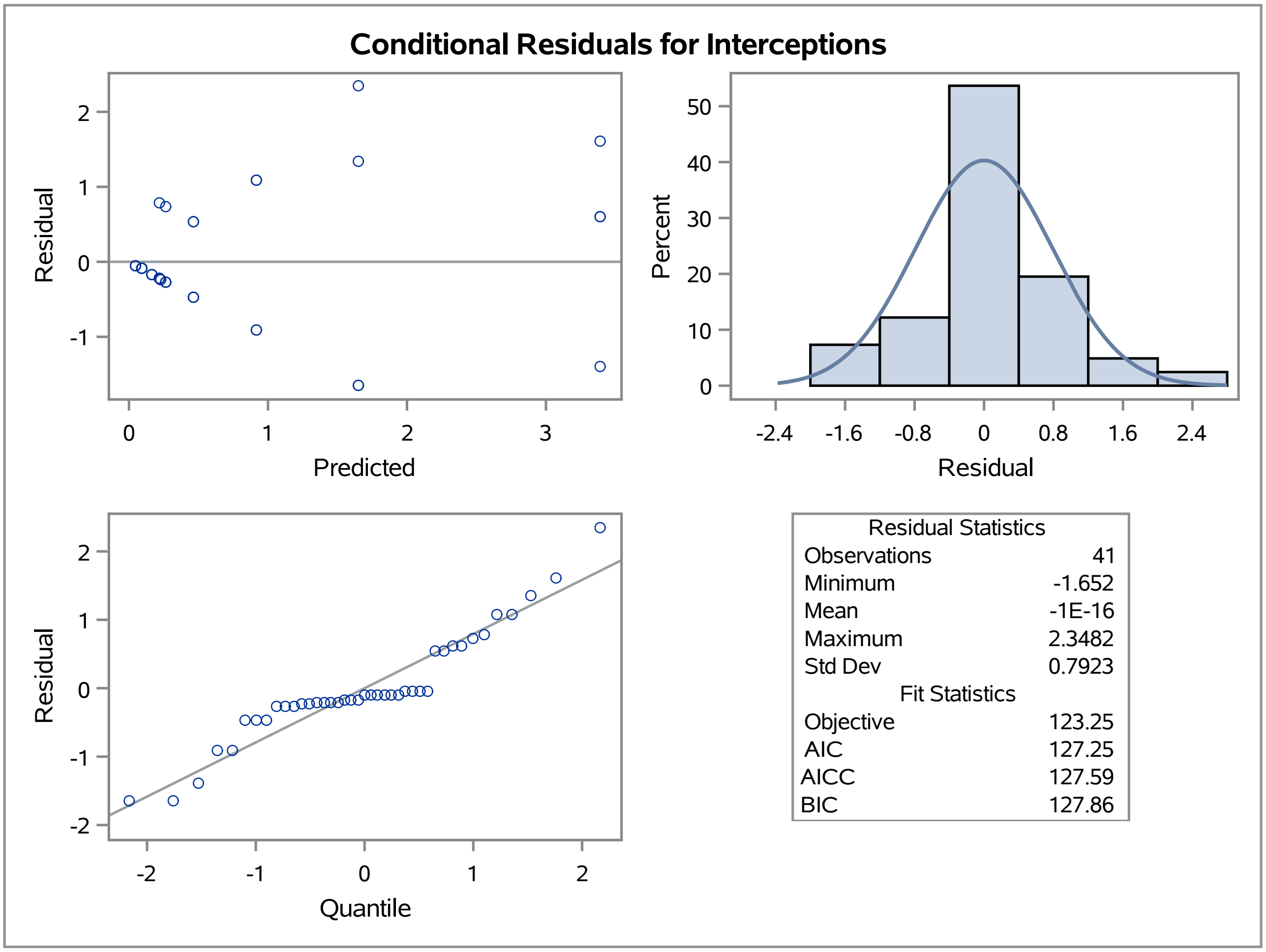

DistSoma=432

Model Information	
**Data Set**	WORK.TEMPDATASORTED
**Dependent Variable**	Interceptions
**Covariance Structure**	Variance Components
**Estimation Method**	REML
**Residual Variance Method**	Profile
**Fixed Effects SE Method**	Model-Based
**Degrees of Freedom Method**	Containment

Class Level Information		
**Class**	**Levels**	**Values**
**Treatment**	2	Meg TTR GFP MsTTR
**Culture**	10	1 2 3 4 5 6 7 8 9 10

Dimensions	
**Covariance Parameters**	2
**Columns in X**	3
**Columns in Z**	10
**Subjects**	1
**Max Obs per Subject**	41

Number of Observations	
**Number of Observations Read**	41
**Number of Observations Used**	41
**Number of Observations Not Used**	0

Iteration History			
**Iteration**	**Evaluations**	**-2 Res Log Like**	**Criterion**
**0**	1	137.69973544	
**1**	2	122.63352071	0.00000456
**2**	1	122.63340380	0.00000000

Convergence criteria met.

Covariance Parameter Estimates				
**Cov Parm**	**Estimate**	**Alpha**	**Lower**	**Upper**
**Culture**	1.1300	0.05	0.4604	5.8293
**Residual**	0.7904	0.05	0.5080	1.3971

DistSoma=432

Fit Statistics	
-2 Res Log Likelihood	122.6
AIC (Smaller is Better)	126.6
AICC (Smaller is Better)	127.0
BIC (Smaller is Better)	127.2

Solution for Fixed Effects

Effect	Treatment	Estimate	Standard Error	DF	t Value	Pr > |t|	Alpha	Lower	Upper
Intercept		0.3613	0.5739	8	0.63	0.5466	0.05	-0.9622	1.6847
Treatment	Meg TTR	0.5975	0.7444	31	0.80	0.4283	0.05	-0.9208	2.1157
Treatment	GFP MsTTR	0	.	.	.	.	.	.	.

Solution for Random Effects

Effect	Culture	Estimate	Std Err Pred	DF	t Value	Pr > |t|	Alpha	Lower	Upper
Culture	1	-0.1415	0.6262	31	-0.23	0.8228	0.05	-1.4187	1.1357
Culture	2	0.5437	0.6378	31	0.85	0.4005	0.05	-0.7572	1.8445
Culture	3	-0.3075	0.6378	31	-0.48	0.6331	0.05	-1.6083	0.9933
Culture	4	-0.09470	0.6378	31	-0.15	0.8829	0.05	-1.3955	1.2061
Culture	5	-0.4901	0.5583	31	-0.88	0.3867	0.05	-1.6288	0.6485
Culture	6	0.6735	0.5754	31	1.17	0.2507	0.05	-0.5000	1.8469
Culture	7	2.1630	0.5754	31	3.76	0.0007	0.05	0.9896	3.3365
Culture	8	-0.8586	0.5461	31	-1.57	0.1261	0.05	-1.9725	0.2553
Culture	9	-0.7103	0.6451	31	-1.10	0.2794	0.05	-2.0260	0.6054
Culture	10	-0.7774	0.6012	31	-1.29	0.2055	0.05	-2.0036	0.4488

Type 3 Tests of Fixed Effects

Effect	Num DF	Den DF	F Value	Pr > F
Treatment	1	31	0.64	0.4283

Least Squares Means

Effect	Treatment	Estimate	Standard Error	DF	t Value	Pr > |t|	Alpha	Lower	Upper
Treatment	Meg TTR	0.9587	0.4741	31	2.02	0.0519	0.05	-0.00823	1.9257
Treatment	GFP MsTTR	0.3613	0.5739	31	0.63	0.5336	0.05	-0.8092	1.5317

DistSoma=432

Differences of Least Squares Means												
Effect	Treatment	Treatment	Estimate	Standard Error	DF	t Value	Pr > |t|	Adjustment	Adj P	Alpha	Lower	Upper
Treatment	Meg TTR	GFP MsTTR	0.5975	0.7444	31	0.80	0.4283	Tukey-Kramer	0.4283	0.05	-0.9208	2.1157

Differences of Least Squares Means				
Effect	Treatment	Treatment	Adj Lower	Adj Upper
Treatment	Meg TTR	GFP MsTTR	-0.9208	2.1157

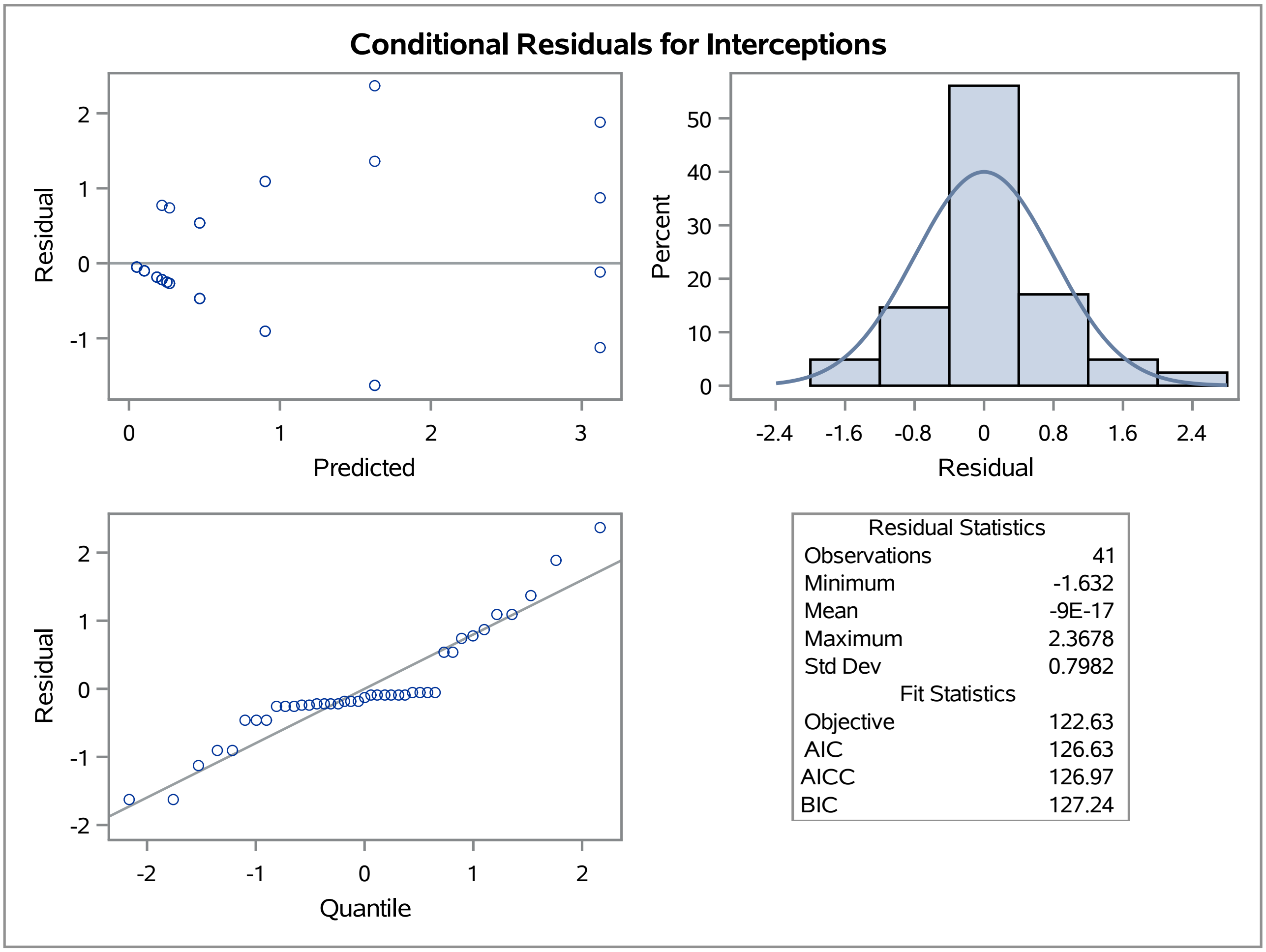

DistSoma=438

Model Information	
**Data Set**	WORK.TEMPDATASORTED
**Dependent Variable**	Interceptions
**Covariance Structure**	Variance Components
**Estimation Method**	REML
**Residual Variance Method**	Profile
**Fixed Effects SE Method**	Model-Based
**Degrees of Freedom Method**	Containment

Class Level Information		
**Class**	**Levels**	**Values**
**Treatment**	2	Meg TTR GFP MsTTR
**Culture**	10	1 2 3 4 5 6 7 8 9 10

Dimensions	
**Covariance Parameters**	2
**Columns in X**	3
**Columns in Z**	10
**Subjects**	1
**Max Obs per Subject**	41

Number of Observations	
**Number of Observations Read**	41
**Number of Observations Used**	41
**Number of Observations Not Used**	0

Iteration History			
**Iteration**	**Evaluations**	**-2 Res Log Like**	**Criterion**
**0**	1	133.07257543	
**1**	2	117.69672253	0.00000570
**2**	1	117.69659037	0.00000000

Convergence criteria met.

Covariance Parameter Estimates				
**Cov Parm**	**Estimate**	**Alpha**	**Lower**	**Upper**
**Culture**	1.0145	0.05	0.4143	5.1947
**Residual**	0.6937	0.05	0.4459	1.2262

DistSoma=438

Fit Statistics	
**-2 Res Log Likelihood**	117.7
**AIC (Smaller is Better)**	121.7
**AICC (Smaller is Better)**	122.0
**BIC (Smaller is Better)**	122.3

Solution for Fixed Effects

Effect	Treatment	Estimate	Standard Error	DF	t Value	Pr > |t|	Alpha	Lower	Upper
**Intercept**		0.2993	0.5429	8	0.55	0.5966	0.05	-0.9528	1.5513
**Treatment**	Meg TTR	0.6169	0.7042	31	0.88	0.3878	0.05	-0.8193	2.0531
**Treatment**	GFP MsTTR	0	.	.	.	.	.	.	.

Solution for Random Effects

Effect	Culture	Estimate	Std Err Pred	DF	t Value	Pr > |t|	Alpha	Lower	Upper
**Culture**	1	-0.08731	0.5918	31	-0.15	0.8837	0.05	-1.2942	1.1196
**Culture**	2	0.5984	0.6026	31	0.99	0.3283	0.05	-0.6305	1.8274
**Culture**	3	-0.2556	0.6026	31	-0.42	0.6744	0.05	-1.4845	0.9734
**Culture**	4	-0.2556	0.6026	31	-0.42	0.6744	0.05	-1.4845	0.9734
**Culture**	5	-0.4540	0.5269	31	-0.86	0.3955	0.05	-1.5288	0.6207
**Culture**	6	0.7121	0.5429	31	1.31	0.1993	0.05	-0.3952	1.8195
**Culture**	7	1.9932	0.5429	31	3.67	0.0009	0.05	0.8858	3.1005
**Culture**	8	-0.8224	0.5156	31	-1.60	0.1208	0.05	-1.8740	0.2292
**Culture**	9	-0.6827	0.6084	31	-1.12	0.2704	0.05	-1.9236	0.5581
**Culture**	10	-0.7461	0.5672	31	-1.32	0.1980	0.05	-1.9029	0.4107

Type 3 Tests of Fixed Effects

Effect	Num DF	Den DF	F Value	Pr > F
**Treatment**	1	31	0.77	0.3878

Least Squares Means

Effect	Treatment	Estimate	Standard Error	DF	t Value	Pr > |t|	Alpha	Lower	Upper
**Treatment**	Meg TTR	0.9161	0.4484	31	2.04	0.0496	0.05	0.001540	1.8307
**Treatment**	GFP MsTTR	0.2993	0.5429	31	0.55	0.5855	0.05	-0.8081	1.4066

DistSoma=438

Differences of Least Squares Means												
Effect	Treatment	Treatment	Estimate	Standard Error	DF	t Value	Pr > |t|	Adjustment	Adj P	Alpha	Lower	Upper
**Treatment**	Meg TTR	GFP MsTTR	0.6169	0.7042	31	0.88	0.3878	Tukey-Kramer	0.3878	0.05	-0.8193	2.0531

Differences of Least Squares Means				
Effect	Treatment	Treatment	Adj Lower	Adj Upper
**Treatment**	Meg TTR	GFP MsTTR	-0.8193	2.0531

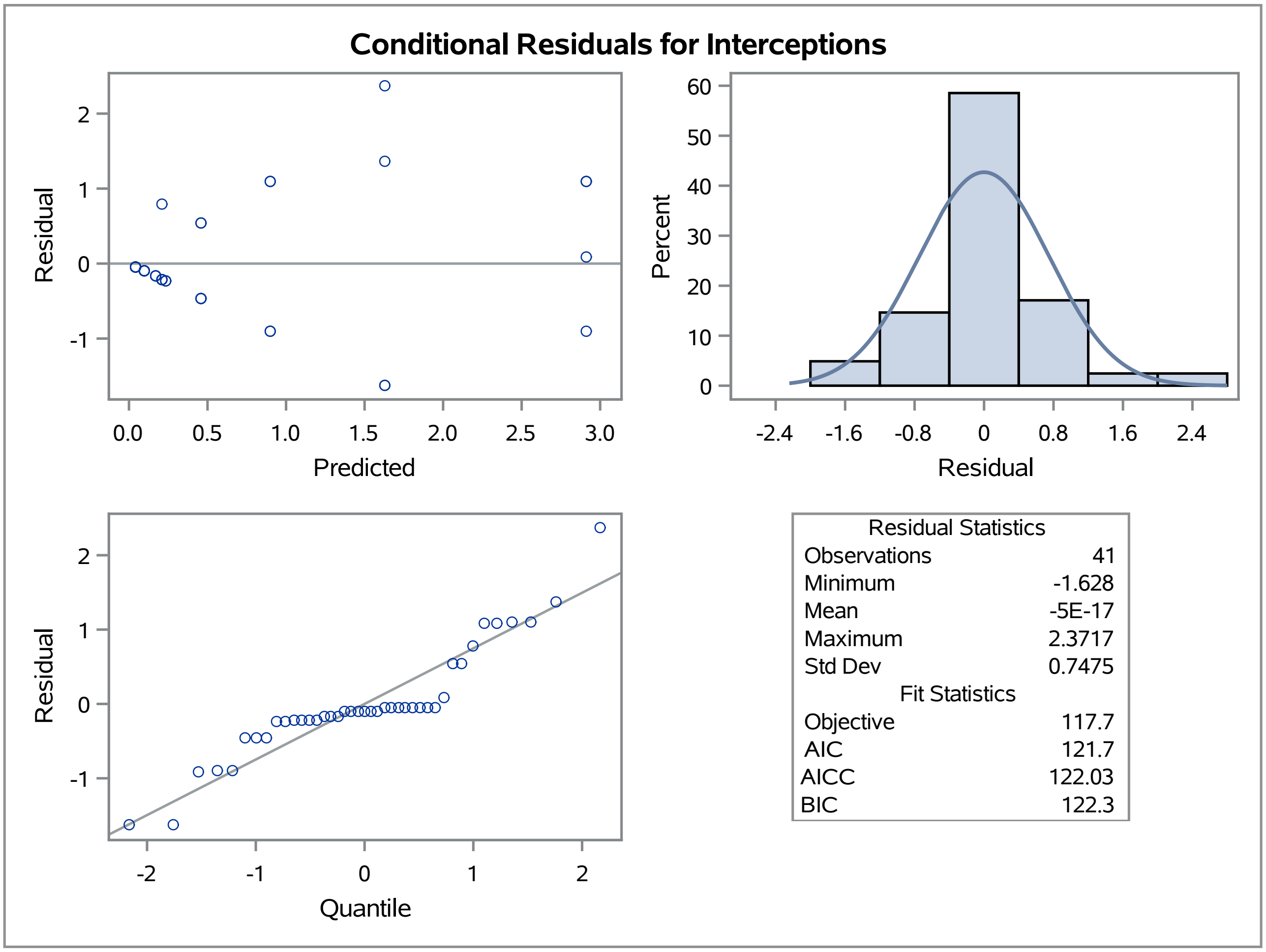

DistSoma=444

Model Information	
**Data Set**	WORK.TEMPDATASORTED
**Dependent Variable**	Interceptions
**Covariance Structure**	Variance Components
**Estimation Method**	REML
**Residual Variance Method**	Profile
**Fixed Effects SE Method**	Model-Based
**Degrees of Freedom Method**	Containment

Class Level Information		
**Class**	**Levels**	**Values**
**Treatment**	2	Meg TTR GFP MsTTR
**Culture**	10	1 2 3 4 5 6 7 8 9 10

Dimensions	
**Covariance Parameters**	2
**Columns in X**	3
**Columns in Z**	10
**Subjects**	1
**Max Obs per Subject**	41

Number of Observations	
**Number of Observations Read**	41
**Number of Observations Used**	41
**Number of Observations Not Used**	0

Iteration History			
**Iteration**	**Evaluations**	**-2 Res Log Like**	**Criterion**
**0**	1	142.37977775	
**1**	2	130.54979249	0.00000106
**2**	1	130.54976121	0.00000000

Convergence criteria met.

Covariance Parameter Estimates				
**Cov Parm**	**Estimate**	**Alpha**	**Lower**	**Upper**
**Culture**	1.1214	0.05	0.4434	6.4025
**Residual**	1.0109	0.05	0.6499	1.7862

DistSoma=444

Fit Statistics	
-2 Res Log Likelihood	130.5
AIC (Smaller is Better)	134.5
AICC (Smaller is Better)	134.9
BIC (Smaller is Better)	135.2

Solution for Fixed Effects

Effect	Treatment	Estimate	Standard Error	DF	t Value	Pr > |t|	Alpha	Lower	Upper
Intercept		0.2991	0.5833	8	0.51	0.6220	0.05	-1.0461	1.6442
Treatment	Meg TTR	0.6275	0.7573	31	0.83	0.4137	0.05	-0.9171	2.1721
Treatment	GFP MsTTR	0	.	.	.	.	.	.	.

Solution for Random Effects

Effect	Culture	Estimate	Std Err Pred	DF	t Value	Pr > |t|	Alpha	Lower	Upper
Culture	1	-0.08392	0.6446	31	-0.13	0.8973	0.05	-1.3987	1.2308
Culture	2	0.5720	0.6579	31	0.87	0.3913	0.05	-0.7698	1.9139
Culture	3	-0.2441	0.6579	31	-0.37	0.7132	0.05	-1.5859	1.0978
Culture	4	-0.2441	0.6579	31	-0.37	0.7132	0.05	-1.5859	1.0978
Culture	5	-0.6156	0.5820	31	-1.06	0.2984	0.05	-1.8026	0.5715
Culture	6	0.6720	0.6013	31	1.12	0.2724	0.05	-0.5544	1.8985
Culture	7	2.1002	0.6013	31	3.49	0.0015	0.05	0.8737	3.3266
Culture	8	-0.8055	0.5682	31	-1.42	0.1662	0.05	-1.9643	0.3533
Culture	9	-0.6387	0.6777	31	-0.94	0.3533	0.05	-2.0208	0.7435
Culture	10	-0.7124	0.6301	31	-1.13	0.2669	0.05	-1.9976	0.5727

Type 3 Tests of Fixed Effects

Effect	Num DF	Den DF	F Value	Pr > F
Treatment	1	31	0.69	0.4137

Least Squares Means

Effect	Treatment	Estimate	Standard Error	DF	t Value	Pr > |t|	Alpha	Lower	Upper
Treatment	Meg TTR	0.9265	0.4830	31	1.92	0.0643	0.05	-0.05854	1.9116
Treatment	GFP MsTTR	0.2991	0.5833	31	0.51	0.6118	0.05	-0.8907	1.4888

DistSoma=444

Differences of Least Squares Means												
**Effect**	**Treatment**	**Treatment**	**Estimate**	**Standard Error**	**DF**	**t Value**	**Pr > |t|**	**Adjustment**	**Adj P**	**Alpha**	**Lower**	**Upper**
**Treatment**	Meg TTR	GFP MsTTR	0.6275	0.7573	31	0.83	0.4137	Tukey-Kramer	0.4137	0.05	-0.9171	2.1721

Differences of Least Squares Means				
**Effect**	**Treatment**	**Treatment**	**Adj Lower**	**Adj Upper**
**Treatment**	Meg TTR	GFP MsTTR	-0.9171	2.1721

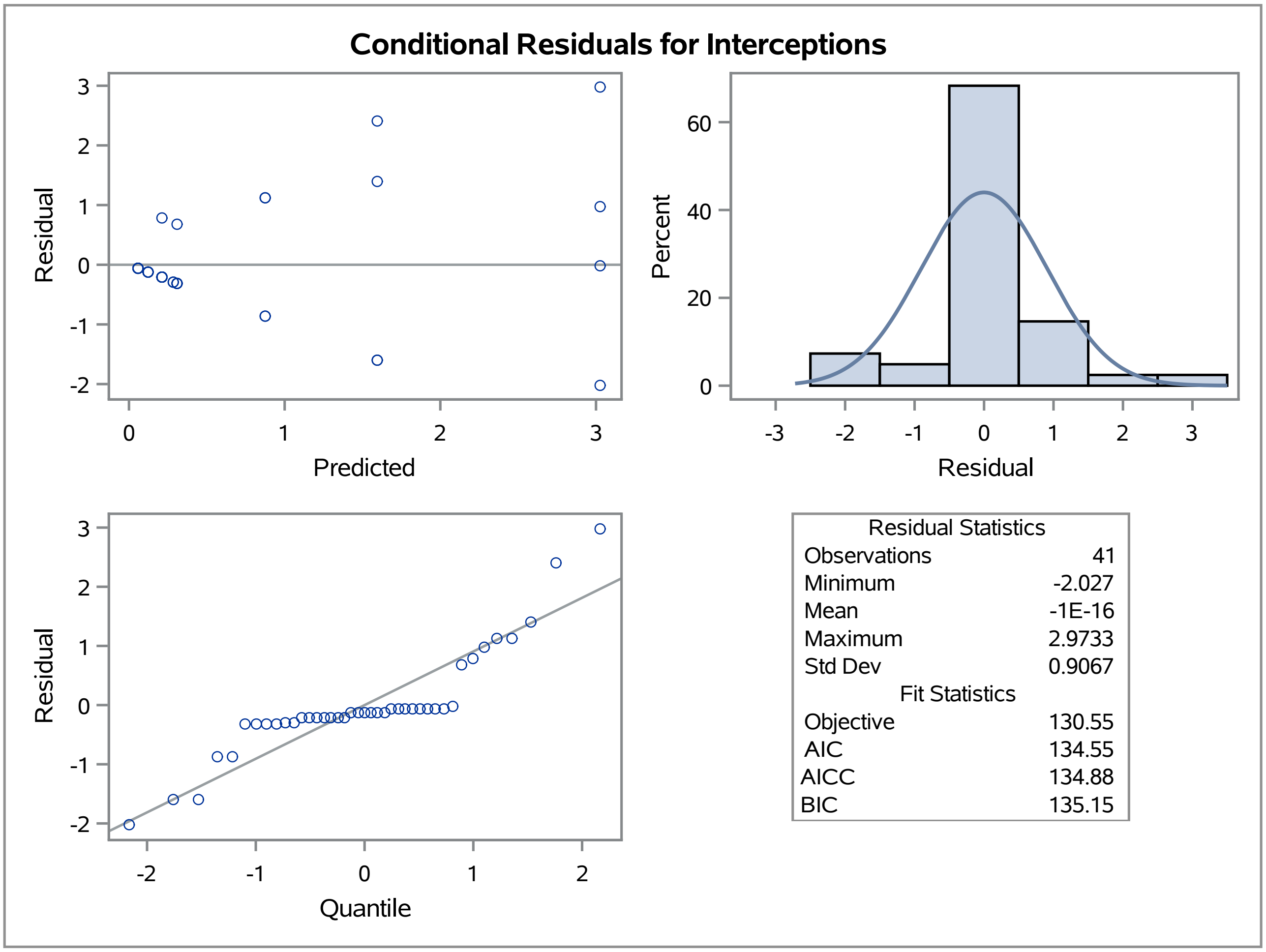

DistSoma=450

Model Information	
**Data Set**	WORK.TEMPDATASORTED
**Dependent Variable**	Interceptions
**Covariance Structure**	Variance Components
**Estimation Method**	REML
**Residual Variance Method**	Profile
**Fixed Effects SE Method**	Model-Based
**Degrees of Freedom Method**	Containment

Class Level Information		
**Class**	**Levels**	**Values**
**Treatment**	2	Meg TTR GFP MsTTR
**Culture**	10	1 2 3 4 5 6 7 8 9 10

Dimensions	
**Covariance Parameters**	2
**Columns in X**	3
**Columns in Z**	10
**Subjects**	1
**Max Obs per Subject**	41

Number of Observations	
**Number of Observations Read**	41
**Number of Observations Used**	41
**Number of Observations Not Used**	0

Iteration History			
**Iteration**	**Evaluations**	**-2 Res Log Like**	**Criterion**
**0**	1	141.07982934	
**1**	2	129.06405625	0.00000095
**2**	1	129.06402902	0.00000000

Convergence criteria met.

Covariance Parameter Estimates				
**Cov Parm**	**Estimate**	**Alpha**	**Lower**	**Upper**
**Culture**	1.0932	0.05	0.4331	6.2002
**Residual**	0.9707	0.05	0.6240	1.7152

DistSoma=450

Fit Statistics	
-2 Res Log Likelihood	129.1
AIC (Smaller is Better)	133.1
AICC (Smaller is Better)	133.4
BIC (Smaller is Better)	133.7

Solution for Fixed Effects

Effect	Treatment	Estimate	Standard Error	DF	t Value	Pr > |t|	Alpha	Lower	Upper
Intercept		0.2371	0.5752	8	0.41	0.6910	0.05	-1.0893	1.5636
Treatment	Meg TTR	0.6892	0.7467	31	0.92	0.3632	0.05	-0.8338	2.2122
Treatment	GFP MsTTR	0	.	.	.	.	.	.	.

Solution for Random Effects

Effect	Culture	Estimate	Std Err Pred	DF	t Value	Pr > |t|	Alpha	Lower	Upper
Culture	1	-0.03155	0.6352	31	-0.05	0.9607	0.05	-1.3270	1.2639
Culture	2	0.4197	0.6482	31	0.65	0.5221	0.05	-0.9023	1.7417
Culture	3	-0.1941	0.6482	31	-0.30	0.7666	0.05	-1.5161	1.1279
Culture	4	-0.1941	0.6482	31	-0.30	0.7666	0.05	-1.5161	1.1279
Culture	5	-0.6168	0.5731	31	-1.08	0.2901	0.05	-1.7856	0.5519
Culture	6	0.6740	0.5920	31	1.14	0.2636	0.05	-0.5333	1.8814
Culture	7	2.1061	0.5920	31	3.56	0.0012	0.05	0.8988	3.3135
Culture	8	-0.8070	0.5595	31	-1.44	0.1592	0.05	-1.9480	0.3341
Culture	9	-0.6416	0.6670	31	-0.96	0.3436	0.05	-2.0019	0.7188
Culture	10	-0.7148	0.6202	31	-1.15	0.2579	0.05	-1.9798	0.5502

Type 3 Tests of Fixed Effects

Effect	Num DF	Den DF	F Value	Pr > F
Treatment	1	31	0.85	0.3632

Least Squares Means

Effect	Treatment	Estimate	Standard Error	DF	t Value	Pr > |t|	Alpha	Lower	Upper
Treatment	Meg TTR	0.9264	0.4762	31	1.95	0.0608	0.05	-0.04483	1.8976
Treatment	GFP MsTTR	0.2371	0.5752	31	0.41	0.6830	0.05	-0.9360	1.4103

DistSoma=450

Differences of Least Squares Means												
**Effect**	**Treatment**	**Treatment**	**Estimate**	**Standard Error**	**DF**	**t Value**	**Pr > |t|**	**Adjustment**	**Adj P**	**Alpha**	**Lower**	**Upper**
**Treatment**	Meg TTR	GFP MsTTR	0.6892	0.7467	31	0.92	0.3632	Tukey-Kramer	0.3632	0.05	-0.8338	2.2122

Differences of Least Squares Means				
**Effect**	**Treatment**	**Treatment**	**Adj Lower**	**Adj Upper**
**Treatment**	Meg TTR	GFP MsTTR	-0.8338	2.2122

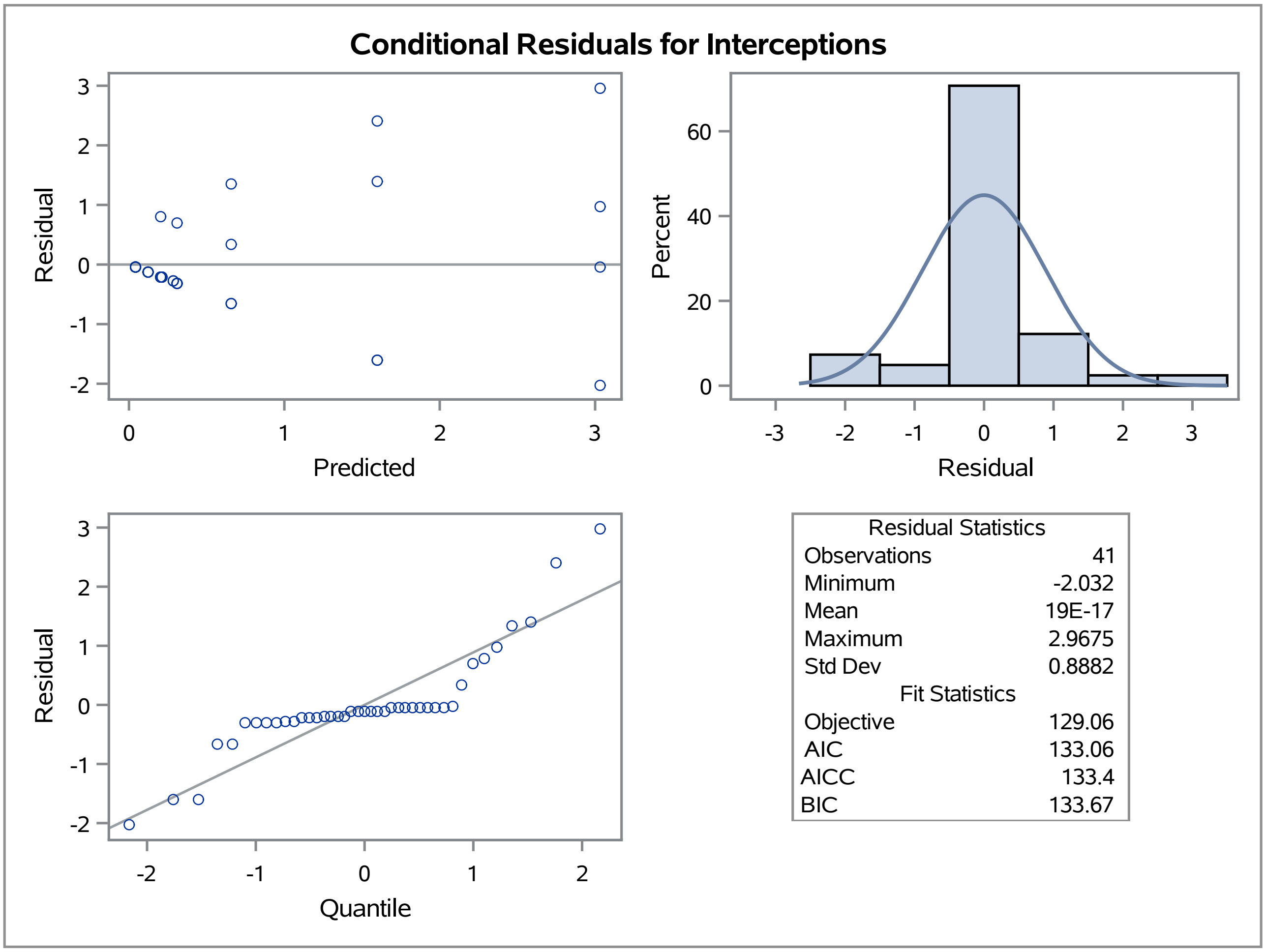

Residual Statistics	
Observations	41
Minimum	-2.032
Mean	19E-17
Maximum	2.9675
Std Dev	0.8882
Fit Statistics	
Objective	129.06
AIC	133.06
AICC	133.4
BIC	133.67

DistSoma=456

Model Information	
**Data Set**	WORK.TEMPDATASORTED
**Dependent Variable**	Interceptions
**Covariance Structure**	Variance Components
**Estimation Method**	REML
**Residual Variance Method**	Profile
**Fixed Effects SE Method**	Model-Based
**Degrees of Freedom Method**	Containment

Class Level Information		
**Class**	**Levels**	**Values**
**Treatment**	2	Meg TTR GFP MsTTR
**Culture**	10	1 2 3 4 5 6 7 8 9 10

Dimensions	
**Covariance Parameters**	2
**Columns in X**	3
**Columns in Z**	10
**Subjects**	1
**Max Obs per Subject**	41

Number of Observations	
**Number of Observations Read**	41
**Number of Observations Used**	41
**Number of Observations Not Used**	0

Iteration History			
**Iteration**	**Evaluations**	**-2 Res Log Like**	**Criterion**
**0**	1	131.86905883	
**1**	2	125.52878757	0.00000009
**2**	1	125.52878507	0.00000000

Convergence criteria met.

Covariance Parameter Estimates				
**Cov Parm**	**Estimate**	**Alpha**	**Lower**	**Upper**
**Culture**	0.6134	0.05	0.2195	5.1291
**Residual**	0.9704	0.05	0.6240	1.7141

DistSoma=456

Fit Statistics	
-2 Res Log Likelihood	125.5
AIC (Smaller is Better)	129.5
AICC (Smaller is Better)	129.9
BIC (Smaller is Better)	130.1

Solution for Fixed Effects

Effect	Treatment	Estimate	Standard Error	DF	t Value	Pr > |t|	Alpha	Lower	Upper
Intercept		0.2369	0.4592	8	0.52	0.6198	0.05	-0.8219	1.2958
Treatment	Meg TTR	0.5248	0.5975	31	0.88	0.3865	0.05	-0.6937	1.7434
Treatment	GFP MsTTR	0	.	.	.	.	.	.	.

Solution for Random Effects

Effect	Culture	Estimate	Std Err Pred	DF	t Value	Pr > |t|	Alpha	Lower	Upper
Culture	1	-0.02806	0.5187	31	-0.05	0.9572	0.05	-1.0860	1.0299
Culture	2	0.3676	0.5311	31	0.69	0.4940	0.05	-0.7156	1.4509
Culture	3	-0.1698	0.5311	31	-0.32	0.7514	0.05	-1.2530	0.9135
Culture	4	-0.1698	0.5311	31	-0.32	0.7514	0.05	-1.2530	0.9135
Culture	5	-0.4268	0.4814	31	-0.89	0.3822	0.05	-1.4086	0.5551
Culture	6	0.5290	0.4989	31	1.06	0.2972	0.05	-0.4885	1.5465
Culture	7	1.4247	0.4989	31	2.86	0.0076	0.05	0.4072	2.4422
Culture	8	-0.6028	0.4685	31	-1.29	0.2077	0.05	-1.5584	0.3527
Culture	9	-0.4253	0.5626	31	-0.76	0.4553	0.05	-1.5727	0.7220
Culture	10	-0.4988	0.5239	31	-0.95	0.3484	0.05	-1.5672	0.5697

Type 3 Tests of Fixed Effects

Effect	Num DF	Den DF	F Value	Pr > F
Treatment	1	31	0.77	0.3865

Least Squares Means

Effect	Treatment	Estimate	Standard Error	DF	t Value	Pr > |t|	Alpha	Lower	Upper
Treatment	Meg TTR	0.7618	0.3823	31	1.99	0.0552	0.05	-0.01788	1.5415
Treatment	GFP MsTTR	0.2369	0.4592	31	0.52	0.6095	0.05	-0.6995	1.1734

DistSoma=456

Differences of Least Squares Means												
Effect	Treatment	Treatment	Estimate	Standard Error	DF	t Value	Pr > |t|	Adjustment	Adj P	Alpha	Lower	Upper
Treatment	Meg TTR	GFP MsTTR	0.5248	0.5975	31	0.88	0.3865	Tukey-Kramer	0.3865	0.05	-0.6937	1.7434

Differences of Least Squares Means				
Effect	Treatment	Treatment	Adj Lower	Adj Upper
Treatment	Meg TTR	GFP MsTTR	-0.6937	1.7434

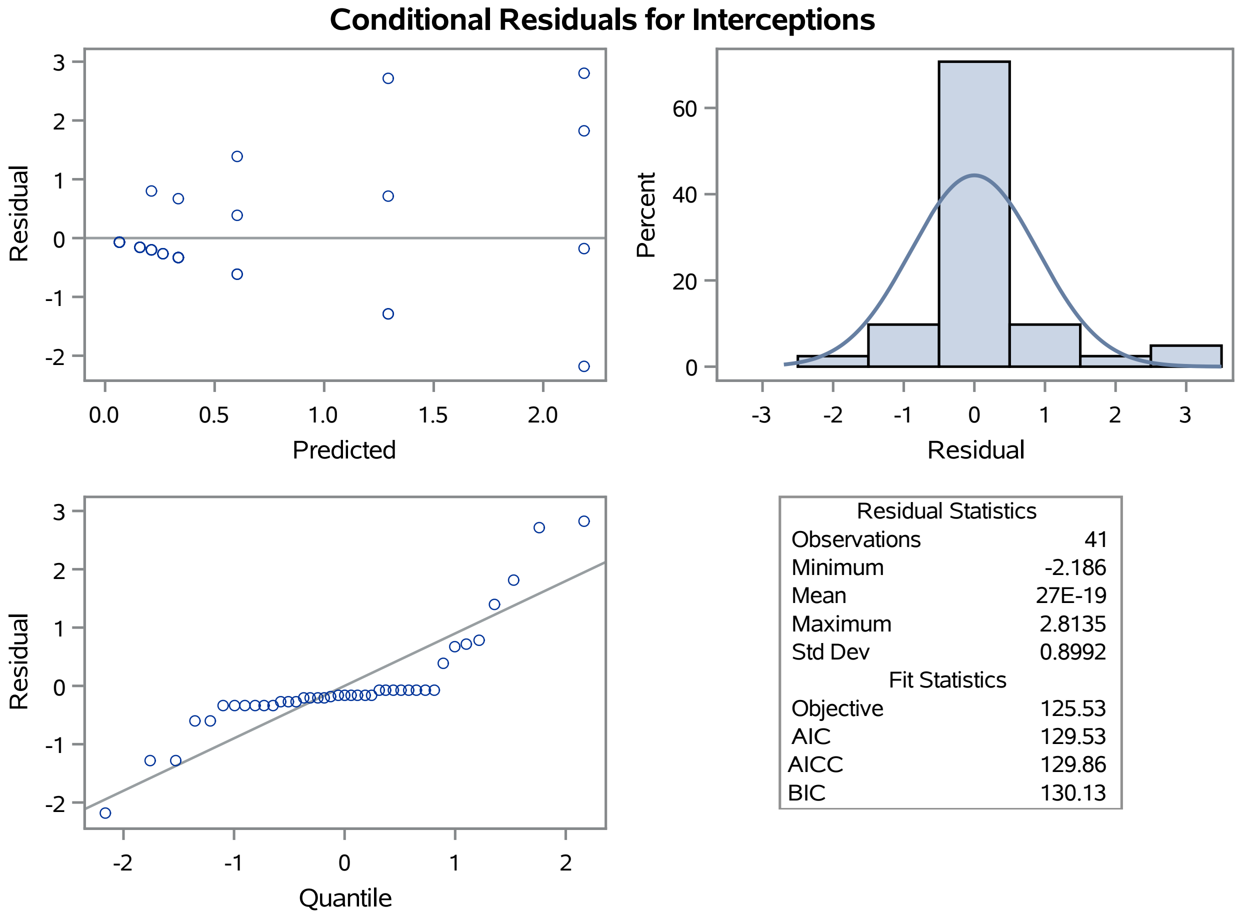

Residual Statistics	
Observations	41
Minimum	-2.186
Mean	27E-19
Maximum	2.8135
Std Dev	0.8992
Fit Statistics	
Objective	125.53
AIC	129.53
AICC	129.86
BIC	130.13

DistSoma=462

Model Information	
**Data Set**	WORK.TEMPDATASORTED
**Dependent Variable**	Interceptions
**Covariance Structure**	Variance Components
**Estimation Method**	REML
**Residual Variance Method**	Profile
**Fixed Effects SE Method**	Model-Based
**Degrees of Freedom Method**	Containment

Class Level Information		
**Class**	**Levels**	**Values**
**Treatment**	2	Meg TTR GFP MsTTR
**Culture**	10	1 2 3 4 5 6 7 8 9 10

Dimensions	
**Covariance Parameters**	2
**Columns in X**	3
**Columns in Z**	10
**Subjects**	1
**Max Obs per Subject**	41

Number of Observations	
**Number of Observations Read**	41
**Number of Observations Used**	41
**Number of Observations Not Used**	0

Iteration History			
**Iteration**	**Evaluations**	**-2 Res Log Like**	**Criterion**
**0**	1	132.17838078	
**1**	2	125.00428516	0.00000002
**2**	1	125.00428454	0.00000000

Convergence criteria met.

Covariance Parameter Estimates				
**Cov Parm**	**Estimate**	**Alpha**	**Lower**	**Upper**
**Culture**	0.6570	0.05	0.2406	5.0021
**Residual**	0.9440	0.05	0.6071	1.6668

DistSoma=462

Fit Statistics	
**-2 Res Log Likelihood**	125.0
**AIC (Smaller is Better)**	129.0
**AICC (Smaller is Better)**	129.3
**BIC (Smaller is Better)**	129.6

Solution for Fixed Effects

Effect	Treatment	Estimate	Standard Error	DF	t Value	Pr > |t|	Alpha	Lower	Upper
**Intercept**		0.2370	0.4692	8	0.51	0.6271	0.05	-0.8450	1.3190
**Treatment**	Meg TTR	0.4880	0.6103	31	0.80	0.4301	0.05	-0.7568	1.7327
**Treatment**	GFP MsTTR	0	.	.	.	.	.	.	.

Solution for Random Effects

Effect	Culture	Estimate	Std Err Pred	DF	t Value	Pr > |t|	Alpha	Lower	Upper
**Culture**	1	-0.02873	0.5287	31	-0.05	0.9570	0.05	-1.1070	1.0495
**Culture**	2	0.3774	0.5411	31	0.70	0.4907	0.05	-0.7262	1.4810
**Culture**	3	-0.1744	0.5411	31	-0.32	0.7495	0.05	-1.2780	0.9292
**Culture**	4	-0.1744	0.5411	31	-0.32	0.7495	0.05	-1.2780	0.9292
**Culture**	5	-0.5631	0.4884	31	-1.15	0.2577	0.05	-1.5593	0.4330
**Culture**	6	0.5702	0.5060	31	1.13	0.2685	0.05	-0.4618	1.6023
**Culture**	7	1.4899	0.5060	31	2.94	0.0061	0.05	0.4578	2.5219
**Culture**	8	-0.5849	0.4755	31	-1.23	0.2279	0.05	-1.5546	0.3848
**Culture**	9	-0.4219	0.5712	31	-0.74	0.4657	0.05	-1.5868	0.7430
**Culture**	10	-0.4902	0.5314	31	-0.92	0.3634	0.05	-1.5740	0.5936

Type 3 Tests of Fixed Effects

Effect	Num DF	Den DF	F Value	Pr > F
**Treatment**	1	31	0.64	0.4301

Least Squares Means

Effect	Treatment	Estimate	Standard Error	DF	t Value	Pr > |t|	Alpha	Lower	Upper
**Treatment**	Meg TTR	0.7250	0.3903	31	1.86	0.0728	0.05	-0.07103	1.5209
**Treatment**	GFP MsTTR	0.2370	0.4692	31	0.51	0.6171	0.05	-0.7200	1.1940

DistSoma=462

Differences of Least Squares Means												
**Effect**	**Treatment**	**Treatment**	**Estimate**	**Standard Error**	**DF**	**t Value**	**Pr > |t|**	**Adjustment**	**Adj P**	**Alpha**	**Lower**	**Upper**
**Treatment**	Meg TTR	GFP MsTTR	0.4880	0.6103	31	0.80	0.4301	Tukey-Kramer	0.4301	0.05	-0.7568	1.7327

Differences of Least Squares Means				
**Effect**	**Treatment**	**Treatment**	**Adj Lower**	**Adj Upper**
**Treatment**	Meg TTR	GFP MsTTR	-0.7568	1.7327

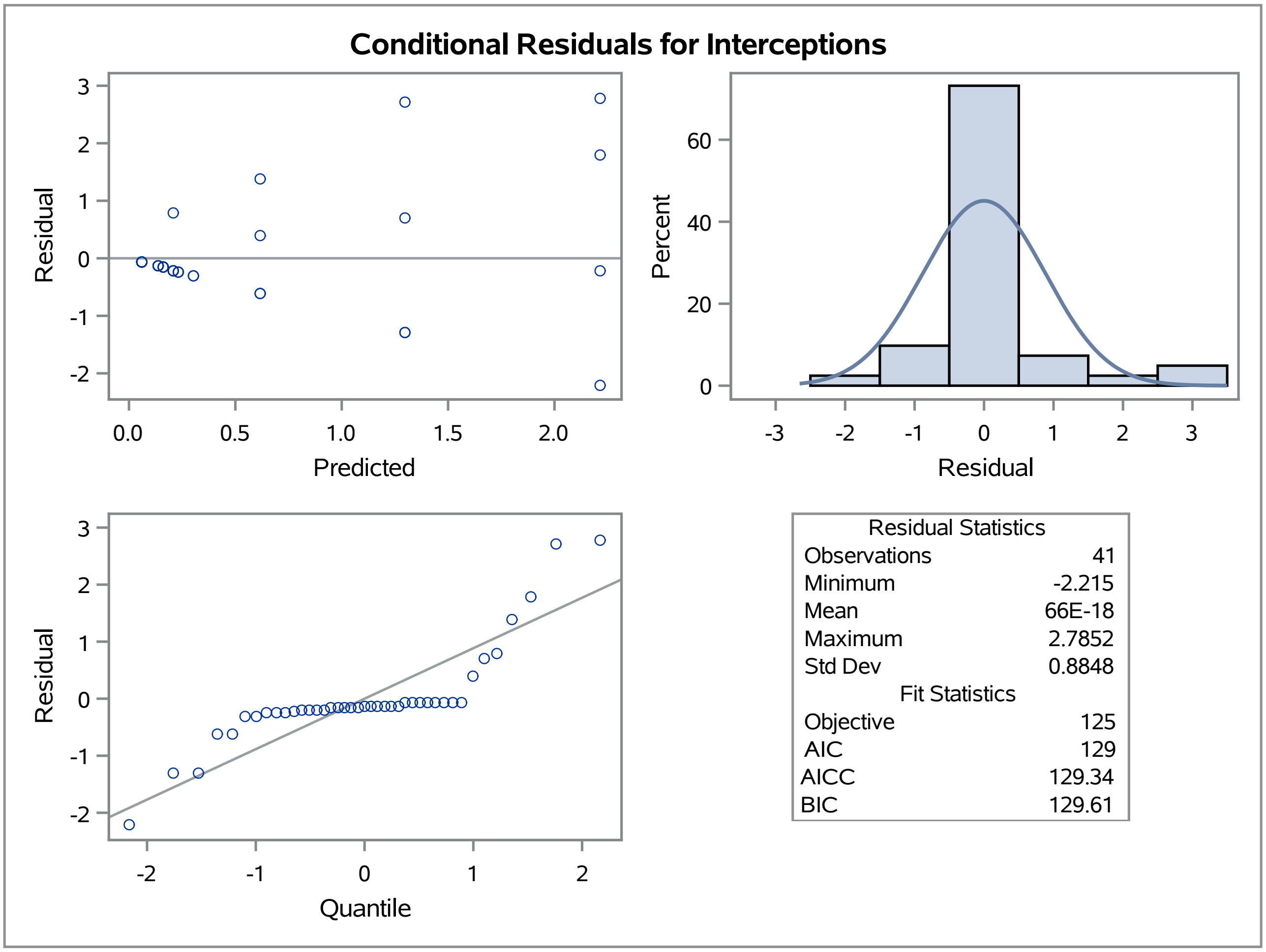

DistSoma=468

Model Information	
**Data Set**	WORK.TEMPDATASORTED
**Dependent Variable**	Interceptions
**Covariance Structure**	Variance Components
**Estimation Method**	REML
**Residual Variance Method**	Profile
**Fixed Effects SE Method**	Model-Based
**Degrees of Freedom Method**	Containment

Class Level Information		
**Class**	**Levels**	**Values**
**Treatment**	2	Meg TTR GFP MsTTR
**Culture**	10	1 2 3 4 5 6 7 8 9 10

Dimensions	
**Covariance Parameters**	2
**Columns in X**	3
**Columns in Z**	10
**Subjects**	1
**Max Obs per Subject**	41

Number of Observations	
**Number of Observations Read**	41
**Number of Observations Used**	41
**Number of Observations Not Used**	0

Iteration History			
**Iteration**	**Evaluations**	**-2 Res Log Like**	**Criterion**
**0**	1	131.07044192	
**1**	2	126.14623062	0.00000000

Convergence criteria met.

Covariance Parameter Estimates				
**Cov Parm**	**Estimate**	**Alpha**	**Lower**	**Upper**
**Culture**	0.5187	0.05	0.1767	5.3850
**Residual**	1.0160	0.05	0.6536	1.7934

DistSoma=468

Fit Statistics	
-2 Res Log Likelihood	126.1
AIC (Smaller is Better)	130.1
AICC (Smaller is Better)	130.5
BIC (Smaller is Better)	130.8

Solution for Fixed Effects

Effect	Treatment	Estimate	Standard Error	DF	t Value	Pr > |t|	Alpha	Lower	Upper
Intercept		0.2369	0.4357	8	0.54	0.6015	0.05	-0.7678	1.2416
Treatment	Meg TTR	0.4472	0.5674	31	0.79	0.4366	0.05	-0.7100	1.6045
Treatment	GFP MsTTR	0	.	.	.	.	.	.	.

Solution for Random Effects

Effect	Culture	Estimate	Std Err Pred	DF	t Value	Pr > |t|	Alpha	Lower	Upper
Culture	1	-0.02648	0.4940	31	-0.05	0.9576	0.05	-1.0339	0.9810
Culture	2	0.3445	0.5060	31	0.68	0.5011	0.05	-0.6875	1.3764
Culture	3	-0.1590	0.5060	31	-0.31	0.7555	0.05	-1.1910	0.8730
Culture	4	-0.1590	0.5060	31	-0.31	0.7555	0.05	-1.1910	0.8730
Culture	5	-0.4915	0.4628	31	-1.06	0.2965	0.05	-1.4355	0.4525
Culture	6	0.5477	0.4796	31	1.14	0.2622	0.05	-0.4305	1.5259
Culture	7	1.2189	0.4796	31	2.54	0.0163	0.05	0.2407	2.1972
Culture	8	-0.5157	0.4503	31	-1.15	0.2609	0.05	-1.4341	0.4027
Culture	9	-0.3456	0.5389	31	-0.64	0.5260	0.05	-1.4446	0.7534
Culture	10	-0.4138	0.5032	31	-0.82	0.4171	0.05	-1.4402	0.6125

Type 3 Tests of Fixed Effects

Effect	Num DF	Den DF	F Value	Pr > F
Treatment	1	31	0.62	0.4366

Least Squares Means

Effect	Treatment	Estimate	Standard Error	DF	t Value	Pr > |t|	Alpha	Lower	Upper
Treatment	Meg TTR	0.6841	0.3635	31	1.88	0.0693	0.05	-0.05730	1.4255
Treatment	GFP MsTTR	0.2369	0.4357	31	0.54	0.5906	0.05	-0.6517	1.1254

DistSoma=468

Differences of Least Squares Means												
**Effect**	**Treatment**	**Treatment**	**Estimate**	**Standard Error**	**DF**	**t Value**	**Pr > |t|**	**Adjustment**	**Adj P**	**Alpha**	**Lower**	**Upper**
**Treatment**	Meg TTR	GFP MsTTR	0.4472	0.5674	31	0.79	0.4366	Tukey-Kramer	0.4366	0.05	-0.7100	1.6045

Differences of Least Squares Means				
**Effect**	**Treatment**	**Treatment**	**Adj Lower**	**Adj Upper**
**Treatment**	Meg TTR	GFP MsTTR	-0.7100	1.6045

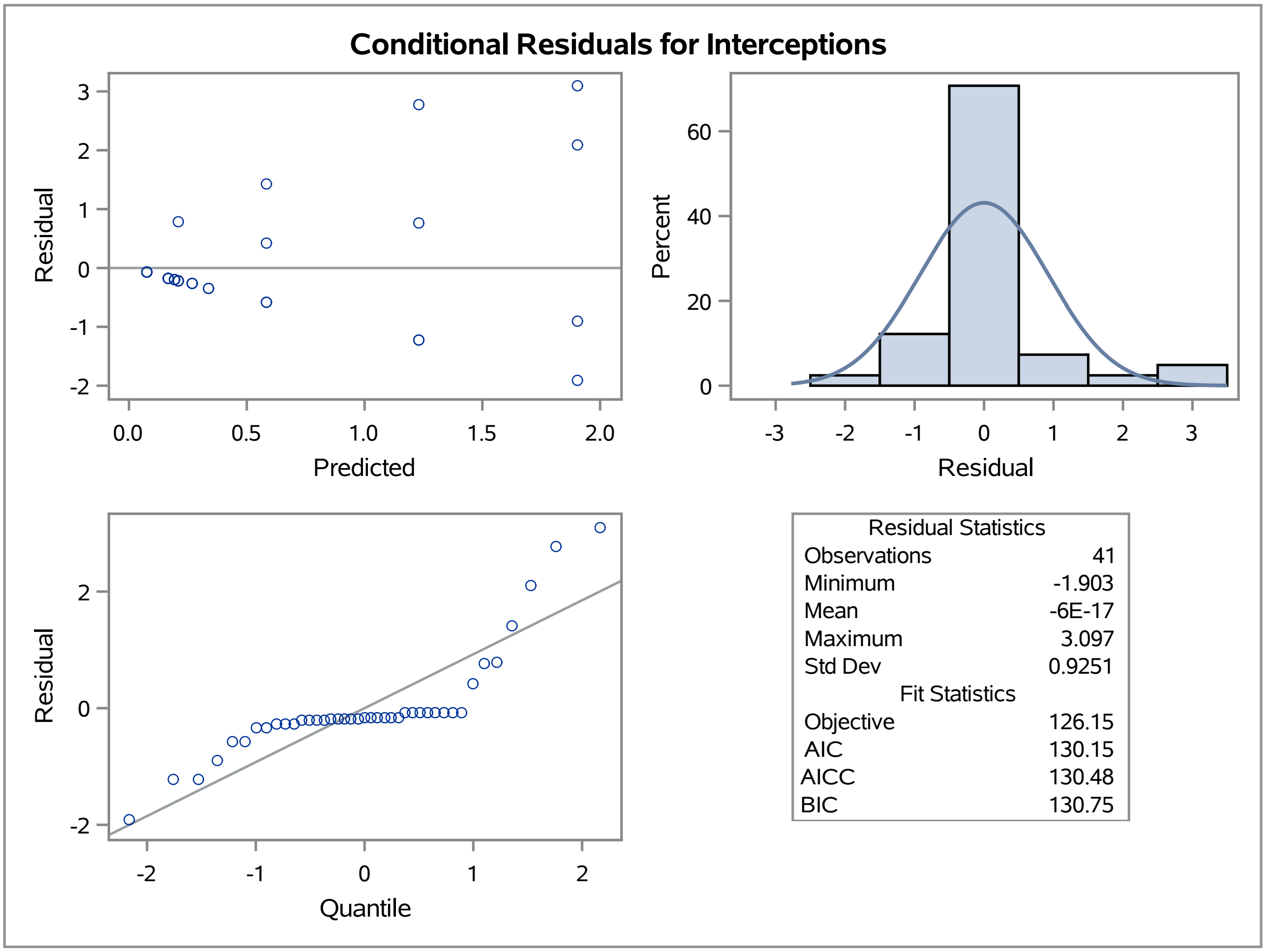

DistSoma=474

Model Information	
**Data Set**	WORK.TEMPDATASORTED
**Dependent Variable**	Interceptions
**Covariance Structure**	Variance Components
**Estimation Method**	REML
**Residual Variance Method**	Profile
**Fixed Effects SE Method**	Model-Based
**Degrees of Freedom Method**	Containment

Class Level Information		
**Class**	**Levels**	**Values**
**Treatment**	2	Meg TTR GFP MsTTR
**Culture**	10	1 2 3 4 5 6 7 8 9 10

Dimensions	
**Covariance Parameters**	2
**Columns in X**	3
**Columns in Z**	10
**Subjects**	1
**Max Obs per Subject**	41

Number of Observations	
**Number of Observations Read**	41
**Number of Observations Used**	41
**Number of Observations Not Used**	0

Iteration History			
**Iteration**	**Evaluations**	**-2 Res Log Like**	**Criterion**
**0**	1	119.97494801	
**1**	2	117.23184619	0.00000000

Convergence criteria met.

Covariance Parameter Estimates				
**Cov Parm**	**Estimate**	**Alpha**	**Lower**	**Upper**
**Culture**	0.2813	0.05	0.08351	6.0542
**Residual**	0.8543	0.05	0.5498	1.5068

DistSoma=474

Fit Statistics	
-2 Res Log Likelihood	117.2
AIC (Smaller is Better)	121.2
AICC (Smaller is Better)	121.6
BIC (Smaller is Better)	121.8

Solution for Fixed Effects

Effect	Treatment	Estimate	Standard Error	DF	t Value	Pr > |t|	Alpha	Lower	Upper
Intercept		0.1756	0.3477	8	0.51	0.6272	0.05	-0.6261	0.9773
Treatment	Meg TTR	0.3814	0.4535	31	0.84	0.4068	0.05	-0.5436	1.3063
Treatment	GFP MsTTR	0	.	.	.	.	.	.	.

Solution for Random Effects

Effect	Culture	Estimate	Std Err Pred	DF	t Value	Pr > |t|	Alpha	Lower	Upper
Culture	1	0.01519	0.3912	31	0.04	0.9693	0.05	-0.7828	0.8132
Culture	2	0.1844	0.4006	31	0.46	0.6485	0.05	-0.6325	1.0014
Culture	3	-0.09980	0.4006	31	-0.25	0.8049	0.05	-0.9168	0.7172
Culture	4	-0.09980	0.4006	31	-0.25	0.8049	0.05	-0.9168	0.7172
Culture	5	-0.3465	0.3730	31	-0.93	0.3601	0.05	-1.1072	0.4142
Culture	6	0.3940	0.3858	31	1.02	0.3150	0.05	-0.3928	1.1807
Culture	7	0.8203	0.3858	31	2.13	0.0415	0.05	0.03353	1.6070
Culture	8	-0.3698	0.3632	31	-1.02	0.3165	0.05	-1.1106	0.3710
Culture	9	-0.2212	0.4278	31	-0.52	0.6088	0.05	-1.0936	0.6513
Culture	10	-0.2768	0.4031	31	-0.69	0.4974	0.05	-1.0988	0.5453

Type 3 Tests of Fixed Effects

Effect	Num DF	Den DF	F Value	Pr > F
Treatment	1	31	0.71	0.4068

Least Squares Means

Effect	Treatment	Estimate	Standard Error	DF	t Value	Pr > |t|	Alpha	Lower	Upper
Treatment	Meg TTR	0.5570	0.2912	31	1.91	0.0651	0.05	-0.03698	1.1509
Treatment	GFP MsTTR	0.1756	0.3477	31	0.51	0.6171	0.05	-0.5335	0.8846

DistSoma=474

Differences of Least Squares Means												
Effect	Treatment	Treatment	Estimate	Standard Error	DF	t Value	Pr > |t|	Adjustment	Adj P	Alpha	Lower	Upper
Treatment	Meg TTR	GFP MsTTR	0.3814	0.4535	31	0.84	0.4068	Tukey-Kramer	0.4068	0.05	-0.5436	1.3063

Differences of Least Squares Means				
Effect	Treatment	Treatment	Adj Lower	Adj Upper
Treatment	Meg TTR	GFP MsTTR	-0.5435	1.3063

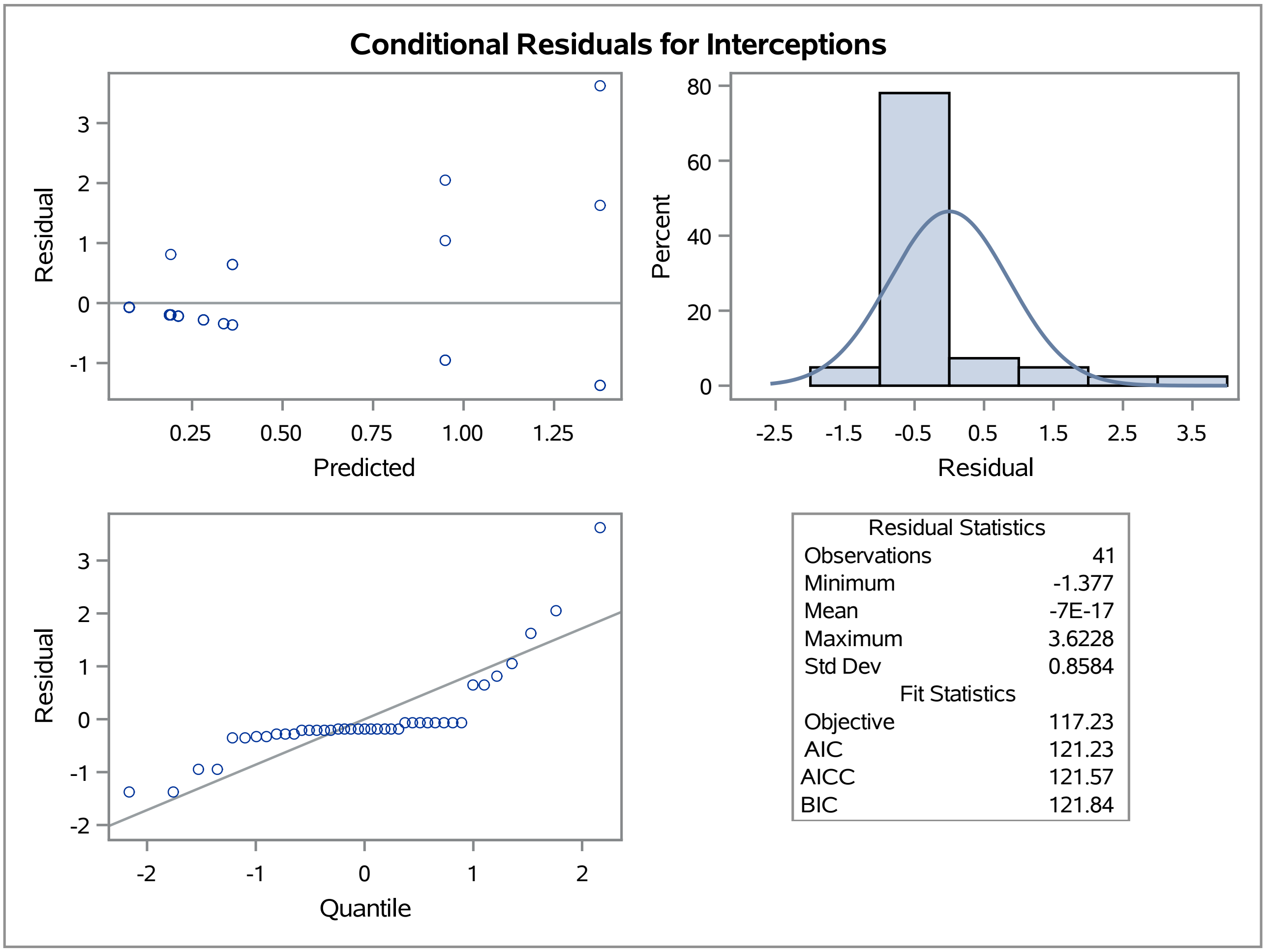

DistSoma=480

Model Information	
**Data Set**	WORK.TEMPDATASORTED
**Dependent Variable**	Interceptions
**Covariance Structure**	Variance Components
**Estimation Method**	REML
**Residual Variance Method**	Profile
**Fixed Effects SE Method**	Model-Based
**Degrees of Freedom Method**	Containment

Class Level Information		
**Class**	**Levels**	**Values**
**Treatment**	2	Meg TTR GFP MsTTR
**Culture**	10	1 2 3 4 5 6 7 8 9 10

Dimensions	
**Covariance Parameters**	2
**Columns in X**	3
**Columns in Z**	10
**Subjects**	1
**Max Obs per Subject**	41

Number of Observations	
**Number of Observations Read**	41
**Number of Observations Used**	41
**Number of Observations Not Used**	0

Iteration History			
**Iteration**	**Evaluations**	**-2 Res Log Like**	**Criterion**
**0**	1	118.13201283	
**1**	2	115.72231496	0.00000000

Convergence criteria met.

Covariance Parameter Estimates				
**Cov Parm**	**Estimate**	**Alpha**	**Lower**	**Upper**
**Culture**	0.2505	0.05	0.07190	6.6662
**Residual**	0.8300	0.05	0.5341	1.4639

DistSoma=480

Fit Statistics	
-2 Res Log Likelihood	115.7
AIC (Smaller is Better)	119.7
AICC (Smaller is Better)	120.1
BIC (Smaller is Better)	120.3

Solution for Fixed Effects

Effect	Treatment	Estimate	Standard Error	DF	t Value	Pr > |t|	Alpha	Lower	Upper
Intercept		0.1756	0.3342	8	0.53	0.6136	0.05	-0.5952	0.9464
Treatment	Meg TTR	0.3386	0.4361	31	0.78	0.4435	0.05	-0.5510	1.2281
Treatment	GFP MsTTR	0	.	.	.	.	.	.	.

Solution for Random Effects

Effect	Culture	Estimate	Std Err Pred	DF	t Value	Pr > |t|	Alpha	Lower	Upper
Culture	1	0.01467	0.3745	31	0.04	0.9690	0.05	-0.7492	0.7785
Culture	2	0.1774	0.3833	31	0.46	0.6467	0.05	-0.6043	0.9592
Culture	3	-0.09605	0.3833	31	-0.25	0.8038	0.05	-0.8778	0.6857
Culture	4	-0.09605	0.3833	31	-0.25	0.8038	0.05	-0.8778	0.6857
Culture	5	-0.3093	0.3581	31	-0.86	0.3944	0.05	-1.0396	0.4211
Culture	6	0.2657	0.3701	31	0.72	0.4781	0.05	-0.4891	1.0206
Culture	7	0.8127	0.3701	31	2.20	0.0357	0.05	0.05787	1.5676
Culture	8	-0.3313	0.3489	31	-0.95	0.3497	0.05	-1.0428	0.3802
Culture	9	-0.1936	0.4091	31	-0.47	0.6394	0.05	-1.0279	0.6408
Culture	10	-0.2443	0.3863	31	-0.63	0.5316	0.05	-1.0321	0.5434

Type 3 Tests of Fixed Effects

Effect	Num DF	Den DF	F Value	Pr > F
Treatment	1	31	0.60	0.4435

Least Squares Means

Effect	Treatment	Estimate	Standard Error	DF	t Value	Pr > |t|	Alpha	Lower	Upper
Treatment	Meg TTR	0.5142	0.2802	31	1.84	0.0761	0.05	-0.05725	1.0856
Treatment	GFP MsTTR	0.1756	0.3342	31	0.53	0.6031	0.05	-0.5061	0.8573

DistSoma=480

Differences of Least Squares Means												
**Effect**	**Treatment**	**Treatment**	**Estimate**	**Standard Error**	**DF**	**t Value**	**Pr > |t|**	**Adjustment**	**Adj P**	**Alpha**	**Lower**	**Upper**
**Treatment**	Meg TTR	GFP MsTTR	0.3386	0.4361	31	0.78	0.4435	Tukey-Kramer	0.4435	0.05	-0.5510	1.2281

Differences of Least Squares Means				
**Effect**	**Treatment**	**Treatment**	**Adj Lower**	**Adj Upper**
**Treatment**	Meg TTR	GFP MsTTR	-0.5509	1.2281

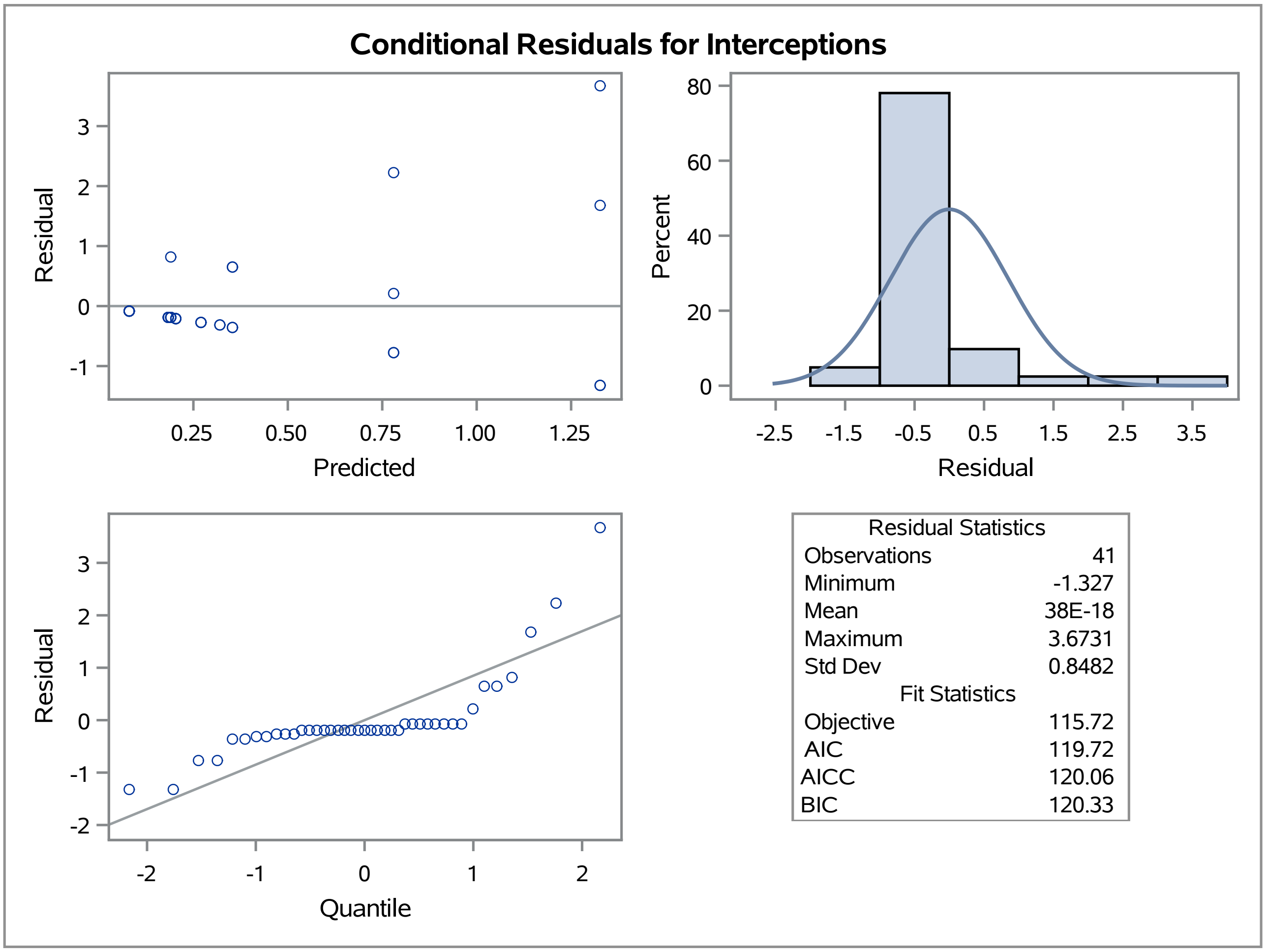

DistSoma=486

Model Information	
**Data Set**	WORK.TEMPDATASORTED
**Dependent Variable**	Interceptions
**Covariance Structure**	Variance Components
**Estimation Method**	REML
**Residual Variance Method**	Profile
**Fixed Effects SE Method**	Model-Based
**Degrees of Freedom Method**	Containment

Class Level Information		
**Class**	**Levels**	**Values**
**Treatment**	2	Meg TTR GFP MsTTR
**Culture**	10	1 2 3 4 5 6 7 8 9 10

Dimensions	
**Covariance Parameters**	2
**Columns in X**	3
**Columns in Z**	10
**Subjects**	1
**Max Obs per Subject**	41

Number of Observations	
**Number of Observations Read**	41
**Number of Observations Used**	41
**Number of Observations Not Used**	0

Iteration History			
**Iteration**	**Evaluations**	**-2 Res Log Like**	**Criterion**
**0**	1	118.13201283	
**1**	2	115.72231496	0.00000000

Convergence criteria met.

Covariance Parameter Estimates				
**Cov Parm**	**Estimate**	**Alpha**	**Lower**	**Upper**
**Culture**	0.2505	0.05	0.07190	6.6662
**Residual**	0.8300	0.05	0.5341	1.4639

DistSoma=486

Fit Statistics	
-2 Res Log Likelihood	115.7
AIC (Smaller is Better)	119.7
AICC (Smaller is Better)	120.1
BIC (Smaller is Better)	120.3

Solution for Fixed Effects

Effect	Treatment	Estimate	Standard Error	DF	t Value	Pr > |t|	Alpha	Lower	Upper
Intercept		0.1756	0.3342	8	0.53	0.6136	0.05	-0.5952	0.9464
Treatment	Meg TTR	0.3386	0.4361	31	0.78	0.4435	0.05	-0.5510	1.2281
Treatment	GFP MsTTR	0	.	.	.	.	.	.	.

Solution for Random Effects

Effect	Culture	Estimate	Std Err Pred	DF	t Value	Pr > |t|	Alpha	Lower	Upper
Culture	1	0.01467	0.3745	31	0.04	0.9690	0.05	-0.7492	0.7785
Culture	2	0.1774	0.3833	31	0.46	0.6467	0.05	-0.6043	0.9592
Culture	3	-0.09605	0.3833	31	-0.25	0.8038	0.05	-0.8778	0.6857
Culture	4	-0.09605	0.3833	31	-0.25	0.8038	0.05	-0.8778	0.6857
Culture	5	-0.3093	0.3581	31	-0.86	0.3944	0.05	-1.0396	0.4211
Culture	6	0.2657	0.3701	31	0.72	0.4781	0.05	-0.4891	1.0206
Culture	7	0.8127	0.3701	31	2.20	0.0357	0.05	0.05787	1.5676
Culture	8	-0.3313	0.3489	31	-0.95	0.3497	0.05	-1.0428	0.3802
Culture	9	-0.1936	0.4091	31	-0.47	0.6394	0.05	-1.0279	0.6408
Culture	10	-0.2443	0.3863	31	-0.63	0.5316	0.05	-1.0321	0.5434

Type 3 Tests of Fixed Effects

Effect	Num DF	Den DF	F Value	Pr > F
Treatment	1	31	0.60	0.4435

Least Squares Means

Effect	Treatment	Estimate	Standard Error	DF	t Value	Pr > |t|	Alpha	Lower	Upper
Treatment	Meg TTR	0.5142	0.2802	31	1.84	0.0761	0.05	-0.05725	1.0856
Treatment	GFP MsTTR	0.1756	0.3342	31	0.53	0.6031	0.05	-0.5061	0.8573

DistSoma=486

Differences of Least Squares Means												
**Effect**	**Treatment**	**Treatment**	**Estimate**	**Standard Error**	**DF**	**t Value**	**Pr > |t|**	**Adjustment**	**Adj P**	**Alpha**	**Lower**	**Upper**
**Treatment**	Meg TTR	GFP MsTTR	0.3386	0.4361	31	0.78	0.4435	Tukey-Kramer	0.4435	0.05	-0.5510	1.2281

Differences of Least Squares Means				
**Effect**	**Treatment**	**Treatment**	**Adj Lower**	**Adj Upper**
**Treatment**	Meg TTR	GFP MsTTR	-0.5509	1.2281

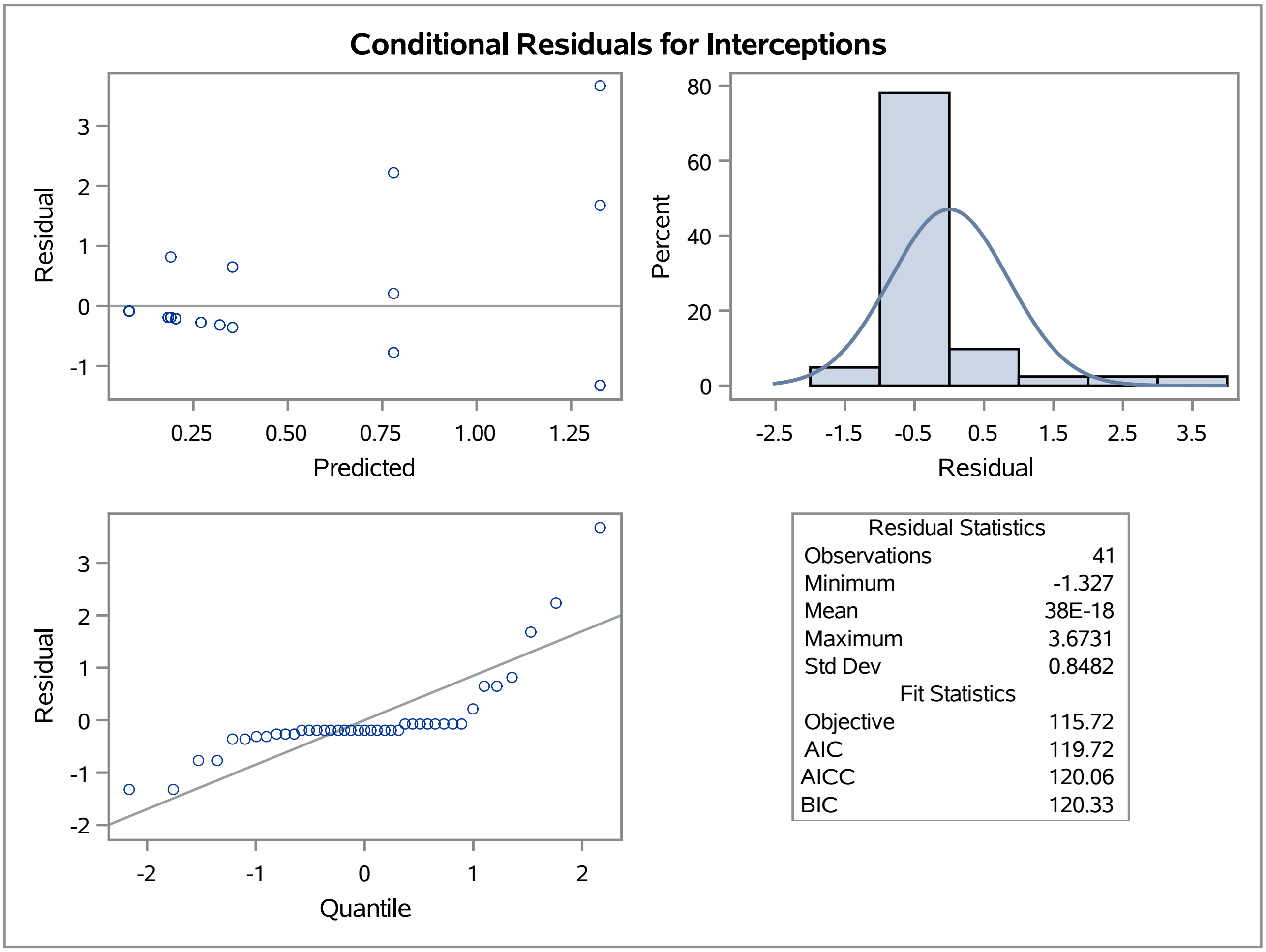

DistSoma=492

Model Information	
**Data Set**	WORK.TEMPDATASORTED
**Dependent Variable**	Interceptions
**Covariance Structure**	Variance Components
**Estimation Method**	REML
**Residual Variance Method**	Profile
**Fixed Effects SE Method**	Model-Based
**Degrees of Freedom Method**	Containment

Class Level Information		
**Class**	**Levels**	**Values**
**Treatment**	2	Meg TTR GFP MsTTR
**Culture**	10	1 2 3 4 5 6 7 8 9 10

Dimensions	
**Covariance Parameters**	2
**Columns in X**	3
**Columns in Z**	10
**Subjects**	1
**Max Obs per Subject**	41

Number of Observations	
**Number of Observations Read**	41
**Number of Observations Used**	41
**Number of Observations Not Used**	0

Iteration History			
**Iteration**	**Evaluations**	**-2 Res Log Like**	**Criterion**
**0**	1	118.13201283	
**1**	2	115.72231496	0.00000000

Convergence criteria met.

Covariance Parameter Estimates				
**Cov Parm**	**Estimate**	**Alpha**	**Lower**	**Upper**
**Culture**	0.2505	0.05	0.07190	6.6662
**Residual**	0.8300	0.05	0.5341	1.4639

DistSoma=492

Fit Statistics	
-2 Res Log Likelihood	115.7
AIC (Smaller is Better)	119.7
AICC (Smaller is Better)	120.1
BIC (Smaller is Better)	120.3

Solution for Fixed Effects

Effect	Treatment	Estimate	Standard Error	DF	t Value	Pr > |t|	Alpha	Lower	Upper
Intercept		0.1756	0.3342	8	0.53	0.6136	0.05	-0.5952	0.9464
Treatment	Meg TTR	0.3386	0.4361	31	0.78	0.4435	0.05	-0.5510	1.2281
Treatment	GFP MsTTR	0	.	.	.	.	.	.	.

Solution for Random Effects

Effect	Culture	Estimate	Std Err Pred	DF	t Value	Pr > |t|	Alpha	Lower	Upper
Culture	1	0.01467	0.3745	31	0.04	0.9690	0.05	-0.7492	0.7785
Culture	2	0.1774	0.3833	31	0.46	0.6467	0.05	-0.6043	0.9592
Culture	3	-0.09605	0.3833	31	-0.25	0.8038	0.05	-0.8778	0.6857
Culture	4	-0.09605	0.3833	31	-0.25	0.8038	0.05	-0.8778	0.6857
Culture	5	-0.3093	0.3581	31	-0.86	0.3944	0.05	-1.0396	0.4211
Culture	6	0.2657	0.3701	31	0.72	0.4781	0.05	-0.4891	1.0206
Culture	7	0.8127	0.3701	31	2.20	0.0357	0.05	0.05787	1.5676
Culture	8	-0.3313	0.3489	31	-0.95	0.3497	0.05	-1.0428	0.3802
Culture	9	-0.1936	0.4091	31	-0.47	0.6394	0.05	-1.0279	0.6408
Culture	10	-0.2443	0.3863	31	-0.63	0.5316	0.05	-1.0321	0.5434

Type 3 Tests of Fixed Effects

Effect	Num DF	Den DF	F Value	Pr > F
Treatment	1	31	0.60	0.4435

Least Squares Means

Effect	Treatment	Estimate	Standard Error	DF	t Value	Pr > |t|	Alpha	Lower	Upper
Treatment	Meg TTR	0.5142	0.2802	31	1.84	0.0761	0.05	-0.05725	1.0856
Treatment	GFP MsTTR	0.1756	0.3342	31	0.53	0.6031	0.05	-0.5061	0.8573

DistSoma=492

Differences of Least Squares Means												
**Effect**	**Treatment**	**Treatment**	**Estimate**	**Standard Error**	**DF**	**t Value**	**Pr > |t|**	**Adjustment**	**Adj P**	**Alpha**	**Lower**	**Upper**
**Treatment**	Meg TTR	GFP MsTTR	0.3386	0.4361	31	0.78	0.4435	Tukey-Kramer	0.4435	0.05	-0.5510	1.2281

Differences of Least Squares Means				
**Effect**	**Treatment**	**Treatment**	**Adj Lower**	**Adj Upper**
**Treatment**	Meg TTR	GFP MsTTR	-0.5509	1.2281

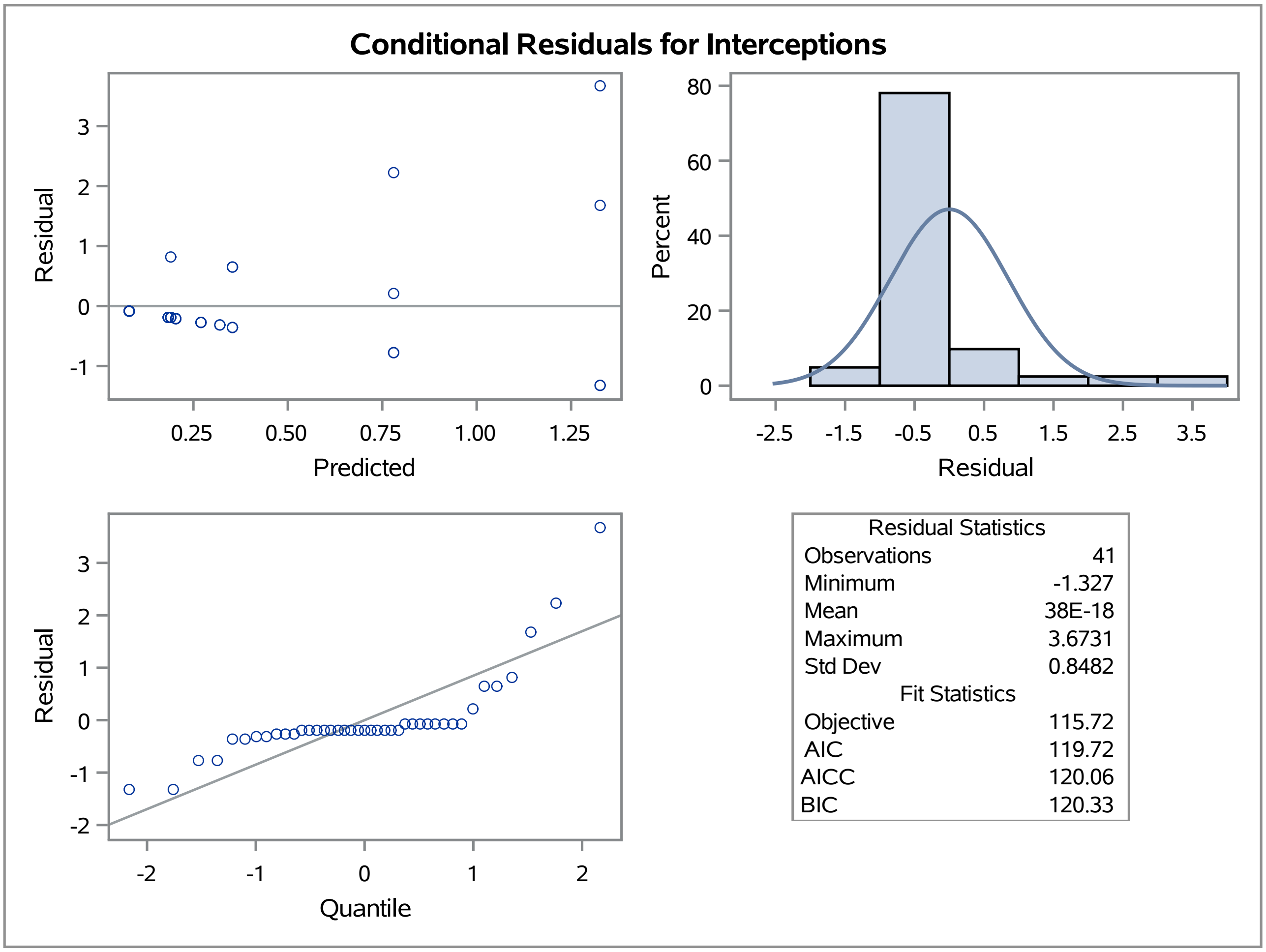

DistSoma=498

Model Information	
**Data Set**	WORK.TEMPDATASORTED
**Dependent Variable**	Interceptions
**Covariance Structure**	Variance Components
**Estimation Method**	REML
**Residual Variance Method**	Profile
**Fixed Effects SE Method**	Model-Based
**Degrees of Freedom Method**	Containment

Class Level Information		
**Class**	**Levels**	**Values**
**Treatment**	2	Meg TTR GFP MsTTR
**Culture**	10	1 2 3 4 5 6 7 8 9 10

Dimensions	
**Covariance Parameters**	2
**Columns in X**	3
**Columns in Z**	10
**Subjects**	1
**Max Obs per Subject**	41

Number of Observations	
**Number of Observations Read**	41
**Number of Observations Used**	41
**Number of Observations Not Used**	0

Iteration History			
**Iteration**	**Evaluations**	**-2 Res Log Like**	**Criterion**
**0**	1	106.58033360	
**1**	2	104.50663065	0.00000006
**2**	1	104.50662974	0.00000000

Convergence criteria met.

Covariance Parameter Estimates				
**Cov Parm**	**Estimate**	**Alpha**	**Lower**	**Upper**
**Culture**	0.1731	0.05	0.04776	6.0281
**Residual**	0.6288	0.05	0.4046	1.1093

DistSoma=498

Fit Statistics	
**-2 Res Log Likelihood**	104.5
**AIC (Smaller is Better)**	108.5
**AICC (Smaller is Better)**	108.8
**BIC (Smaller is Better)**	109.1

Solution for Fixed Effects

Effect	Treatment	Estimate	Standard Error	DF	t Value	Pr > |t|	Alpha	Lower	Upper
**Intercept**		0.1756	0.2837	8	0.62	0.5530	0.05	-0.4785	0.8298
**Treatment**	Meg TTR	0.2100	0.3702	31	0.57	0.5747	0.05	-0.5451	0.9651
**Treatment**	GFP MsTTR	0	.	.	.	.	.	.	.

Solution for Random Effects

Effect	Culture	Estimate	Std Err Pred	DF	t Value	Pr > |t|	Alpha	Lower	Upper
**Culture**	1	0.01411	0.3160	31	0.04	0.9647	0.05	-0.6303	0.6585
**Culture**	2	0.1700	0.3232	31	0.53	0.6027	0.05	-0.4893	0.8293
**Culture**	3	-0.09205	0.3232	31	-0.28	0.7777	0.05	-0.7513	0.5672
**Culture**	4	-0.09205	0.3232	31	-0.28	0.7777	0.05	-0.7513	0.5672
**Culture**	5	-0.2234	0.3030	31	-0.74	0.4666	0.05	-0.8414	0.3947
**Culture**	6	0.05993	0.3129	31	0.19	0.8494	0.05	-0.5783	0.6982
**Culture**	7	0.7150	0.3129	31	2.28	0.0293	0.05	0.07678	1.3533
**Culture**	8	-0.2402	0.2954	31	-0.81	0.4223	0.05	-0.8426	0.3622
**Culture**	9	-0.1369	0.3446	31	-0.40	0.6938	0.05	-0.8399	0.5660
**Culture**	10	-0.1744	0.3262	31	-0.53	0.5966	0.05	-0.8397	0.4908

Type 3 Tests of Fixed Effects

Effect	Num DF	Den DF	F Value	Pr > F
**Treatment**	1	31	0.32	0.5747

Least Squares Means

Effect	Treatment	Estimate	Standard Error	DF	t Value	Pr > |t|	Alpha	Lower	Upper
**Treatment**	Meg TTR	0.3856	0.2379	31	1.62	0.1152	0.05	-0.09963	0.8709
**Treatment**	GFP MsTTR	0.1756	0.2837	31	0.62	0.5403	0.05	-0.4029	0.7542

DistSoma=498

Differences of Least Squares Means												
**Effect**	**Treatment**	**Treatment**	**Estimate**	**Standard Error**	**DF**	**t Value**	**Pr > |t|**	**Adjustment**	**Adj P**	**Alpha**	**Lower**	**Upper**
**Treatment**	Meg TTR	GFP MsTTR	0.2100	0.3702	31	0.57	0.5747	Tukey-Kramer	0.5747	0.05	-0.5451	0.9651

Differences of Least Squares Means				
**Effect**	**Treatment**	**Treatment**	**Adj Lower**	**Adj Upper**
**Treatment**	Meg TTR	GFP MsTTR	-0.5451	0.9651

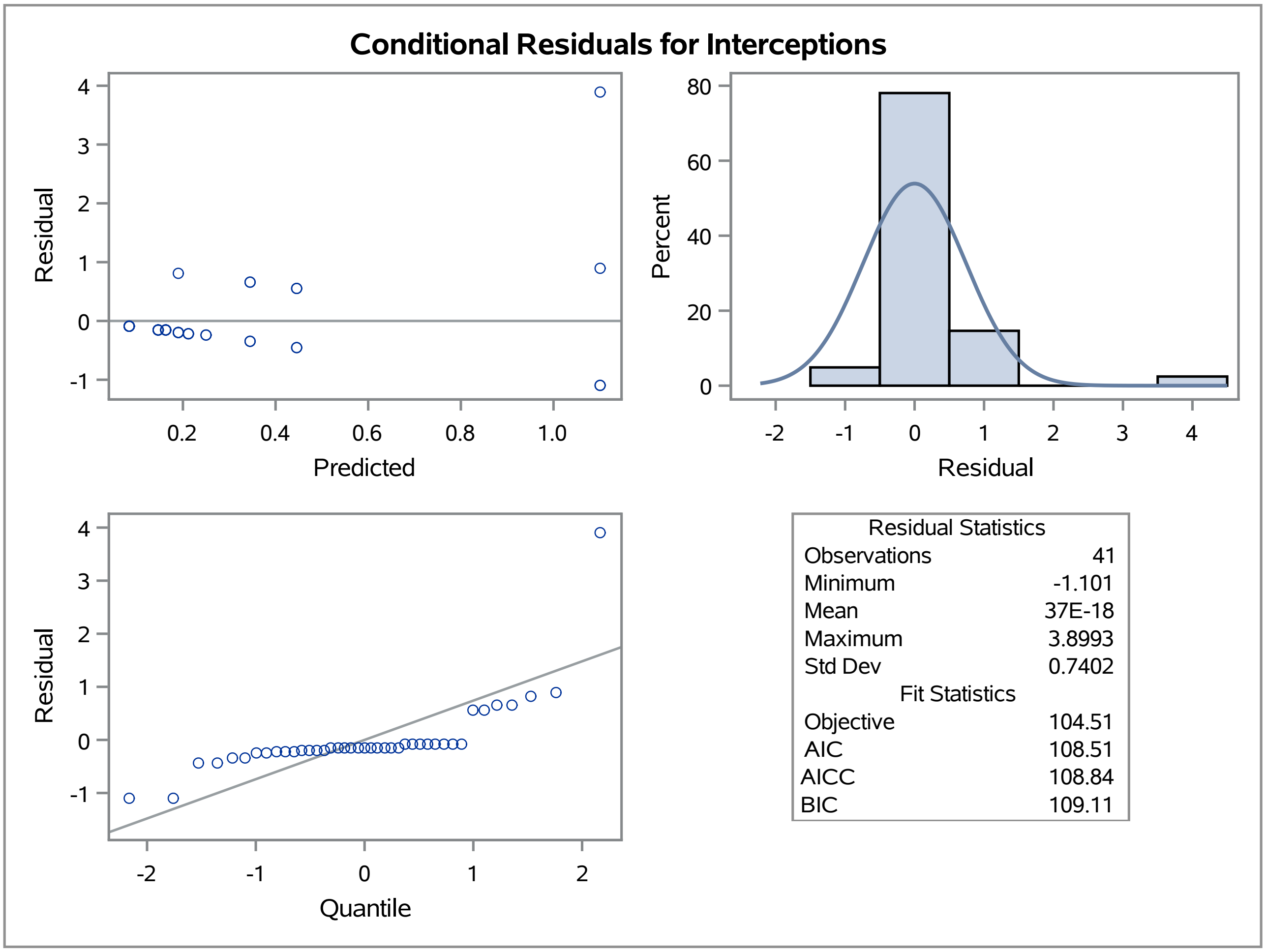

DistSoma=504

Model Information	
**Data Set**	WORK.TEMPDATASORTED
**Dependent Variable**	Interceptions
**Covariance Structure**	Variance Components
**Estimation Method**	REML
**Residual Variance Method**	Profile
**Fixed Effects SE Method**	Model-Based
**Degrees of Freedom Method**	Containment

Class Level Information		
**Class**	**Levels**	**Values**
**Treatment**	2	Meg TTR GFP MsTTR
**Culture**	10	1 2 3 4 5 6 7 8 9 10

Dimensions	
**Covariance Parameters**	2
**Columns in X**	3
**Columns in Z**	10
**Subjects**	1
**Max Obs per Subject**	41

Number of Observations	
**Number of Observations Read**	41
**Number of Observations Used**	41
**Number of Observations Not Used**	0

Iteration History			
**Iteration**	**Evaluations**	**-2 Res Log Like**	**Criterion**
**0**	1	88.58227046	
**1**	2	88.53576361	0.00000000

Convergence criteria met.

Covariance Parameter Estimates				
**Cov Parm**	**Estimate**	**Alpha**	**Lower**	**Upper**
**Culture**	0.01443	0.05	0.001260	1.847E35
**Residual**	0.4745	0.05	0.3057	0.8351

DistSoma=504

Fit Statistics	
-2 Res Log Likelihood	88.5
AIC (Smaller is Better)	92.5
AICC (Smaller is Better)	92.9
BIC (Smaller is Better)	93.1

Solution for Fixed Effects

Effect	Treatment	Estimate	Standard Error	DF	t Value	Pr > |t|	Alpha	Lower	Upper
Intercept		0.1763	0.1776	8	0.99	0.3500	0.05	-0.2333	0.5859
Treatment	Meg TTR	0.07626	0.2323	31	0.33	0.7449	0.05	-0.3975	0.5500
Treatment	GFP MsTTR	0	.	.	.	.	.	.	.

Solution for Random Effects

Effect	Culture	Estimate	Std Err Pred	DF	t Value	Pr > |t|	Alpha	Lower	Upper
Culture	1	0.003130	0.1143	31	0.03	0.9783	0.05	-0.2301	0.2363
Culture	2	0.03511	0.1150	31	0.31	0.7623	0.05	-0.1995	0.2697
Culture	3	-0.01912	0.1150	31	-0.17	0.8691	0.05	-0.2538	0.2155
Culture	4	-0.01912	0.1150	31	-0.17	0.8691	0.05	-0.2538	0.2155
Culture	5	-0.03333	0.1136	31	-0.29	0.7712	0.05	-0.2651	0.1984
Culture	6	0.02684	0.1146	31	0.23	0.8164	0.05	-0.2068	0.2605
Culture	7	0.08106	0.1146	31	0.71	0.4845	0.05	-0.1526	0.3147
Culture	8	-0.03897	0.1129	31	-0.35	0.7322	0.05	-0.2691	0.1912
Culture	9	-0.01448	0.1169	31	-0.12	0.9023	0.05	-0.2530	0.2240
Culture	10	-0.02111	0.1157	31	-0.18	0.8563	0.05	-0.2570	0.2148

Type 3 Tests of Fixed Effects

Effect	Num DF	Den DF	F Value	Pr > F
Treatment	1	31	0.11	0.7449

Least Squares Means

Effect	Treatment	Estimate	Standard Error	DF	t Value	Pr > |t|	Alpha	Lower	Upper
Treatment	Meg TTR	0.2526	0.1497	31	1.69	0.1016	0.05	-0.05270	0.5578
Treatment	GFP MsTTR	0.1763	0.1776	31	0.99	0.3287	0.05	-0.1860	0.5386

DistSoma=504

Differences of Least Squares Means												
Effect	Treatment	Treatment	Estimate	Standard Error	DF	t Value	Pr > |t|	Adjustment	Adj P	Alpha	Lower	Upper
Treatment	Meg TTR	GFP MsTTR	0.07626	0.2323	31	0.33	0.7449	Tukey-Kramer	0.7449	0.05	-0.3975	0.5500

Differences of Least Squares Means				
Effect	Treatment	Treatment	Adj Lower	Adj Upper
Treatment	Meg TTR	GFP MsTTR	-0.3975	0.5500

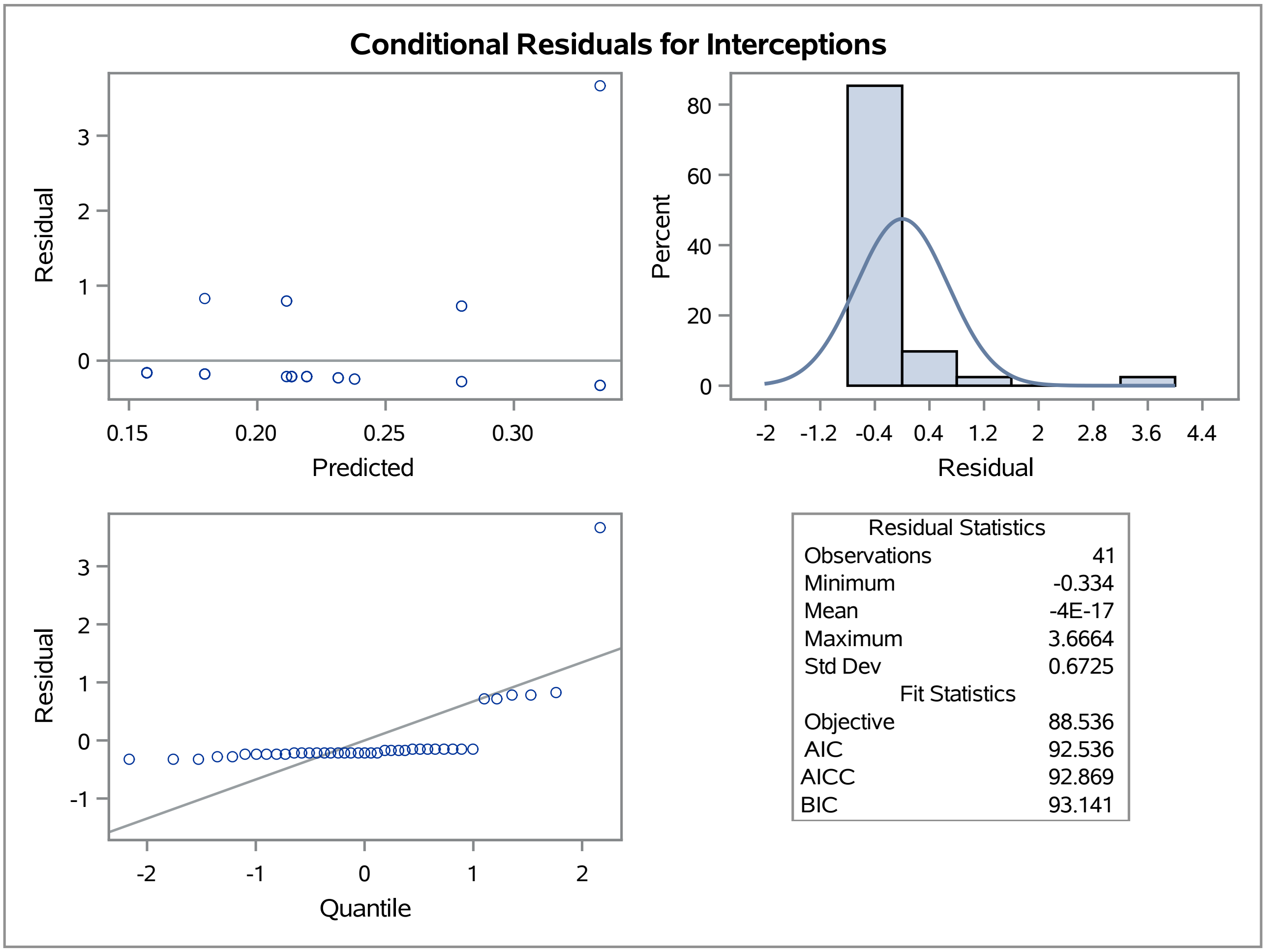

DistSoma=510

Model Information	
**Data Set**	WORK.TEMPDATASORTED
**Dependent Variable**	Interceptions
**Covariance Structure**	Variance Components
**Estimation Method**	REML
**Residual Variance Method**	Profile
**Fixed Effects SE Method**	Model-Based
**Degrees of Freedom Method**	Containment

Class Level Information		
**Class**	**Levels**	**Values**
**Treatment**	2	Meg TTR GFP MsTTR
**Culture**	10	1 2 3 4 5 6 7 8 9 10

Dimensions	
**Covariance Parameters**	2
**Columns in X**	3
**Columns in Z**	10
**Subjects**	1
**Max Obs per Subject**	41

Number of Observations	
**Number of Observations Read**	41
**Number of Observations Used**	41
**Number of Observations Not Used**	0

Iteration History			
**Iteration**	**Evaluations**	**-2 Res Log Like**	**Criterion**
**0**	1	102.96189011	
**1**	2	102.93763455	0.00000000

Convergence criteria met.

Covariance Parameter Estimates				
**Cov Parm**	**Estimate**	**Alpha**	**Lower**	**Upper**
**Culture**	0.01499	0.05	0.001470	9.643E67
**Residual**	0.6909	0.05	0.4452	1.2162

DistSoma=510

Fit Statistics	
-2 Res Log Likelihood	102.9
AIC (Smaller is Better)	106.9
AICC (Smaller is Better)	107.3
BIC (Smaller is Better)	107.5

Solution for Fixed Effects

Effect	Treatment	Estimate	Standard Error	DF	t Value	Pr > |t|	Alpha	Lower	Upper
Intercept		0.1763	0.2108	8	0.84	0.4271	0.05	-0.3097	0.6624
Treatment	Meg TTR	0.1176	0.2756	31	0.43	0.6726	0.05	-0.4445	0.6797
Treatment	GFP MsTTR	0	.	.	.	.	.	.	.

Solution for Random Effects

Effect	Culture	Estimate	Std Err Pred	DF	t Value	Pr > |t|	Alpha	Lower	Upper
Culture	1	0.002317	0.1181	31	0.02	0.9845	0.05	-0.2386	0.2432
Culture	2	0.02585	0.1187	31	0.22	0.8290	0.05	-0.2161	0.2679
Culture	3	-0.01408	0.1187	31	-0.12	0.9063	0.05	-0.2561	0.2279
Culture	4	-0.01408	0.1187	31	-0.12	0.9063	0.05	-0.2561	0.2279
Culture	5	-0.02877	0.1176	31	-0.24	0.8083	0.05	-0.2686	0.2111
Culture	6	0.01646	0.1183	31	0.14	0.8902	0.05	-0.2248	0.2578
Culture	7	0.07636	0.1183	31	0.65	0.5234	0.05	-0.1649	0.3177
Culture	8	-0.03386	0.1170	31	-0.29	0.7742	0.05	-0.2724	0.2047
Culture	9	-0.01223	0.1201	31	-0.10	0.9196	0.05	-0.2572	0.2327
Culture	10	-0.01797	0.1191	31	-0.15	0.8811	0.05	-0.2610	0.2250

Type 3 Tests of Fixed Effects

Effect	Num DF	Den DF	F Value	Pr > F
Treatment	1	31	0.18	0.6726

Least Squares Means

Effect	Treatment	Estimate	Standard Error	DF	t Value	Pr > |t|	Alpha	Lower	Upper
Treatment	Meg TTR	0.2939	0.1776	31	1.66	0.1079	0.05	-0.06822	0.6561
Treatment	GFP MsTTR	0.1763	0.2108	31	0.84	0.4092	0.05	-0.2535	0.6062

DistSoma=510

Differences of Least Squares Means												
Effect	Treatment	Treatment	Estimate	Standard Error	DF	t Value	Pr > |t|	Adjustment	Adj P	Alpha	Lower	Upper
Treatment	Meg TTR	GFP MsTTR	0.1176	0.2756	31	0.43	0.6726	Tukey-Kramer	0.6726	0.05	-0.4445	0.6797

Differences of Least Squares Means				
Effect	Treatment	Treatment	Adj Lower	Adj Upper
Treatment	Meg TTR	GFP MsTTR	-0.4445	0.6797

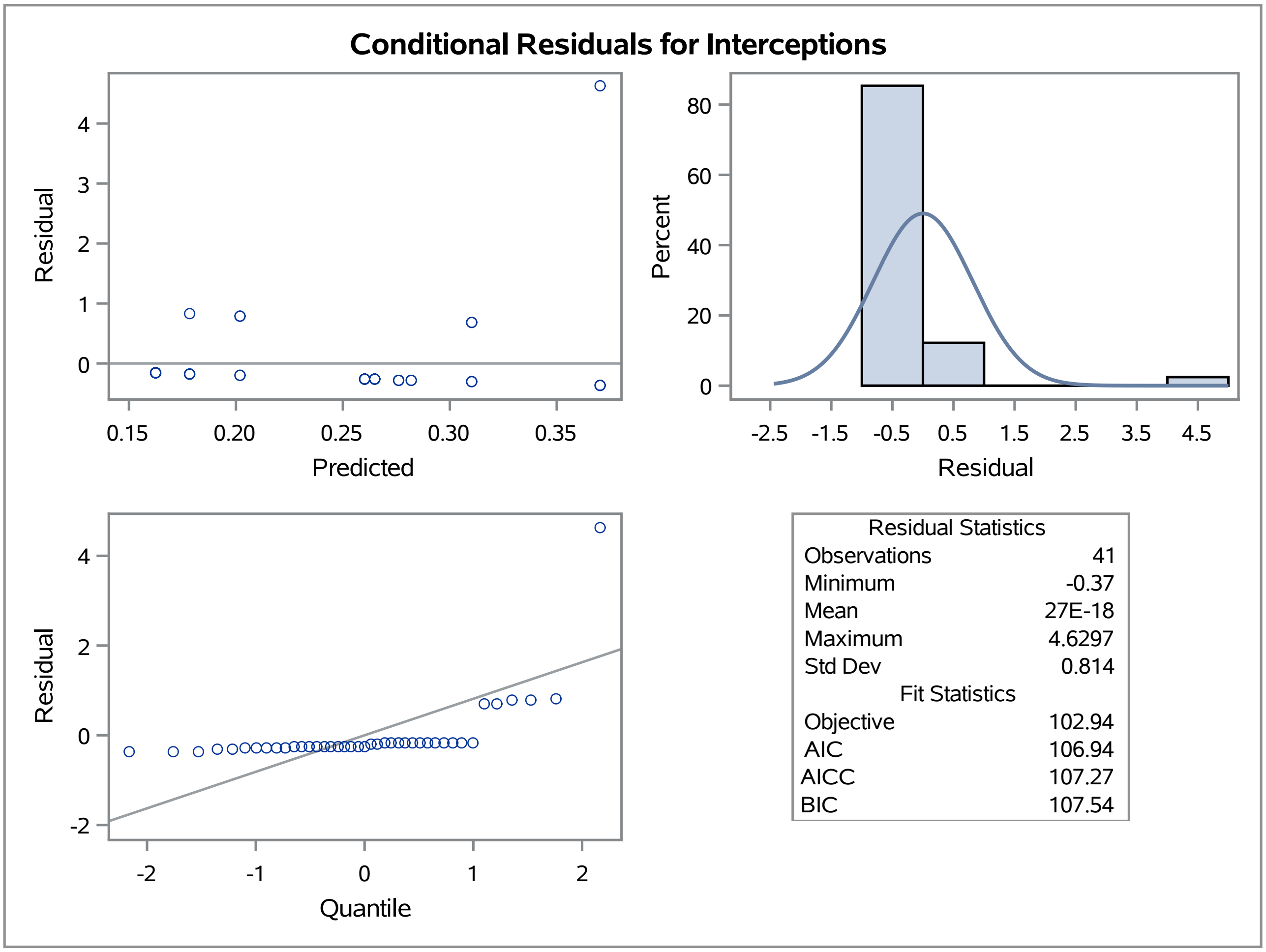

DistSoma=516

Model Information	
**Data Set**	WORK.TEMPDATASORTED
**Dependent Variable**	Interceptions
**Covariance Structure**	Variance Components
**Estimation Method**	REML
**Residual Variance Method**	Profile
**Fixed Effects SE Method**	Model-Based
**Degrees of Freedom Method**	Containment

Class Level Information		
**Class**	**Levels**	**Values**
**Treatment**	2	Meg TTR GFP MsTTR
**Culture**	10	1 2 3 4 5 6 7 8 9 10

Dimensions	
**Covariance Parameters**	2
**Columns in X**	3
**Columns in Z**	10
**Subjects**	1
**Max Obs per Subject**	41

Number of Observations	
**Number of Observations Read**	41
**Number of Observations Used**	41
**Number of Observations Not Used**	0

Iteration History			
**Iteration**	**Evaluations**	**-2 Res Log Like**	**Criterion**
**0**	1	102.30469952	
**1**	2	102.29551471	0.00000000

Convergence criteria met.

Covariance Parameter Estimates				
**Cov Parm**	**Estimate**	**Alpha**	**Lower**	**Upper**
**Culture**	0.009161	0.05	0.002465	5.01E178
**Residual**	0.6840	0.05	0.4402	1.2063

DistSoma=516

Fit Statistics	
**-2 Res Log Likelihood**	102.3
**AIC (Smaller is Better)**	106.3
**AICC (Smaller is Better)**	106.6
**BIC (Smaller is Better)**	106.9

Solution for Fixed Effects

Effect	Treatment	Estimate	Standard Error	DF	t Value	Pr > |t|	Alpha	Lower	Upper
**Intercept**		0.1764	0.2063	8	0.86	0.4174	0.05	-0.2993	0.6520
**Treatment**	Meg TTR	0.07488	0.2697	31	0.28	0.7831	0.05	-0.4751	0.6249
**Treatment**	GFP MsTTR	0	.	.	.	.	.	.	.

Solution for Random Effects

Effect	Culture	Estimate	Std Err Pred	DF	t Value	Pr > |t|	Alpha	Lower	Upper
**Culture**	1	0.001482	0.09356	31	0.02	0.9875	0.05	-0.1893	0.1923
**Culture**	2	0.01646	0.09384	31	0.18	0.8619	0.05	-0.1749	0.2078
**Culture**	3	-0.00897	0.09384	31	-0.10	0.9245	0.05	-0.2003	0.1824
**Culture**	4	-0.00897	0.09384	31	-0.10	0.9245	0.05	-0.2003	0.1824
**Culture**	5	-0.01577	0.09330	31	-0.17	0.8669	0.05	-0.2061	0.1745
**Culture**	6	-0.00006	0.09366	31	-0.00	0.9995	0.05	-0.1911	0.1910
**Culture**	7	0.05079	0.09366	31	0.54	0.5916	0.05	-0.1402	0.2418
**Culture**	8	-0.01869	0.09299	31	-0.20	0.8420	0.05	-0.2083	0.1710
**Culture**	9	-0.00656	0.09456	31	-0.07	0.9452	0.05	-0.1994	0.1863
**Culture**	10	-0.00971	0.09409	31	-0.10	0.9185	0.05	-0.2016	0.1822

Type 3 Tests of Fixed Effects

Effect	Num DF	Den DF	F Value	Pr > F
**Treatment**	1	31	0.08	0.7831

Least Squares Means

Effect	Treatment	Estimate	Standard Error	DF	t Value	Pr > |t|	Alpha	Lower	Upper
**Treatment**	Meg TTR	0.2513	0.1737	31	1.45	0.1581	0.05	-0.1030	0.6056
**Treatment**	GFP MsTTR	0.1764	0.2063	31	0.86	0.3990	0.05	-0.2443	0.5971

DistSoma=516

Differences of Least Squares Means												
Effect	Treatment	Treatment	Estimate	Standard Error	DF	t Value	Pr > |t|	Adjustment	Adj P	Alpha	Lower	Upper
Treatment	Meg TTR	GFP MsTTR	0.07488	0.2697	31	0.28	0.7831	Tukey-Kramer	0.7831	0.05	-0.4751	0.6249

Differences of Least Squares Means				
Effect	Treatment	Treatment	Adj Lower	Adj Upper
Treatment	Meg TTR	GFP MsTTR	-0.4751	0.6249

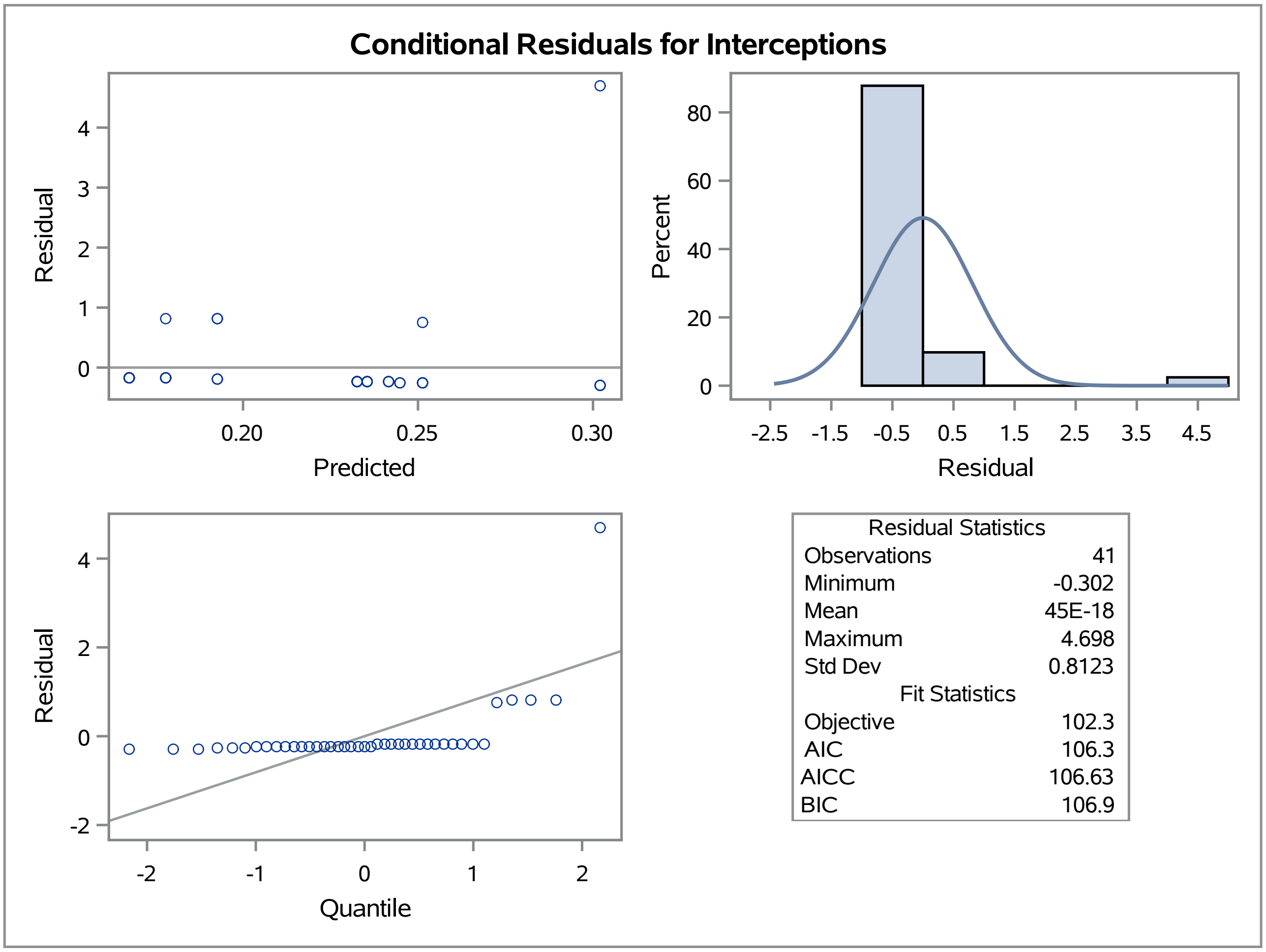

DistSoma=522

Model Information	
**Data Set**	WORK.TEMPDATASORTED
**Dependent Variable**	Interceptions
**Covariance Structure**	Variance Components
**Estimation Method**	REML
**Residual Variance Method**	Profile
**Fixed Effects SE Method**	Model-Based
**Degrees of Freedom Method**	Containment

Class Level Information		
**Class**	**Levels**	**Values**
**Treatment**	2	Meg TTR GFP MsTTR
**Culture**	10	1 2 3 4 5 6 7 8 9 10

Dimensions	
**Covariance Parameters**	2
**Columns in X**	3
**Columns in Z**	10
**Subjects**	1
**Max Obs per Subject**	41

Number of Observations	
**Number of Observations Read**	41
**Number of Observations Used**	41
**Number of Observations Not Used**	0

Iteration History			
**Iteration**	**Evaluations**	**-2 Res Log Like**	**Criterion**
**0**	1	101.27038476	
**1**	1	101.27038476	0.00000000

Convergence criteria met.

Estimated G matrix is not positive definite.

Covariance Parameter Estimates				
**Cov Parm**	**Estimate**	**Alpha**	**Lower**	**Upper**
**Culture**	0	.	.	.
**Residual**	0.6735	0.05	0.4519	1.1104

DistSoma=522

Fit Statistics	
-2 Res Log Likelihood	101.3
AIC (Smaller is Better)	103.3
AICC (Smaller is Better)	103.4
BIC (Smaller is Better)	103.6

Solution for Fixed Effects

Effect	Treatment	Estimate	Standard Error	DF	t Value	Pr > |t|	Alpha	Lower	Upper
Intercept		0.1176	0.1990	8	0.59	0.5708	0.05	-0.3413	0.5766
Treatment	Meg TTR	0.1324	0.2601	31	0.51	0.6145	0.05	-0.3982	0.6629
Treatment	GFP MsTTR	0	.	.	.	.	.	.	.

Solution for Random Effects

Effect	Culture	Estimate	Std Err Pred	DF	t Value	Pr > |t|	Alpha	Lower	Upper
Culture	1	0	.	.	.	.	.	.	.
Culture	2	0	.	.	.	.	.	.	.
Culture	3	0	.	.	.	.	.	.	.
Culture	4	0	.	.	.	.	.	.	.
Culture	5	0	.	.	.	.	.	.	.
Culture	6	0	.	.	.	.	.	.	.
Culture	7	0	.	.	.	.	.	.	.
Culture	8	0	.	.	.	.	.	.	.
Culture	9	0	.	.	.	.	.	.	.
Culture	10	0	.	.	.	.	.	.	.

Type 3 Tests of Fixed Effects

Effect	Num DF	Den DF	F Value	Pr > F
Treatment	1	31	0.26	0.6145

Least Squares Means

Effect	Treatment	Estimate	Standard Error	DF	t Value	Pr > |t|	Alpha	Lower	Upper
Treatment	Meg TTR	0.2500	0.1675	31	1.49	0.1457	0.05	-0.09164	0.5916
Treatment	GFP MsTTR	0.1176	0.1990	31	0.59	0.5587	0.05	-0.2883	0.5236

DistSoma=522

Differences of Least Squares Means												
Effect	Treatment	Treatment	Estimate	Standard Error	DF	t Value	Pr > |t|	Adjustment	Adj P	Alpha	Lower	Upper
Treatment	Meg TTR	GFP MsTTR	0.1324	0.2601	31	0.51	0.6145	Tukey-Kramer	0.6145	0.05	-0.3982	0.6629

Differences of Least Squares Means				
Effect	Treatment	Treatment	Adj Lower	Adj Upper
Treatment	Meg TTR	GFP MsTTR	-0.3982	0.6629

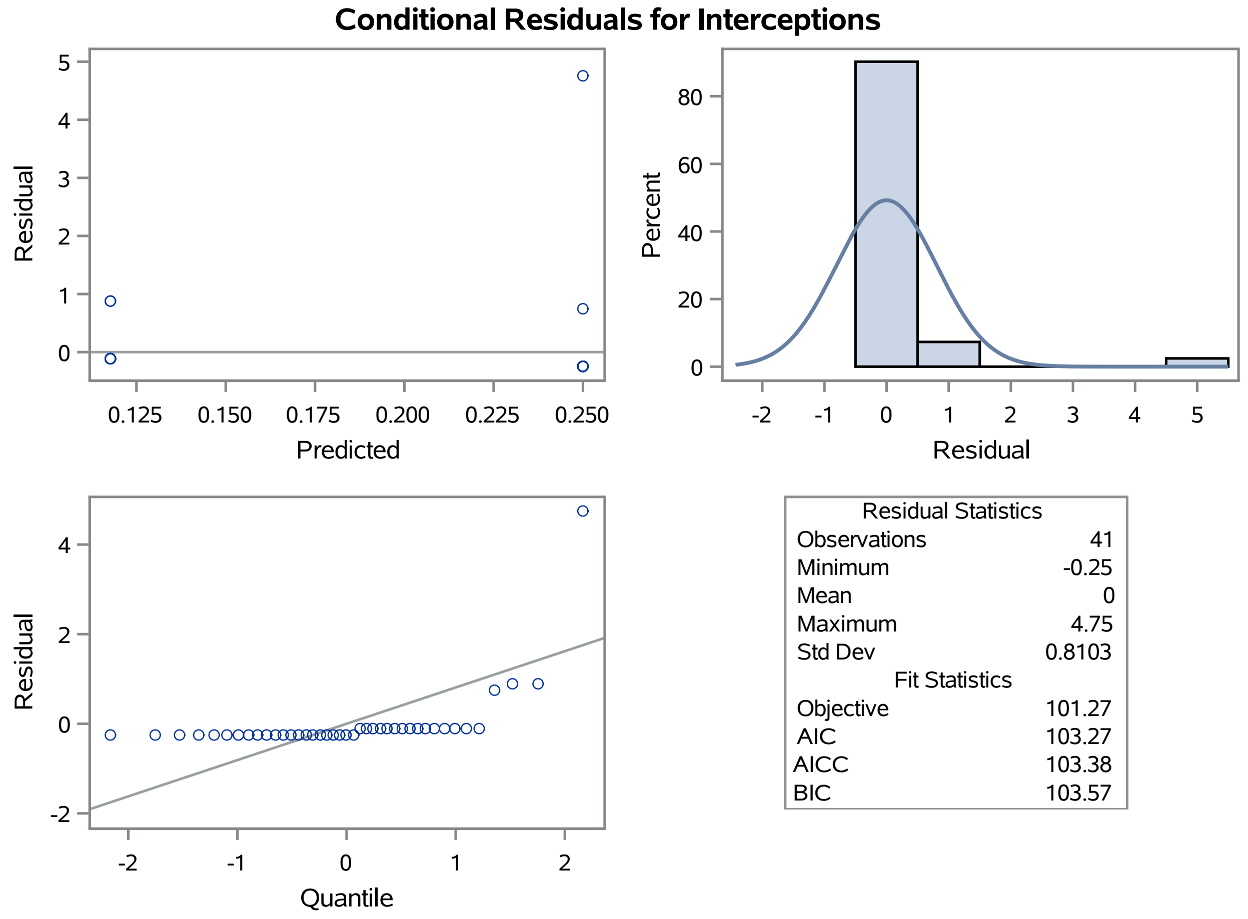

Residual Statistics	
Observations	41
Minimum	-0.25
Mean	0
Maximum	4.75
Std Dev	0.8103
**Fit Statistics**	
Objective	101.27
AIC	103.27
AICC	103.38
BIC	103.57

DistSoma=528

Model Information	
**Data Set**	WORK.TEMPDATASORTED
**Dependent Variable**	Interceptions
**Covariance Structure**	Variance Components
**Estimation Method**	REML
**Residual Variance Method**	Profile
**Fixed Effects SE Method**	Model-Based
**Degrees of Freedom Method**	Containment

Class Level Information		
**Class**	**Levels**	**Values**
**Treatment**	2	Meg TTR GFP MsTTR
**Culture**	10	1 2 3 4 5 6 7 8 9 10

Dimensions	
**Covariance Parameters**	2
**Columns in X**	3
**Columns in Z**	10
**Subjects**	1
**Max Obs per Subject**	41

Number of Observations	
**Number of Observations Read**	41
**Number of Observations Used**	41
**Number of Observations Not Used**	0

Iteration History			
**Iteration**	**Evaluations**	**-2 Res Log Like**	**Criterion**
**0**	1	85.92932087	
**1**	1	85.92932087	0.00000000

Convergence criteria met.

Estimated G matrix is not positive definite.

Covariance Parameter Estimates				
**Cov Parm**	**Estimate**	**Alpha**	**Lower**	**Upper**
**Culture**	0	.	.	.
**Residual**	0.4544	0.05	0.3049	0.7493

DistSoma=528

Fit Statistics	
-2 Res Log Likelihood	85.9
AIC (Smaller is Better)	87.9
AICC (Smaller is Better)	88.0
BIC (Smaller is Better)	88.2

Solution for Fixed Effects

Effect	Treatment	Estimate	Standard Error	DF	t Value	Pr > |t|	Alpha	Lower	Upper
Intercept		0.1176	0.1635	8	0.72	0.4923	0.05	-0.2594	0.4947
Treatment	Meg TTR	0.09069	0.2137	31	0.42	0.6742	0.05	-0.3452	0.5265
Treatment	GFP MsTTR	0	.	.	.	.	.	.	.

Solution for Random Effects

Effect	Culture	Estimate	Std Err Pred	DF	t Value	Pr > |t|	Alpha	Lower	Upper
Culture	1	0	.	.	.	.	.	.	.
Culture	2	0	.	.	.	.	.	.	.
Culture	3	0	.	.	.	.	.	.	.
Culture	4	0	.	.	.	.	.	.	.
Culture	5	0	.	.	.	.	.	.	.
Culture	6	0	.	.	.	.	.	.	.
Culture	7	0	.	.	.	.	.	.	.
Culture	8	0	.	.	.	.	.	.	.
Culture	9	0	.	.	.	.	.	.	.
Culture	10	0	.	.	.	.	.	.	.

Type 3 Tests of Fixed Effects

Effect	Num DF	Den DF	F Value	Pr > F
Treatment	1	31	0.18	0.6742

Least Squares Means

Effect	Treatment	Estimate	Standard Error	DF	t Value	Pr > |t|	Alpha	Lower	Upper
Treatment	Meg TTR	0.2083	0.1376	31	1.51	0.1402	0.05	-0.07231	0.4890
Treatment	GFP MsTTR	0.1176	0.1635	31	0.72	0.4772	0.05	-0.2158	0.4511

DistSoma=528

Differences of Least Squares Means												
**Effect**	**Treatment**	**Treatment**	**Estimate**	**Standard Error**	**DF**	**t Value**	**Pr > |t|**	**Adjustment**	**Adj P**	**Alpha**	**Lower**	**Upper**
**Treatment**	Meg TTR	GFP MsTTR	0.09069	0.2137	31	0.42	0.6742	Tukey-Kramer	0.6742	0.05	-0.3452	0.5265

Differences of Least Squares Means				
**Effect**	**Treatment**	**Treatment**	**Adj Lower**	**Adj Upper**
**Treatment**	Meg TTR	GFP MsTTR	-0.3451	0.5265

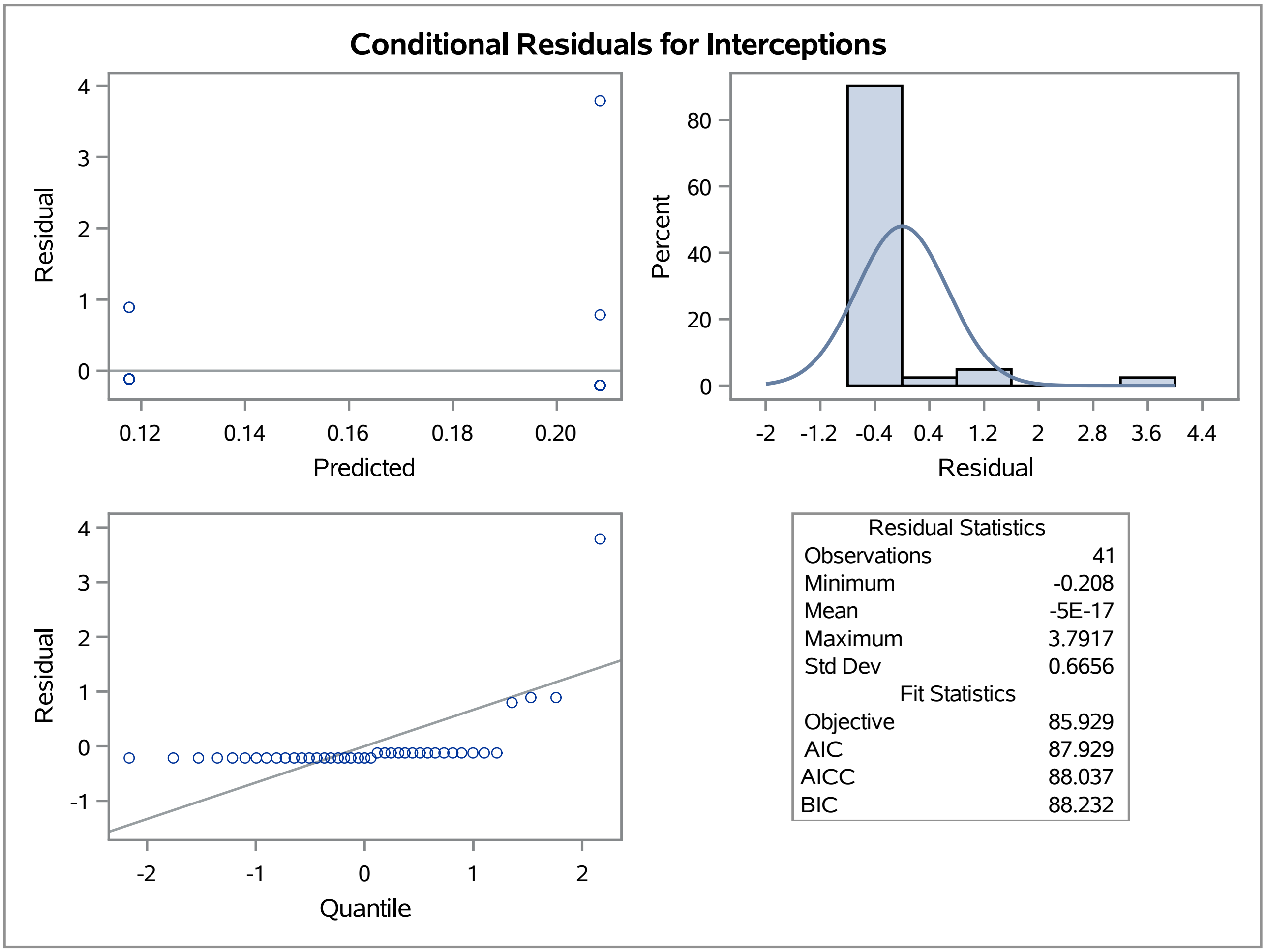

**Data Set Name**	WORK.FIG7B	**Observations**	35
**Member Type**	DATA	**Variables**	8
**Engine**	V9	**Indexes**	0
**Created**	07/03/2020 01:01:02	**Observation Length**	48
**Last Modified**	07/03/2020 01:01:02	**Deleted Observations**	0
**Protection**		**Compressed**	NO
**Data Set Type**		**Sorted**	NO
**Label**			
**Data Representation**	SOLARIS_X86_64, LINUX_X86_64, ALPHA_TRU64, LINUX_IA64		
**Encoding**	utf-8 Unicode (UTF-8)		

Engine/Host Dependent Information	
**Data Set Page Size**	65536
**Number of Data Set Pages**	1
**First Data Page**	1
**Max Obs per Page**	1360
**Obs in First Data Page**	35
**Number of Data Set Repairs**	0
**Filename**	/tmp/SAS_work110900000989_localhost.localdomain/SAS_work03CC00000989_localhost.localdomain/fig7b.sas7bdat
**Release Created**	9.0401M6
**Host Created**	Linux
**Inode Number**	672509
**Access Permission**	rw-rw-r--
**Owner Name**	sasdemo
**File Size**	128KB
**File Size (bytes)**	131072

Alphabetic List of Variables and Attributes

#	Variable	Type	Len	Format	Informat	Label
2	Animal	Num	8	BEST.		Animal
5	E	Char	1	$1.	$1.	E
6	F	Char	1	$1.	$1.	F
7	G	Char	1	$1.	$1.	G
1	Genotype	Char	5	$5.	$5.	Genotype
8	H	Char	20	$20.	$20.	H
3	NeuriteNumber	Char	3	$3.	$3.	NeuriteNumber
4	Neurite_number	Num	8	BEST.		Neurite_number

Model Information	
**Data Set**	WORK.FIG7B
**Dependent Variable**	Neurite_number
**Covariance Structure**	Variance Components
**Estimation Method**	REML
**Residual Variance Method**	Profile
**Fixed Effects SE Method**	Model-Based
**Degrees of Freedom Method**	Containment

Class Level Information		
**Class**	**Levels**	**Values**
**Genotype**	2	Meg++ Meg+-
**Animal**	6	1 2 3 4 5 6

Dimensions	
**Covariance Parameters**	2
**Columns in X**	3
**Columns in Z**	6
**Subjects**	1
**Max Obs per Subject**	33

Number of Observations	
**Number of Observations Read**	35
**Number of Observations Used**	33
**Number of Observations Not Used**	2

Iteration History			
**Iteration**	**Evaluations**	**-2 Res Log Like**	**Criterion**
**0**	1	172.34126833	
**1**	3	171.88200371	0.00000449
**2**	1	171.88174128	0.00000000

Convergence criteria met.

Covariance Parameter Estimates							
**Cov Parm**	**Estimate**	**Standard Error**	**Z Value**	**Pr > Z**	**Alpha**	**Lower**	**Upper**
**Animal**	1.2738	2.5542	0.50	0.3090	0.05	0.1851	1297915
**Residual**	11.8978	3.1756	3.75	<.0001	0.05	7.4969	21.7427

Fit Statistics	
-2 Res Log Likelihood	171.9
AIC (Smaller is Better)	175.9
AICC (Smaller is Better)	176.3
BIC (Smaller is Better)	175.5

Solution for Fixed Effects

Effect	Genotype	Estimate	Standard Error	DF	t Value	Pr > |t|	Alpha	Lower	Upper
Intercept		6.6688	1.1558	4	5.77	0.0045	0.05	3.4597	9.8779
Genotype	Meg++	5.1238	1.5845	27	3.23	0.0032	0.05	1.8728	8.3749
Genotype	Meg+-	0	.	.	.	.	.	.	.

Solution for Random Effects

Effect	Animal	Estimate	Std Err Pred	DF	t Value	Pr > |t|	Alpha	Lower	Upper
Animal	1	0.5726	0.9639	27	0.59	0.5575	0.05	-1.4053	2.5504
Animal	2	-0.9619	0.9774	27	-0.98	0.3338	0.05	-2.9673	1.0435
Animal	3	0.3893	1.0420	27	0.37	0.7116	0.05	-1.7486	2.5273
Animal	4	-0.1614	1.0785	27	-0.15	0.8822	0.05	-2.3743	2.0515
Animal	5	0.3255	0.9866	27	0.33	0.7440	0.05	-1.6988	2.3498
Animal	6	-0.1641	0.9866	27	-0.17	0.8691	0.05	-2.1884	1.8602

Type 3 Tests of Fixed Effects

Effect	Num DF	Den DF	F Value	Pr > F
Genotype	1	27	10.46	0.0032

Least Squares Means

Effect	Genotype	Estimate	Standard Error	DF	t Value	Pr > |t|	Alpha	Lower	Upper
Genotype	Meg++	11.7926	1.0838	27	10.88	<.0001	0.05	9.5689	14.0163
Genotype	Meg+-	6.6688	1.1558	27	5.77	<.0001	0.05	4.2972	9.0404

Differences of Least Squares Means												
Effect	Genotype	Genotype	Estimate	Standard Error	DF	t Value	Pr > |t|	Adjustment	Adj P	Alpha	Lower	Upper
Genotype	Meg++	Meg+-	5.1238	1.5845	27	3.23	0.0032	Tukey-Kramer	0.0032	0.05	1.8728	8.3749

Differences of Least Squares Means				
Effect	Genotype	Genotype	Adj Lower	Adj Upper
Genotype	Meg++	Meg+-	1.8728	8.3748

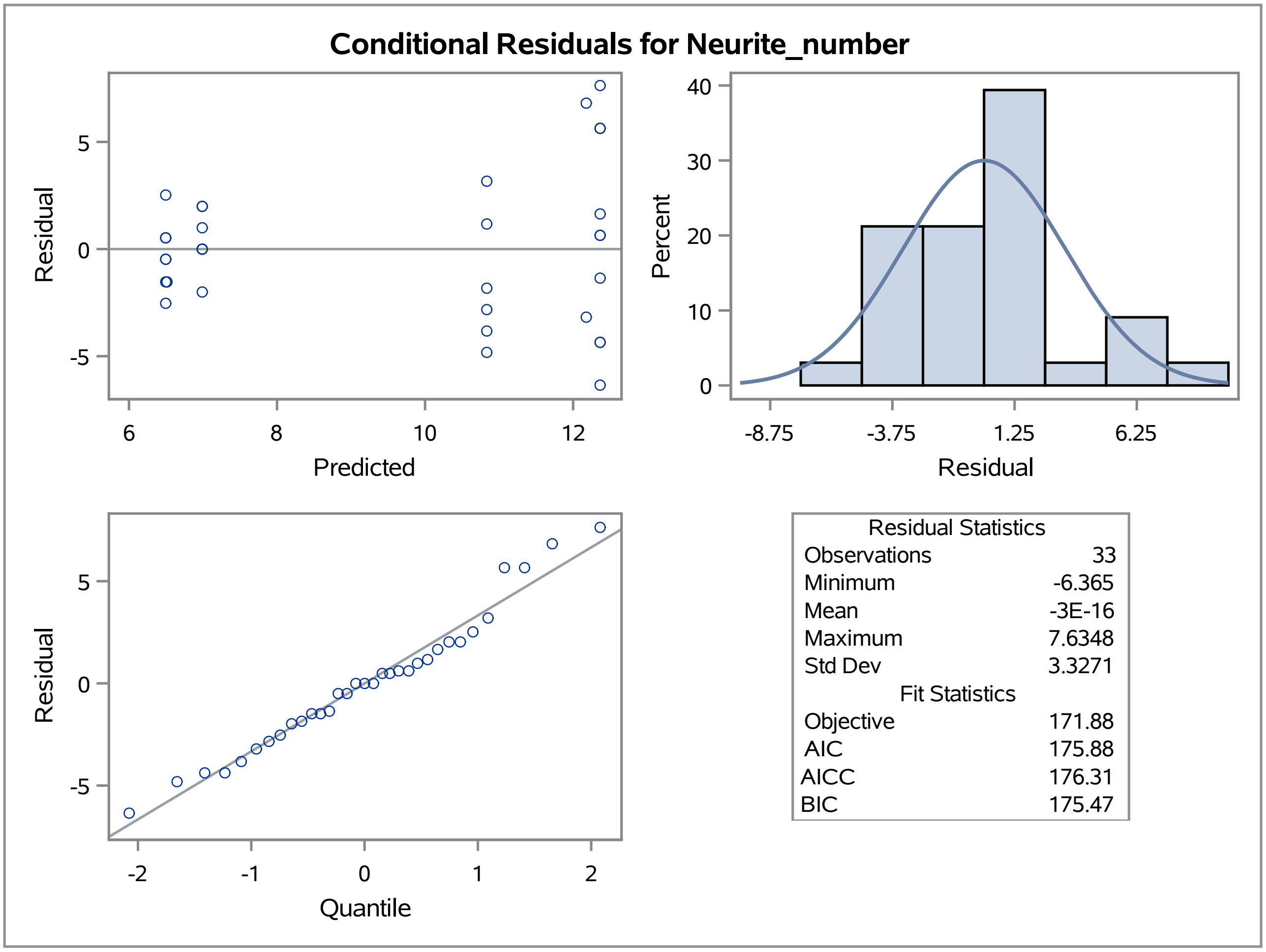

**Data Set Name**	WORK.FIG7C	**Observations**	35
**Member Type**	DATA	**Variables**	8
**Engine**	V9	**Indexes**	0
**Created**	07/03/2020 01:01:03	**Observation Length**	56
**Last Modified**	07/03/2020 01:01:03	**Deleted Observations**	0
**Protection**		**Compressed**	NO
**Data Set Type**		**Sorted**	NO
**Label**			
**Data Representation**	SOLARIS_X86_64, LINUX_X86_64, ALPHA_TRU64, LINUX_IA64		
**Encoding**	utf-8 Unicode (UTF-8)		

**Engine/Host Dependent Information**	
**Data Set Page Size**	65536
**Number of Data Set Pages**	1
**First Data Page**	1
**Max Obs per Page**	1166
**Obs in First Data Page**	35
**Number of Data Set Repairs**	0
**Filename**	/tmp/SAS_work110900000989_localhost.localdomain/SAS_work03CC00000989_localhost.localdomain/fig7c.sas7bdat
**Release Created**	9.0401M6
**Host Created**	Linux
**Inode Number**	672510
**Access Permission**	rw-rw-r--
**Owner Name**	sasdemo
**File Size**	128KB
**File Size (bytes)**	131072

**Alphabetic List of Variables and Attributes**						
**#**	**Variable**	**Type**	**Len**	**Format**	**Informat**	**Label**
**2**	Animal	Num	8	BEST.		Animal
**5**	E	Char	1	$1.	$1.	E
**6**	F	Char	1	$1.	$1.	F
**7**	G	Char	1	$1.	$1.	G
**1**	Genotype	Char	5	$5.	$5.	Genotype
**8**	H	Char	17	$17.	$17.	H
**4**	Neurite_length	Num	8	COMMA15.2		Neurite length
**3**	Neurite_length__um_	Char	8	$8.	$8.	Neurite length (um)

Model Information	
**Data Set**	WORK.FIG7C
**Dependent Variable**	Neurite_length
**Covariance Structure**	Variance Components
**Estimation Method**	REML
**Residual Variance Method**	Profile
**Fixed Effects SE Method**	Model-Based
**Degrees of Freedom Method**	Containment

Class Level Information		
**Class**	**Levels**	**Values**
**Genotype**	2	Meg++ Meg+-
**Animal**	6	1 2 3 4 5 6

Dimensions	
**Covariance Parameters**	2
**Columns in X**	3
**Columns in Z**	6
**Subjects**	1
**Max Obs per Subject**	33

Number of Observations	
**Number of Observations Read**	35
**Number of Observations Used**	33
**Number of Observations Not Used**	2

Iteration History			
**Iteration**	**Evaluations**	**-2 Res Log Like**	**Criterion**
**0**	1	435.23941320	
**1**	3	433.97182180	0.00019350
**2**	1	433.93027713	0.00001219
**3**	1	433.92787733	0.00000006
**4**	1	433.92786595	0.00000000

Convergence criteria met.

Covariance Parameter Estimates							
Cov Parm	Estimate	Standard Error	Z Value	Pr > Z	Alpha	Lower	Upper
Animal	10207	14123	0.72	0.2349	0.05	2072.67	7818974
Residual	54342	14450	3.76	<.0001	0.05	34294	99047

Fit Statistics	
-2 Res Log Likelihood	433.9
AIC (Smaller is Better)	437.9
AICC (Smaller is Better)	438.4
BIC (Smaller is Better)	437.5

Solution for Fixed Effects									
Effect	Genotype	Estimate	Standard Error	DF	t Value	Pr > |t|	Alpha	Lower	Upper
Intercept		746.00	88.8106	4	8.40	0.0011	0.05	499.42	992.58
Genotype	Meg++	436.24	122.07	27	3.57	0.0014	0.05	185.78	686.70
Genotype	Meg+-	0	.	.	.	.	.	.	.

Solution for Random Effects									
Effect	Animal	Estimate	Std Err Pred	DF	t Value	Pr > |t|	Alpha	Lower	Upper
Animal	1	67.4574	80.8254	27	0.83	0.4113	0.05	-98.3827	233.30
Animal	2	-95.1914	82.2665	27	-1.16	0.2574	0.05	-263.99	73.6056
Animal	3	27.7339	89.1230	27	0.31	0.7580	0.05	-155.13	210.60
Animal	4	9.1463	93.7576	27	0.10	0.9230	0.05	-183.23	201.52
Animal	5	32.8149	83.3920	27	0.39	0.6970	0.05	-138.29	203.92
Animal	6	-41.9612	83.3920	27	-0.50	0.6189	0.05	-213.07	129.15

Type 3 Tests of Fixed Effects				
Effect	Num DF	Den DF	F Value	Pr > F
Genotype	1	27	12.77	0.0014

Least Squares Means									
Effect	Genotype	Estimate	Standard Error	DF	t Value	Pr > |t|	Alpha	Lower	Upper
Genotype	Meg++	1182.24	83.7428	27	14.12	<.0001	0.05	1010.42	1354.07
Genotype	Meg+-	746.00	88.8106	27	8.40	<.0001	0.05	563.78	928.23

Differences of Least Squares Means												
Effect	Genotype	Genotype	Estimate	Standard Error	DF	t Value	Pr > |t|	Adjustment	Adj P	Alpha	Lower	Upper
Genotype	Meg++	Meg+-	436.24	122.07	27	3.57	0.0014	Tukey-Kramer	0.0014	0.05	185.78	686.70

Differences of Least Squares Means				
Effect	Genotype	Genotype	Adj Lower	Adj Upper
Genotype	Meg++	Meg+-	185.78	686.70

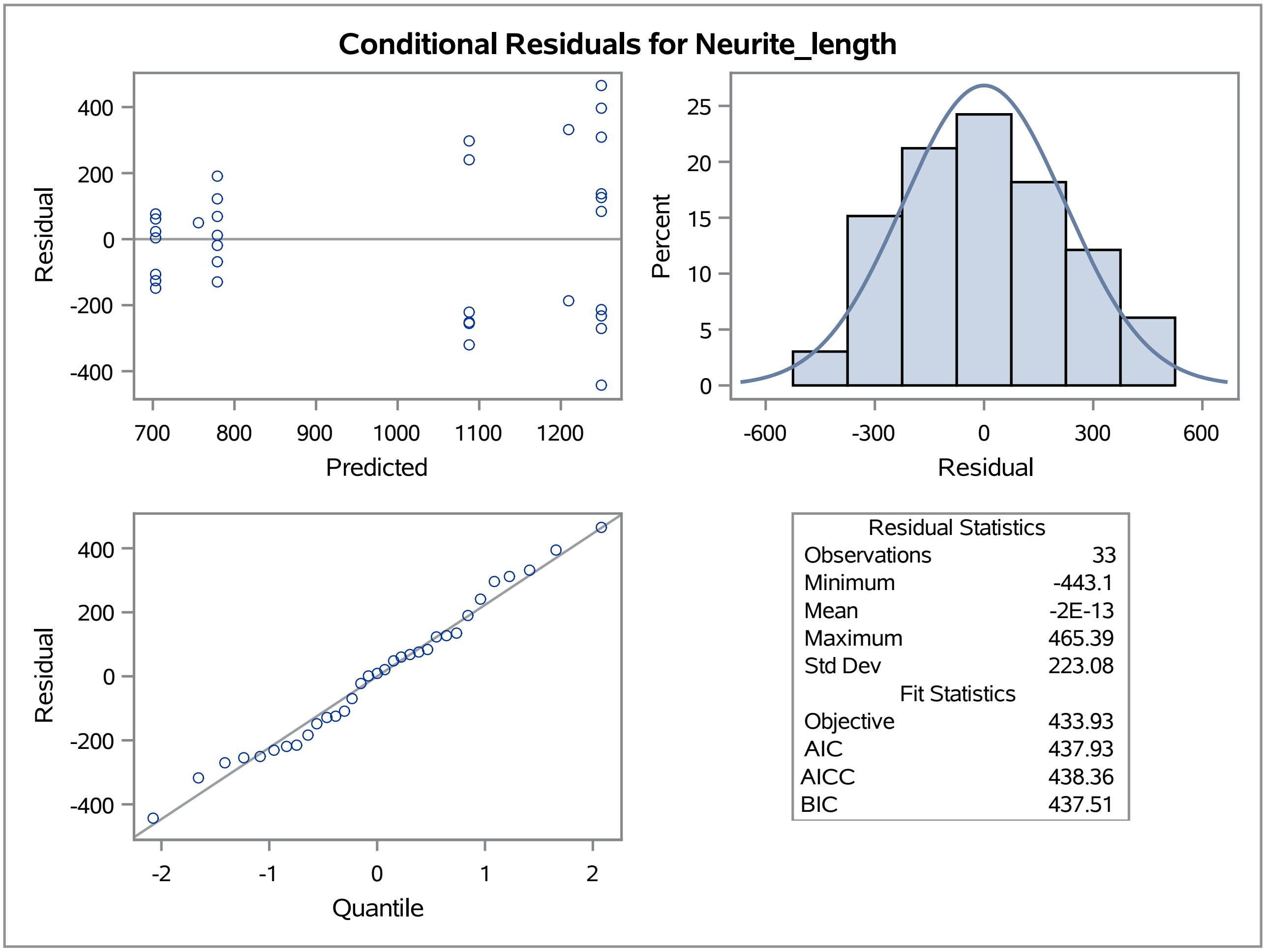

Residual Statistics	
Observations	33
Minimum	-443.1
Mean	-2E-13
Maximum	465.39
Std Dev	223.08
Fit Statistics	
Objective	433.93
AIC	437.93
AICC	438.36
BIC	437.51

The CONTENTS Procedure

**Data Set Name**	WORK.FIG7D	**Observations**	3115
**Member Type**	DATA	**Variables**	4
**Engine**	V9	**Indexes**	0
**Created**	07/10/2020 16:45:20	**Observation Length**	32
**Last Modified**	07/10/2020 16:45:20	**Deleted Observations**	0
**Protection**		**Compressed**	NO
**Data Set Type**		**Sorted**	NO
**Label**			
**Data Representation**	SOLARIS_X86_64, LINUX_X86_64, ALPHA_TRU64, LINUX_IA64		
**Encoding**	utf-8 Unicode (UTF-8)		

Engine/Host Dependent Information	
**Data Set Page Size**	65536
**Number of Data Set Pages**	2
**First Data Page**	1
**Max Obs per Page**	2038
**Obs in First Data Page**	1965
**Number of Data Set Repairs**	0
**Filename**	/tmp/SAS_workBFFD00006523_localhost.localdomain/SAS_workC2B200006523_localhost.localdomain/fig7d.sas7bdat
**Release Created**	9.0401M6
**Host Created**	Linux
**Inode Number**	672678
**Access Permission**	rw-rw-r--
**Owner Name**	sasdemo
**File Size**	192KB
**File Size (bytes)**	196608

Alphabetic List of Variables and Attributes

#	Variable	Type	Len	Format	Informat	Label
**3**	Animal	Num	8	BEST.		Animal
**1**	DistSoma	Num	8	BEST.		DistSoma
**2**	Genotype	Char	7	$7.	$7.	Genotype
**4**	interceptions	Num	8	BEST.		interceptions

DistSoma=0

Model Information	
**Data Set**	WORK.TEMPDATASORTED
**Dependent Variable**	interceptions
**Covariance Structure**	Variance Components
**Estimation Method**	REML
**Residual Variance Method**	Profile
**Fixed Effects SE Method**	Model-Based
**Degrees of Freedom Method**	Containment

Class Level Information		
**Class**	**Levels**	**Values**
**Genotype**	2	Meg +/+ Meg +/-
**Animal**	6	1 2 3 4 5 6

Dimensions	
**Covariance Parameters**	2
**Columns in X**	3
**Columns in Z**	6
**Subjects**	1
**Max Obs per Subject**	35

Number of Observations	
**Number of Observations Read**	35
**Number of Observations Used**	35
**Number of Observations Not Used**	0

Iteration History			
**Iteration**	**Evaluations**	**-2 Res Log Like**	**Criterion**
**0**	1	114.84318208	
**1**	3	114.07076370	0.00004605
**2**	1	114.06949218	0.00000013
**3**	1	114.06948877	0.00000000

Convergence criteria met.

DistSoma=0

Covariance Parameter Estimates				
Cov Parm	Estimate	Alpha	Lower	Upper
Animal	0.2006	0.05	0.03490	2079.83
Residual	1.4678	0.05	0.9365	2.6262

Fit Statistics	
-2 Res Log Likelihood	114.1
AIC (Smaller is Better)	118.1
AICC (Smaller is Better)	118.5
BIC (Smaller is Better)	117.7

Solution for Fixed Effects									
Effect	Genotype	Estimate	Standard Error	DF	t Value	Pr > |t|	Alpha	Lower	Upper
Intercept		3.5777	0.4045	4	8.84	0.0009	0.05	2.4545	4.7009
Genotype	Meg +/+	-0.1714	0.5702	29	-0.30	0.7658	0.05	-1.3376	0.9947
Genotype	Meg +/-	0	.	.	.	.	.	.	.

Solution for Random Effects									
Effect	Animal	Estimate	Std Err Pred	DF	t Value	Pr > |t|	Alpha	Lower	Upper
Animal	1	0.2274	0.3723	29	0.61	0.5461	0.05	-0.5340	0.9889
Animal	2	-0.03285	0.3782	29	-0.09	0.9314	0.05	-0.8063	0.7406
Animal	3	-0.1946	0.4062	29	-0.48	0.6355	0.05	-1.0254	0.6362
Animal	4	0.09067	0.4063	29	0.22	0.8250	0.05	-0.7404	0.9217
Animal	5	0.2764	0.3764	29	0.73	0.4687	0.05	-0.4934	1.0462
Animal	6	-0.3670	0.3748	29	-0.98	0.3356	0.05	-1.1336	0.3996

Type 3 Tests of Fixed Effects				
Effect	Num DF	Den DF	F Value	Pr > F
Genotype	1	29	0.09	0.7658

Least Squares Means									
Effect	Genotype	Estimate	Standard Error	DF	t Value	Pr > |t|	Alpha	Lower	Upper
Genotype	Meg +/+	3.4062	0.4018	29	8.48	<.0001	0.05	2.5845	4.2280
Genotype	Meg +/-	3.5777	0.4045	29	8.84	<.0001	0.05	2.7503	4.4051

DistSoma=0

Differences of Least Squares Means												
Effect	Genotype	Genotype	Estimate	Standard Error	DF	t Value	Pr > |t|	Adjustment	Adj P	Alpha	Lower	Upper
**Genotype**	Meg +/+	Meg +/-	-0.1714	0.5702	29	-0.30	0.7658	Tukey-Kramer	0.7658	0.05	-1.3376	0.9947

Differences of Least Squares Means				
Effect	Genotype	Genotype	Adj Lower	Adj Upper
**Genotype**	Meg +/+	Meg +/-	-1.3376	0.9947

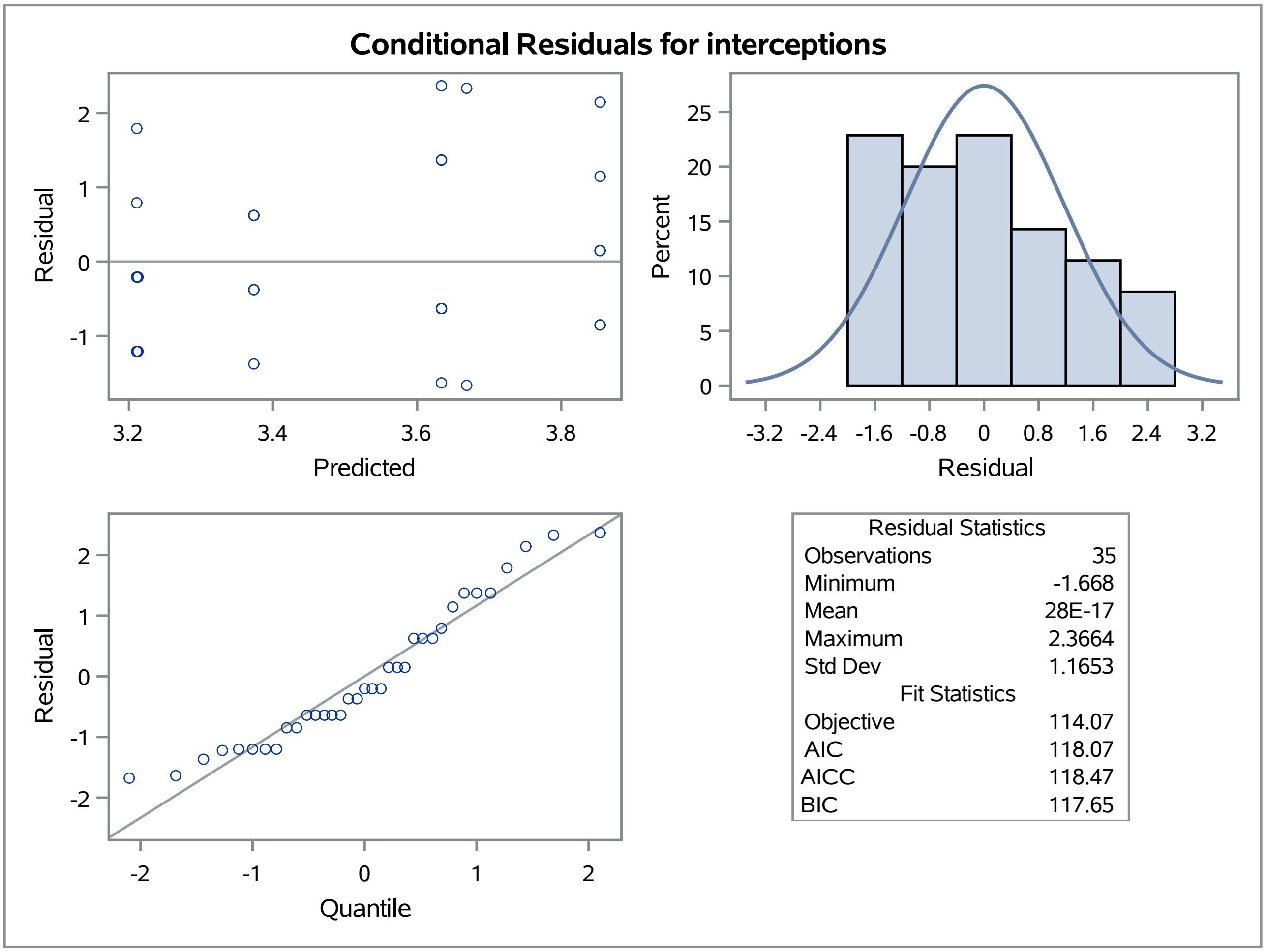

DistSoma=6

Model Information	
**Data Set**	WORK.TEMPDATASORTED
**Dependent Variable**	interceptions
**Covariance Structure**	Variance Components
**Estimation Method**	REML
**Residual Variance Method**	Profile
**Fixed Effects SE Method**	Model-Based
**Degrees of Freedom Method**	Containment

Class Level Information		
**Class**	**Levels**	**Values**
**Genotype**	2	Meg +/+ Meg +/-
**Animal**	6	1 2 3 4 5 6

Dimensions	
**Covariance Parameters**	2
**Columns in X**	3
**Columns in Z**	6
**Subjects**	1
**Max Obs per Subject**	35

Number of Observations	
**Number of Observations Read**	35
**Number of Observations Used**	35
**Number of Observations Not Used**	0

Iteration History			
**Iteration**	**Evaluations**	**-2 Res Log Like**	**Criterion**
**0**	1	113.25978300	
**1**	1	113.25978300	0.00000000

Convergence criteria met.

Estimated G matrix is not positive definite.

Covariance Parameter Estimates				
**Cov Parm**	**Estimate**	**Alpha**	**Lower**	**Upper**
**Animal**	0	.	.	.
**Residual**	1.5232	0.05	0.9909	2.6390

DistSoma=6

Fit Statistics	
-2 Res Log Likelihood	113.3
AIC (Smaller is Better)	115.3
AICC (Smaller is Better)	115.4
BIC (Smaller is Better)	115.1

Solution for Fixed Effects

Effect	Genotype	Estimate	Standard Error	DF	t Value	Pr > |t|	Alpha	Lower	Upper
Intercept		3.8824	0.2993	4	12.97	0.0002	0.05	3.0513	4.7134
Genotype	Meg +/+	-0.04902	0.4174	29	-0.12	0.9073	0.05	-0.9027	0.8047
Genotype	Meg +/-	0	.	.	.	.	.	.	.

Solution for Random Effects

Effect	Animal	Estimate	Std Err Pred	DF	t Value	Pr > |t|	Alpha	Lower	Upper
Animal	1	0	.	.	.	.	.	.	.
Animal	2	0	.	.	.	.	.	.	.
Animal	3	0	.	.	.	.	.	.	.
Animal	4	0	.	.	.	.	.	.	.
Animal	5	0	.	.	.	.	.	.	.
Animal	6	0	.	.	.	.	.	.	.

Type 3 Tests of Fixed Effects

Effect	Num DF	Den DF	F Value	Pr > F
Genotype	1	29	0.01	0.9073

Least Squares Means

Effect	Genotype	Estimate	Standard Error	DF	t Value	Pr > |t|	Alpha	Lower	Upper
Genotype	Meg +/+	3.8333	0.2909	29	13.18	<.0001	0.05	3.2384	4.4283
Genotype	Meg +/-	3.8824	0.2993	29	12.97	<.0001	0.05	3.2702	4.4946

DistSoma=6

Differences of Least Squares Means												
**Effect**	**Genotype**	**Genotype**	**Estimate**	**Standard Error**	**DF**	**t Value**	**Pr > |t|**	**Adjustment**	**Adj P**	**Alpha**	**Lower**	**Upper**
**Genotype**	Meg +/+	Meg +/-	-0.04902	0.4174	29	-0.12	0.9073	Tukey-Kramer	0.9073	0.05	-0.9027	0.8047

Differences of Least Squares Means				
**Effect**	**Genotype**	**Genotype**	**Adj Lower**	**Adj Upper**
**Genotype**	Meg +/+	Meg +/-	-0.9027	0.8046

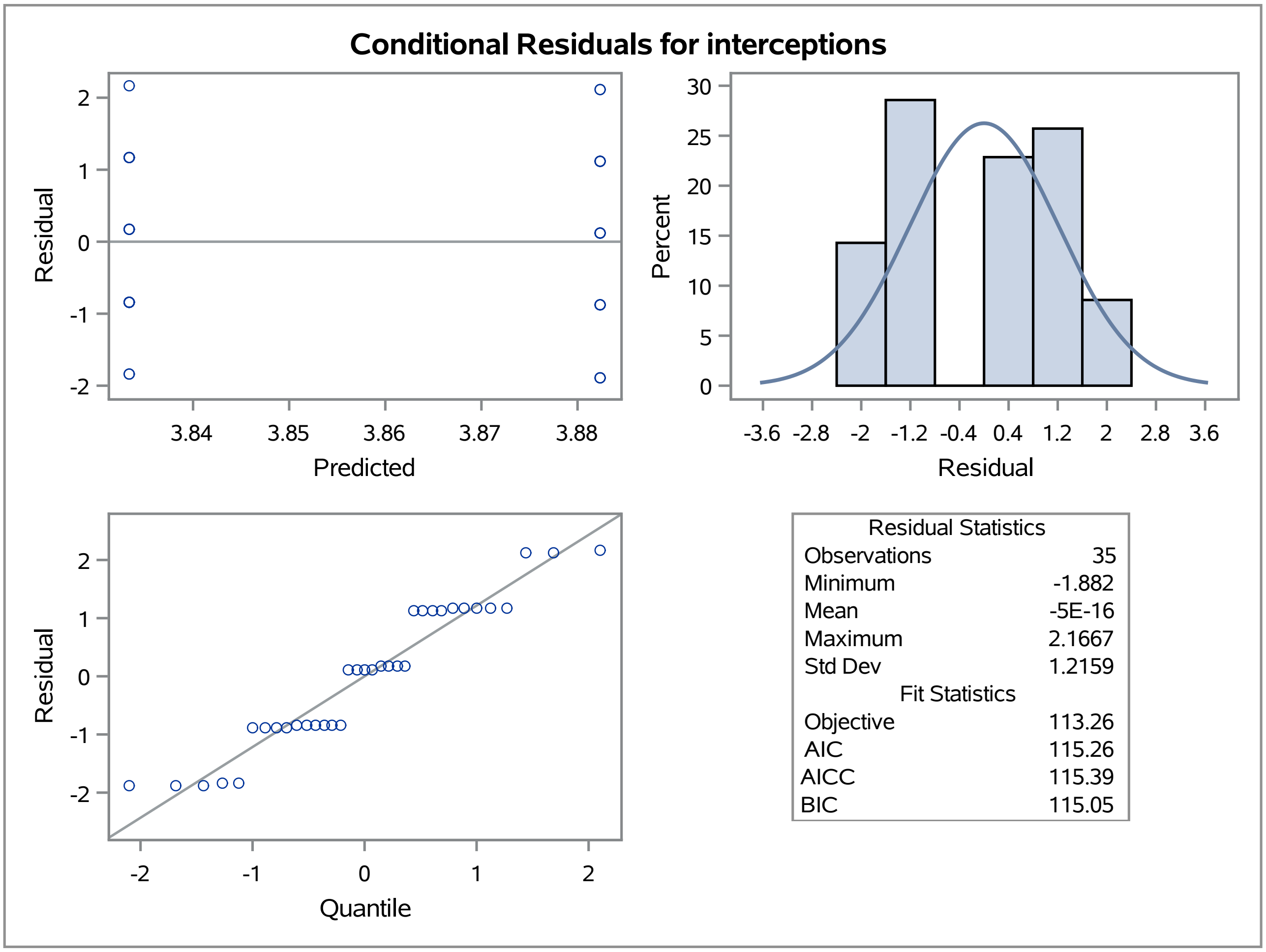

DistSoma=12

Model Information	
**Data Set**	WORK.TEMPDATASORTED
**Dependent Variable**	interceptions
**Covariance Structure**	Variance Components
**Estimation Method**	REML
**Residual Variance Method**	Profile
**Fixed Effects SE Method**	Model-Based
**Degrees of Freedom Method**	Containment

Class Level Information		
**Class**	**Levels**	**Values**
**Genotype**	2	Meg +/+ Meg +/-
**Animal**	6	1 2 3 4 5 6

Dimensions	
**Covariance Parameters**	2
**Columns in X**	3
**Columns in Z**	6
**Subjects**	1
**Max Obs per Subject**	35

Number of Observations	
**Number of Observations Read**	35
**Number of Observations Used**	35
**Number of Observations Not Used**	0

Iteration History			
**Iteration**	**Evaluations**	**-2 Res Log Like**	**Criterion**
**0**	1	118.97248314	
**1**	1	118.97248314	0.00000000

Convergence criteria met.

Estimated G matrix is not positive definite.

Covariance Parameter Estimates				
**Cov Parm**	**Estimate**	**Alpha**	**Lower**	**Upper**
**Animal**	0	.	.	.
**Residual**	1.8111	0.05	1.1782	3.1378

DistSoma=12

Fit Statistics	
**-2 Res Log Likelihood**	119.0
**AIC (Smaller is Better)**	121.0
**AICC (Smaller is Better)**	121.1
**BIC (Smaller is Better)**	120.8

Solution for Fixed Effects

Effect	Genotype	Estimate	Standard Error	DF	t Value	Pr > |t|	Alpha	Lower	Upper
**Intercept**		4.1176	0.3264	4	12.62	0.0002	0.05	3.2114	5.0239
**Genotype**	Meg +/+	0.2157	0.4551	29	0.47	0.6391	0.05	-0.7152	1.1465
**Genotype**	Meg +/-	0	.	.	.	.	.	.	.

Solution for Random Effects

Effect	Animal	Estimate	Std Err Pred	DF	t Value	Pr > |t|	Alpha	Lower	Upper
**Animal**	1	0	.	.	.	.	.	.	.
**Animal**	2	0	.	.	.	.	.	.	.
**Animal**	3	0	.	.	.	.	.	.	.
**Animal**	4	0	.	.	.	.	.	.	.
**Animal**	5	0	.	.	.	.	.	.	.
**Animal**	6	0	.	.	.	.	.	.	.

Type 3 Tests of Fixed Effects

Effect	Num DF	Den DF	F Value	Pr > F
**Genotype**	1	29	0.22	0.6391

Least Squares Means

Effect	Genotype	Estimate	Standard Error	DF	t Value	Pr > |t|	Alpha	Lower	Upper
**Genotype**	Meg +/+	4.3333	0.3172	29	13.66	<.0001	0.05	3.6846	4.9821
**Genotype**	Meg +/-	4.1176	0.3264	29	12.62	<.0001	0.05	3.4501	4.7852

DistSoma=12

Differences of Least Squares Means												
Effect	Genotype	Genotype	Estimate	Standard Error	DF	t Value	Pr > |t|	Adjustment	Adj P	Alpha	Lower	Upper
**Genotype**	Meg +/+	Meg +/-	0.2157	0.4551	29	0.47	0.6391	Tukey-Kramer	0.6391	0.05	-0.7152	1.1465

Differences of Least Squares Means				
Effect	Genotype	Genotype	Adj Lower	Adj Upper
**Genotype**	Meg +/+	Meg +/-	-0.7152	1.1465

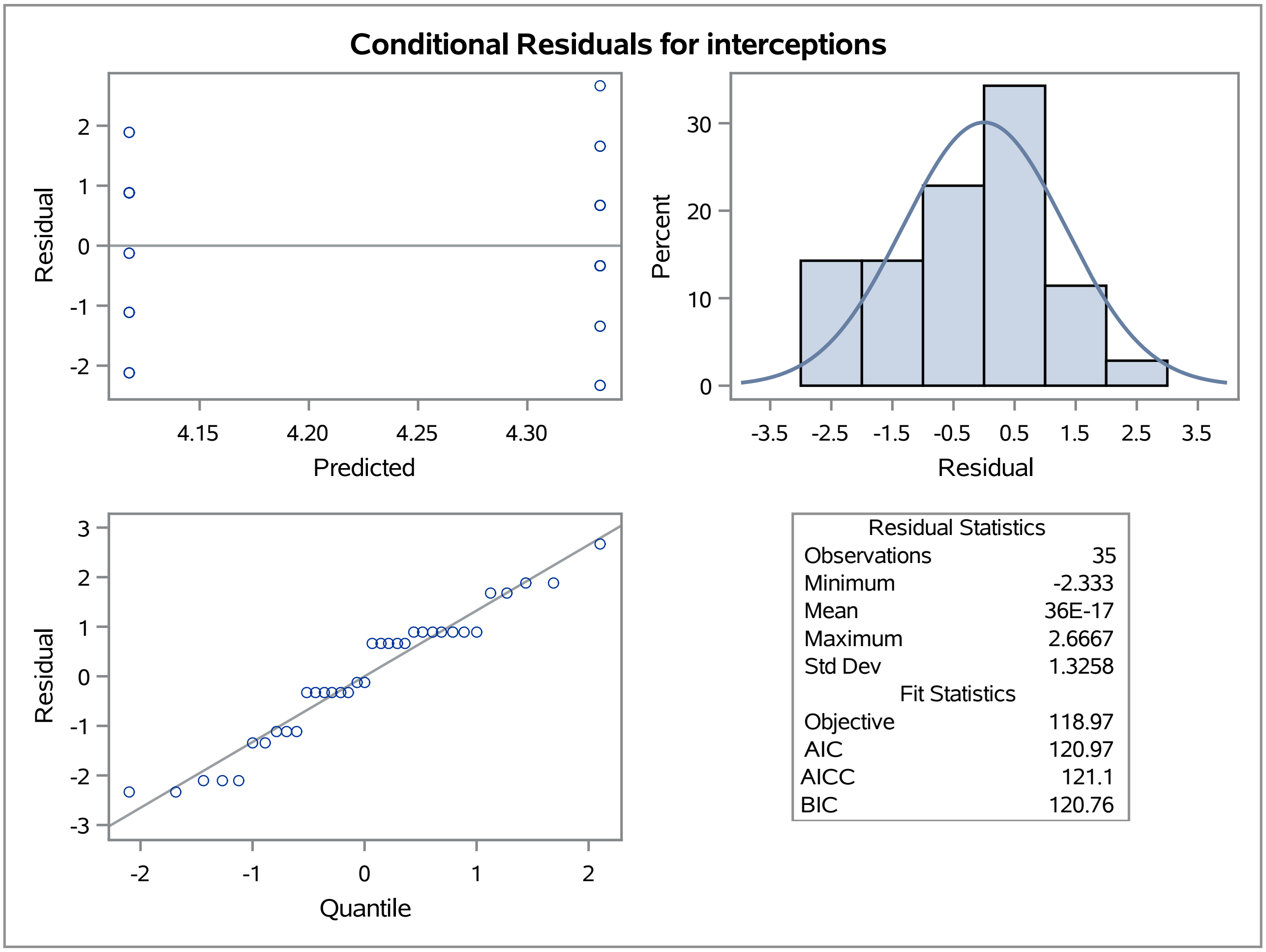

DistSoma=18

Model Information	
**Data Set**	WORK.TEMPDATASORTED
**Dependent Variable**	interceptions
**Covariance Structure**	Variance Components
**Estimation Method**	REML
**Residual Variance Method**	Profile
**Fixed Effects SE Method**	Model-Based
**Degrees of Freedom Method**	Containment

Class Level Information		
**Class**	**Levels**	**Values**
**Genotype**	2	Meg +/+ Meg +/-
**Animal**	6	1 2 3 4 5 6

Dimensions	
**Covariance Parameters**	2
**Columns in X**	3
**Columns in Z**	6
**Subjects**	1
**Max Obs per Subject**	35

Number of Observations	
**Number of Observations Read**	35
**Number of Observations Used**	35
**Number of Observations Not Used**	0

Iteration History			
**Iteration**	**Evaluations**	**-2 Res Log Like**	**Criterion**
**0**	1	133.34933503	
**1**	1	133.34933503	0.00000000

Convergence criteria met.

Estimated G matrix is not positive definite.

Covariance Parameter Estimates				
**Cov Parm**	**Estimate**	**Alpha**	**Lower**	**Upper**
**Animal**	0	.	.	.
**Residual**	2.7999	0.05	1.8215	4.8510

DistSoma=18

Fit Statistics	
-2 Res Log Likelihood	133.3
AIC (Smaller is Better)	135.3
AICC (Smaller is Better)	135.5
BIC (Smaller is Better)	135.1

Solution for Fixed Effects									
Effect	Genotype	Estimate	Standard Error	DF	t Value	Pr > |t|	Alpha	Lower	Upper
Intercept		4.5882	0.4058	4	11.31	0.0003	0.05	3.4615	5.7150
Genotype	Meg +/+	0.02288	0.5659	29	0.04	0.9680	0.05	-1.1345	1.1803
Genotype	Meg +/-	0	.	.	.	.	.	.	.

Solution for Random Effects									
Effect	Animal	Estimate	Std Err Pred	DF	t Value	Pr > |t|	Alpha	Lower	Upper
Animal	1	0	.	.	.	.	.	.	.
Animal	2	0	.	.	.	.	.	.	.
Animal	3	0	.	.	.	.	.	.	.
Animal	4	0	.	.	.	.	.	.	.
Animal	5	0	.	.	.	.	.	.	.
Animal	6	0	.	.	.	.	.	.	.

Type 3 Tests of Fixed Effects				
Effect	Num DF	Den DF	F Value	Pr > F
Genotype	1	29	0.00	0.9680

Least Squares Means									
Effect	Genotype	Estimate	Standard Error	DF	t Value	Pr > |t|	Alpha	Lower	Upper
Genotype	Meg +/+	4.6111	0.3944	29	11.69	<.0001	0.05	3.8045	5.4177
Genotype	Meg +/-	4.5882	0.4058	29	11.31	<.0001	0.05	3.7582	5.4183

DistSoma=18

Differences of Least Squares Means												
Effect	Genotype	Genotype	Estimate	Standard Error	DF	t Value	Pr > |t|	Adjustment	Adj P	Alpha	Lower	Upper
**Genotype**	Meg +/+	Meg +/-	0.02288	0.5659	29	0.04	0.9680	Tukey-Kramer	0.9680	0.05	-1.1345	1.1803

Differences of Least Squares Means				
Effect	Genotype	Genotype	Adj Lower	Adj Upper
**Genotype**	Meg +/+	Meg +/-	-1.1345	1.1803

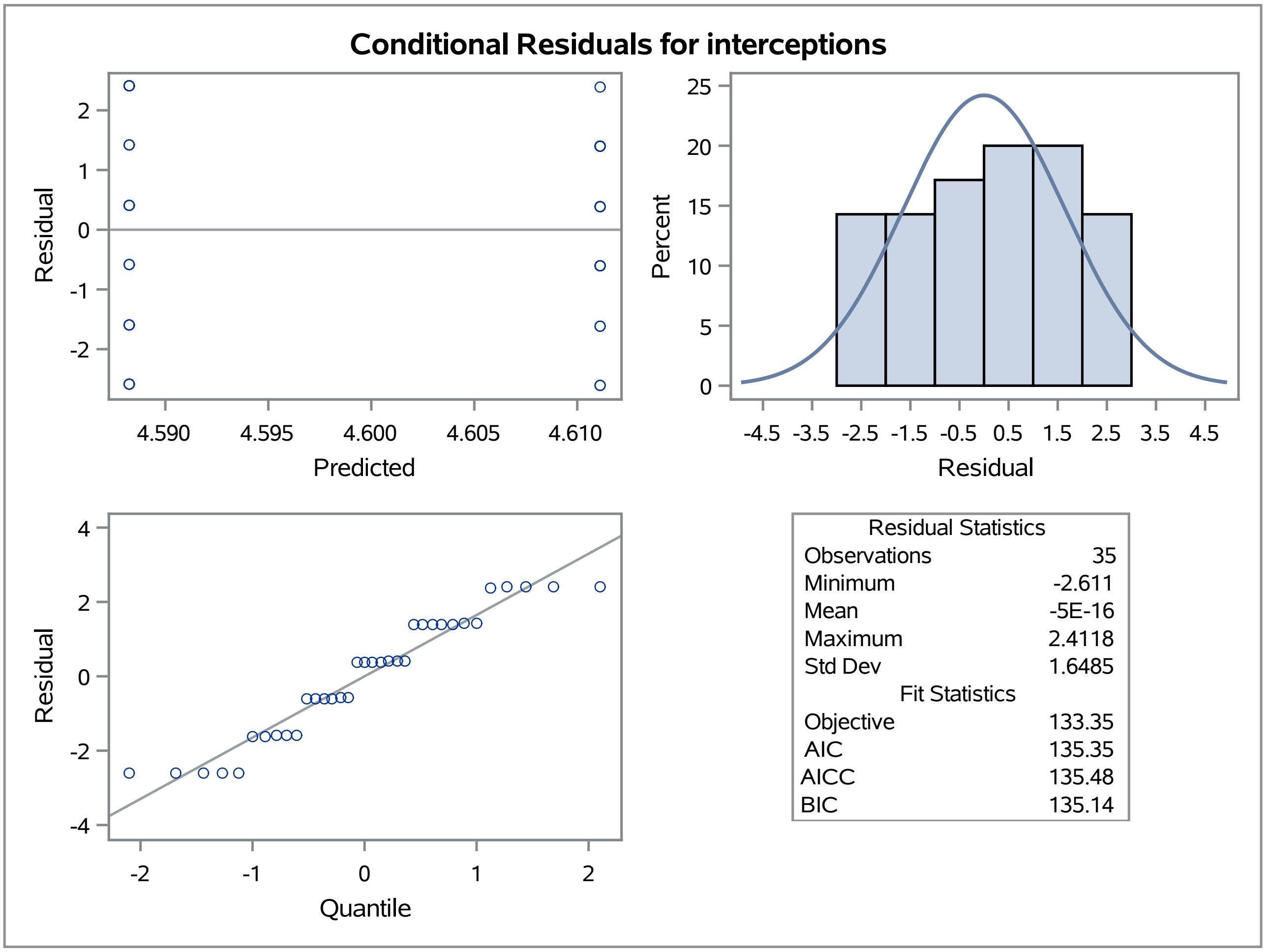

DistSoma=24

Model Information	
**Data Set**	WORK.TEMPDATASORTED
**Dependent Variable**	interceptions
**Covariance Structure**	Variance Components
**Estimation Method**	REML
**Residual Variance Method**	Profile
**Fixed Effects SE Method**	Model-Based
**Degrees of Freedom Method**	Containment

Class Level Information		
**Class**	**Levels**	**Values**
**Genotype**	2	Meg +/+ Meg +/-
**Animal**	6	1 2 3 4 5 6

Dimensions	
**Covariance Parameters**	2
**Columns in X**	3
**Columns in Z**	6
**Subjects**	1
**Max Obs per Subject**	35

Number of Observations	
**Number of Observations Read**	35
**Number of Observations Used**	35
**Number of Observations Not Used**	0

Iteration History			
**Iteration**	**Evaluations**	**-2 Res Log Like**	**Criterion**
**0**	1	134.13363085	
**1**	1	134.13363085	0.00000000

Convergence criteria met.

Estimated G matrix is not positive definite.

Covariance Parameter Estimates				
**Cov Parm**	**Estimate**	**Alpha**	**Lower**	**Upper**
**Animal**	0	.	.	.
**Residual**	2.8672	0.05	1.8653	4.9677

DistSoma=24

Fit Statistics	
-2 Res Log Likelihood	134.1
AIC (Smaller is Better)	136.1
AICC (Smaller is Better)	136.3
BIC (Smaller is Better)	135.9

Solution for Fixed Effects									
Effect	Genotype	Estimate	Standard Error	DF	t Value	Pr > |t|	Alpha	Lower	Upper
Intercept		4.5882	0.4107	4	11.17	0.0004	0.05	3.4480	5.7285
Genotype	Meg +/+	0.5784	0.5727	29	1.01	0.3208	0.05	-0.5928	1.7497
Genotype	Meg +/-	0	.	.	.	.	.	.	.

Solution for Random Effects									
Effect	Animal	Estimate	Std Err Pred	DF	t Value	Pr > |t|	Alpha	Lower	Upper
Animal	1	0	.	.	.	.	.	.	.
Animal	2	0	.	.	.	.	.	.	.
Animal	3	0	.	.	.	.	.	.	.
Animal	4	0	.	.	.	.	.	.	.
Animal	5	0	.	.	.	.	.	.	.
Animal	6	0	.	.	.	.	.	.	.

Type 3 Tests of Fixed Effects				
Effect	Num DF	Den DF	F Value	Pr > F
Genotype	1	29	1.02	0.3208

Least Squares Means									
Effect	Genotype	Estimate	Standard Error	DF	t Value	Pr > |t|	Alpha	Lower	Upper
Genotype	Meg +/+	5.1667	0.3991	29	12.95	<.0001	0.05	4.3504	5.9829
Genotype	Meg +/-	4.5882	0.4107	29	11.17	<.0001	0.05	3.7483	5.4282

DistSoma=24

Differences of Least Squares Means												
Effect	Genotype	Genotype	Estimate	Standard Error	DF	t Value	Pr > |t|	Adjustment	Adj P	Alpha	Lower	Upper
Genotype	Meg +/+	Meg +/-	0.5784	0.5727	29	1.01	0.3208	Tukey-Kramer	0.3208	0.05	-0.5928	1.7497

Differences of Least Squares Means				
Effect	Genotype	Genotype	Adj Lower	Adj Upper
Genotype	Meg +/+	Meg +/-	-0.5928	1.7497

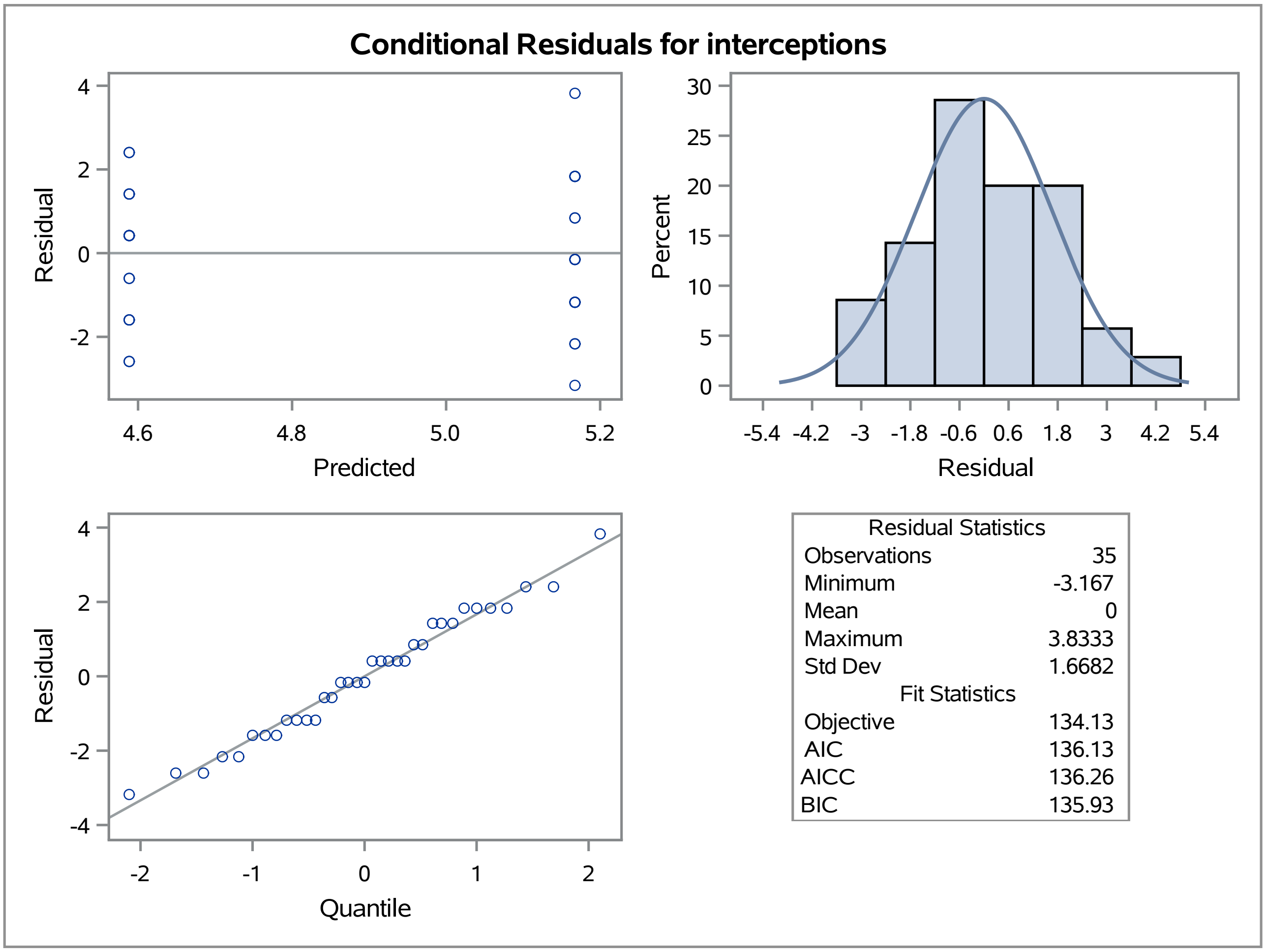

DistSoma=30

Model Information	
**Data Set**	WORK.TEMPDATASORTED
**Dependent Variable**	interceptions
**Covariance Structure**	Variance Components
**Estimation Method**	REML
**Residual Variance Method**	Profile
**Fixed Effects SE Method**	Model-Based
**Degrees of Freedom Method**	Containment

Class Level Information		
**Class**	**Levels**	**Values**
**Genotype**	2	Meg +/+ Meg +/-
**Animal**	6	1 2 3 4 5 6

Dimensions	
**Covariance Parameters**	2
**Columns in X**	3
**Columns in Z**	6
**Subjects**	1
**Max Obs per Subject**	35

Number of Observations	
**Number of Observations Read**	35
**Number of Observations Used**	35
**Number of Observations Not Used**	0

Iteration History			
**Iteration**	**Evaluations**	**-2 Res Log Like**	**Criterion**
**0**	1	138.09320997	
**1**	1	138.09320997	0.00000000

Convergence criteria met.

Estimated G matrix is not positive definite.

Covariance Parameter Estimates				
**Cov Parm**	**Estimate**	**Alpha**	**Lower**	**Upper**
**Animal**	0	.	.	.
**Residual**	3.2327	0.05	2.1031	5.6010

DistSoma=30

Fit Statistics	
-2 Res Log Likelihood	138.1
AIC (Smaller is Better)	140.1
AICC (Smaller is Better)	140.2
BIC (Smaller is Better)	139.9

Solution for Fixed Effects									
Effect	Genotype	Estimate	Standard Error	DF	t Value	Pr > |t|	Alpha	Lower	Upper
Intercept		4.5294	0.4361	4	10.39	0.0005	0.05	3.3187	5.7401
Genotype	Meg +/+	0.9150	0.6081	29	1.50	0.1432	0.05	-0.3286	2.1587
Genotype	Meg +/-	0	.	.	.	.	.	.	.

Solution for Random Effects									
Effect	Animal	Estimate	Std Err Pred	DF	t Value	Pr > |t|	Alpha	Lower	Upper
Animal	1	0	.	.	.	.	.	.	.
Animal	2	0	.	.	.	.	.	.	.
Animal	3	0	.	.	.	.	.	.	.
Animal	4	0	.	.	.	.	.	.	.
Animal	5	0	.	.	.	.	.	.	.
Animal	6	0	.	.	.	.	.	.	.

Type 3 Tests of Fixed Effects				
Effect	Num DF	Den DF	F Value	Pr > F
Genotype	1	29	2.26	0.1432

Least Squares Means									
Effect	Genotype	Estimate	Standard Error	DF	t Value	Pr > |t|	Alpha	Lower	Upper
Genotype	Meg +/+	5.4444	0.4238	29	12.85	<.0001	0.05	4.5777	6.3112
Genotype	Meg +/-	4.5294	0.4361	29	10.39	<.0001	0.05	3.6375	5.4213

DistSoma=30

Differences of Least Squares Means												
**Effect**	**Genotype**	**Genotype**	**Estimate**	**Standard Error**	**DF**	**t Value**	**Pr > |t|**	**Adjustment**	**Adj P**	**Alpha**	**Lower**	**Upper**
**Genotype**	Meg +/+	Meg +/-	0.9150	0.6081	29	1.50	0.1432	Tukey-Kramer	0.1432	0.05	-0.3286	2.1587

Differences of Least Squares Means				
**Effect**	**Genotype**	**Genotype**	**Adj Lower**	**Adj Upper**
**Genotype**	Meg +/+	Meg +/-	-0.3286	2.1587

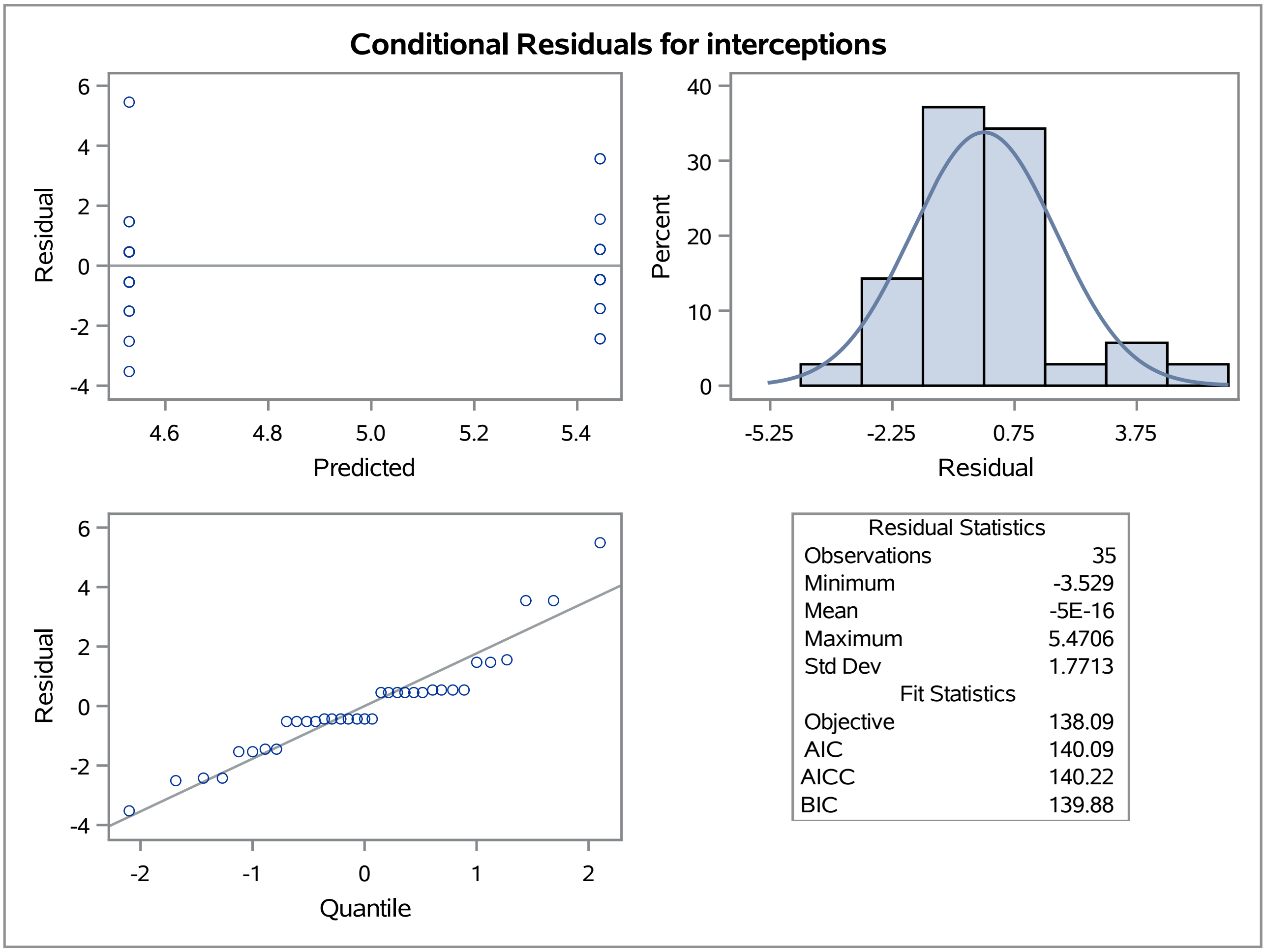

DistSoma=36

Model Information	
**Data Set**	WORK.TEMPDATASORTED
**Dependent Variable**	interceptions
**Covariance Structure**	Variance Components
**Estimation Method**	REML
**Residual Variance Method**	Profile
**Fixed Effects SE Method**	Model-Based
**Degrees of Freedom Method**	Containment

Class Level Information		
**Class**	**Levels**	**Values**
**Genotype**	2	Meg +/+ Meg +/-
**Animal**	6	1 2 3 4 5 6

Dimensions	
**Covariance Parameters**	2
**Columns in X**	3
**Columns in Z**	6
**Subjects**	1
**Max Obs per Subject**	35

Number of Observations	
**Number of Observations Read**	35
**Number of Observations Used**	35
**Number of Observations Not Used**	0

Iteration History			
**Iteration**	**Evaluations**	**-2 Res Log Like**	**Criterion**
**0**	1	134.46026513	
**1**	1	134.46026513	0.00000000

Convergence criteria met.

Estimated G matrix is not positive definite.

Covariance Parameter Estimates				
**Cov Parm**	**Estimate**	**Alpha**	**Lower**	**Upper**
**Animal**	0	.	.	.
**Residual**	2.8957	0.05	1.8839	5.0171

DistSoma=36

Fit Statistics	
-2 Res Log Likelihood	134.5
AIC (Smaller is Better)	136.5
AICC (Smaller is Better)	136.6
BIC (Smaller is Better)	136.3

Solution for Fixed Effects

Effect	Genotype	Estimate	Standard Error	DF	t Value	Pr > |t|	Alpha	Lower	Upper
Intercept		4.2353	0.4127	4	10.26	0.0005	0.05	3.0894	5.3812
Genotype	Meg +/+	1.2647	0.5755	29	2.20	0.0361	0.05	0.08766	2.4418
Genotype	Meg +/-	0	.	.	.	.	.	.	.

Solution for Random Effects

Effect	Animal	Estimate	Std Err Pred	DF	t Value	Pr > |t|	Alpha	Lower	Upper
Animal	1	0	.	.	.	.	.	.	.
Animal	2	0	.	.	.	.	.	.	.
Animal	3	0	.	.	.	.	.	.	.
Animal	4	0	.	.	.	.	.	.	.
Animal	5	0	.	.	.	.	.	.	.
Animal	6	0	.	.	.	.	.	.	.

Type 3 Tests of Fixed Effects

Effect	Num DF	Den DF	F Value	Pr > F
Genotype	1	29	4.83	0.0361

Least Squares Means

Effect	Genotype	Estimate	Standard Error	DF	t Value	Pr > |t|	Alpha	Lower	Upper
Genotype	Meg +/+	5.5000	0.4011	29	13.71	<.0001	0.05	4.6797	6.3203
Genotype	Meg +/-	4.2353	0.4127	29	10.26	<.0001	0.05	3.3912	5.0794

DistSoma=36

Differences of Least Squares Means												
**Effect**	**Genotype**	**Genotype**	**Estimate**	**Standard Error**	**DF**	**t Value**	**Pr > |t|**	**Adjustment**	**Adj P**	**Alpha**	**Lower**	**Upper**
**Genotype**	Meg +/+	Meg +/-	1.2647	0.5755	29	2.20	0.0361	Tukey-Kramer	0.0361	0.05	0.08766	2.4418

Differences of Least Squares Means				
**Effect**	**Genotype**	**Genotype**	**Adj Lower**	**Adj Upper**
**Genotype**	Meg +/+	Meg +/-	0.08767	2.4417

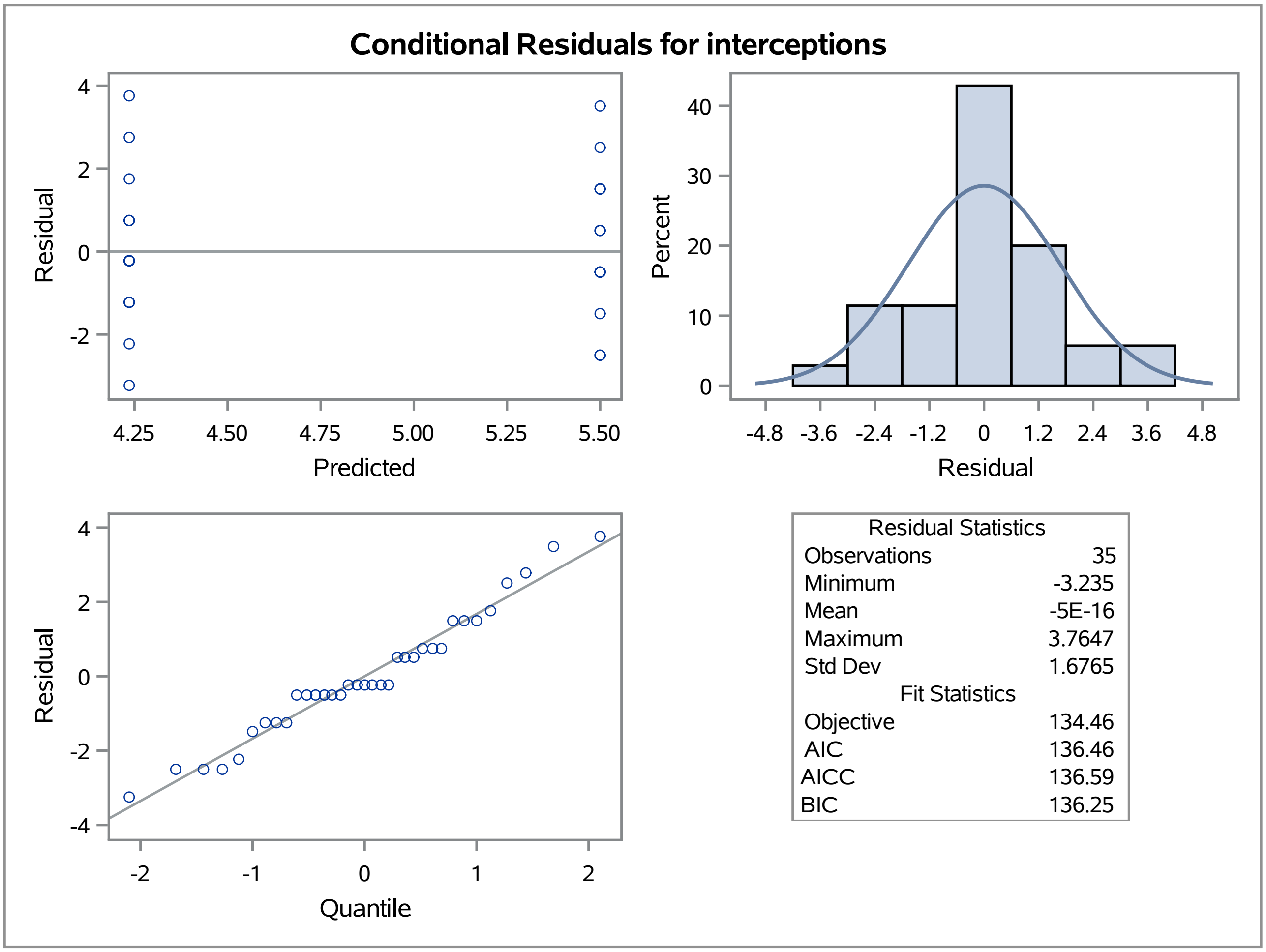

DistSoma=42

Model Information	
**Data Set**	WORK.TEMPDATASORTED
**Dependent Variable**	interceptions
**Covariance Structure**	Variance Components
**Estimation Method**	REML
**Residual Variance Method**	Profile
**Fixed Effects SE Method**	Model-Based
**Degrees of Freedom Method**	Containment

Class Level Information		
**Class**	**Levels**	**Values**
**Genotype**	2	Meg +/+ Meg +/-
**Animal**	6	1 2 3 4 5 6

Dimensions	
**Covariance Parameters**	2
**Columns in X**	3
**Columns in Z**	6
**Subjects**	1
**Max Obs per Subject**	35

Number of Observations	
**Number of Observations Read**	35
**Number of Observations Used**	35
**Number of Observations Not Used**	0

Iteration History			
**Iteration**	**Evaluations**	**-2 Res Log Like**	**Criterion**
**0**	1	139.11737347	
**1**	1	139.11737347	0.00000000

Convergence criteria met.

Estimated G matrix is not positive definite.

Covariance Parameter Estimates				
**Cov Parm**	**Estimate**	**Alpha**	**Lower**	**Upper**
**Animal**	0	.	.	.
**Residual**	3.3346	0.05	2.1694	5.7775

DistSoma=42

Fit Statistics	
-2 Res Log Likelihood	139.1
AIC (Smaller is Better)	141.1
AICC (Smaller is Better)	141.2
BIC (Smaller is Better)	140.9

Solution for Fixed Effects

Effect	Genotype	Estimate	Standard Error	DF	t Value	Pr > |t|	Alpha	Lower	Upper
Intercept		4.1176	0.4429	4	9.30	0.0007	0.05	2.8880	5.3473
Genotype	Meg +/+	1.4935	0.6176	29	2.42	0.0221	0.05	0.2304	2.7566
Genotype	Meg +/-	0	.	.	.	.	.	.	.

Solution for Random Effects

Effect	Animal	Estimate	Std Err Pred	DF	t Value	Pr > |t|	Alpha	Lower	Upper
Animal	1	0	.	.	.	.	.	.	.
Animal	2	0	.	.	.	.	.	.	.
Animal	3	0	.	.	.	.	.	.	.
Animal	4	0	.	.	.	.	.	.	.
Animal	5	0	.	.	.	.	.	.	.
Animal	6	0	.	.	.	.	.	.	.

Type 3 Tests of Fixed Effects

Effect	Num DF	Den DF	F Value	Pr > F
Genotype	1	29	5.85	0.0221

Least Squares Means

Effect	Genotype	Estimate	Standard Error	DF	t Value	Pr > |t|	Alpha	Lower	Upper
Genotype	Meg +/+	5.6111	0.4304	29	13.04	<.0001	0.05	4.7308	6.4914
Genotype	Meg +/-	4.1176	0.4429	29	9.30	<.0001	0.05	3.2118	5.0235

DistSoma=42

Differences of Least Squares Means												
**Effect**	**Genotype**	**Genotype**	**Estimate**	**Standard Error**	**DF**	**t Value**	**Pr > |t|**	**Adjustment**	**Adj P**	**Alpha**	**Lower**	**Upper**
**Genotype**	Meg +/+	Meg +/-	1.4935	0.6176	29	2.42	0.0221	Tukey-Kramer	0.0221	0.05	0.2304	2.7566

Differences of Least Squares Means				
**Effect**	**Genotype**	**Genotype**	**Adj Lower**	**Adj Upper**
**Genotype**	Meg +/+	Meg +/-	0.2304	2.7566

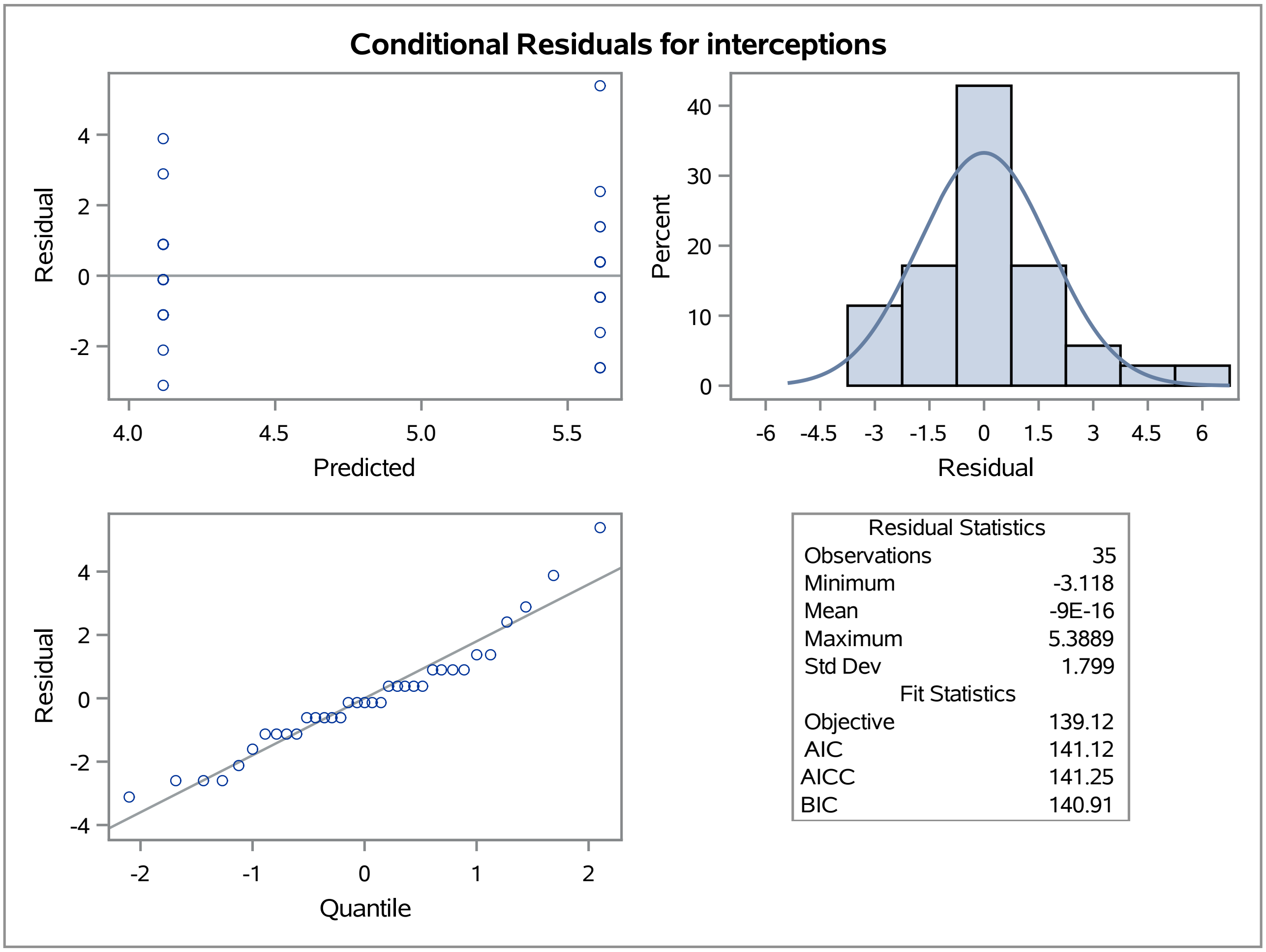

DistSoma=48

Model Information	
**Data Set**	WORK.TEMPDATASORTED
**Dependent Variable**	interceptions
**Covariance Structure**	Variance Components
**Estimation Method**	REML
**Residual Variance Method**	Profile
**Fixed Effects SE Method**	Model-Based
**Degrees of Freedom Method**	Containment

Class Level Information		
**Class**	**Levels**	**Values**
**Genotype**	2	Meg +/+ Meg +/-
**Animal**	6	1 2 3 4 5 6

Dimensions	
**Covariance Parameters**	2
**Columns in X**	3
**Columns in Z**	6
**Subjects**	1
**Max Obs per Subject**	35

Number of Observations	
**Number of Observations Read**	35
**Number of Observations Used**	35
**Number of Observations Not Used**	0

Iteration History			
**Iteration**	**Evaluations**	**-2 Res Log Like**	**Criterion**
**0**	1	150.58421567	
**1**	3	150.38999941	0.00007874
**2**	1	150.38628041	0.00000056
**3**	1	150.38625509	0.00000000

Convergence criteria met.

DistSoma=48

Covariance Parameter Estimates				
Cov Parm	Estimate	Alpha	Lower	Upper
Animal	0.2484	0.05	0.02826	2.915E10
Residual	4.5579	0.05	2.9209	8.0953

Fit Statistics	
-2 Res Log Likelihood	150.4
AIC (Smaller is Better)	154.4
AICC (Smaller is Better)	154.8
BIC (Smaller is Better)	154.0

Solution for Fixed Effects									
Effect	Genotype	Estimate	Standard Error	DF	t Value	Pr > |t|	Alpha	Lower	Upper
Intercept		4.1262	0.6053	4	6.82	0.0024	0.05	2.4458	5.8067
Genotype	Meg +/+	1.4673	0.8500	29	1.73	0.0949	0.05	-0.2711	3.2057
Genotype	Meg +/-	0	.	.	.	.	.	.	.

Solution for Random Effects									
Effect	Animal	Estimate	Std Err Pred	DF	t Value	Pr > |t|	Alpha	Lower	Upper
Animal	1	0.3197	0.4528	29	0.71	0.4858	0.05	-0.6065	1.2459
Animal	2	-0.3105	0.4569	29	-0.68	0.5022	0.05	-1.2450	0.6240
Animal	3	-0.00920	0.4769	29	-0.02	0.9847	0.05	-0.9845	0.9661
Animal	4	0.03673	0.4770	29	0.08	0.9391	0.05	-0.9388	1.0122
Animal	5	-0.07430	0.4558	29	-0.16	0.8716	0.05	-1.0064	0.8578
Animal	6	0.03757	0.4547	29	0.08	0.9347	0.05	-0.8923	0.9675

Type 3 Tests of Fixed Effects				
Effect	Num DF	Den DF	F Value	Pr > F
Genotype	1	29	2.98	0.0949

Least Squares Means									
Effect	Genotype	Estimate	Standard Error	DF	t Value	Pr > |t|	Alpha	Lower	Upper
Genotype	Meg +/+	5.5936	0.5968	29	9.37	<.0001	0.05	4.3730	6.8141
Genotype	Meg +/-	4.1262	0.6053	29	6.82	<.0001	0.05	2.8884	5.3641

DistSoma=48

Differences of Least Squares Means												
Effect	Genotype	Genotype	Estimate	Standard Error	DF	t Value	Pr > |t|	Adjustment	Adj P	Alpha	Lower	Upper
**Genotype**	Meg +/+	Meg +/-	1.4673	0.8500	29	1.73	0.0949	Tukey-Kramer	0.0949	0.05	-0.2711	3.2057

Differences of Least Squares Means				
Effect	Genotype	Genotype	Adj Lower	Adj Upper
**Genotype**	Meg +/+	Meg +/-	-0.2711	3.2057

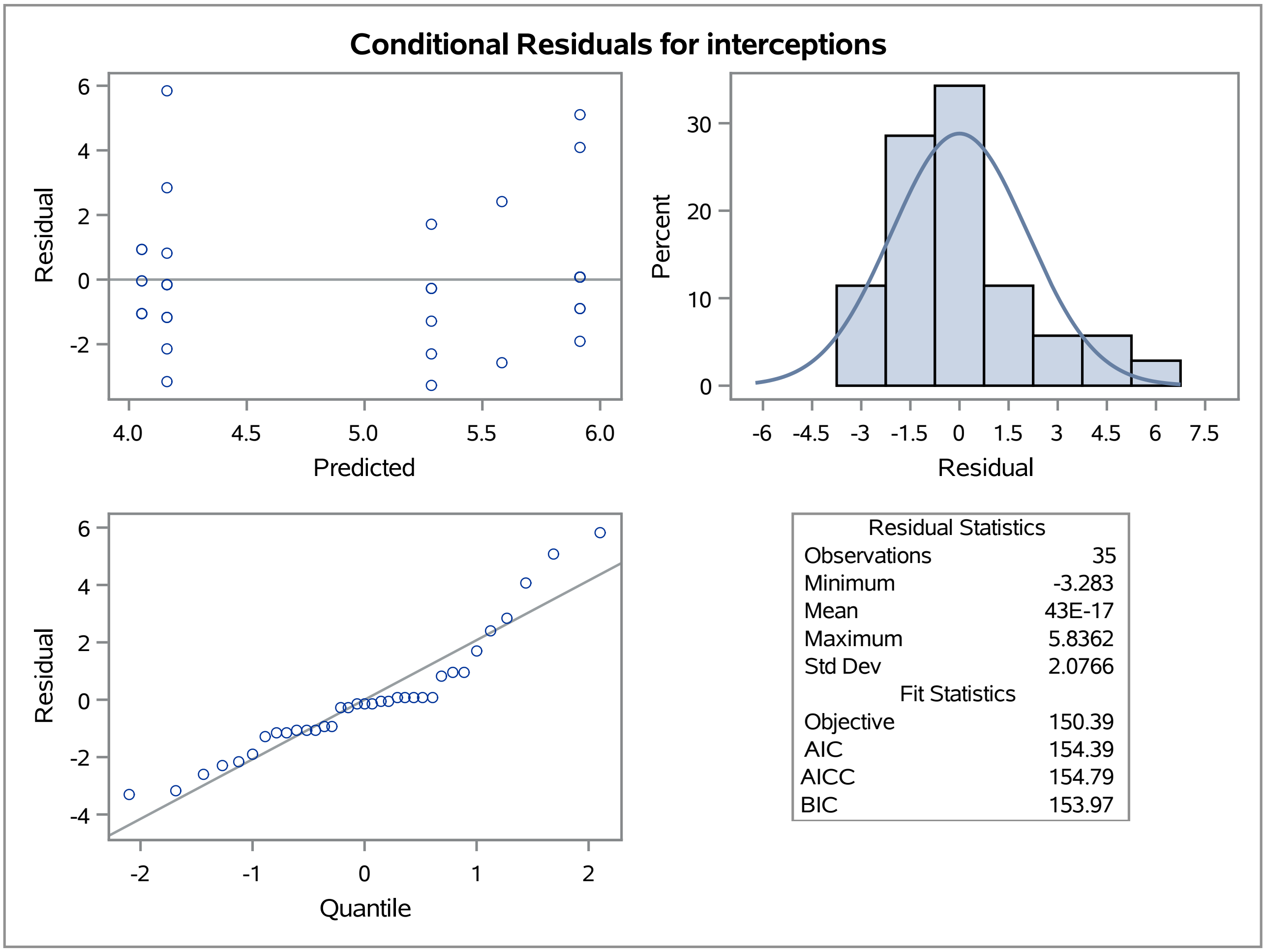

DistSoma=54

Model Information	
**Data Set**	WORK.TEMPDATASORTED
**Dependent Variable**	interceptions
**Covariance Structure**	Variance Components
**Estimation Method**	REML
**Residual Variance Method**	Profile
**Fixed Effects SE Method**	Model-Based
**Degrees of Freedom Method**	Containment

Class Level Information		
**Class**	**Levels**	**Values**
**Genotype**	2	Meg +/+ Meg +/-
**Animal**	6	1 2 3 4 5 6

Dimensions	
**Covariance Parameters**	2
**Columns in X**	3
**Columns in Z**	6
**Subjects**	1
**Max Obs per Subject**	35

Number of Observations	
**Number of Observations Read**	35
**Number of Observations Used**	35
**Number of Observations Not Used**	0

Iteration History			
**Iteration**	**Evaluations**	**-2 Res Log Like**	**Criterion**
**0**	1	156.03943227	
**1**	3	155.99575174	0.00000081
**2**	1	155.99571279	0.00000000

Convergence criteria met.

Covariance Parameter Estimates				
**Cov Parm**	**Estimate**	**Alpha**	**Lower**	**Upper**
**Animal**	0.1400	0.05	0.01228	1.372E42
**Residual**	5.4806	0.05	3.5084	9.7519

DistSoma=54

Fit Statistics	
-2 Res Log Likelihood	156.0
AIC (Smaller is Better)	160.0
AICC (Smaller is Better)	160.4
BIC (Smaller is Better)	159.6

Solution for Fixed Effects

Effect	Genotype	Estimate	Standard Error	DF	t Value	Pr > |t|	Alpha	Lower	Upper
Intercept		3.8875	0.6151	4	6.32	0.0032	0.05	2.1796	5.5954
Genotype	Meg +/+	1.7588	0.8614	29	2.04	0.0504	0.05	-0.00298	3.5207
Genotype	Meg +/-	0	.	.	.	.	.	.	.

Solution for Random Effects

Effect	Animal	Estimate	Std Err Pred	DF	t Value	Pr > |t|	Alpha	Lower	Upper
Animal	1	0.1330	0.3558	29	0.37	0.7112	0.05	-0.5946	0.8606
Animal	2	-0.1745	0.3575	29	-0.49	0.6292	0.05	-0.9057	0.5567
Animal	3	0.04149	0.3661	29	0.11	0.9105	0.05	-0.7073	0.7903
Animal	4	0.02977	0.3662	29	0.08	0.9358	0.05	-0.7192	0.7787
Animal	5	-0.09128	0.3570	29	-0.26	0.8000	0.05	-0.8215	0.6389
Animal	6	0.06151	0.3566	29	0.17	0.8642	0.05	-0.6678	0.7908

Type 3 Tests of Fixed Effects

Effect	Num DF	Den DF	F Value	Pr > F
Genotype	1	29	4.17	0.0504

Least Squares Means

Effect	Genotype	Estimate	Standard Error	DF	t Value	Pr > |t|	Alpha	Lower	Upper
Genotype	Meg +/+	5.6463	0.6030	29	9.36	<.0001	0.05	4.4130	6.8797
Genotype	Meg +/-	3.8875	0.6151	29	6.32	<.0001	0.05	2.6294	5.1456

DistSoma=54

Differences of Least Squares Means												
**Effect**	**Genotype**	**Genotype**	**Estimate**	**Standard Error**	**DF**	**t Value**	**Pr > |t|**	**Adjustment**	**Adj P**	**Alpha**	**Lower**	**Upper**
**Genotype**	Meg +/+	Meg +/-	1.7588	0.8614	29	2.04	0.0504	Tukey-Kramer	0.0504	0.05	-0.00298	3.5207

Differences of Least Squares Means				
**Effect**	**Genotype**	**Genotype**	**Adj Lower**	**Adj Upper**
**Genotype**	Meg +/+	Meg +/-	-0.00296	3.5206

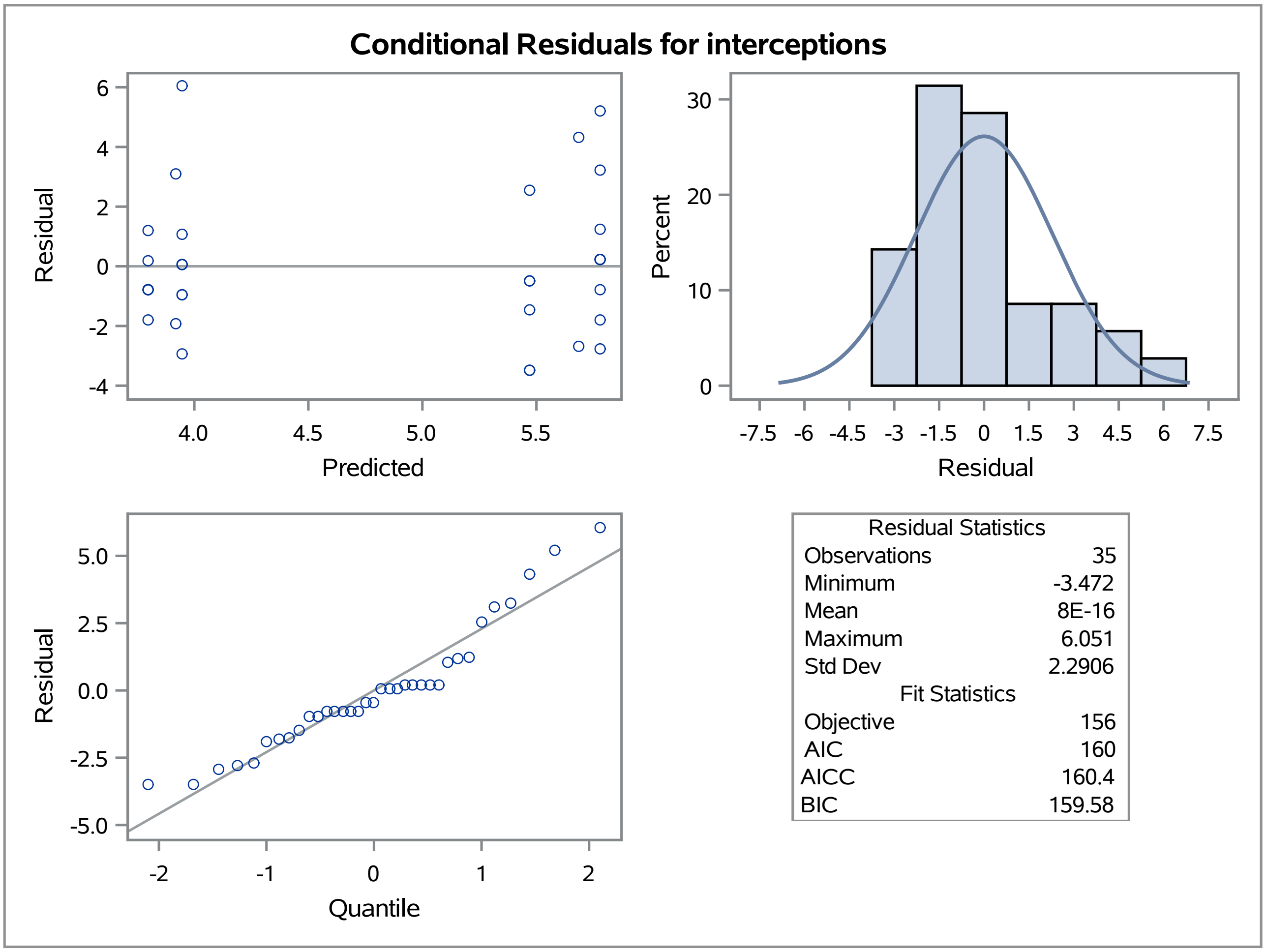

DistSoma=60

Model Information	
**Data Set**	WORK.TEMPDATASORTED
**Dependent Variable**	interceptions
**Covariance Structure**	Variance Components
**Estimation Method**	REML
**Residual Variance Method**	Profile
**Fixed Effects SE Method**	Model-Based
**Degrees of Freedom Method**	Containment

Class Level Information		
**Class**	**Levels**	**Values**
**Genotype**	2	Meg +/+ Meg +/-
**Animal**	6	1 2 3 4 5 6

Dimensions	
**Covariance Parameters**	2
**Columns in X**	3
**Columns in Z**	6
**Subjects**	1
**Max Obs per Subject**	35

Number of Observations	
**Number of Observations Read**	35
**Number of Observations Used**	35
**Number of Observations Not Used**	0

Iteration History			
**Iteration**	**Evaluations**	**-2 Res Log Like**	**Criterion**
**0**	1	155.99245049	
**1**	2	155.69490481	0.00000003
**2**	1	155.69490346	0.00000000

Convergence criteria met.

Covariance Parameter Estimates				
**Cov Parm**	**Estimate**	**Alpha**	**Lower**	**Upper**
**Animal**	0.4407	0.05	0.05590	93116454
**Residual**	5.2863	0.05	3.3672	9.4854

DistSoma=60

Fit Statistics	
**-2 Res Log Likelihood**	155.7
**AIC (Smaller is Better)**	159.7
**AICC (Smaller is Better)**	160.1
**BIC (Smaller is Better)**	159.3

Solution for Fixed Effects

Effect	Genotype	Estimate	Standard Error	DF	t Value	Pr > |t|	Alpha	Lower	Upper
**Intercept**		3.8309	0.6953	4	5.51	0.0053	0.05	1.9003	5.7614
**Genotype**	Meg +/+	1.5784	0.9782	29	1.61	0.1174	0.05	-0.4222	3.5791
**Genotype**	Meg +/-	0	.	.	.	.	.	.	.

Solution for Random Effects

Effect	Animal	Estimate	Std Err Pred	DF	t Value	Pr > |t|	Alpha	Lower	Upper
**Animal**	1	0.3140	0.5815	29	0.54	0.5933	0.05	-0.8754	1.5034
**Animal**	2	-0.4699	0.5886	29	-0.80	0.4311	0.05	-1.6736	0.7338
**Animal**	3	0.1559	0.6224	29	0.25	0.8040	0.05	-1.1171	1.4289
**Animal**	4	0.2385	0.6226	29	0.38	0.7044	0.05	-1.0348	1.5119
**Animal**	5	-0.3062	0.5865	29	-0.52	0.6056	0.05	-1.5057	0.8933
**Animal**	6	0.06766	0.5846	29	0.12	0.9087	0.05	-1.1280	1.2634

Type 3 Tests of Fixed Effects

Effect	Num DF	Den DF	F Value	Pr > F
**Genotype**	1	29	2.60	0.1174

Least Squares Means

Effect	Genotype	Estimate	Standard Error	DF	t Value	Pr > |t|	Alpha	Lower	Upper
**Genotype**	Meg +/+	5.4093	0.6880	29	7.86	<.0001	0.05	4.0021	6.8165
**Genotype**	Meg +/-	3.8309	0.6953	29	5.51	<.0001	0.05	2.4088	5.2530

DistSoma=60

Differences of Least Squares Means												
**Effect**	**Genotype**	**Genotype**	**Estimate**	**Standard Error**	**DF**	**t Value**	**Pr > |t|**	**Adjustment**	**Adj P**	**Alpha**	**Lower**	**Upper**
**Genotype**	Meg +/+	Meg +/-	1.5784	0.9782	29	1.61	0.1174	Tukey-Kramer	0.1174	0.05	-0.4222	3.5791

Differences of Least Squares Means				
**Effect**	**Genotype**	**Genotype**	**Adj Lower**	**Adj Upper**
**Genotype**	Meg +/+	Meg +/-	-0.4222	3.5790

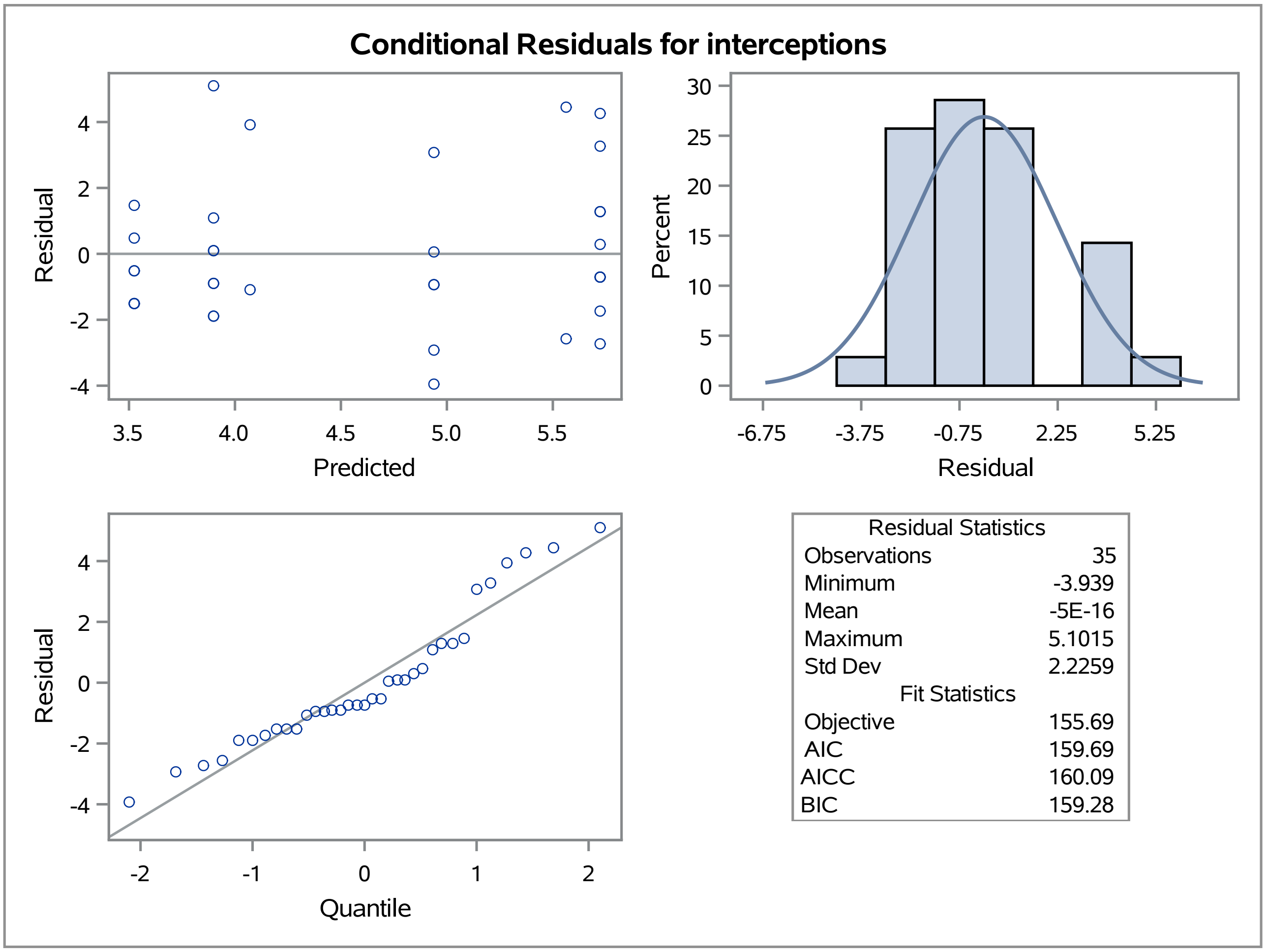

DistSoma=66

Model Information	
**Data Set**	WORK.TEMPDATASORTED
**Dependent Variable**	interceptions
**Covariance Structure**	Variance Components
**Estimation Method**	REML
**Residual Variance Method**	Profile
**Fixed Effects SE Method**	Model-Based
**Degrees of Freedom Method**	Containment

Class Level Information		
**Class**	**Levels**	**Values**
**Genotype**	2	Meg +/+ Meg +/-
**Animal**	6	1 2 3 4 5 6

Dimensions	
**Covariance Parameters**	2
**Columns in X**	3
**Columns in Z**	6
**Subjects**	1
**Max Obs per Subject**	35

Number of Observations	
**Number of Observations Read**	35
**Number of Observations Used**	35
**Number of Observations Not Used**	0

Iteration History			
**Iteration**	**Evaluations**	**-2 Res Log Like**	**Criterion**
**0**	1	159.56277546	
**1**	2	158.87986370	0.00000000

Convergence criteria met.

Covariance Parameter Estimates				
**Cov Parm**	**Estimate**	**Alpha**	**Lower**	**Upper**
**Animal**	0.8244	0.05	0.1355	29130
**Residual**	5.6911	0.05	3.6163	10.2527

DistSoma=66

Fit Statistics	
-2 Res Log Likelihood	158.9
AIC (Smaller is Better)	162.9
AICC (Smaller is Better)	163.3
BIC (Smaller is Better)	162.5

Solution for Fixed Effects

Effect	Genotype	Estimate	Standard Error	DF	t Value	Pr > |t|	Alpha	Lower	Upper
Intercept		3.9571	0.8073	4	4.90	0.0080	0.05	1.7157	6.1986
Genotype	Meg +/+	1.3299	1.1380	29	1.17	0.2521	0.05	-0.9977	3.6574
Genotype	Meg +/-	0	.	.	.	.	.	.	.

Solution for Random Effects

Effect	Animal	Estimate	Std Err Pred	DF	t Value	Pr > |t|	Alpha	Lower	Upper
Animal	1	0.4810	0.7496	29	0.64	0.5261	0.05	-1.0521	2.0140
Animal	2	-0.7534	0.7617	29	-0.99	0.3308	0.05	-2.3112	0.8044
Animal	3	0.2725	0.8195	29	0.33	0.7419	0.05	-1.4037	1.9486
Animal	4	0.4589	0.8198	29	0.56	0.5799	0.05	-1.2178	2.1356
Animal	5	-0.4819	0.7580	29	-0.64	0.5299	0.05	-2.0321	1.0683
Animal	6	0.02301	0.7547	29	0.03	0.9759	0.05	-1.5206	1.5666

Type 3 Tests of Fixed Effects

Effect	Num DF	Den DF	F Value	Pr > F
Genotype	1	29	1.37	0.2521

Least Squares Means

Effect	Genotype	Estimate	Standard Error	DF	t Value	Pr > |t|	Alpha	Lower	Upper
Genotype	Meg +/+	5.2870	0.8021	29	6.59	<.0001	0.05	3.6464	6.9276
Genotype	Meg +/-	3.9571	0.8073	29	4.90	<.0001	0.05	2.3060	5.6083

DistSoma=66

Differences of Least Squares Means												
Effect	Genotype	Genotype	Estimate	Standard Error	DF	t Value	Pr > |t|	Adjustment	Adj P	Alpha	Lower	Upper
**Genotype**	Meg +/+	Meg +/-	1.3299	1.1380	29	1.17	0.2521	Tukey-Kramer	0.2521	0.05	-0.9977	3.6574

Differences of Least Squares Means				
Effect	Genotype	Genotype	Adj Lower	Adj Upper
**Genotype**	Meg +/+	Meg +/-	-0.9977	3.6574

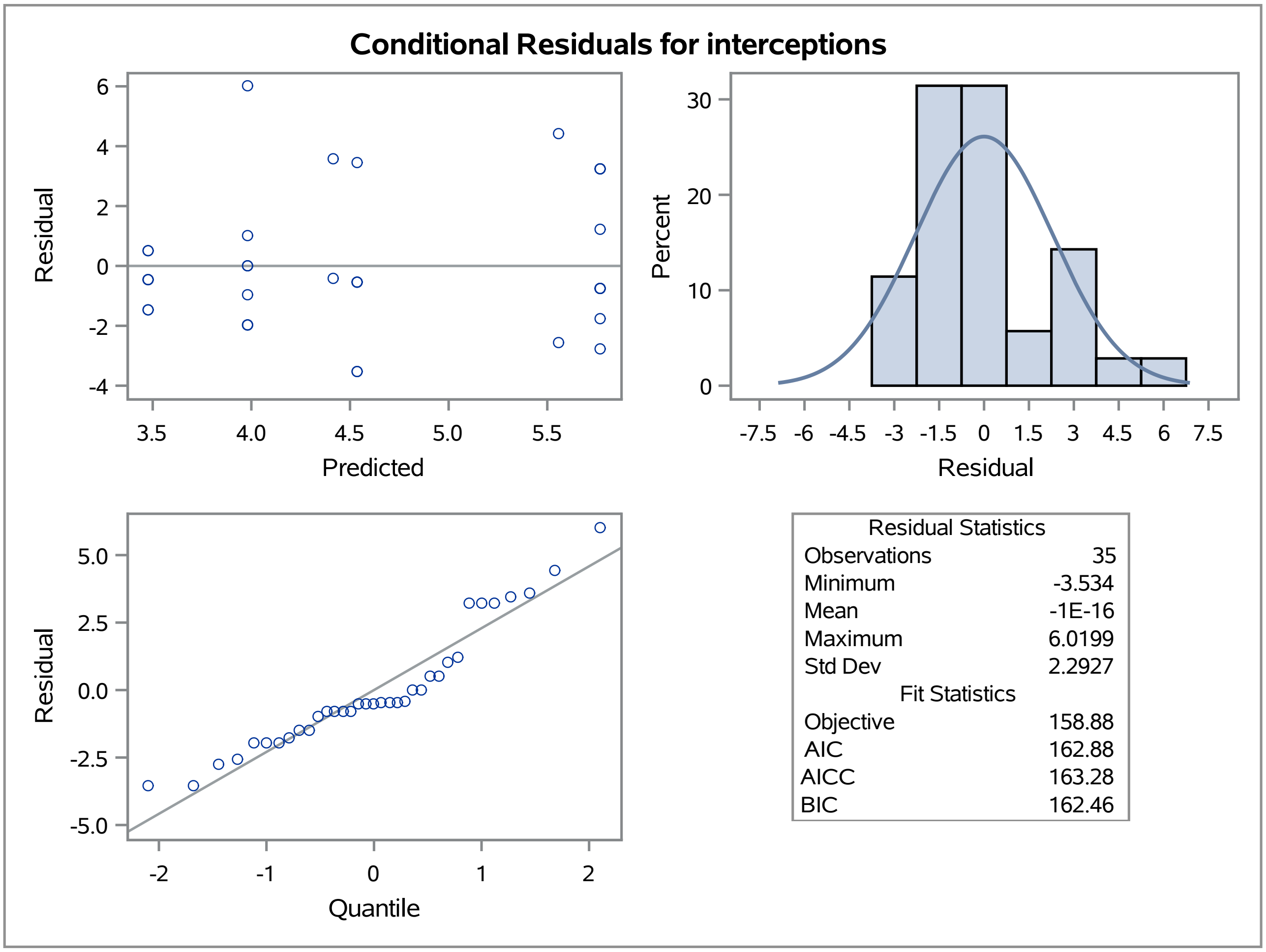

DistSoma=72

Model Information	
**Data Set**	WORK.TEMPDATASORTED
**Dependent Variable**	interceptions
**Covariance Structure**	Variance Components
**Estimation Method**	REML
**Residual Variance Method**	Profile
**Fixed Effects SE Method**	Model-Based
**Degrees of Freedom Method**	Containment

Class Level Information		
**Class**	**Levels**	**Values**
**Genotype**	2	Meg +/+ Meg +/-
**Animal**	6	1 2 3 4 5 6

Dimensions	
**Covariance Parameters**	2
**Columns in X**	3
**Columns in Z**	6
**Subjects**	1
**Max Obs per Subject**	35

Number of Observations	
**Number of Observations Read**	35
**Number of Observations Used**	35
**Number of Observations Not Used**	0

Iteration History			
**Iteration**	**Evaluations**	**-2 Res Log Like**	**Criterion**
**0**	1	159.95911995	
**1**	2	159.81042124	0.00000001
**2**	1	159.81042050	0.00000000

Convergence criteria met.

Covariance Parameter Estimates				
**Cov Parm**	**Estimate**	**Alpha**	**Lower**	**Upper**
**Animal**	0.3643	0.05	0.03747	3.305E14
**Residual**	6.0485	0.05	3.8444	10.8923

DistSoma=72

Fit Statistics	
-2 Res Log Likelihood	159.8
AIC (Smaller is Better)	163.8
AICC (Smaller is Better)	164.2
BIC (Smaller is Better)	163.4

Solution for Fixed Effects

Effect	Genotype	Estimate	Standard Error	DF	t Value	Pr > |t|	Alpha	Lower	Upper
Intercept		4.0044	0.7068	4	5.67	0.0048	0.05	2.0420	5.9667
Genotype	Meg +/+	0.6399	0.9930	29	0.64	0.5244	0.05	-1.3910	2.6708
Genotype	Meg +/-	0	.	.	.	.	.	.	.

Solution for Random Effects

Effect	Animal	Estimate	Std Err Pred	DF	t Value	Pr > |t|	Alpha	Lower	Upper
Animal	1	0.2465	0.5442	29	0.45	0.6539	0.05	-0.8664	1.3594
Animal	2	-0.3922	0.5494	29	-0.71	0.4810	0.05	-1.5160	0.7315
Animal	3	0.1458	0.5751	29	0.25	0.8017	0.05	-1.0305	1.3220
Animal	4	0.2146	0.5753	29	0.37	0.7119	0.05	-0.9620	1.3911
Animal	5	-0.2131	0.5479	29	-0.39	0.7001	0.05	-1.3337	0.9075
Animal	6	-0.00142	0.5465	29	-0.00	0.9979	0.05	-1.1191	1.1163

Type 3 Tests of Fixed Effects

Effect	Num DF	Den DF	F Value	Pr > F
Genotype	1	29	0.42	0.5244

Least Squares Means

Effect	Genotype	Estimate	Standard Error	DF	t Value	Pr > |t|	Alpha	Lower	Upper
Genotype	Meg +/+	4.6443	0.6975	29	6.66	<.0001	0.05	3.2178	6.0708
Genotype	Meg +/-	4.0044	0.7068	29	5.67	<.0001	0.05	2.5588	5.4499

DistSoma=72

Differences of Least Squares Means												
**Effect**	**Genotype**	**Genotype**	**Estimate**	**Standard Error**	**DF**	**t Value**	**Pr > |t|**	**Adjustment**	**Adj P**	**Alpha**	**Lower**	**Upper**
**Genotype**	Meg +/+	Meg +/-	0.6399	0.9930	29	0.64	0.5244	Tukey-Kramer	0.5244	0.05	-1.3910	2.6708

Differences of Least Squares Means				
**Effect**	**Genotype**	**Genotype**	**Adj Lower**	**Adj Upper**
**Genotype**	Meg +/+	Meg +/-	-1.3910	2.6708

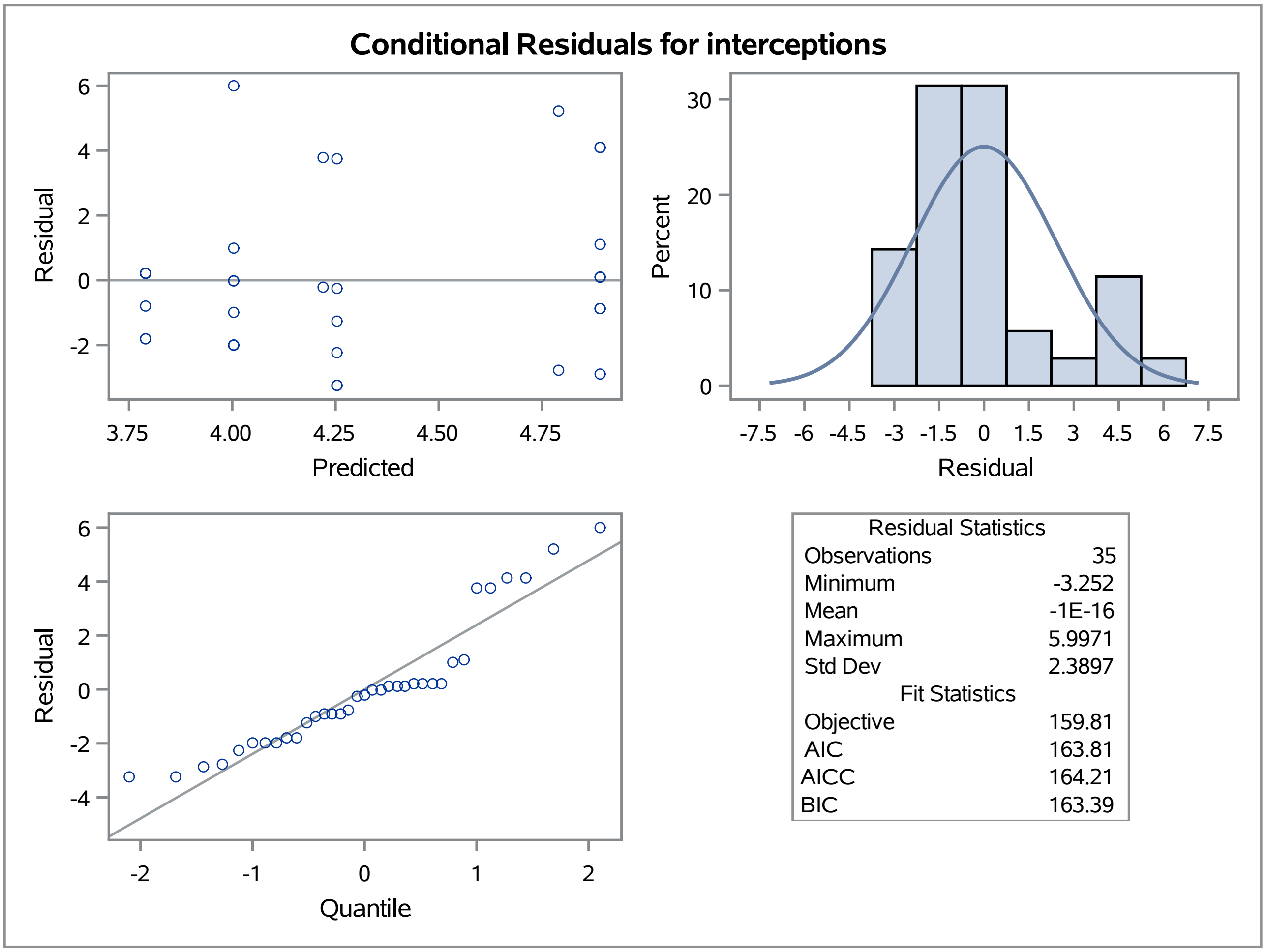

DistSoma=78

Model Information	
**Data Set**	WORK.TEMPDATASORTED
**Dependent Variable**	interceptions
**Covariance Structure**	Variance Components
**Estimation Method**	REML
**Residual Variance Method**	Profile
**Fixed Effects SE Method**	Model-Based
**Degrees of Freedom Method**	Containment

Class Level Information		
**Class**	**Levels**	**Values**
**Genotype**	2	Meg +/+ Meg +/-
**Animal**	6	1 2 3 4 5 6

Dimensions	
**Covariance Parameters**	2
**Columns in X**	3
**Columns in Z**	6
**Subjects**	1
**Max Obs per Subject**	35

Number of Observations	
**Number of Observations Read**	35
**Number of Observations Used**	35
**Number of Observations Not Used**	0

Iteration History			
**Iteration**	**Evaluations**	**-2 Res Log Like**	**Criterion**
**0**	1	165.45891403	
**1**	1	165.45891403	0.00000000

Convergence criteria met.

Estimated G matrix is not positive definite.

Covariance Parameter Estimates				
**Cov Parm**	**Estimate**	**Alpha**	**Lower**	**Upper**
**Animal**	0	.	.	.
**Residual**	7.4082	0.05	4.8195	12.8354

DistSoma=78

Fit Statistics	
-2 Res Log Likelihood	165.5
AIC (Smaller is Better)	167.5
AICC (Smaller is Better)	167.6
BIC (Smaller is Better)	167.3

Solution for Fixed Effects									
Effect	Genotype	Estimate	Standard Error	DF	t Value	Pr > |t|	Alpha	Lower	Upper
Intercept		3.8235	0.6601	4	5.79	0.0044	0.05	1.9907	5.6564
Genotype	Meg +/+	0.5098	0.9205	29	0.55	0.5839	0.05	-1.3729	2.3925
Genotype	Meg +/-	0	.	.	.	.	.	.	.

Solution for Random Effects									
Effect	Animal	Estimate	Std Err Pred	DF	t Value	Pr > |t|	Alpha	Lower	Upper
Animal	1	0	.	.	.	.	.	.	.
Animal	2	0	.	.	.	.	.	.	.
Animal	3	0	.	.	.	.	.	.	.
Animal	4	0	.	.	.	.	.	.	.
Animal	5	0	.	.	.	.	.	.	.
Animal	6	0	.	.	.	.	.	.	.

Type 3 Tests of Fixed Effects				
Effect	Num DF	Den DF	F Value	Pr > F
Genotype	1	29	0.31	0.5839

Least Squares Means									
Effect	Genotype	Estimate	Standard Error	DF	t Value	Pr > |t|	Alpha	Lower	Upper
Genotype	Meg +/+	4.3333	0.6415	29	6.75	<.0001	0.05	3.0212	5.6454
Genotype	Meg +/-	3.8235	0.6601	29	5.79	<.0001	0.05	2.4734	5.1737

DistSoma=78

Differences of Least Squares Means												
Effect	Genotype	Genotype	Estimate	Standard Error	DF	t Value	Pr > |t|	Adjustment	Adj P	Alpha	Lower	Upper
**Genotype**	Meg +/+	Meg +/-	0.5098	0.9205	29	0.55	0.5839	Tukey-Kramer	0.5839	0.05	-1.3729	2.3925

Differences of Least Squares Means				
Effect	Genotype	Genotype	Adj Lower	Adj Upper
**Genotype**	Meg +/+	Meg +/-	-1.3728	2.3924

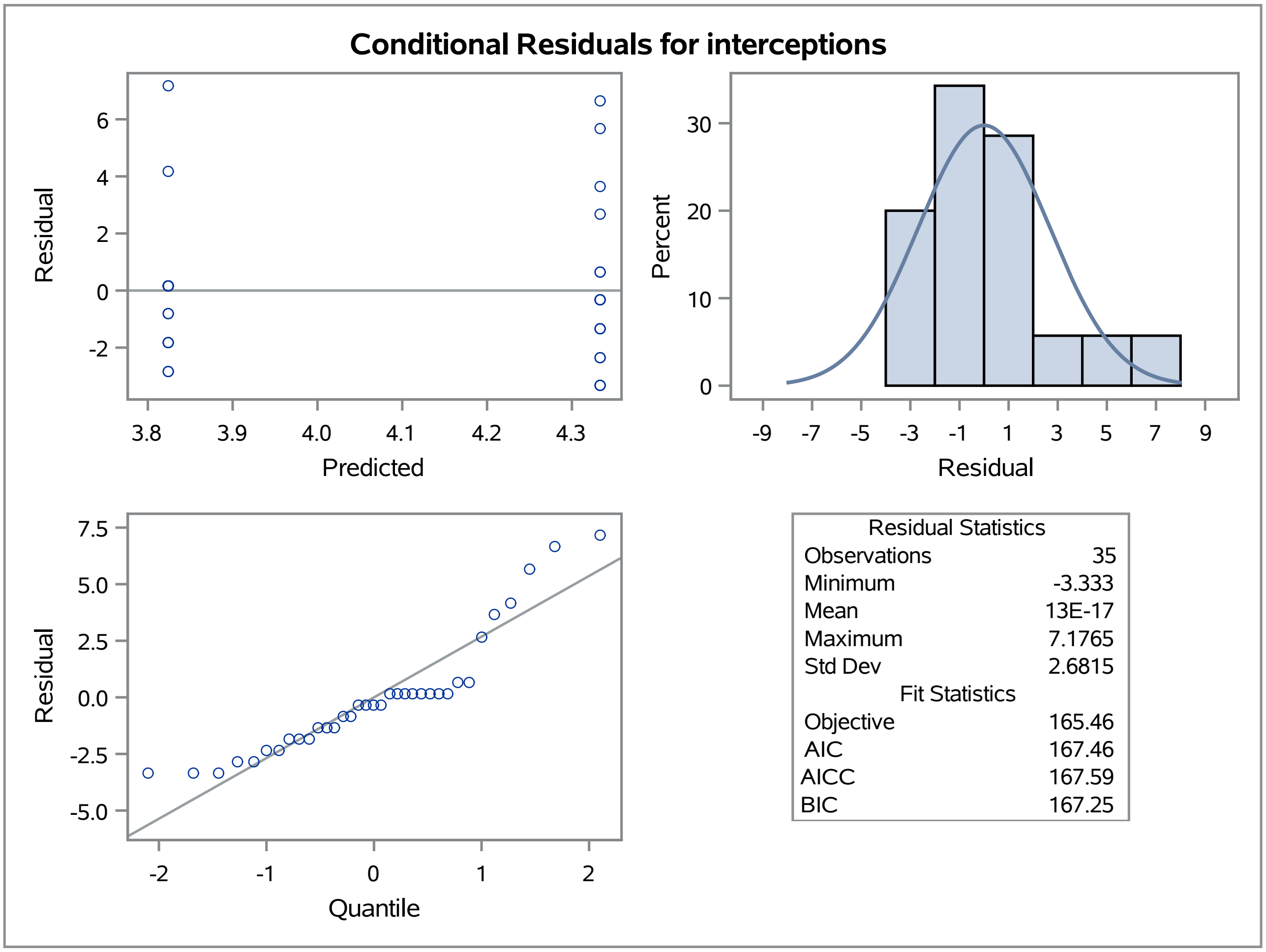

DistSoma=84

Model Information	
**Data Set**	WORK.TEMPDATASORTED
**Dependent Variable**	interceptions
**Covariance Structure**	Variance Components
**Estimation Method**	REML
**Residual Variance Method**	Profile
**Fixed Effects SE Method**	Model-Based
**Degrees of Freedom Method**	Containment

Class Level Information		
**Class**	**Levels**	**Values**
**Genotype**	2	Meg +/+ Meg +/-
**Animal**	6	1 2 3 4 5 6

Dimensions	
**Covariance Parameters**	2
**Columns in X**	3
**Columns in Z**	6
**Subjects**	1
**Max Obs per Subject**	35

Number of Observations	
**Number of Observations Read**	35
**Number of Observations Used**	35
**Number of Observations Not Used**	0

Iteration History			
**Iteration**	**Evaluations**	**-2 Res Log Like**	**Criterion**
**0**	1	168.49778689	
**1**	1	168.49778689	0.00000000

Convergence criteria met.

Estimated G matrix is not positive definite.

Covariance Parameter Estimates				
**Cov Parm**	**Estimate**	**Alpha**	**Lower**	**Upper**
**Animal**	0	.	.	.
**Residual**	8.1228	0.05	5.2844	14.0735

DistSoma=84

Fit Statistics	
-2 Res Log Likelihood	168.5
AIC (Smaller is Better)	170.5
AICC (Smaller is Better)	170.6
BIC (Smaller is Better)	170.3

Solution for Fixed Effects

Effect	Genotype	Estimate	Standard Error	DF	t Value	Pr > |t|	Alpha	Lower	Upper
Intercept		3.9412	0.6912	4	5.70	0.0047	0.05	2.0220	5.8604
Genotype	Meg +/+	0.2810	0.9639	29	0.29	0.7727	0.05	-1.6903	2.2524
Genotype	Meg +/-	0	.	.	.	.	.	.	.

Solution for Random Effects

Effect	Animal	Estimate	Std Err Pred	DF	t Value	Pr > |t|	Alpha	Lower	Upper
Animal	1	0	.	.	.	.	.	.	.
Animal	2	0	.	.	.	.	.	.	.
Animal	3	0	.	.	.	.	.	.	.
Animal	4	0	.	.	.	.	.	.	.
Animal	5	0	.	.	.	.	.	.	.
Animal	6	0	.	.	.	.	.	.	.

Type 3 Tests of Fixed Effects

Effect	Num DF	Den DF	F Value	Pr > F
Genotype	1	29	0.09	0.7727

Least Squares Means

Effect	Genotype	Estimate	Standard Error	DF	t Value	Pr > |t|	Alpha	Lower	Upper
Genotype	Meg +/+	4.2222	0.6718	29	6.29	<.0001	0.05	2.8483	5.5961
Genotype	Meg +/-	3.9412	0.6912	29	5.70	<.0001	0.05	2.5274	5.3549

DistSoma=84

Differences of Least Squares Means												
Effect	Genotype	Genotype	Estimate	Standard Error	DF	t Value	Pr > |t|	Adjustment	Adj P	Alpha	Lower	Upper
**Genotype**	Meg +/+	Meg +/-	0.2810	0.9639	29	0.29	0.7727	Tukey-Kramer	0.7727	0.05	-1.6903	2.2524

Differences of Least Squares Means				
Effect	Genotype	Genotype	Adj Lower	Adj Upper
**Genotype**	Meg +/+	Meg +/-	-1.6903	2.2524

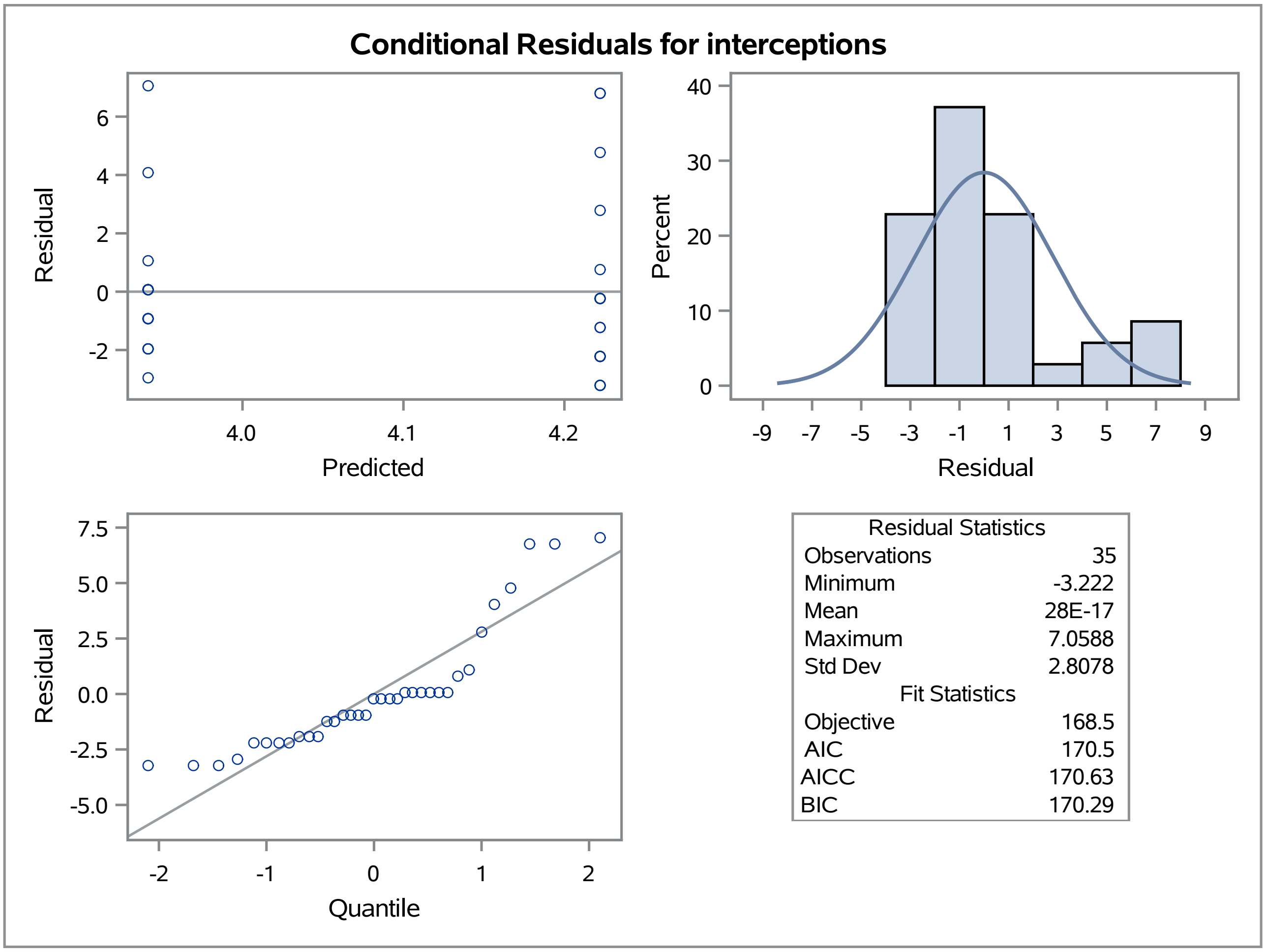

DistSoma=90

Model Information	
Data Set	WORK.TEMPDATASORTED
Dependent Variable	interceptions
Covariance Structure	Variance Components
Estimation Method	REML
Residual Variance Method	Profile
Fixed Effects SE Method	Model-Based
Degrees of Freedom Method	Containment

Class Level Information		
Class	Levels	Values
Genotype	2	Meg +/+ Meg +/-
Animal	6	1 2 3 4 5 6

Dimensions	
Covariance Parameters	2
Columns in X	3
Columns in Z	6
Subjects	1
Max Obs per Subject	35

Number of Observations	
Number of Observations Read	35
Number of Observations Used	35
Number of Observations Not Used	0

Iteration History			
Iteration	Evaluations	-2 Res Log Like	Criterion
0	1	170.98757566	
1	1	170.98757566	0.00000000

Convergence criteria met.

Estimated G matrix is not positive definite.

Covariance Parameter Estimates				
Cov Parm	Estimate	Alpha	Lower	Upper
Animal	0	.	.	.
Residual	8.7594	0.05	5.6985	15.1764

DistSoma=90

Fit Statistics	
-2 Res Log Likelihood	171.0
AIC (Smaller is Better)	173.0
AICC (Smaller is Better)	173.1
BIC (Smaller is Better)	172.8

Solution for Fixed Effects

Effect	Genotype	Estimate	Standard Error	DF	t Value	Pr > |t|	Alpha	Lower	Upper
Intercept		3.7647	0.7178	4	5.24	0.0063	0.05	1.7717	5.7577
Genotype	Meg +/+	0.2353	1.0009	29	0.24	0.8158	0.05	-1.8119	2.2825
Genotype	Meg +/-	0	.	.	.	.	.	.	.

Solution for Random Effects

Effect	Animal	Estimate	Std Err Pred	DF	t Value	Pr > |t|	Alpha	Lower	Upper
Animal	1	0	.	.	.	.	.	.	.
Animal	2	0	.	.	.	.	.	.	.
Animal	3	0	.	.	.	.	.	.	.
Animal	4	0	.	.	.	.	.	.	.
Animal	5	0	.	.	.	.	.	.	.
Animal	6	0	.	.	.	.	.	.	.

Type 3 Tests of Fixed Effects

Effect	Num DF	Den DF	F Value	Pr > F
Genotype	1	29	0.06	0.8158

Least Squares Means

Effect	Genotype	Estimate	Standard Error	DF	t Value	Pr > |t|	Alpha	Lower	Upper
Genotype	Meg +/+	4.0000	0.6976	29	5.73	<.0001	0.05	2.5733	5.4267
Genotype	Meg +/-	3.7647	0.7178	29	5.24	<.0001	0.05	2.2966	5.2328

DistSoma=90

Differences of Least Squares Means												
Effect	Genotype	Genotype	Estimate	Standard Error	DF	t Value	Pr > |t|	Adjustment	Adj P	Alpha	Lower	Upper
**Genotype**	Meg +/+	Meg +/-	0.2353	1.0009	29	0.24	0.8158	Tukey-Kramer	0.8158	0.05	-1.8119	2.2825

Differences of Least Squares Means				
Effect	Genotype	Genotype	Adj Lower	Adj Upper
**Genotype**	Meg +/+	Meg +/-	-1.8118	2.2824

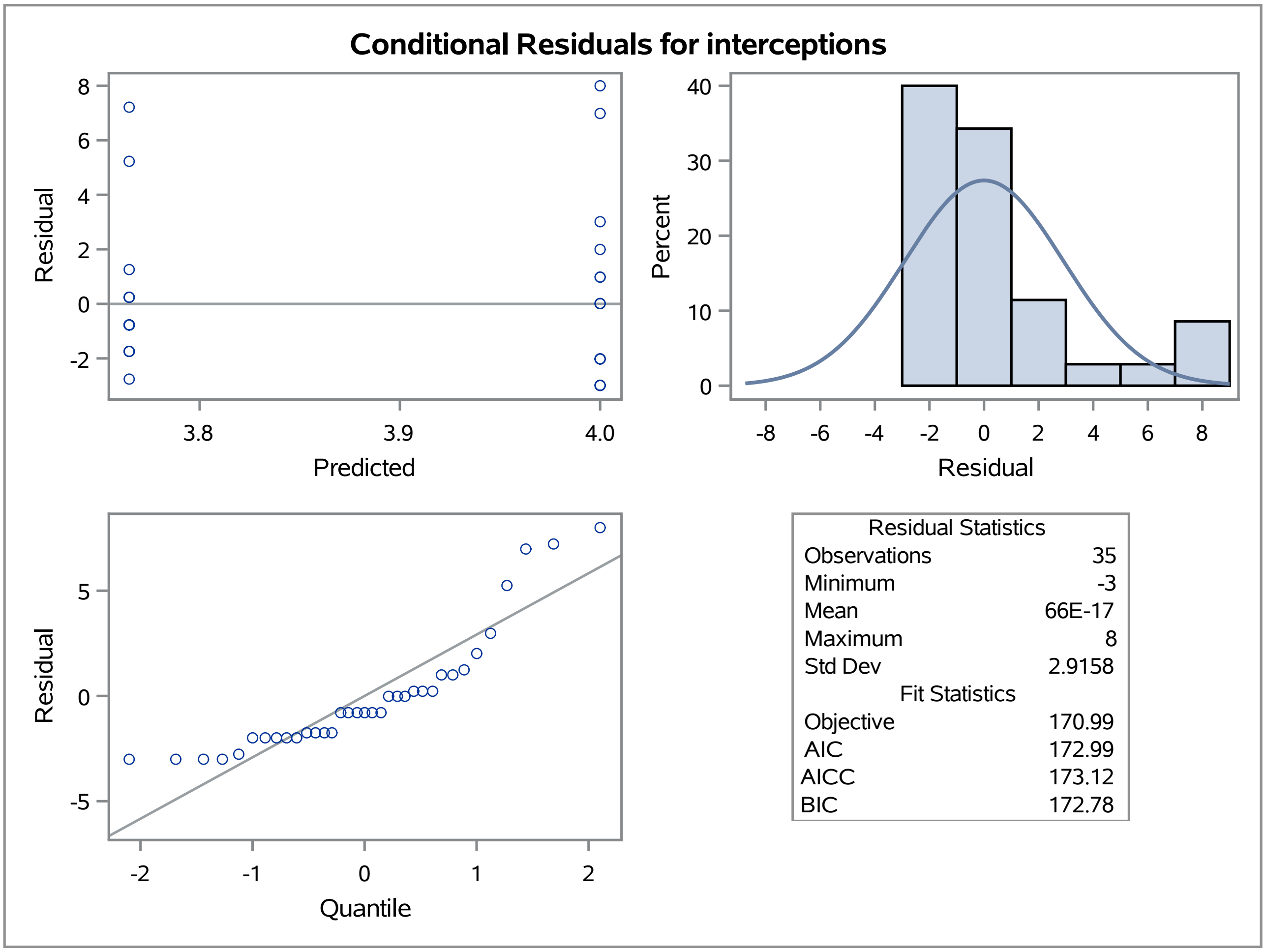

DistSoma=96

Model Information	
**Data Set**	WORK.TEMPDATASORTED
**Dependent Variable**	interceptions
**Covariance Structure**	Variance Components
**Estimation Method**	REML
**Residual Variance Method**	Profile
**Fixed Effects SE Method**	Model-Based
**Degrees of Freedom Method**	Containment

Class Level Information		
**Class**	**Levels**	**Values**
**Genotype**	2	Meg +/+ Meg +/-
**Animal**	6	1 2 3 4 5 6

Dimensions	
**Covariance Parameters**	2
**Columns in X**	3
**Columns in Z**	6
**Subjects**	1
**Max Obs per Subject**	35

Number of Observations	
**Number of Observations Read**	35
**Number of Observations Used**	35
**Number of Observations Not Used**	0

Iteration History			
**Iteration**	**Evaluations**	**-2 Res Log Like**	**Criterion**
**0**	1	169.54980603	
**1**	1	169.54980603	0.00000000

Convergence criteria met.

Estimated G matrix is not positive definite.

Covariance Parameter Estimates				
**Cov Parm**	**Estimate**	**Alpha**	**Lower**	**Upper**
**Animal**	0	.	.	.
**Residual**	8.3859	0.05	5.4556	14.5293

DistSoma=96

Fit Statistics	
-2 Res Log Likelihood	169.5
AIC (Smaller is Better)	171.5
AICC (Smaller is Better)	171.7
BIC (Smaller is Better)	171.3

Solution for Fixed Effects

Effect	Genotype	Estimate	Standard Error	DF	t Value	Pr > |t|	Alpha	Lower	Upper
Intercept		3.4706	0.7023	4	4.94	0.0078	0.05	1.5206	5.4206
Genotype	Meg +/+	0.02941	0.9794	29	0.03	0.9762	0.05	-1.9736	2.0325
Genotype	Meg +/-	0	.	.	.	.	.	.	.

Solution for Random Effects

Effect	Animal	Estimate	Std Err Pred	DF	t Value	Pr > |t|	Alpha	Lower	Upper
Animal	1	0	.	.	.	.	.	.	.
Animal	2	0	.	.	.	.	.	.	.
Animal	3	0	.	.	.	.	.	.	.
Animal	4	0	.	.	.	.	.	.	.
Animal	5	0	.	.	.	.	.	.	.
Animal	6	0	.	.	.	.	.	.	.

Type 3 Tests of Fixed Effects

Effect	Num DF	Den DF	F Value	Pr > F
Genotype	1	29	0.00	0.9762

Least Squares Means

Effect	Genotype	Estimate	Standard Error	DF	t Value	Pr > |t|	Alpha	Lower	Upper
Genotype	Meg +/+	3.5000	0.6826	29	5.13	<.0001	0.05	2.1040	4.8960
Genotype	Meg +/-	3.4706	0.7023	29	4.94	<.0001	0.05	2.0341	4.9070

DistSoma=96

Differences of Least Squares Means												
**Effect**	**Genotype**	**Genotype**	**Estimate**	**Standard Error**	**DF**	**t Value**	**Pr > |t|**	**Adjustment**	**Adj P**	**Alpha**	**Lower**	**Upper**
**Genotype**	Meg +/+	Meg +/-	0.02941	0.9794	29	0.03	0.9762	Tukey-Kramer	0.9762	0.05	-1.9736	2.0325

Differences of Least Squares Means				
**Effect**	**Genotype**	**Genotype**	**Adj Lower**	**Adj Upper**
**Genotype**	Meg +/+	Meg +/-	-1.9736	2.0324

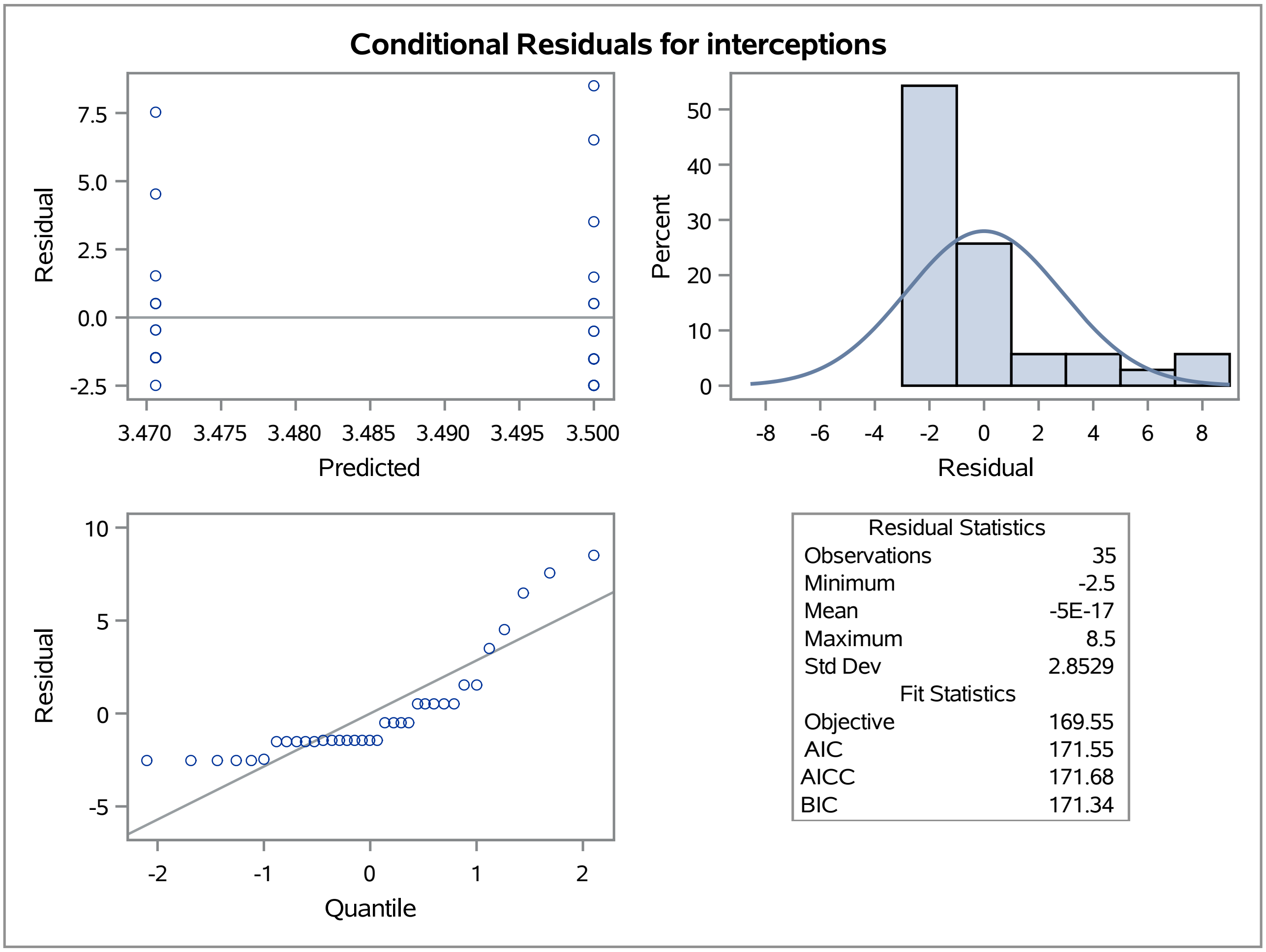

DistSoma=102

Model Information	
**Data Set**	WORK.TEMPDATASORTED
**Dependent Variable**	interceptions
**Covariance Structure**	Variance Components
**Estimation Method**	REML
**Residual Variance Method**	Profile
**Fixed Effects SE Method**	Model-Based
**Degrees of Freedom Method**	Containment

Class Level Information		
**Class**	**Levels**	**Values**
**Genotype**	2	Meg +/+ Meg +/-
**Animal**	6	1 2 3 4 5 6

Dimensions	
**Covariance Parameters**	2
**Columns in X**	3
**Columns in Z**	6
**Subjects**	1
**Max Obs per Subject**	35

Number of Observations	
**Number of Observations Read**	35
**Number of Observations Used**	35
**Number of Observations Not Used**	0

Iteration History			
**Iteration**	**Evaluations**	**-2 Res Log Like**	**Criterion**
**0**	1	166.77044800	
**1**	1	166.77044800	0.00000000

Convergence criteria met.

Estimated G matrix is not positive definite.

Covariance Parameter Estimates				
**Cov Parm**	**Estimate**	**Alpha**	**Lower**	**Upper**
**Animal**	0	.	.	.
**Residual**	7.7086	0.05	5.0149	13.3557

DistSoma=102

Fit Statistics	
**-2 Res Log Likelihood**	166.8
**AIC (Smaller is Better)**	168.8
**AICC (Smaller is Better)**	168.9
**BIC (Smaller is Better)**	168.6

Solution for Fixed Effects

Effect	Genotype	Estimate	Standard Error	DF	t Value	Pr > |t|	Alpha	Lower	Upper
**Intercept**		3.3529	0.6734	4	4.98	0.0076	0.05	1.4833	5.2226
**Genotype**	Meg +/+	-0.1863	0.9390	29	-0.20	0.8441	0.05	-2.1067	1.7342
**Genotype**	Meg +/-	0	.	.	.	.	.	.	.

Solution for Random Effects

Effect	Animal	Estimate	Std Err Pred	DF	t Value	Pr > |t|	Alpha	Lower	Upper
**Animal**	1	0	.	.	.	.	.	.	.
**Animal**	2	0	.	.	.	.	.	.	.
**Animal**	3	0	.	.	.	.	.	.	.
**Animal**	4	0	.	.	.	.	.	.	.
**Animal**	5	0	.	.	.	.	.	.	.
**Animal**	6	0	.	.	.	.	.	.	.

Type 3 Tests of Fixed Effects

Effect	Num DF	Den DF	F Value	Pr > F
**Genotype**	1	29	0.04	0.8441

Least Squares Means

Effect	Genotype	Estimate	Standard Error	DF	t Value	Pr > |t|	Alpha	Lower	Upper
**Genotype**	Meg +/+	3.1667	0.6544	29	4.84	<.0001	0.05	1.8282	4.5051
**Genotype**	Meg +/-	3.3529	0.6734	29	4.98	<.0001	0.05	1.9757	4.7302

DistSoma=102

Differences of Least Squares Means												
**Effect**	**Genotype**	**Genotype**	**Estimate**	**Standard Error**	**DF**	**t Value**	**Pr > |t|**	**Adjustment**	**Adj P**	**Alpha**	**Lower**	**Upper**
**Genotype**	Meg +/+	Meg +/-	-0.1863	0.9390	29	-0.20	0.8441	Tukey-Kramer	0.8441	0.05	-2.1067	1.7342

Differences of Least Squares Means				
**Effect**	**Genotype**	**Genotype**	**Adj Lower**	**Adj Upper**
**Genotype**	Meg +/+	Meg +/-	-2.1067	1.7342

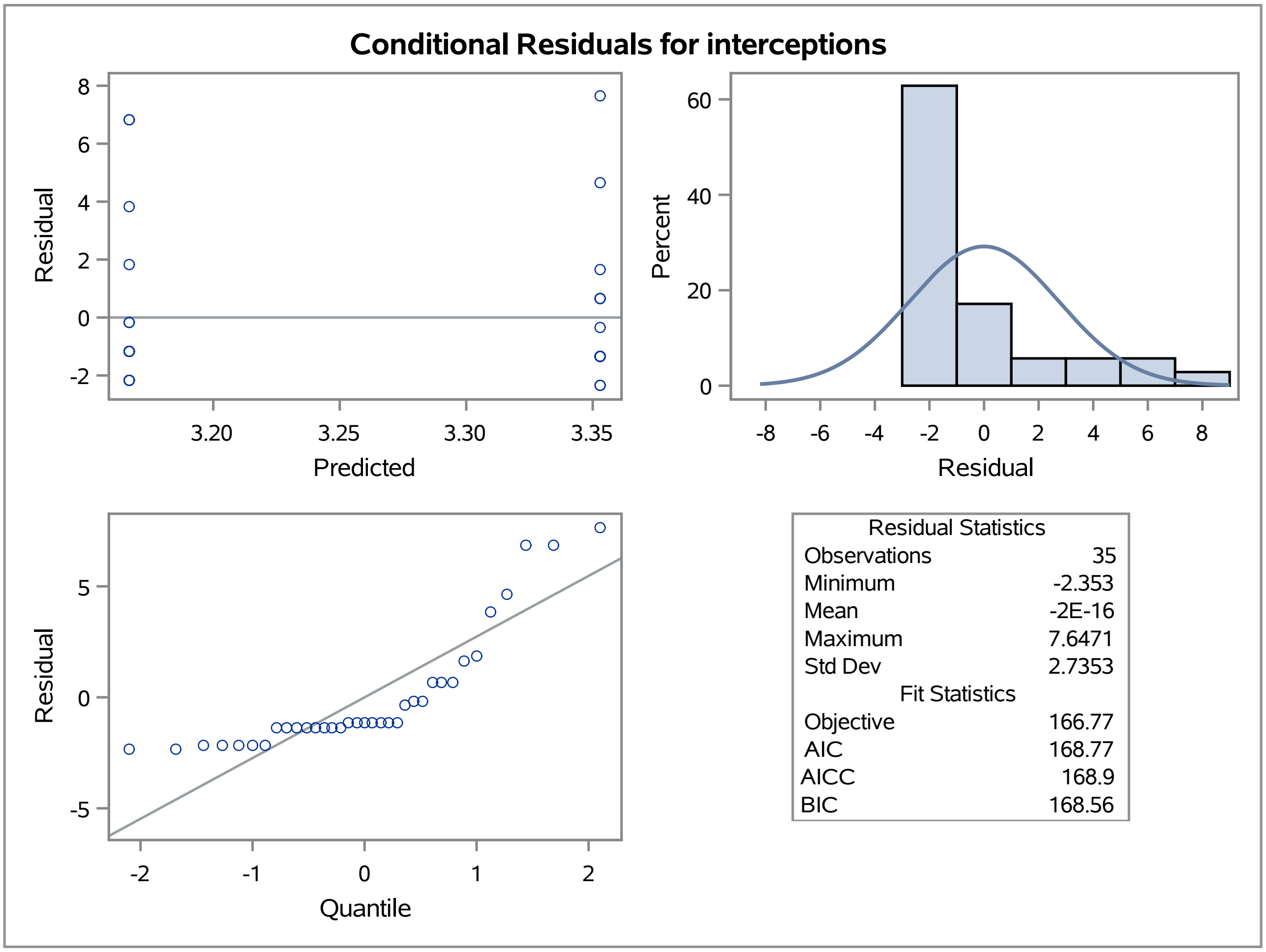

DistSoma=108

Model Information	
**Data Set**	WORK.TEMPDATASORTED
**Dependent Variable**	interceptions
**Covariance Structure**	Variance Components
**Estimation Method**	REML
**Residual Variance Method**	Profile
**Fixed Effects SE Method**	Model-Based
**Degrees of Freedom Method**	Containment

Class Level Information		
**Class**	**Levels**	**Values**
**Genotype**	2	Meg +/+ Meg +/-
**Animal**	6	1 2 3 4 5 6

Dimensions	
**Covariance Parameters**	2
**Columns in X**	3
**Columns in Z**	6
**Subjects**	1
**Max Obs per Subject**	35

Number of Observations	
**Number of Observations Read**	35
**Number of Observations Used**	35
**Number of Observations Not Used**	0

Iteration History			
**Iteration**	**Evaluations**	**-2 Res Log Like**	**Criterion**
**0**	1	166.14172712	
**1**	1	166.14172712	0.00000000

Convergence criteria met.

Estimated G matrix is not positive definite.

Covariance Parameter Estimates				
**Cov Parm**	**Estimate**	**Alpha**	**Lower**	**Upper**
**Animal**	0	.	.	.
**Residual**	7.5631	0.05	4.9203	13.1037

DistSoma=108

Fit Statistics	
-2 Res Log Likelihood	166.1
AIC (Smaller is Better)	168.1
AICC (Smaller is Better)	168.3
BIC (Smaller is Better)	167.9

Solution for Fixed Effects

Effect	Genotype	Estimate	Standard Error	DF	t Value	Pr > |t|	Alpha	Lower	Upper
Intercept		3.1765	0.6670	4	4.76	0.0089	0.05	1.3246	5.0284
Genotype	Meg +/+	0.04575	0.9301	29	0.05	0.9611	0.05	-1.8565	1.9480
Genotype	Meg +/-	0	.	.	.	.	.	.	.

Solution for Random Effects

Effect	Animal	Estimate	Std Err Pred	DF	t Value	Pr > |t|	Alpha	Lower	Upper
Animal	1	0	.	.	.	.	.	.	.
Animal	2	0	.	.	.	.	.	.	.
Animal	3	0	.	.	.	.	.	.	.
Animal	4	0	.	.	.	.	.	.	.
Animal	5	0	.	.	.	.	.	.	.
Animal	6	0	.	.	.	.	.	.	.

Type 3 Tests of Fixed Effects

Effect	Num DF	Den DF	F Value	Pr > F
Genotype	1	29	0.00	0.9611

Least Squares Means

Effect	Genotype	Estimate	Standard Error	DF	t Value	Pr > |t|	Alpha	Lower	Upper
Genotype	Meg +/+	3.2222	0.6482	29	4.97	<.0001	0.05	1.8965	4.5480
Genotype	Meg +/-	3.1765	0.6670	29	4.76	<.0001	0.05	1.8123	4.5406

DistSoma=108

Differences of Least Squares Means												
**Effect**	**Genotype**	**Genotype**	**Estimate**	**Standard Error**	**DF**	**t Value**	**Pr > |t|**	**Adjustment**	**Adj P**	**Alpha**	**Lower**	**Upper**
**Genotype**	Meg +/+	Meg +/-	0.04575	0.9301	29	0.05	0.9611	Tukey-Kramer	0.9611	0.05	-1.8565	1.9480

Differences of Least Squares Means				
**Effect**	**Genotype**	**Genotype**	**Adj Lower**	**Adj Upper**
**Genotype**	Meg +/+	Meg +/-	-1.8565	1.9480

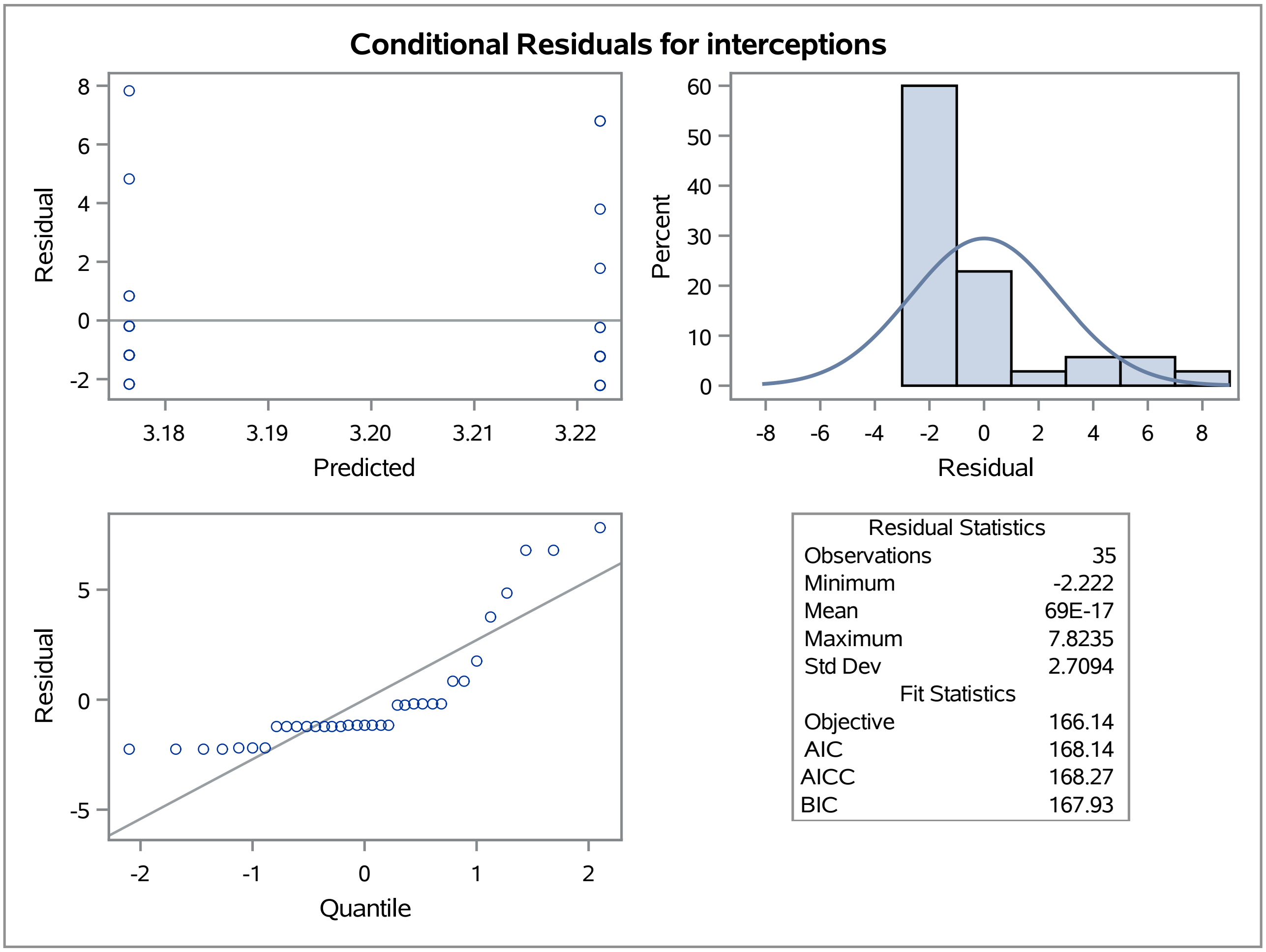

DistSoma=114

Model Information	
Data Set	WORK.TEMPDATASORTED
Dependent Variable	interceptions
Covariance Structure	Variance Components
Estimation Method	REML
Residual Variance Method	Profile
Fixed Effects SE Method	Model-Based
Degrees of Freedom Method	Containment

Class Level Information		
Class	Levels	Values
Genotype	2	Meg +/+ Meg +/-
Animal	6	1 2 3 4 5 6

Dimensions	
Covariance Parameters	2
Columns in X	3
Columns in Z	6
Subjects	1
Max Obs per Subject	35

Number of Observations	
Number of Observations Read	35
Number of Observations Used	35
Number of Observations Not Used	0

Iteration History			
Iteration	Evaluations	-2 Res Log Like	Criterion
0	1	158.87689945	
1	1	158.87689945	0.00000000

Convergence criteria met.

Estimated G matrix is not positive definite.

Covariance Parameter Estimates				
Cov Parm	Estimate	Alpha	Lower	Upper
Animal	0	.	.	.
Residual	6.0686	0.05	3.9480	10.5144

DistSoma=114

Fit Statistics	
-2 Res Log Likelihood	158.9
AIC (Smaller is Better)	160.9
AICC (Smaller is Better)	161.0
BIC (Smaller is Better)	160.7

Solution for Fixed Effects

Effect	Genotype	Estimate	Standard Error	DF	t Value	Pr > |t|	Alpha	Lower	Upper
Intercept		3.1176	0.5975	4	5.22	0.0064	0.05	1.4588	4.7765
Genotype	Meg +/+	-0.2843	0.8331	29	-0.34	0.7354	0.05	-1.9883	1.4197
Genotype	Meg +/-	0	.	.	.	.	.	.	.

Solution for Random Effects

Effect	Animal	Estimate	Std Err Pred	DF	t Value	Pr > |t|	Alpha	Lower	Upper
Animal	1	0	.	.	.	.	.	.	.
Animal	2	0	.	.	.	.	.	.	.
Animal	3	0	.	.	.	.	.	.	.
Animal	4	0	.	.	.	.	.	.	.
Animal	5	0	.	.	.	.	.	.	.
Animal	6	0	.	.	.	.	.	.	.

Type 3 Tests of Fixed Effects

Effect	Num DF	Den DF	F Value	Pr > F
Genotype	1	29	0.12	0.7354

Least Squares Means

Effect	Genotype	Estimate	Standard Error	DF	t Value	Pr > |t|	Alpha	Lower	Upper
Genotype	Meg +/+	2.8333	0.5806	29	4.88	<.0001	0.05	1.6458	4.0209
Genotype	Meg +/-	3.1176	0.5975	29	5.22	<.0001	0.05	1.8957	4.3396

DistSoma=114

Differences of Least Squares Means

Effect	Genotype	Genotype	Estimate	Standard Error	DF	t Value	Pr > |t|	Adjustment	Adj P	Alpha	Lower	Upper
**Genotype**	Meg +/+	Meg +/-	-0.2843	0.8331	29	-0.34	0.7354	Tukey-Kramer	0.7354	0.05	-1.9883	1.4197

Differences of Least Squares Means

Effect	Genotype	Genotype	Adj Lower	Adj Upper
**Genotype**	Meg +/+	Meg +/-	-1.9883	1.4196

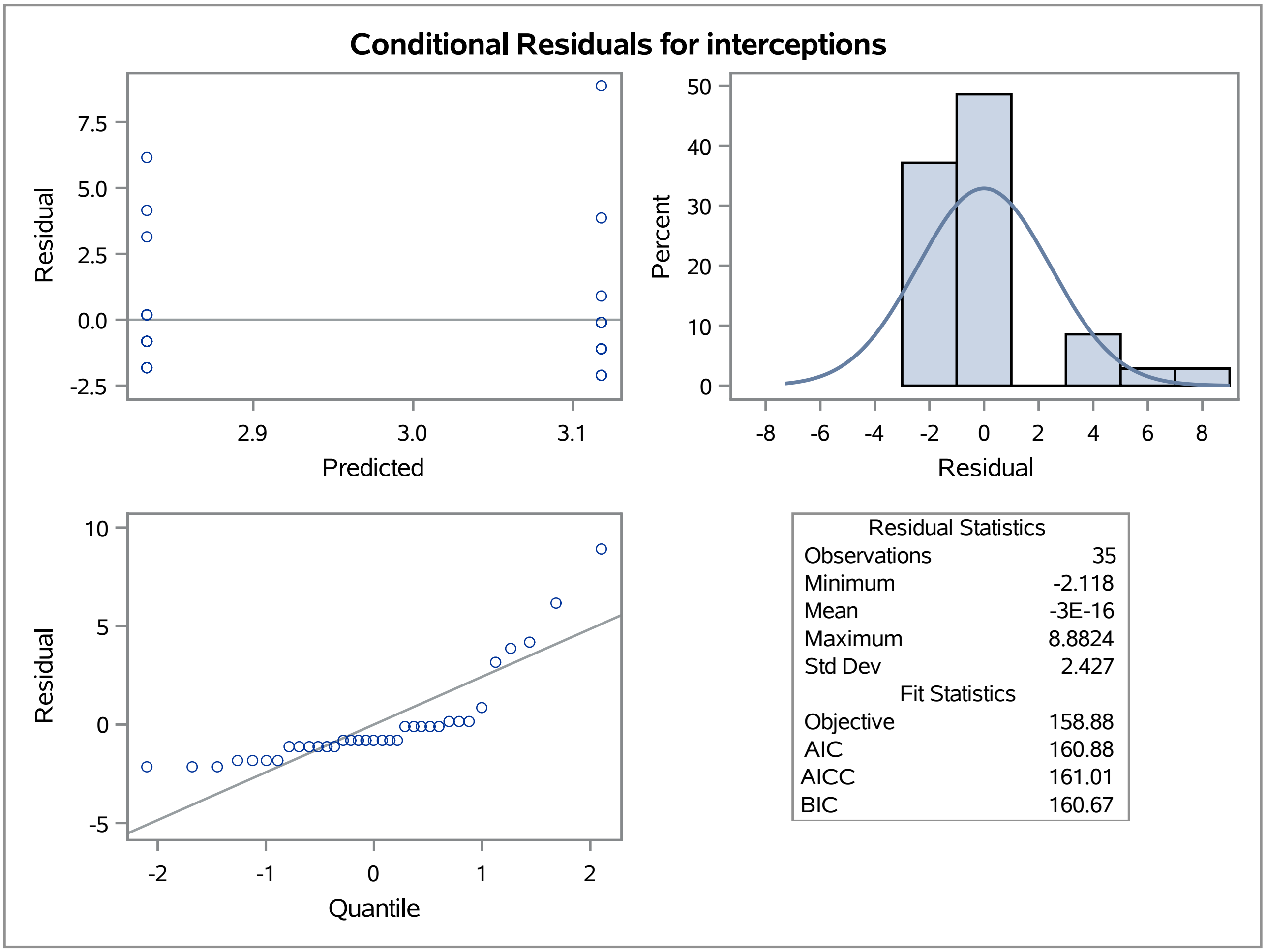

DistSoma=120

Model Information	
**Data Set**	WORK.TEMPDATASORTED
**Dependent Variable**	interceptions
**Covariance Structure**	Variance Components
**Estimation Method**	REML
**Residual Variance Method**	Profile
**Fixed Effects SE Method**	Model-Based
**Degrees of Freedom Method**	Containment

Class Level Information		
**Class**	**Levels**	**Values**
**Genotype**	2	Meg +/+ Meg +/-
**Animal**	6	1 2 3 4 5 6

Dimensions	
**Covariance Parameters**	2
**Columns in X**	3
**Columns in Z**	6
**Subjects**	1
**Max Obs per Subject**	35

Number of Observations	
**Number of Observations Read**	35
**Number of Observations Used**	35
**Number of Observations Not Used**	0

Iteration History			
**Iteration**	**Evaluations**	**-2 Res Log Like**	**Criterion**
**0**	1	154.66434567	
**1**	1	154.66434567	0.00000000

Convergence criteria met.

Estimated G matrix is not positive definite.

Covariance Parameter Estimates				
**Cov Parm**	**Estimate**	**Alpha**	**Lower**	**Upper**
**Animal**	0	.	.	.
**Residual**	5.3414	0.05	3.4749	9.2544

DistSoma=120

Fit Statistics	
**-2 Res Log Likelihood**	154.7
**AIC (Smaller is Better)**	156.7
**AICC (Smaller is Better)**	156.8
**BIC (Smaller is Better)**	156.5

Solution for Fixed Effects									
**Effect**	**Genotype**	**Estimate**	**Standard Error**	**DF**	**t Value**	**Pr > |t|**	**Alpha**	**Lower**	**Upper**
**Intercept**		3.1176	0.5605	4	5.56	0.0051	0.05	1.5614	4.6739
**Genotype**	Meg +/+	-0.2843	0.7816	29	-0.36	0.7187	0.05	-1.8829	1.3143
**Genotype**	Meg +/-	0	.	.	.	.	.	.	.

Solution for Random Effects									
**Effect**	**Animal**	**Estimate**	**Std Err Pred**	**DF**	**t Value**	**Pr > |t|**	**Alpha**	**Lower**	**Upper**
**Animal**	1	0	.	.	.	.	.	.	.
**Animal**	2	0	.	.	.	.	.	.	.
**Animal**	3	0	.	.	.	.	.	.	.
**Animal**	4	0	.	.	.	.	.	.	.
**Animal**	5	0	.	.	.	.	.	.	.
**Animal**	6	0	.	.	.	.	.	.	.

Type 3 Tests of Fixed Effects				
**Effect**	**Num DF**	**Den DF**	**F Value**	**Pr > F**
**Genotype**	1	29	0.13	0.7187

Least Squares Means									
**Effect**	**Genotype**	**Estimate**	**Standard Error**	**DF**	**t Value**	**Pr > |t|**	**Alpha**	**Lower**	**Upper**
**Genotype**	Meg +/+	2.8333	0.5447	29	5.20	<.0001	0.05	1.7192	3.9475
**Genotype**	Meg +/-	3.1176	0.5605	29	5.56	<.0001	0.05	1.9712	4.2641

DistSoma=120

Differences of Least Squares Means												
Effect	Genotype	Genotype	Estimate	Standard Error	DF	t Value	Pr > |t|	Adjustment	Adj P	Alpha	Lower	Upper
**Genotype**	Meg +/+	Meg +/-	-0.2843	0.7816	29	-0.36	0.7187	Tukey-Kramer	0.7187	0.05	-1.8829	1.3143

Differences of Least Squares Means				
Effect	Genotype	Genotype	Adj Lower	Adj Upper
**Genotype**	Meg +/+	Meg +/-	-1.8829	1.3143

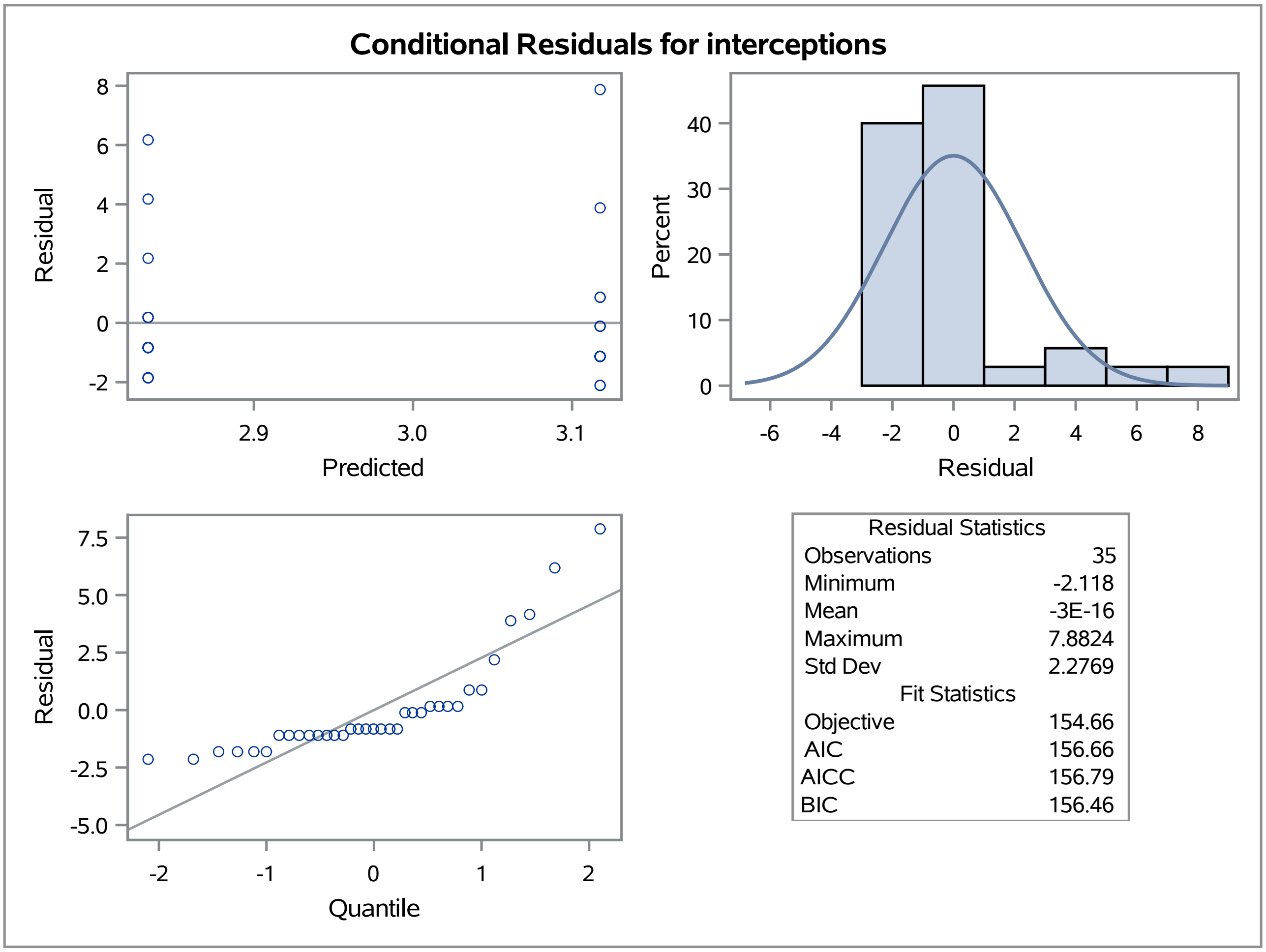

DistSoma=126

Model Information	
**Data Set**	WORK.TEMPDATASORTED
**Dependent Variable**	interceptions
**Covariance Structure**	Variance Components
**Estimation Method**	REML
**Residual Variance Method**	Profile
**Fixed Effects SE Method**	Model-Based
**Degrees of Freedom Method**	Containment

Class Level Information		
**Class**	**Levels**	**Values**
**Genotype**	2	Meg +/+ Meg +/-
**Animal**	6	1 2 3 4 5 6

Dimensions	
**Covariance Parameters**	2
**Columns in X**	3
**Columns in Z**	6
**Subjects**	1
**Max Obs per Subject**	35

Number of Observations	
**Number of Observations Read**	35
**Number of Observations Used**	35
**Number of Observations Not Used**	0

Iteration History			
**Iteration**	**Evaluations**	**-2 Res Log Like**	**Criterion**
**0**	1	150.36819040	
**1**	1	150.36819040	0.00000000

Convergence criteria met.

Estimated G matrix is not positive definite.

Covariance Parameter Estimates				
**Cov Parm**	**Estimate**	**Alpha**	**Lower**	**Upper**
**Animal**	0	.	.	.
**Residual**	4.6893	0.05	3.0507	8.1247

DistSoma=126

Fit Statistics	
-2 Res Log Likelihood	150.4
AIC (Smaller is Better)	152.4
AICC (Smaller is Better)	152.5
BIC (Smaller is Better)	152.2

Solution for Fixed Effects

Effect	Genotype	Estimate	Standard Error	DF	t Value	Pr > |t|	Alpha	Lower	Upper
Intercept		2.8235	0.5252	4	5.38	0.0058	0.05	1.3653	4.2817
Genotype	Meg +/+	-0.2124	0.7324	29	-0.29	0.7738	0.05	-1.7103	1.2854
Genotype	Meg +/-	0	.	.	.	.	.	.	.

Solution for Random Effects

Effect	Animal	Estimate	Std Err Pred	DF	t Value	Pr > |t|	Alpha	Lower	Upper
Animal	1	0	.	.	.	.	.	.	.
Animal	2	0	.	.	.	.	.	.	.
Animal	3	0	.	.	.	.	.	.	.
Animal	4	0	.	.	.	.	.	.	.
Animal	5	0	.	.	.	.	.	.	.
Animal	6	0	.	.	.	.	.	.	.

Type 3 Tests of Fixed Effects

Effect	Num DF	Den DF	F Value	Pr > F
Genotype	1	29	0.08	0.7738

Least Squares Means

Effect	Genotype	Estimate	Standard Error	DF	t Value	Pr > |t|	Alpha	Lower	Upper
Genotype	Meg +/+	2.6111	0.5104	29	5.12	<.0001	0.05	1.5672	3.6550
Genotype	Meg +/-	2.8235	0.5252	29	5.38	<.0001	0.05	1.7494	3.8977

DistSoma=126

Differences of Least Squares Means												
Effect	Genotype	Genotype	Estimate	Standard Error	DF	t Value	Pr > |t|	Adjustment	Adj P	Alpha	Lower	Upper
**Genotype**	Meg +/+	Meg +/-	-0.2124	0.7324	29	-0.29	0.7738	Tukey-Kramer	0.7738	0.05	-1.7103	1.2854

Differences of Least Squares Means				
Effect	Genotype	Genotype	Adj Lower	Adj Upper
**Genotype**	Meg +/+	Meg +/-	-1.7103	1.2854

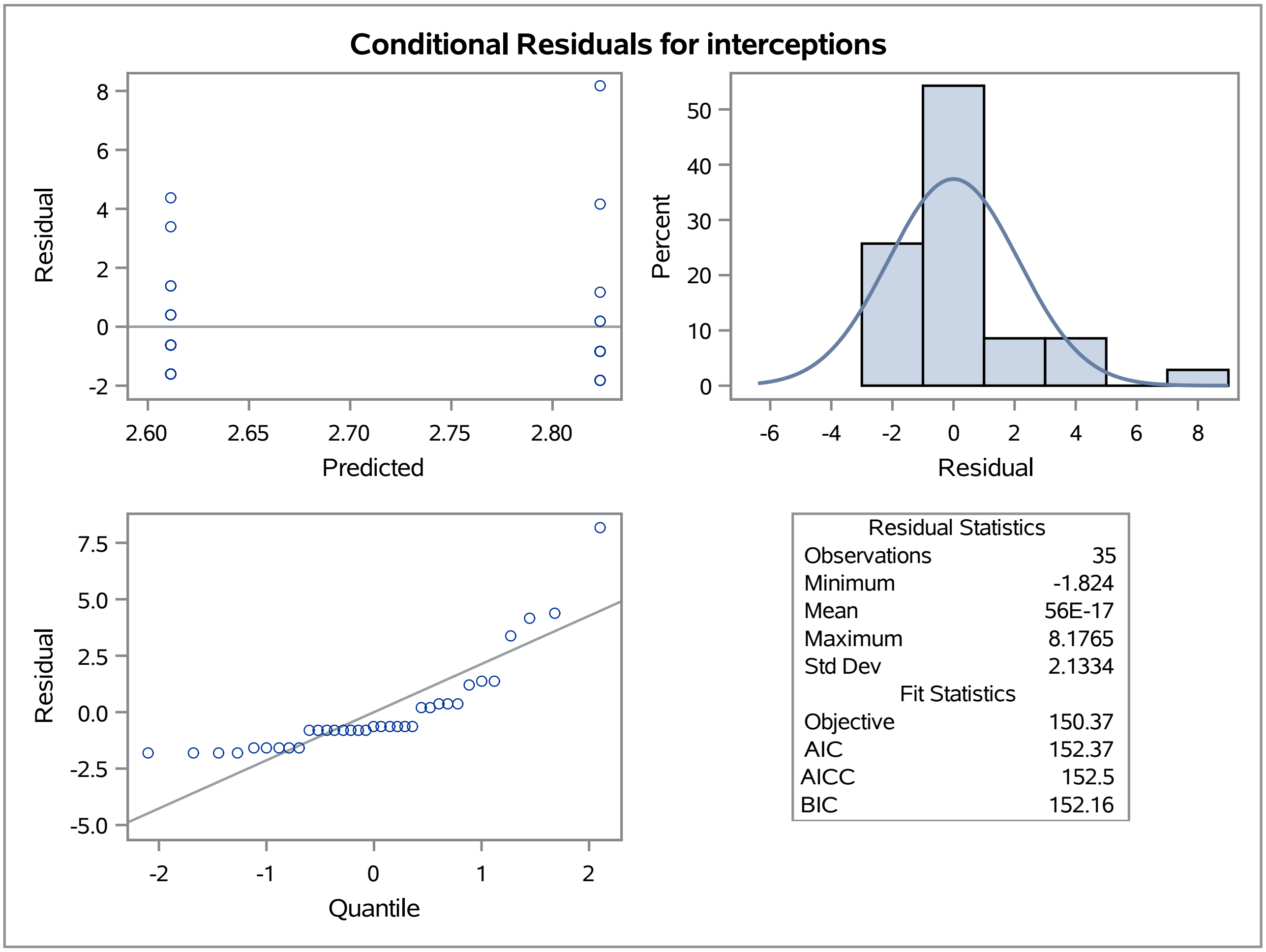

DistSoma=132

Model Information	
**Data Set**	WORK.TEMPDATASORTED
**Dependent Variable**	interceptions
**Covariance Structure**	Variance Components
**Estimation Method**	REML
**Residual Variance Method**	Profile
**Fixed Effects SE Method**	Model-Based
**Degrees of Freedom Method**	Containment

Class Level Information		
**Class**	**Levels**	**Values**
**Genotype**	2	Meg +/+ Meg +/-
**Animal**	6	1 2 3 4 5 6

Dimensions	
**Covariance Parameters**	2
**Columns in X**	3
**Columns in Z**	6
**Subjects**	1
**Max Obs per Subject**	35

Number of Observations	
**Number of Observations Read**	35
**Number of Observations Used**	35
**Number of Observations Not Used**	0

Iteration History			
**Iteration**	**Evaluations**	**-2 Res Log Like**	**Criterion**
**0**	1	142.02001287	
**1**	1	142.02001287	0.00000000

Convergence criteria met.

Estimated G matrix is not positive definite.

Covariance Parameter Estimates				
**Cov Parm**	**Estimate**	**Alpha**	**Lower**	**Upper**
**Animal**	0	.	.	.
**Residual**	3.6412	0.05	2.3689	6.3087

DistSoma=132

Fit Statistics	
**-2 Res Log Likelihood**	142.0
**AIC (Smaller is Better)**	144.0
**AICC (Smaller is Better)**	144.1
**BIC (Smaller is Better)**	143.8

Solution for Fixed Effects

Effect	Genotype	Estimate	Standard Error	DF	t Value	Pr > |t|	Alpha	Lower	Upper
**Intercept**		2.6471	0.4628	4	5.72	0.0046	0.05	1.3621	3.9320
**Genotype**	Meg +/+	-0.2582	0.6454	29	-0.40	0.6921	0.05	-1.5781	1.0617
**Genotype**	Meg +/-	0	.	.	.	.	.	.	.

Solution for Random Effects

Effect	Animal	Estimate	Std Err Pred	DF	t Value	Pr > |t|	Alpha	Lower	Upper
**Animal**	1	0	.	.	.	.	.	.	.
**Animal**	2	0	.	.	.	.	.	.	.
**Animal**	3	0	.	.	.	.	.	.	.
**Animal**	4	0	.	.	.	.	.	.	.
**Animal**	5	0	.	.	.	.	.	.	.
**Animal**	6	0	.	.	.	.	.	.	.

Type 3 Tests of Fixed Effects

Effect	Num DF	Den DF	F Value	Pr > F
**Genotype**	1	29	0.16	0.6921

Least Squares Means

Effect	Genotype	Estimate	Standard Error	DF	t Value	Pr > |t|	Alpha	Lower	Upper
**Genotype**	Meg +/+	2.3889	0.4498	29	5.31	<.0001	0.05	1.4690	3.3088
**Genotype**	Meg +/-	2.6471	0.4628	29	5.72	<.0001	0.05	1.7005	3.5936

DistSoma=132

Differences of Least Squares Means												
Effect	Genotype	Genotype	Estimate	Standard Error	DF	t Value	Pr > |t|	Adjustment	Adj P	Alpha	Lower	Upper
Genotype	Meg +/+	Meg +/-	-0.2582	0.6454	29	-0.40	0.6921	Tukey-Kramer	0.6921	0.05	-1.5781	1.0617

Differences of Least Squares Means				
Effect	Genotype	Genotype	Adj Lower	Adj Upper
Genotype	Meg +/+	Meg +/-	-1.5781	1.0617

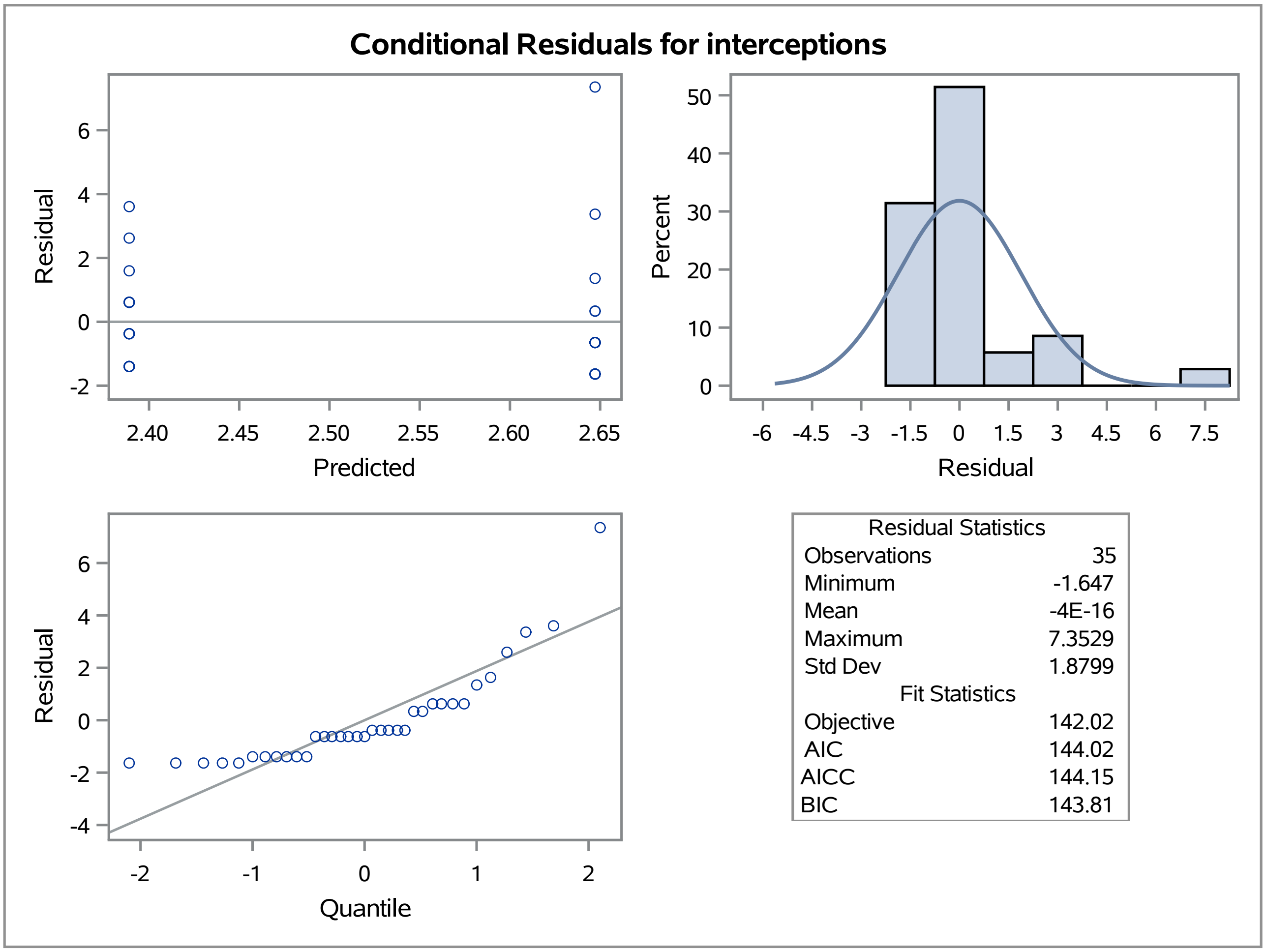

DistSoma=138

Model Information	
**Data Set**	WORK.TEMPDATASORTED
**Dependent Variable**	interceptions
**Covariance Structure**	Variance Components
**Estimation Method**	REML
**Residual Variance Method**	Profile
**Fixed Effects SE Method**	Model-Based
**Degrees of Freedom Method**	Containment

Class Level Information		
**Class**	**Levels**	**Values**
**Genotype**	2	Meg +/+ Meg +/-
**Animal**	6	1 2 3 4 5 6

Dimensions	
**Covariance Parameters**	2
**Columns in X**	3
**Columns in Z**	6
**Subjects**	1
**Max Obs per Subject**	35

Number of Observations	
**Number of Observations Read**	35
**Number of Observations Used**	35
**Number of Observations Not Used**	0

Iteration History			
**Iteration**	**Evaluations**	**-2 Res Log Like**	**Criterion**
**0**	1	135.73974395	
**1**	1	135.73974395	0.00000000

Convergence criteria met.

Estimated G matrix is not positive definite.

Covariance Parameter Estimates				
**Cov Parm**	**Estimate**	**Alpha**	**Lower**	**Upper**
**Animal**	0	.	.	.
**Residual**	3.0102	0.05	1.9583	5.2154

DistSoma=138

Fit Statistics	
-2 Res Log Likelihood	135.7
AIC (Smaller is Better)	137.7
AICC (Smaller is Better)	137.9
BIC (Smaller is Better)	137.5

Solution for Fixed Effects

Effect	Genotype	Estimate	Standard Error	DF	t Value	Pr > |t|	Alpha	Lower	Upper
Intercept		2.7647	0.4208	4	6.57	0.0028	0.05	1.5964	3.9330
Genotype	Meg +/+	-0.1536	0.5868	29	-0.26	0.7954	0.05	-1.3537	1.0465
Genotype	Meg +/-	0	.	.	.	.	.	.	.

Solution for Random Effects

Effect	Animal	Estimate	Std Err Pred	DF	t Value	Pr > |t|	Alpha	Lower	Upper
Animal	1	0	.	.	.	.	.	.	.
Animal	2	0	.	.	.	.	.	.	.
Animal	3	0	.	.	.	.	.	.	.
Animal	4	0	.	.	.	.	.	.	.
Animal	5	0	.	.	.	.	.	.	.
Animal	6	0	.	.	.	.	.	.	.

Type 3 Tests of Fixed Effects

Effect	Num DF	Den DF	F Value	Pr > F
Genotype	1	29	0.07	0.7954

Least Squares Means

Effect	Genotype	Estimate	Standard Error	DF	t Value	Pr > |t|	Alpha	Lower	Upper
Genotype	Meg +/+	2.6111	0.4089	29	6.39	<.0001	0.05	1.7747	3.4475
Genotype	Meg +/-	2.7647	0.4208	29	6.57	<.0001	0.05	1.9041	3.6253

DistSoma=138

Differences of Least Squares Means												
**Effect**	**Genotype**	**Genotype**	**Estimate**	**Standard Error**	**DF**	**t Value**	**Pr > |t|**	**Adjustment**	**Adj P**	**Alpha**	**Lower**	**Upper**
**Genotype**	Meg +/+	Meg +/-	-0.1536	0.5868	29	-0.26	0.7954	Tukey-Kramer	0.7954	0.05	-1.3537	1.0465

Differences of Least Squares Means				
**Effect**	**Genotype**	**Genotype**	**Adj Lower**	**Adj Upper**
**Genotype**	Meg +/+	Meg +/-	-1.3537	1.0465

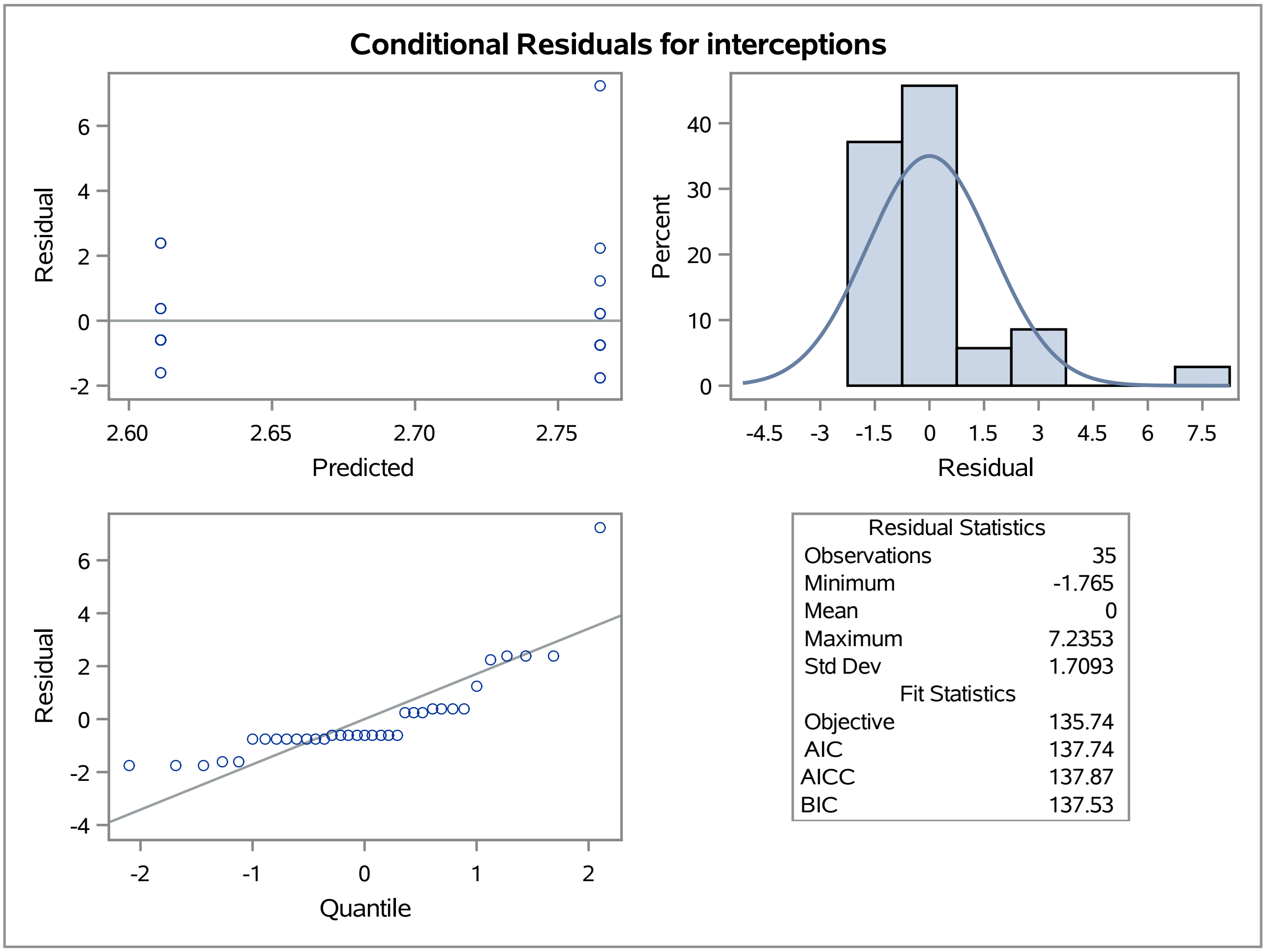

DistSoma=144

Model Information	
**Data Set**	WORK.TEMPDATASORTED
**Dependent Variable**	interceptions
**Covariance Structure**	Variance Components
**Estimation Method**	REML
**Residual Variance Method**	Profile
**Fixed Effects SE Method**	Model-Based
**Degrees of Freedom Method**	Containment

Class Level Information		
**Class**	**Levels**	**Values**
**Genotype**	2	Meg +/+ Meg +/-
**Animal**	6	1 2 3 4 5 6

Dimensions	
**Covariance Parameters**	2
**Columns in X**	3
**Columns in Z**	6
**Subjects**	1
**Max Obs per Subject**	35

Number of Observations	
**Number of Observations Read**	35
**Number of Observations Used**	35
**Number of Observations Not Used**	0

Iteration History			
**Iteration**	**Evaluations**	**-2 Res Log Like**	**Criterion**
**0**	1	128.07527720	
**1**	1	128.07527720	0.00000000

Convergence criteria met.

Estimated G matrix is not positive definite.

Covariance Parameter Estimates				
**Cov Parm**	**Estimate**	**Alpha**	**Lower**	**Upper**
**Animal**	0	.	.	.
**Residual**	2.3863	0.05	1.5525	4.1345

DistSoma=144

Fit Statistics	
-2 Res Log Likelihood	128.1
AIC (Smaller is Better)	130.1
AICC (Smaller is Better)	130.2
BIC (Smaller is Better)	129.9

Solution for Fixed Effects

Effect	Genotype	Estimate	Standard Error	DF	t Value	Pr > |t|	Alpha	Lower	Upper
Intercept		2.8235	0.3747	4	7.54	0.0017	0.05	1.7833	3.8638
Genotype	Meg +/+	-0.4346	0.5224	29	-0.83	0.4122	0.05	-1.5032	0.6339
Genotype	Meg +/-	0	.	.	.	.	.	.	.

Solution for Random Effects

Effect	Animal	Estimate	Std Err Pred	DF	t Value	Pr > |t|	Alpha	Lower	Upper
Animal	1	0	.	.	.	.	.	.	.
Animal	2	0	.	.	.	.	.	.	.
Animal	3	0	.	.	.	.	.	.	.
Animal	4	0	.	.	.	.	.	.	.
Animal	5	0	.	.	.	.	.	.	.
Animal	6	0	.	.	.	.	.	.	.

Type 3 Tests of Fixed Effects

Effect	Num DF	Den DF	F Value	Pr > F
Genotype	1	29	0.69	0.4122

Least Squares Means

Effect	Genotype	Estimate	Standard Error	DF	t Value	Pr > |t|	Alpha	Lower	Upper
Genotype	Meg +/+	2.3889	0.3641	29	6.56	<.0001	0.05	1.6442	3.1336
Genotype	Meg +/-	2.8235	0.3747	29	7.54	<.0001	0.05	2.0573	3.5898

DistSoma=144

Differences of Least Squares Means												
**Effect**	**Genotype**	**Genotype**	**Estimate**	**Standard Error**	**DF**	**t Value**	**Pr > |t|**	**Adjustment**	**Adj P**	**Alpha**	**Lower**	**Upper**
**Genotype**	Meg +/+	Meg +/-	-0.4346	0.5224	29	-0.83	0.4122	Tukey-Kramer	0.4122	0.05	-1.5032	0.6339

Differences of Least Squares Means				
**Effect**	**Genotype**	**Genotype**	**Adj Lower**	**Adj Upper**
**Genotype**	Meg +/+	Meg +/-	-1.5031	0.6339

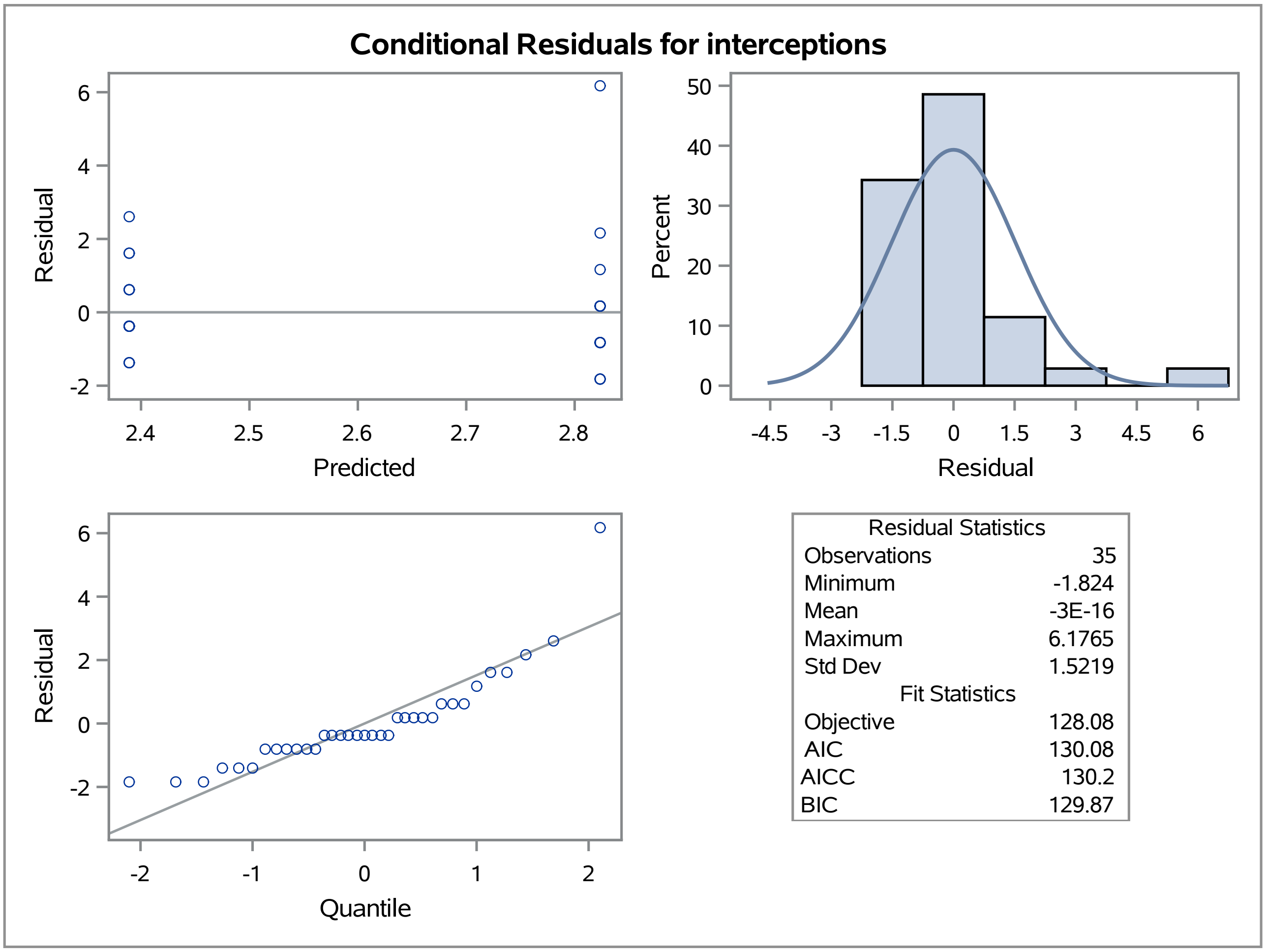

DistSoma=150

Model Information	
**Data Set**	WORK.TEMPDATASORTED
**Dependent Variable**	interceptions
**Covariance Structure**	Variance Components
**Estimation Method**	REML
**Residual Variance Method**	Profile
**Fixed Effects SE Method**	Model-Based
**Degrees of Freedom Method**	Containment

Class Level Information		
**Class**	**Levels**	**Values**
**Genotype**	2	Meg +/+ Meg +/-
**Animal**	6	1 2 3 4 5 6

Dimensions	
**Covariance Parameters**	2
**Columns in X**	3
**Columns in Z**	6
**Subjects**	1
**Max Obs per Subject**	35

Number of Observations	
**Number of Observations Read**	35
**Number of Observations Used**	35
**Number of Observations Not Used**	0

Iteration History			
**Iteration**	**Evaluations**	**-2 Res Log Like**	**Criterion**
**0**	1	132.49807155	
**1**	1	132.49807155	0.00000000

Convergence criteria met.

Estimated G matrix is not positive definite.

Covariance Parameter Estimates				
**Cov Parm**	**Estimate**	**Alpha**	**Lower**	**Upper**
**Animal**	0	.	.	.
**Residual**	2.7286	0.05	1.7751	4.7275

DistSoma=150

Fit Statistics	
-2 Res Log Likelihood	132.5
AIC (Smaller is Better)	134.5
AICC (Smaller is Better)	134.6
BIC (Smaller is Better)	134.3

Solution for Fixed Effects

Effect	Genotype	Estimate	Standard Error	DF	t Value	Pr > |t|	Alpha	Lower	Upper
Intercept		2.8824	0.4006	4	7.19	0.0020	0.05	1.7700	3.9947
Genotype	Meg +/+	-0.4935	0.5587	29	-0.88	0.3843	0.05	-1.6360	0.6491
Genotype	Meg +/-	0	.	.	.	.	.	.	.

Solution for Random Effects

Effect	Animal	Estimate	Std Err Pred	DF	t Value	Pr > |t|	Alpha	Lower	Upper
Animal	1	0	.	.	.	.	.	.	.
Animal	2	0	.	.	.	.	.	.	.
Animal	3	0	.	.	.	.	.	.	.
Animal	4	0	.	.	.	.	.	.	.
Animal	5	0	.	.	.	.	.	.	.
Animal	6	0	.	.	.	.	.	.	.

Type 3 Tests of Fixed Effects

Effect	Num DF	Den DF	F Value	Pr > F
Genotype	1	29	0.78	0.3843

Least Squares Means

Effect	Genotype	Estimate	Standard Error	DF	t Value	Pr > |t|	Alpha	Lower	Upper
Genotype	Meg +/+	2.3889	0.3893	29	6.14	<.0001	0.05	1.5926	3.1852
Genotype	Meg +/-	2.8824	0.4006	29	7.19	<.0001	0.05	2.0630	3.7017

DistSoma=150

Differences of Least Squares Means												
**Effect**	**Genotype**	**Genotype**	**Estimate**	**Standard Error**	**DF**	**t Value**	**Pr > |t|**	**Adjustment**	**Adj P**	**Alpha**	**Lower**	**Upper**
**Genotype**	Meg +/+	Meg +/-	-0.4935	0.5587	29	-0.88	0.3843	Tukey-Kramer	0.3843	0.05	-1.6360	0.6491

Differences of Least Squares Means				
**Effect**	**Genotype**	**Genotype**	**Adj Lower**	**Adj Upper**
**Genotype**	Meg +/+	Meg +/-	-1.6360	0.6491

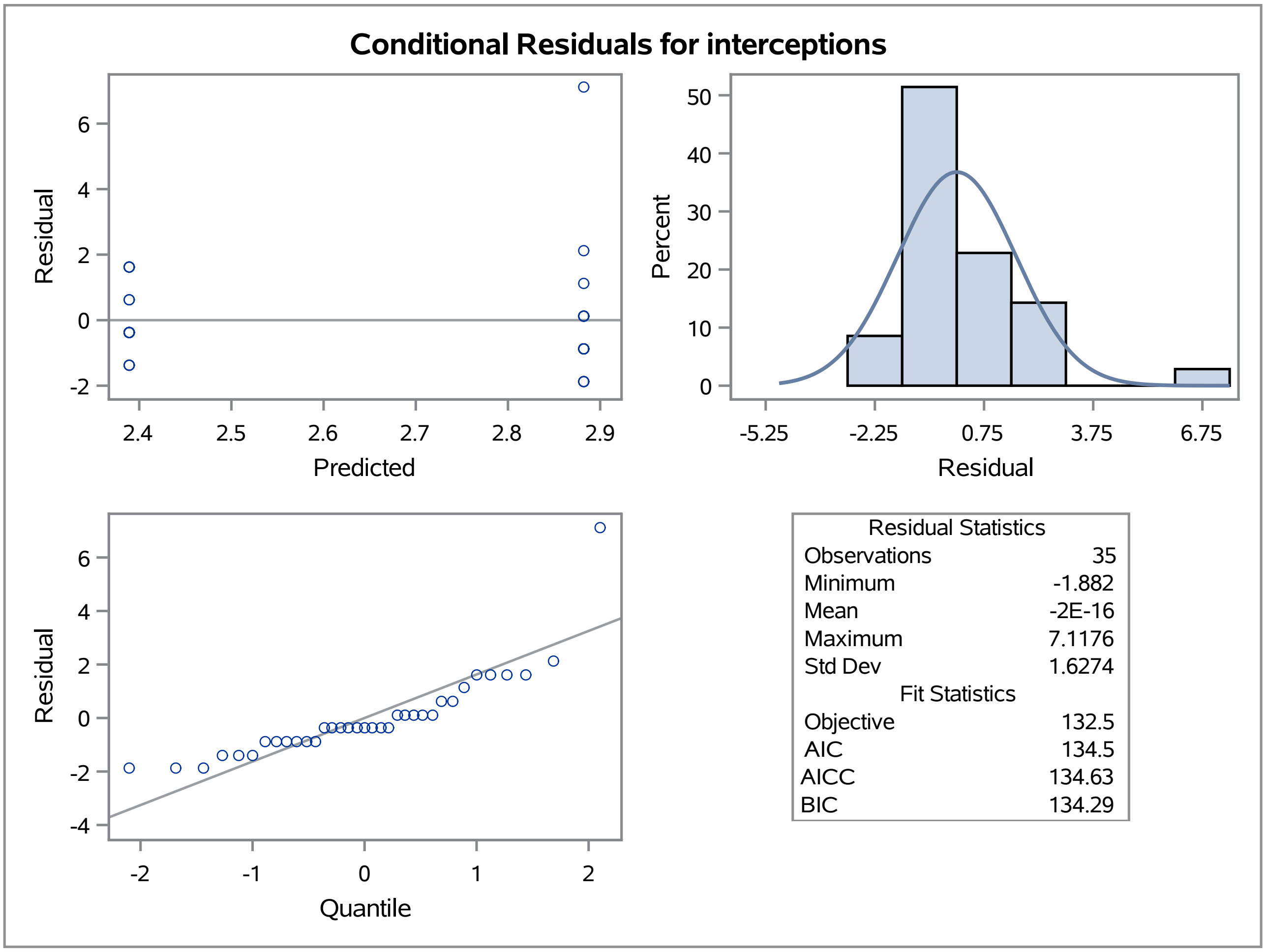

DistSoma=156

Model Information	
**Data Set**	WORK.TEMPDATASORTED
**Dependent Variable**	interceptions
**Covariance Structure**	Variance Components
**Estimation Method**	REML
**Residual Variance Method**	Profile
**Fixed Effects SE Method**	Model-Based
**Degrees of Freedom Method**	Containment

Class Level Information		
**Class**	**Levels**	**Values**
**Genotype**	2	Meg +/+ Meg +/-
**Animal**	6	1 2 3 4 5 6

Dimensions	
**Covariance Parameters**	2
**Columns in X**	3
**Columns in Z**	6
**Subjects**	1
**Max Obs per Subject**	35

Number of Observations	
**Number of Observations Read**	35
**Number of Observations Used**	35
**Number of Observations Not Used**	0

Iteration History			
**Iteration**	**Evaluations**	**-2 Res Log Like**	**Criterion**
**0**	1	126.11606502	
**1**	1	126.11606502	0.00000000

Convergence criteria met.

Estimated G matrix is not positive definite.

Covariance Parameter Estimates				
**Cov Parm**	**Estimate**	**Alpha**	**Lower**	**Upper**
**Animal**	0	.	.	.
**Residual**	2.2488	0.05	1.4630	3.8962

DistSoma=156

Fit Statistics	
-2 Res Log Likelihood	126.1
AIC (Smaller is Better)	128.1
AICC (Smaller is Better)	128.2
BIC (Smaller is Better)	127.9

Solution for Fixed Effects

Effect	Genotype	Estimate	Standard Error	DF	t Value	Pr > |t|	Alpha	Lower	Upper
Intercept		2.8824	0.3637	4	7.93	0.0014	0.05	1.8726	3.8922
Genotype	Meg +/+	-0.4379	0.5072	29	-0.86	0.3950	0.05	-1.4752	0.5994
Genotype	Meg +/-	0	.	.	.	.	.	.	.

Solution for Random Effects

Effect	Animal	Estimate	Std Err Pred	DF	t Value	Pr > |t|	Alpha	Lower	Upper
Animal	1	0	.	.	.	.	.	.	.
Animal	2	0	.	.	.	.	.	.	.
Animal	3	0	.	.	.	.	.	.	.
Animal	4	0	.	.	.	.	.	.	.
Animal	5	0	.	.	.	.	.	.	.
Animal	6	0	.	.	.	.	.	.	.

Type 3 Tests of Fixed Effects

Effect	Num DF	Den DF	F Value	Pr > F
Genotype	1	29	0.75	0.3950

Least Squares Means

Effect	Genotype	Estimate	Standard Error	DF	t Value	Pr > |t|	Alpha	Lower	Upper
Genotype	Meg +/+	2.4444	0.3535	29	6.92	<.0001	0.05	1.7215	3.1673
Genotype	Meg +/-	2.8824	0.3637	29	7.93	<.0001	0.05	2.1385	3.6262

DistSoma=156

Differences of Least Squares Means												
**Effect**	**Genotype**	**Genotype**	**Estimate**	**Standard Error**	**DF**	**t Value**	**Pr > |t|**	**Adjustment**	**Adj P**	**Alpha**	**Lower**	**Upper**
**Genotype**	Meg +/+	Meg +/-	-0.4379	0.5072	29	-0.86	0.3950	Tukey-Kramer	0.3950	0.05	-1.4752	0.5994

Differences of Least Squares Means				
**Effect**	**Genotype**	**Genotype**	**Adj Lower**	**Adj Upper**
**Genotype**	Meg +/+	Meg +/-	-1.4752	0.5993

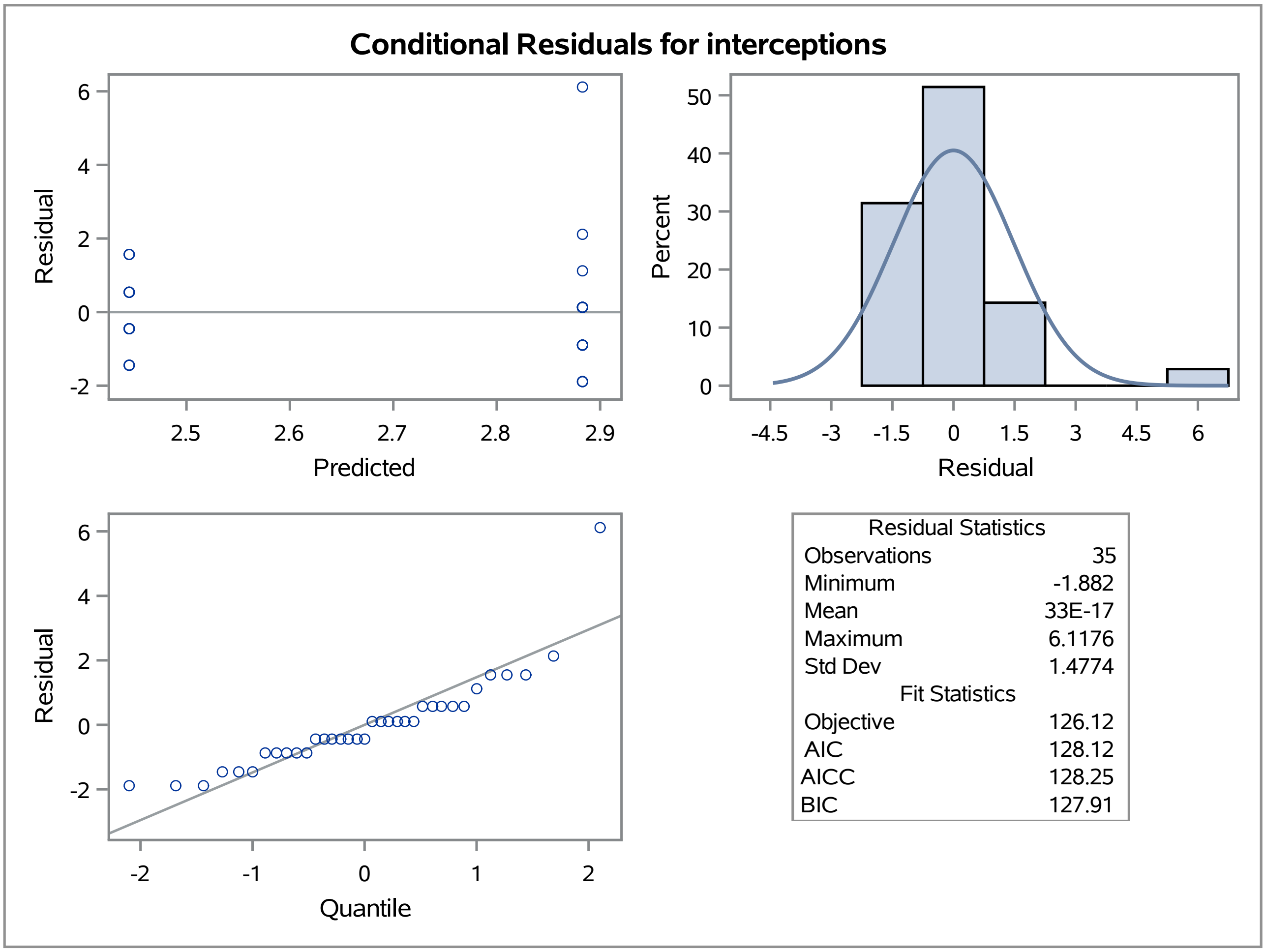

DistSoma=162

Model Information	
**Data Set**	WORK.TEMPDATASORTED
**Dependent Variable**	interceptions
**Covariance Structure**	Variance Components
**Estimation Method**	REML
**Residual Variance Method**	Profile
**Fixed Effects SE Method**	Model-Based
**Degrees of Freedom Method**	Containment

Class Level Information		
**Class**	**Levels**	**Values**
**Genotype**	2	Meg +/+ Meg +/-
**Animal**	6	1 2 3 4 5 6

Dimensions	
**Covariance Parameters**	2
**Columns in X**	3
**Columns in Z**	6
**Subjects**	1
**Max Obs per Subject**	35

Number of Observations	
**Number of Observations Read**	35
**Number of Observations Used**	35
**Number of Observations Not Used**	0

Iteration History			
**Iteration**	**Evaluations**	**-2 Res Log Like**	**Criterion**
**0**	1	109.28593530	
**1**	1	109.28593530	0.00000000

Convergence criteria met.

Estimated G matrix is not positive definite.

Covariance Parameter Estimates				
**Cov Parm**	**Estimate**	**Alpha**	**Lower**	**Upper**
**Animal**	0	.	.	.
**Residual**	1.3504	0.05	0.8785	2.3396

DistSoma=162

Fit Statistics	
-2 Res Log Likelihood	109.3
AIC (Smaller is Better)	111.3
AICC (Smaller is Better)	111.4
BIC (Smaller is Better)	111.1

Solution for Fixed Effects

Effect	Genotype	Estimate	Standard Error	DF	t Value	Pr > |t|	Alpha	Lower	Upper
Intercept		2.5882	0.2818	4	9.18	0.0008	0.05	1.8057	3.3707
Genotype	Meg +/+	-0.1438	0.3930	29	-0.37	0.7171	0.05	-0.9476	0.6600
Genotype	Meg +/-	0	.	.	.	.	.	.	.

Solution for Random Effects

Effect	Animal	Estimate	Std Err Pred	DF	t Value	Pr > |t|	Alpha	Lower	Upper
Animal	1	0	.	.	.	.	.	.	.
Animal	2	0	.	.	.	.	.	.	.
Animal	3	0	.	.	.	.	.	.	.
Animal	4	0	.	.	.	.	.	.	.
Animal	5	0	.	.	.	.	.	.	.
Animal	6	0	.	.	.	.	.	.	.

Type 3 Tests of Fixed Effects

Effect	Num DF	Den DF	F Value	Pr > F
Genotype	1	29	0.13	0.7171

Least Squares Means

Effect	Genotype	Estimate	Standard Error	DF	t Value	Pr > |t|	Alpha	Lower	Upper
Genotype	Meg +/+	2.4444	0.2739	29	8.92	<.0001	0.05	1.8843	3.0046
Genotype	Meg +/-	2.5882	0.2818	29	9.18	<.0001	0.05	2.0118	3.1647

DistSoma=162

Differences of Least Squares Means												
**Effect**	**Genotype**	**Genotype**	**Estimate**	**Standard Error**	**DF**	**t Value**	**Pr > |t|**	**Adjustment**	**Adj P**	**Alpha**	**Lower**	**Upper**
**Genotype**	Meg +/+	Meg +/-	-0.1438	0.3930	29	-0.37	0.7171	Tukey-Kramer	0.7171	0.05	-0.9476	0.6600

Differences of Least Squares Means				
**Effect**	**Genotype**	**Genotype**	**Adj Lower**	**Adj Upper**
**Genotype**	Meg +/+	Meg +/-	-0.9476	0.6600

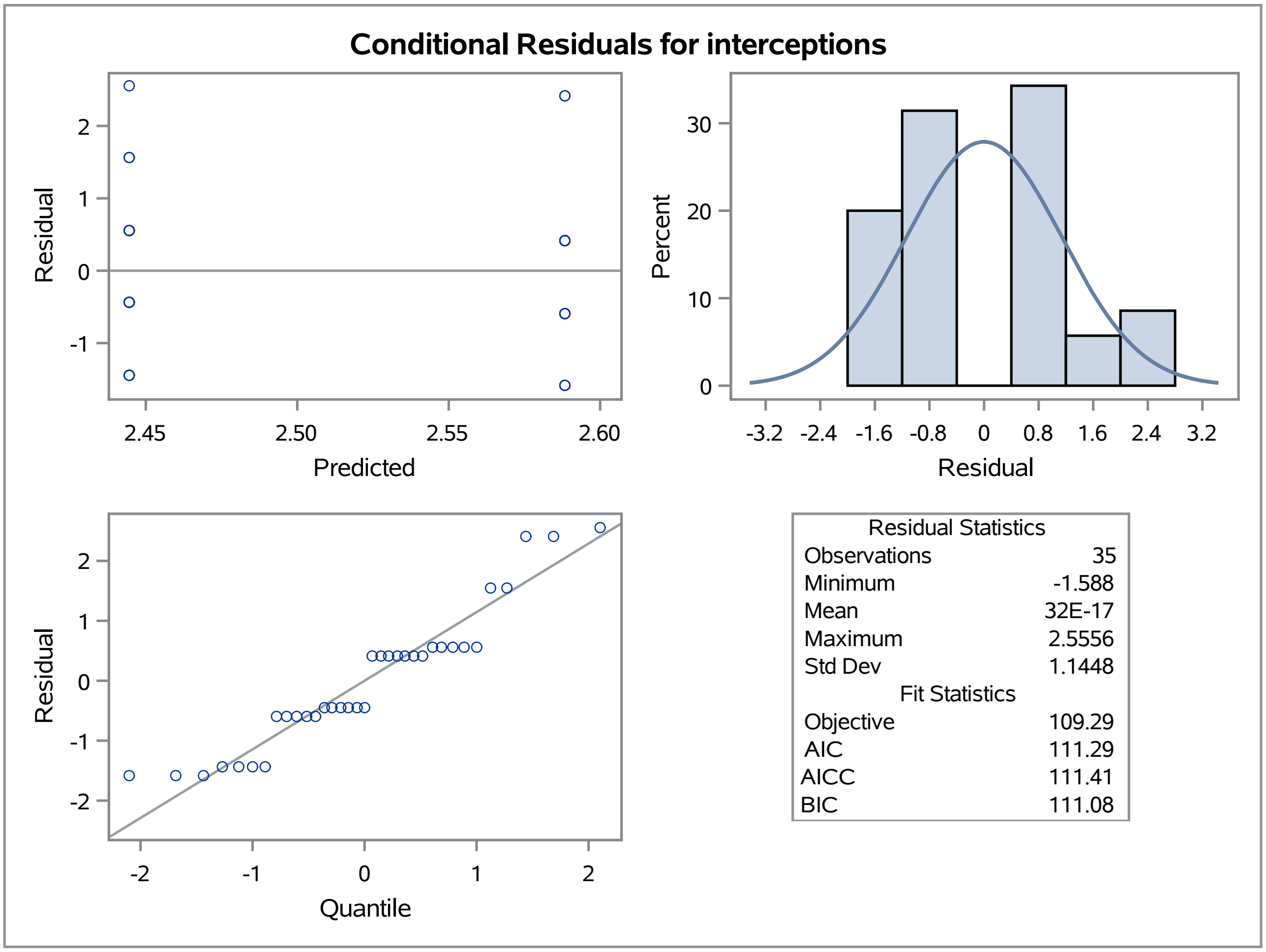

DistSoma=168

Model Information	
**Data Set**	WORK.TEMPDATASORTED
**Dependent Variable**	interceptions
**Covariance Structure**	Variance Components
**Estimation Method**	REML
**Residual Variance Method**	Profile
**Fixed Effects SE Method**	Model-Based
**Degrees of Freedom Method**	Containment

Class Level Information		
**Class**	**Levels**	**Values**
**Genotype**	2	Meg +/+ Meg +/-
**Animal**	6	1 2 3 4 5 6

Dimensions	
**Covariance Parameters**	2
**Columns in X**	3
**Columns in Z**	6
**Subjects**	1
**Max Obs per Subject**	35

Number of Observations	
**Number of Observations Read**	35
**Number of Observations Used**	35
**Number of Observations Not Used**	0

Iteration History			
**Iteration**	**Evaluations**	**-2 Res Log Like**	**Criterion**
**0**	1	106.14239337	
**1**	2	106.08743850	0.00000735
**2**	1	106.08726881	0.00000000

Convergence criteria met.

Covariance Parameter Estimates				
**Cov Parm**	**Estimate**	**Alpha**	**Lower**	**Upper**
**Animal**	0.05270	0.05	0.004602	5.298E35
**Residual**	1.1965	0.05	0.7524	2.1944

DistSoma=168

Fit Statistics	
-2 Res Log Likelihood	106.1
AIC (Smaller is Better)	110.1
AICC (Smaller is Better)	110.5
BIC (Smaller is Better)	109.7

Solution for Fixed Effects

Effect	Genotype	Estimate	Standard Error	DF	t Value	Pr > |t|	Alpha	Lower	Upper
Intercept		2.5665	0.3022	4	8.49	0.0011	0.05	1.7275	3.4054
Genotype	Meg +/+	0.02187	0.4240	29	0.05	0.9592	0.05	-0.8453	0.8890
Genotype	Meg +/-	0	.	.	.	.	.	.	.

Solution for Random Effects

Effect	Animal	Estimate	Std Err Pred	DF	t Value	Pr > |t|	Alpha	Lower	Upper
Animal	1	0.09529	0.2118	29	0.45	0.6561	0.05	-0.3379	0.5284
Animal	2	-0.08814	0.2134	29	-0.41	0.6827	0.05	-0.5246	0.3483
Animal	3	-0.00715	0.2214	29	-0.03	0.9745	0.05	-0.4599	0.4456
Animal	4	0.1160	0.2214	29	0.52	0.6042	0.05	-0.3368	0.5689
Animal	5	-0.06616	0.2130	29	-0.31	0.7583	0.05	-0.5017	0.3694
Animal	6	-0.04989	0.2125	29	-0.23	0.8161	0.05	-0.4845	0.3848

Type 3 Tests of Fixed Effects

Effect	Num DF	Den DF	F Value	Pr > F
Genotype	1	29	0.00	0.9592

Least Squares Means

Effect	Genotype	Estimate	Standard Error	DF	t Value	Pr > |t|	Alpha	Lower	Upper
Genotype	Meg +/+	2.5883	0.2974	29	8.70	<.0001	0.05	1.9801	3.1966
Genotype	Meg +/-	2.5665	0.3022	29	8.49	<.0001	0.05	1.9485	3.1845

DistSoma=168

Differences of Least Squares Means												
**Effect**	**Genotype**	**Genotype**	**Estimate**	**Standard Error**	**DF**	**t Value**	**Pr > |t|**	**Adjustment**	**Adj P**	**Alpha**	**Lower**	**Upper**
**Genotype**	Meg +/+	Meg +/-	0.02187	0.4240	29	0.05	0.9592	Tukey-Kramer	0.9592	0.05	-0.8453	0.8890

Differences of Least Squares Means				
**Effect**	**Genotype**	**Genotype**	**Adj Lower**	**Adj Upper**
**Genotype**	Meg +/+	Meg +/-	-0.8453	0.8890

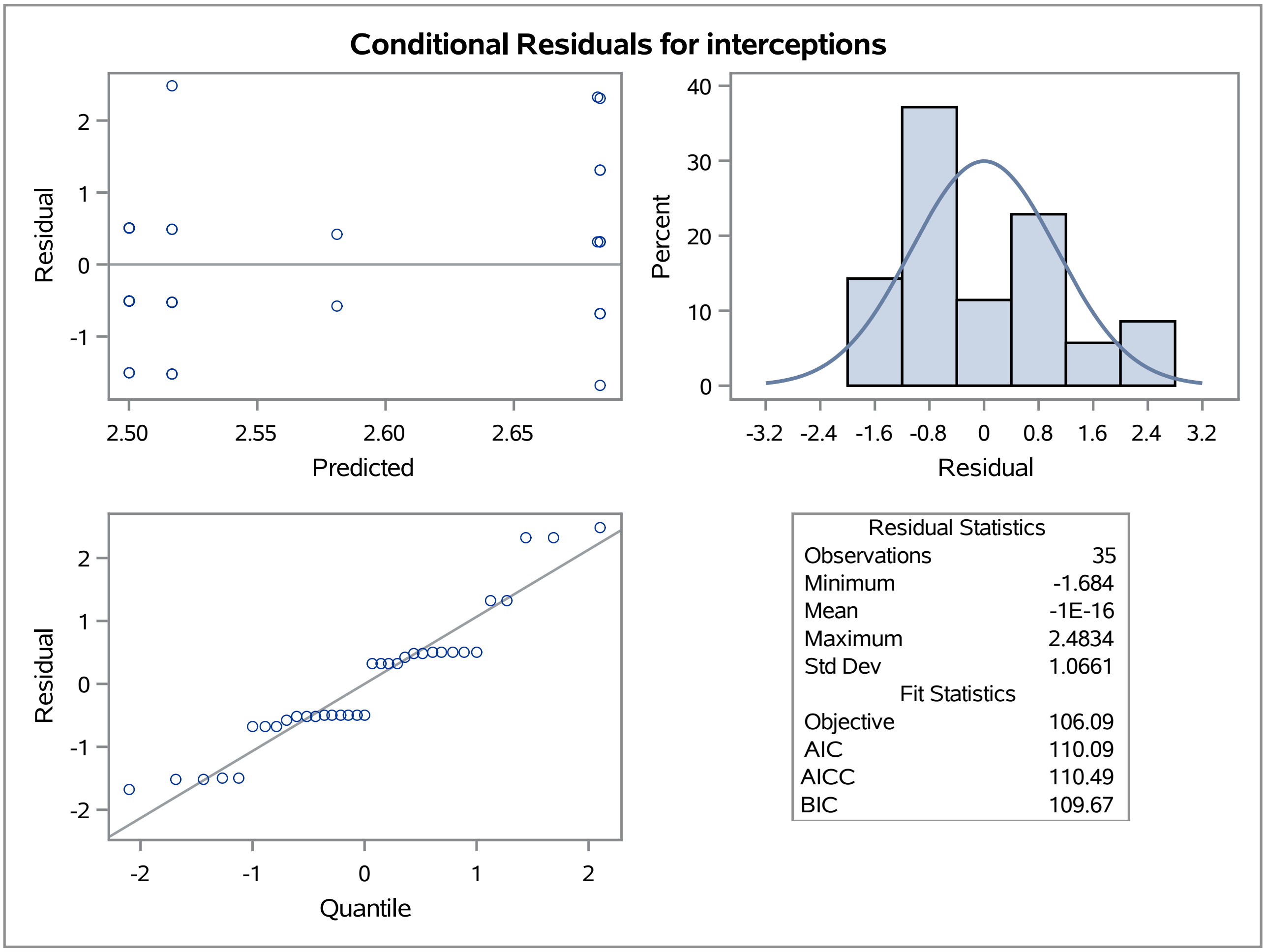

DistSoma=174

Model Information	
**Data Set**	WORK.TEMPDATASORTED
**Dependent Variable**	interceptions
**Covariance Structure**	Variance Components
**Estimation Method**	REML
**Residual Variance Method**	Profile
**Fixed Effects SE Method**	Model-Based
**Degrees of Freedom Method**	Containment

Class Level Information		
**Class**	**Levels**	**Values**
**Genotype**	2	Meg +/+ Meg +/-
**Animal**	6	1 2 3 4 5 6

Dimensions	
**Covariance Parameters**	2
**Columns in X**	3
**Columns in Z**	6
**Subjects**	1
**Max Obs per Subject**	35

Number of Observations	
**Number of Observations Read**	35
**Number of Observations Used**	35
**Number of Observations Not Used**	0

Iteration History			
**Iteration**	**Evaluations**	**-2 Res Log Like**	**Criterion**
**0**	1	96.96765978	
**1**	1	96.96765978	0.00000000

Convergence criteria met.

Estimated G matrix is not positive definite.

Covariance Parameter Estimates				
**Cov Parm**	**Estimate**	**Alpha**	**Lower**	**Upper**
**Animal**	0	.	.	.
**Residual**	0.9297	0.05	0.6048	1.6108

DistSoma=174

Fit Statistics	
-2 Res Log Likelihood	97.0
AIC (Smaller is Better)	99.0
AICC (Smaller is Better)	99.1
BIC (Smaller is Better)	98.8

Solution for Fixed Effects

Effect	Genotype	Estimate	Standard Error	DF	t Value	Pr > |t|	Alpha	Lower	Upper
Intercept		2.4706	0.2339	4	10.56	0.0005	0.05	1.8213	3.1199
Genotype	Meg +/+	0.08497	0.3261	29	0.26	0.7963	0.05	-0.5820	0.7519
Genotype	Meg +/-	0	.	.	.	.	.	.	.

Solution for Random Effects

Effect	Animal	Estimate	Std Err Pred	DF	t Value	Pr > |t|	Alpha	Lower	Upper
Animal	1	0	.	.	.	.	.	.	.
Animal	2	0	.	.	.	.	.	.	.
Animal	3	0	.	.	.	.	.	.	.
Animal	4	0	.	.	.	.	.	.	.
Animal	5	0	.	.	.	.	.	.	.
Animal	6	0	.	.	.	.	.	.	.

Type 3 Tests of Fixed Effects

Effect	Num DF	Den DF	F Value	Pr > F
Genotype	1	29	0.07	0.7963

Least Squares Means

Effect	Genotype	Estimate	Standard Error	DF	t Value	Pr > |t|	Alpha	Lower	Upper
Genotype	Meg +/+	2.5556	0.2273	29	11.24	<.0001	0.05	2.0907	3.0204
Genotype	Meg +/-	2.4706	0.2339	29	10.56	<.0001	0.05	1.9923	2.9489

DistSoma=174

Differences of Least Squares Means												
**Effect**	**Genotype**	**Genotype**	**Estimate**	**Standard Error**	**DF**	**t Value**	**Pr > |t|**	**Adjustment**	**Adj P**	**Alpha**	**Lower**	**Upper**
**Genotype**	Meg +/+	Meg +/-	0.08497	0.3261	29	0.26	0.7963	Tukey-Kramer	0.7963	0.05	-0.5820	0.7519

Differences of Least Squares Means				
**Effect**	**Genotype**	**Genotype**	**Adj Lower**	**Adj Upper**
**Genotype**	Meg +/+	Meg +/-	-0.5820	0.7519

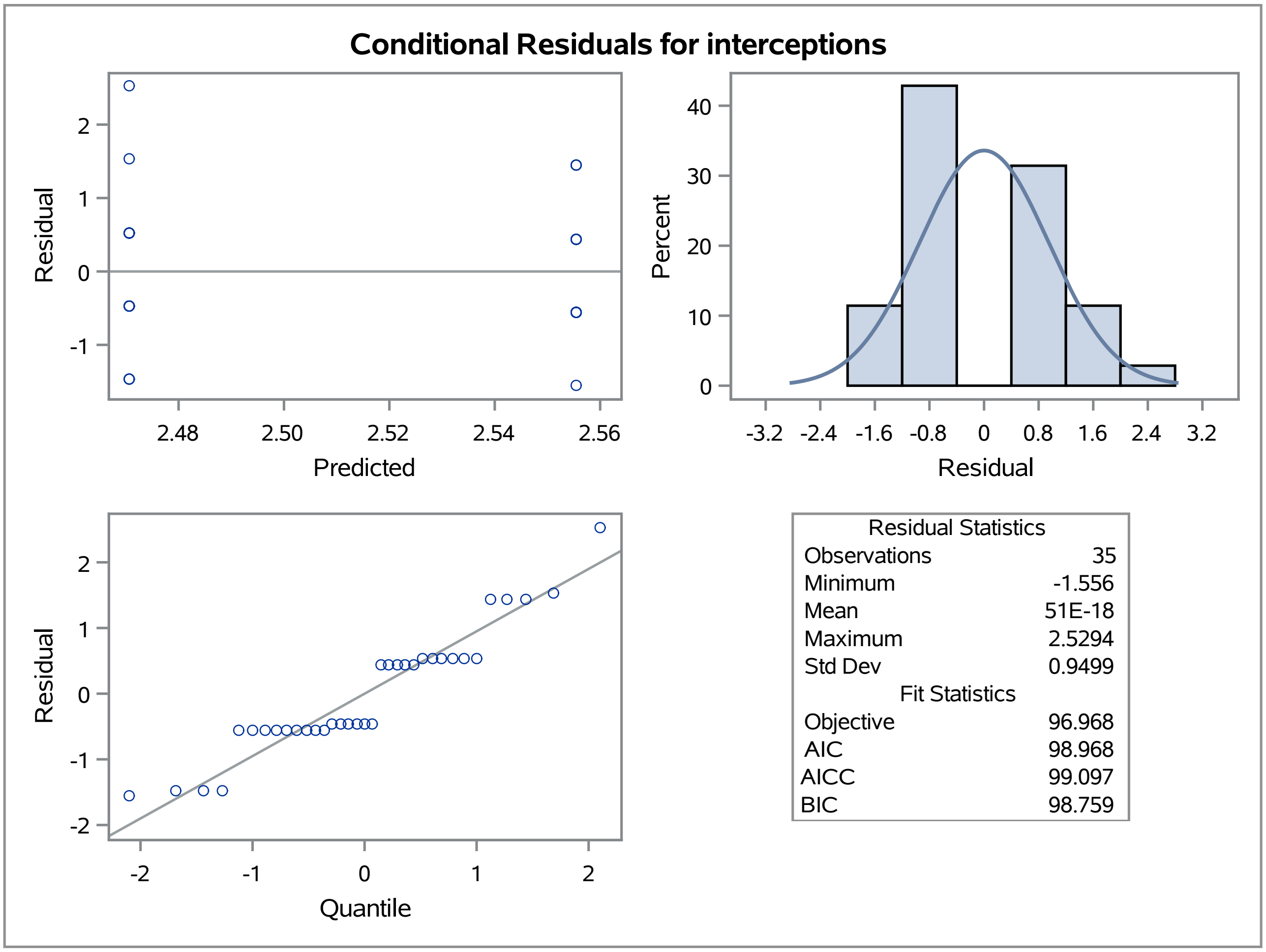

DistSoma=180

Model Information	
**Data Set**	WORK.TEMPDATASORTED
**Dependent Variable**	interceptions
**Covariance Structure**	Variance Components
**Estimation Method**	REML
**Residual Variance Method**	Profile
**Fixed Effects SE Method**	Model-Based
**Degrees of Freedom Method**	Containment

Class Level Information		
**Class**	**Levels**	**Values**
**Genotype**	2	Meg +/+ Meg +/-
**Animal**	6	1 2 3 4 5 6

Dimensions	
**Covariance Parameters**	2
**Columns in X**	3
**Columns in Z**	6
**Subjects**	1
**Max Obs per Subject**	35

Number of Observations	
**Number of Observations Read**	35
**Number of Observations Used**	35
**Number of Observations Not Used**	0

Iteration History			
**Iteration**	**Evaluations**	**-2 Res Log Like**	**Criterion**
**0**	1	91.65494897	
**1**	2	91.18089039	0.00014386
**2**	1	91.17858693	0.00000087
**3**	1	91.17857356	0.00000000

Convergence criteria met.

DistSoma=180

Covariance Parameter Estimates				
Cov Parm	Estimate	Alpha	Lower	Upper
Animal	0.1069	0.05	0.01545	131595
Residual	0.7312	0.05	0.4609	1.3353

Fit Statistics	
-2 Res Log Likelihood	91.2
AIC (Smaller is Better)	95.2
AICC (Smaller is Better)	95.6
BIC (Smaller is Better)	94.8

Solution for Fixed Effects									
Effect	Genotype	Estimate	Standard Error	DF	t Value	Pr > |t|	Alpha	Lower	Upper
Intercept		2.4867	0.2900	4	8.57	0.0010	0.05	1.6815	3.2919
Genotype	Meg +/+	0.09553	0.4089	29	0.23	0.8169	0.05	-0.7407	0.9317
Genotype	Meg +/-	0	.	.	.	.	.	.	.

Solution for Random Effects									
Effect	Animal	Estimate	Std Err Pred	DF	t Value	Pr > |t|	Alpha	Lower	Upper
Animal	1	0.2481	0.2697	29	0.92	0.3651	0.05	-0.3034	0.7996
Animal	2	-0.1163	0.2740	29	-0.42	0.6743	0.05	-0.6768	0.4441
Animal	3	-0.1318	0.2949	29	-0.45	0.6584	0.05	-0.7350	0.4714
Animal	4	0.2293	0.2950	29	0.78	0.4433	0.05	-0.3741	0.8327
Animal	5	-0.1017	0.2727	29	-0.37	0.7120	0.05	-0.6594	0.4560
Animal	6	-0.1276	0.2715	29	-0.47	0.6418	0.05	-0.6830	0.4277

Type 3 Tests of Fixed Effects				
Effect	Num DF	Den DF	F Value	Pr > F
Genotype	1	29	0.05	0.8169

Least Squares Means									
Effect	Genotype	Estimate	Standard Error	DF	t Value	Pr > |t|	Alpha	Lower	Upper
Genotype	Meg +/+	2.5822	0.2882	29	8.96	<.0001	0.05	1.9929	3.1716
Genotype	Meg +/-	2.4867	0.2900	29	8.57	<.0001	0.05	1.8936	3.0799

DistSoma=180

Differences of Least Squares Means												
Effect	Genotype	Genotype	Estimate	Standard Error	DF	t Value	Pr > |t|	Adjustment	Adj P	Alpha	Lower	Upper
Genotype	Meg +/+	Meg +/-	0.09553	0.4089	29	0.23	0.8169	Tukey-Kramer	0.8169	0.05	-0.7407	0.9317

Differences of Least Squares Means				
Effect	Genotype	Genotype	Adj Lower	Adj Upper
Genotype	Meg +/+	Meg +/-	-0.7407	0.9317

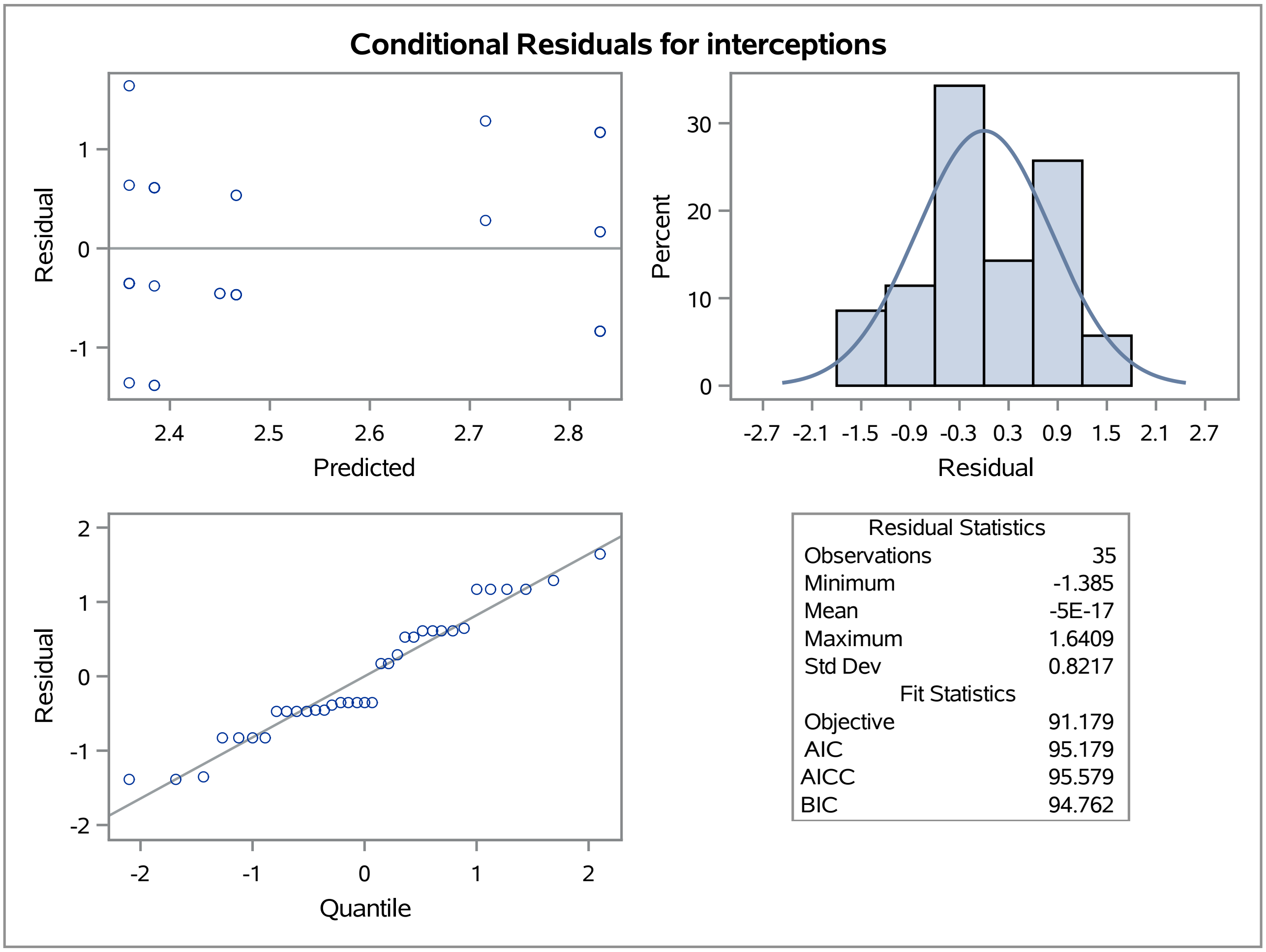

DistSoma=186

Model Information	
Data Set	WORK.TEMPDATASORTED
Dependent Variable	interceptions
Covariance Structure	Variance Components
Estimation Method	REML
Residual Variance Method	Profile
Fixed Effects SE Method	Model-Based
Degrees of Freedom Method	Containment

Class Level Information		
Class	Levels	Values
Genotype	2	Meg +/+ Meg +/-
Animal	6	1 2 3 4 5 6

Dimensions	
Covariance Parameters	2
Columns in X	3
Columns in Z	6
Subjects	1
Max Obs per Subject	35

Number of Observations	
Number of Observations Read	35
Number of Observations Used	35
Number of Observations Not Used	0

Iteration History			
Iteration	Evaluations	-2 Res Log Like	Criterion
0	1	83.76260112	
1	1	83.76260112	0.00000000

Convergence criteria met.

Estimated G matrix is not positive definite.

Covariance Parameter Estimates				
Cov Parm	Estimate	Alpha	Lower	Upper
Animal	0	.	.	.
Residual	0.6231	0.05	0.4054	1.0796

DistSoma=186

Fit Statistics	
-2 Res Log Likelihood	83.8
AIC (Smaller is Better)	85.8
AICC (Smaller is Better)	85.9
BIC (Smaller is Better)	85.6

Solution for Fixed Effects

Effect	Genotype	Estimate	Standard Error	DF	t Value	Pr > |t|	Alpha	Lower	Upper
Intercept		2.4118	0.1914	4	12.60	0.0002	0.05	1.8802	2.9433
Genotype	Meg +/+	0.03268	0.2670	29	0.12	0.9034	0.05	-0.5133	0.5787
Genotype	Meg +/-	0	.	.	.	.	.	.	.

Solution for Random Effects

Effect	Animal	Estimate	Std Err Pred	DF	t Value	Pr > |t|	Alpha	Lower	Upper
Animal	1	0	.	.	.	.	.	.	.
Animal	2	0	.	.	.	.	.	.	.
Animal	3	0	.	.	.	.	.	.	.
Animal	4	0	.	.	.	.	.	.	.
Animal	5	0	.	.	.	.	.	.	.
Animal	6	0	.	.	.	.	.	.	.

Type 3 Tests of Fixed Effects

Effect	Num DF	Den DF	F Value	Pr > F
Genotype	1	29	0.01	0.9034

Least Squares Means

Effect	Genotype	Estimate	Standard Error	DF	t Value	Pr > |t|	Alpha	Lower	Upper
Genotype	Meg +/+	2.4444	0.1861	29	13.14	<.0001	0.05	2.0639	2.8250
Genotype	Meg +/-	2.4118	0.1914	29	12.60	<.0001	0.05	2.0202	2.8033

DistSoma=186

Differences of Least Squares Means

Effect	Genotype	Genotype	Estimate	Standard Error	DF	t Value	Pr > |t|	Adjustment	Adj P	Alpha	Lower	Upper
**Genotype**	Meg +/+	Meg +/-	0.03268	0.2670	29	0.12	0.9034	Tukey-Kramer	0.9034	0.05	-0.5133	0.5787

Differences of Least Squares Means

Effect	Genotype	Genotype	Adj Lower	Adj Upper
**Genotype**	Meg +/+	Meg +/-	-0.5133	0.5787

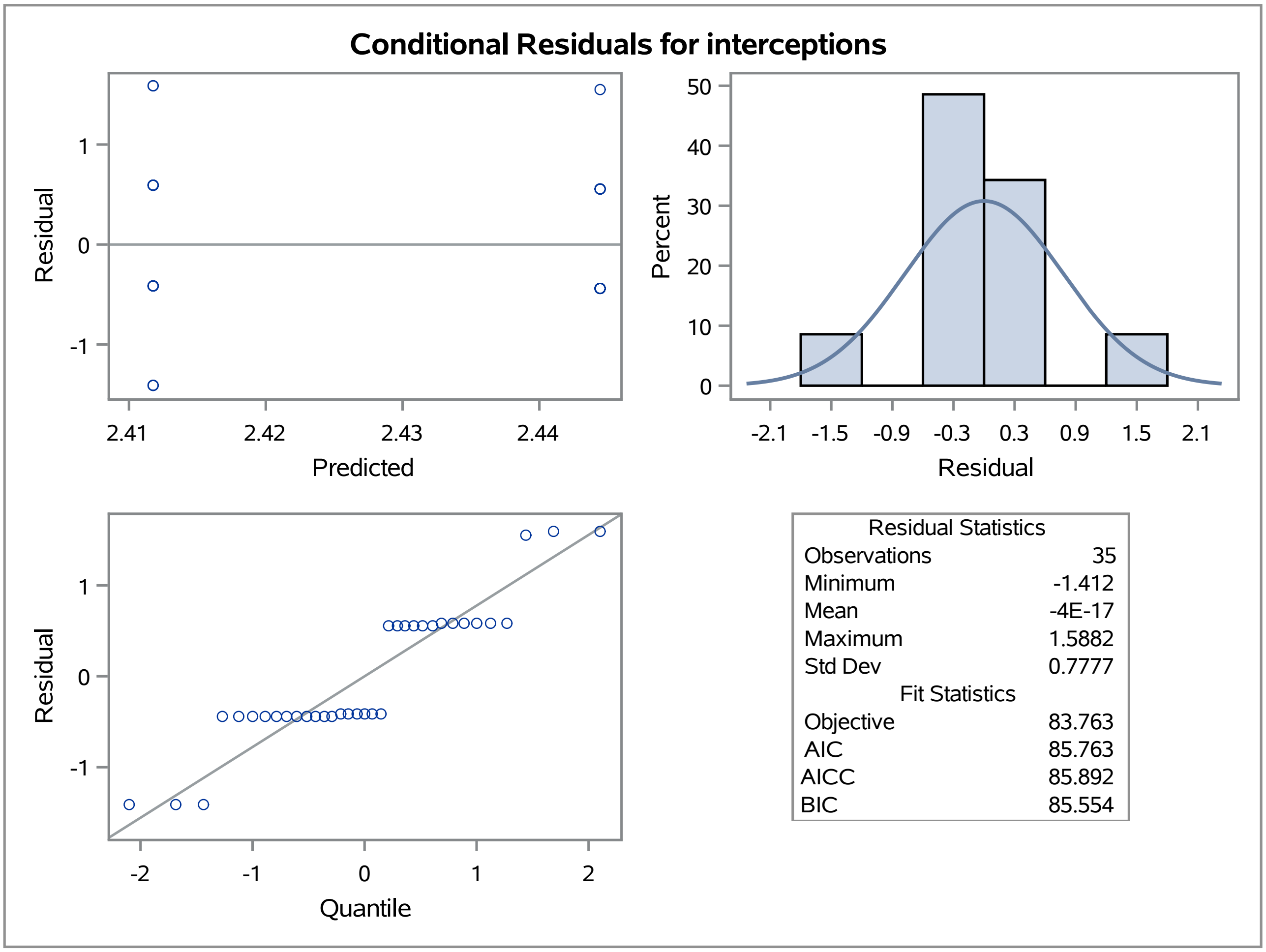

DistSoma=192

Model Information	
**Data Set**	WORK.TEMPDATASORTED
**Dependent Variable**	interceptions
**Covariance Structure**	Variance Components
**Estimation Method**	REML
**Residual Variance Method**	Profile
**Fixed Effects SE Method**	Model-Based
**Degrees of Freedom Method**	Containment

Class Level Information		
**Class**	**Levels**	**Values**
**Genotype**	2	Meg +/+ Meg +/-
**Animal**	6	1 2 3 4 5 6

Dimensions	
**Covariance Parameters**	2
**Columns in X**	3
**Columns in Z**	6
**Subjects**	1
**Max Obs per Subject**	35

Number of Observations	
**Number of Observations Read**	35
**Number of Observations Used**	35
**Number of Observations Not Used**	0

Iteration History			
**Iteration**	**Evaluations**	**-2 Res Log Like**	**Criterion**
**0**	1	83.76260112	
**1**	1	83.76260112	0.00000000

Convergence criteria met.

Estimated G matrix is not positive definite.

Covariance Parameter Estimates				
**Cov Parm**	**Estimate**	**Alpha**	**Lower**	**Upper**
**Animal**	0	.	.	.
**Residual**	0.6231	0.05	0.4054	1.0796

DistSoma=192

Fit Statistics	
-2 Res Log Likelihood	83.8
AIC (Smaller is Better)	85.8
AICC (Smaller is Better)	85.9
BIC (Smaller is Better)	85.6

Solution for Fixed Effects

Effect	Genotype	Estimate	Standard Error	DF	t Value	Pr > |t|	Alpha	Lower	Upper
Intercept		2.4118	0.1914	4	12.60	0.0002	0.05	1.8802	2.9433
Genotype	Meg +/+	0.03268	0.2670	29	0.12	0.9034	0.05	-0.5133	0.5787
Genotype	Meg +/-	0	.	.	.	.	.	.	.

Solution for Random Effects

Effect	Animal	Estimate	Std Err Pred	DF	t Value	Pr > |t|	Alpha	Lower	Upper
Animal	1	0	.	.	.	.	.	.	.
Animal	2	0	.	.	.	.	.	.	.
Animal	3	0	.	.	.	.	.	.	.
Animal	4	0	.	.	.	.	.	.	.
Animal	5	0	.	.	.	.	.	.	.
Animal	6	0	.	.	.	.	.	.	.

Type 3 Tests of Fixed Effects

Effect	Num DF	Den DF	F Value	Pr > F
Genotype	1	29	0.01	0.9034

Least Squares Means

Effect	Genotype	Estimate	Standard Error	DF	t Value	Pr > |t|	Alpha	Lower	Upper
Genotype	Meg +/+	2.4444	0.1861	29	13.14	<.0001	0.05	2.0639	2.8250
Genotype	Meg +/-	2.4118	0.1914	29	12.60	<.0001	0.05	2.0202	2.8033

DistSoma=192

Differences of Least Squares Means												
Effect	Genotype	Genotype	Estimate	Standard Error	DF	t Value	Pr > |t|	Adjustment	Adj P	Alpha	Lower	Upper
**Genotype**	Meg +/+	Meg +/-	0.03268	0.2670	29	0.12	0.9034	Tukey-Kramer	0.9034	0.05	-0.5133	0.5787

Differences of Least Squares Means				
Effect	Genotype	Genotype	Adj Lower	Adj Upper
**Genotype**	Meg +/+	Meg +/-	-0.5133	0.5787

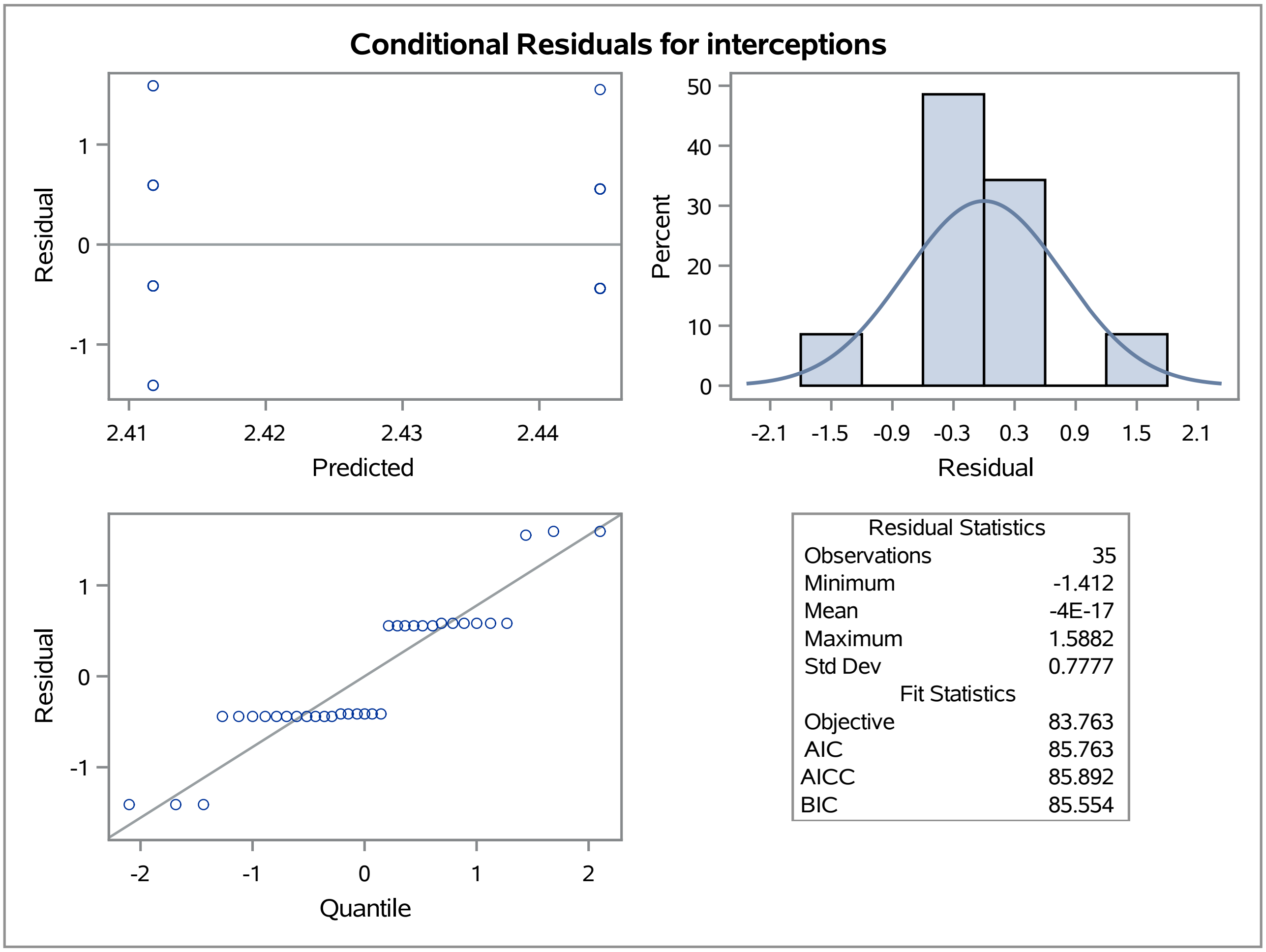

DistSoma=198

Model Information	
**Data Set**	WORK.TEMPDATASORTED
**Dependent Variable**	interceptions
**Covariance Structure**	Variance Components
**Estimation Method**	REML
**Residual Variance Method**	Profile
**Fixed Effects SE Method**	Model-Based
**Degrees of Freedom Method**	Containment

Class Level Information		
**Class**	**Levels**	**Values**
**Genotype**	2	Meg +/+ Meg +/-
**Animal**	6	1 2 3 4 5 6

Dimensions	
**Covariance Parameters**	2
**Columns in X**	3
**Columns in Z**	6
**Subjects**	1
**Max Obs per Subject**	35

Number of Observations	
**Number of Observations Read**	35
**Number of Observations Used**	35
**Number of Observations Not Used**	0

Iteration History			
**Iteration**	**Evaluations**	**-2 Res Log Like**	**Criterion**
**0**	1	89.38614657	
**1**	2	87.84213289	0.01353211
**2**	1	87.61863515	0.00259157
**3**	1	87.57918201	0.00013708
**4**	1	87.57726717	0.00000048
**5**	1	87.57726075	0.00000000

Convergence criteria met.

DistSoma=198

Covariance Parameter Estimates				
**Cov Parm**	**Estimate**	**Alpha**	**Lower**	**Upper**
**Animal**	0.3529	0.05	0.08238	51.0659
**Residual**	0.5961	0.05	0.3741	1.0969

Fit Statistics	
**-2 Res Log Likelihood**	87.6
**AIC (Smaller is Better)**	91.6
**AICC (Smaller is Better)**	92.0
**BIC (Smaller is Better)**	91.2

Solution for Fixed Effects									
**Effect**	**Genotype**	**Estimate**	**Standard Error**	**DF**	**t Value**	**Pr > |t|**	**Alpha**	**Lower**	**Upper**
**Intercept**		2.5939	0.4030	4	6.44	0.0030	0.05	1.4751	3.7127
**Genotype**	Meg +/+	-0.1995	0.5696	29	-0.35	0.7287	0.05	-1.3644	0.9654
**Genotype**	Meg +/-	0	.	.	.	.	.	.	.

Solution for Random Effects									
**Effect**	**Animal**	**Estimate**	**Std Err Pred**	**DF**	**t Value**	**Pr > |t|**	**Alpha**	**Lower**	**Upper**
**Animal**	1	0.2615	0.4118	29	0.63	0.5305	0.05	-0.5808	1.1037
**Animal**	2	-0.04764	0.4198	29	-0.11	0.9104	0.05	-0.9061	0.8109
**Animal**	3	-0.2138	0.4574	29	-0.47	0.6437	0.05	-1.1493	0.7217
**Animal**	4	0.7623	0.4575	29	1.67	0.1064	0.05	-0.1734	1.6980
**Animal**	5	-0.4784	0.4171	29	-1.15	0.2608	0.05	-1.3315	0.3747
**Animal**	6	-0.2839	0.4150	29	-0.68	0.4993	0.05	-1.1327	0.5648

Type 3 Tests of Fixed Effects				
**Effect**	**Num DF**	**Den DF**	**F Value**	**Pr > F**
**Genotype**	1	29	0.12	0.7287

Least Squares Means									
**Effect**	**Genotype**	**Estimate**	**Standard Error**	**DF**	**t Value**	**Pr > |t|**	**Alpha**	**Lower**	**Upper**
**Genotype**	Meg +/+	2.3944	0.4025	29	5.95	<.0001	0.05	1.5711	3.2177
**Genotype**	Meg +/-	2.5939	0.4030	29	6.44	<.0001	0.05	1.7697	3.4180

DistSoma=198

Differences of Least Squares Means												
**Effect**	**Genotype**	**Genotype**	**Estimate**	**Standard Error**	**DF**	**t Value**	**Pr > |t|**	**Adjustment**	**Adj P**	**Alpha**	**Lower**	**Upper**
**Genotype**	Meg +/+	Meg +/-	-0.1995	0.5696	29	-0.35	0.7287	Tukey-Kramer	0.7287	0.05	-1.3644	0.9654

Differences of Least Squares Means				
**Effect**	**Genotype**	**Genotype**	**Adj Lower**	**Adj Upper**
**Genotype**	Meg +/+	Meg +/-	-1.3644	0.9654

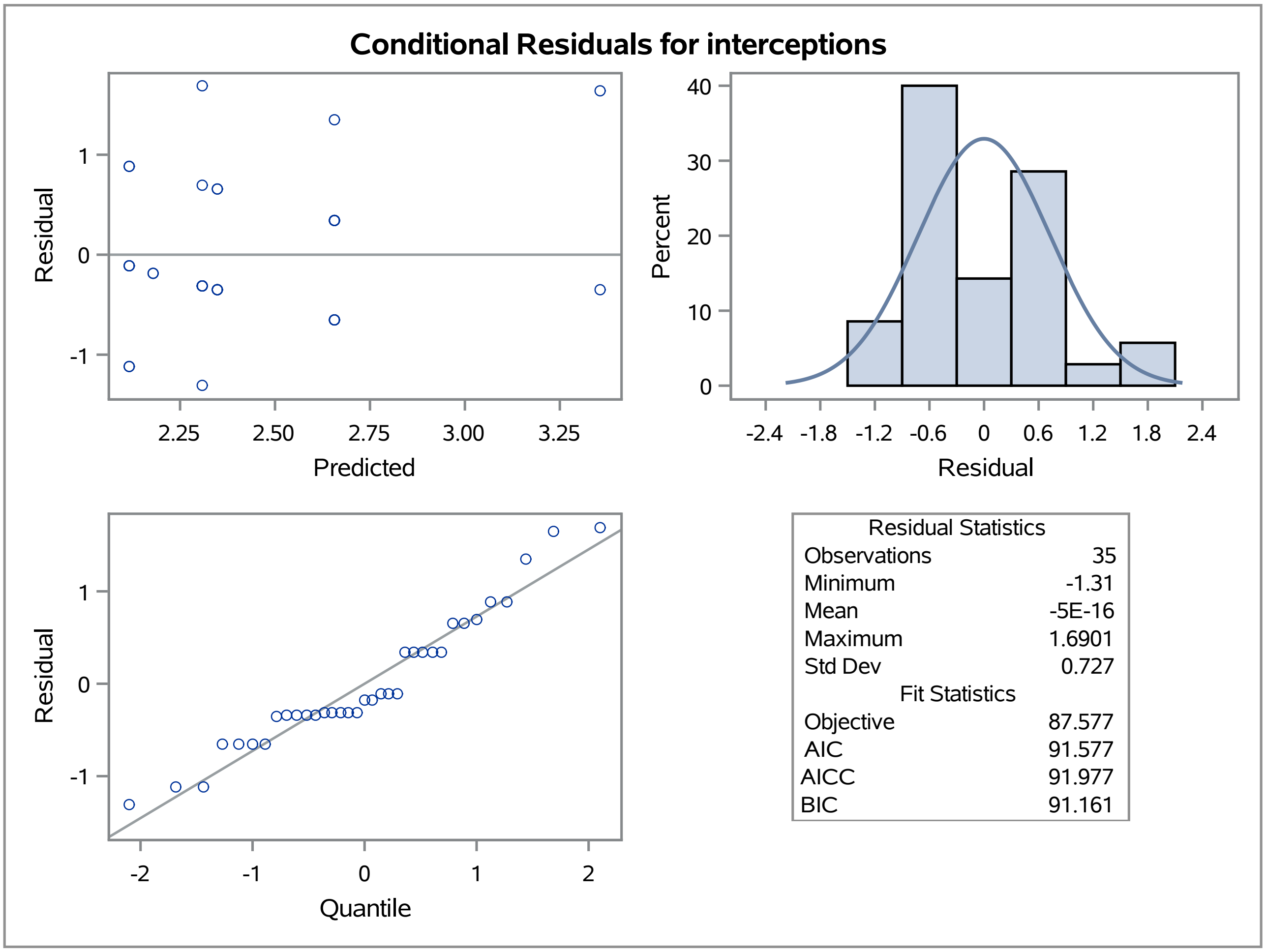

DistSoma=204

Model Information	
**Data Set**	WORK.TEMPDATASORTED
**Dependent Variable**	interceptions
**Covariance Structure**	Variance Components
**Estimation Method**	REML
**Residual Variance Method**	Profile
**Fixed Effects SE Method**	Model-Based
**Degrees of Freedom Method**	Containment

Class Level Information		
**Class**	**Levels**	**Values**
**Genotype**	2	Meg +/+ Meg +/-
**Animal**	6	1 2 3 4 5 6

Dimensions	
**Covariance Parameters**	2
**Columns in X**	3
**Columns in Z**	6
**Subjects**	1
**Max Obs per Subject**	35

Number of Observations	
**Number of Observations Read**	35
**Number of Observations Used**	35
**Number of Observations Not Used**	0

Iteration History			
**Iteration**	**Evaluations**	**-2 Res Log Like**	**Criterion**
**0**	1	83.61542024	
**1**	2	83.03095293	0.00526852
**2**	1	82.96341733	0.00037794
**3**	1	82.95898023	0.00000266
**4**	1	82.95895041	0.00000000

Convergence criteria met.

DistSoma=204

Covariance Parameter Estimates				
Cov Parm	Estimate	Alpha	Lower	Upper
Animal	0.1910	0.05	0.03426	1091.07
Residual	0.5401	0.05	0.3358	1.0097

Fit Statistics	
-2 Res Log Likelihood	83.0
AIC (Smaller is Better)	87.0
AICC (Smaller is Better)	87.4
BIC (Smaller is Better)	86.5

Solution for Fixed Effects									
Effect	Genotype	Estimate	Standard Error	DF	t Value	Pr > |t|	Alpha	Lower	Upper
Intercept		2.3281	0.3200	4	7.28	0.0019	0.05	1.4397	3.2165
Genotype	Meg +/+	0.2466	0.4520	29	0.55	0.5895	0.05	-0.6779	1.1712
Genotype	Meg +/-	0	.	.	.	.	.	.	.

Solution for Random Effects									
Effect	Animal	Estimate	Std Err Pred	DF	t Value	Pr > |t|	Alpha	Lower	Upper
Animal	1	0.1756	0.3226	29	0.54	0.5903	0.05	-0.4841	0.8354
Animal	2	0.06249	0.3291	29	0.19	0.8507	0.05	-0.6105	0.7355
Animal	3	-0.2381	0.3597	29	-0.66	0.5132	0.05	-0.9738	0.4975
Animal	4	0.4855	0.3598	29	1.35	0.1876	0.05	-0.2503	1.2214
Animal	5	-0.3355	0.3270	29	-1.03	0.3134	0.05	-1.0042	0.3333
Animal	6	-0.1501	0.3252	29	-0.46	0.6480	0.05	-0.8152	0.5151

Type 3 Tests of Fixed Effects				
Effect	Num DF	Den DF	F Value	Pr > F
Genotype	1	29	0.30	0.5895

Least Squares Means									
Effect	Genotype	Estimate	Standard Error	DF	t Value	Pr > |t|	Alpha	Lower	Upper
Genotype	Meg +/+	2.5747	0.3193	29	8.06	<.0001	0.05	1.9217	3.2278
Genotype	Meg +/-	2.3281	0.3200	29	7.28	<.0001	0.05	1.6736	2.9826

DistSoma=204

Differences of Least Squares Means												
Effect	Genotype	Genotype	Estimate	Standard Error	DF	t Value	Pr > |t|	Adjustment	Adj P	Alpha	Lower	Upper
**Genotype**	Meg +/+	Meg +/-	0.2466	0.4520	29	0.55	0.5895	Tukey-Kramer	0.5895	0.05	-0.6779	1.1712

Differences of Least Squares Means				
Effect	Genotype	Genotype	Adj Lower	Adj Upper
**Genotype**	Meg +/+	Meg +/-	-0.6779	1.1712

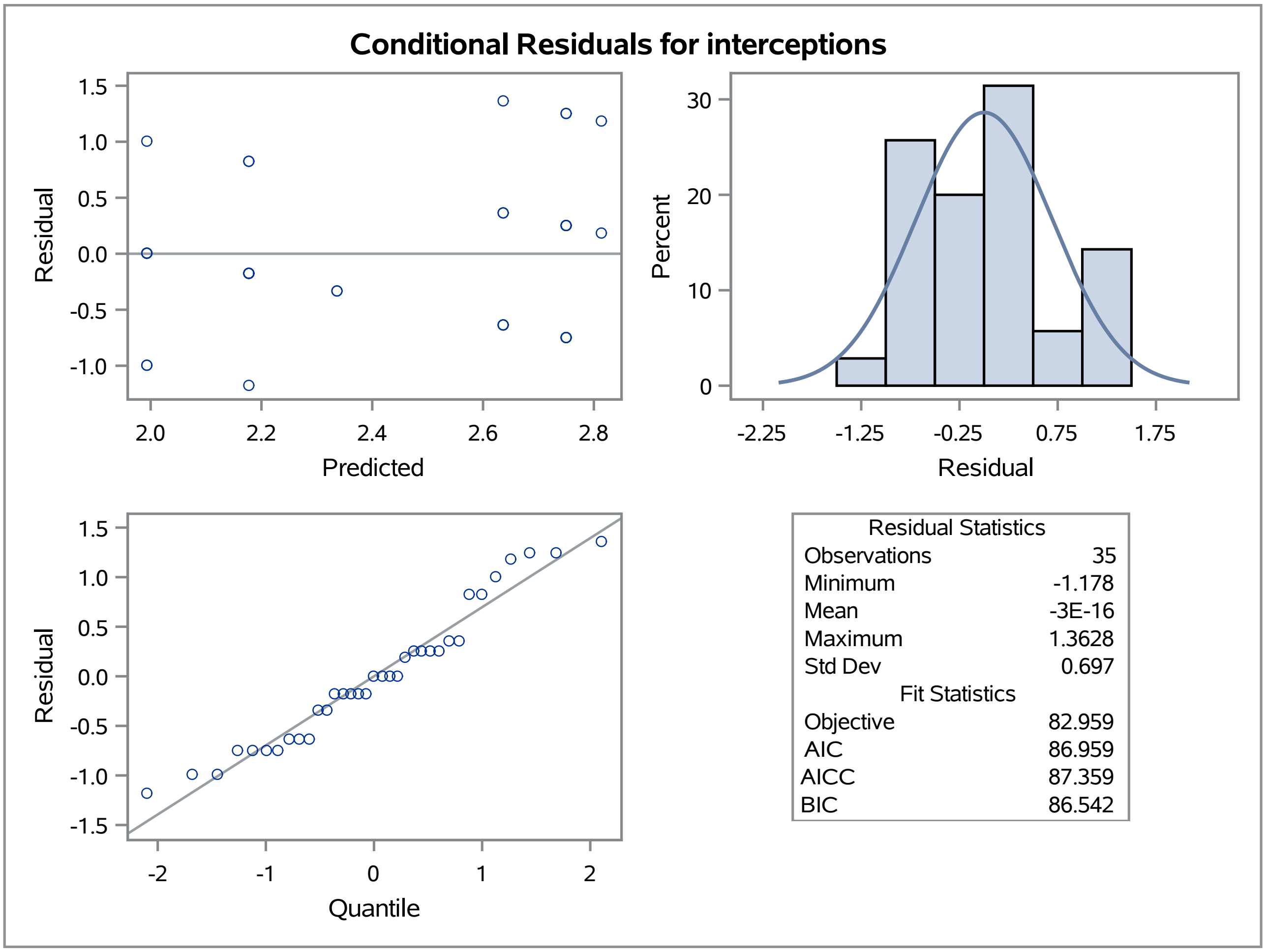

DistSoma=210

Model Information	
**Data Set**	WORK.TEMPDATASORTED
**Dependent Variable**	interceptions
**Covariance Structure**	Variance Components
**Estimation Method**	REML
**Residual Variance Method**	Profile
**Fixed Effects SE Method**	Model-Based
**Degrees of Freedom Method**	Containment

Class Level Information		
**Class**	**Levels**	**Values**
**Genotype**	2	Meg +/+ Meg +/-
**Animal**	6	1 2 3 4 5 6

Dimensions	
**Covariance Parameters**	2
**Columns in X**	3
**Columns in Z**	6
**Subjects**	1
**Max Obs per Subject**	35

Number of Observations	
**Number of Observations Read**	35
**Number of Observations Used**	35
**Number of Observations Not Used**	0

Iteration History			
**Iteration**	**Evaluations**	**-2 Res Log Like**	**Criterion**
**0**	1	87.33795252	
**1**	2	86.54423007	0.00352049
**2**	1	86.49162669	0.00028169
**3**	1	86.48778915	0.00000215
**4**	1	86.48776129	0.00000000

Convergence criteria met.

DistSoma=210

Covariance Parameter Estimates				
Cov Parm	Estimate	Alpha	Lower	Upper
Animal	0.1809	0.05	0.03244	1034.42
Residual	0.6085	0.05	0.3805	1.1263

Fit Statistics	
-2 Res Log Likelihood	86.5
AIC (Smaller is Better)	90.5
AICC (Smaller is Better)	90.9
BIC (Smaller is Better)	90.1

Solution for Fixed Effects									
Effect	Genotype	Estimate	Standard Error	DF	t Value	Pr > |t|	Alpha	Lower	Upper
Intercept		2.3143	0.3213	4	7.20	0.0020	0.05	1.4223	3.2064
Genotype	Meg +/+	0.1466	0.4538	29	0.32	0.7489	0.05	-0.7814	1.0747
Genotype	Meg +/-	0	.	.	.	.	.	.	.

Solution for Random Effects									
Effect	Animal	Estimate	Std Err Pred	DF	t Value	Pr > |t|	Alpha	Lower	Upper
Animal	1	0.2537	0.3210	29	0.79	0.4357	0.05	-0.4028	0.9101
Animal	2	-0.08179	0.3273	29	-0.25	0.8044	0.05	-0.7512	0.5876
Animal	3	-0.1719	0.3574	29	-0.48	0.6341	0.05	-0.9028	0.5590
Animal	4	0.4421	0.3575	29	1.24	0.2261	0.05	-0.2890	1.1732
Animal	5	-0.3088	0.3253	29	-0.95	0.3503	0.05	-0.9741	0.3565
Animal	6	-0.1333	0.3236	29	-0.41	0.6834	0.05	-0.7951	0.5285

Type 3 Tests of Fixed Effects				
Effect	Num DF	Den DF	F Value	Pr > F
Genotype	1	29	0.10	0.7489

Least Squares Means									
Effect	Genotype	Estimate	Standard Error	DF	t Value	Pr > |t|	Alpha	Lower	Upper
Genotype	Meg +/+	2.4610	0.3204	29	7.68	<.0001	0.05	1.8057	3.1163
Genotype	Meg +/-	2.3143	0.3213	29	7.20	<.0001	0.05	1.6572	2.9715

DistSoma=210

Differences of Least Squares Means												
**Effect**	**Genotype**	**Genotype**	**Estimate**	**Standard Error**	**DF**	**t Value**	**Pr > |t|**	**Adjustment**	**Adj P**	**Alpha**	**Lower**	**Upper**
**Genotype**	Meg +/+	Meg +/-	0.1466	0.4538	29	0.32	0.7489	Tukey-Kramer	0.7489	0.05	-0.7814	1.0747

Differences of Least Squares Means				
**Effect**	**Genotype**	**Genotype**	**Adj Lower**	**Adj Upper**
**Genotype**	Meg +/+	Meg +/-	-0.7814	1.0747

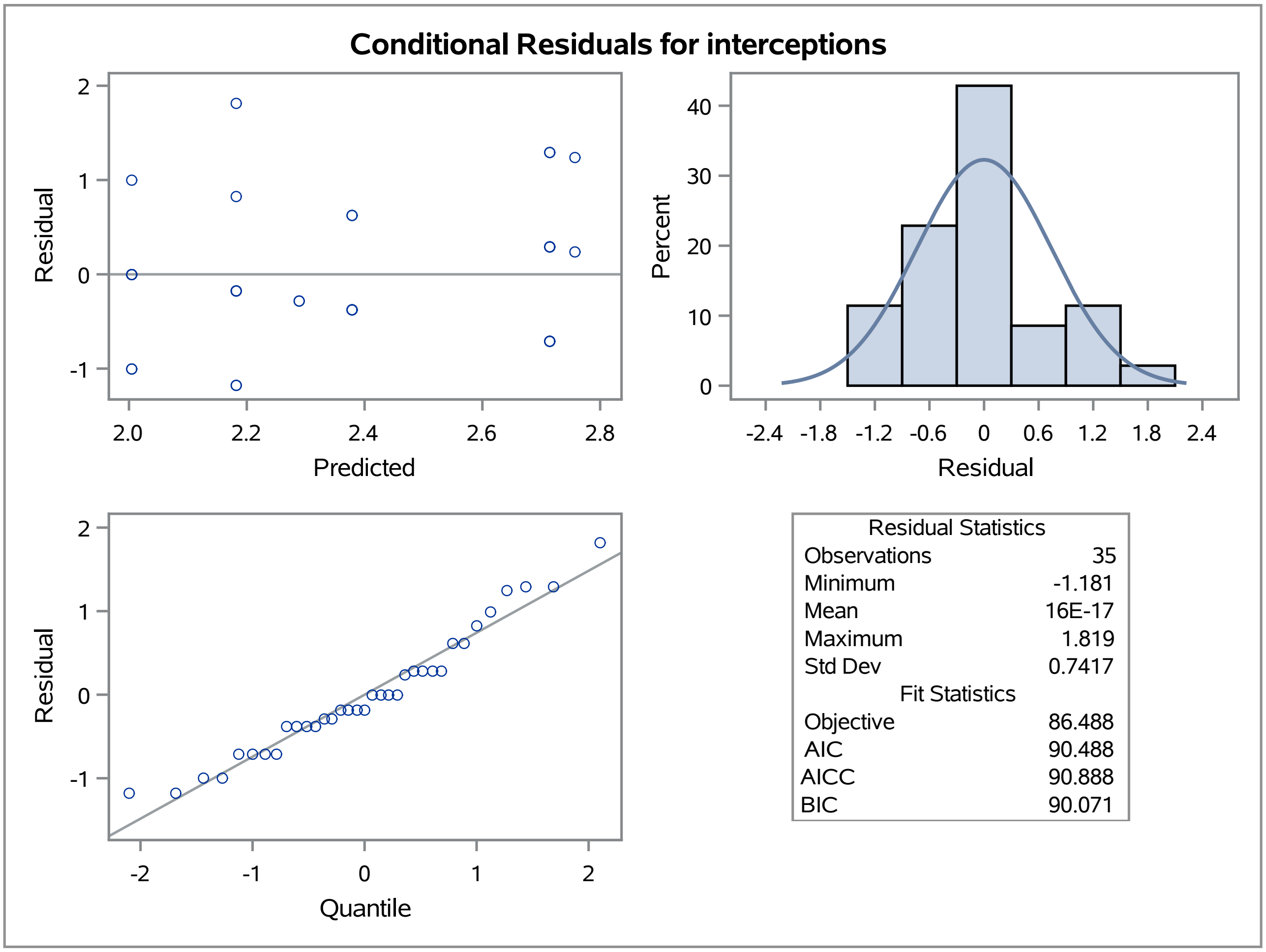

DistSoma=216

Model Information	
**Data Set**	WORK.TEMPDATASORTED
**Dependent Variable**	interceptions
**Covariance Structure**	Variance Components
**Estimation Method**	REML
**Residual Variance Method**	Profile
**Fixed Effects SE Method**	Model-Based
**Degrees of Freedom Method**	Containment

Class Level Information		
**Class**	**Levels**	**Values**
**Genotype**	2	Meg +/+ Meg +/-
**Animal**	6	1 2 3 4 5 6

Dimensions	
**Covariance Parameters**	2
**Columns in X**	3
**Columns in Z**	6
**Subjects**	1
**Max Obs per Subject**	35

Number of Observations	
**Number of Observations Read**	35
**Number of Observations Used**	35
**Number of Observations Not Used**	0

Iteration History			
**Iteration**	**Evaluations**	**-2 Res Log Like**	**Criterion**
**0**	1	88.17438141	
**1**	2	87.28351411	0.00159515
**2**	1	87.25972683	0.00007007
**3**	1	87.25876649	0.00000015
**4**	1	87.25876443	0.00000000

Convergence criteria met.

DistSoma=216

Covariance Parameter Estimates				
**Cov Parm**	**Estimate**	**Alpha**	**Lower**	**Upper**
**Animal**	0.1651	0.05	0.02963	933.43
**Residual**	0.6280	0.05	0.3943	1.1546

Fit Statistics	
**-2 Res Log Likelihood**	87.3
**AIC (Smaller is Better)**	91.3
**AICC (Smaller is Better)**	91.7
**BIC (Smaller is Better)**	90.8

Solution for Fixed Effects									
**Effect**	**Genotype**	**Estimate**	**Standard Error**	**DF**	**t Value**	**Pr > |t|**	**Alpha**	**Lower**	**Upper**
**Intercept**		2.3555	0.3144	4	7.49	0.0017	0.05	1.4826	3.2285
**Genotype**	Meg +/+	0.006814	0.4439	29	0.02	0.9879	0.05	-0.9011	0.9148
**Genotype**	Meg +/-	0	.	.	.	.	.	.	.

Solution for Random Effects									
**Effect**	**Animal**	**Estimate**	**Std Err Pred**	**DF**	**t Value**	**Pr > |t|**	**Alpha**	**Lower**	**Upper**
**Animal**	1	0.2446	0.3115	29	0.79	0.4387	0.05	-0.3925	0.8818
**Animal**	2	-0.1198	0.3176	29	-0.38	0.7088	0.05	-0.7693	0.5298
**Animal**	3	-0.1249	0.3462	29	-0.36	0.7210	0.05	-0.8330	0.5833
**Animal**	4	0.3945	0.3464	29	1.14	0.2641	0.05	-0.3139	1.1028
**Animal**	5	-0.3229	0.3157	29	-1.02	0.3147	0.05	-0.9685	0.3227
**Animal**	6	-0.07152	0.3140	29	-0.23	0.8214	0.05	-0.7138	0.5708

Type 3 Tests of Fixed Effects				
**Effect**	**Num DF**	**Den DF**	**F Value**	**Pr > F**
**Genotype**	1	29	0.00	0.9879

Least Squares Means									
**Effect**	**Genotype**	**Estimate**	**Standard Error**	**DF**	**t Value**	**Pr > |t|**	**Alpha**	**Lower**	**Upper**
**Genotype**	Meg +/+	2.3623	0.3134	29	7.54	<.0001	0.05	1.7213	3.0033
**Genotype**	Meg +/-	2.3555	0.3144	29	7.49	<.0001	0.05	1.7125	2.9986

DistSoma=216

Differences of Least Squares Means												
Effect	Genotype	Genotype	Estimate	Standard Error	DF	t Value	Pr > |t|	Adjustment	Adj P	Alpha	Lower	Upper
**Genotype**	Meg +/+	Meg +/-	0.006814	0.4439	29	0.02	0.9879	Tukey-Kramer	0.9879	0.05	-0.9011	0.9148

Differences of Least Squares Means				
Effect	Genotype	Genotype	Adj Lower	Adj Upper
**Genotype**	Meg +/+	Meg +/-	-0.9011	0.9148

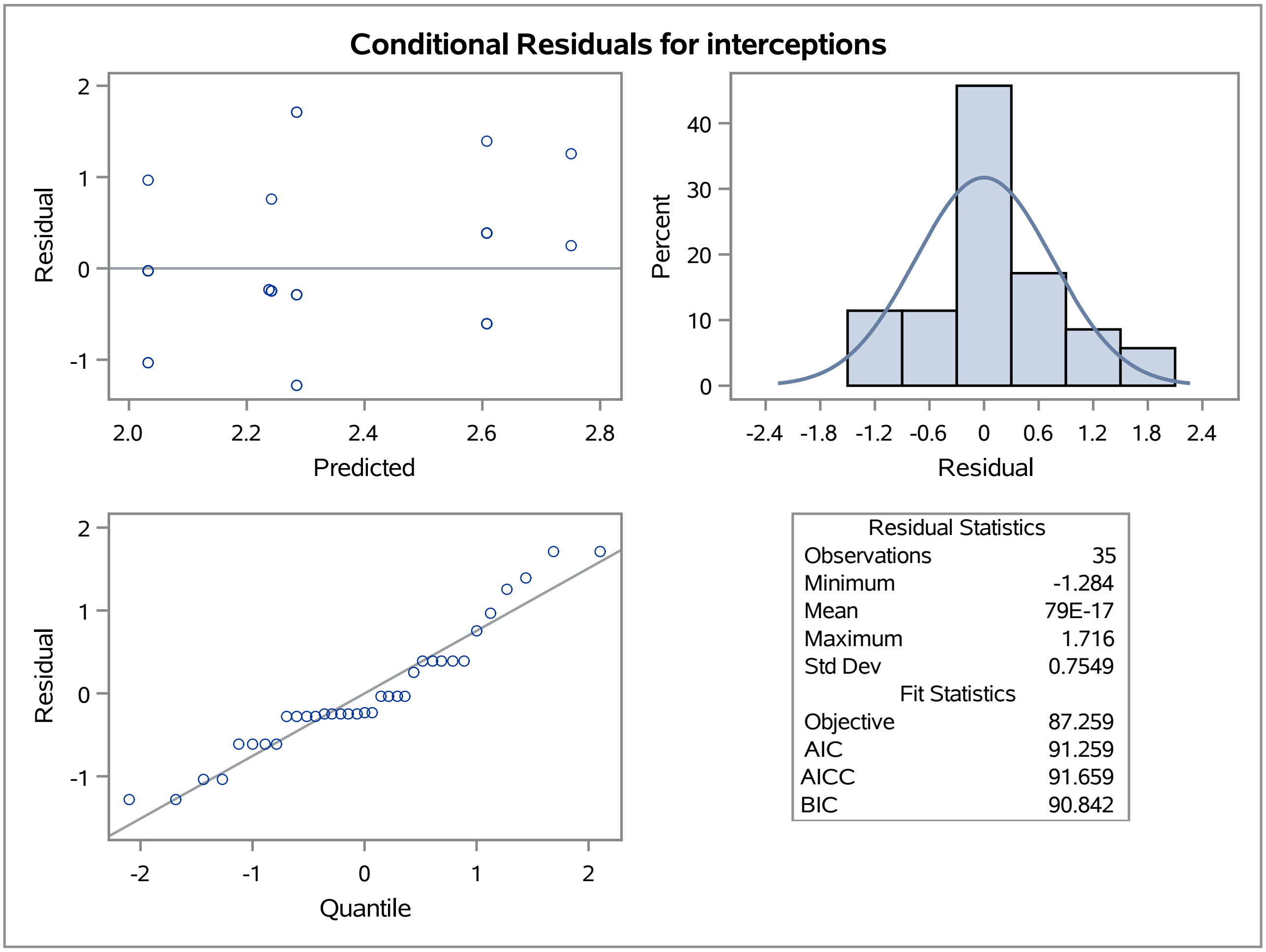

DistSoma=222

Model Information	
**Data Set**	WORK.TEMPDATASORTED
**Dependent Variable**	interceptions
**Covariance Structure**	Variance Components
**Estimation Method**	REML
**Residual Variance Method**	Profile
**Fixed Effects SE Method**	Model-Based
**Degrees of Freedom Method**	Containment

Class Level Information		
**Class**	**Levels**	**Values**
**Genotype**	2	Meg +/+ Meg +/-
**Animal**	6	1 2 3 4 5 6

Dimensions	
**Covariance Parameters**	2
**Columns in X**	3
**Columns in Z**	6
**Subjects**	1
**Max Obs per Subject**	35

Number of Observations	
**Number of Observations Read**	35
**Number of Observations Used**	35
**Number of Observations Not Used**	0

Iteration History			
**Iteration**	**Evaluations**	**-2 Res Log Like**	**Criterion**
**0**	1	98.15862633	
**1**	2	97.32672268	0.00007460
**2**	1	97.32530325	0.00000026
**3**	1	97.32529849	0.00000000

Convergence criteria met.

DistSoma=222

Covariance Parameter Estimates				
**Cov Parm**	**Estimate**	**Alpha**	**Lower**	**Upper**
**Animal**	0.1676	0.05	0.02898	1962.41
**Residual**	0.8681	0.05	0.5488	1.5777

Fit Statistics	
**-2 Res Log Likelihood**	97.3
**AIC (Smaller is Better)**	101.3
**AICC (Smaller is Better)**	101.7
**BIC (Smaller is Better)**	100.9

Solution for Fixed Effects									
**Effect**	**Genotype**	**Estimate**	**Standard Error**	**DF**	**t Value**	**Pr > |t|**	**Alpha**	**Lower**	**Upper**
**Intercept**		2.3901	0.3388	4	7.05	0.0021	0.05	1.4494	3.3307
**Genotype**	Meg +/+	0.1175	0.4780	29	0.25	0.8075	0.05	-0.8601	1.0952
**Genotype**	Meg +/-	0	.	.	.	.	.	.	.

Solution for Random Effects									
**Effect**	**Animal**	**Estimate**	**Std Err Pred**	**DF**	**t Value**	**Pr > |t|**	**Alpha**	**Lower**	**Upper**
**Animal**	1	0.3244	0.3264	29	0.99	0.3285	0.05	-0.3432	0.9920
**Animal**	2	-0.1830	0.3323	29	-0.55	0.5861	0.05	-0.8626	0.4966
**Animal**	3	-0.1414	0.3602	29	-0.39	0.6975	0.05	-0.8781	0.5953
**Animal**	4	0.3092	0.3603	29	0.86	0.3978	0.05	-0.4277	1.0461
**Animal**	5	-0.2242	0.3304	29	-0.68	0.5029	0.05	-0.9000	0.4516
**Animal**	6	-0.08502	0.3289	29	-0.26	0.7978	0.05	-0.7576	0.5876

Type 3 Tests of Fixed Effects				
**Effect**	**Num DF**	**Den DF**	**F Value**	**Pr > F**
**Genotype**	1	29	0.06	0.8075

Least Squares Means									
**Effect**	**Genotype**	**Estimate**	**Standard Error**	**DF**	**t Value**	**Pr > |t|**	**Alpha**	**Lower**	**Upper**
**Genotype**	Meg +/+	2.5076	0.3372	29	7.44	<.0001	0.05	1.8179	3.1973
**Genotype**	Meg +/-	2.3901	0.3388	29	7.05	<.0001	0.05	1.6972	3.0830

DistSoma=222

Differences of Least Squares Means												
Effect	Genotype	Genotype	Estimate	Standard Error	DF	t Value	Pr > |t|	Adjustment	Adj P	Alpha	Lower	Upper
Genotype	Meg +/+	Meg +/-	0.1175	0.4780	29	0.25	0.8075	Tukey-Kramer	0.8075	0.05	-0.8601	1.0952

Differences of Least Squares Means				
Effect	Genotype	Genotype	Adj Lower	Adj Upper
Genotype	Meg +/+	Meg +/-	-0.8601	1.0951

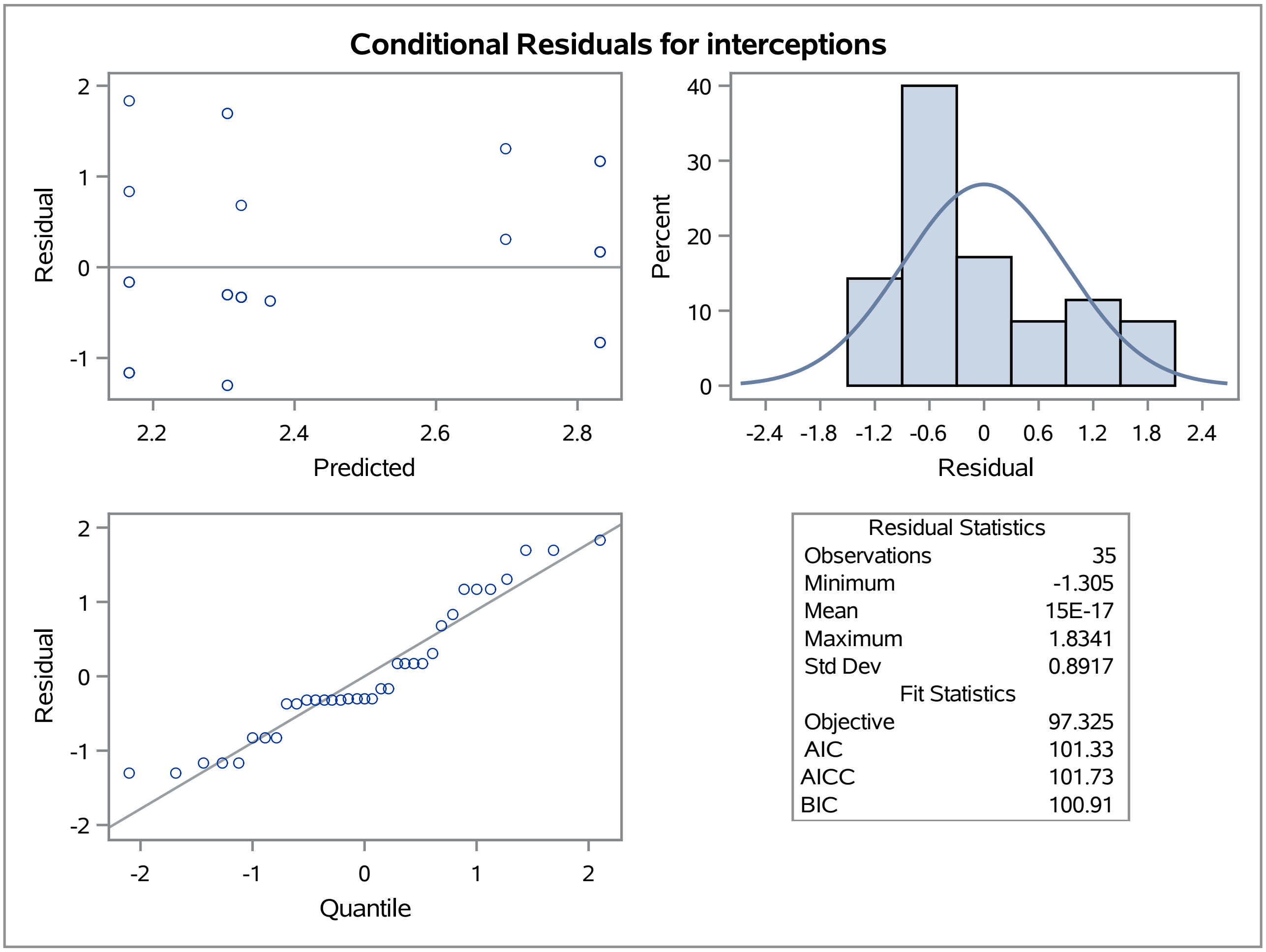

DistSoma=228

Model Information	
**Data Set**	WORK.TEMPDATASORTED
**Dependent Variable**	interceptions
**Covariance Structure**	Variance Components
**Estimation Method**	REML
**Residual Variance Method**	Profile
**Fixed Effects SE Method**	Model-Based
**Degrees of Freedom Method**	Containment

Class Level Information		
**Class**	**Levels**	**Values**
**Genotype**	2	Meg +/+ Meg +/-
**Animal**	6	1 2 3 4 5 6

Dimensions	
**Covariance Parameters**	2
**Columns in X**	3
**Columns in Z**	6
**Subjects**	1
**Max Obs per Subject**	35

Number of Observations	
**Number of Observations Read**	35
**Number of Observations Used**	35
**Number of Observations Not Used**	0

Iteration History			
**Iteration**	**Evaluations**	**-2 Res Log Like**	**Criterion**
**0**	1	93.72350069	
**1**	2	92.30388594	0.00027712
**2**	1	92.29923138	0.00000272
**3**	1	92.29918798	0.00000000

Convergence criteria met.

DistSoma=228

Covariance Parameter Estimates				
Cov Parm	Estimate	Alpha	Lower	Upper
Animal	0.2077	0.05	0.04212	162.24
Residual	0.7276	0.05	0.4597	1.3238

Fit Statistics	
-2 Res Log Likelihood	92.3
AIC (Smaller is Better)	96.3
AICC (Smaller is Better)	96.7
BIC (Smaller is Better)	95.9

Solution for Fixed Effects									
Effect	Genotype	Estimate	Standard Error	DF	t Value	Pr > |t|	Alpha	Lower	Upper
Intercept		2.4167	0.3470	4	6.97	0.0022	0.05	1.4534	3.3800
Genotype	Meg +/+	0.07067	0.4900	29	0.14	0.8863	0.05	-0.9314	1.0728
Genotype	Meg +/-	0	.	.	.	.	.	.	.

Solution for Random Effects									
Effect	Animal	Estimate	Std Err Pred	DF	t Value	Pr > |t|	Alpha	Lower	Upper
Animal	1	0.3796	0.3457	29	1.10	0.2812	0.05	-0.3275	1.0867
Animal	2	-0.2025	0.3525	29	-0.57	0.5701	0.05	-0.9235	0.5185
Animal	3	-0.1771	0.3847	29	-0.46	0.6487	0.05	-0.9640	0.6098
Animal	4	0.3937	0.3849	29	1.02	0.3148	0.05	-0.3934	1.1808
Animal	5	-0.2777	0.3504	29	-0.79	0.4344	0.05	-0.9943	0.4388
Animal	6	-0.1159	0.3485	29	-0.33	0.7418	0.05	-0.8288	0.5969

Type 3 Tests of Fixed Effects				
Effect	Num DF	Den DF	F Value	Pr > F
Genotype	1	29	0.02	0.8863

Least Squares Means									
Effect	Genotype	Estimate	Standard Error	DF	t Value	Pr > |t|	Alpha	Lower	Upper
Genotype	Meg +/+	2.4874	0.3460	29	7.19	<.0001	0.05	1.7798	3.1950
Genotype	Meg +/-	2.4167	0.3470	29	6.97	<.0001	0.05	1.7071	3.1263

DistSoma=228

Differences of Least Squares Means												
Effect	Genotype	Genotype	Estimate	Standard Error	DF	t Value	Pr > |t|	Adjustment	Adj P	Alpha	Lower	Upper
Genotype	Meg +/+	Meg +/-	0.07067	0.4900	29	0.14	0.8863	Tukey-Kramer	0.8863	0.05	-0.9314	1.0728

Differences of Least Squares Means				
Effect	Genotype	Genotype	Adj Lower	Adj Upper
Genotype	Meg +/+	Meg +/-	-0.9314	1.0728

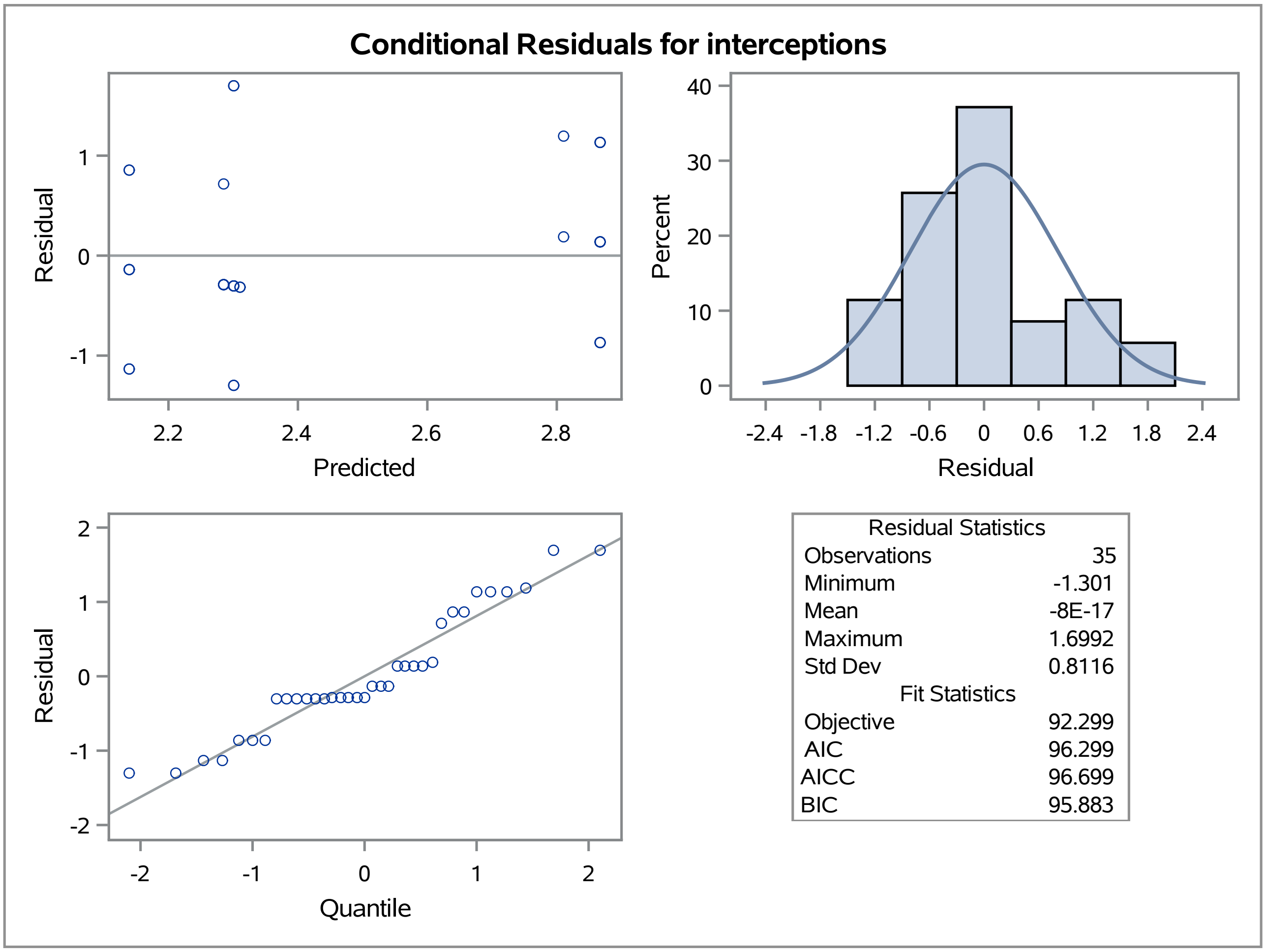

DistSoma=234

Model Information	
**Data Set**	WORK.TEMPDATASORTED
**Dependent Variable**	interceptions
**Covariance Structure**	Variance Components
**Estimation Method**	REML
**Residual Variance Method**	Profile
**Fixed Effects SE Method**	Model-Based
**Degrees of Freedom Method**	Containment

Class Level Information		
**Class**	**Levels**	**Values**
**Genotype**	2	Meg +/+ Meg +/-
**Animal**	6	1 2 3 4 5 6

Dimensions	
**Covariance Parameters**	2
**Columns in X**	3
**Columns in Z**	6
**Subjects**	1
**Max Obs per Subject**	35

Number of Observations	
**Number of Observations Read**	35
**Number of Observations Used**	35
**Number of Observations Not Used**	0

Iteration History			
**Iteration**	**Evaluations**	**-2 Res Log Like**	**Criterion**
**0**	1	88.82858786	
**1**	2	88.36506208	0.00032829
**2**	1	88.36020423	0.00000402
**3**	1	88.36014812	0.00000000

Convergence criteria met.

DistSoma=234

Covariance Parameter Estimates				
**Cov Parm**	**Estimate**	**Alpha**	**Lower**	**Upper**
**Animal**	0.1039	0.05	0.01493	153954
**Residual**	0.6693	0.05	0.4208	1.2277

Fit Statistics	
**-2 Res Log Likelihood**	88.4
**AIC (Smaller is Better)**	92.4
**AICC (Smaller is Better)**	92.8
**BIC (Smaller is Better)**	91.9

Solution for Fixed Effects									
**Effect**	**Genotype**	**Estimate**	**Standard Error**	**DF**	**t Value**	**Pr > |t|**	**Alpha**	**Lower**	**Upper**
**Intercept**		2.3765	0.2815	4	8.44	0.0011	0.05	1.5951	3.1580
**Genotype**	Meg +/+	0.1398	0.3969	29	0.35	0.7271	0.05	-0.6718	0.9515
**Genotype**	Meg +/-	0	.	.	.	.	.	.	.

Solution for Random Effects									
**Effect**	**Animal**	**Estimate**	**Std Err Pred**	**DF**	**t Value**	**Pr > |t|**	**Alpha**	**Lower**	**Upper**
**Animal**	1	0.1725	0.2639	29	0.65	0.5185	0.05	-0.3673	0.7123
**Animal**	2	-0.1686	0.2683	29	-0.63	0.5346	0.05	-0.7174	0.3802
**Animal**	3	-0.00388	0.2893	29	-0.01	0.9894	0.05	-0.5955	0.5878
**Animal**	4	0.2662	0.2894	29	0.92	0.3652	0.05	-0.3257	0.8580
**Animal**	5	-0.1961	0.2670	29	-0.73	0.4686	0.05	-0.7421	0.3500
**Animal**	6	-0.07009	0.2658	29	-0.26	0.7939	0.05	-0.6137	0.4736

Type 3 Tests of Fixed Effects				
**Effect**	**Num DF**	**Den DF**	**F Value**	**Pr > F**
**Genotype**	1	29	0.12	0.7271

Least Squares Means									
**Effect**	**Genotype**	**Estimate**	**Standard Error**	**DF**	**t Value**	**Pr > |t|**	**Alpha**	**Lower**	**Upper**
**Genotype**	Meg +/+	2.5164	0.2798	29	8.99	<.0001	0.05	1.9441	3.0886
**Genotype**	Meg +/-	2.3765	0.2815	29	8.44	<.0001	0.05	1.8009	2.9522

DistSoma=234

Differences of Least Squares Means												
**Effect**	**Genotype**	**Genotype**	**Estimate**	**Standard Error**	**DF**	**t Value**	**Pr > |t|**	**Adjustment**	**Adj P**	**Alpha**	**Lower**	**Upper**
**Genotype**	Meg +/+	Meg +/-	0.1398	0.3969	29	0.35	0.7271	Tukey-Kramer	0.7271	0.05	-0.6718	0.9515

Differences of Least Squares Means				
**Effect**	**Genotype**	**Genotype**	**Adj Lower**	**Adj Upper**
**Genotype**	Meg +/+	Meg +/-	-0.6718	0.9515

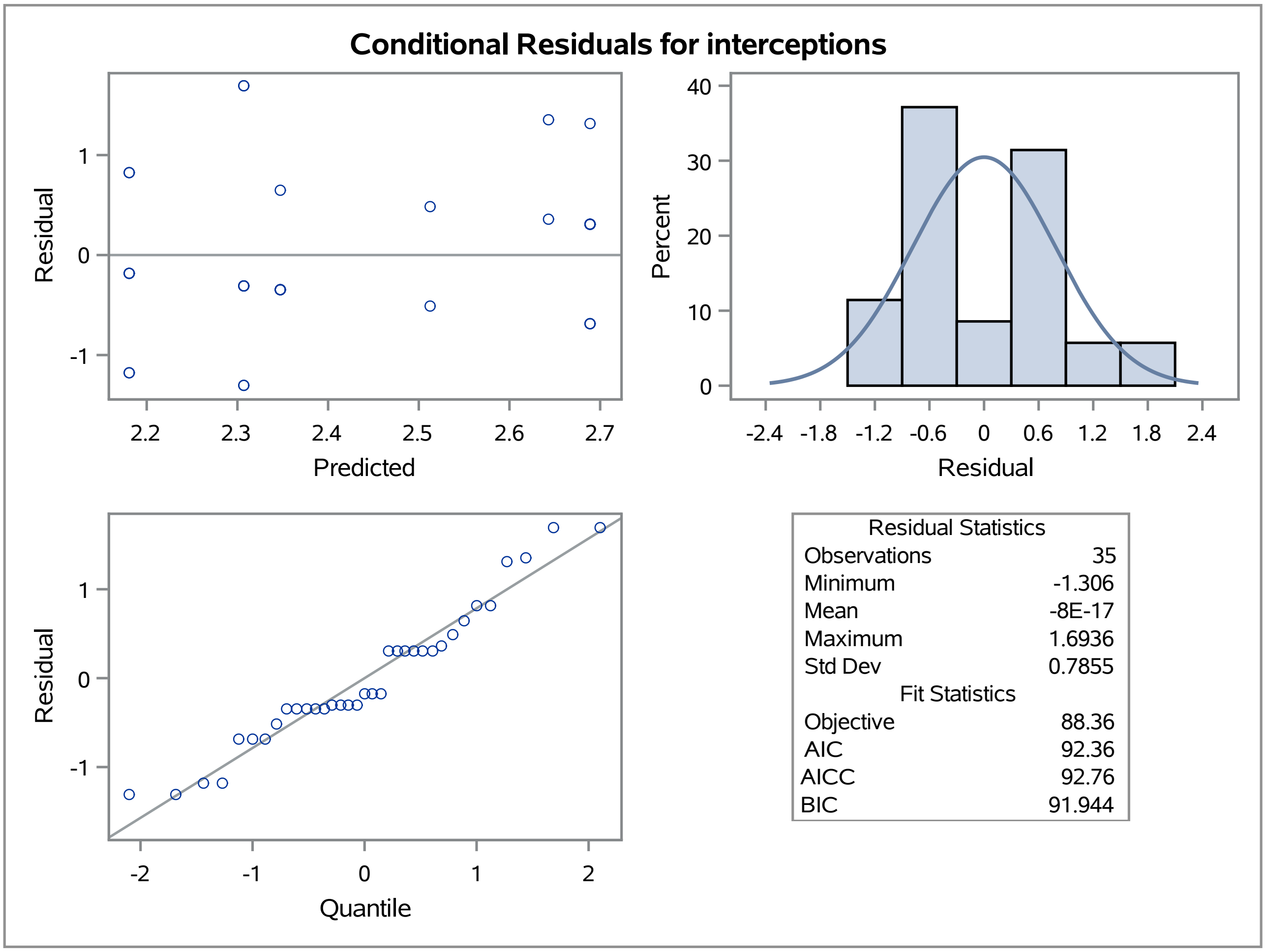

DistSoma=240

Model Information	
**Data Set**	WORK.TEMPDATASORTED
**Dependent Variable**	interceptions
**Covariance Structure**	Variance Components
**Estimation Method**	REML
**Residual Variance Method**	Profile
**Fixed Effects SE Method**	Model-Based
**Degrees of Freedom Method**	Containment

Class Level Information		
**Class**	**Levels**	**Values**
**Genotype**	2	Meg +/+ Meg +/-
**Animal**	6	1 2 3 4 5 6

Dimensions	
**Covariance Parameters**	2
**Columns in X**	3
**Columns in Z**	6
**Subjects**	1
**Max Obs per Subject**	35

Number of Observations	
**Number of Observations Read**	35
**Number of Observations Used**	35
**Number of Observations Not Used**	0

Iteration History			
**Iteration**	**Evaluations**	**-2 Res Log Like**	**Criterion**
**0**	1	90.51226862	
**1**	2	89.16373181	0.00005941
**2**	1	89.16286036	0.00000012
**3**	1	89.16285864	0.00000000

Convergence criteria met.

DistSoma=240

Covariance Parameter Estimates				
Cov Parm	Estimate	Alpha	Lower	Upper
Animal	0.1693	0.05	0.03384	162.68
Residual	0.6668	0.05	0.4221	1.2089

Fit Statistics	
-2 Res Log Likelihood	89.2
AIC (Smaller is Better)	93.2
AICC (Smaller is Better)	93.6
BIC (Smaller is Better)	92.7

Solution for Fixed Effects									
Effect	Genotype	Estimate	Standard Error	DF	t Value	Pr > |t|	Alpha	Lower	Upper
Intercept		2.5195	0.3206	4	7.86	0.0014	0.05	1.6293	3.4097
Genotype	Meg +/+	0.2203	0.4527	29	0.49	0.6301	0.05	-0.7055	1.1462
Genotype	Meg +/-	0	.	.	.	.	.	.	.

Solution for Random Effects									
Effect	Animal	Estimate	Std Err Pred	DF	t Value	Pr > |t|	Alpha	Lower	Upper
Animal	1	0.2584	0.3169	29	0.82	0.4214	0.05	-0.3897	0.9065
Animal	2	-0.3460	0.3230	29	-1.07	0.2928	0.05	-1.0066	0.3145
Animal	3	0.08763	0.3520	29	0.25	0.8051	0.05	-0.6322	0.8075
Animal	4	0.3302	0.3521	29	0.94	0.3560	0.05	-0.3898	1.0503
Animal	5	-0.1496	0.3210	29	-0.47	0.6447	0.05	-0.8062	0.5070
Animal	6	-0.1806	0.3194	29	-0.57	0.5761	0.05	-0.8339	0.4726

Type 3 Tests of Fixed Effects				
Effect	Num DF	Den DF	F Value	Pr > F
Genotype	1	29	0.24	0.6301

Least Squares Means									
Effect	Genotype	Estimate	Standard Error	DF	t Value	Pr > |t|	Alpha	Lower	Upper
Genotype	Meg +/+	2.7398	0.3196	29	8.57	<.0001	0.05	2.0863	3.3934
Genotype	Meg +/-	2.5195	0.3206	29	7.86	<.0001	0.05	1.8637	3.1753

DistSoma=240

Differences of Least Squares Means												
Effect	Genotype	Genotype	Estimate	Standard Error	DF	t Value	Pr > |t|	Adjustment	Adj P	Alpha	Lower	Upper
Genotype	Meg +/+	Meg +/-	0.2203	0.4527	29	0.49	0.6301	Tukey-Kramer	0.6301	0.05	-0.7055	1.1462

Differences of Least Squares Means				
Effect	Genotype	Genotype	Adj Lower	Adj Upper
Genotype	Meg +/+	Meg +/-	-0.7055	1.1462

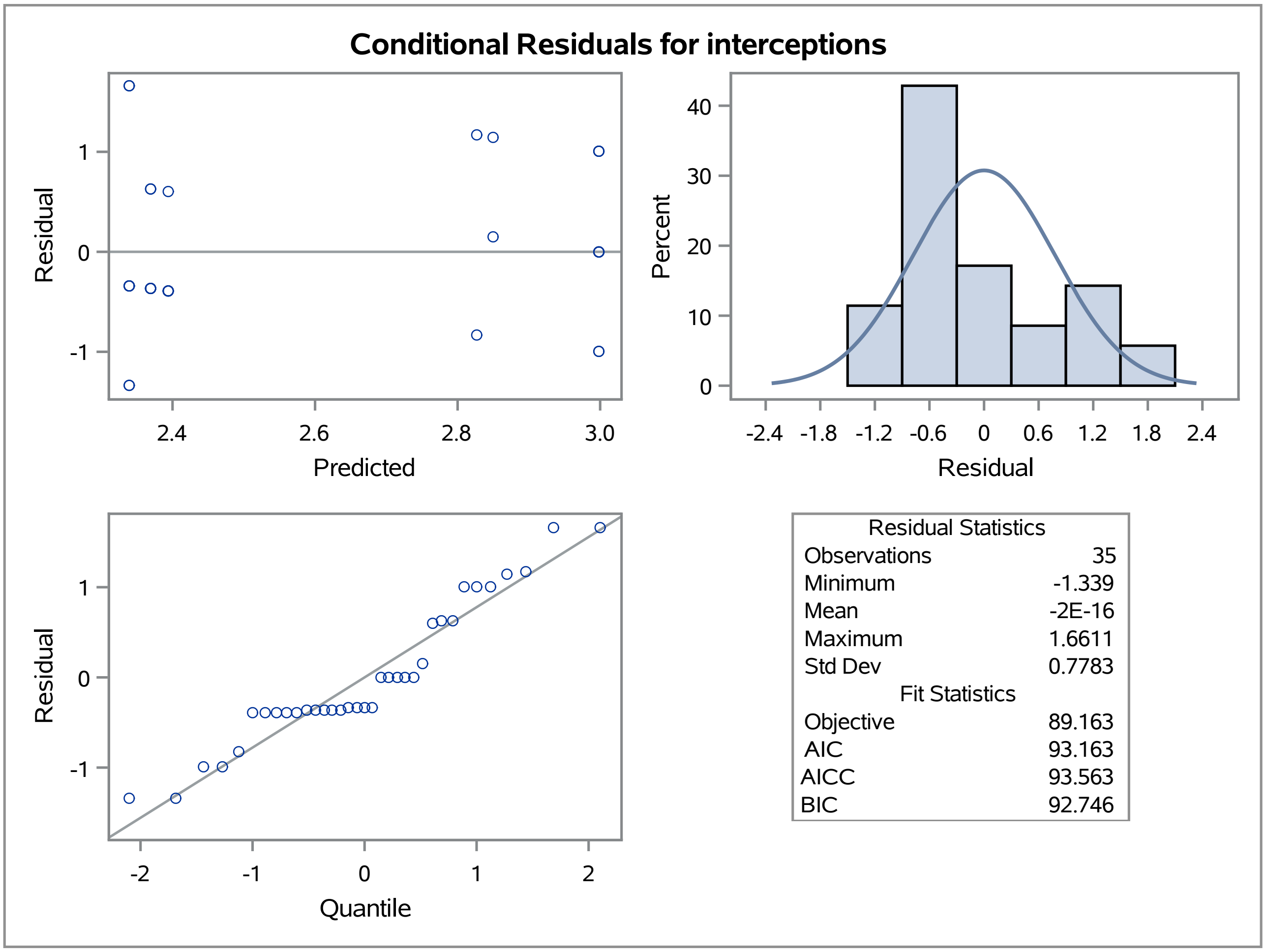

DistSoma=246

Model Information	
**Data Set**	WORK.TEMPDATASORTED
**Dependent Variable**	interceptions
**Covariance Structure**	Variance Components
**Estimation Method**	REML
**Residual Variance Method**	Profile
**Fixed Effects SE Method**	Model-Based
**Degrees of Freedom Method**	Containment

Class Level Information		
**Class**	**Levels**	**Values**
**Genotype**	2	Meg +/+ Meg +/-
**Animal**	6	1 2 3 4 5 6

Dimensions	
**Covariance Parameters**	2
**Columns in X**	3
**Columns in Z**	6
**Subjects**	1
**Max Obs per Subject**	35

Number of Observations	
**Number of Observations Read**	35
**Number of Observations Used**	35
**Number of Observations Not Used**	0

Iteration History			
**Iteration**	**Evaluations**	**-2 Res Log Like**	**Criterion**
**0**	1	102.99422918	
**1**	2	102.99402088	0.00000000

Convergence criteria met.

Covariance Parameter Estimates				
**Cov Parm**	**Estimate**	**Alpha**	**Lower**	**Upper**
**Animal**	0.002649	0.05	.	.
**Residual**	1.1143	0.05	0.7028	2.0330

DistSoma=246

Fit Statistics	
-2 Res Log Likelihood	103.0
AIC (Smaller is Better)	107.0
AICC (Smaller is Better)	107.4
BIC (Smaller is Better)	106.6

Solution for Fixed Effects

Effect	Genotype	Estimate	Standard Error	DF	t Value	Pr > |t|	Alpha	Lower	Upper
Intercept		2.3540	0.2581	4	9.12	0.0008	0.05	1.6373	3.0706
Genotype	Meg +/+	0.5908	0.3601	29	1.64	0.1117	0.05	-0.1457	1.3273
Genotype	Meg +/-	0	.	.	.	.	.	.	.

Solution for Random Effects

Effect	Animal	Estimate	Std Err Pred	DF	t Value	Pr > |t|	Alpha	Lower	Upper
Animal	1	0.003605	0.05120	29	0.07	0.9444	0.05	-0.1011	0.1083
Animal	2	-0.00860	0.05123	29	-0.17	0.8679	0.05	-0.1134	0.09618
Animal	3	0.004994	0.05136	29	0.10	0.9232	0.05	-0.1001	0.1100
Animal	4	0.003057	0.05136	29	0.06	0.9529	0.05	-0.1020	0.1081
Animal	5	-0.00112	0.05122	29	-0.02	0.9828	0.05	-0.1059	0.1036
Animal	6	-0.00194	0.05122	29	-0.04	0.9700	0.05	-0.1067	0.1028

Type 3 Tests of Fixed Effects

Effect	Num DF	Den DF	F Value	Pr > F
Genotype	1	29	2.69	0.1117

Least Squares Means

Effect	Genotype	Estimate	Standard Error	DF	t Value	Pr > |t|	Alpha	Lower	Upper
Genotype	Meg +/+	2.9448	0.2511	29	11.73	<.0001	0.05	2.4312	3.4583
Genotype	Meg +/-	2.3540	0.2581	29	9.12	<.0001	0.05	1.8261	2.8818

DistSoma=246

Differences of Least Squares Means												
**Effect**	**Genotype**	**Genotype**	**Estimate**	**Standard Error**	**DF**	**t Value**	**Pr > |t|**	**Adjustment**	**Adj P**	**Alpha**	**Lower**	**Upper**
**Genotype**	Meg +/+	Meg +/-	0.5908	0.3601	29	1.64	0.1117	Tukey-Kramer	0.1117	0.05	-0.1457	1.3273

Differences of Least Squares Means				
**Effect**	**Genotype**	**Genotype**	**Adj Lower**	**Adj Upper**
**Genotype**	Meg +/+	Meg +/-	-0.1457	1.3273

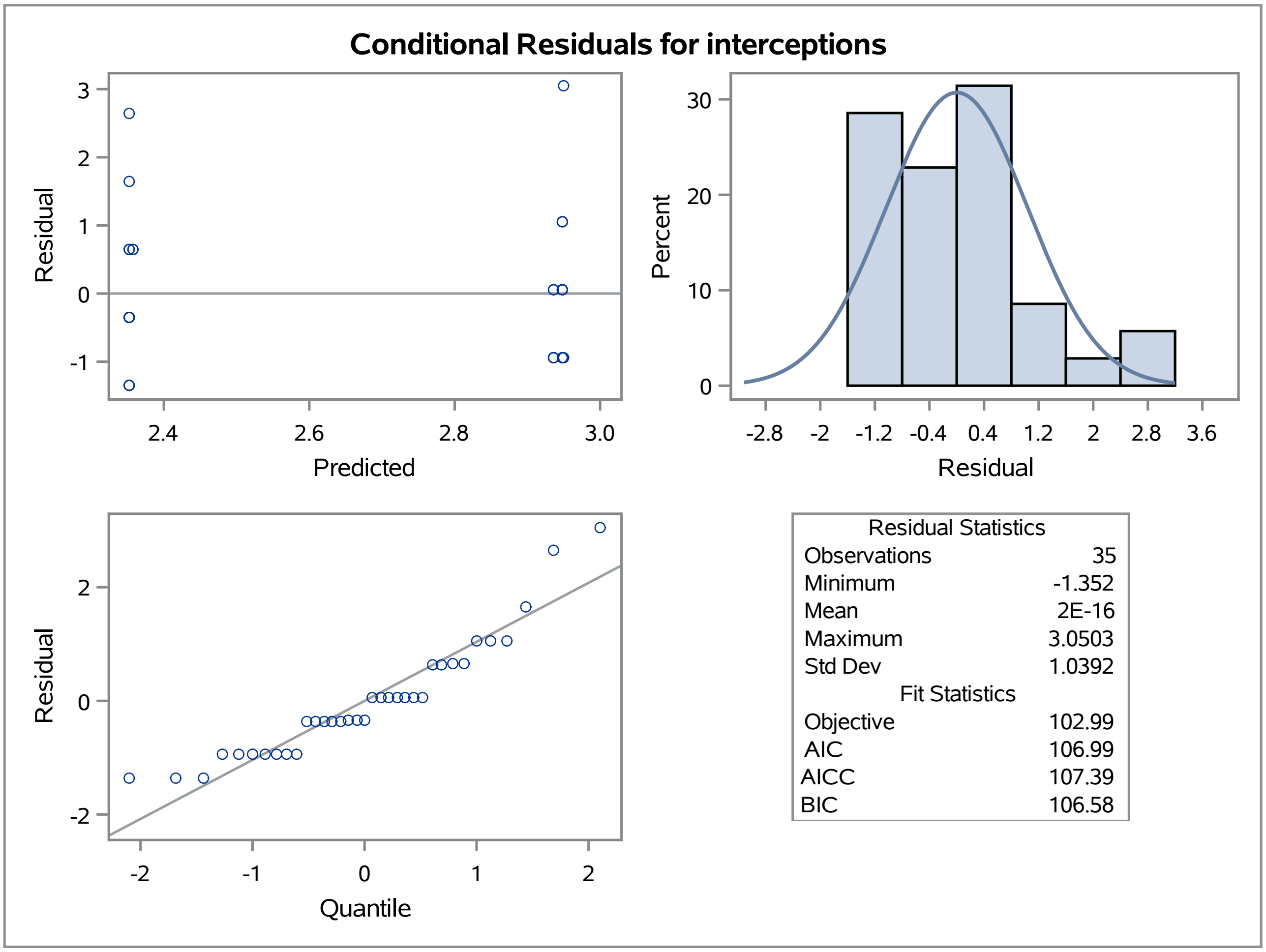

DistSoma=252

Model Information	
**Data Set**	WORK.TEMPDATASORTED
**Dependent Variable**	interceptions
**Covariance Structure**	Variance Components
**Estimation Method**	REML
**Residual Variance Method**	Profile
**Fixed Effects SE Method**	Model-Based
**Degrees of Freedom Method**	Containment

Class Level Information		
**Class**	**Levels**	**Values**
**Genotype**	2	Meg +/+ Meg +/-
**Animal**	6	1 2 3 4 5 6

Dimensions	
**Covariance Parameters**	2
**Columns in X**	3
**Columns in Z**	6
**Subjects**	1
**Max Obs per Subject**	35

Number of Observations	
**Number of Observations Read**	35
**Number of Observations Used**	35
**Number of Observations Not Used**	0

Iteration History			
**Iteration**	**Evaluations**	**-2 Res Log Like**	**Criterion**
**0**	1	95.36883085	
**1**	1	95.36883085	0.00000000

Convergence criteria met.

Estimated G matrix is not positive definite.

Covariance Parameter Estimates				
**Cov Parm**	**Estimate**	**Alpha**	**Lower**	**Upper**
**Animal**	0	.	.	.
**Residual**	0.8857	0.05	0.5762	1.5346

DistSoma=252

Fit Statistics	
-2 Res Log Likelihood	95.4
AIC (Smaller is Better)	97.4
AICC (Smaller is Better)	97.5
BIC (Smaller is Better)	97.2

Solution for Fixed Effects

Effect	Genotype	Estimate	Standard Error	DF	t Value	Pr > |t|	Alpha	Lower	Upper
Intercept		2.4118	0.2283	4	10.57	0.0005	0.05	1.7780	3.0455
Genotype	Meg +/+	0.3660	0.3183	29	1.15	0.2596	0.05	-0.2850	1.0170
Genotype	Meg +/-	0	.	.	.	.	.	.	.

Solution for Random Effects

Effect	Animal	Estimate	Std Err Pred	DF	t Value	Pr > |t|	Alpha	Lower	Upper
Animal	1	0	.	.	.	.	.	.	.
Animal	2	0	.	.	.	.	.	.	.
Animal	3	0	.	.	.	.	.	.	.
Animal	4	0	.	.	.	.	.	.	.
Animal	5	0	.	.	.	.	.	.	.
Animal	6	0	.	.	.	.	.	.	.

Type 3 Tests of Fixed Effects

Effect	Num DF	Den DF	F Value	Pr > F
Genotype	1	29	1.32	0.2596

Least Squares Means

Effect	Genotype	Estimate	Standard Error	DF	t Value	Pr > |t|	Alpha	Lower	Upper
Genotype	Meg +/+	2.7778	0.2218	29	12.52	<.0001	0.05	2.3241	3.2315
Genotype	Meg +/-	2.4118	0.2283	29	10.57	<.0001	0.05	1.9449	2.8786

DistSoma=252

Differences of Least Squares Means

Effect	Genotype	Genotype	Estimate	Standard Error	DF	t Value	Pr > |t|	Adjustment	Adj P	Alpha	Lower	Upper
**Genotype**	Meg +/+	Meg +/-	0.3660	0.3183	29	1.15	0.2596	Tukey-Kramer	0.2596	0.05	-0.2850	1.0170

Differences of Least Squares Means

Effect	Genotype	Genotype	Adj Lower	Adj Upper
**Genotype**	Meg +/+	Meg +/-	-0.2850	1.0170

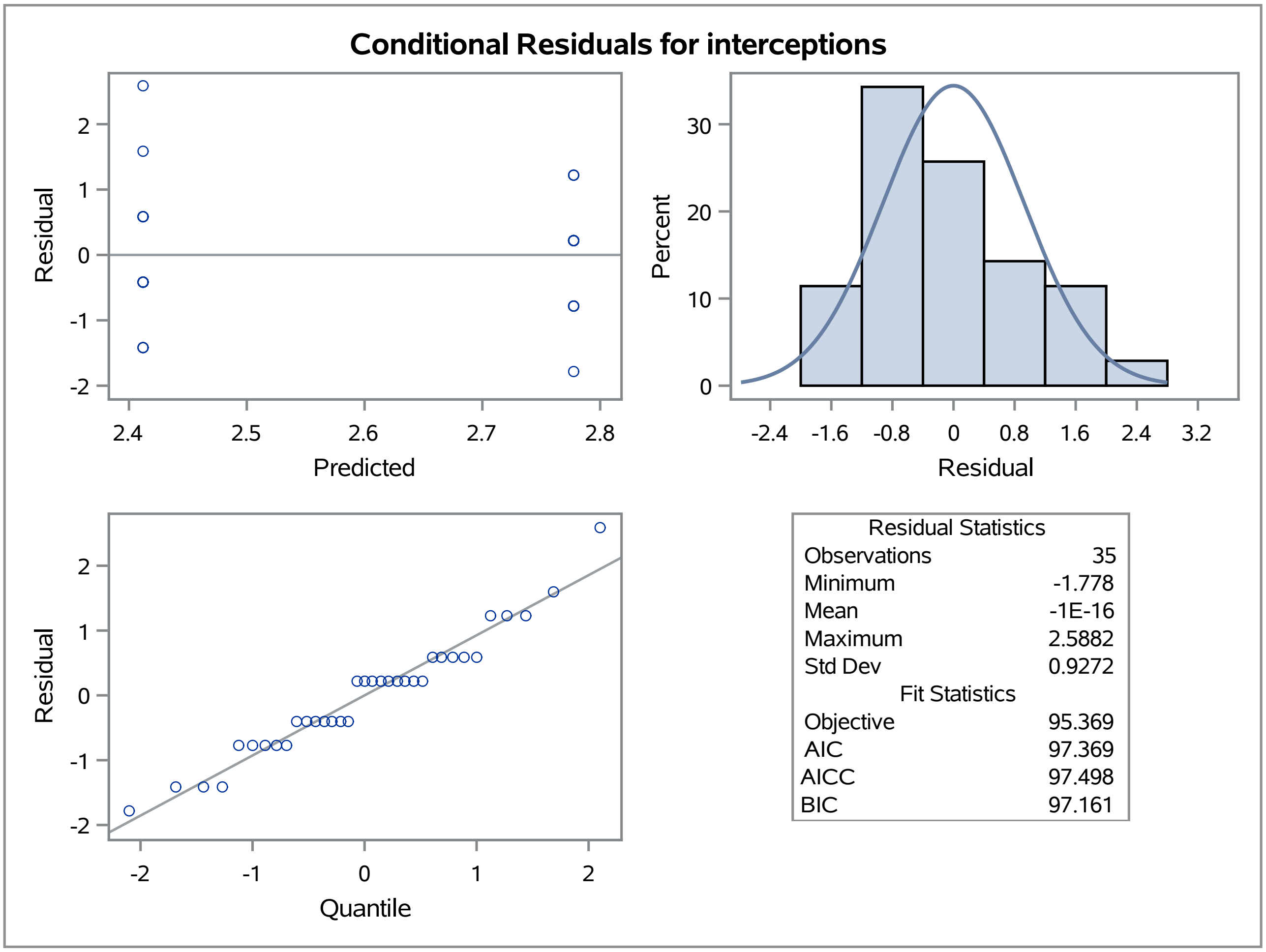

DistSoma=258

Model Information	
**Data Set**	WORK.TEMPDATASORTED
**Dependent Variable**	interceptions
**Covariance Structure**	Variance Components
**Estimation Method**	REML
**Residual Variance Method**	Profile
**Fixed Effects SE Method**	Model-Based
**Degrees of Freedom Method**	Containment

Class Level Information		
**Class**	**Levels**	**Values**
**Genotype**	2	Meg +/+ Meg +/-
**Animal**	6	1 2 3 4 5 6

Dimensions	
**Covariance Parameters**	2
**Columns in X**	3
**Columns in Z**	6
**Subjects**	1
**Max Obs per Subject**	35

Number of Observations	
**Number of Observations Read**	35
**Number of Observations Used**	35
**Number of Observations Not Used**	0

Iteration History			
**Iteration**	**Evaluations**	**-2 Res Log Like**	**Criterion**
**0**	1	89.08400362	
**1**	1	89.08400362	0.00000000

Convergence criteria met.

Estimated G matrix is not positive definite.

Covariance Parameter Estimates				
**Cov Parm**	**Estimate**	**Alpha**	**Lower**	**Upper**
**Animal**	0	.	.	.
**Residual**	0.7321	0.05	0.4763	1.2685

DistSoma=258

Fit Statistics	
-2 Res Log Likelihood	89.1
AIC (Smaller is Better)	91.1
AICC (Smaller is Better)	91.2
BIC (Smaller is Better)	90.9

Solution for Fixed Effects

Effect	Genotype	Estimate	Standard Error	DF	t Value	Pr > |t|	Alpha	Lower	Upper
Intercept		2.3529	0.2075	4	11.34	0.0003	0.05	1.7768	2.9291
Genotype	Meg +/+	0.2582	0.2894	29	0.89	0.3797	0.05	-0.3337	0.8500
Genotype	Meg +/-	0	.	.	.	.	.	.	.

Solution for Random Effects

Effect	Animal	Estimate	Std Err Pred	DF	t Value	Pr > |t|	Alpha	Lower	Upper
Animal	1	0	.	.	.	.	.	.	.
Animal	2	0	.	.	.	.	.	.	.
Animal	3	0	.	.	.	.	.	.	.
Animal	4	0	.	.	.	.	.	.	.
Animal	5	0	.	.	.	.	.	.	.
Animal	6	0	.	.	.	.	.	.	.

Type 3 Tests of Fixed Effects

Effect	Num DF	Den DF	F Value	Pr > F
Genotype	1	29	0.80	0.3797

Least Squares Means

Effect	Genotype	Estimate	Standard Error	DF	t Value	Pr > |t|	Alpha	Lower	Upper
Genotype	Meg +/+	2.6111	0.2017	29	12.95	<.0001	0.05	2.1986	3.0236
Genotype	Meg +/-	2.3529	0.2075	29	11.34	<.0001	0.05	1.9285	2.7774

DistSoma=258

Differences of Least Squares Means												
Effect	Genotype	Genotype	Estimate	Standard Error	DF	t Value	Pr > |t|	Adjustment	Adj P	Alpha	Lower	Upper
Genotype	Meg +/+	Meg +/-	0.2582	0.2894	29	0.89	0.3797	Tukey-Kramer	0.3797	0.05	-0.3337	0.8500

Differences of Least Squares Means				
Effect	Genotype	Genotype	Adj Lower	Adj Upper
Genotype	Meg +/+	Meg +/-	-0.3337	0.8500

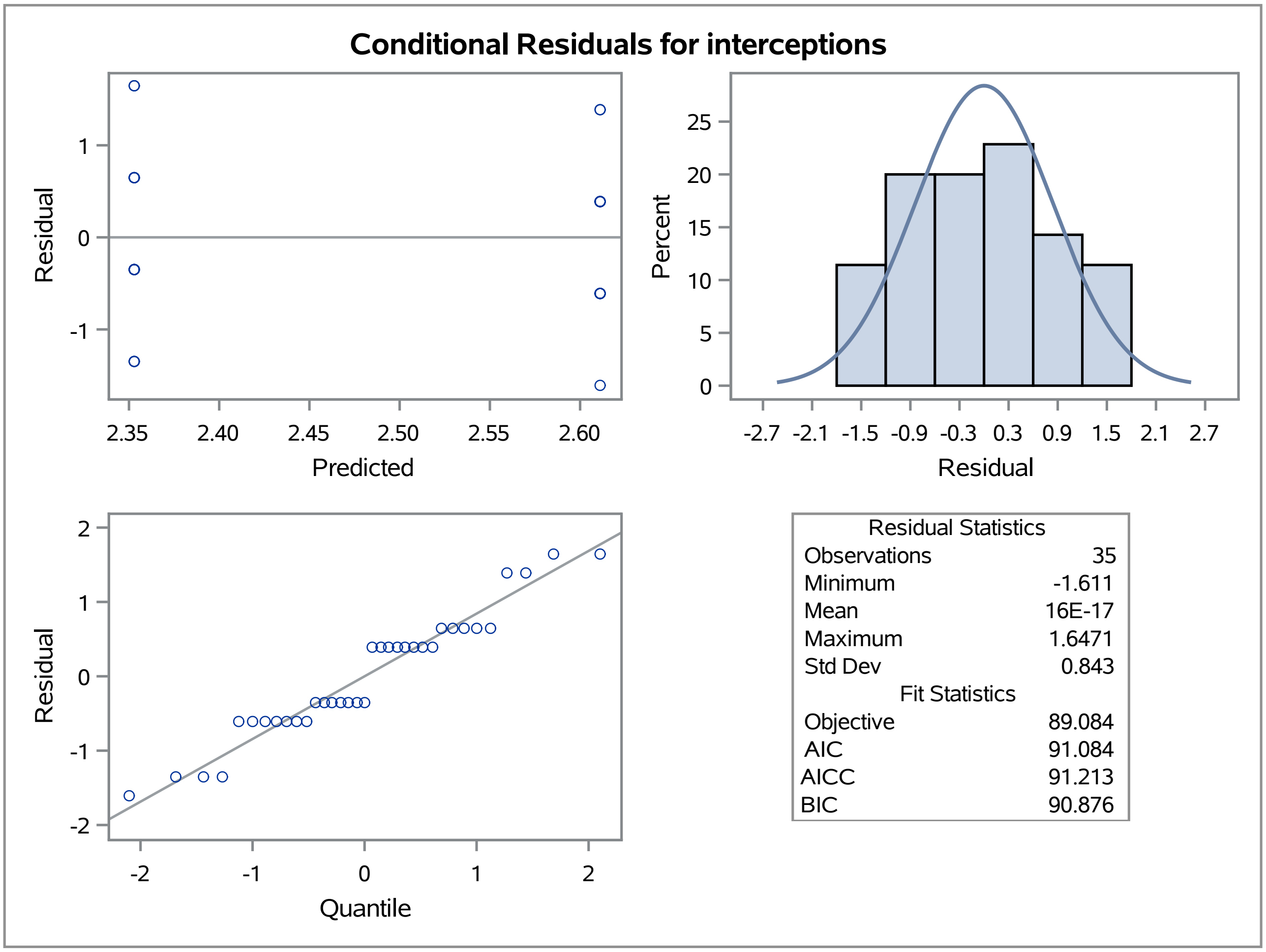

DistSoma=264

Model Information	
**Data Set**	WORK.TEMPDATASORTED
**Dependent Variable**	interceptions
**Covariance Structure**	Variance Components
**Estimation Method**	REML
**Residual Variance Method**	Profile
**Fixed Effects SE Method**	Model-Based
**Degrees of Freedom Method**	Containment

Class Level Information		
**Class**	**Levels**	**Values**
**Genotype**	2	Meg +/+ Meg +/-
**Animal**	6	1 2 3 4 5 6

Dimensions	
**Covariance Parameters**	2
**Columns in X**	3
**Columns in Z**	6
**Subjects**	1
**Max Obs per Subject**	35

Number of Observations	
**Number of Observations Read**	35
**Number of Observations Used**	35
**Number of Observations Not Used**	0

Iteration History			
**Iteration**	**Evaluations**	**-2 Res Log Like**	**Criterion**
**0**	1	100.33329146	
**1**	2	100.04286568	0.00137231
**2**	1	100.01253211	0.00006303
**3**	1	100.01125244	0.00000015
**4**	1	100.01124944	0.00000000

Convergence criteria met.

DistSoma=264

Covariance Parameter Estimates				
Cov Parm	Estimate	Alpha	Lower	Upper
Animal	0.1938	0.05	0.02623	2446108
Residual	0.9381	0.05	0.5793	1.7752

Fit Statistics	
-2 Res Log Likelihood	100.0
AIC (Smaller is Better)	104.0
AICC (Smaller is Better)	104.4
BIC (Smaller is Better)	103.6

Solution for Fixed Effects									
Effect	Genotype	Estimate	Standard Error	DF	t Value	Pr > |t|	Alpha	Lower	Upper
Intercept		2.3622	0.3586	4	6.59	0.0028	0.05	1.3665	3.3580
Genotype	Meg +/+	0.1999	0.5061	29	0.39	0.6957	0.05	-0.8352	1.2350
Genotype	Meg +/-	0	.	.	.	.	.	.	.

Solution for Random Effects									
Effect	Animal	Estimate	Std Err Pred	DF	t Value	Pr > |t|	Alpha	Lower	Upper
Animal	1	-0.1092	0.3480	29	-0.31	0.7558	0.05	-0.8209	0.6025
Animal	2	-0.3111	0.3544	29	-0.88	0.3872	0.05	-1.0359	0.4137
Animal	3	0.4203	0.3847	29	1.09	0.2836	0.05	-0.3665	1.2072
Animal	4	0.1864	0.3848	29	0.48	0.6317	0.05	-0.6007	0.9735
Animal	5	0.03922	0.3524	29	0.11	0.9121	0.05	-0.6815	0.7599
Animal	6	-0.2257	0.3507	29	-0.64	0.5249	0.05	-0.9429	0.4915

Type 3 Tests of Fixed Effects				
Effect	Num DF	Den DF	F Value	Pr > F
Genotype	1	29	0.16	0.6957

Least Squares Means									
Effect	Genotype	Estimate	Standard Error	DF	t Value	Pr > |t|	Alpha	Lower	Upper
Genotype	Meg +/+	2.5621	0.3571	29	7.18	<.0001	0.05	1.8318	3.2924
Genotype	Meg +/-	2.3622	0.3586	29	6.59	<.0001	0.05	1.6287	3.0957

DistSoma=264

Differences of Least Squares Means												
Effect	Genotype	Genotype	Estimate	Standard Error	DF	t Value	Pr > |t|	Adjustment	Adj P	Alpha	Lower	Upper
**Genotype**	Meg +/+	Meg +/-	0.1999	0.5061	29	0.39	0.6957	Tukey-Kramer	0.6957	0.05	-0.8352	1.2350

Differences of Least Squares Means				
Effect	Genotype	Genotype	Adj Lower	Adj Upper
**Genotype**	Meg +/+	Meg +/-	-0.8352	1.2350

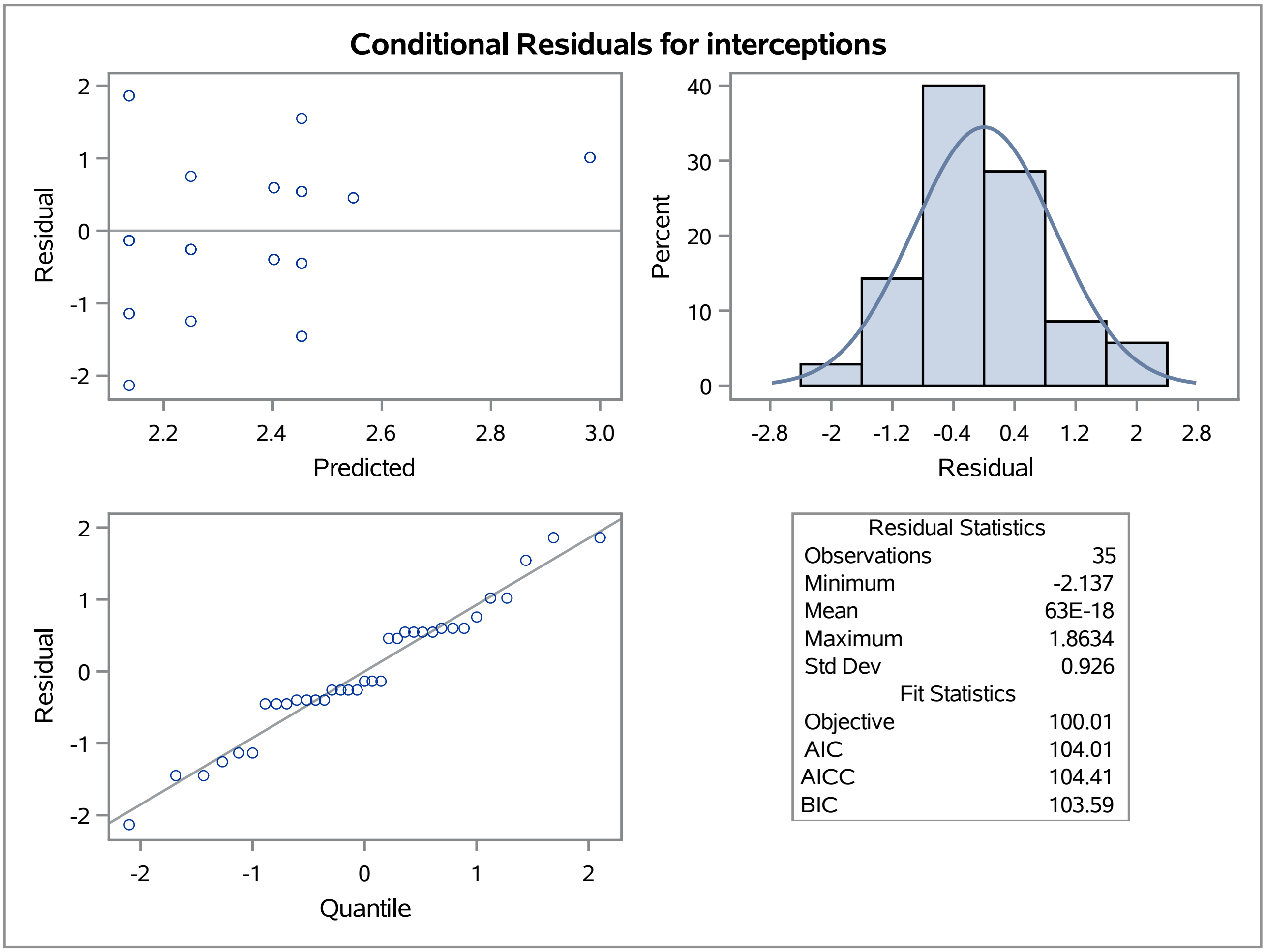

DistSoma=270

Model Information	
**Data Set**	WORK.TEMPDATASORTED
**Dependent Variable**	interceptions
**Covariance Structure**	Variance Components
**Estimation Method**	REML
**Residual Variance Method**	Profile
**Fixed Effects SE Method**	Model-Based
**Degrees of Freedom Method**	Containment

Class Level Information		
**Class**	**Levels**	**Values**
**Genotype**	2	Meg +/+ Meg +/-
**Animal**	6	1 2 3 4 5 6

Dimensions	
**Covariance Parameters**	2
**Columns in X**	3
**Columns in Z**	6
**Subjects**	1
**Max Obs per Subject**	35

Number of Observations	
**Number of Observations Read**	35
**Number of Observations Used**	35
**Number of Observations Not Used**	0

Iteration History			
**Iteration**	**Evaluations**	**-2 Res Log Like**	**Criterion**
**0**	1	95.35776012	
**1**	2	94.50756876	0.00447451
**2**	1	94.41857637	0.00049269
**3**	1	94.40963285	0.00000757
**4**	1	94.40950368	0.00000000

Convergence criteria met.

DistSoma=270

Covariance Parameter Estimates				
**Cov Parm**	**Estimate**	**Alpha**	**Lower**	**Upper**
**Animal**	0.2730	0.05	0.05191	563.77
**Residual**	0.7635	0.05	0.4771	1.4153

Fit Statistics	
**-2 Res Log Likelihood**	94.4
**AIC (Smaller is Better)**	98.4
**AICC (Smaller is Better)**	98.8
**BIC (Smaller is Better)**	98.0

Solution for Fixed Effects									
**Effect**	**Genotype**	**Estimate**	**Standard Error**	**DF**	**t Value**	**Pr > |t|**	**Alpha**	**Lower**	**Upper**
**Intercept**		2.0596	0.3819	4	5.39	0.0057	0.05	0.9994	3.1198
**Genotype**	Meg +/+	0.4897	0.5394	29	0.91	0.3715	0.05	-0.6136	1.5930
**Genotype**	Meg +/-	0	.	.	.	.	.	.	.

Solution for Random Effects									
**Effect**	**Animal**	**Estimate**	**Std Err Pred**	**DF**	**t Value**	**Pr > |t|**	**Alpha**	**Lower**	**Upper**
**Animal**	1	-0.1166	0.3851	29	-0.30	0.7642	0.05	-0.9043	0.6711
**Animal**	2	-0.4883	0.3929	29	-1.24	0.2238	0.05	-1.2919	0.3152
**Animal**	3	0.6050	0.4295	29	1.41	0.1696	0.05	-0.2734	1.4833
**Animal**	4	-0.02484	0.4296	29	-0.06	0.9543	0.05	-0.9034	0.8538
**Animal**	5	0.1616	0.3904	29	0.41	0.6819	0.05	-0.6368	0.9600
**Animal**	6	-0.1368	0.3883	29	-0.35	0.7272	0.05	-0.9309	0.6574

Type 3 Tests of Fixed Effects				
**Effect**	**Num DF**	**Den DF**	**F Value**	**Pr > F**
**Genotype**	1	29	0.82	0.3715

Least Squares Means									
**Effect**	**Genotype**	**Estimate**	**Standard Error**	**DF**	**t Value**	**Pr > |t|**	**Alpha**	**Lower**	**Upper**
**Genotype**	Meg +/+	2.5492	0.3810	29	6.69	<.0001	0.05	1.7699	3.3286
**Genotype**	Meg +/-	2.0596	0.3819	29	5.39	<.0001	0.05	1.2786	2.8405

DistSoma=270

Differences of Least Squares Means												
**Effect**	**Genotype**	**Genotype**	**Estimate**	**Standard Error**	**DF**	**t Value**	**Pr > |t|**	**Adjustment**	**Adj P**	**Alpha**	**Lower**	**Upper**
**Genotype**	Meg +/+	Meg +/-	0.4897	0.5394	29	0.91	0.3715	Tukey-Kramer	0.3715	0.05	-0.6136	1.5930

Differences of Least Squares Means				
**Effect**	**Genotype**	**Genotype**	**Adj Lower**	**Adj Upper**
**Genotype**	Meg +/+	Meg +/-	-0.6136	1.5930

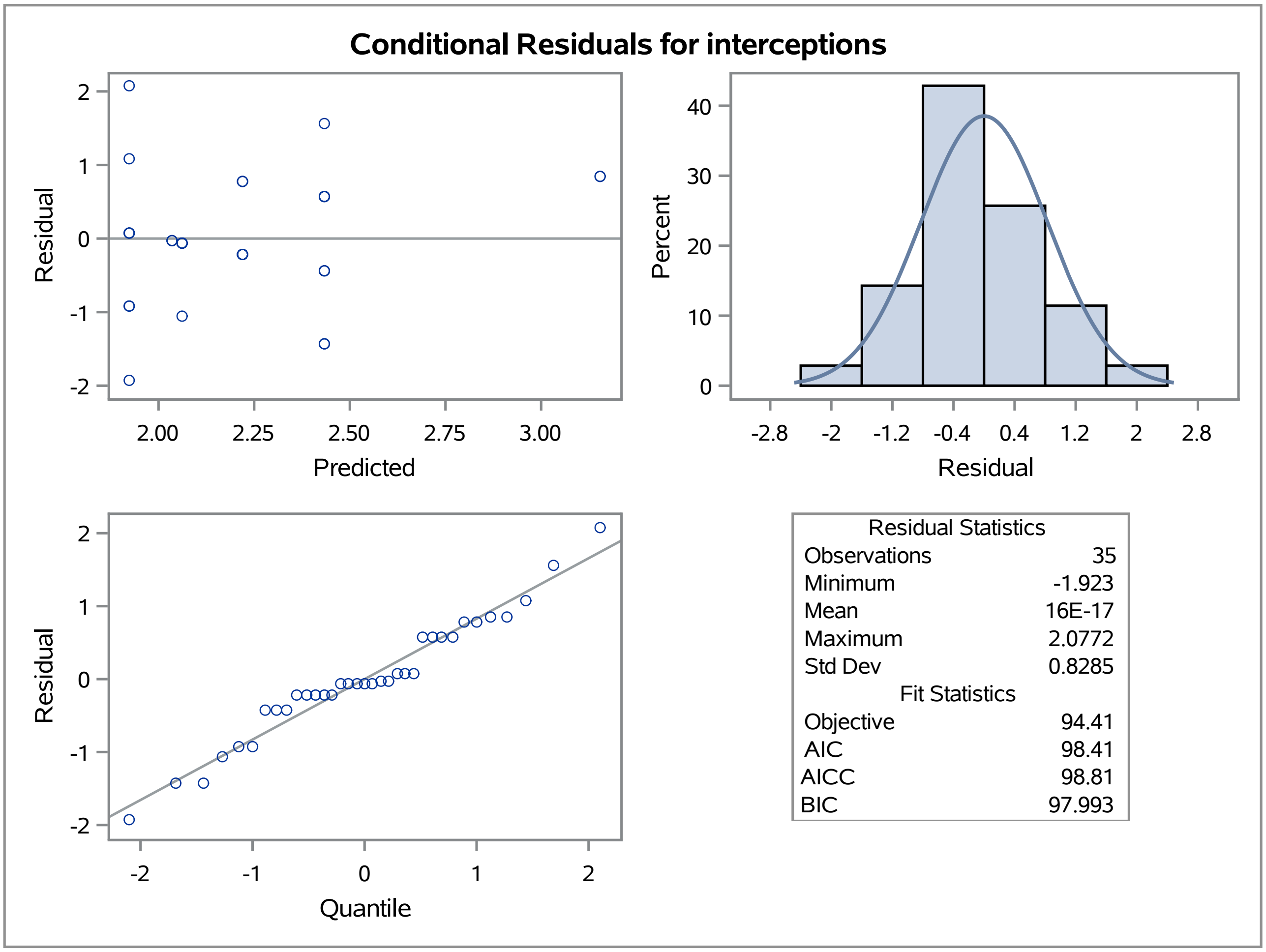

DistSoma=276

Model Information	
**Data Set**	WORK.TEMPDATASORTED
**Dependent Variable**	interceptions
**Covariance Structure**	Variance Components
**Estimation Method**	REML
**Residual Variance Method**	Profile
**Fixed Effects SE Method**	Model-Based
**Degrees of Freedom Method**	Containment

Class Level Information		
**Class**	**Levels**	**Values**
**Genotype**	2	Meg +/+ Meg +/-
**Animal**	6	1 2 3 4 5 6

Dimensions	
**Covariance Parameters**	2
**Columns in X**	3
**Columns in Z**	6
**Subjects**	1
**Max Obs per Subject**	35

Number of Observations	
**Number of Observations Read**	35
**Number of Observations Used**	35
**Number of Observations Not Used**	0

Iteration History			
**Iteration**	**Evaluations**	**-2 Res Log Like**	**Criterion**
**0**	1	95.04253606	
**1**	2	93.12578750	0.00824136
**2**	1	92.96472517	0.00135306
**3**	1	92.94044729	0.00004967
**4**	1	92.93962460	0.00000008
**5**	1	92.93962332	0.00000000

Convergence criteria met.

DistSoma=276

Covariance Parameter Estimates				
**Cov Parm**	**Estimate**	**Alpha**	**Lower**	**Upper**
**Animal**	0.3987	0.05	0.09391	52.6592
**Residual**	0.7039	0.05	0.4425	1.2918

Fit Statistics	
**-2 Res Log Likelihood**	92.9
**AIC (Smaller is Better)**	96.9
**AICC (Smaller is Better)**	97.3
**BIC (Smaller is Better)**	96.5

Solution for Fixed Effects									
**Effect**	**Genotype**	**Estimate**	**Standard Error**	**DF**	**t Value**	**Pr > |t|**	**Alpha**	**Lower**	**Upper**
**Intercept**		2.0585	0.4307	4	4.78	0.0088	0.05	0.8626	3.2544
**Genotype**	Meg +/+	0.4730	0.6088	29	0.78	0.4435	0.05	-0.7722	1.7181
**Genotype**	Meg +/-	0	.	.	.	.	.	.	.

Solution for Random Effects									
**Effect**	**Animal**	**Estimate**	**Std Err Pred**	**DF**	**t Value**	**Pr > |t|**	**Alpha**	**Lower**	**Upper**
**Animal**	1	-0.1118	0.4400	29	-0.25	0.8013	0.05	-1.0116	0.7880
**Animal**	2	-0.6682	0.4485	29	-1.49	0.1471	0.05	-1.5855	0.2491
**Animal**	3	0.7800	0.4890	29	1.59	0.1216	0.05	-0.2202	1.7802
**Animal**	4	-0.03108	0.4892	29	-0.06	0.9498	0.05	-1.0315	0.9694
**Animal**	5	0.1814	0.4457	29	0.41	0.6869	0.05	-0.7301	1.0929
**Animal**	6	-0.1503	0.4434	29	-0.34	0.7370	0.05	-1.0572	0.7565

Type 3 Tests of Fixed Effects				
**Effect**	**Num DF**	**Den DF**	**F Value**	**Pr > F**
**Genotype**	1	29	0.60	0.4435

Least Squares Means									
**Effect**	**Genotype**	**Estimate**	**Standard Error**	**DF**	**t Value**	**Pr > |t|**	**Alpha**	**Lower**	**Upper**
**Genotype**	Meg +/+	2.5315	0.4303	29	5.88	<.0001	0.05	1.6515	3.4115
**Genotype**	Meg +/-	2.0585	0.4307	29	4.78	<.0001	0.05	1.1776	2.9395

DistSoma=276

Differences of Least Squares Means												
Effect	Genotype	Genotype	Estimate	Standard Error	DF	t Value	Pr > |t|	Adjustment	Adj P	Alpha	Lower	Upper
**Genotype**	Meg +/+	Meg +/-	0.4730	0.6088	29	0.78	0.4435	Tukey-Kramer	0.4435	0.05	-0.7722	1.7181

Differences of Least Squares Means				
Effect	Genotype	Genotype	Adj Lower	Adj Upper
**Genotype**	Meg +/+	Meg +/-	-0.7722	1.7181

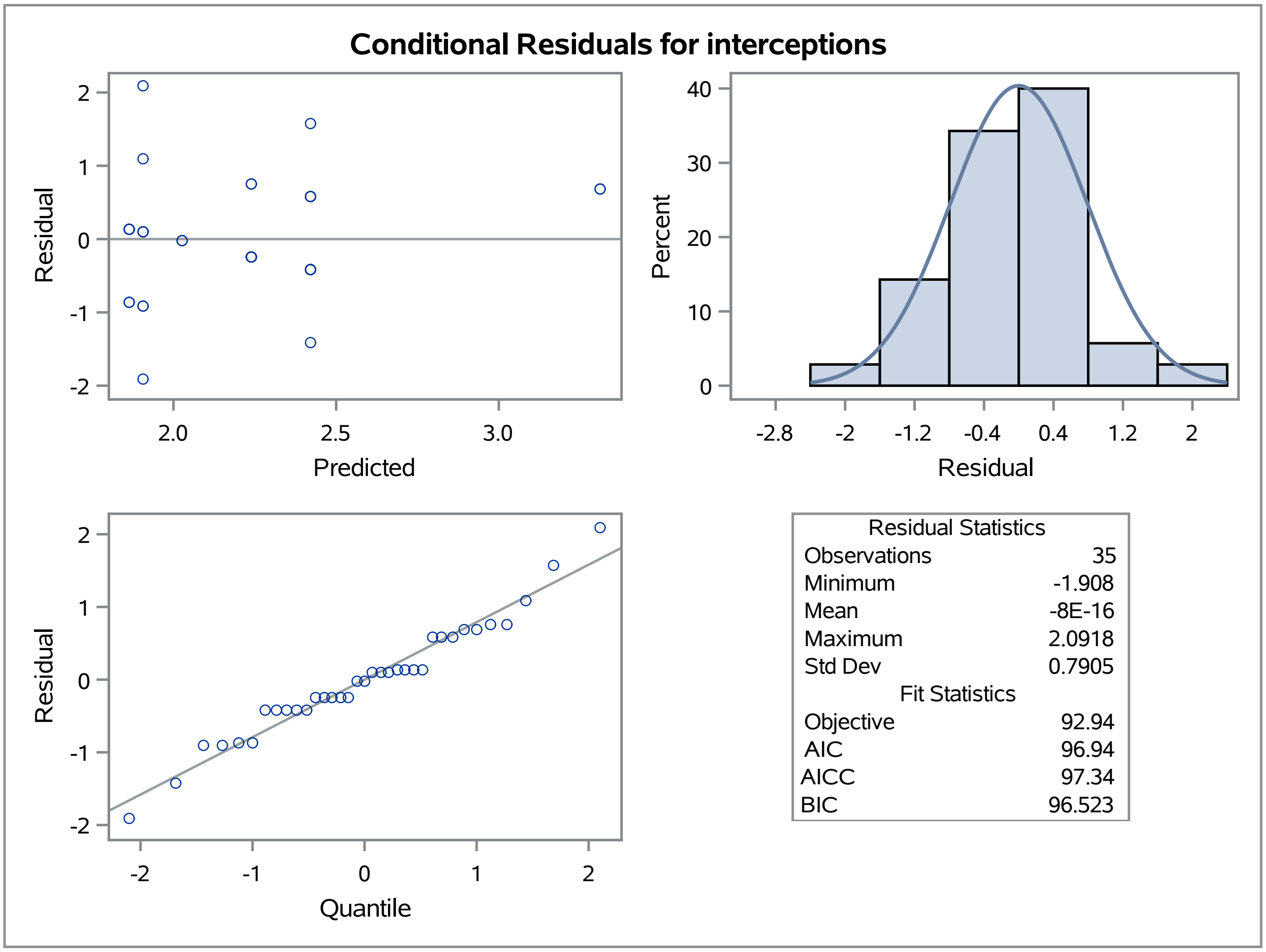

DistSoma=282

Model Information	
**Data Set**	WORK.TEMPDATASORTED
**Dependent Variable**	interceptions
**Covariance Structure**	Variance Components
**Estimation Method**	REML
**Residual Variance Method**	Profile
**Fixed Effects SE Method**	Model-Based
**Degrees of Freedom Method**	Containment

Class Level Information		
**Class**	**Levels**	**Values**
**Genotype**	2	Meg +/+ Meg +/-
**Animal**	6	1 2 3 4 5 6

Dimensions	
**Covariance Parameters**	2
**Columns in X**	3
**Columns in Z**	6
**Subjects**	1
**Max Obs per Subject**	35

Number of Observations	
**Number of Observations Read**	35
**Number of Observations Used**	35
**Number of Observations Not Used**	0

Iteration History			
**Iteration**	**Evaluations**	**-2 Res Log Like**	**Criterion**
**0**	1	95.23573634	
**1**	2	92.73161436	0.01307557
**2**	1	92.47472348	0.00281874
**3**	1	92.42349019	0.00018829
**4**	1	92.42035789	0.00000106
**5**	1	92.42034100	0.00000000

Convergence criteria met.

DistSoma=282

Covariance Parameter Estimates				
**Cov Parm**	**Estimate**	**Alpha**	**Lower**	**Upper**
**Animal**	0.4957	0.05	0.1263	31.9344
**Residual**	0.6774	0.05	0.4264	1.2403

Fit Statistics	
**-2 Res Log Likelihood**	92.4
**AIC (Smaller is Better)**	96.4
**AICC (Smaller is Better)**	96.8
**BIC (Smaller is Better)**	96.0

Solution for Fixed Effects									
**Effect**	**Genotype**	**Estimate**	**Standard Error**	**DF**	**t Value**	**Pr > |t|**	**Alpha**	**Lower**	**Upper**
**Intercept**		2.0056	0.4658	4	4.31	0.0126	0.05	0.7124	3.2988
**Genotype**	Meg +/+	0.4510	0.6584	29	0.68	0.4988	0.05	-0.8957	1.7977
**Genotype**	Meg +/-	0	.	.	.	.	.	.	.

Solution for Random Effects									
**Effect**	**Animal**	**Estimate**	**Std Err Pred**	**DF**	**t Value**	**Pr > |t|**	**Alpha**	**Lower**	**Upper**
**Animal**	1	-0.1378	0.4767	29	-0.29	0.7747	0.05	-1.1127	0.8372
**Animal**	2	-0.7791	0.4854	29	-1.60	0.1193	0.05	-1.7719	0.2137
**Animal**	3	0.9169	0.5269	29	1.74	0.0925	0.05	-0.1608	1.9945
**Animal**	4	-0.00332	0.5270	29	-0.01	0.9950	0.05	-1.0812	1.0746
**Animal**	5	0.1148	0.4825	29	0.24	0.8135	0.05	-0.8720	1.1017
**Animal**	6	-0.1115	0.4802	29	-0.23	0.8180	0.05	-1.0936	0.8705

Type 3 Tests of Fixed Effects				
**Effect**	**Num DF**	**Den DF**	**F Value**	**Pr > F**
**Genotype**	1	29	0.47	0.4988

Least Squares Means									
**Effect**	**Genotype**	**Estimate**	**Standard Error**	**DF**	**t Value**	**Pr > |t|**	**Alpha**	**Lower**	**Upper**
**Genotype**	Meg +/+	2.4566	0.4654	29	5.28	<.0001	0.05	1.5047	3.4085
**Genotype**	Meg +/-	2.0056	0.4658	29	4.31	0.0002	0.05	1.0530	2.9582

DistSoma=282

Differences of Least Squares Means												
**Effect**	**Genotype**	**Genotype**	**Estimate**	**Standard Error**	**DF**	**t Value**	**Pr > |t|**	**Adjustment**	**Adj P**	**Alpha**	**Lower**	**Upper**
**Genotype**	Meg +/+	Meg +/-	0.4510	0.6584	29	0.68	0.4988	Tukey-Kramer	0.4988	0.05	-0.8957	1.7977

Differences of Least Squares Means				
**Effect**	**Genotype**	**Genotype**	**Adj Lower**	**Adj Upper**
**Genotype**	Meg +/+	Meg +/-	-0.8957	1.7976

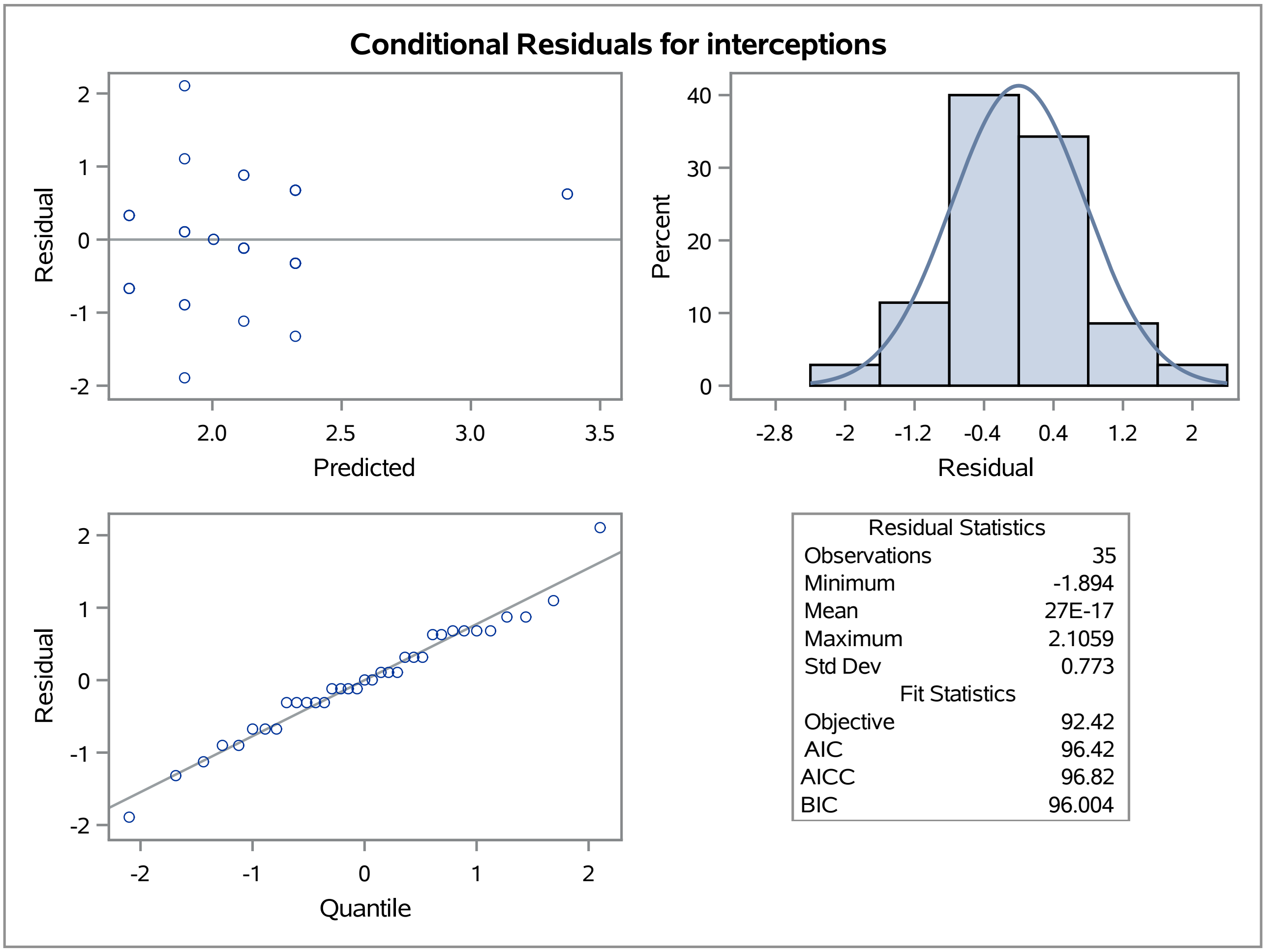

DistSoma=288

Model Information	
**Data Set**	WORK.TEMPDATASORTED
**Dependent Variable**	interceptions
**Covariance Structure**	Variance Components
**Estimation Method**	REML
**Residual Variance Method**	Profile
**Fixed Effects SE Method**	Model-Based
**Degrees of Freedom Method**	Containment

Class Level Information		
**Class**	**Levels**	**Values**
**Genotype**	2	Meg +/+ Meg +/-
**Animal**	6	1 2 3 4 5 6

Dimensions	
**Covariance Parameters**	2
**Columns in X**	3
**Columns in Z**	6
**Subjects**	1
**Max Obs per Subject**	35

Number of Observations	
**Number of Observations Read**	35
**Number of Observations Used**	35
**Number of Observations Not Used**	0

Iteration History			
**Iteration**	**Evaluations**	**-2 Res Log Like**	**Criterion**
**0**	1	99.48445277	
**1**	2	98.79796850	0.00371952
**2**	1	98.71603630	0.00031084
**3**	1	98.70975973	0.00000304
**4**	1	98.70970152	0.00000000

Convergence criteria met.

DistSoma=288

Covariance Parameter Estimates				
**Cov Parm**	**Estimate**	**Alpha**	**Lower**	**Upper**
**Animal**	0.3404	0.05	0.06450	741.67
**Residual**	0.8634	0.05	0.5377	1.6096

Fit Statistics	
**-2 Res Log Likelihood**	98.7
**AIC (Smaller is Better)**	102.7
**AICC (Smaller is Better)**	103.1
**BIC (Smaller is Better)**	102.3

Solution for Fixed Effects									
**Effect**	**Genotype**	**Estimate**	**Standard Error**	**DF**	**t Value**	**Pr > |t|**	**Alpha**	**Lower**	**Upper**
**Intercept**		1.9402	0.4195	4	4.63	0.0098	0.05	0.7755	3.1050
**Genotype**	Meg +/+	0.4754	0.5927	29	0.80	0.4290	0.05	-0.7368	1.6876
**Genotype**	Meg +/-	0	.	.	.	.	.	.	.

Solution for Random Effects									
**Effect**	**Animal**	**Estimate**	**Std Err Pred**	**DF**	**t Value**	**Pr > |t|**	**Alpha**	**Lower**	**Upper**
**Animal**	1	-0.1720	0.4248	29	-0.40	0.6885	0.05	-1.0408	0.6967
**Animal**	2	-0.5264	0.4333	29	-1.21	0.2342	0.05	-1.4126	0.3598
**Animal**	3	0.6985	0.4737	29	1.47	0.1511	0.05	-0.2704	1.6673
**Animal**	4	-0.1941	0.4738	29	-0.41	0.6851	0.05	-1.1632	0.7750
**Animal**	5	0.1487	0.4305	29	0.35	0.7323	0.05	-0.7318	1.0292
**Animal**	6	0.04537	0.4282	29	0.11	0.9164	0.05	-0.8305	0.9212

Type 3 Tests of Fixed Effects				
**Effect**	**Num DF**	**Den DF**	**F Value**	**Pr > F**
**Genotype**	1	29	0.64	0.4290

Least Squares Means									
**Effect**	**Genotype**	**Estimate**	**Standard Error**	**DF**	**t Value**	**Pr > |t|**	**Alpha**	**Lower**	**Upper**
**Genotype**	Meg +/+	2.4157	0.4187	29	5.77	<.0001	0.05	1.5593	3.2720
**Genotype**	Meg +/-	1.9402	0.4195	29	4.63	<.0001	0.05	1.0823	2.7982

DistSoma=288

Differences of Least Squares Means												
**Effect**	**Genotype**	**Genotype**	**Estimate**	**Standard Error**	**DF**	**t Value**	**Pr > |t|**	**Adjustment**	**Adj P**	**Alpha**	**Lower**	**Upper**
**Genotype**	Meg +/+	Meg +/-	0.4754	0.5927	29	0.80	0.4290	Tukey-Kramer	0.4290	0.05	-0.7368	1.6876

Differences of Least Squares Means				
**Effect**	**Genotype**	**Genotype**	**Adj Lower**	**Adj Upper**
**Genotype**	Meg +/+	Meg +/-	-0.7368	1.6876

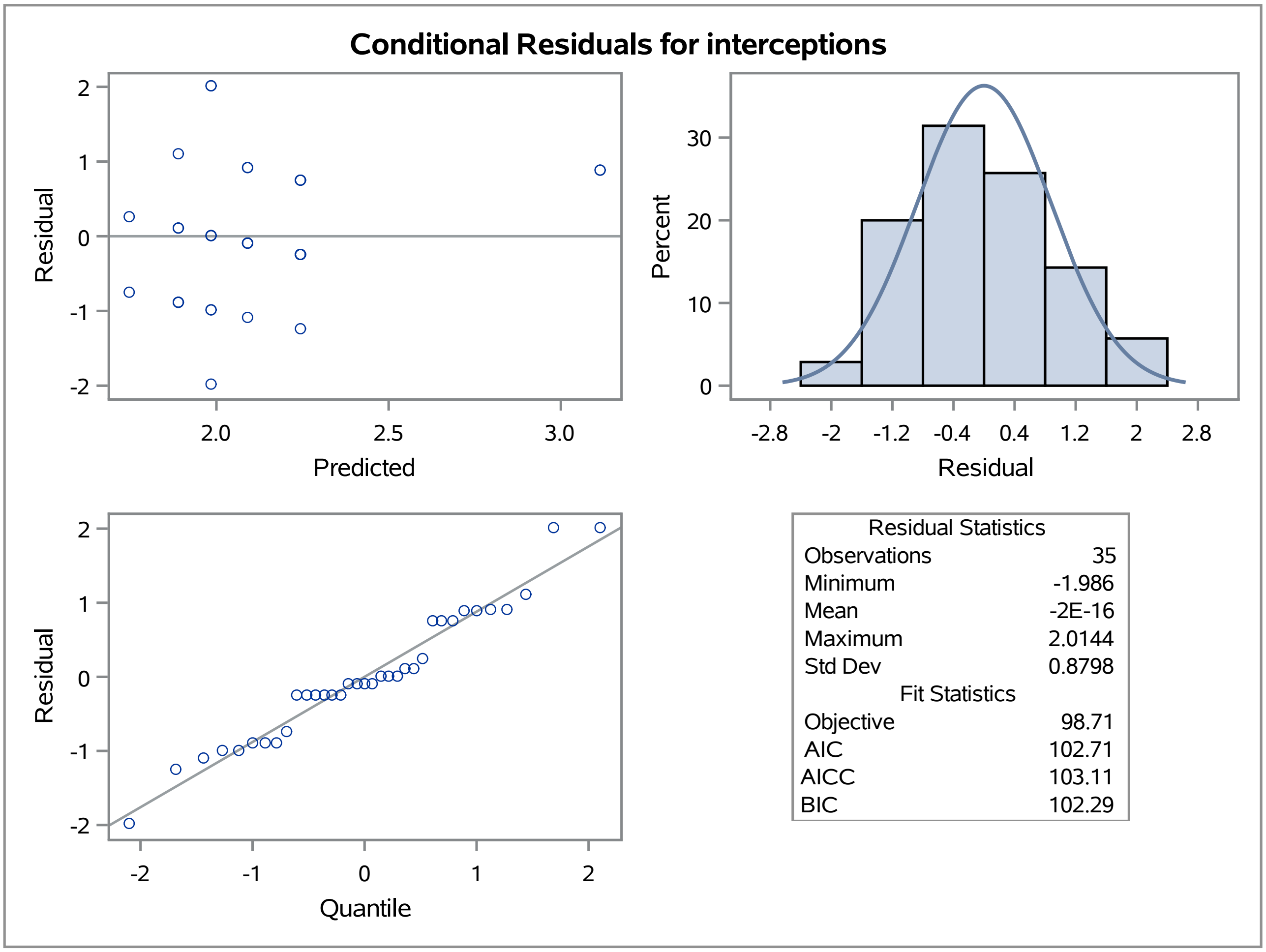

DistSoma=294

Model Information	
**Data Set**	WORK.TEMPDATASORTED
**Dependent Variable**	interceptions
**Covariance Structure**	Variance Components
**Estimation Method**	REML
**Residual Variance Method**	Profile
**Fixed Effects SE Method**	Model-Based
**Degrees of Freedom Method**	Containment

Class Level Information

Class	Levels	Values
**Genotype**	2	Meg +/+ Meg +/-
**Animal**	6	1 2 3 4 5 6

Dimensions	
**Covariance Parameters**	2
**Columns in X**	3
**Columns in Z**	6
**Subjects**	1
**Max Obs per Subject**	35

Number of Observations	
**Number of Observations Read**	35
**Number of Observations Used**	35
**Number of Observations Not Used**	0

Iteration History

Iteration	Evaluations	-2 Res Log Like	Criterion
**0**	1	94.77690342	
**1**	2	94.56367941	0.00107218
**2**	1	94.54352572	0.00003714
**3**	1	94.54288168	0.00000005
**4**	1	94.54288086	0.00000000

Convergence criteria met.

DistSoma=294

Covariance Parameter Estimates				
**Cov Parm**	**Estimate**	**Alpha**	**Lower**	**Upper**
**Animal**	0.1371	0.05	0.01658	3.4547E8
**Residual**	0.8033	0.05	0.4942	1.5300

Fit Statistics	
**-2 Res Log Likelihood**	94.5
**AIC (Smaller is Better)**	98.5
**AICC (Smaller is Better)**	98.9
**BIC (Smaller is Better)**	98.1

Solution for Fixed Effects									
**Effect**	**Genotype**	**Estimate**	**Standard Error**	**DF**	**t Value**	**Pr > |t|**	**Alpha**	**Lower**	**Upper**
**Intercept**		1.8551	0.3157	4	5.88	0.0042	0.05	0.9787	2.7315
**Genotype**	Meg +/+	0.2828	0.4452	29	0.64	0.5303	0.05	-0.6278	1.1934
**Genotype**	Meg +/-	0	.	.	.	.	.	.	.

Solution for Random Effects									
**Effect**	**Animal**	**Estimate**	**Std Err Pred**	**DF**	**t Value**	**Pr > |t|**	**Alpha**	**Lower**	**Upper**
**Animal**	1	-0.02390	0.2998	29	-0.08	0.9370	0.05	-0.6370	0.5892
**Animal**	2	-0.3228	0.3049	29	-1.06	0.2986	0.05	-0.9464	0.3009
**Animal**	3	0.3467	0.3296	29	1.05	0.3015	0.05	-0.3274	1.0207
**Animal**	4	-0.09038	0.3297	29	-0.27	0.7859	0.05	-0.7646	0.5839
**Animal**	5	0.07889	0.3033	29	0.26	0.7966	0.05	-0.5415	0.6993
**Animal**	6	0.01149	0.3019	29	0.04	0.9699	0.05	-0.6061	0.6290

Type 3 Tests of Fixed Effects				
**Effect**	**Num DF**	**Den DF**	**F Value**	**Pr > F**
**Genotype**	1	29	0.40	0.5303

Least Squares Means									
**Effect**	**Genotype**	**Estimate**	**Standard Error**	**DF**	**t Value**	**Pr > |t|**	**Alpha**	**Lower**	**Upper**
**Genotype**	Meg +/+	2.1379	0.3140	29	6.81	<.0001	0.05	1.4958	2.7800
**Genotype**	Meg +/-	1.8551	0.3157	29	5.88	<.0001	0.05	1.2095	2.5007

DistSoma=294

Differences of Least Squares Means												
Effect	Genotype	Genotype	Estimate	Standard Error	DF	t Value	Pr > |t|	Adjustment	Adj P	Alpha	Lower	Upper
**Genotype**	Meg +/+	Meg +/-	0.2828	0.4452	29	0.64	0.5303	Tukey-Kramer	0.5303	0.05	-0.6278	1.1934

Differences of Least Squares Means				
Effect	Genotype	Genotype	Adj Lower	Adj Upper
**Genotype**	Meg +/+	Meg +/-	-0.6277	1.1934

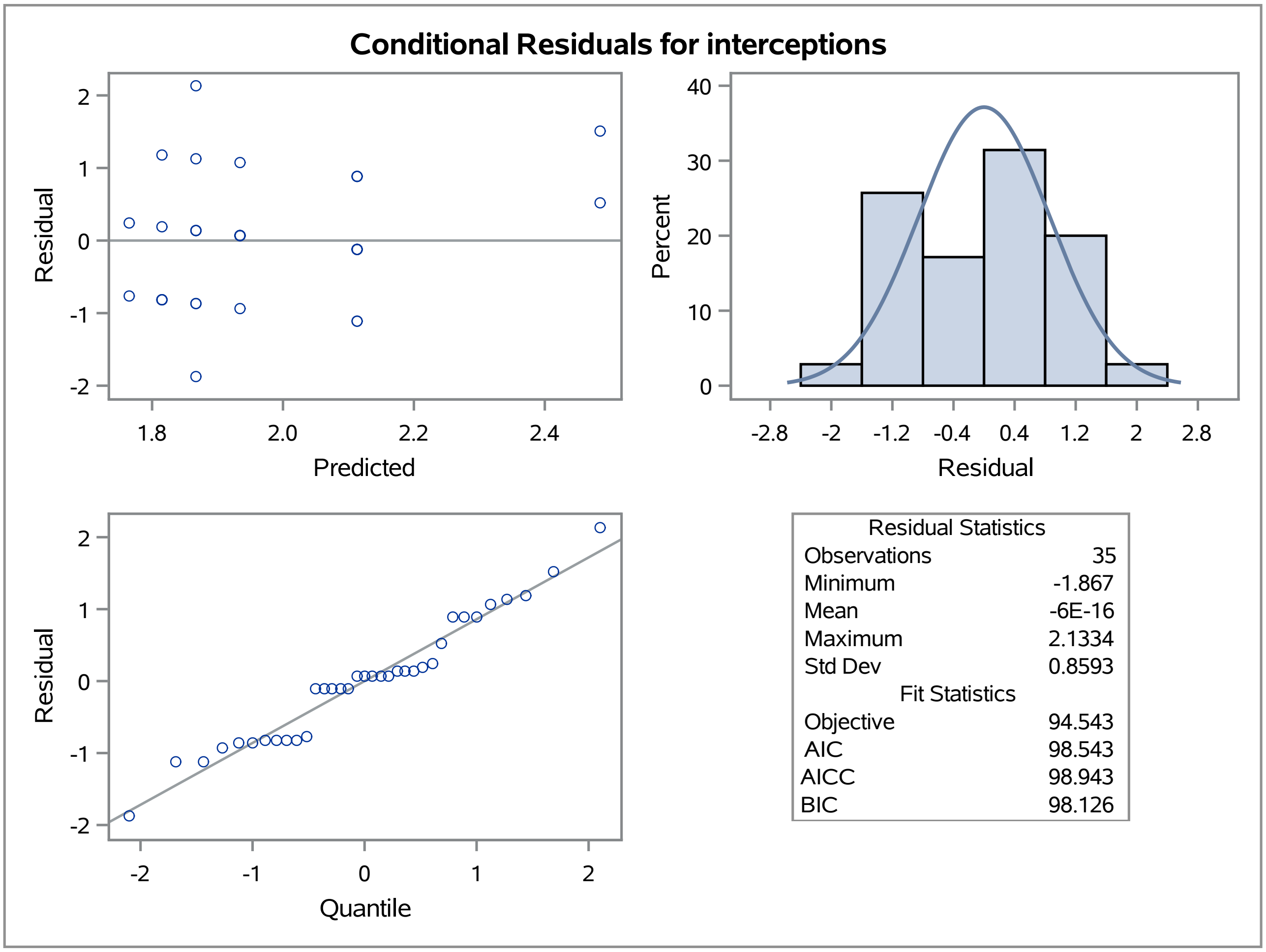

DistSoma=300

Model Information	
**Data Set**	WORK.TEMPDATASORTED
**Dependent Variable**	interceptions
**Covariance Structure**	Variance Components
**Estimation Method**	REML
**Residual Variance Method**	Profile
**Fixed Effects SE Method**	Model-Based
**Degrees of Freedom Method**	Containment

Class Level Information		
**Class**	**Levels**	**Values**
**Genotype**	2	Meg +/+ Meg +/-
**Animal**	6	1 2 3 4 5 6

Dimensions	
**Covariance Parameters**	2
**Columns in X**	3
**Columns in Z**	6
**Subjects**	1
**Max Obs per Subject**	35

Number of Observations	
**Number of Observations Read**	35
**Number of Observations Used**	35
**Number of Observations Not Used**	0

Iteration History			
**Iteration**	**Evaluations**	**-2 Res Log Like**	**Criterion**
**0**	1	95.57847212	
**1**	1	95.57847212	0.00000000

Convergence criteria met.

Estimated G matrix is not positive definite.

Covariance Parameter Estimates				
**Cov Parm**	**Estimate**	**Alpha**	**Lower**	**Upper**
**Animal**	0	.	.	.
**Residual**	0.8914	0.05	0.5799	1.5444

DistSoma=300

Fit Statistics	
-2 Res Log Likelihood	95.6
AIC (Smaller is Better)	97.6
AICC (Smaller is Better)	97.7
BIC (Smaller is Better)	97.4

Solution for Fixed Effects

Effect	Genotype	Estimate	Standard Error	DF	t Value	Pr > |t|	Alpha	Lower	Upper
Intercept		1.8235	0.2290	4	7.96	0.0013	0.05	1.1878	2.4593
Genotype	Meg +/+	0.2320	0.3193	29	0.73	0.4733	0.05	-0.4210	0.8851
Genotype	Meg +/-	0	.	.	.	.	.	.	.

Solution for Random Effects

Effect	Animal	Estimate	Std Err Pred	DF	t Value	Pr > |t|	Alpha	Lower	Upper
Animal	1	0	.	.	.	.	.	.	.
Animal	2	0	.	.	.	.	.	.	.
Animal	3	0	.	.	.	.	.	.	.
Animal	4	0	.	.	.	.	.	.	.
Animal	5	0	.	.	.	.	.	.	.
Animal	6	0	.	.	.	.	.	.	.

Type 3 Tests of Fixed Effects

Effect	Num DF	Den DF	F Value	Pr > F
Genotype	1	29	0.53	0.4733

Least Squares Means

Effect	Genotype	Estimate	Standard Error	DF	t Value	Pr > |t|	Alpha	Lower	Upper
Genotype	Meg +/+	2.0556	0.2225	29	9.24	<.0001	0.05	1.6004	2.5107
Genotype	Meg +/-	1.8235	0.2290	29	7.96	<.0001	0.05	1.3552	2.2919

DistSoma=300

Differences of Least Squares Means												
**Effect**	**Genotype**	**Genotype**	**Estimate**	**Standard Error**	**DF**	**t Value**	**Pr > |t|**	**Adjustment**	**Adj P**	**Alpha**	**Lower**	**Upper**
**Genotype**	Meg +/+	Meg +/-	0.2320	0.3193	29	0.73	0.4733	Tukey-Kramer	0.4733	0.05	-0.4210	0.8851

Differences of Least Squares Means				
**Effect**	**Genotype**	**Genotype**	**Adj Lower**	**Adj Upper**
**Genotype**	Meg +/+	Meg +/-	-0.4210	0.8851

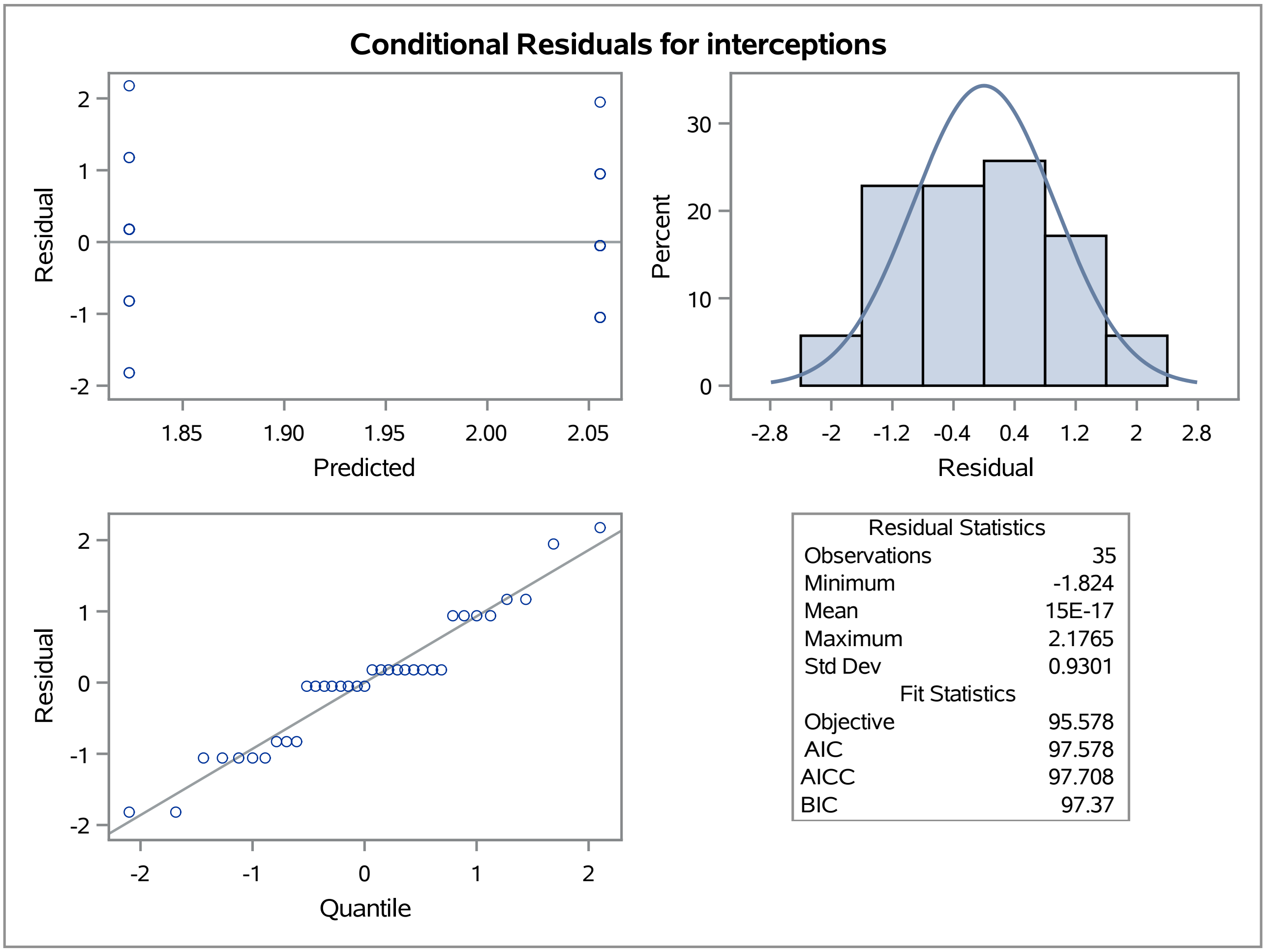

DistSoma=306

Model Information	
**Data Set**	WORK.TEMPDATASORTED
**Dependent Variable**	interceptions
**Covariance Structure**	Variance Components
**Estimation Method**	REML
**Residual Variance Method**	Profile
**Fixed Effects SE Method**	Model-Based
**Degrees of Freedom Method**	Containment

Class Level Information		
**Class**	**Levels**	**Values**
**Genotype**	2	Meg +/+ Meg +/-
**Animal**	6	1 2 3 4 5 6

Dimensions	
**Covariance Parameters**	2
**Columns in X**	3
**Columns in Z**	6
**Subjects**	1
**Max Obs per Subject**	35

Number of Observations	
**Number of Observations Read**	35
**Number of Observations Used**	35
**Number of Observations Not Used**	0

Iteration History			
**Iteration**	**Evaluations**	**-2 Res Log Like**	**Criterion**
**0**	1	102.67348119	
**1**	1	102.67348119	0.00000000

Convergence criteria met.

Estimated G matrix is not positive definite.

Covariance Parameter Estimates				
**Cov Parm**	**Estimate**	**Alpha**	**Lower**	**Upper**
**Animal**	0	.	.	.
**Residual**	1.1052	0.05	0.7190	1.9148

DistSoma=306

Fit Statistics	
-2 Res Log Likelihood	102.7
AIC (Smaller is Better)	104.7
AICC (Smaller is Better)	104.8
BIC (Smaller is Better)	104.5

Solution for Fixed Effects

Effect	Genotype	Estimate	Standard Error	DF	t Value	Pr > |t|	Alpha	Lower	Upper
Intercept		1.8235	0.2550	4	7.15	0.0020	0.05	1.1156	2.5314
Genotype	Meg +/+	0.1765	0.3555	29	0.50	0.6234	0.05	-0.5507	0.9036
Genotype	Meg +/-	0	.	.	.	.	.	.	.

Solution for Random Effects

Effect	Animal	Estimate	Std Err Pred	DF	t Value	Pr > |t|	Alpha	Lower	Upper
Animal	1	0	.	.	.	.	.	.	.
Animal	2	0	.	.	.	.	.	.	.
Animal	3	0	.	.	.	.	.	.	.
Animal	4	0	.	.	.	.	.	.	.
Animal	5	0	.	.	.	.	.	.	.
Animal	6	0	.	.	.	.	.	.	.

Type 3 Tests of Fixed Effects

Effect	Num DF	Den DF	F Value	Pr > F
Genotype	1	29	0.25	0.6234

Least Squares Means

Effect	Genotype	Estimate	Standard Error	DF	t Value	Pr > |t|	Alpha	Lower	Upper
Genotype	Meg +/+	2.0000	0.2478	29	8.07	<.0001	0.05	1.4932	2.5068
Genotype	Meg +/-	1.8235	0.2550	29	7.15	<.0001	0.05	1.3021	2.3450

DistSoma=306

Differences of Least Squares Means												
Effect	Genotype	Genotype	Estimate	Standard Error	DF	t Value	Pr > |t|	Adjustment	Adj P	Alpha	Lower	Upper
Genotype	Meg +/+	Meg +/-	0.1765	0.3555	29	0.50	0.6234	Tukey-Kramer	0.6234	0.05	-0.5507	0.9036

Differences of Least Squares Means				
Effect	Genotype	Genotype	Adj Lower	Adj Upper
Genotype	Meg +/+	Meg +/-	-0.5507	0.9036

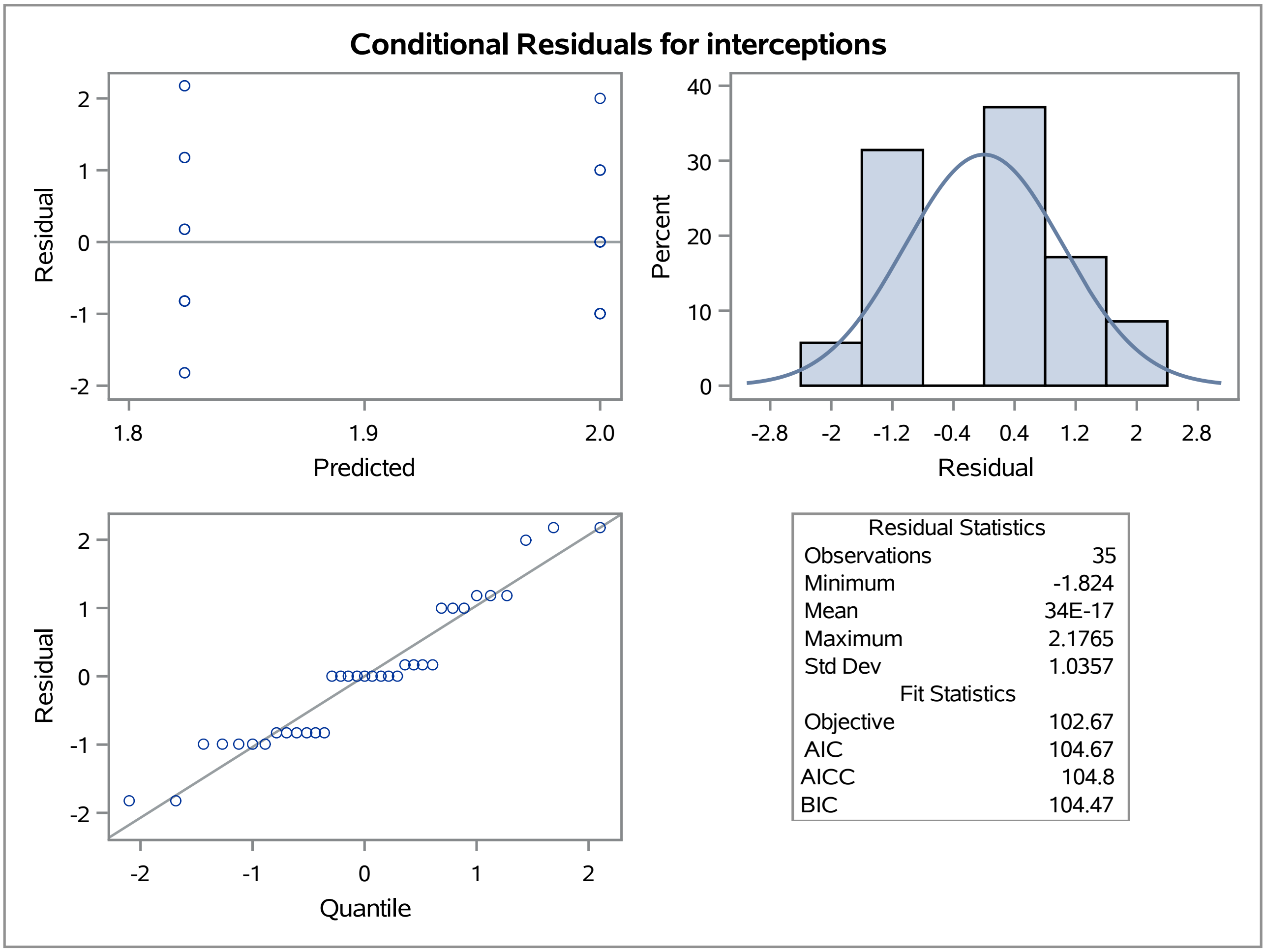

DistSoma=312

Model Information	
Data Set	WORK.TEMPDATASORTED
Dependent Variable	interceptions
Covariance Structure	Variance Components
Estimation Method	REML
Residual Variance Method	Profile
Fixed Effects SE Method	Model-Based
Degrees of Freedom Method	Containment

Class Level Information		
Class	Levels	Values
Genotype	2	Meg +/+ Meg +/-
Animal	6	1 2 3 4 5 6

Dimensions	
Covariance Parameters	2
Columns in X	3
Columns in Z	6
Subjects	1
Max Obs per Subject	35

Number of Observations	
Number of Observations Read	35
Number of Observations Used	35
Number of Observations Not Used	0

Iteration History			
Iteration	Evaluations	-2 Res Log Like	Criterion
0	1	100.81541987	
1	2	100.78228413	0.00000461
2	1	100.78219052	0.00000000

Convergence criteria met.

Covariance Parameter Estimates				
Cov Parm	Estimate	Alpha	Lower	Upper
Animal	0.03506	0.05	0.003236	1.583E57
Residual	1.0238	0.05	0.6430	1.8815

DistSoma=312

Fit Statistics	
-2 Res Log Likelihood	100.8
AIC (Smaller is Better)	104.8
AICC (Smaller is Better)	105.2
BIC (Smaller is Better)	104.4

Solution for Fixed Effects

Effect	Genotype	Estimate	Standard Error	DF	t Value	Pr > |t|	Alpha	Lower	Upper
Intercept		1.6921	0.2724	4	6.21	0.0034	0.05	0.9358	2.4484
Genotype	Meg +/+	0.2588	0.3818	29	0.68	0.5032	0.05	-0.5221	1.0398
Genotype	Meg +/-	0	.	.	.	.	.	.	.

Solution for Random Effects

Effect	Animal	Estimate	Std Err Pred	DF	t Value	Pr > |t|	Alpha	Lower	Upper
Animal	1	0.03802	0.1754	29	0.22	0.8299	0.05	-0.3208	0.3968
Animal	2	-0.1053	0.1765	29	-0.60	0.5556	0.05	-0.4663	0.2558
Animal	3	0.06724	0.1819	29	0.37	0.7144	0.05	-0.3049	0.4394
Animal	4	-0.04436	0.1820	29	-0.24	0.8091	0.05	-0.4165	0.3278
Animal	5	0.03191	0.1762	29	0.18	0.8576	0.05	-0.3285	0.3923
Animal	6	0.01245	0.1759	29	0.07	0.9441	0.05	-0.3474	0.3723

Type 3 Tests of Fixed Effects

Effect	Num DF	Den DF	F Value	Pr > F
Genotype	1	29	0.46	0.5032

Least Squares Means

Effect	Genotype	Estimate	Standard Error	DF	t Value	Pr > |t|	Alpha	Lower	Upper
Genotype	Meg +/+	1.9509	0.2676	29	7.29	<.0001	0.05	1.4037	2.4982
Genotype	Meg +/-	1.6921	0.2724	29	6.21	<.0001	0.05	1.1350	2.2492

DistSoma=312

Differences of Least Squares Means												
**Effect**	**Genotype**	**Genotype**	**Estimate**	**Standard Error**	**DF**	**t Value**	**Pr > |t|**	**Adjustment**	**Adj P**	**Alpha**	**Lower**	**Upper**
**Genotype**	Meg +/+	Meg +/-	0.2588	0.3818	29	0.68	0.5032	Tukey-Kramer	0.5032	0.05	-0.5221	1.0398

Differences of Least Squares Means				
**Effect**	**Genotype**	**Genotype**	**Adj Lower**	**Adj Upper**
**Genotype**	Meg +/+	Meg +/-	-0.5221	1.0398

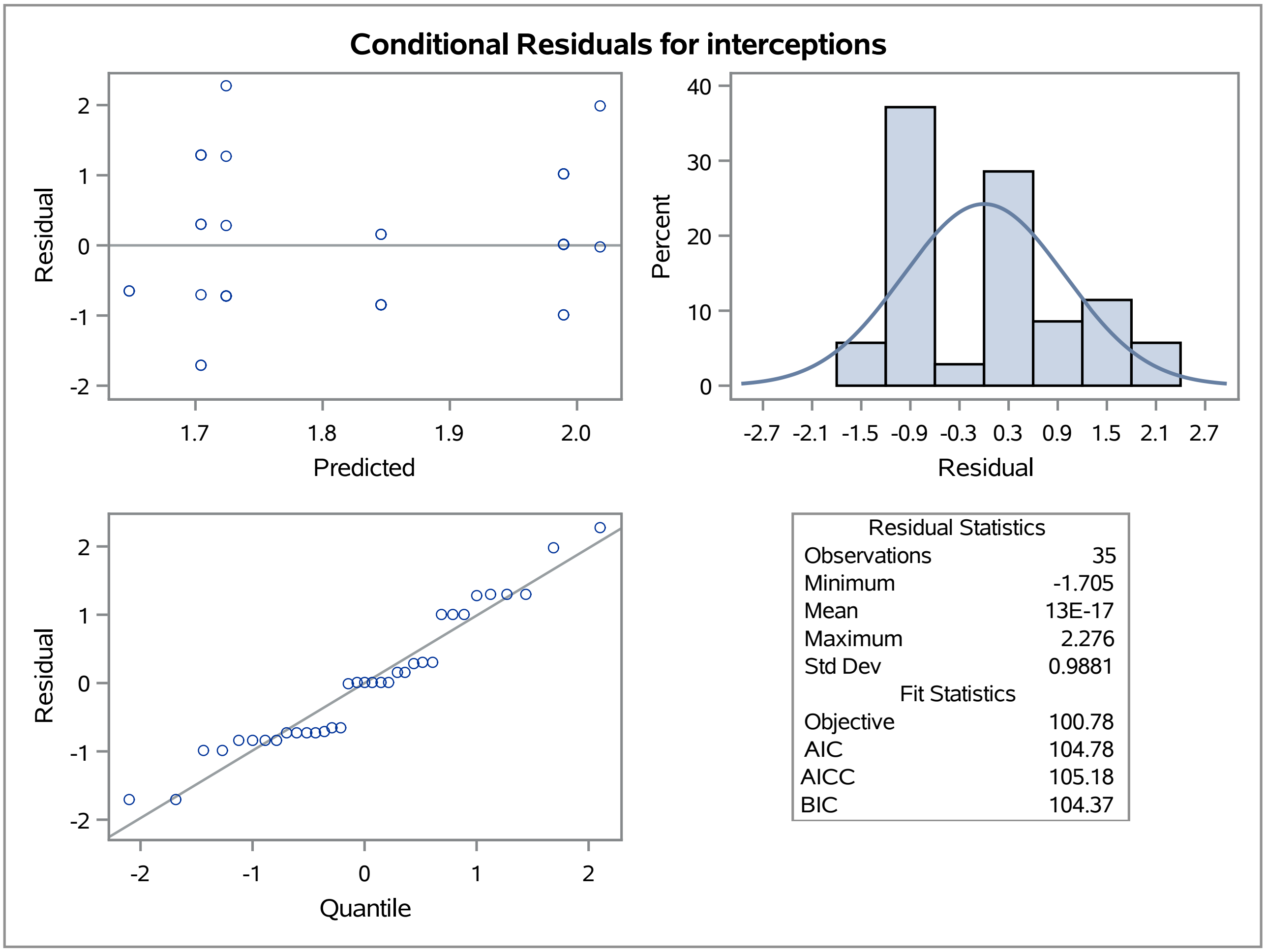

DistSoma=318

Model Information	
**Data Set**	WORK.TEMPDATASORTED
**Dependent Variable**	interceptions
**Covariance Structure**	Variance Components
**Estimation Method**	REML
**Residual Variance Method**	Profile
**Fixed Effects SE Method**	Model-Based
**Degrees of Freedom Method**	Containment

Class Level Information		
**Class**	**Levels**	**Values**
**Genotype**	2	Meg +/+ Meg +/-
**Animal**	6	1 2 3 4 5 6

Dimensions	
**Covariance Parameters**	2
**Columns in X**	3
**Columns in Z**	6
**Subjects**	1
**Max Obs per Subject**	35

Number of Observations	
**Number of Observations Read**	35
**Number of Observations Used**	35
**Number of Observations Not Used**	0

Iteration History			
**Iteration**	**Evaluations**	**-2 Res Log Like**	**Criterion**
**0**	1	103.30898913	
**1**	2	103.04880196	0.00024154
**2**	1	103.04329805	0.00000366
**3**	1	103.04321968	0.00000000

Convergence criteria met.

DistSoma=318

Covariance Parameter Estimates				
Cov Parm	Estimate	Alpha	Lower	Upper
Animal	0.1300	0.05	0.01559	5.2274E8
Residual	1.0559	0.05	0.6602	1.9555

Fit Statistics	
-2 Res Log Likelihood	103.0
AIC (Smaller is Better)	107.0
AICC (Smaller is Better)	107.4
BIC (Smaller is Better)	106.6

Solution for Fixed Effects									
Effect	Genotype	Estimate	Standard Error	DF	t Value	Pr > |t|	Alpha	Lower	Upper
Intercept		1.4721	0.3352	4	4.39	0.0118	0.05	0.5413	2.4029
Genotype	Meg +/+	0.5019	0.4723	29	1.06	0.2967	0.05	-0.4641	1.4679
Genotype	Meg +/-	0	.	.	.	.	.	.	.

Solution for Random Effects									
Effect	Animal	Estimate	Std Err Pred	DF	t Value	Pr > |t|	Alpha	Lower	Upper
Animal	1	0.06950	0.3033	29	0.23	0.8203	0.05	-0.5507	0.6897
Animal	2	-0.2722	0.3078	29	-0.88	0.3838	0.05	-0.9017	0.3573
Animal	3	0.2027	0.3296	29	0.62	0.5434	0.05	-0.4714	0.8768
Animal	4	-0.1921	0.3297	29	-0.58	0.5647	0.05	-0.8663	0.4822
Animal	5	0.1782	0.3064	29	0.58	0.5653	0.05	-0.4485	0.8049
Animal	6	0.01384	0.3052	29	0.05	0.9641	0.05	-0.6104	0.6381

Type 3 Tests of Fixed Effects				
Effect	Num DF	Den DF	F Value	Pr > F
Genotype	1	29	1.13	0.2967

Least Squares Means									
Effect	Genotype	Estimate	Standard Error	DF	t Value	Pr > |t|	Alpha	Lower	Upper
Genotype	Meg +/+	1.9740	0.3327	29	5.93	<.0001	0.05	1.2936	2.6545
Genotype	Meg +/-	1.4721	0.3352	29	4.39	0.0001	0.05	0.7865	2.1578

DistSoma=318

Differences of Least Squares Means												
**Effect**	**Genotype**	**Genotype**	**Estimate**	**Standard Error**	**DF**	**t Value**	**Pr > |t|**	**Adjustment**	**Adj P**	**Alpha**	**Lower**	**Upper**
**Genotype**	Meg +/+	Meg +/-	0.5019	0.4723	29	1.06	0.2967	Tukey-Kramer	0.2967	0.05	-0.4641	1.4679

Differences of Least Squares Means				
**Effect**	**Genotype**	**Genotype**	**Adj Lower**	**Adj Upper**
**Genotype**	Meg +/+	Meg +/-	-0.4641	1.4679

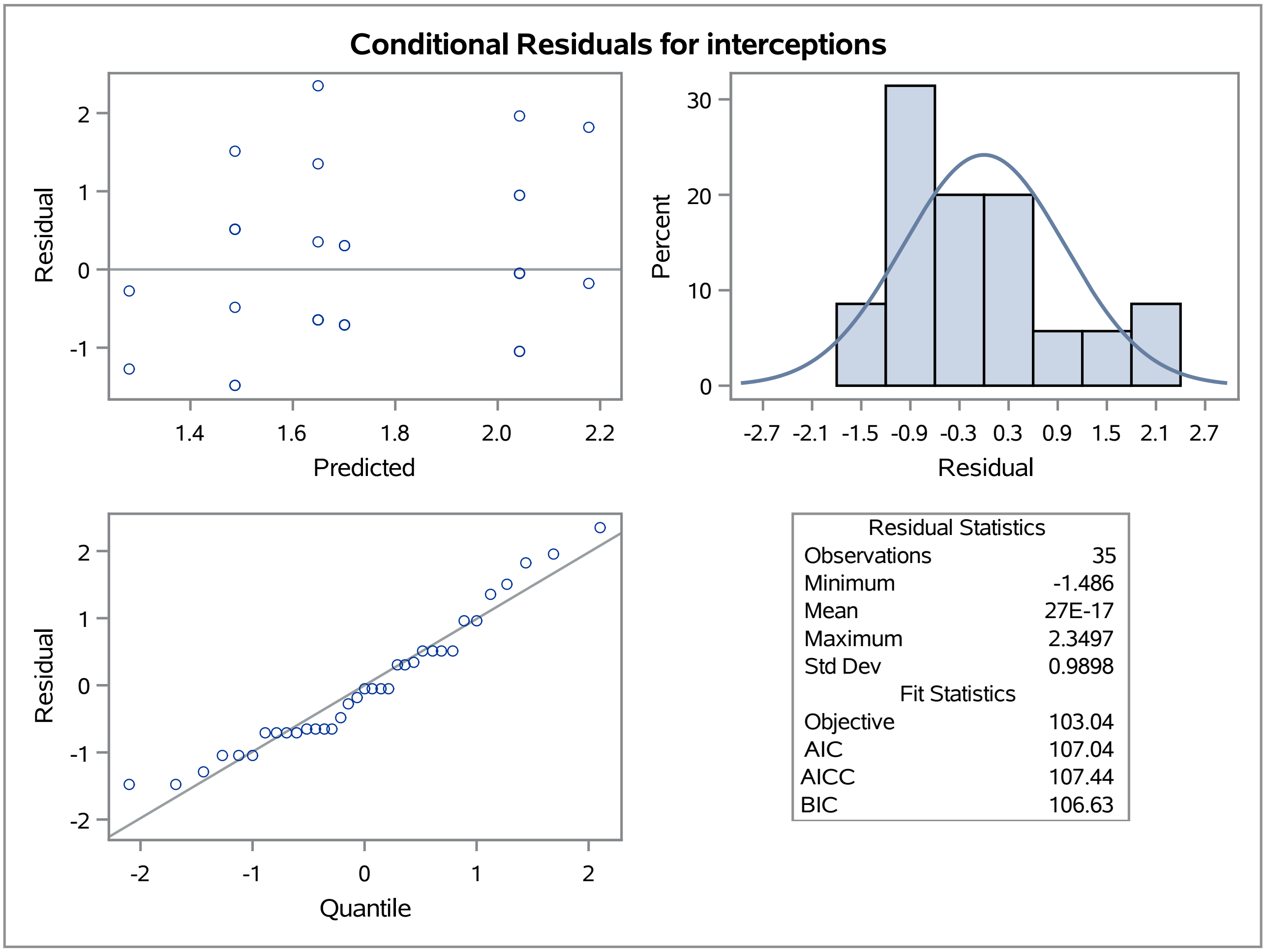

DistSoma=324

Model Information	
**Data Set**	WORK.TEMPDATASORTED
**Dependent Variable**	interceptions
**Covariance Structure**	Variance Components
**Estimation Method**	REML
**Residual Variance Method**	Profile
**Fixed Effects SE Method**	Model-Based
**Degrees of Freedom Method**	Containment

Class Level Information		
**Class**	**Levels**	**Values**
**Genotype**	2	Meg +/+ Meg +/-
**Animal**	6	1 2 3 4 5 6

Dimensions	
**Covariance Parameters**	2
**Columns in X**	3
**Columns in Z**	6
**Subjects**	1
**Max Obs per Subject**	35

Number of Observations	
**Number of Observations Read**	35
**Number of Observations Used**	35
**Number of Observations Not Used**	0

Iteration History			
**Iteration**	**Evaluations**	**-2 Res Log Like**	**Criterion**
**0**	1	101.48429892	
**1**	2	101.29724495	0.00018688
**2**	1	101.29318917	0.00000228
**3**	1	101.29314248	0.00000000

Convergence criteria met.

DistSoma=324

Covariance Parameter Estimates				
Cov Parm	Estimate	Alpha	Lower	Upper
Animal	0.1047	0.05	0.01137	4.372E11
Residual	1.0085	0.05	0.6294	1.8738

Fit Statistics	
-2 Res Log Likelihood	101.3
AIC (Smaller is Better)	105.3
AICC (Smaller is Better)	105.7
BIC (Smaller is Better)	104.9

Solution for Fixed Effects									
Effect	Genotype	Estimate	Standard Error	DF	t Value	Pr > |t|	Alpha	Lower	Upper
Intercept		1.4219	0.3163	4	4.50	0.0109	0.05	0.5437	2.3001
Genotype	Meg +/+	0.5471	0.4453	29	1.23	0.2291	0.05	-0.3637	1.4579
Genotype	Meg +/-	0	.	.	.	.	.	.	.

Solution for Random Effects									
Effect	Animal	Estimate	Std Err Pred	DF	t Value	Pr > |t|	Alpha	Lower	Upper
Animal	1	0.06672	0.2772	29	0.24	0.8115	0.05	-0.5003	0.6337
Animal	2	-0.2440	0.2810	29	-0.87	0.3925	0.05	-0.8187	0.3308
Animal	3	0.1772	0.2993	29	0.59	0.5584	0.05	-0.4349	0.7894
Animal	4	-0.1585	0.2994	29	-0.53	0.6006	0.05	-0.7708	0.4539
Animal	5	0.1230	0.2799	29	0.44	0.6635	0.05	-0.4494	0.6955
Animal	6	0.03543	0.2789	29	0.13	0.8998	0.05	-0.5350	0.6058

Type 3 Tests of Fixed Effects				
Effect	Num DF	Den DF	F Value	Pr > F
Genotype	1	29	1.51	0.2291

Least Squares Means									
Effect	Genotype	Estimate	Standard Error	DF	t Value	Pr > |t|	Alpha	Lower	Upper
Genotype	Meg +/+	1.9690	0.3135	29	6.28	<.0001	0.05	1.3278	2.6102
Genotype	Meg +/-	1.4219	0.3163	29	4.50	0.0001	0.05	0.7750	2.0688

DistSoma=324

Differences of Least Squares Means												
Effect	Genotype	Genotype	Estimate	Standard Error	DF	t Value	Pr > |t|	Adjustment	Adj P	Alpha	Lower	Upper
Genotype	Meg +/+	Meg +/-	0.5471	0.4453	29	1.23	0.2291	Tukey-Kramer	0.2291	0.05	-0.3637	1.4579

Differences of Least Squares Means				
Effect	Genotype	Genotype	Adj Lower	Adj Upper
Genotype	Meg +/+	Meg +/-	-0.3637	1.4579

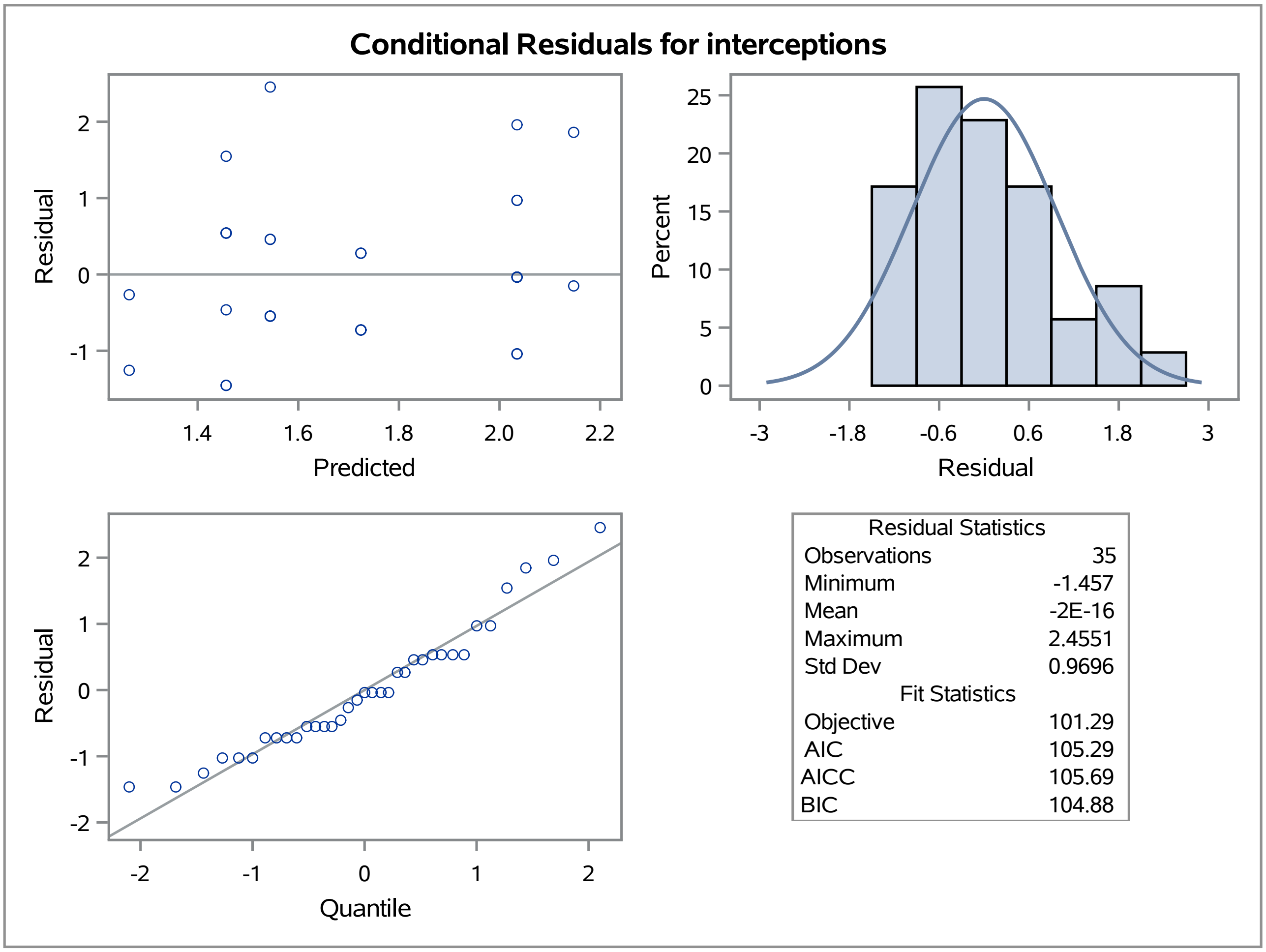

Residual Statistics	
Observations	35
Minimum	-1.457
Mean	-2E-16
Maximum	2.4551
Std Dev	0.9696
Fit Statistics	
Objective	101.29
AIC	105.29
AICC	105.69
BIC	104.88

DistSoma=330

Model Information	
**Data Set**	WORK.TEMPDATASORTED
**Dependent Variable**	interceptions
**Covariance Structure**	Variance Components
**Estimation Method**	REML
**Residual Variance Method**	Profile
**Fixed Effects SE Method**	Model-Based
**Degrees of Freedom Method**	Containment

Class Level Information		
**Class**	**Levels**	**Values**
**Genotype**	2	Meg +/+ Meg +/-
**Animal**	6	1 2 3 4 5 6

Dimensions	
**Covariance Parameters**	2
**Columns in X**	3
**Columns in Z**	6
**Subjects**	1
**Max Obs per Subject**	35

Number of Observations	
**Number of Observations Read**	35
**Number of Observations Used**	35
**Number of Observations Not Used**	0

Iteration History			
**Iteration**	**Evaluations**	**-2 Res Log Like**	**Criterion**
**0**	1	102.13688400	
**1**	2	101.88852313	0.00000000

Convergence criteria met.

Covariance Parameter Estimates				
**Cov Parm**	**Estimate**	**Alpha**	**Lower**	**Upper**
**Animal**	0.08136	0.05	0.009711	4.3432E8
**Residual**	1.0374	0.05	0.6598	1.8658

DistSoma=330

Fit Statistics	
-2 Res Log Likelihood	101.9
AIC (Smaller is Better)	105.9
AICC (Smaller is Better)	106.3
BIC (Smaller is Better)	105.5

Solution for Fixed Effects

Effect	Genotype	Estimate	Standard Error	DF	t Value	Pr > |t|	Alpha	Lower	Upper
Intercept		1.3190	0.3048	4	4.33	0.0124	0.05	0.4727	2.1654
Genotype	Meg +/+	0.6657	0.4287	29	1.55	0.1313	0.05	-0.2111	1.5426
Genotype	Meg +/-	0	.	.	.	.	.	.	.

Solution for Random Effects

Effect	Animal	Estimate	Std Err Pred	DF	t Value	Pr > |t|	Alpha	Lower	Upper
Animal	1	0.1386	0.2513	29	0.55	0.5856	0.05	-0.3754	0.6526
Animal	2	-0.2084	0.2542	29	-0.82	0.4190	0.05	-0.7284	0.3115
Animal	3	0.06986	0.2683	29	0.26	0.7964	0.05	-0.4789	0.6186
Animal	4	-0.1110	0.2684	29	-0.41	0.6821	0.05	-0.6600	0.4379
Animal	5	0.08946	0.2534	29	0.35	0.7266	0.05	-0.4287	0.6076
Animal	6	0.02158	0.2526	29	0.09	0.9325	0.05	-0.4950	0.5382

Type 3 Tests of Fixed Effects

Effect	Num DF	Den DF	F Value	Pr > F
Genotype	1	29	2.41	0.1313

Least Squares Means

Effect	Genotype	Estimate	Standard Error	DF	t Value	Pr > |t|	Alpha	Lower	Upper
Genotype	Meg +/+	1.9847	0.3015	29	6.58	<.0001	0.05	1.3681	2.6013
Genotype	Meg +/-	1.3190	0.3048	29	4.33	0.0002	0.05	0.6956	1.9425

DistSoma=330

Differences of Least Squares Means												
**Effect**	**Genotype**	**Genotype**	**Estimate**	**Standard Error**	**DF**	**t Value**	**Pr > |t|**	**Adjustment**	**Adj P**	**Alpha**	**Lower**	**Upper**
**Genotype**	Meg +/+	Meg +/-	0.6657	0.4287	29	1.55	0.1313	Tukey-Kramer	0.1313	0.05	-0.2111	1.5426

Differences of Least Squares Means				
**Effect**	**Genotype**	**Genotype**	**Adj Lower**	**Adj Upper**
**Genotype**	Meg +/+	Meg +/-	-0.2111	1.5426

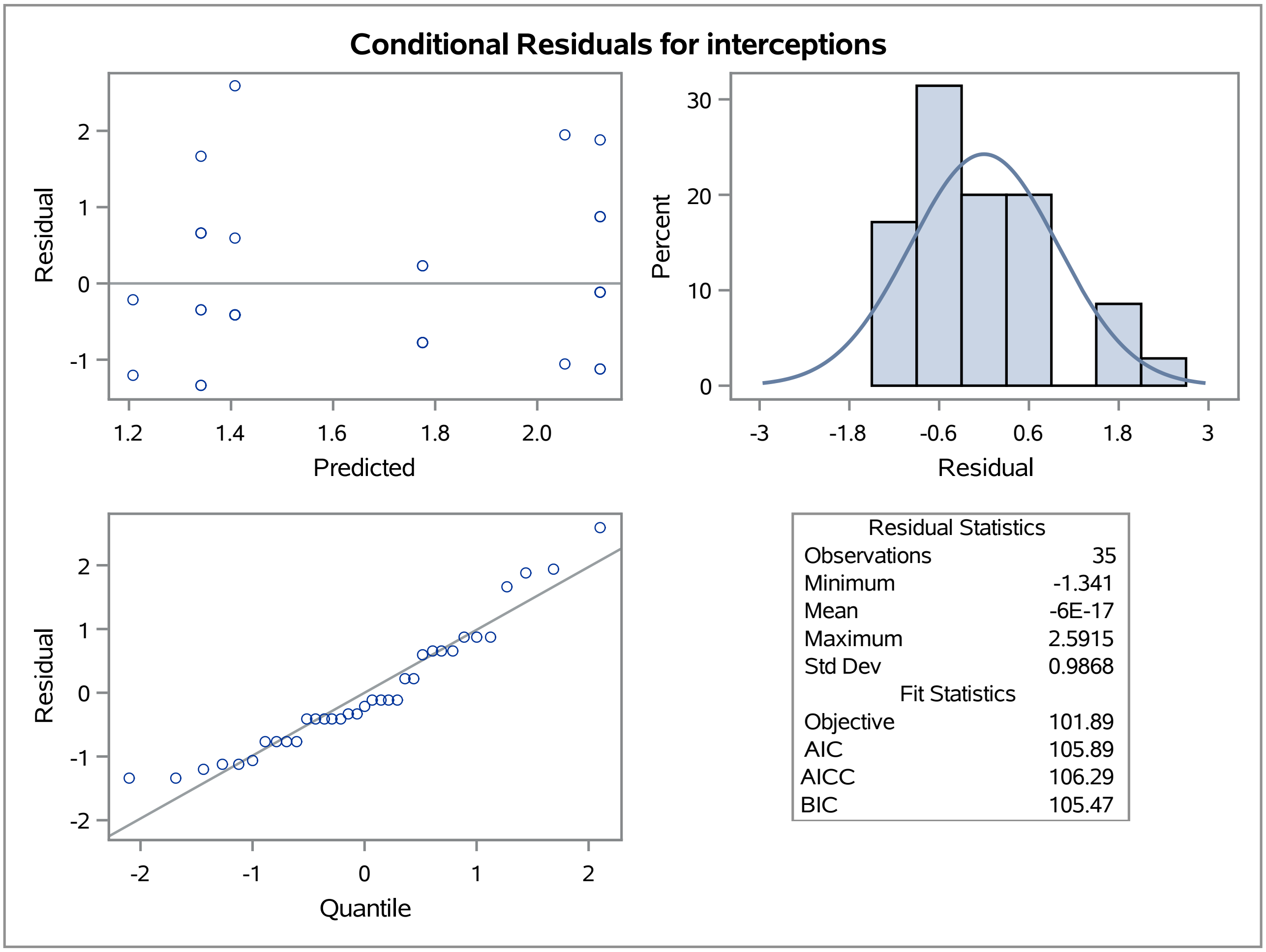

DistSoma=336

Model Information	
**Data Set**	WORK.TEMPDATASORTED
**Dependent Variable**	interceptions
**Covariance Structure**	Variance Components
**Estimation Method**	REML
**Residual Variance Method**	Profile
**Fixed Effects SE Method**	Model-Based
**Degrees of Freedom Method**	Containment

Class Level Information		
**Class**	**Levels**	**Values**
**Genotype**	2	Meg +/+ Meg +/-
**Animal**	6	1 2 3 4 5 6

Dimensions	
**Covariance Parameters**	2
**Columns in X**	3
**Columns in Z**	6
**Subjects**	1
**Max Obs per Subject**	35

Number of Observations	
**Number of Observations Read**	35
**Number of Observations Used**	35
**Number of Observations Not Used**	0

Iteration History			
**Iteration**	**Evaluations**	**-2 Res Log Like**	**Criterion**
**0**	1	98.36143348	
**1**	2	98.00888878	0.00000000

Convergence criteria met.

Covariance Parameter Estimates				
**Cov Parm**	**Estimate**	**Alpha**	**Lower**	**Upper**
**Animal**	0.08793	0.05	0.01172	2032579
**Residual**	0.9157	0.05	0.5824	1.6474

DistSoma=336

Fit Statistics	
-2 Res Log Likelihood	98.0
AIC (Smaller is Better)	102.0
AICC (Smaller is Better)	102.4
BIC (Smaller is Better)	101.6

Solution for Fixed Effects

Effect	Genotype	Estimate	Standard Error	DF	t Value	Pr > |t|	Alpha	Lower	Upper
Intercept		1.2040	0.2969	4	4.06	0.0154	0.05	0.3796	2.0283
Genotype	Meg +/+	0.7319	0.4179	29	1.75	0.0904	0.05	-0.1228	1.5867
Genotype	Meg +/-	0	.	.	.	.	.	.	.

Solution for Random Effects

Effect	Animal	Estimate	Std Err Pred	DF	t Value	Pr > |t|	Alpha	Lower	Upper
Animal	1	0.1294	0.2561	29	0.51	0.6173	0.05	-0.3945	0.6533
Animal	2	-0.2202	0.2595	29	-0.85	0.4030	0.05	-0.7510	0.3105
Animal	3	0.09088	0.2757	29	0.33	0.7441	0.05	-0.4730	0.6547
Animal	4	-0.1134	0.2758	29	-0.41	0.6839	0.05	-0.6774	0.4506
Animal	5	0.1477	0.2585	29	0.57	0.5721	0.05	-0.3810	0.6764
Animal	6	-0.03430	0.2576	29	-0.13	0.8950	0.05	-0.5612	0.4926

Type 3 Tests of Fixed Effects

Effect	Num DF	Den DF	F Value	Pr > F
Genotype	1	29	3.07	0.0904

Least Squares Means

Effect	Genotype	Estimate	Standard Error	DF	t Value	Pr > |t|	Alpha	Lower	Upper
Genotype	Meg +/+	1.9359	0.2941	29	6.58	<.0001	0.05	1.3344	2.5374
Genotype	Meg +/-	1.2040	0.2969	29	4.06	0.0003	0.05	0.5967	1.8112

DistSoma=336

Differences of Least Squares Means												
Effect	Genotype	Genotype	Estimate	Standard Error	DF	t Value	Pr > |t|	Adjustment	Adj P	Alpha	Lower	Upper
Genotype	Meg +/+	Meg +/-	0.7319	0.4179	29	1.75	0.0904	Tukey-Kramer	0.0904	0.05	-0.1228	1.5867

Differences of Least Squares Means				
Effect	Genotype	Genotype	Adj Lower	Adj Upper
Genotype	Meg +/+	Meg +/-	-0.1228	1.5867

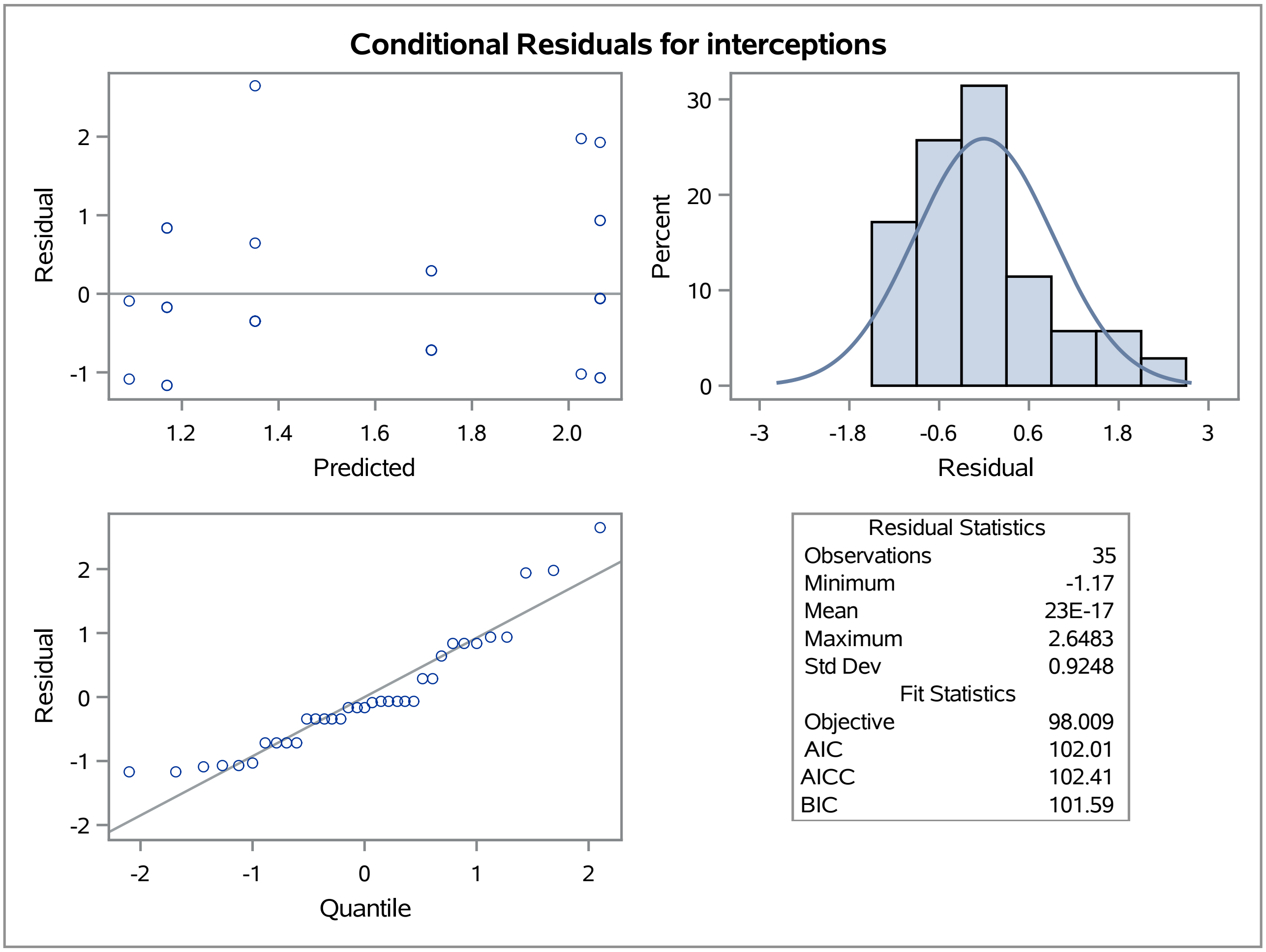

DistSoma=342

Model Information	
**Data Set**	WORK.TEMPDATASORTED
**Dependent Variable**	interceptions
**Covariance Structure**	Variance Components
**Estimation Method**	REML
**Residual Variance Method**	Profile
**Fixed Effects SE Method**	Model-Based
**Degrees of Freedom Method**	Containment

Class Level Information		
**Class**	**Levels**	**Values**
**Genotype**	2	Meg +/+ Meg +/-
**Animal**	6	1 2 3 4 5 6

Dimensions	
**Covariance Parameters**	2
**Columns in X**	3
**Columns in Z**	6
**Subjects**	1
**Max Obs per Subject**	35

Number of Observations	
**Number of Observations Read**	35
**Number of Observations Used**	35
**Number of Observations Not Used**	0

Iteration History			
**Iteration**	**Evaluations**	**-2 Res Log Like**	**Criterion**
**0**	1	101.03988933	
**1**	1	101.03988933	0.00000000

Convergence criteria met.

Estimated G matrix is not positive definite.

Covariance Parameter Estimates				
**Cov Parm**	**Estimate**	**Alpha**	**Lower**	**Upper**
**Animal**	0	.	.	.
**Residual**	1.0518	0.05	0.6843	1.8223

DistSoma=342

Fit Statistics	
**-2 Res Log Likelihood**	101.0
**AIC (Smaller is Better)**	103.0
**AICC (Smaller is Better)**	103.2
**BIC (Smaller is Better)**	102.8

Solution for Fixed Effects

Effect	Genotype	Estimate	Standard Error	DF	t Value	Pr > |t|	Alpha	Lower	Upper
**Intercept**		1.1176	0.2487	4	4.49	0.0109	0.05	0.4270	1.8083
**Genotype**	Meg +/+	0.9379	0.3468	29	2.70	0.0113	0.05	0.2285	1.6473
**Genotype**	Meg +/-	0	.	.	.	.	.	.	.

Solution for Random Effects

Effect	Animal	Estimate	Std Err Pred	DF	t Value	Pr > |t|	Alpha	Lower	Upper
**Animal**	1	0	.	.	.	.	.	.	.
**Animal**	2	0	.	.	.	.	.	.	.
**Animal**	3	0	.	.	.	.	.	.	.
**Animal**	4	0	.	.	.	.	.	.	.
**Animal**	5	0	.	.	.	.	.	.	.
**Animal**	6	0	.	.	.	.	.	.	.

Type 3 Tests of Fixed Effects

Effect	Num DF	Den DF	F Value	Pr > F
**Genotype**	1	29	7.31	0.0113

Least Squares Means

Effect	Genotype	Estimate	Standard Error	DF	t Value	Pr > |t|	Alpha	Lower	Upper
**Genotype**	Meg +/+	2.0556	0.2417	29	8.50	<.0001	0.05	1.5612	2.5499
**Genotype**	Meg +/-	1.1176	0.2487	29	4.49	0.0001	0.05	0.6089	1.6264

DistSoma=342

Differences of Least Squares Means												
**Effect**	**Genotype**	**Genotype**	**Estimate**	**Standard Error**	**DF**	**t Value**	**Pr > |t|**	**Adjustment**	**Adj P**	**Alpha**	**Lower**	**Upper**
**Genotype**	Meg +/+	Meg +/-	0.9379	0.3468	29	2.70	0.0113	Tukey-Kramer	0.0113	0.05	0.2285	1.6473

Differences of Least Squares Means				
**Effect**	**Genotype**	**Genotype**	**Adj Lower**	**Adj Upper**
**Genotype**	Meg +/+	Meg +/-	0.2285	1.6473

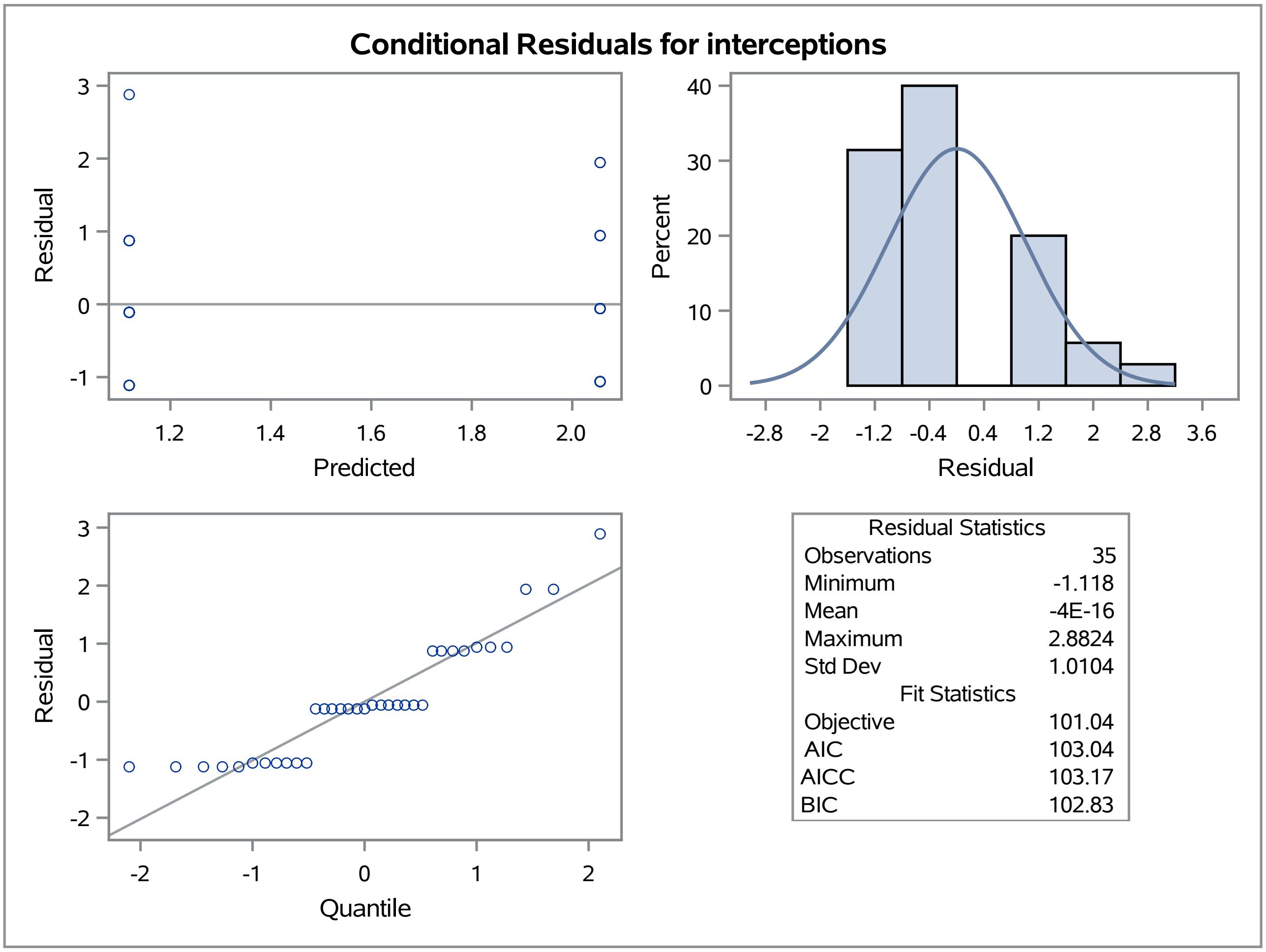

DistSoma=348

Model Information	
**Data Set**	WORK.TEMPDATASORTED
**Dependent Variable**	interceptions
**Covariance Structure**	Variance Components
**Estimation Method**	REML
**Residual Variance Method**	Profile
**Fixed Effects SE Method**	Model-Based
**Degrees of Freedom Method**	Containment

Class Level Information		
**Class**	**Levels**	**Values**
**Genotype**	2	Meg +/+ Meg +/-
**Animal**	6	1 2 3 4 5 6

Dimensions	
**Covariance Parameters**	2
**Columns in X**	3
**Columns in Z**	6
**Subjects**	1
**Max Obs per Subject**	35

Number of Observations	
**Number of Observations Read**	35
**Number of Observations Used**	35
**Number of Observations Not Used**	0

Iteration History			
**Iteration**	**Evaluations**	**-2 Res Log Like**	**Criterion**
**0**	1	103.51717348	
**1**	1	103.51717348	0.00000000

Convergence criteria met.

Estimated G matrix is not positive definite.

Covariance Parameter Estimates				
**Cov Parm**	**Estimate**	**Alpha**	**Lower**	**Upper**
**Animal**	0	.	.	.
**Residual**	1.1338	0.05	0.7376	1.9644

DistSoma=348

Fit Statistics	
-2 Res Log Likelihood	103.5
AIC (Smaller is Better)	105.5
AICC (Smaller is Better)	105.6
BIC (Smaller is Better)	105.3

Solution for Fixed Effects

Effect	Genotype	Estimate	Standard Error	DF	t Value	Pr > |t|	Alpha	Lower	Upper
Intercept		0.8235	0.2583	4	3.19	0.0333	0.05	0.1065	1.5405
Genotype	Meg +/+	1.1209	0.3601	29	3.11	0.0041	0.05	0.3844	1.8574
Genotype	Meg +/-	0	.	.	.	.	.	.	.

Solution for Random Effects

Effect	Animal	Estimate	Std Err Pred	DF	t Value	Pr > |t|	Alpha	Lower	Upper
Animal	1	0	.	.	.	.	.	.	.
Animal	2	0	.	.	.	.	.	.	.
Animal	3	0	.	.	.	.	.	.	.
Animal	4	0	.	.	.	.	.	.	.
Animal	5	0	.	.	.	.	.	.	.
Animal	6	0	.	.	.	.	.	.	.

Type 3 Tests of Fixed Effects

Effect	Num DF	Den DF	F Value	Pr > F
Genotype	1	29	9.69	0.0041

Least Squares Means

Effect	Genotype	Estimate	Standard Error	DF	t Value	Pr > |t|	Alpha	Lower	Upper
Genotype	Meg +/+	1.9444	0.2510	29	7.75	<.0001	0.05	1.4311	2.4577
Genotype	Meg +/-	0.8235	0.2583	29	3.19	0.0034	0.05	0.2953	1.3517

DistSoma=348

Differences of Least Squares Means

Effect	Genotype	Genotype	Estimate	Standard Error	DF	t Value	Pr > |t|	Adjustment	Adj P	Alpha	Lower	Upper
**Genotype**	Meg +/+	Meg +/-	1.1209	0.3601	29	3.11	0.0041	Tukey-Kramer	0.0041	0.05	0.3844	1.8574

Differences of Least Squares Means

Effect	Genotype	Genotype	Adj Lower	Adj Upper
**Genotype**	Meg +/+	Meg +/-	0.3844	1.8574

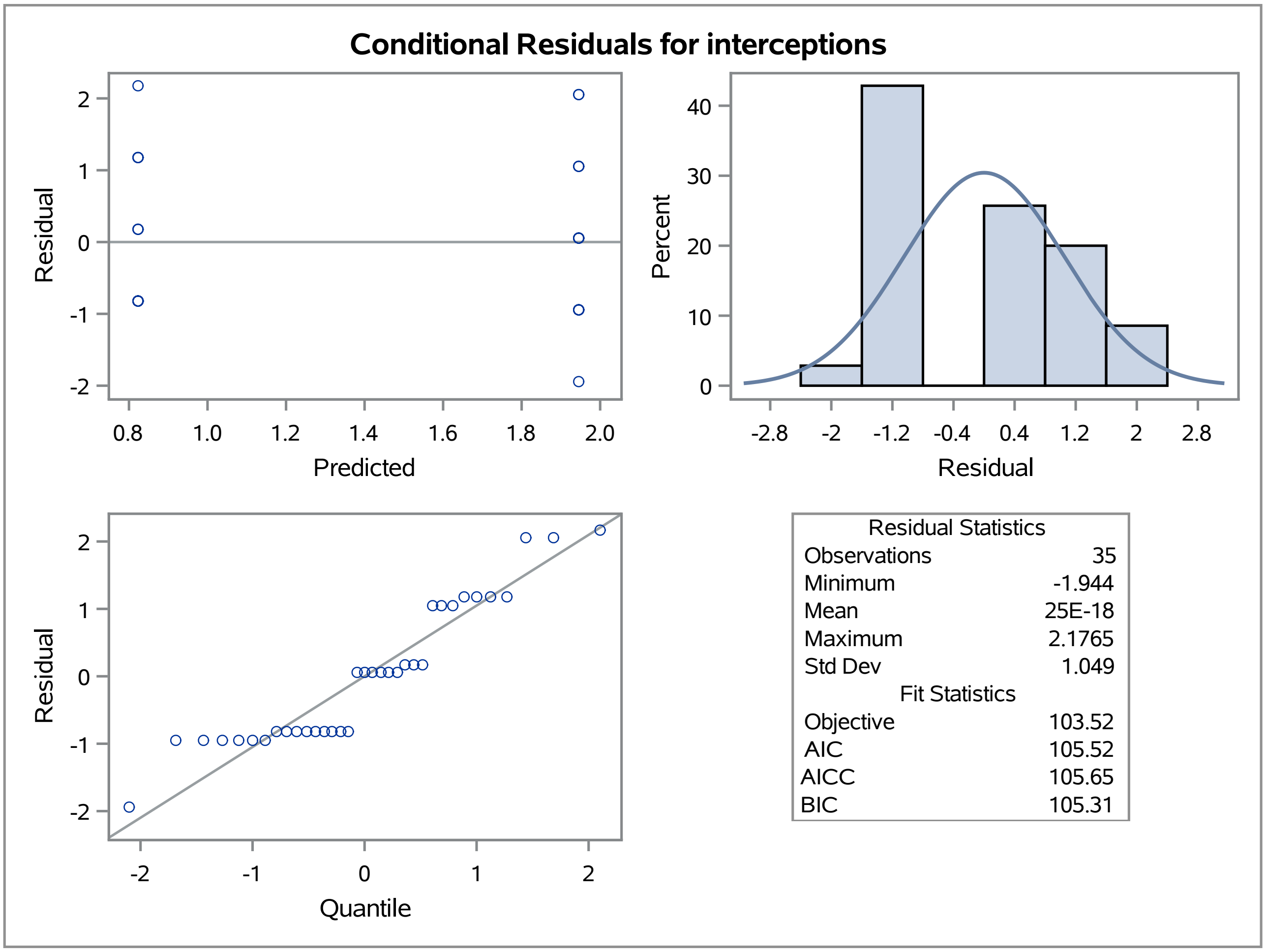

DistSoma=354

Model Information	
**Data Set**	WORK.TEMPDATASORTED
**Dependent Variable**	interceptions
**Covariance Structure**	Variance Components
**Estimation Method**	REML
**Residual Variance Method**	Profile
**Fixed Effects SE Method**	Model-Based
**Degrees of Freedom Method**	Containment

Class Level Information		
**Class**	**Levels**	**Values**
**Genotype**	2	Meg +/+ Meg +/-
**Animal**	6	1 2 3 4 5 6

Dimensions	
**Covariance Parameters**	2
**Columns in X**	3
**Columns in Z**	6
**Subjects**	1
**Max Obs per Subject**	35

Number of Observations	
**Number of Observations Read**	35
**Number of Observations Used**	35
**Number of Observations Not Used**	0

Iteration History			
**Iteration**	**Evaluations**	**-2 Res Log Like**	**Criterion**
**0**	1	100.81541987	
**1**	1	100.81541987	0.00000000

Convergence criteria met.

Estimated G matrix is not positive definite.

Covariance Parameter Estimates				
**Cov Parm**	**Estimate**	**Alpha**	**Lower**	**Upper**
**Animal**	0	.	.	.
**Residual**	1.0447	0.05	0.6796	1.8100

DistSoma=354

Fit Statistics	
-2 Res Log Likelihood	100.8
AIC (Smaller is Better)	102.8
AICC (Smaller is Better)	102.9
BIC (Smaller is Better)	102.6

Solution for Fixed Effects

Effect	Genotype	Estimate	Standard Error	DF	t Value	Pr > |t|	Alpha	Lower	Upper
Intercept		0.7059	0.2479	4	2.85	0.0465	0.05	0.01762	1.3941
Genotype	Meg +/+	1.2386	0.3457	29	3.58	0.0012	0.05	0.5316	1.9455
Genotype	Meg +/-	0	.	.	.	.	.	.	.

Solution for Random Effects

Effect	Animal	Estimate	Std Err Pred	DF	t Value	Pr > |t|	Alpha	Lower	Upper
Animal	1	0	.	.	.	.	.	.	.
Animal	2	0	.	.	.	.	.	.	.
Animal	3	0	.	.	.	.	.	.	.
Animal	4	0	.	.	.	.	.	.	.
Animal	5	0	.	.	.	.	.	.	.
Animal	6	0	.	.	.	.	.	.	.

Type 3 Tests of Fixed Effects

Effect	Num DF	Den DF	F Value	Pr > F
Genotype	1	29	12.84	0.0012

Least Squares Means

Effect	Genotype	Estimate	Standard Error	DF	t Value	Pr > |t|	Alpha	Lower	Upper
Genotype	Meg +/+	1.9444	0.2409	29	8.07	<.0001	0.05	1.4517	2.4372
Genotype	Meg +/-	0.7059	0.2479	29	2.85	0.0080	0.05	0.1989	1.2129

DistSoma=354

Differences of Least Squares Means												
**Effect**	**Genotype**	**Genotype**	**Estimate**	**Standard Error**	**DF**	**t Value**	**Pr > |t|**	**Adjustment**	**Adj P**	**Alpha**	**Lower**	**Upper**
**Genotype**	Meg +/+	Meg +/-	1.2386	0.3457	29	3.58	0.0012	Tukey-Kramer	0.0012	0.05	0.5316	1.9455

Differences of Least Squares Means				
**Effect**	**Genotype**	**Genotype**	**Adj Lower**	**Adj Upper**
**Genotype**	Meg +/+	Meg +/-	0.5316	1.9455

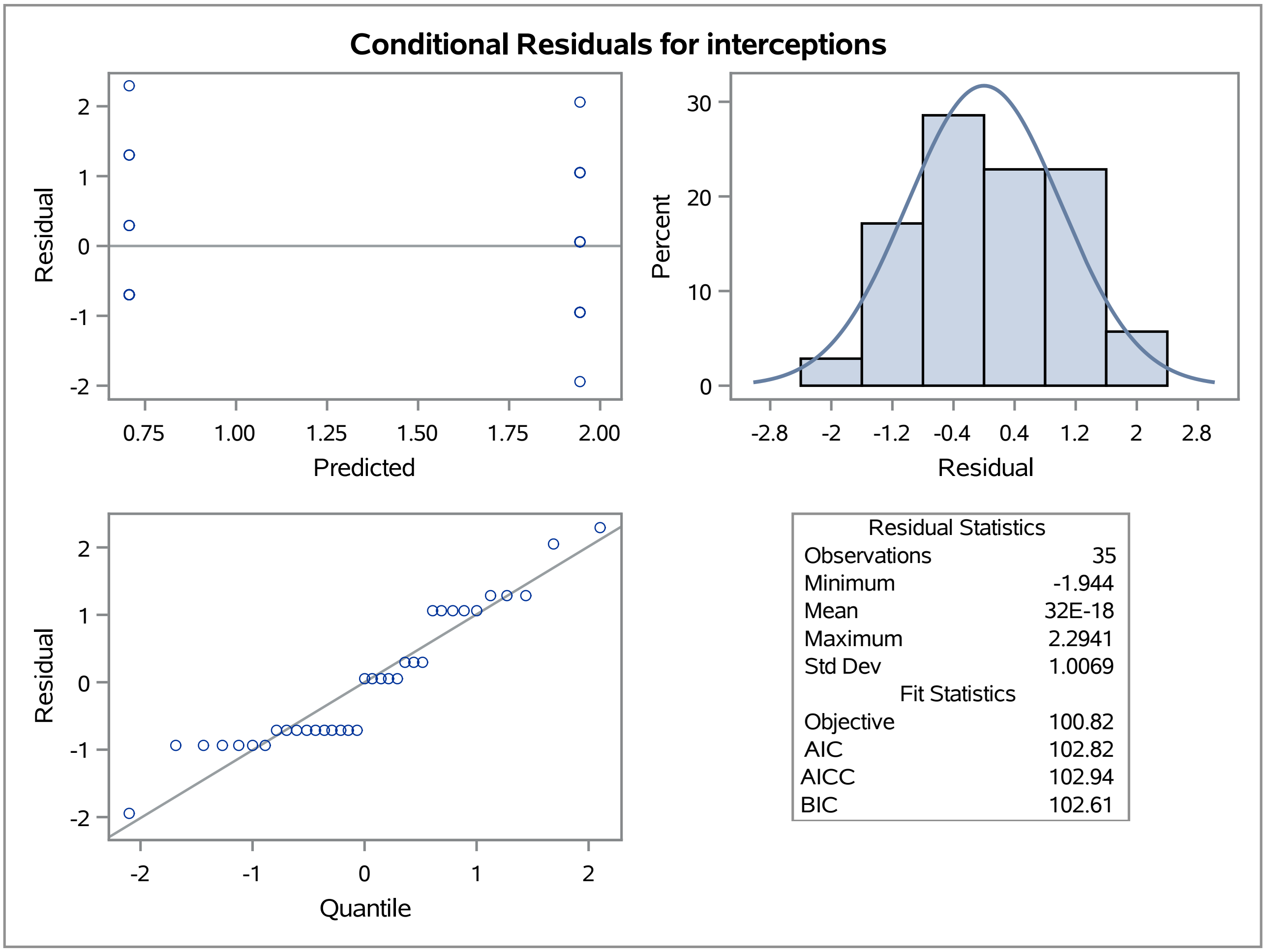

DistSoma=360

Model Information	
**Data Set**	WORK.TEMPDATASORTED
**Dependent Variable**	interceptions
**Covariance Structure**	Variance Components
**Estimation Method**	REML
**Residual Variance Method**	Profile
**Fixed Effects SE Method**	Model-Based
**Degrees of Freedom Method**	Containment

Class Level Information		
**Class**	**Levels**	**Values**
**Genotype**	2	Meg +/+ Meg +/-
**Animal**	6	1 2 3 4 5 6

Dimensions	
**Covariance Parameters**	2
**Columns in X**	3
**Columns in Z**	6
**Subjects**	1
**Max Obs per Subject**	35

Number of Observations	
**Number of Observations Read**	35
**Number of Observations Used**	35
**Number of Observations Not Used**	0

Iteration History			
**Iteration**	**Evaluations**	**-2 Res Log Like**	**Criterion**
**0**	1	97.12546254	
**1**	1	97.12546254	0.00000000

Convergence criteria met.

Estimated G matrix is not positive definite.

Covariance Parameter Estimates				
**Cov Parm**	**Estimate**	**Alpha**	**Lower**	**Upper**
**Animal**	0	.	.	.
**Residual**	0.9341	0.05	0.6077	1.6185

DistSoma=360

Fit Statistics	
-2 Res Log Likelihood	97.1
AIC (Smaller is Better)	99.1
AICC (Smaller is Better)	99.3
BIC (Smaller is Better)	98.9

Solution for Fixed Effects

Effect	Genotype	Estimate	Standard Error	DF	t Value	Pr > |t|	Alpha	Lower	Upper
Intercept		0.6471	0.2344	4	2.76	0.0508	0.05	-0.00378	1.2979
Genotype	Meg +/+	1.2974	0.3269	29	3.97	0.0004	0.05	0.6289	1.9659
Genotype	Meg +/-	0	.	.	.	.	.	.	.

Solution for Random Effects

Effect	Animal	Estimate	Std Err Pred	DF	t Value	Pr > |t|	Alpha	Lower	Upper
Animal	1	0	.	.	.	.	.	.	.
Animal	2	0	.	.	.	.	.	.	.
Animal	3	0	.	.	.	.	.	.	.
Animal	4	0	.	.	.	.	.	.	.
Animal	5	0	.	.	.	.	.	.	.
Animal	6	0	.	.	.	.	.	.	.

Type 3 Tests of Fixed Effects

Effect	Num DF	Den DF	F Value	Pr > F
Genotype	1	29	15.75	0.0004

Least Squares Means

Effect	Genotype	Estimate	Standard Error	DF	t Value	Pr > |t|	Alpha	Lower	Upper
Genotype	Meg +/+	1.9444	0.2278	29	8.54	<.0001	0.05	1.4785	2.4104
Genotype	Meg +/-	0.6471	0.2344	29	2.76	0.0099	0.05	0.1676	1.1265

DistSoma=360

Differences of Least Squares Means												
Effect	Genotype	Genotype	Estimate	Standard Error	DF	t Value	Pr > |t|	Adjustment	Adj P	Alpha	Lower	Upper
**Genotype**	Meg +/+	Meg +/-	1.2974	0.3269	29	3.97	0.0004	Tukey-Kramer	0.0004	0.05	0.6289	1.9659

Differences of Least Squares Means				
Effect	Genotype	Genotype	Adj Lower	Adj Upper
**Genotype**	Meg +/+	Meg +/-	0.6289	1.9659

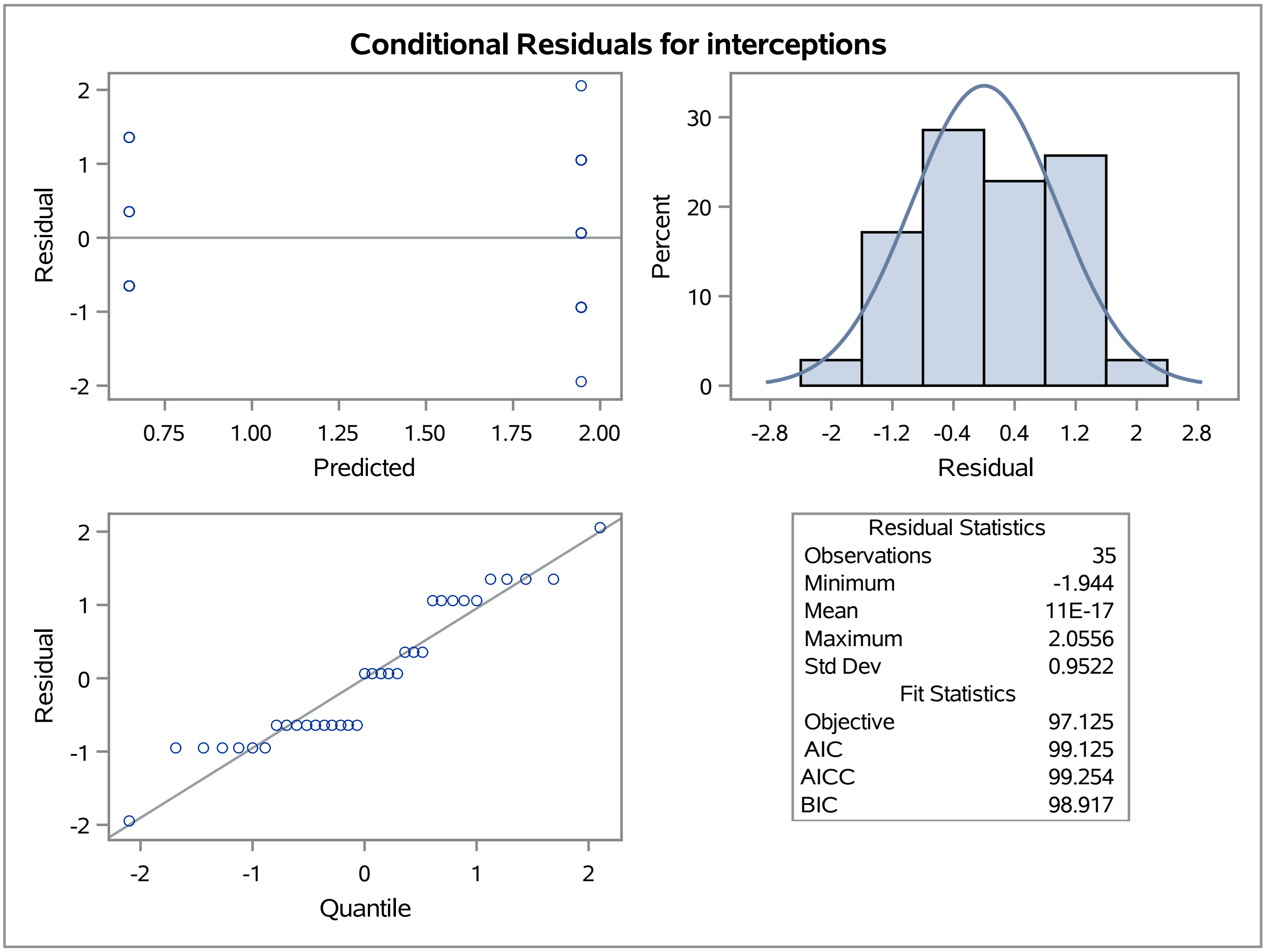

DistSoma=366

Model Information	
**Data Set**	WORK.TEMPDATASORTED
**Dependent Variable**	interceptions
**Covariance Structure**	Variance Components
**Estimation Method**	REML
**Residual Variance Method**	Profile
**Fixed Effects SE Method**	Model-Based
**Degrees of Freedom Method**	Containment

Class Level Information		
**Class**	**Levels**	**Values**
**Genotype**	2	Meg +/+ Meg +/-
**Animal**	6	1 2 3 4 5 6

Dimensions	
**Covariance Parameters**	2
**Columns in X**	3
**Columns in Z**	6
**Subjects**	1
**Max Obs per Subject**	35

Number of Observations	
**Number of Observations Read**	35
**Number of Observations Used**	35
**Number of Observations Not Used**	0

Iteration History			
**Iteration**	**Evaluations**	**-2 Res Log Like**	**Criterion**
**0**	1	95.85230232	
**1**	1	95.85230232	0.00000000

Convergence criteria met.

Estimated G matrix is not positive definite.

Covariance Parameter Estimates				
**Cov Parm**	**Estimate**	**Alpha**	**Lower**	**Upper**
**Animal**	0	.	.	.
**Residual**	0.8988	0.05	0.5847	1.5572

DistSoma=366

Fit Statistics	
-2 Res Log Likelihood	95.9
AIC (Smaller is Better)	97.9
AICC (Smaller is Better)	98.0
BIC (Smaller is Better)	97.6

Solution for Fixed Effects

Effect	Genotype	Estimate	Standard Error	DF	t Value	Pr > |t|	Alpha	Lower	Upper
Intercept		0.6471	0.2299	4	2.81	0.0481	0.05	0.008657	1.2855
Genotype	Meg +/+	1.2418	0.3206	29	3.87	0.0006	0.05	0.5861	1.8976
Genotype	Meg +/-	0	.	.	.	.	.	.	.

Solution for Random Effects

Effect	Animal	Estimate	Std Err Pred	DF	t Value	Pr > |t|	Alpha	Lower	Upper
Animal	1	0	.	.	.	.	.	.	.
Animal	2	0	.	.	.	.	.	.	.
Animal	3	0	.	.	.	.	.	.	.
Animal	4	0	.	.	.	.	.	.	.
Animal	5	0	.	.	.	.	.	.	.
Animal	6	0	.	.	.	.	.	.	.

Type 3 Tests of Fixed Effects

Effect	Num DF	Den DF	F Value	Pr > F
Genotype	1	29	15.00	0.0006

Least Squares Means

Effect	Genotype	Estimate	Standard Error	DF	t Value	Pr > |t|	Alpha	Lower	Upper
Genotype	Meg +/+	1.8889	0.2235	29	8.45	<.0001	0.05	1.4319	2.3459
Genotype	Meg +/-	0.6471	0.2299	29	2.81	0.0087	0.05	0.1768	1.1173

DistSoma=366

Differences of Least Squares Means												
Effect	Genotype	Genotype	Estimate	Standard Error	DF	t Value	Pr > |t|	Adjustment	Adj P	Alpha	Lower	Upper
Genotype	Meg +/+	Meg +/-	1.2418	0.3206	29	3.87	0.0006	Tukey-Kramer	0.0006	0.05	0.5861	1.8976

Differences of Least Squares Means				
Effect	Genotype	Genotype	Adj Lower	Adj Upper
Genotype	Meg +/+	Meg +/-	0.5861	1.8976

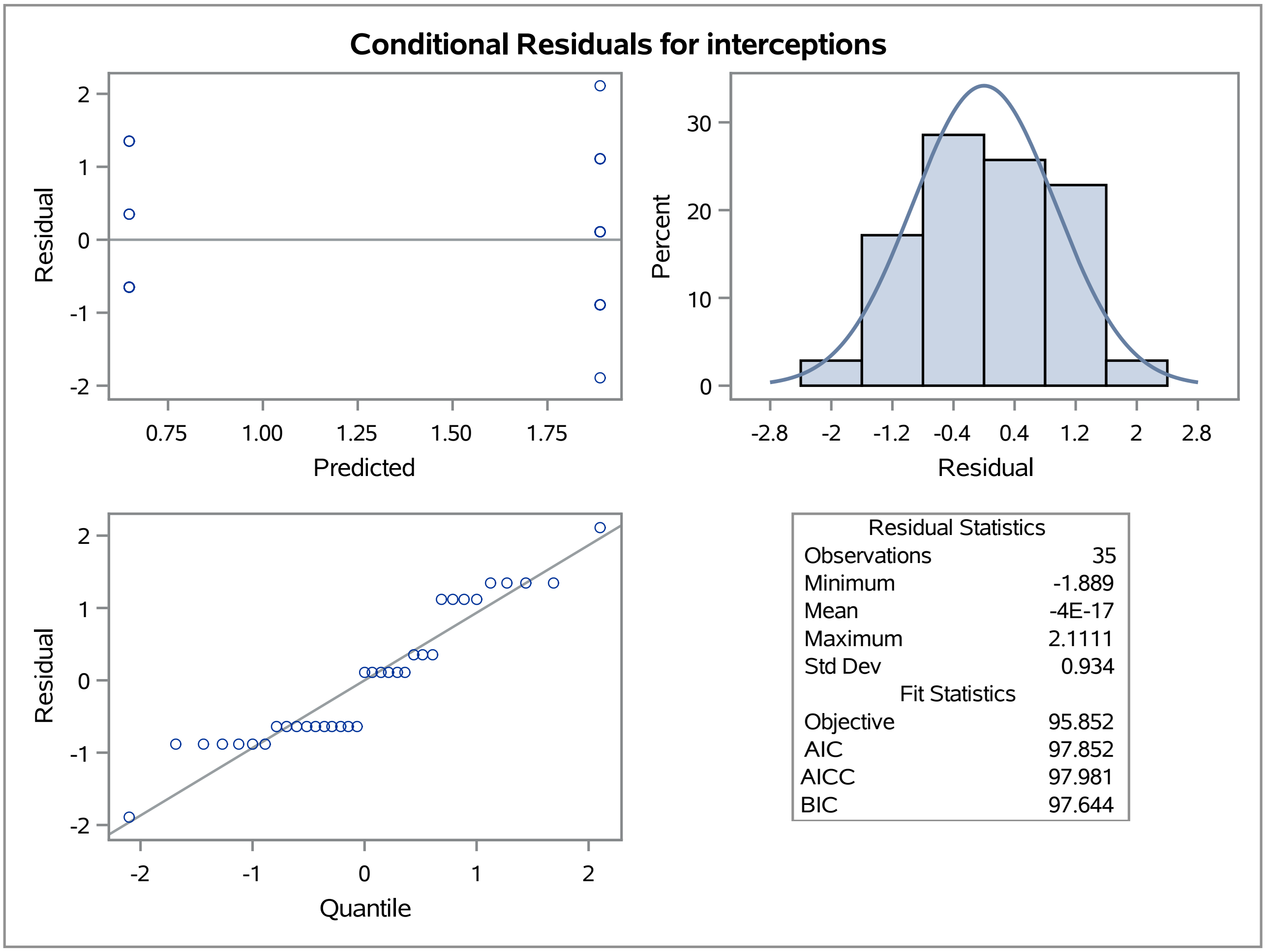

DistSoma=372

Model Information	
**Data Set**	WORK.TEMPDATASORTED
**Dependent Variable**	interceptions
**Covariance Structure**	Variance Components
**Estimation Method**	REML
**Residual Variance Method**	Profile
**Fixed Effects SE Method**	Model-Based
**Degrees of Freedom Method**	Containment

Class Level Information		
**Class**	**Levels**	**Values**
**Genotype**	2	Meg +/+ Meg +/-
**Animal**	6	1 2 3 4 5 6

Dimensions	
**Covariance Parameters**	2
**Columns in X**	3
**Columns in Z**	6
**Subjects**	1
**Max Obs per Subject**	35

Number of Observations	
**Number of Observations Read**	35
**Number of Observations Used**	35
**Number of Observations Not Used**	0

Iteration History			
**Iteration**	**Evaluations**	**-2 Res Log Like**	**Criterion**
**0**	1	100.24429121	
**1**	1	100.24429121	0.00000000

Convergence criteria met.

Estimated G matrix is not positive definite.

Covariance Parameter Estimates				
**Cov Parm**	**Estimate**	**Alpha**	**Lower**	**Upper**
**Animal**	0	.	.	.
**Residual**	1.0267	0.05	0.6680	1.7789

DistSoma=372

Fit Statistics	
-2 Res Log Likelihood	100.2
AIC (Smaller is Better)	102.2
AICC (Smaller is Better)	102.4
BIC (Smaller is Better)	102.0

Solution for Fixed Effects

Effect	Genotype	Estimate	Standard Error	DF	t Value	Pr > |t|	Alpha	Lower	Upper
Intercept		0.6471	0.2458	4	2.63	0.0580	0.05	-0.03527	1.3294
Genotype	Meg +/+	1.3529	0.3427	29	3.95	0.0005	0.05	0.6521	2.0538
Genotype	Meg +/-	0	.	.	.	.	.	.	.

Solution for Random Effects

Effect	Animal	Estimate	Std Err Pred	DF	t Value	Pr > |t|	Alpha	Lower	Upper
Animal	1	0	.	.	.	.	.	.	.
Animal	2	0	.	.	.	.	.	.	.
Animal	3	0	.	.	.	.	.	.	.
Animal	4	0	.	.	.	.	.	.	.
Animal	5	0	.	.	.	.	.	.	.
Animal	6	0	.	.	.	.	.	.	.

Type 3 Tests of Fixed Effects

Effect	Num DF	Den DF	F Value	Pr > F
Genotype	1	29	15.59	0.0005

Least Squares Means

Effect	Genotype	Estimate	Standard Error	DF	t Value	Pr > |t|	Alpha	Lower	Upper
Genotype	Meg +/+	2.0000	0.2388	29	8.37	<.0001	0.05	1.5115	2.4885
Genotype	Meg +/-	0.6471	0.2458	29	2.63	0.0134	0.05	0.1444	1.1497

DistSoma=372

Differences of Least Squares Means												
Effect	Genotype	Genotype	Estimate	Standard Error	DF	t Value	Pr > |t|	Adjustment	Adj P	Alpha	Lower	Upper
Genotype	Meg +/+	Meg +/-	1.3529	0.3427	29	3.95	0.0005	Tukey-Kramer	0.0005	0.05	0.6521	2.0538

Differences of Least Squares Means				
Effect	Genotype	Genotype	Adj Lower	Adj Upper
Genotype	Meg +/+	Meg +/-	0.6521	2.0538

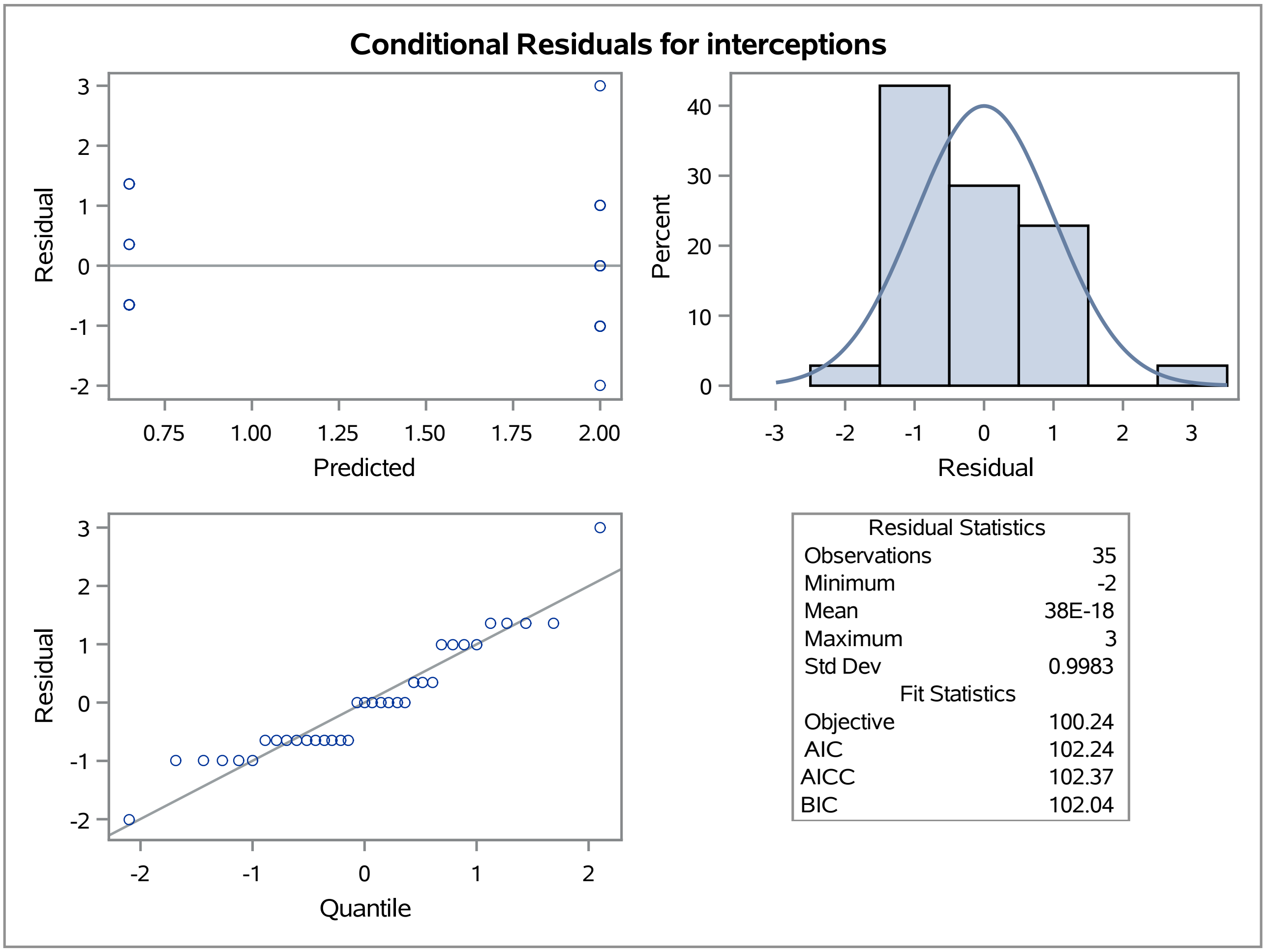

DistSoma=378

Model Information	
**Data Set**	WORK.TEMPDATASORTED
**Dependent Variable**	interceptions
**Covariance Structure**	Variance Components
**Estimation Method**	REML
**Residual Variance Method**	Profile
**Fixed Effects SE Method**	Model-Based
**Degrees of Freedom Method**	Containment

Class Level Information		
**Class**	**Levels**	**Values**
**Genotype**	2	Meg +/+ Meg +/-
**Animal**	6	1 2 3 4 5 6

Dimensions	
**Covariance Parameters**	2
**Columns in X**	3
**Columns in Z**	6
**Subjects**	1
**Max Obs per Subject**	35

Number of Observations	
**Number of Observations Read**	35
**Number of Observations Used**	35
**Number of Observations Not Used**	0

Iteration History			
**Iteration**	**Evaluations**	**-2 Res Log Like**	**Criterion**
**0**	1	100.81541987	
**1**	2	100.73482559	0.00000006
**2**	1	100.73482441	0.00000000

Convergence criteria met.

Covariance Parameter Estimates				
**Cov Parm**	**Estimate**	**Alpha**	**Lower**	**Upper**
**Animal**	0.03990	0.05	0.003618	4.235E23
**Residual**	1.0198	0.05	0.6506	1.8250

DistSoma=378

Fit Statistics	
-2 Res Log Likelihood	100.7
AIC (Smaller is Better)	104.7
AICC (Smaller is Better)	105.1
BIC (Smaller is Better)	104.3

Solution for Fixed Effects

Effect	Genotype	Estimate	Standard Error	DF	t Value	Pr > |t|	Alpha	Lower	Upper
Intercept		0.6868	0.2754	4	2.49	0.0672	0.05	-0.07789	1.4516
Genotype	Meg +/+	1.3602	0.3863	29	3.52	0.0014	0.05	0.5702	2.1503
Genotype	Meg +/-	0	.	.	.	.	.	.	.

Solution for Random Effects

Effect	Animal	Estimate	Std Err Pred	DF	t Value	Pr > |t|	Alpha	Lower	Upper
Animal	1	0.07113	0.1857	29	0.38	0.7045	0.05	-0.3086	0.4509
Animal	2	-0.1040	0.1870	29	-0.56	0.5824	0.05	-0.4864	0.2784
Animal	3	0.03287	0.1934	29	0.17	0.8662	0.05	-0.3626	0.4283
Animal	4	-0.04984	0.1934	29	-0.26	0.7985	0.05	-0.4454	0.3457
Animal	5	-0.02481	0.1866	29	-0.13	0.8952	0.05	-0.4065	0.3569
Animal	6	0.07465	0.1863	29	0.40	0.6915	0.05	-0.3063	0.4556

Type 3 Tests of Fixed Effects

Effect	Num DF	Den DF	F Value	Pr > F
Genotype	1	29	12.40	0.0014

Least Squares Means

Effect	Genotype	Estimate	Standard Error	DF	t Value	Pr > |t|	Alpha	Lower	Upper
Genotype	Meg +/+	2.0471	0.2708	29	7.56	<.0001	0.05	1.4931	2.6010
Genotype	Meg +/-	0.6868	0.2754	29	2.49	0.0186	0.05	0.1235	1.2502

DistSoma=378

Differences of Least Squares Means												
**Effect**	**Genotype**	**Genotype**	**Estimate**	**Standard Error**	**DF**	**t Value**	**Pr > |t|**	**Adjustment**	**Adj P**	**Alpha**	**Lower**	**Upper**
**Genotype**	Meg +/+	Meg +/-	1.3602	0.3863	29	3.52	0.0014	Tukey-Kramer	0.0014	0.05	0.5702	2.1503

Differences of Least Squares Means				
**Effect**	**Genotype**	**Genotype**	**Adj Lower**	**Adj Upper**
**Genotype**	Meg +/+	Meg +/-	0.5702	2.1503

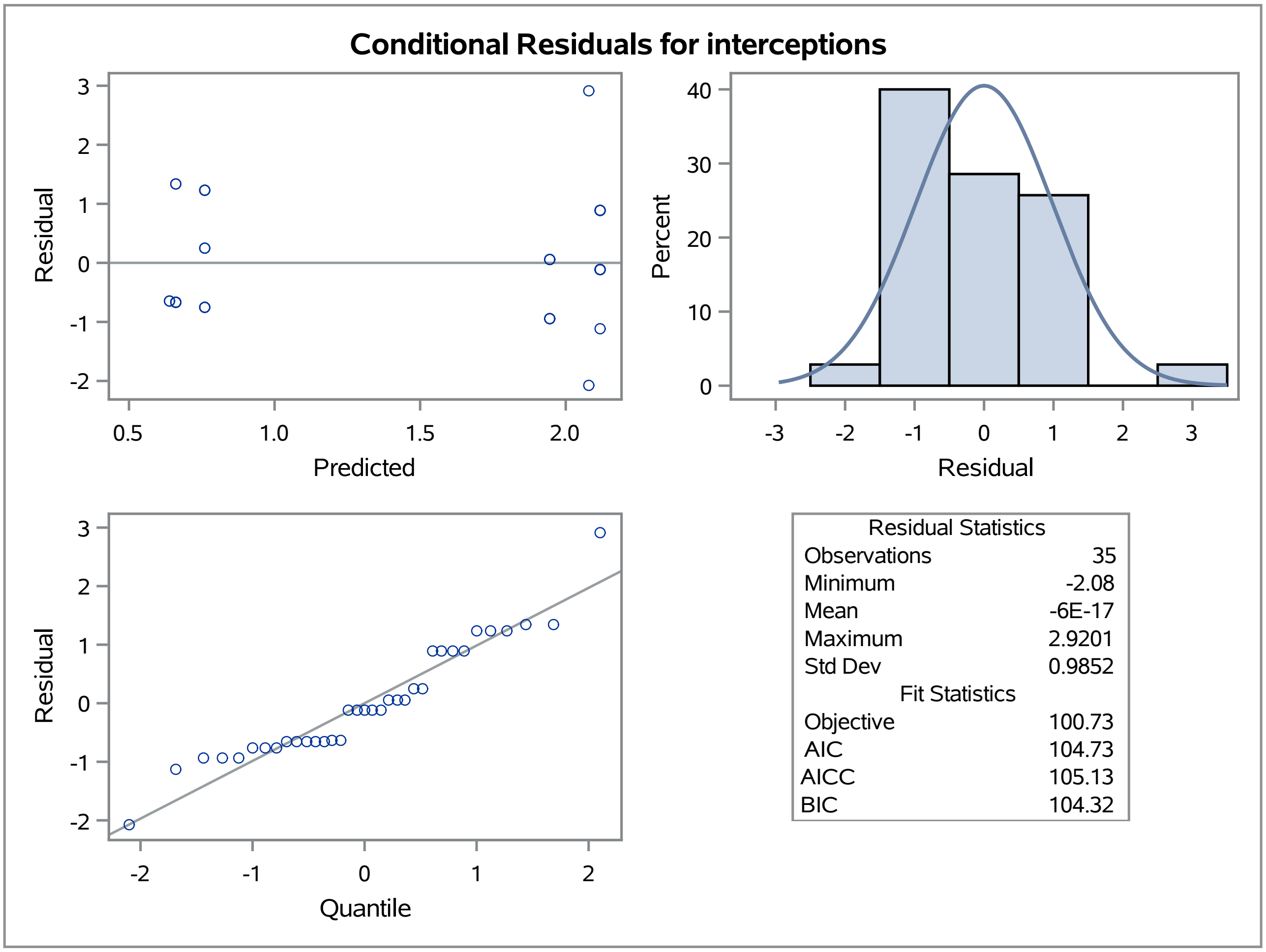

DistSoma=384

Model Information	
**Data Set**	WORK.TEMPDATASORTED
**Dependent Variable**	interceptions
**Covariance Structure**	Variance Components
**Estimation Method**	REML
**Residual Variance Method**	Profile
**Fixed Effects SE Method**	Model-Based
**Degrees of Freedom Method**	Containment

Class Level Information		
**Class**	**Levels**	**Values**
**Genotype**	2	Meg +/+ Meg +/-
**Animal**	6	1 2 3 4 5 6

Dimensions	
**Covariance Parameters**	2
**Columns in X**	3
**Columns in Z**	6
**Subjects**	1
**Max Obs per Subject**	35

Number of Observations	
**Number of Observations Read**	35
**Number of Observations Used**	35
**Number of Observations Not Used**	0

Iteration History			
**Iteration**	**Evaluations**	**-2 Res Log Like**	**Criterion**
**0**	1	102.99422918	
**1**	3	102.99358728	0.00000000

Convergence criteria met.

Covariance Parameter Estimates				
**Cov Parm**	**Estimate**	**Alpha**	**Lower**	**Upper**
**Animal**	0.003530	0.05	.	.
**Residual**	1.1138	0.05	0.7116	1.9883

DistSoma=384

Fit Statistics	
-2 Res Log Likelihood	103.0
AIC (Smaller is Better)	107.0
AICC (Smaller is Better)	107.4
BIC (Smaller is Better)	106.6

Solution for Fixed Effects

Effect	Genotype	Estimate	Standard Error	DF	t Value	Pr > |t|	Alpha	Lower	Upper
Intercept		0.6455	0.2587	4	2.49	0.0671	0.05	-0.07283	1.3639
Genotype	Meg +/+	1.4090	0.3610	29	3.90	0.0005	0.05	0.6706	2.1474
Genotype	Meg +/-	0	.	.	.	.	.	.	.

Solution for Random Effects

Effect	Animal	Estimate	Std Err Pred	DF	t Value	Pr > |t|	Alpha	Lower	Upper
Animal	1	0.007541	0.05900	29	0.13	0.8992	0.05	-0.1131	0.1282
Animal	2	-0.01035	0.05904	29	-0.18	0.8621	0.05	-0.1311	0.1104
Animal	3	0.002806	0.05924	29	0.05	0.9626	0.05	-0.1184	0.1240
Animal	4	-0.00407	0.05925	29	-0.07	0.9458	0.05	-0.1252	0.1171
Animal	5	-0.00161	0.05903	29	-0.03	0.9785	0.05	-0.1223	0.1191
Animal	6	0.005674	0.05902	29	0.10	0.9241	0.05	-0.1150	0.1264

Type 3 Tests of Fixed Effects

Effect	Num DF	Den DF	F Value	Pr > F
Genotype	1	29	15.23	0.0005

Least Squares Means

Effect	Genotype	Estimate	Standard Error	DF	t Value	Pr > |t|	Alpha	Lower	Upper
Genotype	Meg +/+	2.0545	0.2518	29	8.16	<.0001	0.05	1.5395	2.5695
Genotype	Meg +/-	0.6455	0.2587	29	2.49	0.0185	0.05	0.1164	1.1747

DistSoma=384

Differences of Least Squares Means

Effect	Genotype	Genotype	Estimate	Standard Error	DF	t Value	Pr > |t|	Adjustment	Adj P	Alpha	Lower	Upper
**Genotype**	Meg +/+	Meg +/-	1.4090	0.3610	29	3.90	0.0005	Tukey-Kramer	0.0005	0.05	0.6706	2.1474

Differences of Least Squares Means

Effect	Genotype	Genotype	Adj Lower	Adj Upper
**Genotype**	Meg +/+	Meg +/-	0.6706	2.1474

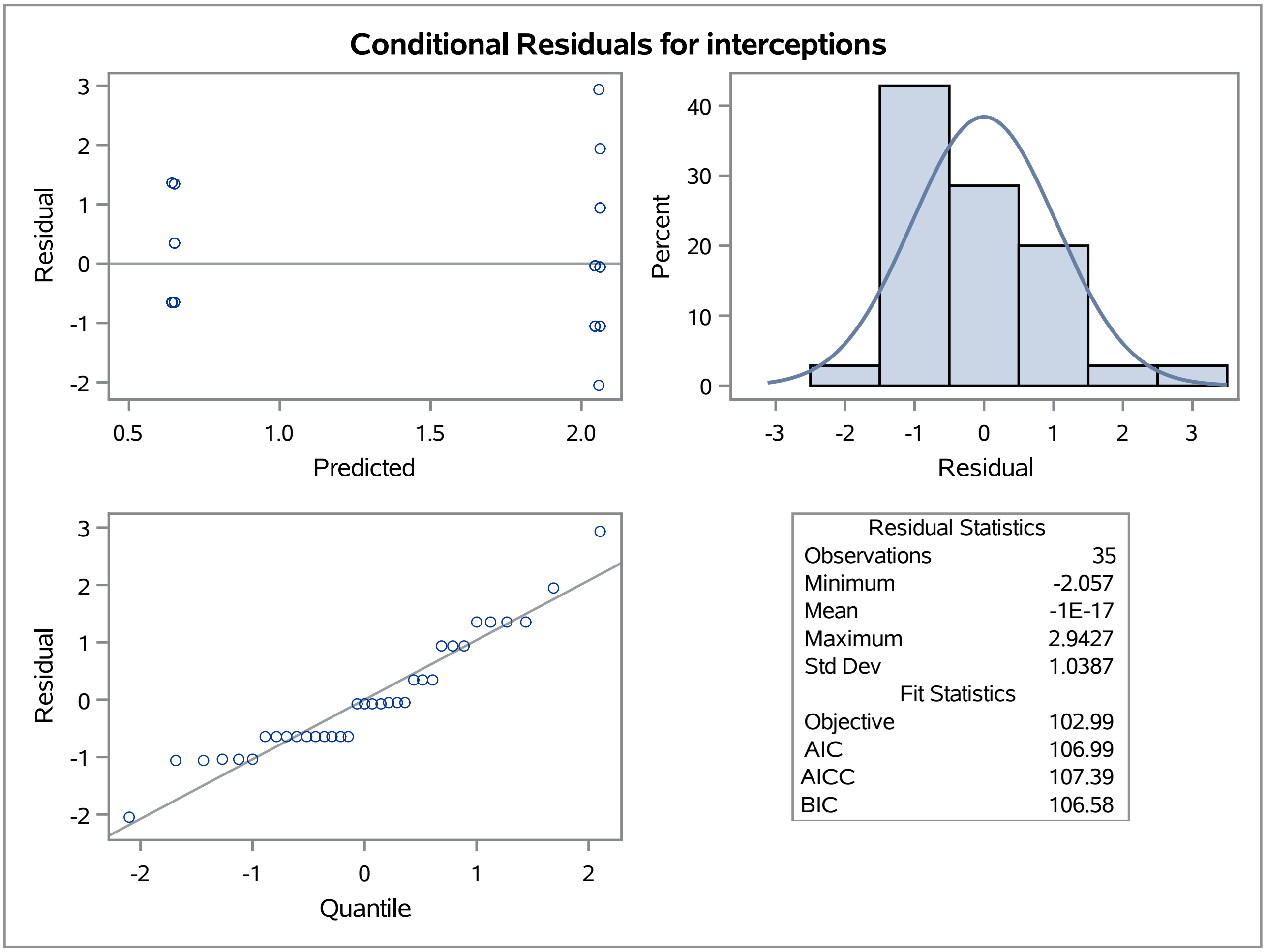

DistSoma=390

Model Information	
**Data Set**	WORK.TEMPDATASORTED
**Dependent Variable**	interceptions
**Covariance Structure**	Variance Components
**Estimation Method**	REML
**Residual Variance Method**	Profile
**Fixed Effects SE Method**	Model-Based
**Degrees of Freedom Method**	Containment

Class Level Information		
**Class**	**Levels**	**Values**
**Genotype**	2	Meg +/+ Meg +/-
**Animal**	6	1 2 3 4 5 6

Dimensions	
**Covariance Parameters**	2
**Columns in X**	3
**Columns in Z**	6
**Subjects**	1
**Max Obs per Subject**	35

Number of Observations	
**Number of Observations Read**	35
**Number of Observations Used**	35
**Number of Observations Not Used**	0

Iteration History			
**Iteration**	**Evaluations**	**-2 Res Log Like**	**Criterion**
**0**	1	95.18012147	
**1**	1	95.18012147	0.00000000

Convergence criteria met.

Estimated G matrix is not positive definite.

Covariance Parameter Estimates				
**Cov Parm**	**Estimate**	**Alpha**	**Lower**	**Upper**
**Animal**	0	.	.	.
**Residual**	0.8807	0.05	0.5729	1.5258

DistSoma=390

Fit Statistics	
-2 Res Log Likelihood	95.2
AIC (Smaller is Better)	97.2
AICC (Smaller is Better)	97.3
BIC (Smaller is Better)	97.0

Solution for Fixed Effects

Effect	Genotype	Estimate	Standard Error	DF	t Value	Pr > |t|	Alpha	Lower	Upper
Intercept		0.5882	0.2276	4	2.58	0.0610	0.05	-0.04370	1.2202
Genotype	Meg +/+	1.3562	0.3174	29	4.27	0.0002	0.05	0.7071	2.0053
Genotype	Meg +/-	0	.	.	.	.	.	.	.

Solution for Random Effects

Effect	Animal	Estimate	Std Err Pred	DF	t Value	Pr > |t|	Alpha	Lower	Upper
Animal	1	0	.	.	.	.	.	.	.
Animal	2	0	.	.	.	.	.	.	.
Animal	3	0	.	.	.	.	.	.	.
Animal	4	0	.	.	.	.	.	.	.
Animal	5	0	.	.	.	.	.	.	.
Animal	6	0	.	.	.	.	.	.	.

Type 3 Tests of Fixed Effects

Effect	Num DF	Den DF	F Value	Pr > F
Genotype	1	29	18.26	0.0002

Least Squares Means

Effect	Genotype	Estimate	Standard Error	DF	t Value	Pr > |t|	Alpha	Lower	Upper
Genotype	Meg +/+	1.9444	0.2212	29	8.79	<.0001	0.05	1.4921	2.3968
Genotype	Meg +/-	0.5882	0.2276	29	2.58	0.0151	0.05	0.1227	1.0537

DistSoma=390

Differences of Least Squares Means

Effect	Genotype	Genotype	Estimate	Standard Error	DF	t Value	Pr > |t|	Adjustment	Adj P	Alpha	Lower	Upper
**Genotype**	Meg +/+	Meg +/-	1.3562	0.3174	29	4.27	0.0002	Tukey-Kramer	0.0002	0.05	0.7071	2.0053

Differences of Least Squares Means

Effect	Genotype	Genotype	Adj Lower	Adj Upper
**Genotype**	Meg +/+	Meg +/-	0.7071	2.0053

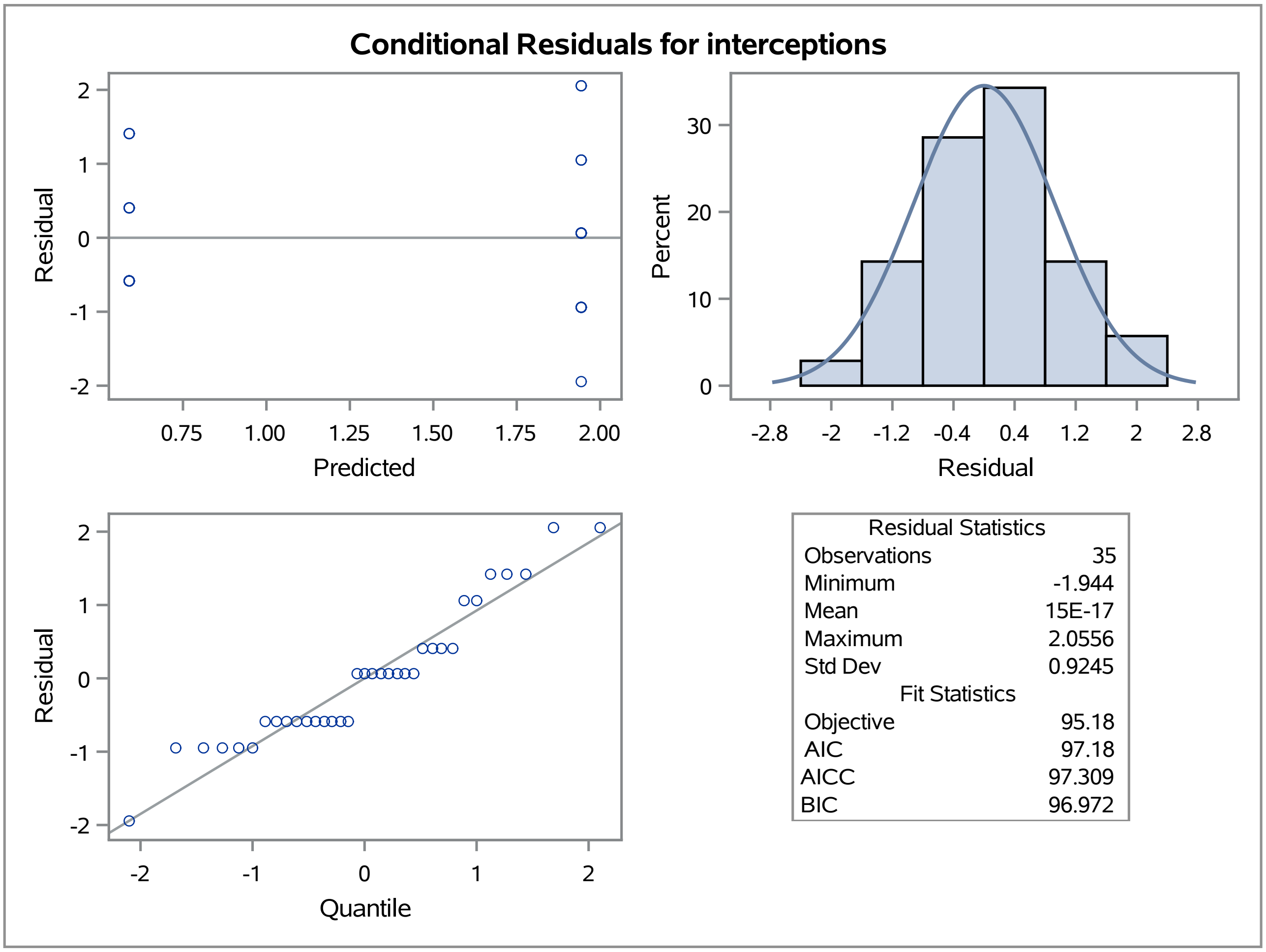

DistSoma=396

Model Information	
**Data Set**	WORK.TEMPDATASORTED
**Dependent Variable**	interceptions
**Covariance Structure**	Variance Components
**Estimation Method**	REML
**Residual Variance Method**	Profile
**Fixed Effects SE Method**	Model-Based
**Degrees of Freedom Method**	Containment

Class Level Information		
**Class**	**Levels**	**Values**
**Genotype**	2	Meg +/+ Meg +/-
**Animal**	6	1 2 3 4 5 6

Dimensions	
**Covariance Parameters**	2
**Columns in X**	3
**Columns in Z**	6
**Subjects**	1
**Max Obs per Subject**	35

Number of Observations	
**Number of Observations Read**	35
**Number of Observations Used**	35
**Number of Observations Not Used**	0

Iteration History			
**Iteration**	**Evaluations**	**-2 Res Log Like**	**Criterion**
**0**	1	89.86040623	
**1**	3	89.22950525	0.00042523
**2**	1	89.22301405	0.00000530
**3**	1	89.22293764	0.00000000

Convergence criteria met.

DistSoma=396

Covariance Parameter Estimates				
**Cov Parm**	**Estimate**	**Alpha**	**Lower**	**Upper**
**Animal**	0.07986	0.05	0.01315	2706.16
**Residual**	0.6968	0.05	0.4453	1.2430

Fit Statistics	
**-2 Res Log Likelihood**	89.2
**AIC (Smaller is Better)**	93.2
**AICC (Smaller is Better)**	93.6
**BIC (Smaller is Better)**	92.8

Solution for Fixed Effects									
**Effect**	**Genotype**	**Estimate**	**Standard Error**	**DF**	**t Value**	**Pr > |t|**	**Alpha**	**Lower**	**Upper**
**Intercept**		0.4951	0.2682	4	1.85	0.1387	0.05	-0.2496	1.2398
**Genotype**	Meg +/+	1.2761	0.3778	29	3.38	0.0021	0.05	0.5034	2.0488
**Genotype**	Meg +/-	0	.	.	.	.	.	.	.

Solution for Random Effects									
**Effect**	**Animal**	**Estimate**	**Std Err Pred**	**DF**	**t Value**	**Pr > |t|**	**Alpha**	**Lower**	**Upper**
**Animal**	1	0.2290	0.2396	29	0.96	0.3471	0.05	-0.2610	0.7190
**Animal**	2	-0.1784	0.2430	29	-0.73	0.4688	0.05	-0.6755	0.3187
**Animal**	3	-0.05057	0.2597	29	-0.19	0.8469	0.05	-0.5816	0.4805
**Animal**	4	-0.09232	0.2597	29	-0.36	0.7248	0.05	-0.6236	0.4389
**Animal**	5	-0.02961	0.2420	29	-0.12	0.9035	0.05	-0.5246	0.4653
**Animal**	6	0.1219	0.2411	29	0.51	0.6168	0.05	-0.3711	0.6150

Type 3 Tests of Fixed Effects				
**Effect**	**Num DF**	**Den DF**	**F Value**	**Pr > F**
**Genotype**	1	29	11.41	0.0021

Least Squares Means									
**Effect**	**Genotype**	**Estimate**	**Standard Error**	**DF**	**t Value**	**Pr > |t|**	**Alpha**	**Lower**	**Upper**
**Genotype**	Meg +/+	1.7712	0.2661	29	6.66	<.0001	0.05	1.2270	2.3154
**Genotype**	Meg +/-	0.4951	0.2682	29	1.85	0.0752	0.05	-0.05350	1.0437

DistSoma=396

Differences of Least Squares Means												
**Effect**	**Genotype**	**Genotype**	**Estimate**	**Standard Error**	**DF**	**t Value**	**Pr > |t|**	**Adjustment**	**Adj P**	**Alpha**	**Lower**	**Upper**
**Genotype**	Meg +/+	Meg +/-	1.2761	0.3778	29	3.38	0.0021	Tukey-Kramer	0.0021	0.05	0.5034	2.0488

Differences of Least Squares Means				
**Effect**	**Genotype**	**Genotype**	**Adj Lower**	**Adj Upper**
**Genotype**	Meg +/+	Meg +/-	0.5034	2.0488

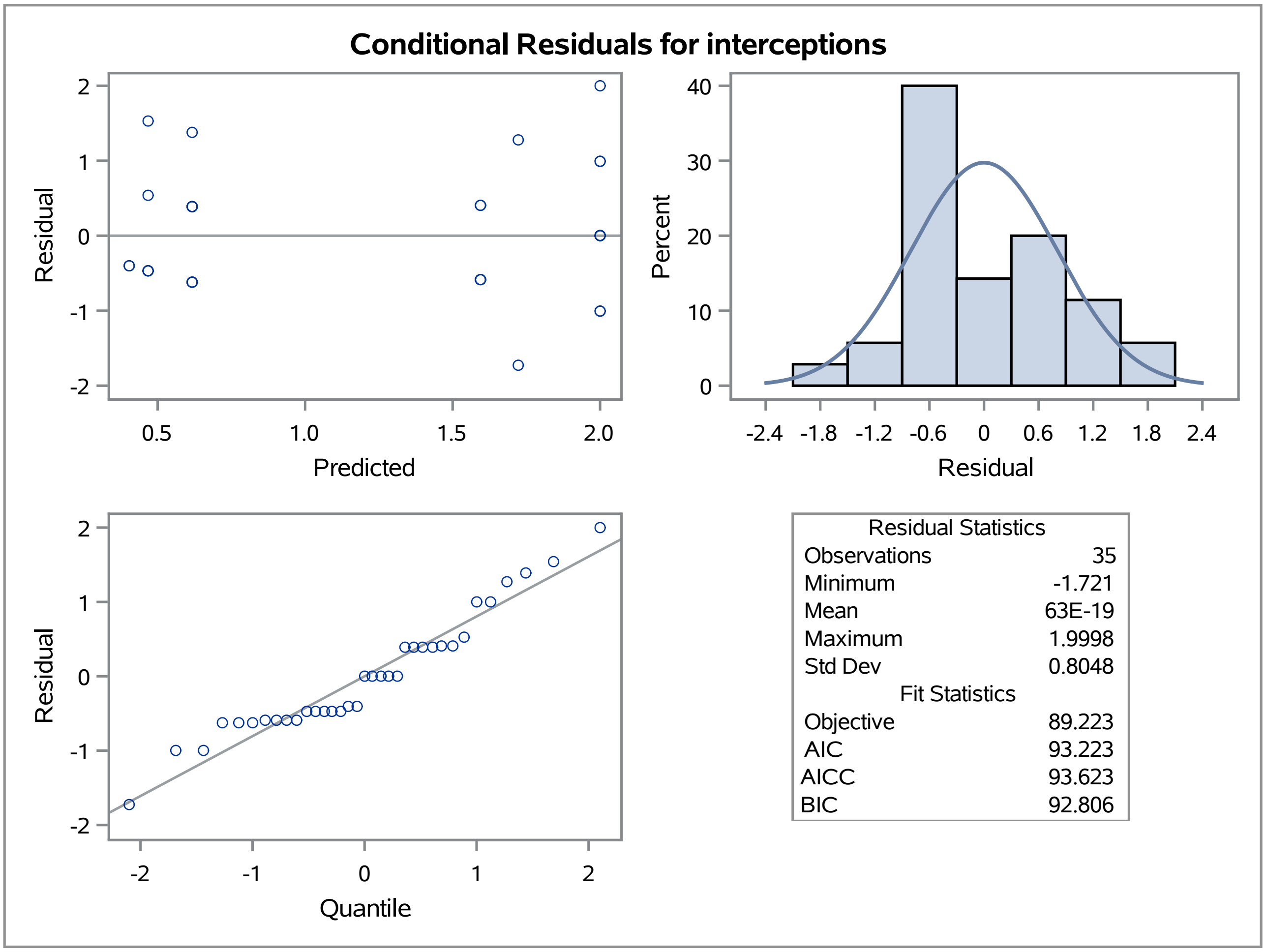

DistSoma=402

Model Information	
**Data Set**	WORK.TEMPDATASORTED
**Dependent Variable**	interceptions
**Covariance Structure**	Variance Components
**Estimation Method**	REML
**Residual Variance Method**	Profile
**Fixed Effects SE Method**	Model-Based
**Degrees of Freedom Method**	Containment

Class Level Information		
**Class**	**Levels**	**Values**
**Genotype**	2	Meg +/+ Meg +/-
**Animal**	6	1 2 3 4 5 6

Dimensions	
**Covariance Parameters**	2
**Columns in X**	3
**Columns in Z**	6
**Subjects**	1
**Max Obs per Subject**	35

Number of Observations	
**Number of Observations Read**	35
**Number of Observations Used**	35
**Number of Observations Not Used**	0

Iteration History			
**Iteration**	**Evaluations**	**-2 Res Log Like**	**Criterion**
**0**	1	81.55020910	
**1**	3	81.18254533	0.00031024
**2**	1	81.17919626	0.00000211
**3**	1	81.17917452	0.00000000

Convergence criteria met.

DistSoma=402

Covariance Parameter Estimates				
**Cov Parm**	**Estimate**	**Alpha**	**Lower**	**Upper**
**Animal**	0.04538	0.05	0.006277	251401
**Residual**	0.5530	0.05	0.3537	0.9852

Fit Statistics	
**-2 Res Log Likelihood**	81.2
**AIC (Smaller is Better)**	85.2
**AICC (Smaller is Better)**	85.6
**BIC (Smaller is Better)**	84.8

Solution for Fixed Effects									
**Effect**	**Genotype**	**Estimate**	**Standard Error**	**DF**	**t Value**	**Pr > |t|**	**Alpha**	**Lower**	**Upper**
**Intercept**		0.3930	0.2243	4	1.75	0.1546	0.05	-0.2297	1.0157
**Genotype**	Meg +/+	1.3408	0.3155	29	4.25	0.0002	0.05	0.6955	1.9861
**Genotype**	Meg +/-	0	.	.	.	.	.	.	.

Solution for Random Effects									
**Effect**	**Animal**	**Estimate**	**Std Err Pred**	**DF**	**t Value**	**Pr > |t|**	**Alpha**	**Lower**	**Upper**
**Animal**	1	0.1651	0.1869	29	0.88	0.3844	0.05	-0.2172	0.5473
**Animal**	2	-0.1321	0.1891	29	-0.70	0.4904	0.05	-0.5189	0.2547
**Animal**	3	-0.03296	0.1999	29	-0.16	0.8702	0.05	-0.4418	0.3759
**Animal**	4	-0.05540	0.2000	29	-0.28	0.7837	0.05	-0.4643	0.3535
**Animal**	5	0.01299	0.1885	29	0.07	0.9455	0.05	-0.3725	0.3984
**Animal**	6	0.04241	0.1879	29	0.23	0.8230	0.05	-0.3418	0.4267

Type 3 Tests of Fixed Effects				
**Effect**	**Num DF**	**Den DF**	**F Value**	**Pr > F**
**Genotype**	1	29	18.06	0.0002

Least Squares Means									
**Effect**	**Genotype**	**Estimate**	**Standard Error**	**DF**	**t Value**	**Pr > |t|**	**Alpha**	**Lower**	**Upper**
**Genotype**	Meg +/+	1.7338	0.2219	29	7.81	<.0001	0.05	1.2799	2.1876
**Genotype**	Meg +/-	0.3930	0.2243	29	1.75	0.0903	0.05	-0.06573	0.8517

DistSoma=402

Differences of Least Squares Means												
Effect	Genotype	Genotype	Estimate	Standard Error	DF	t Value	Pr > |t|	Adjustment	Adj P	Alpha	Lower	Upper
**Genotype**	Meg +/+	Meg +/-	1.3408	0.3155	29	4.25	0.0002	Tukey-Kramer	0.0002	0.05	0.6955	1.9861

Differences of Least Squares Means				
Effect	Genotype	Genotype	Adj Lower	Adj Upper
**Genotype**	Meg +/+	Meg +/-	0.6955	1.9861

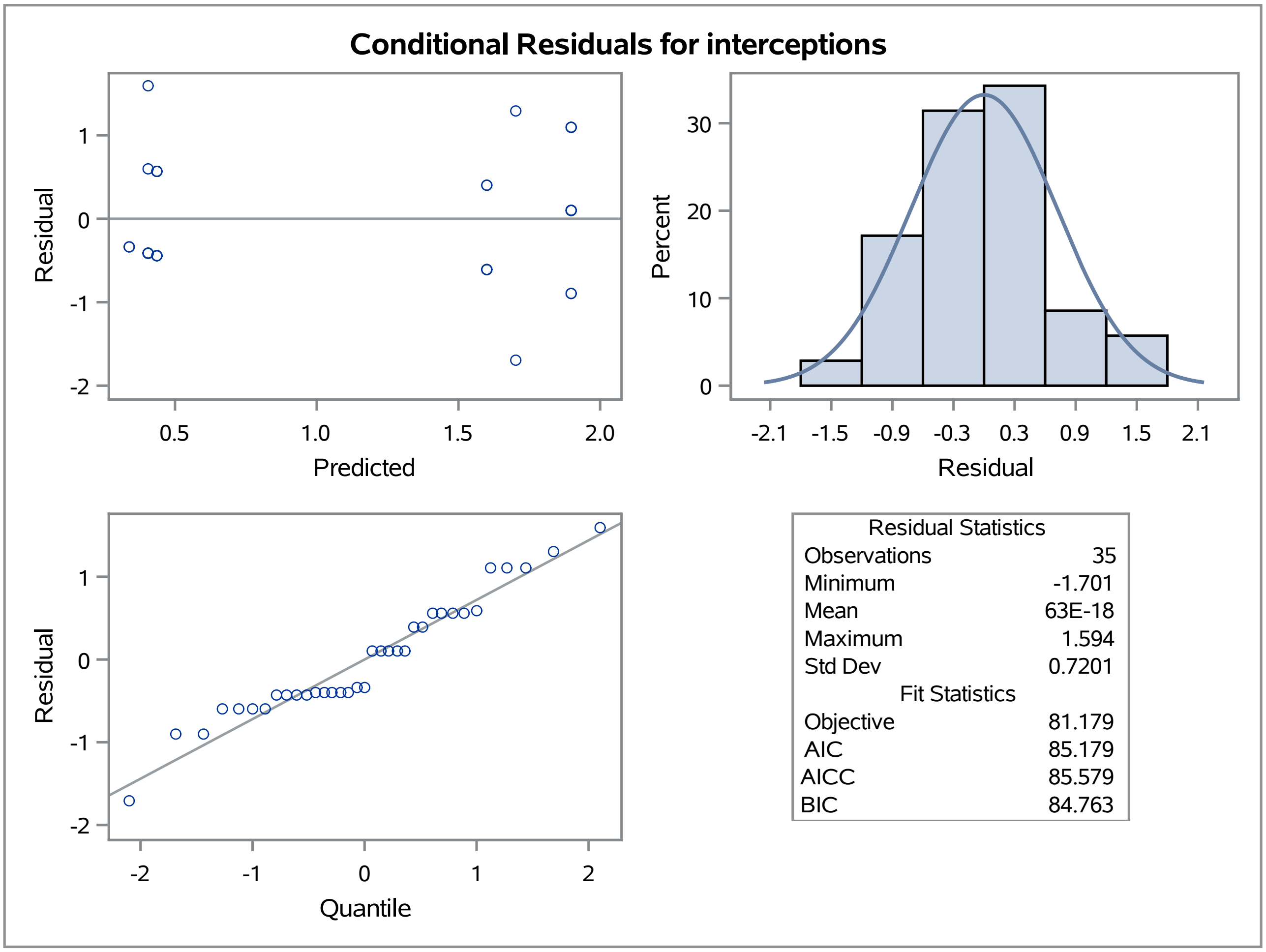

DistSoma=408

Model Information	
**Data Set**	WORK.TEMPDATASORTED
**Dependent Variable**	interceptions
**Covariance Structure**	Variance Components
**Estimation Method**	REML
**Residual Variance Method**	Profile
**Fixed Effects SE Method**	Model-Based
**Degrees of Freedom Method**	Containment

Class Level Information		
**Class**	**Levels**	**Values**
**Genotype**	2	Meg +/+ Meg +/-
**Animal**	6	1 2 3 4 5 6

Dimensions	
**Covariance Parameters**	2
**Columns in X**	3
**Columns in Z**	6
**Subjects**	1
**Max Obs per Subject**	35

Number of Observations	
**Number of Observations Read**	35
**Number of Observations Used**	35
**Number of Observations Not Used**	0

Iteration History			
**Iteration**	**Evaluations**	**-2 Res Log Like**	**Criterion**
**0**	1	84.99792939	
**1**	3	84.68700668	0.00014338
**2**	1	84.68521588	0.00000055
**3**	1	84.68520928	0.00000000

Convergence criteria met.

DistSoma=408

Covariance Parameter Estimates				
**Cov Parm**	**Estimate**	**Alpha**	**Lower**	**Upper**
**Animal**	0.04632	0.05	0.006053	2501857
**Residual**	0.6168	0.05	0.3945	1.0991

Fit Statistics	
**-2 Res Log Likelihood**	84.7
**AIC (Smaller is Better)**	88.7
**AICC (Smaller is Better)**	89.1
**BIC (Smaller is Better)**	88.3

Solution for Fixed Effects									
**Effect**	**Genotype**	**Estimate**	**Standard Error**	**DF**	**t Value**	**Pr > |t|**	**Alpha**	**Lower**	**Upper**
**Intercept**		0.4495	0.2334	4	1.93	0.1264	0.05	-0.1985	1.0974
**Genotype**	Meg +/+	1.2868	0.3282	29	3.92	0.0005	0.05	0.6156	1.9580
**Genotype**	Meg +/-	0	.	.	.	.	.	.	.

Solution for Random Effects									
**Effect**	**Animal**	**Estimate**	**Std Err Pred**	**DF**	**t Value**	**Pr > |t|**	**Alpha**	**Lower**	**Upper**
**Animal**	1	0.1560	0.1904	29	0.82	0.4192	0.05	-0.2334	0.5454
**Animal**	2	-0.1252	0.1925	29	-0.65	0.5208	0.05	-0.5189	0.2686
**Animal**	3	-0.03085	0.2029	29	-0.15	0.8802	0.05	-0.4459	0.3842
**Animal**	4	-0.05869	0.2030	29	-0.29	0.7745	0.05	-0.4738	0.3564
**Animal**	5	-0.00719	0.1919	29	-0.04	0.9704	0.05	-0.3997	0.3853
**Animal**	6	0.06588	0.1913	29	0.34	0.7331	0.05	-0.3254	0.4572

Type 3 Tests of Fixed Effects				
**Effect**	**Num DF**	**Den DF**	**F Value**	**Pr > F**
**Genotype**	1	29	15.37	0.0005

Least Squares Means									
**Effect**	**Genotype**	**Estimate**	**Standard Error**	**DF**	**t Value**	**Pr > |t|**	**Alpha**	**Lower**	**Upper**
**Genotype**	Meg +/+	1.7363	0.2307	29	7.53	<.0001	0.05	1.2644	2.2081
**Genotype**	Meg +/-	0.4495	0.2334	29	1.93	0.0640	0.05	-0.02786	0.9268

DistSoma=408

Differences of Least Squares Means												
Effect	Genotype	Genotype	Estimate	Standard Error	DF	t Value	Pr > |t|	Adjustment	Adj P	Alpha	Lower	Upper
**Genotype**	Meg +/+	Meg +/-	1.2868	0.3282	29	3.92	0.0005	Tukey-Kramer	0.0005	0.05	0.6156	1.9580

Differences of Least Squares Means				
Effect	Genotype	Genotype	Adj Lower	Adj Upper
**Genotype**	Meg +/+	Meg +/-	0.6156	1.9580

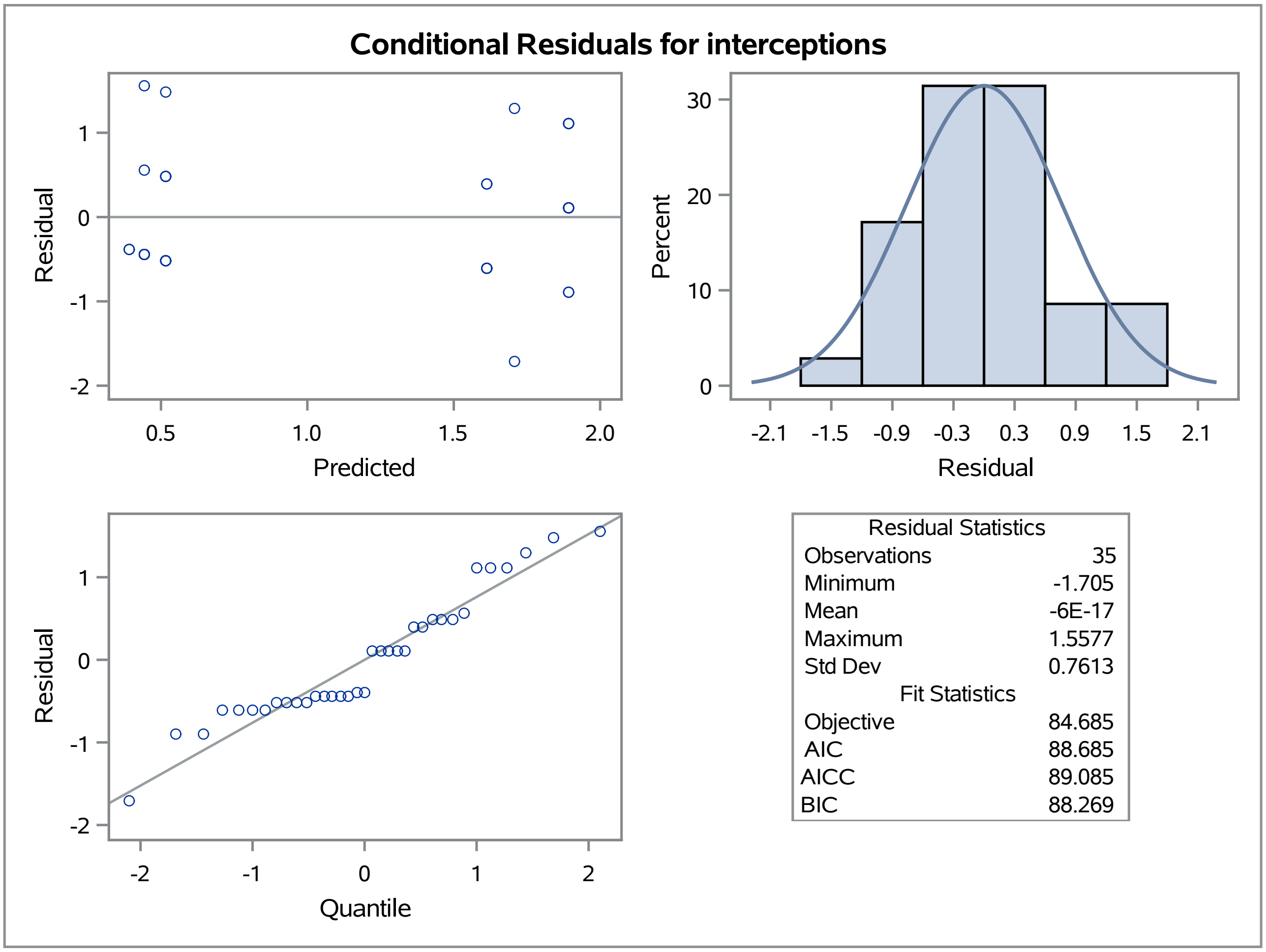

DistSoma=414

Model Information	
**Data Set**	WORK.TEMPDATASORTED
**Dependent Variable**	interceptions
**Covariance Structure**	Variance Components
**Estimation Method**	REML
**Residual Variance Method**	Profile
**Fixed Effects SE Method**	Model-Based
**Degrees of Freedom Method**	Containment

Class Level Information		
**Class**	**Levels**	**Values**
**Genotype**	2	Meg +/+ Meg +/-
**Animal**	6	1 2 3 4 5 6

Dimensions	
**Covariance Parameters**	2
**Columns in X**	3
**Columns in Z**	6
**Subjects**	1
**Max Obs per Subject**	35

Number of Observations	
**Number of Observations Read**	35
**Number of Observations Used**	35
**Number of Observations Not Used**	0

Iteration History			
**Iteration**	**Evaluations**	**-2 Res Log Like**	**Criterion**
**0**	1	86.99735317	
**1**	1	86.99735317	0.00000000

Convergence criteria met.

Estimated G matrix is not positive definite.

Covariance Parameter Estimates				
**Cov Parm**	**Estimate**	**Alpha**	**Lower**	**Upper**
**Animal**	0	.	.	.
**Residual**	0.6873	0.05	0.4471	1.1907

DistSoma=414

Fit Statistics	
-2 Res Log Likelihood	87.0
AIC (Smaller is Better)	89.0
AICC (Smaller is Better)	89.1
BIC (Smaller is Better)	88.8

Solution for Fixed Effects									
Effect	Genotype	Estimate	Standard Error	DF	t Value	Pr > |t|	Alpha	Lower	Upper
Intercept		0.4706	0.2011	4	2.34	0.0793	0.05	-0.08766	1.0288
Genotype	Meg +/+	1.0850	0.2804	29	3.87	0.0006	0.05	0.5115	1.6584
Genotype	Meg +/-	0	.	.	.	.	.	.	.

Solution for Random Effects									
Effect	Animal	Estimate	Std Err Pred	DF	t Value	Pr > |t|	Alpha	Lower	Upper
Animal	1	0	.	.	.	.	.	.	.
Animal	2	0	.	.	.	.	.	.	.
Animal	3	0	.	.	.	.	.	.	.
Animal	4	0	.	.	.	.	.	.	.
Animal	5	0	.	.	.	.	.	.	.
Animal	6	0	.	.	.	.	.	.	.

Type 3 Tests of Fixed Effects				
Effect	Num DF	Den DF	F Value	Pr > F
Genotype	1	29	14.97	0.0006

Least Squares Means									
Effect	Genotype	Estimate	Standard Error	DF	t Value	Pr > |t|	Alpha	Lower	Upper
Genotype	Meg +/+	1.5556	0.1954	29	7.96	<.0001	0.05	1.1559	1.9552
Genotype	Meg +/-	0.4706	0.2011	29	2.34	0.0263	0.05	0.05936	0.8818

DistSoma=414

Differences of Least Squares Means												
Effect	Genotype	Genotype	Estimate	Standard Error	DF	t Value	Pr > |t|	Adjustment	Adj P	Alpha	Lower	Upper
**Genotype**	Meg +/+	Meg +/-	1.0850	0.2804	29	3.87	0.0006	Tukey-Kramer	0.0006	0.05	0.5115	1.6584

Differences of Least Squares Means				
Effect	Genotype	Genotype	Adj Lower	Adj Upper
**Genotype**	Meg +/+	Meg +/-	0.5115	1.6584

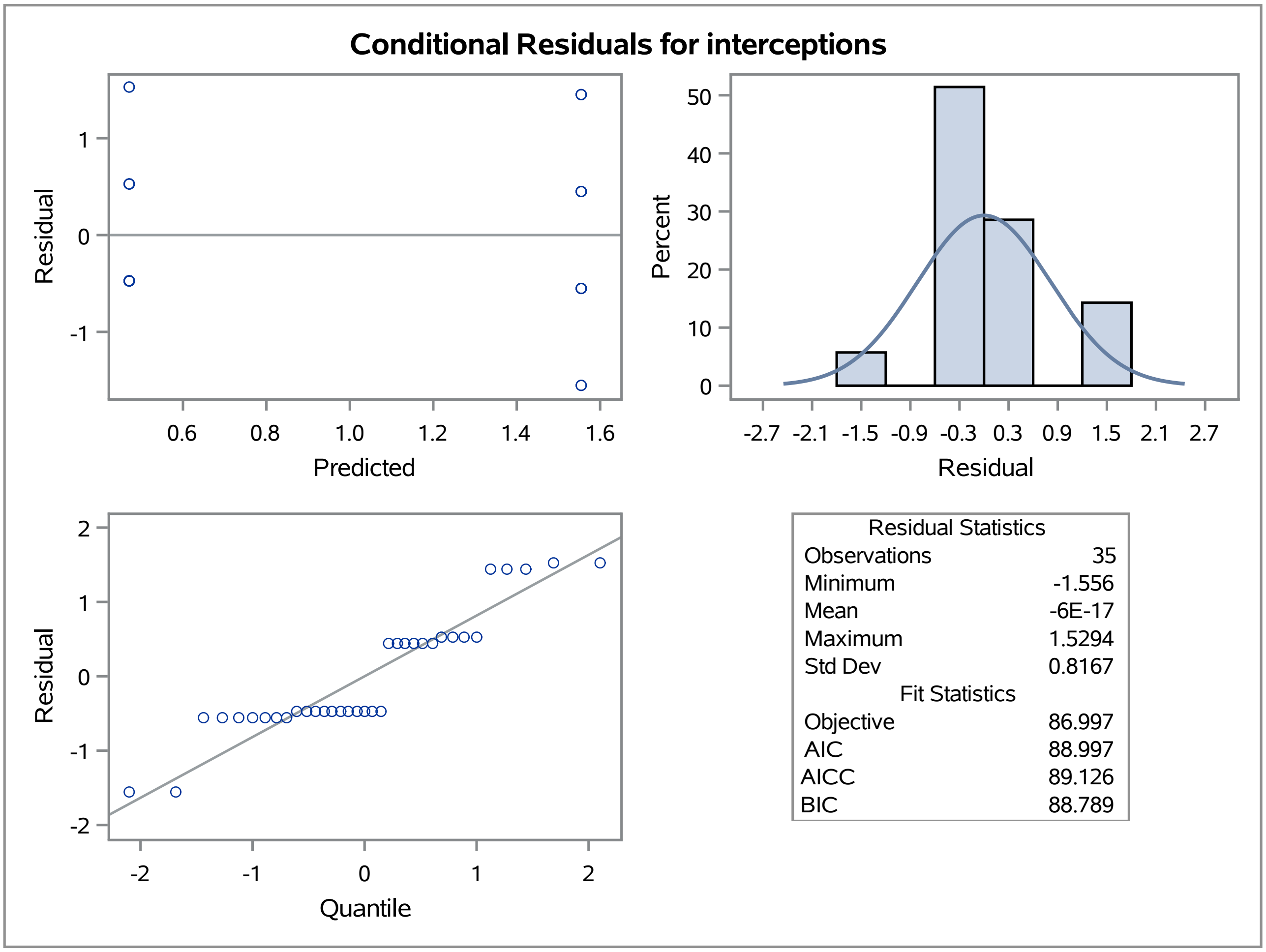

DistSoma=420

Model Information	
**Data Set**	WORK.TEMPDATASORTED
**Dependent Variable**	interceptions
**Covariance Structure**	Variance Components
**Estimation Method**	REML
**Residual Variance Method**	Profile
**Fixed Effects SE Method**	Model-Based
**Degrees of Freedom Method**	Containment

Class Level Information		
**Class**	**Levels**	**Values**
**Genotype**	2	Meg +/+ Meg +/-
**Animal**	6	1 2 3 4 5 6

Dimensions	
**Covariance Parameters**	2
**Columns in X**	3
**Columns in Z**	6
**Subjects**	1
**Max Obs per Subject**	35

Number of Observations	
**Number of Observations Read**	35
**Number of Observations Used**	35
**Number of Observations Not Used**	0

Iteration History			
**Iteration**	**Evaluations**	**-2 Res Log Like**	**Criterion**
**0**	1	79.32308165	
**1**	2	79.32192764	0.00000001

Convergence criteria met.

Covariance Parameter Estimates				
**Cov Parm**	**Estimate**	**Alpha**	**Lower**	**Upper**
**Animal**	0.002978	0.05	.	.
**Residual**	0.5428	0.05	0.3428	0.9882

DistSoma=420

Fit Statistics	
-2 Res Log Likelihood	79.3
AIC (Smaller is Better)	83.3
AICC (Smaller is Better)	83.7
BIC (Smaller is Better)	82.9

Solution for Fixed Effects

Effect	Genotype	Estimate	Standard Error	DF	t Value	Pr > |t|	Alpha	Lower	Upper
Intercept		0.2930	0.1820	4	1.61	0.1828	0.05	-0.2124	0.7984
Genotype	Meg +/+	1.1462	0.2541	29	4.51	<.0001	0.05	0.6265	1.6659
Genotype	Meg +/-	0	.	.	.	.	.	.	.

Solution for Random Effects

Effect	Animal	Estimate	Std Err Pred	DF	t Value	Pr > |t|	Alpha	Lower	Upper
Animal	1	0.01356	0.05393	29	0.25	0.8032	0.05	-0.09673	0.1239
Animal	2	-0.00337	0.05399	29	-0.06	0.9506	0.05	-0.1138	0.1070
Animal	3	-0.01019	0.05431	29	-0.19	0.8524	0.05	-0.1213	0.1009
Animal	4	-0.00318	0.05431	29	-0.06	0.9537	0.05	-0.1143	0.1079
Animal	5	-0.00027	0.05397	29	-0.00	0.9961	0.05	-0.1107	0.1101
Animal	6	0.003448	0.05396	29	0.06	0.9495	0.05	-0.1069	0.1138

Type 3 Tests of Fixed Effects

Effect	Num DF	Den DF	F Value	Pr > F
Genotype	1	29	20.35	<.0001

Least Squares Means

Effect	Genotype	Estimate	Standard Error	DF	t Value	Pr > |t|	Alpha	Lower	Upper
Genotype	Meg +/+	1.4392	0.1773	29	8.12	<.0001	0.05	1.0765	1.8018
Genotype	Meg +/-	0.2930	0.1820	29	1.61	0.1183	0.05	-0.07931	0.6653

DistSoma=420

Differences of Least Squares Means												
Effect	Genotype	Genotype	Estimate	Standard Error	DF	t Value	Pr > |t|	Adjustment	Adj P	Alpha	Lower	Upper
Genotype	Meg +/+	Meg +/-	1.1462	0.2541	29	4.51	<.0001	Tukey-Kramer	<.0001	0.05	0.6265	1.6659

Differences of Least Squares Means				
Effect	Genotype	Genotype	Adj Lower	Adj Upper
Genotype	Meg +/+	Meg +/-	0.6265	1.6659

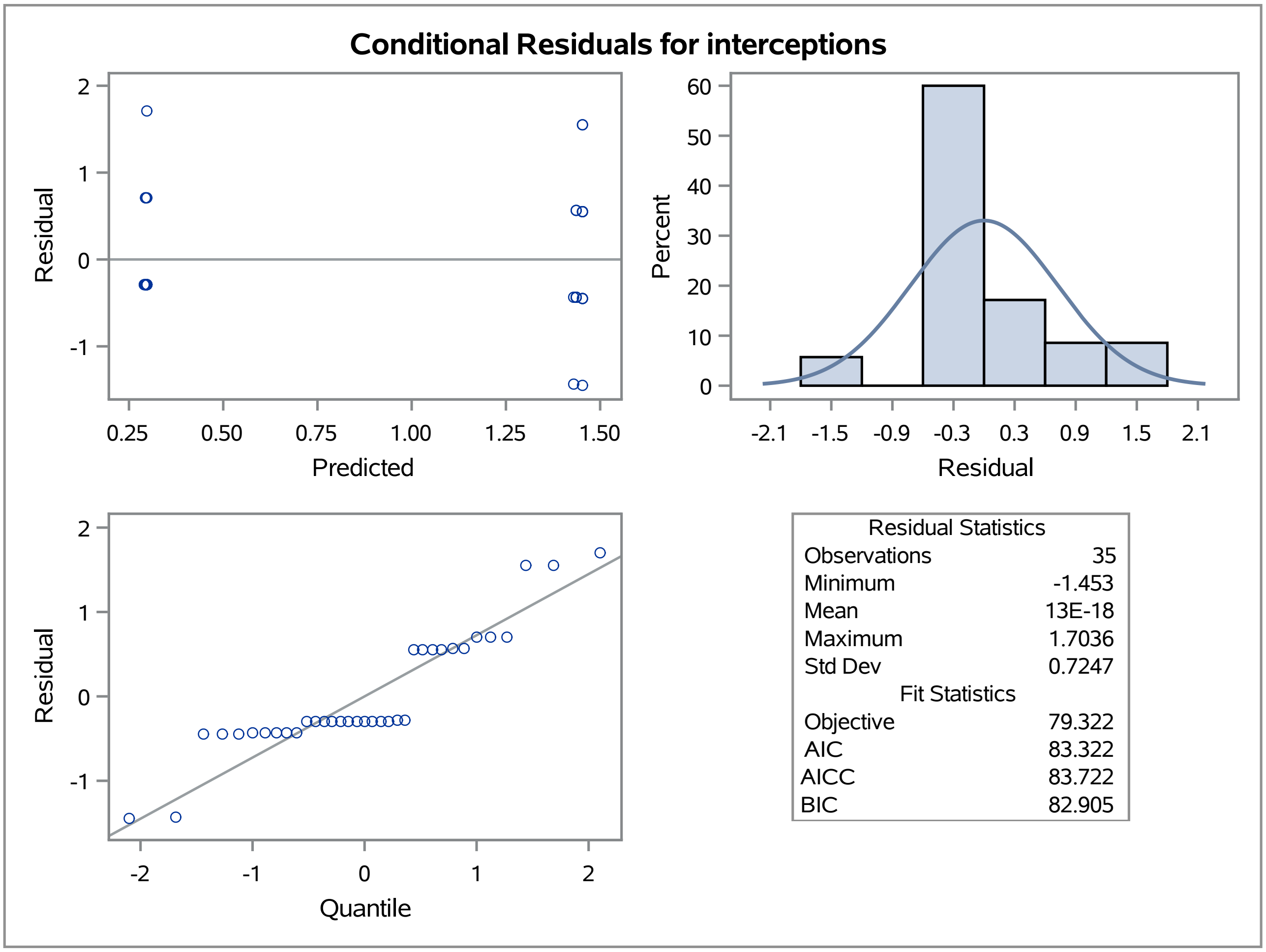

DistSoma=426

Model Information	
Data Set	WORK.TEMPDATASORTED
Dependent Variable	interceptions
Covariance Structure	Variance Components
Estimation Method	REML
Residual Variance Method	Profile
Fixed Effects SE Method	Model-Based
Degrees of Freedom Method	Containment

Class Level Information		
Class	Levels	Values
Genotype	2	Meg +/+ Meg +/-
Animal	6	1 2 3 4 5 6

Dimensions	
Covariance Parameters	2
Columns in X	3
Columns in Z	6
Subjects	1
Max Obs per Subject	35

Number of Observations	
Number of Observations Read	35
Number of Observations Used	35
Number of Observations Not Used	0

Iteration History			
Iteration	Evaluations	-2 Res Log Like	Criterion
0	1	88.53942938	
1	2	88.49758732	0.00005851
2	1	88.49674136	0.00000021
3	1	88.49673838	0.00000000

Convergence criteria met.

DistSoma=426

Covariance Parameter Estimates				
**Cov Parm**	**Estimate**	**Alpha**	**Lower**	**Upper**
**Animal**	0.03557	0.05	0.003147	4.709E45
**Residual**	0.6999	0.05	0.4319	1.3257

Fit Statistics	
**-2 Res Log Likelihood**	88.5
**AIC (Smaller is Better)**	92.5
**AICC (Smaller is Better)**	92.9
**BIC (Smaller is Better)**	92.1

Solution for Fixed Effects									
**Effect**	**Genotype**	**Estimate**	**Standard Error**	**DF**	**t Value**	**Pr > |t|**	**Alpha**	**Lower**	**Upper**
**Intercept**		0.1123	0.2351	4	0.48	0.6579	0.05	-0.5404	0.7649
**Genotype**	Meg +/+	1.1710	0.3300	29	3.55	0.0013	0.05	0.4961	1.8460
**Genotype**	Meg +/-	0	.	.	.	.	.	.	.

Solution for Random Effects									
**Effect**	**Animal**	**Estimate**	**Std Err Pred**	**DF**	**t Value**	**Pr > |t|**	**Alpha**	**Lower**	**Upper**
**Animal**	1	0.1067	0.1723	29	0.62	0.5404	0.05	-0.2456	0.4591
**Animal**	2	0.01169	0.1738	29	0.07	0.9468	0.05	-0.3437	0.3671
**Animal**	3	-0.1184	0.1810	29	-0.65	0.5180	0.05	-0.4885	0.2517
**Animal**	4	-0.01036	0.1810	29	-0.06	0.9548	0.05	-0.3805	0.3598
**Animal**	5	-0.02946	0.1733	29	-0.17	0.8662	0.05	-0.3840	0.3250
**Animal**	6	0.03982	0.1729	29	0.23	0.8195	0.05	-0.3139	0.3935

Type 3 Tests of Fixed Effects				
**Effect**	**Num DF**	**Den DF**	**F Value**	**Pr > F**
**Genotype**	1	29	12.59	0.0013

Least Squares Means									
**Effect**	**Genotype**	**Estimate**	**Standard Error**	**DF**	**t Value**	**Pr > |t|**	**Alpha**	**Lower**	**Upper**
**Genotype**	Meg +/+	1.2833	0.2316	29	5.54	<.0001	0.05	0.8095	1.7571
**Genotype**	Meg +/-	0.1123	0.2351	29	0.48	0.6365	0.05	-0.3685	0.5930

DistSoma=426

Differences of Least Squares Means												
Effect	Genotype	Genotype	Estimate	Standard Error	DF	t Value	Pr > |t|	Adjustment	Adj P	Alpha	Lower	Upper
Genotype	Meg +/+	Meg +/-	1.1710	0.3300	29	3.55	0.0013	Tukey-Kramer	0.0013	0.05	0.4961	1.8460

Differences of Least Squares Means				
Effect	Genotype	Genotype	Adj Lower	Adj Upper
Genotype	Meg +/+	Meg +/-	0.4961	1.8460

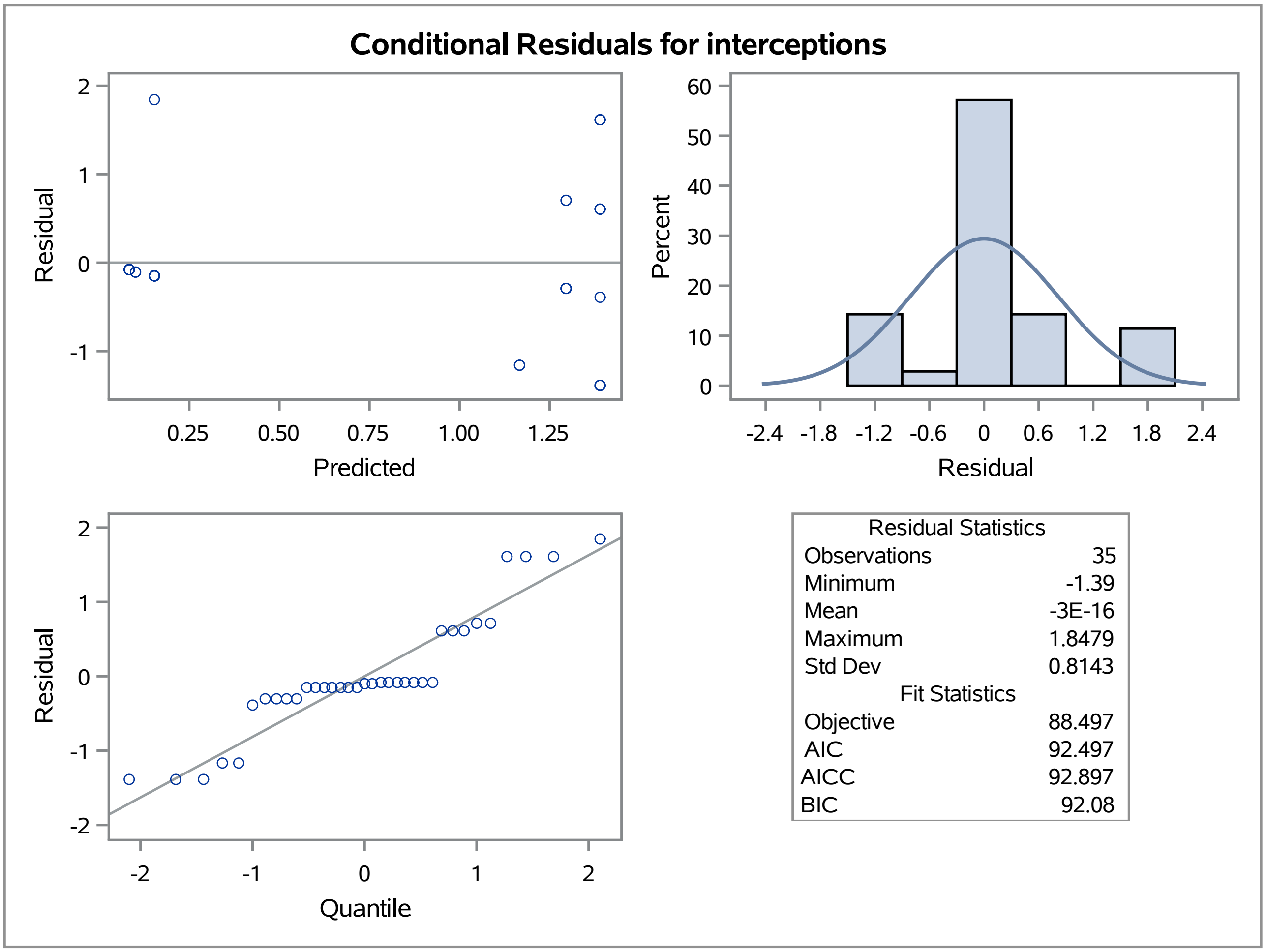

DistSoma=432

Model Information	
**Data Set**	WORK.TEMPDATASORTED
**Dependent Variable**	interceptions
**Covariance Structure**	Variance Components
**Estimation Method**	REML
**Residual Variance Method**	Profile
**Fixed Effects SE Method**	Model-Based
**Degrees of Freedom Method**	Containment

Class Level Information		
**Class**	**Levels**	**Values**
**Genotype**	2	Meg +/+ Meg +/-
**Animal**	6	1 2 3 4 5 6

Dimensions	
**Covariance Parameters**	2
**Columns in X**	3
**Columns in Z**	6
**Subjects**	1
**Max Obs per Subject**	35

Number of Observations	
**Number of Observations Read**	35
**Number of Observations Used**	35
**Number of Observations Not Used**	0

Iteration History			
**Iteration**	**Evaluations**	**-2 Res Log Like**	**Criterion**
**0**	1	84.36545253	
**1**	2	84.26337864	0.00044677
**2**	1	84.25769613	0.00000718
**3**	1	84.25761050	0.00000000

Convergence criteria met.

DistSoma=432

Covariance Parameter Estimates				
**Cov Parm**	**Estimate**	**Alpha**	**Lower**	**Upper**
**Animal**	0.05941	0.05	0.005652	4.09E18
**Residual**	0.6031	0.05	0.3699	1.1549

Fit Statistics	
**-2 Res Log Likelihood**	84.3
**AIC (Smaller is Better)**	88.3
**AICC (Smaller is Better)**	88.7
**BIC (Smaller is Better)**	87.8

Solution for Fixed Effects									
**Effect**	**Genotype**	**Estimate**	**Standard Error**	**DF**	**t Value**	**Pr > |t|**	**Alpha**	**Lower**	**Upper**
**Intercept**		0.05436	0.2421	4	0.22	0.8334	0.05	-0.6179	0.7266
**Genotype**	Meg +/+	1.1946	0.3408	29	3.50	0.0015	0.05	0.4975	1.8917
**Genotype**	Meg +/-	0	.	.	.	.	.	.	.

Solution for Random Effects									
**Effect**	**Animal**	**Estimate**	**Std Err Pred**	**DF**	**t Value**	**Pr > |t|**	**Alpha**	**Lower**	**Upper**
**Animal**	1	0.1742	0.2100	29	0.83	0.4135	0.05	-0.2553	0.6037
**Animal**	2	0.03135	0.2128	29	0.15	0.8839	0.05	-0.4039	0.4666
**Animal**	3	-0.2056	0.2263	29	-0.91	0.3711	0.05	-0.6683	0.2572
**Animal**	4	-0.00895	0.2263	29	-0.04	0.9687	0.05	-0.4718	0.4539
**Animal**	5	-0.02219	0.2120	29	-0.10	0.9174	0.05	-0.4557	0.4113
**Animal**	6	0.03113	0.2112	29	0.15	0.8838	0.05	-0.4009	0.4631

Type 3 Tests of Fixed Effects				
**Effect**	**Num DF**	**Den DF**	**F Value**	**Pr > F**
**Genotype**	1	29	12.28	0.0015

Least Squares Means									
**Effect**	**Genotype**	**Estimate**	**Standard Error**	**DF**	**t Value**	**Pr > |t|**	**Alpha**	**Lower**	**Upper**
**Genotype**	Meg +/+	1.2489	0.2399	29	5.21	<.0001	0.05	0.7583	1.7396
**Genotype**	Meg +/-	0.05436	0.2421	29	0.22	0.8239	0.05	-0.4408	0.5495

DistSoma=432

Differences of Least Squares Means												
Effect	Genotype	Genotype	Estimate	Standard Error	DF	t Value	Pr > |t|	Adjustment	Adj P	Alpha	Lower	Upper
**Genotype**	Meg +/+	Meg +/-	1.1946	0.3408	29	3.50	0.0015	Tukey-Kramer	0.0015	0.05	0.4975	1.8917

Differences of Least Squares Means				
Effect	Genotype	Genotype	Adj Lower	Adj Upper
**Genotype**	Meg +/+	Meg +/-	0.4975	1.8917

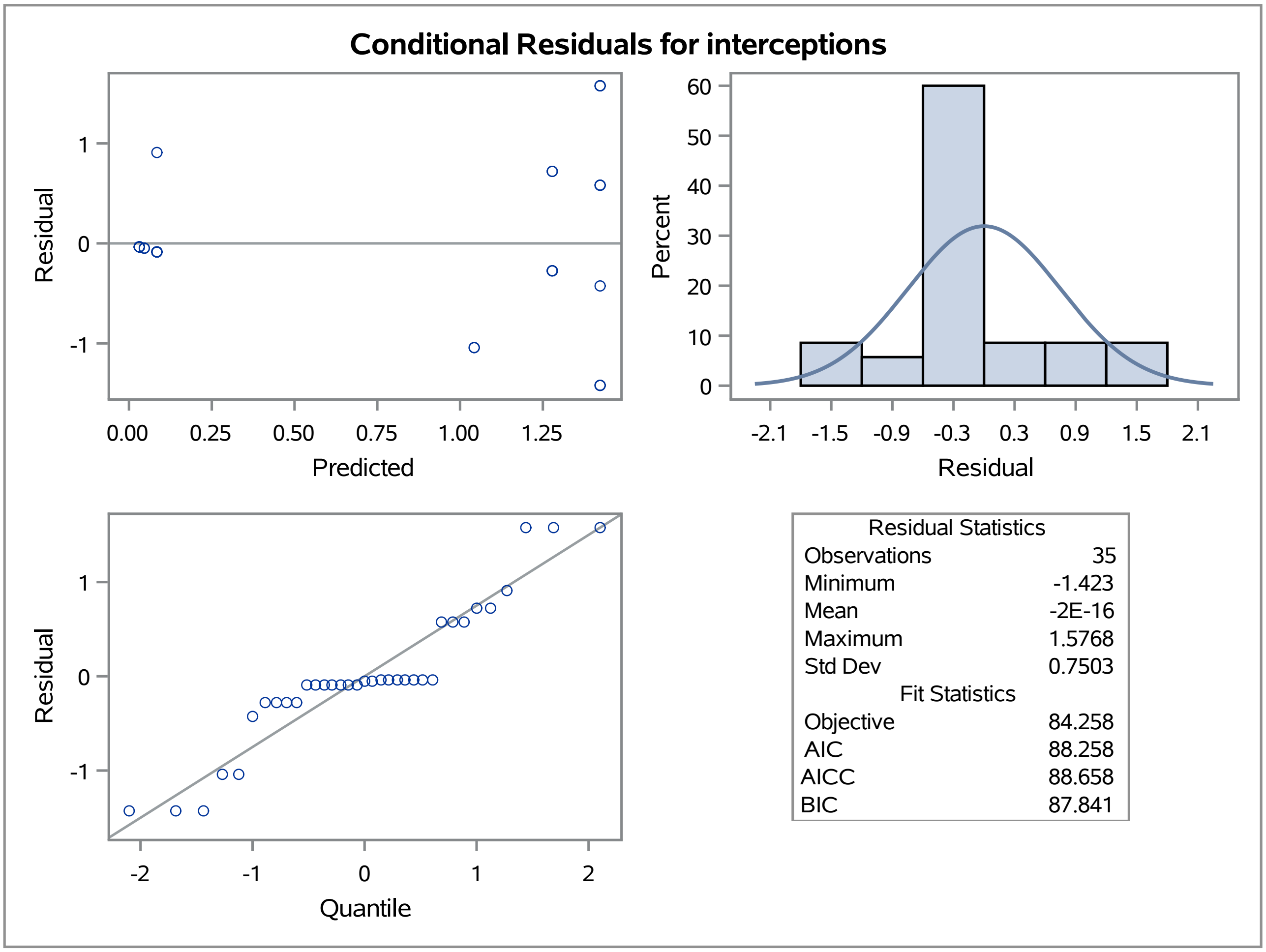

DistSoma=438

Model Information	
**Data Set**	WORK.TEMPDATASORTED
**Dependent Variable**	interceptions
**Covariance Structure**	Variance Components
**Estimation Method**	REML
**Residual Variance Method**	Profile
**Fixed Effects SE Method**	Model-Based
**Degrees of Freedom Method**	Containment

Class Level Information		
**Class**	**Levels**	**Values**
**Genotype**	2	Meg +/+ Meg +/-
**Animal**	6	1 2 3 4 5 6

Dimensions	
**Covariance Parameters**	2
**Columns in X**	3
**Columns in Z**	6
**Subjects**	1
**Max Obs per Subject**	35

Number of Observations	
**Number of Observations Read**	35
**Number of Observations Used**	35
**Number of Observations Not Used**	0

Iteration History			
**Iteration**	**Evaluations**	**-2 Res Log Like**	**Criterion**
**0**	1	86.81138345	
**1**	2	86.59800765	0.00022309
**2**	1	86.59494084	0.00000206
**3**	1	86.59491396	0.00000000

Convergence criteria met.

DistSoma=438

Covariance Parameter Estimates				
**Cov Parm**	**Estimate**	**Alpha**	**Lower**	**Upper**
**Animal**	0.06743	0.05	0.007590	1.712E10
**Residual**	0.6459	0.05	0.4045	1.1929

Fit Statistics	
**-2 Res Log Likelihood**	86.6
**AIC (Smaller is Better)**	90.6
**AICC (Smaller is Better)**	91.0
**BIC (Smaller is Better)**	90.2

Solution for Fixed Effects									
**Effect**	**Genotype**	**Estimate**	**Standard Error**	**DF**	**t Value**	**Pr > |t|**	**Alpha**	**Lower**	**Upper**
**Intercept**		0.05418	0.2534	4	0.21	0.8412	0.05	-0.6494	0.7577
**Genotype**	Meg +/+	1.1310	0.3568	29	3.17	0.0036	0.05	0.4013	1.8608
**Genotype**	Meg +/-	0	.	.	.	.	.	.	.

Solution for Random Effects									
**Effect**	**Animal**	**Estimate**	**Std Err Pred**	**DF**	**t Value**	**Pr > |t|**	**Alpha**	**Lower**	**Upper**
**Animal**	1	0.2119	0.2224	29	0.95	0.3486	0.05	-0.2429	0.6667
**Animal**	2	-0.00714	0.2254	29	-0.03	0.9750	0.05	-0.4682	0.4539
**Animal**	3	-0.2047	0.2401	29	-0.85	0.4009	0.05	-0.6959	0.2864
**Animal**	4	-0.00936	0.2402	29	-0.04	0.9692	0.05	-0.5006	0.4819
**Animal**	5	-0.02287	0.2245	29	-0.10	0.9196	0.05	-0.4821	0.4363
**Animal**	6	0.03223	0.2237	29	0.14	0.8864	0.05	-0.4253	0.4898

Type 3 Tests of Fixed Effects				
**Effect**	**Num DF**	**Den DF**	**F Value**	**Pr > F**
**Genotype**	1	29	10.05	0.0036

Least Squares Means									
**Effect**	**Genotype**	**Estimate**	**Standard Error**	**DF**	**t Value**	**Pr > |t|**	**Alpha**	**Lower**	**Upper**
**Genotype**	Meg +/+	1.1852	0.2512	29	4.72	<.0001	0.05	0.6715	1.6990
**Genotype**	Meg +/-	0.05418	0.2534	29	0.21	0.8322	0.05	-0.4641	0.5724

DistSoma=438

Differences of Least Squares Means												
Effect	Genotype	Genotype	Estimate	Standard Error	DF	t Value	Pr > |t|	Adjustment	Adj P	Alpha	Lower	Upper
**Genotype**	Meg +/+	Meg +/-	1.1310	0.3568	29	3.17	0.0036	Tukey-Kramer	0.0036	0.05	0.4013	1.8608

Differences of Least Squares Means				
Effect	Genotype	Genotype	Adj Lower	Adj Upper
**Genotype**	Meg +/+	Meg +/-	0.4013	1.8608

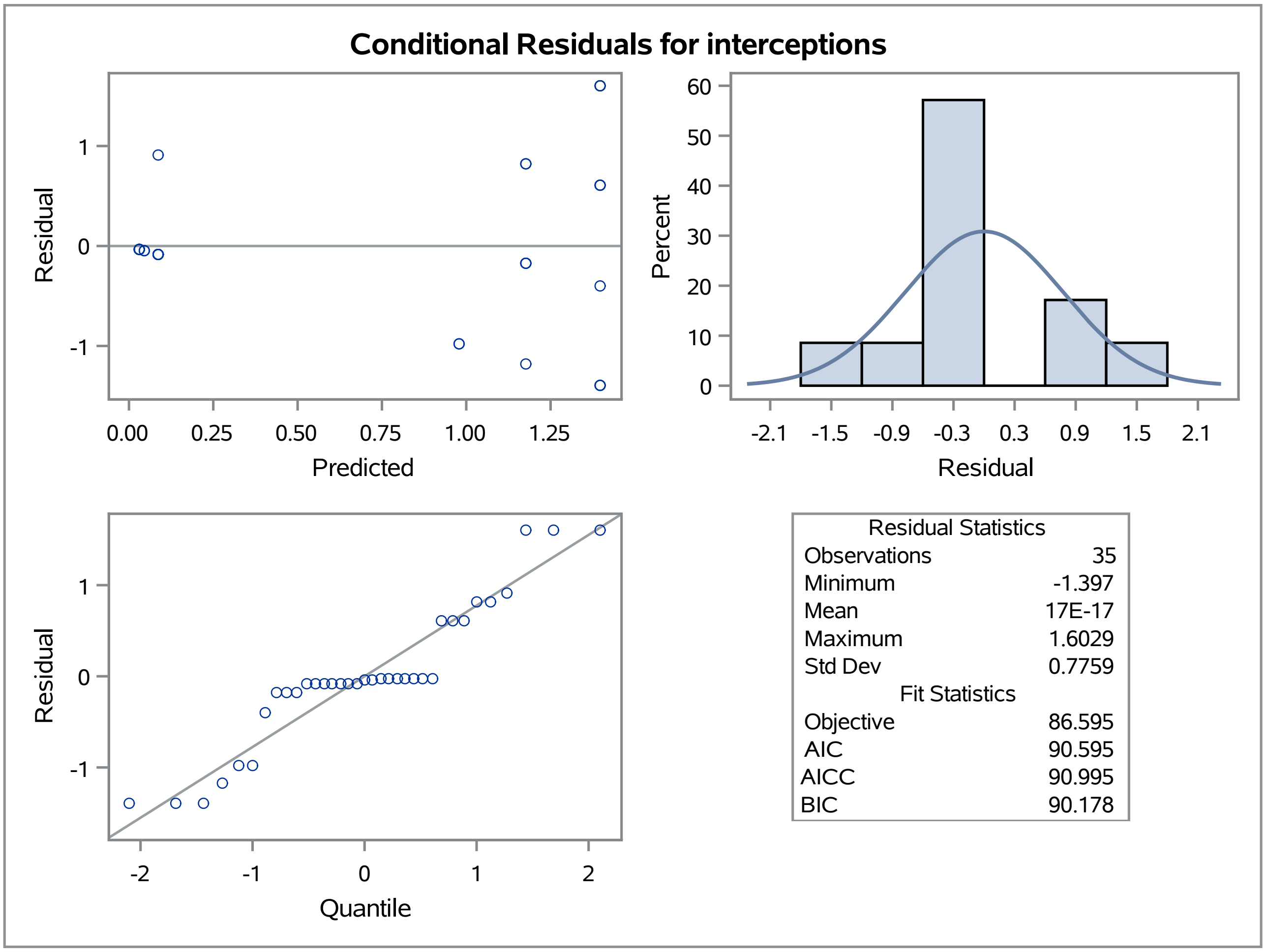

DistSoma=444

Model Information	
**Data Set**	WORK.TEMPDATASORTED
**Dependent Variable**	interceptions
**Covariance Structure**	Variance Components
**Estimation Method**	REML
**Residual Variance Method**	Profile
**Fixed Effects SE Method**	Model-Based
**Degrees of Freedom Method**	Containment

Class Level Information		
**Class**	**Levels**	**Values**
**Genotype**	2	Meg +/+ Meg +/-
**Animal**	6	1 2 3 4 5 6

Dimensions	
**Covariance Parameters**	2
**Columns in X**	3
**Columns in Z**	6
**Subjects**	1
**Max Obs per Subject**	35

Number of Observations	
**Number of Observations Read**	35
**Number of Observations Used**	35
**Number of Observations Not Used**	0

Iteration History			
**Iteration**	**Evaluations**	**-2 Res Log Like**	**Criterion**
**0**	1	75.24745248	
**1**	2	75.19573057	0.00007162
**2**	1	75.19519385	0.00000016
**3**	1	75.19519269	0.00000000

Convergence criteria met.

DistSoma=444

Covariance Parameter Estimates				
**Cov Parm**	**Estimate**	**Alpha**	**Lower**	**Upper**
**Animal**	0.02306	0.05	0.002014	3.583E37
**Residual**	0.4680	0.05	0.2918	0.8710

Fit Statistics	
**-2 Res Log Likelihood**	75.2
**AIC (Smaller is Better)**	79.2
**AICC (Smaller is Better)**	79.6
**BIC (Smaller is Better)**	78.8

Solution for Fixed Effects									
**Effect**	**Genotype**	**Estimate**	**Standard Error**	**DF**	**t Value**	**Pr > |t|**	**Alpha**	**Lower**	**Upper**
**Intercept**		0.05620	0.1915	4	0.29	0.7838	0.05	-0.4755	0.5879
**Genotype**	Meg +/+	0.9582	0.2688	29	3.56	0.0013	0.05	0.4084	1.5080
**Genotype**	Meg +/-	0	.	.	.	.	.	.	.

Solution for Random Effects									
**Effect**	**Animal**	**Estimate**	**Std Err Pred**	**DF**	**t Value**	**Pr > |t|**	**Alpha**	**Lower**	**Upper**
**Animal**	1	0.09427	0.1390	29	0.68	0.5031	0.05	-0.1901	0.3786
**Animal**	2	-0.00328	0.1402	29	-0.02	0.9815	0.05	-0.2900	0.2834
**Animal**	3	-0.09099	0.1459	29	-0.62	0.5376	0.05	-0.3893	0.2073
**Animal**	4	-0.00504	0.1459	29	-0.03	0.9727	0.05	-0.3034	0.2934
**Animal**	5	-0.01441	0.1399	29	-0.10	0.9186	0.05	-0.3004	0.2716
**Animal**	6	0.01945	0.1395	29	0.14	0.8901	0.05	-0.2659	0.3048

Type 3 Tests of Fixed Effects				
**Effect**	**Num DF**	**Den DF**	**F Value**	**Pr > F**
**Genotype**	1	29	12.71	0.0013

Least Squares Means									
**Effect**	**Genotype**	**Estimate**	**Standard Error**	**DF**	**t Value**	**Pr > |t|**	**Alpha**	**Lower**	**Upper**
**Genotype**	Meg +/+	1.0144	0.1887	29	5.38	<.0001	0.05	0.6285	1.4002
**Genotype**	Meg +/-	0.05620	0.1915	29	0.29	0.7713	0.05	-0.3354	0.4478

DistSoma=444

Differences of Least Squares Means												
Effect	Genotype	Genotype	Estimate	Standard Error	DF	t Value	Pr > |t|	Adjustment	Adj P	Alpha	Lower	Upper
Genotype	Meg +/+	Meg +/-	0.9582	0.2688	29	3.56	0.0013	Tukey-Kramer	0.0013	0.05	0.4084	1.5080

Differences of Least Squares Means				
Effect	Genotype	Genotype	Adj Lower	Adj Upper
Genotype	Meg +/+	Meg +/-	0.4084	1.5080

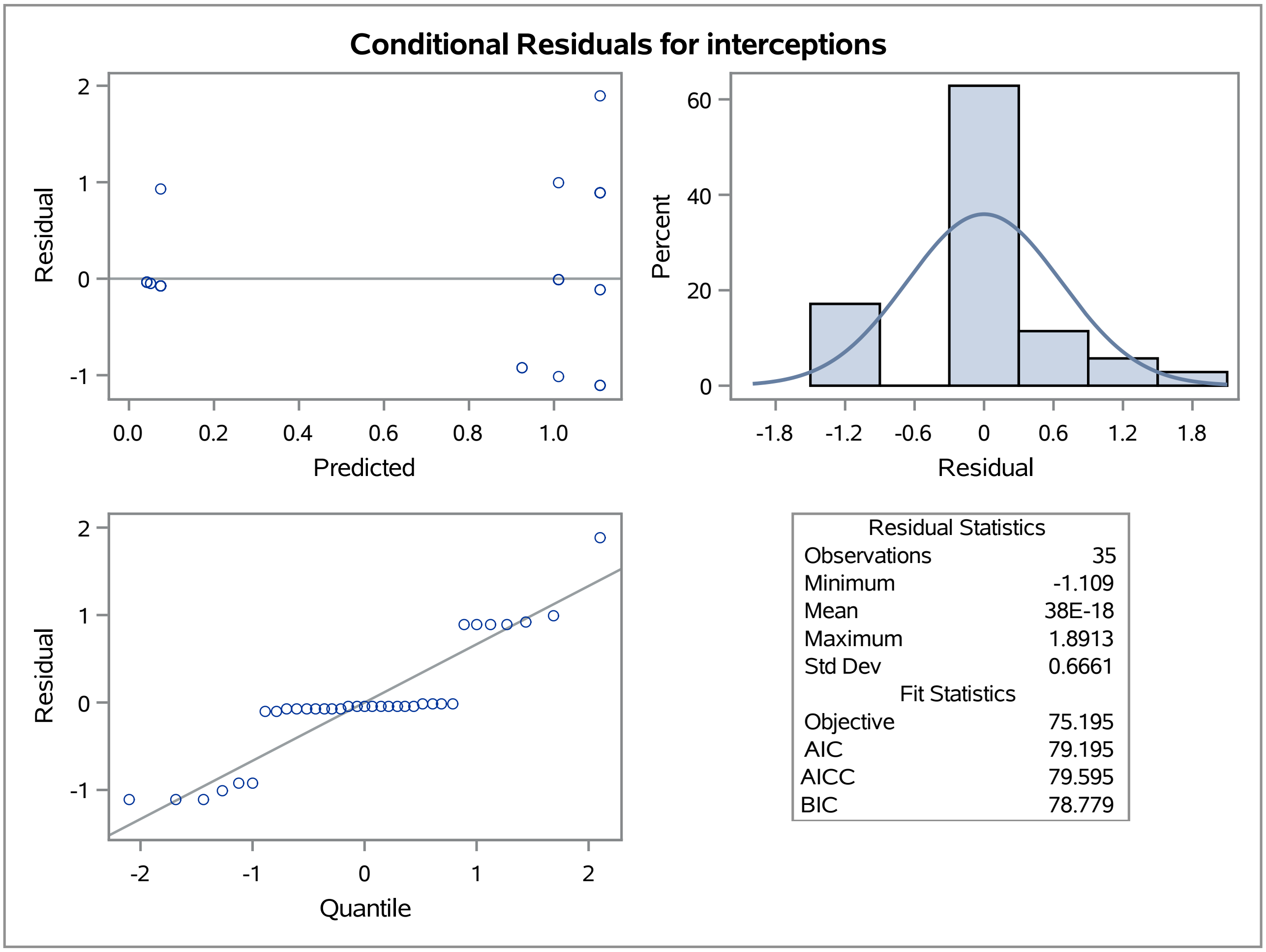

DistSoma=450

Model Information	
**Data Set**	WORK.TEMPDATASORTED
**Dependent Variable**	interceptions
**Covariance Structure**	Variance Components
**Estimation Method**	REML
**Residual Variance Method**	Profile
**Fixed Effects SE Method**	Model-Based
**Degrees of Freedom Method**	Containment

Class Level Information		
**Class**	**Levels**	**Values**
**Genotype**	2	Meg +/+ Meg +/-
**Animal**	6	1 2 3 4 5 6

Dimensions	
**Covariance Parameters**	2
**Columns in X**	3
**Columns in Z**	6
**Subjects**	1
**Max Obs per Subject**	35

Number of Observations	
**Number of Observations Read**	35
**Number of Observations Used**	35
**Number of Observations Not Used**	0

Iteration History			
**Iteration**	**Evaluations**	**-2 Res Log Like**	**Criterion**
**0**	1	75.24745248	
**1**	2	75.19573057	0.00007162
**2**	1	75.19519385	0.00000016
**3**	1	75.19519269	0.00000000

Convergence criteria met.

DistSoma=450

Covariance Parameter Estimates				
**Cov Parm**	**Estimate**	**Alpha**	**Lower**	**Upper**
**Animal**	0.02306	0.05	0.002014	3.583E37
**Residual**	0.4680	0.05	0.2918	0.8710

Fit Statistics	
**-2 Res Log Likelihood**	75.2
**AIC (Smaller is Better)**	79.2
**AICC (Smaller is Better)**	79.6
**BIC (Smaller is Better)**	78.8

Solution for Fixed Effects									
**Effect**	**Genotype**	**Estimate**	**Standard Error**	**DF**	**t Value**	**Pr > |t|**	**Alpha**	**Lower**	**Upper**
**Intercept**		0.05620	0.1915	4	0.29	0.7838	0.05	-0.4755	0.5879
**Genotype**	Meg +/+	0.9582	0.2688	29	3.56	0.0013	0.05	0.4084	1.5080
**Genotype**	Meg +/-	0	.	.	.	.	.	.	.

Solution for Random Effects									
**Effect**	**Animal**	**Estimate**	**Std Err Pred**	**DF**	**t Value**	**Pr > |t|**	**Alpha**	**Lower**	**Upper**
**Animal**	1	0.09427	0.1390	29	0.68	0.5031	0.05	-0.1901	0.3786
**Animal**	2	-0.00328	0.1402	29	-0.02	0.9815	0.05	-0.2900	0.2834
**Animal**	3	-0.09099	0.1459	29	-0.62	0.5376	0.05	-0.3893	0.2073
**Animal**	4	-0.00504	0.1459	29	-0.03	0.9727	0.05	-0.3034	0.2934
**Animal**	5	-0.01441	0.1399	29	-0.10	0.9186	0.05	-0.3004	0.2716
**Animal**	6	0.01945	0.1395	29	0.14	0.8901	0.05	-0.2659	0.3048

Type 3 Tests of Fixed Effects				
**Effect**	**Num DF**	**Den DF**	**F Value**	**Pr > F**
**Genotype**	1	29	12.71	0.0013

Least Squares Means									
**Effect**	**Genotype**	**Estimate**	**Standard Error**	**DF**	**t Value**	**Pr > |t|**	**Alpha**	**Lower**	**Upper**
**Genotype**	Meg +/+	1.0144	0.1887	29	5.38	<.0001	0.05	0.6285	1.4002
**Genotype**	Meg +/-	0.05620	0.1915	29	0.29	0.7713	0.05	-0.3354	0.4478

DistSoma=450

Differences of Least Squares Means												
**Effect**	**Genotype**	**Genotype**	**Estimate**	**Standard Error**	**DF**	**t Value**	**Pr > |t|**	**Adjustment**	**Adj P**	**Alpha**	**Lower**	**Upper**
**Genotype**	Meg +/+	Meg +/-	0.9582	0.2688	29	3.56	0.0013	Tukey-Kramer	0.0013	0.05	0.4084	1.5080

Differences of Least Squares Means				
**Effect**	**Genotype**	**Genotype**	**Adj Lower**	**Adj Upper**
**Genotype**	Meg +/+	Meg +/-	0.4084	1.5080

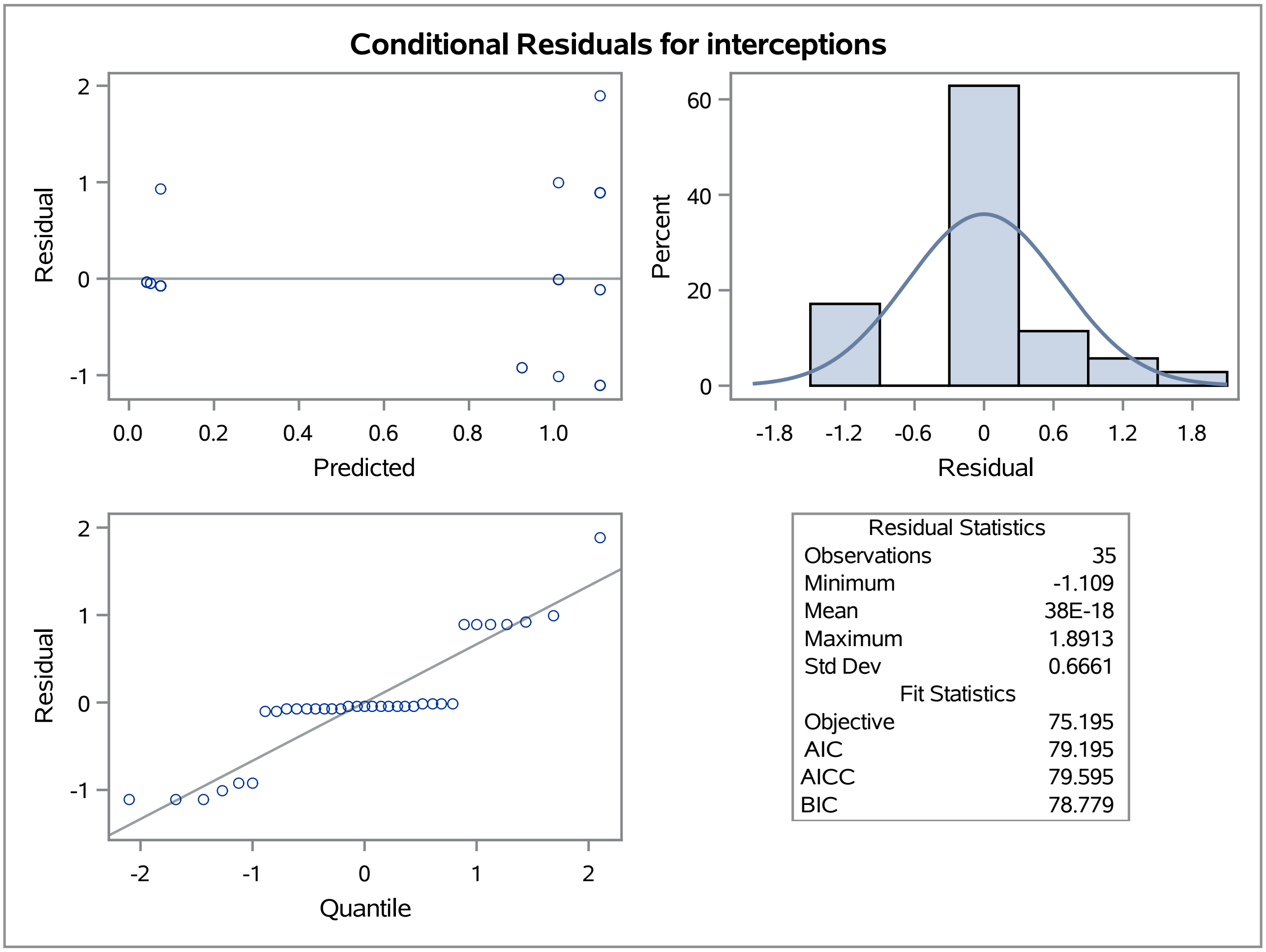

DistSoma=456

Model Information	
**Data Set**	WORK.TEMPDATASORTED
**Dependent Variable**	interceptions
**Covariance Structure**	Variance Components
**Estimation Method**	REML
**Residual Variance Method**	Profile
**Fixed Effects SE Method**	Model-Based
**Degrees of Freedom Method**	Containment

Class Level Information		
**Class**	**Levels**	**Values**
**Genotype**	2	Meg +/+ Meg +/-
**Animal**	6	1 2 3 4 5 6

Dimensions	
**Covariance Parameters**	2
**Columns in X**	3
**Columns in Z**	6
**Subjects**	1
**Max Obs per Subject**	35

Number of Observations	
**Number of Observations Read**	35
**Number of Observations Used**	35
**Number of Observations Not Used**	0

Iteration History			
**Iteration**	**Evaluations**	**-2 Res Log Like**	**Criterion**
**0**	1	75.48420660	
**1**	2	75.15007278	0.00010722
**2**	1	75.14927137	0.00000025
**3**	1	75.14926957	0.00000000

Convergence criteria met.

DistSoma=456

Covariance Parameter Estimates				
**Cov Parm**	**Estimate**	**Alpha**	**Lower**	**Upper**
**Animal**	0.05155	0.05	0.006662	4572500
**Residual**	0.4551	0.05	0.2873	0.8290

Fit Statistics	
**-2 Res Log Likelihood**	75.1
**AIC (Smaller is Better)**	79.1
**AICC (Smaller is Better)**	79.5
**BIC (Smaller is Better)**	78.7

Solution for Fixed Effects									
**Effect**	**Genotype**	**Estimate**	**Standard Error**	**DF**	**t Value**	**Pr > |t|**	**Alpha**	**Lower**	**Upper**
**Intercept**		-686E-19	0.2162	4	-0.00	1.0000	0.05	-0.6004	0.6004
**Genotype**	Meg +/+	0.9172	0.3046	29	3.01	0.0053	0.05	0.2942	1.5401
**Genotype**	Meg +/-	0	.	.	.	.	.	.	.

Solution for Random Effects									
**Effect**	**Animal**	**Estimate**	**Std Err Pred**	**DF**	**t Value**	**Pr > |t|**	**Alpha**	**Lower**	**Upper**
**Animal**	1	0.2033	0.1927	29	1.05	0.3002	0.05	-0.1909	0.5975
**Animal**	2	-0.03392	0.1955	29	-0.17	0.8634	0.05	-0.4338	0.3659
**Animal**	3	-0.1694	0.2088	29	-0.81	0.4238	0.05	-0.5964	0.2576
**Animal**	4	2.97E-17	0.2089	29	0.00	1.0000	0.05	-0.4272	0.4272
**Animal**	5	5.14E-17	0.1947	29	0.00	1.0000	0.05	-0.3981	0.3981
**Animal**	6	7.64E-17	0.1939	29	0.00	1.0000	0.05	-0.3966	0.3966

Type 3 Tests of Fixed Effects				
**Effect**	**Num DF**	**Den DF**	**F Value**	**Pr > F**
**Genotype**	1	29	9.07	0.0053

Least Squares Means									
**Effect**	**Genotype**	**Estimate**	**Standard Error**	**DF**	**t Value**	**Pr > |t|**	**Alpha**	**Lower**	**Upper**
**Genotype**	Meg +/+	0.9172	0.2145	29	4.28	0.0002	0.05	0.4785	1.3559
**Genotype**	Meg +/-	-686E-19	0.2162	29	-0.00	1.0000	0.05	-0.4423	0.4423

DistSoma=456

Differences of Least Squares Means

Effect	Genotype	Genotype	Estimate	Standard Error	DF	t Value	Pr > |t|	Adjustment	Adj P	Alpha	Lower	Upper
**Genotype**	Meg +/+	Meg +/-	0.9172	0.3046	29	3.01	0.0053	Tukey-Kramer	0.0053	0.05	0.2942	1.5401

Differences of Least Squares Means

Effect	Genotype	Genotype	Adj Lower	Adj Upper
**Genotype**	Meg +/+	Meg +/-	0.2942	1.5401

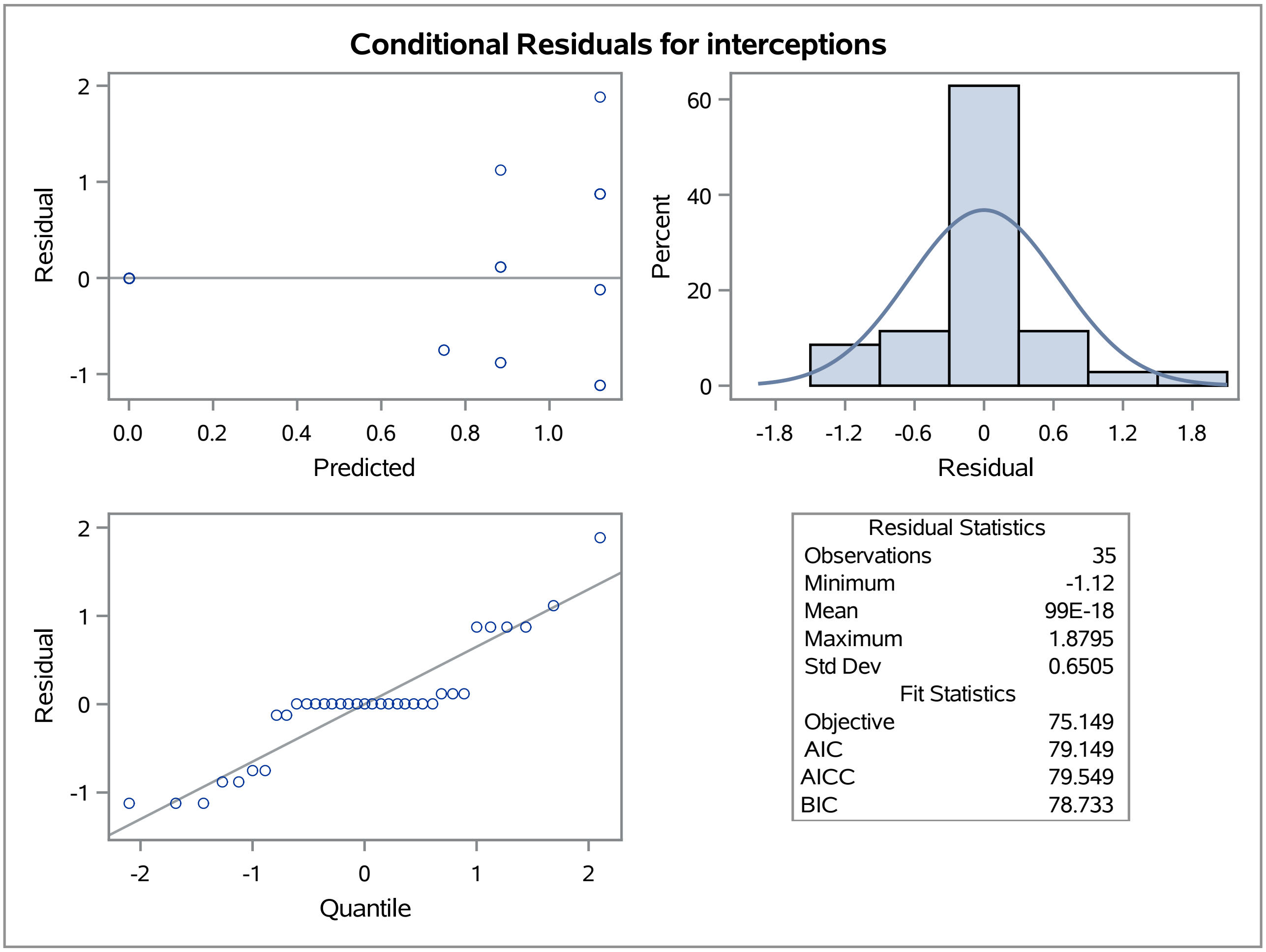

DistSoma=462

Model Information	
**Data Set**	WORK.TEMPDATASORTED
**Dependent Variable**	interceptions
**Covariance Structure**	Variance Components
**Estimation Method**	REML
**Residual Variance Method**	Profile
**Fixed Effects SE Method**	Model-Based
**Degrees of Freedom Method**	Containment

Class Level Information		
**Class**	**Levels**	**Values**
**Genotype**	2	Meg +/+ Meg +/-
**Animal**	6	1 2 3 4 5 6

Dimensions	
**Covariance Parameters**	2
**Columns in X**	3
**Columns in Z**	6
**Subjects**	1
**Max Obs per Subject**	35

Number of Observations	
**Number of Observations Read**	35
**Number of Observations Used**	35
**Number of Observations Not Used**	0

Iteration History			
**Iteration**	**Evaluations**	**-2 Res Log Like**	**Criterion**
**0**	1	77.37679938	
**1**	2	76.52463556	0.00000101
**2**	1	76.52462753	0.00000000

Convergence criteria met.

Covariance Parameter Estimates				
**Cov Parm**	**Estimate**	**Alpha**	**Lower**	**Upper**
**Animal**	0.07979	0.05	0.01400	698.93
**Residual**	0.4652	0.05	0.2953	0.8396

DistSoma=462

Fit Statistics	
-2 Res Log Likelihood	76.5
AIC (Smaller is Better)	80.5
AICC (Smaller is Better)	80.9
BIC (Smaller is Better)	80.1

Solution for Fixed Effects									
Effect	Genotype	Estimate	Standard Error	DF	t Value	Pr > |t|	Alpha	Lower	Upper
Intercept		3.56E-17	0.2405	4	0.00	1.0000	0.05	-0.6677	0.6677
Genotype	Meg +/+	0.8315	0.3392	29	2.45	0.0205	0.05	0.1377	1.5253
Genotype	Meg +/-	0	.	.	.	.	.	.	.

Solution for Random Effects									
Effect	Animal	Estimate	Std Err Pred	DF	t Value	Pr > |t|	Alpha	Lower	Upper
Animal	1	0.2960	0.2285	29	1.30	0.2055	0.05	-0.1714	0.7633
Animal	2	-0.08359	0.2325	29	-0.36	0.7218	0.05	-0.5590	0.3919
Animal	3	-0.2124	0.2513	29	-0.85	0.4049	0.05	-0.7263	0.3016
Animal	4	-909E-20	0.2514	29	-0.00	1.0000	0.05	-0.5141	0.5141
Animal	5	-603E-19	0.2312	29	-0.00	1.0000	0.05	-0.4730	0.4730
Animal	6	-206E-19	0.2302	29	-0.00	1.0000	0.05	-0.4708	0.4708

Type 3 Tests of Fixed Effects				
Effect	Num DF	Den DF	F Value	Pr > F
Genotype	1	29	6.01	0.0205

Least Squares Means									
Effect	Genotype	Estimate	Standard Error	DF	t Value	Pr > |t|	Alpha	Lower	Upper
Genotype	Meg +/+	0.8315	0.2392	29	3.48	0.0016	0.05	0.3422	1.3207
Genotype	Meg +/-	3.56E-17	0.2405	29	0.00	1.0000	0.05	-0.4919	0.4919

DistSoma=462

Differences of Least Squares Means												
**Effect**	**Genotype**	**Genotype**	**Estimate**	**Standard Error**	**DF**	**t Value**	**Pr > |t|**	**Adjustment**	**Adj P**	**Alpha**	**Lower**	**Upper**
**Genotype**	Meg +/+	Meg +/-	0.8315	0.3392	29	2.45	0.0205	Tukey-Kramer	0.0205	0.05	0.1377	1.5253

Differences of Least Squares Means				
**Effect**	**Genotype**	**Genotype**	**Adj Lower**	**Adj Upper**
**Genotype**	Meg +/+	Meg +/-	0.1377	1.5253

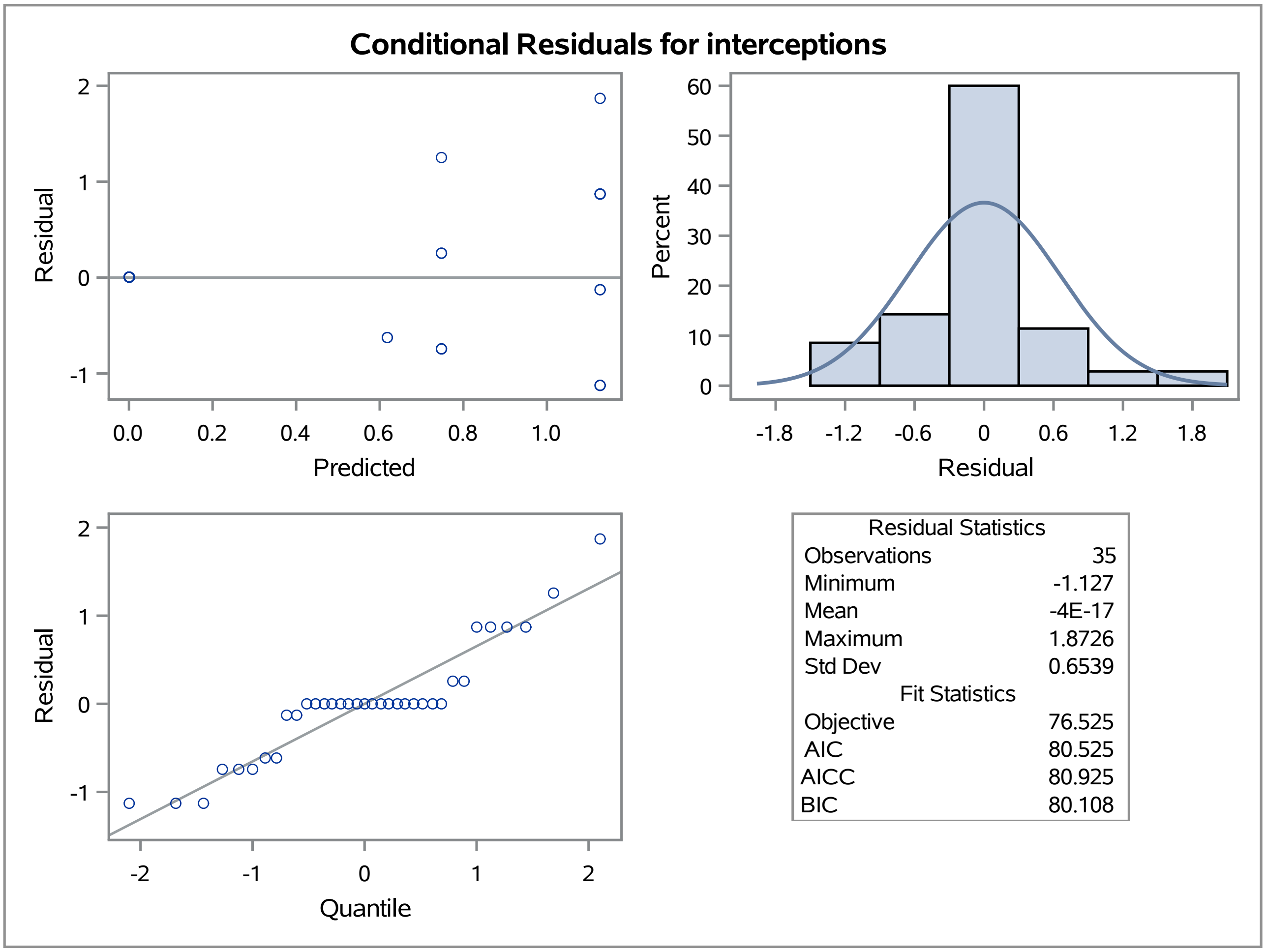

DistSoma=468

Model Information	
**Data Set**	WORK.TEMPDATASORTED
**Dependent Variable**	interceptions
**Covariance Structure**	Variance Components
**Estimation Method**	REML
**Residual Variance Method**	Profile
**Fixed Effects SE Method**	Model-Based
**Degrees of Freedom Method**	Containment

Class Level Information		
**Class**	**Levels**	**Values**
**Genotype**	2	Meg +/+ Meg +/-
**Animal**	6	1 2 3 4 5 6

Dimensions	
**Covariance Parameters**	2
**Columns in X**	3
**Columns in Z**	6
**Subjects**	1
**Max Obs per Subject**	35

Number of Observations	
**Number of Observations Read**	35
**Number of Observations Used**	35
**Number of Observations Not Used**	0

Iteration History			
**Iteration**	**Evaluations**	**-2 Res Log Like**	**Criterion**
**0**	1	68.91296032	
**1**	1	68.91296032	0.00000000

Convergence criteria met.

Estimated G matrix is not positive definite.

Covariance Parameter Estimates				
**Cov Parm**	**Estimate**	**Alpha**	**Lower**	**Upper**
**Animal**	0	.	.	.
**Residual**	0.3973	0.05	0.2585	0.6884

DistSoma=468

Fit Statistics	
-2 Res Log Likelihood	68.9
AIC (Smaller is Better)	70.9
AICC (Smaller is Better)	71.0
BIC (Smaller is Better)	70.7

Solution for Fixed Effects

Effect	Genotype	Estimate	Standard Error	DF	t Value	Pr > |t|	Alpha	Lower	Upper
Intercept		-555E-19	0.1529	4	-0.00	1.0000	0.05	-0.4245	0.4245
Genotype	Meg +/+	0.7778	0.2132	29	3.65	0.0010	0.05	0.3418	1.2138
Genotype	Meg +/-	0	.	.	.	.	.	.	.

Solution for Random Effects

Effect	Animal	Estimate	Std Err Pred	DF	t Value	Pr > |t|	Alpha	Lower	Upper
Animal	1	0	.	.	.	.	.	.	.
Animal	2	0	.	.	.	.	.	.	.
Animal	3	0	.	.	.	.	.	.	.
Animal	4	0	.	.	.	.	.	.	.
Animal	5	0	.	.	.	.	.	.	.
Animal	6	0	.	.	.	.	.	.	.

Type 3 Tests of Fixed Effects

Effect	Num DF	Den DF	F Value	Pr > F
Genotype	1	29	13.31	0.0010

Least Squares Means

Effect	Genotype	Estimate	Standard Error	DF	t Value	Pr > |t|	Alpha	Lower	Upper
Genotype	Meg +/+	0.7778	0.1486	29	5.24	<.0001	0.05	0.4739	1.0816
Genotype	Meg +/-	-555E-19	0.1529	29	-0.00	1.0000	0.05	-0.3127	0.3127

DistSoma=468

Differences of Least Squares Means												
**Effect**	**Genotype**	**Genotype**	**Estimate**	**Standard Error**	**DF**	**t Value**	**Pr > |t|**	**Adjustment**	**Adj P**	**Alpha**	**Lower**	**Upper**
**Genotype**	Meg +/+	Meg +/-	0.7778	0.2132	29	3.65	0.0010	Tukey-Kramer	0.0010	0.05	0.3418	1.2138

Differences of Least Squares Means				
**Effect**	**Genotype**	**Genotype**	**Adj Lower**	**Adj Upper**
**Genotype**	Meg +/+	Meg +/-	0.3418	1.2138

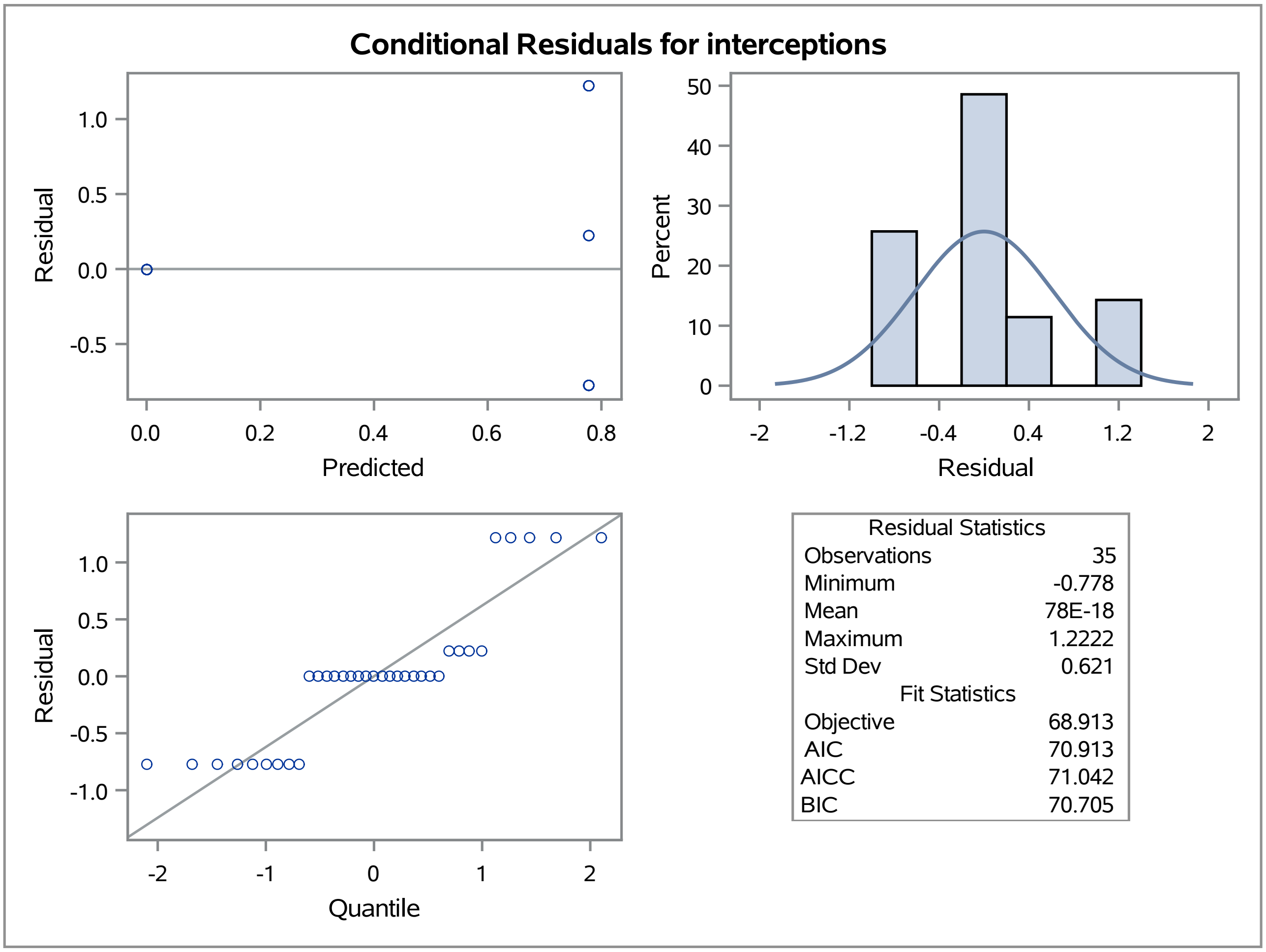

DistSoma=474

Model Information	
**Data Set**	WORK.TEMPDATASORTED
**Dependent Variable**	interceptions
**Covariance Structure**	Variance Components
**Estimation Method**	REML
**Residual Variance Method**	Profile
**Fixed Effects SE Method**	Model-Based
**Degrees of Freedom Method**	Containment

Class Level Information		
**Class**	**Levels**	**Values**
**Genotype**	2	Meg +/+ Meg +/-
**Animal**	6	1 2 3 4 5 6

Dimensions	
**Covariance Parameters**	2
**Columns in X**	3
**Columns in Z**	6
**Subjects**	1
**Max Obs per Subject**	35

Number of Observations	
**Number of Observations Read**	35
**Number of Observations Used**	35
**Number of Observations Not Used**	0

Iteration History			
**Iteration**	**Evaluations**	**-2 Res Log Like**	**Criterion**
**0**	1	70.14803170	
**1**	1	70.14803170	0.00000000

Convergence criteria met.

Estimated G matrix is not positive definite.

Covariance Parameter Estimates				
**Cov Parm**	**Estimate**	**Alpha**	**Lower**	**Upper**
**Animal**	0	.	.	.
**Residual**	0.4125	0.05	0.2683	0.7146

DistSoma=474

Fit Statistics	
-2 Res Log Likelihood	70.1
AIC (Smaller is Better)	72.1
AICC (Smaller is Better)	72.3
BIC (Smaller is Better)	71.9

Solution for Fixed Effects

Effect	Genotype	Estimate	Standard Error	DF	t Value	Pr > |t|	Alpha	Lower	Upper
Intercept		5.55E-17	0.1558	4	0.00	1.0000	0.05	-0.4325	0.4325
Genotype	Meg +/+	0.7222	0.2172	29	3.33	0.0024	0.05	0.2780	1.1664
Genotype	Meg +/-	0	.	.	.	.	.	.	.

Solution for Random Effects

Effect	Animal	Estimate	Std Err Pred	DF	t Value	Pr > |t|	Alpha	Lower	Upper
Animal	1	0	.	.	.	.	.	.	.
Animal	2	0	.	.	.	.	.	.	.
Animal	3	0	.	.	.	.	.	.	.
Animal	4	0	.	.	.	.	.	.	.
Animal	5	0	.	.	.	.	.	.	.
Animal	6	0	.	.	.	.	.	.	.

Type 3 Tests of Fixed Effects

Effect	Num DF	Den DF	F Value	Pr > F
Genotype	1	29	11.06	0.0024

Least Squares Means

Effect	Genotype	Estimate	Standard Error	DF	t Value	Pr > |t|	Alpha	Lower	Upper
Genotype	Meg +/+	0.7222	0.1514	29	4.77	<.0001	0.05	0.4126	1.0318
Genotype	Meg +/-	5.55E-17	0.1558	29	0.00	1.0000	0.05	-0.3186	0.3186

DistSoma=474

Differences of Least Squares Means												
**Effect**	**Genotype**	**Genotype**	**Estimate**	**Standard Error**	**DF**	**t Value**	**Pr > |t|**	**Adjustment**	**Adj P**	**Alpha**	**Lower**	**Upper**
**Genotype**	Meg +/+	Meg +/-	0.7222	0.2172	29	3.33	0.0024	Tukey-Kramer	0.0024	0.05	0.2780	1.1664

Differences of Least Squares Means				
**Effect**	**Genotype**	**Genotype**	**Adj Lower**	**Adj Upper**
**Genotype**	Meg +/+	Meg +/-	0.2780	1.1664

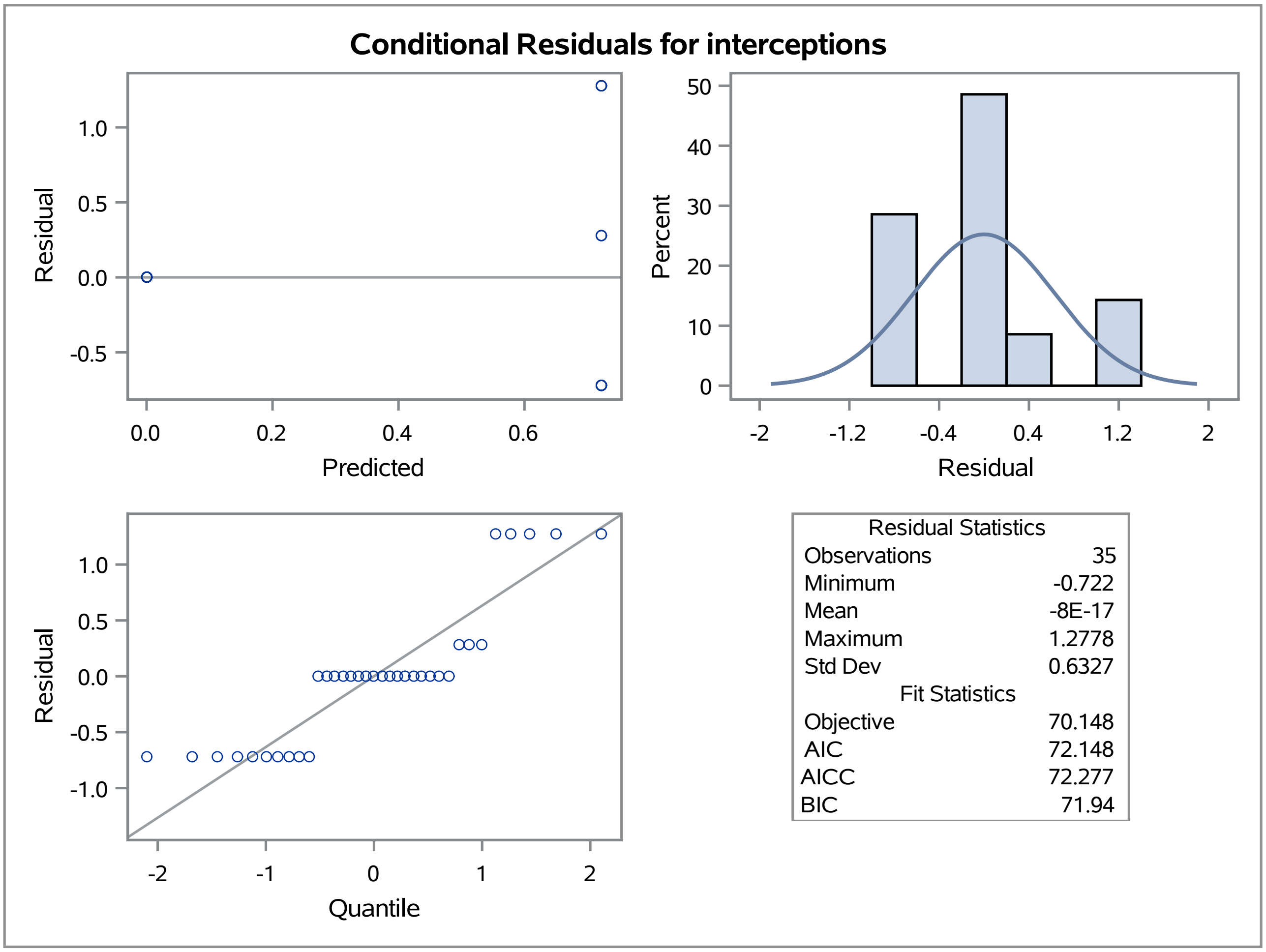

DistSoma=480

Model Information	
**Data Set**	WORK.TEMPDATASORTED
**Dependent Variable**	interceptions
**Covariance Structure**	Variance Components
**Estimation Method**	REML
**Residual Variance Method**	Profile
**Fixed Effects SE Method**	Model-Based
**Degrees of Freedom Method**	Containment

Class Level Information		
**Class**	**Levels**	**Values**
**Genotype**	2	Meg +/+ Meg +/-
**Animal**	6	1 2 3 4 5 6

Dimensions	
**Covariance Parameters**	2
**Columns in X**	3
**Columns in Z**	6
**Subjects**	1
**Max Obs per Subject**	35

Number of Observations	
**Number of Observations Read**	35
**Number of Observations Used**	35
**Number of Observations Not Used**	0

Iteration History			
**Iteration**	**Evaluations**	**-2 Res Log Like**	**Criterion**
**0**	1	70.14803170	
**1**	1	70.14803170	0.00000000

Convergence criteria met.

Estimated G matrix is not positive definite.

Covariance Parameter Estimates				
**Cov Parm**	**Estimate**	**Alpha**	**Lower**	**Upper**
**Animal**	0	.	.	.
**Residual**	0.4125	0.05	0.2683	0.7146

DistSoma=480

Fit Statistics	
-2 Res Log Likelihood	70.1
AIC (Smaller is Better)	72.1
AICC (Smaller is Better)	72.3
BIC (Smaller is Better)	71.9

Solution for Fixed Effects

Effect	Genotype	Estimate	Standard Error	DF	t Value	Pr > |t|	Alpha	Lower	Upper
Intercept		5.55E-17	0.1558	4	0.00	1.0000	0.05	-0.4325	0.4325
Genotype	Meg +/+	0.7222	0.2172	29	3.33	0.0024	0.05	0.2780	1.1664
Genotype	Meg +/-	0	.	.	.	.	.	.	.

Solution for Random Effects

Effect	Animal	Estimate	Std Err Pred	DF	t Value	Pr > |t|	Alpha	Lower	Upper
Animal	1	0	.	.	.	.	.	.	.
Animal	2	0	.	.	.	.	.	.	.
Animal	3	0	.	.	.	.	.	.	.
Animal	4	0	.	.	.	.	.	.	.
Animal	5	0	.	.	.	.	.	.	.
Animal	6	0	.	.	.	.	.	.	.

Type 3 Tests of Fixed Effects

Effect	Num DF	Den DF	F Value	Pr > F
Genotype	1	29	11.06	0.0024

Least Squares Means

Effect	Genotype	Estimate	Standard Error	DF	t Value	Pr > |t|	Alpha	Lower	Upper
Genotype	Meg +/+	0.7222	0.1514	29	4.77	<.0001	0.05	0.4126	1.0318
Genotype	Meg +/-	5.55E-17	0.1558	29	0.00	1.0000	0.05	-0.3186	0.3186

DistSoma=480

Differences of Least Squares Means												
**Effect**	**Genotype**	**Genotype**	**Estimate**	**Standard Error**	**DF**	**t Value**	**Pr > |t|**	**Adjustment**	**Adj P**	**Alpha**	**Lower**	**Upper**
**Genotype**	Meg +/+	Meg +/-	0.7222	0.2172	29	3.33	0.0024	Tukey-Kramer	0.0024	0.05	0.2780	1.1664

Differences of Least Squares Means				
**Effect**	**Genotype**	**Genotype**	**Adj Lower**	**Adj Upper**
**Genotype**	Meg +/+	Meg +/-	0.2780	1.1664

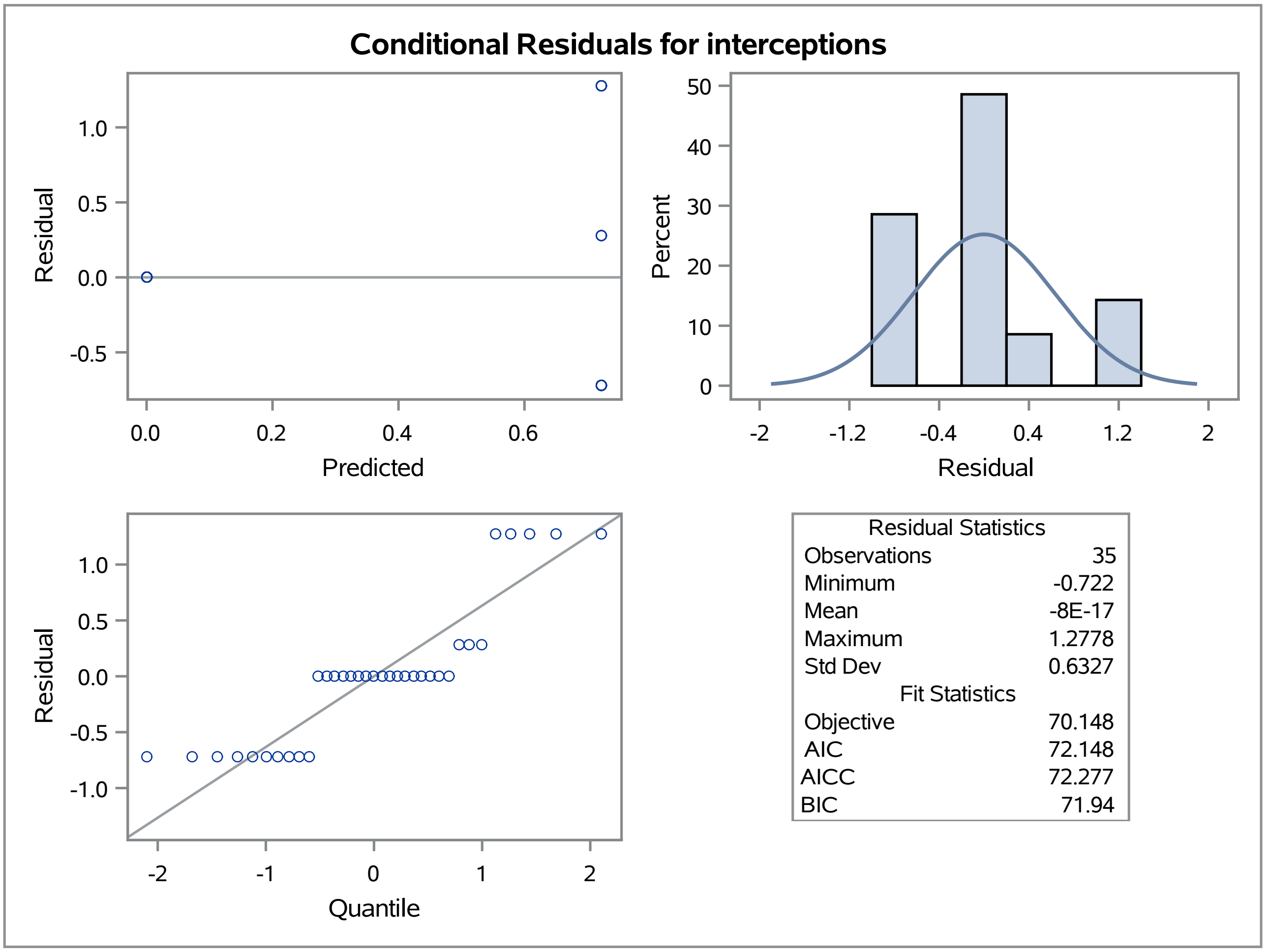

DistSoma=486

Model Information	
**Data Set**	WORK.TEMPDATASORTED
**Dependent Variable**	interceptions
**Covariance Structure**	Variance Components
**Estimation Method**	REML
**Residual Variance Method**	Profile
**Fixed Effects SE Method**	Model-Based
**Degrees of Freedom Method**	Containment

Class Level Information		
**Class**	**Levels**	**Values**
**Genotype**	2	Meg +/+ Meg +/-
**Animal**	6	1 2 3 4 5 6

Dimensions	
**Covariance Parameters**	2
**Columns in X**	3
**Columns in Z**	6
**Subjects**	1
**Max Obs per Subject**	35

Number of Observations	
**Number of Observations Read**	35
**Number of Observations Used**	35
**Number of Observations Not Used**	0

Iteration History			
**Iteration**	**Evaluations**	**-2 Res Log Like**	**Criterion**
**0**	1	70.14803170	
**1**	1	70.14803170	0.00000000

Convergence criteria met.

Estimated G matrix is not positive definite.

Covariance Parameter Estimates				
**Cov Parm**	**Estimate**	**Alpha**	**Lower**	**Upper**
**Animal**	0	.	.	.
**Residual**	0.4125	0.05	0.2683	0.7146

DistSoma=486

Fit Statistics	
-2 Res Log Likelihood	70.1
AIC (Smaller is Better)	72.1
AICC (Smaller is Better)	72.3
BIC (Smaller is Better)	71.9

Solution for Fixed Effects

Effect	Genotype	Estimate	Standard Error	DF	t Value	Pr > |t|	Alpha	Lower	Upper
Intercept		5.55E-17	0.1558	4	0.00	1.0000	0.05	-0.4325	0.4325
Genotype	Meg +/+	0.7222	0.2172	29	3.33	0.0024	0.05	0.2780	1.1664
Genotype	Meg +/-	0	.	.	.	.	.	.	.

Solution for Random Effects

Effect	Animal	Estimate	Std Err Pred	DF	t Value	Pr > |t|	Alpha	Lower	Upper
Animal	1	0	.	.	.	.	.	.	.
Animal	2	0	.	.	.	.	.	.	.
Animal	3	0	.	.	.	.	.	.	.
Animal	4	0	.	.	.	.	.	.	.
Animal	5	0	.	.	.	.	.	.	.
Animal	6	0	.	.	.	.	.	.	.

Type 3 Tests of Fixed Effects

Effect	Num DF	Den DF	F Value	Pr > F
Genotype	1	29	11.06	0.0024

Least Squares Means

Effect	Genotype	Estimate	Standard Error	DF	t Value	Pr > |t|	Alpha	Lower	Upper
Genotype	Meg +/+	0.7222	0.1514	29	4.77	<.0001	0.05	0.4126	1.0318
Genotype	Meg +/-	5.55E-17	0.1558	29	0.00	1.0000	0.05	-0.3186	0.3186

DistSoma=486

Differences of Least Squares Means												
**Effect**	**Genotype**	**Genotype**	**Estimate**	**Standard Error**	**DF**	**t Value**	**Pr > |t|**	**Adjustment**	**Adj P**	**Alpha**	**Lower**	**Upper**
**Genotype**	Meg +/+	Meg +/-	0.7222	0.2172	29	3.33	0.0024	Tukey-Kramer	0.0024	0.05	0.2780	1.1664

Differences of Least Squares Means				
**Effect**	**Genotype**	**Genotype**	**Adj Lower**	**Adj Upper**
**Genotype**	Meg +/+	Meg +/-	0.2780	1.1664

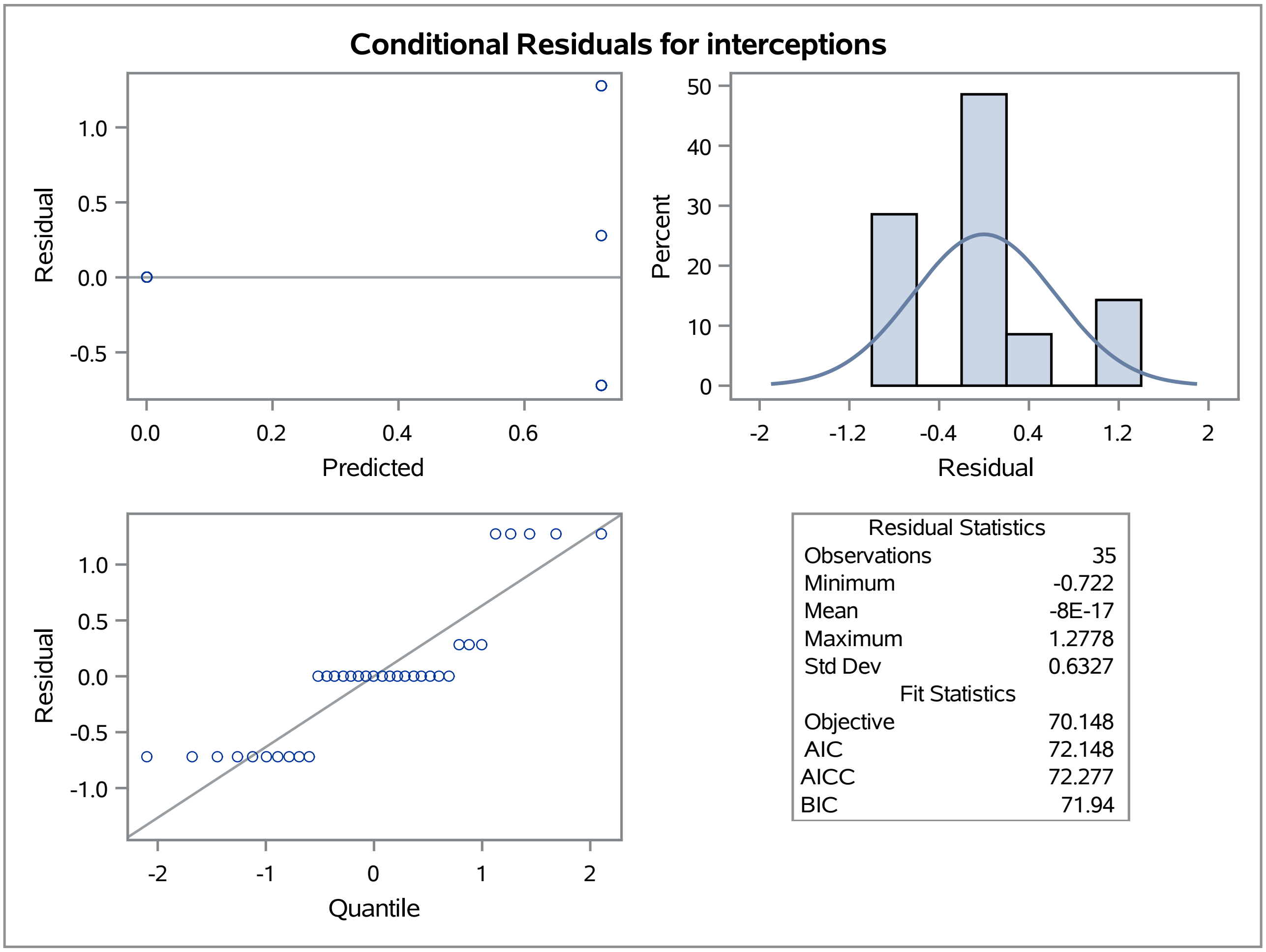

DistSoma=492

Model Information	
**Data Set**	WORK.TEMPDATASORTED
**Dependent Variable**	interceptions
**Covariance Structure**	Variance Components
**Estimation Method**	REML
**Residual Variance Method**	Profile
**Fixed Effects SE Method**	Model-Based
**Degrees of Freedom Method**	Containment

Class Level Information		
**Class**	**Levels**	**Values**
**Genotype**	2	Meg +/+ Meg +/-
**Animal**	6	1 2 3 4 5 6

Dimensions	
**Covariance Parameters**	2
**Columns in X**	3
**Columns in Z**	6
**Subjects**	1
**Max Obs per Subject**	35

Number of Observations	
**Number of Observations Read**	35
**Number of Observations Used**	35
**Number of Observations Not Used**	0

Iteration History			
**Iteration**	**Evaluations**	**-2 Res Log Like**	**Criterion**
**0**	1	70.14803170	
**1**	1	70.14803170	0.00000000

Convergence criteria met.

Estimated G matrix is not positive definite.

Covariance Parameter Estimates				
**Cov Parm**	**Estimate**	**Alpha**	**Lower**	**Upper**
**Animal**	0	.	.	.
**Residual**	0.4125	0.05	0.2683	0.7146

DistSoma=492

Fit Statistics	
-2 Res Log Likelihood	70.1
AIC (Smaller is Better)	72.1
AICC (Smaller is Better)	72.3
BIC (Smaller is Better)	71.9

Solution for Fixed Effects

Effect	Genotype	Estimate	Standard Error	DF	t Value	Pr > |t|	Alpha	Lower	Upper
Intercept		5.55E-17	0.1558	4	0.00	1.0000	0.05	-0.4325	0.4325
Genotype	Meg +/+	0.7222	0.2172	29	3.33	0.0024	0.05	0.2780	1.1664
Genotype	Meg +/-	0	.	.	.	.	.	.	.

Solution for Random Effects

Effect	Animal	Estimate	Std Err Pred	DF	t Value	Pr > |t|	Alpha	Lower	Upper
Animal	1	0	.	.	.	.	.	.	.
Animal	2	0	.	.	.	.	.	.	.
Animal	3	0	.	.	.	.	.	.	.
Animal	4	0	.	.	.	.	.	.	.
Animal	5	0	.	.	.	.	.	.	.
Animal	6	0	.	.	.	.	.	.	.

Type 3 Tests of Fixed Effects

Effect	Num DF	Den DF	F Value	Pr > F
Genotype	1	29	11.06	0.0024

Least Squares Means

Effect	Genotype	Estimate	Standard Error	DF	t Value	Pr > |t|	Alpha	Lower	Upper
Genotype	Meg +/+	0.7222	0.1514	29	4.77	<.0001	0.05	0.4126	1.0318
Genotype	Meg +/-	5.55E-17	0.1558	29	0.00	1.0000	0.05	-0.3186	0.3186

DistSoma=492

Differences of Least Squares Means												
Effect	Genotype	Genotype	Estimate	Standard Error	DF	t Value	Pr > |t|	Adjustment	Adj P	Alpha	Lower	Upper
**Genotype**	Meg +/+	Meg +/-	0.7222	0.2172	29	3.33	0.0024	Tukey-Kramer	0.0024	0.05	0.2780	1.1664

Differences of Least Squares Means				
Effect	Genotype	Genotype	Adj Lower	Adj Upper
**Genotype**	Meg +/+	Meg +/-	0.2780	1.1664

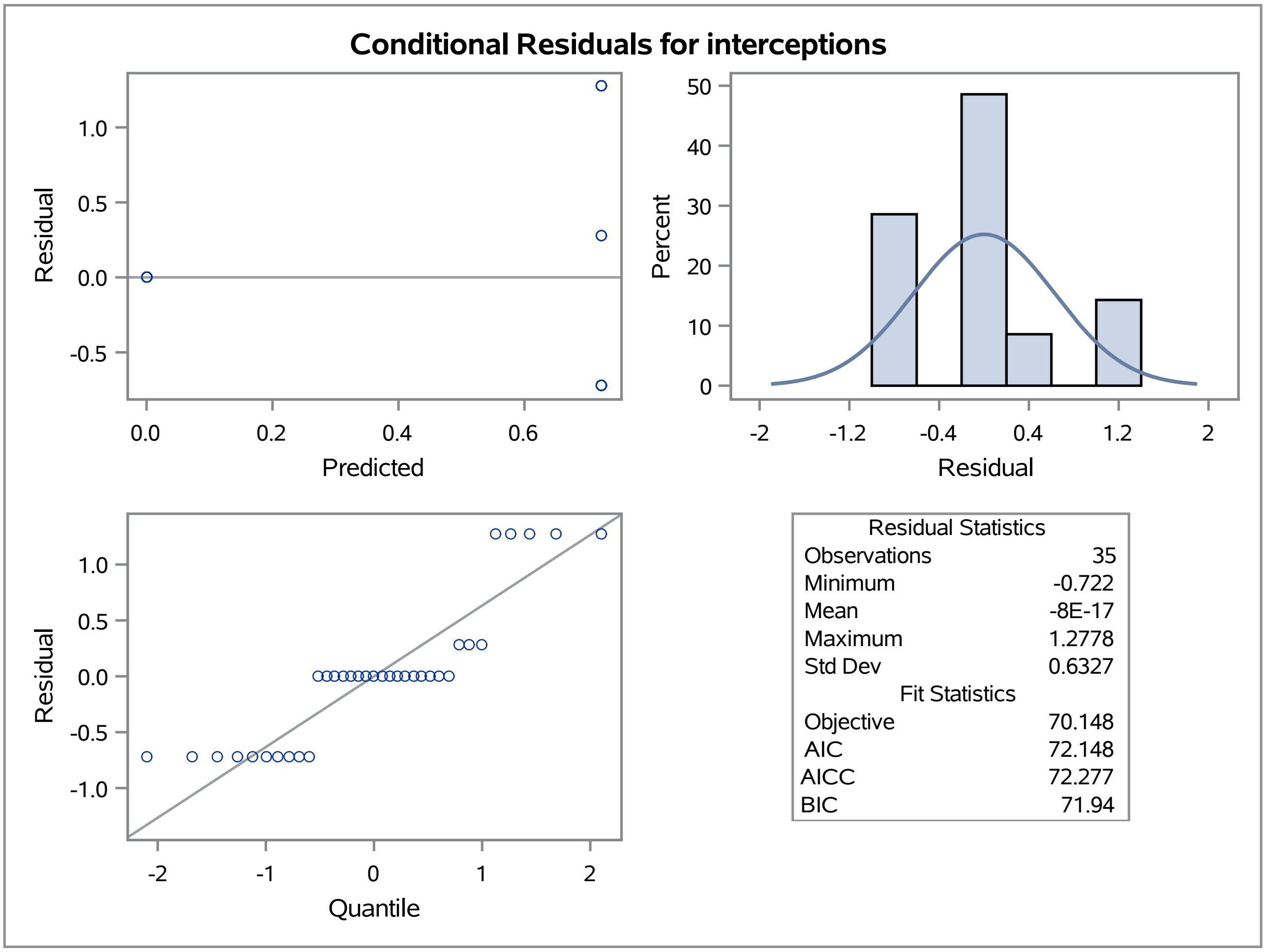

DistSoma=498

Model Information	
**Data Set**	WORK.TEMPDATASORTED
**Dependent Variable**	interceptions
**Covariance Structure**	Variance Components
**Estimation Method**	REML
**Residual Variance Method**	Profile
**Fixed Effects SE Method**	Model-Based
**Degrees of Freedom Method**	Containment

Class Level Information		
**Class**	**Levels**	**Values**
**Genotype**	2	Meg +/+ Meg +/-
**Animal**	6	1 2 3 4 5 6

Dimensions	
**Covariance Parameters**	2
**Columns in X**	3
**Columns in Z**	6
**Subjects**	1
**Max Obs per Subject**	35

Number of Observations	
**Number of Observations Read**	35
**Number of Observations Used**	35
**Number of Observations Not Used**	0

Iteration History			
**Iteration**	**Evaluations**	**-2 Res Log Like**	**Criterion**
**0**	1	70.14803170	
**1**	1	70.14803170	0.00000000

Convergence criteria met.

Estimated G matrix is not positive definite.

Covariance Parameter Estimates				
**Cov Parm**	**Estimate**	**Alpha**	**Lower**	**Upper**
**Animal**	0	.	.	.
**Residual**	0.4125	0.05	0.2683	0.7146

DistSoma=498

Fit Statistics	
-2 Res Log Likelihood	70.1
AIC (Smaller is Better)	72.1
AICC (Smaller is Better)	72.3
BIC (Smaller is Better)	71.9

Solution for Fixed Effects

Effect	Genotype	Estimate	Standard Error	DF	t Value	Pr > |t|	Alpha	Lower	Upper
Intercept		5.55E-17	0.1558	4	0.00	1.0000	0.05	-0.4325	0.4325
Genotype	Meg +/+	0.7222	0.2172	29	3.33	0.0024	0.05	0.2780	1.1664
Genotype	Meg +/-	0	.	.	.	.	.	.	.

Solution for Random Effects

Effect	Animal	Estimate	Std Err Pred	DF	t Value	Pr > |t|	Alpha	Lower	Upper
Animal	1	0	.	.	.	.	.	.	.
Animal	2	0	.	.	.	.	.	.	.
Animal	3	0	.	.	.	.	.	.	.
Animal	4	0	.	.	.	.	.	.	.
Animal	5	0	.	.	.	.	.	.	.
Animal	6	0	.	.	.	.	.	.	.

Type 3 Tests of Fixed Effects

Effect	Num DF	Den DF	F Value	Pr > F
Genotype	1	29	11.06	0.0024

Least Squares Means

Effect	Genotype	Estimate	Standard Error	DF	t Value	Pr > |t|	Alpha	Lower	Upper
Genotype	Meg +/+	0.7222	0.1514	29	4.77	<.0001	0.05	0.4126	1.0318
Genotype	Meg +/-	5.55E-17	0.1558	29	0.00	1.0000	0.05	-0.3186	0.3186

DistSoma=498

Differences of Least Squares Means												
**Effect**	**Genotype**	**Genotype**	**Estimate**	**Standard Error**	**DF**	**t Value**	**Pr > |t|**	**Adjustment**	**Adj P**	**Alpha**	**Lower**	**Upper**
**Genotype**	Meg +/+	Meg +/-	0.7222	0.2172	29	3.33	0.0024	Tukey-Kramer	0.0024	0.05	0.2780	1.1664

Differences of Least Squares Means				
**Effect**	**Genotype**	**Genotype**	**Adj Lower**	**Adj Upper**
**Genotype**	Meg +/+	Meg +/-	0.2780	1.1664

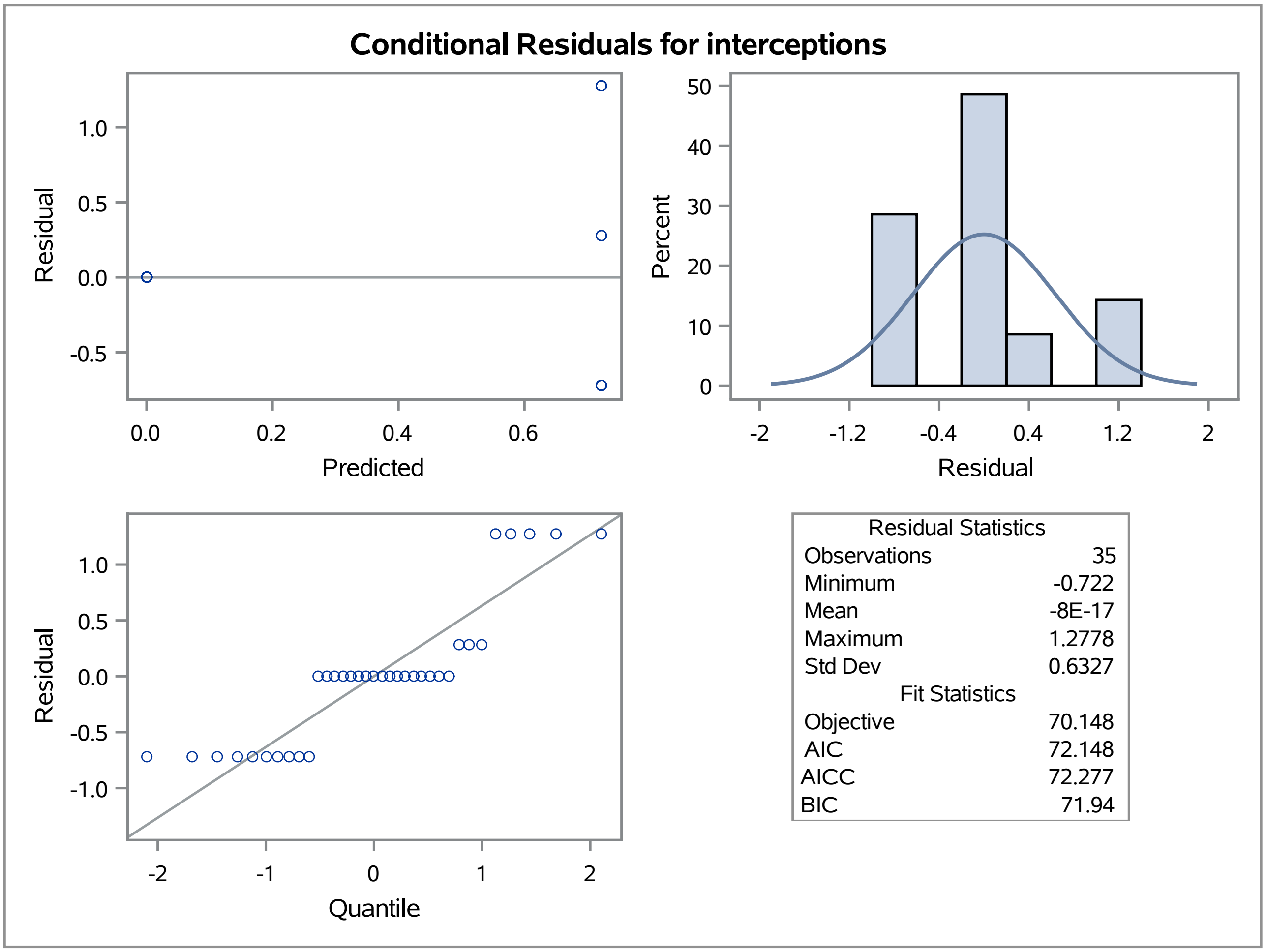

DistSoma=504

Model Information	
**Data Set**	WORK.TEMPDATASORTED
**Dependent Variable**	interceptions
**Covariance Structure**	Variance Components
**Estimation Method**	REML
**Residual Variance Method**	Profile
**Fixed Effects SE Method**	Model-Based
**Degrees of Freedom Method**	Containment

Class Level Information		
**Class**	**Levels**	**Values**
**Genotype**	2	Meg +/+ Meg +/-
**Animal**	6	1 2 3 4 5 6

Dimensions	
**Covariance Parameters**	2
**Columns in X**	3
**Columns in Z**	6
**Subjects**	1
**Max Obs per Subject**	35

Number of Observations	
**Number of Observations Read**	35
**Number of Observations Used**	35
**Number of Observations Not Used**	0

Iteration History			
**Iteration**	**Evaluations**	**-2 Res Log Like**	**Criterion**
**0**	1	66.74587990	
**1**	1	66.74587990	0.00000000

Convergence criteria met.

Estimated G matrix is not positive definite.

Covariance Parameter Estimates				
**Cov Parm**	**Estimate**	**Alpha**	**Lower**	**Upper**
**Animal**	0	.	.	.
**Residual**	0.3721	0.05	0.2420	0.6446

DistSoma=504

Fit Statistics	
-2 Res Log Likelihood	66.7
AIC (Smaller is Better)	68.7
AICC (Smaller is Better)	68.9
BIC (Smaller is Better)	68.5

Solution for Fixed Effects

Effect	Genotype	Estimate	Standard Error	DF	t Value	Pr > |t|	Alpha	Lower	Upper
Intercept		5.55E-17	0.1479	4	0.00	1.0000	0.05	-0.4107	0.4107
Genotype	Meg +/+	0.6111	0.2063	29	2.96	0.0060	0.05	0.1892	1.0330
Genotype	Meg +/-	0	.	.	.	.	.	.	.

Solution for Random Effects

Effect	Animal	Estimate	Std Err Pred	DF	t Value	Pr > |t|	Alpha	Lower	Upper
Animal	1	0	.	.	.	.	.	.	.
Animal	2	0	.	.	.	.	.	.	.
Animal	3	0	.	.	.	.	.	.	.
Animal	4	0	.	.	.	.	.	.	.
Animal	5	0	.	.	.	.	.	.	.
Animal	6	0	.	.	.	.	.	.	.

Type 3 Tests of Fixed Effects

Effect	Num DF	Den DF	F Value	Pr > F
Genotype	1	29	8.78	0.0060

Least Squares Means

Effect	Genotype	Estimate	Standard Error	DF	t Value	Pr > |t|	Alpha	Lower	Upper
Genotype	Meg +/+	0.6111	0.1438	29	4.25	0.0002	0.05	0.3171	0.9052
Genotype	Meg +/-	5.55E-17	0.1479	29	0.00	1.0000	0.05	-0.3026	0.3026

DistSoma=504

Differences of Least Squares Means												
Effect	Genotype	Genotype	Estimate	Standard Error	DF	t Value	Pr > |t|	Adjustment	Adj P	Alpha	Lower	Upper
Genotype	Meg +/+	Meg +/-	0.6111	0.2063	29	2.96	0.0060	Tukey-Kramer	0.0060	0.05	0.1892	1.0330

Differences of Least Squares Means				
Effect	Genotype	Genotype	Adj Lower	Adj Upper
Genotype	Meg +/+	Meg +/-	0.1892	1.0330

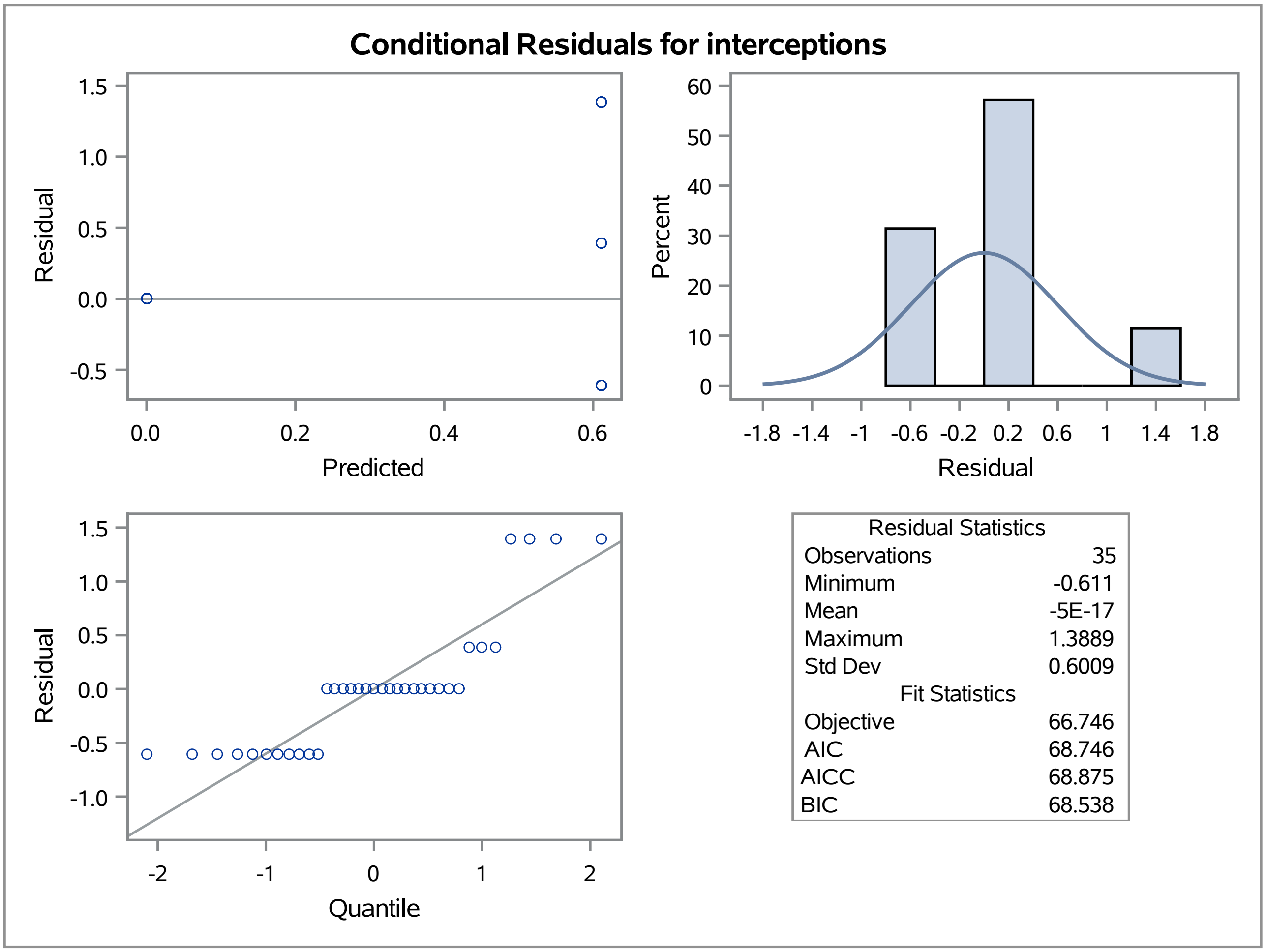

DistSoma=510

Model Information	
**Data Set**	WORK.TEMPDATASORTED
**Dependent Variable**	interceptions
**Covariance Structure**	Variance Components
**Estimation Method**	REML
**Residual Variance Method**	Profile
**Fixed Effects SE Method**	Model-Based
**Degrees of Freedom Method**	Containment

Class Level Information		
**Class**	**Levels**	**Values**
**Genotype**	2	Meg +/+ Meg +/-
**Animal**	6	1 2 3 4 5 6

Dimensions	
**Covariance Parameters**	2
**Columns in X**	3
**Columns in Z**	6
**Subjects**	1
**Max Obs per Subject**	35

Number of Observations	
**Number of Observations Read**	35
**Number of Observations Used**	35
**Number of Observations Not Used**	0

Iteration History			
**Iteration**	**Evaluations**	**-2 Res Log Like**	**Criterion**
**0**	1	61.40909553	
**1**	1	61.40909553	0.00000000

Convergence criteria met.

Estimated G matrix is not positive definite.

Covariance Parameter Estimates				
**Cov Parm**	**Estimate**	**Alpha**	**Lower**	**Upper**
**Animal**	0	.	.	.
**Residual**	0.3165	0.05	0.2059	0.5484

DistSoma=510

Fit Statistics	
-2 Res Log Likelihood	61.4
AIC (Smaller is Better)	63.4
AICC (Smaller is Better)	63.5
BIC (Smaller is Better)	63.2

Solution for Fixed Effects									
Effect	Genotype	Estimate	Standard Error	DF	t Value	Pr > |t|	Alpha	Lower	Upper
Intercept		0	0.1364	4	0.00	1.0000	0.05	-0.3788	0.3788
Genotype	Meg +/+	0.5556	0.1903	29	2.92	0.0067	0.05	0.1664	0.9447
Genotype	Meg +/-	0	.	.	.	.	.	.	.

Solution for Random Effects									
Effect	Animal	Estimate	Std Err Pred	DF	t Value	Pr > |t|	Alpha	Lower	Upper
Animal	1	0	.	.	.	.	.	.	.
Animal	2	0	.	.	.	.	.	.	.
Animal	3	0	.	.	.	.	.	.	.
Animal	4	0	.	.	.	.	.	.	.
Animal	5	0	.	.	.	.	.	.	.
Animal	6	0	.	.	.	.	.	.	.

Type 3 Tests of Fixed Effects				
Effect	Num DF	Den DF	F Value	Pr > F
Genotype	1	29	8.53	0.0067

Least Squares Means									
Effect	Genotype	Estimate	Standard Error	DF	t Value	Pr > |t|	Alpha	Lower	Upper
Genotype	Meg +/+	0.5556	0.1326	29	4.19	0.0002	0.05	0.2844	0.8268
Genotype	Meg +/-	0	0.1364	29	0.00	1.0000	0.05	-0.2791	0.2791

DistSoma=510

Differences of Least Squares Means												
**Effect**	**Genotype**	**Genotype**	**Estimate**	**Standard Error**	**DF**	**t Value**	**Pr > |t|**	**Adjustment**	**Adj P**	**Alpha**	**Lower**	**Upper**
**Genotype**	Meg +/+	Meg +/-	0.5556	0.1903	29	2.92	0.0067	Tukey-Kramer	0.0067	0.05	0.1664	0.9447

Differences of Least Squares Means				
**Effect**	**Genotype**	**Genotype**	**Adj Lower**	**Adj Upper**
**Genotype**	Meg +/+	Meg +/-	0.1664	0.9447

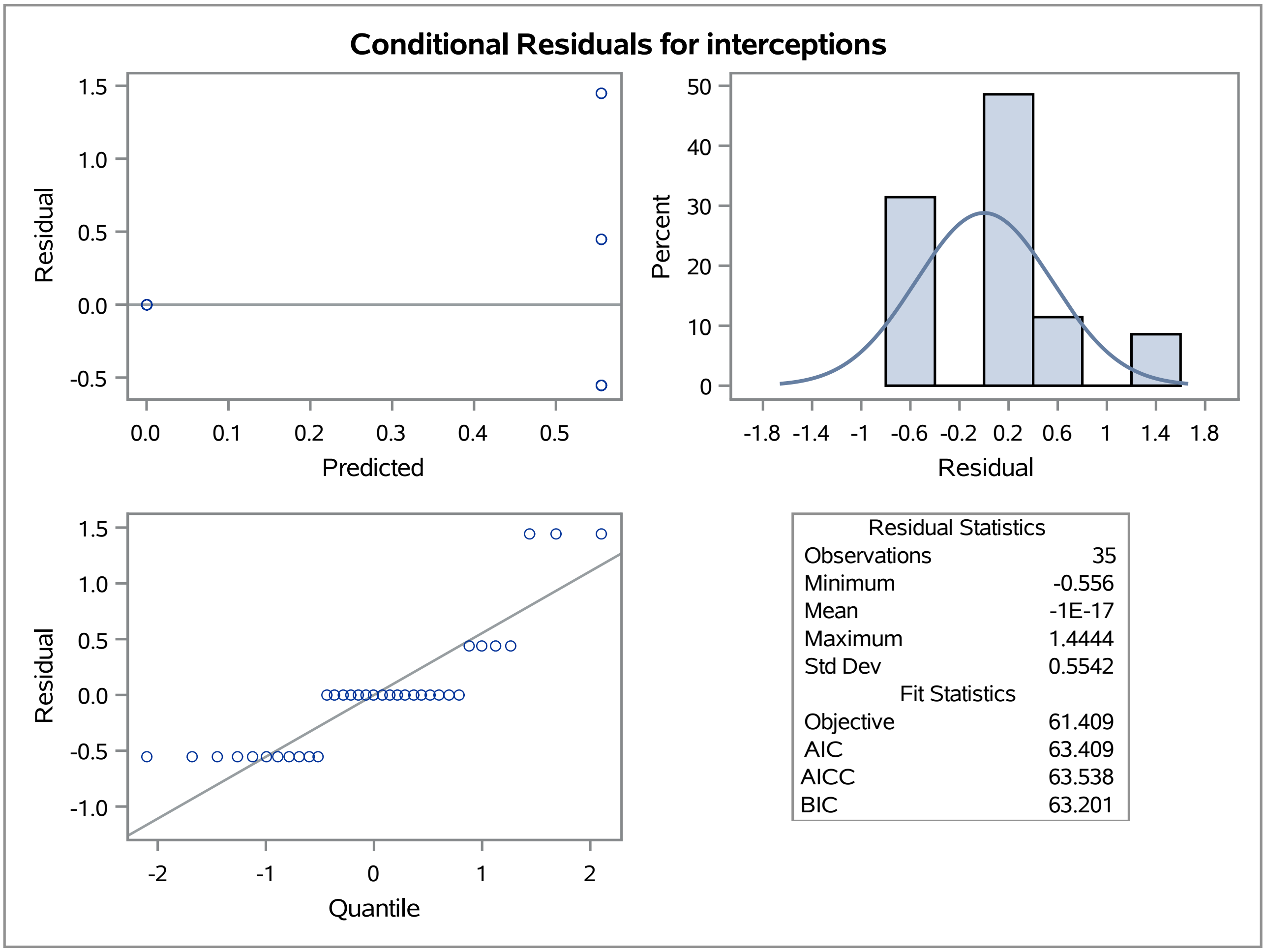

DistSoma=516

Model Information	
**Data Set**	WORK.TEMPDATASORTED
**Dependent Variable**	interceptions
**Covariance Structure**	Variance Components
**Estimation Method**	REML
**Residual Variance Method**	Profile
**Fixed Effects SE Method**	Model-Based
**Degrees of Freedom Method**	Containment

Class Level Information		
**Class**	**Levels**	**Values**
**Genotype**	2	Meg +/+ Meg +/-
**Animal**	6	1 2 3 4 5 6

Dimensions	
**Covariance Parameters**	2
**Columns in X**	3
**Columns in Z**	6
**Subjects**	1
**Max Obs per Subject**	35

Number of Observations	
**Number of Observations Read**	35
**Number of Observations Used**	35
**Number of Observations Not Used**	0

Iteration History			
**Iteration**	**Evaluations**	**-2 Res Log Like**	**Criterion**
**0**	1	61.58416225	
**1**	1	61.58416225	0.00000000

Convergence criteria met.

Estimated G matrix is not positive definite.

Covariance Parameter Estimates				
**Cov Parm**	**Estimate**	**Alpha**	**Lower**	**Upper**
**Animal**	0	.	.	.
**Residual**	0.3182	0.05	0.2070	0.5513

DistSoma=516

Fit Statistics	
-2 Res Log Likelihood	61.6
AIC (Smaller is Better)	63.6
AICC (Smaller is Better)	63.7
BIC (Smaller is Better)	63.4

Solution for Fixed Effects

Effect	Genotype	Estimate	Standard Error	DF	t Value	Pr > |t|	Alpha	Lower	Upper
Intercept		0	0.1368	4	0.00	1.0000	0.05	-0.3798	0.3798
Genotype	Meg +/+	0.5000	0.1908	29	2.62	0.0138	0.05	0.1098	0.8902
Genotype	Meg +/-	0	.	.	.	.	.	.	.

Solution for Random Effects

Effect	Animal	Estimate	Std Err Pred	DF	t Value	Pr > |t|	Alpha	Lower	Upper
Animal	1	0	.	.	.	.	.	.	.
Animal	2	0	.	.	.	.	.	.	.
Animal	3	0	.	.	.	.	.	.	.
Animal	4	0	.	.	.	.	.	.	.
Animal	5	0	.	.	.	.	.	.	.
Animal	6	0	.	.	.	.	.	.	.

Type 3 Tests of Fixed Effects

Effect	Num DF	Den DF	F Value	Pr > F
Genotype	1	29	6.87	0.0138

Least Squares Means

Effect	Genotype	Estimate	Standard Error	DF	t Value	Pr > |t|	Alpha	Lower	Upper
Genotype	Meg +/+	0.5000	0.1330	29	3.76	0.0008	0.05	0.2281	0.7719
Genotype	Meg +/-	0	0.1368	29	0.00	1.0000	0.05	-0.2798	0.2798

DistSoma=516

Differences of Least Squares Means												
Effect	Genotype	Genotype	Estimate	Standard Error	DF	t Value	Pr > |t|	Adjustment	Adj P	Alpha	Lower	Upper
Genotype	Meg +/+	Meg +/-	0.5000	0.1908	29	2.62	0.0138	Tukey-Kramer	0.0138	0.05	0.1098	0.8902

Differences of Least Squares Means				
Effect	Genotype	Genotype	Adj Lower	Adj Upper
Genotype	Meg +/+	Meg +/-	0.1098	0.8902

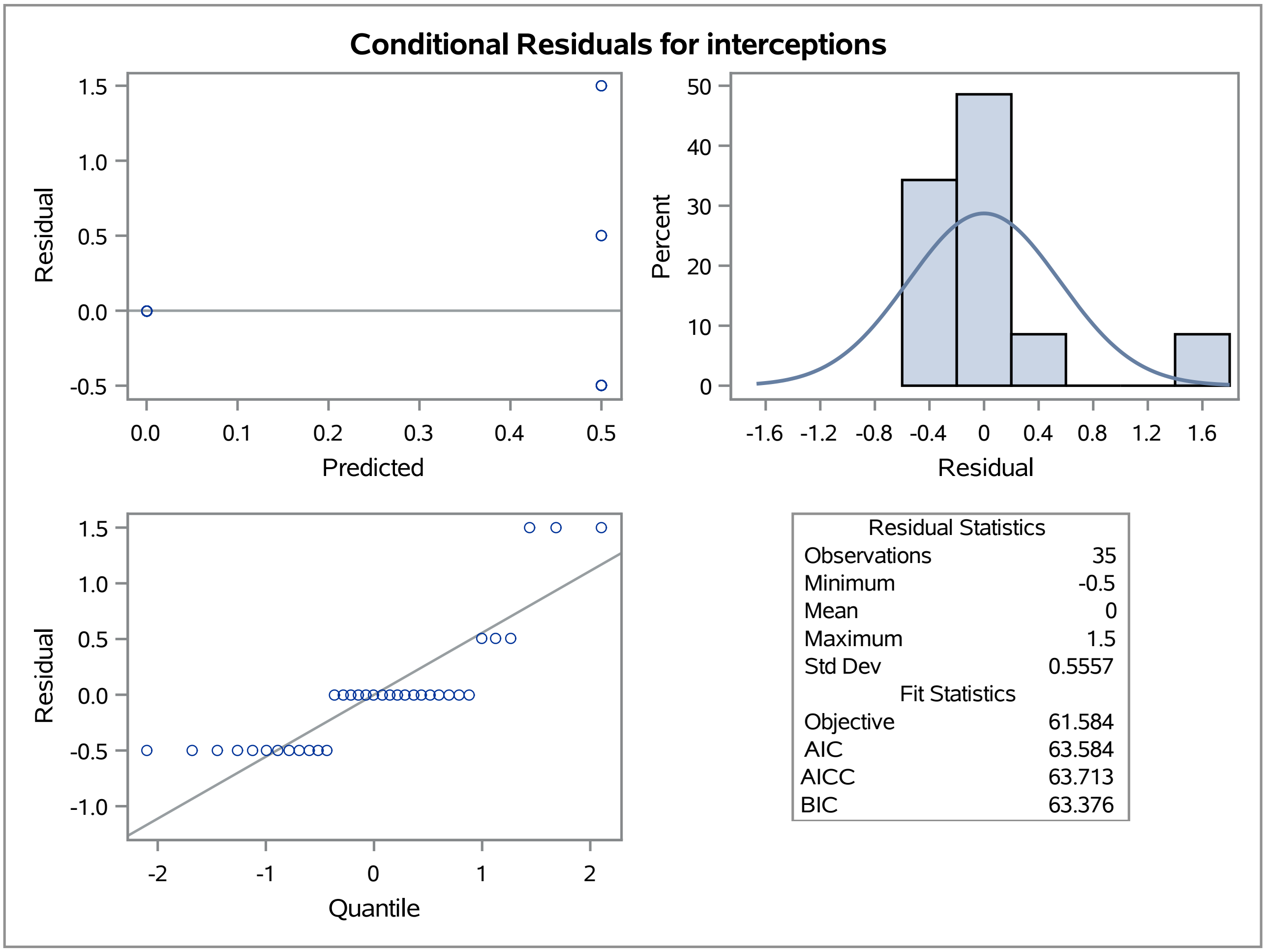

DistSoma=522

Model Information	
**Data Set**	WORK.TEMPDATASORTED
**Dependent Variable**	interceptions
**Covariance Structure**	Variance Components
**Estimation Method**	REML
**Residual Variance Method**	Profile
**Fixed Effects SE Method**	Model-Based
**Degrees of Freedom Method**	Containment

Class Level Information		
**Class**	**Levels**	**Values**
**Genotype**	2	Meg +/+ Meg +/-
**Animal**	6	1 2 3 4 5 6

Dimensions	
**Covariance Parameters**	2
**Columns in X**	3
**Columns in Z**	6
**Subjects**	1
**Max Obs per Subject**	35

Number of Observations	
**Number of Observations Read**	35
**Number of Observations Used**	35
**Number of Observations Not Used**	0

Iteration History			
**Iteration**	**Evaluations**	**-2 Res Log Like**	**Criterion**
**0**	1	61.58416225	
**1**	1	61.58416225	0.00000000

Convergence criteria met.

Estimated G matrix is not positive definite.

Covariance Parameter Estimates				
**Cov Parm**	**Estimate**	**Alpha**	**Lower**	**Upper**
**Animal**	0	.	.	.
**Residual**	0.3182	0.05	0.2070	0.5513

DistSoma=522

Fit Statistics	
-2 Res Log Likelihood	61.6
AIC (Smaller is Better)	63.6
AICC (Smaller is Better)	63.7
BIC (Smaller is Better)	63.4

Solution for Fixed Effects

Effect	Genotype	Estimate	Standard Error	DF	t Value	Pr > |t|	Alpha	Lower	Upper
Intercept		0	0.1368	4	0.00	1.0000	0.05	-0.3798	0.3798
Genotype	Meg +/+	0.5000	0.1908	29	2.62	0.0138	0.05	0.1098	0.8902
Genotype	Meg +/-	0	.	.	.	.	.	.	.

Solution for Random Effects

Effect	Animal	Estimate	Std Err Pred	DF	t Value	Pr > |t|	Alpha	Lower	Upper
Animal	1	0	.	.	.	.	.	.	.
Animal	2	0	.	.	.	.	.	.	.
Animal	3	0	.	.	.	.	.	.	.
Animal	4	0	.	.	.	.	.	.	.
Animal	5	0	.	.	.	.	.	.	.
Animal	6	0	.	.	.	.	.	.	.

Type 3 Tests of Fixed Effects

Effect	Num DF	Den DF	F Value	Pr > F
Genotype	1	29	6.87	0.0138

Least Squares Means

Effect	Genotype	Estimate	Standard Error	DF	t Value	Pr > |t|	Alpha	Lower	Upper
Genotype	Meg +/+	0.5000	0.1330	29	3.76	0.0008	0.05	0.2281	0.7719
Genotype	Meg +/-	0	0.1368	29	0.00	1.0000	0.05	-0.2798	0.2798

DistSoma=522

Differences of Least Squares Means												
Effect	Genotype	Genotype	Estimate	Standard Error	DF	t Value	Pr > |t|	Adjustment	Adj P	Alpha	Lower	Upper
Genotype	Meg +/+	Meg +/-	0.5000	0.1908	29	2.62	0.0138	Tukey-Kramer	0.0138	0.05	0.1098	0.8902

Differences of Least Squares Means				
Effect	Genotype	Genotype	Adj Lower	Adj Upper
Genotype	Meg +/+	Meg +/-	0.1098	0.8902

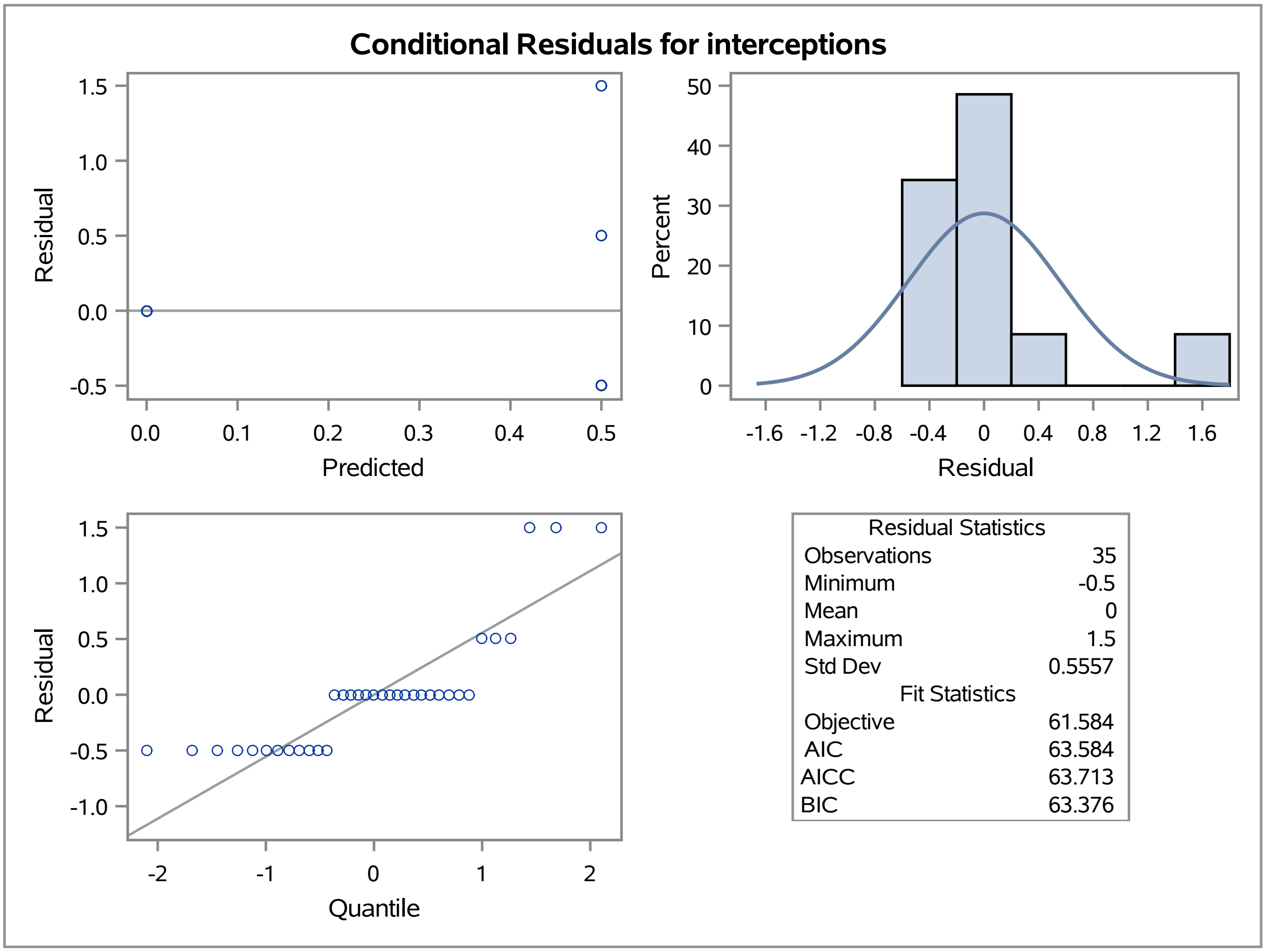

DistSoma=528

Model Information	
**Data Set**	WORK.TEMPDATASORTED
**Dependent Variable**	interceptions
**Covariance Structure**	Variance Components
**Estimation Method**	REML
**Residual Variance Method**	Profile
**Fixed Effects SE Method**	Model-Based
**Degrees of Freedom Method**	Containment

Class Level Information		
**Class**	**Levels**	**Values**
**Genotype**	2	Meg +/+ Meg +/-
**Animal**	6	1 2 3 4 5 6

Dimensions	
**Covariance Parameters**	2
**Columns in X**	3
**Columns in Z**	6
**Subjects**	1
**Max Obs per Subject**	35

Number of Observations	
**Number of Observations Read**	35
**Number of Observations Used**	35
**Number of Observations Not Used**	0

Iteration History			
**Iteration**	**Evaluations**	**-2 Res Log Like**	**Criterion**
**0**	1	54.39456794	
**1**	1	54.39456794	0.00000000

Convergence criteria met.

Estimated G matrix is not positive definite.

Covariance Parameter Estimates				
**Cov Parm**	**Estimate**	**Alpha**	**Lower**	**Upper**
**Animal**	0	.	.	.
**Residual**	0.2559	0.05	0.1665	0.4434

DistSoma=528

Fit Statistics	
-2 Res Log Likelihood	54.4
AIC (Smaller is Better)	56.4
AICC (Smaller is Better)	56.5
BIC (Smaller is Better)	56.2

Solution for Fixed Effects

Effect	Genotype	Estimate	Standard Error	DF	t Value	Pr > |t|	Alpha	Lower	Upper
Intercept		-278E-19	0.1227	4	-0.00	1.0000	0.05	-0.3406	0.3406
Genotype	Meg +/+	0.4444	0.1711	29	2.60	0.0146	0.05	0.09454	0.7943
Genotype	Meg +/-	0	.	.	.	.	.	.	.

Solution for Random Effects

Effect	Animal	Estimate	Std Err Pred	DF	t Value	Pr > |t|	Alpha	Lower	Upper
Animal	1	0	.	.	.	.	.	.	.
Animal	2	0	.	.	.	.	.	.	.
Animal	3	0	.	.	.	.	.	.	.
Animal	4	0	.	.	.	.	.	.	.
Animal	5	0	.	.	.	.	.	.	.
Animal	6	0	.	.	.	.	.	.	.

Type 3 Tests of Fixed Effects

Effect	Num DF	Den DF	F Value	Pr > F
Genotype	1	29	6.75	0.0146

Least Squares Means

Effect	Genotype	Estimate	Standard Error	DF	t Value	Pr > |t|	Alpha	Lower	Upper
Genotype	Meg +/+	0.4444	0.1192	29	3.73	0.0008	0.05	0.2006	0.6883
Genotype	Meg +/-	-278E-19	0.1227	29	-0.00	1.0000	0.05	-0.2509	0.2509

DistSoma=528

Differences of Least Squares Means												
Effect	Genotype	Genotype	Estimate	Standard Error	DF	t Value	Pr > |t|	Adjustment	Adj P	Alpha	Lower	Upper
Genotype	Meg +/+	Meg +/-	0.4444	0.1711	29	2.60	0.0146	Tukey-Kramer	0.0146	0.05	0.09454	0.7943

Differences of Least Squares Means				
Effect	Genotype	Genotype	Adj Lower	Adj Upper
Genotype	Meg +/+	Meg +/-	0.09455	0.7943

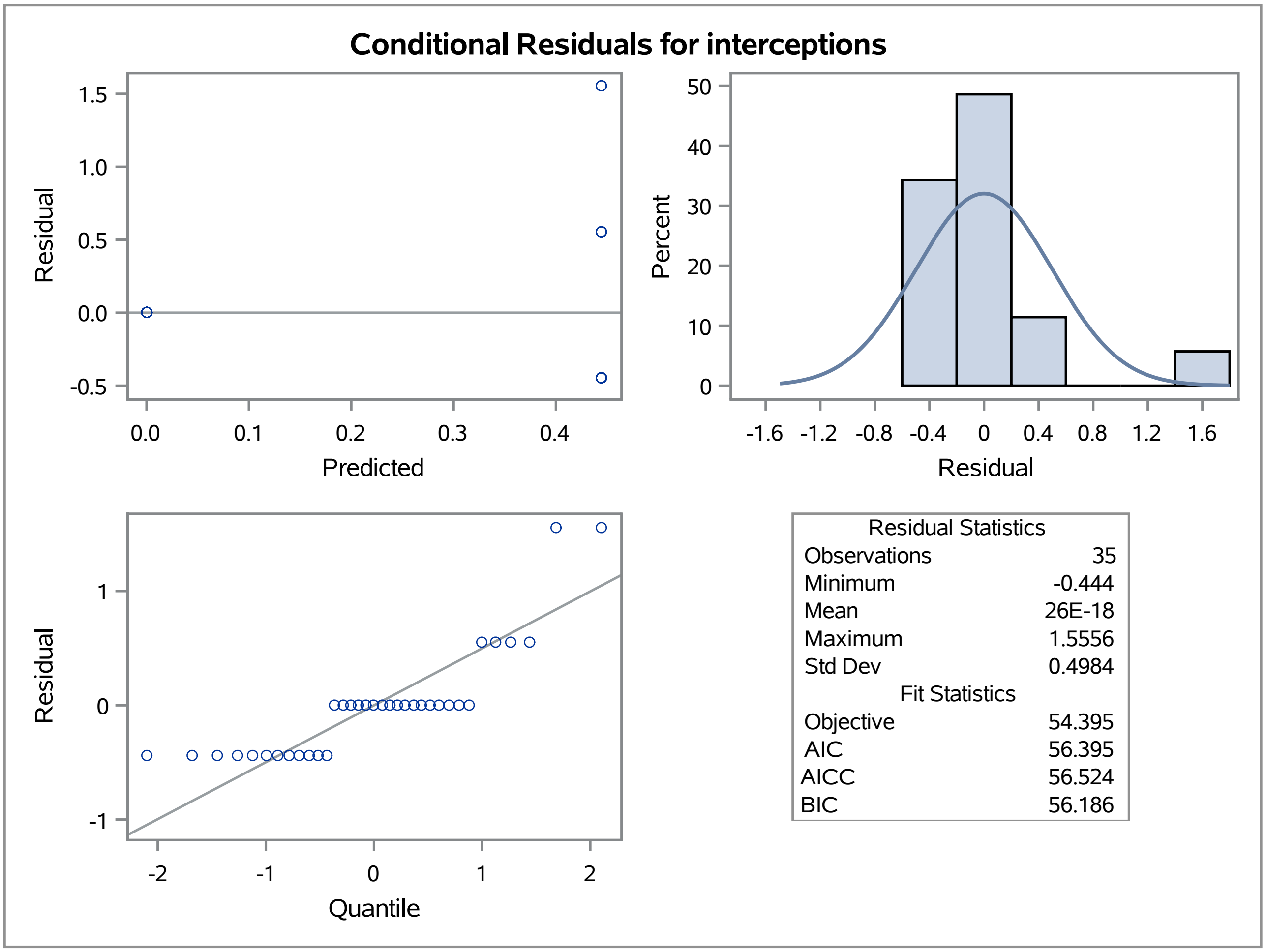

**Data Set Name**	WORK.FIG7F	**Observations**	53
**Member Type**	DATA	**Variables**	6
**Engine**	V9	**Indexes**	0
**Created**	07/03/2020 01:01:04	**Observation Length**	56
**Last Modified**	07/03/2020 01:01:04	**Deleted Observations**	0
**Protection**		**Compressed**	NO
**Data Set Type**		**Sorted**	NO
**Label**			
**Data Representation**	SOLARIS_X86_64, LINUX_X86_64, ALPHA_TRU64, LINUX_IA64		
**Encoding**	utf-8 Unicode (UTF-8)		

Engine/Host Dependent Information	
**Data Set Page Size**	65536
**Number of Data Set Pages**	1
**First Data Page**	1
**Max Obs per Page**	1166
**Obs in First Data Page**	53
**Number of Data Set Repairs**	0
**Filename**	/tmp/SAS_work110900000989_localhost.localdomain/SAS_work03CC00000989_localhost.localdomain/fig7f.sas7bdat
**Release Created**	9.0401M6
**Host Created**	Linux
**Inode Number**	672511
**Access Permission**	rw-rw-r--
**Owner Name**	sasdemo
**File Size**	128KB
**File Size (bytes)**	131072

Alphabetic List of Variables and Attributes

#	Variable	Type	Len	Format	Informat	Label
2	Animal	Num	8	BEST.		Animal
5	E	Char	1	$1.	$1.	E
6	F	Char	30	$30.	$30.	F
1	Genotype	Char	5	$5.	$5.	Genotype
3	NeuriteNumber	Char	3	$3.	$3.	NeuriteNumber
4	Neurite_number	Num	8	BEST.		Neurite number

Model Information	
**Data Set**	WORK.FIG7F
**Dependent Variable**	Neurite_number
**Covariance Structure**	Variance Components
**Estimation Method**	REML
**Residual Variance Method**	Profile
**Fixed Effects SE Method**	Model-Based
**Degrees of Freedom Method**	Containment

Class Level Information		
**Class**	**Levels**	**Values**
**Genotype**	2	Meg++ Meg+-
**Animal**	6	1 2 3 4 5 6

Dimensions	
**Covariance Parameters**	2
**Columns in X**	3
**Columns in Z**	6
**Subjects**	1
**Max Obs per Subject**	52

Number of Observations	
**Number of Observations Read**	53
**Number of Observations Used**	52
**Number of Observations Not Used**	1

Iteration History			
**Iteration**	**Evaluations**	**-2 Res Log Like**	**Criterion**
**0**	1	243.73880358	
**1**	2	243.28887622	0.00000356
**2**	1	243.28860226	0.00000000

Convergence criteria met.

Covariance Parameter Estimates							
**Cov Parm**	**Estimate**	**Standard Error**	**Z Value**	**Pr > Z**	**Alpha**	**Lower**	**Upper**
**Animal**	0.5097	0.9977	0.51	0.3047	0.05	0.07564	269318
**Residual**	6.4069	1.3439	4.77	<.0001	0.05	4.4153	10.1376

Fit Statistics	
**-2 Res Log Likelihood**	243.3
**AIC (Smaller is Better)**	247.3
**AICC (Smaller is Better)**	247.5
**BIC (Smaller is Better)**	246.9

Solution for Fixed Effects

Effect	Genotype	Estimate	Standard Error	DF	t Value	Pr > |t|	Alpha	Lower	Upper
**Intercept**		7.9515	0.6498	4	12.24	0.0003	0.05	6.1473	9.7557
**Genotype**	Meg++	2.8512	0.9224	46	3.09	0.0034	0.05	0.9945	4.7079
**Genotype**	Meg+-	0	.	.	.	.	.	.	.

Solution for Random Effects

Effect	Animal	Estimate	Std Err Pred	DF	t Value	Pr > |t|	Alpha	Lower	Upper
**Animal**	1	-0.2871	0.6182	46	-0.46	0.6446	0.05	-1.5315	0.9573
**Animal**	2	-0.3690	0.6011	46	-0.61	0.5424	0.05	-1.5789	0.8410
**Animal**	3	0.6561	0.6243	46	1.05	0.2988	0.05	-0.6006	1.9127
**Animal**	4	-0.3043	0.6087	46	-0.50	0.6195	0.05	-1.5295	0.9210
**Animal**	5	0.2348	0.6031	46	0.39	0.6989	0.05	-0.9793	1.4488
**Animal**	6	0.06952	0.6238	46	0.11	0.9117	0.05	-1.1860	1.3251

Type 3 Tests of Fixed Effects

Effect	Num DF	Den DF	F Value	Pr > F
**Genotype**	1	46	9.55	0.0034

Least Squares Means

Effect	Genotype	Estimate	Standard Error	DF	t Value	Pr > |t|	Alpha	Lower	Upper
**Genotype**	Meg++	10.8027	0.6547	46	16.50	<.0001	0.05	9.4849	12.1205
**Genotype**	Meg+-	7.9515	0.6498	46	12.24	<.0001	0.05	6.6435	9.2595

Differences of Least Squares Means												
**Effect**	**Genotype**	**Genotype**	**Estimate**	**Standard Error**	**DF**	**t Value**	**Pr > |t|**	**Adjustment**	**Adj P**	**Alpha**	**Lower**	**Upper**
**Genotype**	Meg++	Meg+-	2.8512	0.9224	46	3.09	0.0034	Tukey-Kramer	0.0034	0.05	0.9945	4.7079

Differences of Least Squares Means				
**Effect**	**Genotype**	**Genotype**	**Adj Lower**	**Adj Upper**
**Genotype**	Meg++	Meg+-	0.9945	4.7079

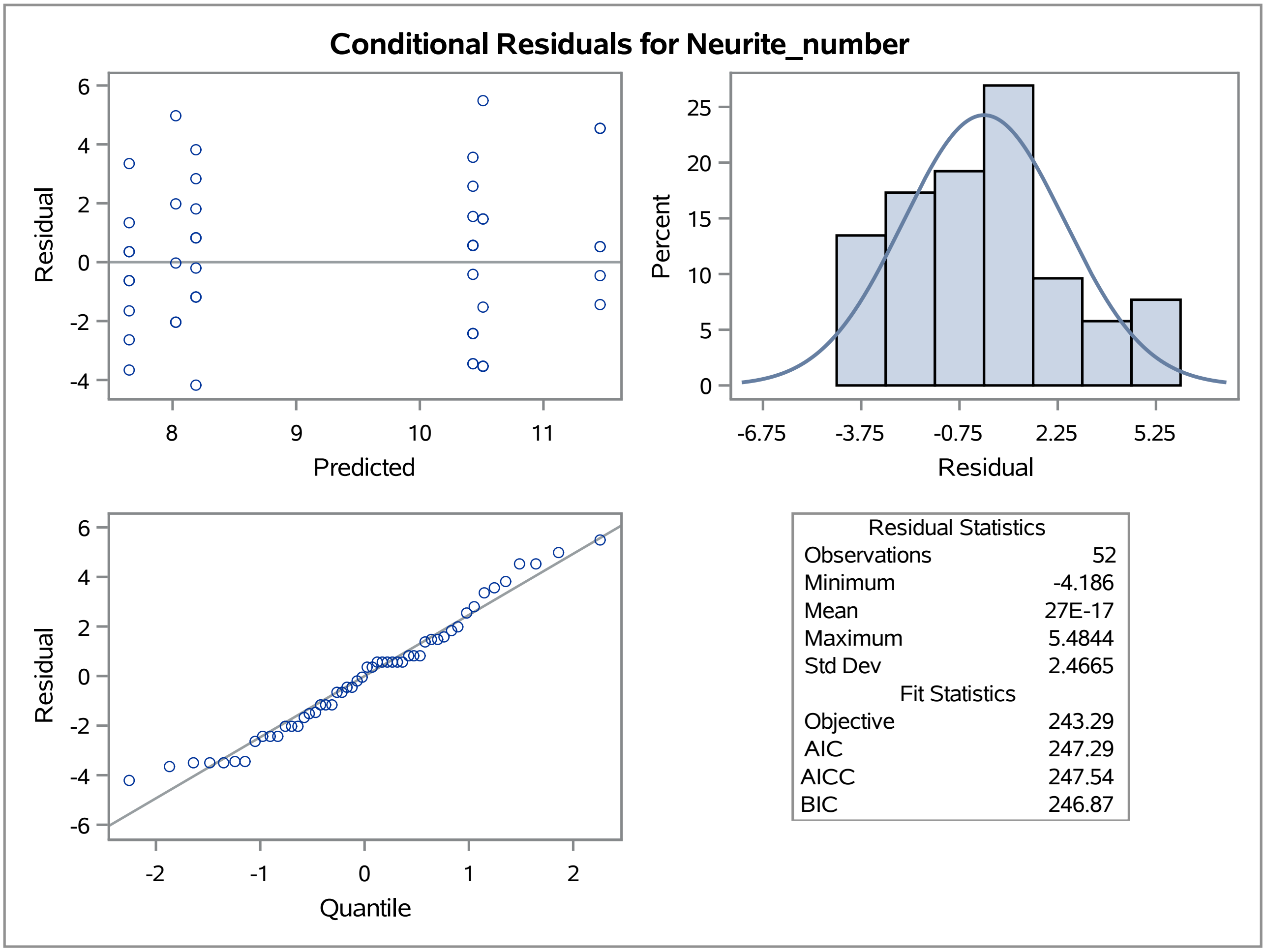

**Data Set Name**	WORK.FIG7G	**Observations**	53
**Member Type**	DATA	**Variables**	6
**Engine**	V9	**Indexes**	0
**Created**	07/03/2020 01:01:05	**Observation Length**	64
**Last Modified**	07/03/2020 01:01:05	**Deleted Observations**	0
**Protection**		**Compressed**	NO
**Data Set Type**		**Sorted**	NO
**Label**			
**Data Representation**	SOLARIS_X86_64, LINUX_X86_64, ALPHA_TRU64, LINUX_IA64		
**Encoding**	utf-8 Unicode (UTF-8)		

**Engine/Host Dependent Information**	
**Data Set Page Size**	65536
**Number of Data Set Pages**	1
**First Data Page**	1
**Max Obs per Page**	1021
**Obs in First Data Page**	53
**Number of Data Set Repairs**	0
**Filename**	/tmp/SAS_work110900000989_localhost.localdomain/SAS_work03CC00000989_localhost.localdomain/fig7g.sas7bdat
**Release Created**	9.0401M6
**Host Created**	Linux
**Inode Number**	672512
**Access Permission**	rw-rw-r--
**Owner Name**	sasdemo
**File Size**	128KB
**File Size (bytes)**	131072

Alphabetic List of Variables and Attributes

#	Variable	Type	Len	Format	Informat	Label
2	Animal	Num	8	BEST.		Animal
5	E	Char	1	$1.	$1.	E
6	F	Char	30	$30.	$30.	F
1	Genotype	Char	5	$5.	$5.	Genotype
4	Neurite_length	Num	8	COMMA15.2		Neurite length
3	Neurite_length__um_	Char	8	$8.	$8.	Neurite length (um)

Model Information	
**Data Set**	WORK.FIG7G
**Dependent Variable**	Neurite_length
**Covariance Structure**	Variance Components
**Estimation Method**	REML
**Residual Variance Method**	Profile
**Fixed Effects SE Method**	Model-Based
**Degrees of Freedom Method**	Containment

Class Level Information		
**Class**	**Levels**	**Values**
**Genotype**	2	Meg++ Meg+-
**Animal**	6	1 2 3 4 5 6

Dimensions	
**Covariance Parameters**	2
**Columns in X**	3
**Columns in Z**	6
**Subjects**	1
**Max Obs per Subject**	50

Number of Observations	
**Number of Observations Read**	53
**Number of Observations Used**	50
**Number of Observations Not Used**	3

Iteration History			
**Iteration**	**Evaluations**	**-2 Res Log Like**	**Criterion**
**0**	1	689.59711254	
**1**	5	658.53890585	0.00002135
**2**	1	658.53333892	0.00000029
**3**	1	658.53325543	0.00000000

Convergence criteria met.

Covariance Parameter Estimates							
Cov Parm	Estimate	Standard Error	Z Value	Pr > Z	Alpha	Lower	Upper
Animal	72532	54517	1.33	0.0917	0.05	24848	734126
Residual	37048	7891.97	4.69	<.0001	0.05	25398	59091

Fit Statistics	
-2 Res Log Likelihood	658.5
AIC (Smaller is Better)	662.5
AICC (Smaller is Better)	662.8
BIC (Smaller is Better)	662.1

Solution for Fixed Effects									
Effect	Genotype	Estimate	Standard Error	DF	t Value	Pr > |t|	Alpha	Lower	Upper
Intercept		517.10	161.17	4	3.21	0.0326	0.05	69.6120	964.58
Genotype	Meg++	297.19	227.74	44	1.30	0.1987	0.05	-161.79	756.18
Genotype	Meg+-	0	.	.	.	.	.	.	.

Solution for Random Effects									
Effect	Animal	Estimate	Std Err Pred	DF	t Value	Pr > |t|	Alpha	Lower	Upper
Animal	1	275.96	165.59	44	1.67	0.1027	0.05	-57.7777	609.69
Animal	2	-352.80	163.44	44	-2.16	0.0364	0.05	-682.19	-23.4152
Animal	3	76.8475	167.44	44	0.46	0.6485	0.05	-260.61	414.30
Animal	4	-174.68	164.79	44	-1.06	0.2949	0.05	-506.80	157.44
Animal	5	-7.7702	163.89	44	-0.05	0.9624	0.05	-338.07	322.53
Animal	6	182.45	169.23	44	1.08	0.2869	0.05	-158.62	523.52

Type 3 Tests of Fixed Effects				
Effect	Num DF	Den DF	F Value	Pr > F
Genotype	1	44	1.70	0.1987

Least Squares Means									
Effect	Genotype	Estimate	Standard Error	DF	t Value	Pr > |t|	Alpha	Lower	Upper
Genotype	Meg++	814.29	160.91	44	5.06	<.0001	0.05	490.01	1138.57
Genotype	Meg+-	517.10	161.17	44	3.21	0.0025	0.05	192.28	841.92

Differences of Least Squares Means

Effect	Genotype	Genotype	Estimate	Standard Error	DF	t Value	Pr > |t|	Adjustment	Adj P	Alpha	Lower	Upper
**Genotype**	Meg++	Meg+-	297.19	227.74	44	1.30	0.1987	Tukey-Kramer	0.1987	0.05	-161.79	756.18

Differences of Least Squares Means

Effect	Genotype	Genotype	Adj Lower	Adj Upper
**Genotype**	Meg++	Meg+-	-161.78	756.17

Conditional Residuals for Neurite_length

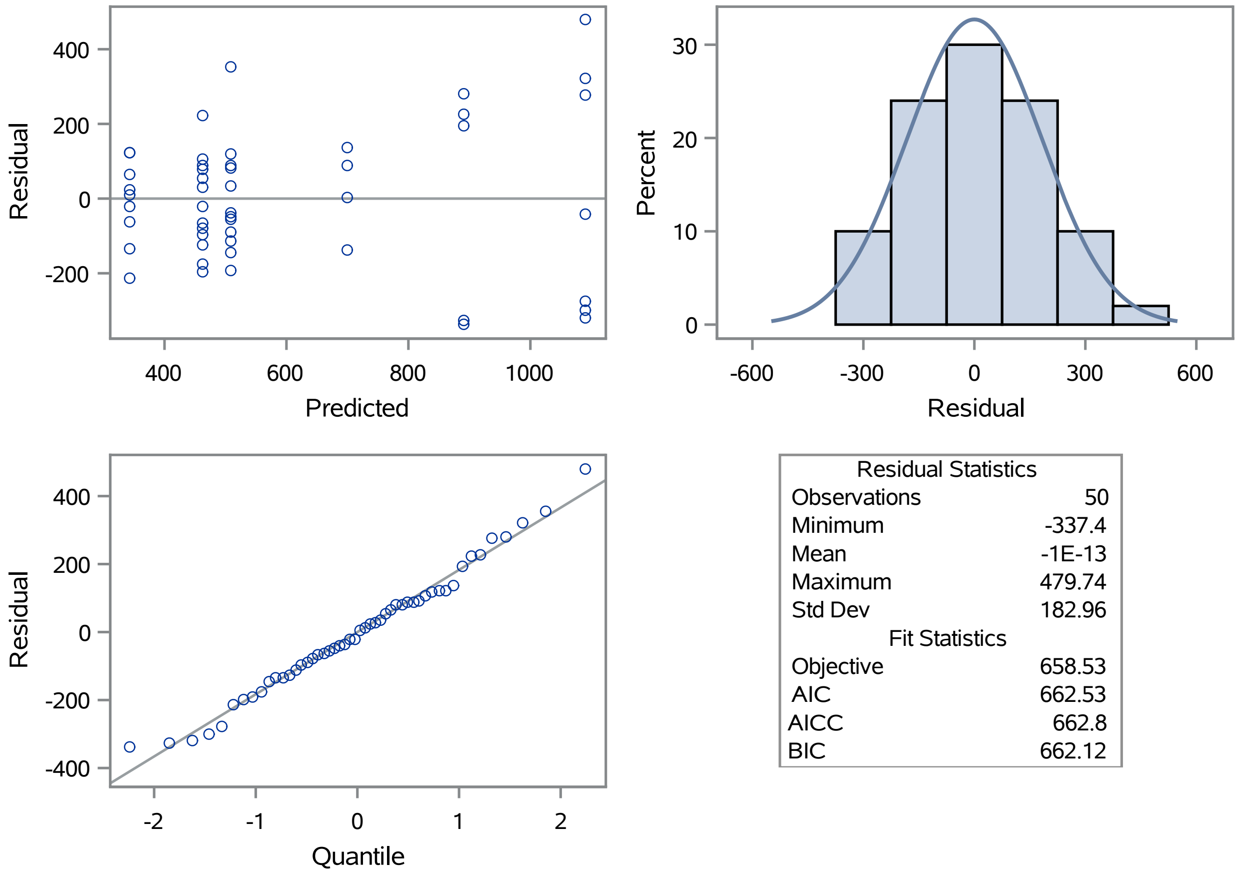

Residual Statistics	
Observations	50
Minimum	-337.4
Mean	-1E-13
Maximum	479.74
Std Dev	182.96
**Fit Statistics**	
Objective	658.53
AIC	662.53
AICC	662.8
BIC	662.12

**Data Set Name**	WORK.FIG7H	**Observations**	4806
**Member Type**	DATA	**Variables**	4
**Engine**	V9	**Indexes**	0
**Created**	07/10/2020 17:16:33	**Observation Length**	32
**Last Modified**	07/10/2020 17:16:33	**Deleted Observations**	0
**Protection**		**Compressed**	NO
**Data Set Type**		**Sorted**	NO
**Label**			
**Data Representation**	SOLARIS_X86_64, LINUX_X86_64, ALPHA_TRU64, LINUX_IA64		
**Encoding**	utf-8 Unicode (UTF-8)		

**Engine/Host Dependent Information**	
**Data Set Page Size**	65536
**Number of Data Set Pages**	3
**First Data Page**	1
**Max Obs per Page**	2038
**Obs in First Data Page**	1965
**Number of Data Set Repairs**	0
**Filename**	/tmp/SAS_workBFFD00006523_localhost.localdomain/SAS_workC2B200006523_localhost.localdomain/fig7h.sas7bdat
**Release Created**	9.0401M6
**Host Created**	Linux
**Inode Number**	672680
**Access Permission**	rw-rw-r--
**Owner Name**	sasdemo
**File Size**	256KB
**File Size (bytes)**	262144

Alphabetic List of Variables and Attributes

#	Variable	Type	Len	Format	Informat	Label
**3**	Animal	Num	8	BEST.		Animal
**1**	DistSoma	Num	8	BEST.		DistSoma
**2**	Genotype	Char	7	$7.	$7.	Genotype
**4**	Interceptions	Num	8	BEST.		Interceptions

DistSoma=0

Model Information	
**Data Set**	WORK.TEMPDATASORTED
**Dependent Variable**	Interceptions
**Covariance Structure**	Variance Components
**Estimation Method**	REML
**Residual Variance Method**	Profile
**Fixed Effects SE Method**	Model-Based
**Degrees of Freedom Method**	Containment

Class Level Information		
**Class**	**Levels**	**Values**
**Genotype**	2	Meg +/+ Meg +/-
**Animal**	6	1 2 3 4 5 6

Dimensions	
**Covariance Parameters**	2
**Columns in X**	3
**Columns in Z**	6
**Subjects**	1
**Max Obs per Subject**	54

Number of Observations	
**Number of Observations Read**	54
**Number of Observations Used**	54
**Number of Observations Not Used**	0

Iteration History			
**Iteration**	**Evaluations**	**-2 Res Log Like**	**Criterion**
**0**	1	146.97395410	
**1**	2	144.76583185	0.00003345
**2**	1	144.76498775	0.00000005
**3**	1	144.76498642	0.00000000

Convergence criteria met.

DistSoma=0

Covariance Parameter Estimates				
Cov Parm	Estimate	Alpha	Lower	Upper
Animal	0.1570	0.05	0.03713	20.0088
Residual	0.7727	0.05	0.5368	1.2077

Fit Statistics	
-2 Res Log Likelihood	144.8
AIC (Smaller is Better)	148.8
AICC (Smaller is Better)	149.0
BIC (Smaller is Better)	148.3

Solution for Fixed Effects									
Effect	Genotype	Estimate	Standard Error	DF	t Value	Pr > |t|	Alpha	Lower	Upper
Intercept		1.8060	0.2843	4	6.35	0.0031	0.05	1.0167	2.5954
Genotype	Meg +/+	0.04142	0.4064	48	0.10	0.9193	0.05	-0.7757	0.8585
Genotype	Meg +/-	0	.	.	.	.	.	.	.

Solution for Random Effects									
Effect	Animal	Estimate	Std Err Pred	DF	t Value	Pr > |t|	Alpha	Lower	Upper
Animal	1	-0.1126	0.2958	48	-0.38	0.7053	0.05	-0.7073	0.4822
Animal	2	0.3585	0.3102	48	1.16	0.2534	0.05	-0.2651	0.9822
Animal	3	-0.2460	0.3064	48	-0.80	0.4261	0.05	-0.8621	0.3701
Animal	4	0.05357	0.2988	48	0.18	0.8585	0.05	-0.5473	0.6544
Animal	5	-0.3353	0.2938	48	-1.14	0.2595	0.05	-0.9260	0.2555
Animal	6	0.2817	0.3045	48	0.93	0.3595	0.05	-0.3305	0.8938

Type 3 Tests of Fixed Effects				
Effect	Num DF	Den DF	F Value	Pr > F
Genotype	1	48	0.01	0.9193

Least Squares Means									
Effect	Genotype	Estimate	Standard Error	DF	t Value	Pr > |t|	Alpha	Lower	Upper
Genotype	Meg +/+	1.8475	0.2904	48	6.36	<.0001	0.05	1.2636	2.4314
Genotype	Meg +/-	1.8060	0.2843	48	6.35	<.0001	0.05	1.2344	2.3777

DistSoma=0

Differences of Least Squares Means												
**Effect**	**Genotype**	**Genotype**	**Estimate**	**Standard Error**	**DF**	**t Value**	**Pr > |t|**	**Adjustment**	**Adj P**	**Alpha**	**Lower**	**Upper**
**Genotype**	Meg +/+	Meg +/-	0.04142	0.4064	48	0.10	0.9193	Tukey-Kramer	0.9193	0.05	-0.7757	0.8585

Differences of Least Squares Means				
**Effect**	**Genotype**	**Genotype**	**Adj Lower**	**Adj Upper**
**Genotype**	Meg +/+	Meg +/-	-0.7757	0.8585

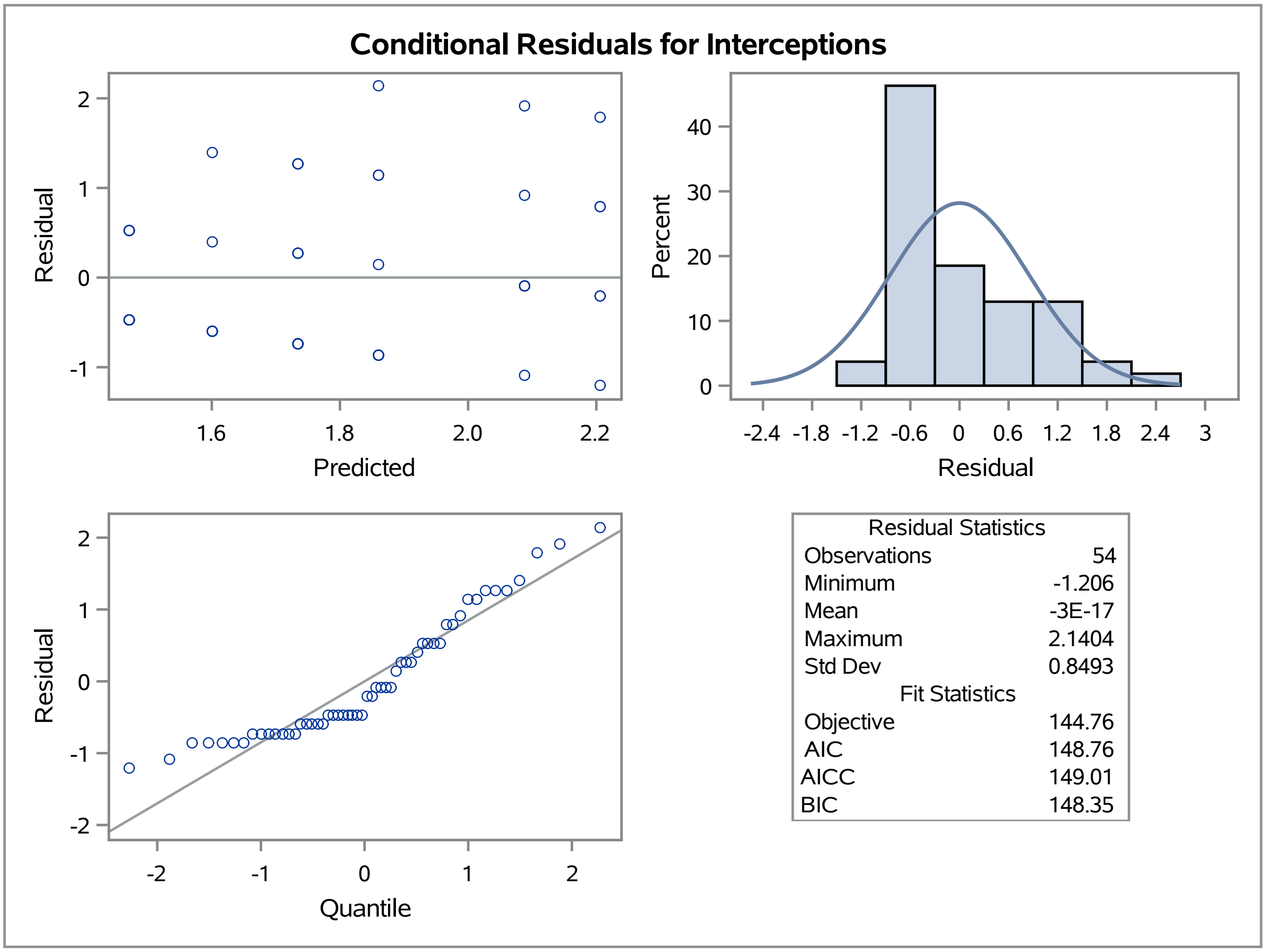

DistSoma=6

Model Information	
**Data Set**	WORK.TEMPDATASORTED
**Dependent Variable**	Interceptions
**Covariance Structure**	Variance Components
**Estimation Method**	REML
**Residual Variance Method**	Profile
**Fixed Effects SE Method**	Model-Based
**Degrees of Freedom Method**	Containment

Class Level Information		
**Class**	**Levels**	**Values**
**Genotype**	2	Meg +/+ Meg +/-
**Animal**	6	1 2 3 4 5 6

Dimensions	
**Covariance Parameters**	2
**Columns in X**	3
**Columns in Z**	6
**Subjects**	1
**Max Obs per Subject**	54

Number of Observations	
**Number of Observations Read**	54
**Number of Observations Used**	54
**Number of Observations Not Used**	0

Iteration History			
**Iteration**	**Evaluations**	**-2 Res Log Like**	**Criterion**
**0**	1	151.52272369	
**1**	2	151.46144741	0.00000068
**2**	1	151.46142824	0.00000000

Convergence criteria met.

Covariance Parameter Estimates				
**Cov Parm**	**Estimate**	**Alpha**	**Lower**	**Upper**
**Animal**	0.02401	0.05	0.002114	4.661E29
**Residual**	0.9350	0.05	0.6482	1.4660

DistSoma=6

Fit Statistics	
**-2 Res Log Likelihood**	151.5
**AIC (Smaller is Better)**	155.5
**AICC (Smaller is Better)**	155.7
**BIC (Smaller is Better)**	155.0

Solution for Fixed Effects

Effect	Genotype	Estimate	Standard Error	DF	t Value	Pr > |t|	Alpha	Lower	Upper
**Intercept**		1.8689	0.2042	4	9.15	0.0008	0.05	1.3019	2.4359
**Genotype**	Meg +/+	0.1338	0.2941	48	0.46	0.6511	0.05	-0.4575	0.7251
**Genotype**	Meg +/-	0	.	.	.	.	.	.	.

Solution for Random Effects

Effect	Animal	Estimate	Std Err Pred	DF	t Value	Pr > |t|	Alpha	Lower	Upper
**Animal**	1	-0.00068	0.1442	48	-0.00	0.9963	0.05	-0.2907	0.2893
**Animal**	2	0.06638	0.1470	48	0.45	0.6535	0.05	-0.2291	0.3619
**Animal**	3	-0.06570	0.1462	48	-0.45	0.6553	0.05	-0.3597	0.2283
**Animal**	4	0.003755	0.1448	48	0.03	0.9794	0.05	-0.2874	0.2949
**Animal**	5	-0.06726	0.1438	48	-0.47	0.6420	0.05	-0.3563	0.2218
**Animal**	6	0.06350	0.1460	48	0.43	0.6656	0.05	-0.2301	0.3571

Type 3 Tests of Fixed Effects

Effect	Num DF	Den DF	F Value	Pr > F
**Genotype**	1	48	0.21	0.6511

Least Squares Means

Effect	Genotype	Estimate	Standard Error	DF	t Value	Pr > |t|	Alpha	Lower	Upper
**Genotype**	Meg +/+	2.0027	0.2116	48	9.46	<.0001	0.05	1.5772	2.4282
**Genotype**	Meg +/-	1.8689	0.2042	48	9.15	<.0001	0.05	1.4583	2.2795

DistSoma=6

Differences of Least Squares Means												
**Effect**	**Genotype**	**Genotype**	**Estimate**	**Standard Error**	**DF**	**t Value**	**Pr > |t|**	**Adjustment**	**Adj P**	**Alpha**	**Lower**	**Upper**
**Genotype**	Meg +/+	Meg +/-	0.1338	0.2941	48	0.46	0.6511	Tukey-Kramer	0.6511	0.05	-0.4575	0.7251

Differences of Least Squares Means				
**Effect**	**Genotype**	**Genotype**	**Adj Lower**	**Adj Upper**
**Genotype**	Meg +/+	Meg +/-	-0.4575	0.7251

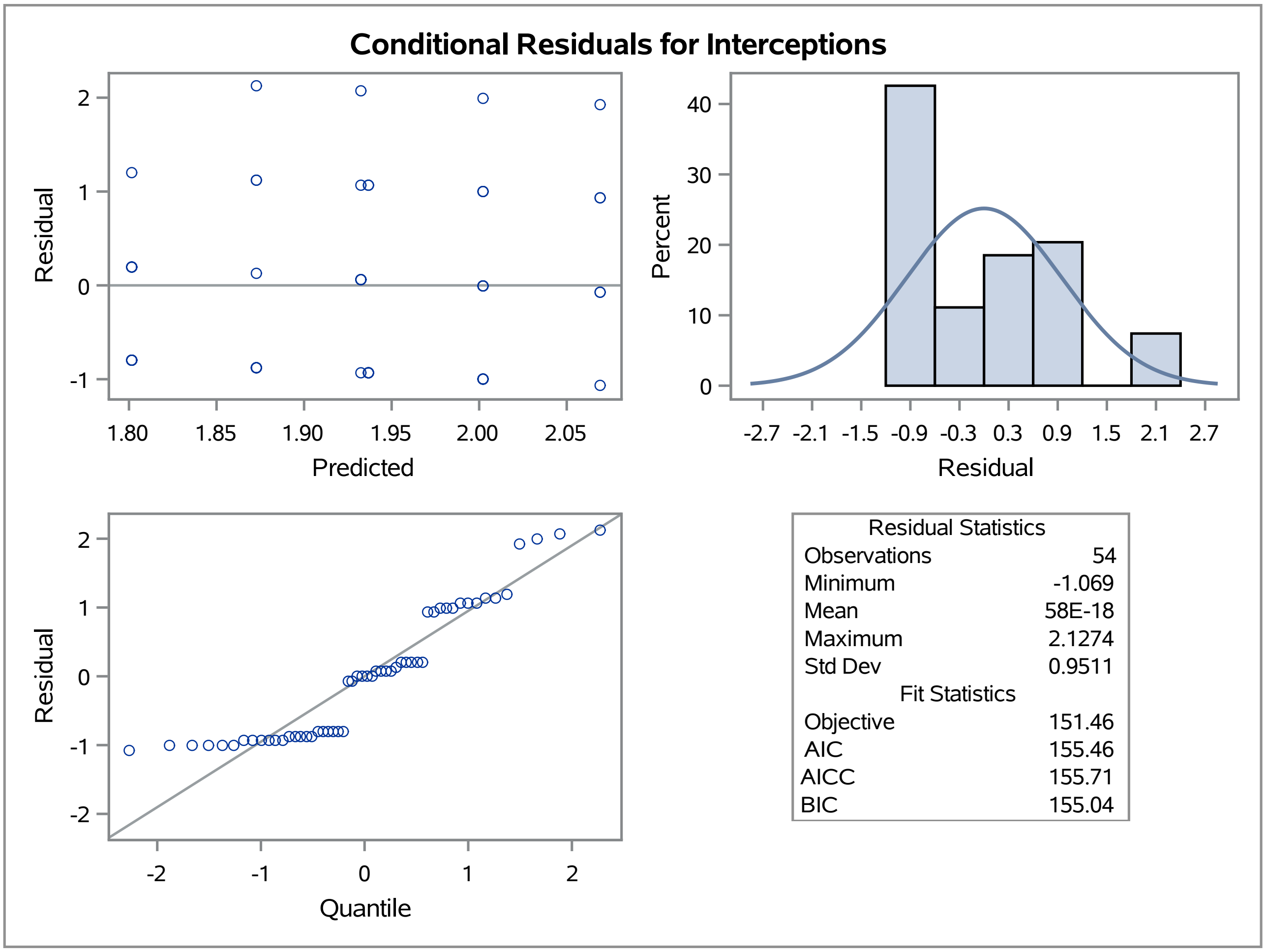

DistSoma=12

Model Information	
**Data Set**	WORK.TEMPDATASORTED
**Dependent Variable**	Interceptions
**Covariance Structure**	Variance Components
**Estimation Method**	REML
**Residual Variance Method**	Profile
**Fixed Effects SE Method**	Model-Based
**Degrees of Freedom Method**	Containment

Class Level Information		
**Class**	**Levels**	**Values**
**Genotype**	2	Meg +/+ Meg +/-
**Animal**	6	1 2 3 4 5 6

Dimensions	
**Covariance Parameters**	2
**Columns in X**	3
**Columns in Z**	6
**Subjects**	1
**Max Obs per Subject**	54

Number of Observations	
**Number of Observations Read**	54
**Number of Observations Used**	54
**Number of Observations Not Used**	0

Iteration History			
**Iteration**	**Evaluations**	**-2 Res Log Like**	**Criterion**
**0**	1	163.34307645	
**1**	2	163.07294198	0.00007303
**2**	1	163.07037089	0.00000034
**3**	1	163.07035938	0.00000000

Convergence criteria met.

DistSoma=12

Covariance Parameter Estimates				
**Cov Parm**	**Estimate**	**Alpha**	**Lower**	**Upper**
**Animal**	0.07948	0.05	0.01009	16186663
**Residual**	1.1451	0.05	0.7916	1.8035

Fit Statistics	
**-2 Res Log Likelihood**	163.1
**AIC (Smaller is Better)**	167.1
**AICC (Smaller is Better)**	167.3
**BIC (Smaller is Better)**	166.7

Solution for Fixed Effects									
**Effect**	**Genotype**	**Estimate**	**Standard Error**	**DF**	**t Value**	**Pr > |t|**	**Alpha**	**Lower**	**Upper**
**Intercept**		2.1534	0.2611	4	8.25	0.0012	0.05	1.4285	2.8783
**Genotype**	Meg +/+	0.1078	0.3751	48	0.29	0.7752	0.05	-0.6465	0.8620
**Genotype**	Meg +/-	0	.	.	.	.	.	.	.

Solution for Random Effects									
**Effect**	**Animal**	**Estimate**	**Std Err Pred**	**DF**	**t Value**	**Pr > |t|**	**Alpha**	**Lower**	**Upper**
**Animal**	1	-0.08740	0.2411	48	-0.36	0.7185	0.05	-0.5721	0.3973
**Animal**	2	0.2662	0.2498	48	1.07	0.2918	0.05	-0.2360	0.7684
**Animal**	3	-0.1788	0.2475	48	-0.72	0.4735	0.05	-0.6764	0.3188
**Animal**	4	0.02646	0.2429	48	0.11	0.9137	0.05	-0.4619	0.5148
**Animal**	5	-0.06971	0.2397	48	-0.29	0.7724	0.05	-0.5516	0.4122
**Animal**	6	0.04326	0.2465	48	0.18	0.8614	0.05	-0.4524	0.5390

Type 3 Tests of Fixed Effects				
**Effect**	**Num DF**	**Den DF**	**F Value**	**Pr > F**
**Genotype**	1	48	0.08	0.7752

Least Squares Means									
**Effect**	**Genotype**	**Estimate**	**Standard Error**	**DF**	**t Value**	**Pr > |t|**	**Alpha**	**Lower**	**Upper**
**Genotype**	Meg +/+	2.2612	0.2694	48	8.39	<.0001	0.05	1.7196	2.8028
**Genotype**	Meg +/-	2.1534	0.2611	48	8.25	<.0001	0.05	1.6284	2.6784

DistSoma=12

Differences of Least Squares Means												
**Effect**	**Genotype**	**Genotype**	**Estimate**	**Standard Error**	**DF**	**t Value**	**Pr > |t|**	**Adjustment**	**Adj P**	**Alpha**	**Lower**	**Upper**
**Genotype**	Meg +/+	Meg +/-	0.1078	0.3751	48	0.29	0.7752	Tukey-Kramer	0.7752	0.05	-0.6465	0.8620

Differences of Least Squares Means				
**Effect**	**Genotype**	**Genotype**	**Adj Lower**	**Adj Upper**
**Genotype**	Meg +/+	Meg +/-	-0.6465	0.8620

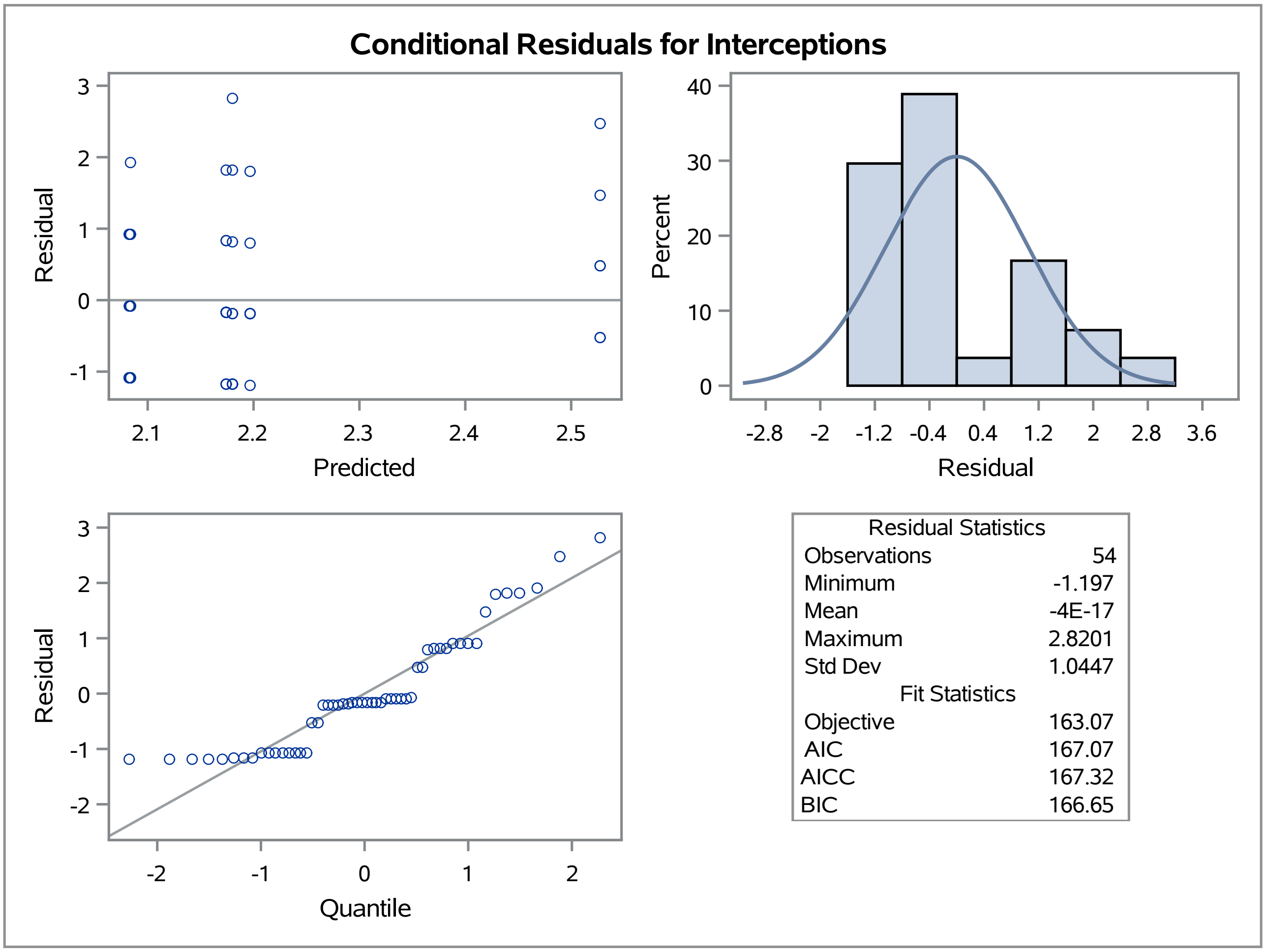

DistSoma=18

Model Information	
**Data Set**	WORK.TEMPDATASORTED
**Dependent Variable**	Interceptions
**Covariance Structure**	Variance Components
**Estimation Method**	REML
**Residual Variance Method**	Profile
**Fixed Effects SE Method**	Model-Based
**Degrees of Freedom Method**	Containment

Class Level Information		
**Class**	**Levels**	**Values**
**Genotype**	2	Meg +/+ Meg +/-
**Animal**	6	1 2 3 4 5 6

Dimensions	
**Covariance Parameters**	2
**Columns in X**	3
**Columns in Z**	6
**Subjects**	1
**Max Obs per Subject**	54

Number of Observations	
**Number of Observations Read**	54
**Number of Observations Used**	54
**Number of Observations Not Used**	0

Iteration History			
**Iteration**	**Evaluations**	**-2 Res Log Like**	**Criterion**
**0**	1	170.66552846	
**1**	2	169.66451711	0.00032059
**2**	1	169.65162461	0.00000658
**3**	1	169.65137739	0.00000000

Convergence criteria met.

DistSoma=18

Covariance Parameter Estimates				
**Cov Parm**	**Estimate**	**Alpha**	**Lower**	**Upper**
**Animal**	0.1874	0.05	0.03629	288.49
**Residual**	1.2650	0.05	0.8765	1.9856

Fit Statistics	
**-2 Res Log Likelihood**	169.7
**AIC (Smaller is Better)**	173.7
**AICC (Smaller is Better)**	173.9
**BIC (Smaller is Better)**	173.2

Solution for Fixed Effects									
**Effect**	**Genotype**	**Estimate**	**Standard Error**	**DF**	**t Value**	**Pr > |t|**	**Alpha**	**Lower**	**Upper**
**Intercept**		2.4835	0.3300	4	7.53	0.0017	0.05	1.5672	3.3998
**Genotype**	Meg +/+	0.07506	0.4726	48	0.16	0.8745	0.05	-0.8751	1.0252
**Genotype**	Meg +/-	0	.	.	.	.	.	.	.

Solution for Random Effects									
**Effect**	**Animal**	**Estimate**	**Std Err Pred**	**DF**	**t Value**	**Pr > |t|**	**Alpha**	**Lower**	**Upper**
**Animal**	1	-0.01322	0.3371	48	-0.04	0.9689	0.05	-0.6909	0.6645
**Animal**	2	0.4430	0.3529	48	1.26	0.2155	0.05	-0.2666	1.1526
**Animal**	3	-0.4298	0.3488	48	-1.23	0.2239	0.05	-1.1310	0.2715
**Animal**	4	0.1047	0.3404	48	0.31	0.7598	0.05	-0.5798	0.7891
**Animal**	5	-0.1494	0.3348	48	-0.45	0.6573	0.05	-0.8225	0.5237
**Animal**	6	0.04476	0.3467	48	0.13	0.8978	0.05	-0.6524	0.7419

Type 3 Tests of Fixed Effects				
**Effect**	**Num DF**	**Den DF**	**F Value**	**Pr > F**
**Genotype**	1	48	0.03	0.8745

Least Squares Means									
**Effect**	**Genotype**	**Estimate**	**Standard Error**	**DF**	**t Value**	**Pr > |t|**	**Alpha**	**Lower**	**Upper**
**Genotype**	Meg +/+	2.5586	0.3382	48	7.56	<.0001	0.05	1.8785	3.2386
**Genotype**	Meg +/-	2.4835	0.3300	48	7.53	<.0001	0.05	1.8199	3.1471

DistSoma=18

Differences of Least Squares Means												
Effect	Genotype	Genotype	Estimate	Standard Error	DF	t Value	Pr > |t|	Adjustment	Adj P	Alpha	Lower	Upper
Genotype	Meg +/+	Meg +/-	0.07506	0.4726	48	0.16	0.8745	Tukey-Kramer	0.8745	0.05	-0.8751	1.0252

Differences of Least Squares Means				
Effect	Genotype	Genotype	Adj Lower	Adj Upper
Genotype	Meg +/+	Meg +/-	-0.8751	1.0252

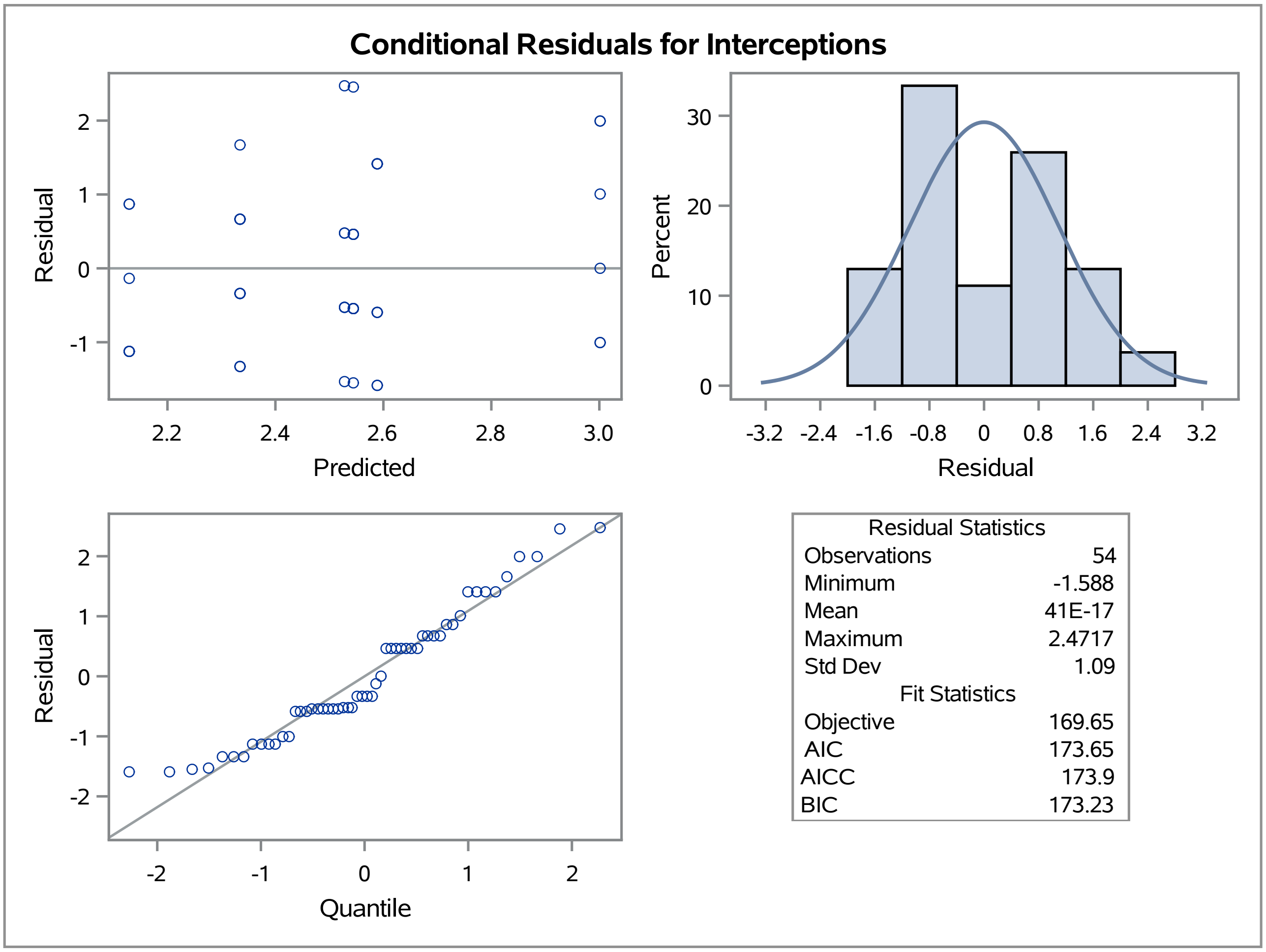

DistSoma=24

Model Information	
**Data Set**	WORK.TEMPDATASORTED
**Dependent Variable**	Interceptions
**Covariance Structure**	Variance Components
**Estimation Method**	REML
**Residual Variance Method**	Profile
**Fixed Effects SE Method**	Model-Based
**Degrees of Freedom Method**	Containment

Class Level Information		
**Class**	**Levels**	**Values**
**Genotype**	2	Meg +/+ Meg +/-
**Animal**	6	1 2 3 4 5 6

Dimensions	
**Covariance Parameters**	2
**Columns in X**	3
**Columns in Z**	6
**Subjects**	1
**Max Obs per Subject**	54

Number of Observations	
**Number of Observations Read**	54
**Number of Observations Used**	54
**Number of Observations Not Used**	0

Iteration History			
**Iteration**	**Evaluations**	**-2 Res Log Like**	**Criterion**
**0**	1	186.23888207	
**1**	2	186.17599522	0.00000751
**2**	1	186.17564933	0.00000000

Convergence criteria met.

Covariance Parameter Estimates				
**Cov Parm**	**Estimate**	**Alpha**	**Lower**	**Upper**
**Animal**	0.05965	0.05	0.005280	2.999E28
**Residual**	1.8159	0.05	1.2510	2.8749

DistSoma=24

Fit Statistics	
-2 Res Log Likelihood	186.2
AIC (Smaller is Better)	190.2
AICC (Smaller is Better)	190.4
BIC (Smaller is Better)	189.8

Solution for Fixed Effects

Effect	Genotype	Estimate	Standard Error	DF	t Value	Pr > |t|	Alpha	Lower	Upper
Intercept		2.7450	0.2924	4	9.39	0.0007	0.05	1.9333	3.5567
Genotype	Meg +/+	-0.01075	0.4209	48	-0.03	0.9797	0.05	-0.8570	0.8355
Genotype	Meg +/-	0	.	.	.	.	.	.	.

Solution for Random Effects

Effect	Animal	Estimate	Std Err Pred	DF	t Value	Pr > |t|	Alpha	Lower	Upper
Animal	1	0.01047	0.2236	48	0.05	0.9629	0.05	-0.4392	0.4601
Animal	2	0.1535	0.2287	48	0.67	0.5053	0.05	-0.3064	0.6134
Animal	3	-0.1640	0.2274	48	-0.72	0.4743	0.05	-0.6211	0.2932
Animal	4	0.007478	0.2247	48	0.03	0.9736	0.05	-0.4443	0.4593
Animal	5	0.02497	0.2227	48	0.11	0.9112	0.05	-0.4229	0.4728
Animal	6	-0.03245	0.2269	48	-0.14	0.8869	0.05	-0.4887	0.4238

Type 3 Tests of Fixed Effects

Effect	Num DF	Den DF	F Value	Pr > F
Genotype	1	48	0.00	0.9797

Least Squares Means

Effect	Genotype	Estimate	Standard Error	DF	t Value	Pr > |t|	Alpha	Lower	Upper
Genotype	Meg +/+	2.7343	0.3027	48	9.03	<.0001	0.05	2.1256	3.3430
Genotype	Meg +/-	2.7450	0.2924	48	9.39	<.0001	0.05	2.1572	3.3328

DistSoma=24

Differences of Least Squares Means												
**Effect**	**Genotype**	**Genotype**	**Estimate**	**Standard Error**	**DF**	**t Value**	**Pr > |t|**	**Adjustment**	**Adj P**	**Alpha**	**Lower**	**Upper**
**Genotype**	Meg +/+	Meg +/-	-0.01075	0.4209	48	-0.03	0.9797	Tukey-Kramer	0.9797	0.05	-0.8570	0.8355

Differences of Least Squares Means				
**Effect**	**Genotype**	**Genotype**	**Adj Lower**	**Adj Upper**
**Genotype**	Meg +/+	Meg +/-	-0.8569	0.8354

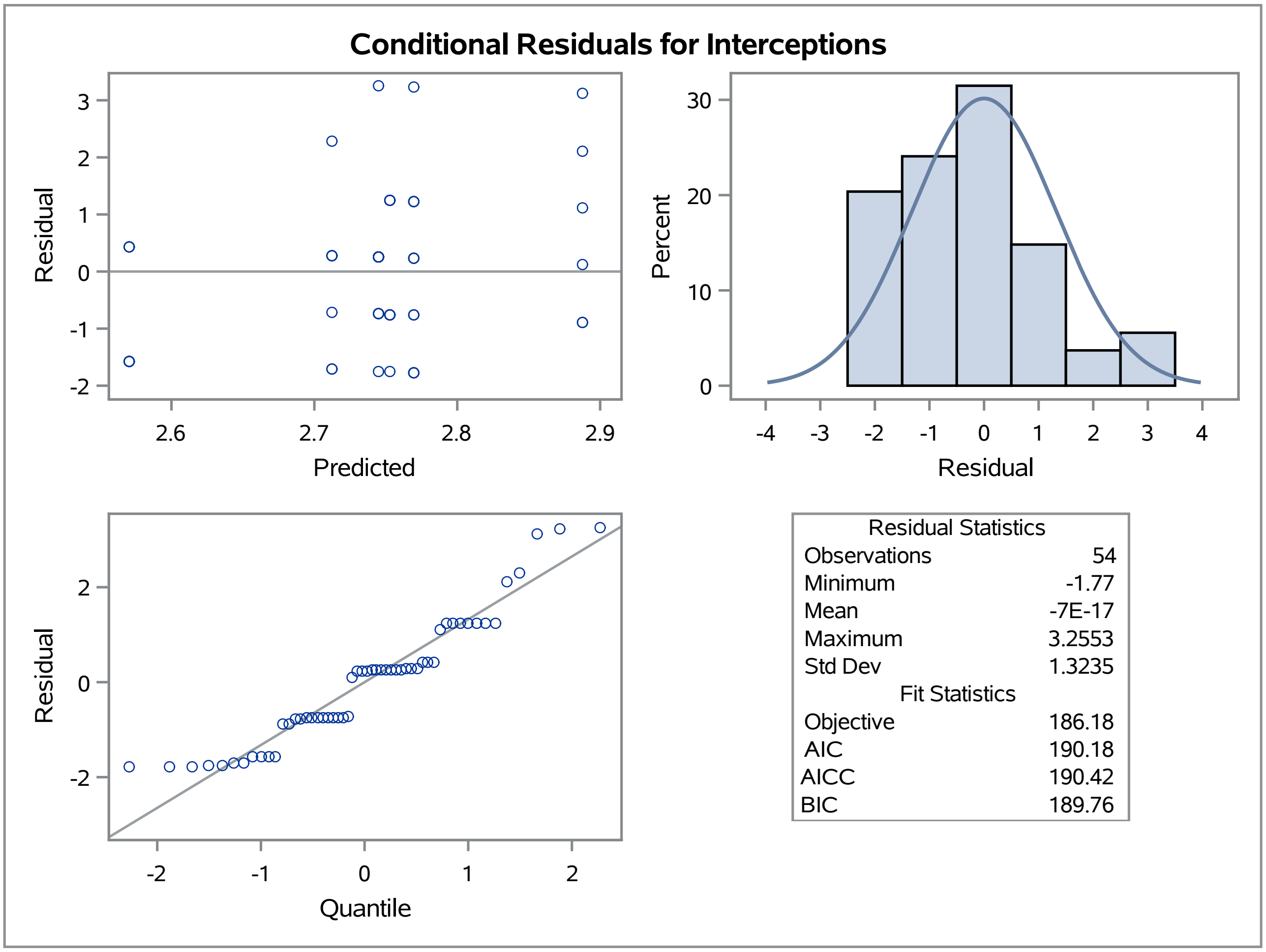

DistSoma=30

Model Information	
**Data Set**	WORK.TEMPDATASORTED
**Dependent Variable**	Interceptions
**Covariance Structure**	Variance Components
**Estimation Method**	REML
**Residual Variance Method**	Profile
**Fixed Effects SE Method**	Model-Based
**Degrees of Freedom Method**	Containment

Class Level Information		
**Class**	**Levels**	**Values**
**Genotype**	2	Meg +/+ Meg +/-
**Animal**	6	1 2 3 4 5 6

Dimensions	
**Covariance Parameters**	2
**Columns in X**	3
**Columns in Z**	6
**Subjects**	1
**Max Obs per Subject**	54

Number of Observations	
**Number of Observations Read**	54
**Number of Observations Used**	54
**Number of Observations Not Used**	0

Iteration History			
**Iteration**	**Evaluations**	**-2 Res Log Like**	**Criterion**
**0**	1	192.55875233	
**1**	2	190.53372012	0.00004341
**2**	1	190.53157678	0.00000017
**3**	1	190.53156861	0.00000000

Convergence criteria met.

DistSoma=30

Covariance Parameter Estimates				
**Cov Parm**	**Estimate**	**Alpha**	**Lower**	**Upper**
**Animal**	0.3693	0.05	0.08559	57.3965
**Residual**	1.8654	0.05	1.2955	2.9173

Fit Statistics	
**-2 Res Log Likelihood**	190.5
**AIC (Smaller is Better)**	194.5
**AICC (Smaller is Better)**	194.8
**BIC (Smaller is Better)**	194.1

Solution for Fixed Effects									
**Effect**	**Genotype**	**Estimate**	**Standard Error**	**DF**	**t Value**	**Pr > |t|**	**Alpha**	**Lower**	**Upper**
**Intercept**		2.8502	0.4380	4	6.51	0.0029	0.05	1.6342	4.0662
**Genotype**	Meg +/+	0.1102	0.6262	48	0.18	0.8611	0.05	-1.1488	1.3691
**Genotype**	Meg +/-	0	.	.	.	.	.	.	.

Solution for Random Effects									
**Effect**	**Animal**	**Estimate**	**Std Err Pred**	**DF**	**t Value**	**Pr > |t|**	**Alpha**	**Lower**	**Upper**
**Animal**	1	0.08391	0.4552	48	0.18	0.8545	0.05	-0.8313	0.9991
**Animal**	2	0.4739	0.4773	48	0.99	0.3257	0.05	-0.4858	1.4336
**Animal**	3	-0.5578	0.4715	48	-1.18	0.2426	0.05	-1.5059	0.3903
**Animal**	4	-0.04641	0.4598	48	-0.10	0.9200	0.05	-0.9709	0.8781
**Animal**	5	0.4573	0.4521	48	1.01	0.3169	0.05	-0.4517	1.3663
**Animal**	6	-0.4109	0.4685	48	-0.88	0.3849	0.05	-1.3528	0.5311

Type 3 Tests of Fixed Effects				
**Effect**	**Num DF**	**Den DF**	**F Value**	**Pr > F**
**Genotype**	1	48	0.03	0.8611

Least Squares Means									
**Effect**	**Genotype**	**Estimate**	**Standard Error**	**DF**	**t Value**	**Pr > |t|**	**Alpha**	**Lower**	**Upper**
**Genotype**	Meg +/+	2.9604	0.4475	48	6.62	<.0001	0.05	2.0606	3.8602
**Genotype**	Meg +/-	2.8502	0.4380	48	6.51	<.0001	0.05	1.9696	3.7308

DistSoma=30

Differences of Least Squares Means												
**Effect**	**Genotype**	**Genotype**	**Estimate**	**Standard Error**	**DF**	**t Value**	**Pr > |t|**	**Adjustment**	**Adj P**	**Alpha**	**Lower**	**Upper**
**Genotype**	Meg +/+	Meg +/-	0.1102	0.6262	48	0.18	0.8611	Tukey-Kramer	0.8611	0.05	-1.1488	1.3691

Differences of Least Squares Means				
**Effect**	**Genotype**	**Genotype**	**Adj Lower**	**Adj Upper**
**Genotype**	Meg +/+	Meg +/-	-1.1488	1.3691

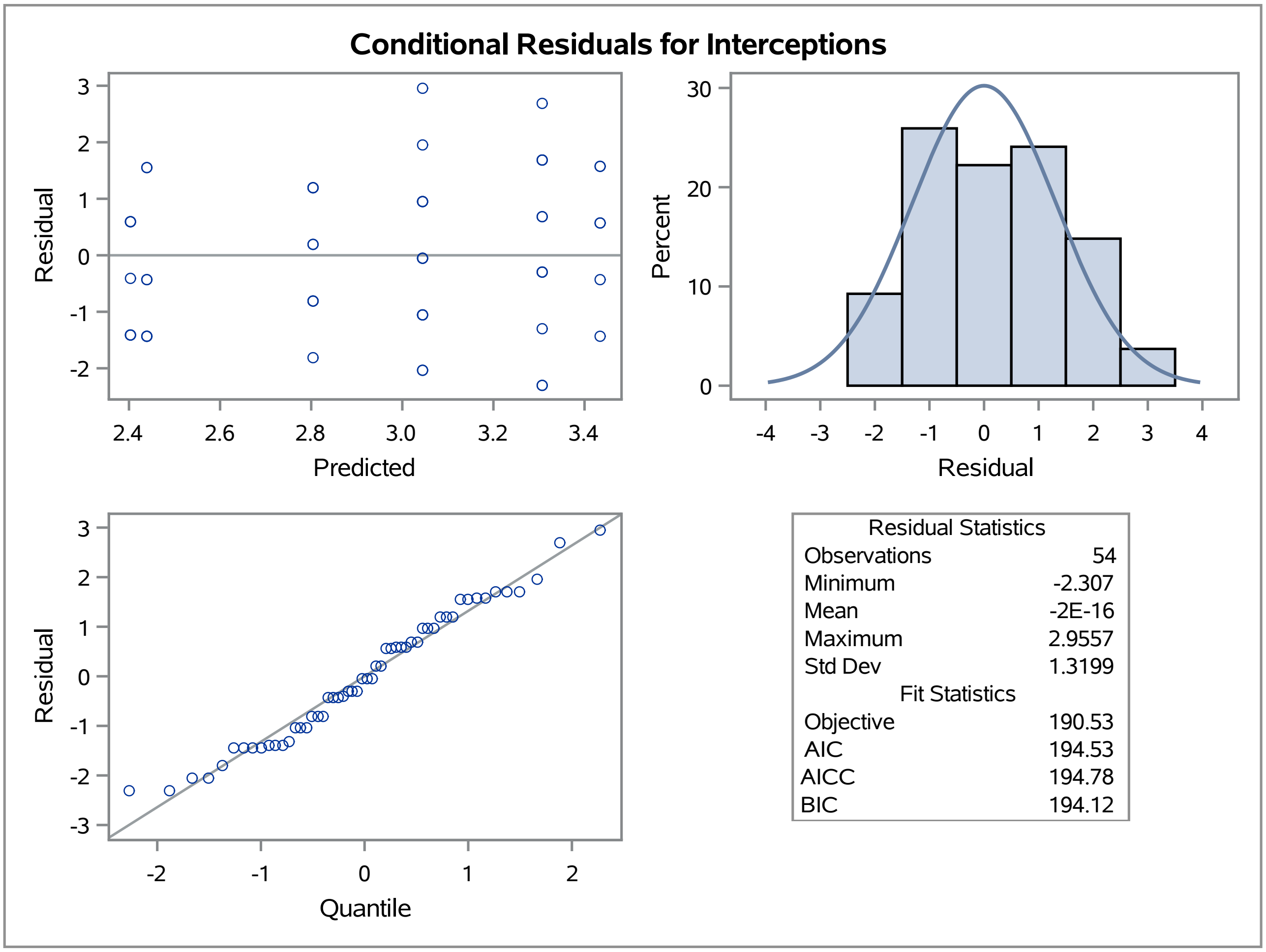

DistSoma=36

Model Information	
**Data Set**	WORK.TEMPDATASORTED
**Dependent Variable**	Interceptions
**Covariance Structure**	Variance Components
**Estimation Method**	REML
**Residual Variance Method**	Profile
**Fixed Effects SE Method**	Model-Based
**Degrees of Freedom Method**	Containment

Class Level Information		
**Class**	**Levels**	**Values**
**Genotype**	2	Meg +/+ Meg +/-
**Animal**	6	1 2 3 4 5 6

Dimensions	
**Covariance Parameters**	2
**Columns in X**	3
**Columns in Z**	6
**Subjects**	1
**Max Obs per Subject**	54

Number of Observations	
**Number of Observations Read**	54
**Number of Observations Used**	54
**Number of Observations Not Used**	0

Iteration History			
**Iteration**	**Evaluations**	**-2 Res Log Like**	**Criterion**
**0**	1	193.99591588	
**1**	2	190.91716299	0.00000013
**2**	1	190.91715693	0.00000000

Convergence criteria met.

Covariance Parameter Estimates				
**Cov Parm**	**Estimate**	**Alpha**	**Lower**	**Upper**
**Animal**	0.4431	0.05	0.1130	28.2142
**Residual**	1.8622	0.05	1.2948	2.9071

DistSoma=36

Fit Statistics	
-2 Res Log Likelihood	190.9
AIC (Smaller is Better)	194.9
AICC (Smaller is Better)	195.2
BIC (Smaller is Better)	194.5

Solution for Fixed Effects

Effect	Genotype	Estimate	Standard Error	DF	t Value	Pr > |t|	Alpha	Lower	Upper
Intercept		3.1673	0.4652	4	6.81	0.0024	0.05	1.8756	4.4590
Genotype	Meg +/+	0.2179	0.6645	48	0.33	0.7444	0.05	-1.1181	1.5539
Genotype	Meg +/-	0	.	.	.	.	.	.	.

Solution for Random Effects

Effect	Animal	Estimate	Std Err Pred	DF	t Value	Pr > |t|	Alpha	Lower	Upper
Animal	1	0.05768	0.4866	48	0.12	0.9061	0.05	-0.9207	1.0361
Animal	2	0.3615	0.5102	48	0.71	0.4820	0.05	-0.6644	1.3875
Animal	3	-0.4192	0.5041	48	-0.83	0.4097	0.05	-1.4328	0.5943
Animal	4	-0.03830	0.4915	48	-0.08	0.9382	0.05	-1.0266	0.9500
Animal	5	0.6784	0.4834	48	1.40	0.1669	0.05	-0.2935	1.6503
Animal	6	-0.6401	0.5007	48	-1.28	0.2073	0.05	-1.6469	0.3667

Type 3 Tests of Fixed Effects

Effect	Num DF	Den DF	F Value	Pr > F
Genotype	1	48	0.11	0.7444

Least Squares Means

Effect	Genotype	Estimate	Standard Error	DF	t Value	Pr > |t|	Alpha	Lower	Upper
Genotype	Meg +/+	3.3852	0.4744	48	7.14	<.0001	0.05	2.4313	4.3391
Genotype	Meg +/-	3.1673	0.4652	48	6.81	<.0001	0.05	2.2319	4.1027

DistSoma=36

Differences of Least Squares Means

Effect	Genotype	Genotype	Estimate	Standard Error	DF	t Value	Pr > |t|	Adjustment	Adj P	Alpha	Lower	Upper
**Genotype**	Meg +/+	Meg +/-	0.2179	0.6645	48	0.33	0.7444	Tukey-Kramer	0.7444	0.05	-1.1181	1.5539

Differences of Least Squares Means

Effect	Genotype	Genotype	Adj Lower	Adj Upper
**Genotype**	Meg +/+	Meg +/-	-1.1180	1.5539

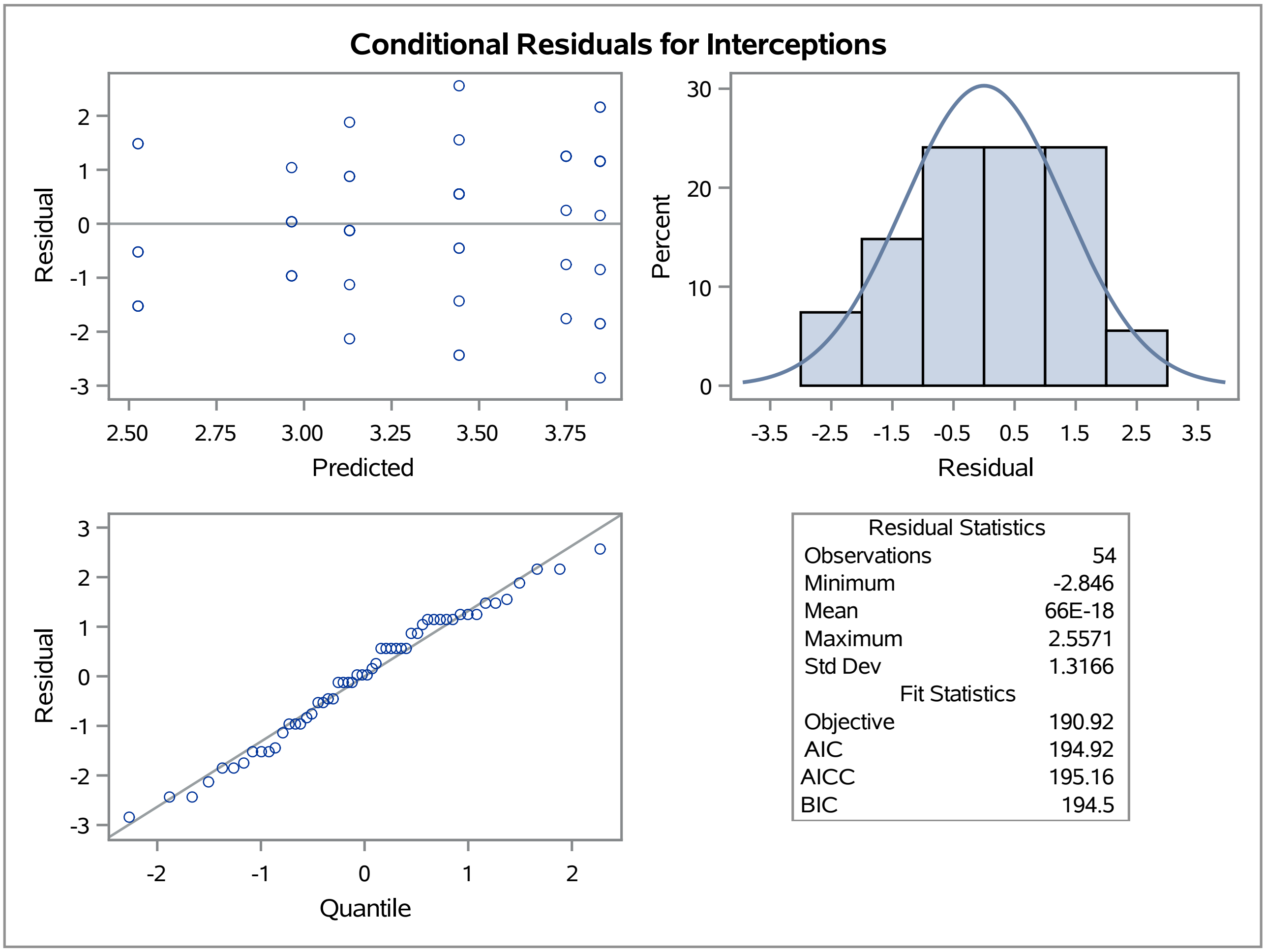

DistSoma=42

Model Information	
Data Set	WORK.TEMPDATASORTED
Dependent Variable	Interceptions
Covariance Structure	Variance Components
Estimation Method	REML
Residual Variance Method	Profile
Fixed Effects SE Method	Model-Based
Degrees of Freedom Method	Containment

Class Level Information		
Class	Levels	Values
Genotype	2	Meg +/+ Meg +/-
Animal	6	1 2 3 4 5 6

Dimensions	
Covariance Parameters	2
Columns in X	3
Columns in Z	6
Subjects	1
Max Obs per Subject	54

Number of Observations	
Number of Observations Read	54
Number of Observations Used	54
Number of Observations Not Used	0

Iteration History			
Iteration	Evaluations	-2 Res Log Like	Criterion
0	1	198.93927353	
1	2	197.08381918	0.00000001

Convergence criteria met.

Covariance Parameter Estimates				
Cov Parm	Estimate	Alpha	Lower	Upper
Animal	0.3449	0.05	0.07809	68.7427
Residual	2.1359	0.05	1.4856	3.3327

DistSoma=42

Fit Statistics	
-2 Res Log Likelihood	197.1
AIC (Smaller is Better)	201.1
AICC (Smaller is Better)	201.3
BIC (Smaller is Better)	200.7

Solution for Fixed Effects

Effect	Genotype	Estimate	Standard Error	DF	t Value	Pr > |t|	Alpha	Lower	Upper
Intercept		3.5074	0.4399	4	7.97	0.0013	0.05	2.2861	4.7287
Genotype	Meg +/+	0.07548	0.6296	48	0.12	0.9051	0.05	-1.1904	1.3413
Genotype	Meg +/-	0	.	.	.	.	.	.	.

Solution for Random Effects

Effect	Animal	Estimate	Std Err Pred	DF	t Value	Pr > |t|	Alpha	Lower	Upper
Animal	1	0.02201	0.4521	48	0.05	0.9614	0.05	-0.8869	0.9309
Animal	2	0.2873	0.4736	48	0.61	0.5470	0.05	-0.6650	1.2395
Animal	3	-0.3093	0.4680	48	-0.66	0.5119	0.05	-1.2502	0.6317
Animal	4	-0.1031	0.4566	48	-0.23	0.8223	0.05	-1.0212	0.8149
Animal	5	0.5997	0.4490	48	1.34	0.1879	0.05	-0.3030	1.5025
Animal	6	-0.4966	0.4652	48	-1.07	0.2910	0.05	-1.4319	0.4386

Type 3 Tests of Fixed Effects

Effect	Num DF	Den DF	F Value	Pr > F
Genotype	1	48	0.01	0.9051

Least Squares Means

Effect	Genotype	Estimate	Standard Error	DF	t Value	Pr > |t|	Alpha	Lower	Upper
Genotype	Meg +/+	3.5829	0.4504	48	7.95	<.0001	0.05	2.6773	4.4885
Genotype	Meg +/-	3.5074	0.4399	48	7.97	<.0001	0.05	2.6230	4.3918

DistSoma=42

Differences of Least Squares Means												
**Effect**	**Genotype**	**Genotype**	**Estimate**	**Standard Error**	**DF**	**t Value**	**Pr > |t|**	**Adjustment**	**Adj P**	**Alpha**	**Lower**	**Upper**
**Genotype**	Meg +/+	Meg +/-	0.07548	0.6296	48	0.12	0.9051	Tukey-Kramer	0.9051	0.05	-1.1904	1.3413

Differences of Least Squares Means				
**Effect**	**Genotype**	**Genotype**	**Adj Lower**	**Adj Upper**
**Genotype**	Meg +/+	Meg +/-	-1.1903	1.3413

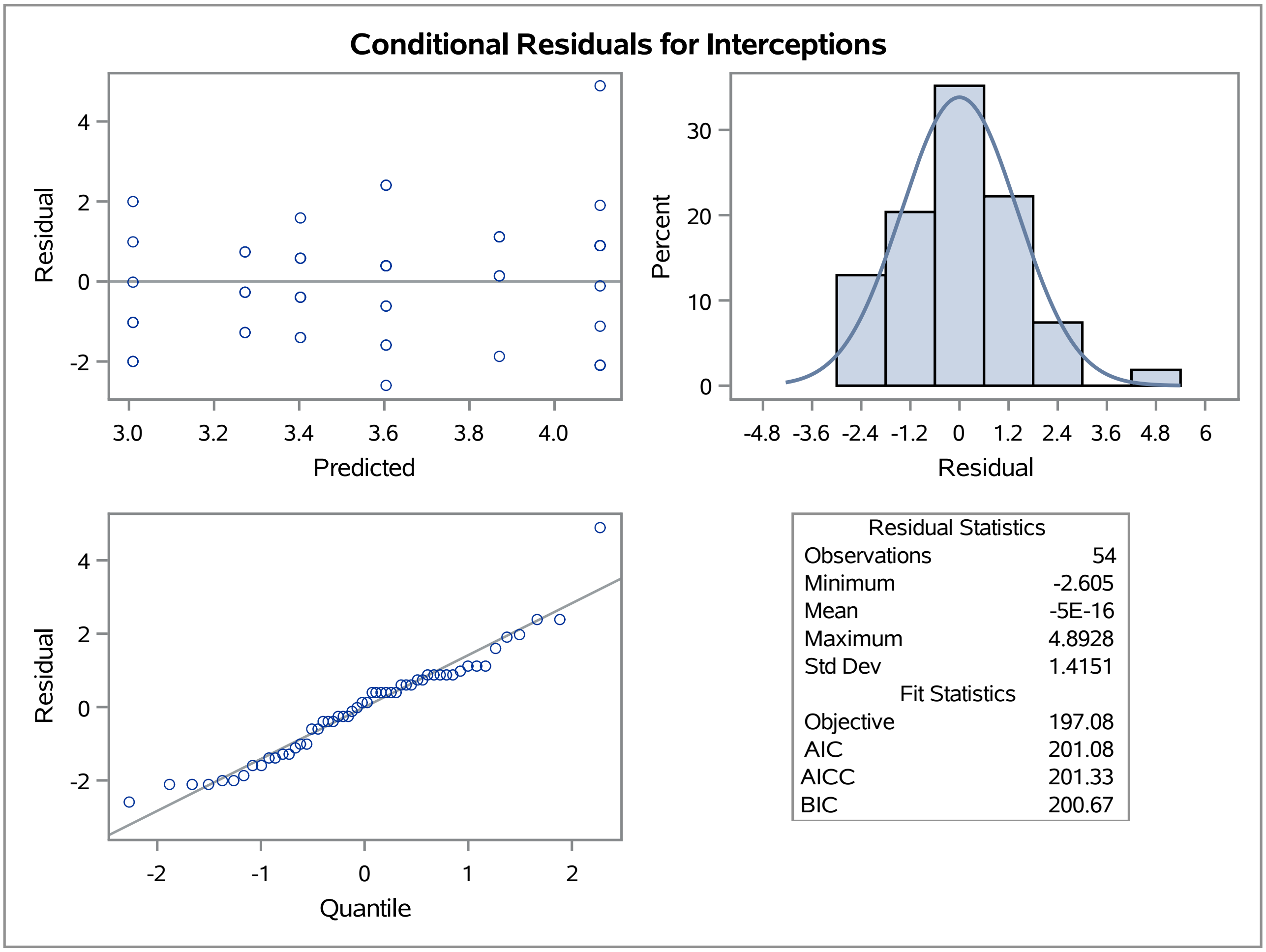

DistSoma=48

Model Information	
**Data Set**	WORK.TEMPDATASORTED
**Dependent Variable**	Interceptions
**Covariance Structure**	Variance Components
**Estimation Method**	REML
**Residual Variance Method**	Profile
**Fixed Effects SE Method**	Model-Based
**Degrees of Freedom Method**	Containment

Class Level Information		
**Class**	**Levels**	**Values**
**Genotype**	2	Meg +/+ Meg +/-
**Animal**	6	1 2 3 4 5 6

Dimensions	
**Covariance Parameters**	2
**Columns in X**	3
**Columns in Z**	6
**Subjects**	1
**Max Obs per Subject**	54

Number of Observations	
**Number of Observations Read**	54
**Number of Observations Used**	54
**Number of Observations Not Used**	0

Iteration History			
**Iteration**	**Evaluations**	**-2 Res Log Like**	**Criterion**
**0**	1	203.81500636	
**1**	3	198.63501104	0.00002918
**2**	1	198.63345592	0.00000008
**3**	1	198.63345159	0.00000000

Convergence criteria met.

DistSoma=48

Covariance Parameter Estimates				
Cov Parm	Estimate	Alpha	Lower	Upper
Animal	0.6820	0.05	0.1922	20.4870
Residual	2.1257	0.05	1.4789	3.3153

Fit Statistics	
-2 Res Log Likelihood	198.6
AIC (Smaller is Better)	202.6
AICC (Smaller is Better)	202.9
BIC (Smaller is Better)	202.2

Solution for Fixed Effects									
Effect	Genotype	Estimate	Standard Error	DF	t Value	Pr > |t|	Alpha	Lower	Upper
Intercept		3.8015	0.5532	4	6.87	0.0023	0.05	2.2656	5.3375
Genotype	Meg +/+	-0.1866	0.7888	48	-0.24	0.8140	0.05	-1.7727	1.3995
Genotype	Meg +/-	0	.	.	.	.	.	.	.

Solution for Random Effects									
Effect	Animal	Estimate	Std Err Pred	DF	t Value	Pr > |t|	Alpha	Lower	Upper
Animal	1	0.06240	0.5811	48	0.11	0.9149	0.05	-1.1059	1.2307
Animal	2	0.3631	0.6084	48	0.60	0.5534	0.05	-0.8601	1.5863
Animal	3	-0.4255	0.6013	48	-0.71	0.4826	0.05	-1.6344	0.7834
Animal	4	-0.2652	0.5868	48	-0.45	0.6533	0.05	-1.4450	0.9145
Animal	5	1.0175	0.5775	48	1.76	0.0844	0.05	-0.1436	2.1786
Animal	6	-0.7523	0.5972	48	-1.26	0.2139	0.05	-1.9530	0.4484

Type 3 Tests of Fixed Effects				
Effect	Num DF	Den DF	F Value	Pr > F
Genotype	1	48	0.06	0.8140

Least Squares Means									
Effect	Genotype	Estimate	Standard Error	DF	t Value	Pr > |t|	Alpha	Lower	Upper
Genotype	Meg +/+	3.6150	0.5623	48	6.43	<.0001	0.05	2.4843	4.7456
Genotype	Meg +/-	3.8015	0.5532	48	6.87	<.0001	0.05	2.6892	4.9138

DistSoma=48

Differences of Least Squares Means												
**Effect**	**Genotype**	**Genotype**	**Estimate**	**Standard Error**	**DF**	**t Value**	**Pr > |t|**	**Adjustment**	**Adj P**	**Alpha**	**Lower**	**Upper**
**Genotype**	Meg +/+	Meg +/-	-0.1866	0.7888	48	-0.24	0.8140	Tukey-Kramer	0.8140	0.05	-1.7727	1.3995

Differences of Least Squares Means				
**Effect**	**Genotype**	**Genotype**	**Adj Lower**	**Adj Upper**
**Genotype**	Meg +/+	Meg +/-	-1.7726	1.3994

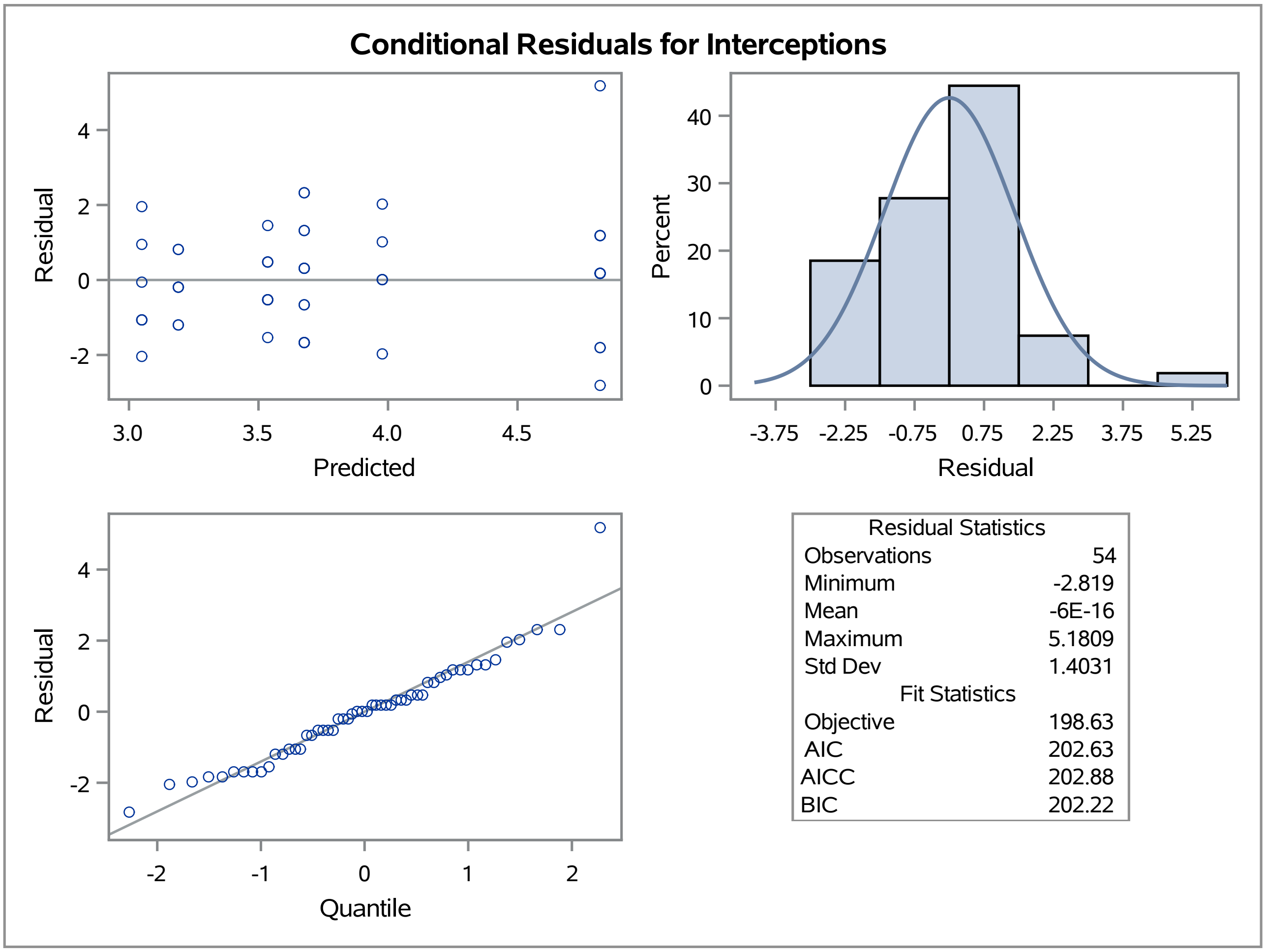

DistSoma=54

Model Information	
**Data Set**	WORK.TEMPDATASORTED
**Dependent Variable**	Interceptions
**Covariance Structure**	Variance Components
**Estimation Method**	REML
**Residual Variance Method**	Profile
**Fixed Effects SE Method**	Model-Based
**Degrees of Freedom Method**	Containment

Class Level Information		
**Class**	**Levels**	**Values**
**Genotype**	2	Meg +/+ Meg +/-
**Animal**	6	1 2 3 4 5 6

Dimensions	
**Covariance Parameters**	2
**Columns in X**	3
**Columns in Z**	6
**Subjects**	1
**Max Obs per Subject**	54

Number of Observations	
**Number of Observations Read**	54
**Number of Observations Used**	54
**Number of Observations Not Used**	0

Iteration History			
**Iteration**	**Evaluations**	**-2 Res Log Like**	**Criterion**
**0**	1	208.51647861	
**1**	3	203.17688870	0.00051559
**2**	1	203.14584843	0.00002250
**3**	1	203.14460106	0.00000005
**4**	1	203.14459825	0.00000000

Convergence criteria met.

DistSoma=54

Covariance Parameter Estimates				
**Cov Parm**	**Estimate**	**Alpha**	**Lower**	**Upper**
**Animal**	0.7238	0.05	0.2055	20.6696
**Residual**	2.3220	0.05	1.6163	3.6193

Fit Statistics	
**-2 Res Log Likelihood**	203.1
**AIC (Smaller is Better)**	207.1
**AICC (Smaller is Better)**	207.4
**BIC (Smaller is Better)**	206.7

Solution for Fixed Effects									
**Effect**	**Genotype**	**Estimate**	**Standard Error**	**DF**	**t Value**	**Pr > |t|**	**Alpha**	**Lower**	**Upper**
**Intercept**		4.0769	0.5720	4	7.13	0.0020	0.05	2.4887	5.6652
**Genotype**	Meg +/+	-0.2093	0.8158	48	-0.26	0.7986	0.05	-1.8497	1.4310
**Genotype**	Meg +/-	0	.	.	.	.	.	.	.

Solution for Random Effects									
**Effect**	**Animal**	**Estimate**	**Std Err Pred**	**DF**	**t Value**	**Pr > |t|**	**Alpha**	**Lower**	**Upper**
**Animal**	1	0.1062	0.6008	48	0.18	0.8604	0.05	-1.1017	1.3141
**Animal**	2	0.1949	0.6292	48	0.31	0.7581	0.05	-1.0701	1.4599
**Animal**	3	-0.3011	0.6217	48	-0.48	0.6304	0.05	-1.5512	0.9490
**Animal**	4	-0.3844	0.6067	48	-0.63	0.5294	0.05	-1.6042	0.8354
**Animal**	5	1.1229	0.5970	48	1.88	0.0661	0.05	-0.07750	2.3233
**Animal**	6	-0.7385	0.6175	48	-1.20	0.2376	0.05	-1.9801	0.5031

Type 3 Tests of Fixed Effects				
**Effect**	**Num DF**	**Den DF**	**F Value**	**Pr > F**
**Genotype**	1	48	0.07	0.7986

Least Squares Means									
**Effect**	**Genotype**	**Estimate**	**Standard Error**	**DF**	**t Value**	**Pr > |t|**	**Alpha**	**Lower**	**Upper**
**Genotype**	Meg +/+	3.8676	0.5817	48	6.65	<.0001	0.05	2.6981	5.0371
**Genotype**	Meg +/-	4.0769	0.5720	48	7.13	<.0001	0.05	2.9268	5.2271

DistSoma=54

Differences of Least Squares Means												
Effect	Genotype	Genotype	Estimate	Standard Error	DF	t Value	Pr > |t|	Adjustment	Adj P	Alpha	Lower	Upper
**Genotype**	Meg +/+	Meg +/-	-0.2093	0.8158	48	-0.26	0.7986	Tukey-Kramer	0.7986	0.05	-1.8497	1.4310

Differences of Least Squares Means				
Effect	Genotype	Genotype	Adj Lower	Adj Upper
**Genotype**	Meg +/+	Meg +/-	-1.8496	1.4309

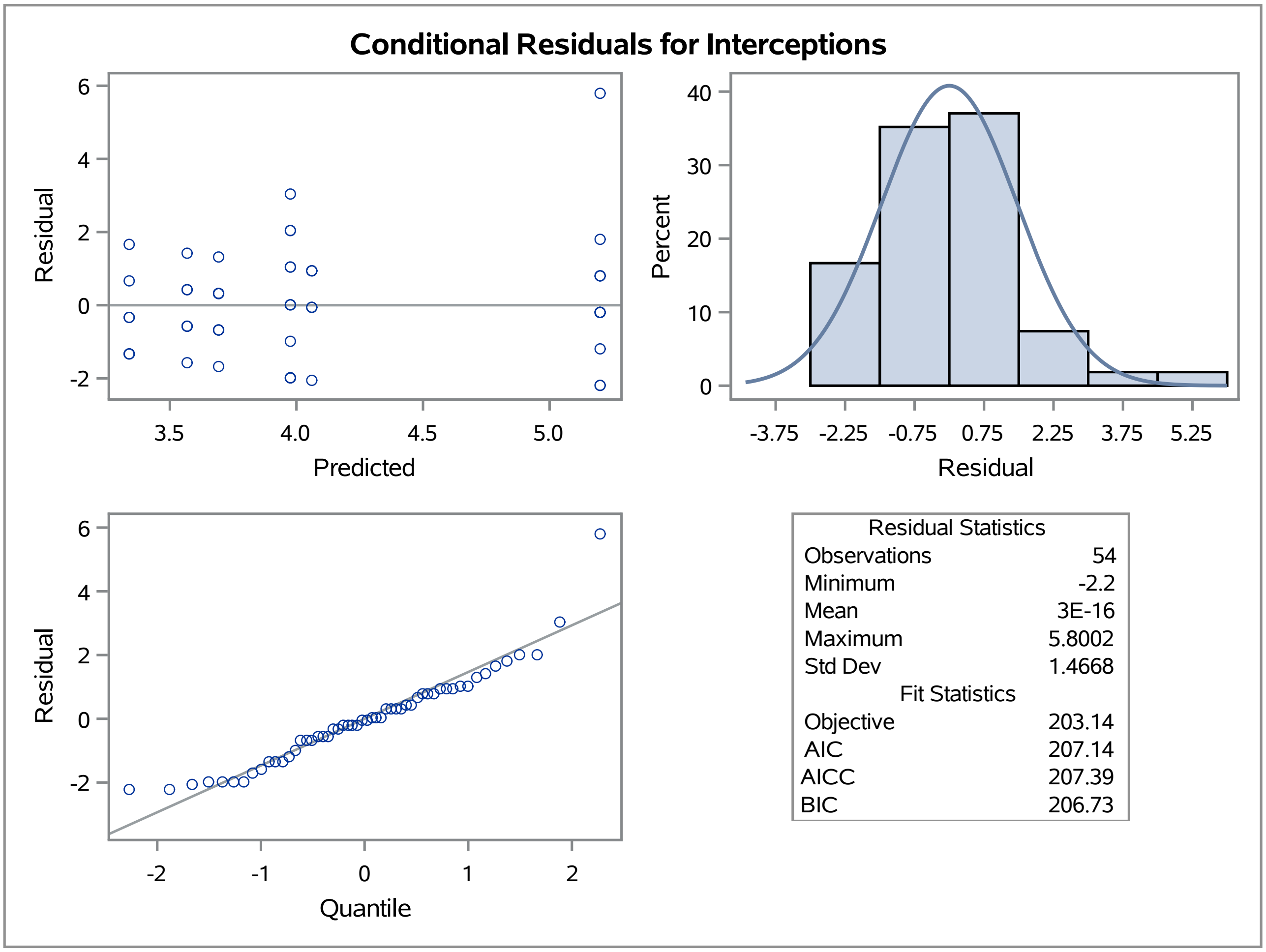

DistSoma=60

Model Information	
**Data Set**	WORK.TEMPDATASORTED
**Dependent Variable**	Interceptions
**Covariance Structure**	Variance Components
**Estimation Method**	REML
**Residual Variance Method**	Profile
**Fixed Effects SE Method**	Model-Based
**Degrees of Freedom Method**	Containment

Class Level Information		
**Class**	**Levels**	**Values**
**Genotype**	2	Meg +/+ Meg +/-
**Animal**	6	1 2 3 4 5 6

Dimensions	
**Covariance Parameters**	2
**Columns in X**	3
**Columns in Z**	6
**Subjects**	1
**Max Obs per Subject**	54

Number of Observations	
**Number of Observations Read**	54
**Number of Observations Used**	54
**Number of Observations Not Used**	0

Iteration History			
**Iteration**	**Evaluations**	**-2 Res Log Like**	**Criterion**
**0**	1	208.91386413	
**1**	3	205.02535292	0.00051526
**2**	1	204.99377441	0.00002288
**3**	1	204.99248302	0.00000006
**4**	1	204.99248001	0.00000000

Convergence criteria met.

DistSoma=60

Covariance Parameter Estimates				
**Cov Parm**	**Estimate**	**Alpha**	**Lower**	**Upper**
**Animal**	0.5924	0.05	0.1588	25.3738
**Residual**	2.4385	0.05	1.6976	3.7995

Fit Statistics	
**-2 Res Log Likelihood**	205.0
**AIC (Smaller is Better)**	209.0
**AICC (Smaller is Better)**	209.2
**BIC (Smaller is Better)**	208.6

Solution for Fixed Effects									
**Effect**	**Genotype**	**Estimate**	**Standard Error**	**DF**	**t Value**	**Pr > |t|**	**Alpha**	**Lower**	**Upper**
**Intercept**		4.3603	0.5362	4	8.13	0.0012	0.05	2.8716	5.8490
**Genotype**	Meg +/+	-0.1632	0.7657	48	-0.21	0.8321	0.05	-1.7028	1.3764
**Genotype**	Meg +/-	0	.	.	.	.	.	.	.

Solution for Random Effects									
**Effect**	**Animal**	**Estimate**	**Std Err Pred**	**DF**	**t Value**	**Pr > |t|**	**Alpha**	**Lower**	**Upper**
**Animal**	1	0.02556	0.5611	48	0.05	0.9639	0.05	-1.1026	1.1537
**Animal**	2	0.2785	0.5884	48	0.47	0.6381	0.05	-0.9045	1.4614
**Animal**	3	-0.3040	0.5812	48	-0.52	0.6033	0.05	-1.4727	0.8646
**Animal**	4	-0.4759	0.5668	48	-0.84	0.4052	0.05	-1.6156	0.6637
**Animal**	5	0.9727	0.5574	48	1.75	0.0874	0.05	-0.1480	2.0934
**Animal**	6	-0.4968	0.5774	48	-0.86	0.3939	0.05	-1.6576	0.6641

Type 3 Tests of Fixed Effects				
**Effect**	**Num DF**	**Den DF**	**F Value**	**Pr > F**
**Genotype**	1	48	0.05	0.8321

Least Squares Means									
**Effect**	**Genotype**	**Estimate**	**Standard Error**	**DF**	**t Value**	**Pr > |t|**	**Alpha**	**Lower**	**Upper**
**Genotype**	Meg +/+	4.1971	0.5467	48	7.68	<.0001	0.05	3.0980	5.2962
**Genotype**	Meg +/-	4.3603	0.5362	48	8.13	<.0001	0.05	3.2822	5.4384

DistSoma=60

Differences of Least Squares Means												
**Effect**	**Genotype**	**Genotype**	**Estimate**	**Standard Error**	**DF**	**t Value**	**Pr > |t|**	**Adjustment**	**Adj P**	**Alpha**	**Lower**	**Upper**
**Genotype**	Meg +/+	Meg +/-	-0.1632	0.7657	48	-0.21	0.8321	Tukey-Kramer	0.8321	0.05	-1.7028	1.3764

Differences of Least Squares Means				
**Effect**	**Genotype**	**Genotype**	**Adj Lower**	**Adj Upper**
**Genotype**	Meg +/+	Meg +/-	-1.7027	1.3764

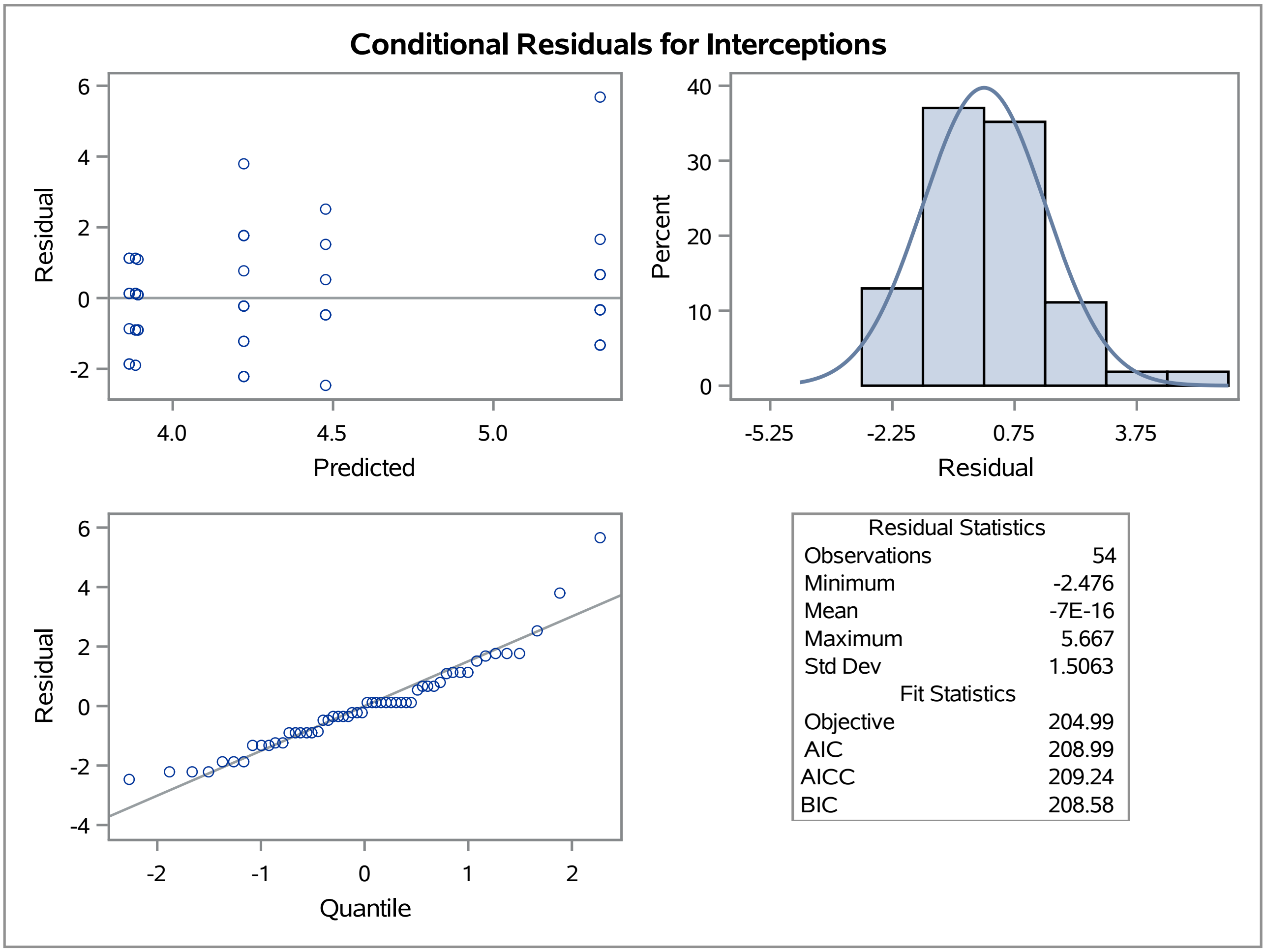

DistSoma=66

Model Information	
**Data Set**	WORK.TEMPDATASORTED
**Dependent Variable**	Interceptions
**Covariance Structure**	Variance Components
**Estimation Method**	REML
**Residual Variance Method**	Profile
**Fixed Effects SE Method**	Model-Based
**Degrees of Freedom Method**	Containment

Class Level Information		
**Class**	**Levels**	**Values**
**Genotype**	2	Meg +/+ Meg +/-
**Animal**	6	1 2 3 4 5 6

Dimensions	
**Covariance Parameters**	2
**Columns in X**	3
**Columns in Z**	6
**Subjects**	1
**Max Obs per Subject**	54

Number of Observations	
**Number of Observations Read**	54
**Number of Observations Used**	54
**Number of Observations Not Used**	0

Iteration History			
**Iteration**	**Evaluations**	**-2 Res Log Like**	**Criterion**
**0**	1	214.07783896	
**1**	5	210.75188853	0.00005389
**2**	1	210.74896660	0.00000036
**3**	1	210.74894560	0.00000000

Convergence criteria met.

DistSoma=66

Covariance Parameter Estimates				
**Cov Parm**	**Estimate**	**Alpha**	**Lower**	**Upper**
**Animal**	0.5693	0.05	0.1481	30.8470
**Residual**	2.7457	0.05	1.9124	4.2751

Fit Statistics	
**-2 Res Log Likelihood**	210.7
**AIC (Smaller is Better)**	214.7
**AICC (Smaller is Better)**	215.0
**BIC (Smaller is Better)**	214.3

Solution for Fixed Effects									
**Effect**	**Genotype**	**Estimate**	**Standard Error**	**DF**	**t Value**	**Pr > |t|**	**Alpha**	**Lower**	**Upper**
**Intercept**		4.6490	0.5394	4	8.62	0.0010	0.05	3.1513	6.1467
**Genotype**	Meg +/+	-0.2569	0.7710	48	-0.33	0.7405	0.05	-1.8071	1.2934
**Genotype**	Meg +/-	0	.	.	.	.	.	.	.

Solution for Random Effects									
**Effect**	**Animal**	**Estimate**	**Std Err Pred**	**DF**	**t Value**	**Pr > |t|**	**Alpha**	**Lower**	**Upper**
**Animal**	1	-0.1738	0.5617	48	-0.31	0.7584	0.05	-1.3032	0.9556
**Animal**	2	0.1522	0.5890	48	0.26	0.7972	0.05	-1.0321	1.3366
**Animal**	3	0.02159	0.5819	48	0.04	0.9706	0.05	-1.1484	1.1916
**Animal**	4	-0.4949	0.5674	48	-0.87	0.3875	0.05	-1.6358	0.6460
**Animal**	5	0.9637	0.5579	48	1.73	0.0905	0.05	-0.1581	2.0855
**Animal**	6	-0.4688	0.5781	48	-0.81	0.4214	0.05	-1.6313	0.6936

Type 3 Tests of Fixed Effects				
**Effect**	**Num DF**	**Den DF**	**F Value**	**Pr > F**
**Genotype**	1	48	0.11	0.7405

Least Squares Means									
**Effect**	**Genotype**	**Estimate**	**Standard Error**	**DF**	**t Value**	**Pr > |t|**	**Alpha**	**Lower**	**Upper**
**Genotype**	Meg +/+	4.3921	0.5509	48	7.97	<.0001	0.05	3.2844	5.4998
**Genotype**	Meg +/-	4.6490	0.5394	48	8.62	<.0001	0.05	3.5644	5.7336

DistSoma=66

Differences of Least Squares Means												
Effect	Genotype	Genotype	Estimate	Standard Error	DF	t Value	Pr > |t|	Adjustment	Adj P	Alpha	Lower	Upper
**Genotype**	Meg +/+	Meg +/-	-0.2569	0.7710	48	-0.33	0.7405	Tukey-Kramer	0.7405	0.05	-1.8071	1.2934

Differences of Least Squares Means				
Effect	Genotype	Genotype	Adj Lower	Adj Upper
**Genotype**	Meg +/+	Meg +/-	-1.8071	1.2933

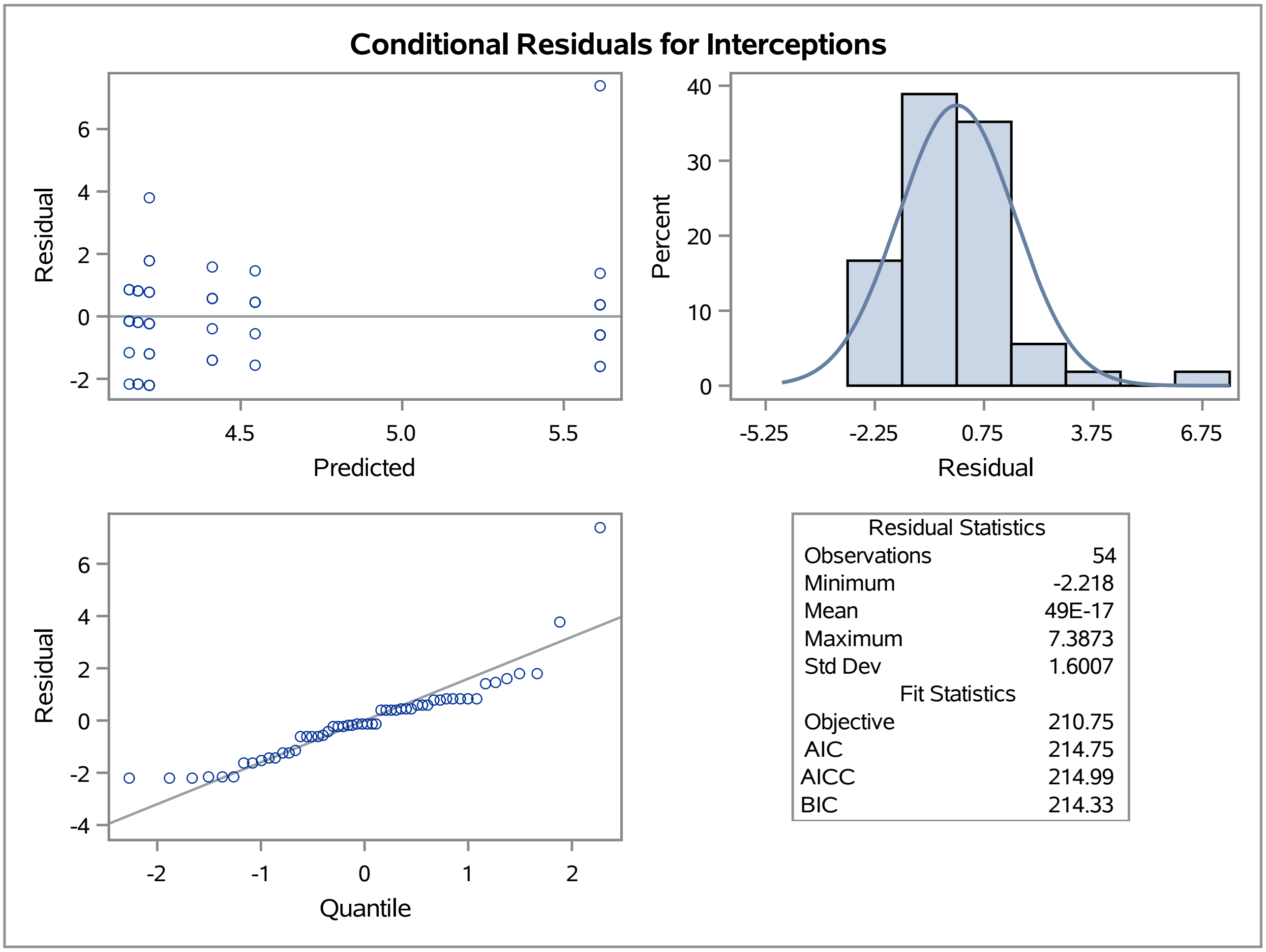

DistSoma=72

Model Information	
**Data Set**	WORK.TEMPDATASORTED
**Dependent Variable**	Interceptions
**Covariance Structure**	Variance Components
**Estimation Method**	REML
**Residual Variance Method**	Profile
**Fixed Effects SE Method**	Model-Based
**Degrees of Freedom Method**	Containment

Class Level Information		
**Class**	**Levels**	**Values**
**Genotype**	2	Meg +/+ Meg +/-
**Animal**	6	1 2 3 4 5 6

Dimensions	
**Covariance Parameters**	2
**Columns in X**	3
**Columns in Z**	6
**Subjects**	1
**Max Obs per Subject**	54

Number of Observations	
**Number of Observations Read**	54
**Number of Observations Used**	54
**Number of Observations Not Used**	0

Iteration History			
**Iteration**	**Evaluations**	**-2 Res Log Like**	**Criterion**
**0**	1	206.18755432	
**1**	5	200.03373957	0.00003599
**2**	1	200.03194357	0.00000014
**3**	1	200.03193601	0.00000000

Convergence criteria met.

DistSoma=72

Covariance Parameter Estimates				
**Cov Parm**	**Estimate**	**Alpha**	**Lower**	**Upper**
**Animal**	0.7350	0.05	0.2135	18.0885
**Residual**	2.1774	0.05	1.5158	3.3930

Fit Statistics	
**-2 Res Log Likelihood**	200.0
**AIC (Smaller is Better)**	204.0
**AICC (Smaller is Better)**	204.3
**BIC (Smaller is Better)**	203.6

Solution for Fixed Effects									
**Effect**	**Genotype**	**Estimate**	**Standard Error**	**DF**	**t Value**	**Pr > |t|**	**Alpha**	**Lower**	**Upper**
**Intercept**		4.8587	0.5707	4	8.51	0.0010	0.05	3.2743	6.4431
**Genotype**	Meg +/+	-0.2940	0.8135	48	-0.36	0.7194	0.05	-1.9296	1.3417
**Genotype**	Meg +/-	0	.	.	.	.	.	.	.

Solution for Random Effects									
**Effect**	**Animal**	**Estimate**	**Std Err Pred**	**DF**	**t Value**	**Pr > |t|**	**Alpha**	**Lower**	**Upper**
**Animal**	1	-0.08408	0.5995	48	-0.14	0.8890	0.05	-1.2894	1.1212
**Animal**	2	0.1798	0.6274	48	0.29	0.7757	0.05	-1.0816	1.4412
**Animal**	3	-0.09570	0.6201	48	-0.15	0.8780	0.05	-1.3425	1.1511
**Animal**	4	-0.4789	0.6053	48	-0.79	0.4327	0.05	-1.6958	0.7381
**Animal**	5	1.1826	0.5958	48	1.98	0.0529	0.05	-0.01530	2.3806
**Animal**	6	-0.7038	0.6159	48	-1.14	0.2588	0.05	-1.9421	0.5346

Type 3 Tests of Fixed Effects				
**Effect**	**Num DF**	**Den DF**	**F Value**	**Pr > F**
**Genotype**	1	48	0.13	0.7194

Least Squares Means									
**Effect**	**Genotype**	**Estimate**	**Standard Error**	**DF**	**t Value**	**Pr > |t|**	**Alpha**	**Lower**	**Upper**
**Genotype**	Meg +/+	4.5648	0.5798	48	7.87	<.0001	0.05	3.3991	5.7305
**Genotype**	Meg +/-	4.8587	0.5707	48	8.51	<.0001	0.05	3.7114	6.0061

DistSoma=72

Differences of Least Squares Means												
**Effect**	**Genotype**	**Genotype**	**Estimate**	**Standard Error**	**DF**	**t Value**	**Pr > |t|**	**Adjustment**	**Adj P**	**Alpha**	**Lower**	**Upper**
**Genotype**	Meg +/+	Meg +/-	-0.2940	0.8135	48	-0.36	0.7194	Tukey-Kramer	0.7194	0.05	-1.9296	1.3417

Differences of Least Squares Means				
**Effect**	**Genotype**	**Genotype**	**Adj Lower**	**Adj Upper**
**Genotype**	Meg +/+	Meg +/-	-1.9296	1.3416

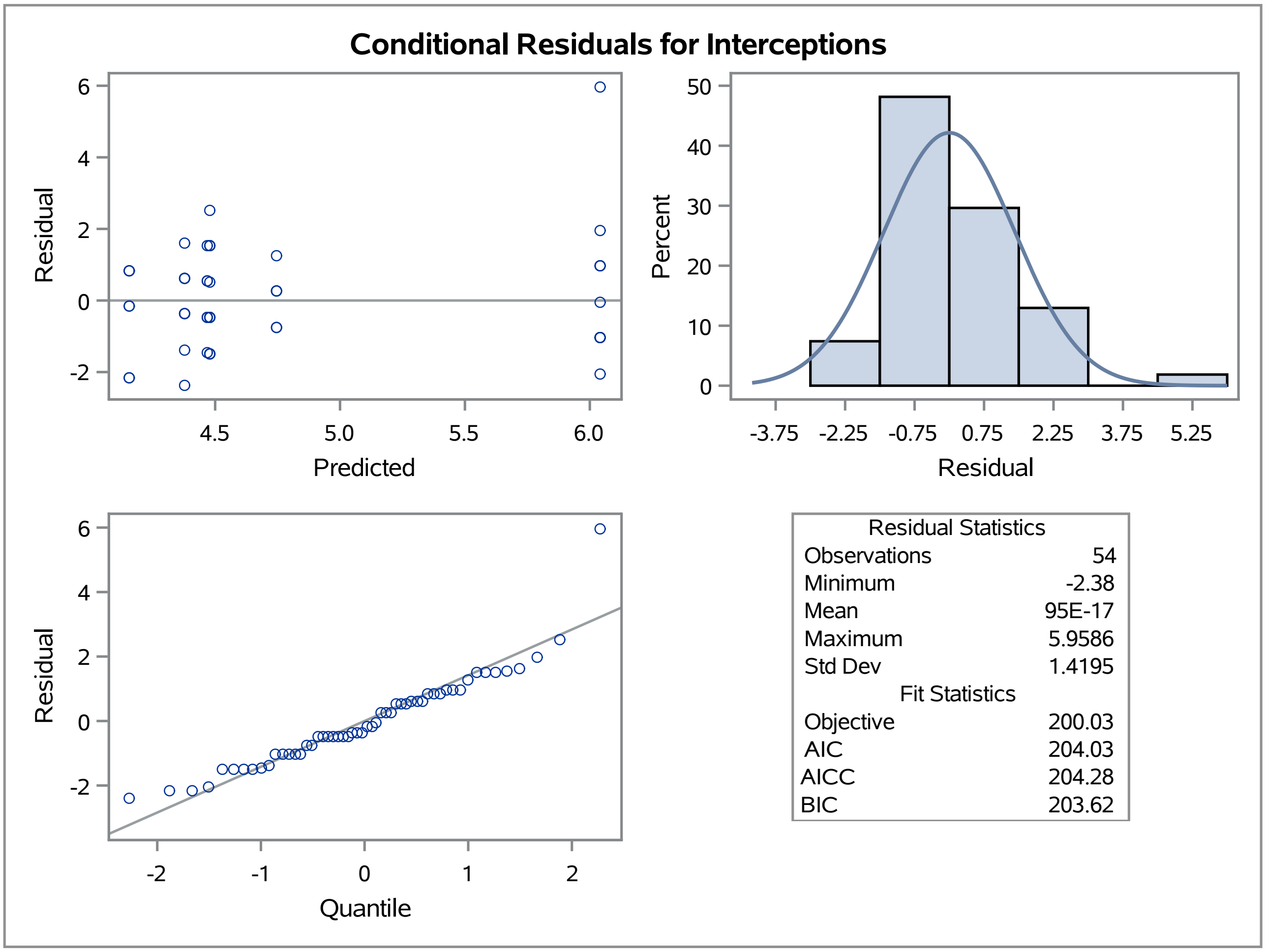

DistSoma=78

Model Information	
**Data Set**	WORK.TEMPDATASORTED
**Dependent Variable**	Interceptions
**Covariance Structure**	Variance Components
**Estimation Method**	REML
**Residual Variance Method**	Profile
**Fixed Effects SE Method**	Model-Based
**Degrees of Freedom Method**	Containment

Class Level Information		
**Class**	**Levels**	**Values**
**Genotype**	2	Meg +/+ Meg +/-
**Animal**	6	1 2 3 4 5 6

Dimensions	
**Covariance Parameters**	2
**Columns in X**	3
**Columns in Z**	6
**Subjects**	1
**Max Obs per Subject**	54

Number of Observations	
**Number of Observations Read**	54
**Number of Observations Used**	54
**Number of Observations Not Used**	0

Iteration History			
**Iteration**	**Evaluations**	**-2 Res Log Like**	**Criterion**
**0**	1	211.39203846	
**1**	3	208.39705493	0.00062777
**2**	1	208.35703108	0.00003388
**3**	1	208.35504743	0.00000012
**4**	1	208.35504049	0.00000000

Convergence criteria met.

DistSoma=78

Covariance Parameter Estimates				
**Cov Parm**	**Estimate**	**Alpha**	**Lower**	**Upper**
**Animal**	0.5212	0.05	0.1329	33.4124
**Residual**	2.6278	0.05	1.8300	4.0925

Fit Statistics	
**-2 Res Log Likelihood**	208.4
**AIC (Smaller is Better)**	212.4
**AICC (Smaller is Better)**	212.6
**BIC (Smaller is Better)**	211.9

Solution for Fixed Effects									
**Effect**	**Genotype**	**Estimate**	**Standard Error**	**DF**	**t Value**	**Pr > |t|**	**Alpha**	**Lower**	**Upper**
**Intercept**		5.2280	0.5202	4	10.05	0.0006	0.05	3.7838	6.6722
**Genotype**	Meg +/+	-0.5242	0.7437	48	-0.70	0.4843	0.05	-2.0194	0.9711
**Genotype**	Meg +/-	0	.	.	.	.	.	.	.

Solution for Random Effects									
**Effect**	**Animal**	**Estimate**	**Std Err Pred**	**DF**	**t Value**	**Pr > |t|**	**Alpha**	**Lower**	**Upper**
**Animal**	1	-0.1746	0.5407	48	-0.32	0.7482	0.05	-1.2616	0.9125
**Animal**	2	0.2515	0.5669	48	0.44	0.6593	0.05	-0.8883	1.3914
**Animal**	3	-0.07695	0.5601	48	-0.14	0.8913	0.05	-1.2031	1.0491
**Animal**	4	-0.4310	0.5462	48	-0.79	0.4339	0.05	-1.5291	0.6671
**Animal**	5	0.8957	0.5370	48	1.67	0.1018	0.05	-0.1840	1.9754
**Animal**	6	-0.4647	0.5565	48	-0.84	0.4078	0.05	-1.5836	0.6541

Type 3 Tests of Fixed Effects				
**Effect**	**Num DF**	**Den DF**	**F Value**	**Pr > F**
**Genotype**	1	48	0.50	0.4843

Least Squares Means									
**Effect**	**Genotype**	**Estimate**	**Standard Error**	**DF**	**t Value**	**Pr > |t|**	**Alpha**	**Lower**	**Upper**
**Genotype**	Meg +/+	4.7038	0.5315	48	8.85	<.0001	0.05	3.6352	5.7724
**Genotype**	Meg +/-	5.2280	0.5202	48	10.05	<.0001	0.05	4.1821	6.2738

DistSoma=78

Differences of Least Squares Means												
Effect	Genotype	Genotype	Estimate	Standard Error	DF	t Value	Pr > |t|	Adjustment	Adj P	Alpha	Lower	Upper
**Genotype**	Meg +/+	Meg +/-	-0.5242	0.7437	48	-0.70	0.4843	Tukey-Kramer	0.4843	0.05	-2.0194	0.9711

Differences of Least Squares Means				
Effect	Genotype	Genotype	Adj Lower	Adj Upper
**Genotype**	Meg +/+	Meg +/-	-2.0194	0.9710

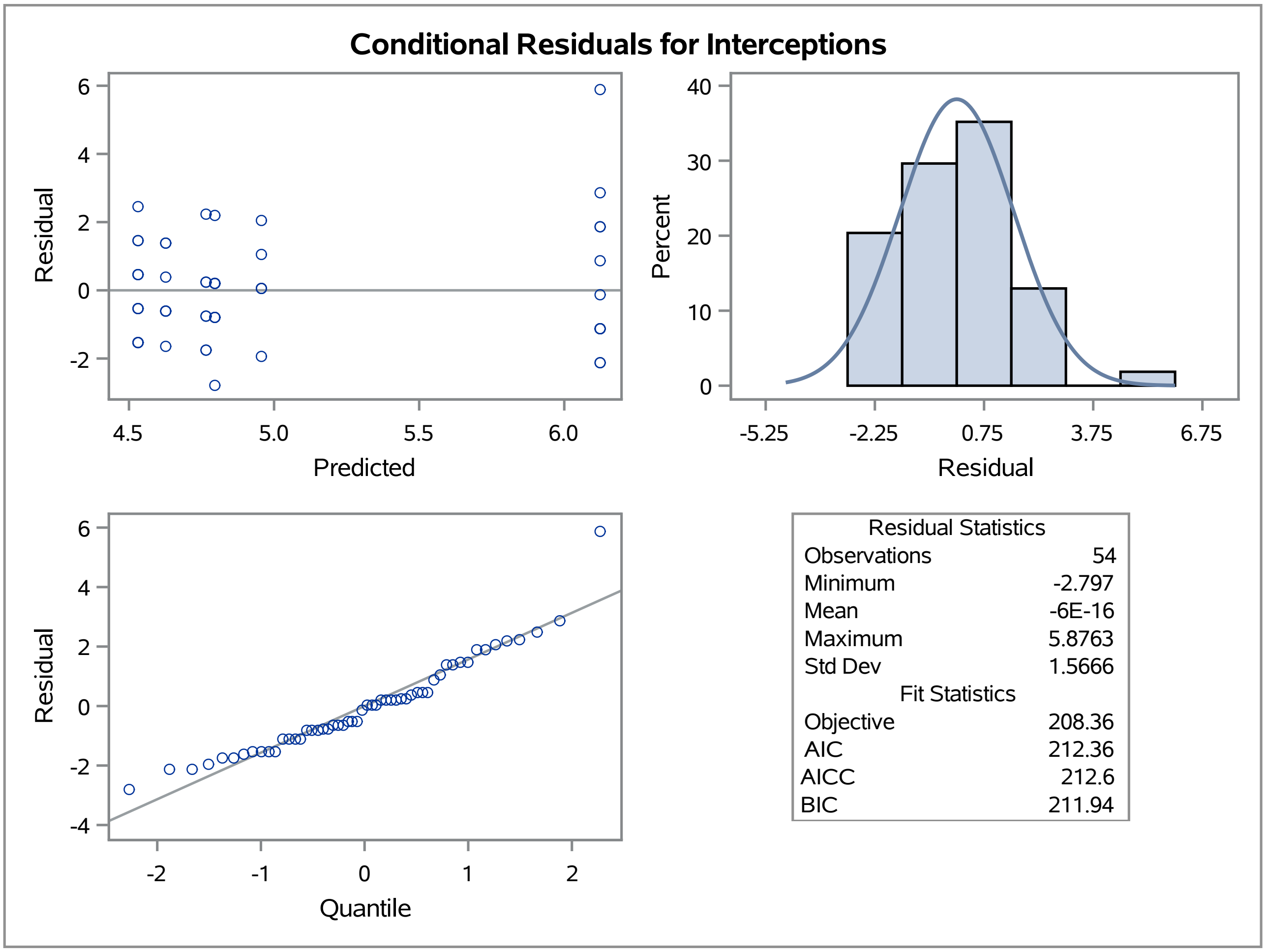

DistSoma=84

Model Information	
**Data Set**	WORK.TEMPDATASORTED
**Dependent Variable**	Interceptions
**Covariance Structure**	Variance Components
**Estimation Method**	REML
**Residual Variance Method**	Profile
**Fixed Effects SE Method**	Model-Based
**Degrees of Freedom Method**	Containment

Class Level Information		
**Class**	**Levels**	**Values**
**Genotype**	2	Meg +/+ Meg +/-
**Animal**	6	1 2 3 4 5 6

Dimensions	
**Covariance Parameters**	2
**Columns in X**	3
**Columns in Z**	6
**Subjects**	1
**Max Obs per Subject**	54

Number of Observations	
**Number of Observations Read**	54
**Number of Observations Used**	54
**Number of Observations Not Used**	0

Iteration History			
**Iteration**	**Evaluations**	**-2 Res Log Like**	**Criterion**
**0**	1	213.02134645	
**1**	3	209.24341977	0.00121727
**2**	1	209.16297136	0.00011332
**3**	1	209.15611235	0.00000130
**4**	1	209.15603780	0.00000000

Convergence criteria met.

DistSoma=84

Covariance Parameter Estimates				
Cov Parm	Estimate	Alpha	Lower	Upper
Animal	0.6044	0.05	0.1621	25.8742
Residual	2.6503	0.05	1.8460	4.1264

Fit Statistics	
-2 Res Log Likelihood	209.2
AIC (Smaller is Better)	213.2
AICC (Smaller is Better)	213.4
BIC (Smaller is Better)	212.7

Solution for Fixed Effects									
Effect	Genotype	Estimate	Standard Error	DF	t Value	Pr > |t|	Alpha	Lower	Upper
Intercept		5.1110	0.5471	4	9.34	0.0007	0.05	3.5922	6.6299
Genotype	Meg +/+	-0.2749	0.7815	48	-0.35	0.7266	0.05	-1.8462	1.2965
Genotype	Meg +/-	0	.	.	.	.	.	.	.

Solution for Random Effects									
Effect	Animal	Estimate	Std Err Pred	DF	t Value	Pr > |t|	Alpha	Lower	Upper
Animal	1	-0.1076	0.5715	48	-0.19	0.8515	0.05	-1.2567	1.0415
Animal	2	0.09466	0.5993	48	0.16	0.8752	0.05	-1.1104	1.2997
Animal	3	0.01290	0.5921	48	0.02	0.9827	0.05	-1.1775	1.2033
Animal	4	-0.5976	0.5773	48	-1.04	0.3058	0.05	-1.7585	0.5632
Animal	5	1.0173	0.5677	48	1.79	0.0795	0.05	-0.1242	2.1587
Animal	6	-0.4196	0.5882	48	-0.71	0.4790	0.05	-1.6022	0.7630

Type 3 Tests of Fixed Effects				
Effect	Num DF	Den DF	F Value	Pr > F
Genotype	1	48	0.12	0.7266

Least Squares Means									
Effect	Genotype	Estimate	Standard Error	DF	t Value	Pr > |t|	Alpha	Lower	Upper
Genotype	Meg +/+	4.8362	0.5581	48	8.67	<.0001	0.05	3.7140	5.9583
Genotype	Meg +/-	5.1110	0.5471	48	9.34	<.0001	0.05	4.0111	6.2110

DistSoma=84

Differences of Least Squares Means												
**Effect**	**Genotype**	**Genotype**	**Estimate**	**Standard Error**	**DF**	**t Value**	**Pr > |t|**	**Adjustment**	**Adj P**	**Alpha**	**Lower**	**Upper**
**Genotype**	Meg +/+	Meg +/-	-0.2749	0.7815	48	-0.35	0.7266	Tukey-Kramer	0.7266	0.05	-1.8462	1.2965

Differences of Least Squares Means				
**Effect**	**Genotype**	**Genotype**	**Adj Lower**	**Adj Upper**
**Genotype**	Meg +/+	Meg +/-	-1.8462	1.2964

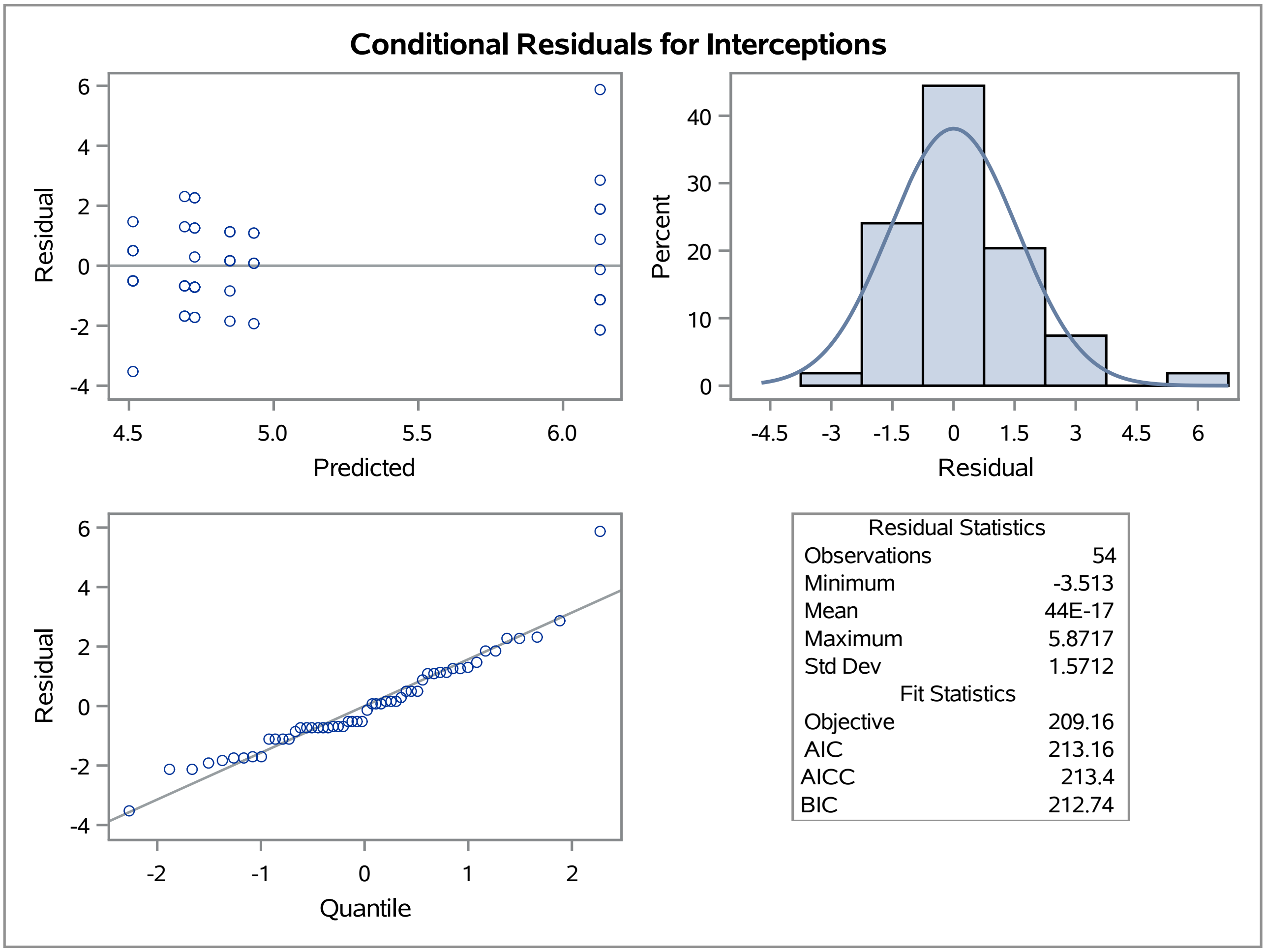

Residual Statistics	
Observations	54
Minimum	-3.513
Mean	44E-17
Maximum	5.8717
Std Dev	1.5712
Fit Statistics	
Objective	209.16
AIC	213.16
AICC	213.4
BIC	212.74

DistSoma=90

Model Information	
**Data Set**	WORK.TEMPDATASORTED
**Dependent Variable**	Interceptions
**Covariance Structure**	Variance Components
**Estimation Method**	REML
**Residual Variance Method**	Profile
**Fixed Effects SE Method**	Model-Based
**Degrees of Freedom Method**	Containment

Class Level Information		
**Class**	**Levels**	**Values**
**Genotype**	2	Meg +/+ Meg +/-
**Animal**	6	1 2 3 4 5 6

Dimensions	
**Covariance Parameters**	2
**Columns in X**	3
**Columns in Z**	6
**Subjects**	1
**Max Obs per Subject**	54

Number of Observations	
**Number of Observations Read**	54
**Number of Observations Used**	54
**Number of Observations Not Used**	0

Iteration History			
**Iteration**	**Evaluations**	**-2 Res Log Like**	**Criterion**
**0**	1	198.54646695	
**1**	3	194.09965353	0.00162242
**2**	1	194.00610848	0.00016929
**3**	1	193.99716452	0.00000248
**4**	1	193.99704122	0.00000000

Convergence criteria met.

DistSoma=90

Covariance Parameter Estimates				
**Cov Parm**	**Estimate**	**Alpha**	**Lower**	**Upper**
**Animal**	0.5114	0.05	0.1413	17.5986
**Residual**	1.9667	0.05	1.3696	3.0630

Fit Statistics	
**-2 Res Log Likelihood**	194.0
**AIC (Smaller is Better)**	198.0
**AICC (Smaller is Better)**	198.2
**BIC (Smaller is Better)**	197.6

Solution for Fixed Effects									
**Effect**	**Genotype**	**Estimate**	**Standard Error**	**DF**	**t Value**	**Pr > |t|**	**Alpha**	**Lower**	**Upper**
**Intercept**		5.0827	0.4931	4	10.31	0.0005	0.05	3.7137	6.4517
**Genotype**	Meg +/+	-0.1952	0.7039	48	-0.28	0.7827	0.05	-1.6105	1.2201
**Genotype**	Meg +/-	0	.	.	.	.	.	.	.

Solution for Random Effects									
**Effect**	**Animal**	**Estimate**	**Std Err Pred**	**DF**	**t Value**	**Pr > |t|**	**Alpha**	**Lower**	**Upper**
**Animal**	1	-0.1506	0.5167	48	-0.29	0.7720	0.05	-1.1896	0.8884
**Animal**	2	0.1702	0.5417	48	0.31	0.7548	0.05	-0.9190	1.2593
**Animal**	3	-0.01956	0.5352	48	-0.04	0.9710	0.05	-1.0957	1.0565
**Animal**	4	-0.5250	0.5220	48	-1.01	0.3196	0.05	-1.5744	0.5245
**Animal**	5	0.9471	0.5134	48	1.84	0.0712	0.05	-0.08507	1.9793
**Animal**	6	-0.4221	0.5316	48	-0.79	0.4310	0.05	-1.4910	0.6467

Type 3 Tests of Fixed Effects				
**Effect**	**Num DF**	**Den DF**	**F Value**	**Pr > F**
**Genotype**	1	48	0.08	0.7827

Least Squares Means									
**Effect**	**Genotype**	**Estimate**	**Standard Error**	**DF**	**t Value**	**Pr > |t|**	**Alpha**	**Lower**	**Upper**
**Genotype**	Meg +/+	4.8874	0.5023	48	9.73	<.0001	0.05	3.8774	5.8975
**Genotype**	Meg +/-	5.0827	0.4931	48	10.31	<.0001	0.05	4.0913	6.0741

DistSoma=90

Differences of Least Squares Means												
**Effect**	**Genotype**	**Genotype**	**Estimate**	**Standard Error**	**DF**	**t Value**	**Pr > |t|**	**Adjustment**	**Adj P**	**Alpha**	**Lower**	**Upper**
**Genotype**	Meg +/+	Meg +/-	-0.1952	0.7039	48	-0.28	0.7827	Tukey-Kramer	0.7827	0.05	-1.6105	1.2201

Differences of Least Squares Means				
**Effect**	**Genotype**	**Genotype**	**Adj Lower**	**Adj Upper**
**Genotype**	Meg +/+	Meg +/-	-1.6104	1.2200

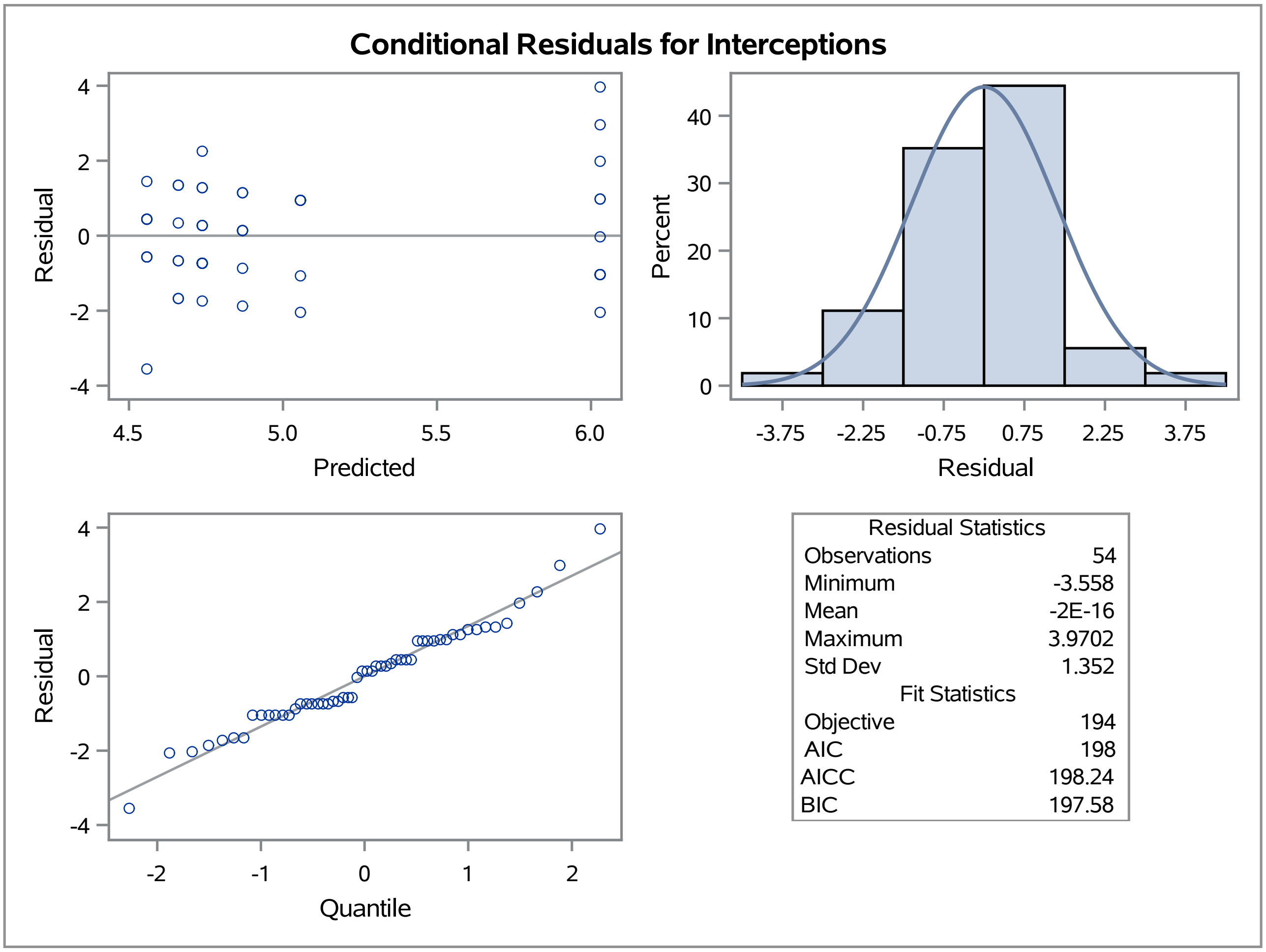

DistSoma=96

Model Information	
**Data Set**	WORK.TEMPDATASORTED
**Dependent Variable**	Interceptions
**Covariance Structure**	Variance Components
**Estimation Method**	REML
**Residual Variance Method**	Profile
**Fixed Effects SE Method**	Model-Based
**Degrees of Freedom Method**	Containment

Class Level Information		
**Class**	**Levels**	**Values**
**Genotype**	2	Meg +/+ Meg +/-
**Animal**	6	1 2 3 4 5 6

Dimensions	
**Covariance Parameters**	2
**Columns in X**	3
**Columns in Z**	6
**Subjects**	1
**Max Obs per Subject**	54

Number of Observations	
**Number of Observations Read**	54
**Number of Observations Used**	54
**Number of Observations Not Used**	0

Iteration History			
**Iteration**	**Evaluations**	**-2 Res Log Like**	**Criterion**
**0**	1	197.47906572	
**1**	3	192.50446879	0.00176134
**2**	1	192.40425893	0.00019350
**3**	1	192.39416715	0.00000316
**4**	1	192.39401226	0.00000000

Convergence criteria met.

DistSoma=96

Covariance Parameter Estimates				
**Cov Parm**	**Estimate**	**Alpha**	**Lower**	**Upper**
**Animal**	0.5396	0.05	0.1520	16.2690
**Residual**	1.8979	0.05	1.3216	2.9564

Fit Statistics	
**-2 Res Log Likelihood**	192.4
**AIC (Smaller is Better)**	196.4
**AICC (Smaller is Better)**	196.6
**BIC (Smaller is Better)**	196.0

Solution for Fixed Effects									
**Effect**	**Genotype**	**Estimate**	**Standard Error**	**DF**	**t Value**	**Pr > |t|**	**Alpha**	**Lower**	**Upper**
**Intercept**		4.9016	0.5000	4	9.80	0.0006	0.05	3.5133	6.2900
**Genotype**	Meg +/+	0.2497	0.7135	48	0.35	0.7279	0.05	-1.1849	1.6843
**Genotype**	Meg +/-	0	.	.	.	.	.	.	.

Solution for Random Effects									
**Effect**	**Animal**	**Estimate**	**Std Err Pred**	**DF**	**t Value**	**Pr > |t|**	**Alpha**	**Lower**	**Upper**
**Animal**	1	-0.1191	0.5247	48	-0.23	0.8214	0.05	-1.1742	0.9360
**Animal**	2	0.2198	0.5499	48	0.40	0.6911	0.05	-0.8858	1.3254
**Animal**	3	-0.1007	0.5433	48	-0.19	0.8537	0.05	-1.1931	0.9917
**Animal**	4	-0.5684	0.5300	48	-1.07	0.2889	0.05	-1.6340	0.4972
**Animal**	5	0.9783	0.5214	48	1.88	0.0667	0.05	-0.07003	2.0266
**Animal**	6	-0.4099	0.5396	48	-0.76	0.4512	0.05	-1.4949	0.6751

Type 3 Tests of Fixed Effects				
**Effect**	**Num DF**	**Den DF**	**F Value**	**Pr > F**
**Genotype**	1	48	0.12	0.7279

Least Squares Means									
**Effect**	**Genotype**	**Estimate**	**Standard Error**	**DF**	**t Value**	**Pr > |t|**	**Alpha**	**Lower**	**Upper**
**Genotype**	Meg +/+	5.1513	0.5089	48	10.12	<.0001	0.05	4.1280	6.1746
**Genotype**	Meg +/-	4.9016	0.5000	48	9.80	<.0001	0.05	3.8962	5.9070

DistSoma=96

Differences of Least Squares Means												
**Effect**	**Genotype**	**Genotype**	**Estimate**	**Standard Error**	**DF**	**t Value**	**Pr > |t|**	**Adjustment**	**Adj P**	**Alpha**	**Lower**	**Upper**
**Genotype**	Meg +/+	Meg +/-	0.2497	0.7135	48	0.35	0.7279	Tukey-Kramer	0.7279	0.05	-1.1849	1.6843

Differences of Least Squares Means				
**Effect**	**Genotype**	**Genotype**	**Adj Lower**	**Adj Upper**
**Genotype**	Meg +/+	Meg +/-	-1.1848	1.6842

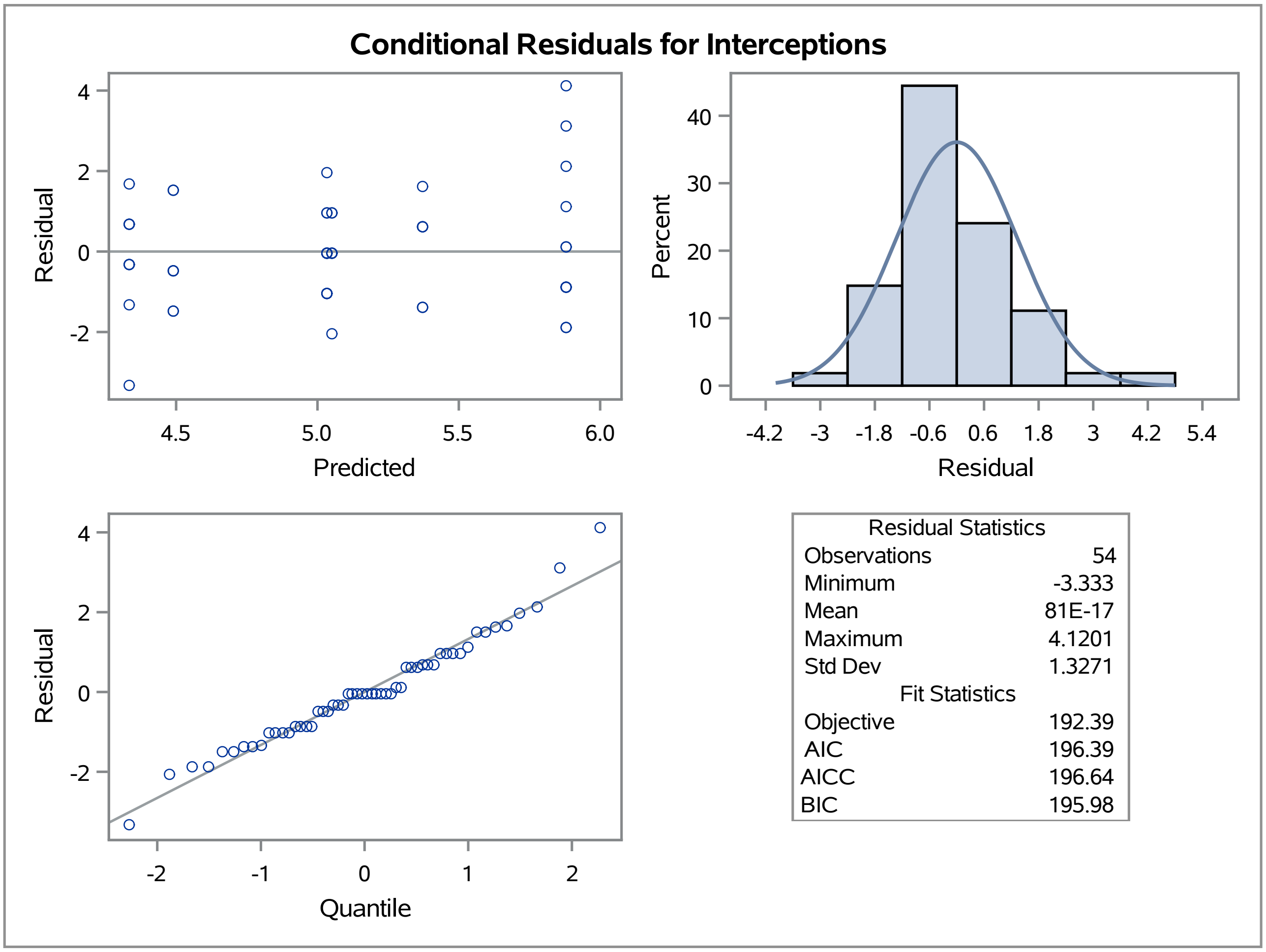

DistSoma=102

Model Information	
**Data Set**	WORK.TEMPDATASORTED
**Dependent Variable**	Interceptions
**Covariance Structure**	Variance Components
**Estimation Method**	REML
**Residual Variance Method**	Profile
**Fixed Effects SE Method**	Model-Based
**Degrees of Freedom Method**	Containment

Class Level Information		
**Class**	**Levels**	**Values**
**Genotype**	2	Meg +/+ Meg +/-
**Animal**	6	1 2 3 4 5 6

Dimensions	
**Covariance Parameters**	2
**Columns in X**	3
**Columns in Z**	6
**Subjects**	1
**Max Obs per Subject**	54

Number of Observations	
**Number of Observations Read**	54
**Number of Observations Used**	54
**Number of Observations Not Used**	0

Iteration History			
**Iteration**	**Evaluations**	**-2 Res Log Like**	**Criterion**
**0**	1	203.17566931	
**1**	3	199.81908358	0.00126281
**2**	1	199.74271389	0.00011300
**3**	1	199.73645619	0.00000119
**4**	1	199.73639356	0.00000000

Convergence criteria met.

DistSoma=102

Covariance Parameter Estimates				
Cov Parm	Estimate	Alpha	Lower	Upper
Animal	0.4644	0.05	0.1217	23.7437
Residual	2.2207	0.05	1.5470	3.4571

Fit Statistics	
-2 Res Log Likelihood	199.7
AIC (Smaller is Better)	203.7
AICC (Smaller is Better)	204.0
BIC (Smaller is Better)	203.3

Solution for Fixed Effects									
Effect	Genotype	Estimate	Standard Error	DF	t Value	Pr > |t|	Alpha	Lower	Upper
Intercept		5.0236	0.4865	4	10.33	0.0005	0.05	3.6729	6.3744
Genotype	Meg +/+	0.1950	0.6953	48	0.28	0.7803	0.05	-1.2030	1.5931
Genotype	Meg +/-	0	.	.	.	.	.	.	.

Solution for Random Effects									
Effect	Animal	Estimate	Std Err Pred	DF	t Value	Pr > |t|	Alpha	Lower	Upper
Animal	1	-0.1036	0.5068	48	-0.20	0.8388	0.05	-1.1225	0.9153
Animal	2	0.06381	0.5314	48	0.12	0.9049	0.05	-1.0047	1.1323
Animal	3	0.03983	0.5250	48	0.08	0.9398	0.05	-1.0157	1.0954
Animal	4	-0.5234	0.5119	48	-1.02	0.3117	0.05	-1.5526	0.5059
Animal	5	0.8769	0.5033	48	1.74	0.0879	0.05	-0.1351	1.8889
Animal	6	-0.3536	0.5216	48	-0.68	0.5011	0.05	-1.4022	0.6951

Type 3 Tests of Fixed Effects				
Effect	Num DF	Den DF	F Value	Pr > F
Genotype	1	48	0.08	0.7803

Least Squares Means									
Effect	Genotype	Estimate	Standard Error	DF	t Value	Pr > |t|	Alpha	Lower	Upper
Genotype	Meg +/+	5.2187	0.4968	48	10.50	<.0001	0.05	4.2198	6.2175
Genotype	Meg +/-	5.0236	0.4865	48	10.33	<.0001	0.05	4.0455	6.0018

DistSoma=102

Differences of Least Squares Means												
**Effect**	**Genotype**	**Genotype**	**Estimate**	**Standard Error**	**DF**	**t Value**	**Pr > |t|**	**Adjustment**	**Adj P**	**Alpha**	**Lower**	**Upper**
**Genotype**	Meg +/+	Meg +/-	0.1950	0.6953	48	0.28	0.7803	Tukey-Kramer	0.7803	0.05	-1.2030	1.5931

Differences of Least Squares Means				
**Effect**	**Genotype**	**Genotype**	**Adj Lower**	**Adj Upper**
**Genotype**	Meg +/+	Meg +/-	-1.2030	1.5930

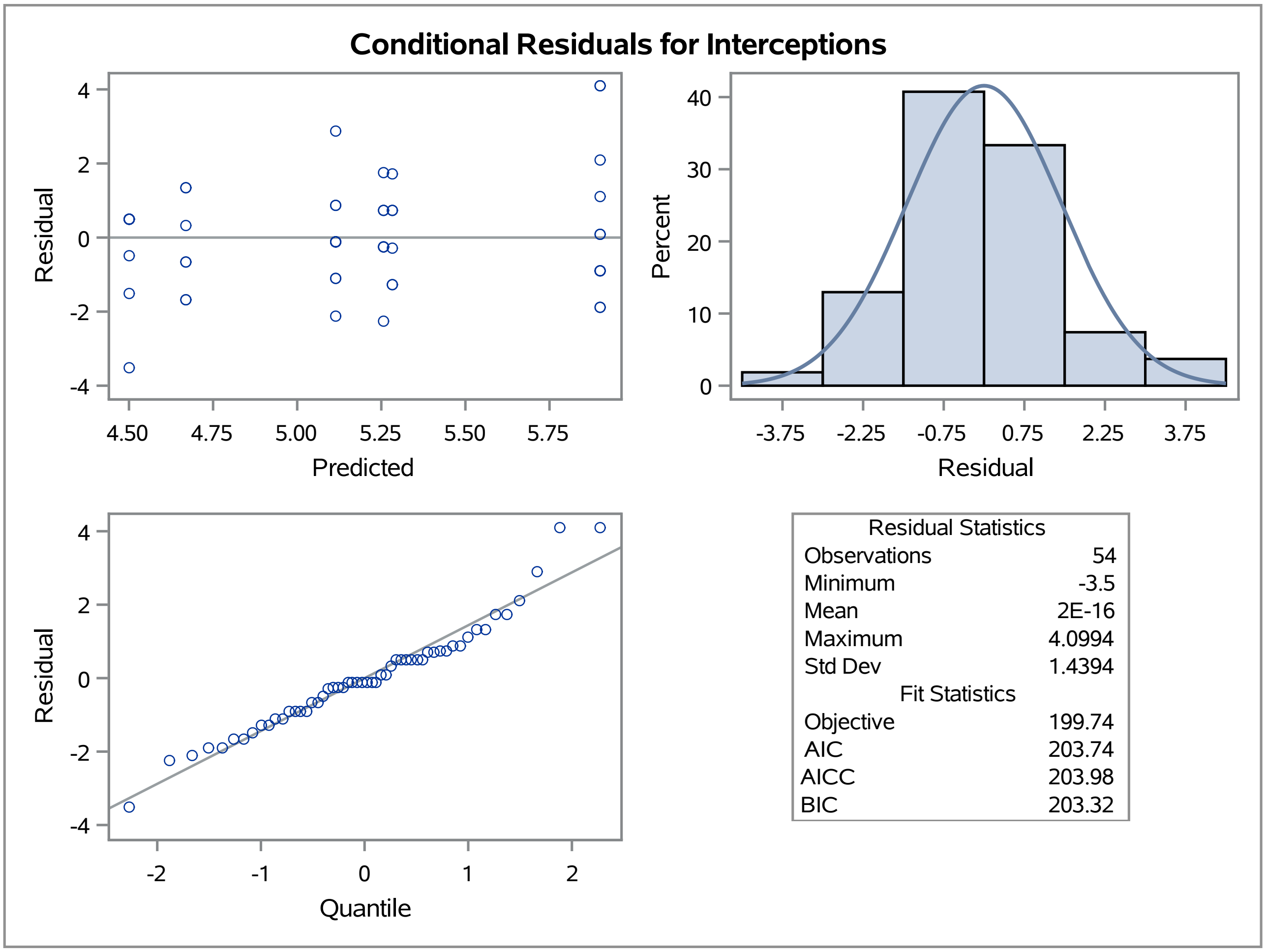

DistSoma=108

Model Information	
**Data Set**	WORK.TEMPDATASORTED
**Dependent Variable**	Interceptions
**Covariance Structure**	Variance Components
**Estimation Method**	REML
**Residual Variance Method**	Profile
**Fixed Effects SE Method**	Model-Based
**Degrees of Freedom Method**	Containment

Class Level Information		
**Class**	**Levels**	**Values**
**Genotype**	2	Meg +/+ Meg +/-
**Animal**	6	1 2 3 4 5 6

Dimensions	
**Covariance Parameters**	2
**Columns in X**	3
**Columns in Z**	6
**Subjects**	1
**Max Obs per Subject**	54

Number of Observations	
**Number of Observations Read**	54
**Number of Observations Used**	54
**Number of Observations Not Used**	0

Iteration History			
**Iteration**	**Evaluations**	**-2 Res Log Like**	**Criterion**
**0**	1	214.77784953	
**1**	5	210.17493784	0.00003961
**2**	1	210.17277996	0.00000019
**3**	1	210.17276888	0.00000000

Convergence criteria met.

DistSoma=108

Covariance Parameter Estimates				
Cov Parm	Estimate	Alpha	Lower	Upper
Animal	0.7149	0.05	0.1980	24.2154
Residual	2.6808	0.05	1.8667	4.1758

Fit Statistics	
-2 Res Log Likelihood	210.2
AIC (Smaller is Better)	214.2
AICC (Smaller is Better)	214.4
BIC (Smaller is Better)	213.8

Solution for Fixed Effects									
Effect	Genotype	Estimate	Standard Error	DF	t Value	Pr > |t|	Alpha	Lower	Upper
Intercept		5.1348	0.5808	4	8.84	0.0009	0.05	3.5221	6.7475
Genotype	Meg +/+	0.2563	0.8291	48	0.31	0.7586	0.05	-1.4107	1.9233
Genotype	Meg +/-	0	.	.	.	.	.	.	.

Solution for Random Effects									
Effect	Animal	Estimate	Std Err Pred	DF	t Value	Pr > |t|	Alpha	Lower	Upper
Animal	1	-0.4826	0.6090	48	-0.79	0.4320	0.05	-1.7071	0.7418
Animal	2	0.2722	0.6384	48	0.43	0.6718	0.05	-1.0113	1.5557
Animal	3	0.2105	0.6307	48	0.33	0.7401	0.05	-1.0576	1.4786
Animal	4	-0.4873	0.6151	48	-0.79	0.4321	0.05	-1.7241	0.7495
Animal	5	1.0402	0.6050	48	1.72	0.0920	0.05	-0.1763	2.2567
Animal	6	-0.5529	0.6264	48	-0.88	0.3819	0.05	-1.8124	0.7067

Type 3 Tests of Fixed Effects				
Effect	Num DF	Den DF	F Value	Pr > F
Genotype	1	48	0.10	0.7586

Least Squares Means									
Effect	Genotype	Estimate	Standard Error	DF	t Value	Pr > |t|	Alpha	Lower	Upper
Genotype	Meg +/+	5.3911	0.5916	48	9.11	<.0001	0.05	4.2016	6.5805
Genotype	Meg +/-	5.1348	0.5808	48	8.84	<.0001	0.05	3.9669	6.3026

DistSoma=108

Differences of Least Squares Means												
**Effect**	**Genotype**	**Genotype**	**Estimate**	**Standard Error**	**DF**	**t Value**	**Pr > |t|**	**Adjustment**	**Adj P**	**Alpha**	**Lower**	**Upper**
**Genotype**	Meg +/+	Meg +/-	0.2563	0.8291	48	0.31	0.7586	Tukey-Kramer	0.7586	0.05	-1.4107	1.9233

Differences of Least Squares Means				
**Effect**	**Genotype**	**Genotype**	**Adj Lower**	**Adj Upper**
**Genotype**	Meg +/+	Meg +/-	-1.4106	1.9232

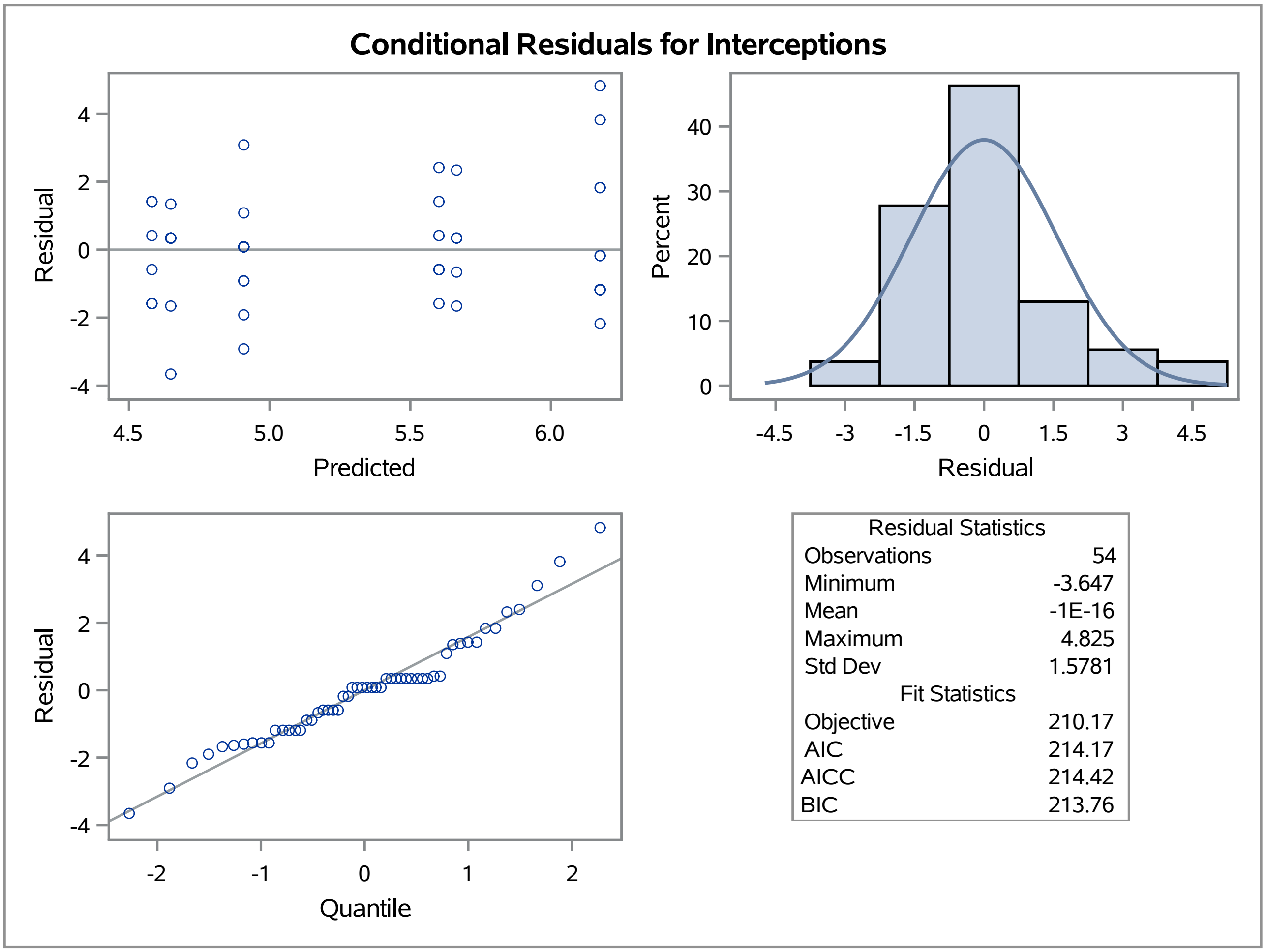

DistSoma=114

Model Information	
**Data Set**	WORK.TEMPDATASORTED
**Dependent Variable**	Interceptions
**Covariance Structure**	Variance Components
**Estimation Method**	REML
**Residual Variance Method**	Profile
**Fixed Effects SE Method**	Model-Based
**Degrees of Freedom Method**	Containment

Class Level Information		
**Class**	**Levels**	**Values**
**Genotype**	2	Meg +/+ Meg +/-
**Animal**	6	1 2 3 4 5 6

Dimensions	
**Covariance Parameters**	2
**Columns in X**	3
**Columns in Z**	6
**Subjects**	1
**Max Obs per Subject**	54

Number of Observations	
**Number of Observations Read**	54
**Number of Observations Used**	54
**Number of Observations Not Used**	0

Iteration History			
**Iteration**	**Evaluations**	**-2 Res Log Like**	**Criterion**
**0**	1	217.55796776	
**1**	3	212.23950943	0.00109135
**2**	1	212.16574758	0.00009523
**3**	1	212.15985363	0.00000095
**4**	1	212.15979767	0.00000000

Convergence criteria met.

DistSoma=114

Covariance Parameter Estimates				
Cov Parm	Estimate	Alpha	Lower	Upper
Animal	0.8457	0.05	0.2407	23.7923
Residual	2.7645	0.05	1.9246	4.3077

Fit Statistics	
-2 Res Log Likelihood	212.2
AIC (Smaller is Better)	216.2
AICC (Smaller is Better)	216.4
BIC (Smaller is Better)	215.7

Solution for Fixed Effects									
Effect	Genotype	Estimate	Standard Error	DF	t Value	Pr > |t|	Alpha	Lower	Upper
Intercept		5.1518	0.6198	4	8.31	0.0011	0.05	3.4308	6.8727
Genotype	Meg +/+	0.3403	0.8841	48	0.38	0.7020	0.05	-1.4372	2.1179
Genotype	Meg +/-	0	.	.	.	.	.	.	.

Solution for Random Effects									
Effect	Animal	Estimate	Std Err Pred	DF	t Value	Pr > |t|	Alpha	Lower	Upper
Animal	1	-0.5776	0.6509	48	-0.89	0.3793	0.05	-1.8863	0.7311
Animal	2	0.3288	0.6817	48	0.48	0.6318	0.05	-1.0420	1.6995
Animal	3	0.2488	0.6737	48	0.37	0.7135	0.05	-1.1057	1.6034
Animal	4	-0.4373	0.6573	48	-0.67	0.5090	0.05	-1.7590	0.8843
Animal	5	1.1251	0.6468	48	1.74	0.0884	0.05	-0.1754	2.4256
Animal	6	-0.6877	0.6691	48	-1.03	0.3092	0.05	-2.0331	0.6576

Type 3 Tests of Fixed Effects				
Effect	Num DF	Den DF	F Value	Pr > F
Genotype	1	48	0.15	0.7020

Least Squares Means									
Effect	Genotype	Estimate	Standard Error	DF	t Value	Pr > |t|	Alpha	Lower	Upper
Genotype	Meg +/+	5.4921	0.6304	48	8.71	<.0001	0.05	4.2246	6.7596
Genotype	Meg +/-	5.1518	0.6198	48	8.31	<.0001	0.05	3.9055	6.3980

DistSoma=114

Differences of Least Squares Means												
**Effect**	**Genotype**	**Genotype**	**Estimate**	**Standard Error**	**DF**	**t Value**	**Pr > |t|**	**Adjustment**	**Adj P**	**Alpha**	**Lower**	**Upper**
**Genotype**	Meg +/+	Meg +/-	0.3403	0.8841	48	0.38	0.7020	Tukey-Kramer	0.7020	0.05	-1.4372	2.1179

Differences of Least Squares Means				
**Effect**	**Genotype**	**Genotype**	**Adj Lower**	**Adj Upper**
**Genotype**	Meg +/+	Meg +/-	-1.4372	2.1178

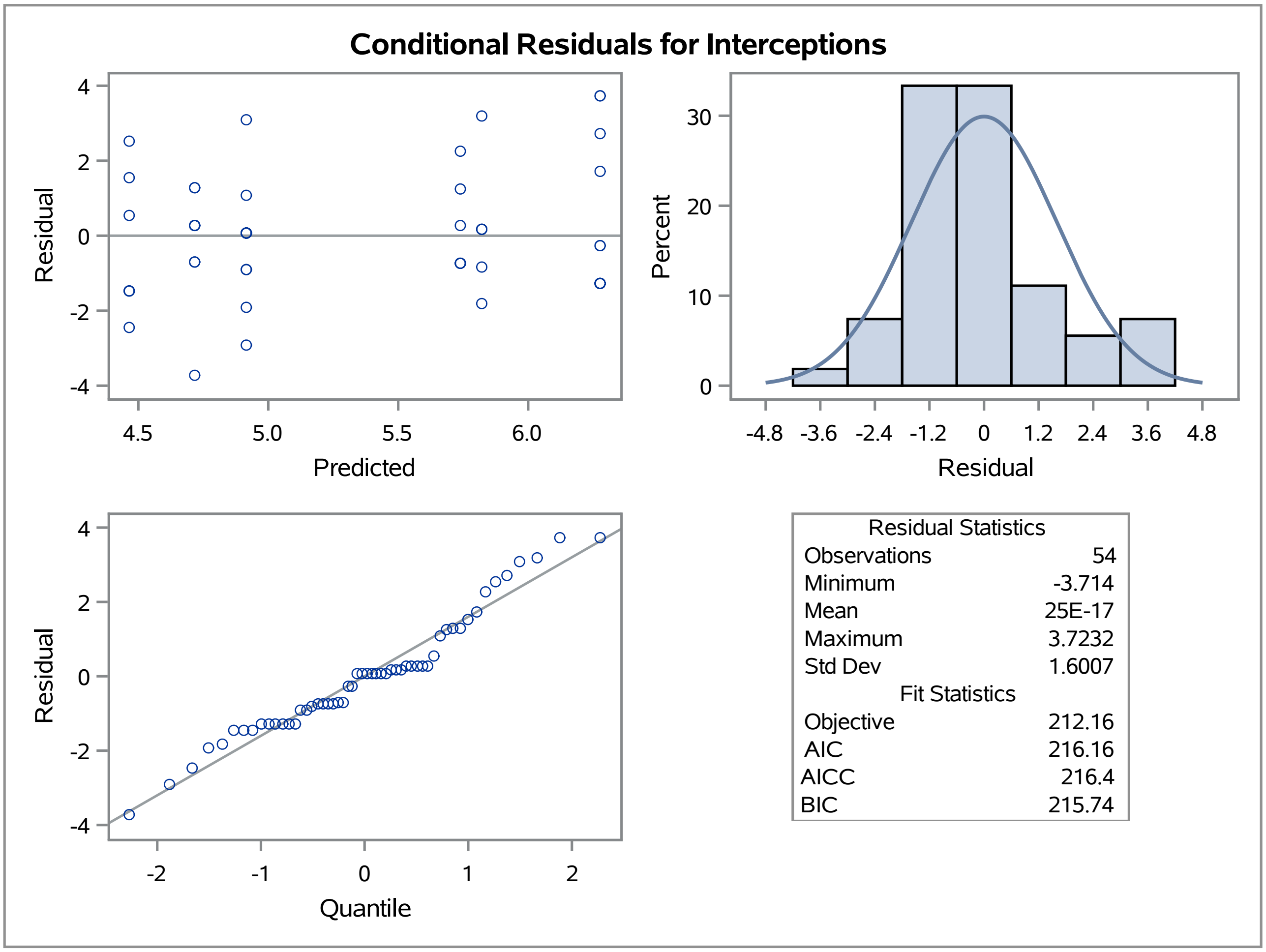

DistSoma=120

Model Information	
**Data Set**	WORK.TEMPDATASORTED
**Dependent Variable**	Interceptions
**Covariance Structure**	Variance Components
**Estimation Method**	REML
**Residual Variance Method**	Profile
**Fixed Effects SE Method**	Model-Based
**Degrees of Freedom Method**	Containment

Class Level Information		
**Class**	**Levels**	**Values**
**Genotype**	2	Meg +/+ Meg +/-
**Animal**	6	1 2 3 4 5 6

Dimensions	
**Covariance Parameters**	2
**Columns in X**	3
**Columns in Z**	6
**Subjects**	1
**Max Obs per Subject**	54

Number of Observations	
**Number of Observations Read**	54
**Number of Observations Used**	54
**Number of Observations Not Used**	0

Iteration History			
**Iteration**	**Evaluations**	**-2 Res Log Like**	**Criterion**
**0**	1	224.47348655	
**1**	5	219.42194303	0.00004837
**2**	1	219.41911955	0.00000031
**3**	1	219.41910005	0.00000000

Convergence criteria met.

DistSoma=120

Covariance Parameter Estimates				
Cov Parm	Estimate	Alpha	Lower	Upper
Animal	0.9098	0.05	0.2562	27.4918
Residual	3.1909	0.05	2.2219	4.9707

Fit Statistics	
-2 Res Log Likelihood	219.4
AIC (Smaller is Better)	223.4
AICC (Smaller is Better)	223.7
BIC (Smaller is Better)	223.0

Solution for Fixed Effects									
Effect	Genotype	Estimate	Standard Error	DF	t Value	Pr > |t|	Alpha	Lower	Upper
Intercept		4.9470	0.6491	4	7.62	0.0016	0.05	3.1449	6.7491
Genotype	Meg +/+	0.6385	0.9261	48	0.69	0.4939	0.05	-1.2236	2.5006
Genotype	Meg +/-	0	.	.	.	.	.	.	.

Solution for Random Effects									
Effect	Animal	Estimate	Std Err Pred	DF	t Value	Pr > |t|	Alpha	Lower	Upper
Animal	1	-0.6429	0.6812	48	-0.94	0.3500	0.05	-2.0124	0.7267
Animal	2	0.3668	0.7138	48	0.51	0.6097	0.05	-1.0684	1.8019
Animal	3	0.2761	0.7053	48	0.39	0.6971	0.05	-1.1419	1.6941
Animal	4	-0.6015	0.6879	48	-0.87	0.3863	0.05	-1.9847	0.7817
Animal	5	1.1373	0.6768	48	1.68	0.0994	0.05	-0.2235	2.4980
Animal	6	-0.5357	0.7005	48	-0.76	0.4481	0.05	-1.9442	0.8727

Type 3 Tests of Fixed Effects				
Effect	Num DF	Den DF	F Value	Pr > F
Genotype	1	48	0.48	0.4939

Least Squares Means									
Effect	Genotype	Estimate	Standard Error	DF	t Value	Pr > |t|	Alpha	Lower	Upper
Genotype	Meg +/+	5.5855	0.6606	48	8.46	<.0001	0.05	4.2573	6.9138
Genotype	Meg +/-	4.9470	0.6491	48	7.62	<.0001	0.05	3.6420	6.2521

DistSoma=120

Differences of Least Squares Means												
Effect	Genotype	Genotype	Estimate	Standard Error	DF	t Value	Pr > |t|	Adjustment	Adj P	Alpha	Lower	Upper
**Genotype**	Meg +/+	Meg +/-	0.6385	0.9261	48	0.69	0.4939	Tukey-Kramer	0.4939	0.05	-1.2236	2.5006

Differences of Least Squares Means				
Effect	Genotype	Genotype	Adj Lower	Adj Upper
**Genotype**	Meg +/+	Meg +/-	-1.2235	2.5005

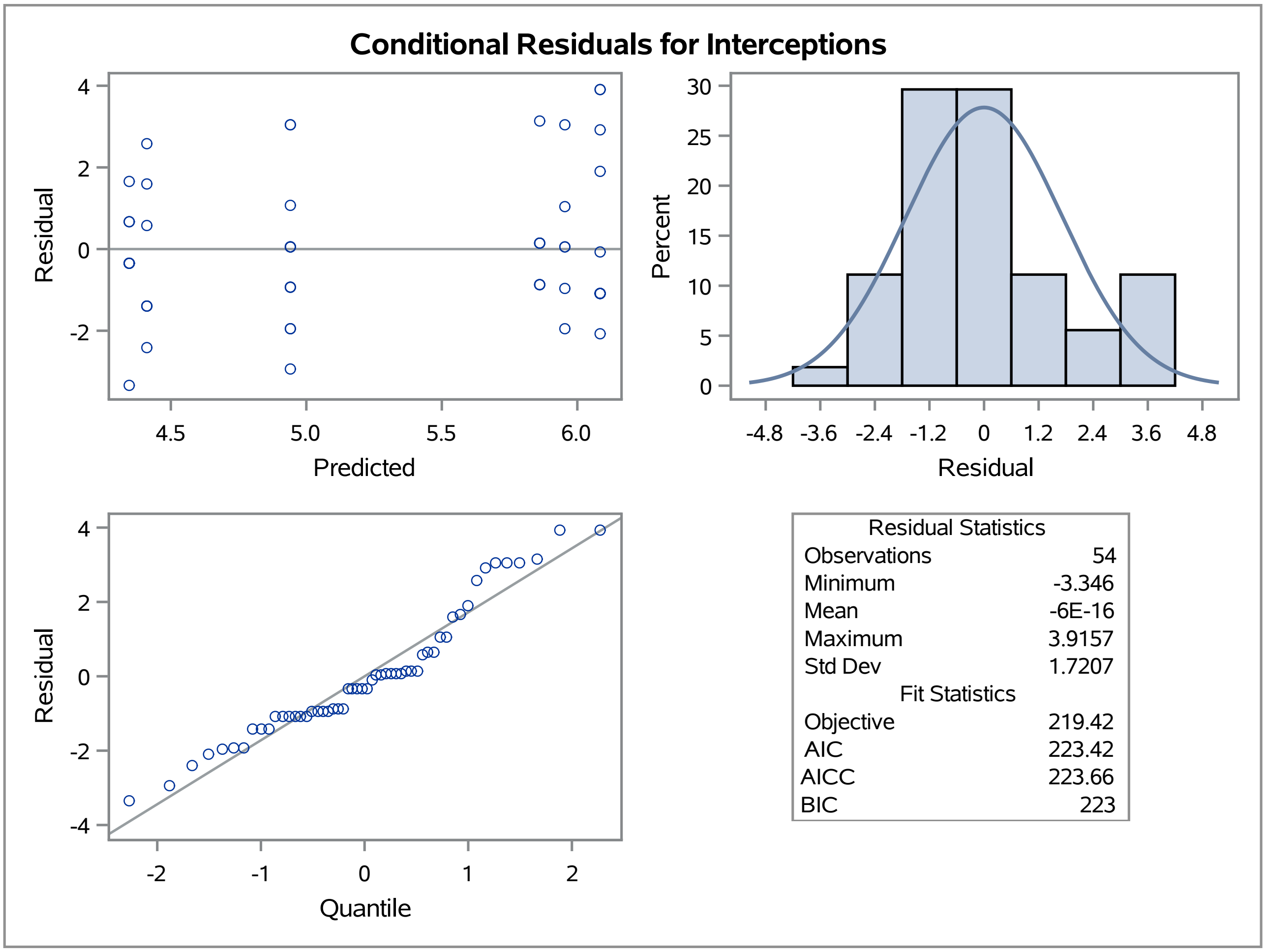

DistSoma=126

Model Information	
**Data Set**	WORK.TEMPDATASORTED
**Dependent Variable**	Interceptions
**Covariance Structure**	Variance Components
**Estimation Method**	REML
**Residual Variance Method**	Profile
**Fixed Effects SE Method**	Model-Based
**Degrees of Freedom Method**	Containment

Class Level Information		
**Class**	**Levels**	**Values**
**Genotype**	2	Meg +/+ Meg +/-
**Animal**	6	1 2 3 4 5 6

Dimensions	
**Covariance Parameters**	2
**Columns in X**	3
**Columns in Z**	6
**Subjects**	1
**Max Obs per Subject**	54

Number of Observations	
**Number of Observations Read**	54
**Number of Observations Used**	54
**Number of Observations Not Used**	0

Iteration History			
**Iteration**	**Evaluations**	**-2 Res Log Like**	**Criterion**
**0**	1	224.61754767	
**1**	5	219.26465839	0.00007814
**2**	1	219.26018852	0.00000083
**3**	1	219.26013663	0.00000000

Convergence criteria met.

DistSoma=126

Covariance Parameter Estimates				
**Cov Parm**	**Estimate**	**Alpha**	**Lower**	**Upper**
**Animal**	0.9417	0.05	0.2678	26.5948
**Residual**	3.1743	0.05	2.2104	4.9448

Fit Statistics	
**-2 Res Log Likelihood**	219.3
**AIC (Smaller is Better)**	223.3
**AICC (Smaller is Better)**	223.5
**BIC (Smaller is Better)**	222.8

Solution for Fixed Effects									
**Effect**	**Genotype**	**Estimate**	**Standard Error**	**DF**	**t Value**	**Pr > |t|**	**Alpha**	**Lower**	**Upper**
**Intercept**		4.9752	0.6568	4	7.58	0.0016	0.05	3.1517	6.7986
**Genotype**	Meg +/+	0.7904	0.9369	48	0.84	0.4031	0.05	-1.0934	2.6741
**Genotype**	Meg +/-	0	.	.	.	.	.	.	.

Solution for Random Effects									
**Effect**	**Animal**	**Estimate**	**Std Err Pred**	**DF**	**t Value**	**Pr > |t|**	**Alpha**	**Lower**	**Upper**
**Animal**	1	-0.6079	0.6895	48	-0.88	0.3823	0.05	-1.9942	0.7784
**Animal**	2	0.2568	0.7223	48	0.36	0.7237	0.05	-1.1955	1.7092
**Animal**	3	0.3511	0.7138	48	0.49	0.6250	0.05	-1.0840	1.7862
**Animal**	4	-0.6286	0.6963	48	-0.90	0.3712	0.05	-2.0287	0.7715
**Animal**	5	1.1904	0.6851	48	1.74	0.0887	0.05	-0.1871	2.5680
**Animal**	6	-0.5618	0.7089	48	-0.79	0.4320	0.05	-1.9872	0.8636

Type 3 Tests of Fixed Effects				
**Effect**	**Num DF**	**Den DF**	**F Value**	**Pr > F**
**Genotype**	1	48	0.71	0.4031

Least Squares Means									
**Effect**	**Genotype**	**Estimate**	**Standard Error**	**DF**	**t Value**	**Pr > |t|**	**Alpha**	**Lower**	**Upper**
**Genotype**	Meg +/+	5.7656	0.6682	48	8.63	<.0001	0.05	4.4221	7.1090
**Genotype**	Meg +/-	4.9752	0.6568	48	7.58	<.0001	0.05	3.6547	6.2957

DistSoma=126

Differences of Least Squares Means												
**Effect**	**Genotype**	**Genotype**	**Estimate**	**Standard Error**	**DF**	**t Value**	**Pr > |t|**	**Adjustment**	**Adj P**	**Alpha**	**Lower**	**Upper**
**Genotype**	Meg +/+	Meg +/-	0.7904	0.9369	48	0.84	0.4031	Tukey-Kramer	0.4031	0.05	-1.0934	2.6741

Differences of Least Squares Means				
**Effect**	**Genotype**	**Genotype**	**Adj Lower**	**Adj Upper**
**Genotype**	Meg +/+	Meg +/-	-1.0933	2.6740

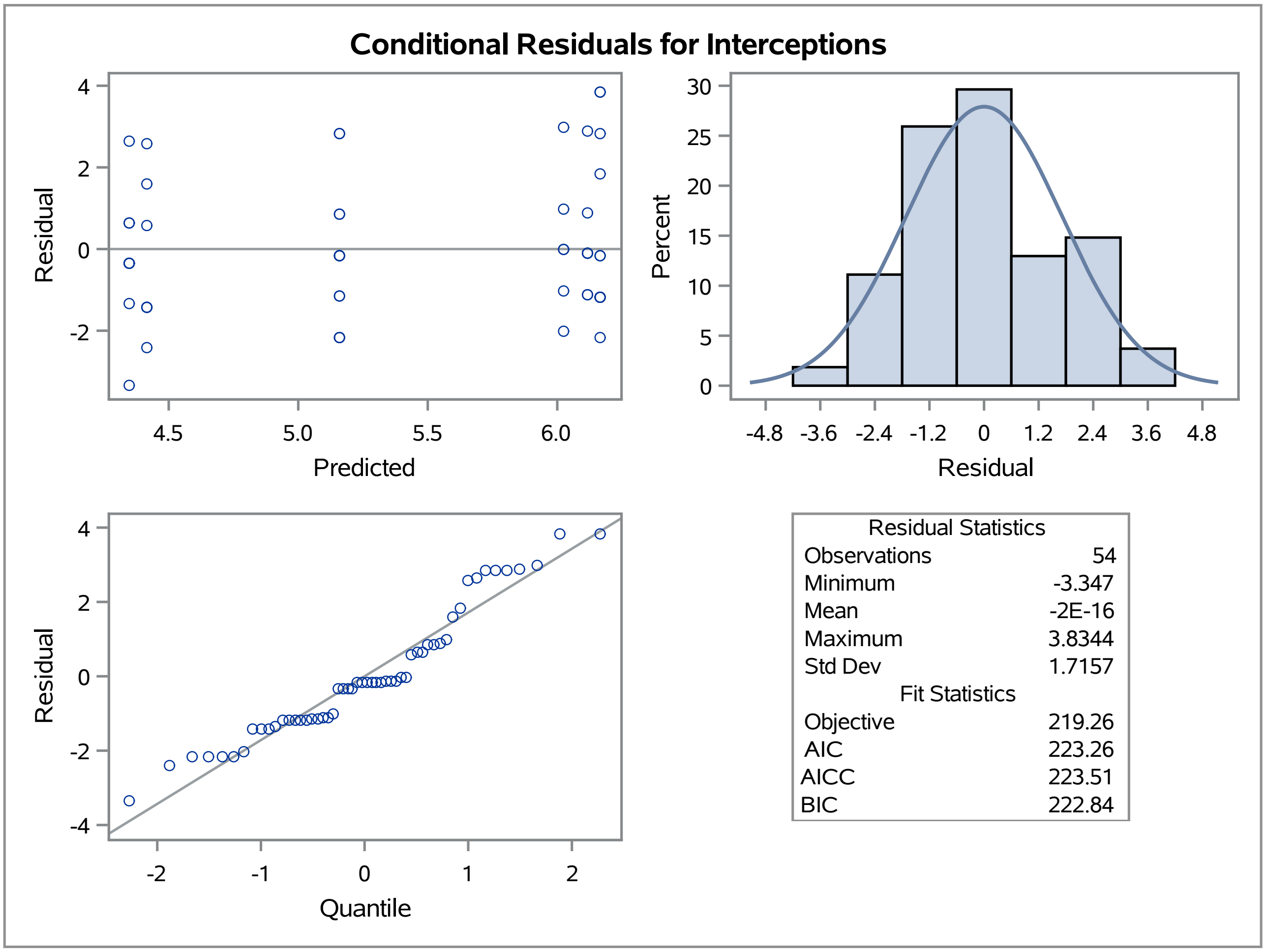

DistSoma=132

Model Information	
**Data Set**	WORK.TEMPDATASORTED
**Dependent Variable**	Interceptions
**Covariance Structure**	Variance Components
**Estimation Method**	REML
**Residual Variance Method**	Profile
**Fixed Effects SE Method**	Model-Based
**Degrees of Freedom Method**	Containment

Class Level Information		
**Class**	**Levels**	**Values**
**Genotype**	2	Meg +/+ Meg +/-
**Animal**	6	1 2 3 4 5 6

Dimensions	
**Covariance Parameters**	2
**Columns in X**	3
**Columns in Z**	6
**Subjects**	1
**Max Obs per Subject**	54

Number of Observations	
**Number of Observations Read**	54
**Number of Observations Used**	54
**Number of Observations Not Used**	0

Iteration History			
**Iteration**	**Evaluations**	**-2 Res Log Like**	**Criterion**
**0**	1	222.35586515	
**1**	5	218.79668952	0.00004157
**2**	1	218.79426345	0.00000023
**3**	1	218.79424927	0.00000000

Convergence criteria met.

DistSoma=132

Covariance Parameter Estimates				
Cov Parm	Estimate	Alpha	Lower	Upper
Animal	0.6988	0.05	0.1843	33.9293
Residual	3.1968	0.05	2.2265	4.9780

Fit Statistics	
-2 Res Log Likelihood	218.8
AIC (Smaller is Better)	222.8
AICC (Smaller is Better)	223.0
BIC (Smaller is Better)	222.4

Solution for Fixed Effects									
Effect	Genotype	Estimate	Standard Error	DF	t Value	Pr > |t|	Alpha	Lower	Upper
Intercept		4.8851	0.5923	4	8.25	0.0012	0.05	3.2406	6.5297
Genotype	Meg +/+	0.9969	0.8464	48	1.18	0.2447	0.05	-0.7048	2.6986
Genotype	Meg +/-	0	.	.	.	.	.	.	.

Solution for Random Effects									
Effect	Animal	Estimate	Std Err Pred	DF	t Value	Pr > |t|	Alpha	Lower	Upper
Animal	1	-0.3110	0.6180	48	-0.50	0.6170	0.05	-1.5535	0.9315
Animal	2	0.06693	0.6480	48	0.10	0.9182	0.05	-1.2361	1.3699
Animal	3	0.2441	0.6402	48	0.38	0.7046	0.05	-1.0431	1.5313
Animal	4	-0.5132	0.6243	48	-0.82	0.4151	0.05	-1.7683	0.7420
Animal	5	1.0485	0.6138	48	1.71	0.0941	0.05	-0.1857	2.2826
Animal	6	-0.5353	0.6360	48	-0.84	0.4041	0.05	-1.8141	0.7435

Type 3 Tests of Fixed Effects				
Effect	Num DF	Den DF	F Value	Pr > F
Genotype	1	48	1.39	0.2447

Least Squares Means									
Effect	Genotype	Estimate	Standard Error	DF	t Value	Pr > |t|	Alpha	Lower	Upper
Genotype	Meg +/+	5.8820	0.6046	48	9.73	<.0001	0.05	4.6665	7.0976
Genotype	Meg +/-	4.8851	0.5923	48	8.25	<.0001	0.05	3.6942	6.0761

DistSoma=132

Differences of Least Squares Means												
**Effect**	**Genotype**	**Genotype**	**Estimate**	**Standard Error**	**DF**	**t Value**	**Pr > |t|**	**Adjustment**	**Adj P**	**Alpha**	**Lower**	**Upper**
**Genotype**	Meg +/+	Meg +/-	0.9969	0.8464	48	1.18	0.2447	Tukey-Kramer	0.2447	0.05	-0.7048	2.6986

Differences of Least Squares Means				
**Effect**	**Genotype**	**Genotype**	**Adj Lower**	**Adj Upper**
**Genotype**	Meg +/+	Meg +/-	-0.7048	2.6986

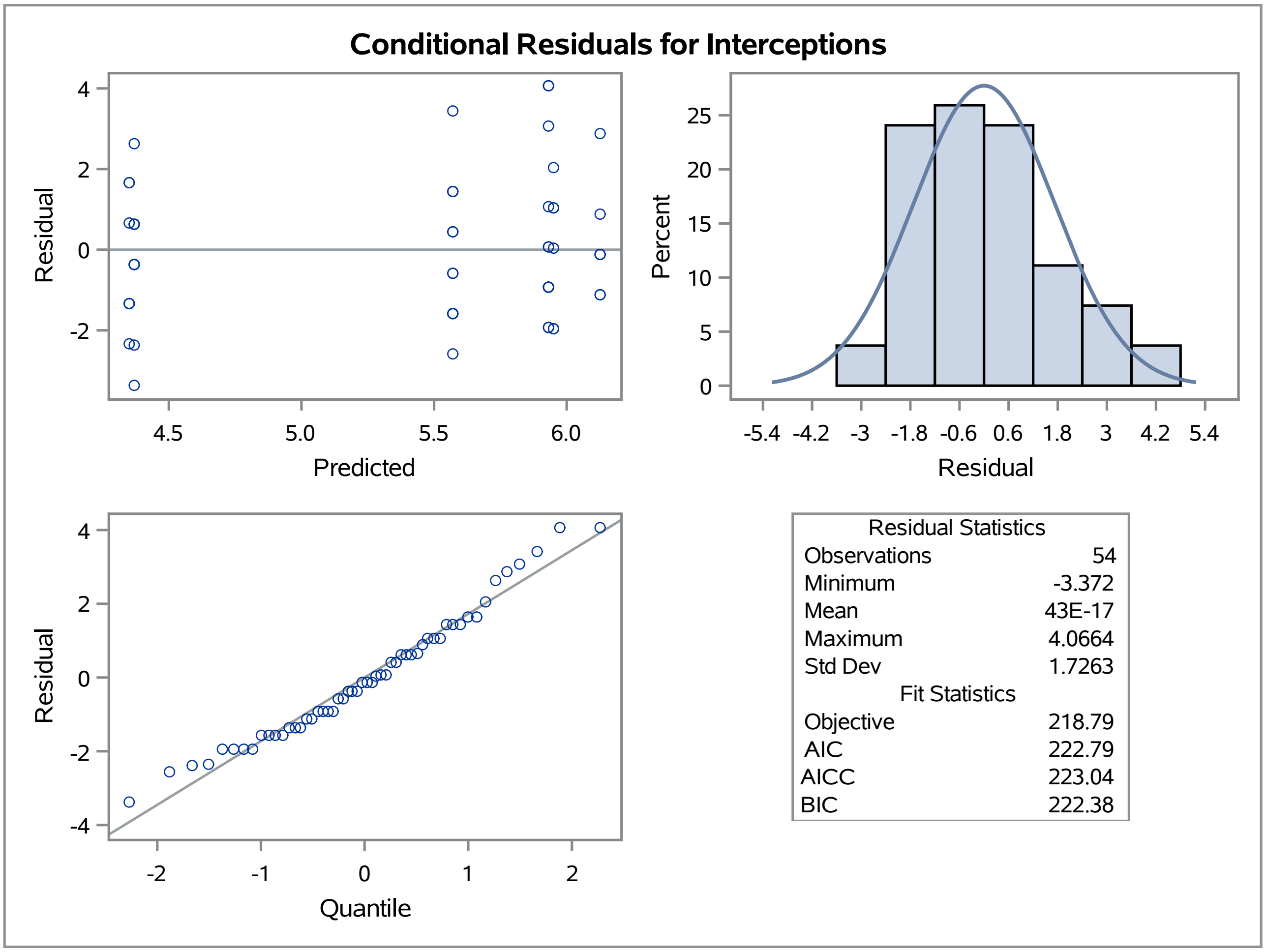

DistSoma=138

Model Information	
**Data Set**	WORK.TEMPDATASORTED
**Dependent Variable**	Interceptions
**Covariance Structure**	Variance Components
**Estimation Method**	REML
**Residual Variance Method**	Profile
**Fixed Effects SE Method**	Model-Based
**Degrees of Freedom Method**	Containment

Class Level Information		
**Class**	**Levels**	**Values**
**Genotype**	2	Meg +/+ Meg +/-
**Animal**	6	1 2 3 4 5 6

Dimensions	
**Covariance Parameters**	2
**Columns in X**	3
**Columns in Z**	6
**Subjects**	1
**Max Obs per Subject**	54

Number of Observations	
**Number of Observations Read**	54
**Number of Observations Used**	54
**Number of Observations Not Used**	0

Iteration History			
**Iteration**	**Evaluations**	**-2 Res Log Like**	**Criterion**
**0**	1	230.93906279	
**1**	5	225.82261092	0.00003679
**2**	1	225.82033679	0.00000019
**3**	1	225.82032441	0.00000000

Convergence criteria met.

DistSoma=138

Covariance Parameter Estimates				
**Cov Parm**	**Estimate**	**Alpha**	**Lower**	**Upper**
**Animal**	1.0421	0.05	0.2940	31.1026
**Residual**	3.6063	0.05	2.5110	5.6182

Fit Statistics	
**-2 Res Log Likelihood**	225.8
**AIC (Smaller is Better)**	229.8
**AICC (Smaller is Better)**	230.1
**BIC (Smaller is Better)**	229.4

Solution for Fixed Effects									
**Effect**	**Genotype**	**Estimate**	**Standard Error**	**DF**	**t Value**	**Pr > |t|**	**Alpha**	**Lower**	**Upper**
**Intercept**		4.6960	0.6934	4	6.77	0.0025	0.05	2.7709	6.6211
**Genotype**	Meg +/+	1.3679	0.9892	48	1.38	0.1731	0.05	-0.6212	3.3569
**Genotype**	Meg +/-	0	.	.	.	.	.	.	.

Solution for Random Effects									
**Effect**	**Animal**	**Estimate**	**Std Err Pred**	**DF**	**t Value**	**Pr > |t|**	**Alpha**	**Lower**	**Upper**
**Animal**	1	-0.4149	0.7277	48	-0.57	0.5712	0.05	-1.8782	1.0483
**Animal**	2	0.1709	0.7625	48	0.22	0.8236	0.05	-1.3623	1.7040
**Animal**	3	0.2440	0.7534	48	0.32	0.7474	0.05	-1.2709	1.7590
**Animal**	4	-0.5830	0.7350	48	-0.79	0.4316	0.05	-2.0608	0.8948
**Animal**	5	1.3355	0.7231	48	1.85	0.0709	0.05	-0.1184	2.7894
**Animal**	6	-0.7525	0.7483	48	-1.01	0.3197	0.05	-2.2572	0.7521

Type 3 Tests of Fixed Effects				
**Effect**	**Num DF**	**Den DF**	**F Value**	**Pr > F**
**Genotype**	1	48	1.91	0.1731

Least Squares Means									
**Effect**	**Genotype**	**Estimate**	**Standard Error**	**DF**	**t Value**	**Pr > |t|**	**Alpha**	**Lower**	**Upper**
**Genotype**	Meg +/+	6.0639	0.7056	48	8.59	<.0001	0.05	4.6452	7.4826
**Genotype**	Meg +/-	4.6960	0.6934	48	6.77	<.0001	0.05	3.3019	6.0901

DistSoma=138

Differences of Least Squares Means												
**Effect**	**Genotype**	**Genotype**	**Estimate**	**Standard Error**	**DF**	**t Value**	**Pr > |t|**	**Adjustment**	**Adj P**	**Alpha**	**Lower**	**Upper**
**Genotype**	Meg +/+	Meg +/-	1.3679	0.9892	48	1.38	0.1731	Tukey-Kramer	0.1731	0.05	-0.6212	3.3569

Differences of Least Squares Means				
**Effect**	**Genotype**	**Genotype**	**Adj Lower**	**Adj Upper**
**Genotype**	Meg +/+	Meg +/-	-0.6211	3.3568

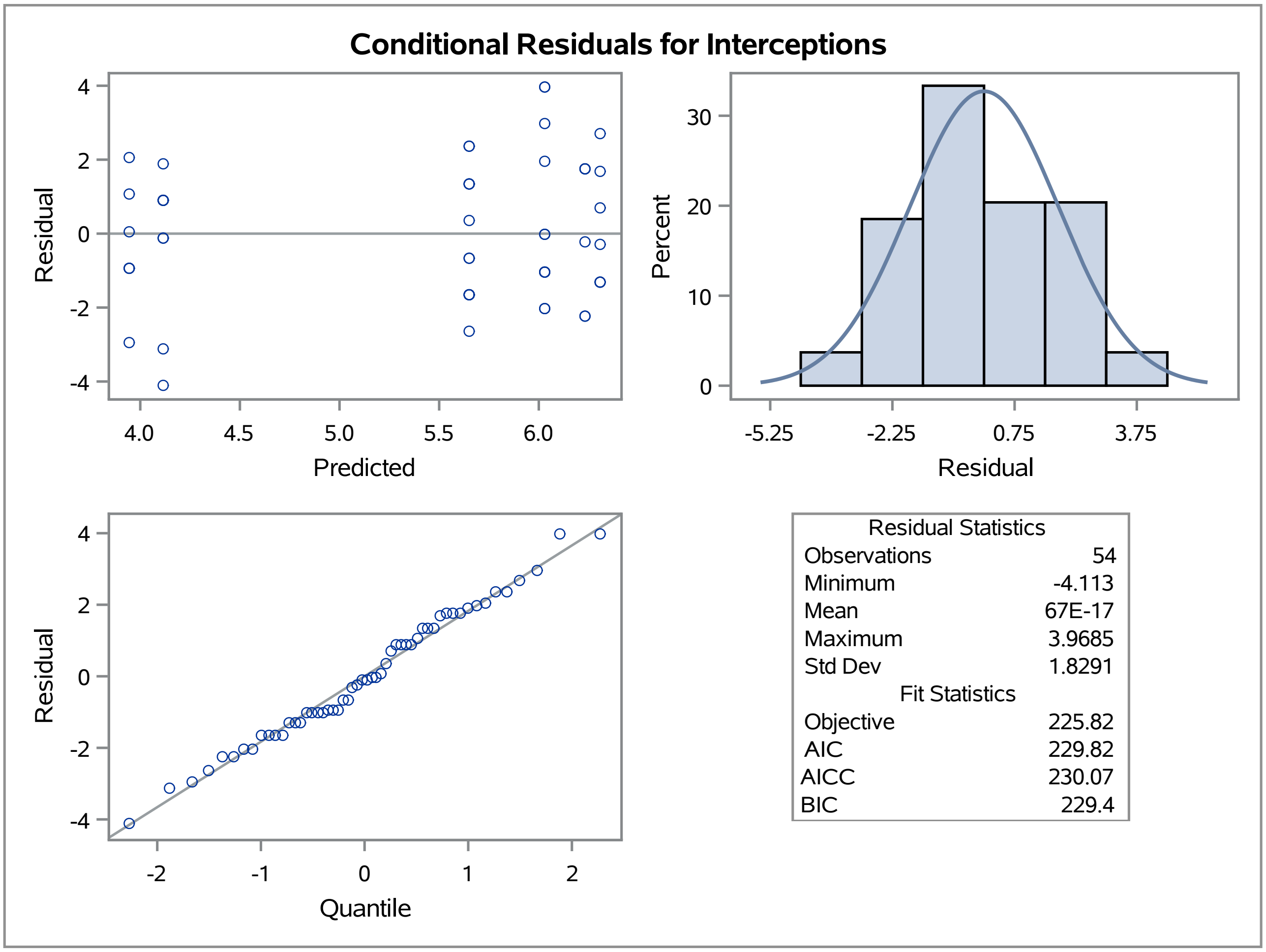

DistSoma=144

Model Information	
**Data Set**	WORK.TEMPDATASORTED
**Dependent Variable**	Interceptions
**Covariance Structure**	Variance Components
**Estimation Method**	REML
**Residual Variance Method**	Profile
**Fixed Effects SE Method**	Model-Based
**Degrees of Freedom Method**	Containment

Class Level Information		
**Class**	**Levels**	**Values**
**Genotype**	2	Meg +/+ Meg +/-
**Animal**	6	1 2 3 4 5 6

Dimensions	
**Covariance Parameters**	2
**Columns in X**	3
**Columns in Z**	6
**Subjects**	1
**Max Obs per Subject**	54

Number of Observations	
**Number of Observations Read**	54
**Number of Observations Used**	54
**Number of Observations Not Used**	0

Iteration History			
**Iteration**	**Evaluations**	**-2 Res Log Like**	**Criterion**
**0**	1	229.81047992	
**1**	5	224.89362939	0.00004151
**2**	1	224.89109060	0.00000024
**3**	1	224.89107501	0.00000000

Convergence criteria met.

DistSoma=144

Covariance Parameter Estimates				
**Cov Parm**	**Estimate**	**Alpha**	**Lower**	**Upper**
**Animal**	0.9923	0.05	0.2780	31.0014
**Residual**	3.5488	0.05	2.4711	5.5281

Fit Statistics	
**-2 Res Log Likelihood**	224.9
**AIC (Smaller is Better)**	228.9
**AICC (Smaller is Better)**	229.1
**BIC (Smaller is Better)**	228.5

Solution for Fixed Effects									
**Effect**	**Genotype**	**Estimate**	**Standard Error**	**DF**	**t Value**	**Pr > |t|**	**Alpha**	**Lower**	**Upper**
**Intercept**		4.6009	0.6797	4	6.77	0.0025	0.05	2.7138	6.4881
**Genotype**	Meg +/+	1.3291	0.9699	48	1.37	0.1770	0.05	-0.6210	3.2793
**Genotype**	Meg +/-	0	.	.	.	.	.	.	.

Solution for Random Effects									
**Effect**	**Animal**	**Estimate**	**Std Err Pred**	**DF**	**t Value**	**Pr > |t|**	**Alpha**	**Lower**	**Upper**
**Animal**	1	-0.4881	0.7131	48	-0.68	0.4970	0.05	-1.9219	0.9458
**Animal**	2	0.2527	0.7473	48	0.34	0.7368	0.05	-1.2500	1.7553
**Animal**	3	0.2354	0.7384	48	0.32	0.7513	0.05	-1.2493	1.7201
**Animal**	4	-0.5891	0.7203	48	-0.82	0.4175	0.05	-2.0373	0.8591
**Animal**	5	1.2704	0.7085	48	1.79	0.0793	0.05	-0.1542	2.6951
**Animal**	6	-0.6814	0.7334	48	-0.93	0.3575	0.05	-2.1560	0.7933

Type 3 Tests of Fixed Effects				
**Effect**	**Num DF**	**Den DF**	**F Value**	**Pr > F**
**Genotype**	1	48	1.88	0.1770

Least Squares Means									
**Effect**	**Genotype**	**Estimate**	**Standard Error**	**DF**	**t Value**	**Pr > |t|**	**Alpha**	**Lower**	**Upper**
**Genotype**	Meg +/+	5.9300	0.6919	48	8.57	<.0001	0.05	4.5388	7.3213
**Genotype**	Meg +/-	4.6009	0.6797	48	6.77	<.0001	0.05	3.2343	5.9676

DistSoma=144

Differences of Least Squares Means												
Effect	Genotype	Genotype	Estimate	Standard Error	DF	t Value	Pr > |t|	Adjustment	Adj P	Alpha	Lower	Upper
**Genotype**	Meg +/+	Meg +/-	1.3291	0.9699	48	1.37	0.1770	Tukey-Kramer	0.1770	0.05	-0.6210	3.2793

Differences of Least Squares Means				
Effect	Genotype	Genotype	Adj Lower	Adj Upper
**Genotype**	Meg +/+	Meg +/-	-0.6210	3.2792

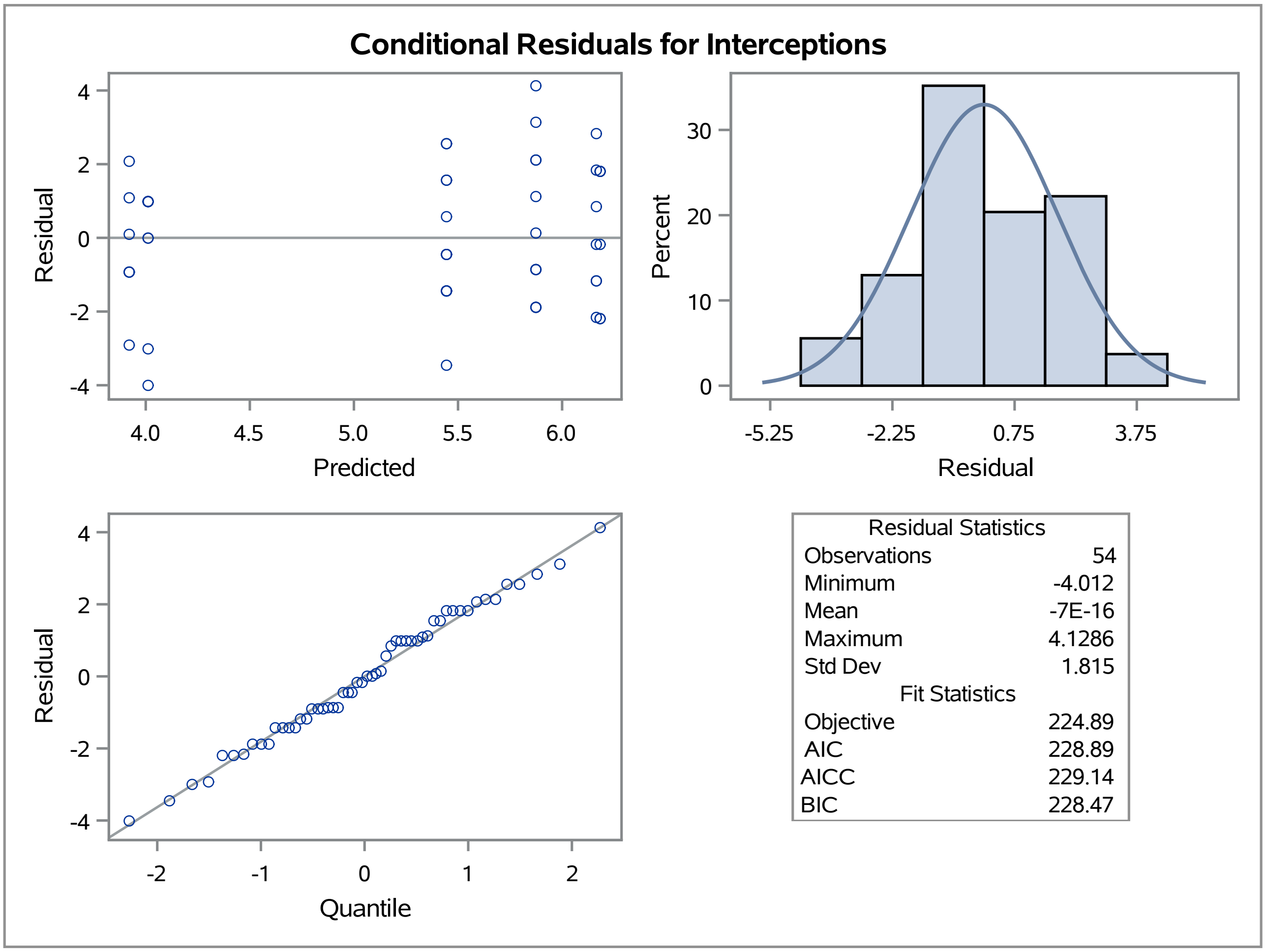

DistSoma=150

Model Information	
**Data Set**	WORK.TEMPDATASORTED
**Dependent Variable**	Interceptions
**Covariance Structure**	Variance Components
**Estimation Method**	REML
**Residual Variance Method**	Profile
**Fixed Effects SE Method**	Model-Based
**Degrees of Freedom Method**	Containment

Class Level Information		
**Class**	**Levels**	**Values**
**Genotype**	2	Meg +/+ Meg +/-
**Animal**	6	1 2 3 4 5 6

Dimensions	
**Covariance Parameters**	2
**Columns in X**	3
**Columns in Z**	6
**Subjects**	1
**Max Obs per Subject**	54

Number of Observations	
**Number of Observations Read**	54
**Number of Observations Used**	54
**Number of Observations Not Used**	0

Iteration History			
**Iteration**	**Evaluations**	**-2 Res Log Like**	**Criterion**
**0**	1	229.51527489	
**1**	5	225.01775230	0.00003573
**2**	1	225.01555476	0.00000018
**3**	1	225.01554324	0.00000000

Convergence criteria met.

DistSoma=150

Covariance Parameter Estimates				
Cov Parm	Estimate	Alpha	Lower	Upper
Animal	0.9347	0.05	0.2578	32.5866
Residual	3.5698	0.05	2.4858	5.5605

Fit Statistics	
-2 Res Log Likelihood	225.0
AIC (Smaller is Better)	229.0
AICC (Smaller is Better)	229.3
BIC (Smaller is Better)	228.6

Solution for Fixed Effects									
Effect	Genotype	Estimate	Standard Error	DF	t Value	Pr > |t|	Alpha	Lower	Upper
Intercept		4.5211	0.6659	4	6.79	0.0025	0.05	2.6721	6.3701
Genotype	Meg +/+	1.8024	0.9506	48	1.90	0.0640	0.05	-0.1090	3.7138
Genotype	Meg +/-	0	.	.	.	.	.	.	.

Solution for Random Effects									
Effect	Animal	Estimate	Std Err Pred	DF	t Value	Pr > |t|	Alpha	Lower	Upper
Animal	1	-0.6662	0.6980	48	-0.95	0.3446	0.05	-2.0696	0.7372
Animal	2	0.4134	0.7317	48	0.56	0.5747	0.05	-1.0578	1.8846
Animal	3	0.2528	0.7229	48	0.35	0.7281	0.05	-1.2007	1.7064
Animal	4	-0.5999	0.7050	48	-0.85	0.3991	0.05	-2.0174	0.8177
Animal	5	1.1219	0.6934	48	1.62	0.1123	0.05	-0.2724	2.5161
Animal	6	-0.5220	0.7181	48	-0.73	0.4708	0.05	-1.9657	0.9217

Type 3 Tests of Fixed Effects				
Effect	Num DF	Den DF	F Value	Pr > F
Genotype	1	48	3.59	0.0640

Least Squares Means									
Effect	Genotype	Estimate	Standard Error	DF	t Value	Pr > |t|	Alpha	Lower	Upper
Genotype	Meg +/+	6.3235	0.6784	48	9.32	<.0001	0.05	4.9595	7.6875
Genotype	Meg +/-	4.5211	0.6659	48	6.79	<.0001	0.05	3.1821	5.8601

DistSoma=150

Differences of Least Squares Means												
**Effect**	**Genotype**	**Genotype**	**Estimate**	**Standard Error**	**DF**	**t Value**	**Pr > |t|**	**Adjustment**	**Adj P**	**Alpha**	**Lower**	**Upper**
**Genotype**	Meg +/+	Meg +/-	1.8024	0.9506	48	1.90	0.0640	Tukey-Kramer	0.0640	0.05	-0.1090	3.7138

Differences of Least Squares Means				
**Effect**	**Genotype**	**Genotype**	**Adj Lower**	**Adj Upper**
**Genotype**	Meg +/+	Meg +/-	-0.1089	3.7137

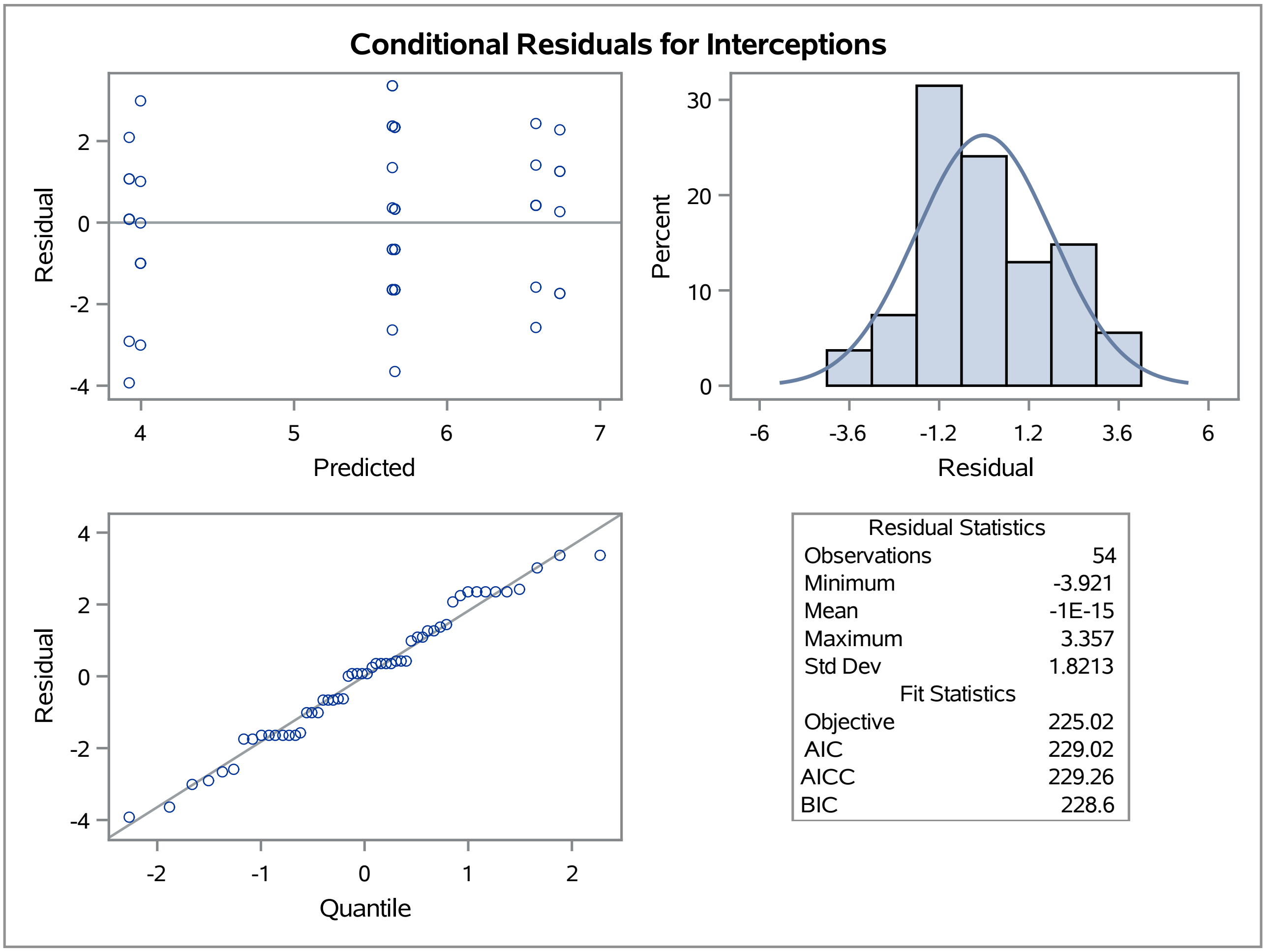

DistSoma=156

Model Information	
**Data Set**	WORK.TEMPDATASORTED
**Dependent Variable**	Interceptions
**Covariance Structure**	Variance Components
**Estimation Method**	REML
**Residual Variance Method**	Profile
**Fixed Effects SE Method**	Model-Based
**Degrees of Freedom Method**	Containment

Class Level Information		
**Class**	**Levels**	**Values**
**Genotype**	2	Meg +/+ Meg +/-
**Animal**	6	1 2 3 4 5 6

Dimensions	
**Covariance Parameters**	2
**Columns in X**	3
**Columns in Z**	6
**Subjects**	1
**Max Obs per Subject**	54

Number of Observations	
**Number of Observations Read**	54
**Number of Observations Used**	54
**Number of Observations Not Used**	0

Iteration History			
**Iteration**	**Evaluations**	**-2 Res Log Like**	**Criterion**
**0**	1	234.60649792	
**1**	5	230.11493082	0.00003232
**2**	1	230.11286100	0.00000015
**3**	1	230.11285085	0.00000000

Convergence criteria met.

DistSoma=156

Covariance Parameter Estimates				
**Cov Parm**	**Estimate**	**Alpha**	**Lower**	**Upper**
**Animal**	1.0285	0.05	0.2836	35.8855
**Residual**	3.9380	0.05	2.7422	6.1339

Fit Statistics	
**-2 Res Log Likelihood**	230.1
**AIC (Smaller is Better)**	234.1
**AICC (Smaller is Better)**	234.4
**BIC (Smaller is Better)**	233.7

Solution for Fixed Effects									
**Effect**	**Genotype**	**Estimate**	**Standard Error**	**DF**	**t Value**	**Pr > |t|**	**Alpha**	**Lower**	**Upper**
**Intercept**		4.4842	0.6988	4	6.42	0.0030	0.05	2.5440	6.4244
**Genotype**	Meg +/+	1.7806	0.9976	48	1.78	0.0806	0.05	-0.2251	3.7864
**Genotype**	Meg +/-	0	.	.	.	.	.	.	.

Solution for Random Effects									
**Effect**	**Animal**	**Estimate**	**Std Err Pred**	**DF**	**t Value**	**Pr > |t|**	**Alpha**	**Lower**	**Upper**
**Animal**	1	-0.7394	0.7324	48	-1.01	0.3178	0.05	-2.2120	0.7333
**Animal**	2	0.4488	0.7678	48	0.58	0.5616	0.05	-1.0950	1.9926
**Animal**	3	0.2906	0.7586	48	0.38	0.7034	0.05	-1.2347	1.8158
**Animal**	4	-0.6515	0.7398	48	-0.88	0.3829	0.05	-2.1389	0.8360
**Animal**	5	1.1491	0.7276	48	1.58	0.1208	0.05	-0.3139	2.6122
**Animal**	6	-0.4977	0.7535	48	-0.66	0.5121	0.05	-2.0126	1.0173

Type 3 Tests of Fixed Effects				
**Effect**	**Num DF**	**Den DF**	**F Value**	**Pr > F**
**Genotype**	1	48	3.19	0.0806

Least Squares Means									
**Effect**	**Genotype**	**Estimate**	**Standard Error**	**DF**	**t Value**	**Pr > |t|**	**Alpha**	**Lower**	**Upper**
**Genotype**	Meg +/+	6.2648	0.7119	48	8.80	<.0001	0.05	4.8334	7.6962
**Genotype**	Meg +/-	4.4842	0.6988	48	6.42	<.0001	0.05	3.0791	5.8893

DistSoma=156

Differences of Least Squares Means												
**Effect**	**Genotype**	**Genotype**	**Estimate**	**Standard Error**	**DF**	**t Value**	**Pr > |t|**	**Adjustment**	**Adj P**	**Alpha**	**Lower**	**Upper**
**Genotype**	Meg +/+	Meg +/-	1.7806	0.9976	48	1.78	0.0806	Tukey-Kramer	0.0806	0.05	-0.2251	3.7864

Differences of Least Squares Means				
**Effect**	**Genotype**	**Genotype**	**Adj Lower**	**Adj Upper**
**Genotype**	Meg +/+	Meg +/-	-0.2251	3.7863

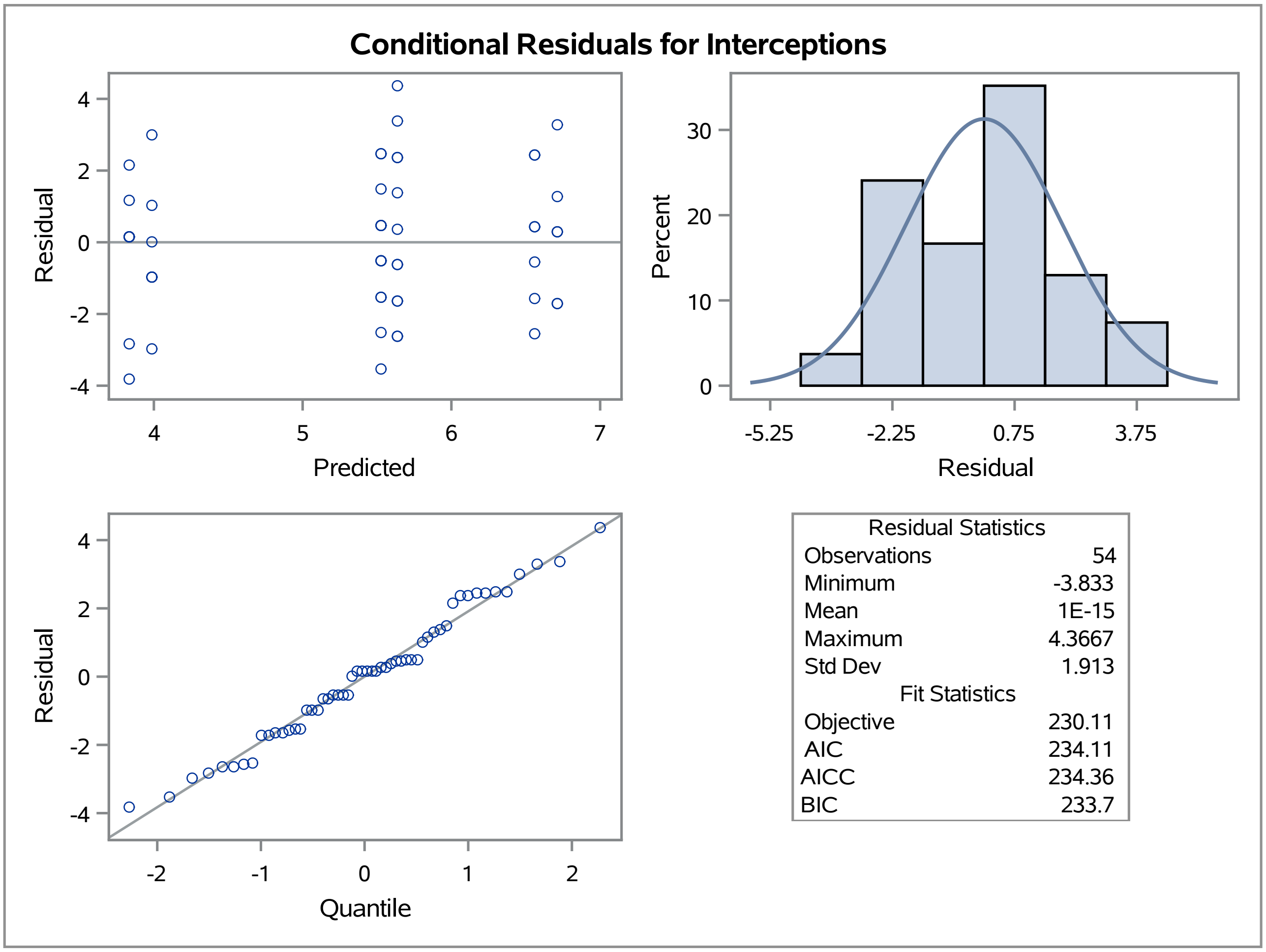

DistSoma=162

Model Information	
**Data Set**	WORK.TEMPDATASORTED
**Dependent Variable**	Interceptions
**Covariance Structure**	Variance Components
**Estimation Method**	REML
**Residual Variance Method**	Profile
**Fixed Effects SE Method**	Model-Based
**Degrees of Freedom Method**	Containment

Class Level Information		
**Class**	**Levels**	**Values**
**Genotype**	2	Meg +/+ Meg +/-
**Animal**	6	1 2 3 4 5 6

Dimensions	
**Covariance Parameters**	2
**Columns in X**	3
**Columns in Z**	6
**Subjects**	1
**Max Obs per Subject**	54

Number of Observations	
**Number of Observations Read**	54
**Number of Observations Used**	54
**Number of Observations Not Used**	0

Iteration History			
**Iteration**	**Evaluations**	**-2 Res Log Like**	**Criterion**
**0**	1	232.11734266	
**1**	3	227.81602815	0.00092204
**2**	1	227.74552834	0.00007763
**3**	1	227.74009277	0.00000072
**4**	1	227.74004485	0.00000000

Convergence criteria met.

DistSoma=162

Covariance Parameter Estimates				
Cov Parm	Estimate	Alpha	Lower	Upper
Animal	0.9726	0.05	0.2670	35.0303
Residual	3.7645	0.05	2.6213	5.8640

Fit Statistics	
-2 Res Log Likelihood	227.7
AIC (Smaller is Better)	231.7
AICC (Smaller is Better)	232.0
BIC (Smaller is Better)	231.3

Solution for Fixed Effects									
Effect	Genotype	Estimate	Standard Error	DF	t Value	Pr > |t|	Alpha	Lower	Upper
Intercept		4.4316	0.6806	4	6.51	0.0029	0.05	2.5418	6.3214
Genotype	Meg +/+	1.7901	0.9717	48	1.84	0.0716	0.05	-0.1636	3.7439
Genotype	Meg +/-	0	.	.	.	.	.	.	.

Solution for Random Effects									
Effect	Animal	Estimate	Std Err Pred	DF	t Value	Pr > |t|	Alpha	Lower	Upper
Animal	1	-0.8822	0.7132	48	-1.24	0.2221	0.05	-2.3162	0.5518
Animal	2	0.4731	0.7477	48	0.63	0.5300	0.05	-1.0303	1.9764
Animal	3	0.4091	0.7387	48	0.55	0.5823	0.05	-1.0762	1.8944
Animal	4	-0.5349	0.7204	48	-0.74	0.4614	0.05	-1.9834	0.9136
Animal	5	0.9968	0.7086	48	1.41	0.1659	0.05	-0.4278	2.4215
Animal	6	-0.4619	0.7338	48	-0.63	0.5320	0.05	-1.9372	1.0134

Type 3 Tests of Fixed Effects				
Effect	Num DF	Den DF	F Value	Pr > F
Genotype	1	48	3.39	0.0716

Least Squares Means									
Effect	Genotype	Estimate	Standard Error	DF	t Value	Pr > |t|	Alpha	Lower	Upper
Genotype	Meg +/+	6.2218	0.6935	48	8.97	<.0001	0.05	4.8275	7.6161
Genotype	Meg +/-	4.4316	0.6806	48	6.51	<.0001	0.05	3.0631	5.8002

DistSoma=162

Differences of Least Squares Means												
**Effect**	**Genotype**	**Genotype**	**Estimate**	**Standard Error**	**DF**	**t Value**	**Pr > |t|**	**Adjustment**	**Adj P**	**Alpha**	**Lower**	**Upper**
**Genotype**	Meg +/+	Meg +/-	1.7901	0.9717	48	1.84	0.0716	Tukey-Kramer	0.0716	0.05	-0.1636	3.7439

Differences of Least Squares Means				
**Effect**	**Genotype**	**Genotype**	**Adj Lower**	**Adj Upper**
**Genotype**	Meg +/+	Meg +/-	-0.1635	3.7438

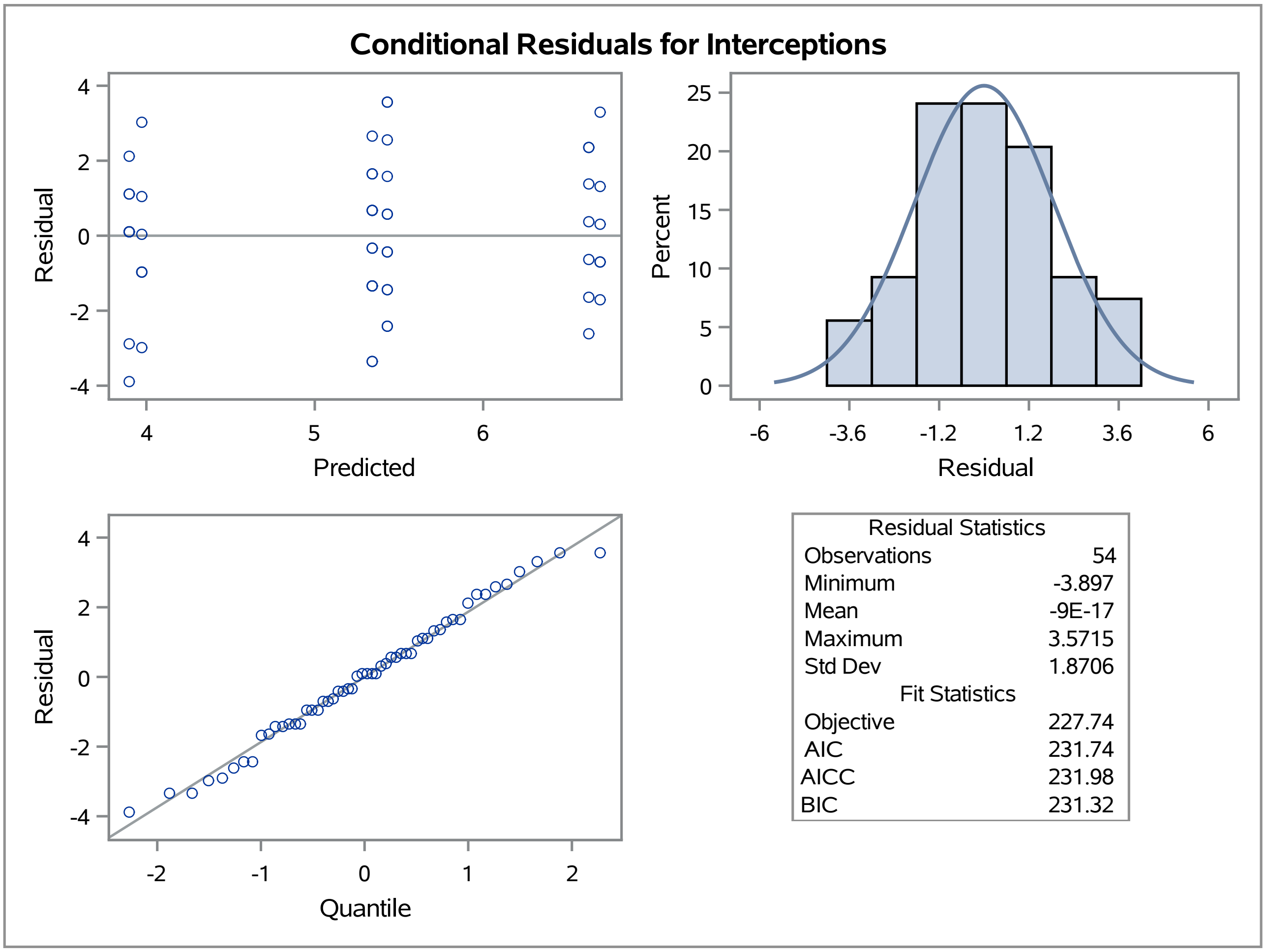

DistSoma=168

Model Information	
**Data Set**	WORK.TEMPDATASORTED
**Dependent Variable**	Interceptions
**Covariance Structure**	Variance Components
**Estimation Method**	REML
**Residual Variance Method**	Profile
**Fixed Effects SE Method**	Model-Based
**Degrees of Freedom Method**	Containment

Class Level Information		
**Class**	**Levels**	**Values**
**Genotype**	2	Meg +/+ Meg +/-
**Animal**	6	1 2 3 4 5 6

Dimensions	
**Covariance Parameters**	2
**Columns in X**	3
**Columns in Z**	6
**Subjects**	1
**Max Obs per Subject**	54

Number of Observations	
**Number of Observations Read**	54
**Number of Observations Used**	54
**Number of Observations Not Used**	0

Iteration History			
**Iteration**	**Evaluations**	**-2 Res Log Like**	**Criterion**
**0**	1	235.54078667	
**1**	3	231.17738673	0.00073559
**2**	1	231.12021514	0.00005260
**3**	1	231.11646950	0.00000035
**4**	1	231.11644599	0.00000000

Convergence criteria met.

DistSoma=168

Covariance Parameter Estimates				
Cov Parm	Estimate	Alpha	Lower	Upper
Animal	1.0512	0.05	0.2892	37.3421
Residual	4.0141	0.05	2.7950	6.2534

Fit Statistics	
-2 Res Log Likelihood	231.1
AIC (Smaller is Better)	235.1
AICC (Smaller is Better)	235.4
BIC (Smaller is Better)	234.7

Solution for Fixed Effects									
Effect	Genotype	Estimate	Standard Error	DF	t Value	Pr > |t|	Alpha	Lower	Upper
Intercept		4.3110	0.7062	4	6.10	0.0036	0.05	2.3502	6.2717
Genotype	Meg +/+	1.8740	1.0081	48	1.86	0.0692	0.05	-0.1529	3.9009
Genotype	Meg +/-	0	.	.	.	.	.	.	.

Solution for Random Effects									
Effect	Animal	Estimate	Std Err Pred	DF	t Value	Pr > |t|	Alpha	Lower	Upper
Animal	1	-1.0348	0.7402	48	-1.40	0.1685	0.05	-2.5231	0.4534
Animal	2	0.5999	0.7759	48	0.77	0.4432	0.05	-0.9602	2.1600
Animal	3	0.4349	0.7666	48	0.57	0.5731	0.05	-1.1065	1.9763
Animal	4	-0.6084	0.7477	48	-0.81	0.4198	0.05	-2.1116	0.8949
Animal	5	0.9020	0.7354	48	1.23	0.2260	0.05	-0.5765	2.3805
Animal	6	-0.2936	0.7615	48	-0.39	0.7015	0.05	-1.8246	1.2374

Type 3 Tests of Fixed Effects				
Effect	Num DF	Den DF	F Value	Pr > F
Genotype	1	48	3.46	0.0692

Least Squares Means									
Effect	Genotype	Estimate	Standard Error	DF	t Value	Pr > |t|	Alpha	Lower	Upper
Genotype	Meg +/+	6.1850	0.7194	48	8.60	<.0001	0.05	4.7385	7.6314
Genotype	Meg +/-	4.3110	0.7062	48	6.10	<.0001	0.05	2.8910	5.7309

DistSoma=168

Differences of Least Squares Means												
**Effect**	**Genotype**	**Genotype**	**Estimate**	**Standard Error**	**DF**	**t Value**	**Pr > |t|**	**Adjustment**	**Adj P**	**Alpha**	**Lower**	**Upper**
**Genotype**	Meg +/+	Meg +/-	1.8740	1.0081	48	1.86	0.0692	Tukey-Kramer	0.0692	0.05	-0.1529	3.9009

Differences of Least Squares Means				
**Effect**	**Genotype**	**Genotype**	**Adj Lower**	**Adj Upper**
**Genotype**	Meg +/+	Meg +/-	-0.1529	3.9009

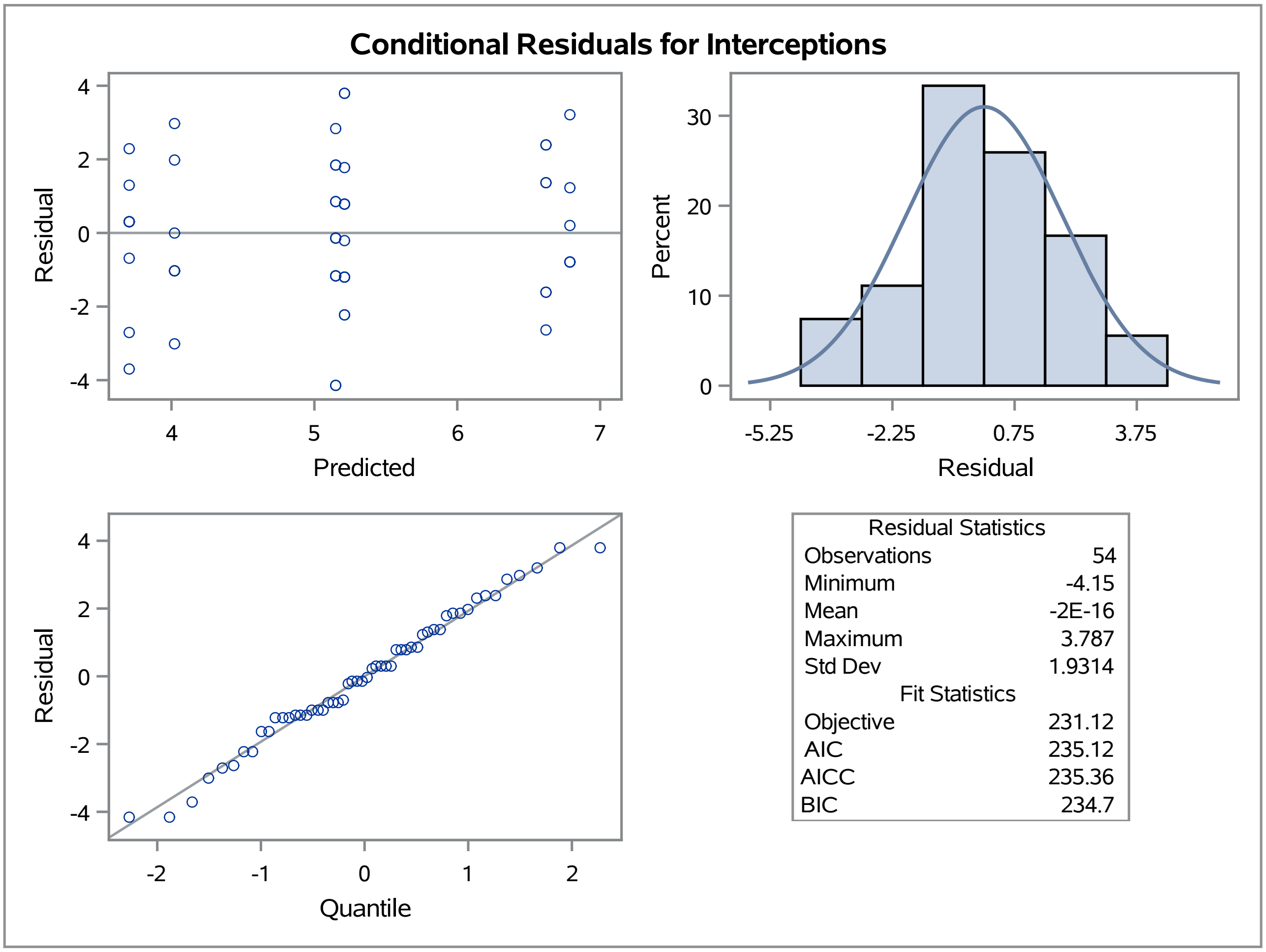

DistSoma=174

Model Information	
**Data Set**	WORK.TEMPDATASORTED
**Dependent Variable**	Interceptions
**Covariance Structure**	Variance Components
**Estimation Method**	REML
**Residual Variance Method**	Profile
**Fixed Effects SE Method**	Model-Based
**Degrees of Freedom Method**	Containment

Class Level Information		
**Class**	**Levels**	**Values**
**Genotype**	2	Meg +/+ Meg +/-
**Animal**	6	1 2 3 4 5 6

Dimensions	
**Covariance Parameters**	2
**Columns in X**	3
**Columns in Z**	6
**Subjects**	1
**Max Obs per Subject**	54

Number of Observations	
**Number of Observations Read**	54
**Number of Observations Used**	54
**Number of Observations Not Used**	0

Iteration History			
**Iteration**	**Evaluations**	**-2 Res Log Like**	**Criterion**
**0**	1	235.00324406	
**1**	3	231.90488304	0.00041809
**2**	1	231.87296188	0.00001864
**3**	1	231.87165144	0.00000005
**4**	1	231.87164835	0.00000000

Convergence criteria met.

DistSoma=174

Covariance Parameter Estimates				
Cov Parm	Estimate	Alpha	Lower	Upper
Animal	0.8400	0.05	0.2158	50.3461
Residual	4.1252	0.05	2.8728	6.4247

Fit Statistics	
-2 Res Log Likelihood	231.9
AIC (Smaller is Better)	235.9
AICC (Smaller is Better)	236.1
BIC (Smaller is Better)	235.5

Solution for Fixed Effects									
Effect	Genotype	Estimate	Standard Error	DF	t Value	Pr > |t|	Alpha	Lower	Upper
Intercept		4.2216	0.6573	4	6.42	0.0030	0.05	2.3966	6.0466
Genotype	Meg +/+	1.8043	0.9396	48	1.92	0.0608	0.05	-0.08489	3.6935
Genotype	Meg +/-	0	.	.	.	.	.	.	.

Solution for Random Effects									
Effect	Animal	Estimate	Std Err Pred	DF	t Value	Pr > |t|	Alpha	Lower	Upper
Animal	1	-0.8563	0.6840	48	-1.25	0.2167	0.05	-2.2315	0.5189
Animal	2	0.5357	0.7172	48	0.75	0.4588	0.05	-0.9064	1.9777
Animal	3	0.3206	0.7085	48	0.45	0.6530	0.05	-1.1040	1.7452
Animal	4	-0.5747	0.6909	48	-0.83	0.4097	0.05	-1.9639	0.8145
Animal	5	0.7889	0.6793	48	1.16	0.2513	0.05	-0.5770	2.1548
Animal	6	-0.2142	0.7040	48	-0.30	0.7622	0.05	-1.6296	1.2012

Type 3 Tests of Fixed Effects				
Effect	Num DF	Den DF	F Value	Pr > F
Genotype	1	48	3.69	0.0608

Least Squares Means									
Effect	Genotype	Estimate	Standard Error	DF	t Value	Pr > |t|	Alpha	Lower	Upper
Genotype	Meg +/+	6.0259	0.6714	48	8.97	<.0001	0.05	4.6759	7.3759
Genotype	Meg +/-	4.2216	0.6573	48	6.42	<.0001	0.05	2.9000	5.5432

DistSoma=174

Differences of Least Squares Means												
**Effect**	**Genotype**	**Genotype**	**Estimate**	**Standard Error**	**DF**	**t Value**	**Pr > |t|**	**Adjustment**	**Adj P**	**Alpha**	**Lower**	**Upper**
**Genotype**	Meg +/+	Meg +/-	1.8043	0.9396	48	1.92	0.0608	Tukey-Kramer	0.0608	0.05	-0.08489	3.6935

Differences of Least Squares Means				
**Effect**	**Genotype**	**Genotype**	**Adj Lower**	**Adj Upper**
**Genotype**	Meg +/+	Meg +/-	-0.08483	3.6934

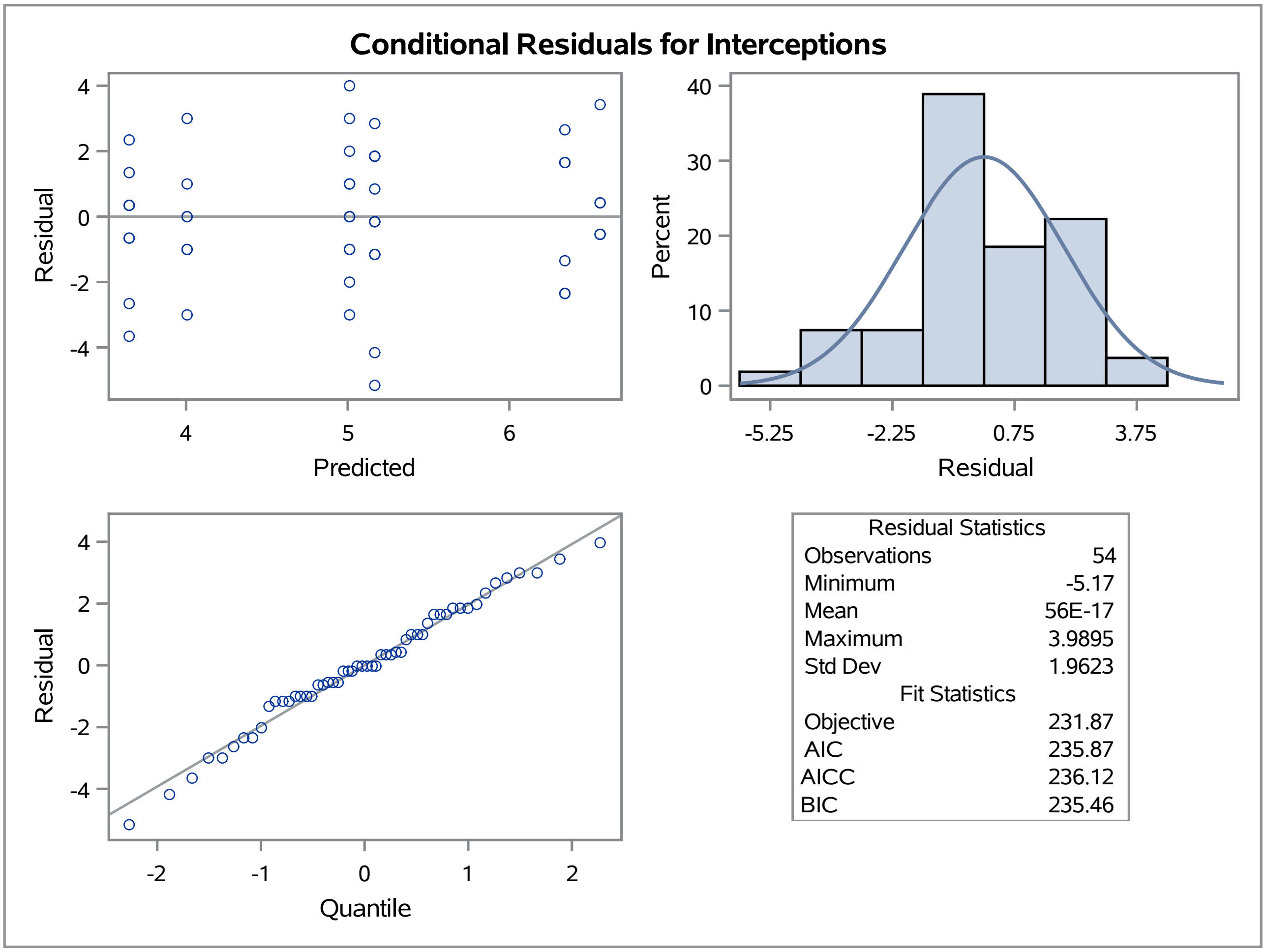

DistSoma=180

Model Information	
**Data Set**	WORK.TEMPDATASORTED
**Dependent Variable**	Interceptions
**Covariance Structure**	Variance Components
**Estimation Method**	REML
**Residual Variance Method**	Profile
**Fixed Effects SE Method**	Model-Based
**Degrees of Freedom Method**	Containment

Class Level Information		
**Class**	**Levels**	**Values**
**Genotype**	2	Meg +/+ Meg +/-
**Animal**	6	1 2 3 4 5 6

Dimensions	
**Covariance Parameters**	2
**Columns in X**	3
**Columns in Z**	6
**Subjects**	1
**Max Obs per Subject**	54

Number of Observations	
**Number of Observations Read**	54
**Number of Observations Used**	54
**Number of Observations Not Used**	0

Iteration History			
**Iteration**	**Evaluations**	**-2 Res Log Like**	**Criterion**
**0**	1	238.37495123	
**1**	3	236.62930467	0.00011962
**2**	1	236.62024394	0.00000178
**3**	1	236.62011706	0.00000000

Convergence criteria met.

DistSoma=180

Covariance Parameter Estimates				
**Cov Parm**	**Estimate**	**Alpha**	**Lower**	**Upper**
**Animal**	0.6348	0.05	0.1424	140.47
**Residual**	4.5994	0.05	3.2038	7.1611

Fit Statistics	
**-2 Res Log Likelihood**	236.6
**AIC (Smaller is Better)**	240.6
**AICC (Smaller is Better)**	240.9
**BIC (Smaller is Better)**	240.2

Solution for Fixed Effects									
**Effect**	**Genotype**	**Estimate**	**Standard Error**	**DF**	**t Value**	**Pr > |t|**	**Alpha**	**Lower**	**Upper**
**Intercept**		4.1886	0.6167	4	6.79	0.0025	0.05	2.4763	5.9010
**Genotype**	Meg +/+	1.8380	0.8835	48	2.08	0.0428	0.05	0.06169	3.6144
**Genotype**	Meg +/-	0	.	.	.	.	.	.	.

Solution for Random Effects									
**Effect**	**Animal**	**Estimate**	**Std Err Pred**	**DF**	**t Value**	**Pr > |t|**	**Alpha**	**Lower**	**Upper**
**Animal**	1	-0.7086	0.6262	48	-1.13	0.2634	0.05	-1.9678	0.5505
**Animal**	2	0.4409	0.6553	48	0.67	0.5042	0.05	-0.8766	1.7584
**Animal**	3	0.2677	0.6477	48	0.41	0.6812	0.05	-1.0346	1.5700
**Animal**	4	-0.5354	0.6324	48	-0.85	0.4014	0.05	-1.8068	0.7360
**Animal**	5	0.5579	0.6220	48	0.90	0.3742	0.05	-0.6927	1.8085
**Animal**	6	-0.02249	0.6440	48	-0.03	0.9723	0.05	-1.3173	1.2723

Type 3 Tests of Fixed Effects				
**Effect**	**Num DF**	**Den DF**	**F Value**	**Pr > F**
**Genotype**	1	48	4.33	0.0428

Least Squares Means									
**Effect**	**Genotype**	**Estimate**	**Standard Error**	**DF**	**t Value**	**Pr > |t|**	**Alpha**	**Lower**	**Upper**
**Genotype**	Meg +/+	6.0266	0.6326	48	9.53	<.0001	0.05	4.7548	7.2985
**Genotype**	Meg +/-	4.1886	0.6167	48	6.79	<.0001	0.05	2.9486	5.4287

DistSoma=180

Differences of Least Squares Means												
**Effect**	**Genotype**	**Genotype**	**Estimate**	**Standard Error**	**DF**	**t Value**	**Pr > |t|**	**Adjustment**	**Adj P**	**Alpha**	**Lower**	**Upper**
**Genotype**	Meg +/+	Meg +/-	1.8380	0.8835	48	2.08	0.0428	Tukey-Kramer	0.0428	0.05	0.06169	3.6144

Differences of Least Squares Means				
**Effect**	**Genotype**	**Genotype**	**Adj Lower**	**Adj Upper**
**Genotype**	Meg +/+	Meg +/-	0.06175	3.6143

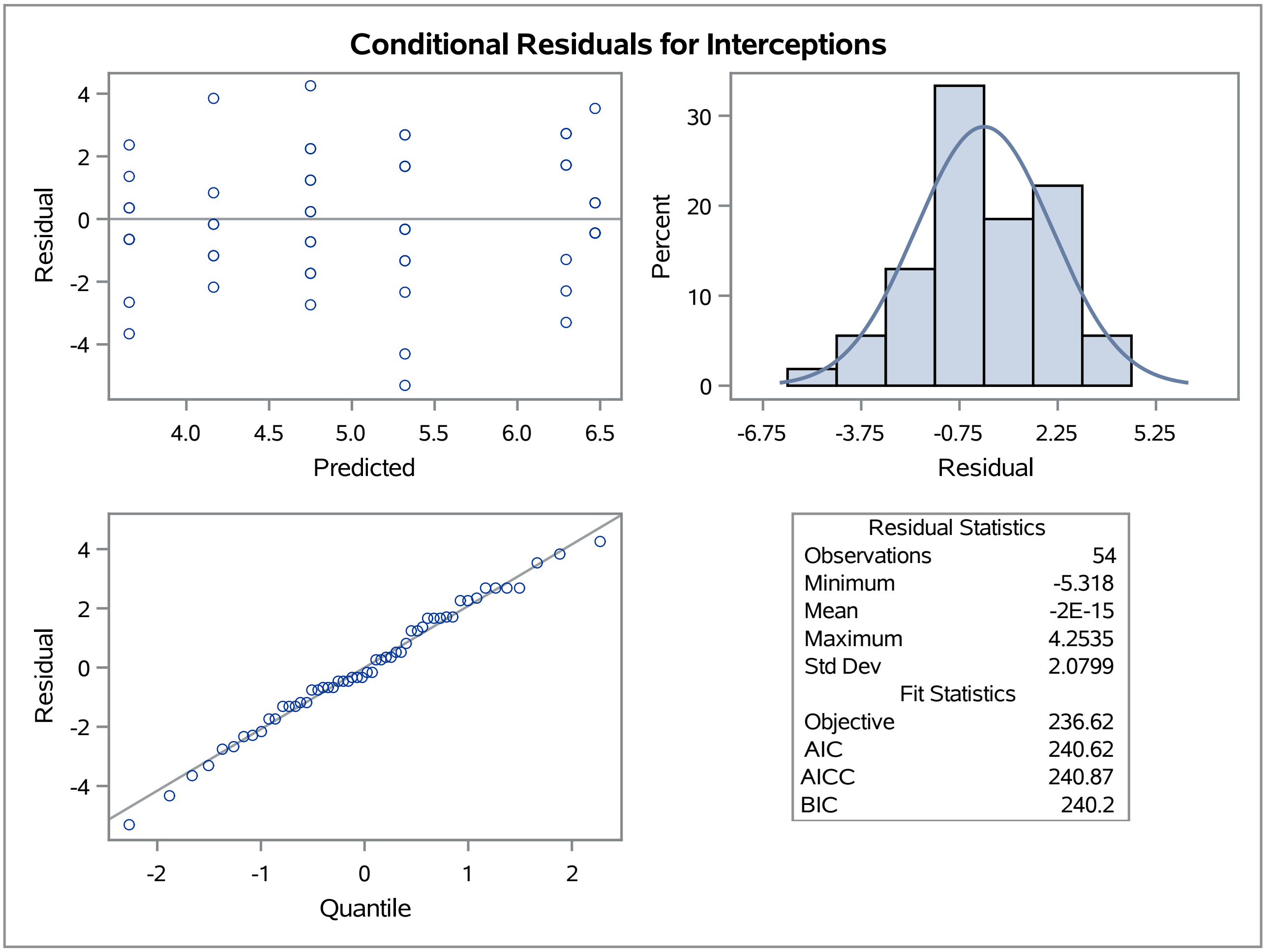

DistSoma=186

Model Information	
**Data Set**	WORK.TEMPDATASORTED
**Dependent Variable**	Interceptions
**Covariance Structure**	Variance Components
**Estimation Method**	REML
**Residual Variance Method**	Profile
**Fixed Effects SE Method**	Model-Based
**Degrees of Freedom Method**	Containment

Class Level Information		
**Class**	**Levels**	**Values**
**Genotype**	2	Meg +/+ Meg +/-
**Animal**	6	1 2 3 4 5 6

Dimensions	
**Covariance Parameters**	2
**Columns in X**	3
**Columns in Z**	6
**Subjects**	1
**Max Obs per Subject**	54

Number of Observations	
**Number of Observations Read**	54
**Number of Observations Used**	54
**Number of Observations Not Used**	0

Iteration History			
**Iteration**	**Evaluations**	**-2 Res Log Like**	**Criterion**
**0**	1	237.92370557	
**1**	3	235.59070049	0.00012053
**2**	1	235.58163801	0.00000179
**3**	1	235.58151121	0.00000000

Convergence criteria met.

DistSoma=186

Covariance Parameter Estimates				
Cov Parm	Estimate	Alpha	Lower	Upper
Animal	0.7571	0.05	0.1823	80.4918
Residual	4.4686	0.05	3.1119	6.9599

Fit Statistics	
-2 Res Log Likelihood	235.6
AIC (Smaller is Better)	239.6
AICC (Smaller is Better)	239.8
BIC (Smaller is Better)	239.2

Solution for Fixed Effects									
Effect	Genotype	Estimate	Standard Error	DF	t Value	Pr > |t|	Alpha	Lower	Upper
Intercept		4.1957	0.6455	4	6.50	0.0029	0.05	2.4034	5.9880
Genotype	Meg +/+	1.7551	0.9237	48	1.90	0.0634	0.05	-0.1021	3.6123
Genotype	Meg +/-	0	.	.	.	.	.	.	.

Solution for Random Effects									
Effect	Animal	Estimate	Std Err Pred	DF	t Value	Pr > |t|	Alpha	Lower	Upper
Animal	1	-0.8655	0.6655	48	-1.30	0.1996	0.05	-2.2036	0.4725
Animal	2	0.5289	0.6974	48	0.76	0.4519	0.05	-0.8733	1.9311
Animal	3	0.3367	0.6891	48	0.49	0.6273	0.05	-1.0488	1.7221
Animal	4	-0.5879	0.6722	48	-0.87	0.3861	0.05	-1.9394	0.7636
Animal	5	0.5391	0.6609	48	0.82	0.4187	0.05	-0.7898	1.8680
Animal	6	0.04882	0.6848	48	0.07	0.9435	0.05	-1.3281	1.4258

Type 3 Tests of Fixed Effects				
Effect	Num DF	Den DF	F Value	Pr > F
Genotype	1	48	3.61	0.0634

Least Squares Means									
Effect	Genotype	Estimate	Standard Error	DF	t Value	Pr > |t|	Alpha	Lower	Upper
Genotype	Meg +/+	5.9509	0.6607	48	9.01	<.0001	0.05	4.6225	7.2792
Genotype	Meg +/-	4.1957	0.6455	48	6.50	<.0001	0.05	2.8978	5.4937

DistSoma=186

Differences of Least Squares Means												
**Effect**	**Genotype**	**Genotype**	**Estimate**	**Standard Error**	**DF**	**t Value**	**Pr > |t|**	**Adjustment**	**Adj P**	**Alpha**	**Lower**	**Upper**
**Genotype**	Meg +/+	Meg +/-	1.7551	0.9237	48	1.90	0.0634	Tukey-Kramer	0.0634	0.05	-0.1021	3.6123

Differences of Least Squares Means				
**Effect**	**Genotype**	**Genotype**	**Adj Lower**	**Adj Upper**
**Genotype**	Meg +/+	Meg +/-	-0.1020	3.6123

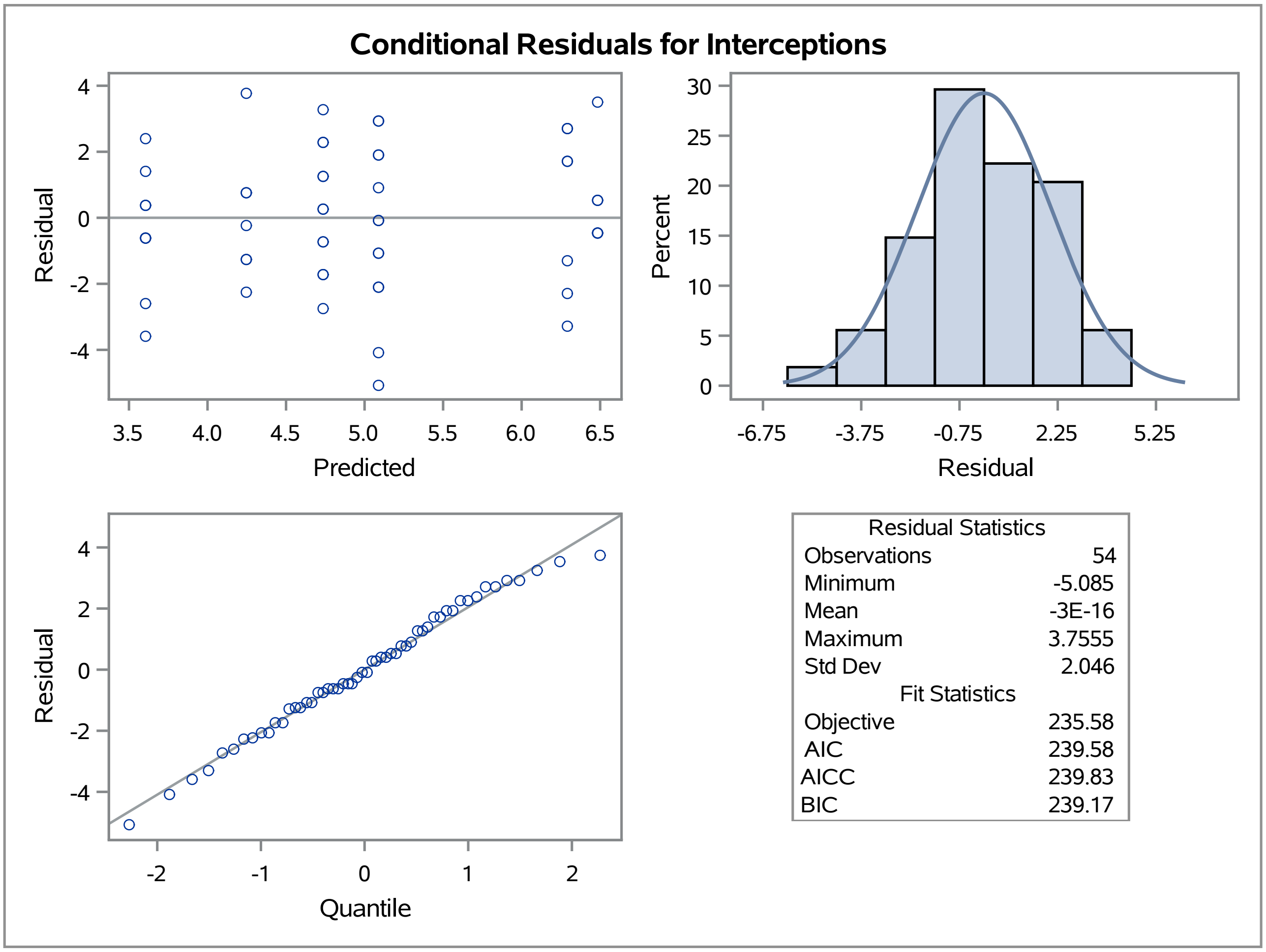

DistSoma=192

Model Information	
**Data Set**	WORK.TEMPDATASORTED
**Dependent Variable**	Interceptions
**Covariance Structure**	Variance Components
**Estimation Method**	REML
**Residual Variance Method**	Profile
**Fixed Effects SE Method**	Model-Based
**Degrees of Freedom Method**	Containment

Class Level Information		
**Class**	**Levels**	**Values**
**Genotype**	2	Meg +/+ Meg +/-
**Animal**	6	1 2 3 4 5 6

Dimensions	
**Covariance Parameters**	2
**Columns in X**	3
**Columns in Z**	6
**Subjects**	1
**Max Obs per Subject**	54

Number of Observations	
**Number of Observations Read**	54
**Number of Observations Used**	54
**Number of Observations Not Used**	0

Iteration History			
**Iteration**	**Evaluations**	**-2 Res Log Like**	**Criterion**
**0**	1	240.24681362	
**1**	3	238.37184728	0.00008062
**2**	1	238.36572987	0.00000084
**3**	1	238.36566956	0.00000000

Convergence criteria met.

DistSoma=192

Covariance Parameter Estimates				
**Cov Parm**	**Estimate**	**Alpha**	**Lower**	**Upper**
**Animal**	0.6941	0.05	0.1585	126.57
**Residual**	4.7449	0.05	3.3046	7.3892

Fit Statistics	
**-2 Res Log Likelihood**	238.4
**AIC (Smaller is Better)**	242.4
**AICC (Smaller is Better)**	242.6
**BIC (Smaller is Better)**	241.9

Solution for Fixed Effects									
**Effect**	**Genotype**	**Estimate**	**Standard Error**	**DF**	**t Value**	**Pr > |t|**	**Alpha**	**Lower**	**Upper**
**Intercept**		4.1305	0.6369	4	6.49	0.0029	0.05	2.3623	5.8987
**Genotype**	Meg +/+	1.6846	0.9120	48	1.85	0.0709	0.05	-0.1491	3.5183
**Genotype**	Meg +/-	0	.	.	.	.	.	.	.

Solution for Random Effects									
**Effect**	**Animal**	**Estimate**	**Std Err Pred**	**DF**	**t Value**	**Pr > |t|**	**Alpha**	**Lower**	**Upper**
**Animal**	1	-0.7863	0.6498	48	-1.21	0.2322	0.05	-2.0928	0.5202
**Animal**	2	0.4759	0.6803	48	0.70	0.4875	0.05	-0.8918	1.8437
**Animal**	3	0.3103	0.6723	48	0.46	0.6464	0.05	-1.0414	1.6621
**Animal**	4	-0.5793	0.6562	48	-0.88	0.3817	0.05	-1.8988	0.7401
**Animal**	5	0.5008	0.6454	48	0.78	0.4415	0.05	-0.7968	1.7985
**Animal**	6	0.07852	0.6684	48	0.12	0.9070	0.05	-1.2654	1.4224

Type 3 Tests of Fixed Effects				
**Effect**	**Num DF**	**Den DF**	**F Value**	**Pr > F**
**Genotype**	1	48	3.41	0.0709

Least Squares Means									
**Effect**	**Genotype**	**Estimate**	**Standard Error**	**DF**	**t Value**	**Pr > |t|**	**Alpha**	**Lower**	**Upper**
**Genotype**	Meg +/+	5.8151	0.6528	48	8.91	<.0001	0.05	4.5026	7.1277
**Genotype**	Meg +/-	4.1305	0.6369	48	6.49	<.0001	0.05	2.8500	5.4110

DistSoma=192

Differences of Least Squares Means												
Effect	Genotype	Genotype	Estimate	Standard Error	DF	t Value	Pr > |t|	Adjustment	Adj P	Alpha	Lower	Upper
Genotype	Meg +/+	Meg +/-	1.6846	0.9120	48	1.85	0.0709	Tukey-Kramer	0.0709	0.05	-0.1491	3.5183

Differences of Least Squares Means				
Effect	Genotype	Genotype	Adj Lower	Adj Upper
Genotype	Meg +/+	Meg +/-	-0.1490	3.5183

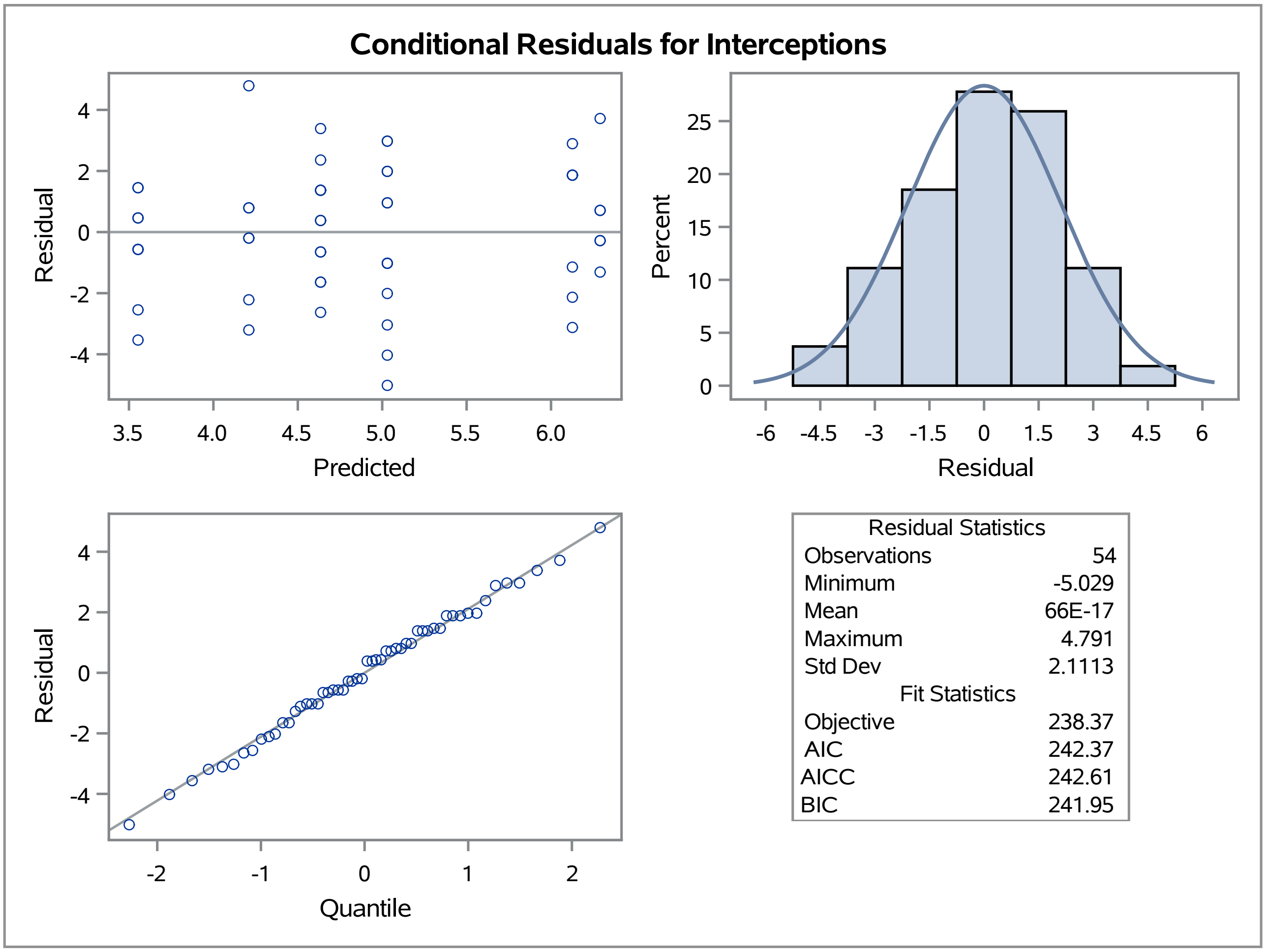

DistSoma=198

Model Information	
**Data Set**	WORK.TEMPDATASORTED
**Dependent Variable**	Interceptions
**Covariance Structure**	Variance Components
**Estimation Method**	REML
**Residual Variance Method**	Profile
**Fixed Effects SE Method**	Model-Based
**Degrees of Freedom Method**	Containment

Class Level Information		
**Class**	**Levels**	**Values**
**Genotype**	2	Meg +/+ Meg +/-
**Animal**	6	1 2 3 4 5 6

Dimensions	
**Covariance Parameters**	2
**Columns in X**	3
**Columns in Z**	6
**Subjects**	1
**Max Obs per Subject**	54

Number of Observations	
**Number of Observations Read**	54
**Number of Observations Used**	54
**Number of Observations Not Used**	0

Iteration History			
**Iteration**	**Evaluations**	**-2 Res Log Like**	**Criterion**
**0**	1	242.03639502	
**1**	3	239.32316927	0.00002677
**2**	1	239.32117103	0.00000010
**3**	1	239.32116400	0.00000000

Convergence criteria met.

DistSoma=198

Covariance Parameter Estimates				
Cov Parm	Estimate	Alpha	Lower	Upper
Animal	0.9282	0.05	0.2311	72.7983
Residual	4.7711	0.05	3.3211	7.4356

Fit Statistics	
-2 Res Log Likelihood	239.3
AIC (Smaller is Better)	243.3
AICC (Smaller is Better)	243.6
BIC (Smaller is Better)	242.9

Solution for Fixed Effects									
Effect	Genotype	Estimate	Standard Error	DF	t Value	Pr > |t|	Alpha	Lower	Upper
Intercept		4.0831	0.6965	4	5.86	0.0042	0.05	2.1493	6.0170
Genotype	Meg +/+	1.8792	0.9959	48	1.89	0.0652	0.05	-0.1232	3.8816
Genotype	Meg +/-	0	.	.	.	.	.	.	.

Solution for Random Effects									
Effect	Animal	Estimate	Std Err Pred	DF	t Value	Pr > |t|	Alpha	Lower	Upper
Animal	1	-0.9101	0.7233	48	-1.26	0.2144	0.05	-2.3645	0.5443
Animal	2	0.5589	0.7585	48	0.74	0.4648	0.05	-0.9661	2.0839
Animal	3	0.3512	0.7493	48	0.47	0.6414	0.05	-1.1553	1.8578
Animal	4	-0.8308	0.7307	48	-1.14	0.2612	0.05	-2.3000	0.6384
Animal	5	0.4669	0.7184	48	0.65	0.5189	0.05	-0.9776	1.9114
Animal	6	0.3639	0.7445	48	0.49	0.6272	0.05	-1.1330	1.8609

Type 3 Tests of Fixed Effects				
Effect	Num DF	Den DF	F Value	Pr > F
Genotype	1	48	3.56	0.0652

Least Squares Means									
Effect	Genotype	Estimate	Standard Error	DF	t Value	Pr > |t|	Alpha	Lower	Upper
Genotype	Meg +/+	5.9623	0.7118	48	8.38	<.0001	0.05	4.5311	7.3935
Genotype	Meg +/-	4.0831	0.6965	48	5.86	<.0001	0.05	2.6827	5.4836

DistSoma=198

Differences of Least Squares Means												
Effect	Genotype	Genotype	Estimate	Standard Error	DF	t Value	Pr > |t|	Adjustment	Adj P	Alpha	Lower	Upper
**Genotype**	Meg +/+	Meg +/-	1.8792	0.9959	48	1.89	0.0652	Tukey-Kramer	0.0652	0.05	-0.1232	3.8816

Differences of Least Squares Means				
Effect	Genotype	Genotype	Adj Lower	Adj Upper
**Genotype**	Meg +/+	Meg +/-	-0.1232	3.8815

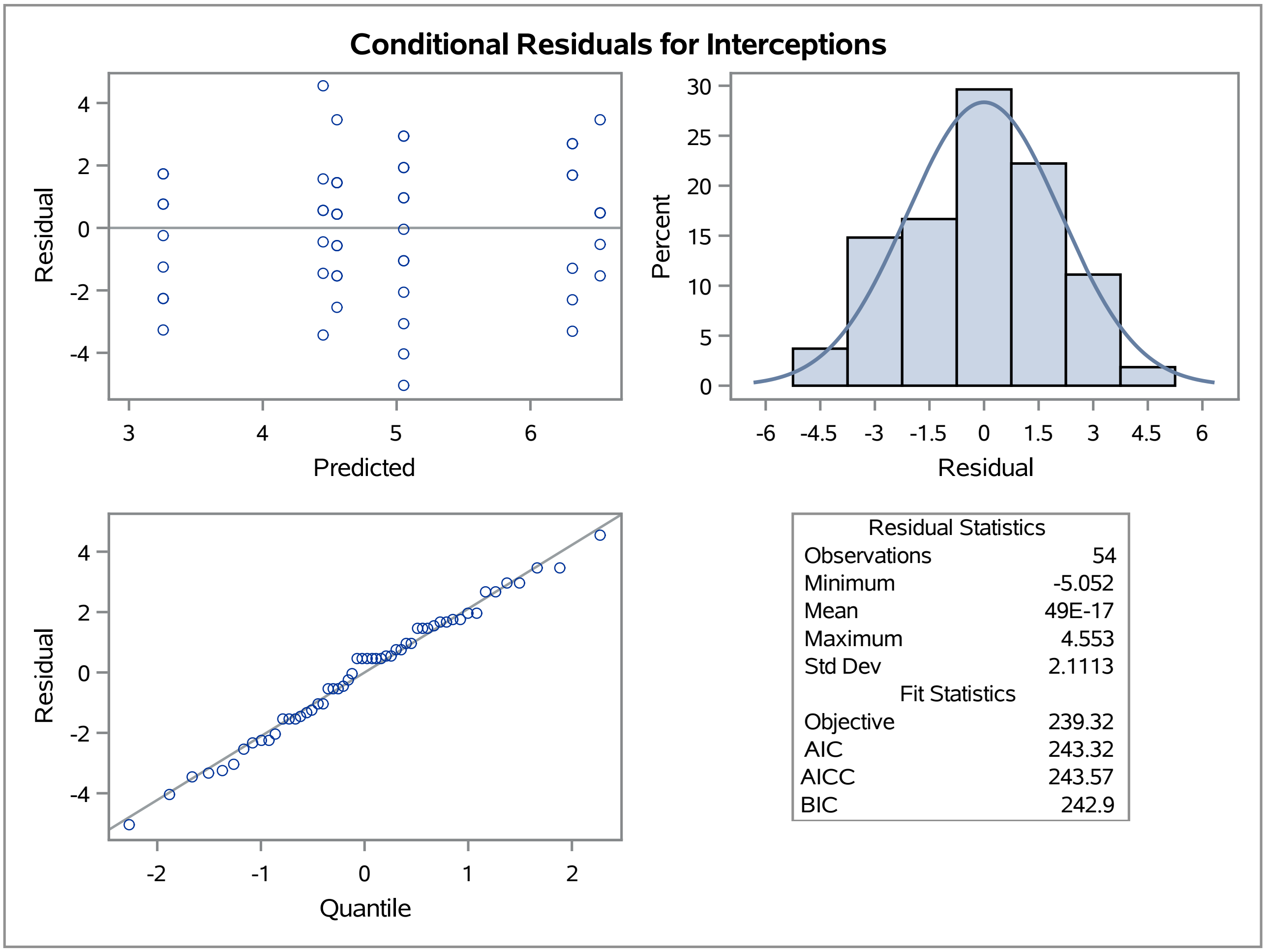

DistSoma=204

Model Information	
**Data Set**	WORK.TEMPDATASORTED
**Dependent Variable**	Interceptions
**Covariance Structure**	Variance Components
**Estimation Method**	REML
**Residual Variance Method**	Profile
**Fixed Effects SE Method**	Model-Based
**Degrees of Freedom Method**	Containment

Class Level Information		
**Class**	**Levels**	**Values**
**Genotype**	2	Meg +/+ Meg +/-
**Animal**	6	1 2 3 4 5 6

Dimensions	
**Covariance Parameters**	2
**Columns in X**	3
**Columns in Z**	6
**Subjects**	1
**Max Obs per Subject**	54

Number of Observations	
**Number of Observations Read**	54
**Number of Observations Used**	54
**Number of Observations Not Used**	0

Iteration History			
**Iteration**	**Evaluations**	**-2 Res Log Like**	**Criterion**
**0**	1	240.80108749	
**1**	3	237.34089513	0.00006302
**2**	1	237.33617724	0.00000051
**3**	1	237.33614057	0.00000000

Convergence criteria met.

DistSoma=204

Covariance Parameter Estimates				
**Cov Parm**	**Estimate**	**Alpha**	**Lower**	**Upper**
**Animal**	1.0437	0.05	0.2739	52.8479
**Residual**	4.5556	0.05	3.1712	7.0996

Fit Statistics	
**-2 Res Log Likelihood**	237.3
**AIC (Smaller is Better)**	241.3
**AICC (Smaller is Better)**	241.6
**BIC (Smaller is Better)**	240.9

Solution for Fixed Effects									
**Effect**	**Genotype**	**Estimate**	**Standard Error**	**DF**	**t Value**	**Pr > |t|**	**Alpha**	**Lower**	**Upper**
**Intercept**		4.0825	0.7183	4	5.68	0.0047	0.05	2.0881	6.0769
**Genotype**	Meg +/+	1.6749	1.0262	48	1.63	0.1092	0.05	-0.3884	3.7382
**Genotype**	Meg +/-	0	.	.	.	.	.	.	.

Solution for Random Effects									
**Effect**	**Animal**	**Estimate**	**Std Err Pred**	**DF**	**t Value**	**Pr > |t|**	**Alpha**	**Lower**	**Upper**
**Animal**	1	-1.0277	0.7506	48	-1.37	0.1773	0.05	-2.5368	0.4814
**Animal**	2	0.5264	0.7871	48	0.67	0.5069	0.05	-1.0562	2.1089
**Animal**	3	0.5014	0.7776	48	0.64	0.5221	0.05	-1.0620	2.0648
**Animal**	4	-0.8786	0.7582	48	-1.16	0.2523	0.05	-2.4031	0.6459
**Animal**	5	0.4895	0.7455	48	0.66	0.5146	0.05	-1.0096	1.9885
**Animal**	6	0.3891	0.7724	48	0.50	0.6167	0.05	-1.1639	1.9422

Type 3 Tests of Fixed Effects				
**Effect**	**Num DF**	**Den DF**	**F Value**	**Pr > F**
**Genotype**	1	48	2.66	0.1092

Least Squares Means									
**Effect**	**Genotype**	**Estimate**	**Standard Error**	**DF**	**t Value**	**Pr > |t|**	**Alpha**	**Lower**	**Upper**
**Genotype**	Meg +/+	5.7574	0.7328	48	7.86	<.0001	0.05	4.2840	7.2309
**Genotype**	Meg +/-	4.0825	0.7183	48	5.68	<.0001	0.05	2.6382	5.5268

DistSoma=204

Differences of Least Squares Means												
**Effect**	**Genotype**	**Genotype**	**Estimate**	**Standard Error**	**DF**	**t Value**	**Pr > |t|**	**Adjustment**	**Adj P**	**Alpha**	**Lower**	**Upper**
**Genotype**	Meg +/+	Meg +/-	1.6749	1.0262	48	1.63	0.1092	Tukey-Kramer	0.1092	0.05	-0.3884	3.7382

Differences of Least Squares Means				
**Effect**	**Genotype**	**Genotype**	**Adj Lower**	**Adj Upper**
**Genotype**	Meg +/+	Meg +/-	-0.3883	3.7381

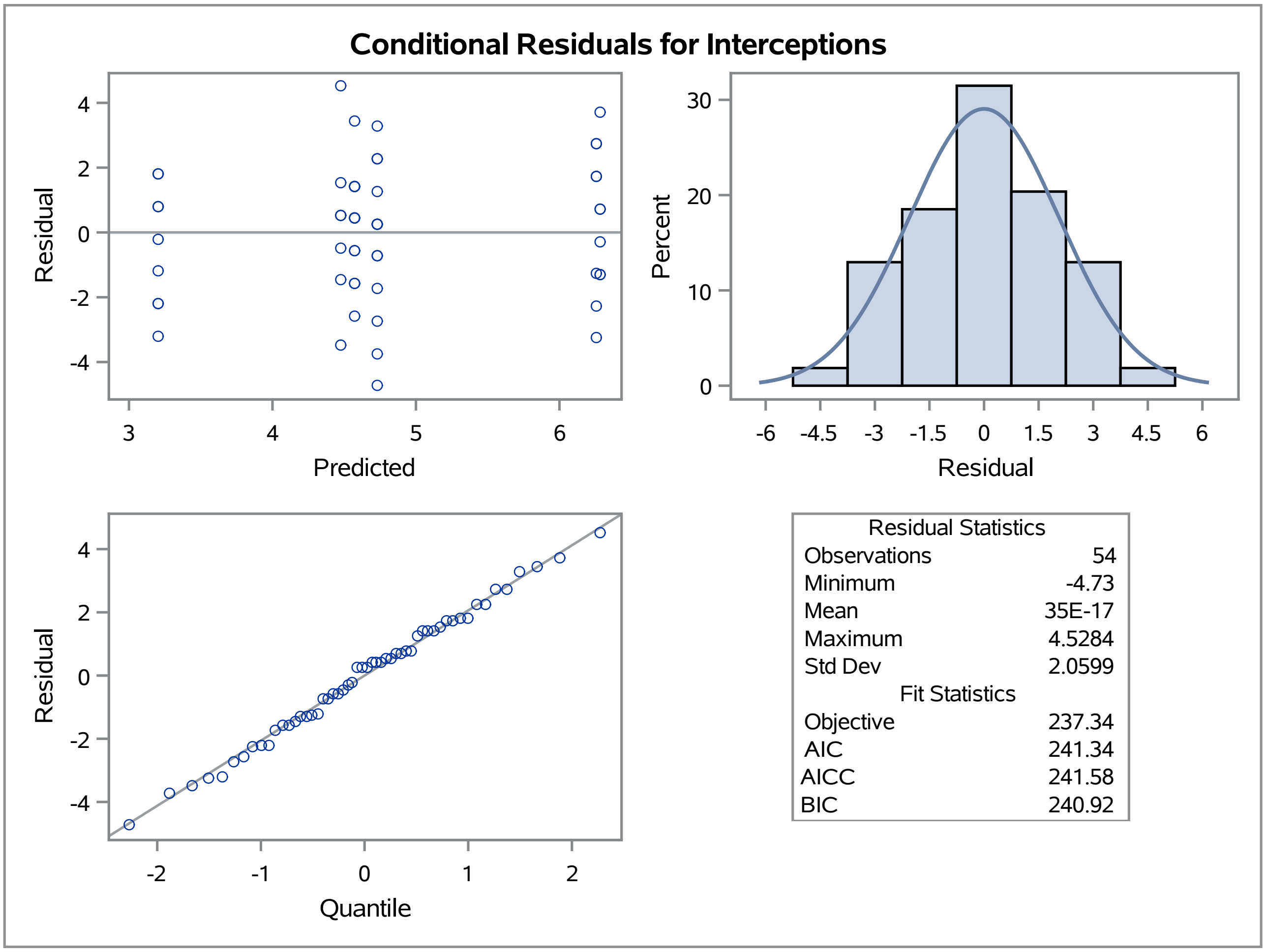

DistSoma=210

Model Information	
**Data Set**	WORK.TEMPDATASORTED
**Dependent Variable**	Interceptions
**Covariance Structure**	Variance Components
**Estimation Method**	REML
**Residual Variance Method**	Profile
**Fixed Effects SE Method**	Model-Based
**Degrees of Freedom Method**	Containment

Class Level Information		
**Class**	**Levels**	**Values**
**Genotype**	2	Meg +/+ Meg +/-
**Animal**	6	1 2 3 4 5 6

Dimensions	
**Covariance Parameters**	2
**Columns in X**	3
**Columns in Z**	6
**Subjects**	1
**Max Obs per Subject**	54

Number of Observations	
**Number of Observations Read**	54
**Number of Observations Used**	54
**Number of Observations Not Used**	0

Iteration History			
**Iteration**	**Evaluations**	**-2 Res Log Like**	**Criterion**
**0**	1	242.47910554	
**1**	3	239.43947353	0.00006812
**2**	1	239.43428671	0.00000061
**3**	1	239.43424279	0.00000000

Convergence criteria met.

DistSoma=210

Covariance Parameter Estimates				
**Cov Parm**	**Estimate**	**Alpha**	**Lower**	**Upper**
**Animal**	0.9893	0.05	0.2528	62.1181
**Residual**	4.7665	0.05	3.3184	7.4271

Fit Statistics	
**-2 Res Log Likelihood**	239.4
**AIC (Smaller is Better)**	243.4
**AICC (Smaller is Better)**	243.7
**BIC (Smaller is Better)**	243.0

Solution for Fixed Effects									
**Effect**	**Genotype**	**Estimate**	**Standard Error**	**DF**	**t Value**	**Pr > |t|**	**Alpha**	**Lower**	**Upper**
**Intercept**		3.9052	0.7110	4	5.49	0.0054	0.05	1.9313	5.8792
**Genotype**	Meg +/+	1.9384	1.0162	48	1.91	0.0624	0.05	-0.1048	3.9817
**Genotype**	Meg +/-	0	.	.	.	.	.	.	.

Solution for Random Effects									
**Effect**	**Animal**	**Estimate**	**Std Err Pred**	**DF**	**t Value**	**Pr > |t|**	**Alpha**	**Lower**	**Upper**
**Animal**	1	-1.0645	0.7404	48	-1.44	0.1570	0.05	-2.5531	0.4241
**Animal**	2	0.5489	0.7764	48	0.71	0.4830	0.05	-1.0121	2.1099
**Animal**	3	0.5157	0.7670	48	0.67	0.5046	0.05	-1.0265	2.0578
**Animal**	4	-0.7343	0.7479	48	-0.98	0.3311	0.05	-2.2381	0.7694
**Animal**	5	0.4244	0.7354	48	0.58	0.5666	0.05	-1.0542	1.9029
**Animal**	6	0.3100	0.7620	48	0.41	0.6860	0.05	-1.2221	1.8421

Type 3 Tests of Fixed Effects				
**Effect**	**Num DF**	**Den DF**	**F Value**	**Pr > F**
**Genotype**	1	48	3.64	0.0624

Least Squares Means									
**Effect**	**Genotype**	**Estimate**	**Standard Error**	**DF**	**t Value**	**Pr > |t|**	**Alpha**	**Lower**	**Upper**
**Genotype**	Meg +/+	5.8437	0.7261	48	8.05	<.0001	0.05	4.3838	7.3036
**Genotype**	Meg +/-	3.9052	0.7110	48	5.49	<.0001	0.05	2.4758	5.3347

DistSoma=210

Differences of Least Squares Means												
Effect	Genotype	Genotype	Estimate	Standard Error	DF	t Value	Pr > |t|	Adjustment	Adj P	Alpha	Lower	Upper
**Genotype**	Meg +/+	Meg +/-	1.9384	1.0162	48	1.91	0.0624	Tukey-Kramer	0.0624	0.05	-0.1048	3.9817

Differences of Least Squares Means				
Effect	Genotype	Genotype	Adj Lower	Adj Upper
**Genotype**	Meg +/+	Meg +/-	-0.1047	3.9816

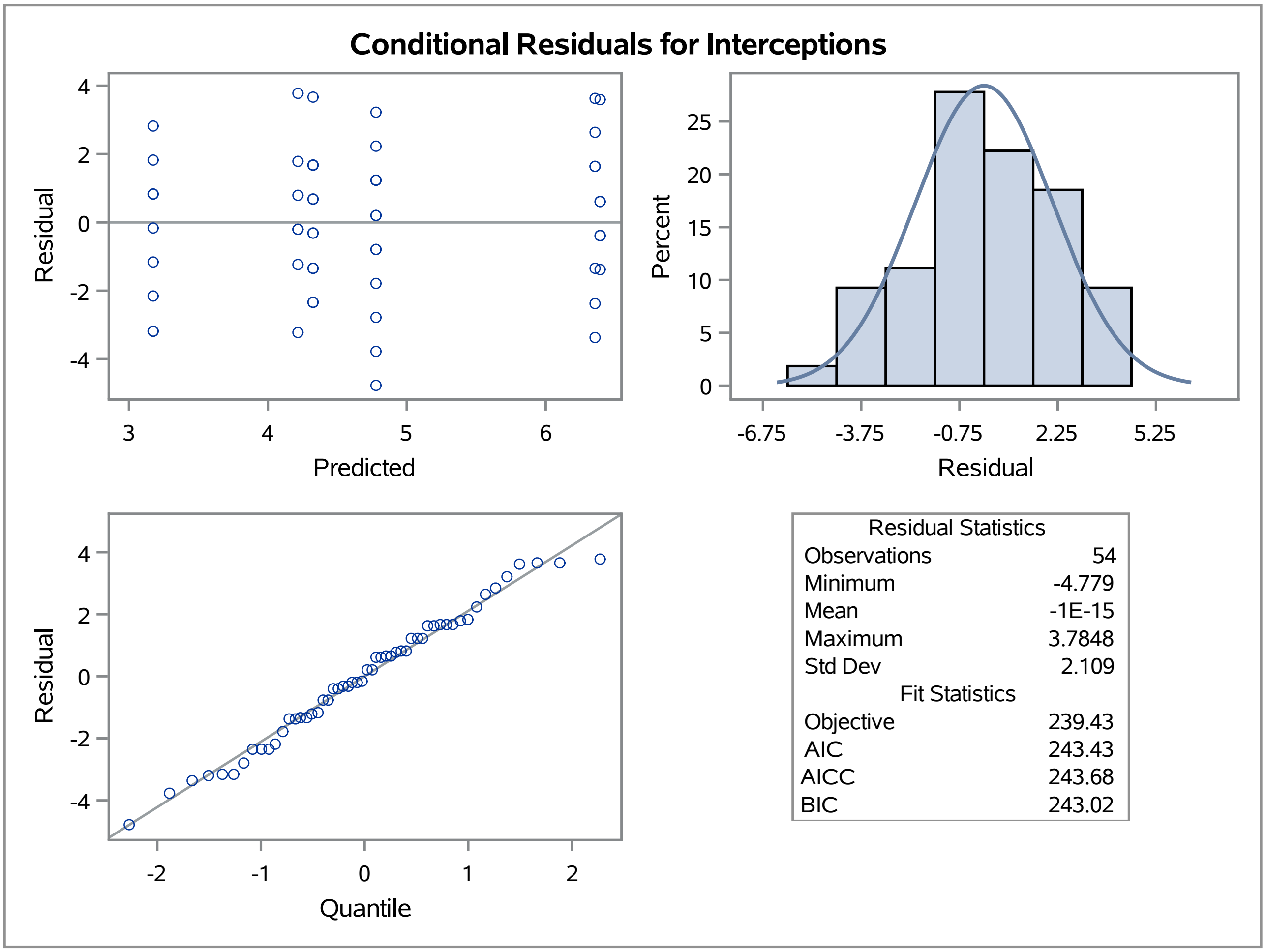

DistSoma=216

Model Information	
**Data Set**	WORK.TEMPDATASORTED
**Dependent Variable**	Interceptions
**Covariance Structure**	Variance Components
**Estimation Method**	REML
**Residual Variance Method**	Profile
**Fixed Effects SE Method**	Model-Based
**Degrees of Freedom Method**	Containment

Class Level Information		
**Class**	**Levels**	**Values**
**Genotype**	2	Meg +/+ Meg +/-
**Animal**	6	1 2 3 4 5 6

Dimensions	
**Covariance Parameters**	2
**Columns in X**	3
**Columns in Z**	6
**Subjects**	1
**Max Obs per Subject**	54

Number of Observations	
**Number of Observations Read**	54
**Number of Observations Used**	54
**Number of Observations Not Used**	0

Iteration History			
**Iteration**	**Evaluations**	**-2 Res Log Like**	**Criterion**
**0**	1	245.93131008	
**1**	3	241.29789357	0.00006275
**2**	1	241.29306227	0.00000052
**3**	1	241.29302391	0.00000000

Convergence criteria met.

DistSoma=216

Covariance Parameter Estimates				
Cov Parm	Estimate	Alpha	Lower	Upper
Animal	1.3923	0.05	0.3861	46.7701
Residual	4.8583	0.05	3.3813	7.5735

Fit Statistics	
-2 Res Log Likelihood	241.3
AIC (Smaller is Better)	245.3
AICC (Smaller is Better)	245.5
BIC (Smaller is Better)	244.9

Solution for Fixed Effects									
Effect	Genotype	Estimate	Standard Error	DF	t Value	Pr > |t|	Alpha	Lower	Upper
Intercept		3.7263	0.8024	4	4.64	0.0097	0.05	1.4985	5.9540
Genotype	Meg +/+	2.0835	1.1448	48	1.82	0.0750	0.05	-0.2183	4.3854
Genotype	Meg +/-	0	.	.	.	.	.	.	.

Solution for Random Effects									
Effect	Animal	Estimate	Std Err Pred	DF	t Value	Pr > |t|	Alpha	Lower	Upper
Animal	1	-1.3662	0.8421	48	-1.62	0.1113	0.05	-3.0593	0.3269
Animal	2	0.8579	0.8824	48	0.97	0.3358	0.05	-0.9162	2.6320
Animal	3	0.5083	0.8719	48	0.58	0.5626	0.05	-1.2447	2.2613
Animal	4	-0.8436	0.8505	48	-0.99	0.3262	0.05	-2.5537	0.8664
Animal	5	0.4703	0.8367	48	0.56	0.5767	0.05	-1.2120	2.1526
Animal	6	0.3733	0.8660	48	0.43	0.6683	0.05	-1.3678	2.1145

Type 3 Tests of Fixed Effects				
Effect	Num DF	Den DF	F Value	Pr > F
Genotype	1	48	3.31	0.0750

Least Squares Means									
Effect	Genotype	Estimate	Standard Error	DF	t Value	Pr > |t|	Alpha	Lower	Upper
Genotype	Meg +/+	5.8098	0.8166	48	7.11	<.0001	0.05	4.1679	7.4517
Genotype	Meg +/-	3.7263	0.8024	48	4.64	<.0001	0.05	2.1130	5.3396

DistSoma=216

Differences of Least Squares Means

Effect	Genotype	Genotype	Estimate	Standard Error	DF	t Value	Pr > |t|	Adjustment	Adj P	Alpha	Lower	Upper
**Genotype**	Meg +/+	Meg +/-	2.0835	1.1448	48	1.82	0.0750	Tukey-Kramer	0.0750	0.05	-0.2183	4.3854

Differences of Least Squares Means

Effect	Genotype	Genotype	Adj Lower	Adj Upper
**Genotype**	Meg +/+	Meg +/-	-0.2182	4.3853

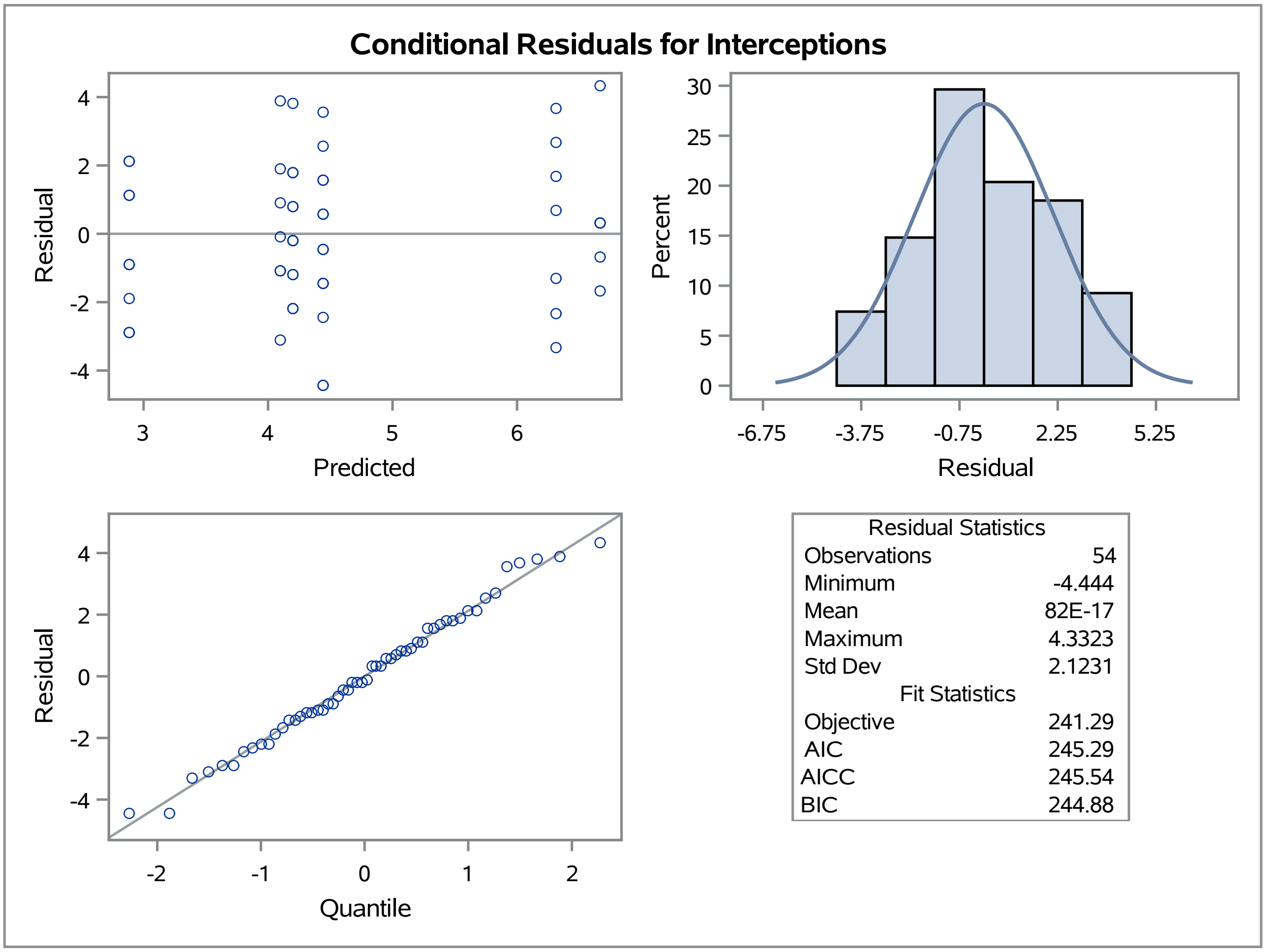

DistSoma=222

Model Information	
**Data Set**	WORK.TEMPDATASORTED
**Dependent Variable**	Interceptions
**Covariance Structure**	Variance Components
**Estimation Method**	REML
**Residual Variance Method**	Profile
**Fixed Effects SE Method**	Model-Based
**Degrees of Freedom Method**	Containment

Class Level Information		
**Class**	**Levels**	**Values**
**Genotype**	2	Meg +/+ Meg +/-
**Animal**	6	1 2 3 4 5 6

Dimensions	
**Covariance Parameters**	2
**Columns in X**	3
**Columns in Z**	6
**Subjects**	1
**Max Obs per Subject**	54

Number of Observations	
**Number of Observations Read**	54
**Number of Observations Used**	54
**Number of Observations Not Used**	0

Iteration History			
**Iteration**	**Evaluations**	**-2 Res Log Like**	**Criterion**
**0**	1	243.81152570	
**1**	3	238.51847801	0.00003556
**2**	1	238.51582616	0.00000017
**3**	1	238.51581405	0.00000000

Convergence criteria met.

DistSoma=222

Covariance Parameter Estimates				
**Cov Parm**	**Estimate**	**Alpha**	**Lower**	**Upper**
**Animal**	1.4708	0.05	0.4174	42.1724
**Residual**	4.5766	0.05	3.1848	7.1358

Fit Statistics	
**-2 Res Log Likelihood**	238.5
**AIC (Smaller is Better)**	242.5
**AICC (Smaller is Better)**	242.8
**BIC (Smaller is Better)**	242.1

Solution for Fixed Effects									
**Effect**	**Genotype**	**Estimate**	**Standard Error**	**DF**	**t Value**	**Pr > |t|**	**Alpha**	**Lower**	**Upper**
**Intercept**		3.4665	0.8122	4	4.27	0.0130	0.05	1.2114	5.7216
**Genotype**	Meg +/+	2.1199	1.1582	48	1.83	0.0734	0.05	-0.2088	4.4486
**Genotype**	Meg +/-	0	.	.	.	.	.	.	.

Solution for Random Effects									
**Effect**	**Animal**	**Estimate**	**Std Err Pred**	**DF**	**t Value**	**Pr > |t|**	**Alpha**	**Lower**	**Upper**
**Animal**	1	-1.4041	0.8531	48	-1.65	0.1063	0.05	-3.1195	0.3112
**Animal**	2	0.8211	0.8932	48	0.92	0.3626	0.05	-0.9749	2.6171
**Animal**	3	0.5830	0.8828	48	0.66	0.5121	0.05	-1.1919	2.3580
**Animal**	4	-0.9246	0.8615	48	-1.07	0.2885	0.05	-2.6567	0.8075
**Animal**	5	0.3575	0.8479	48	0.42	0.6752	0.05	-1.3472	2.0622
**Animal**	6	0.5671	0.8768	48	0.65	0.5208	0.05	-1.1958	2.3300

Type 3 Tests of Fixed Effects				
**Effect**	**Num DF**	**Den DF**	**F Value**	**Pr > F**
**Genotype**	1	48	3.35	0.0734

Least Squares Means									
**Effect**	**Genotype**	**Estimate**	**Standard Error**	**DF**	**t Value**	**Pr > |t|**	**Alpha**	**Lower**	**Upper**
**Genotype**	Meg +/+	5.5864	0.8256	48	6.77	<.0001	0.05	3.9263	7.2464
**Genotype**	Meg +/-	3.4665	0.8122	48	4.27	<.0001	0.05	1.8334	5.0996

DistSoma=222

Differences of Least Squares Means												
Effect	Genotype	Genotype	Estimate	Standard Error	DF	t Value	Pr > |t|	Adjustment	Adj P	Alpha	Lower	Upper
Genotype	Meg +/+	Meg +/-	2.1199	1.1582	48	1.83	0.0734	Tukey-Kramer	0.0734	0.05	-0.2088	4.4486

Differences of Least Squares Means				
Effect	Genotype	Genotype	Adj Lower	Adj Upper
Genotype	Meg +/+	Meg +/-	-0.2087	4.4485

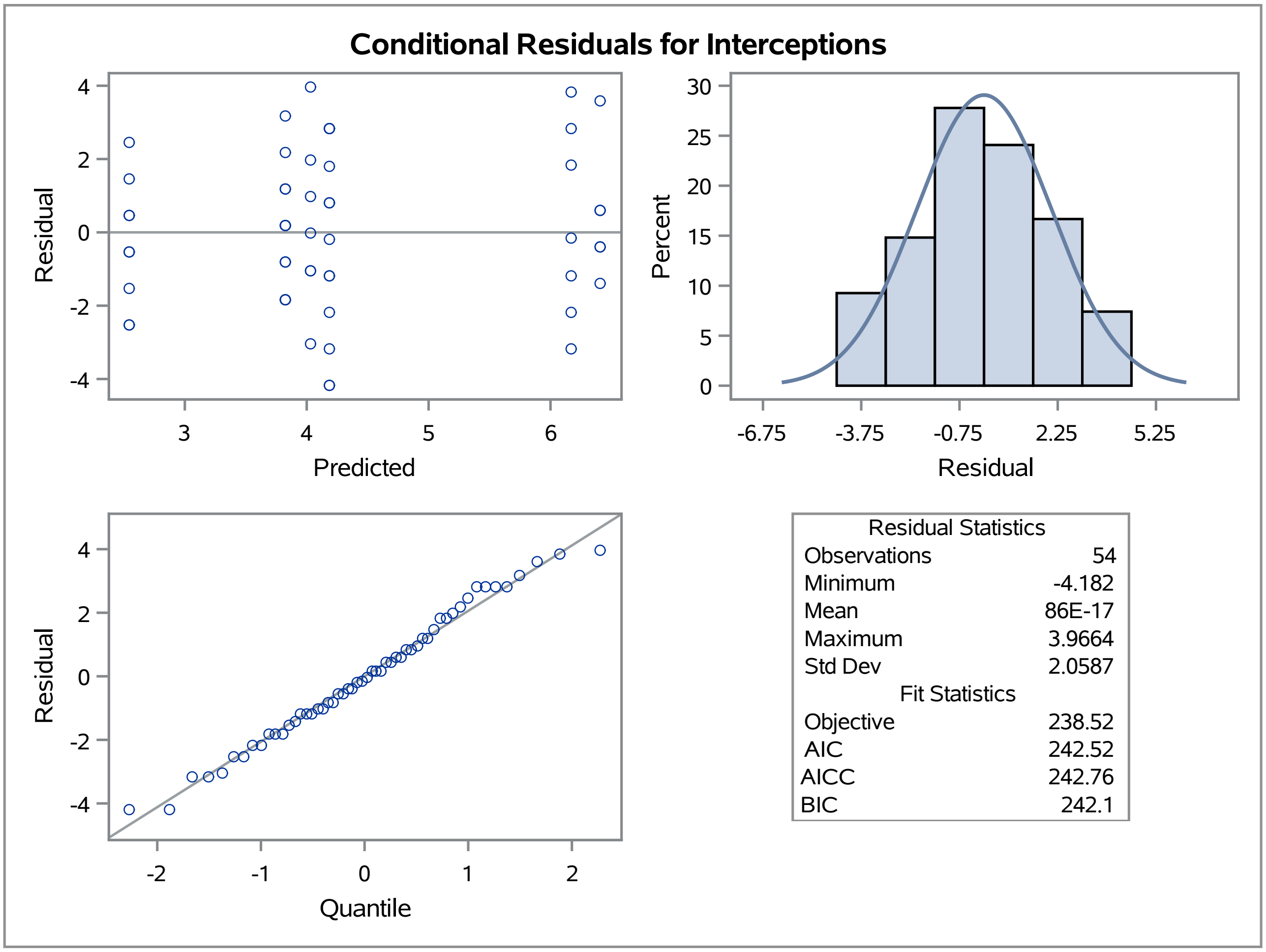

DistSoma=228

Model Information	
**Data Set**	WORK.TEMPDATASORTED
**Dependent Variable**	Interceptions
**Covariance Structure**	Variance Components
**Estimation Method**	REML
**Residual Variance Method**	Profile
**Fixed Effects SE Method**	Model-Based
**Degrees of Freedom Method**	Containment

Class Level Information		
**Class**	**Levels**	**Values**
**Genotype**	2	Meg +/+ Meg +/-
**Animal**	6	1 2 3 4 5 6

Dimensions	
**Covariance Parameters**	2
**Columns in X**	3
**Columns in Z**	6
**Subjects**	1
**Max Obs per Subject**	54

Number of Observations	
**Number of Observations Read**	54
**Number of Observations Used**	54
**Number of Observations Not Used**	0

Iteration History			
**Iteration**	**Evaluations**	**-2 Res Log Like**	**Criterion**
**0**	1	248.45739973	
**1**	3	243.32562684	0.00001139
**2**	1	243.32476329	0.00000002
**3**	1	243.32476192	0.00000000

Convergence criteria met.

DistSoma=228

Covariance Parameter Estimates				
Cov Parm	Estimate	Alpha	Lower	Upper
Animal	1.6004	0.05	0.4517	47.6044
Residual	5.0224	0.05	3.4947	7.8321

Fit Statistics	
-2 Res Log Likelihood	243.3
AIC (Smaller is Better)	247.3
AICC (Smaller is Better)	247.6
BIC (Smaller is Better)	246.9

Solution for Fixed Effects									
Effect	Genotype	Estimate	Standard Error	DF	t Value	Pr > |t|	Alpha	Lower	Upper
Intercept		3.3033	0.8482	4	3.89	0.0176	0.05	0.9484	5.6582
Genotype	Meg +/+	2.3332	1.2095	48	1.93	0.0597	0.05	-0.09870	4.7650
Genotype	Meg +/-	0	.	.	.	.	.	.	.

Solution for Random Effects									
Effect	Animal	Estimate	Std Err Pred	DF	t Value	Pr > |t|	Alpha	Lower	Upper
Animal	1	-1.4422	0.8909	48	-1.62	0.1120	0.05	-3.2334	0.3490
Animal	2	0.8953	0.9328	48	0.96	0.3420	0.05	-0.9803	2.7708
Animal	3	0.5469	0.9219	48	0.59	0.5558	0.05	-1.3066	2.4004
Animal	4	-0.9663	0.8996	48	-1.07	0.2881	0.05	-2.7751	0.8424
Animal	5	0.2880	0.8853	48	0.33	0.7463	0.05	-1.4921	2.0681
Animal	6	0.6783	0.9156	48	0.74	0.4624	0.05	-1.1627	2.5193

Type 3 Tests of Fixed Effects				
Effect	Num DF	Den DF	F Value	Pr > F
Genotype	1	48	3.72	0.0597

Least Squares Means									
Effect	Genotype	Estimate	Standard Error	DF	t Value	Pr > |t|	Alpha	Lower	Upper
Genotype	Meg +/+	5.6365	0.8623	48	6.54	<.0001	0.05	3.9028	7.3702
Genotype	Meg +/-	3.3033	0.8482	48	3.89	0.0003	0.05	1.5979	5.0087

DistSoma=228

Differences of Least Squares Means												
**Effect**	**Genotype**	**Genotype**	**Estimate**	**Standard Error**	**DF**	**t Value**	**Pr > |t|**	**Adjustment**	**Adj P**	**Alpha**	**Lower**	**Upper**
**Genotype**	Meg +/+	Meg +/-	2.3332	1.2095	48	1.93	0.0597	Tukey-Kramer	0.0597	0.05	-0.09870	4.7650

Differences of Least Squares Means				
**Effect**	**Genotype**	**Genotype**	**Adj Lower**	**Adj Upper**
**Genotype**	Meg +/+	Meg +/-	-0.09861	4.7650

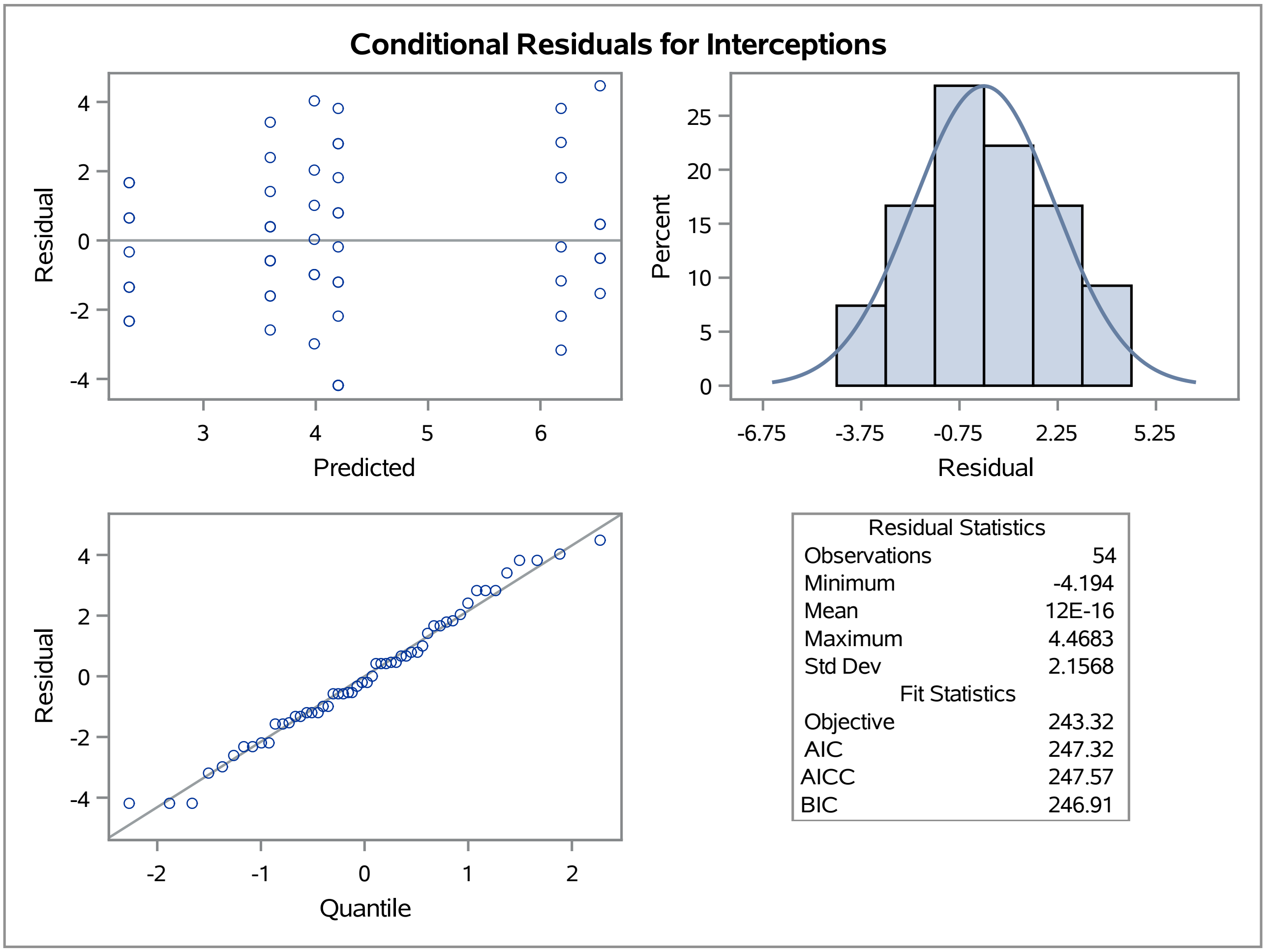

DistSoma=234

Model Information	
**Data Set**	WORK.TEMPDATASORTED
**Dependent Variable**	Interceptions
**Covariance Structure**	Variance Components
**Estimation Method**	REML
**Residual Variance Method**	Profile
**Fixed Effects SE Method**	Model-Based
**Degrees of Freedom Method**	Containment

Class Level Information		
**Class**	**Levels**	**Values**
**Genotype**	2	Meg +/+ Meg +/-
**Animal**	6	1 2 3 4 5 6

Dimensions	
**Covariance Parameters**	2
**Columns in X**	3
**Columns in Z**	6
**Subjects**	1
**Max Obs per Subject**	54

Number of Observations	
**Number of Observations Read**	54
**Number of Observations Used**	54
**Number of Observations Not Used**	0

Iteration History			
**Iteration**	**Evaluations**	**-2 Res Log Like**	**Criterion**
**0**	1	249.35294731	
**1**	3	242.63119634	0.00000651
**2**	1	242.63070835	0.00000001

Convergence criteria met.

Covariance Parameter Estimates				
**Cov Parm**	**Estimate**	**Alpha**	**Lower**	**Upper**
**Animal**	1.9497	0.05	0.5748	43.7812
**Residual**	4.8924	0.05	3.4038	7.6309

DistSoma=234

Fit Statistics	
-2 Res Log Likelihood	242.6
AIC (Smaller is Better)	246.6
AICC (Smaller is Better)	246.9
BIC (Smaller is Better)	246.2

Solution for Fixed Effects

Effect	Genotype	Estimate	Standard Error	DF	t Value	Pr > |t|	Alpha	Lower	Upper
Intercept		3.3285	0.9118	4	3.65	0.0218	0.05	0.7969	5.8601
Genotype	Meg +/+	2.1908	1.2987	48	1.69	0.0981	0.05	-0.4205	4.8021
Genotype	Meg +/-	0	.	.	.	.	.	.	.

Solution for Random Effects

Effect	Animal	Estimate	Std Err Pred	DF	t Value	Pr > |t|	Alpha	Lower	Upper
Animal	1	-1.5959	0.9573	48	-1.67	0.1020	0.05	-3.5206	0.3289
Animal	2	0.9265	1.0001	48	0.93	0.3589	0.05	-1.0843	2.9374
Animal	3	0.6693	0.9889	48	0.68	0.5018	0.05	-1.3190	2.6577
Animal	4	-1.1257	0.9662	48	-1.17	0.2497	0.05	-3.0683	0.8169
Animal	5	0.2108	0.9518	48	0.22	0.8257	0.05	-1.7029	2.1244
Animal	6	0.9150	0.9823	48	0.93	0.3563	0.05	-1.0602	2.8901

Type 3 Tests of Fixed Effects

Effect	Num DF	Den DF	F Value	Pr > F
Genotype	1	48	2.85	0.0981

Least Squares Means

Effect	Genotype	Estimate	Standard Error	DF	t Value	Pr > |t|	Alpha	Lower	Upper
Genotype	Meg +/+	5.5193	0.9249	48	5.97	<.0001	0.05	3.6598	7.3788
Genotype	Meg +/-	3.3285	0.9118	48	3.65	0.0006	0.05	1.4952	5.1618

DistSoma=234

Differences of Least Squares Means

Effect	Genotype	Genotype	Estimate	Standard Error	DF	t Value	Pr > |t|	Adjustment	Adj P	Alpha	Lower	Upper
**Genotype**	Meg +/+	Meg +/-	2.1908	1.2987	48	1.69	0.0981	Tukey-Kramer	0.0981	0.05	-0.4205	4.8021

Differences of Least Squares Means

Effect	Genotype	Genotype	Adj Lower	Adj Upper
**Genotype**	Meg +/+	Meg +/-	-0.4204	4.8020

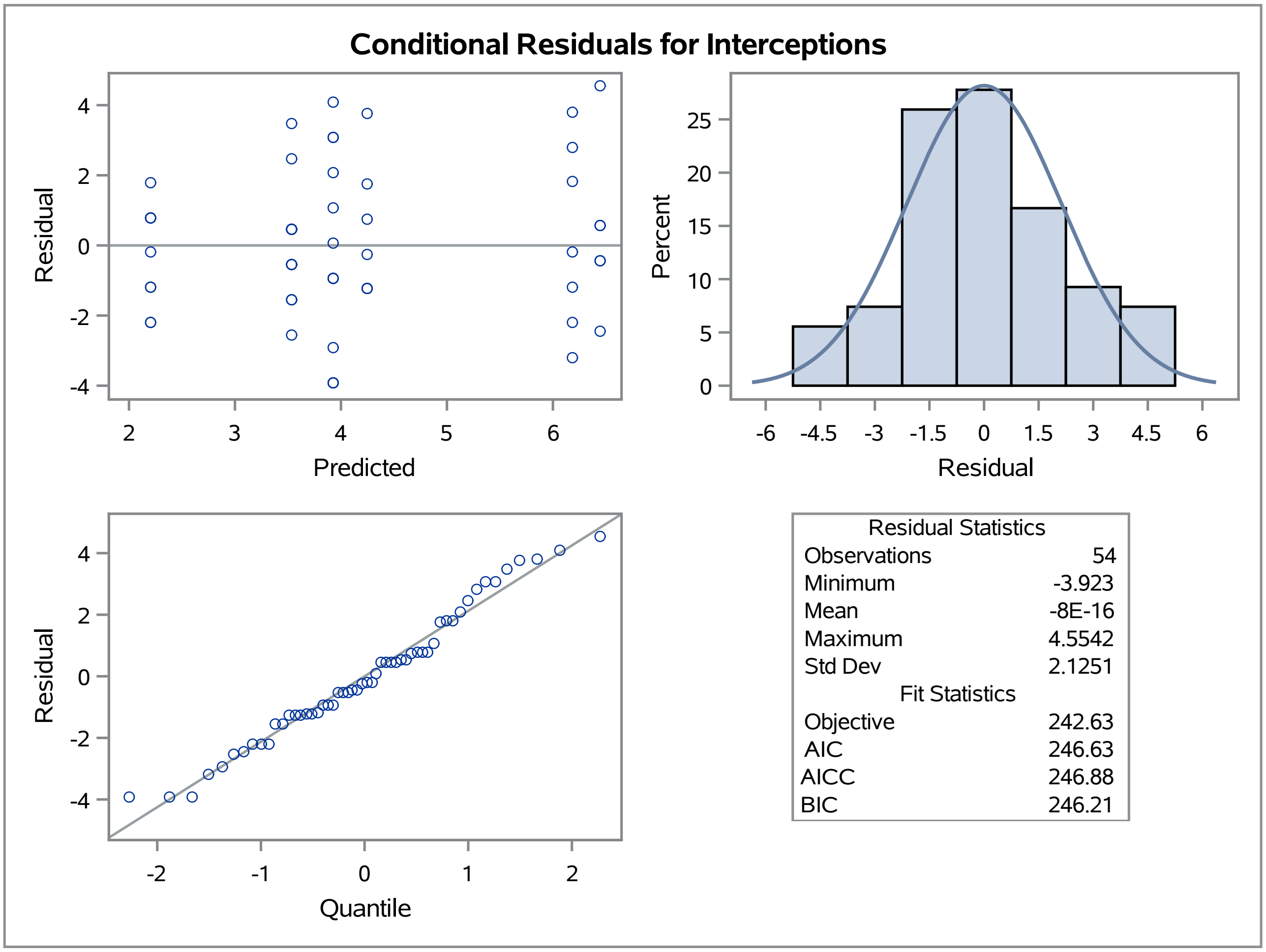

DistSoma=240

Model Information	
**Data Set**	WORK.TEMPDATASORTED
**Dependent Variable**	Interceptions
**Covariance Structure**	Variance Components
**Estimation Method**	REML
**Residual Variance Method**	Profile
**Fixed Effects SE Method**	Model-Based
**Degrees of Freedom Method**	Containment

Class Level Information		
**Class**	**Levels**	**Values**
**Genotype**	2	Meg +/+ Meg +/-
**Animal**	6	1 2 3 4 5 6

Dimensions	
**Covariance Parameters**	2
**Columns in X**	3
**Columns in Z**	6
**Subjects**	1
**Max Obs per Subject**	54

Number of Observations	
**Number of Observations Read**	54
**Number of Observations Used**	54
**Number of Observations Not Used**	0

Iteration History			
**Iteration**	**Evaluations**	**-2 Res Log Like**	**Criterion**
**0**	1	250.86301716	
**1**	3	243.40421971	0.00000377
**2**	1	243.40393715	0.00000000

Convergence criteria met.

Covariance Parameter Estimates				
**Cov Parm**	**Estimate**	**Alpha**	**Lower**	**Upper**
**Animal**	2.1589	0.05	0.6461	44.2717
**Residual**	4.9383	0.05	3.4355	7.7032

DistSoma=240

Fit Statistics	
-2 Res Log Likelihood	243.4
AIC (Smaller is Better)	247.4
AICC (Smaller is Better)	247.6
BIC (Smaller is Better)	247.0

Solution for Fixed Effects

Effect	Genotype	Estimate	Standard Error	DF	t Value	Pr > |t|	Alpha	Lower	Upper
Intercept		3.0935	0.9502	4	3.26	0.0312	0.05	0.4552	5.7318
Genotype	Meg +/+	2.3028	1.3529	48	1.70	0.0952	0.05	-0.4173	5.0230
Genotype	Meg +/-	0	.	.	.	.	.	.	.

Solution for Random Effects

Effect	Animal	Estimate	Std Err Pred	DF	t Value	Pr > |t|	Alpha	Lower	Upper
Animal	1	-1.7107	0.9968	48	-1.72	0.0926	0.05	-3.7149	0.2935
Animal	2	1.0404	1.0402	48	1.00	0.3222	0.05	-1.0510	3.1318
Animal	3	0.6703	1.0288	48	0.65	0.5178	0.05	-1.3983	2.7390
Animal	4	-1.1377	1.0058	48	-1.13	0.2636	0.05	-3.1599	0.8845
Animal	5	0.1314	0.9913	48	0.13	0.8951	0.05	-1.8616	2.1245
Animal	6	1.0063	1.0221	48	0.98	0.3298	0.05	-1.0488	3.0613

Type 3 Tests of Fixed Effects

Effect	Num DF	Den DF	F Value	Pr > F
Genotype	1	48	2.90	0.0952

Least Squares Means

Effect	Genotype	Estimate	Standard Error	DF	t Value	Pr > |t|	Alpha	Lower	Upper
Genotype	Meg +/+	5.3963	0.9630	48	5.60	<.0001	0.05	3.4601	7.3326
Genotype	Meg +/-	3.0935	0.9502	48	3.26	0.0021	0.05	1.1829	5.0041

DistSoma=240

Differences of Least Squares Means												
Effect	Genotype	Genotype	Estimate	Standard Error	DF	t Value	Pr > |t|	Adjustment	Adj P	Alpha	Lower	Upper
Genotype	Meg +/+	Meg +/-	2.3028	1.3529	48	1.70	0.0952	Tukey-Kramer	0.0952	0.05	-0.4173	5.0230

Differences of Least Squares Means				
Effect	Genotype	Genotype	Adj Lower	Adj Upper
Genotype	Meg +/+	Meg +/-	-0.4172	5.0229

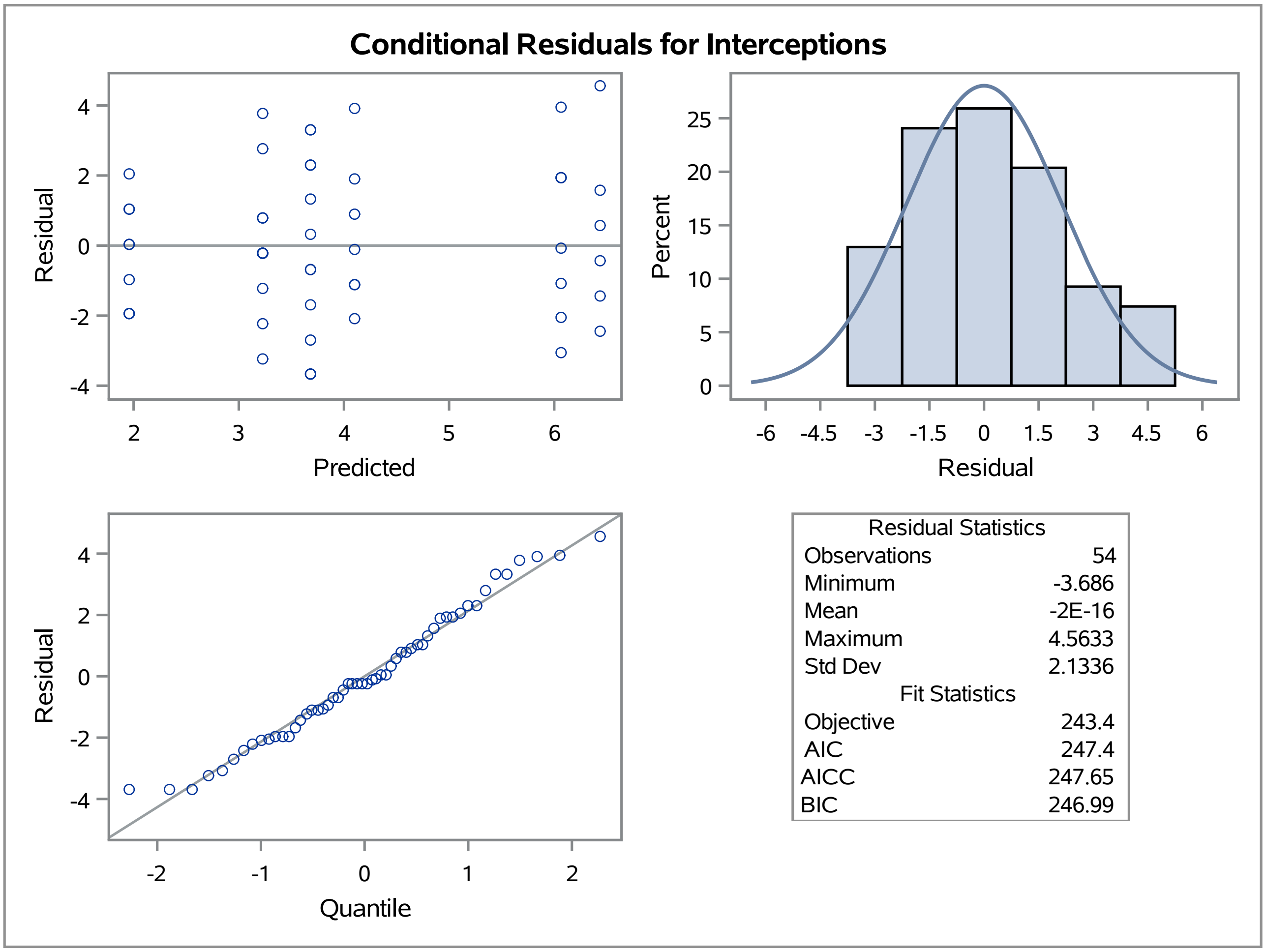

DistSoma=246

Model Information	
**Data Set**	WORK.TEMPDATASORTED
**Dependent Variable**	Interceptions
**Covariance Structure**	Variance Components
**Estimation Method**	REML
**Residual Variance Method**	Profile
**Fixed Effects SE Method**	Model-Based
**Degrees of Freedom Method**	Containment

Class Level Information		
**Class**	**Levels**	**Values**
**Genotype**	2	Meg +/+ Meg +/-
**Animal**	6	1 2 3 4 5 6

Dimensions	
**Covariance Parameters**	2
**Columns in X**	3
**Columns in Z**	6
**Subjects**	1
**Max Obs per Subject**	54

Number of Observations	
**Number of Observations Read**	54
**Number of Observations Used**	54
**Number of Observations Not Used**	0

Iteration History			
**Iteration**	**Evaluations**	**-2 Res Log Like**	**Criterion**
**0**	1	256.74201968	
**1**	3	247.71055386	0.00001100
**2**	1	247.70969549	0.00000002
**3**	1	247.70969414	0.00000000

Convergence criteria met.

DistSoma=246

Covariance Parameter Estimates				
Cov Parm	Estimate	Alpha	Lower	Upper
Animal	2.7067	0.05	0.8311	47.7911
Residual	5.3146	0.05	3.6973	8.2900

Fit Statistics	
-2 Res Log Likelihood	247.7
AIC (Smaller is Better)	251.7
AICC (Smaller is Better)	252.0
BIC (Smaller is Better)	251.3

Solution for Fixed Effects									
Effect	Genotype	Estimate	Standard Error	DF	t Value	Pr > |t|	Alpha	Lower	Upper
Intercept		3.0006	1.0487	4	2.86	0.0459	0.05	0.08898	5.9122
Genotype	Meg +/+	2.3154	1.4920	48	1.55	0.1273	0.05	-0.6845	5.3153
Genotype	Meg +/-	0	.	.	.	.	.	.	.

Solution for Random Effects									
Effect	Animal	Estimate	Std Err Pred	DF	t Value	Pr > |t|	Alpha	Lower	Upper
Animal	1	-2.0121	1.0979	48	-1.83	0.0730	0.05	-4.2195	0.1953
Animal	2	1.1432	1.1431	48	1.00	0.3223	0.05	-1.1552	3.4417
Animal	3	0.8689	1.1313	48	0.77	0.4462	0.05	-1.4058	3.1435
Animal	4	-1.1863	1.1072	48	-1.07	0.2893	0.05	-3.4124	1.0399
Animal	5	0.07109	1.0922	48	0.07	0.9484	0.05	-2.1248	2.2670
Animal	6	1.1152	1.1241	48	0.99	0.3262	0.05	-1.1450	3.3753

Type 3 Tests of Fixed Effects				
Effect	Num DF	Den DF	F Value	Pr > F
Genotype	1	48	2.41	0.1273

Least Squares Means									
Effect	Genotype	Estimate	Standard Error	DF	t Value	Pr > |t|	Alpha	Lower	Upper
Genotype	Meg +/+	5.3160	1.0613	48	5.01	<.0001	0.05	3.1821	7.4498
Genotype	Meg +/-	3.0006	1.0487	48	2.86	0.0062	0.05	0.8921	5.1091

DistSoma=246

Differences of Least Squares Means												
**Effect**	**Genotype**	**Genotype**	**Estimate**	**Standard Error**	**DF**	**t Value**	**Pr > |t|**	**Adjustment**	**Adj P**	**Alpha**	**Lower**	**Upper**
**Genotype**	Meg +/+	Meg +/-	2.3154	1.4920	48	1.55	0.1273	Tukey-Kramer	0.1273	0.05	-0.6845	5.3153

Differences of Least Squares Means				
**Effect**	**Genotype**	**Genotype**	**Adj Lower**	**Adj Upper**
**Genotype**	Meg +/+	Meg +/-	-0.6844	5.3152

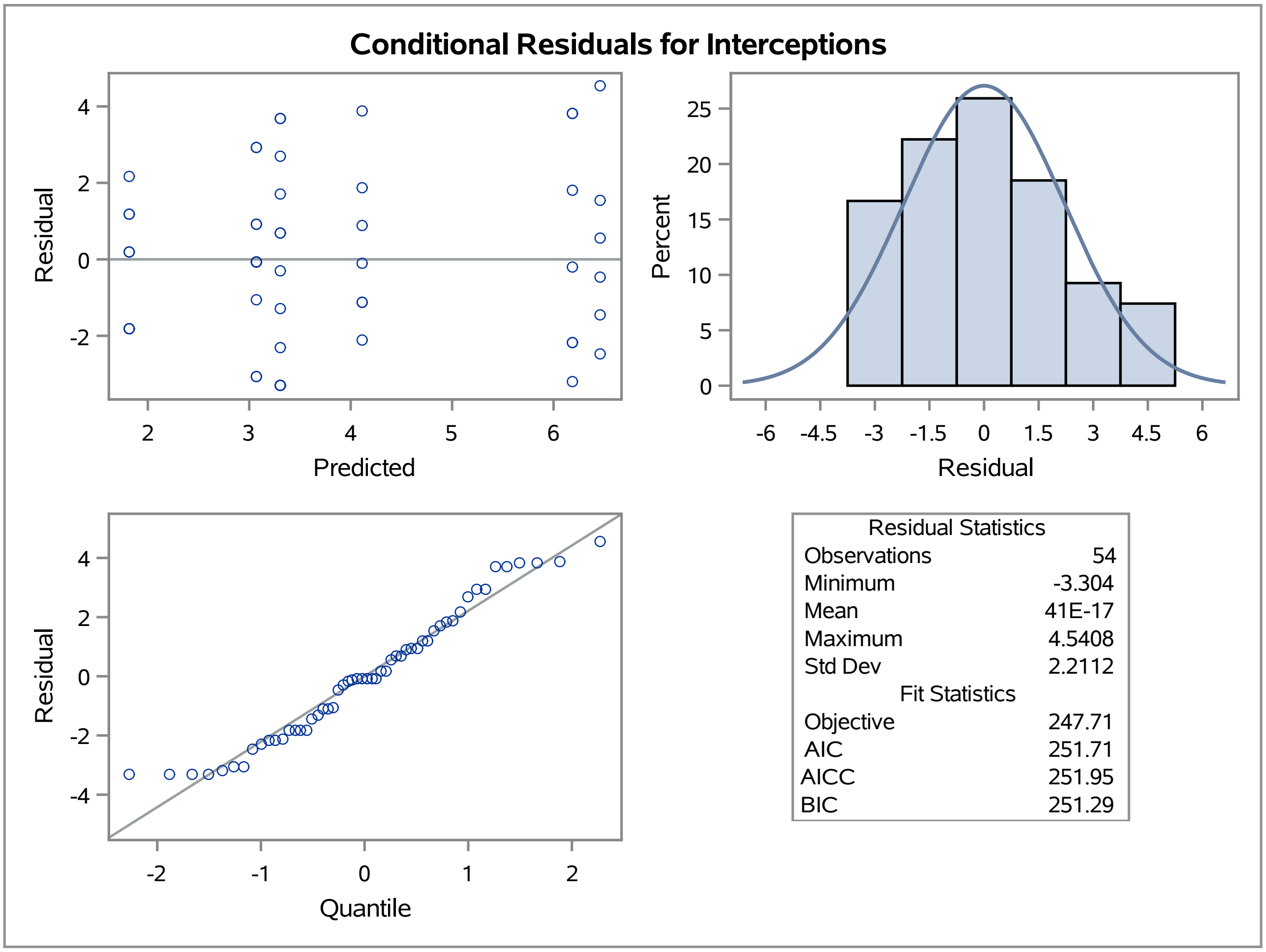

DistSoma=252

Model Information	
**Data Set**	WORK.TEMPDATASORTED
**Dependent Variable**	Interceptions
**Covariance Structure**	Variance Components
**Estimation Method**	REML
**Residual Variance Method**	Profile
**Fixed Effects SE Method**	Model-Based
**Degrees of Freedom Method**	Containment

Class Level Information		
**Class**	**Levels**	**Values**
**Genotype**	2	Meg +/+ Meg +/-
**Animal**	6	1 2 3 4 5 6

Dimensions	
**Covariance Parameters**	2
**Columns in X**	3
**Columns in Z**	6
**Subjects**	1
**Max Obs per Subject**	54

Number of Observations	
**Number of Observations Read**	54
**Number of Observations Used**	54
**Number of Observations Not Used**	0

Iteration History			
**Iteration**	**Evaluations**	**-2 Res Log Like**	**Criterion**
**0**	1	257.01563031	
**1**	3	246.38066532	0.00001233
**2**	1	246.37971057	0.00000002
**3**	1	246.37970891	0.00000000

Convergence criteria met.

DistSoma=252

Covariance Parameter Estimates				
**Cov Parm**	**Estimate**	**Alpha**	**Lower**	**Upper**
**Animal**	3.0197	0.05	0.9460	47.7017
**Residual**	5.1335	0.05	3.5712	8.0080

Fit Statistics	
**-2 Res Log Likelihood**	246.4
**AIC (Smaller is Better)**	250.4
**AICC (Smaller is Better)**	250.6
**BIC (Smaller is Better)**	250.0

Solution for Fixed Effects									
**Effect**	**Genotype**	**Estimate**	**Standard Error**	**DF**	**t Value**	**Pr > |t|**	**Alpha**	**Lower**	**Upper**
**Intercept**		2.8293	1.0943	4	2.59	0.0610	0.05	-0.2090	5.8676
**Genotype**	Meg +/+	2.3357	1.5559	48	1.50	0.1399	0.05	-0.7927	5.4642
**Genotype**	Meg +/-	0	.	.	.	.	.	.	.

Solution for Random Effects									
**Effect**	**Animal**	**Estimate**	**Std Err Pred**	**DF**	**t Value**	**Pr > |t|**	**Alpha**	**Lower**	**Upper**
**Animal**	1	-2.1868	1.1428	48	-1.91	0.0617	0.05	-4.4846	0.1111
**Animal**	2	1.1701	1.1872	48	0.99	0.3293	0.05	-1.2170	3.5572
**Animal**	3	1.0166	1.1756	48	0.86	0.3915	0.05	-1.3471	3.3804
**Animal**	4	-1.0714	1.1519	48	-0.93	0.3570	0.05	-3.3875	1.2448
**Animal**	5	-0.2154	1.1373	48	-0.19	0.8506	0.05	-2.5021	2.0713
**Animal**	6	1.2868	1.1685	48	1.10	0.2763	0.05	-1.0626	3.6361

Type 3 Tests of Fixed Effects				
**Effect**	**Num DF**	**Den DF**	**F Value**	**Pr > F**
**Genotype**	1	48	2.25	0.1399

Least Squares Means									
**Effect**	**Genotype**	**Estimate**	**Standard Error**	**DF**	**t Value**	**Pr > |t|**	**Alpha**	**Lower**	**Upper**
**Genotype**	Meg +/+	5.1650	1.1061	48	4.67	<.0001	0.05	2.9411	7.3890
**Genotype**	Meg +/-	2.8293	1.0943	48	2.59	0.0128	0.05	0.6290	5.0295

DistSoma=252

Differences of Least Squares Means												
Effect	Genotype	Genotype	Estimate	Standard Error	DF	t Value	Pr > |t|	Adjustment	Adj P	Alpha	Lower	Upper
**Genotype**	Meg +/+	Meg +/-	2.3357	1.5559	48	1.50	0.1399	Tukey-Kramer	0.1399	0.05	-0.7927	5.4642

Differences of Least Squares Means				
Effect	Genotype	Genotype	Adj Lower	Adj Upper
**Genotype**	Meg +/+	Meg +/-	-0.7926	5.4641

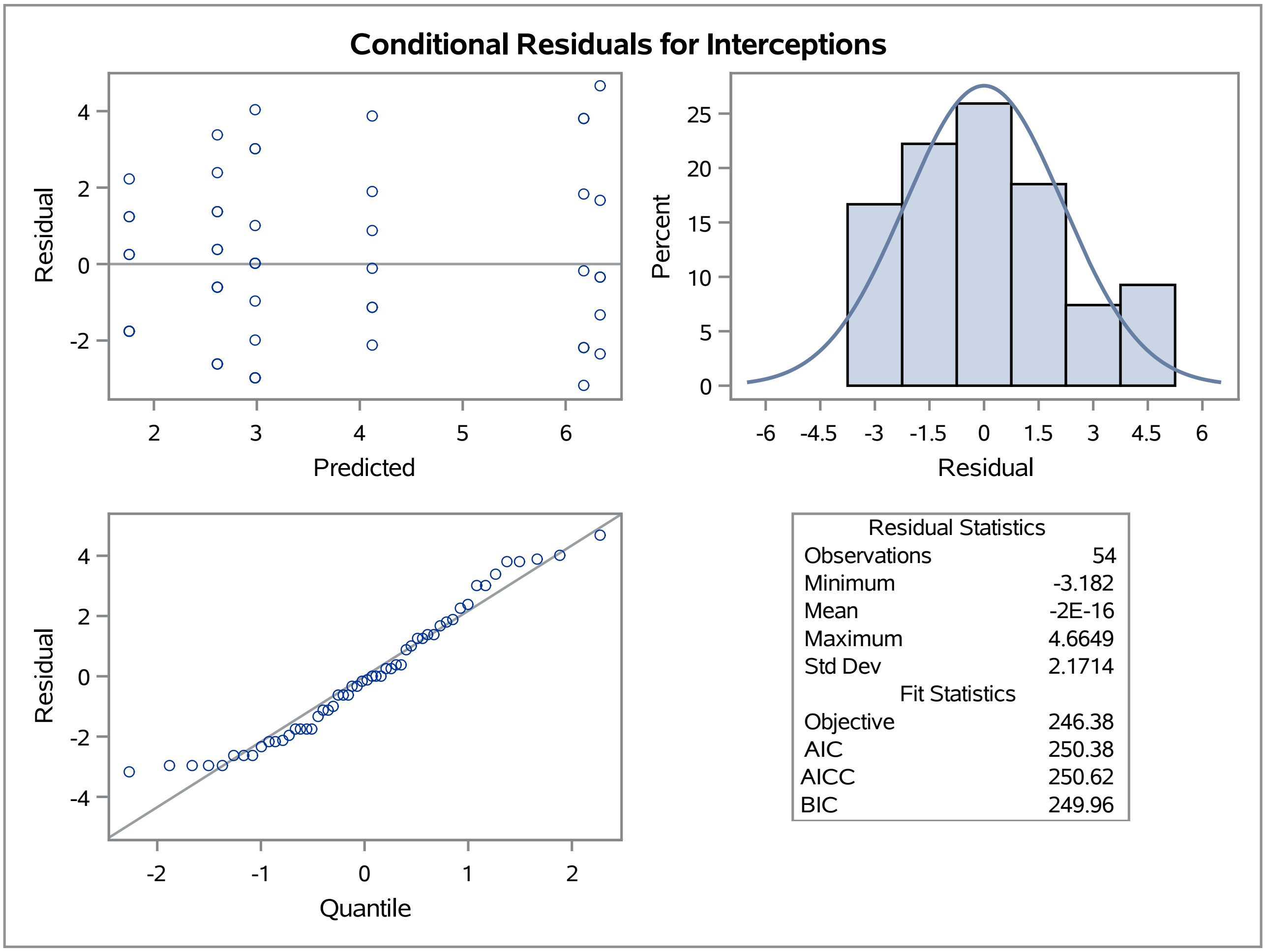

DistSoma=258

Model Information	
**Data Set**	WORK.TEMPDATASORTED
**Dependent Variable**	Interceptions
**Covariance Structure**	Variance Components
**Estimation Method**	REML
**Residual Variance Method**	Profile
**Fixed Effects SE Method**	Model-Based
**Degrees of Freedom Method**	Containment

Class Level Information		
**Class**	**Levels**	**Values**
**Genotype**	2	Meg +/+ Meg +/-
**Animal**	6	1 2 3 4 5 6

Dimensions	
**Covariance Parameters**	2
**Columns in X**	3
**Columns in Z**	6
**Subjects**	1
**Max Obs per Subject**	54

Number of Observations	
**Number of Observations Read**	54
**Number of Observations Used**	54
**Number of Observations Not Used**	0

Iteration History			
**Iteration**	**Evaluations**	**-2 Res Log Like**	**Criterion**
**0**	1	256.93956020	
**1**	3	245.04962238	0.00002870
**2**	1	245.04739092	0.00000012
**3**	1	245.04738226	0.00000000

Convergence criteria met.

DistSoma=258

Covariance Parameter Estimates				
**Cov Parm**	**Estimate**	**Alpha**	**Lower**	**Upper**
**Animal**	3.2094	0.05	1.0182	47.3664
**Residual**	4.9739	0.05	3.4602	7.7589

Fit Statistics	
**-2 Res Log Likelihood**	245.0
**AIC (Smaller is Better)**	249.0
**AICC (Smaller is Better)**	249.3
**BIC (Smaller is Better)**	248.6

Solution for Fixed Effects									
**Effect**	**Genotype**	**Estimate**	**Standard Error**	**DF**	**t Value**	**Pr > |t|**	**Alpha**	**Lower**	**Upper**
**Intercept**		2.6907	1.1202	4	2.40	0.0742	0.05	-0.4195	5.8010
**Genotype**	Meg +/+	2.4207	1.5922	48	1.52	0.1350	0.05	-0.7806	5.6220
**Genotype**	Meg +/-	0	.	.	.	.	.	.	.

Solution for Random Effects									
**Effect**	**Animal**	**Estimate**	**Std Err Pred**	**DF**	**t Value**	**Pr > |t|**	**Alpha**	**Lower**	**Upper**
**Animal**	1	-2.2989	1.1678	48	-1.97	0.0548	0.05	-4.6470	0.04921
**Animal**	2	1.1035	1.2113	48	0.91	0.3668	0.05	-1.3320	3.5391
**Animal**	3	1.1954	1.2000	48	1.00	0.3242	0.05	-1.2173	3.6080
**Animal**	4	-1.0632	1.1768	48	-0.90	0.3708	0.05	-3.4292	1.3028
**Animal**	5	-0.2427	1.1625	48	-0.21	0.8355	0.05	-2.5800	2.0946
**Animal**	6	1.3059	1.1929	48	1.09	0.2791	0.05	-1.0926	3.7043

Type 3 Tests of Fixed Effects				
**Effect**	**Num DF**	**Den DF**	**F Value**	**Pr > F**
**Genotype**	1	48	2.31	0.1350

Least Squares Means									
**Effect**	**Genotype**	**Estimate**	**Standard Error**	**DF**	**t Value**	**Pr > |t|**	**Alpha**	**Lower**	**Upper**
**Genotype**	Meg +/+	5.1114	1.1315	48	4.52	<.0001	0.05	2.8365	7.3864
**Genotype**	Meg +/-	2.6907	1.1202	48	2.40	0.0202	0.05	0.4383	4.9431

DistSoma=258

Differences of Least Squares Means												
**Effect**	**Genotype**	**Genotype**	**Estimate**	**Standard Error**	**DF**	**t Value**	**Pr > |t|**	**Adjustment**	**Adj P**	**Alpha**	**Lower**	**Upper**
**Genotype**	Meg +/+	Meg +/-	2.4207	1.5922	48	1.52	0.1350	Tukey-Kramer	0.1350	0.05	-0.7806	5.6220

Differences of Least Squares Means				
**Effect**	**Genotype**	**Genotype**	**Adj Lower**	**Adj Upper**
**Genotype**	Meg +/+	Meg +/-	-0.7805	5.6219

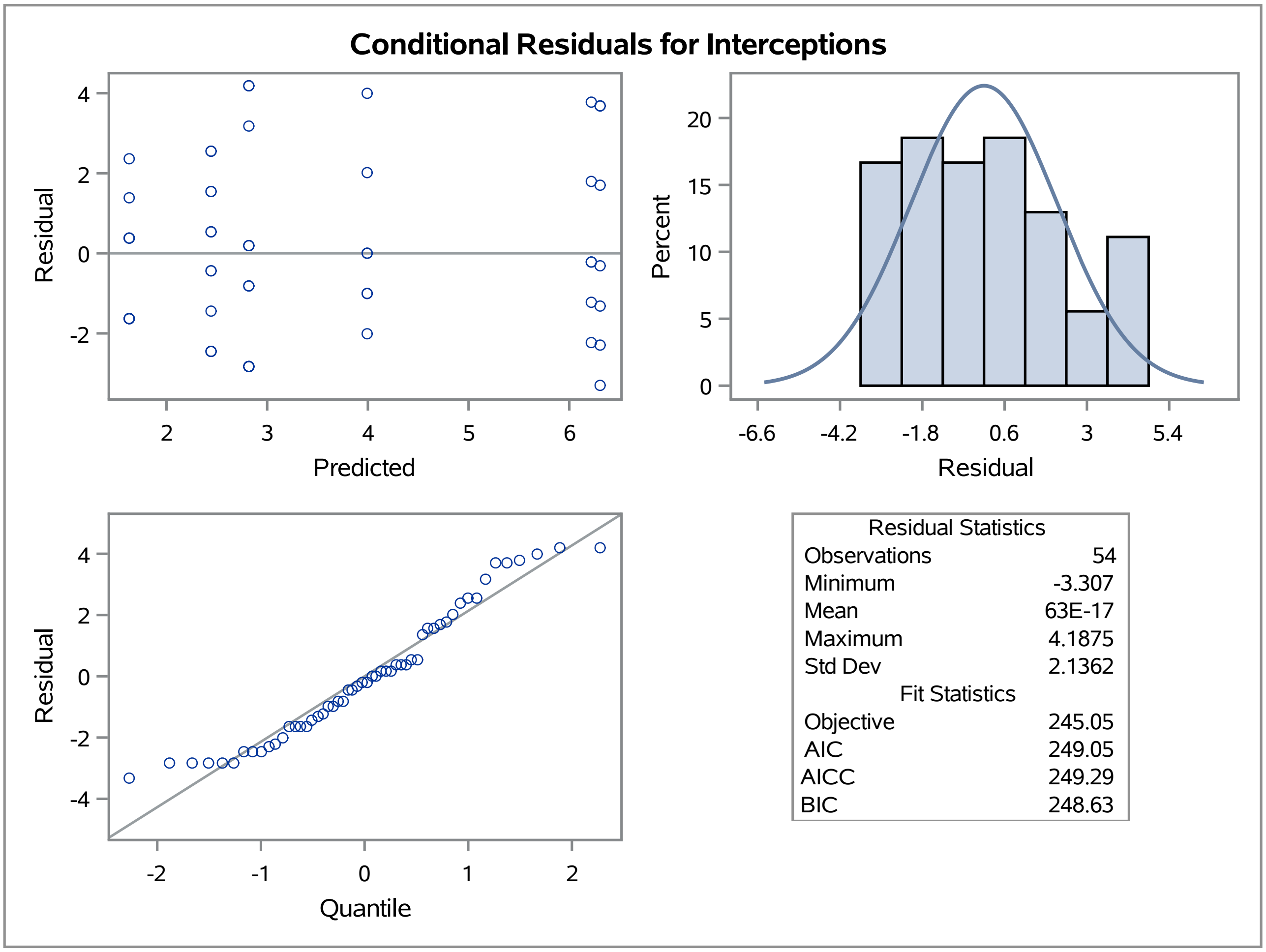

DistSoma=264

Model Information	
**Data Set**	WORK.TEMPDATASORTED
**Dependent Variable**	Interceptions
**Covariance Structure**	Variance Components
**Estimation Method**	REML
**Residual Variance Method**	Profile
**Fixed Effects SE Method**	Model-Based
**Degrees of Freedom Method**	Containment

Class Level Information		
**Class**	**Levels**	**Values**
**Genotype**	2	Meg +/+ Meg +/-
**Animal**	6	1 2 3 4 5 6

Dimensions	
**Covariance Parameters**	2
**Columns in X**	3
**Columns in Z**	6
**Subjects**	1
**Max Obs per Subject**	54

Number of Observations	
**Number of Observations Read**	54
**Number of Observations Used**	54
**Number of Observations Not Used**	0

Iteration History			
**Iteration**	**Evaluations**	**-2 Res Log Like**	**Criterion**
**0**	1	257.52169678	
**1**	3	243.74501286	0.00001850
**2**	1	243.74359778	0.00000005
**3**	1	243.74359420	0.00000000

Convergence criteria met.

DistSoma=264

Covariance Parameter Estimates				
Cov Parm	Estimate	Alpha	Lower	Upper
Animal	3.5845	0.05	1.1548	48.8400
Residual	4.8048	0.05	3.3424	7.4958

Fit Statistics	
-2 Res Log Likelihood	243.7
AIC (Smaller is Better)	247.7
AICC (Smaller is Better)	248.0
BIC (Smaller is Better)	247.3

Solution for Fixed Effects									
Effect	Genotype	Estimate	Standard Error	DF	t Value	Pr > |t|	Alpha	Lower	Upper
Intercept		2.4962	1.1721	4	2.13	0.1002	0.05	-0.7581	5.7505
Genotype	Meg +/+	2.3642	1.6650	48	1.42	0.1621	0.05	-0.9835	5.7120
Genotype	Meg +/-	0	.	.	.	.	.	.	.

Solution for Random Effects									
Effect	Animal	Estimate	Std Err Pred	DF	t Value	Pr > |t|	Alpha	Lower	Upper
Animal	1	-2.3838	1.2183	48	-1.96	0.0562	0.05	-4.8334	0.06574
Animal	2	1.0677	1.2605	48	0.85	0.4012	0.05	-1.4667	3.6022
Animal	3	1.3161	1.2495	48	1.05	0.2975	0.05	-1.1961	3.8283
Animal	4	-1.2055	1.2269	48	-0.98	0.3308	0.05	-3.6725	1.2614
Animal	5	-0.2964	1.2132	48	-0.24	0.8080	0.05	-2.7356	2.1428
Animal	6	1.5019	1.2425	48	1.21	0.2327	0.05	-0.9963	4.0001

Type 3 Tests of Fixed Effects				
Effect	Num DF	Den DF	F Value	Pr > F
Genotype	1	48	2.02	0.1621

Least Squares Means									
Effect	Genotype	Estimate	Standard Error	DF	t Value	Pr > |t|	Alpha	Lower	Upper
Genotype	Meg +/+	4.8604	1.1826	48	4.11	0.0002	0.05	2.4827	7.2381
Genotype	Meg +/-	2.4962	1.1721	48	2.13	0.0384	0.05	0.1395	4.8528

DistSoma=264

Differences of Least Squares Means												
Effect	Genotype	Genotype	Estimate	Standard Error	DF	t Value	Pr > |t|	Adjustment	Adj P	Alpha	Lower	Upper
Genotype	Meg +/+	Meg +/-	2.3642	1.6650	48	1.42	0.1621	Tukey-Kramer	0.1621	0.05	-0.9835	5.7120

Differences of Least Squares Means				
Effect	Genotype	Genotype	Adj Lower	Adj Upper
Genotype	Meg +/+	Meg +/-	-0.9834	5.7119

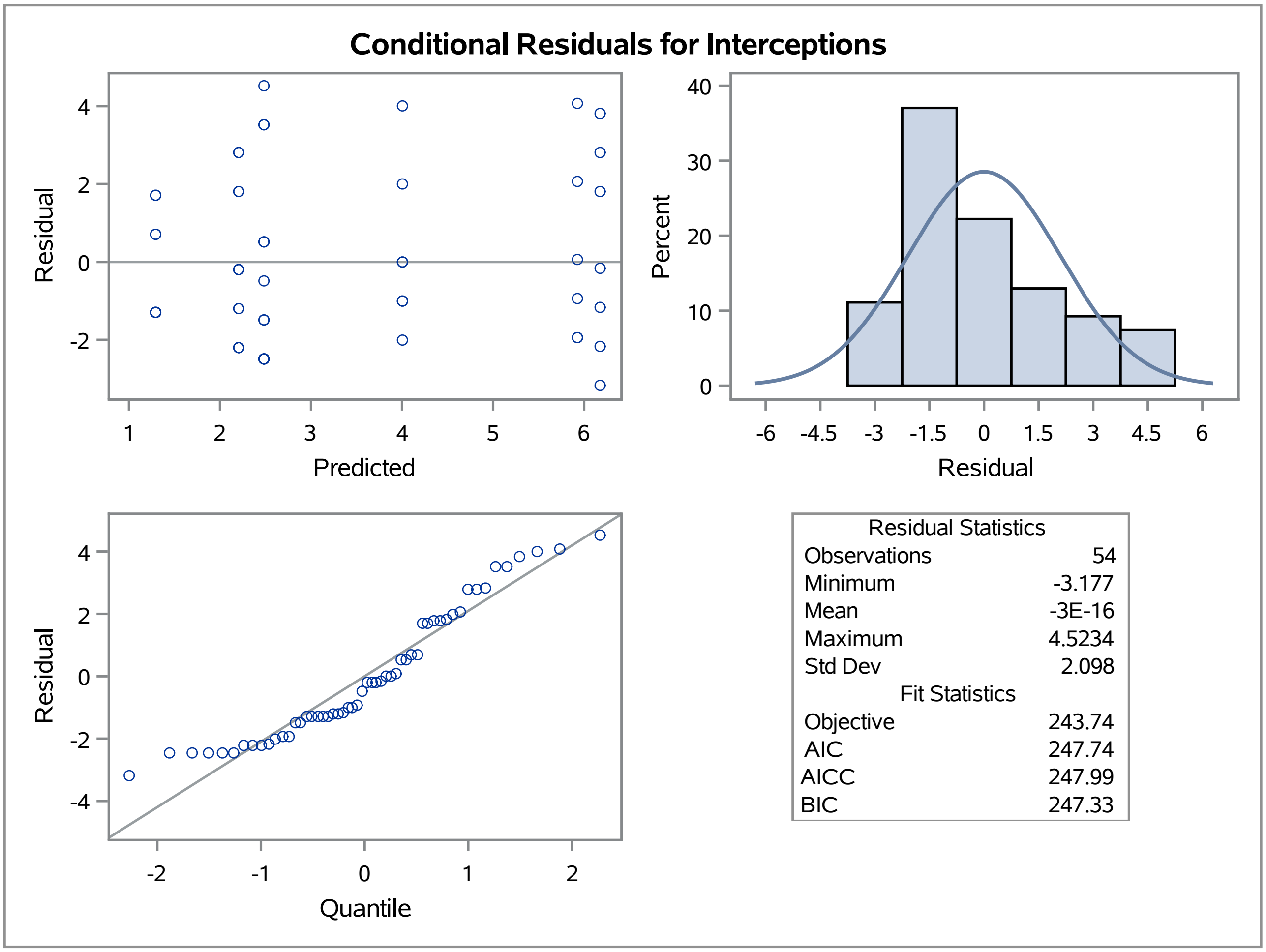

DistSoma=270

Model Information	
**Data Set**	WORK.TEMPDATASORTED
**Dependent Variable**	Interceptions
**Covariance Structure**	Variance Components
**Estimation Method**	REML
**Residual Variance Method**	Profile
**Fixed Effects SE Method**	Model-Based
**Degrees of Freedom Method**	Containment

Class Level Information		
**Class**	**Levels**	**Values**
**Genotype**	2	Meg +/+ Meg +/-
**Animal**	6	1 2 3 4 5 6

Dimensions	
**Covariance Parameters**	2
**Columns in X**	3
**Columns in Z**	6
**Subjects**	1
**Max Obs per Subject**	54

Number of Observations	
**Number of Observations Read**	54
**Number of Observations Used**	54
**Number of Observations Not Used**	0

Iteration History			
**Iteration**	**Evaluations**	**-2 Res Log Like**	**Criterion**
**0**	1	260.11693218	
**1**	3	245.63877968	0.00003172
**2**	1	245.63629955	0.00000014
**3**	1	245.63628893	0.00000000

Convergence criteria met.

DistSoma=270

Covariance Parameter Estimates				
**Cov Parm**	**Estimate**	**Alpha**	**Lower**	**Upper**
**Animal**	3.8660	0.05	1.2516	51.3791
**Residual**	4.9690	0.05	3.4566	7.7519

Fit Statistics	
**-2 Res Log Likelihood**	245.6
**AIC (Smaller is Better)**	249.6
**AICC (Smaller is Better)**	249.9
**BIC (Smaller is Better)**	249.2

Solution for Fixed Effects									
**Effect**	**Genotype**	**Estimate**	**Standard Error**	**DF**	**t Value**	**Pr > |t|**	**Alpha**	**Lower**	**Upper**
**Intercept**		2.3076	1.2140	4	1.90	0.1301	0.05	-1.0630	5.6782
**Genotype**	Meg +/+	2.6287	1.7243	48	1.52	0.1339	0.05	-0.8382	6.0956
**Genotype**	Meg +/-	0	.	.	.	.	.	.	.

Solution for Random Effects									
**Effect**	**Animal**	**Estimate**	**Std Err Pred**	**DF**	**t Value**	**Pr > |t|**	**Alpha**	**Lower**	**Upper**
**Animal**	1	-2.5321	1.2608	48	-2.01	0.0502	0.05	-5.0670	0.002846
**Animal**	2	1.1506	1.3035	48	0.88	0.3818	0.05	-1.4702	3.7714
**Animal**	3	1.3815	1.2923	48	1.07	0.2904	0.05	-1.2168	3.9798
**Animal**	4	-1.0469	1.2695	48	-0.82	0.4136	0.05	-3.5994	1.5055
**Animal**	5	-0.5036	1.2556	48	-0.40	0.6901	0.05	-3.0281	2.0209
**Animal**	6	1.5506	1.2852	48	1.21	0.2335	0.05	-1.0335	4.1346

Type 3 Tests of Fixed Effects				
**Effect**	**Num DF**	**Den DF**	**F Value**	**Pr > F**
**Genotype**	1	48	2.32	0.1339

Least Squares Means									
**Effect**	**Genotype**	**Estimate**	**Standard Error**	**DF**	**t Value**	**Pr > |t|**	**Alpha**	**Lower**	**Upper**
**Genotype**	Meg +/+	4.9363	1.2245	48	4.03	0.0002	0.05	2.4743	7.3982
**Genotype**	Meg +/-	2.3076	1.2140	48	1.90	0.0633	0.05	-0.1333	4.7485

DistSoma=270

Differences of Least Squares Means												
Effect	Genotype	Genotype	Estimate	Standard Error	DF	t Value	Pr > |t|	Adjustment	Adj P	Alpha	Lower	Upper
**Genotype**	Meg +/+	Meg +/-	2.6287	1.7243	48	1.52	0.1339	Tukey-Kramer	0.1339	0.05	-0.8382	6.0956

Differences of Least Squares Means				
Effect	Genotype	Genotype	Adj Lower	Adj Upper
**Genotype**	Meg +/+	Meg +/-	-0.8381	6.0955

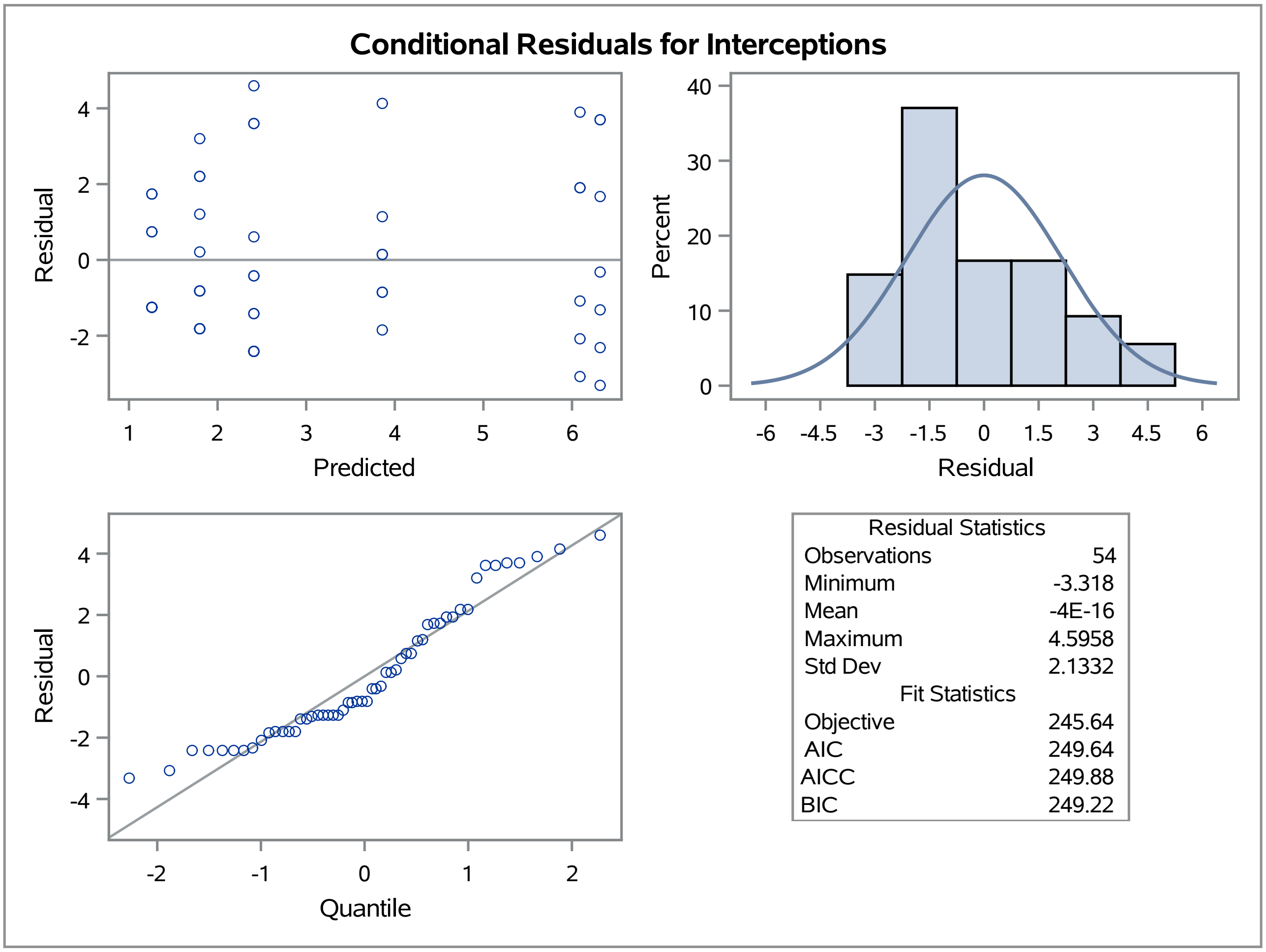

DistSoma=276

Model Information	
**Data Set**	WORK.TEMPDATASORTED
**Dependent Variable**	Interceptions
**Covariance Structure**	Variance Components
**Estimation Method**	REML
**Residual Variance Method**	Profile
**Fixed Effects SE Method**	Model-Based
**Degrees of Freedom Method**	Containment

Class Level Information		
**Class**	**Levels**	**Values**
**Genotype**	2	Meg +/+ Meg +/-
**Animal**	6	1 2 3 4 5 6

Dimensions	
**Covariance Parameters**	2
**Columns in X**	3
**Columns in Z**	6
**Subjects**	1
**Max Obs per Subject**	54

Number of Observations	
**Number of Observations Read**	54
**Number of Observations Used**	54
**Number of Observations Not Used**	0

Iteration History			
**Iteration**	**Evaluations**	**-2 Res Log Like**	**Criterion**
**0**	1	260.59028487	
**1**	3	242.28661074	0.00017576
**2**	1	242.27258308	0.00000384
**3**	1	242.27229673	0.00000000

Convergence criteria met.

DistSoma=276

Covariance Parameter Estimates				
**Cov Parm**	**Estimate**	**Alpha**	**Lower**	**Upper**
**Animal**	4.4297	0.05	1.4648	52.9647
**Residual**	4.5905	0.05	3.1933	7.1613

Fit Statistics	
**-2 Res Log Likelihood**	242.3
**AIC (Smaller is Better)**	246.3
**AICC (Smaller is Better)**	246.5
**BIC (Smaller is Better)**	245.9

Solution for Fixed Effects									
**Effect**	**Genotype**	**Estimate**	**Standard Error**	**DF**	**t Value**	**Pr > |t|**	**Alpha**	**Lower**	**Upper**
**Intercept**		2.1626	1.2837	4	1.68	0.1673	0.05	-1.4014	5.7266
**Genotype**	Meg +/+	2.7747	1.8219	48	1.52	0.1343	0.05	-0.8886	6.4379
**Genotype**	Meg +/-	0	.	.	.	.	.	.	.

Solution for Random Effects									
**Effect**	**Animal**	**Estimate**	**Std Err Pred**	**DF**	**t Value**	**Pr > |t|**	**Alpha**	**Lower**	**Upper**
**Animal**	1	-2.8629	1.3270	48	-2.16	0.0360	0.05	-5.5310	-0.1947
**Animal**	2	1.1905	1.3667	48	0.87	0.3881	0.05	-1.5575	3.9385
**Animal**	3	1.6724	1.3563	48	1.23	0.2236	0.05	-1.0547	4.3994
**Animal**	4	-0.9429	1.3351	48	-0.71	0.4835	0.05	-3.6273	1.7415
**Animal**	5	-0.5332	1.3223	48	-0.40	0.6886	0.05	-3.1917	2.1254
**Animal**	6	1.4761	1.3496	48	1.09	0.2795	0.05	-1.2375	4.1896

Type 3 Tests of Fixed Effects				
**Effect**	**Num DF**	**Den DF**	**F Value**	**Pr > F**
**Genotype**	1	48	2.32	0.1343

Least Squares Means									
**Effect**	**Genotype**	**Estimate**	**Standard Error**	**DF**	**t Value**	**Pr > |t|**	**Alpha**	**Lower**	**Upper**
**Genotype**	Meg +/+	4.9372	1.2929	48	3.82	0.0004	0.05	2.3376	7.5368
**Genotype**	Meg +/-	2.1626	1.2837	48	1.68	0.0985	0.05	-0.4184	4.7435

DistSoma=276

Differences of Least Squares Means												
**Effect**	**Genotype**	**Genotype**	**Estimate**	**Standard Error**	**DF**	**t Value**	**Pr > |t|**	**Adjustment**	**Adj P**	**Alpha**	**Lower**	**Upper**
**Genotype**	Meg +/+	Meg +/-	2.7747	1.8219	48	1.52	0.1343	Tukey-Kramer	0.1343	0.05	-0.8886	6.4379

Differences of Least Squares Means				
**Effect**	**Genotype**	**Genotype**	**Adj Lower**	**Adj Upper**
**Genotype**	Meg +/+	Meg +/-	-0.8884	6.4378

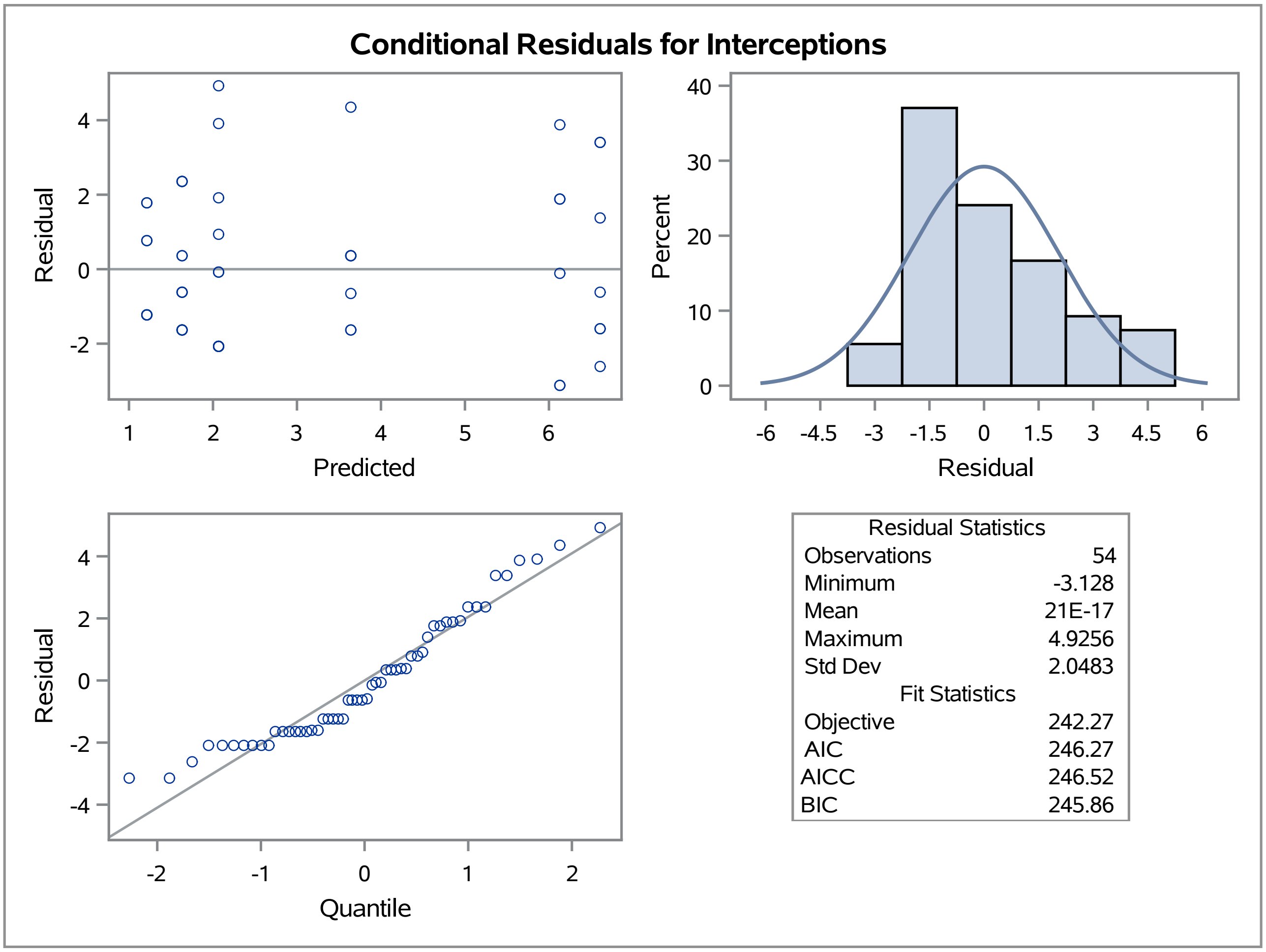

DistSoma=282

Model Information	
**Data Set**	WORK.TEMPDATASORTED
**Dependent Variable**	Interceptions
**Covariance Structure**	Variance Components
**Estimation Method**	REML
**Residual Variance Method**	Profile
**Fixed Effects SE Method**	Model-Based
**Degrees of Freedom Method**	Containment

Class Level Information		
**Class**	**Levels**	**Values**
**Genotype**	2	Meg +/+ Meg +/-
**Animal**	6	1 2 3 4 5 6

Dimensions	
**Covariance Parameters**	2
**Columns in X**	3
**Columns in Z**	6
**Subjects**	1
**Max Obs per Subject**	54

Number of Observations	
**Number of Observations Read**	54
**Number of Observations Used**	54
**Number of Observations Not Used**	0

Iteration History			
**Iteration**	**Evaluations**	**-2 Res Log Like**	**Criterion**
**0**	1	261.87367762	
**1**	3	241.36003585	0.00011827
**2**	1	241.35077288	0.00000178
**3**	1	241.35064142	0.00000000

Convergence criteria met.

DistSoma=282

Covariance Parameter Estimates				
Cov Parm	Estimate	Alpha	Lower	Upper
Animal	4.9018	0.05	1.6354	56.1462
Residual	4.4703	0.05	3.1095	6.9744

Fit Statistics	
-2 Res Log Likelihood	241.4
AIC (Smaller is Better)	245.4
AICC (Smaller is Better)	245.6
BIC (Smaller is Better)	244.9

Solution for Fixed Effects									
Effect	Genotype	Estimate	Standard Error	DF	t Value	Pr > |t|	Alpha	Lower	Upper
Intercept		1.9114	1.3419	4	1.42	0.2274	0.05	-1.8144	5.6372
Genotype	Meg +/+	2.7723	1.9039	48	1.46	0.1519	0.05	-1.0557	6.6004
Genotype	Meg +/-	0	.	.	.	.	.	.	.

Solution for Random Effects									
Effect	Animal	Estimate	Std Err Pred	DF	t Value	Pr > |t|	Alpha	Lower	Upper
Animal	1	-2.9391	1.3835	48	-2.12	0.0388	0.05	-5.7208	-0.1573
Animal	2	1.1426	1.4217	48	0.80	0.4255	0.05	-1.7160	4.0011
Animal	3	1.7965	1.4117	48	1.27	0.2093	0.05	-1.0419	4.6349
Animal	4	-1.0293	1.3913	48	-0.74	0.4630	0.05	-3.8267	1.7680
Animal	5	-0.6921	1.3790	48	-0.50	0.6180	0.05	-3.4648	2.0805
Animal	6	1.7215	1.4052	48	1.23	0.2265	0.05	-1.1039	4.5468

Type 3 Tests of Fixed Effects				
Effect	Num DF	Den DF	F Value	Pr > F
Genotype	1	48	2.12	0.1519

Least Squares Means									
Effect	Genotype	Estimate	Standard Error	DF	t Value	Pr > |t|	Alpha	Lower	Upper
Genotype	Meg +/+	4.6837	1.3506	48	3.47	0.0011	0.05	1.9682	7.3993
Genotype	Meg +/-	1.9114	1.3419	48	1.42	0.1608	0.05	-0.7867	4.6095

DistSoma=282

Differences of Least Squares Means

Effect	Genotype	Genotype	Estimate	Standard Error	DF	t Value	Pr > |t|	Adjustment	Adj P	Alpha	Lower	Upper
**Genotype**	Meg +/+	Meg +/-	2.7723	1.9039	48	1.46	0.1519	Tukey-Kramer	0.1519	0.05	-1.0557	6.6004

Differences of Least Squares Means

Effect	Genotype	Genotype	Adj Lower	Adj Upper
**Genotype**	Meg +/+	Meg +/-	-1.0556	6.6003

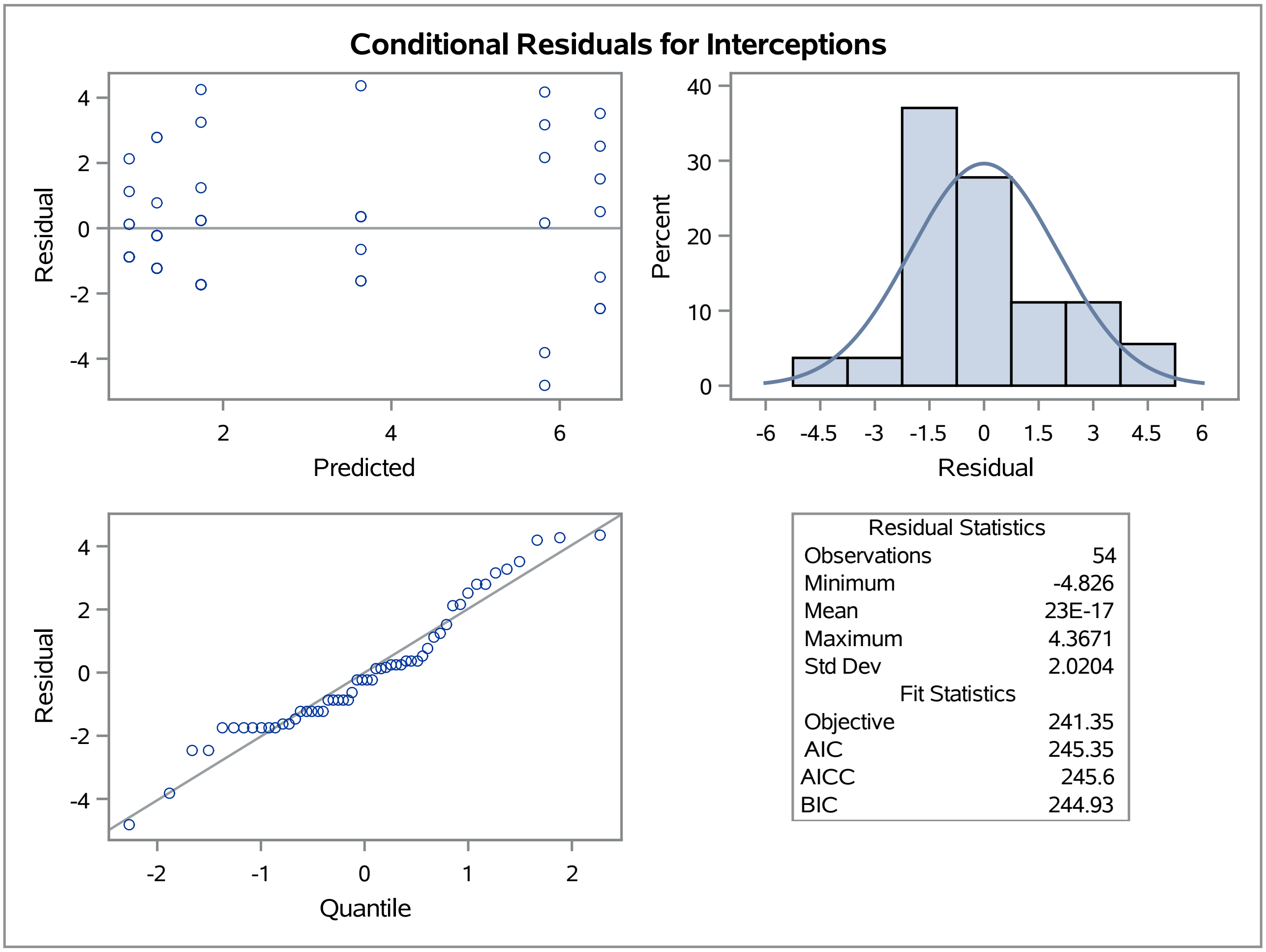

DistSoma=288

Model Information	
**Data Set**	WORK.TEMPDATASORTED
**Dependent Variable**	Interceptions
**Covariance Structure**	Variance Components
**Estimation Method**	REML
**Residual Variance Method**	Profile
**Fixed Effects SE Method**	Model-Based
**Degrees of Freedom Method**	Containment

Class Level Information		
**Class**	**Levels**	**Values**
**Genotype**	2	Meg +/+ Meg +/-
**Animal**	6	1 2 3 4 5 6

Dimensions	
**Covariance Parameters**	2
**Columns in X**	3
**Columns in Z**	6
**Subjects**	1
**Max Obs per Subject**	54

Number of Observations	
**Number of Observations Read**	54
**Number of Observations Used**	54
**Number of Observations Not Used**	0

Iteration History			
**Iteration**	**Evaluations**	**-2 Res Log Like**	**Criterion**
**0**	1	262.60721216	
**1**	3	239.29007132	0.00014235
**2**	1	239.27902436	0.00000252
**3**	1	239.27884127	0.00000000

Convergence criteria met.

DistSoma=288

Covariance Parameter Estimates				
Cov Parm	Estimate	Alpha	Lower	Upper
Animal	5.3680	0.05	1.8077	58.8239
Residual	4.2538	0.05	2.9588	6.6369

Fit Statistics	
-2 Res Log Likelihood	239.3
AIC (Smaller is Better)	243.3
AICC (Smaller is Better)	243.5
BIC (Smaller is Better)	242.9

Solution for Fixed Effects									
Effect	Genotype	Estimate	Standard Error	DF	t Value	Pr > |t|	Alpha	Lower	Upper
Intercept		1.7439	1.3958	4	1.25	0.2796	0.05	-2.1314	5.6191
Genotype	Meg +/+	2.6794	1.9796	48	1.35	0.1822	0.05	-1.3007	6.6596
Genotype	Meg +/-	0	.	.	.	.	.	.	.

Solution for Random Effects									
Effect	Animal	Estimate	Std Err Pred	DF	t Value	Pr > |t|	Alpha	Lower	Upper
Animal	1	-3.0091	1.4349	48	-2.10	0.0413	0.05	-5.8942	-0.1240
Animal	2	0.9511	1.4710	48	0.65	0.5210	0.05	-2.0065	3.9087
Animal	3	2.0580	1.4615	48	1.41	0.1655	0.05	-0.8805	4.9966
Animal	4	-0.8879	1.4422	48	-0.62	0.5410	0.05	-3.7877	2.0119
Animal	5	-1.0105	1.4307	48	-0.71	0.4834	0.05	-3.8870	1.8661
Animal	6	1.8984	1.4553	48	1.30	0.1983	0.05	-1.0277	4.8245

Type 3 Tests of Fixed Effects				
Effect	Num DF	Den DF	F Value	Pr > F
Genotype	1	48	1.83	0.1822

Least Squares Means									
Effect	Genotype	Estimate	Standard Error	DF	t Value	Pr > |t|	Alpha	Lower	Upper
Genotype	Meg +/+	4.4233	1.4037	48	3.15	0.0028	0.05	1.6009	7.2457
Genotype	Meg +/-	1.7439	1.3958	48	1.25	0.2176	0.05	-1.0625	4.5502

DistSoma=288

Differences of Least Squares Means												
**Effect**	**Genotype**	**Genotype**	**Estimate**	**Standard Error**	**DF**	**t Value**	**Pr > |t|**	**Adjustment**	**Adj P**	**Alpha**	**Lower**	**Upper**
**Genotype**	Meg +/+	Meg +/-	2.6794	1.9796	48	1.35	0.1822	Tukey-Kramer	0.1822	0.05	-1.3007	6.6596

Differences of Least Squares Means				
**Effect**	**Genotype**	**Genotype**	**Adj Lower**	**Adj Upper**
**Genotype**	Meg +/+	Meg +/-	-1.3006	6.6595

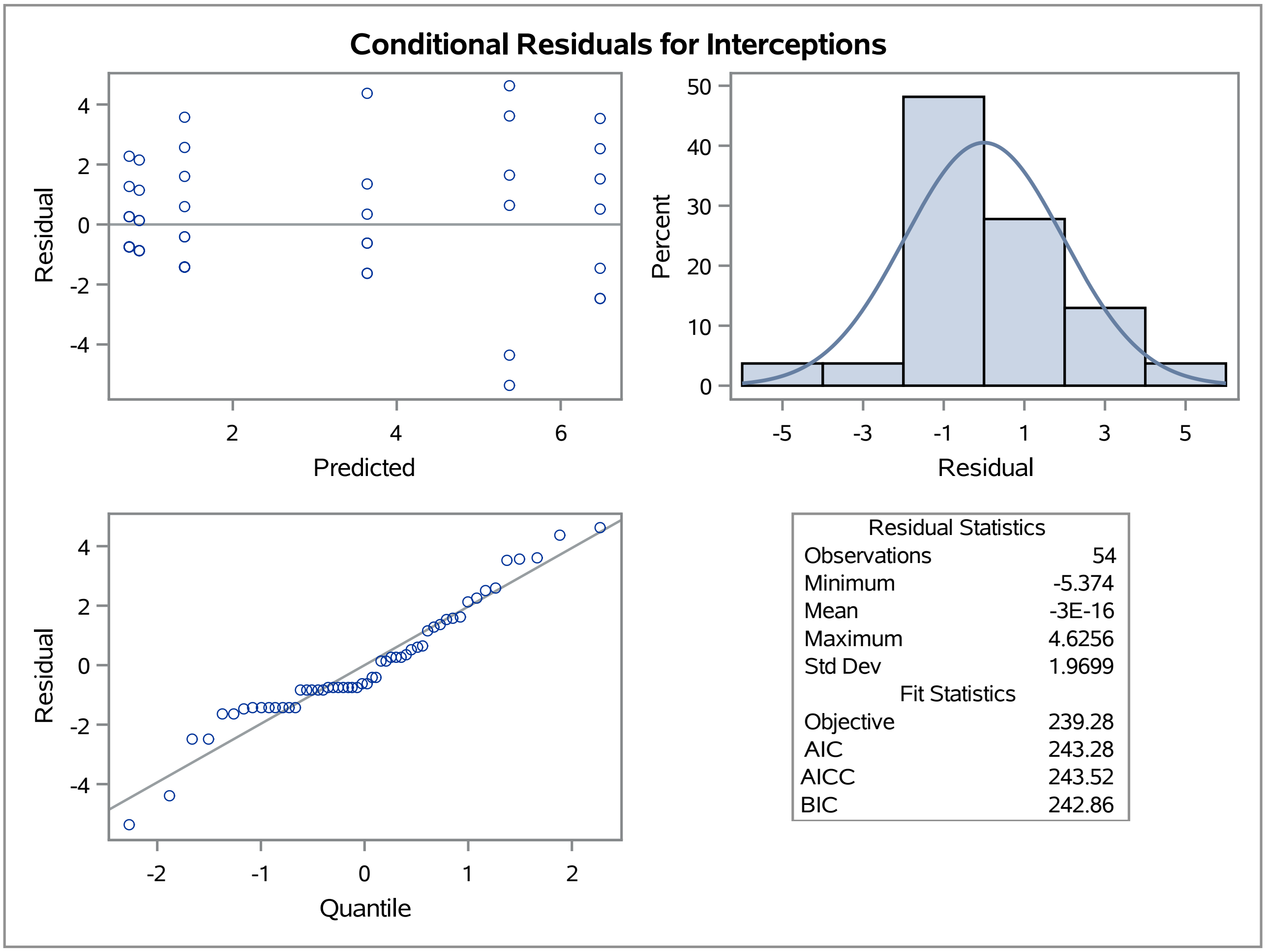

DistSoma=294

Model Information	
**Data Set**	WORK.TEMPDATASORTED
**Dependent Variable**	Interceptions
**Covariance Structure**	Variance Components
**Estimation Method**	REML
**Residual Variance Method**	Profile
**Fixed Effects SE Method**	Model-Based
**Degrees of Freedom Method**	Containment

Class Level Information		
**Class**	**Levels**	**Values**
**Genotype**	2	Meg +/+ Meg +/-
**Animal**	6	1 2 3 4 5 6

Dimensions	
**Covariance Parameters**	2
**Columns in X**	3
**Columns in Z**	6
**Subjects**	1
**Max Obs per Subject**	54

Number of Observations	
**Number of Observations Read**	54
**Number of Observations Used**	54
**Number of Observations Not Used**	0

Iteration History			
**Iteration**	**Evaluations**	**-2 Res Log Like**	**Criterion**
**0**	1	266.34276542	
**1**	3	229.69483343	0.00015054
**2**	1	229.68393527	0.00000263
**3**	1	229.68375681	0.00000000

Convergence criteria met.

DistSoma=294

Covariance Parameter Estimates				
Cov Parm	Estimate	Alpha	Lower	Upper
Animal	7.4883	0.05	2.5894	72.6542
Residual	3.3985	0.05	2.3637	5.3033

Fit Statistics	
-2 Res Log Likelihood	229.7
AIC (Smaller is Better)	233.7
AICC (Smaller is Better)	233.9
BIC (Smaller is Better)	233.3

Solution for Fixed Effects									
Effect	Genotype	Estimate	Standard Error	DF	t Value	Pr > |t|	Alpha	Lower	Upper
Intercept		1.5495	1.6196	4	0.96	0.3929	0.05	-2.9473	6.0463
Genotype	Meg +/+	2.7541	2.2944	48	1.20	0.2359	0.05	-1.8592	7.3674
Genotype	Meg +/-	0	.	.	.	.	.	.	.

Solution for Random Effects									
Effect	Animal	Estimate	Std Err Pred	DF	t Value	Pr > |t|	Alpha	Lower	Upper
Animal	1	-3.4895	1.6489	48	-2.12	0.0395	0.05	-6.8047	-0.1742
Animal	2	0.9573	1.6762	48	0.57	0.5706	0.05	-2.4128	4.3274
Animal	3	2.5322	1.6690	48	1.52	0.1358	0.05	-0.8235	5.8879
Animal	4	-1.2636	1.6543	48	-0.76	0.4487	0.05	-4.5899	2.0627
Animal	5	-1.1719	1.6457	48	-0.71	0.4799	0.05	-4.4808	2.1371
Animal	6	2.4354	1.6642	48	1.46	0.1499	0.05	-0.9106	5.7814

Type 3 Tests of Fixed Effects				
Effect	Num DF	Den DF	F Value	Pr > F
Genotype	1	48	1.44	0.2359

Least Squares Means									
Effect	Genotype	Estimate	Standard Error	DF	t Value	Pr > |t|	Alpha	Lower	Upper
Genotype	Meg +/+	4.3036	1.6252	48	2.65	0.0109	0.05	1.0359	7.5713
Genotype	Meg +/-	1.5495	1.6196	48	0.96	0.3435	0.05	-1.7070	4.8060

DistSoma=294

Differences of Least Squares Means												
Effect	Genotype	Genotype	Estimate	Standard Error	DF	t Value	Pr > |t|	Adjustment	Adj P	Alpha	Lower	Upper
**Genotype**	Meg +/+	Meg +/-	2.7541	2.2944	48	1.20	0.2359	Tukey-Kramer	0.2359	0.05	-1.8592	7.3674

Differences of Least Squares Means				
Effect	Genotype	Genotype	Adj Lower	Adj Upper
**Genotype**	Meg +/+	Meg +/-	-1.8590	7.3672

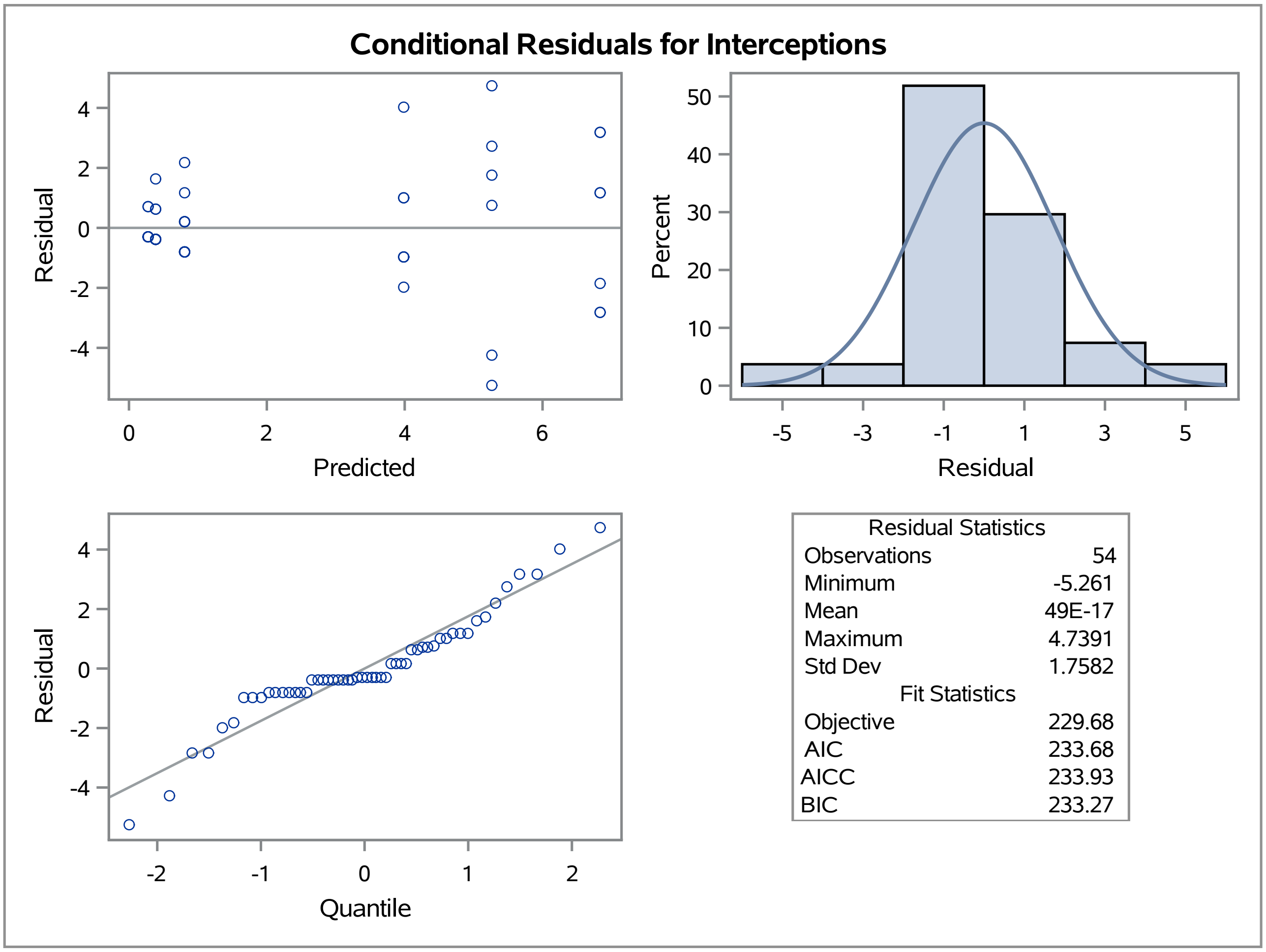

DistSoma=300

Model Information	
**Data Set**	WORK.TEMPDATASORTED
**Dependent Variable**	Interceptions
**Covariance Structure**	Variance Components
**Estimation Method**	REML
**Residual Variance Method**	Profile
**Fixed Effects SE Method**	Model-Based
**Degrees of Freedom Method**	Containment

Class Level Information		
**Class**	**Levels**	**Values**
**Genotype**	2	Meg +/+ Meg +/-
**Animal**	6	1 2 3 4 5 6

Dimensions	
**Covariance Parameters**	2
**Columns in X**	3
**Columns in Z**	6
**Subjects**	1
**Max Obs per Subject**	54

Number of Observations	
**Number of Observations Read**	54
**Number of Observations Used**	54
**Number of Observations Not Used**	0

Iteration History			
**Iteration**	**Evaluations**	**-2 Res Log Like**	**Criterion**
**0**	1	269.95307100	
**1**	3	230.63343146	0.00022278
**2**	1	230.61697843	0.00000561
**3**	1	230.61659312	0.00000000

Convergence criteria met.

DistSoma=300

Covariance Parameter Estimates				
**Cov Parm**	**Estimate**	**Alpha**	**Lower**	**Upper**
**Animal**	8.3027	0.05	2.8810	79.3221
**Residual**	3.4368	0.05	2.3903	5.3631

Fit Statistics	
**-2 Res Log Likelihood**	230.6
**AIC (Smaller is Better)**	234.6
**AICC (Smaller is Better)**	234.9
**BIC (Smaller is Better)**	234.2

Solution for Fixed Effects									
**Effect**	**Genotype**	**Estimate**	**Standard Error**	**DF**	**t Value**	**Pr > |t|**	**Alpha**	**Lower**	**Upper**
**Intercept**		1.4577	1.7018	4	0.86	0.4400	0.05	-3.2673	6.1826
**Genotype**	Meg +/+	2.6636	2.4105	48	1.11	0.2747	0.05	-2.1831	7.5103
**Genotype**	Meg +/-	0	.	.	.	.	.	.	.

Solution for Random Effects									
**Effect**	**Animal**	**Estimate**	**Std Err Pred**	**DF**	**t Value**	**Pr > |t|**	**Alpha**	**Lower**	**Upper**
**Animal**	1	-3.6959	1.7302	48	-2.14	0.0378	0.05	-7.1747	-0.2171
**Animal**	2	0.9779	1.7568	48	0.56	0.5804	0.05	-2.5543	4.5102
**Animal**	3	2.7180	1.7498	48	1.55	0.1269	0.05	-0.8002	6.2361
**Animal**	4	-1.2873	1.7355	48	-0.74	0.4619	0.05	-4.7769	2.2022
**Animal**	5	-1.2479	1.7272	48	-0.72	0.4735	0.05	-4.7206	2.2247
**Animal**	6	2.5353	1.7451	48	1.45	0.1528	0.05	-0.9734	6.0440

Type 3 Tests of Fixed Effects				
**Effect**	**Num DF**	**Den DF**	**F Value**	**Pr > F**
**Genotype**	1	48	1.22	0.2747

Least Squares Means									
**Effect**	**Genotype**	**Estimate**	**Standard Error**	**DF**	**t Value**	**Pr > |t|**	**Alpha**	**Lower**	**Upper**
**Genotype**	Meg +/+	4.1213	1.7072	48	2.41	0.0196	0.05	0.6887	7.5538
**Genotype**	Meg +/-	1.4577	1.7018	48	0.86	0.3960	0.05	-1.9640	4.8794

DistSoma=300

Differences of Least Squares Means												
Effect	Genotype	Genotype	Estimate	Standard Error	DF	t Value	Pr > |t|	Adjustment	Adj P	Alpha	Lower	Upper
**Genotype**	Meg +/+	Meg +/-	2.6636	2.4105	48	1.11	0.2747	Tukey-Kramer	0.2747	0.05	-2.1831	7.5103

Differences of Least Squares Means				
Effect	Genotype	Genotype	Adj Lower	Adj Upper
**Genotype**	Meg +/+	Meg +/-	-2.1829	7.5101

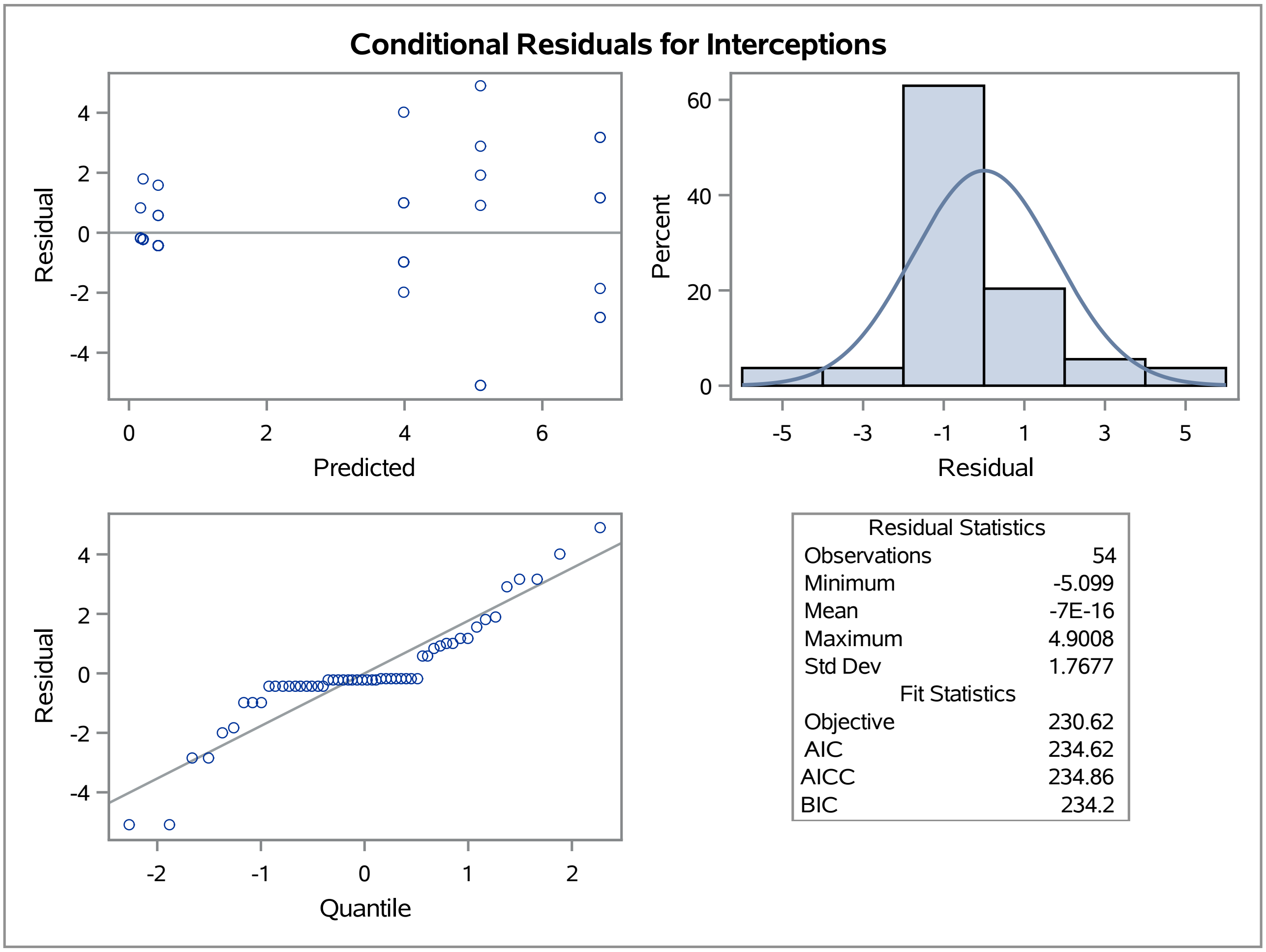

DistSoma=306

Model Information	
**Data Set**	WORK.TEMPDATASORTED
**Dependent Variable**	Interceptions
**Covariance Structure**	Variance Components
**Estimation Method**	REML
**Residual Variance Method**	Profile
**Fixed Effects SE Method**	Model-Based
**Degrees of Freedom Method**	Containment

Class Level Information		
**Class**	**Levels**	**Values**
**Genotype**	2	Meg +/+ Meg +/-
**Animal**	6	1 2 3 4 5 6

Dimensions	
**Covariance Parameters**	2
**Columns in X**	3
**Columns in Z**	6
**Subjects**	1
**Max Obs per Subject**	54

Number of Observations	
**Number of Observations Read**	54
**Number of Observations Used**	54
**Number of Observations Not Used**	0

Iteration History			
**Iteration**	**Evaluations**	**-2 Res Log Like**	**Criterion**
**0**	1	272.73170728	
**1**	3	233.52894309	0.00044548
**2**	1	233.49442790	0.00002112
**3**	1	233.49292262	0.00000006
**4**	1	233.49291859	0.00000000

Convergence criteria met.

DistSoma=306

Covariance Parameter Estimates				
**Cov Parm**	**Estimate**	**Alpha**	**Lower**	**Upper**
**Animal**	8.6827	0.05	3.0118	83.0777
**Residual**	3.6353	0.05	2.5284	5.6728

Fit Statistics	
**-2 Res Log Likelihood**	233.5
**AIC (Smaller is Better)**	237.5
**AICC (Smaller is Better)**	237.7
**BIC (Smaller is Better)**	237.1

Solution for Fixed Effects									
**Effect**	**Genotype**	**Estimate**	**Standard Error**	**DF**	**t Value**	**Pr > |t|**	**Alpha**	**Lower**	**Upper**
**Intercept**		1.3453	1.7407	4	0.77	0.4828	0.05	-3.4878	6.1784
**Genotype**	Meg +/+	2.9136	2.4657	48	1.18	0.2432	0.05	-2.0441	7.8713
**Genotype**	Meg +/-	0	.	.	.	.	.	.	.

Solution for Random Effects									
**Effect**	**Animal**	**Estimate**	**Std Err Pred**	**DF**	**t Value**	**Pr > |t|**	**Alpha**	**Lower**	**Upper**
**Animal**	1	-3.9024	1.7701	48	-2.20	0.0323	0.05	-7.4614	-0.3434
**Animal**	2	1.3160	1.7975	48	0.73	0.4677	0.05	-2.2982	4.9301
**Animal**	3	2.5864	1.7903	48	1.44	0.1550	0.05	-1.0132	6.1861
**Animal**	4	-1.2855	1.7756	48	-0.72	0.4726	0.05	-4.8556	2.2846
**Animal**	5	-1.2194	1.7669	48	-0.69	0.4934	0.05	-4.7721	2.3332
**Animal**	6	2.5049	1.7854	48	1.40	0.1671	0.05	-1.0850	6.0948

Type 3 Tests of Fixed Effects				
**Effect**	**Num DF**	**Den DF**	**F Value**	**Pr > F**
**Genotype**	1	48	1.40	0.2432

Least Squares Means									
**Effect**	**Genotype**	**Estimate**	**Standard Error**	**DF**	**t Value**	**Pr > |t|**	**Alpha**	**Lower**	**Upper**
**Genotype**	Meg +/+	4.2589	1.7463	48	2.44	0.0185	0.05	0.7477	7.7701
**Genotype**	Meg +/-	1.3453	1.7407	48	0.77	0.4434	0.05	-2.1547	4.8453

DistSoma=306

Differences of Least Squares Means												
Effect	Genotype	Genotype	Estimate	Standard Error	DF	t Value	Pr > |t|	Adjustment	Adj P	Alpha	Lower	Upper
**Genotype**	Meg +/+	Meg +/-	2.9136	2.4657	48	1.18	0.2432	Tukey-Kramer	0.2432	0.05	-2.0441	7.8713

Differences of Least Squares Means				
Effect	Genotype	Genotype	Adj Lower	Adj Upper
**Genotype**	Meg +/+	Meg +/-	-2.0439	7.8711

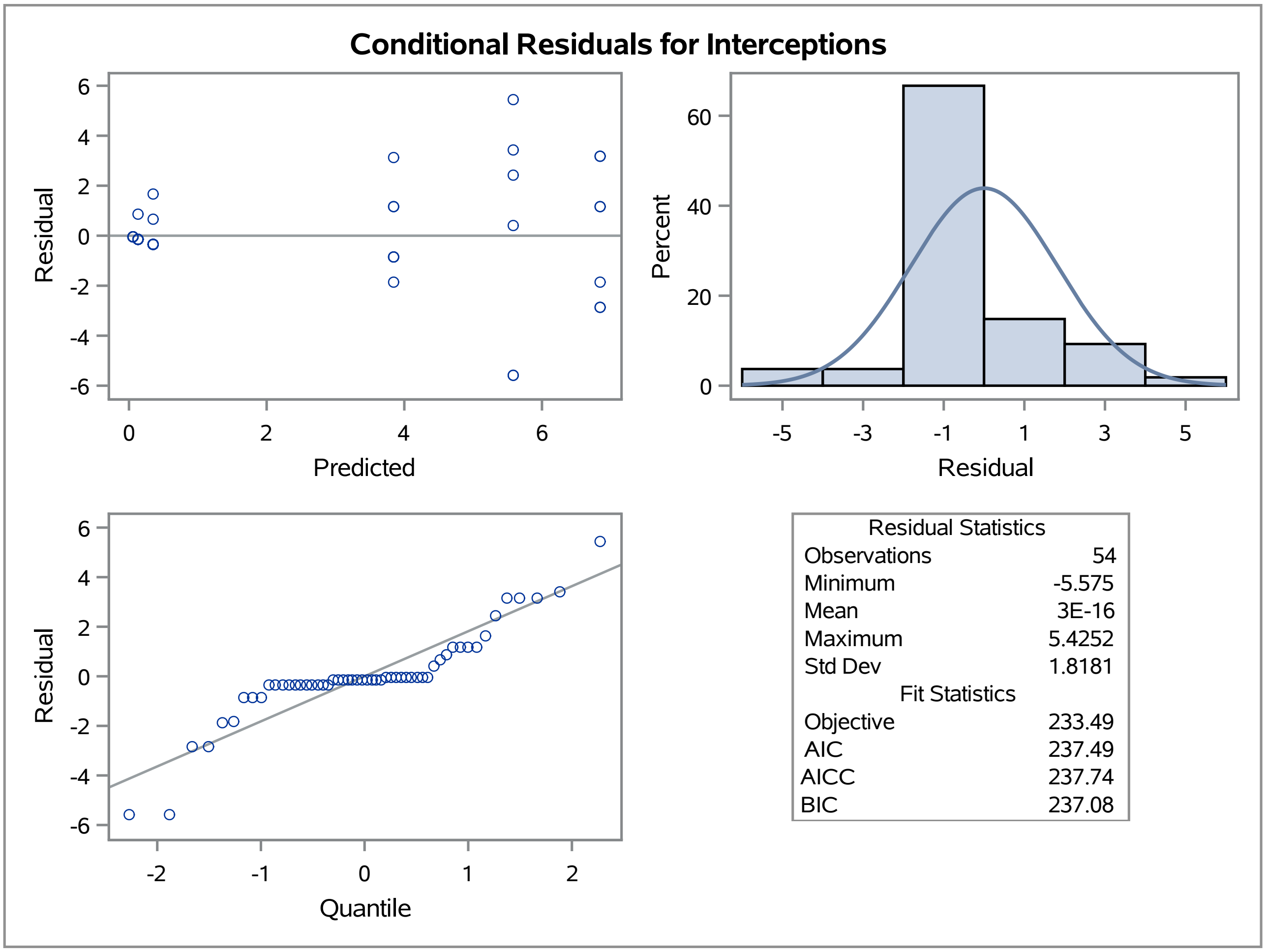

DistSoma=312

Model Information	
**Data Set**	WORK.TEMPDATASORTED
**Dependent Variable**	Interceptions
**Covariance Structure**	Variance Components
**Estimation Method**	REML
**Residual Variance Method**	Profile
**Fixed Effects SE Method**	Model-Based
**Degrees of Freedom Method**	Containment

Class Level Information		
**Class**	**Levels**	**Values**
**Genotype**	2	Meg +/+ Meg +/-
**Animal**	6	1 2 3 4 5 6

Dimensions	
**Covariance Parameters**	2
**Columns in X**	3
**Columns in Z**	6
**Subjects**	1
**Max Obs per Subject**	54

Number of Observations	
**Number of Observations Read**	54
**Number of Observations Used**	54
**Number of Observations Not Used**	0

Iteration History			
**Iteration**	**Evaluations**	**-2 Res Log Like**	**Criterion**
**0**	1	270.27127201	
**1**	3	231.59160840	0.00068056
**2**	1	231.53872824	0.00004565
**3**	1	231.53547691	0.00000026
**4**	1	231.53545903	0.00000000

Convergence criteria met.

DistSoma=312

Covariance Parameter Estimates				
Cov Parm	Estimate	Alpha	Lower	Upper
Animal	8.1868	0.05	2.8382	78.5352
Residual	3.5069	0.05	2.4391	5.4723

Fit Statistics	
-2 Res Log Likelihood	231.5
AIC (Smaller is Better)	235.5
AICC (Smaller is Better)	235.8
BIC (Smaller is Better)	235.1

Solution for Fixed Effects									
Effect	Genotype	Estimate	Standard Error	DF	t Value	Pr > |t|	Alpha	Lower	Upper
Intercept		1.2509	1.6912	4	0.74	0.5006	0.05	-3.4446	5.9463
Genotype	Meg +/+	2.8789	2.3956	48	1.20	0.2354	0.05	-1.9378	7.6955
Genotype	Meg +/-	0	.	.	.	.	.	.	.

Solution for Random Effects									
Effect	Animal	Estimate	Std Err Pred	DF	t Value	Pr > |t|	Alpha	Lower	Upper
Animal	1	-3.8491	1.7202	48	-2.24	0.0299	0.05	-7.3079	-0.3903
Animal	2	1.2789	1.7474	48	0.73	0.4678	0.05	-2.2345	4.7924
Animal	3	2.5701	1.7403	48	1.48	0.1462	0.05	-0.9289	6.0691
Animal	4	-1.1940	1.7257	48	-0.69	0.4923	0.05	-4.6638	2.2757
Animal	5	-1.1273	1.7171	48	-0.66	0.5146	0.05	-4.5798	2.3252
Animal	6	2.3214	1.7355	48	1.34	0.1873	0.05	-1.1680	5.8107

Type 3 Tests of Fixed Effects				
Effect	Num DF	Den DF	F Value	Pr > F
Genotype	1	48	1.44	0.2354

Least Squares Means									
Effect	Genotype	Estimate	Standard Error	DF	t Value	Pr > |t|	Alpha	Lower	Upper
Genotype	Meg +/+	4.1297	1.6967	48	2.43	0.0187	0.05	0.7183	7.5412
Genotype	Meg +/-	1.2509	1.6912	48	0.74	0.4631	0.05	-2.1495	4.6512

DistSoma=312

Differences of Least Squares Means												
**Effect**	**Genotype**	**Genotype**	**Estimate**	**Standard Error**	**DF**	**t Value**	**Pr > |t|**	**Adjustment**	**Adj P**	**Alpha**	**Lower**	**Upper**
**Genotype**	Meg +/+	Meg +/-	2.8789	2.3956	48	1.20	0.2354	Tukey-Kramer	0.2354	0.05	-1.9378	7.6955

Differences of Least Squares Means				
**Effect**	**Genotype**	**Genotype**	**Adj Lower**	**Adj Upper**
**Genotype**	Meg +/+	Meg +/-	-1.9377	7.6954

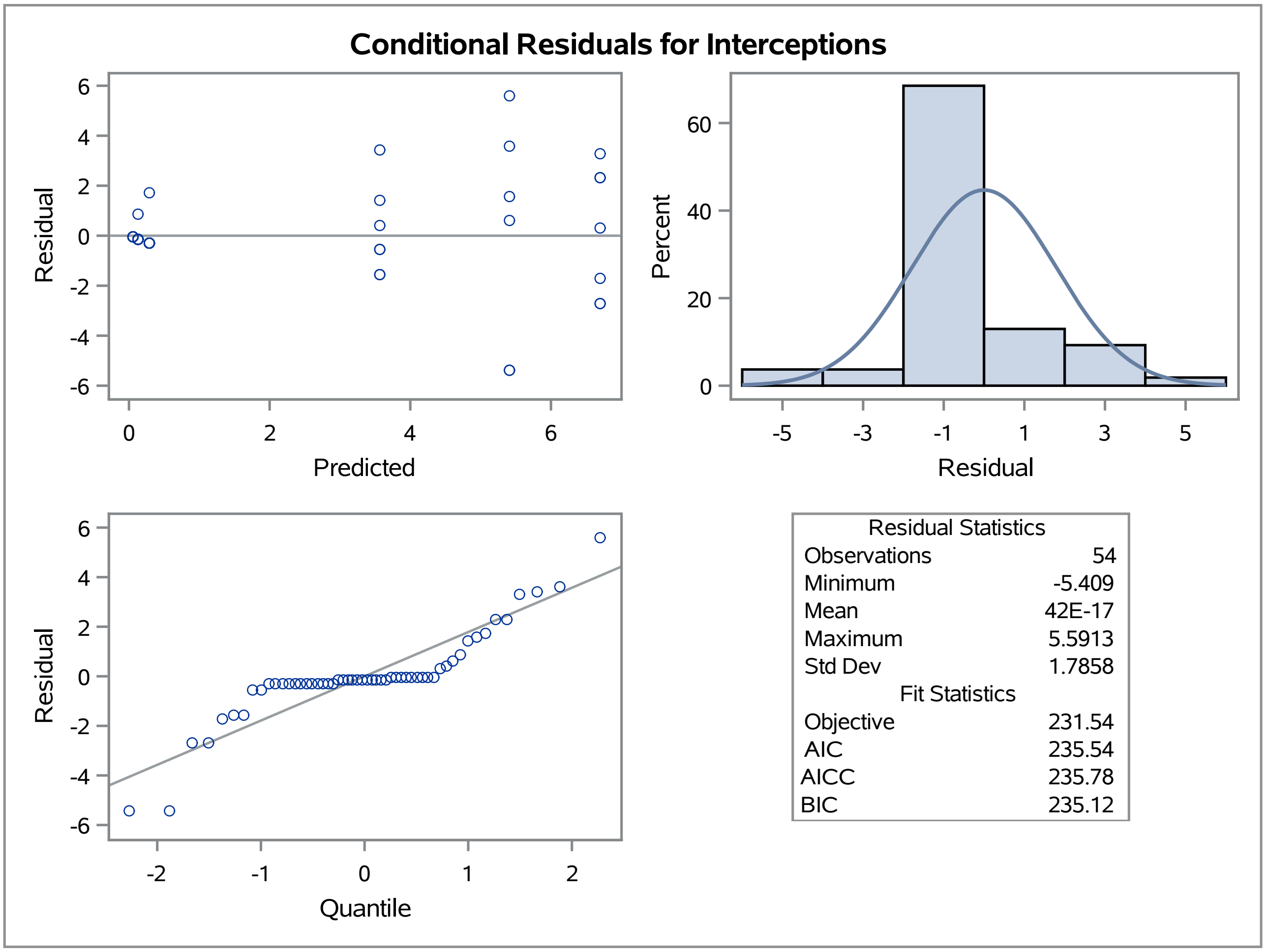

DistSoma=318

Model Information	
**Data Set**	WORK.TEMPDATASORTED
**Dependent Variable**	Interceptions
**Covariance Structure**	Variance Components
**Estimation Method**	REML
**Residual Variance Method**	Profile
**Fixed Effects SE Method**	Model-Based
**Degrees of Freedom Method**	Containment

Class Level Information		
**Class**	**Levels**	**Values**
**Genotype**	2	Meg +/+ Meg +/-
**Animal**	6	1 2 3 4 5 6

Dimensions	
**Covariance Parameters**	2
**Columns in X**	3
**Columns in Z**	6
**Subjects**	1
**Max Obs per Subject**	54

Number of Observations	
**Number of Observations Read**	54
**Number of Observations Used**	54
**Number of Observations Not Used**	0

Iteration History			
**Iteration**	**Evaluations**	**-2 Res Log Like**	**Criterion**
**0**	1	270.97575268	
**1**	3	231.01025060	0.00054798
**2**	1	230.96824671	0.00003053
**3**	1	230.96609798	0.00000012
**4**	1	230.96608994	0.00000000

Convergence criteria met.

DistSoma=318

Covariance Parameter Estimates				
Cov Parm	Estimate	Alpha	Lower	Upper
Animal	8.4662	0.05	2.9393	80.6906
Residual	3.4565	0.05	2.4040	5.3938

Fit Statistics	
-2 Res Log Likelihood	231.0
AIC (Smaller is Better)	235.0
AICC (Smaller is Better)	235.2
BIC (Smaller is Better)	234.5

Solution for Fixed Effects									
Effect	Genotype	Estimate	Standard Error	DF	t Value	Pr > |t|	Alpha	Lower	Upper
Intercept		1.2705	1.7179	4	0.74	0.5006	0.05	-3.4993	6.0403
Genotype	Meg +/+	2.7802	2.4333	48	1.14	0.2589	0.05	-2.1124	7.6727
Genotype	Meg +/-	0	.	.	.	.	.	.	.

Solution for Random Effects									
Effect	Animal	Estimate	Std Err Pred	DF	t Value	Pr > |t|	Alpha	Lower	Upper
Animal	1	-3.8528	1.7463	48	-2.21	0.0322	0.05	-7.3639	-0.3416
Animal	2	1.2009	1.7728	48	0.68	0.5014	0.05	-2.3635	4.7654
Animal	3	2.6518	1.7658	48	1.50	0.1397	0.05	-0.8986	6.2022
Animal	4	-1.2154	1.7516	48	-0.69	0.4911	0.05	-4.7372	2.3065
Animal	5	-1.2287	1.7432	48	-0.70	0.4843	0.05	-4.7337	2.2763
Animal	6	2.4441	1.7611	48	1.39	0.1716	0.05	-1.0969	5.9851

Type 3 Tests of Fixed Effects				
Effect	Num DF	Den DF	F Value	Pr > F
Genotype	1	48	1.31	0.2589

Least Squares Means									
Effect	Genotype	Estimate	Standard Error	DF	t Value	Pr > |t|	Alpha	Lower	Upper
Genotype	Meg +/+	4.0507	1.7233	48	2.35	0.0229	0.05	0.5857	7.5156
Genotype	Meg +/-	1.2705	1.7179	48	0.74	0.4632	0.05	-2.1837	4.7247

DistSoma=318

Differences of Least Squares Means												
Effect	Genotype	Genotype	Estimate	Standard Error	DF	t Value	Pr > |t|	Adjustment	Adj P	Alpha	Lower	Upper
Genotype	Meg +/+	Meg +/-	2.7802	2.4333	48	1.14	0.2589	Tukey-Kramer	0.2589	0.05	-2.1124	7.6727

Differences of Least Squares Means				
Effect	Genotype	Genotype	Adj Lower	Adj Upper
Genotype	Meg +/+	Meg +/-	-2.1122	7.6726

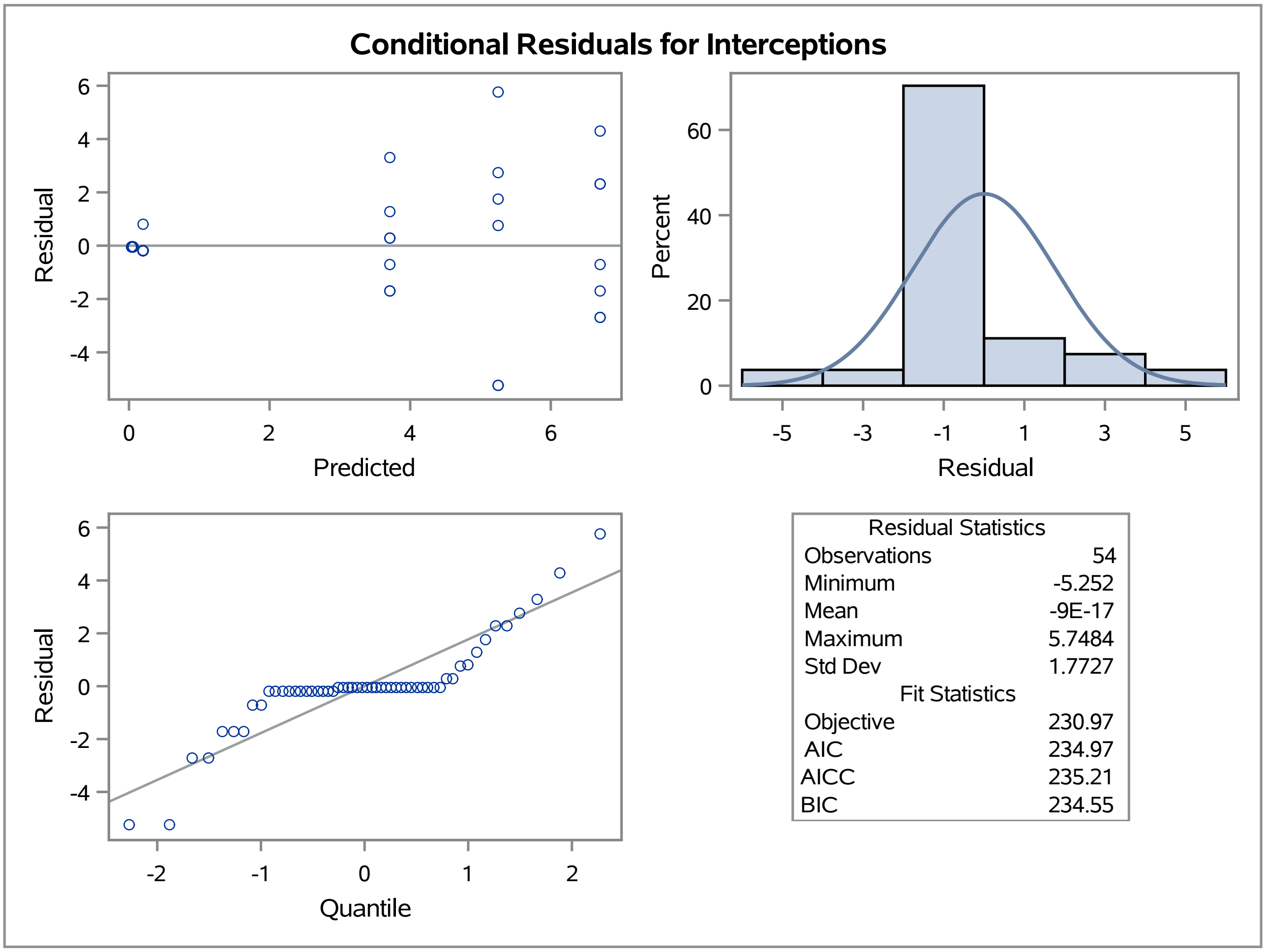

DistSoma=324

Model Information	
**Data Set**	WORK.TEMPDATASORTED
**Dependent Variable**	Interceptions
**Covariance Structure**	Variance Components
**Estimation Method**	REML
**Residual Variance Method**	Profile
**Fixed Effects SE Method**	Model-Based
**Degrees of Freedom Method**	Containment

Class Level Information		
**Class**	**Levels**	**Values**
**Genotype**	2	Meg +/+ Meg +/-
**Animal**	6	1 2 3 4 5 6

Dimensions	
**Covariance Parameters**	2
**Columns in X**	3
**Columns in Z**	6
**Subjects**	1
**Max Obs per Subject**	54

Number of Observations	
**Number of Observations Read**	54
**Number of Observations Used**	54
**Number of Observations Not Used**	0

Iteration History			
**Iteration**	**Evaluations**	**-2 Res Log Like**	**Criterion**
**0**	1	269.07801586	
**1**	3	229.52860776	0.00029450
**2**	1	229.50682692	0.00000946
**3**	1	229.50617920	0.00000001
**4**	1	229.50617842	0.00000000

Convergence criteria met.

DistSoma=324

Covariance Parameter Estimates				
**Cov Parm**	**Estimate**	**Alpha**	**Lower**	**Upper**
**Animal**	8.1687	0.05	2.8346	78.0308
**Residual**	3.3628	0.05	2.3389	5.2477

Fit Statistics	
**-2 Res Log Likelihood**	229.5
**AIC (Smaller is Better)**	233.5
**AICC (Smaller is Better)**	233.8
**BIC (Smaller is Better)**	233.1

Solution for Fixed Effects									
**Effect**	**Genotype**	**Estimate**	**Standard Error**	**DF**	**t Value**	**Pr > |t|**	**Alpha**	**Lower**	**Upper**
**Intercept**		1.3174	1.6878	4	0.78	0.4787	0.05	-3.3687	6.0035
**Genotype**	Meg +/+	2.5835	2.3907	48	1.08	0.2853	0.05	-2.2233	7.3903
**Genotype**	Meg +/-	0	.	.	.	.	.	.	.

Solution for Random Effects									
**Effect**	**Animal**	**Estimate**	**Std Err Pred**	**DF**	**t Value**	**Pr > |t|**	**Alpha**	**Lower**	**Upper**
**Animal**	1	-3.7066	1.7158	48	-2.16	0.0358	0.05	-7.1566	-0.2567
**Animal**	2	1.1845	1.7421	48	0.68	0.4998	0.05	-2.3182	4.6872
**Animal**	3	2.5222	1.7352	48	1.45	0.1526	0.05	-0.9666	6.0109
**Animal**	4	-1.2598	1.7211	48	-0.73	0.4677	0.05	-4.7203	2.2007
**Animal**	5	-1.2737	1.7128	48	-0.74	0.4607	0.05	-4.7176	2.1701
**Animal**	6	2.5336	1.7305	48	1.46	0.1497	0.05	-0.9459	6.0130

Type 3 Tests of Fixed Effects				
**Effect**	**Num DF**	**Den DF**	**F Value**	**Pr > F**
**Genotype**	1	48	1.17	0.2853

Least Squares Means									
**Effect**	**Genotype**	**Estimate**	**Standard Error**	**DF**	**t Value**	**Pr > |t|**	**Alpha**	**Lower**	**Upper**
**Genotype**	Meg +/+	3.9009	1.6931	48	2.30	0.0256	0.05	0.4967	7.3052
**Genotype**	Meg +/-	1.3174	1.6878	48	0.78	0.4389	0.05	-2.0761	4.7110

DistSoma=324

Differences of Least Squares Means												
Effect	Genotype	Genotype	Estimate	Standard Error	DF	t Value	Pr > |t|	Adjustment	Adj P	Alpha	Lower	Upper
**Genotype**	Meg +/+	Meg +/-	2.5835	2.3907	48	1.08	0.2853	Tukey-Kramer	0.2853	0.05	-2.2233	7.3903

Differences of Least Squares Means				
Effect	Genotype	Genotype	Adj Lower	Adj Upper
**Genotype**	Meg +/+	Meg +/-	-2.2231	7.3901

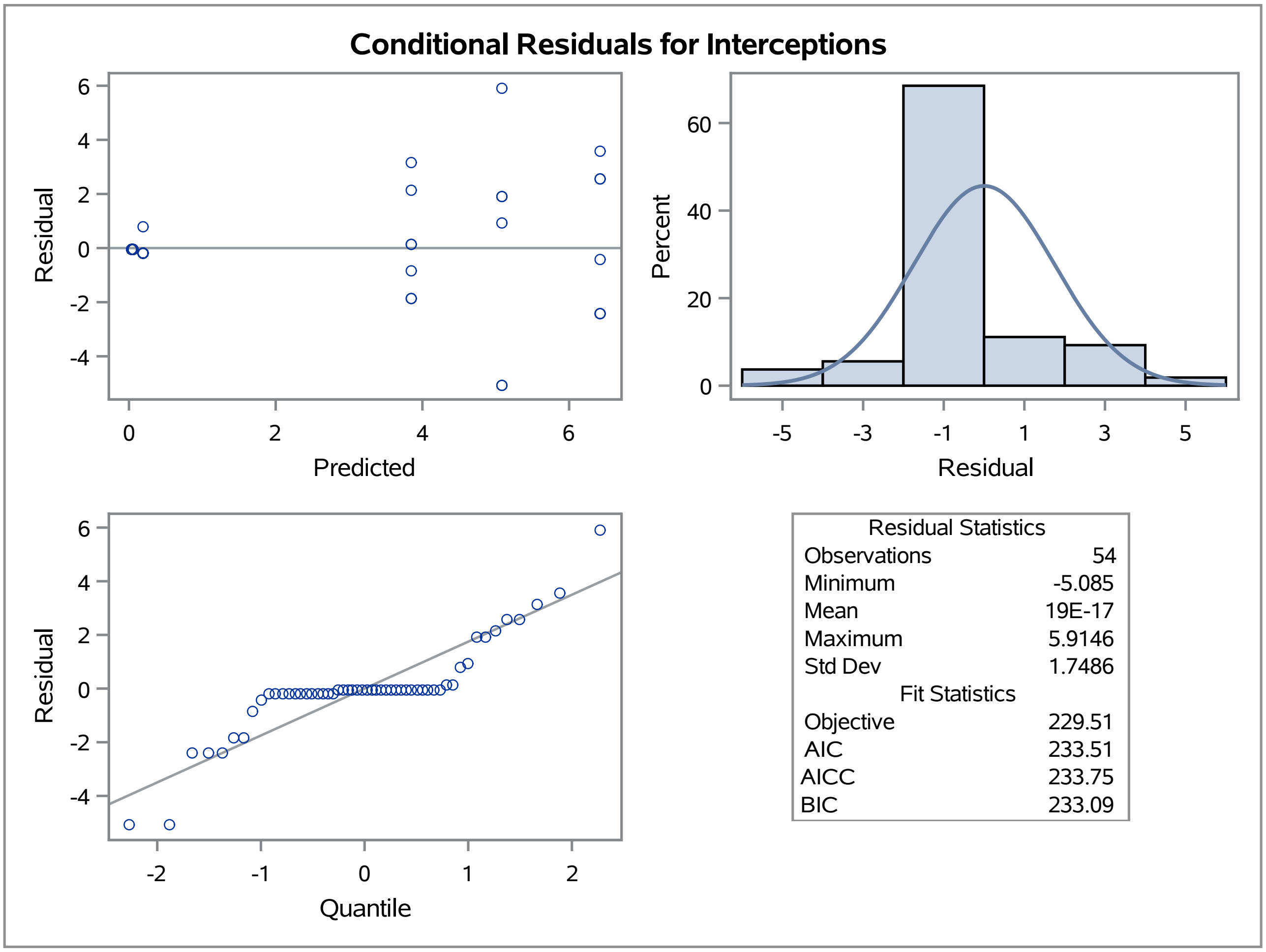

DistSoma=330

Model Information	
**Data Set**	WORK.TEMPDATASORTED
**Dependent Variable**	Interceptions
**Covariance Structure**	Variance Components
**Estimation Method**	REML
**Residual Variance Method**	Profile
**Fixed Effects SE Method**	Model-Based
**Degrees of Freedom Method**	Containment

Class Level Information		
**Class**	**Levels**	**Values**
**Genotype**	2	Meg +/+ Meg +/-
**Animal**	6	1 2 3 4 5 6

Dimensions	
**Covariance Parameters**	2
**Columns in X**	3
**Columns in Z**	6
**Subjects**	1
**Max Obs per Subject**	54

Number of Observations	
**Number of Observations Read**	54
**Number of Observations Used**	54
**Number of Observations Not Used**	0

Iteration History			
**Iteration**	**Evaluations**	**-2 Res Log Like**	**Criterion**
**0**	1	269.06113549	
**1**	3	229.83067374	0.00029573
**2**	1	229.80874711	0.00000955
**3**	1	229.80809137	0.00000001
**4**	1	229.80809058	0.00000000

Convergence criteria met.

DistSoma=330

Covariance Parameter Estimates				
**Cov Parm**	**Estimate**	**Alpha**	**Lower**	**Upper**
**Animal**	8.1299	0.05	2.8201	77.7860
**Residual**	3.3853	0.05	2.3544	5.2826

Fit Statistics	
**-2 Res Log Likelihood**	229.8
**AIC (Smaller is Better)**	233.8
**AICC (Smaller is Better)**	234.1
**BIC (Smaller is Better)**	233.4

Solution for Fixed Effects									
**Effect**	**Genotype**	**Estimate**	**Standard Error**	**DF**	**t Value**	**Pr > |t|**	**Alpha**	**Lower**	**Upper**
**Intercept**		1.3173	1.6842	4	0.78	0.4778	0.05	-3.3589	5.9934
**Genotype**	Meg +/+	2.5097	2.3856	48	1.05	0.2981	0.05	-2.2869	7.3064
**Genotype**	Meg +/-	0	.	.	.	.	.	.	.

Solution for Random Effects									
**Effect**	**Animal**	**Estimate**	**Std Err Pred**	**DF**	**t Value**	**Pr > |t|**	**Alpha**	**Lower**	**Upper**
**Animal**	1	-3.7082	1.7125	48	-2.17	0.0354	0.05	-7.1514	-0.2651
**Animal**	2	1.2527	1.7389	48	0.72	0.4748	0.05	-2.2436	4.7490
**Animal**	3	2.4555	1.7319	48	1.42	0.1627	0.05	-1.0268	5.9378
**Animal**	4	-1.2590	1.7178	48	-0.73	0.4672	0.05	-4.7128	2.1948
**Animal**	5	-1.2731	1.7094	48	-0.74	0.4601	0.05	-4.7101	2.1640
**Animal**	6	2.5321	1.7273	48	1.47	0.1492	0.05	-0.9408	6.0050

Type 3 Tests of Fixed Effects				
**Effect**	**Num DF**	**Den DF**	**F Value**	**Pr > F**
**Genotype**	1	48	1.11	0.2981

Least Squares Means									
**Effect**	**Genotype**	**Estimate**	**Standard Error**	**DF**	**t Value**	**Pr > |t|**	**Alpha**	**Lower**	**Upper**
**Genotype**	Meg +/+	3.8270	1.6896	48	2.27	0.0281	0.05	0.4299	7.2241
**Genotype**	Meg +/-	1.3173	1.6842	48	0.78	0.4380	0.05	-2.0691	4.7036

DistSoma=330

Differences of Least Squares Means												
Effect	Genotype	Genotype	Estimate	Standard Error	DF	t Value	Pr > |t|	Adjustment	Adj P	Alpha	Lower	Upper
**Genotype**	Meg +/+	Meg +/-	2.5097	2.3856	48	1.05	0.2981	Tukey-Kramer	0.2981	0.05	-2.2869	7.3064

Differences of Least Squares Means				
Effect	Genotype	Genotype	Adj Lower	Adj Upper
**Genotype**	Meg +/+	Meg +/-	-2.2867	7.3062

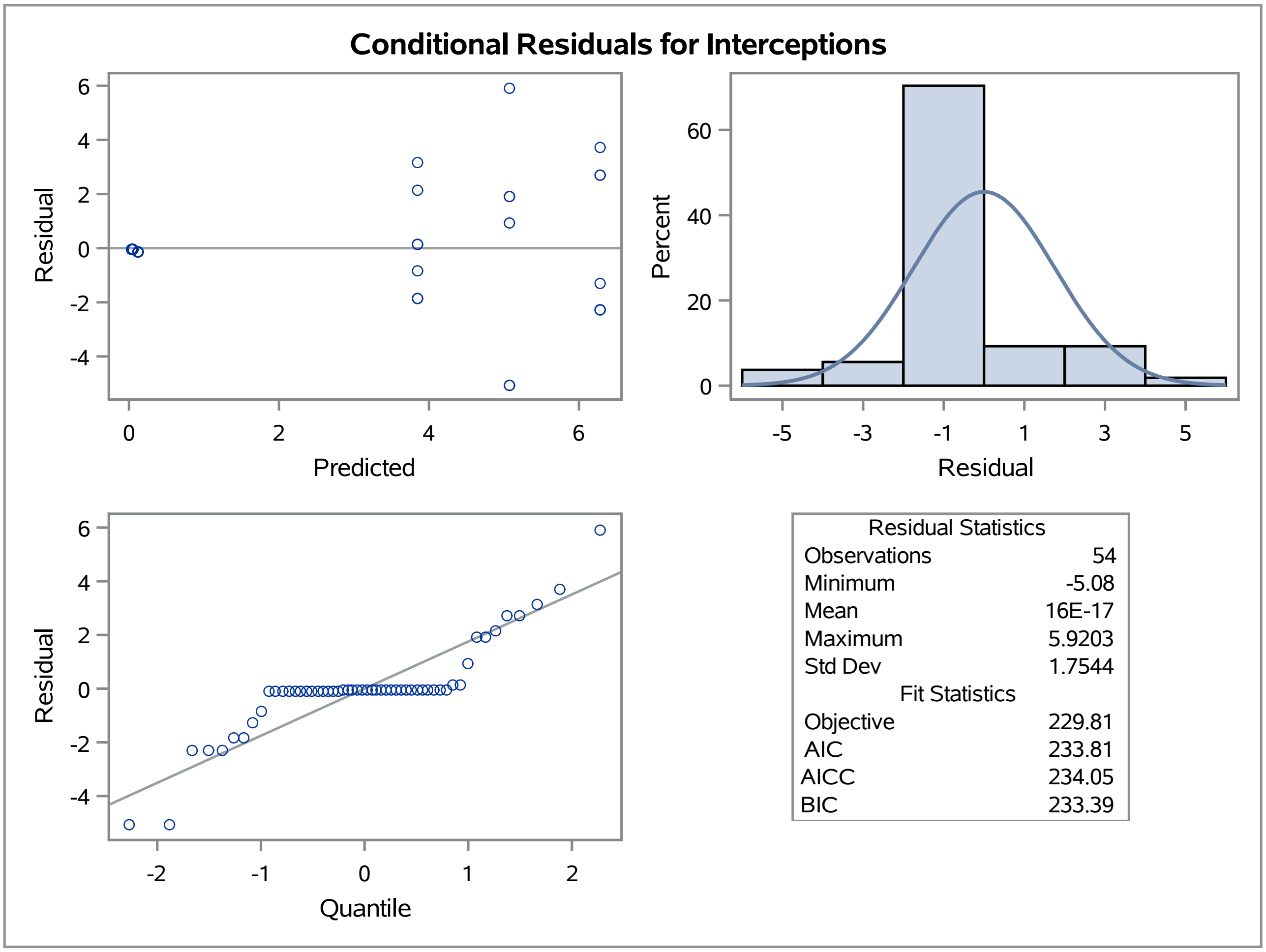

DistSoma=336

Model Information	
**Data Set**	WORK.TEMPDATASORTED
**Dependent Variable**	Interceptions
**Covariance Structure**	Variance Components
**Estimation Method**	REML
**Residual Variance Method**	Profile
**Fixed Effects SE Method**	Model-Based
**Degrees of Freedom Method**	Containment

Class Level Information		
**Class**	**Levels**	**Values**
**Genotype**	2	Meg +/+ Meg +/-
**Animal**	6	1 2 3 4 5 6

Dimensions	
**Covariance Parameters**	2
**Columns in X**	3
**Columns in Z**	6
**Subjects**	1
**Max Obs per Subject**	54

Number of Observations	
**Number of Observations Read**	54
**Number of Observations Used**	54
**Number of Observations Not Used**	0

Iteration History			
**Iteration**	**Evaluations**	**-2 Res Log Like**	**Criterion**
**0**	1	267.67529923	
**1**	3	229.99037109	0.00020926
**2**	1	229.97502481	0.00000495
**3**	1	229.97468632	0.00000000

Convergence criteria met.

DistSoma=336

Covariance Parameter Estimates				
**Cov Parm**	**Estimate**	**Alpha**	**Lower**	**Upper**
**Animal**	7.7707	0.05	2.6911	74.8966
**Residual**	3.4091	0.05	2.3711	5.3198

Fit Statistics	
**-2 Res Log Likelihood**	230.0
**AIC (Smaller is Better)**	234.0
**AICC (Smaller is Better)**	234.2
**BIC (Smaller is Better)**	233.6

Solution for Fixed Effects									
**Effect**	**Genotype**	**Estimate**	**Standard Error**	**DF**	**t Value**	**Pr > |t|**	**Alpha**	**Lower**	**Upper**
**Intercept**		1.3165	1.6485	4	0.80	0.4693	0.05	-3.2607	5.8936
**Genotype**	Meg +/+	2.4139	2.3353	48	1.03	0.3065	0.05	-2.2815	7.1094
**Genotype**	Meg +/-	0	.	.	.	.	.	.	.

Solution for Random Effects									
**Effect**	**Animal**	**Estimate**	**Std Err Pred**	**DF**	**t Value**	**Pr > |t|**	**Alpha**	**Lower**	**Upper**
**Animal**	1	-3.6086	1.6775	48	-2.15	0.0365	0.05	-6.9814	-0.2358
**Animal**	2	1.3384	1.7045	48	0.79	0.4362	0.05	-2.0887	4.7655
**Animal**	3	2.2702	1.6974	48	1.34	0.1874	0.05	-1.1426	5.6830
**Animal**	4	-1.2553	1.6829	48	-0.75	0.4594	0.05	-4.6389	2.1284
**Animal**	5	-1.2700	1.6744	48	-0.76	0.4519	0.05	-4.6365	2.0965
**Animal**	6	2.5253	1.6926	48	1.49	0.1423	0.05	-0.8779	5.9285

Type 3 Tests of Fixed Effects				
**Effect**	**Num DF**	**Den DF**	**F Value**	**Pr > F**
**Genotype**	1	48	1.07	0.3065

Least Squares Means									
**Effect**	**Genotype**	**Estimate**	**Standard Error**	**DF**	**t Value**	**Pr > |t|**	**Alpha**	**Lower**	**Upper**
**Genotype**	Meg +/+	3.7304	1.6541	48	2.26	0.0287	0.05	0.4047	7.0561
**Genotype**	Meg +/-	1.3165	1.6485	48	0.80	0.4285	0.05	-1.9982	4.6311

DistSoma=336

Differences of Least Squares Means												
**Effect**	**Genotype**	**Genotype**	**Estimate**	**Standard Error**	**DF**	**t Value**	**Pr > |t|**	**Adjustment**	**Adj P**	**Alpha**	**Lower**	**Upper**
**Genotype**	Meg +/+	Meg +/-	2.4139	2.3353	48	1.03	0.3065	Tukey-Kramer	0.3065	0.05	-2.2815	7.1094

Differences of Least Squares Means				
**Effect**	**Genotype**	**Genotype**	**Adj Lower**	**Adj Upper**
**Genotype**	Meg +/+	Meg +/-	-2.2813	7.1092

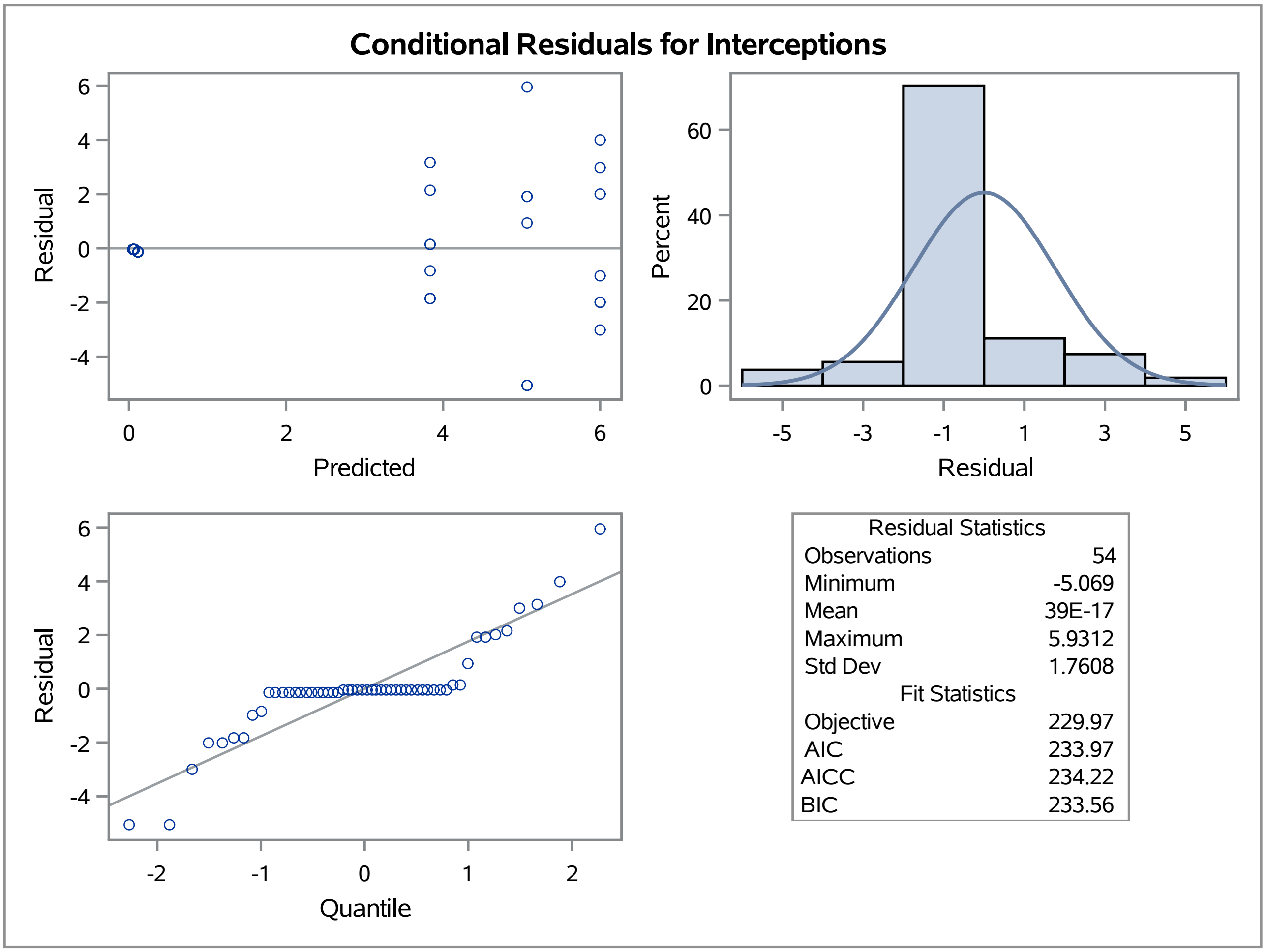

DistSoma=342

Model Information	
**Data Set**	WORK.TEMPDATASORTED
**Dependent Variable**	Interceptions
**Covariance Structure**	Variance Components
**Estimation Method**	REML
**Residual Variance Method**	Profile
**Fixed Effects SE Method**	Model-Based
**Degrees of Freedom Method**	Containment

Class Level Information		
**Class**	**Levels**	**Values**
**Genotype**	2	Meg +/+ Meg +/-
**Animal**	6	1 2 3 4 5 6

Dimensions	
**Covariance Parameters**	2
**Columns in X**	3
**Columns in Z**	6
**Subjects**	1
**Max Obs per Subject**	54

Number of Observations	
**Number of Observations Read**	54
**Number of Observations Used**	54
**Number of Observations Not Used**	0

Iteration History			
**Iteration**	**Evaluations**	**-2 Res Log Like**	**Criterion**
**0**	1	267.67529923	
**1**	3	229.99037109	0.00020926
**2**	1	229.97502481	0.00000495
**3**	1	229.97468632	0.00000000

Convergence criteria met.

DistSoma=342

Covariance Parameter Estimates				
Cov Parm	Estimate	Alpha	Lower	Upper
Animal	7.7707	0.05	2.6911	74.8966
Residual	3.4091	0.05	2.3711	5.3198

Fit Statistics	
-2 Res Log Likelihood	230.0
AIC (Smaller is Better)	234.0
AICC (Smaller is Better)	234.2
BIC (Smaller is Better)	233.6

Solution for Fixed Effects									
Effect	Genotype	Estimate	Standard Error	DF	t Value	Pr > |t|	Alpha	Lower	Upper
Intercept		1.3165	1.6485	4	0.80	0.4693	0.05	-3.2607	5.8936
Genotype	Meg +/+	2.4139	2.3353	48	1.03	0.3065	0.05	-2.2815	7.1094
Genotype	Meg +/-	0	.	.	.	.	.	.	.

Solution for Random Effects									
Effect	Animal	Estimate	Std Err Pred	DF	t Value	Pr > |t|	Alpha	Lower	Upper
Animal	1	-3.6086	1.6775	48	-2.15	0.0365	0.05	-6.9814	-0.2358
Animal	2	1.3384	1.7045	48	0.79	0.4362	0.05	-2.0887	4.7655
Animal	3	2.2702	1.6974	48	1.34	0.1874	0.05	-1.1426	5.6830
Animal	4	-1.2553	1.6829	48	-0.75	0.4594	0.05	-4.6389	2.1284
Animal	5	-1.2700	1.6744	48	-0.76	0.4519	0.05	-4.6365	2.0965
Animal	6	2.5253	1.6926	48	1.49	0.1423	0.05	-0.8779	5.9285

Type 3 Tests of Fixed Effects				
Effect	Num DF	Den DF	F Value	Pr > F
Genotype	1	48	1.07	0.3065

Least Squares Means									
Effect	Genotype	Estimate	Standard Error	DF	t Value	Pr > |t|	Alpha	Lower	Upper
Genotype	Meg +/+	3.7304	1.6541	48	2.26	0.0287	0.05	0.4047	7.0561
Genotype	Meg +/-	1.3165	1.6485	48	0.80	0.4285	0.05	-1.9982	4.6311

DistSoma=342

Differences of Least Squares Means												
**Effect**	**Genotype**	**Genotype**	**Estimate**	**Standard Error**	**DF**	**t Value**	**Pr > |t|**	**Adjustment**	**Adj P**	**Alpha**	**Lower**	**Upper**
**Genotype**	Meg +/+	Meg +/-	2.4139	2.3353	48	1.03	0.3065	Tukey-Kramer	0.3065	0.05	-2.2815	7.1094

Differences of Least Squares Means				
**Effect**	**Genotype**	**Genotype**	**Adj Lower**	**Adj Upper**
**Genotype**	Meg +/+	Meg +/-	-2.2813	7.1092

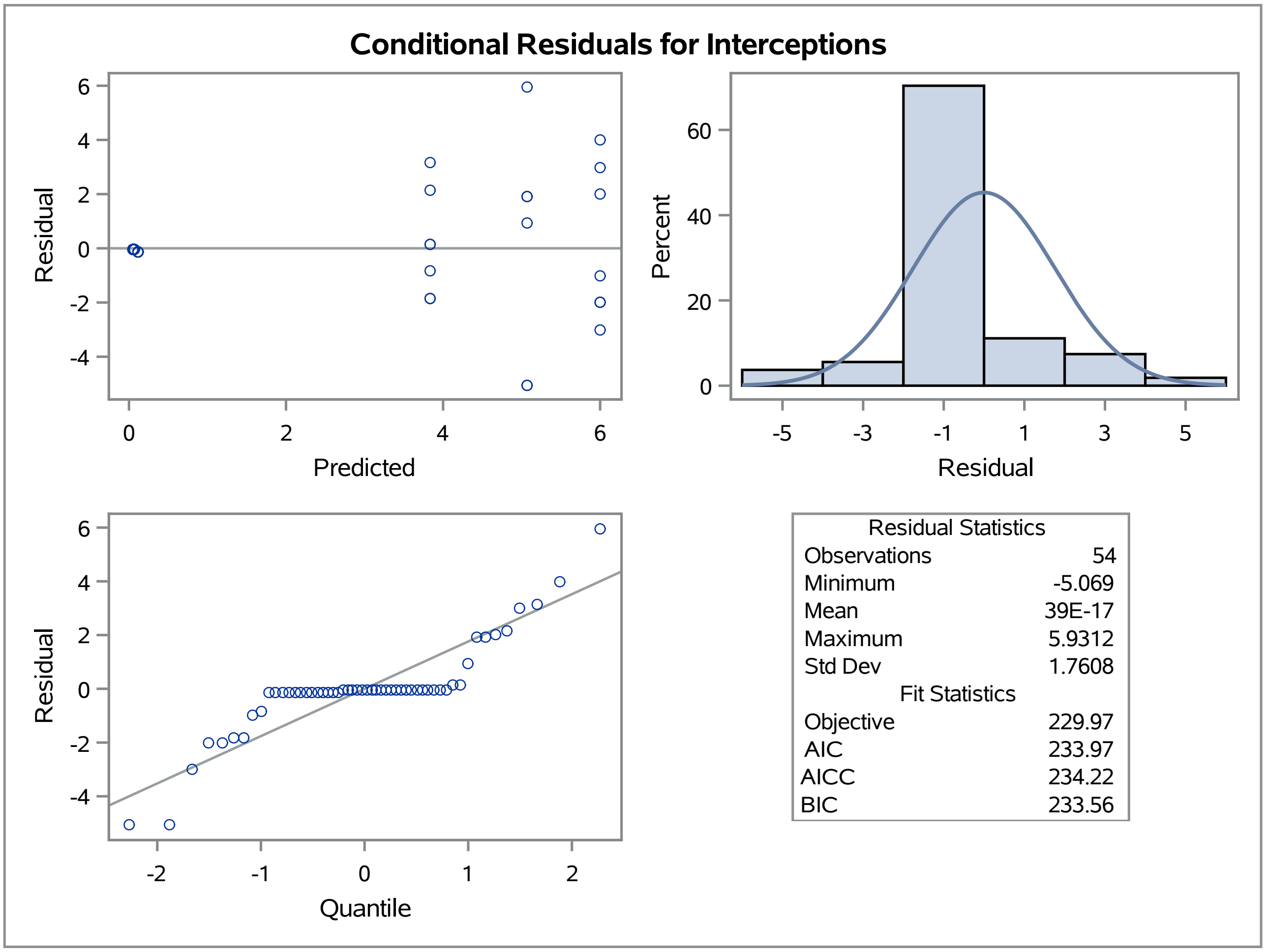

DistSoma=348

Model Information	
**Data Set**	WORK.TEMPDATASORTED
**Dependent Variable**	Interceptions
**Covariance Structure**	Variance Components
**Estimation Method**	REML
**Residual Variance Method**	Profile
**Fixed Effects SE Method**	Model-Based
**Degrees of Freedom Method**	Containment

Class Level Information		
**Class**	**Levels**	**Values**
**Genotype**	2	Meg +/+ Meg +/-
**Animal**	6	1 2 3 4 5 6

Dimensions	
**Covariance Parameters**	2
**Columns in X**	3
**Columns in Z**	6
**Subjects**	1
**Max Obs per Subject**	54

Number of Observations	
**Number of Observations Read**	54
**Number of Observations Used**	54
**Number of Observations Not Used**	0

Iteration History			
**Iteration**	**Evaluations**	**-2 Res Log Like**	**Criterion**
**0**	1	267.22251673	
**1**	3	228.15054549	0.00022966
**2**	1	228.13388916	0.00000584
**3**	1	228.13349496	0.00000000

Convergence criteria met.

DistSoma=348

Covariance Parameter Estimates				
Cov Parm	Estimate	Alpha	Lower	Upper
Animal	7.8480	0.05	2.7225	75.0638
Residual	3.2788	0.05	2.2804	5.1165

Fit Statistics	
-2 Res Log Likelihood	228.1
AIC (Smaller is Better)	232.1
AICC (Smaller is Better)	232.4
BIC (Smaller is Better)	231.7

Solution for Fixed Effects									
Effect	Genotype	Estimate	Standard Error	DF	t Value	Pr > |t|	Alpha	Lower	Upper
Intercept		1.3172	1.6549	4	0.80	0.4706	0.05	-3.2775	5.9119
Genotype	Meg +/+	2.4076	2.3441	48	1.03	0.3095	0.05	-2.3055	7.1207
Genotype	Meg +/-	0	.	.	.	.	.	.	.

Solution for Random Effects									
Effect	Animal	Estimate	Std Err Pred	DF	t Value	Pr > |t|	Alpha	Lower	Upper
Animal	1	-3.6088	1.6827	48	-2.14	0.0371	0.05	-6.9922	-0.2255
Animal	2	1.1922	1.7088	48	0.70	0.4887	0.05	-2.2435	4.6279
Animal	3	2.4167	1.7019	48	1.42	0.1621	0.05	-1.0052	5.8385
Animal	4	-1.2588	1.6879	48	-0.75	0.4595	0.05	-4.6526	2.1351
Animal	5	-1.2729	1.6797	48	-0.76	0.4523	0.05	-4.6502	2.1044
Animal	6	2.5317	1.6973	48	1.49	0.1423	0.05	-0.8810	5.9443

Type 3 Tests of Fixed Effects				
Effect	Num DF	Den DF	F Value	Pr > F
Genotype	1	48	1.05	0.3095

Least Squares Means									
Effect	Genotype	Estimate	Standard Error	DF	t Value	Pr > |t|	Alpha	Lower	Upper
Genotype	Meg +/+	3.7248	1.6602	48	2.24	0.0295	0.05	0.3868	7.0628
Genotype	Meg +/-	1.3172	1.6549	48	0.80	0.4300	0.05	-2.0101	4.6446

DistSoma=348

Differences of Least Squares Means												
Effect	Genotype	Genotype	Estimate	Standard Error	DF	t Value	Pr > |t|	Adjustment	Adj P	Alpha	Lower	Upper
**Genotype**	Meg +/+	Meg +/-	2.4076	2.3441	48	1.03	0.3095	Tukey-Kramer	0.3095	0.05	-2.3055	7.1207

Differences of Least Squares Means				
Effect	Genotype	Genotype	Adj Lower	Adj Upper
**Genotype**	Meg +/+	Meg +/-	-2.3053	7.1205

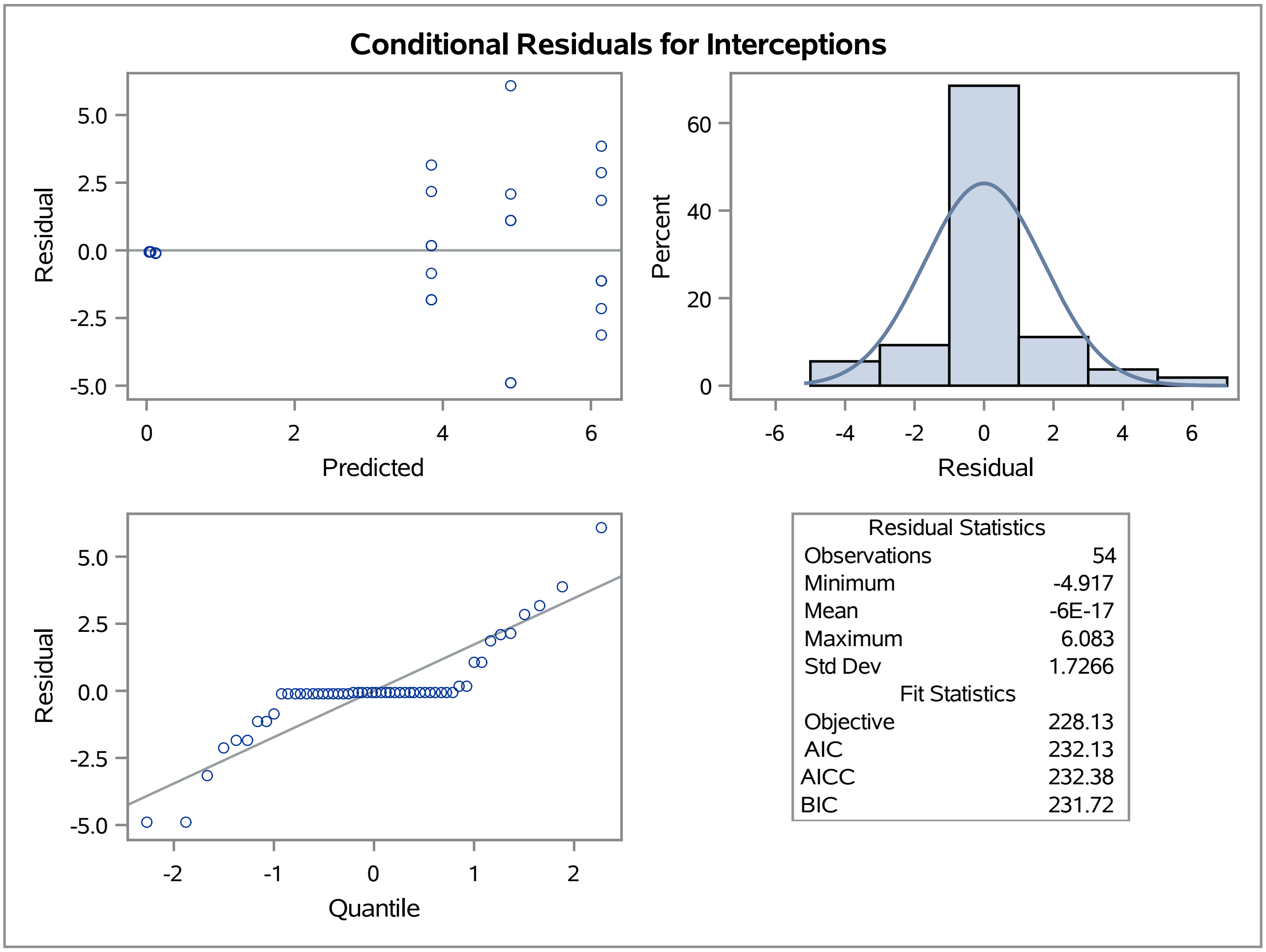

DistSoma=354

Model Information	
**Data Set**	WORK.TEMPDATASORTED
**Dependent Variable**	Interceptions
**Covariance Structure**	Variance Components
**Estimation Method**	REML
**Residual Variance Method**	Profile
**Fixed Effects SE Method**	Model-Based
**Degrees of Freedom Method**	Containment

Class Level Information		
**Class**	**Levels**	**Values**
**Genotype**	2	Meg +/+ Meg +/-
**Animal**	6	1 2 3 4 5 6

Dimensions	
**Covariance Parameters**	2
**Columns in X**	3
**Columns in Z**	6
**Subjects**	1
**Max Obs per Subject**	54

Number of Observations	
**Number of Observations Read**	54
**Number of Observations Used**	54
**Number of Observations Not Used**	0

Iteration History			
**Iteration**	**Evaluations**	**-2 Res Log Like**	**Criterion**
**0**	1	267.10463298	
**1**	3	229.52221477	0.00027871
**2**	1	229.50164404	0.00000852
**3**	1	229.50106118	0.00000001

Convergence criteria met.

DistSoma=354

Covariance Parameter Estimates				
**Cov Parm**	**Estimate**	**Alpha**	**Lower**	**Upper**
**Animal**	7.6521	0.05	2.6501	73.7413
**Residual**	3.3799	0.05	2.3507	5.2742

Fit Statistics	
**-2 Res Log Likelihood**	229.5
**AIC (Smaller is Better)**	233.5
**AICC (Smaller is Better)**	233.7
**BIC (Smaller is Better)**	233.1

Solution for Fixed Effects									
**Effect**	**Genotype**	**Estimate**	**Standard Error**	**DF**	**t Value**	**Pr > |t|**	**Alpha**	**Lower**	**Upper**
**Intercept**		1.2693	1.6362	4	0.78	0.4812	0.05	-3.2734	5.8121
**Genotype**	Meg +/+	2.4535	2.3178	48	1.06	0.2951	0.05	-2.2068	7.1137
**Genotype**	Meg +/-	0	.	.	.	.	.	.	.

Solution for Random Effects									
**Effect**	**Animal**	**Estimate**	**Std Err Pred**	**DF**	**t Value**	**Pr > |t|**	**Alpha**	**Lower**	**Upper**
**Animal**	1	-3.6005	1.6650	48	-2.16	0.0356	0.05	-6.9482	-0.2527
**Animal**	2	1.1896	1.6920	48	0.70	0.4854	0.05	-2.2124	4.5917
**Animal**	3	2.4108	1.6849	48	1.43	0.1590	0.05	-0.9769	5.7985
**Animal**	4	-1.2099	1.6705	48	-0.72	0.4724	0.05	-4.5686	2.1487
**Animal**	5	-1.2243	1.6619	48	-0.74	0.4649	0.05	-4.5658	2.1173
**Animal**	6	2.4342	1.6802	48	1.45	0.1539	0.05	-0.9440	5.8124

Type 3 Tests of Fixed Effects				
**Effect**	**Num DF**	**Den DF**	**F Value**	**Pr > F**
**Genotype**	1	48	1.12	0.2951

Least Squares Means									
**Effect**	**Genotype**	**Estimate**	**Standard Error**	**DF**	**t Value**	**Pr > |t|**	**Alpha**	**Lower**	**Upper**
**Genotype**	Meg +/+	3.7228	1.6417	48	2.27	0.0279	0.05	0.4219	7.0236
**Genotype**	Meg +/-	1.2693	1.6362	48	0.78	0.4417	0.05	-2.0204	4.5591

DistSoma=354

Differences of Least Squares Means												
**Effect**	**Genotype**	**Genotype**	**Estimate**	**Standard Error**	**DF**	**t Value**	**Pr > |t|**	**Adjustment**	**Adj P**	**Alpha**	**Lower**	**Upper**
**Genotype**	Meg +/+	Meg +/-	2.4535	2.3178	48	1.06	0.2951	Tukey-Kramer	0.2951	0.05	-2.2068	7.1137

Differences of Least Squares Means				
**Effect**	**Genotype**	**Genotype**	**Adj Lower**	**Adj Upper**
**Genotype**	Meg +/+	Meg +/-	-2.2066	7.1136

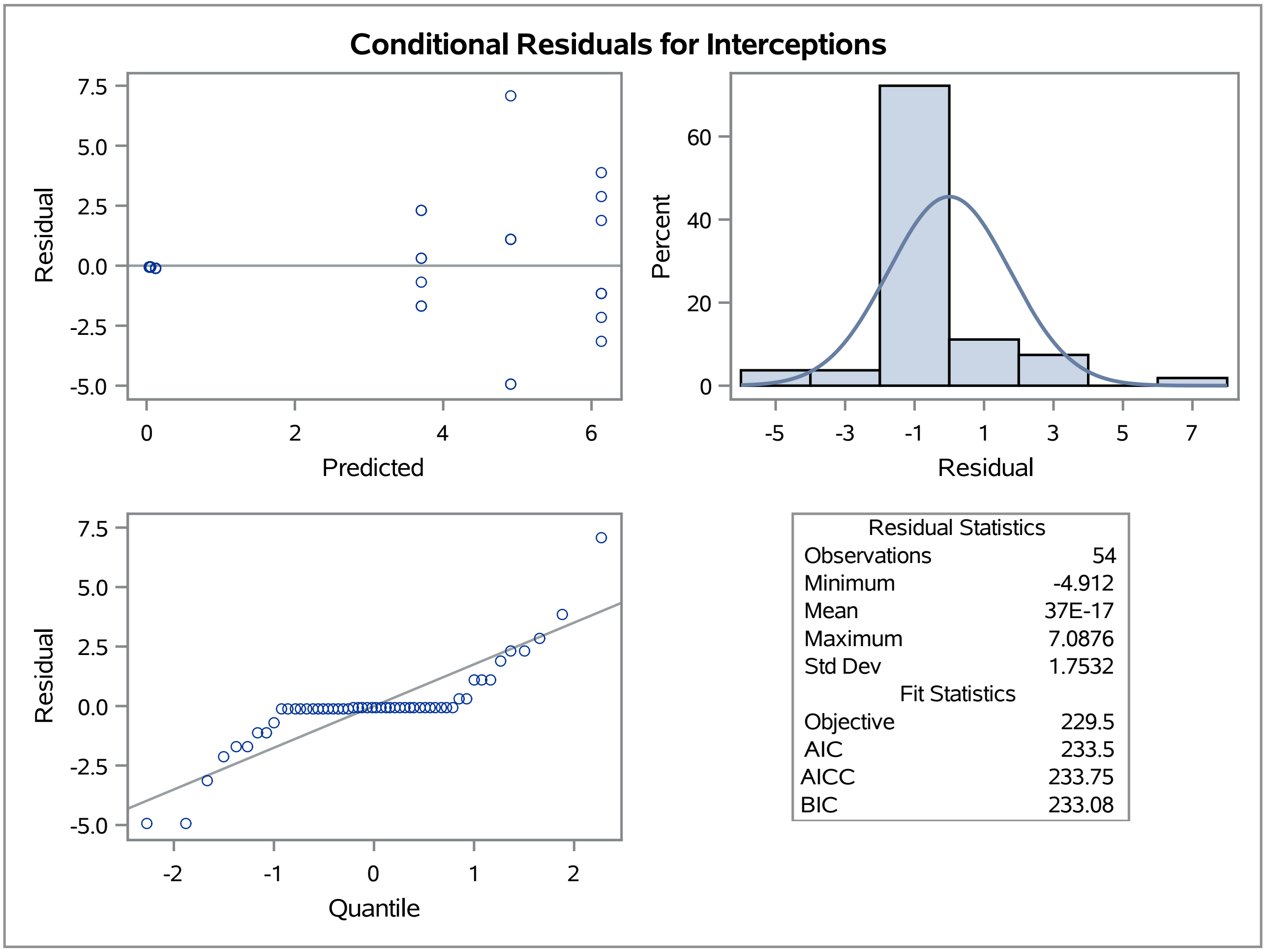

DistSoma=360

Model Information	
**Data Set**	WORK.TEMPDATASORTED
**Dependent Variable**	Interceptions
**Covariance Structure**	Variance Components
**Estimation Method**	REML
**Residual Variance Method**	Profile
**Fixed Effects SE Method**	Model-Based
**Degrees of Freedom Method**	Containment

Class Level Information		
**Class**	**Levels**	**Values**
**Genotype**	2	Meg +/+ Meg +/-
**Animal**	6	1 2 3 4 5 6

Dimensions	
**Covariance Parameters**	2
**Columns in X**	3
**Columns in Z**	6
**Subjects**	1
**Max Obs per Subject**	54

Number of Observations	
**Number of Observations Read**	54
**Number of Observations Used**	54
**Number of Observations Not Used**	0

Iteration History			
**Iteration**	**Evaluations**	**-2 Res Log Like**	**Criterion**
**0**	1	266.72194102	
**1**	3	230.43280955	0.00022685
**2**	1	230.41607073	0.00000580
**3**	1	230.41567289	0.00000000

Convergence criteria met.

DistSoma=360

Covariance Parameter Estimates				
**Cov Parm**	**Estimate**	**Alpha**	**Lower**	**Upper**
**Animal**	7.4688	0.05	2.5819	72.5620
**Residual**	3.4512	0.05	2.4003	5.3854

Fit Statistics	
**-2 Res Log Likelihood**	230.4
**AIC (Smaller is Better)**	234.4
**AICC (Smaller is Better)**	234.7
**BIC (Smaller is Better)**	234.0

Solution for Fixed Effects									
**Effect**	**Genotype**	**Estimate**	**Standard Error**	**DF**	**t Value**	**Pr > |t|**	**Alpha**	**Lower**	**Upper**
**Intercept**		1.2686	1.6182	4	0.78	0.4769	0.05	-3.2243	5.7615
**Genotype**	Meg +/+	2.4051	2.2925	48	1.05	0.2994	0.05	-2.2043	7.0146
**Genotype**	Meg +/-	0	.	.	.	.	.	.	.

Solution for Random Effects									
**Effect**	**Animal**	**Estimate**	**Std Err Pred**	**DF**	**t Value**	**Pr > |t|**	**Alpha**	**Lower**	**Upper**
**Animal**	1	-3.5476	1.6479	48	-2.15	0.0364	0.05	-6.8609	-0.2344
**Animal**	2	1.2314	1.6756	48	0.73	0.4660	0.05	-2.1375	4.6004
**Animal**	3	2.3162	1.6683	48	1.39	0.1714	0.05	-1.0380	5.6705
**Animal**	4	-1.2067	1.6534	48	-0.73	0.4691	0.05	-4.5311	2.1178
**Animal**	5	-1.2216	1.6447	48	-0.74	0.4613	0.05	-4.5284	2.0853
**Animal**	6	2.4282	1.6634	48	1.46	0.1509	0.05	-0.9162	5.7727

Type 3 Tests of Fixed Effects				
**Effect**	**Num DF**	**Den DF**	**F Value**	**Pr > F**
**Genotype**	1	48	1.10	0.2994

Least Squares Means									
**Effect**	**Genotype**	**Estimate**	**Standard Error**	**DF**	**t Value**	**Pr > |t|**	**Alpha**	**Lower**	**Upper**
**Genotype**	Meg +/+	3.6737	1.6239	48	2.26	0.0282	0.05	0.4087	6.9388
**Genotype**	Meg +/-	1.2686	1.6182	48	0.78	0.4369	0.05	-1.9850	4.5223

DistSoma=360

Differences of Least Squares Means

Effect	Genotype	Genotype	Estimate	Standard Error	DF	t Value	Pr > |t|	Adjustment	Adj P	Alpha	Lower	Upper
**Genotype**	Meg +/+	Meg +/-	2.4051	2.2925	48	1.05	0.2994	Tukey-Kramer	0.2994	0.05	-2.2043	7.0146

Differences of Least Squares Means

Effect	Genotype	Genotype	Adj Lower	Adj Upper
**Genotype**	Meg +/+	Meg +/-	-2.2042	7.0144

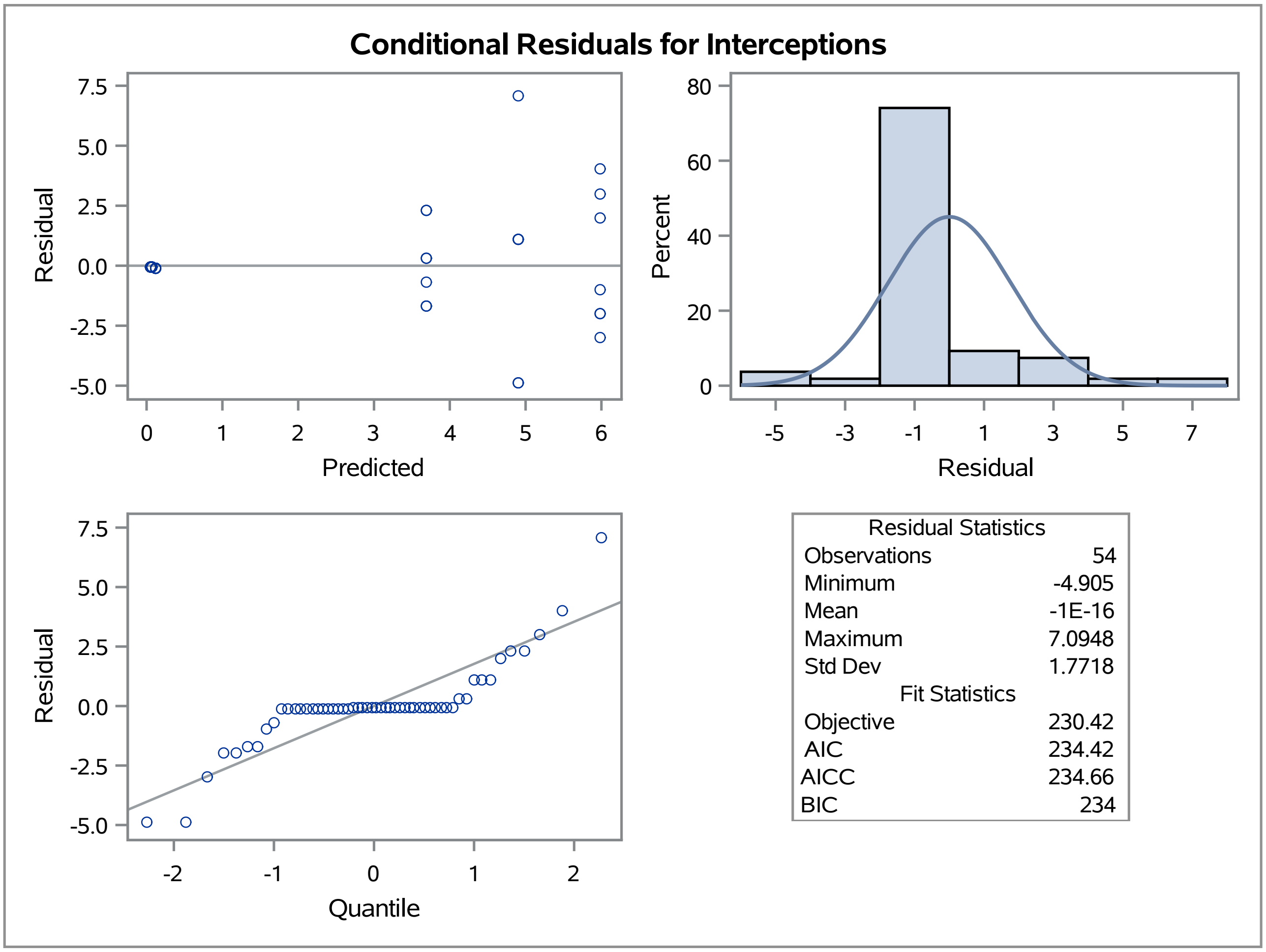

DistSoma=366

Model Information	
**Data Set**	WORK.TEMPDATASORTED
**Dependent Variable**	Interceptions
**Covariance Structure**	Variance Components
**Estimation Method**	REML
**Residual Variance Method**	Profile
**Fixed Effects SE Method**	Model-Based
**Degrees of Freedom Method**	Containment

Class Level Information		
**Class**	**Levels**	**Values**
**Genotype**	2	Meg +/+ Meg +/-
**Animal**	6	1 2 3 4 5 6

Dimensions	
**Covariance Parameters**	2
**Columns in X**	3
**Columns in Z**	6
**Subjects**	1
**Max Obs per Subject**	54

Number of Observations	
**Number of Observations Read**	54
**Number of Observations Used**	54
**Number of Observations Not Used**	0

Iteration History			
**Iteration**	**Evaluations**	**-2 Res Log Like**	**Criterion**
**0**	1	265.62886127	
**1**	3	229.50774616	0.00020145
**2**	1	229.49304642	0.00000459
**3**	1	229.49273401	0.00000000

Convergence criteria met.

DistSoma=366

Covariance Parameter Estimates				
**Cov Parm**	**Estimate**	**Alpha**	**Lower**	**Upper**
**Animal**	7.3045	0.05	2.5244	71.0567
**Residual**	3.3917	0.05	2.3589	5.2926

Fit Statistics	
**-2 Res Log Likelihood**	229.5
**AIC (Smaller is Better)**	233.5
**AICC (Smaller is Better)**	233.7
**BIC (Smaller is Better)**	233.1

Solution for Fixed Effects									
**Effect**	**Genotype**	**Estimate**	**Standard Error**	**DF**	**t Value**	**Pr > |t|**	**Alpha**	**Lower**	**Upper**
**Intercept**		1.2685	1.6005	4	0.79	0.4724	0.05	-3.1752	5.7123
**Genotype**	Meg +/+	2.3577	2.2675	48	1.04	0.3036	0.05	-2.2013	6.9167
**Genotype**	Meg +/-	0	.	.	.	.	.	.	.

Solution for Random Effects									
**Effect**	**Animal**	**Estimate**	**Std Err Pred**	**DF**	**t Value**	**Pr > |t|**	**Alpha**	**Lower**	**Upper**
**Animal**	1	-3.5012	1.6300	48	-2.15	0.0368	0.05	-6.7784	-0.2239
**Animal**	2	1.2751	1.6574	48	0.77	0.4455	0.05	-2.0574	4.6076
**Animal**	3	2.2261	1.6502	48	1.35	0.1837	0.05	-1.0919	5.5440
**Animal**	4	-1.2063	1.6355	48	-0.74	0.4644	0.05	-4.4947	2.0821
**Animal**	5	-1.2213	1.6268	48	-0.75	0.4565	0.05	-4.4922	2.0496
**Animal**	6	2.4276	1.6454	48	1.48	0.1466	0.05	-0.8807	5.7358

Type 3 Tests of Fixed Effects				
**Effect**	**Num DF**	**Den DF**	**F Value**	**Pr > F**
**Genotype**	1	48	1.08	0.3036

Least Squares Means									
**Effect**	**Genotype**	**Estimate**	**Standard Error**	**DF**	**t Value**	**Pr > |t|**	**Alpha**	**Lower**	**Upper**
**Genotype**	Meg +/+	3.6262	1.6062	48	2.26	0.0285	0.05	0.3968	6.8556
**Genotype**	Meg +/-	1.2685	1.6005	48	0.79	0.4319	0.05	-1.9495	4.4866

DistSoma=366

Differences of Least Squares Means												
**Effect**	**Genotype**	**Genotype**	**Estimate**	**Standard Error**	**DF**	**t Value**	**Pr > |t|**	**Adjustment**	**Adj P**	**Alpha**	**Lower**	**Upper**
**Genotype**	Meg +/+	Meg +/-	2.3577	2.2675	48	1.04	0.3036	Tukey-Kramer	0.3036	0.05	-2.2013	6.9167

Differences of Least Squares Means				
**Effect**	**Genotype**	**Genotype**	**Adj Lower**	**Adj Upper**
**Genotype**	Meg +/+	Meg +/-	-2.2012	6.9166

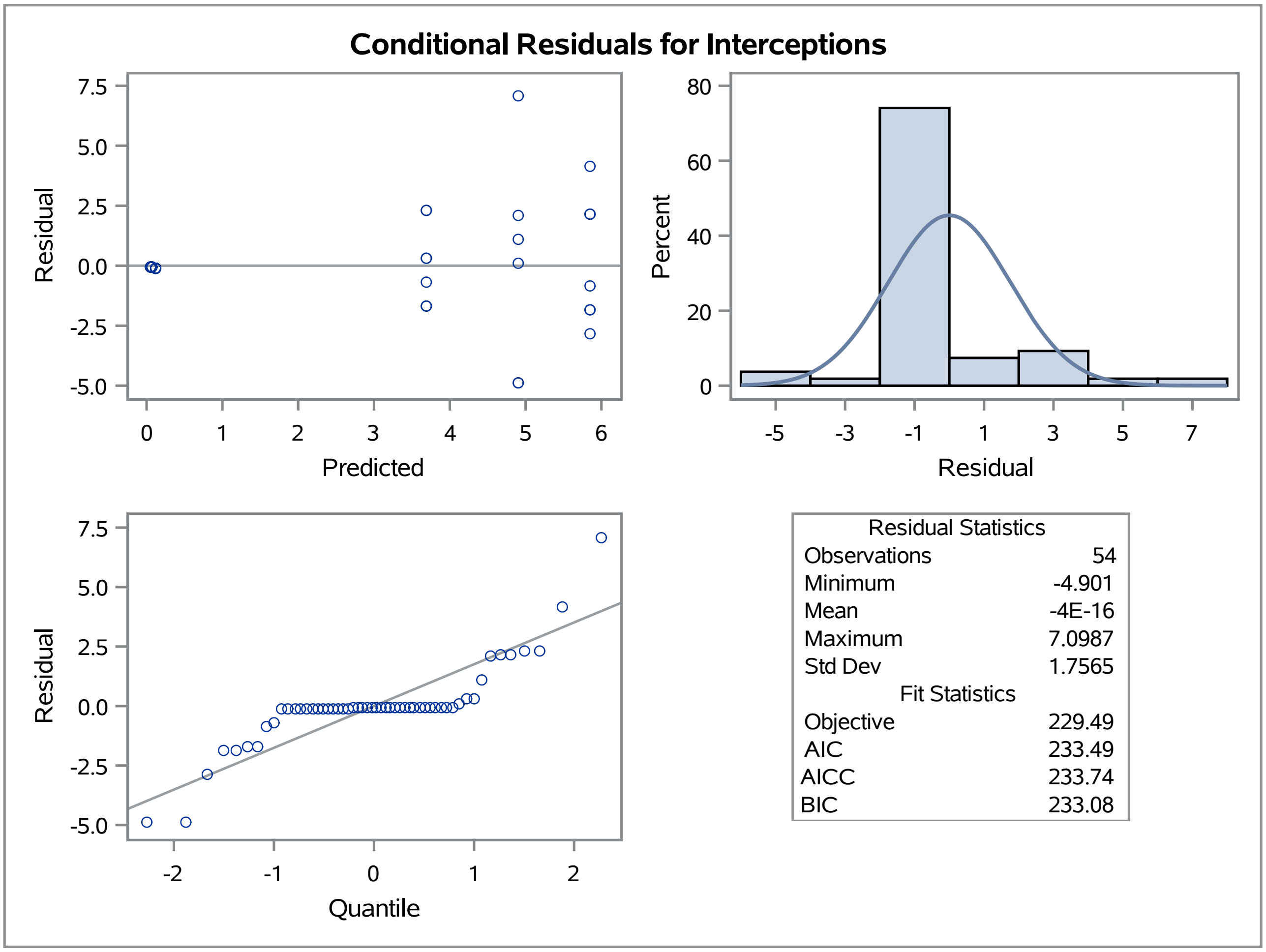

DistSoma=372

Model Information	
**Data Set**	WORK.TEMPDATASORTED
**Dependent Variable**	Interceptions
**Covariance Structure**	Variance Components
**Estimation Method**	REML
**Residual Variance Method**	Profile
**Fixed Effects SE Method**	Model-Based
**Degrees of Freedom Method**	Containment

Class Level Information		
**Class**	**Levels**	**Values**
**Genotype**	2	Meg +/+ Meg +/-
**Animal**	6	1 2 3 4 5 6

Dimensions	
**Covariance Parameters**	2
**Columns in X**	3
**Columns in Z**	6
**Subjects**	1
**Max Obs per Subject**	54

Number of Observations	
**Number of Observations Read**	54
**Number of Observations Used**	54
**Number of Observations Not Used**	0

Iteration History			
**Iteration**	**Evaluations**	**-2 Res Log Like**	**Criterion**
**0**	1	261.38920908	
**1**	3	223.07541351	0.00016318
**2**	1	223.06417292	0.00000293
**3**	1	223.06398379	0.00000000

Convergence criteria met.

DistSoma=372

Covariance Parameter Estimates				
**Cov Parm**	**Estimate**	**Alpha**	**Lower**	**Upper**
**Animal**	6.9686	0.05	2.4149	66.9672
**Residual**	2.9792	0.05	2.0720	4.6491

Fit Statistics	
**-2 Res Log Likelihood**	223.1
**AIC (Smaller is Better)**	227.1
**AICC (Smaller is Better)**	227.3
**BIC (Smaller is Better)**	226.6

Solution for Fixed Effects									
**Effect**	**Genotype**	**Estimate**	**Standard Error**	**DF**	**t Value**	**Pr > |t|**	**Alpha**	**Lower**	**Upper**
**Intercept**		1.2698	1.5602	4	0.81	0.4614	0.05	-3.0620	5.6017
**Genotype**	Meg +/+	2.2027	2.2101	48	1.00	0.3239	0.05	-2.2409	6.6464
**Genotype**	Meg +/-	0	.	.	.	.	.	.	.

Solution for Random Effects									
**Effect**	**Animal**	**Estimate**	**Std Err Pred**	**DF**	**t Value**	**Pr > |t|**	**Alpha**	**Lower**	**Upper**
**Animal**	1	-3.3620	1.5870	48	-2.12	0.0393	0.05	-6.5529	-0.1711
**Animal**	2	1.1147	1.6120	48	0.69	0.4926	0.05	-2.1265	4.3559
**Animal**	3	2.2473	1.6054	48	1.40	0.1680	0.05	-0.9806	5.4752
**Animal**	4	-1.2122	1.5920	48	-0.76	0.4501	0.05	-4.4132	1.9887
**Animal**	5	-1.2261	1.5841	48	-0.77	0.4427	0.05	-4.4112	1.9589
**Animal**	6	2.4384	1.6010	48	1.52	0.1343	0.05	-0.7807	5.6574

Type 3 Tests of Fixed Effects				
**Effect**	**Num DF**	**Den DF**	**F Value**	**Pr > F**
**Genotype**	1	48	0.99	0.3239

Least Squares Means									
**Effect**	**Genotype**	**Estimate**	**Standard Error**	**DF**	**t Value**	**Pr > |t|**	**Alpha**	**Lower**	**Upper**
**Genotype**	Meg +/+	3.4726	1.5653	48	2.22	0.0313	0.05	0.3253	6.6198
**Genotype**	Meg +/-	1.2698	1.5602	48	0.81	0.4197	0.05	-1.8672	4.4069

DistSoma=372

Differences of Least Squares Means												
**Effect**	**Genotype**	**Genotype**	**Estimate**	**Standard Error**	**DF**	**t Value**	**Pr > |t|**	**Adjustment**	**Adj P**	**Alpha**	**Lower**	**Upper**
**Genotype**	Meg +/+	Meg +/-	2.2027	2.2101	48	1.00	0.3239	Tukey-Kramer	0.3239	0.05	-2.2409	6.6464

Differences of Least Squares Means				
**Effect**	**Genotype**	**Genotype**	**Adj Lower**	**Adj Upper**
**Genotype**	Meg +/+	Meg +/-	-2.2408	6.6463

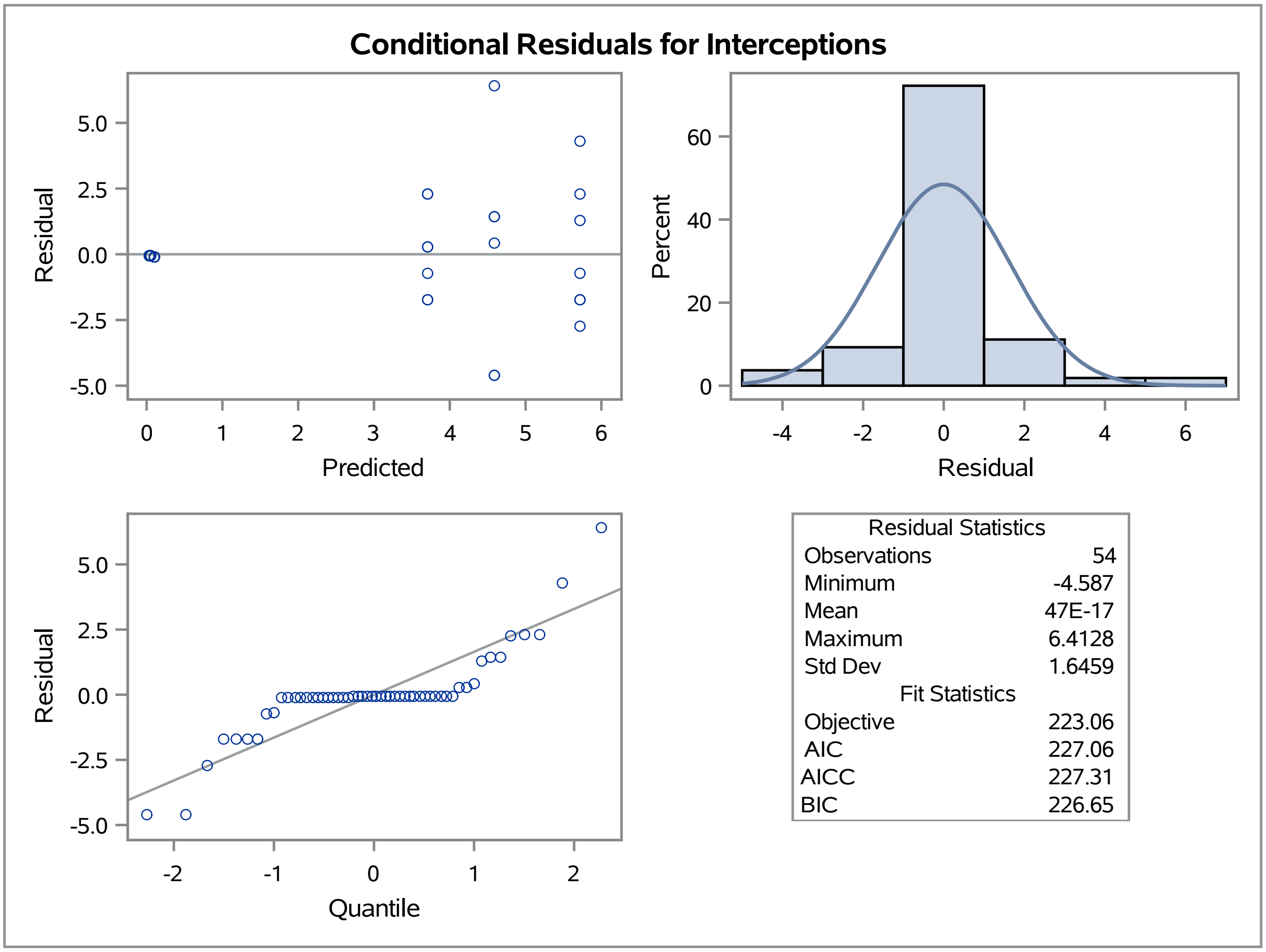

DistSoma=378

Model Information	
**Data Set**	WORK.TEMPDATASORTED
**Dependent Variable**	Interceptions
**Covariance Structure**	Variance Components
**Estimation Method**	REML
**Residual Variance Method**	Profile
**Fixed Effects SE Method**	Model-Based
**Degrees of Freedom Method**	Containment

Class Level Information		
**Class**	**Levels**	**Values**
**Genotype**	2	Meg +/+ Meg +/-
**Animal**	6	1 2 3 4 5 6

Dimensions	
**Covariance Parameters**	2
**Columns in X**	3
**Columns in Z**	6
**Subjects**	1
**Max Obs per Subject**	54

Number of Observations	
**Number of Observations Read**	54
**Number of Observations Used**	54
**Number of Observations Not Used**	0

Iteration History			
**Iteration**	**Evaluations**	**-2 Res Log Like**	**Criterion**
**0**	1	261.38920908	
**1**	3	223.07541351	0.00016318
**2**	1	223.06417292	0.00000293
**3**	1	223.06398379	0.00000000

Convergence criteria met.

DistSoma=378

Covariance Parameter Estimates				
Cov Parm	Estimate	Alpha	Lower	Upper
Animal	6.9686	0.05	2.4149	66.9672
Residual	2.9792	0.05	2.0720	4.6491

Fit Statistics	
-2 Res Log Likelihood	223.1
AIC (Smaller is Better)	227.1
AICC (Smaller is Better)	227.3
BIC (Smaller is Better)	226.6

Solution for Fixed Effects									
Effect	Genotype	Estimate	Standard Error	DF	t Value	Pr > |t|	Alpha	Lower	Upper
Intercept		1.2698	1.5602	4	0.81	0.4614	0.05	-3.0620	5.6017
Genotype	Meg +/+	2.2027	2.2101	48	1.00	0.3239	0.05	-2.2409	6.6464
Genotype	Meg +/-	0	.	.	.	.	.	.	.

Solution for Random Effects									
Effect	Animal	Estimate	Std Err Pred	DF	t Value	Pr > |t|	Alpha	Lower	Upper
Animal	1	-3.3620	1.5870	48	-2.12	0.0393	0.05	-6.5529	-0.1711
Animal	2	1.1147	1.6120	48	0.69	0.4926	0.05	-2.1265	4.3559
Animal	3	2.2473	1.6054	48	1.40	0.1680	0.05	-0.9806	5.4752
Animal	4	-1.2122	1.5920	48	-0.76	0.4501	0.05	-4.4132	1.9887
Animal	5	-1.2261	1.5841	48	-0.77	0.4427	0.05	-4.4112	1.9589
Animal	6	2.4384	1.6010	48	1.52	0.1343	0.05	-0.7807	5.6574

Type 3 Tests of Fixed Effects				
Effect	Num DF	Den DF	F Value	Pr > F
Genotype	1	48	0.99	0.3239

Least Squares Means									
Effect	Genotype	Estimate	Standard Error	DF	t Value	Pr > |t|	Alpha	Lower	Upper
Genotype	Meg +/+	3.4726	1.5653	48	2.22	0.0313	0.05	0.3253	6.6198
Genotype	Meg +/-	1.2698	1.5602	48	0.81	0.4197	0.05	-1.8672	4.4069

DistSoma=378

Differences of Least Squares Means												
**Effect**	**Genotype**	**Genotype**	**Estimate**	**Standard Error**	**DF**	**t Value**	**Pr > |t|**	**Adjustment**	**Adj P**	**Alpha**	**Lower**	**Upper**
**Genotype**	Meg +/+	Meg +/-	2.2027	2.2101	48	1.00	0.3239	Tukey-Kramer	0.3239	0.05	-2.2409	6.6464

Differences of Least Squares Means				
**Effect**	**Genotype**	**Genotype**	**Adj Lower**	**Adj Upper**
**Genotype**	Meg +/+	Meg +/-	-2.2408	6.6463

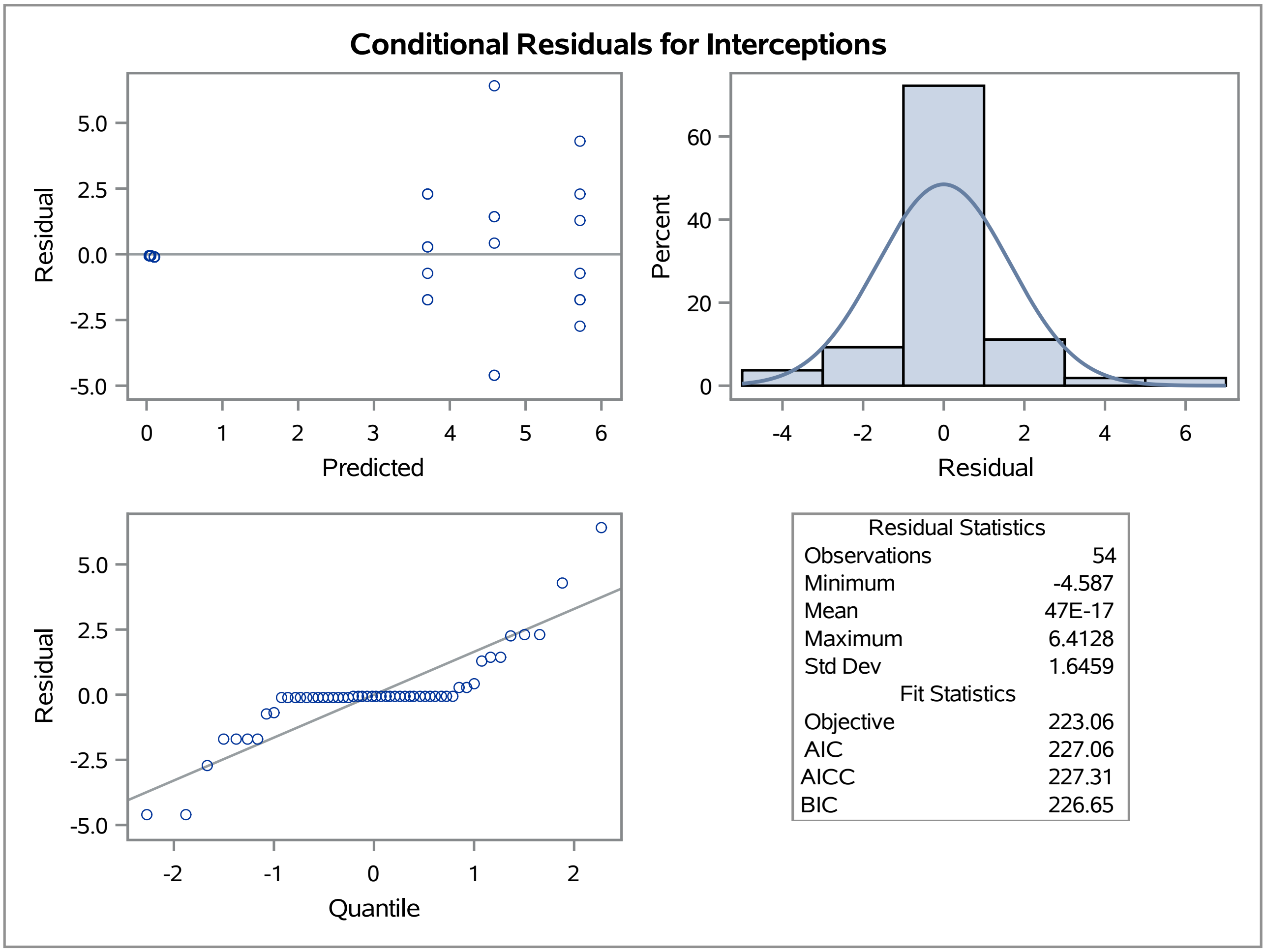

DistSoma=384

Model Information	
**Data Set**	WORK.TEMPDATASORTED
**Dependent Variable**	Interceptions
**Covariance Structure**	Variance Components
**Estimation Method**	REML
**Residual Variance Method**	Profile
**Fixed Effects SE Method**	Model-Based
**Degrees of Freedom Method**	Containment

Class Level Information		
**Class**	**Levels**	**Values**
**Genotype**	2	Meg +/+ Meg +/-
**Animal**	6	1 2 3 4 5 6

Dimensions	
**Covariance Parameters**	2
**Columns in X**	3
**Columns in Z**	6
**Subjects**	1
**Max Obs per Subject**	54

Number of Observations	
**Number of Observations Read**	54
**Number of Observations Used**	54
**Number of Observations Not Used**	0

Iteration History			
**Iteration**	**Evaluations**	**-2 Res Log Like**	**Criterion**
**0**	1	258.17178473	
**1**	3	221.91466228	0.00013346
**2**	1	221.90560966	0.00000197
**3**	1	221.90548380	0.00000000

Convergence criteria met.

DistSoma=384

Covariance Parameter Estimates				
**Cov Parm**	**Estimate**	**Alpha**	**Lower**	**Upper**
**Animal**	6.3718	0.05	2.2021	61.9712
**Residual**	2.9291	0.05	2.0372	4.5708

Fit Statistics	
**-2 Res Log Likelihood**	221.9
**AIC (Smaller is Better)**	225.9
**AICC (Smaller is Better)**	226.2
**BIC (Smaller is Better)**	225.5

Solution for Fixed Effects									
**Effect**	**Genotype**	**Estimate**	**Standard Error**	**DF**	**t Value**	**Pr > |t|**	**Alpha**	**Lower**	**Upper**
**Intercept**		1.2217	1.4945	4	0.82	0.4595	0.05	-2.9276	5.3710
**Genotype**	Meg +/+	2.0990	2.1172	48	0.99	0.3265	0.05	-2.1579	6.3559
**Genotype**	Meg +/-	0	.	.	.	.	.	.	.

Solution for Random Effects									
**Effect**	**Animal**	**Estimate**	**Std Err Pred**	**DF**	**t Value**	**Pr > |t|**	**Alpha**	**Lower**	**Upper**
**Animal**	1	-3.2073	1.5217	48	-2.11	0.0403	0.05	-6.2670	-0.1477
**Animal**	2	1.0953	1.5472	48	0.71	0.4824	0.05	-2.0155	4.2062
**Animal**	3	2.1120	1.5405	48	1.37	0.1768	0.05	-0.9853	5.2093
**Animal**	4	-1.1623	1.5269	48	-0.76	0.4502	0.05	-4.2323	1.9076
**Animal**	5	-1.1766	1.5188	48	-0.77	0.4423	0.05	-4.2304	1.8771
**Animal**	6	2.3390	1.5360	48	1.52	0.1344	0.05	-0.7494	5.4273

Type 3 Tests of Fixed Effects				
**Effect**	**Num DF**	**Den DF**	**F Value**	**Pr > F**
**Genotype**	1	48	0.98	0.3265

Least Squares Means									
**Effect**	**Genotype**	**Estimate**	**Standard Error**	**DF**	**t Value**	**Pr > |t|**	**Alpha**	**Lower**	**Upper**
**Genotype**	Meg +/+	3.3207	1.4997	48	2.21	0.0316	0.05	0.3054	6.3361
**Genotype**	Meg +/-	1.2217	1.4945	48	0.82	0.4177	0.05	-1.7831	4.2265

DistSoma=384

Differences of Least Squares Means												
Effect	Genotype	Genotype	Estimate	Standard Error	DF	t Value	Pr > |t|	Adjustment	Adj P	Alpha	Lower	Upper
**Genotype**	Meg +/+	Meg +/-	2.0990	2.1172	48	0.99	0.3265	Tukey-Kramer	0.3265	0.05	-2.1579	6.3559

Differences of Least Squares Means				
Effect	Genotype	Genotype	Adj Lower	Adj Upper
**Genotype**	Meg +/+	Meg +/-	-2.1577	6.3558

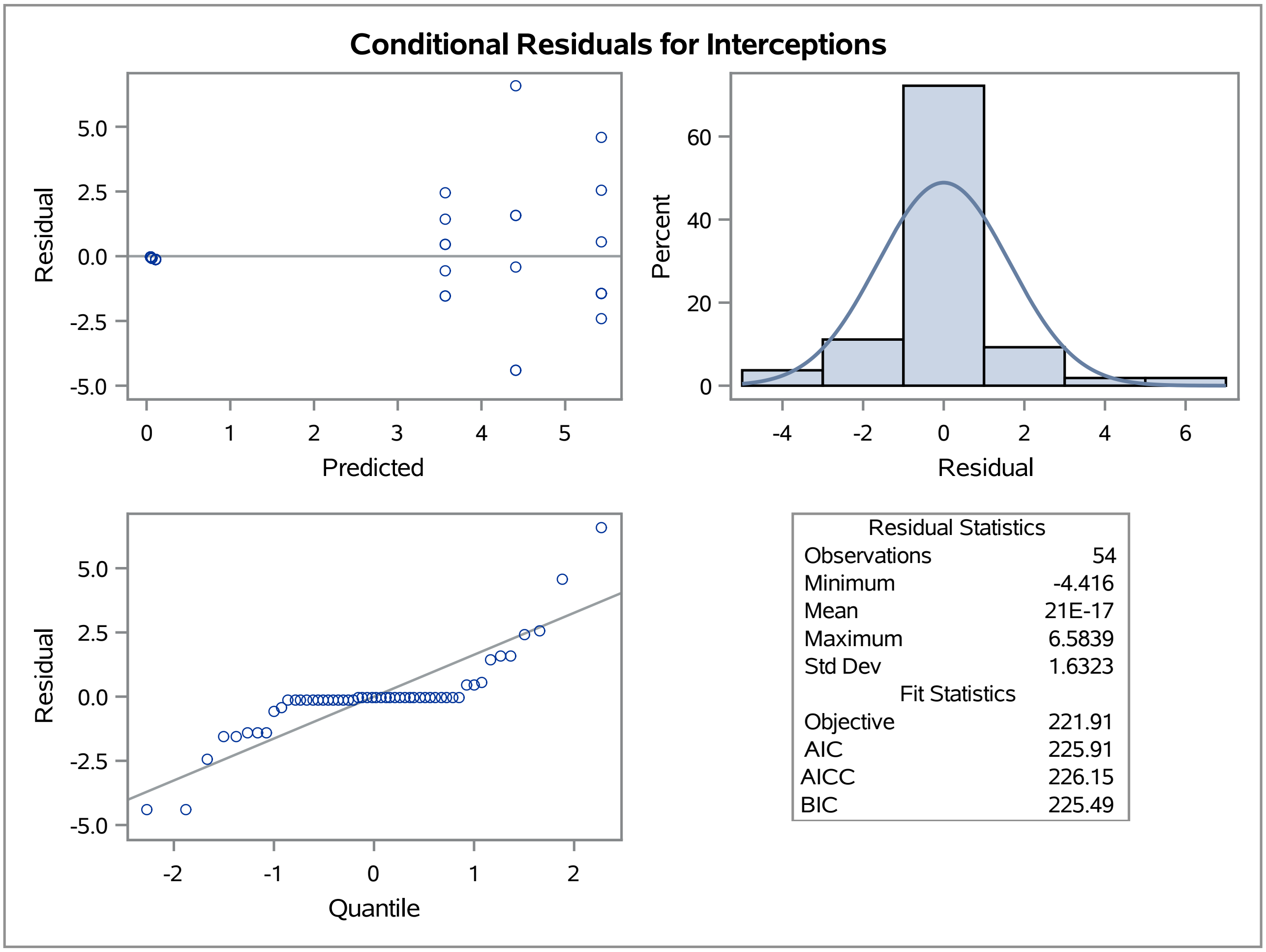

DistSoma=390

Model Information	
**Data Set**	WORK.TEMPDATASORTED
**Dependent Variable**	Interceptions
**Covariance Structure**	Variance Components
**Estimation Method**	REML
**Residual Variance Method**	Profile
**Fixed Effects SE Method**	Model-Based
**Degrees of Freedom Method**	Containment

Class Level Information		
**Class**	**Levels**	**Values**
**Genotype**	2	Meg +/+ Meg +/-
**Animal**	6	1 2 3 4 5 6

Dimensions	
**Covariance Parameters**	2
**Columns in X**	3
**Columns in Z**	6
**Subjects**	1
**Max Obs per Subject**	54

Number of Observations	
**Number of Observations Read**	54
**Number of Observations Used**	54
**Number of Observations Not Used**	0

Iteration History			
**Iteration**	**Evaluations**	**-2 Res Log Like**	**Criterion**
**0**	1	257.90570038	
**1**	3	222.47937082	0.00010198
**2**	1	222.47247384	0.00000118
**3**	1	222.47239860	0.00000000

Convergence criteria met.

DistSoma=390

Covariance Parameter Estimates				
**Cov Parm**	**Estimate**	**Alpha**	**Lower**	**Upper**
**Animal**	6.2760	0.05	2.1665	61.3682
**Residual**	2.9672	0.05	2.0637	4.6303

Fit Statistics	
**-2 Res Log Likelihood**	222.5
**AIC (Smaller is Better)**	226.5
**AICC (Smaller is Better)**	226.7
**BIC (Smaller is Better)**	226.1

Solution for Fixed Effects									
**Effect**	**Genotype**	**Estimate**	**Standard Error**	**DF**	**t Value**	**Pr > |t|**	**Alpha**	**Lower**	**Upper**
**Intercept**		1.2213	1.4842	4	0.82	0.4568	0.05	-2.8996	5.3422
**Genotype**	Meg +/+	2.0439	2.1028	48	0.97	0.3359	0.05	-2.1841	6.2718
**Genotype**	Meg +/-	0	.	.	.	.	.	.	.

Solution for Random Effects									
**Effect**	**Animal**	**Estimate**	**Std Err Pred**	**DF**	**t Value**	**Pr > |t|**	**Alpha**	**Lower**	**Upper**
**Animal**	1	-3.1506	1.5120	48	-2.08	0.0425	0.05	-6.1906	-0.1105
**Animal**	2	0.9902	1.5378	48	0.64	0.5227	0.05	-2.1018	4.0822
**Animal**	3	2.1604	1.5310	48	1.41	0.1647	0.05	-0.9179	5.2387
**Animal**	4	-1.1603	1.5172	48	-0.76	0.4481	0.05	-4.2108	1.8901
**Animal**	5	-1.1750	1.5090	48	-0.78	0.4400	0.05	-4.2090	1.8590
**Animal**	6	2.3353	1.5265	48	1.53	0.1326	0.05	-0.7339	5.4045

Type 3 Tests of Fixed Effects				
**Effect**	**Num DF**	**Den DF**	**F Value**	**Pr > F**
**Genotype**	1	48	0.94	0.3359

Least Squares Means									
**Effect**	**Genotype**	**Estimate**	**Standard Error**	**DF**	**t Value**	**Pr > |t|**	**Alpha**	**Lower**	**Upper**
**Genotype**	Meg +/+	3.2651	1.4896	48	2.19	0.0333	0.05	0.2702	6.2601
**Genotype**	Meg +/-	1.2213	1.4842	48	0.82	0.4147	0.05	-1.7630	4.2055

DistSoma=390

Differences of Least Squares Means												
**Effect**	**Genotype**	**Genotype**	**Estimate**	**Standard Error**	**DF**	**t Value**	**Pr > |t|**	**Adjustment**	**Adj P**	**Alpha**	**Lower**	**Upper**
**Genotype**	Meg +/+	Meg +/-	2.0439	2.1028	48	0.97	0.3359	Tukey-Kramer	0.3359	0.05	-2.1841	6.2718

Differences of Least Squares Means				
**Effect**	**Genotype**	**Genotype**	**Adj Lower**	**Adj Upper**
**Genotype**	Meg +/+	Meg +/-	-2.1839	6.2717

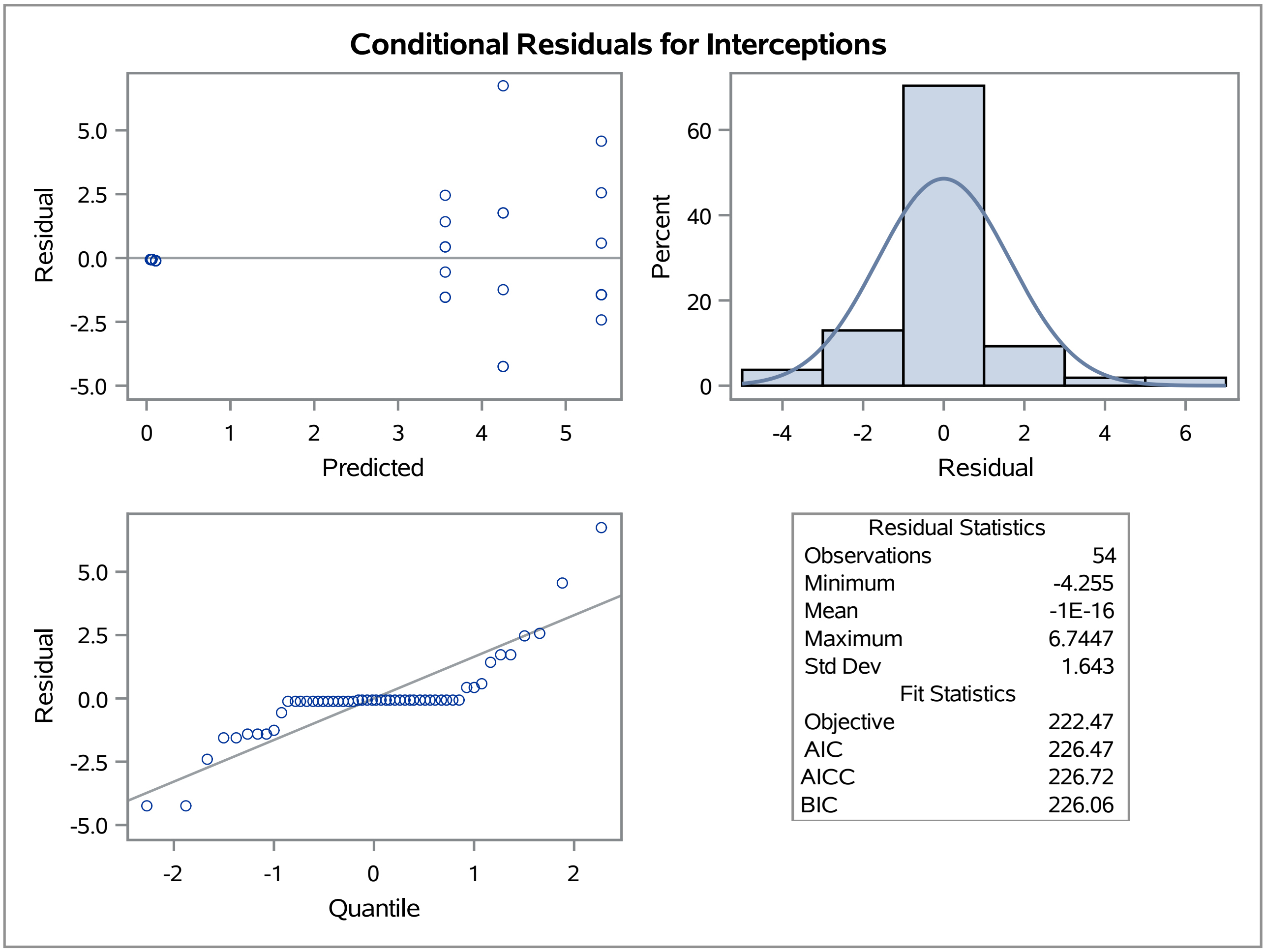

DistSoma=396

Model Information	
**Data Set**	WORK.TEMPDATASORTED
**Dependent Variable**	Interceptions
**Covariance Structure**	Variance Components
**Estimation Method**	REML
**Residual Variance Method**	Profile
**Fixed Effects SE Method**	Model-Based
**Degrees of Freedom Method**	Containment

Class Level Information		
**Class**	**Levels**	**Values**
**Genotype**	2	Meg +/+ Meg +/-
**Animal**	6	1 2 3 4 5 6

Dimensions	
**Covariance Parameters**	2
**Columns in X**	3
**Columns in Z**	6
**Subjects**	1
**Max Obs per Subject**	54

Number of Observations	
**Number of Observations Read**	54
**Number of Observations Used**	54
**Number of Observations Not Used**	0

Iteration History			
**Iteration**	**Evaluations**	**-2 Res Log Like**	**Criterion**
**0**	1	257.90570038	
**1**	3	222.47937082	0.00010198
**2**	1	222.47247384	0.00000118
**3**	1	222.47239860	0.00000000

Convergence criteria met.

DistSoma=396

Covariance Parameter Estimates				
Cov Parm	Estimate	Alpha	Lower	Upper
Animal	6.2760	0.05	2.1665	61.3682
Residual	2.9672	0.05	2.0637	4.6303

Fit Statistics	
-2 Res Log Likelihood	222.5
AIC (Smaller is Better)	226.5
AICC (Smaller is Better)	226.7
BIC (Smaller is Better)	226.1

Solution for Fixed Effects									
Effect	Genotype	Estimate	Standard Error	DF	t Value	Pr > |t|	Alpha	Lower	Upper
Intercept		1.2213	1.4842	4	0.82	0.4568	0.05	-2.8996	5.3422
Genotype	Meg +/+	2.0439	2.1028	48	0.97	0.3359	0.05	-2.1841	6.2718
Genotype	Meg +/-	0	.	.	.	.	.	.	.

Solution for Random Effects									
Effect	Animal	Estimate	Std Err Pred	DF	t Value	Pr > |t|	Alpha	Lower	Upper
Animal	1	-3.1506	1.5120	48	-2.08	0.0425	0.05	-6.1906	-0.1105
Animal	2	0.9902	1.5378	48	0.64	0.5227	0.05	-2.1018	4.0822
Animal	3	2.1604	1.5310	48	1.41	0.1647	0.05	-0.9179	5.2387
Animal	4	-1.1603	1.5172	48	-0.76	0.4481	0.05	-4.2108	1.8901
Animal	5	-1.1750	1.5090	48	-0.78	0.4400	0.05	-4.2090	1.8590
Animal	6	2.3353	1.5265	48	1.53	0.1326	0.05	-0.7339	5.4045

Type 3 Tests of Fixed Effects				
Effect	Num DF	Den DF	F Value	Pr > F
Genotype	1	48	0.94	0.3359

Least Squares Means									
Effect	Genotype	Estimate	Standard Error	DF	t Value	Pr > |t|	Alpha	Lower	Upper
Genotype	Meg +/+	3.2651	1.4896	48	2.19	0.0333	0.05	0.2702	6.2601
Genotype	Meg +/-	1.2213	1.4842	48	0.82	0.4147	0.05	-1.7630	4.2055

DistSoma=396

Differences of Least Squares Means												
Effect	Genotype	Genotype	Estimate	Standard Error	DF	t Value	Pr > |t|	Adjustment	Adj P	Alpha	Lower	Upper
Genotype	Meg +/+	Meg +/-	2.0439	2.1028	48	0.97	0.3359	Tukey-Kramer	0.3359	0.05	-2.1841	6.2718

Differences of Least Squares Means				
Effect	Genotype	Genotype	Adj Lower	Adj Upper
Genotype	Meg +/+	Meg +/-	-2.1839	6.2717

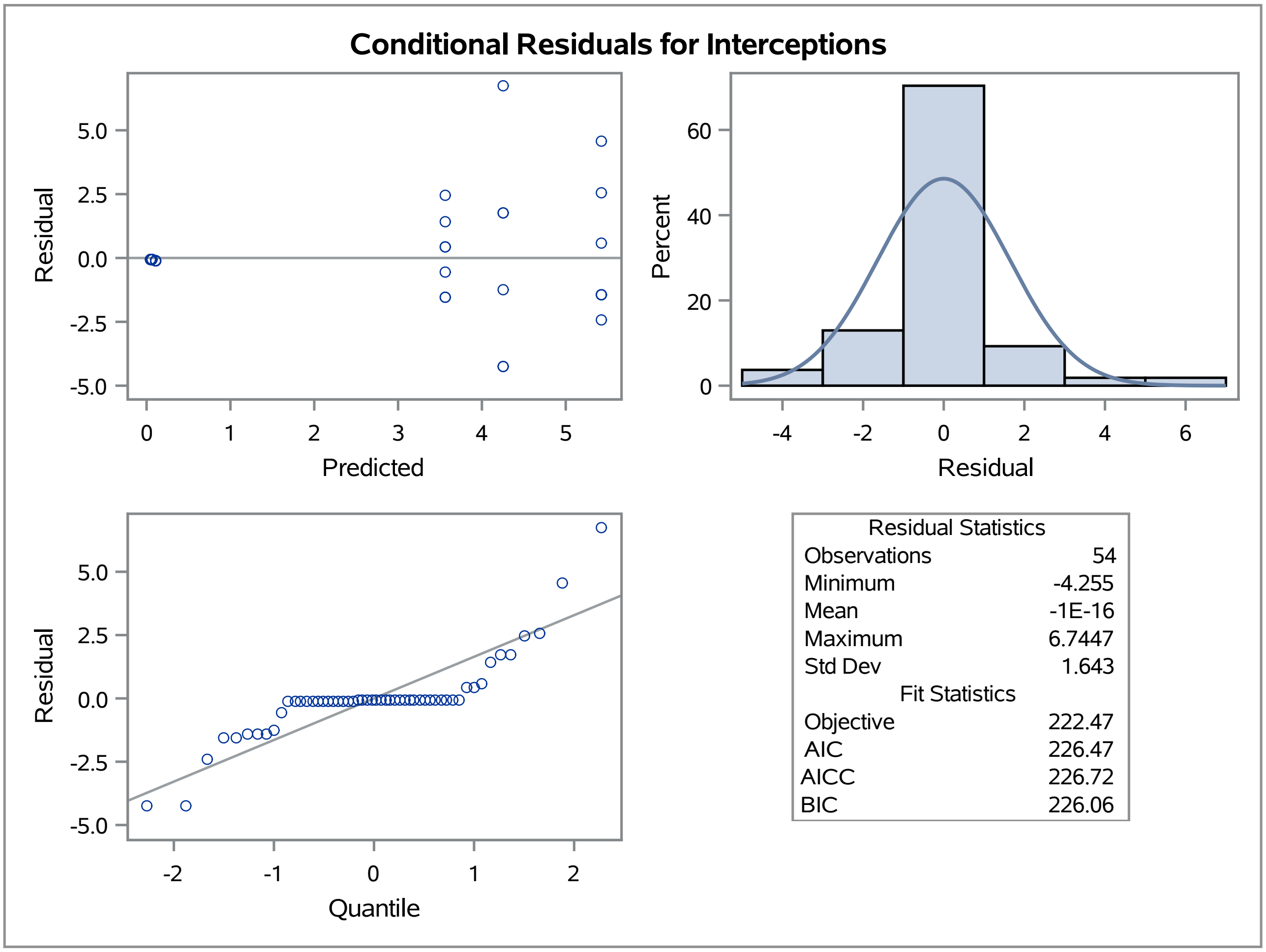

DistSoma=402

Model Information	
**Data Set**	WORK.TEMPDATASORTED
**Dependent Variable**	Interceptions
**Covariance Structure**	Variance Components
**Estimation Method**	REML
**Residual Variance Method**	Profile
**Fixed Effects SE Method**	Model-Based
**Degrees of Freedom Method**	Containment

Class Level Information		
**Class**	**Levels**	**Values**
**Genotype**	2	Meg +/+ Meg +/-
**Animal**	6	1 2 3 4 5 6

Dimensions	
**Covariance Parameters**	2
**Columns in X**	3
**Columns in Z**	6
**Subjects**	1
**Max Obs per Subject**	54

Number of Observations	
**Number of Observations Read**	54
**Number of Observations Used**	54
**Number of Observations Not Used**	0

Iteration History			
**Iteration**	**Evaluations**	**-2 Res Log Like**	**Criterion**
**0**	1	257.74516889	
**1**	3	223.79913700	0.00013264
**2**	1	223.79000391	0.00000198
**3**	1	223.78987593	0.00000000

Convergence criteria met.

DistSoma=402

Covariance Parameter Estimates				
**Cov Parm**	**Estimate**	**Alpha**	**Lower**	**Upper**
**Animal**	6.0959	0.05	2.0998	60.1815
**Residual**	3.0564	0.05	2.1258	4.7694

Fit Statistics	
**-2 Res Log Likelihood**	223.8
**AIC (Smaller is Better)**	227.8
**AICC (Smaller is Better)**	228.0
**BIC (Smaller is Better)**	227.4

Solution for Fixed Effects									
**Effect**	**Genotype**	**Estimate**	**Standard Error**	**DF**	**t Value**	**Pr > |t|**	**Alpha**	**Lower**	**Upper**
**Intercept**		1.1734	1.4650	4	0.80	0.4680	0.05	-2.8941	5.2409
**Genotype**	Meg +/+	2.0897	2.0757	48	1.01	0.3191	0.05	-2.0839	6.2632
**Genotype**	Meg +/-	0	.	.	.	.	.	.	.

Solution for Random Effects									
**Effect**	**Animal**	**Estimate**	**Std Err Pred**	**DF**	**t Value**	**Pr > |t|**	**Alpha**	**Lower**	**Upper**
**Animal**	1	-3.1419	1.4937	48	-2.10	0.0407	0.05	-6.1452	-0.1385
**Animal**	2	0.9878	1.5205	48	0.65	0.5190	0.05	-2.0694	4.0449
**Animal**	3	2.1541	1.5134	48	1.42	0.1611	0.05	-0.8889	5.1971
**Animal**	4	-1.1115	1.4991	48	-0.74	0.4621	0.05	-4.1256	1.9027
**Animal**	5	-1.1263	1.4906	48	-0.76	0.4536	0.05	-4.1234	1.8708
**Animal**	6	2.2378	1.5088	48	1.48	0.1446	0.05	-0.7958	5.2713

Type 3 Tests of Fixed Effects				
**Effect**	**Num DF**	**Den DF**	**F Value**	**Pr > F**
**Genotype**	1	48	1.01	0.3191

Least Squares Means									
**Effect**	**Genotype**	**Estimate**	**Standard Error**	**DF**	**t Value**	**Pr > |t|**	**Alpha**	**Lower**	**Upper**
**Genotype**	Meg +/+	3.2630	1.4705	48	2.22	0.0313	0.05	0.3063	6.2198
**Genotype**	Meg +/-	1.1734	1.4650	48	0.80	0.4271	0.05	-1.7722	4.1190

DistSoma=402

Differences of Least Squares Means												
Effect	Genotype	Genotype	Estimate	Standard Error	DF	t Value	Pr > |t|	Adjustment	Adj P	Alpha	Lower	Upper
**Genotype**	Meg +/+	Meg +/-	2.0897	2.0757	48	1.01	0.3191	Tukey-Kramer	0.3191	0.05	-2.0839	6.2632

Differences of Least Squares Means				
Effect	Genotype	Genotype	Adj Lower	Adj Upper
**Genotype**	Meg +/+	Meg +/-	-2.0838	6.2631

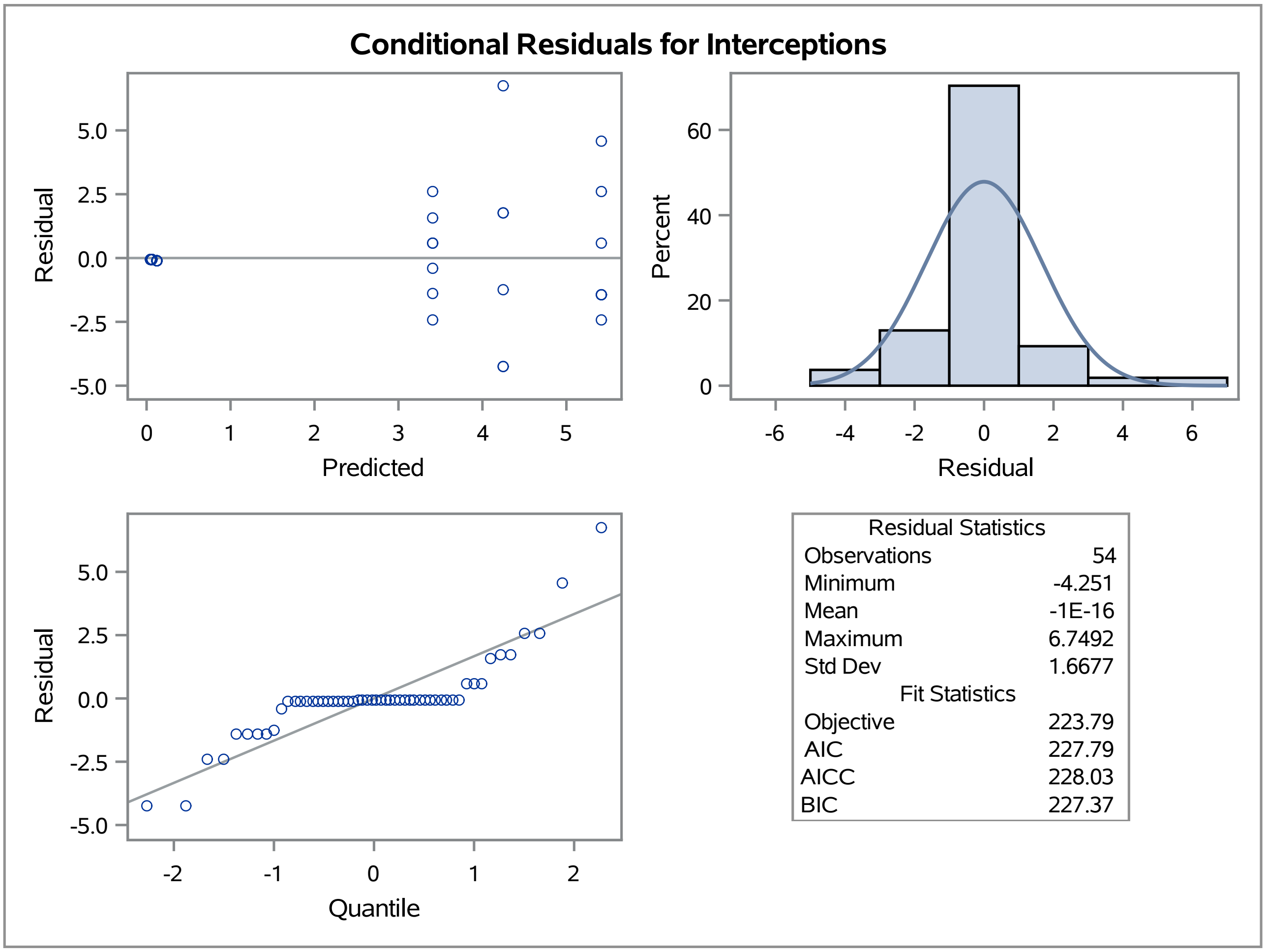

DistSoma=408

Model Information	
**Data Set**	WORK.TEMPDATASORTED
**Dependent Variable**	Interceptions
**Covariance Structure**	Variance Components
**Estimation Method**	REML
**Residual Variance Method**	Profile
**Fixed Effects SE Method**	Model-Based
**Degrees of Freedom Method**	Containment

Class Level Information		
**Class**	**Levels**	**Values**
**Genotype**	2	Meg +/+ Meg +/-
**Animal**	6	1 2 3 4 5 6

Dimensions	
**Covariance Parameters**	2
**Columns in X**	3
**Columns in Z**	6
**Subjects**	1
**Max Obs per Subject**	54

Number of Observations	
**Number of Observations Read**	54
**Number of Observations Used**	54
**Number of Observations Not Used**	0

Iteration History			
**Iteration**	**Evaluations**	**-2 Res Log Like**	**Criterion**
**0**	1	258.82008697	
**1**	3	225.20156305	0.00015423
**2**	1	225.19077460	0.00000267
**3**	1	225.19059949	0.00000000

Convergence criteria met.

DistSoma=408

Covariance Parameter Estimates				
**Cov Parm**	**Estimate**	**Alpha**	**Lower**	**Upper**
**Animal**	6.1791	0.05	2.1275	61.1298
**Residual**	3.1431	0.05	2.1861	4.9047

Fit Statistics	
**-2 Res Log Likelihood**	225.2
**AIC (Smaller is Better)**	229.2
**AICC (Smaller is Better)**	229.4
**BIC (Smaller is Better)**	228.8

Solution for Fixed Effects									
**Effect**	**Genotype**	**Estimate**	**Standard Error**	**DF**	**t Value**	**Pr > |t|**	**Alpha**	**Lower**	**Upper**
**Intercept**		1.1731	1.4755	4	0.80	0.4711	0.05	-2.9236	5.2699
**Genotype**	Meg +/+	2.1439	2.0907	48	1.03	0.3103	0.05	-2.0598	6.3476
**Genotype**	Meg +/-	0	.	.	.	.	.	.	.

Solution for Random Effects									
**Effect**	**Animal**	**Estimate**	**Std Err Pred**	**DF**	**t Value**	**Pr > |t|**	**Alpha**	**Lower**	**Upper**
**Animal**	1	-3.1921	1.5048	48	-2.12	0.0391	0.05	-6.2178	-0.1665
**Animal**	2	1.0905	1.5321	48	0.71	0.4801	0.05	-1.9900	4.1710
**Animal**	3	2.1017	1.5249	48	1.38	0.1745	0.05	-0.9644	5.1677
**Animal**	4	-1.1104	1.5103	48	-0.74	0.4658	0.05	-4.1471	1.9263
**Animal**	5	-1.1254	1.5017	48	-0.75	0.4572	0.05	-4.1447	1.8939
**Animal**	6	2.2358	1.5201	48	1.47	0.1479	0.05	-0.8206	5.2922

Type 3 Tests of Fixed Effects				
**Effect**	**Num DF**	**Den DF**	**F Value**	**Pr > F**
**Genotype**	1	48	1.05	0.3103

Least Squares Means									
**Effect**	**Genotype**	**Estimate**	**Standard Error**	**DF**	**t Value**	**Pr > |t|**	**Alpha**	**Lower**	**Upper**
**Genotype**	Meg +/+	3.3171	1.4812	48	2.24	0.0298	0.05	0.3389	6.2952
**Genotype**	Meg +/-	1.1731	1.4755	48	0.80	0.4305	0.05	-1.7936	4.1399

DistSoma=408

Differences of Least Squares Means												
**Effect**	**Genotype**	**Genotype**	**Estimate**	**Standard Error**	**DF**	**t Value**	**Pr > |t|**	**Adjustment**	**Adj P**	**Alpha**	**Lower**	**Upper**
**Genotype**	Meg +/+	Meg +/-	2.1439	2.0907	48	1.03	0.3103	Tukey-Kramer	0.3103	0.05	-2.0598	6.3476

Differences of Least Squares Means				
**Effect**	**Genotype**	**Genotype**	**Adj Lower**	**Adj Upper**
**Genotype**	Meg +/+	Meg +/-	-2.0596	6.3474

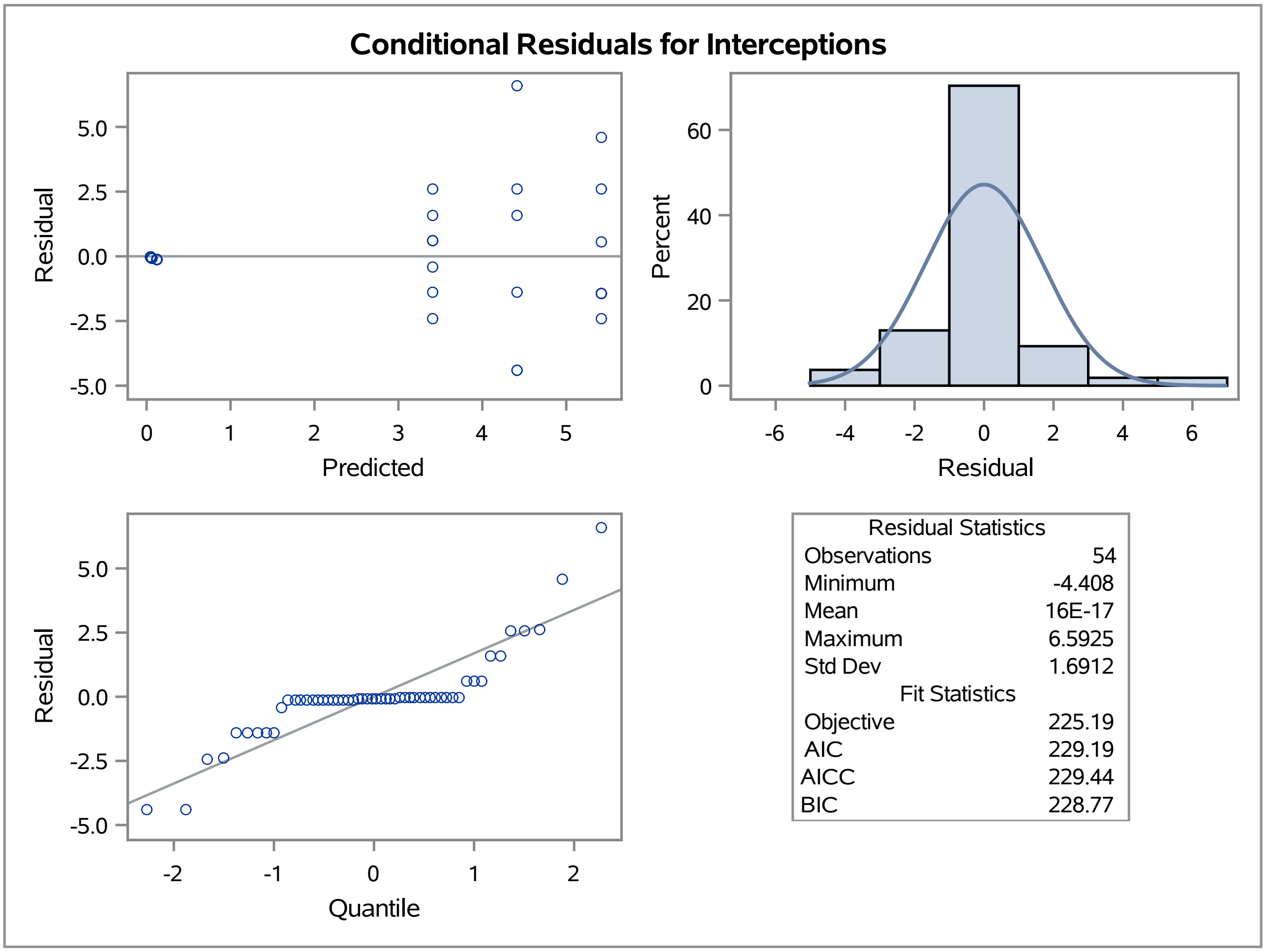

DistSoma=414

Model Information	
**Data Set**	WORK.TEMPDATASORTED
**Dependent Variable**	Interceptions
**Covariance Structure**	Variance Components
**Estimation Method**	REML
**Residual Variance Method**	Profile
**Fixed Effects SE Method**	Model-Based
**Degrees of Freedom Method**	Containment

Class Level Information		
**Class**	**Levels**	**Values**
**Genotype**	2	Meg +/+ Meg +/-
**Animal**	6	1 2 3 4 5 6

Dimensions	
**Covariance Parameters**	2
**Columns in X**	3
**Columns in Z**	6
**Subjects**	1
**Max Obs per Subject**	54

Number of Observations	
**Number of Observations Read**	54
**Number of Observations Used**	54
**Number of Observations Not Used**	0

Iteration History			
**Iteration**	**Evaluations**	**-2 Res Log Like**	**Criterion**
**0**	1	258.65278317	
**1**	3	226.89066721	0.00018820
**2**	1	226.87724198	0.00000396
**3**	1	226.87697821	0.00000000

Convergence criteria met.

DistSoma=414

Covariance Parameter Estimates				
**Cov Parm**	**Estimate**	**Alpha**	**Lower**	**Upper**
**Animal**	5.9519	0.05	2.0432	59.6781
**Residual**	3.2645	0.05	2.2705	5.0939

Fit Statistics	
**-2 Res Log Likelihood**	226.9
**AIC (Smaller is Better)**	230.9
**AICC (Smaller is Better)**	231.1
**BIC (Smaller is Better)**	230.5

Solution for Fixed Effects									
**Effect**	**Genotype**	**Estimate**	**Standard Error**	**DF**	**t Value**	**Pr > |t|**	**Alpha**	**Lower**	**Upper**
**Intercept**		1.1250	1.4512	4	0.78	0.4815	0.05	-2.9042	5.1542
**Genotype**	Meg +/+	2.1963	2.0565	48	1.07	0.2909	0.05	-1.9386	6.3312
**Genotype**	Meg +/-	0	.	.	.	.	.	.	.

Solution for Random Effects									
**Effect**	**Animal**	**Estimate**	**Std Err Pred**	**DF**	**t Value**	**Pr > |t|**	**Alpha**	**Lower**	**Upper**
**Animal**	1	-3.1868	1.4818	48	-2.15	0.0366	0.05	-6.1663	-0.2074
**Animal**	2	1.2327	1.5103	48	0.82	0.4184	0.05	-1.8040	4.2694
**Animal**	3	1.9542	1.5028	48	1.30	0.1997	0.05	-1.0675	4.9758
**Animal**	4	-1.0604	1.4876	48	-0.71	0.4794	0.05	-4.0514	1.9306
**Animal**	5	-1.0758	1.4785	48	-0.73	0.4704	0.05	-4.0486	1.8970
**Animal**	6	2.1362	1.4978	48	1.43	0.1603	0.05	-0.8754	5.1478

Type 3 Tests of Fixed Effects				
**Effect**	**Num DF**	**Den DF**	**F Value**	**Pr > F**
**Genotype**	1	48	1.14	0.2909

Least Squares Means									
**Effect**	**Genotype**	**Estimate**	**Standard Error**	**DF**	**t Value**	**Pr > |t|**	**Alpha**	**Lower**	**Upper**
**Genotype**	Meg +/+	3.3213	1.4572	48	2.28	0.0271	0.05	0.3915	6.2511
**Genotype**	Meg +/-	1.1250	1.4512	48	0.78	0.4420	0.05	-1.7928	4.0428

DistSoma=414

Differences of Least Squares Means												
**Effect**	**Genotype**	**Genotype**	**Estimate**	**Standard Error**	**DF**	**t Value**	**Pr > |t|**	**Adjustment**	**Adj P**	**Alpha**	**Lower**	**Upper**
**Genotype**	Meg +/+	Meg +/-	2.1963	2.0565	48	1.07	0.2909	Tukey-Kramer	0.2909	0.05	-1.9386	6.3312

Differences of Least Squares Means				
**Effect**	**Genotype**	**Genotype**	**Adj Lower**	**Adj Upper**
**Genotype**	Meg +/+	Meg +/-	-1.9385	6.3311

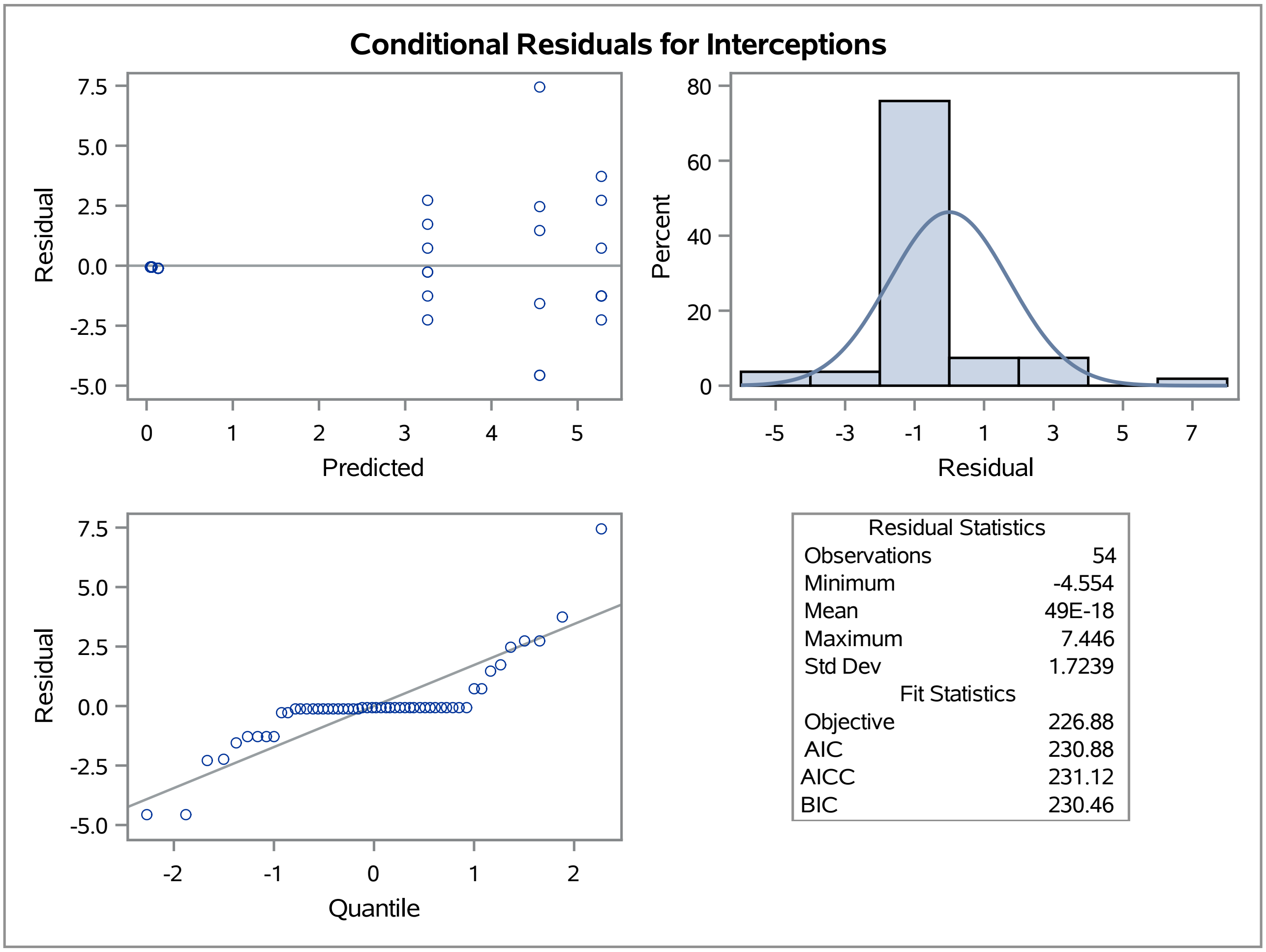

Residual Statistics	
Observations	54
Minimum	-4.554
Mean	49E-18
Maximum	7.446
Std Dev	1.7239
**Fit Statistics**	
Objective	226.88
AIC	230.88
AICC	231.12
BIC	230.46

DistSoma=420

Model Information	
Data Set	WORK.TEMPDATASORTED
Dependent Variable	Interceptions
Covariance Structure	Variance Components
Estimation Method	REML
Residual Variance Method	Profile
Fixed Effects SE Method	Model-Based
Degrees of Freedom Method	Containment

Class Level Information		
Class	Levels	Values
Genotype	2	Meg +/+ Meg +/-
Animal	6	1 2 3 4 5 6

Dimensions	
Covariance Parameters	2
Columns in X	3
Columns in Z	6
Subjects	1
Max Obs per Subject	54

Number of Observations	
Number of Observations Read	54
Number of Observations Used	54
Number of Observations Not Used	0

Iteration History			
Iteration	Evaluations	-2 Res Log Like	Criterion
0	1	257.29461395	
1	3	224.10406343	0.00019838
2	1	224.09019781	0.00000429
3	1	224.08991766	0.00000000

Convergence criteria met.

DistSoma=420

Covariance Parameter Estimates				
**Cov Parm**	**Estimate**	**Alpha**	**Lower**	**Upper**
**Animal**	5.9423	0.05	2.0447	58.9455
**Residual**	3.0816	0.05	2.1433	4.8086

Fit Statistics	
**-2 Res Log Likelihood**	224.1
**AIC (Smaller is Better)**	228.1
**AICC (Smaller is Better)**	228.3
**BIC (Smaller is Better)**	227.7

Solution for Fixed Effects									
**Effect**	**Genotype**	**Estimate**	**Standard Error**	**DF**	**t Value**	**Pr > |t|**	**Alpha**	**Lower**	**Upper**
**Intercept**		1.1259	1.4477	4	0.78	0.4802	0.05	-2.8937	5.1455
**Genotype**	Meg +/+	2.2049	2.0514	48	1.07	0.2878	0.05	-1.9198	6.3296
**Genotype**	Meg +/-	0	.	.	.	.	.	.	.

Solution for Random Effects									
**Effect**	**Animal**	**Estimate**	**Std Err Pred**	**DF**	**t Value**	**Pr > |t|**	**Alpha**	**Lower**	**Upper**
**Animal**	1	-3.2030	1.4769	48	-2.17	0.0351	0.05	-6.1726	-0.2334
**Animal**	2	1.3830	1.5041	48	0.92	0.3625	0.05	-1.6413	4.4072
**Animal**	3	1.8201	1.4970	48	1.22	0.2300	0.05	-1.1898	4.8299
**Animal**	4	-1.0646	1.4824	48	-0.72	0.4762	0.05	-4.0452	1.9160
**Animal**	5	-1.0793	1.4738	48	-0.73	0.4675	0.05	-4.0425	1.8840
**Animal**	6	2.1438	1.4922	48	1.44	0.1573	0.05	-0.8564	5.1441

Type 3 Tests of Fixed Effects				
**Effect**	**Num DF**	**Den DF**	**F Value**	**Pr > F**
**Genotype**	1	48	1.16	0.2878

Least Squares Means									
**Effect**	**Genotype**	**Estimate**	**Standard Error**	**DF**	**t Value**	**Pr > |t|**	**Alpha**	**Lower**	**Upper**
**Genotype**	Meg +/+	3.3308	1.4534	48	2.29	0.0263	0.05	0.4086	6.2531
**Genotype**	Meg +/-	1.1259	1.4477	48	0.78	0.4406	0.05	-1.7850	4.0368

DistSoma=420

Differences of Least Squares Means												
**Effect**	**Genotype**	**Genotype**	**Estimate**	**Standard Error**	**DF**	**t Value**	**Pr > |t|**	**Adjustment**	**Adj P**	**Alpha**	**Lower**	**Upper**
**Genotype**	Meg +/+	Meg +/-	2.2049	2.0514	48	1.07	0.2878	Tukey-Kramer	0.2878	0.05	-1.9198	6.3296

Differences of Least Squares Means				
**Effect**	**Genotype**	**Genotype**	**Adj Lower**	**Adj Upper**
**Genotype**	Meg +/+	Meg +/-	-1.9196	6.3294

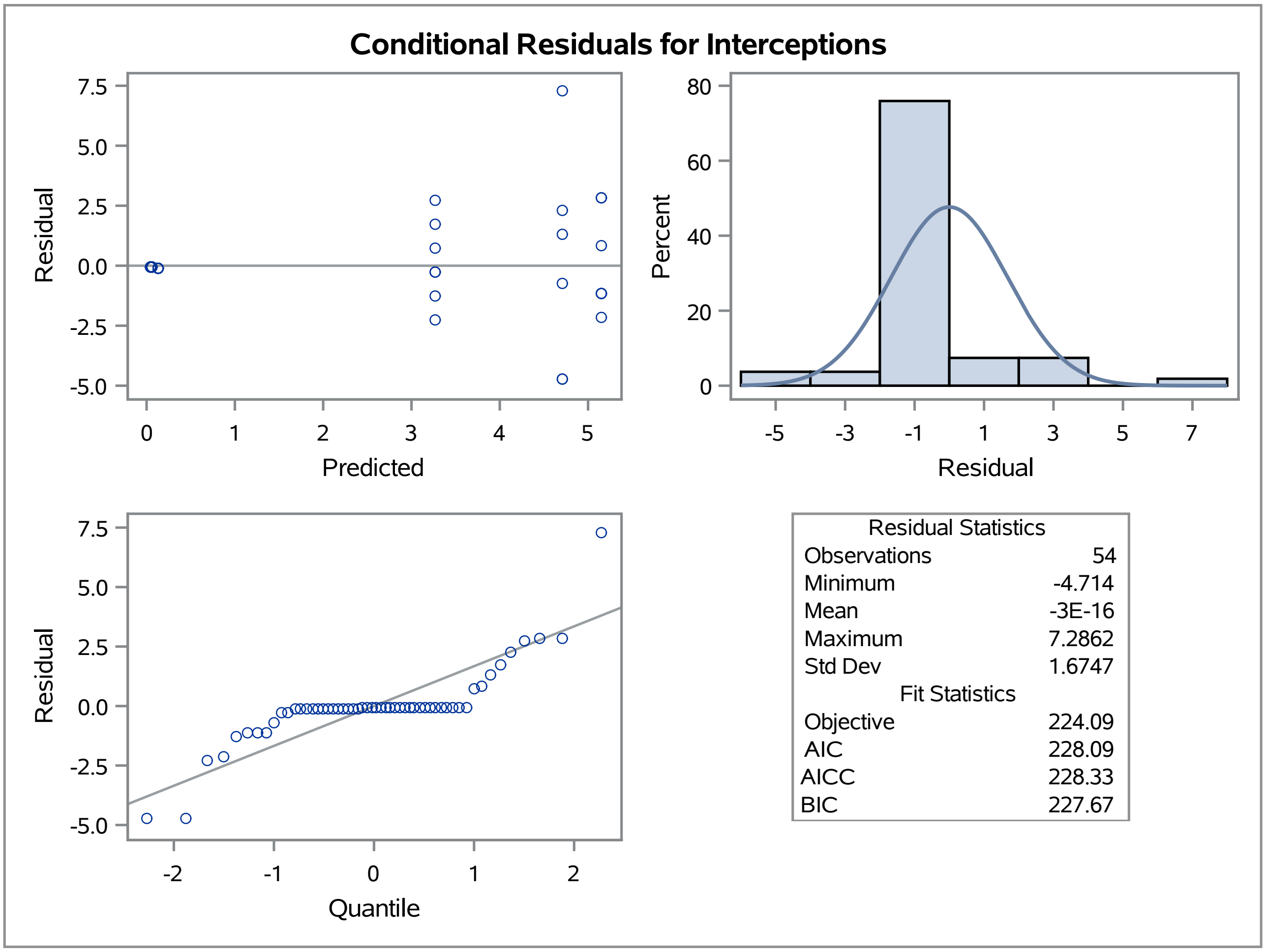

DistSoma=426

Model Information	
**Data Set**	WORK.TEMPDATASORTED
**Dependent Variable**	Interceptions
**Covariance Structure**	Variance Components
**Estimation Method**	REML
**Residual Variance Method**	Profile
**Fixed Effects SE Method**	Model-Based
**Degrees of Freedom Method**	Containment

Class Level Information		
**Class**	**Levels**	**Values**
**Genotype**	2	Meg +/+ Meg +/-
**Animal**	6	1 2 3 4 5 6

Dimensions	
**Covariance Parameters**	2
**Columns in X**	3
**Columns in Z**	6
**Subjects**	1
**Max Obs per Subject**	54

Number of Observations	
**Number of Observations Read**	54
**Number of Observations Used**	54
**Number of Observations Not Used**	0

Iteration History			
**Iteration**	**Evaluations**	**-2 Res Log Like**	**Criterion**
**0**	1	256.74660441	
**1**	3	224.47568034	0.00015126
**2**	1	224.46516684	0.00000256
**3**	1	224.46500002	0.00000000

Convergence criteria met.

DistSoma=426

Covariance Parameter Estimates				
Cov Parm	Estimate	Alpha	Lower	Upper
Animal	5.8051	0.05	1.9943	58.0004
Residual	3.1113	0.05	2.1640	4.8550

Fit Statistics	
-2 Res Log Likelihood	224.5
AIC (Smaller is Better)	228.5
AICC (Smaller is Better)	228.7
BIC (Smaller is Better)	228.0

Solution for Fixed Effects									
Effect	Genotype	Estimate	Standard Error	DF	t Value	Pr > |t|	Alpha	Lower	Upper
Intercept		1.1254	1.4322	4	0.79	0.4760	0.05	-2.8512	5.1019
Genotype	Meg +/+	2.1568	2.0296	48	1.06	0.2932	0.05	-1.9239	6.2376
Genotype	Meg +/-	0	.	.	.	.	.	.	.

Solution for Random Effects									
Effect	Animal	Estimate	Std Err Pred	DF	t Value	Pr > |t|	Alpha	Lower	Upper
Animal	1	-3.1522	1.4619	48	-2.16	0.0361	0.05	-6.0916	-0.2129
Animal	2	1.4239	1.4895	48	0.96	0.3439	0.05	-1.5709	4.4188
Animal	3	1.7283	1.4822	48	1.17	0.2494	0.05	-1.2519	4.7086
Animal	4	-1.0621	1.4675	48	-0.72	0.4727	0.05	-4.0127	1.8884
Animal	5	-1.0773	1.4587	48	-0.74	0.4638	0.05	-4.0102	1.8557
Animal	6	2.1394	1.4774	48	1.45	0.1541	0.05	-0.8312	5.1099

Type 3 Tests of Fixed Effects				
Effect	Num DF	Den DF	F Value	Pr > F
Genotype	1	48	1.13	0.2932

Least Squares Means									
Effect	Genotype	Estimate	Standard Error	DF	t Value	Pr > |t|	Alpha	Lower	Upper
Genotype	Meg +/+	3.2822	1.4380	48	2.28	0.0269	0.05	0.3909	6.1735
Genotype	Meg +/-	1.1254	1.4322	48	0.79	0.4359	0.05	-1.7543	4.0051

DistSoma=426

Differences of Least Squares Means												
Effect	Genotype	Genotype	Estimate	Standard Error	DF	t Value	Pr > |t|	Adjustment	Adj P	Alpha	Lower	Upper
Genotype	Meg +/+	Meg +/-	2.1568	2.0296	48	1.06	0.2932	Tukey-Kramer	0.2932	0.05	-1.9239	6.2376

Differences of Least Squares Means				
Effect	Genotype	Genotype	Adj Lower	Adj Upper
Genotype	Meg +/+	Meg +/-	-1.9238	6.2374

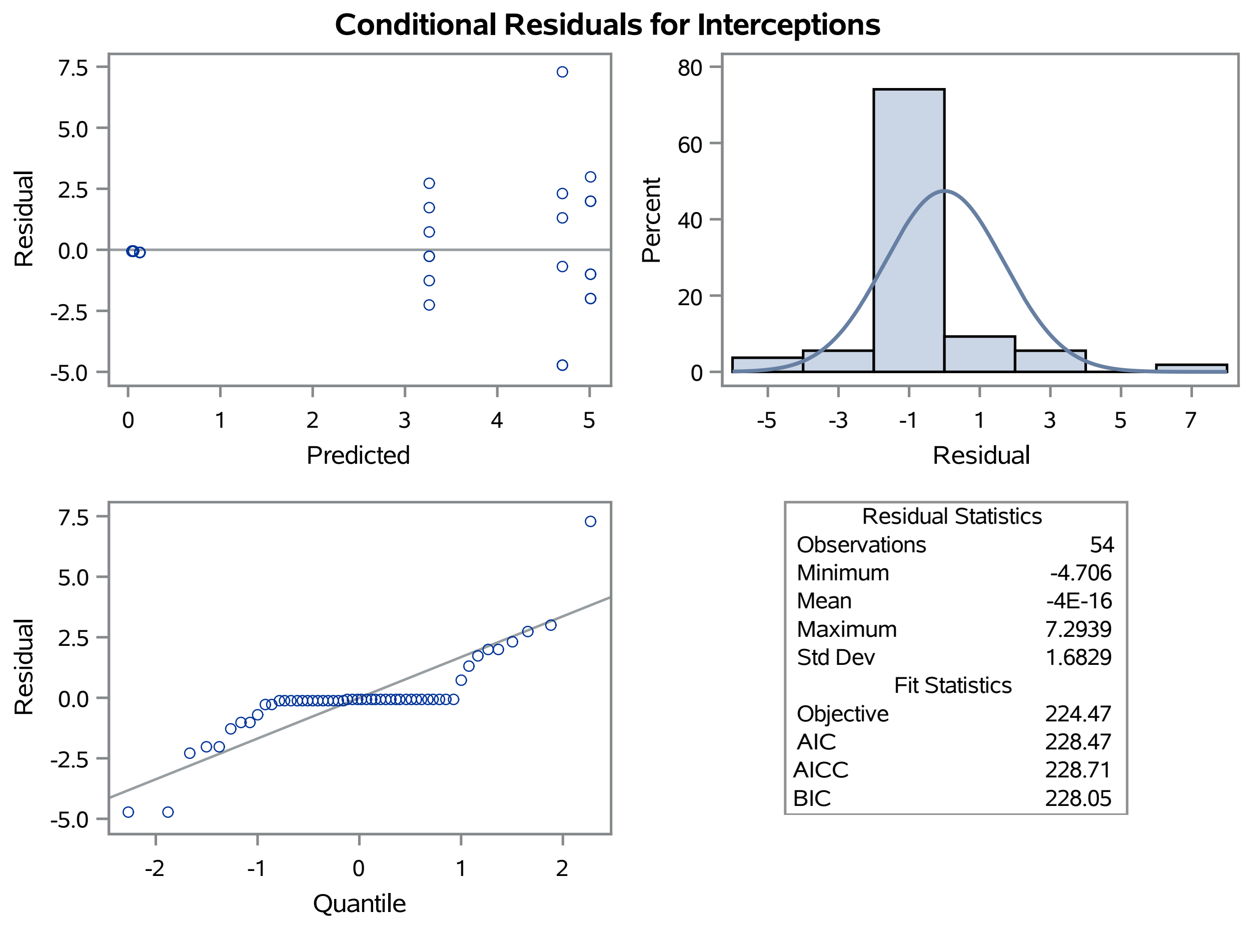

Residual Statistics	
Observations	54
Minimum	-4.706
Mean	-4E-16
Maximum	7.2939
Std Dev	1.6829
Fit Statistics	
Objective	224.47
AIC	228.47
AICC	228.71
BIC	228.05

DistSoma=432

Model Information	
**Data Set**	WORK.TEMPDATASORTED
**Dependent Variable**	Interceptions
**Covariance Structure**	Variance Components
**Estimation Method**	REML
**Residual Variance Method**	Profile
**Fixed Effects SE Method**	Model-Based
**Degrees of Freedom Method**	Containment

Class Level Information		
**Class**	**Levels**	**Values**
**Genotype**	2	Meg +/+ Meg +/-
**Animal**	6	1 2 3 4 5 6

Dimensions	
**Covariance Parameters**	2
**Columns in X**	3
**Columns in Z**	6
**Subjects**	1
**Max Obs per Subject**	54

Number of Observations	
**Number of Observations Read**	54
**Number of Observations Used**	54
**Number of Observations Not Used**	0

Iteration History			
**Iteration**	**Evaluations**	**-2 Res Log Like**	**Criterion**
**0**	1	253.39218040	
**1**	3	219.64667558	0.00024032
**2**	1	219.63037593	0.00000600
**3**	1	219.62999726	0.00000000

Convergence criteria met.

DistSoma=432

Covariance Parameter Estimates				
**Cov Parm**	**Estimate**	**Alpha**	**Lower**	**Upper**
**Animal**	5.5543	0.05	1.9130	54.8626
**Residual**	2.8243	0.05	1.9644	4.4071

Fit Statistics	
**-2 Res Log Likelihood**	219.6
**AIC (Smaller is Better)**	223.6
**AICC (Smaller is Better)**	223.9
**BIC (Smaller is Better)**	223.2

Solution for Fixed Effects									
**Effect**	**Genotype**	**Estimate**	**Standard Error**	**DF**	**t Value**	**Pr > |t|**	**Alpha**	**Lower**	**Upper**
**Intercept**		1.0793	1.3989	4	0.77	0.4835	0.05	-2.8048	4.9634
**Genotype**	Meg +/+	2.1505	1.9822	48	1.08	0.2834	0.05	-1.8350	6.1359
**Genotype**	Meg +/-	0	.	.	.	.	.	.	.

Solution for Random Effects									
**Effect**	**Animal**	**Estimate**	**Std Err Pred**	**DF**	**t Value**	**Pr > |t|**	**Alpha**	**Lower**	**Upper**
**Animal**	1	-3.1082	1.4267	48	-2.18	0.0343	0.05	-5.9767	-0.2396
**Animal**	2	1.3246	1.4525	48	0.91	0.3664	0.05	-1.5959	4.2452
**Animal**	3	1.7835	1.4457	48	1.23	0.2233	0.05	-1.1233	4.6904
**Animal**	4	-1.0216	1.4319	48	-0.71	0.4790	0.05	-3.9006	1.8574
**Animal**	5	-1.0354	1.4237	48	-0.73	0.4706	0.05	-3.8980	1.8271
**Animal**	6	2.0570	1.4412	48	1.43	0.1600	0.05	-0.8407	4.9547

Type 3 Tests of Fixed Effects				
**Effect**	**Num DF**	**Den DF**	**F Value**	**Pr > F**
**Genotype**	1	48	1.18	0.2834

Least Squares Means									
**Effect**	**Genotype**	**Estimate**	**Standard Error**	**DF**	**t Value**	**Pr > |t|**	**Alpha**	**Lower**	**Upper**
**Genotype**	Meg +/+	3.2298	1.4043	48	2.30	0.0258	0.05	0.4062	6.0533
**Genotype**	Meg +/-	1.0793	1.3989	48	0.77	0.4442	0.05	-1.7335	3.8920

DistSoma=432

Differences of Least Squares Means												
Effect	Genotype	Genotype	Estimate	Standard Error	DF	t Value	Pr > |t|	Adjustment	Adj P	Alpha	Lower	Upper
**Genotype**	Meg +/+	Meg +/-	2.1505	1.9822	48	1.08	0.2834	Tukey-Kramer	0.2834	0.05	-1.8350	6.1359

Differences of Least Squares Means				
Effect	Genotype	Genotype	Adj Lower	Adj Upper
**Genotype**	Meg +/+	Meg +/-	-1.8349	6.1358

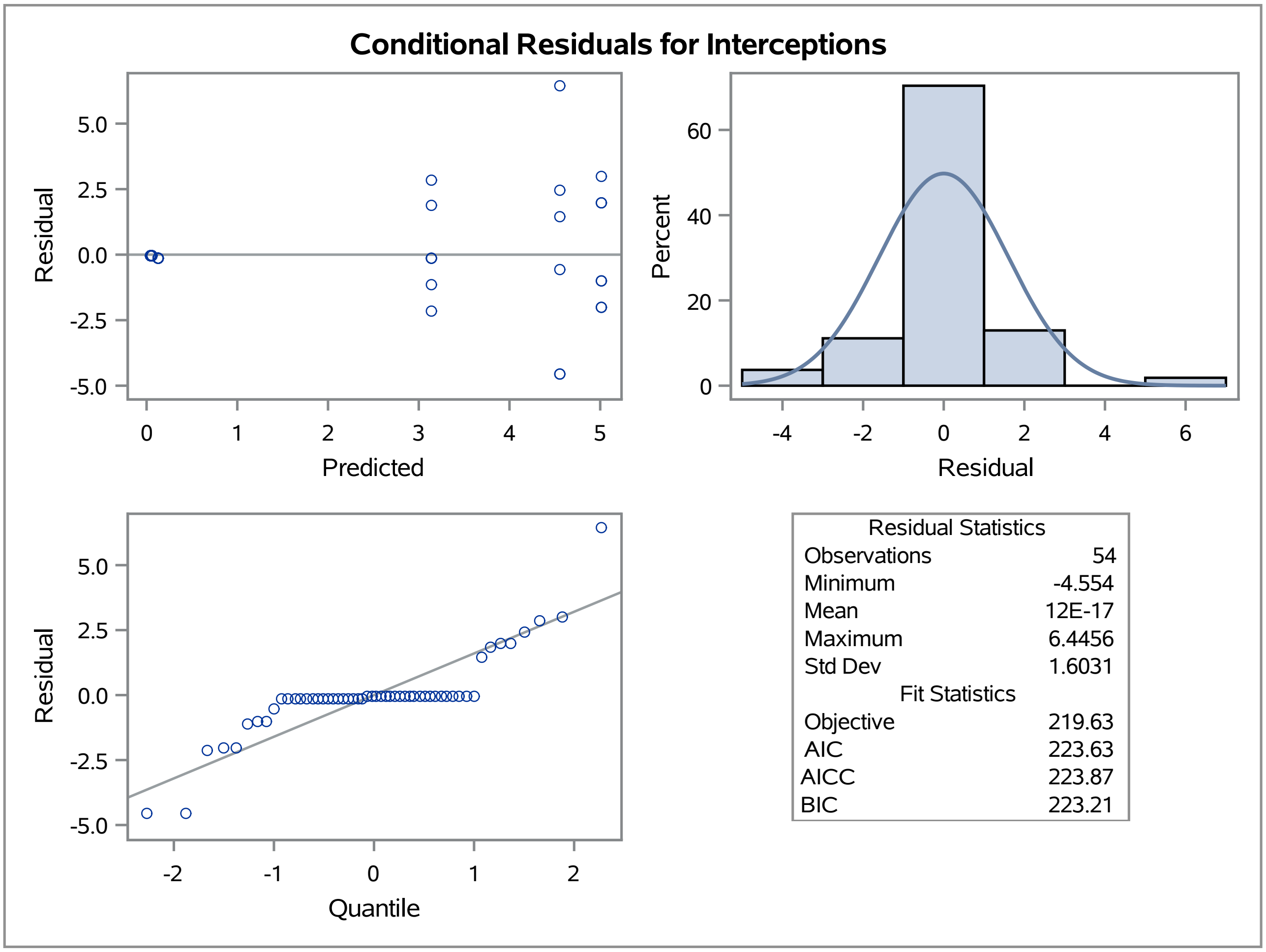

DistSoma=438

Model Information	
**Data Set**	WORK.TEMPDATASORTED
**Dependent Variable**	Interceptions
**Covariance Structure**	Variance Components
**Estimation Method**	REML
**Residual Variance Method**	Profile
**Fixed Effects SE Method**	Model-Based
**Degrees of Freedom Method**	Containment

Class Level Information		
**Class**	**Levels**	**Values**
**Genotype**	2	Meg +/+ Meg +/-
**Animal**	6	1 2 3 4 5 6

Dimensions	
**Covariance Parameters**	2
**Columns in X**	3
**Columns in Z**	6
**Subjects**	1
**Max Obs per Subject**	54

Number of Observations	
**Number of Observations Read**	54
**Number of Observations Used**	54
**Number of Observations Not Used**	0

Iteration History			
**Iteration**	**Evaluations**	**-2 Res Log Like**	**Criterion**
**0**	1	251.22539093	
**1**	3	217.32159082	0.00020469
**2**	1	217.30804943	0.00000434
**3**	1	217.30778139	0.00000000

Convergence criteria met.

DistSoma=438

Covariance Parameter Estimates				
**Cov Parm**	**Estimate**	**Alpha**	**Lower**	**Upper**
**Animal**	5.3518	0.05	1.8436	52.8232
**Residual**	2.6994	0.05	1.8774	4.2122

Fit Statistics	
**-2 Res Log Likelihood**	217.3
**AIC (Smaller is Better)**	221.3
**AICC (Smaller is Better)**	221.6
**BIC (Smaller is Better)**	220.9

Solution for Fixed Effects									
**Effect**	**Genotype**	**Estimate**	**Standard Error**	**DF**	**t Value**	**Pr > |t|**	**Alpha**	**Lower**	**Upper**
**Intercept**		1.0794	1.3729	4	0.79	0.4757	0.05	-2.7324	4.8912
**Genotype**	Meg +/+	2.0415	1.9453	48	1.05	0.2992	0.05	-1.8697	5.9528
**Genotype**	Meg +/-	0	.	.	.	.	.	.	.

Solution for Random Effects									
**Effect**	**Animal**	**Estimate**	**Std Err Pred**	**DF**	**t Value**	**Pr > |t|**	**Alpha**	**Lower**	**Upper**
**Animal**	1	-3.0044	1.4000	48	-2.15	0.0370	0.05	-5.8192	-0.1896
**Animal**	2	1.1184	1.4252	48	0.78	0.4365	0.05	-1.7471	3.9839
**Animal**	3	1.8860	1.4185	48	1.33	0.1899	0.05	-0.9661	4.7382
**Animal**	4	-1.0221	1.4050	48	-0.73	0.4705	0.05	-3.8471	1.8029
**Animal**	5	-1.0359	1.3970	48	-0.74	0.4620	0.05	-3.8448	1.7731
**Animal**	6	2.0580	1.4141	48	1.46	0.1521	0.05	-0.7853	4.9013

Type 3 Tests of Fixed Effects				
**Effect**	**Num DF**	**Den DF**	**F Value**	**Pr > F**
**Genotype**	1	48	1.10	0.2992

Least Squares Means									
**Effect**	**Genotype**	**Estimate**	**Standard Error**	**DF**	**t Value**	**Pr > |t|**	**Alpha**	**Lower**	**Upper**
**Genotype**	Meg +/+	3.1209	1.3781	48	2.26	0.0281	0.05	0.3500	5.8919
**Genotype**	Meg +/-	1.0794	1.3729	48	0.79	0.4356	0.05	-1.6810	3.8398

DistSoma=438

Differences of Least Squares Means												
**Effect**	**Genotype**	**Genotype**	**Estimate**	**Standard Error**	**DF**	**t Value**	**Pr > |t|**	**Adjustment**	**Adj P**	**Alpha**	**Lower**	**Upper**
**Genotype**	Meg +/+	Meg +/-	2.0415	1.9453	48	1.05	0.2992	Tukey-Kramer	0.2992	0.05	-1.8697	5.9528

Differences of Least Squares Means				
**Effect**	**Genotype**	**Genotype**	**Adj Lower**	**Adj Upper**
**Genotype**	Meg +/+	Meg +/-	-1.8696	5.9526

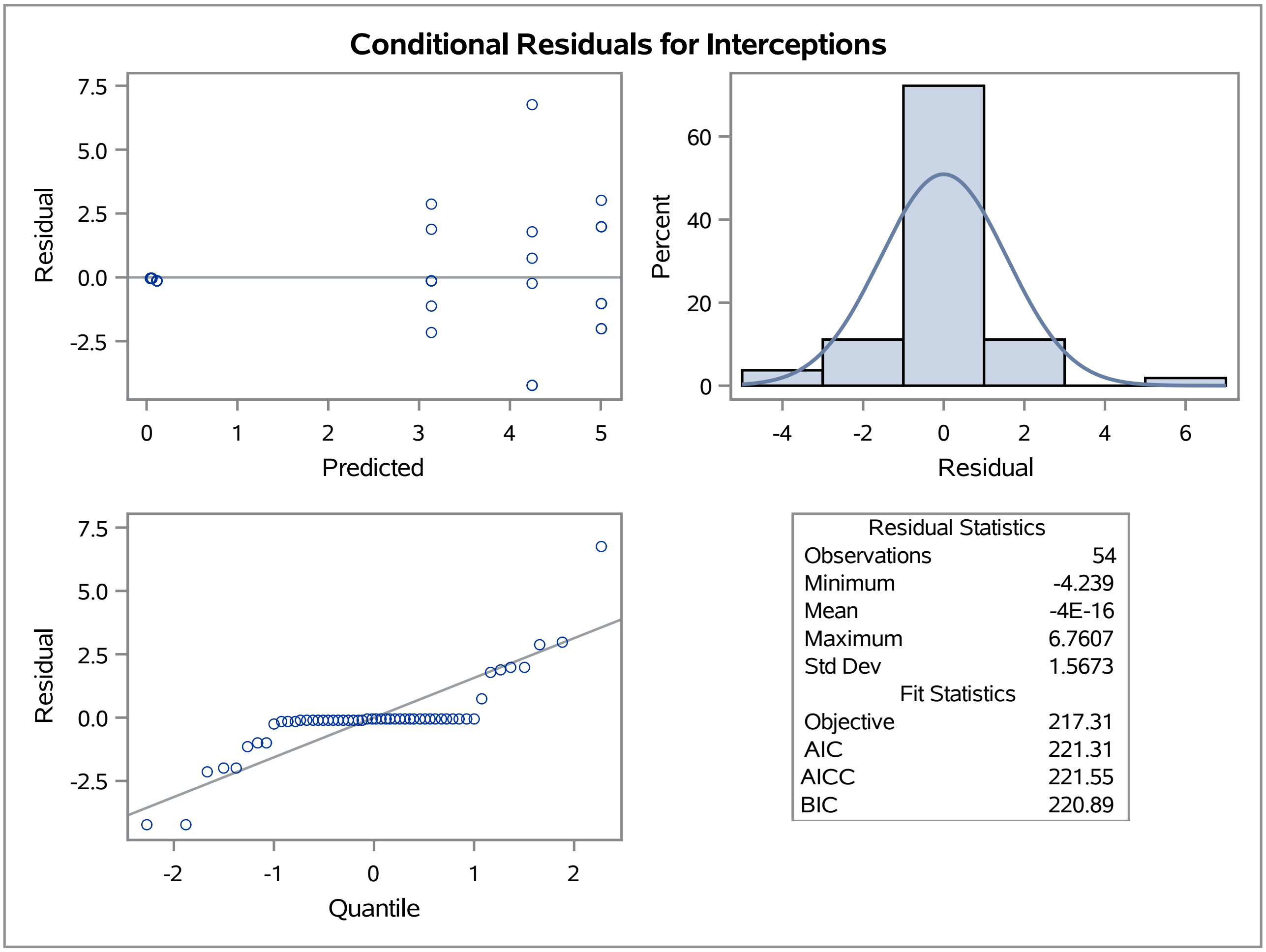

DistSoma=444

Model Information	
**Data Set**	WORK.TEMPDATASORTED
**Dependent Variable**	Interceptions
**Covariance Structure**	Variance Components
**Estimation Method**	REML
**Residual Variance Method**	Profile
**Fixed Effects SE Method**	Model-Based
**Degrees of Freedom Method**	Containment

Class Level Information		
**Class**	**Levels**	**Values**
**Genotype**	2	Meg +/+ Meg +/-
**Animal**	6	1 2 3 4 5 6

Dimensions	
**Covariance Parameters**	2
**Columns in X**	3
**Columns in Z**	6
**Subjects**	1
**Max Obs per Subject**	54

Number of Observations	
**Number of Observations Read**	54
**Number of Observations Used**	54
**Number of Observations Not Used**	0

Iteration History			
**Iteration**	**Evaluations**	**-2 Res Log Like**	**Criterion**
**0**	1	248.84620911	
**1**	3	216.85671881	0.00011238
**2**	1	216.84944491	0.00000136
**3**	1	216.84936168	0.00000000

Convergence criteria met.

DistSoma=444

Covariance Parameter Estimates				
**Cov Parm**	**Estimate**	**Alpha**	**Lower**	**Upper**
**Animal**	4.9798	0.05	1.7099	49.8743
**Residual**	2.6889	0.05	1.8702	4.1958

Fit Statistics	
**-2 Res Log Likelihood**	216.8
**AIC (Smaller is Better)**	220.8
**AICC (Smaller is Better)**	221.1
**BIC (Smaller is Better)**	220.4

Solution for Fixed Effects									
**Effect**	**Genotype**	**Estimate**	**Standard Error**	**DF**	**t Value**	**Pr > |t|**	**Alpha**	**Lower**	**Upper**
**Intercept**		1.0784	1.3268	4	0.81	0.4620	0.05	-2.6055	4.7622
**Genotype**	Meg +/+	1.8910	1.8802	48	1.01	0.3196	0.05	-1.8894	5.6714
**Genotype**	Meg +/-	0	.	.	.	.	.	.	.

Solution for Random Effects									
**Effect**	**Animal**	**Estimate**	**Std Err Pred**	**DF**	**t Value**	**Pr > |t|**	**Alpha**	**Lower**	**Upper**
**Animal**	1	-2.8510	1.3545	48	-2.10	0.0406	0.05	-5.5743	-0.1276
**Animal**	2	1.0984	1.3802	48	0.80	0.4300	0.05	-1.6766	3.8735
**Animal**	3	1.7526	1.3734	48	1.28	0.2081	0.05	-1.0089	4.5140
**Animal**	4	-1.0173	1.3597	48	-0.75	0.4580	0.05	-3.7511	1.7164
**Animal**	5	-1.0319	1.3515	48	-0.76	0.4489	0.05	-3.7493	1.6854
**Animal**	6	2.0493	1.3689	48	1.50	0.1409	0.05	-0.7031	4.8017

Type 3 Tests of Fixed Effects				
**Effect**	**Num DF**	**Den DF**	**F Value**	**Pr > F**
**Genotype**	1	48	1.01	0.3196

Least Squares Means									
**Effect**	**Genotype**	**Estimate**	**Standard Error**	**DF**	**t Value**	**Pr > |t|**	**Alpha**	**Lower**	**Upper**
**Genotype**	Meg +/+	2.9694	1.3322	48	2.23	0.0305	0.05	0.2908	5.6479
**Genotype**	Meg +/-	1.0784	1.3268	48	0.81	0.4204	0.05	-1.5894	3.7461

DistSoma=444

Differences of Least Squares Means												
Effect	Genotype	Genotype	Estimate	Standard Error	DF	t Value	Pr > |t|	Adjustment	Adj P	Alpha	Lower	Upper
Genotype	Meg +/+	Meg +/-	1.8910	1.8802	48	1.01	0.3196	Tukey-Kramer	0.3196	0.05	-1.8894	5.6714

Differences of Least Squares Means				
Effect	Genotype	Genotype	Adj Lower	Adj Upper
Genotype	Meg +/+	Meg +/-	-1.8893	5.6713

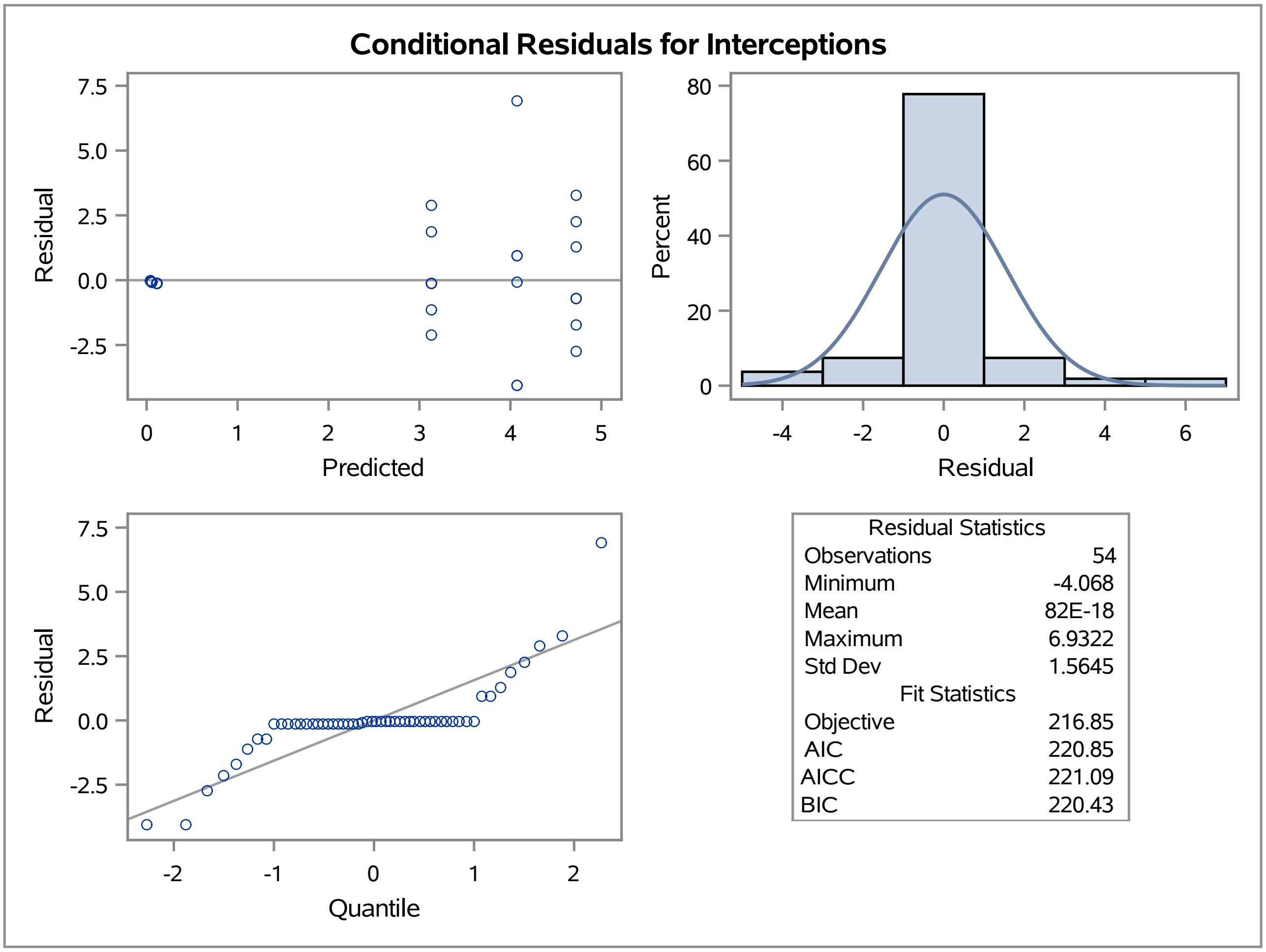

DistSoma=450

Model Information	
**Data Set**	WORK.TEMPDATASORTED
**Dependent Variable**	Interceptions
**Covariance Structure**	Variance Components
**Estimation Method**	REML
**Residual Variance Method**	Profile
**Fixed Effects SE Method**	Model-Based
**Degrees of Freedom Method**	Containment

Class Level Information		
**Class**	**Levels**	**Values**
**Genotype**	2	Meg +/+ Meg +/-
**Animal**	6	1 2 3 4 5 6

Dimensions	
**Covariance Parameters**	2
**Columns in X**	3
**Columns in Z**	6
**Subjects**	1
**Max Obs per Subject**	54

Number of Observations	
**Number of Observations Read**	54
**Number of Observations Used**	54
**Number of Observations Not Used**	0

Iteration History			
**Iteration**	**Evaluations**	**-2 Res Log Like**	**Criterion**
**0**	1	241.80827211	
**1**	3	211.22460214	0.00005870
**2**	1	211.22103972	0.00000037
**3**	1	211.22101840	0.00000000

Convergence criteria met.

DistSoma=450

Covariance Parameter Estimates				
**Cov Parm**	**Estimate**	**Alpha**	**Lower**	**Upper**
**Animal**	4.2689	0.05	1.4621	43.2535
**Residual**	2.4217	0.05	1.6843	3.7789

Fit Statistics	
**-2 Res Log Likelihood**	211.2
**AIC (Smaller is Better)**	215.2
**AICC (Smaller is Better)**	215.5
**BIC (Smaller is Better)**	214.8

Solution for Fixed Effects									
**Effect**	**Genotype**	**Estimate**	**Standard Error**	**DF**	**t Value**	**Pr > |t|**	**Alpha**	**Lower**	**Upper**
**Intercept**		1.0307	1.2302	4	0.84	0.4493	0.05	-2.3849	4.4464
**Genotype**	Meg +/+	1.6934	1.7435	48	0.97	0.3363	0.05	-1.8121	5.1989
**Genotype**	Meg +/-	0	.	.	.	.	.	.	.

Solution for Random Effects									
**Effect**	**Animal**	**Estimate**	**Std Err Pred**	**DF**	**t Value**	**Pr > |t|**	**Alpha**	**Lower**	**Upper**
**Animal**	1	-2.6102	1.2569	48	-2.08	0.0432	0.05	-5.1374	-0.08301
**Animal**	2	1.1657	1.2817	48	0.91	0.3676	0.05	-1.4114	3.7427
**Animal**	3	1.4445	1.2752	48	1.13	0.2629	0.05	-1.1194	4.0084
**Animal**	4	-0.9696	1.2619	48	-0.77	0.4460	0.05	-3.5069	1.5676
**Animal**	5	-0.9842	1.2540	48	-0.78	0.4364	0.05	-3.5056	1.5372
**Animal**	6	1.9538	1.2709	48	1.54	0.1308	0.05	-0.6014	4.5090

Type 3 Tests of Fixed Effects				
**Effect**	**Num DF**	**Den DF**	**F Value**	**Pr > F**
**Genotype**	1	48	0.94	0.3363

Least Squares Means									
**Effect**	**Genotype**	**Estimate**	**Standard Error**	**DF**	**t Value**	**Pr > |t|**	**Alpha**	**Lower**	**Upper**
**Genotype**	Meg +/+	2.7241	1.2354	48	2.20	0.0323	0.05	0.2401	5.2081
**Genotype**	Meg +/-	1.0307	1.2302	48	0.84	0.4063	0.05	-1.4428	3.5043

DistSoma=450

Differences of Least Squares Means												
**Effect**	**Genotype**	**Genotype**	**Estimate**	**Standard Error**	**DF**	**t Value**	**Pr > |t|**	**Adjustment**	**Adj P**	**Alpha**	**Lower**	**Upper**
**Genotype**	Meg +/+	Meg +/-	1.6934	1.7435	48	0.97	0.3363	Tukey-Kramer	0.3363	0.05	-1.8121	5.1989

Differences of Least Squares Means				
**Effect**	**Genotype**	**Genotype**	**Adj Lower**	**Adj Upper**
**Genotype**	Meg +/+	Meg +/-	-1.8120	5.1988

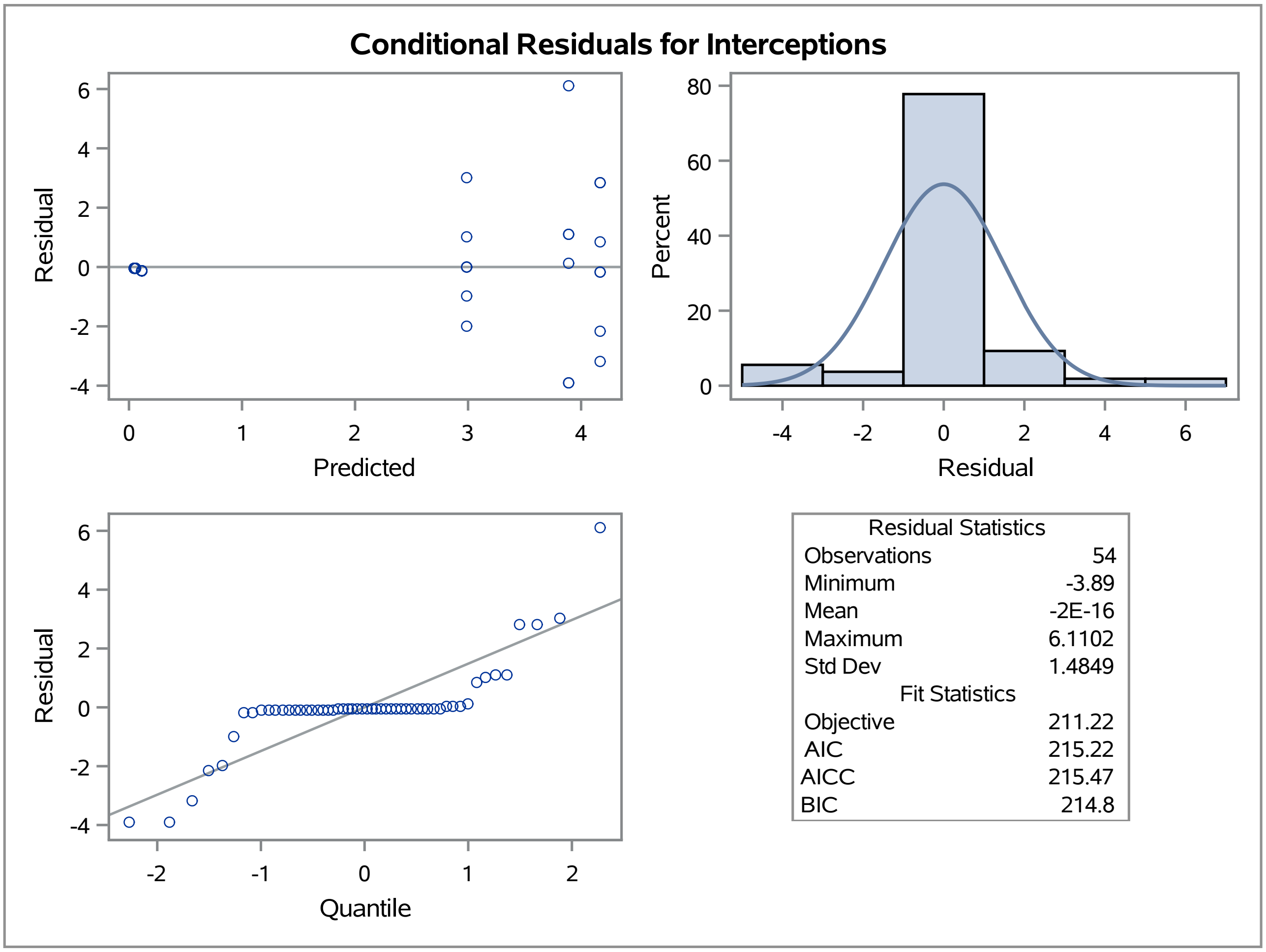

DistSoma=456

Model Information	
**Data Set**	WORK.TEMPDATASORTED
**Dependent Variable**	Interceptions
**Covariance Structure**	Variance Components
**Estimation Method**	REML
**Residual Variance Method**	Profile
**Fixed Effects SE Method**	Model-Based
**Degrees of Freedom Method**	Containment

Class Level Information		
**Class**	**Levels**	**Values**
**Genotype**	2	Meg +/+ Meg +/-
**Animal**	6	1 2 3 4 5 6

Dimensions	
**Covariance Parameters**	2
**Columns in X**	3
**Columns in Z**	6
**Subjects**	1
**Max Obs per Subject**	54

Number of Observations	
**Number of Observations Read**	54
**Number of Observations Used**	54
**Number of Observations Not Used**	0

Iteration History			
**Iteration**	**Evaluations**	**-2 Res Log Like**	**Criterion**
**0**	1	240.89496765	
**1**	3	210.89207832	0.00004080
**2**	1	210.88962721	0.00000018
**3**	1	210.88961684	0.00000000

Convergence criteria met.

DistSoma=456

Covariance Parameter Estimates				
**Cov Parm**	**Estimate**	**Alpha**	**Lower**	**Upper**
**Animal**	4.1629	0.05	1.4242	42.3938
**Residual**	2.4098	0.05	1.6760	3.7603

Fit Statistics	
**-2 Res Log Likelihood**	210.9
**AIC (Smaller is Better)**	214.9
**AICC (Smaller is Better)**	215.1
**BIC (Smaller is Better)**	214.5

Solution for Fixed Effects									
**Effect**	**Genotype**	**Estimate**	**Standard Error**	**DF**	**t Value**	**Pr > |t|**	**Alpha**	**Lower**	**Upper**
**Intercept**		1.0304	1.2156	4	0.85	0.4444	0.05	-2.3446	4.4054
**Genotype**	Meg +/+	1.6457	1.7228	48	0.96	0.3442	0.05	-1.8182	5.1097
**Genotype**	Meg +/-	0	.	.	.	.	.	.	.

Solution for Random Effects									
**Effect**	**Animal**	**Estimate**	**Std Err Pred**	**DF**	**t Value**	**Pr > |t|**	**Alpha**	**Lower**	**Upper**
**Animal**	1	-2.5621	1.2424	48	-2.06	0.0446	0.05	-5.0601	-0.06404
**Animal**	2	1.2074	1.2673	48	0.95	0.3455	0.05	-1.3407	3.7554
**Animal**	3	1.3547	1.2607	48	1.07	0.2880	0.05	-1.1802	3.8896
**Animal**	4	-0.9681	1.2474	48	-0.78	0.4415	0.05	-3.4762	1.5400
**Animal**	5	-0.9830	1.2395	48	-0.79	0.4317	0.05	-3.4752	1.5092
**Animal**	6	1.9511	1.2564	48	1.55	0.1270	0.05	-0.5750	4.4773

Type 3 Tests of Fixed Effects				
**Effect**	**Num DF**	**Den DF**	**F Value**	**Pr > F**
**Genotype**	1	48	0.91	0.3442

Least Squares Means									
**Effect**	**Genotype**	**Estimate**	**Standard Error**	**DF**	**t Value**	**Pr > |t|**	**Alpha**	**Lower**	**Upper**
**Genotype**	Meg +/+	2.6761	1.2208	48	2.19	0.0333	0.05	0.2215	5.1308
**Genotype**	Meg +/-	1.0304	1.2156	48	0.85	0.4008	0.05	-1.4137	3.4745

DistSoma=456

Differences of Least Squares Means												
Effect	Genotype	Genotype	Estimate	Standard Error	DF	t Value	Pr > |t|	Adjustment	Adj P	Alpha	Lower	Upper
**Genotype**	Meg +/+	Meg +/-	1.6457	1.7228	48	0.96	0.3442	Tukey-Kramer	0.3442	0.05	-1.8182	5.1097

Differences of Least Squares Means				
Effect	Genotype	Genotype	Adj Lower	Adj Upper
**Genotype**	Meg +/+	Meg +/-	-1.8181	5.1096

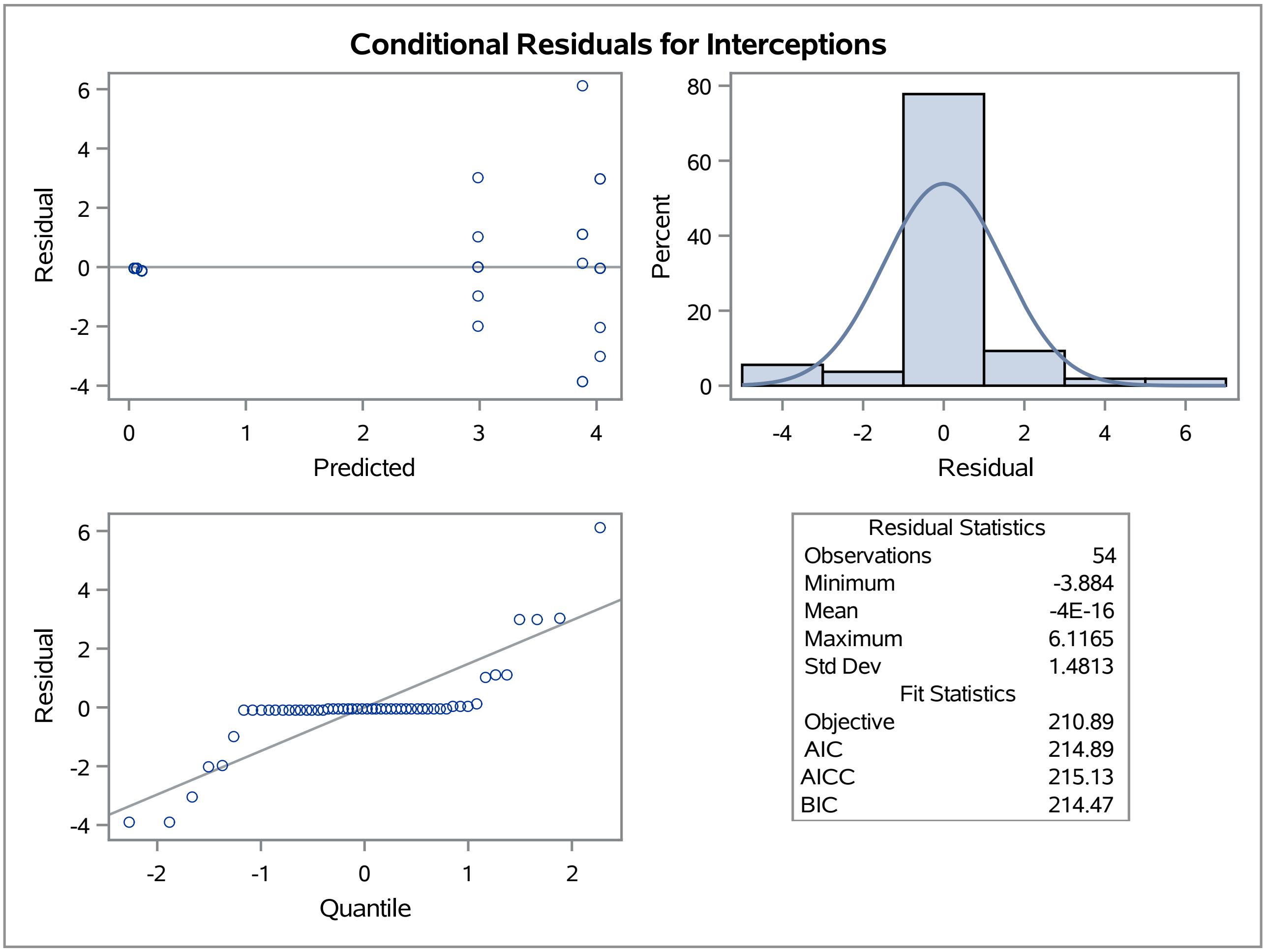

DistSoma=462

Model Information	
**Data Set**	WORK.TEMPDATASORTED
**Dependent Variable**	Interceptions
**Covariance Structure**	Variance Components
**Estimation Method**	REML
**Residual Variance Method**	Profile
**Fixed Effects SE Method**	Model-Based
**Degrees of Freedom Method**	Containment

Class Level Information		
**Class**	**Levels**	**Values**
**Genotype**	2	Meg +/+ Meg +/-
**Animal**	6	1 2 3 4 5 6

Dimensions	
**Covariance Parameters**	2
**Columns in X**	3
**Columns in Z**	6
**Subjects**	1
**Max Obs per Subject**	54

Number of Observations	
**Number of Observations Read**	54
**Number of Observations Used**	54
**Number of Observations Not Used**	0

Iteration History			
**Iteration**	**Evaluations**	**-2 Res Log Like**	**Criterion**
**0**	1	239.92100164	
**1**	3	213.28221582	0.00000766
**2**	1	213.28175611	0.00000001

Convergence criteria met.

Covariance Parameter Estimates				
**Cov Parm**	**Estimate**	**Alpha**	**Lower**	**Upper**
**Animal**	3.8640	0.05	1.3128	40.6199
**Residual**	2.5468	0.05	1.7713	3.9742

DistSoma=462

Fit Statistics	
-2 Res Log Likelihood	213.3
AIC (Smaller is Better)	217.3
AICC (Smaller is Better)	217.5
BIC (Smaller is Better)	216.9

Solution for Fixed Effects

Effect	Genotype	Estimate	Standard Error	DF	t Value	Pr > |t|	Alpha	Lower	Upper
Intercept		1.0282	1.1761	4	0.87	0.4313	0.05	-2.2371	4.2935
Genotype	Meg +/+	1.5014	1.6673	48	0.90	0.3723	0.05	-1.8508	4.8537
Genotype	Meg +/-	0	.	.	.	.	.	.	.

Solution for Random Effects

Effect	Animal	Estimate	Std Err Pred	DF	t Value	Pr > |t|	Alpha	Lower	Upper
Animal	1	-2.4076	1.2048	48	-2.00	0.0514	0.05	-4.8300	0.01484
Animal	2	1.3248	1.2314	48	1.08	0.2873	0.05	-1.1510	3.8007
Animal	3	1.0827	1.2244	48	0.88	0.3809	0.05	-1.3791	3.5445
Animal	4	-0.9580	1.2102	48	-0.79	0.4325	0.05	-3.3912	1.4752
Animal	5	-0.9747	1.2017	48	-0.81	0.4213	0.05	-3.3908	1.4415
Animal	6	1.9327	1.2198	48	1.58	0.1197	0.05	-0.5198	4.3852

Type 3 Tests of Fixed Effects

Effect	Num DF	Den DF	F Value	Pr > F
Genotype	1	48	0.81	0.3723

Least Squares Means

Effect	Genotype	Estimate	Standard Error	DF	t Value	Pr > |t|	Alpha	Lower	Upper
Genotype	Meg +/+	2.5296	1.1818	48	2.14	0.0374	0.05	0.1535	4.9058
Genotype	Meg +/-	1.0282	1.1761	48	0.87	0.3863	0.05	-1.3365	3.3928

DistSoma=462

Differences of Least Squares Means												
Effect	Genotype	Genotype	Estimate	Standard Error	DF	t Value	Pr > |t|	Adjustment	Adj P	Alpha	Lower	Upper
Genotype	Meg +/+	Meg +/-	1.5014	1.6673	48	0.90	0.3723	Tukey-Kramer	0.3723	0.05	-1.8508	4.8537

Differences of Least Squares Means				
Effect	Genotype	Genotype	Adj Lower	Adj Upper
Genotype	Meg +/+	Meg +/-	-1.8507	4.8536

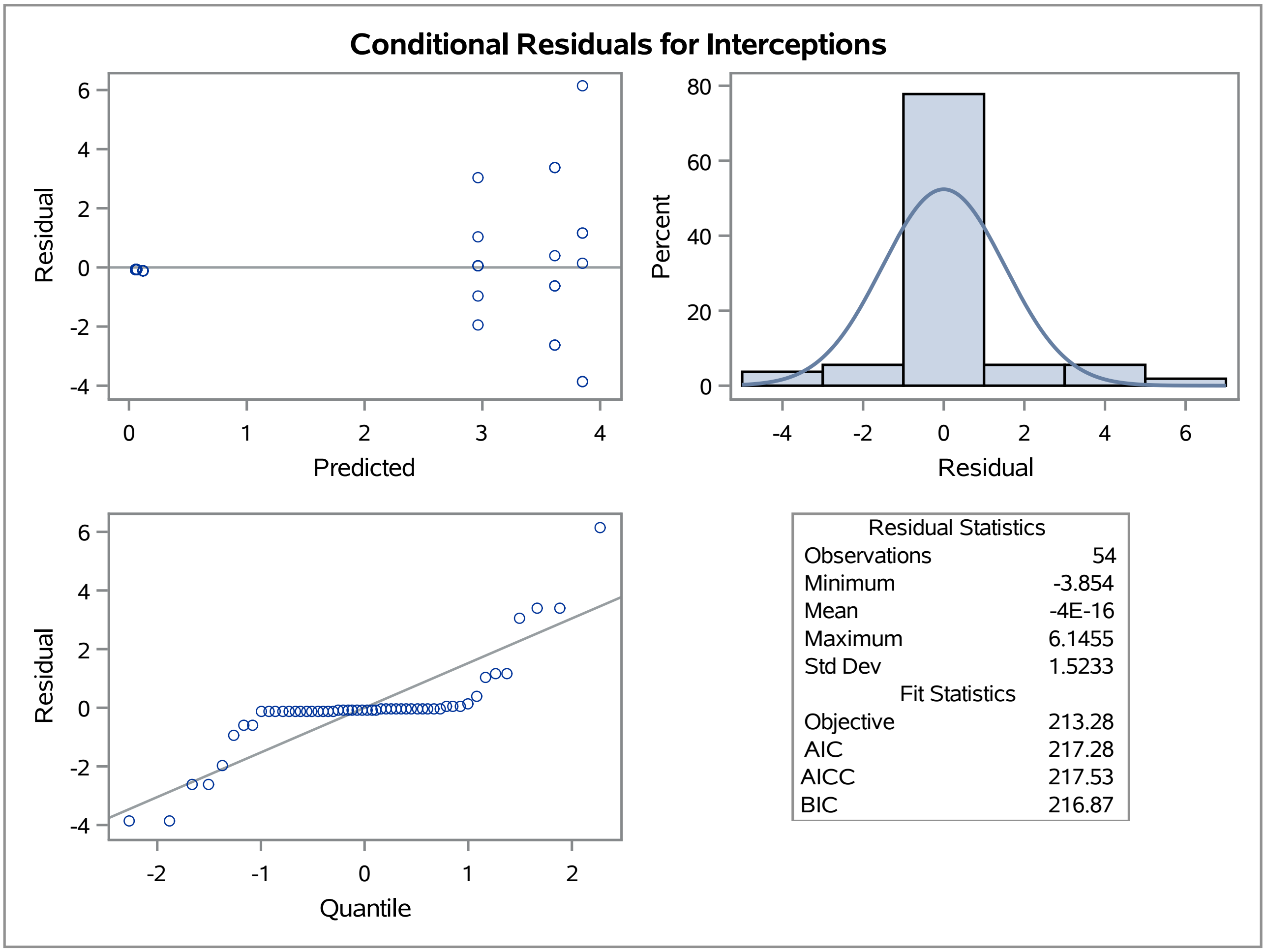

DistSoma=468

Model Information	
**Data Set**	WORK.TEMPDATASORTED
**Dependent Variable**	Interceptions
**Covariance Structure**	Variance Components
**Estimation Method**	REML
**Residual Variance Method**	Profile
**Fixed Effects SE Method**	Model-Based
**Degrees of Freedom Method**	Containment

Class Level Information		
**Class**	**Levels**	**Values**
**Genotype**	2	Meg +/+ Meg +/-
**Animal**	6	1 2 3 4 5 6

Dimensions	
**Covariance Parameters**	2
**Columns in X**	3
**Columns in Z**	6
**Subjects**	1
**Max Obs per Subject**	54

Number of Observations	
**Number of Observations Read**	54
**Number of Observations Used**	54
**Number of Observations Not Used**	0

Iteration History			
**Iteration**	**Evaluations**	**-2 Res Log Like**	**Criterion**
**0**	1	238.79264592	
**1**	2	214.97728604	0.00000147
**2**	1	214.97719756	0.00000000

Convergence criteria met.

Covariance Parameter Estimates				
**Cov Parm**	**Estimate**	**Alpha**	**Lower**	**Upper**
**Animal**	3.5766	0.05	1.2059	38.9592
**Residual**	2.6534	0.05	1.8454	4.1405

DistSoma=468

Fit Statistics	
**-2 Res Log Likelihood**	215.0
**AIC (Smaller is Better)**	219.0
**AICC (Smaller is Better)**	219.2
**BIC (Smaller is Better)**	218.6

Solution for Fixed Effects

Effect	Genotype	Estimate	Standard Error	DF	t Value	Pr > |t|	Alpha	Lower	Upper
**Intercept**		1.0260	1.1363	4	0.90	0.4177	0.05	-2.1290	4.1809
**Genotype**	Meg +/+	1.3504	1.6114	48	0.84	0.4062	0.05	-1.8895	4.5903
**Genotype**	Meg +/-	0	.	.	.	.	.	.	.

Solution for Random Effects

Effect	Animal	Estimate	Std Err Pred	DF	t Value	Pr > |t|	Alpha	Lower	Upper
**Animal**	1	-2.2481	1.1667	48	-1.93	0.0599	0.05	-4.5939	0.09777
**Animal**	2	1.2967	1.1947	48	1.09	0.2832	0.05	-1.1055	3.6988
**Animal**	3	0.9514	1.1874	48	0.80	0.4269	0.05	-1.4359	3.3387
**Animal**	4	-0.9478	1.1724	48	-0.81	0.4228	0.05	-3.3050	1.4094
**Animal**	5	-0.9662	1.1634	48	-0.83	0.4104	0.05	-3.3054	1.3730
**Animal**	6	1.9140	1.1825	48	1.62	0.1121	0.05	-0.4636	4.2917

Type 3 Tests of Fixed Effects

Effect	Num DF	Den DF	F Value	Pr > F
**Genotype**	1	48	0.70	0.4062

Least Squares Means

Effect	Genotype	Estimate	Standard Error	DF	t Value	Pr > |t|	Alpha	Lower	Upper
**Genotype**	Meg +/+	2.3763	1.1425	48	2.08	0.0429	0.05	0.07925	4.6734
**Genotype**	Meg +/-	1.0260	1.1363	48	0.90	0.3711	0.05	-1.2588	3.3107

DistSoma=468

Differences of Least Squares Means												
**Effect**	**Genotype**	**Genotype**	**Estimate**	**Standard Error**	**DF**	**t Value**	**Pr > |t|**	**Adjustment**	**Adj P**	**Alpha**	**Lower**	**Upper**
**Genotype**	Meg +/+	Meg +/-	1.3504	1.6114	48	0.84	0.4062	Tukey-Kramer	0.4062	0.05	-1.8895	4.5903

Differences of Least Squares Means				
**Effect**	**Genotype**	**Genotype**	**Adj Lower**	**Adj Upper**
**Genotype**	Meg +/+	Meg +/-	-1.8894	4.5902

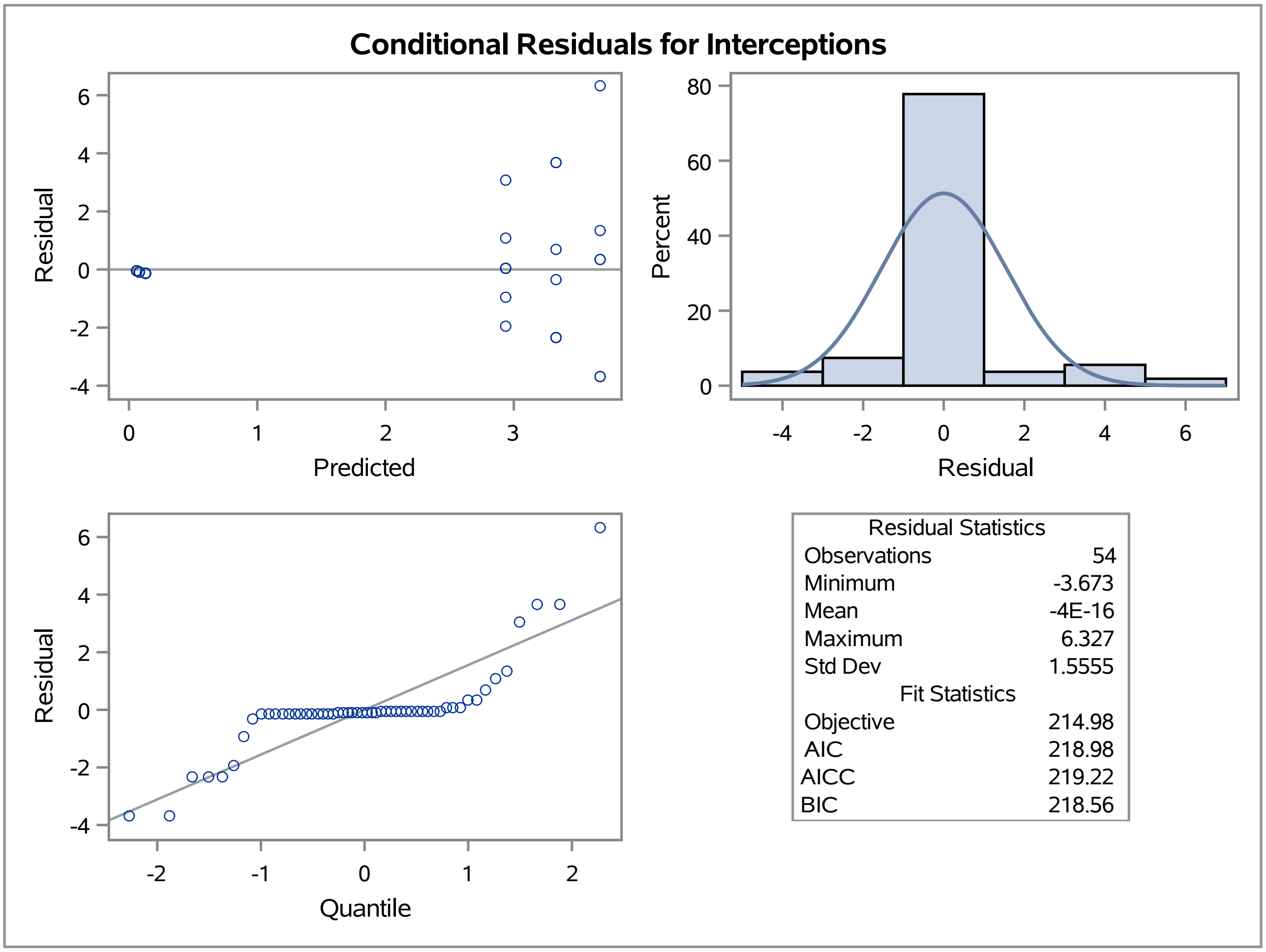

DistSoma=474

Model Information	
**Data Set**	WORK.TEMPDATASORTED
**Dependent Variable**	Interceptions
**Covariance Structure**	Variance Components
**Estimation Method**	REML
**Residual Variance Method**	Profile
**Fixed Effects SE Method**	Model-Based
**Degrees of Freedom Method**	Containment

Class Level Information		
**Class**	**Levels**	**Values**
**Genotype**	2	Meg +/+ Meg +/-
**Animal**	6	1 2 3 4 5 6

Dimensions	
**Covariance Parameters**	2
**Columns in X**	3
**Columns in Z**	6
**Subjects**	1
**Max Obs per Subject**	54

Number of Observations	
**Number of Observations Read**	54
**Number of Observations Used**	54
**Number of Observations Not Used**	0

Iteration History			
**Iteration**	**Evaluations**	**-2 Res Log Like**	**Criterion**
**0**	1	240.44268453	
**1**	2	219.21387719	0.00000088
**2**	1	219.21382211	0.00000000

Convergence criteria met.

Covariance Parameter Estimates				
**Cov Parm**	**Estimate**	**Alpha**	**Lower**	**Upper**
**Animal**	3.4584	0.05	1.1564	39.1893
**Residual**	2.9037	0.05	2.0196	4.5312

DistSoma=474

Fit Statistics	
-2 Res Log Likelihood	219.2
AIC (Smaller is Better)	223.2
AICC (Smaller is Better)	223.5
BIC (Smaller is Better)	222.8

Solution for Fixed Effects

Effect	Genotype	Estimate	Standard Error	DF	t Value	Pr > |t|	Alpha	Lower	Upper
Intercept		0.9769	1.1230	4	0.87	0.4335	0.05	-2.1412	4.0949
Genotype	Meg +/+	1.4010	1.5930	48	0.88	0.3835	0.05	-1.8019	4.6039
Genotype	Meg +/-	0	.	.	.	.	.	.	.

Solution for Random Effects

Effect	Animal	Estimate	Std Err Pred	DF	t Value	Pr > |t|	Alpha	Lower	Upper
Animal	1	-2.2336	1.1559	48	-1.93	0.0592	0.05	-4.5577	0.09043
Animal	2	1.4230	1.1861	48	1.20	0.2361	0.05	-0.9618	3.8078
Animal	3	0.8106	1.1782	48	0.69	0.4947	0.05	-1.5582	3.1794
Animal	4	-0.8935	1.1620	48	-0.77	0.4457	0.05	-3.2299	1.4429
Animal	5	-0.9130	1.1523	48	-0.79	0.4321	0.05	-3.2298	1.4039
Animal	6	1.8065	1.1730	48	1.54	0.1301	0.05	-0.5520	4.1649

Type 3 Tests of Fixed Effects

Effect	Num DF	Den DF	F Value	Pr > F
Genotype	1	48	0.77	0.3835

Least Squares Means
[truncated: 521,083 more chars]
