# Supplementary material for: Neuronal megalin mediates synaptic plasticity—a novel mechanism underlying intellectual disabilities in megalin gene pathologies
Source: Brain Commun. 2020 Aug 25;2(2):fcaa135. doi: 10.1093/braincomms/fcaa135 (PMC7667529; doi:10.1093/braincomms/fcaa135)

Whole original membranes in Western blot analysis of Figure 1

- Fig. 1B (Kidney)

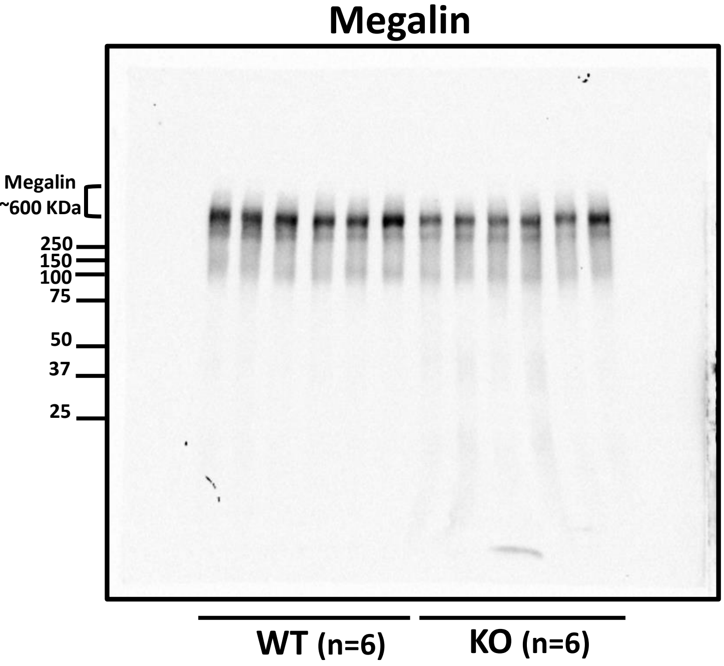

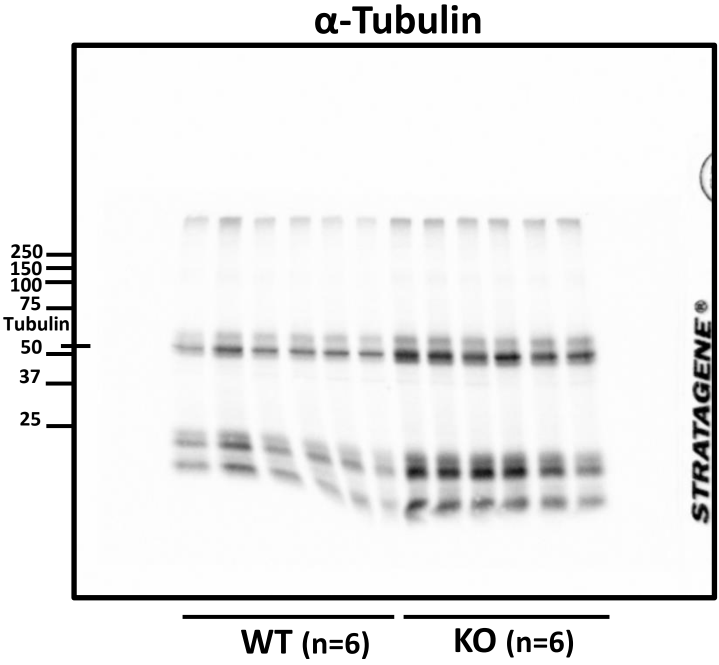

- Fig. 1D (Chroid Plexus)

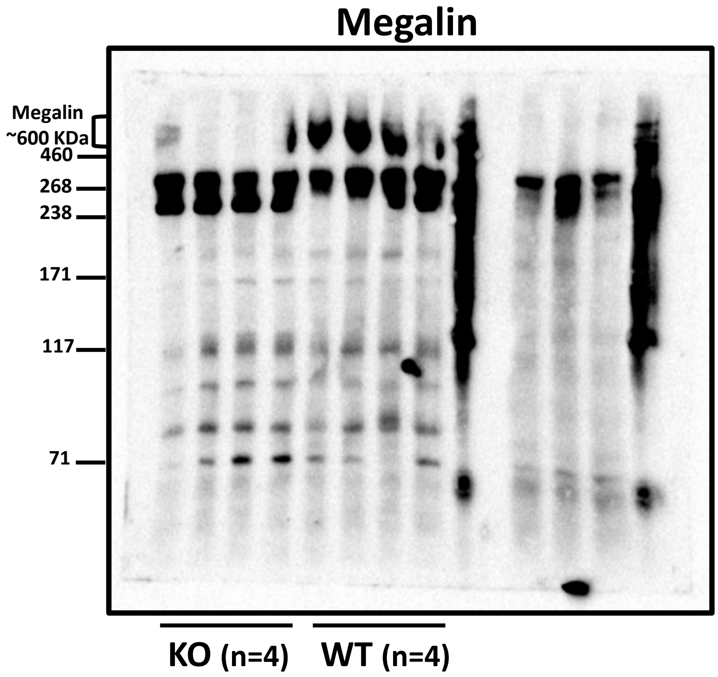

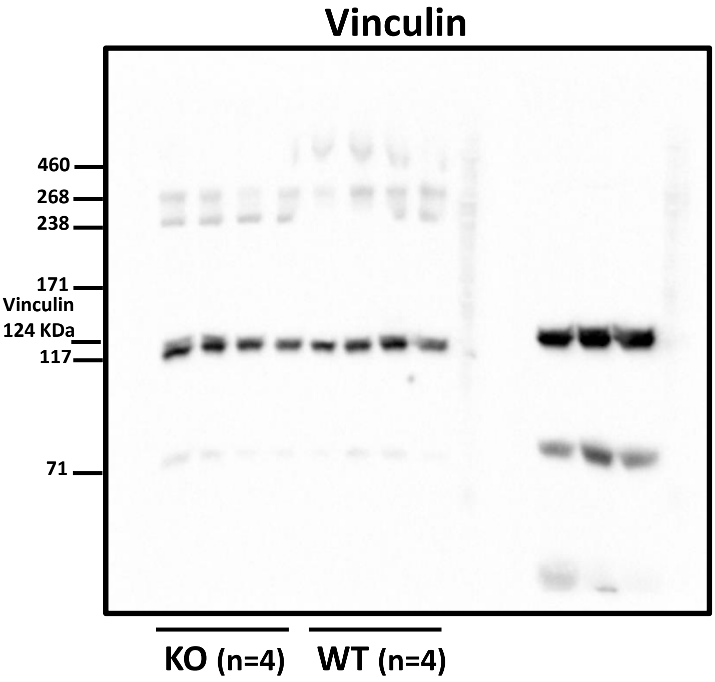

- Fig. 1F (Cerebal Cortex)

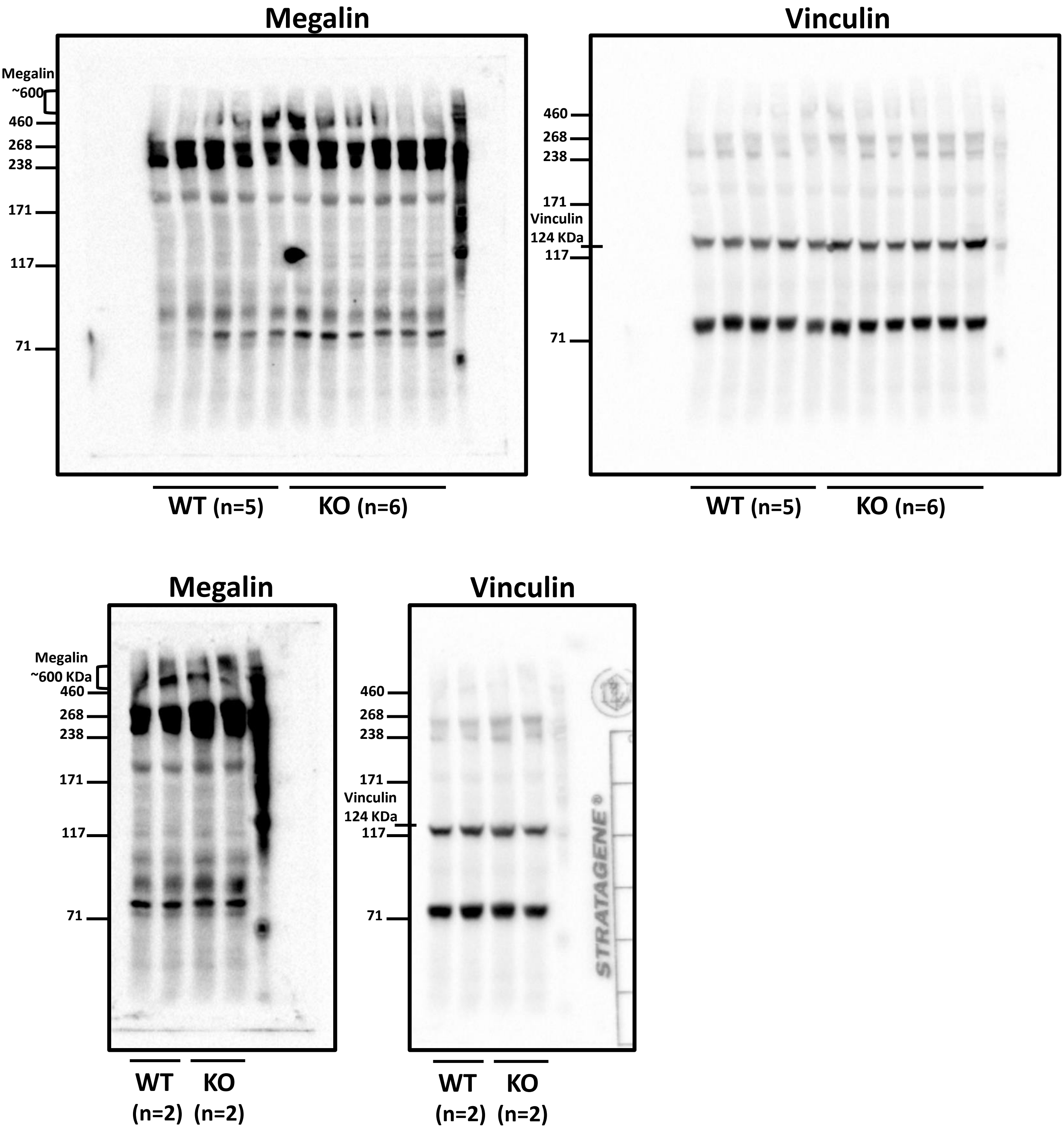

- Fig. 1G (Striatum)

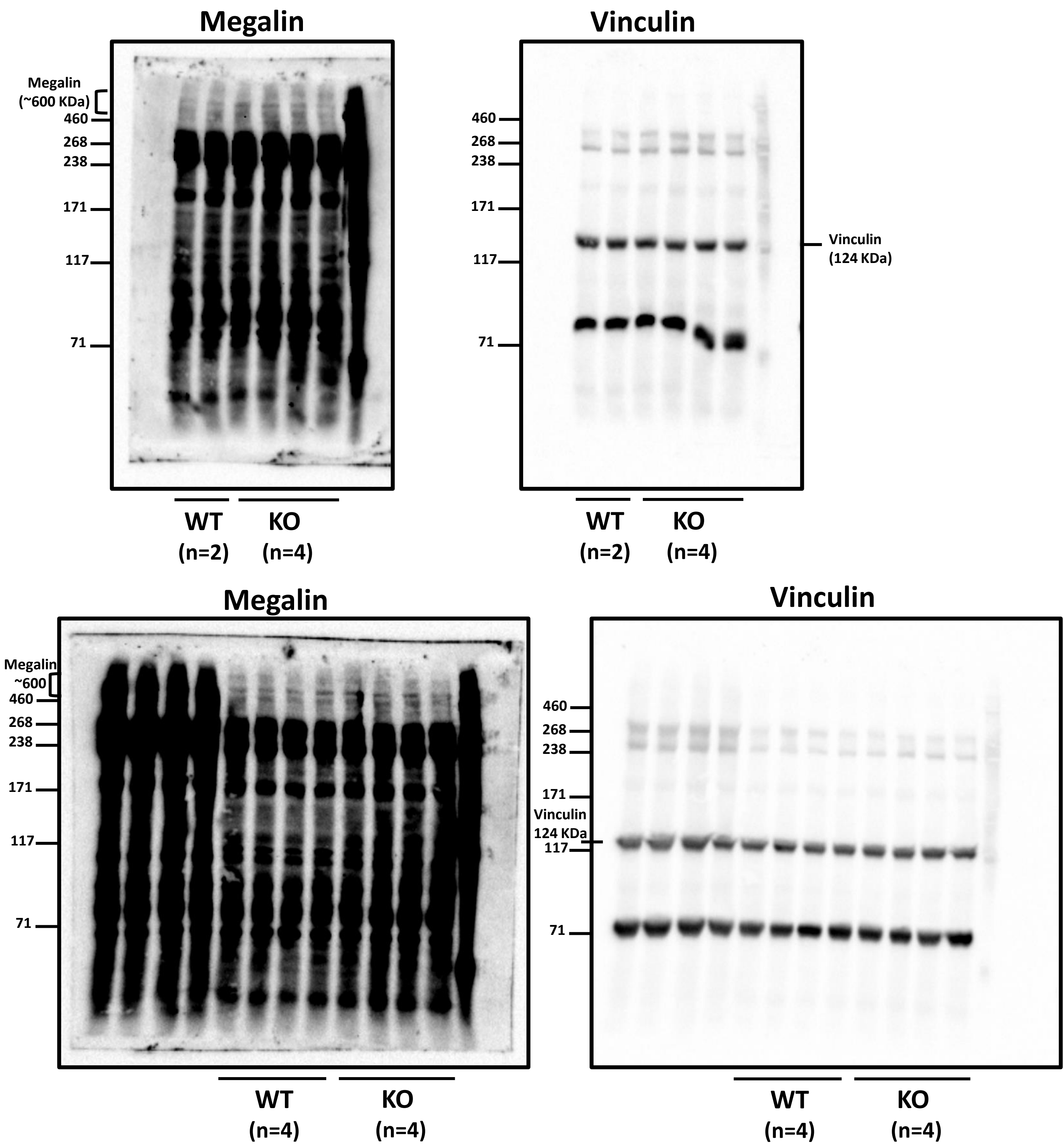

- Fig. 1H (Cerebelum)

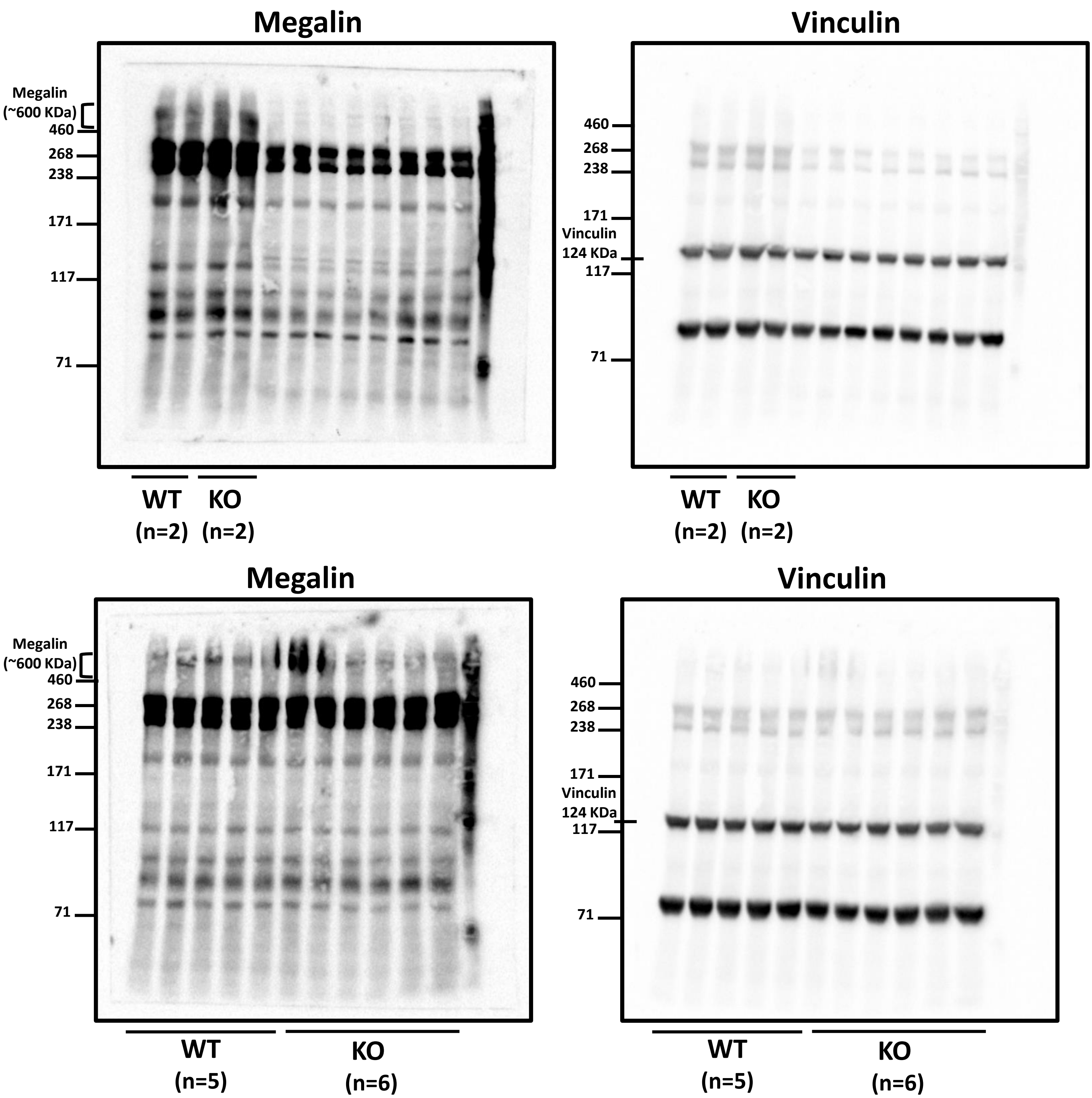

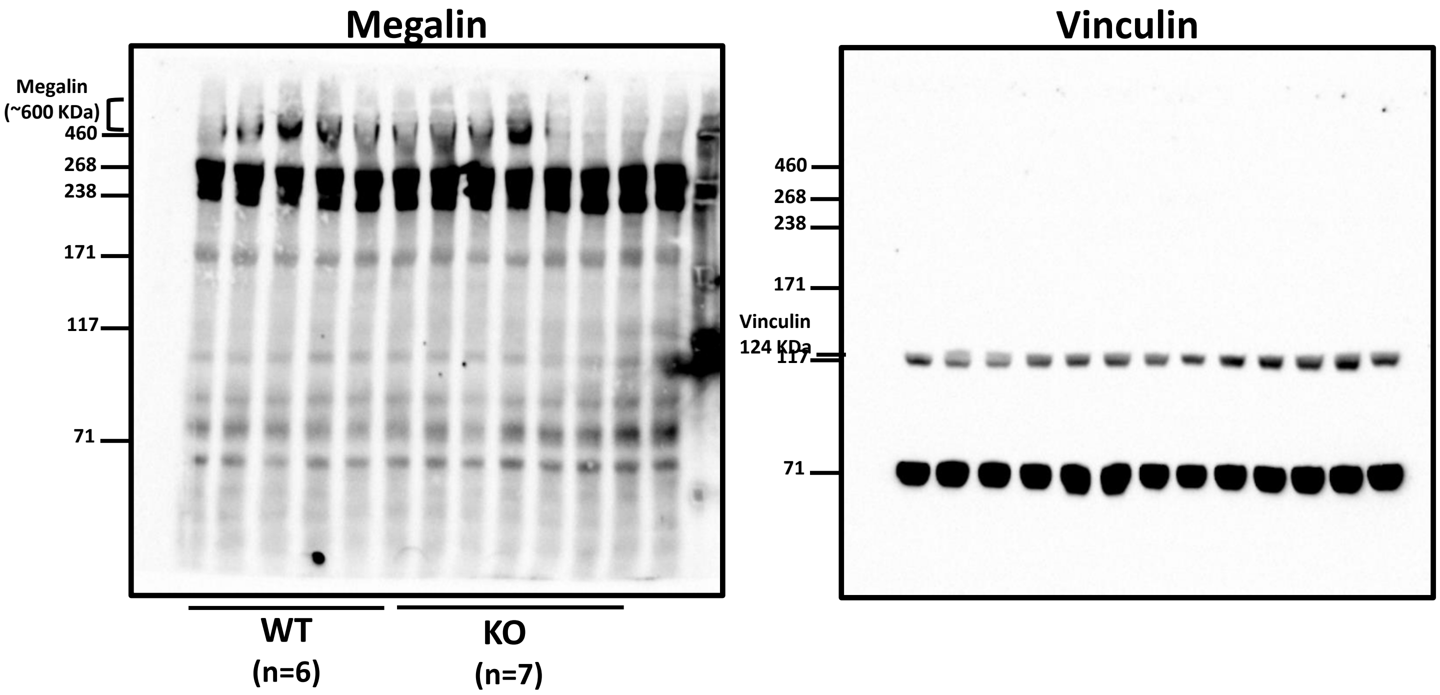

- Fig. 1I (Brain Stem)

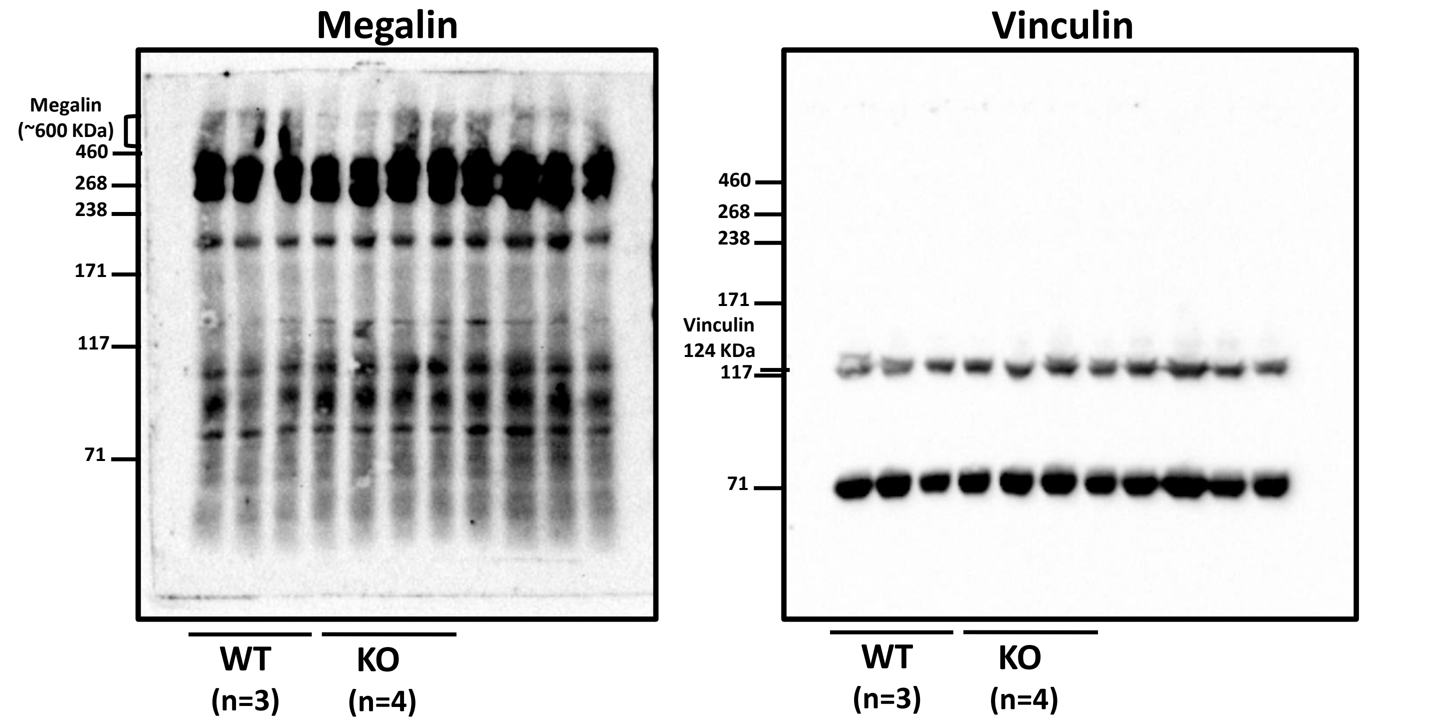

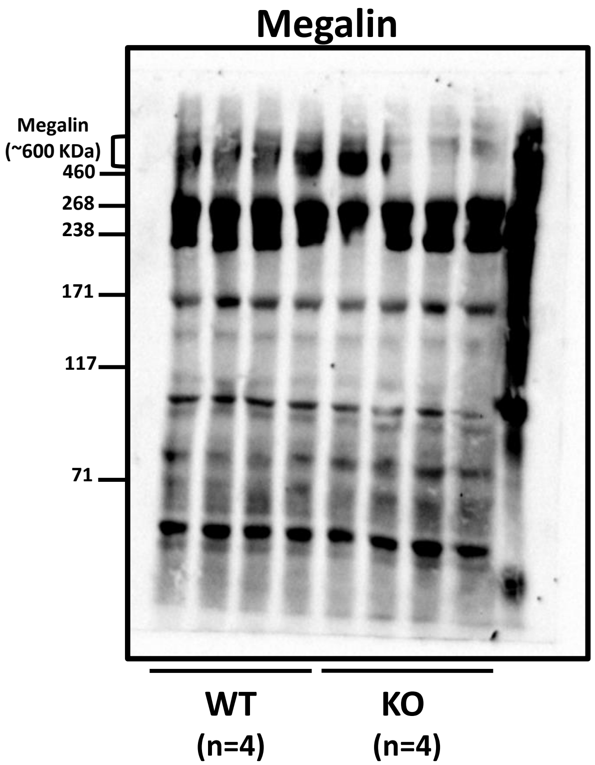

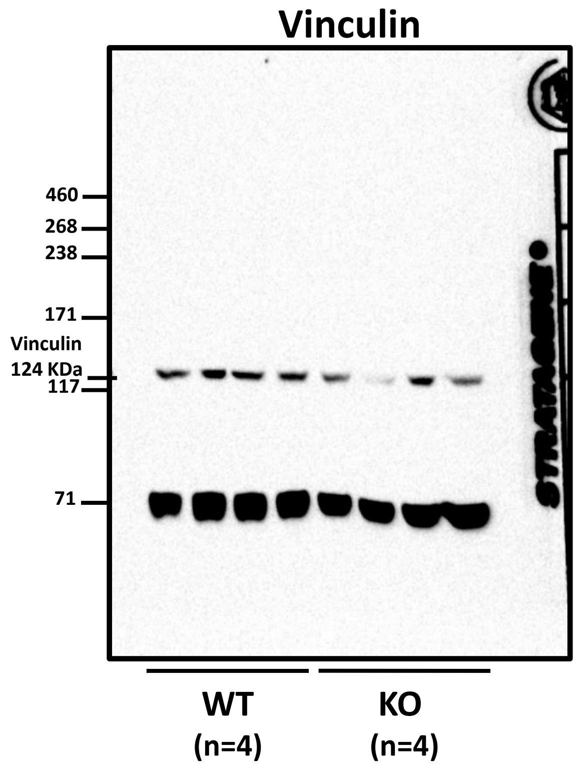

- Fig. 1K (Hippocampus)

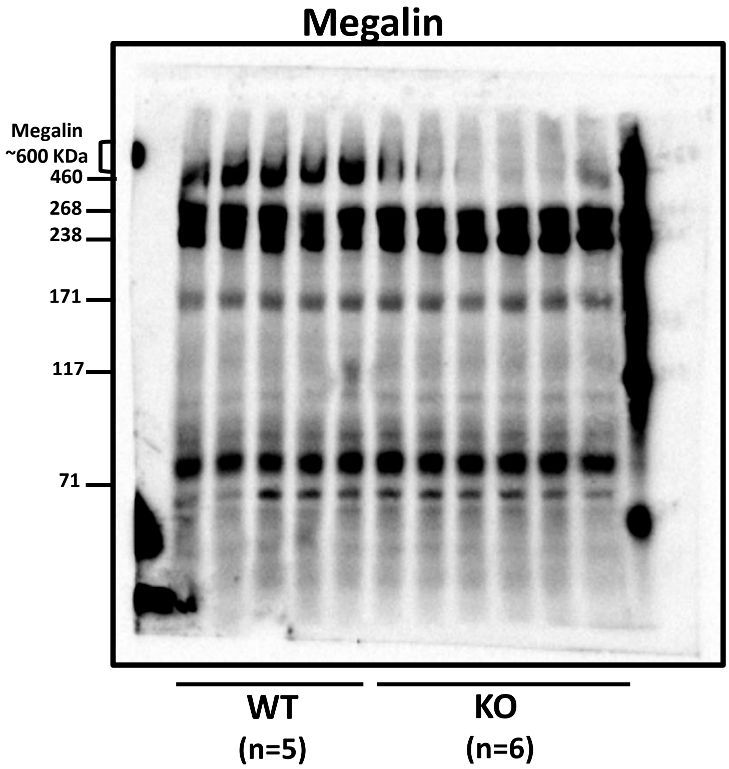

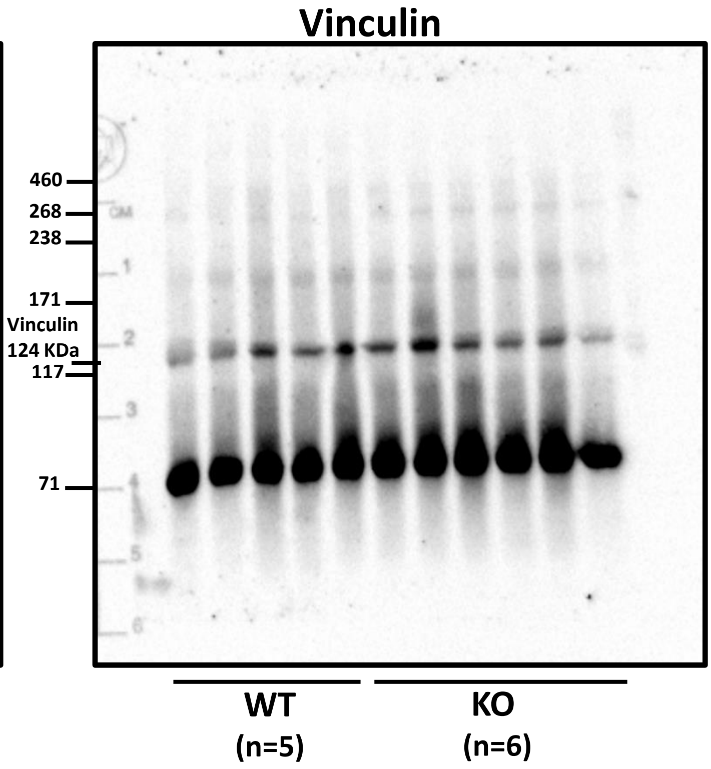

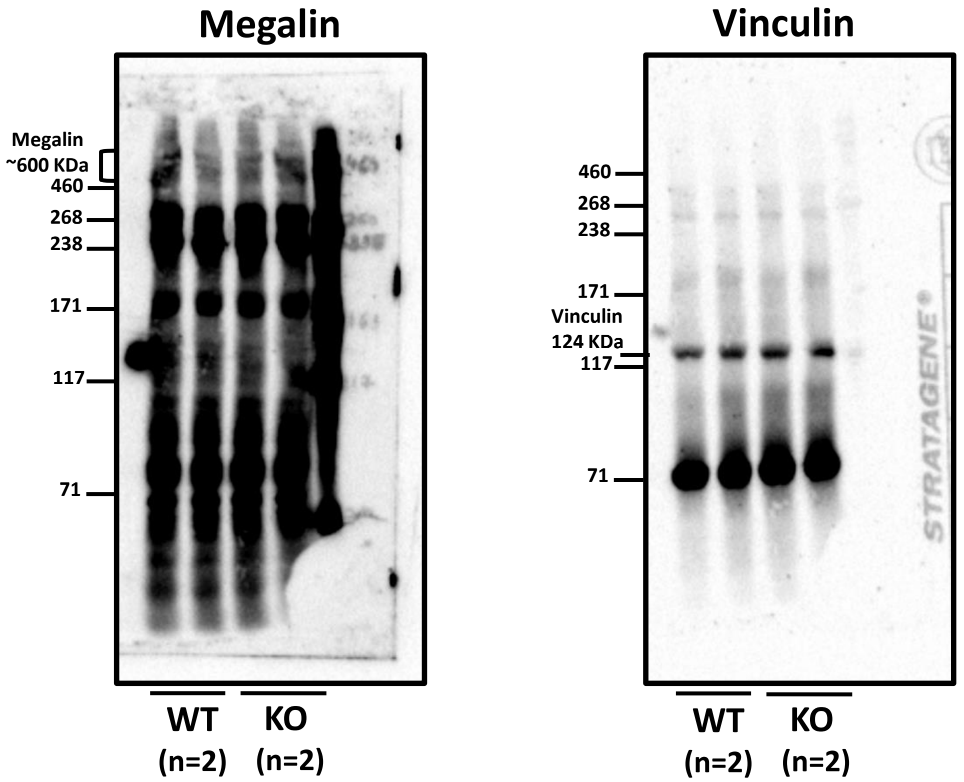

- Fig. 1M (Spinal Cord)

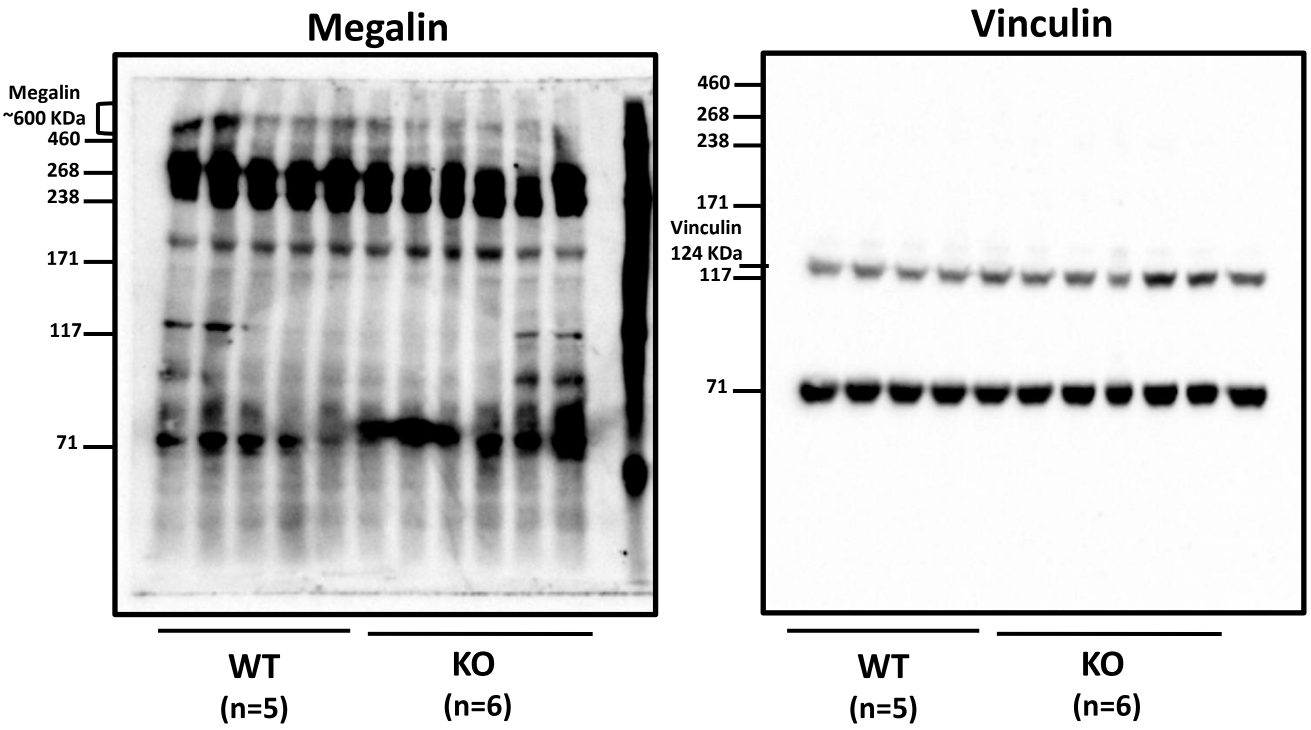

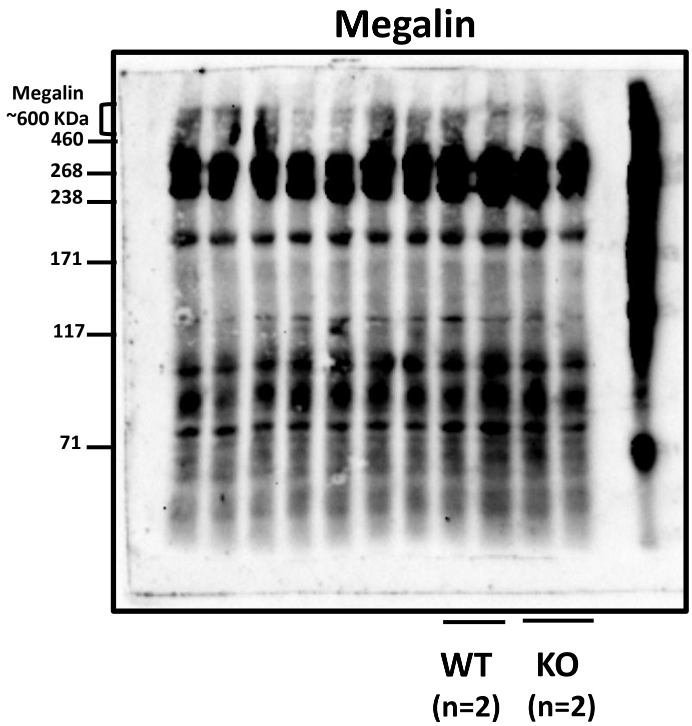
Megalin
Megalin
~600 KDa
460
268
238
171
117
71
WT
(n=2)
KO
(n=2)

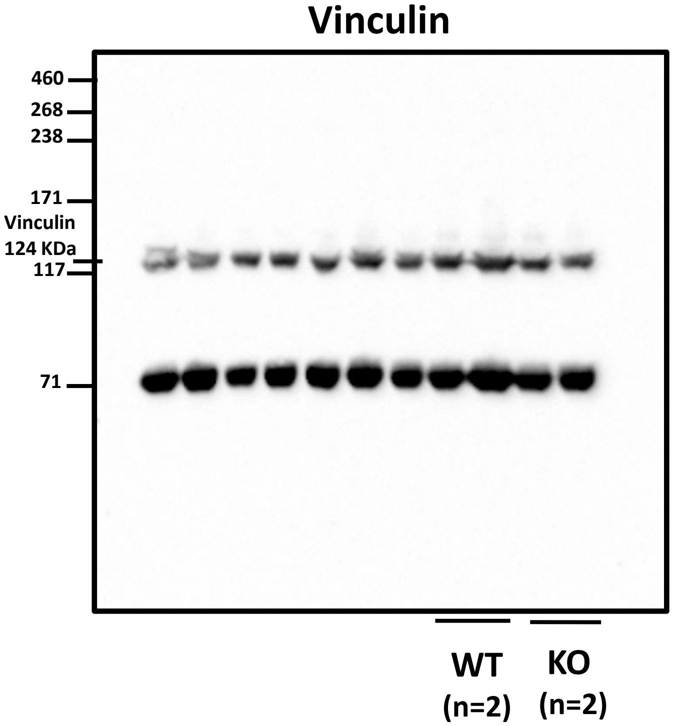
Vinculin
460
268
238
171
Vinculin
124 KDa
117
71
WT
(n=2)
KO
(n=2)

Whole original membranes in Western blot analysis of Figure 2

- Fig. 2B

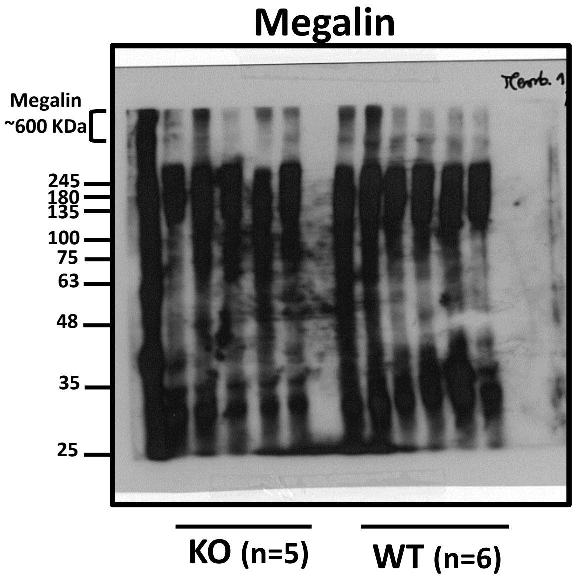

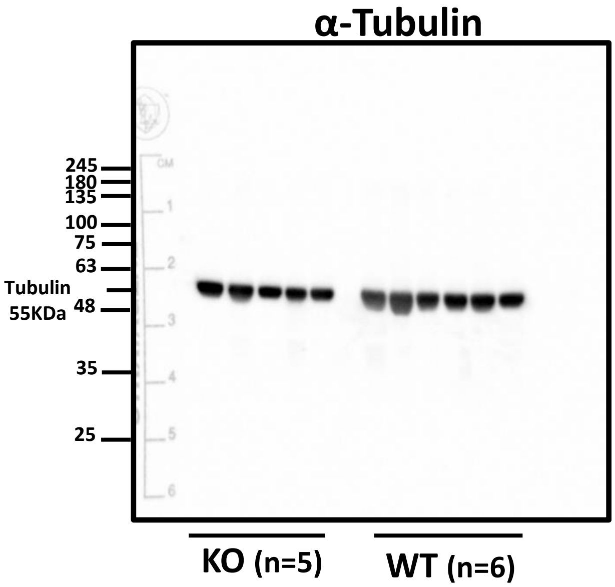

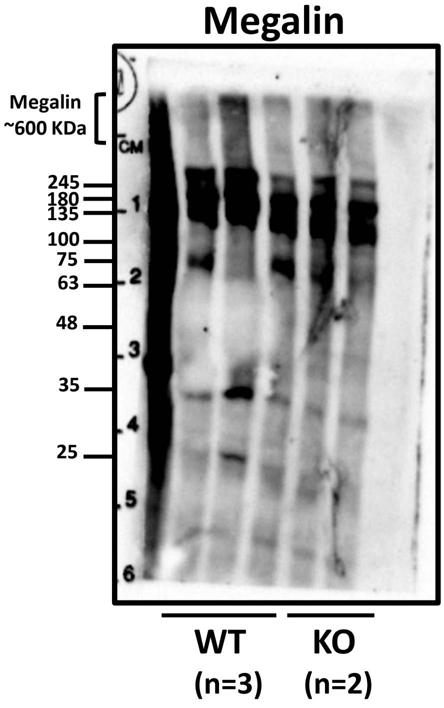

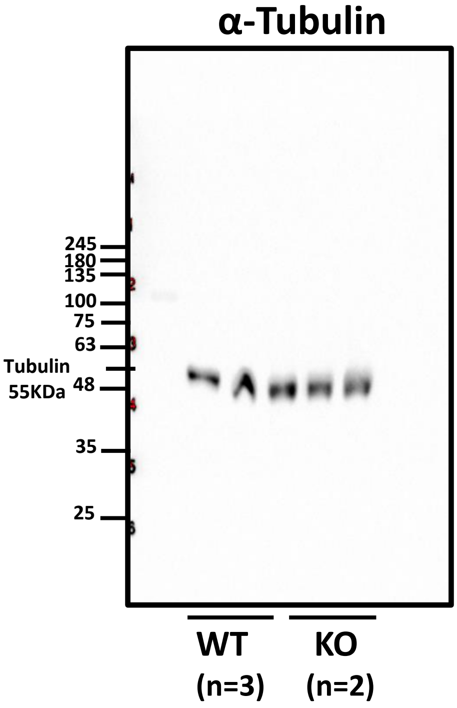

- Fig. 2D

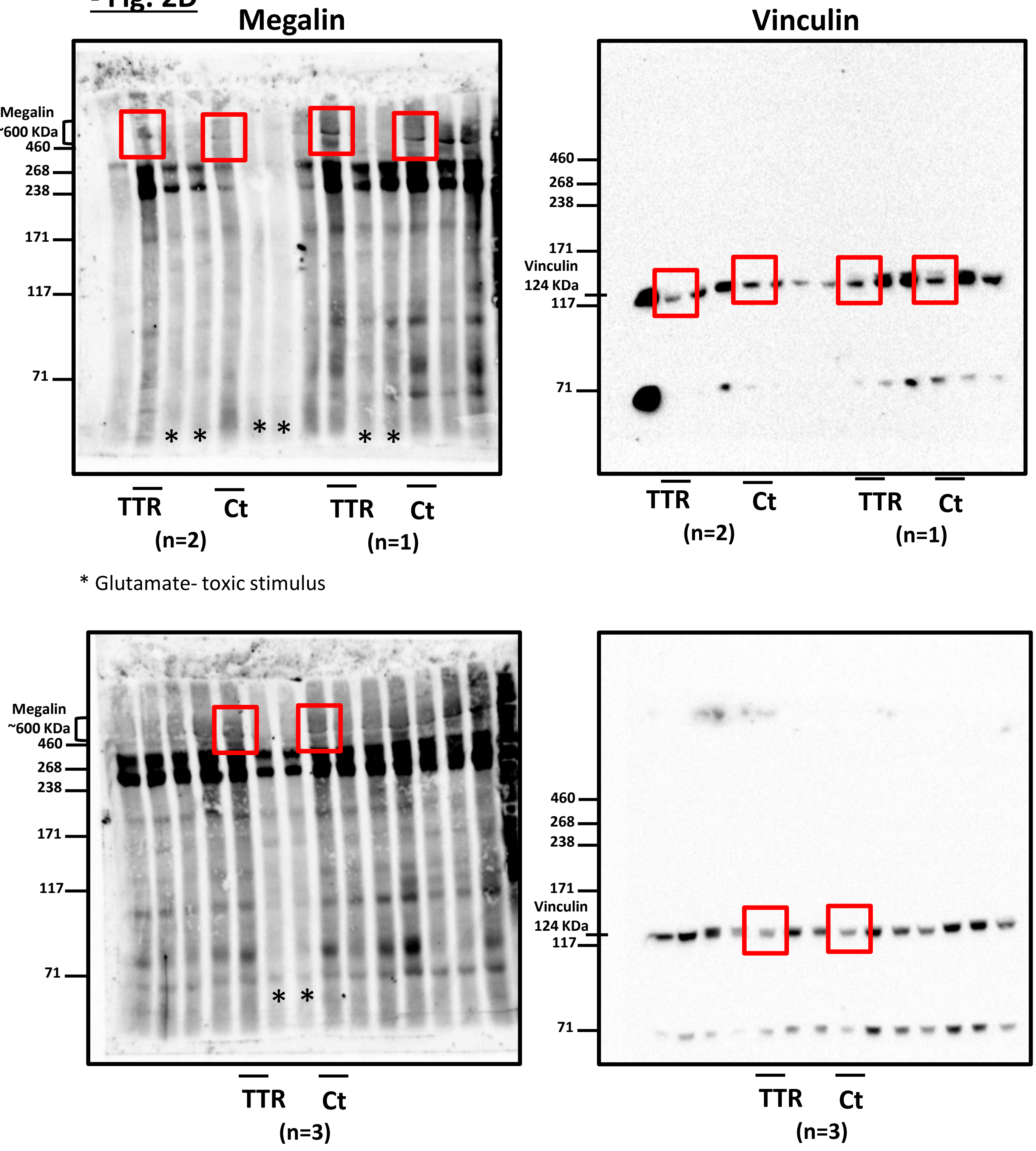
Megalin
Vinculin
Megalin
~600 KDa
460
268
238
171
117
71
* * * * * *
TTR
Ct
(n=2)
TTR
Ct
(n=1)
460
268
238
171
Vinculin
124 KDa
117
71
TTR
Ct
(n=2)
TTR
Ct
(n=1)
* Glutamate- toxic stimulus
Megalin
~600 KDa
460
268
238
171
117
71
* *
TTR
Ct
(n=3)
460
268
238
171
Vinculin
124 KDa
117
71
TTR
Ct
(n=3)

Whole original membranes in Western blot analysis of Figure 3

- Fig. 3D

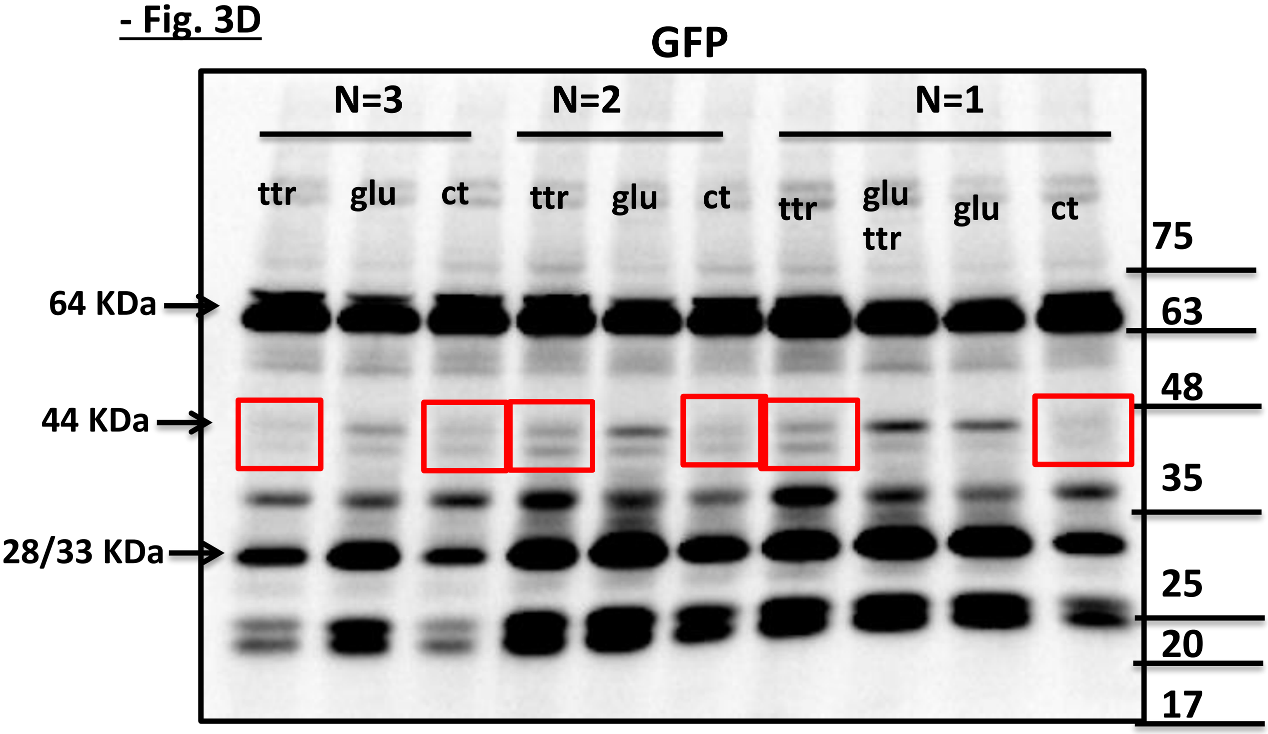

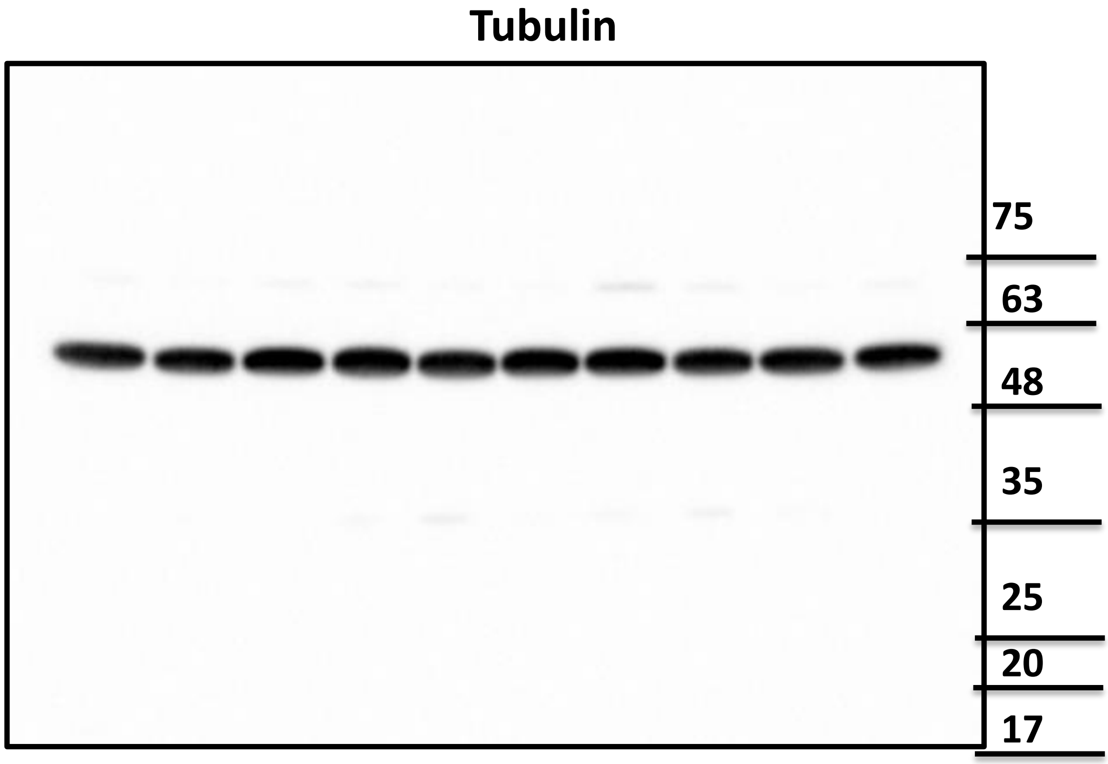

- Fig. 3F

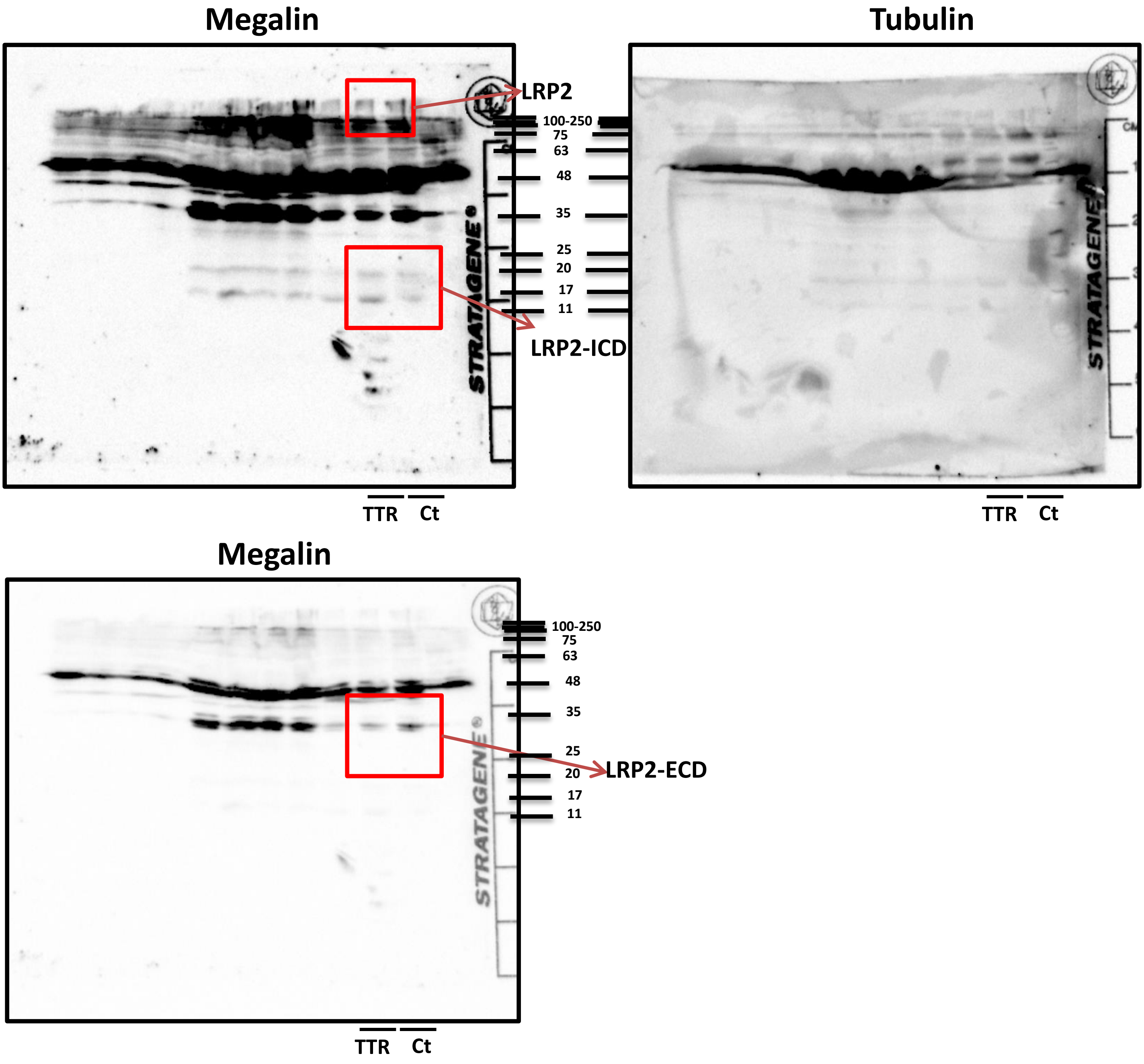
Megalin
Tubulin
LRP2
100-250
75
63
48
35
25
20
17
11
LRP2-ICD
STRATAGENE
TTR
Ct
TTR
Ct
Megalin
100-250
75
63
48
35
25
20
17
11
LRP2-ECD
STRATAGENE
TTR
Ct

Whole original membranes in Western blot analysis of Figure 4

- Fig. 4D

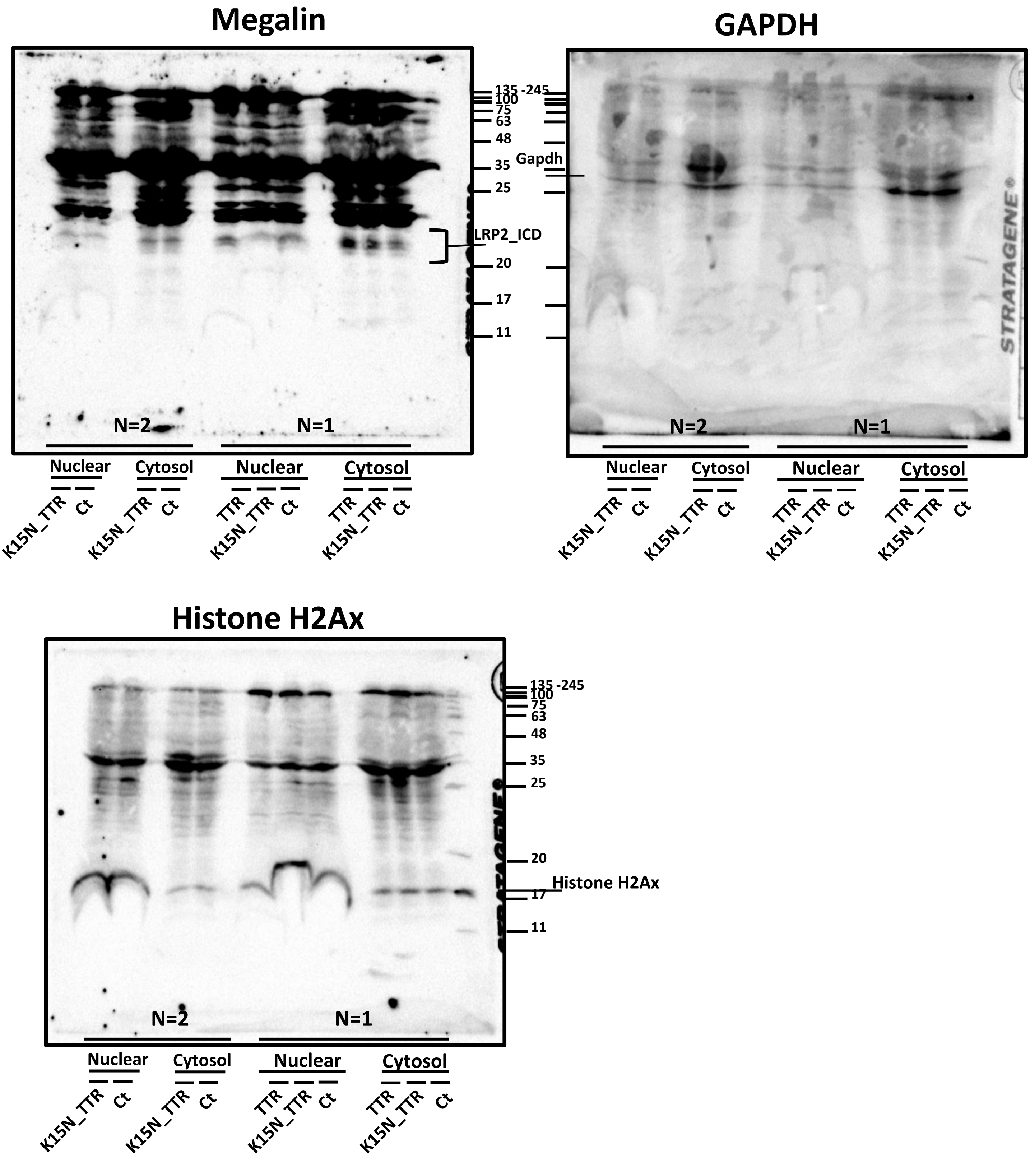

Whole original membranes in Western blot analysis of Figure 6

- Fig. 6A

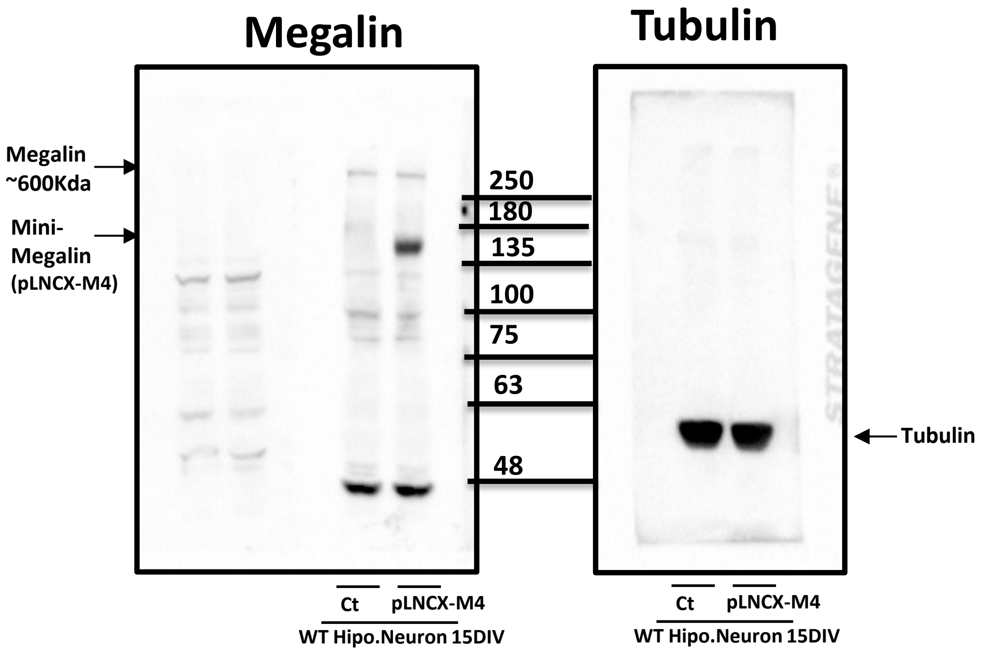

Whole original membranes in Western blot analysis of Figure 7

- Fig. 7N

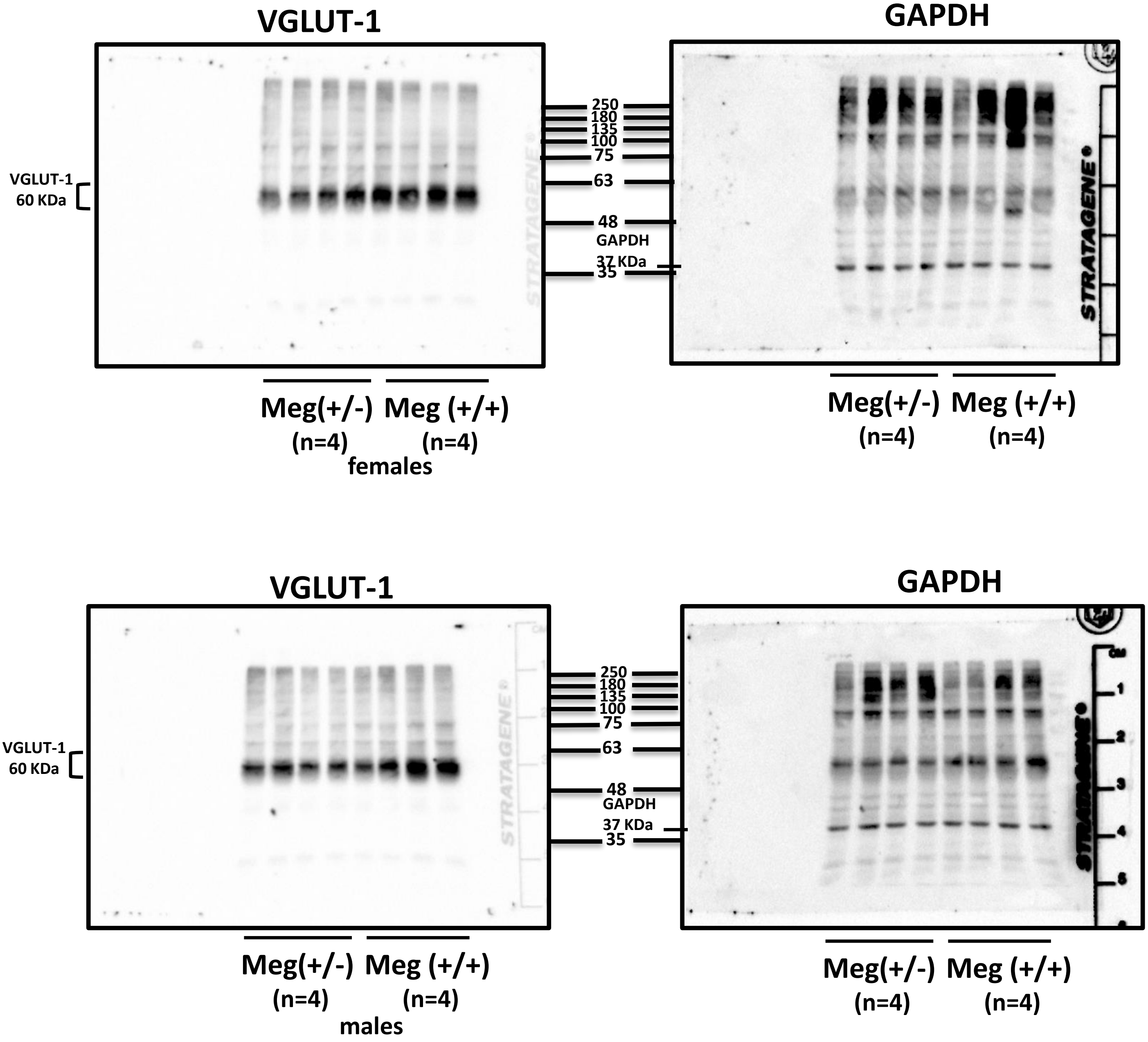

- Fig. 7O Same membrane used for VGLUT-1 (after sucessfull rebloting)

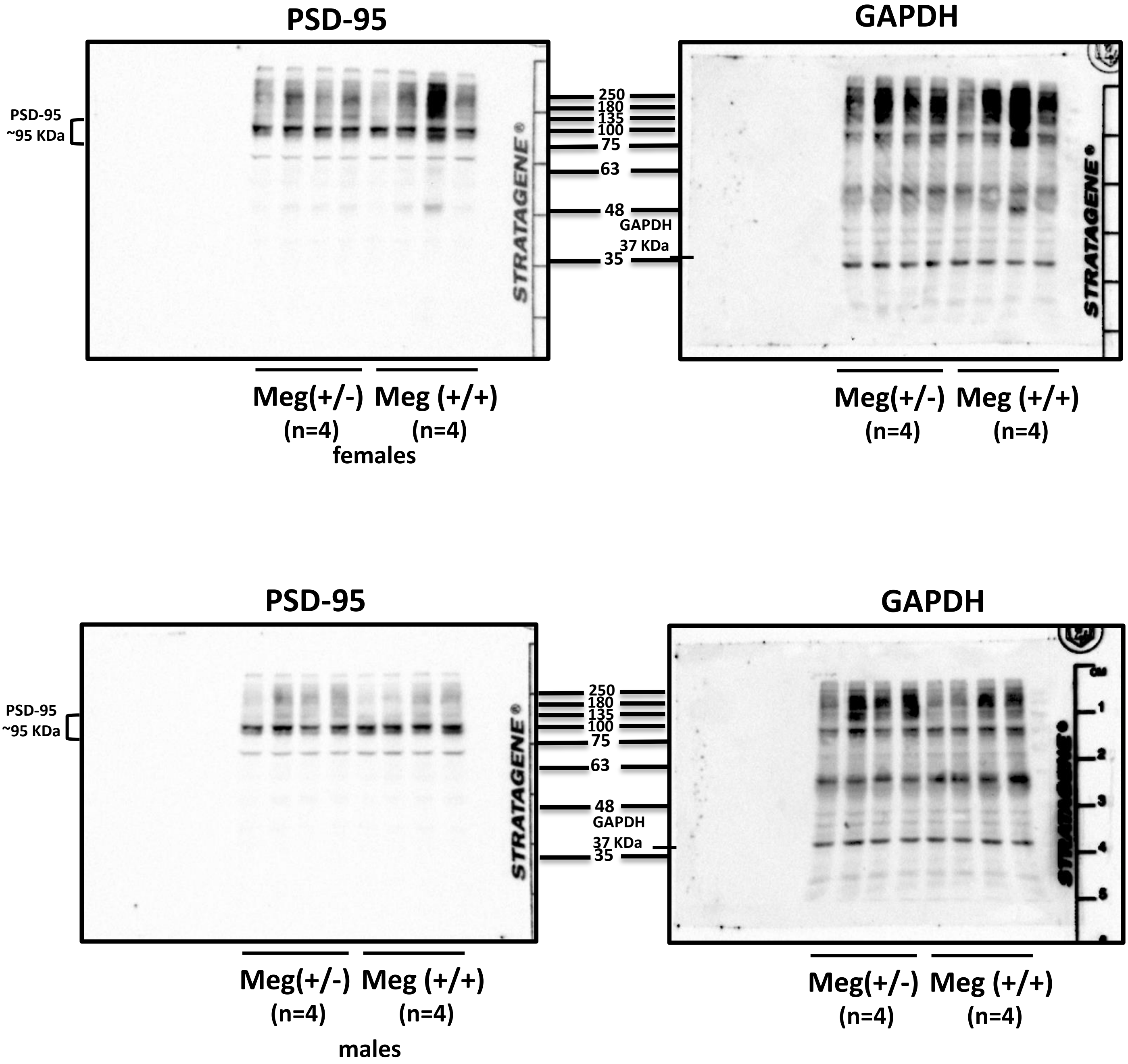

Whole original membranes in Western blot analysis of Supplemental Fig. 1 and 2

- Supplem. Fig. 1

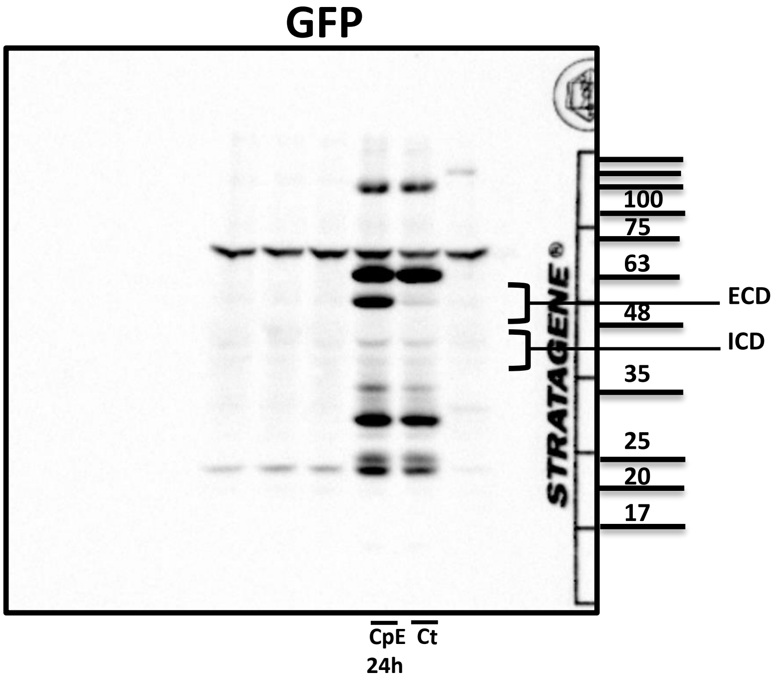

- Supplem. Fig. 2

Megalin

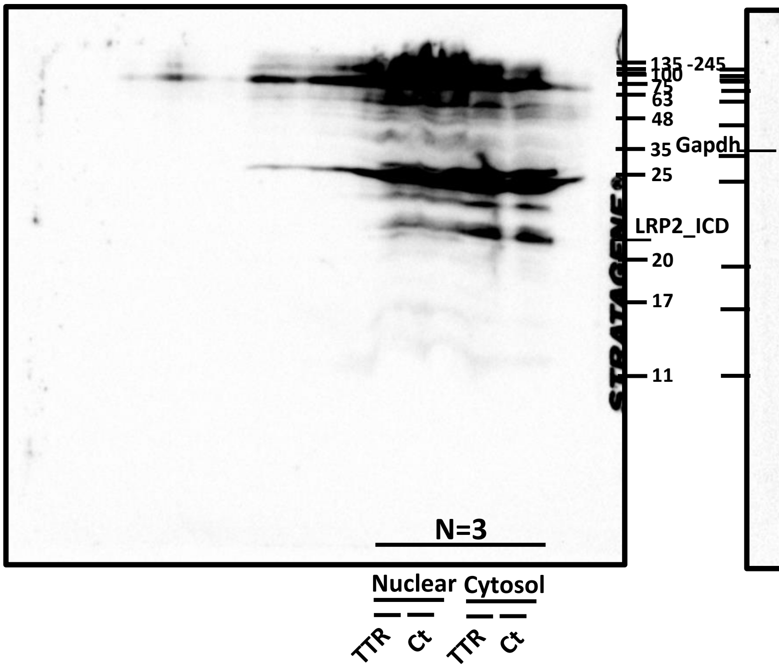

GAPDH

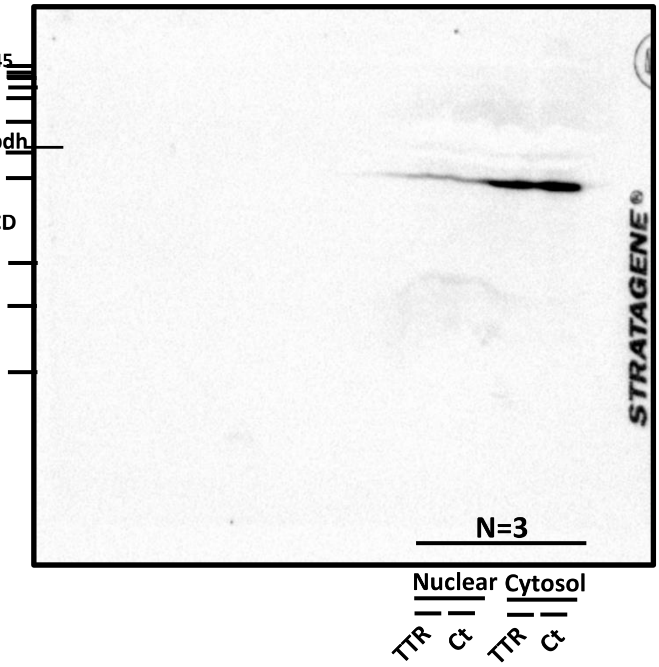

Histone H2Ax

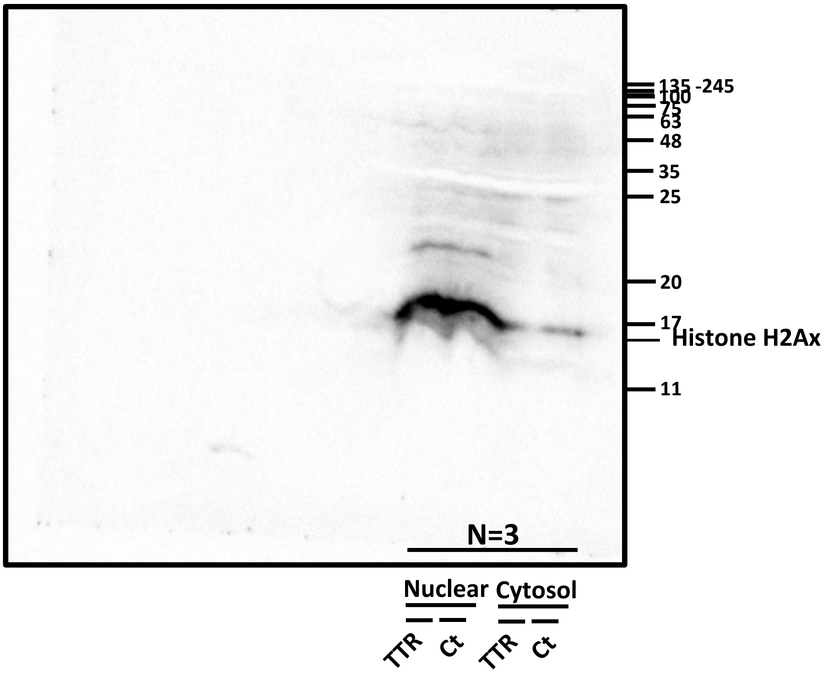

Supplement: fcaa135_Supplementary_Data [file fcaa135_supplementary_data.zip › Original membranes WB of Figure 1_7_Suppl1_2.pdf]
