# Supplementary material for: Neuronal megalin mediates synaptic plasticity—a novel mechanism underlying intellectual disabilities in megalin gene pathologies
Source: Brain Commun. 2020 Aug 25;2(2):fcaa135. doi: 10.1093/braincomms/fcaa135 (PMC7667529; doi:10.1093/braincomms/fcaa135)
Supplement: fcaa135_Supplementary_Data [file fcaa135_supplementary_data.zip › Original Submission.pdf]

OXFORD UNIVERSITY PRESS | Brain Communications

Neuronal megalin mediates synaptic plasticity - a novel mechanism underlying intellectual disabilities in megalin gene-pathologies

Journal:	*Brain Communications*
Manuscript ID	BRAINCOM-2020-050
Manuscript Type:	Original Article
Date Submitted by the Author:	18-Feb-2020
Complete List of Authors:	Gomes, Joao; IBMC - Instituto de Biologia Molecular e Celular, Porto, Portugal, Molecular Neurobiology<br>Lobo, Andrea; Universidade do Porto Instituto de Biologia Molecular e Celular, Addiction Biology<br>Nogueira, Renata; Universidade do Porto Instituto de Biologia Molecular e Celular, Molecular Neurobiology group<br>Terceiro, Ana; Universidade do Porto Instituto de Biologia Molecular e Celular, Addiction Biology<br>Costelha, Susete; Universidade do Porto Instituto de Biologia Molecular e Celular, Molecular Neurobiology group<br>Magalhães, Ana; Universidade do Porto Instituto de Biologia Molecular e Celular, Addiction Biology<br>Summavielle, Teresa; IBMC - Instituto de Biologia Molecular e Celular, Universidade do Porto, Rua do Campo Alegre, 823, 4150-180 Porto,<br>Saraiva, Maria Joao; Instituto Biologoia Molecular e Celular, Molecular Neurobiology
Keywords:	Megalin, LRP2, synaptic plasticity, Learning, hippocampus, Donnai-Barrow syndrome

SCHOLARONE™
Manuscripts

Neuronal megalin mediates synaptic plasticity - a novel mechanism underlying intellectual disabilities in megalin gene-pathologies

João R. Gomes[1,3*], Andrea Lobo [2,3*], Renata Nogueira [1,3], Ana F. Terceiro [2,3], Susete Costelha [1,3], Ana Magalhães [2,3], Teresa Summavielle [1,3#], Maria J. Saraiva [1,3#]

[1] Molecular Neurobiology Unit, IBMC- Instituto de Biologia Molecular e Celular, Porto, Portugal;

[2] Addiction Biology Group, IBMC- Instituto de Biologia Molecular e Celular, Porto, Portugal;

[3] I3S – Instituto de Investigação e Inovação em Saúde, Universidade do Porto, Portugal;

* - contributed equally to the work

# - senior authors

Address correspondence and reprint requests to:

João Carlos Gomes, i3S – Instituto de Investigação e Inovação em Saúde da Universidade do Porto

Rua Alfredo Allen, 208, 4200 – 135 Porto, Portugal; Tel +351 220 408 800

E-mail:. joao.gomes@ibmc.up.pt

For Review Only

Acknowledgements: This work was supported by FEDER funds through the Operational Competitiveness Programme – COMPETE, by national funding from the Portuguese Foundation for Science and Technology (FCT) under the projects PEST-c/SAU/LA0002/2011, FCT- FEDER for Unit 4293 in partnership with PT2020, and by the project Norte-01-0145-FEDER-000008 - Porto Neurosciences and Neurologic Disease Research Initiative at I3S, supported by Norte Portugal Regional Operational Programme (NORTE 2020), under the PORTUGAL 2020 Partnership Agreement, to Maria Saraiva and a post-doctoral fellowship (SFRH/BPD/84178/2012) and associate researcher contract to João Gomes, and a research contract to Andrea Lobo, under the project PTDC/SAU-TOX/30647/2017 The authors acknowledge Anabela Teixeira from IBMC for support in recombinant protein production and Paula Gonçalves from IBMC, for tissue processing. The authors also acknowledge the support of the i3S Scientific Platform, Advanced Light

Microscopy (ALM), Bioimaging, and Histology and Electron Microscopy (HEMS), member of the national infrastructure PPBI - Portuguese Platform of Bioimaging (PPBI-POCI-01-0145-FEDER-022122).

ABSTRACT

Donnai-barrow syndrome, a genetic disorder associated to LRP2 (megalin) mutations, is characterized by unexplained neurological symptoms and intellectual deficits. Megalin is a multifunctional endocytic clearance cell-surface receptor, mostly described in epithelial cells. This receptor is also expressed in the CNS, mainly in neurons, being involved in neurite outgrowth and neuroprotective mechanisms. Yet, the mechanisms involved in the regulation of megalin in the CNS are poorly understood. Using Transthyretin (TTR) KO mice, a megalin ligand, we found that TTR positively regulates neuronal megalin levels in different CNS areas, particularly in the hippocampus. TTR is even able to rescue megalin downregulation in TTR KO hippocampal neuronal cultures, in a positive feedback mechanism via megalin. Importantly, TTR activates a regulated intracellular proteolysis (RIP) mechanism of neuronal megalin, producing an intracellular domain (LRP2-ICD), which is translocated to the nucleus, unveiling megalin C-terminal as a potential transcription factor, able to regulate gene expression. We unveil that neuronal megalin reduction affects physiological neuronal activity, leading to decreased neurite number, length and branching, and increasing neuronal susceptibility to a toxic insult. Finally, we unravel a new unexpected role of megalin in synaptic plasticity, by promoting the formation and maturation of dendritic spines, and contributing for the establishment of active synapses, both in in vitro and in vivo hippocampal neurons. Moreover, these structural and synaptic roles of megalin impact on learning and memory mechanisms, since megalin heterozygous mice show hippocampal-related memory and learning deficits in several behavior tests. Altogether, we unveil a complete novel role of megalin in the physiological neuronal activity, mainly in synaptic plasticity with impact in learning and memory. Importantly, we contribute to disclose the molecular mechanisms underlying the cognitive and intellectual disabilities associated to megalin gene pathologies.

KEY-WORDS: Transthyretin; Megalin; LRP2; hippocampal neurons; hippocampus; neurite outgrowth; ICD; RIP; nuclear translocation; Learning; synaptic plasticity; Donnai-Barrow syndrome

Abbreviations:

TTR, transthyretin; CNS (central nervous system); LRP (low density lipoprotein receptor); RIP (regulated intramembrane proteolysis); ICD (intracellular domain); MCTF (membrane-bound megalin C-terminal fragment);

INTRODUCTION

Low-density lipoprotein receptor related protein 2 (LRP2), aka megalin, mutations are associated to the protein loss-of-function, underlying an autosomal recessive disorder, the Donnai-Barrow syndrome (DBS) (Kantarci et al., 2007), characterized by several CNS functional defects, particularly myopia and ocular complications, sensorineural hearing loss, mild to moderate intellectual disability, development delay and, in some cases, agenesis of the corpus callosum (Pober et al., 2009), and other symptoms such as specific craniofacial features, intestinal and heart abnormalities. Moreover, LRP2 polymorphisms were recently associated to non-syndromic cognitive impairment and intellectual disabilities(Beydoun et al., 2012; Dietrich et al., 2014b; Vasli et al., 2016; Beydoun et al., 2017). Animal models of megalin deficiency recapitulate several DBS symptoms, such as forebrain abnormalities, agenesis of corpus callosum, holoprosencephaly, eye defects, defects in cardiovascular development (Willnow et al., 1996; Kantarci et al., 2007).

Megalin is a membrane glycoprotein with a molecular weight of 600 kDa (517kDa non-glycosylated), standing as one of the largest glycoproteins in vertebrates. It belongs to the low density lipoprotein receptor family (Saito et al., 1994) and is constituted by a large extracellular domain (490 kDa) with four cysteine rich complement-type ligand binding repeats, involved in ligand binding (Russell et al., 1989; Fass et al., 1997), one transmembrane domain and an intracellular C-terminal tail of about 23kDa (Saito et al., 1994; Hjalm et al., 1996).The cytoplasmatic domain contains several motifs that regulate receptor trafficking and endocytosis, ranging from NPXY sequences, which mediate internalization through clathrin-dependent mechanisms, to SH2/SH3 domains involved in tyrosine kinases signaling (Marzolo and Farfan, 2011).

Megalin was firstly identified as an auto-antigen in a kidney pathology denominated Heyman nephritis (Kerjaschki and Farquhar, 1982), and is currently recognized as a multiligand receptor,

https://mc.manuscriptcentral.com/braincom

For Review Only

which binds and internalizes a wide variety of molecules, such as hormones, carrier proteins, lipoproteins, drugs and enzymes, mostly in the epithelial cells of the renal tubules (Christensen et al., 2012). Moreover, megalin is also expressed in the epithelial cells of the choroid plexus (Carro et al., 2005) and lateral ventricles (Gajera et al., 2010), and in different CNS cells, ranging from oligodendrocytes (Wicher et al., 2006), retinal ganglion cells (Fitzgerald et al., 2007), cerebellar granule neurons (Ambjorn et al., 2008), astrocytes (Bento-Abreu et al., 2008), to hippocampal neurons (Gomes et al., 2016). Megalin complete knockout led to abnormal development of the forebrain (Willnow et al., 1996), and conditional knockouts of this protein unveiled its role in adult brain neurogenesis, and presented cognitive impairment (Spoelgen et al., 2005; Gajera et al., 2010; Dietrich et al., 2014a). Megalin's major role in the CNS, through the binding of several ligands, has been as a mediator of neuronal survival and regeneration (Marzolo and Farfan, 2011). However, among its ligands, only metallothioneins (Pedersen et al., 2009) and transthyretin (Fleming et al., 2009; Gomes et al., 2016) have shown a neuroprotective and neuroregenerative role in the nervous system. Many other ligands have shown regenerative processes involving megalin, but not in the nervous system (Min et al., 2003; Zang et al., 2014).

Megalin signal transduction in CNS is still poorly understood. After extracellular ligand binding, phosphorylation of the receptor in the cytoplasmic domain seems to occur (Yuseff et al., 2007), followed by either receptor mediated endocytosis, direct activation of signaling pathways and/or regulation of gene expression via regulated intramembrane proteolysis (RIP) of the intracellular domain (LRP2-ICD). Metallothioneins have shown to promote neurite outgrowth and neuronal survival involving different signaling pathways, such as ERK, Akt and CREB, via megalin receptor (Fitzgerald et al., 2007; Ambjorn et al., 2008; Chung et al., 2008). Transthyretin has proven its neurotrophic properties independently of its ligands, through megalin binding (Gomes et al., 2019) and activation of signaling pathways Src/Erk/Akt/CREB, leading to neurite outgrowth and neuroprotection, both in-vitro and in-vivo, in the CNS and PNS (Sousa et al., 2000; Sousa and Saraiva, 2001; Gomes et al., 2016). APP (amyloid precursor protein) was also shown to interact with megalin in neurons, leading to changes in neurite outgrowth and Aβ-neurotoxicity (LaFerla et al., 1997; Alvira-Botero et al., 2010). However, megalin RIP processing and gene expression controlled by megalin remains understudied. It has been demonstrated in kidney cells and rat yolk sac cells that megalin, like Notch (Schroeter et al., 1998), is subjected to RIP, with ectodomain shedding mediated by a metalloprotease, producing a membrane-bound megalin C-terminal fragment (MCTF or LRP-2_ECD). Subsequently, a γ-secretase cleaves these MCTF to a soluble

For Review Only

megalin intracellular domain (LRP-2_ICD) (Biemesderfer, 2006; Li et al., 2008; Shah et al., 2013). However, the RIP processing remains to be addressed in neurons.

In the present study, we identify TTR, a megalin ligand, as a fine regulator of megalin expression, mostly in CNS hippocampal neurons. We show that TTR regulates megalin mRNA/protein levels in the hippocampus/hippocampal neuronal cultures, in a megalin dependent positive feedback mechanism. Additionally, we unveil that TTR does not only activate signaling pathways via megalin, but also through RIP, producing a LRP2-ICD, which is translocated to the nucleus. Importantly, we describe that megalin, in a mechanism independent of TTR, promotes neurite outgrowth, increases dendritic spine density, and improves neuronal survival, in neuronal cultures. In addition, we unclose a new role of megalin in synaptic plasticity, by increasing dendritic spine number and maturation, and increasing the number of active synapses, in megalin heterozygous mice. Accordingly, these mice present learning and memory deficits in several behavioral tests associated to hippocampal function. These findings suggest novel mechanisms that underlie the cognitive and intellectual disabilities associated to megalin gene pathologies, particularly the Donnai-Barrow syndrome.

RESULTS

TTR regulates megalin levels: TTR KO vs WT mice. Megalin is described as a TTR receptor, and the interaction between TTR and megalin is associated to TTR neuroprotective role of TTR in in-vitro and in-vivo cerebral ischemia (Santos et al., 2010; Gomes et al., 2016). Moreover, megalin is the receptor associated to TTR-induced neurite outgrowth, in physiological conditions (Gomes et al., 2016). Thus, in order to explore the interplay between megalin and TTR, we assessed megalin expression levels in the CNS of WT mice versus TTR KO mice, in organs where megalin is highly expressed: kidney and epithelial cells of choroid plexus. We found that both megalin mRNA (Fig. 1A) and protein (Fig. 1B) levels were downregulated in the kidney of TTR KO mice, compared to WT mice. Regarding the choroid plexus, we observed a downregulation of megalin protein levels (Fig. 1D,E) in TTR KO mice, but no differences in mRNA levels (Fig. 1C). When comparing megalin protein expression in the CNS of TTR KO vs WT mice, levels were reduced in the brainstem, hippocampus and spinal cord of TTR KO mice (Fig.1I,K,M), whereas the cerebral cortex, striatum and cerebellum had no significant differences (Fig.1F-H). In the hippocampus and spinal cord, despite the downregulation of megalin protein levels, no changes in mRNA were observed (Fig.

https://mc.manuscriptcentral.com/braincom

For Review Only

1J,L). Hence, the hippocampus stood out as the brain region where megalin expression is mostly affected TTR absence.

TTR rescues megalin downregulation in hippocampal neuronal cultures, in a megalin dependent way. In order to explore how TTR regulates megalin levels in the hippocampus, we compared megalin expression in TTR KO vs WT hippocampal neuronal cultures at 7DIV. We found that in TTR KO cultures, both mRNA (Fig. 2A) and protein (Fig. 2B) megalin levels were downregulated, compared to WT cultures, suggesting that hippocampal megalin levels in these mice models derive from neuronal cells (Fig. 1K). For this reason, cultured hippocampal neurons are a suitable model to study the signaling pathways involved in the regulation of megalin levels. Since in TTR KO neurons the absence of TTR resulted in a downregulation of megalin levels, we hypothesized that treating neurons with recombinant TTR could revert this effect. For that, TTR KO hippocampal neuronal cultures were incubated with recombinant mouse TTR (55µg/ml– physiological concentration of TTR in CSF (Ribeiro et al., 2014)), which significantly upregulated megalin mRNA and protein levels (Fig. 2C,D). In addition, we observed that a specific-TTR nanobody (Nb) (Gomes et al., 2018)- 169F7 (described to bind/block the epitope responsible for TTR-megalin interaction (Gomes et al., 2019)), was able to block TTR-induced megalin upregulation (Fig. 2C). Accordingly, a mouse TTR mutated in the epitope tagged by the 169F7Nb (K15N TTR) could not trigger megalin upregulation (Fig. 2E). However, another TTR Nb-165C6, described to bind to TTR without blocking interaction with megalin (Gomes et al., 2019), did not affect megalin upregulation promoted by TTR (Fig. 2C). When TTR KO neurons were incubated with a TTR variant (I84S) with low affinity for TTR ligands (RBP/T4) (Refetoff et al., 1986; Berni et al., 1994), megalin mRNA upregulation was still observed, indicating that this effect is independent of TTR ligands (Fig. 2E). Moreover, we observed that TTR-induced megalin upregulation was dependent on megalin signaling, since in TTR KO megalin heterozygous neurons, TTR stimulation was not able to upregulate megalin mRNA levels (Fig. 2E). This transcription control of megalin levels by TTR was also observed in WT cultured hippocampal neurons (Fig. 2F), and specific for megalin (LRP2) receptor, since LRP1 mRNA levels were not affected, in TTR KO neurons (Fig. 2G). Megalin levels regulation is not dependent on its internalization, since when neurons were treated with Dynasore, to block clathrin-mediated endocytosis (Macia et al., 2006; Kirchhausen et al., 2008; Vieira et al., 2015), megalin upregulation triggered by TTR still occurred(Fig. 2F). The effects observed are probably associated to megalin

https://mc.manuscriptcentral.com/braincom

triggered intracellular signaling pathways and/or C-terminus nuclear translocation, as demonstrated for other LRP members (Derocq et al., 2012).

TTR leads to LRP2- ICD formation, probably via γ-secretase activity. Previous studies identified several mechanisms for megalin signal transduction, including the regulation of gene expression involving regulated intramembrane proteolysis (RIP) of megalin intracellular domain (ICD-LRP2)(Biemesderfer, 2006; Li et al., 2008), but never in the CNS. We hypothesized that megalin RIP was also occurring in neurons, with megalin C-terminal translocation to the nucleus (Zou et al., 2004; Li et al., 2008). To assess this hypothesis, we used a plasmid encoding a short form of megalin (short-megalin-GFP), constituted by megalin C-terminal region fused with GFP, the transmembrane region, and only a short part of the N-terminal domain (Bolos et al., 2010). This short-megalin mimics native megalin (and mini-megalin), e.g. enhances IGF-I internalization (Bolos et al., 2010), presenting subcellular localization in neurons, with punctate distribution along neurites (Fig. 3A,B). We observed that in TTR KO hippocampal neurons expressing short-megalin-GFP, and stimulated with recombinant mouse TTR (55µg/ml, 40min), TTR stimulation of endogenous megalin (since short-megalin does not contain the ligand binding domain, working as a decoy) promoted a translocation of short-megalin-GFP from neurites towards cell body. Furthermore, we detected increased levels of megalin intracellular domain (ICD) in TTR KO neurons, upon TTR stimulation (50% increase compared to control neurons, not exposed to TTR; Fig. 3D,E). In, non-transfected WT neurons (15DIV), we also observed the upregulation of LRP2-ICD (~5 fold), after 5min incubation with recombinant mouse TTR (Fig. 3F). This effect was previously demonstrated in the kidney, where upon ligand binding, MMP and γ-secretase were activated, creating ECD, and then ICD megalin domains (Li et al., 2008) (Fig. 3G, supplemental Fig. 4). Taken together, these results indicate that TTR regulates megalin RIP processing (Fig. 3G), a novel signal transduction mechanism in neurons.

TTR induces LRP2-ICD nuclear translocation, as a putative transcription regulation mechanism. Given that TTR leads to megalin RIP in hippocampal neurons, with LRP2-ICD formation, we assessed if LRP2-ICD participates in a similar regulatory pathway as other LRP-family members, such as LRP1, which has RIP activity with ICD translocation to the nucleus, affecting transcriptional activity (May et al., 2002; Liu et al., 2007; Polavarapu et al., 2008; Zurhove et al., 2008). Using several bioinformatics tools to predict nuclear localization of proteins, we looked for short binding

For Review Only

sites on the C-terminus of mouse megalin, described to mediate transport to the nucleus (NLS-nuclear localizing signals), and for motifs associated with transport from the nucleus to the cytoplasm (NES-nuclear exporting signals), as it would be expected in a nucleo-cytoplasmatic shuttling (Kosugi et al., 2009). All the platforms (described in methods section) identified a putative NLS and NES sequence, with high probability, in the C-terminal region of megalin (Fig. 4A, methods section and supplemental information). In addition, to understand if megalin C-terminal region could interact with DNA and regulate gene transcription, we looked for protein-DNA binding residues, and identified two sequences in the C-terminal region of mouse megalin that display a high probability of binding to DNA (Fig. 4A, methods and supplemental information). In agreement with these data, a metal ion binding site, in the first protein-DNA binding region, was identified (Fig. 4A methods and supplemental information). The identification of histidine residue that possibly binds zinc ions indicates that megalin C-terminal might be a metalloprotein with a Zinc finger, potentially functioning as a transcription factor in the nucleus. ICD as a transcription factor, regulating gene expression paradigm has been described for other receptors (Neuhaus-Follini and Bashaw, 2015; Scholer et al., 2015; Gil-Yarom et al., 2017). To investigate if this bioinformatic predictions corresponded to a real translocation of megalin to the nucleus, TTR KO hippocampal neurons (11DIV) were transfected with short-megalin-GFP (Fig. 3A,B), and treated with recombinant mouse TTR for 5min. In fact, we observed that TTR induced a translocation and accumulation in the nucleus of megalin C-terminal fused with GFP (Fig. 4B,C). In a complementary approach, TTR KO neurons were transfected with the plasmid encoding the mini-megalin, and treated with recombinant mouse TTR, 48h later, during 20 min (Fig. 6A,4B). Total neuronal extracts were subjected to a nuclear fractionation protocol, allowing separation of cytoplasm from nuclear fractions. TTR stimulation lead to a translocation of megalin C-terminal (LRP2-ICD) to the nucleus, an effect that was abolished when mutated TTR form (K15N), in the epitope responsible for TTR interaction with megalin (Gomes et al., 2019), was used (Fig. 4D,E, supplemental figure 1). Thus, the experimental data confirms that megalin C-terminal is translocated to the nucleus upon TTR stimuli, in hippocampal neurons.

Reduction of megalin levels impairs neurite outgrowth and survival of hippocampal neurons. Megalin was shown to be involved in neurite outgrowth, not only through TTR signaling, but also by metallothioneins (Fitzgerald et al., 2007). Therefore, due to its prominent role in the CNS, we analyzed megalin's effect in neurite outgrowth using cultured hippocampal neurons from WT mice

For Review Only

and mice with reduced expression of TTR and/or megalin. To assess neurite effects, we quantified neurite number and length per neuron in neuronal cultures at 1DIV. We observed that TTR absence led to a decrease in neurite number (Fig. 5B - WT vs TTR KO), which was further decreased when megalin was also reduced (Fig. 5B – TTR KO vs $Meg^{+/-}$ TTR KO). Neurite length was affected by megalin reduction (TTR KO vs $Meg^{+/-}$ TTR KO), but not by TTR (WT vs TTR KO) (Fig. 5C). Additionally, to address the role of megalin in pathological toxic conditions, we subjected TTR KO and $Meg^{+/-}$ TTR KO neurons to excitotoxic conditions, consisting of a transient incubation with a high glutamate concentration (125µM, 20min), an in-vitro model of neuronal death in cerebral ischemia/stroke (Dwyer et al., 2008), and further incubation in culture-conditioned medium for 14h (40-50% apoptotic neuronal death)(Almeida et al., 2005). Neuronal death/survival was assessed using Hoechst 33342 staining to analyze nuclear morphology. $Meg^{+/-}$ TTR KO hippocampal neuronal cultures have increased neuronal death compared to TTR KO neuronal cultures, in physiological control conditions (53 vs 67% of neuronal survival, respectively (Fig. 5D)). Importantly, $Meg^{+/-}$ TTR KO hippocampal neuronal cultures are clearly more sensitive to an excitotoxic glutamate insult than TTR KO cultures (23% neuronal survival, compared to 46% in TTR KO neurons (Fig. 5D)). Thus, other megalin ligands, besides TTR, present in culture media, seem to be crucial to induce neurite outgrowth and survival via megalin (Fig. 5F). In addition, since megalin affects the neurite outgrowth, we wondered whether intracellular signaling dynamics and neuronal activity were also being affected. For that, we used an ultrasensitive Ca^{2+} sensitive indicator -yellow Cameleon-nano Fret probe (Horikawa et al., 2010) – used in Gomes et al to address TTR signaling via megalin (Fig. 2 in (Gomes et al., 2016)), that can detect action potentials, and allows the visualization of spontaneous neuronal activity (Yamada et al., 2011; Kanemaru et al., 2014). We found that in physiologic conditions, $Meg^{+/-}$ TTR KO neuronal cultures have decreased neuronal activity (blue line), meaning less Ca^{2+} transients, than full megalin neuronal cultures $Meg^{+/+}$ TTR KO (red line), observed by the number of intracellular calcium fluctuations. Moreover as expected and previously shown (Gomes et al., 2016), the absence of calcium in the cell culture medium abolishes Ca^{2+} fluctuations, associated to neuronal activity (black line)(Fig.5E). Together these data indicate that megalin seems to be required for physiological neuronal activity, and for neuronal survival.

Megalin overexpression rescues neurite outgrowth and increases dendritic spine density. To further explore the role of megalin in neurite outgrowth in the absence of TTR, we overexpressed

megalin in TTR KO (to avoid potential TTR synthesis/presence in culture) hippocampal neurons, by transfecting neurons with fully functional megalin receptor (mini-megalin - pLNCX-M4 plasmid) (Fig. 3B) (Takeda et al., 2003). Mini-megalin has the expected size of approximately 150KDa (Fig. 6A), and since is under the CMV promoter, overexpression is observed, when compared to full-length megalin endogenous expression (Fig. 6A). In WT neurons co-transfected with a plasmid encoding GFP (pEGFP) and mini-megalin, megalin overexpression was also observed when compared to non-transfected neighbor neurons, both at the cell body and neurites (Fig. 6A), as previously described (Gomes et al., 2016). Cultured hippocampal neurons from TTR KO mice expressing only GFP, or GFP plus mini-megalin, stimulated with recombinant mouse TTR, both neurite number and total length were increased, in a TTR-dependent effect (Fig. 6B- D). This is the first time that TTR neuritogenic activity is described in mature neurons, but it was previously observed in immature TTR KO (1DIV) hippocampal neurons (Gomes et al., 2016). Of relevance is the fact that, when we compare the control conditions of GFP and GFP plus mini-megalin expressing neurons, there is a clear increase of total neurite length (~50%), indicating that megalin has a role in neurite outgrowth independent of TTR, which probably depends on other endogenous megalin ligands, present in neuronal cultures under physiologic conditions (Fig. 6B, C). Moreover, a cumulative effect of TTR and megalin is observed, since total neurite length is significantly upregulated in TTR stimulated neurons that express mini-megalin and GFP vs GFP expressing neurons. Additionally, we observed a TTR-induced increase in distal neurite branching (100-320 μm from cell body), in GFP transfected neurons (Fig. 6E). On the other hand, when we compare TTR KO neurons expressing GFP vs GFP plus mini-megalin an increase in neuronal proximal branching is observed (Fig. 6F) (0-200 μm from cell body). Finally, when comparing neurons expressing GFP vs GFP plus mini-megalin, in the presence of TTR, there is a slight cumulative effect of megalin and TTR in areas closer to the cell body (proximal branching, until 100 μm) (Fig. 6G). However, in GFP plus mini-megalin expressing neurons, TTR was unable to further increase neuronal branching (Supplemental figure 2). Importantly, we also demonstrate that overexpressing megalin significantly increases the density of dendritic spines (Fig. 6H). In WT GFP transfected neurons (supplemental figure 3), the density of dendritic spines is approximately 8 spines/10 μm dendritic length, showing that in TTR KO mice spine density is significantly reduced, even though megalin is still able to increase dendritic spines density (Fig. 6H). A pictorial figure summarizes this effects triggered by overexpression of megalin vs TTR stimulation (Fig. 6I).

For Review Only

Megalin heterozygous mice show structural alterations in hippocampal neurons. To address whether neurite network changes triggered by megalin in cultured hippocampal neurons where also occurring in-vivo, we evaluated hippocampal neuronal morphology from $Meg^{+/-}$ mice vs $Meg^{+/+}$ corresponding littermates, for neurite length and branching both in CA1 and DG regions of the hippocampus, and dendritic spine density in CA1 region. For that, mice were injected intravenously with AAV produced with PHP.eB serotype for efficient transduction of CNS neurons (Chan et al., 2017), with GFP under the CAG promoter, for high levels of gene expression (Fig. 7A). We observed that $Meg^{+/-}$ mice show reduced neurite number, neurite length, and decreased dendritic arborization, when compared to $Meg^{+/+}$ mice littermates, in neurons from both the CA1 (Fig. 7B-E) and DG (Fig. 7F-I) regions. In CA1 neurons, the effect in dendritic ramification was more pronounced in proximal (between 36-60 µm from cell body) and distal neurites (340-530 µm from cell body) (Fig. 7D), with no effect on intermediate neurites; whereas for DG neurons the effect was only on more distal dendrites (150-440 µm from cell body) (Fig. 7H). Moreover, to understand if megalin is required for dendritic spine formation and maturation in vivo, we analyzed spine density in GFP-labeled CA1 pyramidal neurons. We found that $Meg^{+/-}$ mice have decreased dendritic spine density, and this effect was due to mature spines, whereas immature spines were not altered (Fig. 7K-M). This indicates that megalin is probably mainly required for dendritic spine maturation, and not for the formation of novel dendritic spines. Up on that, we evaluated if the effects at dendritic spines were associated to changes in synaptic activity, by analyzing the expression levels of synaptic proteins in the hippocampus (presynaptic-VGLUT1 and postsynaptic-PSD95), as well as the number of synapses, defined as the colocalization of pre- and post-synaptic proteins that appear at a punctate distribution (McLeod et al., 2017). In total hippocampal extracts, VGLUT1 levels are decreased in $Meg^{+/-}$ mice compared to $Meg^{+/+}$ littermates, and PSD95 show a tendency to decrease (p=0.08) (Fig.7N and O, respectively). More relevant is the reduction in synapse number observed in $Meg^{+/-}$ hippocampus, indicating that brain functions such as memory and learning might be impaired (Fig. 7 P,Q, supplemental figure 5).

Megalin heterozygous mice show cognitive deficits, but no effects in anxiety-like behavior or locomotor activity

Considering the in vivo effects observed in hippocampal neuronal morphology, dendritic spines and synaptic number for $Meg^{+/-}$ mice, we addressed if these might impact on mice behaviors particularly learning and memory, anxiety-like and locomotor behavior. We found that reduced

For Review Only

levels of megalin did not affect anxiety-like behavior, as determined by the percentage of time spent in the open arms using the elevated plus maze (EPM) test, and distance travelled in the center of the arena in the open field (OF) test (Fig. 8B and D, respectively). Moreover, megalin deficits did not affect locomotor activity, as determined by the total distance travelled in the arena of the OF test (Fig. 8E). Importantly, when performing the Morris water maze (MWM) test, the megalin heterozygous animals showed impairment in the learning process, with increased latency to find the platform at learning sessions 2 and 4 (Fig. 8G), when compared to $Meg^{+/+}$ littermates. Moreover, $Meg^{+/-}$ mice show an impairment in reference memory, presenting an higher latency to the target (Fig.8I), and a tendency to an increase in the average distance to the target (Fig. 8H, p=0.09) in the probe session. Representative images of the track performed by the mice in the MWM clearly demonstrate the differences between the two genotypes (Fig. 8J). In the Novel Object Recognition (NOR) test, to assess memory and cognitive capabilities, $Meg^{+/-}$ mice showed a reduction in time exploring the novel object, as assessed by the discrimination index, that evaluates the preference to explore the novel object vs the familiar object (Fig. 8L). Moreover, total exploration time of the objects by both genotypes was not different, indicating that the differences in exploring novel objects are not associated to distinct locomotion or motivation to explore the objects (Fig. 8M). Together, our data suggests that $Meg^{+/-}$ mice display learning and memory deficits that correlate with structural and functional hippocampal synaptic dysfunction, reveling a relevant role of megalin in these mechanisms (see also, supplemental Fig. 6-8).

For Review Only

DISCUSSION

In this work, we report that transthyretin is a fine regulator of megalin expression, affecting both its mRNA and protein levels in areas characterized by high megalin expression, such as the kidney and the choroid plexus, and, importantly, in the CNS, the hippocampus. We describe that megalin levels in hippocampal neuronal cultures are fundamental for neurite outgrowth and neuronal survival, as decreasing megalin levels results in neurons with impaired neurite length and branching, with reduced number of neurites, and , consequently, more susceptible to insults, such as excitotoxic conditions. Moreover, we found that TTR controls megalin levels through a regulated intracellular proteolysis (RIP) processing, and the ICD domain formed is translocated to the nucleus. In addition, we disclose a novel role for megalin in learning and memory mechanisms, as megalin heterozygous mice show hippocampal-related memory deficits, in several behavior

tests, that were in agreement with the neurite, dendritic spine density/maturation and synaptic impairments observed in megalin deficient hippocampal neurons, both in-vitro and in-vivo.

The regulation of megalin expression by TTR described in this work (Fig. 1,2) is in agreement with the effect of other megalin ligands that also regulate the receptor expression. These megalin ligands control mRNA and/or protein levels of megalin, via the receptor itself (Marzolo and Farfan, 2011), in a positive feedback mechanism. For instance, exposure to vitamin A and D, both megalin ligands, leads to increased megalin protein and mRNA levels (Liu et al., 1998). Clusterin overexpression also upregulates megalin levels (mRNA and protein), which in turn confers clusterin an antiapoptotic role in prostate cancer cells (Ammar and Closset, 2008).

Regarding the expression levels of megalin in CNS, our results are also in agreement with several reports: choroid plexus (Chun et al., 1999; Carro et al., 2005); spinal cord in either embryonic (Wicher et al., 2005) or adult stages (Wicher et al., 2006); different brain areas such as the cerebral cortex, hippocampus, striatum and the cerebellum (Alvira-Botero et al., 2010). Moreover, megalin is mainly expressed in neurons in the CNS (Fass et al., 1997; LaFerla et al., 1997; Gomes et al., 2016), although it was also shown to be present in astrocytes (Bento-Abreu et al., 2008) and olygodendrocytes in the spinal cord (Wicher et al., 2006). Nonetheless, in this study, we demonstrate that TTR controls megalin levels, besides kidney and CP, in the CNS, including hippocampus, brainstem and spinal cord.

The fact that TTR is affecting/regulating megalin levels mainly in the hippocampus and spinal cord, and not in other CNS areas, such as the cerebral cortex or the cerebellum (Fig. 1F-I), is probably associated to the fact that TTR has been described to be either synthetized by hippocampal/motor neurons, or uptaken from the CSF TTR pool, making these regions more prone to the effects of TTR over megalin (Stein and Johnson, 2002; Sousa et al., 2007a; Buxbaum et al., 2008; Li et al., 2011; Gomes et al., 2018). TTR does not seem to affect megalin mRNA expression levels in vivo, although it affects megalin protein levels (Fig.1). This could be associated to megalin's long half-life period (Perez Bay et al., 2016), and also to the fact that proteins in the brain usually have almost triple average lifetimes compared to proteins in other tissues (Alvarez-Castelao and Schuman, 2015). Proteins may also display different turnover rates depending on their location in the neuron (synapses, dendritic branch or axons). Supporting this idea is data in cultured hippocampal neurons (Fig. 2A, C), where TTR was able to regulate megalin mRNA in cell body and dendrites, and to a lower extent in axons (early mature neurons) (Gomes et al., 2016). In a study by Alvira-Botero, the presence of megalin in neurons throughout the brain was observed, with synaptic localization

For Review Only

in axon terminals near synaptic vesicles (Alvira-Botero et al., 2010). Moreover, it described a possible role of megalin in neurite development, independent of supplemented ligands, in agreement with our results. Furthermore, the authors reported that knocking down megalin in hippocampal neuronal cultures significantly increased neurite branching. This goes in the opposite direction of our results, as we describe that decreasing levels of megalin led to decrease in number and length of neurites per cell, either in developing (1DIV, Fig. 5A-C)) or in mature hippocampal neurons (13DIV) (Fig. 6B-G). Megalin was also shown to be the receptor mediating the neuritogenic activity induced by different molecules, such as metallothioneins (Fitzgerald et al., 2007), α2-macroglobulin(Qiu et al., 2004) and TTR (Gomes et al., 2016). Altogether, these data highlight the important role of megalin in neurite outgrowth, which we have now explored in more detail.

After establishing this critical role of megalin in neurite development, we assessed whether neuronal cultures could be susceptible to stressful conditions, such as excitotoxicity. We observed that megalin heterozygous TTR KO neurons are more sensitive to neuronal death induced by an excitotoxic insult compared to TTR KO neurons. A previous work (Gomes et al., 2016), also found that TTR KO neurons were more sensitive to this toxic stimuli than WT neurons; we can now indicate that decreased megalin receptor levels are likely associated to this increased neuronal death (Gomes et al., 2016). In addition, we found that decreasing megalin levels in neuronal cultures also decreases their neuronal activity, using an ultrasensitive Ca^{2+} indicator. Taken together, we clearly indicate that reduced levels of megalin impair neuronal viability and reduce neurite outgrowth, making neurons more susceptible to toxic insults. In agreement with our results, Cases et al established a link between megalin and congenital high myopia, in a megalin conditional KO in the mouse forebrain, including neural retina, which resulted in increased cell death in the retinal ganglion cell layer, and decreased axon number (Cases et al., 2015).

The signaling functions of megalin were addressed for several ligands, including TTR in physiologic and pathologic conditions (Gomes et al., 2016), TTR (or other megalin ligands) binding to megalin initiates/activates adaptor proteins in the cytoplasm that recognize specific motifs within megalin C-terminal, activating different signaling pathways (Spuch et al., 2012). However, as we describe here, megalin is also able to initiate signaling events through a different mechanism, RIP, already widely described for APP and Notch (Ebinu and Yankner, 2002). In this mechanism, upon ligand binding, the receptor is proteolytically cleaved, by a metalloprotease, producing membrane-bound megalin C-terminal fragment (LRP2-ECD), followed by an internal cleavage of the C-terminus by a

For Review Only

γ-secretase enzyme, forming a soluble megalin intracellular domain (LRP2-ICD). This intracellular domain will target the nucleus where it regulates gene expression. Li et all have shown that megalin is subjected to RIP processing in opossum kidney cells, demonstrating that megalin C-terminal regulates gene expression (Biemesderfer, 2006; Li et al., 2008). However, details of this signaling are completely unknown, and in neurons are not even described (Carlos and Carmen, 2010). Here, we demonstrated that, upon TTR binding, megalin C-terminal domain translocates from neurites to the cell body, specifically to the nucleus of hippocampal neurons (Fig. 3C). Moreover, using TTR ligand we demonstrate megalin RIP processing and it's ICD formation (using several approaches (Fig. 3D-F)).

Using several bioinformatics tools, we identified several putative specific motifs associated to nucleo-cytoplasmatic shuttling in megalin C-terminus , and also protein-DNA binding residues, as well as a metal binding site, indicating that LRP2-ICD (megalin C-terminal) could regulate directly gene expression, as a transcription factor (Fig. 4A). We validate this bioinformatic data, by a biochemical approach, and found that LRP2-ICD translocates to the nucleus (Fig. 4B-E). These results shed more light into recent works where megalin was shown to be an extremely important receptor for cell proliferation and survival, e.g, in melanoma cells, in which sustained megalin expression was crucial for cell maintenance and proliferation (mainly overexpressed), being a target for therapy (Andersen et al., 2015); in non-Hodgkin-lymphoma, in which LRP2 has increased expression mainly in neurons (Pedersen et al., 2010). This role of megalin C-terminal as a transcription factor might be the source of this sustained support of survival. Likewise, Notch , upon RIP, the ICD domain also translocates to the nucleus, where it acts as a transcriptional co-factor to modulate gene expressions (De Strooper et al., 1999; Bray, 2006).

We found that megalin heterozygous mice show a clear hippocampal neuronal deficits, particularly reduced neurite complexity, dendritic spine density/maturation and synaptic density (Fig. 7), associated to learning and memory deficits, as assessed by the Morris water maze and novel object recognition tests (Fig. 8F-N). Additionally, in an in vitro approach, overexpressing megalin in hippocampal neuronal cultures resulted in increased dendritic spines density (Fig. 6H). Consistent with our results, some reports establish a link between megalin and learning/memory processes. In an exome sequencing screening for non-syndromic intellectual disability (ID) mutations, megalin was detected in a consanguineous family of two boys with mild ID (Vasli et al., 2016). Also, there is both impaired learning ability and recognition memory (Dietrich et al., 2014b), in an endothelial-specific megalin null mice where megalin expression is blocked only in

For Review Only

endothelial cells, also affecting the choroid plexus through the Tie-Cre promoter (Theis et al., 2003). Moreover, degenerating neurons in the cerebral cortex were observed in these mice, that also display cortical and hippocampal exacerbated inflammation processes (Bartolome et al., 2017). LRP8 (Low-density–lipoprotein-receptor-related protein 8), which is another member of the LDL receptor family, is also RIP processed, triggered by Reelin, with intracellular domain cross talking with NMDA receptor, in a similar way that we have described for LRP2 (Gomes et al., 2016); and after going from the synapse to the nucleus, it regulates epigenetic events that culminate in the regulation of memory formation in vivo (Telese et al., 2015). TTR KO mice show some spatial learning and memory deficits(Sousa et al., 2007b; Brouillette and Quirion, 2008; Buxbaum et al., 2014). Given the fact that these TTR KO mice have downregulated megalin levels in the hippocampus (Fig. 1), these data should now be re-analyzed taking into account megalin's role in these effects. However, in megalin gene pathologies in which megalin mutations lead to reduced expression of the protein, and contribute to cognitive deficits, as we demonstrate here, TTR supplementation/overexpression could potentially be used as a therapeutic strategy, based in our results showing that TTR is able to revert megalin downregulation. An increase in megalin levels and function induced by TTR treatment, or other strategies, could potentially mitigate some of the pathology symptoms.

In conclusion (Thumbnail cartoon), our data demonstrate that: 1) TTR is a fine and positive regulator of megalin levels, mainly in hippocampal neurons; 2) TTR signaling through neuronal megalin does not only rely on the signaling pathways Src/Erk/Akt, but also in a RIP process, with ICD formation and translocation to the nucleus; 3) megalin C-terminal has full potential for being considered a transcription factor, with all the motifs associated; 4) megalin regulates neuronal activity and neuronal survival; 5) megalin has a role in synaptic plasticity, regulating neurite outgrowth, dendritic spine maturation and synaptic density; 6) we disclose possible megalin role in learning and memory mechanisms; 7) finally, we have contributed to unveil the molecular route underlying the cognitive and intellectual disabilities associated to megalin gene pathologies, particularly the Donnai-Barrow syndrome; 8) neuronal megalin should now be considered as a relevant player in the mechanisms regulating synaptic neurotransmission and learning and memory mechanisms. In summary, upon TTR (or other ligand) binding to megalin, a signaling pathway involving Src, NMDARs activation, Erk1/2, CREB and Akt and/or a pathway involving RIP and formation of LRP2-ICD can contribute to increase megalin levels, in a mechanism important for physiological structural and synaptic plasticity. First and foremost these mechanisms shed a

For Review Only

light on the knowledge of cognitive and intellectual disabilities that occur in megalin gene-pathologies, and on potential therapeutic targets.

FIGURE LEGENDS

Fig. 1 – TTR regulates megalin levels: TTR KO vs WT mice. Total RNA was extracted from kidney **(A**, n=12 mice), choroid plexus (**C**, n=12 mice), hippocampus **(J**, n=5-6 mice**)** and spinal cord **(L**, n=5-6 mice**)** of WT and TTR KO mice, and megalin and GAPDH mRNA levels were semi-quantified by real-time PCR. Megalin mRNA levels are reduced in the kidney of TTR KO mice. Megalin protein levels were determined by western blot in the Kidney (n=6 mice) (**B**), Choroid Plexus (n=4 mice) (**D**), and in different brain regions of WT and TTR KO mice: Cerebral cortex (n=7-8 mice) (**F**), Striatum (n=6-8 mice) (**G**), Cerebellum (n=13-15 mice) (**H**), Brainstem (n=6-7 mice) (**I**), Hippocampus (n=8 mice) (**K**) and spinal cord (n=7-8 mice) **(M)**. Megalin protein levels are reduced in the kidney, choroid plexus, hippocampus and spinal cord of TTR KO mice. Statistical analysis was performed using Student's unpaired t-test. *** P<0.001, *P<0.05. **(E)**, Representative images of immunofluorescence of choroid plexus from WT and TTR KO mice stained for TTR and megalin (3 mice/genotype), showing a reduction of both TTR and megalin expression in TTR KO mice. Scale bar corresponds to 50 µm.

Fig. 2 – TTR rescues megalin downregulation in TTR KO hippocampal neuronal cultures, in a megalin dependent way. (A) Total RNA was extracted from WT (n=11) and TTR KO (n=10) hippocampal neuronal cultures (7DIV), and megalin and 18S mRNA levels were semi-quantified through real-time PCR, showing a reduction in megalin mRNA levels in hippocampal neurons of TTR KO mice. **(B)** Megalin and tubulin protein levels were determined by western blot in TTR KO (n=6) and WT (n=8) hippocampal neuronal cultures, with a decrease in megalin protein levels observed in TTR KO neurons. TTR KO cultured hippocampal neurons were stimulated with recombinant mouse TTR (55µg/ml (1µM)) for 4h (mRNA) **(C)** or 14h (protein) **(D)**. When indicated, neurons were treated with TTR, in presence or absence of anti-TTR Nanobodies 169F7 and 165C6 (2µM), or treated only with the nanobodies **(C)**. TTR treatment rescued megalin mRNA (**C**, n=5-7 independent cultures) and protein levels (**D**, n=3-4 independent cultures) in TTR KO neurons, and the effect was abolished in the presence of the 169F7 nanobody, specific for TTR-megalin interaction epitope (**C**). Megalin (+/-) TTR KO and TTR KO cultured hippocampal neurons (7DIV) were stimulated with recombinant mutated forms of mouse TTR, I84S (a TTR with low affinity for

For Review Only

its ligands) and K15N (55µg/ml, for 4h; a TTR mutated in the 169F7 nanobody epitope, affecting TTR-megalin interaction), and megalin and 18S mRNA levels were assessed (**E**, n=6-10 neuronal cultures). TTR induced effect in increasing megalin levels is independent of TTR ligands, but depends on TTR binding to megalin and on megalin levels. **(F)** WT hippocampal neuronal cultures were stimulated with recombinant mouse TTR (55µg/ml, for 4h) in the presence or absence of the inhibitor clathrin-mediated endocytosis dynasore (80 µM) and megalin mRNA levels were assessed (n=6 neuronal cultures). No effect was observed in the presence of the inhibitor, indicating that TTR-induced increase in megalin levels does not involve receptor internalization. **(G)** LRP1 and 18S mRNA levels were determined in TTR KO hippocampal neurons (n=4 neuronal cultures), in the presence or absence of recombinant mouse TTR (55µg/ml, for 4h). LRP1 levels are not affected by TTR stimulation, indicating a specific effect for LRP2 (megalin). Statistical analysis was performed using Student's unpaired t-test or One-Way ANOVA followed by Bonferroni's multiple comparison test. *P<0.05, ** P<0.01, *** P<0.001.

Fig. 3 – TTR leads to LRP2-ICD formation, probably via γ-secretase activity. (A) Cartoons describing mouse megalin and megalin plasmids, representing its structural differences and predicted molecular weights. **(B)** Representative images of TTR KO cultured hippocampal neurons 11DIV transfected with either mini-megalin plasmid (pLNCX-M4) or with short-megalin –fused with GFP (Short-megalin-GFP) for 48h, stained with megalin and GFP antibodies, respectively, showing both plasmids with overlapping intracellular distribution. **(C)** Representative images of TTR KO cultured hippocampal neurons transfected with short-megalin-GFP for 48h, and stimulated with recombinant mouse TTR in the presence or absence of specific-TTR nanobody 169F7. Quantification of GFP fluorescence intensity in neurites in the different experimental conditions is shown. TTR stimulation reduces the expression of megalin in neurites, and this effect is abolished in the presence of the nanobody. The results represent 3 independent neuronal cultures. **(D)** TTR KO neurons transfected with short-megalin-GFP were stimulated, or not, with recombinant mouse TTR (55µg/ml) for 4h. Western blot was performed, using an antibody for GFP. The LRP2-ICD immunoreactivity was quantified in Control and TTR exposed neurons, and show the increase in LRP2-ICD upon TTR stimulation. The results represent 2 independent neuronal cultures **(E)**. **(F)** The same result was observed in WT cultured hippocampal neurons (15DIV) stimulated, or not, with recombinant mouse TTR (55µg/ml) for 5 minutes. Western blot was performed to assess megalin

For Review Only

protein levels using an antibody for megalin. (**G)**, Cartoon describing mouse megalin and megalin constructs being RIP processed, upon ligand (TTR) binding.

Fig. 4 – TTR leads to LRP2-ICD nuclear translocation, as a putative transcription regulation mechanism. (A) Mouse megalin C-terminal with highlighted consensus sequences for NLS (nuclear localizing signals), NES (nuclear exporting signals), protein-DNA binding residues and metal binding sites, predicted by different bioinformatics tools. **(B)** Representative images of TTR KO cultured hippocampal neurons (11DIV) transfected with short-megalin-GFP, and stimulated, or not, with recombinant mouse TTR (55µg/ml) for 5 minutes. **(C)**, Quantification of GFP fluorescence intensity in the nucleus (DAPI colocalization) indicates the nuclear translocation of megalin. The results are the average ±SEM of 12-13 neurons obtained in 3 independent cultures. Statistical analysis was performed using Unpaired Student's t test. ** P<0.01 **(D,E)** Nuclear and cytosolic fractions isolated from TTR KO cultured hippocampal neurons (11DIV) transfected with mini-megalin plasmid (pLNCX-M4) for 48h and stimulated with recombinant mouse TTR or K15N TTR (55µg/ml) for 20 minutes, and analyzed by western blot showing the nuclear translocation of megalin. The effect was blocked when neurons were treated with the TTR mutated form that targets the site for TTR-megalin interaction. Histone H2Ax (nuclear fraction) and GAPDH (cytosolic fraction) were used to confirm cytosolic and nuclear fraction separation. The results are representative of 2 independent neuronal cultures**.**

For Review Only

Fig. 5 – Reduction of megalin levels impairs neurite outgrowth and survival of hippocampal neurons. (A) Representative MAP2 staining of WT, TTR KO and Meg$^{+/-}$ cultured hippocampal neurons (1 DIV). **(B)** Neurite number and **(C)** total neurite length of hippocampal neurons were determined (n=220-603 neurons, obtained from 3 independent cultures), showing that decreased megalin expression reduces neurite number and length. **(D)** TTR KO and Meg$^{+/-}$ TTR KO cultured hippocampal neurons (7DIV) were subjected to excitotoxic stimulation with glutamate. Neuronal survival was assessed 14h after the excitotoxic insult through nuclear condensation (n=3-6 independent cultures), demonstrating that megalin deficiency reduces neuronal survival of hippocampal neurons in physiological and toxic conditions. (**E**) Time course of normalized FRET/Donor values in the cell body of YC-Nano15 transfected TTR KO or Meg$^{+/-}$ TTR KO cultured hippocampal neurons (7DIV) under physiologic conditions, or in absence of extracellular calcium (extended results from (Gomes et al., 2016) (n=4-6 neurons obtained in 2-3 independent

experiments), indicating that megalin seems to be required for physiological neuronal activity. **(F)** Pictorial figure summarizing the association between megalin expression levels and hippocampal neuronal viability. Statistical analysis was performed using one-way ANOVA followed by Bonferroni's multiple comparison test (B-E). ** $P<0.01$, *** $P<0.001$, **** $P<0.0001$.

Fig. 6 – Megalin overexpression rescues neurite outgrowth and increases dendritic spine density. **(A)** WT cultured hippocampal neurons (11DIV) were transfected with a functional mini-megalin plasmid (pLNCX-M4) for 48/72h. Megalin protein levels in transfected and untransfected neurons were assessed by immunocytochemistry and western blot. **(B-H)** TTR KO cultured hippocampal neurons (11DIV) were transfected with either GFP plasmid (pEGFP) or cotransfected with GFP (pEGFP) and mini-megalin plasmid (pLNCX-M4) for 48h, and stimulated, or not, as indicated, with recombinant mouse TTR (55µg/ml) for 24h. An immunocytochemistry was performed using GFP antibody. **(B)** Representative images of neurons expressing GFP. **(C)** Total neurite length, **(D)** neurite number and (**E-G**) branching were increased when megalin is overexpressed and or neurons are treated with TTR. (n=12-23 neurons, from 3 independent cultures). **(H)** Dendritic spine density is increased in neurons overexpressing megalin (n= 1-3 dendrites, from 8-10 neurons, from 1 independent culture). **(I)** Pictorial figure summarizes the effects in neurite outgrowth triggered by overexpression of megalin vs TTR stimulation. Scale bar in A corresponds to 20 µm and to 50 µm in B. Statistical analysis was performed using one-way ANOVA followed by Bonferroni's multiple comparison test (B,C) or by two-way ANOVA followed by Fisher's LSD multiple comparison test. . *$P<0.05$, ** $P<0.01$, *** $P<0.001$, **** $P<0.0001$.

Fig. 7 – Megalin heterozygous mice show structural alterations in hippocampal neurons. (A) Schematic representation of mice intravenously injected with AAV.PHP.eB_GFP with representative images of the hippocampus (Scale bar: 100 µm), and high-magnification images of CA1 and DG neurons (Scale bar: 50 µm). Neurite number **(B,F)**, total neurite length **(C,G)** and branching (sholl) **(D,H)** analysis in neurons from CA1 and DG regions of the hippocampus, respectively, show a decrease in neuronal complexity in $Meg^{+/-}$ mice, compared to WT littermates ($Meg^{+/+}$ n=18/4 neurons/mice, $Meg^{+/-}$ n=15/4 neurons/mice). Schematic representations of CA1 **(E)** and DG **(I)** neurons are shown. **(L)** Representative images from secondary dendrites of CA1 pyramidal neurons from $Meg^{+/-}$ and WT littermates, and schematic representation of mature and

immature spines (Meg$^{+/+}$ n=37/13/3 neurites/neurons/mice, Meg$^{+/-}$ n=37/15/4 neurites/neurons/mice) **(J)**. The values are represented by 10 µm of dendritic length. Scale bar: 1 µm. **(J-M)** The density of dendritic spines in secondary branches of hippocampal CA1 neurons of Meg$^{+/-}$ was reduced, when compared to WT littermates **(K)**. This effect was observed only in mature spines, whereas immatures spines were not affected. **(L,M)** Levels of VGLUT1 are decreased in whole tissue hippocampal extracts in Meg$^{+/-}$ mice **(N)**, and PSD95 levels show a statistical tendency to decrease (p=0.09) (Meg$^{+/+}$ n=8 mice, Meg$^{+/-}$ n=8 mice **(O)**, as determined by western blot analysis. **(P,Q)** The number of excitatory synapses, defined by the colocalization of VGLUT1 and PSD95 puncta, is decreased in Meg$^{+/-}$ mice (Meg$^{+/+}$ n=42/7 images/mice, Meg$^{+/-}$ n=45/8 images/mice). Values are normalized to Meg$^{+/+}$ mice. Representative images are shown in **(R)**. Scale bar: 5 µm

Fig. 8 – Megalin heterozygous mice show cognitive deficits, but no effects in anxiety-like behavior or locomotor activity. (A) Schematic representation of the EPM test. **(B)** Anxiety-like behavior was not altered in Meg^{+-} mice, compared to WT littermates, as shown by the % of time spent in open arms in EPM test. Meg$^{+/+}$= 13, Meg$^{+/-}$=11 mice. **(C)** Schematic design of the open field test. **(D,E)** Open field reinforces the absence of effects in anxiety in Meg$^{+/-}$ mice compared to Meg$^{+/+}$ mice (% of distance in center-**D**), and demonstrates no impairment in locomotor activity in Meg$^{+/-}$ (total distance travelled-**E**). Meg$^{+/+}$ n=15, Meg$^{+/-}$ n=15 mice. **(F)** Schematic diagram of Morris Water Maze. **(G)** Meg$^{+/-}$ mice display increased latency to reach the platform during training sessions 2 and 4. **(H,I)** During trial session, Meg$^{+/-}$ mice show a tendency for an increased average distance travelled **(H)** and a higher latency to reach the platform **(I)**. **(J)** Representative tracing of probe trials of Meg$^{+/-}$ and WT littermates. Meg$^{+/+}$ n=15 ; Meg$^{+/-}$ n=13 mice. **(K)** Schematic representation of Novel Object Recognition test. **(L)** Meg$^{+/-}$ mice display a reduction of the discrimination index of the novel object versus the familiar object, compared to WT littermates. **(M)** No differences were observed for the objects total exploration time between genotypes. **(N)** Representative tracing of choice session of Meg$^{+/-}$ and WT littermates (Meg$^{+/+}$ n=12, Meg$^{+/-}$ n=14). Statistical analysis was performed using Student's t-test, except in G, where Two-way ANOVA was used. *p<0.05.

For Review Only

Thumbnail - Cartoon representing Neuronal Megalin (LRP2) unraveled properties. Previously we have demonstrated that TTR, a megalin ligand, promotes neurite outgrowth, in a megalin dependent way (Gomes et al., 2016). Now, we describe that TTR promotes megalin mRNA and protein upregulation through a RIP process, involving megalin ICD nuclear translocation, regulating megalin expression in a positive feedback mechanism. Moreover, we unravel that megalin, independently of TTR, and probably through other endogenous ligands, can be neuritogenic and promote neurite branching. In addition, megalin contributes to physiological neuronal activity and survival, since decreasing its levels leads to a decrease in neurite number, length and branching, and neurons become more sensitive to toxic stimuli. Finally, we unravel a new unexpected role of megalin in synaptic plasticity, since it promotes an increase in dendritic spine density and maturation, and in the number of active synapses. All of these mechanisms impact in learning and memory mechanisms in mice with altered levels of megalin. These findings explain how Donnai-Barrow syndrome, and other megalin gene pathologies, present cognitive and intellectual disabilities, and contribute to address TTR, or other mechanisms that increase megalin expression, as potential therapeutic strategies

Material and Methods

Mice

The number of mice handled for this research was approved by the Institutional and National General Veterinary Board Ethical Committees (approval reference number 003424), according to National and European Union rules. Three to six-month-old TTR wild type ($TTR^{+/+}$), TTR KO ($TTR^{-/-}$) (Episkopou et al., 1993), Megalin heterozygous ($Meg^{+/-}$) and $Meg^{+/-}$ TTR KO mice, in a 129/svJ background were used to access megalin levels in different areas and for hippocampal neuronal cultures. Megalin heterozygous mice were kindly provided by Dr. Thomas Willnow, Max-Delbrueck Center for Molecular Medicine, Berlin, Germany (Willnow et al., 1996). The animals were reproduced, maintained (regular rodents chow and tap water ad libitum) and experimentally manipulated under a 12h light/dark cycle in type II cages in specific pathogen free conditions in the animal facility (microbiological heath status available). The method of euthanasia used was cervical displacement. Charles River Laboratories is the external animal facility used to acquire

For Review Only

animals. Genotypes were determined from tail extracted genomic DNA, using primers for the detection of exon 2 of TTR (which is disrupted in $TTR^{-/-}$ by insertion of a neomycin resistance gene), Megalin and neomycin as previously described (Episkopou et al., 1993; Willnow et al., 1996). Physical randomization, for group selection, was performed by doing a lottery with the ear plug numbers, using small papers. The order by which the animals in the different experimental groups were assessed was random, testing all the groups in each experimental session. ARRIVE guidelines were taken into consideration in experimental reporting. This study was not pre-registered.

Recombinant TTR production and purification

Recombinant mouse and human TTR were produced in a bacterial expression system using Escherichia coli BL21 (Furuya et al., 1991) and purified as previously described (Almeida et al., 1997). Briefly, after growing the bacteria, the protein was isolated and purified by preparative gel electrophoresis after ion-exchange chromatography. Protein concentration was determined using the Lowry method (Lowry et al., 1951). To remove endotoxins from recombinant TTR, a polymixin B column (Thermo Scientific, Detoxi-Gel™ Endotoxin Removing Gel, #20339) was used. Briefly, the column was regenerated with 1% sodium deoxycholate (Sigma) and washed with pyrogen-free buffer to remove detergent. Recombinant TTR was applied to the column and incubated during one hour at room temperature. Aliquots of pyrogen-free buffer were added and the flow-through was collected. Protein concentration was determined using the Bradford method (Hammond and Kruger, 1988).

Primary hippocampal neuronal cultures

Primary cultures of mouse hippocampal neurons were prepared from the hippocampus of E17-E18 WT, TTR KO, and $Megalin^{+/-}$ or $Megalin^{+/+}$ mice embryos (129/svJ background), as previously described (Almeida et al., 2005; Gomes et al., 2011). Neuronal cultures were maintained in serum-free Neurobasal medium (Gibco Life Technologies), supplemented with B27 (Gibco Life Technologies), glutamate (25 μM), glutamine (0.5 mM) and gentamicin (0.12mg/mL). Cells were kept at 37°C in a humidified incubator with 5% CO2/95% air, for 1DIV/13DIV, for the neurite outgrowth experiments or 7 days for the western blot experiments, the time required for maturation of hippocampal neurons . Cells were cultured at a density of $9x10^4$ cells/cm^2 or $8x10^4$ cells/cm^2 on poly-D-lysine-coated 6-well microplates (MW6) (for western blot and real-time PCR experiments) or glass coverslips (for immunocytochemistry studies), respectively. Megalin mice

For Review Only

embryos were genotyped (5h express protocol) to separate the $Meg^{+/-}$ from the $Meg^{+/+}$ animals. Meanwhile, hippocampi were hibernated using Hibernate E medium (GIBCO, Life Technologies) supplemented with diluted 1:10 B27 (Gibco, Life Technologies) and kept at 4ºC. These hippocampal neuronal cultures were performed using a serum free medium (B27), which does not contain T4 or RBP, TTR ligands. The following pre-established exclusion criteria were used: cell cultures with high % of cell death (> 30%) and/or neurite network undeveloped or damaged.

Western blot analysis

For cultured hippocampal neurons, cells were homogenized in lysis buffer containing 20 mM MOPS, 2mM EGTA, 5mM EDTA, 30mM sodium fluoride, 60mM β-glycerophosphate, 20mM sodium pyrophosphate, 1mM sodium orthovanadate, 1mM phenylmethylsulphonyl fluoride, 1% Triton X-100 and 1x protease inhibitors mixture (GE Healthcare). For whole tissue hippocampal extracts, tissue was dissociated in the same lysis buffer used for cultures, using a Eppendorf shape tissue homogeneizer at 3000rpm, with samples frozen. Total protein concentration was determined using the Bradford method. 50-150µg of protein was applied and separated by 10% SDS-PAGE and transferred to a nitrocellulose Hybond-C membrane (GE Healthcare), using a wet system, with Tris/Glycine/methanol buffer (Biorad). Membranes were blocked at least one hour at room temperature in blocking buffer, 5% BSA in phosphate-buffered saline Tween-20 (PBST), and then incubated overnight at 4°C with primary antibodies diluted in blocking buffer, namely sheep polyclonal anti-Megalin (1:1000; custom made), rabbit monoclonal C-terminal anti-Megalin (1:750, Abcam, ab24640), rabbit polyclonal Cterminal anti-Megalin (1:500, Abcam, ab76969), rabbit anti-vinculin (1:1500, Abcam, ab129002), mouse anti-α-tubulin (1:10000, Sigma, T8203), rabbit anti-MAP2 (1:800, Abcam, ab24640), mouse anti-GFP (1:2000, 11814460001, Roche, Sigma), mouse anti-Gapdh (1:20000, Abcam, ab9484), goat anti-Histone H2A.X (1:500, Sta. Cruz, sc-54606), rabbit VGLUT1 (1:5000, Sysy, 135 303), mouse PSD95 (1:2000, ThermoFisher Scientific, MA1-045), GAPDH (1:20000, Abcam, ab9484). Membranes were then incubated with anti-rabbit IgG-HRP (1:10 000; Binding Site), anti-mouse IgG-HRP (1:5000; Binding Site) and anti-goat IgG-HRP (1:5000; Binding Site), for 1 hour at room temperature. Blots were developed using Immun-Star WesternC Chemiluminescent kit (BioRad) and exposed to Bio-Rad ChemiDoc XRS system or ECL Hyperfilm (GE Healthcare), if the signal was too low. Quantitative analyses were performed using the Quantity One software or ImageLab from Biorad® Laboratories. The experimental unit in western

https://mc.manuscriptcentral.com/braincom

blot assays was each individual culture (always performed with different breeding females in independent neuronal culture isolation procedures or different animals).

Subcellular fractionation protocol

In order to separate nuclear and cytoplasmatic fractions from TTR KO hippocampal neurons cellular extracts (13 DIV), cultured neurons were homogenized in buffer containing 10mM HEPS, 1.5mM MgCl2, 10mM KCl, 0.5mM DTT and 0.05% NP40 at pH 7.9, supplemented with 1x protease inhibitors mixture (GE Healthcare). Neurons were extracted in 80µl of chilled supplemented buffer, using a cell scraper, and kept on ice for 10minutes. Then, neurons were centrifuged at 720xg, 4°C for 5 minutes. The pellet containing the nuclear fraction was resuspended in TBS (Tris-buffered saline) with 0.1% SDS, while the supernatant was centrifuged again for 10 000 xg for 5min. The obtained supernatant contained mostly the cytoplasmatic fraction (with some membranes). Both fractions were then sonicated on ice, which is particularly important for nuclear fractions, in order to shear genomic DNA and homogenize the lysate. Western blot confirmed nuclear fraction enrichment by Histone H2Ax in opposition to Gapdh. This protocol was based on Abcam "Nuclear Extraction and fractionation protocol" and "Subcellular fra ctionation protocol".

mRNA semi-quantification through Real-time PCR

Total RNA was extracted from either 7DIV cultured hippocampal neurons or dissected brain/organ areas from WT or TTR KO mice using TRIzol Reagent (Invitrogen), as previously described (Santos and Duarte, 2008). RNA quality and integrity was assessed using the Experion automated gel-electrophoresis system (Bio-Rad, Portugal), as previously described (Santos and Duarte, 2008). Samples showing RNA degradation or contamination by DNA were discarded. RNA concentration was determined using NanoDrop 1000 (Thermo Scientific). The samples were aliquoted and stored at -80 °C until further use. cDNA synthesis was performed using 1µg of total RNA and the SuperScript® cDNA synthesis (random primers) (Invitrogen, Portugal), as previously described (Santos and Duarte, 2008). Samples were stored at -80 °C until further use (1 week – 1 month). Primers used for Real time PCR were designed using "Beacon Designer" software (Premier Biosoft International) as described previously (Santos and Duarte, 2008). Oligonucleotides used for Megalin real-time PCR were: forward, 5'GGCTCACTCAAGTCCGCATCTTCC3' and reverse 5'ACTCAACGGTGCTGCCAGTTACG3'. For LRP1: forward, 5'CGAGGAGCAGGTTGTTAG3' and reverse

For Review Only

5’CAGAAGCAGCAGGAGAAG3’. 18s RNA was used as reference gene with the following primers: forward, 5’AAATCAGTTATGGTTCCTTTGGTC3’ and reverse 5’GCTCTAGAATTACCACAGTTATCCAA3’; for β-actin, forward, 5’CTAAGGCCAACCGTGAAAAG3’, and reverse, 5’ACCAGAGGCATACAGGGACA3’; and for Gapdh, forward, 5’GCCTTCCGTGTTCCTACC3’, and reverse, 5’AGAGTGGGAGTTGCTGTTG3’. The annealing temperature was 60°C. For gene expression analysis, 1 µL of stock to 1:1000 diluted cDNA was added to 10 µL of 2× SYBR Green Master Mix (Bio-Rad) and the final concentration of each primer was 250 nM in 20 µL total volume. The thermocycling reaction was initiated through activation of Taq DNA Polymerase by heating at 95°C during 3 min, followed by 45 cycles of a 15s denaturation step at 95°C and a 20s annealing/elongation step at 60°C. The fluorescence was measured after the extension step, using the iQ5 Multicolor Real-Time PCR Detection System (Bio-Rad). After the thermocycling reaction, the melting step was performed with slow heating, starting at 55°C and with a rate of 0.5°C per 10s, up to 95°C, with continuous measurement of fluorescence. Data analysis was performed using Pfaff method for efficiency correction (Pfaffl, 2001). Results were normalized with either 18S, Gapdh or β-actin RNA, as internal reference gene, since these genes show a stable expression in the conditions tested (we tested the different reference genes). We took into consideration MIQE guidelines, for minimum information for Publication of *Q*uantitative Real-Time PCR *E*xperiments (Bustin et al., 2009).

Immunocytochemistry

Neurons were fixed in 4% sucrose/4% paraformaldehyde, and permeabilized with 0.3% Triton X-100 in PBS for 10 min. Neurons were then incubated with 5% bovine serum albumin (BSA) (Sigma) in PBS with 0.1% Tween 20 (PBST), for 1h at 37°C, to block nonspecific binding, and incubated with primary antibodies, overnight at 4°C. Cells were then washed 5 times with 0.5% BSA in PBST, and incubated with the appropriate secondary antibodies, for 1h at 37°C. The coverslips were mounted in a fluorescent mounting medium (DAKO, Denmark) and imaging was performed on a laser scanning Confocal Microscope Leica SP5 AOBS SE, using the 40x water or 63x oil objective. Primary antibodies used were anti-MAP2 (1:800; Abcam, ab24640), mouse anti-GFP (1:500, 11814460001, Roche, Sigma) and rabbit polyclonal C-terminal anti-Megalin (1:500, Abcam, ab76969); the secondary antibodies were Alexa Fluor 488 or 568 (1:750, Life technologies). The fluorescent dye Hoechst 33342 (0.5 µg/ml, 10minutes room temperature) was used to stain nuclei. In each set of experiments, the same batch of antibodies (primary and secondary) was used, and imagens were

For Review Only

taken using the same settings. The quantification of nuclear megalin C-terminus fused with GFP was performed by quantifying GFP nuclear fluorescence, by colocalizing with Hoechst stain, using ImageJ software. At least 12-13 transfected neurons were counted for each experimental condition in a blind way for the condition, from 3 independent preparations. The experimental unit in these assays was each individual neuron.

Transfection

Transfection of cultured hippocampal neurons was performed through the calcium phosphate co-precipitation method as previously described, with minor modifications (Dudek et al., 2001; Gomes et al., 2011). Briefly, 2µg of single plasmid DNA (or mixture of equal amounts of 2 plasmids) were diluted in Tris-EDTA (TE) pH 7.3 and mixed with HEPES calcium chloride pH 7.2 (2.5M CaCl2, 10mM HEPES). This DNA/TE/Calcium mix was added to 2xHEPES Buffered Saline solution (270mM NaCl, 10mM KCl, 1.4mM Na2HPO4, 11mM Dextrose, 42mM HEPES), pH 7.2. The precipitates were allowed to form for 30 minutes, with vortex mixing every 5 minutes, to ensure that the precipitates had similar small sizes. Meanwhile, coverslips with cultured neurons were incubated with cultured conditioned medium with 2mM of Kynurenic acid. The precipitate was added drop wise to each coverslip and incubated at 37°C, 5% CO_2, for 3h. Cells were then washed with acidic (10% CO_2) equilibrated culture medium containing 2 mM Kynurenic acid and returned to the 37°C/5% CO_2 incubator for 15 minutes. Finally, the medium was replaced with the initial culture conditioned medium, and the cells were further incubated in a 37°C/5% CO_2 incubator for 48h to allow protein expression.

For Review Only

cDNA Constructs

Three plasmids were used: pEGFP-N1, the expression vector containing the green fluorescent protein (GFP) gene only (BD Biosciences, Clontech); pLNCX-M4, that generates functional megalin mini-receptor; and pEGFP_N1-Cterminal Megalin, that briefly contains the entire cytoplasmatic region of the megalin gene fused with GFP. The megalin mini-receptor pLNCX-M4 was a kind gift from Akihiko Saito (Takeda et al., 2003), and was generated by a PCR fragment containing the fourth ligand-binding motif till the end of the C-terminal of the megalin gene (rat origin – 95% identity with mouse) (see cartoon in Fig. 3), and cloned into the pLNCX2 retroviral vector. In this work, no retroviruses were produced as a transient transfection was the objective, and a more physiological over-expression of the protein (CMV promoter). Regarding the pEGFP_N1- Cterminal Megalin, it was a gift from Ignacio Torres-Aleman (Bolos et al., 2010). The cDNA of the plasmid

contains a small extracellular region with two perimembrane extracellular cysteine rich domains, the transmembrane part and the complete cytoplasmatic region of megalin gene (from human origin – C-terminal with 75% identity with mouse), fused Cterminally with the GFP cDNA of the pEGFP vector. All the plasmids were sequenced by DNA sequencing reactions to confirm their identity and integrity.

In vitro neuronal morphological analysis

Cultured hippocampal neurons from WT, TTR KO and $Meg^{+/-}$ mice embryos were isolated under the above described conditions, plated at a density of $5x10^4$ cells/cm^2 (Fig. 5), or $8x10^4$ cells/cm^2 (Fig. 3,4,6). Cells were maintained in culture 24 hours, approximately, in order to allow the precise tracing of all the neurites per neuron. For the analysis of mature neurons (10-15DIV), transfection with a GFP plasmid was performed, to allow the analysis of individual neurons. In these experiments (Fig. 6), TTR KO cultured hippocampal neurons (11DIV) were transfected with either GFP plasmid (pEGFP), or cotransfected with GFP (pEGFP) and mini-megalin plasmid (pLNCX-M4), protein expression was allowed for 48h, and then neurons were stimulated, or not, with recombinant mouse TTR (55µg/ml) for 24h, in cultured conditioned medium. Cells were fixed with 4% paraformaldehyde and immunofluorescence was performed using rabbit anti-MAP_2 (1:800, ab24640, Abcam) or mouse anti-GFP (1:500, 11814460001, Roche, Sigma). The coverslips were mounted in a fluorescent mounting medium (DAKO, Denmark) and imaging was performed on a Zeiss AxioImager Z1microscope, using a 20× oil objective, for the 1DIV cultures, or Confocal Microscope Leica SP5 AOBS SE, for mature neurons. For 1DIV neurons, 15 to 20 images were randomly acquired throughout the coverslip, in a blind way for the condition; in the case of mature transfected neurons, pictures were taken from GFP transfected neurons that were alive, in a blind way for the condition. Regarding the 1DIV neurons, at least 60-80 cells were analyzed per experimental condition in a blind way, and the experiments were repeated in at least 3 independent preparations; for the transfected neurons (14DIV), a total of 15-20 neurons were analyzed in each preparation, from 3 independent preparations. The experimental unit in these assays was each individual neuron.

Morphological measurements of neurite outgrowth (Number of neurites and total neurite length per cell) were performed using the plugin NeuronJ from the ImageJ software (Meijering et al., 2004). The analysis of neurite branching (sholl analysis) was performed as previously described (Braz et al., 2017), using Bonfire scripts for MATLAB (Mathworks). After neurites were defined in

For Review Only

NeuronJ, data was converted to SWC using Bonfire, and the dendritic arbor of each neuron was determined by defining the tracings connectivity using NeuronStudio software. Then, sholl analysis was performed using Bonfire, by drawing concentric circles around the cell body with incremental radii, separated from each other by 6.0 µm, and counting the number of times each circle crosses a neurite. The number of intersections was represented in function of the distance from the cell body. For the analysis of total dendritic spine density, dendritic segments were defined using ImageJ software, and the total number of dendritic spines was defined by the user, blind to the treatment. The number of dendritic spines was represented in function of 10 µm of dendritic length.

Immunofluorescence of Brain Choroid plexus

Tissue samples for immunohistochemistry were collected after mice were perfused with PBS and 4% paraformaldehyde. 5mm thick tissue sections were deparaffinated in Histoclear and hydrated in a descending alcohol concentration series. Then, slides were incubated with Tris-buffer saline (TBS), and permeabilized with 0.2% Triton X-100 in TBS solution for 10 minutes and rinsed in TBS 0.025% Triton X-100. Blocking was performed with 10% fetal bovine serum, plus 1% bovine serum albumin and 0.3M glycine, in TBS, for 2h at room temperature. Primary antibodies were always incubated overnight at 4°C, in 1% BSA in TBS. Secondary antibodies were incubated 1h at room temperature. The slides were mounted in a fluorescent mounting medium (DAKO, Denmark) and imaging was performed on a laser scanning Confocal Microscope Leica SP5 AOBS SE, using the 40x/63x oil objective. In each set of experiments the same batch of antibodies (primary and secondary) was used, and images were taken using the same settings, such as camera exposure times. Primary antibodies used were rabbit monoclonal C-terminal anti-Megalin (1:750, Abcam, ab24640) and anti-mouse recombinant TTR (1:250, custom made, Quantum Appligene, Illkirch, France). As secondary antibodies Alexa Fluor 488 and 568 (1:750, Life Technologies) were employed.

Cell Death Assay

Hippocampal neurons from TTR KO and Meg$^{+/-}$ TTR KO were cultured for 7 days on poly-D-lysine coated glass coverslips as previously described. After excitotoxic stimulation with glutamate (125µM glutamate, 20min), and further incubation in cultured conditioned medium (14h), cells

For Review Only

were fixed in 4% sucrose/4% paraformaldehyde (in PBS) and the nucleus was stained with Hoechst as previously described. Analysis of the nuclear morphology was performed on Zeiss AxioImager Z1 florescence microscope, under a 40x oil objective. Live and dead cells were counted blind, using ImageJ. The experimental unit in these assays was each individual culture (always performed with different breeding females in independent neuronal cultures).

FRET assay – intracellular calcium concentration

TTR KO cultured neurons ($8x10^4$ cells/cm^2) were plated in poly-D-lysine-coated glass-bottom dishes (ibidi GmbH), and transfected at 7DIV and imaged 48h later, with yellow cameleon-Nano 15 (YC-Nano15), an ultrasensitive Ca^{2+} FRET probe (Horikawa et al., 2010), as previously well described (Gomes et al., 2016). Fluorescence imaging of cells was performed using an epifluorescence inverted microscope (DMI 6000B, Leica Microsystems) with a PlanApo 63x (N.A. 1.4) glycerol immersion objective. Data acquisition and processing was based on (Ferraz-Nogueira et al., 2014). The FRET/donor change was calculated using ImageJ software. The experiments performed without calcium used a Krebs-Ringer solution: 119 mM NaCl, 2.5 mM KCl, 1.0 mM NaH_2PO_4, 2 mM EGTA, 1.3 mM $MgCl_2.6H_20$, 20 mM HEPES and 11 mM D-glucose, pH 7.4. The experimental unit in FRET assays was each individual neuron (4-6 neurons were analyzed in each individual culture).

AAV *in vivo* delivery

To achieve a sparse labelling of neurons in the CNS, we performed injections in the tail vein of mice-$Meg^{+/+}$ and $Meg^{+/-}$ - with AAV_(PHP.eB)-CAG-GFP (Addgene viral prep # 37825-PHPeB) in a final titer of $0.5x10^{11}$, diluted in sterile PBS to a final volume of 150 µl. 21 days post-injection, mice were deeply anesthetized with ketamine/medetomidine 170 mg/kg (ketamine) + 2 mg/kg (medetomidine), intraperitoneal , and perfused transcardially with PBS followed by 4% PFA with 4% sucrose in PBS, pH 7.4. Whole brain was removed and fixed with 4% PFA with 4% sucrose for 48 hours, followed by transfer to 20% sucrose in PBS, until they moved to the bottom of the tube (around 24 h). 60-80 µm serial coronal sections of the hippocampus were obtained using a Cryostat (Leica CM 3050S, Leica Microsystems, USA), and kept in PBS with 0.2% sodium azide at 4°C until further use.

Immunocytochemistry of hippocampal slices

For Review Only

For the analysis of neuronal morphology and synapse quantification (colocalization of PSD95 and VGLUT1) in brain slices, we proceed as following. Brain slices from different parts of the hippocampus (anterior/posterior) were selected and processed two slices per well in a 48-well plate. Unspecific staining was blocked by incubating with 10% horse serum in PBS containing 0.2% Triton X-100, and primary antibodies (GFP 1:500 for neuronal morphological analysis; PSD95 1:500 and VGLUT1 1:1000 for synapse analysis) were incubated in blocking solution for 72 h at 4°C with agitation. After washing with PBS (3 times, 15 min each), slices were incubated with secondary antibodies (Alexa 488 for GFP, Alexa 594 for PSD95 and Alexa 647 for VGLUT1) 24h at 4°C with agitation. Slices were then washed in PBS, and mounted in gelatinized slides in DAKO mounting medium, and sealed with nail polish. Slices were kept at 4°C protected from light.

Neuronal morphological analysis *in vivo*

Images of neurons in the CA1 and DG regions of the hippocampus were acquired in Leica TCS SP5II confocal microscope (Leica microsystems, Germany), with a 40x/1.10 NA Water objective, using an argon 488 nm laser, keeping the settings equal for all images and animals. Neurons expressing GFP were randomly chosen for quantification from at least four different sections from the region of interest, and three animals were analyzed per genotype, with at least ten neurons per region (CA1 or DG) analyzed per animal. Neuron tracing was performed using NeuronJ plugin for ImageJ software, and sholl analysis as described for the in vitro analysis of neuronal morphology in vitro in hippocampal neuronal cultures. For the analysis of dendritic spine density, images from secondary dendrites of hippocampal neurons were acquired in Leica TCS SP5II confocal microscope (Leica microsystems, Germany), with a 63x/1.3 NA Glycerol objective, using Ar 488 nm laser. Spine density and morphology were defined as immature spines, which included filopodia (without a defined head), or mature, which included thin (with a long neck, and a small head), mushroom (with a small neck and a large head) and stubby (without a defined neck) using ImageJ software. The number of dendritic spines was represented in function of 10 µm of dendritic length. Experiments were performed by a user blind to the genotype, both in image acquisition and analysis.

Synapse quantification *in vivo*

https://mc.manuscriptcentral.com/braincom

For Review Only

The quantification of protein colocalization at dendrites was performed as previously described (Catarino et al., 2013). Briefly, images from the CA1 region of the hippocampus were acquired in a confocal microscope Leica TCS SP5II confocal microscope (Leica microsystems, Germany), with a 63x/1.3 NA Glycerol objective, maintaing the settings between slices and animals. Seven animals were analyzed per genotype, and two to ten images were acquired per animal. The acquired images had 20 to 40 µm of depth, and were analyzed using ImageJ software. In each image, 2 to 4 regions of interest were selected as rectangles (19.26 width x 15.89 high µm) in regions containing staining of both PSD95 and VGLUT1, and multiplied by the stack depth. The signal was subjected to a user-defined intensity threshold to select recognizable puncta in the region selected. The thresholded signals were then used to determine the colocalization. For this, PSD95 signal was set as binary, whereas VGLUT1 signal was used to define the total synaptic puncta, and the puncta positive for PSD95 were defined as colocalized. Finally, all the values acquired were normalized to the mean values of the control group. Experiments were performed by a user blind to the genotype, both in image acquisition and analysis.

For Review Only

Behavioral tests

Prior to the beginning of the behavioral tests, mice were allowed a 2-week adaptation period to the behavioral tests facilities. Tests were conducted in the dark (active) phase. All material was cleaned with a neutral detergent without smell between animals. The same groups of animals performed the EPM and Morris Water Maze test, and different groups performed the Open field followed by the NOR test (a 3R strategy).

Elevated Plus Maze

EPM apparatus was placed at 50 cm from the ground, composed of a cross from of two open arms (30x5 cm) and two closed arms (30x5 cm, surrounded by 15 cm-high wall, opaque), with the two pairs of identical arms in opposite positions to each other, with the arms emerging from a central platform (5x5 cm). The test was initiated by placing the mouse in center of the apparatus facing an open arm, and allowed to move freely during 5 min. The lights were on and placed above the apparatus. Mouse behavior was continuously videotaped by a camera placed above the apparatus. We evaluated the number of entries, time spent in open or closed arms, time spent in the central platform, and total distance travelled, using a tracking system (Smart Video Tracking Software v 2.5, Panlab).

Open Field

Each mouse was placed in the center of an opaque arena (43x43 cm) and allowed to move freely for 10 min. The total distance travelled, peripheral activity (locomotion along the walls) and center activity (locomotion in the central zone) were automatically obtained through video tracking (Smart Video Tracking Software v 2.5, Panlab).

Morris Water Maze

Method has been as previously described, with minor modifications (Ribeiro et al., 2014). A circular pool (110 cm in diameter) filled with water (27±2◦C) to a depth of 18.5 cm was placed in a quiet room decorated with contrast visual cues. Water was made opaque by the addition of white non-toxic ink. Abstractly, the pool was divided into four quadrants, and eight start locations were defined—north (N), south (S), east (E), west (W), northeast (NE), southeast (SE), northwest (NW), and southwest (SW)—at equal distances to the center. An escape platform (10 × 10 cm) was immersed 0.5 cm bellow the water line. On the first two days, mice were subjected to cue learning in order to test them for their ability to learn to swim to a cued goal. For this procedure, curtains were closed around the maze to reduce the availability of distal cues, and a flag was attached to the hidden platform. Animals were given four 60 sec trials per day, each trial with different start and goal positions. Between each trial, mice were allowed to stay on the platform for 15 sec. After the cued learning, mice were tested for their visual acuity. For this, a large plastic cue was placed on the platform and each mouse was scored for its latency to reach the platform. 24 hours before this test, an identical plastic cue was placed in each of the mouse housing cages to minimize the possible effects of exposure to a novel object. After the cued learning, a 7-day hidden-platform learning phase was initiated. The platform was placed in the SW quadrant and the animals were scored for their latency to find the hidden platform. Mice were given four 60 sec trials per day, each trial with different start locations, and inter-trial intervals on the platform of 30 and 15 sec on days 1 and 2–7, respectively. Twenty-four hours following day 7 of the hidden-platform learning phase, the platform was removed and each mouse was subjected to a 30 sec probe trial starting 180◦ (NE) from the original platform position (SW). The number of platform-site crossovers, the latency to first target-site crossover, the percent time spent in the target quadrant (and also in the opposite quadrant) compared with the other quadrants were evaluated using SMART software.

Novel Object Recognition

For Review Only

The NOR test was performed in an arena (43x43 cm), and consisted in three phases: the habituation phase, in which mice were allowed explored the apparatus for 10 min; the acquisition/sample phase 24h after the habituation, where mice were placed in the apparatus with two identical objects (familiar object) for 10 min; and the retention/choice session, performed 6 hours after (inter-trial interval), in which a novel object and a familiar object were used, and mice were allowed to explore the objects for 3 min (Figure 8L). The objects chosen for this experiment were approximately the same height and weight, with different shapes, colors or materials (legos, glass cup or toy). Object exploration was defined as mice nose touching or directed towards the object at a distance shorter than 2 cm, and the duration of time mice spent exploring each object was recorded by the observer, blind to the genotype, using Observer 7 XT software (Noldus Information Technology, Wageningen, The Netherlands). The discrimination index (DI) was calculated as the ratio TN/(TN+TF) [TN=time exploring the novel (N) object; TF=time exploring the familiar (F) object]. As exclusion criteria, mice exploring the objects for periods lower than 7 sec at 3 min of choice session were removed.

For Review Only

NLS, NES, protein-DNA and metal binding residues - bioinformatic tools

The following bioinformatics tools were used to access nuclear localizing signals (NLS) and nuclear exporting signals (NES), in the mouse megalin C terminal. For NLS: : NucPred, website: https://nucpred.bioinfo.se/cgi-bin/single.cgi (Brameier et al., 2007) , cNLS Mapper, website http://nls-mapper.iab.keio.ac.jp/cgi-bin/NLS_Mapper_form.cgi (Kosugi et al., 2009), PSORT II server, website https://psort.hgc.jp/helpwww2.html (Reinhardt and Hubbard, 1998), NLStradamus, website http://www.moseslab.csb.utoronto.ca/NLStradamus/ (Nguyen Ba et al., 2009). NLStradamus; cNLS Mapper identify classical NLS's, sequences enriched in basic residues K (lysine) and R (arginine); NucPred, PSORTII identify sequences that to be nuclear/non-nuclear, and have increased potential to unveil novel NLS's.

Regarding NES we used the computational tools Nespredictor NetNES, website http://www.cbs.dtu.dk/services/NetNES/ (la Cour et al., 2004) and LocNES, website http://prodata.swmed.edu/LocNES/LocNES.php (Xu et al., 2015). These tools identify potential leucine-rich nuclear exporting signals, but also take into account the accessibility and flexibility of the protein sequence, to allow exporting proteins to interact with the predicted NES region.

Concerning protein-DNA binding residues we used, DRNApred (Yan and Kurgan, 2017), website http://biomine.cs.vcu.edu/servers/DRNApred/ and DP-Bind (Hwang et al., 2007), website

http://lcg.rit.albany.edu/dp-bind/ . For the metal-binding residues Metaldetector (Passerini et al., 2011), website http://metaldetector.dsi.unifi.it/v2.0/ and ZincBinder (Srivastava and Kumar, 2018), website http://proteininformatics.org/cgi-bin/znbinder/, were used. The results are in the supplemental information.

Statistical Analysis

Data presentation and "n" description is described in figure legends. A previous power analysis was performed in order to obtain a 25% difference (10% SD) among two groups, with 90-95% power and we obtain sample sizes between 3-6 animals or individual cultures. Statistical analysis of the results was performed using one-way analysis of variance (ANOVA) followed by Bonferroni multiple comparison test, when 3 groups were present. Unpaired Student's *t*-test was used when the comparisons were only between two groups. For both statistical analysis: ***p<0.001, ** p<0.01, * p<0.05, ns (not significant). Identification and removal of outliers was performed using automatic Graphpad prism software 7.0/8.0, using the ROUT (robust nonlinear regression) method, with a Q=5%, for removing likely outliers (Motulsky and Brown, 2006).

For Review Only

Almeida MR, Damas AM, Lans MC, Brouwer A, Saraiva MJ (1997) Thyroxine binding to transthyretin Met 119. Comparative studies of different heterozygotic carriers and structural analysis. Endocrine 6:309-315.

Almeida RD, Manadas BJ, Melo CV, Gomes JR, Mendes CS, Graos MM, Carvalho RF, Carvalho AP, Duarte CB (2005) Neuroprotection by BDNF against glutamate-induced apoptotic cell death is mediated by ERK and PI3-kinase pathways. Cell Death Differ 12:1329-1343.

Alvarez-Castelao B, Schuman EM (2015) The Regulation of Synaptic Protein Turnover. J Biol Chem 290:28623-28630.

Alvira-Botero X, Perez-Gonzalez R, Spuch C, Vargas T, Antequera D, Garzon M, Bermejo-Pareja F, Carro E (2010) Megalin interacts with APP and the intracellular adapter protein FE65 in neurons. Mol Cell Neurosci 45:306-315.

Ambjorn M, Asmussen JW, Lindstam M, Gotfryd K, Jacobsen C, Kiselyov VV, Moestrup SK, Penkowa M, Bock E, Berezin V (2008) Metallothionein and a peptide modeled after metallothionein, EmtinB, induce neuronal differentiation and survival through binding to receptors of the low-density lipoprotein receptor family. J Neurochem 104:21-37.

Ammar H, Closset JL (2008) Clusterin activates survival through the phosphatidylinositol 3-kinase/Akt pathway. J Biol Chem 283:12851-12861.

Andersen RK, Hammer K, Hager H, Christensen JN, Ludvigsen M, Honore B, Thomsen MB, Madsen M (2015) Melanoma tumors frequently acquire LRP2/megalin expression, which modulates melanoma cell proliferation and survival rates. Pigment Cell Melanoma Res 28:267-280.

Bartolome F, Antequera D, Tavares E, Pascual C, Maldonado R, Camins A, Carro E (2017) Obesity and neuroinflammatory phenotype in mice lacking endothelial megalin. J Neuroinflammation 14:26.

Bento-Abreu A, Velasco A, Polo-Hernandez E, Perez-Reyes PL, Tabernero A, Medina JM (2008) Megalin is a receptor for albumin in astrocytes and is required for the synthesis of the neurotrophic factor oleic acid. J Neurochem 106:1149-1159.

Berni R, Malpeli G, Folli C, Murrell JR, Liepnieks JJ, Benson MD (1994) The Ile-84-->Ser amino acid substitution in transthyretin interferes with the interaction with plasma retinol-binding protein. J Biol Chem 269:23395-23398.

Beydoun MA, Ding EL, Beydoun HA, Tanaka T, Ferrucci L, Zonderman AB (2012) Vitamin D receptor and megalin gene polymorphisms and their associations with longitudinal cognitive change in US adults. Am J Clin Nutr 95:163-178.

Beydoun MA, Tajuddin SM, Dore GA, Canas JA, Beydoun HA, Evans MK, Zonderman AB (2017) Vitamin D Receptor and Megalin Gene Polymorphisms Are Associated with Longitudinal Cognitive Change among African-American Urban Adults. J Nutr 147:1048-1062.

Biemesderfer D (2006) Regulated intramembrane proteolysis of megalin: linking urinary protein and gene regulation in proximal tubule? Kidney Int 69:1717-1721.

Bolos M, Fernandez S, Torres-Aleman I (2010) Oral administration of a GSK3 inhibitor increases brain insulin-like growth factor I levels. J Biol Chem 285:17693-17700.

Brameier M, Krings A, MacCallum RM (2007) NucPred--predicting nuclear localization of proteins. Bioinformatics 23:1159-1160.

Bray SJ (2006) Notch signalling: a simple pathway becomes complex. Nat Rev Mol Cell Biol 7:678-689.

Braz SO, Cruz A, Lobo A, Bravo J, Moreira-Ribeiro J, Pereira-Castro I, Freitas J, Relvas JB, Summavielle T, Moreira A (2017) Expression of Rac1 alternative 3' UTRs is a cell specific mechanism with a function in dendrite outgrowth in cortical neurons. Biochim Biophys Acta Gene Regul Mech 1860:685-694.

Brouillette J, Quirion R (2008) Transthyretin: a key gene involved in the maintenance of memory capacities during aging. Neurobiol Aging 29:1721-1732.

Bustin SA, Benes V, Garson JA, Hellemans J, Huggett J, Kubista M, Mueller R, Nolan T, Pfaffl MW, Shipley GL, Vandesompele J, Wittwer CT (2009) The MIQE guidelines: minimum information for publication of quantitative real-time PCR experiments. Clin Chem 55:611-622.

Buxbaum JN, Roberts AJ, Adame A, Masliah E (2014) Silencing of murine transthyretin and retinol binding protein genes has distinct and shared behavioral and neuropathologic effects. Neuroscience 275:352-364.

Buxbaum JN, Ye Z, Reixach N, Friske L, Levy C, Das P, Golde T, Masliah E, Roberts AR, Bartfai T (2008) Transthyretin protects Alzheimer's mice from the behavioral and biochemical effects of Abeta toxicity. Proc Natl Acad Sci U S A 105:2681-2686.

Carlos S, Carmen N (2010) Expression and Functions of LRP-2 in Central Nervous System: Progress in Understanding its Regulation and the Potential Use for Treatment of Neurodegenerative Diseases. Immunology, Endocrine & Metabolic Agents in Medicinal Chemistry 10:249-254.

Carro E, Spuch C, Trejo JL, Antequera D, Torres-Aleman I (2005) Choroid plexus megalin is involved in neuroprotection by serum insulin-like growth factor I. J Neurosci 25:10884-10893.

Cases O, Joseph A, Obry A, Santin MD, Ben-Yacoub S, Paques M, Amsellem-Levera S, Bribian A, Simonutti M, Augustin S, Debeir T, Sahel JA, Christ A, de Castro F, Lehericy S, Cosette P, Kozyraki R (2015) Foxg1-Cre Mediated Lrp2 Inactivation in the Developing Mouse Neural

For Review Only

Retina, Ciliary and Retinal Pigment Epithelia Models Congenital High Myopia. PLoS One 10:e0129518.
Catarino T, Ribeiro L, Santos SD, Carvalho AL (2013) Regulation of synapse composition by protein acetylation: the role of acetylated cortactin. J Cell Sci 126:149-162.
Chan KY, Jang MJ, Yoo BB, Greenbaum A, Ravi N, Wu WL, Sanchez-Guardado L, Lois C, Mazmanian SK, Deverman BE, Gradinaru V (2017) Engineered AAVs for efficient noninvasive gene delivery to the central and peripheral nervous systems. Nat Neurosci 20:1172-1179.
Christensen EI, Birn H, Storm T, Weyer K, Nielsen R (2012) Endocytic receptors in the renal proximal tubule. Physiology (Bethesda) 27:223-236.
Chun JT, Wang L, Pasinetti GM, Finch CE, Zlokovic BV (1999) Glycoprotein 330/megalin (LRP-2) has low prevalence as mRNA and protein in brain microvessels and choroid plexus. Exp Neurol 157:194-201.
Chung RS, Hidalgo J, West AK (2008) New insight into the molecular pathways of metallothionein-mediated neuroprotection and regeneration. J Neurochem 104:14-20.
De Strooper B, Annaert W, Cupers P, Saftig P, Craessaerts K, Mumm JS, Schroeter EH, Schrijvers V, Wolfe MS, Ray WJ, Goate A, Kopan R (1999) A presenilin-1-dependent gamma-secretase-like protease mediates release of Notch intracellular domain. Nature 398:518-522.
Derocq D, Prebois C, Beaujouin M, Laurent-Matha V, Pattingre S, Smith GK, Liaudet-Coopman E (2012) Cathepsin D is partly endocytosed by the LRP1 receptor and inhibits LRP1-regulated intramembrane proteolysis. Oncogene 31:3202-3212.
Dietrich M, Antequera D, Pascual C, Castro N, Bolos M, Carro E (2014a) Alzheimer's disease-like impaired cognition in endothelial-specific megalin-null mice. J Alzheimers Dis 39:711-717.
Dietrich M, Antequera D, Pascual C, Castro N, Bolos M, Carro E (2014b) Alzheimer's disease-like impaired cognition in endothelial-specific megalin-null mice. J Alzheimers Dis 39:711-717.
Dudek H, Ghosh A, Greenberg ME (2001) Calcium phosphate transfection of DNA into neurons in primary culture. Curr Protoc Neurosci Chapter 3:Unit 3 11.
Dwyer TA, Earl DE, Wang L (2008) The utility of a new in vitro model of the stroke penumbra. J Neurosci 28:6537-6538.
Ebinu JO, Yankner BA (2002) A RIP tide in neuronal signal transduction. Neuron 34:499-502.
Episkopou V, Maeda S, Nishiguchi S, Shimada K, Gaitanaris GA, Gottesman ME, Robertson EJ (1993) Disruption of the transthyretin gene results in mice with depressed levels of plasma retinol and thyroid hormone. Proc Natl Acad Sci U S A 90:2375-2379.
Fass D, Blacklow S, Kim PS, Berger JM (1997) Molecular basis of familial hypercholesterolaemia from structure of LDL receptor module. Nature 388:691-693.
Ferraz-Nogueira JP, Diez-Guerra FJ, Llopis J (2014) Visualization of phosphatidic acid fluctuations in the plasma membrane of living cells. PLoS One 9:e102526.
Fitzgerald M, Nairn P, Bartlett CA, Chung RS, West AK, Beazley LD (2007) Metallothionein-IIA promotes neurite growth via the megalin receptor. Exp Brain Res 183:171-180.
Fleming CE, Mar FM, Franquinho F, Saraiva MJ, Sousa MM (2009) Transthyretin internalization by sensory neurons is megalin mediated and necessary for its neuritogenic activity. J Neurosci 29:3220-3232.
Furuya H, Saraiva MJ, Gawinowicz MA, Alves IL, Costa PP, Sasaki H, Goto I, Sakaki Y (1991) Production of recombinant human transthyretin with biological activities toward the understanding of the molecular basis of familial amyloidotic polyneuropathy (FAP). Biochemistry 30:2415-2421.
Gajera CR, Emich H, Lioubinski O, Christ A, Beckervordersandforth-Bonk R, Yoshikawa K, Bachmann S, Christensen EI, Gotz M, Kempermann G, Peterson AS, Willnow TE, Hammes A (2010)

For Review Only

LRP2 in ependymal cells regulates BMP signaling in the adult neurogenic niche. J Cell Sci 123:1922-1930.

Gil-Yarom N, Radomir L, Sever L, Kramer MP, Lewinsky H, Bornstein C, Blecher-Gonen R, Barnett-Itzhaki Z, Mirkin V, Friedlander G, Shvidel L, Herishanu Y, Lolis EJ, Becker-Herman S, Amit I, Shachar I (2017) CD74 is a novel transcription regulator. Proc Natl Acad Sci U S A 114:562-567.

Gomes JR, Nogueira RS, Vieira M, Santos SD, Ferraz-Nogueira JP, Relvas JB, Saraiva MJ (2016) Transthyretin provides trophic support via megalin by promoting neurite outgrowth and neuroprotection in cerebral ischemia. Cell Death Differ 23:1749-1764.

Gomes JR, Cabrito I, Soares HR, Costelha S, Teixeira A, Wittelsberger A, Stortelers C, Vanlandschoot P, Saraiva MJ (2018) Delivery of an anti-transthyretin Nanobody to the brain through intranasal administration reveals transthyretin expression and secretion by motor neurons. J Neurochem 145:393-408.

Gomes JR, Lobo AC, Melo CV, Inacio AR, Takano J, Iwata N, Saido TC, de Almeida LP, Wieloch T, Duarte CB (2011) Cleavage of the vesicular GABA transporter under excitotoxic conditions is followed by accumulation of the truncated transporter in nonsynaptic sites. J Neurosci 31:4622-4635.

Gomes JR, Sarkany Z, Teixeira A, Nogueira R, Cabrito I, Soares H, Wittelsberger A, Stortelers C, Macedo-Ribeiro S, Vanlandschoot P, Saraiva MJ (2019) Anti-TTR Nanobodies Allow the Identification of TTR Neuritogenic Epitope Associated with TTR-Megalin Neurotrophic Activities. ACS Chem Neurosci 10:704-715.

Hammond JB, Kruger NJ (1988) The bradford method for protein quantitation. Methods Mol Biol 3:25-32.

Hjalm G, Murray E, Crumley G, Harazim W, Lundgren S, Onyango I, Ek B, Larsson M, Juhlin C, Hellman P, Davis H, Akerstrom G, Rask L, Morse B (1996) Cloning and sequencing of human gp330, a Ca(2+)-binding receptor with potential intracellular signaling properties. Eur J Biochem 239:132-137.

Horikawa K, Yamada Y, Matsuda T, Kobayashi K, Hashimoto M, Matsu-ura T, Miyawaki A, Michikawa T, Mikoshiba K, Nagai T (2010) Spontaneous network activity visualized by ultrasensitive Ca(2+) indicators, yellow Cameleon-Nano. Nat Methods 7:729-732.

Hwang S, Gou Z, Kuznetsov IB (2007) DP-Bind: a web server for sequence-based prediction of DNA-binding residues in DNA-binding proteins. Bioinformatics 23:634-636.

Kanemaru K, Sekiya H, Xu M, Satoh K, Kitajima N, Yoshida K, Okubo Y, Sasaki T, Moritoh S, Hasuwa H, Mimura M, Horikawa K, Matsui K, Nagai T, Iino M, Tanaka KF (2014) In vivo visualization of subtle, transient, and local activity of astrocytes using an ultrasensitive Ca(2+) indicator. Cell Rep 8:311-318.

Kantarci S, Al-Gazali L, Hill RS, Donnai D, Black GC, Bieth E, Chassaing N, Lacombe D, Devriendt K, Teebi A, Loscertales M, Robson C, Liu T, MacLaughlin DT, Noonan KM, Russell MK, Walsh CA, Donahoe PK, Pober BR (2007) Mutations in LRP2, which encodes the multiligand receptor megalin, cause Donnai-Barrow and facio-oculo-acoustico-renal syndromes. Nat Genet 39:957-959.

Kerjaschki D, Farquhar MG (1982) The pathogenic antigen of Heymann nephritis is a membrane glycoprotein of the renal proximal tubule brush border. Proc Natl Acad Sci U S A 79:5557-5561.

Kirchhausen T, Macia E, Pelish HE (2008) Use of dynasore, the small molecule inhibitor of dynamin, in the regulation of endocytosis. Methods Enzymol 438:77-93.

For Review Only

Kosugi S, Hasebe M, Tomita M, Yanagawa H (2009) Systematic identification of cell cycle-dependent yeast nucleocytoplasmic shuttling proteins by prediction of composite motifs. Proc Natl Acad Sci U S A 106:10171-10176.

la Cour T, Kiemer L, Molgaard A, Gupta R, Skriver K, Brunak S (2004) Analysis and prediction of leucine-rich nuclear export signals. Protein Eng Des Sel 17:527-536.

LaFerla FM, Troncoso JC, Strickland DK, Kawas CH, Jay G (1997) Neuronal cell death in Alzheimer's disease correlates with apoE uptake and intracellular Abeta stabilization. J Clin Invest 100:310-320.

Li X, Masliah E, Reixach N, Buxbaum JN (2011) Neuronal production of transthyretin in human and murine Alzheimer's disease: is it protective? J Neurosci 31:12483-12490.

Li Y, Cong R, Biemesderfer D (2008) The COOH terminus of megalin regulates gene expression in opossum kidney proximal tubule cells. Am J Physiol Cell Physiol 295:C529-537.

Liu CX, Ranganathan S, Robinson S, Strickland DK (2007) gamma-Secretase-mediated release of the low density lipoprotein receptor-related protein 1B intracellular domain suppresses anchorage-independent growth of neuroglioma cells. J Biol Chem 282:7504-7511.

Liu W, Yu WR, Carling T, Juhlin C, Rastad J, Ridefelt P, Akerstrom G, Hellman P (1998) Regulation of gp330/megalin expression by vitamins A and D. Eur J Clin Invest 28:100-107.

Lowry OH, Rosebrough NJ, Farr AL, Randall RJ (1951) Protein measurement with the Folin phenol reagent. J Biol Chem 193:265-275.

Macia E, Ehrlich M, Massol R, Boucrot E, Brunner C, Kirchhausen T (2006) Dynasore, a cell-permeable inhibitor of dynamin. Dev Cell 10:839-850.

Marzolo MP, Farfan P (2011) New insights into the roles of megalin/LRP2 and the regulation of its functional expression. Biol Res 44:89-105.

May P, Reddy YK, Herz J (2002) Proteolytic processing of low density lipoprotein receptor-related protein mediates regulated release of its intracellular domain. J Biol Chem 277:18736-18743.

McLeod F, Marzo A, Podpolny M, Galli S, Salinas P (2017) Evaluation of Synapse Density in Hippocampal Rodent Brain Slices. J Vis Exp.

Meijering E, Jacob M, Sarria JC, Steiner P, Hirling H, Unser M (2004) Design and validation of a tool for neurite tracing and analysis in fluorescence microscopy images. Cytometry A 58:167-176.

Min BH, Kim BM, Lee SH, Kang SW, Bendayan M, Park IS (2003) Clusterin expression in the early process of pancreas regeneration in the pancreatectomized rat. J Histochem Cytochem 51:1355-1365.

Motulsky HJ, Brown RE (2006) Detecting outliers when fitting data with nonlinear regression - a new method based on robust nonlinear regression and the false discovery rate. BMC Bioinformatics 7:123.

Neuhaus-Follini A, Bashaw GJ (2015) The Intracellular Domain of the Frazzled/DCC Receptor Is a Transcription Factor Required for Commissural Axon Guidance. Neuron 87:751-763.

Nguyen Ba AN, Pogoutse A, Provart N, Moses AM (2009) NLStradamus: a simple Hidden Markov Model for nuclear localization signal prediction. BMC Bioinformatics 10:202.

Passerini A, Lippi M, Frasconi P (2011) MetalDetector v2.0: predicting the geometry of metal binding sites from protein sequence. Nucleic Acids Res 39:W288-292.

Pedersen MO, Hansen PB, Nielsen SL, Penkowa M (2010) Metallothionein-I + II and receptor megalin are altered in relation to oxidative stress in cerebral lymphomas. Leuk Lymphoma 51:314-328.

Pedersen MO, Jensen R, Pedersen DS, Skjolding AD, Hempel C, Maretty L, Penkowa M (2009) Metallothionein-I+II in neuroprotection. Biofactors 35:315-325.

For Review Only

Perez Bay AE, Schreiner R, Benedicto I, Paz Marzolo M, Banfelder J, Weinstein AM, Rodriguez-Boulan EJ (2016) The fast-recycling receptor Megalin defines the apical recycling pathway of epithelial cells. Nat Commun 7:11550.

Pfaffl MW (2001) A new mathematical model for relative quantification in real-time RT-PCR. Nucleic Acids Res 29:e45.

Pober BR, Longoni M, Noonan KM (2009) A review of Donnai-Barrow and facio-oculo-acoustico-renal (DB/FOAR) syndrome: clinical features and differential diagnosis. Birth Defects Res A Clin Mol Teratol 85:76-81.

Polavarapu R, An J, Zhang C, Yepes M (2008) Regulated intramembrane proteolysis of the low-density lipoprotein receptor-related protein mediates ischemic cell death. Am J Pathol 172:1355-1362.

Qiu Z, Hyman BT, Rebeck GW (2004) Apolipoprotein E receptors mediate neurite outgrowth through activation of p44/42 mitogen-activated protein kinase in primary neurons. J Biol Chem 279:34948-34956.

Refetoff S, Dwulet FE, Benson MD (1986) Reduced affinity for thyroxine in two of three structural thyroxine-binding prealbumin variants associated with familial amyloidotic polyneuropathy. J Clin Endocrinol Metab 63:1432-1437.

Reinhardt A, Hubbard T (1998) Using neural networks for prediction of the subcellular location of proteins. Nucleic Acids Res 26:2230-2236.

Ribeiro CA, Oliveira SM, Guido LF, Magalhaes A, Valencia G, Arsequell G, Saraiva MJ, Cardoso I (2014) Transthyretin stabilization by iododiflunisal promotes amyloid-beta peptide clearance, decreases its deposition, and ameliorates cognitive deficits in an Alzheimer's disease mouse model. J Alzheimers Dis 39:357-370.

Russell DW, Brown MS, Goldstein JL (1989) Different combinations of cysteine-rich repeats mediate binding of low density lipoprotein receptor to two different proteins. J Biol Chem 264:21682-21688.

Saito A, Pietromonaco S, Loo AK, Farquhar MG (1994) Complete cloning and sequencing of rat gp330/"megalin," a distinctive member of the low density lipoprotein receptor gene family. Proc Natl Acad Sci U S A 91:9725-9729.

Santos AR, Duarte CB (2008) Validation of internal control genes for expression studies: effects of the neurotrophin BDNF on hippocampal neurons. Journal of neuroscience research 86:3684-3692.

Santos SD, Lambertsen KL, Clausen BH, Akinc A, Alvarez R, Finsen B, Saraiva MJ (2010) CSF transthyretin neuroprotection in a mouse model of brain ischemia. J Neurochem 115:1434-1444.

Scholer J, Ferralli J, Thiry S, Chiquet-Ehrismann R (2015) The intracellular domain of teneurin-1 induces the activity of microphthalmia-associated transcription factor (MITF) by binding to transcriptional repressor HINT1. J Biol Chem 290:8154-8165.

Schroeter EH, Kisslinger JA, Kopan R (1998) Notch-1 signalling requires ligand-induced proteolytic release of intracellular domain. Nature 393:382-386.

Shah M, Baterina OY, Jr., Taupin V, Farquhar MG (2013) ARH directs megalin to the endocytic recycling compartment to regulate its proteolysis and gene expression. J Cell Biol 202:113-127.

Sousa JC, Cardoso I, Marques F, Saraiva MJ, Palha JA (2007a) Transthyretin and Alzheimer's disease: where in the brain? Neurobiol Aging 28:713-718.

Sousa JC, Marques F, Dias-Ferreira E, Cerqueira JJ, Sousa N, Palha JA (2007b) Transthyretin influences spatial reference memory. Neurobiol Learn Mem 88:381-385.

For Review Only

Sousa MM, Saraiva MJ (2001) Internalization of transthyretin. Evidence of a novel yet unidentified receptor-associated protein (RAP)-sensitive receptor. J Biol Chem 276:14420-14425.
Sousa MM, Norden AG, Jacobsen C, Willnow TE, Christensen EI, Thakker RV, Verroust PJ, Moestrup SK, Saraiva MJ (2000) Evidence for the role of megalin in renal uptake of transthyretin. J Biol Chem 275:38176-38181.
Spoelgen R, Hammes A, Anzenberger U, Zechner D, Andersen OM, Jerchow B, Willnow TE (2005) LRP2/megalin is required for patterning of the ventral telencephalon. Development 132:405-414.
Spuch C, Ortolano S, Navarro C (2012) LRP-1 and LRP-2 receptors function in the membrane neuron. Trafficking mechanisms and proteolytic processing in Alzheimer's disease. Front Physiol 3:269.
Srivastava A, Kumar M (2018) Prediction of zinc binding sites in proteins using sequence derived information. J Biomol Struct Dyn:1-11.
Stein TD, Johnson JA (2002) Lack of neurodegeneration in transgenic mice overexpressing mutant amyloid precursor protein is associated with increased levels of transthyretin and the activation of cell survival pathways. J Neurosci 22:7380-7388.
Takeda T, Yamazaki H, Farquhar MG (2003) Identification of an apical sorting determinant in the cytoplasmic tail of megalin. Am J Physiol Cell Physiol 284:C1105-1113.
Telese F, Ma Q, Perez PM, Notani D, Oh S, Li W, Comoletti D, Ohgi KA, Taylor H, Rosenfeld MG (2015) LRP8-Reelin-Regulated Neuronal Enhancer Signature Underlying Learning and Memory Formation. Neuron 86:696-710.
Theis M, Jauch R, Zhuo L, Speidel D, Wallraff A, Doring B, Frisch C, Sohl G, Teubner B, Euwens C, Huston J, Steinhauser C, Messing A, Heinemann U, Willecke K (2003) Accelerated hippocampal spreading depression and enhanced locomotory activity in mice with astrocyte-directed inactivation of connexin43. J Neurosci 23:766-776.
Vasli N, Ahmed I, Mittal K, Ohadi M, Mikhailov A, Rafiq MA, Bhatti A, Carter MT, Andrade DM, Ayub M, Vincent JB, John P (2016) Identification of a homozygous missense mutation in LRP2 and a hemizygous missense mutation in TSPYL2 in a family with mild intellectual disability. Psychiatr Genet 26:66-73.
Vieira M, Gomes JR, Saraiva MJ (2015) Transthyretin Induces Insulin-like Growth Factor I Nuclear Translocation Regulating Its Levels in the Hippocampus. Mol Neurobiol 51:1468-1479.
Wicher G, Larsson M, Rask L, Aldskogius H (2005) Low-density lipoprotein receptor-related protein (LRP)-2/megalin is transiently expressed in a subpopulation of neural progenitors in the embryonic mouse spinal cord. J Comp Neurol 492:123-131.
Wicher G, Larsson M, Fex Svenningsen A, Gyllencreutz E, Rask L, Aldskogius H (2006) Low density lipoprotein receptor-related protein-2/megalin is expressed in oligodendrocytes in the mouse spinal cord white matter. Journal of neuroscience research 83:864-873.
Willnow TE, Hilpert J, Armstrong SA, Rohlmann A, Hammer RE, Burns DK, Herz J (1996) Defective forebrain development in mice lacking gp330/megalin. Proc Natl Acad Sci U S A 93:8460-8464.
Xu D, Marquis K, Pei J, Fu SC, Cagatay T, Grishin NV, Chook YM (2015) LocNES: a computational tool for locating classical NESs in CRM1 cargo proteins. Bioinformatics 31:1357-1365.
Yamada Y, Michikawa T, Hashimoto M, Horikawa K, Nagai T, Miyawaki A, Hausser M, Mikoshiba K (2011) Quantitative comparison of genetically encoded Ca indicators in cortical pyramidal cells and cerebellar Purkinje cells. Front Cell Neurosci 5:18.
Yan J, Kurgan L (2017) DRNApred, fast sequence-based method that accurately predicts and discriminates DNA- and RNA-binding residues. Nucleic Acids Res 45:e84.

For Review Only

Yuseff MI, Farfan P, Bu G, Marzolo MP (2007) A cytoplasmic PPPSP motif determines megalin's phosphorylation and regulates receptor's recycling and surface expression. Traffic 8:1215-1230.
Zang X, Zheng F, Hong HJ, Jiang Y, Song Y, Xia Y (2014) Neutrophil gelatinase-associated lipocalin protects renal tubular epithelial cells in hypoxia-reperfusion by reducing apoptosis. Int Urol Nephrol 46:1673-1679.
Zou Z, Chung B, Nguyen T, Mentone S, Thomson B, Biemesderfer D (2004) Linking receptor-mediated endocytosis and cell signaling: evidence for regulated intramembrane proteolysis of megalin in proximal tubule. J Biol Chem 279:34302-34310.
Zurhove K, Nakajima C, Herz J, Bock HH, May P (2008) Gamma-secretase limits the inflammatory response through the processing of LRP1. Sci Signal 1:ra15.

For Review Only

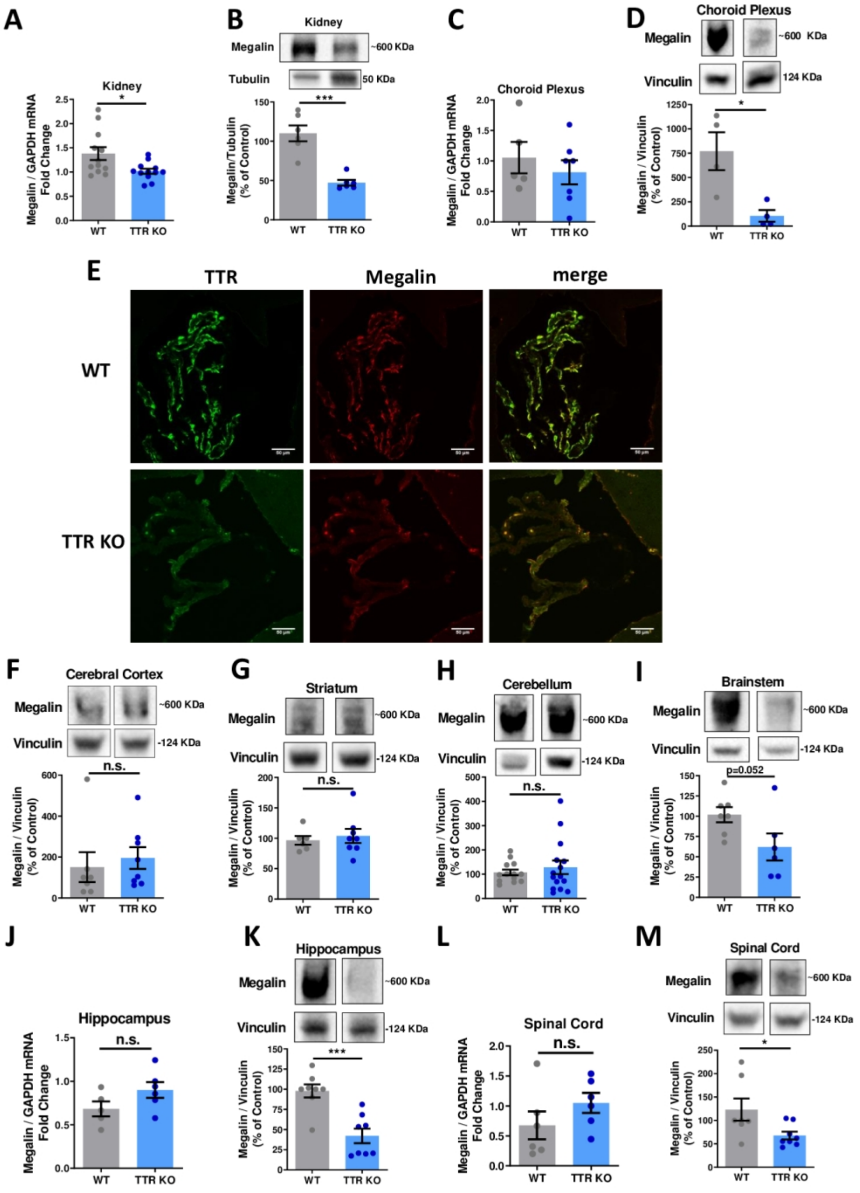

Fig. 1 – TTR regulates megalin levels: TTR KO vs WT mice.

176x240mm (200 x 200 DPI)

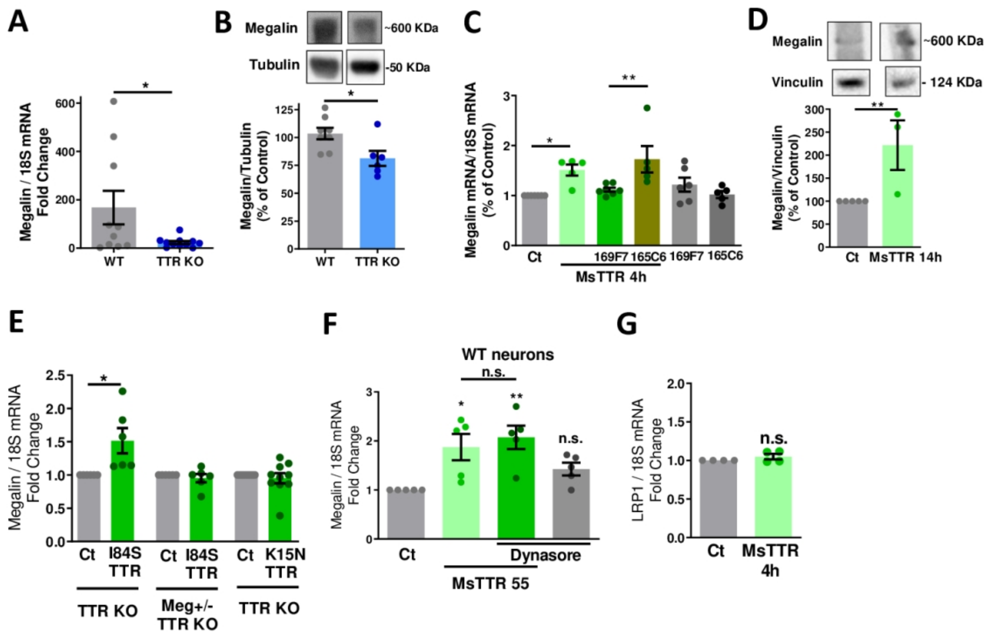

Fig. 2 – TTR rescues megalin downregulation in TTR KO hippocampal neuronal cultures, in a megalin dependent way.

183x119mm (200 x 200 DPI)

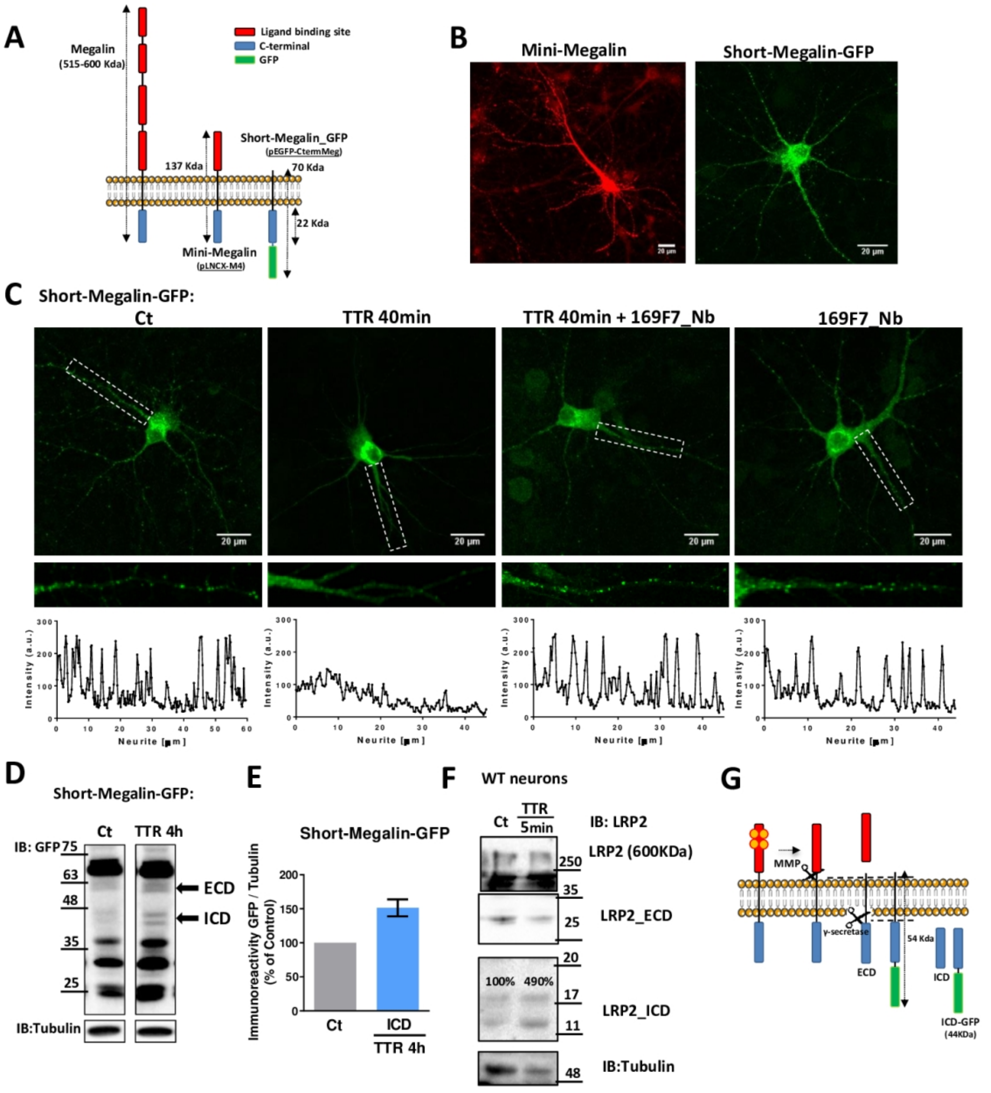

Fig. 3 – TTR leads to LRP2-ICD formation, probably via γ-secretase activity.

184x202mm (200 x 200 DPI)

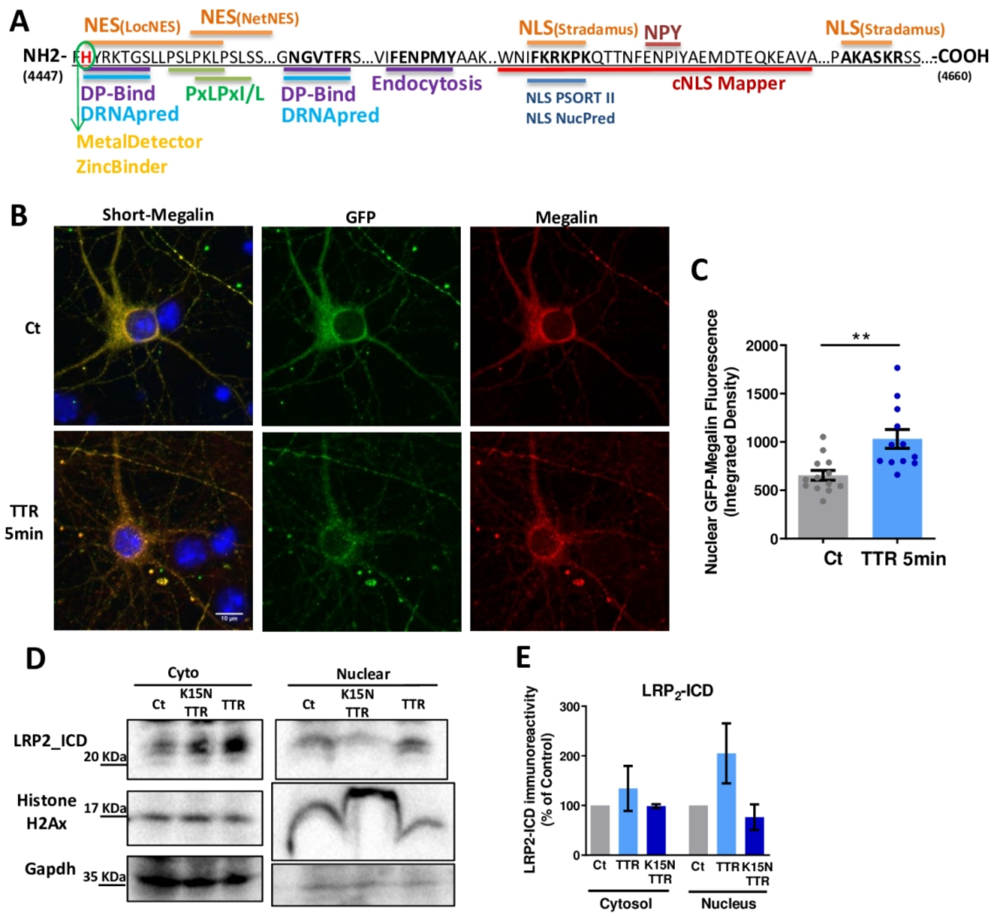

Fig. 4 – TTR leads to LRP2-ICD nuclear translocation, as a putative transcription regulation mechanism.

187x175mm (200 x 200 DPI)

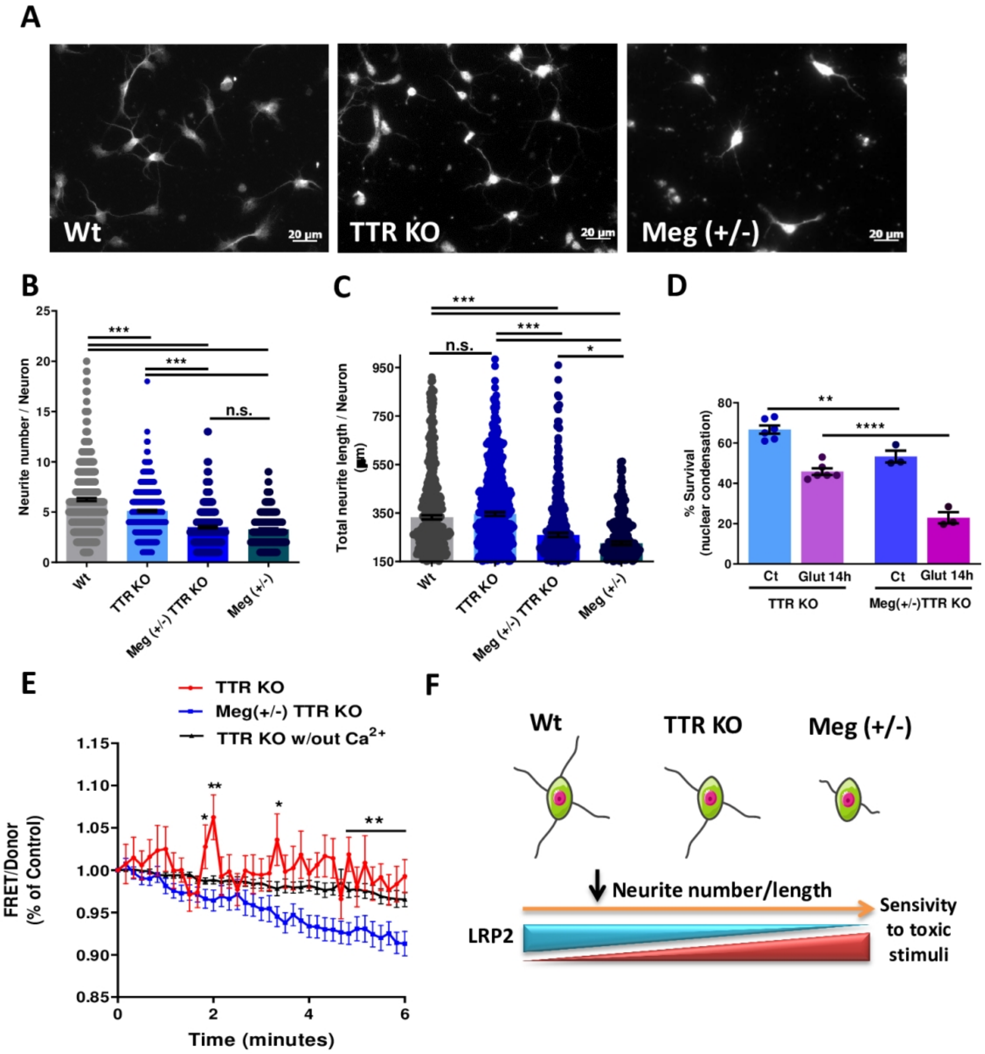

Fig. 5 – Reduction of megalin levels impairs neurite outgrowth and survival of hippocampal neurons.

186x198mm (200 x 200 DPI)

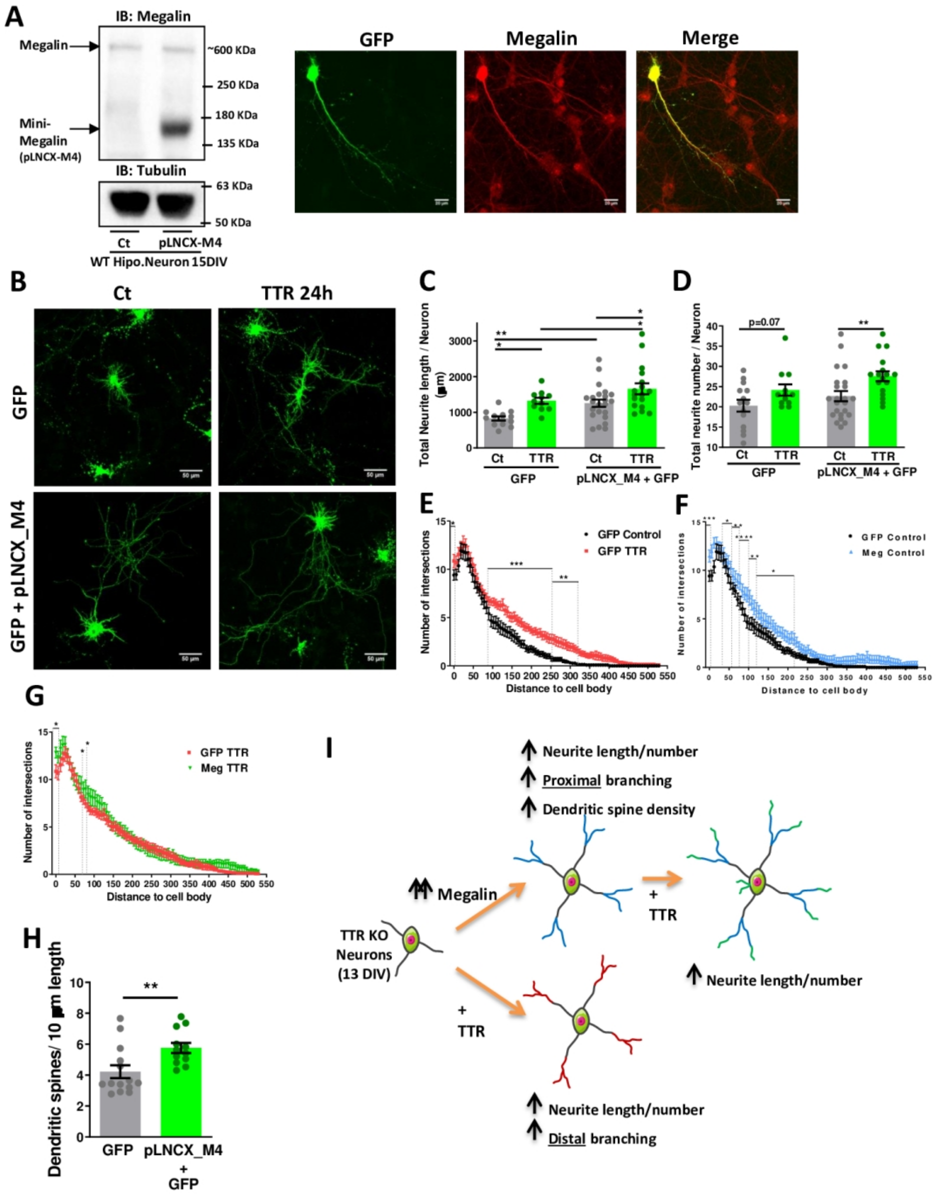

Fig. 6 – Megalin overexpression rescues neurite outgrowth and increases dendritic spine density.

188x237mm (200 x 200 DPI)

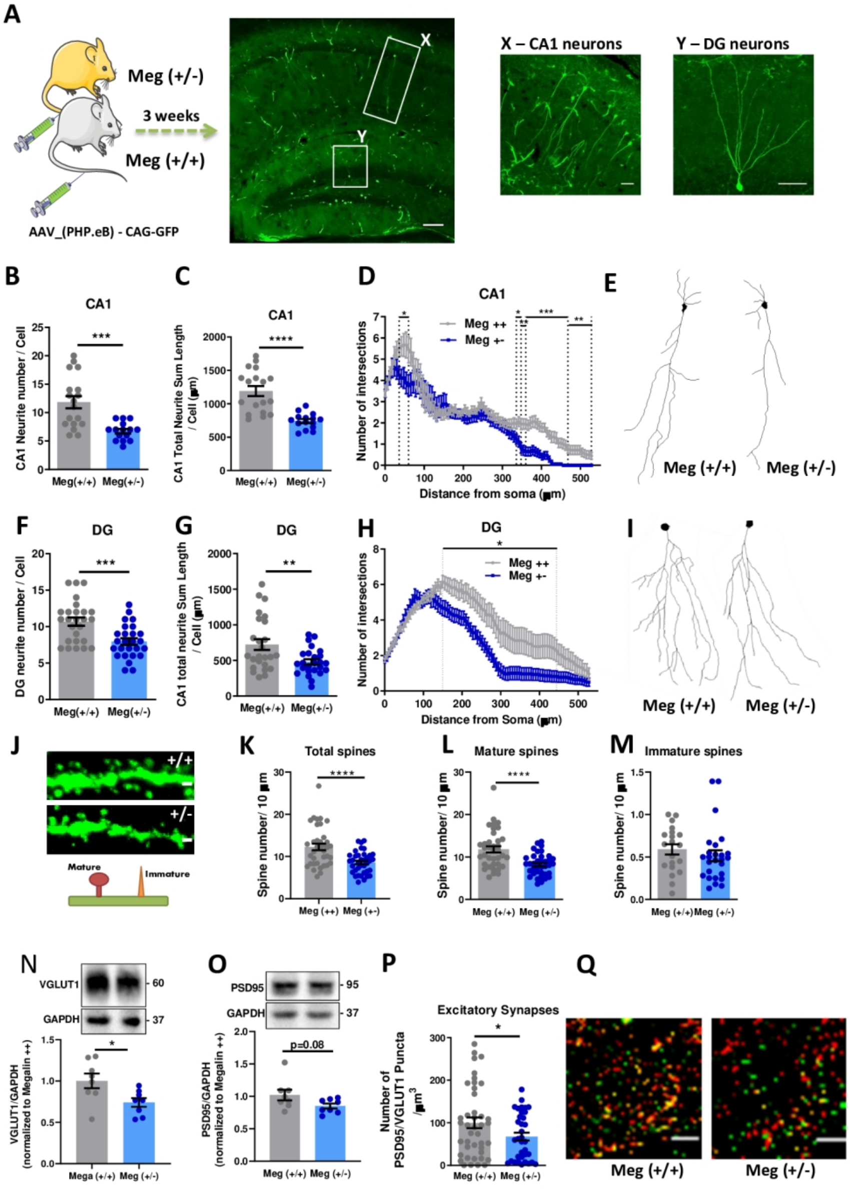

Fig. 7 – Megalin heterozygous mice show structural alterations in hippocampal neurons.

190x263mm (200 x 200 DPI)

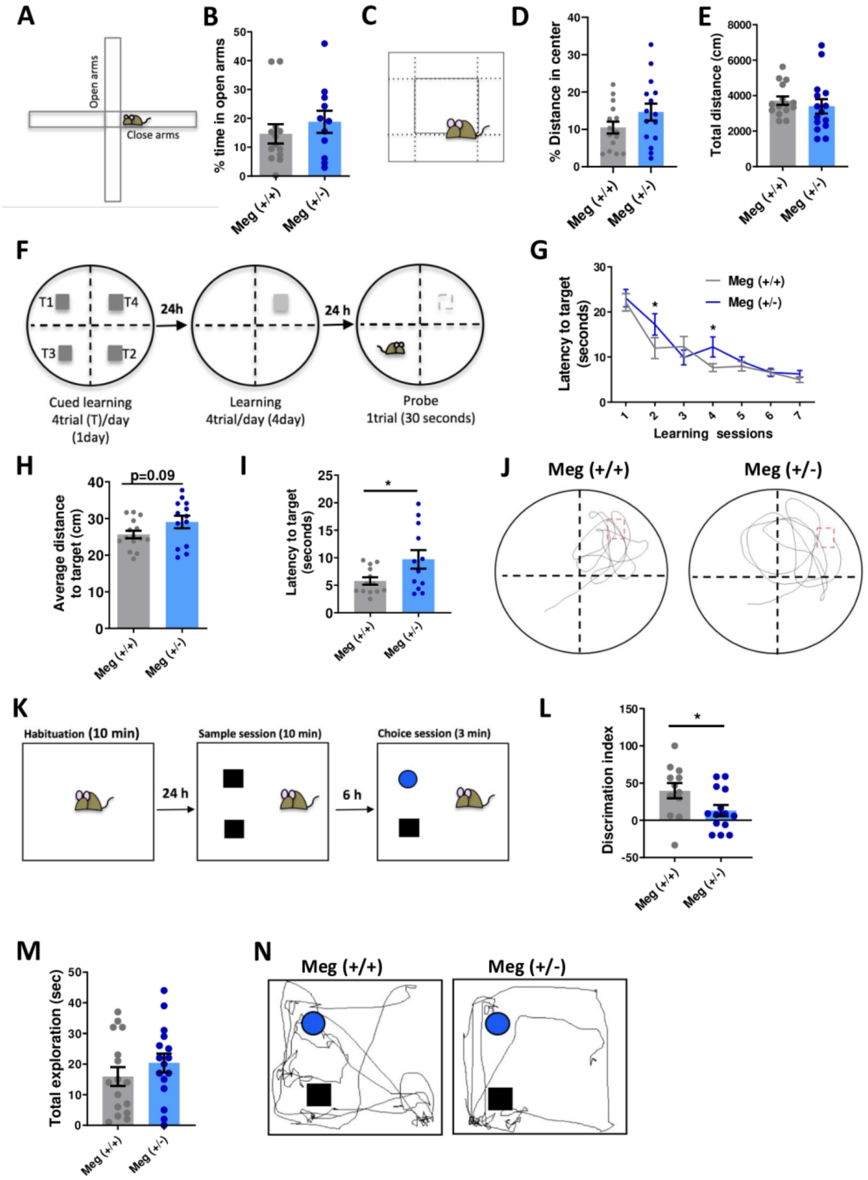

Fig. 8 – Megalin heterozygous mice show cognitive deficits, but no effects in anxiety-like behavior or locomotor activity.

188x256mm (200 x 200 DPI)

Neuronal megalin mediates synaptic plasticity - a novel mechanism underlying intellectual disabilities in megalin gene-pathologies

João R. Gomes[1,3*], Andrea Lobo [2,3*], Renata Nogueira [1,3], Ana F. Terceiro [2,3], Susete Costelha [1,3], Ana Magalhães [2,3], Teresa Summavielle [1,3], Maria J. Saraiva [1,3]

[1] Molecular Neurobiology Unit, IBMC- Instituto de Biologia Molecular e Celular, Porto, Portugal;

[2] Addiction Biology Group, IBMC- Instituto de Biologia Molecular e Celular, Porto, Portugal;

[3] I3S – Instituto de Investigação e Inovação em Saúde, Universidade do Porto, Portugal;

* - contributed equally to the work

For Review Only

Supporting information:

1. **NLS, NES, protein-DNA and metal binding residues – supplemental data:**

The following bioinformatics tools were used to assess nuclear localizing signals (NLS) and nuclear exporting signals (NES), in the mouse megalin C-terminal region.

For NLS, we used the following platforms:

- NucPred, website: https://nucpred.bioinfo.se/cgi-bin/single.cgi, Score 0.32;

- cNLS Mapper, website http://nls-mapper.iab.keio.ac.jp/cgi-bin/NLS_Mapper_form.cgi, Position: 142-172, Sequence: WNIF…QKEAVA , Score 4.1 ;

- PSORT II server, website https://psort.hgc.jp/helpwww2.html, Nuclear – 95% reliability, NLS – KRKPK (position 146 of the C-terminal region);

- NLStradamus, website http://www.moseslab.csb.utoronto.ca/NLStradamus/, 2 state Hidden Markov Model , Position 145-150, Score 184-188 (0.1 Cutoff).

Regarding NES, we used the following computational tools:

- Nespredictor NetNES, website http://www.cbs.dtu.dk/services/NetNES/ ,

#Seq-Pos-Residue	ANN	HMM	NES
Sequence-12-S	0.097	0.200	0.000
Sequence-13-L	0.232	0.683	0.451
Sequence-14-P	0.084	0.683	0.449
Sequence-15-K	0.155	0.685	0.459
Sequence-16-L	0.100	0.685	0.437
Sequence-17-P	0.071	0.676	0.423
Sequence-18-S	0.249	0.676	0.454

Sequence-19-L	0.187	0.677	0.446
Sequence-20-S	0.088	0.618	0.388
Sequence-21-S	0.128	0.618	0.382
Sequence-22-L	0.217	0.619	0.407
Sequence-23-A	0.084	0.003	0.000

- LocNES, website http://prodata.swmed.edu/LocNES/LocNES.php, Position 2-16, Sequence HYRKTGSLLPSLPKL, Score: 0.045.

Concerning protein-DNA binding residues, the bioinformatics tools used pointed the results (aminoacid residues) shown in Figure 4A.

2. LRP2-ICD nuclear localization upon TTR stimulus (n=3) Fig.4D

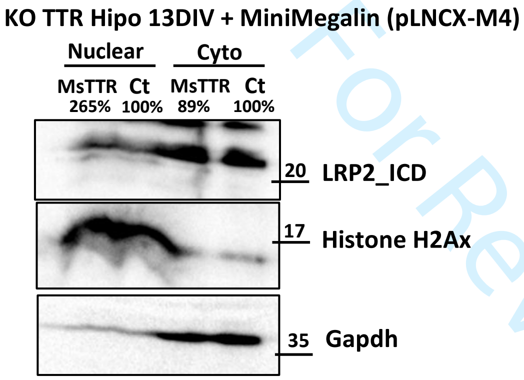

Supplementary Fig. 1. LRP2-ICD translocates to the nucleus, upon TTR stimulus. Nuclear and cytosolic fractions isolated from TTR KO cultured hippocampal neurons (11DIV) transfected with mini-megalin plasmid (pLNCX-M4) for 48h and stimulated with recombinant mouse TTR (55µg/ml) for 20 minutes, and analyzed by western blot showing the nuclear translocation of megalin. Histone H2Ax (nuclear fraction) and GAPDH (cytosolic fraction) were used to confirm cytosolic and nuclear fraction separation. The result is one of the 3 independent neuronal cultures. The other 2 experiments are shown in Fig. 4D.

For Review Only

3. <u>**Neurite branching (Fig. 6)**</u>

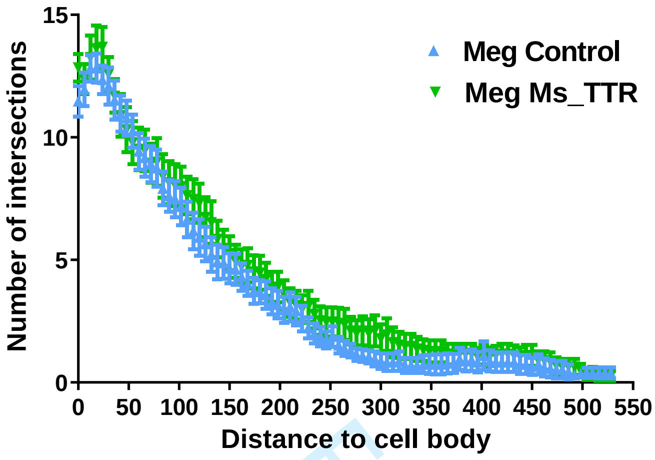

<u>**Supplementary Fig. 2**</u>**. Neurite branching in GFP plus mini-megalin expressing neurons, TTR was unable to further increase neuronal branching.** TTR KO cultured hippocampal neurons (11DIV) were cotransfected with GFP (pEGFP) and mini-megalin plasmid (pLNCX-M4) for 48h, and stimulated, or not, as indicated, with recombinant mouse TTR (55µg/ml) for 24h. An immunocytochemistry was performed using GFP antibody. (n=12-23 neurons, from 3 independent cultures). Neuronal branching was not increased in neurons overexpressing megalin vs neurons overexpressing megalin and treated with TTR. Statistical analysis was performed by two-way ANOVA followed by Fisher's LSD multiple comparison test.

For Review Only

4. **Dendritic spine density**

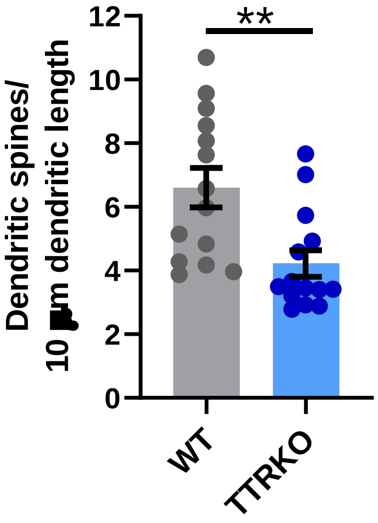

Supplementary Fig. 3. The dendritic spine density is significantly reduced in TTR KO cultured neurons, compared with WT neurons. WT or TTR KO cultured hippocampal neurons (11DIV) were transfected with GFP plasmid (pEGFP) for 48h. An immunocytochemistry was performed using GFP antibody. In WT GFP transfected neurons the density of dendritic spines is approximately 6 spines/10 µm dendritic length, whereas in TTR KO neuronal cultures is 4 spines/10 µm, showing a significantly reduction (n= 1-3 dendrites, from 8-10 neurons, from 1-2 independent neuronal cultures). Statistical analysis was performed using Student's unpaired t-test, ** P<0.01.

5. **Megalin(LRP2)-ECD formation upon γ-secretase inhibition**

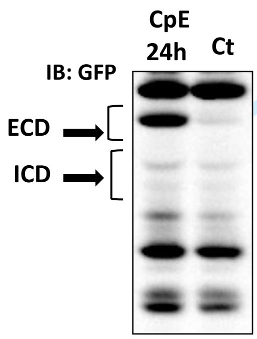

Supplementary Fig. 4. γ-secretase inhibitor leads to an accumulation of megalin-ECD in TTR KO cultured hippocampal neurons. TTR KO cultured hippocampal neurons (11DIV) transfected with short-megalin fused with GFP (pEGFP- Cterminal Megalin) for 48h were further incubated with γ-secretase inhibitor (Compound E (CpE) 500nM, in the culture conditioned medium for 24h. (n=2, n=independent cultures)

6. **Excitatory synapses – Fig 7. Q - colocalization of VGLUT1 and PSD95 puncta:**

Megalin$^{+/+}$ mice:

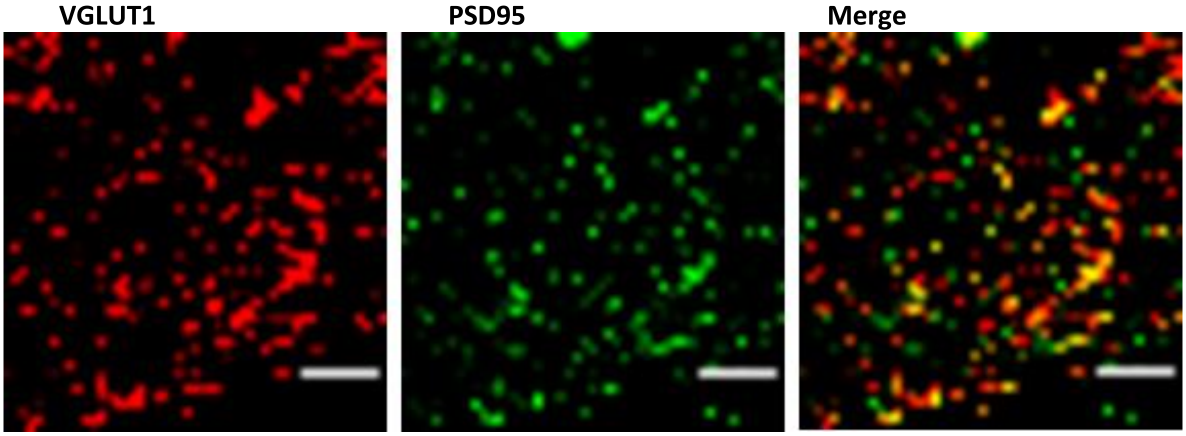

For Review Only

Megalin$^{+/-}$ mice

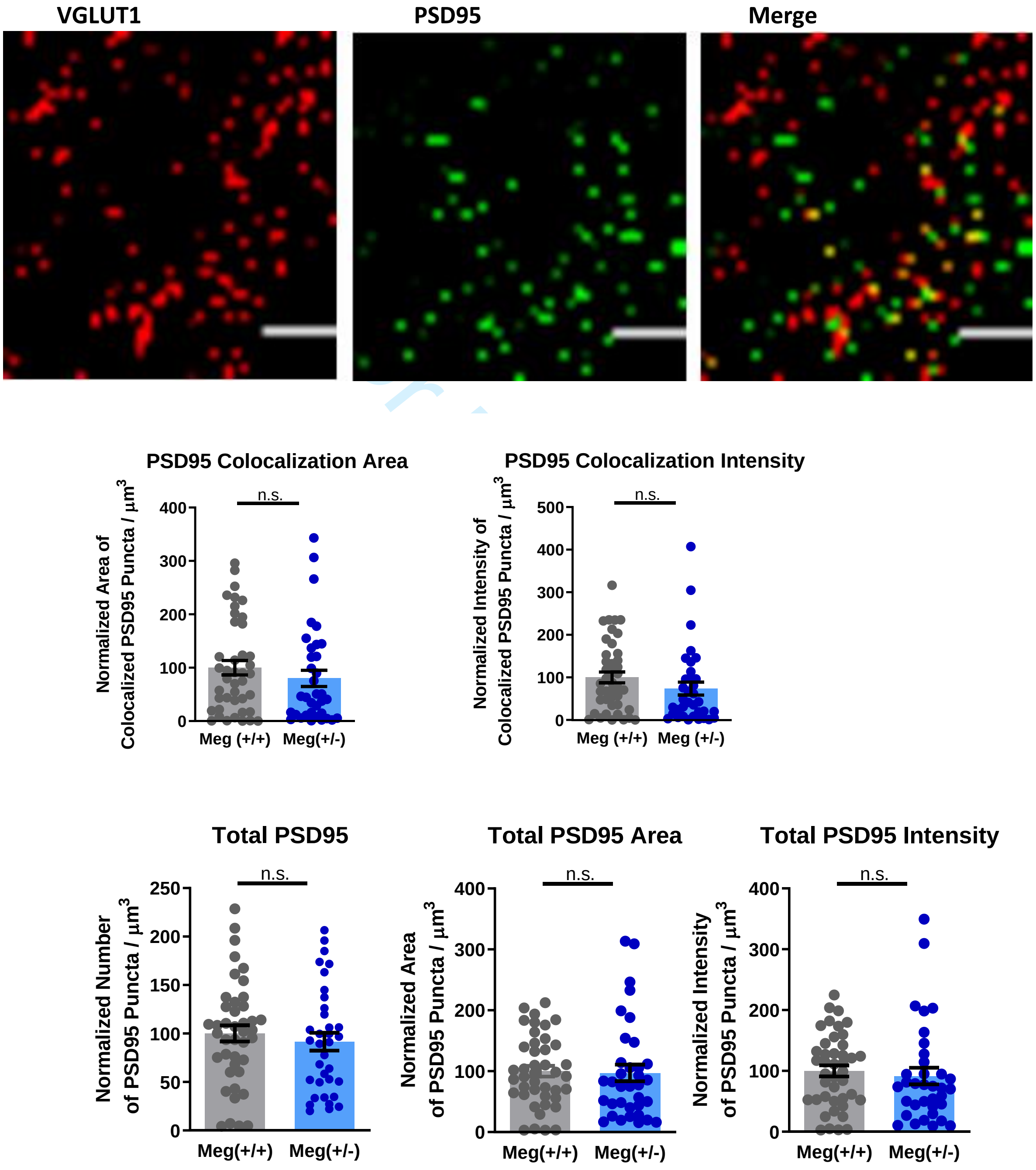

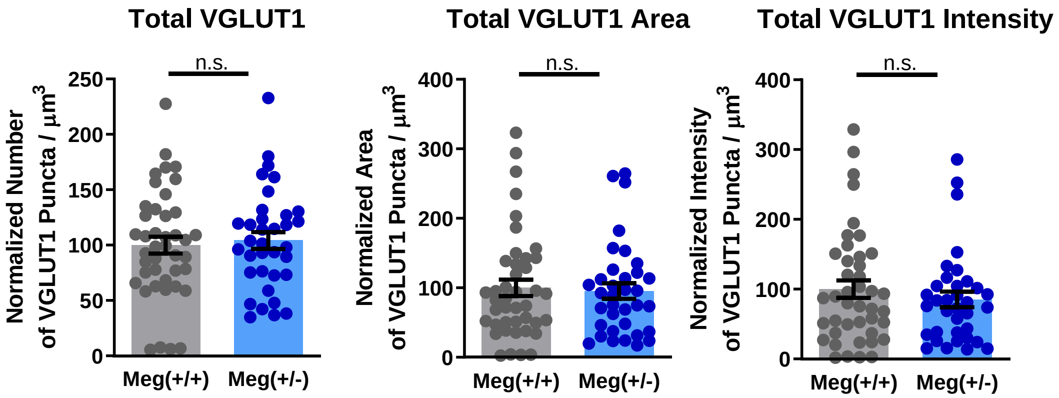

Supplementary Fig. 5. The area and intensity of excitatory synaptic puncta, defined by the colocalization of VGLUT1 and PSD95 puncta, is not altered in $Meg^{+/+}$ vs $Meg^{+/-}$ mice. The total number, area and fluorescence intensity of PSD95 and VGLUT1 puncta are not altered in $Meg^{+/+}$ vs $Meg^{+/-}$ mice. Values are normalized to $Meg^{+/+}$ mice. Supplementary representative images from Fig.7 are presented here. Scale bar: 5 µm. Statistical analysis was performed using Student's unpaired t-test.

For Review

Behavioral tests

7. EPM supplemental data

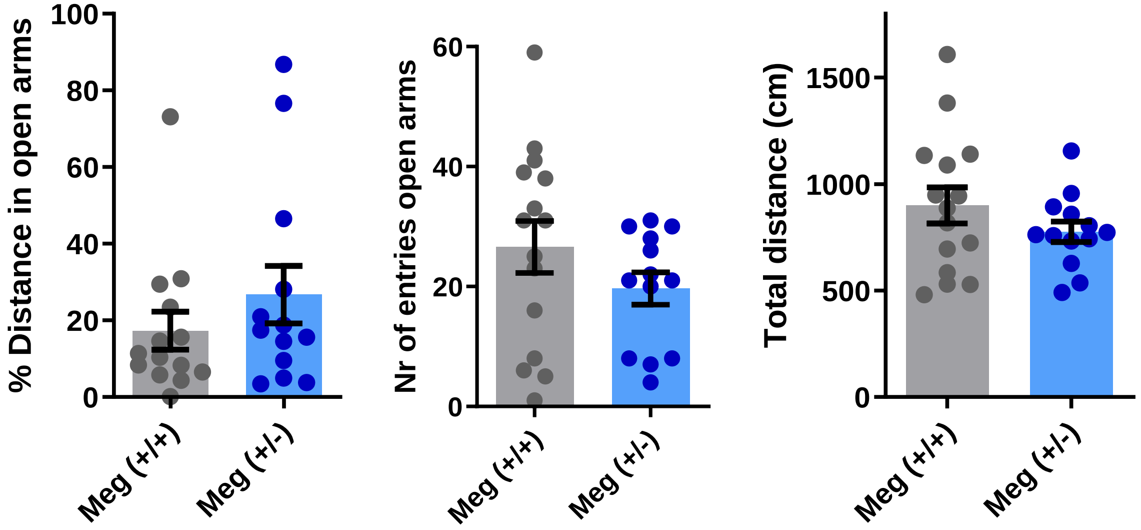

Supplementary Fig. 6. Elevated plus maze test (supplemental data). No differences were observed between $Meg^{+/+}$ and $Meg^{+/-}$ mice in the % of distance travelled and number of entries in the open

arms, as well as no changes in total distance travelled. Statistical analysis was performed using Student's unpaired t-test.

8. Open Field supplemental data

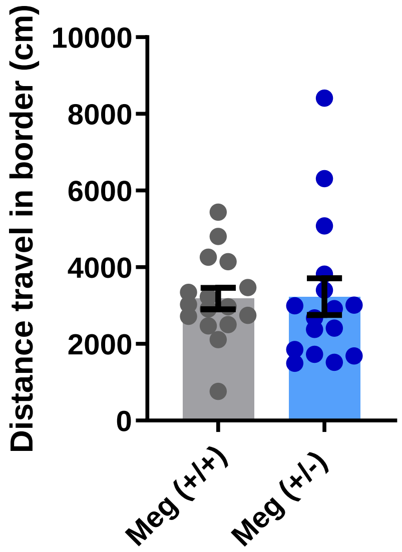

Supplementary Fig. 7. Open field test (supplemental data). No differences were observed between Meg$^{+/+}$ and Meg$^{+/-}$ mice in the distance travelled in the border of the apparatus. Statistical analysis was performed using Student's unpaired t-test.

9. Water maze supplemental data:

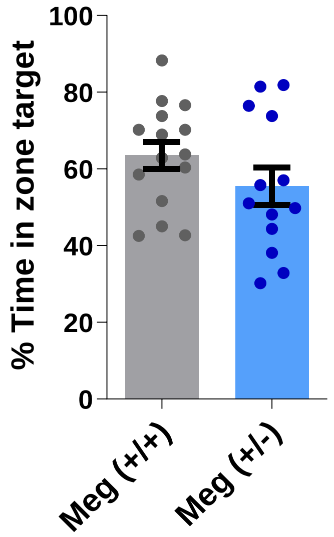

Supplementary Fig. 8. Morris water maze test (supplemental data). No differences were observed between Meg+/+ and Meg+/- mice in the % of time in the zone of the maze where the platform was located during trial session. Statistical analysis was performed using Student's unpaired t-test.

For Review Only
