# Supplementary material for: Neuronal megalin mediates synaptic plasticity—a novel mechanism underlying intellectual disabilities in megalin gene pathologies
Source: Brain Commun. 2020 Aug 25;2(2):fcaa135. doi: 10.1093/braincomms/fcaa135 (PMC7667529; doi:10.1093/braincomms/fcaa135)
Supplement: fcaa135_Supplementary_Data [file fcaa135_supplementary_data.zip › Supplementary_information.pdf]

Neuronal megalin mediates synaptic plasticity - a novel mechanism underlying intellectual disabilities in megalin gene-pathologies

João R. Gomes[1,3*], Andrea Lobo [2,3*], Renata Nogueira [1,3], Ana F. Terceiro [2,3], Susete Costelha [1,3], Igor M. Lopes [2,3] , Ana Magalhães [2,3], Teresa Summavielle [2,3], Maria J. Saraiva [1,3]

[1] Molecular Neurobiology Unit, IBMC- Instituto de Biologia Molecular e Celular, Porto, Portugal;

[2] Addiction Biology Group, IBMC- Instituto de Biologia Molecular e Celular, Porto, Portugal;

[3] I3S – Instituto de Investigação e Inovação em Saúde, Universidade do Porto, Portugal;

* - contributed equally to the work

Supporting information:

- **NLS, NES, protein-DNA and metal binding residues – supplementary data:**

The following bioinformatics tools were used to assess nuclear localizing signals (NLS) and nuclear exporting signals (NES), in the mouse megalin C-terminal region.

For NLS, we used the following platforms:

- NucPred, website: https://nucpred.bioinfo.se/cgi-bin/single.cgi, Score 0.32;

- cNLS Mapper, website http://nls-mapper.iab.keio.ac.jp/cgi-bin/NLS_Mapper_form.cgi, Position: 142-172, Sequence: WNIF…QKEAVA , Score 4.1 ;

- PSORT II server, website https://psort.hgc.jp/helpwww2.html, Nuclear – 95% reliability, NLS – KRKPK (position 146 of the C-terminal region);

- NLStradamus, website http://www.moseslab.csb.utoronto.ca/NLStradamus/, 2 state Hidden Markov Model , Position 145-150, Score 184-188 (0.1 Cutoff).

Regarding NES, we used the following computational tools:

- Nespredictor NetNES, website http://www.cbs.dtu.dk/services/NetNES/ ,

#Seq-Pos-Residue	ANN	HMM	NES
Sequence-12-S	0.097	0.200	0.000
Sequence-13-L	0.232	0.683	0.451
Sequence-14-P	0.084	0.683	0.449
Sequence-15-K	0.155	0.685	0.459
Sequence-16-L	0.100	0.685	0.437
Sequence-17-P	0.071	0.676	0.423
Sequence-18-S	0.249	0.676	0.454

Sequence-19-L	0.187	0.677	0.446
Sequence-20-S	0.088	0.618	0.388
Sequence-21-S	0.128	0.618	0.382
Sequence-22-L	0.217	0.619	0.407
Sequence-23-A	0.084	0.003	0.000

- LocNES, website http://prodata.swmed.edu/LocNES/LocNES.php, Position 2-16, Sequence HYRKTGSLLPSLPKL, Score: 0.045.

Concerning protein-DNA binding residues, the bioinformatics tools used pointed the results (aminoacid residues) shown in Figure 4A.

- **Megalin(LRP2)-ECD formation upon γ-secretase inhibition**

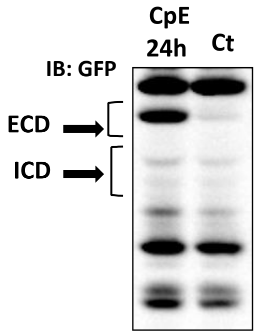

Supplementary Figure 1. γ-secretase inhibitor leads to an accumulation of megalin-ECD in TTR KO cultured hippocampal neurons. TTR KO cultured hippocampal neurons (11DIV) transfected with short-megalin fused with GFP (pEGFP- Cterminal Megalin) for 48h were further incubated with γ-secretase inhibitor (Compound E (CpE) 500nM, in the culture conditioned medium for 24h. (n=2 independent cultures). This data relates to the results of Figure 3 of the article.

- **LRP2-ICD nuclear localization upon TTR stimulus (n=3)**

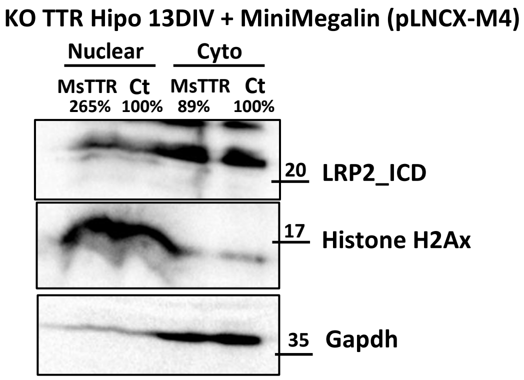

Supplementary Figure 2. LRP2-ICD translocates to the nucleus, upon TTR stimulus. Nuclear and cytosolic fractions isolated from TTR KO cultured hippocampal neurons (11DIV) transfected with mini-megalin

plasmid (pLNCX-M4) for 48h and stimulated with recombinant mouse TTR (55µg/ml) for 20 minutes, and analyzed by western blot showing the nuclear translocation of megalin. Histone H2Ax (nuclear fraction) and GAPDH (cytosolic fraction) were used to confirm cytosolic and nuclear fraction separation. The result is one of the 3 independent neuronal cultures**.** The other 2 experiments are shown in Fig. 4D.

- **Neuronal morphological representative tracings associated to Figure 5A**

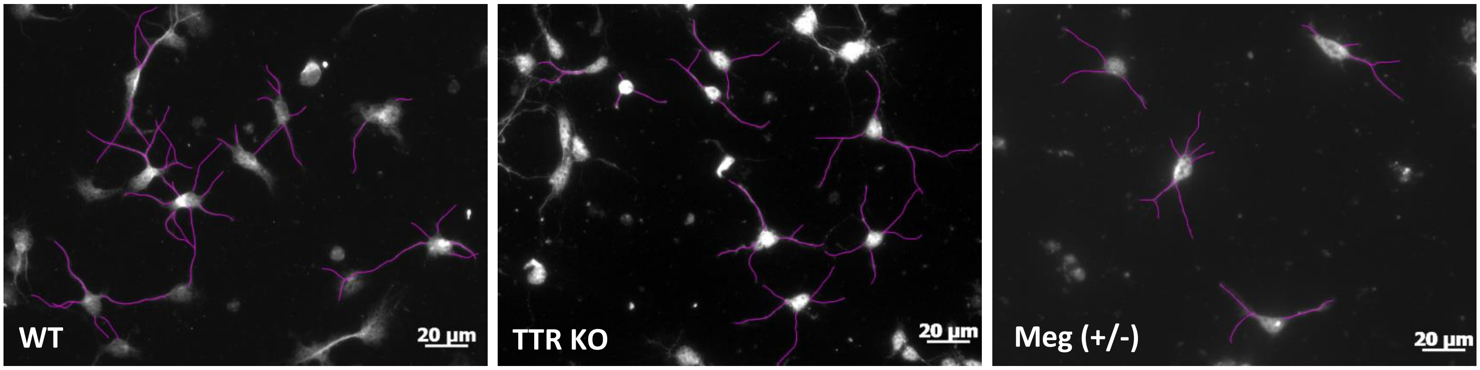

Supplementary Figure 3. **Morphological tracings used for neurite outgrowth on Fig.5A**. The morphological measurements of neurite outgrowth (number of neurites and total neurite length per neuron) were performed using the plugin NeuronJ for ImageJ software (Meijering et al.,2004 – Methods section). These are the morphological tracings from the representative images on Fig. 5A, from cultured hippocampal neurons (1DIV) from WT, TTR KO and Meg+/- mice embryos.

- **Neurite number and total neurite length – Different experimental Unit (each neuronal culture), associated to Fig. 5**:

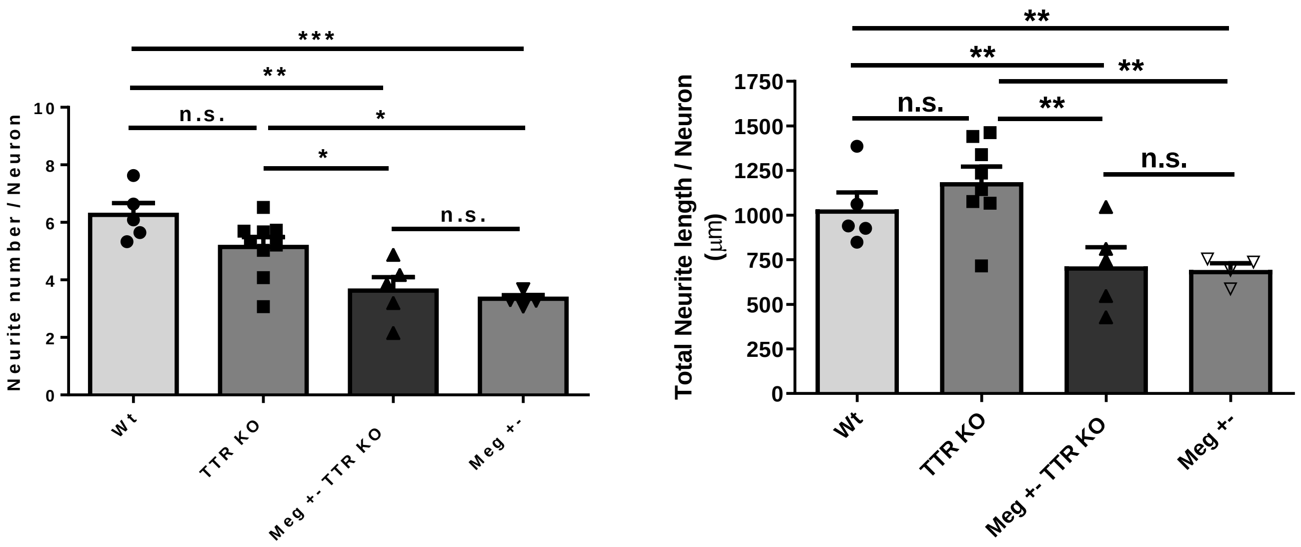

Supplementary Figure 4. Neurite number and total neurite length of hippocampal neurons were determined using a different experimental unit, the culture (4-8 independent cultures), instead of the neuron. The results are similar, indicating that a decrease in megalin expression reduces neurite number and length. Statistical analysis was performed using one-way ANOVA followed by Bonferroni's multiple comparison test. N.S.-non-significant, $^{*}P<0.05$, $^{**} P<0.01$, $^{***} P<0.001$. These results are now also shown in 5B and 5C, respectively, after second manuscript revision, with different statistical analysis, linear mixed model approach, that takes into consideration both culture and neuron (replicates).

- **In vitro neuronal morphological representative tracings of Figure 6B**

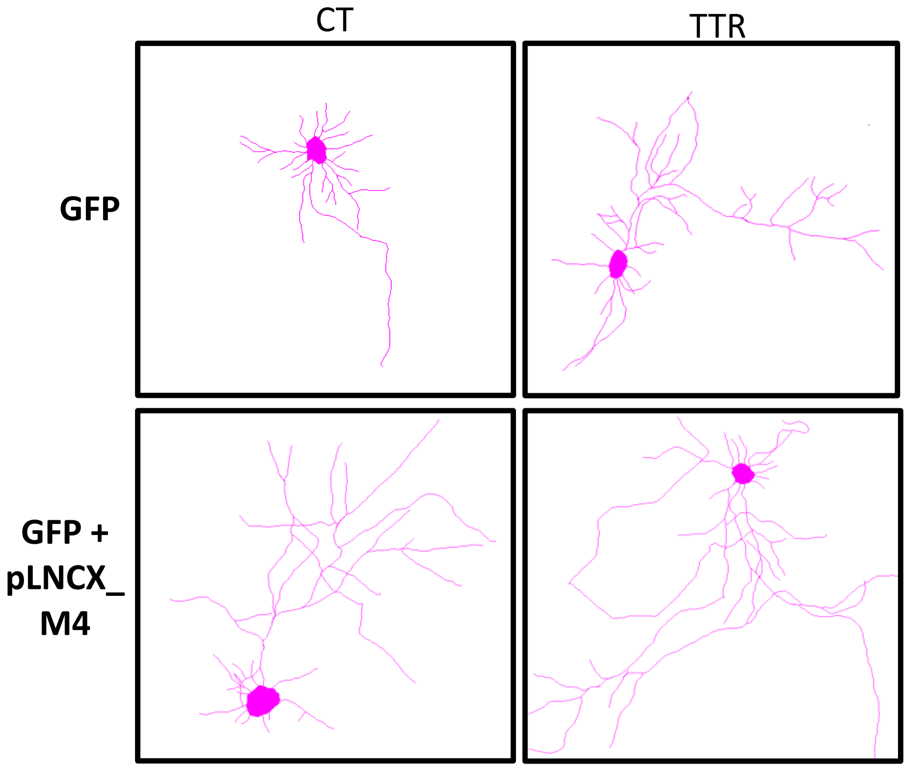

Supplementary Figure 5. Morphological tracings used for neurite outgrowth on Fig.6B (supplementary data). Cultured hippocampal neurons with 14DIV, from TTR KO were transfected with either GFP plasmid (pEGFP), or cotransfected with GFP (pEGFP) and mini-megalin plasmid (pLNCX-M4). 48h later neurons were stimulated, or not, with recombinant mouse TTR (55µg/ml) for 24h, in cultured conditioned medium. The morphological measurements of neurite outgrowth (Number of neurites and total neurite length per cell) were performed using the plugin NeuronJ for the ImageJ software (Meijering et al.,2004 – Methods section). These are the morphological tracings from the representative images on Fig. 6B.

- **Neurite branching TTR KO neurons expressing mini-megalin Control vs TTR-treated neurons**

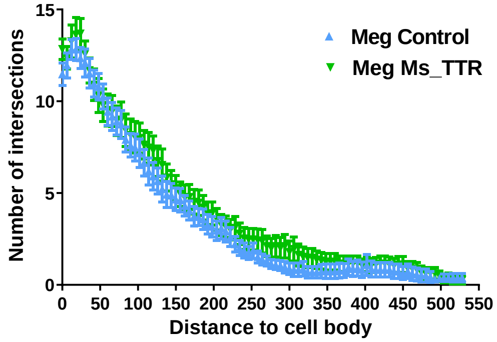

<u>Supplementary Figure 6</u>. Neurite branching in GFP plus mini-megalin expressing neurons, TTR was unable to further increase neuronal branching. TTR KO cultured hippocampal neurons (11DIV) were cotransfected with GFP (pEGFP) and mini-megalin plasmid (pLNCX-M4) for 48h, and stimulated, or not, as indicated, with recombinant mouse TTR (55µg/ml) for 24h. An immunocytochemistry was performed using GFP antibody. (n=12-23 neurons, from 3 independent cultures). Neuronal branching was not increased in neurons overexpressing megalin vs neurons overexpressing megalin and treated with TTR. Statistical analysis was performed using linear mixed model followed by Tukey-Kramer multiple comparison test.

- **Dendritic spine density WT vs TTR KO neurons**

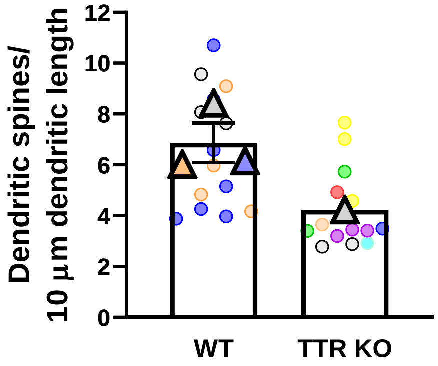

<u>Supplementary Figure 7</u>. The dendritic spine density is reduced in TTR KO cultured neurons, compared with WT neurons. WT or TTR KO cultured hippocampal neurons (11DIV) were transfected with GFP plasmid (pEGFP) for 48h. An immunocytochemistry was performed using GFP antibody. In WT GFP transfected neurons the density of dendritic spines is approximately 7 spines/10 µm dendritic length, whereas in TTR KO neuronal cultures is 4 spines/10 µm, showing a significantly reduction (WT, 3 cultures: culture 1, 3 neurons/3 dendrites; culture 2, 4 neurons/ 4dendrites; culture 3, 7 neurons/ 7 dendrites; TTR KO: 1 culture, 8 neurons/14 dendrites). Symbol triangle represents the average of each culture, and circles represent each neuron of each mice, each color represents each group culture/neuron.

- **Excitatory synapses in vivo –VGLUT1 and PSD95 puncta immununofluorescence and co-localization :**

<u>Megalin$^{+/+}$ mice:</u>

VGLUT1 **PSD95** **Merge**

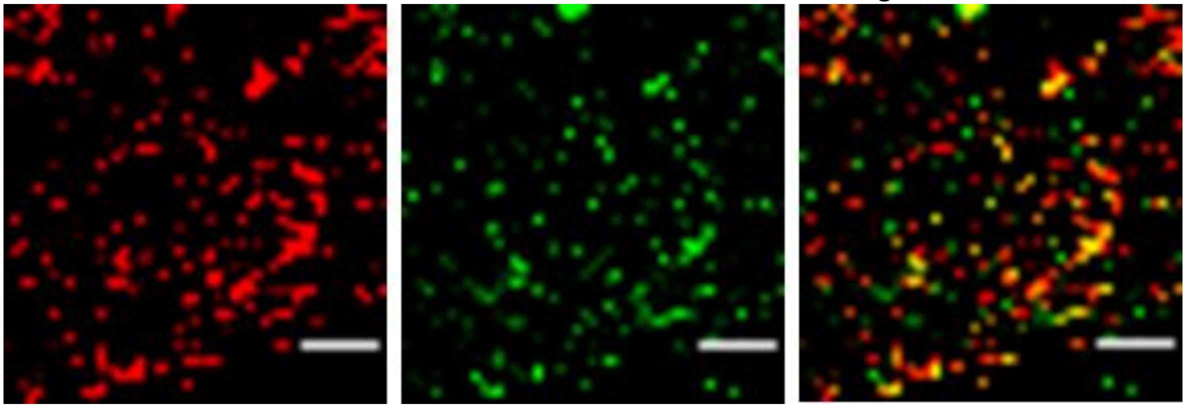

<u>Megalin$^{+/-}$ mice</u>

VGLUT1 **PSD95** **Merge**

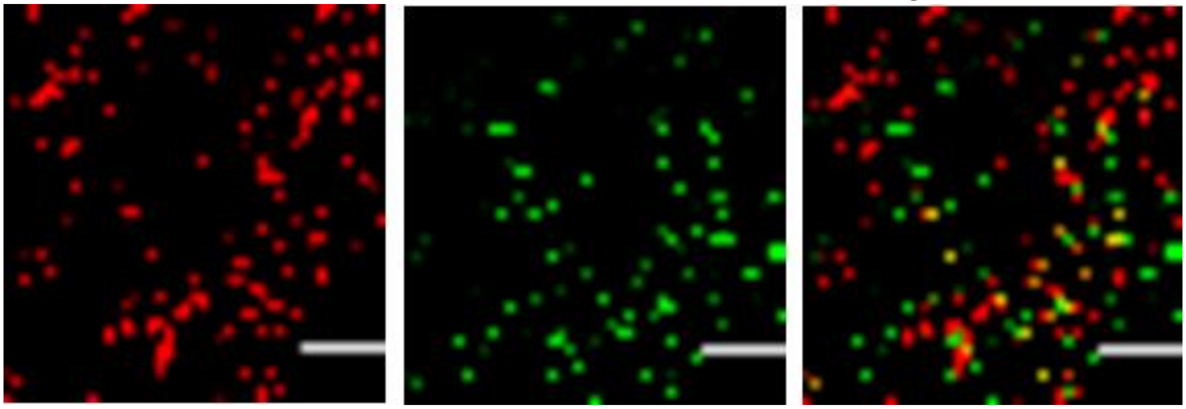

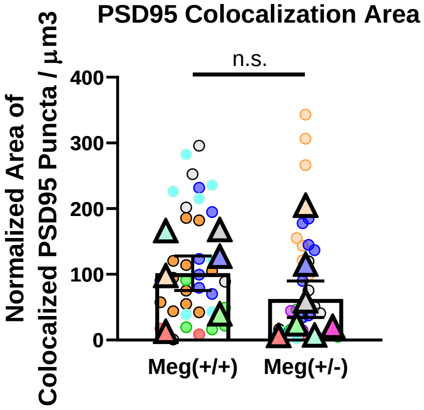

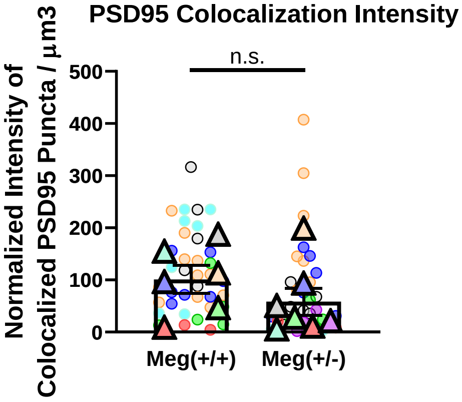

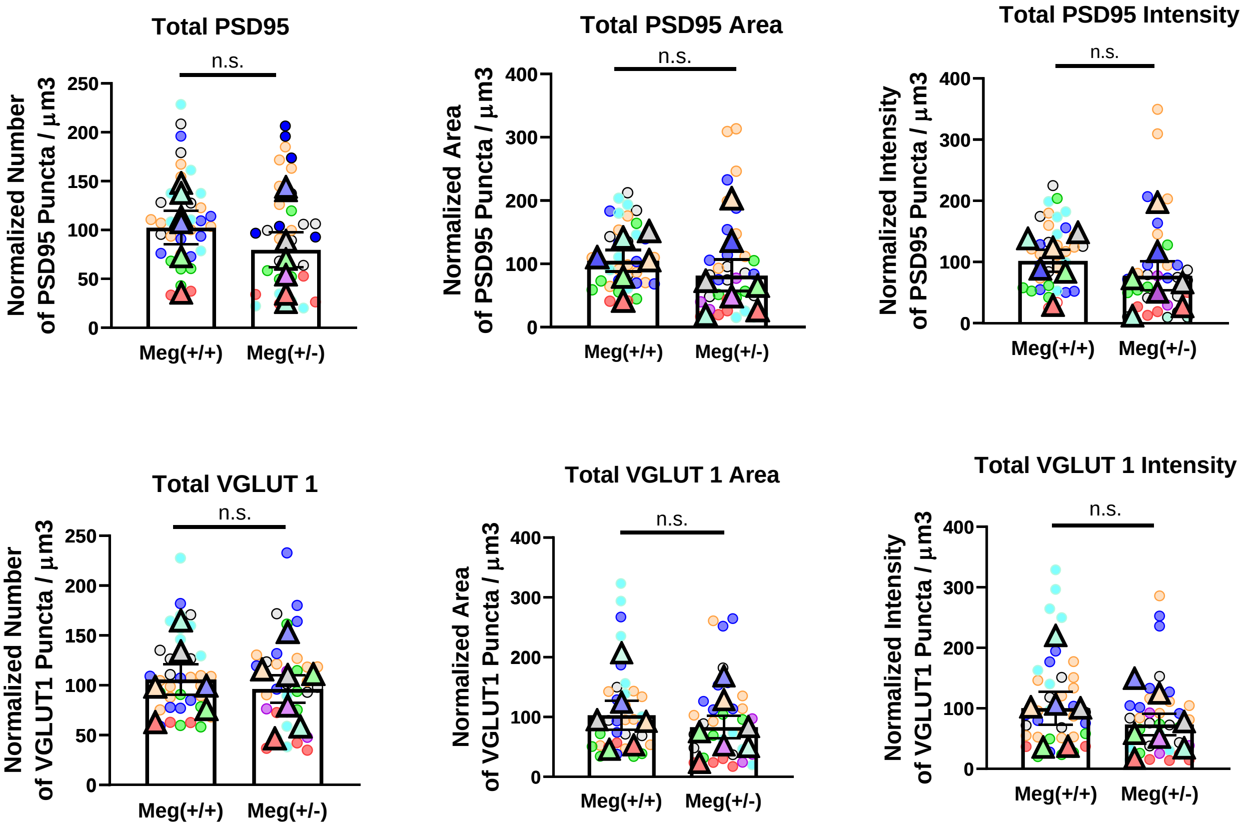

Supplementary Figure 8. The area and intensity of excitatory synaptic puncta, defined by the colocalization of VGLUT1 and PSD95 puncta, is not altered in $Meg^{+/+}$ vs $Meg^{+/-}$ mice. The total number, area and fluorescence intensity of PSD95 and VGLUT1 puncta are not altered in $Meg^{+/+}$ vs $Meg^{+/-}$ mice. Values are normalized to $Meg^{+/+}$ mice. Supplementary representative images from Fig.7 are presented here. Scale bar: 5 µm. Statistical analysis was performed using linear mixed model followed by Tukey-Kramer multiple comparison test. Symbol triangle represents the average of each mice, and circles represent each hippocampal section of each individual mouse, each color represents each group hippocampal section/mice.

Behavioral tests

- **EPM supplementary data**

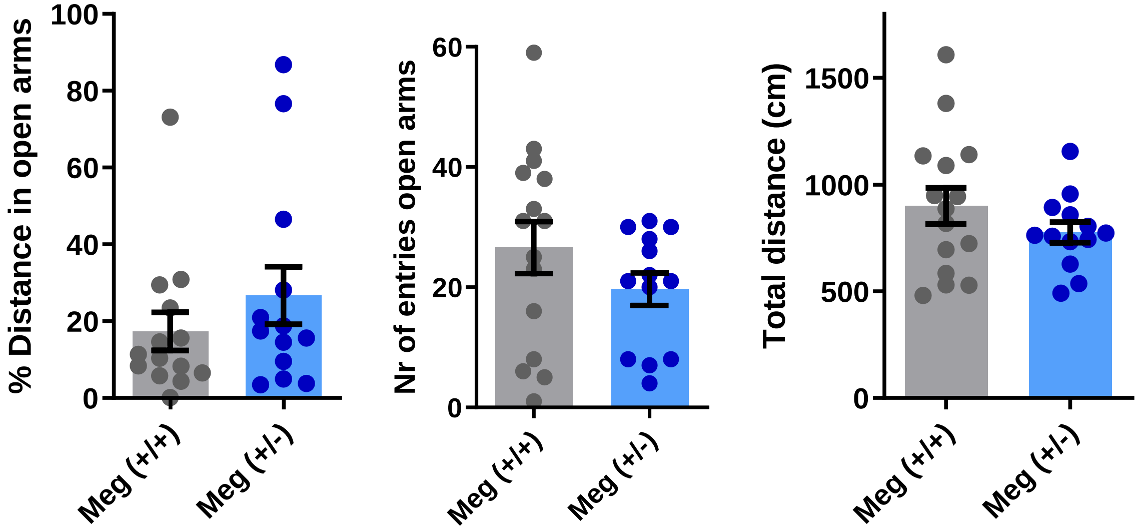

Supplementary Figure 9. Elevated plus maze test (supplemental data). No differences were observed between $Meg^{+/+}$ and $Meg^{+/-}$ mice in the % of distance travelled and number of entries in the open arms, as well as no changes in total distance travelled. Statistical analysis was performed using Student's unpaired t-test.

- **Open Field supplementary data**

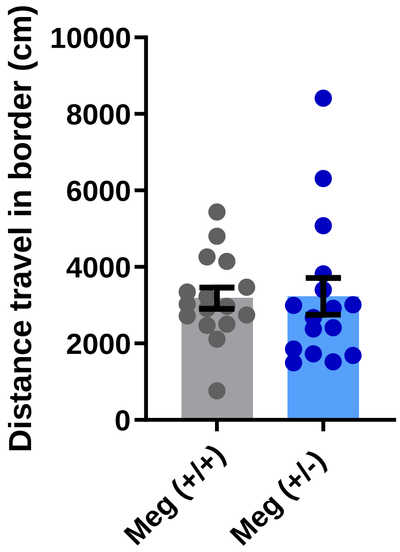

Supplementary Figure 10. Open field test (supplementary data). No differences were observed between Meg$^{+/+}$ and Meg$^{+/-}$ mice in the distance travelled in the border of the apparatus. Statistical analysis was performed using Student's unpaired t-test.

- **Water maze supplementary data:**

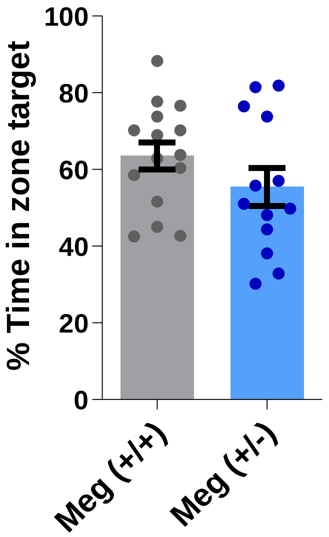

<u>**Supplementary Figure 11**</u>. **Morris water maze** test (supplemental data). No differences were observed between Meg+/+ and Meg+/- mice in the % of time in the zone of the maze where the platform was located during trial session. Statistical analysis was performed using Student's unpaired t-test.

- **SDS-PAGE of recombinant TTR proteins used (purity of protein preparations):**

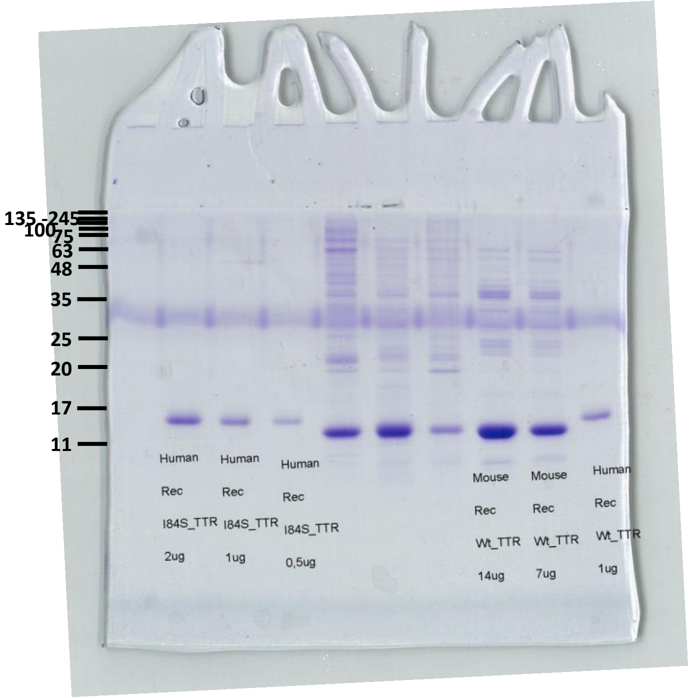

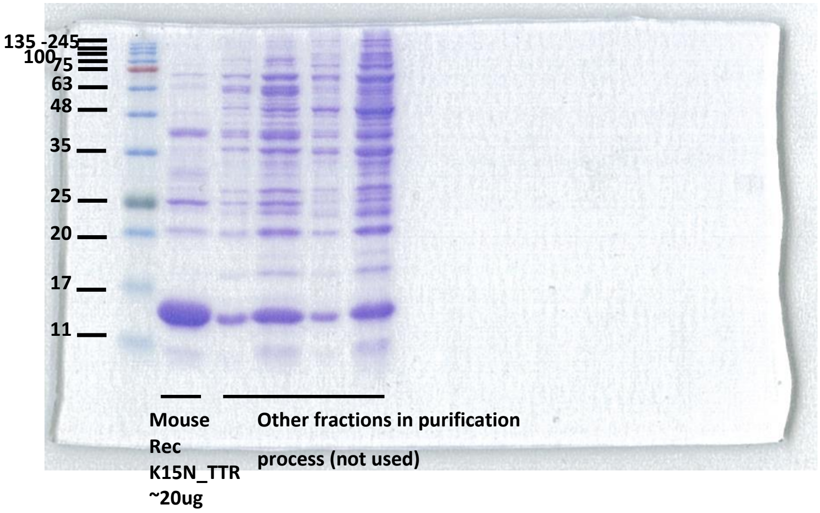

Supplementary Figure 12. **SDS-PAGE recombinant TTR proteins** (supplemental data). The SDS-PAGE gels (4%/10%) were run to confirm the purity of protein preparations after TTR production and purification, as described in methods. Then they were stained with Commassie brilliant blue (R250). Results show a high degree of purity for all the recombinant TTR proteins: Mouse Wt TTR, Mouse K15N TTR, Human Wt TTR, Human I84S TTR. In our paper, ACS Chem. Neurosci. 2019, 10, 1, 704-715, in the supplemental information, Mass spectrometry characterization of mouse Wt and K15N TTR can be found (suppl. Fig.7), as well as the Gel filtration chromatography (suppl. Fig.8).
