# Supplementary material for: Neuronal megalin mediates synaptic plasticity—a novel mechanism underlying intellectual disabilities in megalin gene pathologies
Source: Brain Commun. 2020 Aug 25;2(2):fcaa135. doi: 10.1093/braincomms/fcaa135 (PMC7667529; doi:10.1093/braincomms/fcaa135)
Supplement: fcaa135_Supplementary_Data [file Original Submission Authors Response.pdf › Original Submission Authors Response.pdf]

Answers to the Reviewers:

Answers to the comments of **reviewer #1**:

In the overall comment, the reviewer comments that in the article several aspects of megalin function are not followed up, such as the signal involved in megalin nuclear translocation, the transcription factors, genes downstream and regulation of neuronal survival.

We understand the questions raised by the reviewer, however, we have already addressed megalin-TTR signaling in a previous paper (Gomes J. et al, Cell Death Diff, 2016, 23:1749-1764). In that study, we described the signaling pathways activated by TTR, via megalin, to promote neuritogenic activity and neuronal survival. This signaling involves the mitogen-activated protein kinase (MAPK) pathways (ERK1/2) and Akt through Src, leading to the phosphorylation of transcription factor CREB. In addition, TTR promoted a transient rise in intracellular calcium through NMDA receptors, in a Src/megalin-dependent manner. CREB was also activated by TTR under excitotoxic conditions, contributing to changes in the balance between Bcl2 protein family members, toward anti-apoptotic proteins (Bcl2/BclXL versus Bax). In the present work, we expanded the information on megalin signaling, by demonstrating that megalin C-terminal is translocated to the nucleus, being an important player in the signaling. Therefore, we did not explore further the downstream signaling, and instead, we focused on the effect of megalin (and TTR) signaling in neuronal remodeling and function, both in vitro and in vivo. The signaling and its effects, are referred in the discussion and schematically represented in the thumbnail, the cartoon joining the novel data described in this work, and the data previously described by us in Gomes J. et al, Cell Death Diff,2016.

1. **The reviewer states the reason why individual bands on western blots were cut out, and refer that complete images with loading controls from the same blot should be included for comparison. Same applies for Figure 2.**
   The individual bands were cut out for the sake of space in each figure, but it is important to refer that in all figures where western blot data and images are presented, the loading controls always refer to the same blot. In order to clarify this, we include in supplementary data all membranes of the western blot shown in the article (**Fig1b,d,f-i + Fig2b,d + Fig3d,f + Fig4d +Fig6a +Fig7n-o + Supplementary Fig2,5).** This includes the proteins of interest, and loading controls of the same blots. The proteins molecular weights are indicated, as well as the molecular weights of protein ladder.

2. **The reviewer states that Immunoblot in Fig. 2D is not convincing. There are only 3 data points with high variability. Since western blot data are not very convincing here, the reviewer recommends the inclusion of additional data, for example immunostainings with anti-megalin antibodies.**
   We agree about the quality of the image, but the fact that megalin is a high molecular weight protein (~600 KDa) makes it difficult to obtain good western blots, particularly in regions/cells with megalin reduced expression. In the kidney and choroid plexus, for instance, where megalin has high expression levels, the western blot signal looks better, as shown in Figure 1 B and D. In supplementary data we show all the independent experiments regarding the data presented in Fig.2D. We agree with the reviewer that we could have performed more independent experiments, but we rather prefer to explore different approaches to demonstrate megalin signaling in neurons. Importantly, the data regarding megalin protein levels in Fig. 2D is corroborated by the mRNA levels shown in Fig. 2C in TTRKO neurons, and in Fig.2F in WT neurons.

Regarding immunostaining, we considered doing so, but given the fact that the antibody stains different megalin forms, from post-translational modifications such as glycosylation, to cleavage products, with different molecular weights, it is difficult to evaluate the signal in immunofluorescence. We tested megalin immunostaining in cultured neurons in our paper in Cell Death and Differentiation (Gomes J. et al, Cell Death Diff, 2016, 23:1749-1764), in TTR KO neurons (Fig.1m), and in fact megalin is present in neurons, both at cell body and neurites, but the signal is difficult to quantify given the high density of neuronal cultures, particularly in neurites.

3. **The reviewer refers that in Figure 3F quantification should be provided.**
   In fact the quantification of the western blot in Figure 3F is provided as % of control in the image, but we agree it was not very clear. Now it is presented in the Figure 3F right above the image of the band for LRP2-ICD.

4. **The reviewer states that in Figure 4 the nuclear translocation of megalin effect is barely noticeable. Different way of image presentation (for example choosing other LUT) can help. Additionally, to stringent the significance on nuclear translocation part, NLS mutant of megalin should be included. What are the functional consequences of translocation-deficient mutant? 4D – quality of the immunoblot is very low.**
   Regarding the signal in the nuclear translocation of megalin presented in Fig. 4B-C in a immunofluorescent staining, we agree that is difficult to observe the translocation as the image was presented. So, we altered the Figure 4B using images with higher magnification of the nuclear region, and we believe that the nuclear translocation of megalin induced by TTR treatment is clearly shown now in the image, and represents the data obtained in the quantification (Fig. 4C).
   Considering the experiment using a mutant form of megalin without NLS sequences, and exploring its functional consequences, we agree it is a very interesting experience to further explore this signaling, and in fact we are at the moment pursuing those type of studies to further explore this signaling pathway, but this will be part of a different study.
   Concerning the immunoblot presented in Fig. 4D, we agree that the quality is not the best, but it is affected by the amount of protein loaded in the gel, which was high (150 µg of total protein) to allow the detection of megalin C-terminal region, but impaired the histone staining. Moreover, the method for nuclear fraction isolation, based on high salt concentration nuclear breakage (referred in methods section) affects the running of the samples in the gel, particularly in low molecular weight proteins such as histone H2Ax (~17KDa).

5. **The reviewer points that Figure 5A is not referred in the text. Moreover, he says that in given example images it is hard to judge about neurites, the resolution is too low. Higher magnification or traces of measured neurons should be included.**
   We agree, and added to the text the reference to Figure 5A. Moreover, we agree that it is difficult to judge the neurites in the representative images. Therefore, we improved the image quality, and added the images with the neurites tracing as supplementary data.

6. **The reviewer states that in the neuronal survival assay in Figure 5 no images are provided. Hoechst will stain all nuclei. The definition of dead cells is not clarified in the methods. There are more reliable ways to analyze the cell death, such as propidium iodide staining or Apoptag.**

We agree that representative images are needed, so we added images for each condition to Figure 5 regarding Hoechst 33342 staining.

In fact Hoechst stains all nuclei, but it is possible to distinguish between nuclei of alive and apoptotic neurons. We also added a magnified image of nuclei pointing a live and a dead neuron. We also added to the results regarding this figure section the criteria to distinguish the nuclei from live and dead neurons. Hoechst 33342 allows the assessment of chromatin condensation, in dead neurons, which appear smaller and brighter (pointed in red in the magnified image in Figure 5), whereas nuclei from live cells appear bigger and have a more diffuse staining (pointed in blue) (as described by Crowley et al, Cold Spring Harbor Protocols 2016, doi:10.1101/pdb.prot087205). However, we agree with the reviewer that other methods might be used to assess neuronal death. In fact, in a paper published by us in 2005 (Almeida RD, 2005, Cell Death Diff, 12, 1329-1343), we characterized neuronal death elicited by the same glutamate insult protocol in cultured hippocampal neurons used in the present manuscript. In that work, we demonstrated, using propidium iodide staining, that cell death induced by glutamate is in fact apoptotic (~40-50% neuronal death), and the % of cell death is the same when quantification was performed using Hoechst staining.

7. **The reviewer states that in the images provided in Figure 6, there are many dead fragmented cells and it is difficult to see neurites, traces need to be included.**

   We agree with the reviewer that it might be difficult to assess neurites in the images shown, therefore we added in supplementary data the tracings of the representative neuron in each condition. Importantly, we only quantified neurites in healthy transfected neurons, using Hoechst 33342 to assess neuronal viability. We should say that the transfection process induces some neuronal death, although we use kynurenic acid to reduce toxicity. In the case of figure 6, it refers to TTRKO neurons, which are more sensitive to procedures that cause toxicity, and therefore neuronal death associated to transfection is higher than in WT neurons.

8. **The reviewer refers that in Figure 7 the quality of the representative images for spine analysis is too low for an appropriate spine morphology analysis. It is hard to say if they are mature or not. Another marker, for example staining with PSD proteins should be included in the analysis.**

   We agree that the images presented were not very clear, so we not only improved the image quality, but also included the original images of the neurites from which the representative magnification area was selected. Moreover, we must refer that the images presented represent the maximum projection of the Z-stack images obtained in the confocal microscope, but for the quantification we used the different images of the Z-stack, that allows a more accurate assessment of spines morphology.

   Moreover, the effect in maturation is confirmed with the significant reduction in Meg +/- mice in the active synapses, measured by the co-localization of the presynaptic marker VGLUT1 with postsynaptic PSD95 (Fig. 7 P-Q), as well as the almost statistically significant reduction in PSD95 protein levels in the western blot analysis of the hippocampus (Fig. 7O, p=0.08).

9. **The reviewer states that we should present SDS-PAGE demonstrating the purity of TTR protein preparation.**

   We agree with the suggestion, and added in supplementary data the SDS-PAGE stained with Coomassie Blue showing the purity of Mouse TTR, and the mutated TTR forms used I84S and K15N mutated. Moreover, in our previous paper ACS Chem. Neurosci. 2019, 10, 1, 704-715, in the supplementary information, Mass spectrometry characterization of Mouse WT and K15N TTR can be found (suppl.

Fig.7), as well as the Gel filtration chromatography (suppl. Fig.8). These TTR proteins were obtained using the same protocol for protein production.

Answers to the other comments of **reviewer #2**:

1. **According to the reviewer, the main weakness of the study is the pseudoreplication of data in some experiments. In some experiments, the culture replicate or mouse was appropriately used as the experimental unit for analysis. However, in others, individual neurons or neurites was used, which is clear pseudoreplication and may lead to innacurate conclusions. I suggest re-analysis of these data using the appropriate experimental unit (biological replicate) or using more advanced statistic such as linear mixed effects models that allow use of the mouse/culture replicate as a random variable to account for biological variability. Alternatively, there is so much data in this paper that some of this could be removed without detracting much from the central message. Of particular concern in this regard are the analysis of neurite outgrowth and dendritic spines both in vitro and in vivo. The FRET calcium analysis was also not very robust with only a few neurons per condition.**

We understand the concerns of the reviewer, however, the use of each neuron as an independent experimental unit is adequate in morphological analysis, and commonly used in several papers presenting this type of study, for instance in Cahill et al, 2016, Neuron, 89:566-582. Moreover, the sholl analysis is performed per neuron, by tracing concentric circles around the cell body with increased radii, and determining the number of times each circle crosses a neurite segment. The value in each circle is not an independent measure, and data was analyzed by two way ANOVA, in which we have a dependent variable (the number of neurite crossings), and two independent variables (distance to cell body and treatment/genotype). This criterion was also considered in the paper describing the methodology we used for sholl analysis, involving the Bonfire script for MatLab (Langhammer et al, Cytometry Part A, 2010, 77A: 1160). So, we believe the more accurate methodology is to use the neuron as the experimental unit in all the analysis presented. For the dendritic spine density analysis, in papers analyzing this parameter, such as the one referred above (Neuron, 2016), the experimental unit used is the dendrite. The same applies to the neurons analyzed in vitro. However, despite in the analysis we consider the experimental unit the neuron for neurite length/branching or the dendrite for dendritic spine density, we use different independent cultures (at least 3) and at least 3 mice per group. Nevertheless, we analyzed the data presented in Figure 5B-C as suggested by the reviewer, in this case using the neuronal culture as the experimental unit. The results obtained are similar to the data presented in Fig.5 using the neuron as the experimental unit, and were added as supplementary data.
Moreover, we corrected the legend of Figure 5 regarding the number of independent cultures, referred as 3 independent cultures, but in fact we analyzed the number of neurons referred in the legend (n=220-613) from 4 to 8 independent neuronal cultures for each condition.
Regarding neurons analyzed in Figure 6 B-G, we analyzed neurite number/length/branching in transfected neurons using the physiological method of calcium phosphate co-precipitation, which is the most adequate transfection method for morphological analysis, although its low rate of transfection. This transfection method also induces some neuronal death, although we use kynurenic acid to reduce toxicity. In the case of figure 6 B-G, it refers to TTRKO neurons, which are more sensitive to procedures that cause toxicity. Therefore, the number of neurons analyzed per culture is low. Here we applied the same criteria as for the neurons in the in vivo experiments, considering the neuron as the experimental unit.

Concerning the FRET experiment, we present a Supplementary data table with the FRET raw data for all the neurons analyzed, added in supplementary information. We also clarified the number of neurons analyzed, referred in the Figure 5, since we analyzed 4 to 6 neurons for each independent neuronal culture (2-3 independent cultures), in a total of 12 to 16 neurons per condition.

2. **Another concern is the use of nuclear morphology to assess neuron death/survival. Typically these studies would also use propidium iodide to mark nuclei of dying cells or LDH measurements in the medium. At a minimum, more details are needed about how cells were classified as alive/dead.**

   We agree with the reviewer that the details to classify neurons as alive or dead should be referred clearly. For that, we added to the results section the criteria to distinguish the nuclei from alive and dead neurons, referring that Hoechst allows the assessment of chromatin condensation, in dead neurons, that appear smaller and brighter, whereas nuclei from live cells appear bigger and have a more difuse staining. Moreover, we added in Figure 5D an image with higher magnification pointing a live (blue arrow) and a dead neuron (red arrow), as well as representative images for each condition.

   Regarding other complementary methods used to assess neuronal death, we understand the reviewer concerns, however, in a paper published by us in 2005 (Almeida RD, 2005, Cell Death Diff, 12, 1329-1343), we characterized neuronal death elicited by the same glutamate insult protocol in cultured hippocampal neurons used in the present manuscript. In that work, we demonstrated, using propidium iodide staining, that cell death induced by glutamate is in fact apoptotic (~40-50% neuronal death), and the % of cell death is the same when quantification was performed using Hoechst staining.

3. **In line with Brain Communications policy, the reviewer suggests showing full (uncropped) blots in supplemental data. The histone blot in fig 4D looks very odd which may have affected results.**

   We agree with the reviewer, and have included in supplementary data all membranes of the western blot shown in the article (Fig1b,d,f-i + Fig2b,d + Fig3d,f + Fig4d +Fig6a +Fig7n-o + Supp Fig2,5).

   Regarding the immunoblot presented in Fig. 4D, we agree that the quality is not the best, but it is affected by the amount of protein loaded in the gel, which was high (150 µg of total protein) to allow the detection of megalin C-terminal region, but impaired the histone staining. Moreover, the method for nuclear fraction isolation, based on high salt concentration nuclear breakage (referred in methods section) affects the running of the samples in the gel, particularly in low molecular weight proteins such as histone H2Ax (~17KDa).

4. **The reviewer suggests showing individual data points on all bar charts. This is done for most graphs but not fig 4E.**

   We agree with the reviewer suggestion, and added the data points to the graph presented in Figure 4E.

5. **The reviewer asks what sex of mice were used, and if sexes were mixed were there any sexes effects**

   We agree with the reviewer that this information should have been provided. In this work both sexes were used for the in vivo experiments presented in Fig. 7 and 8. In the water maze test, the first behavioral test performed to compare Meg (+/+) mice with Meg (+/-), we performed a two way ANOVA analysis for genotype and sex effects, and there were no significant sex effects. The same applies for the other behavioral tests (EPM, Open field and NOR). So, we decided to present the final data considering the two sexes together, and perform the morphological analysis also using both sexes

together. Information regarding the number of mice for each sex used has been added to Figure 7 and Figure 8 legends.

6. **The reviewer refers that in supplemental information, there is a confusing numbering system and in Fig S3, the y axis seems to have an error as it is difficult to read the mu symbol in microns.**
   We agree with the reviewer comments, and corrected the figures numbering in supplemental information, and the Fig S3 (now S7) graphic was corrected.

7. **The reviewer also refers a very minor point in the paper, which is the phrase "associated to" is not correct and should be replaced with other phrasing, perhaps "due to".**

   We agree, and replace the expression referred "associated to" for a different one when applicable.

Editorial Office comments:

-please, make sure that the figures and supplementary figures are numbered in the order in which they are first mentioned in the text. We corrected the figure numbering according to the order they appear in the text.

-Fig1b,d,f-i + Fig2b,d + Fig3d,f + Fig4d +Fig6a +Fig7n-o + Supp Fig2,5 : Full- size blots/gels are to be made available as Supplementary material. We added as supplementary material, in a separate file, all the full size blots for the figures mentioned, including the proteins of interest, and loading controls of the same blots. The proteins molecular weights are indicated, as well as the protein ladder molecular weights.

-Please, show individual data points for the following figures as the n<10: Fig3e , Fig4e , Fig5e , Fig6e-g , Fig7d,h. The individual data points were added to Fi.3e and Fig.4e. Regarding Fig5e , Fig6e-g , Fig7d,h, it is not possible to present in the graphic, so we made available as supplementary data an excel file with all the individual data points.

-Please provide the genders of the animals in the study. We provide in the legend of figure 7 and 8 the sexes of the animals used for each behavioral test and for neuronal morphological analysis.

-Please supply an abbreviated summary of up to 50 words (no abbreviations except gene names). This will be used in the table of contents alongside the graphical abstract. Please upload as a separate word document. We supply the abbreviate summary in a separate word document.

- Please a paragraph about data availability analysis must be included within the 'Materials and Methods' section. We added to the manuscript the following information in the material and methods section: Raw data were generated at I3S. The authors confirm that the data supporting the findings of this study are available within the article and supplementary data. Derived data supporting the findings of this study are available from the corresponding author João Gomes upon request.
