# Supplementary material for: Neuronal megalin mediates synaptic plasticity—a novel mechanism underlying intellectual disabilities in megalin gene pathologies
Source: Brain Commun. 2020 Aug 25;2(2):fcaa135. doi: 10.1093/braincomms/fcaa135 (PMC7667529; doi:10.1093/braincomms/fcaa135)
Supplement: fcaa135_Supplementary_Data [file Original Submission Decision Letter.pdf › Original Submission Decision Letter.pdf]

Original Submission Decision Letter

16-Apr-2020

Dear Dr. Gomes,

Thank you for your recent submission BRAINCOM-2020-050 entitled "Neuronal megalin mediates synaptic plasticity - a novel mechanism underlying intellectual disabilities in megalin gene-pathologies" to Brain Communications.

Your manuscript has been reviewed, and while the reviewers and editorial team appreciate many aspects of the paper, they raise some concerns that you will need to address in order for the paper to be considered for publication.

Therefore, I invite you to respond to the reviewer(s)' comments and revise your manuscript. The reviewer(s)' comments are included at the bottom of this message.

In addition to the reviewers' comments, please note our editorial comments added below to comply with our requirements for presentation of scientific data at Brain Communications.

Please format your revised manuscript according to our Instructions to Authors https://academic.oup.com/braincomms/pages/General_Instructions – a summary of key points is attached to help with this.

To revise your manuscript, click the link below or log into https://mc.manuscriptcentral.com/braincom and enter your Author Centre, where you will find your manuscript title listed under "Manuscripts with Decisions." Under "Actions," click on "Create a Revision." Your manuscript number has been appended to denote a revision.

*** PLEASE NOTE: This is a two-step process. After clicking on the link, you will be directed to a webpage to confirm. ***

https://mc.manuscriptcentral.com/braincom?URL_MASK=80f15a97d8104f7dbdc1c4e4252dae31

Please upload a revised manuscript with changes tracked with track changes or in coloured text.

When submitting your revised manuscript, you will be asked to respond to the reviewer(s) comments. Please be as specific as possible in your response to all of the comments.

The editorial team at Brain Communications aims to increase transparency in scientific publishing and to demonstrate the value that peer review brings to the scientific process. Please therefore consider whether you are willing to have reviewer(s)' comments on your paper and your response published as supplementary data with the paper if it is accepted. This is entirely optional and will only be done if both the authors and reviewers agree. While submitting your revision there will be an opportunity to review or update the answer you gave previously, if necessary.

IMPORTANT: Your original files are available to you when you upload your revised manuscript. Please delete any redundant files before completing the submission.

Because we are trying to facilitate timely publication of manuscripts submitted to Brain Communications, we would appreciate your revised manuscript as soon as possible. If it is not possible for you to resubmit within 3 months, the option to submit a revision will expire, and we would then consider your revised paper as a new submission. However, if you need a little more time, please get in touch and we can extend this deadline for you.

Once again, thank you for submitting your manuscript to Brain Communications and I look forward to receiving your revision.

Yours sincerely,
Dr. Ileana Hanganu-Opatz
Editor, Brain Communications

Reviewer(s)' Comments to Author:

Reviewer: 1

Comments to the Author

The manuscript by Gomes et al, provides detailed characterization of megalin function in neurons. Megalin is a multi-functional cell surface receptor which so far has been mostly studied in non-neuronal cells. In this novel work, the authors demonstrate that TTR serves as a ligand and a fine regulator of megalin levels in neurons. TTR triggers proteolytic cleavage of megalin and a nuclear translocation of the C-terminal cytosolic tail, which in terns can be involved in the gene transcription. Furthermore, they show that megalin is involved in neuronal survival, neurite branching, spine maturation and synaptic plasticity which is at the end contributes to learning and memory formation.
This is a well-designed study with a multitude of different biochemical, molecular and cell biology techniques as well as behavioral approaches. The finding could be important for broad neuroscience community. However, I have some criticism related to the quality of presented data which limits my enthusiasm for the manuscript. Moreover, in course of the study, the authors discover interesting aspects of megalin function which they do not follow up but instead move to the next point. One of such examples is nuclear translocation of megalin. It is not clear what signaling is involved, what transcription factors, what genes are downstream and how does it feed-back to neuronal survival?

Please find specific comments below:
1. Figure 1: Why individual bands on western blots are cut out? Complete images with loading controls from the same blot should be included for comparison. Same applies for Figure 2.
2. Figure 2: Immunoblot in 2D is not convincing. There are only 3 data points with high variability. Since western blot data are not very convincing here, I recommend to include additional data for example immunostainings with anti-megalin antibodies.
3. Figure 3F: quantification should be provided
4. Figure 4: nuclear translocation of megalin - the effect is very minor and barely noticeable. Different way of image presentation (for example choosing other LUT) can help. Additionally, to stringent the significance on nuclear translocation part, NLS mutant of megalin should be included. What are the functional consequences of translocation-deficient mutant? 4D – quality of the immunoblot is very low.
5. Figure 5: 5A is not referred in the text. In given example images it is hard to judge about neurites, the resolution is too low. Higher magnification or traces of measured neurons should be included.
6. Neuronal survival assay in Figure 5: No images provided! Hoerst will stain all nuclei. The definition of dead cells is not clarified in the methods. There are more reliable ways to analyze the cell death, such as propidium iodide staining or Apoptag.
7. Figure 6: Judging from the provided images there are many dead fragmented cells and it is difficult to see neurites, traces need to be included.
8. Figure 7: spine analysis – the image quality is too low for an appropriate spine morphology analysis. It is hard to say if they are mature or not. Another marker, for example staining with PSD proteins should be included in the analysis.
9. For TTR production SDS-PAGE demonstrating the purity of protein preparation should be included.

Reviewer: 2

Comments to the Author
Gomes and colleagues present a study of the role of megalin in hippocampal neurons. They oberve that transthyretin (TTR), a megalin ligand, regulates megalin levels in the hippocampus and that TTR activates regulated intracellular proteolysis in. acascade dependent on megalin. Neuronal megalin was important for neurite outgrowth, dendritic spine density, and normal learning and memory in mice.

This study addresses an important topic and the results may have implications for future therapeutic development for people with Donnai-barrow syndrome and similar megalin-linked diseases.

The strengths of the study are the use of multiple approaches in cultured neurons, in vivo in mice, and in silico. Overall the study is well-conducted and most of the results are solid and well interpreted. There was excellent use of randomisation and bliniding in the study. Many experiments have very solid controls (in particular the TTR nanobody blocking TTR effects in culture). I appreciate the hard work, nice presentation, and importance of this study, but have a few suggestions that I think should be addressed before publication.

Critiques for improvement:
1. The main weakness of the study in my view is the pseudoreplication of data in some experiments. In some experiments, the culture replicate or mouse was appropriately used as the experimental unit for analysis. However, in others, individual neurons or neurites was used, which is clear pseudoreplication and may lead to innacurate conclusions. I suggest re-analysis of these data using the appropriate experimental unit (biological replicate) or using more advanced statistic such as linear mixed effects models that allow use of the mouse/culture replicate as a random variable to account for biological variability. Alternatively, there is so much data in this paper that some of this could be removed without detracting much from the central message. Of particular concern in this regard are the analysis of

neurite outgrowth and dendritic spines both in vitro and in vivo. The FRET calcium analysis was also not very robust with only a few neurons per condition.

2. Another concern is the use of nuclear morphology to assess neuron death/survival. Typically these studies would also use propoidium iodide to mark nuclei of dying cells or LDH measurements in the medium. At a minimum, more details are needed about how cells were classified as alive/dead.

3. In line with Brain Communications policy, I suggest showing full (un cropped) blots in supplemental data. The histone blot in fig 4D looks very odd which may have affected results.

4. I also suggest showing individual data points on all bar charts. This is done for most graphs but not fig 4E.

5. What sex of mice were used? If mixed were there any sex effects? If not, why was one sex chosen?

6. In supplemental information, there is a confusing numbering system and in Fig S3, the y axis seems to havce an error as it is difficult to read the mu symbol in microns.

7. A very minor point – while the English is excellent for the most part in this paper, the phrase "associated to" is not correct and should be replaced with other phrasing, perhaps "due to".

Editorial Office comments:
-please, make sure that the figures and supplementary figures are numbered in the order in which they are first mentioned in the text.
-Fig1b,d,f-i + Fig2b,d + Fig3d,f + Fig4d +Fig6a +Fig7n-o + Supp Fig2,5 : Full- size blots/gels are to be made available as Supplementary material.
-Please, show individual data points for the following figures as the n<10: Fig3e , Fig4e , Fig5e , Fig6e-g , Fig7d,h
-Please provide the genders of the animals in the study.
-Please supply an abbreviated summary of up to 50 words (no abbreviations except gene names). This will be used in the table of contents alongside the graphical abstract. Please upload as a separate word document.
- Please a paragraph about data availability analysis must be included within the 'Materials and Methods' section.
