# Supplementary material for: Neuronal megalin mediates synaptic plasticity—a novel mechanism underlying intellectual disabilities in megalin gene pathologies
Source: Brain Commun. 2020 Aug 25;2(2):fcaa135. doi: 10.1093/braincomms/fcaa135 (PMC7667529; doi:10.1093/braincomms/fcaa135)
Supplement: fcaa135_Supplementary_Data [file Revision 1 Decision Letter.pdf › Revision 1 Decision Letter.pdf]

Revision 1 Decision Letter

12-Jun-2020

Dear Dr. Gomes,

Thank you for the submission of your manuscript BRAINCOM-2020-050.R1 entitled "Neuronal megalin mediates synaptic plasticity - a novel mechanism underlying intellectual disabilities in megalin gene-pathologies" to Brain Communications.

The peer reviews of your manuscript are included below. You will see that while the reviewers appreciated some parts of the paper, they raise substantial criticisms and we have decided not to accept the manuscript. Especially the pseudoreplicated data represent a major drawback of the study that persisted even after revision. They are not in line with the Brain Communications policy.

I appreciate that this news will be disappointing after all of the hard work you and your colleagues put into the paper. I hope the reviewer(s)' constructive comments will be helpful as you consider the next steps for the study.

Many thanks for submitting your work to Brain Communications - I hope you will consider Brain Communications for your future work.

Yours sincerely,
Dr. Ileana Hanganu-Opatz
Editor, Brain Communications

Reviewer(s)' Comments to Author:

Reviewer: 2

Comments to the Author
The authors have improved the manuscript based on previous suggestions, however there is still pseudoreplication of data in the paper. Although authors cite previous work using these statistical techniques, they are still not robust, and the field is moving towards consensus that pseudoreplication should be avoided (please see for example https://bmcneurosci.biomedcentral.com/articles/10.1186/1471-2202-11-5). As such, the conclusions drawn in the paper are not robustly supported by the data.

Reviewer: 1

Comments to the Author
The revised version of the manuscript of Gomes and colleagues has significantly improved. The authors adequately addressed the most critical comments and I would recommend this work to be published.
