# Supplementary material for: Neuronal megalin mediates synaptic plasticity—a novel mechanism underlying intellectual disabilities in megalin gene pathologies
Source: Brain Commun. 2020 Aug 25;2(2):fcaa135. doi: 10.1093/braincomms/fcaa135 (PMC7667529; doi:10.1093/braincomms/fcaa135)
Supplement: fcaa135_Supplementary_Data [file Revision 2 Decision Letter.pdf › Revision 2 Decision Letter.pdf]

Revision 2 Decision Letter

16-Jul-2020

Dear Dr. Gomes,

Thank you for your careful revision of statistical approaches based on our reiews.

It is a pleasure to accept your manuscript entitled "Neuronal megalin mediates synaptic plasticity - a novel mechanism underlying intellectual disabilities in megalin gene-pathologies" for publication in Brain Communications. Any comments made by the reviewer(s) are included below.

The next step is for your paper to go through “First Look”. We will contact you again shortly if any final files or formatting changes are required for publication or if we have any queries.

If no changes are needed, your submission will be transferred to our Production Department and you will hear from the Production Editor in due course.

Video abstracts - Brain Communications encourages authors to provide a video abstract to accompany their article. These are hosted on the Brain/Brain Communications YouTube channel. If you are interested in producing a 5-min video abstract to promote your article, then please contact the Editorial Office (brcoms.editorialoffice@oup.com) for further details and guidelines.

Thank you very much for your contribution. On behalf of the Editors of Brain Communications, we look forward to your future contributions to the Journal.

Yours sincerely,

Professor Tara Spires-Jones
Editor
Brain Communications
