# Supplementary material for: Neuronal megalin mediates synaptic plasticity—a novel mechanism underlying intellectual disabilities in megalin gene pathologies
Source: Brain Commun. 2020 Aug 25;2(2):fcaa135. doi: 10.1093/braincomms/fcaa135 (PMC7667529; doi:10.1093/braincomms/fcaa135)
Supplement: fcaa135_Supplementary_Data [file Revision_1_authors_response.pdf › Revision_1_authors_response.pdf]

INSTITUTO DE INVESTIGAÇÃO
E INOVAÇÃO EM SAÚDE
UNIVERSIDADE DO PORTO

Porto, 14 July, 2020

Prof. Tara Spires-Jones
Editor of BRAIN Communications

Dear Prof. Tara,

We would like to once again acknowledge the second opportunity to have our work considered in BRAIN Communications, after a complete reanalysis of a significant part of the data presented in this work, in order to improve rigor and reproducibility, by avoiding data pseudo-replication. We also thank you for letting us realize the improvement of data quality this analysis provides, since plots showing the replicates and the animals/cultures clearly show if we are in the presence of a significant biological effect, and together with the linear mixed model statistical analysis, we can clearly understand if the data is robust or not. From now on, we will use these statistical models for sure, in all our research work.

Regarding the paper data, we performed the statistical analysis using the linear mixed effects model (SAS software) with culture/mouse as a random variable, and, as pointed, we changed all the graphs in figures 5, 6 and 7, which did not have the animal or culture as the experimental unit, and convert them in plots where both replicates of neurons and animal/culture, as the experimental unit, are presented (the same for figure 4C). Moreover, each animal or culture as its own replicates in the same colour, to understand/visualize diversity within each group/condition. We present the plots in all the graphics re-analysed, with the exception of dendritic branching analysis (sholl analysis) (Figures 6 E-G, 7 D and H, and supplementary figure 6) and FRET assay (Figure 5F), since if in these graphics we presented both neurons and culture averages at the same time, it would be a very confusing presentation of data, so we kept the same graphics (single neuron analysis), but the statistical analysis was performed using the linear mixed model, with culture/animal as a random factor. For the majority of the re-analysed data, the statistical significance is similar as the analysis presented before, with slight punctual changes that do not impact on the paper conclusions. Moreover, we describe the statistical analysis in the graphs, figure legends, in a table (Table S1) presented as supplementary information, which combines all the statistical results (including ANOVAs and t-test details,) and a pdf that combines all the statistical results from the linear mixed effects model (similar to the R scripts).

Concerning the linear mixed effects statistical model, we used, instead of the LME4 and lmerTest for R, the SAS® program with the REML function, which stands for linear mixed models, where we got support from the research colleague Igor Lopes, now author, in due time.

We are submitting the revised manuscript with the previous changes tracked in yellow (first revision), and the changes concerning the present revision in green. We send the revised Figures 2, 4, 5, 6 and 7; a revised supplementary information file with 12 supplemental figures; a table (S1) in supplementary information that combines all the statistical results; and a pdf file that combines all the statistical results from the linear mixed effects model.

We now hope that you will find the article suitable to be published in BRAIN Communications.

Looking forward to hear from you.

Yours sincerely

João Carlos Gomes/Andrea Lobo and Maria João Saraiva
Institute for Molecular and Cell Biology (I3S-IBMC)
University of Porto, Portugal
